# A transcriptome-wide, organ-specific regulatory map of *Dendrobium officinale*, an important traditional Chinese orchid herb

**DOI:** 10.1038/srep18864

**Published:** 2016-01-06

**Authors:** Yijun Meng, Dongliang Yu, Jie Xue, Jiangjie Lu, Shangguo Feng, Chenjia Shen, Huizhong Wang

**Affiliations:** 1 College of Life and Environmental Sciences, Hangzhou Normal University, Hangzhou 310036, PR China; 2 Zhejiang Provincial Key Laboratory for Genetic Improvement and Quality Control of Medicinal Plants, Hangzhou Normal University, Hangzhou 310036, China

## Abstract

*Dendrobium officinale* is an important traditional Chinese herb. Here, we did a transcriptome-wide, organ-specific study on this valuable plant by combining RNA, small RNA (sRNA) and degradome sequencing. RNA sequencing of four organs (flower, root, leaf and stem) of *Dendrobium officinale* enabled us to obtain 536,558 assembled transcripts, from which 2,645, 256, 42 and 54 were identified to be highly expressed in the four organs respectively. Based on sRNA sequencing, 2,038, 2, 21 and 24 sRNAs were identified to be specifically accumulated in the four organs respectively. A total of 1,047 mature microRNA (miRNA) candidates were detected. Based on secondary structure predictions and sequencing, tens of potential miRNA precursors were identified from the assembled transcripts. Interestingly, phase-distributed sRNAs with degradome-based processing evidences were discovered on the long-stem structures of two precursors. Target identification was performed for the 1,047 miRNA candidates, resulting in the discovery of 1,257 miRNA--target pairs. Finally, some biological meaningful subnetworks involving hormone signaling, development, secondary metabolism and Argonaute 1-related regulation were established. All of the sequencing data sets are available at NCBI Sequence Read Archive (http://www.ncbi.nlm.nih.gov/sra/). Summarily, our study provides a valuable resource for the in-depth molecular and functional studies on this important Chinese orchid herb.

The *Dendrobium* genus plants, especially as *Dendrobium officinale*, are highly prized folk medicinal herbs in China and the Southeast Asian countries. The dried stems of the *Dendrobium* species, named as “Fengdou”, have been used as a crude drug in traditional Chinese medicine. To date, several bioactive constituents have been identified and validated to make great contributions to the excellent tonic efficacy of “Fengdou”. Interestingly, most of the reported medicinal ingredients from “Fengdou” are secondary metabolites, or are involved in secondary metabolite biosynthesis, transport and catabolism, such as polysaccharides, lectins, alkaloids, flavones, chalcone synthases and sucrose synthases^1^. Genetic studies at molecular level are one of the efficient approaches for thoroughly deciphering the complex regulatory networks underlying the production and accumulation of the medicinal constituents of “Fengdou”. However, this kind of research was greatly hampered due to the lack of the omic data of *Dendrobium*, even for the closely related orchid species. Limited studies on *Dendrobium*, including SSR (simple sequence repeat)-based marker development and functional gene cloning, were carried out mainly based on the small public EST (expressed sequence tag) data set^2^,^3^. The recent advent of high-throughput sequencing (HTS) technology has significantly enhanced our ability for transcriptome- or genome-wide studies through a cost-effective way^4^,^5^. Relying on Roche 454 GS FLX Titanium sequencing platform, Chen’s group provided the first transcriptome map of *Dendrobium officinale*. A total of 553,084 ESTs, which could be assembled into 36,407 unique putative transcripts, were generated from the RNA sample prepared from the stems of *Dendrobium*. Functional analysis revealed that 69 unique transcripts encoded by 25 genes were potentially involved in alkaloid backbone biosynthesis^6^. By combining the second-generation Illumina Hiseq 2000 and the third-generation PacBio sequencing technologies, Yan and his colleagues reported the *de novo* assembled genome of *Dendrobium officinale*^7^. Currently, a total of 23,914 scaffolds are available online (http://202.203.187.112/herbalplant/genome.html#). However, the reported genome has not been completely assembled and not been well-annotated. For example, users could not obtain any information of annotated gene models on either scaffold through the online tool “Gbrowse” (http://202.203.187.112/herbalplant/scaffold.html).

MicroRNAs (miRNAs), a small RNA (sRNA) species of about 21 nt in length, have been uncovered to be a post-transcriptional regulator of genes functioning in diverse biological processes^8^,^9^,^10^. In plants, the miRNAs play essential regulatory roles in nearly all biological processes along the plant lifecycle, such as organ development, stress response and metabolism^9^,^11^. The plant miRNAs are processed from the primary transcripts (pri-miRNAs) of the miRNA genes. Through Dicer-like 1 (DCL1)-mediated cropping, the pri-miRNAs are processed into the pre-miRNAs (the secondary miRNA precursors), and then into the miRNA/miRNA* duplexes with 2-nt 3’ overhangs at both ends^10^. In most cases, the mature miRNAs are incorporated into Argonaute 1 (AGO1)-associated silencing complexes while the miRNA*s are subjected to rapid degradation. Recently, increasing evidences point to the activities of miRNA*s in gene regulation^12^,^13^,^14^,^15^,^16^,^17^. Thus, the current miRBase registries have renamed the highly expressed miRNA*s as miRNA-3ps or miRNA-5ps^18^. The association with the plant miRNAs leads to the binding of AGO1 silencing complexes onto specific target transcripts containing highly complementary recognition sites of the miRNAs. Post-transcriptional regulation of the targets by the plant miRNAs is performed through transcript cleavages by AGO1 complexes. In this regard, identification of the miRNAs and their targets is a critical step for understanding the molecular mechanisms and the corresponding biological essentiality underlying miRNA-implicated physiological and biochemical processes of plants.

In this study, we did a transcriptome-wide study on different organs of *Dendrobium officinale* by combining RNA, sRNA and degradome sequencing (RNA-seq, sRNA-seq and degradome-seq for short). It is a highly integrative approach for organ-specific investigation of the *Dendrobium* transcriptome, which is different from the previous transcriptome analysis of a single organ (stem) of *Dendrobium officinale*^6^, and also different from the recent genome study^7^. First, by using the RNA-seq data of four different organs (root, leaf, stem and flower), a total of 536,558 transcripts assigned to 299,107 unique genes were *de novo* assembled. The expression patterns of these transcripts were investigated through an organ-specific way. As a result, 2,645, 256, 42 and 54 transcripts were identified to be highly expressed in the flowers, the roots, the leaves and the stems, respectively. Based on the sRNA-seq data, 2,038, 2, 21 and 24 sRNAs were identified to be specifically accumulated in the above four organs respectively. A total of 1,047 miRNA candidates were detected by sRNA-seq. Based on secondary structure predictions and sequencing evidences, tens of potential miRNA precursors were identified from the assembled transcripts. Interestingly, phase-distributed sRNAs with degradome-based processing evidences were discovered on the long-stem structures of two miRNA precursors. Then, target identification was performed for the 1,047 miRNA candidates, which results in the discovery of 1,257 miRNA--target pairs involving 147 miRNA candidates and 276 target transcripts. Finally, some biologically meaningful subnetworks involving hormone signaling, development, secondary metabolism and AGO1-related regulation were established. Summarily, our study provides a valuable resource for the in-depth molecular and functional studies on the important traditional Chinese herb *Dendrobium officinale*. All of the RNA, sRNA and degradome sequencing data sets are available at NCBI SRA (Sequence Read Archive; http://www.ncbi.nlm.nih.gov/sra/).

## Results and Discussion

### Transcriptome sequencing and *de novo* assembly

Four organs including root, stem, leaf and flower were collected from *Dendrobium officinale* for paired-end transcriptome sequencing (called RNA-seq hereafter). Two biological replicates were performed for each organ (Table S1-1). Summarily, for roots, a total of 54,469,054 and 71,462,678 raw reads were obtained from the two replicates respectively. After removing the low-quality reads, such as those containing ambiguous base “N” or partial sequences of sequencing adaptors, a total of 54,433,348 (99.93% of the raw reads) and 71,358,490 (99.85% of the raw reads) valid reads were retained for the two replicates respectively. For stems, 50,076,260 and 64,920,086 raw reads, and 50,076,260 (100.00%) and 64,826,004 (99.86%) valid reads were obtained from the two replicates respectively. For leaves, a total of 73,647,052 and 53,904,216 raw reads, and 73,534,024 (99.85%) and 53,862,708 (99.92%) valid reads were obtained from the two replicates respectively. For flowers, 38,776,952 and 38,669,310 raw reads, and 38,736,660 (99.90%) and 38,602,508 (99.83%) valid reads were obtained from the two replicates.

All of the valid reads from the eight RNA-seq data sets were combined and used for transcript assembly (Table S1-2). As a result, a total of 536,558 transcripts which could be assigned to 299,107 unique genes were assembled. The length of these transcripts range from 201 nt to 21,555 nt, with the average length of 931 nt. The number of the assembled transcripts is an order of magnitude higher than that reported by Chen’s group^6^. This discrepancy could result from the sequencing depth of Illumina Hiseq2500 platform employed by our study, which is much deeper than that of Roche 454 GS FLX Titanium platform used in Chen *et al.*’s study.

Next, BLAST algorithm was utilized to identify the transcripts of other organisms homologous to the assembled unique genes of *Dendrobium officinale*. NCBI non-redundant (NR) protein database, the Swissprot database, Clusters of Orthologous Groups of proteins (COG), and Kyoto Encyclopedia of Genes and Genomes (KEGG) were included for functional annotations of the Dendrobium transcriptome (see Materials and Methods for details). As a result, 38,765 (12.96%), 65,286 (21.83%), 52,928 (17.70%) and 70,146 (23.45%) out of 299,107 unique genes of *Dendrobium officinale were annotated by the Swissprot, KEGG, KOG and NR annotation systems, respectively. We noticed that a large portion of* the unique genes were not annotated. In Yan *et al.*’s genome-wide study, only 35,567 protein-coding genes were obtained for *Dendrobium officinale* by employing an approach combining *de novo* and homology-based gene prediction^7^. In this consideration, we deduced that the low annotation rate showed above might be caused by the high percentage of the non-protein-coding genes within the 299,107 unique genes of *Dendrobium.* And, the feature of Illumina sequencing that it could capture small reads in depth might contribute to the large numbers of non-protein-coding genes obtained in this study. Summarily, our study provides a much more comprehensive transcriptome map of *Dendrobium officinale*.

### Organ-specifically expressed transcripts in *Dendrobium officinale*

**Figure 1:**
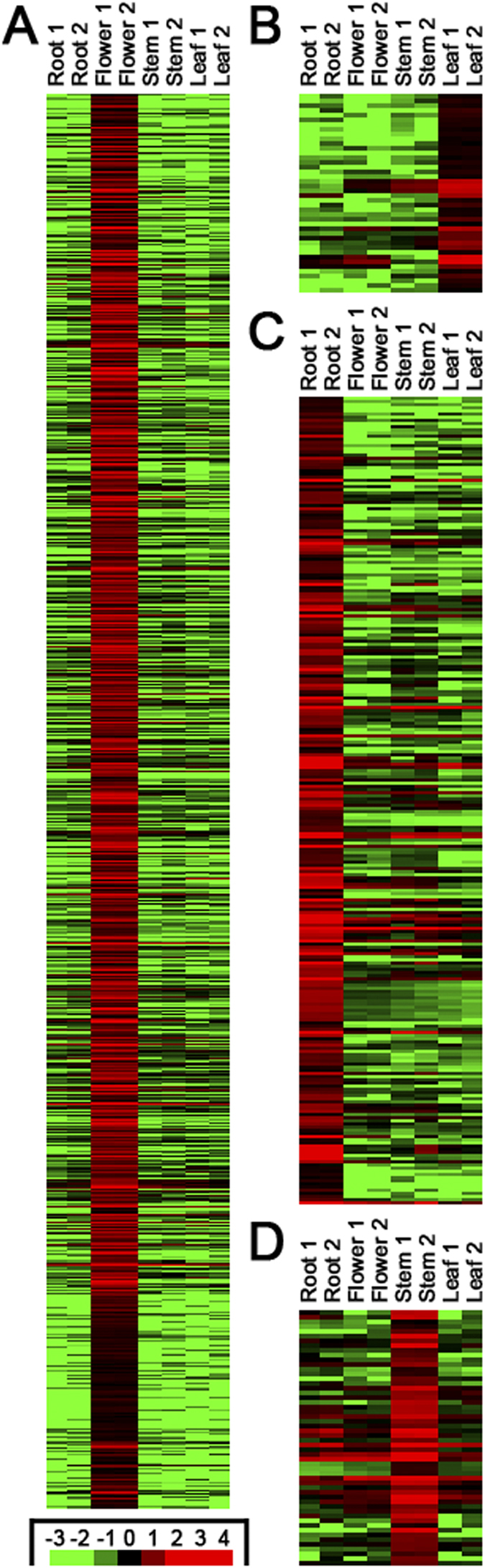
Heatmaps showing the expression patterns of the transcripts highly accumulated in specific organs of *Dendrobium officinale*. (**A**) The expression patterns of the transcripts highly expressed in the flowers. (**B**) The expression patterns of the transcripts highly expressed in the leaves. (**C**) The expression patterns of the transcripts highly expressed in the roots. (**D**) The expression patterns of the transcripts highly expressed in the stems. The expression levels (normalized in RPKM, reads per kilobase of exon model per million mapped reads; please refer to Materials and Methods for RPKM calculation) of all the transcripts were rescaled by log10. For each organ, there are two biological replicates (“Root1” and “Root2”, for example). The heatmaps were drawn by using Treeview^41^. Please refer to Table S2 for the detailed list of all the transcripts.

Based on the RNA-seq data, we intended to investigate the transcriptome-wide discrepancy among the four organs of *Dendrobium officinale*. To this end, we set out to extract the transcripts highly expressed in a specific organ. First, RPKM-based algorithm (see details in Materials and Methods) was employed for calculating the expression levels of the assembled transcripts in each organ. Second, an expression-level-based comparison was made for each two-organ combination. A total of six combinations (“root vs. stem”, “root vs. leaf”, “root vs. flower”, “stem vs. leaf”, “stem vs. flower” and “leaf vs. flower”) were included. Taken “root vs. stem” as an example, a transcript is considered to be highly expressed in the roots when compared to the stems, if its expression level satisfies the following criteria: (1) “Average_root” (the average expression level of the two biological replicates of the roots) of the transcript should be five times or more than “average_stem” (the average expression level of the two biological replicates of the stems) of this transcript. (2) The *p*-value of t-test should be smaller than 0.05. (3) “Average_root” should be 1 RPKM or higher. Third, further identification of the transcripts highly expressed in one organ compared to the other three organs was carried out. For example, the transcripts included in the intersection among the results of “root vs. stem”, “root vs. leaf” and “root vs. flower” were considered to be highly expressed in the roots of *Dendrobium officinale*. As a result, 256 transcripts were identified to be highly expressed in the roots, 42 transcripts were highly expressed in the leaves, and 54 transcripts were highly expressed in the stems. Strikingly, a total of 2,645 transcripts were identified to be highly expressed in the floral organ, which exhibited a great difference compared to the transcript numbers identified from the other three vegetative organs (Fig. 1, Table S2 and Data S1).

To further investigate the potential involvement of the above transcripts in the organ-specific biological processes, the annotations of the transcripts highly expressed in specific organs were manually screened. Fortunately, functional implications were obtained for 45, 4, 12 and 8 transcripts highly expressed in the flowers, the leaves, the roots and the stems, respectively (Table S3). For examples, the transcripts comp133870_c0_seq1, comp137025_c0_seq1, comp138834_c0_seq2, comp152995_c0_seq1 and comp76010_c0_seq1 are highly expressed in the flowers, and they are involved in “carpel development”, “ripening”, “petal development”, “specification of floral organ identity” and “reproduction”, respectively. The transcripts comp156118_c0_seq20, comp158397_c0_seq5 and comp171332_c1_seq2 are highly expressed in the leaves. Based on the GO (Gene Ontology) annotations, comp156118_c0_seq20 and comp158397_c0_seq5 are implicated in “red/far-red light phototransduction” and “shoot development” respectively, and comp171332_c1_seq2 encodes a protein possessing blue light photoreceptor activity. The transcripts comp148295_c0_seq2 and comp167541_c0_seq11 are highly accumulated in the roots, and they are involved in “radial pattern formation” and “lateral root development” respectively. The below-ground interactions between fungi and plant root systems are the widespread phenomenon for the orchids (including *Dendrobium officinale*) during their lifetime^19^,^20^. Notably, ten transcripts highly enriched in the roots of *Dendrobium officinale* play a role in “defense response” (Table S3). As mentioned above, the dried stems of the *Dendrobium* species, named “Fengdou”, are used as a crude drug in traditional Chinese medicine. Several bioactive constituents identified from “Fengdou”, such as polysaccharides, lectins, alkaloids, flavones, chalcone synthases and sucrose synthases are secondary metabolites, or are involved in secondary metabolite biosynthesis, transport or catabolism^1^. Interestingly, eight transcripts highly expressed in the stems of *Dendrobium officinale* are involved in secondary metabolic pathways or carbohydrate transport and metabolism (Table S3). Taken together, the expression level- and functional annotation-based analysis of the *Dendrobium* transcriptome indicates that several organ-specifically expressed transcripts encode protein products with organ-specific functions.

### Sequencing of the sRNAs from four different organs

Small RNAs (sRNAs) including miRNAs play essential roles in transcriptional or post-transcriptional regulation of gene expression in plants^11^. In this study, we performed organ-specific sRNA sequencing (sRNA-seq) to obtain a global view of the sRNA expression patterns in the four organs (root, stem, leaf and flower) of *Dendrobium officinale*. Two biological replicates were performed for each organ. Briefly, 2,289,842 and 1,348,544 different sRNA sequences were obtained from the two biological replicates of roots respectively, 3,982,881 and 5,238,458 sRNAs were obtained from the two replicates of stems respectively, 2,512,564 and 2,664,190 sRNAs were obtained from the two replicates of leaves respectively, and 2,800,831 and 2,142,620 were obtained from the two replicates of flowers respectively (Fig. S1). Surprisingly, for each organ, only a small intersection was observed between the two replicates. Specifically, the numbers of the sRNAs included in the intersections between the two replicates of root, stem, leaf and flower are 193,576, 699,781, 317,921 and 216,801, all of which contribute less than 20% of the eight sRNA-seq data sets. On the other hand, it indicates that the complexity of the sRNA population existing in *Dendrobium officinale* could not be thoroughly uncovered by HTS with frequently used sequencing depth (about 5 to 10 million of reads). For the following analysis, the sRNAs from the two replicates of each organ were combined respectively. As a result, a total of 3,444,810, 8,521,558, 4,858,833 and 4,726,650 non-redundant sRNAs were cloned from the roots, the stems, the leaves and the flowers of *Dendrobium*, respectively (Fig. S1). Based on these non-redundant sequences, the numbers of the sRNAs included in the intersections between two different organs were calculated. As shown in Fig. S1, the most abundant intersection was detected between the stems and the leaves, indicating the homogeneity of the sRNA populations between the two organs is higher than those of the other organ combinations.

Next, we set out to search for the sRNAs specifically accumulated in only one organ. An organ-specific sRNA is defined as follows: (1) The accumulation level of the sRNA should be 10 RPM or higher, which could be detected from at least one sequencing replicate of the organ. (2) This sRNA could not be detected from any of the sequencing data sets of the other three organs. Based on the above criteria, a total of 2,038, 21, 2 and 24 sRNAs were identified to be specifically accumulated in the flowers, the leaves, the roots and the stems of *Dendrobium officinale*, respectively (Table S4). A striking contrast exists between the large number of the sRNAs specifically enriched in the floral organ and the small numbers of the sRNAs specifically accumulated in the other three organs. This observation might be explained by the discrepancy between the reproductive organ and the vegetative organs. In other words, a peculiar population of highly expressed sRNAs might be required for flowering or other reproductive programs.

### Secondary structure- and sequencing-evidenced identification of miRNA precursors

To date, limited studies have been reported for miRNA identification in *Dendrobium*. In Yan *et al.*’s study, 1,005 miRNA genes were predicted on the assembled genome of *Dendrobium officinale*^7^. However, when seeking for the related information of these miRNAs, no detailed data was available in the supplementary materials of that publication, and the miRNA annotations were also not available in the online database (http://202.203.187.112/herbalplant/genome.html#). Thus, we were not clear the way they got evidences for miRNA annotations. In the present analysis, based on the sRNA-seq data, we intended to do a comprehensive search for the miRNA genes in *Dendrobium officinale*. First, the full list of the miRNAs registered in miRBase (release 21), including 35,828 miRNAs from 223 organisms, was obtained. Then, the accumulation levels of the 35,828 miRNAs in four organs of *Dendrobium officinale* were extracted from our sRNA-seq data sets. The miRNAs undetectable in the eight sequencing data sets were discarded. As a result, a total of 6,804 miRNAs from 163 organisms were detectable in at least one of the eight data sets. After removing the redundant miRNA sequences (in some cases, one miRNA could be shared by several organisms), a total of 1,047 miRNAs were retained, and were renamed from dof-miR-1 to dof-miR-1047 for *Dendrobium officinale* (Table S5). The number of miRNA candidates is quite equivalent to that reported by Yan and his colleagues^7^. However, we noticed that among the 1,047 miRNA candidates, a portion of them were reported in non-plant organisms according to miRBase. To date, only two miRNAs in *Arabidopsis thaliana*, miR854 and miR855, have been reported to be conserved in *Caenorhabditis elegans*, *Mus musculus* and *Homo sapiens*^21^. Thus, whether the non-plant sRNAs expressed in *Dendrobium officinale* are reliable miRNA candidates remains ambiguous.

As introduced above, mature miRNAs are processed from the stem-loop structured precursors through DCL1-mediated two-step cropping in plants^9^,^10^. In this regard, a secondary structure-based approach was employed to identify the precursors encoding strong miRNA candidates in *Dendrobium officinale*. First, the 1,047 miRNA candidates were mapped onto the 536,558 assembled transcripts, and the transcripts with perfectly mapped miRNAs were retained. As a result, a total of 186 transcripts with 82 different miRNA loci were identified. To see whether these transcripts could serve as the miRNA precursors, secondary structure prediction was performed by using the online server RNAfold^22^. After prediction, the first-step screening was performed by developing an in-house script obeying the following rule: on a transcript, 50% or more of the miRNA candidate loci should be paired with another region. Specifically, according to the dot-bracket formula depicting the predicted structure, 50% or more of the miRNA loci should be covered by “(” or “)” (a bracket indicates that the corresponding base will be paired with the other base when the secondary structure is formed), and the brackets should be in the same direction. After the first screening, a total of 158 transcripts were retained. Then, the predicted secondary structures of these transcripts were subjected to manual screening. As mentioned above, the canonical miRNA precursors could form featured stem-loop structures. Thus, the transcripts failed to form structures containing stem-loop regions were discarded. As a result, 110 transcripts with miRNA candidate loci on the stem regions of the stem-loop structures were identified.

**Figure 2:**
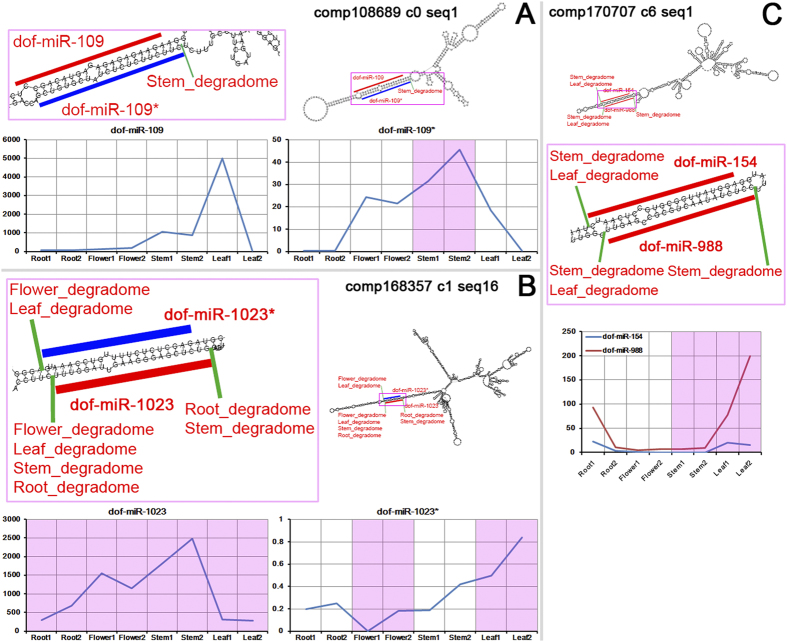
Examples of highly structured microRNA (miRNA) precursor candidates identified from *Dendrobium officinale*. (**A**) The transcript comp108689_c0_seq1 assembled by RNA-seq reads could form an internal hairpin structure with a long-stem region (partially delineated by a pink box) encoding dof-miR-109 (red line) and dof-miR-109* (blue line). The accumulation levels (normalized in RPM, reads per million; please refer to Materials and Methods for RPM calculation) of dof-miR-109 and dof-miR-109* in the eight small RNA (sRNA) sequencing libraries (roots, flowers, stems and leaves, two biological replicates for each organ) are shown in the two diagrams respectively. The expression levels of dof-miR-109* in the two libraries from the stems were highlighted in pink background color since degradome signatures from the stems of *Dendrobium officinale* were detected at the 3’ end of dof-miR-109*. (**B**) The transcript comp168357_c1_seq16 assembled by RNA-seq reads could form an internal hairpin structure with a long-stem region (partially marked by a pink box) encoding dof-miR-1023 (red line) and dof-miR-1023* (blue line). The accumulation levels (in RPM) of dof-miR-1023 and dof-miR-1023* in the eight sRNA libraries are shown in the two diagrams respectively. The expression levels of dof-miR-1023 in the eight libraries were highlighted in pink background color since degradome signatures from the four organs of *Dendrobium officinale* were detected at the 5’ end of dof-miR-1023. The expression levels of dof-miR-1023* in flowers and leaves were also highlighted because degradome signatures from the two organs were detected at the 3’ end of dof-miR-1023*. (**C**) The transcript comp170707_c6_seq1 assembled by RNA-seq reads could form an internal hairpin structure with a long-stem region (partially delineated by a pink box) encoding two miRNA dof-miR-154 and dof-miR-988 (both denoted by red lines). The accumulation levels (in RPM) of the two miRNAs in the eight sRNA libraries are shown in the diagram together. And, their expression levels in the stems and leaves were highlighted in pink background color because degradome signatures from the two organs were detected at the 3’ end of dof-miR-154 and at the 5’ end of dof-miR-988. The secondary structures of the three transcripts were predicted by using RNAfold (http://rna.tbi.univie.ac.at/cgi-bin/RNAfold.cgi)^22^.

It has been reported that the intermediates produced from DCL1-mediated processing of the miRNA precursors could be partially detected by degradome sequencing^23^. And, the cloning of the mature miRNA partner, miRNA*, is one of the favorable prerequisites for defining a standard miRNA gene^24^,^25^. In this regard, based on the sRNA and degradome sequencing data, we set out to obtain further supports for the above identified miRNA precursors. In this analysis, we focused on the precursors encoding the highly accumulated miRNAs. Based on the sRNA-seq data, the accumulation levels of the mature miRNAs should be 5 RPM or higher in at least one organ of *Dendrobium officinale* (values averaged from the two replicates of an organ). As a result, a total of 94 precursors were identified (Fig. S2 to S4, and Data S2 to S4). Then, we searched for the miRNA*s on the 94 precursors. For a miRNA*, it should form a short RNA duplex with the mature miRNA on the secondary structure of their precursor. And, the duplex should possess 2-nt 3’ overhangs at both ends, which are resulted from DCL1-mediated cleavages^9^,^10^. Besides, the miRNA* should be detectable in at least one of the eight sRNA-seq data sets. As a result, 79 out of the 94 precursors possess miRNA* loci (Fig. S3 and S4, and Data S3 and S4). Finally, degradome signals supporting the processing of the precursors by DCL1 were identified for 18 out of the 94 precursors (Fig. S4 and Data S4). Among the 18 precursors, 14 have detectable miRNA* loci. For examples, the predicted secondary structure formed by the transcript comp108689_c0_seq1 contains a large stem-loop substructure (Fig. 2A). Dof-miR109 (homologous to miR529-5p in *Brachypodium distachyon* and *Zea mays*) and dof-miR109* were identified on the stem region of the substructure. A degradome signature from the stems of *Dendrobium officinale* was detected at the 3’ end of dof-miR109*, supporting the processing of the miR109/miR109* duplex from the precursor by DCL1. Interestingly, both miR109 and miR109* (especially for miR109*) are highly accumulated in the stems of *Dendrobium officinale*, which correlates well with the degradome signal. Dof-miR1023 (homologous to miR159 family members in various plant species, see Table S5) and dof-miR1023* were identified on the stem-loop substructure formed by comp168357_c1_seq16 (Fig. 2B). Several degradome signals from the four organs of *Dendrobium officinale* were detected at the 3’ ends of dof-miR1023 and dof-miR1023*, and at the 5’ end of dof-miR1023, supporting the processing of the miR1023/miR1023* duplex from the precursor. Dof-miR154 (homologous to miR171i-5p in *Oryza sativa*) and dof-miR988 (homologous to miR171 family members in *Malus domestica*, *Prunus persica* and *Citrus trifoliata*) were identified on the stem-loop substructure formed by comp170707_c6_seq1 (Fig. 2C). Notably, these two miRNAs could form a short duplex with 2-nt 3’ overhangs at both ends. Degradome signatures supporting the processing of the short duplex from its precursor were detected in the leaves and the stems of *Dendrobium officinale*. Interestingly, both miRNAs are highly accumulated in the leaves.

### Phase-distributed sRNAs identified on the long stems of the miRNA precursors

**Figure 3:**
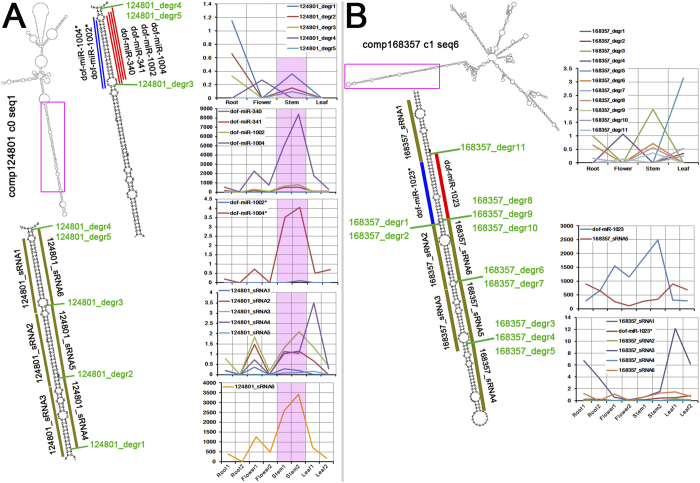
Tandemly distributed small RNAs (sRNAs) identified on the highly structured microRNA (miRNA) precursor candidates in *Dendrobium officinale*. (**A**) The transcript comp124801_c0_seq1 assembled by RNA-seq reads could form an internal hairpin structure with a long-stem region (partially delineated by a pink box). In addition to generating miRNAs (dof-miR340, dof-miR341, dof-miR1002 and dof-miR1004) and miRNA*s (dof-miR1002* and dof-miR1004*), the long-stem region potentially encodes three pairs of tandemly distributed sRNAs (124801_sRNA1 and 124801_sRNA6, 124801_sRNA2 and 124801_sRNA5, and 124801_sRNA3 and 124801_sRNA4). Each pair possesses 2-nt 3’ overhangs. Five degradome signatures (124801_degr1 to 124801_degr5) were detected at the ends of certain tandemly distributed sRNAs. And, 124801_degr3 also appeared at the 5’ ends of dof-miR-340 and dof-miR-341, and 124801_degr4 and 124801_degr5 are present at the 3’ ends of dof-miR-1004. The accumulation levels (normalized in RPM, reads per million; please refer to Materials and Methods for RPM calculation) of the degradome signatures, the miRNAs, the miRNA*s and the tandemly distributed sRNAs are shown in the diagrams on the right of the panel. Their accumulation levels in the stems of *Dendrobium officinale* were highlighted in pink background color. (**B**) The transcript comp168357_c1_seq6 assembled by RNA-seq reads could form an internal hairpin structure with a long-stem region (partially included in a pink box). Within this region, three pairs of sRNAs (including 168357_sRNA2 and 168357_sRNA6, 168357_sRNA3 and 168357_sRNA5, and the dof-miR-1023/dof-miR-1023* duplex) along with two unpaired sRNAs (168357_sRNA1 and 168357_sRNA4) were identified to be distributed tandemly. Each pair possesses 2-nt 3’ overhangs. Eleven degradome signatures (168357_degr1 to 168357_degr11) were detected at the ends of certain tandemly distributed sRNAs. The accumulation levels (in RPM) of the degradome signatures, the miRNAs, the miRNA*s and the tandemly distributed sRNAs are shown in the diagrams on the right of the panel. The secondary structures of the two transcripts were predicted by using RNAfold (http://rna.tbi.univie.ac.at/cgi-bin/RNAfold.cgi)^22^.

Previous reports showed that in addition to the miRNAs and the miRNA*s, multiple distinct sRNAs, also called miRNA-like RNAs, were originated from the stem regions of the miRNA precursors in *Arabidopsis thaliana*^26^,^27^. These miRNA-like RNAs are often arranged in phase, and can form duplexes with 2-nt 3’ overhangs just as the miRNA/miRNA* duplexes. Besides, these miRNA-like RNAs might share the same biogenesis pathway with their sibling miRNAs. Systematic examination of the public sRNA-seq data from four additional plant species and four animals showed that such miRNA-like RNAs were widespread in the eukaryotes^27^. During the identification of the miRNA precursors in *Dendrobium officinale*, we noticed that some of the transcripts, such as comp44555_c0_seq1, comp124801_c0_seq1, comp164879_c1_seq2, comp168357_c1_seq6 and comp169101_c1_seq1, could form structured precursors with long-stem regions (Fig. S2 to S4). In this regard, we mapped the sRNAs from the eight sRNA-seq data sets onto the long-stem regions of the above mentioned precursors, and the perfectly mapped sRNAs were retained to search for the miRNA-like RNAs. Fortunately, we identified phase-distributed miRNA-like RNAs on the long-stem regions of two transcripts (Fig. 3). Specifically, on the long stem formed by comp124801_c0_seq1, in addition to the four miRNAs (dof-miR340, dof-miR341, dof-miR1002 and dof-miR1004) and the two miRNA*s (dof-miR1002* and dof-miR1004*), six phase-distributed miRNA-like RNAs were discovered (Fig. 3A). Notably, the six miRNA-like RNAs form three short duplexes with 2-nt 3’ overhangs. Degradome signatures supporting the processing of these duplexes from the precursor were detected at the 5’ end of the miRNA-like RNA 124801_sRNA4, and at the 3’ ends of 124801_sRNA4, 124801_sRNA5 and 124801_sRNA6. Among the degradome signatures, the ones (12408_degr1 and 12408_degr2) detected at both ends of 124801_sRNA4, and the one (12408_degr5) at the 3’ end of 124801_sRNA6 (also at the 3’ end of dof-miR1004) have strong signal intensity in the stems of *Dendrobium officinale*. Interestingly, dof-miR1004 and its partner dof-miR1004*, 124801_sRNA4 and its partner 124801_sRNA3, 124801_sRNA2, and 124801_sRNA6 are also highly accumulated in the stems. Although no such expression correlationship was observed between the sRNAs and the degradome signatures on another long stem formed by the transcript comp168357_c1_seq6, phase-distributed sRNA duplexes were discovered (Fig. 3B). The duplexes 168357_sRNA3/168357_sRNA5 and 168357_sRNA2/168357_sRNA6 along with dof-miR1023/dof-miR1023* are arranged in phase on the long-stem region. Besides, 168357_sRNA1 and 168357_sRNA4 without detectable partners appear at the 5’ ends of dof-miR1023* and 168357_sRNA5, respectively. Degradome signatures supporting the processing of these phase-distributed sRNAs from the precursor were detected at the 3’ ends of dof-miR1023, dof-miR1023*, 168357_sRNA4, 168357_sRNA5 and 168357_sRNA6. Taken together, the above results indicate that the miRNA-like RNAs might be produced from the long-stem regions of certain miRNA precursors in *Dendrobium officinale*.

### Transcriptome-wide identification of the miRNA--target pairs in different organs of *Dendrobium officinale*

It has been recognized that miRNAs play an essential role during plant growth and development through post-transcriptional regulation of their target genes^9^,^11^. In plants, the miRNAs are frequently incorporated into the AGO1 proteins. Guided by the miRNAs, the AGO1-containing silencing complexes bind onto the specific target transcripts with the regions highly complementary to the miRNAs. It results in the cleavages of the targets by AGO1 complexes between 10^th^ and 11^th^ nucleotides of the regulatory miRNAs. Degradome-seq has been widely used for high-throughput cleavage site mapping, thus for the identification or confirmation of the miRNA--target pairs. In this study, the above identified 1,047 miRNA candidates (Table S5) were included for target prediction. Obviously, based on sequencing data and structure prediction, a portion of these miRNAs are not highly accumulated (less than 5 RPM) in the four organs of *Dendrobium officinale*, and only a small portion of the miRNAs could find their precursors. However, we considered that the limited biological samples investigated could result in the failure of detecting the highly accumulated miRNAs and their precursors. On the other hand, we should acknowledge that the 1,047 miRNA candidates were detectable in *Dendrobium officinale*, and were conserved in some other organisms. In this regard, target prediction was performed for the 1,047 miRNA candidates (see details in Materials and Methods). Based on the degradome-seq data, a total of 1,257 miRNA--target pairs involving 147 miRNA candidates and 276 transcripts were identified (Fig. S5 and Data S5 to S6).

Among the above identified miRNA--target pairs, organ-specific regulation was observed for several pairs based on degradome-seq data (Fig. S6). For examples, the transcript comp150463_c0_seq1 was specifically regulated by dof-miR522 and dof-miR523 in leaves. Also in leaves, comp169140_c0_seq5 was specifically regulated by the above two miRNAs. In stems, comp153832_c0_seq1 was specifically cleaved by dof-miR992, and comp86146_c0_seq1 was specifically cleaved by seven miRNA candidates. In roots, comp163430_c1_seq2 was specifically regulated by dof-miR992, and comp168564_c0_seq2 was specifically regulated by dof-miR1004. In flowers, comp172594_c3_seq3 was specifically regulated by dof-miR831, and comp126829_c0_seq1 was specifically cleaved by dof-miR1047. Interestingly, some regulatory signals were specifically detected in the vegetative organs including root, stem and leaf. For example, the cleavages of comp162234_c0_seq2 by dof-miR804, and the cleavages of comp167551_c0_seq1 by dof-miR992 were specifically detected in the vegetative organs. Besides, some cleavage signals were specifically detected in the leaves and the stems, suggesting the regulatory homogeneity of certain miRNA--target pairs between the two organs. For example, the cleavages of comp165418_c0_seq1 by three miRNAs, and the cleavages of comp166416_c0_seq1 by six miRNAs were specifically detected in the leaves and the stems. It is a reasonable observation since both leaf and stem of *Dendrobium officinale* are “green” organs.

**Figure 4:**
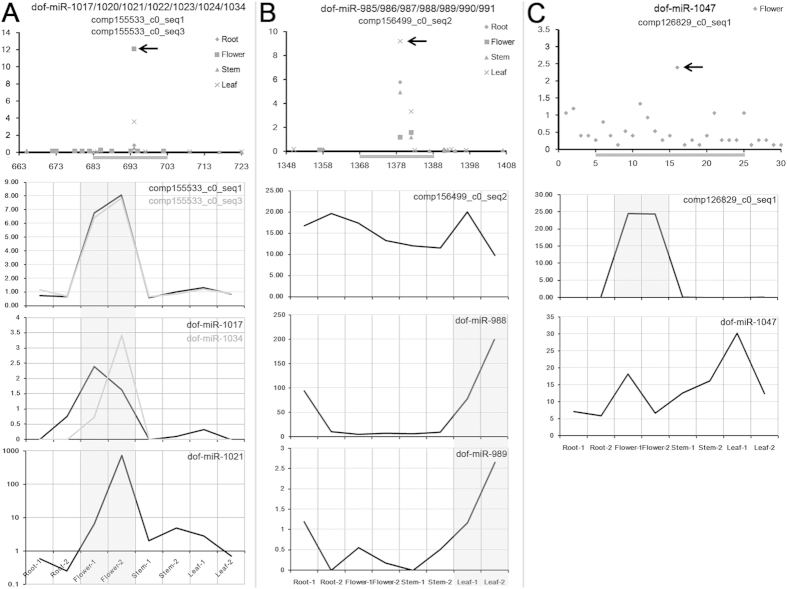
MicroRNA--target pairs supported by degradome signatures, and comparison of expression patterns between the microRNAs and their targets. (**A**) Comp155533_c0_seq1 and comp155533_c0_seq3 were predicted to be targeted by seven microRNAs (dof-miR-1017/1020/1021/1022/1023/1024/1034). The scatter diagram shows the degradome-seq data supporting the predicted regulation. *Y* axis measures the levels (in RPM, reads per million) of the degradome signatures, and *X* axis represents the region surrounding the microRNA binding site (grey horizontal line). Compared with the signatures from the other organs, the most abundant one was identified from the flowers (arrowhead). The first line chart shows the levels (in RPKM, reads per kilobase of exon model per million mapped reads) of the two targets, and the other two charts show the levels (in RPM) of three microRNAs (dof-miR-1017/1021/1034). Consistent with the degradome intensity, both the targets and the microRNAs are highly accumulated in the flowers (grey background color). (**B**) Comp156499_c0_seq2 was predicted to be targeted by seven microRNAs (dof-miR-985/986/987/988/989/990/991). The scatter diagram shows the degradome-seq data supporting the predicted regulation. *Y* axis measures the levels of the degradome signatures, and *X* axis represents the region surrounding the predicted microRNA binding site (grey horizontal line). Compared with the signatures from the other organs, the most abundant one was identified from the leaves (arrowhead). The first line chart shows the level of the target, and the other two show the levels of two microRNAs (dof-miR-988/989). Consistent with the degradome intensity, the two microRNAs are highly accumulated in the leaves (grey background color). (**C**) Comp126829_c0_seq1 was predicted to be targeted by dof-miR-1047. The scatter diagram shows the degradome-seq data supporting the predicted regulation. *Y* axis measures the levels of the degradome signatures. *X* axis represents the region surrounding the predicted microRNA binding site (grey horizontal line). Within this region, the degradome signals were only detectable in the flowers, and the most abundant cleavage signal was denoted by an arrowhead. The first line chart shows the level of the target, and the other chart shows the level of dof-miR-1047. Consistent with the degradome intensity, the target is highly expressed in the flowers (grey background color).

In our study, sRNA-seq, RNA-seq and degradome-seq were all performed with an organ-specific manner. Thus, deeper investigation of the organ-specific interactions of the miRNA--target pairs was carried out. Distinct cases were observed for different miRNA--target pairs. For examples, the transcripts comp155533_c0_seq1 and comp155533_c0_seq3 were regulated by seven miRNA candidates sharing common recognition sites on the targets (Fig. 4A). The most prominent degradome signal supporting the cleavages of the two targets by the miRNA candidates was detected in the flowers of *Dendrobium officinale*. Notably, both the targets and three regulatory miRNAs (dof-miR1017, dof-miR1021 and dof-miR1034) were highly accumulated in the floral organ. That is, high expression levels of the regulatory miRNAs and the targets in a specific organ might result in abundant cleavage signals in the same organ. A quite different scene was observed for the transcript comp156499_c0_seq2, which was regulated by seven miRNA candidates (Fig. 4B). The intensity of the cleavage signals was higher in leaves than in the other three organs. However, no obviously high expression level was detected for comp156499_c0_seq2 in leaves. Instead, two regulatory miRNAs, dof-miR988 and dof-miR989, were enriched in the leaves. Thus, we proposed that in some cases, higher activity of a miRNA in a specific organ might result in relatively intensive repression of the corresponding target(s) in this organ. Another different scene was uncovered between comp126829_c0_seq1 and dof-miR1047 (Fig. 4C). The cleavage signal was exclusively detected from the floral organ. Consistently, the target transcript is specifically expressed in the flowers. However, although dof-miR1047 was detectable in flowers, it was not specifically expressed. Thus, we proposed another case that if the target was organ-specifically expressed and the regulatory miRNA was detectable in this organ, then an organ-specific regulatory pattern might exist.

### Biological indications inferred from regulatory subnetworks

**Figure 5:**
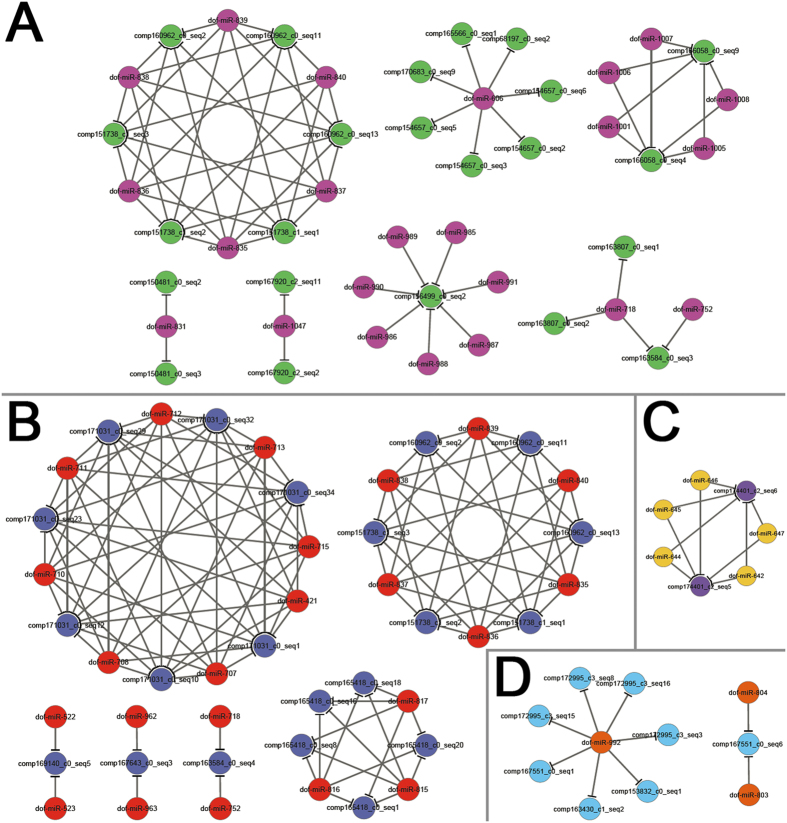
Degradome-seq data-supported, microRNA-mediated regulatory networks in *Dendrobium officinale*. (**A**) MicroRNA-mediated network implicated in plant development. (**B**) MicroRNA-mediated network involved in hormone signaling. (**C**) Transcripts encoding Argonaute 1 are targeted by dof-miR-642, dof-miR-644, dof-miR-645, dof-miR-646 and dof-miR-647. (**D**) MicroRNA-mediated network involved in secondary metabolism. For the above networks, all of the microRNA–target regulatory relationships are supported by degradome signatures (please refer to Figure S5 and Table S6). The functional involvement of the networks was deduced based on the annotations of the target transcripts. The networks were drawn by using Cytoscape^42^.

Next, we intended to dig out some biological indications from the above identified miRNA--target pairs. Based on the target annotations, four groups of miRNA--target pairs were identified to be involved in plant development, hormone signaling, AGO1-related regulation and secondary metabolism, which enabled us to establish four regulatory subnetworks. Specifically, within the subnetwork implicated in plant development, there are 17 miRNA candidates regulating 17 transcripts encoding floral homeotic protein AP2 (involved in specification of floral organ identity and seed development), MADS-box transcription factors (involved in flower development), transcription factor TCP2 (involved in leaf development), growth-regulating factors, scarecrow-like protein 6 (involved in cell division and root hair cell tip growth), and expansin-like B1 (Fig. 5A and Table S6). Within the subnetwork implicated in hormone signaling, there are 23 miRNA candidates regulating 21 transcripts encoding NAC domain-containing proteins (involved in auxin signaling), auxin response factors (involved in auxin signaling), transport inhibitor response 1 (involved in auxin binding), and ethylene-responsive transcription factor (involved in ethylene signaling) (Fig. 5B and Table S6). The action of AGO1 in the miRNA pathway and its regulation by miR168 have been reported to form a homeostatic regulatory circuit crucial for plant development^28^,^29^. In our study, five miRNA candidates including dof-miR642, dof-miR644, dof-miR645, dof-miR646 and dof-miR647 were confirmed to regulate two transcript isoforms (comp174401_c2_seq5 and comp174401_c2_seq6) encoding AGO1 (Fig. 5C and Table S6). Notably, the five miRNAs are homologous to the miR168 family members in various plant species (Table S5). Thus, the AGO1-related regulatory subnetwork is conserved in *Dendrobium officinale*. As introduced above, several bioactive constituents identified from “Fengdou” are secondary metabolites, or are involved in secondary metabolite biosynthesis, transport and catabolism^1^. Our study revealed that three miRNAs of *Dendrobium officinale*, including dof-miR-803, dof-miR-804 (both miRNAs are homologous to the miR408 family members of several plant species) and dof-miR-992 (homologous to miR397 of *Malus domestica*), regulated eight transcripts encoding laccases involved in secondary metabolism (Fig. 5D and Table S6). Summarily, the above identified subnetworks provide us with some biological indications for further genetic and functional studies on the miRNA-mediated regulation in *Dendrobium officinale*.

## Materials and Methods

### Sample collection, RNA preparation, library construction and sequencing

The seeds of *Dendrobium officinale* were germinated and grown in a growth chamber with 25 °C from day (12 hours) to night (12 hours). Leaves, stems and roots were collected from the six-month-old seedlings, and flowers were collected from the plants at the reproductive stage.

RNA preparation, library construction and high-throughput sequencing were performed by LC Sciences (Houston, TX, USA). The experimental procedure is introduced as follows: Total RNAs were extracted using Trizol reagent (Invitrogen, CA, USA) following the manufacturer’s procedure. The total RNA quantity and purity were analyzed by using Bioanalyzer 2100 and RNA 6000 Nano LabChip Kit (Agilent, CA, USA) with RIN number higher than 7.0. For sRNA-seq experiment, approximately 1 μg of total RNAs were used to construct sRNA library according to the protocol of TruSeq™ Small RNA Sample Prep Kits (Illumina, San Diego, USA). Then, single-end sequencing (50 bp) was performed on the Illumina Hiseq2500 platform following the vendor’s recommended protocol. For RNA-seq experiment, approximately 10 μg of total RNAs were subjected to enrichment of poly(A)-tailed mRNAs with poly(T) oligo-attached magnetic beads (Thermo Fisher Scientific, MA, USA). After purification, the mRNAs were fragmented into small pieces using divalent cations under elevated temperature. Then the cleaved RNA fragments were reverse-transcribed to produce the final cDNA library according to the protocol of the mRNA-seq sample preparation kit (Illumina, San Diego, USA). The average insert size for the paired-end sequencing library was 300 bp ( ± 50 bp). The paired-end sequencing was performed on the Illumina Hiseq2500 platform following the vendor’s recommended protocol. For degradome-seq experiment, approximately 20 μg of total RNAs were used to prepare the degradome library: (1) Approximately 150 ng of poly(A)-tailed RNAs were used as input RNAs and annealed with biotinylated random primers. (2) Strapavidin capture of RNA fragments through biotinylated random primers. (3) 5’ adaptor ligation to only those RNAs containing 5’-monophosphates. (4) Reverse transcription and PCR amplification. (5) Libraries were sequenced using the 5’ adapter only, resulting in the sequencing of the first 36 nucleotides of the inserts that represented the 5’ ends of the original RNAs. The single-end sequencing (36 bp) was performed on the Illumina Hiseq2500 platform following the vendor’s recommended protocol.

### Pre-treatment of the sRNA-seq and degradome-seq data

First, the sequencing adapters were removed and the short reads containing ambiguous base “N” were discarded. Then, in order to allow cross-library comparison, read count normalization was performed. Specifically, the normalized read count (in RPM, reads per million) of a short sequence from a sequencing library was calculated by dividing the raw count of this sequence by the total counts of the library, and then multiplied by 10^6^.

### *De novo* assembly and expression level calculation of the transcripts

By using Trinity^30^, a *de novo* strategy was adopted to assemble the transcriptome of *Dendrobium officinale* based on a total of 34.3 GB RNA-seq data (four organs with two biological replicates for each). To identify transcripts homologous to those in the other species, a locally installed BLASTall program^31^ was utilized to search the assembled transcripts against the sequences in NCBI NR protein database (http://www.ncbi.nlm.nih.gov/protein/)^32^ and the Swissprot database (http://www.uniprot.org/)^33^ with E-values lower than 1e-10. Genes were tentatively annotated according to the best hits against known sequences. COG^34^ and KEGG^35^ annotation systems were employed to analyze the biological pathways that the assembled transcripts were involved in.

For expression level calculation, Bowtie (version 0.12.7)^36^ was utilized to map RNA-seq reads to all the assembled transcripts by using the “single-end” method and the parameter “-v 3 -a --phred64-quals” (allowing one read to be mapped to multiple transcripts). The perfectly mapped reads were retained for expression level calculation by using the following formula:

*Expression level of a transcript* (RPKM; reads per kilobase of exon model per million mapped reads) = *Number of the reads mapped to the transcript*/[*Total number of the reads mapped to all the transcripts* (in million) × *The length of the transcript* (in kilobases)]

### Prediction and validation of the miRNA targets

Target prediction was performed by using miRU algorithm^37^,^38^ with default parameters. The degradome-seq data was utilized to validate the predicted miRNA—target pairs. First, all of the degradome signatures were mapped onto the predicted target transcripts. Then, the previously proposed criteria^39^ were applied to detect the cleavage sites. Specifically, (1) “Average_Read count_Cleavage site” is the averaged read count (in RPM) of all the degradome signatures (belonging to one library) with their 5’ ends mapped to a potential cleavage site; “Average_Read count_Surrounding” is the averaged read count of all the degradome signatures (also belonging to this library) that mapped to the target transcript except for the cleavage site; “Average_Read count_Cleavage site” should be five times or more than “Average_Read count_Surrounding”. (2) Also for this degradome library, among the degradome signatures mapped to a potential cleavage site, the most abundant signature should be amongst the top 12-most-abundant ones that perfectly mapped to the corresponding transcript. (3) The cleavage site should reside within 10 to 11 nt region of the regulatory miRNA. For any degradome library, if the three rules were fulfilled, the potential slicing sites were retained. Finally, both global and local target plots were drawn to perform manual screening, referring to our previous study^40^. Only the transcripts with prominent cleavage signals were considered to be regulated by specific miRNAs.

### Data availability

The availability of the HTS data sets generated in this study are described as follows: The eight RNA-seq data sets could be retrieved from NCBI SRA (Sequence Read Archive; http://www.ncbi.nlm.nih.gov/sra/) under accession IDs SRR2014227 (root_repeat 1), SRR2014230 (root_repeat 2), SRR2014236 (stem_repeat 1), SRR2014246 (stem_repeat 2), SRR2014297 (leaf_repeat 1), SRR2014325 (leaf_repeat 2), SRR2014396 (flower_repeat 1) and SRR2014476 (flower_repeat 2). The eight sRNA-seq data sets are under SRA accession IDs SRR2014142 (root_repeat 1), SRR2014143 (root_repeat 2), SRR2014477 (stem_repeat 1), SRR2014478 (stem_repeat 2), SRR2014146 (leaf_repeat 1), SRR2014147 (leaf_repeat 2), SRR2014148 (flower_repeat 1) and SRR2014149 (flower_repeat 2). The four degradome-seq data sets are under SRA accession IDs SRR2012529 (root), SRR2012531 (stem), SRR2012580 (leaf) and SRR2012592 (flower).

## Additional Information

**How to cite this article**: Meng, Y. *et al.* A transcriptome-wide, organ-specific regulatory map of *Dendrobium officinale*, an important traditional Chinese orchid herb. *Sci. Rep.* **6**, 18864; doi: 10.1038/srep18864 (2016).

## Supplementary Material

Supplementary Information
