# Supplementary material for: A transcriptome-wide, organ-specific regulatory map of Dendrobium officinale, an important traditional Chinese orchid herb
Source: Sci Rep. 2016 Jan 6;6:18864. doi: 10.1038/srep18864 (PMC4702150; doi:10.1038/srep18864)
Supplement: Supplementary Information [file srep18864-s1.pdf]

A transcriptome-wide, organ-specific regulatory map of *Dendrobium officinale*, an important traditional Chinese orchid herb

Yijun Meng[1, 2, &, *], Dongliang Yu[1, &], Jie Xue[1, 2], Jiangjie Lu[1, 2], Shangguo Feng[1, 2], Chenjia Shen[1, 2], Huizhong Wang[1, 2, *]

1 College of Life and Environmental Sciences, Hangzhou Normal University, Hangzhou 310036, PR China

2 Zhejiang Provincial Key Laboratory for Genetic Improvement and Quality Control of Medicinal Plants, Hangzhou Normal University, Hangzhou 310036, China

&These authors contributed equally to this work.

*Corresponding authors:

Huizhong Wang

Zhejiang Provincial Key Laboratory for Genetic Improvement and Quality Control of Medicinal Plants, College of Life and Environmental Sciences, Hangzhou Normal University. Xuelin Street 16#, Xiasha, Hangzhou 310036, P. R. China

Tel: +86-571-28865330

E-mail: whz62@163.com

Yijun Meng

Zhejiang Provincial Key Laboratory for Genetic Improvement and Quality Control of Medicinal Plants, College of Life and Environmental Sciences, Hangzhou Normal University. Xuelin Street 16#, Xiasha, Hangzhou 310036, P. R. China

Tel: +86-571-28865198

E-mail: mengyijun@zju.edu.cn

Supplementary data introduction

Table S1 Statistical results of the RNA-seq experiment.

Table S2 List of the transcripts highly accumulated in specific organs (root, flower, stem or leaf) of *Dendrobium officinale*. The transcript IDs along with their expression levels (normalized in RPKM and rescaled by log10) in four organs are included in this table. For each organ, there are two biological replicates ("Root1" and "Root2", for example). ND: not detected.

Table S3 List of organ-specific transcripts potentially involved in specific organ-related biological functions.

Table S4 Organ-specific sRNAs identified in *Dendrobium officinale*. The sRNA sequences along with their accumulation levels (normalized in RPM) in sRNA-seq libraries prepared from specific organs were included in this table. Seven miRNAs were identified to be specifically accumulated in the floral organ.

Table S5 List of 1,047 miRNA candidates identified from *Dendrobium officinale*. The miRNA candidates from *Dendrobium officinale* along with the homologous miRNAs from other species sharing identical sequences according to miRBase (release 21) are provided. The accumulation levels of the miRNA candidates in the sRNA-seq libraries prepared from four organs are also included in this table. For each organ, there are two biological replicates ("Root1" and "Root2", for example).

Table S6 A partial list of miRNA--target pairs identified from *Dendrobium officinale*. The miRNA IDs, target transcript IDs, target length and target annotations are included in this table. Based on the functional annotations of the target transcripts, the miRNA--target pairs were classified into four functional groups (hormone signaling-, plant development-, secondary metabolism- and Argonaute 1-related groups) for

network construction.

Figure S1 Statistical results of the sRNA-seq experiment.

Figure S2 Certain highly structured transcripts might serve as miRNA precursors. The transcripts were assembled by RNA-seq reads, and the transcript IDs were provided in the figure. The mature miRNA-coding regions on the long stems of the miRNA precursors were marked by red lines, and the mature miRNA IDs were provided. Note, for each mature miRNA, its average accumulation level in either organ (flower, leaf, root or stem; two biological replicates for each organ) of *Dendrobium officinale* should be 5 RPM or higher. The secondary structures of the transcripts were predicted by using RNAfold (http://rna.tbi.univie.ac.at/cgi-bin/RNAfold.cgi).

Figure S3 Certain highly structured transcripts might serve as miRNA precursors. The transcripts were assembled by RNA-seq reads, and the transcript IDs were provided in the figure. The mature miRNA- and miRNA*-coding regions on the long stems of the miRNA precursors were marked by red and blue lines respectively, and their IDs were also provided. Note, for each mature miRNA, its average accumulation level in either organ (flower, leaf, root or stem; two biological replicates for each organ; thus, there are a total of eight libraries) of *Dendrobium officinale* should be 5 RPM or higher. Besides, the accumulation level of each miRNA* should be detectable in at least one sRNA-seq library. The secondary structures of the transcripts were predicted by using RNAfold (http://rna.tbi.univie.ac.at/cgi-bin/RNAfold.cgi).

Figure S4 Certain highly structured transcripts might serve as miRNA precursors. The transcripts were assembled by RNA-seq reads, and the transcript IDs were provided in the figure. The mature miRNA- and miRNA*-coding regions on the long stems of the miRNA precursors were marked by red and blue lines respectively, and their IDs were also provided. The 5’ ends of the degradome signatures supporting the

processing of miRNAs and/or miRNA*s from their precursors are represented by green arrows. Note, for each mature miRNA, its average accumulation level in either organ (flower, leaf, root or stem; two biological replicates for each organ; thus, there are a total of eight libraries) of *Dendrobium officinale* should be 5 RPM or higher. Besides, the accumulation level of each miRNA* should be detectable in at least one sRNA-seq library. The secondary structures of the transcripts were predicted by using RNAfold (http://rna.tbi.univie.ac.at/cgi-bin/RNAfold.cgi).

Figure S5 T-plots providing degradome-seq data-based evidences for cleavage actions between specific miRNA--target pairs identified from *Dendrobium officinale*. Four degradome-seq libraries prepared from four organs (root, flower, stem and leaf; represented by four different signs) of *Dendrobium officinale* were included for drawing t-plots. For each miRNA--target pair, two scatter diagrams are provided. The left diagram with target ID provides a global view of degradome signals distributed all along the full-length target transcript. The right diagram with ID(s) of regulatory miRNA(s) provides a detailed view of degradome signals within about 60-nt region surrounding the predicted miRNA binding site (marked by a green horizontal line) on the target transcript. The *y* axis of each diagram measures the intensity (in RPM) of degradome signals. Please note: only the miRNA--target pairs supported by prominent degradome signatures resided within 10th to 11th nt from the 5' ends of the regulatory miRNAs are included in this figure.

Figure S6 Examples of miRNA--target pairs showing organ-specific regulation.

Data S1 Sequences of the transcripts highly accumulated in specific organs in Table S2.

Data S2 Sequences of the transcripts predicted to be miRNA precursors in Figure S2.

Data S3 Sequences of the transcripts predicted to be miRNA precursors in Figure S3.

Data S4 Sequences of the transcripts predicted to be miRNA precursors in Figure S4.

Data S5 List of miRNA--target pairs identified based on degradome-seq data.

Data S6 Transcript sequences for Data S5 and Table S5.

Data S1 Sequences of the transcripts highly accumulated in specific organs in Table S2.

>comp100062_c0_seq1

GACGATTTGTCCTCTACCACGTGACGTTCGTCATGCAAAGTATGAGATCATCCTTAAGGATTGTATATAAATACGTCGTAGGGTTGTTTGTGCTTACTTTCATATTCACTGATCACTTTGATG
CTGTTGCACAGAACTTCTTTTAGTCCCCTCTTAAGGGTTGGCGCTCCCCAGTTTCACTTGTCACCTTCAAGTGTGGTCTGAGATACCAAAACTACTAGCTCGAAT

>comp100156_c0_seq1

TTATAGAATTAAATTTTGCATCTCAATGATCATGCATTGTAGAATAGACACCATATCAGTTTTTAAACCTTAGAGAAAATTGAATTTTTGTGTTTTTCATAATGCTACAACCATTATTTGTTCA
CTCTCCTGCAATTAAGCCTTATCTCTCCTTCTTTCCCAGTTAGCACATTTACCTCACCCAATTTTATCATGGACTGTGAAAACTGTTTCCTAAACAACTTTGGTCTGTCGACATATCGGCTTA
CTATTTTATTTGTAACATTGGTGAATGCTAATTGCTGGTCTAGCTGGAGAATAGCCTTTGCATCGAGCACATTTTTGAAGAATGATTGATCCAGCTTGAAAGGAGTTGAAGTGCCTTCGTTC
ATGAAAATATTCGGATCTATACTAATATTTTGGAGTGTTGAAGCAGGGCATTTCTGTCTTAACCTGTTGAGCAAGTTACTGTCCATCTCATGATCAGGTAGGCCAGTGCC

>comp100311_c0_seq1

AAGAATAAATTGGTAATGTATAGTTTGCTCACTTGCTTACGGAAATAAAGAAGTCGATAATATATATTCTAAACGAACGTATCTACCTCACTATCTCTTTCTTCGACAAAGTAAACACAGGTT
GGAACCCTTCAAAGCCCTTGGTGACTGCAAAAATGACGAAGGCGAACAAGTGAAGAGAGTGGCTATAGTCGGACGTACCGGGCTTAAAATAGGGCAATCACTTGCTCGGTTGGGCTCG
CCTTGTCACAGAAATGACAAATAGTCGAAGAGAAGAACTGACGAAGAAGACAAGAGAAGGAGGAGGTTTCGAGCTTCAATCGTCGCTTTCTCCGCTCGGTCGGCTCGCTTGGTCGCAT
AAACGAAGAAGACGAGGGAGAACGATGGTGTAAAGGCAAACCGTAAGGCATACGAGCAGTCGGGCATGTATTCACGAGATATACAGTTTGAATGGTCACCTAAACACCTTTTATTTGATT
AAATAATATAAAATTGTAAAATTTATAATTTAAATAAATTTAATTCTAAAT

>comp100722_c0_seq1

AATAAATGGCAGTTTTATGTTAAATGAGAAAAAAAATCTAAATTAGAAGTATTTTCCTCATATAACTAGAATTTTCCCTTTTATTATCATTAAAAAGAAGCAAAGTTCACACTTCTCACTCACA
TTCCATGAAATCATACTCAGACAATTACGTAGTCCTGAACCTTGCTTGTTGCGCTCTCCTTTGTCAAAAACTCCAAAACTTCACCTCTAAACTAAAATGCCACAAGTACCTCAAACAAAAA
CTACTCCAATATGCCTTTCTAACACCACCTCACCTCTTCACATAAACTTCACTAGGCACCACACACTTACAACATATCATTACATATCACTTTCCAATGAAAAAAAGAATGGAAAATGCTAC
CTTCCTTACTCCACCATCTCCCGAACTCCACTCCATTGTACATCCTAACACTTTCTTAGTTCCCTAATCTCCCTCACAACACAACCTCTTCCCCTCCCGCATGCCACGCCTCTCCAAACTACT
GCAACTAAGAATCATACCCCAACAATCAAACGCTGATCAGCCCACCGACAACCTCAGCTAGGATTCCCAACTGGTGCTCCTTCATAAAAGTATTTATGGGGGTAAGATCAATTCAGTCCTC
TGTTTGCTTCTTGAAGGGCACGATATCACATGCATCATTTTTGAAGGGAAAAACAATCTATGCTCACATAGCTTTTCCATCTTTTGTTTCTCCTAAAGCTTGTTCTGTACTCTCTGCGGAAAC
TGCTTCACCTAATATGACAACATCAATGTTCCATCCAATTGCTCTGAATGAACCAGTTGTTTCTCCTGATTTGTTCCGATGCTAAATCTAAGGGA

>comp100781_c1_seq1

GAGGTCACTTAGTACCTTCATAAGGAAACATTGACTAGGGTAGGGCCTTCTTACACGAGGGAAGCTAGGAGTTTATATATTATCGAAGCCTATATGTGTTTGTGACTTGAATTAATTGAATA
CCACTATGTTGATACATAATCAAGATCTCGATCTCAGGAGATCAAAATCTGTTTCAATAGTAGAATCTGGACATACTTTAAGTATAATGTTATAAATATGAACACCAAAACCATGTTGATATTA
CCGCAAAGATTGATCTTGATCACAACCTAGTGGATGCATACATGAACCATAATGATTTTTATCTGTTCAACAAGAGTTCCTAGCGAAAAAATAGAAGGTGATACACCATCAACAACTTTAG
CATGCCATCCTTATGAAGAGCTCTAGCTTCTAGATTTTAGCCTAGCA

>comp100850_c0_seq1

TTCTGATAGAAATGCATCTTGATTTGTTTGCCAACTTTTGTAAAGGATGCAATGTTGCACATCAGCTATCGGATTCTCAACATTTACCACGACAGAACCATCTTTTGCTCGTGCGATTTCAC

CAATTCTTGTTCTAAGAATACTTATAGGTGCAAAATGACCATTATGATCCCTAAATCTTTGTCGTACAAATTTCCAACCCATTTCAGTAATTGTTTCTCCATCAACATACATTGGATAACGACT
TTTGATAACTTCATGATATACGTCCCATAAATTATCGTCCTCATACCAAGACCATTCATGTAAACAACAGCGAAAAATTTCATTCGCTCTTGCTTTCTCCTCCAAGAAATCATCCATGGAATA
TGAGATAGAGTTTGCCAATCGAATCGTACCATCATAGATTAAATTCTTTCTTGGAATAATATGTAAGAAATCCCGTGAAGGTGAGCGCTTGATTGAGGATGTCCTTGGTGCGGATGACATGA
TATG
>comp101251_c0_seq1
TGTTTCCATTCAAATTAATATGGAAATATATTAAAAAAAAGTTGAAGAAAATGATCAAAAAATTTTATATGGAGAAAAACAATACATAAATCAAACAAAGCCGTGCATAAACAAATTTTGC
AAATATCCCAGTCGATCCTGAATTGAGAAAAAAAAAGTTTAGATTATCATCCTAATAAGAGAGATAGTGCTCAAAGACATTACTTATTGAAAGGGCCATTTCAATCACGTGATCATGAATTCC
CCCACACAACTTGGAGCTAAATTTTGTAGATTTAATATTTGCATGGTTTGATGATCACAAAGCTTGGTTAGAATATAGTATAACCAAGAATGCAACTTATTATTTATTTTTTATCTGTATAGAC
CTCATATTGGAGAACATGCTAGTGGTGATAGTTTTGAACAAAAAAGTTTTAAAATTGAAAAAAAAGATAGATTTCGTATTCATGTTGGTAATATTAACAGTGCACATAACATTGCTAGACAG
AAATGTGAAGATTTAATGAATCAAGAAAGAGATATAAGATTTTCTTTCAAAAGACCTTCTAATCAAAACCACAAAAATTATCGTACATGATTAAATGCAAGTGTAGATTGTGTATGTTTCCT
TTTGCAGCAGGGATTAGCATTCCGTGGACATAATGAATCAGAGGATTTTGAGAATCAGAGGATTTTGAGAATCAATGAATTTTTCTTAAAATTTTATGCTTTCTTGCTAGCCATATTGAAGA
CATAAGTAAAGTTGTTTTGGATAATTCACCTTGAAATAATAAATTAATAGCTCTAAAAATTCAGAAACAAATCACAAAAGCTACTACAATTGAGGTTTCAAATTTATAATGACTGAATTAAA
AAAATGATGTATTCGTAATTTTACATGATGAATCTCAGGATATATCAGTTAAACAACAAGTTGTTGTTGCATTATGTTATGTTGATACACAAAGATATGTTATTGAGCATTTTATTGGGATTATT
CATATAGGAGACACAACAGCTCCAACACTAAAGATGGTTATTGAAGAACTTTTTGCGAAGTATGAGTTGAGTGTGACACAGTTACGATAGAGCAAGCAATATACGAGAATAAGTTTAATG
GACTAAAAACATGAATCATGAAAGAAAATTGTTTAGCTTATTGTGCATTGTTTTGCACACCAATTCCAACTAGCATTAGTTACAATCGTAAAAAATTATGTCCAAGATGTTTTCTTCTTTAAT
ATTGTGACTAGCTTCTTAAATTTTATTGGTAGCTCATGCAAAAGAAGAGATTTACTAAAAGGAAAACATTATGAGAAAATTGTTGAGTTAACTATAAAGTGGAGGAGTTACAAAAGGTCGA
GGTTTAAATAAAAAAAAATTGTCTTTCAAAGACCAAGAGACGCTCATTGGGATTTCCACTATAATTCACTCATTAGCTTAATCCTTTTGTTTGGGTCTATAACAGAGGTTCTTGAGATAGTGA
TACATGAGGGATCGAGTAGTGAGAAAAAAGGTGAAGCATATTTCCTACTTGAATCGGTGCAAAATTTTAAATTTGCATATAATGAAAACTAAATTAGGAGTTACAAATGAGTTATCATAAGT
ACTACAAAGAAAGGATCAAGACATTATAAATGTCATGAGTTTGGTTAAAGTTTCGAAACAACGACTCCAAAATATGAGAAATGATG
>comp101378_c0_seq1
GCTATTGTTAACTACCTCATAAATAGACTTCCTTCCAAATACACCTCTCTACAATCTCCCTGTCAAAATCTTCACGGGATATCCCCCACTTATACTCACCTCCGCTCCTTCGGTTGCCTCTGCT
ATCCTTGGTTACAACCTTATACCTCAAGCAAGTTCTCTCCTCGGTCGCACGATTGTATCATCATCGGTTACTCACCTACCCACAAAGTTTACAAATGTTACAACCTTGACACACACAAGACT
CACATTTCCCATCACATTGTGTTGCACGAACACATTTTCCCTTACAATCCTTCACCGCTTACACCCTACTCAACTACTGCTTCCCCCACTCACAACACCTCCCCTCTTCTCCTGATTCCTACT
TTCGTCGCATCATATTCAGCACCAAATCCTGGCCCATAATCTGCTACCAATTCAAACAAAGAAAACCATCTCACTTTGCACCAATTGCCTTCTAACTTGCCGTCCACCATAAGCACCGTGTC
TCAATCTGTTCCCACACCCTTCCTCATCCAATTCACCACATGCAGACCAGATCAAAATCTGGTATATCAAAACCCAAAAGCATCTTCACTCTTCTTAGCAATACGGCCTCACCCTCCACTCT
GACAACATACAAACAAGCGGCTAAATACGAACACTGGCAGGCAGCCATTACAGCTGAATTCAATGCTTTACAGTAACAAGGGACCATGTCTCTTGTTCCCACCCCTCCAAACAAGCTCAT
TGCCAATGAAAA
>comp101455_c0_seq1
GCTCTTCCGATCTCTGGCACTGGCAGCGGCACGCTCGCGGGCGGCAGCGGCACGCGGTACGGCAGCGCGGGTGGCAGCGGCACGCGGGCAGGGGACAGGGCAGCGGCCGGGCAGGG
GGCAGGGCAGAGCGGGCAGCGGTCAGGCAGCAAAAAATTCATTTTTTTTTTTAAATAAACGGGTTATTTGGTCTCGTTCGGTCATTTTGTTGCTAGGGAATCGGATTTGAGTATGTATTTTA
CACTCACTTATTAAACACGTCAGGTTTAGATTGACATTTGAGTCGTGTCGATTTTGAGTTTGATGTGAACCTGCTCTATTGACCTATTTTAACACCGCTATAAGTACGGGTGCAAACCAAGT

CATGTTGACTCGTGAGCTTGCTCGAGCTCAACTCGATAACAAGC
>comp101476_c0_seq1
ACGTTTCCTGGCAGACACACCATGGAATATTTCGGCGCAGAATTTTCTGTTGTTCATCATCAGCATTTCAAGCTCCCTTTTGTTACGGACTACAAACTTCTTGAACCCGTTGGGTAACAGAT
GTCTGGTCTTTTTGTTGCTACCATAACCAATGTTTGGCATCCTCATCATACCCTTGAACCTACGCCGGACTCTGTTGTCGATACCCTTTGGTTTTCTCCAGCTTGGCTTGACACGCATAAACC
TGTCACTCTGAT
>comp101736_c0_seq1
ATGAGGTAATCTTTGAAATGGATTCACAGCAATGAGAATATTTCCTGTGTATGTGTAAATTTCATTCAATTCATATCTTGCTGACAAATTCTGCAGAACCCCAGGTTCATGTAGATAGGATAG
CTTTGTCATGTCATCAACTCCACCTGGAGGAGCTTCCATATCTTTGGGATATATTTTTGAAAGTTTTTCAATAACTGTTTTCCCATTAGTCGTTTGAATCTCGGCCTCTTGCCCGGTGATTTTA
GTAACTCGTCCATCAGTCCAGGAAACAGCAGAATCTTCAATCCAGACATGAGAACCAACAATGATATTAATTGTTGTTCCCATGCCTTAAGAGCCTGGTAGATAAATTTATGCACCTTGCA
AGCAGGGCAAGCGATTCAAAAGAAAATAACCCTAGAAGACCGACGCCAACTCCAACATTACTCAGGCATT
>comp101775_c0_seq1
AAATTCTAGAATTTATGTAATACTTCATGAGAACGGTTGGGACCTTCCAGCCGATTTATCGGCTGACCTCTTGTGTGCGTTATCCACAATCTTTGTTAATTCGACAAATAATGATAGCATTATT
TGGGGAAATGAGGAGGTTAAACACAGCAGCTATATTAAATATCTTTATAGAAATGAGCTTATGGTTACTTGGCACCAATTTGTTTGGCACAAACATTTCTCTCTTAGATATTCTACCTTTTGC
AGGATGGCCTTGGTTGGTGGCCTTAAGATGGCTAATGCTTTGAATATAAGGGGCATTACTATAGATTCATGCTGCTGTTTTTGTCATTCGGAATATAAAAGTGTCACACATCTGTTTTTTGAA
TGCACCTACACCTTTTCTGTGCTCACCCAGCTCATTCCTCGGATGAAGAATCATCTACTTAGACCCAATATTCTTCAAGCTTTTGAATGCACTAGTGGGACTAATAAGTGGACTATGACCTT
TTACCATCTTATCATATGCTGTACGATATATCACATTTGGAGGAAAAGAAATGATAGGAAGTTTGGAAACTCCTTTGCATCTTCCACTACTATATGCCTCAAAATCAAGTGTTGTTTTTGCTA
AGATGTTGAAATGGAAACGTGGAAGCTCTCTTTTGGATATGATATAGTTTTTGGCTGGAGGTTAATTCGGGTGCTATTTTCAGCAGTTGCGATGGTTTCATCGATATGGATGAGTTTGTTTAT
ATGAGCAAGGATGATAGAATTGCCTTCTTTCTCCCTTTGGATGGAAATACTGGGGGCTACTGCAAACAAGTTAGAGCACCAGCAATTCCTAAGCCCGTGATGGGGGAAGTATCTATCTGAA
TGTATTTCTGGTTTTTTATGGTTGTTTTTCTGAGGTTATTTTTTGTGGTGGTATAATTTTGTTGGAGTTATAGCTGTTTTTTCCTCAACTCCTTGTAGGGTTTTGTAGCCTTTTTTGC
>comp102949_c0_seq1
TTTTTCTTCAGCTAAAACAGCAGATAATTTATTGTACACGTGTTACATTCAGCCACAACTGAGAACAGAACTAGTCCAGAAAGTCACAGGTCCAGGGCAAAAAACCAAAAGGGACCATT
TTGATATGAGCAAGGTGGGTCTCAGAGTGGTCCAGGCGATCAGAACGGCGAAAAGTTGTTATAGATCCATTGAGACAAGTTCTAGACAGAAGGCATGCAGTCCAACAAATTGTACCAAC
GTCCCTGGCCTCTGGGATTTCGTTTCTGCTCCTGTGGCTTCCACGGTGTACAAGTTTACTTGGACCTCTGCCTCATCTTTCTTCTTTTGCGCTTCAGCCTGCGCATTCGCTTCTTCCTCCACT
TCGCTCTCATGGCGCGGAGGTTTCTCAGAAGATGGCGCTAAGGCCGAGAGAGCGCT
>comp102949_c0_seq2
CCTGCGCATTCGCTTCTTCCTCCCCTTCGCTCTCGGCCTTAGCGCCATCTTCTGAGAAACCTCCGCGCCATGAGAGCGAAGTGGAGGAAGAAGCGAATGCGCAGGCTGAAGCGCAAAAG
AAGAAAGATGAGGCAGAGGTCCAAGTAAACTTGTACACCCATGGAAGCCACAGAAGCAGAAACAAGGGAAGCCAGAGGCCAGGGACGCTGGTACAAAGTGTGGACTGCATGCCTACT
ATCTAGAACTTATCAATGGATCTGGAACATCTATGGCCATTCTGATCACCTTGACCACCTTTGCGAGACCTACCTTGCTCATATCAAAGCCGTCCCTTTTGGTCCATTGCCCTGGACCTGTG
ATAACTATGGACTAGTTCTCTTCTCAGTTGTGGCTGAATGTAACGTGTACAATAAATCACCTCTTTTGCTGTCTTAGCGGAAGAGATGATTTATTGTACACGTTACATTCAGCCACAACTGA
GAAGAGAACTAGTCCATAGTTATCACAGGTCCAGGGCAATGGACCAAAAGGGACGGCTTTGATATGAGCAAGGTAGGTCTCGCAAAGGTGGTCAAGGTGATCAGAATGGCCATAGATGT
TCCAGATCCATTGATAAGTTCTAGATAGTAGGCATGCAGTCCACACTTTGTACCAGCGTCCCTGGCCTCTGGCTTCCCTTGTTTCTGCTTCTGTGGCTTCCATGGGTGTACAAGTTTACTTG

GACCTCTGCCTCATCTTTCTTCTTTTGCGCTTCAGCCTGCGCATTCGCTTCTTCCTCCACTTCGCTCTCATGGCGCGGAGGTTTCTCAGAAGATGGCGCTAAGGCCGAGAGAGCGCT
>comp103765_c0_seq1
CACCAGATTGAGTTGAACCCTGAAAAGTTAAAATTAAAGCACATCACTATAAAGATATAATAATATGTGATTGAAGTATAATTGGTATGCAATTGAAACCAAGAAGATGTAAAAGGCTTTA
GAGGAAGTGCTGATAAAATAAAATCGCAAAACCACGGAACTCGGGAAGAATATTTTCCATCATTCACAATTTTCTTTTATAATAGCCAGACATGAAACTGCTAATTTCTTTCCTCCAGTATC
CTCTCAACAACAAGAGGTTCTAGTTATGACATTGCTTTACACCAAAAGCCTAGCCTGCTCCCCAATCAAAAATAAAACTCAGAAAGCAACTTCAATAACAATGAAAATTATGTTACTCCA
ACTCAATCTAGCGTTCCTACATAAAACATCTCCTAACAACAATCCCTCACTCAAATGTAGAAGCATATGATCGATTATCCACAGCTCCACAAATAACAGAAACTTAAAAATAGCCAGGATCA
TCAAGTGTTTTGAGACATAAGCCAAATATTTGATGGGATAAAGTCAACATTTTATATGGGAAGGAATTTATTTTGTAACCTAAAACTACCATATAAATTTTCTACTGATGGTGGGCAATTCTA
ATCACAGAGAAACACTACCTCTATCTT
>comp103898_c0_seq2
AGGATTTGCTTATTCAATAAGCGCATTGAGAGAAAATTAAGAAAAAAGTTGCTACGAATTGTGCGCATGTGTGGCACTAAGCTATAGAATATATATATCCATTTAGTAGTAATAAAGAACAT
GTCTATTACGATGCCAATTTACTTATCTTTAAATTGTTCAAAGTTGCGATTCAGGAGATCAGAGAGCCAACTGTTACCTTCGAAAATCTATAGATGATGATATTATTAAGAGGGCTTAGTCAC
GGTTTACTCTACGTCTATTTACATGCGTCATTCTTGTTTATATCCATAAATAAACGACCCCATGTCAATGAGCTGACCCTTCGCGGTCTTCCGAGTTCCCAATATGGAATCCGGAGTTGTAAA
CGCGCCAAAAGCATGAGCCGAATCCTTGCTTCCATTGGGAGTGACAGCATCATGAATATTCATTCGAAAGTATATGTGTTCTGTT
>comp103965_c0_seq1
CTGACCGTCCTGCATTCTTATCTGATGAGAACAAAGAAAAAAATCCAACTAATTTTCACAAAACAATAAAACAATTAAGAATAGTTGGACAGGGAATATAAAAATATTGATTGTGATGATA
AGGTATGTAGTAAGTTATATGTTACCACAAGGCTTAATAAGGTTTTATTTATTTAGTATAATGAAAATATGATATCCAGAATGAACATACTGATAATATTCAAATAAGTTGGCCATAAATTTGA
GACAGATTGTAAATGGAGTTGAGAGACAACTATGACATAAATACTGCTCAAGTTATTGCAAGAAATTAACTGCCGAATGCAAAATTCCAATAAACAAATTGAGATAAAAAAGACTTTTGT
GGATACCATGAAAAACCTGCAAAGGAAGAGTATTGAGATAGTCCTCTACTTAAGGGGTTGCTAGGTTCAATGTTTTCTTTGATCTAATGTTGTTTTATTTATGATAAGAGATGTTTAGTTTGA
TGTCATTTCTCATATTGACATAATCTGAATAGCATTAAAAGCTAAGATTGCGGAACTCTAAATAACCAAATTTGATGCAATCTAAATTACCTCAATCTTGATAGCTTAAAAAATGATAAAATG
AAATAAATATCTTTATACCCCTTAATTTTAAAAAAAAAATATTATATTCTTTA
>comp104594_c0_seq1
GTCTAGGGTTCGCCGTTCATCTTCCCTCTTCATCGTTGTTGTTTGGCTTCAGTTCGCAGGAGGGGAAGGATCACACGAGCCGATCCTTCGACTCCTTATTGTCGTTCGACTTCACCCGCAT
CTGACGCCACCGAGCCCGACGACACAACCTCCGCCCGTCTCCACCTCCCACGCCTGACCCACCTCCGACGCTGCCGATCCTGTCGCCATTCAACTTCGCCCCTCCGACTCCTTGTCGCTG
CCCGATCCCATCACTCCGCAGGAGGTATAGGGATTGCACGGTCGCTTCTTTGGCTCTTTGTTATCATTCGGCTCCGTTGCTTCACAAAGAGCAGAGATTTCACCGTCACTTCTTAGGCTCCT
TCGT
>comp104812_c1_seq1
TAAATTTATTAGCAGTTTGTTAACAATTTTTAATTTATTAGCAGTTTGATTGTTTGTTAGTAGTTGATTAAGTGGTTGTTAGTAGTTTGTTATGTCATTCATCGATAAATATGAAATTTGAAATC
AAATTAGTTTTATAGCATGAACAAAATTTTTCTTTGGATGTCAAGATTTTTCTTCATGCAGTTCGTGATTTGAACTTGATTAGATAGCCTTGAAATCCTTCTGGGACAGATCTCCCTTTATATA
AGCACGAGGTTTGCTTATTTCCTTACTTTTATTAATCTTTTTATCTTTTTCAATGTTTTTATCTATTGCATTTTATCCTCTATTTTCATGACTATCCTGCATCAATTGACATGCCATAAAAAATATG
TAAGCGGTAGCCGAGATGGGGTGATTGAAAAAATGGTTGACTACCTCCTACCAAACCTCATTCATATTGCAGGAGTGTTAAACAAATGCATCAATA
>comp105124_c0_seq1

AAAGAATAAGTATACTGGATCATATAAACCAAATGTTGAATAGAATTGAACTCAAATTCCACACACAGCGAAACAAGATAGGAAACACCAAAAAATCTGGTCCTCCCATGCCAGCAATAA
CGAACTTCCATACCATTGATCCTAGTAATTACACAGCCAACAACCATATAGAAAAACCATAGAATAACTCTAAAAGAACAGCAAAACAAAAACATATTCCTCCAGAGCCGGTAATCAACA
CCCAAACTAATACAAAACACCAAATCATCATCCAAAAGAGTCCCAACCACAAAAAGCTTAATAAAAATTAGACCAATCCTGAAACAAAATACAGTAGGAACAACCCAATCTGAGAGGAA
TGAAGCAACACCAATCCAAAACATAGCTCCGTATTGAATAACAAGAAACATAAAACTCCAGGAAAACAAAAAGCCACATCCATCACAGCAAAATCAGAAAGGGTAAACAGCAAAATCA
AGAATAACACTAAAGTAACCGGATATAAGGAAAACCCAAACGATCTAATCTAGCAAGCCCTTTAACTTCTTTAGGAAGGTTATGTTCCTTAAACTCCACAAAGTTATCTAATTTACAACCC
CTTTTTGTAAGAAAATCGGCCACCGCATTCCCTTCTGTTAAAATATGAGAAATTTTAAAGGACGTAAGAGACAGGAGATGTTTAATATCTCTAAGACTATAGAAGTTATCTAAATTAGTAGA
AGATTTACCTTTAATGTAATGAATAATAAGCATAGCGTCCACTTCCACCCATAGGTTATTAATCCCAAGAGAATTACAAAGATGAATCCTATATAGAAGGTTATCCAACTCAGGTTGAATAAC
ATTAGTAGCATTAGAGGGACCCGCATATGCTTTAATGACAAAACCCCTAGTATCCCTAATAATGCCACCACAACCTGCCTCAGTAATGTTTTTAGCACCGTCTGTATTGAGTTTGAAGCACC
CCACCATAGGTTTAGTCCAGTAAATAATCCTGTTGTTAGAAACAACAATAGTAGTAGAGAAATTCAAACCAAATTCCTCATGCAGATGATGGTAACCTTTAAAAGAGTCATGTTTTAAAAG
CTTCGCAACATACAATTGAAAAATTTTATGCTTAATTTTGTAAATAATCGTGCTCGCATCCATATAGACATTCTCATGTTTAGAAGCATTTCTACTAACCCAGATATACCAACATATCAAGACA
GGAATGCTATTACGAATATGGCCTTTCAGTTTAGTAAACCATTCACTTAACACCTCATTGATAGAAGTAAAATGAAAAGTTGAGAACACTTTAAACAAGTTGTCAAAGTGAATCCAAACTT
TCATAGCAATAGGACCTTAAAAGAAAACATGGAGTAAATTTTCAGTGTGATAGCAACACTGACACTTGGAAACAATATAGAGACCTTTCTTGATAAGCATTGAGTCCACAGGAACATAAC
TATG
>comp105424_c0_seq1
CATTTCTATATTAAATGAATTGTACAATATATTTATATGTATGTCGAATGGGTATGCATCAGTCTGTTGGAGCAGCACAAGTAATGAACAGTGCTGATTTAATTAGCTCTGCAAAAAATGACA
GATGCAGCGAGTGGCTCCTAGTGACATGGGCTAGGCGCAGTGCTACTAGCTGGGTTTGCTAACAATACCCAATAAGTGAATGGGATAGGTGATATCGTGAGCTTGTCACAAATAAAGAAA
AATCACTGCTAAGGGAGGATCTTTGTAAATATATCAACAATTTGATCTTGGGTGTTGATATGATGAATATTGACTACATTGTGCTTGATACATTCTCTAATAAAATGATAATCCATTTCAATATG
TTTTGTCCGAAGATGGAAGATAGGATTATGAGCAAGTTTTATTGCGGAAACATTGTCACAGTATACTGAAGTTGGTTGAGATACATTTAATTGTAGATCACTAAGTAAGCGGCATATTTAAA
TGATTTCAGTGGTTGTGGTGGCG
>comp105470_c0_seq1
CGACTGTGAAGGTTTATTAAAGGAATTTATTTGCCCAATAATACAAGGAAAATTTCCCATTCCTCCCTGAAACTATGATCAAAATCAATTCTCATCATCTTCGTCACGATCTCATCATCAACA
ATAACAACAATTGTGTACAAAGTAAATAAAAGGAGGCACCGCTACACAAGAGTAGGTATACACATCTTTCTTGATGCAACAGGGAAATACTATATTTGTATATTTTCTTGTGCCAGCGACA
CACGCCATTCTTATAATGATGAGGGAGAACTAATCATCAAAAAAGTGTGATTGTCCTGTAAGTACATGTAGGTGTTCTTGATGTAACGAGAAAATCATATATTGTCAAATTTTTCATCCAAG
CAAATCCATACACGCTTTTCTTGTAAGATAGAAGACTTAGCCATGGTGTCATGTATATCACCCATATATGCCTTTCTTGGCATGGTAGTTGGGCTTAGCATTCCCACAAATGCCCCCTATGCT
ACCAAA
>comp105735_c0_seq1
CTTATTGAGGTGTTTGATACAAGTTTTTTAGGTATAATTTCTTCTAAAATCCAATAAGCCATAGTATTAGAAATTATGAATTTCAAATATGACACCCTAACAGCAAGGGAGAGATATCTTCTTT
TATGCCCTTCTAATGAAGAAGTGACTTTGAAAACTAACATCTAAAAGTAAAAAATATTAAGCCTTTTGAAAAAAATAGTAATGTCAAAGTATCTCATGATAGAAGTTTAAGAAAAAATATTA
AGATTGATAGAAAGAGTAGTATTATTACCATGACACCTAGATACATAATGGAGCTCTTGGAGGCATTGAATTTGAGGCCTAAACCTTCACCAAAAGCAATATGAATAGTGTTCATGGCTTGA
AGATTTCTAATAGTAGCTTTTCAAAAGACCATAAGATCATCAACATATAAAAGATAAGCAATACAAGAGTTACGAATTTGAATTTTAGAGAAAGAGCCATCCATCACAACCTTGTTAAACA
AAGTAGAAAGTTCATCCATAGCAATGTAAAAGAGGCAAGTTGATAATGGGAAACCTTGTTTAAGGCCAAATGTAGAGTTAAAAACCCCCTCCAAAGAATCATTAAGACAGATAAAGAAA

CGAGCATTCTCAATGCAAGACTTTATCCAAATAATAAAAATATTAGGTATACCTTTCTCATAAAGCCTAGAAAGAATGAATTTTCTGCAAACAGTGTTATAGGCTTTAAAGTTGTTGAATTTG
GCATAAAAAAATTTTGAATTTTTATCTTGCCTGAAGTCAGATAAAAGTTCATCAGCTAGAAGAATATTAAAAGTAGACACCATATTCTTAATAAAGCCTGACTGAGGCAAGTCAATGAGGT
GAGGCATACAATCTCTAAGTCTATTAGTAAGAAGTTTATAATTGAATTTATAAAACAAATTACAAAGAGAAATCAATCTGTGATACTTTATGTTACCCGCAAAAGAGCTTTTAAGGATAAGA
GATAACGTAATTGCTTTAAATTTCTTATAAAAGTATCTCTTTTAAAGGACATGATAATAGCCTCATATATTGCTTTCCAATAACATTCCATGTGGAGGAATAAAATTCAGCAGGATAGTCTTCA
GGTCTTAGAGCCGAGCATTTAGGAGAATTAAAACCCACTAGTTTAATTTCCTCATCAGAAAAAGGAACCGTTAAGGAAGATAGTAGATTTAATGGATGGAGCTATCAATAGGAATTAACTG
AATGGAAGCAAGAGCTGGAGTATTGAGAAAGCACTGGTAGTATCTAATAGTAGCTGAAGCAATATCATAATGAGAATAGAAAGTCCATAGGTCATCAAAAAGCTCCCTAATAAAATAATTA
TTCTTTCTACCTTTAATTTTATCATATACGAATTTATGATCTTCTTCACTTTGAGAAAGATGACATTTACCTATTTGAATCACTCAAGAGGCCCAAGCATCATATAAATCAGATAAATTCTAAA
AGATGAGTTTTGCAGAAGAATTGAGATCCATATTAGGGGGGTCAAA
>comp105857_c0_seq1
ATAACATAATTAAATTTAAGAAAAAAATTAACTCATCTGATTGTTTGAATCCGCATTCCTTCTCCTTTTGGATCATCTTAATGACATTGAGATTGTCACTTTCAATGATAGCTCCCTTGTATTT
GAACATCCAATCCTTCATTATAATCTTCAAGTATTTAATTGCAATAACTTCCAATTGGTATATATCCCAATGAATGCAAGATTTCCCAAAGGCCAACAAGAATCTT
>comp105922_c0_seq1
TGATTTGTACAGGAATTGTGTTGGATTTGAATAGAGATTTACACAAAATTCTGTTAATAGGATTTATACAAAGTTCGGGCATGATTTTTGCTAAATCCTATTAACAAGAGGTCGTCTGAATGA
ATGCAAGATTGGAGTATGATTTAGGCGTTGTTGGAAACAGGATTTAGGGAAATGTTAAATCTCACTATTAGTTAATTCCTCTACTTCCCTATTTTTACTTCAGCAGATTTACATGATTTGTCC
GAAGAAGCTTGTGAAAGGGAGATGGTTACCGTCGCAAGTAAGCGAGTTCCCATCCTCACTCCCAATAGGGGTGTCAATTTCGGGTCACA
>comp106085_c1_seq1
TGCTGGAAACATGTTAAAGGAATCATGGTGTTGAGGAAAAAAAGAAAACTCTTTTAAAAGCTGCCATCTGAGGTGATGGCTTCTCTGTACTTACGCCATACCCCAGAATACAATAAATAA
GCAATTAGAAAATGTTCAAGTATGAAGGGATTTCCTCCTCCCCGCCAAAAGCACTGCTCTCTGAAGGAAGCTGGTTTCTCTGTAGCTACACCAGCTGTTCAGAAAGCTCATTGGACCTGG
TTTTGAAAACAAAACAAAGTTAAAACCCTGGGAGGAATTATTGTGCAGTGTGGAGTACTCAGTCTTTCTTATAAAGAAAAAAAAAAGTTATCTGGTACCAAAGTGTGCAACCTACAGAC
CCTCAGGTACTGCCCTGTGACTTCTCTGTATGATATCACAAGGGTGCCAAGTGCCTGTTTTTCTAGAGCTAGGAGTTGGTGGTTTGGCTAGTGCTGAAACCATGCATAGGATTGGTTTACTA
AATAAAAACCTTATTACGTACGTCCTCCAAAAGACAGCTGAAAAGTGGAGAGATTTTGATTTGGTGCTGTTTAAAAGAGATGGCAATTAAGAGCATAAGCACCAGGGAGATGGTTTAATG
GTAACTGCCTTTTTTTTTTCTTTCTTTCTTATGTCAGGCTAGAGCTCCAAGTGGAACTTGCGTTACATGCAAGAATGACTGGATAGGGAACACTCCCTGACAAAGAGCTACATAATTAGAA
ATGGATCAAGTTATAGGGAGGGTAAGGGAAGGCTTCACATTCATAATAGAGCTCAAGACATTACAAATTCATGCTGACAGGGCTGTGGCATTGCCAGACAGAGGCTATAATGGAACTACA
AGTACTTTACGTGCACATTACAAGGACATTTTCAGCATTTGGTGGGAAAGTCAATGCAATTAGGATTTTAGAAGGTTGGGTGATAGGGCACAAAGGTTCAATTTCAAAGCATTATGTA
>comp106264_c0_seq1
TTTTTATATGTGTTAATTTATTAATTAATGTGCACATACATTACATTCATGCATACATATTTATTTGATTTACTTTCTGTTGCAAAAAAGAAAGAAAAGCAAAGTAGAATGTATCTAGGATGCTT
GTAAAAAAGAAGACTTCAAGTAGAATGTACCTAGGTGCAAATGTATGTGCAAAGGGTCAAATATAAATGTAAGGTGTTAAGAGTTATAAAGGGGGTTGAAAATATTTGCTTATATATATACA
CATAGGGATCCAAAAGATAATACCCTATCTCGATGGTGTTGGATTTTGGAGTCCTATAGAAGATGCGGAGCTAAAAGATAATACACAAATATATTCAAATCCATGTTTCATAAGAGTAGAAA
ACTAGGTTTGTTGTGGTGGATCCTAGCCACA
>comp107141_c0_seq1
GAAATTGGAGTTCGGATGGTCCCAATTATAATTTTGGAAGTTTCCAACTTGATTTTGCATTGAGATTGCCAGAGGTCCCCACCTTCAAAAATTAGTCATAAAAAATTACAAAATAACAGTA

GAAGCATTAAAAATGTCGGAACTCTTGCATCAAGAGGGTTGAAGCTGGAGGTGTTGGCTGCGGCTAGATGATGGATGTAGTCGACGCGGTATTGGTCCTGTTGCATCATCAGATCATACT
GTTCCGCAAGGGACAGATTATATTGCCGCCCCAAGGGTGAGCACGGGCCATATCGCTGGACCAACCTGAGCTTGTGAGGGCTCGTGCCATTATGAGGCACGGAGCAGGAGCTGCTCGGT
ATTAGTGACTTGACGTCGATGAGATCATATTCTCCGGAAAGCCCACGTACTGATACTAGAGAAATCTTCTCTATAAGAAGCAAAAAGTAGAAGAGTTTGAGAAGAGTATAACATGGGAAC
AGAAAATAAGAAGTCGCCATGAGAATAAAGCTTCCAATTTGCCCTTTGTAGAACTTGA
>comp107441_c0_seq1
AGCTTTTTGATTTATTATCTTTCATAGCCAAGGATAAAATCTTGCACTACTCAGAAACCTATCTGACATGATTATGTTAGATGAATTATTTGAGCATTGCTGCAAATTGATAATTCTAAACCCT
AAAGCTCAAGAGAAATGACATATTTAAGTGGATTGGTGAGGGCATTATATACATCATTGGAGAGAACGTACAAGAAGTTCTTGAAGTTATAGGCGAATGTATAATTCACATATTTCCAAATG
AACTTGAATAAATTTTTTTTGATGATATGTGAAACATCGCTTCATAAAAAGGTCCTGATGTTGCATGTCTCTCTCAACCTTCTTGTACCTGAATGAAGACCTTGTAAGCAACTATTAGTTCAT
TTTGAAAGTGACTCCCTAGATTGATTAAGATGTTATGGTTATTTAATAAGCAACATAAACTTTTCCTCCCCAAATCTCCTTGGAAGTGGAGGAGGGCCACTACAAATTAGGTTTGAATGACT
ATAATAAGGTAAGTTATTTGATATTTTAATAAAATTTTATACATATTACTATCTTATATAACTTCATGAGCGTATTATACTGCATTTTCA
>comp108850_c0_seq1
GATTCCGCACAGAATAATTTCTCCTTGTTCAGACTCCTGAATTTCGTTCTGAATGATTTGCTCATTATTTCTAGCTCTGTTAGGGACAATTATTAGGTCTAAGCGTAGTCAAAAAACGCAGTC
AAAAAGCACTTAAAGCAAAGTTAAAGCGTAGTCAAAACGTAGTCCACATGGAAAATTTTAATTGAATATTTGAACCTATTTTTAGGTCTGGTGTCCGGGCCATATAATTTTCCCTCGTTAGC
CAATAAATATCTGTAGAAACAAAATATAGAGAACACCCATAGGATTGTGGTCACAAGTTTTAAATAGAAACCGACACTAGATGCTCATCAATTTTATTTTTAAAAATGTTTGTTTATTGTGTT
TTTTTTTTTTT
>comp109949_c0_seq1
AAAAACTAAAAAATTTTAAATTTTTATAAAATGCATAATTGTATCATGAAAATTACTTAAGAGATTTATGAAAGTTAGACATAGATTATCGTATTAAAAATTATCAGAAATAAATTCAACACAT
TTGAAAAAATTACATAAGAGGTTTAGGACTCTCATTCCCCACCCTTCAAACTTCAAAGGCAGGCTTCACATTCATCAGTGCATTGATCCGAAGCTGCTACTCATCAATGTGTTGCGAGAGA
GCTTTGGCAATAAAGCCAGACCTACAAAGCCACCAACAAATGCAAGAGCTGCTGCGAGCCCGAAAGCTGGCACATTTCCCTTGCCAAATAGTGAATCCCAAGGACCAGCACTGAGTGC
AATGATCACCTGAGGAACCACAATGGAGACATTCAGAACTCCTGTGCATAACCCTTGTTGAGTGCTTCCTTCGTTCGCAACCAGCTGTGCAGCAGTTGCAAAAGGAATGCTATACAGAAT
AGCTAGAGGAAACCCAA
>comp110240_c0_seq1
CTTAAAAGAGGTACCCATCCCAGAAACCACCTGGCCCGCACTTGATTGAGCGCTCGAGGGGAATCAAACACGTTTGTATAGAATTTCAAAAGCAAAAATATAAAGGTAGGTAGATTGAGA
TAAGAGAGAGAGCGCGAGAGTGAGATGACCAATGGCGTGTGAAGGTTGGACGCTAACGAGAATTATTATTTTTAATTGTATTTTATTGTTTTGAGTGCAATATGTATACATAATGAAGTTTA
GGTGTTGAATATGTAAATCTTCTTTTTTTATATGCT
>comp110466_c0_seq1
AGGGGGGGGAGGAGGGGGCGGGGGAGCCCCGGGGGAGGGGGACACGGAGACAACTCAATACAACTGCAGACGAGGCGGCCCGGCCGGCGAGGTCCCGCGGCGGCCTCCGGAGTCC
TCAGTGTGGCGGCGGCGTGGCGGGTGGCGGCGGCGACGGTGGCTTCTGGGCTCGGGGGCCGAGGGCAGGGCACAGTCTCTAGGCGGCTCCCCTCGCCCCAAACACTGAGGCCCCGGA
GGGCTGGCAACAAGAGTCTGTGGAGCGGCAGGCGGGCGGCGGGCAGCGGGCATGCCCCCACCGCAGGCGGGCGCGGGCGGAGCGGCCTGTCTTCTTGCCCTGCCCCCCGGCATGGGA
CGGGCCAGGGTGCTGGGTCGGTACCGGAGTACGAGCTCG
>comp111039_c0_seq1

TATTTCAAGCCTTGTTTCATTTGGACTATAAAAGTCGTGCTAATGAATGGCAATAAAATATCAGAGTCGAGATTAAGGAGGCCACAATCATGGAGGTTCTCTACACTTCCCTCTTCCTGATC
TCATTAATCATCCTCTTCCTCACGCTCAAGATCTCCCATGCGAAGCAGCGTGGCAGAGAACCCCCAGGGCCATGGAACCTCCCCATCCTCGGCAGTCTCCACCGCGTCGTCGGCCGGCAA
CCTCCTCACCACTCCTTCCGCAACCTCTCCGCCGTCTACGGCCCCATCATGCGCCTCAAGCTCGGGGAAATCCAAGCCGTCATCATCTCCTCCGCCGAAGCCGCCAGAGAAATCTTGAAG
ACCCACGAAATCTACTTCGCCTCACGCCCCGTCAACGACTCCGTTAACGCCATCTTCTACAACGCCAACAACATCTTATTCGGCCGCCACGGCGAATTCTGGCGCGAGATGCGGCGGCTA
TGCACCATGGAGTTGCTCAGCACGAAGCGCGTTCGGTCGTTCAGGGCTGTGAGGGAGGAGGAGGTATCTCATCTCGTCGAATTCTTAGCGGCTTCTTCCGGTGATCTTGTCAATATTAGC
AGAAAGATGGCAGAGACTGGCAATAACATCATGGCTCGGGCGGCGATTGGGGGTCGATGCGATGACCAGAAGATGTTCTTATCGGCGCTGGGCGATGCGGTAGAATTTTTGTCGGGATTC
ACCATTGTGGATTTGTTCCCCTCCATGCCGTTCATAGGTTTGCATTTCAATCAGAAACTTATTTTT
>comp111775_c0_seq1
GTGAGGGAAATGAACCTATGCAAATCTCTTGACTTAGGGAAGGCCACTTACCTACAAAAAACTCATGAGCCTTTGTTTGGCCATGGCATTCTCAAGTCTAGTGGAGAGTCATGGGCTCAT
CAAAGAAAAATTATTGCACCAGAGTTCTACATGGATAAGGTCAAGGGAATGGTTGAATTGATGGTGGATTCTGCCATCCCACTGTTAGAAACATGGGAGAAAAGAATTGAGAAGGAAGG
AGGAACAGCTGAGATAAATATTGATGAGGATTTGAAGAGCTTTTCTGCAGATGTAATTTCAAGAGCAAGTTTTGGAAGTAGCTATTCCAAAGGAAAGCAAATGTTTTTGAAGCTTTATGAA
TTGAAGAAAGCTGTTTCTAAACCAAATCTCCTCGTCGAAATCACTGGAATAAGATATATCCCTACGAGAAGCAACCGAGAGGTGTGGAGACTAAACAAAGAGATCCGCTCCCTCATACTC
GAACTAGTGAAAGAAGGAAGCATTAGCGAAGGGCGAAGCTCGCCGGAGAACAACCTCCTTCAGGCCATCCTCCGAAGTGCAACCGACGCTGGCGGTGGCGATGCGGAGGACTTCGTT
GTGGATAACTGTAAGAATATATACTTCGCGGGGCATGAGACCACCGCCGTGGCTGCCACCTGGTGCCTCCTCCTCCTCGCCCTTCATCCAGAGTGGCAAGATCGTGCCCGTGCTGAGGTC
ACGGAGCTCTGCGCTGGCCTAGCACCCGACGCTCACTCCCTCTCCAAGATGAAAACCGTGACAATGGTGATCCAAGAGGTACTTAGGCTCTATCCGCCTGGACCAGTGGTCTCAAGAGA
AGCTCTCCAAGACATGAAAGTTGGAGGCATTCAGCTACCAAAAGGCATCAATATTTTTA
>comp111971_c0_seq1
AAGCTGCCGCAGCGTCTCCCCCTTTGCGCCGGCAGCCGTCAGGCTCAGAGCAGCGCGGATAGAGAGCGGTGAGAAGACGAAATTGGAGCCTTCATCCGCAGCCGGAATCCCTTTCAAC
CTAGCTATTTGAAGGCACGAGTCCATTTGTGTGTGAGTAGGTTCTTGGGCGATATACTCCGGAAGGAGACGATGATACTGTCGTTGGTGTTCAATTCCATGGTAGCGAAAAAAGGAGGGG
ATCTGATGTTTAGAAGAATGAGAGAAATGCTGAAACAGATGGCTGCAAGGGGTCAAAGCTCTGCTCTTATACATGGTGGATTCCAAATCCTTGCAGGCGAACTCCTGGCACTTCCGATCC
GACAGGAACAACG
>comp111987_c0_seq1
GGGGTTATGTTGCCATGTAGAAATGTAGCATGGTATATTTTATGAGTTGTGATAATAATAATAAGATTTTTAGGTGATGGGGGCATGGATACGGGTTCATTGGAGAGAGGCATGGTCCTGAAT
TCGCCTACAACCCATAATTAATTAAATTTAATTTTTTAATATTAAAGTACATTAAATAAGAGGTCTTAGTTTCTAACGAACGTATAACCAAATGCTCTCGATTTGAAATACAATTCAATCTACA
AGAAGGCACTTATACTCATTCATTGAGATAAGCCTACGGAACTGTCTTTAGTGTATTGATTGTGAGTTTTTACTATCAACTAAGAGATCTTATTTAATGTACTTTAATATTAAAAAATTAAATT
TAATTAATTATGGGTTGTAGGCGAATTCAGGACCATGCCTCTCTCCAATGAACCCGTATCCATGCCCCCATCACCTAAAAATCTTATTATTATTATCACAACTCATAAAATATACCATGCTACA
TTTCTACATGGCAACATAACCCCAAAAAGCCCTCTCTATTAAGGGAGGAATAACATGTGGAGTTTAAATGTAATTTTATGTGTGGACTCTGAGTACTATACACAATGTGTATATATTATGTAC
AAATACATACCATATACACGTTGTGTATACTC
>comp112394_c0_seq1
AAAACTCAAAATTGATTTTGCAAGTGAACTTTGATCATAGCCAAGAGTTCATTGGCCATATCAACCACATTTCTTGTCATATAATCTCGAAAGTCTAATTGTTCTGACAACAAAACATGTTT
GGCAAAAAGCCAAATGAAAGATAAGAGGTCACCTCCTCTACTCAGTTGCTTTATATGCATAATTGCTTTGGTATTTCTCGCCCCTTGCAATAAGAGCTCCACCCATGCCATGGTCACAACTT

TCCATCTCTCTTCGTCTTCAATAATAAGCATCAGATGAGCAAGCACTCGTGCTATCTCAAATAAAGATAAATATCCATGCCCCTCTATCGGAGTAGTCATCATCTTTTTGCATGCCTCCTCAA
CTTTTGCATTTGGCATCAACAAGTGATGGTCATCACCTCTTCTACGAATGAATTGAACCATATCATAAATGCAAGCCAGAACGCCAACGTGCTTGCTCCTCCCTTAGTAGACATCATATCTA
GTCGCATGAGCACCATATACATCATGTAGTTTGATAAGTACTTGCAAACTTCTATCTCTAAGCTCTTCCTCTTTGCTCCTCTTGGTATGTTTTCCTCTGTTGTAATCTCTATTTGATGAGGATG
CAATGGATGTAGAGACTCCGACTTCCAATGGAAACAAAGATCGGTGCAGATGTGCCATACCAGAACTTGTTGATCAAATGATAACCCCTCTATAAGCTTAACAAATGATGGGACAACCCA
AGCAGGAGTGTTTGTTACTTGTTGGAGAACAGTCAGGCCTGCAGATTCATTAAATTTCTCCCATGATACATGCAAGGGACTGCGTACATATGTAGCAATGATATTCATAAGTTCTTTAGTTG
CTTGTATTGGTTGTTGTGTTTGCAATGTGTAAGTTTCCCTATCATCAATCCAACTCATGTATAACTTTATCTCTTGCACTAAGCCCAGTTTCTCTTTAATCCAATCAATCATGTTTCGTCGAGG
AGCTTTATTTGGTGCAAAATTATTAAGACAATTACTAATAAGATTCAATTGTGGCAGCTCTCCTGATCAATACCTTCTCATCAGCAACGACCTTTTAATTTTAGCAATGTGCTCAGCAAGCAA
CTTGCTCCAATTACCCAGCTTCTTAACATTGAGAAGAGTAACTATCTTTCAATCTGAGAACATGAGCATTATAGTAGCCATGGTGTCGAGGAATATGGAAGCTACAAGTAGGACATA

>comp112619_c0_seq1

GGCAGATGAAGGGTAGGCAGCGAGAAACCAAACCCTAGCAAAGTGGGTTTTCAAAAAAAAGTTTATGTTAGGAAAACATATTCCTATCTTGATAGCTACTATTTCATCATTGAATAAAAA
AGAATATTGTATGCTATATTATTAAATTTTCTTATTATGAATTAAAGAAAAAAAATAATCACTTTTATCAGAGATTAAAGCATACAAGCCCACAGTCATTAATAACCTAATGAAGTCCCATCTTG
AAAAATACAAACCAAATTAAAAAATATTTTTGAATGGAAAAGTAAGAGTAGAAAGACAGAAATGGCTGGAAGTCCAATTCCAGAATTTAATTAAAGAGCTCGGCCTTTTATGATATCCAA
GCTCATCTCACTGGGATCAGTTGCTTGGAGCTCCACCTGAATGCCATCGAGCTCTCTCTTGAATCTATCCTTTGAGCTGGCATACAGCATTTTACTCCTCACCCTTGATGTATCAGGAGACC
AGGCAATGAAGAAG

>comp112628_c0_seq1

CGCGTGAAGCTCAAGGACATCACGGCTCCAGAAAAGGAAGAATGGGGCAGCCTGCTGGATGCTTTCAAGGTCGCCTTGGAACTGGAAAAGAAGGTGAACCAGAGTCTGTTGGACCTT
CACGGTCTGGCAGACTCCAAGAAGGATGCGCAGATGTGCGACTTCATCGAGACCCACTACCTGACGGAACAGGTGGAGGCCATCAAGGAGATCGGTGACCACATCACCAATCTGAAGC
GCGTCGGCACCGGCCTGGGCGAATACATCTACGATAAGGAGAACCTGAAGGAGGATTAAGACCCGGACCTCCCTCGGATGCCGTGGAACGGTCGGACTACTTCCTTAGGAAAGAATAAA
AAGAAACATTTTAAAACATTTTCGAAAACAAAATCTAGGCACCGATGAAAAAAATGAAAAAAAGAATTATTGTTCAGTACAATTGCCAGAGACCAACCTGGGAATTATTCGATAAACCTT
TGAGATTAAGATACTGTCTGTGCAAATTCTGTCATTCCAATGCTGT

>comp112712_c0_seq1

TTTTGTTCAATTTTTAATAGCTTAAGTAAGTTGAAATCTCACAATAAGCTAAAAATGTATTTATTTTTACTATTTTAATCATCCTTTAAATGTTAAAACACATAAAGTAAGAAACAGTGTCCTT
ATATATTACATCAAATGGAGCGGCCATGTTTGGAGCTGCTCAATTATCAAGTATCGCTTGCCTCATCATGTAAATGAACTAATATGAAAATATTGTTGTAAGAAAAATAAAATATACGATTGTA
TAACAAATTCATATTTCCCCCTTTTTGGCATCACCATTTTTCCTACAAGCAAACTCTACATAGAACACGATATATTATACTCCTGTTTCATTGGCTTCTTGCATAGCAAAATGACAACCGGCG
ACTGCATCGTTTATTGAGATGAAAAACCACTCTTTTCCAACCGCTTCAATGAAATTTGCCGCAGTAAACTTCTCCACCACTTTTAAACTTGGGTTTGTTAGTGCCACCTTGATCCCCTTCTT
TTGAAGCATTTTATGAGCTTCCAACATCATTGCAATGCCACTGTTGTCAATGGCAGACACCCCGCTCATATCAAATATAACATACTGTAAATCCTGGCTTCTTGTCTCCTTAATTCTATTCTCT
TCAGCTTCTACCCACCTTGCGATCCTATAATTCATTTGATACTTATCAAGTGAAGAAGATTTGAAGTATTTGGTTCCATTCTTGTACGTCAGCTTAGTCAAATGTGGAATGACTTTTTTTTCTT
TTTACCTCTCTCTAAGGTAACTGGCATTAGCGAAATAGATGGGAGAGCCAAGCTGCAAGATTAGGATACCAGAAATGGTCTTGGAATCTGGATATTGATCAATGTCGCGGTAGAGATCGGT
TCCAGGCAAGTTTCCAAGCTTATATGTGACGGGTCTTGCCACGTGTAGCAATGCTCTTATGATCGACAATAAAAACGATATGGCAAGGCCTGTCTTCATGTTAATGAAGAGAACGCCGAGA
AGGGCAGCCATGCAAAGGCAGAAATCGAACTTATCAACTTTAAAGAGATGGCGAAGCTCATGAAGCTCGATGACGCCGATCATGGCAACGATGATAATGACCGCAAGAGCCACAAGTGG
TGTGTAC

>comp112712_c0_seq2
TTTTGTTCAATTTTTAATAGCTTAAGTAAGTTGAAATCTCACAATAAGCTAAAAATGTATTTATTTTTACTATTTTAATCATCCTTTAAATGTTAAAACACATAAAGTAAGAAACAGTGTCCTT
ATATATTACATCAAATGGAGCGGCCATGTTTGGAGCTGCTCAATTATCAAGTATCGCTTGCCTCATCATGTAAATGAACTAATATGAAAATATTGTTGTAAGAAAAATAAAATATACGATTGTA
TAACAAATTCATATTTCCCCCTTTTTGGCATCACCATTTTTCCTACAAGCAAACTCTACATAGAACACGATATATTATACTCCTGTTTCATTGGCTTCTTGCATAGCAAAATGACAACCGGCG
ACTGCATCGTTTATTGAGATGAAAAACCACTCTTTTCCAACCGCTTCAATGAAATTTGCCGCAGTAAACTTCTCCACCACTTTTAAACTTGGGTTTGTTAGTGCCACCTTGATCCCCTTCTT
TTGAAGCATTTTATGAGCTTCCAACATCATTGCAATGCCACTGTTGTCAATGGCAGACACCCCGCTCATATCAAATATAACATACTGTAAATCCTGGCTTCTTGTCTCCTTAATTCTATTCTCT
TCAGCTTCTACCCACCTTGCGATCCTCTCTCTAAGGTAACTGGCATTAGCGAAATAGATGGGAGAGCCAAGCTGCAAGATTAGGATACCAGAAATGGTCTTGGAATCTGGATATTGATCAA
TGTCGCGGTAGAGATCGGTTCCAGGCAAGTTTCCAAGCTTATATGTGACGGGTCTTGCCACGTGTAGCAATGCTCTTATGATCGACAATAAAAACGATATGGCAAGGCCTGTCTTCATGTT
AATGAAGAGAACGCCGAGAAGGGCAGCCATGCAAAGGCAGAAATCGAACTTATCAACTTTAAAGAGATGGCGAAGCTCATGAAGCTCGATGACGCCGATCATGGCAACGATGATAATG
ACCGCAAGAGCCACAAGTGGTGTGTAC
>comp112990_c0_seq1
AAAGATGATGGACCACAGGGAATACATACAACAGTCCGCCTAATGACATGTAAATAGAGCCATAAAACCAGTGCTACCGTGGTTTCGCTGCCGTGAATAAAGCCATAAAACCAGTGCTTG
TGCGGAAGTGAATAAAAATGCAGAAAAATCCAAAAATCTCACCGGTTCTCCGTGAATAAAGCTAGTGCTTATGCGGAGTGAAGGCCACAGGCGGAAGAGCAGCTCAGAGGCATACACA
AGGTTTCGTTGAAACTTGTTCTCCCGCGAAAATGGGAAGGAAAAAAAGAACGGAGTGTGTTAATAGATCATATACGCCATTCAACTTATACAAGCACACCTGCTTATATTCGGCTACTGCT
CCACTTCCTCCTCAGCGAATCATTACAAAAAATGGTTTTCTGCCATCGCCGCGTCTGGGCACCTGCTCCTTCTGAAACTCTAACAACCTGTTCCCTTCTTCTTAATATATACCAATGTATACA
GGAATTGTTGACTAAGGGAATATATGAAACCTTCAACCAAACCTCTTCTTCTTCCTCTGTTTTTCTAACATCTATTTTGCAGATCATTTCATGGTATCAGAGCTCCAGGCTCAATAGTGTGAC
TGAGATTCCTCTATGGTAGACAGCCAAAGCTCTTCGGCTCCTCAAGCCGCTTCTACAGTTACAGCCGCAACAGATATCATCATTCCCGTGTCTCTGAAGTTCATAATCTCAAATATCAAGCA
TATCATCAACATCCAGCTGAACAGCGACAAACACGCCATTTGGAAGCTCCAGGTTTTTAAAATCTTTTCCGCCAACGGTTTTGATGGTTTTCTCACCGGATCTGATGTTTGTCCAGTAAAA
CACATTTCCGATTCATCTGGAACCATTAGTTTGAATCCAAACTATTAACTATGGCAACTCACTGATCAAAATCTTGCTGCGGCATTGTTCTCCACTATCTCACCGAATATCCTTTCTTATGTAT
TGACGCTGCAAACTACACACGAAATCTGGGACACTTTGGAAAAGAGGCTTCAACCAACCAATCGCTCAAGAGTCATTCAACTCAAGAACGAGTTGTATCGCATCGACATGAAAGATCTT
ATGGTAAATCAATACCTAGCTCATGTCAAATCTTTGGTGGACAATATTGCAGCAGCGGGATCAAAGGTGGAGCAAGAAGATATAATTCTACACATTTTGAATGGCCTACCTTCATCGTATAA
TGCCTTTAAAACAGCTATTCGTACACGTCCCTCCAACATCCCACTGGACGAGCTCTATTCCTTACTACTCAGTGAGGAAATTAATATTCAGAATGATCTTTCCAAGGATGCAACGTCTGCTG
CTGACTCAGTTGCCCTGAATGCCTTCAAAAACACTACATCCAGGAATATATCAACTGTGCAACCCAAATCAAACCGTGGCAGATATTCAACAAACAGGGGATCCTCACAAAGGCCATTCA
ATTCAGGCCCCAGACCGCCCAGAAATTATGCTTCTCCACGGATTACTTGTCAAATCTGTAACAAACCGGGCCACCTAGATGCACAATGCTGGCATCGAGCCAACCTGCAGTATGTTCCCAC
AATGCCGTCTCCGCAACGCAGCTTTTTCACCAATCAGCAACCAGGATCACCAACGGATTGGTATCTGGATACCGGCGCCTCTTCTCATCTCACCTCAGATCCACTTCAATTGTAGTATTCTC
AACCATATACTGGCACCGACAGCATTTCCATTGCCAATGGCACATCACTTCCCATTCAATCTACTGGGCAAGGGATCCTACCTCTCCCCAACACACCCCGACAATCTGACCAACCACACTA
TCCTTAGAGGCAGAAGTCGTGACGGTCTCTATGTCATTCCCTCCAACTCCATTTCAGCTCTTCACAATCAGCAAAAATCTACCTCCACTTGGCATGCTCGGCTGGGACATCCTCACATTCA
GCTTCTACAACATTTATCTAGGCACATCTCTAATATATGTATCTCTACTAGTTATACTTTTCAATGTGACTCATGTAATGTGGCCAAAAGCCACAAATTACCTTTAAATTCTAATGCTATAAAAA
TGACTGTTCCATTTGCTTTAATTCATTCTGACGTGTGGGGTCCCGCACCCATTTCTGCACTAAATGGCTATAACTACTATGTGCTCTTCATAGAT
>comp113103_c0_seq1
ACTCCCCGAAAAATACCCTACGAGTATTACACTAAACTTTTAATGACTATTCTTTAACCCCCACAATTAGACGAAAGAATCCTTACAAAATGACTTTCACCTTCCCCGGTGCAGCGAAAAG

GTCGGGTCCTTTGACACCAAAGATATCTTCTTCTTCCCAAAGCTTCACTCAAGACGAACGTTGAAATCGATAATACTAAGAAGCAAGCTCGCCAAAACAACTGTTTGAAATCATCTCCAC
ACTCCCTTGAAACCCCCCTCCAAAGACCTCTCTCTCTCAAATTCCAAACTTTCCTCTCCCTTTCAAATTCTC
>comp113219_c0_seq1
AAGCATCAACCTATACACATAAATAATAAAAATTTTCAGGAGGCATAACACATCTCCACATTTATTTAGACAACTAAAACATTCCTTCGCGAATCTCAAAGCACATATTACAAATATTAGAA
ACATAAAGCATATATAAACTAAGTTTAGAAAAAAAATAGCAATTTAACTACGAAAAGCTGGAGAAGGGCAGCAGCATAAGGAAAGGCCACCTCATTCTAGCTCAGCAAATCAGCCGGAC
CAGTAGCAAGTTCAAAAGAATCTGGTTCCGTGGGCTCCTCAGTGGTTATTGTAGGCACAAGGAAGTCACAATAATCTGTTTCGTTCACTGCAGCAATGATATCTTCCTTTTTGATAGTCCG
ACGACACCTCTGGTCGGCGTGAGCCCATCCCTTGTGGGTTAGCTCGAGGATGAACATCTCGCAGGCACGTGCCAATAGCACAGGCGCCTCGTTGGAGACTATATCCACGTCGTCGTCAGC
CTTCATAATCCTCTTGATGGTGCCTAAAGGGATCCTTTGCTTCTCGAAATCTGTGGTTTCCTCCATCTCGCGGTATTGATCGGCCCAGTAGCATCGACCGGGCTGTGGTTGATGCACTACCT
GCTCGGGATAGAGATTGGGGTCTGCGTCGGCGGCGAAGTTGGGCAAATGGGGAACGTCGCCGGAGGGTGGCGGCGGTCTGTTGTGGCCTTGTCCATCCATTGAGATCGATGCAAGTGGG
AGTCGCTTTAAATGGAGGTTTCAATGGAATGTAGA
>comp113411_c0_seq1
ACATCCACTACCTTTTACTACTTTCAGTTATTACCTTTACTTATTAGCTTTATTTTATTTTTGCAAGATATAGCAATAGGGAGTATTCCAGAATACTGATATAGCCATGAGCGGCGGGAGGCAT
CGAGCAGTTTTTTGCAAAAATCATTTTAGAGGAGTTAATACAAACACCTGAATATACAGGTTTATACTTCATTCCAACGATCTCATTGTTCTTGTGATTACTCTGTTTTTTTTTGCAGGTTTC
CAGTAGGACTTCAGCTGAGTGCACGGGCTGCAACAGGTTGGCATCAGAGCCTCGCCTGGGAAAGAAATGACAGATCGCGGCAAGGAACCCATCTCCGAAGGAGCGCGAAGTATTGACG
CGCTCTAGGCAAACCAGGAAAATCTCAACCGCCGAATAGATGAGCTATTGGCCGACGTCCAGAGGCTTACCGTGGAGATTCGCCACGAGTTCAATCTCATCAGGGCGAGACAACCACCA
CAGCTGCCACCACAAGTCGATCAACAAGGAATCAGGCCAATGCGGCGAAGGGGTTTGGTAGCGAATCGACCTTTC
>comp113485_c0_seq2
CCACACAGAATAATTAATCCCATCGTAGGATGTGTGCATGCTTATGTTTGTTTATCTTCAGTTTCGTTTGAGACAACTTTGCATGCTTAATTGTTTGTTTATCTTCAGCTTCGTTTGAGACAAC
TTTTTTACGGTTCTAAAACAGCTATTTAGTACAAAAATTTCTAATTTTAAAATTTTTTGTTCAAGAATGCTGCTTTAAAAAGCCATAAAAAGCTGTGCCAAAAGAAACCCGAGTCGAGCCA
TCATATGAACGAGCGATCATGATTTGAATGAGATTTTTACAGCATAATTGGTAAGAAAAATTAGATCAATTAGTTGGGAATTTGAAAGCAAATTTGTCTAATTTCTGTTGTGCAACGAAATT
GGATCAAGCTGAATCCGTGATCTGTGATGCTTTATTGTTCTATGTAATTTCGGGAAAATGGTTTTAATTTTGTTATATATTTTCATTTTTATTTGCCTCTT
>comp113635_c0_seq1
TAAAATATCATCTTGCTCTTCGCTTCTTTTTTTCTTTTTACAGGCTCTGTAGCAAAAAACAAACACAAAAGCAAAAAAAAACAAAAAACAAAAAAGAAAAAAAAACATAAAAAAATAGGGG
TTTGGTATACTATACGAGAAACCCCTTTTTTTTTTTGGGAATTTTGAGAAACCGCATTGATCATAAGGAACGTGACGCCGGAGATGATTGCGACGATGAAGAGGAAGAGGAGGTAGAAGA
GGAAGCGCTTTGCCGTCATCTCTAAATCCTTATAAATTCTGTTGTGGAGAAAGATAGGCTTTGTTTCCTTACCCAATAATGGAAGGAACTTATATGTGCAAGGAAATAAACAATAGAAAAG
TTCAAAGTTTTTTTTTTAATTTAATTTTTGGGAAAAGTAAGGCTGACGCCTACAAACTAATTTGAAAAATATTTAGTAAGCGTGCTTTTTAATGTTGTTTTTTTAGACATTAAAGATTACTAG
TTTTATTGCACGT
>comp114874_c0_seq1
ACTTTTTGGAAATGTCTCGGGCCAAAAGATAACGTTTGTCATGGGGAAGATGAGATGCATACTTGTCTACAAACTCAAAGAAATTCCCATGATTAAGGCATTGGAATTCTTTCACAGAAAA
TTGCAACCTCAACCACATCTCACAAGCACGCAAAACCAGTTGCTTTTCTAGCGGTATACCTCTTCCACTAAGTATGAGATCAGGTTCAATAAAGTTGCATATCTGCTTAATAATATCTTCGA
CGCTAACAGGAGCACCTTGCTCCACTTCTTTAGAGCAATTATCATCATCTGAAGCTAATTTTCTCTCAGTTGAAGGTTCTATCTTGAATATTTTTGTTGACCCGGTAGAATTTTCCTCAAAAC

TTGTCACCTCAGCAGCATCTTGTCTAATCGGTTTCTTTTCATTCCCAATTATGACTTCATCATCACCCAACTGAGTAACCATATGTAGCTCTTTTTCACTCAAATTTGCATCGTTAAAATGTGA
CTCGTTGTTATAATCAGATGGCTCCTGTTTCCTGGCTTCTCTATTGATAGAATTGTCAGCATAGTCAACATCATCTAGGGGAACAAATTGCTCACAGTGGTCACCGCTTTCATCATCTATGAG
AGAAGTTAACTTAAGATGACCTGGAATTAATTGTGATGGCATTTCTGGATCTTCCTTTGGCAATTCTCTTTCTTTAATAAATTTAACAACAACTTTTTTTGCTCTTCTCCCTGCCTTAATGTAC
TTGATAAACAATCCCATGCTCCGAATCCGAACACCTAGATGCCCCCTTGAAGCAACTGAGCACTTTAGCACCAGAAAATCAAAGAACTCGTCCAACTTGATTTTCTCGTTTCTCTTTTCAT
AATGGAATTCAAAGAGGTGGCTGACTAATCCCTCAGTTGTGATCTCACAGATTTCCTGTGCATCAGGAGACACTGAAAAGTAGTCTCGAACTAAGGGATGTTGTAGTAGAGGCCCAAGCC
TTAGCTCATCAAACTTGTCAACCCCTTCATTCTTGCAAATACTCAATCCCAGTTCATATATCGAAGTCATTTTTCGAGTATGCACAAAGCAGTGGATGAATGCATTCACCTTTCCTTCTACTG
GTTGGAGATTACGAAGAGTGGGTGCATCAACTGTCCTAAACCCTAGAACCTCCCAAGAGCTCACTTTCAAAGAGCGAGCTACGGATTGGGAGATCTTCCACACTGATGCATTCTCTCCTG
CCTCTAGGAGGTCCTTATGAGCTTTAATAACTGCTGATTCAACCTCTTCTATGGAGAAGTGCTGGGACTGATCTATGTAGTTCTCGTTTTTTGGGAATTGAGGAGAATAGAACCTAGGGTTT
TGGAAATTAGAATTTACCCTCTGCCAATAGGGAGAGTTCGAAGATGAGGCGTACGACGAGGAGTTAAGATTTTTCGGTAAAAGATCTGTCACAGAACCAATTGGAGGGCCTCGGCCGCC
TCGAGGCCGGAAGTTTGCCGGAGAGCCATACATGTTGACGAAGGCGGGAAGATAATTGTGACCGTTGG
>comp115195_c0_seq1
CAGATAGGGAGAAGCCGACGGCGACGAGCACGGCGACGGCGATTTTGCCAGTGTCGAAATTCTTGGACGGATGTGTTTTTTTTTTTTAGTTGTAGCAGCTCACAAGTTCGCATGCAGCGA
TGAGGAGAGCAACCTCAGCCCAAACATATTGGAAAGCTTTACTTCCGCAAACTCCCATCCCTGCCAGCATCCAGGAGCTACTGCGCTATCAGAAAGAAATGAAGTCGTTTAAGTTTGAA
AGAGGCCAAAAAATTTCGAAATATTTCGGCTGCTACAAATGCGTAGACAATGAGACGCCAAACCATAACGACCTCATTGCTCAGTTCTTCTTCCTGAAGGAGAGCCTTAACCGGGGCACA
AAGATGACTCTCGAACTCAGCAAGTCAATCTCAATGCAACCCTTCCTTTCAGTCGAGATGGCCAAGGCCACGCCTTTCTCCTCCACCAAACTCACTAAAATCCTCGGACTCTTCAACATC
ACACGGGGTTCAATGGAAGCCAAAGCCATCCAGCAAACATTGGAAGACTGTGAAGAGCAGCCTATTGAGG
>comp115336_c0_seq4
TATTTGAGTACGAGAGTGATTTTCGTAAAAGCTTTGGCTATTTTTTTGGGAAGAACCGGGGCATAGCCCATTTTATTAAGATAAAAGGAACAAAAAACCATACAAAAAAATAAAGGCAAA
CAAAGACAAAAGACAAAGACTATTGTTCGACAGAAGCAGCGCCCATCATAGAGCTAAACAAAGACTGAAACTAAAACTTTGGAAAACCAACAAATACAAACAAAAACATCTATAACAG
CAACAGAAGCAACAGGATGAATCAAAACAGCAATCTTGAGATATCCCTCTCCAGCCCGGTGAGGCAAGGCTAATATTTCAGTTGAATTGAAATTTTTCAATGAGGTGATGGTAATGCTTC
CAGGTGCAAGTTTTGACCCGAATAGCATTTTTTATAGTAATGATGATGTTCTCCGAATCATTCCATTTGTTGGCAAATTTTCGATTATTCCTTTCTCTCCAAAGATGATACACGGTGCATCCTA
TGATTAGCCAGCAGAAATTCTTTTCCCATTTGGACATAAGTAGCTGGTTATCCATGTAATCAAAGACCAAGCGGAACATATTGTTGAGCCGACCGAGCGAGAGGAAGAGAAGGTGAAGG
CGTAGTGAGACGGGCGTAGTTAGCCGACCGAGCAAGAAGATGAGGATGCGAAGGCGTAGCAGACGGTTGGGTTTC
>comp115519_c0_seq1
TTTTTTTAGTTATAGTCAGATCTTTATTTAAAAGTCTGATCTTCCAACTTAGCGTTTTCCACCAACTCGGGGAGCAGAAACCTTCACAGGCTTCACAATCTTTTGCTTAGGTGCTGCCTTTG
TGGGAGCCTTTGCAGCAGCCATTGCTGTCTTTTTAGATGCTTGCTTAGCTTTTTTTGCTTCCTTGGCAGCCCTGATAGCTTGTTCTCTTTGAGCCTTCCTAACTTCAGGTTTCTGATTCCTCT
TGGCCATTATGTCAGCAAGGGATGCACCAGTTATGGCCCTCTGGAATTTGACTGCACGACGGGTTCTTTTCTTCTGAATCTCTTCCGACTGTCCCTTTTTGTGCTTCCTTCTGTAGAGAACA
GTCCAGTTGATCTGCCGAGGATTCCTCTTGGAAAGGAACGCGGACTCACATTTTGCATTAAGAAATTGGAAAACCTTCCCGTCGGTCCTGGCGTAGCGCCTCCCGTGTCCTGGGTAGATC
TTGTACCCGCTGAAACTGCACAGCTCGACCTTCATGGCTGCTACTGCCGGGGAGAAAG
>comp116270_c0_seq1
AAAAACACTAAATTTTGTGTGTGAAGTCAAAGAGGGAAAAAAAAAACTACAGGATCTGGCTTAGATGAAATGATCCACAATGGACGTAAAATAATATAAATTCACTCTTCCCAACAATTG

GAGAATAAATGATTACAAATTTGTAAACTCAATTTTGGTCTTTTGTAATTGTAGTGGAGTTTTCTTAGTAGTAGAGGAAAAATGATGGCAATCGGACGACGGTACTCGGGCAAGGACATTC
AACAACAATAGTTTGGATAACTACAGTTAGGCAATTGCAATTGGACACAACAGTAGGTCGATGACAATCAGAAAACGGTGAATGAACTATGATGACCAAACAATGATGGTCAAACGATGA
TTGCCGGGTGATGACAGTTGAACGACGATAGTAGTGGCTAGCGATGATCATCAACATTGATTTTAGGTAAAGCTTCAAAGTTCTTTATATATCCAAGCTCTCTCCAAACTCAAGTCCAAGAT
CACTTCATGTAAAAAGTGTCTCAATTTATAATTATTTTTAGAGTGTCATCTTGATAAATCTAGCCACAATCTCATAACAGTCTATAGGACGGTCCATATTGAACACTTTAATCATGCAGTCAAT
TTATTTAACATTTAAGTGTCTTAATAACATAAAAATTAAATGATTTAAACTCATTGAATTTCATCTCATCGAGACACGCGATAATACTATAAGCACTCGGAGAACATTTGCTTCCTCAATTAAT
ACTACAAAATTTTCAATAATCTTCAACTGTTTGATTCAGGCTCATTTGAACTTGAATCGATCATTTCAAGTGTAGCTAGATTCATCTTGTTGAAACATTAAGCTCAATTATAAATTTGCTGAA
ATCAAACGTTAATTTTTAAAGATTCTTCTCAAATGAGCTAACCTCTAGAATTAAGTGATTAAGATATTGGTGTTGTATGAATGCGCTTTCATTTTATACATGAAAAAATCTTCTCTTATTCAAC
TAATTGATATCTACATCTCTTTTCTTTTGTATTTAAATTTAACGAATATTTTGGCATGATTATGACAAGCATCGTAGATTTTATAATTTTAATCA
>comp117054_c0_seq1
TCTCGCATAAATGTGCATTCCAGCCTAGAGAGGCAACACTGACATAAATTATTACCCATCGTGTGGCAATAATCAAATTAAAAAATTTAGAACAGAATAGGAAGCCGTCAAAATATTTGTTT
CATCTGATAATATCTAATAATATCTACTTGCAATAATTTAGTCATAGGAAGCAGTCTTACATTCCACTTCTAGTTATCATCTAATAGCAGCTGAGAAAATCTATAAAAATTAAACTTGTTAAAA
CTTTTAATTATCCATAGCCGCTGCTATAAACATCATAATCCTTAACACAAAACTAGAATATGATGGATTTCCTTGACATTAATAGCATTTTATAACACTTTTCACCTCAAAATAAGTACACTTA
CAATGCTAAAACTCAAGACCCGGACATGAGTGATTGAACAATATACAACTTTTAGGGATTAGCTCTCCCTTTTATCTGGAAGGCATTATAAAATGCCAAATTCAAGAAACGGTATGACTGA
ACAAAAGCAAGCCCTCTCTTTCACATCAGTCCATATTTAGGGCCCAAGATTTTCTGCCTTGTCCGGAATTGGACTCCCTTGACAAATGGAAAGGACAACAGAATGCCCGTGAGATTATCT
GGGCATGTCAGAGAACTGAAACACGAATCTATTCAAAAATTGGGTACCAAAAAGTTGGAATATACACATTGCATGAGAACCGTGGGGACAACCATTCACTACTTCCTGTACCATGTGGCA
TGCAGGAGCATAGCAAAACAAAATTCTAATTAGACAAACTAAAAGTTAAAACCATCTCTCAAACAGGGGAAGAGTTGCTTACTGTGTTTGAACCAACAAAAGATA
>comp117315_c0_seq2
TTAACGCTTCTCCAATCTCGATTTGCCTCCCCACTTTCTCATAAAATAAATCCGGTCTTACGTCTCTTCGTATTTTCAGCTAAGCATCTTCCTCTTCCTATTCCTGCTGAATCGGAGGTCACGC
GAGAGTCGCTATTAAAGACATTCTTCACTCATTATTTATGTCAGATCAGAGGCCACGAGAGAGCTCGCCGTGATTGTTCATTTCTTCCTTGGCTATCAAGTCAGTTCGCCTTCTTCCATGAC
ATCGTCGGTTTCACGCCCTTCTCTTCCTCAATTGCTAGCCTGGATGCATTTCTTCTCTTCATAATTCACTCATCTTTTAGAAATTAAATTATTCAAGTAATCTTCTTTTTTTTTTTCTG
>comp117397_c0_seq1
GCAAATTATCCATCAATAGGTCTCATGTTTCTTCTCCTCCTGCTTCTATCACAACATAAGACATCAAGTACAACAACTATTGCCATCGACCCTCCAAATCCTACTCCAAATAATATTCCCAAT
ACAATAATATAACTTTTATTCTTTGATGTTTGGCCACTTAAACCAGCTGATGTTATAGTCTTATTCTCGGCTGTAAATGTTATGTTGCAAGGAAACAAATCTAATCCCTTATTTCCTTCAAATG
ACGTGTATGAGAAAGTGCATAATTGGCCTCCAACTGGAA
>comp117454_c0_seq2
CATATATAGTATCAAATAAAAAATGCATATCTCATTTTCTAGCTCTCCTCTCTCTCTCTCTCTCTCAGCTTCTTTTTCTATCTCTCTCTAAAAGTCTCTTCCTTCTAAATTTGCCTTCTTGAACT
GTATCTCTCGTCGCCGGACGAGTTTGCCGGTTCGGAGGGCGCGAGCGCAAAAGGCCCCTACTGCATGAGAAGGCCACCGGTCCTCTCAGTTAACCTTTTAACGAGAACGGGATTTAGAT
CTCCGCAGAAACCGGCTAGTCTCATTCTAACGGTAGCATAATAAGATACTTTTTT
>comp117552_c1_seq1
GACTATGATTATTCTGACCTCCGTCTCTATACAACGATCCTTTTGATTTCTATCATCTGGCAATGATCTTTCTAATCATCGTAACCCGCCGACTACTATCCTTCTAACGTCCATCGCCATACAA
CGATCCTTTTGACCTCCGTCGCTAGACAATTATCTTTCTGACCATTGTCATCGATGCGGTCCTTCTAATCTCTGTCGTCATACAACGGCCTTTTTTTACCTCCATCACCTTGCAACTATGATCC

TTCTGACCTCCATCGTCATGCAATGATCTTTCTGACCACTGTCACCCGCTG
>comp117641_c0_seq1
AAAAATTATTGTGTGTGAAATTCGAATGCATTTGGAGATTATCTAAAAGAGTGAAATTGAGATATTTGCATCCTGTCGAAATAAGGAACCATGACATATATGTTTGAAACAGATTTCATTTTG
AGAGATTTGAAATCTGAATGGGCCAAGTCAGTGAATTTATTGGGCCTCGGTGACGTCGCATGTCAGAATTCACCTCCGAACTGCACAAAATAATTTCTGGTGATGGTGGCGCGTAGCTCC
GTGTGGGAAACTAGATCTTGTCAATATTCTCGTCCTCGAAGTCGATGTAGTCGTCTCCGGCTCCTTCGTCCTCATCGTCTACGCCACCAGCGATGCCCTCGATGAGGCGAGTGGTTTCTGG
GAGCTCGCCATAGGCTTTGAGAAGGCGGGCCTCGTCAGGCATGTATTTGAAGATGACATCAGCCTTGTCGTTTTGGAAATCGCGGAGACCGACGAGAATTATATCGCCGGCGGCAATCCA
AATCTTCTTGTGCATCTTGCCGCGGATGTGACAGAGGCGCTTGGTTCCGTCGATGCACATGGCCTCGCAACGGCCATTTCCGAGCATGCGGAGGACCTGCGCGTAGTCCTGTCCGTCTTC
CTTGAAGACGAGCTCGCGCTTATCGTCGTCGGCCTCGTTCTTCCCTCTCTTCCGATTCTTACCGCCCTTTCCCTTGTTCTTAGGCATGGCGTGTGATTGAGGACTCTTCTCTTCGACTGAGA
GTTTTGATCATGAGAAGTTTCTTGAATTTAATTCTGCAAGCAATAAGAAAGACACAGGAAAACAGCCGCTTCGATTGAGAAAATGAATGTGAAGCTTCGGTAATTGACTAAAATTCGA
>comp117912_c0_seq1
GTAAACAAGCTGCCCCTATACAAACATTAGTAAACATTACAGATTAATTAGTAATTACTTTGCTGGTAGTTACATTATATCAAATTTTCATCTAACCAAGCTGCCCCTATATAAAAACATAAAC
ATGAATAACGTAAGTAAACGTACAAAACTAACAGAACCATAACCACATTCAATCAGAATATTAACCTACCTGATTAATGGAACTAACATACATTTTATGCATGCTGGTAACTTAAGCTGAGA
CAAAAGTTTTATTTTGAACCGTAATTGTGAGTTGAAGCATAAAAGGTTGATCATGCTAACTTAAGTTGAGAGATAAAAGTTTTATCATGCAAGTAACTGGAGTGTAAACAATGCATGCAAC
TAGATTGTGAGTTTAATCAAAGGGGAAATTAATCTAAGGTCCTCTGATTGATCAATATTTATTTTTTAGTCATTGAATTTCCTGTAGTTTTTCAGATAAATTAAATTTTACTGTTTAAAAAAAA
TTAATATCAAATGCATCTTTATTTTCCACCTCATAGAAGTGCCCTGAGATCTACCCACAATCCACCCACTGCCTCTCTCTCTCTCTAATTATCGACTTAAGACAACTGGTCATAGCTTCCTTAT
AAATGATTATTATTTTATGTGTGGTAATAATAAGATATAATTTTTTAATAAGCAGATGACGTTAGATGATAAAAATAAGATTTTAGGAAATCAACAGACTTCTATTAATAACCAGTTCTAACTTT
TTGTTAATCATATGCTAACAGAAAATAACCCCATTGAATGAAAGAAAAAAGATATGATAGGACTA
>comp117914_c0_seq2
GAAGACCAGGTGCAATTTGGGAGCTGTGACAGGTATGAAAAACACCAGTTAAGGCAAGCCTTAAGGTGGTTGACTGTTAGGAAACAGCAGTTTTTCTCGAAACGAGTGAGAAAATCAG
GAAGAAAACACTTGAAATTCAATGGTAGAATTGAAAAGAAACAACCCGATAGAATGTATCTTCAATTCGAAAGGAAGATTTAACTCTTCAAATCTCTAGCCAAAGCCTAGAGTCCAAAA
GCAATACCTATATCTCAAATTCA
>comp118193_c1_seq1
TGTATTCATTAGAATGCAAAAATTATTTTTCTATAACCATTATCTATCAGTATCAAGGATTACAGTGCACAATTTTGAAGCAGCTAAAAAGACACCCAGAATACAACCAAATCTCATATTACA
TGCAAGCACTTAGGGGATGAAGATGACCAACCTAATTCGAGCATCTTTAGCTTTCGAACATTGAAATTTTTTTTTATGAAAATGGAGCGATCAGTCTACTTAAAGACTAACATTGAATCAC
CACCATTACTAGACAACTATCATCAAACTTTTAGCACCCAAGCATTATTATCAAAGATAGTTGACATCAAAAATAATCATATTCATCTCCAATCAAAGTCCATTTTGTCAAAATCCATTTTGC
ACCCACATCTTTCTCCTTGCACATTTCTTAAGAAATAAATCTGATAGAGACAAAAATAACTCAAATAAACAGTAAAACTTCAATATTGACATCGCAATGCTCACCCCAAACCAAAATTTTAT
CATTGGTTCAAGTTAAGTTGGAAAAGGCATGATCTCCTAGCAACTCTAGGATATTATTTATTAATCATTCATACTTTTATAACCTCATCTTCACCAAATACTATGAACAATAAATCATTTTAATC
AAAATTCAAAAATTATAATTATTTGATCTAGATCATAACTTATGATTTTTTTTCCCTTAAGGAGAGTTT
>comp118346_c0_seq1
CCCTTTAAGATTTTTATTTATATCGTACTTTCGACAAAAAAAAATTTGCATACAAATTAGATCCTCACTCACAGATTGGCAAATAACTTGTAACTCTTTGCATCGAGTGCTATTTGCCTCACA
GAGGAAATGCAGCCGAGCACAGTCAACCCCGAAAAGACTGTGATGATGACCATGTTGACCCAGTAAATTGCCTTCCTCACAGATGATCCAAATGTGATGTTATACATAATAGCTGGTATCA

TAAAGTCCATTGGCAAGATTGCGAATGCTCCTATCAATGCAGTGAGATCCCCAAAGAATGGAATCATGGCAGCAATGAGGGTACTAAAGGCGACAACTAATGAGCGAGAAATGGTTCTTG
GAATCACATTGCGTGGAGAGTATTGATGTTTTTTTGGGTCGGCGAGGAGCTGCTCGAGCACTTCATTGGTTGGTTGTAGGTAGACCACTGAGACAGCAAAAATTTGAACGATACAGAAGA
AGTTGATCATCATGTAGAGCGACTTAGGAACAAGGACACTGCCATCAGAAGCGAAGAAGTTTGAAAGGATGTTGCTTTGTGCTTGGTTTCCAAATGCCCAATATCCTGCTATAGCAACAC
TAAAGAAGGTGCTGATCACCACCACATAGCAGAGGCAGAGTCCCTTAAACATTTTGCCTTTTACTGGCGGAGCAA
>comp118699_c1_seq1
CTGCCATTTCATCAGGGATTGCATCAAGAAACATCATATATCAGTTCATCACATATGTACAACAGATCAACTAGCAGATCTCTTCACTAAGGCACTATCAAATCCCAGATTCAAACTTTTATC
CAGCAAACTTACAGCAACTACATAGCCACAAGTTTGAAGGGGGATGTTAACAGCATTACACGTGTAAAGCCATTAGTGGTTATTAAAAGTATACCGTTGTTTAGCAATGTACTTTCTTTATT
CTTCACCATTACATATACCCTGTAATAGATATGTTAGAATTAATGAAATACAACAATTGTTTTCTCTGGTCTCTTCCCTGTTCCGTCA
>comp118815_c0_seq1
AGGTTATTTCTTCCGACCAAAAAAGAATTAGATGCTAGTTACTCCTAGCATAAGCCAGTGGGACAGGTGGAACATGCCCTCGATCTTTCCACTCACCAATTTAAGTTAAATTAAATTGAGA
TTGGAAAAAATATCTCATACTTCTATTATTTTTTAGTAGTTTGAGGTTATAATTGCGTAATAAGAATAGTATGTGGGAATGATTGGTCAAGAACTGGAGTTGCTATTTGACATGCTCTGCTTG
ACAATACATTTCAAGTATATTGCTCTAAGAAAATTAGTGATTGTTGTCTTAGAAGTTCAAGGAAATAACGTCATTCTAGCCTAATTATCTTCTACGTATAATTTAAGAAAAAAACAGTAATTA
ACAATTCAAATTTTAACGGTAGAAAATAAAATTCGATAGTCCAATGTCCACAATTAGATATGAAAATAAAGAAATTGAGTCTAAAGCTTAAAAGTGTAATGAAGTGTGTTGTATCTATAAAA
AAGAACAACTAACAACCCTCAGCACGAACTAATAAGTAATAACATCTAATAGTCCCAGTATTAAATATATGATTTGTGATGAAATTCACAATGAAATGGAGATCTGTGCAGTTTGATGCTTC
AATGAACAACTTTTTGCATCTCGATTGTTAGGATCTCGAATGAGGAAGAATCTCTTCCTTTTCAGCTTCTTGAGAGAGGGGGAGAGA
>comp118843_c0_seq2
CCGACTGTCCATGCTCCTTCGCCTTGTGCTCTAATGCTCCCTCTCCTTTGCTCTCCAAAGATCACTGTTAGAATGGAGAGGTTCGGCTAGGGCAAATGAGGCGAGCCGATCCAGCGACGG
TATCGGCTAGGGCGGCGGGAGCAAGGAGAAAGGCGAGTGAGGGGGCTTGCGGCTTCCTCTTGGAGTCTTCGAACAGGAAACTGCTGCTATGGAAGGAGGTTGGTCGGCCAGGGCAGA
GATTCTGGCGACTGAATCGCGTCGGGCTCGGCTAGGGTGAGACGACTTGGGCCTTCGGGTTCGGTCCGAACATAGGAGAGGCGGAGGGAGTCGCCGGCCTGGCTGGGGCTTCCTTTTGT
TTCGCGAACCGAGGAAGAACCTCGAGAGGGGGAGGGCGATCCTGGGCTGGATGTCCGAATCTGAGAGGAAATGAGAGAAGCTGGCTTCGGGCCAGTTGAGGGCTTCGCCTCCTCCTG
GTGTGTGCGAAAAAAAAAAAAAGAGCTTGAGGGAGGAGGGGCGGAGGCGTGCTAACAAAACACAGC
>comp119247_c0_seq2
AGAAAATAAAACATTTGAGCTATATAAGTAAAAAAGTGCATGAAAACACTCGAAGATTTCTTGGTGTAAACACTCATGAGAAGTGTTGCAAAAAAAAAACATGAAGATTACAAATGGATT
TGCTCAATTAAGGTTCAATCTATGAGAAGTTGATGGCCGACTTCACTTTAATTTGCTGAACACTATGTGGCGAAATGGCGAATCACTCTTGAAGCAAGTTATTAATTTGAGTACTTGAGCG
ACATTGCCCCATTGATTCTTAGGCCAAATATGCTTTATCATTACATCTTTGTGAGAATTCAATCAGAGTCTTCATGATGAGCTATATTTATGCTCTTCCTACTATTACTTGTCTATTTCTATGATT
TCTCTTCTCTTTGATCTTTGTCTACCTCTTCCTGTCTATAATTTGTTTTTCCTTATGATCATTTCTTCGTTGTTGTTTCAGCTTCACATGATTTTTGAAGCAAAGTTAAGAAAGCGTCGCTAAA
GAAGCTCCTTTGCCTCTTTTAACGTCCAATCTTTGTAGCTTTGGTTTTGCATGCTTTGTGTGGAGTAGTATTTTGAAAGAGTCGCTAAAGAAACTTCTTCACCTCCTTCGACAGGCGACAG
CGTCTATCGAAATAAACAAGAGGAGCTCGCCCGCTGCAAAGGCCGTCATCTTTTTCTCCTTTCACGCACTTCTTCGATTAATCCCGCAAATCCATCGTACTCCTGAATTTGATAAATTTGAT
ATTTACAGCAAAAATATAGTAATATTGGATTTATTTCATCATTAATCCT
>comp119829_c0_seq1
ATTAAAAAAAAAAAAAAAACATGAGAAGACAAGGCGGCAAGCTGATAATTTGTTTTCTGATGAATCACAAAGAATTAAAGCAAATAAAACTGTGGAGAGAGAGAGCTAATTCATTCATG

CTAACCCTAAATAAAACTCGATTGCCCTGGAAGACTTCAGTAACTCCTTTCTTAATATTATAAAGTAATGCATGGAGGAAAGGCCAGCAATAAACTCAGCTGTCTCCACATTAAAAATAAA
GCTAATCAATAAGTTTAACAATGGTTGGAAATGATATATCTTCTTTGAACAAAATAATATAGCTTTGTTCTTATTTTTTCGTAAATTTAACGGTCATATGTTTGTATGAGGTTAATGTCTAAATG
ATAATGAAGGATTCTACAGCAATATCAAACCTGTAAAAGATCATTCGCATAATAATTCCTGAAGTAGGAACTTTGAGTATTTGAAATAGATA
>comp119973_c0_seq1
CACTAGTTCTCCTCATATGCAAGTTGCTTGTCGGAGTTCTAAAGGATTATGGAGGTTTAGGAGCTTTAAAGGTCAACAAATTCACGATTGTTCGCGAGATCTAACGGTCTCTTCAATTCTTG
AAGAAAATTTGAAGAAAATTGAAGCTTGTAAGGCCACATATATGATCATAGTCACCATCGATGCAACAAGACTTACACATCAGGATTTACATTACAGCTATTAATTTTCCTTCAACCTTTGC
AATTATCTTACTACGGCATATAATATCCTCATTATTGAAAATCTTCTATTGAGATT
>comp120258_c0_seq1
TTTTTTTAAATATAAAGTCAGAAGCATATTATTATTTCAACAAAATATTATGGCAATATAATTTTACAACAGTCGTAGTACAATCTGCAGCTTATTTAGCATGCATTCACCAATCACGAATAAG
AAACCAAGTGGATATATAATCGACTGCTGATACGTTTTTCTGGAAATAATCAACCCAGCTTCAATGAATAAGATACGTCAGTACAAGTGCTATCAGCATGAGCATATAAGCAATTCCTTGAT
CTATCGCTTTAGCATCGTTTCTGTATGCTTCTGTTATCTGATTAGAGGTATTCTGCAAGCCCAACGAAAGACATGGATGCAAGAAAATAGAAAGAAGAAAGTAGGCCATGAGCAGCAGAG
CTGGAACTTTCATTGAGCTCATCTTCAAACACACGATTAAATTTCAGAATGCAGAGTG
>comp120574_c0_seq4
TCCCTGGGAGCATTATTAACACCGTGGTAGTTCCTCCTGAACTCACCAGGCTGATTGATTTGTAAATCTCCCAACTTTATCATCCCTTCAACAAAATCAACAAAGAAAGCTTTCTAATCAAC
AATGTATCTTTTCACCATAGCCATGTCCTTGCATCACCAGAAAATAGTGTTTGATCTAAATTCAAGAAAACTCTCTGCATTCTCAATTCTTTGAAATACTGGTTATCAAACTTTGCTTGAGTG
GCCTCTAGTCTTCTTTTGATGTTGCCATCTCCACTTACTAGGCAAAGGCAGTTTAGCTTGTATTGAAATCTAGGATCCATGTATGGGTCAGGTCGACCTATGTCGAATTGATTGTAAAGCCTG
AGTACAATAGAGAAGCATCATCCCATACTGATATAGTGAAAACATTGGATCTAGGACTTAGCAGTATGTTGTCTGAGGTTTCCTGGCTGGCGCTTAGACTATCTTTCCTGCCAAGTTTCACT
TCCCATTTGGGACCTCCACTCTGTTAAAAAGAGAATGAGACTTAATTCTCAACTCTTATTTGAAATCTATTGATGTTACAAATTGTATTCAAGTTCTAATTTATTATTTAATAGATGGAGATGA
ACCCAACCAATCATTGTCCAAATTAATAAAAAATGATTTGTTCAGAATAAATAATAGGTCTCAGTTGTTGGATTTACTTCTAAAATGAGTGCAAAATAAATTTTATCTGATCATCCTTATTTTG
CACTCATCTCAAAATAAATCTAATTATTCTTAAATATAATCATGGTGTAAATGAGCATAATCGCTTGATAGAACACCAACTTGAGCTTAAGTTTGACAAATTAATCAAGTTTGAGTTCAAGCT
AAAACTTAATTTTATTAGTTTAG
>comp120574_c0_seq5
TCCCTGGGAGCATTATTAACACCGTGGTAGTTCCTCCTGAACTCACCAGGCTGATTGATTTGTAAATCTCCCAACTTTATCATCCCTTCAACAAAATCAACAAAGAAAGCTTTCTAATCAAC
AATGTATCTTTTCACCATAGCCATGTCCTTGCATCACCAGAAAATAGTGTTTGATCTAAATTCAAGAAAACTCTCTGCATTCTCAATTCTTTGAAATACTGGTTATCAAACTTTGCTTGAGTG
GCCTCTAGTCTTCTTTTGATGTTGCCATCTCCACTTACTAGGCAAAGGCAGTTTAGCTTGTATTGAAATCTAGGATCCATGTATGGGTCAGGTCGACCTATGTCGAATTGATTGTAAAGCCAT
TGGATCTAGGACTTAGCAGTATGTTGTCTGAGGTTTCCTGGCTGGCGCTTAGACTATCTTTCCTGCCAAGTTTCACTTCCCATTTGGGACCTCCACTCTGTTAAAAAGAGAATGAGACTTAA
TTCTCAACTCTTATTTGAAATCTATTGATGTTACAAATTGTATTCAAGTTCTAATTTATTATTTAATAGATGGAGATGAACCCAACCAATCATTGTCCAAATTAATAAAAAATGATTTGTTCAG
AATAAATAATAGGTCTCAGTTGTTGGATTTACTTCTAAAATGAGTGCAAAATAAATTTTATCTGATCATCCTTATTTTGCACTCATCTCAAAATAAATCTAATTATTCTTAAATATAATCATGGT
GTAAATGAGCATAATCGCTTGATAGAACACCAACTTGAGCTTAAGTTTGACAAATTAATCAAGTTTGAGTTCAAGCTAAAACTTAATTTTATTAGTTTAG
>comp120944_c0_seq1
CAGATAAAGCTTCCACAAAAGAACGCGATTTAAAGGATCCATCCAAAAAACCGGGGTCACACACACGATTAACCACCATAATGGAATGAAAAGGCAAAAAAAATGAGAGGGCAACTTC

CCTGAAAATTTGAAGAAAACGAGCCCCAGATGTCCCTCACGTGACGCCTGAACCACGGCCGAACGCCCGAACCCCAAGCGCAGCCGCCCGCGAACGGCGAGTGGCGGATCCGAAGCG
ACGGCGAGCCGTCGCGAGCGGAATCCCTAGCGGATGCCCTAGC
>comp120967_c0_seq1
CTTAAAGCAGCTTTCGAGTATAAAAATTTAATTTTAGTAGTAATAAGCTGTTTTAAGCAGTAGTAAGAAAACAGTCCCAAACAAAGCCTTAGTTTCATGTTCTAGTCACCAAACCAAATT
CAAACCTCTGCTCAAGTGTAAAGACAAAGAAAGTATGTTACATGGAGTTAACTTTAATGTTTGGTTCAAATGTTTCATTTTCAAGAAATACGAATGAAGAAGTGGAGGGAGAGTCCAGAA
AATTCTTGAATCAATGTAGCTTTCTAAGGAGAGAGAGAGAGAGGTTCCAACCATGAGGCCAGGTTCGTTTAATAGAAAACACAGTAGAACAGAGAGAGACAGTGGGATGAGCAGGGGGGA
GG
>comp120994_c0_seq1
GTGCTAAAAAATTTATAACAATAGACAAAAAATATGCAAATATCAACCTATACACAAAAAATAATAGAAATTTTCAGGAGACGTAAAACATCTCCTCATTTATTTAAACACCTAAAACATTC
CTTCACGAATCTCAAAGCACATATTACTAATATTAGAAACACAAAACATCTCCACATTTATTTAGACACTCAGATATATATAAACTAAGTTTAGATATAAAAAAAAGGCAATAAAACTACGAA
TTCAAGCTGGAGAAGGACAGTGGCAATGTAGCATAAGGTAAGGCCACCTCAATTATTGCCGTGGTACCGCGGTTCAGGCGGATCAAGCGGATCACCGGGGATCGCCGTAGAGGAGCTGG
CTCCATGAGCTCCTCTGTGGTTCTTCCTTTTTCTATCCACAAGGAAGTCACAATGGCCTGTTTCTTTGGCTGCAGCAACGATATCACGCTTTTCGATAGTCTGACGATTCGACTTCTGGGCG
TGAGCCCACGCTTTCTCGGTGAGCTCCTGGATGAACATCTCACAGGCACGTTCACATATCACAGGCGTCTCCTTGGCGGCAGCCGCGTGTTGTGGCGGCAGCGGCTTTTTGTGGCCTTTT
CGATCCATTGAGATCGATGTGAGTGGGCGTTGCTGTGAATCAAGGTTTCAATCAGATCAAGAGGGGAAGCCTTGGCTAGATTTGGATGGAGAGGAGGAGGTAGTTATATATAGAAGGAAA
AAGAGATTTTTTTTGGATAGCGAGATCAAAAACCCAAATACTCAGG
>comp121694_c0_seq1
TAATCCTTGTTTCGTTTTATTTGATTCGTATCAGTAACTTACATTGTTCACGGGTTTATTTTCACAGTGCATTTAGTTTAATGTGACACACTGAAGATGCAGTCGGGTCAATTTATATTCATGA
AGTTAATTAACATTAAAGCACTATTAATCTAAAACAAGTCTACAGACAACTACCAGTAACTTCATTTTGTATACTTAGACTCAAAACGTATCTTCAATACTGGCCGCTGGTCCAGTTCCTTT
GTCTGTAGCAGCTTTTACCTCAAACGAAT
>comp121722_c0_seq1
TATGTTTACCTTCTAGTAGACAACAATTGTCTTATTATAGATAGATGTTCATCATTTGATATATGAAGAGCTTGGCGGAGATTTGTGAGTAACAACTCTTGCTCCCAATTTAAGGAACCAGAC
TCATATAACGACCTCATGGTGGAATAGTATGAATCGAACTTTAAATTATGATTTATCTCTAAAGGTACTTTTGGAATGTTGGAGAAGGATTCAGCTTCATCAACGAGGCCATGGAATTTTTTA
TTCTTTTTTACCTCATGGCAAGCCGAATTATATGAGCCATTGTTAAAGCTACAACTAGCAACAGAAGATAAGCTCACATCATCAAAACTTATCTCTTTTCCCATAATTGTTGTTCTCATCTTC
TTGGAAGCTCTAGTTATTGAAGAAAATGGTCTTTTTTTTCTCCTTCCATATTTTTCTCTGATCATATCATCTCTTGCTCCACATTTTGGCACCTTTAGGCATGAAGGGGGGATTCGACTTCTCA
AGTTTCTCCCTTTTGATTGATTATTTATTGTTGAGTTCTCATTTTTACCAGATTCCATAAGAGGTGTATTCCAATGATTGTCTTTCCAGGTAGATGGCACTCTCATCAGGAAAAGAGGAACAT
TAATCTCCTTGGAGGATCCAAAAAGCCTAACAACAACGATATTTTTGCTGAGAGCACCTAAAATCTCAGCAAGCCTCCAAGAGTAATTTTCAAACACCTCAACAATATCACCAATATTATG
ACTTTTTTGTTGCATAGGAGGAGGGCGTGGCCTGAGCAATTTTCTT
>comp121773_c0_seq1
CTGAATTTGGCTCATCACATCTCATAAAATGGCTCAAAATTGCATTTGAATAGAAAATAAAATATTTAAACTAGTTTAAGCTAAACTAATAAATAAGCCTTAGAGTAAAACAATCATTAGGCT
TTGCATTTAGTAATTCACTTCATTCTTTTTCGATTAGCCCTCAAACTTCTCAATTGCATAATCCGCAATACTTTACATGAAGTTCAAATCTTCAGATTCAGAAGCAGGAGGGAGGAAATATGA
ATCCTTGGGCTTCTGCATTCCAACATGTATACAGGTACTCAAAAGCACAGTCGGCAACAAGCCCGGGCTTGGTGGTAGAGGCCTTAAGTCAATGTTCTTCAGTTTGATATCGCATGGAAAC

GCAGAACTGCACAAGAGGTCGATTGCTACGTCGGTTGCAGAAGTACCCCTAATTTGCTCGAAGGCCGTGTTGCTAATCTTAACCCTAGAAGGAGGCTTGTCATCATCACAAGAACCAGG
ACAATACTCTTGATCTATGATGATAGGATTTCTGACATCCTTCATCACAATATCGTAAAAGTGCAAGTTGGTGGCGTTGATTGCCGGCGAATCCTTCCACGTCTTGATCCGGACGCCATTGG
TTGTCCCCGAGAGCGTGCAATTAGTCACGTAAGCCCCATCGACGCCTAAGTCGTAGTTGTACTTGCCCAAACTCCCAACACTGATTCCATGGCCTGGCCCGCAGAACACATTGCTTACTG
TCACGTTTGTGCTTCCTGGGCCAATGGAGATGCAGTCGTCGCCACATCCAATCGTGGAGTCTATTATTTTGATGTCGGACGAGTCGCCGATGTGGATGCCGTCGGTGTTGGGG
>comp121911_c0_seq1
ACTATTATTTATACTTGGTGAATAAGTAGGTATGGAATTATTTAAAATTCGATTGTTGAAGTCATCAAGAATTTCGCTGCATCTGCTTGGAGTAGAGCTTGCAATATTCTGATTGATCAAGTAT
GAAGAATATGGTGGTGTGTTGGGATAACGGCTAGTTAAAATGGAACTTGTATTGTCCAATTCATGAGTGATGGAGCTAGAATCCAGAGTGGTTAGGCAGGAAGAAGGTGGAGTGTTTTGA
ATCGGATTGAATGGTACAGAGGAGGCCGGGTTGTAATCAAATTGTTTGTGGTTAGGAAAGTCTTGGGGAAAAATGTTCTGATTCCCGTAGTTTTCAGAAACGTGGGGTAGCCATTGTGAG
TTGGGCTCCATCGTGTTCTTTGAAATGAGTGCAATCTAAAGCGAAACGATCGAATTCAGCCAGAGATGAATGCTTGCTTGTTTGATTCTAAGCTTT
>comp122050_c0_seq1
CGATTATTCTGTGCGGACATCGGATGTCGTTCGGAATCTAACCAGAACGTGCAAGGTCATTTTTTTGCTTGGTACCGGTTCTGGGTACTACTGGATATCGGATAACATAGCGCGTTCTGATT
GGCCTCCGGATCGTCCGGTGTCCACACAAAAAAAAATGTCTCCTTTTTTAGGACTGAGCTCTTCAAACTTCTGCGAATGCTGAGAAGCAAAAGAGTAACTAAAGTTTCAGAGATGTGTTT
GAGTGACCTTTCCAAATAGTTGTTGTCATTACGG
>comp122138_c0_seq1
AGTACAAAAATTAAAAATTTTGTGTTAAAATGTTGCTTAGGAAGCAGTAAAACAGCTGTCCCAAACAAAGCCTAAATTTAATCCACATGCTTTAGGGTTTCAATTTTCATTTTTTTTTAATT
TAATTGTAATCTATGGGATCAATTTTTTGGCATGTTTCTTGATTAAATGATACTCTTTGTTATTGTAAGTTGGACATAATTTAAGCTTCAATTGATATAAAAAGTTATTGGTCCTTTTAAAAAAA
AGATCGGAAG
>comp122268_c1_seq1
AAGATGTAATTTTAAAAAATATACATACAAGAAAACTATAAAGATTAAAGCCATCTCGCTCTCATCCCCTAAGATTTCCTCATGGGTTGCCATTCAAGCAAAATTCCTTCCAACCCTCTCATT
CCCCTCCGTTCTCCCTTTAGCTTTGACTCTCTGAAACAAGCCATCACCTTCCTCCTCGAGAAGAACAAGGAGGTCGTCTGCATCATCTTAGACTGCAAAAAGACAGTCAGGAAGAACCCT
AAAGTCTTCCTTCTCGTAAAAGACTACTTTGATAAGGGTCTCCACAACCTCCATTTCTGCACCGCCCTCGAGAACTGCCTCAACAATGCCCAAAACTTCCTTCACGAAGCCGACCCAACA
CTAAAATACGAGAAGGCCAAAGCCGAATTGAAGCGTTTCAAGGAAAGCGTTGATCTATTCGCAAAAGAATTCCTTGACGTCTTCCACTCAGTCAAC
>comp122268_c2_seq1
CACCATAGCCTTAATCCCATCCATTTCATTAATCCCAGCCGAAGTTCCAGCCTTTAATGCGCTAATTATCATCTTCTCCCGCTTAATTGCTTCCCTGTAGTTATTCCACATGGAGTCAAGCCAC
GTGCCCATGGCCGAAAATGCTGCGAAGATTGCTGTCGCAATAGGCGGCGCACCAACAATAGCAGACACAATTGAGGAGATTAGAAGAACGGCAGAGGCCGAGGCAAAAATTATGGAAA
ACAGTTTCCACCATGCCTGAGCTGATTTGAGCTTCTTGTCAATCTTCTTCTTTTCAAGAATCAATCTCTCCAGCATTGAGAGTT
>comp122426_c0_seq1
TTAAGAGAAATGCATCTTATTTGGGGAGTTCAGATGTATGGGAAACTTCTAATGAATGAACAGAATCTCTAACAACTTGATTGATAAATAATGCAAGAAATGGATAACAAAGGTGAAAAGC
TACAAGGAGAACAAACTAATAACCTTATGAGACAACTTAAAAAAGGGGATGTAGGACCATTTATCAAGTGGAACCTGAGTGAAAAGCTACCAAAGAAGCATAAGCTCCATCCTTCATATT
GATCAATCCTTCATGATTTCCTTTCTCTATGATCACTCCATTTTTTACTACTGCAATGAGATCAGCATTTTTTATTGTGGTTAGCCGATGAGCAATCACTATAGTAGTTCGATTGATCATAACCC
TATCCAATGCATCCTGAACAATTCCTTCAGACTCAGCATCAAGTGCACTTGTAGCCTCATCCAGGAGCAAAATTCTTGGCTGCTTGATAATGGCTCGAGCAATAGCTACCCTTTGTTTTTGA

CCTCCAGATAGTTGGACTCCTCGCTCTCCGACCATTGTGTTATAACCCTGCTGTAAGCTACAAATAAACTTGTGGGCGTTTGCTGCCTCTGCAGCTGCTAAAATCTCAGCTTCAGTTGCTTT
CCCTTCCTTCCCATAAGCAATGTTCGCTCTTATTGTATCATTGAACAAGACTGGTTCCTGACTCACCAAACCCATCTGTTTCCTCAACCATTTTAGCTTAAACTTTTCCAGTTCTATTCCATC
AAGCAGAATATGACCCGAATCTGGATCATAAAATCTCTGCAGCAGTGCTATTGCTGTTGACTTACCACTACCGCTCGCACCTACCAATGCAACAGTCTTTCCAGATTGAACAGATAAGCAA
AAGTCCTGGAAAACTTGAACATCTGGTCTTGAAGGGTACTTGAAACTAACATGAGAGAATTCTATGTGGCCTTCCAATGTTTCTAAAGTCATCCCAGAGTTATCGCTTGGATTAATTTTGG
ATTTGCGGTCAAGAATGGCAAAAATAGAAGCTGCAGCAGATTTAGCTTTAGATGAGTCGGTTGCTAGGGAGCTCGACTGAGAAATTCCAATCGCTGTCAATGAAAGGGTGAAGAAAACC
TGAAATACTTTACGAAATGTTGTCTTCCCATCCTGAACTAGGTGAGCTCCAATATAGAAACAAATTGCATACACGCAAAAGAGTATGAAGAAGGAAACTCCATAACCAATCCCGCCAATC
ACTCCTTGTTTGATTCCTGTTTTTATATGACCTTCACACTTATTCATATAAAGTTCCATAACTTTCTCTTCAGCTGAGAATGAAGCAACTGTCCTTATGTTTCTCAGAGCATCATTAGCCACCT
GGTTTGCTTCTTCATACATCATCTTTGCATCAGCACTGAATCCTTTCCTAGAATTCATTCTAATGCATCCTT
>comp122478_c0_seq1
CAAGATGACGTGCCTTGCTTTGCCGTAAAACCGGTCTTCTACCCCAACAAAAAACTGCCTATCTATATATAGCCATTAATCCCAACACCGGCATGGTGCAAGTGCTTTATTCCTCCATGCAT
CAAACTGAACTGAGCTCCCCTCGTTGCTTCTTTATTTCCCCCCATCATCTTCTTTGCTAGAAACAAAGACATCGTACACGAAGCCCATCGATGGATCGTCAGATTTTCCGGCGAGCACAAA
AGAGAGGCCGGACTCTCTCATACTCTCCTTTGAGAATCGAAAGCCGGGCAATGGATGACCAAAGGGAGATCGAGAGAGAAACACACCAGTACCGTATGTTCATGGAGGAGCCTTACATG
CCGGTAGCAGAGATGGCGATGGTGCAGAATACGTTGGAGAGAGCGCTGTCTTCGTGTAAATTAAGGGACGATGCAGCGGCGACGCCGGTGGTTTGCAAGGAGTGTGTTGCAATAATGGA
ACAACGACGGTGGCCTGATTTCCACTGCTGCGTTCATGATGAGGAGATAGTAGGGTTTCGTACTACGGCGGGGCCTATCCAGCGGCCATCGAAGAAGTAATACATCCGAGCTTGGGCATA
GGTTGGATCCACATGGGGTTGTTTAGGCTTCGTTTGGGACAATTT
>comp122568_c0_seq1
AAACTAATAATGGCCATGAAAGCCTCAGAGAAGCAAAGTTGCACAAAGCTGGCGGCCATGGCTCTCGTGAGCCTGCTGCTTCTTTCTAGCAGCCATTATTGCTATGCCGAAGCTTCAGTC
CGCTATACAATATATCTTTCTCCGAGCCAGGAAAACAAAGCCTTGGAAATGGAAGAGAATGGCCATCACCTGGGAGTTTTCGGAACCGCGGCTGCCATAAGATCTTCTACTGCTAGGGCG
GCGAACGGTAGCTGGCAGGAGAGAAACATCGCCGGCGCCACCTATTAATTGTATAGTTGCTATTATTGTTGTTTATGTGATTGATAATTGTCGTCGTTGCTTCAATGCTTGTGATGAAATTGG
GCACTATTTTGAAATAAAACTTGTTATTGATTTATTTTTTATGTGTCACTAAAATAGCTTTGATCAA
>comp122650_c0_seq1
GGGAGACCTTCCTAGGGACCCTGGAGACCCAAATTGGTTTTCTGGCCTAGATTGGAGGTTCTTCTCAATACCTGTGTCCTCCGTCAATTTTGCACTACCTTTAATCTAATTCTAGCTTAGTT
TTAGTTTCATCTTAACTAAGATGGCGAACAATCCTGAGTCCTACATGAACAGGCTTGCAGCAGATGCTGATCTCCAGGACCGTATTACTCTGATAAGAAATAGAGTTCAAACAATTACTGC
TGCGACTCCTGTTGCGGAAGAGTTAGCAAATGGTGCAAGTGCCTATGACTTGGTATCACATCTGGTGCTCCTATCCTTGAACGGCCAGGCTGCGCTTCCTGAGCAACGCGTACAAGTTGA
GTTCCTGAGGAATGAGCAACGACTGATCATCCCAACTGCTATAGCTATGTCGCCACTCCAAGGGGCTATGGGTCGCTGTCGAGCGAACAATATACTGGTTGAAAGGTATTGGCGAGCTACT
ACCATGACGTATGCTGAGATCAAAGCGCTTGTAGATCCGGTAAATGATGCTGCAGCAACCCAGGCGGCCGTAAACCAACGACCATTAGCTAGGGCAGCCCAACTCTTCCAAGGATGGAA
CCAACCTGCATGGAATACACTAAATGACCCTCCTGAATTGGTGGATATGATTATTGGTGCATTTAGTGCTTCACTGCAGTTCAACTGTGATATACGTGATGTGGGCGTACTGAATCTACCAA
ACATGAGCACTTCTGATGTCGCATGGAGGGACGCTAAAGCTGTGCCCCGTGAGCGATTACGTAATGTTCTGAGGGCATACACTATTCAGTGTATCGCTGGGCTGAACCTGCCATGTGTACA
AGGATACGAATTAGCTGCGTATGTGAGAGCATATGGTCTAGGGATAGGATTACTGCCATCTGCTAATTCTAGGTCGCAGATTGAATGGCTGTTTACTGCTAGACAACCGACAACTGCTGATA
TAACTATGACTAATAACTATCAGAAACGGTTTCCTAGGAGTTTAGTATCACTATGCTGCAATGTTCTGTCGATGTTTGGGCTATTTCACTTAGCGAAGGACCATACTTTCAAAACGGGTGAC
CCTGATATGAATAGAATTGCAAATTCATATATCCAGGCACTGAGGACGTGTGTGACACAAGAAGAATTAGAGATCATGGTACAACATGTTGAGCCCTTGGTCAGAACTGGACCACATCCTT

TTGGACTAGCGCAGACATACTGGCTGGCTCAAGCGATGGGCCATCATGGCTTGCTGACATCTCCACTGGCTATTAGAATGGAGGTATCCCCACCTCCTGTACAACGACTTATGATCGCAAG
AGCGACGTCACGCGAGTGGACTAATCTACCCGCTGGCAGAATCATCGACAAGTTATTTGCTGAGGGTTACGTTATAATGGATCAGGAAATCGAGAACATTAGAGCTAATTCTCCAGCATAT
TCAGGCTTGCACAGGTTGTATGGGATTGAGCATCAACTGCATGTTGGCCCTCTAGCTATGGCACATATCGAAGCGATGATGCCAACAGTCTATGGGTACATTATGGTAGCACACACTGAAA
CCAACGATAAGAAAGATGGTCTCGCCTACGCACTGAGTCTGAAGAACGTAAATCGTGATCAGAAAGGGATGACAAACATGTGGACTGTTGTTTGGAACAAGTACTTAGATGAAATGGAA
GGAACAGGCTTGGCAGAGTTCGTAGCGAAGATGCAAGCTACGATAGAAACTACTGTGAAGTCTGCACTAGGTACGTAGATGCTGAGGGCAAGCTATGGTTAGCCAATGATATGGGCATTA
TCTATATATCTAGAACTGTACGCCGTGGTAAATAGTGGTTGATAGCGGACCTTCCAGAGTGCGCGTGTTGGGAAACTATCGCTGCCTCTATTGAAAGCTACAGTAACTAGTACACAATTGA
GGCTAGCCAAAGTGGAGCCCTAAGTATCTCCTGTCAGTCCAAAACCGTGATTATCCTTACATGTTTCGTATATTGCACTGTCTCTCCTTGTGTTTACCCTAGTTGCTCTTACTTAGGTTGTAC
TATGTGTGTTAACCAGTCTGTCGATGTTTTATCTAGTGGTAGCAATCACATCACAGCTGGAAGCCTTCGTTTTAGTCTATCACCCTTCATTTGTATTTACAGTAATTATTGTTATGAGAATGTT
GGTGAAAACTCCGCACCATGTGCATTCGTTTTTGGGGTATCCTAAGGTGATGTAGCTGGAGGGGCCCGGATAGGCTACTGACCAGCACACACCCATAGAGCTTTATGAACCCCTCTAACT
ACGCCTCACCTAGCTTCGTCCCAGCATACCCGGGCGCGTATGATGCGCATAATGCCCATACGTAGTCATTGCATCCATACCAAT
>comp122670_c0_seq1
TTTGAACTTGATGTAGAACTTCGTCGAACATGTGAACTTTGCTCCATTGTCCTTCAGGATCCGAACGATGATCGTTTGTGAGTTCTCCTTGAAGCAAACAGTCCTTTGTCAAAGGATTAAG
GTGCTCGAGGGTGTTCCATGTGGATGGTCAAGGGAGGAGGTTTTATTTCTTCTTCCTTAGGTGACATATCAAGCATATGAGAAGAAGTGGAGAGAGGGTGGTGGGGTAAGTGGCCAGCC
GCCAAAGAAGAGAGAAGGGGCCAGAGTAATCCCGGATTTAGAGGTCAAATTAATGTAGGTGAAACAATTAGAAGAAATATGAATTAATTTTCTTGCCTAGGTAGCATGCAATTTAAATCA
AGAATAAATTCAGATGGATTGAGATTTAATGGCAGCATTAATTTGGGAGATTAATTGGAGAAATTTGAATTTGAATCTCCAAAATAAAGAGGGAAAAGAGGGGCCGCGCATTGATGGTTG
GTGTGATTGGTAACCCAAGTTAGAGAGGAAATAATGGTGGAGAGAAAGGGTAGGGGCCGGCGGCTAGGCTTAGGGGAA
>comp122716_c0_seq1
TTAAAAGAAAAGAAAAAAAAAAACTCTATCCACATATTTTGATTAAGATTCTCATGACACAATCTATTCAGAGCTCATCAATACAATAATAGTCATAAACATCAAAGAACTACCAAGCACG
CTTTAACACCATACACTTTGCCATTAAAATAAAAAAAGGTCTGCTAACAAGCATGCTTTATTCTCGACTCTTCAGTTTTGTGAAAGAACAGAAAACGCCCAAAAAAAAAAAAACAACTTG
GCATTCAATACTTATT
>comp122990_c1_seq1
GGACCTGACCTGTAAGGAATGACCCGACCTGACCCGCGAATGCTTTACTTTGCATAGGGTTGGCGAACGCAATGTGTGAGACCACACGTGAGGCTGGGACTGGCAGTGAGGTGAGGCT
AGGGTTGTTGGGCCGGTGAATGCAGCAGTGTAGGAGACTCGCGTTCTGGGTGTAGTCGTGTGCTGCGGTGTGAGTCTCGTGTGTTTCAAGTATCCTACGTGTAGGTTGGCGGAGACGGG
TCGGCAGTCGATCGTGCGGCGTGTGGTCCAATGGTCCGTGGTGATTGGGTGAGAGTTAGCCGGGTAGGATGACAGGGTAGAGGACGGCGATTGAAATCTGGTTTCGAGTCAACCCAAAC
CCAACTCGTTTTTTAAACAGGTCAAAAACCTGAACCCAAACTCAACTTGTTTTACAAACGTGGTTGACCCGAACCTGGATGTTG
>comp123286_c0_seq1
TACATGATATGAGACTTTCATGAGTACTCTGTCTTTCTTTATATCAAAAAAATGTTCTTAAATTATTATAACATACCAATATTAGATACAGATAAAAGAGTAAGCAAATAAAGGAGTAAATATA
ATTAAAATAAAACATATTAGAGTGCAATTCAATAATCCAACGGGAGTATACATAAATAACTTAAAGGTTAGATTTTACTAGAAAAGAAAGAAAAGACATATATATCCAAGATCCAAATTGAA
TGAAGAAAATCACAATCCAATATCAAAGATCAATAAAGAAAATACAAAGTCA
>comp123968_c0_seq1
TTTAAAGATTTCTTCCATACAATTTTCTAGATACAAAAAGAGAAAATAGGATGCGACCGTGGTTGACAGCCGCCTGTTATTGTAATCGGCTATTCTTACTGAGCTTCAAGCTCTGATGCTAA

TTTAAGGTGGTAGTAAGTGGTAAAAGGAGATGCAAGAAATAGGGTAGTTTTTCAAACTGAGCTTCAAACAGTAACACAAGTTTTCAGAATGCAAACGTGTAGTTTTTCATTCTCTTCCCC
TCATCTTCCTAAGAATATTTATTCAGTATAAAGATACGTACATGATGCGTAGCAAAAGCTTTAAAGTTTAGTGTTATAACAGAAAAAGCAAATAGGAAAGTGGAAAGCAAATATAAAAGAG
TAAAACGTGCA
>comp124031_c0_seq1
GCTTGGCAGATAATTTCAAGATTTAAAATTTAAATGAAGGGAAAAAAAACAGTACAATGAGTAGAAATAGCGTAAATTTCTTAAATCTTACTTCCGCGGGATTTACTAAATCCAAGATTCA
GTCAAAAAAATGGGTGGAAATTAAGATTTAAGGAGGTAGTAGAATTTGTATAGCACAACACAGTTTGGCAAGAGTTCACCTAGATCCTGGCGAAATTTTGTAACCACTTTTTAGCGAAGA
TGTATGCATAACACTGGCTAAATCTCGCAATCACTACTTAACTGGAGATCTAAGCATGACTGAAATTCCCACATTCGTGGCATGATTAGATCTTCTGCATTTATCTAAGATTTTCAACATCTT
TTCACTTTCACTGAAATTCCTGGCATCCAAATGTACCCGTTAACTAACAATACGATGAGATCGGATTATGAACATGCTTGAATGCTATTTCATACACAACTACCAACTACAAAACATACAAC
TCTAGAAAAACTTAACCATCAGCCAGACGAAATAATG
>comp124166_c0_seq1
AGACCATGCGACTGATGACGTTGAAGCTGTGAATGGAGAGGTAGTCCTTAAGGAGGAGGGGTTCACCGGCGGCGTCGAAGAGGCGGCGAAGAAGCCCGCGCAGCTCGGCGGAGCGAA
TGTGCTCGAAGGAGTCGATGCGGCGAGGACTGAAGAGCTCTGATGCACACATCTTACGCGTCAGACGCCAGTGGGGTCCATAGGTTGACCACGTGATGCCACAACGGTTATAGGCCGTG
ATCTTGCCGGAGGAGGTATTAGGCCGGCCAGCGAAGGCTAGGTCGTGGGTCTTTAGAAAGAGG
>comp124442_c0_seq2
ATTTAGGGCATCTCCAACCAAAAAGCTCATTTTCAATTTCAAAATACAATAACAATTCAAAATGCAGTAACTCATCTCCAACCGAATTCCATTTCAAACACACAAAATTTTATATTCTTTCTC
AACCGAACTCCGCCCTGAATTGCTCGCCTTTCCATCATTTCATGATTTTTTTGCGAAAATGCAACCGTAAATGACATTTGGGTGGAGCTTGAGGCAGACAAACAAACAACTATT
>comp124473_c0_seq1
CTCTTCCTCTTCATCTTCCTCCTCCTCATTGCCCGGCGAAACAGCCCCGCCGGAAGCTTTACAACGGTGAGTATGAGGAGATTGACAAGGCTGCATGGACAACAACACACCACCGCTGCA
CATTCGGCCGCCTTCCCGCCGGCGACCTCTGCCAGACGCTTGCTTGGCTTCGGAGGCTCCTTCAAGATCAAAGACCGCTTCGTCGCCAGCGCTCCGCCTTTTGCCGGCGGCCTGCGTGA
CATTGCGAAGGGGTAGTTGCGATTATGCGATTTCTTGGATCATAACATCGCCGGCTTGTTTCTTGTCTCAAATGGCGGAAGGAGAGCTTTTAGCCTTTTACGTTTCGGCCTATCGGGTTCGG
GTCGGTCGGGTCGGTCGATGGGATGTTGACCCGACGGGCCAACAGGCAGCAGTGCAGAATTCCTTCGGAGTTCCGACTCGCCCTTGCCTTCTGCCTTCAGGAAGGCAATTTATTATGCAG
TTATTGTGTCCTCTGAACACG
>comp124605_c0_seq1
AGGACTTGAGGGACGGACACCATTTGAGCATTAGTGGGTCGGGCGGTGGCTCGGTCCATGGGAGCAGCGACGGCATGGAAGCCGAGAAAGGGCCAGGGTCTCCTTGTGAGGACTCAG
CTGCCAGCGACATGGACGACGACAGGGTGAGCAGCCATCGCCATAGTGCAAACTCCGCCATGGGTTCGGACGCTGATTTGTTGTCGGACCAGGAAGAGGAAGCCGCCAGCGATATGGA
GGCTTCGGCTGAGGGTTCCGACCACCAAGCAGACATGGAGGAGAGCATAGCCGTCCAAGCACAAACCCAGCTTGCTGCGTTGGAGGCGGCCGTGTGCCCAGCTGGACGGGGCGGCAT
CTCTTGGGCGGACTGTGCAGAGTTTCAGGAAAATCCAATCTTTGATCAATTTGCTGGTGATTGTGGGAGCTCACCGCCTTTGGTTGGTGTCAATGATGCGAACAATATTGATAGCTCTAGG
TGTGAACCCCCCCATGGTTCAACCCTCCCCCAAACGCTTGATGCAGGTTTCTGCACACAAGATGCTCGGGTTAATGCCCCCAAGGATCCGATTGCCTATTCTAGGCCGGGAAAGGCTGCT
GCCAAGCCCGATGACCACCATGGGGGCACGGCAAGCAATCCCTTCGCTTGGAACAGGGTCCAGCTTGTGCCCATTTGCAAGCTCAACAAGGAGATTTTCTTAGGCAAGGACGGCAAATC
CATGGAACCCGAGATAGAGGCTGTCCATTGCAACATTGCAAAGCTTGATCGTGCGATTATTGCCAGAGTCATGGGGCGTCGCATTGCTTTCCCCTACCTCATGACCGAACTTAGAAAGCGA
TGGTTCCATTTCGGAGAATTTGAGATTGTTACTATCGCACCCAACTCGTTCATTTGCTTGTTTCAAAGCTTGGAGGCTAGGGATGCTGTG

>comp124609_c0_seq1
TTCCTCAAATCAAAACGACGAAAGAGAAAAAGATACGTTCAAATAAAAAAAAAAAGGTTCATACATATTACTTACAAACATTTGACTAACAAAAACTAAAAATAAACTGTAATTCAAAGA
TATTCATGTTACATTCTTCTTAAAAAGATTAGGATAATCAAATTATGAAATTGATGGCATAAACCAGATTGAAGGAGGCCAGTTAGGTTGTGAATCATAGTCAAAAAAATAGTGAAAGCAAT
CATTCTGAAGATCAAAAAACCCAGCATCATGATAAACATCATCTTGTGACGATTCATCCAAATGATCACAGAAGTATATACAGTTTGGTTTAATTTCAGGAACAGTCGTATGATGCAATGAG
AATGTCTGGTTACAACCCACAAATAAGGAAAACCCCCCCAAGTCTTTGATGGTGGTCCATTGAGAGAATGAAGAAGAAGTTGCAACAACATCCTGGACTAACTTCAAAACTGAGATTCT
AATTGTCTTCCCAGCGTACCTTTCATCAACAGGCCGAGTAAAAATATTGATCTCTCTCTCTATTTTTAGAAGAGTTCCCATTAAAAAGAAAATATAGTTCATGCAATATTCTTGTATGGGGCG
TTCATCCTGTATTGGCTGGCATATCATTTCAACATTTTGTCCATTATGGTCGAAGGCTAGCACCCTCGAGAAACGTGTTATGGCGTAGAACCTTTGTTCTTCTTCGTGATAGCACACATCTTC
AAATTCATCATGATCATTGGTGTTGTAAGGAAGCAGCTGTGGGCCAAGAACCCAAGCTCGATCACCAGGCCTTGCTAAAGCCAACAATTTATCATAACCATAGATTACAATTGCAGTTCCA
TGTGGATCATTAGAGGAGATGACAATCTTTTGAAAATATACATTGCAGAAATATTTTGTGTATGTGCAAAGGCCTCGACAAATTTCTTTATAAGATAACTCGATAGAGCCGTCTGAAGCATA
TTCGGGCTTGATCGTATGATATTTATCATCAGGGATGGTGAAGAGCGATGGGAAGGATATTTCAGCTTTTGTGAGAGGGTTGAAGATACGAGGCCGTAGATCTTTCTTATCAAGTGTCACA
AGCCATCCATCTTTGGAGCTGATACATCGACGACCGAAGATATGTCCATTAAGGGAGGAGAAGTGGTGATGGCAGGCAATGTCTTCTTCTTTGATGGTAAGATCCCAGAAGGCAAGAGTG
TCGGAGGCCTCTCCTTTTTTGCTAGGAAGGAGCAGCCATGGCGAGGGATGGAAATGAATGTGTTTGTGTGCATTGTTGGCAAAAGTTGAATTCCATGAGGTGCAGACGACGGAGAAGCG
GAGGAAGTCGACGACATGCAATTTCGATGAGATATAATAGAGGAGATCTACCGGGAGATTCGACCATGCCATATTATGTTTTTGTTAGCTATAATGTCATATGGACCTTATATAGATAGGAGG
AGGAGATGCATGGCCTAATTTAGGGCCATGGCGGTTTTAAAATTTTTAAATCATTTGGTGAAGGATGGTCTGTATTTTCTTAAGTTATAAATAGAAATTTTTTCTTGAATACAACTGGAACTA
TGCAAAAATAGGAGAAAATGGTTGTCCAATATTCATAAATAAATATTCTTTTTATATAGT
>comp124722_c0_seq2
CATTACCAAAATAACAAGGAAAGAGTCTCATTACAACACAAATCTATAAAAAGCTCATTGGGGATGCTTAACATGAGAGAAAAGTACATGATGCTAAACTATTGCACTTCAAATTAACTTG
CCAAAGCGTAGTCATAATGCTTCAAACTTGAATCATTTAACATAATCTACCTGTAACTTAATGTCGTATTCATATCGTTGTTTAGCAACTACAAAACTCAACAACTCATAAGCAACAATAAAA
TAAGAGTATTCATTATGCTTCAAGAAAATTCTTCAATTAT
>comp124826_c0_seq2
AGCATCAGAATACCAGATTCAATGCTTCTTTAATTTCAATACCATTCTTCCATCCCAGTAATTTGATGATAACAGCTTTCGAGATCTTCTTGATCAGAGAATTGGAGCAAACAGCAATAGAC
TTAAACTTTCTGTTATTCCTTTCTATCCATATAAAGTAGATAACAGCATTTAAAACGAGGAGGATGCCATTTTTTAAGCTCTTAGGCTTATCCAAATTATCTATATGTTGAAAGGCTAAAATAG
TTGTAGCTCTGAAGTAAAAAGTTTGAAATGAGGGTATGAGACTATGGAGCACTTTAAAAGAGTAATCACATTCAAACAGAATATGCGAAATGGTCTCCATACTATTGTGACAGAGAGAGC
ATGATTTATCTAAAAGTTGAATGTTTCTTGAAGCTAGCACATCACATGTTTTTAGACCTCCTTTAAGAGCCATCCACGAGTAAACAGAAAATCTCAAAGCATGCTTCTTATGCCAGACCATA
TGATGCCAATCAGTTTGATCATCATTAGTAAAATAGTATAAATAGAAATCCCTAAATTTGGCATTCACAGTGTTTTCCCACCTAATATGATACTTATTATTATGAACAATAGGAATATTCAGAAT
AAAATCCCTTATGGAAGGGATCTCCAATTCCGGGATGTTCCAAGCATCATGTTGAATCCAAGTAGCTAATTGAGAATTCTTAGCAATGTTAATGGGAAAATCATGAAATTTATCCAGGATAG
TTTCCCCAAAACACC
>comp125050_c0_seq4
CTTACTGCAGACGAAGAGAAATCCGCAACAAATAAGCAATAATTAAACAGAAAATGTGCAACATATACGCAACAAATACTCAATAAATACGCAACAAATATGCAATAAAATACTCAATCAA
TACGTACATTTTACGCAACGAATACGCAATAAATACTCAATCAATACGCAAGTCAAATAAATATCGCGGACTTTTAACTTTCCACGTAAGCATCCGAACACAGCTCTATAACCAGATTCCGC
ACAAAATAATTTCTCCTGAACGAGGCCAACATTGCCCTCTGGAAACTGGCTTGAGATGCAGAGGTTGTTTATAGTAATGCTAACTACCAAAATATACTCTTCTTCCACTGCTCTCTGCATGA

TTCCTTCCAAGATTTTGTTGTTAATTGTGTTCTTGCACTGAAAACTTTCTGTTAGATAAACATTTTAATTAATCTTTCACTGCATTGCATCACAATCAGGAAACTAAAAGACTGATACAG
>comp125616_c1_seq1
TACTAAGAAAAAGTAACTCAATGACCAAGCTGGATCGTCAAACCCAAAGAAAGTAATATAAGATTAATCGCTAAGCATTCCAATTGTTGGAGAATACCTATCGAGAAATGATGCAGCATTG
GACCAGAGAAACAATGCTGCCAGAAATAACATAAGTGAGTGGCAGATAAAAGTGAGCAGGTGATAGCCGACCCATTCAAAGAGAAGCCAAATCACTGTCACACTGGAAGTATGCCACC
CGATTTGTGCTTATTCTTCCATAATATGATATCAGCAGATTTCCCTCCGCCGAGAATAGTGTGCACTGGCTCCTTCCGTCCAAAGAGGCGTTTCTTCCTTCCTGCAGGGATCGACGGCTCAT
CCTTTTCTGACTCCGAAGAATAAGATGGTCGCTTGTACTCATGAATTTTATCATGGATTTCCTCATATAGAAAGGCGTGACCCACATCTTCCGACATCTTTGACTCTCAAATCCTTCGTTTTT
CGATCGCCTCGGCGGTGGCTTGATCGATCTCTCGCTTTTCCCACTCGCGAAAAACTGAAACTG
>comp125666_c0_seq1
GTCTTTATCCCACTACGTGGGGTCATACAAGCAGAAAGATAAGCAGTAAGAAATGAAAGAGTGGACAATAATTTAGGAAGCTCTTTGATAGGTCGGTTCATCCAAAACCAAGCTTTGATA
GGTAGGTTCATCCAGAACCGACCCAGCCGGCCTCACCTGGGCCGACAACGGTCTATAGGAGGCGGTTCAAACCGCCTCCCTTGAGTTGCCGCCTAGCATAGGGGCCACACCTATTTGAAT
TAATTTTAAAAAAAATTGTATTTTAGGCAAGGGGAGGGGCGGTTATAGCTAGGGCTGAGGGTTATAAATTGGTATGAAGGGGAAGGGTTATGGTATGCTTTGATTATTGTTTACTTGTAGTC
ACT
>comp125713_c0_seq1
GCACTTGTTCACGCCCAAATTTCATCCTCATATATATTTACGACTATTCCCACTCGTTTGGAAGGACAAAAAAATAAGCTTCTATAAATATTTTTAAGAAAATCGTATAGATGGCGCTGCGAA
CCAATCGCGATTCGTGAGGGCTTGCTCTTCCCGGTTGCAAGTTCTTCCCGGTTGCAAGTTGTCATCCCTCCCATTTCGGAATCCCTCTCAGGCTCACCTCTTCTTCTCTCTGATTCGGCTTT
TCTTTTTATTTAATTTCTCTGTGAGTCGAGTTGATTTGGATTAGCACGGCACTATGGCCGTGAGTGGAGTTGAGAAGTCGAAGAGGGTGGTCGTTGTGGGTGGTGGGATTGCCGGAGGCA
TCATCGCGAAGACGCTGCAGTCGCGGAGCGAAGTAACTCTTATTGATTCCAAGGAATACTTGGAGATACCATGGGCACAGTTGAGG
>comp125854_c0_seq2
GGAACATATATAAAGACAGCAGCTACCAACCTGGAAGTGTTGGAGGAGCGCAAAAACCTCTTAGAAGTGCTCTGCCATAAGCTTCTTCTCCATTGGCTCCTTACCAAATCCGTCAAAGTT
TTTCTTCGATCGTTTCCAGCCTCAGGAGGAGGTGAAAGCGGGGAGATGGCTCACAGACTGGATCATGAGTACGATTACCTCTTCAAGATCGTTCTGATAGGCGATTCCGGAGTTGGGAAA
TCGAACATACTCTCCAGATTTACTCGGAACGAGTTCTGCCTGGAATCGAAATCCACGATCGGTGTAGAGTTCGCCACGAGAACGCTTCAGATTGATGGAAAAACAATCAAGGCTCAGATT
TGGGACACCGCAGGTCAAGAGCGTTATCGCGCCATCACCAGCGCGTATTACAGGGGCGCAGTGGGAGCACTTCTGGTGTACGACATAACTAAGAGGCAAACTTTCAACAACCTCCATAG
GTGGCTTCGCGAACTGAGAGACCACGCCGACTCCAACATAGTCATTATGATGGCTGGCAACAAAGCAGATCTGAACCATCTCAGAGCCATTTCCGAGGATGATGCGCACTTATTAGCTGA
GAAGGAGGGTCTGTCCTTTCTCGAGACCTCGGCTCTCGAAGCCTTCAATGTCGAAAGAGCTTTTCACTCTCTGCTCACAGAGATACACAGCATAATAAGCAAGAAAGCTCTTGCTGCAC
AAGAGGCAACTGCTACTGCTCCAATTCATGGCACAATAATCAATGTTGCTGATTCCAATGGGAGCTCAAAGCGAAGCTGCTGTTATACCTTCACTTAAATGTTGCCAGACTTCTGAAACTA
TGAAAAGCAGAGACCTCTCTCGCACTCTGGATTTATTTTTTGGACTCTGGAGGTTATTTTCTTCACAGTTTTTTTTAGTATTCTCTACAGGAATTACATTTACCCATCTCTCTCTCTCTCTCTC
A
>comp125902_c0_seq1
TTGTTTATACCATTTGCCATTTATGACCATCTTATTCTGACTCAGTTGGGTATTGGGGGCTCAGGGGAGGGGAAACCTTCCGCTCTCGTGCTTTCGGCTTACCGGCGTTACGACTGTTTTGTT
AATCATAGAGGTTGGGCCATCACAGAGCTTCGTCGGCAAGAAGAGGGGAAGATTTCAGGGAAGAAATTCATTCTTCCATTAATTTAAGGATAATCTCCCCACGTTTCTCTTTCTATCAATC
AACAAAGATTTGGGTGGATGAGCGGCCGACTATCCGACTATCCGCTCCGCTTTATTGCCTCGCGTAATTTCCCAACAAAAAGAAACCTCCGTTCGAAATGGAGATGGTGTCATCGGAGTTA

TTCAAAGGGGCGAGCAATGACATCGCCGGACAGATTGGGTTAATGTGGGATCAGGTGAAGGCGCCGGTGATCTTACCATTGCTGCGCGTGGCAGTTTTCCTGTGCCTTGTGATGTCGGTG
ATGCTCTTCGTGGAGAAAATTTACATGACCGTCGTCATCGTTGTCCTCAAGCTCTTCGGAAAGCGCCCTGAGAAGCTATACAAATGGGAGCCCCTCCGTGATGATCTCGAGCTGGGCAGC
TCGGCTTGCCCCATGGTCCTTGTTCAAATCCCCATGTACAATGAAAAAGAGGTTTACAAGCTCTCAATCGGAGCTGCATGCGGGCTTTCATGGCCGTCCGATCGAATCATCATTCAAGTGC
TCGACGATTCTACAGACTCAGTCATCAAGGATCTGGTGGAGATCGAATGCCAGCGGTGGGCGAGCAAGGGCATCAACATTAGGTACGAGATACGAGACAACCGAAATGGTTACAAGGCT
GGTGCGCTCAGGGAGGGCATGAGGCACAGCTATGTGAAGAACTGTGACTTTGTCGCCATCTTTGACGCCGATTTCCAGCCTGAGTCCGACTTCCTCCTCCGCACACTTCCATTTCTCATC
CACAACCCCGAAGTCGGCCTTGTCCAGGCTCGC
>comp125909_c1_seq1
ATGAATAGTGAGAACAGATTTGAACTACCACAGCATCAACCCCCAAAAGATCAAAATTAACATAAACAAATTTCTAACTTCTGCCCAACAAAAACAACATCAAGCCTTCATTACACTACA
TGGGATCAGCTACATTAATCTTTTTTCTCCATTGTATCTTGTTAAGGGGCTATTTGGTATACGGATTTAGGCCGGATTTGAGGCGGTTTTGAGCAAAATTCAGCCAAATGGCCTAAAATGCA
AAGCCAGATCTCACGGCAAATGAAGGCCGCCATATACCAAAAAACTTCATCTTTTCGTGCTAAATCCTGGATTTTTGAAATCTTGTATTTTGCCAAATTTACGAAATCCAACGGCTTGCGA
GGAGTCTGCTCGTTAAATCCACTATATGGCCAAACAATCACATGAGATTTAACAAGAGTTATCATGAATGTTACAAAACAGTTTTCAGGATTTAGGCCC
>comp125914_c0_seq1
TAGATTGAAGAAGTTATTGAGTTCTATTATTAGATTTGGTATTTTTATGAATGAAATTTTGTTCAACCTAGACTCTTAAGAAAAAAGTGAAGATAGTTTCTCTATATGCATAAATGTCTTCAAA
GATAGTTTAGTAGGTAATGGTTGGGTTGTAACGAGGACAAGCACCCTCAAGAGGAGTTCCCTTGGATATTCACTTGAATTAACGAATTATATAACTACAATTAATAAAAATCATATATAATTC
CTCCTTATTCTTTCTATACTTCTCCATCAAATCTCTTAAGAAATGAATAGGTTCCATTGTCGACCTACCCGATATGAATCCAAATTGATTTGAGAAACATTAGTATCATGTCGTAACCTATG
>comp125931_c0_seq2
CCATGCTCCAGAGTATGCAATGACTGGTACACTTAATCAGAAGAGTGATGTCTACAGTTTTGGAGTCATTCTTTTAGAGCTTCTTACTGGAAGAAAGCCAGTGGACCATACAATGCCTAAA
GGTCAACAGAGCCTGGTCACTTGGGCAACTCCCAGATTAAGTGAAGACAAAGTTAAGCAGTGTGTAGATCCAAAACTGAAAGAAAGCTACCCACCTAAGGCAGTAGCAAAGTTGGCAG
CAGTTGCAGCACTTTGCATACAATATGAAGCAGATTTTCGTCCCAATATGACTATTGTTGTGAAGGCTCTCCAACCTCTTCTCATCACGAAGTCCCGACAAGAACACTATTGATGGCCACT
GCTTCTTTCTGCTTGTTTCTCATTCATCAAAACCTCTGCATTTGTTTCAGTGCCACATTTTTATTGATTTTTTCCTCTGTGGACGAAAGTGTTGGTTTATTTCTTGTAGAGCTATAAAATTTGA
GAAATATTCTTTTCATTTACTAAGTAAATATATATATATATTTAGCACTTTG
>comp126018_c0_seq1
AAAAAGTAAGTGTTTGGAATGTCTAATTTTTATATATTTGGCACTACTGATTTTCTCATGTTTACTTACAGGTGGAATGAAGCTAAAGCGAGATAGCTTCTCGTAACAACCATTAGTACTTAT
AAAGATGAGCTAGATGGACTTTTGGATAGTCAAGTCCTTAAATTTAAGCAATTTTCTTATATTGTTAACTAAAAATTCACGATATCAAACACTTACACAAATATTTTAATTTGTATAGATTATT
TGGGAGCCATACACTCTAGATGTTTGTGCACAACTTCCAGAAATATGCCTCATAGGAGAGCAGATCTGGATGGCACATGTGCCTTTGATTAGCTGGACGAGAGTTGAGTAGAACTTTCCTG
ATAAAGTGATAATACAATTTGGGTATGTCCCTTCAATTGATATACTTCCACTGGATATCCTAGCTTCATCCGTTCAGATGGTCACCGGAAGTTTTACATAGATTGGATAATATACTATCATAATC
ATTGGCATGGGGAGGACAATCAGTTTTGTATTCTGCAAGAGACCACATTGCAGCCATATTTGGTAGACATGACTTAGGAGTGATCCAACCAGCTATCGACCATATGGAGGACTTGATTCAT
GATATGTGAAGACATATGCATATCGAAGATGATCATTATACATAAGCTACACATACCTAGTACACCTACTTCAATGGCACCCCATCATTTGTGCAACCACATAGCATGGTTGCGAGCCCATTA
TCTTACAAAGAGCACTGTCATCTCAAGATTCTTGTGCCAGCTGACACCGCTTAACATTCCATCTTGGCCCACATATATATGACTACATTCATACGGTCATTCCATCTACATATGAGGAGGCAG
TTGCGCATCATGCAGAGTTGCACGAGTTGAGGGATAGACCTCTTAGGACGCCACATCACTACATGCAAGGTACAGATGCCATTCCATATAGGTCGAAGAAGAAACATTATGCTATATTTTC
TCTGTATTGGATGGCCTCAACCTTCATTGTTATATCATATTTTCTTTTGAGTAAGAAGACAATGTAATACTTTTTTCCTTACTGTACGTATGCATCTG

>comp126060_c0_seq1
GACCCTGGTGGATACCAGAGGGGAATTCCAATATGGATACCCAAATTAGTAATAATTTTCAATTTTGTTACGTCTTTTTATCGGTCCTTGTCACTTTTATTATGACAATATGTTAATACTAGTC
CAGTCCAATTGATGATTACAATGCACGCGCACTCTGGCATGTAACCACCATTGGAAAACTGGATATAATTACTTACTACATATCGTCTGTAGTCACGACGGTGGTCGCAAAACCACATCGTC
GCATCATCAGACTCTTCTTTGAACCCCATTTCCTCCAGACAGCTCTTTCCTCCCCCTCAAGTATTAAGGGACCAATTCCAGGCTCTAGGTGTTCTGAATCTAAATCTGTTCTGACATCGAGC
CCAAAACCAAGCCGCATTTTATCATCATCCACTTGAGGCATCTCTAGTAGTTTCCAGATCTCACGGTAGGAGTGGTAGCGAGCCCTCACTTCTATTATTTCAGAAGAGAGAGAAGGCACCT
CAGTCATCATAACTGCTCCTAGTGATAGCTCGTCCTCATCGGATAAAAACTCCTCGTTATTGAAGTGTGCTGTCTCTTGAGTCGTGGAAACAGCCACAAACGAGCTAGCTTCTATCTCGATT
GTAGTGGTAAGAGTATTCTTACCTTCCTGCCGTAATACCTGTGAAGTTTTACTGGGCAGGGAATATGGAGGTACGTAGCCGTGAACTGGTAGTACTATCGGATCAGCCCGCAGTAGTGATT
GGTAGGGGGAAGATAATCCAACATCTAATTCCTCCTGAATTTCTAGTTCACCAGAGTGGACTTCTTCTATGTATTTCATGATCGTGTCTAACTCCTGAAACAGGTCTCCCCATAGCTCTGTC
AACGACTTTCTAAACTTATCTTGCAAAACTTTTGGCGATACTGTCCATCCTACCTTGTTAAGCCCATATGTGAACATCATCATCCCTTCAAGATCAGTAGGTAGCTCCTCACACAGCTTTCC
CCATGGGTGTTCTTTTAACTCTACAGGCCCTAGTTTAAAGACCTTCTTCAACTTTTTTGCGGCCTCTATTCTGGTCTCTAAACTCAACCAACGTGCCTTGTAGAACTCTGTTAACATCTCGC
ACATTACTTCATCCGGTAACCCAACACATACTCGATCCCCAACGGAAAACATCTTCAGATGTGAGAATATTTTCGCTTTCGATTGCATTTCCACCATTCTTTCAAAGTCAAATTTCCTGTTCT
CATCATCCGGTGGTGCTAGCTTCTTCTTATCCATACCGTGTTCTATCGTTATCATTCTTCTCACATGTGGGGGGATATACTTCTCGTATGATATTCCAGCCTCCAGTGCAGCATCTAAAGTCTC
CTCGACTGATTCACTAGGCAATTCTACCATTCTTGCTAACCTCTCCTCTATTAGTTTATATTCTGCAGTGTAGTAAGCTGCCCAGTTGCGTTTCCTCTCTTCGAACCCTTTCTTGAACATTGTC
CGTAGATCAACTGATTCACCTGCCGCTATTGGTGCAAAGATGCCCCCACATGACGTATGGTGTTTTCTGCCAGCAGATACACGCATCCAGTAGTAGAGCGACCATCCTTCAGGCAATGTGC
GGAGTTCGAGTATCAGGTCCTTCCAGCTACCAGTTACAGGGCAGTCTCTATGCACTAACGTCCTTGCCGACAGATACTTCCATATTTCAGTCTTGATTGACACCTCAGTCAAATCTGTCGTA
GTCACTAGATTAGACAACAGCATTGTCAATGTAGACATGAAGACATCAGATAGCAGGTACTCCCCTAAGCCTCGCACCTCCTTACTATCATATAGAGGTTGTCTACGTAATCTAATTGATGA
CTTGTGGTGGAATCCATCTTTAGGATCTGGAACTAACCGTACCAGCATGTGTATGAACGGTTGATTGAGCAAGGATGTCGCTGTTATGTAGGATGCCAAGTTGAGAGCTCCAGATACAGTT
AATGGCATTGTCATTTTGGCTGCTCTGAATTCGTCTAGACTGAGATGAAGCGTATTGGACCCTCTAGTAGTCATATCAGGAATAAGATTATCTTTGGATCGAGTGTGTGTTATACCCTTGTTG
AATGTATCCCTCGTTACTAAGTATCTAGGCACTTCAAATGTGGCAGCAAGGTTCGACGATTCTAGTGTATCAAATTCATCTTCCAAGTTCTGCGATATTATTGCACGTTCTCGATCGTGTTCC
AGCATAATTAACCTCGTCTTGTCGTATGCTATTGCAGAAGTTCGGGCTACCATGTTCGCTGTTCTTGTTAGCTTACCAAGTAGGTTCGTCTGCAGCCGTTGTTTGATTTCGTCGAGTGATTTC
TGAGATAGACTGTCAGGCATTGAGACTTCCAGACTCGGTAAAGTGCCAGTATCTGTCTTATCCACTCCCAGCAATTCCAATATCAGTTGCTCCCAGGAGAATGGTCGCTCATACACAGGCT
GAGATCCCACAGTCATATCCATCCGAAATCGCACAGCATGATGGTCAGATACTAATCGCGTATTTGCCCCCAATTGAACATCAGCGTTCAACTGTGAATTTGCTATCATTTTTAACGCAACT
CGCAACCTAGGCAGTTCAAAGGTACATTAGTCCCAAGTGCATCAGAAGCTATTGTCGCTGTGTTCACACGGAACCCCCCAACGTGTCCGGGATGTCCTGACAGTGAGTGGTAAGCCCGG
TCCTCCATTGCTGACAGTCGAGTGGCCTCAGCACCATCTACATGTAAGTGTGGTATCAATCGAGCTTGGTCAACATTAAGACCCCACATTTTTGCAAATAACTGTTCCACAGCTTCAACAT
CTCCATCCCTTGCAGCTACCCGAGCACGTAACTTTGCAAAGTTTAAACTGGCTGCAGGAAAACTGCCCTTTAACTGGTATGTGGATTCCTCTCGCGCCAATGCCCGAGCTGACCTCTCTCC
GAATCCTGCCGGATATGCATCCGCATCTTGAGTAGATGCATATCCAAGCAAGTTAGCATAGTTTTTTCCGTGTTTCCTTTCTAGTTTGGCTCGTATCTCTGAGAATGCTGGATATACAATTTCA
GAGAATGCTCCTTCCTTTCCTGGAGAGGCTACTGTTGAAGCGGATGATTTCACAAATTGTGATGAGAATGCTGGAGCGGACGTCGACCGCACTGATGTACCCCTATTTGCTTTCTGTACTA
GTCGTTTGAATACATCGTCAGCTGACAGAGCTATTGAGTCAACATTCCTCAGACCTCCCACGTCATTATTCATAACTAGTACTACTCTATAGATGCAGACATTGAAGAAGAACTAAATTAAG
GACGATCAAAAAGAACCGG
>comp126086_c0_seq1
TTAATTAAAGGCTCCCTTGTTAGAATACCTTATGGGAAGGGTCTAATTGTTCAAATCTTAGAATTACCAAAAGTTTCTGTAATTTAGATACTGTGATTTTTACGGTGGACGATCCGATCTTGT

GCTTTATTTTGAATCAAGTGACGTTTAGTTTTCTGAGTCAAAATTACGGCCTATATGCAAAATATATTTTAGAAGTTAGAACTCTTTTTTAATACGAACCCCTTTGCCTAAAAGAAATTAAAA
AAACTAAAGTTGAGAAGTATTGCTTAGCCCAAACGGCTTTAAAAACGGCTTTAAAGAAGGGTGGGTTAAAACTTTTTGGTCTTGTAAAACTTGATGAAACTAATATTTAGTTTTGATATGG
CATCAAAATCTCGATCGGGCACAAATCTCAAGGTATATTTGAATTGAAATTTTTCAAAATTAATTCATAATCATATGAACTCTGATTTTAGC
>comp126197_c0_seq1
TCACCCCCCTCTTCAACTTCTCTCTTTAACTTTCTACTTGCTTTTCTTTCTCCTGTAATCAAGCTAGCTTCGTTTCTCCTTCATGATATATAATGGCAAAGACCAAGGCTTTGCGGTTTGCTAC
AGAGGTGGCGCCGCCTCAGCTGGTTTCAGTGGTGAAGAAGAAAAAGGTTTCGAAGTTAATGGAGACTATTGTGGAAGAAGAAAGAGATAATGCCATTTCTTCTTCTTCTTCTTCTTTGTG
TATATATTGCTTCAAGGCCATTTACCAGAGCTTTAATGTTGCAAGTGGCCTGACCTGTCTGCAACTGTACAGTATATAGATTTATAAAGCTTCCATTAAAACCAA
>comp126304_c0_seq1
TATTCTCCATCTCCTCAATGCTTATAACTATTTTTCCCTTGTTGTAAATATTATCCTTTATATTATGAAGCTTCTTTCATTGTTTTAAGACAAAGACTACTCTTTGTTGTTGATATATCATAAAAAT
ATTGTCTTGATCGAGCACGAATCTCAAGGCAAATTCGCAATGAAAACTTTTAAATTTAAATTCAAGACCATTTGAACTCCGATTTCAGTAAACTTATAATCGAACTGAACATCTCAGAAATA
CAGACCCAAC
>comp126393_c0_seq1
CAATATCAATGATTGGATCCCCTTTTCTCCTTCTTCCTCTTCAAATGCTCCAATGAGCTCAAAATAAACGAATTAACATTAGAAAGCAAAAACTTCAAACTTTTTGTTAATTTCACAGTTTCC
AAATTGCATATTGAGCTTCTTCACTTGCTAATCACATATAGAAGATCAGACCTTCCTTTCTCCATATTTCTGTTTCTCCGTAACACATATCAAATTAAGGAAAAAAAAATCTAAGAAAGAACG
ACAAGGAGAACCGGAGGTTCATTTACGGGGGGGTTCGTCGCCGGCGACAGCCGCAGCAATCTGGTTAGGACTGGTACCTTTCTTGGGCTTTGCGTAGAGCGTAAAATACGCAATGCTGG
CGATGAGGGCGAAGCCACCGACAGCCATCACCGCAGGGCTCTGCGGCAGGCGCCGGCGCTGGTGGAGCACCTCGCCGGAGCCGGATGCCGGCCTCTTCGATTCATCAACGCCACCGTA
TTTGTTCTTCAAGCCATCGTTCCCGTTGGTCGGCATCGCTCTCTATTGGTCGACGCAGTAGTATTTCTGGCTCTCTTTCTACCGGTTCTGGTTGTATATAGGAAGGGGGTAATTCAGTCCAGT
GAGGGCAGCTGGAGCATGGACTATGTGCATGTTCAACACGCACACCGT
>comp126446_c1_seq1
GGTAAGTTTTACAGTTAATCCTACAGCAATTTGTGAAATAGAAAATTGCGAACAAACACAAAAATGAAAAAGAAACAAAGGAGATTCTTCTCCTTTCTTAAACATTTTACAGAATATCTCA
ACGATTTGAGTTTTTAACAAACGTTATATATCATATTACTTTGGCGTCTATATTTTGCCTCTCAAACCCTACCAACTATTAGAAGATAATGCGCAATGGGTGAGAATTAGCATCCATGAATGAT
CTTTCTGCATAAGCTTTTAAGAGAATTTACATTTTAGTGTTAAATTCAACAACACAGCCGATCTAAAAACACAGTATTCGTCACGAAGATCTTTAGATTACGCCGAACACAAAATATGAATA
GGTCAGGTGGATTTAAAAGTAGATAACAAACATTAGATCTTCATAGCAGAGAAGAACTAAGAAATTGAGGAAGCGCCATTCCACAAACCTGGAGGAGGAACGAGGGAAGGAAAGCTTG
CTCGC
>comp126675_c0_seq1
GCAATCCAATATATTGTGCATTAGGTGACGTGACACGTTCGGATTAACCTCCGGATGCATTCAGACACCGTGACAAAGGATAATGAAGACATGACAACTTCTCATTAAGAAAGGAATTCAG
TGCGAAGAGCAAAAAGTCAAATATCAGAACATCTTGATACAAATTTGCATCATGATGGATTTAAAAATTATTGAGTTTACTTTAAAAGTAGTGTCATACATATTTTTCATATATATAACACTAA
TCTCAAAATAAGTTCAACAATTTTTAAGGTCATTTGAAATATGAAATCCTAACTTTTTTTTTTTTTTGATTAGATATTTCAATACATTTAAGTGAGCTTCAATTTTATTATATTTATATCAAAATT
AATATTAAATATAATATTTTAAATTATATTAATTACATATAAATTTACAAAATTTGAAGCTCCACCTTAAAAAAAAAAAATCAAAATAGCTTCATACACTCCATTTCCGTCGATCCCTCCGCCTC
CGGCTCGGCGTTCAAATCGATGCGCACGGCCTCCTTCTCGACGGGCAACGCTTCGCCACTCGAAATCTCATTAACCGCCACCGCCGCCTCTTCGAAATCCTCCAACATGAACGTTGGCCG
CCACCTCCCCAACCCTTCCTTCCCCCCTACCGCGGTCCGGTCACCGGTAAGCCGCCCACCGCAACCGGTGGCATCGCGAATACGATGAAGGATCTCACAGAATTCCTTCACCTCGTCCTC

CAAAACCGCCCTTTCCTCGCTCCTCGGCCGTGTTTGTCTCGTCGACTCTCCGTTACCGTCAGAGACGTTTCCCCGACGTTTTTTCTTCTGGTTTTCTGTGTCCA
>comp126694_c0_seq1
TTTTTAAATTTTTTTTTTTTGCCAAACAGCCCCTAATTTCCATCAAATAAATTTGGATCAACCACAAAGTTCATACAAACCATCAACATGGTGAAATTATGACAGTAGTTGAAAACCGAAAC
GGCCATTCATATACAAATCATCATCAGAACAAACCTTAGCGACCACAGCCGTAGGTGAAACCAATCAACAGAGATAAAAAAGAAGAGAAAACAGAGCAGAGAAACCGAAATCAAAATG
CTTTAGAAACATCGTTCATCAATCCGCTATTGTTTCGGCAGAAGGAAGCGAACTCGTGGAAGTCGATGAAACCGTCGCCATTGGTGTCGAGCTCGGCCATAATGTGTTGAACGTCGTCGG
AAGATGTCGATCCGAGAGTTCGCAGCGCCTCCGCGAGCTCAGACTTGGAGACCTTTCCGTCTCCATTGGCGTCGAATTTCTTGAACATTCTCTCCATCTCTGGCGTCAATCCTTCCTCGTC
TTTGCTCATGTTGATCGATTGATTGGCAGAGGGAGAAGGAGATCGAGAAGATGCGGAAATGCTATTTATTGTCGGGGTTCTGCAGTTTGAAAGGGC
>comp126713_c0_seq1
GATTTTTACCGGTGCCTAAAAATTTCAAAAAAATTTGTGTAAGTAAAATACTACAATCAAAATACTACGACAAAGACGGTTTACAATTTAGAGTTATAATGACAACAAAAAAAATTTCAAA
GTCAAAATACAGGCTATTATAGGTATCCGTTCAGAAAAAATGATCTGATAGCCCTGCAAAGAATAGGCCCTAAAATATTCCACCCTATTTTATAAAAATGAAAGTTGAATCTGGTAGGCCCA
TGAGCTGAAGAAGAAGCTCCAAAGAAAACCATAATTTTGATTTCTTCATCAAAAACATGTTGATAAAGGATGCTGCATCAGATGGGGATAATGTGTTACTAGTACGGAACATAACCATATA
CATTAAGTGGGGGGCAGCGGGTTGTATAATGTTTGGAAATGGCTAATAATGTGATTAATAGCTTCATCTCTTATAGAGTTTGACATATAGGCACCTAGATTGACCATAATATTTTTATTATAATT
CTTAGCATGGATTTTAGCATATAAGAACCTCAACTCATCCTCACCCTTAGACAGCCAATTATCTTTTGCTCTCTGGATCACCCAAGAAGCAAGCATATTCGTGAACTCTGAAATTTGGACAT
TTATGGACTATAAAGAAGTATTCAAGATAGAATCTAGGAATCAACATTTATTTTATCCAAACATTATGCTTGTTTTCTATGAAGCCCAACAAGATGAGCATCAATGTTGGTAGAGTTAGCCTA
AGGATACTCCTTAACTTTACCTCTTAAATTTTTAAGTTTAATACAAAAATCCTGAATAGGATTTCCATTAGAAGGAGCAAAAAAGACATCTAGATCAATATTCTAATAACTTCCCAACTTGGT
CCAATAAATTTTAAAGTGGATTTTGTGATTAGAAGGCGGGTGAACACTAGATTATAAGAGGATGGGGTAGTGGTCAGAGTATGAGGGGCTTTGTATGAAGCAAAAGGAGGTATGGTATGA
CAAAAGCTAATCCTCATTCAAAAAGATTTCCCACCGATTGA
>comp126829_c0_seq1
AGGAACTGCGCGGTCTTGAGCAAACTTTGGAAGAGTCTCTAAGAATTGTTAGGCAGAGAAAGTATCATGTGATCGCCACACAAACTGACACTTACAAGAAAAAGCTTAAAAGCACCAG
GGAGACCTACAGAGCTCTAGTACATGAACTGGAGATGAAAGACGACAATCCGAATTATGCTTTCAGTGCAGAAAACCACAACAGAGTCTATGAAAACTCGATTCCAATGGTGAATGATT
GTCCTCAGATGTTTTCTTTCAGGGTTGTTCAGCCAATCCAGCCAAATCTGCTTGGTATAGGATATGAATCACATGATCTTAGCCTCGCATGATATGAAGAAGTCTTATAAACTTTATGATTTG
ATGTTGTGTTTAATAAGATTGGAGAGACTAACGATTTGTGAGTTCATGAGATGAGTGATAGATTCAGAGAACTATCCTTTCATTTGCTTGTTCAGTTGTTTCTATTTCAAGTTTGGTATGTTT
ATGTTTTTCAATGTGTTTTTGAACTTGATAGCTATATGAGTTTTTGTGTTTTGGTATGGATGATAAAGAATATAGTGTATATAAGAATTTTTTTTTTGATTAGTTAAGAAGTTCATATTCAATTT
GGTCGTTATAAGAGCTGATATTGAACAATTTTTTTAAGTCTTTCAGCTAGTTTTCTAAATAAGCCAAATAAAAACAGCACACACACACACATGTGCACACATACGCA
>comp126833_c0_seq2
AGAGCTCGCGGGTCGGCGTCGGTGCAAGAGGTCAGTCGCGAATCCTTTCTCTTCCTTGCGATTTTTTGTGCGTGTATCTCTGTAACCGAATGAAAAGAAGTGTTTTATTTTGTGAGTATGT
CGGTGAATGAGACGATGCGTCGTTTGGGTAGGTCGGTGGGTTTTGGTCGTGCACTGACGTGGAATCGGTCGGTGGTTTTGCCGTTACAGTCAACAATTTTTTTAATAATTTGTTAAATTATA
TATTTTATTAGTTTTTTTAGACACAAATCTATAAGTACCTTATTTCGATTTTCATCTTAAATATTTAAGTATTAGTTTACAAATATATCATATAGTAATGTCCTTTTTAATTATAATATAAGTTAAAA
TTAGTAAAAATAGAATATACAATTGCTTAGTGGCCCAATGGATAATTGTCATCTGTGGAACCCGAAGGTTTGGGTTCAAATCTAGGGATATTCGCATTTCTTCTGGGCGGCAGGTTATTGTT
GGGCTTTTACTGCTATCCACATTTTTGTTAATTGGATAGTGGGGGCCTATGCCCCCCGGGCCCCCCACTATAGCTCCGCTATTGTATAAGAGTTATATTTTGAAATGCGAGCGAAATTTTACT
AAAAAATTTATTCAACATCCCAATAACGA

>comp126969_c0_seq1
CACTTGACCTCAGGATGGCGACTACAATAATGGAACACCTAATATTAGGTTTTAGAAAAGCACATACAATAGGCATTTTTGTGCATTTTATTTATGTAAAACTGAATAAAATATAATATTAATC
AAACAATGAAGTAAATTTTAACCAATGCCATTTAAAATATTTATTTTTAAGTTAGGTCACCAAATCCTTCCACATTAATTCTATTTATTTTTAAATTAGGTCCCCAACTACTTGAAAATAGAAT
TAATAGTTAAAGGTTGTTATTCTCTCCTATTATTAAATAGAAGCTCATGTAAAACAAATACAATCAAATTCAAAATGATATTACAAATCGAAGCATACTACCTCTATATGTTTCTCAAACACAA
TCTATCAATTGAGTGACTCATTCTATCTGTGATCCAAATCCTAAGGAATCAAGCAACTAAGAGTCCTAGATACAAGTTATAAGAGTTGCAGGGAATTATAGACAAGTGCATGGATATTTTTT
TTCTCCTCCACTTTTTCTCTCAGCTGCAATTTCCTCTTTGATGGAAGAATTTTGTTGTACAAACAGTAAAATTACTGGTAATAATATATATATTTTTATCAATCAATACCTTCTAGGAAAATCTG
TAAGCTCTTGGTTTATGGAGAAATTTGAGGTAGAGAGAAAGGAGAAAGGGCCAACAACTGCTATCCCTGGGATAGGAGCTGTCAATTAGGGGAGAAAGACAGAGTGAGGAGAGGAAG
AAAGAAGTCGAGGAAAGAGAAAACTTAGATACGACATTTCAAAACAGGAATTTGATTTCTATTGCAACAAAAATTACGCCAATTACAAAACAAAAAGGGATTCAAAATATTAATTAAAA
GAATATCAACATATCTCCAAAATTCAAAATCTTAAACCAACTTTTCTAGATTTTAGATAAGCAACTCCTAAATACAAGGAAAGATCTATTCATTAGTCTAAAATCTAGGAACAAGATAAGAG
CGTAGA
>comp127026_c0_seq1
AATTCCTTTCTGTTCAAGCCTAGCCAAAACAAATTCCCTTGAAACCGTATCAAAAGCCTTACGAATGTCAAATTTGGCACAGAACAAATTATTCTTTCCTTTATTAGTAAAAGAGAGAATTT
CTTGTGCTAAAATGATGTTGTCCGTAGATATTCTAGAATTGATAAAGCCAGCTTGCGTATCTTTAATAATTAAAGGCATGATCGGTTTTAATCTAGATGCAAGAATTTTGGAGATAATTTTATA
GATCACATTACACAAGGCAATGGGTCTAAAATCTGCAAAAGAAGAGGCATGTTTATTTTTAGGAATTAAAGCTAAAGCAGTGGCCTTAATGCCAGCAGGTAGAAGACCCTTGGAAAAAA
AATATTTGACAGCTTTGCAAACCAAAGGTCCAATAATATGCCAGCTCCGTTTGTAAAAGTGATAATTGAATCCGTCAGGTCCAGGAGATGATGATGCAGCACCATCAAAGACAGAACCCC
TAATTTCATCATTAGAGACAGAGACCGTAAGTGAAGCAGCAAGAGCAGGATGTAAAGTATTACCAAGGGGAAAGTATTCCATATTCAAATCCTCATAGGGGGAAGGGTTATAAAGAGTGT
GAAAATGGTTAGTGATTCCAGAAATAAACTCCGATCTGTTCAAGCTGGAAGAAGCAGCCAAATTGATAGCACCGTTATTCATAGCTCTTCTACGACGAATTTTAGCATAAAGAAACTTGAG
ATCGTCTTCACCCTGAGAGAGCCAAGAAGTCTTGGCTCTCTGAATCACCCAAGAGGCATGATAACTAGAGAGCTCTGAAATTTGGTGATTAATGTTCTTAAGGTTGCTATTGAGGGCAAC
ATTAAGAGGATCCAAGTCTATCATCTGTAAACAGTGGAGTTGCTTACGGTGCAGCAAGTCTAATTGAGATTGAATAGCATTTGAGTTGGCCCAGGATTGAAGTTTAAGACTTCTTTTAAAC
AACCTGAGCTTAGCACAGAAATCTGCAAGAGGATTACCTTTAGCA
>comp127121_c0_seq1
GACGTGGTAATAGATGCGGCCGAGCAATTTTGTGACCAGAGAGATGCAGAAGAGGTCATAAGCATCGGTGAAGAAACCCATTCCGGCGATGACGATCGCCGTGAAGTGGTACCATTGTG
TCTTGGCGACATCGAGGGCGTTGAGTACCTCAAGCTGTTCCTTGGCCATTGTATCCGAGAGTAACAATGAGATCTTTCAACCCCTCTCTGTCTTCTTCCCTGGACTTGCTTCCTCTGCTTTG
TGCCTTCTGAGCTTTTCTTTCGCCGGAGGAGAAAAGGAAGGGCAAGGCAGAGCGGGGGAAAATGTGAGGATTTTCGTGAGGATTGGAAGGAAAGGAGGATGTCGGGGCGTTTAAATGG
AAAGGTCGGTATAAGTGGGCGTCTATTGAAGGAATATTCTCCTAATTTTGGAGTCGATTTGTGGCCACAACCGCACATCGCTGTTCTTCACCTCCTAAATTCTCTTTTGAATTCTTAAAAAA
GGCGTGGATTTGTTCAAGAACGACGATCCCCTCGCGGCTGTAGTGGAAATTTCCCTATTATTCTGCAAATTATTCCATCTCTTGAGAGATTTTGATGGG
>comp127121_c0_seq2
GACGTGGTAATAGATGCGGCCGAGCAATTTTGTGACCAGAGAGATGCAGAAGAGGTCATAAGCATCGGTGAAGAAACCCATTCCGGCGATGACGATCGCCGTGAAGTGGTACCATTGTG
TCTTGGCGACATCGAGGGCGTTGAGTACCTCAAGCTGTTCCTTGGCCATTGTATCCGAGAGTAACAATGAGATCTTTCAACCCCTCTCTGTCTTCTTCCCTGGACTTGCTTCCTCTGCTTTG
TGCCTTCTGAGCTGTTTCAAGATAGGCAAAATTAGAAAATCGGAACGCAACGGAGGAAAAATTAAGGCGGGCTTTAAACTTGTTCAATTTCTATTCAAATTAATATTTTTGATCATTGATTC
CAACACAAATCTAATGCTTTGAACTCCTTCATGATGCAGTGGGGATGCTGGGTTACCTTTTCTTTCGCCGGAGAAGAAAAGGAAGGGCAAGGCAGAGCGGGGGAAAACGTGAGGATTTT

CGAGAGGATTGGAAGGAAAGGAGGATGTCGGGGCGTTTAAATGGAAAGGTCGGTATAAGTGGGCGTCTATTGAAGGAATATTCTCCTAATTTTGGAGTCGATTTGTGGCCACAACCGCAC
ATCGCTGTTCTTCACCTCCTAAATTCTCTTTTGAATTCTTAAAAAAGGCGTGGATTTGTTCAAGAACGACGATCCCCTCGCGGCTGTAGTGGAAATTTCCCTATTATTCTGCAAATTATTCCA
TCTCTTGAGAGATTTTGATGGG
>comp127189_c1_seq1
ATGCATGCAAATGATGAAAATCAATGTATAAGGGTCCCTACAAGTCATAAGGAAGCCCCTACTACTGAGATTGGCTATCTGGTAAATATATGTGGCAGTCCTTACTAATACTAGAAGTTACA
CAAGTAAATACGGCGGCCCACCAATCAAACACATCAATCTCCTGTAAGCAAAAGGGTTTAATGACAATTACAATACCCATACAATCAATCACCATTCAATGATCAGATGAAGACATAATCA
CATGCCTGCAATCAACCACCGTCATGAGCTCAACATGTTGTCATTTGCAGCGTAAATACAGAAGAGAACTGTAGTAGATACGAGCTCTTACAAGATGTTGTAAAAGGCCTGTGGTACGCC
CAATATGTTGTCCACATGCAAAGACACCAAATGACAACACATCAAATGCAAGGCAGGAGATTAGTTAGTGCA
>comp127199_c0_seq2
TGGTCTCCCTATTTGAAGTTGTCTAACCTTCGTAATAGCTCAAACTCATTTCCCCTGATCACGTTGTCTCCACATTCATCATTGTGTGTTAAAAATAGTTCGAACATCCACCCACCTTCGACG
AAGACAGACACATTCATCCCATCATAGTTCCTTCTAGCACTTGAAGCTAGAGCCATGCTATCTCTCCTTCTCCAAGTTTCATTGAAGATGAAAATGTTATGTTGCTCTGAGAAGTCTGAAGC
ATCTACATTCTTCACCATCAAAAGCTTGAGTTTATTTGCATTGTTCACTTCAACCACCCTTTCTTTAAATCCAAATCAACTAGA
>comp127398_c0_seq1
TTTTTACTAAAAAAAAAGCTTCATTAGAAAATAATAAAAAAAAGTTCTCAAACAAAGCCTTAATACATTATTATGATGAGAATATGGATAGAATTAATAATTTATAGAAATACATTAAAAAAA
AACATAAATATATATAGAGCTAGCGGCGGCGGTTGTTGGGCTCTCTCTGGCAATTGAAGTAGACGGCGGCGACGGGGAGGCCAAGCTCAAGTTGTGAAGCGAACAACCGAGTGTTGAAA
TTGGCGCGGCTGGCCAATGGACTCATTCCTTCTATTCTCTTCTCCTGCTGGAACAGAACGAAGACGTAGCGGTGGATGCCCAGCGGTGGCTGAGGCGGCATATATGGAACAATTTCTTCTC
CTTTGAAAGGATCAGTCCCACCTGGAATATTGACCACCAGCCAGTGTATCCATTCCCTCATTCTGGGCTCACTTGGGCTTGGTGCATCTGGATCTGTCATAATCAAGGTATAGAGATCAGAG
ATATTTCCAGAGATGCACAGCGCCGGTGGGTTGACCGCCATTGATGGCTTTACATGGCAGCCATTGTTAATGTGCTTCGCACCATAATTTACTGACATAGTCACTGTAGGAACAAAGAGGT
CGACCACCTCCCCTATCACTCTCCCCACCACCAAAGACTCCACAAAGGTTGAGGCCATGGCCGAAGAGAGAGAGAGAGAGATTCC
>comp127540_c0_seq2
AACCCCAACTATACCACCCAAAATTATACAACAAAAGAAAACTATAAGCTCCAAAATGGTAGTAAGTAAGGAATTTCAAAAAGAATCCCGCCAGCTGAGACGATAAGCCTGCTCCTCTGC
ATCGGAAATAAGAAAACCTGCACAGAGAAACACAAAACCTGCTCCCGGGCTAAACAGGCTGTACTCAGAGGAAAAATTAAGAAAGCAAGTCGTAATCCTGGTTGCCCAGAGGCCATCC
GAGAGATTACAGAGAGCTTCATCATTGTACAGCCAGCCCCAAGCCACCACGAAGGGACCAGCCAACATGCCAAAATCCAGCACAGAGAATGAAAATAAATCCAGATAACCAGCATAACA
GCAAAACCGTCAGTGCCCCATTTAGGCCAGAATTTCTTGAAAGTGAAGTTTAAGGGAATCATAATGCTTCCAATTGATCACTTTAGCTCTAATAGCCTGAGTTATCACCTCTTTAAGTTCAG
AGATATTCTTCGTCAAAGACGCAAATTTCCTCCCATTTCGTTCCCTCCAAATCCAATAAATAGTCACAGAAATAGTAAGATAGCAGAAATTCAACTCCAAACTACTAAAATGCCTCATGCTG
CCCAAAAAAATCATAGACCTGATGCA
>comp127585_c0_seq1
ATTGGTCTTCACATGATGATATTCTTCGTTAATACCAAATAATAGCTAGCAATACCAAGCAAAAACTGAGTTTTTTAGCGGTGGGAGGACGTATCTCTAAGCAGGTGAGAGGACTGAATAG
TTGCGAATTAAATCCAACTTTCCTATTTTTCCCGTTAACACCAGATTTCCTAAATCCTATGGTTCCGGGATTTACGTGGATTTACGGTATTTGGCCGAAGAAGCGCGTGAAAGGGGAGAAA
>comp127594_c0_seq3
CCACATGTCAATATTTTATTAGACACCGGACGTATCTCTACGTCCGGTGCTTGCAGCTAATAATTTTCCCTCATCAAGGCTTGCCTGCCACTGGATCTCGTCCCTCATAAAATATCTGTTGCT

TGTTATACATACGATGCCGTCTTTCACGAGCGTAGATGACCCAGAAAATGATCGACAACATCACAGCAGCTGAAACGAGCGTAAGGCCAATGAAGACCCAGTTTGTGTATTTTCTGAGAC
TTGGCAGTTTTCATGGCTTACGGACCTAAATGCCTCTCTAGCAAACGAGCAGTCCTCGAGCTGAACAAGGAAAGGAGAATAATGGTATAAAGCATAAGCCACACTTACAGCAGAATTCA
TCTGATCGTAGAGAGTTGGAGTTACACGGCCCACAGTTTTACAGATCTCAGCCCCATTGACAATGGTGACATTGCATTCATATTTCCTCCACACCTGCGATGCATTAGTCAAGACCACCTC
ACCAGCAGCACAAGAGCGATTGCTCAGGTCTTGGTTGAATGGATTGCAAAGTGTTGGCATCAAAGGCCCAGACTGGTTGAAGGAGATGGATGGGTTTGTAGGATTGGACACCCCATTGA
TGACATTATTGACCATGTCAGCCAGCTTGTAGGTTACTTGACGGCTGCTGTACAAGGATTGATTTGCGGTGGCCGCATTCACGCAGGGAAGAATGTCATCAAGAGCAGTGTGTTCTTTGG
GGTGGCTCACCCATTCATCCATCGCCACACAAGTGTCTCCAACGGCATTGTGCATAAGAAGGAATACACCTGACAAGATAAACATTGCCGCAACAAGAAACCATCCAATAAGAACTAAGA
TGTAGACGAGGAATTGCAATCCCAATATAGATAAAATAAAGCCCAGAAATGCTAACAGAAGCATCACAGCCGCGACAATGATCAGAATCAATCGCACAGTGTTCAGCACATCTCTGATATT
TTTAGAGTTTTTAGATGCCCGTCTATCTAGATCATTCGCAGAGGTATTAACTTTGTTCACAATGTCATCAATTCTATTCTGTATATCACCTGGAATAAGCAAATTAGTCACTTTGATCCTCTTA
GCCTCTGACAAATTGCCAGAAAATTCCCTAAGGTTTTCCACAGTTAAATTTGCCTGGCTAACAACATAATCCAATGTATCTGAGCTGCTGTTGTGAAACTTTTGCTGTCCATAGTAAAGAA
GAACACATCCAATGCTGGATGCAGGTAAAAAAATCTTCTCTGTTCTCTCCGAAGCCAAGAAAAAAGAAGAATTGTTGATGGAATTACCAGAAAGGGCAACTGGCCCCTCTCCAGCCGAC
CTCCTAAAGGGAACCAGGGCATATCCTCCTCTTCCTGCAGAAACCGGCTTATAAAACTTGAAAAAACTTTCAAGAAAGACGACTCCTTTCTATCAGAAACCAGCAAAAAGATCGG
>comp127720_c0_seq1
CTAATCGGGTCGGGTCGGGTCAATGTGTGCAGAATATAACATTCCTTTGAGAACTGATGGTCTGGTGAGGGGCTAGCAATAACTTCTCCATGACACAAATGACAACATCTCCTTCATTCGC
TTCCAATTCTTCGATATCTGCATCGGAGTCTTCATTACTTAGATCTTCTGTGTTTTCATTCTCCCCTTCCACAAGCTGAATCCTCGGCCGCGTCAGACATTCGTGAGATTTATGACCCGATTA
AAAACACCTGAAGCATTTGTAAGTGGCAACTTTAGAATAAGGATTATCCTTTCCCACAAGAAGGGTTTTAGCAAGATTGGGATCGGGCGTTGAAGAATTAACCGGTCCTAGCACTGTGGG
AATTTGTTGTTGTTTATGGGTTGCTGGTGCAGCTCTTTACTCACTAGATTGATCAGTAATGTGTCGATGGCTCAAATAACTATGAAGTGTCAAGTTACTTGCATTTCGGCCTTCAAAGTGAA
ATTGACAGCTTGAGAAAGTGACCACACCGAGTTTAATTCTAATTTATCTTGTAAG
>comp127818_c0_seq1
GTGTGGATCTCTTAACAATGAGTATATGATCAAAAACAGGTCGGTAAGGATGTCGGCGGGCTTTGGATCGCGCAAGAGAGGGGGAGGCGGAGGGCCTTCGTTCCGTCTGGGGCGTGCAG
AATGAGAGGGAGGAGAGCGCGGTAAAGTGAGAGTAGCGGGAGAAAGTAGGAAGCTTAGGAAAGAAAGGAGGAGGAAATGAAGAAAAGGAAGAAGCAAGAAGAAGAAATGTTTAGA
AGTTATCTGTGCGTGGGAGAGGGCCGGCACCAGGGCGGGTTTCGCGTCGCGCGAGAGAGGTGGAGGCGAGGGTTTCGTTATGTAAACGCCAACCTGAAGATTTA
>comp127884_c0_seq5
TAGTCAGCAGGAGTACTTGAAACTCAAAGCTCGGGTTGAAGCCTTACAAAGATCCCAGAGGAATCTCCTGGGCGAGGACCTTGGTCCCCTCAGCAGCAAGGAACTCGAACAGCTGGAA
CGGCAGCTGGACGCTTCACTTAAGCAGATAAGATCAACACGGACGCAGTTCATGCTCGATCAGCTTGCTGACCTACAGCGAAGGGAGCAAATGCTCTGCGAGGCTAATAAAACGCTGAA
AAGGAGGGTAATTACAATTTAAGTGCTCGCTTCTGTTCAAACTCGCTTCTTTTACTGTAAAAATCGTAAATTTTATATTTGGTCATGTGAGCAGTTGGAAGAGAGTAACCAGGCATCCTCAC
AGCACGTCTGGGATCCCAGCACTGCTCATGCTATGGGGTACGATCGGCAGTCGGCTCAGCCTCATGGTGATGCCTTCTACCACCCCTTGGAGTGCGAACCCACATTGCATATAGGGCATAA
TTACTCTGAATTTTGGAAGTTCGATGATTGCAACATTTTCACGTCTTATAGTCTTCAGAGTTTTTTGAGGTACCATTCTGATTTATCGATTGCACCAATGGCTGCTCCAAATGTCAATAATTAC
ATGCCACCTGGCTGGCTTGCATGAATAACTAGACCACTCATTGCCTCGTATAGTCCTAACAAACTTTGTGTTAATTTTGGCCATGTCTGTATAAGATTTCTATTCTATTTGGTGTTCTCAAGG
TCATTTATATGTAAACATATCTATAAAGCTTCTTAAACAGGAGGGCCTGAAATAGAAGACAATGTTTTATAATCATATGAGACTACAATACATCTTTCTCGGCTTCAATTTTTCAATTATTCCC
ATCTGATTGATAGTTCAAGCCACATTTAATTTTGGAAATGTATGAGTGCCTGATATCCAGTTGTATTTTATACGTGGAATTGCTATGTACATGAAAAATGTGAGAGAACGCAAACTTTCATGT
ATTGTTTTAAGTACAGTACCAAGTGTCTTTTATTTTCCTCTTAAAATCGAACTTGTATCTCTAGAGTTACTTGCTGATGCAGTTCTGGACTAGCTTGTTGGTTAACTTGCCTGAATAGTTCAT

>comp127927_c0_seq1
TGACCCTTATTCAGAACCTAATTGACCTCCTTCAATACAGTTTTGTTGAATAAAAAAAATGAAACAAAAAAAATTTGAAACAACCAACAAACCCAAACGGCACGGCGATTCAACCACCG
CCATTCACGATACAAAATAAAACCCCTCTCATTCGGTTGAGATAGGCACATACGTACAAAAAAAACTTAAAACCAAAGAGAAGGAAAGTCTAGAACTCACATCTCGGCTTCCACCGAAC
TCCTGCACTCGACTGTCGCCGAAGAAAAAACGTCGCCTCTGCACTCGACCGCACCGGCCAATCTCGTTGTAGACCTCCTACTCCGGCGTCGACCCCTCCGGCCTCCTTCTTCGACGTCGA
CACCGTACCTATTCGCCGTCGTGTCCTGAGCCGACGCCGATCACACCGCCGACTACAACCAAGCTTGCTAACCTCCTCCGACTCTGGCCAACCCCGTCCTCCTGCCTGAGCCGACGACGC
CTCCGCCGCCTCCATCGCCGAGCAGTGTCAGAGCCCCCAGCGCGTGTGACCTATGACGCCTTGCCCTAGCCCGATCGGGGAGAAATTATTGTGTACGCAGTCCGAACGCAGGGATAGGTT
CGGATGCTGCGTATTTACT
>comp128139_c0_seq1
ATAAAAAAACCGTCCCAAATGAAGTATTAGTATTGTTTCTCGCCCTCTTTCTATAGCATTCTTATTTTCATCGTTTCATGTTATAAACATGTCATTTGTGTTTGCAATTGTACAGATAACACTTT
AATCTAGCTTTAGTCAAGTAGAATTTGATATAGGATGTCTGTGATTTAAACATATAGCCTTATTATTAAACGTAATGTACTTATAAAATATCATGAAAATTA
>comp128365_c0_seq1
ACAAATAGTTAAATCTATTTAAAAAAAAAAAATCACGCATTTAGTGTTGTAAATTAATCTATATTTCTTTGAATTTTGGCCTCATTTTATGAATTGAAGAAATATTGGGCCAATAACAGCTCC
GATTTTGATCTCTCTTAGAATTATAAAAACAACGCTTTAAAAAAAAAACATATGATATATGTATATATATGCACACACACGAAAGTCAATTATAAAAATTTCATACATTTTTAATAAAAAAGTT
ATTGTAATATTACTAATGGACTGGTCATCGCTGTCATTATTTGTCTTACATTTAATGCCAATGAAATTTGTATTGGTTGTTTTGTAGGACTTTGAATGATCAGGTTTATTTCCAAAAGAAGATA
ATCAGGTTTGTTTGGGGAATGATCATATTTGTTGCGGACTAATAAATTTGTGGCGGTTGAATGGGAGAATCTTTTGTCTAGTAATGCAAGTTTTGCCTAAGTGAAAATATTCAACTATATTTG
TGTATTTTACAAAGATTGACCCGTCCCTTCGCTTGAAGAAGCTCCTCTACCAATAGATTGGGCAACAGTCAAACTTCGTAAGGGTATTCAGGAGTTTCTGGAGACCGTGGTCGAATGACG
AAGGTTGAAAAAATGGTGGCAGCAACCGGCGAAGGTCTGGCGGCAATCTACGGCCAGACAGAGTTCTAATTGGTAGTCGGTTGCCGGGTGGAGATTCGGCGGTGGTTGGCGGCCCGGT
TGAAACCTAGGGATGGTCGGCGGTCATGCGGAGTTCCAGCTGCGATCAATGATCATGCGAAGGTCTATGGTTGATGGTTGGGTAGAGGTCTGATTGCGATCAACAATCGAGCGGAGGTTT
GATGGCGATCGGTGGTCAAGCAGTGGTTCGATGGTCAACAACAGAGGGCATGTCCGACGACGGTTAGTGGCCATGCAAAGGTTTGTGATGGCAATGTTCGGGTGGAGATCCAGCAGTGG
TAAAGTGATTGGTAGGAGATTCGGGGGCCGTTCGTCAACAGAAGATCATCTGCGATTGTGCTTAAGTAAAACTTCAAAATCCTTGAAGCCTAAGAGCTATTTCAAATCTCCAGCCATAGGC
CCCTCTCATGAAGGGAGCGCGGGCTTATTAATAAATATTTGTGAGCAGCCTGCCCCATAATTATATGAGAAATAAGGAGCAATACGGAGCATTCCAACGGATGACCCGTATTGATTCTCTTG
AACATAAATCATTCTTTCAACATTTAAGTGTCTTAATAATGTCATAATTGGGTGATCTTAGGTTTGTTGGATTCGTCTTGTCAAGCCACTTGATTTGACTACAAGCATGTCATATTCGAAAATA
ATTTAATTATCCATGGTAATATTTTGAAACTTAATTCCAAGTATGCTTCGATCAATTGATTTGGGCCCATCTAGGCTCGGATTGGATTGATTTTGGTGTCTTTAGATTAGTCTTATAAGGTCATT
CAGCTTGCTAATAAGTTTGCTAAAATCGGATCTTAGATAGTCCCAAAATTAATTTTGAAAGTTACCACTTCATGCTTGCATCGAGATTTGTGCCTGATCAAGGCGATTTTATGTGACATATCA
AAATTATTAACATAAAATTGACTAATTTGTTGTGCAAGCTCATTGGTGATTATCAATGGGCTGTTTTAAATTAATTTATGTTTTTATTTTATTTTTGTTATA
>comp128479_c0_seq1
TTTTCCTGAACAGCCTCTGGACACTAGGAGGCGTAATCACGGAAAGATGGCTCCTTGTCCACCGGTGCAGGAGGACGACAACGAACGAACGCAATGAAGATACATCTTGAAACAAAAT
AGATTTTAGGTTTGTTTAGATTAGAATAGCATCTAAGCTTCGTCTCCTCTTATTGTTTTGGGGAAATTGATTTCATGGTTTAGGTTTTTCTGTCGCTGAGAAGTGAAGGATTTGAACGCTGCC
TCGTTTTTTGAATTTGTGTAGAAGAATTCTCATGATTAATTGAGGTAAATAATAAAACGGCTAAGGGGAAGGAAAATTTTGGAAATGTCTGAGTCTGATTCTCCGCCTGCTTCTGAAAATTC
CTCTTCTTCGGACAATGCGAACTCGGGCGATTCTTTACCACCCTCGTTGGGATCTAGCTCATCGCCTCCTTCTCAAAACTCTACCTCGTCGCCACCTTCGCCAGCGTCTAGCTCTTCACCG
CCTTCAGCAGGGTCTAGATCAACGCCTCCTTCTCAAAACTCAAGCTCT

>comp128517_c0_seq1

CTTTGTAAAAAATATAGAAGACAATAAAAATTACCGCATTGAGACAATTTCATCAGAATCAATCTCGAAGTATTTTGATATATTTCAAGACAATTATAAAACATATATAATTAATATTACACTA
ATCTCATCAAGTTAATCCTCCTGATCACTCTTTAAATAATCTCACACTTTTTTTCTTGCTGTAACGTACTTTCATTAGTCCATCTTGAAATTGGCTGATCCAAATACTTTTTTTTTTTTTTTGTT
CTCTTTTTTCTCTCTTTTGGGTCCTCTTTACTTGTATTTTTGGGTATAGGGATGTTTTTTGGTTCATTTTGTAACTTTTTTTTTTCATTAATACATGGCTTTTTGCCCCAAATTTCCCCCCCAAA
AAAAATGATAGCCGACTTCATAAAACCTTTAAATATGACAGAAAGGCATGGCAGCTCAGGTTAGAAAAGCCTTGGAACCACGT

>comp128706_c0_seq1

GAGAATGTATTTTATTGAAATATATTCTTTATTACACAGACGAACTAATCATCATGATTACAGTAGAAATAAGAAAAACCAACACGATACAATAATTTCACACCAAACATAAATAATACACAC
CGTCACTCCAAACAAAGTTCAGGGATGCAAAAGCCGACAAGCTTAAATACCAAAGTTTTTCACCCTATTTATTGCATATATAACTATAAAAAGCCATGTTGTAACACACCCGAGGAAGAGC
ATAAAGTGCCTTCCTACAATCCTGTGCCAGTGTGGTGGCCAGGCAAGTGTTTATTAGTGCCTGTAATTGGTGCATTAGTACTGTGTAAGCCAGTTGCGCCACCAGTAGTCTCATCTAAGCC
AGTATGGTGTCCAACTCCAGTGTGGTGCCCACCCAAGCCGGTATGCTGCCCAACTCCTGTGTGGTGCCCGCCCAGGCCAGTTTGGTGCCCGAGTCCTGTGTGATGAGCTTCTCCCTTATG
TATAGCATTCTGCTCGTACGCTTGCTGCTTCTCCAACTCAGCTTGATGAATCTTCTGGTCTTTTCTGTCCTCCGCCACTTCCTTCTGTAGTGGATCCCTTGTCTTCACTTTGTCGACCTTTTCT
TCAGCTGTTGCCTTCGTCTTGTCCAAGCCAGCCTTAGCTGTTGCAGAGATGTTCGAGACCTTCTCCTTAGCGGCTTCCATTATGAACGGATACTTGCAATTTCACTCTAATCACAATTGGTA
GAAACAGAAATGTT

>comp128712_c0_seq1

ATCATAAAATAAGAAGAAAATTATGTTAGAATATTTTGATATAAATTGTATGAGGATGAGCATGGACTTTAGAACCTATGAAGTTACTTTAAGATTAGTGTTGTATACAAAAAGTTTTTGTTG
TATTAATCTCAAAGTAAATCTACCAATTTGTAAGCCCATCCTAATAGAATTTGTATCGAGATATCCTGCAATAATTTTTATTATTAGGATATTTTATAAAAAAAATTTCTCTCTCTCTATATATATA
GGCTCTAAAATAAAATAGGAAGCCAAAAGGGAAAGGTTTGTTCCTATGAAAATGGTTGCCTCTTAATCGCTTCTTCAACATCATACTAATGTAACTTTGTTTTTGGTCACCTTTATGAATCC
TATTAGAGACCAATCTTACCCTTATCTTGCTAAAGATTTCATTGCCGCCGTCTCTCCAACCGCACAAACTTCTATCTACGGGATCCCTACCTGTTTGACTAAGTAGGCTAGGTTCCATTACTT
CCCATGAAGATGGAATCATACAATCATAATTCTGTTTATTATCATTGCCCCAATCATCTTCCCTGAATTTATAGTTGTAAATTCTTTTTTTTTCAATCCAAATAGCTTCTACTAAAGCAATTAAT
CATTAGAGCCACACAAATGAAATTTAATTGAAAGTGTTCTCTTATGATCAAGATAAATAGTCAACTATAGAGGATTGCACTTATAAAAAATCTCAAATTAGAATAAAAAGTCCTCATAAGAC
GAGTAAGACTTCGTTTG

>comp128791_c0_seq1

GCTGAGATGTATGGGCCCCAGTAGTATTGGGAGACCGAAGTGCACCGTGCCCTGAATGTATCGCAGCAGATGTCGTAGCTGATGGAAGTGTAGTTGCTGCGGATCATGCATGTGTTGGCAT
AGGACGTTCACCGCATAGGCGATATCTGGTCTGGTTATAGTAAGGTATTGGAGAGAGCCGGTGATCTGTTTGTAGATGAGTGGGTTATGAAGTATGGGATCCGAGAAGGGTGTGATCGGA
GGTTTGGTAAAATTGGGATTTGAATTTGGTTTGCAGTAGGCTTGCCCGACTTGTTTGAGAAGGGACTTGGCATATTGTGCTTGAGAGAGGAAGTAGGAATTGGGACTTGATTGGATGTTG
ATGCCAAGAAAGTTGTGAGCAAGCCCGAGATACTTCATTGTGAATTGGTGACTGATTTGCATGATGAACTGGGAAATGGCTTGAGCGTTGTCTCCGGTTAAGAGAATGTCGTCAACGTAT
ATAAGAAGATATATGTGAGTGGAGCCGTGAGTGAGGGTAAGCAAGGAGTGATCGGATTGACTATGAAGAAACCCAAGAGAAAGAAGGTATCGGGAGAAGGTGTTGAACCATTGACGAG
GGGCTTGTTTGAGACCGTAGATTGCCTTTTTGAGTAGGCAGACATGATGAGGATGACTGGGGTCTTCGAAGCCCTTTGGTTGTGCCATATACACGGTTTCTTGAAGATCACCGTGGAGGA
AGGCATTGGCCACATCCAATTGATGGATTTTCCATTGGTGATGTAGAGCGATGGTGAGTAGCACGCGGATAGTGGGCATTTTGGCAACC

>comp128947_c0_seq1

AATTTCAATCTTAGATCTCGCTAGTTCTCCAAATACCTTTAATTTCCGGCTTTTTCTTCGCATCTACAACTGCGGCCTCATGATTTCTTTGAAATTTTGTTCCGCGTCGTTGCACTGCGATGAT

TCACTGATCTTCTCATCAAGCCTTACTATAAATTTTATTATATTTCCATGTTATCTTCCGATTGTTAGTTTTGAGTTAATGTCGGTCTTCCTGCAGAGCGAAGATTTGGAGTTCAACTTGTAAG
TAGCATTGCAGGTTTTTTCCATCTATACCTCACTGTGGAGTTAAGTCGATTTTGCTCGAAGAGAAGAAATTGAGTATACAGGATTTCTCCGGCTCGATCAATCAATTGAGCGGAAGACGCC
ATGAATCAGGGTTTTGCTTTGCATGGAATGTGTATCTTGTACTTTTCTCTTATTCATTCATGGGGCTTTGCCAAATTGGTTGTAAGAATTGTAGAAGGAATCTTCTTTACCCACAGAATCTTT
GTAATATTGATACTTCATCAAAATTTGATTTGATTCGGC
>comp128947_c0_seq3
AATTTCAATCTTAGATCTCGCTAGTTCTCCAAATACCTTTAATTTCCGGCTTTTTCTTCGCATCTACAACTGCGGCCTCATGATTTCTTTGAAATTTTGTTCCGCGTCGTTGCACTGCGATGAT
TCACTGATCTTCTCATCAAGCCTTACTATAAATTTTATTATATTTCCATGTTATCTTCCGATTGTTAGTTTTGAGTTAATGTCGGTCTTCCTGCAGAGCGAAGATTTGGAGTTCAACTTGTAAG
TAGCATTGCAGGTTTTTTCCATCTATACCTCACTGTGGAGTTAAGTCGATTTTGCTCGAAGAGAAGAAATTGAGTATACAGGATTTCTCCGGCTCGATCAATCAATTGAGCGGAAGACGCC
ATGAATCAGGTATCTCAGTACTTTATTCAATTACTAAATTGTCCATCTAATGCCAATGATTCCACCGCTAAATCACTGAAACTACACTGCTAGGGTTTTGCTTTGCATGGAATGTGTATCTTGT
ACTTTTCTCTTATTCATTCATGGGGCTTTGCCAAATTGGTTGTAAGAATTGTAGAAGGAATCTTCTTTACCCACAGAATCTTTGTAATATTGATACTTCATCAAAATTTGATTTGATTCGGC
>comp129096_c0_seq1
CAGGGACCCTGGTTTGGAGGAGGAGGCTGATTGAATACTGAAAAATAACTATTCATCATAGCTACATTAATGTTCACCGAGTCGAATTGACCTAGGATCAAACCCATCTCTTGCTGGTACT
GTGCATGCTGCTAGTTTAAGTGAGTTTGAATTTGATCAAAACATTGATTAAAATGTATTTCCTGTCTTTCAAAGCACTAGACCAATCTTTTATCAGGTATAGGTACTGGTGCAGGTGCTGGA
GTTGGTTCCCCGCCTTCTCCCTTGGAATCTTCTCCCTCATAACGTTGATCATTCCCAAAGAGTTCCAGAAAGGATGCATCAATAAATTAAATGGGATGTTCTGTAGTAGGTTTTGGATGGAT
TATATTGCACTTTTTTCTAAGACGTAGTCAATTAGGTGTCCTAAGGCCACATACTTCTTTCTCTTATTATTAGGAAGTCCAATATCAGAGAGTTTGTAAATGTAGTTCATGATAAATTGGGCTT
CATCAAAAGTAGTTTCAGTCATAATCAAGTATGTGATCATGGATAAAAGAGGAGTCAAGTTTATGTAATCACCAACTTTGGGAATGATATAGGTTCTTAAAACATATT
>comp129096_c1_seq1
GGAATGATATAGGTTCTTAAAAAAATATTGGATAATTTTGGCCTTTTGATTAAGAGTGTACACATATGATAAATGTCCTACTTGGGCAACACTTCTAAGAGTCTAATTAATTTCTGACAAGTTA
AAATATGTTCTAGATGTTAAAGTGTGCGCTTAACTCCTTGGGTTCTAAGGTGTAGAAAGTTGCCTAGATCTTCCTTAGTGATTTCTACATACAAGTTTAAGATGAAGGAGGTAATCCTAGTG
TGATCTTAATTAACCTTCATGTTCACAAGGAACATTTGAAAAGTTTCCCGATGGTAGGCATAAATCAGGGTAAGGATGAGGGAAAACTAGGTTGAGGCAAATAGGGGCACGAAAATAAAT
TTTTTTGTCATTTTAAATAAGGAGTCAGGAAGGAGTTACTTTTGCTACACAATACCTATCAAAAGATTCATCAATCTTATTGGAAAGAAAGTTGTTGTCCCTGCCAAATCTAGACGACGAG
CCCTTAGAAATGCGAGGTCATTTACCGGTGCTGGCCATTCTAGATTATTTGGAATGAATGAGAATGTGAAGAAAAACTTATCCAAGACCCTTCTATTTAAAGAAAAAATGACCTATCATACA
GTACGAAATATGGATCATTCGTACTATTCTAAAATTGTACGAGACGAA
>comp129156_c0_seq1
TCTATTCATTTCGACTCCTCAAGCCCTAACTACACTCATCGTGCGGCCTCTCGTCAATCCCGAGTCCCGTCTCTCTTCTCCCTCGGTCGATCGAACAGGTGTCGAGTGCCGAGTCTGCCGG
TCCTCCGTCGTCCTCTCATCCGACCCGAGACAACGTCACGCAAAGACCCTCTCTCCGTCGTTCTCTCTTCGTCATTCTCTCATTGACCGAACAGGTGCCGAGTTTGTAGGTCCGGCCGTCT
CTCACGCGATTGCAACGGTGAACCGACTACAGATTGGAAACACCAAGCCTTTTGGTTAAGGGTTCTGTTGGAGTGAAGAAGAACATTTTTAGC
>comp129275_c0_seq1
TTCACTTTGTATAAGAAATATCTCACGCAATTTATGCAAATAATTAATCGCTTGTAAGATATTTGTTAAGGCTTGAATCTTCAAAGCTTCTGTTATTTTATTAGAAAAATCAGCATTAATTAATT
AGTGCTTTCATTAAGAACAGCTAGAGGAATTTAGCAGAAAGCTTCTCTGAACATTTATCCTTCACCTGCTCCATAGAATATGAGCAGAACAGCTGAGGTGGCTGAGCTAAGAGATCTCTTT
GCAGGAACTCCGTCGACGAACTGTTGAGAGATTCAAAGAGGAGTACGATGGATTTGCGGGATTAATCGAAGAAGTGCGTGAAAGGAGAAAAAGATGACGGCCTTTGCAGCGGGCGAG

CTCCTCTTGTTTATTTCGATAGACGCTGTCGCCTGTCGAAGGAGGTGAAGAAGTTTCTTTAGCGACTCTTTCAAAATACTACTCCACACAAAGCATGCAAAACCAAAGCTACAAAGATTG
GACGTTAAAAGAGGCAAAGGAGCTTCTTTAGCGACGCTTTCTTAACTTTGCTTCAAAAATCATGTGAAGCTGAAACAACAACGAAGAAATGATCATAAGGAAAAACAAATTATAGACAG
GAAGAGGTAGACAAAGATCAAAGAGAAGAGAAATCATAGAAATAGACAAGTAATAGTAGGAAGAGCATAAATATAGCTCATCATGAAGACTCTGATTGAATTCTCACAAAGATGTAATGA
TAAAGCATATTTGGCCTAAGAATCAATGGGGCAATGTCGCTCAAGTACTCAAATTAATAACTTGCTTCAAGAGTGATTCGCCATTTCGCCACATAGTGTTCAGCAAATTAAAGTGAAGTCG
GCCATCAACTTCTCATAGATTGAACCTTAATTGAGCAAATCCATTTGTAATCTTCATGTTTTTTTTTGCAACACTTCTCATGAGTGTTTACACCAAG
>comp129414_c0_seq1
CAATTGTACTGATTAGCACAATGCTACTTATTTAATCTGATTTCGAGCATAACAAGAAATAGCGGTGACCATGAGTGACACAACTCGTCGTCATGCTCTGTTGCACGATGAACAAACAACT
AGTTAGATTCAATCATACTGCTTTATTTTCACTCCAGCGGATGAATGACTGGTTGGTTTTTGTTCAGATGTTTGTTTTGACGAGCAACCTAATCTAAAATTGGTTGTAGTCTTATTAGTAATT
GTTGCTGCATAATTAACCCGTTCATCACCCAACACTCTCCAGCAATCAATGAGACATCGAGCAAGTTTTCGAATCCTGCTGGAGCAGTGCTTTAGCGTAGCACGCACTATTCTTCTAATTTT
AGTTGTCTTTAACACATCTATAGATAATTGCATCTGTTGTAGAGTCCTCAGCGATTCATATAAGATATAATCATATTCAGGCTCATGTAATTGATTGTTGGACAAGATCACCTCCTTTATCCTCA
TCACCTCTCCAACTATTTGATCATTCTCCTCGAGGTTGTCGGACTTGGAGCCTTTTTTCTCCTCATTGAGAGATTCAGCATCACCATTATTAATAGAAAGATGGCAGGTGTAGAGCTT
>comp129509_c0_seq1
TGAAATTAAATTCAGATTTTGAAATTATCTCCCACCACCAACTTCCCCTAACCCAACTCCCCACTAGACCGTGATCTAGAGAGGAATTTCTGCCACATTAACACTGCTGTCCAGCCTCTTTT
AATCCACGAAGTGCTGTCCCAAACCAACTCATGTGCTGTGCAGAAAACTTCAGCAAACTCCCTTGCTGTCCCAAAGTCCGGGAGCTTCCTTTTGCTGACAAGAAGTCTCCCACTATCCGT
CAGCTTCTTCAAATCCGGGATTTCAGCAAGATTACCCAGCTAGTTTGCTGTTCGAGTAGTTTTTCATTGCTGTTCAGAGGATTCCAGCAAGCTTCCTTTGCTGTCCAACAATATTGAAGGCT
TGAAGCTTCCCTGTGCTGTCCAGGAGTCTCCACACAGCGGCCGGCTTCTTCATATTCGGGTAAACTCTCTGTTGATAAAAAACCCTAGTGCCTATTGTCTTTTAGTGGCTTCTTTGTTATTG
TGTAGATTGCTCTCCTGCTTAAGCCTATTTCAAAGAGTATGAGTGAGAGAAATTCAAGGTGAACTATCTATTTGTTAAGCGGTGGATTCTTATTAATGGCTCATAAGTGTTGAACATTTCTTT
GATTATTCTAAATTGTCTCTAATCTAGTTGTAACTGCACCTGTTATAATATTGAAAATTGAGA
>comp129587_c0_seq1
GATTTCTCTCAGCACTGTCGTCCATTCATTTCCTCGTTTATTCCAGATAACTTACTCCTGTCGCTACTACTATTGCCATTGACATCCAAGGACCTCAAGCTTGTACTCCTGATAGAAAGGTCA
TCATAGCTTCCAACAGAAAATCTCTGCAACTGCACGTTGTTGATACTAATACTCTGCAACTCTTCTGCATCTGAGGATTTTTGCATGCTACTTATTGCACGTATTTGAGCCTCCTCCAGCTCT
ATGACAACTTGTCTCATGGTTGGTCTATCCTTGGGCTGCCAAGCTGTGCATTTAATGCCTAACTGTGCCATTTTCAGAACCATTTTCATGTTACATGGTCCGAGTTGGAGACTTGCATCTAG
TATCTCTTCTAACCTCCCTCTCTCCATACTTGCCTTGGCCCAATCACTGACGTGATATTGAGATCTTGGCCTCATATGGTCAAGTGCAGGGCAGGCAGTGACAAGCTGGAGGAGAATGACT
CCAAAGCTGTATACATCGCTTGAAGGAGTAAGGTGGAGGCTGGAGCAGTAGGCAGGGTCCATGTAGCCCGGAGTGCCCTTCACCTGAGTGCTGACATGCGATGCATCGCCATCCGGTCC
CAATTTTACTAGTCCAAAGTCTGAAACCTTAGCCTCAAATTCATCATCTATTAGGACATTGCTTGGCTTGACGTCCCTGTGTATTATATTTGGCTGGGTTTCATGAAGGTGAGCAATGCCTTT
TGCTGATCCAATGGCTATCTTTATTCTTTGCTGCCAAGTTAAAGGCCTTTTTCCTTTTCCAACAAGGTATTCAAGTAGACATCCTTGTGAAACATACTCATACAGAAGTACTCTTTGCCCATC
TTCTGCACAGTAGCCAACCAAACACACAAGGTTCTTGTGCTTGACCCTGTGAAGCAGCTCAACCTCATTTCGAAACTCCTCAACAGTCTGATAAGAATCTAAATGAGCTTTCTTGATTGC
AAGTGTTATTTTACCACCATTAAAAACTCCTTTATAAACATTTCCAAATGCACCTGAGCCAAGCAACCTTTCACCGCTGAAGGAGTTTGTAGCCTTCTCTAGTTCTGCAAGTCGATATTGTC
TCAGCTCTACCTTTGGCCAGTCAGGATCTTTGTTTCCTGCATTTTTTCTTTTGTAGTAATAATATTTCCTGATGTAGAAGAAGAGCAGAAGCGCAAAGATGATTCCTGCAACACTCGCACCT
ACTGCAGCGCAGATTTGTAAAACAGGAAATCCTGCTGTGGATTTGGGAGTTTTGTAATTGTTTGGAATTGGTGTTGCTTCTTGTAATATGAATCTGCTCATGTTTTTTATCTGTACGAGATCC
TAATGGCAAAAAGGTAGCTCTTTTATTCATTTCCTCCTCTTATCCATGGCTGCTTCTGCTATTCCATTCATGGCTTTCTTC

>comp129590_c0_seq1
ATCTTATACATTTAATCACCATAGATTCTTAATAAACAGGCAAATTAGTAGAAATTCCCTTCAACAAAAACAAAAATTATGCATGGTCTAATTTTCTTCCGTTTAACACGTCCAATTTTTTTT
TTTCACAATTTGCAAATTTCTATACTAAAATGAACAAGCATTGAATAAAGTTTAGGGTATCATGAAAGCAGTAATGGTAGCTCAACAATCTTCAGTGGGGGCTCGAAAGTGCCAGGTGTGG
GTACAATGGCATGGGTGGCCTGGTAGGTTAGCCGAGATTTACGGACTGCCAAACGCGCTCGGCGAAGCCAGACATGGCAGCAGATGGTTCCTCGTTATCAAAAAATAAAAAAATAAAAA
GTAAAGCTCAACAACCTAAACAGGTGATGCATGGAGGCTCTACTGTACCAAAAACAGCTTCAACGCACAGATTATGATAGCAGTGGCCACAAGACAATACTGCAGCAGTTGGAAACTCG
TTGAATTGGAATGATTCCGAGAAATTCACAGGGCTGAATGCTAGATCCAGGCTACAGAGTTGGCAGCTTGCTCCAAGTGGTTTTATTCGATGACCAACACAAATATCTTCTTCAGCTCTGA
TATGGCCAACTATATCCTTCTCCAAACATGATTGTAGAGACTTCTCGCGGATCAATTTAGAAGTAACAGGACTTGTAACCTTGTTCTTGTTGTCAACGACGTCACGCACGGTGCAAGAAGT
ATCATTTTCTCCTGCAGTAGCAGTAATACTTGGGCCAGACAATGAAAGTGCTTCACTTTTGCCTTTGCATTTGCTAATTCTCGCAAATGCTAAATCTTGAGTAGTGGCTTTACTCGAACCAG
CATGACTTGATCGAGCAACACTTCTAGTTTGGTAACGATGAGCAAAGATACTTCCGGTCGCATTTTTAAGCCTTCTCGAGGAGGAGTTCGTCATTGCTCTGAATCAATTTCCTAGGTCAGA
GAGAGAGGGGCAGAGATAGAAAGAGAAAAGAAAGGCCTCAAAGAGCGAAAGCCGAAGCGGAAGCC
>comp129769_c0_seq1
ACAAAGAAAGTTCAAAAGTATTTACAAAAAAAAAAAAAGTTTAACATCTCCCGTAACATCCAATGAGCACAAATAGAAAATAAAATTCACATGGTCAATCCCAAATATTTTGGATGATGA
TGCATAGAGAGGAGCCTGAAGTAAAAGTATGCATAATTGGTTTCTAGCTATTTCTTTCCTGTTGTTGTACATCCTTGATCAAGTTTTGTATATGATCCAATTCCTTTCTTGCACATTTGACTAC
ATATGGACAATGTGAAAAATATTCCAAGAGAATGCTTATTTTTTGAAACATTGCTTTAACTCTAATCCAAATTTGATGGAAAGCTTAAAATCACCATCATGATTAGTCAAATCATAGGGAAA
CTTCCAAATCATGCCTTGTCATTCATTCCTAATGAATATTGCATTTCAAGTACAATCTAATTAAAAAAAAAAAAAAAAAGGCATTTGCACTCTAAACCCTTAACCCTAAATGAAATTCCATCA
CAAAACTTTGTTCAAAGCATGTTTCATAATAACTCGAAAATATAAAAAAAGTAACTTTTAAAGGTAAAGATTTGAAATTTAATATTTTGAATTTATTTGCATGTGTTTGAAAGTTTTTTGTGT
ATAAAAAAGGTATGCAAGTGTTTTAGAATGAGTCACAACAGTTTGGATTGCTTTTGGTAAAAAAAATTGCAATTGTACTATTTTAACGCATAATTGTTGGGTGTCCGAGCACCGATGTGCC
AAATTGTGATATTTCAATGCTATAGTCTTGGGCTTTTGGCAAATGAACTAGCATCGCAACGCTAGGTGGTAATGTTGTATGCAGCTCTAGGCCACAATCTGCACTTCAAATATAAAATAAAC
CTACTATTCCACTCATTCAAGCGCCGTATCCAGCTAAGCAGGGGATAGAAGGAGAGGGTTTTGGAGGGGGATAGAAGGATTTTTAAGTTACTTCCAGCAACTTTTATCCATTAGGCCAGTT
GAGAATTAAAGTTGTCAAGATTAAGGTAGGTCACATTATACTTATCTCAACTTTCAAAAATATGAACTTTTCTTTTTTGATTAAATTTATATTTCCGTTACAAAAAAATGGATAATTTTGTACT
GATAAAACTTTTAATGTGTAACTTTTT
>comp129799_c0_seq1
GTTTTTTCTCTCCGTCTAGCTATAATCCACCATTCTTGATATTACAGATCGGGAAAAAAACTTCCATGCATGATCTGTGCAGAATAAAAAAAACATTTAGTTTCTAAATAGAAATTTTATAGG
GCCAAAAGCCGGGGACCAGCCGCCGCACCACCAGGCCAGCGAAATGGCTATCCCAACGGTAAAGTCCGGTAACATTTGCTTCTGGTTCATAAGTTTCCTTTTCTTCCTTCTCATCCTCGC
CGGCGGCGCCATGCTCATACTATACATCACCTTGCCGGAGATGGAGGACTCCACCTGGCTCGCCGTCGCCGGCATTGTCCTCATTGCCGTCCCCTGGCTCTTCTGGATCTTGGCCTGCCTG
TACCTTACACTGTTTCCTTGGCGAAAGCCATGCGTCGTCGTTCCTGTCGCTTCGGCCGATCTGAAGTCAGAGGGGGGCCAGCGATATCGGGCCTAGCTAGTGTCGCCAGGAGAAACAAGC
GGCGGCGACGCCGGGACTTGATGGATAAGTCTTTATTATTTGTAATACATTTTATAATATTTATGATATTTCCGATTTCTTTGCCATTATTGCCTTAAAAACTAAAAGAGATAACTCCTCAATTT
AAAAAAAAAA
>comp129821_c0_seq1
TAACTATACCAACATAATTTAGTCATCATCAAAAAATGGGAGATTGTTGATATTATCTACCTCATGACCTATTTTAGAGATTTTGATGATAACAAATATATGCTTGGATGCTCTAATTTGTGTGC
TAAGTATTTCAGGTGTAATGATGTGCATTATAAATAGCTTATGAGATTTACATTGCACAACTTAAGATCAATCAAATGTTGAGATTAAGATGGACCAAGATGGATAATTTGAAGCTTTTTACA

TTATCGTAGGAATTTGTTAATGCTCCACCAATATTACTTGCATGATTTGATTTGCT
>comp130001_c0_seq3
ATTCCATTCGCCTCCCACCTCTAATTTATCAATCGATAAACATTAACAGCCCAGCATTTTTTAAGAATCCGGAGACCTAATCTCCTTCTGATTCATCGCCTATTCACGCGCTTTCTCTGTCGCT
GCAACTTTGATTTGGATGCGGAGGTTCTCATCTTCACATTGTGCCCCTTGGCCACTGCTGTTCCAAGATCGAACGGTTATCTTCAGCTAATCCATTTCCGAGCGCTTTCGGAGTTGTTCCCG
CTGAGCCGGCTGAAGGCATGGCCTTGATTGGGGATTTCGGCAATGCGCCTGAGGCCACGCATTTGCTCATTGCGATCTTTTTCGCCATGTGTTTCGCTCTGGCCAGGTTTTTGCTAAATTCT
CTCGTATATAAGATTCTGAAGATGAGGAAAGCCGACAGACATGATTTGTAACAGAAGCCAAATATTAATTACTCTCTACTTGCATGTCTTCATTGGGAGTAGCATTCTTTGTTAATTCCTTCT
CTCATTGCAATGTTGTTGACCACTAGTGATTGGAATATATTCTTATAGCATAGCAGGCAGTGTAAATTTGTAGGCTTCTTATTTGTTATCTCTCTTTTTATTTAAGGTTTCGTTT
>comp130262_c1_seq2
TCGGCTGAGTCGCTAGAACGGCGAGGCGGAAGGACTTCGGCTTCCTCCTGCTTGTGCAAACAGAGGGATTGACGAAGAGAGGAAGGACTCGGCGTCGGGTCTTGTCCAAACCAAGGG
GAGACGGGAGCAAAGTCGTTGAAGGAGGTTCGGCCTGGCTGTTGCTCGCGAGCACCGAGGGAGAGATCGTGAGAGAGGGGGGTGTTCGCCTCCTAGTTCGGTCCGAACAGAGGAGAA
AGGAGGGAGGGGTTCCGCCCCTGGCTTGTGCAAACGCAGAGAGACAGAGAGGGAAGGGAGTTCAGACGTCTGCAGCGTGCCAATGGG
>comp130375_c0_seq1
GGAGTATGTGTGGACACATCGTCTGAATTGCATTCAGATACTGTGGCTGGTAAAGGAAGAGATCGATAATGTTATCACGTCCTTTGGGGTGATTGAAGAAAACAAACAGAGACCAGTGAA
TGAGCCATGTTCGCTGCTGAAGAGACTGAAGTGGAGAACTCACAGAATTATTATCTATGGTCTCTTTTAGTCGGGTAAGGTCTTCCATGGCTGCATCCCAATTTTGCATTAAGATTTCAGAG
GCCAGTTTTCCCCAGAGTGAACTCAAAGCATTTCTATCCGTTGCTGGAACCAATACTCTAAAGAAGTAAAGATATTCTGCTGCTCCAGAGTAATTTCCACATTCATACTGGAATTTTGCATA
CCTATAGAGTGTATCTAAATATTCCTGCCTAAAACCATGCTTGTCTGCCAGATAATCAAACAGCATCCGACCATCCCTGGTTGATTGCATTTGCCTGGTAGTTTCTGGATCTTCGAACATCTT
CACAATTGGTTCTGTTTCTGCCTGTAGCTGTTTCAGTTGTGCAACAACAGTGGTCCTTTTTTCTCTCAAAGCATGAGGAATATCATCAGAATAAAGGTTTTTGTATACATCCATAGCAAAAT
CCACCATGTTGGTATCACTAAGAAGATCCAATTTCCCTTGTAATAATTCTTTTTCATTATATATCTCCTTTACGGAGAGAAACTCAAGCAACGGAAAGACTAGATGCCGATCCAAAAA
>comp130473_c0_seq2
TTGATCGCCCAAAATCACAATCCCGTCACTATGCCTATTACTACTAGAATTACACTCCATATATACAGTTTTAGATCTACTCAAGCAAAAACCTTATGCAAGAATAGCCTAAACAAAAATAAA
TAAATAAATGAAGCGAAAGTCAACAAACAAGAGTAATTGTCATAACTGTCAGCATCATCCTCAGCCTTGTCCAACTATTTGGGGCATGTTTCATAAATTATTCTCCTCCATTCAATCCTACC
AAGTATTAGATAATATGTAAACTCTAG
>comp130561_c0_seq1
GGGAAATTATAAAAAATATGAATTTAATAATTATTTGTGTTGCGGGCAAGAATATACGCTGCAGTAAAGTAAGAGAATAAAGAGTCTTAATAATGTATGTATATCTAACACATCAACAAATAT
TTGAGAGAGAGAGAAACACTAGAAGTTTTTTACGTGTAAAACCCAAAAAAGGAAAAACCACGGGGCCTAATCAAGATGAGAAAGTTCCACTAAATTTGACGAAGCCTGATTCGCTCATT
TACTTGGAGATGGTAGAATTACAATAGATTGAAGAATCGGACAAAAATCTAATGATGGTCAACGAACAGAAGTTCAATAGTGGTTGGAAAAAAATTTCGGCGGTGGCCAACGAACATGTG
AAAGAACTTCAGCGGTGTTTGACAAACAGACGAAAGTCCGATCGTGGTCGACGAACGACCGAAAGTCCGACAATGGTCAACAGACGGCAAAAGTCCGACGGTGGCCTAGAGGAGCTC
CAAATTCTCTACTTTTTCAAAAATGCTTTGAGTCTTCGATTTGGGCTCGAATTGAGTCAAATTTTGTGTTGTTTGGTTCGACTTTTTGAGACGTTTTGTTCGACTATAAATTTGTTGAAATCG
GAGTTTGAATGATCCCAAATTTAATTTCAGAAGCTTTCAACTCATTAATTATGCCTCGAGATTCGTGCCTGATCAAGATAACTTTTTGATGATATATCAATATGTATAAGGACCACATGGAAAT
CAAAAGCTTCAAACACCACACATATTTCATTGACAAGCATTCGATGATCAACAAAAAACATAAGTTGGGGTCTATGATTGGTGTATGATGGTGATTTGTATTAATGTCTAGAAAAGATTGGC
CATAGGTGATAATCAACCATTTAAGCCTATCAAATTAATGGCTAATAATGGGCTGTGATTAACGAGAGATAAATCTCTCTGTGACAAATCTCAACATGAGTGATTAGTAAATAAATCTTTTTT

CAAATTTTGTGGGATGACACTATGTTTATTTCTATTAATATTGATACAGTTCATTAACAATTTATCAAAGTGTTTGTAATAATTCTCATTTGAATATTTCATTTTTCCTAATATTTCAAAACATAA
>comp130612_c0_seq1
CGTCAGCCAGCAGAAATGGCTGAAACTAAACATCCTTTTGGTGGATTAACAAATGATTTCAAAGGAAGAGCTAGTTGTTATAAGCAAGATTGGCTTGGTGGATTTCGTTCAGGCTTGAGG
ATATTGGCTCCGACTATGTTCATCTTTTTTGCTTCGGCCCTTCCTGTTATTGCTTTTGGGGAGCAACTGAGCAGAGATACAGATGGAGAACTAAGCGTTTTTGAGACCCTTGCTTCAACTTC
TATTTGTGGCATCGTACACTCAATCTTTGGGGGGCAACCCATGCTGATATTAGGAGTAGCAGAACCCACTGTGATAATGTATTCCTACTTGTACCATTTCGCTAAAGGGCAAGAATTACTTG
GAAGCCGTCTGTTTTTGGCCTGGGCTGGCTGGGTATGCTTCTGGACAGCGTGTATGCTGTTCTTTCTTGCTATTTTCAATGCATGCAACATCATTACTAGGTTTACAAGAGTTGCAGGCGAA
CTTTTTGGCATGTTGATAACTGTTCTGTTCTTGCAAGAAGCCATTAAGCTCCTGTAGGTGAAGATTATAATCAACACAAGTACCAGTTTGAGGGGCTTTACACAAACGGTTTACTGGGTGT
TATTTTCTCACTTGGTGTGCTATTCACTTCTCTGAAGAGCAGACGAGCAAGGGCCTGGAGATACGGCACAGACTGGTTTAGAAGCTTGATAGCAGACTATGGGGTACCTATGATTGTATTG
GTATGGACAGCAGTGTCTTATGCTGTTCCAAAAGATGTTGCTTCAGGAGTTCCCCGAAGGCTTTCTATTCCATTTCCTCGGCAGGGTGAATCATTGCATAATTGGTCTGTTGCAAAGGATAT
GATGCTGGTTCCCTCGTTGTACATCTTTGGTGCAATAGTGCCTGCTATAATGGTTGCAGGCCTTTACTTTTTTGACCACAGCATTGCATCTCAAATGGCTCAACAAAAGGAATTTAATCTCA
AACACCCATCTGCGTATCATTATGACATCTTTGTCCTTGGTGTTCTGGTTCTGATTTGCGGGCTTCTAGGAATCCCTCCATCAAATGGGGTCTTGCCCCAGTCCCCAATGCATACAAAAAGT
CTTGCAGTTCTCAAGAGGCAGTTGATTCGCAAGAAGATGGTTGAGAGTGCAAAGGAAAGCATACAATTGCAGGCTAGCAATTCTGAAATATATGGAAAAATGCAAGAAGTATTCATAGA
AATGGACAAGAGCAACAGTGCTCGAACAGTGGTGAATGAATTGAAGGACTTAAAACAAGCTGTCATGTCTAGTGGAGATTTAAATGGTGAATTTGATCCTGAGAAGCACATTGATGCAC
ACCTGCCTGTTCGGGTGAATGAGCAGAGAATTACCAACCTATGCCAATCCCTAATGGTTGGAGGCTGTCTTGGAGCTATACCATTTATCAGGAAGATTCCTACATCAGTTCTGTGGGGCTA
CTTTGCATACATGGCTATAGATAGCCTTCCTGGAAACCAGTTCTGGGAAAGGATTTTGCTTTTATTCATTACTCCAGGAAGACGTTACAAGGTCCTGGAAGGAATCCATGCTTCATTTGTGG
AATCAGTTCCTTATAGGGTTATAGGGACCTTTACAATTTTCCAATTGGTCTATCTCCTAATATGCTTCGGAGTTACATGGATTCCTATAGCAGGAATTCTCTTTCCACTGCCATTTTACCTGTT
AATTAGCATCAGAGAGCATCTTCTTCCCAAATTCTTTCACCGGCAGGATCTGTGGGAACTGGACGCAGCTGAATATGAAGAAATTGCTGGAGTTCCTATTCACGACCATAACCTCTCATTT
CGGCTTGATGGATCCCCAGCGTGCACTGACAGCAGTCAGGAAGCTTGTGAGGCATTCGGCCATGGTGCTGAAATTTTGGATGAACTCACAACCAGCAGAGGGGAGCTTAAGCTTAGGAC
TAAAAGCATCAATGAGGAGAGGTTCCTTCAGGTTCACCCACTTCCGATGCTGGGCAACTCAATATGTGAGTAATACAAGTGATGATGCATTCATTTAGACGCCAAATCAGGACTACAGATA
CGGACCATACATTAGCCCTTCAATGGGCTTTGCTGAAGTTCTGAAGTACCTAAATAGAGAGCCGAAGACGAGGGCATCCATAGTGCAATTCTTTCTTTATTTTATCCCTTTCTTTTTGTATAA
ATTTTTTTGAAAGATTGAAATATTGCATCAAAGTGAGGAGATTTAGAATGAGAAAAGGCTGCAAGGAATATAAAGTTGAGAGTGCAATAACTATCTTTTATACGGTTGAATATTTTTAGAAA
TTTTGCCAAATTTAAATTGTTCTACAGATTGTTAATTTTAATTTTACATAAAGGCCTAATCGTTTTTTTTTGTAAATAGACAATATATTTGAGAAGAG
>comp130642_c0_seq1
TAAGTAGTGTATTATGAATACGGTAAAATGATGGGGGAGTTAGCAGCTACGCTATATATGTTGTTTGGCCACTTTTTTATATGAAAAATCATCTACTTTACCAATGTAATTTTATATCACATCTG
GATGGACATCAATGTTGGTTATGCTACCACAGAGAAGTTTTATTTTGTATTGGTCATTTAGTTATTGTTCTTCCTTTAACGCTAGACAAATACTGTGAAAGGCAATGAGTGTTAGTTTTGTGT
GGAATATGATTTTGTTGACCAATCGGGTTGTATTGTTGACACAAATCTCAATCGAGCTTGTTGGGGGTTGCAAGGGGTCCTCCCCCTGCTTAGCTTTGAGTGCTCTATGTGATGCCTTCAGG
TTAAACATCTAGATTGACCTGTTCTAACCTAATTGGACAAAAAATAAATATTTAAACTTTCGAGTTTTGAACCCATTTACATATGTTTGAAAACTGATCTCAAATCTCCAGAACTAGAAAGC
CAAAATCTATCCTTGCAAAAAATAATACTAGTATCAGATACTAGCAATGCAAATTTCTTACCCTCTTTAATTAGACCAATCATCAAAGAAGGTTGAATAATCATTTGAGCTAATATACTAGAA
TCCCGATCGGTAGAAGAAATCTCATATCCCACATCAAACACTCATCGGAAGTTAACTAAACACATGAAACTCTAAAAGATATCATGCTCAGAGCATATGCTGCAACAT
>comp130659_c0_seq1
TAAATGTGGACGGTGCATTACACAAACCAAATGGCATTACCTTCCACTCAAAGAGACCGTCTCTGGTCTTAAAAGCAGTCTTCCACTCGTCGCCGGGCCGAATTCGTATCTGGTGATAAC

CGCTACGGAGGTCTAATTTGGAGAAAATTGTCGCACCAGCCAAGTGATCTAGCATATCCTCAATCCTCGGCACCGGAAAACGATACTTAATAGTTATCTTGTTGATTGACCGACTATCTATG
CAGATTCGCCACGATCCATCTTTCTTTGGCACCAACAGAGCGGGCACGGCACAGGGGCTCAAACTGGGCTGAATCATTTGTTTCTGAATTAGCTCGTCAATCAACTGTTGCATAGCTTTAT
GTTCTGTTGGGCTCATTTTATAATGCGGCAAATTGGGTAATGTGGCTCCTGGTACCAAATCAATTTGGTGTTGTATGGTCCTCAGCGGCAGCAATTCCTGTGGTAGTTCAGTTGGAGCAATA
TCCTCAAATTCGATCAGTAGTTGTTGTAGTTTTGTTGGAGGCGAAGTGTTTTGGGGTCTTGAGTTCTCCTCGGCTACAAAGACAGCTAAAATTCCCTTAGCTTCATGCCCAAAACGGATGA
GGGACTTTCCTGTGACTATATGCAAAGCTGGCCGAGTTCCTGAGACAGAAGGTGGTGTGCTGTGAGAATTTGGAAGTAGCCGCAACTTCC
>comp130670_c0_seq1
ATAGATATATCTGTTGTTAATACTTACACAGATCGTGTATTCACAAAGTTACGGGCTTGTGGATTATAGAAACAGGTGCAAGTTCCGAAGACAAAACGGAAACGGCACAGAAAAAATTTC
GCGTATGAAGTAGCAGGAAGACGGGCGTTCGCGTGAGCAAGGTTTCGGCTTTGACTTACGTGCTGGACGTTTGTAGTTGGGGATCAATCGCAAGCAGCAAGAGCCGTCAACGGTAGGC
GTTTGTGATTGGAAGGAGGCAAAAATCCGGCGAGAAGGAACCAGATTTGAGAAAATGCAAACCATGAGAAATGATCAACCCGTTAGTTCGCTCGAGCACGCCATC
>comp130849_c0_seq2
CAAATGCTTCACCAGCTGGAATGATTGATGTACCCCTTCTTCGTAGTTCCTCCTCCAAAAGTTCAGCAAGTTCCCTATGAAGCCTCATCCTCATTCTTCCTTTTGTCTTTGCATGTGTCATTA
GAGGTTGAAGACCAGTGTACCAGTCAATAGCTCCCGGACCACCCTGGCAGGCAGGGCAGTGCCATTGTCTAGCTTGTTCATTTATTTGCTCCAAATTCAGATTATCCAAAGATTCAAAAAA
TGGCTTCAACCACTTATTCTTCTTACGTGTATCATGGCTCTTTTGACTATCATCAGAGTCAAAGCCATCACTAAAATCATCCTCACTATCATCTAGCAACTCATCATCATCTTCAATATCGTTA
TCAACATCTTGAGGAAGTTCATCCTGAGGATGCATATTGCAGTTTTTAACTGGAGTATTGGTTTGCAGGCCAGGCTTACCAGCTCTTGCAGACCACTGCCACCCATTTTGTAGTGGTGGAT
GAATGGCTGCGGATGGTACCTTGTAAGCAGTTCCCCAAGAGCCCCAACTGCTATCTAATTGTTGAGCCTTAGAGCTTTCTCCCCCAACTGATTCTTTGGAATTAATGACTTTAGGCCAACT
GTCGTGAGATATTCTTCCTGCACCAGTATCGCCCAAACTTTGCATCTGTGTGCCTTGTGAAGGGCCCCAAGCTTTAGGAGCAACAGATGAAGCATTCATTCCTTTACCGCCAGTAACACCT
AGTTGATTAATAGAATGATTCCAAGTCAAACCCCTATCAGCATCTTGATTAGGCTCTATTTCTAAATCTTTCATCTGATGGTTCAAACGAACAACATTCCCCCCTTCATAACTATATTCTTCAT
ATTGGTTTACTTTTACTGATGATGTGCCACCAGCATCAGCTGCAGCATTGGTAGACTTGCTAAGAGCTTTTCTCGAATTCATTTAAGATAGCTATGAAAATTCCAACAGAGGAAATGAGAG
AGCCAACCAGAAGACAGAATACGGAGAGCTATGCAGTTCCGGCGACGCTGCGATATGGCATCCCAAAACTTCACGTGATGCGACTGCTTCTGCAGGACAGAGTGA
>comp131128_c0_seq2
AGGATCCAACAACAGTTCCTTGTTCCCAACAAAAATAAGAATCAAAATCCGGTAATGTAAAAGAAACTTCATCTCCCTTAATCTGGCTCTAAATGTCAAATATATGGTAATTTTTCCACCTC
TACCTCTTTAACTCCCTCTCATCTAGAGAGGCATTGTGTCCATATAACATAGGAAACAACCATCAATACATAGAAAGACAAATTGAAATGCATATATACACAGTGAAATTGAAATGCATATA
>comp131222_c0_seq1
TCATTTTCTCTATATAAAGTGCTTATTGGTCATTCATAGAATTCACAGATTCACAACCATTGATCAAGCAATGGCAACTAGCGCTACCACCCAATGCATAGCCATGGTTACAGTGCTTGTGAT
GGTCTCAAGTGGCGCCATTATTACTGTAACTGAGTCTACTATTCCTTGCAGCGAAGTAGCAAATAAGCTTCTGAGCTGCGTCGACTACGCACAGCACGGCGGTGAGCTGCCGGCGGCGTG
TTGCAGCGGCGTGAAGGAACTGAAGGCAGAGGCTATTACTGCAGAGGACCGACAAACTGTCTGTAACTGTATAAAATCACTCGCCAAGCAGATCGGAGGGCTCAACCAAAACCTTCTG
GCGACCATTCCTGGCAAGTGCGGAGTCGACGTTGGCTACCCTATCAGCTTCTCGGTCGACTGCTCCAAGGTGAAGTGATATGCTGAGGTGTTCATGAGTCAGGCCTATTAGAAAAATAATA
TCTTTCAGCTTTGAAGATGACGACTATTAATAATTTGCTTAGCCAAATTACTATTATGCAATAATTAATTATCATTAGTGCTATGTAAGAGAGCTGCTTAATCTGAACCGTTAATGGAATAAAT
CCTTTGTTATAATTAAGAAAAATTACAGCGCTCGAATTTAATTAAAA
>comp131256_c0_seq1
AATCAAGGCATTCCATGAGAAAAGATTGTTCTTAGTCAATTCATCAAATACCTTACAAGTTGATTCTAAGCAACCTTTGGCATTACACCACAGAAGGGAGTTCTCTATGCCATCTGGTGAG

AGCATGAATGATGACATCCCCACACCTGCATTGTTAATTAAAATATTTAAAGGAAGATTTAGAGAATTGAAATCTGCTGCAATTTTGCTCACAGAAGCCATTGAACAAAGGTCTAATTCCA
AGGCATCAACTTTAGCAAATGGAATTTCCTTCAGAATCATTTCCTTCACATTGTTATCAGCAGAAACATTTCTGATCCCTAGGATGACATGAACACC
>comp131314_c0_seq2
AAAACTCCCACAGATTCTACGCAAACTACCCGGCTTCGTCTCTCCTCTGTGTTTCCCTTCCTCTATAAAACCCGTCACTGTGGCCGGTTGCCGTGGAACAAATCCCCGTTTGCTTATATGA
GGACGGCGAAGTTGGTGGAAGAGGTACGTCAGCGGAGAGAGGATTCGGTTAAAATGGCTTCTTTGACTGCGCGGCAGGGAAGGGAGCTTCAGCGCTACACCAGGACCGGAGGGCGCC
TCGTTGCCGGATGCATTCCTTATAAGTTCAACGTGAACTGTTCAAGTAGTGATTTGCTTAATTCATTGGAGGTTCTTGTTATCAGTTCTCAGAAGGGACATGGAATGATGTTTCCCAAGGGT
GGTTGGGAAAGCGATGAGACAATAAAACAAGCAGCTTCTAGAGAAGCTTTAGAGGAAGCTGGCGTTCAAGGAAATGTAGAGAACCAGCTTACAAGTTGTAAATGAGGGAGTAATGTTT
CCACTGAATGTAACAGATGAGCTTCTCCAATGGCCTGAGATGAGTGTGAGGAAGCGCAGCTGGGTCTCTGTGGCAGAGGCAATGGAAGGATGTGAGCATCAATGGATGAGAGAGGCTTT
G
>comp131320_c0_seq2
AAAAATTATTCTGTGCGGATGCCGGAAGCTGTCCGGAGACCAATCAGAATGTTTCGTGTCATCCTCTTACTGTACCGTGGCATCTGGACGTAGAGCTGTGTCTGACATCCGCACAGAATAA
TTTCTCATTTACTTGCCGCAAATAAATCATAGAATATAAAAAACTATTATAGAAACTGAATCCCCATCAAGCCAAGCAATTTGAAATGTTATCTGAATATGGAAAGAACCAAATCTGAATCA
CTTGAACCCTCTCCTTCTCTGTTCCCAACTCACAAACCAAACCAAATCTATTCTTACAAAGATCATAAGACACCAACTTGCCCAAAACCCATAAGTAAACCACATCCTCCTTCGGATGAA
>comp131416_c0_seq1
AATTTTTATTTTATTATTATATAACTTGATTCTACCAACTCCAAAACACAATAAAAACAATCTAATAACTGCTCGTAGCACAAAATCATAAGAAAAGGAGCAGCTTTGGGAGACACTCACAT
CGTAGATAACAGTGGAATATATGCCAGTCCTTATCCCGGAGCCAAAAATCATTTCAAACGATACCCACACAGCTCATAACATTCCAAAAATCAAAACAGGGATGGTCCAATGCAGCTGTAA
ATTCTTCAACAGCCAAAGGGGACCTGCCACCTATCCCACCCACAGTCTCTTTACCCTTTCCTGATTTGTCTCGGCTATAGGTACACTACAGGAACTGGAAACCGTAAGTACGCATCAAAGA
AAGCAGAATTGCAGGAGAACAAACACAGCGAAAGCAAACAAAAAGTAGAAAGAACCCAACACCTATGACCGGTGAGATTAAAAAGAAGATAGCTTCGCCACTTTCCCTTCCCCATCTC
ATCACTCTACTTTTAGTTCAAAGAATTTAAAACCTTGCAGAGAAATATCTTAATATAAAATACAGTTAAAATGGATCATCAGTCGAGACAAATATCAGCAATGTGCCTGTCAGTTCAACAAA
CCCAGTGTTTCATAACCACCACTCTAACATTAACCAAGAAGTGGTGGTGGTGGTGGGTTCCATATGCCATCAAATAGTGTTCTCATTCAGGGAATCAAAGGACAAGACAGAGGATTACAA
GCTTAACTATCCTTGCAGCAGAAACTAAATTATGGGGGTATTGGAAACCTGATAGGGAAACCACAGCAGATGAGCCTAACGCTTTCTTTTGAGAAGTGCTAATAATATAAGGTATGGATGT
GACTTATTTAACGTTAGAGATGGAGATTTAAGGGAAAAAAAATGATTATGTAGTAGCTCTAGTAGAAGATAAAGATTAGAGAGAGTCGTCTTAGA
>comp131454_c1_seq1
GCAAGGGGAGGTCGGCTTCAGGCTGGCCATGCGAGAGGGAGAGAAGAGAGCGGATCGGCTTGCGGCTTCTCCTGAAACCCACGAGGCAGAGGGAAATCGGGAGGGGGGCTCGGCTT
CTCCTGCTCGCGCGAACTGAGGGAGCTCGAGAGGGAGGCGTCCGGTTTCCTTTCGAGCAGAGGAGGAAAGGGATCGGGCTCGAGATCTCCCTGAAGAAAATGAGAGGGAGCGTCGGT
GCCTTGCAGTGAGGTCCATGGCTAGGGTTCCGCGAACCAGTCCGGCCAGAGGGGTCAAGGGATTTCCGTCTTGGAAAAAAAAAAAAATCGGGAGAGAGTGTCGGTTTCTGCCAAAATCC
AGAGAGAGCCAGCGAATCGGGTCTCCTGGCAACGGTCGAATACGGATTT
>comp131673_c0_seq1
TGGCATCGAGTCGAGCGCAGCAGGGGTAAGTTCCTCCGCCGATTCCGACTGCCGGAGAATGTGAAGGTGGATCAGGCGAAAGCGTCCTTGAAGAATGGGGTGCTTACTGTTGTCGTGCC
CAAAGAGGAGGTGAAGAAGCCTTACATGAAGTCTATTGAGATTTCTTGAGAGGATAAAATAGAAGAATATGATTTGCATCTCAAATCGCCCGTGTTCTTAAGTTGCTTGTTTTGTTGTTGG
CGAAGATTTTTTGATTGCTCTGCTTTGTGAAGTTTTTGATGTTTGATGTTGGCTTGGTTTGTGATATTTTTTTTTTTCTAAGGTAGTTTTTTAGTACAATATTTTTATCAAAAAATTTGTATGCT

AAAATATTATTTTAAATAATATTGAAAAACGGTGAAGTGGTGAAATTGA
>comp131861_c0_seq1
TAATTTTGACATCATTAAAGTACTAAAATGTTGATCAAATACGCTATAATCAAGGGGATCAATATGAGTCGTTCGATGGATTGCTCCAAATTTACCCTGGATTTTCTCCATGTTTACGGGGCA
TGCCACTCTTAAAATCTATAAATAGATCCCAAGCCATCTTCATCCACAAACCTGACCAGGCCCAATTATCACTTGCATTAAGCAGCCCTTAACTTCAACCTTCCTCCCCCATCAATCCCATG
ACTTGACTCAACCCTTGCAACACCCAAATGGACCAAAACCGTAAAAGTTCGAGTCAACCCGAGCCCATTCATTCAATTGGTCTGCCTCATGTTCAATTGCCTAACTAATGAGTTTTATAAA
CACACATCGATTATTGATTATTTCCCTGTAAGCATGACCATTCACATAATGTTGAACAGGTACAAAGTAGCTATTGTAATTCTTTGTGTTGAACGAA
>comp132603_c0_seq1
AGAGAGAATGGGGGGTTGCGCCACGAAGCCGCTGGTTTTGAAGGAGGAGACGGAGGCTCTTAAACCGGTAGAGGACGTTGCTGAACCCGTCAAGGCTGAGGAAGCCGGCTCCGGGGA
GGAGCAGGAGGCGGCCAAGTCAGAGGAGGCTGAAGGCCAAAATTCACTCAGCAATTACTTCCAGGAGAAGGAGGCCGAGGAGGCCGCCGGTGCTGCGGGGGAGGAGAATGTGAAAG
CCGCGGCCGAAGAGGCAGAATCGAAGCCGGAAGTCTCTGAATTCAATGTTGAAGATGCTCCTGCTGAAGAGAAGCAGCAGTAATGAGAGCTCGAGAAGGGAATGTTTTTTGCTTGTTCT
TTGATGTTTGTGGATTATAAGTGATCGAATGATCATTTGATGCTTTTGTTTTGAACGGTTGATTTTCGTTTCTTTGAATTTCATTTTGTTAAGAACTAAATGATGTGATTCTTTATTTGGTCGA
ACAGCTTATTTTGTTTGGTTGAGATGATCGGCAAGTACCGGTGGACATATATATTGATAAGAAAATTTGCTTGCTGAGTATGATAAAAAGCGTTCTTTCTGGCTTGAATTTGACGATATTATT
TTCAGCTTTTGGACAAAATTTTAGGACAGATTTCTCTTTAACATTTTTAGGGGAATAGAATTTTTTTTTTTTAATTTATTGAAGCCGGCAAAATTTTATGTGAATCATTTTTTTTTCGGCTAAC
TGTGGCGGTTTAAATAGCTGTATGTCTTTTCCCCAACGGC
>comp132723_c0_seq1
ACGACCTCCTCACGAGAACATGCCTCTCGCAAAGGATCTTCTTCATCCCTCTCCAGAAGAGGAGAAGAGGAAACACAAGAAGAAGCGCCTGGTGCAGAGCCCCAATTCCTATTTCATGG
ATGTAAAATGCCCAGGATGCTATAAAATCACCACCGTCTTTAGCCATGCACAAACAGTAGTCTTGTGTGTTGGCTGCTCTACTGTCCTCTGCCAGCCTACAGGAGGAAAAGCGAGGCTTA
CAGAAGGATGCTCTTTCAGACGGAAGCAGCACTAAAAGTACCCTGTATCAAGATGAACGGGAAACCATCCCAATAAAC
>comp132740_c0_seq1
TGCCCCGTCGCTCGGCAGTTCCCATCTCTGAAAACTAGAGTCCTGAAAACTACCTCTCGATCCATCGTTGTTGTTCGCGCAAAAGCTCTGATTCCCGTTGCAGAGATGGAAGGAAAACTT
CAAAGGCCAGATCACGAATCGCTAACCCAATCGCCTCTCAGGCGAAAGTCTGATGAGGCGGTGAAGCAGCAGAAGCCCGACTTTATTCTCGAAGAGTCGGGCCAAGATGACAAGAAGG
GGAAGGGGAAAATGGCGGTGGATGAGGAAGAGGGCGGTGATGCCCAGGAGAGTGATTCTAGCGATGATGGTCACGGAAGCGATGAAATCAGTGACAGTGACTGCACTGATGATCCTCT
TACAGAGATGGATGTTGACAACATCATACCCGGAAGGGTACGGCCGCGCATTGCAGTGAAGGCCGGGACTTATCGATTAGAGGGCGAGAGCGATGAGGATGATCATACAACGGAT
>comp132749_c0_seq1
AAAATATAAAATTATAAATACACTAACGCAAAGAAATGAGTTCAAACCCTCCACAAATGCCAGTTTCCAAGATATTCAGTAATAAAACAATCAAACTATCCCATGATAAGATAAAAAAATA
ATCCAGTATAAAGCCATAGAGTTGAAAATACAGATCATGTAGATACATTATATGAGAAGACTATTCCAGAAATATCCTCAAATCCTAAAAAAATTACATAATTACCTCATTAAGGAGAAATGAA
GGGTAGCAAATCATAGGCATTATCATTTTCAAACGAAATTACATGTCGTAGAATCCCATGTATTCCATCTCCATATCCCAAATCAAAACATTCAAAATAAACATTATCATCACCATTAGCCCCT
TGCCAAACTATTTAGTATTGGATTTATGAATCCTCATTCTACATTCTCCTGTTCAAGACTCATTAAGATGTTAAATTTTTTATAGGTAAACTCATTGTACGCAAATTCTATCCATATAACCTTTG
GTTAATCCTTTTCCTCATTAATCCTTTTTTTTATACAAATATGATCTACCAATCTCATTGATGCATCTTCTATCATTTTGTTGTACATACAAATCACATAATCTCTCATATAATCATTTCACAAATC
TAAATTTTGGGACATATACAACTTAAAATTTTCATCTTTGCCTCGTTCAACTAAGCTATACTACACTCGATGCTTGAATCAATAATATCCTAAATATATTTCTTCCTTTTGACCCCTCAATCTTA
ATTCCATAAGTTATAGCCCACATTTAAATGCTTAATCACCAATAAAATAAATCCTAAATACTTAAACCTATCACACACATCACTTAATCTTCTATCAATATCAACTGATAATTATATAAATTCTA

CACTCATTAAAATCTTGTATATACTAATACAAACCCTTTATAAGTAAATTACAGCCTCCATGATAGGTACAGGTTAATCGCATAGGCCCCATTTGAAAGTGTGATGCAATAGAAAAATGCCCC
ATTATACAACATCTGTTGCTGCCTATTAAAGCTTGCACAAGGCAAATGTTTAATTACTTAACAATTCCAGGAAACAATTCTCCCAGGAGGACGCATGCCTGGTTTTCACCATGCTATAGAGG
CTTCAGCTACATTTACAAACCAGGCCTTACCACCAATTCTGCAACGACATGGATTATATCATTGGAACGCTCATTTGGCTAACTTCGTCTTTTTAGCCGTCAGAAAGAGGAAGCAGACATC
TATGATTTTCTGCTAAGCTTGCGATGTTGATATTGTGATAGTCCAAGTGACCAACAGATAAAACAAATCAGCTCACAATACATC
>comp132856_c0_seq2
GAAAACCCGCTAATTCTGTTCCTCCGCTCACTTCATTTCCTCAAATATTCTTCCTCTTCTTTGGAGAAATTACCAGTAATCAAAACAATCCAATGTCTCTCTTCCTTTCATTTGTCGCCCAAG
TCATTTTCTTTCTCTTTCTTTTTAACTTCCTACAATTCCCACCCCACAGCGTCAAACCCCCTTCCTGTATTTTATTTTTGTGCCCTAACAGATTTTGTTCTTTTAGAGTTTTGCGTGGGTTTTT
GCGGCTAAAACTCCCTTTTTATTCGATTTTGGAAGAGAACATTGAATTGAGTTGATATTTTCTTGTAGATTTAGGGATTACTTATTGGAGTGATTATTTGGAAAAGCTTGTGATCAAGAGATG
AGCTTCTTTTCTTGTTTTTCATTCGACGGCAGGAAGAAATCAAAGAAGCATAATGCAAGGAGGGTGCCATTACGGGCTCGATTAGTTCAGGTTTCACATCAGGAGAGCGAATCCCGTTCC
AAGCAAGGAGTAGCAAAACAGAGGGCTGTAGCTAAAACAGTGGCAGAGAAAAGAGACACGGGAAATGGAAACATTGCAGCACAAACATTCACATTCCGTGAGCTCGCTGCGGCTACC
AAAAACTTTCGACCAGAATGTCTTATTGGTGAAGGAGGATTTGGAAGAGTTTATAAGGGCCGACTTGAAAAAACTGGCCAGGTTGTAGCTATTAAGCAGCTAGATAGGAATGGCGTGCA
AGGAAACAGAGAGTTCCTTGTGGAGGTCCTCATGCTCAGCCTGCTCCATCATGAGCACCTTGTCAACCTCATTGGATATTGTGCTGATGGAGAGCAAAGGCTTTTGGTTTATGAGTACATG
GCTCTGGGTTCTTTAGAAGACCATTTGCTTGAGATATCACCTAATCAAACGCCCCTTAGTTGGTTCACAAGAATGAAGATAGCATTCGGCGCAGCCAAAGGTCTCGAGTATTTGCATGAGA
AAGCTACTCCTCCTGTAATATACCGTGATCTCAAATCATCAAACATCTTACTCAATGAGGACTACATCCCAAAGCTCTCAGATTTTGGCCTTGCTAAGCTCAGTTCTCTTGATGAAAAGGCC
AATTTATCATCGAGAGTGATGGGTACCTATGGTTATTGTGCACCAGAGTATGCCAGGACTGGTCAACTCACTATTAAGTCAGATGTATATAGCTTTGGAGTGGTGCTCCTAGAGCTTATCAC
AGGCAAAAGAGTCATTGACACAAGGAGGCCGATTAAGGAGCAAAATCTAGTTGCCTTGGCACAACCCATATTCAAGGATCAAAAGAGATATCCAGAGTTAGTGGATCCTCTTCTGGGAG
AGAACTTCCCATCAAGGGGCCTAAACCAGGCAATGGCAGTTGCCGCTATGTGCCTCCAAGAGGATGCTTCAGTTCGTCCAGTGATGGCAGATGTAGTTGTAGCTCTATGCTACCTTCAAG
AATCACCATGCAGTGTGGTTGACGGGAGCTCCAACCATCGTCAGCCTCTCTTAGACTCACAAGAAGATGATATACCCAACATCCATGGAATACTTGTTGATAAGACTACCGTGAATCGCCA
ACACGCTGTTGCAGAGGCTATCCGGTGGGGCTCAAATAATTCAAGAAATGGTCAGAGGTCACGACGTGGAATTGGTCCTTTGTAAGCTCTTTACATGGAAGAGAAGCTTAGTATACATCT
CTTCACAATTCACATCTATGTTGATGCTAATATGAGGATTGTATTTAAACAATAGGTAATGAGGTCATTGGAAAATGCAAGTGGTAAAAATATTGGATGATGGACTGTGGTTTTCCTTACAAA
CCACATGTATCTCATTTTATTTTTACTATTTATGCAAGAAGATGATTCTGATAATCAAAGAATATATGTAATAACATTTTCTGTGGCTGAGTGACATGCAATTTTTATGAGGAATATACACAATA
TTACTAATTATGCATATATTGA
>comp132936_c0_seq1
ATTATTTCTCACACATATCACCTTTTCAAAACAAAACACTTTTCATTCTCCAACAATCAAGCTTTACTGTACCATTCACAACCTACAAGTTCACTGTACCATTCTCCAACAATCAAGCTTCA
CTGTACCCTTCGTTTTTTAAAACATACTCATGCTACTATACTAAAGAAAACATCGTTGCAACATACAAGTTCCCATAAACCCTTACAACAGTAATAATGAGAAAGAAT
>comp133048_c0_seq2
ACACTATCCTCAATCAACCAAAAGAGATGAATCACTCAAAAAGAATACAAGCTTCTAAAGAACAAATTAAATAAAAATCCTGCTTCCATTATGCCCCATGCAAAATGCAAGCATCACATAT
AGCTCTTGGGTGTAATTGGGAGAAAGATAATTATTATTCAGGCAGTTGTAAGAATTATCCAGCTCCCTTCCATTATACCCCATGCATATAATATCCTATAAGTTTGATGTATTGAATGTTGGAC
TAATATCCATCCTACTAGACACTTCAACTTGATATGTAGTTAGACCAAATTTTTTCAAGGCATGACAAACCCATCTTCTGTTGCATGAGTTTGGGATATGTCAATACAGGGAATCTCTCCTAG
CATAAATATGCCATTATCATGTTACCTTCCCAGTGCCCATGTCGGCAATGTGTGTTGATGTCTGTAGGTGTTGTTCATATGATTTTTTAAGAGTCCTCATTTGTTCTATTCATCTGTAATATTGC
TTACTAGCTTCTCCTCTTCAAAACCTCTTGTGAAATTTGGAATTGGTGCATAATCTATGCTGTGTCATAGGTTTCTTCCAGAAACTATTTCAGGACAACTTTAAGTTGACCGACATTTCCAG

AAGTTGTACTGTTGTAGTAACCTTGGCTTGGGACATGCCCGTCTCTCATTATCTCGACTCCACATAGCTTTCAACA
>comp133216_c0_seq1
CCTCTTAACAAACATAACTAAAATAAACACCAGTAGGACACAACTATAAGAGAGAATTTGAACAAGCATCTTTCTTGACTTAATTAAACTATATACTTTTCCAAACATAAATGAGAATGATC
TTATAGACTGATTATCACATTATGATCTTATATCCGTTAATTGGGCCAAATTCTAAGTACAGACTCAAATTATCTCACATTTTTCAAGCCTTAGCATATGATTCCCGGTTTCAGGAAAGTGTCA
AGATCTACATACAAAACTAAATCTCTAGATTTGTGTGATCTAGGAATGCACACCTGTCAAGTTTTCTTAGATGGTGTGCAATGGTGCCAATAAGAAGAGGTGTTCGATCTAGGGGTTGAGG
CAAACAATTTGAAGATCTCTCTTGTTTTTCATTGAGTAGATAAAGTGAACTTTTGATGAAAAAAATCAAGGAAATAATATTATTTTATATAAAGGATATAGGAGAATGTATTATGAGTGATTT
GGGGCTTCAAAGGATTTGGGAAGGTGAATTTCAATGAATATTCAAGAGCAATAGTGAGGGTGCAGGGATTTGGGGTCTATTTGAGTGGTCGATTTTGTGAGGAGCTGAAGGGAAAGTTA
AGTTTGTAGTTATGAGGGGTGTGAGTACACTCTGGTAAGCTAAGGATGTCAGGACTAAGAATTTGAAGTTGTTTAGCATGATTATATGGAGATTTTGGCGTGTAACATGAAAAGAAGGTTG
GTTGGCTAAGTTTTTGAGCATAACTAAGTGTATAAGTGTGAGAACTTAGAAAGAATCTTCGTAACACTAAAATG
>comp133273_c0_seq2
CATTGGGGATCAGTTCAGTCTGGGTATTCCAAATTTAAACTCTCCCAATGAGGTGGCTTCCTCATCCTCCACTAACCTTCTTTCTCAGTCGACCTCTGATGGGAAAGGTTATGTTTCTCCTA
ATAGGTTTGATTTATTAAATGTTGAGGATGATAACAAGGACTCTACCATTATCTCGGTGGTAAATTTAGAAGGGGATAATATTTTGGCTGCTGATTCTGGGCAGAATTTGAATGTTAAAGTTC
ATTCCAGTAAACATACTGAGTCTTCTACAGTCAAGAAAACTGCTAAGGGGAAGCAGGGGAAAAAGCCCCAATCTAAACATAAATCCTGATGTCTATTCCACCTATGGCTGCGTGGAATGT
GCGGGGTTTTAATCGCCCTGACAAAGTGCTTTGTTGTAAGCGTCTCATTGAGTCATTCAAACTTGACTTAGTTTGTATATTGGAAAACAGGATTCATCAACAATCTCTTGCGGACCCGTTCT
TTGAAAGCACTCATTCTCTATTTTCTAATGAGTCCAGCTGCAATAATTTCTCCCATTCTGCCTCGGGGAGGATTTGGGTGAAGTGGAATTCTTCTAAATTGTCTTTTATCCCTTCAGTCATTA
ATTCTCAACTTATCTCTGGTTTGGTTTCTGTCTCTAATCATCCTCTCTTTCAGCTTTCTGTTGTGTATGCCTCTAACAGTATGACTGAACGCAAGACTCTGTGGAACTCTCTCTCTCTCTGGC
TGCTCCAGAGAGCAATTTTCCTTGGGTCATTATTGGTGATTTCAATTGTTGCAGATTTGCAACAGAAAAGCTTGGTGGATCCCCTCTCACTCTTTCTGCCATGAAAGACTTTAATGATTTCA
TTTTCCATAATGGGCTGCTGGACCTTAACTCTGTTGGGGGAAAATTCACTTGGTATAATCAACAGTTAGACAATCCTATTCACATTAAGTTGGATAGAGTTCTTGTGAATGTTGTTTGGGGG
AATGTCTTCTCTGATTCTTTCTGCTCCATTCAAAGCCCTTCCTGTTCGGACCACTGCCCTGTTATCTTACATCCTGGGAATGCTACGCAGGTTTTTCACCGATTTCTATTCAAAAATTATTGG
ACTAAGATTGATTCTTACTGGTCAATATTATTGGAGGTATTCTCTAGGCCTTGTTCAGGGAACCCCCTCTCTCATCTCTGTCACTCTCTTAGGATGTTGAAGAGTGGTATCAAAAAGGAATAT
TGGGCATCTTCTAATAGTGGTACAAGACACCTTGCTTATTTACACAATAAACAAAGTACATTGCTGGAACAAATGCAACTAGATCACTGTAATGCTGACACAAACATGGCCTACAAAAATT
GCAACTCTGAGCTAGCTAAATTCAACTCACTGCATGCTTCTTGGATTATTCAGAGAGCTAAGGTTAAATGGCTCAAATTTGGGGAAGATGATCTGAAATTTTTATATTCCAATATCAATATTC
GCCACAACTGCAACCATATTAAGGGTATTGATGTTAATGGGGTTTTACACACTTCCAGTCAGAGCATTTCTAATGCTTTTATTGATCATTTTCAAAGATTATTCAATACCCCTCAACCTCCTAT
GCATTCCAGCCTTCCTACTGGAAACACCATTCCCTCCGGGATGCTGGATTCGCTTATTGCCCCGTTCTCGGTTGAGGAAATTAAGCAGGTTGTCTTCTCTTCACCCTCCAATGCTTCCCCGG
GACCTGATGGCTTCACTTTTGAATTTTATAAGGCTACTTGGCACATTCTTGGCAACAAAATTTGCTCTGCTGTTCAATCCTTTTTCGCCACTTCTTTTATGCCTAAACATGCTAAAAATACTG
CTATTGCTTTGATTCCAAAACACCCCCATGCTTCCACTGTTAGTGACTTCCGAG
>comp133298_c0_seq1
CATATGTTACATTCATAAGAAATGATTGGTTTCAAACTTTATAGGTAGGTGGAAATGCTCGTAAAAACATATTTGGGCAATAATAACATGAAATTATTTTATTAATCATATATTATGATGAAAAT
CTTATCCTTAAAGTGGATTGAGACTTGAAGGTTTACTATTAGAGAGATACACTTTGGAGAACGATGGTTTCGATGGTTACACCTGGCCCGAATCCCAAAAGAACACCCCACTCAAGACCC
TCACCGGTTGTAGTGTTTCCTTCATCTATAGACCTTTTACGCATCTCATCAAGAACAAAATGCACACATACACCCATTAAATTTCCATACTCAGCAAGCACATGCCTTGAAATCATCAACTT
CTCTGGTTTTAGTCCAACCCTCTCTTCTAATTGGTCTAAAATGGCTCGACCACCTGGATGAACCACCCAAAAGATAGAGTTCCAATTTGAAATACCAAGAGGGGTGAAGGCCTCTTCTAA

GATCTTTCCAATATTATTGGAAAGGAGGTTCGGGACATCTTTATGGATTGTGGCTTTGATACCTCCTTCACTTAAATGGCCTGTAATAGCATGTGATGAGTTCGGCAAAATGACTTGAGATG
CAGATGTTATGACAAAGCTTGCCCGCTCACCAATAGTCTCATTTGGGTTGACACCAACTATAAGAGCAGAGGCACCATCAGCAAATAGGGCTTGGGTAATAAGGTCTTCTTGGTGCTCCTC
AGATGGTCCACGGAACAACATGGTGGTGGTCTCTGCAGAAACAACAAGAACACGTGCACCTTTATGGCTTTCAGCAAGACACTTTGCAAGACGAAGCATGGTTCCGCCAGCGAAACAC
CCATGCTGAAGGATCACAACACGTTCAATATTTGGGCTGAGGCCAAGGGTTTGTAAGAGGTGGTAGTCTCCACAAGGTAGGCCAGGGTCACTCAAGGTGGAGAAGATGAGGTGAGTGAT
ACAAGACTTAGGCTGCCCCCATTCCTGAATTGCCTTGGTGGCTGCCTCCACGGCTAACTTTTGTATCTCCTTCGCATAAATTTCAAGCCTTGTGTTTATAGAGTTTTCCATAAACTTAGAAA
GGCAAGGGTTTTTTGTAATAAGCTCCTCATTCAAAATGAAATGTCGCTTTCTAATTGAAGTTTTATCACAAATGCGTTGGAATTTCTTCTTGAGATCGATCAAGTGCTGGCTGTTGGTGACA
CGAAAGAAATAGTCCGGGTAAGTGCTCTGTTCTAGGAAGTTTTCGGGGGTTGCCTACCAATGGCCAATATGGAGGCAGAGCCGTCTGCTAGGAAGTTTTCGGGGGTTGCCATCTTGGTGG
ATTCAGAACCCAGCAATGTCAGAACCTTAGGCTCTACAACTCCAAATTTTTGTTGAGTAAATAGAGCTTGGCTTGCTGCTCTACTAGGCTGATGATTAATTTATATTCTCAGGAGGAGAAA
AGAGGGCTTGATATTGTTAGTTGTACTTTGTTTTGCTATACAAATCTGCCATAGACATTAAAATTTGTCTTCTACTCTTATATCTTCGCAATACTCTGAATGGACTCTGTACAGTTTCCACCGA
TTAT
>comp133298_c0_seq2
TTCTAATTGAAGTTTTATCACCTACGGATGGAGTTCAATGAATTTATTAATATTTCATCCCATTTATGATAAGATGTTGCAAATCAAAGAGAAGTATTACACTATAATGGAGATTAGATATCGA
TGACTCACAAATGCGTTGGAATTTCTTCTTGAGATCGATCAAGTGCTGGCTGTTGGTGACACGAAAGAAATAGTCCGGGTAAGTGCTCTGTTCTAGGAAGTTTTCGGGGGTTGCCTACCA
ATGGCCAATATGGAGGCAGAGCCGTCTGCTAGGAAGTTTTCGGGGGTTGCCATCTTGGTGGATTCAGAACCCAGCAATGTCAGAACCTTAGGCTCTACAACTCCAAATTTTTGTTGAGTA
AATAGAGCTTGGCTTGCTGCTCTACTAGGCTGATGATTAATTTATATTCTCAGGAGGAGAAAAGAGGGCTTGATATTGTTAGTTGTACTTTGTTTTGCTATACAAATCTGCCATAGACATTAA
AATTTGTCTTCTACTCTTATATCTTCGCAATACTCTGAATGGACTCTGTACAGTTTCCACCGATTAT
>comp133517_c0_seq1
TCCCACTCTTTTTTAAAAAAAATAAATATATAATAACAAACATATATATATATATATAGTATACTGGCAAGAAAACAACAAATAAAAATAAATATATATTTTCATGGCTTATATGATTACATGAGCT
GGCAATTTCTATCTCCATCATCACAATCACTTTAAACTGAATGATAAATATGCTATAGCTCAGCTAGCCAACAAAAAGCATGAGAGATATATATTTTTGGCATGGCATGGCATGGATTGTGAC
AAGTAACGTTCATTGGTCTTTTGAATCATTAGAACACTGAAGAAATTTTCATATGAAGATATATATAAAATGGAACAAGCAGACAAACGACTTCTCCCATTATTTTGGTATAAAATATCGATC
AAATGACTCACACTGCAGCCACTTGTTCATCATTTTACTCCCAACTGAAGCAAAATACTCACCCAATTAA
>comp133818_c0_seq2
GACTTCCTACCGAACAGTTCCACGTTCACCTCCATTCTCTTCTTTGTTCCTTATGCGTCTTTAGACGAAAATAAAGTCTCAAATCCATATCAGTTACAGACTTTGAGCCCAGGGGCATAACC
TCGACCGATCTCTCCTTCCCCATTTCCCATCCCAGAGCTCTTCAAGGCATAAATCATGGCTGAAGGAGAGGATATTCAGCCACTCGTCTGCGATAACGGGACTGGAATGGTCAAGGCTGGA
TTT
>comp133870_c0_seq1
CAAAGTCAGTACTAGTTATGACATCAAACATATGAACTAACATAGTGAAAGTAGATGTTCTTGTAATTAAGGAAACTACGTAGAAATAAACATGAAGTGGATAGATTCTTCTTCAAATAAA
ACATCACAACAAGTAATTAGGAATTATAGAAAGGTTTCAATGTTTTGAAATTAACTAAGAAAGAAACTTAAGTAGTTGGCTAAGTAGTTCAGTTCTGACGCTCCATTTGCTTGAAAACATC
AAAGGCTTCAACTGGAAATCCATTACCTCTTTCATGTTGAAACATGATAGATTTCATGGCTTCGTTGCTGTCAGATGTTGAGTTCAGAGGGTTTACACCACGAGGATCACACTTAGCCCAA
TTCTTTGCTGCTGTGCTGAAGGCTTCTCTGTGTGGGATGTCAGGCTTTGCTGCTTTAATTCGTTGTATCTCATCCCTCATGAAGCGATTATAAGCTGATGGTAAGCGATGCTTCTTTTCTGGA
GGTTTGACAACAAAAGGTGCTTTCGGTGCTTGCTCCATAGAAGTTGATGAAGATGCTGCTGAAAGCTGTCCCTTCCTACAATCATTACATGGATCCTGAAATCCTAACTGGTGATCAGAAT

AATGAGCTTGAAGCAAGCGCCTAGGGTTTAGGAAGGAGAGATGGTTACAGTGCCCACACTTTACAGTGACAGTATCCATTAACCTCTTGCATGGAACTCCAACCGCAAGAACAGTGCTG
CAATATGCACAGCGAACATAGCAGAGATGCTCCCTTGAAGAAACCAGATCCATAAGAAGAGGAAGAAGGAGGAGAAGAGGGAAAGAAGGAGAAGAAGAGGAAAGAGATAGAGGGAG
GAGGAACGAATGAGTGGAAGAAATGAACGTTTCTATTCTAGCAATACTGCCCGCCGGAGGCCTACTCATGAACGTACGGCCATGGAGTAGAAATTATATAACACAAAAA
>comp133938_c0_seq1
TAGTTTATAATATAACCAAGATTTATATATATAATACCATATATTATTAATATTTTGATTAAGATAGCAACCATATTTAAGATAGAAATACATTCCTTATATATATAAGAGCATAGAGCGCTTTATTT
AAGCTAGTAAGTAAATAGGTAATCGGTGGTGCTTTGAAAATGAGGTTTGTAGAATACGTCGCAATTGTATTCAACTACGACATAATGAGACGCTTTATCTTCATTGAAGTTGCGGAGTAAG
CAATATCCTCGAGCAGAGCGGAGCAATCCTGTCCCACCAACAATAGGCAATTCACGCACTTTTAATGACAACTCATTGCGTCCCAAGACTGAGAGCGTGCATCCTCTGAATTCCCCTTGG
GCAAATACAAAGGTCATTGCCATGAGGGAGGCAATCTTGTTTTGGTCTGACAATATATACGTGCCTTGGGACCGACCTAATAAGTGAGACGATTCCTTTGGCCCGATGGTTATCGGATCGT
CGAATACAATCACGAGTCCGAAGCCGGTGGAAGAGGCATTAGCTATGGGGTTTCGAGCGACTGACACAGCCGTGGGATTTGGCTCGCCAAACTTGTTGTGCCAGAAGAAATGAATTTTG
ATATGCCTCTCCTTAATTGGAAAGGTAGCGTAGTTGTTGGAGGTTTGGGTGTCGGCGGTGGTGGAAGTGTGGGAGAGGAGGGGAAGGAGTAGGATGATCAGTAGTAGTATTTTCATTGT
>comp134180_c0_seq1
TAAAGATAAATTTCATTGCCCACAAATTTATGATGATAAGATATCTTAACAATAAGATTTTCAACAGAAATCTAGATTTCTTTAAATCCAACTATACATTTCACATCACGATTAACCCAATGCG
ATTTTTTATAGCAAAAAAATAATATATGTTTTTATAATGTACTGAATAAATATGAGTCAAATTTGTAGAAAATAAAATAATAACCCTAAAATCATAGCAAAACCGAGAAACTTCATACAGGTG
CAAATTCAATATTAATCACCCGCGCATCCAGGCTCGCTATCCGCCTGTACGCGGCCTGTGAAAGTGCGATGGTAGTCCCCGCCATTGACTGCTGCGACCCAAGCCTGGCGGCCTGGTCGA
CGACGGTGACCTCCACGGTGGCGTCGCTAATGCAAGCCGCCGCCGGCTGCGAGCTTATGCAACGCACAACATACCGACGGCTGCACGCCCCACCGTTGTTCCATATCCCGGAGCCGGCG
GCAGCTATGGTGCCGTCGGAGGGGAATTGCGACCGGTCGTTGCCGAAGCACCTTGTTGGAATGTACGGTGGGCTGTAAGAGGAGGCTGTTCCGACGTCAGCGTTGGAGAGGGGGAGGA
GGAAGGAAAAGAGGAGGAGGAGGAAGGGAGGGAGATGGCTCATGAAGAAGATAGGGGAGAGATTTGCTGATTTGGTATGTTCTACAGCTTTTATTTTTTCTTTAATTATTGGAGATTTTT
GTGGTGTTTAAATAGCTGCGACTCGCCGGACTAATTTTAATGGGTCAAATGAAGAAAATATTAATACGCCGGTTAGATGCTAAATAAAAATTTATTGGCTTCTATAGTAAAATATCTAACGAC
GAAGTTGTAACGAAGCGGACGGCTATATCCAATGAATTTTTTCTTCAATTATGCATATTTTATAAATTTGTATTTTTCCTTTTATAAAA
>comp134220_c0_seq2
TTTTTTTTTTTAAAACCAGAACATTTATTGCGTGACTTTGCTGAAATCCTTAAGATGAACTGGATGCCGCAACAGCTGCCCTCTTGGGTTTAGGTGTTGTTCCTTCACGGAATCCATGCCTG
AATCTGCGATATACAATTTTTAGGTGCCTCATTCGACCAGTCCCGGTGGTGTTTCGTCTTTTAGCTTTCGCACTCCAGTTATACTTCCTCTTTCGCTTGGCAGGGTAGCCACACTTGCCACA
GGTCGACTTCTGGAGATGGTAGGCCTTAGAGCCACAGCGGCGGCACAACGTGTGCGTCTTATTCCGACGCTTTCCAAACGATGACGTTCCCTTCGTCATCTCGCCTCCGCCACCGTGACC
AA
>comp134281_c0_seq3
GAAGGACAATTTATCAATTAACTTGATAAAAAAAAATCTATTACAAGATTTTGCAATTTGCTCATAAACTTCCTCATAACGTAAATACATAGGATTTCTATATATCGAGCAAACAAATCACAA
AAGTAAGATATCATATTTCTGGCAATCACTATCTATTACAGATTTTGAATAGTAATAATAGTTCTTCATCAAGGCAAGCAAGCATTGCATTATTCTCCTATAATTGCAGAGCCATTCAGATGTC
AAAAGAATACCATAAAATTCTAAACCCTAAACATTCTCTTTGCTTACAGCAGCAAAATCAGCTTTAACATCTGCTGGTATTGCATGTTCAATAGCCACAGATTGATCTATGGGAATGTGTTT
CTCAGCCATTTCCAAATCCAAATTTGAATTTGTAATATTCTCTACTTCAATATCTTGGTTTGTATTCTGTTCTAATCTGTCGTTCTTCACATCCATGTAGATGAAATATAAAATTATTTGTGCAA
CTCCGAATAAAATCCCAAGAACATTTGGTAACGCTACAAAGAAATCCCTTAGAAGAAGCCCATAAAAGAACCATGCAATTGCACTAAGAGTGAGAAAGAGGGAGAGTGAAAATGGCATA
TATTCCACGCTTTTTGTTCTTATCACCAGCCTCATGATGCTTAAAGGAGCAACAAACACTGCGACGGCAAAGGAAGCACAAATCCAACCAGTGATTTCGGAACGCTTTCTCTCATTTAGA

AAACCAAGTGTGATGAGCAAGACCGAACCAAACAGGCCAACGTTGAAGAAGAATATCATCTTGACGGTTGAAAGCCTTATTTTCTTGGAAGCATAGAAAAGGTAAATGGCTATGTAGAC
AGATTCAAGGGCACATGTGCATGTGTTGATGGTGAGCAGGAGCACGTTATGGTTAAGAAAGGCATAGTAGAGCCACAACATTGCACTGGACAAAGCAACCACATATGGCGTTGATTGGA
AGGAACCAGTAGACTTCTTCCGGTACACCTTATAGAATGTAGTCATTGGCGTTAAGGCCACCAAGAATGATAAAATATTTCCGAGAAGGCCAGCACCAAAAGCCAAAGGGTTCTCAATGC
TGATGGACACCATCTTTATTTATGAGAGGTTCCTCAGTTCTACTAAGAAATTATCTCTAAG
>comp134312_c0_seq3
CAAAAACAAAGAACGACAATGCTAGAAGACAAAAAAGAGATGACATTGGGTACATAAGCAGAAGAGCTAAGATTCAAAATTGATGATTCTTAGAATGAAATTGGTGGTAACCAAGAAA
GACGACAATGTTGATAAGATTAACTAAAGTTGTCGAGAACTAACCAACTCTGATACTATGTTAAAAATCATAAAGGACTACACTTTGCTTGATTAATCCTAATGCTAGTGTAAATA
>comp134389_c0_seq1
AATTATTCTGTGCGGATACCAGACATAATTTTATGTACGGATGCCACGGTACAGAACGGTACCGAGTGGTGCTGAGCAGTACCGAGCTGCGTTACAGTAAAAGGGTGACATGATGCATTTT
TATTGGTCTACGGATGGCGTTCGGTGTCCATACATAATAATTTTTATTAGCTTGACAAGTTAAATGTGAAAGAAGGTGCTTGGCCTCGCCACAAACAGAAGAAGCCTTCTTTTTAGAATGA
AGTACAGAATGGAGCATAACGAAGAGGATTAAAAATAACAATTAGAGAGCAACACAGTAATAAAGTAGGGAAATGAAAGAAAAACCAGAGTAGCATATATTCATCTAAAGAAAACGGTA
AAAGGAACCAGTGGAGCTCAGCTGTTAAATTACTAGAAAAAGAAGACACGAAAAGAAGCTGTTTATTTTCTTATGCTGCCAACTCTTACAAGGAAGAATAAAGAAAGAGAATACAAGG
ACAGGGAATAAAAGCTTCCAAACTTAGCAGAGCATAGTAGATTTTAGATATTTCAGAATTATCATCAAACCGTATTTCTTTTTTGGAGTTTTGAGCAAATTAGCCAAAATTCTCTACAAAGA
GATCAAGGTGAGCTGTGTGTCATCTAACAGTATTAAAAATCTCTTATGGAGTTCGTTTGGGGCGGCTTTCTTAAT
>comp134506_c0_seq1
TTAATAAATGCTCAATCCAATTACCCTATACAATTATCCCAATCAATCAACCGGCAATGGATGGTGCTCACCCCGTCATAATAATCGGCGCTGGGCCGGCCGGCCTCGCCACCGCCGCCTGC
CTCTCCGTCCGATCCATCCCCTACCTCATCCTCGAACGGGAGGACTGCATCGCTTCCTTATGGCGCCACCGCACGTACAGCCGCCTGAAACTCCACCTCGACAAACGCTTCTGCCAACTC
CCTCACATGCCTCCCCCCGCCGACTCACCGACGTACATGCCCAGGGACAGGTTCATTAGGTACCTGGACGACTACGCGGCTAAGTTCGACGTCGTGCCGGAGTTCGGGTCGGACGTGGA
GTCGGCGGTTTTCAGGGAAGGGGATGGGAAGTGGGTGGTGAAGGTGAGGAGGGTGGAGAATGGAGAGGAGAGAGAGTACGTGGGAAGATATTTGGTTGCTGCCACGGGGGAGAACG
CGGAGCCAAACGTGCCGAAGATTGAAGGGATTGAAGGTTTTACTGGAGAGTTGATTCATTCGGCGGATTATAAGAACGGGACTAAGTACGAAGGTAAGAGAGTTCTGGTTGTTGGTAGC
GGGAATTCGGGCATGGAGATAGCTTACGATCTTTCCAGCCACGGCGCCGTCACCTCCATTTGTGTGAGAGGCGAGGTTCAGGTGATGAGCAAAGAGATGATTAATTTTGGAATGCATCTT
TTGAAGTACAATATGCCACTGACTATTGTTGATTCGTTTTTAATTTTGCTTAGCAACATCAAGTATGGAGATTTATCCAAGTTTGGGATTAAAAAACCAAAGGATGGGCCATTTTTCCTCAA
GGCCACTGTGGGAAGATCTCCTGTTATAGATGTCGGGACCATTCACAAAATCAAGAAAGGTGAAATTCAGGTTTTACCAGCACTTTCATTTATAGAAGGCGATTGGGTGAAATTCACCAA
CGGAGAATCGCACTACTTTGATACAATATTGTTTGCCACCGGATATAAAAGCTTAGCAAACAAATGGCTTAAGGTGATATTTTGTTTATATATCTACACACTATATGTGGTTGAAAATTAGGA
GTGTCAACATTTGACACAATACGAAAATTCGATTTGAATCCGATCCGAAAATTTTGGATTAGATCCAACCCAATTTCAAAACGGGTCATATTTGAATTCTCATATTTGACCTGCTTGAAAAA
CGGGTCGGATTGGAGTTGACCCGAAAACCCTTTTAAAACGAGCTGACCACGCTGCCCAAGCATGCTCGCTCGTCACGTCCGAGCTACCCACCCG
>comp134506_c0_seq3
TTAATAAATGCTCAATCCAATTACCCTATACAATTATCCCAATCAATCAACCGGCAATGGATGGTGCTCACCCCGTCATAATAATCGGCGCTGGGCCGGCCGGCCTCGCCACCGCCGCCTGC
CTCTCCGTCCGATCCATCCCCTACCTCATCCTCGAACGGGAGGACTGCATCGCTTCCTTATGGCGCCACCGCACGTACAGCCGCCTGAAACTCCACCTCGACAAACGCTTCTGCCAACTC
CCTCACATGCCTCCCCCCGCCGACTCACCGACGTACATGCCCAGGGACAGGTTCATTAGGTACCTGGACGACTACGCGGCTAAGTTCGACGTCGTGCCGGAGTTCGGGTCGGACGTGGA
GTCGGCGGTTTTCAGGGAAGGGGATGGGAAGTGGGTGGTGAAGGTGAGGAGGGTGGAGAATGGAGAGGAGAGAGAGTACGTGGGAAGATATTTGGTTGCTGCCACGGGGGAGAACG

CGGAGCCAAACGTGCCGAAGATTGAAGGGATTGAAGGTTTTACTGGAGAGTTGATTCATTCGGCGGATTATAAGAACGGGACTAAGTACGAAGGTAAGAGAGTTCTGGTTGTTGGTAGC
GGGAATTCGGGCATGGAGATAGCTTACGATCTTTCCAGCCACGGCGCCGTCACCTCCATTTGTGTGAGAGGCGAGGTTCAGCAACATCAAGTATGGAGATTTATCCAAGTTTGGGATTAA
AAAACCAAAGGATGGGCCATTTTTCCTCAAGGCCACTGTGGGAAGATCTCCTGTTATAGATGTCGGGACCATTCACAAAATCAAGAAAGGTGAAATTCAGGTTTTACCAGCACTTTCATT
TATAGAAGGCGATTGGGTGAAATTCACCAACGGAGAATCGCACTACTTTGATACAATATTGTTTGCCACCGGATATAAAAGCTTAGCAAACAAATGGCTTAAGGTGATATTTTGTTTATATAT
CTACACACTATATGTGGTTGAAAATTAGGAGTGTCAACATTTGACACAATACGAAAATTCGATTTGAATCCGATCCGAAAATTTTGGATTAGATCCAACCCAATTTCAAAACGGGTCATATT
TGAATTCTCATATTTGACCTGCTTGAAAAACGGGTCGGATTGGAGTTGACCCGAAAACCCTTTTAAAACGAGCTGACCACGCTGCCCAAGCATGCTCGCTCGTCACGTCCGAGCTACCCA
CCCG
>comp134661_c0_seq3
AATTTGAATTTCAAATACTTTCCTAAATTAAGAAAGCTAAATTAGGAAAGTCATGCAAATCTGGTTTTAACTTCTTAAAATCTGAAATTGATTTCCTAATTGAGATTAGTTAATTTAGGAAAT
GTCCCACTATTCAAATTCATTTTAAATTTGAAACAGAGATCTTCTAAATCTCCTTTCTTAATTTAAATATACAATTTTAGTAACCCCTTTTAAGCACCCAATCAAATCCCCATCAAGGACCGG
CCCACTTTCCCTCTCTAACAATTGGATGGGTTCAAATTCAAATTAACCTTCCCTAATTAAACCACCAAGCTAGCTCTTCCTACAGCTGCTGTGCCCTCTCTCCCTCCCATACCTAAATAAGAT
ACTCCACTAAGTTTCCAACTCAGCCCCACTCTAACCCCATCCACCCTAGTCGTCACCCTCAATTTCCCTCTATTTTCCTGAAGCTCCTACTTGCTATCAAGGGAATTTTGGCTAGGAAAAGG
GGCTGGCCTGTTGTTTGAACCCCCACACTGCTGTCCAGAGCCTCCATTGGAAGAATTTTCCTGCTGTCCAGGAAATTCCCGTCCAGATTTTGCTATCCAGAAAATTCCAGCCAAGGTTCTT
GCTGTCCAGAAATTTCCACCCAGGACCTTTGCTGTTCCAGCCCTCCTAACGCTAGGAAACTGCTGTCCTACAAAACCAGAGCTGCCCTCTTGCTGTCCAATGTCCCCAGAAGCTACCGTT
TTCGTCCAAAACTTTCTGGAATTGTTGTCCCAGATATTCTACTTGTTGTCCAAGGGTTCGAGAAGGAATGTGTTGCTATCCAGACGTCCAAGGAGCCACCACTGGCTGTCTACTTCCTCTC
AAGCCCTCA
>comp134723_c0_seq2
GCTTTATTTGATCTCAACAAAGAAGCATCTATATTAACTTTTAATTAATTCAGGAGGAGAAGGGTGCCAATAATTGTAAAACAGCTGAGAAAGCTGATTAACACCCCAATTTTTCGATATGA
GATTATAGCGATATGAAGTAACAACAAAGCTAATATTTTAGTTATTTATAAGCATTTATGCTGTTTTTTTGTTTGTAATATGGGATGCAGGTGTGGCATTAGGAGGTGGATGGCAAGCTCTTGT
GGCTTATATAAACTTGGCTTGCTATTATGTCTTTGGCCTTCCTTTGGGATTCCTACTGGGGTATGGATTGCAGTGGGGAGTTCAGGGTATATGGGTTGGAATGCTTTTGGGAACTGCACTGC
AGACTTTGATTCTGTGCTATGTGCTTTGGAAGACCAACTGGAATGATGAGGCTGCACAGGCATGCGAACGAGTGAGATTGTGGGGTGGAGAACAAAAACTTATAGAGATGGGAAAGTGA
GCTTCTTTTATTGGAATTTAATTGTTTTTTTAGGTGGATTTTGTGAATTGTTTGGTGGGTGGAAAGTAAAAATCTACCAAGAAAGAAATAGCATAAAGATTTTTTCTTTTTCTTTTTGGTTTT
AGGAGATATGAGCCTCTGCATAAAAACATATGCAATTTTTTTTTTTTTTTAATGGCATTCAATCCATCATTCTCTAAGTCAAATCTGTGGGGTAATCCTCCTTCGGCCTTTTCTCCTATAATGA
TTTCTTTGATGGTAATGGTTACTCATCTTCCCCTAAATCTAATTTTTTCATGATGTGTTTGTC
>comp134727_c0_seq1
GCTTTTCGAGCATTTTCTTCACCCTTACCCAAGTGACAATGAAAAGCTTTTCTTGGCTTCGCAAACAGGCTTGACAAGAAACCAAATTTCGAATTGGTTCATAAATGCTCGTGTTCGGTTA
TGGAAACCCATGATAGAAGAGATGTACAGAGAAGAGTTTGCAGAGGACCAAGGAGACTCCAATAGTTCTCCTTAATAGTTGACATGGCAATGCTATCAGTAGCAAGTAGTGGCACAAAC
TAAATGTTCACAAAATTGGGTAGCTGAGAGGTTTGGGGATGTGCTGAATGTAGTTTTTGTAGAAATTGAACTTTTTGCCATGCTGAGAGATCATATCTAAATAGGTAAAAGCGGCTGATTC
AGTTCAAGTGTTCGTGAAAATCTTCAACTAAATGAATATAAGAGAATTTGTTCCATGACTTGAGGCCTAAACAGTTCGATGTTTTTGGA
>comp134807_c0_seq1
ATACTGATGGTTCTGTGGGTATTCAAAAAGCTGGTATGGGGGGGGCATTATTTGCAACCATGTGGGCTACGTTCTTAATGCTTTTGCTGCTCGTTTAAACCATTGTTCTGTGATTTGTGCTG

AGTTGCTGAGTCTCTGTGGTTGTCTGTCCATTTGCCATCGAAAAGGTTTTTACAATGTGAATATTGAGGTTGACACCATCCTGGTTATTCATATTATTCAAAGGGACAATATTGGCCATCCTA
ATTTGTTTTATATTATTAGGAAAATTAAAATGCTTCTTGCTAATTTGAATTATATTATCAACCATATTTATCGGGAAGGTAATACTTGTGCTGATTGGTTGGCTAAAATGGGTTGTATTGTGCCT
TCTTTGCAAGAATTTGAGGAAGATAATATTCCCCTGCTGTTGAGAGGGTTAACGAGACTTGATAAAATTGGGCTTCCTTATATTAGAACTCCTTAAGTTAAAGAGATGATGCTTTTTGCTGG
GTGTTCCCGCCCTCCTAGATGTCTTCTGGTTCCTTGGTGTTACTGCTACTGTTATCACTTGTTTAGGTTGGGTTTTATTCTGTCATTCTATTTGGGAAGTTCTCGGGTGGTGTCACCGACTGC
TTACTCTTCTTGGTTAAATGTTCTTGGGGTCTTTGCTGCTGCTTCCTTGTTCTGTTTTGCAGAGATGCGAGAATCCTGGGTTCTCTATTCGTAACCAGGTTTTTTCTAAACTTTTCTTTATTTT
TGTTAGAGTTTTTGCTGATTTTTTTTTTTTTTTTTGCTAATGGAGGCTACT
>comp134926_c0_seq1
TTAATGATACAAATGGCTGGAGAAATGTAGATTTACCTTTTCCATTACCTTGCGTGATAATCGCCTTTTTTAAAGTGCCTGCACATCTTTGCATGCCTTTATTTGGTCCTTCACAGTGATTGC
GGATCCTGGAGGGGGCGTGTTCATATCGGATCCTAAGAAGCCAGCAGGTGGTCACGTGCTGGCGCATGCAGCTACCATCCGGCTTATGCTGCGTAAAGGCAAGGGCGAGCAGCGCGTTT
GCAAGATATTCGACGCCCCTAACCTCCCAGAGGCTGAAGCTGTGTTCCAGATAACACCAGGTGGCATTGCGGATGCCAAGGATTGAGGCCTTCACCTACAGTGGAGTAGGATAAAGAGA
GTTATTTGAGAATTTTGGTGGATATACGATGGTGTAAATGATTGCACTTTTGGGCCTATCGGCCTGAAAATTATTTCAACTGACACTCTTTTTAATATTACATGGATACTTTTAGGGGTATGGG
CTGC
>comp135074_c0_seq2
AGTCTTTCTTAGAGCAAGATCATGATTAGCGTATGGATTATCTTTTCATTAATTCACATTTGTTTCTAAAAGATAAGTGTTGGGGTTTTAAAGCTTTAGTAGTAGCTTTGTGTTAGAAAGCCT
TTAGTGTCTTCTTGGAGTAGGTAACCCTCACTTAAGTTAGCTTTCTTTAAGGTTAACTTCTAAAATATATATAATGTCGGATAAACATAAAATAAAAGAGAGTCTAGCCTTAAGTTTAATTTT
AGAGGTTTGTCTCCCCTAGTTGCTTTAGGCTCTGTTTGTAGTGTGTGGTAGTTAGAGGTTCCAAACGTATCAAGTACCTCATTTGGATTGTGGGAACATT
>comp135095_c0_seq1
AAGGGGATTTTGCAAATTTTGGAAATCTGGAGTAAAGAAATTTTGATAAATTATTTTGCTGTTTAGATCTTTTGTTGTACGAACATTAAGTCATGAAGATGTTTCTATAATCGGAGGCACATG
CAAGTGCACAATGAAATCGAGCTTTCTGCTCTTGGAATGGCCATTGCAACTACTGTTTCCGTTGCGGAGATTCTGAAGAACAGTGGATTTGCTACTGTGAAGCAGATATTGACATCTACTG
TGGATATCAAAGATGACTACAGGGTCCGACCATTGCAGAAAGCAAAGATTTCAATATTACTGGGCAAGACTGAAGAGTTCGATGAGTTGATGGCTGCTGCTGCTGCAGAAGCGAGCGGT
GGTGCTCAAGGGGATGAACAGAACTAAATGATTATATAACTACTGTTCATTTTTCATCTTTTTATTTGTATGCACACATTTACGGTGTTATTTTATTGTGAGATCTGAACAGTAATGATGCTCC
GATTTGCGCACCTATAATTCAAATGCTTATTAATTTGGCTTGGAGCCGTATATAATCTATCTTTGTTGAAAAA
>comp135147_c0_seq2
ATTCAGGTTCTACCTGAATGTGTGGGTCACCTATATCATCAGGTGGATGGTAGGGAAAGATATCAGCAACATTGAAGGTCTTGGACGTGCGCATGGTATCTGGTAAGTCAACGACATAAGC
ATTGTCGTTGATCTTTTTACTGATAGAAAATGGCCCCCATTTCCGCCTACTTAATTTCGACTGCGGCCCCGGTGGCAGTCTTTCCTTGCGAATACGAACATAAACTAGATCTCCTGGCTTGA
AGATCTTTTCTCTTCGGTGTACATCCGCTGCAGCTTTATACGTCTGAGCGGAATCCTGTAGTTTGAGGCGAACTTCAGAGAGCATCTTGACAAATTGGTCTGCGAATGCATAAGCGGCTTT
ACTCTGGCTTTTGGGAAGAACTGCAACGTCAAGAACAGTGTTAGGCAATTTGGTATAAACAATACTAAACA
>comp135167_c0_seq1
CGCATTGAAAATTTTCTTCAACTATTATTTATTACAATTAAAATTCATGTTCATCATAACGAATCAGCCTTAAAATCAAGAAAAAAAAATCAAGATACATCCGAACCACCACCGTCTTCATGAA
TTATTGGATTCTTTTATTTCAAAATCGCAACCGCCGATCTCGTTGCGCAACCACCGGATCAGCCGGCGAGCCACATTCCCGCTCGATGAGCCCCCCTTGACATAAAAAATACCAGCAGCCA
ATTCTCCCTTCTTGTCCCACTTAGCCCCCAGCACCAACATTCCATTCTCCCGTATGACCCTCGTGATCTCCCATAACAAACTGTCTCGGTACTCGAGAACTCGCACCTCCAGTCTCATCCAC

CAAGGCCCCGGCCTTCGTTCTATAGCCGCCATGAGGCACTCGACGAGCTTCCCCTTCTCCATCTCCGTCAGCCTCGACCAACCACCCCTTTTCTTGATGTAGTATTCCTGAACAGCAATGG
TTTCTTCCGTGCTAACAGAGGCATGAACCACGTCATAATCCAAGTCCTCCAGCGTGCACACCACATCAAAAACCAGCTTTGGACGGTCCCTACTCCTCACATTCACCTCCACCATGTAATT
ATTTCTCACCGACACCTCCATCTCCACAGCCGTCACAACAGCTTCTGATGCCAATGGAAAATCAGTCCTTCCGGCCATCCCCTCCCAGTCATTGTCCTCTCGCATTATCTGATGAAGCCGTC
GCTCAGCACTGTGCGCACGATCAGGCGACGGCCCCGAAATCTCCAACTGCCATTTCTCAATACCCCTAATACAATGGGCTTCAACCACACGCCTCAATTGCACCTTCAACCCGGTCGTCG
GGGCACGCGAATTCGGGCCGTAGAAAATGAAACCAGCGCGGCCATTGTGCGCCCATGCTACGGCGGAGTTAACCTCAAAGGAGAAATCGAGGAGCGTAGCCAACACCTCGGAGAGAA
GGCCGGGGTGGTCGGTTACCGTCATTTCGAGCACGGCGTGTTCGGAGGAAAGGTGGCCTGCACCGAGCAGGTTGTTCAGGCATGTCCTCACCTCATCCGGCGGACGGCCGCTTCCTCCT
CCGCGCCTTTCGGCGAACATCAATGACTGTTCTATGTAGCGGATGTGCGCCGGGTCCGTCATTCTGTTGCCGAGTTCATCCGTCACGTGGAATACATTCATGATCCATTCCCCATCGGCTGA
GACGGTGGATTTGGAGATGATGAGGTCGAGATCGGTGATGGCCTGAACTATGTCAAGCAGTATTCCATGCCTGTTCGCACCGTTCACCTGCATGCAAAGTAATTGGCCATGCAAGCGTTAT
ATGTAATGTCAATATATATATATGCAGTA
>comp135203_c0_seq2
TTGTTGTCAACCATTACTAGTCACAACCATAGCCTGACAACCATCGCCCACCACCACTGATGTCCTCCATGACTAACTTGCATAATACTTAATAAAAATAAAAACAACAATTGTTTAGAATA
CTTACTTGTTTTTATTTCATATTAAATATAAACCAAACAAAAATAAAAAATATCAATAAAAAAAGAAATACATAACGGCACCTAAGCTTTATAAGGCCAACAAACTGACAGATGGATTGAAT
AACAAAAAAATAATAATAATACAACAAGATAATGTGAAGAGCAATAATACGAGGTATAATCTTGCCGATAAATTTGGAAAAAAAGTATATAAAATGAAAGAAAATTTCAAATGTGTTGGTG
AATAAAGAGTTCATTAGCTTGATTTCCGGATGCTGATTGTCCACAAGAGATCTGTATCAGAATGAACTGTAACTTTCTTCTCTCGCACAATATTGGATGTACTCACCAATCCACCAAAATGC
ATAGCCGTTTCAGAAAGATCCCATTGGAGACCAGTGGTGGTAGTACTAGCTGATGATGTACCAACAGGAATCAGACCACAATGTGGGCCTTCTACAGATGACTCGATGTAGATCTCATGAC
GATGTGTTTTTGGAAGAAGATAAATTAAGCAATCATCTGACAGGAGAATGATGCGCATGTTTGAAAAGTAATAAAGCACATTGATGTTGCCAATTTCATGATCAAATCTTCCACCAAGCGC
GCCGACAACAAGAATATGTAGATTTGATTTCTCCAAGATGGGTGTGTAATCACGAATAAAAGCTACACACTTGTGTAAGTCG
>comp135365_c0_seq2
ATCTCAAGACAACCAGGATGGTGCTCGTGAGATCACTGGTACTGGTGAGAAATGCGGCTGTCTTAAATTCTCCAAGGCAAAGAACTATAGTTCTTCATGCAATATGTAGTTTCTCTTTTCA
GGTACGGAAAACTGACGTTCTTCAAGAAGTTTGTCAAAGATAGAAATGAATTCTTCATGCAATAGTTTTGTTGCTATATTTCCTACCTGGTCTTCAATACCTAAACCTATAGTCATTGTCTTC
AAGAATATTCATTGAGATTAAAAATAGATTGTGCCAGAACTATGTGAACATATGTTTTTCTTAGATAGTTAAAAATCACGAATATTATTTTTTCCTCATTCAAGATATCACCGGATCATGCTAA
TGATTCAAGCCAACTTAAGTGCACATTTCTTTCTTGAATGATAAGATTTCTGCATTTAATTCT
>comp135675_c0_seq1
TAAACATATTAACTCAAACTCAAATTTTTTAGATCAAACCCACATCAAATTATCATCCTTTCCACATATAATATAATAGTACTTAGAATATATATAATACTTGCATATATGAAATATGTCTACAGT
GAATATTGCAGTTATCACTCATAAAGTTTTATTAAAATATTAAAATATATTAAATAAATATTTATAAACGAATCATGAGCAAATGCGCAACAAGATTCCAGCTCCTATAAAAATCGAAGGTCG
GTGGTCCCCACAAATACTTTAAGCAGAAGGCCTCGAAATCGCCGTACCCGATGACCCCGTCGCCGTCGCCGTCTAACTCCGCCAGCAGCCACTGGATCTCCTCCGCCGATATCGTATTCA
GCTTGATCATCGCCTCACTGATCTTCTCCGACGAGAGCTTGCCGTCGCCATTGGTGTCCAAGTGGCGGAAGACCTGGTCCACCTCCGGTGTAAGCTTGGACCCGTTCATCTTTCCTTCCTC
TTTCTCTTCCTCTGCCTCTTTTTCCTC
>comp135726_c0_seq1
CAAATGAAACCTAAATCTCAGTAAAGGTGGAACAAAGAAGAATGACCATTGTATGGATGTTCTTTACATGTTGGGTACTTGGAATTCTATTTAATTGGGAATCTTTTTTTTTCTTCGGTTTTA
CTGCACAAGCTCTCTGAAATCTCTGTTGCTTTGCTTCTGTATAAGCTGCTCGTTGAATCCAACGCATGTAGAGTTTCGTATTCTGCTTTGGTTCTACTTCATCATTGTGTATACCATCACATTA

TTACTGAGTCTTCCAGCTCTACCAAGAGGGAGCTGGAAGAGCAGTCCTTGAAAACCACCTTCACATTCCAACTTAATTGATTTCTAAAGTAGATTGTAAAACAAAGTACAGCTCAACA

>comp135759_c0_seq2

CGCCAACAACAAAACATGACAAAGACCGCGGCTATTTTAGATGCAAACTAGATTCGTCTATTTATCCTCTCCAAAAATCGAGAGTGTACGTACACGGGATGGGAGAATCGTCTAATTTAGG

CTTAGGTACCGTACCGCTCGGTATTAGCTCGACACCGGAAGTGGCACATTTTAATTTTTCCAAGAAATTTCAATAGACTTAACGTTAGGCTTCTTCACCTCCTCTTTGGGTACGACAACACT

AAGCACCCCATTCTTCAAGGACGCTTTCGCCTGATCCACCTTCACGTTCCCCGACAGTGGGAATTGGCGGACGAACTTACCATTGCCTCGCTCATGTAGATGCCAGCAATGGGTCTTCTCC

TCCTGCTCCTTCCGCTCGCCGTTGATTTAGAGGACCCAGCCTTCCACAACCTCGACCTTAACATCCTCCTTTTTGACGTCAGGGAGATCGGCCTCAACGATGTGAGCCTCCAATGTCTCCT

TCCAGTCGAAGCAGGGGTTGGCAAAAGTTAAAACCTCGGTCACAAAATTGGAGCCGGGAGCATAGATGGCGTTGTCGGAGAAGGTGTTGAAGCTCTTTAATGGATCCCATTGGTCAAAT

AATAAAGGCTCAAAAACTTTGCCTCCGCTAATTGTCGACATCAAACCTTTAATTGATTGAACAAGATTCGATTCGGCGAAGTACTTGAGAATCGAAAAGTGCTCGCTTGATTTTGCAGGA

AAGAATTGCAATGAACATTTATAGATGAGGAGGAAAGAGGACAGCACCTTCTCG

>comp135972_c0_seq3

ATTTCCTTCCCTATAAATATGAGATATAGTAACATTCATCTCCTTAACTCTATTTTTAACCTTTCTAATCAGATAAAAATAATTAGCATTACCCGTAATCCCCTCAGTAATACTTTGAACAATAA

AAAGAGCATCTAATTCAACCCACACATCATTATACCCATGTTTAATGCACAAATCAAGACCCAGAAGTAAAGCATTGAGCTCAGCAGAAATAACATCAGAGGTTTGCAAAGGAGAAGCA

AAAGCAGCCAAGAAGTCACCACAATAGTCCCTAATGATTCCCCCAGCACCGGCTTTCACCACACTCACCGAACAATCCGTATTCAGCTTAACAAAAGGAAGTTTAGGCTTTCTCCAATAC

ACATAAATATCATCACTAGTTCCCGAAACCACCTCCAGCTTAATCCCAAAGAAAACAGCCAAATGTCGGCATCTATAAAAGGTTTTAGCAGAAAGGATGTTGTAGTTAAAAAGCTGCAGC

ACTTTACATTTAACATTTTCAATAATAGAAATAGCATCCATCTTACAATTCTCATGTTTGGCCAGATTTCTAGCATTCCAGATAAACCAAATAATGAGAATGGGGATCAGATTAAATAAATGC

CCTTTTTTGGGATGACACCAATTATGAAACATTAAGTTAAGAGAATCAATATACAAGGAGTTTTTATCAGCAATATTATTGAAATAGCTCC

>comp136186_c0_seq1

TAAAAAAAAAAAAAAATTAAAAGCCGAGTTTGACTCCTTGCGCGGAAAGAGCACAAAAGACGGCGAGACGCTGGTATCTCTCGCCCCATAAATCAAAGCCAAATCGCTCCATCTCAGTA

TTAGATATTCGCAAAACCATAAAGACAATCCATTCAACCAAAAAACTACCCAAATGTGACATAAATCAGAAGTGAAATTAACTTACAATGTGTTAGAGTGCATGCAGCACGGAGAGCCCT

GCCTTCTTCTCCACCTTTTGGGGTTGTGGTTTATGATTCTGCTGCTATTGATGGCTGAAGGGATTTTGGCGAGAGGGCCGAGAAGCGGAGAAAAGAAGTGACGAAGAGGCCGCGGTGTG

AACCAATGATGTGCTGAATAAAGCAATATGTCGTTGATCAAATCCTTTCAAATTATAGGGTGACGGAAATGAGAGAGAGAAAAAAGTTTACAAAATCCTTTCAAAATGTGAGAAGAAAAG

TGTTGGGGCAGTGCTGAGAGTGATAAAAGTAATGTAGGGGAACGAACTTTTACGCCAAGCTCAGGATGGAGAATGGTTGGAAATTTAAGATGTCGTGAAGAGGAACCGTTATAGAAGTT

GAATAGGCTAAAATAATTGGATAAGCCACCGGAGAAGAAGATTGAAGGTCAAGCCGAAGAATCAAGACCGAGCAAATCGTGAATAATAGATAAATGAGGCTTAAGTGAGCATTAATTTGT

TTAAATGAAATTTGAACATATAAACGCGCATTAATTGGCAATTGGTGATTCTACCGGAGAAGAAGGCGGAACGTCAAAGGCCATGCCGAAACAGAGCGATATAATAGGTAACCGACGAGG

GCTTTATTTTGTTTATTTAAAAGAAATTAACACGTACAAATGGACATTAATTAGTTTGTATTAGAATTAATTGTGGCAGTAAATA

>comp136257_c0_seq1

CAATCTTCAAACACTCTTCTGCGGCTCTACAATCAAAAACCTGACTACACTCACCTCAAGATGTTCGGCTGTGCTTGCTACCCGTGGCTGCCACCCTCCAAACGACACAAACTTGCCCCC

CGGGCAGCCTCCTGCATCTTCCTAGGCTATCATGATGCCTACAAGGGATACAAATGTCTCGATGTCACAACTCGCTAGCTTCTCATCTCTCGTCACGTCACCTTTGACGAAGATACATTCCC

AAACATCTCAACTCCATCAGCTCCCTCATCCACAATTCATCAATCCACCACTACAACTCCTCCCTCTCTACTTGTCCCAATCTCCATTGCCACAAACCCAATCACACCACCGTCCTCCAACC

AATCACTACAAAAATCACCCTCTGCCATCTCTCATACTCAACCAACCACCCCATCTCTCAATTCCACTCCTCCCAACCACCCAACTCCACCTCTAAAACACCACCACATGATAACCAGGGC

TCAAACAGGACACCTCAAACCCAAATAATTACTCAACCTCTTTCACCAACTCAATGCCACAGACAAATCCATCCAGTCCGATCCCACCTCATTCACCGTAGCCTACAAATCGCCTCACTG

GCGTGCAGCCATGGCATCCGAATTCCTAGCACTTCAACAACAAGGAACGTGGATCCTTGTCCCACCACCTCCCAAACACACCATTCAAGGATGTCGTTGGACCTACAAAACCAAACATC
ACTCCAACGGTTCTATCGCCAAATACAAGGCTCGGCTTGTTGCTCAA
>comp136458_c0_seq1
GAAAGTAACACGAGGGGTCCTGTTCAGAGGCGGCGACCACGGCGACCCCCTTTCCTGCGGGTGCTGTCGGAGGGGATGGGGGTGACGTCCTCAATCCGCCCAATCTTCATTCCCGAGC
GGGCGAGGGCTCTGAGGGCTGACTGGGCACCTGGCCCAGGGGTCTTGGTCCGGTTCCCGCCCGTGGCCCGGAGCTTGATGTGGAGGGCAGTGATGCCCAGCTCCTTGCAGCGCTGGGC
CACGTCCTGGGCAGCCAGCATGGCGGCGTACGGAGAGGACTCATCCCGGTCGGCCTTCACCTTCATCCCGCCCGTCACGCGACAGATGGTCTCCTTGCCCGAGAGATCGGTGACATGGA
CGAAAGTGTCATTGAAAGACGCGAAGATGTGACACACGCCGAACACGTTTTCCCCTTCAGCCACCTGCGGGCCGAGGCTGATGACCTGCTCCTCCTTCTTCTCCTTCCCCTTGCGCGGT
GCCATTTCTGCGCGTCGCCTTCAG
>comp136606_c0_seq1
CTAATTTCAAACTCCAAAAGGGGCAATAAAGTTTTTCTCTTCACTAACACAAGACCTTAAAATCAAAGGGTATAATTACGCTATATAATAAAGAGAAGTGCACAAAAAGTTTTATACATTAC
AACAAAACTGTTTGTTTAGCAAATAAATAAATAAATAACAAACAAGTGTATGCCAAAATTTTGACAACAAAGAATAACATATATGGATGATTATTATCAAGAATGCCTCGCACAGTGAGTTT
GGTAAATCTAAGGCTTCGTTTGGGACG
>comp136658_c1_seq2
ATGATATTGCCACCATAGTTTGTTTCTTGAATGACCAAGATATAAGAGTTGTACCAAGAAATGAATAGTATCCCGACGTTAATTTTCGAGAGAGCGGATCTCCAGCCCAGTCAGCATCAGA
AAAAGTTCTGAGTTGATGATCAGACTTGGTAATAGGTAGACCAAAATCTTGTGTTCCATGTATGTATCTAAGAAGGCGTTTGAGCATATACGCGTGGTATGTGGATGGCTCGTGCATATGCT
GCGAGAGAAAATTAACTTCAAATTCAATATCAGGGCGTGTTAACGTCAAATATTGAAGTGAGCCTGTGATACACATGTAAAGATTTAGATCAGATACCAAAGGATCTAAAGTAATTTTGGT
AGGCATCTTGGTACACATAGGATTGGATAAGGGATTACACCGAACTAGATTCGCCTGCTGAAGAATGGAATTGGCATATGCACGTTGAGAGAAAAAGTGTTGATGATCTGGACTTGATGTA
ATTTGGATTCCGAGAAAGTTATGGGGTAGACCAAGATTTTTCATGGTAAATCTTGATTCAAGCTGTTTAATGAGATGATTGATGGCTGATTAGTTGTTGCATGTTAAGAGGATGTCATCGAC
GTATACAAGTAAATATATTCTAGTTTCGTCTTGCTTGAAAGTGATC
>comp136704_c0_seq2
TACTGTATGAAATTATATAATATTTTGTCTTTTATCTCTACATAGAATATATATATAAACCAACCCAAAATCTCCCACATACCAAAGCTCTTCTCTCACATTCTTCTTTCTTTTTCTCATCATCTC
CTCCTCTTCTTTGTTCTTCTTCCACCTCTTCTTCTTCCATGATAAAAAACGCTTCTTTACATTCACAATGCCCTCCCATTGATCCCGAACGCTTACTCTGGATTTTCATTGGTGAATTTGGATC
ATCTCTGACTGATGAAATATTTGTAATTGCTACGTGTTGACAGGAGAGCAGGGAATTCAGGGAGGTCGCCCAGAGGAGCATAGGGGGGTGGGAG
>comp136738_c0_seq1
TTGATCTAAAGTAACTTTCACATGTATTTCTTGTTTTTTTTAAAGCATTAAGAGATATTCTTCTGGATTAATAGAATAATATGTTGGGTCAACATAAACTATGGTGGCCCATTAGTAAGAGTTC
GAAGAATAAAGTCAATAATTATAATTTGGTTGGTATACCAATAAATGTGAATGCATCTTGTGGTATATTATTAAAAATCCCAAAATACTTGGGACGAGTGCCCTATGATGCTTCAGAAGTGCT
GGCTAATAAATATGTTCAATGAATAGATATTTGTTTAATTTTAATTGAGAAAATGATGAAAATGGTCCCTTGACATTTTATGAAAGGTCAAAATGGTTCCCAACTATTTTAAAAGACCATTTT
GGTCCGTCAACGCTTTAAAGAGGAATTAAAATAATTCCACCCTGAGACTATTTCAGTTCTTTTAAGTATTTGAGGGACCAAAATGATAATTTAATATAATTGGATATTATTTTAATCTTTCCTA
AAGAATTAAGGGGACCATTTTAATAATTTCTCAATTTTAATCGTCTCAACAAATGTGTATATATAAACATGTATATGTATATGCCTTCCACACATATACATGCAATTGATCTATTCATATTTAGAA
AAAATAATAATACACATGCTAAATTAAATTACAATTTTTTAAATAATTTTAAAAATAATAGGCTTATCTTGAATCAGATTTCAACTTGAAATATATTATTTGTTGAACATGACTTGAACTTGACT
AGAGTCAAGATTGCAACCTTCAATTTCAATTTGAGGTTGAATCACTCTAACTGG

>comp136751_c0_seq1
CCGACAAATCTATCAAAGGAGACAAGAAAAAGAAGTCTTTGCTTCCAAGTATCTTTGGTAAAAAGGGAAGAAGTGGAAGAGAGTCAGATGGAGATGTATTACCAGATTTGAATCGACAA
TATGCGGCGAGAAGTTTTTTCTTGGATGCATCTCCTCTTATCAGAAAAAGCTTTTCAGATAGACCTTCGAATTCACGTATAGAAAGCCTGAATCTTTGCACCTTTGAACGACCGTTGGAGG
AACAAATCATAGAGAAGAAAGAGTACAGGATATTTGTTGGGACATGGAATGTAGGGGGTAGACCTCCACACGTTGGCCTCAATCTTGAAGATTTTCTACAAGTTGAAGAAGGCTTAGATA
TATATGTGATAGGGTTTCAGGAAATAGTTCCTCTGAATGCAGGCAATGTGCTTGTCATTGAGGACAACGAACCCGCTACAAAGTGGCTCGCGCTCATCGGTCGAGCCCTTAATAGGCCGA
AATGCAGATCCTTGAGTGATAGGGAAGAGCCTTCATCAGATTCCAACTCATCAAAGCACATCAACCAAGTGAAAGAGGAGAAGACAGGAAGAACCACCTTCCAGAGGTCCTCACTGAA
GGTTCTAAGCAAACAAAATAGAGCAAATAGTGATTTGGCCAAGATCTGTAACTGCTCCCTCGACCCATCTAGCGTGCACTGGAGGGCGAGGGAGATGAGGGAGTTCATAAAGAGGATTG
AATCAATCGCTGTTGATGAGAATTCTGCCTTACCATGCTGTGGTGGTCAAGAGAAAGTGATTGGAAATGAATATTGTTTGATTTCCAACAAACAGATGGTGGGCATCTTCTTGTCTGTGTG
GGTGAGAAATGCTCTTGTACAACACATTGGCCATCTTAGGATTTCCTCTACAGGGAGAGGGATCATGGGTTACCTTGGAAACAAGGGATGCATCGCAATGAGCATGACAATTCATGAGAC
CAGCTTCTGCTTTATCTGTAGCCATTTGGCTTCAGGAGAGAAAGAAGGTGACGAGTTGAGGAGGAACTCCGATGTCACCGAGATCTTGAAGAACACGCAGTTCCCTAAGATATGCAAGA
GAACAACTCGTCGAGTACCTGAAAGAATCATAGATCATGATCGAGCATTTTGGCTTGGTGATTTGAATTATCGAGTTGCTTTGAGCTATGAGGACACAAGAACATTGCTAGATGATAATGA
TTGGGATGCTTTACTAGAGAAGGATCAGCTAAATATGGAGAAGGAAGCAGGAAGAGTATTTCATGGCTGGAAAGAGGGAAAGATCTACTTTGCACCAACTTACAAGTACTCTCACAACT
CTGATGAATATGCTGGAGAGACAGTAAAGTCCAAGAAGAACCGTCGAACCCCGGCATGGTGCGATCGTATATTATGGTCTGGAGATGGTATTGAGCAACTACAATATAACAGAGGAGAGT
CGCGGTTCTCCGACCACCGGCCGGTATGTGCAGTGTTCAAAACTCAGGTGGAGGTTAGCAACAGTGGCTGTTACAGGAAGGGCCACTCCAGTTCAGGAGGAAGGAGTTCCATTGATGA
AACCATACCTCAAAGGCACAGCTTCTATGAGTTCTGAAAGGAAGAGAAGTACATTTATCAGCTTCATTTTGTATATATAATTAGTCAAATATTAGGCACCCTTTATTGCTTGTTTTTGGAACA
ACTAACAACTATGGC
>comp136787_c0_seq1
CTAACCCTCTAGACATTCATACATGTATAAGCAAGTTTAGGGATTTATGAATGAAAGTACGATAATAAATAGAAGAGCGGGAAGTGAAAAAGAAAGACGCTTAAACAAGATAGAAAGAGA
AGAATGAAAATCATACATAAAAGGAATATGGGCATTTTGCTATTGGACATGATTCTTTGAAGCCAAACATGTCTCTAAAATGCCCCTCCCTCCTATTCAATGCAATATGGATAGGAGAGGAA
GTGTTTTAATGCCATTTTACTGTCCCCATACTCCAAACTCAGCACTTGATACAATACTCAACTCAAATAAACTTAACTACCCTCTAAATAAAGCTCCAATCGACAAA
>comp136843_c0_seq1
AGCTATTCCAAACGAAACCTAAATCTTTGAAGTTGATGACTGCATATCCATCAATGTAAATTACTAGTATTTTGCATTAGGTCATACTAAATGGCATCTATTTGAAGATTTGGCCCTCTAATTA
TTGAACTCAGCCATGAAAGCAAAGAGCATCTCACATAAAAAGTCTTTTTACTCTTTTGTACTAATCTGGTGCCAAACTTACTGCCCTACACTTAATAGAATGCATTCTTTGGATTCTATGAA
TTTGAAATGATATAAAGTTGCATGAATAATGATTAAACGACAACTACTTTATGAAAAATGTCAATGTCAATTGCCGAAAATAAATACAATATTTCCAAATGATATTACCCAAAAAGGCTCAA
CACATATTAAAGACACCTTTAATCTATTCCTTTCCTAATACTTCCTTTCTTCTTTCCATTACGAGTTCTCCCCTCAGTGAATGAAATTGCCTACTTACACATGATAAGTAACAGAACTAGCGG
CACTATAATGAGTTTGCGTTTCATTATATCTATGTGTTAAAATAGAGCGAAATGAATGAAAGACTAGCAATGATTTGGATGGGTACAAAATTGGCTCAGAATATCTTCACCAAATTTTTGGTA
AGGATGGATCTCAAAGCTGTTAGATTGCTTTGAGATGAACAGAATATAAATTTTGATTGATTCTCCATGATATGTTGCATCCATCTCAAAGTAAATCCAACGACTCTGAGATCCATTCGGATA
AAAAATTAATCAGGAGTTCTTAATACATAAATTTTCTGTCTTGAATTTGGATCCTCTCCAATTACCAGGTTGCAGTTACCAATCCAAGACTTCCAATCCAAATTGGATGGTTGGGATTAAAAT
TTTCAAATCAAGAGCGGTTTTCATAAAATAAGCTCGAATTAATCCCAGTCATCCAAGCTTGTGCACTTACCTTGGAAATTGGAGATAATCTGAATCCGGCATTTTTAAGATCTACAAGATTG
GCAGTATCAATTCATGCACAACTCAAAACCAAGCAACTGAATTTTCTATCTCACAAGCTTCATCCAATCCAAGCAATCACAAGATACAGATCTTTCAGCTCAAAATCCATAAACCTACACA
TTAATTCCTAAAATCTCTGCATTTTCTATCTGGGTAGGAAAAAATAACATAACCATTCAAGTAAAGGAATGGATCCACTGAATACAGTAATCAATTAAAGCATAAGAAATCAAACCCTCGAT

TCCAAACCGTTCGCATTGTACATGAAGAATGAACAAAGAGAGAATCAATCCTCTTATTCCCAGGTCACATTACAAAATACATATGGAAAAATTTAA

>comp136986_c0_seq1

TTTTATTAATAGTTAAAAATCTCAATAAAAAAATCAAAATGAGGTGAGTATAACAGCAACTGCCTTTGACAATGCAGCGCAGCAAAACCCAATTCATGACATGTTGACAAGAGCAATAATG
CCGAAAAGGTCAGAATCCTTCCTGCCAAAATATTCAAGCAGGACAAAAAACAATTCCACCCGTTCCTTTATAAGAAGACCAATACTTGCCATTAATTAAGATCAAGCAATGTTACTTCGCG
CGCAACCTCTGCGATCGCAGCAAAGGACGACCCACCCGTCCGCAACGATACCATTCCGCCGACGCGAGCAGCCGCGACCCGTTCTCTCAGTTCCTCACCTTCCTCAGAGTTCATCAGCC
AACGGACTCGTTCCGCTATTTCATCGGAGTGTACCAGCCCGTCTGTTAAAGCCCGCATTGCCACCGCGAGCGCCGCCACCTCAACCAAGAAGTTTTTGTTCACCATATGTTCCTTCTGCTG
AGGCCACGCAATCATCGGCACTCCGGCTACGATCGACTCCAGCACTGAGGTCCAACCACAGTGTGTCACGAATCCCCCAACCGCCGGGTGTTCTAGCACGGCCACCTGCGGCACCCACG
ACTTCACCACCAAACCTCTCTTTTTCGTCCGTTCTAGAAACCCCTTAGGAAGCGCCGCCTCCAGTTCCTGTGAGACCCGCACAACCCACAGGAAACTGTGCCCGGTACGCTCCAGGCCG
ACAGCGATCTCCCTGATCTGCTCACCTGGGAGGGCTGATCCCCTGCGCTCAAAGCTGAGAAAAACTACGCTGTTCCTCGGTCGGCCGCTTAGCCACGCAAGACTTTCGTGTTGAAGTTC
GTCGCTGCTGTATATTTCTTGGCAATCTTTGGCTGTTATGGGACCGACGCAGTAGACAGGGGCCAGGGCGCTGCTAGGATCGCGGTTGCGTTCCGCAATAATCTCGAGTGCAACCGGCTC
CAAAATCTTGAAGGTGTTTACGATAATTCCCGCAGACTGGGTTAGTCGTTTATACTGAACAATCATTGCGTCCGTCTCGGTTGTTTGACCAGTGATCCGAATCATGTCCGATGCCGGGATCG
GGCACAGTGAACGAGGCAATTGTATCGAGGTGCCGCCGGCGTTCAATTGGTTGTGGAGAGCAGGGAGTTGGAGATAGACGGCGGAAAGGATGGCAGCGCTAGAGGGGAAAAAGAAGT
AGAACCGGAGATGGACCTCGGCGGCGACTTCAAGGGCGGCGCTTAAGAAAAAGTCGAGGATGATTGCACGGGCAGATGAGGTGTAGAGGTAAATTTTGAGATGGGGGAGGACGAGGT
GGAAGTGGTCGAGTAAGGTGGCGGAGCCGGAGTTCGACGGTGGGTTTACTGGTGGCAGGTGGTTGAAGGAAAGGTAAGGGTGGGCGGTGGAGACGCGGGAGATGTACGTGTCGATGT
GTGAGTTGTCGACGGAGATGATCCGGGCAATGAGGATGGTGACGGAGACGCCGGAGCGGTGCAAGAGCTTAGCCAGCTCCACCATGACGTCAAGGTGGCCCCTTTCAGCCAGCGGGAA
AATTATGATCTCCTTCTCCATCGCTCATAGGTAATTCGGCTATGGCACGATTACTATCTCTCTGCGGATTGGTGCGTTCTATAGCTGTGGAAACTCACGATG

>comp137012_c0_seq1

GCTCCAACTTCCACAAAACCCGCCGGCTCAGGAAACATGTTTCAAATCCATTTATCGTATTCCCAAAATCCTCATCCACGTCGATAACCAACCGTACGAACCCAAACTCGCTTCCTTCGGC
CCTTACCACCACAACAGCCGCCATCTCCTCCCCACGGAGGAGAGCAAAAACAGTGCCCTATTTCAATTCCACCGCCGCATTGCTAGAGCCTCCAAAACCCCCTATGACGTCCTCATCGCC
ATGAGAAGGGTGGAGTCCTGCCTCATTGCTTACTACGATGGCCTTCAAGTCGAGGAGGACAACAAGGATTGCTTAAAACGTGGCCAATTTCGCCGCCGTGATTGGCTCAAGGAGAAGGA
CGAGTTCCTCAAGATTATGATCACGGACAGCTGTTTCGCCTTAGAAATCATGC

>comp137022_c0_seq1

ATTGTACACATATCGCCTCACCTAAATTTAATTTTACAAAAAAAAATTGCATAATTATTTAGTTCAGTTATACACTTATACTATGTGAATTATGAAAATTTTATACATAAAACCTATATTTTACAC
AATCGAAAATTTGAATAAAATCCTGCAAAAAGTTATTTCCAAATAACCCCCGGCAAGAGAATCTAACAAGTTTTGGAGTGATTAGGTAATAATTGTATCTGATTTAGTCATTTAGTATTTATT
ACCACAATTTTTTTGACCGGACTGATTCTCATCTGGATGGGGACAAAGGCCGTAGCGCTAAGACGTTTTTACCCCTCTGACGACGGGTACAGACATCGAGGAAAGGGAATTTACTAGAGG
GCAGAGCGGTAATCCTGTACCTTTAGATAAGCTGTAAAGTGGAAATTTTTTTTAAAACATTTTTTGACCTCGAAAAAAGAGGGAAAACGGCGAGCCGACTTCATTTATGAATGGGAATGG
CATCGACGCTTTGGAAAAAGCATTAAAAAGTCTAAAAACTTTTGCTAATATTACAATTTTCTTGCATCGGGGTAGATTTGAAAATTGAAAAGTTATTACAAGGAAATAAAACGAAATAATT
ACAGATTTTCTCGATTAATTAATAATTTGAAAGATAAAAAGAAAAAAAAATATTATATATAATCCCAAAAGAGAGACCAGAAAGCACCGGTAAGGGCGGTAAGCAAAAGATATTTCCATAA
AAAGCAAAACAGGTCGGAAAAACCCTGCCACAAGAAGCAAGCATCGACATGAAAAAGGAATGATCTATTGCAGAGCCAATTTATTTTGTAAAAATTAAGGAAAATTTAAATTTATTTGCA
AAAAAAAATCAAATATGTAAATTTCAAATTTTAAATTGCAAAATTAATTTTATTAAAGTGATGAGCAAATTCAAGAAATAATAAAAAAAAAGGAAATTGAAGTACTCCCTCTGCCTAAACCC
TAACGCGAAGCAACGCAGGAGAGAACAAGGTAGAGAGAG

>comp137025_c0_seq1
CATTAATCATCATCGTCCTCATCGTCTTCATCAACACCTTTCTCTCCTCATAACTCAACTTCTTCAAATTGTACCTGACAAGTAAAAAGAAGAACGCAGTAAATAAAAGTAAATAACAGAA
CAAACTAAGAAGCTTTTGGTAGCCAATTCCGATTCAAGCCACAAATGAAGGAAGACGTGCTTTAAGCTTGCAGCAGCCAGTACCTTATAGAACTAAGTCATAAATGGAAGATAGTAATTT
AAGTTGCGTTTGGAGCAGCTTTAACACAGTTTTGCTTTTCATATAAAAAATAAATTTCTATTTAGCTATTGTTTTCCTGCTATATATATATATATATATATAGGGGGGGCGCAATTACTCCCCAGGC
TTTAGGTGGATCCGCCCATGATATATATCACAAAAAATTATAATATTTCATACCTACACATATAAACAACTTTAATATCGATTATGCATACTTATATCTCATTCATAACATCCACCACCCTATAAG
CTTAAATATCAAATATTTATACTTTTTTTGGCCAAACTCACACACACATCACATATGCAATACTCGAATCCAAAGCATCTTGGTTAGTAGCCAAAGCTCTCAACCAACATACTAAAATGAGTT
GCATATTTTATTTCAAATTAACTTATCTGATTTTTTAAGTCAATCCAAAATTAGTATATATATATATATATAATTTTTTAATAAAATGATAATTACCTCTCTTTTATAGGATCTAATTATAGCAGGAAC
AGATACATCTGCAGCAACTATTGAATGGGCGATGGCAAATCTAATAAGAAATGCATGTGCCTTAAAAAGGGCACAAGAGGAGGTTTGGCGAGTAGTTACTGGCAAAGAAAAAGTGCGAG
TGAACGACCTACAACATCTTCATTATCTCAAGTTAGTAATAAAGGAGACATTGAGGCTTAATCCGCCCATCCCATTGCTACCACCCCGAGAGTCCATGAAAGATACCAAGATAAATGGTTAT
GATATTCCGGCAAAGACGATGGTATATGTTAACGTATGGTCTATTGGAAGGGATCCTAAGTACTGGAAAGATCCTGAGCTTTTTATGCCTGAGAGATTCGAGAACAGCCTTGTTAATTTTAA
AGGGAGCGACTTTGAGCTCATACCATTTGGTGGAGGAAGAAGGATCTGCCCTGGATTGGCACTTGGCCTGGCTAATATTGAGCTGTGTTTGGCAAATCTACTCCATGAATTTGATTGGGAA
TTTCCCAATGGAATGACCAAAGAAGATATTGAAATGGGGTCTGCACCTGGGATGGTTTCTCCAAAGAGAACGCCACTTATTTTGGTGGCAAAACCGGCTTTGAACAAGTGACATATGGTT
CATATGTAATAATAGAAATATAAATAAAAGGTCACTTGATCAAAATGCACTTATAAAATTATATATGAAAAAATAATTTTATATATACTATGAGCCATATTATCATGATTTTTAATTCCATAAGAA
AATTCAAATTGAATTGTTCTATATGAGAGACCAATGATTTCTCTCCCTAGAAAAACATTTTAACACTCGACAAATTTCTAATGACTAATCAGCCTTG
>comp137082_c1_seq1
ATTATTTTTTTAATCTTTTAATAAATAATAATAAGAGGTAACTAATATCATTTATCAGTAATAAATAGCAAAAAAGATTCTATAATTTAAAATATCAATATACAAATCTATATTTATTTTTAGAATT
TAGATATAATACTAAAAACTGCACAATCAAATCATAACTATTATATTATGAGTATAAAAAAATAATTAGAACAAAAAAGAAACAAAACCTAAAGAATTCTGACTAGCCTCACACCCCTATTC
GCTAGCCCTGACATGAATTCATTTAATCATATATAATGAGCTTAAAAAGCCAAACAAGTCACCAAAACCTACAACTGAATTCAAATCACGGCAAATGAGCAATATGAATGCCAACAGTCAA
ATTATAACAATTGGATCCATATGATAATTGAATCAATA
>comp137107_c0_seq1
AACACCATGTTAAACTATTCATATAAGAAATGAGAGAAAAAAAAATTTATTCATCTAATACAAAACAGTAAGAGAGGTGCGTATTTATAAGAGCTAAATAACCTATATTTTTGGAACAATGAG
CTATTAAGTCAAATTAACACATACAACACATAAGCACTTAGCATTGGCCTGAAACACGTAATTTCTTTCACATCTCTCTTCATTGTATAATGGCCAAATGATGGATAAAACATGTAAATACAT
ATTTTCTCCCAATCACCTTAGCTTGATGACTCTGAAGACTCTGCTACTTCGAAACCCTCATCCATTACTTTGGTGGAGTGTGTCAACAAACGGTCTAATTTATGGTGAGACGAACACTCAG
ATTCACCTTCAGAAATCTCATCGATGTCATCAGAGCCGCCGCTGCCAGTTTCCGGCTGTAGCCGGCGGTACAACTTCTCAACAAAGTTATGCTCCAACCAGCTGATGTACAAGTTGTGCCT
CTCCTCTGTCCATGCCTCCCCCATCTTCTTCTTGGATTCCATCAACCGTTGCTTTCTTCCCAAACCCAGCAAGTCCCATTTATACAAGCAATAAACGTAGATCTTTCACCATTGTTCCATTAT
GAAATTCTATTATGAAAGTAGATAAAATATCTCAGATATTTTTGTGCTGCAGGAAGGGAGCAGCAAAAAATCCATAGCAG
>comp137129_c0_seq2
GAAGAAGAAGAAGAAGAAGACGGTGGTGAAAAGAAACGCAGAAATGAAAAATTCATTGGCTAAGAGCGTTAGCCGCCCTCTTCATCCATATGTTTTGCTGGCTTTGCTTCTGTCACTCT
TAAAGCCTTTTCTACACTACCTGAGCTTTTTAATGCATACAGATCAAATGAAAACCCGGAATTTTATCGCCTCAACGGAAGAAGAAGACGATGAGAGTGATGGCCCTCAAAAGGACCCGC
CACCTGGTGGAGGTGGTCAGACTAACTGATCAATTTTGTTCCTCCCAATCGAGTTAACGTTCCATGCACATATATCGATCTAGCTTAGTTGATCAAGTTCCTTAGAAAGTTATGAATTCTTTC
TGATAATTTGTCTTGTAATAATAGTTGCGCACGTCGGTTAAGTTTATCGTCGATATGCCCTCATTTTCTTATGAAATTTATTAGTTTTCTGATCTCTGAATTTCTGCGAGTTGCAACTATATATT

GCGCAAATGAACAGTAGAAAGCAATAAAAATGATACAACCAAACACAATCAACACGATGTGATTAATGTGGTCCAG
>comp137319_c0_seq1
ATAAGTTACCGTGGCCAAATCCTCCACCGAGGTAGAATACTGTGCCTTGTCATCTGCAACCTCTGATGTTATCTGGCTCCGCCGTCTTTTAGCTGACTTCCACATCCAGCAAACTACACCTA
CTCCCCTATACTGTGACAACACCTCCGCCATGGCTCTTGCTCACAACCCGGTCTTCCACGCATGAACCAAACATACAGATTGAATACCATTCTATTCACCAACATATCTCCTCGGCTACTGT
AATCATCAACCACATCAACTCAGAAGATCAACCAGCAGACATTCTCACTAAACAATTACCTCTAGCTCGCTTCGCTCAGCTCAGGGACAAGTTAAGCAATCGACCATTGAAGCCTCAACT
TGAGGGGAGCTGTTAGGACATCCAATATGCACCGATTGCACATAACAATTTATGTGCTCTCACCACGTGAGCAATTTTATGCCTTCCATTACTCTGTTCTCTGGCAGTGGCTAACGACCTAA
GCTCCATTACCACTTTACACTTTATCAACTATATATATCACATGTAATAGGATACAAAGATATGGAGGAAATACTTTAGCTCAGTCCCTTCTCATCTTTCTCCTTCTTTCTTTAGTTGCTACTAA
CCTTTTGCAGGTACTTTCATGGTATCAGAGCATTGGTAATCGCCATCTTCCCTTACAATGGCGGAATAGGAATCCTCTTCAGCTACTCAACTGATGTCACTCCAGGCCTCAAGACCGGATCT
ACCCTCCTCCATGGCCACGACGGAGGTTACTATAACTCCCTCCCTTAAGTTCTTGATGACGAATCTTAAGCTCATCATACCGAATCAACTCACCAGCGGAAACTATCCAATATGGAGGCTCT
GGATTTTCAACTGTTCACAACGAATGGGTATGAAGGGCATCTAACAGGCAACATCACCAAACTGTCCCCGATAATAGCAGAATCTAACCCAATGGAGTTTCACATATGGAACCTAGTTGA
ACAAAATCTGGTATCTGCCCTTCTCTCCACCATTTCTCCATTAGTACTGCCATACATACTCACCCTCACGTTAGCCAGAGAGATCTAGAGTACACTGGAACTTCGACTACAACCCACGAAC
AGATCTCGAGTAATCCAGCTTAAGAATGAACTGTATCACATTCAGATGAAGGATCTCTCCATGATGCAGTATTTAGCCAAGATCAAATCCCTCGTCGACAACATCGTCGTGGCTGGTTCCCT
TCTAGATCCCGAAGAAATTATACTGCACATTCTAAACGGGATGCCTCCCTCTTACAACCCTTTTAAAACAGCCATCCACACCAGTCAACTCCCCATCAACCTTGACGTACTATATACACTAC
TATGCATTGAGGAGATTCATCAGTAGACAGAGATTCAATGGGACACCACACCAGCCTCCGACAATGTGGCCTTCTAAACTACTCGGGGCAGCTCCTCCCGCACTCGCTCCTACTATTGT
>comp137324_c0_seq2
AGAGACCTCCAGCTAGCTATTGCTCAAAAGAGCTACAAGGAAATTCAAAATAAAACCTAAAAGCCGATGGTCCCTTTTTCTACGATTATAAAATAAATAAAAGCAAAAGCAAGGGCTGCA
AGAAGACAACACCAAAAAAAAGAAGAGCAGATTGATCTCATTCAACACAATCTTCCGGAAAAAAATAGCAAGCAAACCTCAAAAAACTCTGAAGACTATAAGACGTGAAAATGTTGAAA
TCATCGAACTTCCAAAATTCAGAGTAATTATGCCCTATATGCAATGTGGGTTCGCACTCCAAGGGGTGGTAGAAGGCATCACCATGAGGCTGAGCCGACTGCCGATCGTACCCCATAGCAT
GAGCAGTGCTGGGATCCCAGACGTGCTGTGAGGATGCCTGGTTACTCTCTTCCAACCTCCTTTTCAGCGTTTTATTAGCCTCGCAGAGCATTTGCTCCCTTCGCTGTAGGTCAGCAAGCTG
ATCGAGCATGAACTGCGTCCGTGTTGATCTTATCTGCTTAAGTGAAGCGTCCAGCTGCCGTTCCAGCTGTTCGAGTTCCTTGCTGCTGAGGGGACCAAGGTCCTCGCCCAGGAGATTCCT
CTGGGATCTTTGTAAGGCTTCAACCCGAGCTTTGAGTTTCAAGTACTCCTGCTGACTA
>comp137324_c0_seq3
TAGAAAATATATCAATCCATTTCTCATGACGCGCTTCAGTTGGAAGAGACTGTTTACATATTACTAAAAAATTTTCGAAATTTTGATCACATGAACTATTCAGGCAAGTTAACCAACAAGCT
AGTCCAGAACTGCATCAGCAAGTAACTCTAGAGATACAAGTTCGATTTTAAGAGGAAAATAAAAGACACTTGGTACTGTACTTAAAACAATACATGAAAGTTTGCGTTCTCTCACATTTTT
CATGTACATAGCAATTCCACGTATAAAATACAACTGGATATCAGGCACTCATACATTTCCAAAATTAAATGTGGCTTGAACTATCAATCAGATGGGAATAATTGAAAAATTGAAGCCGAGAA
AGATGTATTGTAGTCTCATATGATTATAAAACATTGTCTTCTATTTCAGGCCCTCCTGTTTAAGAAGCTTTATAGATATGTTTACATATAAATGACCTTGAGAACACCAAATAGAATAGAAATC
TTATACAGACATGGCCAAAATTAACACAAAGTTTGTTAGGACTATACGAGGCAATGAGTGGTCTAGTTATTCATGCAAGCCAGCCAGGTGGCATGTAATTATTGACATTTGGAGCAGCCAT
TGGTGCAATCGATAAATCAGAATGGTACCTCAAAAAACTCTGAAGACTATAAGACGTGAAAATGTTGAAATCATCGAACTTCCAAAATTCAGAGTAATTATGCCCTATATGCAATGTGGGT
TCGCACTCCAAGGGGTGGTAGAAGGCATCACCATGAGGCTGAGCCGACTGCCGATCGTACCCCATAGCATGAGCAGTGCTGGGATCCCAGACGTGCTGTGAGGATGCCTGGTTACTCTC
TTCCAACCTCCTTTTCAGCGTTTTATTAGCCTCGCAGAGCATTTGCTCCCTTCGCTGTAGGTCAGCAAGCTGATCGAGCATGAACTGCGTCCGTGTTGATCTTATCTGCTTAAGTGAAGCGT
CCAGCTGCCGTTCCAGCTGTTCGAGTTCCTTGCTGCTGAGGGGACCAAGGTCCTCGCCCAGGAGATTCCTCTGGGATCTTTGTAAGGCTTCAACCCGAGCTTTGAGTTTCAAGTACTCCT

GCTGACTA
>comp137324_c0_seq5
TAGAAAATATATCAATCCATTTCTCATGACGCGCTTCAGTTGGAAGAGACTGTTTACATATTACTAAAAAATTTTCGAAATTTTGATCACATGAACTATTCAGGCAAGTTAACCAACAAGCT
AGTCCAGAACTGCATCAGCAAGTAACTCTAGAGATACAAGTTCGATTTTAAGAGGAAAATAAAAGACACTTGGTACTGTACTTAAAACAATACATGAAAGTTTGCGTTCTCTCACATTTTT
CATGTACATAGCAATTCCACGTATAAAATACAACTGGATATCAGGCACTCATACATTTCCAAAATTAAATGTGGCTTGAACTATCAATCAGATGGGAATAATTGAAAAATTGAAGCCGAGAA
AGATGTATTGTAGTCTCATATGATTATAAAACATTGTCTTCTATTTCAGGCCCTCCTGTTTAAGAAGCTTTATAGATATGTTTACATATAAATGACCTTGAGAACACCAAATAGAATAGAAATC
TTATACAGACATGGCCAAAATTAACACAAAGTTTGTTAGGACTATACGAGGCAATGAGTGGTCTAGTTATTCATGCAAGCCAGCCAGGTGGCATGTAATTATTGACATTTGGAGCAGCCAT
TGGTGCAATCGATAAATCAGAATGGTACCCTATATGCAATGTGGGTTCGCACTCCAAGGGGTGGTAGAAGGCATCACCATGAGGCTGAGCCGACTGCCGATCGTACCCCATAGCATGAGC
AGTGCTGGGATCCCAGACGTGCTGTGAGGATGCCTGGTTACTCTCTTCCAACCTCCTTTTCAGCGTTTTATTAGCCTCGCAGAGCATTTGCTCCCTTCGCTGTAGGTCAGCAAGCTGATCG
AGCATGAACTGCGTCCGTGTTGATCTTATCTGCTTAAGTGAAGCGTCCAGCTGCCGTTCCAGCTGTTCGAGTTCCTTGCTGCTGAGGGGACCAAGGTCCTCGCCCAGGAGATTCCTCTGG
GATCTTTGTAAGGCTTCAACCCGAGCTTTGAGTTTCAAGTACTCCTGCTGACTA
>comp137386_c0_seq1
AAGAAAGTCATCCCAAATGAAGTCTAAATCTTCAGTAATTCAAAGTTGATAGGTCTGTGAATCTCAAATGAAAGTCATGTATTTATGTAGGCCTTGATACTTCTATGGAGTTTTCACATAGC
AAATTTGCATTCATGGCATGTGAATCGCCCTTATTATCTTGTTTCCCCTCATTTAGATTTCTCCTGCTATGTTTAGCAATGTATTAATAAAGTTCAGAAGTTCGTGTTACTGCTGCTACTGCAA
GGCAATAAAGTATATTAAGT
>comp137441_c0_seq1
CCGACGACTCAATTTCAGTAGTCGCTCCATCCCTGGGATGGTAATGGCTAATATCGGCCACGTTGAAGGTGGCGGATGTTAGGATGTCTGGTGGAATATCTATAACATAAGCGTTGTCACTG
ATTTTATGCTTGATTGCCACTGGACCGATCTTCCTTGGACTGAGCTTTGAGTATGTTCCAACTGGAAATCTTTCTCTCCTCAAGCATACCATAACTAGTTCTCCCGGTTGGAAACTCATAGA
GCGTCGGTGTACATCGGTTGTTTGCTTGTATGAGTCATTGGAGGCTTGCAAATTGTCACGAACAGTATCCAGCATGGTCTTGAAGTCTCCAGCTAACTTTGTTGCCGCAGAATTGTGGCAT
CTTGGAAGAACAGTGATATCTACAAGATGGTTAGGAGGTTTAGTGTAGACGATATTAAAGGGGCACATACCGGTGGATCGGTTCGGCATTGAGTTAAATGCAAACTCAGCTTGGCTCAAA
GTTTCGTCCCACTTCTTCGGGTTGTCTTGTACTAAGCAACGCAACATGTTGCCTAGAGTGCGATTCACTACTTCCGTTTGCCCATCTGTTTGGGGGTGGTATGCCGAGCTTAATTTAATGTC
AGTCTGCAACTGCTTCCACAGTTCCCGACAGAAGTGGCTCACAAACTTGACATCTCGGTCTAACGTAATGCTCCGGGGCAATCCGTGGAGGCGTACGATTTCTCTGAAAAACAAACGGG
CAACTCCAAGTGCATCAGAAGTTTTTTTACAAGCTATGAAGTGGGCCATCTTGGAGAATCGATCGATGACAACCTTGATTGAGTCATTTCCCTTATGCGTGCGGTGTAGGCCGAGGACGA
AG
>comp137464_c0_seq1
CTAATGTAGTTTTTTCATTACTTTCCGGTTTATCTCATTGATGGCTAATAACAAATCACTATAATTTCAAATTAAGACTGGTGAATATATTATAATTGTACAAGACCTTATATATGAATTTTCATT
AAAAAAATTTATTTTCACTGTAGATTGTTGAACTTCTCTTTATTTGTACGCGTGCAGGATGTTCATACAAGCTAAGAAATATTCTTAAGATATTAAACTTTAGGTTTGAACAAAGACCGGAG
TTTTTCTTGTTGGAATTCCCTTAGAAAAGTAAACGATGGTTTCCTCTGGGTATGAATAATTGCCCCGTCCTCTGTGTCAATGCCGAACGTCATCCATAGACCAACCAGAATGCGCCACATC
>comp137530_c0_seq1
TAATTCATTATATTTGAGGAGTGCTCATTAGTTACTAATTATGTGTGTGCAAACAAGATAACAGGAATCAATCCATTCTCAATTAGACACAAACATTTAAACATCAATTACAGTTACATAGAA
TCTCAAATGTAAACATTTCACACTGTCAAAAGTCAAAAGGAAATTTCGACACGTGATTTGCAGTAACAAATCACCATCTATATTATTTGCAAAAATACTTAAAAAAAAAAAAAGGATGTG

AGAACTAAATGGGAATTGAACTAGACACACGGGGGTACACAACATTAACCGGATGAGCCATGATGCGCCGGCCCTTCATGTGCCGGAAAATATACACCACCGCACCTATGGGCCACTCCA
CCACTGCCGCCACCGCACATGCTAGAATTCCCACCGTGGGCCCTACCACCTTGCACCGGCACGGGTTACTCCTTGTCCCACACTGCACGCACAACATTTTCCTCCAACGTAAATTTGATAT
CGTAAAGTTTCACAGAGTATCGGCCGAGTTCTAAGATTAGAGAAGAAATGATTACTATAAGTTCACCAGTTAAGCTCCAACCCCCTGCAATTTAAGGAAATCGAACAGGACGCGTGGCGA
TTTCCGCTTACGCTGAAGTTTT
>comp137601_c0_seq3
ATGCCTTGATCCGAGTCCAAATCGTCCTATTTGACAAATCAAATATTTTTCAAAATCTATGTACAATTATTTATTTATTTCAATTTAAATATTTTTAACCGTTCAATTATAAATCTTTTACATAA
ATTTAAAACATCAGCGCCTGGGCAGTTTCCTTTCATTTTCGACTTTAACGACCCCCCAAACCTCCGCTGGAAAAAATCTCTGAACATCCTGTAAATAATTTGCAAAATTGAGTATTTTGTCC
CAAAAATTTGCATTTAGATGAATACAAATTGATGTAAGAACTACAAAGCTTCCACCCCTCCGAATTTAAATTTATCTGAGAATATCCTTACGGAATTTTTTTCCACTTACCAGTACCACTAAA
GTAAACAAGTAGTTCTCATCGGGAAATGCTGCGAGTACTGCTGATCAGAAGCCATGGCCTCTTCCCAGCTTGCCAACACTCCTCATAACTCCCTCTTCTCTTTATAAATATCCTTTGCGCTG
CTTACCCCCTCTCACTCTCTCTCTCTCTCTCTCAACAGGATGGAGCTCAGCGTAAACGAGAAGCTGAAGCTGGCGAAGAAGGGAAACAGAAAAGCTTCTGCGGCCGGCAAAGTGCAAA
CAAGGGAGAGGAGGCCGAGCGCGGCAGCAGGGCGGTCATTCGCGCGGCGGTGCGCGAGCTTAGTGAAAGAGCAGAGGGCGAGATTCTATATCATGCGGCGCTGCGTTGTGATGCTTAT
TTGCTGGCGAGACTTAGCTTAATCACCGGCCGGCCGTCTACCTCTTTCTCTCTCTCCAGATTTGTTTCTTCTTTAAGCCAGATCGTCTTCTCCGCTTGTACAGATGGGAGCGAACAGAGCA
AGGAGAAGATGAAGAAATTAGCCATCTTGCTTAAAAATTTTCTTTTTTTCTCTTTAGCTGGCTATGTATAATAAAAAGCTGTGAGCTTTAGGTAGTAGCTTGTACTTTGTTGTTGTTGGATCT
AGGATCTAATGTTGGGGCATGCAGTGCATGACTGAAACTCTGGAATCTGTACTTGGATTTGAGTAAGGGAAAGGAGATTAATTTTAATAAAAATTTGTTTTTGCGAAAAA
>comp137654_c0_seq1
CGCCAGGGGCCTGCCTAGGCTGCGGATTTTCTATGATTTTTACATAAGGTTAGCTGCCTGCATATACTCTGCAATATTGTTCTTTTGCTGCTCCTGATTCCTTGTTGTTTACCACTGGAATAA
GAGTTTTAGGTTTTAAAAGCTGATGTAACACTATTTTCTTTAACATTCATGTCAATTTTGTTGAACTATGTAGTCTAAATGAGATGAGGCTTTTACCTGTATATTATTTATGGTTCCCGAAAAG
AACACTGTTTTGAGTTTGTTTGAAATTTAGCTATCTAATCTGCCGTTTTGATGCGATCAC
>comp137728_c0_seq1
TTAATGGCCCTCCGCGGCCGGCCTCCTCCTTTATTCCTTATATAGCTCGGAACTGAATTCCCTCCTTCCTCCATTCTGTTTCCCGTAAATCGAAACCCTAACCGAGTTCTCCGAGCGATCAG
GGGAAAAATGATGGCGTGGAACCGGCCGCCGTATAAAGGCCCCGATGTCGAGGCCGGCGGAGGAGCGCGGTCTCTCTATCCGTCGATGTTGGAAAGCCCGGAACTCCGGTGGGCGTTC
ATCAGAAAGATCTACAGCATTCTATCCATTCAGATGCTTCTTACTATTGCAGTTTCATCCGTTGTCATCTTTGTCCGACCCATCTCCCACTTTTTCGTGTCTTCCGGTGCCGGCCTCGGCCTC
TATATTTTCCTCGTCATCTTGCCATTCCTTGTGTTGATCCCTCTATATTTCTACCACCAGCGCCATCCATTAAATCTGTTTCTCCTCGCCCTTTTCACTGTATCCATCAGCTTCGCGGTGGGATT
GTCATGCGCATTCACCAAAGGGAAGGTTATTCTTGAGGCAGCCATTCTCACCGCAGTGGTTGTCTTCAGCCTTACTCTTTACACCTTCTGGGCAGCAAAGAGAGGCCATGATTTCAGCTTT
CTCGGCCCGATATTATTTTCCTCACTCATTGTCTTGATTGTTTTTGCCTTCATCCAGATCTTTTTCCCAATGGGTAGAATCTCACATATGATCTATGGCGGCTTGGCTGCTCTAATCTTCAGCG
GCTATATCATCTATGACACAGATAATTTGATTAAACGCTACTCCTATGATGAGTACATATGGGCTGCAGTCGCTCTATACCTCGACATTATCAACTTGTTTTTAGCTCTTCTTACTCTTTTCCGA
GCTGCTGATGACTGAGTCATCGTAAATATTACAAGTTGTTATTTTGTTTCTGCTACAAGCTTTGGATTTCAGTATTAAGAATGTTTTTTTTTCCTTTCAATATAGGTTACAGTGCTTTTTTTTG
GGGGTTGGTTCTTTGTAAAGTTGCTTGAACTTATCCATGAATGAAGTTTGATTTCATGATATTTGATTTTTTATTGTATTACTTTGAGGTCAAGTATTCTTGACATGGCATGGTTGGAGAATGT
TTGAAATTAGTGTTTGCTTAATTGCTAGGTGATTCCAAGG
>comp137750_c0_seq1
TTGATTGAAGAAGAAAAAATATTTCTTAGATCTAATGCATCGGGATTCTCCTCCGAATGAATCCGGACGCTGCACCTAATAATTCTCCTTATGATGATGTTGTATCTGTCAGAACTTATACTC

TGAACATTTATAAAGTTCCATGCTGATTATAAGTTGATTGAAGAGAAAAAATATTTCATAGAACTAATCAAGGTGGGCAGCTGACTATGTGAACCGGAGTCACAGGTATTGTCTCCCACCA
CTCCTTCAGCCTTCTATTTCCTTGCTTGCTCTTCTCATGCCTTGTGTTCTGAAACTTAGGCATGAAAATATATGTAACCAATCCAATCAGAACAAATTTGAAGGGCACAATCATCAACAGAA
GTGCAACTACACTCAGCCCACTCATTGCCATGTTTGAATGCTTTGGAGTCTTTGAAACAAAGATGGATTTGATTTTTAGTATGCTGATGTTTACTGTCTTAACCATTTCATCAAAGTTTTTGA
GGCCATATTGGGCTGATACTATGCTTTCCACTGTTGTTTTATCTGAGGGAGGGGAAACCACAACCTCTTCAGATACCTCTCCAAGTCTTTTGTGCCTTGCCCACATCATCATTCCGACGACA
AACAGTAGGAGCGCGGCGAGGGCCGGACCAAACCATTCCTTGTATATAGTGATGAGAGTTATGATAAATGAAGCTAAAGAGACTGTTGGCCTTTCCCATGAAATGATTCTTTGGAACCAA
GGTATTAGATTTCTAAGTGGGCTCAGTAGCTCTGTAAGAAAAATAATGCTATCAGTGATTCCCTCTTCTTTAATCCCCCCAACAGTAGCATTGGCGATGCTAGCCTCCTTGGCTTCCTCTCTC
ACCAATTCAACGGTCGTCTCCAGTGATGCTAATGATTCTGCTTCTGGGCTGCTTGGTGTTATCTCACCACCTTTTATATTCGTTGCATCCACCATTGGAATTGCCAGATTCAGACTCTTAAGT
ATGGAGGCAGCACTGCAACAGCTTATTCCTTTTGTCTTCTTCAAACTATTATACAGTTCTTCCAACACATAATCCCCTTTGGGAACTTCATCAAGCAAAGTGAAGATTAAGAAGTTGATGG
GAGGAGGTGGAGCCATTCTAAGCATTTCCCTTGCTGCATGAAGCCTCATTACTCCCAAAATTGTTCTGGCCTGCACTTCCCAAGCTTGCAAGAAGGCTTCTGTTTCAGGGTACTTTGCATT
GAATTTTGACATGAATCTATGCATTAATATCACTTCTTTTATGAGTGCAAACCAATGATCTCTACGGGTGGAGCTGGTCATTTCTGGGAACTCTAATGTCACTGGTTCTGCGAGTAGTGAAG
ACTCGTATGTTATGGCTTTATCAAACAAGGGAGCACCAAAAGGTCCAGTAGATGTTGCTTTCACTTGATGATCTATATCAGAACTCGAAAGATCAATTTTAAAGGCGTTCTCATATGAAATT
GATTTAGAAGCCTCAAAATAGAGTGCAAGATTTGTTAGAGTAAGTCGGCCTGGCCAACTTGTTGAGCCTATGTGACGAACAACTCTTGGAGTCATTCCATCTCCTTCTACATGCAAGATGA
ATTCATCTTCTGCAAGTTCAAATCCTGTTGGTGATCTT
>comp137809_c0_seq2
TTATATTAAGTTTGAGCTATAAAATGATTGCAATCATTTCCATAGAAAAATTATCAATCTTTTTTAAAATAATCTATACAAGATATTTCTATAAACCTCAAGGACTTATAATGGTGTTTAAAAAT
AGTGAATCCAAATGTTCTTTTCTCAATGGTTTAGAATTTTGATGCAATTTACCTTTTGATCATTACTACTTGCTTTCTTGAAAGCAATCAAAGTATCATTGAGGAAAAACCCTATCCCTAGTA
CGATTAAGGCATTAATAATCCTACTTCTCCTTTGGCTATTGATCTATGCTCTTGTTAAATAGGTTGAATTAGGTAGGTTCAAATCCATGTGATATTCCTTACATAGCAGGTAACCTTCTATGGTT
AATTTTATTATGTTAGGTTTGGAATTTGCCAATCCACCCCTCCCAAGTTTTGAATTGAGGCTTCGTTTGGGACAGCTTTTGTATTGCTTTTTAAAATATTTATTAGTACAAAAATTAAAAACT
GCTTTAAAAA
>comp138007_c0_seq1
TTCCTCAACCTTCAGACAAAGCCTCTCTCTCTCTCTCCTTCCCTTTCATGACTTCCAGATTTGAGTTCATATTACGGATTCTGCTGCCACTCTTTCTGCTACTGAAAGCAGGAGTGATAGCT
GTTAATGGCGATGCGTTGGTGACTGGTACAGTGTTCTGTGATCAGTGCAAGGATGGCTCTCGTGGCTTCTTTGATTATCCATTATCTGGCGCTAAGGTGGCTGTTTCGTGCGGCGACCAGT
ACAAGGAGGAGAGCTCCAACTTCATGGGATCCTATTCGGTCCGGTTCGACGGCAACCCGGACCTGAGCGGCTGTTACGCGCGGGTGGTCCGCGGGCCGGACGATTGTGGGGCGGCGGC
TGGCCCGGCCCAAGGGCTTAAGTTCATGTTCCGAATGTTTGATATGGAGGCTTATACCGTCGACCCGTTGCTGTCCCAGCCCAGCAAGCCAATGAGTTTCTGCTCCCAGTCCGGGGACCC
GACGAAGCCCAAACCGACCCCACCTGCGATCAACGTGCCGCCGCCGGTTGCTTTGTCTCGGCCGCCTCCAGCACCGCCACGTTCGCGATCGCCGGCCATCCCCTTTTTGGAAGCTTCCG
CTTGCTCTTATGACAAATGGCTAATGCCGGCATTCAAATGCCACTGGAGGGTGGTCTCACCGGAGACGCCGGTGGCCTTAGCTTTCGGGCCGGTGGCAGCCGGGAAATACGGCCCGGATT
TGACGCTATGGAATGGGCTTTACGGCCGCGGCGACGTATACCGAACTCTTCTCCGGGAATCGACGGCGGCGCTGCTTAACTCTTACAACAGCATAAGGTTCCCTTACCCGACGCTAACTG
TGGTCAGTAATATGAATTCAGCTCTCCTTGGCTCTCAAAGGGAAGCCTTGATGACGGCGTTAAGATTCCGGCGAGCTAACTCCGGCATCTCCGGCCGTTCAACGGTCGCCTGCAATTTTAC
TCCTTGTTCTTGAGGACGGATCTCTGTTTTATGGTTATTATATGCTTATTATACTGTGTTGCTTCCATCTTCGGTTTATCAATGAGAGTATATTGTGGTCATTAGAGCTTATAATCATGATTAGC
>comp138023_c0_seq5
CGTATCCGGGCCTTTATAAGGGGCATGGACTTCTTAAAAAAATACCATTCATCCATTCAAATCACGCTGTCACGGAGATGGCGGAGAAGGCGGAGGCATTGGAGTCGAGCAGGGAGAGTT

CGCCTGCTGATGAGACGGCAATTCATATGGCACCGGCAGAGGAGGACTTCGCCGGAGATCAGACTTTCACTGACGCAGAGGCTTCAACGGTGACGGAAGGAAATCCTCAAAGTACTATG
CAGAAGCACGAGCTGGAGCATTCATGGACATTTTGGTTCGACAATCCCTCAGGCAAGTCCAAGCAAGAAACCTGGGGAAGCTCCATCCGCCCCATCTATACATTCTCAACGGTTGAGGA
CTTTTGGGGTCTTTACAATAACATTCAACCGCCAAGTAAACTGCCTGTTGGGGCAGACTTCCACTGTTTTAAACATGGAATCGAGCCAAAATGGGAAGATCCAGCCTGTGAAAATGGAGG
AAAGTGGACAATCATCTGCTCATCAGGAGAAGTTGATAATTGGTGGTTGTACACATTACTTGCAATGATTGGTGAGCAGTTTGACCATTGGAATGAAATATGTGGAGCAGTCATTGCAAGT
AATGTGTACAACCACCAATTATCAACTTCTCCTGATGAGCAGATGATTGTCCACTTTCCTCCATTTTCACAGGCTGGATCTTCCCATTTTGGCTCGATTCCATGTTTAAAACAGTGGAAGTC
TGCCCCAACAGGCAGTTTACTTGGCGGTTGAATGTTATTGTAAAGACCCCAAAAGTCCTCAACCGTTGAGAATGTATAGATGGGGCGGATGGAGCTTCCCCAGGTTTCTTGCTTGGACTT
GCCTGAGGGATTGTCGAACCAAAATGTCCATGAATGCTCCAGCTCGTGCTTCTGCATAGTACTTTGAGGATTTCCTTCCGTCACCGTTGAAGCCTCTGCGTCAGTGAAAGTCTGATCTCCG
GCGAAGTCCTCCTCTGCCGGTGCCATATGAATTGCCGTCTCATCAGCAGGCGAACTCTCCCTGCTCGACTCCAATGCCTCCGCCTTCTCCGCCATCTCCGTGACAGCGTGATTTGAATGGA
TGAATGGTATTTTTTTAAGAAGTCCATGCCCGTTATAAAGGCCCGGATACGCTCAAGGT
>comp138159_c0_seq1
ATCTCAAGTGTACAAATCCAGTCTGCACAATCTCCCCCTGCTTCGCAATACAAGAAAAAGATGGATTTAACGAAAACAACAACAAAAATCACTAAAACACGATATACTTTAGTATACAATT
ATCAAAACAAAATTGTTCTAATCAAATCCAAGCTTTAGCTCCAAAACCGGACTGTTTCCACCTTCAGCACTCTCCAATGGCGGCGGCAAGTTTAAATCCAGCAAGCACTCGTCTTCCGAA
CAAAAACAAGAAGAAGAGGAAGAAGGAGAAAATTGATCTCGAAAACCCTTTCTTTTCTTACTTTTGATTGAGTCATCAAAGGAAGAAGACAATGGCTGGATAATGGCGGCACCGCCGA
GTTCGCCGGAGGAGGTTTCCTCAGGTGGGCCGTTGGCGAGTAGAACGAGTGAGGAGGCGACTTGGTGTTCCTCGTCGGTGAACTGGGCCCGGGGAGAATGCGGCGGCGGAATAATTCC
TCGCCACTGGCGCTCTGGATGGCATCGCATGTGACCGAACAGAGCTTTCCATGAGGGGAAGAGCTTCCCGCATTCAGTGCAAGGCGGAATGGCTTCGGGCATGCCGGAGAACTCGGGCT
TTTTTGGGGCCCGTCGCTTTGTGGCTCCGGCGGGTGGTTTTGGTGGTGGAAAGCGTTGGGGATGATTGGAAGGAGGAAGAGGAGGCTGTGAAACGGGAGGATCCGCTGGAGGAGGAG
AAGAAGGATTTGAGGAGTTCATCATGGTGAAGTTCGAGATTTCATTATCGAATTCTTCGTGCGTGAAGATCGAGCGAGCGGCAAATAGCGAACAAGAGAGCGGGAGGGGCTCTGTGTGG
GTGAATTCGAACGAGGCGAGAGGG
>comp138208_c0_seq1
GCATTGAAAATAGTTTTTTTATTGAATGTTTTATTCAAAAGAAACAGAGATTAATTAGCACATAAAAATACCACCAAACAAACAAAAATCAAACAGGATAACAACAACAAATAGTATATGA
ATTTTTACCCTTTACAACTCATTTTTACTAAGCTACGAACAGTTATTGATCGAATAACTTAACTCATAGCTAGTCCGCCATGCGAAGAAAAGCTCGGCAGTCTTCAGATCCTCACGAAGAAA
ATACTGAGCTCGGGGCCGCTGTTAGTCTCCGGATTAAGCATGTAGAAAGTCTCCGAATCCTTGCTGCCAACAACGCGGTCAAAATGAGCCCGCATATACGCAAGCTCGCCGTCATTTGCG
TCGGCGAGGTGCGGCGGAAGAACCCCGGCGCCCATCGACACCGCGTGGAGAAGCTGCAGAACGTGGAGATCCGATTGGTTCGGTTCGCGCCGCTGCGCATAGCCGATCCTCCGCCCGT
TTATGAATGCGGACCAAATCTTCTCTTCTAATAGCCGGACGCGGTCCCCCGGTTTTTTCTCGCATTCGAGCGCCACGCGCAGCAGCCCGGTCCCCATATCCTGCATGAACCGGGCGGTCTG
GACCGCGAGCTCGAGGACCAGAATGGGGATGCTACGGGAGTTCTCTTGGATGGCGATGGAGATGCGGGCCTTGCGGTAGCCGAAGAGAGTGCCGGTGGTGCGGGTGGCGCCGTGGATG
TGGCCGTCGTGGTGGGCGCTGGGGGCGGATCGGGGGATGGGGGAGTGGAAGCCGCAGGCGGGGCTGATGATGGGGAAAGTGCGGAAGACGGAGCGAAAGACGCGGAAGACTTTGGA
TGGATTGGGGCCGCGCTTGCGGTGCGAAGGGGGTTGGAGGATGGTGGTGGGGGAGGCAACGGTAGGGCTTGTGGAGCTTCCGGCAGGTTTTGGAGGAAGGGGAGGCGGCGGCGGCGG
CGGCGGCTTTGTCATGTGGAAGATGGGCAGCGGAGGACGTTAGGGGTTGTTGAATTGAACGTATGTGTATACGAAGCTTAGCTTTGGGCGGGTTAATGCGTTGTACGCACGTCGTCGGAA
GAGGAAGGTAGGAAGGGATCAAAGACGCAGTAGGAGAGAGAGAGAGAG
>comp138323_c0_seq1
CGTGTCGACTCTCACTGCTCAATTCAATTTCTATTGCCTCTTAAAACCCTAACAACCCCAAGATCTCGAAGAAGTTGAAAGGGAGGAGTAAATTGCTGATTTGCATTTGCCAATCAGAAAC

GGAGATTGAGTGACGATCTTTTTTTAAGGGCAATGAGTCGGTGTTGTGGGCTTTTCTGCAGCGACGACGAGCTTCAATGGCACTTAGCGCTCAAGACACACAACTATGGAGACTTCTCAT
TCGCTATTTCTCAGGCAAACTCTCAGTTCGAGGATCAGGGTCAGGTTTTCAATACTCCTTACGCGACCTTCATTGGCGTTTACGACGGTCATGGCGGCCCCGATGCCTCACGATTCCTCAA
CTCCCGGATGTTCTCTAATCTCCACAGGTTTGTTACGGAGGATGGAGGGGTGTCAATCGAAGTGATAAAGAAGGCCTTCCGTGCCTCTGAGGAGGAGTTCTTGCAAATGGTGGGGCGTTC
TTGGCAGTCTTGTCCGCATTTTGTTTCGGTTGGTTCGTGTTGCCTCGTTGGGGTGATAGTGGAGGATATGCTTTATTTGGGGAATTTAGGAGATTCCAGGGCGGTGCTCGGCCGTAACAGA
ATACCTGGGGAAAATGAGGTGGTGGCGGAGAGATTAACTTCTGATCACAATGTGATGGACGAGGAAGTCAGGAATGAGGTGATCAACCAACACCCAGATGACTCCCGTATACTAGTTCAT
ATAAGAGGAGCGTGGCGGATTAAGGGGATCATCCAGGTATCACGAACCATTGGGGACTACTACCTGAAAAACCCTGAATTCAGCAGAAGCCCACTGTACCAACAGCTCTTCCCTTCCGC
CACCCCATTCAACCGGCCTGTTATCACATCCGAGCCATCTGTTGGGAGGCGCAGGCTGAGACCTCATGACTTGTTCGTGATATTCGCTTCAGATGGCCTTTGGGAACAACTAAGTGATGAA
>comp138461_c0_seq2
AGGGTTAGGGTTAGGGTTAGGGTTGGGCATAGGAAGAAAAGCAACAAGATTAGGGTTTGAGTTGTAAGATGGATTCCAGCTGTTGGCGAAGGAAGGGAAGAGAGGGTTGTGGAGGAG
AGGATGGGAGAGGGTATTATTAATGGTTGCTGGATCGGAATTTGCGATCAGCCTCGCACTTTCTTCTGCAAGGGCATCACAGAAGGCTCTGTGAGTGATGAAGCTGTCCTTCCTGCATGCA
AAAATTGAATTTTTTTAGCATTTCTATTATACAATTCGATGATCAGAAACGACAATTTTTTTAAAGAGCCTAACTCGCTTATATGGTTTGGGTCTGCATGTAAGAAATTTAAGAACTTTGCTG
AATTGTGGAGTTAATGTGTTACGAAATTTGGATCTATAAAATGAGCAAATTTGCTGAACATGTCTTGATTTTGTAGGTGTAAACATTTCTCTTCCAACACAATCTATATAGAGCTTCAAGAGC
CTTTCTAACTAGTTAATACTTCTCTCTTTTGTTTTGACTTCGCTTCTGAAGAATATGTATAAGAAGAATAAAAAAAACTTGGCTGCATGGCTGCGATGGAGTTTTTTCTAAGATCAAACCAG
GTTTTTCATCAGTAAT
>comp138484_c0_seq4
ACGATGCTCGAATGACCTCCGATTGACGAACGGACTACGTCATTGCGACCGGGACATTGTCAATACTCTAAATGCACTACCAATTTGTCTAATATCTATGTTCAACAAAATTGACACGTAA
AACAAGACAATTACACATTAATCGAGAGGTTTGCACACAAAACGGAGCGACTTTGGGTGATGCATTGCAGTCATCTCAAATTAATGAGGATACCAACAATAGTATATCATTATTAAAATTTC
AAATCTCTTTGGCTGAAATGGAAAGTCAAATGAAGGATCAAATCAAAGTTTAATGCAAACTGATGTTATCTCGAATGCTTCTACCTCAAATCCCAATTCAAATCCCATAAATCCAGCTGAAT
ACTCTGCTTTGCGTCCCTTCCTTGGCCTAGATTTTGTTGTTCTCTTGCGTTAAAATGAGAAGACTAGGCAGCTCGCGTTGGTGCTGGGTCACTGCCTGCTCTCTGAATCTTCTCCCGGACG
GACGAGTTCTGCCGGAATTACTTCTCCAGCTCTTCGATCTGTACCTTAAAACAAGATCGTCGAAGCCTGTTCTTCTTCTCTTCCTGGTGGTCCGAACCAGCAACCCCTCCGCCAACTTCCC
AGCTCTATAGTGAAGTCCCTCCAAACTGTATTATAACAGCA
>comp138500_c0_seq2
AACAACAGCGTGAGCTCCTTATTCTCATCGATTTCTACATTCCATGCGAGGGAGAATGGTTTGTCTCAGTGACGGAAGCTTCGAAGAATCTCAATCCCCCAAGGTAATTTGGTTTCAAATG
CTCACTTGTGTGCGCTATACTGCGCGGCAATAGCATATATTGTTTCTGTGTTTGGTATCTCTATTGTCTGGCTTTCAGAGTGGAAAATGCAATATCAGATTCTGAATT
>comp138501_c0_seq1
GAGAGTTGCCAACCAATAGGATATTGAGGAAGCAACATATGCCCGCAGTGTTGGGTGTGTTGGGAGACAAGATAAATGTGCTTTTGGTATGATTGATATGAAGCCCAGACAAAGAGGCTA
AACCGTCAATATGTTATTAAGGGCAATAGTAGTATTCTGAGCATTGGTTTTGCCAAACACAAGGAGATAATTAGCATAAGGCAAATGTGTGAATTGTGTACTATCCATATAAGCCCTCAAAA
TTCTGACCTATAACAAATGAATCTAAAAGATTTGAAGGTCATCCATAATAATGCAAAATAGGTATGGAGAGAGGGGACATCCTTGTCTCAATCTAGATGAGGAACTAAAAACCTTTTAGAC
CACCATTAATACAAACTGAATAATAAATCTCATTGGTGCAAGCTTTAATCCGAGAAATAAAAGCAGAAGGGAAACTTTTTTGTTCCAGTCGTTTTAGTAGAAAGCCTCTTGAAACCGTGTT
CAAACCTTAATAAATATCAAGTTTAGAATTTTTTTTTTCATAACAGAATGATTAAACTTCCCCCAACAACTCAGAAGCAAGAATAATATTATTTGTTGAAATCCTCTTTTGAATAAAGCCAAA
TTGATTGGGATGAATGATATGAGACATAATAGGTTTGAGCCTATTAGCTAAAATTTTTGCAATCACTTTATAAAATATATTGCAAATAGAAATAGGCATGAAGTTAGAAATATTATTAGCATGA

TTGGTTTTGAGAATCAAAATTATAGCTGTGGCTTTGTCTCTCATTGATAAATAGCAATGTTGAAAAAAGAATTTAACAGCCTTGTAAACTTGAGGTCCAATAATATTCCAGTCACTTTTATAG
AATTCAAATGTGTATCAATCAAGACCAAGGACTGAAGAGGTAGGATTAGAGAGAATGATATATTGGATTTCATCATCTGCACTGGGGAAGTAAGAGAAGCAATGAGGTGGGGGGGGGATT
TATTGAGCCTAGTGGAATGA
>comp138534_c0_seq2
CAAATACAAATACAAAAGTGTCAATAATCAAAAGATAGTCCACCCTTTAAATATTAAAAAAGAAAAGATTTGGTATATCTATATTTAATTTTGAATACCGTGCGCACATTTGCTGCTTTTCTA
AACATATAAAGAAAAAGCATGAAGATTAATGACCCAGCAGTAGAGCTCCTCTCGAGCTTTTATGTTGCTTTACCTCAGTACTGTGTCAATAGCTCTTCACAATCTTTAGTATTGAACTGTAG
GTCCCCAAAATTGCACTGATGATCCCAATAGCTACAATAACGATGGATACAGCAACCTGGAACTTTGTGGCTTTGTTCTTTGATAACTTCAAGAAGCATAGGGTTGGCGCTATGATCGCCAT
TAAAAACAGCCTGGGTGAAACTGCAACCATGAGAAGCAAGTTTTCCATGAATTTGTACATGACAAGAAATTGCTTTGTCTCCAGTTAAACTGATCATTTTCTTGAAGTTCTTGTCCAAACC
ATCTTTCTCTATAATACTTCCCGAAAGGAGAGGTTGCTTCAAGTTTAGATCATGTCCACTTTGTGAGCCAAGGTCAAGGCTTGAATATCT
>comp138541_c0_seq1
CCACAATTCGCGCCAAAAGTGACTAATAAACTTCACGTCGCGATCAGACGTGATGCTTCGATGGAGGCCATGGAGTCGTACTATCTCCCTGAAGAACAGTCGAGCAACACCGAGGGGGT
CGCAAGTCTTCCTGCAAGCTATAAAATGTGACATTTTAGAAAACCTATCTACTACAACCATGATGGAATCGGCACCTTTTTTTGTACGAGGTAGCCCCAGTATGAAGTCCATACTCAAATCC
TCCCAAATAGACATGGGCACGGGTAGAGGTGTATATAAGCCGGCATTTTGTCGAGCACCTTTATACACCTGGCACACCTGACATCTCTCAACAAACCGAGAGATATCTCGCCGCAAATGA
GGCCAATAAAAACGTTGTTGCATTTGCTCCACTGTCTTTTCCCTTCCCAGGTGAGCCGCCAGTCCTCCTCCATGCACTTCTTGTATTAATTGCAACCCGACCGAAGAGAAGGGAATACATA
AAGAATTACCTTTGAACAGGTAACCGTGGCGTATGAGATAGTCTCCCCAAGACCTGTTGATGGAGCAAATTTCCCAAGCCTTAGCAAAGTCTGGATCTTGAGGATACAGGTCTTTAAGAG
ACTCTAAGCCTGTGAGTTCGGTCTGCAGCTTGGTGATCAAGGCTGACCGCCGGCTAAGTGCATCAGCCACCCTATTTTGACTGCCCGATTTGTGCTTTAGAACAAAGCTAAATTTTTGTAA
GAATATTATCCACCGCGCATGCATTCGTTTAATATTCTTTTGGGTATTTAGATATTGTAGCGATTGGTGATCACTACACAAGATAAAGTCTTGGTGAAGTAAGTAATGCTCCCATTGCTTCAA
AGCTCGAACAACTGCGTACAGTTCCTGTTCGTAAGTGGACCATTTTTGTCT
>comp138627_c0_seq1
AGGGTGTGCTGAATCAGGCCAGACGGAAGTAAGGAGGAGGTCGTTCGAACATGCCTATGAGATCCGGATCAGGTACACGAAGAAGTCGGCCTGAGGTAGGGTTGGTCGGCTTCATTTG
CTTAAGGATCAAGCGTAGGGGTCTCACATGGAATCGAGCCATGCATATGCAGGCCAGGCGCTAGGAAAGCGAGCTTCTCGTGCGAATTGGAGAATGTAGTCGGGCTGGAGAGTTGCAGG
TGACAGGCTGGATCAGAGGTCACGAGCGGAAGCAGTTGGGGTTCGTGCGTCTGGGATCGGAAGAGGGGGATCAAAATGATGCTTTTGCATGCGGTCTCTGTGTATGACAAAGAGGGAG
TTCGATATCCCTTGTTTGTGTGTGCGCCAAAATTCGAATGCATGTGTTTTGGTCATTGTCGTGTGAGGGATGGTTGTGAGATCGTGGGATCATGCGTGAGTGTCATGGATCGTGTGTGGGG
GCTGGTCGGCTAATCAACAATCAACATACTCTTACACCTTCGCAGATAGCTCTATGATATACACAATGGTATGTACAATATCAGTCATTTCCAATGAAGTGCGAGATGTCAACAGTACATTTC
AAAACTAAGAGTGAGATATTGGAAAGCTATAAACTAGATTCTACAATACCTAAAAGATACGATCAACAATTCATGAAGGTTTGGAAGAACAGCCTTAAAACTACAGGGATATGTTGCCGG
TGATTTAGGAGGCCTATATACTTGAATAAATAGTATTAGATACATCTACACTTTGGGTGACACTAGCATCAAGCTACATGTTAACTTAAGAACAAAGTTGCATTATTAAAAGGCCAA
>comp138670_c0_seq1
TAATGCAAAAGCTTCTACTTCAGTTAAGGCTTTCACTGTTCTTGTGGAAGAAGGGAGATTGGCTCCAGCTTTTGGATCTAAAGCCCAGGTCAACAGCTCCTCGCCACAGAAATCTCCTTC
CTTGAGAATACTTCTGTTGAAGAAACCACTTCTGCCACCGTCAGTGGTGATGCTCTCGAGTCGGCCGCGAATGATGAAGAGCATTTCATCAACTGGATCTCCTTCTCTGACAACATAGATG
CTCTCAGTGTACAGGCTTGGTTTCAGCCGTTCACATATGGCATCTAGTAATCGCTCATCCATGTTTTCAAACAAAGGAACCCTTCTCACCAAAGCTAAACAGAGATGGCGTTTGATATCCC
TCCTAAGATCCTTGGGAAGGCTCTGGACCAAGTTCTCTTCATCAACACCTCTCGTTTCCAGCCACTTATATTGATCATAGCGCCTAACACGTTCCCTGAGTTCCTGTGGCAGTAATCTATGA

TGCATCCACTGCTCTGAGTCGCGCCTTTTCACTCTCATCTCTTCCAGTCTTATGGTCAGTGACTGAAGATAAGTCTGCATGTTCCCAATTAGAAGGGCAAAGAGAATGAGTCCAAGTATAG
AAAGAGCAATGGAGAAAACAACCTCCCCAGGATAAATACTCGTTGTGAGACCTTGGCCAAGGGTACTCAGGTTTTGTAAACCAAACCATAAACAATAACAAAATTTGGAGACGAACTTA
TTTGATGTAATGATTCCAGAAGTTACAGCCTGAGTGTAGATGCCAAAATTAAATTGCGGATTATCAACATCCACAGAGCATTGTTTCTTAAGAACTGCACTGCTGACATTGGACCATTGATC
AAAACCATCAAGATGATGATCACTGCAATACAAGAAATCCCTGAGGCATCCATTATACTTTGAACAAGCAAGATGCCAACAATCATCCTGACGTTCAACAGATAACAGATACCAAAATGC
ACCCACTATATGACTAGCAAGTAGAAACCACAATAGGTAGTATGCAGCGCCAGCCCAAGCAGTTTCAGCAAACACACCAGTAGTTCGTTTAAGCTCTGATGTCAAAGGAAAAATTCTGAT
AAATCTGGGAATATACTGAAGGAATATAAGGAAGAGCAATATATTTTTTGTGGCCAGCACATCATAACCTTCTGATCGTTGGAGAAACCTCCAAACAACAATCTGT
>comp138722_c0_seq1
AATTTTCTTCAAAAATATGTTGGGATTAAAATACCAATCATCAGCAAAATCAGATACGAAATTTGTTAATCTTCATTGAAATCCCAATGAGAACTAATACAACTGCAGATCAAATTCTGTGTT
TGATCTCCTGCTTCGAATTGATCAACACAAGAAGCAGGGCTCAAAACTTCCATATCATAATTCATTGAAGAGCAACAGGAATCCATGTCCTCGTCACAATAGAAATCAAAATCGAAATCCC
TCCCACTGCATTCTCCTGCTTCGCCGGAGAACAAGTTTCGACACCCAGTTTCCGGCAGAATTGCCGCCTTTCTGTCGTCGATAGTTGCTCGGACTTCGGTCCAGTTGTGATCCCAAGTCCA
GTTTGCTGGTGGCGACACCAGCTGCTGAATTCCCTCCATCGAATGCTCCATTGGGCTTGGGAGTTCTTCTTCTTCTTCTTCCTCATCGATTTCCGCATCCCATGAATCAAAATCCAGCGTTG
TTTTAGGAGAGATCCACTTCCACTGTTCCAAATTAAAACGGGGCAGAACAGAGCATTCCTCGCCCTTCTCTGCTTCTGCCACAAATGGATGGCTTAATAGCTGCTCCGCTGTCCATCTCTC
CTTCGGATCTCGCTGGAAGCACTTCGTAACGAAGTCCTTCGCTTGCTCCGAAACCCACTCCGGCATCGCCGGCACGTCGGAGGTAAATCCAATTCGATGAATTGCCGCAACGGGATCATC
GGCGTCCGGCCACGGCGACTTCCCTGTCGCCATCTCCACCACAGTACACCCCAGCGACCACACATCTCCGGCAATCCCCTGCTCCTCCCCTCGCACCACCTCCGGTGCCATGAACGCCGG
TGTCCCCATCGCCAACCCCTTTCCAATACGGCGAGCGCAGCCAAAGTCCCCGAGCTTAATCTTGCCATCAGAGCCCACGAGAACGTTCCTTCCCTTGACGTCGCAGTGGACAATGCCGTT
GGAATGGAGGTAGCGGAGTGCGAGAAGGATCTGAAGAGTGAAAGATCGGATTTTGTGCTCTTCGAGATGTCCGGCGTGGTCGGACAAGGCACCGCCGCCGGCCAGCTCGAGAAAGAG
GTGATAAAATTCGCCAGAAACGCTGGAGCCGAGGCATGATATGATGTGAGGGGAGGAAAGCGAGGAAAGGATGCTCCGCTCCTTTTGAAGGAGAGCAGATCGAGAGACGTGGGAGGA
CTTGACAGCGAATAGATCACCGGAGTTGTCGTGAGCCGCCAGGGAGACGATGGCAGTGGCGCCGCGGCCAATGACGGGGCCAAGTGTCCATTCGCCCTGCATGGCCATTGATCCAAATT
CTTCTTAGAGCTCCGCACAAAAGGAATCGTAACTCCTCGACATCGCTTCCTGGCTCTATATAAAGGCCGCAGGCAGAGAAGGTAGAAATGCAGAGAGACGTTGA
>comp138756_c0_seq1
GTCCCTTGGAAAATATTGTTTTCCCATCTTATCCAACTTGTTAATAAAAATTATCACCAAGCTCTCTTTGTCTATCAATTTGGTTGGCTTCCACTTCGATAGAGGATCAAGCAAATTTTTTCCA
TTCCTTAGATATATCCTTCCATTATACACATGGGATGAATCCAGCGTACAACCACGCCCCAAGTGCAAGTCTTTGTATTTCCATTCTATATACCATTTTATGCCAACATCTGCTCCACCATCAG
CAGCTGGTCTTATAATAAACTCTATATGCTTCGTAAGGAATGTAATGAAATGATTTATCATTTCATAAATTCG
>comp138834_c0_seq2
TCAATCCGTTCATGCTAAAGAGGCCAAACACTCTAAAAAGCCATAGACAATAAACTTTATCTTACAAGAGGAGAAGAAGAAGAAGAAATTTACCAACACCAGAAATAAAAATGTAGCAA
CTGCATCTTTCTAAAAGTTAGCAGTATTTTTCTACTAGTACCAATTCAACATTATTATTTTTTTTTTTCTAAGTAGGACTTACTTGTTTGCATGGAGATAGAAAACACTATTTATTTAACATACG
TGGAGAACATTTTTCTTCTGCAAGTGGCTTCGGAGATGCAGCCAAATATATATCCAAAACCCTTTCTTCTTGCTGGTCAACTTGCCACTCAAGAAGACTTCTCAGCTCATCATCTTCAGCA
AAATCATATTGACTTTGGAATGCACCTACGTCCTTCTCCCTTGTAGAAGTCTCTTTATCCACCACCATACTGAAATCATAAACCTGACTCTTGACTACACCCTCCAACGCCGACCCATCACA
TCCGTTATTAATCATCATCACATTCTGCTGTTCTTCCATCTTCAACTTCCGTTCTTCGAGAGCCGCCATTGCCGATTCCCATCGGGATAGCTCGGAAAAAGCGTAGGATAGCTCAAACTGGA
TCTGCTCCAGTTGCTGCTGGAGTCCAGAGATTGTTCTAACGCAGCCGTAGACAGGATCTATGAAATACATTTTGGACTCATAGACAAGTGAAATTACTGCATCTTGCCGCTCTCTGGGATG
TAGATCAGTCAAGATCTTGGCCACGTTGCTGGCACCGAAGACGCGGTGAACTAGGGCAAACATCTGCTGTTGTTCCGGGAGGAAATAGGGTGCGAAGATGCAATCAGGATGACATTTTC

GGCGTAGATATTTGCATGCAGCGCATGCGGTATTTTGTACAGACCCCCATTCCGCTGCCATTGATGAGGATGATGATGATAAGACAGAAGATGGGGTAGATAGGATTGGCGATTGTTGATCG
TCCATCGATGCGAGGGTCATTGTCGTCATCTCAGATAGATTCATCAATCATCTATCGATCAACAGAAGAAGAGCACGGAGGGATTGTACTGTAAATCATCAAAGTGAAGGAGAAAAAAGA
AATTGCAAGAAGGATTGGCATAAACAGATCATCAACGAAGAGATCGA

>comp138837_c0_seq1

AATAGGTAGCTGTAAAAGTTAAAAGCGAATAAATAAAAGATTTTAAAGTATTATTATATAATATAAGACAGTACCGCAAGGGAAAGCTGAAATATAATGAAAGTTAAAAAAAGGATAAGAG
TTAATCGTACCTTTTGTATTATGGTTTAACAAGATCTGATGATAAACAGAACAGCACCCGAAATCTGGAGAGCTAACCAAACCCTCTCAAAGGAGAACTACACAACCACTGTTGCAAGAG
TGGTAAAAGAGGTTTGGTTAGAGATGATATGTTTAACGCGCCAGATGATAGCTGGTTTTCTTAGAAAAAAGTTTAAGCTTTTCTCCCTTATTCACAGTTTTTACCTTAAAGGAAAATATTAG
GAAAGAAAAAAGGGAAGAAAGAGAAAAGAAGATAAGTTCTTTTCTCAAAAAGGAAACAACCAGGAAAGAAGGAAAGACCAAAAACAAGTGAAAAGGGAAAGGATCAAAGTAGGCC
TAAAAGCGGCCATCTAAAAGAAAGCGTTAAAGCTCAAATCCTCTTAACCCGAAAATTTTTGATACTAGATCCAACCTCTTTTAAACTAAAGGAAAATAATAATGTTAAAACGAGTAAGAA
AAAGACTTTGTTACCCACTAAAGGAAAACTAAAACAAAACCAAGAACGCCTAAAACCACAAGAGTAAGCATCAAGGAATCCTTCCCAACACAGGAAGTGGCCAAACAAGGAAAAAGG
GGAAGAAAGGAACTAGGCAAATAAAAAGGGGGACTGTTTACCAAAAACATAGCCCCACGAACTTCATATGTGGGGTGCAGCCTGCCCAGTGGAATTTATTCTAAACGGCCGCGGTATTT
TGACCGTGCAAAGGTAGCATAATCACTTGTCTCTTAATTGGGGACCTGTATGAATGGCTTTTCACTCTTTCACTGTCTCTCTTCCCTCCCTCCTAATCTTCTAATCACGTGAAGAAGCGTGA
ACAAATAAGAAAGACGAGAAGACCCTGTCGAGCTTAAGCTAACCCTGAAATGTCGTCTACCTTTTTTCTTAGAAAGAGGTAAATTTCAGATAAAGTTTTGGTTGGGGCAACCGTGGAGA
AAAAGAATCCTCCAGTTAGTTAGGAAGAAAACCTTTCACCTAAAAAATTTAGCGACCCAGTTACTCTGGTAAACGGAAAAAGTTACCGCAGGGATAACAGCGTTATCTTCTCTAAGAGCC
CATATTGACGAGAAGGATTGCGACCTCGATGTTGGATTGGGGTAACCAAAGGGTGTAGCAGCTCTTTAAGGTTGGACTGTTCGTCCATTAACTCCCTACATGATCTGAGTTCAGACCGAC
GTAAGTCAGGTCAGCTTCTATCTTCTAAATAACCCTTCTAGTACGAAAGGACCGAAGGGAGGCCTTCAATGACCAACGTTAAAAGGTAAAAAAACAAGAAAAAAAGTTAATAAAAAAAA
AA

>comp138882_c0_seq4

CACTTTTGACACAGAAGGAAAAAAAAAACTTAAGATAATACTCAAGAAATACTTAAAATCCATTCAGGAGTAAAAACAGGTTGTCATTTGAAATCTTTATTCAGCTTTTCCACTGCATTGC
TTCTATGGAAATCAGTCTAGAAGTGATGAAGTAAATATCATTTGCCCTGGAGTCAAAATTGCCTTAGGATCATACTTCTTCTTCAGCTTCACAAGTCTCTCCCATTTTGATCCAAAATAGCTC
TTCCACTCCTCTGTAGTTTTGTAGCATGGCGTGTACTCCTTTGTCCCATTGCCATTCTCATTACAGAAGCTCCTGATCTCCTTAATGCGACCTAAAACTCGCTCCACATCATTGGCATCAGCG
GTGATCAGAAGTCCGATTTTGTAAATGATTTCTTCTTCCTCCGGAAACATAAGAGACCATCTCTCATCCCACTTGTTTCTATTCATGGGATAAACAAGATAGACACCCAAGGGCTTCTCCTC
TTGGAAAATGCCCTTAAAGAGCTTCTCATTGAAAATGTTTATCTTTGATTTGGGTACGATCAGGTTTAGCCATGGATGATGAAGGTTCCACAGGTTACGACGACGGAGCTTCTGCTCCTCC
CTGCGAACCCGGTCTATGAATGTGGTGTAAGGGAGGTCTCTGGTAAACTTGACCCGTAAGCCATTGATCACGCCACTGATAATTGTTTCAATCTCCTGGTCGACAGTGGATGCAGAGCTTT
CACTATAATGAAATGCTGCTTCAATGAGGTAGATCCCGGCATTGTGGTCAGCTAAGGCGCCAGCGATCTCTCGATCCTGTACAGACAGAAATGGAGAAATAAAGTTTTCTACATGACTGCT
GTTTGAGAAGAAGAAGCCTTCGATGTAGTCGAATCCTTTGTTGCTCTCTTTCATTCCATCCAAGGAAATCAGTCACCTGGCCGCGTGGAGTGACAGAGAAAGAGCTTCCAGAAGACTTAT
AAGAAAACTCGATGATCTTCACAATATCAAGCTCGGAGTTGGGGCAAAGAACGCCGCCCGGCCTGGCTCTGACGATGCCGCCGAAGTCGGTGGAAACCTCCTCAGCAGCTTCGTCGTCG
AATCTGATCCGCGCTGCAATGTCCAGTTCCTGAAACATTTCCATCACAGTCTTCTTCGTCTCGGAGGAACAAACTTCCCATAATGAGTTATTTATAGGACTGTAAATTATATATGAGATATTG
TAAATTAAGTATTTCTTGTTAGATTGAAGGCAGCGAAGCAGAGATGTTATGTACTGTATTGAGAGA

>comp138887_c0_seq1

CATCGAAGAAGGTTCCTTCAAGGAGCCCGGTGAATTAGGCCGAGCGTTCCTTCTATTGAATTCCTAAACCTAGATTTCTTTCCGATGGCCGGAAGAGGGAAAGTGATCGGTGCGGCGGCA

TCGAAGAAGGTTACTTCAAGGAGCAGCAAGGCAGGGCTTCAGTTTCCTGTTGGTAGGATTGCTCGATTCCTCAAGACTGGCAAGTATTCCGAGCGCGTTGGTGCTGGTGCGCCGGTTTA
CATGGCCGCTGTGTTGGAATATCTTGCTGCTGAGGTTCTAGAGCTTGCCGGGAACGCTGCCCGGGACAACAAGAAAACGAGGATAGTTCCAAGGCACATTCAGCTTGCTGTGAGAAACG
ACGAGGAACTTTCAAAGCTCCTTGGTTCTGTAACCATTGCTAATGGCGGTGTGATGCCCAACATTCACAGCCTTCTCCTTCCAAAGAAGTCCGGATCTTCAAAGGCCGGTGCTGATGCCG
ACAACTAGATTGTGCAATTTCTCATTCTTTGTTCTTTCCGTTTAGAAATCTCGGGATTTGTATATGAGTAACCTTCTGATATAGTCCATCAACTTGGAACTATTATTGCTAACGTTGTTTGTTT
TCTTGTCCAAGTGTTACAGAAATTGTAGGTCGTCTTGTCTGACTATTTCATGGTGTTTGGTTCTGTGATTACAGTGATCTTTTTAATTAAATTGTTAGTTTGTTAATATATTTTTGGAGATTATA
ATCATGTT
>comp138965_c0_seq1
AATGTAAAAAAGTAGTTTAATAAATCAGCAAATTAAATTTAAAATGTATAAAAGCAATCTTTTTTGTTTATAGAAATTGAAATAAAATGCAATGAAACATCTGAGGTTTTTAATGAAAAAAA
GAGAGCTTTGTTAAACCAAATGGAGGATAAATATGTAAAATTTGGGTATAGGGAAGACAATTAGCCCCCTCAAATCTAGACATGATGAACTCATGCATGCAATGTTTCTCATGTGCACGTG
AAATTAGCTAGGGTTCATATGCATGCATTGTATTTTGTGCATGCTTTATTAATTACTTAATTCTATTTTCTTTAATCAATTAAGTATAGAGAGAGAGAGGTGAGAGAGAAAGAGAGAGGCAG
AGA
>comp139069_c0_seq1
TTTTTTATTATGATATATATTGATCCTAAACATGGATCGACACCTAGTTAGACTCGATAGTACCTTTGGGCACGCGCACTCTGGCCCTCAGGTACACTTAGCTATTTAAAGTACCTAAACTTGC
TATCAGCTCTGGATTATTGGAGGCAAAACGCAGTGTCTCACGAGAGAAACATCTTTTGGGGTAATAATCCAGAGCTTCTATTAGATCTTTCTTCACCATTCGCCGTAACCGCTGCAATTCAT
ACTTATCCAGGAACTCTGCGAGAGTAGCACTTGATGCTAGCTTCTGAATGAATTCGTCTATAAACATCACCGGCGACGAAGCATATATCAGCCGAATTTCTTCAGCGCTAGTCGACGGTGT
TGCTTGAAAGACCTCACCCATAGCTGTCAAGAATTCTTCTCCCAGTTTATCCTGTTCTAAATACTGCCTACTCAGGGTATTGCTAGCCAGACTCCTCAGTTTACGCCTCATTGCAGATACAA
GACGTTGAGTCTTGATTCTCGGACCAGTGACATGAAATTGCTCAGGCTCACGCATAAAGTCTAGAGGACTCAGTGTTTCAACTTCTTGCTCCATTACAGCATTCAAACAGTGACTCAACA
GTGGATACTCATAACCAAGTCTAATCATCATGCCTATACCCTCCTTGGTTACATTGGCTACCTCATTACACCCCATCTGTGATATACTTCTAATCCCAGCACCGCCAAAGCTTAGGGGAGTAA
TACAGAATAGCCTATAGTCTATAGGGTATAGGCCATGATTGTCCTGCAGACGAACCCCCATTCGGTAGTGTGCACACAACAGTTCAAATATGTAAACATAGTGGCAGATATCTATTGGTGCA
CCAACACCATGGGCTCCACGCATCTTTCCAAAGTATAACTGCTCATAGTCCGAAGCAGACATAACCTCCTTCAATTCCATGTCACCGATCGTACAGAAGCTTTTCAGTCCACCAGATATGA
AACTACCATCGACGACTAGTTCGTTCAGCATTACAAACATATCTCTACTGATGAATGTTTTATCCCAGCTTAACTCTCTTCCTAATACACGTAGTCCAAAGATAACGTCATCTATCACATCAC
GCGCAGCTTTCCAGCATTCGCCGTCCTCTCGTTTATCAGACTCTATTGTAAACTTTCTTAATCCGTCATCGATGAATGATAGAAAGTGCGATTTTCCTTTTAGTAGCCCTTTTTCCCGGCAGA
TACGGGTCGAGAGTCCCTGCGCAACTATTTCCAGCCATGTGTTACTGGATCCTCGCATGCCTTCCCTATCTGTGCCATTACTGTTGTAGTGTACCAAGTGACCATGTACTCGATAATAGAGA
TCACAGTGATACATGATTGGACCATAGGCCTTCATCCCAGGAATGTCAAACAATTCAGCGAAGAAGTCAGCTGGCAGTTGCTGTGACATGGGATCCATCCTCGGACTATACTTTGCTTTAT
CATCACTCATGTAGAAACAATAGCGTTCCAGATCATCCTTCTCACTTGCACTTTGTAAGTCCATGAACTTCTGCCGCGTAGACTTCCCCAACATGCTTTGAGGGACGTGGTTCAGAAAGTC
TCGAGTTAGGCCGTCGAAGTAGCTCAGAATACATCCCCATGGTGGACTGGCAGCATAGAATGGCCGAGGCGCATCTTTATGTCTCTCACATCTGGAGCCTGTGACTATATCAGTGGTAAAT
TCAAAGTCAGTAGAAACCTCTCCAACATACTGATCAGGAAGCTTACGCCCAATCTCATCCAACTTAGCAAGTACACCAGGTAGATCTGGTAGCTCTGGTTCCTGGATCGCGTATAGCAGTT
TGCGTTTGTGCCTACGAGGAGCTTTCCGCATCGCTTCATAATCCCGGTACGCGCTAGGCATTGGGGGGGCACATGCAGAATCTGCTTGCTTGAAATATTGCTCGTTAGTTATATTTGCTACTA
TAATCGACCCAGTCAGGTCTACATCATGTGCATCCCGCCAGTATTCACCCCCAATGCCATCAATCTTATACTTGGCGTGCCATGTTTTCTCCTCGACACCTTCTCGTATTCGGCCAGGCCAAA
TCCCTAATCTTTTATGGAGGACTCTAATGCCCAAGTACCACATATACGACTTTGTGTCCATGAAACGCTCAGATTCAGCAGACCCCAGTAACCACTCAGTTTGAAACCTGGACATATGCAA
TTTATACTGTTCACCAACTACCAAAAAAGGGTCCACCTCAGGGTAGATACTAGCCTTATACATTCCCAGTAAATCCACAGCCCAGTCCACTGGTAAGTTCTTCAGATATTGCAACGCTAGTT

CCTGGCAGGGCATCTGCAGTAGTCCCTTACTCTTGAGCTTTTGTCTCATAATACTAGAGTGATCATAGAAAGTGTCTCCGCTTTTCCAGGCCAAGTACCTCCACTGCAGCACATCAAAGTA
AACTCCCAACTTATTCCTACAGCTAAACTCGTTTCCACATGTCTCCATTGCATCCAGGTATAGTGCCCACATCATTTGTTGTGCCTTTTGTAGTCGTCCATCCTTCCAGTCGCGTCGTAGGTC
AAAGGTCCGACGCTTGTGATAGAGCTTTATGGCTGAGCACGCATTCAGTAGATATGATATATCCTGTAAATCACGCGAACTCAAGACATAAAGCTCATGCTCACTCTTCACCTTCCTCTTCC
TAAATTGAGATGTGCGCGATTGGAGACGACAGGTACGCGGTTCGATGAGACTGCTGGGCGATTCGTGTGTATGTTTCTTGTAGACCTTGATGTAGTAGAGGTCGCTGCAGTGGTAGATCTT
GACGTCATAGCTCGATGACTTCTCAGTTCTAATCCACGATTGGTTGAGATGCGGGGGATCTGGGGCCTTGTGGATACCACTGGGGTCGAACGAGTTATAGTGATCGACGGCCTTCTTGGTG
TCCCGATATCTACCCTGGAAACTGGTGCAGTTGCGATACTTGATGCACCACTGTCTAACCATTCTTGCATTCTTGTGGTGGTGGAAGCTTCTGGATAGTTGGTCAAAGAAGTCTGCTGCTC
CTCTATAGTATCGTCTATCAGCGGTGGCATTGAATCGTCCAACGTTGTGTTCTGTTCTGGCAGAGACTCCGTAGGCGGCTGGGGCAAGTAAGTTCGATATCTGCGCGACATGGCGATAAGC
ATGCTTAATGGCACCAGACTCGCTAAAATGCAGACCCCACTTCCTCCTAGATATAGCACTGCAGAGTCTGAGAACAGCAGCGTTTCCACTATAGGTGGTAGCATCTGTCGTAAGCCGGAGT
AGGAAAGGCGCGAGTCTCGTGCATACAGGAGCACGATGCAGATTAGGAAACAACTTATTAGCAAGTTGTACAGGATCATCTGAACTGCGTAGGCGCATCCCGAGATTTTCAGTTTTCCCG
AGCTCCTCAAAAGGCGGCATATCAGTAGCAAGTTGATAATTGTCAAACACCCCAGTAAGTATAGTAGGCAGTATTCCATAAACATAGCAGTTACTGACGAATGGTTGAGAGACATTCTTCT
GTTCAGTAGAGCTCATAAAACAGCTAGGCGAATCAGTGAAAAATCTAAGTACAGCTAACTAAACCGGATGCACAGGAAGTGAAGCAGAACTCCTAATCTAGGTGGAAAACTAATTTGAG
>comp139089_c0_seq2
GAACCTTAAATCCATAATCATTCATAATAAGAACATAATGAAAGAGAAAAATTGAGTGAGGATATCTGAACTATAATCAGATAATGACAAATTTTTAAAATTTGAATTTATTTTTTATTCTATA
TTAATTTTAAAATAAACTCAAATTTTAAAAATTTATTATGATATCCTCACCTAATTTTTATTCTGTTTACGTTAGGACGAAACAAAATAATAATAATAATACATGTTAGAAAACCTCTGAGAAA
AGATTGCCGTTCAGCTGCCAGAGTCTTTAGTAAAATAGACGAATAAAAGTGGTTGCTGCAGAATGCTGCTCCAGATGCAATCGGTATCTTCGGGATCCTCGTCGGACACCTGCGGGAGGT
TGGACGGCGAAGCCATCGGCAGGGAGAGGCGGTCGCCGGGAAATCCGATGGCGGCGGAGCAGCCATCGACGAGGGAAGAGAAGAAGACGTCTGTGAAGCTGCAGCCCTCCAATTCCG
GCGCCAGGGATCCCTCTTCTTTCTTCCATGGAAACTCGAAAATCCCAAGCGGCATTTTACACAAATCGTCGCAGCTGCCGGAGACATCGTCTTCTGGTTTCGCCACCGCCGGCGCCTTGG
CCTTTTTGGTAATTGGCTCCGGCGCCAGCAATACATTAGTCTTTACCGCTGAGTTTTTCACATTAGCTCTGCTTCTTTTCTTCTTTAATTCTATCTTCATTTTGTGATCGAAAAGTTCAAAGCC
GAGATGCATGAAAGCCAATGGGAGATTCAAAAATAGAAATTCCATATAAAAACGAAGCGAAGATCAGAAGTCATAACGGGATGGAAATTTCAGTAGGCAAGAAAAATGGAAACCTCAGC
GTCTTGGATTCAACAAAAATGGGCCAAACGAATTCTTCTTCAAATTTCCTTCTCAAATGAATCAAACTCCCATGATTAAAACCGAGCTATGATCAATTCAGAGAAATTAGAGCTAAACGGA
TCAAGCAACGAAACTCAAACCAGATCGAAAATTTAATATAAATAAATCGTGATCTGAAGCTCAGGCGAAGTAACATGATCCTAAACTTTCGTGCTTCCTGCTTACACGTTCTTCTCTTCCC
CTCCAAGGGCAATCA
>comp139089_c0_seq3
GAACCTTAAATCCATAATCATTCATAATAAGAACATAATGAAAGAGAAAAATTGAGTGAGGATATCTGAACTATAATCAGATAATGACAAATTTTTAAAATTTGAATTTATTTTTTATTCTATA
TTAATTTTAAAATAAACTCAAATTTTAAAAATTTATTATGATATCCTCACCTAATTTTTATTCTGTTTACGTTAGGACGAAACAAAATAATAATAATAATACATGTTAGAAAACCTCTGAGAAA
AGATTGCCGTTCAGCTGCCAGAGTCTTTAGTAAAATAGACGAATAAAAGTGGTTGCTGCAGAATGCTGCTCCAGATGCAATCGGTATCTTCGGGATCCTCGTCGGACACCTGCGGGAGGT
TGGACGGCGAAGCCATCGGCAGGGAGAGGCGGTCGCCGGGAAATCCGATGGCGGCGGAGCAGCCATCGACGAGGGAAGAGAAGAAGACGTCTGTGAAGCTGCAGCCCTCCAATTCCG
GCGCCAGGGATCCCTCTTCTTTCTTCCATGGAAACTCGAAAATCCCAAGCGGCATTTTACACAAATCGTCGCAGCTGCCGGAGACATCGTCTTCTGGTTTCGCCACCGCCGGCGCCTTGG
CCTTTTTGGTAATTGGCTCCGGCGCCAGCAATACATTAGTCTTTACCGCTGAGTTTTTCACATTAGCTCTGCTTCTTTTCTTCTTTAATTCTATCTTCATCTGGGCGGAAAGAAAAGAAACTA
GAAAATGATTGAATTTATCGGAGAATTTAGAAGGGAAGAGGATGTTCAAATCTTTTACTTTGTGATCGAAAAGTTCAAAGCCGAGATGCATGAAAGCCAATGGGAGATTCAAAAATAGAA
ATTCCATATAAAAACGAAGCGAAGATCAGAAGTCATAACGGGATGGAAATTTCAGTAGGCAAGAAAAATGGAAACCTCAGCGTCTTGGATTCAACAAAAATGGGCCAAACGAATTCTTC

TTCAAATTTCCTTCTCAAATGAATCAAACTCCCATGATTAAAACCGAGCTATGATCAATTCAGAGAAATTAGAGCTAAACGGATCAAGCAACGAAACTCAAACCAGATCGAAAATTTAATA
TAAATAAATCGTGATCTGAAGCTCAGGCGAAGTAACATGATCCTAAACTTTCGTGCTTCCTGCTTACACGTTCTTCTCTTCCCCTCCAAGGGCAATCA
>comp139089_c0_seq4
GAACCTTAAATCCATAATCATTCATAATAAGAACATAATGAAAGAGAAAAATTGAGTGAGGATATCTGAACTATAATCAGATAATGACAAATTTTTAAAATTTGAATTTATTTTTATTCTATA
TTAATTTTAAAATAAACTCAAATTTTAAAAATTTATTATGATATCCTCACCTAATTTTTATTCTGTTTACGTTAGGACGAAACAAAATAATAATAATAATACATGTTAGAAAACCTCTGAGAAA
AGATTGCCGTTCAGCTGCCAGAGTCTTTAGTAAAATAGACGAATAAAAGTGGTTGCTGCAGAATGCTGCTCCAGATGCAATCGGTATCTTCGGGATCCTCGTCGGACACCTGCGGGAGGT
TGGACGGCGAAGCCATCGGCAGGGAGAGGCGGTCGCCGGGAAATCCGATGGCGGCGGAGCAGCCATCGACGAGGGAAGAGAAGAAGACGTCTGTGAAGCTGCAGCCCTCCAATTCCG
GCGCCAGGGATCCCTCTTCTTTCTTCCATGGAAACTCGAAAATCCCAAGCGGCATTTTACACAAATCGTCGCAGCTGCCGGAGACATCGTCTTCTGGTTTCGCCACCGCCGGCGCCTTGG
CCTTTTTGGTAATTGGCTCCGGCGCCAGCAATACATTAGTCTTTACCGCTGAGTTTTTCACATTAGCTCTGCTTCTTTTCTTCTTTAATTCTATCTTCATCTCAGCGTCTTGGATTCAACAAAA
ATGGGCCAAACGAATTCTTCTTCAAATTTCCTTCTCAAATGAATCAAACTCCCATGATTAAAACCGAGCTATGATCAATTCAGAGAAATTAGAGCTAAACGGATCAAGCAACGAAACTCA
AACCAGATCGAAAATTTAATATAAATAAATCGTGATCTGAAGCTCAGGCGAAGTAACATGATCCTAAACTTTCGTGCTTCCTGCTTACACGTTCTTCTCTTCCCCTCCAAGGGCAATCA
>comp139123_c0_seq1
TTTTTATTAAAAAAATATTAATGCATAATTCATTTATAATATATTAACTTTTAAGAGTTTACATTACAGCATTATTAAAAATATTTAAATTATATCTCACTTCCTTATATCAAATAATTTCTTATTTT
TATCATTTTATTACATATCATGAACCAGTAGAATTAAGCTATGCCCTTTACTTCCAACCAGTCATCAACAATTCACTTGCCTTCTCCTGAACCAGCTTCCAAGTTGCTTCCACATGTTGTTTCT
CCGTCAGCGTCCCACCAACCGCAAACCGCAACACGAATTCCCCACCAACGACCGCATGTGTCATAAATACTTTCCCGCTTCCATTCACCGAATCAAGCAACTTCATGTTCATCTCCCTACA
CTTCTTCCCCTCATTTTTATCAACCCTCAATTTAAAGCAAACAAGCGTCAATCGTCGTGGCACAACCAAATCGAACCTCTCATCTTCAGCGACAAGTTTCTCGAAATGCTTTGCCATGTCC
ACATCACTCCTCACGTGCTCCATCAAATTAGCCTGGCCGAAACGCCTAATGACCACCCACAATTTCATGGCGCGAAACCTCCGGCTCAGTGCGATCTGCCAATCTTTGTAGTCGATCACAT
TGCCGACTTCAGTTGCGGTGTTCCGCAATATCTCTGGCTTGGTTGACAGCGAGTCTATCAATTTCGTCGTGCACCTTACCCAAAGGCAGCAGCAGTCCATATTCGCAAGCAGCCACTTGTG
TGGATTCATACTAAACGAATCCGCCGTCTCCAATCCATCAAAGTACCACCTGAACTCCGGGCAAATAGCAGCGCTCCCGGCGCACGCTGCGTCCACGTGGAACCACACCCCGAATTCCCT
CGCCACTTCCCCGATCTCCCTTAATGGGTCCACCGCCCCCACCGCCGTTGTCCCCACCGTTCCACACAAATACAACGGCACCATCCCCGCCACTACATCAGCATCCATCGCCGCCCTCACG
CTCTCCGCCGTCAACGCATACCCTGTCTCCGCCGACGTCGATATCACCCGAAAGTTTCTGGTCGGAATTCCAACCAGCTTCGCTGCCTTTTGAAATGTAAAATGCGTTTGATCAGATGCAT
ATACCGTCAGCTTTGTGATCCCTTCGCCATTGCTCTGGCTCAGAGCATTATCTCTTGCCGCGGCAAGCGTGCAGACCACGGCTTCGCACGTGCTGCCGTGGATGACGCCGCCGCCGAGTC
CGCCAGAAAGAAAAGTTGGTGGGAGTTTGAGCATATTGGCCATCCAGTCCATAACGATGGTTTCAAGCTCGGTGGCGGCAGGGGAAGCGATCCAGCTGAATCCGACGACGTTGAGGCC
GGAACAGAGCATCTCTCCGGCGAAGCCGGCAGAGCTAGCGTTGGCTTGGTAATAGGCATAAAAATTGGGGCTTTGCCAGTGGGTGAGGCCGGGAAAGATGTCTGTTTTGATGTCGTGGA
GAATGGTGGTGATGGGTGAGGGGAAGAGGGGAGGGGTGTCGGGAATGAGGTTGCGGAGATAGCCCGGCTTTACTTGGCTGCGAACGGGGAAGCGTTCTATGTCGCGGTAGTAGTCGGA
GATGAAGTCGACGACCGCTTTGGATTCTTTGGAGAAGAGGTCTAGGTCTAGTGGCTGGAATGGTTCGATGGGGAGGTTTCCCATTGCGTCTTGAGTTTGGAGGAAGTAGAAACAAGAAA
GGGGCTTGCTTGTGCTTTTGTTGTCTG
>comp139258_c0_seq1
CAACTGTGATGAGTTCTTAGATCATGATCGTCTACTTTCGACAAAATTTCTAATAAACCGACGTGTTTGAGATGAATCGATTGGATTACTTGGATTAAATCGACTAGATTTAGCTTGAATCGG
GTTTTTGGATATTTAGGTGTACATCATATGCGGATAATCTCATCAAACAATAAAAAAATAATTTAAATTATTTTGGTCGAATTGGAATATCATTACACATTAAAAAATCTTTTATAAATCACCAT
AAGCCACAGTAATTACTAATTAGTCAAAACAACCAAACAAATTACAAATTCTTACAACATAAAAATAGGGCGAAAAATGCTCTTTTTTTTATTTTTTTTCAAACTGCACCTTTAAAAACAA

CTTATTACATAACACTACACTGCGGAGGAGCAGGAGCCCCTTCACAATCAAAGGAGAATTGATAGGTGACCGGCTTACCGACAACTGGCAGTGAGCAGGGCCCCACCCCTCCACACCCG
CAGTTGCAGCTTCGACAGATGTCATTGCACCCCACATAACACGGTCGGCGATGGCAAACCGGAACCGGTATGGGTGGCCCAACCGGCCAGAATGGCAAGTAAACCGGTTCAGGGTTCCT
CGGCGGCGTGGGCGGCGGGGGCGGCGGCGTGGGCTCTATTATGTTGATATTCTTAATGGCTTCACAAGCCTTGCAACAGAGCTTATTCTTCAGCTTGACGGGATCGAACGGCCCAGAAAT
CGTCACTGTATTCTTCTTCTCATCGTATTCTATTTTCTGAATGCGCTCTCGTTCTAGATTTAACTTAATTAGCCATTTAACAAAATAATTATTAATTAGAGCAATTAATGAATATTAATTAAGATT
TTCAATTTTAATTTACCTTGGAGTTTGCAAATGGCCTTTTTTATCTTCTTGCTGCAAAGACGGCACTCCAGATCAACCGTCAAGATTAGTGTTGGGGTCTCCCCTGCCATTCTTTGAGATAAT
GAGCGAGAGAGAGAGAGAGAGAGCGAGGGAGGAAGAGACCAACTCGCTCCTTTCGCATTGGATCAGAAGCAGACATTTTAATACTCGCTGTGACTCCC
>comp139258_c0_seq2
CAACTGTGATGAGTTCTTAGATCATGATCGTCTACTTTCGACAAAATTTCTAATAAACCGACGTGTTTGAGATGAATCGATTGGATTACTTGGATTAAATCGACTAGATTTAGCTTGAATCGG
GTTTTTGGATATTTAGGTGTACATCATATGCGGATAATCTCATCAAACAATAAAAAAATAATTTAAATTATTTTGGTCGAATTGGAATATCATTACACATTAAAAAATCTTTTATAAATCACCAT
AAGCCACAGTAATTACTAATTAGTCAAAACAACCAAACAAATTACAAATTCTTACAACATAAAAATAGGGCGAAAAATGCTCTTTTTTTTATTTTTTTTCAAACTGCACCTTTAAAAACAA
CTTATTACATAACACTACACTGCGGAGGAGCAGGAGCCCCTTCACAATCAAAGGAGAATTGATAGGTGACCGGCTTACCGACAACTGGCAGTGAGCAGGGCCCCACCCCTCCACACCCG
CAGTTGCAGCTTCGACAGATGTCATTGCACCCCACATAACACGGTCGGCGATGGCAAACCGGAACCGGTATGGGTGGCCCAACCGGCCAGAATGGCAAGTAAACCGGTTCAGGGTTCCT
CGGCGGCGTGGGCGGCGGGGGCGGCGGCGTGGGCTCTATTATGTTGATATTCTTAATGGCTTCACAAGCCTTGCAACAGAGCTTATTCTTCAGCTTGACGGGATCGAACGGCCCAGAAAT
CGTCACTGTATTCTTCTTCTCATCGTATTCTATTTTCTGAATGCGCTCTCGTTCTTGGAGTTTGCAAATGGCCTTTTTTATCTTCTTGCTGCAAAGACGGCACTCCAGATCAACCGTCAAGAT
TAGTGTTGGGGTCTCCCCTGCCATTCTTTGAGATAATGAGCGAGAGAGAGAGAGAGAGAGCGAGGGAGGAAGAGACCAACTCGCTCCTTTCGCATTGGATCAGAAGCAGACATTTTAAT
ACTCGCTGTGACTCCC
>comp139262_c1_seq1
GTGTTGTACCTTACAGTCACTACTCATCCATTTCCTCATTTGCACTGAGAAGAGAGACGATCATCTAGCCAGTCCAAACATCTTGGAAACAGGAAAGTCACACACAACGCAGCATTATGA
AGCAAACCACAGTAAACCAGATACTTATGTCGGTTATGGCCATGCTTTTGGTCGTCAACATAGCGACAGCACAAACTAATAGCCCTGTGGATAGAGCTGATGTCATGAGAGAGTTGATCG
GAAAACCATTCCGCACATTCGACAGAAACAGCGATGGAAACGACTGTGCCAGTAGATACGGCGGCGCCTGGTGGTTCGGAGACGATTGCGCGAGGGTGAACCTACATGGCGATTTCGA
AGAGAGCGGCAGACCAGGTGTCTGGTATGGAGTACCTCTGGACGCTAACAAGATTGTCAAGATGGAAATGAAAATTAGACCAATGGAATAAACTCCCCAATCAAAGAGAAGGAAGATT
GTACAAATTTATTGCCCTGCAGGTTGCTGCTTTGATTTTTAAACTTGCCAAAGTCGACTTAACGAATAGTCTATATGTTAACAGCGATTTTCTATACCGTTGAACTATACCTTATCACTGAAC
TTTTCTGATAAAAAACAATTAAAAGTCTATATCCCAGACAGCTTATAAATGTAAGTCGTAGCACGTGTGCATGTATTTATAGCTTGCAGTTTAATTACGTTAAATAATAAACTTCCCAAAGCT
GATTTTAACGAGTATAGATTTAGAGAGATAATTCAATCATTTCCTCAACCTATCTCATATATCGACTTGCTTGATTAGTCTATATAGCCAATGTAAAGTGTATTGTATGTCACCATATTGCATGT
AGTTTAGTACGTTCAATTCTAAACTTGCCCGAAAACCGGTTGCCAAGGCGATTTATCGTTACTAATCGTTCACAATCGCACATGACTTTACTTTCCTTTTCATTTCTAGCGTCCAGTCCATAA
GGTTATTAAGATGATCAGATCACATAGACCTACATCATGACATAGTGTACACCTTCCTGTTGAAACAGTTATCATGGTTATTGAATTAAAATAACAATCACATATATCCATAGACGTTATTTTA
AAGTGTTTCTTTTCTTTCTTTCTTTCACTGTAAAAGAGAGAGCCCGCTAAGTTGCATATTTAGCAGGCCTATAGAAAAGCTTTGGACTTGCCAAAATCGATTTATCCCGAC
>comp139287_c0_seq1
TACATAACAACATATTAAAGTAAATGCTAACGTCCTTAAAATCTGAAAAAGGTATTCTGATCATGCATATCATATAAATCAGTACGTATAGCTAACAAAAGCTGACTTCAATTAATGAATCAG
CACATGTAAAAAAACTCTTGTTTTTACAATTATTTCTTTATCCTGATAGCATATTTCATAAGGAAATTAAACAGTTACAATTACTAATGCTCAAAAACACAAGCTTAATTTGCATTACAAACA
TGTTATAAGCTCAAAGAAATTTGCCATTGGATTTATTGGATGCTTGGAGATACGAAAGAAGGGCTTTGTTCTCTTGGCTGAGCAGAAGAGCTTTCTTTCTAAGCATCATATTCTCCTCCATG

ATGCATTTGTTCTTCAAGTAAAGCTTCAAGTTCATCATCTTCATCTTCTCTCCCTTCAAGCTTTGGCTTCTCTTCATTGACCTTCTCGTCATCGAAGAAGCTGCCTTTGAATTGAGGAAGCG
TGTAAGCTTTGCCTTCTTTAAGAATCTCTTCAGCAGAAGAGGAGAAGAGCGAACTCTGAAGGAGATTAACTGGGAAGAACACATGTTTGCTTTCTTGTTGAAGCTTGAGTTTTGAATGAT
AGAAGATAGATAGGATCTTAGGATGAAGGAGAATAACTATTATCATTTGGTATTTATAAAGATGGATGGAAGCTTAAATTAATGGTTTACTGACCAATAAGGTCGAATTTGCAGTGATAGAC
CCCACGTGAAAAAAATGTAGTTGAACATATCCTGCACTAAATTTAATGTAATAGATATTGTCATTAAATTTCTAAACATACCTTTTATATTTTGCAGGACTAAGATCAAGACATTAATAAATAG
TTGTTCTTTGCAATGAATTGTTTTATAATTCAAGAATCATTATCTTGTATCACTGGAAATATTTAGCAAGAGA
>comp139301_c0_seq1
CACCCGCACACAACTAATTACTATATATAGGCCTCCTCCGCACTGCCACCCAACTTACACAAGACGTCTTCAACCCAAATTTACCGAAACATAGTAAATTAATAAATCTATAAGCCACAACC
ACAAACCAACATAAAAAAAAAAAAAAAGAATTTCATACATAGTAATCCATTGTCTCCCTCTTTCGCCCATCTCACATCAAACATAAACAATAAAACAAATACCTTAGTTCCTTTCCCCACAG
CTTTCCATCGTCCTCCTTCCTCACCATATGGGAAGAATACTACTACTCGTCCCTACAAAAGAATTAAATCTCTCACCTCTTCACCTTCCCCTTCTCTCAGCACTGTTTCTTCTCTCTATCATCA
CCCTCATCTCCGCCCTCTGCGCAACCCATAATCGCAAGTCACGTGCGCGTTCATTGCCTCCTAAATCAATATCTTCATCAGAGGAACAAGAAGAAAAGGCAACAGTTGTGCCCGCAACCC
ACGGGCCAATCGGCCCAACGGACCCGCCCAGCCCAACCAACCCCATTATACCGCGACGGAAGCTGTCCATGAACCTGAGCCTGAAGCTTCCCGAAGGGCTGACGCGTGTGCGGACAGG
GAGAAGGGAGGCTCATGAGGATAAGGAGGGGACGGCGACCGACGATTCGACGTGGATGAAGACTATTATTCTCGGAGAGAAGTGTAAGGTTCCTGAGAATGGTGAGCGAGAGGATGAG
GAGATTATTGTCTATGATGAGAAGGGGAACCGGCAGAGGAATTATCATCCAAGGACTCCGAGATCGATGCCTGTATCGAGGACTAATTCTTTCACCATCCCTTCTTCTTGATTCATCAATCG
ATAATGTGAGAATAATCTGGCTCATGAAAATATCTTTTTTTTTTAAATGAAAAATAAGGTTTTCTTATTAACTAATTTCGATCGGTGTAGCTTGTTGTAATGTTATGCAAGATACGAGATAGAG
ATGGTGGCGGGTCATCGTCCACTGCCTCTTTCATTGATTAGTTTTTCTTTTGGAACAATTTTGTATCTGTTTCTTTTAAATAAATATTGATTAGAATTAAATTTCTTTTGAGAGACTATAATGTC
CCACTAAGGGTCCGTTTAGATTTTCATAAATGTTATTGTAATGCTGCTG
>comp139349_c0_seq1
TTATAGAGGCAAAAGTATGAGAGCTTAAATAGCTATCAATAGAATAAGGCCCAATTCACTTGTCCTGCCAAAAGAAAATATTACATTGACCACCCCATTGAATATTCTGCTCTGCCTTCCAC
TTAAATGAGCAAAGCCTTTTTCACAGCCTGGATTCAAAGGCTTTATAATTGCATAAAACTAATTATAGTTTAAGGACAACATAACCGTGATTAATATATATAAACCACACAAGTCACTGAGA
AAAAAAAAACAAATTAGTCGTGGCGCTTCATTTGTCTGAAACCACCATAGCTTTCCTCAGCAGAGCCATTTCCACTTTCATGTTTGATTGAGACTGATTCCACTGATTCATTATCGTCAGA
AATTGTATTCAAAGGGTTTAAACTATAATTTAAAGGACCACCATACCTTGCCCAATTCTTTGATGCCATGCTGAAGGCCTCTGTATGTGTAATCTCTGGCATTTCAGCTTTTATTCGCTGTATC
TCCTGCCTCACGAAGACATTGTAAGCTGTTGGTTGCTTCTGCTTCTTCTTTGGAGGTTTTGCAACAGGAGGAGCTTCAGGTTTTTGTTCAATAGAAGTTGATGAAGAAGCATCCGATTGCT
GTCCCATAGTGAAATCCATTTGATAGTCAAAGTATTGAGAATTAGGTTTAGGTTCAGGGCTAATGTAGGAGATATTGTTGCAGTGCCCACACTTCACTGTAACAGTCTCCATCATTCTACTG
CATGGAACTTCAACCGCAAGAACAATGTTGCAGTGTGAACAGTGAACATAACACAGCTTATCACTTGAAGAAGACAGATCCATAAGTAGAGGAGAAAATCAGAGGACGAAGAAGAAGC
AGTAAGGATAGAAACCGTAGAAACAAAAGAGAGCAGGAAGAAGGAGAGAGTT
>comp139353_c0_seq1
AAGTTTTGTCGGATGACCTGATAAAGTAGTTCCGCTGGCTTTTCAGGTCTTTACAACCTGTTAGACAATCTGTTCTCCCAAAGAACCCGGCCCGGCCCATTTCAAGAACCGTCCAATCTAT
TTAAACTCATCCCCAGCAAGAACGGGTCGCCATTTTGCCTCCCTCCTCCTTCCTCAGCGGCCCATACACCCCAATCTCGAAACCCTAATACGAGCTATGGACCACGCATCCTCCTCCATCC
CCTCCTCTGTTCGATCCCCTCGCTCTAAATATACCCAAAACGCCCAAAACACCTCGCAGCAGCCCAACTCCGCCGGCTCCTCGCCGCGTCATTCTCCCATTGACCCCCGATCCTCCCCCAA
ATCCATCTCCTCCACCAGCTCCAGTAGTGGAGGGGGATCGGTCATCGTCCACGACTCTGAAAGTAGTGAGGAGTGGGGAAGCTCGGATTCGTCCGAACATAAAGCCTTCGAGTCGTTCTT
GAGGAAAAACAGATGCATGAGGTTCTACCTGGAATGGAATGCGTGCGTCGACCAGACCATTAGGAATGGGCAAGACCATAGGATTCAGTGTTGCAGGCTTTCCAAAGTTCTCAGAAATT

GCGTCAGGCTTTCCTAGATTTCTTCTTCATTTCCCTATCTCTTCTACTTATCAGATGGCAATCTGGAAGACTTTCTTGTTTCTTCTCTCTCCATCTTTTAATAATTAGTTTCAGTGTTTGAGAA
CGGTAATTGTGTCAGTTTTTCTAGGTTTTTTCTTGAATGCTTTGTCTCTTCTCTTTATTAGGTAGTAATCAGGTCTTCGATGATCTTTTAAGAATAAGTTTCAGTGTTTGGTCACTGAAATTGT
AGTGCTGCCGTGTTAGAATCTGTAAATTTGAGATTCTTGAATGG
>comp139353_c0_seq2
AAGTTTTGTCGGATGACCTGATAAAGTAGTTCCGCTGGCTTTTCAGGTCTTTACAACCTGTTAGACAATCTGTTCTCCCAAAGAACCCGGCCCGGCCCATTTCAAGAACCGTCCAATCTAT
TTAAACTCATCCCCAGCAAGAACGGGTCGCCATTTTGCCTCCCTCCTCCTTCCTCAGCGGCCCATACACCCCAATCTCGAAACCCTAATACGAGCTATGGACCACGCATCCTCGTCCACCA
GCTCCAGTAATGGAGGGGGATCGGTCATCGTCCACGACTCTGAAAGTAGTGAGGAGTGGGGAAGCTCGGATTCGTCCGAACATAAAGCCTTCGAGTCGTTCTTGAGGAAAAACAGATGC
ATGAGGTTCTACCTGGAATGGAATGCGTGCGTCGACCAGACCATTAGGAATGGGCAAGACCATAGGATTCAGTGTTGCAGGCTTTCCAAAGTTCTCAGAAATTGCGTCAGGCTTTCCTAG
ATTTCTTCTTCATTTCCCTATCTCTTCTACTTATCAGATGGCAATCTGGAAGACTTTCTTGTTTCTTCTCTCTCCATCTTTTAATAATTAGTTTCAGTGTTTGAGAACGGTAATTGTGTCAGTTT
TTCTAGGTTTTTTCTTGAATGCTTTGTCTCTTCTCTTTATTAGGTAGTAATCAGGTCTTCGATGATCTTTTAAGAATAAGTTTCAGTGTTTGGTCACTGAAATTGTAGTGCTGCCGTGTTAGAA
TCTGTAAATTTGAGATTCTTGAATGG
>comp139414_c0_seq1
GACAATGTGTCGAGCATAATTACAAGAATGAAAACCCTACTGATGACTAACATGTTTATTTTATTTTGGCTAACTCGATCTCCATCCAGATCTCAGTCGACAGTTACTAAACCAAGCTAGCT
AGCACTTCATCTTCTTCATCATTATCCTTCTCCTCCTTAATTAAGCAAGTACTATCAAATATCCATCATCTTCCTCCTCCTACTACTAGCCTCCTCCACCTCTTGATTATGGACTTGGGATCTCC
TTCAGAGCATCTTTGCTATGTTCGCTGTGCCTACTGCACCACTGTTCTCGCGGTTGGAGTTCCATGCAAGAGAATGACAGACACTGTGACAGTAAAGTGTGGCCACTGCAACTCCCTCTC
CTTTCTCAATCCCAAACCTCCGTTCCAAACACTGTGCTCTGATCATCATCAAACGCTGCTTCAGAGGCCATGCAACTTTCTGTGGAAAGGGCAACATTCACTAGCTTCTTCATCATCTACA
GTTGCTGAAGACATGAACCCAAAAGCAGTGCCCTTTGTCGTTAAACCTCCGGAGAAGAAGCACCGCCTCCCATCTGCCTATAATCGCTTTATGAGGGAAGAGATACAGAGAATCAAAGC
TGCACAGCCTGACATTCCACACCGTGAAGCATTTAGTACTGCTGCCAAGAATTGGGCTAAATGCGACCCTCGCGCCCTTTTCTATTCTGCTACCGGTACCAGCGGAGCAAGGAGGGCAAC
AACAGCCATTCAGCAAGAGAAAAGAAGTGGCGTCCCGGTGGAAAGCTTCGAAGTCTCCAAGCATGGGCAACTGCATCGCATGGAATAATTAATAAACCACAACTACATGTGCTATTTCAT
CCGTATATATATGACTGGGACTTTCTTAATTTTATGTACGTTTAATGGATCTAAGACTCTTAAAAACAACTGAAG
>comp139428_c0_seq3
TCTCTCTCACTCGCTCAAAGCATATTCGAAGAATTGAAGCCGGACGGCAGCGGCGGAGGCCATGAATCCAGTAAAATTTTGCCCCGAATGCAACAACCTGCTCTACCCATTTGAATATAG
GGAGCTCAGGACCTTCTGCTTTTTCTGCCCCACCTGCTACCATCAGGTGAAAGCCGATTAAGGAAAAAAATTTATGAGGCCTTTCAGGGAGGAGACAATACACCTTTCTATGTACGAGCA
GCCTCAGGTCCTGCAAGATGCTTATGCAAATCGAACACTTCCCCGCGCATGGGGTGTCGAATGTCCTGTATGTCATTATCCAGAAGTTGTATTCTTTCTGGTCAGCTCCTCTTTTTTTATACA
CTTAATTTTCTTTTTAATTTTATTCTAGCTCATGTATTCTTTTTATTCAAAGTGTT
>comp139479_c0_seq1
CGAGTTCTTATTGTTTAATTTATTCACCCTCAAGTTTCTATTTAAGTACTTAGCCATTTGGGGTGAATTAATTAAAGTTTGACATGAGAGTAGAATGACATGTTATCTATAGAAATGTATATGG
AATCTACCTGATCTAAGTTATAACACTTGGGTGGCTAAGATAGTTGGTTTAAGTCCCTAAAGATGGATTTATTTTTTTCTAAAAAAGGGGCAATTTACATAGACGTTTGCATAGCACAAAGG
TGAATGTAAATTCC
>comp139640_c1_seq1
GTACGCCGGTCACCTCACCGGCGCTTCTACCTCCCCCACCGATCACACCACCTCCGCATTCACCCGATGGCAACTGGTGGATAACAATCTGCTCTCCGCCCTATTCTCCACCATATCACCCT

CTATCTTACCCTATGTCATCACGGCCAAGACCGCCCAAGAAGTTTGGCAAGTTCTTGAACGTCGGCTCCAACCAACATGCCGCTCTCGCGTCATTCAGCTCAAAAACGAGCTCCACCGCG
TTCAGATGAAAGATCTTACGATGCAACAGTACCTCAGCCACATCAAAAGCATCGTCGACAACATTGCGGCCTCCGGCTCCACCGTCGATCCGGAAGACATCATCATCCACATTCTTAATGG
CCTACCCACTTCATACAACCCTTTCAAAGCGGCGATCCGCACCTATCCCGTAAAACTTGAATTAGATAATCTTTACTCTCTTTTGTGCAGCGAAGAAATAAATGTCTCTCAAGAACAAGCC
AAAGAAACACTTCTGACTGCCAGCAACACCGCCCTTTACACCTCTTCCTACAACACTCAACGCGGCAACAAATCCAAGAAATACCTCAAGCCCAAACAGCCTACGCAAAATCAGCATTT
ACCTATGACCTCTCAGGCTCCGCAGACTCA
>comp139644_c0_seq1
CGCTTTTGATGCGCTCTCGATTCAACATCAAATCCAAGTTTTTTTACATTGCTCCATAAAAGGCCTACAAGTGTGATATGTCCCATTTTTCGCAGCTTCCGCATTTATACATTATTAAATGGA
TATGTAGCATTAAGATAGAAACATAACATAAAGTTTTTATTATCCTGAGAAAAAAGATCATTGAACGAATGTTTCTAACCTAGTTTGTCATATCGATGAACTGTGGCTGCAGGAATGCTTAAT
GCTTTATTCATTGGTTGATGAGCTAAATAGAATTGTCGCTCTCCCAAGATGTCAAAAAAACTTTGTAAATGAAGTTCATCTCAACATATAAGCCGCTTTCATATCCATCCAGTGCTAGCTGC
AAGATGCATGATGCTTAGCACAGGCTATGATTACTTGGTAATGGTCAAGATCAAGGGTAAACAAATTATTAAGAATTGAACTGAGTTTGATATATTTAACTAAAGTCCATAGCAAAGACATC
GCACACGATGTGTTATAGCATTTCCACGCCCATCATAA
>comp139728_c0_seq1
GATTTCAACGCTTATGTTAACTTCCCTCTTCTCCTTCGCGGTTGCGACCGCGATCGCAGATGCGGCGACATTCAATGTGACGGAAATGGCTTTCGATGATGAATACCTACCATTGTTCGGCG
AGCGCAACCTCCTGCGCTCCGCCGACGGACGGACAGTCAAGATTCTCCTGAATCAACATTCAGGGTCAGGATTCATTTCTTCGAACTTGTATTATCACGGTTTGTTCAGTGCGTCGATAAA
GCTCCCGTCTGCATACACTGCCGGCGTCGTAGTCGCATTCTACATGTCGAATGGGGATATATTTGAGAAGAATCATGATGAACTGGACTTCGAGTTTTTGGGCAATCTTTGGGGAAGAGAA
TGGAAGGTTCAGACCAACGTGTACGGGAATGGGAGCACAAGTCGGGGACGAGAGGAACGGTATCGACTTCCTTTTGATCCGACGGCTGAGGCTCACCGTTACTCTATTGTTTGGACTAA
TACCACAATCATATTTTACGTCGATGAGATTCCGATAAGAGAGGTAGTGCGAAGTCCGGCGATGGGCGGCGACTACCCATCAAAGCCCATGCTAATCTACGCCACCATCTGGGATGGCTCC
AACTGGGCCACTTCCGGCGGAAAGCACAAAGTCGACTACAAATACGCACCTTTCGCCAGTGATTTCTCTGAGCTCATCCTCAAGGGCTGTCGTCTTGATCCCATACAGGAGTTGCCTGCG
CATGCGCAGTGCCATGAGCAGGAGCTCGATATTGCTGTTGCAGACTACTCGGTGATGACAGAGCAGAAGCAGAAGGCCATGATGCAGTTCAGACGAGAGTACATGATCTACAGCTTCTG
CTACGATGAGTTGAGATATCCATCCACCTTTCCTGACTGCAAAATCGTGGAGGAGGAGAAGGAGAAGTTTGGAAGGACAGGGCATGTTATGCGACGTCCGTCGCCAAAGCCTGAAGTAA
TGAAGGGAGCTCTGCCTGTGCGCAAACGAAGGCCGAAGAGACGCAATCAAGCCAGGAGTAGAATTATCATTGGTGAAACAGCCGAAATATGATTATATGTTTGTAGATGTCTAGCTTTTA
TTTGAGATTGTTTACCAATTAATGTTTTATGGTATGATGATTATTGATAATTATAATTAATTGTTCAATAATGATTTTCTTTTTGAATAATTTGTTATCTTGTTTAACTCATTTAATGTAGAGAAAA
CATAGTTTGCTGTATAAATTGGTGATTACTATATCATACCGTGAC
>comp139824_c0_seq1
ATTTATTATATTGATATTTACTATTCTATATACCAATACTACTATAACATCGATCAATACATAATAATAAGACTAACACTGATAAAAATCATAATTTCAATCCGAACAAGAACAAGAAAACATAA
ATCGATCATTGAATGAAACATTCATAAATGTGTATTTAGGTCCACTTCTCTATGATCTTCTTCAAAAATCCTTTCTTCTCCTTTCTTTCCTGCAGCGATCCTTCCATCTTCTCCTCCCACATCG
CCTCCTCCATTGCCACCCTCTCGATCTCCACATTTGTGTCTTCCTCCTCAATCGCCATTTCCTTCACATCTCCCTTCTTCTTATGTTTTTCTGCATACATTTTATCCTCGATCTCATACTTCAAA
TTCATCATATTTTTCTTCTCATTTTGCTCCTCTGTCCCTCCCTCTTCGATTTCATCATCTTCATCATTAGAGTAACAATAAATTCCATGCTTCTCCTCATCTTCGTTCCTCCGGCCATTGTTGAG
GTTGATCTTGTTGACGACGGTGACCAGAACCTCCGGCTTCTGTTTCGGCTGCTTGGTTTCACCCTCTTCCATGTTTTCCTTGAAATAATCGAACAACTCCCGGTCGATTACCTCCCCGTCTC
CCTCCTGCACGCTAACAACCCGTTCGTTGAGCTCCTTGCCCGCCATTGCC
>comp139845_c0_seq1

AAATATTACTTAAAAAATTATAAATATATCCACATTCACCATATAAAGGGGGAAGCTTGATTTAAAATTTGAAGAACTTTGAGATTTGAAAAATTTTAGAGTTCAACCTGATATTCAATGTTG
ATGATCAATATGAATAATTGTTACTTGACGGTCGACAACCATCCACTAATGCCCAACCACAATCACCCAACTTCCATTGTAAGATCACTATGCAAGATCACTATCACTTGCCTATCCTTGTCA
TCATCAACAACGTCAATGCAGAACATTGCTAGTAAGATTGACGCACGTTATGGACATAAACGCTAATACTTGTTGGTGCTTCCCCCGCCGTCATGACTAACATTCATCATCCACTGTCAAGA
TCTATTATACTATATTAATTCTTTAGCCAATTACAGCTAATGATATGCAAGTTGTCTAAACGCCTATGATTTTTGAATATCTTTATGTATTTTATGGATGCATAATATTTTTTATTCTATTTTTTTTA
TGTTTCAAAGTTTTGCCTTAAAGTCAAATGGTGATACTTAATATTTAATACCATCATGCATTACTTTGATCTATACAATTTCATCAATGC
>comp139869_c0_seq2
GTCCACTACCTTCATTGCAGATTTCACTATGAAGTTTTTAACACCCTTATTTATCATATTCAACAAAAAGATGATGAATCTTTCTTTATCAAGAAAAAGGTACAGCAGTGGAAAAGTAAATT
GAATTCCAAAAAAAATCTTAATGAAATAACCCTCAAACATAAAGACGATTCCCCTTCTTTACAACAAAAAAACTAAGACTAAATCTATGCAGCACAAATCTTTATCCCACAGTGGGAAAAT
TTTCTTATAAAAATTAAAATTCATAACTTAAATAAATTTACATAAAGATGAGTTTCGTGCCACCATTTATGTTGCATTTATAACTGTTGAAGAGTATGTGAAAGGATCCGCAACCTATGTGTT
GAAAAGGTTCTCTCCACATCACATATTTGAGCCACACTTTGATATTTATTGCTCATCATTTAAGAAAAACAAAAAGGTGCATTTGGCTTGCCATAATATTTCATCCTAAGCCTGAAACTGCT
GTATCTGACACAAATTAATAAGAATTTCAAACTAAGAGATGGTTAATCTGGCTTGCAGATGAGAGAAATTGGTAACTGAACACAGAAGAGCCATCGAAAAAGATATGTTTTTGGTCGAGG
TCGGCCAGTCTCCCTTTATATTATTTCCAATCAAATTCTTGCATAATATAGCCTG
>comp139899_c0_seq1
TAAATGTAATTTCAAATTTGAAGCAATCAAAATTAGCACTACAAATCATGAAACTCTATTAGCACATTGGATTTCATCAGCTACATAACTGCAGCCATTAAACATACATCTTAGATTAAAACT
CCATACATAACTACCCCATACAATTGGTTAAGGACATTCTGAAACTACAAAACAGCTTCATTACAGAAGAGCAATATCTTCGATCTTGGAAGAACAATTATGGCATGGTAGGTTCTGCTTTA
TTAGACATTAATGGGCTAGAATTCAAGTCAGGGAGTGAATGAGAGTTGGCTTTAGCAAGCCTTTGCTTCATCAGCTGGATTAGCAGAGCTTGATTCTCTTGGTTCAGAAGCTGCGCTTTCA
TCTTCAACCTCTCGTTTTCTTTTATGATGTAGTAGTTTTGAATGTACAGGTTGAAGTTGATCCTCTCCATTTACTTGATCGTTTTTTCTTCCTTCTATACAGCTTCTCCAGTTCTTCAAGTTTC
AGTGAAGGAAGCAACAGATAAGGAACCAAGCTGATCAGAAGAGGGAGATTCCAGTTATTGAAGAGGGTCAATATGATCTGTGTGATCTATGGGGTTAATTATAAGCCATTTACATATAAAT
ATATGCATATATATAGTGCAGACAGAGCATAGGGAGACAGAAAGAAAGAGAAGAAAGAATGACTGCAGCACCAGTTTCATGCATGTGTTGTGTGCTCTCTGGCAGATCTT
>comp140048_c0_seq2
TGTATTATCACATTTAATAGGGATATTTTTAAGCATAATTCCAAAATCTTCTATTTGTTGCTTTAGCCATAAAACTTGAGCAACACAACTACCTGTGGCTATGTACTCTGCTTCAGCAGTGGAT
AAAGCTACTAAATTTTGTTTTCTAGAAAACCAAGAAATAAGCGATGATCCTAAAAATTGACAGGTTCCAGATGTACTTTTTCTATCAACCTTACAACCAGCATAATCTGCATCAGAATATGC
AACTAAACTACATGATAATAAATTTTTAGGATACCATAAGCCTAACTTATGAGTTCCAATCAAATAGCAAAATATTCTCTTAACTGCTATTAAGTGTGATTCCTTAGGATTTGTTTGAAATCTT
GCACATAAACATACGACAAACATAATATCAGGCCGACTAGCAGTAAGATACAAAAGTGAACCTATCATACTCCTATAAAGTTTTTGATCTATTGATTTTCCTGTTTCATCTAAACCAAGTCTA
GTTGATGGGCTTATAGGAGTTTTATTCAATTTGAATCTCTTCATATCAAACTTTTCTAATAATTCATTTATGTATTTGGTTTGACATATGAATGTGCCAGACTCTAATTGTTTTACTTGAAGTCC
TAAGAAAAAGGTGAGTTCTCCCATCATACTCATTTCAAACTCTTTTGACATTAATGAGGAAAACTCATGACATAAACTTTCATTTGTACTTCCAAATAATATATCATCATCATAAATCTGTATA
ATAATCAAATCATCTCCAATATTTTTGATAAAAAGAGTTGTATCTACCTTACCTTTTCTAAAACTATGTTCAATTAAAAAGTTACTTAACCTATCATACCAAGCACGTGGAGCCTGCTTTAAA
CCATATAATGCTTTCTTTAATTTATATACATGATTAGGAAAATTAATACTTTCAAAACCTGGTGGTTGTTCTACAAAAACATCTTGATCAATATATCCATTCAAAAATGCGCTTTTGACATCCAT
TTGAAAAAGTTTGAAGTCCTTAAAACAAGCGAAGGCAAGGAGTAATCTAATAGCTTCTAGTCTTGCTACGGGTGCAAAGATTCTTCAAAGTCTATTCCT
>comp140067_c0_seq1
TAGAGCCATTTTTATTTACTTTTTACAAATGCACTGAAGAATCACGCAATGCTACCAGCATTGGATGCAATTCTGGGCCACAAGTCTGCACATTCCTTTGCAACTGGTCCTGTGATGGCAGA

ACCTTTCATCTCGCCTTTATTATTTACTATGACCCCTGCATTATCTTCAAAATATAGAAACACTCCATCTTTTCTCCGGTATGACTTTCGTTGTCGAATTACCACTGCTGGATGTACCTTCTTTC
TGAGCTCTGGTTTCCCTTTCTTGACTGTGGCCATCACCATGTCACCCACACCAGCAGCAGGAAGTCTATTCAATCGTCCCTTGATCCCCTTCACAGAAATGATATAGAGGTTTTTGGCTCCC
GTGTTATCAGCACAGTTGATCACTGCTCCAACCGGAAGACCCAAGGAAATCCGGAACTTAGCACCAGAGGACCCACCACGTCCTCGCTTCGACATCTTGAAGGCC
>comp140160_c0_seq1
CCTGCCAACCATTACATTGGCTTTCGGCAATAAGTTTTATGATAATACTCAAATCATTTAAAAAAAAATATTCTCATTCAGAAACTCCAAAGCATATCGCCACAACAAGTCTAGAACTTACA
GATCTTCATCTCCTTCTGTATTCTTATCGCTGGTTAAAGTTTTTCCATTGACAGCTACAAGTTCAGTATCAAAAACCAGTGTGGCTCCTCCAGGGATCTTGGGAGGGGATCCTTGGGCGCCA
TAGCCAAGCTTTGCAGGGATCTTTAGCTTCCTTTTCTCACCAATGCACATACCAAGCAGCCCTTGGTCCCAACCTTTGATTACTTGACCGCTGCCAAGTTCAAACTCAAAAGGGTCACCTC
TTTCATAACTGGAGTCGAATACACTCCCATCAACAAGCATCCCATGATAATGCACTTTAACTTTATCTCCTTTGTGAGCTTGAATGCTACAAGTCTCTGGTTTGTACTTGACACCGATTTGCA
GTTCCGTAACATCGCGGGATTTCTTAGCAGAAACAAATATAAGTAAAACGGCGAAGAATGCAACGCAAATTAAAATCCTCCCCATCGTTCTCCTCTTGGCCGGCACCGATCGCTTGGTCTG
GCTTTCGATCTTCTTACACGGAATATGAATCCGGTATCGTCGAAGTGAACAAATCGA
>comp140224_c0_seq1
ATTGCGTGGACAAAGTTAAAAATAAAATAAAATTAATTATAATATGATAGACTATTTTTGAGATTTTATATTTATTTTAAAATTGATTATTTTTTTGGCTTCCGTTTCCAGGCGTTCTCATTCTTC
CCCCTCTCCGCCTATAAATAACAGGCTCGGTGAACCCTACGGAAGAAACCCCCAAAAGCCTTTCTTTCTCCTTGAGGTCTCTTTCTTTTATATTTGATCTCTCATTTTTCTCCTCGAGGTCTC
TTTCTTTCCTATTTGATCTTTCACTTTTCATGGGCTCAAGTTCTATAATGTCACTCTCTCTCCTCCTCCTCCTCCTCCT
>comp140235_c0_seq1
CCAACCTAGGAACGTGTGTGATTCCAAATACATACCTGCCATTGAAGCAAAACAAAAAAAAAAACAAAAAGATTATAACTGTGCATGAAAAAAACTTCACGAGATGGGCAAACTTTCAG
AAGAATTAGATGCAAAACTAAGCTGAGAATGAACTTAAAATATTGCTTATGTTGCAGTCACTCCAGAAACAGGGTGGGTAAAACAAAGTTAACATATTAAAATGGATACAATGAGAAAGA
GAGATCAATCATGGAAGTTCAATACTTTGCCTGCCTAACTTAGGAGGAAGTTGACCAAAGTAGTTCTAGTAAGGAGAGTTCCTTGTCGAGTGGACCGACTTATTAGCGAGTATAGAACTT
GTTCGAAATACATAAGGCAATTAGTTTAATAGGTATGCTGATCCGGTTTATATCAATCCTAGGGTGGGTCTTTCTATCATTTAAGAGTGACGAGTCCCATTAACATGACAAATAATTGGAGCA
TAGCTAGGACAGACTAGTATGTTGTAGCCACCATTAGGAGGATTAAGGGTTGACCATACTTATTGGATGATAGAAGACCTTATATTCTTTTTGAAGGCAGCAAATGCAATTTGTGTTGGTAG
CTCTTATCGTTGGATTTCACAAACCATAATGAGTCTTTCTTGAATGTAGGGGCAATGCAAATACATATAACATCGAAACTCGGCATCATTGCGTTAACCATCCATGTGGGCGCGACTCCACT
TGCCATACCCACATCATAAACAGATACATGCGTCATTGTAAAGGTGATTTCATCCAGCTCTTTTGGATTCACCATGAGGCAAAAGCAAAGTCGGCAGGATAACATCATGTTGGTTGTCCTAT
TGGCTTACTAAAAAATCTGAAATACATGAACCAACATCTGTGGAACATGGCTGCATGTGGCCTGTAGGTGTGTAGACAAAGGCTATGGTAGGTAGCCAAAGTGATTGGAAACTACTCAAA
TGAAAAAGAAAAAAAAAAAACAAATACTTAGCACATTGACTTTGGTTTCGCGACTAAGAGAGCGATGTGTTGACAAGTTCTTGAAATCCAATCTCCAGTCAATATGTAAAGGCATTTATCT
TTTTGGGTTGAGTTATTGAGAAGTTATAGGGAAGGATGACTTTTGAGGTTGCCTATAAAAGGCAAGCTAAGGAAAGAGGTGGGGTTGACTTTCCATAAAGAGTTGGTTTGTTTTTGGAAG
GGGGTGAAAAGAAGTTTGACTTATTAGGAGAAAGCATCAAGTACTCAAAAAAAGGAGAGATTATTGTGTCCGGACA
>comp140250_c0_seq1
TGAGCACAAAGAAGTCTTTAGATTACCATGAACTTATAATCATAAAGGGTAGATAATATTAATCATAATAATAAAACACACAAGAAAGTAGAATACAAAGTACGAAATAGAAAAAACAAAA
TACAAGCTTGGCCAAAAAACAATGGCACATGCACATCAAGCACATAGAACACATGACGATTATTCTATTTCGTACTTTGTATTCTACTTTCTTGTGTGTTTTATTATTATGATTAATATTATCTA
CCCTTTATGATTATAAGTTCATGGTAATCTAAAGACTTATTTGTGCG
>comp140256_c0_seq1

CCTCTCCAATCCAATTAGGCCGCTGCCCGAGCTTTCTAATGTCCGTGAACATGTCCTTAAGACGGATCGCTCCTCTCCTCTCAAAATTTGATCTGATCCTCGTGCTGTACTCAGGCAACCAC
CTAAATTCTTTCTCCTCGACCACCGCCGTCGGAGTTTTTCCTGCCGCCTGAAGACGCTGGAGCTCTGCCGGATTTTCAGGAAACACAGGAATTCTGCACAACTTCCACATGATGCTGAAA
TATTTTCCAATTAACAACAGAGTTCCCTCTTAACGCCAAAACTCCGCCCAAGTGATACTGGAGTTCTCCTCGGCCACCAACAAATGTCGGACTTCTACCCGACGCCAGACCTTCACTCATC
GTCAAGTTTCTACCCGATGTCGTACTTTTACTGGTTATCGAACTTTTGCTGGACATTTATCTGAAGATGGATTTC
>comp140264_c0_seq2
CCAAGCTAATTTAGCTCCAAATGCCATTATCCTTTTTACTCATCAAAATTCCATAAATTATTATCTTCCAAGACTTCTACCATCTAATCTTATTCTGGATGGCCAAATCTTGGTGGACTCACCA
TCAACCCCTCTTCCATCTCAAGCAAAATATGTGGCATATTTTATAGTTCCTCTTTATCAAAATACTCACGTCTACTGTGGTGGCACCTCAGATGTGTCTATAGGCTCAGGCCTCGTACCAACC
TCAGATGTGTCCTTTGGCTCAGGCCTCACACCAAACTGTGCTGCTGCTACCGCTGCTGCTGCTCGAATATCAGTAATTGAATTCGATTTTGGCACGGGATGTGATCGTATTATGTCAGGAAA
ATTAAACGCTCCATTTTTCCCATGGATTGTAAGCACGGCCACATCATAAGCAATGGCGGC
>comp140341_c0_seq1
GAGTTATTGAGGAAAAGAAGCAAAGTAGAGGACGAGCTTAACTATAGGTAATCTCATTGCTGTCGAAGAAGCTCGCCAAATTCTAAGCTTTTTTTTATCTTCTGATCGATTCAAAATGAGA
GGATAGAAAGAAATTCGTTGGATCGTAGGGATTAGTTTGAGTTCTGGTTTTTTTCTCAACTTAAACTCTGCTTCCCTGATCAGTTTTGGTCTGATTTTCTTATTTGAAATGGAGACGGTCCG
CTGTGAAGTTTGCGGACGAAGCTTCCCGAGTACTAGATCGCTTGCAGTGCACACGAGGTCTCATGCTGTGCTGAAATCTGAGGAGGAGCCAATGTGTACGTATGGACTTAGAGAGAATC
CGAGAAAATCATGGAGGTTGAAGGAGAAAGAGAGGGATATGCTTTGTAATAATTGCGGGGAAGGATTCGGTTCATGGAGTTCTTTTTTTAAGCATACGAAATGTCATTCTGAGACGAGAA
GAGATGTTCTTAGCGTGGAGGTTGATGAAGATGATGAGGATGAGGAATTCGAGGAAAAAGAGGAGGATTTACAGAGTAATGAAAGTTATGAGGAGGGAAAAGATGAAATGCAGAGCGA
ATCTGAAAGCCAAGTTACTGCAGTTGTTTTTCCGGCTAGAGAGCGGAGGAAGAGGAGGAGATCACCGGATTTATCGCCGGAAGATGAGAATGAGATAAAGTTGGGTGCTTTTTGTCTGC
TGATGCTATCAAGAGGCACCGAGTCTGTTACAAATTCGTCTGATAAAGTTTCAATTGTAATAGAAGAGGAGGTGGAGAAGCAGAGAAACAATTCAGGTTTCCCAGCATCCGATTTTGAAA
TGAATGTGCTAAAAAAATGTTCAATCTTTTCAAAAATATGATTTTGGAATGAATGAGCTCAAAAAGGTCCAATCTTTTCAGTCTGAATCTGAGTTTTCCCATAAACTAAAGAAGTCAAAGGC
CTCTCCCGATAAGAAGCATCTCAATTCTGTTGTTTCAGAGCTTGGAAAGAGAAGCACATATAATTGTACGTTATGCAACAAGAGCTTTCATTCTTACCAAGCTCTTGGAGGTCACATGGCA
AGCCATAAGAAGATCACAGAAAGTTCCTTATCTGAAATTCAAGGAACTGGAGAACTCAAAATTCAGAATCAAAGATCAAAGACTACTAAGAACAGTCATATGTGCTCAATTTGTGGCAG
AATTTTTGCATCTGGTCAAGCTCTTGGTGGTCATAAGAGATCACACTTGGTTCCAGCAAACAATGCTGCTTCTGCTGATACTGTTCAAGCTTCACCTGCAGTAATTGAGATAGAACAGAAG
AGTTCTATCCTTGATTCACTTGATCTCAACCTTCCTGCTCTTGATGATGAAATTTATGATAATTCTAGTGGAAGTATTGAAGATTGCGTCTGAGGCATTTGAAATTTACGTAGTCTCATGCGA
AATCTGTTCGAATTCTCCTTTGAATTTGAATCGGCTATTCATTCTTTTCATCCTCAGTTCAGGACATTTGAGGTGTTCTTACAAAAATGATCCAGTGAAGTAGGTGATTACTGGATTAAGAA
GCTAAATTGCTGCTGCACATTCTTGTTACTGCTAATCTGTTCTTTAATTCGAAATTTTTATTATTTTTTCTTAGAAACA
>comp140438_c1_seq1
TTTTTAGAAGAAAAAACATTTATTATCAAATATAAAAGTAGACTGACATTGTTCTGCATCTGATGACAATTTCACAAGGTTACATAGTATTATTTTAAAGACTATTGCACAATACGAGACAAT
ATTGACCATTAGTTGGTCTTATTCTTCAGTATCTTTAGCTATGAGGCTTTCACATGAAACTTTTATTTTTGACTAAGGACAATAATTGAAAGCAGCCACTTCCACAGCCCTCTACTGTGTACT
AAACCACCACCACAAGGATGGTGGTCGTGTCCGTGGCAACCTCCTGCTTCTACGAAGCCTTTAGCGGCTGCCCCCTCCGTCGCCACCGCCATCTCCA
>comp140455_c0_seq1
AAAACGAAACATAGAGTTTGATTAATTAATATATAACAATTTTTCAAATCCCTCTCAGAAAAGATCATAAAGTCATATAGTTTTTAACAAAGCAAGGGGTCTCTACATTGAACTAACATGGC
AAGTCAAAGAGTTTCATGAACTATAATGTAGAAACATCTATTTTCTATCATTCCAATTCACTCCAGATTTAGTTCCCATGAATAAAAGCTCTTCTCCATTTCTTAACTCTCTTCAGCTCCAGG

ACGCAGCACTACCACCCTAATTGGAGGGCAGTCTGCTGTTGTAAAGAATAGTGATGGCTGGGATCCATTAGATTTACTTGTAGAAAGTTTCTGGAATCAAAGGAAGGCATGACTTCATACT
CAGTGCTTGTTGATGGCATCATGTTTATGTGTTGCTGCTGCTGTGTTCTTTCATTATCAGCTATCTTATTTCGGAGGTACATGTTATCATTTTGCAGTTCCATCTCCCGCTTCTGCATGTACTCA
ATCTCAGCATATAACAATTCATTCTTTTTAGATCTGATTTTGTTGATGCCTTTCTCTAGTCTTGTTTCCAGTTGCTTGAGGTCCCTTAAGCTCATAGTACTTAAAGCCTCGCCCATCAGATTCC
TGTTTGAGTTCTGTAAGTTGGTGATTTGCTGACGAAGTTTTGAGGCTTCTTGCAGGTAATACTGAGCATTTGTTTCAGATATTGATCCAGAATTGGAATTATCAGCACTAGCTTTCTTGTATC
GCTCGATCGTTCCCTTTACACTGTTATTGGCATACTCGTAGAGGCGGCCACGGCTGGAGAAGATGACAAGGGCGACTTCAGCATCGCAGAGAACGGAAAGCTCGTAAGCCTTTTTGAGG
AGGCCATTACGACGCTTACAGAAGGTGACTTGCCTGTTCGTAGTGTTTTCTATCCTCTTGATCTCTATCTTTCCTCTTCCCATCTTTTCCTTTGGCTCCATCATTTTCGGTTTCGTTGCTCTCT
GCAACTGCTGCTGTGCTTCTTCCCTTGTGCCTGCGTGAAAATAAAGCCAGCTTTTGCTAAGAAAACGAGTCAGCTTGCAGGTTAAGAAGCTGATGACTTGAAGCTTTCCCTCTCTTTCTA
CAAAGGAAGGACTGGGTAATAACTCAGGCAACCATTGCCGTCGGTTTCAGAAATTTAGAATGTGGGGGGCATTAGAGAGAGAAAGGGAGAGAGAAAAGAGTTAGAGGGAGAAAGAGG
GGAAAGCGTCTTTTTGAAAGGGATTATTAATGGTGTTTGAATACGACGTTGGACTGAGTTGCACTCAAACTAAAATGCTAGCAAAATAAAACAAAAAGAACGGGGGAAAGATGAAAGCA
ACGAGTGCGCCATTGTCCTTCAACATCCTCTTCTACCCTT
>comp140493_c0_seq9
CGACTTGGAGCAAAGGAGTATTGCGTCCTCTTCTTCTTCTAAACTTCATGATGTATGTTACTGTGCTAGGTCTGGCTGGTTGGTCCACTGATAAGTATATCAACCACAACACTGCTCATCAA
CGTATGAGAGGCAACATTACTACGATGTACTTACTGATATTCTCGCTAATCGCCGGAGCGACGGGGGTGTGCGCGGTGGTGGCCGGACTGGTACATGTTTGTGCATGGCGTGGGATAGTT
TGGCTTCTGCTGTGTCCTCCGCCCTCGTCTCTTGGGCCATGACAACCATTGCTTTTGGTCTTGCATGCAAGCAAATCAGTATGAGAAATCGACATACAGACTTGAAAGCTTTGGAGGCATT
CATCATAATTTTGACAGTGACCCAACTGATCTATCTCATAATGCTCCATTTGGGCATCTTCAGCAGCAGGTATGGTGTGGGTTATAGGAACTACTGCATCAATGCTCCTATGATGATGGAGGA
GGAGACTCCAAAGGATTCAGCAACTGTTGCAAGGCCTTATGCTGCTTAGAGAAGATTTATTAGTTATATAATATTTGTTAGGCATGCAGTATGTTCTATTGATTTCACTTCTAGCTTTTATTTT
GGAAGACATTCTTGCTTAATAAAAGCTAGAAGTGAAATCAATAGAACATACTGCATGCCTAACAAATATTATATAACTAATAAATCTTCTCTAAGCAGCATAAGGCCTTGCAACAGTTGCTG
AATCCTTTGGAGTCTCCTCCTCCATCATCATAGGAGCATTGATGCAGTAGTTCCTATAACCCACACCATACCTGCTGCTGAAGATGCCCAAATGGAGCATTATGAGATAGATCAGTTGGGTC
ACAGTCAAAATTATGATGAAGGCCTCCAAAGCTTTCTGAAAAAGAATAGAAATATAATTTCTTGTAATGAATTTGCAATTTTATATTGCATTATTGCTATGTAAATTTTATCAAGTCTGTATGT
CGATTTCTCATACTGATTTGCTTGCATGCAAGACCAAAAGCAATGGTTGTCATGGCCCAAGAGACGAGGGCGGAGGACACAGCAGAAGCCAAACTATCCCCACGCCATGCACAAACATG
TACCAGTCCGGCCACCACCGCGCACACCCCCGTCGCTCCGGCGATTAGCGAGAATATCAGTAAGTACATCGTAGTAATGTTGCCTCTCATACGTTGATGAGCAGTGTTGTGGTTGATATAC
TTATCAGTGGACCAACCAGCCAGACCTAGCACAGTAACATACATCATGAAGTTTAGAAGAAGAAGAGGACGCAATACTCCTTTGCTCCAAGTCGCCACCATTTTCTCCAAATTACTTAGTT
TTTTTTTCTTGAAGATGTCTGCTTGCGTTTATATAAATGGTTAGGAAGAGAGAAGTGTGGCC
>comp140582_c0_seq1
AAAAAGTAGCGGGTACAGTTGTCCTAAGACCGCTGCAAGGAAAAAAGTAGCGGTTATAACCGTAGATATAGGTCTAAATTTATTCTACGGTAGCCCTATTTTTGGATCTTTAGGCAAATTTT
ATTGATATCAAGGGAGAAAATTCAAGTAAACATATATAAAAGGATTTGACTATTAGCCAACCCTTCAAAAACTGGGGATAGTTCCAGTTAAAGATACATGGATCATACAAGCTTCTTTGGC
AGCCAACTTAGAAAAAACAGGTCAGAGAGTGCCTTGTCATGCTCCTTCCCAAAGGCCGAAGAGCTGCTCTTGCAAGAAGAACACAAAGAAGAAGAAGAAGAAATGCCATTATCTCTTT
CTTCTTCCACAATAGTCTCCATTAACTTCGAAACCTTTTTCTTCTTCACCACTGAAACCAGCTGAGGCGGCGCCACCTCTGTAGCAAACCGCAAAGCCTTGGTCTTTGCCATTATATATCAT
GAAGGAGAAACGAAGCTAGCTTGATTACAGGAGAAAGAAAAGCAAGTAGAAAGTTAAAGAGAGAAGTTGAAGAGGGGGGTGA
>comp140610_c0_seq2
AAAGCAGTAAAAAAGCTGTCTCAACCAACACCTTAATACAGTTTGCTTAAATTTGATGACAATAATGACTTTGGTCGTATCGAAGCCATTGTTGATTGCAGGAACCTCAAAATGCAACTAC

TTCTAATATCCTCTGTCCTCATCTGCACCAGATTTATCACCACTATTTGGTCAGCTTTCAGTACTGGAAATCCGCCAAACGCGAATCTCTCAAGTCCTTTTCTCAGAATCAACGGCCAGGAT
CCGTAGCAGATCATCGAATCCGACGCCGGCGGATTCGTCGCACCCGCCGGAACGGATCATCTCGCGCACATCCTCGTTGCTGGCGGAAATCCCCGCCCATTTCAAATACGCCTTCAGGTC
GTCGAACCCCACCCTTCCGTCTCCGTCACGGTCCATCAAGCGGAAGGCCTCCTCCATTATCTCGCCGTCACTAGCGGCGTCGGGTAGGATGCGCTCAAACTCGTCGAACTCGACAAAGCC
GCTGCAATCGGCGTCGGCAGCGGAGATCATGGCAGCGATGACATCGTCTGAGGCAGCGGGGACGCGAGAGAAGAAGGCGCGGAGATCGTCGCCGCTGATTCGGCCGTCACGGTCGGC
GTCGAGGACGTCGAAGGCCGGCCGGATTTGCGTCGATTTAGCGGCAGGGTTGCGGTGGGGATGGCGGGCGGTGGGGCACATTGTGCGGTTTCTTTTCTGTGTTGGGGGATTTGATTACTA
CTTTAGAGTTTTTAGAAAGGGGAATGAGAGGGGAGTAAAGAAGAAAGGTTGAAATTATGGCGTTGGGGAGGGGCATTTTTGGGAAAAAAATTAAGGTTGGG
>comp140650_c0_seq1
AAGAAGTATAATATTTTAAATAGCAAAAGCATGTAAATGAAGGACATAACAATAACAATTAATGCTCAGATCACAAAGATAAAGAAAGGCAAATTTAATTTGTTTCCAAATCCAATATTATC
TTACTATGTTATAAATATTTTCTTATTTAGTAACAGGTTTCTGTGCATTATCTACACCAAGGCCATTCAACACACCAGCCTGCTTCTCAGTTAAGCCATTTGGAAAGTAAGAGTCACAGAGCT
TCCTGAAGGTGCAGGAGGTGACGAGGCTGTCAGAGCCCGCCTGATGGCTGATCCCAACTCTCTGCACACCAACCATCTCCGCAATCTTACTGAGCCCCCCATAAAGATTGTTGCAGAAC
TTTATAAGATGTTTAATGTCATACAGAGTCGGGAAGTAGATCTTCATGAGCTGAAAAAAGCCTTCCCGGGTATCAGGAAGCTCTTTGCAAGTTAGTAGCTTTAGGAGGTAGCCGAAGTCAT
AGCTGCCGTGGAAGGCGATCCAATGCACATCCTCGTTGAGAACAATGCCGGAGGACATGAAGAGCTCACCGAAACGAAGGGCGTCGACGCCGTGCTCTCTGTTCTTCTTGAAGTTGATT
CCACACTGGCGGAGGAGCTCAATGGAATCGGAAGCGCAAATGTCGCGCTCGTCGTCGAACTCCCGGAAATTGAACTGCCAGACGCAGCCGTAGCCGGTATTGCAGGTGGGGAGGTTTC
CGCGCTCGTCGGCGAACGTGAGGCCGAGCTGGATCAGCTTCAACATATCGACGTTAGCCTTTAGCATCGCATATTGATAGTCGAATGACGACTTGAAGTTTCCTACCGGCCGGCAAACGA
CCCCCGGGAACTCCGTGTCCATGGCGACG
>comp140680_c0_seq1
AAACTATTTTTAAAATTTTTTTTTGCCAATTTCAGCACATGAACGAACACCAAAATTGATGTCAAAGTAAGAAAAAAAAACCTTTAAAAAAAAATGCAGATCAACTGCCCTCCATTATTGCC
ACACGGGTACCTGGCGTTGCAGCTCTTTCACCAAACAAGAAAAACTTAGTTATTTTACAGAGCAGATAGCTGAGCATGTTGTGAGGGATGTCATCGCTGCTCTACAATCTTCAGTTCATCA
GAACTCAGGGAAAAAAGCCATGGCCATAATCCTGTGGTTCATGAGAGAACATATCCACAGGAGCGTTGGTGCAATGGAAATTCTCACCAGTAAATTGATGATCAAGATATATCACAATAA
CTGTGATGTCGTCATGAATTTGACGCCTTTCTCCTTTGGGTGTTCCTTTCAGGCTACCGTAACTCATCTTGCGCTTCTTTGCGGCCTCATTAAGCGCTGCTCTGACCAACCTCTTCGCAATT
CCCTGGATGATCCCCTTAATCCGCCACGCTCCTCTTATATGAACTAGTATACGGGAGTCATCTGGGTGTTGGTTGATCACCTCATTCCTGACTTCCTCGTCCTTCACATTGTGATCAGAAGTT
AATCTCTCCGCCACCACCTCATTTTCCCCAGGTATTCTGTTACGGCCGAGCACCGCCCTGGAATCTCCTAAATTCCCCAAATAAAGCATATCCTCCACTATCACCCCAACGAGGCAACACG
AACCAACCGAAACAAAATGCGGACAAGACTGCCAAGAACGCCCCACCATTTGCAAGAACTCCTCCTCAGAGGCACGGAAGGCCTTCTTTATCACTTCGATTGACACCCCTCCATCCTCC
GTAACAAACCTGTGGAGATTAGAGAACATCCGGGAGTTGAGGAATCGTGAGGCATCGGGGCCGCCATGACCGTCGTAAACGCCAATGAAGGTCGCGTAAGGAGTATTGAAAACCTGAC
CCTGATCCTCGAACTGAGAGTTTGCCTGAGAAATAGCGAATGAGAAGTCTCCATAGTTGTGTGTCTTGAGCGCTAAGTGCCATTGAAGCTCGTCGTCGCTGCAGAAAAGCCCACAACAC
CGACTCATTGCCCTTAAAAAAAGATCGTCACTCAATCTCCGTTTCTGATTGGCAAATGCAAATCAGCAATTTACTCCTCCCTTTCAACTTCTTCGAGATCTTGGGGTTGTTAGGGTTTTAAG
AGGCAATAGAAATTGAATTGAGCAGTGAG
>comp140692_c0_seq3
GTGGACAGCAGTTGTTCTTGAGATTTGTTGCCAAAGTGATTAGGAAATACCAATCGTGGGCTGTTTTTGACAAATTTCGGCGGCTGAAATTTGTGCCGGCGAAGAGGAAATTGCTGAAAT
TGTTTTTCAGATTTAGAGATCGTCACCAAATTTTCAAGCCGTAAGAGATGGCGGCTAAGAAGTTTGTTGATGCTTTGGAGGAGCGAATTGAAGGGGAAATAAATCAAATCAAGG
>comp140756_c0_seq1

TTAATCGAATATCTGCAATAAATAAGATACCTCAATCATATCATAAAAAGATTTGATGGGAGGAAATTTTGAGGTCATAATTTTTAACACATTGTACATCTAAAAACAGAAAACTAATTGACA
CTTTATGGAACATGGCCTTTTTGCCCTTTAGATTGATATGCTTTGTAAACACTTCACTGTCTTTATCTCTCTCTCACTCACTCACTCTGGGTTAGTATCTTCTGATGCAAAAGATCGATAGATG
TCTGAATTTGAAAATCTGCTCAGTACATACATATAATGATGTAATTTCCAACTTTGATTGCTAAGGCCTCCCTAAGGTGAACTCGATAATGGGCTTCTTCGAGCTGGTCTCTGATAGGCAGG
ATGAGTTAAGATCCGAAATCATCTCAAAGCTTCTGGATTGCCTCTCACACTGATCAAAAGGGGACATCCTCCCTCTCCCTGATGAGGTGTATTCCTTTGGTTTGTCATGAAACAGCTCTTC
CCTGGAGGAATTGCTCCATAAACCATAAGAATTCGTGCTATTTTGTCCTGCTGACTTTCCATTTTCAACTGAAGATTCGGGTCCATGCACAATATTGTTTAAATCGAGCAAGTTGTCATCA
>comp140812_c0_seq1
CCTAAAAAAAAAAAAAAAAAAAACTTCTTTCTCAACAGGAAGAATGGATAGAAGTTCCTCTTCAAGTCATTGAATCAAGAAAAAGACAAACTACTACAGAGATGGCATCAAGAACTATAA
ATCCGAGGTGTCATAGTTTGTAAGCGTGAAGAAGGAAGCAGCCCTTTTCAAATCATTTTCCTCCCCTTGCTCTATAGTTTTTGCTTCCACTTCAATGTCGGAGCTGCTCAGTGCCATGACG
GTGGAGCCGACGTTAACTTTCGATTCTACGTCCTCATAGTCTGCACTAATGCCGAATGCATGGCGCCCCATTTCAGACATGTCAAGAGGCGGCCGCTCGTCCACATCTTCCTCACCCTCTG
CTTTCTGTTGAATGCTCAATATCTGCTCCTGCGTCAGCTCTTCGTCTGCATTAATCGTATTAATATCGATACCAATGAGGTTGTGTTCATGAAAGTCATGCAGGTCTTGTTGCCCATCACAGG
AGAAGTAGTGATCATTATAATACTGATGCTGCTGTTGGCTGAGGAGAAGATTTGGTTGAATATCGGAACTTGCGTCGAATTGAGCTCGAAAACTGGCGATTTGGCGGAGGACGAATTGCA
GCTCGGCCTGTGCAGCGTTAATCCTTCGTTGGAGATCAACGATGATGCGGTAGCAGCCGCCGGCGGGGTCTTGGGCACGAACATTCGATTGGAAGATGATGGAATTCATTGCCTCTTGAC
GGCGGTCTGGCTCGATGGATTTGAGTATTTTGAGGATGTTGGAGACGCCGAAGAGGCGATGGACGTTGAGAAACTGGCTTTGTTTATCGGCGGGGAAGAACGGGGCGAGAGTGCAATC
GGAGGTGCATTTGCGGCGTTGGAATTTGCAGGCGGCGCAGGCGGTGCAGGCGGAAGCAGTGGGGATGGAGTAAGAAAGGTCAGACTCGGGGGTCAAGGGGTTCATTTTTCGGAGGAC
GGGTGATGGGGAGGTGAAGGGGGAATTGGGATGGACGAATAAGGCGTAGCGAATGCGAAGGCTTAGGCGGGGAATTCTGATGCTTTTTCTTTTTTTCGCTTGATCTTGGTCATGCTTAGA
GAGCAGTGGTAGTTTAGAAATGAGGGAAGGAGGAGGCGGAGGG
>comp140820_c1_seq1
GGCCCAAACGAAACCTTAATTTCGGTGAAAAAATAAAGAAGAAAAATTCAAATGATCATTATTAAATGATGGATAGTAAAAACACTTCATCATTTATGTCTTTTAGTGCCTTCGATCATTAT
AATGGATAAATATCATACATGAACTCTACGATAAACTCTTATTGTTTTGACGACCGTGTCATTTTTCTTCGATGGGACTTAACGAGAATAGACCATAATACATGCACATCTTCGTATGGGCAT
GACTTCACATCTTCATAAAGGATGTATATCCCTCGCTCTTCTTGTTGCCATAATTCATGTGAAACCAGAGCTTCTTGAAGGATTGAAGCCAGCTCATTGAGAGGAAGAGGAAGTGAAGAG
ACTGCTGGGTCAAGTTGAAGGAAATAAAGATG
>comp140895_c0_seq1
TTTTTAACAATCTTAAGAGTTCTATTTTGCATTTATAAGCATCAATAAAATAATTAAATACATTCACCGCAAAGAACACAGATAAAGCATATTGAAAAATATCATAAAATCAGTGAATCCTCT
TTAAATCATACCCAAAGAAACGAAAATGGAGCCATGCATTCACAAGACAAATGAGGAATTTAATGAGATGAATGAATTAGTAATGCCGGTGGCGATGAAGAAGGCTTGTTGATCTTCTTG
GCAAGCTTTTGAACCTTTTTCCATCCCTTCTTGCAGGTGGAACCGACAACCTTTGCCCCCTGCTGCACAAGACGTTTCAGCTCCTTCCCCCTCTTCTTCAGCTGCAGCTTCTTCTCCAACT
CCACCATCTTCTCCCCCATCTCACCCATCTCTCAGACAAAGACAGAGAGAGAGAGAAAAGTAGCAACAGCTGCGAGAAAAAAAGAAAGGGCTTGTATGGCTACGGCCTCTGATGGCCTGAT
TTATAGCAAGAAAATGAGAGGAGGGGATTGATTTCTCTGGGTTTTAGTTGTTTTTTTTGCTTTGTTTAGAAAACATCTTCGCTTTCGGTGGATACGCGTAGACAGATATGAAATAATGAAAA
TCGTATTCTTCATAAGACCTAAATATGAGAAAGAGAGAGGGAGAGATAAGAAAGAAGATCGTTGTGTTGGATTGCAACAAGAATAGGGTGGAATGGTTACAGCCGTGCATGGTTATGACT
TCGTCAATGGCTTCCCGTGCAGTTTTGAACTCTAACGGTCGGATTCTATTGCTTGAACTCTGACAATTACAGCAAAAATAACTAGCACTTGCAACAGTTGCCATAATGGGGTTGATTCTTA
AGCTGTAATCCATCCGGTTCCGCTAGGTGACAAGTT
>comp140916_c0_seq1

AAATTGTTCTGATGAGATGGAATTGGAAAAAGAATTTAATTAATAATAACATCTGGGAAAATAAGAAAAAGAATTAAATGATATTGTTGTAAAAACACTAATTTCATGCACCATATGGTTTT
GATGTTACATTTATACAAATCATTTTTATTATTCTGATAACATATATTCAACGCTTAAACCAACAAAGGGATCGATAGTACTCCTACAGTCCTACAGCACAATTTAAATAAACCAAATGAATAC
TAAAAGAACAAATCGAACTCTGCGATACAATCACTACGTCATGAGGAAGAAGATGAATCAGAGTGTAGATTCTGTTCCTTCTGAGTGGGATACGGAGTGGAGCTTTCACAAGCTCTAGTC
TTCCTCTTCTGTAAGAAACTCTGCAGCGATCTTTTCATTGACAACCTCGAATTCAGTACCTGGTGGTCCGGCGCCGGCGCCGGCTCCGGCCATGAACCACCAAGAGCAGAAGCTGACTG
GCCGCTTCTCTTCATCTTCTCCTCCATCTCCCTCTTTGCCATGCATATTATTGCCCTCGCCTGAATCTCAGTGACGTCGCAGATGCAAACCTGGCCGCCATAGAAAATACTAATTTTCTGCTG
CAGATCGCCGGCGGCGGGGGTCGCCGGTGGACCTCTTACCGACACCCCCGGCCTTCCATCACCACTTTGGCTCCTGCTACTGCTCGACCGGCCGCCAGGCGTTAGAGAGAGCTCTGTGT
CACAATCGATATTGCCGGACGCCATTCTTCACCTTAATCTTCCTTCAAATTTTATACCAGTTTATATCACTGAAATTCAAATATCCTTCTCCTTTGTGGATTCCACAAGAACTAGAGAGAGAA
ATAGGATTGGAGATAGAGAGAGAAATAGGGAGAAAGGGCGCACGTGATTTATAAATTGCCTC
>comp140923_c0_seq1
CATTCTTTATATCATTCTCTTGCTCATCTCTTCCATCGATACCCATTGCTTCTCTTGCTTTAAGGGTTTCCTTTTTTCTTTCTTTTAAGCAGTTGAGGAGAAGAAGTGATCAGGATGGGGAGG
GGGAAGATAGAGATTAAGAAAATAGAGAATCCGACTAATCGGCAGGTGACTTACTCGAAGAGGAGGGCTGGGATTATGAAGAAGGCGAAGGAGATCACTGTTCTCTGCGATGCTGAGGT
TTCTCTGATCATGTTCTCGAGTACCGGGAAGTTTTCTGAGTACTGTAGTCCGTCGACGGAAACGAAGAAGGTTTTTGATCGTTACCAGCAGTTATCTGGTATTAACTTGTGGAGCGCGCAG
TACGAGAAGATGCAGAATACGCTGAACCATTTGAAGGAGATCAATCATAACCTGAGGAGGGAAGTAAGGTGGGTGGTGGGAGATTGAAGGGCTAAGGGAGAAGAGTACTGGAGTGATA
AAGCTACTAACGCGTTTACTTGTTTACTTTTGTTTTTTGGCTTTGGGAATTAAGTGTTAATTTATGTGATTTTTTTTTGGGTTGTCGTGAGAAGGCAGAGGATGGGGGAAGATCTGGAGGGA
CTGGACATCAAGGAACTGCGCGGTCTTGAGCAAAACATGGATGAGGCATTAAAGCTTGTACGGAATAGAAAATATCATGTAATCAGTACCCAAACGGACACATACAAGAAGAAGTTGAA
GAACTCGCAAGAAACACACCGAAACTTAATGCACGAAATGGAAGTTGTTGAGGACCATACAGTCTTTGGGTTCGACGATGATTCGAGCAATTATGAGGGCGTTCTTGCCCTCGCAAATG
GAGGGTCTCACATGTACGCCTTTCGCGTGCAGCCCAGCCATCCAAACCTTCATGAAATGGGATATGGCCCGCATGATCTTCGCCTCGCTTGATATATGCGTAGTAAGTGCACACGAACAAA
TGCATTAATTGCTCCATGTTCACAAAGCTATATGATCTTTGTTCTTGACATCCAATTATTGCAGAACTAACTGTAGCTTGTTCTTATTTAAGATATAATTCTGTGACTAAGTGCTTATGAAACA
TGCAATAAAGGTTTGTCCCAGACAAATTTATTTCACTTTATTTGCTTTTTTATGTGTTTATTGTGCATTTGATAGATAATTGAAAG
>comp140923_c0_seq4
CATTCTTTATATCATTCTCTTGCTCATCTCTTCCATCGATACCCATTGCTTCTCTTGCTTTAAGGGTTTCCTTTTTTCTTTCTTTTAAGCAGTTGAGGAGAAGAAGTGATCAGGATGGGGAGG
GGGAAGATAGAGATTAAGAAAATAGAGAATCCGACTAATCGGCAGGTGACTTACTCGAAGAGGAGGGCTGGGATTATGAAGAAGGCGAAGGAGATCACTGTTCTCTGCGATGCTGAGGT
TTCTCTGATCATGTTCTCGAGTACCGGGAAGTTTTCTGAGTACTGTAGTCCGTCGACGGAAACGAAGAAGGTTTTTGATCGTTACCAGCAGTTATCTGGTATTAACTTGTGGAGCGCGCAG
TACGAGAAGATGCAGAATACGCTGAACCATTTGAAGGAGATCAATCATAACCTGAGGAGGGAAGTAAGGCAGAGGATGGGGGAAGATCTGGAGGGACTGGACATCAAGGAACTGCGCG
GTCTTGAGCAAAACATGGATGAGGCATTAAAGCTTGTACGGAATAGAAAATATCATGTAATCAGTACCCAAACGGACACATACAAGAAGAAGTTGAAGAACTCGCAAGAAACACACCGA
AACTTAATGCACGAAATGGAAGTTGTTGAGGACCATACAGTCTTTGGGTTCGACGATGATTCGAGCAATTATGAGGGCGTTCTTGCCCTCGCAAATGGAGGGTCTCACATGTACGCCTTTC
GCGTGCAGCCCAGCCATCCAAACCTTCATGAAATGGGATATGGCCCGCATGATCTTCGCCTCGCTTGATATATGCGTAGTAAGTGCACACGAACAAATGCATTAATTGCTCCATGTTCACAA
AGCTATATGATCTTTGTTCTTGACATCCAATTATTGCAGAACTAACTGTAGCTTGTTCTTATTTAAGATATAATTCTGTGACTAAGTGCTTATGAAACATGCAATAAAGGTTTGTCCCAGACA
AATTTATTTCACTTTATTTGCTTTTTTATGTGTTTATTGTGCATTTGATAGATAATTGAAAG
>comp140926_c0_seq1
ATAAACATGTTCAGTTGTGGAGTTTAATTTGGTTAACTAAACTATGAAGAAAATTTTTATGATAACTCAATAGTGAATCGATTAAAGTTTTGTTTGAGACGGCTTTCTTATCACTTCTTAAAT

CAATTTTCTATTACAAAAATTTTAATTTTAATACTAGAAAGCTACATTAGAAAATAATAAGAAAGTTGTTTCAAACGAAGTCTAAGATTTCGTTTGGTAAGGCTTTCTTTCTGTAAAATTTTT
GTCCTAAGAAGCTGCATTATGAAGCAATAAAAAGCCGTCACAAAGGAAGCATAGGTATATGCCAATGTTTTCAACATTTGCAAGTTGTTTTGGGAATAGTACCGAATTTTTTTATTGTCTTG
GTTTCTTTTTTATGGGGGTTCTTTGGTTGATATTTAAGATATTACGATCATAAAAGCTTCCCGATTGAAACTATAATAAATTCTATAGTGATGGGAATAGAACATAGCTATTAATTGGATGAGC
CTATGTAAAATTATTGTTGTGTT
>comp140927_c0_seq2
GTAGACGCGTACTCCGCTTCCTCCGCCGCCTTAGCATCCACCGGCTTAGCTGCGTCACCATCCCCCCTCCCCAGACTCCGAGCTGAATAAAGCAGTTTTTCGCCAGTAGAACTCCTTGAA
GGCTCGAAACAAAGGGCTCTTCTCGAGGGCGCCATTGAAAGCGCGGTCAGGGAACCTGCTGCATGGGATTCACAATATCCCTTGACGGCAGGAGGAAGACAGTAATTTAAGCATGGGTT
ACGGAGCACGATCTGCAGTAGAAAAGTCGCAATCTATATCCCCTTCCACCTTGATTTCTGTCTCAATCACCTTTTTGGTTATGCAGTTTGGGTAATTCAGCCCAGCGGGCTTAGAACTTCAG
ATGCGCGAATTTCTTGAAGCTTAACAGCCAAAAAAAACCCCACAAAACCCTCTACAATGTGGGAAATGCGATACAGATGCAGTGAGAATACTCGGGGGCTGTTTGGCAGCTAAAGGAGT
CATGTACGCAGTTTGCTAAATCCTGCCAAACAGAATTTGAACGCCTCGTAAATCTCACGGCGGAAGGGAATAAATCCTCCGTTTTGCTCTTTTTGCCGTAAATCCTGGATTGGTTGTAATC
AGAAAAATTAAAATTGTTGCGCTTAAACTACTGCAGATCCCTTTACTTGGAACGCTTGATGAAATAAATTCTTATAATTGATTATTGGTTTATATTTAAATGCATGCCGG
>comp140942_c0_seq2
GTGTCCGAACACAATAATTCCTCCAAAAAAAGTCCAAAAATATTTTATATTCTGTAATACAAAATCTTTCCTCATAGTAATATCTCATCGCTGCAAGCAGATTGGGCTTGCTCATCCTAACTC
AACCTTCGAAGGAAAAATTGTCCTCTCTTCTCTATAGGCCTCATCTGTTTAACAAAAATCACAAGTAAAATCACAGCGACAGTTATCAAATATTGTCAGGAGGAATAACGGAAAGTTTTCG
TCCGGATTGTGAGTATATACACACTGTGTATAAACAGTGTCTACACAATTTGAATCAAATTATGTATAAAAATTATACACAGCGTATACACTCACAGTACGAACGAGAAGTTCCCGTTCAGA
CTCCGCACGGAATATTTTCTCCATGAATTGAGAACATAATTCTGAGTACTAACAACTAGGCTTTGTCATGGATCTCTCAGACTTTAGAGTACTATCAATATCATTTGATATAAGCCGAGCATC
ACTAGCTATTCCTTCTAGTCCTCGGCGAGACAATCCTGCACTGTAAATTCCATTCTCTCCTTTCCAGTGATCAGGAAATTTTCGAGCAGGAAGTCCTTTTTCATTCAGGAAGTTCTTCTCGT
CCTGAATTTCACCTTTCTTGATTTTGTGAATGGTCCCGACATCTATAACAGGAGATCTTCCCACAGTGGCCTTGAGGAAAAATGGCCCATCCTTTGGTTTTTTAATCCCAAACTTGGATAAA
TCTCCATACTTGATGTTGCTAAGCAAAATTAAAACGAATCAACAATAGTCAGTGGCATATTGTACTTCAAAAGATGCATTCCAAAATTAATCATCTCTTTGCTCATCACCTGAACCTCGCCT
CTCACACAAATGGAGGTGACGGCGCCGTGGCTGGAAAGATCGTAAGCTATCTCCATGCCCGAATTCCCGCTACCAACAACCAGAACTCTCTTACCTTCGTACTTAGTCCCGTTCTTATAAT
CCGCCGAATGAATCAACTCTCCAGTAAAACCTTCAATCCCTTCAATCTTCGGCACGTTTGGCTCCGCGTTCTCCCCCGTGGCAGCAACCAAATATCTTCCCACGTACTCTCTCTCCTCTCCA
TTCTCCACCCTCCTCACCTTCACCACCCACTTCCCATCCCCTTCCCTGAAAACCGCCGACTCCACGTCCGACCCGAACTCCGGCACGACGTCGAACTTAGCCGCGTAGTCGTCCAGGTAC
CTAATGAACCTGTCCCTGGGCATGTACGTCGGTGAGTCGGCGGGGGGAGGCATGTGAGGGAGTTGGCAGAAGCGTTTGTCGAGGTGGAGTTTCAGGCGGCTGTACGTGCGGTGGCGCC
ATAAGGAAGCGATGCAGTCCTCCCGTTCGAGGATGAGGTAGGGGATGGATCGGACGGAGAGGCAGGCGGCGGTGGCGAGGCCGGCCGGCCCAGCGCCGATTATTATGACGGGGTGAGC
ACCATCCATTGCCGGTTGATTGATTGGGATAATTGTATAGGGTAATTGGATTGAGCATTTATTAATGGAGGGAGGA
>comp140942_c0_seq3
GTGTCCGAACACAATAATTCCTCCAAAAAAAGTCCAAAAATATTTTATATTCTGTAATACAAAATCTTTCCTCATAGTAATATCTCATCGCTGCAAGCAGATTGGGCTTGCTCATCCTAACTC
AACCTTCGAAGGAAAAATTGTCCTCTCTTCTCTATAGGCCTCATCTGTTTAACAAAAATCACAAGTAAAATCACAGCGACAGTTATCAAATATTGTCAGGAGGAATAACGGAAAGTTTTCG
TCCGGATTGTGAGTATATACACACTGTGTATAAACAGTGTCTACACAATTTGAATCAAATTATGTATAAAAATTATACACAGCGTATACACTCACAGTACGAACGAGAAGTTCCCGTTCAGA
CTCCGCACGGAATATTTTCTCCATGAATTGAGAACATAATTCTGAGTACTAACAACTAGGCTTTGTCATGGATCTCTCAGACTTTAGAGTACTATCAATATCATTTGATATAAGCCGAGCATC
ACTAGCTATTCCTTCTAGTCCTCGGCGAGACAATCCTGCACTGTAAATTCCATTCTCTCCTTTCCAGTGATCAGGAAATTTTCGAGCAGGAAGTCCTTTTTCATTCAGGAAGTTCTTCTCGT

CCTTAAGCCATTTGTTTGCTAAGCTTTTATATCCGGTGGCAAACAATATTGTATCAAAGTAGTGCGATTCTCCGTTGGTGAATTTCACCCAATCGCCTTCTATAAATGAAAGTGCTGGTAAAA
CCTGAATTTCACCTTTCTTGATTTTGTGAATGGTCCCGACATCTATAACAGGAGATCTTCCCACAGTGGCCTTGAGGAAAAATGGCCCATCCTTTGGTTTTTAATCCCAAACTTGGATAAA
TCTCCATACTTGATGTTGCTAAGCAAAATTAAAACGAATCAACAATAGTCAGTGGCATATTGTACTTCAAAAGATGCATTCCAAAATTAATCATCTCTTTGCTCATCACCTGAACCTCGCCT
CTCACACAAATGGAGGTGACGGCGCCGTGGCTGGAAAGATCGTAAGCTATCTCCATGCCCGAATTCCCGCTACCAACAACCAGAACTCTCTTACCTTCGTACTTAGTCCCGTTCTTATAAT
CCGCCGAATGAATCAACTCTCCAGTAAAACCTTCAATCCCTTCAATCTTCGGCACGTTTGGCTCCGCGTTCTCCCCCGTGGCAGCAACCAAATATCTTCCCACGTACTCTCTCTCCTCTCCA
TTCTCCACCCTCCTCACCTTCACCACCCACTTCCCATCCCCTTCCCTGAAAACCGCCGACTCCACGTCCGACCCGAACTCCGGCACGACGTCGAACTTAGCCGCGTAGTCGTCCAGGTAC
CTAATGAACCTGTCCCTGGGCATGTACGTCGGTGAGTCGGCGGGGGGAGGCATGTGAGGGAGTTGGCAGAAGCGTTTGTCGAGGTGGAGTTTCAGGCGGCTGTACGTGCGGTGGCGCC
ATAAGGAAGCGATGCAGTCCTCCCGTTCGAGGATGAGGTAGGGGATGGATCGGACGGAGAGGCAGGCGGCGGTGGCGAGGCCGGCCGGCCCAGCGCCGATTATTATGACGGGGTGAGC
ACCATCCATTGCCGGTTGATTGATTGGGATAATTGTATAGGGTAATTGGATTGAGCATTTATTAATGGAGGGAGGA
>comp140949_c0_seq1
GCCGTCGGTGTTGGGGCTGTTACCTGGAGCACTGATGTTGATGTTCTTGATTGTGACGCGGGCTGACTGGCCGATGAGGATGTGGAAGAACTTGCTGTCTATGAAGGAGATGGACTGGA
GAAGTGCGTTGCTCACGCTTAGCAACTTAAGGGACGGTGGGAGTCTGCTGCCCTTCTTCTTCTTCTGCCATGCAGTAGCTCCCTGACCATTAATCTTTCCGCTCCCAGTGATCAGCAAGCC
ATTAACGTGCCTGAAAGCAATCCATTCCTTGTGAAAAACCTGTAGATCTGTCGGAGCAAGAATCACACCATTCACTTCCACCACCATTGCACTCTTGCAAAGCCCTTGAAAACTGACCGG
GCCGATCAAGAATGTTCCTTCCGGTATCAACACTTTCGCCCGTCCGAGCAATCCACAATCTGTTGAACATGCTTCGCTCCAAGCTTTGAGGAAAGCTGGCACGCTATCATTTTTGCCTTCT
GGGGACGCGCTGTAGCTAGTCACATCAAACACTTTTTCGGTAAAATTTTGGCCGCAACAACTATTTTCACGTGCTACGATGAGGACGAGGAGCAATAACAAGATCTTGGAATCCATTTGC
GATCGTTGTTATGCTTGTCTTCTTTTGGCGCCCCCTCGGAACCCTCTCTCTCTCTCCTCAATCTATCTGATCTTTCATTTTTGGATGCTTTTATGTTGATTAA
>comp141002_c0_seq1
TTTTTCTTAATCTACAAATCAATCATCATATCATACGCTAAGCAAGTTGTCATAAATGCGTACCTATTAACATCTTAAAAAATATACATAACATCGGAAAAAAAAAAAAATAAACATAATTTTTA
TTTAGGAGGAGAATAAAAAGCGTTGAAACAGTACTAAACAAAACTCCAATGAATCTTCCTCGATCGCTAAACTCCGCCGATGCCATCCCCGGCGCGATGGCCGCACGAGCGCAGGCGTT
TGTCGTTGGCACCCACGGCGGTGAGCGAGTCGACGACGGGTTGGACGACGAGGATGGCCTCACCACCGACGACAGCGGAGAGCAGTGCGAAGAAGGAGGCGAGGTGCAGCGCCTGG
CTGACAGGGGACGTTGGGACCCACAGCGCAGCAAGGGCTCGCGCCGCTATCTGGACTGAGAGGGCGGCTGTTACGGTGGCGGAGAGGGCGTAGAGGCGGATTCTGAGGCGTTTGTTGA
TGACGATAGCAACGGCGCTCCATATCGCGGAGATGAAGAGGGGTACGTAGACGGCTGCGATGGCGGCAAGGAGTATTGTGGAGAAAAGTGATACACCGCAGGGGATGCGACGGTGAGC
GCGGGCATGCCGTTGGGAGAAGGCGTCGGCAGCGGCGGCGGCGTGGCCGAGGAAATAGGGAGGGAGGCCGAGGCTGAGGAGGAAAGAGTTAGGGAGGACCAGGAAAAGGGTGTGG
AGGAAGAGGAAGGGGAGGGAGGTGGCGAAGGCTGAAGCGGAGGCAGAGAGGAGGGAGAGGGAGGTCGGGATATTGTGGGGATGGCTGGAGGCGCGGAGGAGAAAGAGGAGAGTG
GAAAAAAATGCGGGCTCGGCTAGGCAGGGGGAAAGGAAAGAGTAGGCGGCACAAAGACGGGGAGAAAGAGAGGACGGGAATTGGTGGCGGAGGAGTGGGAGACGGAGGAGTTCAA
GGAGGGAGAGGAGGGAAGCGAGGAGGACGAGGAAGAGGCGAATGGACCAAAGGGAGTTGAGGCCGTGGAGGCAGCGGAGCCGACAAGGGGAGGCGGAGGCACGAAATTGGAAAT
GCAGGACAATGACAGCGGAAGCAACAGAGAAGGCTAGCTCGCCCGCGACGAGGGTTGCCACGATTAAATCGAAGGGAGATTCCGCCGGTCCCTTCTGTTTCATCTCACATCTTTAATCT
TCTGGCTCGTTTTGTTTTATGCTGCTTCCCTTTTCTGCATCAAAATAGTTCACCTCCGCATCTCCACATCTCCGCATCTTCTTCTTTGATTGTTCCGATCTCCCTATCTCTCTCCACTTCTTTGA
TATTTTAATTTTAATTAAAAAGAGAAATACTTGTTTGGCAGAAAGGATGGATTGAATTGCGGTGGGAGTCGGGCACCCGGC
>comp141109_c0_seq1
GCTGGATATGGTGTGGCTTTGGCAGTTAAGCCAATATTTGCTTCTTCCTTCAAAGGGCGTACATTTGAGACAGATCTTCTTGAGTTGATGATACAAAGTGGTCGAAATGGGAAAGCAAATG

GAAAGGGTTACTACTTGTATCAATCAGGAAGCAAACCAAAACCTGACCCCTCCATACAAACTGTCATTGATGAATCTAGAAAGCGTACTCTCATCACATCTGGTGTAAAGGTTGCCTTTTA
TCTTAGCTCCCTAGCAAAGATTCAGCTTAGAAAAGAAACAAAAGAGATTATATAAAAAATGAGACACTAGTTGCCTTTTATGATTATACTAGTTGTATACTGTCAAATCTATGCAGCAAATC
CCTAACATGTTTAAATAATGCTTCTTTTAGCCTGTGAGTGTTTCCGACCAAGAAATTGTGGAGATGATA
>comp141112_c0_seq3
TAATTCTTTTAATTTATTATTATCAGTCTTCTAGATACCAGACGTACATGATGAACAAAAAAGAGTCGATAAAACAAAAGAAGAAATAGAAGGTAAAATTTAGTTCTTGATGGTCTTCTGAA
TCCACTTGGAGAATGCGTCCATGGTGTTCATATCGGCTCTGTAATGCTTCTGGGTGAATTCAAGCAGGGCCGACCAAGTGAAGTCCGGATAAATCCTCGGGTTGTTGGAATCAACAAGCT
CTGTCGGAGGCTGCACCAACTTATCCATCTCTGGGCTAAGGAAGAAAGCCAGCGACTTCCTCGGCTTTTGACTGTTCACCACCGCGCGGTGAAGGCAGCTTTTGTACCTCTGGTTTGACA
GTGCCATGAAGGTGTCGCCGATGTTAACGATAAAGGCGTTGGTTTTGGGGCTGACGGTGCGCCACTTGCTGTCAACGAAGACCTGAAGGCCACCGACGTCGTCCTGGTGGAGAATAGTC
AATGAGGTTGGGTCGCAGTGGGGGCCCGTCCCCAGCGTGAGCACCGGCATCTGGCATGGCGGGTAGTAGTTGAGCCTCATTATGGAATCGTTGCCTTGGAAGAAGTCACTGAAGTGTGC
GCGGCCAACGCCGAGGCTTATTCCAAGTATTTCCATTATCTTCAGCGACAGCGTGTTCATGGCCTCGCAGTAGTTCTGGTACACCTCCCCGAAGTGTCGGAATTGTTCTCCGAGCTTGCCG
ACAAAATAGTTGAGGACGATGGACGAAGAGGAAGGGGAGAAGCGGAAGGAGAGCGTTTCCTTCCAAGGGAGTTTGGAAGAGAAGCGGCCGGTGAAGCTGCTCGCGTAGCCGCAGCT
CTCACCGGGATTCCTCTGCGTTTTCTGCTTTTCGCTTAGCGGCATCGAGAAGAAGGCTTCGACGCATCGGTGGGCATCGGCAACTAGCGTTGGGTCGATGCGATGGTTCACTACTTGGAA
GAACCCGTGCTTTGCGCAGGCTTCCCCTACAAGCCGGGCCACCTCGGCCTCGGCCTCCGGCTGGCCTGAGAGAAACATGTCAAGGTCGATAAGGGGAAGGGAGAGCTCGTCCTGAGCG
TCCGACTTTGGCGTCTCCTCTTCAGGCCATACGAATTGGGAAGGTATTTCTGCCTTTTGGCTCATAACCGCGGCGTCGAACACCAGTGGAAGTTGTGACGGCGACGGATTGCACTTATGTA
AATTGTCGGTGGCTTCCATGGGCCGTTGAGGCGATTCGGGAGGCCCGTCGGCGGCCCGACGGTGTACCATTGCTCCTGCTATACCGCGTTTCAGGTCAATTTC
>comp141156_c0_seq1
CTTTGCAACTTGAAGGCAACGTGTATTGGGAGAGATCTTGGTGCTAAGGTAAGTCTCAGCAGCCTTGTACATCTCATTTGGCAAGATGCCACTGAATTCTTGGACGACGATGGTGAGCTT
AGAGGAGAATCTTGTGAAGAAACTGGAGGTAAAGGAGAATATGTAGTCTTTCATAGGGTGGGGGAGAAATTCATTAACCATGGTGCGGATGAGCACTGCAGAGGCCGCTACGGAGGCTG
CAGCAGAGAGTATTGTCTTGGTTGTAGCCAATGATGGGATTTGAATTCCTGACATTGTTAGAGAAAGAGAGAAAGAGAGAGATTATGAGTGATGGATATGGAGTAAGGATTTCTTTCGAG
CTTGCTGATTATGTAGTATAGCCTGCTCGCAGTCCGGTGGGTGGGCGGTGGCTTATGTTGTCGAAAATTAAGACCATTGGCGACGCTCAAGAAAAATTGCCTCTAAAACGGTGATTGCAGA
TGCGAACTGGGACTGGGAGGAAATGGCTGCAGCGAGCCGGCGACCTCTTCACGAAGACAAGATGATGAAGGCTACGGGAAGCGACTGCGAACAAGAGTGCTCCGTCTGTCTGGCGAT
CGCTGAAATTTTCACGAAGGT
>comp141267_c0_seq2
AACTGAACCAAAATACTCACTCAATTAAACTTCCTTATTAATATATAATGATTCAATATCTAGCTAGCTAATAGTTCATATATACATATATATCGTTTGTCCGTTCTTTTGGGAACTGTTTTGGTG
CTACTGTTGTTGCAGATGCAGTCTTTTTGGATGTTATTTCCCTCTTTACTTTGATTGGTATCGGGTCGTTTGACTGCTCCGGGTAGCTATTAATCGAGGTGCTGGTCTTGATGCTTGCTGCCG
TTGGTGCTTTTCCTTATTGGATTTTTGTTGTATTCCTTATTTGTTTTGCTTTTATTTTCTTGTAATAGATAGGATATATAATTCTCAGGTTTTATTTTAAATACTGTTCTTTGAGTGAGCTTATGAT
CCTCTAGGACCTGTAATTTTTAACTTTATTTTATCTATCTATATATATATTGTGATATATCTCATCATCATCACCTCGATGGAGTTCTTCGATCTTAAAAATATATTATGTAATTTCAAATCCTTTTG
AGCAATAAAATTAGTTCAAATTTGCAATTTTTTTTTTTTAAAGAAAATGTTAATTAGTTGAATGTACAGTATTGTACACGCAAGAACTGATCAGGAAAATCCTATTGCATATAACTCATAACT
CGTTAAATAAACAAGTTCCAAGAACAAAAATAAAAGACAGAACAAAATGTTAAGAGGTTTTACCTGTTCATTAGATCAGATGGAAGAAAGGAGAAAAGATCATATAGAATATGCGCGCG
ATTGATATTAAAGTCATTTATTGCCGAAGCAGCATCATCAAGATTCTCAATCCTTAAGGAGTAGCTAGGACTATATATATTATAAATAAATTGGCCCACGACAATGCTCATGATCTATATATAA
GAAAAAGAAAAGAAGAATAATATTCTATTTATATGAAAATATATAAAAGAGTTAACTCCTTTCAAAGTTTGTGAATCTTGATGGTGCCACACCAGCAAACAGTGACTGGCTAAGCATCTTT

CTTCAGCTAATTCAGCAGAACTCGTAACCTAATTAAACCTGAAATCATGAAGAAGAATAAGCAGCAGCAGCAGTGGAGGAAGAAGACGAAGAAGAAGAAGGGTGCGCAA

>comp141319_c1_seq1

AGACTGTCGACGACATTCAAATCCTACACCAGTCGGCAACCATACTGGCGAATCTACACATCCCCCGTTGGCAACCATACCAGTGATTCAATCTTTTGGCAACCATACCAGAATGAATCCA
CCACCGATTGCAACTATACCATCGGTTATCTTTCGGCACGAATTGCCATATGCAAACAACACCTCTCAATAATCGACATGCATCGTTGATCAGAGCTTGTGTCTTGCCTTGTCCAGTCCTCA
CGAACAACCATGACGGTATCCACGACAATCGCGATTACAGGCATCCACTCCAATATCGCTATCTACGAGCACCTCTACCTCTGATCAACTTTTCGCACACCCCCGCCGACACCTCCTTTGC
AAAAACAGATGCTCCGCTACTCTCATTTTCTCTTCATCTCCCACCGTCCAAAACATGACCAAACAAATCAGCCTCATTACAGAGTTCCCCGAACAGCACTTCCGGTGCTGATTCACTCGAT
CGTCCCCGACCGCACCATACCACTTGAGCATCCAGCATCCACCTCCGTGTGCAACACCAGCCCTCATCAACCTTCATCTACATAGCAACGTTTTTCCTCCATCACTTCTCTGTCTCTTGCTA
CATCTCCCTCTCAGTTGATGCTGACATTGCAGCTTTTAGCAGGAGCAGGGGTGATCCCAGACTCTACCAGGAACAAGTGATCGGCTGTAATATAACTTGGCTATTTGCTCCCTCCAGCTATT
TGCTCGAAAACAAGTTAGTGGCGCCTTTTTAGATCCATCCCAAGTCCGCGACCAAATCTCGCTTTCATCGACTCCGCC

>comp141400_c0_seq1

AAGATTAAGGAAAATAAGTAATCTAACATATGAACATGACAGATTCCACCAGAAAATAACATAGAAAGAAAGATGAAGGAAATGCCTTAGAGTGTATTTTAGCATAATTTCTTTACTTTGG
GAATAAAATTTTAGTGAGAAAAAGTGAGAGTTGGGTACTTGTCTATAAAGGATGTTGGATCTTGAGAGGAAGCTTGAGCTTCTTGATTAAGAGAGAGAGGGAGAGAGAAAGGGAAGAA
TGGAAGAGATGGAGAGGATTTTGGAGGAAAATAATACTATAGTTTTATTGAATTTTCCCCTAGATTTACAACAAGTCATCAACCTATAGCATCTATACATAAGTATCGAACACGGGTGTGGT
GTCTTGTAAAATAACCTAATCTAAAATTAAACCAATTCCTTACAAAGGATTAATCGACTTTATCAGACTAATAAATAAAAATCAACTAAATAATACTAAATAATTTTCATAAATCAACTTTTAT
GAAGTGAAAACTATATATTGATGTTTAATTTATTTTAACATGCAGGAACTCAAGCAAGGATTCTCAGCTCAGCAGCACGCAACGGTAGCCGCAAAGAACCTGAAGATTTTGATGAGGAAA
GGTAAAGAAGAAAAGCTATAAGTACAGGCCATTTCCCAAAATGGAAGTAATTTCTCTAGGTAGGAGGAATGCTTTGATGCAGATTACATTCTTCAGAGCTATTGGATTTCTACCTGGAGTT
ATCAAATCAAGAGATTTTAAGACCAGAAGATTAATGGGTCTAGATCACCATTCCAGAATCTCGGTTACCTCTGCTTCTGATGAGATATCAGAAGGTTCAATTGATTAATCTGTGGTGGTTGT
TTTAGTTTCTTAGGATATTCTGACATAATTATTTTAAGTTTAAATTTTTACCCCATGATGAAATATTCAAACTGCATTTTAGGACAACTTGTTTGAACAAATTAGCATTATTCATATTTGAGCAA
TTCAAATTT

>comp141422_c0_seq1

GTGGCCGTTCATATCGCAAAATCTTTCTTGTAACAACTTCCAACCACTTGCTCGAAGTTCATAGCCAGACCTATATTGCCTTAATTTCATAATTTCATCATTTTCATGCCACAGGACAACATC
CTCGTCTTCCCCCCAAGTATGTAGAAGACATCTAAAAATTTCATTTGTAGTTGCTTTTGCTTCTAGGATCTCATCTGGGCTATAAGCCAAACTTTCATGTATACGGCTACTTCCTTCTTCTATT
AATTGGTTTGTAGGTGCGCCTCTCATATTTTCATTGACCTCTGAGAATATTTTGTAATAATAAACTTCTTCTTGCTTAAAATGTGGATTCCTATGAACAAGGAGCGAAAAATGTTGTGAAATG
GCTTGGAATAATATTCAAGTCAAGTATGAAAGAACGTATAGAATACTATAATTTGCATGCTGAATATAAATAAGAGAGCATGAATAGGAGGTATTATGTTGTC

>comp141491_c1_seq1

CGGAAGTATGGTCGACGAGAGTGGAAGCAGAGGTTGGAGAGGAGGCTTCGGTTGGATGTGGGAAGGTCGGAGCAGAGTGTAGGATCGGGAGTGAGCTTTTTTCGTGGAAGGAGAAGA
GCGGTAGCTCGGGCGTGTAAGGAGGTCGGAGCTCGGTCTGCTCTCTGGACGGGATCGGACGGAGTAGTGAAGCTCGGGTTTGACGGGAGGAAGAGGGATTTCGTGTGAGAGGAGTCC
GTGAGTGGAACCAGCACCCGAAGCCTTCATCTCCTTCGGTATTTAAAGTAGCGAAAGGCGTCTAGTTGGCACGGGATTTGGGCAGAGTTTCCGGAGAAGCGACGCAACGGCGCCGAGCT
ATAGGAATTCAGCGGGATTTCTTGCT

>comp141510_c0_seq1

AGCCCCTTTCCTCTCAAATGGTAGAAACAAAACTTAATGACGTCGGTGCCACCACCTCCATTGAAGAAATTCATCCTGACATCCTTATTCAAATCCTGCGCCACCTCGACGGCTCATCACT

CGCTTCGGTGAATTCTGCCACCTCTTACCTCCATTCTATCTCCTCCGATCCTGACCTCTGGCGCCGCCTTTGCCTCTCCACATGGCCATCCCTCCATCACCCTCACATCCTCCCTCTCATCAA
CTCCTCCCCCCACCGCTTCTTCTCCGACGCCTTTCCCTACCCCACCTCGTTCACACCAGCCAACTACTCAGACATCCCAGATAAACTCATCTCTGCCGTCGATCTCTACCAGCATGACATCC
CAATATCTTCCTTCGTTGTTGAGAGTGACACCTCCTACCCCACCCCTTTAAATGTTCCCTTCGGAATCAAAGGACTAGATATTATAACGGACATGTTGCCAGAAGAGCTAACATTGAGTTGG
ATTGTGATAGACCCAACGAAGGGACGGGCGGTGAACTTATCGAGCCGAAAGGCAATGGTGGTGAGGCGACATTATTGGGGCTGGGAAGCGGTGGCACGATTCACATTGGCGAGAGATG
GTTGGGTAGTGGGAGTGCTGGGAAAATGGTTGGATGAAGGATTGGGATTGGTTGAGTTGGGTCTGTATATTTGGGACGAGGGTGGTGTGCCTGTGTACAAGATGGAGGGATTGAGAGTC
GTGATGGCAGCAATGGAGGCGGAGAGAAGGCGATGGGAGAGAAAGAGGTGAAGGAAGGATATTTTGAGTATTGCAATATGAAGATAAATGAAGAAGAGCTAAGACAAAAGAGGGAGT
GGCTGGGGATCTGAGCTTTGTCATCGTAATCGCCATCTTTCTAGCTTTTATTTTTCTGTTAATTTTCGATGACATTTGGTGAATTATAAAGTCACCTTTTTTTTTAATGTTGAATGTTCTTCGCT
GCTTGTTGTCGTGTGCAAACAATGAGAATAAAATCTTCATAGCGTATTGTCTTGTGCATTTAGAGATGTAAATGTACTGAGCCGAGCGAATATTTATTCATATTTGAGCTTGTTTATTAATACT
TTGCTCAATCTCTGTTCA
>comp141602_c0_seq1
TGTCATGATTATTCAACAAGTTTTACGCTGGCAGTCGACTGCCTAACGCAGCCCTCCACTTTTCTCATCTAGGACTTGAGACCTGACAACAACAGTGTTTAAAATATACTGGTGAAGAATA
AACTTAAGCATATAATAAAAATAAAAAAATACACCCAAATAGATAAAATTGAAGTAAAAGAATTAAAAAAATAAGTTGTTGGAACAGATAAAAATGAATAAGAGTTGAAGAAAGATATAA
CTGAAGAATAAGGACTCATAAAATCAAATCCAAATAGTTGGAGAGGTAAGAGGTTGCAGCCACCATGCTTTGCAGACTAGCCAAGAGTAGAGTCGGGCAAAACACTATTTTATTGTTCAT
AATAGTGTACACTTGTATTGCAACCTATATTGTTCATAAAATTCACTATCCTATAACAAGGCAGTACTGGTACACTTGACAATATAAAAAACTGATGCAGAAGGATGAGGAAGGACCTCAA
AGG
>comp141749_c0_seq1
GCAACAATCTCCTGACATTGCTCAAGGTGTTCATGGCCACTTCACCAAGCGCCCGGAGGAGATTGGTGCTGGTGACCAGGGACACATGTTTGGCTATGCCACAGACGAGACCCCCGAGT
ATATGCCTCTTAGCCATGTCCTTGCTACCAAGCTTGGTGCTCGCCTTACTGAGGTTCGAAAGAATGGAACATGCTCTTGGTTAAGGCCTGATGGCAAGACCCAAGTGACCGTCGAGTATCG
CAATGAAAATGGTGCAATGATTCCTATCAGAGTCCATACAGTCCTCATCTCTACACAGCATGATGAGACTGTCACAAATGATGAGATAGCTGCTGACTTGAAGCAGCATGTTATCAAGCCT
GTGATTCCTGAGCAATACTTGGATGAGAAAACCATCTTTCATCTAAACCCATCGGGTCGTTTCGTCATCGGTGGACCTCATGGCGATGCTGGTCTTACTGGTAGGAAGATCATTATCGATAC
ATATGGCGGATGGGGAGCACACGGGGGTGGTGCATTCTCTGGAAAGGACCCGACCAAGGTCGATCGTAGCGGTGCCTACATTGTCAGGCAGGCTGCGAAGAGCATTGTCGCCAATGGAC
TTGCAAGACGCTGCATTGTTCAAGTGTCATACGCAATTGGTGTGCCGGAGCCATTGTCAGTCTTTGTCGATACTTATGGTACTGGCAAGATTTCTGATAAAGAGATACTTGAGATTGTTAAG
GAGAATTTTGATTTCAGGCCTGGAATGATTAGTATCAACCTTGATCTGAAGCGTGGTGGCAATGGGAGATTCTTGAAGACTGCAGCTTACGGGCATTTTGGAAGGGATGATGCTGACTTCA
CCTGGGAGGTAGTTAAGCCCCTCAAATATGAGAAATCTGCAGCTTGAGTTTTGAAGAATGGAAATCTATCCATGGAGGGATGCAAGGGCAATTGTTTTGTATTCTCTGCTTTTTTCTCTTTT
ATCTTCATTGTTATCACTTTTTCCTTTTCTACGGGGTTTGTTCCTCTTATGATTTTATGTAGGTCTGCCATTTTTTTTGTTTGATCTGAAGCTGGCCTGAATGTTAAGGATGCTTTATCTGTAAC
CAGGATATATAGGAGAAGACAAACCTGGAAGGTCTGCTTTGAGTCAAATACTCGGTGTAATGCTTAAGAATATTGGATTTCAGTAAAATGAACTTTTGTTTCTTCTATTATTGTAACACATG
GTACTGTTCTGGATTTCGATTTGCCT
>comp141777_c1_seq1
ACAATTACTTTTCTTTAACCAGACATAATAGGAACCAAATCACCATTCTGCACAGGATAACAAATAAACAAAGCTCCAACAACACATCTTAAATAAATAAGATCTTTAAAACTTTATAGACA
GCAGTAGTAACCAGGAACATAAAAAATCCAATTCCAGCATCATAGCTCCCAAATCATCAATCCCTCCAAACAACATATACATAAATATACTTCCAGGATTGACAACATATCCATCCACCAAA
TATTCAAACTTCTAAGAACGTCCAGAGAAATAAAAAGGAGAAATAAATGGATCGTGGGGGAAAATCCTACATACCAGAGCCGCGGCTTCCTTATTTCCTCCCCCACGTCTAAAAATAATAT

CCATATTCACGAAAATAACAGCAATCCATCAAGAGACATAGCTTACAGCTTGAGGGATTCAGATTCATCGAGAAAGCCCGTCTTCCGAAATACGGCGTTCCGCACCCGTCGGAGATCCCT
TCCCTTCAGGGTTCTCCCCGCTCCCTCGAACACAGAGAACGACGTCGCGTGCGATCTCTTCACAATCAAGTGCGGCGGTAGCATCTCATCATCCGCCTCGCCTTCCTCGTCATCGTCATCC
ACCTCAATCAACGCTCCAACAGGTTTCCTCATCCCACTCGGCCACGTCGGCACATTCACCGGCGCCGACTGATGGAATATCGTACTTGTCTCCTCCGCCTCCGGTCTCCCCGCTGGCACTT
TGATTGCCGGTCGCCGTTTGTACACCGGTTGCTCACCTTCCTCCGCTAGGGCAGCCGAGAGCCCAAATCTCCCCGGCTGGAAAGAGCGGCGCCGGATGCTGGAGTCGCTGTCGCCGGAA
CCTGGCCGGTTGGAGATTGGCCAGTTTCCACCGCCGGATGAAGGATCAGATTTAACGTCCGGTGCCCAAACGACGTCGCATTCATCGAGATCGAAGTGGTTGTCAGGAGTCGGAGAAGA
ATTTGAGTGGTCGGCGAAGCGGAGAAAGCGGAACGAAGAGGAGAAGGGAGAGGTGTTTTTGTGGATGGAGGAGGAAGGTGATCTTATCGTTGTGGTACCGTCCTCCGCCATGGCCAGT
CGCTTCTTGCCTTCTTTCCCCCCATTTTCGGCCCGCGGCTTCATAATATAACCAGTTGACGGTGTAGTTTAGCTTATGTCATTCCTTGGGCTCAGTATAACGCCTTAAGAATCTACGGGCTAC
TGCATCTGAGCCGATCTCGTCGGGCGGCCAAGGATTCCTCGGACGGTCAGAGCCGTTGGTGTCAACGAT
>comp142060_c1_seq1
CTACCTAAAACTATTAGTTGCCGAATCACATAACCTGTTGAATTAGTCAAAATAAAGTTTTCTAAACATAGAGAACGTAACCCATCAACACACGAAAAATAAGGATAAAGCAAAGATTCCA
TTGATGGGAGAAAAAGCAGGTGAGCATGTTGTAATTCATAAGGTGATGGGAGGCCCAATGAATCAGGCGTTCAAAATTTAGAGCAGACGGAGGCTTAAGAGGGCATTGTTGGAGAAGC
GATTCCATTTAGAGCATTAATCGACCAAATAAGATAGAAGTGCAACAGTAGTATGACAACTATGTAATAGTAATATGGCAGCTACGTAAATCACTAGTGAGATGTGTGTTGCTGTATTATTAT
TATTCAAGCACGAATAATAACCGGGGGTTTATTAGGCTATTTTGATTTTTTTTTTATTAATAATTGCTGTAGTATCTAGTTTATACATGAAACACCTATTATTATGTAATGGAACTTTGTCTTTTT
GATCCAAGGAAAATAAAACAATGGCAGATTTATCTCAATTCCTTTGCTTGGTCATTATAAAGACTAATTGTTTTGTGCTTTTTTTCGACTTATATAAGTTTCTTAAGAATGTTCAATTCGGAA
GAAGTCTATATCCCATTCTCTCAGGACTATCTAGATCTCTGCTGTAAAGAACAATCTTAGATTGTGTGGTCGCTTTTCAAAGATAAAGATGATTGATCCTTTAAAATAGGATGCATGGGGCTT
TTGGAATTGGTAGGAAGTAAATAGGGCAAGAAAATTAGATTTTCTAGGAAATACCTCGAGTTTTTACTATTTTTTGGACCTCAACGGTAACCATTAAACAGGGAGCAAAGAACGTATAGTT
TTACTTTTAGATGCATTGAAATAGGTCCCAGAAGTGGAAGGTGCTAGACTGACTCTACGAATGTAGAAGGTGAAGGCCGGCGGGCGTTCATTAATGGAGAGAGGGAGAGGGAAACAGA
AGATATTGGAGGAAAGGAGGAAGTTTTGAAAGAGGGAGTTCAGATTTTCATTCAATCTAAACTTGATTTCATGTCACAAACTTGATATCTACTCTTAGAGATCCCAAAAAAAATCTAGAAGA
ATCTATTTAAATTCAAATGAAACTAGAAGATAAACTACCCCACAAAAGGGCTTACTTTTAGATTCC
>comp142111_c0_seq1
CAAAGAAAAAACAAATTAAATAGCTGCAGAATCCGCAGTTGATTCCTTACTTTGTTAAAAAGACGATGATTCTAACGCTATAGTAAGAGTGCTTTAGAACATCCAAAACGCACAGAATAC
CCTAAATTCCTAATTACAAATAACTACGGTACCAGTTCAGATCCATAACAAATGCAACAAAGATTTACAAATTGCACGACGATATAGTTCAATGTGTTGAACGGATGAAGCGACGAAGTAA
GGAAAGATGCTCCGTACCTATTTCTCTTTAGCCTAAACCAAGCCTCCGCGCAGCGGCGGTGCGCGTGGCCCAATTCTCCCTTGCAGCCGCACCCAATCTGGATCAGCTTCCAACCCGCCG
CCGCTGATTCTCCTATCTCTGGTCTCAAATGACAGATCCGACAAACCTTGTCCTCACCGCTGCACTCCACGATAAGTTCTATAAAATTTTCTTTATCGTTTCCTTTTCCAGCATCCCGCTTGT
CCTGCTTAAAGCTCGCCGCCGACGGTGCATTTCCCTCATTCGATGCCGCCCTGGCTCCGAAGACCTTCAAGCTCCCATCTCCGTCGGAAGAACAATCATTCTCGACGGAGTTCACCGTGA
TAACAACGGATTCCTCCGGCGATATTCTAAATGATCCGGTCATTCCTGCGTTGTTGTCGCCGGAGTGCATTACCGCCTCTCAGTGCATCGCATCAGAATCGTATGGTTTCAATTTAATCACA
AAAAGAAGAAGGGCATACGTTTCGATCGAAAAGCAGATGGAAATCCG
>comp142151_c0_seq2
AATCCTAACAAGAAGCAAGCAAAATCCCACAAATTCAGATCTCAAGCCAGATTCTCCTTCTCCTCATCCACGATAAATTACACAAACACACATAACACGAAAAGGTTGAAAATAGTGGAT
ATCCCCAAACGACTAAAGTTAGAGAAAAAGGTTAAGATTGGAGACCTTTCTTGTCCAGCGGTGTCCCAAATCTGAGCTTTGACAATCTTTTCGGAGACTTGGATGCTCCTGGTAGCGAAC
TCCACTCCAATGGTGGACTTGGATTCGAGGCTGAACTCGTTCTTCGTGAACCGCGATAGGAGATTCGACTTCCCCACTCCCGAATCTCCGATCAACACCACCTTGAAAAGGTAGTCGTAG

TCGTCATCTGACATATACGCCATTGCCAAGCTGCAGTCTCCGCCGCCTCCTAATGGCGACAGGTTCTCTAACGTTTCCTCCTCTCAATATCGCAACTTCGTTTCTTCAAAAGAGGAAAGAA
GAATCGCGGTGGTGAGATTGCTCGTCATTGCCGTCGAGCACAGAGATGAACGATAATGGCGGAGAATCGAAGGATCC
>comp142153_c0_seq10
CACCACCTGAGGCCCTCGCCTTCGTCGGACCACCACCTGAGGCACTCACACTTGCTGGATCTTCGCCTAAAGGCTTGAACTCCACCTGACCAACACTTGAGACCCTAAACTTTGCGCGC
CATACCATCAACTGCCGCCAGAATTCTGCCAAGATGCTGACATACTACTAACTAACGCCCAAACTTCCAAGGAACACCATATTTTACACTAGATTTCAACCATACTGCTGACATTTGTAATC
TGATCGTCCTTTGGGACGAGTCATCCCACTGTAATCAACTCAAATTCTAGTGAAATCTTTTCAACTGGAAGTTGGTGCGTCAGTTAGTAGTCTGACAATGAATTGGTAGAATTCTGGCGTC
AATTGATAGTCTTGCAGAGTTTAGGTCCTCAAGGGTTGGTATGAC
>comp142272_c0_seq1
AAAGAGTGATATCGGCTGTTGATCTTCGTCTCTTTCATTACCGTCGGATGTCCGCATCAGCTATCTCTTTGTTCAAGCATGAGGCTTATCTTTGCACTATCATTATCCCTTTATCTTTTCCTTCT
TCTTTATCACACTTTGTGTATTATTTTCAAGTTCTTGCATTAGCATTGTCATTCATGTTCTATCAAGGATCGTTGTGTATAACTACATATAGATTAACAACTTTTTCATGATAGTAAGAAGGCTG
AGTACTGCATTTAAGAAAAGAAATAGATGGGATGCCAACTTGGACTTATAGGCACCACATCTTTTATTATTTGTGTGTTTTATTGACACTTCTGTAGTATTATTGGGTTATTCTAAAGCTGGG
GCATATTGTCCCAAGTGGAAATAAAAAATAATTTTTTTTTGGAAGGTGGAGCAATCAATTCACCTAGTGCTGCTGCTATTTATATCATGACATGAACCTAAAAAATTTTGAATTTCAACTTAA
GCTAATGTTAGACTGCTAAATGGGTATGCTGGTAAAGGTCTTCTGGTGATCAGAGGTGCATAAGCCTTAACTAATTAGATTTTTTTTTATGTTTGTTAATAACTACTAATTCGTGGAATATTTG
CTGGAGATCTATACATCATAGGTGCTTGAGTAGCTCAGCACTGAGGCCTAGATTATTTCTTCCATTACACTGAATTGTGCATTTTACATTATGATACACTTTGACATTCTTTTCGGCTCACATT
GCCTGGGCTAACTTTTTACTCATTTGCTCATATATTTACATGCGCTTGTATTACTTTATAAAATTTTGGTGACTATTAAAATAATATTTAACACACATATTTTATTCATGTCCATATTTATTTTAGT
GTTGTTGGTAAAAAATTTGCACCATTAACGATGGCAACCACAACATTTTGAGTGTTAAGTTTGATTTTGTAGTGTGCAGAAAAGAAGGTGTGGGTGTTCTGGCAATATGCTAAAGTTTGTG
CAAGTTTATATTATCTATTATTTTGCTATTTTTTTTCATTCAGTTTGGGTGTTTTATTTTTTTATCCTTTATGTTTACACTTTTTTTTTTCATCCTTTTGATTTCATGTCATATCTTTTACATTTTAGC
GGACTTATTTTTGATCTTGAAATGTTCTAGTAAAATTCAACTACACGTTTGCATTCTCCTGCAAGGGTGTAGCTAAAGTTGGCTATTCCTCCCTCGCAATGTAGATAAAGATGTTTTGGGTG
CATTGCATATTTGCTTGGGTCACTATGGCTTGAAAGGCTATCCCATCACACCAATAAAGGTGCTTAGGGGGCCATTTGAAAAAACATTTTTGTGCATGTTTTTGAAAAAAAGGGGAGGTGG
GCAAAAGTTCTTAAAAATTAATCATGGAATTCCTAACTAGTGCTTGCAAGGTGTAGGAGCTAAGCTTGAGTGGTTTTGTAAGAAAGGAGTGGCAATGAGTGACAGTGAGGATGATAGAA
AGGGAGTGAGAGTAGCAAAGAGCCATAGTTGTCTAGTGGGAAGGAGGAAATAGGGAGATGAGAGAGGATTTTTAAATGTTGCTTTTTTTTTATATTATTTGTATGGCCTCCCATGTGCCTT
TGCTTACGAAAGTCAAGAAAATGCCCACCTTTAACAAATTGATGGCCGAGCGACCCTCTGCTTTTATGCATCATTTTGTGCTTGCCCATTCAAGGCACATGAAAAGGGGAAGGGCGACCC
CATTAAGTCAAAATATGTTAAACTTCTTAATTTTTTTTACTTACCCCTTCAGCATAGTTGGTATAAGTACATCACGGCTCATTAGGCTACAATAA
>comp142309_c0_seq2
CGTCCCAAACGAAGTCTAAATAAAGCAAATCCAGTAAAATTTGGAGTTGATGTGAGGAGTGCCAGTCGAGAATGTATCAAATAGGCTCGGCTAAATTGAGAATGATAGAACTGCCTCCAT
TATTGAGACAAGTGAGGATTCATCCACATGAGAGTTCTTCCTGGATGTTGACCTCTTTATATGAGTCTCTATTGAATTCAGCTGCTTTAACATTTCCTTCAACTGCTTCTTTCCTCTTTCAGA
ATCCTCATGCAAGATAATCCTCTCATCCTCAAGCTTCTGCAGTCTTCTTTTAAGCTGTTCAATATCAGAATGGGAAATAACATTTCCATTTCCAGGATCGATGAGATCCCATTGATAATTAAC
AGAAGTACCCTTTTTCGGTGTGCACATCTTGCCCAACTTTGCTATGATAGGCTCTTTAGCAGGCAAAGAGAACTTGGCCTCATTGTGATTGCCAATGAATGTTTTATTAAGGCCCTCATCAT
GTTTGATAAGATAATCTGGAACTTCCTTTAGCATCATATTTGGCATTTCAATCATGGAATTAGTCTCCGAGATTGCATCCTGCAATAATATCTTACAAAGTTCACCAGGCTTGCAATCTTTGTG
GCTCTCTGGAACCCCAAATATGTCATGGAATAAGGCAGAATACCTTGCTTTCCCACTAAAATTTCTTGTTAGGCTAGAAGCATTTTCATTATCTTCATATTGAGAAGGTGAACTAGAAACGG
CTTCATTATCTGACTTCATGTCTTCCACTCGTTTAGAAGAATCAGTAGAAACATCACTAATAGGTGCAGAAAACCAGGAAGCCATGGAACCAATAGGTGAAGAAGTGCTTTGAACCTCAT

TGCCGGCCACACTGGAACAAGAACCAAATGCTGAATTTTTAGCCTTCAAAAACCCTTTATCCCTCCTGCTTACTTCTATAGCTTCATATTGCCTTCCAGGCTTTCCTTCGCTAGCTACCAGA
TCATCAAACAAGTCATCTACTTCATTCTCACTCATCCCGATATGTTGATTTGTGAAATCACCAATCTTCCTCCATACAGATAACTTAGAGGATAATATAGGACTTTCATCATCAAGGAATCTCT
TATCGAAACTAAACTCATCCCCACGAAGTGAAGGCAAAGAAGCATTCCTTCTAAAAATACCATTACAACATGATTTGTTCTCACAAAGCATCATTTCTCCGATGTCTGGAATGCTGATGCC
ATGATTTGTCTGTTTTTGCTTGTTTGCCTTGAGTTGGAATCGGAGAGATTCAATCTCCAAGTCTTTCTGCTGAACGACTTCTTCAAGCACCTCCAAGGTCTCTTCAGCATGGTTTATCCGTT
CCTCTGCCATCCTCTTGTATTGACAAGCTTCCATCTTCTCTGCTGCTTTCTCTCTCTGCAGGCGAAGGATCATAGATAGAGCTTCAGTAGCTGCAGTTGCTGAAGCTTCTCTTTCTTCCTCTA
ACTCAACGTACAGCTTCTTTATGATAAGATGTTGGTTGCGGAGAGCTTCCTTTAAAGCAGCAATTTCTCCTTTAGCCATATCTGGATATGGTTTTCTTGCTCCTAATTACCCTTAGTCACTCA
GACACAGAAGATCCTGCCTCAACGCTTTCTACTCTATCAGGCCTTTACCTTTTCTTGTAATGTCAGAAAGAATGGAATGAGCACAAATAGCTCAGCAAACTCCCTCCCTTACCTTTCTTTC
ACGGAACAAAACATCTTCCCCACGAGCCGACAATATGCTTGCTTCGGTCTCCATCTTTTGTTTCCGCGTTTTATTGTTCTTTCTCCTCCGATAAAATAGGCGACAGACAGACACAGAGAGA
GAGAGAGACAGCTAAACAAAGAGAGTTAATCCATATTCAAAAAACAAAAATTAAATAATAATATACATACACACACAAAAAAAGGATAACTAGCGCCTCCCTCTCTCCATCCTCAGATTTT
>comp142412_c0_seq1
GTAGGAATGTAGACATAATCGCTAAGAAAGCAAAAACATTAATTGACTTTGAAAAAGCATATAATGATTAATAGAAAATATGATAAATTCTCACAGAGAATGAGAACGTTAATTTTGGCTA
GTATACACAACAATTGGGCTTCTAACATTATGAGTACCATCACTCCAAATCAAAGCACCCGAGATAATGGTCTCGTAAGCAACTCCTTCAAGTTTGACTGAAAATGATTTCTTCTCCATAAG
AGCTTCAAAAGATAAAACTTTCGGCTCAACGTTGATCTTGAGGCTAGAAGGAGCATTAATAATAGCGTAATAAGTGGAATTTGGCACTCCCACATTAGTGACTATTCTTCGGAAAGAAGTT
GAAAAAGACTTTCCATGTTGAGTTTGCAGAGCAAATGTAGGATAATTGAGATCTAAAACTGTTCCATTATTTGTAGAGGCACAACTGCTCTGATCTCCAGTGACAAGTCTTAGAATTGTAG
TGATGTAGCCTTCACCACATAGCATTTTGATGTAGTCTATCTCTTGAGCATCATATATAAGACCAGGACTCAAAGCTGCGAGTGGGTTTATGTGACCGGCCCCATAAGCAAGTTCTGCCTCA
GCATTCTTTGTTGAGCTCATCACATAGGCAGTGGTGATGAGAGCAGACTTTATGGCTGCTGGAGACCAAGATGGATTAAATGATTTGACATAAGCGGCAACACCTGTTGTATGAGGGCAA
GCCATTGATGTGCCTGATATTATGTTATATGGTACTTTAGGGTTACTAAGAGGTGACCATGCTGCTAAAATATCAACCCCTGGTGCTGTTAGGTCCGGCTTTAGTATGTTCGGGGTAAGTGGA
TTTGGACCGCGTGATGAGAATGATACCACATGTGGTGCTGCAGCATCGAAGACACCTTCACTTTTGTAAATGCTTGCCATAGGATTGCTTGTGTTGTTTGCATAATTTAGAACTTTTATGCTA
TTGTTTGATTCTAGCACCGAGACAGGAAGCACGAACTCAAAAGATACATCATTGGTCCAATTTGTGCCCATAATTGCTCCAACGGCACCTGCAAGTAGTGGCCCAGAACCATAACGTATC
CCATCACATAAAACAACTTTTCCTTTCACCAATTCTTCATCCAACGTCCCAACTTTGCAATATCTTGAGTATTTTGCATCATCACCTGCTGAAATATTGGGTGCATTTCCGCCGAATATCAAA
GGATGCATCTCACCATCCAAGCCATCGATGTTCACTGCCTTCCCCTGAAATTCTTCTCCATTACCAAGCTTAGTTCGGGCAACAATCTTGCGATCAATTGTGCTTGCTGCCACTGTGAGTAT
CCATGGTGCGTAATTCTCAACGGTAAAATTATCAGGACCACCATTCCCAGCCGAGCTAGATGTCAGAATGTTTTCCTTCATTGCATGGAAAGATCCTATAGCTATGGAGTCCTCGAAGTAGT
TATTGGAATAACCACCAATGGAAATAGAGATGATGTCGACGCCATCTTGGATGGCATCTTCGAAGGCGGCAAGTATATCAATGTCA
>comp142512_c0_seq1
TTGGATTAGCGATTATTTCTTTGTACAGAATGATTGGGCCTCCAAAAAAGAGGGGAAGAGTTGAGGGAGCTACCTATTCCCCTTCATATAGGAGCGGAAGACTTGTTGAGTATGTTGAAGC
TTCCTGATATCGAAGCAACGCTATGACTGATAGTATTTGCGAAGGTTGCCCACGTTCCAAGTTCGGGTAAGCATTCTTCCGTTGGCATCTTTTAATTTGAAGGTTCCATGCTTGACCACAAC
CGCCACCAAGTAAGGACCTTCCCATAGTGGGGAGAGCTTATTGGGTTGAGGTCCTTTTCCAGCAGCTTCGATGCTTCGCAGAATTAACTCCCCAATGGAAATAGTTCGAGATTTTACTCTT
CGATTGTAGTGACGAGCGACCCGCTGGTGGTATGCAGCGATCCTGAGTGAGGCTATATCTCTTGCCTCGGAGAGGAGGTCAACTCACCAACTCTTCGGCTCGTCGACGACCGATGCACTT
ATGTTTCAGAACATCCTCTGGAATGTATATCCTGGACGGAGAAAAG
>comp142592_c0_seq1
CATAATAAATAATTAACTTCCTGTTCACAATGATATTTAGACCATTCTGTATTACAGATTTTTATTCAAATATTATAACACCTTAAAAACCAAATAGGATGAGCTTCTTTTTTATTTTAACAACA

TCCCTCCATTCTTCCAAACACTTGGCAAAAATAAAATAAGGAGATCTGAATTGTTACTTTCCGCACAGTTCTCTATCCATCATTCTAATATGTTCAAGCTTAGTCAACAACCAAACCATGTG
ACCCCCAATATATCTATCACATTCTCATGTTCTCTAGATCTAATTGAGCTTCCTACTCCATCTTCATTTCCTTCTTGACCACAATGCTCTTGTTGTTGGTATTAAAAGTATTCTACGTAATTATG
GCCGTAGTGGTAGCGGTCAAGGTGGTGGACATGATAGTAGAAACGATTCCTAACTTATCATCACTCTAAAAAAATTAATCATTATCTTATCATTATTAAAGAATGTTCATATATCATCCCACTA
CTAAGCATGTCACTAGGAATAAACTAACTCAAATAATATGCCTTCTAAAACATTTTCCCAAAATAACTATGACTTGTCTACTACAAAACACAAATAACTATTATCTCCCATGTGATTACCTCT
ATCCAACACTCCAATCAATTAAAGACAATTTAATGGTCAACTTTGCGATTCCAAATTCATTCATCACTCTCTCACACATTATTTAGGGCTTACGTTAACTACTCCTTTTACCCTTGATCATAGT
GCCATTATTGCTAGCACTAAAGATAGTCGTGGTACTCATGGACATG
>comp142676_c0_seq1
ATGCTTCAATTCATCCCAGGCTGAGCCTGCATACTCATCAGTAGCTTTATAGCACCAATGTTCTAGTTCAGCAAGTCCTGCTTTAACATATTCTCCATTACTGAATGAGCAGCACTCTCTCCT
CAGCAGAAGGCTATTAAATAGCTGCACGTTGATGAAGGAAAATATCTGTGTGAACACCTTTCTAACTAAAAACGGGGGAACATGGTTTCCTTTTAATGTATTCAAGAAACTCCAAAGACT
CCTTACAATCCCTTGCCAATGAGCAATTGGGGTTGATAGTCCTTGTGGACGTATAGCACCTTTGATTAGAATTGCTCTTGATGTTCTCGGTGCCTGAATGCACAAGCCAAGCAAGGGAGAG
ATATCTTTTTTCAAATTATCCCGAATCATTCCATAAATTTTCTCCACATATGCTGTAAGTTGTTGTTTGAATAGCAGAGCAGGATATTTGGCTTCCACTTGCCGCAAAGTATCAACTCCACCA
ACTAAGCCGCCATTGGCAAAAGAAAGGTTCACTCCCTGAGGAGTTCCCCTGAAACTTTGAGCCATCCTCCCAAATAAACTAGCTGATGAAGATCGACTGCGCTGTGGAGCCATCCCAGC
TGCACCACTTGCCTTTAAAGTCCGTTGTAATAGTAATAGCAATGTTGAGGCATTGGAAAGCCAATAAGCTAACACATCATTGTCTTCTTGGGTCTCAATAGCATGCCCAATTGTCTGAATAA
TTCGATCAAAAACACTAGTTCGCTCAACTTCAAATGATCTCCACTGGAGAAGACATTTGTATATGATACATGCTGCAACAGGCCTGCTCCCTGCAAATCCCAGGTGCTGTGCAATACACCT
GATTAAAAGCTCCTGGTTCTCCTGTTGCTTCTCATTTAGAGATCTCTGTGGTTTGTCCTCTAACTCGGAAAGTTCCTTTACAGAAGAGGAGGAATGAATATCTGCAGAAGACTTTGGATCA
ACATTGTTATGTCCGCTATCCGAACTCCTATGTACAATCGATCTAGAACGTCCTGACAAAAATTTGTTAGGAGCCATGGACACAGCCTGCTGACGAAGAACCTGATTTTCAGATTCTAGATT
TGACAGCTTTTCCTCTAGCCTATTCAAATGGTCCTGTAGATGCCGAGATTTTTCTTCAATTCCCTCCAACTTTCTTTGTTTTTCTTCACTTTTCTTCTGTTCTTCGCCATATTTTCGCAAAGAA
TCATCAGCTCGTTCCTTTTCTGAGCCTAAAGAAGTCTTCAGCTGTTCAACCTCAGCTGTCAATGAATCAACTTTCTGAGTATCCTGAACAAGAACAGTAGTTTCCTTGATGATAGGTGGCG
CTTCTTCAATTGCTTTTTTCATCGCCTCTCTTTCCTTAGCAAGTAACTCATTAGTCTCATTAACCTTATTCTGTAGCACTTGCAAAGAGCTCTGCAACTTTGCAATCTCCTGCGCTTTTGCTTC
CTCCAAGTCTGTCCTTAAGCGCTTCTCCAACTGCAAACGCCATGTAAGTTCTTCCACCTTTTTTTCAAGTTTGTCCTTCGCTTCTTTGAGTGCCCCAGTTTCTCTGGCTGCCATTTTAAGCT
TCCTCAATTCTTTCCTTGCAACCCTTCCCCTCCACTTAGTTTGTGTGACAATTGAAGCTCTCCTTAACTTTTTGTGATATGAATGGGCTTTGTGGCAACGCCATTGAGCCTGAATCTCTGTTG
ATGCTTTTGTTTGCTTCCTAAATCTGAAATCATCTCGGGCAGCCATAGCCCTCAAACCAGTTTGAAGGACAAGAACTGACACCCTTAGACCTGTGTACTTATTTTTTGCCCAGTAAGATCG
AAAATTTTTCTGCATTTTAATTGCAGCCGACTCCTTTCTCATGTGCTCATACAGTTTTTGAGCCAACCTTCTTCTCCATGATGACTGCACTAGAATGCTAGCCTTCCTTGTTGCGACAAACC
GTTTATGAGTAATGTGCGTTCGTATCTGGCGCTGAATAGTCTTTGCAGCATTGCCAAGTACTTCAGCCCTCCTAGCATCTAATTCAGCCATCTGACCAGCTCTAAGGAAAACTTTTGTTTTT
CCCATCTGCCAACCCTTTAGCCCCTTCTTCTCTAAAATCTTTCCACAAGCAACCTTCTCATCGTAGTTTCCTTCTAGAACTTCTGGAGCAAGAATGCCAAATCGATGAAGAAATTCATAAAA
TGTACGTCGAGTAGGATATCCAGCACAACTAATTCTGATAGCTTCCAGAACACCACCACAACGTAATTGTTGCATGACATTAACATTCTCAAAAATGGCAGGTTTAAGAAGATTGTTTGGC
TTCACACATCTAATATAATGTGGTTCAGTTGAATTCAGTATCTCCATCAAAGATTGTAGTTGTAGCTTAAAACGAGCACCAATTGATGAAAATTTAGAAGACTTTGATGTATCCTCGGGTAG
TGGAGGGAAGAGGCCCGAGACAAATGGGCATTTTGAGTCACTCAGCAATTCTTGGTGTTCTGCTACAACGTAGTCCTTATTTTTATCTAGGAATTGTTCAGATTGATATTGAACCTCTCCA
GCATAATGGGAAATGGTAAAGTCAGTTGGAGACAACTTTGGTTTGACAAAACGCTTGTGCGTTTTAAATGTCTGATAAAGCTTTTGAGAAAATGTCTCATATGTTGATTTTGGAAACATAC
ATGCTTCATCAAGC
>comp142678_c0_seq1

ATTATATAATTTGATAAATGTTATTGTTAACACTCATAATTTCATGTAGAAATCATCTATAAAGTACTATAGACCTATTTCTCTATGCAGTTTTTTAACAACTAGAAGCCATCTATAAATTATTC
TATATATTTCTTCTTTTCAAGGTGCTTTTATCACTTATAGGCTCCTAGAAGAAAACATTCTTCTTGCCACAAAACAAATTGGAGACTTTCACAATAAATGGGCTTAAAGTTTATTATACTAATA
TTGATAGCTCCAATTAGAGGTGTCAATTTTGGGCTCGGGTCGAACCCGAACTGACCCGAGTTCAAACGGGTTGACCCTAACCTGACCCGGGTAGGAAAACAGGTTGGAAATCAGAACCC
GGACCCGACCCGGCGGGTTGCCGGGTTGACCCGACCTGACCTGCGCGGCAAAGAAGGAGCTCGGGTTTTGCGAGAGCGAGCGCGGCAGCAGGCGACGGCACGAGGCGCGAGCGTGG
AACGACGACAGCGTAGGTTCGGCAGCAAGCGTAGGCGCGGGCGCGGCAGCGGGCGCGCGGGGCAGCAGCGCGGGGCAGCGGGGCAGTAGCGCGGGGCGCACGAGGTAGCGGGTAGC
TTGGGCGGGGGCAGCGGGCAGCGAGC
>comp142726_c0_seq1
AAAATTTAGGAATCAAAATAAGTCATATTATATAACATCAATAAAGGATTTTAACACACATCATCAAAATTACAAGCGCAATAATTAATATATTTTCAACAATAAATAAACAATATAAAAAAG
CAAGGCCAAAATGTGCAGCAGCTCCTGTCAATAGATCCATCTGCATTCACCTTGCCCGTCACCATTAACACTATTCATTTTAAGAAGAAAGCCGTGACCGACTGCGACCAGCTTGAAGAG
AAGAGCTAGCGGGTGGAGGGCTCAGGATTGCCTTCATTGGAATCACATGGCGGCAGAGGACCATTTGAACTTGGAAAAGAAGGTGTCGCTTCATTGACAGGCCTCATGCTGATACAAAA
ATTGGAGAATCTACACCTCCAAATATTTTCATCATCTGTAACACTCAAAGAACTTGTCGACGCCGGTGGTTGGCTTCCCGGTGGTGATTGGTTCTCTCTTGGCATAGCGAATGATTGGTCA
GCCTCGATGGCTTCATTAAAGGCTCTTCTCCACGCCGCCGGTTGGTTTCCTTCGCCAGACGCGTTGCCGTTGCCGGAGGTAGAGTGGCAGTCTGAAGCTGAATGGCTCCCGCCGCTTGAC
ATTCACCGAGATTTGATTGTCAGACCGCCACGACGACAGAGATGAAGACGACGGGACGACGGGAGGGAGGCAGCTTGAGCTTGTTTGGATCGTTCGGTGGAAGGAATGGAAGGAGGA
GTAATGTAGAGAAGAATTGCGTTATAATGTGCAAAAGAAGCCA
>comp142836_c1_seq1
CAAACGAAGCCTAAATATCTCCAGATTTTTTGTCATTCTTAGCAGCCATTAATGTGTTCCGAGTTAGAACAAGAAGCAGATTCACTAAACTGCGATGCCAATAACTTCTCCACCATCTTCCA
GTCAATTGAGTTGCTGGTTGCATGAGAACCTTTAGCCAGCAAATCACTTGAAGATGAGAAGCCTGCAGGGTTCTCAAGCTGTGGGATCGCAAAGACATTGTGGCACTCCTTTGATTCATT
TCCAATTCCAAATCCATGCAATGGTTCATAGCTAAAGCTTGCGTTTGTTTCCATTTGAACAATTTCGTAATGGTTTTGATCAGCTCTGAGGAAATAGCTTGGATTGTTAGTGCTGAAGCAGT
GTCTTTCATGAATTGGACTTTGTTTTTTGAATGCTCGGCATACTACCCAACCTTCTTCCTGAGAATTAGGCGCTAGTTGATCACTTGAATGAAGGCGATACTCATGCATGATCCA
>comp142957_c0_seq1
TTTCTCTCTCTCTCTCTCTCCCACCCCTTCATCTCTAGTCATACCATCATCCTTGAAACCGATGGCCATGGCTGGCTGAGTTCATAAACTTCAGATTTCCGGCCACTCCAGAAGTGGAAAGA
GAGAGAAGGTGTAGCTCTTGAGTTCATTAATAAATCTCCGTAGATTACTGATAAGCTTTCCTGCAATACAAAGAGAGGAAAAAATAAGCAGATATATATAACTGATCAGCTATTGCAGGAG
CCATGGAAAGGAACATGAAGGGAAGAGGTAAGATAGAGATCAAGAAGATAGAGAACACCACAAACAGGCAAGTTACCTTCTGTAAGCGTCGTAACGGTCTTCTTAAGAAGGCTTACGA
GCTCTCCGTCCTCTGCGACGCTGAGGTGGCGCTGGTCATCTTCTCCAGCCGCGGTAGACTCTACGAATATGCCAACAATAGCGTAAAGGGAACTATTGAGAGATACAAGAAGGCTAGCTC
TGATCATTCCAATACCAGAACGATTTCTCAAACAAATTCACAGTATTACGAGCAAGAAGCCTCAAAACTTCATCAGCAAATCACTAATTTGCAGAACTCAAACAGGAATTTGATGGGTGA
GGCTCTCAGCACAATGAACTTAAGGGACCTCAAACAACTAGAAACTAGACTGGAGAAAGGCATCAGCAAAATAAGATCTAAAAAGAATGAGTTGCTGTATAATGAGATCGAGTACATGC
AGAAGAAGGAAATGGAGCTGCAAAATGATAACATGTACTTACGAAATAAGATTGCTGAGAATGAGAGAGTGCAGCAGCATCAGATGAACATGCTGCCTTCAACAAGAAATGATTGTGAA
GTTATGCCTCCATTTGATTCTCGAAGCTTTCTGCAAGTAAATCTTCTTCCTAGCAACAGGCAGCCTATACCTTAGTGGAAGAGATGTCAGGTACTGGAGATCAGGTTTTCTTCATGGAAATA
AACCATAAATATGCATGAATTGTCAAGAATAGACTTTTTGTCCAGGAGCTTAATTTGTTGTATATATATTGCCAACTCTTTGTTTTATAGCTTGTGATGGCTTTGTATTGTTACAAATAAACATA
ATTTTATAACAAAAATTTGGTCTTAGCAATTCCACATATGTAACCTTAACAATTTGATTTTTCACATTGAGAATTCTAAATTGACCTGAAAACACAAGACTT
>comp142957_c0_seq2

TTTCTCTCTCTCTCTCTCTCCCACCCCTTCATCTCTAGTCATACCATCATCCTTGAAACCGATGGCCATGGCTGGCTGAGTTCATAAACTTCAGATTTCCGGCCACTCCAGAAGTGGAAAGA
GAGAGAAGGTGTAGCTCTTGAGTTCATTAATAAATCTCCGTAGATTACTGATAAGCTTTCCTGCAATACAAAGAGAGGAAAAAATAAGCAGATATATATAACTGATCAGCTATTGCAGGAG
CCATGGAAAGGAACATGAAGGGAAGAGGTAAGATAGAGATCAAGAAGATAGAGAACACCACAAACAGGCAAGTTACCTTCTGTAAGCGTCGTAACGGTCTTCTTAAGAAGGCTTACGA
GCTCTCCGTCCTCTGCGACGCTGAGGTGGCGCTGGTCATCTTCTCCAGCCGCGGTAGACTCTACGAATATGCCAACAATAGCGTAAAGGGAACTATTGAGAGATACAAGAAGGCTAGCTC
TGATCATTCCAATACCAGAACGATTTCTCAAACAAATTCACAGTATTACGAGCAAGAAGCCTCAAAACTTCATCAGCAAATCACTAATTTGCAGAACTCAAACAGGTAATAAATTAACATA
CAGTCCACTTCACTATTCTCCGTTTATAAATTCCATATGTTACAGAATCCTTATATGCCTATAGGAATTTGATGGGTGAGGCTCTCAGCACAATGAACTTAAGGGACCTCAAACAACTAGAA
ACTAGACTGGAGAAAGGCATCAGCAAAATAAGATCTAAAAAGAATGAGTTGCTGTATAATGAGATCGAGTACATGCAGAAGAAGGAAATGGAGCTGCAAAATGATAACATGTACTTACG
AAATAAGATTGCTGAGAATGAGAGAGTGCAGCAGCATCAGATGAACATGCTGCCTTCAACAAGAAATGATTGTGAAGTTATGCCTCCATTTGATTCTCGAAGCTTTCTGCAAGTAAATCTT
CTTCCTAGCAACAGGCAGCCTATACCTTAGTGGAAGAGATGTCAGGTACTGGAGATCAGGTTTTCTTCATGGAAATAAACCATAAATATGCATGAATTGTCAAGAATAGACTTTTTGTCCA
GGAGCTTAATTTGTTGTATATATATTGCCAACTCTTTGTTTTATAGCTTGTGATGGCTTTGTATTGTTACAAATAAACATAATTTTATAACAAAAATTTGGTCTTAGCAATTCCACATATGTAAC
CTTAACAATTTGATTTTTCACATTGAGAATTCTAAATTGACCTGAAAACACAAGACTT
>comp142988_c0_seq1
AAATTAAAATTTTTATATTAAAAAATTGTTCTAAGAAGCACTAAAAAAGCGGTCGTAAACAAAGCCTAAGTCAACACACTTCAAATAGTAGGGTAGGAAGGCTCCATGGCTAAGCCACAT
AATCCTTCAACCGCAGACACGTCCCTCTCGATCCTCATATATCCGTTCTCCCCCCAGCTAGTCCCCCATGAGTTCTTCACTAGCCAGTACTTGGTGCCGTCGGTATCGGTGCCGTACCCGAT
GGCGGCCACACCGTGGTCAAGTTCGGTGCCACATGGCCCAGTGAACAGTCCTCCACTGTACAGCTGGAACCTGAAGTCACCGCCGTCGATGGCGACTGACACTGGCTGCGCGGTCACC
GCCTTGAGCAGCGCGGCCTCGTTGTTGGCCGGCACATCCTCAAAGCCGGAGATCTTAACAGCGCTGGTGGACGACTTCTTGGTGTTGCAGGAGCCGTCGGCGGATTTGTAAGGGTAGTT
TGCTTCGGTTGTTAGGCCGCCGTTCTCGATTATGAACTTGAAAGCATCGTCCATGAGGCCGCCGCTGCATCCTTCATCTTCCCCGTTCACATCACAGTCGACCAGCTCCTGCTCCGAGAGC
GATACCAACTTACCGTTCTTTATTTGGTTGATGCTCTCCGTAGCCGCCACAGACGAGAAAGCCCAGCAGGCGCCGCATTGGCCTTGGTCCTTGACGGCAGTGACGGCCCCTTTGGTCCTC
CAGTCTAAGCTGTTTGCGGCTGTGATATTCTCGTACCTGAAATTCTTAGGCGCGACGCGATTCGAGCTGGACTTCTTACAGCCGGTGTAGAAAGTTCTGAATTCCTGATTGGTTAAATCAG
CGAAGCGGTTGGCTCCCAGCTGAAACTTGTTGTTGCCGGCATTGAAGCTCTCTATGTGCTCCATGTTGGCTTTAAATATTTCAAACCGTCGCGCTTTCTCTGCCGCGTCACCATACACTTTC
CCGTGCTTCGCCATCCACCGTTCGTGCTGCTCAGTTATGGACATCTCGACGGGTAGGAGTCCAGATAATGACGCAACAATGGCCACAGCCCCGAAGACTGCGAGAGCAAGAAGAAGGCT
TCTCGTAGGAGCCATGCGAAAGGATAGTTAATTAATTAAGCTTGGTATGCTCAACGGGTATGTTTTGGGAGGGTTTATATAGAGGATTTGAACGAGAGTTGGACACTCAATTCGACTAATAA
TGGAGGCTGTAGAATTGCGTGCTGCTTGCCGTGGGGATTGAGCAGATTGAGACCTTCATCAGGAACTTTTATTAGGTCAGTTCACTGGACGTAGAACTACGTTCGGCACCCATTCTTATAG
CGCCACGTAGTAGCCCGGAGACCCGCCCAAACCCGTAGTGATTTGTCGCTTGGTCACGTGTTTTACAACATTTCGGGCCACCCCGACTTATTTTAT
>comp143124_c0_seq1
TCCGAACAACTCATCCACCTACATCATAATACATGAATAGTAGTATCAACTTTATTTTTTTCTCATAAGATAATGCAAAAAGCCAAAATACAATCCACGGAGCAGCAAAGCACACAGTGCGT
ACGTAGTAACGACGTTACCGGCCAGAAGGAGCAAGTGAAAATAAACGAAATTATAAAACAAATTATGCAAACTAATTTTGGGTGAAATTTTCAAACGCCAGCTAAAGTACAATACCATTC
CCAAGCTTATTTATTAGATAAAGGATTCAAGTAACAGTATTGTTCTAATAAAATCGCAGTATCTCCCACAAAGCACAAAGTAATCAGCAAATGCCATCCCTAATCCACATTAATTAATTTATC
TTACAGAATCTCATAATTATCCATTCTTAACTCGGCACGTGACTACAAATAAGCGGGTTCAGATCTGGAGACGCTGTAGTGTACGTAGGCCCGTCCGTCTATATATACGTCGCCCGACACCA
CATGCATGCATGGAATAAGAAACTACTTAGGAGTATAGTGCACGGCTGGACACGTGTCAGCTACCCAATCCAATCGGAGAAAGAACGGTCCGACAATGCTACTCTTCTTTTCCAGATAAA
TTAAAATATTAAATAACTAATGTGGAGAGTTGATTTATAGCAGGCATTATTAATAATTTCTACTGCCTCAGTGTCCCTTACAACGAACGGGCAGAAGGAAAAGGAATTAGCTAACATTTTGT

CTCTTGTCTTCTTCCTTCGTTCCCGTTTTACCCACCCTCAAAAAAGAAATATCATATACTAACAATCATATTTCCAATCTTTTTGTCTCTCTGTTTTTTTTTTTTTCTCTTTTTTTTGTAACTCTC
ACTTTTTTTAACTCTTTTTTTCTCTTTTTTTTTG

>comp143130_c1_seq1

AAAACCTCCACGAAACCTGCAACCGAAGACCCTGTTTTCAAAGGATTAAGCAATGGAACAGTAACGATAGCTTAATTTCATTAAGTGGTAAAAACTACTGTGCCTCCTCTATCGACAAGG
CATATCACAGGAGAAAAATTAACAATGGCAATATCGAAAATAACTAAGTATTGAGTAAAAGCTAATGATGAAGTCGTTAGGAGCAGAGGTGGAGAATAGGAATTCCTTCAGGGTAGTGGC
CACGGTGGCAATCGTCCAGACAGCGGAAGTGGCCACGGTGGCAAGCCGTGAGCTGCTTATTAACTGGAACCTGGAACCACATCAGGTACCTCCCGCTATAGAAATTTCAGGTTCCACCT
GAATATGTGAACTGCCTGCATCATCAGGAGGATGATACGGAAAGAGATCCGCGACATTAAAGGTCTTGGAAGTATGCATAGTGTCTGGGAGATCGACGACATAAGCATTGTCGTTAATACG
TTTGAGAATAGGCACGGGACCAAATTTACGAGGACTCAATTTTGAATTTGCACCGGTGGGAAAACGGTCCTTGCGAAGTCGTATGAGAACGAGGTCGCCAGGTTGAAAAGAAACATGAT
GTCGGTGAAGATCAGCGGCTTTCTTGTAGGTGGAATTGGACGTGCTGAGCTTCATGCGAACTTCATCAAGAACAGATTGGCATTGGTCCGTCAAAGCTGTAGCGGACTTGTTAGCTCATT
TGGGAAGAATAGCTATATCGAATAAGTGGTTCGGAGCCTTAGTATAGACGATAGCAAAAGGACATTGTCCAGTTGATCGGTTGGGCATAGAGTTATAAGCAAACTCCGCCTGGCTTAATGC
ATTTTCCCACTGTTTCGGCTGGTGCTGTACAAGGCAACGCAGCATATTGCCAAGAGTCCTATTGACGACCTCCGTCTGTCCATCAGACTGGGGGTGATAAGCAGAGCTAAGGTTCAAGGA
GGTATGAAGTCGCTTCCACAATTCGCGCCAA

>comp143142_c0_seq1

TAATCAACCAAATAATGAATTCAACAAACTTCGTCACTTTTAAAAACAAGAAGTACTTTAAAAAACAAAATAAGCCTCAACTACAACTGAAAACAAAACAAAGTTTCATACAAGATGAA
ATTATAACTTATAAGAAATGCTTCAGTAAGCCATTAATTTGTCATCCATTAAATAACTTCAACTTTGAACACGTTAAGGAGCACAGTTAAAAGAACATATTTTGTTCATTTTCTCATTCAGTAT
TGATCACTGAACATAATCATCTACTTTCTCCTTATTGCCACCAATATCATTCCCTACATTTGAACCATGAGAGTCTGCGCTTTGATCAGTGACAGGTTCCTTTGATCTATCTTTATCAATTTTT
GGAAGGGCAAGAAACTCAAGAGCAGTTACAACATCTGTAATGAGAGGACGAGTACTTGCCTCTTCCTGAAGACACATTGCTGCCACTGCAAGAGCTTGATATAGACTTTTTATGGGATAC
TTTTCTTCAAGCAAAGGATCTGCCATCTCTATAAATCTCTTTTTATTTTTGAAAAGTGGTTCTGCCCATTGGACTAGATTTTGTTCACTTGAAGGCCTGGAGGTGTCGATGACTCTCCTTCCT
GTTATTATTTCCAAGAAGACAACACCAAAGCTATATACGTCGGACATAGTAGTTAACTGGCCTGTCATTGCGTACTCAGGTGCACAATAGCCATAAGTGCCCATCACCCTTGTTGATACATG
GCTTTTATCTCCAATGGGACCAACCTTAGCTAAGCCAAAATCAGATAACTTTGGTTCGTAGTTTTCATCAAGAAGAATATTTGATGCTTTAAAATCACGATAAATAACTGGTGGTTTTGCAA
TATCATGTAGGTATTCAAGGCCCTTAGCTGCACCTTGAGCTATCTTCATTCTTGTAGTCCAATCTAAAGGCTTCTTGTTTGGTGACAGGTCAAGAAGATGATCCTCCAATGAACCCAATGGC
ATATATTCATAAACCAAAATTCTTTGATCTCCATCAGCACAATATCCAATTAACTTGACAAGGTTTGGGTGGTGAAGAAGGCTCAACATTAATACTTCAACAATAAACTCTCTATTTCCTTGA
AGTCCATTTCTGTCGAGCTGTTTTACTGCTATGTCCTGATTTGTGTCTTCTAGATGCCCTTTATATACTCTTCCAAATCCACCTTCGCCTAGTAAACAGTCTAATTTGAAGTTGTTGGTTGCAG
CACACAATTCAGCAAAGGAGAAGACTCGAGCGGTGGGATTTCCATTGCCCATCTTTAAAATCTCTTCAGCTACAGCTCGATGCTTTTTACTTTCAGCTTGGAAAGAGATGCTGTGAACGA
GGCTGGAAAGAGGGTGATTGCTTCTAGACTCCAATGTCTTCTTCCATGATTCCATTACTTCTTTATCTTTAATTGGTTTAAAGCAACAGAAGCAGCCCATCACCCTTCTTTCCCACCTTCCG
TTTCCTCTTCTTGATCCACCTTCCTTTTTCTTCTCCGTCTTCTTTTCCTTCTTGTTCTTCTTTTTATTCTTCTATAACTATTTGTTTAATTATAATGCCTCTTTGGATTGATCCAATTGAGTGGATC
AAAGAAATCATAGAAATCATATAAATGGTCAAAGAAATCATACAATGCCAACAAAAGAAATTGGATCTATAAATGATCGAAGTTTAAGAGCTTACCAGAGAAAATTTATTCTCCAATATTTT
TTTGCTGTTCGTGTTTTTCATTAGGAGGAGTTTCTATTTCGGCTTCTTTGGATGATAGGATTATCAATGGAGAGAAGAAAACCACGCGGTCCTCTTGAAAA

>comp143205_c0_seq2

TTTAAATTCTAGAAAAGCATATCTAATAGAAATTCAAATATATAACAAATATTAAATACATAGATTTCACCAATAATAAAAATAAGAAAAAACAGAATTAGGAAAAATATATTAATTTCCTAAT
CTTAAAGTCAGCAACTTGTTGACTCTCCATTTAAGTAGTCTAAGATTGCAATTACTCCAACAACAAATGCTTGATCAAAGCCAGGCTGCACCACAAGAGAATATAAATCCTTGCTTCCATT

CATCTCCTTGAATCCCACCTGAGCAGCAACAATTCCCTTGGAAGTTACAATAGAGCAATTTCTCTGAGGAAAGGAGCCAATGACCTCAAAGTAGTAGTCTTTGTTCATCCTCTTAGGTTCA
ATGTAAATCTTTGTAGACCTTCTCAGTGCAAATAATGGCTTTGAGTCACTCAGACTAAAAACTTTGTCTTTAGCCCCCTCATAATTCATTGTATATCCATTCCATTGAGAGCAGCTGCTAATT
GCTTGAATGATGCCCCCCTTCTTATGAATTAGAAGTATTAAGGATCCATCACTGTCTCTCACAACAAGTTCTCCCTTAGTTCCCAGAATTCCACAGCCATCAACACTGAAAGCTACCTTCTG
GTTGCAATCAGAGACTACAAATCCACCTCCATTGATTACATGAGGCCTTCTGCGAACCACCAATGTTGAGAGGGAAGGAGAACAGAAGATCTTACTAACAATAGGCATAAGATTTGATGA
TCCTTCAGCCATGACTAAGAGATGAGAAGGATTTGGGTTTCAGTTGAAGTGATGAAGAAGGAAGAGAGCAAATGAAGAGCTTAAGAAGCAAAGCGGGGAGGAGAAG
>comp143235_c0_seq1
AAGTTGAAAGAATAGCACGATACAGATATGTTTTACCTGTACCTCCAGGACCATCTATGAAGAACATGCCATGACGTTTGATTAACACGATTGATAATTTCTTCAAATGCGTTCTTCTGATTT
GAATTGAGTGTATGTATTATATTTAAATCTTCAGCTGGAGTAATGACATTTCGCTCTTCAGATATTTCTCTATTATTGAGCTCATCATTTAAATCATGAAAGATGTCTTGTACATGTGGCAATTC
AGGCAAGTCAAAATCATCGATGGACCTTCCCATTCTCTCAAGAAATAAGT
>comp143259_c0_seq1
GCGATATGAACGGCCACCTAGTATTATTAAATTTTATAAAGGATCGTATTCGAGAATTTGGGTGCGATAGACACGGTAATGTTGAGCCGGGGATAGGAATATCAATGGTTGATATGGATCACT
GTATTCTATATCAACATTGGTCGCATAAAGAAGATAAATGGCTCTTAGATAACTACAAAATGCTAATGCAATGTTCTCAGGGCGGAAAGGAGAGAAAATTTTTAGATGGCGAAGATTGGAA
CCACCTGCAACACGGATTTATGTACCACTTCAAATGTTGTCCAACGCAAACACAACTTCAAGGTCGTGTACGAAATCTGCTTAGGAAATGACTATGATGTGTTCTGAAATGTAAGCAAATA
TTAATATTTTATGTATAAGATTATGGTGCTAACTATCTAAAATTCTTATCGGCCTTTACATATAAATTTGTATTATTTTCATAAATAATAACTCATTCCGCATTTGCTTAAATTTCAGCACATAATG
TCATCATCTTCATCAAAGACCTCAGCAATAAAGCGCTCACCAGCTAGGGATTTCCTTCATATAATTCCTAGAAAGAAGTTGATATATGATGGAACAATTCGATTGTCAGAATCAATGTCGTA
CCCTATGGATGAATTCTTGGAGGAAAAAGCAAGAACAAATGAATTTTTTCGATGTTGTTTACATGAATGATCTTGGTATGAAGACGATAGTTTATGGGAT
>comp143270_c0_seq1
GATACCAAACAGCCCCTTAAGGATCCCGAACCGTTTAGAGGGGTAATTTCGTCATTGCTTCTCGCAACAAACTCTTGCCCTCAATGCGCGCGGCCTTTTCGATGGTGGACAGCGATCCTAA
AAAGAATTCAAAATCAGATACCAGAGAAAGGGTTAGGGGAGATAAATTATGCCGCTTGGATAGGAAGGACAGAAAAAACGTATATCTAAGGTTGAACGGAGCTGGTTCCTTCGGCCGTC
GACAATCATCGGCGATGTATGGTGTGAAGATACAAGAGGTATCGAAGAAGCTGGCGCTCTGGCACACGCCGACCTTCAGTCCAATCATGAGCCATGATGACCTCGAGCCCATCTTGTGTT
CGGTTGGATTCATCTCCCCCGCAAATGTGCCCCAGCCATCGACATCGACTAACCTGAACCAGCCGTCATCACCATCAAGAAGCCCGCCGGCTGATGTTAAATGGAAAGAGTACGCGTTCC
CTTCGTCGGCGGCGGCGGTTTCCGGCTTTTTTCCGCCCCGCCCAAGGCTTCCATTTCCGATGATATGGGGGCTCCATCTCATTGCCTACAAGGCTTTTCTCAGCGCCTTAGAATGCTACATA
GACCCCACAGACGTCTCCAACCTATTTCATGTGAGTCCTTTCAATGATTCAGAGCTATGTCGATAACCCCCATGCCTGAGGAAGAATTCGAGAAGGAATACCGACCAATGAAAGACATGG
ACGCCGATGACTGTACAATTTATGTGTACCGAAAAGGCACCGTCTATAACTGGATGTTAGAACAGATTACAGCTTTGGAGTACAGAACTCAACGCTACGAAGAAGATGATGATCCTAGAA
TCAAAAGCGGAGGTAGCAACACCGATCAAATCTTTATGGTTTCATGGGACAACCTTCTACCAGAAAGGTATGTTACCAGTCCATGTCCTTCGCCTGAAAATGCCGAGAAATAGAGCAACC
TTTTTTTTTTTTTTTAATTTTTTAAAATTGGGTTAAATATACTAGTGGTTAAATCTTATTTTTCAAGCCAAAAGATTAAACTTGTGGGTGAGGGCCAACCATATAGTATTTCAACACAACAATG
TTTTTTTTTTTTCCTATTATCTTATATGGGAGACTTATTTACCAACGATTTTACCTGAAGAGACTTATTTACCAACGATTGCGAAATTATTCAGGCCCTTAGTACCAATGATGCTGTCCTCTCA
CAAAAATCACTTTTTGGTCAGGTAAGGTTCGTCTTTCCCTAAATTCATACCAAGCCCATCAACATATTTGCAAAATTAAAAACAATTTTCACCCCTCAACAAAAGCATAATAAAGCAAGCA
AAGGCTCCTATTTTACTAAACTATCAAATTTCACCGTCT
>comp143284_c0_seq1
GTAAAAAACTTTCTTTTTATTACTTTGTAAAATTATTTTAGTATAAAAACTTTTTAAAAACAGGAAAAAAAACAGTGGTCTAAAACTGTCCTAAACCATATAAGAATCAAAGCACAATTG

TTTTCCTTTTAACTCATGGAATAAACAGTCATTTAGTTTGAAACCATTCATGCAAAAGGTGAATTTTAGCATGAAGAATTAAGGATACAGAGCCCATGAATGTAACTTGTTTCACCATGCAA
AAGGTCGACGCCGTAGATATTGTCGCCTCTATAGGAGGGATGGTTCAAGGGCTTTTCATAGAGAAGAAATATGGAAGTGTTGTTCTAAGCAGAAGGTACAGAGGCCTTAATGGGCGGGAG
AAGGTTGCCTTTCTGCATGGATGACAAGAAAGGCTTCTTCTTCTTCTTCCTCGTATCTTCAGAAATCAGCTTATCTCTGCACCTTTTGCTGCAGAACGCAACTTCCCTATACATGAATGTAT
CAATGCCGGCGCCAATGCTGCGCTTGCAGAAGCCGCAAGCATTGAGGAAGGGGTCGGCATCGACGAAATGGGCCGCATTCACCGATGATAGATCAGTCGAGTGCCTCTGTTTGACGTCT
TTCCGTTTCGGGGCCGGTCGAATCTTTTCCGAGTGATTCTCATCCGACCAAGTCGGGCGGCTCGACGGCCGAAGAAATGACGAGAACTCCGACAAGCTCGTCGTCCTCCTCATCGGACG
TCAAGTTTACCTTCCTCAACCTCGATCGCTGGTCCCGCGTTCCCGTCAAAAACGAAAATGTGTAAGGAAATAGAAAGGTACGTAGAGTTCGTTAAGCAGGATTGA
>comp143284_c0_seq2
GTAAAAAACTTTCTTTTTTATTACTTTGTAAAATTATTTTTAGTATAAAAACTTTTTAAAAACAGGAAAAAAAACAGTGGTCTAAAACTGTCCTAAACCATATAAGAATCAAAGCACAATTG
TTTTCCTTTTAACTCATGGAATAAACAGTCATTTAGTTTGAAACCATTCATGCAAAAGGTGAATTTTAGCATGAAGAATTAAGGATACAGAGCCCATGAATGTAACTTGTTTCACCATGCAA
AAGGTCGACGCCGTAGATATTGTCGCCTCTATAGGAGGGATGGTTCAAGGGCTATACATGAATGTATCAATGCCGGCGCCAATGCTGCGCTTGCAGAAGCCGCAAGCATTGAGGAAGGGG
TCGGCATCGACGAAATGGGCCGCATTCACCGATGATAGATCAGTCGAGTGCCTCTGTTTGACGTCTTTCCGTTTCGGGGCCGGTCGAATCTTTTCCGAGTGATTCTCATCCGACCAAGTCG
GGCGGCTCGACGGCCGAAGAAATGACGAGAACTCCGACAAGCTCGTCGTCCTCCTCATCGGACGTCAAGTTTACCTTCCTCAACCTCGATCGCTGGTCCCGCGTTCCCGTCAAAAACGA
AAATGTGTAAGGAAATAGAAAGGTACGTAGAGTTCGTTAAGCAGGATTGA
>comp143389_c0_seq1
TTATGTGATTCGTAAGAGAATCAAATTGAGTGCAGAGAAAGCCATTTTTATATTTGTCGATAACGTGCTTCCACCAACAGGTTTTTGTTTTCCTCTATCAAAAATTTGAAGTTTGTTTTAGCA
GAAAGGCATCAATAAATTTTTTTTTTTAATTCTTTAGGTTCTGTTATGTCAAAAATATATGAAGAAAAGAAAGATGAAGATGGATTTCTTTATGTCACATACAGTGGAGAGAATACATTTGGT
ATTCTCACTTCAAACCTTCATTAAATAGATTGCAGTAGAAGTTCAAAATTTATTTCTGCTGTTTTCATGTCTTGCCACAATTAATAAGTTGTACAGCTTGATTTCTGTAAATAATTTAATGGGA
TCGCCTGTACTGTTGAACACTAATTTCCTGAATAATCAGATCTTCTGTCGATCTTTATGTTCTTTGTGATTTCTGTAAATAGTTTAATGAGATCTTCTAATACTGTTGAACACTAGTCTTTTGA
ATAATC
>comp143445_c0_seq1
AAGGCATGACATGAGCAGGGCTGCAAAATTCATATTTTCAGAATAGCAAGTCCACATGTAGAGATAACATACAAGAATTTGGTTAAGACGGTGGGTTGATGGTTAGTCTGTGCATTAGTTA
TTTGGAGCAGCTTCAACAATGATAACATTTGATGATTAAATACTGTACTACAATTAACATAGAAATGGGAAAATAGTCTTTCAGAAATACTTTTCTAGCTTTATGAAGAAGGCAATAAATAA
TTATCTATCTATAATAATAACTGGTTGTTTATCTTTCCGGCATTAAAATAAATGCTTTAATTCTCCAAAAATGCTTTGTAAGTCCTCATAAAAACAATTGCAGAGTAAGTGGCATACTGCTGCT
CTAAGTTGAACTTCCATAATTCCTATGTTTAAATTTCAGTTTCTTGGAAAGTCGGCCAAAAAAGTTTTAGGATCATTATTCGAATATTGTTGTCAAATGACATACATATCACGTTTAAAAATA
AATGAAATACAAGTTTGTTTCAATGGTATAATCGCACCAAAATTTAAGCCTCACATTATGCAAATTTTTAAGAAGCTAAACAATCTGTCTATGAGTTTTTGTGATTTTCCGGGGAATATTTAA
TTTACAACAAGAGATATTATCCATATTACATTTGAAACAATTGATATCAGATATTTTTTGAATGTTTTCCATCAAGTTAGGCATGAATGAAGTGCTTTGGAGCTCATAAATCCAAAGATTAATG
CTAATAGGGTCAAATAAGTTTAACTACAGATCGAATTTATTTGCCTAAACTTGTTTTTGTGTT
>comp143544_c0_seq1
AAAAAAAAAAAAAAAAAAAAAATGGTTTGTTTTGTTGGTCAAGTTTTTCCAACTTTGTCAGTAAAAACAAAAGAACCAAAATCAAAAACTAAAAACGAAATTCTGATGTAAATTCAAG
CTGCATCTGTTTATGAGCTACTTGAAATTAAGAGTTTCGAATTTCAGGCATTATGAGCTACTTGAAATTTGAGAGGAAGGAAGAGAGTACTTTTCAATCAATTTGGCAAGCTCAGCTTCAA
AGTTCTTTCCTAATTCAATCTCCTCTGTCAAATGCTTTTGCTTCAGCTCAATCTTCTTCTCTTCATGAGATAGTATCAGTTTTGCCCTCTCAGCTTCAAACTCCTCAACACCTTTTAACTGGC

TGGGAATGAACTTTTGCATCTCCTCTTCCCTGCTTTGCCTGCAGTCTACAATGGTTCCAGAGTTGCTGACATCATACAAATTGACCTTTGCTCGCTCCTGTGACAAATTCTTAGATGTCTTT
TCCTTATCTTCTGTTATTTATGTATTTTGTCCATTTGATCCTTGACAAATTTCTCTTGAAAATCCATCTCTTCTTTATTTTCTTCATGTTGAATCTTTGTTCTGAGCCTCACAATCCGGTTTTCT
TCCACTGTATCACGCAATTTCTGGCTCAACATGTCAAATGACACCTCAAGAGCCTTTGAGCGCTGCTCTTGTTTTACAACTTTGTTCTTAAGCCAAATCAGCTTCTGGTTATCTTCACTCAT
CTGCTTCATTGGGTTCACAACCATTTCTCGGTAAGATCTCATGTCAAACTTCAGGCATGCTTTCCCATGAGAATGTTGGTTGAAGATGATCAGATCTTCTTTGGATGCCAAGAAACCATATA
GCTGACGCTTACCCCCTGGATAGAACAGTGTAGGGCGCTTTTCCCAAGCATCCCGATCTCTTCCCTCACCTCTAAATTGCCTGTGCAATCGTTCAGCTTCGAAATATCCCAATGCAGAGGA
TTCAAAGATTAGAACACTCATGCCTCGATGCCCATTTGGACCATAGGAATGACGAGCTTTTACTGCTTCATAAGTATTAAAATATTCAAGAAGCTCTTGATTACCCATGCCTTTCCACTTATC
TTTCTCATCTTTTTCTAGTAGTGTATTCATCACAACCACCATTGGAGGCCAGACAATCTCATGATCAGGTGTTGTCTCACACAGACCCATCCATTTTCCAAATGCTTCACCAGCTGGAATG
>comp143550_c0_seq1
AAGAATACCTAGTGTTCTTTGGATTTGCATATAGGACAGCAAGATTCAAACTTATCTTACCGAGGAATTGAATTCAACAACAAGAACAAAAAAAAAAAAAATCAGCAATTCATTATTATTCT
GAAACTACCATCAATTCTAACAAATCCTTATTTCCCTCGATCAGCACCAAAAAGAAAAAAAAAAATGAAAGAAAAAAGTTAAGAAGATGGATCATATCTCAGAGAATGTTGGCGTTGCACC
ATTGATATTAGGCAAAGGAGAGCTTCCAGGCAGCTCGGTGTATTTGTATAATGCATTGGGCAAGTCGAAGAACTCTGTCGTAAACCCTTGCATGAACAACGGTACATCTGACAGCGGCGG
ATTGAGCTCAAAGTTGGTTGTCGCCGGCGTAACGGAGGGCCGTTGCTCCGGCGTCAATTGCAGTGTGTCATCCAATGATGGTTCGCTTAATTCTTCCTTTTTCTTTTCAACAGAGATCTTT
CTTTTCTTGTTCAGATGGATGCTATCATCATTGTTAGAAGAAGATGTGGTTGGATTGTCGTTAATGTCATCATTGGAAGAGCGGGAGAAGCCGGTGAGCTTTTGGACAAGGGCCATAAAAT
CACAAGCCCGTGTGTGGATGATTTTGGGGGACTGTGTGTAGATTATGACCGGCGGACGATATGAGACAGAAGAAACGGAGGTGGATGAGGTGGAGGTGATGGTGGTGGTGGAGACTTTA
TTGAGCTTCATAATTGGTGAAGTTCGGACGAAGCCATTGATCTCCGGAGATGGATTCATTGTGGAGGCAAAATTGGTAGAAGTAGAGGAGAGGAGATGAGAAGATGAAAACGAAGGGAT
GGGGGAGAGAGAGGGAGAGAGAGGTGGGTACGGACGGGAGTTTGGAATGGCTTGAAATAGAAGAGGGGAGAGAGATAAATATGAGCGGGGAGAGGG
>comp143651_c0_seq1
AAAATAAATGCATGAGATTATTTTTCTATTTAGTTTGAAATAAAATGAGTGTAATGTAAAGTCCAAAGGTGTTGATGGTTGGTGATAGTGATGGTGGGTAGCGGTGGCCAACGTTGGTGGC
CTGCACCGATCTACAACATGTTGCGATGGTGGCGACGGTGGGGTGACTGGTGGGTTGTGGCCGACGATGTTTGGCGGTGGTGGTGGCCTATAATGGGTGGCGGCGACAACTGGCAATGG
TCAGTGGTCATAGGCAGTGGTCGATTGTCATGACAATGCTAGGTGATGACAGTGACTGGCAGCGGTGGCGGCCCAAATATATAAAAAATAAACTATAGGAAGAATTCTAAATAAACCAAA
ATTTTACACTATAATTGTTTTACTTCAGATCAAACACTAATATTCTTAAAACCCCTATGTTTAGAATATTTTATACCTGATTTACTTCAAACCAAACAATTCCTAAATTGGAGCACTTTACCCAT
TAAAAGGTGAAGGCTTTCTTTAAAAATAAATAAAAGGTGAGGGTTTTCCTTCCTTGAAAGTAAAAAAGTTGTGAAAAGCATTTCTCCATCAACTTGTGGACAAAAAGAGTGATTATAGTA
AAATCCTTAATAAGATGGTCTTTAGGTGTCGAGGTGGCTCAATTTGAGCCAACAAATGTAAATATCCTAGCTTCCATTTGCTCTATAACAAGATTTGGATTACTAGCATTATTATTTGCCTAGT
TTACATAGAGATGAATATGATGTCTTGAACGGCTATGTAATCACATATTATTGTAGATATATAATATTTACAATTATTTCTTTATGATCATCATTAACTTTGTGTCATTATCATTAGCATCATATTTT
GTATATTAATCAACTAAATCTTGCTATACATATTTGTGTGATGATAAAAGTATTAAATATTCATATGCTTATGTGCTAGGAAAACCTTTGTGCTTGCACTTTAAATTATTCAAAAATACTAATCAT
AGCATTATATATATATATCCATAATTATGTTAATATTG
>comp143735_c0_seq1
ATAATAGATGTAAATCTATGTCACCTTAAAGAAGTAAAATATATGCTTTGCTAAACTTTATGGATTTAAAAAATATGATACATGAAACTTTAATATAAAATTTCCAATAAGTATTTCACCATAA
AATAAAAATTTTCAAGAAAGAAATATATGGTGAAAGCTAAAAGAACAGCAAGTAAAGATTTAGAAATGGAAAGCAAACTCAAGAACTTAAGAGATGCTACTCGGCTTTGTGTGTTGAAG
CTTTCCAAAAATGATTTCGATCTCACAAATGAAAAGAACTCTTGGAATAAACTCAAGGCTTCTCCTCTCAAGATTTAGCATGTAAAGAAGAAAGGAAGTATGAAAGAATTGCTGGAGTTT
TTCTTGTGTAAGCAGCCCTTATATGAGGAAGAAGAGAGGTATTTAAAGAGAAAAGGCAAGTAAAGGCTTATTTGAATAAAAATATTATTCTAATATGTTTTCTATCAACTAATAATATTATATT

TCATTAAGAATTCATTTATATTAATTATATATGAGATTCAAACGGCTAGCCACATGGCAAGGTCAAAAAGTCATCATATGCATCCAAAATAGGGCTAAAACGGCTAGTAGCAGCTTTTATTTA
AATCAGATAATGTTTGGAAATTCATTATAAATTCAAATGAAATCCAAATTAGTCATATAATATATCAATTTACATCTACTGAATCCAAGC
>comp143892_c0_seq1
CACATTTCTATTCAAAATATCACATCAAATTTTATTGCAGACATTGTTGATTCATTGTTTACACCATAACTACACCAACATAATCCAAATTAATGCAACATCTTAACTATAATAGTCACAAAAA
TAAGTAGCTCATCTCCCCCACAATCTTCATTCTAATATGTATATTTGAAACACATTGAATTGCCTCATGATCTCATTAATTTATTGAGAGAGGATTGCACTTAGACTCATCATAGAGCTCAACA
TTTCTTTCATTTGATGACTGAATTGGCCTGGCATTGTTCTGAAACTCTGGAGCAAGAGAAGATTTGATCAAAGCAACTTGCTCCTTGGAGACCTTCCTCACCTTGCCAAGAATATACCATG
TGACCTTCTCGGTACAAGGAGGAGTTGTGAGCGAGCCAACATATCTGAAGAACCTTCGACTATGACGCTTCAATGACTTGGTTCGAACTGGACCAATTGGCACTTGAATCTCTTCATCCA
GCGAGCAATGTTCCTTAGCCAATTGAGCCACCCAATCCTTCAACTGATAGAGAAAAGGGTCAGTGTTACCAAAATCATATAGAATAGCTACAATTGCAACATTGCCATCTTCATCTTCACTC
TCATGAACCATTTGAAGCTCCAATGGATATTTCTGATTGTCAATGGTGTGCTCTGATGGTGCATGCCAATGCAATTCCTTTAAAGAATAGTTCTTCCCATCTTGCAACAAGAGTCCCCACTT
TTCTCAAATTTCAACATTACATTGAAGCCATTGTTAATAAGAGTGGCATTTGCATCAACATAAAGCCTACTGAGGTCATGCAATTTGGAGTTTAGAGTGGCATTATCCTGCAAAATGTTGAT
GGGAGATTGAATGGTTCCGCTGGAGCAAGATTTGTACTCAGCAGCGAGATTTCCCCAATTGGATGGGCCATTTCCAGCATAATTGAAGCTAACAAATCTTCAAATATGGTTCGAACAATTC
ATCATTCCTTCAAACAAGCACTATATGAGCTCTATCATGAACCCCGCTCCTTAACATATCGTCGGTGTCGTTAAGCCTGTAGCCAAGAAGCTCCTTTTGCATGCAGTACTCGATCATATTGAT
GCCTTACCTCATTAACTTCGTTGCAAACTATTCTTGTCCTTTACACACCATCAATCCTAATGCCTTCCCCAATTAAGCTCCCTTCATACACAATTAGAGCATATAGATGTCTTTTCCGAACAA
GTGTCATTGTGAACTCATCTTCTCTTCGCATGTCATTCACGCCAATGCCTTCCCCTAATGAGCTCTCATCACTTGCGGCTGGTTTCATCAAGGCCTTCTCAATTATTTTCATTCTGAAACCTT
CCCTTAGCTCACGGTATGAGCCTTCACATGGAAGAGAAGAAGATAAGCTAATCCCAAAGCTAAGAAAACCTCTTTGATAGACATTCCTTAAATTCTCCTATTGGCCTCTCCAACTCTCTCA
AAAATTTTTGTTTCTCCCTTCTCAATCTTACCTTTTAAAAAGTAATGGGGGAAGAAGAA
>comp143892_c0_seq3
CACATTTCTATTCAAAATATCACATCAAATTTTATTGCAGACATTGTTGATTCATTGTTTACACCATAACTACACCAACATAATCCAAATTAATGCAACATCTTAACTATAATAGTCACAAAAA
TAAGTAGCTCATCTCCCCCACAATCTTCATTCTAATATGTATATTTGAAACACATTGAATTGCCTCATGATCTCATTAATTTATTGAGAGAGGATTGCACTTAGACTCATCATAGAGCTCAACA
TTTCTTTCATTTGATGACTGAATTGGCCTGGCATTGTTCTGAAACTCTGGAGCAAGAGAAGATTTGATCAAAGCAACTTGCTCCTTGGAGACCTTCCTCACCTTGCCAAGAATATACCATG
TGACCTTCTCGGTACAAGGAGGAGTTGTGAGCGAGCCAACATATCTGAAGAACCTTCGACTATGACGCTTCAATGACTTGGTTCGAACTGGACCAATTGGCACTTGAATCTCTTCATCCA
GCGAGCAATGTTCCTTAGCCAATTGAGCCACCCAATCCTTCAACTGATAGAGAAAAGGGTCAGTGTTACCAAAATCATATAGAATAGCTACAATTGCAACATTGCCATCTTCATCTTCACTC
TCATGAACCATTTGAAGCTCCAATGGATATCATTACATTGAAGCCATTGTTAATAAGAGTGGCATTTGCATCAACATAAAGCCTACTGAGGTCATGCAATTTGGAGTTTAGAGTGGCATTAT
CCTGCAAAATGTTGATGGGAGATTGAATGGTTCCGCTGGAGCAAGATTTGTACTCAGCAGCGAGATTTCCCCAATTGGATGGGCCATTTCCAGCATAATTGAAGCTAACAAATCTTCAAAT
ATGGTTCGAACAATTCATCATTCCTTCAAACAAGCACTATATGAGCTCTATCATGAACCCCGCTCCTTAACATATCGTCGGTGTCGTTAAGCCTGTAGCCAAGAAGCTCCTTTTGCATGCAG
TACTCGATCATATTGATGCCTTACCTCATTAACTTCGTTGCAAACTATTCTTGTCCTTTACACACCATCAATCCTAATGCCTTCCCCAATTAAGCTCCCTTCATACACAATTAGAGCATATAGA
TGTCTTTTCCGAACAAGTGTCATTGTGAACTCATCTTCTCTTCGCATGTCATTCACGCCAATGCCTTCCCCTAATGAGCTCTCATCACTTGCGGCTGGTTTCATCAAGGCCTTCTCAATTATT
TTCATTCTGAAACCTTCCCTTAGCTCACGGTATGAGCCTTCACATGGAAGAGAAGAAGATAAGCTAATCCCAAAGCTAAGAAAACCTCTTTGATAGACATTCCTTAAATTCTCCTATTGGC
CTCTCCAACTCTCTCAAAAATTTTTGTTTCTCCCTTCTCAATCTTACCTTTTAAAAAGTAATGGGGGAAGAAGAA
>comp143893_c0_seq2
TTGGTAATGTGAAGTTACCACTATGGTTGCATGAAAACCATATATCTTCATCTGAAACATCTGAAACACATTTAACATCAGAAACAACCTGAAAATTTGGGTAAATATGGTGTATCTGTCAT

AGTAAGTTGTGAATACTTTCATTAAACTTGGCTAATCTAGAAGAAAATTTGGTATTCCAGATCTGTTAATTCTGTTTAAATAATTCAAAACCAGAGTAATATAAAGTACATGATCATATTCCT
GTGAGACTAACAGCGACTGTAACAACTAAAGTTTCATGGTGGTTTCCTTTATCTCCAGGCTTGAAGATTCCGCTTACTATTGCATCCTTCTTCACCTTCTCAATCTCTTCAAAAGCATAGCA
TACATGAGCTCATTCCATGCGTCAGGGACCCCGGGAAGGCACCAGTGGCTACAATCCTGGCTCTTCACTACCCTCTGCTTCGTCTCTGGGAAGCGGTACATGGAAGGATGCGCTTCCTTC
CTGTAATCTGTCATTCTTGTTATGTTAAGATAGAGAACAGGTGTCTTCATCTTCGATATGACCAACTCGAGTATGTTCAAGAGGTTTGGGTATTTTGCCAGATGCTTGTCGTCGAGGATCGG
TAAAGTCTCTCCATCACAGCTGCCACCAGAGTTCCATTGCCCACCTCTGAAGTGAGACCATGAGTAACCCCTAAAGAAGACTGTGGTGCGGTTAGAGTCAATATGCTCATCCAGCCACCG
ACCCCAGGTCGTCAAGGCTTTGTGATAAGCGTCATCAGCACTGAGCTTTCTATGAACATAGTCACCTTCCTGGTAGAAGTCCTCCCCTTTAGAAGTCTTCTCATGCGTCCACCAGTGAGCT
GTGTTGAATATAATCACATCTGCTGTTTTATACATGGAAGAAGTTTTGCCCATTATATCAAGCCGAAGGGTCTCCTTTCGAGTGCCATCTGAGCTCCAAATCTCCCATTCTTGAACTAGAAAT
GGTGCTCGAAAGAATTCAATTGTGCAATTGTATTCAGTGAATCTGTAAGCATAGTAGTTCTCATTTCTGAATTCATTCTTGCCCGTTATGTCATATACTCCATTTTTGTTGTTCAAAGAATTTC
TCAGGATGCAGACCAGTGATTCCCACATGTTTCGGTTCAGCGAGTCGCCAACGAAAGCAAGTCTCTTCCCTCTCAGTATCTCAAGCAGGTTTCTTCCTTCCAACCTGGGCATGGAACAAG
CCTTAGGCTGCCACCGTAGCCTGGTGTAGTTTAAATCCGGTCGTCCATTCTTAAAGCAGTTAAAAGCCTCTTCAACAAACGGGCAAGACCCTGCCTCATACAAAGGCATCGTCTCTCCATC
CTCCACCCATCTCCCATCAAACAGATCACAATGATACTTATCATTACTGCTTCTCCTCCTCCTCCTTTCTCCCCATCCCTTCTTCATCTTCCCCTTATTCTTCGCCGACCTTCGAAGCTCTTCG
TCACTGGTCAGCGTCGGGGAAGAAGAAGATGAGACCATGAGGTTGGCGTTGAGATCAATGGAAGTGGAAGTAGTACATGAAGAGGAGGAGAAGTTGGTCATGAGAGGGAGAGGAGTT
TGTTGGAGAACAATTATCTTAGTGGAAGGGTAACGGGCGGTGCATATGAAGAAGAAAGTGGTGAGGACTGTGATGAACGTTAGAGATATTTGGAAGTAGGTGACTCCTTGCAACATGCA
GTTCTTGCTCAGTTTCTTAACTTCATTTGAAGACGCCATGGGAG
>comp143916_c0_seq1
AGGAGGAGATTTATAGTGATATGGTGGCGGCGGAGACGAGTATATATATTTATTAGAGTCTGCCTCTACAGTCATGGCGGTCAAGCTAACCACCAATGCCAGTAATAGGCATGGCAACAGC
CTCACCTGGCCTTGATCAGCCATGGTTGGCTGTTTGGGAGCTTATCGTAAAGCCACTTCCTCCTCCCACCTCAATCTAATGAGATAACAAACTAACTGTGTTTATGCTTTGCAAGGCTTGA
AGCCATCCCTTTATATAGCCCAACTTACCTTTGGATAGCCCAACTTATTTTTTATTTTTCAAATAGAACCTCCTAAAGCTCTCTCATTACTCCATCTTTTTTTCTCCAAGTTCTGTAGTTGGTTC
CCATTAAGCAGGTTCTCCTTACCCACGTTGTATGTGAAAGAATCCAAAGTCTGAACAAAGAGTTGTAAAGTTTGAATCGCTGAACAGAAAAAGATTCTCAGAGCACCGAATAAAAATTAT
TATCTATATATGGCCCGAGTTCAGTAGGTAGTATTCAATATTGTCCCTCAAGTTGATGAACATTTTTGGCCTTA
>comp143946_c0_seq3
AAAATCAAAACCATACATAGGATAACCACAAGAAAATTAAACAGCTTCAAGTCTGAAATCTCAAACAAAGGCCACCATTTTCCGACCAAAATGAGAGAATATAAATAGAAATGAGAAATT
GAGACTCACAAAATGACGACTTGCGCACAAAAGAAGGCGCCGTACATAATCATTTCTTTATTAGTTAGCTTGCAGATAACTGTTAAATTTCATAGGGATAATAACAAATTAGGCCATTATTC
TGCAATCATTATTCTCTAGCTCTAACGGGAAAAAAAACATAAATGAAATGAAATTATGTAAAAGAAGAAACAGTGGAAACTCTTTGCATGCCAGAAAACGATGAGGTACGTAGATGCAAT
GCAACAGTTGGTGCCTAAATAAAACGCACCACTCACTGTCACTGCTATACTTCAACCATGAAGAATCTTCTTGGGATCCAATTCCCCACTGCAGTAGCTAGACGGAACACAAGAGATAAC
CAGACTGCCTGTCCATTCTTCGCCTGGTTTCAGTTTAAGAGGTTCCTCAATTACAGCCGGCGCCACGCACAGCATACGCTTGTATTCATCGTCGCCAAAATCCGCAATGGCTTTGGCTTTC
TTGTCCCAAGGATTCCAAACAACCGCATCTGGCATTCCCTCTTTCCACAGAACATAAGTTCTCCTCTTTTCATGATCTATTATTGCAATCTTGTTAGGCGTACCAACGTAACTCCTGTCAATT
TCTGATTCAAATGTAAGAGAATCTCCCTGATCTGTAAAACGCTCCTTATTCTTCAAATTGTCGAGATAATAATATGTCTCCAACCCCTCAACCCGCACTTCACAAATATCAGAAACAGAGAA
GTACGCATAGAACGCAAATGTAAATGTAAATGGCTTTCCATTTCTATTGATATTTCTTATGAGTTCCGACTGAGGGTTGCTCTGACACCTAGAGGGGATCTTATACTTGTATCACGCATAAGA
AATATCAATAGAAATGGAAAGCCATTTACATTTACATTTGCGTTCTATGCGTACTTCTCTGTTTCTGATATTTGTGAAGTGCGGGTTGAGGGGTTGGAGACATATTATTATCTCGACAATTTG
AAGAATAAGGAGCGTTTTACAGATCAGGGAGATTCTCTTACATTTGAATCAGAAATTGACAGGAGTTACGTTGGTACGCCTAACAAGATTGCAATAATAGATCATGAAAAGAGGAGAACT

TATGTTCTGTGGAAAGAGGGAATGCCAGATGCGGTTGTTTGGAATCCTTGGGACAAGAAAGCCAAAGCCATTGCGGATTTTGGTGACGATGAATACAAGCGTATGCTGTGCGTGGCGCC
GGCTGTAATTGAGGAACCTCTTAAACTGAAACCAGGCGAAGAATGGACAGGCAGTCTGGTTATCTCTTGTGTTCCGTCTAGCTACTGCAGTGGGGAATTGGATCCCAAGAAGATTCTTCA
TGGTTGAAGTATAGCAGTGACAGTGAGTGGTGCGTTTTATTTAGGCACCAACTGTTGCATTGCATCTACGTACCTCATCGTTTTCTGGCATGCAAAGAGTTTCCACTGTTTCTTCTTTTACA
TAATTTCATTTCATTTATGTTTTTTTTTCCCGTTAGAGCTAGAGAATAATGATTGCAGAATAATGGCCTAATTTGTTATTATCCCTATGAAATTTAACAGTTATCTGCAAGCTAACTAATAAAGA
AATGATTATGTACGGCGCCTTCTTTTGTGCGCAAGTCGTCATTTTGTGAGTCTCAATTTCTCATTTCTATTTATATTCTCTCATTTTGGTCGGAAAATGGTGGCCTTTGTTTGGAATTTGGAAC
TTCTTGCTGCTTGATTTTGTCGTGGTTATCCTCTTCATGGTTT
>comp143946_c0_seq4
AAAATCAAAACCATACATAGGATAACCACAAGAAAATTAAACAGCTTCAAGTCTGAAATCTCAAACAAAGGCCACCATTTTCCGACCAAAATGAGAGAATATAAATAGAAATGAGAAATT
GAGACTCACAAAATGACGACTTGCGCACAAAAGAAGGCGCCGTACATAATCATTTCTTTATTAGTTAGCTTGCAGATAACTGTTAAATTTCATAGGGATAATAACAAATTAGGCCATTATTC
TGCAATCATTATTCTCTAGCTCTAACGGGAAAAAAAAACATAAATGAAATGAAATTATGTAAAAGAAGAAACAGTGGAAACTCTTTGCATGCCAGAAAACGATGAGGTACGTAGATGCAAT
GCAACAGTTGGTGCCTAAATAAAACGCACCACTCACTGTCACTGCTATACTTCAACCATGAAGAATCTTCTTGGGATCCAATTCCCCACTGCAGTAGCTAGACGGAACACAAGAGATAAC
CAGACTGCCTGTCCATTCTTCGCCTGGTTTCAGTTTAAGAGGTTCCTCAATTACAGCCGGCGCCACGCACAGCATACGCTTGTATTCATCGTCGCCAAAATCCGCAATGGCTTTGGCTTTC
TTGTCCCAAGGATTCCAAACAACCGCATCTGGCATTCCCTCTTTCCACAGAACATAAGTTCTCCTCTTTTCATGATCTATTATTGCAATCTTGTTAGGCGTACCAACGTAACTCCTGTCAATT
TCTGATTCAAATGTAAGAGAATCTCCCTGATCTGTAAAACGCTCCTTATTCTTCAAATTGTCGAGATAATAATATGTCTCCAACCCCTCAACCCGCACTTCACAAATATCAGAAACAGAGAA
GTACGCATAGAACGCAAATGTAAATGTAAATGGCTTTCCATTTCTATTGATATTTCTTATGCGTGATACAAGTATAAGATCCCCTCTAGGTGTCAGAGCAACCCTCAGTCGGAACTCATAAGT
CTGTGGCCAATATTTGTAGAAACCTTCAGGTGGCTTAAGAACTAAATCAATAAAAGTCCTATAACTGCTTTCTTCCAAAGGTGGTGGAGTTTGATCAATAGCCCAAAATAGTTTCCTCACA
AAACCATGGTTTTCATAGTTTTCAAGTCCTCCGACTTGTGGAAAACATATAGGAATGCCGCCCCGTATTCTATTTGGAGGTTCGAATAAAGACTTTCTACTGAGAAAAAGAAGCTCTTCAC
CGCGATCATTTTTCCATGATGTAACATGTCCACCATATAGATAAACCTCGACGGAGCTTCCCCTAGCCTCCTTTAACACGAACTTGTCCAGGCCATTGTTGCCTTTGCATTGCTGAATGTATG
GCTTCGCCACTGCCATCTCTCAAACAAATTTCGTCGATATTTCCCCACTTCCTTCGACGTAATTGCCAATCTCTATTTCTCACCTTTCTTAGTTCCGGTCACAGCACCAGTTAA
>comp143947_c0_seq1
TAATCTCATATTAGATCATTCAAAGTATGTTACTACAAATCTTATGACAAATAGTTGATAATCTGAGTTGTAAATTTATCAACACCTAAACAATCTCCCCTTGTTCACCCCAATCCAAGTAAAT
GTCAAGAAAATATAACACCACAACCATAACAAGAGAAGAAGCCATTGTAGCCATCGCACCCAAAACCCATAGGAAGAGAGGAAACGAGAAGTAACAAGCTCTGAGCCCCAACGACCA
GCAGAAACTCCCTCTGTTAACCATGCTCGCCACATACTCCGACGCTCTACCCCTTTCCCCATGGCACTTGGGCGGCACGTTAATCAAAAAACTTGCATGACTGTAGTACCTCACCGACTGG
ATGTTGAGGAGAAAGGAAACGAGAAAACATAAGAGTATGGAGAAGTAGTTTGCTCTGGCAAGTTCTTTATTTTCGTTGTTGGTGTAATTTGTCATGAGAACCGCAATCAAAGAGCAGAG
CATGATCGATGTTGAGGCCATTAGTGTTGATGCCATGATGTTGTTCCTTAGCGTTTGGACTGCTAGGACTCCGTTCTTTATTGGGTCCTCCATCATGATCTGAACCCATAATCGACGGTTAAT
AGCGTTGATGCCTATGACGGTCTTGGTGGGGCGGTGAAGGATGTAGAGGAGAAGCCATAGATGATAACTTGCCAAAATTAGAAGCCCGATAAAAACGAGAATATAATCTAGCACCATTGC
TAAAATAAGAGTTGAGAGAC
>comp143955_c0_seq1
TCCTTCGTCTTCTCCTTGGTTGACTCCGCGGACTCCTTTGTCTTCTCCCACGCTGAATCCGCAGACTCCTTCGTCTTCTCCTTCGCGGACTCCGCAGACTCCCTCGCATCATCCCCTATATTT
CTTGCCTTCTCTGAAGCACTGTTCGTTGCATCCTTGGCCCAGTCTGCTGCAGACTTCGCCCTATCCTCTGCCGTATCCTTCACATTCGAGGCCCTCTCTTTGGCGCTCTCTGATCCTTCCTTC
AGCACCTCCTTTGCTCGGTCGGCAGCCTCTGCTGGCTTTTCCCGGATCTGTCTGGCCCTAGCATCTTCATGATATGATGCTGATGCCTTGCAGTAGTTCTGAAAAGTTTTCATGAAAAAATC

AGGTAGAAAGAAGATAGAAACTAGAGCCATAGATTTCATAGCTGTATTAAACATTTCACCTGCGTAGATTTGAGAGGGAACAAGATGTGCTTGGCCTTGAGTGCTGAAGGAGAGCGAAC
TGGATAAGAGAGCTTGAGAAACAAGCCTCGGCTTCCGTTGAGCATGACAGCCATTTGCGTACGATTTGGAATTCCAACTTCTGTTGCTGGAAGATGAAGATATTTGTGTAGGAGGGTTGG
GCAAATTTGGCTGTTAGAAATAGGCGAGGAAGATTGAGGATAGAGAACGATGACAGGTTGGGGATAGTGATGACACGTTGCATGCATGCACGGAGACTGCAGCTTAGTCACCGGCAGAG
GAACGGGTAACGAACACCTGTGATGTTATGCATGTACTGTTAAGTGAAATATGCTCGGCCTTATTTTTTGTTAATAAAATGTCTGTTTGAGCGTGTAGTTTGAACTGTTTTT
>comp143965_c0_seq1
ATACTCTGGAGCATGGTAGCCAAATGTTCCTAAAACCCGAGTTGAATGTAGACGGGCAGCTGTATCAGGAGACTGATTGGTTAAGTTAAAATCAGCTATCTTGGCCTCATAATCACCAAAC
AAGAGTACATTGCTTGATCTGACATCCCTATGTACAATTGATGGCTGAACTTTCTCATGAAGATACTCAAGACCTCTAGCTGCACCATAAGCAATTTTCACTCTTTGGCTCCAGCTCAAAAC
AGGACCAGGTTCTGCACCTTCTACTCCTTTCCTCCCATGTAAGATATTATGCAAGGAACCTAAAGTTGCTAATTGGTAAACAAGGATTCTATTGCCATCCTCCAAGCAATATCCCAGCAGCT
CCAGAAAATATTCATTTTTGAGTCGGGAAACCAGTGATAACTGGGCAGAAAAGTCAGTATCAGTTTCTTGTGAGGCTGCGGGATCCAATTTCTTTATTGCCACAGCTTGCCCTGTGCTCAA
AATTGCTTGGTAAACCTTCCCATAAGAGCCCTCCCCAATCAAAGCCTTAGGACCAAAGTTTTTTGTCAGTTTGTTTAGCTCAGCCAAGGGTATGACCGGTATCTCAATAGGTAAGACCTTT
TGGGGAGCTCCACTTTTGGCTGCCGTAGGACCTCTTGGTTCTTTGTCATGTGCGGTGACATTACGAGTTGCAGGGTGAACAGAAGGATTAATGGGTTGGCTATAGATATCCTCCTCTCCAT
CACAACAGCAAATCATCTTCCTGGTCACGAGCGCCCAAATCCTTTGCTCATCAACAACCTCCGCCTCCTCCCTCCTTATTCCTTATCTCCAATTTACGGCTGTTCTTGAGCAATTCAAATAG
GCAAAAAGCAACCCTAGAAAACTATAACTTTCAACTTCTCTGTATCGATTAAAAAACACATGAACAAAAGGGTGAAAGATATTCAGAATTTTGATCGGATAATTATTTCGAGATCTAAATG
GGCATCTGAATGATTATTTCCAGACCTTTTCTCTTTTCCCACAGCGGCAGGTGTAAAGAGCGATGAACTTTGCCAGTTTGGCGGGAGAGCGAGAAGGATTGGGGAAGAAAGGCAATGGC
ACAGCTGAGCAGGAA
>comp144105_c1_seq4
AGATAAAAAAAATAAATATACACTCTTACCTGCTGAAATCCTACGCCCATACCCGATTTCCTGCCGAAAATCCCGTTGCTACAGTAACCATGCTCTGTCATTTGGCAATTTGGTGCCGCAGT
GATCAAATCCCATAATTCCTCCTTAGAAGCCTGACAACTTCCTCTATATCTGCTTATAAAATGAGAGCCAAATGGCTCTCTATTTTGGATGGTTTGCTGCTTCTCTCCCTCCAACTCTAAGTT
GCAGAGCTCCCGTCGTTGCCTCCGACCCGACCTTCATTACTGATCAACCTCAAGCCGATCTCCTCTTATTCTCCATCTAAATCTACCGAGACCTCCTAGAGCTAAGGTAGGCACTTTTGTTC
CCTGCTTCCTTGAT
>comp144216_c0_seq1
TCCGCATCCACTCTCATAATAACCGCACGGTCCACCCATTAACCACCACGACCCCTTCGCTTCATATAATGCAACCCGCCGCTCTCCTTCTCTCTCTCTCTTCTTAGCTTCATCTTTCTCTCT
TGGTTTCTTGGGGACGATAATGGAGGGTCAGAAGCGGGGGAAGGTGAATCTGGCGAGCCGAAGGCCCTTCGTAACGTTGTTGAAGTCTCAGCTAAAGGAGACTTTCTTTCCCGACGATC
CGTTCAGGCATTTTTCGGGGCAGCCCGCGGCCCGACGGTTTTGGAGCACGATTTCTTATTTTGTTCCAATTCTCCAATGGATGCCCAAATACTCTCTCGCCTTCTTTGGATGCGATTTCTTC
GCCGGCGTTACCATTGCCAGTCTCGCCATTCCGCAGGGAATAAGCTACGCCAAGCTCGCCCAGATTCCACCAATTATCGGCCTTTATTCGAGCTCCGTTCCTCCGCTGGTGTACGCCATGT
TTGGGAGTTCAAACAACATGGCGGTGGGGCCGGTGGCAGCGGCGTCGCTTCTGATGGCTGCGATAATACAAGAGATCGTCTCGCCGGAGGAGGAGCCGGAGAGATACGTTTACACCATT
ATCACCGCGGCGTTCTTTACCGGCCTTCTGCAGCTGACACTAGGCATTTTCCGGCTGGGAATAATAGTGGATTTTCTGGCGAGGTCTACCATCACGGGATTCATGGAGGGACGGCTATGA
TCATAATATTACAGCAACTAAAGGGCTTTCTTGGCCTCAAGCATTTCACCAACAAAACAGATGTCATCTCCGTGCTTCATGCAATCATCCAACACCGATCCGAGTGTAAATGGCAGAGTGC
GGTGTTAGGCATCGGCCTTATGATCTTTCTACTTTTCGCTCGGCAGTTGAAAAAAAAAATCCCAAAACTTTTTTGGGTAAACGCTGTCGCTCCGCTCGTTGTGGTAATAGTTGGAGGAGTA
ATCGCATATTTGGTAGATGGAGAGACCCACGGAATTCCTATTGTGGGTGATTTAAAGAGGGGACTTAACCCTATTACGATATCAAACCTTCAATTCAAATCTCCATATGTTAGTGTTGCCATG
AAAGCTGGAATCATCACTGGATTCATTGCTCTTGCGGAAGGAGTAGCTGTAGGTAGGAGCTTGGCTTTGCTAAAAAACGAGCAGATAGATGGGAACAAGGAGATGATTGCTTTTGGATTG

ATGAACATAGCTGGTTCCTTCACCTCATGTTATTTAACCACTGGTCCCTTCTCAAAATCAGCAGTTAATTACCACGCTGGATGCAAAACGCCAATGTCAAATGCAGTGATGGCAGTATGTAT
GATGCTGGTTCTTTTATTCTTAGCTCCACTATTCAAGTACACACCACTTGTGGCT
>comp144216_c0_seq2
TCCGCATCCACTCTCATAATAACCGCACGGTCCACCCATTAACCACCACGACCCCTTCGCTTCATATAATGCAACCCGCCGCTCTCCTTCTCTCTCTCTCTTCTTAGCTTCATCTTTCTCTCT
TGGTTTCTTGGGGACGATAATGGAGGGTCAGAAGCGGGGGAAGGTGAATCTGGCGAGCCGAAGGCCCTTCGTAACGTTGTTGAAGTCTCAGCTAAAGGAGACTTTCTTTCCCGACGATC
CGTTCAGGCATTTTTCGGGGCAGCCCGCGGCCCGACGGTTTTGGAGCACGATTTCTTATTTTGTTCCAATTCTCCAATGGATGCCCAAATACTCTCTCGCCTTCTTTGGATGCGATTTCTTC
GCCGGCGTTACCATTGCCAGTCTCGCCATTCCGCAGGGAATAAGCTACGCCAAGCTCGCCCAGATTCCACCAATTATCGGCCTTTATTCGAGCTCCGTTCCTCCGCTGGTGTACGCCATGT
TTGGGAGTTCAAACAACATGGCGGTGGGGCCGGTGGCAGCGGCGTCGCTTCTGATGGCTGCGATAATACAAGAGATCGTCTCGCCGGAGGAGGAGCCGGAGAGATACGTTTACACCATT
ATCACCGCGGCGTTCTTTACCGGCCTTCTGCAGCTGACACTAGGCATTTTCCGGCTGGGAATAATAGTGGATTTTCTGGCGAGGTCTACCATCACGGGATTCATGGGAGGGACGGCTATGA
TCATAATATTACAGCAACTAAAGGGCTTTCTTGGCCTCAAGCATTTCACCAACAAAACAGATGTCATCTCCGTGCTTCATGCAATCATCCAACACCGATCCGAGTGTAAATGGCAGAGTGC
GGTGTTAGGCATCGGCCTTATGATCTTTCTACTTTTCGCTCGGCAGTTGGTAAAAATTATTATTTTTTTTTTTTTCATTCACTTTATTTAAATAAGCTGAAACTGGTCATTATTGCAGAAAAAA
AAAATCCCAAAACTTTTTTGGGTAAACGCTGTCGCTCCGCTCGTTGTGGTAATAGTTGGAGGAGTAATCGCATATTTGGTAGATGGAGAGACCCACGGAATTCCTATTGTGGGTGATTTAA
AGAGGGGACTTAACCCTATTACGATATCAAACCTTCAATTCAAATCTCCATATGTTAGTGTTGCCATGAAAGCTGGAATCATCACTGGATTCATTGCTCTTGCGGAAGGAGTAGCTGTAGGT
AGGAGCTTGGCTTTGCTAAAAAACGAGCAGATAGATGGGAACAAGGAGATGATTGCTTTTGGATTGATGAACATAGCTGGTTCCTTCACCTCATGTTATTTAACCACTGGTCCCTTCTCAA
AATCAGCAGTTAATTACCACGCTGGATGCAAAACGCCAATGTCAAATGCAGTGATGGCAGTATGTATGATGCTGGTTCTTTTATTCTTAGCTCCACTATTCAAGTACACACCACTTGTGGCT
>comp144228_c0_seq1
TCCTTTGGGTTGGGATGGAAAAAATTTAAATTTGGCCAATTTTTGGTAAAACCGGATTAAATTTTTCAAAAAAAGATTTTTTTTTTCATAATAGCTTCACGACTACATTTATAAGACGCTATA
GCTATTTGACCCTCAGCTCCTCTTCAAAACCATATATGCACAAAAAAATACCACATACATTTCTCCTCTTATATAATATAAATAATTTATCGGCAGAAAATCTGCAATCGAACAACATCAGCT
CTTAATAAATATAAATAAAAAATAAAAAAATAAAAAAACACCAAAGAGTAGAAGGAACAAGATAAATGGGGACATTCTTCTGTATTTGATGCCTAAACCACTCTTCAGCTGGTCCATAAA
GCTCAAATAAGAACACATTCTTGATGTTGGAACTAAGCACTCATCTTCAACCTCTGAATTCAGATTTGGGTCTTCTTCGAAGACGATTTCGAGACCATTTCTTGCTCGACGGTGCTGTTGC
ATCCATATTCTCTGAAGCTGCCGCCGCTGCCTCGGCGAGTACTGTGGAGAAGCAATGAGAGCCCCATCAGCCTCACCTTCAAAGTTGGCCACCATCTCTTGGGCCCTTGACATGTCTCTCT
GCTGCT
>comp144354_c1_seq1
TCCACTTAATTACAAGAAATCAAATAAGCCTATAACAGAAAAATCATTGGTAACTTCATTTTAATCTTCCATATGCAATATTTAAAACACTTCAAAATCCAAACTATAGAGATCAATAATTTC
CAAACTATATTAAAGATACAGGACAAAGCAAGAAATATAAAGTAAAACCAAATATTCGATCATCAATTAACTGTAGCTACTGCCTCCTCACCAGAAAGGAAGTGCCGCAGTCACAGAATC
CCTGATCACACTGCATAACTATGCGATGGAAGCTCCGATGACAACCGCAGGCCGCACACTTGAGCTCGTCTTCTTCTCCTGCCATGAACTCCCTGCATCCATCCACCACATACCCGCCTGC
GCTCGCTGCATGGTTCTTCCTGCACTCGCCGTAGCTCTCCGCCGCACTGAAAGCCCGTTCACATCTTCGAAGCACCACATACAACTTCTTCATGGAAACTCCTAGCAGTTACTTTGCCGTT
GGAAGCCC
>comp144364_c0_seq1
AAAACTGTAAATTACAAAAACAACTAAAATTTACAACATTACCAACAAAATCTATTTTATTAAGTCTAAAGCATATCTTAAAGAAGCTTCATGCATATTTACAACCATAAACAATACATAAA
GCAGTGCAACTTCATGATCTCCATAAAAGCAATATCAGCTTGAGTTCTGAACAAATAACATTGCACATCAACTAACATCATATGACCCATCATAGCTTTACTGTCTTCCCATGTGGCCATTCT

GAAGAAGAAAATGCAAAAGACAAACAAAATCCAAATCAGGAAAACAAACATGGACATACCTTAGGTCTCATGAAAAGGTAGTGAAGCCCAGTTATCAAAAGTGTTTGACTAAATGCCT
AAACCACTCTCATGTGACCAAATAGATGGGGCCAAATGCATGAGAACAAGAAGAGTAATTTCTACTGCCAAGGCACATGTAAGGAGTCTCTGTCTCCTCTTCCTTTGTCTGTGCAAACCA
AGAAGCAAGTGGTCCCGACGCTGGCTTCCGAATGCTCCGGCGAAGGTGGCTGGAGGCTTCTGCTTTTCCACCAGTTATTGGTCCTGAATGTGAACCATTGATTCTTCCATGTTCTTCTTTC
TGGGCTGAGCTCTTCTTATCAGATTTCCTCCACAAGAATGATAGCGATCTCTTCCATGACTTCTTCCCCTTACGAGTTTCGACTTCATCCGTCTTCGCGGGCGACGAATGGAATTTGGTTTC
AATTTCATTCTTTCTTGCTTCCTTCAAGATCTGATTCCAAAAGGAGCAATGAATCCAAAAGTCCATAGTCATACATGAATTGAGCATAATTTTGAAACTGTGCTTCAACTGATAAGCTTATAA
TTTAAATCTATAAAAGTAAAAGATAACTCATGAGAAAATCTATAAAAGGTGTCTATCCAATTGCTTTGATTTATCAGTGCATTTACAGAGAAGCTATTTCTGAGACAATAAAGACAACCAGA
CTACTATTTCAGATCCACACTCCTGTTGAGAAAATTGTCTGTTCAGTCAATTCAGTTTTATAGAACATCACAAAGGACAAAAAAAAAAAAAGGATTCTGAA
>comp144411_c0_seq1
GCAGATAAACGGATCGGTGCATTCGCGACGTGGTAAAAGCGTTAAGGCATATTTGGTTCAGAACATCACTGAAACATCAATAAAGGTTTGTAAAAAAAAGAAATACTAGTAGTTATAGAA
CTTAAACTTGAGTGCCTACAGAGAAAAAAAAGAGATATTTGACGTTTACAAATATAAACTTTCTTTGACCTCAGCACCGTTCCACTGCTCGTTCATTTCAGAAATCAGCAATTTCAAAGCC
TGCTCTTCATTTCAGAAGACCAATAACCGTAGCTGGTTCTTTTCATCATAGTCATGAAATCTTGCTCATTCACATCTCCATCACCATCATGATCTGCTTCTTCTATCATTTCCATAATCTCTTCG
TAAGTGAAGCTTTCACCCAACTCTTTTGAGATGATCATAATGTCTGCCGCAGAAATTTTACCATCACTGTCGTAATCAATAATTTGAAAAGCTTTCTGTAGGTCTTGCTGAATTTCCCTTTCT
TCGATTTTTTCAGTCATCATGCATAGAAATTCATCAAATTCAATTGTTCCACTTCCATCTTTGTCCACTTCAGTAATCATCTCATCAATTTGCTCATCTGAAATTTCAAATCCCAAGGCCCTCA
TGGCAATATTTAATTCTTTGGCATCAATAGTTCCAGATCCATCTGTATCAAATAGCTCAAAAGCTGCTTTGATTTCTTGCCTTTTCTGCAATGATAAACCATAGTTACGACCTCTATTGTTGTC
TCTCTTGGACCATGTCCTGGGACCTAAAGTTGCCATTTGATCTCGGAGATTGAATTCGAGTCGAATCGCTCTCTCGCAAGCTTGCTTTCACCTCGAGTTTTCACGCACTGCATGCAATGATT
AAAAAAATGCGGGGAACCGTTTATATAGCGTCAGAGCGCAAGAGAG
>comp144418_c0_seq1
GTCCGGATTCGTCCGGAGGCGAATATGGACGTGCCACGTCATCCAGGGCCCGATTCATCCGAATGACGTGACGTATCCGAATTCGCCTCCTTACGATATGGACACCACAAGTAATAATCTC
TCTAGAGACTGTGAGACCCGGCTCTACCAGCATTTCACATGCAACTATTTTAATATGATTTAGATATTAAGATATTTTATAATTAATATATGACAATTTAAGCATTATATATATATAATAGAAGTATA
AGTATTCTCTGCAATTTAATATAATTTATAACAATCTGATTACGACAATTGAAACAACTCCCGTCGAAGTTCAGTTAGCCCCCGGCGCGGAGCGGAGGTAGTCGACGAAAAAGCGCAGTGT
CTCGTCGTCTACGCGGTGGCGAGTCTGAGTGAACTCCAGCAGCTGCAACCAGGTGAAGTCTGGGAACTTCCTACGATCTCCGTTGCCGTCGCCATCGACGCCGGCGATGAGCTCCGGCG
GTGGGCGGATGACCTTATCAAGCCTCGGGCTCACGAAGTAGGCGATTGACCTTCTTTCTTTGTCTGGGTTCACCACCGCCCGGTGGAGGCAGCTCTTGTACTCTCCGTTGCTCAGCGCCT
CGAAGGTGTCGCCGACGTTGATGACGAGGGCGCCGGGGACGGGAGGAACGGAGCTCCACCGCCCGCCGGTGAACACATCGAGGCCGGAGACTTCATCCTGCTGCAGGATCGTGAATG
CGTTGGGATCGGCGTGTGGGCCCGTCCCCAGCGTGAGCTCCGACTGCTTGCATGGAGGATAGTAATTACAGCGTGCGATCGCCTTCCCGTCCTCGAAGAACTCGCGGTACCATTTCCGGT
CTCCGGCGAGACTCATCCCTAGCAGCTCTGCTATCACCAACGACAACTCCTGCATTGCCTCGCAGTAGCTCTGGTAGATCCTCCCCATTTGCTTGAAATCCGGCCCCAGCGCCGACTGAA
AATATTTCTCCACCGTGTCCGCTTCGTCCCCCTTAAAGCCATGAACGAACGTGAAGGTCTCCTTCCACGGAAGCAGCGCCAAGAACCGGTCGGAATGCGCCACCGCATATCCGTCTACTC
TCCCCTTCCCCTGTCCTGCCTCCATCTTCTTTTCAAACGGCAAATCAAATAGCCCATTAATGGCGTTCATGGCCGCCGCGTAGATCTCGGCGTCAACTCCGTGGTTTATCACCTGAAAGAA
CCCGTGCTCACGGCACGCGGCTTCGACTTGCTTAGCTGCTCTGCGTGCGGCGGCTTCATCGCCGTGAAGGAAGCCGGATAGGTCAATGACGGGGACTTCGAGGTTGCCAACAGCGGCCT
GACGGTCGATGGCCGGCCAAGCGAAAGCAAGAGGTATTTTGGGCTGCTTGATGAGGATGGAAGGGTTGTAGAGCCTCATTCTGGAGACCGTATTGTCGAGGACGAAATTGCCGGGGAG
GCATTGGGTGGCGTCGCCGGAATCCATTGGTGGTTTTTGGTAAGATGTGAGAAAGGAAGAGGAGATT
>comp144432_c0_seq2

AGAAAAAAAAAAAAAAAAAATGAGATCTTTAATTCATTTGAAGCAGAAGCGCTTCTCAACTTCCCCGCTAAACTCCGGAGGCCTATTAAGCTTTTCCATTATCGCACTCCGGAAATTGAA
GGTCTCACTAACCGGAGAGCTTGGTCTTCCTCTCGACTGCTCGACGACGACCAGATCACTGAGGGGAACTCGCTTCCCCAAACCATGGCCCTCTGCCCTCTGCTTTAATCCTTTATTCCAC
CGCACCTTCGCCTCAAACGACTTCGTACTAGACATGAAACTCGGACTATTATCCGCAGAGTTCAATAAACGCTGGAAAACCCCATTAGAAGCACCGCGGGCGCTCTTCGTCGGAGAAGG
CGGGGCAAAGCTTAGACCTGTGTTCAACCACCGCGGTGTGTTCTGTGCTGTGGAGGAGCACCTCCGCGTCTCAATAGGGCAGGGGATAGCAGAGGACATTGAATTGAAGAAGCTCTCAT
CCATATCGGAAAGGCTTGTTCTTCGAGACGATCTCGATTTAAGACGGTAAGTATCGATGCCGTTGAGTCTCTGGCTTTGGAACGATGCCTCGTCAAATCTTTCCTGTAATGGCTTATAAGAA
CGAGATTTTCGGGGAAGAGATCGGAACTTTTTGGTTCTAACTGTTGCCTGAGCTCTCATGAGAGCTTGTATACTACGAAGAGTGGCCGCGGCTTTTTTCCGAACTATATAGCCTCGAACTA
AAGCTTGCAGCTTAACCAGAGCTTTCAAAGCTCTCAAAGCCTTCTTTGCTAGATAACCTCTGAATACTGTTTGGATCTTAAGGGCTGCAAGCTGCTCATGAATGCCGCATAAAATGACACG
ATGGCTTTGATTCGTCAGACGTACCACCGCCACTGCCGCCTGAGCTGCAGCCACCGCTGCTTCGGCAGCTGCAGCTGTCGCGGCTGCGACTGCAATAGCGTGCTTTCTCTGCTCCTTCTC
TTTTTCTGCATACATTGAACGGAGCCACGCCATGTCGCAGGAAATCCTCCCGGACATGTAACTCCACCTCTTCTTCTCCGTTTGGTTATCAATGCTTGGAGCTAAAGCGGCAGCGAACGCC
ATTGTTTCTTTGTGTTCTCTGCCTTCTTTTTTGCCTCCCAATAAGTTAGTAAGCCATCTTGTAGCTCGACCCATTGTTGCTTTCTGGTTGATGCTTCAGATCTTTCGCTGCTCGTGCGCTACA
GTTTTGAACTTTGCGGCGATTACAAGAATCGGAGAGAATTGACTGATCCTCTCAGATCACGAAACTGTGGAAAGAACTGAGAAGCAGGAAGCTGTAAGGAATGACAGATAGCAAGAGA
AGGCAGATTTT
>comp144446_c0_seq4
CAGGTTTTTTTTTCAACCCTTTTTTCCCCTTAATTTTTTTTTTTCCTTTCTTTTGCTTGATATTTACACCAAAGATTATAGATTTTTACAATGAAGCTCCATGGTAACCGGAAGATTAGTATATA
AATATAGTGAGATAGTCACAATCTAAAGCTTGCTTAATTGCTCTTCTTTCATCACTCAACTGTTGTTATTCTGTATATCATGTAAGCTAGTAGCTTCTTTTGTCAGTTGATTTTAAGATCAACA
TTATGACTGTTTATTATCCAACACCATCATATGCTGATAGCTTCTCCTCTTTAACAAAGCTCCCAACTGTTACAAATCTTGAAGATTGGAGAAATTAATAGCGCAGCCCCCTGACTAACAGCT
ATCTTTCATCATTTCCTCTGTTGAAGTTCTTCCGACTTTCTAATATTAACTCATCCATCACTTTTTATTTTGAATTTGTGTGTCAGCCTTACATTAACCTAAGGCATCCATCTAAAGGGCCGCA
TTAACTTCACAGAAACTAATAGATATGCTACCATCTCAAAGAATAGAAAAATTATTCTGTGCCGACCAGGGATGGAGTCCGAAGACCAGTCAGAATGACTCACATCACTTTTTTGCTTGGT
ACCACTGTTTTCTGGATGATTTGGCACTTTCTGATTTGTCTCCGAACCGTCC
>comp144477_c0_seq3
TTTAAAAAGTTGAGAGACCAAATTACTCTCTCAAAAGAGTTGGGGATCATTTTGGTCTCTCACAAAGAATTGAGGGACCATTTTGGTATTCTCAAATCTAATGTTACTGTTCATGATAAGTT
TTTTTTTAATGGTTCATTTCTTACCAACACCTCCCATGTGTTGACCTTATTTGATTACCTACCAAAATAACTATTGCAACCACAACCTTGGCAAATCCTTACGTTCATATCACATATCAGTACT
TAAAGATAGGGCATATCTATAATGCGCTTGATCTTTAAATGTTTTTGAAGGTTAATAGTTTGTTCCTTAGTAGAGAATCTTACAAGCCTGCATTTGGGATTGTAGGTGTTCCTGTAAATAATGC
TAATTTTCCCTGCCGACCAGGATTAGCCAACCTT
>comp144581_c0_seq1
CAAATAGAAGATTCCATAGAGCATGAACAGGAAAGAAAAATTTTCAACAAGCTGTCAAAGCATGGAAGAGTAAAAACTGGACAAGTTTCATGGTTTTATTAACACAGTAACAATGTGCA
CACGAGCCCACTATTCATTTCAACTTGGGGTCTAAACCACATCTGCCACAACTCGTTGCAACACTGGATCCTTAACTCACTGAGGCCAGGGATCATGGCATCACCATGGATACTAGCTGGG
TGCTTAATCTGCTGAGCCACAACTGGAACTCCTGTCTATTCATTTTCTTCGCTGCGCAGCCTGGCATTGGGATTGGTGACCCTGATGGCCAGTTGGGCCGCTCTCTCGACGATGGCTTTGC
GGTTCTTGGAAGAGACGTTGTGAGCAATCTCAGCACAGTATGATTTGTTGCACATTAGCAGCACTTCAAGCTCTTTTACATTGTGTACCAGAAACTTCCGGAAGCCACTGGGCAGCATGT
GCTTTGTTTTCTTGTTGCTCCCATAACCAATGTTGGGCATCAAGATCTGGCCCTTGAACCTTCTCCGCACCCTGTTGTCAATGCCTCTGGGTTTCCGCCAGTTACGCTTAATTTTGACATATC
GGTCTGACTGGTGCCGGATGAACTTCTTGGTCCTCTTTTTGACGATCTTGGGCTTCACAAGGGGTCTGAGAGCAGCCATGATGCCAGGCGGGCGATGGCTGCCACCTCCGTAAGGCAGC

GCCGAGGAAGAAGAGGACCAAGAAGTTCA
>comp144625_c0_seq1
GCCATACTCCATATAATTACTCCTGTTAGACACCATTTAGCATAAAACGCCTCTCGATCACATCCGACTGGAGAAGAGAACTATCTACGTAAACCGGCCGCTAAACTGTCTCGTGCACGGC
TGCTGTTCTCCTCCATTCCTTTCCTTCACGCGTTTCACCGCATGCGTTCTGTTTCTCTCCCTTTCCTCTGTTATCTTCCAAATACTTTTATCCATTTTCTTCCTTCTTTTATATCTTAGTAGTTAT
TTGCTTGCTTCCATTTGTTATAAATACGACCACATTCAGAAGAGGCGGAGATATTTGTTTCGGCAGGCTTTTCCGGCGAGGGGTTCTGCATCGCGTCACCTTCCTCTCCCACAAGATCTTAA
TGGAATCCTCGTCGGTGCCGCCCACCACCTCCGGCGAAGCCCCGTCAGCTTCCAACCCGCCGCCCACCACCTACGTTCAAGCCGACGCCAACACCTTCAAGGAACTCGTTCAGCGCCTC
ACCGGACCCCACGAAGACCCCCCAACTACTTCCACTCCCATCCCAAAGCTCGCCGGCGTCCGCCGCCCCGCCTTCAAACTCCATGAACGGCGGCAAGCCTCCCGGTCCAAGCTCGCAGT
TTTGAAGCCCCTAGTCAGCCCCCGCCCTCCTGCTGCCGAGTTAATGTTCTCCTCGCCCGGCCGCCTCAGCCCCATCGTCTCCCCGTCCACACAATTGTCGGTGCTTTCTATCTCTGAGGGA
TGCAAGTCACCACCGCCTCCGCAACCTCCGCCACCTCCGCCACTTCCACCAGCAGCTGCGGCCCCCGAACTGAACGAGGATGAAGAGGAGAAGGCGATTAGGGAGAGGAGGTTCTATT
TACATCCGTCGCCCCGGTCTCGAACGAGAAGCGCTGAGCCGGAGCTGCTGACGTTGTTCCCACTAAACTCGCCGAGTGCCAAGCGAGGAAGGTGATGATTTGCCGATGCTTTAACAACC
CAAAAATCAATAATATCATTCAACATATAACATAAAATATTACCTTTGTGCTATTTCTTTGCAATTTTTTTGTATGTTATATGTTGAATTTACGTGTCAACGTATTGGGTGATTAAAGTAATATTA
ATATTGTTTTTGCGATCGGATTGATAGATATGTAAGCTGTTGGGTTTTTATGTAATTGTTTCATTTGCATTGTTATTTTATTATGGTAGGGGGAGTACTTGCATTGTTTCATTCAAAAGGAAAA
AATAAAATTGGATGATTGAATTTAGATGTCAGAGTGTATATATGCAAATATCATACAAAAAGTGAAGTTAGGTCGGTTTGAAGGCATTCATTGTAAAGATATTTTTGGCAGTCAAATTAATTG
CATAAACTGTTGAGGGTTTATGTTAATTAAATTAAGG
>comp144672_c0_seq1
TGGAGGGGAAGGGATGGGGGGAAGGGGGAGGTCGTGGAAGTGGACAGAGGAGAGGGAGGAGAGGGGCCAGCCTTGGAGTTGGAGGCGGAGCTTCGCTTGGCGGTTGTGGGAAGCC
AAGGATGCAAAGTGGACGGCTAAGCCGGAGCGGGTGGAGAGAAGGAGGGACAAATGGAGGAGTTGGTTGAGGTGGCCCTGTACTGGGAAGGGAACTATCACTACGGTTACTTCGCCG
GTGTTCGACATTTCGCTGGCGAGTGGAGTTGGAAGCTTTAGGGGCCGTTTGGATATAGCATTTGTTGCAGGATTTGGTTTCAGGGCGAGTTAAAACCTTCAATTTTTCAAAAGTTCGGCG
AATTTAGGGGATTTCGCTGAAGAAGCGCAGGAAAGAGGGCGAAAGATGGTTACTTCGCAGAGCGCGCCTATTGCGGCTTCCGATCCGACGGGGTCAG
>comp144724_c1_seq1
ATTATTCTGTGCGGAGTCCGGACGAGAAGAACCGTTCGGATTATGAGTGTAAACACAGTATGGACGCTAATTTGATACATATTGTGTACACTCACATATCCGAACGGTTTTTCCCGTCCGTA
CTCCGCGCAGAATAATTTCTCCAAGTCTAGTGCTATATGTATCATTATTTCCTAAATTAAAATCATTGAAACTTTATTTTATTTTGATTTTATTTCCTTCTTTCATTTTTTTCCTTTCTTTGATGA
AATTATTTCTCAGTACATGTAGTACTTAAACAGTTAAACTAGTTACCTTGCCTGTTTTCTTAATATTCGTGAACCATTATTTTCTTCCTTAG
>comp144747_c2_seq1
TAGTATGAAATAAAGTGGTATGTACATAAAGCCCCCAACGTCAAAGTTCAAAAGGTTTGGTCCTCAAGTTTCTGAGCCTGAAACTAACTTTGAAAGCCCATACAAGTATACAACTACAAG
TTGTTCATTTTGCCTTCCTGCTTCTCCACTCATGAATGATGAACCCGTGAAAGCTGGATATAAAATGTGGGAAATGGATAACTTTATTGAATGTTTCAAGGCGGAGGTCAGCATACTCATCT
CCATAAAATCCAAAGATATAAACGAACAAGATGCAAGTTTCATCAGATTATATTGAAGGTTGCCAAGTCAAATAATCTGATGAAAATTCACCAAAAACAATGTCATTATCTCTTCAGAAAC
TCCATAGGCAATCAAGATGTCACTTTTCTAACTTGTAATGCATGATGGGGAAAAAATAATGAACGAGACAAAGGAAAGAAATTGCCGGGACAAATAAAGGGCCGAAAACTCCTCAACGA
CCATTTTTTCTCAGAAACAAAAGGCAAAATCGAACAACATCAAACCGTAATAAGAACCAAAACCACAATCTTCATCTCAACCACAAAATTGAGAAAGACCAGTAGGTCAGTTAGTGATC
GAACACAAATACAAGCGATTTAATGGAGGTAATGGAGAATCTAAACTTCAAAGTAGTTCACCTCATCTT
>comp144868_c0_seq2

AAACACTTCTCCGTGTGCATTACTGTAAATAGACTTCTCGTTTAATTACTTTGCATGAGATAGTTGTTCTTAGCAGAAAGTATATAAACTTGTATTGATATTGCGATTATCCTGTGCACTTATG
TACTTTCTCATCACTGGATATCTGTGGAATGCGGACATGCAGCAGAAACCTACTGTACCAAAAAGAAGATAAGAAGCAGAAAACTCGCGTCATTGCCTGCAGAATCTGCCCTTACCAGGA
GAAGGTAGAAGGAAATGACAAGATGAGACATGAAATAAATCTGACAGAGAAAACTTTTGGCTTACCAGACGCTTACGAAGATCCAACACTTCCTCGCGCAAAGGGTGTTTCATGTCCTG
TTTGTAGCTATCCAAGAGTTGTATGGTTCTATGGTAATAGGGAAAGCATGGAACAAACGTTTGCTTGCTGCAACCCAGGGTGCCGTCAAAACTGGAGGAAAGAAGATAAAAATAATGGTT
GAAGATTGTTTATCAATTGAAGATCATGCCTAGATTAGATTTGACAGCTTTAAAAAATTAAATAAAATGGTGAAGATATTCATCATTCCTTTTTATCTCTTTTAACTTTTCCATGTGAGAACTA
AAACTAAAGAATTCATTTTTATATTTTTTATGGTGATTTTTGGTCGAAATAATTTTGTTTTTCACATTGGGAAGCCAACGTACAATTATCTCACATAGAGATAATTCTCCCTTTTCATCTTTTTT
>comp144876_c0_seq3
TTATTTTTCAACTTCCAAATTTATATAACATAATTGGTCAACAAATCAAATATTCATGGAAATAGAAGCAGCAAGCAGATTCCAAAACATCAAAAAAAAAAAAAAATTCCAGTGTTCTTGGA
AGAAATTAAAGAGGATGAAGCCAAGATAAGCTACAAATAGAATGAGAAAAGTGTAATAAAACTTGAAAAGATTCTGCAGTTACCGGATCAGTTTCCTCCAAATGGTTCTCCCTCTGTTTA
TTTCCAGGCCGGTTCCTGAAAGTCTCAAGAAGTGCAGGAACTGAAGATGATCACTTCCATGTCCATGCTGCTTGGAGGGATATCCATTGGTTCTCTCTGCAAGTAAGATTTCTTCTGGTTC
TGAGCTGGTAAGTGAATCAACCATGTCATCGTGCTGGCATTCTCGTCGATAATCCAAGGTAATGGTTTCGAGTTCGTGGGTGTCGATGATTTCTTGCGGCATGCTCTCAAGGACCCAGCCG
ATATAGGTAACATTGTTTACATGTTGATTCATGTCTAAATCAGCTCTTCTGGGCACCAGTCCTAGCCGTGAATAATCTGCTGGATCTTTAAGTTTGGTAATTTTCTTCAAGCTATCGTTCTCCT
CCGGGAATGCTAATCTGGAGGTGTTTCAGAGATTTATTTACATGAGTTGAGAGGAGAAAGTATAGATTGGTAAGCTATATGGTTGATACAATAAATGGCTCGCATTCAAGCACAAATTCAA
GACAAAATTTCAATACATAGTGAGCTCAGATCAAACTAAGCAATGATTTGTACCTAATTT
>comp145011_c0_seq1
GAGGCTTAATAAAAGGCATGGAGGGATGAGGAAATAAGGGAAAAGCCAAAGTCGTGGTCTTTCTTTCTCCTAACACAGATCCCTAAGGATTTCAGGAGTGGCGTAGCAGTAGTAGTATC
AATGTTTCAATGCTCTGTAGTTTGGTAGATAAAGCTCATTCTTTTTGCGTTGTTTAAGGGCCCGTCCTTGAGCTCTCTCCCAACTTCTTTTGCTTTCCATTCTCCCCCTGAAACAGTTCAAG
GCATTGAGTTTCTTGAGAACACAGAAACCCAGGCTTCAATAAAGATGGTGAAGTAGCGTGTAGGATGTATGATGAAATTTAGTACTCTCCAATGATTGAGCTCCCCTGAGTTTAGTTTGTG
TGTGAGAAAGATGAAGTCCATGAACAATACCAACTGGTTGGGCTTCTCTCTCTCCCCTCAGCTGGGAATGGAAGTTGCTCAAGATCCCCTTAAGCAGACAAATCTTCAAGCTACTCATCA
ATCTTCACCGGCTAACGCTTCCTCTTTTGTTCCAAGCAACTTTCTTCTTTCTTATCCCCATTTCAGCAGCTCTGGCATTTGCTATGGAGCTGATGGAGAGAATGTAGGGTTTTACTCTCAGCT
TTCATCAGTCATGCCAGTTAAGTCTGATGGTTCTCTCTGCATTATGGAAGCCTTCAGCAGATCACAAGAAGGAATGGTGCCATCTCCATCCCCAAAACTGGAAGACTTCCTGGGCTGTGGT
CCTAACATGGGGACCCATCCGTTTAGCAACAGTGACAGGGGAGCAATGGCTCTAAGCTTGGACAGTATGTATTATGGACAAAACATAGAGCACGAGAGCAATGAAAACCATTCTCTTGAG
CTCTTTCAACAACCTTTCAGGCACCAAAACTACTTTCAGCCATTGCATGAGATGTATCAGGGTTCATTGGTCGGAGATCAAATGTCCGATTGTAGATTCCAAAGCAATGAAGGAGTTAGTG
ATGAAAGAGAGATGGGTTTTAGCTCTCTTGGATTACTGAGGCATGGTGATCTCAAGCCCTTGAGCTTATCCATGAGCCCTGGATCTCAATCTAGCTGTATAACTGCTCCACAGCAGCAGAT
CACTTCTGCTTCAGAAAATGAGCAGTTGGCTTTGGAGCATTCAAAGAAGAGAGCAAATGGAAAAGGAGCTGAGAAGCAGCCAGTTCACAGGAAGTCCATTGATACATTTGGCCAGAGA
ACTTCTCAATACAGAGGAGTTACAAGGCATAGATGGACAGGTAGATATGAAGCTCACCTTTGGGATAACAGTTGCAAGAAGGAAGGGCAGACAAGGAAGGGAAGACAAGTTTATTTGG
GGGGTTATGATTTGGAAGAGAAAGCTGCTAGGGCCTATGACTTGGCTGCATTAAAGTATTGGGGACTTGCCACCCACATCAATTTCTCTCTGGAGGATTACCAGGAGGAGCTTGTACAAAT
GAAGAACATGAGTAGGCAGGAATATGTTGCACACTTAAGAAGGAAAAGCAGTGGATTTTCTAGAGGGGCTTCTATATATCGCGGAGTCACTAGGCATCACCAACATGGAAGATGGCAAG
CTCGCATTGGGAGAGTCTCAGGAAACAAAGATCTTTACCTTGGAACCTTTGGTTAGCTCCTCTAATTCTTTTTACAATACATTCCATTTAATTTTCTCAAATGCTT
>comp145027_c0_seq2
ATAGGGAATGGATCAATAATAGCACCTTTCATCATTGTCTAGTTAAAGACATAAAACCAAGCCTTTTCAATAGAAGAACTTCAGGCTTCGTTAAAAAAGGCAACGTTGAGATAAAAAGGA

AACAATGACAAAATCCAAGAAATCAGCCACAACCGCAATACAAAAACTCCAACAAAGAAACCTCCTTAGTATAACAAAGCTTGCACTAGGGACTTAAATTCCATCCAGGTGAAACAGAA
TTATTTTAAAGCAAAATGTTCATGCCAAACCAATAGCATGGTACAATGAGAGACCTTCCCTGGAAGTTATAAATCAACCTTGTATAGTACTCGAAGAATTTGAATCTTAAAAGCAAACACA
AATTTGAGTGAAAAATGAATATAATGAATTTTTACATTCCTCAATATCCCTGTTACAATCAAAGTGAGCCCAAAATTTAGCAGTCTCATAAATTCTCATAATGAACCATAATGTAAAAATCTA
ACGTTGCAATATCAATATCCCTGAATTCATCAAAGTTCAAATCTCTCTATAAGTTGCAATTTTAGCCGATATGGAAACCATGAAGCGACCACCAGTAAACTTCATATCACAAAACGCATTAA
ATGTAGGATTCTCAATTACAAAGCATGTTAGTCTATCACCTACCCAAATCTACTTTTAGCAGCCAGTGGATCAGCAATTTAAACAATTTCATTGAATCAACTTTAGGGATTTAACACAGTTGT
AGGATAACAACAGAGAAAATAATATAGCCTACTTGTGAGCCACTCAATTGACCTATTACTAGAACTATTATAGAAAAACTCAGTAGTAGGGAGAATAAGATCCAACTAGCAGTATAATGATT
TACTAAGCACCTAAATAAGTCTCATAAGCTTCTGGTGATGACTTCAGTATGACCATCAATTTGAGTTTGATAGGATGTTGCAAGTAAAGTTTAGTGCTAAATTTTTTTCATATGTTTTTTCAA
AAATAGCTCATAAATATCACATTATAATCAAAGATAATTATCTAGGGAAGCCCATGTAAAATATTCTCTCTAAAGAAGATGAATGTCGTATGTGATGCATCTGATGTCCTATAACATTTTTAAT
AAAATGTGTCATTTTAGAAAGGTGATCAACAGCTACTAAGCATTAAAGCGTTGTACAGTATGGGACAATCCTACGATAAAGTTCATACTAAGATCATCTCATGATTTATGAGGGATTGGAAG
TTGCCTGTATAAACCAGAGTTGAGTTTTTCATATTATGTCATAAGGAAGCTATGCCATTGAGCAACAACACGGATTTTATATCTTGTAAATCTTTGCAAATAAAATTTATTCCTTGTCATAGCT
ATTATCTTATCACGGTCTAAGTGACCAGACACACCTCGTAAATGTATTTTCTCAATGAGAAAATCTCACAATGAAAAATCAGAAATACAAAGAGCTTTTTTTGAAAGAGTTAGCCATCCCTT
AATAGTAGGTGCATTAGAATTTAAGAACTGAAAGTTTTGATGAAAATCTTTGCAATTAGGGTTCTCACTCCTAACTTTATCAAATCCAATGCCATTTATACTCATAGTCTGGTTGCTATAAGA
TTTTCAG
>comp145077_c1_seq1
TTGAGTGGTCGGTTGATGCATGTGTTGGCAGACCTGATTTGTAGCAAAGGCGATGTCCGGCCTGGTGATGGAGAGATATTGGAGTGAGCCAGCGAGTCGGCGGTATAGAGTCGGGTCATG
AAATGGCTGGCTAGTTGAGACGTTGTGTTTTGATTTGGGAGATAATGGTGTGACAGCAGGTTTGCATTCAGCTAGTCCGGCGTCCTTTAGTAGTCTCTCGGCATAATGTTGCTGTGAAAGG
AAGAGACCGTTGGGTTGCTGAGAGATCTTGATGCCAAGAAACAAAGATAGCTGATTTGATTGTTTGAGAGAAAACTGAGAGTGTAGTTTGGCTAGTAGAGCGGTGATGGCAGCGGAGTT
GTTCCCTGTAATTAAAATGTCGTCTACATAGATTAAAAAGAAAATTTGTGTATGATTAAGTCGATAAGATAATAGAGACGGATCGGAGCGAGTAAACCTGAAGCCTTGTGTCTGAAGAAAG
GAGGTTAGTTTTTCAAACCATTGTCGCGGAGCTTGCTTGAGACCGTAGAGAGATTTATGCAATTTGCAGACGTAGGAAGGTGATGCGGCATTGATAAAGCCCGGTGGCTGTCGCATATAGA
TGTTCTCGGTCAGGTCACCGTGGAGGAATGCATTTG
>comp145085_c0_seq1
GAGAATATAAGCTCTATTTTTAGATGTTATATATAACAAAGACAAAAAAAAAAAAAGCAAAACAAATTAAGTTTTCCTTGAAAGCAAATTTTGACTTTATCATAGAGCAGATGGATCGCGA
ATGCACAGAAAAAAAAAATCAACAAAACACAGAAAAAAAAAGTAGAAATTATTTAGAAAATGGCTTAACTATCTTCTGTATGTTGGGGATCTTGGAGTGGTTCCTTGGCCGCACTGGCCT
CCTCCTCCTCCTTCTTCATTGAAGCAGTTTTAATCAAATTCTTATAGTTACCTTTCTCCATAAACAACTGAGAAAAATGGTGTTTGCAGTAAAGAATGCCGTTTAGAGCAGCGTAGCTTGAT
GGTGTGAGAGTGCATCCGCCATGAGAGCACTTGAAGCAGGTCTTGTGGTATGATTCTCCCTCCACATTCAGCTTCTCTAGAGGATAAGCTGTCTTCTTACATACAGCACATTTGTCTTGTGT
ACCAGAGAACATCTGTGAGACCTTGCTTGCTGTTCTGTTCAATTCAAGCTTCTCTGCTGATTTGCTTGGGCTGTGAAATTTCTTGGTGAAGGAGCCCGTTTCCTTGAAGAGCTGCTCAAAA
TGGGGTTTACAGTAGAGCACACCGTCCATGGATGAGTAGTTACACATAGTGAGAGTGCCACTGCAATGGCTGCATTTGAAGCAGGTCTTATGATAAACCACTCCATCGGCGGTGAGCAGG
TCGATGAAATGAACCGTCTTCCCGCACGCCTTGCACTTATCCTGAGTCCCACTGAAAGACATTGCGGTTGCTTTATCGACGACGGCAGACGTAAACCCTATCTCAGAAGCAGAAGAACAA
GAAGATGATCCAGACGCTCTCCGATCCCTTCGGCAACAATTTGATGGGGAAAGGGGCGATTCGGAAGAATGAATTAATGCTACGAGATTTAAGTTTCGGTCTGTTGAGGAGAAGAGGATG
GAGAGGGAACTGTTGAGGAGAGGAGG
>comp145154_c0_seq1

TTTAACCAACGATTAACTCACTAGCATTTGAATAAAAAACATGAAATACTTCAACATTTTAAGTAGAGGGAACATCACTAAGAGAAAAACCATCAGTGTTGGGGTTACTATTAATCATAGTA
TTAACCACATGAGTTCTATGATCCATGCCATCCATAAAAGAACCAATAACAGCATCATTAACATGTGGGGGTGAGGACAAAGTGCCCACCAGATTATCACCTAAATGAGGGTGAAGTTTGT
GATATTCCACAGCTGAATGTCTTAATGCTTTACACAAATCGCAAAATGAAGGAGAAGCTTTCATTTCAACAGATTGAATGAAACCAAATGTATTTGGGCCAACACATATACGATCGGGATA
ATGCTCGGAGATATCCAGCTCAACCAGTATGCGGGCAACAGAAGGTCTCGATCCAACGGATATTGCACTGTCAATTTTTAAAGGTTTACCGGACAAAGAACCCAAACCATGAAGAACAAA
TGAGGACGAAGATTAGGGAAGGATACCCATATCGGAATAACATGGGAATCCACGTTGACATGTAAGAAAGGCGACCATTTTGTAAGTTTCATGTAGCAGTTATGCACAAAATACAATCGAT
GGGAGAAGTTGCGACTGTAATTCAAATCATTCTCAAGTTTAATTAACACATGCTTGGGATTCAGAAGGGTAACAGAGAAAACTCTCTTAAGCTTAAGATTGAAAAAGAAATTCCTAATTG
AATCA
>comp145349_c0_seq1
CACAGGTAATTCTACAACCGAATTAGATAAACTCCTTTCTAATCTTCATCTTCATTTTCAGATGCGCAACCTTGGTTCTCTGTCACAATTTCTTGGCATCCAAACGTTCCAAACCGAGTCGG
GCATCATCCTTCATCAAGCAAACTACGCAAAGAAAATTATAGATCGAGCCGGCATGACTCAATCAAAACCCGTCTCCACGCCACTCTCAACAAAAACAACTGTTTCAACAAAATCGCAA
GAACCGTATGATAACCCTCATCTATACAGACAAATCATTGGCTCGTTACAATATCTTACACTCACGAGACCGGACATCCAGTTCGCCGTCCAACAATTGTGCCAACACATGCAGAACCCGC
TGAACCTACATCAAGAAGCACTCAAACGACTTATCAGATACATTCACGGCACCACCCAAACAGGTATCCCGCTATCTCGTAATTCTCTTTTACTAACCGGGTACGTCGATGCAGATTGGGC
AAGCAATCAACAGGACCGGTCATCCATATCCGGCTTTTGCAACTTTCTCGGAACATCTCCCATTTCGTGGCAAGTCAAGAAGCAAAGCACCATCGCGCGATCTTCCACTGAAGCCGAATA
CAGGGCCTTGGCATCCGAAGCAGCAGAAATCCTATGGCTCCGAAGACTACTCGAAGATCTTCACATACCGCAAATTACCCCGACCACCGTTTATTGTGACAACACGTCGGCAATAGCCCT
CGCCAACAATCCAGTTTTCCATGCACGTACCAAACACATAGAAGTCGACTGCCACTTTATCAGGGATTGCATCAAGAAACAT
>comp145373_c0_seq2
AAATGAGAGTTTCGGTTGGAGATGCCCTTATTTGATAGGACTTAAGACCTTATTAAGCATAGAAAAGAATACATATAATATAAAAAAAATAAAAAAATAGAAAAGAAAGCTATAATATGACC
AAAGTTCAGAATCTGATAATTCAACATTGTTCAAAAACAGAAATTAAATGCACAACAAAAGTCATTATTTTTACATATTCTCAACAAAGAATAAAGAAAGAAAAACAACAATGAAATCCT
TTGAAGAACTAATTACTTGCAATGGAATAATAAATATTTAAGCCTTGGAAGCCTCAGAAGGCTCCTCCTTCAAGCTCTCCTCGCTCTTCACATTCTTCTCTGCTTCATCTTCCTTCTCCTTCT
TGTCGCTCTCACCTTCTTCTCCTTCAATGGCTTTGGACTCCTCCGGCTTTGTTTCCGACTCGTCGGTCGTCGCTGCTTCCTTCTTCTCCTTCTCCTCCTCCTCTACTGTCTCCGTCGGCTCTG
CTGCTTCAGCCTCTTGGGGAGCGACGGGCTCGGTGGCCTTGTCGGTCTCCTCGGGGGCAACAACGGTGTCGACTACAGGCTCCTCTGGAGCCTGTCCCTTAATGTCCTTGGGTATGCTTG
CACAGGCACCCATGGACTTTTACTTGGAGGAAAAGAATTAGAGGATTTGAAGTAATATTGAGACGATTCTCAATAGTAT
>comp145373_c0_seq3
AAATGAGAGTTTCGGTTGGAGATGCCCTTATTTGATAGGACTTAAGACCTTATTAAGCATAGAAAAGAATACATATAATATAAAAAAAATAAAAAAATAGAAAAGAAAGCTATAATATGACC
AAAGTTCAGAATCTGATAATTCAACATTGTTCAAAAACAGAAATTAAATGCACAACAAAAGTCATTATTTTTACATATTCTCAACAAAGAATAAAGAAAGAAAAACAACAATGAAATCCT
TTGAAGAACTAATTACTTGCAATGGAATAATAAATATTTAAGCCTTGGAAGCCTCAGAAGGCTCCTCCTTCAAGCTCTCCTCGCTCTTCACATTCTTCTCTGCTTCATCTTCCTTCTCCTTCT
TGTCGCTCTCACCTTCTTCTCCTTCAATGGCTTTGGACTCCTCCGGCTTTGTTTCCGACTCGTCGGTCGTCGCTGCTTCCTTCTTCTCCTTCTCCTCCTCCTCTACTGTCTCCGTCGGCTCTG
CTGCTTCAGCCTGTCAAGCAATAATGAAGGCATTTGATGCACAATATAAGAGAAAATAGCAAAGCATTGAGACATATAAGTATAATTAATAGAACAATTGTCATAGAAAGTGAAATATAAAT
TTAGAATAAATAAAAAATCAAAATTTATAATACTTGCCCTTACTTTAGAATAAATAAAACTCGATTTCTCAATAACTGATCAAAAATTAAAATTCTATTTTGCTAAACTAGACTACTTTTTATT
GTGTGCAGATATTGGACAGAGAACCGTTCGGATTTCTCTCTGTCTACAATTAAAGCGTAGCTGCTACAGATTACAGTAACTGCAATTTGATTATAGACACAGAGAAATCCGGACGTTTCTC
TACTGTTTGGTGTCCGCACATAGTAATATCTCTGACTAGACATATTATAGTTATGAGTAACCTCTTGGGGAGCGACGGGCTCA

>comp145409_c0_seq2
TTTAATAAAAAATATATTATTAGTAGTCAATGAACCAATTGTTAAAAATAGTGCATGCATGACAGCAAGTCATCTTCCACATTTGAAACTAATTCAACAAGCTGTTATGGTTCCAATAGGTTT
GATTCTCCATAGAGAAAATTCTATACATTCATTCTAAGTGCATCAATGTTCTCACAAAGTTGGAGCTATTTTGTCCATGAGCTCGAACTAGGTTCTCTATTGTTCGAATCAAGTTTGAATTTT
GTTCGGATGATTTGAACTAGATTCAAGCAAGATTTTGACTAATTTAATTTTAAAATTAAACACAGTAAAATTTCTTAAAATTAGAAAATTTTGGTTGAATAGAAAAAAATACAATGAATACA
AACATATGATTGAATAGAAAATAAAAATTTTAGCATGAAAAGCGGTTCATTCGAAGGCCCA
>comp145436_c1_seq1
CTTCCTCCCTTTCTCTGTCGATAACCAGACGTAAAATTCCAATGGCTTCTCGCTCTCCTCTTCACTCTGTGAAGCTCTTCGTCTTCCTCATTCTCCTCTCCGCTTCTGCTTTTCTGAAAGACG
CCGCAACGGCTACCCAGTTTCGGGTCGGTGGACCGCTGGGATGGAGGAAACCGACCGGCTTCGAGTCGGAAACGTACAGCCAGTGGGCCGGCCGGAACCGGTTCCACGTCGGCGACT
CCCTTTATTTCAAGTACGAGAAAGACTCGTTGTTGGTCGTCGACAAAGGCCATTACGACGAATGCAACACTATGAACCCGCTCTCGAGGTTCACTGATGGCAACACTACGTTTAGGTTCG
ATAGATTCGGCTATTTCTACTTCATAAGCGGTCAATCGGGCCATTGTAAGGCTGGACAGAAACTCATTGTGCGTGTCATGGTTCACCCAGAGATGATATCAGCGCCGGCGCCGGCCCCAAT
GAGTGTACCATTTGTTGGATCAAGCCCAACATCTTGGTCGGGATCGCCAGACCAGCCTGAGTCCAGTGGTTCTTGGAGAATTGGAAGGACTGATTATGCGTTGGCCATTGGCCTAATTGG
AGTACTGGCTTGGCAGATGGTTTAATTCTTGATTAATTGTTTGCTTTTATACTTCCGAAGTTTGGTTTTGTTGCTGTGAATGTTATGGTCATTTTGTTTTGGATTAGATGCTTCACTGTGAG
>comp145442_c0_seq2
AGCAGCAGCAGCAGCAGCAGCAGATGATGGTGGAAATACAGCAGCTCGCAGAGGTTGTGGCAGCGGAGAGAGAGCATGAGATGCTGAGGAGGAGAGAGCATGAGATACTGAGGAGCA
TAGAGCAGCAACATGTATTAGCGAGGCTTATCGGAGATGGAAGCAGTGGTGGGGCGGCGGCGGCTGAGGCTTCTCCGCCGCTGGCGTTGGTTTCCCCGCACATTAGTGGGTTTGAAGGC
TCGTTTGCGGAGCTGCAGCATCAACAAGGGTTGCAGTTCCAGGAAGGGAGTGGCCTTCGTCTTCATCATAATTTTCATCAGAGGAGAAGGTTCGATGAGGAGAGAAGTGGAGAGGGTCC
TTCTTCTTGACTTCTTTAACTATTCGTTGAATTTTAGACCGCGCTTGATCTTCTTTTGGTGGTTCAGCTTTCAGCTTTTCAGCGTAGAGATTGCTGCATTCATCCTAAGCTCCGACAGACTGC
AGAGGTGGTCATTTGTTCATCTGACACCACAATTAAATTCATATGCTGACAGATTAATTCTCTGTGCACGATGGATCACGTTTCAGGCCTATATTTCAAGCGCAAGAAAGGAACATATGGCG
GGAGAGACCTGAAAGTTTTTTTATTGATTCTTAAAGCAGTTTTTGAATACAAAAAATTTAAATTTTGTACTAAAA
>comp145469_c0_seq2
TAAGAATTCTACATGCATTTCGTGGGCTTCCTTCCCTATGGATTTCCGAAGAGGAAATTTTAGCTTTAGCAGCTCCATTTGAATTTTCTTTAGTTGGTAAATTCCCTGATCGTAGGCCATCAC
TTGATTCAATTAGGAATTTCTTTTTCAATCTTAAGCTTAAGAGAGTTTTCTTTGTTACCCTTCTGAATCCCAAGCATGTGTTAATTAAACTTGAGAATGATTTGAATTACAGTCGCAACTTCT
CCCATCGATTGTATTTTGTGCATAACTGCTACATGAAACTTACAAAATGGTCGCCTTTCTTACATGTCAACGTGGATTCCCATGTTATTCCGATATGGGTATCCTTCCATAATCTTTGTCCTTAT
TTGTTCTTCATGGTTTTGGTTCTTTGTCCGATAAACCTTTAAAAATTGACAGTGCAATATCCGTTGGATCGAGACCTTCTGTTGCCCGCATACTGGTTGAGCTGGATATCTCCGAGCATTATC
CCGATCGTATATGTGTTGGCCCAAATACATTTGGTTTCATTCAATCTGTTGAAATGAAAGCTTCTCCTTCATTTTGCGATTTGTGTAAAGCATTAAGACATTCAGCTGTGGAATATCACAAAC
TTCACCCTCATTTAGGTGATAATCTGGTGGGCACTTTGTCCTCACCCCCACATGTTAATGATGTTGTTATTGGTTATTTTATGGATGGCATGGATGAGAGAATTCATGTGGTTAATACTGTGAT
TAATAGTAGCCCCAACTCTGATT
>comp145495_c0_seq1
GGCGGCCACCAGCAGAGGACAATATTAGGCAGAATTTGTTTACATTCTGCCTAAAAATAGGGATGCCAAACAACGCCTAAATTTAATTAGAGATCTGGGAAGGGAGAGAAGAACGCGCA
TTAGGGTTTGTCGGAGATGGGTGGAGAGGAGGAAGACAAGAAGAGGGCTGATCGTGCGAACCCTAATCGGCGACACGGGTCGACAGCTTTCTTCGTGATGTTGGACTACACGTTCATTT
TCATCTTCTTCTGCTTTCTCTGCTACTTCCTCGCCAAGATTTTGCCCGGATTTAATCTGTTCTTCTCTATTTGATCGCATTGTTTCTGAGAGGCTAGCGAATATATCCCGGTAGAACGTTCTCA

TCCTTTTTTCTACAGTACTATGCTTTATCTTGGCTTTCCCCATTTTTTCTGTTTTGGAATTGTATTAGGGGGTTTGGTATTTAGCCATTAAGTGAAAATGGGGGTTTCATCTTGTTAGTCTGAA
GGGCAAACTGTACCCTCTGACATAGATTAAATAAGATACTATTTTTTGTGATGTTCCTTTTAAATCGTCGATGACCAATTTCCCAAAGCAAGACATTTTTTAGAAGAATCAG
>comp145506_c1_seq1
TCTACATCATTTAACATGCAATGAAAGATTTTCTCCACACTGAAGGCTATGAAAAATTTCAAATAGAACAGTTCTAAGAACATATTTGCTATAGGCAGCCATGACTAGTTCAATGCTCATTG
GCTCTCAAATTTTATTAAAATACAAACAAGCTCCAGAGGGATAGACAAGAGTAAGAAAAAGGAGACCACCAACAAGAATGTTGATGGCAAGAACAGACATAAACGGAATAGAAGGCGG
CTATGATAAGCTCTTCTTATTTGAAAAGTCTTGCAAGATCAAGAAAATAACGCGAAGCTTAAGAATGACAAAGACAAGAGGAACGAAAGACGGGGAGAAAAAAGCTCGATAAGGATAG
CGCCCTGTGAAAGTAACACGTTCTCCGCTATTATTGTGATGCTTCAATCTCTTCTTATATCCCTTCGATGCAAAAATCATCGAAGACCAATCTCTAGCAACCAAGAGGAAGAATGGGATCAT
GATTCTCTAAGTCTCCCTCCCTGAGGCCATCTCCAAAAGCATAGGCAGATGGCACAAGAAAGGGCAAAGGAAGCCCTCCATTGAAGGCTTAAAGCCATAGAGAG
>comp145556_c0_seq1
AAAAAAAAAAAAAAAAATCAAATTAGAAAAATTTAGGTTAGAATATCCAAACTTCAATGTACTTTAAAATCATTAAATATTTTGAGATTGATGTAATACAATTATCTAATCATATTTTATCCATC
TCAAAATAAATCTAACTCATTTTTATCCTATGTTTGACACTGAACGTACATTGAATGATGGCCTAAATTTATAACTGTTGAGACACTCCAACCATTCAAGGTCATCTTTTTCGGTCGAGCTTT
CAAGGTCATCTTCGCTCCAGCTTGCATCGCATGCAACTTCAATGTTATTACTATTACCATGGAGTTCTGCCAAATTTATTTTCAAAGATATAGTCAGCTGTCCAGGTCTGTATTTCACACAAG
ACCCACAAATATTTAGATTTGTTTCTCCACTTGCTTTCATGCTGTCAAATTCGATAGGTCGTAGACGTTTAGGAGGTGGGCTGATGTCAGAACCGGACTCAAAAATGTGCTTTGGCAACCA
TAATTTGATTCTCTTTAATTTGATGAATGGAGGCCTTTCATCGAGAATATAGAACTTACAATCTCTGCTAATAGCATAAAAGCGGCCATCACCAGCAAGGGAGATATCTATTATGCTTTTGCT
TTCTTTCATCACAAGGCTCCACCTTTGACAATCATCGTCTGCATGCCTGCAGAATACAAATATGTTATCTAGAAAACTCATTGTTTTATCCTTTATCGTAACATTGCAGGCCACACCAATGCT
TGCGTCAATGCTCGAAGAGACAACTACCTTCCAGACGTGTATGCAATCTGCATTAATGTGAACGGTTGGTTTTACCTTTTTATAATGACGGAGTAACTTTGGGTATAAAGGGGGGAATATG
AGTTTGCGTTTTGATACAGGATCAAAAAGAAGAATAATTCCATCGTCGTGGGTTAGAAAGATAGACCAACCATTTGAACAACCAACAAATGACATGGGATCAAATGATGAAAAAGGAAA
GGAGATGACGTGAGGACGACTTTTATCATTGATGAAGTCCGAGTCCAAATAAAAAATGACTTCGGTTCTATGTCTGAAATGTACTAAGTAAAGCGGACAAGGATTCGGGGTAGGTGAGGC
ATAACGCCATTGGGAGCAAACTAAGCGAAAAGACAAAAGATCGGCATTTTGTAGCTTATTTCTAATGATTATCAATAGGTCAAGAGGAAGATCAGACCATGATGGAGACATTGAATATGGG
AAATAGAAACTTCGTATGAAATTGTTTAATCTCGAATAAAGAATAATCATCATTT
>comp145556_c0_seq3
AAAAAAAAAAAAAAAAATCAAATTAGAAAAATTTAGGTTAGAATATCCAAACTTCAATGTACTTTAAAATCATTAAATATTTTGAGATTGATGTAATACAATTATCTAATCATATTTTATCCATC
TCAAAATAAATCTAACTCATTTTTATCCTATGTTTGACACTGAACGTACATTGAATGATGGCCTAAATTTATAACTGTTGAGACACTCCAACCATTCAAGGTCATCTTTTTCGGTCGAGCTTT
CAAGGTCATCTTCGCTCCAGCTTGCATCGCATGCAACTTCAATGTTATTACTATTACCATGGAGTTCTGCCAAATTTATTTTCAAAGATATAGTCAGCTGTCCAGGTCTGTATTTCACACAAG
ACCCACAAATATTTAGATTTGTTTCTCCACTTGCTTTCATGCTGTCAAATTCGATAGGTCGTAGACGTTTAGGAGGTGGGCTGATGTCAGAACCGGACTCAAAAATGTGCTTTGGCAACCA
TAATTTGATTCTCTTTAATTTGATGAATGGAGGCCTTTCATCGAGAATATAGAACTTACAATCTCTGCTAATAGCATAAAAGCGGCCATCACCAGCAAGGGAGATATCTATTATGCTTTTGCT
TTCTTTCATCACAAGGCTCCACCTTTGACAATCATCGTCTGCATGCCTTCCAGACGTGTATGCAATCTGCATTAATGTGAACGGTTGGTTTTACCTTTTTATAATGACGGAGTAACTTTGGGT
ATAAAGGGGGGAATATGAGTTTGCGTTTTGATACAGGATCAAAAAGAAGAATAATTCCATCGTCGTGGGTTAGAAAGATAGACCAACCATTTGAACAACCAACAAATGACATGGGATCAA
ATGATGAAAAAGGAAAGGAGATGACGTGAGGACGACTTTTATCATTGATGAAGTCCGAGTCCAAATAAAAAATGACTTCGGTTCTATGTCTGAAATGTACTAAGTAAAGCGGACAAGGAT
TCGGGGTAGGTGAGGCATAACGCCATTGGGAGCAAACTAAGCGAAAAGACAAAAGATCGGCATTTTGTAGCTTATTTCTAATGATTATCAATAGGTCAAGAGGAAGATCAGACCATGATG
GAGACATTGAATATGGGAAATAGAAACTTCGTATGAAATTGTTTAATCTCGAATAAAGAATAATCATCATTT

>comp145556_c0_seq4
TTTTTATTAAATTTACTTTGAGATGAATACAATAGAAGATAATTAATCACAATAAGTATCGAATCCAAAATGTAACTCATTTTTATCTTTTGTTTGACACTGAATATTGAATGATGTTCCCGATA
GTGATGGCCTAAAAATATCTGGGTCAATGTCATTATGCAAACGTCTAGGGCCATAGCTCCGGCTAGCATCGCATACACTAGCATCGCATACAACATATATGTTATTACAATTAGCATGGAGTT
CTGGCAAATTTATTTTCAAAGCTATGTGCGGCATTTCATATCTATGTTGCTGAGCAGCCGCATAAATATCTAGATTTGTTTCTCCACTTGCTTTCAAGCCATGAAATGAGGTAAGTACACGTT
TAGTAGGTGGGTTGTCGTCAAATTCGGAATCTAAAATGTGCCTTGGCAACCATAATTTGATTTTCATTAATTTGATGATTGGAGGTCTTTCATCGAGCATATAGAACTTACAATCTTTGCCAA
CAGCATAAAATCGGCCATCACCAGCAAGGGCGATATCCATTATGCCTTTACTTTCTTCCATCAGAAAGCTCCACCTTAGACAATCACCATGTGCATGCCTGCAGAATACAAATCTTCTATCT
GGATACACCGATGCTTTTTCCTTTATTTCAACAATGCAGGCCACAGCGATGCTTGCGTCGCTGCTCGGAGAGGCAACTACCTTCCAGAGGGATACGCGATCTACATCGTGAGCAGTTGGTT
TTACCCTTCTATAATAACGAAGAAACTTTGGACATAGAGGGGGGAATATGAGTTTGCGGTTTGATACAGGATCAAAAAGATGAAGAATTTCATTGTGGCGGGTTTTGAAGATAAGCCACCC
GTTTGAACGAGAAAAAAGTGATATGGGAAAAGATGATGAAAAAGGCAAGGAGACGAAGTGTTCACGATATATATCATCATTGATGCCCAAGGTGAACCGAATGATATTGTTTACTTCGTT
GAATTTACTTGGTAAAGTGGACAGGGATGTGGGATAGGTGATGCATAATGCCATTGGGAGCAAACTGAGCGAAAAGAAAAAAGATTGGCATGAAGTGGTAGCTTCTTTCTAATGCTTAT
CAATAGGTCAAGAGGAAGATCAGACCATGATGAAGACATTGAATATGGGAATAGTAACTCTGTATGAAATTGT
>comp145582_c0_seq2
TTTTTAAAATTTCTATTAAAAAAAATTAAAATTAATCGTCAAAAGTAGGATGGAACACCGTCCACATTAACTATGCATTTGATTTGACACTAAAAATAAAAAAATAATAATAACTAAAAAAA
ATAAAAACAAAAGGACGGCGTCGCTGCATTGTAGGCGCAATTTACGTGGCTCTCTCGCTGCAAAGCAAACCGCGGTTCACAGGATCCAAAATTTTGAAAGACTATAATACCCTCACCTTT
TCGCTCCTTCCATATTTCGAACAACGTTCGCACCCCTCGCTCTCATCCATGGACAAAATTGTAATCTCAAATAAACCTCCTAACAAAGCAACAAACTAAACCCATTAACCCCAACCCTTTC
TGTCTTTCTTTGAGTCTCTCTGTAGCCCTCTTCATTCTTATAACCCTAAATCCTTCCTTTTTTCATTCTGATAGCTGGAATCTCTCATCACAAAGAATCATTTTCTTCTACAGTAATCGGATCTC
TTACCTTGCTCCTCACCATCTCCCTTACCCTACCAACAGGTTGCAACGGTGGCGTGGAAGGCGCGACTGGAATGACAGATGGAGGAAACAAACTCTCCAGTGCCCGGAAACAATAAGCC
GGAGGAATCTACGGCAACCGGCAATTCTGGCGGAAAAACAACCCCGCAATGCTCTGTGATCATCGTGTACAAAGCTAAGATCGGTAACAAGCTCCGCAGGGTAACGGTAGTGTGGAACA
AGACTCTCATCAACCACTCCCTCTCCATCTCCGTCGACGGTTCCGGCGGAAGTTGCGATCCACCCTCCACCTGCAGAGTCGATCTCAAGCCATGGCCCTTCTGGAGCAAGAAGGGTTTCA
AATCCGTCGACGTTGGCGATCACCGAGTCGACCTTTTCTGGAACCTTCGCCACGCCAAATTCTCCGGTGGAGGTAGCCCGGAACCCACCGGAAGCTACTACGTCGCTCTCGTCAGCGGC
GAGGAGGTCGTCCTCCTTCTCGGCGACTTGAAGAAGAAAGCCTATAAACGGACCAAGGCGCGGCCGTCGCTCGACGACGCGAAAATGGTTTTGAAGAAGGAAAATGTGTTTGGGAAA
AGGTCTTTCTCGACGAAGGCGAGGTTTGATGAGAGGAAAAGAGAGCATGAAATTGTGGTGGAGAATTCGATATCAGGGGTGAATGGGAAGGAGGATGCGGAAATGTGGATCAGAATTG
ATGGGATTGTAATGATGCATGTGAGTAATTTGCAGTGGAAGTTCAGGGGAAATGAAACGGTGATGGTGGAGGGTTCGCCGGTTCAGGTTTTCTGGGATGTTCATGATTGGTTTTTTGGGG
GGCCGGGGATGGGGCAAGCGTTGTTTATATTTAAGCCTGGGGCGGCGGCTGCAACGGCGATGGCGACGGCACTGGCGGAGATGGCCTTGATGGAGGAGGAGGAGGAGAATGAGGAGA
GGAGCGAGAGCGATTTGAGTTGGTCGAGTGAGAACGAGGGAAAGCAGCAGGTGGTTGCAGAGCAGAATGAGTGCGGCGATGGGTCGTCGGAGTTTTGCTTTATGCTCTGTGCGTGGAG
AGTGGAGAAATGAAAAGGAACTATGTAAGATAATTTGATATATATTTTATATTAGTATGAGATTATTTTGATAGAAATTTATTTTCATAGAATTATTGGGAAAGTATATGTGGCCCAATATTTGA
AGTCTGAAGATGCTTATTTCTTCAAAACACATACTTTTTGATTTCTTAATCATTAATTCATAAAAAAAATTAATCCA
>comp145597_c0_seq1
TGGATCTATATATTAAGCAATGTCATATTTGCAATATAACGACTCATGGACAAGGCACGAGACTATATTTATCTCAGTCAGTGTCCAATACTTCTTGGACCAACATTATAGCATTAATTTTCTTA
TAAGTCTTCCATACACTCAGCAAAATATAATAAAGATACAATAATTATAGTTATGGATCAATTCTTCAAAATGGCACACTTCATGTTGTGCAACGTCCCATGCCATCGAGTTCTATTCTCCAG
GAAGTGGTGTGGCTACCCGACATTCCAAGAATGATTATGTCCAATCATGATTTGAGTAATCTCTTGCGGTGTTTTGTTATGTCCGAGTACTTAGGGATTAATTATTATGTACAGACACCGAAG

TTCATGCAGAGATCAATCAGAATTTGCCATGTCAGCCCTTCGCTCCGTGCTATGGCTCGGTAGTGCATATACCGTTGATGTAGCGAATTCTAATTGCTCTCCAGATGTCGTTCTAGATCCGAT
CTCCCCTCATAATAATTTCTGCGGCATGCGAGACTTGCAGCTTTTCAATCCATAATTCCATATACTTCTCCGTCTTATACATGTAGATATAGCGCGATCAATGTCGTCTCAATTAATTGGTGTTC
TACTTTCTCTTCACCTAATCCACACTCCAAAAAGCTTCGTCTCCTTTTTCCCCTCTCCGCTATCCTCCTCCTCCATCAAATGCTTCGACTTCCCATCCTCCTCGTCCGACCCAGTAAGGTTCT
CAACCCCTTCTACATCCTCCACAGGGTTGTCGGCGGCTGCGCCGCCAATGTTTCCGACAGCGCGAACGATATCAATCATGCAAAACGATCTTCTCATCAACCCTCCCTTCTCTTCGCAGCG
ATAGAATACTACCGTATCCCTTGCATTCAAATCCATCTCCTTCACAAACTTGTTCCATCCTTTAGTGAATACGTAACTTTGACTACTCCTCCAATAGCAATATCGAAACACCCATGTCTTATTC
ATCCGGTCACGAAAATCCAGCAGAACTTCCTCCACTGCGTCAGCAGTCACCGCCGGCAAGTATCGAGTGGCATGCTTCTTTGGTATCACCAGTCTATTCAGCTTTCCGACATCACTCGGG
GTCAGCTCCTTCTGAAACATTTCTCTACATATAACGCCTTCGATGAGGACCGGCGGAGCCTCCAATGCGGGTGCGCCGGCGCCACCGCCGCTACCTGTGTTGGAGGATTGAATAGAACGG
ACGAATTCAATGAATTTGGATCCGTAGGAGCCATCCCTGATCATTTCGAGAATGACTTGGGTGGTGAAGTTGTCCTGAAACGCGGGCTCATTGATAGTGATTTCATCCCACGGGAAGTTGC
GGTGGCCGGAGTCATCGCGATGGAGTTTCATGGCCGCGCTGTCATAAGCACGGGCTGCAGCTATTTCGGACTTGAAGGTTCCGAGCCATATGCGACTGTGGTTAACGTAGATCTGAGCGC
CCCAGTTGCCGTTTTGTTGGAGCACTACACCTTTGTACCTCGCTATCACCGGTTCGAGCTCGCGTTGCTCTGCCTCTGTCGATGGAATCTTAGAAGACGAGCCCTCATCGAACAGCTGTAG
CGTCTCCAAAGATTTGGAAGGCTTCAGTGGAAAAAACTCTTTCTCCCGAGGTGAATTCATTGCTCGACTCGAGGTCTTCGATGAATGAAATTTAGGTTTTTGACGAAGACAAGGCGAGC
AATTATATTTTTTGGCTTGCGAAAATGCTGGGGATTGTTGAAGAAATGAGTGGCAAAATTAAAGGCTCAGCTGTGTGGGAATCTCTATTATGGGAAATATTGGTGAGCAAGTAACTTAATTT
AGAAAAATAAAATAAATAAATAAATAAATA
>comp145599_c0_seq2
ATCAACCTGCAACTGGAGCATATTCACCTCGAATTGGAGCATATCGAGGATATCCATCTCTAACTGGAGAAAATCAACCTCCAACTGGAGCATATCCAACATATTCACCTCCAACTGGAGC
ATATCCACCTGCAACTGGAGCATACCGAGCATATCCACCTCCAACTGGAGCAAATCAACCTCGAATTGAAGCATATCTACCTGCAACTGGAGCTTATCAATCTCGAACTGGATCATATCGA
GCATATCCACCTTCAACTGGAGCATATCAACGTCCAACTTGAGCTTATCAACGTCAAACTGGATTCATAGCTTACCCATACTATTGTGCTTATTAAGCTATCAATCATGTTATTTAGCCTCTGT
GTGTAGAATAATGGCTTGCAACAGAGATGTGTAGCATAGCTACCACAGTTTTTGGGTTATTTATCAGATTTGTTTTTATTTTGCTTTGTGATTGGATTTCTTATTTGTTATGTGATTGTGTGTAA
TTAAGGTAGTTTTGGACAGCTTTTTTTAATGCTTTTTAAAGCAATTTTTT
>comp145634_c0_seq1
TTGAATTTAAGCAAACACAATAGAACTAACCATAAGAAGCTGAATCCACTTACAAAATATGAACCACAAAGAAGTAGCAAGGAATACTACGATACATAACATCTTAAAAGTACAAAACCA
GAAGAAACCGGACGACTTTCAGTGCTTCAAATGGCCTTCGTTAGCCTTCTGGAAGATCTTTCCCTGCTCTGCGGCCATCTTCTCCGATTGAATGGCTGTATTGAAGGCTTCCCTGACCTGC
TGAGTGTTAAAATTAAAGTTTTTACTCGCCATTGAGTTAAGAAGCGAAGCTGTTCCTTCACGCAGTAGGGCTCCATAGCCATCTTCACGAGTGTTTCCCAAAGCTTCAAGTACGCTGAGGT
TTCTTCCGAAGAGGATGCCGGCTGCCGGTGACGTGCCAAAGACTTCGCCTATTGTTCCCCAATAGCCGAAGAGAACCCAAATGGCGGTTGGGTGAGTGAACCAGTAGTTGCATGAGAAG
GGTGGGTGGTTCGGGTCAGGGATTAGAGGAGATGGATTGGTGGGGGAGGGGGGAGTGAGGGGAACTAGAGGAGTTGGTGGAGTTTGAGAGATTGGTGTGGTTGGGCTTGGTGGATTCT
CAGGGAGGGAAGGAGTAGGAGTAATGTAGGGTGGAGTGTTGCAGTTACAGCCGCCGCCACCTCCACCGCCACCGCCGGAGCCCCCGTGGGATGGGGTGGTAGGCGGAGAGTAATGGAT
TCCACCGCCATGAGACGGCGTCGTTGGAGGAGAATAATGGATTCCACCGCCGTGGGATGGAGTTGTGCCAGAGCTAGGAGTTTGGCATAACGGGCTGTCATGGGAATGACTTGGCGAGC
TCCATGCATGGGGATCATTGCCATAGTTGTTCTTCTGCTCTTCAACCTCAGCAAGATTTCTGCTTGTGACCAAGCAGAAATGGGTAGCAGACAGAACCCACAAGAGAAGCGAAATCATCA
TCTTCTTCCTCTCCATCCCAACAAAAACGCAAAGCTCAGAAGCTCAGAAGCTAGAAATTAAATTCTCTATCTTAGATGAACCGAAGCAATGGCGCCATTACAGCATTGGCTCTTAAATACA
GAGAAGGAAACAAAGATTTGATTGTAGCCATTACATG
>comp145661_c0_seq2

GAAGATTTCGTAGGGGAGAAACAGCGAGAATATCTGCTTCGTGATATCTGTGCTTTTACTCGCTTGTGGGGAAGGGTGAAGACTGTGAGTCGTTGGCCAAATGAGGCGGTTCTGTTGCTT
CTTGTCTTCTCGAGCCGGTTCCTTTTTCTCCTCTTCCTCTTTCTTCAACACTTTCTCTCTCCTCCCCTCGCTGTTGGCTTTAACTCCTCTCGCTTTCTCGAGCAGAGAGCGCTCTCCTCTCTC
CTCCACCAAACAGTCCAAAATCCTTTTCTTTCTTCTCTGCAACACCTGCCACTATAAATAGGTTCCTCCCTGATTATTTTTTCTCCAAGCTCTCTGTTTTCTTCCTTCTCAGTAGCTCATAGC
CAAAGAAGAGGAGCTTCTTCTTGTTCCCTTGTATGCTCGTGGCAATGTCAGCTTGGAAGGACAGGGAAAATGATCCTCGTATTGAAGGGATTGCTTCCTCAATGCGCGTCATTTCCGACTT
CCCTAAGCCAGGGTTTAAGTTCAAAGACATTACCACGCTGCTCCTCAATCCTAAAGCATTCAAGAACACTGTGGATTTGTTCGTGGAGCGCTACACCGGCAAGGGCATTTCCGTTGTTGC
AGGAGTTGAGGCTAGAGGTTTTATATTTGGCCCCCCTATTGCCTTGGCAATAGGTGCTAAGTTTGTTCCTTTACGAAAACCAAGGAAGCTGCCAGGTACAGTTATATCAGAAAAATATGTT
CTTGAATATGGAACTGATTGTCTTGAAATGCATATTGAGGCTGTCCAACCTGGTGAACGTTCATTAGTAGTTGATGATGTGGTTGCAACTGGTGGAACACTTTGTGCAGCCATAAAATTACT
TGAACGTGCTGGTGCAGAAGTTGTGGAATGCGCTTGTGTCATCGAAGTACCCCATCTTAAGGGCCGTGAAAAGCTGAATGGAAAGCCATTGTACGTGCTTGTGGAAACTGACTGATGCT
GTTTATCTTCTCAAAATCTGCTACTGATAAATTAATATTTGAAGCTTGAAGGGTTAATTAATATCTAGCATAGATTTAGATGGAAGCTGGCCTCATTTTGCCAGAGAATCAAGTAATTTTTTTA
TTATTCTGACTGCTTTTCTGCCATATTTCAACTATGTTACTATGCAATGTAGTCTTTCAAAATTTTTTTGCTTTGCATTGTGGTGTTTTAAGATGCATTATGCTTGCAATATTCAGCTGAACAGA
AATGAATGAGTTGGCTATGCGGATGATCGAGACAAATATATACTCATTTTAAAT
>comp145779_c0_seq3
CTTCATTACCTCTGCAAAAAGTTCAGATGAGATGCCCCTTCGACTGATTCTGATAGCCTCAGCCAATTCACCAATCTGGGAAGCAAGGTGATCAATCTTTTCAGCAAGATTATGCTTTCTCC
TACTACTCTGCCCAGTTCTTTCCGTTGAAGCAGAAGATGAAGGGACCTGTTGGGACCTGGTAGGCGTTTCGTCCACATCCTCTTCAATCTCGAAGTCTTCATCCACGACGACGTTTACATC
GTCAGGAGCTACGATAGCGATCCCATCAGGATCCTCCTTTAGGTAGTTAGTAAAATTCTCAGTAAGATTGTCTTCACTGCAAATGATAGCCATCTCATCGTACATTTCAATAGGTTTGTTCAA
AAATGGCTCATCTTTAGGATTCGCCGCAATATGTGCACCATATCCGACATCATCCATGATGATTTTCTTTTCCTTTTCATCAAATTTTGTGTTGCTCAAACTCTGAAGTCTCTTTATCTGCAAG
AAGCGAGTCTTGATTGTTCTCAGGTGATTTTCAACATTTGAGTCACTACAAAGAAGCTTGAACCTTTCTGTCACAGCCTCAGCAGCAGCAATGAATGCTTGCCTTTTAAAGGTTTTATTGA
CTTTCAATCCAAGCTTAGCCTGTTCTATGAGACATGAGAGAAGGAAACGACTCATTGATGGGCTCCACCGAAAGTTGGTGCAACCTTTCTTATTTTGGCGTTGATACAGTTGTGGAGAATA
GGTCCCTCTACAGCTGATGGGAGAAACTCTTTGAGGCGCTCCTTCATCTCCATCAATTCTCAGTCTTTTATTCATTCCTCCACTGACCAGCTCAGAACAAACTTTCTCAAGATTCTTTACAT
TCTTCGAAGAAAGGGTAATCCTTACACGGTGAACTTTATCTTGTGCCTTTTCCTCACAAGATATAGATTTCATTGCTGGTAAATGCTACTTCTCAGGAGTAAAAATTCATCAATGCTGCCCAT
ATTCCAGGAATGAGATGGCTCCACAGAAACCGATATTAGCTCCAGAAAAACCCGAAGCACGCGTCTTGGAAAATGGTTTCCGCTGCAAGTTCGTAATCCGTTTCACGTTGAAACAGACG
CGCAAGTTGAGCTGAGATTTGGCCCTAGAAAATTTCATGAGGAGAAATTCAGTTTCGTTAAACCTAACTGATAAATGTAAGCCGCGACCTGATGCTTCGACAAGGGGCTGATATCTTCATG
GGAGGTGGATCAATTGTGCCATTTTCTCCATTTATATTGTCCACTAGCAGGAAAATCGTGATGGCAGAGCAATTTGGCAGCCTTCTGCACAAGAAATGGAAGACCGAAGAATG
>comp145806_c0_seq1
AAATCTTTTTTTTTTTTTTAATTTTTCATTATGATTGATTAAATGAGAATAACTACACATGTATAATCGAGTACAGTTGTTTTCATGGGATCGCCTAAATTAGATAAAAGAGTTTAGATATGGTA
AAAGTATTATGCAATATGTATTTATCATCCATCAACTGTTTTAGCTAAGTTATAATTTGCTCTTTGTTGCAGGGCATCTCAGAGTATATTTTTAAAAAGATATACAACGGGATGTACGATATGCA
GACAAACCTTTTAATGCTTATGAAGATGCCAAACTTGAAGGTGTTTGAATCTGATCTGACAGCTATCCCTGTGCCCTACATCATATTAGAGATCTGACTCCTACAGTGGAAGAGAATGTAA
CTGTATTAACCACCTTCTTTATGCTTCCATTCAAGCACCATAAAGAGCGAAGGCATAAATCACACACGCACACGCTTATACCTATCTTTGAACCTTAGATCTCCTAATGGGTGACAAAGTCT
TCAACTAACCAGCCTAAGGAGAGGGCAACATGACTTGTTATCTAAGTTCTATGTATTATTAAACATTTCTCATGTATATTTTATACTTCTATACCCTGTTCATTTTTCTCCCTGCTAAGAATATG
ATTGACCATTCCTTCAAAAAAATATTTAGTAAAATACAGCAGCTTTAGCTTGTAACTTGAGAAGAGGTGCTGATTACTTTGCACTCGCTGAGGTTTGTCATTCCTTTAACAAGCTAAGTAAAG
CCAGCTGTAAATCGAAATTGTGCCCATTGACTATCATGAATTCTGGTCCAGTGGTTTTGTTATCTATGATAGTGGTCTAAAGCTTCTACATGAAGCTTTACAAGGTCTCCGCAACTTTCAGC

GTTAATGATGGAATTTTGGCAGAAAGTTATGCAGATTGAATTCTATGGTGATTGATGATGAAATCATTGTTCAGCTTTGACCTTTGGACAATTAGTCTTCTCTTTTGCACATTGTTGTGGAGT
AATCCCAAAAAGTTGCGTCAGATTGGCGTTTTCAAATGCTAAAGTGGCATCAGTACTGTGCTCTTCATATCAATGTGATTATGTATTTCTGGAATTACGATTTCTTTCTTTCCTTTTTTTTATT
TGTTTATATTTTATCTATCGTGCTTACTTTAAGTTGTCTTCAAGGGTCATCTGCTGCACCTTTGAATGTTGTTAAACTATGAGGTCATTCCTGTCTTATCTGTCTCTCCAGACATTATTGCCAAC
>comp145827_c0_seq2
GCCTATGGTATGAATATGTTTGATTTAGTTGAATGGAAGAAGAAAAACATAACAGGCATCTATCATAAATGGCAAACATTGAATAAAGACAGAGCCCTTTGGAAACTTGGAACTCTGCCAC
CTGGTTTACTGACATTCTATAATTTAACGCACCCACTGAACAAGTCATGGCATGTTCTTGGTTTGGGCTATAATCCGAGCATCGATCGCTCGAAGATAGAAAATGCTGCTGTTATACATTACA
ATGGTAACATGAAACCTTGGTTGGAGCTGGCAATGACAAAGTACAGGCCTTACTGGACCACATATATCAACTATGATCATCCTTACATTAGAAACTGCAAATTGAGAAGATAACTATAGCT
CAACAAAGGCATGATTAAATTGCATAAGAAGGAGTTTTTGATCCTATTGAGGCCTTTCCTTACTCTGTCCAGTTCTAATTTTGATGATGTACATTACATCTTTGTAAATTTATTCATCCTAATA
GGGCACATGATTTCCTTTGTTGAACAATTTACTGGTGCCTGATAGTTGTAGATATAACAATTTAACAGTTACCAGATTGTTATTTTATTCCTTGTAGCTTGTTTATAAAAGTAATGCATAGTTG
GGATCTTCAGCTTGGCATTGTCATGGATATAGTTTTGTTTGTTTGTTTGTTTGTTGTTATTTTTTTTGGCTTGAAGATGATTGTCTGCTAGTTTTC
>comp145843_c0_seq1
CAAGTGTTCACAATCAACTAAGAAAAAAATCTCCATAAATTGAAAAAACTTATAAAAATCATTAAAAAAAAAACAGTAAATCCTGGTACTATAATCTCAGAACAAAAAATCAATAAAAAA
ATCTCTCACATGAATTCAAATAAATGGCAGATCAGTAGAACTAACAACCGCTTCTATTAATCACCTATAAAAGAATAATATGCTTCTTCAAATTAGAAAAACAACTGAAACAAGACTTCATT
TCAGCGCTCTGCTTCACATCATGAATGCTGCAAATCTGCTACTTTCCTGAAAAGCTGTAAACTTTTTTTCTTCAATGCTTCTATTAGAATCAAAACTGTCACAAATTTCATAATCTGAAGCA
GGAAGGTTGAGATCCAGTGAGTTCAGCTCTGAATTCTTCTGCTCAATCTCAATTACTGCCGCAGAAACAGTGGCAGCCGAAGCTGCAGAATTTCCTGCAATCAAATGTGATCTCTTATGA
CCACCCAGAGCTTGCCCTGAAGGAAAAACTTTTGCACAAATTGGGCACTCATAACTTCTAAATTTCTGAGAGATAAGATTTTCGGCAACCGCTGCTAAAACAGAGGAAGAATCCACTCT
GATCTTTTTATGGCTTGCACAGTGGCCTCCTAGAGCTTGATAGGAATGAAAGCTTTTGTTGCAGCTTGCGCACTCATACTTACTTCTCTTGCTAAAATTCTCAATTCCAGCAGCGAAATACC
TTTTTCTTGATTTCCTCGTGTTCCCAGATTCAAAAACAGAAGCAAAATTCACATCTTCCTTGAATTCAGCTTCATCATCATCATCATCATCTCCGTTATGAAACCCAGATTCAGAAGGAAAG
GGGTCAACTTTCTTGATCCCAATCTTCAAAATATCAGATTCAGATCTCTTCACCTCATTTCCGTCTGAAAAAGAGCCTGCGTCTCTCGAGAGCATCATCAGACACATGGCACCATACTCTG
GTGAGGAAGGTGCCGGGGAAGAAGAAGCAGAGATTGCTTCAACCAGCTTTAATCTCATTCCCTGTCTCTTGTTTCTAGGCTCTTTGCATTCTGTTATCACCTCCAGTTCTTCTTCTTGATCA
TCATCAACTCCAGTGCTCCATGTCTCTTTCTCCTCCAGCTCCACATCCTCATCATCAAACCACCGCGATTTTTTCGGTTTCTCTCTGAGTCCATATCTGCTCTCCGAGCTTCCCTTGGAATTG
AAGGAAGATTTGGTCATGATATGAGACCTCATATGGCCTCCAAGGGATCTACCATTGGGGAAAGCTCGTTTGCAAATTTTGCAGAAGTGCGTTTCCATGGAAAGAAACAGGGGAAGAGA
GAAGGACGTGAGAGAAGAACAGATTTTACTCAAATAGGTTCTAGTAATCTGGAGAAATTCGTATCTGTCTTGTTGTTTGAATCGATCTGATACGAACACATTTGGTTTTTGATGGAAAACA
ATCTCACGCGTTTCAGTCCTAACTCTGCTCAGCTTCTTGTGTTCCACTGTTCGAG
>comp145874_c0_seq2
AAAAAAAAAAAAAAAAAACATCATGTACACTCTAGAAATTGATTTAGTACTGCAGTGTTTCTCAAAACCTGAAGTTCTTGCCCTCGAATGTCTGGCCAAACTGCCACTTTGAAGGAACA
GCATGCCAAGAGGTGGAGCGGCGGCCATCACCGCCGGTGACTCTGAAGGTGAGAGAACGACCGACGAGGATGGCATCGGTTTGCCAGTATTGGCCCCAGTTATGGCTTAGTGTTATCCA
TGGCAGCCTGTGCCCTTTCACCTGAACTCGGCGAACATCACCAGCACCGGCGACATTCCATACTAGTATCAAGTTGAAGTAACGGTTGCCGGTAAGTGTGAATCGGATTCCTCCCTCCTTC
TTGCATGGAACCCTGCGGTAAGAGACGGGAACAATGCCGGCTTGGTACTGTGCGATATGGAGAAAAGCCGGCTGCGTGAGATCGAAGTGCGGCCGCGGCGGGTTGCACCAGCCGCCGT
TGTCGCCGGGGAGGTTGTAGTTTGGCGGGCAGAGGTTGGTGGCGGTGACGAAGAGCGAGGGCTCGCCGGCGTTGCACCACTTGGGATCATCCACGCACTTTATCTCATAACAGGCGCCG
CAAGAGAGCCCATCGTTGAATAGCGCCGAGCTCAGCGCCGCCGTCTGTAAGCCGTAGCCTTGCGCTACCGTGTCCCAATACCCGCATGCGCCCTCTCTTGTTTGAGAACCGTCACTGCCT

CCATAGAATGTGGCGTGTGCTGGGTACCATGGGCCGGGCTCGAACCTGCCATGGTACCTGTGCCTTGGGCGGGCCGAATAGGGCCCTGGCTTCCTGTTCAGAAAGGCTCGCCGGAGCTC
GCCGGTCCGGCCATGATGGTGGTGCTGGCCAGAAGCTTTCGTTAAAAAAAGCAACAAAAGGAACAAAAATGCCCGCGTAGCTCTGTAGAAAACTCGCAACGATTTTGCCATGGCGTTGT
TGGTGAATAGTTTCTGTGTTCTTGGATGAAATTGATAAAGCTGTAGTTATATATATAAGCGTATAGGCTTAGTGGTCAGAATTGGTTA
>comp145874_c0_seq3
TTCATGCACAAAAAAAAAAAAAGTTAATGCTCAGAATACAAGGCGTTTATTTCATAATTGTAATTTTTAATTATTTTGTAAAACATTTTTTGCAAGGAACAATTTTCTCGAAATGTCCAAAAA
AAAAAAACAATTTTCTCGAGTACTACATTAATGAGTTGTACCATTTGATTACCTCTTGTTTGAGAACCGTCACTGCCTCCATAGAATGTGGCGTGTGCTGGGTACCATGGGCCGGGCTCGA
ACCTGCCATGGTACCTGTGCCTTGGGCGGGCCGAATAGGGCCCTGGCTTCCTGTTCAGAAAGGCTCGCCGGAGCTCGCCGGTCCGGCCATGATGGTGGTGCTGGCCAGAAGCTTTCGTT
AAAAAAAGCAACAAAAGGAACAAAAATGCCCGCGTAGCTCTGTAGAAAACTCGCAACGATTTTGCCATGGCGTTGTTGGTGAATAGTTTCTGTGTTCTTGGATGAAATTGATAAAGCTGT
AGTTATATATATAAGCGTATAGGCTTAGTGGTCAGAATTGGTTA
>comp145893_c0_seq2
CTGCAACAACAAAGAAAAACATTCTCAAACAAGAGAACAAAATTGTTCTCTAAAGATCAACAAATCAGAAGTTAAAGTTCAATTGAAACTAATCAATCTGCTCTAAGAATTACTAAGCAT
TAAAGAGGAAAACTTGGCAAGAAATAACAACAAACATAAAAAAAGGAACTAAATATATCCTCTTGCCTCTTAACCCACAGGCCCCCACTTTGCAACATCATTTCCCCACATGGCACAAAA
GCAGAACTACCAAAATTTGAAACCACTGGAGAAAAGGTGGAAGAGGCCTGATTCCTCCAGTCTTTTTCCCCACAATACACAAACCTATCTCCTACTTTCTCTTTTTCACCTTCTCCTTTAC
TTCATATCATCTTTGGCCTGCTCTCAACATAAGAAAAAAGATTCAAGCATCATTCCATGCAGCATAAACAGGCGTTTGAGCCATAGGAACTGAACTCCTAAGCTGGTTCGATGTCGGCCCC
CAGGAAATCAGTTGCGTTGTAGGACCGACAAACTTGGTCGACAGAGGCGGCATCGAGGCCATAGGAAGAACACCATAGCTTGTGCTTCGTTCCCTTGAGCTGCTCGAGTCACCAGTCGC
CAAAGATACAGTATTCATTCTGTTGGGATTATTCTCCATGCCTTGCTCTTCTGAACTCCAACTTTCCATTCCAAAAAACCCATGGAAATTTGCCAAGAATGAACTGTTCTGATGATTGCCATT
AGCTGATATAGATTGGTGATCCAGTGGTTCTGAGTTTGCTGGAGCTTGCTGTGGTGACTGGTAGAGTGTCATTCTCCAATCAGATGCACTGGTGTTGTCTTCAGTAGAATCAAGTTCTCTA
CTGTTGTGTTGATCTGTCAATGCTACATTGTCGATAATTTCGATAACTCCATTTCTTCGAGCAAGTTCGCCGGACAATAGAGTGCTACTTGCCATGATCTTATCAACATCATATCTTGACATGA
TGAAATTTGTTACAGCATTCGTTCCGCGAAATTTGATTGCAGCAATGTCATAAGCTTCAGCTGCTTCTTCTTGTGTACCAAAGGTTCCAAGGTAAAGATCTTTGTTTCCTGAGACTCTCCCA
ATGCGAGCTTGCCATCTTCCATGTTGGTGATGCCTAGTGACTCCGCGATATATAGAAGCCCCTCTAGAAAATCCACTGCTTTTCCTTCTTAAGTGTGCAACATATTCCTGCCTACTCATGTTC
TTCATTTGTACAAGCTCCTCCTGGTAATCCTCCAGAGAGAAATTGATGTGGGTGGCAAGTCCCCAATACTTTAATGCAGCCAAGTCATAGGCCCTAGCAGCTTTCTCTTCCAAATCATAACC
CCCTAAAGTTTACAAAATGCAAAGAATAGGAGTTTGAGCATTGAGGAGGAAGTTTAATGAACAAGTCATGAAAATCTCAAATAGAAAAGAAAGGCGCATATTTTTGGTAAAGAAAGTAG
AGGCATACCCAAATAAACTATTGTTGAAGTTTTTTGTTTCATGGTACCCCAAGGAAAGAACATGACAAGGAACCAAAGTTAGATAAAATATTAACAAACTTTATAAAGGGAAATAAAAAA
AAAAACAGACCACAAAGCTTCAAACAAAGATTTTTCTTAACCAACCTTGTCTTCCCTTCCT
>comp145893_c0_seq3
CCTGCAGCCTACCAACTAGCTGGTCATTATTGGGTTGTTAGCAGTTTATTTTATATAAAATCTCATCAAATCATTATTGGGTTCTTAGCAGGTTTAGGGTCTGCTGGTTGGTTTACTGTTGCAA
TGATCATTTTTTTTTTTGAAACTAATCAATCTGCTCTAAGAATTACTAAGCATTAAAGAGGAAAACTTGGCAAGAAATAACAACAAACATAAAAAAAGGAACTAAATATATCCTCTTGCCT
CTTAACCCACAGGCCCCCACTTTGCAACATCATTTCCCCACATGGCACAAAAGCAGAACTACCAAAATTTGAAACCACTGGAGAAAAGGTGGAAGAGGCCTGATTCCTCCAGTCTTTTT
CCCCACAATACACAAACCTATCTCCTACTTTCTCTTTTTCACCTTCTCCTTTACTTCATATCATCTTTGGCCTGCTCTCAACATAAGAAAAAAGATTCAAGCATCATTCCATGCAGCATAAAC
AGGCGTTTGAGCCATAGGAACTGAACTCCTAAGCTGGTTCGATGTCGGCCCCCAGGAAATCAGTTGCGTTGTAGGACCGACAAACTTGGTCGACAGAGGCGGCATCGAGGCCATAGGA
AGAACACCATAGCTTGTGCTTCGTTCCCTTGAGCTGCTCGAGTCACCAGTCGCCAAAGATACAGTATTCATTCTGTTGGGATTATTCTCCATGCCTTGCTCTTCTGAACTCCAACTTTCCAT

TCCAAAAAACCCATGGAAATTTGCCAAGAATGAACTGTTCTGATGATTGCCATTAGCTGATATAGATTGGTGATCCAGTGGTTCTGAGTTTGCTGGAGCTTGCTGTGGTGACTGGTAGAGT
GTCATTCTCCAATCAGATGCACTGGTGTTGTCTTCAGTAGAATCAAGTTCTCTACTGTTGTGTTGATCTGTCAATGCTACATTGTCGATAATTTCGATAACTCCATTTCTTCGAGCAAGTTCG
CCGGACAATAGAGTGCTACTTGCCATGATCTTATCAACATCATATCTTGACATGATGAAATTTGTTACAGCATTCGTTCCGCGAAATTTGATTGCAGCAATGTCATAAGCTTCAGCTGCTTCT
TCTTGTGTACCTGGGATTATAATTTACTTAAAGTCAGCAAGATCTGGTAATATGGACATGGTGGATTAGTCAAACAAGAGTTTTGTAGCCACAGAAGAATCAGCCTTATAAAAAGGAGATG
TACTGTTGAGAGAGAATTAAAGAATTATGAAAAAGCAGTTGTTAGTCTGAAATGGCTTATTCTGAATGTTCTTTTTGAATGACAGGTAAGGTACATTGATAAATTTTGAGAATAAGCCAGA
AACAAAATAGCATCAAGCATTTGAGAAAATTAAATGGAATGTATTGTAAAAAGAATTAGAGGAGCTAACCAAAGGTTCCAAGGTA
>comp145893_c0_seq4
CCTGCAGCCTACCAACTAGCTGGTCATTATTGGGTTGTTAGCAGTTTATTTTATATAAAATCTCATCAAATCATTATTGGGTTCTTAGCAGGTTTAGGGTCTGCTGGTTGGTTTACTGTTGCAA
TGATCATTTTTTTTTTTGAAACTAATCAATCTGCTCTAAGAATTACTAAGCATTAAAGAGGAAAACTTGGCAAGAAATAACAACAAACATAAAAAAAGGAACTAAATATATCCTCTTGCCT
CTTAACCCACAGGCCCCCACTTTGCAACATCATTTCCCCACATGGCACAAAAGCAGAACTACCAAAATTTGAAACCACTGGAGAAAAGGTGGAAGAGGCCTGATTCCTCCAGTCTTTTT
CCCCACAATACACAAACCTATCTCCTACTTTCTCTTTTTCACCTTCTCCTTTACTTCATATCATCTTTGGCCTGCTCTCAACATAAGAAAAAAGATTCAAGCATCATTCCATGCAGCATAAAC
AGGCGTTTGAGCCATAGGAACTGAACTCCTAAGCTGGTTCGATGTCGGCCCCCAGGAAATCAGTTGCGTTGTAGGACCGACAAACTTGGTCGACAGAGGCGGCATCGAGGCCATAGGA
AGAACACCATAGCTTGTGCTTCGTTCCCTTGAGCTGCTCGAGTCACCAGTCGCCAAAGATACAGTATTCATTCTGTTGGGATTATTCTCCATGCCTTGCTCTTCTGAACTCCAACTTTCCAT
TCCAAAAAACCCATGGAAATTTGCCAAGAATGAACTGTTCTGATGATTGCCATTAGCTGATATAGATTGGTGATCCAGTGGTTCTGAGTTTGCTGGAGCTTGCTGTGGTGACTGGTAGAGT
GTCATTCTCCAATCAGATGCACTGGTGTTGTCTTCAGTAGAATCAAGTTCTCTACTGTTGTGTTGATCTGTCAATGCTACATTGTCGATAATTTCGATAACTCCATTTCTTCGAGCAAGTTCG
CCGGACAATAGAGTGCTACTTGCCATGATCTTATCAACATCATATCTTGACATGATGAAATTTGTTACAGCATTCGTTCCGCGAAATTTGATTGCAGCAATGTCATAAGCTTCAGCTGCTTCT
TCTTGTGTACCAAAGGTTCCAAGGTAAAGATCTTTGTTTCCTGAGACTCTCCCAATGCGAGCTTGCCATCTTCCATGTTGGTGATGCCTAGTGACTCCGCGATATATAGAAGCCCCTCTAGA
AAATCCACTGCTTTTCCTTCTTAAGTGTGCAACATATTCCTGCCTACTCATGTTCTTCATTTGTACAAGCTCCTCCTGGTAATCCTCCAGAGAGAAATTGATGTGGGTGGCAAGTCCCCAAT
ACTTTAATGCAGCCAAGTCATAGGCCCTAGCAGCTTTCTCTTCCAAATCATAACCCCCCAAATAAACTTGTCTTCCCTTCCTTGTCTGCCCTTCCTTCTTGCAACTGTTATCCCAAAGGTGA
GCTTCATATCTACCTGTCCATCTATGCCTTGTAACTCCTCTGTATTGAGAAGTTCTCTGGCCAAATGTATCAATGGACTTCCTGTGAACTGGCTGCTTCTCAGCTCCTTTTCCATTTGCTCTCT
TCTTTGAATGCTCCAAAGCCAACTGCTCATTTTCTGAAGCAGAAGTGATCTGCTGCTGTGGAGCAGTTATACAGCTAGATTGAGATCCAGGGCTCATGGATAAGCTCAAGGGCTTGAGAT
CACCATGCCTCAGTAATCCAAGAGAGCTAAAACCCATCTCTCTTTCATCACTAACTCCTTCATTGCTTTGGAATCTACAATCGGACATTTGATCTCCGACCAATGAACCCTGATACATCTCA
TGCAATGGCTGAAAGTAGTTTTGGTGCCTGAAAGGTTGTTGAAAGAGCTCAAGAGAATGGTTTTCATTGCTCTCGTGCTCTATGTTTTGTCCATAATACATACTGTCCAAGCTTAGAGCCA
TTGCTCCCCTGTCACTGTTGCTAAACGGATGGGTCCCCATGTTAGGACCACAGCCCAGGAAGTCTTCCAGTTTTGGGGATGGAGATGGCACCATTCCTTCTTGTGATCTGCTGAAGGCTTC
CATAATGCAGAGAGAACCATCAGACTTAACTGGCATGACTGATGAAAGCTGAGAGTAAAACCCTACATTCTCTCCATCAGCTCCATAGCAAATGCCAGAGCTGCTGAAATGGGGATAAGA
AAGAAGAAAGTTGCTTGGAACAAAAGAGGAAGCGTTAGCCGGTGAAGATTGATGAGTAGCTTGAAGATTTGTCTGCTTAAGGGGATCTTGAGCAACTTCCATTCCCAGCTGAGGGGAG
AGAGAGAAGCCCAACCAGTTGGTATTGTTCATGGACTTCATCTTTCTCACACACAAACTAAACTCAGGGGAGCTCAATCATTGGAGAGTACTAAATTTCATCATACATCCTACACGCTACT
TCACCATCTTTATTGAAGCCTGGGTTTCTGTGTTCTCAACTTGAAGAAACTCAATGCCTTGAACTGTTTCAGGGGGAGAATGGAAAGCAAAAGAAGTTGGGAGAG
>comp145911_c0_seq1
CTCTCTCTCTATATATAGGTGTGTGAAGACAGAAGACTGTAGAGCATCGATATCCCTTCGTAAATCTCAAGAAGGATGACAGAGAAAGTAGCAGCGATGGGCCAGCAAAACAACAACGA
GACAAAGGCGTCTCATAAGAAGAAGAAGAGCAGCAGGTTTATGGGTTGCATGAAGGCCCCGCTCAGAGCCTTACGATGCGCTCGTGACATGTACGTGAAAAGCCTTGGTGGCTGCGCA

GGAGCAGGTGGAGGTGGGCGTGGAGCCCCAATGACGGGAGTTACTGCAGGGATGGTGGCGATGATGCCGCGCGCTCGCAGCGTCAACGCATTTGGGAACTCAAATGGCTTTGGAGGAG
GGAGTGGCCGGGATGATGATTTTAAGGAGCTTATTCGCGCGAATTCTAAGCAAGATCATGATGATGCAGCTGCAGGGATGGCACAAACGATTAAAAGGAGCCAAAGCGTAGCGCCTGCTA
TTGGGAGGATTGATGAGGATGCTCCATGCGATGACTTTGATGGGGATATTATTAAGTTGGGGATTCCAAGGAGCAAGAGTTGCGCGGTGGTGCCAACTGCAAGAAGGATTAATGTCCTCG
GTGTTACGCCCACGGCTTGATTTTGTTTCTTTTTTTTTTCCCTCATGTGATCGAGGGTGTGTGCGTGTGTGTCATGGGGTGTGGCGGGCGCAAAGATTTCTCGTCTAACGTTTTGTTTTGA
GGTATGTGCTATGTGCATGTAAAGCTTGCTTTCAAGTAGTGTTTTCTATTATTTTAAACTTCTAATGATTTTGCTGTGTGCTAACTTGCCCTCCGTTGGCGTTCTTTTGTTATAAAATGAATCA
ATCACAAATATCTCAAGGATTTTTATCAAGACATATGCCACTATTGACTATTTAAGTTCTTTTAATATATATATATACGTTTAGTGCATTTAAAAATATACTATATACAATTATTATTATTAATTCCT
ACATCAAGCTTTCTTGAAAATTTTAGTTTGGGTCCTATTTCTTCTATGAATTTTAGGACATGCAATTTAACGTTTTCTTGTAAATGTAATTTTGTTATTTCAGACACTACTAAAAAAAT
>comp145934_c0_seq1
TGGCTGATCCAAATACTTTTTTTTGTTCTCTTTAGTTAACTGATCATCAAGCAACCACCGCTACTTCCTTCGGCACCACCACCGGAAACTGATCAATCTCCCCCACCCACGGATTTCCCTTC
CTCCCAACCTCCCCGCATCCCTCCACCGTCCTCATCGCCTTCCAGACCGCGCTATTACACTTGAATCCCGATCCGAGCGCAATCTGCCACACCTTATCCCCCTCCTTTATCCTCCCCTTGGC
CTCACAATACGCCAATTCATACCACAAAGAACTACTCGAAGTATTCCCGAACCTATAAAGCGTCATCCTCGACGGCTCCATATGCCACTCACTCAACCCCATGCTCTTCTCCAGCTGATCC
AGCACCGCTCTGCCTCCGGCGTGGATACAGAAATGTTTGACTGCTTTTCGAAAGTTTGGAACATAGGTACTCTGTTTTTTGTTTTGGAGCAGGACGCGGAAGGAGAGGAAGGCGAGAAG
AAAGCGAAGCTGTTCGGAGAAGGGGAGGATGAGGGGGCCGAGGGAGGAGATGTTGGTGCGGAGTGCGGTTGCGGCAACGGACATGAGGTCTTTGGAGAGG
>comp145941_c1_seq1
CTGCCAGAATACTCTGGATAACTGCACAACACCACCTATTAGAGCAACATAACATACAAGGCATAAAATGTAGAAAGAATTTTAGTGTTTGCCAATGGTAGCCTCCAAATACCAAAAGTTC
AATAGGTATTAACGACTAACCTTCAAATGTTAATAAAAAGTACAAAAAAAGTTAGGTAATTGATGTAATGAGTAGTTAATAGGTTCATTTTAGGTATTACATAGTTAATAGGTGTTTGATAGC
TCCTTATGGGTCTTATAATGCTATAAATGCCCCACTTGCGGGGGAGTTAGAAAATGGAAAATGAATGAACTAAGCTTTAGCTGCA
>comp145944_c0_seq1
TCTGCTTTAATGCTTTAAAAAATAGGGAACACCTTTTTTTTTTAATGCGGCTATAATTTTGCTATTCTTAAGAACCTTATTCCTCAAATGGACAGTATGCATTTACATCCCAACCTTTTGCAAG
CTTTTAGTTGTATTGAAGATTGGGATATTCATAAGGATTGGAGAAGCTTCTACTTCTTTTTGCTATGTGTTGCCCCTTTTACTAATTTTGGAGAGCTAGGAATGACATATTATTTGGCAACTCT
TTTGACTATCATGCTACCACTGCTGTCAAAATTAGGAGAGTTGTCTCTTATAAATTCTACAAATGGAAAAACTATCAACTTTTTCAATGTCTGCTTCTCTGATCTTATCGGATGGTTGTGCTAT
ATTTCTCAATCCTACTTTTGCTGATTGGTTTTGGCAGGCTGGAGGCTTAATTTGTGATCTCTAGATGATTTTTGGCTCGATGAATGGTCTTTTCTTGACAACCAGGTATCGGTTTTGAAGGAC
AATGATGAATGCATGTTTATCTTTGCTTCCCCTTGGACAGTCGGCTCTGGCATCCAGGATCTTTTGCCCATCTCTTTAGATAATTTTGCTGCTGGGTGATCTCGACATGCTATGCATCGTAAG
TCCTTCATTCCTTTTGTTCATTCTATTCTTTTTAGCTTCTGGTTTTGTATTTTCTTTTTCAGCAGCACTTGATGTATGTTTCTTTCATTTGCTGCACATTTTTGTTGTGCCTGCATATGGCTTCGG
CCCCAAACTTTCCCTGAAAAAAAATTGTACAAATATACATATAACATTATTGAATTT
>comp145985_c0_seq2
AAAACAAAGAAAAAGAAATTAATTAGACAAAATGTTGGACTCAAACGATAATAAAAACATCGAGGAACAAGAAATTTGTTATCAGTAGCACTCCTGATTTCCATATAGGAGAATTCAAGA
CCATCCAGGTAGGGAATCTAGTTGACATAAAATGGTCGTCATTATCTTGAAGCTAGAAAAAATAAAGCATACACATGCTGTCAGGAAGAAGAAGAGCCACCCAGTTTAATACCTGAGAAG
CAACTAAAAGTTTTACCAAAGTTTTCATCTGAAAAGTTTGAGTTAAATAGCCTTAAAAATGCTAGCCCGAATATTATTAAGACTACAAAATTATTGTATGAACTTTCTCCCATTCCTTTCTTT
TGATATATAATAAACAAAGGCCCAAATAGATAAATATAAATAAAATTATATGTCAGTAGGTATGTCCATATGGTCAGCCACAAAATTCAAAATTTGCTGCAGGTTTGGCCTAAAAAGATAAG
AAGAGCATCAAGACAAGATATTGCAAAGCAAATGAAAAGTAAAATCACATTTAAAGTAAAGTTGACTGGTGGTTTTATTCGAATTAAAGCATAAAACACATTTGTGATACACCTGAATGTT

TCTCTTAATCAAATTATTAGTAGTTTTCAAACCCCCCATTAATGC

>comp146092_c0_seq1

TATGATCAAACCAGCAGCAGTTGATGATTCAATTCAATTAATCATCCCAAAATGAAAGTCAAAAAACTACTTGCTAAAATTGACTCCCATCGTTACGAAAAATACTAGCCGTTAGAACAGC
AAAGAAACAGCCTTTGTTTAAATAAGAGCGAAGGAAAAGATGGTGGCCATCCATCTTAATTCTTCCTCTCCATTCTGTCTCAACAGAGCGCCAATGGTGGGAATGTCGCTGGATTCAGGC
AAAGCTCAGGCGGTGGTGGCAATAGACAGAGACAAAAACAGCCTACAAGCCGTGAAATGGACCGTCGACCACCTCCTGATCAATAATTACACCATTGTCTTCCTCCATGTTCGACGGCC
TTCCATTAACAGCCATTATGGGTTTGCCATGGCAGAGACCACTGATGATGCGGCAAGAGAGCAGTTTGAGGCTGAGATGAACCAGCTTTTTCAACCTTACAGAGGTTTCTGCGTCAAAAA
AGGGGTGCAGGTGAGGGAAGTAGTGCTAGAAGACATGGATATCTCTAAAGCCATTGTGGACTACATAGCAACTAACAAAGTCCACAATGTTGTTGTTGGTGCCTCCAGTAGGAATGCTAT
CACAAGAAAATTTAAAAATCCTGATGTGCCTTCGAATGTGGCCAAATCAGCTCCAGATTTCTGTACAGTATGTCATATCCAAAGGGAAGCAAGTAAGTGTTCGATCAGCAAGAAGCCCTG
CCCCTGCTAATCACACACCACCGAGGCAAGCACAGCCGCTTACTGTAGCGCCGAGCAATCATCTTGGCTCGGATGATTCTATCAAAGCACCATTTGTAAGAGGAAATTGGAGAGGAACA
GTTCCACCACCACAAATTCCAGAGAGGAGGTCTTTTGAGAAAAATGAGCTGATGAAACCTCCAACAAGAGAAAGATTTGTAAATCCAATGAAAGGCTCACCATGTCATGCTTTGCATGA
CAGTTTTGACGTCTACTGTTCCCAAAGGCCTGCAACTTCAACGCCTAGGGAATCCTTTTCAGAGGATTTTGATGTTCAAAGAAATATGGGATTCAGAGTTCCTGATTTCAGTGAGTCATTT
GAGATGAATATGAGCAGCATCACTGACATATCCTCAACAAGTACTAATGCAAATAGAGACATAGAAGCTGAGATGAAAAGACTGAAGCTTGAGCTGAAGCAAACCATGGACATGTACAG
CACAGCGTGCAAGGAAGCAATCACATCTAAACTGAAGGCAAAAGAGCTTCATGATAGGAAGATGGAGGAAGCTAGAAAGGTAGAGGAAGCAAAGTATGCAGAAGAAGCAGCTCTTGC
TCTTGTAGAAAGGGAAAAGGCCAGATGCAAGGCTGCTATTGAAGCAGCTGAAGCAGCTCAAACCCTTGCAGAATTTGAGGCACAGAGAAGGTTAAATGCAGAGCTTAAGGCCAAGCAT
GAAGCTGAGGAGAAGTTGAGAGCTCTGAATGAACTGGCACTTAAGGATCAACGCTACAGGAAATACAATATTGATGAGATTGAGATTGCCACTGAATACTTCTCAGAATCACTAAAGATT
GGGGAAGGTGGATATGGGCCTGTTTATAAAGCTTCACTAGATCACACTCCAGTTGCCATTAAAGTTCTGAGACCAGATGCAGCTCAAGGGAGGAAGCAGTTCCAACAAGAGGTTGAGGT
TCTGAGTTGTATAAGACATCCAAATATGGTCCTACTACTTGGTGCCTGCCCCGAGTATGGATGCTTGGTGTATGAGTTCATGGATAATGGAAGCTTGGAAGACAGACTATTCCGACGTGGG
CACAGCCCTCCTATCCCATGGCCGGTGAGATTCAAAATTGCAGCAGAGATAGCCACTGCTTTACTCTTCCTCCACCAGGCTAAGCCAGAGCCATTAGTCCATAGAGACCTCAAACCAGCT
AATATACTCCTTGACCACAACTATACCAGCAAGATAAGTGATGTAGGCCTCTCAAGGCTTGTTCCTCCTTCAGTAGCTGACAGCGTAACCCAATACAGAATGACTTCAACTGCTGGAACCT
TCTGTTATATTGATCCTGAGTATCAGCAAACAGGAATGCTTGGTACTAAGTCTGATGTTTACTCACTTGGGGTTCTTCTGCTTCAAATTATTACTGCTAAAGGCCCAATGGGACTAACTCATA
TTGTAGAAAGAGCTTTAGAAAGGGGAACCTTAGTTGATTTGCTTGATCCTACGATAACTAATTGGCCAATGGAAGATGTTATAAGGTATACAAGGCTTGCACTGAAATGCTCAGAGTTGAG
GAAGAAGGACAGACCTGATCTTGGGACTGTTATATTGCCAGAACTTAATAGGTTGAGAAATCTTGGTTTTGAATATCAAGCTAGTTGTAATAGTAGTAGCAACGGTAGCACTGATGGTGGA
AGTTTTGGAGTGCATCAAAGAGCTAAATCTGCTTGTCAAATAGGTTTACCAACGGCCAAAAAAAATATTATTTGAGAATGGAGATTTAAAAATGTAAGGCTTTTTTTTTAATTATTTTTGAC
AGATTTTGTCAGATTTTTTAATCTATGATGTAACTCTTTTTCTTATCTTCTCATCTTTTCAGCACAAAAGCTATTAATGACGATTAATTCATATGGTATTTTACTATTTC

>comp146112_c0_seq1

TGAGAAAAAATACATTTTGAATGTGTGTCCGAAAGTTAATAAGTTTTAATTGTTTGGCGTTTGCAAATTAATGAGATGAAACAAGTAACTAAAGAGTTTGTAAAGGGGATAGGAAACGATT
CGTTTTGATCCATGAACAGAATGTTGCGAGTTAAATTTAATCTGAAAAGATTTCAAAGAATCAATAAACAATGGTGGATGCTGAAAGACTTAACATAGTTCACAAAGCCCCAGTTATGTTT
TGAGGAAACCAATGAGATGATGCACAAAATGAAAATTTTAAGAAAAGAAAAGAAAAAAGAGATTAGTGACGCATAATTCAATTAGAATCTGATCAGTAATGGCAATCAAAGATCGCAGA
TGTCAGCAACAGAATCGGTGCTCGGCTCTCAAAGGAGAAAGGCGGTGTCTTTGTCGCGGAAACAGAGGAAGGAGACTTCGCTGGATTGACATCTTCATCGCAGGGAGCAGGGAGGTTG
AGATCCAGGGAAAGACCGTTCGAATTCCTCTCCTTCTTCGCCGGTTCCGGTAGGTTTGCAGGGCGGTGCCGCCGCATATGTCCGCCGAGAGCCTGACCGGAACTGAACTCCGCGCCGCAT
ATCGAGCATTGGTGAGTTCTGGCCTTTGCGGCGACTTTCGAAGGGAATGAATTCATGCTTAGCTGAATCGAATCCTCGTCCATCACCATCGTCTTCGTCGCATTAACCAGAAGCTTGGGTT

TTTTATGGCTCGCGCGATGCCCGCCGAGCGCCTGAAAGGAGGGAAAGCATTTACCGCAACTCTTGCATATGTAGATGTAAATTCCAGCGCGGCCGCCGGTGGTCGTCGGCGCCTCTACGA
ATCGCTTGCTGTCAGATTTCATGCTGGTAGTCGGGTCCTCTGTTATGTCAAGATCTGGCCTTTGATCGCCTTGGGCAAGGAGCATGAGACATTTAGCCATTTCTTCGTCCTCCTCAGTTACG
CAATCAGAGAACTCCGCGGAGGTCGACGTATACGACACAGCCGACGCCGCCACCGGCGCAGCCTCCGCCAATGGAAGCTTTAACCATTGCCGCTTGGTTCTCTTCCTTTTCACCATGGGC
AAATCTTCCATTATACCTTCCATTGATGGGTAAGAAACAACCTTTTGTCGAGAGAAGTGAAGGGTGAGGAAGGTTAAGGGCTGTTATATATACTGAATTGGATTAGAAGCTGTGGACTTTA
ATTTGCGAGTCAAAACCGGACATAAAAAGCCGCAAAATCACTCCTTTTTCCCTCCCCCTCTCTTTAAAAACCGCAAATGTTCGCTATCTTTCCGTTTTCAAGTTCACTTACTTCTCTCCTAT
GGTTATTAATGGAACA
>comp146147_c1_seq1
AACTTTCAGAACTACACATTCTAGTAGATTTTGAATGTGAAAAGCTTATTCTCACTGTAAAAACTACAAGAAAAATGATGAAATAAATCTAACAAAATATAGAGAGCAATGACTTCCATGT
CTTACCTCTCTAACAAGTTTTACCAAAACAGATGCATATTTATTTTAATGTAAAAATATTTACAGCATGATTACAAAGGTGGAGCTCATAGTTCCCCTACCAAGTTACATAAAATAATAATAA
GGAATAATGATGACTCCCCCTTATACAGTCCCTGAATTACTATTTTAAATAAGAGGGAGAAACAAATTATCTAAAATTTAAGGTGAAATTTCTATAATATAGATAATTATATAAAAAAGGAGT
TTAAGTTCTTTTCTATAGATAATCACATAATTTTTTTTTCTTGAATTTAGAAGAGATCACCACTATACCTTTTTGTATTCTACCAAAGATCTTATGAATGATTAAGGCTTCATTTGAGACGACTT
TCTTATTGCTTCATAAAGCAGCTATATAGTATGAAAATTAAAATTTTTATACTGGAAATCTGCTTTAAGAAGCAAAAAAAAAGTTGTCCGAAACGAAGCCTAAATATGATAAAGCTACTATT
GATGATGATGATACCTTCACAGCATCTTCTCAAAATTGTCAAGCTTCTTAACTCTTTGGAATGATCTTGCCAACAGCAACAAGAAAACACCAGCACCAATGAAAGCT
>comp146179_c1_seq1
TCCTGTTGTTGACGCTGCTCCGTCGGGATGGATCCTGAGGTCGACGCTGGAGCAACAGGCTTACTACTTTCTGGTTTATTTTTAAGAGAATCCTGTTGAACCTGCTTCTTCGGGTCCTCAC
GGCTTTTAGCAACAGTACCAGTAACAACACGTTTAGTCTTTCTCTTTTTAGGCATGGCTTCAAGTCTGCAATACGCCGGAAAGTGTTCTGGGTATTAGCCAAGACCACCTCTTCCTCGCCG
GAAAATCCCTTGGCCTTTTCTCAGTGGGACTATATATATAAAGAGTAGGGAGGGGAAACCGGGAGCTGTCACGATCAGTTTCGCTCCCCTCCAGGTTGGATATGCTAAAAACGATGGCCG
CA
>comp146180_c0_seq1
ACCACCCAAATCCCCCTCTTCTCTCCAACAACCATTTCCCGCCTCCCAACCGCCTCCTCTGCGCCGCCTCTCCCCTCCAACCTCCACCCGCTACTTCACCGGGCAAACCACTCATCCCCTC
GCTTTCACCAAACGTAAAAGCTATAAAATCCTCCCCGCTTCTCCCATCTCAGAACCCGCCCCAAGCCCTAACTCGATAGACAAATCGAGTTAAAGAAAAAACCTACAATCTCTTCGCCGC
CTCCCCGTCGCCGGATCGCACAATGTATCGCGCAGTGGCCCTCGCCTTCCTCCTCGTGGCTTCAGCCACCCTCGGAGAAGCCCAAGCCCCCGCGGCTTCCCCCAAGTCCTCGAAGGCCG
CCGCTCCCGCCCCCGTGAAAGCCAAGTCCCCCACCACCGCCGCTCCGGCTAAATCCCCCTCCGCGTCCGCCAAACCCCCTGTTTCACCAGCCCCCAAATCCAAGTCCCCGCCTGTCTCGA
TCGCTCCCTCCACCTCTCCCGCCGCCTCCCCAGCTGTCTTTCCCGATCCCGCCGACTCTCCCGCGGCATCCGGCCCAGTTGCTCCAGCTCCCGCCCCTGAATCCGCCGGAGTTGCCGACC
AGAACTCTGGCGCTGTGAAAATTGGAGCTTTTATTGGTGGGGCTTTAGCTGCCTCTGCCTGGATCGCCGTCTCTGCTTTCTAAGTCAAGAACATGAAAGAAGAAATGGAATACCGGAAAA
AAGAAAACATAAACTGATGGGTAGCGGTTTCCTTTCTGCAATCGTTTGTTTGATTTTTTTTTTTTTTTTTTTCTTTTAGTTTGTAAAGAAACGAGGGAATTTGATTCTCATAGCTTGTTGTTT
TTCTATTGAATTTTGGTGCTTTTCTGATATAACAAGTTGGATTGATCTTGATAGTAACAGAGATGTTTTGAACTAGCAATTGTATTATTTTGGTTTGGAGGTTAGGGGCGATAGAGAAGGAA
AA
>comp146183_c0_seq1
TTTATTTTTTCAACTAGAAAATTATTTTAAGAATCAATAAGAAAACCGTCTCAAGCCTTAACTATTTGAAAATAAAGTTGATTAATTGAACTATAAACACACATGAACATCATATGATAACAA
TAATAGCATGCCTATACAAGATTTTCTAAATTATAGGGAGGGAGAGCAAGAGCCACCCCTGCTCCTCTTCTTCACTCACTCCTACTAAGCAAACTATAACTTCTCTAAGTGAACAATTCTTC

TCAATGACACACTTAGTCAATCACCGATCCAATGCGGCGAAATTCACCGAACCCACGGCCGTTGTCGACTTCTCGCGCATATGCAACCCACCCATCACATCATCTTCAAATTCCTCCTTCC
GCCGCCGTCGAAACCGGCGAAGATAGACATAAAAGAGAAGACATAGGAGAAAGATAGCGAAAACTGACACAATTACAAACAACATGATCTCTCCATGAACAGTATACCCTTTGTCTTGA
GTCTGGCTGATGGCATCCAACGAGGAAGGGATGGAGACTGAGTCATTTGTCATTGCCATAGAGAAGAAAGAAAAGGAGATCGAACAGAAAAGAAGGATCAGCCTCAACTTCAAAATCT
CCATCTTTATCAATCTCTATATAGCTACAGGAAGATAAAAGGCCAGCGCTTGTGATGGAGCTGAGGGACAATGATTGCTTGAGAAAGAGGACTTACTGTTTCTTATTCAGAGGCAGACAGC
CAAATATTCTGTGTGATGTATTATTGTACTAAGTTCACATTTC
>comp146216_c0_seq1
TCAAGTTAACAGTAAAACCAAATACCCTGATTTTATTCCCATATCTATAGAGGATGAAGTAATGAATGTCAATGTTCAATACGAATGGAGGCCTAATCCGTGTGAGTTTTGTGAATCCCTCTT
TCATTCGACTGCTACATGCCCTACTAATCCCAAAAACACAGTTGAGGACAATTCAAAGAAAGTTGTTACCTTTAGAGGAAGAAGCACCTCCCGTGCTCCTCATAACGCCAGGGATAGAAG
TTATATTCCGCCTGGACGATCTAAGCAACTACCTTCACAGCGAAATCACAAGACTTCTGCTAGTCCGCAAAAACCACCAGCCAAAGTCACCACTTCCGATATCCCCTCATCTTCTTCCACC
TCAAAATCTCCCCAACATAATTATGTAGCCGATCAGATCTCGGCTTCAAAACCTCCTCCACCACAAACCAACCCACAAACCGACCCAATACCTAACCTTAACTTCCCTACCAATGAATCTG
TTATCAGATCCATTAACACATACTCAAACCAAAATCACAAAGAGAACATTGTCTCCCCCAACAAGTTTGATGTGTTATCAGACAACCAAAATGATATTGGTTCTACCAATTCAGATCCTATT
AAAGTGGCAGAGGATCCATTTCCTCCTAAAACAAAAATCCACAGCATAATACAGCCACCCCCCTCATCCTCAGCCAAAAACACTAGAGGCAAGAACAAAAAGCCTCAAACCTCCAATTC
AAAATCTCAATGACCCTTCCCTCAATCGCTTCGTGGAACATTAGGGGATTTAATCACCCTAGCAAAGTTAGTGCTTGTAAGCATCTTATCAAAGCTCATAACCTGGATTTGATTTGTATTATT
GAAAATAGGATTCATTCTAGCTCCTTGGATGATCCTTGGTTTACTGCCGCTCACTCGGTTTTTGATTCTGAAGTTAGCTGTAACAACTTCAATCTCAATTCCTCAGGGAGAATTTGGATTAA
ATGGGCCGGGGATCGCATCTCTTTTACCCCCACTTTCTTCTCTTCTCAGCTTATTTGTGGTTTTGTTTCTATTGGATTGGCAAATTCTTTTTTCCTCAGTGCTGTTTATGGTTCTAATTCGGTG
GGTGAAAGGTTGTTGTTGTGGCAACAACTCTGTTCCAATGCTCCCTCTTCTGATATTCCGTGGATTATTTTGGGAGATTTTAATTGCTGCAGGTATCAAAATGAGAAATCTGGGGGAACAC
CGATCCACTTCAGCAGGTTGGGGGATTTTAATTCCTTCATCTTTGACAATCATCTCATTGATCTTTCCTCTACTGGACTTTTTTACACGTGGTTCAACCAGCAAACTGATAATGCTATACATAT
CAAGCTAGATAGGGCTCTTGTGAATGAAGCCTGGATTGGTACTTACCCTCTTTCTTACTACTTCGTGGACAAACCTTTTTGTTCTGATCATAGCCCTATGGTTCTTAAGAATGGGGAATTAA
ATAATAGCAGCAAACGGTTCCTTTTTAAAAACCTCTGGACTTCTAGGAATGATTATTGGGGACTTTTACTGGACACTTTTATGGAGCCTGTTAGAGGTAATCCACTGATGGATTTCTCTAAT
AAGCTTAGGAAGCTCAAAGCTTCTATCAAAACTAGAGAATGGGCTTCTGCTAATGCCATTCAATCTGATTTGTATAGTCTTCACGACAAGCAACAGTCAATTTTGAATTTACTGGACCAAG
ATCCTTCTAATCCCAACCTTAATGTTGCTTTGAAGGGCATCAATGGGGAAATTGCTGATTTAACTTATTCCTGGACTTCTTGGGTTTCTCAAAGAGCAAAAGCAAAATGGCTTAAAGATGG
GGAAGATGACCTCAAATTTCTCTACTCTAAAATCCGCATGAGAAATTCCTTTAATAGTGCTGCTCTTCATAATGTCATTTGTGATAGTTCTGGGAATTCAAATTATGCTAAGCAGAAGGTCAT
CAATCATTTCCATGATCTGTTAAACCCCACGGCTAGTCACTCTCTACCTCAAATTTCTCTTTTCCCACTGGGCAGAACCGCTTCTCAATCTGTGCTTTCAAATCTGATTTTGCCCATTTCTGA
TCAGGAAATTAAGACAGCCGTTTTCTCTGGGGCTTCTTCCTCTTCTCCAGGACCTGATGGGTTTAACTTTCACTTTTATAAATCGGGATGGCATATCCTTGGCCCCTCTCTTTGCTTGGCTGT
GCAATCCTTTTTTACTAAAGGATATATTCCTTCGGGGGTGAAATCTTCTGCGATTTTCTTAATTCCCAAAAAGACCCATGTTAATTCCATTTCTGATTTCAGGCCCATCTCGTTATGTAATACC
TTATACAAGATTATAGCAAAAATTTTGGCTAATAGGCTGAAAAGCATTATGCCTCTCATCATTCACACGGCTCAGGCCGGGTTTATTAAGCACCGAGTTGCTACGGATAATGTCATTTTAGCT
TCTGAAATTTTAAGGTATTTCAAAAAATGTACTAAAGTCCCTTACATGTGTGCCAAGTTAGATATCAAGAAGGCCTTTGATTCTCTCTCTAGAGAATTCCTTTTGCAAAGAATGGCTCAGAA
GGGTATCCCTACTTTATTCATTAATTGGGTTAAAGCATGTATTACTAATGTGAATTTTTCTATTTGCATGGACGGATGTCTGGAAGGGTATTTTCCTTCTTCTGTCGGGCTGCGTCAGGGCTGC
CCCTTAAGCCCTTACCTATTCTGTATTGCCATGGATGCGCTCTCAAGTTTATTTGATAATTCCAACGACAGTCATTCTTTCAAGCCGGTTGATATTAATGCTTTTAAAATCTCCCATCTTCTTTA
TGCGGATGATGTTCTGGTTTTTGGGGAGGCTACTACTTCGAATAGTCAGGTGCTGAATGACATTCTTACCAAATTTTCCAAGTCTTCTGGCCTCGTGATTAATCCTGAAAAGAGTTCTATTA
TCCTTCAAGGGAATCCAACTCTTTTCAGGATTAATCACGAGGCCAGAAGACTTGGAAAATTTGGTAAGAATGTCATTCAGCACCTGACTATTCGAAGTAGTAGCCTCCCCAAAAACCAGA

ACATCATCCGCATAAAGAAGATGGGAGATTTTAAAAGCATTAATATCAACCGGCTTGAAAGAATGACTGTCGTTGGAATTATCAAATAAACTTGAGAGCGCATCCATGGCAATACAGAATA
GGTAAGGGCTTAAGGGGCAGCCCTGACGCAGCCCGACAGAAGAAGGAAAATACCCTTCCAGACATCCGTCCATGCAAATAGAAAAATTCACATTAGTAATACATGCTTTAACCCAATTAA
TGAATAAAGTAGGGATACCCTTCTGAGCCATTCTTTGCAAAAGGAATTCTCTAGAGAGAGAATCAAAGGCCTTCTTGATATCTAACTTGGCACACATGTAAGGGACTTTAGTACATTTTTT
GAAATACCTTAAAATTTCAGAAGCTAAAATGACATTATCCGTAGCAACTCGGTGCTTAATAAACCCGGCCTGAGCCGTGTGAATGATGAGAGGCATAATGCTTTTCAGCCTATAGCCAAAA
TTTTTGCTATAATCTTGTATAAGGTATTACATAACGAGATGGGCCTGAAATCAGAAATGGAATTAACATGGGTCTTTTTGGGAATTAAGAAAATCGCAGAAGATTTCACCCCCGAAGGAATA
TATCCTTTAGTAAAAAAGGATTGCACAGCCAAGCAAAGAGAGGGGCCAAGGATATGCCATCCCGATTTATAAAAGTGAAAGTTAAACCCATCAGGTCCTGGAGAAGAGGAAGAAGCCCC
AGAGAAAACGGCTGTCTTAATTTCCTGATCAGAAATGGGCAAAATCAGATTTGAAAGCACAGATTGAGAAGCGGTTCTGCCCAGTGGGAAAAGAGAAATTTGAGGTAGAGAGTGACTA
GCCGTGGGGTTTAACAGATCATGGAAATGATTGATGACCTTCTGCTTAGCATAATTTGAATTCCCAGAACTATCACAAATGACATTATGAAGAGCAGCACTATTAAAGGAATTTCTCATGCG
GATTTTAGAGTAGAGAAATTTGAGGTCATCTTCCCCATCTTTAAGCCATTTTGCTTTTGCTCTTTGAGAAACCCAAGAAGTCCAGGAATAAGTTAAATCAGCAATTTCCCCATTGATGCCCT
TCAAAGCAACATTAAGGTTGGGATTAGAAGGATCTTGGTCCAGTAAATTCAAAATTGACTGTTGCTTGTCGTGAAGACTATACAAATCAGATTGAATGGCATTAGCAGAAGCCCATTCTCT
AGTTTTGATAGAAGCTTTGAGCTTCCTAAGCTTATTAGAGAAATCCATCAGTGGATTACCTCTAACAGGCTCCATAAAAGTGTCCAGTAAAAGTCCCCAATAATCATTCCTAGAAGTCCAG
AGGTTTTTAAAAAGGAACCGTTTGCTGCTATTATTTAATTCCCCATTCTTAAGAACCATAGGGCTATGATCAGAACAAAAAGGTTTGTCCACGAAGTAGTAAGAAAGAGGGTAAGTACCAA
TCCAGGCTTCATTCACAAGAGCCCTATCTAGCTTGATATGTATAGCATTATCAGTTTGCTGGTTGAACCACGTGTAAAAAAGTCCAGTAGAGGAAAGATCAATGAGATGATTGTCAAAGAT
GAAGGAATTAAAATCCCCCAACCTGCTGAAGTGGATCGGTGTTCCCCCAGATTTCTCATTTTGATACCTGCAGCAATTAAAATCTCCCAAAATAATCCACGGAATATCAGAAGAGGGAGCA
TTGGAACAGAGTTGTTGCCACAACAACAACCTTTCACCCACCGAATTAGAACCATAAACAGCACTGAGGAAAAAAGAATTTGCCAATCCAATAGAAACAAAACCACAAATAAGCTGAG
AAGAGAAGAAAGTGGGGGTAAAAGAGATGCGATCCCCGGCCCATTTAATCCAAATTCTCCCTGAGGAATTGAGATTGAAGTTGTTACAGCTAACTTCAGAATCAAAAACCGAGTGAGCG
GCAGTAAACCAAGGATCATCCAAGGAGCTAGAATGAATCCTATTTTCAATAATACAAATCAAATCCAGGTTATGAGCTTTGATAAGATGCTTACAAGCACTAACTTTGCTAGGGTGATTAAA
TCCCCTAATGTTCCACGAAGCGATTGAGGGAAGGGTCATTGAGATTTTGAATTGGAGGTTTGAGGCTTTTTGTTCTTGCCTCTAGTGTTTTTGGCTGAGGATGAGGGGGGTGGCTGTATTA
TGCTGTGGATTTTTGTTTTAGGAGGAAATGGATCCTCTGCCACTTTAATAGGATCTGAATTGGTAGAACCAATATCATTTTGGTTGTCTGATAACACATCAAACTTGTTGGGGGAGACAATG
TTCTCTTTGTGATTTTGGTTTGAGTATGTGTTAATGGATCTGATAACAGATTCATTGGTAGGGAAGTTAAGGTTAGGTATTGGGTCGGTTTGTGGGTTGGTTTGTGGTGGAGGAGGTTTTGA
AGCCGAGATCTGATCGGCTACATAATTATGTTGGGGAGATTTTGAGGTGGAAGAAGATGAGGGGATATCGGAAGTGGTGACTTTGGCTGGTGGTTTTTGCGGACTAGCAGAAGTCTTGTG
ATTTCGCTGTGAAGGTAGTTGCTTAGATCGTCCAGGCGGAATATAACTTCTATCCCTGGCGTTATGAGGAGCACGGGAGGTGCTTCTTCCTCTAAAGGTAACAACTTTCTTTGAATTGTCCT
CAACTGTGTTTTTGGGATTAGTAGGGCATGTAGCAGTCGAATGAAAGAGGGATTCACAAAACTCACACGGATTAGGCCTCCATTCGTATTGAACATTGACATTCATTACTTCATCCTCTATA
GATATGGGAATAAAATCAGGGTATTTGGTTTTACTGTTAACTTGAATACACACACGAGCGTATGTTAATCTCGTTTTCTGAGCAGTGAGGGCATCAACCGCTAGGGGGATACCTATTTTGCT
AGCAATACGCGAAATACCTTCCGAATTCCAGACACAAAGCGGAAGGTCAATGAGCTTAACCCAAATCGGAACAGTAGTGAAATTCTCTTTTTGTGGCTTAAACCTAGGATGCCATTTCTG
GAGGATGAATGGCCGACCTAGGAGAAACCACACCCCACCATTCCACGCCATTTCATAATCCACGGGCGAGGAAAACTTGATGAGGAAAAAACCATCGCTCAAGTTAAAGAGATGGATAG
TACCTTGGAGCTTCCAAGTTTTCTTAAGAGCAGCAAGAAGAGCCTCGTAGTAGGGTCTCCTGCCAACGGAGAAACCTACCAGAGAAAGATTCCACTCCTCAGCAGCGGCATCGGTCTTA
GTACGCTGAAAAATGAGAACTTCGTTTGGATCTTCCTGAAAATCCACCGGGAGATCTTTAGACGACAGACAGTCCGGAGTCACAATGTTCGTCCAGCTCCGAGGAGGAGGGATGAACGC
AGAAACAGGGATAGAGGAGAGATGAGGGAAATCTTGTTCATTGAGAGGGGTTCCCATGGAGGAGGGAGCCCCTCACGGAGAAAGGAAAAAGGCGGCAAGGCAGAGCGGCGGGAGAA
AGCTCTGCGAAACCCTAGCAGAGCGTAAGCTTAAATAATAATAATAAATTTGAATT
>comp146216_c0_seq2

CCCTCTTCAAATTGTTGCCTAAGTTTCCTTCTTTGGCCTCACTTGCTGCCGGCCCAGTAAACAAATTCCAGGGAATTGTTTCCTACTGGAAAGGAAATTAATTTCCTCCTCCTCATACAGTC
GGATTCTAGCCCTCTCTACATCCCGCCGTTTCCCCCCTTCATTGGCTTGGATTTAAACTTATCTCCTCTGCATTAATTGAGTAACTATTTTTTTGGGAAAAGTAGGGCAAAGCCCAATTTCCA
TTGATATAAAGAAACCTAGTTACATGTGTACATCAAAAGCAAAATTGTGAAGACCATAAAGGAAAAGGAGCACACACAAGGTGCCAAAATTACACATGATTGCAAAAACCTGAAAAAAC
CGGGAAAAAAACCCCTCAGAGCAGCTCAGAGCAGCCAACAGAATAGCATCCCGTATTTGGTTGGAAAATTGATTTGGCTCAGGAAATCAGTCCACATCCTCAAAAAGATCGACCCCCGGT
TGCCAATAATGCCATCAGGTGTAGACAAGTTGCAGTATTCGCCGTCCAGGGGGGAGGATGCAGACTGCACATCATCGCCCCTAGCCAACAGAATAATGAACAACTTCTCGAGACAGAAG
AAAAATAACGTCACTGGATCATAAAACCATGAAGTCATCATCCCAGAAAATCGAACTTCAAGCACCAGAGCAACCATAAAATTCAAAATTTTTTCCATCTTCTTAGCTTGGTAACCACCGC
CTTATTAATCAGGAGTTTGAGAGTAGAGATAGATCTTGTACGATGTGCAAATTTACGTTCATTTCGCTCGCGCCAGATTGAATAAACAGCAGCCCAAAGCGTCAGCAGATTAACAGATAAA
CTACCATAGTTCAATAAAACTGAATTGGAAAGAAAGTCAGAAATCTGATGTAAATTTGGCCTCAAGAGGAATGAGTTAATAACAGGTAGCAGTTTTGAAATAATGCAAAAGGTGTAATCA
CACTCAAAGAAAAGGTGGGATACACTTTCCTCATGGCAATGACAGAAAACGCATTTTGGATCAACCTCTATATTTCTCTTTCTTAAAGCGTCTGATGTCTTCAACCCGCCAACAAAACATA
ACCAAGCATAGATAGAGAATCTTATAGCATGATGTTTATGCCAAACCACCTTGCTCCACATCACATCCTCCTGAAAACTGTAAAATTCCTCAAGGAAAGAACCAAATTTATGAATGCTATTC
GATCCCCAAGCAATACATCTATCTCTACCATCACCAGAAATAGGAATAGAATTAAAAGCGTTTTGTATGATAGAGTTCCAGGTAGTAGGGATGTTCCAGTGATCACCAGCCATGAGAACATT
TAAAGTTATATTTTCAGGGAAGAAATTGATAAGAGAAGAGAGCCCATGAACTTCATGTATGCTCTGGCCATTAAACCAATGATCCCACCGAAAATTAATGGGACAGCTTTCAGTAATTTTA
AAAAGAATTTATGCTTAGCTTTAGACGCAGCATAACAAATGCTTTTCCACATACCCGAGGATGAAGGGGAATGGGGCTTCCAAGGGGAGCAATGGGTGGAGAAGTACCAAGAAGAGAG
GGGTGAAGACCACTTGTAGATTCTGAGAATTAGAGAGCAATCATAGGCAAATCTAATGGCTTGAAAACAAGGAATACCAAGACCACCTTTAATTTTAGGCTTGCACGTATTAGCCCAAGA
AACCATGTGAAGTTTCCTCGCCTCTAAGTTCCCAAAAAACAGAAACTTGGCAGAAATTTTTGCCATCTTCTTAAGACAGGATTTAGGTATAATCGCACCGCGGATCCAATATGCTATGGTG
TTCCATATAGTAAATCTCAAAAACTGAAGCTTGCCTGCAAAAGACAGAGACTTAGCTTTCCATCCAGAAAGCTTATTAGTAATAGTGTCAAGAAGCGGGGAGAAGTGAGAGACAAGAAC
TCTTTTAATTGATATAGGGATACCCAAGTAAGTGAATGAATTACAAGTATTGTGAATGTGTAGGGTGTCACATATCTCTTTGGTATTAGTTGGATTCCCTTGAAGGATAATAGAACTCTTTTC
AGGATTAATCACGAGGCCAGAAGACTTGGAAAATTTGGTAAGAATGTCATTCAGCACCTGACTATTCGAAGTAGTAGCCTCCCCAAAAACCAGAACATCATCCGCATAAAGAAGATGGG
AGATTTTAAAAGCATTAATATCAACCGGCTTGAAAGAATGACTGTCGTTGGAATTATCAAATAAACTTGAGAGCGCATCCATGGCAATACAGAATAGGTAAGGGCTTAAGGGGCAGCCCT
GACGCAGCCCGACAGAAGAAGGAAAATACCCTTCCAGACATCCGTCCATGCAAATAGAAAAATTCACATTAGTAATACATGCTTTAACCCAATTAATGAATAAAGTAGGGATACCCTTCTG
AGCCATTCTTTGCAAAAGGAATTCTCTAGAGAGAGAATCAAAGGCCTTCTTGATATCTAACTTGGCACACATGTAAGGGACTTTAGTACATTTTTTGAAATACCTTAAAATTTCAGAAGCT
AAAATGACATTATCCGTAGCAACTCGGTGCTTAATAAACCCGGCCTGAGCCGTGTGAATGATGAGAGGCATAATGCTTTTCAGCCTATAGCCAAAATTTTTGCTATAATCTTGTATAAGGTAT
TACATAACGAGATGGGCCTGAAATCAGAAATGGAATTAACATGGGTCTTTTTGGGAATTAAGAAAATCGCAGAAGATTTCACCCCCGAAGGAATATATCCTTTAGTAAAAAAGGATTGCAC
AGCCAAGCAAAGAGAGGGGCCAAGGATATGCCATCCCGATTTATAAAAGTGAAAGTTAAACCCATCAGGTCCTGGAGAAGAGGAAGAAGCCCCAGAGAAAACGGCTGTCTTAATTTCC
TGATCAGAAATGGGCAAAATCAGATTTGAAAGCACAGATTGAGAAGCGGTTCTGCCCAGTGGGAAAAGAGAAATTTGAGGTAGAGAGTGACTAGCCGTGGGGTTTAACAGATCATGGA
AATGATTGATGACCTTCTGCTTAGCATAATTTGAATTCCCAGAACTATCACAAATGACATTATGAAGAGCAGCACTATTAAAGGAATTTCTCATGCGGATTTTAGAGTAGAGAAATTTGAGG
TCATCTTCCCCATCTTTAAGCCATTTTGCTTTTGCTCTTTGAGAAACCCAAGAAGTCCAGGAATAAGTTAAATCAGCAATTTCCCCATTGATGCCCTTCAAAGCAACATTAAGGTTGGGATT
AGAAGGATCTTGGTCCAGTAAATTCAAAATTGACTGTTGCTTGTCGTGAAGACTATACAAATCAGATTGAATGGCATTAGCAGAAGCCCATTCTCTAGTTTTGATAGAAGCTTTGAGCTTC
CTAAGCTTATTAGAGAAATCCATCAGTGGATTACCTCTAACAGGCTCCATAAAAGTGTCCAGTAAAAGTCCCCAATAATCATTCCTAGAAGTCCAGAGGTTTTAAAAAGGAACCGTTTGC
TGCTATTATTTAATTCCCCATTCTTAAGAACCATAGGGCTATGATCAGAACAAAAAGGTTTGTCCACGAAGTAGTAAGAAAGAGGGTAAGTACCAATCCAGGCTTCATTCACAAGAGCCCT
ATCTAGCTTGATATGTATAGCATTATCAGTTTGCTGGTTGAACCACGTGTAAAAAAGTCCAGTAGAGGAAAGATCAATGAGATGATTGTCAAAGATGAAGGAATTAAAATCCCCCAACCTG

CTGAAGTGGATCGGTGTTCCCCCAGATTTCTCATTTTGATACCTGCAGCAATTAAAATCTCCCAAAATAATCCACGGAATATCAGAAGAGGGAGCATTGGAACAGAGTTGTTGCCACAAC
AACAACCTTTCACCCACCGAATTAGAACCATAAACAGCACTGAGGAAAAAAGAATTTGCCAATCCAATAGAAACAAAACCACAAATAAGCTGAGAAGAGAAGAAAGTGGGGGTAAAA
GAGATGCGATCCCCGGCCCATTTAATCCAAATTCTCCCTGAGGAATTGAGATTGAAGTTGTTACAGCTAACTTCAGAATCAAAAACCGAGTGAGCGGCAGTAAACCAAGGATCATCCAAG
GAGCTAGAATGAATCCTATTTTCAATAATACAAATCAAATCCAGGTTATGAGCTTTGATAAGATGCTTACAAGCACTAACTTTGCTAGGGTGATTAAATCCCCTAATGTTCCACGAAGCGAT
TGAGGGAAGGGTCATTGAGATTTTGAATTGGAGGTTTGAGGCTTTTTGTTCTTGCCTCTAGTGTTTTTGGCTGAGGATGAGGGGGGTGGCTGTATTATGCTGTGGATTTTTGTTTTAGGAG
GAAATGGATCCTCTGCCACTTTAATAGGATCTGAATTGGTAGAACCAATATCATTTTGGTTGTCTGATAACACATCAAACTTGTTGGGGGAGACAATGTTCTCTTTGTGATTTTGGTTTGAG
TATGTGTTAATGGATCTGATAACAGATTCATTGGTAGGGAAGTTAAGGTTAGGTATTGGGTCGGTTTGTGGGTTGGTTTGTGGTGGAGGAGGTTTTGAAGCCGAGATCTGATCGGCTACAT
AATTATGTTGGGGAGATTTTGAGGTGGAAGAAGATGAGGGGATATCGGAAGTGGTGACTTTGGCTGGTGGTTTTTGCGGACTAGCAGAAGTCTTGTGATTTCGCTGTGAAGGTAGTTGCT
TAGATCGTCCAGGCGGAATATAACTTCTATCCCTGGCGTTATGAGGAGCACGGGAGGTGCTTCTTCCTCTAAAGGTAACAACTTTCTTTGAATTGTCCTCAACTGTGTTTTTGGGATTAGTA
GGGCATGTAGCAGTCGAATGAAAGAGGGATTCACAAAACTCACACGGATTAGGCCTCCATTCGTATTGAACATTGACATTCATTACTTCATCCTCTATAGATATGGGAATAAAATCAGGGTA
TTTGGTTTTACTGTTAACTTGAATACACACACGAGCGTATGTTAATCTCGTTTTCTGAGCAGTGAGGGCATCAACCGCTAGGGGGATACCTATTTTGCTAGCAATACGCGAAATACCTTCCG
AATTCCAGACACAAAGCGGAAGGTCAATGAGCTTAACCCAAATCGGAACAGTAGTGAAATTCTCTTTTTGTGGCTTAAACCTAGGATGCCATTTCTGGAGGATGAATGGCCGACCTAGGA
GAAACCACACCCCACCATTCCACGCCATTTCATAATCCACGGGCGAGGAAAACTTGATGAGGAAAAAACCATCGCTCAAGTTAAAGAGATGGATAGTACCTTGGAGCTTCCAAGTTTTCT
TAAGAGCAGCAAGAAGAGCCTCGTAGTAGGGTCTCCTGCCAACGGAGAAACCTACCAGAGAAAGATTCCACTCCTCAGCAGCGGCATCGGTCTTAGTACGCTGAAAAATGAGAACTTC
GTTTGGATCTTCCTGAAAATCCACCGGGAGATCTTTAGACGACAGACAGTCCGGAGTCACAATGTTCGTCCAGCTCCGAGGAGGAGGGATGAACGCAGAAACAGGGATAGAGGAGAGA
TGAGGGAAATCTTGTTCATTGAGAGGGGTTCCCATGGAGGAGGGAGCCCCTCACGGAGAAAGGAAAAAGGCGGCAAGGCAGAGCGGCGGGAGAAAGCTCTGCGAAACCCTAGCAGA
GCGTAAGCTTAAATAATAATAATAAATTTGAATT
>comp146311_c0_seq3
AAAATTTCTTTTTCACCGCTTCTCCAACATCCAAACATTTGTTGACAAATTAGACCAAAAAGAAGAAAAACAACTCAATCACAACTCAGAAAGCCATATTGCTTAGTGAATATAATATCTAT
AAGCTTTCAAACGTTTTAAATACATAAAGGGAGCAAAACAATAATCATACCAGTTTGAATCTACCTCCCTATAATGATTTCTCTACAAAAAATGCCACAGGTTATGACTGAACTTGATCATC
CTATATCTGAGTTGGTGTAATCAAAAGCTTGTATATACTATAATATTTGATTATATTCAGTTGGTAACAAAATAGAGAAGAAAAGCATGAAGGAGCTGACTCAGGAAGAAGCAGAAGCCAT
TCCAGGTTCCATCTCAGCCACAAAGCTTGCTGCTTCTTGATGATCTGCTGCCTCCCAACACAACTTTGAAACAAAATCATCAGGTTGAGGAACATACTCCTCTAGTTTCCTGACCAGCTCA
TTCGCTGTTGGAGCAGAAACAATGATGTGACGGGCCTTCTGAGAAATAAATTCCTCCATGACAGCCATGTCAATGAATGAGAGCAATGAATTATAGTATCCATCCACGTTAAGAAGACCA
ACCGGCTTCTGATGAATTCCAAGCTGTGCCCATGTGATGACTTCAAGCAATTCTTCTAATGTCCCATAGCCACCAGGCAAAGCAATGAAGGCATCAGCTTGGCGAGCCATCTCAGCTTTCC
TCTCGTGCATATCTGAGACTGCTCTAACTTCCCCAACAGTCTCTCCAGTTATCTCCCTCGGCATCAAAGACTTCGGGATGACCCCTAAGACATGGCCGCCACCATCATGAACAGCGTGAGC
GACGAGGCCCATCAATCCAACGCTACCACCTCCATAAACCAAATCAATTCCTCTCGCCACCAGTTCCTTTCCGAGCTCGACGGCCGCTTCCCTGTAACCCGCCTTCTTCCCGGTGCTACTC
CCGCAGAAGACGCAGATCCTCTTGAAACAGGACAGGGAACCCACCTTCTCTCCGGCCGCCGTGAACTCGACATTCGGCTTCTCTTCCATTCTTCGAGCTCTCTATTCTCCGTGGAAGAAA
CAACGTGGAGATAACGAGAAGATGCGCAGGGCCCCTACCCTCGGAGTCCTTTGGGGGTTTTTATAGACGATGGATTAGCGGTGATTCAGAATTTGGACGGCTGTGATTCGTTGCTTAGAG
CTAATCGCCGACATAAATAAGGAGTGATTCGGTTTTTTTGTTTTAAATATTATTATTTTTTTATTTTTTGGTATC
>comp146322_c2_seq3
ATAAATAGAGCCCTCATTTCTCATGAGAGGGGGACAGAAGAAAGAAGAAGAAGAAGAGAAGAATAAAAGGGAAAAAATTCCTCCTCGACCACTGCGGGATTCTACCCGACTTCGACGT

GGCACCGGAATTCCACCCAACTTCCAAGTGACACTGAAATTTCGCCTGACTTCCAAGTGACATCGGAATTCCACACAACTTCCAAGTGACACCGGAACTCTGCCCGACTTCTAAGTGAC
GCCGAAATTCTTCCTGACTTTTCAGTGACGCCAGAACTCTGCCCGATCTCCAAGTGACTTTGGAGTTTTGCCCGAATACCATCTAAAACAGAGTTCTCCTCGGCCGCCGACTGAATTCAG
ACTTCTACCCAACGACGGACCACCGCTCGTCGCCAGATTTCTACCTAACACCGTACTTCTACCCGTTGTTGGATTTTCGTCGAATAACTATCCAATGTGGGTTTTCTATCTATAGCTGGACTT
CTACCTGACGCCGCCCTTCTGATCTTCGTCGTCAGACAATGGTCCTTCTAACCTTTGTCATCCAATGACTATAGTCCTTCTGACCTTTGTCGCCCGCCGACTACGATCCTTCTGGCTTGCATC
GCTCACCTACTACTGTCCTTCCGACCTCAGTCGCCAGAATAGGGTCTTTATGACCTTCGTCGCCCGTCGACTACGGTCATTCTGACTTATGTCGCCTGCCAACTACGGTCCTTCTGACTTAC
TTCGCCCACCGACTACTGTCCTTCTGACCTCCGTCGCCTATTGACTACGGTTTTTTCGACTTACGTTGTCCATCGACTACACTCATTCTGACCAAACGAATACGTCAACAGTAACTCTTTGT
ATTCTCACATTGTAATGTACTGCATATGATGTACATAAAGCCAAAGCCTTCCCCCCAAGGAAGGGCCTCATTTGTAA
>comp146369_c0_seq2
TAATATGCTTTGGCTTCCACTTCTATAGGGATCTGTAACGTGAAACCCTCTCTCTCTCTCTCTTCTATCTCTCTTTCTCTCTCTTCAGCGGACCGGAAATCCATGCGGCTATGGCGGTCTCCC
TCCCTTCTTCTCCTCCTTGTTTTTCTCCCAATTCCGTCATTTCCAAATTATTTTCTCTCGTTTTACCTTTATACCATTTCTAAGCTGAGCTAAATCCAAAAAACTGAAAGCTACTTCTCCGCCG
CCGCCGCCTCCTCCTCAGCCGCCGCCGGCTTAGGGGGAAATTTTTCGCGAGATCTTCTCCAGCCGCGGCGGAGATCGCCTGCGAAACGATCTCCGATTTCCCGCTATCCTCCTCCGATCCG
ATTGAGCTCCCGCCGCCTGAGATCTAATCTGATCAGAGTTTCCGGGAACCCTAACCCTAGGTCAAGGATCAGCCGCCATCGCCCCCCTATTTCGAAATAATTAGGACTAGAAAAAATCATA
CTGTCCACGCTTACGATGCTGCTCCATTAATTCTCATCTAACCGTTTGTTCTTTTTGTCTTCTTGTGGATAGCGAGAGCGCGACAAGGAAAGGAGACAAAAGGCCGACAGAGAAATATAG
AAATAGGGAGAGGAAAAGAAAGAGAGAGATATTGAAATCGAACGAAGTGGATGTCGTCGTCGTCGAACTCGCCGTGCGCGGCGTGCAAATTTCTCCGACGAAAATGCATGCCGACCGG
TTGCATATTCGCGCCATACTTCCCGCCAGACAATCCGACAAAATTCATTCATGTTCACCGGGTGTTCGGCGCCAGCAACGTCTCCAAGCTTCTCAACGAACTTTCGCCGGCGCAGCGCGA
AGACGCCGTCAACTCGCTCGCCTACGAGGCCGAGGCGCGCCTTCACGATCCTGTGCTTGGATGCGTCGGCTATATCTCCCTGCTTCAGCATCGCCTAATGCAGCTCCAGCGCGATCTATCC
GCTGCTAAGAGGGAGCTTTCGCTCTGCATCTGCCCGGGCCCACAAAACCAATTCTATGCCGAGGCCATGGCCGTGAGGGCTGGGCTCGCCGGTCAGGGATCGGCTTCAACATCTGCGGC
GCCACCGATGTATAAAGATCAACAACATCATCAATATCAACATCATCACCATCAGCAGCAGCAGCAGCAGCAGCAG
>comp146470_c0_seq2
TTAATATTGCATATAATATGTAAGAGCTACACTATTCTCAGTACAAATGCAAGAATTTAAAACTTCATTCTAACATAAAATACATACAGTATGCTCTATATAAATTATACACAATATACTTTCCTT
AATTTAGAGAGAGAAATTAATTTCTCAAACCCCTTTACAAAAAAAATAAAAAATCCTTTAATCTAAATGCAAGATGGAGGGATCATAAGGCCAAATGAGCGGCCCTTCACATTAACACAA
GAAGCAACAGCAAAATCTCCAGCAACACTGTACTTCAAATCTATGTTGCTCAGCTCTATATCTTCACATGGCAATCTATCGCTGCAAACCAGATTTATAGCATTCTTTGTTCTTGTTTCTCCT
GTTATGTTTCTGAACTTCACATTACTGATCTTAACCAGAGATGGAGCCTTACTACCGCAGAGATTATGCGGGCAATATTTCTGATCAATAACAATTGGATTATCCACATTGTTCATCACCAAT
TCCTCAAACACAAGATCATATGCTGAAGAGAAAGTAGGAGACTCCTGCCAAGTCTTAATTCTAACACCATTCTCTGTATCACTAAAAGTGCAATTTCTAACGCTAATTCCAACAACATCAC
CTTCATCTTTGCCTCCTCCAAGGCTTCCAATGCTTATGCCATGGCCGGGCCCACACCGCACCCCTGATATCGATAGGTTCGCCGTCCCCGGCCCGATCGATATGCAGTCGTCGCCGGTGCTG
ATGATGGATTGGTTTATACTAATATTTTTTGATTCTCCCACGTGAATTCCGTCGGTGTTGGGGCTATTTTC
>comp146487_c0_seq1
CAAATTGAATATGAACTTCTTAACTAATCAAAAAAAAAATTCTTATATACACTATATTCTTTATCATCCATACCAAAACACAAAAACTCATATAGCTATCAAGTTCAAAAACACATTGAAAAAC
ATAAACATACCAAACTTGAAATAGAAACAACTGAACAAGCAAATGAAAGGATAGTTCTCTGAATCTATCACTCATCTCATGAACTCACAAATCGTTAGTCTCTCCAATCTTATTAAACACA
ACATCAAATCATAAAGTTTATAAGACTTCTTCATATCATGCGAGGCTAAGATCATGTGATTCATATCCTATACCAAGCAGATTTGGCTGGATTGGCTGAACAACCCTGAAAGAAAACATCTG
AGGACAATCATTCACCATTGGAATCGAGTTTTCATAGACTCTGTTGTGGTTTTCTGCACTGAAAGCATAATTCGGATTGTCGTCTTTCATCTCCTGCTTTCATGGAAGCTTGAAGCTTTAAA

TATTGAGGCTTTTTCTTAAAAGGGAATTGTAAGGGAAGTGTAGAATACTAACCAGTTCATGTACTAGAGCTCTGTAGGTCTCCCTGGTGCTTTTAAGCTTTTTCTTGTAAGTGTCAGTTTGT
GTGGCGATCACATGATACTTTCTCTGCCTAACAATTCTTAGAGACTCTTCCAAAGTTTGCTCAAGACCGCGCAGTTCCTTTATTTCCATCCCCTCCAGTTCTTCTCCCTTCCTCTGCCTAATC
TCCTTGCGAAGGTTTTGGTTGATCTCGTTGAGATGCTTCAGTGTATTTTGCATCCTCTCATATTGAGCATTCCATAGATCCATGCCAGTAACAACCTGGTACCTCTCATATACCCCCTTAATAT
CAGTAGAAGGGCTACAGTAATCAGCCAGCTTGCCAGTGCTGGAGAACATGATGAGAGAGACTTGAGCGTCGCAGAGAACAGTGAGCTCCTTGGCCTTCTTCAAGATCCCAACCCTCCTC
TTAGAGTATGTCACTTGCCTGTTCGTTGGGTTCTCTATCTTTTTAATCTCTATCTTCCCCCTCCCCATCTCTGTCTTGCTCTGTTTTGCTCTGTTTTGCTTTATTCTCAATCGTTCTTCTTCTTCC
TCCTCTCATATAAATAGAAAGAGAAAGATCAGTTTTTTAGAGAGAGAGAAAGGGGGTGGTTAATACAAGTGAGAATATTCTTCTATGCTTAAATTTTTACTAAAAATTCAGTTTTGATTGTT
CCATTGAAATGCACTTACAGTAACTATCTATCAGAGCTTAATTT
>comp146487_c0_seq5
CAAATTGAATATGAACTTCTTAACTAATCAAAAAAAAAATTCTTATATACACTATATTCTTTATCATCCATACCAAAACACAAAAACTCATATAGCTATCAAGTTCAAAAACACATTGAAAAAC
ATAAACATACCAAACTTGAAATAGAAACAACTGAACAAGCAAATGAAAGGATAGTTCTCTGAATCTATCACTCATCTCATGAACTCACAAATCGTTAGTCTCTCCAATCTTATTAAACACA
ACATCAAATCATAAAGTTTATAAGACTTCTTCATATCATGCGAGGCTAAGATCATGTGATTCATATCCTATACCAAGCAGATTTGGCTGGATTGGCTGAACAACCCTGAAAGAAAACATCTG
AGGACAATCATTCACCATTGGAATCGAGTTTTCATAGACTCTGTTGTGGTTTTCTGCACTGAAAGCATAATTCGGATTGTCGTCTTTCATCTCCAGTTCATGTACTAGAGCTCTGTAGGTCT
CCCTGGTGCTTTTAAGCTTTTTCTTGTAAGTGTCAGTTTGTGTGGCGATCACATGATACTTTCTCTGCCTAACAATTCTTAGAGACTCTTCCAAAGTTTGCTCAAGACCGCGCAGTTCCTTT
ATTTCCATCCCCTCCAGTTCTTCTCCCTTCCTCTGCCTAATCTCCTTGCGAAGGTTTTGGTTGATCTCGTTGAGATGCTTCAGTGTATTTTGCATCCTCTCATATTGAGCATTCCATAGATCCA
TGCCAGTAACAACCTGGTACCTCTCATATACCCCCTTAATATCAGTAGAAGGGCTACAGTAATCAGCCAGCTTGCCAGTGCTGGAGAACATGATGAGAGAGACTTGAGCGTCGCAGAGAA
CAGTGAGCTCCTTGGCCTTCTTCAAGATCCCAACCCTCCTCTTAGAGTATGTCACTTGCCTGTTCGTTGGGTTCTCTATCTTTTTAATCTCTATCTTCCCCCTCCCCATCTCTGTCTTGCTCTG
TTTTGCTCTGTTTTGCTTTATTCTCAATCGTTCTTCTTCTTCCTCCTCTCATATAAATAGAAAGAGAAAGATCAGTTTTTTAGAGAGAGAGAAAGGGGGTGGTTAATACAAGTGAGAATATT
CTTCTATGCTTAAATTTTTACTAAAAATTCAGTTTTGATTGTTCCATTGAAATGCACTTACAGTAACTATCTATCAGAGCTTAATTT
>comp146487_c0_seq7
TTCTCTGCCTAACAATTCTTAGAGACTCTTCCAAAGTTTGCTCAAGACCGCGCAGTTCCTTGATGTCCAGTCCCTCCAGATCTTCCCCCATCCTCTGCCTTCTCACGACAACCCAAAAAAA
AATCACATAAATTAACACTTAATTCCCAAAGCCAAAAAACAAAAGTAAACAAGTAAACGCGTTAGTAGCTTTATCACTCCAGTACTCTTCTCCCTTAGCCCTTCAATCTCCCACCACCCAC
CTTACTTCCCTCCTCAGTTTATGATTGATCTCCTTCAAATGGTTCAGCGTATTCTGCATCTTCTCGTACTGCGCGCTCCACAGATTGATGCCGGACACCTGCTGGTAACGATCATATATACTCT
TCGAGTCAGTGGACGGGCTGCAATACTCAGAGAACTTGCCCGTGCTCGAGAACATTACAAGTGAGAGCTGAGCGTCACAGAGAACTGTCAATTCGCTCGCCTTCTTCATGATCCCAGCT
CTCCTCTTCGAGTAAGTAACCTGCCGGTTCGTAGGGTTCTCTATCTTCTTTATCTCTATCTTCCCCCTCCCCATGGATCTCTCTCGTTCTTTCTCTCCTTCTCCGAAGCAACAGAAGCAAAAG
AGATGAGATTGATAAGGGTAGGAAAAGGTTCAAAGTAAGCTTATAAAAATGGAGGCAGAAGAGACGAATTGAAGGCATGTAAGGCTGGGGAGGAAAAAAGTAGCAGCTGGGTTTCCAT
TATCGGGCAATGGCCAAAGGGTTGTGCGTACTACCTCGCGTGCCATGTCTGGCCG
>comp146487_c0_seq9
CAAATTGAATATGAACTTCTTAACTAATCAAAAAAAAAATTCTTATATACACTATATTCTTTATCATCCATACCAAAACACAAAAACTCATATAGCTATCAAGTTCAAAAACACATTGAAAAAC
ATAAACATACCAAACTTGAAATAGAAACAACTGAACAAGCAAATGAAAGGATAGTTCTCTGAATCTATCACTCATCTCATGAACTCACAAATCGTTAGTCTCTCCAATCTTATTAAACACA
ACATCAAATCATAAAGTTTATAAGACTTCTTCATATCATGCGAGGCTAAGATCATGTGATTCATATCCTATACCAAGCAGATTTGGCTGGATTGGCTGAACAACCCTGAAAGAAAACATCTG
AGGACAATCATTCACCATTGGAATCGAGTTTTCATAGACTCTGTTGTGGTTTTCTGCACTGAAAGCATAATTCGGATTGTCGTCTTTCATCTCCTGCTTTCATGGAAGCTTGAAGCTTTAAA

TATTGAGGCTTTTTCTTAAAAGGGAATTGTAAGGGAAGTGTAGAATACTAACCAGTTCATGTACTAGAGCTCTGTAGGTCTCCCTGGTGCTTTTAAGCTGCAAATACATGTAAGTGATTGCG
ATTACTAGAATTCACTTTGAAAAAATTGAAGTTTTTTTTTCTGTGAGAATAATTGTGATATTGAAATCATATGAAATATAATTCTTACAAAAATTTTTGAGAGCATGTATATAAGT
>comp146569_c0_seq1
AGTTGGCAAGCTTGAAGCGTTCGTGGCCCTTTCAATCTGGTTCAGCTCTACAGTCTGCTACAGATCACAAAAGAATTGCTTCGTCGAGGTAATACGATTAAAAATTTCAAATTTTCTTTTC
TTATTTTGATGTATTGCACACAAAAGCTGCTGATTCGACGTGTTTGAACGAATGCCAAGGGACGATTCTTCGAGTTCGAGCTGTCCTCGACTCAATATCGGAGCTGAAGGTGAAGCTTCTA
AGCTTGATGGAAGCTTACTCAGCCTTGGTCTTCTGCCTGTTTCAGAACCTTTCTCCATGCAAACAATACCGTTTCAGAAACATTTTGTTCATTGTTTGCAACATATTCAGAAAAACACAGA
AAAAAAGAATTCGGAAGCTCTTGGCCCACCAACTCTTTACAATTTCATGGGTCTGCCTCATGATCTCATTGGAAGCAATCCTTGTGATGCAGATACTGAATCTAAAGATTAATGCAAATGC
AGATCATTGAAGCAGCAAGGTTGAAATAGAGATTATTTGATATAGTTTTCTTCATCATGCAGTAATTTCTTCTCGTTTCCCTGCTTCAATATTGTATGTTTCAGGCTTTCAGCTTCATTGTTGT
TTCCATTTACAGGCATCAATGAATGCATACCCGTTTCTTTTCCCCTCTATTTTT
>comp146585_c0_seq1
TTTATTAAGTAACAACTCGAGAATTTCAAAACAAATAGAACTATAAATTTCCTCATATAAATTTTTTGAGCTTTCTAGCCTTATTTTTTTCTATTTGGAAGATGATGAAGGTTTAGCTGACCTT
AAAGATGAGTCATTTGTGTCTCATTGCTTGTAGACTCATTCATGAAGATGGATATAACGTCCCACTCTATCAATCATAACCTATAAAAACCTTGGTTATAAGTGAAAATCATATATAAATTAAC
ATCGCAAAGTATGTGCCTCATTGCTTGTAGAATCATTCATAAAAGATGGATACAATGTCCCACTCTGTCTGTTAGAACCTATAAAAATCTTGGTTGGAAGTGAAAATCATATTTAAATTAAAA
TACAAAAGTTTAATTTGTATAAAATGTCACAAGAGAGTGAAATAATGCTTAATTTACCAAAGAAATTATAAGTGTTGAGCCACTAATAAGAGTAAGTTGAGGCCTGGATTTGAATCATAACA
TGTTCATCATCATATTGAGTGTAAGAGAAAAAGATCGCTATCTTCATTGCGAGCTCCCCTTGTTACCTTCGTGGCAAGCTCCTGTTGTGCCTTCCAATATAACTAGAAGAACATTGTAGAAG
AGGGTGAAGAAGCTTCTTTTCTTCTGATTATAAAATGGCTACAGTTGACTTAATAGTTGTTTTGTTTTGGATCAATCTTAATTCTTAAAATATAGTCACTTAATAATTGTAATTGAACACTCAA
ATCTCTCTCCACTGTATTAATCTTATAGTTTGATATGTTCAAGATATTAAATCACACTGTATTGTATCTATCATGTTCGAATCTTATTCAAACAGTGTCTGATCCTGCTTCAAACATATTCAATCA
TTGTTCTATTCGTGTTTGAACATTTCTGAAGTAATTTTATATTAAATTTGAATAAAATTTAAACATTTTGAACAAATTTGAATATATATTTTTTTAATATAGCTACACTAGTATTTTGCTCAATGT
TTATTCTTTTTTTTTTAATTATTGGAGGTTGAGATT
>comp146615_c0_seq2
ATTTCGTGCGTCATTTATTTGAAAGCCGAGGGCACACACTGTACCTGCATTAGAAGAATCTGTCGCTGCGTGCCCGACTTTTCCAGCTTTATATGTCACCTTCACCTTCTTCTTCGCATTCC
CCACCACAGCCACCTTGAATCCATTTACAATGACGACGACAAAAACACTAGCCGGCTCCGGCGCCGCCCGCATCAATGGTACCCATCCCCTTCTCTCCCGTCGTGTTTGCTTCTCCTTTGC
CGCATATTCTAAATCGATCATCAACCATCTCCGATCATGCGGCGTCCCCATCGACGCCGGCCTGTCCGACGAAGAATTCACCGCCATCGAATCCACCCACCGCTTCCAGTTCCCGCCCGAC
CTCCGTTCCATCCTTCGTGAAGGCCTGCCTGTCGGACCCGGGTTCCCCAATTGGCGCTCCGCCTCGCCAAATCAGCTCCAGATACTCCTCTCCCTCCCTACATCCGGCCTACTCCATGAAA
TTTCCCGTCCCGGCGGCGATTTCTGGCCATCGGCGTGGGGACCTCGGCCCGAATCCCTTACCCAATCAATCGCAATGGCCCGATCCTTTCTATCCAGAGCGGCGCAATTGGTTCCAGTGTA
CCGCCAGTTCTACATCGCCGCGAACCCTAATGTGGCCGGTAACCCCCTTTTCTATATCCACGGCGGCGATGTACGATGCTGCGGGTTCGATCTCGCCGATTTCTATCGACGCGAGGAGATAT
GTTTACTGCCGGCGGCCGCGCCGGCGTGGACTGCGAAGGAGCCGAGGAGGGTTGAGGTGTGGTCAGAGCTCGCTGGAAGCGGAGGCGGCGGTGGCGGAGAGAGCAGAATGGATAAG
TTGATGAAGGAGGTGGGGAGGAGATTGAGGGAGGGAGGGTGGTGTGAGGAGGAGGTAAGAGAGATGCTGATGGCGGGAGGATCGAACGGTTACGATGAGAGTATGGATGGACATCGA
AAGGGTGTGAAGGATCGAGAAGGAATGATGTGGCACGTGCGGGGCCTCGCACAGGTGATGCTTCGTGCAGGGTGGAGTAAGGAAGACGTCGCATACGCTTTAGGCGGGAGCGGACGG
AATGCCGGGAGCTTAACTGTCGCTGGCCACGTGGACCCAACAGTCTTCTCTTCAACGTCTAATTAGAAATTAGTAATTACTAATTACAATTAATAATTAATTATCTGCTAAACTAACATTTAC
TTGTCTAAAAAATAAGCGGAAATTAAAATTTCCTTCACCTCTACGTTGTTATTTATTTATTTATTTAGGTGAAGAGGTGAGGGAAATCAAAGCGTCACTTGTTTTTTATTTATTTTTATTATTT

TCAACAAGTGTCAATAATATAAACCGAAAGATTTTATTTATTTATCAATCATCAAATTAAATGAAGTAGAAAAT
>comp146616_c3_seq4
TTTTCCTTTTTATACCAATTAAAAAACGAGTTTTCTAAGGACAAAAATTACTAAATAAATCAAAAAAGAGGGAGTTTTAATGGAAATAGGGAAACAAGATGTTTGTTAAAAAATATTTGCA
CAAAATAACTAAAGTTTTTTATGAAAAGAAAAGGGGTTTAGTTTGTAATTATGAAAAGGGGAAGGGGCGGAACGCAGAAGGCGCGCAGTAAAATCCCTGCACCTTCACTACGCCCTCCC
ATGTGCCTGTCACTGGCGGGCAGAGCCCTAACAGCGCAAGACAGATGTGGGGCCCAC
>comp146694_c1_seq1
GTTTTTTTTTTTTTTTTTCCTTAGATTTTTATTATCAAGTCGCGGGTGCACCAACTAGCGCATGTGCTTCACAGAGCAAGCCACGATTTGTGCCCATTGCCTGATCCTTGCCTTCACGCGTT
CCGGGCTGTCCCCCGGTTTCATGACCCTCCAAGTATCATCATCAGAACTTATAGGGCGTCCCTGCTGATCCAGTTGCGGCTGCGCATGCGGAGTTCTCGGGCTGGGTGTCCCGTTAAGCGT
CGCCGACGATGAAGTCGCCGTGGTTGTCGTCGGCGTCGATGCGGGACTCGGTGTCTGGCTCAGCTTCGGGTCAGACAGCTGCCTATTAATAGCAAAGTACAGTTTCAGCGCCGGCAGCG
TCTTGCACAATTCCTTCCCTCCGTTCTCCTCACTGAAGCCAAAGCCGAGATCTAAGGAGCCCTTCAACTCATCCAAGTCCTCATCCGTCAAGCTCCTGACCCTCTCCCTCCTCTCCTTCAC
TGTCACCTCCTTCCCATCCTCGTCTCTCTCACTCTCCTCCACCAATTCATCGCTGAGACTCCGCAAATACATCAGCCTCTTTTTCCTCTCCCATTTCTCCTCCCTCATCATATCTCTCATCGAA
AGCTGCTTTGACAGAACCTTCTTTTCCACTCTTCTGGCCAACGTTGAAGGCGTAGGGAGGTTCGTCATGACTTTCTCATCCTCTGTTTCACTTTCAGAGGAGGAATGCGGCGGCGACGGG
TGTTGCAGCAATAGTTTATGCTGTTCCATCCCCCTGTTCATGGTTCTTCTATTGCTCAGCATCGCCGAGCCGGCGGTAGCCAGAATGGCCGCCTCGCTGCGACGAGAGAGTGGCGGTGAA
GGAATAAGGAAATGCAAGATAAATGGTAGGGGTTAGAAAAAGGAGGAAAGAGTCGGTATA
>comp146729_c1_seq1
CGACTCGTAGTGATGGAAAGGAAATCAAAGCAAATAAACTTCACTTCAAAAGAGCAATGACAAGTGTTCATATAAACATGCTTCTTCATTATCGCCTTCCTCTTGAGGGCTTGCGCTTGAT
GAATCTAGCCCTTTTTGGCCTGTTTACTCAACAATACAACACATATTGGAGGGAATATAAGCGTGTGATGCGCCTTGCTGATTTCTATGCACCATATGCATTCTTTAAAGGAAGCTTTGATG
>comp146741_c1_seq1
TGCCTGTCGACCTGGAATTCGCCAATTCGGCCTTAAGAATTGAAACCAACACAGCTTTTCTCATTTCCCGACAGCTCACCGTTAGCTTCATCGTCATCGCCACCTGAAAAATGTCCTCCGC
CGCCAATCGCACCGGCGGTGCCGTCACCTCGCTGTTGTATTTTTGCCACCGGTGTGAAGCAACAGTGAACATCGTCTCCCCTGTCGCCGCTGACCCCGTCTGCCCTATCTGCGGCGACAG
CTTCGTCGAGGAGCTCGAGGTACCGGAATCAGGGTCCTTCATCTCCAACCAAGCCGGTGTTGAAGATTCTACGATCGCCCCAACGTCGGCTCTCCCGTTCAACTCCTCTTCGGGCAGCGT
GGAAATTAGAAATCCAGGAGACTTTGTCGGTCTCCTCGGCATGAACTCCACTCCTCAATATTCGCGGCCATCCGAAAACCCTGGCTTCGATCCTCAGCTCTTCTTCAACGATTACATTCAA
TCGATTTTCTCCGGCGGAGGAAGCATCCAAATCATGTTTGATGGCGCCGGCGGCAGCTCTGCCGAAAACATCGGCGAGTACGTATACGGCCCGGGCCTCGAGCATGTCGTTCAACAACTT
TTTGAGAACGATCCAAACCGGTACGGTACTCCGCCGGCGTCGAAATCGGCGGTGGAGGCTTTACCGATCATCGAGATTTCAGAGGAAATGGTTGCTGGAGACGTCGAATCGCAGTGCGC
AGTTTGTAAGGATGAGTTTGAGGTAGGTGCTGAGGCGATGCAGATGCCTTGCAAGCACATCTACCATCAAGATTGTATCGTTCGTTGGCTCGAGCTGCATAATTCGTGTCCGGTATGCAGG
TTCGAGCTTCCCACGGAGGATCCTGATCACCTGCAGCGGGTTGCGAGAGCGCCGGCTGCTCGAGGAGGGCGATCGGGGGGAACGTGGGATTTGAGTGGCCTGGCTTCAACAATTGGGG
AAGAACAAAGATCTTCGGGAAACAGGAATAGGCGAAGGAGGGTAAGAATCTCGCTTCCAGGGCCTTTCAGCGGTCTCCTCGGTAATGCTGGTGGTGTGAACGATGGTTCCAGTAGTGCA
AGCTCTGATGAGAATTTTGAAGGTCGATAGATTAATGGTCCGGAGGATTAGGACTGGGCGATGAAGCTCCAATTTGGGAGTAATTATTATGTCCGGACATAGAACGCCTCCAGAGATTAAT
ACTCATGGGTCATATCACCTGGAGCTCGGTATTGAGCACTCGGTATTGTTCGGTATACGTTCAAATTTCCATTTTTTTACCAGATGTAATAATTTTTCCAATTTTGAAGCT
>comp146788_c0_seq1
TTGTGCCCGTTATTCTCCTTTCCTCACCTCTCCGCTGTCTTTTCCCAAATAAAACAAACTCGCCCTAGAAAGACTCTACTCCAAATCCTTGCCGCTTAACCCCAACAATCCGGGGCTACCCT

TCTCCCTCTCGGGATCAAACGCTCTATCTCTGGCCTCCCATTCTCTCCCTCCTCTGCTTTCCAATGAGCGATTTCCGCAGGGCCTATCGTATCTATAACACCACCAATGGCGATCGCCGCCAT
CTCGAGGTCGTCTGCGGCAAGGCGTATAGCGCTGCTCAAACCTCTTACCTAACCTACGCACGCTCGTTGGACCACCCTCCTCCCCCAAGGAGAGGGACAAGCCGGCCAGTGGCGTCGAA
GTGGTGCTTCAAAGATCCAGAGATGAAACGGCGGCGAAGGGTTGCAGGTTATAAAATGTATTCGGTGGAGGGAAAGGTGAAGTCTTCATTGCGTAAAGGGATGCGATGGATTAAAGGTA
AGTGCTCCAAGCTCGTTCATGGATGCTAGGGAGCGACGGTGAAAGGAGACGACATGGAAGAGGAGAATTTGTTTGGTTTATGCTCAACTTTTTAAGTTCACAATAGTGGTGGAATTTTTA
AGATTGTTGTTTGATTGATGGATTTTGTTTTGAATCTTCTATGCTCTTTATTTTAAATATAATCGTGATTTATTTGGTAATAAGTTTGTTTCTCCATATTGTTGTAATTTTTCGGCTTCCTTTTTTT
TTTTTTTGCATTTTTCTTAAATTTTCTTTGTTATTCTCCGCGTCTTAGGTCTTGTTATACGGTTTTTGTAGTCTCTAGGGTTTATATTTTGATTTTGTTTTCTTGGTTGGATATTTGATGTGGTAG
GGTTTCTAATTCATTGTTGATTTGAATCGTATATGCAGCATGGTCTACTATGTTAACACAGAGAGGGAGAGAGTATATTTCTGACAGATTTTATAGTCTCATTGCTGAATTAAATTTCGCTGG
AATTTTCTTACAACATATGAGAATCCATCTGTTGGATACATCTTTTACGTATTCAAAGCGGAAATTAAGATGAATTTTTGACAATTTGAGCTT
>comp146795_c0_seq1
ACAACAGGGTTTATCTTCTGCATCTTGCACCGATGAATAAAAACATGAACATCAACAAATGACCATACAAAAATAAAATCTAAGTACCTTACAATATAAAACTCCAATCATTAAAGCAATA
GAAAACAGACACCACAATTCAAATTAAACGGTCGACAGCCAACGGTAAAAAGTCATAAAATACTCAAAAATAAAATAACAATATCAATAGCATCATCATCCCTTCTATCTTCTCTTCCATAT
ATCTCTTCTTCTATATGCCCATATATAGAATCAATGAAGGTCTCTGGCACACCTGGTGATAAAACTGCAGCCGCGGCGATAAGGGTTAGCTCTGCCATGGCGGTAACAGTTGTAGTAAGAA
ACTCCGCGGCGGTTGCAGGGGACTTGGTTTCTCCTTAGCATTGCATAGCTGAGGAATCGGCGGCGTCTCGCCATGAGCTGGCGGCGTTCGTCTGTTTCCTCAGCCTCGTTGTCATTGAGG
AAACAATTATTCTCCAAACAATTGAAGTCGTTAATGGCGTATGAGGATATGGAGGTCGTGAGATCATCGATGGGGGAGGTGGCTGCGGTTGTGAGGGGGAGGAGGAAAATGAGGAGGAG
GAGGAGGATTGTGAAGGGAGAGGGAGGCGTAGCCATGGCTTTGGTTTGTTTTCTTTCAGAGGAGCGAATAAAGAGCTGGGGAGAAGAAGAAGAAGGAGGCCGGGGACAAG
>comp146864_c1_seq13
CATGTGGTCAGTCCCAAGCCATAGGTACTGTAAAGAAGAAGGTACTCAAGTTCAACCTTAATATGCTTCATCTATTAAATCTCTCCATTTGGTTATTCCAATGACGAGGACATTGATTAAGG
CCTCAACTCCATCAATCTTAGTAAGTAGATGATTCAAAATACCGTCTTGAACACCAGTACCATCCCGCGTTGACCCTCTAGACTCGTGCCCTTTGTTCCTGTTCGGCATCGACAAATAAATC
CCTAATATTTTTCTCCATTTCTCCAACGTATTTGCTCAAAACTTCAGGTCCATTAACAATCTTTATGGCAGCAGCCTTGGTCAATGCCGGGACATGAGAAGTAAAAGAAACTTGCTTCATCT
AGTAAATCTCTCCTACTGGTTATTCCAATGAGGATGACATTATTTAAGGCCTCAACCTCATCAATCTTAGTGAGTAACTAGTTCATAATACCATTTTGAACACCAAGACCATCTCACGTTGAC
CCTCTAGACTTACAAAT
>comp146864_c1_seq7
CATGTGGTCAGTCCCAAGCCATAGGTACTGTAAAGAAGAAGGTACTCAAGTTCAACCTTAATATGCTTCATCTATTAAATCTCTCCATTTGGTTATTCCAATGACGAGGACATTGATTAAGG
CCTCAACTCCATCAATCTTAGTAAGTAGATGATTCAAAATACCGTCTTGAACACCAGTACCATCCCGCGTTGACCCTCTAGATTGTCAAGGAAAAAATAATAGCCTCGTGCCCTTTGTTCCT
GTTCGGCATCGACAAATAAATCCCTAATATTTTTCTCCATTTCTCCAACGTATTTGCTCAAAACTTCAGGTCCATTAACAATCTTTATGGCAGCAGCCTTGGTCAATGCCGGGACATGAGAA
GTAAAAGAAACTTGCTTCATCTAGTAAATCTCTCCTACTGGTTATTCCAATGAGGATGACATTATTTAAGGCCTCAACCTCATCAATCTTAGTGAGTAACTAGTTCATAATACCATTTTGAAC
ACCAAGACCATCTCACGTTGACCCTCTAGACTTACAAAT
>comp146864_c1_seq8
AATTTACTCCATGGGACACGGAGTAAATTGTTGCAATAAATATTTCTTCATTATTGGCTCTTTTGCATTATATTTGAAAATATTTTGTACAATTTAAGTTGTATTCAACTACTAAAATTTTCTTC
TTTTAATCCTTAATTTTATCACCTTTTAAGTGTGATTATTAATTTCATATGATTCATGGTTCCATATATAGCATATCATTAATCATATTTATTCAATACAAGCATATATAATCAATTTTGATACTATTT
AAATTACTATTTTTCAAGGGGAAAAAAGAAAATTTTTCTATTTCTAATTATTATATATAATGTAAAATAATAATATTTTTCAAAGTTTCTATGTAACAAATATTGTTATTTTTTAAAAAATTCGA

GCATTGTAAATGTTAGGTGGATGTTATGTGTAAAATTTTATTTAAAAAAAAAAATTGAGAATTTTTTTAATAAATGATTTTAGGAATTTAATCCTTATTTTAAAAAGGTGAATGCACTGCCTC
TTGTCACAAGTGAATTCAAATTTTAATGGAGTCTTTCAGTTAAATAGGTATAATGTACAAAAGACTACCCAGTCTATGTGGAAGTGACATACCTTAATATGCTTCATCCATTAAATCTCTCCA
TTTGGTTATTCCAATGACGAGGACATTGATTAAGGCCTCAACTCCATCAATCTTAGTAAGTAGATGATTCAAAATACCGTCTTGAACACCAGTACCATCCCGCGTTGACCCTCTAGATTGTC
AAGGAAAAAATAATAGCCTCGTGCCCTTTGTTCCTGTTCGGCATCGACAAATAAATCCCTAATATTTTTCTCCATTTCTCCAACGTATTTGCTCAAAACTTCAGGTCCATTAACAATCTGCAC
ATGTGGAGGCCGATCAGAGGGATCTCGCACGCCTAGAATCGGACGGTTTGGCTAGGGCTCGCGAACGGTAGAGAGGGAGAAGGAGAGGGGATTGACGGGCTCTTGGGGGCTGCTTTCG
CCTGGGGTTTCTTACATGAGAGAGAGGGAGAGGTTAGTTTCTTGCGAATCCAAAGGAAGAGGAGATCATGGCGGCGTTTTGAGCTGGCCCTAAGAGA
>comp146894_c0_seq2
CTGATCAAAACCTTAAGCATAACAATTATTCATTTATGTTTTAAATGAGAGCAATCGTTTATTCTTTGTAAAATGCAACCATTCACTCAACAACATAAAATTGCAAATTAATATACCGCAAAC
ACGCCAAACATTATGTAATAATACTGTTCCTTAGGGGTTTGTTTGCAAATCATTTTGATAAAAAATGGAGGCCGTGTCCTCACCGCCGGCGTCGATGGCTGCCGTCATCGCATCCGTCTGCA
CAAGATTCTGTCGTTGGTAGAGCTGACTTAGCCTCCCTATCTCCTTGTTCAGTGTCTCATAGCAGCCATCTCTAACGAAATTCTCTTGTGCAAGGACAGCTATGCGCTGCTTGATTTGGCTA
TTCTGCATGACGAGCATCGACCGTTGGCAATCGAAGAAGGCAACTCTTGGCGTCAATGCCGCAACGTCCGTCTCCAACTTTCCGACGCGTTTCTGCAGCTCGGAGATGTACTGCAACTTC
CTCTCCCTCGATCTCCGCGCTGATTGTCTGTTCGCCAATATCCTCTTCACCCTCTTGGGATCTTGAATGGGTAATTGAAGGGTATCGTCGGCCGAGGAAATCTCTCCTTCAGAGTGGATAAA
ATGAATTGGCTGGTTTGGATGGGCATGATAGTCAGGTGGGTTTACGTTGGGCTTATAGTTGTCTGCGTTGACATTGTTATGGTCGGAGGGAGGAGCGGACGACATTGTTGGGTTTGCGGCG
GCGGGAGGCTCGAGAGCGACGATAGAGTCGCTAAAAGCGCGACGATGGGAGGCTTTTGCGTATGCAGCGAAGTTGAATAATTTGTCAACCCATGAGTCGCTCTCGGGAGAGAGACCGA
AGGGGGAAGGAGGGGAGGAAGGGAAGGATGGAGAGTTGAAGGGGGGATTGGGGGGGAGTCGATCAGCCATCTTATTTAAAAATAAATGGGTTAAATTAGAGATTTA
>comp146939_c0_seq1
CAAACAAAAAAGCAAGTTGAGACTGTCTCAAGTTTGGCTCGTTCATTCATGAAACTCAGCTCACGAGCCATTTATCAATCAAGCAAGAGACGACTCAACTCAATTACACCTTAGACACA
CCACATCTCATACCCATTCAACTTTTGGCTTGCTCTAACATACATGCATCATATTGATATTTATGTATATCATATAAAAAATCTTACAATGCCATTAAAAAAAAAAAAAATTGCATATGTTTTTAT
GCAGAGGCTCATATCTCCTAAAACCAAAAAGAAAAAGAAAAAATCTTTATGCTATTTCTTTCTTGGTAGATTTTTACTTTCCACCCACCAAACAATTCACAAAATCCACCTAAAAAAACAA
TTAAATTCCAATAAAAGAAGCTCACTTTCCCATCTCTATAAGTTTTTGTTCTCCACCCCACAATCTCACTCGTTCGCATGCCTGTGCAGCCTCATCATTCCAGTTGGTCTTCCAAAGCACAT
AGCACAGAATCAAAGTCTGCAGTGCAGTTCCCAAAAGCATTCCAACCCATATACCCTGAACTCCCCACTGCAATCCATACCCCAGTAGGAATCCCAAAGGAAGGCCAAAGACATAATAG
CAAGCCAAGTTTATATAAGCCACAAGAGCTTGCCATCCACCTCCTACTGCCACACCTGAAATCACTGGCTGTATGCTATTGAGGATCATGGTGATGGCAAGAAGATGAGCAATCTTAGAAA
CGGCATGCTGAAGCTCTTCATCATTTGTAAATAGTACTGGAAAATGGTTACGAGCTGCGAGTATAAGAAACATTGCAAGGAGACCAATTACAAGGGAGGTGGCCACCACCACAGCCACT
GCGTGCTTGGTGGCTCTCGGCCTTCCTGAGCCGAGCTCGTTGGAGACCCGAACGCTTATGGCCGCGTTGATGCCGATGAACAACATTCCTTCCCAGCCGTTGATGTTCATGCAGATAGAA
AGAGAGCCAACGGCTATCTCGGCGTTCTCAAGATGTCCCGTAAGCACAACTAGCACCATCATGTACCAAATCTCCAGGCAAAGCATCACAGCTGAAGCGAGGGAGAGTTTGACGAAAG
CCCACAGATCCCTAAATGCGCCCCAGGAGAGGCCCGCTCCCCATCCGTCCCCACACCATCGAACGACGTACACCACCTGGGCAATGGAGACGATCCACTGCGAAATATCGTATGCGGCG
GCGGCGCCGATGATGCCCCACCCGAATCTGTAGAGGAAGAGGTAGAGTAGAGCGATATGGATGAGCAGTGCGACGAAGCCTATGCAGGCGAGGACTGACACCTTGCTCTGCGCCTGGA
GAAATTTCTGTGCCGGGAAGTTGATGGCGAGGGCGAAGATTTGGGGGATAATGGCGACGGTGAAGCGGCCGGCGGCGTCGGCGATGGCGGGGTCTTGGCCTATCAGCTTCAAGATGGG
GGTAGAGAAGAGGTAGAGGGGGCACATAAAAATGGCGGAGGCCGTGAGGATGATCCATGACCGTTGCATATAGATGCCGAGCTTGTCCACCTGCCCCGCGCCGTAGGCCTGCCCGCATA
GAGTTTCCAGTGCGCTCCCCATGCCTAGAAGAAATCCAAAAGAGAAATTCGATATAACTGAAAGCCCGATTGCGACCGCCGATAACTCCAGATTTCCAAGATGTCCGGCGAAGATCTGCG
TAGTAGAATTAACCCCATATAGACAAATAATGCTGAATGCGATCGGCGTCGCGATCCACCACAGCTTCATCGACTCTCCCACAAAGGTCTGCCAAGCGTCGCGCAAGCTCCTTACCTCCGG

CTCATCGTCTACGTCCGGGAAGCCGGCGGCGACCGCATCGGCCAAATCCGCATCAGCTTCCGTCGTCGCAACAGGTGACGGAGACGGACCCGACCCGATATCGAAAGGGTGAATAGGCA
TTTGGTCCTCGTCGAGTCGTCGTAGTTGGTGAGAAGTGGGGAATCCATTGAAGCAATTGATTCTTTTTCGTCATGGCTCTCCGGGGCTTCGGAAGGCGCAGAGAGGAAAGCGGAGGGTT
TGCAGCTCACGACAGAGGTGCCGAGTGGATGGAGGGACGGAGG
>comp146956_c0_seq3
AACCAAACAATGTTGATTAGGACATATCATTTTTAAATCAATTATTTCTGATAAATGTCCATACCCAACCAATTCTCTGGTCATCTTATAATACAAAGTTCAAACAAAATCAAATAAAGTTTA
CCAATTCATAAACTGTAAAATTATTTCTTCTTATATAGAAACATTCCAAATTGCACCATCACCTTCTCCACAAGCATTATGGACATCTTTCATCTTGTCCACCGATTTGCATATCCCACTGCAG
GACCAATCAAATGATGCCACACAAACATTTCCCGCTTGCATTTTCCACTCGCAATCTGGAGGAGTTCCACAGCACATAGTTCGATCATCTATGTGCTGCACGTCCAAGCCAATTAGCCATG
AACCCAAAGAAACATCCTCATTTGCATATCTATGCAATATTGGCGCATTTATAGAGATATATGCTGCCAGATCCTTTGAGATGGCATATATTTGACCCGTAGCATGCCTGAAATACTTATTGCC
TTCTTCTCCAAATTTCCAGAATTCTGGTTCATGATATTTCACCCCCTTCTGTGATAGAACGGGCCCAGACTTCATGCACCCGACATACACCCTCGGCTTGGTTTTGTATCGTGAAAGAGTTG
TTCCCAGCATTCCTAGATTTACATGCACGTCGTCATCAACCTTTACATAGAAATCTGCATCCCAGATAGAGACAGCCGTGGCGAAGAACACCCTCGTCTTCGTTGACAGCTCGTGATAACC
TTCCACATGCTCAAGCCTTAAAAAATCCTTTGTTTCTGCTTCCTCCGCGTCGATAGCGCGATCCAGTACTCCCCCAGGTGTGGCACTGTGACCGATTACAAATCGGATTATTATACCTTTCT
CCTTCTCCAATCTCCAGAGCTTAGAACCTTTAGGCATCCAAGTCTCTCGAATCGAGTGGCGCCGCCTACTGCTGCTAAAGGCAGTGTTGATGCCGATGACGACGAAAGCCTTTTGGAGGC
CTCTGCCGGAAGAAGGTGGCCCCCAATTTTTGCTAGTTCTAGCCACTGCAAGCTCCATTTCAAGTGAAGAAATAGACTTTTCCAATGACTGAATGGCTTTATGGGTCTTTGACACCTCTCC
GATTATGTCCTTCGAAATGCCCTCTCCCAATCTTCTCTTATGCTGACACTCGATTGAGTTCTTAGATTCATATGTAGTTACAGTTGTGGAAGAGTTTGCTGCTTTTCTGTTCTCCGGCGACGA
TGAAGATGGGAGCACAGCGATTCGGCGGGTGAAGAGAAAGCCGAACATAAAGCAGAGAAAGCACAGCGCCGCCACCAGCCTATGAGGCAACAATCTCCCCCTTGATTTCTTCTCGGAT
GATAGGGATCTCTCCGCCATGGCCTTCATGAAGCCGCGCAGGAGTAGCATTACGATAAATTTAGGGATAATTC
>comp147016_c0_seq1
CATAATGAAATTGAAATAGAATCCAACAAAAAAAAAAAAAAAAGACTCAAAATTTGTCAAAGAGAAAAAATATTTTTCCAACATTATAATGTATGGCAAGGAAGAGCAGTCCCTTGCAGA
CCTGATTGTCCTATGGTCCATCCACATCTTGGAGGCGGAGAAATTATTCCATGTCGAGTTCGGACTGGAAGAATCGTACGAATTGTGAGCTAGCGTATATAATATGTATCAAATTTTTATACA
TACTGTTTGTACATAATGTGTATACTCACAATTCGGATAATTCTTCCTGTCCAGACTTTACATATAATAATTTCTCCTGCAAGTGTGGGTTGAGAATTGGAGATATCTCATTCATCATTGTCCA
GATCCAAAAAAGAAGCCCACAAAAGCAACTTATATTTTTTTGTACTAATTAAGGTAATTTTCCTTAATATAAATATTATGATCTTGCAAAATGAAATCATCACAGAAGAGAAAAATCCCTCA
CAGTCCCTTCGGAGCCATAAGGAGCTTAGTAAAAGAAATTGTTATATTCAGCAGCTTCTTCGCCCCATCATTCCTTCTCCTAATCTCAGCCGTCGCCACCGCACCGCACCCTTTCTCGCAA
GAAATCACATCCGCCATTGCCGCATTCAGCCAGATCATCGCATCCTTATCGGCCTCATCATCAATGGCCGCCGTCGTCACGTCGAGCTGCTCGGCGACATCAACATACAGATCTGTACAATC
GGCGACGCATTGATAGAGCATTGGATCAACGGCGCCGGCGGCCGCGGCGTCCGTCTGTTGGGCGGAGAGAAAAGCGGCGGTTTGGGAGGCGTTATTGGCGGCAATGCGGACGGAGATG
ATGGAGAGTAGGCGGAGGTCGGCGTTGGAAGACGGCGGTTCGGCGGAGAGGGAGGAGATGCAGAGTTTTTGTAGATTGGATGCGGTTTGGCTGCAGGCTTTGTGGATGAGGGAGGGGA
TGGCCGGGTGGGCGGCGGCGGCGGAGATGAGGAGGGAGATGAGGAGGGTGATGAGGAAGGCGAGGATGGAGTATGAAGCCATTAAGGTTTTGAGGTGGCGAACGAGGAAGGAGGGG
GGATTCATTAGAGGAAAAGGGGATTTGTTAATTGTTAGGACTTGGTGGTTATTGAGAGTTTAG
>comp147064_c0_seq1
CATTTTAACAAAAACTTCTTCATAGAGGAAATTCTATCATTTTTTTTATATCTCCTCATTTTGAGCTGCCAATGAAAAATAATTTATTTATCTATTCTCTAAATTTTAACACACAATCATAATGT
GAGTGTGAAACAATCATAATGTGAGTGTGAAGCACTATATTATATAGGAATAAGAGTCTAACTCGTAAATTTTTATCTTCTTAGCTTTGCCCCAAAGGCATAAGTTCTATAAAAATTTTGGAA
GATCCTGTGATTAGATTGTGTTTTCTGGGAAGCAACTATTTTTATTTGCTCCTAGTGATGATGAATGAATACGTCTTTTGCATTCCACCTCAAGAAGTTAGTCAAGTAATTGCATAAAGCTGC

AGAGTTTAATTACTCTTTTAAGGTAGATTCTTTTATTATTTTATTTAGTTATATGCAAACAAAGCCTTAAGCGGCACAAAAAAAAAGTGATATCTTTATTGAGAAAACAGACAGATTCCAAAT
GTTAAAGAATTTTTGAAGTACTGAAACCCTTTGATATAACAAAATTTCAGTTTTATTTTTTAAAAGCAATGTCTTTCATCACAATGGCCATTGATCTTTCATTCATCTAAGGCATTTTGCGTTG
CATTTAAAGCTCAATTTGATAGAGCTATAGTCTTCATAGTAAGTTTTAAGAGTAGTGTTGTAAATATTATTAAAAAAAAAAGCACACCGAGTGATTAGGCTTATGTCAATGCATGCAAGATCC
AGGATCAGGTTGATTTATACAACTGACCCTATTGTAAAGATTCTATTTCTATGATTTGAATTACTGGATTTTAATTCAACAATGGAGGCAACTAGCCATTTCTCTAGGGCCACACTTAAAATA
CCGGTCATTCTTCATTATCTTTATTAAAATTTGCTGCCCATTATGTCCTGGTCCTCCAGACTTAGCTGTACTATTTCCACCAAAATCGTTACCATTTAAACCTGGGCTGTTTGATTTGGCTCTG
TTCTTTCCGGAATTGTTGCTGGTTTCTTTGTACCTTGTTATTAAGGGCTTGAAAAGAATTTCTAATTTTCTTAGTGCTTAAAGAATTTATAGTTTCTAATTTTCTAATTTAATCGCTAGCTTTCA
GCCTTTCAGTACTTTTCTCCAAGATAGCCTGCATATGGCATATTGGCTATCAAAGTCATGCTAGATTCCTTTCTAGTTTTTAATATCCTTTGGCATGATTAATGGATTGTTATGTATGAATGTGT
AAAACTTCTTTTTTTTTGGAAGTTAACAGCTAAAATATAAATTTGATATCAGCGGGATTCGATCTTAGAAAGTTCAAAACAACTTTTAAGATCTTTATTGACTAAGCTAGTTGGCGCCTATAC
TTTGGAATAGAACAATTGCTTGCTACTTTCTTT
>comp147077_c0_seq1
GAAGTGGTGGGAGACGGCCCCCAAATATCTGAATGAACTAAATCAAAAATGTTAGAGGTAGAAGAGGTAGAATTCTTGAATGGTAACCTGCGGCTTTTAGCCAAGTTACAAGAATGACA
ATGATTCTTATTCAGATTGTTACAAATTGCTGGAATGAATTTGCTAAGGACTTGAAGAGTCTGCCGAGCCGGATGGCCAAGACGTCTATGCCAAAGATTTGGTATTTCTTGAGCTGAAAAC
AGAGCAAAACCGGAGGTGGAAGAAGAAGCGCGAATGGGGTAGAGCCCGTTAAGGCAAGGTCCCTTGAGAAGAGTGTGACCCGTCTTCGAGTCCTGGATTTTAAATTCATGAGCAGAAA
ACTTGACAGAGCAGTGATTATCTTTGGTTAGCCGGTAAATGGAAATAAGGTTGAAAGACAGATTTGGTACATGAAGAAGATGTTGAAGACATAATTTACCTGATGGAGTCGGAAGAATAC
CTTTTCCGGTGTGCTGAATAGGAAGTTGATAGCCATTTCCAATTGTGATTTGTTGGGTTCCTGTGTAGGGACGACCGGATTGAAGGTTGGACGGATCGGATGTAAGATGATTTGATGCGCC
ACTATCAAGAAACCAGTCGGTAGTGTTGCAGGTATCGGACGGAGTGATTAAAGCAGTTTTGACAGATTCTTCGTTGTACGATGGATCGTTGCGATACCAGCATTTTATTGATGAATGTCCG
ATTTTAGAGCAGATCTGGCAGGTGATCCCGCTGGATGAGGTTTTATCTGAGCGGCTGCGACCACGGTTGGACGTTCGGCCTCTACCAGAATTGTTACGTCCGCGACCCCTTCCGCGATAA
GCGGCCAGAGCAATTGCAGGATCGTTGAGAGTAAGATTCTGGAGGGCAGTAGTGGTTTCTTGGGCTAGATTCAGTTCTTCACTGCAGAGCAAGGTGTACAAGTCGTCGAGGCTAAGAGG
TTGCAGGTTCGTGCGTATAGCAGTTTTGAAGGACTGATATTGCACCGGAAGCCCGTTAAGAGTGTAGAAGATCACTTCTTCTGGATCTAAGGGAGATCCAGTGGCAGCAATGGCATCAAC
AATGGATTTGACAGCAAGAAGATATTGAGGCATAGACTGATTTTTCATGGACAGA
>comp147110_c0_seq1
TAAAAAAAAAAAAAAGAAGAAAGAAAAATCACACAAATCTAATAATATTAGGATAATTCCATCATACGTGAATCCTTCCCTTGCGTGAATCAAAATTTCCTGAATTTTATTTTACCATTGGT
GCCCAGCTAATTTTTTTCTGGGAACTAATGATTCTTTGAGCAACGGCGGTTGCTGGGCTTGGCCCTGCCGAAACCCCTTGCAGCTTGCACAGTATAACATCCTCCATGTCAGGCCTTTCGT
CTGGCTTTTCCTGAACACAGGCCATTCCCAGCTTCAGCATCCATGCCTTCTCCAAATCTGTTTCAGCCATATCATCTACTTTCTCGAGCCTTTTCGCTTCAATGTCTTCGCAGTATTTTCGCG
CCGACTCAAACAGATTATTGTCCACCGTGTTCCTCTCCCAAAAAACGGTCTCCAGAACGACGATGCCGAAGCTGTACACATCCGCCTTCTCCGTAACATTTTCCCCCTTGGACTGAACGC
ACTCGGGAGCCACATAACCAGACGTCCCAGGTGCTGAGGTCCGCGTTGTCGATGTAGCCTGTAAGCCTCCCACCAAAAGCTTGGAGATGCCGAAGTCGGAGATCTTGACTCTCCAATTC
TCATCAATGAGGATGTTTTCCGGCTTTATGTCACGGTGAATGATCTTGGTCTCGCACTCGCGGTGCAGGTAGTGAATTCCCCGGGCAACACCGAGAAGGATCCTGGATTGCTTCTTCCAGC
TAGGGGGATCAGACTGTAATATCAGATTGGCGAGTGATCCCCTGCTTATGTATTCGTATATCAAGAGACGTTTAGATTCTTCGTAACAGAAACCGAGCAAGCGAACCAGATTCGCGTGGTG
AGCTCTCCCGATGATCCTGATCTCTGCATGGAAATCACTCTCCCCTTTCTCCTCTTCCGCGCTGAGTCTTTTCACTGCAACAGGGACGGGGTTTGGCCTTCCATACATGGTCAGATAACCCT
TGAAGACAATCGCGTGGCCTCCTCTCCCCAATTCCGAGGCGAAGGAATTCGTGGCCTTGGCTAGTTCTTGGAAGGAGAAGCTCCGCGGCGCGACCTCCTCCTGAAACGCCAGCTCCCAT
TTGCGCCATAGCAATCGGTACCTCCCATAAAACAACCAATAACCTCCGGTGCCGGCGACCAGCAAGGCTAGAAAGACGCCGGCGTTGATTGCCACGCACAAGAGCACGGCCTTCAGGTG

TATTAGGCTCCTCTGTTTCTCCTCAGTTTTGCCTATCTTGATGTAAGCAGTGCTAATGCCTGCGGTAAAGTTGGTACCTTTAGCAGCGCGACTGATCGGTAGCTTATGCTTGGCGCAGTTCTT
GTTGAAAAACACGGCAGCCTCGCAGTTGCATTCGGTAAGGCAGGTGCTGCTGCATTCATCCTCGCTCTCGACGGAGATAATGCTGTAGGCCTCTTCATACAACACTAAATTGTCAACGTG
CTCCATCGAGAAGGTGGTGGTAGTATCGGAAGTGCCTGACCAACAGTTTGGTTCGGTAATACTTTTCTGGCATCCATTGTCCGGAGCGCCGGAATCGGTGAAGTTGAATCCAGGCAGGCA
GCGACACTCAGCGCTGCTGTTGCTGATCACGCAGTAGCTGTTCATGCCGCAAAAGCTTTCAGTCTGGCAAGCGTCTTGATGAGTAAGAATTTCGTATACCGGAGTGGACCTGTTGGTGCC
TAAGTTCAGCGCGAACAGCTGGAAGTATTTATTCGAATCTAGCGTCGCATAGTAGAGCATTTTTGTTTTGCTGTCTTTTGGGTTATACTGTGTCAAATTGATCTGATTGTCAGCGGTGCTGA
GGAAGAGACGGCCATCTTCGCCAAGGATCCACTTTGTGTACCTGATCGGCGAGGCCCATGTCTGTAGTTGTATCACCATCCGCAGCAATATTCCTCTTGAACGAGTAAAGGACCAGGTTG
GAGTCGCACTGCATGGTGAGAAAGTAAAATAACTGGTCGGTGCTATTCTGGCGGGAGGTCAGGCCGGTGCCGCAATTCAAAACCTGACCTCCCATCATTGTGTCGGTCGGATTATCAAAA
CTCTGCCAAGTGATACAAGAGTCGCCTTTGACGCCGCCACTCCCGGCCTGGACGGATATTACGCTGCAGTTCTTCGTAAAAGTCCAATTGTTGGAATCATAGACCACGAAGTTGCCGCTG
TCGAGCATGCTTGCACCGGTGGACTCTGTACCGTTGAATTCGATGAGGGTTATGGTTTTGGCTCCGTTAGATGGCGTGAGAAGAAACCCCTGTTTTGTGAGGAATAGAACGGCTTCTTTG
GAGATAAGCTTGATGCCTTGATCTCTTGCCGCTGTCCAAATTACTAACGGTTCCATCGTAGGGGAGACGTACATATTTATGCCAATGGAGAACTCCTTGCCTTCCTGATAGAATCCGAAGGC
GAAGCGGCCGGAAGGAGAGATCCAAGAGCTCGGGCCGGCGGGAGAGTTGAGATGGAGGGAGGAGCCGACGGGGATCTCAGTAAAGGCATGTGTTTTAGCTGCGAAGCCTATTACTGCT
TGCCCGACCGCGACGAAGGTGAGGAACAAGGCGAACATGGCTCAGTTTCTCGGGCCTTTCTGCAAGCTAGGCTTCTTTCTGTTTATCTAAGCAATGGAATGATGATAAATAGCGGAGAGT
TCATTGACTA
>comp147131_c0_seq2
AGATGAACTCACATGATATATGACATCTGAATGTTGATTTGAATGTGCAGCTATTGCTAAAATCATGGCATTAACCACCATGTCTCCAGGAATCTATCAAAGTTAGCATTCTTGAATAAGTTA
TAATAATAGAGCTAATTTTATCTTGACATTGTGATATATTATAAGTGTGTAAATTTGCCTTTATTATGTATATGAAACCAATATTTCATGAAAGGCAGTATTATATCATTATCA
>comp147140_c0_seq2
AGACTTGGATAGGGTGCTTCATGTGTGTAATGCAAGCAAGGCTTCCAGAAATTTGCAATAGAGTTTCCATCTCTGCATAATACACTGTAGCCCACATAAATCCATCAAGAAAAAATTTGCC
GATTAATTTAAGTATTTGTAGTTAGAACATAAGAAAAAAATGAATCAAATCATATCAACATTTATCTGTTTTTTATTGTAATGATAGAAGTCACCTCAGAAAGGTGACACCAACAGTTTTCTT
CAATAATTCTTGAGCAGTGAGATGAGTGCATCTCTAACCCACCCAAGGGAATTCTCATGACTGCTGCAATTGAACTCGTTCCTTATAAGACCCTCCAGTGACCATCCAGTCTTTTCAGAAA
ATACACGGTCAATATTTTTTATGTCGATTCCAGAGCGTGTAACCAACGTTCTGATAAGCACTTCCTGAGAATCTCCAGTCACCATTGCTCTGCGTAACTGCTTTTCGAAATGCTTCTCTGGG
AACTGAATGCATCGGATTACAGCTCGGAGTTGGATTCCGAATTCACCGCTGTTATAATACTTCAGGAACTTCATGAATTCGTGGCCAAAGAGTTGCTTGAATGAGGAAAGAATTGTCTTTA
TCTGGTCTATGTTCCTCAGACTCAGAATAGAAATGACGGAATTATGATCAATGAATTTGCTACTTGTGATTGCTTCATAGAGGATCTTCGCATCACACAGAGCCATACTTGTGTCGAACCTT
TCTCCACTGTAAGTTGAATTCAAGATGGCTGAAATAATCTCTTTCAAGCTACCATTTGCCTTTGTTAAGATATCTTGCTCAAGTAAGGAAATGTATTTCACCCGATATGCTTGCTTAATTGAT
AGTAGCTCTAATGAAGATCGAGTGCATATGATTTCCATGACAAGTTTGAGGTCAGTCTTATTAGCATACATTGCTCTTCGAATTATCTCTGCATCTCGATCACTGGGCTCACAAAGTTGAAG
ATACACCATATACGAGAAAAGATCATTGTTTTGTGATAGGGTGTAAAGAACTTCCTGGTCATAAAGATCACGTAAGGTCGGACGAAAAAGTTGGAGTTCTCCACCATCCCTGTGATTGTTG
ATCTCCATCGCATTTCGTTGGTCACTAGAGCCATTATTACCTGAGATAGTTCTGGAAAAGTCCGTGGGGTAAGTACCGGGATGTAATTGAGACGGTTCATGAAGCCCCAGATTGGAATATA
GAAACTGTCCAGAGGGCATAAAGCGTGAGATTTCGTTGAATGAATCGTAAGATCGCCATTCTCCTTCCATGACTGCTTCCGTCACCGTCGGCACCGTGGCCTGGAAGGCGGAGGGAGTT
CGACGTTTGGTACGTGACGAGCGGTTTTTGTATACAGAGAGAGATAGAGAAGCGAGAATGAGCAGAATAACAACTCCCGTGAAGGATGCTTAGCCTCTCCAAATTTCCTCCAACGCGTC
AGCATCTCCCGTTTCTTCCGGCAGTTGGATGGATGAAGAACGGGCAATTTATTTAGGTGCATAACTTTTAAATTTTTGTTATTTATTAAACTAAAATTTATGTTTT
>comp147166_c0_seq11

TTCTTCTTCCCCCATTACTTTTTAAAAGGGAAGATTGAGAAGGGAGAAACAAATATTTTTGAGAGAGTTGGAGAGGCCAATAGGAGAATTTAAGGAATGTCTATCAAAGAGGTTTTCTTA
GCTTTGGGATTAGCTTATCTTCTTCTCTTCCATGTGAAGGCTCATACCGGATTTGTTAGCTTCAATTATGCTGGAAATGGCCCATCCAATTGGGGAAATCTCGCTGCTGAGTACAAATCTTGC
TCCAGCGGAACCATTCAATCTCCCATCAACATTTTGCAGGATAATGCCACTCTAAACTCCAAATTGCATGACCTCAGTAGGCTTTATGTTGATGCAAATGCCACTCTTATTAACAATGGCTT
CAATGTAATGTTGAAATTTGAGAAAAGTGGGGACTCTTGTTGCAAGATGGGAAGAACTATTCTTTAAAGGAATTGCATTGGCATGCACCATCAGAGCACACCATTGACAATCAGAAATAT
CCATTGGAGCTTCAAATGGTTCATGAGAGTGAAGATGAAGATGGCAATGTTGCAATTGTAGCTATTCTATATGATTTTGGTAACACTGACCCTTTTCTCTATCAGTTGAAGGATTGGGTGGC
TCAATTGGCTAAGGAACATTGCTCGCTGGATGAAGAGATTCAAGTGCCAATTGGTCCAGTTCGAACCAAGTCATTGAAGCGCCATAGTCGAAGGTTCTTCAGATATGTTGGCTCGCTCAC
AACTCCTCCTTGTACCGAGAAGGTCACATGGTATATTCTTGGCAAGGTAATGTGTTGGTATTTAGCCATCAAGTTATGTAGAGTACTCTCTTTTTCTTTGTGTTTTTATGATTTTTAGTATGAA
TTATTGCGGTGTATATGAGATTAAGGTTTTGTTT
>comp147166_c0_seq13
TTCTTCTTCCCCCATTACTTTTTAAAAGGGAAGATTGAGAAGGGAGAAACAAATATTTTTGAGAGAGTTGGAGAGGCCAATAGGAGAATTTAAGGAATGTCTATCAAAGAGGTTTTCTTA
GCTTTGGGATTAGCTTATCTTCTTCTCTTCCATGTGAAGGCTCATACCGGATTTGTTAGCTTCAATTATGCTGGAAATGGCCCATCCAATTGGGGAAATCTCGCTGCTGAGTACAAATCTTGC
TCCAGCGGAACCATTCAATCTCCCATCAACATTTTGCAGGATAATGCCACTCTAAACTCCAAATTGCATGACCTCAGTAGGCTTTATGTTGATGCAAATGCCACTCTTATTAACAATGGCTT
CAATGTAATGATATCCATTGGAGCTTCAAATGGTTCATGAGAGTGAAGATGAAGATGGCAATGTTGCAATTGTAGCTATTCTATATGATTTTGGTAACACTGACCCTTTTCTCTATCAGTTGA
AGGATTGGGTGGCTCAATTGGCTAAGGAACATTGCTCGCTGGATGAAGAGATTCAAGTGCCAATTGGTCCAGTTCGAACCAAGTCATTGAAGCGCCATAGTCGAAGGTTCTTCAGATATG
TTGGCTCGCTCACAACTCCTCCTTGTACCGAGAAGGTCACATGGTATATTCTTGGCAAGGTAATGTGTTGGTATTTAGCCATCAAGTTATGTAGAGTACTCTCTTTTTCTTTGTGTTTTTATG
ATTTTTAGTATGAATTATTGCGGTGTATATGAGATTAAGGTTTTGTTT
>comp147199_c0_seq3
CGTCTTTGTGGATTGTGATGTTGCCTCTGAAATAAATAAATAAATAAAAACTGCATTTCATTTTTCAGCAAATTAAATTCCCAAATAAATCTTACCGATAGAAATTGACCGCTAAAATATCTC
TTCAAAAAAAAAAAAAAACTTGTTCTTCGCATGATCGTCGCCGTCAGCACTTAAACAACAAAAAGCCGGAGAAAATATCAAAAGAAAAGGACGGCGAACGCATCCATCCGTCCATCAA
AAGCCTCAAAACTCATCAAACGGCTCTCATCGCATCATCCGGCTGCACACCCTCGACACATCAGACTGCTTCACCGGCTTCAGGAGAAAATCCTCCGCCCCTTCCTTCATGCACATTGTG
ATTCTAGTGGGGACGTTCTCTGATGACATGATGACCACAGGTATCTTCTTCAGGTCCTTTGATTCCTTGATCCTCTTTAAAAGCTCGAACCCCGTCATCTCTGGCATCCAGTAATCTGTGATT
ATCATGTTCACATTCGGCTCCAAGCCAAGCATCTCCAGCGCCCGCACCCCGCTCTCCACAGTCGTCACTCTATGCTTAAAATTACGCAGTAGCCTCGCGATAACGGCTCGGTCGACGGAG
CTGTCATCGACAGCCAGCACATGCATCTCCCTCTCCCCGTCGCCGCCGCCGGCAGCCGCCATTTCCGGCAATTAGCACGGCCTCAAAAATCCCAAGGAGCTCGCGAACCAAAGAGGGCA
CCAAATCTCGCCGCACCTGCGTGGGCCCCGCAGCTCGATTCTCTTGAGATTTTCCTCCTCTCTCTCACTAAAACGCAATCTACTTCTCTCTCTAAAAAGTTGGAGTAAACGAGCGAATCTT
CCTACGAGGGGACGTTAATAAATGGTTGCTTTTAGGGGGCGAGAAGCGTAAAAGTCCACTCGCCGACGGCGGGTGGCGTTGGGGAAACTCGCGGGGCTAAGATGACGTCAGCGAATCC
ATTGAGATTACAATAAAGACGTAAGATTTTTGGGTTGGGATAATAAAGC
>comp147215_c0_seq1
AGTACATGATTTCGCTCTTCCTATCCAAAACCGAGATTTCTCTTGCTCAGGCTCTTCGGTTTGCCCTAGACTTCCCACTGTTCGCCGTCTTCTCCGCCGATAGGGTCTCCACATAAGGCATG
ATCGGTGGAAGGCGAAACGACAAAGTCACTCAAATCACAATTGTGATTCATTTCAATTTCGGCGTTCCAATCCGTTTTCATTACCAATCCACTTCATCAGCACTTTGTATACCCAATATTCA
CAGTAAAAGCAAGAAGACGAAGCATCATATATGTTGGATAGTTGAATCACAAATGTTAGCATCTTTGAAGTTCTATCGGCAGCTTCAGAAACTGTTGAGAAAGGAATCCTGGAAGGATGT
TTTGATGATTGCAGCAAGTAAGGCAGGAGTAATCCCGTTGTGAAGGGCAAGCTTGAAGCTC

>comp147269_c1_seq1
GGGGTGTCAATATTTGACACGACAGAAAATCCGACCCGGGCCCGACCCGATTTCAAAATGGGTCAGGTTCGGGTTGACCCGGCGGGTTGTCAGGTCATCCCGCGAACCCGTTTAAAACG
TCTGTCAAGCTGCCCGTGGACCTGCCCGACCCTACCCGCCCCCTGCCGACTACCCGAGGACCCCGCCCGACCCTGCCCACGCTGCTGCCCTACCCCCTGCCGCGAAGCTGCCAGCCTGC
CCGCTCCTGCCAGCACGTTTCCGCGTGCCCTAGGCTGCTCGAGCTGCTCGCGTCTACCGCTCGCCTCGCCAGCCCGCGTGCGTTCAGCGTCGGAGCCCGAGTCCGTGCCATCGTGGGCC
CGCGTGAATCCGAGTTCGTGTCGGGTCGGGTCAACCCGACAACCCGCCGGGTCGGGTCCGGGTTCTGATTTCCAACCTGTTTTCCTACCCGGGTCAGGTTAGGGTCAACCCGTTTGAAC
TCGGGTCAGTTCGGGTTCGACCCGAGCCCAAAATTGACACCTCTAATTGGAGCTATCAATATTAGTATAATAAACTTTAAGCCCATTTATTGTGAAAGTCTCCAATTTGTTTTGTGGCAAGA
AGAATGTTTTCTTCTAGGAGCCTATAAGTGATAAAAGCACCTTGAAAAGAAGAAATATATAGAATAATTTATAGATGGCTTCTAGTTGTTAAAAAAACTGCATAGAGAAATAGGTCTATAGT
ACTTTATAGATGATTTCTACATGAAATTATGAGTGTTAACAATAACATTTATCAAATTATATAATTTGGCATTAAAAAAAAGAAGTAGAAGCATAGCTAACACAAGTGAAATAGGCAGGCCT
GTAATATTCTCAAAAGAGTTGAATAATTTTGCAAAAAAAATTGATAATCCCCAAAAATTATTTTATTATTAGTAGCATCAGCTGTAGTATTATAGCACGATCCAAAATGTCACCATCAAAATA
GCAAGCCTATTTTTTAGAAAGATTAGTTCCAACAAGATTGTTATCAAACATAGGTAGAAAAGTTTTAATGTTTTATAGTAGATATTGTTAAAGAAAGCAATAGCCTTTTTAATTATTAATAAG
GAATATTACCAATTTTAGTGGAGTAGGATCTGATATTATTCCCCCCAGATTAAGCTAACAAGCCTTAGCCCATTGTTGTCAGTAGAAATGTTTTTGATATGGGTAAATTCTTTTATATTTTTAG
GAAAGTAATCTCCTCTCAATTATGACATCATAATTAAAGGAATCATTATTAAGAATCATGAAAATTTTTCTATTAACTTAATATATGCGGTCACAACTCATGATAAGAGAGAATAAGGCTTCG
TTTCGGATAACTTTCTTACTGTTTTT
>comp147307_c0_seq1
TGAGCCATATTACTTGCGGGCCTGGTCATGGAATAAGCATTGGAAGCCTTGGAAAAAATGGCGTTGAAGACAATGTTGAGGGAATTCATATCTCCAACAGCCACATTTTTAACACCCTTAC
TGGTATAAGGATTAAGACATGGCAGGGTGGGGCTGGTCATGCCAAGTCTATCTCCTATGAGAACATCAACTTCACTTCAGTTAAAGCAGGTGTCATAGTCATCGATCAGTACTACTGCCCC
GAACTAAACTGTCCAAATAAGACGGGAAATGTGGCGATAAGCGAGGTATCGTATCTGGGTTTGCAAGGAAGCTCAGTCAGTGATGTGGCAATCAAACTGGCTTGCAGCTCGAGCATGCC
GTGCCGCGACATCACCATGAGTGAAGTGAGCTTTACTCCTCAACAAGCCAATATTCGTCTTCAATCTTATTGCATCAATGCACAAGGAAGTGCAAGCGGGCATGTCACTCCTCAAACTCC
CTGCCTGCAACATTAGCTATATTTGTAATATTGATACTATCTGTGGGTTTAACATTTACTTCTTTTGTTTGGAATTCAGTTTTTTTTTTTTCTTCTTGTTGGTGAGTCTGAGCTGTACGCGGAAT
GAGACAATGTGGACAGAATTAGCATCTTTTTGTGATTATCTATAGGTTCTT
>comp147413_c0_seq1
CGAATGAACCAAACAAATTCTTTTCCTTTACAAATCTAGCAAACTAAATAAATTTTCAGTTCTTGTATCGATATCCACTTTCAAATAGAAATAAGTCAACATGCATCCATAACCACGTACATC
AACAACCTCAAAATCATATAAACTCCTCCTAATTAACTCCCCAATAGAACTAAATGTTCATATATCCCTCAAGCAAATACAAATGAAGTGATGAACTCGATCTCATCTATCTTCTACCTCTCA
CATCCTGCTCAATCTCAGACGCCGGGTCATCCTCCTCCTCAAAACAATCAATACCATAAGCTGCATGGCCATGCCCAAAGGCCTTTATATGATCCTTCAGAGACCTCTTGTGCTTGAAATCT
GACCCACAAGTGCAATACCAGAGCTTTCCACAGTTTTTCTCATGGGTCCTCCAATCGCCTCGAACGGCGAAGGCTTTTCCGCATTTTCTGCACATGAAAGGCTTAATGCCGTGCTTGCGCT
TGTAATGGGTCTGAAGCGTGCGGAAGTCTTTGAGGGGTTTGGCTCTCGGGTGGTCAATGTTGTTGCGGCAGCCAGCCGCGCAGCAGTAGCAAGGTAGCCGTAGCATAGCCGTTGGTTGT
GTGCCTCTTAGGGACTCAGGTCCTTTTCTGTATTGTGAGCCGTGTCCCCACATGTGCATCTGCATATATATCATTCTGTAGGCCTTCATTAACGTATATGAAAAGAGTAAAAGCTGCACTTCT
TTTATATGTGAAAGTGTTAATACATTTATAATGTTCTTGCTATAGAATATGGTTAGAAAGATGTTAGCGATAAAGATATTCTTTTGCTTTGGAAGATGCAACGCAGAAACCAGTACAAAAAAT
TTTCACTTCTTAGGTTAGCTAAGCTCTATTATTTTTGCTTGCTCTGGTAATTAATTAGATGCTCAATTTTTGTTTCAACCTATTTTCTAGTCCTTCTCATCCTAATATAAAATGTATATATAAGATA
TCGAATAAGACAGATTAAAATTATAATTAATGGAGTAATAAGTCACTATGAGTTCGGCTAATCTAATAATAAGATTTCTTTTATGAATGAGCTGCCCTATGGACTAGACTTACTAGTGGTTTGT
GTTTAGCTTTGCACGCAAGCAAATTCAACACAATGCAGTTCATCATGCCCTTAATCACATACTCACACGTTTCAATTCGGAGGAAAGAAGAAGCACAAAAATCCCTGCCCGTAATACAGC

TTAACTTGAACTAAGATCATCCAAAATCCCTTAGACAGAGAGAAGACTAAGGCAAGAACAACATACACAAAACTGCACAACAAGGTTCATCTTGACCTGAGACTCACTCTCTCTTTCTCT
TTCTCTTTCTCCATCTCTCGCGTGAGAGAGAGAGAGATGGGGTATGAACAGGTCAAGATGAACAGTATTGTGTAGTTTTTGTAGAGAGTGAGAGGGAGAGAGAGATTAGGCTGGATGAT
GATGATGTTTTGCTTTAAGAAATAGATCTAGAGTGGCAGGGCTGAAAGGGCTGGGGAGGGCTTTATTCCCAGAGAGAAGTGAATTTCTCTACATACATACTGAAGAAGCGCAACAGCCAA
GGAGCAGTAAAAGGAAAACTCCAAAAGTGCCAAAAAGCATGAGAGGAACAAACATACATATCCTCCTTCTGTGTGGTTCAATCACATAAAAAGAAGGGCATGATAGAATCACTGTTTGT
TCTCTTCCTCTTCACAAATAATAGACCAGTACCTTAGTTAAATATAGTTATTGAGAATAAGGAAGAAAGAAGTGAGAAGTGTGACCTGCATGTTATTGTATCTGTTGAAGGTTTTGCAGCA
GACTGGGCATGAGAACTGTGTGGGACCAATGAGGATCTGAGAAGGGGTTGGGATCCAGTACTGGCCTTTCGTAAGCTTGCCAATAGTGGGGCAGCCAAGAGGGATGTTGATGTTGCTGC
TTCCATCCTCATCGTCTTGATCAAGGTTGTGATCTTCGTCGACCGTAGCGGAGGAAATTCTAGAGATGAGATCGGCAGCGGTGGGGCTTGGGAGACCGATGTGGAGGGCGACGGTAACC
GCCCCCTCGGTTTCGTCATCTTCTTCATCATCGTCAAGGGTTATTTTCTTCTTCTTTTTTGAGTCTGCTTCGGCTTCGCCGTCGAATATGGTGAAGGAGGTTTCATCTCCATCTTCAAGTTGA
TGCTTTTGAGGGCTTAGTTTGAGGAGGGGAAGGGCTTCTCTTAGGGGAGGGGAAGGAGGGGGCGATTGGAGGAGATTGTTGTTTGTGTTTTGGAAGTAGTAGCTGTAGTTGTAGTTGTA
ATTGGTGTAGAAGTTGTAGGGCGGAAGAGACGCTGGTGTTGGTTGCGGAGGAGGAGGATGAGGAGGAGGAAGCGGAGGATTTGATGTTGAGAAAGGATAATAAGAAAAGTGTCTCTTG
AAGAAATTGGAATAAGGGTCTGCCATGGCTGGTTGTGGTTGTGTGAGGTTGAAGGGATGCAGAAAACAAGGGTACAAGACTATTATAGATTCGTTTATGGAGAGAGAGAGAGAGA
>comp147432_c1_seq4
TCCACTCCCAGTCTCCCAATATTCAAACAAGTCAATTTACACACAATTTCACCTTCTTTTCCCCACACTCTTAAAGCTGCTACTCATAAATAGAGCTAAAATTCAAAAACAAGCCCATTACT
CTTTCACCCCATTGCCATCTTTACACATGAAACTCCATCAGGTAAAATACAAGAAGGAATGCAATGAAATGAATAACGGGTAAGCTTACATCTAGGAATGACAAGGGCTTTGAACCAAAA
GAACCTAGCTTTCAAGCTCCAAGCCTATTAGTAAATACTCTTCAATGGTTTCTTCGTTGTCTTCATCATCCTTTTTCAAACGTACTGTCAGCTTGAGACTTTGCGATAAAAAAAGCTTCACT
TTCGCTCCCATCGTTTTTATACAGTGAAACTAAAACAGTGAAAGTTCTTCTTTTTCATTCCAAGATAATTACTTACTCCACTAACCATGAGGTGATTGCAGTCTTTTCTTCTTGTTGCTGGTG
GCAGATAAATTGTTGATATCGATGAATTCATTTAAGTTTGGTAGATTAGCACAATTTGATGATATTTCCAATGAAAATAAAACCTTTTGAGATATCGTTTTCTCCTTCTGTGACTCGCGAATCA
TTGCATTATAGCAGGTAAATATATGCTCTCTTTCTGAAGGTTCAGGGAGTGAAAATACATTAGTATTAGATTCGAATAACCTCTTTGTTGATTCCTCACCTGATGCAGCTAAGATAGCCGCGG
CAGCTATGGTTGAAGGTCGGTAATCAATCAAATTCATAGCTTTTACAACCACAAAGATGAATCTGATGGCAATGGGGAGTAGCTTCCTCGAGCAGTTCTCAAAGTGAAATTTTGATGCAAA
ATAGCTAAGGTAAGTAAATGGAGTGACTGAGTTCAGTCTCCATTCCAATGCATCCAAGACAAGAAGTTCCATTCTCTGTATCACTTTGTTGCTGAATCTGTAACCTTCGAATCGATACTCCG
ACAAAAATGGCGGCTTGCATTCTTCCATCTTTGCCGCCAGGGATAAGCAAGCTATCGATAAAAGCTTCATCGCCCAGATCTTCCCCTCATCGATGGTCCGCCGGAGTAGAAACCGGTCTAA
GAAAGTCACAGCGAGGTACGCGGTCTGTGAGCTGAAACCGAAGCCTGACTTCATTTTGAGGATCCAACGGACGGATTCGAATCTCTCGCTGTGCAGACGGTCGCCGGAGGAAGAATGG
GTTGGGCTTGCAAAGGTAGTCTCTTTGGATACGAGAGTTTCTATATATTCGTCCTCTGCTTGCGAGAGAAAAGAGCTGTTCGAAAAGATGAGTTCGTTTGCTTCATCATCTTCTTCGTCGG
AGAAAGCAAGGCGATTGCCGTCTTCCGGACAGAAGAGCTCCATGAAGAAAGCAAACAGAGACTTGAGGAAC
>comp147557_c0_seq1
CCAAAAAAAAAAAAAAAAAAACTCAGCACCTTTCTGCTTCAAAAATACACATGAAAAAAATTTAAAAAACAAAGCTGCTATGGAATCGATAGGGCTCAGGATAATTGTAACATATTGCAG
TCGCCACACAGAGAGGAATGGGAACACCCCCGGTTGCTAGGACCGCCTTCAAGAAGAATTCAGGTTGCAAGAGACCGTCATCCTGGGGGTGAGAGATGCAATCCAGCATCATTGCCCC
CAAAATTCCAGTAGCAAGCAGAGCCATCCAAAAACGGGAAAACAGAGAGAAACGCATGAAAAACCTTCCAGTATGAGCCGTAATAAGAGTCCACACCCAAAAAATAAAAAACTGGGC
ATAGGAGAAGAACCGGCCACCTAAAGTAGTTCCAGCAAGAAGTTTGCATCTTTCCATCTATGTACTTTTGAAAAAATAACTTAATCTTGATCAGAGTCGTAGCATGGCTAGACGAACAATG
AGCAAACCTTCTCTCATTTCTTTCCTTCCACACATGATAAATAGTAGAACAAATGCAGAGGAAATAAAAGTTCTTAGTGTTAGAAGAAGAGGGACTGTCATCTATCCACTCAAACAGTTGC
AACAGACTCGGAGTAAGCAGAATAGAGGAGCTATCATGGATTAAAGAGGTGATGATACTGTGAGAGTAAGAACATTCAAAAAAGAGGTGCGTAGAGCTTTCAGTACATGGGCTATATTTG

ACACTCAACTGAACAAACATAGGAATAAGAACATGCCAATGATAAATATTTCATTTGGTTTAATTAATAGAAGAAGAATAAGACTTTATTTATGATAGTTTTTTTGCCGCTTTTTGCAATAGT
TTTCTAGCATACAATTTTCTATTGTGATAGTTTGTAAATTAAAGTTGTAAAAAAAAATATCTCAAATGCACTTCATATCTAAAACTTTGAATTTTGGGTGCAACTTTTTAAGTAGTTTATGTTTA
ATGCTTCTTCATATCCCATGCTTCC
>comp147578_c0_seq2
TTTTGAATTTTTTTTCCAGAGTATAAAACACCGGCGGCCTCCACCCTCCTCGTTTAAAGCAGCGCTGTTGATCTCGCCGTCGGCGGCCTCCTCCTCTCACTTCCTTCCCAACATTTTCCTGC
CGGAGGAGGCAGTGCCATAAGCTAAGACGAACATATCGTTCTCACCGGCGGCCTCGGGCTTTCTGTCGATCCCCGCGTTTTCTTCCGATACCCTTTTTTTTTTCTTTTACTTTCCTTTGAAG
GCTTTTCATCGATAGAAGAAGGAACCGGAAGAAGTTGAACCTAACCTGGCTTCGGGCATTACTAGAGGAAAATCATGAAGAAGGCAATGCAGAAGGAATCCTTCGCTTGAAGCTGATTT
CTATGGAACTGTATGCCTTTGCTGATCAATATGACGCTTTACATTGTACGAGAGGCTGTGAAGTTATGGAGGAAGATTGCAATGGAGACTTGTGTAGAACTACAAATTTTTATGGAGAAAT
GGAAGCTTCTGCTTGCGGGGCTCTTTTTTCAGTATGTTCATGGGGTTGCTGCTCACGGGATTCATTATTTGCATCGACCTGGACCTATTCTACATGATCTTGGTTTCAGTCTCCTTCCGGAGA
TAGGGCCAGATAAAGCCTATGTCAGCGAAATCTTTTTCTCTGTCATCTTTTTCTCCTTTATATTGTGGACATTCTATCCTTTCATTTATCATAGCAAACGCTTCTACACAGTTCTGCTATGGCG
TAGAGTGTTGGCAATTTTAGTTACTTGCCAGGCTTTGAGGATCATCACATTCTACTCTACACATCTTCCAGGTCCAAACTATCACTGCCGCAAGGGTTCAAAGTATGCCACACTCGCTCCTC
CTGAAAGAATATTGGATCTATTTCTCATTAATGTCCGTGGAGTCTTTTATGGTTGTGGCGATTTGATTTTTTCATCTCATATGATATTTACACTGGTTTTTGTTCTCACATACTATAAATATGGTT
CTAAAAGGTTTATTAAAATGCTTGCATGGTTGATGGCAGTCATACAAAGTCTTCTTATAATAGCTTCACGGAAGCATTACAGTGTTGATGTTGTGGTTGCATGGTATACTACAAACTTGGTG
GTATATTTTATTGATACAAAATTGACAGAGATGCCTGACCGTTCTAATGGAAACCAGCCATTGCTCCCATTAGTCGCTAAAGAGAAAGAAAGCAAATCGGACGAGACCCACAAGCTATTG
AATGAACTATAGGAACCAGGCGAATGGGAAGCATCTAGAAGACCGGAACTATGTACATTCTGAAACAACAAAGAATGGCAGGCAGATAGGTGATGCCACACAGTTGTAATGGCTGATGA
TTCGTAATTCAATGGGAAGCTCCATGCCGAGCATTTGAACTAATAGAAATGGGCGGCAGTTTCTTACTGTTGTGGTAATCTTTCCCTTACCAAACGGATAATTCTGTTTTTGAAGCAGAAAT
CTTTCGTAATTCATTAACATGAAACAGAAGTCAAATGTCTATATGTCCCTTACAACATATATTTTTATATTATTTGATTTC
>comp147603_c0_seq2
CAAATCAAAGGTCTAAAGTATTTTTGAATGTTTAAGAGCATTAAGTGGATGACCAAATAAAATTCAAACCGTCTAGCTGCTTTGGAATTTTTTTTTAAATTTTTTTTTTGAATTTCCGTTGCA
AAAAAAATCAGTCCGAAATATTTTTATAGCGACAAATTCGTCGGAAATATTTCCGGTCAATTATTTTGCCTTGGGAACAGCCTTATCCCTCCCCTTCTCCCTCGGTAGTCGGAACTATATCCA
CAAGAAAGTCAAAAATGTCTGTTTGGCCGATTGCTGCGGCGATATCATTTCTTTGGAGAGTTCGACGCTTGTTCTTCACGGCGTGAGTCCACCCCTTTTGGGTGAGCTGGAGGATGAACA
TCTCGCAGGCCCGTGCGAAGACTACCGGCGCCTCGGCGGCGATCATCTTCACGTCCTCGTCAGCCTTCATTATCTTCTTGATGCGAGCCAGTGGGAGATTCTGATTTTTGAAATTTTTGGT
TTGCTCGATCTCGCGATACTGATTGGCCCATAAGGTTCGCAGTTCTTGTTGTTGTTGCTGGTGGTAGAAATGCTGATAGGTAGCTTGGTAGGGTGTTGCAGCGCCGGCGACCGGATTGGGC
AGAGAGCCGCCATCATCGATTGCTTGGAGCGTGTTGTGGTTTTGTTGATCCATTTAGATTGATTCGAGTGGGCGTCGTTTTCAGTCGAGGTTTTAGTCTGATCTAGAGGGGAAGCGGATGA
TGGATTTGAATGAAGAAGAGAAGCTAGTTATATAGAAGGAAAGAGAGAATTTTTAGGGCAAGGAGA
>comp147603_c1_seq1
CGAACTCTCCAAAGAAATGATATCGCCGCAGCAATCGGCCAAACAGACATTTTTGACTTCCTTGTGGATATAGTTCCGGCTACCGAGGGAGGAGGGGAGGGATAAGGCTGTTCCCAAGG
CAATCCTTTGTGGGGAGATTCATTCGTCGCACTACTACATGCAACAGTAATAGAGGTGGCAAAATGAGTGCCCTGCTGCCTTCTATGATGTTGTTGGCCTTCACTAGTTTTCATAGCCTAAT
TTCTTGAGTGTCTAAATTTAAAAAAAAAATATGACGTGCGGTGTGCGGAGTACAGATACCTTCAGAGGTCAATTCGGATGCGTCAAATTATCCAATACACAATGCATTGTATGATATGATGC
ATCTGAATTGACCTCCAAACACGTTCTGACTCTGTACAGAATAATCTCTCCTAAATTTATGTGGAATTATTTTGCGTTCTCTATTATTATAATATGTGCTTTGGGATTCGTAATTGAAGCAGTT
CGTTAGTTGATTA

>comp147671_c0_seq1
AGAAACGGATGAAATATAGGTAAAATTTCAGAGCATAAAATTGCAAATTATAAAGCTTTTTTTTTAATATTCCGAAATTTATATAAGAATATGGTAAACTGAAGAATCACTCAAACCAAAG
CTACAGGATAACAATAAATGTCGATAGTCTGCACCTTAGAACTCCTATAAATTTCCTCTCCATGAATGTAGTATTGGATGCCCTATTCGCGTCTTCCAGAAAGAAGATCATTTGCTATTAGTC
TAGATTCAAATTATTAGCAGAAATACCTGAAAGAAGAATGAACAATGGCCTCATTCATCTACTTTGTAATCATCCGCTGTAACCTCCGAAAGCCACAGACCAGGGAACAGCGCATCGAGTT
GTTTCTGCACGATCTTAGGCCTCTTTTTAGGTCGTCGACTGGGCTTCGTCCCCTTGAAGGCGTAGAAGTCCTCTTCGATCTCCTCGCGAGATAGGGAAACAGAGAATTCCCTCCGCTTCCC
ATTTTCGCCGACCATCGATTTTAGCATTCCGCCTTTGGCCGGATCGTCTTTTCCCAACAGAGGCTTAGCGTAACAGTTTCTTCCGCTTTCCGATGGGGCGAAGCACGAAGCTCTTCTTGCC
CTAAGATTCCACGGCCTGGCTGGCGCTGCAGCGGCTGCATCAGCGGCGGCAGAAGAAGGTTCATCCTGCTCTGTCTCCTTTTCTTTGAACACTCCTGCTTCAGGAGAATATGAGGCTTGC
CTCTTCTTTCGGAGCTCTATTTGGGTTTCAGGAACCGTCGATCCAGCGGCAGAGCCGTCGATTCCAGCGACGGGGTTCACGCAGCGGAGAAGCCGTTGACTGCCCCAGTTCATCGTTGG
AAAGGAAAAGTTGTGAAAACGAGATTGCCTGGCAGGGGAGCGCGTCGCTACCTTCTCTCCGGCGGCGCCGCCGACTTCTCCCCGACTCTCTCCGGCCACGCCCTGACCTCTCAACGACA
TCGTTAGAGAGAAGCACAACGGGAAGATGAAGAAGAACAAGAGAGGGAAGAAGAAGGTGCAACGTTCGTGTGGGGTATGCTCCACGGCTCCAGGTTATGTATCGATGCTGCAAATAAA
TTAACGTACGGTACCTCGCATTTTAAACTATATGCGGGTAAATCTATCCACCGTCGAAATGAGTTTGTTTATTTGAATTTTGCTTAATTTGGGCCATACGATGCAAGG
>comp147689_c0_seq1
ACAAAATTAGAATAAAAAAATCAGTTAATAGTGCAAAATAAAAAAAAATCAGTTAATAATGCAAATTAAAAAAAAAAATCATATATTTTTACACATTCCTCATAGAATATTCATTCAAAAAT
CTCTTTCCTCCACCTTCCCACACCTCTTCAATTCATCAGGTACTAGCTCTTTATAAAATGATCCAATTGCTTAATGGATGATGAGGCATACAACATAAACCAGCACTACAAAGGCTTCACCA
CCAACATTTATAAAAATAAAATAAAATACATTATTAATCCAGTACTATCACACTTTGAAATATTCCTCACCTTTCTCTTCACCACCACTTCCTTTCCCTTCACACAGCACATCAAGACTATACA
ACACAAAAACCATAACAACCGAACATATAAAGACCAAAACAGGCCCAAATACCCACAACAGTAGAGGAAGAGCAGCATAGAACAATCTATTTCCAAAAGTATTAAGTAAAAAGCCCTTC
TCCAGTAGGTCACAAACATATTCAGCTGTGATAATTACTGGAGAGGTAGAAGAAAGTGGAGTAGTGTTGATGAGAAAGCTAACTTGGCTTGTGAAGCGAACTGAGAGAGAGTAGCAGAT
GAAGGCGAAGAGGAAGATGAGGAGAAGAGAAACGTATTTTAGGGCGACCATGAGCTCACCATGAGCTCCATAAATGGAGTCTGAGATTGGCTTTTTAACACTGTAAGTGCTGCTTAAGA
CTGCAGCGAGAGCTGAGCAGAGGAGAATACAGGTGGTGGCCATTAAGGTTGAGCTCATTATGGCGTTTCGTAGTGTCTGTACTGCTAGCACGTTTTTCTTGTCTGAGTCCTTCATTATCGA
ACGGACCCAAAGCCGGCGGCCGGCTGAGTTCATTCCGATGATGGTCCGGCGAGGCTGTGTCCTAACCTTGTGCCAAAGCCAGCCATGGTAGATGATGAAGAAGAGAAGGCTCACAGGA
ACTAGTACAAGATCCATGTAGCTCTTTCTCCACACCATCCTCCTCAGTCCCTCCTTCTCTCTCTCTCTC
>comp147774_c0_seq1
AATAAGGAGAGATCCTCAAGCAAATTTAAATCGGAATCAAACAACATAAAACTTCCAATCTTGTGTGATGTAAAATAAATAAATGAATAAATTCAAAGCGAGGATAATTACATAAATGGAA
ATAATACAATCAGATATGAGAGATCAATCGATGATCACATAGTGGAGAAAAACATAAAGAAGAAGAAATTAGTGAATGAACAAATAGATAGAGAATGCCTCTTTCGTTGCTCTTTCCATTT
CAGTCCTCGTCATTGATATTGAAGGAGATGACCGTGGCGCCTCCGACAGTGGCGTGTCCGTCAAAGGAGACGCGGGGGTTAACTTCGGCGGTGGCCGCAGCGAGGTTGGTGGAGGATA
AGGAGGAGGTGAGGGATTGCCACGAGGAGGAGCGGCGGCGGATGGGGTAGGAAACGGAGGACTGCGAAGGGCACGGAGCGTCGGCTGCAACGGCGCGGGAGCGATGGAGGGCGCA
ACTGCAGTTACGGTGGTACGGCCTCCGGCGAATGTCGCCGTCGAATGACGGGATGCTCGCTTCCCACATTCCCTGACCTTTGAACCACCCTGGCGTCGCCGCCGACCCCGCCATCGATCT
CTGTGCCTCTTGCTTCCGATCCGATCGAAGCGCTGTAGATCTGAGGATTTGGGTTCTCCTCGATTGATCGATTAGGTTTTGTTCT
>comp147885_c0_seq2
TGCTTTGTAGCTATTCTTAACTACAAAAGAAATAATAGTGCATCAAACAAGTTGAAGTACAATCAATGCCATTCAAAACTTCAAAATTTAGGAACTAATTAGTCTATAAAGTGAAGAGATG
ATGTTTGAAGAACATCTGGAGTGAGCAATTTTGAGAAATTTCATATTCAATATGACTTGAAAGATCATTTTTACAATAAAATTTAACTCATTTATGTGGATTTAAGCCCCAAGGTCTTCCTTG

TCTTGCCCACAAATAGATCCTTCGATTTAATCAACCCTGGAACCCATCCAAAAAGCGTGCCAAAGGGAAACTGAGCAACTGCTTCTTTTCTTCCTAAGGAAACCATTGCAATGGGCGGCG
AAGGAGTATATGTCTCAAGCTTCTTATCAGGAGCTCCCCTCATCAATAGTTGCAGGTTTTTGGCCACCACTAAAGCATGCTTTTCTGCAACGAATCCTTGCTTGAGCTCATGGATGTCAGTA
ATGTCTCCAATGGCAAAAATGTTATTGCGACCTCTCACTCTCAAGTTCGCATCAACCATTAACCGCCCCTTCTTGTCCAAGGACTGCTTCAAGATAGAATCATGTAGCCATGATGAAGCCA
CCGGCTTGCCCACACAAACAAAATGGCAATCAGCAGAGATGGTTTCTCCATCTGATGTCACATAAACTCCTCCTTCTGATATGGACTGTAGATCAACCGTTTGTTCCAAAATCACTTCCAC
TTTCTTTTTAATCAACCAATTGAATGTCTTTCTTGATGCTTTTGGGCCTATGAATTCTATTAACCTAGGCCCTTTATGAACAATGGTCACTTTCTTCTCCGGAAAATCAACAGCAATCTCTCC
GGCAAGCTCAACCCCAGTTGGACCACCACCAATGATTAAAATGGAGTTCGAGGCCTTAATCTTTTCATAATCTTGCTGAAAATGCTTGAGTCTATCCTTCCTGTTTACTTTGGTAGAACCTG
CGTGTCCAGTTGCTACCACAAGATAATCGAATGGAAATGTTTGGCCATCATCTGTTTGGACATGATGATTAGTTACATTGGTTGCATGTGATGTGATGATTGTAGCATTGGTTAGGTATTCAG
AGTGCTTGATCAATGTTCTTTCTGCAAAAGATGGTTCTATCATTGACCTCAACTGTGCCCATGG
>comp147889_c1_seq1
ATCAATTAATGCTGATGCAAACCTATTTAAAAAAGTAGACAGGAGCACAAGAATAGTTAACAGCACACTGAATGCTATAATAGCAAAATAATTTTTTATTTTGTAAACCACAGATAAAGTTT
GTTGATATAACCTACCACTAAAGCTAGTTAAATACAGAATGGTATGTTATATGTAGAACAAATCATGAATAAAAGCAAACTTTCTTAAAGGCGCAGTTGAGATTTATGCCCTCCATACCAGC
TCAAAAAAGAACAAATCACACATGGAAAATTTAACCTGCTCAGTCCAAGTTCCTACGAGTAAGATGTAAAAAAGCAGAAAATCCCAACTATTAAAATGACAATTTAATCAGAAATGTCTT
CCAAGAGAGAGTATCAAAATCCAAGCATTCAAACCAACGGTCTCTACATGATGTTACTCATGACAGCACATAGTTTATTTCAACTCACTCTGTACCGAGTGATATCCTATTCTGTACCGAGT
GTTATCCTACTTTGTACCGAGTATTATTTACTGCTTTACCTCATAGCCTACTATGTACCGAGTGTTATCCTACTCTGTACCGAGTGTTATTTACTGCTTTACCTCATTCCGCTTATATATACTTGT
AACCTGTTCAATTATAATGATACATACGCTTTATCCTTCTTTGTCTTTCTGCTTCGTTTTCCTTTCTTTCTCTCTTGGCTGTGAATCTCTCACATGGTATCAAGAGCCAGGAGATGGGAGATCA
AGAATCAGCCTCTTCAATCCCTCCCGCCTCCACCACTACGCTCGTCGCCCCATCAGATGACTAACAATCCCGGCGTCCCTCAAATTCACATATCCAACATCAAGAATCTAATCGCTCACTCC
CTCACCACCGATAACTACGCAATTCGGCGAATCCAAATCTTTCAGAACCTCTCCACCAATGGGTACGCAGATTACCTCACCGGCAAAGCAACTCCTCCAGCTGATCTGCATTTGCCGAATT
ATGCATGCTAGCGTCTAATCGACAGCAATCTAATATCCGCTCTATTCTCAACTTTCTCCCCCACGCTTCTTCCTTACGTCATTACGTCTACTACCACCCAAGAAGTCTGGGTGACGCTTGAA
CCCCGGCTACAATCCTCTAGTCGCTCTCGAGTAATCCAACTCAAGAACGAACTACACCATGTTCAAATGAACAATCTCACGATGCAGCAGTACCTATCTCAAATCAAGAACATCGTGGATA
ACATTG
>comp147918_c0_seq1
TCTTAGTAATAGTATATCTTAGTTTAATAGAAAAGGAATTCAATTACACTTTTTTTGAAGCTTTTTTCCATGGAAATGTAACAAAGCTGCAACAAAATTTGGTTTCTGGAAACAAAAACAAG
TAAATAACTGAAAAAAACAAATAAAATGAAATGAATCCTCAGAGAGGAAGATTACTAGGTGAGCTTTGCACTCCTCTCTATCATTCTGTATCAATCTTATAATGCGAAAAACAGATTGAAA
ATACAGATTGAGCTATGCTCACCAAATAATCTCTCATTAACTCAGCTTTCAAAGCAGCCGAGAACTTTCTTTGGGACAATTCCAGCAGTTTTGAACTTTGCTGCAGTCTCCTCTGCCTTGA
GTATGCCCTCTCCTTTCTTAGCCTCAATAATGGCTCTCTTCTCTTCTGCTGCCTTGTGAATGGAAGCTATTTCGTTCATCATTTTCTCTGTGCGCTGTGCCTTCTTCTTCTCATAAGCTTCCTC
CATCTTCTTAATCTCAGCTTCAATGGATGCCCTCTTAGAATTTTCCCATGTTGTAATGGATGACATCTTCTTTTGAGCCTTGTTTTTAAGTTTGGCTTTTTGACTGTCTTCCCATGCCTCTATG
AAAGCTGTCTTCTTCTCTCTTAGCAGACGGGCAAGTTCTAATTTGTGGACGATATGGGCTTTGGTGGTGCCATCGGCGGTGCTCGTCTCCGCCGACGGTGGCTTCATCGCAATCTTCACTT
CTCCGGTGACCTTCTCAGCCATTGGTGGCGGCGGAGCAGCCTCGGTCGCGACCGTGGCCTCCACCTCTGCTTTCTCCAACTCCTTCATCTTCCTGATTATATTCCACTTTTCC
>comp147935_c0_seq1
TAAAAAAGTCGTCCCAAATGAAACTTTAGTTCAACTCGTAAATAAGTAAAGCGTTTATTATAACATTCTTCAAATAAAAATAAAAATTGCAGCCCCAAGTCATCAGTAATAACCACAATCC
AAGTAAAAATATTAGACTCCTTATGTGTAGAAGCTCAAATTAGAACCTTATTATTAGGGCCTGCTCAGTTCGCCAATGGCCAGGCAATTGTCGACCATGTTCTTAAGCATTGTAATCCGGTC

CGTCAATGGCGATTTGATGGCGAACTCATTGAATCCATCCTCGCAGGAACCGAAGTTCGAGATCATGGAGCTTAGCATGGCATCTCGAGTGTCGATGTCGCCAGCCTTGATGGCTTTGGTG
GCGGATGCAAGGTTGTCGAGGGCGGTGTCGTAGAAGTCGACGCAGTCGTGAAGGAGCTCGAGGGTTTTGCACGGGAATGAGATTGTTTGGTGAGCCAGAGGGTGAAGGCCTTGCCCT
GGCGAGCCCTAACATCAATGGAATGCAGCAGGATGGCAAGGACGATGTTGATGTTGGCTCTTTTGAGGCGGGGAGCGATTTCTTTAATCGATTTGATGCAGAGACCGGAGTAGTCGGTGT
GGCTACAGAAGAATTTGAGAGAGTTGTTCGAGTTGGTGGCGGTGGCGGAAGGGTGTAATAGGGCGGCGAGGGTGAGGACGACGGCGAGAACGATGAATACCCTCATCTTTGGGGGCGG
CAAGAGAGGTTAGCGAGGAAATGGAGAATGGGGGAGGTTGAAGAGGCAGAGGGTTTAGGGTTATATGGAATGAAGGAGAGTAAATTTAGGGGAATTGATTGGATCTCCGAAGAAAAAG
AATTGGGAGATAGGTCGTGGGTAACTAATTTTTTATATTTTT
>comp147947_c1_seq2
GACCTTAAATCAATATAAGCTGCGACATGAAATCACCCAAAAATGAAATATTATTCTGTGTGGAAGCAAAACCCATTCTTAAACCTAAGCCCGCGGCTCGGTACCGCTCCATACCATGGTA
CCGAGTGTATGTACCACAGCTCGGTATGACTAACGTGACTCATTCTAATTGATATCCGATTGTAAAACTGCATTCGGCGTCCACGTAGAATAATCTCTCCCCAAAAACACACAAAAGCTAG
ATAGAATACATACAAAATGACACCACAACATCTAGACTACAACTAATCCAAAAGGGCAAAAAACCGAAATAGAATTATATATGTTTCTGGAGAGAGAGAGAGAGAGAGACTGAAGAGAGAG
AGAGGACTATACGCTTCTGAAGCTGGGTTCAATCTTTGATCAACACCATGCAAGAGGACAGAGATATGAGCGCCCTTTGAACTATTATTTGTCGAGCATCTCAGCGTCATCTTGCATAGAA
CACTACATCAACAATCCTTGCACTTAAATTACTCGATAATCTCCGTGATATCAGACCAGATTTTCTCAAAAACCTCAACAATCAGATCATGATACTCACTTGAATTCAGTAACAGATAGCAA
GAAAGCAATTCCTCCAACTCCTTCAATGAAAATAGATTGTTCTCAACAATCATGTCCACCATTGATTCTCTGAAATCTCCCTTCGGATCCGAAGAATATTTCACCACTACGAAACTGTCTGT
CAATCCCTTCCTCTTCACCAATCTCTTCTGCACGAGCTTTGGTGACTTTATCCTCAGCTTTATCTGTTGAGTTGCAGAGGGAGACCTGCTCCCCTGTTTCGGACTCTGTTTTCTTCCATTGAT
CAGGCGGCGCTTCGTCAATATAGGAGGAGGAAGAGGATGATTGGGCTCTGTAACGAAAATATCTGATGAAGAGGTGAAGAATTTGGCATCGAAGCGGTCATCGAGGCTGTGTAGGGGAG
TTTCATGGGATGGCTGTGCGTTTAAAACAGCGGAGCTCAACAGCTTCTTCTTTGGTTTTTTCTTTGATTTTTTTGGGTGAGTAGGAGAACGGGAAAGCGGCTCTGCTGCTCTTTCTTCGCT
TGTAATGTAGTGAGAGAATCTATTTGTGGAAAAATCTTGAATTTTAGGAGATTTGGGAGGTTCTGTTCTTGAGATTTGGCTTTTGTCTTTGGAATTATTAGCTTTCTGTACTCTGTTTCCTCT
GCTTCGCATGTTCTTAAGCTTATAGAACCACGAATATGGCATCAGGTCAGATAACCTGAACGTGTTGCTCCCTTTTGCCATGAAAAAATAACTGAAAAGAAGAAGAAGAAGAAGAA
>comp147947_c1_seq3
GACCTTAAATCAATATAAGCTGCGACATGAAATCACCCAAAAATGAAATATTATTCTGTGTGGAAGCAAAACCCATTCTTAAACCTAAGCCCGCGGCTCGGTACCGCTCCATACCATGGTA
CCGAGTGTATGTACCACAGCTCGGTATGACTAACGTGACTCATTCTAATTGATATCCGATTGTAAAACTGCATTCGGCGTCCACGTAGAATAATCTCTCCCCAAAAACACACAAAAGCTAG
ATAGAATACATACAAAATGACACCACAACATCTAGACTACAACTAATCCAAAAGGGCAAAAAACCGAAATAGAATTATATATGTTTCTGGAGAGAGAGAGAGAGAGAGACTGAAGAGAGAG
AGAGGACTATACGCTTCTGAAGCTGGGTTCAATCTTTGATCAACACCATGCAAGAGGACAGAGATATGAGCGCCCTTTGAACTATTATTTGTCGAGCATCTCAGCGTCATCTTTGTCATTG
AAAAAGATCAAAGCTTGATAATTTTATTAAGAAAGAAGGATCATATAAAAATATAAAAATAATTCAAGTTACTACCTTGCATAGAACACTACATCAACAATCCTTGCACTTAAATTACTCGAT
AATCTCCGTGATATCAGACCAGATTTTCTCAAAAACCTCAACAATCAGATCATGATACTCACTTGAATTCAGTAACAGATAGCAAGAAAGCAATTCCTCCAACTCCTTCAATGAAAATAGA
TTGTTCTCAACAATCATGTCCACCATTGATTCTCTGAAATCTCCCTTCGGATCCGAAGAATATTTCACCACTACGAAACTGTCTGTCAATCCCTTCCTCTTCACCAATCTCTTCTGCACGAG
CTTTGGTGACTTTATCCTCAGCTTTATCTGTTGAGTTGCAGAGGGAGACCTGCTCCCCTGTTTCGGACTCTGTTTTCTTCCATTGATCAGGCGGCGCTTCGTCAATATAGGAGGAGGAAGA
GGATGATTGGGCTCTGTAACGAAAATATCTGATGAAGAGGTGAAGAATTTGGCATCGAAGCGGTCATCGAGGCTGTGTAGGGGAGTTTCATGGGATGGCTGTGCGTTTAAAACAGCGGA
GCTCAACAGCTTCTTCTTTGGTTTTTTCTTTGATTTTTTTGGGTGAGTAGGAGAACGGGAAAGCGGCTCTGCTGCTCTTTCTTCGCTTGTAATGTAGTGAGAGAATCTATTTGTGGAAAAAT
CTTGAATTTTAGGAGATTTGGGAGGTTCTGTTCTTGAGATTTGGCTTTTGTCTTTGGAATTATTAGCTTTCTGTACTCTGTTTCCTCTGCTTCGCATGTTCTTAAGCTTATAGAACCACGAATA
TGGCATCAGGTCAGATAACCTGAACGTGTTGCTCCCTTTTGCCATGAAAAAATAACTGAAAAGAAGAAGAAGAAGAAGAA

>comp147965_c0_seq1
GCTGGAGAAGAAGGCGATGATGGCGCTGGACAAAGTTACCAGAAGAGTTGCATGGATGGGCGTACCCGTCTTCGGGTGGACGAGAGCGAAGAAGGGGGGTATCATGTGAGCCCTAGCA
ATCTGCGTCGTGTATCGCGCCTGGCCGAGCGCGCCGACGAGCATTCCCGTGGTCATACCCTTGAGCGCACCAAGCGCTACTAAGTACTTGGCCCATTTCATCCCGGCGGCAGCGAATGCG
ACAGAGTAAGCGGCGTTGGTGTCGAGCTCGGAGTATTTCTGCATCATCACCAGAGCAAGTGCCATGGCGCAGTAAACGGCGGTGATTGCCGACATTGATCCAATTAAGCCAATAGGAATG
TCCCTTGATGGGTTCTTAGTTTCTTCTGCCATGGTCGCCATCATGTCGAAGCCGGTGTATGCCCAATAGACGACTGCGGCGGCACGGAAGACGCCGCTGGCACCGAATGGGAGGAATGGG
GAAAGATTGGAGGGCTTGGCGTTAACAAATCCGGCGGCAATGACGAAGGCGATGACGAAGAGGGTAAGGACTGAGGCGATCCAATTGAGAATGGAGGTTCTGTGGGTGCCTAAGGCGG
CAAAAGTGGAGCAGGAAATGAGGACGACGACAGCGATGGGGTCGAGGAGGTTAAAACCCTCGGCGAGAGATGGAACGTGGATGCGAAGGGCGTCAGGATCGCGGCCAATTAGAGTTG
CGAAATAGGAAGTCCAGGAACGCGCAAGCCCAGCGGCACCGACGAGAGCTTCTAGAAGAATGTTAGCAGCCGCAAGGTAAGCCGCAGTGTCGCCAAGCTCAACGCGGAGGTAAGAGA
ACGATCCACCAGCAGAGGGAATTTCGACGGCGAACTCAGCGTAGCAGAGGACGGAGAGAAGTGCGGAAAGGCCGGAGGCAGCGTATGAGAGGACGATGGCGGGGCCGGCGTTGAGA
CGTGCTTCTTGGCCAGTGAGGACGAAGATGCCCGTACCCACTACGGATCCGAATCCAAGCCAGCCGAGATCCCACCACGTGAGGCAGCGGCGCATGGAGTTCTCGCTGCGGCGGCGAA
GGACGTCTACCTCATCGGAGTCAGAGGAACGGTGTAGAAGTCGGTTGCGGAGACGGGGGAATGTGCGGAAGAGGGCGGAGCGGTAAGAGGTCCAGCTGCTAAAGGAGGGTTCGGGGA
AGAAATCGGATTTGGCGAATCTCCAGTAGCTTCTGGGGTTGAGGGATTCGCCGTCGGCGCCGCTGGCAGAGGTGTATGCCGGAGATTCGCCGGCCATCGTCGTAGATTCTGGAGCTGTGA
GATCAGTTGGGGAAGAAGAAACTGCGCCAAGAAAAGGTTCTGTCAAGCCGGATTCCATTATGTTTAGGAATTCTCCTAAAATTTGGGGAATTCCGAATTTGGCAGCGGAAATTCTGCTAT
TTGGACGGCGGAGAGAAGCGTGTCGGTTTTCATATTTTCTGTACCTGGACGGA
>comp147966_c0_seq1
TGGCGCATTCTGGTTGATCTCCGACTGACGTGGCGCATTCTAGTTGGTCTCCAGATGACGTTCGACGTCCTCAAAGAATATTTTTTGCCAATGAACAAAGATTCAAACAAAATAATATTTTT
TACTTATCCTTCTCTTAGCTTTCCTCTCAGTTGTTTCTTATCAATAACAAAATGAAATAATAGAATTGAAAGAAAAAGTAAAAGGCAGAAGAAAAAGATCACATCTTGATCTCATTTTTGTT
CACCAATGTCTTTCTCAGCCTTCCACCTTATTCCCACCTAATACCTCCTTCACGTAAGCACGTTGAGCAGCTCTAGCTCTGTGCATCTTCATCTTCTCCCTCACAAACCTTTCATATATAAAA
GAAGCCCCTCTGAATTGTGGAAGAACAAGCCAGGCCACCAGCAAAAGCTTTATCTTATACCATATTGGAATCCAGTATAAAATAGGTGCTGCTATCATCTCCATGAGAGTGATGAATGAAT
AGATGATCCAATAAGCTAACCATTGCTCATCATCAAGCTTCGACGTGCTCTCTATTGCTTGAACAGAAGCGTATAAGGGGTAAAGGAGCATGAGGGCTGGCCCAGCGAGAGAGATGAGAT
GAGAGAGCAAAGTCCAGAAGACGTCCATTGTGAAGAACTGGAGATTGAGACCTGGAAATTCAGAATTGATCGTCTCTCTATGGCTCACAGAATGAAGAATTTTATCCTTTTAATGGAAGA
TGAGTAGAAAAAGAGAAAGAGAGAGAAATCTTTATTTATTT
>comp148000_c0_seq2
TTTTTTTGTTTTTTAGTTGTCAAATAATCTTTATTTTTCTTTGCAGTTCTTAGCTAGCTGGGCATTCCACTGCACCACTGTTGATGTCATCTATGATGTCATGAGGGTGGCGGCCATCAACATT
GCATCCCACAGACTGAGCAGTCCCCAGGATCTCTTTAATGGTTCCAGAAAGCTCTCTAGCTAAAGAGCGGTGCCGCATCTGCCGGGCAATGTTGACAATCTCATCAAAAGTGATGTTTCC
ACTGTGCTTAATGTTTTTCTGCTTCTTTCTGTCTCTTGGAGGTTCCTTTAGAGCTTTGATGATCAGGGCAGAGGCAGAAGGTACCACCTCGATCTGGGCTTGTCTGTTCTGAATGGTCAGTT
TCACTGTAATCCTCAGACCCTTCCAATCACCAGTTGCCTTGGCAATGTCATCACCAACCTTTTTTGGAGACAGACCCAGGGGGCCGATCTTGGGGGCCAGGGCAGATGTAGCCCCGACTT
CTCCACCGGTGCATCTCAGATACACGACTTTGATCTCGTTGGGGTCGAACTTAGGCGGCATGGTGGAGGCGGCTGGTGTCGGATGAACCCGGATTCGGGACGACCGAAGAAAGTTGCAC
CGTAGCCTCCTCCGAGCCGAAAGCCGAAAGACCGGAAGCCT
>comp148044_c0_seq2
ATCAATAGGTACAACAGTTTCCACAACAACCACAACTCCATTCTCCGGAAGCAGAGCACCGCACCCCATTATTAATATTACAATGGCCCAAGCCCACAGTCACCATCGCCTCAGGAGGAA

GCAGTTCCCCAGATGAGACCAGATTAATATTACCTAAGCTCTCAACAGACGAATCTGAATTAACCAAACAATAGTTCTCCTTCACAGGATCAATAGGTACAGCTGTTTCCACAAGTCCATT
CTAGAAGTAAGGAACACTTCCCTTAGTGGAGCGAGCTGACGACGAGCAGCAGAAGCGTCCCCAGGAGAGCAGTAGAAGCGCCCCCAGGCGAGTAGCAGAAGCGCCCCCATGGCATGG
GCGTGCAAAAGGAGAATACCCTAGCAGACGGGATGAGGTGGTGTCTTAGCCGCTTAGTTTTTGTTCCATATTCCGATAATTTAATTTCTTCTAAAAGAAATTGCCTAAAAGGGTTATACTTA
TTTTATCTCATATGTTAGTCATTCATGTTTATTTTTTTAATTCTGATTTATGTTACTTTTTAAAAAGGATGTGCTTATTTTATCTCATATTCT
>comp148091_c0_seq2
TTTTTCAAAATCACCAAAAAGAACACGCCTAATTAGTATTATTCATCTATCTTTATCATAAAATTACATCATGTGAATGCATTAATATCGACTAATAATACAAATATTTTCAAAATTTCTTTTTAT
ATTATTTGACCCGGACCAGAAACACACTCAACTCCGGCCCGCCCGTCCCATCCGGGTTAATCATGTACAAGGCAACCGAATCCTTGGACCCCACCACCCTCTCAAACCTGGCCCGCATGT
ACGTCATCTCCCCGTCCAGCCCAACCTCGCACGGCACCACTCCGGCCCCCATTGAAACGCCGCGCAGCAACTGCATTACCTGCCGCAGAACAAAAAAAATAAAAAAAAAATACAGAATTTT
CAGATATTCAGTCATAAAAAATTTAAACTTTGATGGAAGTTTTTCTTTTTGATTGTTAAGTTGAATCACCTGTTGTTCGTCATCGGAAGCCTGGGAATGCTTTCTCCGAATCGAGTATCCAGA
CTTACGGCCGTTGCAGAAAACAGCCCAAACATATTCTTCCATGAGCTTTTTCCTCTGCGTCCTCGTCTCGCTCTCCAAAGCAAGCTTAACGACTCCAGAAGCCATTTCCTTGTGAAAATAG
CTTGTAAGGATGGGAAGTTCTATGAGGAAAATGGGAGGAGATCGAGTGTCGTCGTGGATGGCGAGGGTGACTCTTCCCTTTCGGTGGCCGAAGAGGGTTATGGTGGTGGCGTGGTCGGC
AAGGAAGTTGTTTTTGCTTGAGGATCGGTTGAGGAGAGCTACCATCTTGCAGCCGGAGGAGAGAGCGGGGAGGAGTTTGAACATTCGAAAGATGCCGCCGCCGCCGCCAGAGGCGGTA
GACCGGAAGGACTTTGACGGAGTATGCTTGTTGTGGTGGTTGTGGTTTTCGTCTCCGGAGTGACGAAGGAGGGAGAAGAGGGATGGGGTTTCAGCTAAGGATGGCATTGGTGGAGAGG
GGAGTGATTTTCTAGTTTTTGGAGGAAGGCG
>comp148130_c0_seq1
ATAGTTTTATCAAAAACTTAACTATCAACATGGTGATAATTAAATATTATTGATAATTAAAAAGACATACTTTCCATATTAACAAGTAACATTTGCATGAATTTATTTATGTAGATCAACAAATG
TTTAAGAGCTTCGAATTAAATCTCTTAATTAAAAAGGAGCTGTGCTGATGTCCCGAAATCCTTCAAGCACGCCGAAGCTCCTTTTCTTGCCGATGAGATCGGATAAATCTGTATCGTCCAA
GTTCTGCCTGCCTTCAGTAACGTTGAGCACCGGCTGCAAACCCTCAGTAAAAATACTTAGAGCCTCCTCCATTGTCTTGTTGCTGAGCACATCTAAAGCTGCTTCCCTCGCGTACTGTATC
TGACACAAAGAAAGAGTTCGGGGCTCTTTCTCTAAGGAGAATTTCCTCTCTATTTCCAGCTGATCTGTACCCATTTCAATCACTGCAAATTCTCCCAGCATAAAACAAGAGACGATAGGGA
GGGAAGAGAGACGGAGGCTTCAGAGTAATAATAACAATAAGCAAATCCTTCAGCTTAAAACATCTTCGTACACGTGGCGAGAAAATGTGTTAATAGATGACACGTGGCACGAC
>comp148152_c1_seq10
TTCACTTCAAAGGAGTGACCATCAAAACATAACGAAGCTCTTATCCTCCAAGGACAACCACGATATGAGCATGTAGCAATAACTCGTGATTTGTCACTTGCCTTTATCTTGATATCATAGTT
ATGTATTATTGCGTTGGAACGAATGCATTGTTTGAAACTGAATGAATCCTCAAAACGCGATCCCACAACTAGCAAATTAGCCTCTAAATCTGGAGTCTCAAGAAAGCCATCAGACATTCCA
CCAACTATTGCATCATTGTTTGCCATTTTGCTCCCACTTAAATCATGCGTCTCAACATGATGGCTATCACAAGTAATACCCATATCTTCTTCAAGCTCTTTCACAACAACAACAACCTCCATT
TTGTTGGAATCAGAAGAATATGTAAGCATGTCCACTACATCAGTTTCACTTATAATGGGCCTGTAATTTTTAGGGGATGTTCCGTTAATGTGATAGTGTAGGTCATAGTCATGACCCCGTAGC
CACGAGCATAATGCTTCGGTTCGTTCTTTCAGTAAAATCAAATTAACATCATTTTTCCTAAGCTTTAAAGTTCTCTGATTACCACCAACATATTTTAGCCTGTTCTGTCCCTTTACTGTGCTCC
AATATCCTCCATATTTGAATACAATATGGCACCAATTGTTGGAATCCATATCTACCAAAGAAGTTATTAATGAGAAAATTTTAATGATGAACTATAATGAAGTAAAACTAAAGGAAAAGGAA
AAATTAAAGTTCTGTTAAAATGTAGAAATCTGTAAAAATCACTATTTCT
>comp148157_c0_seq1
TTTATATAATTTATAATGTATTGAAAATGGAAATAATTTATAATTACAAACAAACCCATCCCATAATAATTTTAATAATTTATGCCAAAGAACATACATCATTTAATTTAGAATGATTGAAAATGT
CAAATATATATGATCCTTTACATTTATTCCATTTTCTTCTTAAAAACAAAGCACACGTGTAGCATTTCATTCAAATTAAAAGTTCCAAATTCTTCATTGAGGGCACTCTGGTGGAGGGTTTGG

AAACCTTTTAACATCATTACAGTAATCATAGGTCATATATGTTCTCCTGACGTTCTGATAAGCCGATTTTTCAGAAGCACTTAACTGTTGATATCTTGCTGAGTTCCACCATAATGAGGGAGA
TGAACAGTGAGCATCATGTGAACTTACTGAAACGCATCCATCTACATCAAAACCTTGAAAAATTGCATTAAATGGAGCATAAGACCAGTCTACTTTAGTCTTTCCTCCATCAGTAGCCCAG
CTATCTCCATTCCACAAGCTTGCTATTAATTGCATTGGTTGAGATGGATAACCCACTCCTATATTCTCTTTGTTAGCAAATACTCTAATTGGTGTATCATCCACATAAAACACTATTTGATTAGT
GTTCCATAGTATTTTGTAGTCATGAAGGTCAGCAGAAGGATCAAACCATAATCTGATCCTTTGTTCTCTATTTCCTTTCCCATTTGCAAAGACATTTGTCTGAAGAATTATGGGTTTCCCTTC
AAGGTTGCCAAGGAACTCAAAATCCAGTTCAGCATGGTTGGTACTTGAGTCAGAAGCCAAATAGAAAGCAGTTACAACTCCAGCTGAGTTTTTTCCTGGCAGCTTTATCTTCATATGGAA
GAACCCAGAACCGTATTTAAGCTTTGATCCAAATCCAGAACCTGAAGCTTTGTCCATGAAAAGAGAAGCCTCAGTTTCCTTATTCAATATCTGTATTCGATCTCCACCCCATGATCCTTCAT
AATTTTCATAAAAACTTGTAGCTGGCCGATCACTCTTAGCCCAGTCCATGCAAGCAGCTAAGAGAAGAAGATGAAGAAGAAGAAGAGGAAGAGTAGTAGAAGCCATTGCTTTTGTTGGA
ATCATGAGAGATGAA

>comp148157_c0_seq2

AAAGATGACAACTGTTAGCAGTCCATACTACTGCATAAAAAACCCCAGAATAAAATTGATAAATAAATTTCATGCTAAGAAATAATTTCTCCATAAATTTAAGAACAAGAGTTTGCATATCT
CAAAGCCTTAATTTTAACTATATTGTTATATCAACCCTTTTTTATCCCAGATAAAAATAAAAATATCAAAATATTAAAACCTGAAGCTTTGTCCATGAGAAGAGAAGCCTCAGTTTCCTTATT
CAATATCTGTATTCGATCTCCACCCCATGATCCTTCATAATTTTCATAAAAACTTGTAGCTGGCCGATCACTCTTAGCCCAGTCCATGCAAGCAGCTAAGAGAAGAAGATGAAGAAGAAGA
AGAGGAAGAGTAGTAGAAGCCATTGCTTTTGTTGGAATCATGAGAGATGAA

>comp148188_c0_seq2

TTTAAATAAATAAATCTTTCGTCATTTAATAAAATGTCAAAAATATTTCCAATTCATTAACTGTAACTAAAGTGATATACCCCACTGTAATTCAAAACCTTTCCTTCAATAATGCATTAGGAAT
ATATGGTTACATCTGAGCTCCCTTGTCATAACATTCTTACAACCTTTGATGGTTGAAAAGCCTCAGTATCCAAAAACTTTTAAACAATCTACACATTAAAATCTCATACATAAAAAATTTCAT
ATCACTGGATTTGGCCCTTTTGCATCTCCCAAATAGAAAGCAATGCCATAAAATCAGCTTTAGATGCTGTTTTTTAGTCCATTTTGTCGCTCGACCCTTTTGGGTGGCATGGATGCTTCCTTC
AGCAATTTTCGACTCAGTGTATTGTAAAATGCTCGCGAATACTGCCTCGAAGCATTCCTCCAATCTGTTCCAGACTTCATCATTGCCTCGAATTCTTGGTAACTAATTTTCCCATCCTTGTCT
ATATCAACATCTTTTATGATTTCTCGAATCACTTGATCATTCGGATCGAGGTGACCCTCGCCAAGAGCTGCTCTCAGCTCATCAATCTCAATATATCCACTACCATTCTTGTCAAAGTAGTTG
AATGCTTGAGGCAGATGCTGCTCACTATTGACCTTCCTTAGATGCAAAGAGACAGTCAAAAACTCATCGCAATCCAGCATTCCATTTCCATCTATGTCTGCTGCTTCCAATAGCATCCTGAC
ATCGTGTTCTGGAACAGGCTGACCAATCTTGTGGAACCCTTCCTGAAGTTCTTCAAGGGTCAAGTTCCCATTTTGATCCTTGTCCATCATATGAAACATCTGCATGATTCCATTAATTTCTTC
TGAAGGCAACTGATCAGCTACAACCCTCAATGCTTTCTTCTTGAACTTGTTCATCACTAGGAACTGCTTCAACCTAGACCTGACGGCCTCGCCGAGTGGAATATTTGGAGCTGAACTTGC
ATTCCGCAACCAAGGATGTTCAAGAACTTGTTGAGCAGTAAGCCTTGTATCAGGGTCTGGATCGAGCATGCGCTTCACAAGATCCTTGGCACTTTCTGAGACCTTTGACCATGGCTCCCT
CTTGAAATCAACAGCTGCCCTTATAATCGCCTGTGCAATTCCTTCATCACTATCTGCCCAAAAGGGTGGGACTCCACAGAGGAGGATATACAGGATAATCCCAGCACTCCACACATCAACC
TCTGGTCCATAATTGCGCTTCAGAAGTTCCGGTGCCATGTAAAATGGACTTCCAACAATCTCGCTGAAGCGCTCACCAGGCTCAAAGAACACAGAGAGGCCAAAATCTATCACTTTCAAT
GGAGAGTTTTCTGACTGATCTAGAAACAAAAAGTTCTCAGGTTTTAGGTCCCTGTGTATCACTCCATGCTTATGGCATACCTGGACAACCTCCATGATAGTCTTGGTAACGCTCGCCGCCG
CCCTCTCCGAGTAATGCCCTCGCGCAACGATGCGATCAAATAACTCCCCACCCTCGCATAACTCCATGACAAGATATACGTTGTCCCGATCCTCGTAGGCCTCGATGAACCGGACCAGGTT
AGGGTGAACGGGCAAATGCTGCATGATCTCCACCTCCCGCCGGACATCAGCCAAGTCTACAGTGCACCGCAATTGCCGCTTCGATATTGTCTTGCAAGCCAAAGCCTCCCCTGTCTCCAC
ATTGGAGCAGAGCCGCGTCACGCCGAATTCACCTCGACCTAGCTCCCGGCCGAGCTGGTATTGGTAGAGGAAATCGGCGGCGGAGGCCGGGTTAGAGAGGACGAAGATAGGCGAAACC
GGGGAACCGGAGGAGGCGACGGAGGAGTCGCGGGGAGAGGTTTCGATCTCGTCATCGGAGTCGGCGGCGGCGGGGAGTAATCGGAGGGATTTCGGATGCGCATGATTGTTGGTGATGG
ACGGGGAGGAGTAGCACCCGCCCATTTATTGCCGTTGCCGTCGTCGCCGCCGCCGGCGGCGAATTAATTGCTCGCTTGTTGATTTAGATCGATTCTGATTTAA

>comp148208_c0_seq1

CCATTATAAATTGAAAATGATTATCATTTATGCTCTTCATTCATATAAATTTATAGGATGCTATGCTTCATCTTCTTTTTATCATTCCTGAATCAATTACTAAGAAAAGGACATTTCAGGCTTGA
CTGTATCCCTCTAGTTGATAAAGCTTTCTTCTCCGTCTGGCCGCAGTAAAATATTTATGTTATTGTCGCACTTGTAAGTGTGTGTGGGACCATCATGACCGGGTGATCGCTTTACTCGAGCTT
CTGGCATCGAAGTTGTCATCTGTCTGGAGACTGCCGTCGCTTGATTATGGTTAAGATCAGCGGTGCCTGAGTTTTAATCAACTGTAGAGTAGCCCTGGGCCTCCTGGCCCTCCAGCTAGTC
CTCCTATTAAGGAGTACTTTATATTTTATAGACTCTAATCATTATTTTGGTCAAGGATAAGCCTGATATTACTTCTCATCCCAGTTTCACTATCAAAAGGAACAAAGTTTAAAAGGTTCTTTTT
ATTTTATAATTGAAATGGTTAGATTTTCGTGCTTCTCCACTCCCCCAGTCAGCAGATCAAAGAAAGTAGCTCAACGTTCTATTGGAACATTGCAGACAGATTATCAAAGTGCAATTCAGGAT
GAATCAATAAAGTTTGTTAAAGATTCTGTAAAATCGAACCCTTTGATCGACAACTATAATTCTTCCTATAACTCTGTGGAGAGAAGTACATCTACTTACTCTCTGCAAGGTTGTTGGAGATC
GGATAGCCTGAATGTTAATATTCCTGAGGATAACCATGAGATACCACAGACAGTACTGATACATAAAAGTCATTCTCTACGCAGCATTATGGAGGAAGGAAGTGATTACTCTGGTGATGATA
TCACAGAGGACGATGATATTGATCATGGATTTTCATATGGTTTATTGAACGGTTGGAAATTGAAGGAAGTTCCAGAGTTATCTGATAATGCTAATTCTACAGAGGTTTTTCACCATGAAAAT
GATAGGAAAGATTATCAGAATCAGAAGGAAAATGACTTAATGAAATCCTTTGATGTCCTTACAGATCCCATGCATCATGAAACACTTTTCTCTATAGAAGTTTTGAAGCAACCTGATAGAG
ATTGGCATGCTGATATTGCTGAGAAGGCTGTTGATGATGTTGCTGACTTTGGATGCTCATCAGAACATATTCCTACTTTATCAAGATCATTTTCCGTGGCTAACCTCAGAGTAAATAAGACTG
AATACGGCAAAAATGACTCAAGGCATAGAATCATTTTTCGTAGGTCTCGATCCTTTTCAGATCTAAATAACTTGAATATACTAGAAACAGAACATAAAAGTGGTGAAAAATATTTTGATTCT
GGGGATACTGTTGAGAAAAATGTCATTAGTTATCACTCTAATTTGCCCCTACATGATGATGTTACTGGAAAAGTTCCATGGTATGATGATAAGGAAGTGGGAGGTCTCAGTTTGGTGAGAA
GGAAAATCATTAAGTTTAATGATAGTGATGGTAAATGCTTGAAGTTGAAGTTAGACATGGATAATAAAGCTGCATGCAACTCAAAAGGTAATTTTAGTTTTGCAGACGCCTTGAGAAAATC
TGAAGCAAGTAATGCTAATATCACAAGGGAGTTCCAACAGGAATCTTCTCACGACAATTGGGATCAACTGACCCCTCAAGAATTTAGTATAAGGAGAGTAGAAAATTGGATAAGTCAGAT
TGATATCCATGGTGACCTTATTGTTGAAGAACAAGAAGCGAGTTCTACTACTGTTTCCAAAGAGGAACCCCAAATTTTAGCTGCTGTGGATCCCCCAAAATCTGAGACTCGAAGCAACCT
GGCCATGGAGGTAGCTTACAATTATATCTCTTCCATGACTTCCTCATCTTCATCAGCGCAAATGGCAAATCTTGGGTTGGTTGCAGTCCCTGTTCTTAGTTCATTTATCAGTTTGCGAGTACT
AAACTTATCAGGCAATGCTTTGGTTCAGATAACTCCAGGATGTCTTCCAAAAGGCCTCCACATGTTGAACTTGTCAAAGAATAACATATCAACTATTGAAGGCCTCCGTGATCTATCACGT
CTTCGTGTTTTGGACCTCAACTATAACCGAATAGCTAGAATTGGCCATGGTTTAGCTTCCTGTTCCTCACTCAAGGAACTTTATTTAGCTGGTAATAAAATCAGTGAGGTAGAAGGTCTGCA
CCGTCTCCTCAAACTGAGCACCTTAGACTTGCGGTTCAACAAAATCTCTACTTCTAAAGGCCTTGGACAACTAGCTGCTAACTATGACTCTTTGCAAGCAATAAATCTTGAGGGAAATCCT
GCCCAGAAAAATGTTGGAGATGAGCAGCTGAAAAAGTTTCTTCTTGGTCTTCTTCCTAATCTTGTCTACTACAACAAGCAGGCTATTCGAATGGGCTCAAAGGAAGTTTCAGACAGTGCC
GCTCCGGCTGTTGCTTCACCTCAGTTTGATAGAGGCTTAAGAGTTGAGCACAAGAATTCACAAAGGGGATCTCGTGGCTCAGACCTTAACCTGTTGGCATCAAACAATGGACAGCAAATT
CTTGCTACTCCACCACCATCAAGGAAGCCTTCCAAGGAAAGGCATGTGCGGTTGCAACTGCCACCTGCTTCATCTAAACCAACCAAGCTGCGTTCGGATACTGGCAAAAAACCACTTGG
TATCAACACTAATGATGCTATGAAGCGAAGCTGAAGCACAGATGTACTTCGAGGCTTTTTCAAATTGTAATTGCAAGAGCAGATGAACTATGGCTTGTATGAAAGAGATACTGCTTGCAGT
CTAGTCTGGTCCTTGAAATAAACTTCTGCCAGCACCAGTCTTGTAATTTCCAGGGTCATGGCTATTGCGCAGAAATAAATGATGGTACGTAGCTGTTGATAAATAATTGGAGGTAGGCTTTC
TTTCATTTGTTAGTATTCATTCCTTTCACCCTTTATAAATTGGAGAATTTCAGTTATGTGTATTATCAGACTCAGTTGTAAAGTTTGAACAAGAGTTAAAGTACATACTTGTAGCTCAATTATG
ATAAAACATCTGAGATTATAAATAAAAAA

>comp148323_c0_seq1

TCGTTTAAGACAATAATAATCGAAATGGAATTACAATGTGGTGAGCTAGCTAGGTTTTATGAGATTAGAATTACTAGAATGCCTCTGAGGCTAATGGGCGTGCTGTCGGGTCTGGTGTTCAT
GTAGAGGATGTAGGTGGCGAGGCCGGGTCCGAGCGCGCCGATGTATGACTGGCCGGCGATGTAGACATCGTTGTTCTTGTGTTCAGGAAACCTCTTCATCCACCGCGCAATGAAATGGTA
CATATCGGCGATGATCCAGAACTCCTGGGTTTCATATATGTCCTGATCATTGTCGGAGAGCGAGAACCCAACCCCTACTGGTCCCTCAATGAAGAGAATATTGGCAAATTTATTCCATGAAA

ATGGGTTGGGGATCAGAGTCTGGCCGTCATCGCCAACAGTGTATGGCCCTACTTGCTGGAAGCAGGATAGCAAGGCCGAGGCCAGATCGTTAACAGATGGGGCGCCAATGTAGATTATGA
GGGGCTTGGATAACGGTCGAATATCTTCAGCCTCAACAAAATAGTAGAAGAAGCGTCGATTGCTGACTGGTTCGGCGAGAAGATAACCCGAATACTGACGAAAATCTATGCTTTCAGGCT
GGTTTGGAAGCTCGTCTATAAAATCGGCATCTTTGGCTAACGCGTCGCTTGCGTCGCTGCCGTTGCCGGCGAAGTTCGCCGGAAGAGAAGATGCGGCGACGATGAAGAAAAAGAAGAA
GAAGAAGAGAGCTTTGGCTTCTTCCATTTCTGTGCTTAGGTTGGTAGAAATAGGTGGGAAGGAAAAATGGGATATTTAAGAAACTCAAATGC
>comp148365_c0_seq1
TTAATTCTCCCAAGAAAAAGCATAAATAACTTAGCAAAATTGATTTCATGCACACTATCACACATCATTCCACCCTAAAATGGCAAATCCTCTCCCCCCTTTTGCATTCTTCTTTCTCCTCTG
CCTACTCCTCGCAACCCCCAGCTCCACCTCCTCCCCAACCTTCCCAATCCCCATCAACAGCGACCGGCAATCTCACCTCCAATTTTACATCCACGACAAGATTAGCAGCCCCAACGCAAC
CGCCATCAAGGTCGCCGCCGATCCCAATGGCTCCCGCGCCTCCGCCACTGGCCTCCACTTTGGAAGCATTTATGTCATTGACGATCCCATCACCGAAGGACCCGACCCGAACTCGAAGCC
CTTGGGCCGTGCCCGTGGCTTCTATTCCGTGTCCTCTACGGATGGATCAGATCTCTACTTCACCGTAAATGTGATTTTCATGTATGGCATCTATAACGGGAGCTCTGTAGCTATCGTGGGGA
GGGATCCCATTATTGAGAAGGTAAGGGAGCTGCCGGTGGTGGGCGGAACAGGTGTTTTCAGGCTGGCCCGTGGGTTCGCCTTTATAAAGACGCATGCATTTGATACGGCCACCGGGGATG
CTGTGTTGTTCGTGGAGGCTTACATTCTCCACCATTGATTCATTTCTTTGCTTGTAGGTGTTCGTTCGATATTTCGACAGAGGACGAGGGAAGGAATTAATGTTTTTTATCCTTTGTTTTCGT
TTTGGTTTTAACGAATTAATGTGGTTGAATATTTGTCTCTGATCTTATTTATAAATTTTTATTTTGGATTTATGGATTAATATGATGGAATTCCGATCTCTGTCGTTTTTTTTTTTTTTTAATTTTG
TAGCTCTTTTGAGCATTAGTGAGCTTGAGGTCCCTTTAAGGACTTTGTAACTTTTTCCCGATATTAACGCGTAGGCCCGTAGGAGGGTTTTAACAAA
>comp148438_c0_seq2
ATAAGACAGACCACAACATTGAGTAATCTATAAATAACATTGAAGCAATTTGAATCAGTCAAATCTAAATAGCTAAAAATGCAACTCTTGTCTAGATTCACTCAAAACGGACAGTAGCTGA
GATTCTAGATACATCGAACCTGGACGCTTCTACGAAATTCAGCTTACCAAGACGGCATTAGCCTATAACCATCAGCAGTTAATTGCCCACAAACCATTAATGACTCATTTGTGACATGGCAG
TCCGCCCGCCATCGACCTCGCGCGAATCACGTGCACGTACCTGGACGTCGTATTGAATTATGAAACGGGACGATCGGACGGCTTCAGTTCCGCTTTTGGCCAACGTGTTGCGAAAGTGCA
ATTTTTGCAAAAACACCCTTTAGAAACTAAAACTTCAGTCCCGTGAAGTAAATGGAGAAAAATGTATGAATTATGAAATCAAGCCAGCACCTTGTTTTCTCAATTTGACATAATAAACCCC
CTCAGTTATTATAAATGAAGAGCAATTTTGTCGAGTTTTTTTAAATCAGTAAAACAAAAGTTGATAGGACTGCTATTTTTTATTATTTAAAATCAAATTCTTATTATAAAAATCATTTTATGGTA
AATAAAAAGAACGAGATTCTAAATTACAAATAATTAAAAAAAGAGGGATAACTATGTCTCATGGTATAACGAGGTTCATTCTACAAATTAATAATTCCAAAAGGAGGAAGATGAAAAGTTT
TTCATTTTCATTAATGCACATTTCACTATAAATTATATCTTGAGCCAGGATGGGAGGAAAGGAAAAGAAAGGGAGAGCAATCAGAAACTTTTAAGATGGCGACAAGATACAAGAGGGCTA
TGGCGCCTCTGGACGACAAGGCTCGAGCGCGGCTGTGGGACGGTGGCGGAGCAACCGCGGTGATCGCCGTTGGCAGTGAAGGGTCGACGGGGAAGTTGGAGGAGCTGGTGCACGCGT
TCTACAACGATGAGATGTCCATCAGAGGAGAGAAAATTGAGGAGGAGGAGAGGCCGGCAGCGAAATGGCGGCAAGCACTGGAGTTGGAGCTGAAGGATGATTTGGCAGAGCCGGCGG
TGAAGTGGATACGGGCAAAGGCGGAAGCAGCGGTCGGAGCCGCTGGTTTGGAATGCGAGGAGGTCGTCAGGCGAAGGTTGGTTCGCCTGATGCAGGAGAGGGGCTTGGATGCAGGAA
TTTGCAAGTCCCAGTGGCATAACACCTCCGGCATCTCTGCCTGCTCCCACGAATACATCGACGCGGTCGTCGGCGAGACACGTTACATGGTTGAGATCAACCTGGCCGGAGAATTCAAGA
TCGCAAGGCCAACAGAAGACTACACGGAACTTCTCCGTTCACTACCAAAGCTCTTCGTCGGGAGACCGTCGACACTTGAGGCAGTGATCAGGTTGATGTGCTCGGCTGCTCAAGAGTCC
ATCAAGAAAGCCGGCATGCACGTACCCCCGTGGCGGCGGACGAAATACATGCACGAGAAATGGTTCGGTGATTACCGGCGATACACAGATACGTTTTCTTCTTCGGATTCCATAGGCAGG
ACGGCTCCTACAGCTTACACGGCCAACCAACGCTGCAGAGGAGCCACAGGTAGCAGCAAACTAGTAAATAGCATTTTCCTAAGGGGAATGTGAAAGGGGACGATCATTTAACGGTTATA
GGAAGCTTGTGATGTTTGCTATTTTGCACATATATATATATAACATTAGTTATTCTATTGAGGAGATTGTCATGGAGAGATGTTATTAAATTAACTGGAGGAACTGGGAGGATACAGGAGAAA
GAACAGTTTTGAACTTTCAATAAATGATGTAAGATTTATTTTGGGTTTTTAAATAAAATGCACAAGAATGTCGTGCTAAAGGTTTATATCTTGCAGTTTAAAATTCTTGTGTTGACTTTGAAA
CAGTTTTTGATTTTGACCATGTTCTTGAAGATATTTGCTGTTGAATCTGAGATGTGGATAAAAGACAATGGCAAGTCAATGTGTCCAATAACTGGTTTTAGTTAATATTATTCAATATTTCAAT

ATTAATGGAAGCTGATATTAAAATTGAAAGTTTTAGCTTCAAGATAAATCTT
>comp148451_c1_seq1
TTTTTTGTTGTAGCCGGCTTGATGATGTTGTTGTTGTTGGAAATTAAATTCCAAATATTGGGACCAAGATTTGTATGACAATTTCATTTGGGATAGTACAGCATTTGACAGTTTTCATGCACA
AGGTAGCTTACAAGGAGTTTTGGAATAATAAGCTAGGAAGCAGTTAAGTAAGAACACAATCTTGTTCTTGTTTTCCTTTGAGTTCCATTTTGAATTCATAATAATCAAGAACATGGAGCACT
ACTTGGTAACCTCCAAACCCTAACTCGATACTATTTCACTACGACATGTGAGTCAGAGGAGCAGCAGTCATCACAATTTGCATAGAGCCAGCAAACTTCCTCAATCTCAAGTCACTAATGC
TTCATTAGATCAACTTCAATATATCAATCACTGTAATCTATACGGAGTAAACATTAACATGCAGTTTATCATCTTTACTAGGCAGTGAAGTGATCATTGGTTGTACGAACAAATAGGAGGAAG
GCAAAAAAGATAGCTGAATAGAAACCAGTATAAAGATTAACAGAACAACATAAAAACTGCTTGTTAACGCTGAAGGCTCTTTGAAATTATGAAGAGAATATGTGTCTTATGTCCCTAGCTA
TCATAAAAGATATGAGGATTGGTGAATTCTCTCAAATCAAAAGTGAAACTAGAGTTGGATATCACCAGCCAATTCCCCTAAAGAAAAAAAAGATACTCTTATACTCAGAAAAACGATTAAC
AAGAAAACAGAACCCGGAGGAAACAAAACAAAACTTTATCAACAATTTACTTCCAGACAATGAATTGTAATCAACAAACTCTTTTACAATGAATGCTAACCTATAATTTTTATTTTAAATT
ACACATCTAGTAGTTTATTAGAGTCACATAAGATTGAAAATCAGGACTTAATCAGCAAAATTTCAAAAATATGGCTAAAGTTTTGTTTGGGATG
>comp148474_c0_seq1
CATCTCTCTCTCTCTCTCTCTCGCTGGCTCGTGATATCCCTTCTGTCCATTGATTCCCAATGTCAAAATTCCCATCTTGCATTCAAAATAGTGCTTTAAATCAAAATCAATAGTTTCTACCTCC
CAATCTGAAAAAAAAAAAAGTTTTTTTTTCCAGAATTTCCTTCCTTTCAATGCTCGGCGAACAAGATCCAAACCCATTTCTTGCGTTCTTCACCAATCCCTCATAACTCCCAATCCACAA
CGCGGAGCGTAACGGCGGCCGGAGGAATGGAGGACGAGGACAAGAAGGCGACAGGCTGCTGCCTTTTTGTCCTTTACAACTCCTGCTTCCGTCGCCACCCGGTCCCCGTCTCTCCCCTT
AACCCCATCTCCCCATCAGCCGTCTCCAAAGGTAGCATCCACAATTCCAATGCTCGGCGCCCCATTCCCAACCAAACTTCCGCCGTCTTCCCCTACTCCCACCCCAAGCCCCCGACCGCC
GACCAAACCAAGTTAACACCACCCGGCCGTATTTCGAGGCCGGGAAACGGCGGCATATCCTCTGAACTAGACAGCATGATCTACGACCACCAACGCACCAAGGGGAGCAGCACTCTCGT
CCGCGCCTCCTCCGGCAACTTTATGCTCTACGGTAATTTGGGAAATCTTCGCGCCCCTGGAGCCGTCACGCCCAACCGCAACATTCTTGAATACCTCCCGAAGACGGCCAAGGAGATGGA
GAAGGCGAGGGGTGGAGGAAAGGTTGAGGCGTCGTCCTCCATTTGCCGGGCGCTGTCGAGAAGGCTCGACCCGGAGGAGCTCAAGGAGATGGGTAACGAGGAGTACAAGAACGGGA
GATATGCGGAGGCCGTTGCGCTCTACGAGAGGGCTATCTTGATTGATCCTGAGAAGGCTTCTTACCGGAGTAATAAGGCGGCGGCGCTCATGGGCCTCGGCCTCCTTCTTGACGCCGTGG
AGGACTGCAGAGAGGCTATTAGGATTGATCCTTATTATTTCAGAGCGCATCAGAGACTTGCCAATCTCTATCTCAGATTG
>comp148484_c0_seq1
GTGTTTGGTGCGAGCGTGGAAGACTGGATTGTTGGCCAAGGCAATTGTGGACATGTTGTCGCAGAAAATATCAACTGGTTGATGATGATGAATACGGAAATCAGCTAGCAGCCTTTTTAT
CCAGATCGTATCCGCTGTAGCTGAAGCTAGAGCACGGTATTCAGATTCCGTGGATGATCTTGAAACCGTTTGTTGCTTCTTTACGGTCCAAGAGATGAGTGTATCACCAAGAAATGTACAA
AATCCGGAAGTTGATTTACGGGAGATAGGATCGGACGCCCAATCGGCATCCGAAAATGTGCGTAGCTTGAGGTCACCTTTGGTGATTGGCAGTCCAAATGCAGCGGTTCCTTGTATATACC
GTAACAGTTTCTTCAGCATGAGCATGTGTAGATTGCTTGGTTCGTGCATGTGCTGAGATAAAGCATTAACCGCATAAGAGATGTCTGGTCTCGTGATGGTTAAGTATTGTAAAGAACCTATT
ATTTTGCGGTATCTTGGAGCGTCCAGCCAAGGCTTATCATTTGCATCTGAGACAGGCGATTTGGTAAAAGAAGGATTAGAGACCGAATTGCATTTTTGCAATTCGGCCATTTGTACGATAG
ACATTGCATAAGAGTATTGAGATAGAAAGAACTTATCAGTGGTGGTGTCGATTTGAATGCCGAGGAAATGTTTTGGCTGTCCGAGATCCTTCATCGTGAATTGAAGTTTCATTTGTTCAATT
ATTGTGTGCATGACACTGATATCGTTGCCGGTGATGAGGATATCGTCTACATAGACTAGTAGGTATATTTGCAGATTTCCTTTATGCATAATCATCAACGATGGATCGGCTTGACTGTGACGA
AACCCGACCTGCGTAAGGTAGTTAGTAAATGTAGTGTACCATTGACGAGGTGCTTGTCGGAGTCCGTAGATAGCCTTCCGAAGTCTGCAAACATGTGTTGGATGGATGGAGTCTTCGAAT
CCTTTAGGTTGATGCATATAGACTGTCTCTTTAATATCACCGTGCAAAAATGCATTAGATACATCAAGTTGATGTATTTTCCATTTGTGAAAGATTGCGATTGCTAGTAAGATGCGTATTGTAG
GTAGCTTAGCGACCGGACTAAACGTATCTTGATAATCTAGTCCGTACTCTTGTTGATTTCCTTGGGCTACTAATCTTGCCTTGAATCTTGCAATCGATCCATCTGAATTGTATTTAATTCGATA

TGTCCATTTTGAGCCAAGAATGGAGCCGGTGGGTGGTGGCGAGACTAGTTCCCATGTACCCTGTTTTTGTAGTGCATAAAATTCTTCCGCCATGGCGTGCCGCCATTCAGGGAACTTATTT
GCATCGCTGTAGCATGTTGGTGCTCGAGAGGGTTGTTGATTGTGAATTAGATTCAGTCTTTGAACAGATTTAAGTGAACCGGTTTTGGCTCGCGTGATCATCTGATGGTGAGATGTATAATT
CTGTGTGTGTTGAGAGGTTGTGACGTTTGGAGCTTTACCGTGATCTTCAGTGGAAGCCACTACGTTGTTATTTCCAGCTGAAATCATTGGAGTTGTACTGGCTGTATGAGTATTCATTTGAG
ACGTGTTGCGAGACAGGGAGGCGGGTATGAGCAGATTTGGATGATTAGTGCTGGGCGCCGGAGGAGAGATGTTCGTCCTACTCGTTTGTGGATTTACAGCCAATG
>comp148569_c0_seq2
GAGTTTCCACCAGTAGATGTGGAATACCAAAAGCGTTAAAAGCATAACGTTGAAAATATAATATGATGATCTAGGAAATAGGCCTGGCTTCATCAATGACTCGATAGAGTGGTAACTTGTT
GTTTTGATTATCCAAAAGGGAAAGAATATTAGTCGCAGCAAAAACCAGGATATAGCAAAGCAAGCAAAAAATAGGCTAGCTGCCATCTCCTTCCCTGCGTACTTGGAAACTTTGGCTGCT
TCTAGAAATACATCACTCATATCATGAAGGGCGAGAATTACAGTTCCAATGCGGAAGAAACCTGACAAGTAGGAATATCCAATTAAAACTGAAGTAATTATGTGATGGGAGAACATAATAG
AGAAATCTTTTCTTCGGGTTTCCCATGCCAGAAGAGCACAAATGCTGTAAAGATAAAATCCACACTGGCACATATAGAATAGCTTCATTGGCAACCCCAAATCCTGGTTCGGCCACCCGTT
AAAATACTCCTTTGTATTCAAGGCCCAAGATGTTTTTTTAAGGATTGAAATTACCCACAACTGCACACTAGCATAGTATGTTAACTTCCACATTGACTCCGTGCACTTGATAAACTTTGATTT
TCTAGTCGCATCAGCTATCAAAGGAACAACATGACCGTACAACATTCTGATTGCCAGCCTCTTATATACGAGAGAATTTAGCAAAAACCTGGCCAGAGCGAAACACATGGCGAAAAAGAT
CGCAATGAGCAAATGCGTGGCCTCAGGCGCATTGCCGAAATCCCCAATCAAGGCCATGCCTTCAGCCGGCTCAGCGGGAACAACTCCGAAAGCGCTCGGAAATGGATTAGCTGAAGATA
ACCGTTCGATCTTGGAACAGCAGTGGCCAAGGGGCACAATGTGAAGATGAGAACCTCCGCATCCAAATCAAAGTTGCAGCGACAGAGAAAGCGCGTGAATAGGCGATGAATCAGAAGG
AGATTAGGTCTCCGGATTCTTAAAAAATGCTGGGCTGTTAATGTTTATCGATTGATAAATTAGAGGTGGGAGGCGAATGGAA
>comp148687_c0_seq2
TTTTTCTTTTTTCCGTCCGGCACCGCTCCTTTCCCCCCGCCATAGATCATTTTTCCTTGAAACTAATAGATCTTTATTTCGTTGCTTACGAACCTTACCGAGAGATATGCACGCTTTGTGCCGC
GTTCGTCACCGGCGACGGGCTATAGCTGCCCTGTTGTTGTTTGTCGCCCTCCTCGCCGCCGGGAAGCTAGGGCAATGCCAGGACGACGACGATGATTATGACGACAGCGATGGCGATGAC
GACGATGGCGGAGGAAACTCAGCAGATGTCCCGCTCTTCGCCAACATTGTCTATGACCGTCTAATGAGCCTTACCGGAGCGTTCACTAAAGAGTTTGGCAACCAGCTCAAATATTGCATC
AAGGATTCAGATAAAGATTGGAATGAGGCGTTTAATTTTTCGGCGGATTTGAGATTCTTGGTTAATTGCATCAAGCAGAAAGGGGACCTCACATCACGCTTGTGTAATGCTGCAGAAATAA
AATTTTACTTCAATAGCTTTTATGACAACACTGGAGAAAACGCAGCAAAAATCAATATACTTAAACCAAACAAAAATTGCAACTTGACTTCATGGGTACCTGGATGTGAGCCAGGATGGG
CATGCAACATCAATCCAAATCAGAATGTTAAGCTTAATGATTCCAAACAAATGCCTGCAAGGACTTATAAGTGTCATCCTTGTTGTCCAGGATTCTTCTGCCCCCGTGGTATTACTTGCATG
ATACCTTGTCCTTTGGGTGCCTACTGCCCACTTGCTAAACTTAATAAGACCACTGGTGTTTGTGATCCATATAATTACCAATTACCTCCTGGTCAACCAAATCATACTTGTGGTGGTGCTGAT
ATGTGGGCTGATGTTTTATCTACTAGACAAGTGTTTTGCCCGCCTGGATACTATTGTCCGAGCACTATACAAAAGATTTCTTGCAGTAGTGGGCATTACTGCAGGAAGGGTTCTACATCTGA
AACAAGTTGCTCTGTGTTTGTTGCTCTTAATAATATACAACTTCTCTGGCCAAATCCTTAGTAATCGTGAGAGGAGGCAAGCTAAATCAAGAGAAGCTGCTGCAAGGAGTGCTAGAGAAA
CTGTGCAAGCTCGTGAAAGGTGGAAATCAGCTAAAGATGTTGCCAAGAAACATGCTGTGGGACTTCAGGCACAACTATCAAGAACATTCTCACGCAAAACGTCTAAAGGTTCGGGGCA
ATCTCATGAATCATCTGACGCATCTAGTTCTAAAAATGCAGGCCTTCCTCCTAAGAAAAAAGAATCTAGTAAACTTACAAAAGTTATTCAGTCACTAGAAGAGAATCCTGAAAGTGACCA
AGGTTTTAATCTACAAATTGGAGATAAGAACATAAAGAAAAATGTGCCAAAAGGGAAACAAATGCATACTCGCAGCCAAATCTTCAAGTATGCTTATGGTCAAATTGAGAAAGAAAAGG
CACTGGAACAACAATATAAGAACTTGACATTCTCTGGAGTTATTTCTATGGCTACTGATACGGATTTAAGAACCAGGCCGAGAATTGAGATTGCTTTTAAGGACCTAACACTAACTTTGAG
AGGGAGTAAGAAAAAGCTTCTAAGAAGTGTGACAGGCAAACTCATTCCTGGCCGTGTGGCAGCAGTGATGGGTCCTTCTGGGGCAGGCAAGACTACATTCCTAACTGCTTTGGCTGGA
AAGACAACGGGGTGCGAGATTACAGGCCTGGTTCTTATTAATGAAAAAGTAGAACCAATCCGGTTTTATAAGAAGATTATAGGTTTTGTACCACAGGATGACATTGTTCATGGAAACTTGA
CAGTGGAAGAGAACCTTTGGTTTAGCGCAAGATGCAGACTTTCAGCAGACTTGTCAAAGGCTGACAAGGTTCTTGTTGTTGAAAGAATTATCGAGTCCTTAGGATTGCAGACAATACGT

GAATCCTTAGTTGGTACTGTTGAGAAGCGTGGAATATCTGGTGGGCAAAGAAAGCGAGTTAATGTTGGACTTGAAATGGTTATGGAACCTTCTTTATTAATTTTAGATGAGCCAACCTCTG
GTTTGGATAGTGCTTCATCATCTTTACTTCTTAAAGCCCTACGTCGCGAAGCACTGGAAGGGGTAAATATATGCATGGTGGTTCACCAACCTAGTTATACCCTGTACAGGATGTTTGATGAT
TTGATACTTCTAGCAAAAGGAGGTATAACAGTGTATCATGGATCTGTGAAGAAGGTTGAAGAATACTTTTCAGGCCTGGGTATCAATGTTCCTGAACGTGTTAATCCTCCAGACTATTATAT
TGACATTTTAGAGGGCATAATTAAACCAAACACAACTTCAGGAATAACTTGCAAGCAACTTCCTCTTCGATGGATGTTACATAATAAATATGATGTGCCTCGTGATATGCTTAATGATGATGC
TACTGGAGATGTATCTAGCAGGGATAGTGAAAATACTACTGTTAAGGTCGAAACACCATCTATTGCAGGAGAAGTATGGAACCATGTCAAGGATATTTTAGGGCAGCGAAAAGATGATTTT
AAGTACCACTTTTTACATTCTATAGATTTATCAAATCGGAGCACTCCTGGTGTTATGACGCAATACAAGTATTTCTTGGGAAGGGTAGGTAAACAACGTCTTCGTGAAGCCAGAATACAAG
GAGTGGATTTTCTTATTCTGTGTCTTGCTGGAGTCTGCCTAGGGACGCTTGCAAAAATGAGTGATGAAACATTTGGCTCGTTGGGTTACACATACACAGTTATAGCTGTTTCTCTATTATGT
AAGATTGGAGCTCTCAGATCATTTTCACTTGAGAAGTTGCATTACTGGAGGGAAAGGACTTCTGGCATGAGCTCACTTGCTTATTTCCTTGCAAGGGACACCATTGATCATTTCAATACAA
TCATCAAACCAATAGTTTATCTGTCAATGTTCTATTTTTTCAACAATCCTAGGTCCTCATTTGTAGATAATTACATCATACTGGTTGCTCTTGTTTACTGTGTAACTGGTATTGGTTACTTCTTT
GCCATCTTCTTCCAGCCAGGAACAGCCCAGTTGTGGTCTGCACTTATGCCGGTTGTTTTAACGCTAATAGCAACTGAGGAGACAACTTCAAAATCCCTGGCTAACATATGCTACACAAAGT
GGGCATTGGAAGCATTTGTTGTTGTCAATGCTGAAAGGTATTCTGGAGTATGGCTGATAACTAGGTGTGGTTCTATGATGCAAAGTGATTATGATATCAATGATTGGGCCCTTTGTATAATTG
TTCTCATTTTATATGGCATGCTTTCTCGCTGCATTGCCTTTTTTTGTATGGTCACTGTCTCAAGGAATTAAATGTCTGAAAGCTTATGCTTTAGAATTATTCGTGTAGGGAAAATGCTTGTAGA
ATTCTTGTCTGGATAGTCAGATACAGCAAAGTTCAACAATTAGGAATGCCTAAACAGCATGATTGTATTTTTGTAAATGAGCTTCTTCGTTCAAATAATGTAAAATTAGTACTCATTTATTTAT
TAACTGAGTTTAAATGAGAAGAACTAACATGCTTAACTGCAGTTATTATGCAATAGCCCTATCCATATACGAAAAATTATTCTGTG
>comp148687_c0_seq4
TTTTTCTTTTTTCCGTCCGGCACCGCTCCTTTCCCCCCGCCATAGATCATTTTTCCTTGAAACTAATAGATCTTTATTTCGTTGCTTACGAACCTTACCGAGAGATATGCACGCTTTGTGCCGC
GTTCGTCACCGGCGACGGGCTATAGCTGCCCTGTTGTTGTTTGTCGCCCTCCTCGCCGCCGGGAAGCTAGGGCAATGCCAGGACGACGACGATGATTATGACGACAGCGATGGCGATGAC
GACGATGGCGGAGGAAACTCAGCAGATGTCCCGCTCTTCGCCAACATTGTCTATGACCGTCTAATGAGCCTTACCGGAGCGTTCACTAAAGAGTTTGGCAACCAGCTCAAATATTGCATC
AAGGATTCAGATAAAGATTGGAATGAGGCGTTTAATTTTTCGGCGGATTTGAGATTCTTGGTTAATTGCATCAAGCAGAAAGATATAATTACCAATTACCTCCTGGTCAACCAAATCATACT
TGTGGTGGTGCTGATATGTGGGCTGATGTTTTATCTACTAGACAAGTGTTTTGCCCGCCTGGATACTATTGTCCGAGCACTATACAAAAGATTTCTTGCAGTAGTGGGCATTACTGCAGGAA
GGGTTCTACATCTGAAACAAGTTGCTCTGTGTTTGTTGCTCTTAATAATATACAACTTCTCTGGCCAAATCCTTAGTAATCGTGAGAGGAGGCAAGCTAAATCAAGAGAAGCTGCTGCAAG
GAGTGCTAGAGAAACTGTGCAAGCTCGTGAAAGGTGGAAATCAGCTAAAGATGTTGCCAAGAAACATGCTGTGGGACTTCAGGCACAACTATCAAGAACATTCTCACGCAAAACGTCT
AAAGGTTCGGGGCAATCTCATGAATCATCTGACGCATCTAGTTCTAAAAATGCAGGCCTTCCTCCTAAGAAAAAAGAATCTAGTAAACTTACAAAAGTTATTCAGTCACTAGAAGAGAAT
CCTGAAAGTGACCAAGGTTTTAATCTACAAATTGGAGATAAGAACATAAAGAAAAATGTGCCAAAAGGGAAACAAATGCATACTCGCAGCCAAATCTTCAAGTATGCTTATGGTCAAATT
GAGAAAGAAAAGGCACTGGAACAACAATATAAGAACTTGACATTCTCTGGAGTTATTTCTATGGCTACTGATACGGATTTAAGAACCAGGCCGAGAATTGAGATTGCTTTTAAGGACCTA
ACACTAACTTTGAGAGGGAGTAAGAAAAAGCTTCTAAGAAGTGTGACAGGCAAACTCATTCCTGGCCGTGTGGCAGCAGTGATGGGTCCTTCTGGGGCAGGCAAGACTACATTCCTAAC
TGCTTTGGCTGGAAAGACAACGGGGTGCGAGATTACAGGCCTGGTTCTTATTAATGAAAAAGTAGAACCAATCCGGTTTTATAAGAAGATTATAGGTTTTGTACCACAGGATGACATTGTT
CATGGAAACTTGACAGTGGAAGAGAACCTTTGGTTTAGCGCAAGATGCAGACTTTCAGCAGACTTGTCAAAGGCTGACAAGGTTCTTGTTGTTGAAAGAATTATCGAGTCCTTAGGATT
GCAGACAATACGTGAATCCTTAGTTGGTACTGTTGAGAAGCGTGGAATATCTGGTGGGCAAAGAAAGCGAGTTAATGTTGGACTTGAAATGGTTATGGAACCTTCTTTATTAATTTTAGAT
GAGCCAACCTCTGGTTTGGATAGTGCTTCATCATCTTTACTTCTTAAAGCCCTACGTCGCGAAGCACTGGAAGGGGTAAATATATGCATGGTGGTTCACCAACCTAGTTATACCCTGTACAG
GATGTTTGATGATTTGATACTTCTAGCAAAAGGAGGTATAACAGTGTATCATGGATCTGTGAAGAAGGTTGAAGAATACTTTTCAGGCCTGGGTATCAATGTTCCTGAACGTGTTAATCCTC

CAGACTATTATATTGACATTTTAGAGGGCATAATTAAACCAAACACAACTTCAGGAATAACTTGCAAGCAACTTCCTCTTCGATGGATGTTACATAATAAATATGATGTGCCTCGTGATATGC
TTAATGATGATGCTACTGGAGATGTATCTAGCAGGGATAGTGAAAATACTACTGTTAAGGTCGAAACACCATCTATTGCAGGAGAAGTATGGAACCATGTCAAGGATATTTTAGGGCAGCG
AAAAGATGATTTTAAGTACCACTTTTTACATTCTATAGATTTATCAAATCGGAGCACTCCTGGTGTTATGACGCAATACAAGTATTTCTTGGGAAGGGTAGGTAAACAACGTCTTCGTGAAG
CCAGAATACAAGGAGTGGATTTTCTTATTCTGTGTCTTGCTGGAGTCTGCCTAGGGACGCTTGCAAAAATGAGTGATGAAACATTTGGCTCGTTGGGTTACACATACACAGTTATAGCTGT
TTCTCTATTATGTAAGATTGGAGCTCTCAGATCATTTTCACTTGAGAAGTTGCATTACTGGAGGGAAAGGACTTCTGGCATGAGCTCACTTGCTTATTTCCTTGCAAGGGACACCATTGATC
ATTTCAATACAATCATCAAACCAATAGTTTATCTGTCAATGTTCTATTTTTTCAACAATCCTAGGTCCTCATTTGTAGATAATTACATCATACTGGTTGCTCTTGTTTACTGTGTAACTGGTATT
GGTTACTTCTTTGCCATCTTCTTCCAGCCAGGAACAGCCCAGTTGGTGAGATCAACTCTCTTGAAATTCAATTCTTTCTCATTTAAATATTTTTTAGATTATTAACCTTGAGATTCAC
>comp148782_c0_seq3
ATGGGAAAAAAAAAAAAAAAAAAACCGAGTACTAATTAATGCTTGCCTGGATCAACAGAAATACTGTGAAGGATACCAACAGAGCCAATCCATGGCAGAGGTGGCCATCAGGAGGTGGA
GAAGCAGTGCTACCTCGCGGCAAGGAAGGATATTCATCCGGCGTCGGCATCACACATTCTTGGCCATTGAAAGATATCTTCCTCGGAAATGGCCATCCTCCCTGAAATGTGAAAATAGCA
GGGTCCTTTTTAAGCAATATTTCGGTCTGCACATTCCCAAACTCACCTTGCTGCAGCAGCAGGTCATTGTAGTACTTAATTCCCCAGAACACTCCAGTGTCATTAATAGTATTATACTGGATG
AGAGGTTTATAGTTGAAACTGAAGACTTGAGTTAGGCTTTGCAGATTGGGGTGTTGCACCACTAGAGTCCAGTCACTATAGTTCTTCAGCATATTGAAGTTGTTGATAGTCACTTTAACCC
TCCAATACTCCTTATAACTAAGCTTGAGATGCCAGTGTACACGGATTGGGCACATATGTCTTGTGCACATAACGATATCAGGTACAGGATCATCATAACCTTGAGGCAACTGCAAAAATGTG
GGATGTTGGCCATCGTTGGCACATTTTGGCGCTTCTGGTGATCCTTGACAACCACAGCTACAAGTAGGACATGGAACTATCGTCTCGTTGTAAAAAGTAGACAAAGATACACAGCACTTG
GGTGCTTGAGATTCTCTGAACTGGGAATATGAACAGCTAACCAGCCATGTTAGGAGGGCTTGTGTTTTGCGGCGACCTCCATCATTCTGAAACTTGGTAGGGGGTACCACAGTAGCATTA
CTACATGTATATCCAGGCAAACCAAGACTAAAATTGTAGGGCTTTGGGGTTTCAGCATCGCTAACATTCAGAGAAAGGCCAGTACTTGAAGTCCCAACACTTATTTGGAAAGATGAATGGT
AATTCAAATGGTCCTGAGCCAACGAAGCCAACACACCACCTTTGCAACAATTTTTAGTTTGCATATTATATGGAGCACCTGGAAGCAAGTCCACAATAACTGGACTCTTGTCACAACAAT
GGGGGATGTTGCCTGTGAACTTAGAGCAGTTCCCTTGCTCAGCAGCCTCAGCACCTCTTGCATCCCATATTACTTCTTCCCCTCCCCATGTCCAGCCTAGCTTCCAGCCAGGACTATCAATA
TGACGGTATAGCTGGTAGTTGTATATATCAATCATGACCGTGTAGCCGTCATCACGGAACTGCTGAAAGTCCCACTTGATTGTGATATTCCCATTGGGGTCCATTGGATCGTACGCACCTGC
AAGCATTTCAGGAAGAAAAAGCTTCGAAATCTAATACAAAGGAAACATAGCAGAGATAAAAAAGAAGTACAACTAGCAGACCGGCGAGACAGAGGTGGAGAAGAAGAAAAACGTATG
TGGCCACCCACAGCAGAACTCCGCCACCTCCGCAGCACAAAGACATCGTTGCGTAGCAGCAGATACTATTGGAGCTGTGAATGATTTGGGTTTTCAAGGAGGAGAGAGAAATGGAAATG
GCAAACAGAGAGGGAGAGAGAGAGAGAGACAGAAGTGGAAACGCCTGCGCACGCGTTCCAATTAGGAATTCAGGGGAGACGAAAAAGGAAGGAGATATTGAATTGGTTGTCGCCATA
TTGTTTCCCATAAAACGTCCGTATTGCTGTTCTTCTTATTTTGCGATCTGCTCGGTGTATCTTCGATCCACTTGGCTGGCACCAATTGGCGGTGGAGGAGGAGAAGCGACGGAACAAGGCG
GAGTAGGCGGCGGTGGCGGAAAATTTAAAAGCCAGGATGTTTTCTTGCACTGAACCTTCGTTTCCTTGA
>comp148788_c1_seq1
TTGATAACTTGCTCATAAATGTCAAAGTGAAAACCAAAAGCAGCAGAAGAAACCCTAAGTAGGAGCAGGCAATAGAGACAGTTGAGTGATAGCAGTAATCCTCAGCATGGTTACAAACA
GGCTGCCAAGATGCATTTGCATTCCCTTTATTTCCAATATAGCTCACCGCCGTCGCCGCCGCCGCCGCCGCTATGACCAATCCCATCACTAACGTATCTAAGAAATGCATAGCGAGGTTTGA
TCCGTAGCAAATGATAATGATCATTGAAGCAAAGGCGTAAATGCACGCAAATCCATTGCCGATCACAAAGAACTTGAAAGCTGGTGAGAATTTGTAATTTGCAGTGAGTTGAAAGCCAGA
GAGATGGATCTGCTCTTCGTTGAAGAGCATGATAATGGATGCGGTGAGGGTGGCCATGGCGGCGAGATTCCGTACAATAATCTGCACTACGCGCATTGAGTGTTTCCTGACGTTGAGTGA
ATCTGTAGTTTTTGCCGGCGGCGGGGCGGTGGTGATGATAACTTCCGACGGTACAAGGGTGGTTGCGGAAACCTCCGGCACGGCTGACGTGGTGGCAGAGATTTCCCAATTAGGAATTT
CGACGACTGAGAATTCCGGCGGGGCCGGAGCGGGCGAGGTTTTGACAGCCTTTGACGGCGCAGGCGAGGTGACGGCGGTTTCCGTCAGCTTGAGGCCGGCGATGAGCTTCAGCAGCG

CGAGAATGTTGACGACGAAGTAATCCGGCGGCGGCGCTGGCTTTGCAAGAGGTGTCGTCTTCGAATGAACGGTGAGGGCCCGAGCCGGCTTCGGAAAAGTGTGGAAGGTGAAAGCGAT
ATCTGTTGGTGCCGGCATGGTGACCGCGGTACTCAGCGGAATAGTGATGGAGTGTCGGCCGGCGGCGAAGTCTTCTCCGGCGAGGCGGTTTGTTGGAGGAGCTGCGGAAATGAGAAAA
GAATCTCATTTTATGTTTTATGTGAGTGAGGAGAACAGCGGGAATT
>comp148802_c0_seq1
ATAATTAAAATTATGTTACATCTGATTTTAAATACATGAAAAAAAAAAAAAAACAATTAGGCAAAAGTTTCCCAGCGCGGCGAGCAAAATCGGCCAAAGCCTCCGCTCCGCCACACAGAA
AATAAACAAGCTCAAAACGAAAATAACGACCTCCACCATCCACCTCCAGCCACTATTGCCAATTTGCCATTACTTCCGCATAAATCTTCGGGCTTAGCTTTCGTCTTCAGTCGCCCCCCAA
TTCCTCCCAATTCATCCATGAAGGCATGAACCCGCCGCGTAAAGCGTAAAGCGTAAAGCGTAAAGCACCGACGGGGCCAGCCAAGCGAAGTTTACCGCCACCTTCTTCAATTCCTTCTCT
AAATTAAAAAAAAAATGGCGTCTTTGGAGGACATCAAGAATGAGGCGGTGGATCTTGAAAGCATACCTATTGAGGAAGTCTTCACCCAGCTCAAATGCACCCGCAATGGTCTCACCTCTT
CCGACGGCGAGCTAAGGCTTCAGCTCTTTGGTCCTAACAAGCTCGAGGAGAAGGAGGAG
>comp148865_c0_seq1
TAGGAATAATTAAATCAATAAAAATAAATTTATACAGATTATATATTCAAAACTCTAATCTAAAATATGTTCATTGATAATCAAAACTTCCATCAAAAAAAAAAAAAAATCTAGAATTCCTTTAG
GACTCCAATCCCAAAAAGAAACAAGAAAATGTTCTCAACTCCACACTTTTCCGAGTGCTTCGATAACCTGACGGAAGAGCAGAAAGTCGACGAACTCCGCGCCCGACAGCACCACGAG
GAAGAAATCTCTAGCCTACCTCGGCATGAAGGATGGCGACAGCTGTCTCTCCGCCTCTACAAAGGTTTCTGGTTCCCCGAAGCCATCCTCCCCGGCGTTCTCTCTCTCCGCCGCCACCTA
CTACCCACTTCTTCCGACATCTTCCTCGTCAGCTTCCCAAAATCCGGCACAACCTGGCTCAAAGCCCTCGCCTTTGCCATCAGCACCCGCAAGCCTGAATTACTCCAATCCCGCCACCCGC
AGGACTGCTCCCCTAATCTGGAGACCTTCTTCGCCGGCCCCCGCGTTCCTGACCTGGACGCGTTACCCTCCCCAAGGCAGTTGAGCACCCACATCCCCTACTCTGTCCTGCCCAAATCCG
TCCACGACTCTCCCTGCCGGATTATTTACCTTTGCAGAGAGCCCAAAGACACGCTCGTTTCAACCTACCACTTCGTCAATGGAATGCAACGCCCTGAAGCGGCCCCGCAAATGCCGTTGA
CGGAGGCGGTGGAGCTGTTCTGCAAAGGGCTGACAATGTACGGTCCGGTGTGGGAGCATCAATTGGAATACTGGCAGGAGAGCCAGAGGCGACCGGAGAAGGTGCTGTTCCTCAAGTA
CGAGGATTTGATGGCGGATCCAGAGGCCATTGTGAGAAGGATGGCGGAGTACATGGGGAAGCCCTTCTCGGCGGCGGAGGAGATGAATGGGATGGTTGGGGAGATTGTCAGATTCAGC
AGCTTTGAGAAGCTTAGTGGATTGGAGATTAATAAGGTCGGATTGTGGGGGAAGCAGTTCGATTTGGGCCATTTGGCTATAAAAAAGTCATCTTTTTTTAGAAATGGGAAGGTAGGAGATT
GGACGGAGCATTTCACCGCCGAGATGGCTGAGAGGTTTGACGCCATTGCTCGGGACAAATTTCATCAGAGCGGATTGACCCATTCGCATTCATCTGTTTAATCCCTTTATATTTTTAATTCG
C
>comp148870_c0_seq4
TGTCCAAGTCAAGGCCAACAGCTGTTGCCATGCGATGTAATGGTTCCAACATCTCTTTGGTTGGAGCAGGTTGGTGTCTCAATAAGGAACACGATGGAAAATGCTTCAATATCTTTTTTGC
AACAAAGAAATTGCAAGCCAACATAAATTCTTCAATCATCTGACTGGCTTCTATAATTGGATAATTTCCAATATCCAAAGGATCACGAGTTTCTGTATCCATCTGAAATTTAACTTCTGTTGA
ATTAAATGTGAAAGCTCCTCCATCAAATCGTCGTTGCCTCATAAG
>comp148870_c0_seq7
AAATTACATAATAATTCTATCTTGCATAATAATTCTATCTTTTCTTAATGGACCTATATATCTACATTATTGTGAAATAAATCTATACACATATACTCCATAGTCTCCACTTATGAAAGACTTGGG
AATCTCATATGTGCTGGCACTTCACCACTATCTTCTTCTCCTTCTCCTTCTCTTCCTCATGCCCAACCTCAACAAAATGAATGGATAAATCAGTTGCAAAGACCTCTGAGCTCTCCTTCTTAG
TATTCCTCACTTCTCCATGTATAAAATTCAAGATCAAATTGCTCGAAGCATCCAATTTCTCCTTCAAGCACAATTGAGTATTCTCCTATCGAAGTTGATTGTTCATCAACTCTTGTGTATCAA
AAATAAGTTGTTGGAACTCTTTAGAGGGCATGGAATGGTCGATAAATGATTGTTCTTCAGCTAACTTCTCTTCTATGTGAACATCACACTCTCTTCTAGAACATGTGGTGGTTACCCTCTTC
TTCTGCACCCTAACTGGGATGTGATGAAGAGTATCGACAATGGATTTATAGAAGTCCTCTTTATCAAACTTTATGAACTAGCATTGGCTTCACAATTTCCTTGTTTATATGAGTGTAAATTGA

AGCTTTGGCCTGTATGGCCCACGTTCAACTACCTTCACATGAATCCTAACCATCTGCAATAAACAGTAACTAATATTTGTGCCTTTCTTTGACAGTCTCTGATGTGCCTCATCTAAAATCCAC
TCTTCCCCTTTACTTCCTCTCGATGTCAAGCAAATGAGCCCTTCAATGTCAAACCTGGGCACAAAGACTATGAACCCATAGGGCTTTATTTTGATTATTCTGGCTTCTGCATCCATAGGTCG
TTTCTTGAAGTATATAACATTGTGTAGGTAAATTGAAGCTCGGCTAGCCATCTTAGCATTTATTTGCTGATAATTCAAGTCTGGATATACATTTAACCTATTTACAATCTCTGTACGTCGTAAA
CTATCTAGAAAATTTTCTGGAAGTTCTGATATTTCTAGAGCAGCTGCAAGCAATCTATGAACTATAATATCTGCATATCTCCGCATGGGAGAAGTGAAATGAGTATACAAAGGTGCTGCAAG
TCCAAAGTGGTGAAACTCAGTAGGCTTCATATCTCCACTCCATGAGTAAATAACCTTTGTCATGCACCTAGTAGCCAAAATCTTCATTTGTTTTTTGCAGTTTGGATCAACACCCTCAATAA
GGAACACGATGGAAAATGCTTCAATATCTTTTTTGCAACAAAGAAATTGCAAGCCAACATAAATTCTTCAATCATCTGACTGGCTTCTATAATTGGATAATTTCCTGCCAAAAAGAATTAAA
TCAATATCCAAAGGATCACGAGTTTCTGTATCCATCTGAAATTTAACTTCTGTTGAATTAAATGTGAAAGCTCCTCCATCAAATCGTCGTTGCCTCATAAGCTTTGCTAATGAATTCAAATTT
CGTAGGTCAGTAATTAATGGATCAATCTTGCGACTATCGTCCATTAGGGCTTGAGCTTCGACATAAGATAATGCAGCACAAGATTTGATCGTGCTCTTTGTGAATCTTGTAGAAACTATATTT
GCATCACGTGTCAATGTCAAAATAAAGGAAAATGTGAATCTTTCTACACAGGGCTGAAGAGAACATATGCCTTCAATTAATATTTTTGGAAGCATGTCAATTTTCTTGTCAACAAGATACA
CACATGTGCCCCTCTGCGCAGCCTCCTTATCAAGTGGAGTTCCAGGATGAACAAAATTAGTAACATAAGCAACATGGACTCCCACTGCAAAATTTCCGTTTGGAAGAGGCATA
>comp148945_c1_seq1
GTTTTATGATTCAGCTTATTATCTGCCAAAAAAGGAACGACAACAAAACACACCAACTCTTATCAAATCAAAGATTGTCAAATAAGATGAAACATATGCCCAACAAAATTTGTAAAGAACT
AAGCTCCCCTTGATCCCCAGCCCTTATGTGCATGCATACGAGATTTTTTTAGCGCAAAGAAAAATTTAAAGCCAAATCAGACTAAATTAGGGCGTGGCATGAATATCAAGCTCCGAAAAGC
ATAACAATATTAGCCCACAATAGGGGCTCATTTGGTAGCTGAAGTTAGAGAACATGTTTAACAAAGAAGAATTGATTAAATTCTGTCATTTGAATGAATGAGTTTAGAAATCGATATTAGTT
TTAAACTTAGCAAACATCTTCAAAAT
>comp148978_c0_seq1
TTGAAATTATCTACTATTACCGAATAATTAGGAACCACTGGTACTGTAATCTAAATTAAAATCTCTTCCTGATACAAACTCTAATCGTTGGAAACAAACACGTCGGAAAATTAAAAGAAACA
ACTCAAAAACACCAAATCTTCACTTCACCTTAAACACCAACAATTACGTACAATTCATAACACAAATGCGCTCAATAAAAAAAGCATAGAAAATATCAACTTAAAGTTCTAATTTAAAGAA
CTAACGATTCACTCTGCAGCCTCTTCCTAGTCTTTGATAGCCCATTTGAATATCAAGAGAGTGCTAAACGCTACCGCAAAAACGGGAACAGAACCACCGTAGGAAGTGAAGGCGGCGCT
GGACGCCGGCGAGGGACCAGGTGACCCTGGAAACGGTGGGCGGTGAGAAGCCATGACGTCGATCATAAGCCTCTGACCCTTCTTGCAATGTTCAAAGTTGCTGCTAACGAAGTAGAAA
AGGCCTGGCTTGTCGAAGAGGAAGACGGTTCTTCCGTCCTTGAAAACGGCCCCATTGCCGCTCTCGTTGCAGTGATAGTAGCCTCTTTCTCCACCCGAATCACAGAGTCGTTTTTGTACT
CGAACACAATTGAGTCGCCGACTTGGAATCGGAGTTTGCTAGCCCAATTTGTGTACAAGTCCGTGTTATTGTCGTCTGGAACTCGCCACCCCTCCTCGCCACCAACCTTGTACTGGTTTTC
GGCTTTGACGAATAAGACGCTGAAGGAGGAAATGAGGAGGAAGATGAAGACGTGGATGGAGAGAGACATCTCTTGTTTTTCGATTGGGATAGTTGGAAAGAGAAGGGATGGGAGTTAC
GAGAAAGTGTCTGATTTTTCTCTGGGGTTGGGGGAATGAAGTAACGGGGGTGACTAGAAAATGAGAAGAGTTTAATGAGTTGGTTATAGGAAGCAGTCGAGGCGGTTCTGGGTTGCGAA
GAGATGCGTAGATCATGCAGTTGTGATCCTTCGTAAGTGGAGCCGTTCCGAGTTTTGATTTCTTTTTTTTTTTTTTGGTTTTTTGAAACTGTTCGAATTTGAAAGGAAACGAAAAATTTTAT
GGGACAAATTTTTAAACTAAATCTAATATATAATTAATTTATTTGATTAGGCATTAAAAAATTAGAATAGTCAAGTCATCCATTTAAGGGGGAACTGGCTTTT
>comp149042_c1_seq1
AAAGAATTTCGACAATCAGAAAAGAGCGAAAAGTAAAAATTCGCAAAGAAGAAAAACACTAAAATTAAACGAATAATGCCATCAGATCCAATAGCTGTTGCTGGGAAAACCAAAACCC
TATATTCCCAAACCAAGAATAGCAATTACAGATTTAGAAACAAGATCTCAAAATTACTGGAAGATAAGAACCCTAGAACGGTGCGGGTGCGTAGTAGAAGGGTCTTCCTCCCAAATCCCC
ATATCGATGCACATCGTAGTGCAAACTTTCCTTAATCTTCTCAAATCCATGAGCAAAAATACCGAAACCAAAGATGAATCCAATGGCGATCACCAAAAGAATCAATAGCAAACAGCAGCA
GCAAGTCTTACCCGGACAACACATCTTTTGGTGAGGGGATTGCCGGAGTTCGGAGCGGAGAGACAGAGAGATCAAAAAGAGGTGATGGATTCCGTTGCCGTGAGGAGAGAATGCCTAT

AAAGATGAGGAGAAATGGATCGAAACGAATTTGTTACGGCACTCCAAAGATGGAATAAAATTTTGAACAAAAAACTGCATCAGACGCATTTAGCAGCCTGAATTTGCGTTCGTTTAG
>comp149131_c0_seq2
TCGGGTCATGTAGCAGCTCCGCCATTGCCCATTCTATAACTGTAGCAAGGGAGTCTGTTGAGGCCTCCAAAAGATCCTGCAAAGACAAATAGTTAAATCTTTTTTTTAAATAACGCTTTTA
GTGTTGTAAATTAACCTATATTTTTTTGAATTTTGGCATCATTTTATGAATTGAAGAAAAATTGGGATAATAACAGCTCCGATT
>comp149131_c0_seq3
AACTTGAACAAGCTAATAATAAACAAATTCAAACACGAACACGAACACTCACATACTCGACTCAGTTCAGTTCGTTTGCAGCCCTAACGGTCTGCTTATTAAGTACATTAATGTGCACTCG
TGGCGTGTTCCAATAATAATAGATGGTTCCATAAGTACATATTAAACATATAGCTCTGGTTTGTCCAATCTCGCCACCGGCGCCACCACGAGCTCCTTTGCCAATTTTAAAACAATCCCAAA
CTTCTCCTGAAGCTCCAGCGTCACATTCTCCGGCAACCGCCACTCGAAAGAATGTAGGAGAGAGGCAACCAAGTAAATAACCATCTTCTCTCCCAATTCATACCCTGCGCACATCCTTCTA
CCAGCTCCAAATGGCAAGTAGTAAAATTGATCATTCCCTTTAAGATAAAAGTCGTTCATTTCCTTCCCGGTTGCGAGGAACCTCTCTGGCTTGAACTCCTCGGCGTCCTCCCCCCAGAAAT
CGGGATCCCTATGAATCTTCCAGATGTTGACGATGACGGTGGTTCCGGCCGGAACAGAGAAACCGCGGACAACACATTGAGAGCTTGGGCTATGGGGAACGAGGAAAGGAGCTGGTGG
ATGGAGGCGCAACGTTTCTTTAATGATGGCGCTTACATAGTGAAGCTGAGGAATGTCAGACTCTTCCACGACACGATGCTTTCCGATGACATCGTCAAGCTCTTGTTGAGCTATTCGCATC
TTTTTCGGGTCATGTAGCAGCTCCGCCATTGCCCATTCTATAACTGTAGCAAGGGAGTCTGTTGAGGCCTCCAAAAGATCCAAAATCAATTTAAAAAGGTCATCCATGGTGAACGGCTCCT
GCGGGTTTCCTGTCTCGATGAGATCCAGCAATACTTCCAACATGTCCCTCCTTCGCTCCCCAACCGCCATCTTCTGCTTCTTCTTCTCCACGACCTTTTCCAGATACTTAATTAACCACCCC
GTGGCCTTGTTCACCCGCCGCCCTAACCCCTGCGGGTCGAGCGGTGCCACAACAGGGAAGAAATCTGCAACATTGGGTGCAAAAAAAAAGGAATATATGCTCCCTGACCGCCCGCTGAAG
GTGCTCCTCATCGCATTCGCTGGCCCCTTCGCCCCACAATATGCTCATCAACATGTTAAGCATGGTTAGAAAGGAAACATCCCGCATATGTACTGTCACCCCCATCTTCGCTCGGGCCCATA
CTCTCTCCACTGTGCGTCTCATTTCTCGGCGACGAATTGGGGCCATGTCTTCAAGTCTGGCAGGATGGATCATCTCACGCGCGACTATTTTGCGCATTAGGCGCCAATGACTGTCGTTGGG
GGCCCACAACAAATCGGGTCTGGAGCCATTGTAATTGAAGAGGATTGCCGCAGGGATGGAGCGGTTTGCAAAGGTGGCGTCTTGATCTTTAAGGATCTCGCGGGCGGTGGAGGGGGAG
GAGATAACGACGTAAAGTTTGTAGCCGAGGCGGATGCTGAGGATTGGCCCATAGGTTTGGGCCAAGTCTGAAAAGTACTGGTGTACGTTTGGTTTCACGAAGGGGAGGCTGCCAAGAAT
CGGCAAACCACGCGGGCCTGGCGGGAGGGGGAGGAGTTGTTTTTCTTTTCCTTGTTTGCTTTTTAAGCAGATGAGAAAGAGGAACAAGCGGGTGAGCGAGAGCGTTAGTAACGCGGTT
ACTATTGCTATGCCAACTGAGATGTTTGAAGGAAACATCAAAGCCATCCTACTTCTCAATCTCTTGCTCTGCAATTTGCTTCTCCTCCTCCACCATACCACTAC
>comp149131_c0_seq4
AAGAAATCTGCAACATTGGGTGCCAAAAAAAGCCATACATGCTCCTTCATGGCCCGCTGAAGCTGCTTCTCATCGAAGTCGCTGGCCGCCCGTTCGCCCCACAAGATGCTCGTCACAATG
TTAACCATGGTTAGAAAGGTAGTCTCTCGTACAGGAACTGTCACACCCAACTTCGCTCGGGCCCATATGGTCTCCACTGTGCGTCTCATTTCTCGGCGACGAATTGGGGCCATGGCTTCAA
GGCTGGAAGGACCGAGCATATCACGCCCAACTACTTTGCGCATTATGCGCCAATGACTATTGAAGGGTGCCCAAAACAAATCGGGTTTGGAGCTTTTGAAGTTGTAGATGACTGCCGCAG
GGATGACGTGGTTGGCAAAGACGGCGTCTTGATCTTTAAGGATCTGGCGGGCGGCGGAGGGGGAGGAGACAGCGACGTATAGTTTGCAGCCGAGGCGGATGCTGAGGACTGGCCCATA
GGTTCGGGCCAAGTCCGAGAAGTAGTGGTGTACGTTTGGTTTGAGGAAGGGGAGGCTGCCAAGAATCGGCAAACCACGCGGGCCTGGCGGGAGGGGGAGGAGTTGTTTTTCTTTTCCT
TGTTTGCTTTTTAAGCAGATGAGAAAGAGGAACAAGCGGGTGAGCGAGAGCGTTAGTAACGCGGTTACTATTGCTATGCCAACTGAGATGTTTGAAGGAAACATCAAAGCCATCCTACTT
CTCAATCTCTTGCTCTGCAATTTGCTTCTCCTCCTCCACCATACCACTAC
>comp149144_c0_seq2
ATTTCTGATCGTTAAGAAATCCGCATAGAATTTCATATGCGGATTCCCAGCCACTGCTCTCTGTCGTTGGCGAGAGATGCATTCGGCCACGTACTTTATTCCATTGGGAGGGAAAGAGAAA
GCTTGCAAGAAGAGCGACAAATGACCGAATGAATAATAATCCTCAATCCCCGAACCGTCCGCTCGCCACATATACTGCCGCGAACATCGCGGGCATTCCGACCAGGAGGCGACCATCAG

GCTCGCCGCCACGCCACCCAGCTTCGACGATGGCCCCGACGGGGAGGAAGCCGAATGCGGAGTTCATGGAGGATTTTCTCATCATCTCCGGCCTCATTGCTGTGCAGATCATAGGCGCTT
TCTACATAGTGCTCTTGAAACCTATCCTATCCCTCGGAATCAAGCCTCTGTTCCTCGTAATCATTGGAAGCTTGGCGATGGCCTTCTTCATCTTCCCCTTCGCAATTGCATTGGAGAAGAAG
AAATGGCCATCAAAATTGAGCCTTACTTTGGTGGTTCAGTTTATTCTGATTTCCTTGGGAGGGGTGACTACATTCCAATCTCTAATGTTCGTGGGAATCAAGAAAACTTCCCCAGCTATAGC
CTCGGCCATGCCCAACCTTGCGCCGGGGATTATTTTCATAATCGCGGCTTGTCTCAGGCTTGAAAAACTAGACTTTAAGTGCAACTACACTAGAACAAAGATCCTAGGGACATTGGTTTGC
TTGACTGGAGCAGTAGCCTTGAGCATCTTGCAAAGTCCTACAGATCCAACGGATTTTGGACGCTCTTCTGTCTTGCCTAATCCGTCGCCAGTTAATGTGTACAGGGACTGGATCATTGGTT
GCTGCAGTCTGCTGGGAGCAGTTCTCGTTCTATCCTGTAGCACGGTTTTGCAGGCTGCAACAATGATGGAGTTCCCGGCTCCCTTGTCCTTGTGCTGTGTAACATCCCTGCTGGCCTCAGT
ACTTACTACAATTGTGCAATTTATCATGGAAGGGAAGCTGGATTTTGGTCAGCCAACTATCAGTATCCAAGCCATCATCTCCTTTGTCTTCCTGGGAGGAATGTTGATTGCACTATGTGTTG
CATTCCAAACTTGGGCTGTGCTAAGGAAGGGGCCAGTAATGGTCTCTATGTTTAATCCCATTCAGACCGTTTGCTCCACGGTTCTTTCGACAATAATACTTGGCCAAGTTATCAAGTTTGAA
AGCTTTGTAGGAATGTTGGCAATGTTTTTTGGTCTCTACATGGTTTTGTGGGCAAAGATGAAAGAAGGATTTGGGCTACCAAATACTGATGATCAGGACAAGCTAACAGAATCAACCACC
AGTGAAGAAGAGGAAGAGGACATTGAGAGACCCCTTTTAAGCTAACAAATTGTTTTTATTTTTTAAATTATTTTGGTCATCAATCCATATTCCCTGCACAGTAGAAGTATTGTTGTTGGGGA
GGCGGTTTAGTTATTGGTTTTGCTTTCAATTCTCTTTTGATTATTTAGAATATTTATGTGTATATTTTA
>comp149174_c0_seq2
CATCTGCAACTTTAGTGCTTCAGTAATTTGTAACCCACTGGCAACAAAAAGTATATTAGGATGTTTATCCGCTAAGCAGTATAAGAATAATAAAATAACAAGAAAGAATTTCAAATAGGATA
GAGCAGATCGGCACAAAATTTAGATATCTTCTAGAGTTGATCTTCCTCCTACAGAATTTTCTGATTGTTATTTTCTACAAGAGAGAAACAAGACTTTCAAATGCTATACTATACCATTTTTTG
AAAAGTAAACTAAGAGTTCCACGATCCAAAACGACTAATTGAATAAGGGAACTTCTACCGTTAATTACTTTGAGGCTTGGTAATATAAACAATGTAATTAGAGCATAAAATAATTCTAAAAT
ATTAAAAACATGTAACTAATATTGCAATGCTTCCCTGTGAGGTATCAGAATGTTACCTATAATCCAAAAGATAAATTTAATAGGCTAAATTAAGAAATGTGAGGAACCTTGCTTTGAAAGAG
CTATTGTTCTCTGCCTAAGAATATCAATTGATTACATGAATAAGGAAGTGTGGGCTCCAAAAATTAACATACGACAATTGCTGGGAGAGTTTAATAGTTTTGGGTCATATTTTCAGAAGACA
TTCAAGATTTAAGATAAGACAATACTTAGAGGTTTTGCTCCCGTTTCAAAAAAAAAATTATAATATAGTTCCACATTTCCCATTGGCACAAGTCTTAGTTAATTTCCCTTCTATAATAAGAGA
GAATTTTCAATTAAAGCAAATCGTTAGGAGCTAAATACTTATATCTAGTAGAAGTATACTAGAAGTATTTTGATATTGGGTCTGATGTTTACCATCTTATATTTAAAACAGGTGGTAGAGGAA
GTGATGACGAACCCAATGAAGATATCATGATTATGTCATTTAGTGCTATCATCGACCATAATTTGTTTAGTCAGATGTCAGAACCGGCTGGATTGGTTCAATTGAACCGGTCCCCTAACTAA
AGCACACACAAGCAACAGAGCAAGCGGGCAAGCATGGGCAAGCACACGTGAGCATGAGCGAGTGAGCGAGCGAGCGAGCAAGCTTGGGATAGCACGAACGAGTAGACCAATGCGAT
CGAAAGCATATAAATGTGACTCCTCGTGTGAGATATCAACTCCCGCTCTGTCCATAAATGTCACACGAATGAGAGAAGCCGAGAAAGCAGTGACTGAGAGGAAGAAAAGGAAATTGGGT
GAGAGAGAGAAATTTGTGTGCAAGAAAAAAAATGGGTGGAGAATTTCTCTACTGTTGAGGTAGTCGAAAGATCTAGGATTAGCAAGACGAAGAAGAAGAAGGAAGAGCGGTTTGGAA
GTTTCTATCTCTAACAACAGAGAAACTATAAGTGATTTAAATTTCTAGTTTTCTAATATTTTTCATATATATGTACTTCGAAAATATATATTTGATAAGTCAAAAAGAATTTTTCCAAAATCAAC
TGA
>comp149192_c0_seq4
ATGATGTTTCCAAACAAAACAATCAATAAACAAAGATGAAAAAATGGGAATAAAGCAAGCATATACGATCAATAGAATTTATGAGCACAAATCCTTTTCCAATTCATCTTGTTAAGTTATTG
TTGTATACCAGAAATCAGAAATATGCATATATTAGCTTTGGAAAGTGCAGATGTTGAATTTCATCATGTCTTGGCTATGAAACTGGAGTACTTTGTTCTCTTGACTGAGGAAGAATGCTATCT
ACCTATCCCTCTTGTTCAACCCAATGATGCAATTGTTCTTCAAATAGAGTGTGATCTTCACTATCTTCATCTTCTCCTTCTTTATCTTCTTCCCAACTATAGCTTCTTCTCTTCAACTTTATTTT
CCATATCCTCAGTGATAGCATCCTGCATGGATTCCTTCTGCTTCACAGTCATCAGATACTGAAGAATTCAGACAACAGTGTTCATCACCAGTACAAAAAATGCTCGATCTCATCCATCTGAG
AGCTCTTTCCAGCATCGCTGTACAGCACACCTTTTTTGACATCTGCTTCTCAACGTTCTCTGCACTAGATCTCCATGGCTTTCCTCTCACCATTCTTCTGCTACTATTACTGGATATTTTCAGC

ATCAGTTTCTCAGCTGGAGATGAAATGACTCTGTAGCTGGTGTCGCTTCCTTTGTGCAGTTCGTTGCTTAGCTGGCGGATTTTTGTCTCGAGCAACCGGAGGCGGTCCATGAGCGTGTGCT
TTACGTGAGCGTCTTCCATGGTGGAAGTTTGGGGCTTGTTCCTAGCCCTAAGGTCTCCGGAGAAACAGCTCTCTTGGGGCCTCACTTTTTCCTCCAGCTGCCTCAGTCTAACGTCGATGTG
ATTCAGCCTGCCTAGCAATGACTCGACTGAAGGAAACGAAGCAACAGCCCTGTAAGTTGACTTCTCATGGTTTCGTATACGATAAGCCATGGTTATTTCGATTGCTTCCTTGTTGCAAGTTT
ATACGCGCCTTTGTTGCTTGAGATTGATGAGAGAAAGAATGTGAGAGGATGATGGGTGGTCTATTTCTATCTAACAAATGCAGATAATAGAGATAAAAAAGAATGGGAATGGTTATTATTGT
TCAAT
>comp149192_c0_seq5
ATGATGTTTCCAAACAAAACAATCAATAAACAAAGATGAAAAAATGGGAATAAAGCAAGCATATACGATCAATAGAATTTATGAGCACAAATCCTTTTCCAATTCATCTTGTTAAGTTATTG
TTGTATACCAGAAATCAGAAATATGCATATATTAGCTTTGGAAAGTGCAGATGTTGAATTTCATCATGTCTTGGCTATGAAACTGGAGTACTTTGTTCTCTTGACTGAGGAAGAATGCTATCT
ACCTATCCCTCTTGTTCAACCCAATGATGCAATTGTTCTTCAAATAGAGTGTGATCTTCACTATCTTCATCTTCTCCTTCTTTATCTTCTTCCCAACTATAGCTTCTTCTCTTCAACTTTATTTT
CCATATCCTCAGTGATAGCATCCTGCATGGATTCCTTCTGCTTCACAGTCATCAGATACTGAAGAATTCAGACAACAGTGTTCATCACCAGTACAAAAAATGCTCGATCTCATCCATCTGAG
AGCTCTTTCCAGCATCGCTGTACAGCACACCTTTTTTGACATCTGCTTCTCAACGTTCTCTGCACTAGATCTCCATGGCTTTCCTCTCACCATTCTTCTGCTACTATTACTGGATATTTTCAGC
ATCAGTTTCTCAGCTGGAGATGAAATGACTCTGTAGCTGGTGTCGCTTCCTTTGTGCAGTTCGTTGCTTAGCTGAGAACCAGTAAGCGTCTCTTTAGAAGACGTTTGTTATCTCAGGACAA
AATGATGTAATACAGCTCTGAAAGTTTTATCAGATGCTCTTAATGATTTTCAAGACTGTTTAAAATATGTGCATTAAATACTAAGCAAAATTTTATTTTTTATTTTGATGTTCTGTACATTTTTA
TTTAGCTAATTAACTACATGTTTATTGTCAGGAACCTGGCGGATTTTTGTCTCGAGCAACCGGAGGCGGTCCATGAGCGTGTGCTTTACGTGAGCGTCTTCCATGGTGGAAGTTTGGGGCT
TGTTCCTAGCCCTAAGGTCTCCGGAGAAACAGCTCTCTTGGGGCCTCACTTTTTCCTCCAGCTGCCTCAGTCTAACGTCGATGTGATTCAGCCTGCCTAGCAATGACTCGACTGAAGGAA
ACGAAGCAACAGCCCTGTAAGTTGACTTCTCATGGTTTCGTATACGATAAGCCATGGTTATTTCGATTGCTTCCTTGTTGCAAGTTTATACGCGCCTTTGTTGCTTGAGATTGATGAGAGAA
AGAATGTGAGAGGATGATGGGTGGTCTATTTCTATCTAACAAATGCAGATAATAGAGATAAAAAAGAATGGGAATGGTTATTATTGTTCAAT
>comp149262_c1_seq1
TGGGCTTCCCCAAAATGCTCTGAGGAGATTTTTGGAGAAGTAGGGCATGATCTATTTGGTGAGTGGTGGCAACACTAGTTACATTTATTTTCACCTCCTGTTCTTGTTTATGTTCCTTGTTAT
TCAAAAAATTCATTTGCTGAGCATCAGCGATCTTTGGATGCTGATTTTCTTTATCTGGCGGAATGTGATCATCTGTTAAATTCATCCCTGAATTCCTCGACTCCAAGGCTTGAAAGCTATGAT
GCACCTGATGATGGTGATCACGACCAGCATGTTGGAGAATACCATCCTTGTTTTTCAACATTTTATTTTGCTGAACATTAGCTTTATGTGGATGCTGATTTTCTTTGACTGCTGGAATGCGAT
CACCAGTTAAATTCTTCCTTTTAATCATCATTCCATTTGTCCTTTTTTCCTTCTCCTGTATCCTTATTTCTCTTGAATATCTAGCATGGGCGCCCCTTCCATGCTTCTCTTGCAACACTTTAAAT
CTTTCCCCTTCATCAACCCACCTCTGTCTCATTGTCTTAATCCTATAAAGATTCCTTCCCTTGATGAGAAGCTGACGAAGAGGTGGCAATGGTTTCTCTCCTTTGCTCCAAAGAACTTCAAT
TGTGAATGTGTGTTGCTGCTTGGCAACACCATAACTTTCCTTTACAATACGACCAGCAACTGTCCTGGTGCCACATGGTGGACCAGTAGCACTCCTGGATGCGATGCTGAACATTTCATAT
ACATTCTGTTCAAAGATGACCACATCTCCCAGACAAGCATCACCTTTGCAGTTAAACACAAAGCTCTCTGGTGGATATTTCTTCTCCCCACAGCCATCAATGATCTCTTGGTGTTCTCTAAT
TCTGGAAATTAGGATTCCTTTGTTGCCTGTTAATCTCAAACCATACTTTCTCAGGTAGACCTTGCACTGATCAAGTGTAAGTTTCTCTAAATGGGCACCTTTGATAAGCTGTTCAACCATCT
CAAAGGATTTCTCATCCTTCTTATCCAGCTTGGGGATCTCATCCCCCACAGTTTCCCTCTCGGATTCACTCTCCAAGTTGCCATCAACAATTTTTGATCTCCTGGCTCTAGGTTTTCTTATGG
AGAGCTTGGATATAGCAGAACGGGTTTCCTCCACGATGCCGAAAGAGGGATCGTCGTCAGAATCATCCGCACACTCAAACTCATCGGATCCGGACTCGTCTTCCATCTCGCAGCGGTGTT
CTTGAAAAGCTTCGTCTAAAGCCATTGAATCCATTGTGAAGAAATATC
>comp149276_c0_seq1
ATTCCCAAAATGTCAGCAGGCATTTCATCCATTCAAAATTTTAAGGAAAATGACCACCATAACACTTAACCACAAAGAACCAATTTCTACATGCATTCATCTAAAAGTCAGCCTATGACAA

GCTGAAAAGTTCTGAATCATATGTGAGAGAATACCTAATTCCATATAACAGGAGGGATGTCCGCACTCTCATAGCACAAATAACTACGCCCATCCAGAAATCCTTCCCACTCCAGATTTGA
GCATTTACCAATGTGATTTGTTTCCATCTTCTTCAATCAACATTCCTTCTGATTTCCTCACCAAAGAATTCACCTTCTGTCAACTTCTGAATCCCAAGCCTTCCGCTTCTCTTCCCAGCAAAT
TTACAAATTCATACTTCCAAACCCTTATAAATGGCTCACCAGCATCTGCTAGCTCCCCAATCTAAAGCCGCAGCAATTCTTATAGCTGCTCCAACAAATGTAATTAAAATTAATCGCAATCGC
AGACGAAGAACCTGCCCTAAGAGCCGGGAGCAGGCGCCTAGGGCAGGAAGGTTGACAATACAGTTGAACCCTCCCAGGCTCCGAAGAAGAAGATGGCGATTGAGAACGACCAAAGGT
AAGTAAGAAAGCGAAGGCGGGCATGAGAATGCCGATCGGATTAAAATCCGGGAACAGGTCACCTTCCCTTCTCTCAGCCATAAGTCGTGAGAAAAG
>comp149276_c0_seq3
AAACGAAACATAAATCAAATCAATCATAAGAATGCTTCCACTCTTTAAAAGCTTATAAAAATGAGTATTTGTGTAGAGAACTATTCTGTTCAATTTCTGCAACCACTATGCCTTCAGCTTCC
AAAGCCAACAAAAAATTTTCAGAAGGAAGATTACTGAGGTAGCAGATAACCGATTTTAATGACATAGAAGAAAGTAATACTGTGAACTGCTTGAGAAAAAACATAAATATATTCATTATTA
CTTTTATACTGCATTAGCTGGACCAAAGAAGAGCTCCAAACAGAAGGATTGGCCACATATGAATTTACCCTTATTGCCAACTTTGTCCAGATCTCGAACTGAAAACTGCATCAAGCTGCCT
TTAATGAAAGCTGTATTAAAGCACACGTAAAAAAGGCGACTACCAATCAACTTTTCATAGAAAATGACTCTCACATCTCCTGACACTTTTAAAGGGCTTTCAAAGTAGTAATCGATGCATT
TCTTCTGGTAAAAAGCGGATCTTTCAGTATCCATCTGCATCACAACACGAGGAGACACGTGTTCAGTTTGATCTGTATCATTCCCATCATCAACATAGGAGAGGTAATAGCGAGGACTGGG
GTTTCTGTGATATCCTTGTTTTACTTCTCTGCAACAACCTTTTGCAACTTCTATTGAAGGACGGTAGAGTTGGTTGGGAACATCTTGCATTTCCATGACAACAAAGAACACAGAGTTAATG
TTGATGGTGTCATATAGGCGAATTCGGCGCAATTCTCTGGTAACTGTTTCAGGCAAATTAACAGTGGGAGGTCCACGAAGCTTATCTTTGTGGAATGACAGCACCTGAGACCAGTACCTC
ACATAACGCCTTTGACTTGGTATTGAAACCCCTTCATTGTTAGTAGTCCTTCTATGAGCATACAACTGAAGTGCTTCATCTACAGTCATCCCGCTATAAACAAGATAAGCACAAACCATTAA
GCCAGTCCGACCTTTGCCAGCCATACAATGGACGACAACAATATTCTCTGGATCATTTGAGAGCCATGAATATATACTTTCACAAAAAAGTTTGATCATTGCCAGAGGAGGAACATGGTTG
TCATCAAATGGATAAACCTCCACGCGTCCATGAAAATGTGCCGGATCATAGTTTTCTTCAATGCATAGGTTGTAAACCTTGTACCGACCTGGATGTCTCATATCCAATACATGCTTGACCTG
CCAAAGAGGGTTTCGATACATTGCTCGCACATGCTCGCCCGGGTATGACATTGCCAACAAATGGTTTGTGATATATGACATGTCTAGATCATATCCAGCTACTAGCATGCGCCTCCGGCTTT
TTGATACCAGATTTCGAAGGAAGAAGGATGATGTGACATAGTTGAACATTTTGTCACAGCGATTAGGACCACCAATCTTCTTGGACTTTGAAGAACCTTGTCTTGAGAATTTCATCCCCAT
GCTATTCTGAATTTGACTAAGTTGACAATTTTGATTAGTTTCACCTAAAAACACTGCTAGCTCCCCAATCTAAAGCTGCAGCAATTCGTATAGCTTCTCCAACAAATGTAATTAAAATTAATC
GCAATCGCAGACGAAGAACCTGCCCTAAGAGCCGGGAGCAGGCGCCTAGGGCAGGAAGGTTGACAATACAGTTGAACCCTCCCAGGCTCCGAAGAAGAAGATGGCGATTGAGAACGA
CCAAAGGTAAGTAAGAAAGCGAAGGCGGGCATGAGAATGCCGATCGGATTAAAATCCGGGAACAGGTCACCTTCCCTTCTCTCAGCCATAAGTCGTGAGAAAAG
>comp149276_c0_seq6
AATATAGACACAATGCCCTAATTAGTATAGAATAACCATTAAGAGATTATTTAAATTTACTAAAGAGCAAAAAATAAAGCAAACATGCATAGCTGCACTAAATCAGCACCATTAAAACTAGT
GATTTGAGGTGTCTGTTTAGCTTAACATACCAATGCGCACCCCAAGATCATGTAACAACTTGTTTTTAGTATCAAAATTACCTTGTACCGACCTGGATGTCTCATATCCAATACATGCTTGAC
CTGCCAAAGAGGGTTTCGATACATTGCTCGCACATGCTCGCCCGGGTATGACATTGCCAACAAATGGTTTGTGATATATGACATGTCTAGATCATATCCAGCTACTAGCATGCGCCTCCGGC
TTTTTGATACCAGATTTCGAAGGAAGAAGGATGATGTGACATAGTTGAACATTTTGTCACAGCGATTAGGACCACCAATCTTCTTGGACTTTGAAGAACCTTGTCTTGAGAATTTCATCCC
CATGCTATTCTGAATTTGACTAAGTTGACAATTTTGATTAGTTTCACCTAAAAACACTGCTAGCTCCCCAATCTAAAGCTGCAGCAATTCGTATAGCTTCTCCAACAAATGTAATTAAAATTA
ATCGCAATCGCAGACGAAGAACCTGCCCTAAGAGCCGGGAGCAGGCGCCTAGGGCAGGAAGGTTGACAATACAGTTGAACCCTCCCAGGCTCCGAAGAAGAAGATGGCGATTGAGAA
CGACCAAAGGTAAGTAAGAAAGCGAAGGCGGGCATGAGAATGCCGATCGGATTAAAATCCGGGAACAGGTCACCTTCCCTTCTCTCAGCCATAAGTCGTGAGAAAAG
>comp149276_c0_seq8
AATATAGACACAATGCCCTAATTAGTATAGAATAACCATTAAGAGATTATTTAAATTTACTAAAGAGCAAAAAATAAAGCAAACATGCATAGCTGCACTAAATCAGCACCATTAAAACTAGT

GATTTGAGGTGTCTGTTTAGCTTAACATACCAATGCGCACCCCAAGATCATGTAACAACTTGTTTTTAGTATCAAAATTACCTTGTACCGACCTGGATGTCTCATATCCAATACATGCTTGAC
CTGCCAAAGAGGGTTTCGATACATTGCTCGCACATGCTCGCCCGGGTATGACATTGCCAACAAATGGTTTGTGATATATGACATGTCTAGATCATATCCAGCTACTAGCATGCGCCTCCGGC
TTTTTGATACCAGATTTCGAAGGAAGAAGGATGATGTGACATAGTTGAACATTTTGTCACAGCGATTAGGACCACCAATCTTCTTGGACTTTGAAGAACCTTGTCTTGAGAATTTCATCCC
CATGCTATTCTGAATTTGACTGCTAGCTCCCCAATCTAAAGCTGCAGCAATTCGTATAGCTTCTCCAACAAATGTAATTAAAATTAATCGCAATCGCAGACGAAGAACCTGCCCTAAGAGC
CGGGAGCAGGCGCCTAGGGCAGGAAGGTTGACAATACAGTTGAACCCTCCCAGGCTCCGAAGAAGAAGATGGCGATTGAGAACGACCAAAGGTAAGTAAGAAAGCGAAGGCGGGCAT
GAGAATGCCGATCGGATTAAAATCCGGGAACAGGTCACCTTCCCTTCTCTCAGCCATAAGTCGTGAGAAAAG
>comp149284_c0_seq1
TTTAACATGCAATAATAGTTGGAAATTATTAAAATTGAATCTCATATACCAACCACTTTAAATTCTACATGTATATTTATATTAAATACTTAATCTTTTTAAAAATTTCAGCTTACGCTTGCATAA
AACATGCATAGAAAAAAATAATAATAAATAAAGAAGCAGGTACCACTAAAAAAAACCCACAATCCTATAAAACTTCATTAGAGGTTCCGAGAACGTCCCGTGGATCCCTAGCGAACGTAA
TCCTCGCACGATATGGCTTAAACCAACGAGCGATCGACTCAAAGGGAAGGGAATCCACGAAGTGCCAATTTCATCTTCCTTAAACTTCTTCATCGTTCTCGCCTGAAATTTCCTCAAGCGA
TTTCCCCTTGGATTCCGGCACCAAAAACGTTAATAACATACCGACAAGATTGCAGCCTGCGAGCAGGAAGAGCGAATTCCTAACACCAATACCAGGAGGATAACCGTGGTCAGCCCGCG
CCGGATCGGGATTCTGCGCAAGATAAAGAAAGCCAAATGAGCCAATGATCGCGCCCAGCTTGCCGCTCGCCGCTGATATCCCATGACAGGTTGACCTCAGCCTCGCCGGAAAAATCTCTG
CCGGAACGATAAAGGTAGTGCTGTTAGGCCCAAAATTGGCAAAGAAAAATGTGAATCCATACATTATAATGAAACCAATGTGATGGCCTGGTGTCGTCCAGTAAGTGTAGGGAATGGCTA
GGCCCAGCATGAACAGAGTCATCATCGCAAAACCCAACATTTGAATTGTGAAACGCCCGATGAAGTCGATTAACGCGACGGTGAGCCAATAGCCAGGTACGGTGCCGCAGAGGGCGATG
AGGGTCTGCGCACGCGCGATGCGGAAGACCTCCTCGAGTGCATTCATGGTTGCCCCCTTTGGGATCCACCCAATCGCGCTAAAAATGTCTTTTTGAAAGAGATTCTGGCTGTAGAAAGCG
ATGTCAAGGAGGAACCATGTGGTCGTGGTACCGACGAGGTGGAGGCCATGACGGCGGATGAACTCTTTCGAAAATAGCCCGAAGCTGTTAGCTTCGTTTCGAATGATTCGCACGATCTTG
TCCGGCTCTTCCTCGATCTCCACCTGGAGGACCTTAGACATGTCAGAAGCTGCCTGCTTGGCGTTCTTGGCGATAAGGGCGGTGTAGCGCGCAGTCTCCGGCATCTTCATTCGCCAGTAAT
AGGTGAGCGCGGCGGGGACAGAACCGACCATGACGATTATGCGCCAGACATAATCCGCCTCCGGGACTGTGGAGGCAATAGGGTCGACGGCATAGGCAGGTGCAGGGAAGTGATTTTT
AAAAGCGGCGGAGACAGCGATGGCAACCATACCGCCGGCCAAAATGCCAAACCCCTGCATCGCGAACACGGCGGCAATGAAAGCTCCGCGGGTTTTCTTATTTGCGTATTCGGACATGA
TGGTGGCGGAAAGAGGATAGTCTCCGCCAATGCCGAAGCCGAGCCAGAAGCGG
>comp149333_c0_seq3
AAGAAGAAGAAGAAGAGGAAGAAGTAGAGGAGACTCACAATGAAGCCAGCCGGATGGGTCGTCGCGTGAGGAGAATACCGAGCAAAATGCGAGCGGGATGTGCGGTTTGATCCTCTC
CGACTCTGTTACCTGATCCGCTTGAGTCGCGCGCAGGAAGGAGAAGAGGGATTGGATATGAACCCTTGAATGGTTCGCTTGAGTCGCGCGCAGGTAGGAGAAGAACCCTCGAATGCTCC
AGCGTGCAGGAAGGAGAGAAGATCGACGAGACGATCACAAAGAGTTTAGTTCGCGCGAATTAGAGCTTTGGGCAGAGGCTAGCGGGGGTTAAATGAGGTATTTGCATCAGTTAAAACC
GGTGCAAAAAGACATTTTTAGAAGCGGTTCTTAAACCGCTACTAATAACTGGTGCGAAAAATCCAACTTTCTAGTAGTGTTAGTACCGAGTAGTAGGTGATGTGGCGCATTTTCATTGAGT
TCTGGATGGCGTTCGTCATCCACATAGAATAATTTCACAACCTTTTGCCATCTTTATTGAAGAGTAAGTACTAAAACAACAAACTCTATTAACTAATATTATATGGATAAAAACAGGAAATAA
CATCAATCTTTGGAAAAATTGTTCTGTGTGGGCGTCGGACGGTGTCTAGGGACCAACCAGAA
>comp149400_c0_seq2
AAAACACGATTTACCATTCAGCCTCTCAACCCCGTCCCTGTGATCTCTGCTACGGTTGCAGCTGCCCCTTCTATAAATGTATCACTTCATCAATACTCTCTTCCTCCAAGAAAGTCACACCT
TTTACCTTTAGTTGGCTAACCATCTCTCCTCCACATTCAAGACCCCTCTCTTCAGCCTCTTCTCTCACTTCCCTTCTCCTTCACAAATTTATTTCAAAGTCTTTTTGCCTGCCTCTTTCACCAA
TTTGAAGTCCCCTTTCCTTCAACACAGCCATCTCCGTTGTTTCACAAATTCATTGCCATACCTCTTCCTATATATGTCTCTTACAGCCTAGCTATTTCCTGTGTTGTCTTCCTCATAAGCTGGG

TCATGTGAGAGTGAAGGTTGTGGGTGCATAGTGGCCGGTGTTTTGCTCAAGACCGCGCAACGTTTCCATATTACACCGCATCGCTGTACTGGCCTATCAATTAGAGGAGCTTCTTCTTTGC
AGCAGAAGAGAAGAAAGAGAAGATCGAGTACTGATCAATGGATAGGTCTGCACGCCGATGCGCTGCTTATGGCCTGGCTGCGTGGTCTTGCGCAAGATAACACCGCTTATCCGCTCTTCT
CTGTGCTTTCACACGCCATAGCTGTCTTTCCTCTGCTATCAAAACTTTCTTCTTTCGTTTTTTTTTTTAATATCCTATCATGGCAAAAAAATAGCATCTATTCTTCTCCTTCCTTTACCACAGG
CATTACATGGTTTAACCTCGCAAGTGTTTTATTTATCTCTTAGCTATTTCTTTCTTTTCTGTTATATTTTCATATTACATGGTTTGCTTGAGCCAATATTCTTCGTATCATTCATAGATGCAGCGC
ATTTCTGTAAGAGAAAGGACAAAGACTCGACCTGACGTGGTTTGGAGCAGAAACCTAGCATCTCGCAGGATGGAATTGACGAAGGCAATCGCCGACAAGGAGGTTAGGATTTGGAAGA
CACCGAGGCGGCCACGGCGCGTGCGGAAAGGGAAGGCCCGATCGCCCGGGAATTTTGGCCCGAAGGGGCGGGCAGGTCGATGCTGGCTAGCTGCCGACCCATCGGGTCACATCGGAG
GCGACAACCAGGCCGGTGAGGGACGGCGTGTGCGCCACGCCGAACGGTCCCCTTACGCCCTTCTCTTTCCTCAGTTGGCATGTGGGGGCAAATTCTTTGTATGGTTCGGGTACGAGATG
AGATCGGGTTGGACAAATGTGCGCGTTTGTGCTTCGCCGAGGAAACTCATTCATCCTTACGTTATTTATTTGAGACAAAAGAAAACGATGTGGCTTCCGCATTCAATCCCATCCCTTTGTA
ATGTTGGTAGTATTTATTAGTTTGTCAGATATAGCCGGTGCATAGGTTGAAATCTGCTGCACTGGGAAAGGTCCAGCTCATTATGTGAGTTTTATCTACAGGGAAATGATAACGGTGCTGCTT
ATATCTATATATATATATATATTACTCTCTATATGTTTACCTTCCAGTTTTATATGGCTTGTTTTCAACATGTCGAAAAGAAGTCAGAGAGTGGCCTTCATGAGTCCAAATCTCACAGAGGAGC
AGACTACGCCTCTCAAGTCTCTCAAGTCCTACTTAGATACGCAATCAAGGGAGAACAAGTCATCAATCAAAAACAGAATTTCAGACTTCTTCAGCTCATCGCCAGAAAGATTGTTGAGCT
GTATCAAGAACAGTGTTA
>comp149400_c0_seq4
AAAACACGATTTACCATTCAGCCTCTCAACCCCGTCCCTGTGATCTCTGCTACGGTTGCAGCTGCCCCTTCTATAAATGTATCACTTCATCAATACTCTCTTCCTCCAAGAAAGTCACACCT
TTTACCTTTAGTTGGCTAACCATCTCTCCTCCACATTCAAGACCCCTCTCTTCAGCCTCTTCTCTCACTTCCCTTCTCCTTCACAAATTTATTTCAAAGTCTTTTTGCCTGCCTCTTTCACCAA
TTTGAAGTCCCCTTTCCTTCAACACAGCCATCTCCGTTGTTTCACAAATTCATTGCCATACCTCTTCCTATATATGTCTCTTACAGCCTAGCTATTTCCTGTGTTGTCTTCCTCATAAGCTGGG
TCATGTGAGAGTGAAGGTTGTGGGTGCATAGTGGCCGGTGTTTTGCTCAAGACCGCGCAACGTTTCCATATTACACCGCATCGCTGTACTGGCCTATCAATTAGAGGAGCTTCTTCTTTGC
AGCAGAAGAGAAGAAAGAGAAGATCGAGTACTGATCAATGGATAGGTCTGCACGCCGATGCGCTGCTTATGGCCTGGCTGCGTGGTCTTGCGCAAGATAACACCGCTTATCCGCTCTTCT
CTGTGCTTTCACACGCCATAGCTGTCTTTCCTCTGCTATCAAAACTTTCTTCTTTCGTTTTTTTTTTTAATATCCTATCATGGCAAAAAAATAGCATCTATTCTTCTCCTTCCTTTACCACAGG
CATTACATGGTTTAACCTCGCAAGTGTTTTATTTATCTCTTAGCTATTTCTTTCTTTTCTGTTATATTTTCATATTACATGGTTTGCTTGAGCCAATATTCTTCGTATCATTCATAGATGCAGCGC
ATTTCTGTAAGAGAAAGGACAAAGACTCGACCTGACGTGGTTTGGAGCAGAAACCTAGCATCTCGCAAGTGCGGCGTCATCCTCGCCATCTTTCTTAAATTTTCATGATCATTTTGTTCCT
TTAATTTGATTCCCCCCCCTCTCTCTCTCTAAAAGCTGGTTTTTTATGTATCTATTTATATACATATGTCATGTCGAAATAGTTCTTGGGAAAACGCAGGATGGAATTGACGAAGGCAATCGC
CGACAAGGAGGTTAGGATTTGGAAGACACCGAGGCGGCCACGGCGCGTGCGGAAAGGGAAGGCCCGATCGCCCGGGAATTTTGGCCCGAAGGGGCGGGCAGGTCGATGCTGGCTAGC
TGCCGACCCATCGGGTCACATCGGAGGCGACAACCAGGCCGGTGAGGGACGGCGTGTGCGCCACGCCGAACGGTCCCCTTACGCCCTTCTCTTTCCTCAGTTGGCATGTGGGGGCAAAT
TCTTTGTATGGTTCGGGTACGAGATGAGATCGGGTTGGACAAATGTGCGCGTTTGTGCTTCGCCGAGGAAACTCATTCATCCTTACGTTATTTATTTGAGACAAAAGAAAACGATGTGGCT
TCCGCATTCAATCCCATCCCTTTGTAATGTTGGTAGTATTTATTAGTTTGTCAGATATAGCCGGTGCATAGGTTGAAATCTGCTGCACTGGGAAAGGTCCAGCTCATTATGTGAGTTTTATCT
ACAGGGAAATGATAACGGTGCTGCTTATATCTATATATATATATATATTACTCTCTATATGTTTACCTTCCAGTTTTATATGGCTTGTTTTCAACATGTCGAAAAGAAGTCAGAGAGTGGCCTTC
ATGAGTCCAAATCTCACAGAGGAGCAGACTACGCCTCTCAAGTCTCTCAAGTCCTACTTAGATACGCAATCAAGGGAGAACAAGTCATCAATCAAAAACAGAATTTCAGACTTCTTCAG
CTCATCGCCAGAAAGATTGTTGAGCTGTATCAAGAACAGTGTTA
>comp149420_c0_seq1
CCATATTATTAAATAAAAACTTTATTTTTAAGCCCTCCTACTTAATATATTTACAAATTATTTAATTTCTTACATCTGAAAACAATTCTCTATCTTCAATAGATGCTTGTTTTTTTCAGTTACACAT

AATTTCCTGAAAAATGAGATACAAAAGGTACTTCTGAGCCCATCCTCATAGAATTTTCATGCAGGATTTATGTTCATAAATGTGTTCTATATTCTCACATGAATCCATTTTGTTCTTCTAAGCC
ATAGAGGAGATTTTCTTTATCAGTTCCATTGCCCAAATCTTCCAAATTTAACAACTTAAACACTTATAACGCAAGACCAACAGTAATTGGATTAGATTAGCGCTTATATCTCCAGAACTATAT
ATTCTGAATCAGTTTTGATCCATTTGCATCCATCCTCTACCTTCTCTTCTTCTTTGTTTTTCTGCAAGTACTTCATTGATGGTGGTCGAGGTACAGTTTGGGGATGAATCTTTTTGTGAATTAT
TTTCCTCAAGATCTTAGCTCAGAAGGAATAGTAGAAGATCATTAAGAGATCAAAGAGAAAAAAAAAAAAAGACAAAAATATGCAGCTTATACTAAATTCATACCTTTTCCACTTTTGATTCA
GAAATCGAGTTCCTTAAACTTTGCTGTGTTGATAAGCAGCTTCTGCTTATAAACATTTTCTTGAGAAGGAAAGAGAGAGACCTTTGCCTGATCCCATTGTGATTATCAGAAAGCAGGTCTT
TGAGTTTGTTTAAGGTGATCTTGGAATTGTCAGAATCATCTGTTGATTCCTTACAGTTTTTTAGTGAAGAAATGTTGTCTGCTTCCAGAGTTAAGGAGGTGTTTAGAGAGTCATTCAGCGA
GAAATCTGCTCCATCTGTGCTTGAACTTAGCTGGAGACTTAGTAATTTGCTTATTTCTTGCTGCAATTTGATCACCTCTTCAATGTTTAGGTCTGAAATTTTATGTAATAATTCTAGAGTTTCA
TAAGAACTTTGTGCCATTTGTGAATCTTCCTGCGGGTCATTGTTGCCAAATGTCCCAATTGCCAAAAATGTTTGTTGCCAGTCTTTGAATTCTTGAAGTGAGAAGAGCTTGGGCCAACTTC
AAACCTCCTTCCTTGTCTCCCATTGAGCTTACTTTGAACCCAGCTGAAAATCCTCATAACTCCACCAGTGCTGCAAGCTTCAGTCTTCTTTCAATATATATACAGAGAGAGAAGAACACTA
GTAAACTGCATCGAAAAGGTTTCCAGGACAAGAAAGAAAAGAATTTACAAAATTGGCTCTCTTTGTGGTCCTCTCAATCTCAGAAATGGCTGCAAGTTATAAGAGAATTTATCAAGAGGA
AAGCTATAGAGGATTTCTACCAAAGATACTAAGACATTGAGGGACTTAGGAAGATTAAATAGAACGAAAAGAACATGGCCACAGATTTCAATGAATTTGTTCCACTAGTTTCAGGACATTT
CATCAGCTCAAGATCCCAAGCAAGAGTTCCACCCATGCTTCTTGAACTTTTAAATTTGCCAGA
>comp149420_c0_seq2
CCATATTATTAAATAAAAACTTTATTTTTAAGCCCTCCTACTTAATATATTTACAAATTATTTAATTTCTTACATCTGAAAACAATTCTCTATCTTCAATAGATGCTTGTTTTTTTCAGTTACACAT
AATTTCCTGAAAAATGAGATACAAAAGGTACTTCTGAGCCCATCCTCATAGAATTTTCATGCAGGATTTATGTTCATAAATGTGTTCTATATTCTCACATGAATCCATTTTGTTCTTCTAAGCC
ATAGAGGAGATTTTCTTTATCAGTTCCATTGCCCAAATCTTCCAAATTTAACAACTTAAACACTTATAACGCAAGACCAACAGTAATTGGATTAGATTAGCGCTTATATCTCCAGAACTATAT
ATTCTGAATCAGTTTTGATCCATTTGCATCCATCCTCTACCTTCTCTTCTTCTTTGTTTTTCTGCAAGTACTTCATTGATGGTGGTCGAGGTACAGTTTGGGGATGAATCTTTTTGTGAATTAT
TTTCCTCAAGATCTTTTCCACTTTTGATTCAGAAATCGAGTTCCTTAAACTTTGCTGTGTTGATAAGCAGCTTCTGCTTATAAACATTTTCTTGAGAAGGAAAGAGAGAGACCTTTGCCTGA
TCCCATTGTGATTATCAGAAAGCAGGTCTTTGAGTTTGTTTAAGGTGATCTTGGAATTGTCAGAATCATCTGTTGATTCCTTACAGTTTTTTAGTGAAGAAATGTTGTCTGCTTCCAGAGTT
AAGGAGGTGTTTAGAGAGTCATTCAGCGAGAAATCTGCTCCATCTGTGCTTGAACTTAGCTGGAGACTTAGTAATTTGCTTATTTCTTGCTGCAATTTGATCACCTCTTCAATGTTTAGGTC
TGAAATTTTATGTAATAATTCTAGAGTTTCATAAGAACTTTGTGCCATTTGTGAATCTTCCTGCGGGTCATTGTTGCCAAATGTCCCAATTGCCAAAAATGTTTGTTGCCAGTCTTTGAATTC
TTGACTGCATGAATTTTGCTTTGTAGTATAGTGAGAAGAGCTTGGGCCAACTTCAAACCTCCTTCCTTGTCTCCCATTGAGCTTACTTTGAACCCAGCTGAAAATCCTCATAACTCCACCA
GTGCTGCAAGCTTCAGTCTTCTTTCAATATATATACAGAGAGAGAAGAACACTAGTAAACTGCATCGAAAAGGTTTCCAGGACAAGAAAGAAAAGAATTTACAAAATTGGCTCTCTTTGT
GGTCCTCTCAATCTCAGAAATGGCTGCAAGTTATAAGAGAATTTATCAAGAGGAAAGCTATAGAGGATTTCTACCAAAGATACTAAGACATTGAGGGACTTAGGAAGATTAAATAGAACG
AAAAGAACATGGCCACAGATTTCAATGAATTTGTTCCACTAGTTTCAGGACATTTCATCAGCTCAAGATCCCAAGCAAGAGTTCCACCCATGCTTCTTGAACTTTTAAATTTGCCAGA
>comp149430_c0_seq1
TTTTTTTTCCATGAGATGCCCTCCCTTATAAAAACAAAAAAAAAAAAAAAAAAACATCTTTTAAGCTCTTCACATAAATTGTGTCAAAATGTAATATATTTCATTTGAAATAGCTTTATGCCTC
TTCTCCCATGCCATAGTAATCCCTTAGGAACTTCTCACACATTGGTAAAGGCTCATCCTTGAGGAAGCCAAGAGAGTTGGCAAACCTAGCATTGTTGCTCATGCCGGTGTTTGTCGAGAGC
ACGACTTGCGCCCTATCTCTTCCATCATTGATCTTGTTGCAATTCTCCAATGGGCTCTCGACGAGCACAACCTCACAAATTTCCTCCTCGTGATCATCTGCCACCTCAATTTTGTAGTTGCC
GGTGGAATCCGTCACACCATAGCTGGTGTGCTCGATATTTCCTGTTGCGAAGTGCGTGCACTCGACCTTAATCGTAGCCCCTGTGATGTATTCGGTGATATTTGTTTCAAAGCCGGCATGAC
AATTGTCACAGTAAACACGCCCCACAACAAAGAAGATTGGGCTATTGGCTGAAGCCACCAAGGCGATGGAGGAGAAGAGGCAGAAGGCAACGATGAATACAATCTTCGCCATAACTTC

TTCTTTTCGGATTCTTTTGCGTCTTCTCCTCTAATTTGAGAATGGACAAGA
>comp149435_c1_seq2
CTTTTCTTCCAAAGTGAGTTCAAGGCTGGGGCATCTTTTTAGCTTCAATTCCTTAAGTGAAGGAAGGTTTTGCAAGCCTGGTATTGATGTCAACTCCCAGCAATCTTTGAAACATACAAAT
TGCAACTTAGAAGGAAGTATCTCCTCCGCAGAGATCAAGAGATTATCACACCACTTTATTTCTAACTTTTCAAGTCTATGCAGACTACATAGCCAATGCAATGATGTTATTTGCAGGCAAGA
TCTAATAACTAGTCTTTTAAGTGAGGATGACTGCACTACTTCTATATTTTTTAACATACCACATTGAATGATGGAAATACTTTGAAGACATGGGAGATCTCTATCCAACACTTTACCTGATCC
ATCCCATTTATTACACCCCTGAAGGGTTATATCTTGTAACTTAGAATACGAGGGATCACCAAGCCATGACGGGAATAGCATCCCAGGGTAATTATGTATCTCTAATGCTTTGAGGTTGTTGTG
AGGCCGCAA
>comp149499_c0_seq1
TACCTTCCAAAAAAAAAGGAATCTAGAAGAGATTCTCTTTAAGATGGTGATTTCTTCTCCTCATTCTACTAACACTAAGATTTTCAAAGCCAGTGGTCGCCGCCGTCGACGGAAAAGCTCC
GGCGACCTCCCATCGGCTAGCCGCAGCGCCAAAAGGAGCTCCATTTACAGAGGAGTAACCAGGCATAAATTGACTGGGAGGTATGAAGCTCATATCTGGGACAAGAACTACTTGAATAA
ATCCCAAAACAAGAAAGGAAGACAAGTGTACTTAGGAGCTTACGACACTGAAGAGAGTGCAGCTCGAAACTACGACCTTGCTGCTCTGAAGTACTGGGGAACTGATACGACCCTTAATT
TTCCTGTGGCGAATTACGACGATGACTACAAGGAAATGCAGGCGATGTCGAAGGAGGACTATTTAGCTTCGTTGCGCCGCCGGAGCAGTGGATTCTCCCGCGGAGCCTCCAAGTATCGCG
GAGTAGCAAGGCACCATTATAATGGAAGATGGGAAGCAAGAATTGGTCGCATCAATGGCAACAAATATCTTTATTTGGGCACATTCAATAATGAAGAAGAGGCGGCACAAGCTTACGATC
GTGCTGCATTGATTTATCGTGGCTCTCGAGCGGTGACCAATTTCAACATCAATCAATACACGGAGGTTCGAACACCGTCATCAGAACCGTTGCCACTACCCTCACAACATCAAAACTTAGT
TCATCCCATAACGCTCTCTAATCCAACTTGTTCCTTTGGTGGAACAATGGCGATGGTTGATGACAATAACGAACTCTCGTGGAGCGGCTCGCGAATGGGCTCGAGCTATGAAACCTTCTCC
ATCTCTGATTTGTTCTTTGACTTTAATGTATCTGATGATCATGTTGGAGGATTCATCAATGACATTGGAGAACTCATTGATGACATCGAAAGGCTCTTTGATGAATCATGGGTCGATCATTTC
AGTGTGAATGGTGAGGACAATGAGAAGTCAATGGCAGCTATTTCATCTGGTTCTGATGAAATTATCTCAACTTCCTTAGTTTGAGACCAACATTTAGTGGAAGAAGATATGAGTTGTTGAG
AAGTTTTGGAAGCAAATATGATTAGATATATGAGATTTAAGTTTTATTTG
>comp149515_c0_seq1
TGGCTTAATCCACCACCCCTCTCGCATCCTCAAACAATCACCATCCTCTTTCATTTCTACACACCCTACGTCTCCTTCTTCCTCTTCCTTCTCATTCCATCCCTCTCAACTCAACACCGAAAT
CAGAGAAAAAAGTAATGCTCCTGCGGCTACAACCATTCTGATCTCATGTACTCAGACGCCTCAATCAAATCCTCATTGGAAGAAGAGAGCTCTTCAAACCGCCACAGCAACGTCTCCTTG
CCCATGTCTCCTTACAGAACCTCTCCTTGGACTTCCCACACCAATCCCTTCATCATTCCGCCGGATCAAACCGACCTCTTCCCCTCCGGCACCGGTCTCATCGCCTCTCTCATTCGCGAAG
AAGGCCACATCTACTCCCTCGCCACCTTCAACGGCCTTCTCTACACCGGCTCAGACAGCAAAAACATTCGGGTATGGAAAAACATGAAGGAGTTCACCGGATTCAAATCCAGCAGCGGC
CTCGTCAAAGCCATTGTAATATCCCCCAAAGGTCAGATCTTTACCGGCCATCAAGACGGAAAGATTCGAACTTGGAAAATCTCCCTCCAAAACCCCCAAATCCATGCCCGTCTCGGCACT
CTCCCTCGCCTCAAAGACTACATTAAGAGCACGCTAAGCCGGCGAAAGATTCGGCATTCCGATGCGGTATCCTGCCTCGCCCTAGACGACATCAACGGCTTCCTCTATTCCGGTTCATGGG
ACCGATCGCTCAAGCTGTGGCGGGTTTCGGATTCTCGCTGTCTCGACTCGGTAAACGCGCACGACGACGCGGTTAATTCGGTAATTATCGGGTTTGATGGGTTCGTTTTTTCCGGTTCGGC
CGACGGGACGGTTAAGGTTTGGCGGGTTGAGCCGAGCGGTAAGAGTCAGACCGGGCGCCGGGTGGTGGAGGTGGCGGTGTTGGTGCGGCAGGAGTCGGCGGTGACGGCTTTGGGTGT
GAGCGTGGGTGCGGGCGTGTTGTATTGTGGATCGTCGGATGGGCGGGTCAGTTTTTGGGGGCGGAATTTGGTGCGTTTGGGAATTCTGAACGGTCATAAATTTTCGGTGCTTTGTTTGGCT
TTGGCGGGGGATTTGGTGTTCACGGGATCAGCTGATAAGGCGATCTGCGTGTGGGGGAGGGAAGAGAATGGGACGAGACACGTGTGGCTTTCGGTGCTGACGGGGCACGAGGGGCCG
GTAAAGTGTCTCGCGGTGGAGGAAGATGTTAACGTGGAGGAGGCGGTGGGTGGTCGGAGATGGGTGGTGTATAGCGGGAGTTTGGATAAGTCGATTAAGGTGTGGAAGGTAACGGATTT
TGTGGGTTGGGAAGGAGGGAGGAGGAGTGGGTTCTTATCGTGTTCCAGCAATGATGATCCAATATTCGATGCGTTTTGATAGAATGTTTGTGACTTGTGAGGTAATAACATTCATGATGCA

TTTTTTATTTATTGGAATTTTTTTTAGAGAGCAAAATTTGGTTATTATGATAAATTTTAATGTTATTATGTTTGTGTTAAGATTGATGAAATATTAGTTTTGATTAAA
>comp149551_c0_seq3
AAGAAGTAGTAAAAAAGCCGTACCAAATAAAACTTAAATCTCATATATCTAATCATATTTGCTTCCAAAACTTCTCAACAACTCATCTCTTCTTCCACTAAATGTTGGTCTCAAACTAAGGA
AGTTGAGATAATTTCATCAGAACCAGATGAAATAGCTGCCATTGACTTCTCATTGTCCTCACCATTCACACTGAAATGATCGACCCATGATTCATCAAAGAGCCTTTCGATGTCTTCAATGA
GTTCTCCAATGTCATTGATGAATCCTCCAACATGATCATCAGATACATTAAAGCCGAAGAACAAATCAGAGATGGAGAAGGTTTCATAGCTCGAGCCCATTCGCGAGCCGCTCCACGAGA
GTTCGTTATTGTCATCAACCATCGCCATTGTTCCACCAAAGGAACAAGTTGGATTAGAGAGCGTTATGGGATGAACTAAGTTTTGATGTTGTGAGGGTAGTGGCAACGGTTCTGATGACGG
TGTTCGAACCTCCGTGTATTGATTGATGTTGAAATTGGTCACCGCTCGAGAGCCACGATAAATCAATGCAGCACGATCGTAAGCTTGTGCCGCCTCTTCTTCATTATCTTTTTTTTTAGCAA
ATTTGGGAGGAAATGAATAATATGAGTCTAATCGATAATGGTTAATATAAATATTATTATATGATCAAAGTAATATTATTTTTAAAATAACAATGAACCAATCATCAAAAGCTTTATATCGATAA
CCGCTAGAGAACCATGATAAACCAATGCATCACGATCATAAGCTATATCTGACTATGTGGGGTCACATAAAATGGCGGACTTCCGATGACCCGGTTAAGAAAGTAGTTGTATTAGATTTTTT
TTTTTTTAAGTTGTATTAGATTTG
>comp149595_c1_seq1
TAAATTGAGTTATCATCATAATTTGAAAAAATAAAAACGGATCCAAACAACATATAAAATAGAAAAAATAAAAAAAAATCCTGGATTTTTGTTAGTATTTGACCTTAGAATCAAAAGACAA
CAGCCTCCTCCCCTAGCTTTGAAAACAGAAAAAAATTAGCAAGTAATGATCATTAATGTAATACCCTCTCAGAAACTCAAAGGTAAGATATCCTCTCAGATTATGCTATTCGCTGCCTCTTC
CTCCAATAACCGTAAATTTCTTCTTCTTCCTCCTCGATTCCAACTTCAGCTTCAAGAGGAATATAATCTTCAGAGGCGGAAATATTGTCTTCAAGAGGAATATAGTCTTCAGAGACGGAAAC
ACCCAACAAGTTCTCTGTTTCCGCTGCTTCAAGAACTGCGCCATCTTCATCTCCTCCCACCTCAAGAGGGATATAGTCCTCCGCCTTTGCTGCAGAAAGCTGTCGTTCTTCAACTACACGA
TCATCACAAGTACGGCCGTAGACATTAAGGAACACGATATAGTCGGCTATGGAAGACAACGGACGAAGTCGGAAGACTTTCTCCATGCTTTCTTCTTCTTCTTCTCCTTCTTGGCCGTGGC
AATCCACGGAATCTCCATCATCAAAAACATCCTCCTCTTCTCGTCCATTCAAGCTGTCCAAGGCAAGAGGAGCGCAAGCTTCTTCTTTTGGAGAGATTTTTCCTTCTTTTGTGACTTCCATT
GTAATGTCCTAATAGTTTTCTTTTCCAAGGTGCGTCCTTTATGTTTA
>comp149595_c1_seq2
ATTACATATATTTGACAACAGATAATAAATAGAACTCCTCAATAACCTGTCAGTTCGAAGAAGAACTAAGTTTTACTATCATTACTTTTCGCTTAAATTCTGGTCAGAAGTCCAAAAAGAAT
GGAAATAACTAAAATTTGCAAAGAAACTTCTGAATAGTTTTACAAGAGAATTTTTTAATTGTGCAAACTTGAAATAAAAAAAAAACAATATTATCTAACAAATATTCTAAAATATCAAGCAC
AACACTCATTTTTGTTGAAGCTCTCATAAACTCGAAGGTGCGATCCTTGCAGATTATGCTATTCGCTGCCTCTTCCTCCAATAACCGTAAAGTTCTTCTTCTTCCTCCTCTATTCCAACTTCA
GCTTCAAGAGGAATATAATCTTCAGAGGCGGAAATACTGTCTTCAAGAGGAATGTAGTCTTCAGAGACGGAAACAGCCAACAAGTTCTCTGTTTCCTCTGCTTCAAGAACTGCGCCATCT
TCATCTCCTCCCACCTCAAGAGGGATATAGTCCTCCGCCTTTGCTGCAGAAAGCTGTCGTTCTTCAACTACACGATCATCACAAGTACGGCCGTAGACATTAAGGAACACGATATAGTCGG
CTATGGAAGACAACGGACGAAGTCGGAAGACTTTCTCCATGCTTTCTTCTTCTTCTTCTCCTTCTTGGCCGTGGCAATCCACGGAATCTCCATCATCAAAAACATCCTCCTCTTCTCGTCCA
TTCAAGCTGTCCAAGGCAAGAGGAGCGCAAGCTTCTTCTTTTGGAGAGATTTTTCCTTCTTTTGTGACTTCCATTGTAATGTCCTAATAGTTTTCTTTTCCAAGGTGCGTCCTTTATGTTTA
>comp149670_c0_seq2
TATCTCAATCAAGTAAACTAGATGGAGCTTATAACTTCATATAGTTATGATGTAATGCATCTCAATCATTCACGTATCATCTATCCCATTACATATGAATACACTCTTTTCCATTCCAATTTCAGT
TACGTGTTTGGCATTCTTTGGGCACTAGCCATATGTCTTTTTTCTCCTGCATTTGTAGTTGATGTAGGCAAGGGGGGTCAAAAATTTTCTTTGGTCTTTGGGCTGCTGGTTGCTCTTGGGTT
GGGCTTGGCTGCTTGGTTTTGAGCTGTGTTTTTGGG
>comp149690_c0_seq4

AGGATCTACTAGAGGCAAACTAAGTAACTAACCCTAATCATACGAAATCTCAGCCCATTTTTTTGTTTTTATTTTCCAGCATTTGGCATGAACAAATTTCTTACTAACTCCAAGAAATCAAC
ATCATCATCCGATTAATTCTGTGTGTTTTCGTCTTTTTTGGGCATGAAGCCTGACATTTCCTAACCAAAACCAAATCTCTCACGGTAAGTAATTAGAGTAACAATCTGATTAAGCATCACGAT
CGCAAGAAAGCTTCTCCCAATCGAGCTTATTTCTAGCTTTTCTGCAACAAACTTTCTCAATCTCTGAGTCATTCTATTGCCAAAATGGAGAAAGACGGTCCTTTTGCGCCAAACTGACAAT
GACATTGCCCCTTCGCTTTCACAGAGCGCAGATGGACACGGAGGAAGAGGGCATTGGCTGGGTCAGTGACCTTTTTTAAGTCACCAGCAGCAAAGCACTAAAGATTAAATTTTGATTCCT
TTGTTTGGCCTTTTGGTCTCTTTACTGCTGATCAGTCATGAAAACTTGGTGAGGGTTTTGGTTCTTATGAAGGAGGTTGAAGACGGCTTGGCTGAGGAAGGGCTTCGGTTGAAGTAACTG
CATTTTCTTCTCATTCCTTCTCAGCCATGGTGCCACAAGAGTTACCAAAGGAACCGATTGGTGTCTCAGAAATGAATAACCGACAATCTCATCCTGGAGGAATTTGTTATTGCTCTCACTG
GAATAGTGTAGATCGTGGGTTGGTTCGGCCTCATAGCTTACCATCCAGCTTTCAGCCTCATGACACACAGATCCGTAGATCTGTTGAGCCTTTTTCAGTGAGATGTAAAGAGGATGATTTCT
CAAGCTTTGACCATATACAGGGCCATGGCTTGTCACTAAGCCTTGGACCTGAGCTGGAGCTGGAGCCTAACAATGTTAGGCCATACGATTATAATCAAGCACAGATGGTAGAAAATGTTAT
GAGAAACAAATCTTTGATTGAACTTATGTGTGAGCCCTCTTGTTCTTGCGCCGAACGTTTAAGTGATGACGGCATTATATGTGATCAAGATGTGGATGGTAGTATCTATAATTCAAGATGCT
CAAATACGAAGTTCGATAACATGGCGCCAGTCATGAGTGTTCTTCAGAAGTCTCCATATCTTAAAGCAGCTCAGCAGTTACTTGATGAAGCTGTTTGTGTGTGTAACTTTGTTCCCGAGCC
ATCTTCAGAAAAGGATGCGCACAGGAAGAATCAGATTAAAGAGAACACACACACTGTGCACTTGTCAAGGGAAGAACAAGAGGTCAATAGAATGAATAAACTTTTTACTCTGCAGGAA
GAGCTTGAGAAGCGACAGAAGATATATTTCCATCGAATGGATGAACTAGTAACATCCTTTGAAGCTGTAGGTGGTCCTGGGTCTGCTGCTGCATACACAGCTCTTACCTCACGAGCAATGT
CTAAACATTTTGCTAACCTTAGGGAATCCATTATCAATCATATAGAGTCTTCAAGAGAGGCTTTTTCCAAAGATATGCCAAAAAACCTTAAACCAAAGAGGGAATCACTGCAACACCTGGG
CTTGATCCATGTTGGACAAATCTGGAGGCCACTCAGAGGACTACCGGAAGACTCAGTTATGGTGCTTCGAGCTTGGCTTTTCGAGCATTTTC
>comp149690_c0_seq5
AGGATCTACTAGAGGCAAACTAAGTAACTAACCCTAATCATACGAAATCTCAGCCCATTTTTTTGTTTTTATTTTCCAGCATTTGGCATGAACAAATTTCTTACTAACTCCAAGAAATCAAC
ATCATCATCCGATTAATTCTGTGTGTTTTCGTCTTTTTTGGGCATGAAGCCTGACATTTCCTAACCAAAACCAAATCTCTCACGGTAAGTAATTAGAGTAACAATCTGATTAAGCATCACGAT
CGCAAGAAAGCTTCTCCCAATCGAGCTTATTTCTAGCTTTTCTGCAACAAACTTTCTCAATCTCTGAGTCATTCTATTGCCAAAATGGAGAAAGACGGTCCTTTTGCGCCAAACTGACAAT
GACATTGCCCCTTCGCTTTCACAGAGCGCAGATGGACACGGAGGAAGAGGGCATTGGCTGGGTCAGTGACCTTTTTTAAGTCACCAGCAGCAAAGCACTAAAGATTAAATTTTGATTCCT
TTGTTTGGCCTTTTGGTCTCTTTACTGCTGATCAGTCATGAAAACTTGGTGAGGGTTTTGGTTCTTATGAAGGAGGTTGAAGACGGCTTGGCTGAGGAAGGGCTTCGGTTGAACCATGGT
GCCACAAGAGTTACCAAAGGAACCGATTGGTGTCTCAGAAATGAATAACCGACAATCTCATCCTGGAGGAATTTGTTATTGCTCTCACTGGAATAGTGTAGATCGTGGGTTGGTTCGGCC
TCATAGCTTACCATCCAGCTTTCAGCCTCATGACACACAGATCCGTAGATCTGTTGAGCCTTTTTCAGTGAGATGTAAAGAGGATGATTTCTCAAGCTTTGACCATATACAGGGCCATGGCT
TGTCACTAAGCCTTGGACCTGAGCTGGAGCTGGAGCCTAACAATGTTAGGCCATACGATTATAATCAAGCACAGATGGTAGAAAATGTTATGAGAAACAAATCTTTGATTGAACTTATGTG
TGAGCCCTCTTGTTCTTGCGCCGAACGTTTAAGTGATGACGGCATTATATGTGATCAAGATGTGGATGGTAGTATCTATAATTCAAGATGCTCAAATACGAAGTTCGATAACATGGCGCCAG
TCATGAGTGTTCTTCAGAAGTCTCCATATCTTAAAGCAGCTCAGCAGTTACTTGATGAAGCTGTTTGTGTGTGTAACTTTGTTCCCGAGCCATCTTCAGAAAAGGATGCGCACAGGAAGA
ATCAGATTAAAGAGAACACACACACTGTGCACTTGTCAAGGGAAGAACAAGAGGTCAATAGAATGAATAAACTTTTTACTCTGCAGGAAGAGCTTGAGAAGCGACAGAAGATATATTTC
CATCGAATGGATGAACTAGTAACATCCTTTGAAGCTGTAGGTGGTCCTGGGTCTGCTGCTGCATACACAGCTCTTACCTCACGAGCAATGTCTAAACATTTTGCTAACCTTAGGGAATCCA
TTATCAATCATATAGAGTCTTCAAGAGAGGCTTTTTCCAAAGATATGCCAAAAAACCTTAAACCAAAGAGGGAATCACTGCAACACCTGGGCTTGATCCATGTTGGACAAATCTGGAGGC
CACTCAGAGGACTACCGGAAGACTCAGTTATGGTGCTTCGAGCTTGGCTTTTCGAGCATTTTC
>comp149690_c0_seq8
CTTTAAGAAGCAATAAAAAAGCCATCCGAAATGAAGTCATATGCTGTCCATCTTTTGTAGATGAATTAAAACATAAAGTGAATGGAAGAAACACAATTGTTAAGTTTATATTACCATGGTGT

CTGTTGATGTCGTAGTAAATATACTTATTTACCTCACTTGCATCACCTTTGCTTGATTTAATTTTATTTAAACATTTGAGTGAAAGTAAATTCCTTCTATTACCAAATAATTGACAGATTTAGAT
GTAGATGTGGGGCCTTACTGTTCTAATCGATAGTTCCCACGAAATTAACTCTTAACTATACAACTTTCCAGCTTGAGAAGCGACAGAAGATATATTTCCATCGAATGGATGAACTAGTAACA
TCCTTTGAAGCTGTAGGTGGTCCTGGGTCTGCTGCTGCATACACAGCTCTTACCTCACGAGCAATGTCTAAACATTTTGCTAACCTTAGGGAATCCATTATCAATCATATAGAGTCTTCAAG
AGAGGCTTTTTCCAAAGATATGCCAAAAAACCTTAAACCAAAGAGGGAATCACTGCAACACCTGGGCTTGATCCATGTTGGACAAATCTGGAGGCCACTCAGAGGACTACCGGAAGACT
CAGTTATGGTGCTTCGAGCTTGGCTTTTCGAGCATTTTC
>comp149703_c0_seq1
GCAAATAAAAATATTTCATGGGTCTTTATAAGAAAAATCGAAGAAGCACACAAGTTCTTAATAGTTAATACTTATAATTACATTAAAAAAACAGTTATTTAGATTACAAACCTCAATCACCA
CATTGACAAAATATCTACCGAATACAAAGGTTCAACCTCTCAAAGCAAATGGTAAATTTGTAATATATTTATTCTTAACATCAGATATATAAATAGCGATCTGGTTCTTCAGCGGTGCTTGCT
CCTTGTCTCTCCACTGTACATCCAGTCGTAAACAGTAGGCGAATTTGGCTGAGGCTCGTCAAACAGATCAGCCCCTATGGTCTTGGTGTTTATGTTGCTCCCCGGGTTGAAAACGCTCCGC
CACACGTTGGCCTTCTTCGCCGTCTCCGGCGGCATCACCGGCGTTCGTGGTGTCTCCGGCATCGACATTGATCGTTGGAACGCCTTGCCAGCACTGCTTTCTCCTTCACCACCTTGGTTGA
TGACGAGGGGCTTGGGGGAGGCCTTTCGGCGGAGCTTGCCGAGGCCTCTGTCGGGCTGAGGTCCGGCGAGCACATCGTCCCAAAGCTTTTCGAGAAGAACCATTTTAATAATATATTATT
ATTGTTATTTTCTTTTAATCAAATTGTTTCTCTCTCTACTTTCTCGC
>comp149799_c0_seq1
AAATTTGCAATATGAGAGGTGCATGAGGGAGGGGTGTTTCTGCAATAATATATTTGTTGTTTCCTGCCAGGGGAAGTAGAAAGTACTGGATGGAAGTCGTTTTCCATGTTTCCTCGTACTG
GATGGACGATTTTGCTAATTTCCTTCTTTCCTTCCTTCCTTCTTAAGCTCTAATTCCGCACATTCTGCAATTATTATAAAATTGCAATTAAGCCAACCATTATTTTCTCCATCCTTTGGCCTCGT
CGTTTGTCGCTTAGCACCCCAATTATTTTATAATATCAAAAAGGGTTCCGATGTGGTTCAATTCGAAGAGGTAGAGGGAAATGGGTGATTTTGAGGCTGGAGAGATGGAATTTGATGGAGG
AGAGGCTTCCAGCGTGAACGATAAGAAGGTGGAGGAGTTAGCCGGAGAAGAAGAGAAGTCTATGACACCGTCGCAGGTGGAGGATATGCTGATTGATATGGAAGAATCAGCTCAAAAG
TTGATTTTTTCGGACGAAGAAGACGCCTTTCCTTCACTTGCTGATTTCGGATGCCTTTCTTCTCCATCGGCGTCGGCCTCTTATTCATCGGCGGCTTCCACTCTTCCCAAACAATCGACTTC
CTCCTCTTCTTCTTCCTCCTCTTTCTCATCTTCATCACCGTGGTCGTTCCTCTTCACCCCCACTGAGACTGATGGTGGCATGCACCCAGCGGCGGCGAGTGAAGACCCTCTGGCTCCCATC
ACCGACGCCGGATTTGATATTCTTGGTGATGATATAGATCTATGGGAGACCTGCACGGACGAGCCTATCGAGTCCACGTGGGATTCATCGCGGTCCTTCTTCCCGGACGAAAGAAACTGGG
CCGGTGGCAGCGGCGGTGACCAGCAACCCGGGAATGAAGAAAAGTTGGGATTTTTGTCCGGCGGGGATGATTGCTCGTCGGAGGATTTAGCTAACGTTTTCTTAGAATGGCTGAAGGAA
AACAAAGATTTGATTTCGCCGGAAGATTTGAGGAGCATTAAGCTCAAGCGCTCGACAATTGAGTGCGCGGCTCGCCGTCTCGGCGGGGGAAAACAGGGGCGAGTTCAGCTTCTCAAGC
TCATTCTTGATTGGGTTCAAAATCACCATCTCAAGAAGAGAAGACGACGCAACCGCGGATCAACAAGAGATCAGGAATTGCCAACTTACGCTGCTCCACCGCCTCTCCTTCCTCCTCCAA
CGATTCCTCTTCCACCTACTCATATCGCAACTACTTCTAATCCTAATATTGACTTCTACCAGGAAACTGTGCCCTGCAACTCTTGGATGCCTTATGCAGGGGATTCCTCTTGCCCTCCGGTGA
TGCCCGGTTATAGAGGCGAGGCTCCTGCTGTAGCTGTGCCGTCTTACCATTACTACAATGACCAGGCTTTCTCAGCTCATCCATGGCAGCAGTTATTGTCACCGGCCTCGACATCATCAGT
GATTCATTACGCGCCATTTGGGCAGCAGTCGCCTTCACCAAGCCTGCAAAACTTTCCAGTGCCGGTGCAGGTGCCGGTTCCGGTGCCTGTGCAGGTGCCGGTGCCGGTGCCTGTGCCAG
TGCAGGCACCGCCGCAGTATTTGCCAATGTATTATCCGGGCCAGACGGCATCGGCTACTAAAGAGGCTAGGAAGAAGAGAATGGCTCGGCATAGAAAATCTTCCTCTCTTCACCCCCACA
GAAGCCAACAGCAGCAGAACCAACAGCTACAGAGTTCGGATAATATAATTGTAACAGAGGAAACTTCTTCTTCTCACTCTAGTGTTAATAAAGACAATGCAAGCAACTTGCCATCTCTAC
CCGCCGACATTCCGTCTTCGCCTGCGGCCACATTGGCTGCTGTGGCGCCGTCTCCTCCCACCATAACGCCGATGCATGAGGAGAATTATCTACACAAGCGTACCACTTCACCTGGAGAGA
GACGTCGGGACGGAATCAAGTCGGAAAATAACTATAAATTTTTGCTTCGGAAAGTGCTGAAGCAGAGCGATGTGGGGAGCCTTGGAAGGATTGTTCTTCCCAAAAAAGAAGCAGAGATT
CATCTCCCAATGCTTGATTCAAGGGACGGAATACCAATTGAAATGGAGGATATAGGAGCCTCAAGGGTCTGGAATATGCGTTATAGGTTTTGGCCGAACAACAAGAGTAGAATGTACCTCT

TGGAGAACACAGGGGATTTTGTCAAATCAAATGGACTTCAAGAAGGAGATTTCATAGTAATATACTCCGATGCCAAGTCTGGAAAATATATGATAAGAGGAGTGAAGGTCAGACAGGCAG
TGGATGTGATCAAAGGCACAAGCAATAGGAACCCAGCTGGAAAGGCTCATCAGAAGAAGGAAAAGAGCAGCTCAACCATGGCTGCTGTGAGCCAATTGCTGGCCGATGACAAAGGCA
AATCTCTTTGTACTGATCAGGAATAATAAAGATTGAATATTTTGGCAAATCTCTCATGCATGCCAAGGATGCAAAGAACTCTTAAGAAACAGCATTAAGAATGTATAATTTTGCCCGTTGGC
AATGCCCCCATTCATTTGGATTATTCTGTTTATATATTTATATCATAGAATATTTCCACAAATATAAAAGTACTCACGAATAATATTTTCTTCTGCAGGAGGTAGTTATTTAGCATGCATGAGAA
AGGTAGATATGATTCATCAAATCATATATGCCTGCATGCTTAATTTCAACTTCTATTTTCCTA
>comp149802_c0_seq1
AGGTTGAGAGGGCAGGACACTTAATTATTGTCCACTCATAGCAGGAAGAAGTTAAACCTGGTAGAAATCACAGAATCACTTTACAGATGACTAAGCTATCGTAACAAGCAATTCATATATG
TATAATCATAAATATAATATATGCAAAAAATAATCTCCCCAAATATGCTCTTATAGTATTTTGATTCAACAACAATGATAAATTCTTTGGCGCTAAGCATGTGGCATATATAGAATTAAAAGACC
AACAAAACATAAGGAAAAAATAGTTCCTCCTTACAGAACTAAGGAAGGTTTTAAAATGGATGAGAATACAAGACAAGGCTAGCTTCTCAAGCAATCTGTAAACTCCATGGCTTATGTCAG
CCATGGGTGTTGAGCGCCTCCCACACCAAATTCCTTGCAGCCAAATTCTCAAAATTACGTGCTTGCAGTGCTGCATTCCTCAGAGTTGCCACAGTCATCCTGTTTGCTTCCATGAAACTGA
CGCTCGAATACTGTAACTTATGCTTCTTACAAAGCTCCTTCACAAGAGGAACAATTCTCCGAAGATGGCACCTTGGCAAGCGAGGAAACAAATGGTGCTCCACCTGAAACTGTAATCCGC
CATGAAACCAATCCATCCAAGGAGAACAAGAAATGTCAAGGGTTCCCATTGTCTGCTTCTCGAACCAGTCATTCCCCTGTGGCTGACCAACATAAACACTGGAAGAGAAATGGCTGAGA
CAGAACTGAACATGCTGAAGACCTGTAACTACAAAGCTGGCAACAACAAACATAGCCCTTTCGCCCCAATTCGGAAGGAATGAAATAAGGTAAGGGTACCAAATCCAGAACACGATCAC
CCCAACAATCTCCTGCCATCGGGAGGGGACCTTCTTCTTTGAGAGGAGGAGGACTATGGATTGAGCAAAAAGATTTAATCTTGCGAAGCACATTACAGGGTAGAAGGTTAAATGCTGGTA
GCTGACCATGAACCTTGCAATGGAATCAAAGGTCATCGTTCTTTCGTAGAAGTAGGAAGTGAGGGAGTTGAATAACTTAGAGGAGACGGCGAAGAGGGGAATATGTTGCAAATCTGGAT
CAAAATCGAGACTATTGCAGGCTATATGATGGGCATTGTGTGTTCTCTTCCACCATGCAATACTTATTCCGGTGAGGCAATTTCCTGTGACGATTTGGGCCAATCGATTCAAGTTCGGGTTG
AGCATGACGTTATAATGGCCGGAATCATGCCCTACCCATCCAGATTGGATCCATAGAAAACCCATAATCCCGCCAGCGACAAGGTGGGCCCAGAGGCTGGTTGCGCCGACTACCAAGTAG
ACCGAGGTGAAGAAGAGGGCGAACATAAAACAGAAAGAGAATAAAACCCCATGCCCCTTTTCTTCGAAGAAACCGAGCTTTGTGAACTCGGCGACAAGGCGGCGGTAATCCTTAGATA
CTTCGGAAGTTTGGTAGTCGGATAAGTAATATCCAACGAAGAATCGATCAAGAAGCTTCCAGGCCGGGGCTGGATGGTGAGCGACGAAGGCGTCGGTGACGTCCTGGCCGGCGAGATTG
AGGAGCGGGATTTCGCCGCCGGGATGGTCTTTGACCCAAGTGGTTACATCGTACACCTTGCCCTGGATTGATATCCAAAGATCCGACGGGGCTTTGTGCTTCTCAAGCTCCTCCGACGAA
ATGTACTTCCTTTGCTCCGTTTCCATAACCATGGCGCCGGAAGAAACGGCTTGGCAGCCCTGCTTCCTTCGGCCAAAGAAGCAGAAAAGAAAACCCTAGATCTCCTTCCCTCCTCTCTAG
ACGAGAAAAGAGGGTACGAAATGGAAAAGGAAAAAAAGACGGCCTTTTCTCTTCTCTCCGATCTTCTCTAAATAACTCGTTTTT
>comp149812_c0_seq1
TACATCGACAACCACACAAAGATCAAATACTAAGCTTTAAAAGAGGTGCATATAGTGAGATGACTAAAAACACACATTTTCATTAAATAAGCTACTAAACAGTAACAATTATATAACTATTG
AATAAAATATTAAGACAAGTGCCATGTAAGATAACAATCTTGCCAGGTTAGAAGATTATTAGCTAGACAAACAAAGGCATCAAGAAGCTCATGCTCTCACAAATGGATGCTTAGAAATGGT
AATAGACTGGTGGAAAATATGCTAGATTGGAGGGGGTAAATCATGTAGCTATATGACCGGAAAGCTACAGGCCTTGGTCTATGAGGTAATCTCCCAGCCTCTCCACCACCATATTGCATTGC
CCTGGTGTCATGGGCTCATCATAAATGCCAAAAATAATTGCTTGATTGGTCTTCTTAACAGTGACGCCTCCAGAACCCTTCTTTCCACGTATAACAGATCCTGCCTCTCCCTGGATAACCAT
ATATTTGTTGGGCCCAAGAAACAAGCCAGTAGGAGCTAGGGATCCTGGTTCATTGAAATCATTCATAATAGCAGTGATTTCCTGAGGCTTGAACTGCGGGAAGGAATCGCTCTTGGCCCA
CACGTTGCCATCGTGGCCGACGATCGCAGCCGCGGAGAGGTGGTGACCGTCAATGTCGCACATCAGATGCTCATCAACATACGTTTGCCAAGACATTTTCGCTATCTTCCTCAATTCCTAT
CGATCTCTGTTCTCTCCTCTCCTCTCCTCTTTCCTTCTCTCTCGCTGCTCTTCCTCTAAATTTTGAATTTGGAGCAATTAATATAAATATTAGTACGGAGAAAAAACCGGATCTCAGGCGCTA
GTCAGCCGCTCACGAAGTCATATTAGATTAGAGCCCATCGCGTCCGCTCTTTTTAGTATTTTCTTTTAATTAATGAAATGTGAAAATCTGTACCCATTGGCTTGGAAAGAGTTAGGAATCTCC

GGCCCAACGGAAGAGCAACAGTTTCCTATTTCTGGTTGCCGGTCTATGGACCGGTGGCGGATTGCCGGTTACCGTTAGGCCGTAGCGCGCCAAATTCGTTATGGTGGGGCCCA
>comp150057_c0_seq4
ACACAGATCGTTTCCATTAAGAGCAACAATCAGAAGACTTCCAAATCAATCTCTTCTCACTGTTAAAAGACGACGATACATTTAAATATATATTTATTCATAATGCCCCCCGACATGTATTTC
ACACATAAATTAAATATTATGCAGATAGATTTATATATATTTATGAAGAATGGTTATGAGGTGTTTGCAAAGTTCCTGAGGCCTCGAAAGCATGACCATATGATCTGCCTCAATCTCCATCACT
TCCTTAACCTGATTATTACTAATCATCCAACGCTGGTAATCTTCAGTAATGGCTGCATCTTGCTTGCAGACGATGTATACTTTATCAACTGAGCCATATCCTTCTTCAGAGAGAATGGGAGCC
TTCTTCAGATCCTCTTGGAATAGTGATCCAGTTCTTATCAGTGCATTTGCCAGAGTCAAATCCTTACTTACCATAAAAAAAAATTATCATTTACAGAATTCGAAGAATATAAAACCACAATAA
TTTTCATTAATTAATGATTCACCTCAGGAGCACAAAGTTGGTACAGATGCTTTCCCATGAACTGAGGGCCAAACAACATTGTGATGAATGGTTCTTCTCTTTTTGGAACTCTCACCTCTTCG
AATTCAGTATCCATCCATGCAACAGCTGAAATAGACTCCCCAAGCTGCTTTTGTCACACATGAAAAGGTTAATTTTTCAGCAGAAAAGTAATCAGCAGAAACTATATTTCTGGCATTTTTC
GTCGGAAGAACAGCCGGAAATATTTGTGGCGAAAAAAATTGCCGGTGTTGTTTTCTGACGCGATATTTCCGGCGTATTTTGATTGTCATACCTTACTAAAAACAGATGGAGGGGGAGAAA
GAGTATCTGGCATGAAAGCAGATACGAAGACAGCAACGGCTATTTTTTGAGGAAAAGTTTCCATAGCGAAAGCCAGATTCAGTCCTCCTAAGCTATGACCGACAAGGACGGCTTTTTCTC
CTTCTGGAAGTTGAGCCAAAGTTTCCATCAAAGGCTGGGAGTACTCGGCGAACGTGAAAACATCTTCGACCATTCTCTTTTTGTTGATGCCCGATGCGGCAAGATCTAATGAGAGAACAG
AATGGCCATCGGAGCGCAGAGCGGCCTCCACTTTGTACCAAGTCCAAGCTCCATGGCAAGCACCGTGTACTAAGATGATTTTTAGTTGCTGCTCTGGAATCATCTCTGTTATGCTTCTTGC
ATGAGGAAGAAGATGCTTAATGAGAGAGAAAGAGAGAGGGGGGACAGGGAGAGAAGAGCTGCAGGAATTTCTTATTCCTTTATTCAATAGGCCTAATGGCTTAGTGCAAGAGG
>comp150057_c0_seq7
ACACAGATCGTTTCCATTAAGAGCAACAATCAGAAGACTTCCAAATCAATCTCTTCTCACTGTTAAAAGACGACGATACATTTAAATATATATTTATTCATAATGCCCCCCGACATGTATTTC
ACACATAAATTAAATATTATGCAGATAGATTTATATATATTTATGAAGAATGGTTATGAGGTGTTTGCAAAGTTCCTGAGGCCTCGAAAGCATGACCATATGATCTGCCTCAATCTCCATCACT
TCCTTAACCTGATTATTACTAATCATCCAACGCTGGTAATCTTCAGTAATGGCTGCATCTTGCTTGCAGACGATGTATACTTTATCAACTGAGCCATATCCTTCTTCAGAGAGAATGGGAGCC
TTCTTCAGATCCTCTTGGAATAGTGATCCAGTTCTTATCAGTGCATTTGCCAGAGTCAAATCCTCAGGAGCACAAAGTTGGTACAGATGCTTTCCCATGAACTGAGGGCCAAACAACATTG
TGATGAATGGTTCTTCTCTTTTTGGAACTCTCACCTCTTCGAATTCAGTATCCATCCATGCAACAGCTGAAATAGACTCCCCAAGCTTACTAAAAACAGATGGAGGGGGAGAAAGAGTATC
TGGCATGAAAGCAGATACGAAGACAGCAACGGCTATTTTTTGAGGAAAAGTTTCCATAGCGAAAGCCAGATTCAGTCCTCCTAAGCTATGACCGACAAGGACGGCTTTTTCTCCTTCTGG
AAGTTGAGCCAAAGTTTCCATCAAAGGCTGGGAGTACTCGGCGAACGTGAAAACATCTTCGACCATTCTCTTTTTGTTGATGCCCGATGCGGCAAGATCTAATGAGAGAACAGAATGGC
CATCGGAGCGCAGAGCGGCCTCCACTTTGTACCAAGTCCAAGCTCCATGGCAAGCACCGTGTACTAAGATGATTTTTAGTTGCTGCTCTGGAATCATCTCTGTTATGCTTCTTGCATGAGG
AAGAAGATGCTTAATGAGAGAGAAAGAGAGAGGGGGGACAGGGAGAGAAGAGCTGCAGGAATTTCTTATTCCTTTATTCAATAGGCCTAATGGCTTAGTGCAAGAGG
>comp150075_c0_seq1
TGAAAGGGAAATGATAGGTACGAGCTTTTATATTGGTGAATCCACCTGCAGTCAGGCGCCAATGCCTGAAGCCCAATCCAGGTCTCCAATGGCGGCTTCCTCAGCTTCGTCATCTACAGCT
ACCAGCGCTTCACTCATGGCGTCGAGCAAGGCTCTAATCCATATGCCTCGTCCGGAGTTTGGAATAGCAGGGAAAAAATACTTCCCGATATTTCAATATGAGGTGAGGCAGCTCTTTCCCG
TGCTTGTTTAACAGTGCAATGTAGGATAATGAGCGTGGTTGCTGTGTGTAATCTGCATGTTGCCGATATTTCTCTGGTTTTCTATTACGTTCTATGATTTTTTTTTTCATTGTTTCGGTTATCAT
TCTTTTAATTCTTTAATTAATTTCTTAATCGACTCCTTGATATGGTTATTTTTGAAAATTTAGATCTGATTGAGTTGCTTACTTCGATATGTAGCGATATTTGTT
>comp150076_c0_seq1
CATCAATCATAATATTAACAATAACGAACTATAAAAAATTATCAATGACATAATATTAAAAATTTTAGGATTAGGGCTTTATAAACAATAAATGTTTTAGTGCCTGTGACAGACACTATCTAAT
GCAATAAAACTGTTGTTCATGAAAGCTAAAAGAAAATATATCTTCATTTTAATGGATTGATTACTCATCATCTTCATCCTCATTTTCATCATCCTCACGAGTGCTGAGCCCCTTTCCACGCAA

GCTATTTTGAATTTGATTTGCAATATTTGGCGTTAGCCGATCTGAGAAGGAATCCAAAGCATATCTAAGCATAATCTCCACTGCCTGCTCCACTTTTATCATATGTTGATGTGATAGTCTTGAT
CGAGATTGAGCCTTGGAAGAGTCATCTATGTGCACACCGCGAGTAGTCCTTTTGAAATTGGAGCTAAGATAATTGGATATGTGCGTATACAGTTGGGATTTTCCTGAGTTTGTTGCATCCTT
CATCCATTTTTCAATGTCAACACCACCCTTATATTTACATGAGTCCCCACACCCCCCTGCAAAATTGGCTGCATAATCATCCATCTTTTGATGTAAGTGTTTATCAATAAACTTGTCTGTACCC
TTCTTCATATGTGTCATGTTACATAACTCTGCTTGTGTGGGCAACTTCTCTTGTGACATAGACATGCTGTTACAAGGAGTGATGAAAGAGATGGCATAACCATCATGCTTTGCTAATTCACCT
TCCATAAGGTGGTTTGAATGAGCCACTTGTGATCGATCATTCTGGGCATCTATATTGCACTTATCAATCATTTGTAAGGACATGCATTTTGCAATCCTTTTAGGATGGTTATCCGCCCAAGTA
GAGATATCCTTAGAGCTAGTCCCCATTTTCGCAACATTGTGTGTTTCGCTTAAAATATGACTGACATGACTTTGTATTGTATCCTCCCAAGTACGTCGAAGAAGTGTACCCTGGGGTTCATC
AATGTAGCTATAACAATGCAGAAAATACTTCCATTGCCTCTCCCTCTCATCATGTTGAATGTCTCTCCAAGACATCCACGGTTCCATCAATTCCTGTTTTAGACTTTTGAATGCCCGAAGAG
ATGCCTTTGGATTATTGAACTTCTCAGCTTCTGGTTCATTGAAAATTTCTTTTGGTCCATTGTATCGAATCACATCTTGCTCAAGAGTGAGTTCCTCTTGCTCCTCAATCTCAATTTGCTCGT
CGTCTGGTGTCTTCACTTGTTTATCATGAGATCCATGGGTGGACGCCATATCTTAGAGCAACGAGCGTCTCTTTCCTGTAGGGTTTTACTATCGTTGCTACTCCTATGGTTGCTAATGAGGCT
CATTTCTGACTACCTCCACAGCACCATCGGGTAAAAGTTTATAAGAACCCAAAATAGTAAAAGCAAAGTAAGGTGGGTATTTT
>comp150077_c0_seq2
GTTTGCTCCCCTTTCAGAATTAAGGTTGGTTCCACCCTTGACCGAAGCCAGACAAGAGCACGAGAGAATTTCTGAAGCCACTGACTATCAAGAATATTGACAATAATGAAAAACAGGAG
AGCCAAACGAGTAAAAAATTTAAAATGTGGGAAGCAACAAAGAGGATGATTTTGATACCTGGAACAAATACAGCAGCACAATGATTTTGATCCCCATACCAAGCAATTATATATGATTGAT
GAAGATTCAACCAACAAAAGAATGATAACATATGAATATTAGCCTCTTCAGTCAAATATTGAGCAAAAACAACAACAAAAAAGTGGCAATCCAACTCTCATTATTATCTCCCCTTGTAGAG
ATAAAAGATAAGAGGTTGTCAAGATAAATCCCCATAACTGAGGCGACGAGCCTGAGAACCGCATCCGCCATGGTAACGGTAGATAATGCCAAGCACGCATTCTGTCATGCACGATACATC
GAAAGACTCGATGAAGCTAAGGTCCGAAATACGAGCTTTCGTGCCTGCAAATCGCACGTACTCTTCAAACACTGCAGTCAAGCACCAGTTTTGTAGTTTCCTGAAACAGCCGATAAGGC
TACCCGTCCGGTGCTTTCCACGCTTGCAGTGAACGAGGACAGGATGGTTCCTTATATCAAGCAAAACTCTCAGCGCTCCCATGATGGAACCATTCGGTATGGTCACCAAAGGCCTCTTGCT
ACCGTCAATTCCAAACTGAAAGATCCGAATCCCAACCGAATTCATATATGCCGCCATGTCATCCGGGTAAGGCTCCGGGCACAAATAAACGACGGATCGCAGGTTCAGCGTCTCAAGGAA
TCCAATGTTCATTGTGCCAGGAAATCCAGAACGGTATATACCGTTGTCCACCATTGCAAAATTGGACGGTGGGAGCAACAACGCTTTACCACCGTCGTTGAAGTCCCCGCCGGGAAAGCG
CGAAATTCCATCACGATCGTTTCTTCCTCCCGATTTCAACACTATCATCTCCTTAATCTCATCATCCTCTTTCGCCTCCAGAATCATCCCTCCCCCTCCTCTCCGAATTCTACAGCTTGATCGA
AAGAAACGAGAAGAGGCGACCACTCTCCTCTTCTTTCTTCCTTTTATGCCTTCCCGCTATCGACCCGTCGCACTTTCCG
>comp150083_c1_seq3
GATCAATGTACTCTGGTAACATGTCACTCAAATGATCTATAAAGTATATAAAATTTGGTCTAATACCGAGAACAAGAGTGTGATACAAGGAAAAAGTCTGATTTAAACCCACAAACAAGGA
GAAACCCTGAGATCCTTAACAGGGATCGAATGAAAGGAAGAACAAGAATTGAAAGCAATTAGCACAAGCTTGGAAACAAATATATCCACTATCTTTGCTTTTAAACTATTATCTATCTTCC
TTTCAGTATTATCGATCACCCTCTCTATTTTTAGAAGTACTCGCGACAACAAAGCAATATAACATATATAATCAAAGTTTTTGGTGCGTAAGCATGGGTTTTGCTTTGCCGGGCAGATCAATT
CAACATTTTGTCCATTGAGGTCAAAAGCCAGGACCATCGATAAAGGTGTGATTTTGTAAAATCTTTTATCTTCTTGATGATAGTAAACATCCTCAAATTGCTCCACGTGCATCATGGCGTAA
AGAGGCAACTATGGGAAAAGAACCCATGTTTGATCTCCTTGCTTTGCCAACGGCAGAGATTTTGTACGTCCATAGATATCAATAGCAGTTCCTAATAAGTCATTAGA
>comp150083_c1_seq4
TCCTTGGAGCCAAAGCAACGACGATCGATGATGTAACTATTAAGGGAGGAGAAGCAACGATGGCAATAGACACCAATCTCTTCTTCTTTTATTGTAAAGTCACAGAAGGTGAGTGAATTG
GAGGCTTCTCCAGCTTCACTGGGAAGTAATAACCAAGGCGATGGATGGAATTGAATTCCATTGGGTACATCGTTGAAATGAATAGAATTCCATGAGGTGCAGACAATAGAGAGTCGAAGG
TAGTCTACAATGGGCAAATTCAATGAGATGTAATGGAGGAGATCTATTGGGAGATCAGACCATGTCAAAATATTATTGTGTATGTGGTGG

>comp150083_c1_seq5
TAAAAATGGACTTATAATTTAAAAATATTTATATTACATGGTTAGCAAAGATTAAGATATACAATTTAAGAAACTGATGGTTTAAACCAAATTGAAGGGGGACAATTTGGCTGTGAATCCGA
GTCAAGGAAATAATGAAATTGTTCACTATGAAGATCAAAAAGCCCAGCATCCTGATCAACGTACTCTGGCAACATGTTGCTCAAATGATCGGCAAAGTATATACATTTTGGTTTAATTCTAG
GAGAAAATGTGTGATGCAAGGAAAATGTTTGATTACATCCCACAAATAATGAGCAACGCCCTAGATCTTTAACGGGGGTCCAATGAGATGAAGAACAAGAAGCGATAGCAGTCGGTACA
AACTTGGAAACAAAGATACTCATTGTCTTCATTTTCCAACTATTATCTATCTTCCTTTTTGTAGTATTGATCACCCTCTCTATTTTTAGGAATGCTTCTGACAAAAAAGCAATATAGTACATGT
AATCAGAGTTTTTGGTGCGCAAGCATGGGTCCTGAGTTACCGGGCAGATCAATTCAACATTTTTTCCATTGGCGTCAAAAGCCAGAACCATCGAGAAATGTGTGATTGTGTAAAATATTTT
ATCTTCTTGATGATAGTAAACATCCTCAAATTGCTTCGTATGCATTATGGCATAAAAAGGCAATTGCGGGCCGAGAACCCATGTTTGATCTCCTAGCCTTGCCAAAGCAAGAGATTTTGTAC
GTCCATAGATATCAATAGCAGTTCCTAATAAGTCATTAGAAGAGACAATGATCTTCTGAAAATATATATCACGTAATCCTTCCTCGTCTATGGTAAAATATGAGTAAATTTCATTACAAAAAAA
TCTAATAGACCTGTTAGAGCCATGTTTGGGGGTTATCATATGCCCTTTGTCTTTGGGGATGGTGAAAAGAGACGGGAACGATATTTCAGCCTTTGTGAGAGGG
>comp150083_c1_seq8
TTCCTAATAAGTCATTAGAAGAGACAATGATCTTATAAAAATATATATCACATAAGCATCCCTCGTCTGTGCTAAAGTATGAGTAATTTTCATCACAAAAAAAAAACTGATGGAGTTGTTGGG
GTAATATTGGGGCTTTATCATATGCCCTTGATCTTTAGGGATGGTGAAAAGAGATGGGAGCGATATTTCGGCCTTTGTGAGAGGGTTAAAGAGATGAGGCTGCATATCTTCTTTGTCAAGGG
TAATGAGTCATCCATCCTTAAAGCCAAAGCAGCGACGATTGGTGTTATAACTATCAAGGGAGGAGAAGCGATGATGGCAACTGACACTGGCCTCTTCTTCTTTGATTGTAAAGTCATGGA
AAATGAGTGTGTTGGAGGCCTCTCCAGCTTCATTGGAAAGCATTAGCCATGGCAGGGATGACATTGAATTCCATTGGTTGCATTATTGGAAAGAACATAATTCCATGAGGTGCATACAACG
AAGAGTCGAAGGTAGTCAACAATGGGCAAATGTGATGAGATGTAATGGAGGAGATCTATTGGGAGATCAGACCATGTCAAAATATTATTGTGTATGTGGTGG
>comp150083_c2_seq1
CTGCACCTCATGGAATTATGTTCTTTCCAATAATGCAACCAATGGAATTCAATGTCATCCCTGCCATGGCTAATGCTTTCCAATGAAGCTGGAGAGGCCTCCAACACACTCATTTTCCATGA
CTTTACAATCAAAGAAGAAGAGGCCAGTGTCAGTTGCCATCATCGCTTCTCCTCCCTTGATAGTTATAACACCAATCGTCGCTGCTTTGGCTTTAAGGATGGATGACTCATTACCCTTGACA
AAGAAGATATGCAGCCTCATCTCTTTAACCCTCTCACAAAGGCCGAAATATCGCTCCCATCTCTTTTCACCATCCCTAAAGATCAAGGGCATATGATAAAGCCCCAATATTACCCCAACAAC
TCCATCAGTTTTTTTTTGTGATGAAAATTACTCATACTTTAGCACAGACGAGGGATGCTTATGTGATATATATTTTTATAAGATCATTGTCTCTTCTAATGACTTATTAGGAACAGTTGTTGCTA
TCTATGGACGTCCAAAATATTTGGTTTTGGCAAGGCCAGGAGATCAAACATGGGTTCTCGGCCCACAGTTGCCTCTTTACACCATGATGCACGTGGAGCAATTTAAGGATGTTTAATATCAT
CAAGAAGATAAAATATTTTACACAATCACACATTTCTCGATGATCCTGACTTTTGACCTCAATGGACGAAATGTTGAAGTGATTTGTTCGGCAATGCAGGACCCATACTTGCATTTCAAAA
AGTTTGATTACATGTGCTATATTGCCCTTTTGTCGGGAGCACTTCTAAAAATAGAGAGGGTGATCGATAATACTGAAAGGAAGATAGATAATAGTTTAAAAGCAAAGATAGTGGATATATTT
GTTTCCAAGCTTGTGCTAATTGCTTTCAATTCTTGTTCTTCCTTTCATTCGATCCCTGTTAAGGATCTCAGGGTTTCTCCTTGTTTGTGGGTTTAAATCAGACTTTTT
>comp150091_c0_seq3
GATCGTTGAAGGCATCAGTTCGTTTAATACGTCTGCTCACAAATTTCAAACAAACCTATAAATATCTCTCTCTTCTATCGTCCGTAGACTTCCCCGAGAAGTCCGTGCTTTGTTGCAACCTC
CATTAAACAAAGAGAGGAGAAGCATCTTCCCCTCTCGCTCTCCTCCCTCCTTCACTCAATTTGGAGCAGAGCTCGAGTCGTCGACGCCCAATACATGACGGCCTCCGGCGTCGCCCCGCC
GGAGGCGCCGGCAAAGATCATCTTCGGCAAGTACGAAATTGGCAGGCTCCTCGGCTACGGAGCCTTCGCCAAAGTCTATTACGCCCGCCACATCCTCCAGGGTCACAGTGTAGCGATTA
AGATCATCAGCAAGGCAAAGGTTATCCGCGGCGGCCTCGTCGTTCACACCAAGCGCGAGATCGTCGTTATGCGCCGCCTCCACCACCCAAATATCGTTCGACTCCTCGAAGTCCTCGCCT
CCCGCTCCAATATCTACTTCGTCATGGAGTACGCCAAGGGAGGCGAGCTCTTCTCCCGCGTCGCCCGTGGTCACCTCTCCGAAGACCAATCCCGAAAATTCTTCCACCAGCTTATCTTCGC
TATCGCCTTCTGCCACGCGCGCGGCGTCTTCCACCGTGATCTCAAGCCTGAGAACCTCCTCCTCGATGATTCCGGTAATCTCAAGGTCTCTGATTTCGGCCTCTCCGCTGTGGCCGAACAG

ATTCGCTCCGACGGCCTGTTTCACACGCTTTGCGGTACTCCGGCTTACGTTGCCCCTGAGATTCTCTCCAGGAAGGGCTATGAAGGGGCTAAGGTCGACATTTGGTCCTGCGGGGTCATC
CTATTCGTTCTCAGTGCTGGATATTTACCCTTCAATGATCCCAATTTGATGGCATTGTACCGTAAAATCTACCTCGGTGAGTACCGATGCCCTAAATGGATTTCTCCCGAGCTCCGTCGGCTG
CTTTCCAGGCTTCTTGATACCAATCCGGAGACAAGGATAACCGTGGAGGGAATTCTGAATGACCCCTGGTTCAGAAGGGGGCTGGAAGAGGGAAACATGTTTAGATTGATGAGATTCCG
GGAGGATATCGAAGAACGGATTGCGAGGATCGAGAGGGAAAACGATGAGGACGACGACAGAGATCTCAATGCCTTCGATATAATTTCCCTTTACTCCAGATTTGACCTATCCGGCTTCTT
CAATTCAACCGTTAAGGCGCGGAAGCGTTTCGTCTCGGCCGGGCCTGTCGATGCGGTGCTTGTGAGGGTGGAGACGGTGGGAAGGGAAGAAGGGCTCGTGGTTCGCCGGCGGCTGAG
GAGTTTGAAGGGGCGTGGCGCCGCCGCCGTGGAGGGTTGGATGGGGAACCTCATTGTTTGGGTGGACGTTTACCGTCTATCGCCGGAGTTAGTGATGGTGGAAGTAGAGAGAGGCGGC
GGCTTGGAGGCAGCGCGGTGGAGCTTTGAGTTCTGGAGGGAAAAGCTTACCTTGGGTCTGAGAAGTTCGGTGAGCGAGCCGCCGACACCTGTCTGCGCGACGTCCGGTGAGTTGTCGA
GCGGCCCGTCTACACCGGTTACCGGAGGGGCAAAAAGAATAGGGTCGGCGAGCGAACCGGCGTCGCCGGTCTACGTTGAGGAGAAGGTGATGTTGGAAGGCGAACCAGCGATCAACG
GCAAATGAATGATATTGTTCCCATCTTAGTTCGACCGTTGGAAGGCGCGTGATGGTATCTACACATGCATGGATCAGACTGGCTCATATCCTTGCATTATTCTTTATAAGTGTAAAATTTTATT
ATGCATGTATGTATGCACACTCGATGGTAATGCATTAAATTTTTTTTTTTTTTGTTCTAAGAATAATAGTTCATGTATTCTTAAAACTTCTTGGAAGAAGAGTTGTGGTGCTCTTGATGAAGG
GGGGCTTGGGTGTAAAAGTATTG
>comp150093_c0_seq1
GAAACTAAATGATTTTGTGCAGATAATTTTGGTGCTATATCAACAGTTTTTCTCTCAATAACAAAAGAACGAATCAAAAACTATTACTAGAGTTTCTATTTTGCATAAATTATCAGTCTCATAT
CACTTGTAAATTAGGGTTCATAGCAAACATGGTTCAAAGTATCAACAAACAGGCAATGGAAAGCATAATAAGCAGCTCATCAATTCTAATAGATCAGTTTTTCAGATGCTCCACAGCTTGG
CCAAAACTCACAGCAGAATATTAACATGTTCATTCATGAAAGCCATGTCAAATATACAGAAGATGGCCAAGCCAAGAACACCCCAAAATCAGAAATATAAATTTAGTTACCCGCCGATTTG
GTGAAGCATGGCATCGATTTCATCTCCATCTTCCATCTCAAGCTCATGGGGAGTCTGCTCACCGCCAATCCTACGGCCATCGAAGAGGAAGGCAATGGAGTCCGAACCAATAGACTGGCG
CTCACAGTATGCATTTATTAGCTTCTTCAGTTGAGTATTGCGCTTGATCCTGAAGAAAAGCTCGTTGCCATCCTGCCCTTTCACCTTAAGATTGATATGCGTGCCACCCTGATCTTGCTTCTT
TTCCTCCCCTGCGTTCGTTCCTGACATTCTTTTATCCCCTCAATCGCTCAGAGAGAAACTGAGAGAACCAGTGCGTAATCGAAGGAATAGGAAAGGGCCGATCGAAAACGAGAAGCCTA
AATGGCCGTTTTATAGGGACATTCAAAATTACAGTTAATTGATTTTTGGAGTCAGTATCAAATGAAACGGCCTAAGCAAACCAACACAGGCTGGGCGGGCCCTAAATATGGCCAAATTATT
ACGTTGCGGAACGGAAAGCGTGCCGAGGTCGGATTTAATATAGGAGAATATTATTCTCTTCTAAATTTGTATGGGGAGAATCTATAAAAATTTGATACATATTATGTACAAATATGTACACAG
TTCATATATCCGAACCGTTATTTCCGTCTAGGCTCCGCATAGAATAGTTTCTCCCAATATACAGTGTATAAGCCTGTCGGAATTGGCCTCCAGACAAATTCGACATCCGCACGTAATAATGAT
AGCTTCGCAATTGCAACAAAAAATTGAGCGCCCCCTAAAATCCTTGTTGGGGTGCTGCCCTTGACCCCGCAACTGGACGCCAAGTGG
>comp150105_c0_seq1
AGAAAGTTATTTTCCACATCAGCATTGATAAAAACAAAGTTCCATAATAATCAAAATAACGGTCTACGAACACCAAATCTATCAATGCTGATAAAAAAACAGTCCATCACGAAAAACCTAA
TAATTGGACAAAAGCAGTCAACCTTGATATTTTTTTGACTAATTAAACCCTAAAGCAAGTGCTAGATTTCCAACCGTCAACAAAAAGATAGCGAGTGAAGTAGAATGCAGATCATTGATAA
CAGAGTCTGATGAGTCGGTATTGTTCCTCTGGTCTGGCGGTGATGCATTAGGCCCCGTTATCACCTTAATAATCATTCTCTCTCCCCTTTCGCAATGCCCATTGACTCCGCTAATGAAATAAA
AGAGACCCGCTCGCTCGAACTTGAACTCGGTCTTGCCATTATTGGAAAAGAAGATTGGGTGCATCGATCGACACTTGTCATATTCTGACTCTGTCACTACTAAGACGGAGTCCTTATTGTA
CTTGAAGTTAATGGTGTCGTCAACTTTAAACCGGTTCTTAGCGGCCCACTTGTTATAGAAATCTGGATCTTTGGAAGAGGGAATGGCCCAATCTCCATCGCCGCCGACGGCAAAGTCGGT
GGACAAGGTGAGCAGGGAGGAAATGGAGAAGAGGGAGAAGAAGAGGAGGAGTTTGAAGGAGACGAGAAGTGTGACTTCCATGGCGTAATTACTTGTTTGATTTCTTTCTCTAAGAAAT
GGTAGAGAGAAGAGGACTGTTGGAGAGCGAGACAAATAGGAGAAAGTGAGAATAAGAAGCAGAGGGATTAGCGTAAGCAGTTAACAAGATGAGACCGTGGGGCTGCCGTGGCTTGCG
GTGGAATTTGAATTGGTAATAGCCGTTGGAGATCGAGGCGGGAGGCAAGAAGAGAATCCGGGATGGAAACGTTAGATTTTTTTTTTTTTTAATATCCCTTTTTATAGCTTTCAATTTATTTTT

TGGTATGTTTAAATTATTTCGAATGACTGGTTTTTTTATTTGAAAAAAGAAAATCCAAGTCGTTTAAAATGAATTTAATGAATGGAATTTTGTAGCTTTGCC
>comp150149_c0_seq2
AAAATATTAGCGCCTTCTTCTGTTCGTTGGCTTTCTCTGATCTACAGAGAGGCGCTGAGGCGCAGAGATTTGAAGAAAGGCAATCCTAAACTTACATTTTGTCGCAGCATCGAAGCTCTTC
TCATCACATTGTAGCATCGAAGCCAAGATGTTTCATATCAGCAACAGCGTCATAGGTTTCCTCAACGTTCTCACCCTCCTCATATCCTTCCCCATCATCGGCACCGGCCTCTGGCTTCGCGC
ACACGCCGCCTCCGTGTGCGAAAAGTTCCTCCTATGGCCGATTATAATCATCGGCCTCTTCTTGATGGTGGTGGCTTTGCTCGGGCTCTTCGGATCTTGCTGCAATGTGTCGTGTTTTCTGT
GGATTTATCTCTCTGTTATGTTTCTGATGATTGTCGGGCTCTTTTGTTTCACGATATTCGCCATTGTTGTTACCAATAAAGGGATCGGTGAGGCGGTCTCGGGGAAAGGATATAAGGAGTATA
AGCTTGCGGATTATACAATTTGGTTGCAGAACAGGGTTGGGGATTTCCAAACTTGGGGGCGGATTAAGAGCTGTTTGGTTGATGCTGGGGTTTGTGGAGAAGTTCAGCTCTTGGTTTTTC
AGCAGTCCGATGATTTCTATATGCAAGGCATATCTTCCATACAGGTATTATATTCTATATATTGTCAACGGAATTTGAAAATTGATGCTAGATTTTTATGCTAAGAAGAAAATTTCTTAAGCTCT
CAAAATTCTAAATCTAATATTTATGAGCAATTTTGTTAAGGGTGTTTTTAAAATTGCTCATAAATATTAGATTTAGAATTTTGAGAGCTTAAGAAATTTTCTTCTTAGCATAAAAATCTAGCAT
CAATTTTCAAATTCCGTTGACAATATATAGAATATAATACCTGTATGGAAGATATGCCTTGCATATAGAAATCATCGGACTGCTGAAAAACCAAGAGCTGAACTTCTCCACAAACCCCAGCA
TCAACCAAACAGCTCTTAATCCGCCCCCAAGTTTGGAAATCCCCAACCCTGTTCTGCAACCAAATTGTATAATCCGCAAGCTTATACTCCTTATATCCTTTCCCCGAGACCGCCTCACCGAT
CCCTTTATTGGTAACAACAATGGCGAATATCGTGAAACAAAAGAGCCCGACAATCATCAGAAACATAACAGAGAGATAAATCCACAGAAAACACGACACATTGCAGCAAGATCCGAAGA
GCCCGAGCAAAGCCACCACCATCAAGAAGAGGCCGATGATTATAATCGGCCATAGGAGGAACTTTTCGCACACGGAGGCGGCGTGTGCGCGAAGCCAGAGGCCGGTGCCGATGATGGG
GAAGGATATGAGGAGGGTGAGAACGTTGAGGAAACCTATGACGCTGTTGCTGATATGAAACATCTTGGCTTCGATGCTACAATGTGATGAGAAGAGCTTCGATGCTGCGACAAAATGTAA
GTTTAGGATTGCCTTTCTTCAAATCTCTGCGCCTCAGCGCCTCTCTGTAGATCAGAGAAAGCCAACGAACAGAAGAAGG
>comp150149_c0_seq3
AAAAAAAGTTAGAAAAAATATTGTTACAATAGACAGAAATTTTCAAAGTAGCAATAGCACAACATATGAAATCAAACTCTACTTGTTTTCACCTTCTCACATATACTCATCAATACAATCCA
CTAATCCAAAAATTTTTTAATTATAACCTCTGTAACGTAAATATTGGTTATCTTTACAACTATTCCTAAAGGCACAGCAGCCAATTGAATAAATGACAATGAGAAACACAAGGAGGCCAACA
TTGAAAATTGCAAGTTTCCTCCATTGCATTTTTATATTCGCAAGTACTCCAGCTTTGCATGAGCTGCAGTCAAAGCACAAGCGATTCTGCTCATTGCTCCATGTCTTACAATCAATATCATTT
GAATTAGGATTATCTGATTTTGAGACTGTCCAGTATGTGGCATTCACTTGTTTGTACCCACAATCTGAAGGTGGCTTACAGCAACCTGACTGGAATTAGGTTGATTTGTTTGAATTAGTAAT
GGAAAGAATGGAATTAAAAATGATTAACTAGGACAGTTTAGAGGTAAATATAAGAATTTGCAGACAGTAATTTGGATCAAACTTCATTCCATTTACTTCAAGACAAAGCAAGAATATGGCT
ATTGAAAGAAAACAATTTGTTATTTATGCATTATAATTTTTGCTTGTTAGCTCAGTAAAATCTGCTTTAGTTGAGGTTTTGAAGAAATCAATATTTAGATGGGATACTTGAATAAGTTAATGTT
TTTTTATTACAACAACAACAATAACAATAACAAACTTTGACCCACTATTTGGGGAGCTCTAAGATTTTCTAAATTGAGATTTATAAGATTCGCTAAATCCTACTTCTAAGTGATCAACTAATC
AACCCAGAAGTTTCTGGTAAGTCTTATTTGTACTCTACTATAAACAGAAGTTCTTTCAACGGCTACTAGTCCTGTATCAGAGTTCGGTGATGTTGCTATCAGAAGAATTCCACAGAGGTTTT
TAAGAGGTTCATGTAAATGAGTTTGGATCCAGTAGCTGTTCGATCCTTGTGGCCGTTTGGTTCCATGGCTTTAGTGGTGCTTCTCCTCTGCCTGGATTCACATGTGTAGCTTACGATGTCATT
TCAGCAACCTAAAGATCATTTTATTTACACGATTTCATTCCAGGAAAATCC
>comp150171_c0_seq1
GCTATGGAGTCAATTCCTGTTTAAGAGAAGAAGTTGATAGTGTCATGTAAAGCCTACAATAGGAATAAGTTTGATGGTTTAATCATGCAAAGTTTCATGTTCCAACCATTACCTAATGATAG
ATTGTTATATTCCATCACTCAGTTCCATCATATATCGTTCATCTCATTACGGTAATTGAAATCATCCTTGAAAAAATAGTTAATGAAAAAACAAAGGGGGGAATTATTCAAACTTTAAAGGTA
GAGAAAACCTTAATTTCATATTGTTTTTGGCTACTTAGAGCTTTCAATTTCTAAATAGTTTCGAAATTTGAAGAACTGTATGAAACATCCTACCTTCCATTTGTTTCAACCTGAATCATGTAA
TTTAAAGTATCTCATCTAAGCATTATTTATCATTCATAACAAAGAAAGCATAAATAATTAACAATTAAAATATCATGTAAAAGGAATTCTAAATCTGGCTGGTGTTTTAATTTCTTATTTATCTA

TTTTGTAGAAAATCGATGACTTTTTGTTGCTAGGTATAATTAATTTGACTCTGCTACCCAACTTATCATGACACAGTTGTGGTTTTAGGAACATTCTATATTTATGTTGAAAATTTGAGCTTTC
AGTGCCCCCTTTAGCTTCCTATGCTACGTTGTTAGCATACTGTTACTTGAAAGAATCCTAAATATCTTAAACGCTGGAGTTCTAACTTCACATATAGACTGATAAACACATACTAATCATCAC
TGTTTGAATACTTTCAAGTAAGGAATAAATGATAGGGTTATGGTCTTTAATAGGCAAAACAGTTCAAGCACGCTTTGATATCATGAAACCCCTCAAATTCTCTTCTTGCTTGCTTCCATGGCT
AGGCTCGTTCTAATTTGCTTGCTTTATTTCAACTAGCAATATGTCATATAAACGATTTCCATAAAGGCAACATAATCAAAGCACTAAACTACTATAAATCTGACAACTTTTAGGTCATTTTCTC
TTCAAATATCCACCAGATCCAAATCTAACATATAATAGAAAGTGAAACCACAGAATTTCCAATTAAAAGAATGCTTAAAATATCAGATGAAAAGTCAGGTATTGGGAAAGTACCAAAGATA
TCAGCCACAGCCTCT
>comp150184_c0_seq2
AAACCCCAAACTGGCGCGCTCTTTCCTTGGTTTCCTCATCCATTTCTCCCATGGCCGACTCGAAATGTCCTTCTTTATCTAGGTTTTCTTACATCGATCTACTCTGGTCTTAACAGGGTTCTT
ATTATCTCAAGAATTTGTCTAAATGCCAAGCTCTGTTCCTCTCGGATATTCGCCCTAACCCTATGCGAAGATCGATCGGCAGTTTTTGCCCATTTTAGGCGGTGTGCGCTGTAAGCAGGAGA
AATGCTCATTGGGTCAGATATCGGTAGGCTGAGATGAAGCTTGACGCTCTGCTGACCTCCGCAGGCATAAACATCGGCCTCTGTGTGTTCTTTATCTCGATCTATTCGGTGATCAGGAAAC
AGCCTTCAAATTTGTACGTGTATTTTGGGCGGAGTCTCACCCAGGAGCGGAACAAGCCAAATGATTCCTTCAATATTGAAAATTTTTTGCCTTTCCCAGGTTGGATACTCAAGGCATGGGG
ACCAACCGAAAATGAGATATTGGCATCCGTCGGCCTTGATGCTGTTGTCTTTTTAAGGATGATTGTTTTCAGTATCCGAATATTCTCTATTGCTTTCCTCATCTGCATGTTTGGGGTATTGCCA
CTGAATTATTATGGACAAGAAATGACTCACAATCAAATTCCTCATGAATCTTTGGAAGTTTTTACTATTGCAAATGTTAAGGAAGGTTCTCAATGGTTGTGGGTTCATTGCCTTGCCCTGCA
TATTATCTCTTTCTCAGCTTGCACCCTTCTTTACTTCGAATATCAAAGAATTTCCAAACTAAGACTGGATCATGTTACAGCACGTCCTCCTAAACTTAGCCATTTCAGTATTCTTGTTCGGTC
GATACCGAGATTAAGAGACATGCCACTTGATGATGTCATTAAAAATTTTTTCATAACTTATCATGGAATGAGTTACTTATCACATCAAAGAATACATCGGAGTGGAGCATTTCACAAAATTAT
GACCAATGCGGAGAAGGCGTGTACGAAGTTTGTACGCCTAAGGGCTGCAAGTTTTGATCTTAAAAGAAGACCTGCTTCATATCGATGTGGTCTATGTGGTGGAACTTCTAGATCTTATGAT
CTGTATGACAATGCCTCTGACGTTGAGGAGAGAAGAACTCCACTTAGCCGTTCAGATTCCGGGAAAAAGGAAGAGGATTGTTCAGCTGCATTGGTATTTTTTAAGACACGGTATGCTGCA
GTAGTGGCTTCTCGAGTTCTTCAATCATCAAACCCTATGTTATGGGTTACTGATTTGGCTCCGGAACCTGATGACTTATACTGGTCAAACCTTTCAATACCGTACAGGCAACTTTGGATTCG
CCGAATATCAATAGTGGTGGCTACTATTCTATTTTTGTTTCTGTTTCTCATCCCAGTTACATTTGTGCAAGGTCTTACACAGTTGGAGACATTACAGCAAAAACTCCCATTTCTAAGAGGTAT
ACTAACAAAGAAATACATGATCCAACTGGTAACTGGCTATCTTCCCAGTGTCATTTTGCTTCTATTTTTCTATGCTGTACCACCAACAATGATGCTATTTTCTACTGTGGAAGGAGCTATCTC
CCGCAGTGACAGAAAAAAGAGTGCATGTTGCAAAGTATTATACTTCACGATTTGGAATGTTTTCTTTGTCAACGTTCTATCTGCATCTGTTATCCATCAGCTAATGTTCATTTCCCATCCAAA
GACATCCCTTTTGTTCTTGCTAGAACTTTACCAGGACAGGCTACCTTTTTTATTACTTATGTCTTGACTTCAGGCTGGACAAGTTTATGTGCTGAACTAGTACAATGTTTTCCCCTTACTTAT
AATCTGCTACAAAAGTATGTATTCAGAAAAAAGTTAGATGATCCAAATATGGTGCCAACATTCCCATATCACACTGAAGTTCCGAGAGTCTTGCTTTTTGGGCTTCTCGGATTCACATGTTC
TATTTTGGCACCATTGATATTGCCATTCTTGATGGTGTATTTCTTGCTTGGCTACTTGATATACCGCAACCAGATGCTTAATGTTTATCGTACAAAATATGAGAGTGGGGGACAACTATGGCC
CGTTGTGCACAACTCCACAATATTCTCCCTTGTCCTTGCACAGATAATAGCCCTCGGAGTATTCGGAATAAAGAAATCAACGGTTGCATCAGGTCTAGTCGTCCCTCTTGTGATATTCACCC
TCCTTTTCCATGAGTACTGCAGGAAACGCTTCCTCCCAATATTCAAAAACTTCTCTGCACAGGTTGGCTCCCTGCTTGTTTTGTACAACTCTGCTGCCCTCGTTGAGATGGATAGAGAGGA
TGAGATGTCTGGAAAGATGGAAGAAATCCACCTGCTACTTCAATCTGCTTATTCTCAGCCTGTACCCAAAGATAAAGATGAAGATGAAGATGATGTGGTTGATGGGGGTGGAGACGGTGG
AGCGGAGAACTCTGCTTCAGTAATTTGATTTTACTACAAGCAAATGAGCTGATTTTGTTTTGCGACGTGCTTGGTGCCATTTTTAGTCATATTCTAGCTCATCAATGTAAGGATCTCCTTCCT
CCAGGAGCCACCTGGACAGAACCGTTTCCTTGGCTTGTAAATCTTAGTTGATCCAAGTATTAATTTCTTTTTTTTTAAGTGCAATAATGTTTAAAATTGTCAGCTTACCACAGCATTTTTCTA
GGTTACTGGCAATCTCGCACCATGAATTCTACTAGTGAATAAGAACATGATCCTCGAGCATGAAATCTTCATTGCGCTACTAAGCCTGCCAATGAGTGTTTGGACTGCAGACAGAATGCAA
AACCTCAGGTCTTTTGTTTGTTGCATAACTAGAGGGTAGACTAAGATAGATATAGTCATCAAGCTCAAGTTGTAACTTTGACTTGCCATTTTCTTTTTCTATTGAGTAAATTAGACCAGTTTC

TGCTGTCTGAGATGTTTGCAGCATATATCATAAACCATATGGTCGAATTTGGCTAAACAATTAACCCAATACTACTATGTATGCAAGCCCAA
>comp150184_c0_seq3
AAACCCCAAACTGGCGCGCTCTTTCCTTGGTTTCCTCATCCATTTCTCCCATGGCCGACTCGAAATGTCCTTCTTTATCTAGGTTTTCTTACATCGATCTACTCTGGTCTTAACAGGGTTCTT
ATTATCTCAAGAATTTGTCTAAATGCCAAGCTCTGTTCCTCTCGGATATTCGCCCTAACCCTATGCGAAGATCGATCGGCAGTTTTTGCCCATTTAGGCGGTGTGCGCTGTAAGCAGGAGA
AATGCTCATTGGGTCAGATATCGGTAGGCTGAGATGAAGCTTGACGCTCTGCTGACCTCCGCAGGCATAAACATCGGCCTCTGTGTGTTCTTTATCTCGATCTATTCGGTGATCAGGAAAC
AGCCTTCAAATTTGTACGTGTATTTTGGGCGGAGTCTCACCCAGGAGCGGAACAAGCCAAATGATTCCTTCAATATTGAAAATTTTTTGCCTTTCCCAGGTTGGATACTCAAGGCATGGGG
ACCAACCGAAAATGAGATATTGGCATCCGTCGGCCTTGATGCTGTTGTCTTTTTAAGGATGATTGTTTTCAGTATCCGAATATTCTCTATTGCTTTCCTCATCTGCATGTTTGGGGTATTGCCA
CTGAATTATTATGGACAAGAAATGACTCACAATCAAATTCCTCATGAATCTTTGGAAGTTTTTACTATTGCAAATGTTAAGGAAGGTTCTCAATGGTTGTGGGTTCATTGCCTTGCCCTGCA
TATTATCTCTTTCTCAGCTTGCACCCTTCTTTACTTCGAATATCAAAGAATTTCCAAACTAAGACTGGATCATGTTACAGCACGTCCTCCTAAACTTAGCCATTTCAGTATTCTTGTTCGGTC
GATACCGAGATTAAGAGACATGCCACTTGATGATGTCATTAAAAATTTTTCATAACTTATCATGGAATGAGTTACTTATCACATCAAAGAATACATCGGAGTGGAGCATTTCACAAAATTAT
GACCAATGCGGAGAAGGCGTGTACGAAGTTTGTACGCCTAAGGGCTGCAAGTTTTGATCTTAAAAGAAGACCTGCTTCATATCGATGTGGTCTATGTGGTGGAACTTCTAGATCTTATGAT
CTGTATGACAATGCCTCTGACGTTGAGGAGAGAAGAACTCCACTTAGCCGTTCAGATTCCGGGAAAAAGGAAGAGGATTGTTCAGCTGCATTGGTATTTTTTAAGACACGGTATGCTGCA
GTAGTGGCTTCTCGAGTTCTTCAATCATCAAACCCTATGTTATGGGTTACTGATTTGGCTCCGGAACCTGATGACTTATACTGGTCAAACCTTTCAATACCGTACAGGCAACTTTGGATTCG
CCGAATATCAATAGTGGTGGCTACTATTCTATTTTTGTTTCTGTTTCTCATCCCAGTTACATTTGTGCAAGGTCTTACACAGTTGGAGACATTACAGCAAAAACTCCCATTTCTAAGAGGTAT
ACTAACAAAGAAATACATGATCCAACTGGTAACTGGCTATCTTCCCAGTGTCATTTTGCTTCTATTTTTCTATGCTGTACCACCAACAATGATGCTATTTTCTACTGTGGAAGGAGCTATCTC
CCGCAGTGACAGAAAAAAGAGTGCATGTTGCAAAGTATTATACTTCACGATTTGGAATGTTTTCTTTGTCAACGTTCTATCTGCATCTGTTATCCATCAGCTAATGTTCATTTCCCATCCAAA
GACATCCCTTTTGTTCTTGCTAGAACTTTACCAGGACAGGCTACCTTTTTTATTACTTATGTCTTGACTTCAGGCTGGACAAGTTTATGTGCTGAACTAGTACAATGTTTTCCCCTTACTTAT
AATCTGCTACAAAAGTATGTATTCAGAAAAAAGTTAGATGATCCAAATATGGTGCCAACATTCCCATATCACACTGAAGTTCCGAGAGTCTTGCTTTTTGGGCTTCTCGGATTCACATGTTC
TATTTTGGCACCATTGATATTGCCATTCTTGATGGTGTATTTCTTGCTTGGCTACTTGATATACCGCAACCAGATGCTTAATGTTTATCGTACAAAATATGAGAGTGGGGGACAACTATGGCC
CGTTGTGCACAACTCCACAATATTCTCCCTTGTCCTTGCACAGATAATAGCCCTCGGAGTATTCGGAATAAAGAAATCAACGGTTGCATCAGGTCTAGTCGTCCCTCTTGTGATATTCACCC
TCCTTTTCCATGAGTACTGCAGGAAACGCTTCCTCCCAATATTCAAAAACTTCTCTGCACAGGCCCTCGTTGAGATGGATAGAGAGGATGAGATGTCTGGAAAGATGGAAGAAATCCACC
TGCTACTTCAATCTGCTTATTCTCAGCCTGTACCCAAAGATAAAGATGAAGATGAAGATGATGTGGTTGATGGGGGTGGAGACGGTGGAGCGGAGAACTCTGCTTCAGTAATTTGATTTTA
CTACAAGCAAATGAGCTGATTTTGTTTTGCGACGTGCTTGGTGCCATTTTTAGTCATATTCTAGCTCATCAATGTAAGGATCTCCTTCCTCCAGGAGCCACCTGGACAGAACCGTTTCCTTG
GCTTGTAAATCTTAGTTGATCCAAGTATTAATTTCTTTTTTTTTAAGTGCAATAATGTTTAAAATTGTCAGCTTACCACAGCATTTTTCTAGGTTACTGGCAATCTCGCACCATGAATTCTACT
AGTGAATAAGAACATGATCCTCGAGCATGAAATCTTCATTGCGCTACTAAGCCTGCCAATGAGTGTTTGGACTGCAGACAGAATGCAAAACCTCAGGTCTTTTGTTTGTTGCATAACTAGA
GGGTAGACTAAGATAGATATAGTCATCAAGCTCAAGTTGTAACTTTGACTTGCCATTTTCTTTTTCTATTGAGTAAATTAGACCAGTTTCTGCTGTCTGAGATGTTTGCAGCATATATCATAA
ACCATATGGTCGAATTTGGCTAAACAATTAACCCAATACTACTATGTATGCAAGCCCAA
>comp150213_c0_seq2
ATGTCGTAGATCGCATTTCACGCTCTAGGGTTTTCGCGACCGACTCTCCGCAACTCCCTAACTTCAATCGCAACTCCCCCTTGGTTAACGGCCTCGACTTCAATCGTGCGTGAATATGAGT
CGCCATCGAGTTCGTCCTCTCAGAAGGTTAATAGAGCAATAACAATGCATTCTATACATACTCTCAACTGACAAGAAATGAAGCATTTAATTTATTCAGCATCCAAGCAAATC
>comp150225_c0_seq1

TAGCGAACAATAATCCAACTTTAAAGATCGACAACAATCCTTTCTTATTCCATACTGACACCTTATCTGGAATCTTAGCATGCAAACATATTATTAGACAAGACTTGTAACATAAGGCACAC
ATCAACTGCTTGAGATTAACCCGAACATGTTGATTTGGCATATTCCCTTACATATATGTCGAAAGGAACGATGAAGATCCTCAATCTTAGTTCCTTCTCATCTTCTTCATCTTTTAGTAATTTT
GCTTCAATGTGGATACAAAAAAGAGGCATTGGGTTTAAAACTGAGCACATCTGTGCCCTCATTCAGTTGAGCTTTAGGTATCTCAGCCTCGACTCCTCTTCGGTAACAGTGTTCCTGGCAC
CGGAGGATTTGTAATCTGGGGCTGGGAAATGGCCAGACTCAATGTACTTGAGATCCTCAACAAGAAGTTCGTAGTGGAGTTTCACCTCCTCCGCCGTCTTACCTCCGACGGCTCGGGCCA
CATTTTGCCACCGGTCCGGCGTGTCCTTGTCATACACTGCAAGTGCTCGCTCGAAGAGCTTGTTCTGCTTCTGTGTCCAGGTTGAAGATGAGTTCCTTGAAGCCATTAATTGTGAGTAGTG
AAACTGCGGGAGTGCTTGGATTGATGGTTGAAGAGTGGGAAGCAGGTGGGTTTCTTATATGGAGGAGAAGGGAGTTGAACTGAGCTGGAAATGGCGGTGGAGCCAGTTGGAGAATATC
ACTTAGGGAAAAGCTGGGCGTGGTGGTGGAATAAGGACATACTAGTTTAGTTAATAGCCACAAGATTGAGCGAGAACAATGCGGAAGAATCTGCCTTACCATGGCAGAGAATG
>comp150245_c0_seq1
GGCGAAGGTGGCGAAGAGCTTGAGTTTTGAGAAGGAGGCGTTGATCTAGACCCTGCTGAAGGCGGTGAAGAGCTAGACGCTGGCGAAGGTGGCGACGAGGTAGAGTTTTGAGAAGGA
GGCGATGAGCTAGATCCCAACGAGGGTGGTAAAGAATCGCCCGAGTTCGCATTGTCCGAAGAAGAGGAATTTTCAGAAGCAGGCGGAGAATCAGACTCAGACATTTCCAAAATTTTCCT
TCCCCTTAGCCGTTTTATTATTTACCTCAATTAATCATGAGAATTCTTCTACACAAATTCAAAAAACGAGGCAGCGTTCAAATCCTTCACTTCTCAGCGACAGAAAAACCTAAAATCTATTT
TGTTTCAAGATGTATCTTCATTGCGTTCGTTCGTTGTCGTCCTCCTGCACCGGTGGACAAGGA
>comp150255_c0_seq1
TTTTTGCTCCACAATTACAATTGCTTTGATGTTTAATAAGAATTTTAAGACAAATTGAACAATTTTAAAATTAAACAGAGTAAAATTTCTTAAAATAAGAAAATACTGATTGAATAGAAAAA
ATTACAATGACTATAAACTTATGATTGAATAAAAAATAAAAAATTTAGCATCAAAAGCGGTTCATTCGAAGGCCCATGCGTGTTCCTTCCGGATCTTTTCTTCTTCTTCGGGCGTGAAATCA
TTTTCAATTTTAAATATTTGACGGATTTCCTCTGGTGTTTTCCATCGGATTATATTTGCGACTTTCTTGCAAATCAAATCTATCAAGTCTTTCACGCCCAAATAATTTGCTGCCATGATAAGAT
CATAGAGGGCATCGGTATCAATGTCAACGAATTTGTTATCAAAGTCCTCAAGCTCTTCGAATTCAGGCAAGAAGTCCATGTGCTTCTTGCAGTAATCAAAGATATTAGAGAGGACGGAGG
CGGTGACATTGGGTATTGGAATGACATCTTCTGTGAAATTGTCTTCAATCATGTGACGGATAATCTCAGACTTCATCGCTACCGCCTCTTCCACCACCACCTCCTTTCCATCAGAAGCCATC
ATTCTTATCATCTTCTTCATCTCGTTGGCCATCCTCGTTCTACCATGTAAAGCCGTTGCTCAATTTATAAATGTCGGTTAAAAAAGCCAAGACCCACCCATTCTTATTTATTGCAAAGATTAG
AATTGGTCAAGATTTAAATTTAGAAAAAGTATTTTCTTAATTTATCATACATTTTCGGCACCTCTCATTGAACGCCTAAGGAATAAAATTCCAAACATTGCGCAAGCTAGCATTGACTAAATT
TAGCTTTAAAAAGATGAGGCCATGATTTAAACAAGCAAAATCATAGAAAATTTTGTTATTTTAAGCTAAAACTTAAAAAAAAAAAAAACCTAAAATTAAAAAGAGCCACAAATT
>comp150255_c0_seq3
TTTTTGCTCCACAATTACAATTGCTTTGATGTTTAATAAGAATTTTAAGACAAATTGAACAATTTTAAAATTAAACAGAGTAAAATTTCTTAAAATAAGAAAATACTGATTGAATAGAAAAA
ATTACAATGACTATAAACTTATGATTGAATAAAAAATAAAAAATTTAGCATCAAAAGCGGTTCATTCGAAGGCCCATGCGTGTTCCTTCCGGATCTTTTCTTCTTCTTCGGGCGTGAAATCA
TTTTCAATTTTAAATATTTGACGGATTTCCTCTGGTGTTTTCCATCGGATTATATTTGCGACTTTCTTGCAAATCAAATCTATCAAGTCTTTCACGCCCAAATAATTTGCTGCCTATGATAACAT
TAAACAAAGAACGAAAATATATGATAATGAAACAAAGGACATAACAAAAAAGAAAATACTTGAACTTGGATAATTATTTACCATGATAAGATCATAGAGGGCATCGGTATCAATGTCAACG
AATTTGTTATCAAAGTCCTCAAGCTCTTCGAATTCAGGCAAGAAGTCCATGTGCTTCTTGCAGTAATCAAAGATATTAGAGAGGACGGAGGCGGTGACATTGGGTATTGGAATGACATCTT
CTGTGAAATTGTCTTCAATCATGTGACGGATAATCTCAGACTTCATCGCTACCGCCTCTTCCACCACCACCTCCTTTCCATCAGAAGCCATCATTCTTATCATCTTCTTCATCTCGTTGGCCA
TCCTCGTTCTACCATGTAAAGCCGTTGCTCAATTTATAAATGTCGGTTAAAAAAGCCAAGACCCACCCATTCTTATTTATTGCAAAGATTAGAATTGGTCAAGATTTAAATTTAGAAAAAGT
ATTTTCTTAATTTATCATACATTTTCGGCACCTCTCATTGAACGCCTAAGGAATAAAATTCCAAACATTGCGCAAGCTAGCATTGACTAAATTTAGCTTTAAAAAGATGAGGCCATGATTTAA
ACAAGCAAAATCATAGAAAATTTTGTTATTTTAAGCTAAAACTTAAAAAAAAAAAAAACCTAAAATTAAAAAGAGCCACAAATT

>comp150295_c0_seq1
ATCCTACCACATCCCATCTCCATGTCTTGTTCCCCTTTCTCTCTCTCCTCCCTCTAACCCCTCGCCGTCCCCAGCAGGCCAGCATGGCCGGAGGAGAGAAACATGGGCCGTGGCCCATTTT
CTCCCTCCTCCTCCTCGTCTTCCTTCGCCTTCCTCTTCTCCTCTCCTCCACCACCGCTAAAAATCCCACCTTTACCCCTTCCGACATTTACCTCCTCGACTGCGGTGCCCCTAAAAGCGTCC
AACTTTCCGACGGTCGCACCTTCCGCTCCGAACCCCAATCATCCTCTTTCCTGTCGACCGACGAAGACATCCGCCTCTCCTCCACCTCTCCTTTACCTTCCCTCTCCCCTCTCTACCAAAC
CGCCCGCGTCTTCTCCTCAACCTCCACCTACTCCTTCTTCATCACGAAACTCGGGATCCATTTTATTCGCCTCCATTTCTTCCCCTTCCCTTCCTCTTCCTACAACCTCTCCTCCGCCATCTTC
ACCGTCAACACCGAAGACTTCGTCCTCCTCCGCGACTTCGTCGTCCCATCCCCTGCCTCCCCTGTTCTCAAAGAGTACCTCATCAATGTCGACAATGAACGCCTTTACCTCACTTTCAAAC
CCAAAAAAGGCTCCTTTGCCTTCCTCAACGCCGTCGAAGTTGTGTCCGCGCCGGAAACTCTCATCCCCACCTCCGCCGCGAGCGTCAGCCCGCAGGAGCAGTTCAGCGGGATCAACAA
CTACGCGCTTGAAGTTATCAGTCGGATTAACGTCGGTGGGGATAAGATTTCATCGGAGAATGATACGTTGGCGAGAGAGTGGCTGCCCGATTCGTTTCTGAAGCCGCCGGACACGGTGCA
GAAAGTGGCTGTGGCGCCCGAGAAGGTTAAGTATCCTGAAGACGGAACAGTGACGCCGCTTATTGCGCCGAGGAGCGTATATGCGACGGCGGATAGCATGAGGGATGCGCATGTGTCGG
AGCCGATGTTCAATATTACGTGGGATATTCCGGTCGACGGCGGCGCATATTCGTACCTCGTTCGACTCCATTTCTGCGACATTATTAGCAGGACTCTTAATTCGCTTTACTTCAACGTTTTCA
TCAACAGAATTTTGGCGATTCCCAACCTCGATCTTTCCTCTCTCACAGGTATTGTAAACAAGGCTTATTAGCGCTTGCATTATCATTTCTCATAGCAGTATTAATTGGACAATCCGCCATGTC
TGAAACTCTCAGAAGTGTCATGAGCATTTGACCGTTTGTTGTATCTCGATCTTGTAGGTGGATTCACGCATTTGTTCATCATCTTATTAATTATCTATGCTCTTCACATCTGAGAATACTTCAA
ATCTAATATTATGTATGAAAATATATAAAGATTTTATCAGAGAAATGAATCAAAATTTGAAAGTGAGAAGATGGG
>comp150295_c0_seq2
ATCCTACCACATCCCATCTCCATGTCTTGTTCCCCTTTCTCTCTCTCCTCCCTCTAACCCCTCGCCGTCCCCAGCAGGCCAGCATGGCCGGAGGAGAGAAACATGGGCCGTGGCCCATTTT
CTCCCTCCTCCTCCTCGTCTTCCTTCGCCTTCCTCTTCTCCTCTCCTCCACCACCGCTAAAAATCCCACCTTTACCCCTTCCGACATTTACCTCCTCGACTGCGGTGCCCCTAAAAGCGTCC
AACTTTCCGACGGTCGCACCTTCCGCTCCGAACCCCAATCATCCTCTTTCCTGTCGACCGACGAAGACATCCGCCTCTCCTCCACCTCTCCTTTACCTTCCCTCTCCCCTCTCTACCAAAC
CGCCCGCGTCTTCTCCTCAACCTCCACCTACTCCTTCTTCATCACGAAACTCGGGATCCATTTTATTCGCCTCCATTTCTTCCCCTTCCCTTCCTCTTCCTACAACCTCTCCTCCGCCATCTTC
ACCGTCAACACCGAAGACTTCGTCCTCCTCCGCGACTTCGTCGTCCCATCCCCTGCCTCCCCTGTTCTCAAAGAGTACCTCATCAATGTCGACAATGAACGCCTTTACCTCACTTTCAAAC
CCAAAAAAGGCTCCTTTGCCTTCCTCAACGCCGTCGAAGTTGTGTCCGCGCCGGAAACTCTCATCCCCACCTCCGCCGCGAGCGTCAGCCCGCAGGAGCAGTTCAGCGGGATCAACAA
CTACGCGCTTGAAGTTATCAGTCGGATTAACGTCGGTGGGGATAAGATTTCATCGGAGAATGATACGTTGGCGAGAGAGTGGCTGCCCGATTCGTTTCTGAAGCCGCCGGACACGGTGCA
GAAAGTGGCTGTGGCGCCCGAGAAGGTTAAGTATCCTGAAGACGGAACAGTGACGCCGCTTATTGCGCCGAGGAGCGTATATGCGACGGCGGATAGCATGAGGGATGCGCATGTGTCGG
AGCCGATGTTCAATATTACGTGGGATATTCCGGTCGACGGCGGCGCATATTCGTACCTCGTTCGACTCCATTTCTGCGACATTATTAGCAGGACTCTTAATTCGCTTTACTTCAACGTTTTCA
TCAACAGAATTTTGGCGATTCCCAACCTCGATCTTTCCTCTCTCACAGGTGGGAACCTCTCCATGGCTTACTATAAAGACTTCGTCGTCGCTGGAACCACTCTCATCTCCGGCAACATCTC
CATCCAAATCAGCCCACCAGTGATGAACGACCCCGGTCTCCCCAACGCCATTCTCAACGGCATTGAAGTCATGAAGATGAGCAACACCGCAGGAAGCCTCGACGGTCTGTATTCCGTCG
ACGGCTCCTACCACAGCAATGAACCCGCTGCCACGGCAAAGATAAAGGTTCTGGTAACCGTCTTTGGTTTGGCCATTTTTCTAATGGCATTGGCGGCGATTATGTTTCTCCGGCGTCGCCG
GCGGCCAGACGATTGGGACAAAAAACATAGCTTTTTTTCTTGGTTGTTGCCTCTTCATGTCAGCCATTCAGGCCGGCCGGGGTTTATTAGTAAGTGGAATGGGAAGGGGAGCACCTCTTC
TTCAAAGAATGGAAAGGGGTTTGGGTCGCATAAGAGCAAGAGCGGGTATAAGAGTTATCTTGCATCAGGAGGGCTTGGCCTTGGGCGATTCTTCACCTTCGGTGAGCTCCAGCAAGCCA
CCTCCAACTTCGACGAGAAGAACGTGATCGGCGTCGGTGGATTTGGCAAAGTCTACCTCGGCTTGCTCGACGACGGCACGAAGCTGGCCGTGAAGCGAGGAAATCCTTCGTCCGACCA
AGGCATAAACGAGTTCCATACAGAGATCGAGATGCTGTCAAAGCTCCGGCACCGGCACCTCGTCTCTCTGATCGGCTGCTGCGACGAGAACAACGAGATGATTCTCGTGTACGAGTACAT
GTCCAACGGGCCCCTTCGCGACCACCTCTACCCTTCTTCTCCTTCGTTCACACCTCTGTCATGGAAGCAGCGCCTCGAGATCTGTATCGGCGCCGCCCGCGGCCTTCATTACCTCCACACC

GGCGCTGCCCAGAGCATCATCCACCGGGACGTGAAGAGCACCAACATACTCCTGGACGAAAACCTCGTCGCCAAAATGTCAGATTTCGGTCTCTCCAAAGCCGGCCCTTCTTCCCTTGA
TCAAACGCACGTCAGCACCGCCGTCAAAGGCAGCTTCGGTTACCTCGACCCAGAGTATTTCCGGCGGCAGCAACTGACCGATAAATCGGATGTCTATTCATTCGGGGTGGTGATGTTAGA
GGTTCTCTGCGCGAGGCCGGCGATCAATCCGGCGTTGCCGAGAGATCAGGTCAACTTAGCTGAATGGGCGATGCAGTGGCATCGAAAGGGAAAGGTTCAGATGATCATTGACCCGCATT
TGGTTGCAGGTGTCGGGCCGCCGTCGCTGAACAAGTTTGTTGAGGCGGCCGAGAAGTGCTTGGCGGATTCCGGCGTTCACCGGCCGACGATGGGGGATGTGCTTTGGAACCTCGAGTA
CGCGCTGCAGCTGCAGGAAGCATGGATAGGGCAGTCGTACGAGGACGACGACCCATCTGACATCGGCAACGCCGGCGAAATCCATGTAGAGCACCTCGACAGCTTGCAAAGAAAGGAA
ATTCCGTTCGACGTTGAAGTTGTCTCCGTCGATTTCTCCACCCCGGCGGAGCCGTCGCCTCTGCTCCACGGGAGGTGAGCAAAAGATCATCAAGAAGAAATTGTGATGGAAAAAGGTGA
CGCCTTGGATTTGCATAAGCCTTCCACAGTGTAAAAGATATGGTTTTTTATGATGTGAAGTGAAAGCTGTTTACATTTTATTTTTTGATAATTTGATATCTACAACGATTTTGAATGTATACT
GTTGTAATAATACGCTGAAGGATTTTAGGAACCATTTTTTAAATTAA
>comp150300_c0_seq1
AAAATATTTAAATTTGAAATGAACTTTCAACATATTTCTATAAGAAAGCATACAAGAAAATATTTTAGCTATACGTATTATGAGACATAAAACCGCAATTTTCCTTTTGATACAAAATTTCTAT
CAACATAATTTTTTATTAAATTAACCTCCAAACATAAGTATGAAGTTCGGTTGGAGATGCCCTAACGAGCTTAGATAATGTCTATTTTGTTCTAAGATTTATCCAACATATAATATATATAACAT
CTAAGAGATGCAGTAAAATTGACAACAATCATAAATAATAAGCGTCAATGCAAGAATTAATGATAATAATATTGAAGGGTCAGCCAAAAGAAGAGCCTGTGGAGATGATGCCTTTGCCTTC
TTTTCCTCATCGACATAATCATGGAACCAAATGGATCCAAATACTGAAAGAACCCAAAATTAGCAGATGCGTACTAAATGATTGATGCGTACAGAAATCTGGAAGCATGCAAAAGGGATAA
GAAAAGACTGATGCAAAGATACGAAGACGATTGAGTAACTCTTACTATTAATTAGTTTGATCGTGGGATCATAAAAGGCTGTGGAGGTCGAAGTCGATAGAACAGTTGATGAAGACGACG
AACCTCTCTCTTTAACATGTCATTTTGTATGCATCTCATAAATTGTTGATGCAACATGCTGTTGAGCCGATGCTTCAGGACCTCATTTTCGAAGCTCAATATATAATTTTGTCTATCCACATATT
GCAATTGAGCTAAAACTTCCAATCCTTGAGCCTGTAATGCTTGAACGCAGTTCTCCAGCTCATTAATATATTGAAGATTTTGAACCCGAGACCTTTGAGGAAATTGCTGTTGAGGATTGGA
ATGATGTTGAGGGCGATGCAAGTATAAGTCATTGGAAGAAGGACGATGAGTATCAGGCTCATCAAGTAATTGAATTAACCAAGAAAAATGCTGCTCCATTGAAATGCTCTCAGGGGAGGA
GGGCTGATCTTGTTTCAGCTCCTCTTTTGATCCAGATGGACCACAATCCTCATGTACAGGAGTGCTAACATTGCTTGGGGAGGAGGTTTTTGGTGGGGGTGAAGGGTTTTTCTCAAGGTT
GTTGAGGTTGGCTTCTTCAGTCTTGCGCTCCTCCATTTTTCTAGTTCAACCTAGCCTTAGATGGAAACAAATAGCATTGCGGTGGTCGAGATCAAAATGCAAAGGAATCATCCATGAGGAG
AACCTAGTAGGAGAGGAGGAGGAAGAGAGACGACGATCCGGTTGGAGAGAAGATTAAAAGAGCAATGTATGAAGGAAAAAAAGGAGGAAGGAGAGAGATGGAGCTTGCAAATTTGA
TGGAGCCTCTCCCTCAATCCCTCCCCACCCACAGGCTCAATGCCCAAAGGACGAAACCTAAATATTAGAAAGAGGACTAACTAAGATCGACGAGGTTACCTAAGGTA
>comp150300_c0_seq2
AAAATATTTAAATTTGAAATGAACTTTCAACATATTTCTATAAGAAAGCATACAAGAAAATATTTTAGCTATACGTATTATGAGACATAAAACCGCAATTTTCCTTTTGATACAAAATTTCTAT
CAACATAATTTTTTATTAAATTAACCTCCAAACATAAGTATGAAGTTCGGTTGGAGATGCCCTAACGAGCTTAGATAATGTCTATTTTGTTCTAAGATTTATCCAACATATAATATATATAACAT
CTAAGAGATGCAGTAAAATTGACAACAATCATAAATAATAAGCGTCAATGCAAGAATTAATGATAATAATATTGAAGGGTCAGCCAAAAGAAGAGCCTGTGGAGATGATGCCTTTGCCTTC
TTTTCCTCATCGACATAATCATGGAACCAAATGGATCCAAATACTGAAAGAACCCAAAATTAGCAGATGCGTACTAAATGATTGATGCGTACAGAAATCTGGAAGCATGCAAAAGGGATAA
GAAAAGACTGATGCAAAGATACGAAGACGATTGAGTAACTCTTACTATTAATTAGTTTGATCGTGGGATCATAAAAGGCTGTGGAGGTCGAAGTCGATAGAACAGTTGATGAAGACGACG
AACCTCTCTCTTTAACATGTCATTTTGTATGCATCTCATAAATTGTTGATGCAACATGCTGTTGAGCCGATGCTTCAGGACCTCATTTTCGAAGCTCAATATATAATTTTGTCTATCCACATATT
GCAATTGAGCTAAAACTTCCAATCCTTGAGCCTGTAATGCTTGAACGCAGTTCTCCAGCTCATTAATATATTGAAGATTTTGAACCCGAGACCTTTGAGGAAATTGCCTTAGATGGAAACA
AATAGCATTGCGGTGGTCGAGATCAAAATGCAAAGGAATCATCCATGAGGAGAACCTAGTAGGAGAGGAGGAGGAAGAGAGACGACGATCCGGTTGGAGAGAAGATTAAAAGAGCAA
TGTATGAAGGAAAAAAAGGAGGAAGGAGAGAGATGGAGCTTGCAAATTTGATGGAGCCTCTCCCTCAATCCCTCCCCACCCACAGGCTCAATGCCCAAAGGACGAAACCTAAATATTAG

AAAGAGGACTAACTAAGATCGACGAGGTTACCTAAGGTA
>comp150300_c0_seq3
AAAATATTTAAATTTGAAATGAACTTTCAACATATTTCTATAAGAAAGCATACAAGAAAATATTTTAGCTATACGTATTATGAGACATAAAACCGCAATTTTCCTTTTGATACAAAATTTCTAT
CAACATAATTTTTTATTAAATTAACCTCCAAACATAAGTATGAAGTTCGGTTGGAGATGCCCTAACGAGCTTAGATAATGTCTATTTTGTTCTAAGATTTATCCAACATATAATATATATATAACAT
CTAAGAGATGCAGTAAAATTGACAACAATCATAAATAATAAGCGTCAATGCAAGAATTAATGATAATAATATTGAAGGGTCAGCCAAAAGAAGAGCCTGTGGAGATGATGCCTTTGCCTTC
TTTTCCTCATCGACATAATCATGGAACCAAATGGATCCAAATATATTAATTAGTTTGATCGTGGGATCATAAAAGGCTGTGGAGGTCGAAGTCGATAGAACAGTTGATGAAGACGACGAAC
CTCTCTCTTTAACATGTCATTTTGTATGCATCTCATAAATTGTTGATGCAACATGCTGTTGAGCCGATGCTTCAGGACCTCATTTTCGAAGCTCAATATATAATTTTGTCTATCCACATATTGCA
ATTGAGCTAAAACTTCCAATCCTTGAGCCTGTAATGCTTGAACGCAGTTCTCCAGCTCATTAATATATTGAAGATTTTGAACCCGAGACCTTTGAGGAAATTGCTGTTGAGGATTGGAATGA
TGTTGAGGGCGATGCAAGTATAAGTCATTGGAAGAAGGACGATGAGTATCAGGCTCATCAAGTAATTGAATTAACCAAGAAAAATGCTGCTCCATTGAAATGCTCTCAGGGGAGGAGG
CTGATCTTGTTTCAGCTCCTCTTTTGATCCAGATGGACCACAATCCTCATGTACAGGAGTGCTAACATTGCTTGGGGAGGAGGTTTTTGGTGGGGGTGAAGGGTTTTTCTCAAGGTTGTTG
AGGTTGGCTTCTTCAGTCTTGCGCTCCTCCATTTTTCTAGTTCAACCTAGCCTTAGATGGAAACAAATAGCATTGCGGTGGTCGAGATCAAAATGCAAAGGAATCATCCATGAGGAGAACC
TAGTAGGAGAGGAGGAGGAAGAGAGACGACGATCCGGTTGGAGAGAAGATTAAAAGAGCAATGTATGAAGGAAAAAAAGGAGGAAGGAGAGAGATGGAGCTTGCAAATTTGATGGA
GCCTCTCCCTCAATCCCTCCCCACCCACAGGCTCAATGCCCAAAGGACGAAACCTAAATATTAGAAAGAGGACTAACTAAGATCGACGAGGTTACCTAAGGTA
>comp150300_c0_seq4
AAAATATTTAAATTTGAAATGAACTTTCAACATATTTCTATAAGAAAGCATACAAGAAAATATTTTAGCTATACGTATTATGAGACATAAAACCGCAATTTTCCTTTTGATACAAAATTTCTAT
CAACATAATTTTTTATTAAATTAACCTCCAAACATAAGTATGAAGTTCGGTTGGAGATGCCCTAACGAGCTTAGATAATGTCTATTTTGTTCTAAGATTTATCCAACATATAATATATATATAACAT
CTAAGAGATGCAGTAAAATTGACAACAATCATAAATAATAAGCGTCAATGCAAGAATTAATGATAATAATATTGAAGGGTCAGCCAAAAGAAGAGCCTGTGGAGATGATGCCTTTGCCTTC
TTTTCCTCATCGACATAATCATGGAACCAAATGGATCCAAATATATTAATTAGTTTGATCGTGGGATCATAAAAGGCTGTGGAGGTCGAAGTCGATAGAACAGTTGATGAAGACGACGAAC
CTCTCTCTTTAACATGTCATTTTGTATGCATCTCATAAATTGTTGATGCAACATGCTGTTGAGCCGATGCTTCAGGACCTCATTTTCGAAGCTCAATATATAATTTTGTCTATCCACATATTGCA
ATTGAGCTAAAACTTCCAATCCTTGAGCCTGTAATGCTTGAACGCAGTTCTCCAGCTCATTAATATATTGAAGATTTTGAACCCGAGACCTTTGAGGAAATTGCCTTAGATGGAAACAAAT
AGCATTGCGGTGGTCGAGATCAAAATGCAAAGGAATCATCCATGAGGAGAACCTAGTAGGAGAGGAGGAGGAAGAGAGACGACGATCCGGTTGGAGAGAAGATTAAAAGAGCAATGT
ATGAAGGAAAAAAAGGAGGAAGGAGAGAGATGGAGCTTGCAAATTTGATGGAGCCTCTCCCTCAATCCCTCCCCACCCACAGGCTCAATGCCCAAAGGACGAAACCTAAATATTAGAA
AGAGGACTAACTAAGATCGACGAGGTTACCTAAGGTA
>comp150366_c0_seq1
TGATCCTCCTCCTCCTCCTCCTCCTGCGTCGGCACTGCCACCATCATCACCACCGCCATCTTCACCGCCTCCATCTTCACCGCCTCCATCATCCCCGCCTCCAACGTCACCGCCGCCATCAT
CTCCGCCACCCCTGCATTTCCCGCCGCCTCCTCCCGCTTCATCTCCGCCTCTTCCAGCTTCATCTCCGCCTCCTCCAAATTCAACTCCTCCGCCTCAATATTCATCTCCGCCACCTCCAGATT
CGCCTCAATATTCGTCTCCGCCGCCTCCCGCTCCATCTTCACCGGCTCCATTATTTTTCTCTCCGCCACCATTATCACCTAATGAGTCTCCTCCCCCATCACCCACCTTACCTCCACCCTTACC
GACCTCCCCAACACCGCCCTCATCTTCCAAAGGCTCTCCTCCTCCACCAACAACCATACCTCCACCCTTTCCGCCCTCTCCAACACCGCCTTCATCCTTCAAACCACCTCCTTCCGCCGAT
GTTTTCCTGCCACCTCCACCGCCAAAACGCGCAGCAGAGGCTCCTCCCCATTCTTCACCGTCAACAAATGATTACAATGTTGATTCAAAAAAGTCATCCGGAGGCTCTCATGTCAATCTTC
CAATGGTAGCTGGGATTGCTGCAGGCATCGCACTGTTCTTTCTGATTATCGGCGTTGTAGCATGTGTTTGCTGCAGGAAAAAAAAGGGAAAGGTGGCGAAGAAGAAGAAGACTCCTAAT
TCTATAAACTATTATCCTGATCCGGGGGTCAAAGGCAATAATTACTACAACATTGGGGCCAAGCTCCCACCACCTCCAGGCAAGGCTTGGAATCCACCTCATAGTCCCGCAGCCATCAACA

ACGAAAACAGCGTTGGTTACTCAGAACCAAATGGTCCTCCAATTGGTTTCGTTAAGAGCTCATTCAGCTATGAAGAGTTGGCAGCAGCTACAAATGGGTTCTCTGAGGAAAACATATTAG
GACAGGGTGGTTTTGGCTATGTGCACAAAGGAATTTTACCAAATGGACAAGAGGTTGCTGTGAAGCAGCTTAAAGCAGGAAGTGGGCAAGGTGAGAGAGAGTTCCAGGCAGAGGTTG
AGATCATTAGCAGAGTTCATCATCGCCACTTGGTCTCCCTCGTTGGTTACTGCATTGTTGGGTCTCAACGGTTGCTTGTATATGAGTTTGTTCCTAACAAGACTCTTGAG
>comp150386_c0_seq2
CGGACATCGAATGTCGTCTGAAGATCAACCAAAATGCGCTACATCATCATGTGCTTGGTACTGTGGCTCGGTATTGCTCGGTACTGCGGCTTGATACCGCTTACGTGATGCATTCTGATTGG
TCTCTGAACGGCATTTTTTGTCCGGTCTCCAGACATAATAATTTCTCAGTTAAAGGGGGTTCAAAAAATCCCTTTGAGATCATTTGGTTTAACCATTGCTGTCTGTACAGTTAGTTGAGCAG
AAGGAAATCAGTGGTAATAAGCTGTTGCGAGTTGGGCTACGTTTTGGGAAACTTTTTCAAAACTTCATTGTTTGTATGAATTTTGTCACAAATCTGCTTGGCTATAATTTAGTTCTCATTTTT
CATAAATTTCTGCGGTTAAAAACAACTGTTTAAGAACTTAAATATGTCAGTATAAGAAAGTTGTCCAAAAATTATAATATCCATAACGATTTTCTAAAAATTTGTTTTCCAACAACATTTCTA
ACCACCCCATACAAATTCTTGGTTTCATTTGAGTTGGCATACGTTTTCTACTATTTCAATCGCAGTTTCCATTATGCATACCCAAAAGCAACTTTTTAAGGCAAAATTTAAAAAAGGCTGCA
AAAAATTGTCATCGAAAAGAACACTATTTTAGTTGGATATTTTCAATACCACAAAAACAGTGACAAACGAAAGCCTTAGTTGGAGTAG
>comp150408_c0_seq1
TTTTATCAAACGGAGTTAAGTATTTTGATGTGTATTTATGATTTCTAATAAAAGTTGCTATCGAAAAATAAAAGCCCAGAAATATTATTGTTAACATTATTTGTGAATTCTTTATGATTAGTTAC
TATTAAAGTGTGTGCATTAACTTATCTCAAAATTCGGAATACTTTGCCATTATCTCGAAATTTGGACTACTTTGCCATAAGGGTGAAATTCTAAATATTTATGTGATGTGTCTGTTTCTATTTAT
ATATGTTAAAAGTATTATGTTTATGTATTTGTGATTGAAACTGTGTGAGAAGAAAATATCGGCGGATGTCTATTGATGCTTATGTGTATCCAAGGGGAGATATAAGGGTCCGATTCTAAATGA
ATGAATGATGATATGTATTGAGAGTCATAAGGATCTAATATTGATATGAGCTAGAGTTATAAGAGCTTGATACTGATATGTTGTGCATAAAGAGCCATAAGATTTTGATTCTGTATCTATTCTAA
GTTTGTGAAATATTTGAGAGACATAACTGTAATAGCCTCAAAATTTAATTTCAATGAAATTTTTATAAACTTAATATTTTGGTTAGGTGAGCTGCGGCGCCAAGATCTAAGGTTTATAAGGGT
ATGAGTGAGCACGACGACATTTGGGTCTTGAGCTTTGTAGTACTGAACTAGAAATTAAATAAAAAAGTATTTAAACCCATTTTGATAAAACCTCACCCTAAGCTATCCCTCATTTTCCCTTT
GCTTGGTCAGAAGAAAACAATAGAGAGGAGAAAACATGAAAAGAGAGGAGAGAGAAAGCGGGGTTCGGGACGATATTTTTGTCGCTCCAATTGCGAATGTATTCTGATCGTGGTAGGT
AACAAGAATACTATTTTGTTTTCAATGATTTTATGTTTTGTCAATTTTCCTCCCCCTAAAATTAATAACTATGCAATATCTCTTATTTTAATGATTTTCCACTGCATAACTTTTGATATGTGATATA
TGTAGAGTAAAGGTTACTTACAATGAGTAAAATAACTTTCCTATAAAAATTTATGAATAGAAAATATCCAAA
>comp150472_c0_seq11
TACCTATGGAACCTATAGTTAGAAGTACTGTAATCATGGTTCTTTTAGCAGTGAACCGAATTTTTGTTTGAAAACTAAAAAAAGTTGAAAAAATAGAGGGGAAAAGAAACGGGTATGCAT
TCATTGATGCCTGTAAATGGAAACAACAATGAAGCTGAAAGCCTGAAACATACAATATTGAAGCAGGGAAACGAGAAGAAATTACTGCATGATGAAGAAAACTATATCAAATAATCTCTA
TTTCAACCTTGCTGCTTCAATGATCTGCATTTGCATTAATCTTTAGATTCAGTATCTGCATCACAAGGATTGCTTCCAATGAGATCATGAGGCAGACCCATGAAATTGTAAAGAGTTGGTGG
GCCAAGAGCTTCCGAATTCTTTTTTTCTGTGTTTTTCTGAATATGTTGCAAACAATGAACAAAATGTTTCTGAAACGGTATTGTTTGCATGGAGAAAGGTTCTGAAACAGGCAGAAGACC
AAGGCTGAGTAAGCTTCCATCAAGCTTAGAAGCTTCACCTTCAGCTCCGATATTGAGTCGAGGACAGCTCGAACTCGAAGAATCATCCCTCGACGAAGCAATTCTTTTGTGATCTGTAGC
AGACTGTAGAGCTGAACCAGATTGAAAGGGCCACGAACGCTTCAAGCTTGCCAACTTCTGCTCGGGCATGGAAGGAGGATTAATGCTAACGTAGCTTTGGTTTGAAGGGGGCTCCATCT
GAAGACAAGCTCCAGAAGACAAAGGAGGTGATAGAAAGTTATTGATCACCTGCATGTAGGCATCAGTCAAACACTTGCTGCTCAAATTCGAGCAAAAAAGTGAAATTGATCATTTTTTAA
TTTTTTTTTAAAGTTTTCAAGAACCAGAGAACATCTATGTTGCTCCTGTTGAAGCTTCCTGGGAGGTAGAGGCAACCGCGAGTTTGAATTAGAGGAAATTTCGCTCTGAATTGAGTTCCA
AACTAATGGCGAAGAACCATCGATGGCGTCCGAATGTGGCCGGAACAGAGCATAGTTACCAAAATAGCAAGGTTTTTTGCTTTGCTCGAGCAAGGGGAAAAAGGGGGTTGGCGCGATG
ACGCTAACGGACAAATGCTCGCCGCTTCCGATGCCTCCAGCGCAGGAAGCCGTGGCGGCGATGTTCTCACTGGTTGAGAAGGCGGCGGCGATACTCTTCTGCTGGTCTTCTCTTCGGAG

CTTCTCAAGCGCAGCGATTCCGAGCCCACGCTGCGGAATTTTATTGGGTTTGGGCTTCTTTGGCGATAAACCAGTTCTATCATTGCAGCCTCCATTGTTGGGATCTCCATCTTCTTGGAGTA
CCATTTTGGTTTACAAAGCGATCACAGAGTTGTAGAAGAAATTTTGTCTTTCTTTTAGATACAGGAGAGTACGAAGCTAGGTATTTGTGAGAAAAGTCGCCATCTTTGTATGGAAGTGATT
GGAAGAGGAAGATATGTAAGTTGAAGAAGATGTTGGGGGACTAAAACGAAAGGGATCCAACTCCATGAAACGAAGTTATTGCCGGAAATCTGTAGGATGAGAATGGAAACGAATCCAAT
AGCTATTGCAGATGGAGTCTTTCAGGCTATCGATTGAATCTGCAAATCTTTCAAACATTTTGGAGAGGAATTAATTCAACCGAACGAGACAGAGATCCCCCCCGCTCCCACCTTTGCCTGT
TCATCCGACTGGCCTCCGTTTGTTTTATAAACGAAACATA
>comp150478_c0_seq1
TACTCTTCCATCAGTTCTCTTATTTCCCAATCGAAGGATGTCGACAGTTCCGAAGTTCAACGCCGGCGAGGCCTGCGGCGAAGGCCAGGAGAAAGCTGGCCAGTGGGCTCAATCCGTTC
GTGATACGACCCAAAATGCCGCTCAGTCTGCCCATGACATGGCCCAGAACGCAGCCCAGTCCACCAAACAGTCCGCGGAACATACCGGAGGGCTGATGCAGCAGGCTGGAGAACAAAT
GATGGGAATGGCTTACGGTGCAGTAGAAGCTGTTAAGAATGCTGTCGGAATGGGGAGTGATAATAACACCACTGCAAATAAGACTAGCTCGTCCACTAAGAACTGAGTTCGGCTCGAATC
AGCCTAATTTTGCTTATTTTGATTTAGCACTTAAAATAAACGTAATGGTATCAGAATGACTAATTATGCCTTTGTAATGAGGATTTAGTTTTTCTATTTCTAATTTGGTTAATAATTGAGAAATG
TTCTTGAGTTTTGATAAATTTTCAGCATTGGTCCTTTGTTTTTTTTTTTTAAGAATTTGATTTTGCTAAATTATCAAAAATCAATATTTTTTTTTTAATGAAAGGCAACCCTCTTCTTTCTATTTT
GTCTAATATGAATAAAATTTAATTTAATTATTTTAGTATGTCTCCT
>comp150504_c0_seq1
TGTAAATATGTAAGTTTAATTGAAGCTTATCATAAAAAATTCTTTGCATTTTTTATAACATTTTTTTCCCTTCAAGGGAAATCAATTCATACCTAATAAGGCTTTCACTTCAAGTCTTTTTTGA
CGCCATCATAATAAGATATCAATATCATACCTTAGTTATTGTAGAATAAGAACAACTTGCAGAATAAAACAAAAAAAGCCTTACTTTCTAAGCTAGCTTTAGCCCATAATATATGAAACCAAA
TCTCGTTCCAACTTAGCCAAAGTCTCCAATGCCGGTAGGTATAATTTTTTGTTTTGGCTGCAGAATAGAAACTCCAAAGTTGTCATAATATTCTTAAAACTTTAATTTGTGACTGAGACAGG
CCATCACCTCTTCTTTTATCTTGTGGAATGCCACAGTACTTTCATCTACCTGACTTAACCATCAAGCAAATTAATCAATCCATGGAAGAAAAAAAAATAGGACCTCCCCTTTAGCATAATAA
TTTCCCACTAGCATTTCTTACAGTCATTCTTCCACAAAAGCAAAGTAAAAGCCTAAACAGTCCACCTTCAAGTAAATCTAGCCATTCATACCTCACTCTCAAAAGCTGGATTCCAAAAAGC
TAAAGCAGAGACACCAAAACAGATCAGAGCTAGTGGTAGAAACAGTGGTGCTGATGGTACCAATTCTTTTTCCTCATAAACTAATTATCACTATATGCAAATTTTGATTCAAAATTTAGTTA
AAACTTCGTTCTTTCGAATAAATCTTGATTCTGTAGCTGATAAGAACCTTCTGAAATCTTCAAAAGGAGAGCTCATCCAGTCAGGTAGGCTTCAGATCCTGAAACATGGAGTCCCACCTCA
TTTGGTCTGTATCGTTGTACCCAATCCAAGTAAAGCCATCATCATGGCCTTCTTCCCTTGAAGCAAGCAGTGCCATTGGGCTCATGGTCTGTGAAGCCGGGCTCCGACCGATCTGAACTGC
CTTAACTCCATTGCCGACTACAGGGCTCAAGCTCTGTGAAGCTGGGCTCCGACAGAGCTGAACTGCTTTAACTCCATTGCCGACTACAGGACTCATGCTCTGCGAAGCCGGGCTT
>comp150504_c0_seq5
AGACTACATCAGTGATCTGCAACCTTCCTTTCTTCACCATAGCCAGCCTCCTGATCCCTCCGCTCACCATCTGTCGCCCCCCTCCTCCTGGCCTCGGCATAATCACCACTGCCTTGCTTGTA
TGGTGGACTAGATCATGAACATACCTTTAGTCCCTCTCTGATTCCTCCTATGCTTAGAGATGCGGTCGAAATGTTATAGTTACCTTGTTAAGCAGTTGCAGGCTTCGTGGATGACTGTCAGG
AGAGCGGCGACTGGTCGGCCGGGGCGGTGATAACGGCGGTGGCTCTTAGCAAGCTTGAGGATCTCGAGGCGATGCTTGGCGACATCAATGCCCATGCTGAGGAGAAACTCGTGGTCGA
AGTGGGCGATGTCATCTTCCTCGAGCTCGTTCTGGGAAAGAAGGACAGAGTACTCGTAGACCAGATTCCGGTCGAGCCTCGAAGATTTTAAGAGCCATGAGTACCAGTCCATGGTCAGG
AAGTAATTAAGCTTCTGATGGCAGATTCAGTTTAGTTAGGACTTATATAGTGAAGGGTATAAGGAGACATGTGAACTTTGTTGAGGAATTTTTTTAAAAAAATACTGATTTCGGCCGCTTT
GGCTGTTCTTATGGAAGAAAATAGTTAAGCAAAATCATATTAAGATAACCACATTTGCGGTAGTTAACGGACGATAACTGGACGTCTGCTTTCAGCAC
>comp150506_c0_seq1
ATAATAACCATTCCCATTCTTTTTATCTCTATTATCTGCATTTGTTAGATAGAAATAGACCACCCATCATCCTCTCACATTCTCTCTCTCATCAATCTCAAGCAACAAAGGCGCGTATAAACT

TGCAACAAGGAAGCAATCGAAATAACCATGGCTTATCGTATACGAAACCATGAGAAGTCAACTTACAGGGCTGTTGCTTCGTTTCCTTCAGTCGAGTCATTGCTAGGCAGGCTGAATCAC
ATCGACGTTAGACTGAGGCAGCTGGAGGAAAAAGTGAGGCCCCAAGAGAGCTGTTTCTCCGGAGACCTTAGGGCTAGGAACAAGCCCCAAACTTCCACCATGGAAGACGCTCACGTAA
AGCACACGCTCATGGACCGCCTCCGGTTGCTCGAGACAAAAATCCGCCAGCTAAGCAACGAACTGCACAAAGGAAGCGACACCAGCTACAGAGTCATTTCATCTCCAGCTGAGAAACT
GATGCTGAAAATATCCAGTAATAGTAGCAGAAGAATGGTGAGAGGAAAGCCATGGAGATCTAGTGCAGAGAACGTTGAGAAGCAGATGTCAAAAAAGGTGTGCTGTACAGCGATGCTG
GAAAGAGCTCTCAGATGGATGAGATCGAGCATTTTTTGTACTGGTGATGAACACTGTTGTCTGAATTCTTCAGTATCTGATGACTGTGAAGCAGAAGGAATCCATGCAGGATGCTATCACT
GAGGATATGGAAAATAAAGTAGATCACCTAATTATAACATGTAAAGACGGTTGTTTTTTTTTTTTCTGCCTTAGCTTGACCTTTTTCTTCCTCGCTTTATTACAACGTATTCTTTGTTTAGCCT
ATCCGGTCTGTTTAATGTTGGATCAAGTTTATCATATATTATTTTGATCTCTTAGGATGCAAGAAGCAAGAAGTTAGCAGAACTGGTGAAAGTGAAATGTTCGAGATACATGGTAGGAATAA
ATT
>comp150513_c0_seq10
TTGATATATAATACCAACAATTGCAAGCTGGAGCTCTTCAAATCATCTTCTATTGCTTTTTTATCTTCTCAGGTTGTTTTATTCATATAGATCTGAGGCCTGAAAGGAGATTTGTTACCCATTG
AAGAATGTCTGCTCCTGCTGTTCCAGACCATGTTTGCTATGTTCACTGCAACTTCTGCAATACCATCCTTGTGGTAAGTGTTCCAGGCAGCAATTTGTCCGCGACTGTGACAGTAAGATGC
GGACATTGTGGGAATTTGCTGTCTGTGAATATGGGAGTTCCTCCTCAGTCAATGCCATTTCAAGATCTTCAGGGCGCTCAGCTCTTACGTTGGAACTGCGAATCATCTTCAAATTGTAACA
GGATTTCACTGTTCAATGCCGTTGGCAGTAATCAGCTGGATATGCCTCCAATACATCGTAAGTTTATCACAGTTCTGCAAAGTTGCCATATTTTCCTTGTACAATTTTGACTTGTGAAAACTT
TATGTACTTAAGAAATCATATCGTACTTCCAGCAAGACATTTTAATTTTTTTAACAAAATTTTGAAGTTTTTTTTTTTTTTTTGAACTGTTAGGGATCAAATATGGGAGTCGGGGGAGGAATT
TCTTCGCTCAACCACGGAGCTGCCCCACTAGTCCCTG
>comp150513_c0_seq3
TTGATATATAATACCAACAATTGCAAGCTGGAGCTCTTCAAATCATCTTCTATTGCTTTTTTATCTTCTCAGGTTGTTTTATTCATATAGATCTGAGGCCTGAAAGGAGATTTGTTACCCATTG
AAGAATGTCTGCTCCTGCTGTTCCAGACCATGTTTGCTATGTTCACTGCAACTTCTGCAATACCATCCTTGTGGTAAGTGTTCCAGGCAGCAATTTGTCCGCGACTGTGACAGTAAGATGC
GGACATTGTGGGAATTTGCTGTCTGTGAATATGGGAGTTCCTCCTCAGTCAATGCCATTTCAAGATCTTCAGGGCGCTCAGCTCTTACGTTGGAACTGCGAATCATCTTCAAATTGTAACA
GGATTTCACTGTTCAATGCCGTTGGCAGTAATCAGCTGGATATGCCTCCAATACATCCACAGGAGAAGAGACAGCGTGTTCCTTCTGCATATAACAGATTTATCAAGGAGGAGATACGTAG
GCTAAAGGCAACAAATCCAGATATAAGTCATAGAGAAGCCTTCAGTGCTGCCGCAAAGAATTGGGCACATTTTCCAAATATCCATTTCGGTCTTAACCATGATGGAAACAGTCAGGTGAA
GATTGATGATGCCATTCCTGCTCATGGAGTTCAGAATTCTCAAGGTTACTACTGATCAATCATGGATTATTTGAGTTGATGTTAAAATAAATCTCCTTCTTAGCTACTTTAAATTGAATTTTAT
GAAGCAGAAATCAATGTGTTTGAAAATGATTATGATGCTCTTAATAAAGATTGTAATGTGTTTCATAGTATAATTAATTTGGTTAAA
>comp150513_c0_seq4
GATATTCATATGAAACTCTAGAATAATAAACTAACATTATTTCTTCTGTCTTAACACTGTTATTCCTAAATATATCAATTACGGAGTCATTATAGGTTTACTATCTTGAACCCCAAACCAAGCAT
GAATCTAGTTCTTCCAAGCACTTAATCAATAGCTGATCCAAATTATGCTAAGCTTAATTACACATTATAGGTTTTTCCAAAGCCCAACTATTTTCAAACAATTTCCAGCGTCCACCAAGCACC
TTAGGCAACAAGATCCAGCTGGCATTGCTTTCCTGAAGAAATGGAAGATAAATGGTGTAAGCAGAAAAAGGGAAAACTTAGTGACTGGAGAAAGGAGGGAACCCAGCACAGACGTAA
GTGTCCTTGCTGGCCCCTATTCTTGATCTCTCTGAGAGCCCTCCTCTCTGCAATCATAATCTGTTGCAATGTGGAAGTGAGTGTGGTGCGGTTGGTGTTTGGACCAATGCCTCTCCAGCTAA
ATTAGTGGGAACGAGGTCCCCAACCTGACTTTTATTTATTTATTTATTTATTGTAAGAAATTGCAGGGCGCTCAGCTCTTACGTTGGAACTGCGAATCATCTTCAAATTGTAACAGGATTTCA
CTGTTCAATGCCGTTGGCAGTAATCAGCTGGATATGCCTCCAATACATCCACAGGAGAAGAGACAGCGTGTTCCTTCTGCATATAACAGATTTATCAAGGAGGAGATACGTAGGCTAAAGG
CAACAAATCCAGATATAAGTCATAGAGAAGCCTTCAGTGCTGCCGCAAAGAATTGGGCACATTTTCCAAATATCCATTTCGGTCTTAACCATGATGGAAACAGTCAGGTGAAGATTGATGA

TGCCATTCCTGCTCATGGAGTTCAGAATTCTCAAGGTTACTACTGATCAATCATGGATTATTTGAGTTGATGTTAAAATAAATCTCCTTCTTAGCTACTTTAAATTGAATTTTATGAAGCAGA
AATCAATGTGTTTGAAAATGATTATGATGCTCTTAATAAAGATTGTAATGTGTTTCATAGTATAATTAATTTGGTTAAA
>comp150513_c0_seq9
GATATTCATATGAAACTCTAGAATAATAAACTAACATTATTTCTTCTGTCTTAACACTGTTATTCCTAAATATATCAATTACGGAGTCATTATAGGTTTACTATCTTGAACCCCAAACCAAGCAT
GAATCTAGTTCTTCCAAGCACTTAATCAATAGCTGATCCAAATTATGCTAAGCTTAATTACACATTATAGGTTTTTCCAAAGCCCAACTATTTTCAAACAATTTCCAGCGTCCACCAAGCACC
TTAGGCAACAAGATCCAGCTGGCATTGCTTTCCTGAAGAAATGGAAGATAAATGGTGTAAGCAGAAAAAGGGAAAACTTAGTGACTGGAGAAAGGAGGGAACCCAGCACAGACGTAA
GTGTCCTTGCTGGCCCCTATTCTTGATCTCTCTGAGAGCCCTCCTCTCTGCAATCATAATCTGTTGCAATGTGGAAGTGAGTGTGGTGCGGTTGGTGTTTGGACCAATGCCTCTCCAGCTAA
ATTAGTGGGAACGAGGTCCCCAACCTGACTTTTATTTATTTATTTATTTATTGTAAGAAATTGCAGGGCGCTCAGCTCTTACGTTGGAACTGCGAATCATCTTCAAATTGTAACAGGATTTCA
CTGTTCAATGCCGTTGGCAGTAATCAGCTGGATATGCCTCCAATACATCGTAAGTTTATCACAGTTCTGCAAAGTTGCCATATTTTCCTTGTACAATTTTGACTTGTGAAAACTTTATGTACT
TAAGAAATCATATCGTACTTCCAGCAAGACATTTTAATTTTTTTAACAAAATTTTGAAGTTTTTTTTTTTTTTTTGAACTGTTAGGGATCAAATATGGGAGTCGGGGGAGGAATTTCTTCGCT
CAACCACGGAGCTGCCCCACTAGTCCCTG
>comp150524_c0_seq8
AATGGTGATGGTAAGATATCATCGGCGACTGAGCTGAGAGCAATCTTCGAGAACCCTCGTACAGCCTTTGATGGAGGACAAGCCATTGCGGATGATGGGCGAAGCTGATGCAGATGAGG
ATGGCTTCGTCAGCCTAAAGGAATTCGATCGTAATTTTGAACACTAAAATGGTCAATCCATCGACGACACTCGAGAACCACCTTCTTCTGGTTTTCTCAGTATTCGATTTGGATCATACTGC
GGCCAATTATTACGTCCGGTCACGGACAGCCAGACACCCAATCCAGACACGTCACGTCACCCGAATACATTGTGTTCCGATGTATTTTGGTAATGTGGCACGTCTGGATTGGTATCCGGAC
GAATCCAGACACCGAGTGTAATAATTTTTCTGGTGACGGGGTGTATTTGGCGCAGTGCCGGAAGATGATTGATGGCGTTGATCAAGACGGTGACAGTCTCGTTAACTTTGAGGAGTTTAT
GCAATCATCCCACAGAGTCTTATCTTAGCATTTAATATATATTTCAAATGTTTATGTTGAACTTTCAAAACTAAGTGGCTCCTATTAAGATTTTTTTTTTTTTTATGGAAAAATGTTTGTACTAG
TTGTCAATTTATTTATTGTGTTTTTGAAGCATTTACGATAGTTAAAAAAAAAAAGAATTTATTATCTTTTCTTTTCTTTCAATTTTTACTCCAATCTCATATGACAGTGTATATGTTTTAGGTAG
AAAAGAGTGAACTTAATTTTCAACTTG
>comp150808_c0_seq5
CCACAATTATATTTGGTGCAGATGTGACTCATCCTCAGCCTGGTGAAGACTCAAGCCCTTCAATTGCTGCGGTAAGTTTGATCCACTTATTGTGGAGCTTTTTTTTTTTTCTAAATGTTATTT
GTGGCCCTTTTGGTCTGAATTTCCAGTTATTAAATATTTTTCGACATATGGTAGGTTGTTGCTTCTCAGGACTGGCCAGAAATAACCAAATATGCTGGACTTGTTAGTGCTCAGGCCCATCG
TCAGGAGCTGATTGAGGACTTATATAAGCTTGAATCAGATCCACAGCGTGGAAATGTTTCTGGTGGCATGATCAGGGATTTACTTATTTCATTTAAAAAAGCTACAGGGAGAAAGCCCGAG
CGAATAATATTTTACAGGGATGGTGTTAGCGAGGGACAGTTCAATCAAGTTCTGCTTCGTGAACTTGATTCAATTCGTAAAGTACGTGCATGCATAGTGTTTTTTAATGCTTTATCTTTGTGT
TATTAATGTCAATGATTGTTTTTTTTTTCATTTGTATTGTGTTTTACCAATATGTGATTTATGCACTGCACCACAGGCTTGTGCCTCGTTCCATCTTGATTATCGACCAAAGATTACCTTTGTGA
TTGTTCAGAAAAGGCATCATACCAGGCTGTTTGCAAACAACCATTTAGGCATTGAGTCAGTAGATGGGAACGGAAACATCCTTCCAGGGACTGTGGTTGATTCAGATATCTGTCATCCATT
TGAGTTTGATTTCTACCTATGTAGTCATGCTGGAATCAAGGGGACTAGCCGGCCAACACATTATCATGTTTTAGTGGATGAAAACAATTTCTCTCCTAATGACCTGCAAACACTTACATATA
ACTTATGCTACACATATGCAAGGTG
>comp150877_c5_seq6
CGCCCGCCGACTACAGTCCTTCCGCCGACTATAGTCCTTCTGACTCATGTCGCCGACTATAGTCCTTCTGACCCATGTCGCCCAATTACGATCTTTCTGACCACCTTCACCTGACTACGGTC
TTTATAACCTCTGTCACCCACCGACTACGATCATTCCAACCATCATCACTTGACTACGATCTTTCTGACATCTGTTGTCTGCCGATTATGATCCTGCCAACCAAATGAACATGTCAATACTAA

TTCTTCCAACTCCCACCGTGTAATGCAGTCTATATGATGTATATTTTTATATTGAGCAAAGGCCTTCTTCCAATAAAGGGCGTAATTTGTAATCAAATGCAAATAGGATTCATTTAAACTGGA
CAATGGCCCGTTGTTTTTCTCCTTTTCAGGGATTTTCACATAAAAACTCTTGTATCTCTTATGAAAAAATATCCCTCTTTTATCTATATGCATGTTGGATTTCGTACAAAGCAAAGATGCTAA
ATTTTATATTTTTCTACGGCATCAAATATTCTAGCACATAACAATAATTATTTTTTATCATAATATTTTTATAATTATTTTTATAGAAAGCCACATCCACCCCAATCGAATCCAAGCCACCTGCAA
AAAAATAAGATTATCATGCATCAAAACAAACACCAAACATAGATTGAGAGAATCAAATCTTGACACTCGACAAAGTCTAAGGCCAACAAGATCAATAAAGAAGTTCACCTTGGCTTTAGA
TTCAGCTTTCTAGATTCACCTTTCTTGGGAGAAGAAATTTTATGGATCATAGGCTAGTCCTCAAATTCTTGTTCAAATTCAAATTCGAGTGAAGGCTATAACAAGAAGAAATTTCTTTATTTA
TGGTTTTCTAATAGGAATAGAAGCACAGCAAATGATTTAGACGGCAACCGTCCATGCACACTCCTTCCTTTTTGAAAGTCATGATGACTCGTTTGGGGATGCACTGAGTCTAGTTTTGTGA
GCGCCTATACCGATAATCTTATAGCCATTTATTTTTTATGTTTCGTGCATTTCTCATTTCTTATCATGATTATTCAAGGAAGTGTTATATTAATGGTCTATTATCTAACAAAACCTATCATTTTTCT
CATTCAAAACTTGAAATC
>comp150910_c0_seq9
TGTAGCCAAGATAGAGAGGCCTACCCTCAAAGCAGGAAATGACGGAGGTGCTGAACGTGCAACCGCCAAACTGGATTCTGCCCCGCCGATCGCCTCTTTCCACCAGTGCCAAGGCCGTT
ATTCCTGCTTCTTTGCTGACGGAAGCAAGCAGAATGTTCTAGGGCTTTATGCTTTAGTTTGGTTACTGCCAATTAGTGTGTCAGATGTACTCCAAAAACTTTGTTATTTTATTTCAGAAGCA
GGATTCGTGTTTTGATGACTGAATTTGCACATATCCTTCGCTATAATAACAGCAGATTCCAGATTTCGAAATCTTTGGTTCCAACAGAATCAATATTACTGATCCTAGCAGAATTAAGAAACC
ATGGGGTGAAAACCAGTTCAATAATGGGAAAATTTCTCGACTAAGTACAATTAATACTTGTATTAAGCATTTGAGCCTGTTTAAACAAAATTTAAAGGTCAGCTAGCCATTACCAGAAGAA
AATTAAGTTTTATATAAGTCGCTGAAGTAATCTTTGACTTATGTAAGTCGCTCATTCAACGGGTGACAA
>comp150937_c0_seq1
TTATTCCGTGATCCATTTCTTGAAATCTAACATCTTTTACAAAGTCTATGATAAAAAAATAATACATGATAGTATCTTTTTTTTGGATATTTAGGCCAATTAAGACAAACTCACACAATATCAC
ATACGCAAGACTCTAAGCCAAAACTTCTTGGTTCGTAACCAAAACTCTCAACCATCATACATTTAATCGCCAAACTAAATTCACCTCACTATGAAGATAATGTAATCAACGAATGACTAAG
TTTCTACATTGCTCTTCAAACATCACCCAAACTAAGCTAAATCAACGGCCACCCTCCCCCTTGTCTAGATTCTTAAACATCTGAGACTGTTTATTCTTTTGAATTCTGTAAAAGGATCCTTTT
TATATTTCTATTTAGTTTGTTCCAGCTTACAGAACAACATCCCTTCTCTTCTTTGGATTAGCAGCATCTGAATTTCCTTTCCTCATCATTGCGACAAACTCCTCATAATTGATTCTTCCATCATT
GTCAGAATCAGCTTCGGAGATCATTTCCTTTATGTCATTGGTTTCATAAAATCCCTTTTCTTTTAGGGCTTGCTCAAGCTCTTCTTTTGTAATGAACCCACTGCTATCCTTATCAAAGTATTGG
AATGCCGTAAAAAGATGTTCTTCTCTGTCCATTCTATTCAAGTGCATTGTTGCAGTGATAAATTCATCGTAATCAATAGTTCCATTCCCATCTGCATCAGCGGCTTCCAATAACTGTTTAACT
TCAGCTTCTGAAAGCTTTGTTCCTTGCTTTAGCAGCCCTTGCCTTAACTCTTCGGCTGTTATAGTCCCACTGTTATCAGAATCAATGTTTTTGAACATCTCTTTAAGGCCCCTAATCTCTTCC
TCGGATAAGCATCCTGCAATGACCCTCAAAGCAACTTGCTTGAACTTGTTTAGCTCTCTGAATTGCTTTAGCCTATTGAGAACGACATTATCAAGAGGTGTGTCAGGTGCTTCTCCATCTTC
TTTAATCCATGGATGGTTGAGAACGTCAAATGCTGTTAACCTCTTCTGCGGGTCTTTGCAGAGCATCCTCTTGACAAGGTCCTTTGCACCGTCTGATATTCTTGGCCAGGGTTCACTCTTCA
AATCGATGGATGCTTCGAGGATGGATTTGAAAATTCCATGCTCAGACTCTGACCAGAAAGGAGGAACACCACACAAGAGAATGTAAAGCATAACACCAATACTCCATATATCAACTTCCG
GTCCATATTTCCTTTTCAGTACTTCAGGAGCAATGTAATAAGCACTGCCAACTATATCTTTGAAGACCTCACCTGGCTTGAAGAAAACGGAGAGTCCAAAATCAGTGGCTTGGAGAGGAG
CATCTTCCGCTCTATTGAGCAATAGGAAGTTCTCTGGTTTGAGGTCTCTATGCATCACCCCCATGGAATGACAAGTATGAACAATCTGCACGATGGTTCGCAATAGGGCCGCGGCCGCCCT
CTCAGTGTAATGCCCCTTCGCAATAATCCTATCGAAAAGCTCTCCGCCGGCACAAAGCTCCATAACTAGGTGCACTGAATGTTTGTCCTCATAGGCCCCCTTGAGCTCGACGATGTTGGGC
TGGCCGGTGAGATGGTGCATGATTTGGACCTCGCGGCGCACATCTTCAATGTCCTCAGCATTGACGAGCTTGCGTTTGGCGATTGTCTTGCATGCGAATTGTTGGCCGGTGAATTTATGAG
TACAGAGGTGGGTGATGCCGAATTGGCCGCGGCCGAGCTCCTTGCCAATGGAGTAGGTGGAGCGAACATCTTCCATAGGTCGGCCGAGGACCGGGCCGATGGGGGCGAGCTTGGGGGG
TTTCATGGTGGGTGGAGGGTTGGGGGAGGGGAATTTGGGAATGAAGAGTTGGGGGTTATTGTGGTAGGAGGGGGTGGCATTGGTGATGGCGTGGTCGACGGTGGCGGCCGGGGATTTCT

GAAAGAAGGAGAAGCAGATTCCCATGGAGGGAAGGGTTGGGGTGTGAAAGAAAGGGTGAGGAGGGGAGGAGAAGGAGATGATATATCGATGATTTGTGCAAAGAGGCGGTGCGAATG
TCGGAGTTGAACAGGTGGGAGGAATTTAAGGGAGAAGGAGAGGGGTTTGGGAATCAGTTGCGGCTTGCGAAGGGATTGT
>comp150986_c0_seq1
TTAATCGATCATCAACCCCTTTGTCCCATCATCGGCATCCTATGCATGCCCGAACGAAAGTCGAATCCGATGCGGAGACGTTGCATTACGAATCATTCTAATAAACTCGAATCCGATGTGGA
AAATTCTACGGACGAGGTGACAGTAATACATGGCTTACATGTGTCACATCTCACAGCACCTCTTCCATTGCTTAGAGAACAATCCATCCATTTTTATAAGATAACGGGCCATGGCCGTGCTT
GG
>comp150988_c0_seq3
CCAAGGATCTTCCCTTCCTCAATCCACCCTCTCCTTCTCTCCTCCCTCCTTCAGGAGGCATCTCAATTTCTCTGCAATAGCAATGGCGAACTCAGCTTCGGGGATGGCTGTGGTCGATGAG
TGCAAGCTCAAATTCATGGAGTTAAAGGCGAAGAGAAGCTTCAGGTTTATTGTCTTCAAAATCGATGAGAAGAATTTGCAGATAATGGTGGATAGAGTCGGCCAACCAGATGAGAGCTAC
GATGATTTCACTGCTTGTCTTCCTCCTGATGAATGTCGCTATGCTGTTTTCGACTTCGATTTTGTTACTGATGAGAATTGCCAGAAGAGCAAGATCTTCTTCATTGCCTGGTCTCCTGATACA
TCAAGGGTGAGGAGTAAAATGCTGTATGCCAGCTCAAAGGATAGATTCAAGAGAGAGCTCGATGGCATTCAGGTGGAGCTCCAAGCAACTGATCCCAGTGAGATGAGCTTGGATATCAT
AAAAGGCCGAGCTCTTTAATTAAATTCTGGAATTGGACTTCCAGCCATTTCTGTCTTTCTACTCTTACTTTTCCATTCAAAAATATTTTTTAATTTGGTTTGTATTTTTCAAGATGGGACTTCA
TTAGGTTATTAATGACTGTGGGCTTGTATGCTTTAATCTCTGATAAAAGTGATTATTTTTTTCTTTAATGCATAATAAGATAAT
>comp150988_c1_seq3
TTAAAACAAGATTTTCATGATATTTAACTAATTAAATATTAATTTACGTACATGTGTGAACTAAAGCATAAACATAACCAGGTTAACATATAGTGTGCCATAAATTGGAGATATTACTATTGAG
GAGTCCGAACATAGAGAACCGTTCGATTTTGTAACAGTGAACTGTAAAAATACAGTTACTGTTTACCACTGCACTATTACTATTCACAGTTACAAAACCAAACGATTCTCTATATCCGAACT
CTGGACGTAATAATCTCTCCACGAATTAAACAATCCTAATCTCATAAAATTAAACCTGAAAAGTACTTATGAAACTCCAGTTCTGTCGTTTAGAGAGCCCGACATCTTACAATGTCCAAGCT
CATCTCGCTGGGATCTGTTGCTTGCAACTCCACTTGAATGCCGTCTAGCTCTCTTTTGAATCTGTCTTTGGAGCTAGCATATAACATCTTATTCCTAATTTTTGAACTGTCAGGAGACCAGGC
AATGAAGAAGATCTTGCTCTTCTGGCAATTCTCATCAGTAACAAAATCGAAGTCGAAAACAGCATAGCGACATTCATCAGGAGGAAGACAAGCAGTGAAATCATCGTAGCTCTCATCTGG
TTGGCCGACTCTATCCACCATTATCTGCAAATTCTTCTCATCGATTTTGAAGACAATAAACCTGAAGCTTCTCTTCGCCTTTAACTCCATGAATTTGAGCTTGCACTCATCGACCACAGCCA
TCCCCGAAGCTGAGTTCGCCATTGCTATTGCAGAGAAATTGAGATGCCTCCTGAAGGAGGGAGGAGAGAAGGAGAGGGTGGATTGAGGAAGGGAAGATCCTTGGAATATGGAAGGGAG
AGTGGTTAATTTGGGTACTGATTTGAGGAGGAAAACGTGGGGTGGAGA
>comp151026_c0_seq1
TAGATACTAAATATCTTGTGTATAATTGCAACAAATTTGATATTGGTATTTGAAATTTATCATTCTACACTTAAAGTGAATGGCACGGGAATTATGACGAACTCAATTAAGATTTCATGGTTCC
ATCAACCTAAGTTGTTATGCTCACTGATCTACTTAGTCAAAGGAGTTAGTCAACCAAACTTGACTAAAGGATAATTGTCAGTAGTAATGGGTTGCATCATTCCCCCGGCCTTGAAACGATC
CTGCTATAGTGGTGGGTTCGGTATCCTTGGTTGGAAATTTATGTCCTACTGCTGTCTTTGGTCCTTGAATGTTGTTGGCTGCTACATTGCATTCTTCCTTATCTTCATAATAGTGAGAGAGATC
TGCAACATTAAAAGTAGGTGAGATTTGTAGACTTCCAGGTAATTCTAATTGAAATGCATTATCATTAATGTGTTTAAGGATACGGAAAGGTCCATCTGCTTTGGCTTTGAGTTTGTTGCATCC
TAGTGTTGGAAATCTTTCCTTGCTTAATCTGATTCATACTAAGTCTCCCACT
>comp151031_c0_seq4
ACGCAACAATTTTCAAAGTTCATTTTTTTTTTATTTGAAAGAATTAAAATAAGCAAGAGTCATTGTTGACTGTAACCGTTTATTTCACCGACAAAACCCACGATTCAACCGCTCATTCACCG
ACTTGCCTATGATCGGACACGACGATTCAACCACCGACATACTCACGGCATAAAACACTCTCTCACTCGGACGGTTAGGGAGATACGCACGCACACTCAAGAGAGACATATACAGAGAA

GAGAAAGAAAAGATTTCAGAAAACAGACCTCTTCTCCCGACACCGTCCAAGCCCGACCTCGCCGT

>comp151095_c0_seq12

TTTTTCCAAAAAAAAACGATTAGGCCTTTATGTAAAATTAAAATTAACAATCTGTAGAACAATTTAAATTTGGCAAAATTTCTAAAAATATTCAACCGTATAAAAGATAGTTATTGCACTCTC
AACTTTATATTCCTTGCAGCCTTTTCTCATTCTAAATCTCCTCACTTTGATGCAATATTTCAATCTTTCAAAAAAATTTATACAAAAAGAAAGGGATAAAATAAAGAAAGAATTGCACTATGG
ATGCCCTCGTCTTCGGCTCTCTATTTAGGTACTTCAGAACTTCAGCAAAGCCCATTGAAGGGCTAATGTATGGTCCGTATCTGTAGTCCTGATTTGGCGTCTAAATGAATGCATCATCACTTG
TATTACTCACATATTGAGTTGCCCAGCATCGGAAGTGGGTGAACCTGAAGGAACCTCTCCTCATTGATGCTTTTAGTCCTAAGCTTAAGCTCCCCTCTGCTGGTTGTGAGTTCATCCAAAAT
TTCAGCACCATGGCCGAATGCCTCACAAGCTTCCTGACTGCTGTCAGTGCACGCTGGGGATCCATCAAGCCGAAATGAGAGGTTATGGTCGTGAATAGGAACTCCAGCAATTTCTTCATA
TTCAGCTGCGTCCAGTTCCCACAGATCCTGCCGGTGAAAGAATTTGGGAAGAAGATGCTCTCTGATGCTAATTAACAGGTAAAATGGCAGTGGAAAGAGAATTCCTGCTATAGGAATCCA
TGTAACTCCGAAGCATATTAGGAGATAGACCAATTGGAAAATTGTAAAGGTCCCTATAACCCTATAAGGAACTGATTCCACAAATGAAGCATGGATTCCTTCCAGGACCTTGTAACGTCTT
CCTGGAGTAATGAATAAAAGCAAAATCCTTTCCCAGAACTGGTTTCCAGGAAGGCTATCTATAGCCATGTATGCAAAGTAGCCCCACAGAACTGATGTAGGAATCTTCCTGATAAATGGTA
TAGCTCCAAGACAGCCTCCAACCATTAGGGATTGGCATAGGTTGGTAATTCTCTGCTCATTCACCCGAACAGGCAGGTGTGCATCAATGTGCTTCTCAGGATCAAATTCACCATTTAAATC
TCCACTAGACATGACAGCTTGTTTTAAGTCCTTCAATTCATTCACCACTGTTCGAGCACTGTTGCTCTTGTCCATTTCTATGAATACTTCTTGCATTTTTCCATATATTTCAGAATTGCTAGCC
TGCAATTGTATGCTTTCCTTTGCACTCTCAACCATCTTCTTGCGAATCAACTGCCTCTTGAGAACTGCAAGACTTTTTGTATGCATTGGGGACTGGGGCAAGACCCCATTTGATGGAGGGA
TTCCTAGAAGCCCGCAAATCAGAACCAGAACACCAAGGACAAAGATGTCATAATGATACGCAGATGGGTGTTTGAGATTAAATTCCTTTTGTTGAGCCATTTGAGATGCAATGCTGTGGT
CAAAAAAGTAAAGGCCTGCAACCATTATAGCAGGCACTATTGCACCAAAGATGTACAACGAGGGAACCAGCATCATATCCTTTGCAACAGACCAATTATGCAATGATTCACCCTGCCGAG
GAAATGGAATAGAAAGCCTTCGGGGAACTCCTGAAGCAACATCTTTTGGAACAGCATAAGACACTGCTGTCCATACCAATACAATCATAGGTACCCCATAGTCTGCTATCAAGCTTCTAAA
CCAGTCTGTGCCGTATCTCCAGGCCCTTGCTCGTCTGCTCTTCAGAGAAGTGAATAGCACACCAAGTGAGAAAATAACACCCAGTAAACCGTTTGTGTAAAGCCCCTCAAACTGGTACTT
GTGTTGATTATAATCTTCACCTACAGGAGCTGTAAACTCACTCATTATGCCCTTAATGGCTTCTTGCAAGAACAGAACAGTTATCAACATGCCAAAAAGTTCGCCTGCAACTCTTGTAAAC
CTAGTAATGATGTTGCATGCATTGAAAATAGCAAGAAAGAACAGCATACACGCTGTCCAGAAGCATACCCTGCATGAGTAAGCAGCCAGCCCAGGCCAAAAACAGACGGCTTCCAAGTA
ATTCTTGCCCTTTAGCGAAATGGTACAAGTAGGAATACATTATCACAGTGGGTTCTGCTACTCCTAATATCAGCATGGGTTGCCCCCCAAAGATTGAGTGTACGATGCCACAAATAGAAGTT
GAAGCAAGGGTCTCAAAAACGCTTAGTTCTCCATCTATAACAAAAGCAAAAGATTTTCAAGCAAACAGCCCAATGACATCTCTCTTTTTTTTTATTTTGAAATAGCAGGATGACTTCTAAA
AGATGAGGCATGCACTTCATTTCAAATATCACCTACCTGTATCTCTGCTCAGTTGCTCCCCAAAAGCAATAACAGGAAGGGCCGAAGCAAAAAAGATGAACATAGTCGGAGCCAATATCC
TCAAGCCTGAACGAAATCCACCAAGCCAATCTTGCTTATAACAACTAGCTCTTCCTTTGA

>comp151105_c1_seq1

TTTAAGCATGAAAAAAAAAATTATTAGGATACTCATGTTCAATTAAAACATGGATATACTGTTGATTGTCATTATTTACTCATATGAACGTTCTCTCCATAGCAAAGCAACAATTATTCTTCAT
TCCAAGTAGCCCAAAACATGTAACTAGAAAATATTTTCTTTTTCCACTTAACAGGAAAAAAATAAAATTAAGCTCTACTCATTTTCTCGTTGAGCCGCGCAGCCGCCGTGACAGAGTCGGC
GACACCGCCCGGCACCGTCCTCAAATCGGGCCTATTCCTTAGTTCAGCTCCCATTACTGCTTCAGCGTCTTCCCTGGTCACCTCTTTGTCCGGCGCCAGCTTCGCCGTCGCATTGTTAAGC
ACGTCACCAACCGTAATCTTATCCTCCTCCCTCGCAATCCTAGCATTTATCTCGGCCGCCGACTGAGCCGCCGCGGCCACCCCTCTCGGTGCCGTCATTTTGATCGCCCTCACCTCCGCCGC
CTGTATCGCCGCCGCATCACTCTGTTCCACTGGCCTGTCGCTCTGCGAAAGCCCTGCCGCCTCCAATGCTTCTCCCATAGTTATTTTCTCGCCAGGGACATCCTCTTCCGCCGGCGTCGCCG
TCGGAACTGTGAAATTAACGACGGTCTGGCCGGCAACCCTCTCGCGAACTATGCGGCAGCCAGGAGCCTCAGTTTCAGTAACGGTGACTCCTCGAATGGCTGCGTCGTCGGTGAAGTCG
TCGTGGCCGACAAGGCCGGCGCGCTCATTAAAGGTGGCAGCGGACTGCATGACGGCGGCAGCGCCGTCTTTTTGGGTCTTGCCGGTGATCTTGTTCTCGGCGGTCTGCATCAACGCGGC

GTCGCGAGGTCGGATGGGTTCACCGGCAAGGTCTCCTTGGACCGGGAAAACGTCACCATACTTGATGGGTTCATCCAGATGGTCGGAGCGGCGGCGGGGTTGTTCATTCTTGCTCATGG
CTGATTGGAGCTTCGGAATCGAAGTGAAACATCCCAGAGTACACCGGTGTATTTAAAGGGAGAGGTGAAGAGGAGATTGT
>comp151167_c0_seq3
TTTTTTATGCTCAATGACCAAATGAAGTTATATTAAAGAAATTGCCGTAGTACAAAATTCATCTGCCAAAGAAAAGCACCTATATCTCTTCTTTTAGGCTATCAGTATAAGTTTACAACGTCT
GAAATAAAAAGTGATACAATGGACAGAAAAATATGCCCCAAGCATTATACAAGTTGAATCACATAATCTACACTACCCTTTGAAACATTCTAGCTTGTTAATCTAGGAATCCAACACAGCA
AGCTCATTTGCTATTGTATCCTACAAGCTCGCTTGCAATCAGCCAAAACATATAAAGATATGAGTCTCTCTCTACACTGCTAAATGCGTCTTGCCTTTTTCTGGCTAGTATCCTTACGGAACA
AACAATGGCCAATTCTAGTTACTGGCGCAAAAGAGTGCCAGTCAGCTTAAGAACACAGTTTTTATAATTGAAGGAAAAATGTCTCTATGCCTGCTTGGGAACTCTCCTCTTTCTTCATAGA
CTCGATAAAACAACCCTCACTGAAAGTTAAGTTTAACCATTCAAGGGCATCAAACTCGTTCAGACCAACAATTTTCTCAATCGTAGCACCTTTCATGAAAATATCATGAAAAAATTTGGCT
TCAAGCTCCAGCAAAGGTTCAATTGGCAACTGTTCCAACTTGTTTCTCTGGTTCTTAGATTTTAGCTGAGTCTCTTTCGTGGTGGAGTTGATCCTTTTCTTTGTTAAGGTGTTAAAGGGAA
ACAGATTGGCCGAGGCTTGGGCTATAGAGGTGCTCAACTGTGATGGCAAGCGTTGGGAATTGTTATAATTTTTGCTTGTTGGAGCTATAGATTTCCATCCTGTGGTAGATTTCTTTGGCATT
TGACTCAACTCATGCCTTCTACCACATTGAAGTTGCATAATCTTTCTAATTTCTGATCGATTATTGGTCTTCCTGGGCTTGACCTCAACTGATTTCAACTCATTAACTTCTTTTTTTCCGGCCA
TCTTCTTTTCCTTCCGAGGAGAGACACATAAGAAGCTTTTGCCCAATTCAGAACTCTCATACATGTCCTGATTGTATGGCCAGAAGAGAGGTTCATCATAGTCAAATTCTCCCAACAAACT
GCCGCATGTTTCTGTCAGGTAAGAAGAAGTTATAGAGCTGGGACTAGAAAATGAAGATTCAGATAGAGGATTTACCCTGTAACTACATGAAGGACTTGACAAATCCGAATAAGTGGAATT
TACTCCATCTTTCAGTTGAATCAACTCAGACCTCTCAAGATTGAGATCAAATGTTGAAATCCATGTACCACTTCTATCTAAATCATCCATTGTCGCAAGAGATGTGCATGAACTCTCAAGAG
ATCTATCATAAGAGAAATCTCTTCTCTCATAAGAACAAACAGATTGATCATTCATGACCCCCTCTTCCTTTGAAAGTTCACCACTGAGATCAATTTGCAAAGGAAGTACAGTAGAATCAGC
GTTCTCAGACTTCATCAAAGGAACTGTGTCTATGAAAGACGGAGTCCAGCCATTCAACAGACAAAGATCTGATATATCAGAACTAAATAAGGAGGAAGATTCACAGTCTGAAGACAACC
ACATGCAAGTACTCTCATCAGACACTCCTTCGGCAAGTTTTAGCTGCATCTCCAGATCAATATCTTCAATTTCTAACTCACAAAAAGCATCAGAAGTCTTTTTATTGGGCACAGGTTGCGG
ACTTTCTTCTGGTCGATTACTTGGATTGTTTTTCTTCGAGCTTGTTTTGATTCTAGGCAAAGCCCTGTCCACAGGCTGAGATGACCTATTGTAAGTAAGGAACCCCATTTCTTGCTTTGATAT
AGCTGACTAAACTAGTTCCTCAATGCACAGTTCTAGCTTTCAGAAGTAAGCATTACAAAGAGATTTCTGAACAAGCACACCATCCAAATATCTCCTTCAAGACACAAACCCTCGAACGTT
GGGTACGATGAGGGTTTCTCTTCCTCCGCTCCAAG
>comp151237_c0_seq2
CCATACTTATACTTATAAAGTTACCCCTGAAAATTAAAGTTGATTCTGCCCCTAGATATCCATATGTAGAGCTGCATTACAACCTTCCACTATACGAAACACGAATTTTGTGGCTACTAGATA
GGATCCAAGCAGTTTTAATATTTTTTTTGTAAATGAGAACACGAAGTCATCCTCTGCAATCTAATTAGAATGGAGTGAAGAAAATAGGATACTGGTGATGAAGTGAGATTAGAGTGAAAAG
TCAGGGAGGATGGCGCTGCCACCGTCTTCTTGATCGAGCATGCGGACGACATCAGCCACACCAGAAGACAACTCAGTTGAGAAGGGGCCGACCGGCAGATCGAACTTTCGCCGCCTCG
ATCCCGATTCCCGGTATGGCTGTGGATCCACCACCGAAAGCATGGCTTCCAGCTCGCGGGTCATTGACCTCTTAGCTGAGCGCACCATAAGATCTTGACCCTCTATGGCATCAACAGTGAC
TAGAAGTATGATAATTTTCTCAGAGAACCGTTGCCGTTCTTCTGCATCTTTTAATATCCGATGACGATAAGAAAAATTTGAGAACAAGGCTCTAATCTCCTTCAGCTTGGACTTAGCCACTG
CCAGATCCCTAAGAGAATGAAGAGCTTGCGAGCGTCTTGACTTATGATCTCTGAATGTCTTCTGAATTGCCATTGCTGCATCTTGTGGTGATAGTTCTTTCTTCTTACCCTTACTTCCGTGTA
CAGTTCTCTTGGCTAAAGCCTGTCTAACAATTTGCTGATCGGCTCCATCATCGATCTCCACGGGGCGTGCCATAGCCTTTTTTACTTTCTTTTCTTTCTTAGCCCTCTCCCAAACATCTTGCG
CTGACGCCGAAGCAGCATCGAACGTATACTTTACCTTTCCACGGGACTCTATCGTTCTTCTCGCGCTATTCGTAAAAGCTCCCTTCGTCTCCGCTGTGCACTTGTATTTTTGATCGAATCCTT
CCCGCTTATTTCCCTTGATCTCAGCAGTCCATTTCCATTTGCTCTCTTGGGGGCCAATGTCCCGATCAAACCCAAGCTCGAGCATGGCAAGCCGATCGCCGAGGCCCCGGACGCAAAGCT
CTGCCTCAGCTCGGTTATGAAAGATAAGATCCGAGGCTGCATCAAAGAAGTCCACATTTGGCAGTAACAAGCGAAAAGGATGATATTGATGATGATGATAATGAGGGTGAAGGCAATATG

AACGGGCGCGATCGACGGGAATGGAGCTAGGGTGTAAAATATCGAATTCGAAATCAAGATAGTCTTCCGCTGGTTCCAAGCAGGAGAATCTCGGACTAGTAAAGCCGGCCTTTCGGCAC
AGGTAGTGGAAGTAATGATCAAGAAGCTCAAATGAATTGAACGCACTCATTTCTGGATATTGGGTCATGGGTCGAGACGATGCCTTCCACTGTGGAAAGAAAGCCTAGGGTTTTTTTATT
GCAGAGCGGAACAGAGATTGGAAA

>comp151256_c0_seq4

GCGCTTTAAAGCCAGCAGAGATAATTTTATTAGAGATTAATGTAGCATTCTGCAGTTCATGCAACGTACATAAATTCTGAAAACCGAAACCAAAAGCATATGGTAGCTTTTTATTCATGACC
GAACAGTTTGTTAAAAGCTCCAAATCCACAGAAAAAAAAAAATTCTAAGTAGAGGAAAGCAAAATCGCAAGAGGAAATCCGAGCTATGCACCATTTATCCTTTAATAATTAACAGACTCCA
ATGCATCCCTACGATTGATATAACGTCTCAAACACTCCATGACTTCATCGGCAAATTCTTCATTACTAAGTTCGACGATGTCTTCATAAACATTAAGTTCTCCATGTTGGTTAACAGTATCTT
CAAGTAATTCTCCGAACACATTTCTAGTTTCACGAGCAATTCTTTCAACAGTTTGGCCAAGATCAACCTGCTGCTCAACCTGCCGAGAAACATCGAGATCAGAAAGTCTCATCCCATCATT
GCCTGTGTCTAGTCAAGTGCAACATGAAAAGTGAAAATTAAAGTAAAAAATAGCTCATAGTCAGCAAAATTTTGAAGCTGATAGAAAAATTGCATCTTGTGAGTACCTACAAGTACGTCT
TCTAGGTTAGATAACTCATTCAAATTCAACTGAGTCGTTTGCATTATACGATTGTATAACTCAGGAGGTTTAAGGTGGAATATGACATACCAGATTGTGTTCTGCATTCAGTTGCCTGACCTT
CAAGTGTCGGGACAGTATTAGATCCTGAAGGAGGCCCTGTACGTAGTGCAGAACCAGAAATTTGAGAAGCTGAAACTACAGGTACAAATTCTTCTTGCCTATAATGGACAGGGCGAGGG
CGATGCACCCGAAATGGATGCAATGATTGATGAGGAAATTGCTACATACAAAGTATTAAAATTAGATAGAATCCCCAAAATGACAAGAAATGGGAGTAATAGATACAGATATATGTGATTTA
TAATGGGAAAGATCAACCTCAACCATTATTTGATTTGGCCCTGTTGAAGATATATAAACAGAGCCTCCAGTTTCTACAGATGACTCCCTTGGCCTTCGCCCAAGGCGTAGAGACATCGATG
CTCGGCCAAGCTCTAAGAACAAGGCTCCCAGATGCTGCATTGCTAAACCAAACTGCACTGCTCCAAACTGTGTCTGACCTCGCAAGACTTGGTCATCGCTGTTTTCTATTCTTAGCAAAT
GTGTGGATAGATTTGAAAGAGCCGTGGTTGCATTTTCCCTAAGAAGCTGTGCTGCTCGCCACAAAACAATACTCAAAGTTTGAAGGGCTGGTAAAGTTGCAGTTATTGTGCCCCTCGAAA
GGATCTCATGCTGATCATTGTTCAGTGAAAGCAGCTGATGCTGATCATTGTTCAATGAGTTACAACAACTTTGAAACTCCCCATTCATCCGATCAATGAAATCATTGACAGTCGTCAAAGTA
TCAGGAAATAGCTCCTGTTCATTGGAAATTCGCCAAGGCGATTCAAAAAATGAAAGAGAGGATGCTGGTTGACTCTGAAATATAAGCCCTAGATTTACTGAGTTAGTTGACCGAGTCCTT
CTTGAGTTCTGAGAACTTTCTGCTCCGGCACCTTGCTGGCTGCTACTTCTTGATGTCTCAGCGGCAACAGTTGAGGAAACAGGAACTAGCCGCCTTTCAGCTAAATGCATAGTGTCTCCA
TCTGTCAAATTGAGCTCCGATAGCAATTGGTGATCCTTGAGAACCTTCCCTTTATAGAGTAGCCGCTGTTTTTCCAAGGGAACGCCAATTTTAGTCACTACCTCCTCCCTCAAAGCTAATAT
GGGCATATTTTTATTCACACGGAAGGAATGTGTTTGGAAATCTAGAGTTTTGATTTTGATCTCAAAACTTGAGTCTGAACTTTCACCGGTGCCCTGGATTGAACTAGTGTCCTTCAAGCTG
GATTCATGCGCCATCCTTGACGATTTCGGAAATGGATAATAAGATTGATTTCCAGATGAAAATGCAGAGGATAGCCGTTGGAACTTTTCGTTTGTAGCCTTCTTCGCCTGACTGAAGGCATC
TCAGAGCTCCTTCTTGAAGACTGTAAGCAGAGAAGAGGGCAACGCTTTGCCGCGTAAGTCGTAGACATGTGTGAGAGGATCCGTGTATGGAGCAA

>comp151256_c0_seq6

GCGCTTTAAAGCCAGCAGAGATAATTTTATTAGAGATTAATGTAGCATTCTGCAGTTCATGCAACGTACATAAATTCTGAAAACCGAAACCAAAAGCATATGGTAGCTTTTTATTCATGACC
GAACAGTTTGTTAAAAGCTCCAAATCCACAGAAAAAAAAAAATTCTAAGTAGAGGAAAGCAAAATCGCAAGAGGAAATCCGAGCTATGCACCATTTATCCTTTAATAATTAACAGACTCCA
ATGCATCCCTACGATTGATATAACGTCTCAAACACTCCATGACTTCATCGGCAAATTCTTCATTACTAAGTTCGACGATGTCTTCATAAACATTAAGTTCTCCATGTTGGTTAACAGTATCTT
CAAGTAATTCTCCGAACACATTTCTAGTTTCACGAGCAATTCTTTCAACAGTTTGGCCAAGATCAACCTGCTGCTCAACCTGCCGAGAAACATCGAGATCAGAAAGTCTCATCCCATCATT
GCCTGTGTCAGATTGTGTTCTGCATTCAGTTGCCTGACCTTCAAGTGTCGGGACAGTATTAGATCCTGAAGGAGGCCCTGTACGTAGTGCAGAACCAGAAATTTGAGAAGCTGAAACTAC
AGGTACAAATTCTTCTTGCCTATAATGGACAGGGCGAGGGCGATGCACCCGAAATGGATGCAATGATTGATGAGGAAATTGCTACATACAAAGTATTAAAATTAGATAGAATCCCCAAAAT
GACAAGAAATGGGAGTAATAGATACAGATATATGTGATTTATAATGGGAAAGATCAACCTCAACCATTATTTGATTTGGCCCTGTTGAAGATATATAAACAGAGCCTCCAGTTTCTACAGAT
GACTCCCTTGGCCTTCGCCCAAGGCGTAGAGACATCGATGCTCGGCCAAGCTCTAAGAACAAGGCTCCCAGATGCTGCATTGCTAAACCAAACTGCACTGCTCCAAACTGTGTCTGACC

TCGCAAGACTTGGTCATCGCTGTTTTCTATTCTTAGCAAATGTGTGGATAGATTTGAAAGAGCCGTGGTTGCATTTTCCCTAAGAAGCTGTGCTGCTCGCCACAAAACAATACTCAAAGTT
TGAAGGGCTGGTAAAGTTGCAGTTATTGTGCCCCTCGAAAGGATCTCATGCTGATCATTGTTCAGTGAAAGCAGCTGATGCTGATCATTGTTCAATGAGTTACAACAACTTTGAAACTCCC
CATTCATCCGATCAATGAAATCATTGACAGTCGTCAAAGTATCAGGAAATAGCTCCTGTTCATTGGAAATTCGCCAAGGCGATTCAAAAAATGAAAGAGAGGATGCTGGTTGACTCTGAA
ATATAAGCCCTAGATTTACTGAGTTAGTTGACCGAGTCCTTCTTGAGTTCTGAGAACTTTCTGCTCCGGCACCTTGCTGGCTGCTACTTCTTGATGTCTCAGCGGCAACAGTTGAGGAAAC
AGGAACTAGCCGCCTTTCAGCTAAATGCATAGTGTCTCCATCTGTCAAATTGAGCTCCGATAGCAATTGGTGATCCTTGAGAACCTTCCCTTTATAGAGTAGCCGCTGTTTTTCCAAGGGA
ACGCCAATTTTAGTCACTACCTCCTCCCTCAAAGCTAATATGGGCATATTTTTATTCACACGGAAGGAATGTGTTTGGAAATCTAGAGTTTTGATTTTGATCTCAAAACTTGAGTCTGAACT
TTCACCGGTGCCCTGGATTGAACTAGTGTCCTTCAAGCTGGATTCATGCGCCATCCTTGACGATTTCGGAAATGGATAATAAGATTGATTTCCAGATGAAAATGCAGAGGATAGCCGTTGG
AACTTTTCGTTTGTAGCCTTCTTCGCCTGACTGAAGGCATCTCAGAGCTCCTTCTTGAAGACTGTAAGCAGAGAAGAGGGCAACGCTTTGCCGCGTAAGTCGTAGACATGTGTGAGAGG
ATCCGTGTATGGAGCAA
>comp151256_c0_seq7
GCGCTTTAAAGCCAGCAGAGATAATTTTATTAGAGATTAATGTAGCATTCTGCAGTTCATGCAACGTACATAAATTCTGAAAACCGAAACCAAAAGCATATGGTAGCTTTTTATTCATGACC
GAACAGTTTGTTAAAAGCTCCAAATCCACAGAAAAAAAAAAATTCTAAGTAGAGGAAAGCAAAATCGCAAGAGGAAATCCGAGCTATGCACCATTTATCCTTTAATAATTAACAGACTCCA
ATGCATCCCTACGATTGATATAACGTCTCAAACACTCCATGACTTCATCGGCAAATTCTTCATTACTAAGTTCGACGATGTCTTCATAAACATTAAGTTCTCCATGTTGGTTAACAGTATCTT
CAAGTAATTCTCCGAACACATTTCTAGTTTCACGAGCAATTCTTTCAACAGTTTGGCCAAGATCAACCTGCTGCTCAACCTGCCGAGAAACATCGAGATCAGAAAGTCTCATCCCATCATT
GCCTGTGTCTAGTCAAGTGCAACATGAAAAGTGAAAATTAAAGTAAAAAATAGCTCATAGTCAGCAAAATTTTGAAGCTGATAGAAAAATTGCATCTTGTGAGTACCTACAAGTACGTCT
TCTAGGTTAGATAACTCATTCAAATTCAACTGAGTCGTTTGCATTATACGATTGTATAACTCAGGAGGTTTAAGGTGGAATATGACATACCAGATTGTGTTCTGCATTCAGTTGCCTGACCTT
CAAGTGTCGGGACAGTATTAGATCCTGAAGGAGGCCCTGTACGTAGTGCAGAACCAGAAATTTGAGAAGCTGAAACTACAGGTACAAATTCTTCTTGCCTATAATGGACAGGGCGAGGG
CGATGCACCCGAAATGGATGCAATGATTGATGAGGAAATTGCTCAACCATTATTTGATTTGGCCCTGTTGAAGATATATAAACAGAGCCTCCAGTTTCTACAGATGACTCCCTTGGCCTTCG
CCCAAGGCGTAGAGACATCGATGCTCGGCCAAGCTCTAAGAACAAGGCTCCCAGATGCTGCATTGCTAAACCAAACTGCACTGCTCCAAACTGTGTCTGACCTCGCAAGACTTGGTCAT
CGCTGTTTTCTATTCTTAGCAAATGTGTGGATAGATTTGAAAGAGCCGTGGTTGCATTTTCCCTAAGAAGCTGTGCTGCTCGCCACAAAACAATACTCAAAGTTTGAAGGGCTGGTAAAGT
TGCAGTTATTGTGCCCCTCGAAAGGATCTCATGCTGATCATTGTTCAGTGAAAGCAGCTGATGCTGATCATTGTTCAATGAGTTACAACAACTTTGAAACTCCCCATTCATCCGATCAATGA
AATCATTGACAGTCGTCAAAGTATCAGGAAATAGCTCCTGTTCATTGGAAATTCGCCAAGGCGATTCAAAAAATGAAAGAGAGGATGCTGGTTGACTCTGAAATATAAGCCCTAGATTTA
CTGAGTTAGTTGACCGAGTCCTTCTTGAGTTCTGAGAACTTTCTGCTCCGGCACCTTGCTGGCTGCTACTTCTTGATGTCTCAGCGGCAACAGTTGAGGAAACAGGAACTAGCCGCCTTT
CAGCTAAATGCATAGTGTCTCCATCTGTCAAATTGAGCTCCGATAGCAATTGGTGATCCTTGAGAACCTTCCCTTTATAGAGTAGCCGCTGTTTTTCCAAGGGAACGCCAATTTTAGTCACT
ACCTCCTCCCTCAAAGCTAATATGGGCATATTTTTATTCACACGGAAGGAATGTGTTTGGAAATCTAGAGTTTTGATTTTGATCTCAAAACTTGAGTCTGAACTTTCACCGGTGCCCTGGAT
TGAACTAGTGTCCTTCAAGCTGGATTCATGCGCCATCCTTGACGATTTCGGAAATGGATAATAAGATTGATTTCCAGATGAAAATGCAGAGGATAGCCGTTGGAACTTTTCGTTTGTAGCCT
TCTTCGCCTGACTGAAGGCATCTCAGAGCTCCTTCTTGAAGACTGTAAGCAGAGAAGAGGGCAACGCTTTGCCGCGTAAGTCGTAGACATGTGTGAGAGGATCCGTGTATGGAGCAA
>comp151295_c0_seq1
ATGTTTTACTGTTTTCAAAAACTGGTATGAAGAATAAGTTTATACATACAGCAGATTGGAACTAGAAAGACATACATATTAATAGAAAGAAACTCTTTTTCATGTTACTTCTTCTACTCTGTT
CTTCCTGTTCCATTGCTTAAACAGCAGTTTGATATCCCTTGCTTCAGAAGAGTTTGTGTTTGACGAACTGATGTGTGTAGGTGCTGAATCAGCTGGATGGTATGCTGAAATGTCTGCCACA
CTAAACGTGGATGGAGTATGTCTGAAGGTAATTCGACTGTGTAGGCATTGTCGTGCACCCATTGCTTGATAACAATAGATATGATCTTCCGTGGGCTTAGCTTGGAGTAAGTGCATGAAGG

AAATCATTCCTTTCTCAATCGCACCATAACTAGTTCGCCTGGATGAAAGACTTTGGATGGGCGATGTGCATCTGCCTTTGACTTGTATGCTACATTGGACTGTTCCAGCTTCTTGCGAACTT
TGTCCAGAGTGCATTTAAAGTTTTCGGCCAGTTTATGGGCAGTTGAATTGTGGCACTTTGGCAACACAACTATACCCACAACACGGTTAGGTGGTTTAGTATAGACAATTTGAAAGGGGTA
CATACCAGTATATCGGTTTGTCATCGAATTATAGGCAAACTCCTCATGGTTGAGTACATGATCCCATTGTTTCGGCCTTTCGGGTGATCTTGCACTAAGCATCGCAACATGCTTCTAAGGGCA
CGGTTTACCACCTCCGTTTGGCCGTCAATTTGGAGGTGGTATGCGGAGCTTAACTTGATTTTTGTCTGCAACTATTTCAAAAGCTCTCTTCATAAGTGGGTGATAAACTTGACGTCACGATC
TGAGGTAATGCTGCGAAGCAGGCCATGAAGGCGAAAAATCTCTCTAAACAATAATTTGGCAACTCCCAGTGCATCACAAGTTTTCTTACAAGCTGTGAAGTGAGCCATTTTCGAGAAGTT
GTCAACTACTACCATAATAGAATCAACTCCCTTTTTTGTGTGTGGGAGACCTAAAATGAAATCCATACTTTAAGTCCTCCCAGATTGATATTGTCATCGGTAATGGGGTGTATAATCTAGTGT
TTTAATTGCTTCCCTTGTAAACTTGACATATTGGACACCTCTCAACAAATCTGATAACATCCCTTCGTAAGTGTGGTCAGTAGAAGTGCTCTTGTAGATGGATTGAGGTTTTTTCTCTGCTG
AAATGTGCTGACAGACCTCCAGCATGCATCTCCTTGATCAATTGTTGTCGCACGGATGACAAGGGTATGCAAAGAGCATTGCCTTTAAATAAGAATCCATGGCGTAGTGTGTAGTCTTTAT
ACGATCTCCCCACCTAATAGTTCTCCCATGGTATTAGAAAATCTGCATCACCAATTTATAACACTTTCAAGGATTCCAACCCTGTAATTTCGGTTTGAAGTTTAATAATGAGGGCAGATCGTC
TGCTCAATGCATCAACCACCCTATTTTGAACTTCGAATTTGTGCTTAAGGCCAGCTGAAATTCTGTAGGAAAATCACCCATCTAACATACATCTGGTTAATGGGCTTCTGAGTGTTAATAAA
TTGGACGGTTTAATGATCACTGCATAGTACAAAATCTTGGTGAAGCAAATAATGCTCCCACTATTTGAGGGCTCGATTAATTGTGTATAACTCTTGCTCATACACCGACCACTTCTGTCTTGC
CAGACTCAACTTCTCACTGTAAAACTCAATTGGTCAGTTTTCCCGAGAGAGCACCGCTTCAATTCCCACTGCTGAAGCATCAGTGTCAGCATTAAACGGCTTAGTGAAGTCTGACGTTGT
TAGAATAGCTGCTGAAACTAATGCCGCTTTGATGGTCTCAAAACTACGTTGATGTTCTTCGTTCCAACTGAAGGTGCTCTTCTTAAGACAATGCGCCAAAGGGGTCATAATCATACTGAAA
CCAAGCACGAATCGATGATAGAAATTGGCTAAACTGTGAAAACTTTGAATGTCAGTGATGGAACGAGGCTGCGGCCACTCCTGGATAGCTGCAATTTTGCGTGGATCCACACTAACTCCA
TCTGTCTAGACAATAAAACCCAGAAAATGGACTGCAGTGGTAGAAAACTCATAACTACAATTTTTGACCTTTAGAGGGAGCATTGCACAATCCGAACGGCATCACCCACCATTCATATAA
GCCATAAGCATCTGGAAGGTCGTCGTCCATTCATCTCCAGGGTGGATTCTGATTTGATGGTAGCCACTACGCAAGTCTAATTTTGAGAAAATTTTGACTCCAGACAAGTAATCTACCATATC
TTCCAGCCTAGGGATGGGAAATCGATATTTCGTATTGATCTTGTTGATTGCACGCCTGTCCACACACATTATCCACAACCCATCCTTCTTCGGGACTAATATGGCAAGTACTGCACAGGAGC
TCTGGCTAGGTTGTTTTAGCTTTTTCTGTTATAATTCTTCCACAATCTTCTAAAATTGTTGATGCTTAGTGGGACATAGTTTATAATGCAATAAATTGGGCAAAGTGACGCCAGTACCAGATC
AATTTGATGTTGAATAAAGCGTATGAGAGGGAGTTCATTTGGCAGTTCTTGGGGTGTAAGATCTTCAAAATCAGTCAACAATTTTCGTAGTTCAGGCAGGTAAGTAGAAGTATAAACCTCC
TGACCTGCTTCAATAGTCGCAAGGGCTACCAAGGACTTCATCTCACGGCTGATCTGTAACAGAGACTTCCCTGACACAATACAAAGGGATGGTTTATTGGAGCTAAGGTTAGTTGCATTG
GTTGGTGTGCTGGGTAGTAGTTGAAGTTTTCGTCCCTT
>comp151303_c0_seq2
TCTACTTGAATCATTTGGCATCATCGATCGATCCCCTAACTAGAATATTCCCCTGAGAAGATTAGCTGCAGTAGAGAGAGCTGCTCCAGTTATGGCGCACTGCACCAATTGCTCATGAGTTG
TATCCTGCAGTGTTAAGGCTAGTGCTGCTCCTGTAACAGCCCCAGCAACCACACTGTTCTTCCAATCATGAGTTCCTCTGGCCTCTCTCAGCCCATATGTTATCCCTGAGTAAATGCCAGCA
GTCAGTCCCCATTGGAAAGATTCCCTCCCAGTATACCTCACCAGTGCTTCAAGAGATTTCCCATTATGTTCTCCTCTCAGATCTGGGAACCTGCGCTTCCTTGCACCCGGGAGCTCAGGCA
CAGTGCCTGAATCAATACTAGAACCATCCACTGCACTGAAATAGACCTCTCGGGAAACTGCTTGGAGAGCACCAATCCCAGCGGCCTTGACGAAGCTCTCGGCAACGCGATTGAGGAGG
GGATGGCCAAAGTCGAAGAGCGAGCCCTTGTCAAAGCTTCTCAGCTCGTCCAACAGACTCCTACCCTCCAAGTTCACTTCACTCATTTTCTTTCTTCTTGCTTTAACCAGAACAAAACAC
TTAGAACAATAGAGAACGAAGTGAGAGAAATAAAATGGATGGAGAAGCTTAAGAAGCGAATTG
>comp151306_c0_seq1
ACTTGAAGGGAAAAAAAAAAGAAAGAAATTGAAATCAATGATATCCGAGAAGAGTAAAAGAAAGCAGAGAGTAAGCGGAAACTTTGGATACAAACGAACAAGCAAAATCAACAGCTT
TCCAGATGAATTCTTTTCAGTGCGATTTCATTGTTTTCAGAGTCAGTTACAGTCATCGAACCTTACCTCGAGAACGGCAAACGAAGTCGGTTTCTTCAGTTCAAGCAAGACCGGAAGTCA

TGAACTCAGCTTCGGAGACGGCGGAGAGCTCTGCAGCATCTTCTAATGGAGCGGGGAGATTGAGATCAAATAAGCTCTCGCTAAGCTTAGCAGGGCGTGGCTGCGAAGTGATGGTAATG
GCGGTCGTCGAAGACGAAAGGTGGGATCTTTTGTGCCCGCCGAGGGCTTGACCACAGCCGAACTCTCGGAAGCAGATTGGGCAAACATGAACGCTTTTTCCGTCGGCGTCGGCATTGG
CTTCCGTCGAGCCTTCAACATGGATCGGCGGGACACAGCTCCGCGACTTCTTGTGACTAGCGCGATGCCCGCCGAGAGCTTGGTATGACCGGAATACCTTCTTGCAAGTGCCGCACTGGA
ATCGGCTTCGCCCCTTCGCTTGCAAAGACCGCGGCTTTAAGTCAATCATCATCATCTCCTCTACTCTTTCATCTGCTTTGTTCACTTCCTCATCCTTGCTCCAAACATCACGAGACAGCAAC
ATTAGGCTGATGGCAACGTCCTCCTCCGGCGTGGTGTCGGAGACAGAGCTAAGCAGTTCAGTCTCCGGTAGCTCCGGAGCCGGTCGCTTTTTGCGGCGTCGATTGATTGTGATAGGGGAG
GACTCCGTTTCGCTTTCTCGGTCCTGAACAACTGATGACGAGAACTCCGGATCGACCATGCGGAAGCTTTTTTAGGATTCTCTCGAAGGCCGTAGCCCAGCCCTTTCGCCTCCTCTGCTT
CGCGGGAGGAAGTTGAGGAAGATGAGTAGTCGGAAAGAAGCGATTTCACCGAGGCCGCGGCGGCAGCCGCGACAGCCACAACGTGAGAGCGCATGTGGCCGCCAAGCGCGCGGCCAT
TGGAAAAGCTTCGGTCACAGAGCTTGCAAACATGCCTCTCCATGTCCAAGAAGCAGAGAGATCGAAGAAAGAGATAGGGTAGATTCAGCTATTTCTCTTTCTTCTCCCTCTACTCCATTA
CAAAATTGCTTTGCTGATAGCTCTTCTTCTTCACTCAAAGAGGGGAGAGTGAGAGCGAGTGGGAACTAAGAGGAGTAGGTGACAAGC
>comp151352_c1_seq14
TAAGACATTCAAATGTCCATCAAATGGTTCATGATCAAGGGATTCAAGATCGGCCGTCCATCCAACCACTCCAAACTTACCCTTGATCCTTCATATATTTACAAGTCATGCCACTCTCAAAA
AACTATAAATAGCCCCCCACTCTTCTCATGAGGAGGGGGGAGAAAGAGGAGAAGGAAGGGAGAAATTTGGGAGCTCTCTTGCCGCCGACCATCGCCGGACTTCCACGGGAGTTCCCGC
CGACCACCACTGGGGTTCCTGCCAACCACCGCCAGTATTCCTCTCAACCATCGCCGGATTCCCCGCCGACCACCGCCGGAGTTCTTCCGACCGTCGCCGGACTTCCACCCGTTCTCCAAC
CACCGACGGACTTCTACCAGTTCGTCGACTGCCGCCGGACTTCCACCAGTTCGACGACCACCGTCGGACTTCCACCAGCTCAACGACCGCAGTTGGACTTCAGCCAAACCGTTGCCCAC
CGACGGACTTCCGCCAGTTTGTCTACCGACGCTGGACTTCCACCAATTCGCCAACCACTGACGGACTTCTGCCAGTCAGT
>comp151385_c0_seq1
CTCGTTCAAATTTCCATAATTGCATATAAAATCTGCCTTCTCTTGCTATCTGTCATTCCTTACAGCTTCCTGCTTCTCAGTTCTTTCCACAGTTTCGTGATCTGAGAGGATCAGTCAATTCTCT
CCGATTCTTGTAATCGCCGCAAAGTTCAAAACTGTAGCGCACGAGCAGCGAAAGATCTGAAGCATCAACCAGAAAGCAACAATGGGTCGAGCTACAAGATGGCTTACTAACTTATTGGG
AGGCAAAAAAGAAGGCAGAGAACACAAAGAAACAATGGCGTTCGCTGCCCCTTTAGCTCCAGGCATTGATAACCAAATGGAGAAGAAGAGGTGGAGTTACATGTCCGGGAGGATTTCC
TGCGACATGGCGTGGCTCCGTTCAATGTATGCAGAAAAAGAGAAGGAGCAGAGAAAGCACGCTATTGCAGTCGCAGCCGCGACAGCTGCAGCTGCCGAAGCAGCGGTGGCTGCAGCT
CAGGCGGCAGTGGCGGTGGTACGTCTGACGAATCAAAGCCATCGTGTCATTTTATGCGGCATTCATGAGCAGCTTGCAGCCCTTAAGATCCAAACAGTATTCAGAGGTTATCTAGTAAGTT
TAATTCCCTTATTATATGCCATTCATATTACTTCTCTGCGGTGATTCTGAGCTTCTTCAGCCTAGCTATTCGTTTCAATTTCTCCTTTTGTTGGTTTCTCGGCGTCATTTGCAGGCAAAGAAGG
CTTTGAGAGCTTTGAAAGCTCTTGTTAAGCTGCAAGCTTTAGTTCGAGGCTATATAGTTCGGAAAAAAGCCGCGGCCACTCTTCGTAGTATACAAGCTCTCATGAGAGCTCAGGCAACAG
TTAGAACCAAAAAGTTCCGATCTCTTCCCCGAAAATCTCGTTCTTATAAGCCATTACAGGAAAGATTTGACGAGGCATCGTTCCAAAGCCAGAGACTCAACGGCATCGATACTTACCGTCT
TAAATCGAGATCGTCTCGAAGAACAAGCCTTTCCGATATGGATGAGAGCTTCTTCAATTCAATGTCCTCTGCTATCCCCTGCCCTATTGAGACGCGGAGGTGCTCCTCCACAGCACAGAAC
ACACCGCGGTGGTTGAACACAGGTCTAAGCTTTGCCCCGCCTTCTCCGACGAAGAGCGCCCGCGGTGCTTCTAATGGGGTTTTCCAGCGTTTATTGAACTCTGCGGATAATAGTCCGAGT
TTCATGTCTAGTACGAAGTCGTTTGAGGCGAAGGTGCGGTGGAATAAAGGATTAAAGCAGAGGGCAGAGGGCCATGGTTTGGGGAAGCGAGTTCCCCTCAGTGATCTGGTCGTCGTCGA
GCAGTCGAGAGGAAGACCAAGCTCTCCGGTGAGTGAGACCTTCAATTTCCGGAGTGCGATAATGGAAAAGCTTAATAGGCCTCCGGAGTTTAGCGGGGAAGTTGAGAAGCGCTTCTGTT
TCAAATGAATTAA
>comp151451_c0_seq1
CAGAATAGCTAGAGGAAACCCAAGGGCTGTGAAAATGGCCAAAGCTCCAATTTTATGCCATCAGGTCCCCGGACGGCGGTGCTGATGCTTCCTGTGAAGCTTTTATTGGAAATAGCACTT

AGGACAGTGATGGCAACCATGGCGAGGAACAGACTGAAGTTGCCGATGACCCACACGACTCTCGAAGAAAACTTTCGGCACATTGGCTCGAGCAACGTTGCAGTGATACCAAGAACAA
TCGAATTCAGCAGCAGCCCAAATGCACCTTGTTGAACTCCTTTATTAAAAGCTTCAATTTCTTCTTTTGTGCCATTAGGATCGCCATGGTAGATCTCACGGCCCATCCAGTCAGTGTCGTAG
AGGATGAAAGGGAACCATGCCATCCATGTGAGAGATGTCATAAGAAGAACAGAGAGCATTCCAGAAGGCAAGTTCTTGAAAGCTTTGAAAACAAAAAAGAATCCAGAATTCTCCTTTG
ATGCCTCTGCATCCAACTCCGACAGCGGAACTTCTTTTGCAGATATGAGAGTAATTGCTAAACAAATTCCAAGGAATATCACTGCAATCAAGAAGGCACCCTTCAGATTCGCGCATGCTTC
ACAACAAGAATTTGTCATCAAGAATGGGAACCACTTGTGCCAACTTCCACTGGCACCAGAAGCATAACCGAGGATGTTTCCAAAAGCCATCCAAAATGCATAGATTATATTTCCTGTGCT
GCATCCATGGCTGCCAGAGAGATCAGCCATCATCGCTCGAGCTGGACCTTGAACAGCATTGTTAGAGAAATCCAGCACCCAAAATCCAACCACATAAATAAGAGCTGCTTTCCATCGAGG
ACCAACATAGGTGCTGCAATTTTCCTTTGTGTCACCAAGTGCAGAGCCAATGTCAGATGAGAAACCAATAACCATTACAGCACAACATATAATAAAGCATCCAGCAAGAATGAATGGCTT
TCTCCTTCCAAATCTTGAACCACATCTGTCGCTCCACATACCAATTGTAGGCCCAACAACAAAACCTGCTATGGGGCCACAAAGCCACATGATAGAAGCCAGAGAATGAGGAAGCCCAA
GTGTCTGTACATAAGGTGTAAGGAGAGAGAGCTGAAGCGCCCAGCCATATTGCACTCCTCCGGCGACCATACATGAGAGGATAAGTCGCACTAAGCTTATTTCCGGATCCTGCACTCCATT
TTCTGACACCATTTTGGTACTCGTTTCGATTCGCTCTTTATTTTATATTCCGATAGCCAATTCACGTCGCCGGACACAAAGGTGATGGCGTTGCCCGGAGCTCTGGTTTGTTCTCCTTTTATA
GCCTTCGCTCTGGCTCCCCACCGGTCTGTTGTGGCCGAGGTTGCGCATATTTGTG
>comp151456_c0_seq2
GCAGGCGTCGTCGCACTTCTCGGTAATTATTACTATTTCTGCCATTTTTCATAGAAAATTTGATAATTAGCGAAAGAATTATACTTAATTGAGTAAAAAATTACGGGAAAAGACTATGTGCAG
CTAGCTTAATTGTAATTATGCGATCGAAATTCTCTTTAAGCATTATTAATTAACTGTAATTATTAGTCTTTATTATTGTTATTAGTCTCTTTGGCTTTATTCGCAAAACCGTACGCTTAGACATGT
GCGCCTTCTTTATTAAATGGATACAAGTGTCGTCTGGCATCTTGTGTTGTAGTGTTGTACAGGATAGAATAGTAAGAAATATGTTCATTTCAATTTAATTTTCGGAGATTTTCATTTCATTTCA
TTTCATTTTTTTTTAAATTTCCGTCGATTTTCCATTATTTTTATTGAGAATAATATAAAATAAATAAATTAATTAATTATGCATTAAATTAATCAATAATTTATGTTAATTAATAAATAGTTGTTGA
AATGGGACCGGCTGTAGGCCGGCGAAACTGACGGGGAATGATCATAGGCTTGAGCTGGGCTTTGGGTTTAGAAGAAATAATTCTGTGAGGGCTTAAAATTCTGTCAGGAGATCAATCAG
AATACGCTACGTCATCTTCTTATTTGATACTCAGCTCGATAATGAGGCTCAGTACCACTAGTTACCTTGGCATTTGGTACCGAAATTCGATATCACTGATATGATTCAGTGAAGCGCACTACT
AATTTTATTTTTATTCATTTGACATTAAAGTTGCTGTAAATTTCCTTAAGCACCATCCAAAGTGGAAATAAGGCAGCAATCTTTATTAAGTCGTGGGACCCACATCATGAACCAAATCAACA
AAAAATTTATTCGATGTGGGACCCATCAAGCCAGTTGCACGAATATCAAAGCATCGCAACAAGAAAAATAATACTAAAAAAATGAAAGTGTCTTAAATGAAGCCAAATAACGTCGGTGGA
TTTTGATAGGGTCGCACAGCGTGGGCCGG
>comp151463_c0_seq4
TCCTGGTTGTACATACATTGTAGTGATGGGCACATCAAGACACAACGTGACTTCGGGAAAATCATGTATTGGTGATGGTATGTTCAAGTCTGGAATATAAATTAAAGATCTAGTCCTCAGCT
TGTGGGGTTGTATATCGTTTAATCTACTGCTACTATTCCCTTCTTCCACCTTAGTGGCAACAACGCTCAATGGCTCAACGGTGGCACCACGGGAAGTTGATGCCCTCCAATGACAGCGCAT
GTGCCCGCCAAGTGCCTGGCCAGATGGAAACTGTCGTTTACAAATTGAGCATTCGTGTACTTTGGGTT
>comp151463_c1_seq3
AACCCAAAGTACACGAATGCTCAATTTGTAAACGACAGTTTCCATCTGGCCAGGCACTTGGCGGGCACATGCGCTGTCATTGGAGGGCATCAAGTTCTCATGGTGCCACCGTTGAGCCAT
TAAGCGTTGTTGCCACAGAGCTGCAAGAAGGGAATAGTAGCAGTGGATTAAATGGAATACATCCTCACACACTGATGTTCAGATCTTTAATTTCTACTCCAAACATGAACATGCGAGCACC
AATAAATGATTTTCCTGAAGCCACGTTGCGGCTTGATATGCCCAGCACTACAATGTATGTACAACCAGGATGGGAAAATGCGAGGGAGAATGCATTGAATGTAAATATAGAGGTGGTTGAG
AGTAGGGAGGTGAGATTAAATGATCTTAGTGATCTCCTTGACATAAATGAGAGAAAGGTTGGCTCTACATGGTTGCAGATTGGCTTGGAATCCATATCTAAGCAAGAGACTAGAGCTGATC
AACCTTAACTCATATGCCAATCCTGATTTTGTTGTTGTTTAATTTTGCGGTCATGTTATCGACGTTAAGACTTCTGAATTTATTTTATTATCGAATGAATAAAGAATGATTTATATTTATTTATTC

AA
>comp151480_c0_seq2
AAATTATTCTAAGAAATAATAAAAAAACTATGCCATACCAAACGTTCATCAATTTAAAATCTTGAAAAAATGCTATCGACAAGTACATCTTGTGACAAGAAAGCTTCATTTTGCATTACAGT
GGAAAGATCTGAATCAAGCCTTGGACTCTTATCACTTCTTTAATGCTGCTCTCTACATACAAGTTACTGGAAGTATATCAGAAGCTGCGTATACGCGAGCAATAAGTCAACAGTAGCAATTT
CATGTCATAGATAAAAATTTGTATCAGAAGGAATGGCCATGGTTCAATTTGTCGTCTTCTCAAAGAACAGGATTGCAATTCCTCTAGTAACAATCAAAATATCTACTCCAGCACCTAACCTT
TCTACAAGAAGTTACAGGGCTCATGGAAGCTGACAAAGCCATTCCTTCTACATCTGCTCGCATAGCTTCAGTTGCCTGATTCATGGAAAGCCTCTGCTGCCATATCGATAGTTTCCAACATC
ACTCCTTTCAACTGGTTTGCGAAACATAGCACGAGTCTCTGACTCACCACTGTACTCACCTGAACTTTCTCCAGACCTCTGTGAGTTCGACAAATCTCGATTCTGCGGCACACGTCGATG
ATACTCGCGACTATGACTACGGTACTCTTCGCTGTTTGGTGAGCTCCGGCGATCATATTCATTGGAGTATCCGTATTCAGAAGTGAAATCATCAGTTCCTAGTGCCGTTCTTCGAAATCTCTG
GATATCTGCTGAATGCTGACCGGAATCATAAACTTGACTCTGACCAAATTTCATGCCGTTGCTGAGGTCTTCCATATCTCCTTCACTGTCCAGGGCCCTCAAAACCTGAACCATACGGGGT
CGTTTAGGAGCAGAATGTCGAACACATGCAGCAGCCGCTTCAATCATTCGGAACATCTCATTCTTTGCATAGCAATTCTCAAGTCTTTGGTCGACTAGTTGATCACAGTTTCCAGTTTCAA
GGGCATGAACAAGGAGAGGCCGAGCCCATTCAACCAAGCTCTCATCCCCCATTGGTTGACTAGCATCAACTGGCTTACGTCCAGTTATAAGTTCTAGTAGTACAACACCAAAGGAGAAA
ACATCGGATCTATCAGTCAACTTTCCACTTGATGCATACTCTGGGGCCAAGTATCCAAAGGTTCCCATGACACGAGTAGACACATGCGTATGAGTATCATTTGAAAGTTTTGCAAGGCCAA
AATCAGCAACCTGTGCTTCCCAATCATTATCTATAAGAATGTTAGCAGATTTAATGTCCCTGTGGATGATTCGAGGGTGGCAGTCTTCATGCAAGTATGCCAATCCTTTGGCTGAACCAATT
GCAATCTTTAGCCTTTTTGGCCAGTCCATAACAGGCAGCCACTTTCCATGTAAATGATGCTCCAGAGTTTTATTCGGAACAAATTCATAAACAAGCAATCGCTGAGTTTCAAATATGCTATA
ACCCACAAGAGAAACCAAATGACGATGGTGAACACGACTAATGATCTCAACCTCTGCTCTAAATTCTCTCTCTCCTTGACCACCGCCTGCTTTGAGCTGTTTCACTGCAACGCATTTCCCA
TCAGGAAGGGCACCCTTGTATACACACCCAAAACCACCTTCACCAATGAGATTTTCATGAGAAAACCCATTTGTTATTCCCATCAGCTCTTCATAGTTGAACCATGACTTACTGCCAGATG
GGTCTGGCTGCGAGTAATAGTCTTGTCTCCGCTGACTGCCATACAAGCCAGAGGTATTCGATAATTGTGGTGGAGATCCATAATGTTGTTCTGAAAAGTTGGGATGCCCCATGGGGTTTTC
ATTTCTATGGTAATAATTATCTGACTTAACAGAAAAGCTAGGCGGCTGCCGGTAGTGATCAACCAAAGCATCCAATCTTTTCTTCTTCCTCCTTGAGATCACAAAGAAAATAGTCACCAATG
TAATCGCAACTCCAACAAGGACCAAGCTAACTATAGCTCCAGTGGAAGTACCTCCTTTATCAACAGAAACATTGGATGATCCTATCGAAGGAGGACTCACGTGTGGGGTGGTTTGAGTTG
GTTTTTCATCCAAAGAAAGGGAATCAGAAGGCGTCGCTGGGGAGCCATCTATAGATCCCTGTGAAGAATTAGGCTGAGACGGTTTTGATGGTGATTTCACATCACCACCAGGAGGAGACT
TGTCAGCATTCTGGTTACCCTGCGAAGGTGGAGATGGTAAAGATGAGGAGGAGGAGGAGGATTCATCTGAAGCAATAGAATTTGGGGGTGAAGGAGAAGAGCCAGATTTTGATGAAATG
GGTGGAGACGAATCTCCATCTGAAGATTTGCTCTGTGAAGGAGAATCTGATTGAATAGGGCTTGGAGGTGAAGGCGGGGGTGGTATTAATGATGGAGGAGATGGTGAAGAAGATGATGA
ATTAGAAGTCCCTGGAGTTGACGATGAGCTAGGCTTTGATGGTGGAGGCAATATGCTAGACGGCGATGAGGGTGGTGAGGTCAAGCTAGACCCTGCTGAAGGCGGTG
>comp151537_c1_seq1
CTTATAGAATTTTCCCTTAAGTGTCTATTTGATTCAACATTATTCAGATAATAACATTGAATATTTTGCTTGGCTGCTTGATTACTCTGTAGGGGTTTGTTCTTTATTTGCATTGAATTTTAGGC
TTTTTTCTGTCTTTCTTTTTGTTATATAGAGTAATAGATAGATACCCCAAACATTGATATCTGTGTAGATGAACCAATTGCAACTCCAAGAGAAATGTCTAGTTTGTCTTTCATGGCAAACATA
ATGGCACTT
>comp151593_c0_seq2
AAAAGCAAGCGGGAAACCCGCGGGGAAGAGCGACGGCGCATCGTTCTCCGCTACCGCGCCTCCTTCATTTATTTCATTACCCAGAAGCTTCTACGTTCTCCATCTCTCTCTCTCTCCTTCA
AATCGCCGATTTCGTCACGTAAAACATCTGCGGCGCAGATCGCCTTCCTACTTCAATTTCAGAGGCGAAGCTCCTTCTTATTCCTCTGCTAGGGCTCTATTTCGGCTTTCCTTCCTTGCTTTA
AGATCGTGAATCTATTGTGGTCTTATCGTCTTTTTATTTTATTCCTAGGTTTATCTTCATCTGAAAGCTCAGTGGAGAGAAAGGTTCTTTGGGGATGAAGTTTGGGGAGACTTTTACAGGGT

TTCTTCACGGAGACCAAGAGTGGTTTTTGGACAATTGCTCGCACGTAGAGTACAAGAGGCTGAAGAAGGTTTTGAAAGCCTGCAGGTCGTTGCGGGATTCGAATGGTAATGAGCAGGA
AGAGAGCAATGACGGAAATGATAGTACCGGGTCAGAGCAAGTCTGCCAATGCGATATCTGTCCAGCTTGTGATCAGAAGTTCTTTGATGAGCTATCAAAGGAAGCTTCAGACATTGCTGG
ATGTTTTAGCTCAAGAGTTAGGCGACTTCTCCATTTTCACTTTTCCAATGGATTGCAACGGTATATGCGGCGCCTTAGCCATTGCTTTTCAGATGATCAGCAAACTTCTGTTCAAGAAGTGA
GGATGTTACTTGATTATGTTACTATGAATGCCGTTGCTATAAGAAAAATACTCAAGAAGTACGATAAAGTTCATGGCTCTGTTAATGGTAGAAACTTCAAGATGGTAATGCGTGCTAAACGG
ATAGAACTATTGCAATCACCTTGGCTAATAGAACTGGGTGCATTTTATTTGAACTGCGCTGGTTCAGATGCAGGTGAACCTGGTTCATTTTTCCGAAAGTTCTCTTGTGATCTATCTGATTCC
CAACCTGTAATGACGATGGTTCTATTGGATTCCATTACCTATGAATACAGTTTGAGTTGCCCCATCTGCTTGGATACCGTATTTAATCCTTATGCTTTGGGTTGTGGACATCTTTTTGCAAAA
GCTGTGTTTGTGCTGCAGCATCAGTTCTAATTTGTGATGGTCCCAGATCTGCATCTCGCGAGGCCAAATGTCCTGTATGCAGGGAGGTCGGCGCCTACCGTGACCCAGTGCATATGTTAGA
GCTGGATCATCTACTACGAAGCAGGTGCAAGGAGTACTGGAAAGAGAGGCTAAGAGAAGAACGCGCTGAGATGGTAAAACAAGCCAAGCTGTACTGGGAGAGGCAGACCATAACTGC
GTTGGGATATTGAATCTGCTTATTTTTTTATCTCTAACTGCATTTTTCAGAATCTACAGACAATAAACCTTACTAGCAATGAAAAGGAATATGCGTATATGGTATATATAATGATTTGTTGCCA
CAGAACCTGAAGAAATTATGGCTTTTACTGATTCATATTGTTTCATTTTGTCATCTGGAATTTGGAATTTGGTCTCAGTTAGTTTGGAAATATTCTTAATTTTTAATAATTTTGATTGTTGAAA
AACATAAATTAATATAAAAATGCGTAATTTTTTTTTTAGTTTTAT
>comp151625_c1_seq2
ACCTTTTTCCATATATACCAAAAGCTGGTGAATGAGGAATGAAAGATCTTTCTTTCAATTCAAATAAACTTAAAAACTTGTAAAAGGAAACTCATATAGAATAATTGGTGATTTTTATTCTTT
AATATTTTGAAATATTCTTACATACATGTTTTGGCTTACGATGAGGTTGTTAATAGCTTGGTGATCCTTGCCAGACAGTTATCCGTTGGCCTTGGGTGAGGCAAGTATCTCACTGGACACTCT
GGATTCGCACGTTCAAGTTTGTAACCCAAGAGAAAATAATGAAGGAAAACGGAGATCTCTATCCTGGCAAGGTCATTTCCTGGACAAAGCCTGCTACCAGCACCAAAGGGAAGAAAGG
TCCCTGCCCTTGGTACAAGACCATCCCATCTAGATGGATTGAAACTCTTTGGATCAGGATAAACTTCAGGATCCATGTGGACATTCCGATACCAAGTTTGAACTTTCCAACCTTTTGGAATT
AAGTATCCATTTATGTAAACGTTCGTTGTTGCTTGCCGGAAAGACACAAAGGAGATGTTCACAATCCTCAATGTCTCATCAATAATCTTTGAAAGATATTCCATTTTTCGGACATCCTTGAG
TGTTAATCCCTTCTGTGTGGGTAGCATATTTCGTCTGATTTCTTCCTGTTCTGCCTAAAAAACAATCGCCAAGATAGTTTTTCATTTAGTAGTTTATAGACCGATTAAGGAATGAAAATACCTT
ACATTTCTCAGATGTTTTTCATACTTTGGCTTTTTCAAAATATTCACGATTTTCTTGCAGGAATATGGTGGCCCACATAGTAATATGGCCAGAAGATTCATGACCAGCGTTGAGGTACATGAT
CAATGTGTCAATGATTTCCTCATCGTCCAGTTTCCTGCCATTTTCATCTTCAACATCAATTAAGCCATCCATCATGTCCTTTGCCTTCTTAGAAGTCCCTCTGGCCCTTGAAGCCCTTCTAAT
GTCCAGGGCAGCTTGGAGAACTTCCACCAATTTCTTCCTAGCCTTCAGGGCTCTCTGATATGCAAATCCAGGAAGTTTGATTGCCATTGCTCTCATGCCATAGTTGAGATCAGTGTATACTC
TTTCCAAAGACTCCATCACACTGTCACCCTCCATGCTAAGAAAAAATATGCATTATAATCCTGAATGTAAGCTTCCGAATCTCAGTAAGGAACTCAATTTGTCCCATCTCTGCCCACTTCTCC
AGTGTTGATACAACAGTTTCTTCAATGAACTTCAGATATGTGGAAAGTGCATCGTAACCATTGATAGGAGCTGATGTTAGTTTTCTGAGCCACTTGTGCTCCTCAGGAGATATTCCTATGAA
TGACTTTTTTCCAACAAGTTCAACAGCAGCTTTAGGCCATCCTGTTACAAAGTTCTCATCATCCATCAATACTGGTTTGCAGGTTTCAGGTATAGTAACCAAAATTGTTGGGCTTCCAAACA
TAAATGACTTGTATATCCCAATGCGGCCATATCTTTGAACAAAAGAATCAATAAAGGAATCAGGAACACCCGACTTGAATGCTCGGAGAAACGCCCACATGTTGCCGATGAACGGAAAAC
CTAAATCCCCAGGTGGAAGCCGACGGCGAAGATCATCGTCGAGATTCCTCTCGTACGACCACTCGTGCACCAACCGCACTGCCCTCCACAAGCCCGCCGCAGCCGCCACAGCGACGGC
CAACGTCGCCGCCGCCACGCGCCACCAATCCGATTCAAATAACCCCATCGATGACTTCCTTCACCCCCTTTTCCCTCTTCGGATGACGACGTGGTAATTATGGAGGAAGAAGGAACGGAT
TTTCGAAAATGGAAGGGAAAGGCTGGG
>comp151626_c0_seq5
TATTGTTAGTCTAATCTTGGCAAGCACAAGGGATCCAGGAATTATTCCGAGGTATGAGAGCTCTCCTTCGGAAGATATTGCTTCTGTTAGCAGAGTAAGAAGCAGAAGGATCACCATTGAT
GGCATTGAGATGAAGATTAAGTACTGCAGGATCTGTAAGATATTTCGCCCACCTAGAAGCTGCCATTGCCCTGTTTGTGACAATTGTGTGGAGAAATTTGATCACCATTGTCCATGGCTAA

GCCAGTGTGTTGGGTTGACAGCTCGGGAGAATTTTAAGCAAAGATATTCAAATTCTAGAAATCCTTATGATAAGGGAATGATAAATAACATAAAAGAAGCTCTATTCTCCAGATTGCCTCCT
CCAAAAATCAATTTCAGAGCAGCTGTTGAAGAAGCACAAGATGGTTCAATTAGAAGCACAAAATCTCATGCTAATAGCATGAGAATGGATGGTGAAGGCACTTCTACCATGCAAATCAAT
ATGAACCAAGAAGAAATTTAAAGAAATCAACCAAGATGAACAACTTGTACCTCTCTCTCTCTCTCTGTTTTTCTCACAAGTCATGATTATGTTAATAAAGTTAATAATACAGCGAAGTCTCA
TTGTCAGTAATACTGTATTAAATGTTTCCTTAGGCTGCAGCTGTAGTATCTTCCTGCTATATTTGGATTAAAGAGAAGTTCCTTTCTAATCAGCTTTGAAAAAA
>comp151667_c0_seq2
AAATTCAATCAATAAAACTTATATTTTACTCAATTTTAAGTAGATTTAATCCTAATTAAAAATTAAGTAGATTTTTGTTATTTTATTTTATTTTTCTACTGAAAGCTCACCGGAAGAAGGGAATT
TGGGGATTTCTGCTGGGTCTTCATTGACGGTGGCGGTGAGCGGCGCCGACCGGAAATCGTTCATCCAGCTGGGGGCAGTTTCGGAAGGGCCTTCCCATCCGATGTGTGCCACGTGCTTAA
CGTTGGTTGGGTATCCTATTTGCATCTCTTGCTCCTTCGGATCAAATATATTGGAGAAGTATCTGAAACCCTTGTAGATTCCCTTCTTCATCTTGGCTCCCATGTAAGCACTTGGAAGGAGGA
ATTTCTCTTTGCTGGAGGGGAATTTGGCCTCCTCCAATCTTCTCTGAGGTTCCATCTAGCGAGAAGAATTGGAGAAAAATCTTCTGCGCTCAATTGGCCATCGCAATGCAAAGAGGAAGA
GCAGATGGAAGAATGGCGGAGAGAGAGAAAGGGGCCGTTGAAAGGAGAACACAGCTTGGACGGAGCGGCTGAGAAAATTTACGGAGGAAGGAGAGAGAAGCAGAGAGAGAGAGAG
AGAAGAAGAA
>comp151748_c0_seq1
AATTCACGCTGGAAAATCAAAACATTTGTCCATCCATTATTGCCAAACAGTCTAACACAAAAATTATAATAAATAAACCTCAATATCTGAAATTCCTGAAAATAAAACAATGATTAGTAAAG
TGTTCAAGCAAGAGAAATTCATGCTCAAACTACGAAAATATATATAAATCTGCAAAGAGAAAATCCAAGTTCCTTTAATCTTGAACTCCCCAAACCCGGATGAGGCCATCTGTGTATCCTC
CATAGAGAGTGGTTCCATCAAAACTCCAAGCTAAGCATGTGCAGTGCGTCATCTTGCTGGTACCCTTACGATTCGTGGTGGGAGTTTCCAGCTTGATATCTTGCACAACGGCCCTGTTATC
CAAATCCCAGATCAACACACCGTGCTGTGTGGCAGCGCAAAGCCAATACCTGTTGGGACAAAAGCACACCGCATGGACGGTTGTCATTGTATTTAGCGAATACAACTTCTTCCCCCCATT
AATATCCCACAACAGCGCCTCGTCATCCCTGCCTCCACTGGCGCATACAGATCCATCAGGACTCACAGTAACGGCGTTCACATATCCTGCATGGTCAGTCATCGTGCGTTGGAGCTTACAG
TTTGTCAGATTCCATACCTTCACCGTCCTGTCCCATGATCCGGAGACGATGGTTGGCTGGGCGGTGCGGATAAACCGCACGCTGCTTACCCAATTGCTATGGCCACCACCATCTAAACCAT
TTTGGATGGTGTACTTGCAGGAACCTATCGTATTCCAAAGCTTGATGGTTTTGTCACGGGAACCGGAGACGATCTGGCGGTTATCAAAAGAAAAGGCGACGGAAAGAACGCTATTGGTGT
GGCCAATGAAGCGGCGGGTGGTGCAGCCTTTTTCAAGGTCCCAAAGGCGGAGTTCACCGTCCCAAGATCCCGACAGAGCGAACTGGCCGTCGAAGGAAAGGGCGACGTCTTGGATGA
AGTGGGAGTGGCCAGTGAGGCGACGGCGTGGAAGGCCGACAAGGGAGGGGAGGTCGTGATGATTGGGAAGCCAAGACTTGGTGAGATCCCAGACGACAATGGATTTGTCGCGGGAGG
AGGATACCATCATGTCTCCATGGTCGATTGGAGTGGCGATGGCGGTCACGGCAGCCGAGTGGGCGCTGAGCATGCCTTTAAGAAAGAAAGGTTCCATTATTGCTATGGAGG
>comp151754_c0_seq1
AGCTCCCCGGTCGCCCCTTTCGATCTCCCAAGCTAGCCTCACAAATTCACATATTGCTAGCCCCGGGATCTTCCATGTCTCCCCGCTAAACTCCATCCCTCTCCCTCCTCCTCCCATTCTTCC
CAATTGCATCAATCAAAGAAGGGATTTTGATCAGCTTATAAAGATGTCGAGGGCGGCGACGGTCCTCGCTCTCCTCCTCTCCTTCTTTGCTCTCTCCAATGCCGAAGACCCCTACCTTTTCT
TCACCTGGAATGTCACGTACGGCATCATCTCCCCCATGGGGGTCCCATACCAAGCCATCATGATCAACGGCCAATTCCCCGGCCCTGTCATCAATTGCACCAGCAATAACAATATCGTCATC
AATGTCTTGAACAACTTAGACGAACCCTTCCTTCTTTCATGGAATGGCATCCAGCACCGTAAGAACTCATGGCAGGACGGCATGCCGGGAACCAACTGCCCCATCCCCCCAGGCCAGAAT
TTCACATATCACTGGCAACCCAAAGATCAAATCGGCACCTTTTTCTACTTCCCTTCCATCGGCATGCACAAAGCCGCCGGCGGCTACGGTCTCATCCGCGTCCACAGCCGCCTCCTCATCC
CCGTCCCATTCGACCCCCCGGCTGACGAATTCGACGTCCTCGTCTCCGATTGGTACGGCCTAAGCCACAAAACTCTAACCCGCATTCTCGACTCTAGCCGCCGCCTCAGCCGAGCTCGCG
CCGTTCTCATCAACGGCGCTCAAAACTCCGGCGAGTCTCCTGCCTTCACCTTCGAAGCCGGAAAAGTTTATCGCTTTCGCGTTTGCAACGTCGGCCTGAAGGCTTCTCTTAACCTTCGCA
TCCAAGGCCATAAGCTGAAATTGGTCGAGGTGGACGGCAGCCACACGATGCAGAACGTGTACGATAACCTCGATGTCCACGCCGGGCAATGCTACTCTGTTCTGGTCACCGCCGACCAA

TCTCCCCGCGATTATTACCTCGTCGCCTCCACCCGCTTCCAGAAGACGATTCAATTAGCCACCGCGATTATCCATTACAGCGGCAGCAACCTCCCAGCAGCCGCCGAGCTCCCACCGGCGC
CCATCAGCTGGGCCTGGTCGCTCAACCAATGGCGTTCCTTTAGGTGGAATCTCACCGCAAGCGCGGCTCGGCCCAACCCCCAGGGATCCTACCATTACGGCCAAATTAACATCACCCGCA
CCATAAAGCTCTCCAGCTCGACATCCATCGTCGACGGAAAGCTCCGTTTTGCTCTCAACGGGGTTTCTCACCGCGACTCTGAGCTGCCGCTTAAGCTCGCAGAGTACTTCGGAGTGAACG
GCAGCGTGCCGGAATACAACCAGATCACTGACGACCCAACTTCTCCCGGTACTCCGGTGAAGTTAGTTCCTAATGTCATAACAGCTGAGTTCAGAACGTTTATTGAAATCATTTTTGAGAA
TCCTGAGACTAGCATTCAGTCTTACCATCTCGACGGCTACTCCTTCTTTCCGACGGGAATGGGGCCAGGAAAATGGACGCCGGAGAGTCGGAGCACTTACAACCTGCTCGACTCCGTGA
GCCGACATACTATTCAGGTTTACCCCAAGTCCTGGACGGCGATCATGCTCACATTCGATAACGCTGGCATGTGGAACCTTCGGTCTGAGATTTGGGACCGCCGTTACCTCGGCCAGCAGCT
GTACATCAGTGTTCTGTCTCCAGAGCGATCTCTTAGAGACGAGTATAACATGCCGGAGAATGCACTGCGGTGCGGCGCCGTTCTCGATTTACCTCTTCCAAAGCCTTACTCTTGAACTGAT
TGTTGCAAAAGATGCCATCTTTTGTTTCTCGGGGGTTATGTGATTGTGCTTTGGATTCATTTGATTTTTTTTCTTAATTAACTCTATGTTTTGCGGGATCATTAAATACTTTTTCCTTTAATATG
AAGATGTTGAGAATTGAGATGAATTTAGTTGAAGAGAGAAGGTATAGCAAATATCAAAGGCATGGTGAGTCAATGAAGCAATCCATGTATACCTTTGTATATTTATGATTGTGCTAATAAAT
TTGTTATATATTTATAATTTATTTAAGAAAAGTTACACATCTTGAAGCGGTTGTCTATGATAATCTATGATTTTATGAATTATTTATCTTAAAGTTAGGCTGTATGTTTAAGTTTTATGATGAGCC
CA
>comp151757_c0_seq1
AAAAGACTACAATTATATAGGATTTTGAGGGATTGACTTTGAAATAGACACAATAGAAGAAAAAAAAATTCAACATTACAAGGGAAAGTAGGAGCTAATAAAATTTATTAAATAATTATGGA
AACTTCTATCAATTAGCTTTCCATTTTAATGAGGGCCCATTCATTAGTGTATTAGCTCAACCTAAAGCCCACAATATTTGACTCCCAAAAAATGCTTATAACATACTCAATAAATTGTTGCACT
AATAATGAATCCCTTATCTTCTTTTTATTTTCTCTACCATTTCCACTAATCCCAAACTTAAAGCTTACATCTTCAATCTTCCACTTTTTTCTTGCTCCCTTTATTTTGGTTCATCAACATCATCTG
AAAGTGCACTTCCATTCTGCAACTCAACTATTGTAGGTTCCACCCCCACATCAGTAGTTTCTTTATGTGTAGGAAACTTTCCAATATTTGATGCTTCCGCTTCTTTACCTTTGCCCCATAACA
CCAAGTATAATCCAATCACAATCAACACAGCTCCTATAATACTCCCAATATGTAACTTCTCATCAAGTAATAAGGATCCAAAAATGGCCACAATCACAAGCATGAGAGGGTTAAACACAGA
GGCATAAAGTGGACCCTTTTTATTTATGCACCAAGCGAGTAAAGAAAGTATGGGACCTGAACCAACCGCACCCGTGTAGGCTGCAGAGTAAAGTCGAATTCCAAGGCCCATATGCCAATC
CTCCCAATTCCTCTCCATGCAAAGAGCAAAAATTATAGATTGAACCGAACCCATCAGGCACATGATTGCAACGCTCGAGTAATGATAAGGATAGTCCTTGCTCATTTTAGCCATAATCTTGG
CGCAGTGCAGCCATCTGATTTCCACCATTACTATGTGATTTTAGAAGATTGACATTTGTTGGGAGGTTGATTTCTTTTCCTTTCAAGAAGGTGAGGATCGTTGCACCGGAAACTCCAACGA
GTGTTCCTAGTATCTTTGCTTTCCCTGATTTTGTTCTGATTCTTAAATGCTCTAATCTGAATATGACAGCCATGATGAAAGTTATGGCTGGTACAAGGTTTGTCATGGCTGAGGCAAAAGTTG
CAGATGTTAATTTTATACTAGACACATATAGGTTTTGCGCCAACATAGCCCCAAAGAACCCGCAGAAGAAAGCCTGCATCAAGATTTTCCATGTTAATTTCGGCCAGTTCTTCCTTTCAAGG
ATGAACGCAAACGGGGCCACGGAAACAGTGGCGAAGAGATAGCGATATGCGACGAGAATCCTCATGTTCATTCCATCGTTAATGGCGAGCTTGTTAAAGATATTTATACCGGCGAAGAAT
AATTGGATCACCACCATTGCCATTGCCGGCTTCATCCCTTCCAACAAGGTCGTCCATGAACTTTTTCTCCCTTCTTCATCTCCCATTGCGGCAAGTTTTTGCAACAACTACGAGAAAGAAA
TCCTTAACAAAAAA
>comp151757_c0_seq2
AAAAGACTACAATTATATAGGATTTTGAGGGATTGACTTTGAAATAGACACAATAGAAGAAAAAAAAATTCAACATTACAAGGGAAAGTAGGAGCTAATAAAATTTATTAAATAATTATGGA
AACTTCTATCAATTAGCTTTCCATTTTAATGAGGGCCCATTCATTAGTGTATTAGCTCAACCTAAAGCCCACAATATTTGACTCCCAAAAAATGCTTATAACATACTCAATAAATTGTTGCACT
AATAATGAATCCCTTATCTTCTTTTTATTTTCTCTACCATTTCCACTAATCCCAAACTTAAAGCTTACATCTTCAATCTTCCACTTTTTTCTTGCTCCCTTTATTTTGGTTCATCAACATCATCTG
AAAGTGCACTTCCATTCTGCAACTCAACTATTGTAGGTTCCACCCCCACATCAGTAGTTTCTTTATGTGTAGGAAACTTTCCAATATTTGATGCTTCCGCTTCTTTACCTTTGCCCCATAACA
CCAAGTATAATCCAATCACAATCAACACAGCTCCTATAATACTCCCAATATGTAACTTCTCATCAAGTAATAAGGATCCAAAAATGGCCACAATCACAAGCATGAGAGGGTTAAACACAGA

GGCATAAAGTGGACCCTTTTTATTTATGCACCAAGCGAGTAAAGAAAGTATGGGACCTGAACCAACCGCACCCGTGTAGGCTGCAGAGTAAAGTCGAATTCCAAGGCCCATATGCCAATC
CTCCCAATTCCTCTCCATGCAAAGAGCAAAAATTATAGATTGAACCGAACCCATCAGGCACATGATTGCAACGCTCGAGTAATGATAAGGATAGTCCTTGCTCATTTTAGCCTGAATTATCA
GCCAGATTGCATAAGAGAAACAACTTCCTAGAGACATAATAAAGCCCATGATGCGATTACCATAATCTTGGCGCAGTGCAGCCATCTGATTTCCACCATTACTATGTGATTTTAGAAGATTG
ACATTTGTTGGGAGGTTGATTTCTTTTCCTTTCAAGAAGGTGAGGATCGTTGCACCGGAAACTCCAACGAGTGTTCCTAGTATCTTTGCTTTCCCTGATTTTGTTCTGATTCTTAAATGCTC
TAATCTGAATATGACAGCCATGATGAAAGTTATGGCTGGTACAAGGTTTGTCATGGCTGAGGCAAAAGTTGCAGATGTTAATTTTATACTAGACACATATAGGTTTTGCGCCAACATAGCCC
CAAAGAACCCGCAGAAGAAAGCCTGCATCAAGATTTTCCATGTTAATTTCGGCCAGTTCTTCCTTTCAAGGATGAACGCAAACGGGGCCACGGAAACAGTGGCGAAGAGATAGCGATAT
GCGACGAGAATCCTCATGTTCATTCCATCGTTAATGGCGAGCTTGTTAAAGATATTTATACCGGCGAAGAATAATTGGATCACCACCATTGCCATTGCCGGCTTCATCCCTTCCAACAAGGT
CGTCCATGAACTTTTTCTCCCTTCTTCATCTCCCATTGCGGCAAGTTTTTGCAACAACTACGAGAAAGAAATCCTTAACAAAAAA
>comp151763_c0_seq1
TTTTTGAAAACTGAATCAGAAATTCCCAACAAGGCACCAGAGAGGATGACTATCAAAACATACACAAAAATCAATCCTTTTCACTTAAGAAAAAAAAACATACTGATTTTTCTTAAATTTC
AGACAAGCACTGCTTTCATACAGCATCACCAGTTCGTTCATTCAGAATCAATATATTGCTCGTGAAATTGCCCATTTTCACGAGGATGTAGAATCATACCAGAAATCTTTAATTTTTTCCCCT
AATACAAATGACAGTAGTTACAAATAGAAAAAATATTTATCAGATTTCTATGACCTCAACCACCTCACCAGCCTTTTCCAATGAGCCATAACCAATCTAACGAAACACCTGAAAAAATACC
ACCCAAAAGAAACATAACCAGCACATTTCCCAAAGCATTTGCCTCCGGTCCCTTGTATCCCTTGGGTGCTTCGGTTAGAACACAAACAGTGAGATGACCATTAGTGAGCCCTAACAACGA
TGTTAACATGATCATCCAACCTTGCCCTCCATATTTTGCAGTGAAATAAAATGCAGGGATGAATAGGAAACGCGAGATAATAGCAATCATAAGGCCTTTCCGTGATTCTATTCGAAGACATT
TTATCAGAGGTGTGTATCTTCCAATAAGGTCCAGCACATTATACATTGCAATCAAGACAAGAACATACCATGATCCCAGGGCATGATTTCCAGTATCCTCAGACAAAAAGCCAGGGAAAAT
TGACAGTGTCAGAACATAAATAAGATACAAGCCAATTTCATAATCCAAGTTCTGCATTAGCAGCTGTCTAATGGTCAAGCGTTCGTATTGCTTTGGGTCTTCTTCTGCCTCTTGTTTAATCTG
CAATTGGACGCCAGCAGCTGCTAGGTCAGCAGAAACCGTCTTTGATCCTTCCGAAGCTGCCTTGGATCGATAATACTTGACAATTGGTAGCTTCGGAAAGATGTACGCATAAAGTAGAAC
ACATAGCAGCTCTAAAAAGGATGACACTGCAAAGAACATCATCGCTCCCTTTCGAAGACCATCATGACTGTGTTCAAAACCTGCCTTTGTAATCAGCCTCAAAGCAGATGTTAAAGCTCC
AGAAGCAGCCAAACCAGCCATGAATGACTGAATAACTCTGGTTGCATAAGGGCCAAATCACCAACCATGCCTCCTTGGACATTAGCATCAGCAACACCAAAAGCTGCACTAATAAGGCAT
ATGCCTACGAATGCTCCAACGCCTCCTTTTCCAGATGTTGCCAAATCTAAAATGATTAATAGAAACGAGCTTATGAAAAAGAGGATGTAGCCAAATATGTTCCTCATTCTGGTATTGAGCTT
TGCTTCATGATATGTGAGTATTGCAATAGTCACGAAAGCAAAAGGCTGATAAACTAGCGTAAGTATTCTTGTGGGATGGTACCTCGGGAATACTTCAGCATAATAGTCTACAATTGTTAGCA
TGCTATTCCATGAGATGAGAGAGCCATTTCCTAGGAACCAGCAAACAACAATAGCAGCAAATCTCCCCTTTTTCGGTACTGCTTCCTCATTTTCAGTATCATAAACCATGATTACTCTGCTT
CACCTTCTCCCTGAAGCCGGACTTGATGATTTCTTGAGGATCTCCACACGGTGAGAGATATTCAGGAATCTGAATATCCTATAAATGAATTCCTTACAAAGGAAAGAGAACGGGTCAAATG
TGAAAGTTCAGCTG
>comp151799_c0_seq1
TTATTTGGCCAGGAATTAGACCAAGCTCTCTTCCTTTCTTTAATCTTCTTTCAGTTTTCCTCTCTCCCCCGTTAGCAACCAACGGCCAGAATCCATTCAAAAAATAGTTAGCCCAGAGCTTT
AATAAAGAAGAAAAACAAACCTTCCTTCTTTCTCTCTCCGGCATTCCATTATCTAACTAGAACGAGAAACCAGAGCTTGCTCGGACGCGCCAAGCCTTCACAGCCATGCCGAGCAAGCT
CCGGCGAACCATCGATGCCGTGAAGGACCACACAAGCCTCAGCCTCGCATTAATCTCCGGCTCCTCCGACACCCACGTCGCAGTTCTCCGCGCCACCTCACACGATCCTTCCCCCTCTGA
AGAGAGAACGGTCGCCGAGCTTATCTTCCTCGCCTCCTCCTCGCCGGAACGGGCCGTCCGTTCCATCAACCGCCGACTCTCCCGCACCCGCAATTGGATCGTCGCCGTCAAGTCTATGTT
ACTCATCTCCCGCGGCATCACCGACGCCGGTCCCGACTTCCGCCGCGAAGCCCTCGCTGCCACCGGAGAAGATGGACGGCGCCGCCTCCTCGACCTTTCCAGCTTCCGCGACGAATCCG
GGCAGTCCTCCCCGTGGGACTTCACAGCCTTTGTGCGCACCTTGGCGCTCTACCTTGACGCTCGGCTGGAAGCTTCCGCTGCAGGCAAGCTTTTGCGGCGCCCGCGCCGCTGCCCTGTTT

CAAGCCTCAAGCCTTCTGTTCTCGTGGATCGAATCGGACATTGGCAGAGCCTCCTCGATCGGGCGATGGGGACCCGACCTACCGGTCAAGCCAAGGCGAATCGCCTCGTCCGACTCACA
TTCCATTCCGTCGTCCGCGAAAGCTTCGACCTTTACCGTGACATCGCCGACGGAATCTCATTTCTCCTAGATAGCTTCTTACATCTCCCCATTGACTTATGGCCTCACATTTTCCAAGCGAG
CAAAGCCTCGGCGAAGCAGTTTAAGGAGATTGAAGATTTCTACGCCGGATGCAAGAAAATGGGAATCGGAAGGCAATCTGAATACCCAAGCCTGATCAAGATTACCGATTCTCTTCTAGA
AAAGCTTGAAGAGTTATTGAAGGAGGGTGAAGCGGCAAAGATTAAAAGTCCACCGAAGAAGCTGATCCTGGAGCCGGCAAGGAATGAAGGAGGTTCAATGGACTCGGAAAACTGGGA
GCTTGCTCTGCTTGAAAGCATGAATGAATTGAGCATCAAATGTCAAGGAGAAGGAACGAAGGCAAAAGAATTGGGAAATTTGAACCAGGAATCGCCGATTAATAAGAATCAGGCTTTCA
CTACCAGCTTTCAGAATCCTTTCCTGCGTGCTTCGAACGACAAAAGCTTGATGGCAGTGACGCCGTCTCCGTCGAGCTTTAGAGCGGCGCCACTGAAGAAGGATTCCATGATTGCAGCTA
AAGATCGAAGCTTGATGACGTTGACTACATCACCGTCGCCTTCGCCGTCGCCGATGAGATTCGGGTCCTGGGATTATAGCAATCAAAGGAAGGAGAAGATGATAGAGGAAGAGCAGGAA
GATCCGTTCGATGAGTTGGCGAAACGGGAGCGTCCTCCATTGATGTCGGAAGCTGCCAGAGATTTCCGCGAACAGTATCACAGGCACAGGTCCACGAAACCGTGATTTCTTTTTGAATAT
TTGTCTTCAGCTTTGTCCACAGACCATTGTTTTGCGGCTAATTTTACATTGAGAAAATATGAAAATAATTTTTAAGTAAAAATAAAATTCTGTATATTTATATGAAGATGTTCTTCATTTTAATT
AATTCATAAACCTGCAG
>comp151805_c1_seq3
GAAACCCATTTTATTGATTTACAGAAACTTGTTCACAAAAACAAAAAAAAAATATAGTACATTTACTGTTGATATCATTTTAGGCTTCATTTGAGACAACTTTAAACCATAACATTTTTCCTA
TTTTTAAGATATTTTTTTAACTAAAAAATAGCTTTACAAAATAGTAAACGAAACATTAGTGTGCCGATATACTGGGCCCGCTTGCACAAGAAAAAATTCAATAAGATTATCATGGCCTTTGAT
AAATTAAATTTTATAATTATTAAAATTATTTTAGATAAATATAATATAAAATTTTTAATATTATATTCATCTGAAAAGAAATCTAATAATCTGATAATCGTCATGATTAGATATCCTGTTTTTTTTT
AAACCACTCAGAATCTCCGTCTCGGCAAAGAGGAATCGCCGGACACCTTATTCTTGCCTCGAATGGCCGGGCTGAGGTCAACGAAACTAAGCAACCCATGCCTCTGAAAAGGCAGAGG
CGTTTTTCTCTTCCGCTCCTCCGCCGCCGCCCGATTCGCCGTGTAATGCTTCTCATGCGCCGATACCGGCGGCCGACTTCCTCTTCTAACCGTCTCACCGCCTCCCGTCTTCTTTTCCTCTCC
TGCTCTCTTATTATTCAACAAAGAAGCAGAGGGCAAAACAGTATACTTCTGCAGAGGATCTTTGCTTCCTCTTCCAGTAGCTCGCCCTTCCGACGCGCTCCGAAACAGAAGCAAATCCTT
CAGTCTGGGCCACTTCCTGCTTAACACACACTTCACGGGATTGGCCGATGGGCTTCTATCTTTCGCGAGGAGTCCTTCGTTTCTTAAAGGAGAAAGCGATCTTGATTTCCTTCGCCCGTTA
CTCAGGGAATTTGGGGCCAACAAACTCCGGCCTCTTTCTTGCAGCGGCCGGATTCTGCCCTTCTCAAAGAGCTTATCGGCCGTTGTTATCTCCGGTGACAGGCGATGACGGTAGTTGCTC
GGAGAAAAAGCGAATTCGAACATATCGCCACCGTTATCGTTACGGCTGCAAGGGCTGCACTGGGAGTAGATGGCCGCGGCTTGGGCTGGGCTGCCTGGCGCGCTGGTGTAGTGATATCG
GTAGTTGAATTGGTCGCCGAACGGGAACGGGCTCGACGGAGCGCTT
>comp151836_c0_seq14
TCCCTTCCCACTCTTAAATGCTCATAACCACCACACTTCCATAAACAATAAAGTCCCACTTCATCAGTTCATCCCATAAAATCCAGCAACAATATTTCTATGAAACACCACTAGCTAAACTC
TTCATTCTTCCACTAGCCATGGCTGCTGCTTCTGCTCTTCTGCTAATGGTGGCGGCAGCATGCTTAGTGGTGCGCTTAGCAGAGGGACAAGGACTGGTGCCTGCCGTGATGATCTTCGGCG
ACTCGGTTGTGGACACAGGAAACAATAACTACTTGCTCACTCTCGTCAAATCCAACTTTCCTCCCTATGGCCGAGACTTCACCACCACCCGCGGCGCCCCCACCGGCAGGTTCTGCAACG
GCAAACTAGCCACTGACTTCACCGTGGAAAACCTGGGTTTCAGCACATACCCGCCAGCTTACCTCAGCAGAGAAGCAACTGGGAAGAACCTCCTCACCGGTGCCAACTTTGCCTCTGCT
TCCTCCGGGTACCTGGACAGGACTGCAAACCTTTATGTGCGGTTCATTCCTGAAACATTTGAACATTTTCATTAAAATACATGTAGATGTAATCTGTATATTAACTAACAAATATGATGTGTT
TATGGGTTGCAGCATGCAATAAGCTTAACCCAGCAGCTGGAGAACTATAAGGAGTACCAGAGCAAGGTGGAGAATATTGTAGGTGGAAGAGCAAAAGCTGCCGGAATATTCTCCGGCGC
CATCAACATCCTCAGTGCGGGGAGCAGCGATTACGTACAGAACTACTACATCAACCCCCTCATCAATGAAGCCTACACTCCTGACCAGTTCGCCAACTTGCTCGTCCTCTCCTTCGGTTCG
TTTGTTCAGAACATGTATGGCCTGGGAGCCCGGAGGATTGGTGTGACATCGCTGCCGCCAATCGGTTGCTTGCCCGCCGCAATAACCTTGTTTGGTGGTGGCAGCAATAACTGTGTTGATC
GCCTGAACAATGATGCAGTTGCCTTTAACAAGAAGGTGAGCGCAGCTGCAGAAACGCTTAAGAAAAGCCACTCGGATCTCAAACTTGTCATATTTGATGTGTACAATCCCTTGTTAGACC

TCATCAAGAACCCTGGGAACAATGGTTTCTTCGAGGCACGAAAGGCATGTTGTGGAACAGGCACCATTGAGACTTCTCTTTTGTGCAATGCGAGGTCGCCGGGGACTTGTGCCAATGCT
ACTGGCTATGTTTTCTGGGACAGTTTCCACCCTTCAGAAGCCGCCAACAAAATTTTGGCTGATGCACTACTGCTTCAGGGTATAGAACTCATCTCCTAGATAGACTTAGCTAAGTTACCTA
GCATCCTCTCCCTCCAAAATGGTGTAGGACGATAGATGCCGCAACAGTAACATTCAGTGATGCTGTTCCACAGCCATATTGTGGAATATAAACGAAGAAGTCGCAGATCTCGCACTCCTTA
GCTGAAAGGCCGGTACCCTCGTTGCCGAGAAGGAAAGCGGTGCTCCTTCTGAAAGGCTGCTGAGTGATAGGGACTGCCCCATCAACAATCTCCACGCCACAGATATCGCAGTTCCTCTC
CTCCTTCAGGTATAGGCGGGCTTGAGAGAGTGAATGGAAATGTCGGAATCGGAGATGGGCGGTGGAACCGTGACTGCCAAAGGCGTTGAAGTCACGGCGCCCGACGAGAACCATCTCT
GAGACACCGAAGGCAGTGGCGCTTCGGACCATCGTCCCGATATTGTGTCTCTTCGCTACGTTGTGAATGATCAGCACGCTCTCCGCAGCCGTAGGAGCTGGCGCCGGCGACGGAGAAGC
TTCAGCGGCCGCCATTATTTGGGTCGTTTGTTTGCAAGAAGCGCGGAGCTGGGGTCAGT
>comp151836_c0_seq2
TCCCTTCCCACTCTTAAATGCTCATAACCACCACACTTCCATAAACAATAAAGTCCCACTTCATCAGTTCATCCCATAAAATCCAGCAACAATATTTCTATGAAACACCACTAGCTAAACTC
TTCATTCTTCCACTAGCCATGGCTGCTGCTTCTGCTCTTCTGCTAATGGTGGCGGCAGCATGCTTAGTGGTGCGCTTAGCAGAGGGACAAGGACTGGTGCCTGCCGTGATGATCTTCGGCG
ACTCGGTTGTGGACACAGGAAACAATAACTACTTGCTCACTCTCGTCAAATCCAACTTTCCTCCCTATGGCCGAGACTTCACCACCACCCGCGGCGCCCCCACCGGCAGGTTCTGCAACG
GCAAACTAGCCACTGACTTCACCGTGGAAAACCTGGGTTTCAGCACATACCCGCCAGCTTACCTCAGCAGAGAAGCAACTGGGAAGAACCTCCTCACCGGTGCCAACTTTGCCTCTGCT
TCCTCCGGGTACCTGGACAGGACTGCAAACCTTTATCATGCAATAAGCTTAACCCAGCAGCTGGAGAACTATAAGGAGTACCAGAGCAAGGTGGAGAATATTGTAGGTGGAAGAGCAAA
AGCTGCCGGAATATTCTCCGGCGCCATCAACATCCTCAGTGCGGGGAGCAGCGATTACGTACAGAACTACTACATCAACCCCCTCATCAATGAAGCCTACACTCCTGACCAGTTCGCCAA
CTTGCTCGTCCTCTCCTTCGGTTCGTTTGTTCAGAACATGTATGGCCTGGGAGCCCGGAGGATTGGTGTGACATCGCTGCCGCCAATCGGTTGCTTGCCCGCCGCAATAACCTTGTTTGGT
GGTGGCAGCAATAACTGTGTTGATCGCCTGAACAATGATGCAGTTGCCTTTAACAAGAAGGTGAGCGCAGCTGCAGAAACGCTTAAGAAAAGCCACTCGGATCTCAAACTTGTCATATT
TGATGTGTACAATCCCTTGTTAGACCTCATCAAGAACCCTGGGAACAATGGTTTCTTCGAGGCACGAAAGGCATGTTGTGGAACAGGCACCATTGAGACTTCTCTTTTGTGCAATGCGAG
GTCGCCGGGGACTTGTGCCAATGCTACTGGCTATGTTTTCTGGGACAGTTTCCACCCTTCAGAAGCCGCCAACAAAATTTTGGCTGATGCACTACTGCTTCAGGGTATAGAACTCATCTCC
TAGATAGACTTAGCTAAGTTACCTAGCATCCTCTCCCTCCAAAATGGTGTAGGACGATAGATGCCGCAACAGTAACATTCAGTGATGCTGTTCCACAGCCATATTGTGGAATATAAACGAA
GAAGTCGCAGATCTCGCACTCCTTAGCTGAAAGGCCGGTACCCTCGTTGCCGAGAAGGAAAGCGGTGCTCCTTCTGAAAGGCTGCTGAGTGATAGGGACTGCCCCATCAACAATCTCCA
CGCCACAGATATCGCAGTTCCTCTCCTCCTTCAGGTATAGGCGGGCTTGAGAGAGTGAATGGAAATGTCGGAATCGGAGATGGGCGGTGGAACCGTGACTGCCAAAGGCGTTGAAGTCA
CGGCGCCCGACGAGAACCATCTCTGAGACACCGAAGGCAGTGGCGCTTCGGACCATCGTCCCGATATTGTGTCTCTTCGCTACGTTGTGAATGATCAGCACGCTCTCCGCAGCCGTAGGA
GCTGGCGCCGGCGACGGAGAAGCTTCAGCGGCCGCCATTATTTGGGTCGTTTGTTTGCAAGAAGCGCGGAGCTGGGGTCAGT
>comp151846_c0_seq1
TCATTTTACTCTTACCACATTTTTCGCCGGCGATTCGAAGGCCATAGAGGAAAAAGATGCCGCCGAAGCACGATAGCGCTGAAGGCATTGTTCTCGGCTTCGTGAACGAGCAAAATAGAC
CACTAAATTCCCAAAATGTTGCTGATGCTCTGCAAAAGTATAACCTCAAAAAGTCTGCAATCCAAAAAGCTTTGGATACACTTGCTGACAGTGGGCAGATTTCATTTAAAGAGTATGGCA
AGCAGAAGATTTACCTTGCTCGGCAAGATCAGTTCAAGATACCCAGTAATCCGGAGCTTGACCAGATGAAGAAGGAAAATTGCCAATTACAAGAGCAGATTGATGTCCAAAAGAAGGCT
AGAACTGAGATTGAGGCTGAAATTCGATCTCTGCAATCAAATTTGACACTGGAGGAAATACGGGCAAAACAGTCAATGCTGCAAACAGAGGTTAATGAAATGGAAGCAAAGCTGAAGA
AATTGCGGGATGGAGTAATTTTAGTGAAACCAGAAGAGAAAAAAGTTATTGAACAAACATTCAGTGAAAAGATGAACCATTGGAGGAAGCGGAAGAGGATTTTTAAAGATTTGTGGGAT
GCCATAACAGAGAATTCACCAAAGGATGCCAAAGAATTCAAGGTTTTTTACCTGTGAAATCAACCTTTTCTATTTCTTTAGATTTTACTAACAGCACCATTACTTATCACAGGAGGAGCTCG
GCCTTGAATATGATGAAGATGTTGGAGTTAGCCTGCAATCCTATAGTGAACTGATGAATGCCAATAAGAAAAGAAGAACTGCACAATGAGTTATCCTGCAATCCTTTTAAATTTAGATTTGT

AAAGGGATTTTTAAGCCAAACTCTTCCAATGCATGTATTTTCCCAAACAGTAATTGATCAAAGTTTCACACTTATGTGGACCTGTTACCGTTAATGCTTACTGCTATAGTACTTCCATGTTTA
ATCTTAAAAG
>comp151847_c0_seq2
TCATATGAAGTCAAAATTTCCTCATAATATGCAGTTAAAAAAAAAGGAGAAAAAAAAAACCTTTGTCTTGCACCGACTCTATATACAAAGATTTAGCACAAAATCATAAGGAAAAAAAATA
TATCAGTCCTATCAAGGTTTTCGGCATGCGATTAAACGGCAAGAAAAGGTTCTGTTTGTATACAGATTCAACCTATGAAATGAACGAAAAAAAAAAAAAAATTCAAGAAATGGTAGTTCT
ATATATCTGAAATTGTTCCCCGAGATTAACCAAAATAGAGAGACAGAAAAGAGATTGATCTCATCTTGACAAGGACTTAAGGCATGAAGATCTGGACGGCGCCTTCCTTCGAGCTATTGCA
GATGGGACATATGACAACCGTCTTTTCACATTCCAGGCACAAGCAAATATGGCGGCAAGGAAGAAGGAGAATGGACATTTCATACAACTGGCAAACCTTGCAAACCATCCGTGCGCGAA
CTTTCTCGCTGCTCTCCGGCTTCTCTTCCTCGATCTTTAAGCAGGACTGCTCGTCATTAGCAGGAAATGGCATCCCATCGGTATCGCCGTAACCCTCAACCTCCGGCGGAGGCGCCGTCTC
CGCAGCGGTTGTCTGGATTAAAACTTGTTCGAGGGTTGCCTTGAGCCTGGAGACTAATGCCTCATTTTGACTGGCACGACTAAACAACATCTGGTTCTCGGAGCACAACTGGAAGATCTT
TTCCTCCAGCTCAGAATTTCTCCGATTCACGTTCGCCAGCTCGGCATCCTTATCCAGGACCCGCTTCAACGCAATCTTCTCCAAGATAACAATAAGCGAACTAAAGTACCTCTTTTGCGTCT
CCTCAATTCCCTTACGAATCATATTTCTGTAATGATTTAACAGAGTATCAATTTCGATGTTCTGATCTCTGATATGCGAAAGAAACTCCTGCGATAAAGAACAGCCAGGCTGCGAGGAAACC
CCTCTTGGCGGGACGCTGCTGGTACACGCCCCAGTAGATTCGAACACCCGGATCCTCTGATCAATGGGCATAGGCAATCCAACCGTAGAGGTAACCGGCATAGCCGCAAACGCTAGATTA
GGTAAAACGGTTTGCTGAGGTTGGATTACTGGCGCAGCTGCCCTGGAAGACAAGGGAACATTCTGACTCCCTCTTCCAGAGCCAGAGACATTGTAAGCAGCCCGGGCGGTCGCCGTCG
GCTGCTGATCGCCATTATTGATTTGAGCCTGCTGGCGCATCGAAGGAACAGAGTTTCCAGTCTGATGCTCAGGATTCTGCCACATCCGAAGAACTTCAAGAACACAGCCTTCCTGAGCCC
TGTCAATCTGATAACAGAAATAATAGCGAAAGATCCGAAGGGGATAAATAAATGGTGGAAGAAATGGAGGGAAGGAGCCTGACGAAGCGCTCTTCAAAAAGAAGAAGAAAACGGGGTT
GGCGTTGAGGGGAGTGCGTACCTGGAGCGTAAATATGGGGAAACCGCCATTTTATACAGGACGTGCGTTGTTTCAGTGAATCCGCGCACGCTCTGATTGCCTGACATTTCGTTGGTCGTG
GCGCCGAAGGAAGGTTATATCGGGCTATCGGTTAGGATGGAGAAGGCATTGGAGTTCTGGTTTTGCTGTTGCCGTTCTAAATTTCAGGCGGGGGGATAATTCTAGAGAGGGAGGTAGGGC
GTATTGACGTGAGAAGAGGAGGGGACGGTACCGCTACGTGTGCCGAACGCCGATGGATTTGTATTCATTGTATTAGGGTAAGGGGAAGAGGATTAGGGCTGATTAG
>comp151849_c0_seq1
AAATTAAAAAAAAAAAATTCTATTCCCCTAAAAATGTTAAAGAGAAATCTGTCCTAAAATTTTGTCCAAAAGCTGAAAATAATATCGTCAAATTCAAGCCAGAAAGAACGCTTTTTATCAT
ACTCAGCAAGCAAATTTTCTTATCAATATATATGTCCACCGGTACTTGCCGATCATCTCAACCAAACAAAATAAGCTGTTCGACCAAATAAAGAATCACATCATTTAGTTCTTAACAAAATG
AAATTCAAAGAAACGAAAATCAAACGTTCAAAACAAAAGCATCAAATGATCATTCGATCACTTATAATCCACAAACATCAAAGAACAAGCAAAAAACATTCCCTTCTCGAGCTCTCTCAT
TACTGCTGCTTCTCTTCAGCAGGAGCATCTTCAACATTGAATTCAGAGACTTCCGGCTTCGATTCTGCCTCTTCGGCCGCGGCTTTCACATTCTCCTCCCCCGCAGCACCGGCGGCCTCCT
CGGCCTCCTTCTCCTGGAAGTAATTGCTGAGTGAATTTTGGCCTTCAGCCTCCTCTGACTTGGCCGCCTCCTGCTCCTCCCCGGAGCCGGCTTCCTCAGCCTTGACGGGTTCAGCAACGT
CCTCTACCGGTTTAAGAGCCTCCGTCTCCTCCTTCAAAACCAGCGGCTTCGTGGCGCAACCCCCCATTCTCTCTCTCTTCCGATTCTCTCCGAAGGTTTCGAAATTGATCAGAGAGAGAG
AAAGAAAGAAAGAGAGAGGAGAGACAGAGAATGAGAGATGGAGAAAGAGAATGCGAGAGAACGAAAAGAGCTTATTGAGAAAGCGATGCAGCTCCACAGATTTTATAATAGGAACCT
CCAATTTCTCGCCGATATATGCTCGCCGTTGCCTTATGAATGGGTGGCCAAACGAAATAAATGGGTACAGATGTGAAATTTACATCTA
>comp151890_c0_seq1
TGTTCTGCGAATGAAATTAGCAACAATATTTTTCTTCCAACATGCCTAACATATTTTCAATTGGCAATTTATATCACAAAAGCTCTAAACACATGCAAAATAAAGAGAGAGAAACTAAGACA
TTAAGAAGAAAAGTGTAGAGGTTATGGACACGATCTAGGAGCTTTTGATGCCAAGTTACAGATGTGCACCATGCTTTATTAGATTTCAACACTAAATACATTGCTAACATTAAATACCAGCA
GTGGAATTATCAGCTTATTTGTATGTAACATTAGGCAGTATTATCTTCTCTAACAATGTCAAACTTTGAACTAAGCTAGCCCTTTTCTCTGATGAACATAATCTGAAACAAGTAGATAGTTCT

GCTATAGCATCATCAATGCCCTCATACACTATAGTTCATTCCAACTTGCAGAAGATTCTTGTTAGGTATCTGCAGATCAACAAAACCCTGCCTGCAGTTCCTCCTCAAGAAATCGTAAACAT
AATTGACAATCACTCTCTTGAGCAAAGAAGAATTCTCACTGGCCCTAACCTCACTGTGACCCAGCAAGTAAACCACTCCAGCTTCTCTTGATTTCTCTAAGAACCTAATCTCCTCCCCTTC
CACACTAGAGTCCCCTTCCTCCCAGCTCTCATTCCTTATGAAGTTCTTAAGGTTCTCCATTAACAAAAGCTCAAATTCTTCATTCCCAATTCTCCTGTCTCTATAACCATATCTTGCAATGCA
CCTATACATCTTGTAGTCCTTAGGTCCAACTCTTTGCAGCAGAAACCGCTCCTCGGCCGGCACTGTGTTAACTGGCAAGTACTTAACAGAGACAAAGACAAGAACAGAGTGGATTGCTG
GCAAGTTTGTGAGAAAATGAGGAAATATAGCAGGGATTCCATGAGTGAGCTCAGTATACAGGAGGCCAAGTCCTGGGACCCTTGAGATGCCAAGGTTAGAACCAAGGCTGTTTAAGTAT
TCTGTTGAGACCTTTTGCTCTACTTCAAAGTTATGACGCTTTGTCTGCACATAATTCCATACATACATTACAAAGTATAGAACGGAAGCAAAAGTAAGAGGAAGGTAGCCTCCACTTGTGA
ACTTGTACAGCACGGAAGAGAAGTAGACCAGCTCAAAGGAGCCGAAGCTTAGCACAAAGAGTGCTACTAGTGGGAGGCTTGTCTGCCAGACCATTAACATTATCAGAACTAACAGTGA
AGAGGTGACTAACATCACTCCAACCACAGCGATACCATACGCATTTCCAATATTGGTGGTTTCCTTGAAGGATGCAGTGACCAAAACACAGGCCAACATGAGTAGAAAGTTAATCTCAGG
GATATAAACCTGGCCTTCATGTTTGTGCGATGTGTGAATGATTCTAACTCTTGGAAAGCAGCCTAACGCCATTGACTGCTTTATGATAGCAAAAGTTGCAGAAATCATTGCTTGGCTAGCAA
TGATTGATGCCATGACAGCAGCAACAAACATTGGCCAGTAAACAGACTCTGGAATTGATTTGTAGAAGGCATCGGTGATATTTTCGGGGAATATAGACAAGTAAGCAGCTTGTCCAACAT
AAGCACATATCAAACATGGATAAACTATTCCTGAGAAAGCGATCTGAATTGATCTCACTGAGAAATGCCCTAGATCAGCAAACATAGCTTCAGTTCCCGTCATGCAGAGAACAACACCGC
CAAGTGAGATCCATGCTGCTTTTGGATCTCTCTTGAAGTATGAAATGATGTAGATGGGGTTAAAGGCTTTGAGAACAGTACTATCATGGCGGAGAATATTGAAGATACCAATGCTACCTATT
AACATGAACCACACAAGGATAGAAGGAGCAAAAGTGTAACCGACTTTATCTGTGCCAAACCTTTGGATCGAGAAGAGCATTACTAATATGAAAATTGAGATCATAGCTACAACATCTTTA
CTCATCCCAGTATCAAATTTCCTTACTCCATCCACCGCGGATAGAACTGAAATGCAGGGAGTTAAGATCCCATCTCCAATAACCATACATGTACCAAGAAGTGCCATGGAGAGCAGACCAG
TCTTAGCACAATAGCTACTTTCCAAAGCCTCCTTTATTCTTTGAGCCCGCTTCAAATTCTTGTTTGGAATTTTTAGTTTATAGGCAGATAGCTCCATCTCTTCTTGTCTCTCATTTGGAATCGC
ACTGACCTTGGAATTCCTGCATATCAGTGAGTACAGAGCAAACGTTCCCCCATCTCCATTGTCATTTGCCAAGAGGACAATGAAAACATATTTGATGAGCGGGAAGAGAGTAAGAGTGTA
GATTATGAGAGACAGAGCGCCAACGACGTCGTTCTTGCTGGGAACTCTGTTCGAGAATGTGCTTGAGAACACATAAAGAGGCGAGGTGCCGATGTCCCCGTACACAACTCCAATGCTCT
GAAAAGCAAGTTGTAGAACGGTGGCTATGGATACAACTTTAGAAGCACTGTGTAAACTAGAGATCTTGTTGGCTTCCTTATCGAGGGAATCCAAGCTGTGGAACTGTTTTTTCTTCTGGTT
CTCTTCTTCGTGCACTTCTTCCAATGGTTCTCCATTCTTCTCCATGGGTTCAGATCCAAGCGTTTCTATGTTCAACACTACATCATCTCCCATGGTGAAGTAAACTGCTTAGAGACTAAGAAT
TATCTGCTAAGCGAGCCTGCTCTCCAAGATCACTAGGAGAAAATCAGAGCTTTTAAAAGCGGCAAAGGACGTGAACAATGCGGTAATGTTACCTTATCCCGCCACCTTGGAGGAATGGAC
TCGCCGACTACTCGTACCACGTGAGGGAA

>comp151890_c0_seq5

TGTTCTGCGAATGAAATTAGCAACAATATTTTTCTTCCAACATGCCTAACATATTTTCAATTGGCAATTTATATCACAAAAGCTCTAAACACATGCAAAATAAAGAGAGAGAAACTAAGACA
TTAAGAAGAAAAGTGTAGAGGTTATGGACACGATCTAGGAGCTTTTGATGCCAAGTTACAGATGTGCACCATGCTTTATTAGATTTCAACACTAAATACATTGCTAACATTAAATACCAGCA
GTGGAATTATCAGCTTATTTGTATGTAACATTAGGCAGTATTATCTTCTCTAACAATGTCAAACTTTGAACTAAGCTAGCCCTTTTCTCTGATGAACATAATCTGAAACAAGTAGATAGTTCT
GCTATAGCATCATCAATGCCCTCATACACTATAGTTCATTCCAACTTGCAGAAGATTCTTGTTAGGTATCTGCAGATCAACAAAACCCTGCCTGCAGTTCCTCCTCAAGAAATCGTAAACAT
AATTGACAATCACTCTCTTGAGCAAAGAAGAATTCTCACTGGCCCTAACCTCACTGTGACCCAGCAAGTAAACCACTCCAGCTTCTCTTGATTTCTCTAAGAACCTAATCTCCTCCCCTTC
CACACTAGAGTCCCCTTCCTCCCAGCTCTCATTCCTTATGAAGTTCTTAAGGTTCTCCATTAACAAAAGCTCAAATTCTTCATTCCCAATTCTCCTGTCTCTATAACCATATCTTGCAATGCA
CCTATACATCTTGTAGTCCTTAGGTCCAACTCTTTGCAGCAGAAACCGCTCCTCGGCCGGCACTGTGTTAACTGGCAAGTACTTAACAGAGACAAAGACAAGAACAGAGTGGATTGCTG
GCAAGTTTGTGAGAAAATGAGGAAATATAGCAGGGATTCCATGAGTGAGCTCAGTATACAGGAGGCCAAGTCCTGGGACCCTTGAGATGCCAAGGTTAGAACCAAGGCTGTTTAAGTAT
TCTGTTGAGACCTTTTGCTCTACTTCAAAGTTATGACGCTTTGTCTGCACATAATTCCATACATACATTACAAAGTATAGAACGGAAGCAAAAGTAAGAGGAAGGTAGCCTCCACTTGTGA

ACTTGTACAGCACGGAAGAGAAGTAGACCAGCTCAAAGGAGCCGAAGCTTAGCACAAAGAGTGCTACTAGTGGGAGGCTTGTCTGCCAGACCATTAACATTATCAGAACTAACAGTGA
AGAGGTGACTAACATCACTCCAACCACAGCGATACCATACGCATTTCCAATATTGGTGGTTTCCTTGAAGGATGCAGTGACCAAAACACAGGCCAACATGAGTAGAAAGTTAATCTCAGG
GATATAAACCTGGCCTTCATGTTTGTGCGATGTGTGAATGATTCTAACTCTTGGAAAGCAGCCTAACGCCATTGACTGCTTTATGATAGCAAAAGTTGCAGAAATCATTGCTTGGCTAGCAA
TGATTGATGCCATGACAGCAGCAACAAACATTGGCCAGTAAACAGACTCTGGAATTGATTTGTAGAAGGCATCGGTGATATTTTCGGGGAATATAGACAAGTAAGCAGCTTGTCCAACAT
AAGCACATATCAAACATGGATAAACTATTCCTGAGAAAGCGATCTGAATTGATCTCACTGAGAAATGCCCTAGATCAGCAAACATAGCTTCAGTTCCTGTTCCATTGGAAAAAGACCACTT
TTTTTTTTAGTGGCAAAAAGGTGTAAAGCTCAGAGCACTTCTAAGAAGGTTAGAGAGCTCAGAAAGCATACCCGTCATGCAGAGAACAACACCGCCAAGTGAGATCCATGCTGCTTTTG
GATCTCTCTTGAAGTATGAAATGATGTAGATGGGGTTAAAGGCTTTGAGAACAGTACTATCATGGCGGAGAATATTGAAGATACCAATGCTACCTATTAACATGAACCACACAAGGATAGA
AGGAGCAAAAGTGTAACCGACTTTATCTGTGCCAAACCTTTGGATCGAGAAGAGCATTACTAATATGAAAATTGAGATCATAGCTACAACATCTTTACTCATCCCAGTATCAAATTTCCTTA
CTCCATCCACCGCGGATAGAACTGAAATGCAGGGAGTTAAGATCCCATCTCCAATAACCATACATGTACCAAGAAGTGCCATGGAGAGCAGACCAGTCTTAGCACAATAGCTACTTTCCA
AAGCCTCCTTTATTCTTTGAGCCCGCTTCAAATTCTTGTTTGGAATTTTTAGTTTATAGGCAGATAGCTCCATCTCTTCTTGTCTCTCATTTGGAATCGCACTGACCTTGGAATTCCTGCATAT
CAGTGAGTACAGAGCAAACGTTCCCCCATCTCCATTGTCATTTGCCAAGAGGACAATGAAAACATATTTGATGAGCGGGAAGAGAGTAAGAGTGTAGATTATGAGAGACAGAGCGCCAA
CGACGTCGTTCTTGCTGGGAACTCTGTTCGAGAATGTGCTTGAGAACACATAAAGAGGCGAGGTGCCGATGTCCCCGTACACAACTCCAATGCTCTGAAAAGCAAGTTGTAGAACGGTG
GCTATGGATACAACTTTAGAAGCACTGTGTAAACTAGAGATCTTGTTGGCTTCCTTATCGAGGGAATCCAAGCTGTGGAACTGTTTTTTCTTCTGGTTCTCTTCTTCGTGCACTTCTTCCAA
TGGTTCTCCATTCTTCTCCATGGGTTCAGATCCAAGCGTTTCTATGTTCAACACTACATCATCTCCCATGGTGAAGTAAACTGCTTAGAGACTAAGAATTATCTGCTAAGCGAGCCTGCTCT
CCAAGATCACTAGGAGAAAATCAGAGCTTTTAAAAGCGGCAAAGGACGTGAACAATGCGGTAATGTTACCTTATCCCGCCACCTTGGAGGAATGGACTCGCCGACTACTCGTACCACGT
GAGGGAA
>comp151914_c0_seq3
ATGACAGGCTGATTTCATACATTCCACTTCAGGAGGAAAGCAAAACACATTCTTATAATTTTTACAAAACTTACTTCAAATTCATGCACAGAAAAGCTAGAACTTGAGCAAATCCTGCACA
AAATATAGAAATTTGATACATTCTGTGCTGAGATGTAGTTACATGATGAAAAATCCGAGAACCAGCTCTTGCCATTCATGCGACTGTAGAATGTACATAAGAAGAAATTATTAGGTGCAGAC
ATCAGACGGAGAGAATCATCTGAATTTCTTTTTATCTACAGTAAAAGCACAGTCAAAGCATTGCTGCTACAGTAACTGCGATTTTATTGTAGACAGAAAGAATTCCAAATGATTCTCTATGT
CTGGTGCCACACCTAATAATTTGCCGTACATGAGACCTTTCCCCTCAGCTTTAGCTGGTTTTCACTGGCTTTAAACATGTGGATCATCTCGTTTATGCAGAATCAGGTTGATGAAGATACAA
TGATCTCTGCCTCTTCAAGTCTTACTACCTCATTAGAAGATGGTAGTGCATAAAACAAAGTTTCGCTAGATATCTGCAAGTATTTTTGACATCATGCCTTTGATCTGCTTCGAAGAAAGACC
CAATCCTTTAAGATCATCAATTTCCTTGAAAGGTTCAGGAGTCTCTTCACGAAGTTCAAGTATATATGTTGCTCTCTTCTCCCCAATTCCCTTCAATTGCTTGAGCTCCTCCTTGCTAGCACT
GTTTAAGAAAACCAAGTATTCATCAACCAGCGATTTCTTCAGTCTAGTACTTCTTGTTTTAAACTTCTCTTGCGGGGTTGCAAAACTCTCAAATTTTGAATTACAGTTGATCTTTGAACTTA
ATGAAAATCTTGGAGTTTTAGGCTCCACACTATCCACTGTAAATTGCATGTTACATGAACTACCTGTTTTTGATTCGCACATGTTTATGGGTTGAGAGCTCAGAGTTCTTAGCTGTTCAGTT
AAGGTTGGTAAAAGTTTAACATTTTCAAAGCATGACTTCTGCTGTTCATTTTCCATAGAACTACCTTCCAGGCTATGAGATTCCTTAATGTCGATTGCAAGAGGACTATCAACACTTTGTTC
AATATGATCATCTGGAATGGAAACATCCACTTCCAATGTAGAAGAAGCCAAAACAACATTGGATGCAGCAATCCCCTCTTTTTTGCAAAGATTTGCCAGTTTCTGAGTTTTTCCAAGGGGA
CTAGCAACATTAAACAACTTTCTTTCCTTTGACCAACATGGGGGTGGAGTGGGTTTCTTTATTCTATTACCATGGCCTTTGAATCTTCCATTACTTGTGGATATGCTTTTTTTTATCTTCTTTC
TGCTTGATCCCACTTGATAAATTCCCCCATTTATTATTGTTTTATCAAATGGATGGATTCCACTAACAGAAGAACTTGTCGGAAAATATCTTCTACATTCTTTACTGGTCATTGCAAGGTCGT
GCTTTTGTCTATTGACAAATTGGCATGACCTGGAAGCCAAGCTAATCACAGGAAAAGTTTGTGGGCATAAATTGGGACTCAAACATGTTATCAGCACTGCATTGTTCACGCGACATGAAA
AATCTTGCAACAATCTAGTTAATTTTGTTTCTCGATAGGGAATGTATGTTTCATCACGATTTAGCGCACTCACAACATTATGCAATGCATAGAGTGACTTATTAATTTTAATGCATTCATATAA

AGGAGCTTGTCCATCAGTCCTTTTTCGAACTTCCTCATATCCTGCCAATTCCATGAAGTTTAGCTTTCCAACAAGAGAAACTTTTGATTCCTTGTCAACCGATGTAGCATGCATAATCAAGC
CTTTGTGACTTCTAACTCTGGGATCATTTTCAACCTTTTGTGGATGATTTCGTTGGTTGCTTATGTGAAAATAATATTTCTCAAAATCTGAGAGTGATTTCACAGGGATCTTAGAAAGTCCTT
TCAGTTGAATTGCCTTTTCAGCATCTTCCATAATCTGGACCTCTTTCTCTTTACAATCCAATAGATCATATATATGATCATTATACATTTCATAACATGATATGGTCATAGAGCCACCAACTTCC
TCAATAGAAGAAAAGATTTCACAAAATGCCATCATTGCAAGACCGGGAGTCTCCACAGATCCCTGAATCAATTGGGTTTTCCCACTTCCTTGAACACCATAAGCAATAACACAACAACTA
TCACCATGAAGAATGCCCTCAAGGAGAGGCTTCACCTCATGAGCAAAGAGTTTACTGTTGCTCATCTTGTTCATAGCAGTCATCCAGTATGTAAGTATCTTTGCGACCTTTCGCTTGATCAT
GCAATATAACGGAGGCATGGTTGTTTCCTCTAGAAATGGTAATTTGAGAAGGACTCTCTGAGCAGTGCTTCTCGGATTCCGTAAATGGCCTGATTTTGCAGACGACTCGAACTCCCTTCCT
CGCTGCGGGCTTCGGCCCGCAAATTTCTCGTTGCTCCTCCGAGCTGACGAAGAGTCCATGGCTTGAGGCAGTTTCGCCAGAAAGGCCCCGGAGAGATTGCAGGCGAGGAAAGAAA
>comp151914_c0_seq4
ATGACAGGCTGATTTCATACATTCCACTTCAGGAGGAAAGCAAAACACATTCTTATAATTTTTACAAAACTTACTTCAAATTCATGCACAGAAAAGCTAGAACTTGAGCAAATCCTGCACA
AAATATAGAAATTTGATACATTCTGTGCTGAGATGTAGTTACATGATGAAAAATCCGAGAACCAGCTCTTGCCATTCATGCGACTGTAGAATGTACATAAGAAGAAATTATTAGGTGCAGAC
ATCAGACGGAGAGAATCATCTGAATTTCTTTTTATCTACAGTAAAAGCACAGTCAAAGCATTGCTGCTACAGTAACTGCGATTTTATTGTAGACAGAAAGAATTCCAAATGATTCTCTATGT
CTGGTGCCACACCTAATAATTTGCCGTACATGAGACCTTTCCCCTCAGCTTTAGCTGGTTTTCACTGGCTTTAAACATGTGGATCATCTCGTTTATGCAGAATCAGGTTGATGAAGATACAA
TGATCTCTGCCTCTTCAAGTCTTACTACCTCATTAGAAGATGGTAGTGCATAAAACAAAGTTTCGCTAGATATCTGCAAGTATTTTTGACATCATGCCTTTGATCTGCTTCGAAGAAAGACC
CAATCCTTTAAGATCATCAATTTCCTTGAAAGGTTCAGGAGTCTCTTCACGAAGTTCAAGTATATATGTTGCTCTCTTCTCCCCAATTCCCTTCAATTGCTTGAGCTCCTCCTTGCTAGCACT
GTTTAAGAAAACCAAGTATTCATCAACCAGCGATTTCTTCAGTCTAGTACTTCTTGTTTTAAACTTCTCTTGCGGGGTTGCAAAACTCTCAAATTTTGAATTACAGTTGATCTTTGAACTTA
ATGAAAATCTTGGAGTTTTAGGCTCCACACTATCCACTGTAAATTGCATGTTACATGAACTACCTGTTTTTGATTCGCACATGTTTATGGGTTGAGAGCTCAGAGTTCTTAGCTGTTCAGTT
AAGGTTGGTAAAAGTTTAACATTTTCAAAGCATGACTTCTGCTGTTCATTTTCCATAGAACTACCTTCCAGGCTATGAGATTCCTTAATGTCGATTGCAAGAGGACTATCAACACTTTGTTC
AATATGATCATCTGGAATGGAAACATCCTCTTTTTTGCAAAGATTTGCCAGTTTCTGAGTTTTTCCAAGGGGACTAGCAACATTAAACAACTTTCTTTCCTTTGACCAACATGGGGGTGGA
GTGGGTTTCTTTATTCTATTACCATGGCCTTTGAATCTTCCATTACTTGTGGATATGCTTTTTTTTATCTTCTTTCTGCTTGATCCCACTTGATAAATTCCCCCATTTATTATTGTTTTATCAAATG
GATGGATTCCACTAACAGAAGAACTTGTCGGAAAATATCTTCTACATTCTTTACTGGTCATTGCAAGGTCGTGCTTTTGTCTATTGACAAATTGGCATGACCTGGAAGCCAAGCTAATCAC
AGGAAAAGTTTGTGGGCATAAATTGGGACTCAAACATGTTATCAGCACTGCATTGTTCACGCGACATGAAAAATCTTGCAACAATCTAGTTAATTTTGTTTCTCGATAGGGAATGTATGTTT
CATCACGATTTAGCGCACTCACAACATTATGCAATGCATAGAGTGACTTATTAATTTTAATGCATTCATATAAAGGAGCTTGTCCATCAGTCCTTTTTCGAACTTCCTCATATCCTGCCAATTC
CATGAAGTTTAGCTTTCCAACAAGAGAAACTTTTGATTCCTTGTCAACCGATGTAGCATGCATAATCAAGCCTTTGTGACTTCTAACTCTGGGATCATTTTCAACCTTTTGTGGATGATTTC
GTTGGTTGCTTATGTGAAAATAATATTTCTCAAAATCTGAGAGTGATTTCACAGGGATCTTAGAAAGTCCTTTCAGTTGAATTGCCTTTTCAGCATCTTCCATAATCTGGACCTCTTTCTCTT
TACAATCCAATAGATCATATATATGATCATTATACATTTCATAACATGATATGGTCATAGAGCCACCAACTTCCTCAATAGAAGAAAAGATTTCACAAAATGCCATCATTGCAAGACCGGGAG
TCTCCACAGATCCCTGAATCAATTGGGTTTTCCCACTTCCTTGAACACCATAAGCAATAACACAACAACTATCACCATGAAGAATGCCCTCAAGGAGAGGCTTCACCTCATGAGCAAAGA
GTTTACTGTTGCTCATCTTGTTCATAGCAGTCATCCAGTATGTAAGTATCTTTGCGACCTTTCGCTTGATCATGCAATATAACGGAGGCATGGTTGTTTCCTCTAGAAATGGTAATTTGAGAA
GGACTCTCTGAGCAGTGCTTCTCGGATTCCGTAAATGGCCTGATTTTGCAGACGACTCGAACTCCCTTCCTCGCTGCGGGCTTCGGCCCGCAAATTTCTCGTTGCTCCTCCGAGCTGACG
AAGAGTCCATGGCTTGAGGCAGTTTCGCCAGAAAGGCCCCGGAGAGATTGCAGGCGAGGAAAGAAA
>comp151928_c1_seq1
ATGTGCAGAGTGCTTCAATAATTTCAAGTCAATGATGATAGATAAAATAGACATAGTAAAAGATTTTACACTTGAATTTTTAAGTCAATACTATTATTTGGGTTTGGTCACCATTATGAACAA

AGCGCAACAAATGACAATACAAAAGTTCTGGACGATTTTCAAGTCAATAGATTTTTCCAACAATTATTTTGAAGGAGAGATTCCAATAACCATTGGCCAACTTACTTCTCTTCAAGTGCTC
AACATGTCACATAACTATTTGACAGGAATTGGTATCTTTGCATTTCTTGGATTATTTAAACCTCTCATACAACAAGTTAATAGGAAAAATTCCAGTTGGAGGTCAATTCTCCACTTTCC
>comp151934_c0_seq5
GCAGTGGCCGCCTGAGAGCTTTCGTCTTGCCAGGCTTGAGAGCTTATGCTGCTTCAACATATTCCTTCCTTGGATGTCTGAGGCCATCAATTACATGTAAACCATTAATTAGCTTACATTTG
TTGATTGGTTAGGTATTAGATGGGAGCTCTTTTAATTACGGTAGTCTTTTGATTGGATCAGATGGATGATATGCAGAAATAGCATTGAAGTTCCAAGTATATAAAAAGTTATGATTCAGCGAG
TGATGTGACATTTGTGATCATTATTTATTGTATTGATGAAGGGATTGCGATTTTTGTTAAGGAGAATCATTATTAAATCACTTTTTAAGAAGATTTGTATTAATAATAATGATCAATTGTTTCTT
TGTGTTGTTTAATGGCGATTTCAAGCTTCTTCAAGGGCTCTTTTAGTCTTGGCCAAGATGTAAAGAGAATAACTCAAATGATACTTAGCATGTGTTGGTTTGAGTTCTAATATGCTCGATGA
GATCAATGACTATGAAGTTCTTGATAACAAAGCCATTGAGGTTTGTGAGAGTATGATTAAGTCTTTGTTTGGAGGATTTTCTTATTGCGTCATATAACATTTTAAATTTTTTATATTATAAATTT
GTTTTAAGACTTAGCTTGAATCCTTGAAAACATGTAGCTTATTTTTGTTGTTGGGTTATTGATTGGGGTGCCTTAACATTGGCGCTAAGTATTGCCCAAGTCATTTTTATAAACATGAGCAAC
CTTGTTTAAGTGGTGTGATTGATGTTTGAATAAAAATGAGATCTTTTGTAAGAACGGTATCTATAGCAAGTGTGGTTAGCTTGTGATTAAGTTGTAATGTTGCTGTTCAAATATATTAGCATT
TGTTATAATTCATTAAGCTTTATATGATTTTGTCATTTTGTGTGTATTCAATAAACTTTTTAGTTTCTTTTGGGACAGTTCTTTTGTTATCTTATAAAATTATTTTTTTAGTACAAAATGATGCTG
TCAGATTCAAGATAAGCTTATTGTTGTTTAAATTCATTATGACTTGTTAGAGAGGCATCAGATGCTCTAAGAGCATCTCCAACCAAACGCTAAACTTGCGTATAGCGTTGTTAGGTCGTTCC
AGTCGAATGCCAAATCTTCCGTTGTTTACTTGCGAATGAACAGTGCAACGCTAAATTGGTGTTGTACTATTCACCTACGTTATTTTGCTCTCTCAATCGAAAAA
>comp151955_c0_seq1
CTCATTAATAATATGATATTATGTCAAAAAGCAAATACAAAAAAAAAAAACACCGAACATTAAATAATTCATCAGATGAAAGCATAAAAGAACTTAAATTGCAACCTAAAATATAAATATTT
AAAGAATAATAATAATAATTTCTTCCTAATTACCATTACTTATATTAAAAAATCCCAGCCATCCATCATCGTTGGCCATTTACCGTCATCAAGCGGCGCGGGCCGCTCCAGCGCCGCGCAAG
ACTTCTTCGACCTCATCGCTGGCGAGGTCAATTCTCAGGCGCCATCGCCCTGCGGCCACCGCCGCAACTATCGTCCTCGGAATGAGAACGGTGAGCAAACTATTCGCACTGAGTTGAGTT
CTCGTAAGCCGCCGAGATCCCTTCCACATTGACCATCGCCGGCAATCGGAACTTCTTCATAAACTCCTTCCCTCTCCTATCTCTGTCATCGCCGCCTTCCGCGGAACTCAACTCAGTGCGA
ATCACCGGGTATCGCGAATCCTCCAGCTCCACTCTGATTTCCTCTATTCTCACTCCTGGAAGATCGGCAGACAAGAGGTAGGAATCGGGCGTTTGGATCCAACGAACATTGTTATTAGCAT
GGGATTGGGCGCGGAAGAGCAAGGGCATGGGGAAGAAGATTGGCCATGGCGAACCGTGAATTCTGTAGAGTTCCATGGTAAGGGAGAGCAAGGGAGAAATTGAAGGGTAGCGACAAG
GAAGAGAGAAAGAAGGATCTTTTAAAGCGGAGGAGGATATTGTAATTAACTAATTATAGCAT
>comp152013_c0_seq1
TTTGCTTAGCTTTTTCGCAACGGGTTTGCCGCCAGGACACAGGTGTCGTGAAAACCACCGTTAAACCTAAGCCAAAATGGGAAAGGAGAAGACCCACATCAACATCGTTGTCATTGGGC
ACGTAGATTCAGGGAAGTCTACCACGACTGGCCATCTGATCTACAAATGTGGCGGGATCGACAAGAGAACAATTGAAAAGTTCGAGAAGGAGGCTGCCGAGATGGGAAAGGGCTCCTT
CAAATATGCCTGGGTCTTGGACAAACTTAAAGCTGAACGTGAGCGTGGTATCACCATTGATATCTCCCTGTGGAAATTTGAGACCAGCAAGTACTATGTTACCATCATTGATGCCCCAGGA
CACAGAGACTTCATCAAAAACATGATTACAGGCACATCCCAGGCTGACTGTGCTGTCCTGATCGTTGCTGCTGGTGTTGGTGAATTTGAAGCCGGTATCTCCAAGAACGGGCAGACCCGT
GAGCATGCCCTTTTGGCTTACACCCTGGGTGTGAAACAACTAATTGTTGGCGTTAACAAAATGGATTCCACTGAGCCACCCTATAGCCAGAAGAGATACGAAGAAATTGTTAAGGAAGTC
AGCACCTATATTAAGAAAATTGGCTACAACCCCGACACAGTAGCATTTGTGCCAATTTCTGGCTGGAATGGTGACAACATGCTAGAACCAAGTGCTAATATGCCATGGTTCAAGGGATGGA
AAGTCACCCGTAAGGACGGCAATGCCAGTGGAACCACCCTGCTTGAAGCTCTGGATTGCATTCTGCCACCAACTCGCCCAACTGACAAACCCTTGCGTTTGCCTCTCCAGGATGTCTATA
AAATTGGTGGTATTGGTACTGTCCCTGTGGGTCGTGTGGAGACTGGTGTTCTCAAACCTGGCATGGTGGTCACCTTTGCTCCAGTCAATGTAACAACTGAAGTGAAGTCTGTAGAAATGC
ACCATGAAGCATTGAGTGAAGCCCTTCCTGGGGACAATGTGGGCTTTAATGTCAAAAACGTGTCTGTCAAAGATGTCCGTCGTGGCAATGTGGCTGGTGACAGCAAAAATGATCCACCC

ATGGAAGCTGCTGGCTTCACAGCTCAGGTGATTATTTTGAACCATCCAGGCCAAATCAGTGCTGGATATGCACCTGTGCTGGATTGTCACACAGCTCACATTGCTTGCAAGTTTGCTGAGC
TGAAGGAGAAGATTGATCGTCGTTCTGGGAAAAAGCTGGAAGATGGCCCTAAATTCTTGAAATCTGGTGACGCTGCCATCGTTGATATGGTTCCTGGCAAGCCCATGTGTGTCGAGAGCT
TCTCTGATTATCCTCCCCTGGGCCGTTTTGCTGTGCGTGACATGAGACAGACAGTCGCTGTGGGTGTCATCAAAGCAGTGGACAAGAAGGCAGCTGGAGCTGGCAAGGTCACCAAGTCT
GCCCAGAAAGCTCAGAAGGCTAAATGAATATTATCCCCAATACCTGCCACCCCAGTCTTAATCAGTGGTGGAAGAACGGTCTCAGAACTGTTTGTCTCAATTGGCCATTTAAGTTTAATAG
TAAAAGACTGGTTAATGATAACAATGCATCGTAAAACCTTCAGAAGGAAAGGAGAATGTTTTTGTGGACCATATGTTTTGTGTGTGGCAGTTTAAGTTATTAGTTTTTAAAATCAGTACTTT
TTAATGAAAACAACTTGACCAAAAATCTGTCACAGAATTTGAGACCCG
>comp152041_c1_seq3
CCTATTTTCGGACTTCAAAACTTATAAAGTCCGAAAATAAATATTTTACCTTCTCCTGAATATTCAACAACATAAGCAACAAAAAATGATAAACATAATTATAATTAATTTCTCTAAATTTTCA
AATTATTTGTTAATAAGATGAATATCAATTTCGATATATAAAAAAAAAAAAATTGATTGGTACATGGTATGCTGTTGATCGGGTTTTTGGATTTATTTGTTGGCTTAAGCTATAATAATTTTAAT
GTAAGCCATTAAACCATACATTGCATTAAGCCATGAAGCCATACGTTACAGGATCTATATATAGGCTTCAATATATTAAATGGTGGCCATAATGATACACTAGTTCTCATGCAAGCCTTGATCT
TCCTACGGGACTGCCTTCCTCCCATACGTCCAGAATTTGTTTTATTTCTTATTATTATATTTTTTTTTTTTATGCGGACGTTTGGGCTTCTCAAAGCAGAATTATTTGAGACAGCTGCAATGCC
GGGTCGTTATTTTATTTAATTTTTGCCTTTTAGACTCTCAAAAGTTTTATTAACTAATTAATTTGTATTTTATTTTTAGAATTTTCTAGAAAAACTCTTCATCTTCCCCTCTTGCAACTATAAATA
CCCCTTTTGGGGAGGATGAAAGGCACAATCCCTTGTGCCGGTCCAAGCCCTCTCTATGGATTGGTAGCTCGGGGATTGTCAGCGGCGATCAAAACCTTGCTCTCTTGTGCCGGTCCAAGC
ATTCCCTGGATTGGTAGCTCGGGGAGCGTAGGTGGAGATCAAAGCGAACATTGGCGGCGATCAAAGCCTAGTTCTCTTGTGCCGGTCCAAGTTTTTTACTTGGATTGGTAGCTCGGAGAA
TATTGGTGGTGATCAAAGCTTTGCCCTCTTGTGTCGGTCCTTTTGTGGATTGGTAGCTCGGAGGGTGCCTACGACAATCAAAGCATTGCCCTCTTGTGCCGGTCCTTGTGCGGATTGGTAG
CTCGGAGGGCATTGATGACGATTGAAATTTGCTTTCTTGCAAAAGGTAGAAACAACTCGACGGTGGCTTCCCTTGGGGAGAGATTTGACAATAGAAATGCCTTGAAGAAGATTTGATTAT
AATCAACGGACAGGTAGAACTCTGTAATATGTGGCGACTGGGTTGAAGTTCGATGGCAGTCAGATGAAAGTCTGACATCGATCGAGAAGAAGTACAACGGTGGGAAGAATTCCGACGA
AAGTCCTGTGACGGGTAGAAATCTAACGATGGGAGAAAGTCCAACAACAGGAAGAATTCTAACAAAAGTTATGCGACGAGCAGAAGTCCCAACGACGGGTGGAAGTCCAGTGTCGAG
AGGAAGTTCAGCGTCAGGGAGAATTCAGACAAAAGTTATGTGACAAGTAGAAGTCCAATGATGGGTGGAAGTTCGATGGCAGTCAGGTGAAAGTCTAACAGCGATCGGACTGAAATCC
AACGTTGGTCGAGTAAAAGTTTGAATGCCAATGAATGCCTCTTGGAAGTGGATAGGCGGTATTCCGACGTAAGAGAACAGACGATGAGATGATGATGATAATTAAGCTGCATTTTATTGAA
AATAGAAGGTATGCATGATGACATTAACTTGTATGAAAAATGATGAGAAAATCCATCAAGGATGAATGACCTATCTTAGGTTGTTTGTCATAGCTTCACTCGATGACATTAACCTGTATGGA
AGAGGTCAAGGAAATCCACTAAGGATGTAAGACCTATCCGAGGTCGTATGTCGGAGCCCTGCTCGACGACGTT
>comp152095_c0_seq4
TTTTTTTTAGGAACTACTCAGCAATTTTTCCATATATCATCAAAATTGAAGATTCTTAAAGCAAAGAAATGAACATGTTAAGGCCAAAACAAAATACAAGTTTTATGTCAAACTGGGAAAT
GAAAAATGTAATTAGCATTGAAGAAAATCTTAAGTTTGAAAACCTGCGTTCTGATTGTCCGCAGGAACAACAAATAAAGAATTTAAAATTTCTTAAAACCCTGTTGGTCATTGAAAAGTG
TCAGAAGAACGTAAATATAGGTTGTTGAAAGCCATCACAACTCATCATCCATATAATCATCATCGGAGTCATCACTTTCATCACAGTGCTTGCTAAGGTCTTGACAAAGGACCTTGTTGATT
TGTTCTGGTTTTAAAGAACTGCTTTTTATCAATTCCGTGAGCTTTTACAGAAAGATGAAATATACTTTGAGTATGTCTGTGCCTCTTGTTTATCTTTACCTGCTGGAAGATCGTTCCAGTCGT
GTACTCTGACCCAGATCTCTTCACCTGTGTCCAACTTCTCTAGTGTGTGGTCTAGCATTTTCTCACAAAGACCATCCAGCAGTTCAACAACTGTTTGCGTGCCATTTCTGTAATCAGTGTAT
TTCCCTTCAAGACGACTTATAGAATCCATTCCATTAAAATACTCGATCTCTATCTCGTCCGCAATCGCGTTGCAAGCGGAGCATTCGTCTTCCACGCTAATAACCAAGGAGAGGTTTGCAAG
AATGA
>comp152173_c1_seq1

CTCTCTCTCTCTCTCTCTCTCTTCAACATGCATGCCATCTTGTCCATACATCATTAACATCCGCCATTCCCTTCAGCAACCTACTCTTCAAGAGAAGGCGAAGAAGATCTGTATAAATCATAA
GGAGCCCAGAGATAATCAACCGCGCAGGGCTTCCTAGAATCCAACCTCAACCATGGCTTCCCCTTTCCACTCCAATGAAGCAGGCTAACAGGCCCTGGATGAAGATTCCTACACCGGCCC
TCTATATTATCCCCTCCAAGCCCATGCTGGTTCCATTTATGATCCACAGCCTTGATGTTTCCAGCTAGAACAAGCAGAAACGGAGGTAACGATCCAAGCTGATATATTCTTTTCTTCTTCTGC
ACAGTCATCCAATCCTCCACCATCCTCGTATAACCCGCCGCCCTCCATTTCTCCACGTCCATCACCATCACCCCCGTGTTGAAATAACATGCAGGACGCCCATCGAACGTTCGGCCAGGA
CCGGGTCGGCCCAGAATGCGTCGGTGAAGTATTTGGTGAAGTTGGCGTGGCAGTATTCGGGCGCGGCGACGACGAGGCTTTCGAGGTCGACGGCCCAGAGGGTGCGGATGTCGTCGAC
GACGATGACGTCGGAGTCGAGGTAGATGACGCGATGGACATTGGGAGGGAGGATATCGGCGAGGTAGATGCGGGCGTAGTTGAGGGGTTGGTCGAGGGCATGGCGGACGGAGCGGGA
GATGCGGGAGCGAACGCGGGAGGAGTCAAAGAGGTGGAGTTGGAAGTTGAGAAAAGGGAAGGTGGAGCTGATGATGGCGGAGATAAAGGGGTTGGAGCGGGAGGAGAGGAAGTGG
AAGGCGACATTTTGGGGGCAGGAGGTGTGTTGGAGGATGGAGAAAACGGCGGCGATGGTGCCGCGGAGGTAGTTGGCGTCGAGGGTCATGGAGATGTGGATGAGGATGGGAGGGGAG
CACTCATCGGCGTTGCGGAAGGCGGGGGCTTCACGGAAGTGGGGGAAGGGGGGAGGAGCGGAGGACCGGCGGATGACGTCGATGCGGAGGGGGTGGA
>comp152197_c0_seq1
GTTGTTTAATAATAAACAGTAAATTGAGGCAAATAAAAATTGCACAAACCATCAATATAAGCAAAAATGTATAATGTAGTGGTTTTGTGTCTGTCTACAATACTAAAAGTATAAAAAATGAA
TTCCTTATTGATAGGCTCCAAGCATATAGGAGTTCTGAACGTATATGTTGTTAGCATAGCATTCGAATGTATAAATGGTGCACAATGATCTGATGCTATTGATACAATTGAATAGATACTTCAC
TACTCACAGACCAATAATGCACAATAAGCACCATTCCTAGAAAGCAGCAAGCTATATTAGCATAATCATGAAAGTATAAACTCTTTGACCTCAGATGACAGAAAATCAATCACTAGTTCAG
ATTCCGGCTGTGACTTGAACTCGAGCTCGAAGTCAAAGATCTATGGCTTTCATTATGCTGCAACTTCTCTCTTGTGCACTCCAAGAATGTTGGAAATAATGCTCTTTGATGCTTGGCGATAT
TAACTTCTTGCCTGTTGCACATTCGAATTTGGATTACAAGATGGTTGGTGAGAAGAGTCCTCACTGTTCACTGAATCCATTGAGGAAGAACGGCTAATTTCATTTCCAAAAGAAGACAAC
TTGCTATACATTGAGCTAGAGCTTTGATTGAATTCTAGCAATCTTTGTAACTGCATTGGGCCGCTTGATCTGCTGCTTCCATGGCGGGAATTTTCGCTGTGACTTGAATTTGAATGGCTTCTT
TCGACACCGTCTATTATTATTGGTTCAGGGGGATGGTATTGAGTTTTTTCTTCAGGGACTGGTGGTTTGACTATGTGGGAAGGAATGGAGGAGGCTTCGGAAAGAGGCGGCCCCTCAGCG
AGGAAGCTGAGGGCTACTACAACATCGGCCATGAAAGGGCGAACAGAAGCTTCTTCTTGTAGGCACATGGCGGCAACACCAACAGCTTGGTTAAGGCCAATTACTGGATATGCACGTTT
GAGAAGGGGGTCAACTAGTTCTGGGAATCTCTTCTGATCTTTGAACATTGGTTGTGCCCAAGCAACCAAATTCTGCTCGTTTGTGGATCGCCTTGTGTCGATGACTCTTCTTCCAGTGATC
AGCTCAAGAAGAACTACTCCAAAACTGTAAACATCTGATCTGAGGGTGAGCTCACCAATTCTGGCATATTCAGGTGCACAGTAGCCATAAGTGCCCATTACACAAGAAGCAACATTGAAC
TTATCACCTCCAGGGCCAAGTTTTGCAAGGCCAAAGTCTGAAAGC
>comp152251_c0_seq4
CTCTTCGTTCTTGGCCATGACTGGTATGCCGAAGCTGTTCTCAACCTTTGTTATTTATTTTCACCCCATTCCTTTATCGAAGTCCGCGATAGATTATGCTTTTTTTACCTAAAGATCTTATTTTG
GGCAGTGGACACGGAAGCTTTTCGAATAATCACAAACTGAACAGTGTTGTTGGCCATCTCCTTCATTCCTCCATTCTTGTGCCCTACCATGGATGGAGAATAAGCCACAGAACTCATCATC
AGAACCACGGCAATGTGGACAAGGATGAATCTTGGCACCCTCTGTCTGAAAGTGTTTATAGGAGCTTGGATACAACAACTAGGAATCTTAGGTTCACATTTCCTTTCCCAATGCTCGCCTT
TCCCTTTTATTTGTGGAGGCGAAGTCCAGGAAAGAGTGGGTCACACTTCAACCCAGAGAGTGATTTGTTTGGTCAGAATGACAGGATAGATGTCATCACTTCTACAGCTTGTTGGATGGC
AATGGCTGCTTTGTTAGCTGGATTAACTTTGATTATGGGTCCTCTGCAATTGCTCAAGCTCTATGCAATTCCCTACTTGGTTGACCTTTCCTCTTCTTATTGTAGAGCTTTTTTCTAATTTAAG
TGTCAGGTCATAAACAACTTAGTTATGTGGATCTAATTGCAGATATTTGTTATGTGGCTGGACTTAGTAACTTATTTACATCACCATGGACAT
>comp152301_c0_seq1
ATCCATAAAAGAATCATCTAGCGTTTATCATGGACGATTCTCCTTAAACAACAAATCAACCAGCGCTTTTTTTGCTTTCCTTGAAATGACACCGGCCAGGTAGGGGTTTCGACGAATCCAC
GTCGAGGATCCGGTGACGGGACCAAGAACTCCTTCTTGGGGGAGGGATCACCACCTGAAATCCTTCAGGATACAGAAACCCAAAAGCAGTGTAAAAACTACTCAAATATTATTCATCTC

AATCTCCCCTTATTACAAGACCTTGGCTACCTATTTATAGCCAACAAGGAAGCATCATACTAGGCTACATACAGAATAAGAATGCATTAAACCTTTATATCCCTATACTACCAAATGGCATTAA
TATAGCCTGAAGTTTAATAC
>comp152326_c0_seq2
ACCTATGTTAAATTATGTATATCTATGCTTGTAATCGGTTCATTGGAAGATCAATGATTTATGTGATCAAATCATCTAAGACGACACTCTAATTTTATCTGCCACAGGCTACTTGTACCTTTTCA
CACAGATTTACACTTGCCTCTAAAATTCACCCTCGTTGCCAAACTACCATCTATCTCAAAAAATGATCTAGCACTCCTATGATTTAACCATGCCATAATCAAATCATTTGAATTTGGAGCGAA
AGCCAGAAATATGGGTTGAGACTTGAGATTGAGATTTTATTGCGGAATTCGGTATTTGGTAGAAATTTGATGAATTGAGTTTACAAATCGTCAAGATTGGCAAGGAATGTATGAATTGCCTT
AGTATGATTTAAAGTTAAGGCTGGTATGCTTTTGTATGATTTAGTTAAGGCTGGTATACTTATTCCGTCATGATTTCTCTCTTCACTTTCAGCTTTCTAAAAGAAGGGAAAATTTGCTAAACTA
TATGCCTTTCCTCAGTGTTACTCCAACCTTGCCAAATACCTTTATCAATGTGGCGCGACACCAAATAAGCGAACGTACAAACCCAACAATGGCTAGCCCCCGCCAAGTCGTCGTCTTTTGG
GCGACGGCCCTCCTCGCGGCGGCTACCTTCTTTCTCTCCGGCAACGCCTCTCTCGGCGTCAACTGGGGCACCATCACCTACCACCGCCTCCCTCCCAAAACCGTCGCTCGCCTTCTCCAC
GACAACGGCATCAAAAAAGTCAAGCTTTTTGACGTCGACCCCTACACCATGGAAACTCTCTCCAACACCGACATCCAGGTCATGATCGCCGTCAACAATCTCCTCCTTGCCGCCATGAAC
GACTACGCCACCGCCAAAGCTTGGGTGAACGCTAACGTCACCCATTATCGCACTCATTCGAAGGTCAACATCAAATACGTGGCCGTCGGGAACGAACCTTTTCTCAAAGACTACAAAGA
TCGATTCACAAATGTAACTTTGCCCGCCCTCAAAAACATTCAAAAAGCTCTCGATGAAGCCGGACTGGGCGAGGAAATCAAAGCCACGATTCCGATCAATGCCGACATCTACTACTCGCC
GTGGTATAATCCTGTCCCCTCCGCCGGAAAATGGCGCGACGATGTCGCCGGTCTTATGTCCGACATTGTCAAATACTATAATCAAACCGGCGCGCCTTTTACCGTCAACATTTACCCATTTT
TCAGCCTTTATATCGACGCCAATTTCCCCTCCGACTTCGCCTTCTTCGACGGAAAGGCGAAGCCAGTCGTTGATGGAGATTTAATCTACACGAACGTCTTCGATGCGAATTACGACACGCT
GCTCGCCGCGCTGAAATCGGAGGGCTGCGGCGACGTTCCGATCATCGTGGGCGAAGTTGGTTGGCCGACCGACGGCGATATACACGCCAACGTATCGATAGCCGAGAGATTCTACAAAG
GAATAACGCAAAAGATAGCAAAAAAAACCGGGACGCCGCAGCGGCCTAAGGAGGATATAGAAGTTTATCTCTTCGGGCTATTCGATGAAGAAGCAAAGAGCATACTTCCGGGGCCTTTT
GAAAGAAGCTGGGGCATTTTTTCAACCGACGGAAAGCCGAAGTTTCCGGTGGACTTATCGGGCGGCGGAGTGGCCGGAAAAGCGGCTGTCGCGGCGGTGGAAGTTAATTACTTGGCGA
AGAAATGGTGCGTTTTGAACAAAGAGGCGAAGCAGGCGAAGAATGAAAGCAGAGTGAGTGCGAATATGGATTACGCTTGCGGTCATGGTGCTGATTGTATGCCGTTAAGTTACGGTTCA
TCTTGCTCGGGAATGGATTTGGAAATGCAGGCTTCGTATGTCTTCAATTCGTATTTTCAGGCGCTTGGACAGGGGACTGGGACGTGTGTTTTTGATGGGTTGGCGAAGGAGACGACCAAG
GATTATAGCAGAGGGAGCTGTAAGTTTGAGATTGGGATCAAGGCGTATAATCTTGGTGAAGTGGAGGCGCCGGCCCCGGCCCCGGTGGAACAGGATGAGGATTCTCCGGCGAAGGTACA
TGGTAAACATGCTCATGTTGGAAATGAAGCTTCTGAGGGTAGGAGGAGAAGCATGTGGAAGGTTGGATCATTGATAATGGCGTTTGTTTTGGCTGGATTTTTGTTAGATTAGCTAAGAATG
GAGTTGGAAAGGATTTTTTTTTTGGTTTGGGAGTTTGTTTGTTCTGTGGTTTGTGCCTGTGGTCTGGCAGATATTCCCAAGGAAAGAAACAAAAGGGAGATGACCGGTATTGTTATTTTTA
TTTTTAT
>comp152347_c0_seq1
TCGGAAACTAGCAATATTCCTATAGTGTTGCTTCTATGCATCTACACTTGATGATGAATTATTCAATTTGGGCTAAATTTATGGCTATGTGATTTAGCTTGTGTTGCACGCTTGAAAGTTAAGG
CTAATATTTTGTTTTAAATTTTATCATTTAGAACATCATTTCTAAAATTAGTAGTAATAATGATCGAACAACATCATATTTGAGTACATAAAGAATATAATTAATATTTTTCATTTAATTATATTTT
TATTTTTTTTAAAAATCTTATCAATATACATGATGTATATGTAATGGTAGATTTTGCATGATATTATTTTTTGTAGAAACAGCTTTAATTTTAAGGTTGTTATCTTCTATTTCAACTGTTCTACTAC
ATTGCAGATCCAAAAAATAAAAAAAAGATAAATGTAACAAAGCTATTACACACTCAGCAGTACAATAAATCTTAAACATATAGGATTTAGTTGTATTTTATAGCGAGAGTACCAGGATGCTA
CAGAAGCGGGGATGACGAAGGAGGGTGACCTCGAGACCGGCCTTTATCTCCTCGATGGTTAAGATCTTTCCAAATCCT
>comp152389_c0_seq1
TGAATATCTTGAGCCAATTGTTTTCAATGATTGCATGCACTTGAGAGTAGTATAACTAAATAAAATTATATCAGGAAAAAAAAACTTAACGAAAAATTCCATAAAACAGGTCTGTAATTACGT

GGATTCTGATAGCATTAGCCTAGACTACTAGTCAATAGATATACTACAACGCATAAATAATGAAGCTTGGAAGCTAAACATTTAACACTCAAAGATAGAGGAGTCAGTACCAGCAACTCAT
TTGTGCTACTTGATAGTCACAAACTTGTTAGTGCCATGCCTTGCCCAATGATTCTTCAAATCAACTCCATATGAGAGGTCCTGTCCTTCAGCCACAAGTCTTTGAAGTAGCTTTGTGTAAGC
TCCAATGCTCATTTTCCTTCCAGCCATCCGCGAACCCGGCCTTGTCGGGCTCACTGGGAGGGGATATTCCGACAAGCCTGTGGTGCAACAAGATGACTGCCAACCACCAGCTCCCCACCT
ATAGCATTGCCGCGGCATGCCGGTGCAAGAACATATAGGTGCAGGGACACCTGAAAGATCAATCATAACACCATTGAACATATCATCTTGATTCTTTTCTCTCGCTTTCCTGTTGACATAG
AACCTCCTTTCTTCTTTGTGCCCTTTTCTGCTTTAGGTCTTAGAACCTTGGTAGCGACATTTTTTGCCTTGTTCTTTGAAGATTGTTGCTTTCTGGCATTAATGGGGTTAGCAGCAATGGGAG
ATTCCGGCACTGAACTATTAGATTGAATTGGCATGCTTGACATATTGAACGTTTCTGAAATCACAGGCATTGTCTGTAAGTGATTAATGGTTTTTGTGGGATGAAGAAAGCTTCTTTGTGGA
ACCCATGTGAAGCTTACAGAGGATGCTTCAGAACGGTGTTCATCCATCACAGTAGTGCGATCAGAGTATGCACCAATCTTCAGGAAACCAGGTTGATAAGCTGAAGAATTGCTCTCTGGG
AAGGAGACACCCAAACCGGGCTTGAACATGGATTCGGAATGCCGATTATCAGTGAAGTCCCAATTTCTTAGATTTAATTTGCTCTTTCCATCCATGAATAAGAGAGAACACTGACGCCTGG
CTACAGTTTTAAGATACGGCTTAAGAAATTTCTTCTTGTTCTTTCCAAACTCCTCAGCTACATCTCCTGTCGTTTTGTAAATATTTTTGCTACATCTATATTTTTATTTTTCATTAAGATGTCCA
GCTTTCTCCTTCCTCTAAAGCAGAATGAGTTATACCCTTCAGGGAGACCAAGAAATAAGGCGGGCACTTCTTTTATTGGCGCTGCACCAATTCCATGGAGAATTGATGAAACCTTTATTGC
TCTACAGATGTTGACATTGGCATCAATGATGAGAGTCTGACTTGTT
>comp152402_c0_seq10
CCCCCTGCTTGGATTAAGGCAAACCGGCAATGGCTCGTTGGAGTCCTGTACTACTTCTACTTAGTTTTCTCGTGATATTACAAAAATCTATCTCTTCAACTCCAAATCATGTACAGCAACTA
CCAGGCTTCAAAGGGCAGCTTCCCTTCCAAATGGAAACTGGGTATTTGACGGTGGATGAAGTTAATGGCGTGGAGTTGTTCTACTACTTCATTGAGTCGGAGAGAAACGCCAGCGAGGA
CCCACTCCTCCTCTGGCTTAGTGGAGGCCCCGGTTGTTCAAGCTTCTCAGCTCTCTTCTTCGAAATTGGTCCTGTTAAATTTCAAATAGATGCTTATGATGGGAGTGTGCCAACTCTTCTTT
ATAATCCTTACTCTTGGACCAAGGTATCTAGCATTATCTTTCTGGATTACCCGGCTGGGACTGGATTCTCAGTCCCAACTGTATCTCAAACCTATATGACAGGGGATTCTTCTTCTTCTAATC
AAGTTTATGGGTTTCTAAAAAAGTGGTTAGTAGAGCACCCTCGTTTTCGCTCAAACGGCTTTTATGTTGCTGGAGATTCTTATGGCGGCATGTTAGTCCCCGTCGTTGCTCAATTTATTGCA
GATGGAAATGATGTGAAAGAACAACCCTTCATTAATCTTCAGGGTTATATTATTGGAAATCCAAGAACGGGTGAAAGGATTGATACTAAAGCCCGAGTTCCATTTGCCCTTGGAATGGGCA
TCATCTCTGATGAATTTGCAGAGCTCGTTGAAAAGGATTGTGCTGGACAAGATTATGAAAATCCAAAAACTGCAGAGTGCGCTGCTCGCCTCGATACCTTTAAAAAATACCGTTGGGAGC
TGTCGGAGACAGACGTATTGGAGCCCAAATGTGCGACGGACTTCCCAAGGCCTAAAGACATCGCCAGAGAGTACTATAAGAGATCACTGAATGAGGACAGCTTTGGCCGGCTCTTCCGT
CCACCGAAGGCTCCCGAACTCCGCTGCAAAGGCTATGCTTATTACCTTGGTTATTTCTGGGCAAACGAAAATGTCACCAGAGAGGCCCTTCAAATCAGGAAGTGTGCATGCATGATCGAG
CAGGGAACAGTGAGTGAATGGCAGAGGTGCAACAACTTCGATTACCTGAAAGACATCCCAAGCAGTTTACCTTATCAGCTGAATCTATTAAAAAGAGGTTACAGAGCTCTTGTTTACAGC
GGTGACCATGACATGTTGATCCCATTTGTGGGCACCAAGCTATGGATTAAATCCTTAAATTTAACGGTTGTGGATCCATGGAGATCATGGAGTGTTGATGACCAAGTTGCAGGGTATACAG
TTCTGTATTCAAACGATTTAACTTTTGCGACAGTGAGGGCGGCCGGACACACTGCGCCAGAGTTCAAGCCTCAGCAATGTTTCGCCATGTTCGATAGGTGGACTTCAAAGAAACCTCTCT
GAAATATATTTCAAGGGAGGTGTACTCCAAAATTTTACATTATAATATCTTTATGATTATATGTAATATTATCCGAGTTCAACATTTTCTATTTCTACTATTTTCTACTTTGTAGTAATTCATTGCA
AAAATGATATAAACCTATAGTCAGTGGCAGACTCAA
>comp152510_c0_seq1
CCCGCACTTTATGGTGACCGGTAGTCTGTTGCTTCGCCCAGCTTGAAGCCCAGGGACAATCTGACGGATCTTAACCTACGGTAGCTACGAGCTTCCTCCACGAATGGAACGCCAACCGCA
ATGGACTATCCGCCGCGCGAAAAATTCCTTCGAAATGCTTTGCAGTTACGAGCTATCAATTCGTTGGAACAGCGCATTATGTATAAAATCGGCATTCTGTGTTCTCTCTGGCGGTGGCCGTC
GTTTTCTCTCCGATCCGAATTGAAAAAAAAGACGAGGAGAATTCAAACCCGATTCCAAAACCTAGGACCTGTTGAGATCGCCGGCGAGGAAGAGATGGGGCCCAGGAAAGGAGGAGGG
GTGGCGGCGCAGCCGGAATTTAGCCTGGCTAGGCTTTATTTCTGTTGCATGGTCGCCGGAGGGGTACAGTATGGCTGGGCTCTCCAGCTTTCTCTCCTCACTCCCTATGTCCAGACGCTTG

GGATACCACATTACTTGGCTTCCGTTACGTGGCTTTGTGGACCCATTTCAGGGCTGCTTGTTGCACCTACTGTTGGTTTATTGAGTGATAGATGCAGATCAAGATTTGGCAGAAGGAGGCC
ATTTATTCTTGCTGGATGTATTACTATCTGTGTTGCTGTGATTTTGATTGGGTTTTCATCAGACATAGGATCTGCATTGGGTGATACAATGGAGAATTGCAGCACATATATTGGTCCTCGATGG
AAAGCTGCTATTATCTATGTGGTGGGTTTTTGGGTTCTCGATTTTGCTAACAATGTCGTACAGAGTCCAACACGTGCAATGATGGCTGATCTCTCTGGAAAACATGGATGTAATGCAGGAA
ATATAATCTATGCCTTTTGGATGGCCTTTGGAAATGTCCTAGGCTATTTATCTGGGGCTGTTGGAGGCTGGTATAAATGGTTTCCTTTCCTAATGACAAATTCTTGTTGTGAAGCCTGTGCTA
ATTTAAAGGGTGCCTTCTTGGTTGCAGTTGTTTTCCTGTCTCTTTGCTTAACGGTGAGTCTAACATGTGCAAAAGAAGTTTCTCTCTCTGAATTGGACCCGGCATCTGTGTGGCAGGAAAA
TTATGGATTACTTTCTTTGTTAAAAGCCTTTAAAAGCTTGCCAGTTGGAATGTTGTCTGTGCTTCTTGTGACATCTATTACTTGGATGGCCTGGTTTCCCTTCCTTCTTTATGACACAGACTG
GATGGGACGAGAAATCTACCGTGGTGATCCCAATGGAACAAAGGCTGAAATTGATGCTTACAACAACGGTGTCCAACATGGTGGATTTGGCTTGCTCCTGAATTCGATTGTTCTTGGGGT
CAGCTCAATTTTTCTTGAGCCTATTTGCCGGAAATTCACTTCAAGGATTGTTTGGGTCCTTGGCAACTTCACCGTTTTCATTGCCTTGGTCGCAATTACAATTCTAAGTCTGATTTCTAAGAA
TCTACATGCTGAAAGCATGAGTACTGTATTTGGAGAACTTGGAGTAAAAATTGGAGCTTTGGTTATCTTCGCAGCTCTTGGGTTTCCTCTAGCTATTCTGTTTAGCATTCCCTTCGCCAGTG
CGGCGCAGCTTGCTGTGAACGAGGGAGGCACCAAACAGGGACTGTGCATTGGTATTTTGAACATCTCCATTGTCATTCCCCAGGTGATCGTTGCTCTTGGTGCTGGTCCATGGGATGCTAT
CTTTGGCACTGGTAACATTCCAGCGTTTGTTCTGGCTTCAGCTTTTGCTCTAACAGGAGGCTTTATTGCCCTTGCTGTTCTACCGAAGCCCCCCACATCATCCTTGAACTACGGATATGGGC
CTGTGCAAGATCGTTAAAGAAAAAAACATATAGGAAAAATAACAGCTGTAATCAAAGAGCATAAATTCCTATATGATTTGATGAGAATATTACATAATATATATAGCCAAAAACAAGAATGA
ACTAGCAGACCATCGAACATCCTGGAGGTGAATCTAATATGAATAAAGTTGGAGAGCAAGAACATAACATTCAAACTAACATATTATAGCTTTTATTATGAAATCAATCTCTTTTAATACAGT
AATTCTTTAATTCCCTCTCTCTTGTTTGAGAGTAAGGCTTTGTTTCG
>comp152510_c0_seq3
CGAATGCTCTTAACGGGGGAACATTTGGGTTTCAGAATTCTTTCCCTTTTAAACCAAGCCCTGTCACCCACCACCCTCTTCTTCCAGACGCCGATCGTGAGAAAATTTTTAAAAGAATTCG
TAGAAGATATATCAAGCAGAGAAATCTGGAGATCGTCGACTAGGAAAAATGGATTCAGGAGAGGAAAACGAGAAGTCGGTGAAGCCCCAAATAAGCCTGGTGAGACTAATCTTCGCCTG
TACGGTCGCCGGAGGAGTGCAGTATGGCTGGGCTCTCCAGCTCTCTCTCCTTACACCCTACGTACAGACGCTTGGGCTTCCACATTCATTGGCTTCCATCATGTGGCTCTGTGGCCCGGTA
GCAGGATTTGTTGTTGGACCTTCGATTGGTTATTGGAGTGATAAATGCCAATCAAGATTTGGCAGGAGGAGGCCTTTTATTCTAGCTGGATGCATACTTATATGCTGTTGCGTGCTTGTGGT
TGGATTCTCATCTGACATAGGATCTGTACTTGGTGATACAAAGGAAAACTGCAGGACTTATGTTGGCCCTCGATGGAAAGCAGCTATTATCTATGTGGTTGGCTTTTGGGTGCTGGATTTCT
CAAACAACGCAGTCCAGGGTCCTGCTCGGGCAATGATGGCTGATCTCTCTGGACAACATGGATGCAATGCAGCGAATATAATATATGCCTTTTGGATGGCTTTTGGAAATATCTTAGGCTAC
TCTTCCGGGTCTACTGGAGGCTGGCACAAGTGGTTTCCTTTTCTATTGACAAACTCTTGTTGTGAAACCTGCGCAAACTTAAAGGGCGCCTTTTTGGTTGCAATAGTGTTCCTTCTCATTT
GTTTGACAGTGACACTGCTCACTGCAAAAGAAGTTCCACGATATGAGTTGGACCCAGCTGAAGCAGCACAGGGAAGTTCTGGAATATTTTCTGTTTTCAAAACTCTGAAGAGTTTACCA
ACTGGAATGCCCTCTGTGCTTCTCGTGACATCTCTAACCTGGCTTGCCTGGTTTCCATTCATCCTTTACGACACAGATTGGATGGGTCGTGAAATCTATCATGGTGATCCCAGCGGAACAA
GGGAAGAAATTGATACTTATAACAAAGGTGTGGAAGATGGGGCATTTGGCTTGTTGCTAAATTCGATTGTTCTTGCAATAACCTCATTGCTGCTTGCTCCAATGTGCCGGAAATTGACCTC
AAAAGTTGTGTGGATCATTGGCAACTTTATTTTGTTCCTTGCCATGGCTGCCATAACAGTTCTTAGTGTTATTTCAACTAATGGGTACCGTGGCAAAATCCATTCTGCAGTTGCAGGACACG
ATGAAGTGAAAATCGGAGCTTTAATTATATTTGCTGCCCTTGGATTTCCTTTAGCAATTCTTTATAGCATTCCTTTTGCTAGTGCCTCACAGCTTGCTGTGAATGAGGGAGGCAGCAAACAA
GGACTGTGCATTGGTGTTCTGAACGTATCCATTGTCATTCCCCAGGTTATAGTTGCTCTAGGTGCTGGCCCATGGGATGCCATCTTTGGCAAAGGCAACATCCCAGCCTTTGCTCTTGCTTC
AGGCCTAGCTCTACTTGGTGGTTTCATTGGCTTGGCCATTCTGCCGAAGCTCTCAGGGGATTCTTTAAAGAGCAGCAGCTTTGGTTCCATTCACTAATGCTCTCTCTACATCCGGGAATCAC
AGAAGTATATAATAGTTGCTTCCAAACAAAAGAGTTAGCATTTTATACTCTATATAGTAAACCAAAGAATAGAGTTTTTAGCTTAATTTTATGTCATTTGACTTACTGGGCATTGCAATTTTGT
ACAGTGACTACTGACTTTGGAATTTGAGAAATTTTTTTTGACAGGCGAATAAACATTGCTCTAATTTTTGGACAGCTATAGAGTAGAAATGTTACATCAAATTAAACAATTGTATTTGTAAC

ATGACTTTGTATTTACCCAATTTGGTATCAATTTAATGAATTTATTAATGG
>comp152510_c0_seq4
CGAATGCTCTTAACGGGGGAACATTTGGGTTTCAGAATTCTTTCCCTTTTAAACCAAGCCCTGTCACCCACCACCCTCTTCTTCCAGACGCCGATCGTGAGAAAATTTTTAAAAGAATTCG
TAGAAGATATATCAAGCAGAGAAATCTGGAGATCGTCGACTAGGAAAAATGGATTCAGGAGAGGAAAACGAGAAGTCGGTGAAGCCCCAAATAAGCCTGGTGAGACTAATCTTCGCCTG
TACGGTCGCCGGAGGAGTGCAGTATGGCTGGGCTCTCCAGCTCTCTCTCCTTACACCCTACGTACAGGTTGGACCTTCGATTGGTTATTGGAGTGATAAATGCCAATCAAGATTTGGCAGG
AGGAGGCCTTTTATTCTAGCTGGATGCATACTTATATGCTGTTGCGTGCTTGTGGTTGGATTCTCATCTGACATAGGATCTGTACTTGGTGATACAAAGGAAAACTGCAGGACTTATGTTGG
CCCTCGATGGAAAGCAGCTATTATCTATGTGGTTGGCTTTTGGGTGCTGGATTTCTCAAACAACGCAGTCCAGGGTCCTGCTCGGGCAATGATGGCTGATCTCTCTGGACAACATGGATGC
AATGCAGCGAATATAATATATGCCTTTTGGATGGCTTTTGGAAATATCTTAGGCTACTCTTCCGGGTCTACTGGAGGCTGGCACAAGTGGTTTCCTTTTCTATTGACAAACTCTTGTTGTGAA
ACCTGCGCAAACTTAAAGGGCGCCTTTTTGGTTGCAATAGTGTTCCTTCTCATTTGTTTGACAGTGACACTGCTCACTGCAAAAGAAGTTCCACGATATGAGTTGGACCCAGCTGAAGCA
GCACAGGGAAGTTCTGGAATATTTTCTGTTTTCAAAACTCTGAAGAGTTTACCAACTGGAATGCCCTCTGTGCTTCTCGTGACATCTCTAACCTGGCTTGCCTGGTTTCCATTCATCCTTTA
CGACACAGATTGGATGGGTCGTGAAATCTATCATGGTGATCCCAGCGGAACAAGGGAAGAAATTGATACTTATAACAAAGGTGTGGAAGATGGGGCATTTGGCTTGTTGCTAAATTCGAT
TGTTCTTGCAATAACCTCATTGCTGCTTGCTCCAATGTGCCGGAAATTGACCTCAAAAGTTGTGTGGATCATTGGCAACTTTATTTTGTTCCTTGCCATGGCTGCCATAACAGTTCTTAGTG
TTATTTCAACTAATGGGTACCGTGGCAAAATCCATTCTGCAGTTGCAGGACACGATGAAGTGAAAATCGGAGCTTTAATTATATTTGCTGCCCTTGGATTTCCTTTAGCAATTCTTTATAGCA
TTCCTTTTGCTAGTGCCTCACAGCTTGCTGTGAATGAGGGAGGCAGCAAACAAGGTTAGTTTGATTTGATCCGTTTTTCCCTTCTGTACTGG
>comp152510_c0_seq5
CCCGCACTTTATGGTGACCGGTAGTCTGTTGCTTCGCCCAGCTTGAAGCCCAGGGACAATCTGACGGATCTTAACCTACGGTAGCTACGAGCTTCCTCCACGAATGGAACGCCAACCGCA
ATGGACTATCCGCCGCGCGAAAAATTCCTTCGAAATGCTTTGCAGTTACGAGCTATCAATTCGTTGGAACAGCGCATTATGTATAAAATCGGCATTCTGTGTTCTCTCTGGCGGTGGCCGTC
GTTTTCTCTCCGATCCGAATTGAAAAAAAGACGAGGAGAATTCAAACCCGATTCCAAAACCTAGGACCTGTTGAGATCGCCGGCGAGGAAGAGATGGGGCCCAGGAAAGGAGGAGGG
GTGGCGGCGCAGCCGGAATTTAGCCTGGCTAGGCTTTATTTCTGTTGCATGGTCGCCGGAGGGGTACAGTATGGCTGGGCTCTCCAGCTTTCTCTCCTCACTCCCTATGTCCAGGTTTGTT
TCTTTTCTATTCCCCTTAACAATACTAACCTTTTCTATCCAATCAGCCCCTTTAATTCCCATTGTAGTAGTTGTTATTGATCCAAATAGCACATAGGTGAGGATATCTTGATCAAAATTTGGTG
AAAATAAGTCTT
>comp152510_c0_seq8
CGAATGCTCTTAACGGGGGAACATTTGGGTTTCAGAATTCTTTCCCTTTTAAACCAAGCCCTGTCACCCACCACCCTCTTCTTCCAGACGCCGATCGTGAGAAAATTTTTAAAAGAATTCG
TAGAAGATATATCAAGCAGAGAAATCTGGAGATCGTCGACTAGGAAAAATGGATTCAGGAGAGGAAAACGAGAAGTCGGTGAAGCCCCAAATAAGCCTGGTGAGACTAATCTTCGCCTG
TACGGTCGCCGGAGGAGTGCAGTATGGCTGGGCTCTCCAGCTCTCTCTCCTTACACCCTACGTACAGGTTGGACCTTCGATTGGTTATTGGAGTGATAAATGCCAATCAAGATTTGGCAGG
AGGAGGCCTTTTATTCTAGCTGGATGCATACTTATATGCTGTTGCGTGCTTGTGGTTGGATTCTCATCTGACATAGGATCTGTACTTGGTGATACAAAGGAAAACTGCAGGACTTATGTTGG
CCCTCGATGGAAAGCAGCTATTATCTATGTGGTTGGCTTTTGGGTGCTGGATTTCTCAAACAACGCAGTCCAGGGTCCTGCTCGGGCAATGATGGCTGATCTCTCTGGAAAACATGGATGT
AATGCAGGAAATATAATCTATGCCTTTTGGATGGCCTTTGGAAATGTCCTAGGCTATTTATCTGGGGCTGTTGGAGGCTGGTATAAATGGTTTCCTTTCCTAATGACAAATTCTTGTTGTGAA
GCCTGTGCTAATTTAAAGGGTGCCTTCTTGGTTGCAGTTGTTTTCCTGTCTCTTTGCTTAACGGTGAGTCTAACATGTGCAAAAGAAGTTTCTCTCTCTGAATTGGACCCGGCATCTGTGT
GGCAGGAAAATTATGGATTACTTTCTTTGTTAAAAGCCTTTAAAAGCTTGCCAGTTGGAATGTTGTCTGTGCTTCTTGTGACATCTATTACTTGGATGGCCTGGTTTCCCTTCCTTCTTTATG
ACACAGACTGGATGGGACGAGAAATCTACCGTGGTGATCCCAATGGAACAAAGGCTGAAATTGATGCTTACAACAACGGTGTCCAACATGGTGGATTTGGCTTGCTCCTGAATTCGATTG

TTCTTGGGGTCAGCTCAATTTTTCTTGAGCCTATTTGCCGGAAATTCACTTCAAGGATTGTTTGGGTCCTTGGCAACTTCACCGTTTTCATTGCCTTGGTCGCAATTACAATTCTAAGTCTG
ATTTCTAAGAATCTACATGCTGAAAGCATGAGTACTGTATTTGGAGAACTTGGAGTAAAAATTGGAGCTTTGGTTATCTTCGCAGCTCTTGGGTTTCCTCTAGCTATTCTGTTTAGCATTCCC
TTCGCCAGTGCGGCGCAGCTTGCTGTGAACGAGGGAGGCACCAAACAGGGACTGTGCATTGGTATTTTGAACATCTCCATTGTCATTCCCCAGGTGATCGTTGCTCTTGGTGCTGGTCCA
TGGGATGCTATCTTTGGCACTGGTAACATTCCAGCGTTTGTTCTGGCTTCAGCTTTTGCTCTAACAGGAGGCTTTATTGCCCTTGCTGTTCTACCGAAGCCCCCACATCATCCTTGAACTA
CGGATATGGGCCTGTGCAAGATCGTTAAAGAAAAAAACATATAGGAAAAATAACAGCTGTAATCAAAGAGCATAAATTCCTATATGATTTGATGAGAATATTACATAATATATATAGCCAAA
AACAAGAATGAACTAGCAGACCATCGAACATCCTGGAGGTGAATCTAATATGAATAAAGTTGGAGAGCAAGAACATAACATTCAAACTAACATATTATAGCTTTTATTATGAAATCAATCTC
TTTTAATACAGTAATTCTTTAATTCCCTCTCTCTTGTTTGAGAGTAAGGCTTTGTTTCG
>comp152517_c0_seq2
AGCAATAAGAAAGTCATCCCAAATGAAGCCTTAGTTATCTATAAATTGCTTGATTTACTTGTAGTTTTTTTTTAATGAAGAGAATACCAAATTGTACGTATGGACAATCAATCAGATTTTATC
AATGATTGTATATAAAATTTTGATTGACTGTCAGCAAGTATAGATTCACATATTCCAACCCAAATTTAATGATTTCTAAAAAAAAAATAAGAACTATAATCAGTCAAAGAAATTATCATAAATTA
AAAAGGAAAGTTTGGTCACCCTTTCTTCTTTCCATATGCTCAATTTCTTTCTGCAATTGACATTATAGTCATTATCTTCATTTGAACTTTTCATGAATTAATAGTTATTCTAAAAGCGTCACCA
GCAAATTTAAAACACCAGATATATATTTATCAAAGTCATACTAAAGTACACATGTATTTTCCCTTTCGCTGATTATTGTTTTAGTGCACTAATTATTTGAGATTATAGGGGTGTATTATATTTTAA
TTATTGTATACCTTATATAACTTTTGAATGTTTAATATATGCAAAGTTTTAAATATGTAAAGATGACAGTTTGGCTATATATTCAATTGACTCATATATAAATTTCTGAACATAAAAAGAGAAAA
TAAGAATCATATATTTTTGTGGATTTTGAGCATTTTTTTTTTAAATGATGAAAGATAAATAATATGTGGCTATAATTTATGAACTTAGACAAATTAAGAATATTCACCTTGATCAATTGCTTCTT
CATGAATCCTTTCCATTTAACACTTAGTATAAAAATTTTAATTTATGTACTAAAATTTTGTTTTAATTTTTTAGTAAAAGATTTTTTCAACTAAAAATTTTTATAATGAAAAATTACTTACGAAA
GGAATAAAAAAGTTTTCCCAACAATTTAAGCTTTAATCTCTCTAAGGCTTTG
>comp152527_c0_seq2
TCCAGAGATCCTGTTCTCCCGGAACGTCTCCTGCGGCAGTGGCCCGGATCTCGTCTTAATGCGATTCAAGCCGTGGGAAGAGGACAGATTCGGAGACAGAAAAGACCCCGAAGTCACCC
CTCCCTCCTTGATCGCGCCACCCTTCCAAATTTTACCCGGGGCCGAAGCAGCTGTTTGGGGCGAGTGCTTCGCGGCCTCTTTCCCTTTCTGATCCTTCTCTTTCTCCTTCACCTTCTCTTTC
TCTTTTTCCTTCTCCTTCTCCTTCTCCTTCACTCCCGGCGGCCGCATTTGCCTTCGGAAACCGCTCGCCATCGCCACCAGCGTCTCCCTCAGTGTGCGTCCAGAGTCCTTGCCCTTCACCA
CGAAATCCACCCCTACCTTCTTTTTGGCCACGTCGGTGAAAGAACTGAGGAATTTGGCATTATACGAGCAGTCGTAATTCCCGTCGGTACAGACGGCATAGATTTCTGTGAGTGATAGGAC
GATGTAGGCATCGTCTAACGATTCGCCGAAAAGCCATTCGAAGAAGTTGAGGGAGTCCTGGTCTGGATCGCACTGATCGCGGGAGGATGAAGCCGCGCACCAATAGCGGGAGGATGGA
GGCGCGCGGAAGACGACGAGCTTTGCATCCATCCATTCCGGGCTCTGGTGCTTGCTCCAGTTCGCATAGAAGGATTGCAATCGCTTGGGAAACTTCTTTAATTGTGATTTCTCTTCTGATG
ATCTCATGATGGAATCTCGATCAAAAACAAGAAGAAGAATGCTGTTGCAGACGAACAGAGGTAGATGGGTGTAACAGAGGGAAAAGGCGGTGGTTTCCTATGGATACCCCACAGATCCC
TCCCTTTCTATTTCTCGGCACTTAGG
>comp152533_c0_seq1
ATGACCTAAACAAGCCATTGTGCGTACTGAATGAAGTGTACAACACATTTCATGTAGAATTCCAACCTGTTTATTTAGCTTCGAAGAAACAAATCAAATAGAAAAAATTGCATATTAATTTC
TTCAAAAATATAAGACATTTTTATAATATCTGCAAGAAAAATATGTTGCAGCGCAGAAGAGAATACTCAAAACTCATAAATTTTTGCATGATAAAATTATAATGTCAAATATTTTTGAATCAA
ACGCCGGCCTTTTAATTGTTATCCACTTCAAAAAATATCTTCACAACCTCAATAAATTTATTGCAACCTTGCACTCTTGAGAAATCACCTTTGTCGTCTTCCCTGAACTCTTCATGATCTTCA
CATCACACTCCACCTCCATCTTCATCCGCCACAGTGTCACACCCCAAATCTCCACCCTCACCGGCGTTGCCACCGTCAATCTTAGCAGCACCGCCTGCTTCGATCCCTGTAGACTCTTCTC
CGCTTCCTCCATGACAGCCTTCTTCACCCCATGAAGCGCCATGCTGAAACTTCTCGACTCCCTGGCCTTCAAGTTTAATTGGGGCGACTCTCCATCCGCCAAATTTCTCTCTTTATAACTAA

GCTTCGCCGCTCGCGCAATGCTCTTCTCGATCAAGTATCCCATACCAATACTTGGATTTGTAACATCCATTAAGATCTCGCGCTGAGTTTTCGAGTCAATTACAACGCGATCGATGTTGAAG
ATGGGAGCAGATGGGCGGACGACAATGTAGAAGATGAGGGTGGCGATGAGGAGAGGAAGCGCTATGGAGATCAAGATGCAGAGGAAGCGGAGCAAGAAGTTCAGGCAAGGAGAGCG
CTTTTTTCGCAGTGCGGCGAGATGGGCGCGATATTCCTCTGCGAGACGAGCATTCTCTTTGGGCGGGATGCGGTAGATCTGATCTTTGGGGACCTGCACAATGTAGGTGACGAGGGCGGA
GGAGAAGGCCTTGTCATCGGAGCTAGGAGGGCGAGGAAGCGGGGGACGAGAAGCAGGAGATGGCGTCCCCTCCTCCATCGAGAGAGATAGAGAAG
>comp152550_c0_seq5
CAAAATCTTTATTCCCTTGACTGCTTATTACAAGCCTAAACATATCTATTTAAAGACTCCTAAAGGCTATACAAATAGCCCCACATATGGCTACATATGCCATACATAAAACCTATACAAGAA
AGAATCATAAATCTAGGAACAAAACACAACAATCTCCAACTTGGGCCTAGATTTACAAAAATAAAAGATAACAACTGTTCTTAAAGAACAGCCTGTCGGTCTCCTCCGCCAAAAGCGTCC
ACCGACATTCTAGCTCCACCTCCCGACAAATCTCCTGTCGAACTACGTCCAACGCAGCCAATCCCGAGCACCTCTAGCCCGGCTTCCACGCCCTGTGCATCCACCAGCACCC
>comp152563_c0_seq1
AAAGAGAGAGAGAGAGAGAGGGAGAGTGAGTGCGTGCGTGTGTGAGAGAGAGAGAGGCATTTTGTTCTTAAAGAATTCAAAAAAATTGATGATTTTATTCTCATATCTATAACAGGAAAAG
AAAATAGGCAATTTCTAAGGCACCATGTCAGAAGAAGAGCAACAACGAACCCCTAAGAAGAAACAATCAATATATCATCTCTGAAAACTCTTCCTCCTCAGTATGTCACTGAAACTCCTC
AAAAATTGCTTCTTCTTCTTCTCCTTACTCCATTCAAACTTCCCATCCTCCACCTTCAACCTCCTGTTCTCAAAAAGCACGCATTTTCCATCCTTCCGACTCCTCACCAATGGCGACCCACC
ACCTCCCTCAATCTTCTCAATTCCTTCCATCTCTCCCCATGAATTCTGCCTCCTCACCACCACTGGCTTCTCCACCATCCTCCACTGCTCCATCGAAGAAGTCGCAGCAACCAACGCCTTCA
GCACCTTCACACTATCCGTCGCCGACGCCTCACTCACCTTCAAGCTTTCTAATTCATGCTTTAACCCATTCATCGCGTCATCTTTCTCCCTAACCAAATCCTTGATGCTACAATTCTCTGCTC
TCGCAATTTCCAACGCTTCCTTCACAGCAGTAGCCTCGTTCACCGCTTGCTTTAAAATATCCCTTAGCTTCCATATCTCTTCCCTCGAATCCCGATTCAATTCCATTAATCTCCTGTTCTCTTC
TCTGATTTCCATTATTTCGTTTTCTGATGCCTTTATACAGTTTATAAAGACTGCTTCTTCTTCATCTGCTTCCTTTTTCAGCTTCTGCGATTCTTCACCGGCCTCTCGGAGTTTCTCCTCCGCA
AATCTTAAAGCGGTTTCTGTGCTTCTTAGCATAGCCTTGGTTTGTTTTGAGTCGTTAGAGACTTCTATCAAAGCTAGAGCTAGAACGTCCATTGCTTTCCTGCTCTCCTCCTCGCCCTGCAA
GGCTAACTTGAGCTCGTTCTTGAGATTATGGAGCTCATCGGACAGTTCTTTGTCTAAAGAAAGGATTTTTAGGGATTCACGGAGGGAATCTATTTCGAGCTTGGATTCTTCCAAAGATATCT
TTGTTTGTTCCAGCCGTTTCGTTTGCGAGACGAGGGATTCCAGCATTTTTCTTTCTGATTCCTTTGCTTTATTTGCCTCGTTCTCATGGAATTCGATAAAGCCTCTGGTTTTCGTTCTTATTTT
CTCCAGATCTTTTAGCGCTTGGGAGCGTTCTTCTCGTGCTCTGGTTAGCTCCTGCTGGAGATTAATCAATTGCTGTTGAATCTCTAATGCCCCATCCATTAGATTTCTTTTCTGAGTATTATAT
GAATGAAGCAAAGAGAGATTCAAGGAGTCATGGAACTAAACCCACAAGCTT
>comp152567_c0_seq3
TAAATTTATTGCTTACCCGAAAGCAAAATGTTCTGATTTATAATTGCTATTATCCCACCATAACGTGACCTCAAAATCTAAATGCAACAACTGTTGCATAAACAAGCTGTCTTTGTACATGAA
CAAAGAAAAAGTGAAGCATTAGCAGCTTAAAAGACTATGAAACCAGTGCTATGAGGCATCATCCAATCAGAAATAAATTGCATTACAGCAGAGTTAGTGAGGTTCAGACAGAATCAGTA
CACAAATATGTTTGATCTGCAACATGAAAGGAGATCAGTTGAAAGAACACCAGATGCTTGCATTTGCAGCTTGAATTTGGAATTTTGTTGAAAATTCTTCAAAATGGAAGATGCTGTTGAT
CTGTTCTTGTGTGTCTGTAAGTGGTTTATCAATAAGCATGTAATATTGATTCTACGAGATCATGGAGTGGTGCGAGCTCTCGTTTGTCAATCAACTTAAATTCGTTGGCCCTGCTGACATTGT
TGAGCAGGTAGTAGGTGTGCAAAATTCCGTGAGAGAGCCATACAAAAAGTTTGCCTGATTGAAGAAGTCGATCGTGTTGACAGCAAGCCACTCATTAAGATTTTCTCCAGGGGGTAATC
GAACAGCCTCTTTTATGTTTCCAGTACCCAAGGTTGCATCAATGTACTTTCTGAGCTGAACACCCTTATTTCCAGAAGGAGCATTTTTCTTGGGCCGGAAGGTTTTCAGATTTCTGTTTCCA
AAGCCGAAGAGACTCATCTCTGCAAGCGATGATAAATTAAGACGAGGAGGAGGAGGAGAAGCAGCTGATTTCTTTCTTTTCCTGCCCAGCCGCCCTTCTCCTTCTCTTATTCCGATCAAC
ATTGGAGACGCTCAGGGAATTTACTCTTGTTATTTAGATGGAAAGGAAGAAGAGGACAAGAGGTGGAGAGAAGAGAGAGAGAGAGATGGACGGG
>comp152586_c0_seq6

TTAAATTAAATTGCCATCATTTAGAAAAGAAAAGTTGGAATACATCAGAAGTAAAACCATCCGAAAAATTATGCACACAGATTCACAATTTTAGAAATTAGTAGTGAAAAAGATTTATTTTA
AATAGATGCCAACCTGGTGAGTTAGATAGTGATTAGGATGATTTACAGAGAAGAAATGTTCAACATGCTGGCTAACTGATTCATCGGGATCAGGAGGAACATTCCTTACAAGGACAGTGA
ATTGATCTGGACGGCGACTTTGAGATGCAAGAAAACGCAATCTCATCGATGTTATAAGCTTGTATTCTTTGTATAATGTATAGCATGTCCAAAAGGTAAAGACATAAGCCATGGTTAAATGT
GCCCAAAACCTCTGTGAGCCTGGGGGAATATTCGAGATAGAAAGCTTGTCTAGATCACTAAAAGTTAGATCTTTGCGATGATCCAAATATCGGCCGGTCACATTAACTGGCACCAAAACG
GCAAAGGCCAGCACGGAAAGAGGAACAAATATCTTCAATCCAAGTAGATATATTCTGATATACATAGCCGAATCAAATCCTGCATGATCAATAAGCTCCGGCTCTGGCATTTGTAATGCTGC
TGGCATCCAACTCAAAAATTTTATATATGTCCTGCAGTCCAAATTAACAAATTTTTTTACAGAACCTCCTGAATGCGTTGGGCTGCTTCTTATCCCTTTAAGGTACCACTTGGGAAAGTATAC
TCTATCATTGAATGGCTGAAGCCTTAATATAGCAAACGCCACAAAGAAGGCAAACGCTGATAGAATATTGATAGCCGCAGCCATACAGATATCTCCAATTGTTGCCATATGTTGCTCGGAAA
ACTAGTTCTCCTCGCGGAAAGGCTCGCTCTACCTTGTGAAAACAAAAATGGAGGAGAATCTTTACGGCTTCTTCCGCAGGAAGACAGAAGTGACGACCAACTAGCCAGGTCTTCTACAC
GCCAACCATTTTTGCCCTCTTAAATCGGCCATTTTCTCG
>comp152619_c0_seq2
TATTACCTTGAGAATTTCAGTATAGAGAGGATTCGGACCTCGGGCTGAGAAGGAAGGGATCACAATATTTTTCGTAGTGAAGGCATGTGACTTCTCAATCTTCGCCACAGGTTTCCCTTTA
CTGCCGAGGCTATCAATATACTCTCTAATGATAGCGCCACCTTCCTTGGATACCATCGTCGCCGGAAGCTCGAATGTTTCCGCCCGGTCCACCGGCATTATCATCTCAATAACCAACCCGAC
TCCGCCGGCGGCGTTGACCACCTTGTCGGCCGTTTCTGCCGACCCTGTGATCTTACACACCACGATCTTCCCCTTCACCTTCTCAGGATCCAACGAGTCCGGCCCACACATCTGAGCCGA
GAATGTCATTCCGATGGTGGTCGCATCAATCCCGAGCACCAACGGCCGGAAAACCTTATCGGTCTGGAAAGTATTAACCGATGTCCCCGTAATCCTCTTCCCATTGCCGAGCACAATATCC
GTCCCAAACTGCCGGTCATGCGTGCTGGCCCCGACGGTCAACATCCACGGCGAGTAGTTAATGACGGTGCGATGCATCGGTCCCATATTGCCCGCCGAAGCGACAGTCAGGATTCCGCG
ACGCATGGCATGGTAAGCCCCGACGGCGAGGGCGTCCGTGAAAGACTTCCCGGGATCTTCGCCGACGGATAGGTTGATGATGTCGACGCCGTCGGAGATGGCGGCATCGAAGGCAGCG
AGGATATCCTCGTCGTTGCAGGCGCTGTCAGCGTAACAGGCTTTGTAGGAGGCGATGCGGGCCGACGGGGCGCCGCCGCGGGCTGTGCCTTGGGCTAGCCCGTATATGTTGGCGTTGGAT
ATGGGATTGCCGGCGGCTGTGGAGGCCACGTGGGTTCCGTGGCCTTTGTTGTCTTGAGGAGCCATCACGTCGTTGGAAGGGGCTTTGCGGTCTAGCCGAAACCATTTCGCGCCGATTAGC
TTGTTGTTACATTTGAACACTTTGGTGCCGGTGCATTTGCCCCGCCATCTCGCCGGCGGTGGACCGTAGCCCTTCCCGCTGAAGCTCGCGGAAAAAGGATCGATCCCGGAATCTATGACG
CCAACGATGACGTTGCTCTCAAGTGTATGATTATGCGGGGCGGTTTGAGGGAAGCCTATGAAGTCCCAAGTACGGGTGGTGTGTAGTTTCCGGGTACGGGCGGGGAAAACCGAGACGAC
GCCGTCGAGCTCGGCCAGCTGAGCTGCCTCCTCGTCGGAGAGTTTGGCGGCAAAGGCGTTCATGCTTAGAGTGTAACTGTGGAAGATGGAATCCTTGGCGGCATCATGGCTTCCAAGAA
TATCGGAGAGAAGGCGGTGGTGATGGCCAATGGCGTCGAAGCTGCGGTGAGGCTCTTTGCCGAGGTATACTATGAATTTCTTTTTTTCAGGTATGGTGGCCGCGAGAGCGGCGGAGGAG
AAAAGAAGCACTGAAAAGAGAAGATGGAGAAGAAGAGACGACGCCATGTTTGTATGGCCGCGGAGAAGAGAGGAAGTGAGGTTGGATATCTATATGAAAGTTTTTTTAATAGGGAGTT
TTATATAAATAGAGAGAGAGAGAGAGGATCAAGTGGGATTTGAGATTTGTTAGCTTGTTTATGTTGTTTCTGATGCTGTTCGGGGGAGGAGAAGATGGGCCCTACAAGGAAAAATGCTCTGC
ACCTGCCTCCAATGCCACTAATGGGAGCCCAACTTAGGCAACTACCCGCTATGTGTGCTAAAGGAGAGATTATTTTGTGCGGTGTTTGAACTTGTTTGGAGACTAATTTGGATGGGCCACG
CCATCTAATGTACTGTGCATTGGGTGACTTGACGCATCCAGACACCGTGCATATTATTTTTTTCTCATAAGACAGCGATGGTCCCACATGGCTGGTGAGTTTGC
>comp152657_c0_seq1
GGAAGAGAACTGAACAAAAAAACAAAATTACACAGATAGAACCAAAGGAAAATAGTGGGCATTAAGCTAATAAAAGACTCGGAAAAAGCCTAAAACCATAGAAACAACATGATTTTCA
GGATCCAAAAAAATAGTCGCTGCGGTGGATACAGGTTGCCTGAAGGCGATAAACGAAGCTGAAAGCAGGGGGCGGTCAGACTAAAGGCCATCTCAGGACCACCAGGAAGCGACAAGT
TACCCAGGAGATCGAGCCAGGTTGCCATACTAGCCGTCCGTAGCTCGAGAGGGTAGAGGAAGGCCGAGCTCATCATCGTCAAATAGCTATAATGCCACCACTGTTGCCTTTGGAACACCG
TGAAGTAAACCCAGGAAATGAAGACATAGTTCCCAAAGTCGGATTGGATAGTAGAGGGATCGAAAACCTCAAAACCATCCCAAAAAATGTTCTCCCCAGGTCTGAAAGTAAAGCAATCA

GAAAAATGAGCCAAATGCATAACCCCGAAGTCAGCAGAACCAAACATCAAGTCTAGCCAGTCGTGAAGAGTCCAAAAAATTTCTCCAGAATGTTGGGAACCATGTGATCCCGCAATGAA
ACACAGAAAAACAGCCAAAGTTAAGAGCCAGTAAACCTCCTTAATAATTCTTGTTTGTGTTTCCATTTTGAAATCTTGAAATGAATTGCCATGGAAATATTGCATATGATCTTGGTGACATT
GTTTGAAGAATTAGAGAAACGGCGGGAGTTTCTTTCTTTCCAAACATGGTAAATGATACAGCTAATAATAAGGCAACAGTAATCCTTTTCCACTCTACTAAAAAGTGTATTAGATTCAATAA
AATCATAAATCTGGCCTAAGGTCGGCCTAAATAAAAAGCAGTTTAGGTTCGGCAAAAAAGTTTTAAGGATGTAAAAAGAAAAATCGCATCCAAAAAACAGATGAGTGTGGTTTTCCGCAT
GGGAGGAACAAAAAGAGCAGTCAGTATTAACTTGTATGCCGCGTGATAATAAATTGTCCGCCGTTTTAAGTTTACCCAAGATTGCCATCCAAGTGAAGCAAGCAAATTTGAGAACATGTT
TTTTATGCCAAACAGAAACAGACCAGTCAACTCTAGGTAAAGAGGAGTAAAAATGATCAGTAAAGAGTCTGAACACCCAAATAGTCGAACCTTTCCAAAGAATTTCCGATTTTTCCCCCA
GGTTAATCGCAGAGATGCTATCCAAAATATTATGAGGAATAGAAGCAGGCAGGGACCAATTGGCATTGGTAATGAGATCTCCCACACATTTCCGGTCCAGATCCAGAGAAAGAAAGTATT
CAGATAAACTGGTCCCATTGAGCCATGGATCCCACATGAAAGAGAATGTACAAGCTTGAGACACAGAGAATGAAAAGACTGTACTTAAAAAGAGTAAGTGTCATAAATTATAAATTAAAA
AAGAATAGATTATTTATAAATTACATTCACTTTTAAATAAAATCTAAAAATTT
>comp152680_c1_seq1
GTAAAATAAATATAATATAAACTCCAAATATAAGCCAGTTGTAAGCCGATGGGCTGTGGCTGTCGGCAATGGCAGGTGATTGCCAATGAGCATTAGTCATAGGTGCGGCAATGGCTGGTGG
TGATATGCAGGCGGGAGGTGGCGACGACATAGTTAGCTTACTCATCCAAAATCTAAAGAATTCTAAATACACTAAAATTTTACACTATATTTATTTTACTTCAAATCAAATATCAATATTCTCA
AAATCCCTGCATTTGAAATATTTTACATAGCTTTTACTTCAAACCAAATAGCCCCTAAAACATTTTTCCTCTTACTATAATCCCCTCATCCTCCTACTGTAATCCCCTCAATAGTGTCACTTCC
TCCTACCAATCTTTTAGCCAATATTATACAATTAATACTTTAATAATCATACAAATCTGTACATTATAACTACAGAATTACAAACAATATTATATTTATACACACACGAAAGTGGCCCCTTCTTT
TAACTACTTCAAGCTCTATTCCGCCCGACTAAATTAATGGCCTTTTGGGCCTCCAAATCCCAATCAATCCACCATGTTGCGGCAACGATAGAAACAGCACACGTTATTTGGGCCGACAAAA
GCCCAAAAGAAAGCCCAATGAAACCCACATTTAACCCGAAGGCCAACAAAATGGCCACCGGTGCCCCGACCAAGTAAAAAGAGTAGAGGTTAATGGCGGCGCCTAAAGCCGGGCGGG
CGCACCCGCGCAGCACTCCGCACCCCACCGTCTGCGGGCAGTTTCCAAGCTCACATAACCCGATTAGGGGGAGCGCGACGCGTGTCATACGTAGAACGTCGCTATCGGATGTGAAAACC
CGGGTCCACGCATCTCGGCCCAGCGTGGCCCACGCAAAGCTGAAGCAGGAAGCTAGAAGGGACAAAGCCATCGCGATTGCAGCCGCGGCTCGAGCTCGGGCGGGCCGGCCCGCCCCG
AGTTCTTTGCCCACTCGAGCCGATGCCGCCGCTGATAGAGTCGCCGGAAGAGTGTAGAGCAAGGCGGTGGTTTGGATGACGATTCCTGCGGCAGCAAGGGAAGATTGTGGGTCGGTTAA
ATACCCGGCGGCGATGGTCATGATTAGTTCGTACCACCACCATTCTAAGCAGACGGACAGGCAGCTGGGAATTGCGAGATGGAGAAGCGGCAACCATGTGTCGGATGATGGCGGCGGCG
GCGGAGGAGGAGG
>comp152767_c0_seq1
TAGAACCATTTCTTCTCTCTCCTGTCTTCCCCCTTCGTCGTATGTCACCCTTCAGGGATCAGCGGTGAGGTCGTGGGGAGGATAGCGGGAGACAGAAAGTTGCCAGAGTTTTATCTGCCA
GGCGCTTTCGCTATCGTTACTGAAACTTTCGATTTTTTGATTCGGTGATCAAGCACAATAGCCGTTCGCTTCGGGGGAACAATAAGGAATCAATCTGCGTCGTAGGTTTGTTCCCAATCAGA
TCTTTCTTAATCGATTGGGATTTTATTCAATTATGCATTAATTTTAGCAATTTATGGATTTTGAGGCTTTTAATTAAATGCGTTTCCTTATGGGTAGTTCGCTCCAATTTGCAGCTTTCTAGGTTT
TTAAATGTTTATTGCTCTGGATGAGTTTGTTCAACCTAGATTTTGGGAGAGGTTATTTCACCGGGGTTGGAAGGGATTTTGATCAACGCCGTGGTATATGATTATTCGCAATTTGTTATTGGG
CGCACGGAAGTGTTGATTTTGAAATCGAAGAAAGATGAATGTGTCGAAGAGCAAGATGGGCGAGGAGCACGGGGATTTCTACTCTGTGTCGCCGAAGATGGATTGGAGGATTAGGTTGT
TAAGAATAATCTGGGGTTTGGTGATATTCATGGGAGGGGCTTTTCTGGGCTTGTCGGTAACTTCCCGGTATAATCAATACTTGACTACGCAAACTAATGATCTTTTCTTTCCGACGAAGATG
TTCACTACAAATTGTGATATGCAGAGGTTAAGTATAAAGAGTTTTGTTCATCCCATGCATCTTTTTCATGATTTAACTGATGAAGAGCTGTTCTGGAGGGCAACCTTGGTGCCAAAGATTGA
AGAATATCCATACAAGAGAGTGCCCAAGGTGGCTTTCATGTTCCTTACACGTGGACCGCTTCCGTTTGCTCCCCTTTGGGATAGGTTCTTTAGAGGACATGAACGCTTCTACTCTGTTTATG
TGCATTCTATTCCAAACTACAAGCTCAATGTCTCAGAAACTTCAGCATTCTATGAGCGCCAAATCCCTAGTAAGGAGGTTTCATGGGGATCGATTTCACTTGTGGATTCAGAAAAGAGACT

TTTGGCCAATGCTTTATTGGATTTCTCCAATGAACGGTTTGTTCTTCTCTCAGAAAGCTGCATCCCTGTTTATGATTTTCCCACAGTCTACAAGTACCTTATCAGTTCCAAGCATAGTTTTGT
CGAGTCCTACGATGAAGATTCGCCCCGTGGACGTGGTCGTTACAACCGTCACATGTACCCTAATATCAAGCTCAGCAAGTGGAGGAAAGGATCCCAGTGGTTCGAGCTTAACCGGGAGTT
AGCTGTGAAAATAGTTGCAGATTACAATTACTATGCCATATTTAGAAGATACTGCAAGCCCTCTTGCTACCCGGACGAGCATTACATCCCGACGTACTTGAACATGTTCCATGGAGAATTGA
ATGCTAACCGGACTGTAACTTGGGTCGAT
>comp152767_c0_seq4
TAGAACCATTTCTTCTCTCTCCTGTCTTCCCCCTTCGTCGTATGTCACCCTTCAGGGATCAGCGGTGAGGTCGTGGGGAGGATAGCGGGAGACAGAAAGTTGCCAGAGTTTTATCTGCCA
GGCGCTTTCGCTATCGTTACTGAAACTTTCGATTTTTTGATTCGGTGATCAAGCACAATAGCCGTTCGCTTCGGGGGAACAATAAGGAATCAATCTGCGTCGTAGGTTTGTTCCCAATCAGA
TCTTTCTTAATCGATTGGGATTTTATTCAATTATGCATTAATTTTAGCAATTTATGGATTTTGAGGCTTTTAATTAAATGCGTTTCCTTATGGGTAGTTCGCTCCAATTTGCAGCTTTCTAGGTTT
TTAAATGTTTATTGCTCTGGATGAGTTTGTTCAACCTAGATTTTGGGAGAGGTTATTTCACCGGGGTTGGAAGGGATTTTGATCAACGCCGTGGTATATGATTATTCGCAATTTGTTATTGGG
CGCACGGAAGTGTTGATTTTGAAATCGAAGAAAGATGAATGTGTCGAAGAGCAAGATGGGCGAGGAGCACGGGGATTTCTACTCTGTGTCGCCGAAGATGGATTGGAGGATTAGGTTGT
TAAGAATAATCTGGGGTTTGGTGATATTCATGGGAGGGGCTTTTCTGGGCTTGTCGGTAACTTCCCGAGGTTAAGTATAAAGAGTTTTGTTCATCCCATGCATCTTTTTCATGATTTAACTGA
TGAAGAGCTGTTCTGGAGGGCAACCTTGGTGCCAAAGATTGAAGAATATCCATACAAGAGAGTGCCCAAGGTGGCTTTCATGTTCCTTACACGTGGACCGCTTCCGTTTGCTCCCCTTTG
GGATAGGTTCTTTAGAGGACATGAACGCTTCTACTCTGTTTATGTGCATTCTATTCCAAACTACAAGCTCAATGTCTCAGAAACTTCAGCATTCTATGAGCGCCAAATCCCTAGTAAGGAGG
TTTCATGGGGATCGATTTCACTTGTGGATTCAGAAAAGAGACTTTTGGCCAATGCTTTATTGGATTTCTCCAATGAACGGTTTGTTCTTCTCTCAGAAAGCTGCATCCCTGTTTATGATTTTC
CCACAGTCTACAAGTACCTTATCAGTTCCAAGCATAGTTTTGTCGAGTCCTACGATGAAGATTCGCCCCGTGGACGTGGTCGTTACAACCGTCACATGTACCCTAATATCAAGCTCAGCAA
GTGGAGGAAAGGATCCCAGTGGTTCGAGCTTAACCGGGAGTTAGCTGTGAAAATAGTTGCAGATTACAATTACTATGCCATATTTAGAAGATACTGCAAGCCCTCTTGCTACCCGGACGA
GCATTACATCCCGACGTACTTGAACATGTTCCATGGAGAATTGAATGCTAACCGGACTGTAACTTGGGTCGAT
>comp152771_c0_seq5
ATTATTGATATACCAAGAACATAACAGCGACAATTGATTTGGATTCCTATCACAACATCTTCTAATAATTTGCAACACTGTTGCTTACAAGTCTTACACTCTGCTAACATTCAACATATAGTA
GGAAATACAAATTAAAACAAACACGCAGCGCAGCAGCAGCAACTGCAGTACACAATAAGTGATTAGGCAACAACAAAAAGATTTAAAGCTCACAGAAAAGCTTCCAAACTCGTCATCT
CCGATATTCCAATCCCTTGAAACCTCGAACTTTCCCCCATCAAATCCATCTTGAAACCATAATTACTCGAACCCAGAAAACAGTCGTTATTACGTTGCATCACTCCCTCTGCTCCTCTTTCTT
CTTTTTCCATAACCTCATAATGCAAAAAATTGTTGTTCTCTACTTCTTCAGCTCTAATATCTTCCCTGCTCCCTCCATCAAATTGTGTCATCATGGGCTTGGGCAAAGCTTCCTGCTGTGGAA
GCTCAGGGAGTTGGTCTAAGTATTGATCCGACATAAGAAAGTTTCCACCTATTCTGCCAGACATGTTTTCGAGGGCCTGGTCTGGCCGAAAAGAATTGGATGGCGAAGGAAGAGAAGAA
GAAAGGGTGGAAATAAACTTAGCTGACTTCCCACGACGGGTCTTTCGGCAGCCACCACCAACGGGAATGCTGCGGAGAGAGCCTCCTTTGGTCCAATAGCGGCGACAGCCTTTACATAA
GTAGCGGGGTTGAGCGAGGCAGTAGTTGTTGTAATAACAGAACTTGGTGTTTGGTGAATCGCAGCGGGGGCAATTGGGCGCGACTTCTTCGACTTTGGGGCGCCATCGGCGGTCTGTTAT
ACCAACCCCCAAAATCTGCTCTTATCCTGCTCAAACCCTACTGTACCAGCAGCCCATACCCCTGCCAAGAAAAATCTCTGTCAAATCCATCAAAGATGCACCTGCCCCGTCAAAATTCTAC
TCCATTCCTGCAGTAGCCACCTGTTTTTGTCAGGCAAGTGGAAATAAAAAAAAGACTAGCTTATTATGTTTTCTTAACTGATCCCTCTCATCTCTCTATTCACTCACATAAGCCCCCACTCC
CATTTCTTGAGTTCCTGCAGCTCTCCACGTAAAGCTCCCCACTGCCAGCAGCACCTCCACCTTCCATCCCATTCTCCCTTTTCTTCTTCAGCAAAACCACTCCATAACCCACGGCAGCCCT
CTCCTCTCCACTACAACAACTCACATAAGCAGCCATTCCTTCTTTTCCTTCTACTACTCAACCACCTACAACAGCTCCTATTTCATACAGCAGATCTGAACCCCATACAACCCTCAACCATT
GCTCAATCTCTTGCAACCAAGTAACGTCTCCTCTCTTCCAACTCTA
>comp152784_c0_seq1

AGAAATTGCTCAATCATATAAAACTTGAAAGAAAATAACGTGTATTGTCTCAATATATAGAATATACAACTCAACGCTCAAAAACAGGTTTTTGAGCAAGAATAGTAGAGACAATTAGAAG
TTAGAAATCAGAAATAAAATGTGACATCAAAGAATCAAGAGCAGGAACATAAGGGAGAGCAGAAAATGCATCTCTACACGAAATAAACAAAAGAGCGACGGTCGACTATCGAGTGATTA
AAACAGAGTAAATCCCTCCTATAAAGTTTAACAGTCAGCCAACAGACTAGCTTAATCCTCCTCATCTTCCTCGTGCATGGAAGCTTCCCCTTCGATGACTTCATCCCTGAGCCAATTTTTTT
CCTTGTAAATTACCGGATGAATTAAGTAATGAAAGAGATCGGATATAATAAATTGCCTGCACGTCAGAGGTCCAGAGAGTGAGGTTCTCTCGGAGGAGTTGCATGATCAGAGTGCTGTCCT
TATATGATTCTTCGCCAACAGTGTCCAGCTCAGCGATCGCATCATCAAATGCCTGCTTCGCAAGAGCGCAAGCCCGATCAAGAGCGTTCAATATCTCATAGTAGAACACGGAAAAGTTGA
GCGCGAGTCCCAAGCGAATCGGATTAATCGGTGCAAGCTCTGCCGTAGCAATGTCCTGGGCAGATGTGTAGGCGGAAAGCGTGTTATCGGCGGCCTCCTTGCGTTCAGATCCGGTCTTGA
ACTCGGCAAGATATCGATGGTAGTCGCCCTTCATCTTTAGAAAAAAGACCTTGGAGTCATTGGACAATGCCGAGGGGAGGAGGTGAGATTCCAGCAGCTTGAGTATTCTGCCACAAATGG
ATGTTATCTCCGACTCGATCTTAGCCCTAAATTCCTTGATAGCGGCGAGGTGATCATCATTCCCGCGACTCTCCTCCTTCTGCTCTATCGACGACACGATCCTCCACGAAGCCCGGCGTGCG
CCGATCACGTTTTTGTAGGCTACCGAGAGGAGATTTCGCTCTTCAATGGAAAGCTCCCCATTCTCCGTCGCCATCTCCGTAACCTTCTCCATGAACTCCGCCATTTCCTCATACCTCTCAGC
CTGCTCAGCGAGCTTCGCCATGTAGACGTTATCCTCCCGGGACTCTGCCATCATTACGTTCGCACAAAACAAACCCTAGAAGAGATTGAGAAGAAGAATAGAATAAAACAGGAATAAATC
GAAGGAAGGAAGAAACTTGATTTTGGTGAAGAAGCTTTGTTTCTCTGTAATTTATTCGTTTTGGGAAATGGTTTCTGCGGTGGCGCGTACGAGGAGGAACAGAGACCAGCCGCGAGCGC
ACAGCTGTACAAAC
>comp152784_c0_seq3
AGAAATTGCTCAATCATATAAAACTTGAAAGAAAATAACGTGTATTGTCTCAATATATAGAATATACAACTCAACGCTCAAAAACAGGTTTTTGAGCAAGAATAGTAGAGACAATTAGAAG
TTAGAAATCAGAAATAAAATGTGACATCAAAGAATCAAGAGCAGGAACATAAGGGAGAGCAGAAAATGCATCTCTACACGAAATAAACAAAAGAGCGACGGTCGACTATCGAGTGATTA
AAACAGAGTAAATCCCTCCTATAAAGTTTAACAGTCAGCCAACAGACTAGCTTAATCCTCCTCATCTTCCTCGTGCATGGAAGCTTCCCCTTCGATGACTTCATCCTGCACGTCAGAGGTC
CAGAGAGTGAGGTTCTCTCGGAGGAGTTGCATGATCAGAGTGCTGTCCTTATATGATTCTTCGCCAACAGTGTCCAGCTCAGCGATCGCATCATCAAATGCCTGCTTCGCAAGAGCGCAA
GCCCGATCAAGAGCGTTCAATATCTCATAGTAGAACACGGAAAAGTTGAGCGCGAGTCCCAAGCGAATCGGATTAATCGGTGCAAGCTCTGCCGTAGCAATGTCCTGGGCAGATGTGTAG
GCGGAAAGCGTGTTATCGGCGGCCTCCTTGCGTTCAGATCCGGTCTTGAACTCGGCAAGATATCGATGGTAGTCGCCCTTCATCTTTAGAAAAAAGACCTTGGAGTCATTGGACAATGCC
GAGGGGAGGAGGTGAGATTCCAGCAGCTTGAGTATTCTGCCACAAATGGATGTTATCTCCGACTCGATCTTAGCCCTAAATTCCTTGATAGCGGCGAGGTGATCATCATTCCCGCGACTCT
CCTCCTTCTGCTCTATCGACGACACGATCCTCCACGAAGCCCGGCGTGCGCCGATCACGTTTTTGTAGGCTACCGAGAGGAGATTTCGCTCTTCAATGGAAAGCTCCCCATTCTCCGTCGC
CATCTCCGTAACCTTCTCCATGAACTCCGCCATTTCCTCATACCTCTCAGCCTGCTCAGCGAGCTTCGCCATGTAGACGTTATCCTCCCGGGACTCTGCCATCATTACGTTCGCACAAAACA
AACCCTAGAAGAGATTGAGAAGAAGAATAGAATAAAACAGGAATAAATCGAAGGAAGGAAGAAACTTGATTTTGGTGAAGAAGCTTTGTTTCTCTGTAATTTATTCGTTTTGGGAAATG
GTTTCTGCGGTGGCGCGTACGAGGAGGAACAGAGACCAGCCGCGAGCGCACAGCTGTACAAAC
>comp152807_c0_seq1
ACCCCAAACATCAGTATGAATTAAAGAAAATGGTCTCGTAGTATTGCTAGTAGAGATAGGAGAAGCTAATCGGTGGCTCTTAGCCACTCTACAAGTATTGCAATGCAGAGAATGAAATTTA
GTAAAAAGAGAGGGAATATGCTGTTTGAGATGTTGAATAGTGGGAGCCGCCGGGTGGCCAAGCCGCTTATGCCAGAGATGAGGAATTTCTTGAGCCGAGAACAGCGCAAGTGTGGTAG
GCGAAGTGGTGGGTCGGAGAGGATATAAGCCGTCAAGACATGGACCCTTGAACAGAAGACGTCCCGTCTTCAAATCCTGAGGGGGTGGGTAGAATACCTTTGCCGGAGTGCTGAATAGG
GAGTTGTTGTCCGTTACCTATTGTGATTTGTTGGGAGCCGGTATATGGACGGCTGGATTGAATTTGTGAAGGATCTGATGTGAGATGGCTGGTAGCACCACTGTCAAGAAACCAGTTAGTT
GAGCTTGCTGATTCCGAAGGGGAGAATAGAGCGGTTGCGGTTTGTTCTTCCGTGTAGTTGGGATCATGACGGTGCCAGCATTTTAGCGCCGAATGTCCTGTTTTGAAGCAGATTTGACAC
GTCATGGGAGAGGGCGTGTTCTTGTCGGAGCGGTTGCGATTGTTACGATACGTGTTGGAGGACCGACCGCGGTTGTTGTTGGTGTTACGGCCACGACCACGGCCGCGATAGGCAGCTAG

AGCGGTGGAAGGTTCAGCAATATTCAAATTCTGTAGGTCCTTGATGGTTTCTTGGGCAATATTTTGTTCTTCGCTGCATAACAAGGTGTAGAGGTCGTCCAAACTGATTGGTTGCAGATTT
GTGCGAATGGCGGTTTTGAAGGCTTGGTATTGAGATGGTAGGCCGTTAAGGGTATAAAGAATTACATCTTCGGGGTCGAGTGGGCTGCCGGTGGCGGCAATTGCGTCGACTTTGGATTTG
ATGGCAAGTAGGTACTGAGACATAGTTTGGTTTTTCATGGAGAGATGGTGCAGTTCGTTACGAAGTTGGATTGTGCGAGAGCGGGTAGTAGATTGCAGGCGACGCGCGATGGTTTGCCAA
ATGTCAGAACAATGTTCTAAATAAAGGACGTAGGGAAGGATGCTAGGGGAGATGACCGAGTAGAGGGCTGCGGCCAAATTTTGATCAAGAAGTGTCCAAGTTTGGTGGGAAATTGACGT
CGATGAGTTGTGATCGACCGGGGGACAGGGAAGAGTACCGTCCAAGAAGCCTTCGAATCCATTGGCCTTGAAGATCTTGAAGACTTGTGAGCTCCAGATGGGGTAATTTTCGGTGGTAA
GGGTGATGGAGATGATGCCCTTGATGTTGCTTAGGAGAAATTTGAGCTGAGGGGGAATTGAATGGGCATCAAGAAGATTACCGGTGATGGTGGCGGAGTTCGTGGTAGAGTCCGACGAG
GCCGCTGATGAGGCCATGAAGACGTCGGTAAGGTCGGAGCAAAAGAGGGGGATGGAGGAGGGAGAAGAA
>comp152829_c0_seq6
AAAAGAAAAAGAGATATATATACATAATTATAGTAATATATATGTTGACCTCGTTTTTCGCTCAAATATCGTCGAGCGCATTCGATCAATTCGATTGAACCTCGTTGAGCCAAATCCGACCTC
GAATTCGATCTGAATTGATTTTAATTTGACTCGACCCTGTTCAATCAAGCCCGAATAGTTCGCTTTGACTGCTACGAGTTCAATTTGATCCGAATCGTCTTTAATTTGATTTGATCAAATTGA
TCGAGCCTAATCCGCCTTTAAATCAAGCCGATTTGAATCAAATT
>comp152867_c1_seq1
TGTAATGTGCTCTATATGATGTATGTTTGATACATTGAGTCAAGGCCTTCCCCACGAAGAATAGCCTCATTTGTAATCAACAATAAATACTAGTAGAATCATTTTAACTAGGTCAGACCTCAC
GATTTTTCCCTTTTTCAAGGTTTTTTACATAAAAATCTTGGTGTTTTTTATGACTTTTGCATCCCTCTCTTTTATCTGTCAGTGTGCTGGATCTTCATACAAAGCTTCGATGCTAAATTTTATAT
CTCTACTGTCGTGAATATTCTCGAATGCAACAATCTTTGTCCTTCAAATTTTGTATGAAGAGAAATTGTTAATGTACCCCTATTTTGAAGCTTAGTGAAAATTTTGATTTATGTGTTGGCATTG
ATGTTGGTGGATCCCATCGAACTATGCAAATTTGCGTGCTAAGTTGATTTCACATCTTCTCATGCTTTGCTTTTCACTCATAAATTATTGAGATTTCATTTCATCTCCCCCAAAATATCGTAGA
TTCGGAATTTTAGAGAAATCATCGAAAGCAAACTTTCTGTGCAGATGCGAAGAGATAAAGAAGTTCTGGATCCCTTTGGATCAGAGAATTTATTAACTGCGAGTAGCCGATACAGAAGAA
ATGAAAAAAGGTTTGTACAGTATTTATCTGCACTTATTTGCATAGAAAAGATTAAACCAAACTGACGAAGATAAGTTCTTCATCGATAAAACAGAACGCAACAGAACTGAAATCAGAGAA
CAGTAATTCAATCTCACGTCAAACAAACAATAACTCAACAAACGCTTAAACGACGATAGCAATGCAAACCCTACAATTAAGATTAACTCATGGATTTAGGCAATCTTGACCTCGATCTTCT
TGGTCTTGTTCTGCTCAGGCGGCGGAAGCTTCTCCACGGTAACGGTGAGAACCCCCTCCCGGCAAACTGCAGAGATGACTTCGACATTGACGTTATCCGGGAGGGTGAACTTGCGCATA
AATTTCCCCATCCGGCGTTCAATCCGAAGGTACTTTCCTTCTTCCTCGTCCTTCTCGTCATTCCGCAAGCGCTCACCGGTGATGACGAGCAGGTTTTCGTCCTTGATCTCCACATTGATGTC
GCTATATTTGAGACCAGGCACATCGAGCACGAATTCGTAGGCCGACGGCAGCTCCTTGACGTCGGCCGGCGTTGAAGCCATCGCCCGTGTGTCGCGCACGAATGATTTCATCGGGGAGTT
TACGGAGTTCTCCCATTCCTCCGGCAGATCTACCAGATAATGCAGCGTTGAGATGAGCGGGTTTTCCAAACTGAGACTACGGAAATCCATTGTTGGCGATCGATCTCCCTTCTACTGTGTG
TTTCTCTCTTTCTGATATTCGATTACTTTACCCTATATATAAGAGCGAATCGGGGATGGAAGGCGAATTGTTCTAAGAGTGCGGAGAAGCGGAGAGAAGCTTTCCTTGGTTTTGGATTGAGA
TGGAAAAGCCAGCAAATCGAAGTTCTGAGCTAAAGGATTATATTAAGATTTTATTTGGGGCCGTTTTCCTATGCT
>comp152892_c0_seq5
TATATACACACACATATACATATACATGAAACAAAATTCTTTTAAGGTATACTAATGAAATCCTAAGAAGAATTCTTTTTAATCAGTCTTCCACATGTTGGTTTAATGATCGGTTCTTAGGCCC
ACATTAGTCACAAGTTCGAGTAACAACTGGAACGTACTAAGTATTGTATTCAGCACAAAACTATTCTCCCAAAGAAAAGGAATTAGTACACTGAAAAATAAATCTATATAGAATCATATGT
GGTCTACTTTTTCATGTAGCTCTCTTTAAGTGGGAAAGAATCTCACACTACTATTTGAGAAACGTCTTTATTGGTCCACTTAATCAGCATAAGTTTTTGTGTTAGTACTTTTACCTTAATTTTT
TTTTGTAATGGGTATTTTCCATATATCTTAATTGAATCTTATATCTTGATTAATTGCACTAATTCCAACAAGGATCCAACAACACAAAAGCATTGCTTGATTGAATTTTGTCGCCGAATGTTCT
TCATTTCTAGGCAAAACTTATGAAAGCTGATATTTGGCGCTATTTGTTTTCTGTGGTATTGAATAGAAACCCAGTTAGGGTTTCTCAACAAAATTTTTATGAAAAGCTGTGCAAGTATTCTC

ACCCAAACTCCTATCTGCTACGAGGCAACGTGTTTTGTAATCTTACCAAACGATGCCAACTTAGTAAAAAATTACAAATTATGTACAATATTACTTTTTTGTATTCATCTTATTTCTTTGTGTA
TTTTAGAAGCCTAAGATAATAATTTGTGCTGCCAACTGATCTTCTCTTTTTCCTAGGTATCGCTGTGGTTGGAGTGATGTTAGTCACCTCTTCACTGTTAGTTCTGATAATGTTAATGGTCTG
GCAGACAAGCCTCCCACTAGTAGCACTCTTTGTGCTAAGCTTCGGCTCCTTTGAGCTGGTCTACTTCTCTTCCGTGCTGTACAAGTTCACAAGTGGAGGCTACCTTCCTCTTACTTTTGCT
TCCGTTCTATACTTTGTAATGTATGTATGGAATTATGTGCAGACAAAGCGTCATAACTTTGAAGTAGAGCAAAAGGTCTCAACAGAATACTTAAACAGCCTTGGTTCTAACCTTGGCATCTC
AAGGGTCCCAGGACTTGGCCTCCTGTATACTGAGCTCACTCATGGAATCCCTGCTATATTTCCTCATTTTCTCACAAACTTGCCAGCAATCCACTCTGTTCTTGTCTTTGTCTCTGTTAAGTA
CTTGCCAGTTAACACAGTGCCGGCCGAGGAGCGGTTTCTGCTGCAAAGAGTTGGACCTAAGGACTACAAGATGTATAGGTGCATTGCAAGATATGGTTATAGAGACAGGAGAATTGGGA
ATGAAGAATTTGAGCTTTTGTTAATGGAGAACCTTAAGAACTTCATAAGGAATGAGAGCTGGGAGGAAGGGGACTCTAGTGTGGAAGGGGAGGAGATTAGGTTCTTAGAGAAATCAAGA
GAAGCTGGAGTGGTTTACTTGCTGGGTCACAGTGAGGTTAGGGCCAGTGAGAATTCTTCTTTGCTCAAGAGAGTGATTGTCAATTATGTTTACGATTTCTTGAGGAGGAACTGCAGGCAG
GGTTTTGTTGATCTGCAGATACCTAACAAGAATCTTCTGCAAGTTGGAATGAACTATAGTGTATGAGGGCATTGATGATGCTATAGCAGAACTATCTACTTGTTTCAGATTATGTTCATCAGA
GAAAAGGGCTAGCTTAGTTCAAAGTTTGACATTGTTAGAGAAGATAATACTGCCTAATGTTACATACAAATAAGCTGATAATTCCACTGCTGGTATTTAATGTTAGCAATGTATTTAGTGTTG
AAATCTAATAAAGCATGGTGCACATCTGTAACTTGGCATCAAAAGCTCCTAGATCGTGTCCATAACCTCTACACTTTTCTTCTTAATGTCTTAGTTTCTCTCTCTTTATTTTGCATGTGTTTAG
AGCTTTTGTGATATAAATTGCCAATTGAAAATATGTTAGGCATGTTGGAAGAAAAA
>comp152921_c5_seq1
CATTAACTTCCCTTCCCTGCTTTATACCAGCCTTGGACTCCCATGACCTCCTCACATCATGATGTACATCATTTACAGGCAACAATTCAGAGTTCTGCCCTCTTACCAATCCTTCCATATCAA
CATTCTGAAAACCAATTTGTTCCATGTTACAAATGTTGTTCTGTACCGAAATTTCCATATTAACTTCCTGCAATTGTGGATGCTCCATATTATTTGATTCGTTCTGTACCGAGCCATCCTCAAT
AACATTTCGGTATTGAGTATAATCCATGATATTTGATTCCTTCTGTATCTTGTCTTTCAACTTCCCTCCCAAATCTTGACCACCAGTCTCCTTCTGCACCTTATCAACAACTCTCCAAACAGA
ATTAGAAGAAGGATTTATCTGCTTGCTGCTAACAGATTGAGGTCTCTGTCCAGATTTATTAACAGATCTCTTCCTCCTAGTGACAATTGTCCATTCCCCTTGATCAACATCTACAACTACAGA
GTTTGAGACTCTCCCCTTACCATCACAAGCAGGGTCATGCTTCAATCCCGAACCAGCTGCCTCAATACCTCCCAGTTTCTGTGAATCACCAATCTCTAAGCCCTGCTGCTTAGGTAGAACA
CAAGACTCCTTACACAGCTCAGCTTTATGGCCAACCCTTCCACATCTTAAACATATTTGAGGAATGCCTTCATATTCAATTGGTTGGAAGAACATACCTTCCAAACCATGAGCCCAAATACC
CTTAGGTAATCTCTTAGATAAATCGAGTCTAATACAGATGCGCGCAAATGCACATCTATTCCAAAGCTTAGTTTGCTCATCCATTAATAATGGTTCCCCAATGCCCCTCGCCAACCTAGCCAA
ATTAGCTTCATCCCAATATTCTAACGGTAAGTTTGGAAGTCTTACCCACACAGGAGACGTAAATCCTCTAAAAGATTTAGATGAAAGCCCTGTTGACCACTGCTCCACCCCAATGATCTGA
TCAGCAACATACCAAGGACCACCAATAAGAACATTATCCCCTGCTTCCATATTTACAAACCAACAGAGAAAGCAGTCAGATCCCAATGGCTTAATCTGAGTCAAACCAAG
>comp152922_c0_seq1
TATAAACATAGGCGCGGATCAAATTTACAAAGGACCCCCCAAATAGCCATGCTTCTTCCACAACAAGAACAATAATAATTAAAAAAAAAAAACTAAAACCCCACAAAATATGGCTGAAAA
AAATGAGAGAAAATAAATAATAAGAACAAAAGAAGGCTCTCATCAACCATAAAAATCTGAATGGGGTAGCCTTCAAATAGCTCAAAGTCCATTTGGATTAATGCTAATGAACTTCAAATTA
ACAAGGCCTATAACACATAATTAAATATCAATCAATATAAATAGTCCTCACAACCCAACTCCTTCACTTGTCCCCAACCCTTCTCCTCCATAGCCACAATGGCGATCCACGTCGCTTTCTTCT
TTCTCCTCGCAAGTCTTCTCAAAGCCGTTAGCTCAACAACGTTCAACTTCACCAATTACTGCCCCCACGCCGTGTGGCCCGCCACCCTCGCAGGTGCAAGCTCGCCGCCTCTGTCCACCA
CCGGCTTCCAGCTCGCCGCCAGCGCGTCTTTCTCGCTTCACGCCCCATCCAACTGGTCTGGCCGCATGTGGGCCCGCACTAACTGCTCAACTAACGACTCCGGAGAATTCCACTGCCTCA
CCGGTGACTGTGGTACCGGCAAACTCCAGTGCCACGGCGCGGGCGGCATTCCTCCGGCGAGCCTCGTGGAGTTCACCCTCAACGGCGCTGACGGAAAGGACTTCTATGACGTCAGCCT
CGTGGACGGCTTTAACCTTCCGGTTGCAGTGGCTCCGAGCGGCGGCAGACCGGGCTGCGACGGCGCCACCGGCTGCGCTGCCGATATAAAGTCTTTGTGTCCGCCGGAGCTGAGGGAG
CCGGCGGCGACGGCTGATGGAAGCATGGTGGGGTGCAATAGCGCTTGCTGGGTTTTCGGGCTGGACGAGTACTGTTGTACTGGAAAGTACAACGATCCCGACAAGTGCGGGCCCACTAA

ATATTCCATGGTGTTCAAGAAAGCTTGCCCTTTGGCTTACAGCTACCCTTATGATGATGAGACGAGCACTTTTACTTGTATTGGAGCTGACTATCTTATTACCTTTTGCCCGGCAAAGATTTT
CTAAGTCAATAATTATGTGAATGAATATTTAGGATTCTCGTCCCTAATAAAATTATGAGAATTATAATAATATTAGAAATATATTTGAGAGAAGAAAATAATTTTTATATTATTAATTTTGCTCTT
TTTTTTATATATGTATTATTTTTTTATGTATCGAGCAGTGGCCAAATCAAAAATTTTTAAAGGGGC
>comp152922_c0_seq3
TATAAACATAGGCGCGGATCAAATTTACAAAGGACCCCCCAAATAGCCATGCTTCTTCCACAACAAGAACAATAATAATTAAAAAAAAAAAAACTAAAACCCCACAAAATATGGCTGAAAA
AAATGAGAGAAAATAAATAATAAGAACAAAAGAAGGCTCTCATCAACCATAAAAATCTGAATGGGGTAGCCTTCAAATAGCTCAAAGTCCATTTGGATTAATGCTAATGAACTTCAAATTA
ACAAGGCCTATAACACATAATTAAATATCAATCAATATAAATAGTCCTCACAACCCAACTCCTTCACTTGTCCCCAACCCTTCTCCTCCATAGCCACAATGGCGATCCACGTCGCTTTCTTCT
TTCTCCTCGCAAGTCTTCTCAAAGCCGTTAGCTCACCCTCGCAGGTGCAAGCTCGCCGCCTCTGTCCACCACCGGCTTCCAGCTCGCCGCCAGCGCGTCTTTCTCGCTTCACGCCCCATC
CAACTGGTCTGGCCGCATGTGGGCCCGCACTAACTGCTCAACTAACGACTCCGGAGAATTCCACTGCCTCACCGGTGACTGTGGTACCGGCAAACTCCAGTGCCACGGCGCGGGCGGCA
TTCCTCCGGCGAGCCTCGTGGAGTTCACCCTCAACGGCGCTGACGGAAAGGACTTCTATGACGTCAGCCTCGTGGACGGCTTTAACCTTCCGGTTGCAGTGGCTCCGAGCGGCGGCAGA
CCGGGCTGCGACGGCGCCACCGGCTGCGCTGCCGATATAAAGTCTTTGTGTCCGCCGGAGCTGAGGGAGCCGGCGGCGACGGCTGATGGAAGCATGGTGGGGTGCAATAGCGCTTGCT
GGGTTTTCGGGCTGGACGAGTACTGTTGTACTGGAAAGTACAACGATCCCGACAAGTGCGGGCCCACTAAATATTCCATGGTGTTCAAGAAAGCTTGCCCTTTGGCTTACAGCTACCCTT
ATGATGATGAGACGAGCACTTTTACTTGTATTGGAGCTGACTATCTTATTACCTTTTGCCCGGCAAAGATTTTCTAAGTCAATAATTATGTGAATGAATATTTAGGATTCTCGTCCCTAATAAA
ATTATGAGAATTATAATAATATTAGAAATATATTTGAGAGAAGAAAATAATTTTTATATTATTAATTTTGCTCTTTTTTTTATATATGTATTATTTTTTTATGTATCGAGCAGTGGCCAAATCAAAA
ATTTTTAAAGGGGC
>comp152948_c0_seq1
TAATAAGTTTTCCCCGTTTCCTTTCCCCTGATTTTGTCTAGCATCAAAATCCTATGAGAAATTTCTCACAAGATTTGTAATTAACATGTATAAGCTCATCTCAAACTGATTCAGTAATAAACTT
TCACACAACATACAAACGAAATTCAGTAACATTCAGAACTTCTGCAAGCGTTACGAGTACATGAGGACTAGAGCTGGATATGTACAGATCTCAGCAGGTCCAGAGAAAAGTCCAGAATC
TTTTGAGAAGTCTGAAAGTAACCTCTCGATCGGAAAACCAAAAGAAAATTAAAATAAAAAGTATCTTCAGCTAGAAAGAAGAAACTGAAAAACAAACGACAGAGGAAGAAACAACAC
TTCTTAGCGGGTTGCCATTGCGAATGCCATGTCAGAAGAACTGACGAGTGAAGGTAATTGCAACAGCAAAACACCGGGTAGATCAATTCATGATCTACTCCGCCACCTTGATGCCGCCAT
TCTCGCCCTGAGAGGGGTTTTTGCCGGCGTTGATGCGATTGGCCTTTCCTGTTGCTCCATTCCTGTTGCCCTTTCCCTCGGCCTTCGCGGCTGTCGTAAGCCCGTCGAAGTTCTCCGTTTTC
CCAAGGAAGATCTCAATCTTGGCTTTCCTGATGAGCCGACCCTTTGTTTCGTCCTTGGTTCCGATGGTCGAAGTCAGAATCTTCTTCTCAGTTGCTAGGCCTTTGTTCTTCAGGATCTCCGC
GACCGTAACAACTGTACCGATCGCCATGCCAAGAGCAGAAAGCTCGATCTCGTTGTATTGCTGCATGTACCTCTTGGCAAGATTGACGTAGAAGTAGAGCGGCTTCTTCTTATTAGAGACC
TGGATTCGATTCCGCTTCTGCGGATCGACCACCACATTCGAATCCACCACCGCCAACGGATCATTGATCGCCTCCGCCACCATAGTCGCCGCCATCTCTGTCTTTTTCTCTATGGATGCTCT
GGTTCGATTGCGCTTTTGGATGACATTGTGGTTAGAAGCGACGATTAAATAGCCGGTGAAGCTGCGCGGGGTGGCGAAGCGCGCTTGTAGAGGGAATTGGTGATCGTTTTTTTTTTCTTG
GCTATTGGCGGGTACGAAGCGGCTTTTGCCGTTGAAATCTTGCGTGAGGTGGTGGTCCTCAAGCCCG
>comp152990_c1_seq1
AATAAATAAATTATTAGTTCTTGTGTAAGGGATTCAGATACTCTTTATTTATCATAGTACAATTATCCATTCTAATTATTGAATTCACTTTGGATGGTTAAGATTAACTTTTTTCAAAACTATAGT
AATTTTCAAACCTTGGATATTATTAATCTCAATCATCCAAAGTACATTGGATGACATGGATGGCTCACTGCACAAGGTAAATTCATTATGATAGTTACAGAGAATCTAATCCTTATAAAAGAG
TATTAATAATTATGCAAATTTCAACCCCCTCAATATAAAAAAAACATTTGAATTGTTCTTCAAAATAACAACTCTTCCACTCCCTCACATTTGGACGGTCTGAATTGGACTCAATACCTCGAA
AATAAATATTTATGAAGTGGTACATACTACCCTTTCTCCTGTAACAAGAAAGAGATAGAGAAATGGAAAAACAATAATGTAATAGTGACAACCATCAAACTTTTTTATCTTTTACTCCCTCT

TGTTACTCATTGGATCATACATATATAATTTTTTCGTTATAGAAATAGTGATAAGAAATACAATACACCTATTCACAAAAAGCATAATAATTCTCCATAATTAACAAGAGGGCCAATTTTTTCTT
TCAAAGAAATAATAATTTAAGTGTTGTCTTATAATGCAAGAAAACTTATATTTTAATTTT
>comp152995_c0_seq1
TTTAAGCAGCACAGCAAAAAATATTTTTATTAGGAGTAAGAAAAGAACTAAAAAAAACAAAAAACCCAATAAAACAACTCAAAATGTTGCATTTAGATTCAACCTTTCAACTTGTGCTAA
CACATGTTCCTACCAAGTGCAGGAAAAAATCTAAACCTATTTGCACTCCTTATGTGCTTGAAGTTAACTAATAAAACTGATCAAAGCAACTCCAACCCATCACAGAGGAACCATGAAAAA
GAAAAGCCATGAAGTTTACTATTGATAGTTCAGTTCCTCTGTATTTTGTTGGGCTGAGGCCTCACAGTTTGATAAGAGAATCCTGATGATGCTGCTGTCATGGCAGAAAACAGATGAGGAG
AGCTACTTGCAGTGAAAGCTGCAGATGCTGGCCACTGGATTCCGCCGCCCATTGCAGAAACCGGCAGCCTTCGAGGCTGCTCGTTGCTGCTGACCGATAAAGAAAATAGTCCTAAACCT
TCATCTTTTGTTTTCATTCCATGGCTACTGCTTCGAAATTGCTTATGGTTAGCAAATTCAAGCCGATGGGCCTGGAGGACTGGAGTTTGGATATATATGCTGCTTGAGGGATGATGATTAGTT
CTGTAATGCTCTTGTTCAGGCAGGTTAGGCTTGTCATCGTACTTGCGTCCAGTGCTTCTAATCCATTCAGGTTCATAACTCATTTGAACTCTCTGATCCATGATCATCAAGCCATCATCTTCA
ATCGGTGCAAGACTTCTTTTAGAGCTGAAGCTCCCCAAAGTGAGATCCAAATTATGATCAGCTTCTACATCTTCAGAGTTGAGCTCTTCTCCATATTTCCTTGGGTCGAAGTTGGTCACTG
CATCCCTCCCATTGAGCCTTATTGCAGCTTTATCATAAGCCCTAGCAGCTTCAACTTCTGTATCAAATAAACCCAAATAAACATATTTCTTGCCTAAAAGTTGGCCTAATCTAGCCTCCCATC
TGCCACATTTGTGAAGGGTAACCCCTCTAAACTTAGAACTTCCTCTAGGAAATCCTGTACTTTGCCTTCTAAGAAGAAGAACAAATTCCTCCTTTGTTAGATTACCCATCTGCTTCAGTTCA
TCCTCATAGTCACTCAGGATGAAATTAATGTCTGCTTCAACGCCACGAAACTTTAGGGCAGCTCTGTCATAAGCTCTTGCAGCAGCATGAGCTGTGTCGAATCCTCCTAAATAAACTTGTT
TGCCACAATCCCAGATGTGGGATTCCCAACGACCGGTCCTCCGGTAGAAGGTGACACCGCGGTACTGTGAGCTCCGTGATCGCGGGCCCCGCCGGCTCTTTTTGATTGGCTGCGAGACC
ACCATGTTCTTGACAACGCCGCCGGCCATGGCCGGCTCCGACAGCTTCAATCCGAACCTGGTGTTCTGCTCCGGCGGCATACCACCGGCGCCTAAAGTAAGCAATGGTAACTCCGGCTTC
CGTTCACTAATCGGAAAGAACTGGTGCGTCACTACCGTCTCACTTTCGCCTGAGTAGCTCTCCTCGAACCCAAACAAGTCCTCAACGCCGACTCCACCGTTCGAGTGCTCGATCACGAC
GGTAGAGGAGCTGGAGTTTTCAGTAGACCCCGCTCCAAGCTCCATCACCATGGCTTCGGAACCGCCTGAGGAGATCTCCTCGACTTGGGATATCGCAGCATTTCTCTGGTCCGGCGACTC
ATTGAGATCCCACATCTCTCAGCTTTTCAAGCAAAATCCAATCTTTCTTCCAAAATTTTTTCTCCAGCGAACTTTAGAGAAGAAAGGCGGGCTTCTTTAGAGAACACTTGTGGGCAAGCTT
CCCCCTTTCTCTGCTTCCCCTCTTTCTACAGTAAAATCTAAAGCGCAAAGAGGCACAAACTTTGCCAAACTCGGCCGACGAAATCTAATGAGAGAACTTCAAAGAGAAATCTAAGATATG
AGTAAAAAAAAGACGCAGGAGGAAGAGACCAAGTTGGCCTTCTTCTCTACCGTTATTCTTTCTCTCTTCAAAAATCAAACATCCTCCCCACACGCTCCCCGCTTTGGTTACTTGTAATGG
CAGCTA
>comp152995_c0_seq2
TTTAAGCAGCACAGCAAAAAATATTTTTATTAGGAGTAAGAAAAGAACTAAAAAAAACAAAAAACCCAATAAAACAACTCAAAATGTTGCATTTAGATTCAACCTTTCAACTTGTGCTAA
CACATGTTCCTACCAAGTGCAGGAAAAAATCTAAACCTATTTGCACTCCTTATGTGCTTGAAGTTAACTAATAAAACTGATCAAAGCAACTCCAACCCATCACAGAGGAACCATGAAAAA
GAAAAGCCATGAAGTTTACTATTGATAGTTCAGTTCCTCTGTATTTTGTTGGGCTGAGGCCTCACAGTTTGATAAGAGAATCCTGATGATGCTGCTGTCATGGCAGAAAACAGATGAGGAG
AGCTACTTGCAGTGAAAGCTGCAGATGCTGGCCACTGGATTCCGCCGCCCATTGCAGAAACCGGCAGCCTTCGAGGCTGCTCGTTGCTGCTGACCGATAAAGAAAATAGTCCTAAACCT
TCATCTTTTGTTTTCATTCCATGGCTACTGCTTCGAAATTGCTTATGGTTAGCAAATTCAAGCCGATGGGCCTGGAGGACTGGAGTTTGGATATATATGCTGCTTGAGGGATGATGATTAGTT
CTGTAATGCTCTTGTTCAGGCAGGTTAGGCTTGTCATCGTACTTGCGTCCAGTGCTTCTAATCCATTCAGGTTCATAACTCATTTGAACTCTCTGATCCATGATCATCAAGCCATCATCTTCA
ATCGGTGCAAGACTTCTTTTAGAGCTGAAGCTCCCCAAAGTGAGATCCAAATTATGATCAGCTTCTACATCTTCAGAGTTGAGCTCTTCTCCATATTTCCTTGGGTCGAAGTTGGTCACTG
CATCCCTCCCATTGAGCCTTATTGCAGCTTTATCATAAGCCCTAGCAGCTTCAACTTCTGTATCAAATAAACCCAAATAAACATATTTCTTGCCTAAAAGTTGGCCTAATCTAGCCTCCCATC
TGCCACATTTGTGAAGGGTAACCCCTCTAAACTTAGAACTTCCTCTAGGAAATCCTGTACTTTGCCTTCTAAGAAGAAGAACAAATTCCTCCTTTGTTAGATTACCCATCTGCAAGAAAAA

AATAAATGCATTTTGCTAAACTTTTTCAATAAAATTACTGATCAATTACTTGCCTAAGAACCTATACCTGCTTCAGTTCATCCTCATAGTCACTCAGGATGAAATTAATGTCTGCTTCAACGC
CACGAAACTTTAGGGCAGCTCTGTCATAAGCTCTTGCAGCAGCATGAGCTGTGTCGAATCCTCCTAAATAAACTTGTTTGCCACAATCCCAGATGTGGGATTCCCAACGACCGGTCCTCC
GGTAGAAGGTGACACCGCGGTACTGTGAGCTCCGTGATCGCGGGCCCCGCCGGCTCTTTTTGATTGGCTGCGAGACCACCATGTTCTTGACAACGCCGCCGGCCATGGCCGGCTCCGAC
AGCTTCAATCCGAACCTGGTGTTCTGCTCCGGCGGCATACCACCGGCGCCTAAAGTAAGCAATGGTAACTCCGGCTTCCGTTCACTAATCGGAAAGAACTGGTGCGTCACTACCGTCTCA
CTTTCGCCTGAGTAGCTCTCCTCGAACCCAAACAAGTCCTCAACGCCGACTCCACCGTTCGAGTGCTCGATCACGACGGTAGAGGAGCTGGAGTTTTCAGTAGACCCCGCTCCAAGCTC
CATCACCATGGCTTCGGAACCGCCTGAGGAGATCTCCTCGACTTGGGATATCGCAGCATTTCTCTGGTCCGGCGACTCATTGAGATCCCACATCTCTCAGCTTTTCAAGCAAAATCCAATC
TTTCTTCCAAAATTTTTTCTCCAGCGAACTTTAGAGAAGAAAGGCGGGCTTCTTTAGAGAACACTTGTGGGCAAGCTTCCCCCTTTCTCTGCTTCCCCTCTTTCTACAGTAAAATCTAAAG
CGCAAAGAGGCACAAACTTTGCCAAACTCGGCCGACGAAATCTAATGAGAGAACTTCAAAGAGAAATCTAAGATATGAGTAAAAAAAAGACGCAGGAGGAAGAGACCAAGTTGGCCT
TCTTCTCTACCGTTATTCTTTCTCTCTTCAAAAATCAAACATCCTCCCCACACGCTCCCCGCTTTGGTTACTTGTAATGGCAGCTA
>comp153093_c1_seq1
GATACTTCTGAATCTCATAGCAGTTTTGTCGTGAAGGTGAGGCTGAGATAATAAGCATTAGAATTCTTGAAGAATAGAATGCTCAACAAATGAAAGCTTGCTTTATAGCTGTTAGTGTATGG
TTACACAAAATTATATTTGGAAGGTTCTCCATATGGAGTGGTGTCTGTTAATTTGGATGCAGCATTATTAAAAAAACATTATAGGAGCCTTGTGAAATGATGTGTTGACGTTGTACACCCTTC
ATGGCAAAGTGTGTGTTCCTTTGCCATATCGTGCTGCTGTATTGT
>comp153169_c0_seq1
CATGTCGATGATTCTCCGCCTCCAACGAGATAAAGCAGAGGCCCAGATGGACGCACGTCAGTTCAAGCGCCTATCCGAGGAGAAAATGTCTCATGATCAGCAAGAGATTGCTCTCCTTGA
AGATCTTCTTTTCAAGCGAGAAGAAGCTGTGCAATCCTTGTCCTGCGAGCTCCAGGCTTACCGTCACTGTCTCCTCTCCTATGGCATCGACCCTGCCACTATTGAATCCAATTCCTCCAAC
GCCGTCACCGCTGCGACCACCTCAACAGCCGCTGGGGACGGCGGCAATGCCTATTCCTCTCTCCGCTGTGAATTGGCCGATTTCAGGACAAATGTTGTGGACCCGGAGCTCAAGTATGCC
GCCGGTGACAAACCCCGTAGTCCCATTCAGCGCATGCCTAGTATCATCATGGAAAAGGGTGTAATCGAAAATAGCCATTCACCCAGCTGGGCACAGCGGGCTATGCGCCGGCTTTCCATT
GACAGTTTGGGTTCTGCTTCATCCCGACAGGAGTTTGGAAGCACTGAGTTACCATCCATGGCAGATTGCGCCTCTGATGCATGTACCGGTGTGAGGGATGACATAAGTGATCGAGTGTGC
ACTATTGATGCCGTGCACGACGTGGATGGGATGTATGATAGGGCTGATATGAGCACGGCGAGGGACTTTTTAAGCAGGAGGGATGCGGGTAATAGAACTGTGGATGAAGGAGAGGTTAA
GAAGCTCTATATGAGGCTGCAAGCGTTGGAGGCAGACAGGGAGTCAATGAAGCAGGTTATCATGACGATGGGGACGGATAAGGCACAGAGGATTCTGCTTAAGGAGATTGCTCAGCAGT
TGTCTAAGGAGGTGGTGCCAGAGACGAGGATTGTAAAGAAGCCTTCTTTAATAAGGAGTTTCTCTATAATGTCGGTGGTTAAGTGGGTGGCATCATTTTTCTCTTGGAGGAAGAAATCAC
CAAGGATCAAGTACCCCATTGGTTTATCAAATCAGAAGACCGGCTTATTGATTCTTCTAGAAAAGTCCAAGCGCAAGAGGCTGAAGAGGTGTCTCACCAGACTACCCAGTTGATTATAAT
CATCACCTTCTCCATGGGTACTGTATTTTGCTTCATAAAGTTCATTGGCTTATTATAACTACATATTAATTTCTTTTATTTATAAACCAGAAATAGCTTACTGTAAGAGGCTATTTTGTACATTTT
ATTCTATTCTATTCTATTTTTTGTTTGTTAAGAATTAAGGAAACTTTACCTTAGCACCAGCTGAGTTCAAGAGAAAATTTGTGCTTTGAACGCTAGCAGAATTTGTTTGATTGACTGT
>comp153204_c0_seq1
TCGAAAATTTCAGAAGAGTTTTAATTATTTCCTAAAGAACGTCAATATAAATTTTATTTGTCAAATTAGCTTTCCAACAGTGAACACATTGATGATTTCTTTTTCCGAAAGAATAATTGGGT
TTTCACCTGTGCAGTACTGAGAGGAGATTGAATGTTCAACGCAGATAGATTAATCAGGGACGTGGTAGTCTGCCGGTGAGATGGACGGAAGCAACAAGCCGGGGAAGAGTTGATCTAGC
TGCCGTTGAATGTGTTTTGGCCGCTTGGTAGGCCGCCGGCTAGGGCGGGATCCTGTTATTGCGATGAAGTCCTCTTCGATCTCTTTTCGGGAGAGAGAAATTGAGAACTTTGGCCAAGAA
TCACGCTTCTCCGGTTTTTCAGCCCGGAGTCGGACGCTCCTTCTTTGCACGCTCTTCTCTACCACAGGTGGAGGAGGTGGAGAAGGACTGGAGCACCGCTTTTTGCGGATCGGCACTCTC
CTGCTTCTCATATTATACGTTTGGTTCACATCGGAAGGCGAATTTGAAGAAGGTGTTTGGACCGGCATTGGAGCCGCAGGAGCGCAGGAAAAAGGTGCTCGTGGGACTTCTGGTTGGTGA

TCAGGTTCGGGCTCGTATATCCTGCAATTAGGAGCGGCGGCCTCGCGGAGTCGGGGGAGGAGATTGGCCTTGCCTCCCTCGGAACCGTCGTCATTTTTAATGGTGACGTGATTGTCATCCT
GTCGTAAGTTAGCAGACGGCCCAATTAGGTCCGATTGGCGGTGGCCGGCGATAATCTCGCCCTTGCGATTAACGTTCAGGCAGCGGAGGACCCGCTGCTTTCCCCAGGTCGTGAGAAAC
GGGGAATCGAAGTCATGGAGATTAGATCGGAACGAAACTCGACGCCGCAACCGTGGTTCTGGTGAAACCGGAGGTGCGAGGGGTTCTCTTGGCGACGGAGGAACCTTCTCAGCGGAGA
TTGCCATTGATGGGAAGATAGAGGTGACAGAAATGGCGGATCAGCGGAGGAGAAACAGAGCAGAATGTATAGAATGTGGCTGGCTCGCGGGAGAAGAATGATGGAGCGACGACGCTAA
GAGGCGGAGAAGAAAAGCTCTGGGAATTTTGGGGGCGATGAACAGTTGTGAAACTACGCGCGTCAAAATCTTTTAAGTAGGCAGAGAGCAGACGTCCGCATGGTAAGCCGTCGGGTTT
CGTTCGCATGCGAGTTTGCGATTTTCTCTCTCCCCACTATTCCATTGCCTCTCGCTTTCTCTCTCGTGGATTTGAATGACTGATTAATAGGCGACGTTCTC
>comp153395_c0_seq7
CCGAACTAGAAACCAAATCATTAAAACTTAATTATAATTAAACAATCACTGGGATCAAAGATTACAGAGTAAGATCTCTTAATACACCTACAGGAAACTATAATCAGCTTGTTTTTTCATGT
AACTATAGATGTTGGTACTTCATACAACGCACTGCTAAAAGCTGTATGGAAACTTAAAACTAATTAATTTTCTATATTCACAACTGTTGCATCTGTCTTCATCATATCAATTTCTACTGCAGCA
TTTATAACCTCCTTCCCTAACTCATGTTCTAATTTCTCAATCTTTGCACTCTTAGTAGAGCTTGCAATCTCTTCTGGATTTACAAACTTGACCCTTTGAATATAATCTTCAGCCTCTCTTTTTGT
AATTTCTTGTTTTTCGGTCTTATTGTCCTCAGCAATCCTTGATTCCTTCTCCAAAGTTGTCATCTCCTTTGAACGAGCAATTTCAATCGTATCACTACAATCTTGTATATCTCTTCTAACAACT
ATTTTCGGCAGATCAATCCCCCTTTCAGACTGTTTTGGCTCTAAAAATTTGAACCTCTCCTTATAAATTTGCATCTCTTTTTTGTATCTCTCCTTGTCTCTCCTTGCA
>comp153420_c0_seq1
TAAATAAAGGGCTATATATGAATACAACAAAGGGGGCATATTGGCCCCAAATAGCAAGTTGATTTTATTTTAGAAAAACAGTTGTTCCAAACCAACTATCTTTTGTTATTTTACGGCATTAAA
AACAGACAGCCATACAAAATAACCGACGCCTATAAAGCCCCACCTATTCCTTGCTTACTCCTTCAACATCTCCAAAACAAAGCTTCGAAATTCTATTCTCATGGCAACTTCTTCTCTGCTCT
CCTACTATACTCTTATCAATATCTTCTTCTTTTTGCTTCTAATAGACAAGAATTCTCTAGTATCAGCGCGACGACTTGCCGGAGAATACGATGTCATCGATGTCGAGTCATTGCTGCCGGGCA
AATCCTGCTCCATGTCTGATAAAGTCATGGGCCCGTCCATGATCATGCTGGTCCAACGATGCGGACCGTGTTCACCTCTGGCGTGTGAGGACAAGCTATCCCCTGAGGATATATTTCATCA
GATATTAAAACGTGACCAAGCTCGGATCGACTACATCCACAAGCTCCGGAAGCTCGCCACCAACTCCTCTAGCTTCAACCCACTTGGCAGATCTCTTTCCGCAACAGTTCCAGCAAAAGA
TGTTTTTGTTGGTAGCTAATTACATATTAATGATATTACTTTTGCTAATTACATATTAACGTTATGTTTAAAGATGTTACTTTTTCTAATTACAAATTAACATTTTATTATTTTATTATAAAGATTTG
ATGAAAATATCTTCATTAGTTTT
>comp153449_c0_seq1
TGACACAAAAAATACATGATGAAATATTACTATATTCTTCAAATAGTTGAATAATAATTCTATTAATAGATAAGATTCAACTCTTTCTCACATAACAACCCCTAAAAGCTATAAAACCAACAAT
AAAAATCAAACTCATAATCAATAACAAACATCAATTACGAAAGGGGTATTTACATTCAAGTCACTTTATTTCTTGTTATTTAAAAATCTAACGTCCGAAACATTAATATTACATGCCTATCATT
TATAAAATAAATATGAATGTATGGAATAAAATATTAAAACTTATAGAGATAGTAGGCATATAATATTAATATTTTAAATATTAGATATATAAATATCATAAAATAAAATGATATGCATATAAATCCC
CCATTACATAAACATTTCAACTACTTAATCCATCATCCTCACAATCACTCCCATGCAAACCACCATTGTTCAAACCGATCTCAGACAAGCAAAGCAAAAGCTGAACACAAACTTCTAACAC
AACATATCATCCCTTCTCCTCTGCTGAACAAATTAAACTAGAAGGTACCCGACAGCCCCTGCAACGGTCGCCGCGCCGCCCACAAAGCTCGCTCCAAGAAATGAAGCCGCGCTCTTGAA
CTTAGACTCAGTGTCAATGGCCGGCAGATATGCACTCGCCGGAGCGCCTATAGAAGCCGCATCGCTATCAGCAACTGTATCAGGAGAGGGGCTTTCGCCGGAGGGAACGGCGGCGTATG
GTGTAAGGAAGGAATTGGATGGGGAAGCCAATGGAGCATCGGAAGCTCTGATTATTGTGATTGGAAGGGCTAAGAGGAGAAAAAGTATGGCGAGTTTTGCCATTTTTGTTGATGGAATTG
GGGCCGCGAGCTGTTTTTTTGTAAAGAAGAGGAGGATTTATGGGGTGAA
>comp153473_c0_seq3
TATTGTATGGTTAATTTATTTGCACTGTTACATATGTTTATTAGTAGTTCGTTCATTACTTTTTAATTTGTGCCCAAAAACGTGGATTGCATCGACAACTTGTTGCGGCTCGAAAGCTAGGAAC

TTGGATTGCATCAACAACTTGTTGAGGTTCGGAAGTTAGGAAACTTATAATGGATCGACAACTTGTTGCAGCTTGGAAGCTAGGAAACTTGGAAAGGAACCACTTTTGGCGTCTTTCAAC
CTCAGAAACTCGGAATGCAACAACAATTTTGGGTGGCTTGGAAGCTTGGAAACTTGTAATGCATCTAGTGGCAGCCGCCTGATACAACACAATATTTAGCTTGAAGACAGTGGAGCATAC
ATGGATTTTGTTTTGTTGCTCATAATCAAGGAAACTAAGTGAAGTTTTATTTATTTCCTCCTTTCTATTGTATGGTTAATTT
>comp153488_c0_seq1
GAAAAGTGGTAATAATTGTTTACTTATATATTAATAGATTTATTAAAAAAAAAATATTAATATATAGATTCAAATAAAACTCAAACAATCGGAATTTATTTCAGTGAATAGCAAAATAATAATTA
GAAGATTGAAGACATCCGGTCAATGAAGATATTGAACGTCTCCTCACGTTCCAGCAAGCTGATCCTCACATACTTGCTGTCCGTCCCAAAATGCTTCCCGCTCCGAGTCAACATGCTGTGT
TCTGTCCTCAGGAAGTTCGCGGTGTCCTCAATGTCAAGATTTCCTTCGTACTTCAGCCATGCGAAAGCAGGCAAATGAGAGGTCTCCTCTCCCATGAAGCCACAATAAGCTGGGGGCGG
GTACTCCACCAAGCTGAACGCGCCATTCGCCGTGACAACTTGTCGGAGCCGGTTCCACCGTTCTGTCATCACCTTCCTTCCAAAATGGAAAAGTTTGTAACCGCCGCCCTCTTCGTAGCC
ATCCGCCACCGCCCTCAAAATCGTGGCCGCGCGAAGCTGAGAGTCCTTCGACACGCCGATGGTTCCAAGTTCCACGAACTGGACCATTCTCCTCGCCACCTCCTTATCTTTCACCAGCGC
CCAGCCCAGCCGGCTCCCGGCATGGCCTGTGCACTTGGAGACGCTGAAGAGCATTATATTGTGATCAGCCGCGTTTGTGATTGGCGTGTATTGGGGCCAGTAGTAGGCGAGGTCGTGGAT
ATTATTCGTTATTTGTTTGAAGACGGAGGCGTTCAGGCGGCCGTCGGGGTTGTTTGGCGAGCAGATGACTTCGATAAAAGGGTCTGTTGTATGAGGATCAAAGGAGTTAGCGTCACCGGC
CCATTTGTAGAGCGCGGATTGGAGGACGTCTGTAGCTGCCGGATAACCCTGTAGACGTTTTTCATTATGGAGATGCATAAGTTTACCATTCAATTGTTATAATTTAATTATTGGGGTTTTTATT
GATGTATTATTCTTAAAATGAAGTATTTTAATGTAATAGTATTTAATTTGTTTCTTATCAATATTGTTTATTATGTTTTGCTATTAAACACTTAAAAGTGGTTGGCTATGATGAATTGGCAAAATA
CATATGCTCCAACTCAA
>comp153488_c1_seq2
TTGCACTCTATATATAAGAAGCTGGGACGAGCTAGGAGAGATGCATGGTGCGGCGATTCGAACGTAGCAATTAGATTGGTAGTGAAAGGAATCGGCAGAGGCAGAGGCAGAGGAGGTAG
CTCGTGGCCTCGACGACACCGCAAGCAGTAGAATGGCGGATCCCGTCGTGCCTGTTGACGGACTTGTCGTTCCTGCCTCTAATGGCCTCCCCCCTCACTCCCAGGATTCCGTCATCAATCT
CGACCATGGGGATCCAATGATATATGAGAAGTTTTGGAAGGAAACAGGTGATGTGTCTTCATTTAAAATAGAAGGATGGGAAAACTTGAGCTACTTTTCCAACCGGAATAATCTCTGTTGG
TTTCTCCACCATGAGTTTGCTAGTCAAGTCCGTCGAATCCATAGACTCGTGGGCAATGCTTCTGTCGATGAAAATTGCCACATTGTTGTAGGTAATGGCTCCACACAACTATATCAAGCCGC
CATCTACGCTCTCTCCACTCATCCAAGCTTGGCTGAGACCTGCCGCCCCATACCCGTCGTCTCCGTTGTTCCTCATTACTCGGGTTATCCGGCAGCTACAGACGTCCTCCAATCCGCGCTCT
ACAAATGGGCCGGTGACGCTAACTCCTTTGATCCTCATACAACAGACCCTTTTATCGAAGTCATCTGCTCGCCAAACAACCCCGACGGCCGCCTGAACGCCTCCGTCTTCAAACAAATAA
CGAATAATATCCACGACCTCGCCTACTACTGGCCCCAATACACGCCAATCACAAACGCGGCTGATCACAATATAATGCTCTTCAGCGTCTCCAAGTGCACAGGCCATGCCGGGAGCCGGC
TGGGCTGGGCGCTGGTGAAAGATAAGGAGGTGGCGAGGAGAATGGTCCAGTTCGTGGAACTTGGAACCATCGGCGTGTCGAAGGACTCTCAGCTTCGCGCGGCCACGATTTTGAGGGC
GGTGGCGGATGGCTACGAAGAGGGCGGCGGTTACAAACTTTTCCATTTTGGAAGGAAGGTGATGACAGAACGGTGGAACCGGCTCCGACAAGTTGTCACGGCGAATGGCGCGTTCAGC
TTGGTGGAGTACCCGCCCCCAGCTTATTGTGGCTTCATGGGAGAGGAGACCTCTCATTTGCCTGCTTTCGCATGGCTGAAGTACGAAGGAAATCTTGACATTGAGGACACCGCGAACTTC
CTGAGGACAGAACACAGCATGTTGACTCGGAGCGGGAAGCATTTTGGGACGGACAGCAAGTATGTGAGGATCAGCTTGCTGGAACGTGAGGAGACGTTCAATATCTTCATTGACCGGAT
GTCTTCAATCTTCTAATTATTATTTTGCTATTCACTGAAATAAATTCCGATTGTTTGAGTTTTATTTGAATCTATATATTAATATTTTTTTTAATAAATCTATTAATATATAAGTAAACAATTATTA
CCACTTTTCTGGTATTTCTAAGATTTTAA
>comp153489_c0_seq2
TCGAATGGAGCAAACCAGCTACCCCTTACAATTATTTGGAGTTCGGCTGAAGACGTCCTAACAAGAGATAGGATTGGAATAAAGTACAAGAGAAAGTTGAAGTACAACATCATCTAACAA
AGCATCCAGTATTAGACATTCTAGGTCCAGCTCAACATCTTCAATATCATATTGGATATTTATCCATCCATCATTCTTCGTGAAATTTCGAGACACAATGTCTTCAAAATTTATGCCTGCCTCG

GGTTGGCCTTTCAGATGCCAGCAGATATTTGCCCATATCTCTTCTAAAATATGGTCACCCATAGGCATTGGTCTATTGTTAGTATTGAAACGAGATAGCCAGGGGAAAAAATAACCAAAATA
TTTCTCATATATTTCCACCCAAGTTTCATTTATCAGATCAAACAAAAGCTGGTGTTCCAACAAGATATCATGTTCATCTTCTGTAATGTCAAGTTCATGAGATAAATCTTCTCCTTCATTAAAG
ATTAAGGGATCCATTGGCTGATAGGGAGAGTTCCAAGCTCCAAGAAAACCACTGCGACTAAATCCAGATCTCGTCAGTATCTCTCTCACATAATTGAATGTAGCTTCATCCTTTTGGTCAAT
TCGAAGATGTAAAGAATCAAAGCAATCAATATCTGGGAACGGTTTTGCTTTTGCAAGATCTATTTCATTAGTGTCGACAAGACCATCAGCAGCATTGCTATCAAAAAGACTGGAGTATTCT
GATGGATATTTGTGTTCTAAACCTCGAGCAAGTGCCCCTGTTGCATATGCTAGGGTTGGTTTTGTTGGGTCATATGTGTGTGCTTCATCCAGTAGATTTTCTCCTTGTGGAGTGACAGAACT
GTTTTCTTCTCTTATCAAACCATCTTTCGTCAATTTTTTAAAAATTTGGCATGATTCACTTTGTTCTTTTTCATTACTTTCAAGTGGAAATTTCCAACAATCAGTCACAATCCATTTTGAACTA
TCTTGTGTGGTAAATTCCTCTGATTCTTCTTTTTCTGTTATTTCCTCTGTATGTGAAGTAGTTAAATTCATAGGAGATGAAAAGAGCGCTTCACTTTGAGCATTTTTTCTTGATGAACAATATG
TGATATCGGGAAGAGAGAATACTCTTCCTAAAGATTTTACTTCCCATCTTTCGGGAAGTCTTTCAAATTCTTCTTTTGATATAGAGTCATACAAATGGGAATACTTGTCTAAGGAGTCATTAA
GAGATTGAGATCTATGGATATTTTTCTTGACATTTTTGTTGATCTTGTTCCAACATACAGGCATCAAGCCTTCCCTGTCTGCCTTTTCTACAGTTAACCCATCCCTCCAACCGAATTTACTTTT
CTTCACGGGTTCTATCACCCTTCTTCCAAAAGGGATTTTATGAAGAATCCCATCCATAAAGATGCGATGGTGCTCTTTTTTGTGATGTTTGATGGCCATAAATCGACTAGAATTGGGAGGAT
TATTCCTCACATTTTGCAATATACTTGATGAAGCTAACACTGCAGAATGGTTATTGGACTCTACAAGTTTTTGATTTCTTTCCTCATCGCCATCCATCATTTCTGCATTTGTTGTATCATCTTGA
ATTATATCAGTATATACACGCGAGTTTTCTGAATGAAACTTATAACTCTGCTTGGCAAGAGATTCACTCTCAAAATTTTTATGATTGATTTGAAATTGGCCATCATTTTTCCTTACTAGCAACG
GTGATGATGGGAATGAATAACCCCTTGTTAACTCTGCTTTGGAAGCTTGTTTGGCTTGAAGATAATGGGAAAAAATAAAGCTCGGATCATCAAGAATATTTCGAATTAACTCTATGTTTCCA
TTAAATAGCTTCATCATAGCCAGAAATTCTTGTGATGGATACAAGATAACATCTCCTTGCAGCTCCTTCGCATTTGTATGTTGCTGTGCAAAAGAACCGACCTCAGCTTCATTCTGAGTATC
GCTAAGATGCGGTTGTTTTATCTGTAAAATACTTTCAAGTTCATCAGGTTTTCTTGGACCGAAGGGACCATTTTTACTATAAGCCATATTCTCATCACATTGTTTCTCAAAATTGTGCTCATC
AAAACAACATGCTCGCTCACTGCTTGAAGAAGCTTTATTCAAATCTACATTTTGCTTAATGAATGAAATTTCTAACCCAAAATTCACATTATCCACATGATCCTCACTCCAATGATGAATAG
AGGTAGTTCGAAACAACTTGGGAGCTGGCTGTGAAGCTTTTTGTTTCTTGTTATGCTCTCTTGATTTCCACTTGGCAATAGATATTCTCATTGATGACTTCAGTGGTTGTTTTCTGGTGCTG
GATTTGTCTTCCACCCCAAGGTAGGCATCTTCAGTACAAACATATGGCTGGCTAGTTGAAGGAGCATCTGGATCTGTTTTAGGCTCTTTGTTTCTAGCAGCACGCTTGCCCTCGCTATGCTT
CCTGTATGTCAGCAACTTCATCTTTCTGAGACGAAGATGGTGATGGAGATTTATAAAGTGAAACAGGCCTTTCATGCAACCCGGAAAAGACCGGCCGTAGCTCTCCTCTTCCATTTAATCC
TCGCAACCAGCAACAAACGAACAAATCGTTAGATAGTAAAGGAAGCGGGAGGTTAGGGTGACGAAATCTAGAGTCTTTGGAAAGGCCACCCCTTTTTTATTT
>comp153489_c0_seq3
TCGAATGGAGCAAACCAGCTACCCCTTACAATTATTTGGAGTTCGGCTGAAGACGTCCTAACAAGAGATAGGATTGGAATAAAGTACAAGAGAAAGTTGAAGTACAACATCATCTAACAA
AGCATCCAGTATTAGACATTCTAGGTCCAGCTCAACATCTTCAATATCATATTGGATATTTATCCATCCATCATTCTTCGTGAAATTTCGAGACACAATGTCTTCAAAATTTATGCCTGCCTCG
GGTTGGCCTTTCAGATGCCAGCAGATATTTGCCCATATCTCTTCTAAAATATGGTCACCCATAGGCATTGGTCTATTGTTAGTATTGAAACGAGATAGCCAGGGGAAAAAATAACCAAAATA
TTTCTCATATATTTCCACCCAAGTTTCATTTATCAGATCAAACAAAAGCTGGTGTTCCAACAAGATATCATGTTCATCTTCTGTAATGTCAAGTTCATGAGATAAATCTTCTCCTTCATTAAAG
ATTAAGGGATCCATTGGCTGATAGGGAGAGTTCCAAGCTCCAAGAAAACCACTGCGACTAAATCCAGATCTCGTCAGTATCTCTCTCACATAATTGAATGTAGCTTCATCCTTTTGGTCAAT
TCGAAGATGTAAAGAATCAAAGCAATCAATATCTGGGAACGGTTTTGCTTTTGCAAGATCTATTTCATTAGTGTCGACAAGACCATCAGCAGCATTGCTATCAAAAAGACTGGAGTATTCT
GATGGATATTTGTGTTCTAAACCCCAAGGTAGGCATCTTCAGTACAAACATATGGCTGGCTAGTTGAAGGAGCATCTGGATCTGTTTTAGGCTCTTTGTTTCTAGCAGCACGCTTGCCCTCG
CTATGCTTCCTGTATGTCAGCAACTTCATCTTTCTGAGACGAAGATGGTGATGGAGATTTATAAAGTGAAACAGGCCTTTCATGCAACCCGGAAAAGACCGGCCGTAGCTCTCCTCTTCCA
TTTAATCCTCGCAACCAGCAACAAACGAACAAATCGTTAGATAGTAAAGGAAGCGGGAGGTTAGGGTGACGAAATCTAGAGTCTTTGGAAAGGCCACCCCTTTTTTATTT
>comp153489_c0_seq4

GACATACAGGAAGCATAGCGAGGGCAAGCGTGCTGCTAGAAACAAAGAGCCTAAAACAGATCCAGATGCTCCTTCAACTAGCCAGCAATATGTTTGTATTGAAGATGCCTGCCTTGGGG
TGGAAGACAAAGCCAGCACCAGAAAACAACCAGCGAAGTCATCAATGAGAATATCTATTGCCAAATGGAAATCAAGAGAGCATAACAAGAAACGAAAAGCTTCACTGCCAGCTCCCAG
GTTGCTTCGAACTTCCTCTATTCATCATTGGAGTGAGGATCATGTGGATAATGTGAATTTTGGGTTAGAAATTTCATTCATTAAGCAAAATGTAGATTTGAATAAAGCTTCTTCAAGCAGTG
AGCGAGCATGTTGTTTTGATGAGCACAATTTTGAGAAACAATGTGATGAGAATATGGCTTATAGTAAAAATGGTCCCTTCGGTCCAAGAAAACCTGATGAACTTGAAAGTATTTTACAGAT
AAAACAACCGCATATTAGCAATACTCAGAATAAAGCTGAGGTCGGTTCTTTTGCACAGCAACATACAAATGCGAAGGAGCTGCAAGGAGATGTTATCTTGTATCCATCACAAGAATTTCTG
GCTATGATGAAGCTATTTAATGGAAACATAGAGTTAATTCGAAATATTCTTGATGATCCGAGCTTTATTTTTTCCCATTATCTTCAAGCCAAACAAGCTTCCAAAGCAGAGTTAACAAGGGG
TTATTCATTCCCATCATCACCGTTGCTAGTAAGGAAAAATGATGGCCAATTTCAAATCAATCATAAAAATTTTGAGAGTGAATCTCTTGCCAAGCAGAGTTATAAGTTTCATTCAGAAAACT
CGCGTGTATATACTGATATAATTCAAGATGATACAACAAATGCAGAAATGATGGATGGCGATGAGGAAAGAAATCAAAAACTTGTAGAGTCCAATAACCATTCTGCAGTGTTAGCTTCATC
AAGTATATTGCAAAATGTGAGGAATAATCCTCCCAATTCTAGTCGATTTATGGCCATCAAACATCACAAAAAAGAGCACCATCGCATCTTTATGGATGGGATTCTTCACGGGTTCTATCACC
CTTCTTCCAAAAGGGATTTTATGAAGAATCCCATCCATAAAGATGCGATGGTGCTCTTTTTTGTGATGTTTGATGGCCATAAATCGACTAGAATTGGGAGGATTATTCCTCACATTTTGCAAT
ATACTTGATGAAGCTAACACTGCAGAATGGTTATTGGACTCTACAAGTTTTTGATTTCTTTCCTCATCGCCATCCATCATTTCTGCATTTGTTGTATCATCTTGAATTATATCAGTATATACACG
CGAGTTTTCTGAATGAAACTTATAACTCTGCTTGGCAAGAGATTCACTCTCAAAATTTTTATGATTGATTTGAAATTGGCCATCATTTTTCCTTACTAGCAACGGTGATGATGGGAATGAATA
ACCCCTTGTTAACTCTGCTTTGGAAGCTTGTTTGGCTTGAAGATAATGGGAAAAAATAAAGCTCGGATCATCAAGAATATTTCGAATTAACTCTATGTTTCCATTAAATAGCTTCATCATAGC
CAGAAATTCTTGTGATGGATACAAGATAACATCTCCTTGCAGCTCCTTCGCATTTGTATGTTGCTGTGCAAAAGAACCGACCTCAGCTTCATTCTGAGTATCGCTAAGATGCGGTTGTTTTA
TCTGTAAAATACTTTCAAGTTCATCAGGTTTTCTTGGACCGAAGGGACCATTTTTACTATAAGCCATATTCTCATCACATTGTTTCTCAAAATTGTGCTCATCAAAACAACATGCTCGCTCAC
TGCTTGAAGAAGCTTTATTCAAATCTACATTTTGCTTAATGAATGAAATTTCTAACCCAAAATTCACATTATCCACATGATCCTCACTCCAATGATGAATAGAGGTAGTTCGAAACAACTTG
GGAGCTGGCTGTGAAGCTTTTTGTTTCTTGTTATGCTCTCTTGATTTCCACTTGGCAATAGATATTCTCATTGATGACTTCAGTGGTTGTTTTCTGGTGCTGGATTTGTCTTCCACCCCAAGG
TAGGCATCTTCAGTACAAACATATGGCTGGCTAGTTGAAGGAGCATCTGGATCTGTTTTAGGCTCTTTGTTTCTAGCAGCACGCTTGCCCTCGCTATGCTTCCTGTATGTCAGCAACTTCAT
CTTTCTGAGACGAAGATGGTGATGGAGATTTATAAAGTGAAACAGGCCTTTCATGCAACCCGGAAAAGACCGGCCGTAGCTCTCCTCTTCCATTTAATCCTCGCAACCAGCAACAAACGA
ACAAATCGTTAGATAGTAAAGGAAGCGGGAGGTTAGGGTGACGAAATCTAGAGTCTTTGGAAAGGCCACCCCTTTTTTATTT
>comp153515_c0_seq1
CAGGTCAGTTCCGGATTGGACCCGAACTCGACCCGCGACTCAATTTTAAGAGCCCTAAATAAAATGGTAAGACATACTAATGGCTAGTATAATAATAATGATAATAATAACAATATAATAAG
CATAGGATATCATTCAGGTCGTGTTGATACACATTTGACACAAATTTTATCACAAAGATCCGATAGAAGTCCTTTCTCACTGCCCATTATTATTCATGGAGTGCTAATTGACAATTAAAAAAA
AAGTAATATGGTCCATCAAACAGCAGATAGAAACATCCGTCCATGAAGAACTTGGTCTCCTCAAGCTCATCGTGAGTTAGCAGCGATAGGCGGGCTGCTCGCAGAGGCGCCGGCGAAGT
TCTGCTGCTCCACATCCCCACCTTGCTGCCAAGCCCAAACAATGTCCTGCAACGACGGCTTCAACCCCTTATCACAGAACTGCTGGAACTCAAGCGTATCCCCTGCGGACTTCTTCACCT
CAACAAAATGAAAAGACGGGGTCACCTCAAATATCTCCGCATCAATCGCCAACAGTCCCTTCCTCCCTTCCTTTCTCCCTTCTAATTTCACCTTCCCATCCTTCTTACTCACCCTAAAACTC
CCCGTCTGCGCAATCTCCTCCAACCTTGACACAATCGCAGCCGCCGGCTTCTGAGTCGTAAACCTCGCCTCCGCCGCAATTCCCCTCTTCCCACACCCTTCAAACAACCCCGACAAATCA
AACCCCTTTGACAACGATATGATATCAAATGCATTTAGACTGGTTGGTTTCATCGAGAAAGCCGCGTCCACGACACCCGCCGGGTCGTCGTTCGCTTCGACCGGCTTAAGCCCCTTCTTAA
ACCAGGGGGATTCGACGAGTTTAGGGATTGTGATGCGGGTATTAGGGTTAGGATCAAGGAGGCGGACAATTAGCTTGCGGGCATCGGGAGGGAACCAATTAGGGCATCGGAAGTCGGCT
TTTGACATTTTGCGGTACATTACCATGAGATTGGAGTCGTGGAAAGGGAGGAATCCGGCGAGGAGGACGAAGAGAATAATGCCGCACGACCAGATGTCGGCTTTGGGGCCATCGTAGCC
GCGTTTGTTGATGACTTCCGGGGCGACGTAGGCGGGGGTGCCGCAGGTGGTGTGGAGTAGGCCGTCCTGACGGCGATTGTCGACGAGGGCGCTGAGGCCGAAGTCGGTGATTTTGAGG

TCGCCATTTTCATCAAGGAGAAGGTTTTCGGGTTTTAGGTCGCGGTGGTAGACGCCGCGGCTGTGGCAGAAGTCGACGGCGCTGATTATTTGCTGAAAGTAGCGTCGAGCTGCGTCCTCC
TTGAGCCGGCCGCGGGCGATCTTGTTGAAGAGCTCGCCCCCACGAACATACTCCATGGCAAAATAAATTTTGGTCTTACTCGCCATCACCTCGTAGAGCTGCACAACATTGGGGTGTCGC
ACCAGTCTCATAACCGAGATTTCCCTCTTAATCTGCTCAATCATCCCCACCCTCAATACCTTCTCCTTTTCAATTACCTTAATAGCAACGCTCTGCCCCGTCGTCAGGCTCCGTGCAAAGTA
CACCTTCGCAAAGGTTCCCTGCCCGAGCAGCCTCCCCATCTCATACCGCCGCATCAATATCCAATCTCCCTTCTTCTCTGTCGTCATAGTTACCGGCTAATTCATCTGCAATGAAACTCACA
GAAGGGCTTTGCTGTTGAACAGTGAAGAGGAAGCATGAGATGGGTTTCGCATTGTCGTCGGGGAAGAGAGGAGGAATGCGGATGGAGAGGAAAGAGGAGTTAAGGTTAGGAATAGCG
GTTCATTTATGGCCAAGGAGTAAATGATGGTTGGGCACGTTCAAAGATAGTATGGATGCTATCTTTGAACGT
>comp153535_c0_seq1
ACAAATGAGTAATCTCTCCATATAATACATACATATGTGAAACATATAAATGTTCAGAAAACACCATTGTCATTCTTTAACAAACATTAATGTAATGATTCTATATATAATTCAAACAAATTTCT
CAGTTACTGATCATCAAATGAAACTAATGACTAACAAATGATTTAACAATATTAATAAAATATGCGTTGAATCTTCCCAATGTATCAGTTGCAGACATACTTAAGCACACCTGGTATGTGAAT
GTTGTAAAAGTACTCATCCCAATCTATATTCTTTGGATCAAAATCGAATATTTGACTATCATTGTGCTTCTTTGTTGCCATTCTCAATCTCTCCAAGTTAAAATCATCAAAACTGCATGAAATT
CAGGCTTTCAGTGAAGATATTATAATATATAAATTTGGAGATCGAAATTATCAATGAGGTCCTAATAATTTTACATAATTATATATGCATAATTTAGTTATAACTACAGGTCAAATAGAGCATAC
CATCCCTTGAAGAAGCAGTAAGGTATGTAGAGATCAATGAGAAGCATCAAAAATTTGTACTTTCTGCTAAGCTCGTTGTACTTCTTCGAAAATGGGCAACAAAATATGGCATTGACCAAAC
GCAACATCTGCAAGGGAAACAAATTATTGAAGATTACAATTTTCATCAGGGCTCTATAATTCAATAATAATAATTTATCATGTTCAAAGAACACTGACCTCTAATGGCAGCCAATATCGAATA
AACAAAAAAATACGAAAAATGGCCAATTTCTTGAATACAGGAACTTTTTGCAGGCTGATTTCCTTTCCATTCTTTCCAAATAATGGATTTTTGGAAAAATAACGGTACCCACATTGCTCTAA
AATTGAGTACTTGACAGGATTTCTTACAGATGAGCTTATGTGATAGATGAATTCTGCTTGATGATTTGAGTGAGCGACCACCATTGCTATCAAAGCATTGACAACCATATCTCCAGGAATCA
CATCCATTGGAGATTCAATGTCGGCAACAAAGCAAGGAAGGGTTCCTTTTGCATAACCAACAATTATGTTGTCAATTGTTCTATTTCCTTCAATCCAACCAGGAAAAGGCTCCTTCCATGT
ACTTGTTATAATTGTTGGGCGAGCTATAACAAGAGGCAAGCTTCCTCTCAACTGCCCAAGCAACATCTCACCCATTGCCTTTGTGAACACATATGTATTTGGCCACCCAAAATGTCGAGCC
CTCTCAATGCCCAATTCTTTCATGGCCACTTTCTCAACATCCATTGTTGAATTATCAAGTTGCAAGAGTTCTTTCTTCCTTCTCTCCACCAATTTTATCTCAGCATCTATGTCCAAATACATAT
TGCCTTTGAGTGTTTCACCCTTGCCAAAAGCCTTCTCTAATAGGAGTCCCTCTTTTTCTCCAGTAACATAGGCTGTTGAAACGTGGAGAAAAACTTCAAGTTTACTGCACTTTTTTGCAAA
ATTCATGGCATTCTTTGCACCTATAACGTTAATATTCAAAGCAATATCATACCTGACATAGAAATTGGTTGTTGCTGCTACATGGATGACCATGTTGATATCATTCCAAATTAATTCTCTCAATT
TTATGTCTTCTATTCCCAAATTCTCATGGATAATATCTCCTGGGATAGCACAAATCTTCTCTGTGATAAACGAATGGAATTCCTTTCCATGCTTCTTCCTCAGACAATCAAATAATTCTTTTTCT
ATCACCTCGTTTTGCAAGCGCTTCTCAGCGGACTTGGTATCACTTGCTCTCACAAGAAGGTAGAGCCTCTTCACCTTTGGCTGAACTCTAAGCACCTTCTCCACAAAGAGCTTTGCTAAG
AAGCCAGTTGAACCAGTAATGAGAATGCTCTTGTCTTCCAAGGCATCGACTATTCCCCCGAACTCCATTTCAGTAAGTAGAATTCACAACTGTTAGAGAGA
>comp153535_c0_seq10
ACAAATGAGTAATCTCTCCATATAATACATACATATGTGAAACATATAAATGTTCAGAAAACACCATTGTCATTCTTTAACAAACATTAATGTAATGATTCTATATATAATTCAAACAAATTTCT
CAGTTACTGATCATCAAATGAAACTAATGACTAACAAATGATTTAACAATATTAATAAAATATGCGTTGAATCTTCCCAATGTATCAGTTGCAGACATACTTAAGCACACCTGGTATGTGAAT
GTTGTAAAAGTACTCATCCCAATCTATATTCTTTGGATCAAAATCGAATATTTGACTATCATTGTGCTTCTTTGTTGCCATTCTCAATCTCTCCAAGTTAAAATCATCAAAACTGCATGAAATT
CAGGCTTTCAGTGAAGATATTATAATATATAAATTTGGAGATCGAAATTATCAATGAGGTCCTAATAATTTTACATAATTATATATGCATAATTTAGTTATAACTACAGGTCAAATAGAGCATAC
CATCCCTTGAAGAAGCAGTAAGGTATGTAGAGATCAATGAGAAGCATCAAAAATTTGTACTTTCTGCTAAGCTCGTTGTACTTCTTCGAAAATGGGCAACAAAATATGGCATTGACCAAAC
GCAACATCTCTAATGGCAGCCAATATCGAATAAACAAAAAAATACGAAAAATGGCCAATTTCTTGAATACAGGAACTTTTTGCAGGCTGATTTCCTTTCCATTCTTTCCAAATAATGGATTT
TTGGAAAAATAACGGTACCCACATTGCTCTAAAATTGAGTACTTGACAGGATTTCTTACAGATGAGCTTATGTGATAGATGAATTCTGCTTGATGATTTGAGTGAGCGACCACCATTGCTAT

CAAAGCATTGACAACCATATCTCCAGGAATCACATCCATTGGAGATTCAATGTCGGCAACAAAGCAAGGAAGGGTTCCTTTTGCATAACCAACAATTATGTTGTCAATTGTTCTATTTCCTT
CAATCCAACCAGGAAAAGGCTCCTTCCATGTACTTGTTATAATTGTTGGGCGAGCTATAACAAGAGGCAAGCTTCCTCTCAACTGCCCAAGCAACATCTCACCCATTGCCTTTGTGAACAC
ATATGTATTTGGCCACCCAAAATGTCGAGCCCTCTCAATGCCCAATTCTTTCATGGCCACTTTCTCAACATCCATTGTTGAATTATCAAGTTGCAAGAGTTCTTTCTTCCTTCTCTCCACCAA
TTTTATCTCAGCATCTATGTCCAAATACATATTGCCTTTGAGTGTTTCACCCTTGCCAAAAGCCTTCTCTAATAGGAGTCCCTCTTTTTCTCCAGTAACATAGGCTGTTGAAACGTGGAGAA
AAACTTCAAGTTTACTGCACTTTTTTGCAAAATTCATGGCATTCTTTGCACCTATAACGTTAATATTCAAAGCAATATCATACCTGACATAGAAATTGGTTGTTGCTGCTACATGGATGACCA
TGTTGATATCATTCCAAATTAATTCTCTCAATTTTATGTCTTCTATTCCCAAATTCTCATGGATAATATCTCCTGGGATAGCACAAATCTTCTCTGTGATAAACGAATGGAATTCCTTTCCATGC
TTCTTCCTCAGACAATCAAATAATTCTTTTTCTATCACCTCGTTTTGCAAGCGCTTCTCAGCGGACTTGGTATCACTTGCTCTCACAAGAAGGTAGAGCCTCTTCACCTTTGGCTGAACTCT
AAGCACCTTCTCCACAAAGAGCTTTGCTAAGAAGCCAGTTGAACCAGTAATGAGAATGCTCTTGTCTTCCAAGGCATCGACTATTCCCCCGAACTCCATTTCAGTAAGTAGAATTCACAA
CTGTTAGAGAGA
>comp153535_c0_seq13
ACAAATGAGTAATCTCTCCATATAATACATACATATGTGAAACATATAAATGTTCAGAAAACACCATTGTCATTCTTTAACAAACATTAATGTAATGATTCTATATATAATTCAAACAAATTTCT
CAGTTACTGATCATCAAATGAAACTAATGACTAACAAATGATTTAACAATATTAATAAAATATGCGTTGAATCTTCCCAATGTATCAGTTGCAGACATACTTAAGCACACCTGGTATGTGAAT
GTTGTAAAAGTACTCATCCCAATCTATATTCTTTGGATCAAAATCGAATATTTGACTATCATTGTGCTTCTTTGTTGCCATTCTCAATCTCTCCAAGTTAAAATCATCAAAACATCCCTTGAAG
AAGCAGTAAGGTATGTAGAGATCAATGAGAAGCATCAAAAATTTGTACTTTCTGCTAAGCTCGTTGTACTTCTTCGAAAATGGGCAACAAAATATGGCATTGACCAAACGCAACATCTCTA
ATGGCAGCCAATATCGAATAAACAAAAAAATACGAAAAATGGCCAATTTCTTGAATACAGGAACTTTTTGCAGGCTGATTTCCTTTCCATTCTTTCCAAATAATGGATTTTTGGAAAAATAA
CGGTACCCACATTGCTCTAAAATTGAGTACTTGACAGGATTTCTTACAGATGAGCTTATGTGATAGATGAATTCTGCTTGATGATTTGAGTGAGCGACCACCATTGCTATCAAAGCATTGAC
AACCATATCTCCAGGAATCACATCCATTGGAGATTCAATGTCGGCAACAAAGCAAGGAAGGGTTCCTTTTGCATAACCAACAATTATGTTGTCAATTGTTCTATTTCCTTCAATCCAACCAG
GAAAAGGCTCCTTCCATGTACTTGTTATAATTGTTGGGCGAGCTATAACAAGAGGCAAGCTTCCTCTCAACTGCCCAAGCAACATCTCACCCATTGCCTTTGTGAACACATATGTATTTGGC
CACCCAAAATGTCGAGCCCTCTCAATGCCCAATTCTTTCATGGCCACTTTCTCAACATCCATTGTTGAATTATCAAGTTGCAAGAGTTCTTTCTTCCTTCTCTCCACCAATTTTATCTCAGCA
TCTATGTCCAAATACATATTGCCTTTGAGTGTTTCACCCTTGCCAAAAGCCTTCTCTAATAGGAGTCCCTCTTTTTCTCCAGTAACATAGGCTGTTGAAACGTGGAGAAAAACTTCAAGTTT
ACTGCACTTTTTTGCAAAATTCATGGCATTCTTTGCACCTATAACGTTAATATTCAAAGCAATATCATACCTGACATAGAAATTGGTTGTTGCTGCTACATGGATGACCATGTTGATATCATTC
CAAATTAATTCTCTCAATTTTATGTCTTCTATTCCCAAATTCTCATGGATAATATCTCCTGGGATAGCACAAATCTTCTCTGTGATAAACGAATGGAATTCCTTTCCATGCTTCTTCCTCAGAC
AATCAAATAATTCTTTTTCTATCACCTCGTTTTGCAAGCGCTTCTCAGCGGACTTGGTATCACTTGCTCTCACAAGAAGGTAGAGCCTCTTCACCTTTGGCTGAACTCTAAGCACCTTCTCC
ACAAAGACTTGGAACCGGAAACAACATTAGAAAGGAAAAAATATTATTCATTCCATAAAAATTACACAATGAATAA
>comp153535_c0_seq6
ACAAATGAGTAATCTCTCCATATAATACATACATATGTGAAACATATAAATGTTCAGAAAACACCATTGTCATTCTTTAACAAACATTAATGTAATGATTCTATATATAATTCAAACAAATTTCT
CAGTTACTGATCATCAAATGAAACTAATGACTAACAAATGATTTAACAATATTAATAAAATATGCGTTGAATCTTCCCAATGTATCAGTTGCAGACATACTTAAGCACACCTGGTATGTGAAT
GTTGTAAAAGTACTCATCCCAATCTATATTCTTTGGATCAAAATCGAATATTTGACTATCATTGTGCTTCTTTGTTGCCATTCTCAATCTCTCCAAGTTAAAATCATCAAAACATCCCTTGAAG
AAGCAGTAAGGTATGTAGAGATCAATGAGAAGCATCAAAAATTTGTACTTTCTGCTAAGCTCGTTGTACTTCTTCGAAAATGGGCAACAAAATATGGCATTGACCAAACGCAACATCTGC
AAGGGAAACAAATTATTGAAGATTACAATTTTCATCAGGGCTCTATAATTCAATAATAATAATTTATCATGTTCAAAGAACACTGACCTCTAATGGCAGCCAATATCGAATAAACAAAAAAA
TACGAAAAATGGCCAATTTCTTGAATACAGGAACTTTTTGCAGGCTGATTTCCTTTCCATTCTTTCCAAATAATGGATTTTTGGAAAAATAACGGTACCCACATTGCTCTAAAATTGAGTAC

TTGACAGGATTTCTTACAGATGAGCTTATGTGATAGATGAATTCTGCTTGATGATTTGAGTGAGCGACCACCATTGCTATCAAAGCATTGACAACCATATCTCCAGGAATCACATCCATTGG
AGATTCAATGTCGGCAACAAAGCAAGGAAGGGTTCCTTTTGCATAACCAACAATTATGTTGTCAATTGTTCTATTTCCTTCAATCCAACCAGGAAAAGGCTCCTTCCATGTACTTGTTATAA
TTGTTGGGCGAGCTATAACAAGAGGCAAGCTTCCTCTCAACTGCCCAAGCAACATCTCACCCATTGCCTTTGTGAACACATATGTATTTGGCCACCCAAAATGTCGAGCCCTCTCAATGCC
CAATTCTTTCATGGCCACTTTCTCAACATCCATTGTTGAATTATCAAGTTGCAAGAGTTCTTTCTTCCTTCTCTCCACCAATTTTATCTCAGCATCTATGTCCAAATACATATTGCCTTTGAGT
GTTTCACCCTTGCCAAAAGCCTTCTCTAATAGGAGTCCCTCTTTTTCTCCAGTAACATAGGCTGTTGAAACGTGGAGAAAAACTTCAAGTTTACTGCACTTTTTTGCAAAATTCATGGCAT
TCTTTGCACCTATAACGTTAATATTCAAAGCAATATCATACCTGACATAGAAATTGGTTGTTGCTGCTACATGGATGACCATGTTGATATCATTCCAAATTAATTCTCTCAATTTTATGTCTTCT
ATTCCCAAATTCTCATGGATAATATCTCCTGGGATAGCACAAATCTTCTCTGTGATAAACGAATGGAATTCCTTTCCATGCTTCTTCCTCAGACAATCAAATAATTCTTTTTCTATCACCTCGT
TTTGCAAGCGCTTCTCAGCGGACTTGGTATCACTTGCTCTCACAAGAAGGTAGAGCCTCTTCACCTTTGGCTGAACTCTAAGCACCTTCTCCACAAAGACTTGGAACCGGAAACAACATT
AGAAAGGAAAAAATATTATTCATTCCATAAAAATTACACAATGAATAA
>comp153540_c2_seq13
CCCCACCTGATCCATTAACGCTTGGGAACCAATTTCATTCACTGGAACGTCCACCAACTTTCCCTGACCATCCGTCCTCACAGCAAGATTAGGAACCCGAAACGGTTCAGCGTGCTCTAC
AATCACAGTGCTAGACAGAATAGGCATGCCCGGATTCACATCAGAAATAACAACACCTCCATCAGCATTAGTATGACATCCCGAACTGGCGGTAACATCCACAGCAGGGGATCTATCCTTA
CTAATACAAACAACAACATCCACAGGAGACACAACAGGTTCCGAAATATTAACAAGAATAGGAGTATCAACATCAACCTCATTAATAATAGTATTAACAAGAGGAATATTAAGACCAGGTT
GAAACCCAACCACAGTATCACCAAAACCCGAAGCATCAACCTCAGCATCAGAAGGAGATAACAGATTCACACC
>comp153540_c2_seq5
ATGATTGGTTGGTCATGGTAGGCCGATCTTAGGGCTCATGTGGATAGGTGCTTTTCTATAGTTGGACTGGAATATGTAAAGGTGAAGTCGCTCGCCCGGATGAAAGCGATTAGAAGTGATG
GCCAACCTCCCTTTTTTTTTGTCAAAACATTATAATATATTATAAAAGACAAACAACGGTTACAACAGCTAAAGCCAGGGAAGAAAAAAATCAAAAAGATAAAACCTCTCAAAACAAAGG
AAAAACCCCGGAAACAAAATGGGGGCTCAGTAGATGACCGAGGCTACCAACAACCTCGAAACCGCACAAGGACCATCCAGTAAAGGTTGCCACAAGGAGGCGACAAGGAGAGGAGC
CCTGGTCCCATCATTGCCCTGCAACCAACAACAGAAAAAACCAAATCATCTTATTCTACCACCTCTACCCCAAAATTTTCCACGCGAAGTTCAAGATAAAGGTCTAACACGAACCATACTA
AATTCATTTCCTGGATCAGAATAGGAATCAGAATCAGAATCGCATTCCGACGAATTATTCAGT
>comp153548_c0_seq3
GGTCGTCTAAACATAAATGATATGTTATAAAAATTAACGACAAAAATGTTCATTTTTTAGCTCCACCGACGAAGATTCTGATATATCTCGTTTCCTTCACACCTTTCCCTGAAAATCACCGAG
CCGATGTCCATTATCAGCTTCGGCGAGCACGGCGGCGAGCCGATCGCCGGCATCCACCAGAAGCTCCTTACACAATTCGAAGCCGTCGTTGTCAATGGGGCCCTTAGCCAGCTTATGAAG
CTCGTTAGCTTTTCATTCGCAGCTTCGACGTGAGTTGTGATCACCTTAACGGCGGCGCGGATAATATCGCCGGCGGAAGATGTGTCGTTGATGGCGCCGGTGAGGGTGCGCTCACATGCAT
CTTTGTAGGATGTCGGTGCGCAGAGAACGGAGACAGAGGACTTAACGGTGGTGGCGAGTTGGCTGGAAGCAGTAGGCTTGTTGAAGGATCGGGAGGAGATGGCGATGACGCTGATGAC
GGCGGCGACGACCAGGATGGCGGAGAAGCCGCCGATGGCAGCTTTGTTTACCATTTTTGTTGGGGGTGGAGACCGATGAAGGATTGTGGGGGAAGGGATGGATTGGTTATTTATTGGGC
TTGTTAGAGGCTGTCAACGGTTAG
>comp153557_c0_seq6
TTCTCTCTCTTTGGCTTTCTCTCTCTTTCTCTCTCTCCCATCTTCTTCCTCTAACGTACCTATGGACTATGAAAGGATTCACAAAGTTCAGATGGGCCTTATTTCTCCCAGCAAGTTACGCAT
GAAAATACTTGGAGCTCAATATATGAAAAGGAAGGAAGAAGGGTGTTCCTCAAGAGTTTCCCCTTCCAAGCTTGAGGACATAGAGTATGCTAAGAAAAATTTACTGGCTGGCGATCTTGA
TGATGAAGTTGTTGGCTTTAAGGACTCGCCGGTTTCATCGATTAATGCTAAGTCTCAAAAATCAGAGTTCTGTAACAAAGAACTCTCACGCTATGGTGAAGGCTTTCAAACTCAGTCTTCT

ACTGCAAATTCCCTTTCTAAAGGAAATTCTGAGACAAAATATTCAAGAAACCAGAATAATAGTTCTAATTCTTCTTATCAATCCAAACTTGGAAACAACTACTGCACGGTGCACCCTATGA
AGCATCAAGAAGTGGATGATGATGGTTATGACAGTGGGCATGAGAGTGCTAGCAATTCTAACTTCGAGTTCCATCGTAGAGGAAAAGTTTCCCAACTTCCAATTATGGGTCATTTCTCTAG
GCATATTCCATCAAAATGGAATGATGCAGAAAAATGGCTTGTTAATAAGCAAGTGGTTCATGAAAATGGATTAAAGAACAATTCAGTGCAGTATCAGTGCAATAGGTTGGTGAATTCCAGT
TTGGCAAGGATTGCTCCAGAATCCATTAATATAGATCAGAAACCTTCTGTAATTCAGCCTTCAGCCAGCAATTCTAATTCTCAAAATATGGTGGACAAGTTCTCATTTGTTTCTCAGGGCTT
GCATGCTAATTCAGCTTCATTGAATGATGAAAGTTGCGTGACAGATCCTTCTGAGGGTATAAATTATAAAAATTCATCAGTCTCTGAGGGTTTTCCTTCTGGAACTACAGACATTTCTGCCA
AACAGCTGGTTTCAATGAGAGATGTAGGCACAGAGATGACCCCGATTCCTAGCCAAGAGCCATCAAGAACAGCAACCCCTATTGAAAATTTAACACCGAGCCGCAGCCCATTATCTTCAA
ACCCCTCATCTCCGCGAATTGGATCAGTAGCAGTGGATAGAATGCCGTCCACTGATGAGAATGAGAAGGATTACCAGAAAGATGGTAAAAAGGTAGAATTTTCTGAAAGAGAATTGCAG
CTGAAAACTAGACAGGAAATTGCAGCTCTAGGCTTACAGCTTGGTAAACTGAACATAGCCTCTTGGGCTAGCAAGGAGACAGAGCAAAATTCTCAATTTAATAAAAAAACTGACGAGGA
AGAACAAGTGAAACAGGAATATGAAGCTTGTGCTGCAGCTTGGGAAGAAGCTGAGAAATCCAAGCACTTGTCCAGATATAAACGAGAAGAAATCAAGATTCTAGTATGGGAAAGCCAC
CAAAGAGCAAAATATGAGGCTAGTTTGAGAAAAGTAGAGGTAACACCCCCATTTTTATCATTCATGGTGTTTAATTCATATAGATGACATTAAATACGTTGCAGCATGCAAAGTTTTCTATT
TTTGATTGAAGGGAAGATGAAGAAAGAACCATATTCGGTTCAAATTTAGCTTTTTTTGTCCTCTCCCCTTTCAATTTTGTAAGCCAAAAGCTCTGCTAAATCCATGCACTTCATAAGTGATT
GTAAGCCTCATAATTGGGCCAATTGCCTATTCTTTTCAGTAGCTCAAATCATATAACCAATAAAGTGATCCTAGCGGCAGACCAAGCTTTACTTTGTTTCTTTCACTAACCACCATCTTCTTG
ATAAACTATCAGATCCGAGCAGTAAATATGAGATCTCGCGCAGAGGCAGCCATGGCAGAGAAGCTCCGTCGAGCAGAGCGGAAGGCGGCGGAGAAACGAGCCAATGCCGAAGCCATAA
TGAACTTGCAGACTGCAAGGATATCTGATCAAGCAGACTCGATACGTCGATCCGGCCGAGTTCCTTCTCATTTTAGATGCTGCAATTGGTTTCTTTAAAGCTCAATAGAGAATTTCAGATTC
AGATTTAAGTGTGGTGTGGAATTTTCACCCTTACAGAAGACCAAGCTAGAATGCTCATTGCTCAATTCTTCTTCTTATGTTGCTAGATTCACCCACTTCATAGCTTCACCTTTTGATCCTAAT
GTAACCATCTTACGTTTGAGACGGCTTTCTTACTGT
>comp153568_c0_seq1
CAATGGAGGTTACACCATTGAGGTGGAGATCCACGATGGGCCTTACAATGTGATCAAAAACTGGAACTACACTGCATTGGTGGATGCCATCCACAATGGGGAAGGCAAGTGCTGGACTA
CAAAGGTGCATTGCGAGGAGAAGCTTATTGAGGCAATTGAGACTGCTACGAACGAGAAGGAGGACTGCTTGTGCTTCATTGAGGTTATCGTGCATAAGGATGACACCAGTAAAGAGCTT
CTGGAGTGGGGTTCTAGGGTTTCTGCAGCTAACGCCAGGCCACCGAATCCACAGTAAACGCAATCTTAGTTGGATTGTAGCTTTTTATAATTTATTTCATTTTACATTATTTTATTCATTTTTA
TTCTTTATTGATATTATAAATATCAATATAGAATAAATACGGATCTTTTCGTTTAAAGTATTATCAGACACTCTCATCTAATCATCATCCCCTAAGTAGAGAAATTTTTCCTATCAAGGTACATTT
TGACGAGTTCCTCCATGATTGAATCTTCTCTTTTCTTCTCTGTTTCATTTTCACTATTGCAAGTATTTGCTGTAAAAACATTGGAGTAATATGCAGATCCTTGAAGTTATTTCTGCTAGTAGAA
ATAACTTCAAGGATCTGCATATTACTCCAATGTTTTTACAGCAAATACTTGCAATAGTGAAAATGAAACAGAGAAGAAAAGAGAAGATTCAATCATGGAGGAACTCGTCAAAATGTACCT
TGATAGGAAAAATTTCTCTACTTAGGGGATGATGATTAGATGAGAGTGTCTGATAATACTTTAAACGAAAAGATCCGTATTTATTCTATATTGATATTTATAATATCAATAAAGAATAAAAATG
AATAAAATAATGTAAAATGAAATAAATTATAAAAAGCTACAATCCAACTAAGATTGCGTTTACTGTGGATTCGGTGGCCTGGCGTTAGCTGCAGAAACCCTAGAACCCCACTCCAGAAGCT
CTTTACTGGTGTCATCCTTATGCACGATAACCTCAATGAAGCACAAGCAGTCCTCCTTCTCGTTCGTAGCAGTCTCAATTGCCTCAATAAGCTTCTCCTCGCAATGCACCTTTGTAGTCCAG
CACTTGCCTTCCCCATTGTGGATGGCATCCACCAATGCAGTGTAGTTCCAGTTTTTGATCACATTGTAAGGCCCATCGTGGATCTCCACCTCAATGGTGTAACCTCCATTGTTGATGAGGAA
GATGATACTGTTCTGCCCTTGCCTAATCATCGTTGACACGTCCTGCGCTGTCACCTGAAAGCTTCCATCACCAATGAAGGCGAGGACGCGCTTATCCTTGGCAGCCTGAGCATATCCAAGT
GTTGCTCCGACCGACCAGCCAATAGAGCCATACTGCATTTGGAACTCATAGCCGCAACCTTCCGGCAAGTTGAGCTTCTGGCAGTTGAACCAGGAGTCGCCGGTCTCTGCGATCACCGC
AGTCCTACTTGAAAGCAAATTCTGTATGTGATTGAAGAAGACATTTACCCGCAGAGGTTCCTTGGGATTGGATTCTAGAAGATGTCCATTCGGCACGAAGATGCGGCTGTAATTCTCATAA
GCAGTTGTGTTTTTCTTCAACCTCTTCGATAGCGCCTGGAGGAAATCCTTCATAAGAATGCACCCGAAAGTCTGGCCATTGGCGACCACCACACGGTCTGGTTGGACGATTATGGCCTTCT

CTTTCTTGAGAAGGAGGGAGTAGCCGACGGAGCTGTAATCGTTGAAGATTGGTCCGACGAAGAGATAGGCGTCGGCGGATTCGACGATCTCTGCGCAGAAGGTGGTGCTTACGGCGCCC
CAGTAGGTGCCGATGAACCTGTAGAGTTGTTCGGGAACCAATCCTTTTGCCGAAGGCATCACGGCCACGGGGTACCCACATGCATCAGCAAGCTCCACGAAGGCTTTCCCGGCCTTCGC
CACCCTTAGCTTTGGCCCGCCCACCAGCACTGGTTTCACCGCCTTGTTCAGGAAGTCCGCGGCGGCCTTCACTGCGCACTCGAGACCCATTTTATTGCTCAATCTGTACAAATGGTTTCGC
TAACCTTATATATATAGCATAATTGCGGTACCTTAAATAGTTCGATTAAATACCGTTCTCTAAAAGAGATTTTTTAAACACAAAAGTGTAATCTAATTTTTTAAAAGCTTATTATGTGTTCCGTC
TTTTTCTAGCTATAACATATGGGCATCTTCAACCAAAAAATCTATTTTCAACTTTAAAGTACAATAACAATCATATTTTTAGTATTAATAAAATTTTGAGCTCACGGAAAAAATTTGGAATGAAT
GAATCATTAATTCGTGCTCCTTAATAGAACAGGGAAACGTAGGTGGAGAAGGAACAATTCAGAACCTCGGAGTTAGGAAGAATGGAACTGGCTCATGGCAGAAGGTTGGATGGGGAATG
GCTGGAAGATTACATGCAACGCTGATGTAAACTGGCTTGCTCTCCTTGAGAGCAGTTGATATGGCGGTGTCGATCAGCTCATGAGCGTCATCCAAATTATTCACAACCGCCTGAAAGCAG
GTGACGGTCCGAAAGCATTGAAGCTCTTGGGAGAAATCGGGGAGGCCGATGGTGTGATGCAAGATTCGGTTGGTGCCATAATCATTGGAATTCGGACCACCGACAATGCAGATGACGGG
AAGATTCTCGCTGTAGGCTCCTGCGATGGCGTTGAGGATGCTGAGCCCACCGACGGTAAAGGTGACGACGCACGCTCCGACGCCGTTCGCGCGGGCATACCCATCGGCTGCGTACCCTG
CGTTGAGCTCGTTGCAGCATCCTACATTGGTGAGTCCGGGCTCGGCGATGAGATGGTCAAGAAGCGTGAGATTGAAGTCCCCCGGGACGGAGAAGATGTCGCGGATGCCGATCTGGACG
AGGCGGCGGGCGAGGTGGCGGCCGAGGGTGGCCTTCCCTGCCGGCGCCGCTGGTATCACCGTGTTGCAGGACATCGGAATGCTCCCAACACCGCCGTTTGCTGCTTTCGACGGTACGTC
TACGGATCCTACCTTGGTTTCCATGGGAGGAGTTGAGGGCAGAGAGAAGATTTCGTAGTTTG
>comp153568_c0_seq2
CAATGGAGGTTACACCATTGAGGTGGAGATCCACGATGGGCCTTACAATGTGATCAAAAACTGGAACTACACTGCATTGGTGGATGCCATCCACAATGGGGAAGGCAAGTGCTGGACTA
CAAAGGTGCATTGCGAGGAGAAGCTTATTGAGGCAATTGAGACTGCTACGAACGAGAAGGAGGACTGCTTGTGCTTCATTGAGGTTATCGTGCATAAGGATGACACCAGTAAAGAGCTT
CTGGAGTGGGGTTCTAGGGTTTCTGCAGCTAACGCCAGGCCACCGAATCCACAGTAAACGCAATCTTAGTTGGATTGTAGCTTTTTATAATTTATTTCATTTTACATTATTTTATTCATTTTTA
TTCTTTATTGATATTATAAATATCAATATAGAATAAATACGGATCTTTTCGTTTAAAGTATTATCAGACACTCTCATCTAATCATCATCCCCTAAGTAGAGAAATTTTTCCTATCAAGGTACATTT
TGACGAGTTCCTCCATGATTGAATCTTCTCTTTTCTTCTCTGTTTCATTTTCACTATTGCAAGTATTTGCTGTAAAAACATTGGAGTAATATGCAGATCCTTGAAGTTATTTCTGCTAGTAGAA
ATAACTTCAAGGATCTGCATATTACTCCAATGTTTTTACAGCAAATACTTGCAATAGTGAAAATGAAACAGAGAAGAAAAGAGAAGATTCAATCATGGAGGAACTCGTCAAAATGTACCT
TGATAGGAAAAATTTCTCTACTTAGGGGATGATGATTAGATGAGAGTGTCTGATAATACTTTAAACGAAAAGATCCGTATTTATTCTATATTGATATTTATAATATCAATAAAGAATAAAAATG
AATAAAATAATGTAAAATGAAATAAATTATAAAAAGCTACAATCCAACTAAGATTGCGTTTACTGTGGATTCGGTGGCCTGGCGTTAGCTGCAGAAACCCTAGAACCCCACTCCAGAAGCT
CTTTACTGGTGTCATCCTTATGCACGATAACCTCAATGAAGCACAAGCAGTCCTCCTTCTCGTTCGTAGCAGTCTCAATTGCCTCAATAAGCTTCTCCTCGCAATGCACCTTTGTAGTCCAG
CACTTGCCTTCCCCATTGTGGATGGCATCCACCAATGCAGTGTAGTTCCAGTTTTTGATCACATTGTAAGGCCCATCGTGGATCTCCACCTCAATGGTGTAACCTCCATTGTTGATGAGGAA
GATGATACTGTTCTGCCCTTGCCTAATCATCGTTGACACGTCCTGCGCTGTCACCTGAAAGCTTCCATCACCAATGAAGGCGAGGACGCGCTTATCCTTGGCAGCCTGAGCATATCCAAGT
GTTGCTCCGACCGACCAGCCAATAGAGCCATACTGCATTTGGAACTCATAGCCGCAACCTTCCGGCAAGTTGAGCTTCTGGCAGTTGAACCAGGAGTCGCCGGTCTCTGCGATCACCGC
AGTCCTACTTGAAAGCAAATTCTGTATGTGATTGAAGAAGACATTTACCCGCAGAGGTTCCTTGGGATTGGATTCTAGAAGATGTCCATTCGGCACGAAGATGCGGCTGTAATTCTCATAA
GCAGTTGTGTTTTTCTTCAACCTCTTCGATAGCGCCTGGAGGAAATCCTTCATAAGAATGCACCCGAAAGTCTGGCCATTGGCGACCACCACACGGTCTGGTTGGACGATTATGGCCTTCT
CTTTCTTGAGAAGGAGGGAGTAGCCGACGGAGCTGTAATCGTTGAAGATTGGTCCGACGAAGAGATAGGCGTCGGCGGATTCGACGATCTCTGCGCAGAAGGTGGTGCTTACGGCGCCC
CAGTAGGTGCCGATGAACCTGTAGAGTTGTTCGGGAACCAATCCTTTTGCCGAAGGCATCACGGCCACGGGGTACCCACATGCATCAGCAAGCTCCACGAAGGCTTTCCCGGCCTTCGC
CACCCTTAGCTTTGGCCCGCCCACCAGCACTGGTTTCACCGCCTTGTTCAGGAAGTCCGCGGCGGCCTTCACTGCGCACTCGAGACCCATTTTATTGCTCAATCTCGGAGTTAGGAAGAA
TGGAACTGGCTCATGGCAGAAGGTTGGATGGGGAATGGCTGGAAGATTACATGCAACGCTGATGTAAACTGGCTTGCTCTCCTTGAGAGCAGTTGATATGGCGGTGTCGATCAGCTCATG

AGCGTCATCCAAATTATTCACAACCGCCTGAAAGCAGGTGACGGTCCGAAAGCATTGAAGCTCTTGGGAGAAATCGGGGAGGCCGATGGTGTGATGCAAGATTCGGTTGGTGCCATAAT
CATTGGAATTCGGACCACCGACAATGCAGATGACGGGAAGATTCTCGCTGTAGGCTCCTGCGATGGCGTTGAGGATGCTGAGCCCACCGACGGTAAAGGTGACGACGCACGCTCCGACG
CCGTTCGCGCGGGCATACCCATCGGCTGCGTACCCTGCGTTGAGCTCGTTGCAGCATCCTACATTGGTGAGTCCGGGCTCGGCGATGAGATGGTCAAGAAGCGTGAGATTGAAGTCCCCC
GGGACGGAGAAGATGTCGCGGATGCCGATCTGGACGAGGCGGCGGGCGAGGTGGCGGCCGAGGGTGGCCTTCCCTGCCGGCGCCGCTGGTATCACCGTGTTGCAGGACATCGGAATGC
TCCCAACACCGCCGTTTGCTGCTTTCGACGGTACGTCTACGGATCCTACCTTGGTTTCCATGGGAGGAGTTGAGGGCAGAGAGAAGATTTCGTAGTTTG
>comp153587_c0_seq1
TTTTTCTACTGTTTTTTCTATTTTCTTATTTATTTCTCGTTGAGTGTTTTTTTTTCCAACACAAAATAAGAAATTATGGTAGGTTAAACATATAAAGGCTTTTTAATATTTATATAATTTTAATTAT
TGAGACTGTGCTCCATGGACAGCCATGAAGCCATGAAAATTACAAGTTAATAATAAAAATATAAATCACAGGCTTATCTCTTTATTTTCCCCAAATTCCCAAGAAAAAAAAAAACAAAATT
ACAAAGCTTCATAAGAAAAAAATAAAAATAAAAACCACCCTGCAAGTAAAGCCGACTCCCCACCTAGAAAAAGAACAGACTAACTACTCTTCTCCGTCTAGCCTGAAGACGACGGCGG
AAACTCGTCGGAATCCTCCCATCTCGTCGCCGGGAAACTCGTCCCCCAAATTCCCGACTGCCCCAAATACCCCATCCTCTGCTGCTCTCCTCCTCCCAATCCATAAAACGTATTCAAAACA
GAGGAAGACGATGTTGAAGACGACGACGACGTTGAAGAAGAAGAAAGAGGGGAAGCTGAATTCAAAATATTCTCATTAAGCAAGTTAGCATATTGTCCCTCTCCTCTCAACAATCTTGA
ATACTCCAAGTAATCCCTCGACGCAGTGGAAACAGGGGAGCCAAGAAACTGGCCGCTTGAAACTATCTCCGGCGGCTGGAGACGCGCGTTTTCCGGGAAGTTGAGCTTCGCACGGCTG
CCACGAAAGCGAAGAGCGGCTTCATCGTAAGCTCGTGCGGCGGATTCGGCAGTATCGAAGGTTCCAAGCCAAACGCGAGCAGCTTTATGCGGGTCGCGGATTTCCGCCGCCCATTTTCC
CCATGGGCGCTGCCGTACTCCCCTGTATCGCCGGCGAGGTTGGTTTTCAGTTTCTACTTGGGAAGGAGTTGTTTCTTCCATTGACGGAGCTTGCGAGGAGGATGCGGAGTGAGAGATGGA
GGCTGGTGCTGTAATTGATGGAAGACTATTTGTTAAAGGTTCAATTTTTGAGCGTTTGATTCTTGAAAAAAACGATTTTGAAATGCAAGTCATGCAAAAAAGAAGTCCGATAATGAGAAA
TGCACGTTCAAATATTCTGAGAAAAAAAATTATTTTTTATGTATAAAAATCATTATTAAAATTTTTATTTCTTTGTCTTATTGAAGGAAAGAAAGATAGGATTTTGGCATTCTCATAATATCAG
AGTCTATTGAAGACAAAAAAAAAAGCTCTCTTTTTTGTTTACTGTGCTGCTTGCAGACATGAAATAAAGAAGTTTGTTGA
>comp153587_c0_seq4
TTTTTCTACTGTTTTTTCTATTTTCTTATTTATTTCTCGTTGAGTGTTTTTTTTTCCAACACAAAATAAGAAATTATGGTAGGTTAAACATATAAAGGCTTTTTAATATTTATATAATTTTAATTAT
TGAGACTGTGCTCCATGGACAGCCATGAAGCCATGAAAATTACAAGTTAATAATAAAAATATAAATCACAGGCTTATCTCTTTATTTTCCCCAAATTCCCAAGAAAAAAAAAAACAAAATT
ACAAAGCTTCATAAGAAAAAAATAAAAATAAAAACCACCCTGCAAGTAAAGCCGACTCCCCACCTAGAAAAAGAACAGACTAACTACTCTTCTCCGTCTAGCCTGAAGACGACGGCGG
AAACTCGTCGGAATCCTCCCATCTCGTCGCCGGGAAACTCGTCCCCCAAATTCCCGACTGCCCCAAATACCCCATCCTCTGCTGCTCTCCTCCTCCCAATCCATAAAACGTATTCAAAACA
GAGGAAGACGATGTTGAAGACGACGACGACGTTGAAGAAGAAGAAAGAGGGGAAGCTGAATTCAAAATATTCTCATTAAGCAAGTTAGCATATTGTCCCTCTCCTCTCAACAATCTTGA
ATACTCCAAGTAATCCCTCGACGCAGTGGAAACAGGGGAGCCAAGAAACTGGCCGCTTGAAACTATCTCCGGCGGCTGGAGACGCGCGTTTTCCGGGAAGTTGAGCTTCGCACGGCTG
CCACGAAAGCGAAGAGCGGCTTCATCGTAAGCTCGTGCGGCGGATTCGGCAGTATCGAAGGTTCCAAGCCAAACGCGAGCAGCTTTATGCGGGTCGCGGATTTCCGCCGCCCATTTTCC
CCATGGGCGCTGCCGTACTCCCCTGTATCGCCGGCGAGGTTGGTTTTCAGTTTCTACTTGGGAAGGAGTTGTTTCTTCCATTGACGGAGCTTGCGAGGAGGATGCGGAGTGAGAGATGGA
GGCTGGTGCTCCGGAATAGTATCCTATCACTGACTCCTCGTCTCTCTCTCTTCACTCCGCCACCGCCAACACCCGCACCAAACATAGAATGCGGGGATAACAGAGAAGCATTATACAAAG
AAGACGGTGGTGCTGACTTTGAACCAGCAGAATCCAAATAGCCGCCACCCATCGTCGGCTCTTCGCCGGCGACAACTCGTGTTAAAGCCGAAACCATCGCAGACATCTCTTCCGCCCTC
CACCGACCCGAGAACATTGTTGCTGCTGTCGCCAACTCTTCCTCCTCCCCTTCCGTTGCCGGCGAAAATTCCGGTGGATTCGCCACTTTAAAGCACATTAATAAGAGGATAAGCCGAATTA
ATAACGTAAAATAACTGTTTCCAATTATCTGAATTTGTCTCAATATTTGAAACTTTTTCAAGAAAGAGAAGAGTGGGGATGGCTTTTATAGAAGAAGAAGATGAGTGGCAAAGGCCAATGC
CAACGTGAAGGTCAC

>comp153594_c1_seq1
TGGTTCCTATACTCCTTTCAATCTCAGGAAGCAATGGAGGAATTCTCAATGGAGGTCCTTGGTACATGGGAGGTTTTGTCATTGGACTTGATAAATGGACTCCCAATTTTTCTCCTTATTCCT
TAGATGGGATTTCTTCTCCTATTTGGGTTCGTCTTCCCACTTGCCGTTACATTGCTGGGACAAAACCAACATCACGAGAATTATTTTGAGTATAGGGACTCCATTATTATTGGATGGCAACAT
GTTTATATGCGGGAGGAGGGAGTTCGCTAGAGCATGTGTTCAGGTTAAATTGAACTTGACACTCCCTAGTGGTATTTGGGATGAAGGTATTC
>comp153662_c7_seq11
TAGGAGAAGAGGCGGGCTGTGCGAACTGCAAGGAGGAGATGAGGATTGCTCGGGAGTTGGAGTTCCTTGCCTAGACTTCACGCAGGAGAGGGAGAGAGAGCAGTTGTGCGTAACTGT
TGGGTGGTTTTGCGAAATAGGGCTGGGAGATCGTGGGATGGAGAGAGAGAGAGGGGCTGTGTGAGAGGGTTTTCCTGTGTGTGAATCGGAGATGAGATGAAAGTTTTTTGGGGAGTTG
GAGTTCGGTTCTGCAGAGATGG
>comp153673_c1_seq3
CGGGATACCTAACCCTCCATAGCATTTCAGCTTACACACATCTTTCCAAGCTATAAGATGCATATTCTTTGAGTGAATATTTCCATGAAAGAGAAACTTATAACAACCTTTCCTAATAAACTT
GAAACAATATTTAGGCAGAATCATACCTCTAATCCAGTAAGCAATATAATTAGTAATAGAAAACTCGAGAAATTGCACTCTACCCGCAAATGAGAGCATTTTAGCCTTCCAACCGGAAAAT
AAATCAGTAACTTTATGAAAAAGAGGAGAAAAATCAGCAACACTTAGTCTCATAATAGAGATAGGAATACCCAAATAAGTGGTACAATCCTGAGAGTAAGGAAAGTTCAGAACATTACA
AATAACATCTCTTTCCAGTGTATCTTTTGAGAAGATAACCATACTTTTATGCTGATTGACATGAAGACCAGAGCAACTATAAAAATAATCCAGAATATTTTGAAGCATCTGAGCATTGCAAAT
TGTAGCTTTACCAAAAATAAGCACGTCATCCACATATAATAAATGGGAAATATTAGAATTTCTCATTTTAAGATCCTCAAAGTTAAAATCAGATAAACTGCTATCAATTAAGTTAGAAAAAG
CATCTATGATAATACAAAATAAATAAGGTGAAAGAGGACACCCTTGACGCAATCCTGAAGAAGAGCTAAAGAATCCTTCTAAAGAACCATTAATAATAATAGAAAAATGAGTATCATCAAT
ACAACCTAATCATTTTATGAATTTGTCCGGGAATCCTTTTTGAGCCATTATATGTAGAAGAAAATCCTTTGAAACTGTATCAAAAAAGCTTTTTGAATGTCCAACTTAGCACAAAAATATTTT
TCTTTGCAATAATTAAAACTTCTCAAAGTATCAGAAACAAGAAAAATATTATCAATGGAAATCCTTTTTTTAATAAATCCATATTGACTAGGATGAATGATGATCGGCATCACGTTTTTCATA
CGATTAGCCAAAATTTTTGCAACCATTTTATAAAAAACATTACATAAAGAAATAAGTCTAAAATCAGAAATATGAGAAGCATGAGAACATTTAGGAATAAGGCAATAGCTGTAGCTTTAGTA
GCTTTACTCATATAACCAGTAGAAAAATAGTGACAAATAGCTTTACAGAAAGAAGGGCCAACAATATCCTAAGATTTTTTGAAAAACTCTAAAGTATAACCATCTGGCCTCAGAGCTAAAG
GAGCAGAAAAAATAACATTTTTAATTTCCTCTAAAGATATAGGAGCAATAAGATTATAAATAGCAATTGAAGGAACTTTAATACCACTA
>comp153690_c0_seq1
CTAAAAAGTCGTTTTAAGAAGCAGGAAGACATCCGTCCCAAACGGAGCCTAAAATGACCTACATATTCCTTCGAAAAAAGCATCCTGATGCACCAGGCACAAATTATGTTAGGATATCCT
GATGAAAACCAATCAATCTGAAAGATCTTATGATTTGCAGTGACATGAGTGTTATATAAGATAAGATATCTTATATAACACTAATGTCACTACAAATCATAAGATTTTGAATTCCATTTTGATG
TAATGTGCAGGAGGATAATCCTATATTTAGTGGTGATTCTCGAAGCAGACAAGCCTGTTATCTGCGAGCCTGGATTTAAAGGCAGCGGTCTTGTACTGAGCCCATGTGAAAGCTCTGTACC
TGCTCTGCTCTTGGTCTCCAAGCAGCTGGGGCAGAGGCTCAATCCTCGCAGCTAGCGGCGGGCCCCCGAAATATATCATCGAGAACCTTGACTTCCTCCCGTTGGTCACCACCCTGTGCC
TCACGCTCCGGAATCTCCCATTTGTCAGCACCTGACATCAAAAATTTAACCAATTCAATTGCCCCCATCAATACCCCCACGTTCTTCACATATTTTGCACACTGTACATCTATTAATTATTTTA
TTAATCAATCAAATCGATCGAAAACCCGACCCACAAATCCCATCTTTTCACACATTAAATAAACAGTGACCGCAACGGAGAGATGAGAGAGATAAAGGTAACCAAATTGAAATCTTTCAC
TTCGTTTCTTCTTTTGATTGTGATGCATGCAATCATTAAGCAATGTATATATTGCGCTAATTTAGTCTGATTTTTGTGGGTCAAATTGAGCGATTGTGTTGCCTCGTGTTGACACGAAGTAAA
GCTTTTGACTGCACTAAGTCTTGTAAAGGGTATGGAATGAATGACTAAACTGAGTTTCATGGTATTTTAATGGATCAACCAAAGTAAAGTGGAGCTTAGAGCATCTCCAACCGAACGTC
>comp153690_c0_seq2
CTAAAAAGTCGTTTTAAGAAGCAGGAAGACATCCGTCCCAAACGGAGCCTAAAATGACCTACATATTCCTTCGAAAAAAGCATCCTGATGCACCAGGCACAAATTATGTTAGGATATCCT

GATGAAAACCAATCAATCTGAAAGATCTTATGATTTGCAGTGACATGAGTGTTATATAAGATAAGATATCTTATATAACACTAATGTCACTACAAATCATAAGATTTTGAATTCCATTTTGATG
TAATGTGCAGGAGGATAATCCTATATTTAGTGGTGATTCTCGAAGCAGACAAGCCTGTTATCTGCGAGCCTGGATTTAAAGGCAGCGGTCTTGTACTGAGCCCATGTGAAAGCTCTGTACC
TGCTCTGCTCTTGGTCTCCAAGCAGCTGGGGCAGAGGCTCAATCCTCGCAGCTAGCGGCGGGCCCCCGAAATATATCATCGAGAACCTTGACTTCCTCCCGTTGGTCACCACCCTGTGCC
TCACGCTCCGGAATCTCCCATTTGTCAGCACCTGAAGAGAATCCCCCACGTTAACGAAGAAGGAATCCTGGTCAGGAGGAACCAGGACCCAACTCCCGTCTTTCAGAGCAATCTGAAGC
CCATCCGAGTCATTCGATCTCAAAACGGAAATAATCTGCGGATCCGTATGCTCCCCAAATCCGTTCAAGTTGTTGTTCGATCCCTGCAACATCGGCCATGGCGGGTAGTGATTCAGCCTGA
AGAAAGAATCGCTCTTTTCGTCATTCACAAATTTACTCAACACATTCCTCTGCTCCAATCCCAGTCCTTCCGCCATTAACTCAAGCATTTCCGACGCCAACTTCCTCGTTGCCGAAACATAC
TCCTTCAAAGCAGAGCAAAACGAGCTCGCCCATGACTCCTTAAGGAAGGGAATCGTTAAGGACGGGTTTGAGGTGACTTCGAGAAGGAGATATTCTAACCAACCAACATCTCCATTGCA
GCCAATCTTCTTGTTTCCATAGCCGAAGGGGTTGGCCATGCCGGCCTTCTCCAACTTCTCCCACTCCGGCAAGGAAAAGAAGTCTATTGCAGCGGCCTCCAATCTAGCCGTTAGGCTCTT
GGAGACCCCGTGGTTGGTGACTTTGAAGAAGCCGAAATCGATGCACGCAGCGACGATGTCGGCAGCGGCGGTCGGTGCGGTGATGTCGATTGCCGGCACGTTGGAGAAGGAAGCGCA
GGGCTTGGAGGATTTTACGAGGAATATGTCTTCCAGGGATGGACTAGTGAGGACCCCCATAGTTGTTGGTTGAAGAGCAGAAGAAAGGGTAAGGGGA
>comp153762_c2_seq7
AAAGAAGGATGTCAAAGATAAAAAAATGTAACTATTCATAGATTATAGAAATCAGTTAATGGCAAAACTCAGGCGAACAAATCAAAAGAAAATCTTCTTTGATTTCCATTCAAGAAAACT
TGAATCTTTCCATATCTTTGTTATCAAAATCAGTAGATTAATGATAGAAATGTATATAAAAAAAAAATCAAGTAGATAAGGCAATAATTGGAGAATCTATATAAAAGAAGTATACCTAAACTC
ATCGATGCAAAGAGAGGTTTGATCAGGATTTCTAGGGCTGGAGATCAGATGGCGTCGGGTTCGATTGGCTAGGGCTGGTGACGACATGTGGTATTTGGCCTTAGCATCGTGCAGCACAGA
GAGGCGATGAACAAAGGAGCGACGGGCTGGTTCTTGCAGTTTTTCTTCCGAGCGAACAGAGAGCAGACGAGAGAGAGATGATCGAGCTCAGGGTTTGTCCGTTGGGTTTCTCACACCT
GGATCTGAGAGGAGGAGTTCGGGCTTTTTT
>comp153787_c0_seq2
TATTTAAAAAATCCCAATAGTCAACAAAAGAAATATAAAATGCTTTTTATTATAATGGAAGATGACCAACCGAGGTGCCAAACCTCACGGTAAAAAAGCACAAATAAATCGCAAGTAACC
CGCAGCCCAAAACCCTATCCGGCCTCATCCCCCTCCTCGGCAAAATCACCAATGCCCACATCACCCCGCCGGCCAAAAACCCCACCGTCTCAAACACGCTGGAGTCCATCTCCACCAGT
GCCGGCGCCGGCCACGTCATCCACGCCGACATCCCTATCGGAGCCCCTAACCCCACCAGCACATTAAACGTCGGCCCCGCATAACACCCCGACACCGCCACCTGTATCCCGCCGTCTCCG
CCTCCCTTCGCCATCGCCACCGCCGCCATTAGATCCCCCAAAGAATCCCCCCATGCTAAAACAGTCAACCCAAGCGACGCCGGGCTGATCCCAATCACGGTCCCGATAGAGACCAAAAG
CGAGACGAGCTCCTTCGCCGTGACGTAAGTCCACGTCACGCTCATAAGGAAGCCGCCGGTGAGCCACGGAAGCCGGAATTTGGTCGGAGGATTTTGACTCTCCGTTGTAAAGAACGCG
GCGATGCCGGTGAATAAGCCGACGAGAGCGCCCGTTAGAAGTTGGTTTGAGGAAGAAAGAACTGCTAAGAGGACCGGCGACATTGCGGCGGATGTAACGGCGAAGGGTTTCGACCAA
CGGTCTTCGGAGACGTCAGGAATGGTTATTCGTCTTGGGAGATCGAGCGCGAGCTTGAGAAGACGAAGATATTTTACAGAGATGAGGTTCAGTCGTCGGCCTGTACTATTGGTTTCTTGA
TTTTGTTTTATCGTGGTTAGAAGAGGGGTTTCAAGTTCTTCGGTAGCCGCTTCGGCGTCGAAGATGGTCAGCTTCTCCTCAAAATGCCCAATTCCTCCAAAAAGATGAGTGATGCAGACC
GACAAAACATAGAGCAGGTAGAGCAAAGGGAAAGCCAAGGCAACCCAAACGTTTATCTTCCCAGCAACCAATATGACCGTTAAACTCAACAGAACGAGCAGCAAGAAGCAGGCATCC
CTTACGAAGTCGATCTTCTCCACCGCGACTTTCTTCGGCGCTACTAAAACGCTAACAACGCCAACAACTACAGTAGTCACAAACAGAGCTCCTCCAAGTATGCTATTCAGGCCAAAGTCG
CAGGCGGCGCCGCTGCCGCCCCCTTCCTCGAAGGATACGATGCTTGAGAAAACATCGGGTGCGCCATTTCCGAGCGAGAGAAGGGTCGTTCCGGCGATGGCCGGAGAAAGCTTGAGGA
GTCTTGAGAGACCTTCCAAGCTCGAACAGAAGTAAGTGGCGGCGGTGCTGCCGAGGAGGTAGAAGAGGAAGATGAGCCAGAGAAGTAAGACGGTCAGGCCTAGAGGATGCAGGTGAT
GGCTGAAGAAGCAGTAGTAGAGGTGGAGGTAGTTTATGTAGCCTTTCGGGCTGCATTGGTTGGAGGATTGTAAGAAGTCGCACTTAGCTTTGTCGGATTCAAGATTTATGAAATTACAGG
ACTTATTTTGATCAGTGAGATTGAATCTTCTGAAGATGGGCTTTGCTGATTTTTGAAGGAGAGCTTGAGATGATGAGAACAGGAAGAGGAAGATGATGAAGAGGAGAAAGAAGAGGTTT

GACAGTAGACGAGAAGATGAAGAAATTGATGCGTAGTTTGCCATGAGAGAGGCTGCTAGCTTCTCAAGTAGAAGAAATGGAAGGCTGCATGGGTAGCTGTTAGTTATATCAATGGAGGA
GATAAAGATAGTTTGG
>comp153797_c1_seq2
TTTTTGTAAATAAACTTCCAACTTTATTCAACCGAAACATTGCAAGTAGCTACCACAACTCTATTTAAAATGACAAGAACATGATGAAAAAAACATAGTTCCAACGTAAGACTAGAAGGA
CACATTGAGCCAACTATAGACAAAAACAGTAGGAACCACCATCTAAAATTAACATAAGGCGGTCTTATTCGCATTCAACGTTTAACACTCAATTATGATAACGGCAGGCTCGCTGAGAGCC
TAGCTGGTGGATGCCATGGCAGCTAAGACCTTCTTGCTAGCTTGGCGGGATGTTATGGCATCCCACCAGGCCTTCACATGAGGACGAGAGTTAATGAGGGAGCCATGCGGCGTGTTGTTG
ATGAGGTGGTAGCCATAGGGGAGATGATGGAGGTCAGCGAGGCTGTAAAAATCACCTGCAAGAAACTTTGTCTTTGAAAGCCTGGCCTCATAAACATCAAGTACCTTACCCAGCTTATCC
AGATCTAGTAACACCACATTCTGGTCAGTGTGACCTCCAAATAAGAGTGGAAGGAAGACATGTTGGAAAACAATGGCGGAAATGGGCCCATTGAAGTGTTGTGTTTCCACCTCCATCCA
CTGATCCACTGCAACTGTGTCAACGAAGCTCTTCTGTTTCAGGAGATAAGTCCCCTTGCCATATTTTTGCACCAAGTAGCGAGTAATAACCCGAGATTCAAATAGAACCAGGCCGCCATCC
TGGAGGGCAGGGATTTGACAGAAGGGGTTTCTGTCAGAATGAGGTGGTCTTTGGTGCTCGCCGGCTTTCAAGTCGACGGGGACAAGCTCGTATCGCAAGCCGATCTCTTCCATGCAAAG
CATCACTCGAGCTGTGCAGGTCGAAACAGGGGAGCCGAACAGCTTAACAACCATCTTTCCTATTGCTATTTTTCTCTAAATCTCTTCCTTTACTCTGAGCTGATCAGTGTTTGCGATTTGAT
GA
>comp153812_c2_seq2
CCAATCTTGCAAACTGATTTATTACATCGACATATAGTAACATAGATCAGTATCCTCCACAAACAAACAACATAATAAACATCCAAACCAGACAAGATTGGGTTACCAGCGACCGTAGCAA
AAAAGAATAAAGTACTTCCCTATAGTAGGAGAAATTATTCTGTATGTAATCCGAACACGTTCGGATGCTGCGTATTTACTGC
>comp153816_c0_seq1
AGCATTTACAAATTTAAGCAGTCCACGAGTTGTCTTGGTGGTGAAGAACTGGCTGACCATAGGCCAGGTACCGAGTTCGAATCCCATGGCAATTCGTGCAAAGCCTTAAGTGTTGGCCCG
GATGAGTATGGGGTGGGTTGTCCGCTAGGTCGACCGGAGAGATTAGTGGCCGAAGGTTGATAATCCTTGACTGTAAACGGGATCTTCAAACATGGGACGAACACACGACTTAGTGACAT
CAACCCAATTCTGTAGTTC
>comp153849_c0_seq1
AAAGCATGAGTTAATTAGTAAAAAAAAGATTATGTCACGATATTCTGATGCAAAATATATGAAAATTATTTTACAATCTTATTATATTACCTTAAATTTTAATTTTATAAACAATATAAAAATTT
TAAAATCATTTATATGAAACAATTTTAACTAGTAAATAAGAGAAAGACGAATAAGATAGATTATTCCCACACAACTCTCATGGAGGAACCAGCGATCGCAGGGCCAAGTTGCGACCCTTGG
CTTCCCCTGCGCGGCGGCGATACCTCGGTCAAATGCCCCCACGGCGACCCCCCGGCCGTCGGAGGTGAGGGCACGTATTTCCTCATAAGTTGTGAAGATGAACACACGCACCCACAATT
ACACCCACATCCACAACCAACCTCCTGACGAAACCCTCCACCGCCACCCATTCTCCACCCTGGCCCTCCGCCGTAGCCGAAGGCGGCGTTCGGCCTCTGGTAAGCCATCGGTGATCCTCC
ACCGCTTACTCGGCCGAGGTTCGGGGTGCTCCTCACCCGGTGCATCCCTCCGCAGCCTGCATAGTGCGCCGGTTGGGGGGTGGGAAGACCACCGTTTATGGCATCGACGTATCGTTGATC
GAGAGTGGCGGCGGGTTCCGCTGCCGCGGCAGTCGCCACCGCCGCAGGGGAAGGGAAGAGGAAGAGACGGAAGAGAGGGGAGGAGGCGACGGTGGGGGATCGGGGGCGGTCGTAT
TCGTCGATCATGTGGTAAAGATCTTCGTCGGTTTTGACGGAGACGAGGGTGTCCAGATCCTCTGCCATCAGCTGATACTTCAACACCAAATCCGCGTTGAACATTCCGGCGATCTTCTCCT
TCAGCTCTGCAAAGGTGAAGAGGAAGAAATAAGATAAATTCCTGAAACTTTCATTAAAGATTGAAAATTTGGGGGTCATTGTTCTAGCCTTTAAAGGAGATGAAGCGGGGAACGGATAG
AACGCGAGTATCGCCGCCGACGTAGCGGAGGTGGCCGTCTGAGGGGCGCGGGAGAATACGGCCGCCATAGCTACAGAGAAACTTGAGGCGGTTTCTGGGGGAAGATTGGGTTTTCTCC
ATGGATGGGACTATGAGCGAAGATGCTATTACTAACGAAGGTGATAATGGCGGTGAAGTTGTGATTACGATGATGATTTCCGCTTTGAACGGTAAGAAGTTAGTGGTAATGAGATAAATTT
GCGCGCGTCGGTTCGATTAGGGATAACAGATTGGACAGAATTGAACAAATCTACATAGATATATATTGTGTGGGGCTATTTCTATTAATATTTTTATTCTGATAGG

>comp153849_c0_seq2
AAAGCATGAGTTAATTAGTAAAAAAAAGATTATGTCACGATATTCTGATGCAAAATATATGAAAATTATTTTACAATCTTATTATATTACCTTAAATTTTAATTTTATAAACAATATAAAAATTT
TAAAATCATTTATATGAAACAATTTTAACTAGTAAATAAGAGAAAGACGAATAAGATAGATTATTCCCACACAACTCTCATGGAGGAACCAGCGATCGCAGGGCCAAGTTGCGACCCTTGG
CTTCCCCTGCGCGGCGGCGATACCTCGGTCAAATGCCCCCACGGCGACCCCCCGGCCGTCGGAGGTGAGGGCACGTATTTCCTCATAAGTTGTGAAGATGAACACACGCACCCACAATT
ACACCCACATCCACAACCAACCTCCTGACGAAACCCTCCACCGCCACCCATTCTCCACCCTGGCCCTCCGCCGTAGCCGAAGGCGGCGTTCGGCCTCTGGTAAGCCATCGGTGATCCTCC
ACCGCTTACTCGGCCGAGGTTCGGGGTGCTCCTCACCCGGTGCATCCCTCCGCAGCCTGCATAGTGCGCCGGTTGGGGGGTGGGAAGACCACCGTTTATGGCATCGACGTATCGTTGATC
GAGAGTGGCGGCGGGTTCCGCTGCCGCGGCAGTCGCCACCGCCGCAGGGGAAGGGAAGAGGAAGAGACGGAAGAGAGGGGAGGAGGCGACGGTGGGGGATCGGGGGCGGTCGTAT
TCGTCGATCATGTGGTAAAGATCTTCGTCGGTTTTGACGGAGACGAGGGTGTCCAGATCCTCTGCCATCAGCTGATACTTCAACACCAAATCCGCGTTGAACATTCCGGCGATCTTCTCCT
TCAGCTCTTTAAAGGAGATGAAGCGGGGAACGGATAGAACGCGAGTATCGCCGCCGACGTAGCGGAGGTGGCCGTCTGAGGGGCGCGGGAGAATACGGCCGCCATAGCTACAGAGAAA
CTTGAGGCGGTTTCTGGGGGAAGATTGGGTTTTCTCCATGGATGGGACTATGAGCGAAGATGCTATTACTAACGAAGGTGATAATGGCGGTGAAGTTGTGATTACGATGATGATTTCCGCT
TTGAACGGTAAGAAGTTAGTGGTAATGAGATAAATTTGCGCGCGTCGGTTCGATTAGGGATAACAGATTGGACAGAATTGAACAAATCTACATAGATATATATTGTGTGGGGCTATTTCTAT
TAATATTTTTATTCTGATAGG
>comp153911_c0_seq2
AAAATCTAAATCTGATCATGAATCAAAACTCAACTCATCATAACAGCACAAATTATAACTCTATAGATTACATTCTTCAGGCAACTTTGGGTATCTTTCAGCATCCCTGCAGTAATCATAAAT
AACAAGGTTTCTTCCCACCCAGGTGAAGTCCTCAAGCTGATTATCATCAAGGCTCCAAGCCTCATACTGATCCCACCAATGCTCGGTCGTCGTTGAAACGCAAGCAGGGTAAGGGTCCTC
CCACTGGCAGGCATCGACGCTGAAGTCTTTGTAAGTTGAAACGAAGGGTGAGTTCTTCCAGTTTGTCTTCTCAAGGCCTCCTCTGGTAGCCCAGTCGTCGGCGTTCCATATGCTTGAGAA
TATGTACATTGGCTTCTCATTTGGGAAGAAGCTGTTCAGTTTACCGTTGTTTTTGTACACTCTTATGGCCACTCTGTCCACGAAGAAGCTGCAAGTTTTGGTGCAGGGGAGATGAGGATTT
CGACATTATAAAGAGGCCGTTTGGTTGCAAGTTTTATTTAAAATTTAGTTGGAATTAAGGCCAAGTCTTGCCAAATAATCTTTAGAGAAATTCAAAATTTTAATAGCTGTCACAGCTTTAGT
TCGATCCTTTCAAAATTTATCAAACATTATTTACTAAGGGCATCTCCAACGAAAAATCTCATTTTTTACTCTAAAATGCCCTGAAACTAAAAAAAGAAGTGATTCATCTCTAATCGAACTCC
ATTTTGCGCCCTTCGCAGA
>comp153912_c0_seq2
TAATTTTATGTTTTTCTTTTTAATGACAAATATTATGATCCATAAAACATTACAAATCTCATCATCATCTAATAGAGTTTTACTTTAAATTTGAAGGATACTTTTTTATAACACAAAAGACCTGA
TAAAAAACTGTCATAATCACAGCAAACTCCACAACAAATCAGTATTTATATACGTTCTGCAAGAATTACGCATTCCACCCTCAATTAATTAATTTAAAAACTGCTCAAAAGGCCGAACCGT
CATCGAACCCGGCCTCATAACCTGAGCCGTCGGAGATCATATCGCCAATGAGAAGCCCTCCAAGAGCGCCGCCCATAATCCCCGCTGCCAGCCCCATCCCCGAACTACCTTTATTCCCAGG
CTTCATTGACACCGGTGCCGGAGCCGGTGCTGGAGCTCCATAACCGTATCCAGCCAGCGGGTAACCATACCCAGCAGGCGAATAACCATATCCAGCCGGCATGCCCGGAGCAGCGTTCCC
ATATGGCTGTTGGGCAGGGTAATATCCCGCCGGCGGGTATGCAATCACCGGCTCCTGAGCCGGCTTTTCCGCCGGTGGTGGAGCGACGAAGTGAAGATGAGGGTAGGGGGAAGGCGGCG
TGATAGGATCGCTGATCTTATATGAGAAGTTGAGGACGCCTTTGGGCTTATTGGAGAAGGGGCGGCGGACCTGGTAGCTGAGAAACCGAGTATCTCCGGCGTTGGCGATCATGTCGCGGA
GAGGGATTTGAACGACGCCGACATAGCGGTCTCCGAAGAAGCGATCAGCGCAGAGAAGGATGCGGAGAAAAAGGTTGCCCGGATTCCCGGCGGGGAAGGTGAAGTGGATCGTAGCGT
TCCAATTGGGGTTCCGGCCACCGTCGCGGTCGGGGGCGATGCGCTGCCGCGACAGCGGGCTGTCGGAGTACATGGTGACGATGGCGTAGACGCGCATTCTGGTGAAGAGGTTAACATTT
TTTAGGTCATTTGCGGAGATGAGGGTGAGGTCTAGCGTGCGGTATGCCATTGCCGGACGGTAATTTGGGTTAGGGTTTCGGGATAGCGGACTATAAATTGATGTGAAGAGGTTAGCGAGT
GGTGGCGGGAACCC

>comp153968_c0_seq1
TCAATAATAAATATGCATTAAAAGTAGGTTCTTCATTAAATAGTTATAAATCAGATACAATAGAACCATAGACATTTCAATGAATTAAAAGTTCCAAAGAAAAGCTTTAGAACAAAAAAAA
CCAAAAAATAAAATTAAAAAACCAAGCCATACATATGAGGACTTGAAAGAAAAATAACTAAATAAATAATAAATGGTCCATAAAAGAATATATATATTAAATGTGATTGGCAAACATGTTTT
GAAGCTCCTGTTTCCACGCCATTGTTAGTTCTTAATGTATGTAGTAAGCGAAGAAAGAGATGGTGGAGGCGACAATGGCAACTACGAAGACAGGCGTGACTACGAAAGCATCACTGGAG
GGGCCTGGTGCCGGGGCCTCAGCCGCCAAGGCTGATGTTGCAGAGACAACAACGACCAAAAGGGTGACGAGGAAATTCTCCATCTCCATTAATGCTAGCTATCGAAGGCAAGGGGATTT
GTAATGGAAGATGAAGTTTTAAGCGGTGAAAAGGAGAATGAGGGAGGTGGATTTTATAAGGATGGTGGTTGTTAGGCTTTTATTTTAAGTAGCCAGGGTTGGCTGGCTTTGGCCTAATTTT
AGGGTTTTGTTTTTGTGAGATTAGTGGAGGGGCATATA
>comp153971_c0_seq2
TGGATGCAAAATTCTTTTGAATAAGCAATACATATCCCACAGTAAAAATACATTCGAATTTATGAAAATAGAAGACATGGACTTCATCGTCTTCTACTTATTATCAAATTATTTCATATTTTCA
AGAGCATTACAGATTTTACATAAAATAGTCCATTCTGATACAGTTTCTATATGAGATGTTGATACCAAGATCCTATAATATATTCCCATCTGTTACATAAAAAAGAGAAGAACACATCAACGC
TTAGCAATGATCAAAATCAGGACCCTCAAATATCTCATTCCATGGCCAGTTAGGCTTAAAGCCCTCAGTCTTCTCCACAGCCAGACTTGTTGAGGCCAGAGAGCCAGCAGGATTACTACTA
TTGCCAATACTTCCACTGCTGTTACTCAATTCATTCACTGCGGCACTGCTTCTGTTGCTGCTACTGTTGTTTGTGAGATAGTGTTGAAGAGCAACCCTCACACCAGTGATTAGGAATCTCTG
ATGGTGGGATACAAAAGGGCTTGCTGAGTGTATTGTTAGATCGCAATGCCTGCAGAGCAAAGCTCGATCCTCCAAGCAGAAAAAATAACCAGCTTTTTCCTGGCAGATATCACAACCGGG
CAATTTGGAGGTAGCAGAGGCGGCGCTGGGGAGGAGGAGCGGTACTCTCTGGTGTTTTCCGGCTAGCTTGTTGGCTGCATGCACCTTCTCGTCGCAATCCCAGCAGAGCGCCGCCTCAT
CCGCGCAGCAGAGCACAACCGCCTCTGCCTTCTCGCACGCATCGCACTGTATCTTCATATCTCCGCCACTGCGATCTTCAATCACAATGTAATCTTTGCCGGTCTTCCAGCAAAATCAGATT
CCGGCAATCTACGGACGCCACGACAGCTAACCGGAATAAGATCAACGGCTTCAGAAAGCAAAGATGCAAACCCACCGCTAAGCTTAATTTGGGAATCAAATACGGCTGGCCCGCGGCGA
GGTTTTCTGCTACAGAGAAAGAGAGAGCAGCGCGGAGTTGAGGGGAAGAAGATGCT
>comp153992_c0_seq2
CCACTCGGTCTTGTCTTCATACCTGCCAAACAAAGGAAATAGTTAAGGGATGATCTTCGGCTCAGATAATTGGATTGTAATAAAATTGCAGAATATTCTGATGCCAATGCAATTTTTTATAAA
TATCAGCATAACAAAACAACTACTAAATTAATTCATTGAGACAATTCATCGATACTAATCGTACTTACCAGCAAGATATGACGGAGATTGCTTCTGCGACCGTAGCAGAATCGAGTGGCAC
ACGGGGAACAACAAGGGCGCCGGGAGGCCTTCCATACTTGATAGCCGGATGATCGGCAAGAGGTCGTCCCTGAAACTCCGAAACAGGAATAGATTCCGCGAGCACGGCAGAATTTCCA
AATCTCTTAGAAAGAAGATTGGGATCGAGCTCATCATCAAAATCCGTTGCGGTGAGTGGCCTCTTCTGCATCTCAACATCATCCAGATCCAAAGGGGACTGAATCTCCCGCCGAGAGTAA
TCAGTAATCGAAGGTGGATCAAAGCCATAAAGTGCGAAGCGGCGAAAGAGGCATCCTGTCCCAACATAAACGGGACCTTGGATGCCATCCAAGGCTCGCATGTTGCCGTCGAAGAAGA
CGGTGTTGTGATTGGCGTAGCGGTCGGAGGGATCGATGTTCTCGAAGCGTTGGGGGAATTGAATGTAGCAAATATCTTCGCCGCCACGATCCATCATGAAGCACATGCCTTCACGAACAG
CAAGGGAGTTGTAGATGTAATGATCGCAGTCAAGGTTGAGGATGAAGGGGGCGTTGGACATGACGGCGGAGGCGCGAACGAGAGCATTCATG
>comp154072_c2_seq3
CTCCTCTCCTCTCTGTTCGACAAATTTACAGGATTTGGCCGAAAAGCGCGCGTGAGGGAGAGGGTTACCTTCAGCCAGCGAGCTCCCGTGCGTTGCTTCCGATCAAAATGAGTGACTTCC
GATCCGAATAGGAAGGGAGAGTCGTCAAATGAGCGAAGTCGAGCGGCGACCGAGGAGATGGAGAGGGGCTCGGGTTCGGATCGGCGTCGGCTTGGGGAAGATGTCGGAACAAGAGGG
CTTGGCCGGCTCTGTTCGTGGAGGCAGAGGGAACGAGATCGGGGAGCTCGGCTTCGGACTGGATGGAGGAGCTCGGGATTGAGAGAAATGCAGAACTTGACACTATTGAATTGCTACA
AAATTTACCTGCTTTGCAACAGCTACTTTTCCGCCTGCTTGGCTGCCAGCCACAGGGAGCTGCCATTTACAATACTGTAATCCACCGTGCACTGTCAATTATTGTCGGTGAAAGTATAAAG
ATATACATAGCAATTAATATCTGTATCCTTAATTTGGTTGACAAGTTCTTTGATATGCAGCAACGTGATGCCATTAGAGCACTTGAAATTTACCGAAGAGTTGGGCCACAGGCTAAGAGTCT

GTTGAAGTTTTATGATTTCTGTAGAAGGTCTGATTTTGGGCGTGGCCAAAAATTTGTTAAAATCGAGCAGCCTCCTGCTTCATTTGTTGTAGCAATGGAGGAGTATGTGAAGGCTCCCTCA
CCACAGTTATTGGGTTACAAAAATGTGGTTGAACCTAACAAAAGAATAGTTGGCCCTATGCCAAAGATGGCCTTTGAACATAAGAAAGTAGACCTTCATGACAAAATTCAACGACAGCCT
ACAACAGCACCAATCCAGCTAACTCACTTGGTACCTAATCTACTTGTAGCTGAATATGCCCAATCTCTTGTTCCAATACCAGATCCAGTTAATGATCTCCTGCACTTGGACGATTTTCATGA
CCGCGCAACTAATTTGGAAGAGATAAATTCTTCAATCTTCGCTATAGTTTCCGACGATAATCTGATGAATAATGTAAATTCTTCTGATCGCTCTGGTGGATCAACAGAATGGGAACTTGCTC
TAATGACAGCTTCCAGTTCAAATACTGCATTGGTGGCTCCCTTCAACAAACTAAAGCTTGAAAGCCTGTATGCCGATCAAATTTCAAGAACAACAAACCAAAATTCAAGTTATCCAAATC
CGTTCGAGTCAGATCAGTACACCCATGACCCATTTTATGCTTCAGGTGGCATCGATCTGCCGGCCAATTTGCAGAACCAAGCAATGGGGCTTGAAAACAATCCATTTGGACCAACTGGTG
TTTCATCTTTTCCTGCCCAGGATCCATATACAGGTTTGATGTAATTTTTTTTTTTTTTTTCCAATTTTTGTTCAAGTTCAAAGATGTACATAACTAGACAGAAAGTTATTGAGATTTCATTCAG
>comp154072_c2_seq8
AAAAAAAAAAAAAAAAAATTACATCAAACCTGTATATGGATCCTGGGCAGGAAAAGATGAAACACCAGTTGGTCCAAATGGATTGTTTTCAAGCCCCATTGCTTGGTTCTGCAAATTGGC
CGGCAGATCGATGCCACCTGAAGCATAAAATGGGTCATGGGTGTACTGATCTGACTCGAACGGATTTGGATAACTTGAATTTTGGTTTGTTGTTCTTGAAATTTGATCGGCATACAGGCTTT
CAAGCTTTAGTTTGTTGAAGGGAGCCACCAATGCAGTATTTGAACTGGAAGCTGTCATTAGAGCAAGTTCCCATTCGTCGGAAACTATAGCGAAGATTGAAGAATTTATCTCTTCCAAATT
AGTTGCGCGGTCATGAAAATCGTCCAAGTGCAGGAGATCATTAACTGGATCTGGTATTGGAACAAGAGATTGGGCATATTCAGCTACAAGTAGATTAGGTACCAAGTGAGTTAGCTGGAT
TGGTGCTGTTGTAGGCTGTCGTTGAATTTTGTCATGAAGGTCTACTTTCTTATGTTCAAAGGCCATCTTTGGCATAGGGCCAACTATTCTTTTGTTAGGTTCAACCACATTTTTGTAACCCAA
TAACTGTGGTGAGGGAGCCTTCACATACTCCTCCATTGCTACAACAAATGAAGCAGGAGGCTGCTCGATTTTAACAAATTTTTGGCCACGCCCAAAATCAGACCTTCTACAGAAATCATA
AAACTTCAACAGACTCTTAGCCTGTGGCCCAACTCTTCGGTAAATTTCAAGTGCTCTAATGGCATCACGTTGCTGCATATCAAAGAACTTGTCAACCAAATTAAGGATACAGATATTAATT
GCTATGTATATCTTTATACTTTCACCGACAATAATTGACAGTGCACGGTGGATTACAGTATTGTAAATGGCAGCTCCCTGTGGCTGGCAGCCAAGCAGGCGGAAAAGTAGCTGTTGCAAAG
CAGGTAAATTTTGTAGCAATTCAATAGTGTCAAGTTCTGCATTTCTCTCAATCCCGAGCTCCTCCATCCAGTCCGAAGCCGAGCTC
>comp154080_c0_seq10
AGATTCCACCTTAGTTGGTTCTTCAACCTCCTGAAAGTCTCTTGCTTTTCTGAAAGCTCGAAAGCCGAAGCCTAAATCTAGATATATATGGGGAGAGTTCCTTGTTGCGATAAGAACGGTT
TAAAGAAAGGTCCGTGGACGCCAGAGGAGGACCAGAAGCTCATTGATTATATACAGAAGCACGGCCATGGCACCTGGAGAACCCTCCCGAAGAATGCAGCACGATGTGGAAAGAGTTG
CAGACTCCGATGGGCGAACTATCTTCGACCAGATATAAAGAGAGGAAGGTTCTCCTTTGAAGAAGAGGAGGCCATCATTCAATTACACAGTGTCTTGGGCAACAAGTAAGAACAACATAT
ACCACTAATTAAATATTACAGTAACTTGAATTTTTTATTCCTTCAACTAGCTAAATGAACGATCGATCTTCGCTAATTGATCAGATGGTCAGAAATTGCTGCGCGCTTACCTGGAAGAACTGA
TAATGAGATCAAGAACTACTGGAGGACTAGAATTCAGAAGAAGGTTAAGAATGGCGATGCAGCCGATTACTCGAGCCAAATGATCATTGATGAGGCCAGCACAAGCCATACAAGTAGCA
TTGAAGAAGGAATAGCTCAGCAAAGTTATCCCGTTCACCCAAATACAAATCCTGAAGGTTATCCTCCTTCATACAACACTGGTGAATTGGGAGAGAACTTTTGGAGTGTGGAGGATTTCT
GGTCAATCCAATCTGACCAATCTCTTAATGGTGATTAATTAAGTTAGATATCAAGAAACCATCATGCTTATATGTAAGAACTATTGATGAATGAGGTAAATGGCTTTGATCAGCTTCAGGAAT
ATATATAATTATATGATTGTGCTGGTCGCGCTTGTATTTGTACAAAAATTGCTAATTATCATGGTTACAATGTAATAAATCAAGTGAGAGATAGCATGCGCTTGGTTTGCTTAACTATTACATGT
AGCATCGGGAGCTAAAGATTTCATAAGACTCAAATAATAATTTTGTATAAATGTTCTCTGAGCTTTCCAAGGAATATGGCAAA
>comp154080_c0_seq11
AAAAATAGCCTTTAAACTTCAAACCACAAATTATGGGCCGCGTTACCTCATTGATGAACAACGGATGGCACCAACTCCCTTTGACTCTCTCTCTTCTTCTCCTTCTAACTGTAGTTATATATA
TCCATATTCATGTTCCCTCTTCTTCCATTCCACCTCAGTTTGCTCTTCAACCTCCTGCAAGCCTCTTCCTCCCTCTGTCGCTCTCCTCATCCTTTACTGAAAGCTCTAAAACCGAAGCCAAAA
TCTTGATATATATGGGGAGAGTTCCTTGCTGCGATAAAAATGGCCTTAAGAAAGGCCCGTGGACGCCAGAGGAGGACCAGAAGCTCATTGATTATATACAGAAGCATGGCCATGGCACTT

GGAGAACCCTCCCCAAGAATGCAGGTCTTGCAAGATGTGGGAAGAGCTGCAGGCTTCGATGGGCGAACTATCTTCGACCAGATATAAAGAGAGGAAGGTTCTCCTTTGAAGAAGAGGA
GGCCATCATTCAATTACACAGTGTCTTGGGCAACAAATGGTCAGAAATTGCTGCGCGCTTACCTGGAAGAACTGATAATGAGATCAAGAACTACTGGAGGACTAGAATTCAGAAGAAGG
TTAAGAATGGCGATGCAGCCGATTACTCGAGCCAAATGATCATTGATGAGGCCAGCACAAGCCATACAAGTAGCATTGAAGAAGGAATAGCTCAGCAAAGTTATCCCGTTCACCCAAATA
CAAATCCTGAAGGTTATCCTCCTTCATACAACACTGGTGAATTGGGAGAGAACTTTTGGAGTGTGGAGGATTTCTGGTCAATCCAATCTGACCAATCTCTTAATGGTGATTAATTAAGTTAG
ATATCAAGAAACCATCATGCTTATATGTAAGAACTATTGATGAATGAGGTAAATGGCTTTGATCAGCTTCAGGAATATATATAATTATATGATTGTGCTGGTTAGTAATGCATAAATGTCGCGC
TTGCATTTGTACAAAAGTTGCTAATTATCATGGTTACAATGTAATAAATCAAGTGAGAGATAGCATGCGCTTGGTTTGCTTAACTATTACATGTAGCATCGGGAGCTAAAGATTTCATAAGAC
TCAAATAATAATTTTGTATAAATGTTCTCTGAGCTTTCCAAGGAATATGGCAAA
>comp154080_c0_seq12
CAGTTAAAAATACTTCAAATTTCTTGCTATATATATCTCTTAATTTGCGGGTTAGAAAGTCCTATATGAAAAGAGTTGTAGGTTTGATTAACTAAAAGATCATGACCAATCTTTTTGTAGGTTT
AGCACGATGTGGAAAGAGTTGCAGACTCCGATGGGCGAACTATCTTCGACCAGATATAAAGAGAGGAAGGTTCTCCTTTGAAGAAGAGGAGGCCATCATTCAATTACACAGTGTCTTGG
GCAACAAATGGTCAGAAATTGCTGCGCGCTTACCTGGAAGAACTGATAATGAGATCAAGAACTACTGGAGGACTAGAATTCAGAAGAAGGTTAAGAATGGCGATGCAGCCGATTACTCG
AGCCAAATGATCATTGATGAGGCCAGCACAAGCCATACAAGTAGCATTGAAGAAGGAATAGCTCAGCAAAGTTATCCCGTTCACCCAAATACAAATCCTGAAGGTTATCCTCCTTCATACA
ACACTGGTGAATTGGGAGAGAACTTTTGGAGTGTGGAGGATTTCTGGTCAATCCAATCTGACCAATCTCTTAATGGTGATTAATTAAGTTAGATATCAAGAAACCATCATGCTTATATGTAA
GAACTATTGATGAATGAGGTAAATGGCTTTGATCAGCTTCAGGAATATATATAATTATATGATTGTGCTGGTCGCGCTTG
>comp154080_c0_seq13
AAAAATAGCCTTTAAACTTCAAACCACAAATTATGGGCCGCGTTACCTCATTGATGAACAACGGATGGCACCAACTCCCTTTGACTCTCTCTCTTCTTCTCCTTCTAACTGTAGTTATATATA
TCCATATTCATGTTCCCTCTTCTTCCATTCCACCTCAGTTTGCTCTTCAACCTCCTGCAAGCCTCTTCCTCCCTCTGTCGCTCTCCTCATCCTTTACTGAAAGCTCTAAAACCGAAGCCAAAA
TCTTGATATATATGGGGAGAGTTCCTTGCTGCGATAAAAATGGCCTTAAGAAAGGCCCGTGGACGCCAGAGGAGGACCAGAAGCTCATTGATTATATACAGAAGCATGGCCATGGCACTT
GGAGAACCCTCCCCAAGAATGCAGGTCTTGCAAGATGTGGGAAGAGCTGCAGGCTTCGATGGGCGAACTATCTTCGACCAGATATAAAGAGAGGAAGGTTCTCCTTTGAAGAAGAGGA
GGCCATCATTCAATTACACAGTGTCTTGGGCAACAAGTAAGAACAACATATACCACTAATTAAATATTACAGTAACTTGAATTTTTTATTCCTTCAACTAGCTAAATGAACGATCGATCTTCG
CTAATTGATCAGATGGTCAGAAATTGCTGCGCGCTTACCTGGAAGAACTGATAATGAGATCAAGAACTACTGGAGGACTAGAATTCAGAAGAAGGTTAAGAATGGCGATGCAGCCGATTA
CTCGAGCCAAATGATCATTGATGAGGCCAGCACAAGCCATACAAGTAGCATTGAAGAAGGAATAGCTCAGCAAAGTTATCCCGTTCACCCAAATACAAATCCTGAAGGTTATCCTCCTTC
ATACAACACTGGTGAATTGGGAGAGAACTTTTGGAGTGTGGAGGATTTCTGGTCAATCCAATCTGACCAATCTCTTAATGGTGATTAATTAAGTTAGATATCAAGAAACCATCATGCTTATA
TGTAAGAACTATTGATGAATGAGGTAAATGGCTTTGATCAGCTTCAGGAATATATATAATTATATGATTGTGCTGGTTAGTAATGCATAAATGTCGCGCTTGCATTTGTACAAAAGTTGCTAAT
TATCATGGTTACAATGTAATAAATCAAGTGAGAGATAGCATGCGCTTGGTTTGCTTAACTATTACATGTAGCATCGGGAGCTAAAGATTTCATAAGACTCAAATAATAATTTTGTATAAATGT
TCTCTGAGCTTTCCAAGGAATATGGCAAA
>comp154080_c0_seq14
AGATTCCACCTTAGTTGGTTCTTCAACCTCCTGAAAGTCTCTTGCTTTTCTGAAAGCTCGAAAGCCGAAGCCTAAATCTAGATATATATGGGGAGAGTTCCTTGTTGCGATAAGAACGGTT
TAAAGAAAGGTCCGTGGACGCCAGAGGAGGACCAGAAGCTCATTGATTATATACAGAAGCACGGCCATGGCACCTGGAGAACCCTCCCGAAGAATGCAGCACGATGTGGAAAGAGTTG
CAGACTCCGATGGGCGAACTATCTTCGACCAGATATAAAGAGAGGAAGGTTCTCCTTTGAAGAAGAGGAGGCCATCATTCAATTACACAGTGTCTTGGGCAACAAATGGTCAGAAATTG
CTGCGCGCTTACCTGGAAGAACTGATAATGAGATCAAGAACTACTGGAGGACTAGAATTCAGAAGAAGGTTAAGAATGGCGATGCAGCCGATTACTCGAGCCAAATGATCATTGATGAG

GCCAGCACAAGCCATACAAGTAGCATTGAAGAAGGAATAGCTCAGCAAAGTTATCCCGTTCACCCAAATACAAATCCTGAAGGTTATCCTCCTTCATACAACACTGGTGAATTGGGAGAG
AACTTTTGGAGTGTGGAGGATTTCTGGTCAATCCAATCTGACCAATCTCTTAATGGTGATTAATTAAGTTAGATATCAAGAAACCATCATGCTTATATGTAAGAACTATTGATGAATGAGGTA
AATGGCTTTGATCAGCTTCAGGAATATATATAATTATATGATTGTGCTGGTCGCGCTTGTATTTGTACAAAAATTGCTAATTATCATGGTTACAATGTAATAAATCAAGTGAGAGATAGCATGC
GCTTGGTTTGCTTAACTATTACATGTAGCATCGGGAGCTAAAGATTTCATAAGACTCAAATAATAATTTTGTATAAATGTTCTCTGAGCTTTCCAAGGAATATGGCAAA

>comp154097_c0_seq16

TTTTTTCAAGAAAACAAGCTTCATTATATAATAAGAAAATTAAAGAATAGGAAATAAATTACACAGGCCTCTGTCCCAATAATAGTTCATGTAAACTATTCCTTTCAGAAAAGTTCCATAGG
AAGAAAATCTCCTGCATTTATGGAGAAGCTCACAAATTTCAAGAAATATTATTACAAGCCACATCCACTACACAAAATATACTGGATCAAATATAGGGACAGTGAGATCTCCACAACGGAC
ACATAGCTTGAATTATTCCTCTTATTTCATGTAGGAGAATTCCTTCTTCTGATCGTTTCAGTCCTCCTGATCATATATGAATGTTACTTCTGGGAAATTACATGCTCGAAGGAGAAGAGACCAT
ATAGTGCATGGAAATTTTGTCATATATTTATATAGAAGAAAGTCTTTCTATTAATCTTACTTTTCTTTTCCATTTTTTATTTTGACAGAAATCTGTGTTATTTTAGACACACAAAGGTTCACTGA
TGACAGGTTGGAGTTGATTTGGCATTTCAGGTATAACTTCTTCATAAGTAACTGGAAGATGACTTTGTGTTTCAACAGGTTCTTCGGAGGTTGTGGGGAATGAAGAGCTTGAAGAATATAT
AGATGTCACAGGGGATGAGACCAGAACCTTCTTTCTTTTCTTCTTCTTTAAAGGTTTTGGTATCTCGGGGCACGAGCTTTCTATAGAATTTTCAGATGAAGAAACTGATTGCCATCTTCTGG
GTTGCTTAAACTCAAAATCATTCAAAGAAGCTGAATTGAAACCATTCAGAGATGCAGGGGTAATATCCGATGCAGACTTGTATTTACTCATCCAGCTGGGCGCGTGTATTGAATCGCTGTC
GAAGCCAATGTGTGCAATATGCCTCACATCTGTAGGGTGCCCTATCTCCATCTCTTGTTCCTTCAATGAAAATAAATTATAATCCTGAAAAGAGAAATATAACTCAGAAATTGGCTTTTCCTA
CCCCTTCCAAACGCCATTCTCCATCTTCTCTTTCCTCCATTATTAATCTAACAACAAGGAAAGAAAATGCAGAACATGGCCTGTTTGTCCATATATTAAAAACAAACAAAAAGGCGATGTTA
CAAATAAAAATGTGAAATTTCAGATTCGAAAGAGGAAATGGAGTGAGGAGATAGAGAAACGACGGTAAAAGAAGCGTTTTTACTCAGTTTGGGAGTTG

>comp154097_c0_seq23

AATTATTATGTGCGGCGTTCGGATCCGTCTTGCACCCATCACGTCACTCAGGGCCAGTGCATCCGGGTGACGTGGCGAGTCTGGATTAATCTCCGAAGAGTCCGTACACTGCACGTAATAA
TCTCTCAAGATAAAGAGAGAGAAAACAAGCTGTGAGAAAACCAAGAGGTCTCGATAGACTGACCAAAATCATTCAAAGAAGCTGAATTGAAACCATTCAGAGATGCAGGGGTAATATC
CGATGCAGACTTGTATTTACTCATCCAGCTGGGCGCGTGTATTGAATCGCTGTCGAAGCCAATGTGTGCAATATGCCTCACATCTGTAGGGTGCCCTATCTCCATCTCTTGTTCCTTGTAAAC
AAAGATCTGTGAGATATACTTTAATCCCTTGAACAAACCCTTCACCTTTGGGGTCATCGGAAGAGGCTTCAATGAAAATAAATTATAATCCTGAAAAGAGAAATATAACTCAGAAATTGGC
TTTTCCTACCCCTTCCAAACGCCATTCTCCATCTTCTCTTTCCTCCATTATTAATCTAACAACAAGGAAAGAAAATGCAGAACATGGCCTGTTTGTCCATATATTAAAAACAAACAAAAAGG
CGATGTTACAAATAAAAATGTGAAATTTCAGATTCGAAAGAGGAAATGGAGTGAGGAGATAGAGAAACGACGGTAAAAGAAGCGTTTTTACTCAGTTTGGGAGTTG

>comp154097_c0_seq25

TTTTTTCAAGAAAACAAGCTTCATTATATAATAAGAAAATTAAAGAATAGGAAATAAATTACACAGGCCTCTGTCCCAATAATAGTTCATGTAAACTATTCCTTTCAGAAAAGTTCCATAGG
AAGAAAATCTCCTGCATTTATGGAGAAGCTCACAAATTTCAAGAAATATTATTACAAGCCACATCCACTACACAAAATATACTGGATCAAATATAGGGACAGTGAGATCTCCACAACGGAC
ACATAGCTTGAATTATTCCTCTTATTTCATGTAGGAGAATTCCTTCTTCTGATCGTTTCAGTCCTCCTGATCATATATGAATGTTACTTCTGGGAAATTACATGCTCGAAGGAGAAGAGACCAT
ATAGTGCATGGAAATTTTGTCATATATTTATATAGAAGAAAGTCTTTCTATTAATCTTACTTTTCTCTTCCATTTTTTATCTTAGGCATCCATAGTTCATCAAAATGACAGAAATCTGTGTTATTT
TAGACACACAAAGGTTCACTGATGACAGGTTGGAGTTGATTTGGCATTTCAGGTATAACTTCTTCATAAGTAACTGGAAGATGACTTTGTGTTTCAACAGGTTCTTCGGAGGTTGTGGGG
AATGAAGAGCTTGAAGAATATATAGATGTCACAGGGGATGAGACCAGAACCTTCTTTCTTTTCTTCTTCTTTAAAGGTTTTGGTATCTCGGGGCACGAGCTTTCTATAGAATTTTCAGATGA
AGAAACTGATTGCCATCTTCTGGGTTGCTTAAACTCTGTAATGAGAACAAGCAATAATGTATGAGGATTCTGTCAATATAGGATTTGAAATGCGTTTATATATGTATCCTTAAATTGTATTGCA
ATTAAGTAATTGGATAGATTGAAGGTTTGATCTTCAGAGCATAATAAATAGGCAATCTGATTATATCTTCATGGTTCTCTCTTATGGAAGAAATGCCAAACAACTTTCATTTTTCTTAAAAAT

GAACTGTTGGATATTGAAGGAGTATGATGGAAATACCTCAAGATCATGAGAAAACTGCATTTCACAACTACTGCTTTCTTTATAGTCAACAAAATCATTCAAAGAAGCTGAATTGAAACCA
TTCAGAGATGCAGGGGTAATATCCGATGCAGACTTGTATTTACTCATCCAGCTGGGCGCGTGTATTGAATCGCTGTCGAAGCCAATGTGTGCAATATGCCTCACATCTGTAGGGTGCCCTAT
CTCCATCTCTTGTTCCTTGTAAACAAAGATCTGTGAGATATACTTTAATCCCTTGAACAAACCCTTCACCTTTGGGGTCATCGGAAGAGGCTTCAATGAAAATAAATTATAATCCTGAAAAG
AGAAATATAACTCAGAAATTGGCTTTTCCTACCCCTTCCAAACGCCATTCTCCATCTTCTCTTTCCTCCATTATTAATCTAACAACAAGGAAAGAAAATGCAGAACATGGCCTGTTTGTCCA
TATATTAAAAACAAACAAAAAGGCGATGTTACAAATAAAAATGTGAAATTTCAGATTCGAAAGAGGAAATGGAGTGAGGAGATAGAGAAACGACGGTAAAAGAAGCGTTTTTACTCAGT
TTGGGAGTTG
>comp154097_c0_seq27
TTTTTTCAAGAAAACAAGCTTCATTATATAATAAGAAAATTAAAGAATAGGAAATAAATTACACAGGCCTCTGTCCCAATAATAGTTCATGTAAACTATTCCTTTCAGAAAAGTTCCATAGG
AAGAAAATCTCCTGCATTTATGGAGAAGCTCACAAATTTCAAGAAATATTATTACAAGCCACATCCACTACACAAAATATACTGGATCAAATATAGGGACAGTGAGATCTCCACAACGGAC
ACATAGCTTGAATTATTCCTCTTATTTCATGTAGGAGAATTCCTTCTTCTGATCGTTTCAGTCCTCCTGATCATATATGAATGTTACTTCTGGGAAATTACATGCTCGAAGGAGAAGAGACCAT
ATAGTGCATGGAAATTTTGTCATATATTTATATAGAAGAAAGTCTTTCTATTAATCTTACTTTTCTTTTCCATTTTTTATTTTGACAGAAATCTGTGTTATTTTAGACACACAAAGGTTCACTGA
TGACAGGTTGGAGTTGATTTGGCATTTCAGGTATAACTTCTTCATAAGTAACTGGAAGATGACTTTGTGTTTCAACAGGTTCTTCGGAGGTTGTGGGGAATGAAGAGCTTGAAGAATATAT
AGATGTCACAGGGGATGAGACCAGAACCTTCTTTCTTTTCTTCTTCTTTAAAGGTTTTGGTATCTCGGGGCACGAGCTTTCTATAGAATTTTCAGATGAAGAAACTGATTGCCATCTTCTGG
GTTGCTTAAACTCAAAATCATTCAAAGAAGCTGAATTGAAACCATTCAGAGATGCAGGGGTAATATCCGATGCAGACTTGTATTTACTCATCCAGCTGGGCGCGTGTATTGAATCGCTGTC
GAAGCCAATGTGTGCAATATGCCTCACATCTGTAGGGTGCCCTATCTCCATCTCTTGTTCCTTGTAAACAAAGATCTGTGAGATATACTTTAATCCCTTGAACAAACCCTTCACCTTTGGGG
TCATCGGAAGAGGCTTCAATGAAAATAAATTATAATCCTGAAAAGAGAAATATAACTCAGAAATTGGCTTTTCCTACCCCTTCCAAACGCCATTCTCCATCTTCTCTTTCCTCCATTATTAAT
CTAACAACAAGGAAAGAAAATGCAGAACATGGCCTGTTTGTCCATATATTAAAAACAAACAAAAAGGCGATGTTACAAATAAAAATGTGAAATTTCAGATTCGAAAGAGGAAATGGAGT
GAGGAGATAGAGAAACGACGGTAAAAGAAGCGTTTTTACTCAGTTTGGGAGTTG
>comp154097_c0_seq8
TTTTTTCAAGAAAACAAGCTTCATTATATAATAAGAAAATTAAAGAATAGGAAATAAATTACACAGGCCTCTGTCCCAATAATAGTTCATGTAAACTATTCCTTTCAGAAAAGTTCCATAGG
AAGAAAATCTCCTGCATTTATGGAGAAGCTCACAAATTTCAAGAAATATTATTACAAGCCACATCCACTACACAAAATATACTGGATCAAATATAGGGACAGTGAGATCTCCACAACGGAC
ACATAGCTTGAATTATTCCTCTTATTTCATGTAGGAGAATTCCTTCTTCTGATCGTTTCAGTCCTCCTGATCATATATGAATGTTACTTCTGGGAAATTACATGCTCGAAGGAGAAGAGACCAT
ATAGTGCATGGAAATTTTGTCATATATTTATATAGAAGAAAGTCTTTCTATTAATCTTACTTTTCTCTTCCATTTTTTATCTTAGGCATCCATAGTTCATCAAAATGACAGAAATCTGTGTTATTT
TAGACACACAAAGGTTCACTGATGACAGGTTGGAGTTGATTTGGCATTTCAGGTATAACTTCTTCATAAGTAACTGGAAGATGACTTTGTGTTTCAACAGGTTCTTCGGAGGTTGTGGGG
AATGAAGAGCTTGAAGAATATATAGATGTCACAGGGGATGAGACCAGAACCTTCTTTCTTTTCTTCTTCTTTAAAGGTTTTGGTATCTCGGGGCACGAGCTTTCTATAGAATTTTCAGATGA
AGAAACTGATTGCCATCTTCTGGGTTGCTTAAACTCAAAATCATTCAAAGAAGCTGAATTGAAACCATTCAGAGATGCAGGGGTAATATCCGATGCAGACTTGTATTTACTCATCCAGCTG
GGCGCGTGTATTGAATCGCTGTCGAAGCCAATGTGTGCAATATGCCTCACATCTGTAGGGTGCCCTATCTCCATCTCTTGTTCCTTCAATGAAAATAAATTATAATCCTGAAAAGAGAAATAT
AACTCAGAAATTGGCTTTTCCTACCCCTTCCAAACGCCATTCTCCATCTTCTCTTTCCTCCATTATTAATCTAACAACAAGGAAAGAAAATGCAGAACATGGCCTGTTTGTCCATATATTAA
AAACAAACAAAAAGGCGATGTTACAAATAAAAATGTGAAATTTCAGATTCGAAAGAGGAAATGGAGTGAGGAGATAGAGAAACGACGGTAAAAGAAGCGTTTTTACTCAGTTTGGGAG
TTG
>comp154131_c0_seq4

AAGGTGTTTTGTATCTTGGGATTGTTGTTTTCAGCTGCACCGGGGACTTCCTGCAATTGGAATTTGTGATCGAAGACAGTATAACATGAAACGATATAGCCCTCGCAAAAATTCCACTGAA
CCTCGTAGGTGAATCGGCTTGTGAATTCAGAAAGAGCCTCAGTAAATCAAAATAGTTGATTCTATGATTCCTTTTTTAGTACTACGTGTTGTTCAATGCGTTTGGTTCATTGGGTTCAGAG
TTTGTTGGGCTACGGTTACCCATGTCCATTGTTTTGTGCAGAAGAAAGGTTCCTGAGAGTATTGTAATGAATCCACAAAGTTCAGTTGCAATCTGTGCATTATGTGATGTTTTCAACTTTCA
CAATTCTAGCTAGCATGATCATGTTTAAGGACTGGGACTCGCAGAATGCTACACAGATTGCAACTGAACTTTGTGGATTCATTACAATACTCTCAGGAACCTTTCTTCTGCACAAAACAAT
GGACATGGGTAACCGTAGCCCAACAAACTCTGAACCCAATGAACCAAACGCATTGAACAACACGTAGTACTAAAAAAAGGAATCATAGAATCAACTATTTTGATTTACTGAGGCTCTTTC
TGAATTCACAAGCCGATTCACCTACGAGGTTCAGTGGAATTTTTGCGAGGGCTATATCGTTTCATGTATACTGTCTTCAATCACAAATTTCAATTGCAGGAAGTCGGTGCAGCTGAAAACA
ACAATCCCAAGATACAAAACACCTTGTAATAGTATTTCTTTTTCCTCTTCATTTTTTGTTCTTTGGTAACCTTCATATTGTTCTAGTTCTGTTTTCAAGTAAAATTCGAATTGGAGACTGAATC
GAGCTGATTTGTATATATAAGCCC
>comp154161_c0_seq3
GGAGAAAGCTAGCAGAGACGCGAAACGAACCGTAGTCGTCCGGCGATCTCGCGGGAACAATCGTTGATCGACGGAATCTTCGCCAGTGACGAAGATTAAGAAGCAAGGTAAGAAGATC
GGCGATGGGCCGAAGCGATTGACCATTTTCGTCCACCGCGAGTGGTGAGACGACCTGCACAGATGGGGAACTGCCTCTCTTCCAAGGATCCTAGGGCTCTTGCTAGAAGGAACCCAGTT
ATTCCATCAAATAGATTTGCAGTTGCACCGTCAAGGAGTAAAGCTCCCAGGCCATCAAAGAACAAAGCTGTCGTACCATCAGAAATTGATTTTACTGCACCAAAATATACCAAGGCGGCC
ATATCATCAGTGAAAGCTTCAATTGCACTATCACTTTCTCCAAATGAGGATGCAGACAGAACTGGCTATGTGGAGAGCAAGCCAGTGATGCATGGAGATGTGGAAGACTTTCAACAAATA
ACTAGTTATGGAAATGTTGTCATATTTACATATGATGAGTTGAGATTATCAACCAGGAACTTCCGAGAAGATCTAATCCTAGGAGAGGGTGGCTTTGGGCTTGTTTATAAGGGAGTCATAGA
TGAGAACATTCGGCCAGGTCTCAAGCCCATGTTTGTCGCTGTTAAGGATCTCAACCGAAATGGTCTACAAGGTGATAGGGAGTGGCTGGCTGAAGTTAATTACCTCGGGCAATTGAGCCA
TCCTAATCTTGTCAAACTCATTGGATACTGTTGTCAAGATGATCACCGGATATTGGTTTACGAGTATATGGCTTTGGGTAGCCTAGAGAGGCATCTTTTCCAACGTATGCTGATGAATTCTCA
AAGATATCATGATAATGCGTTGTTATTATTTAAATTTATTTATAAAATAACCATTTTATTTTAACATTCATATTTGGAAAGTGGTTTTTCCTTGGTATCAGCACTTTTATGGAAGCCCGCAAGTA
ATTTCTTCAAGTTAACCAACAAACACCGTTGACACTGAGTTCTTGTGGAGGGTAGTCATAATCATAAATGATGCTTACAAATGAAAATGATTACTGAAGACTGAAGCAGCATGTTATGTAC
TTTGAGTTCAGGAAAAAAATGT
>comp154169_c0_seq2
TTTTTTAAGACCAGCATTAGCAAGTTATTCTCTTTACAACTCTCTCCCTTTCTTCCTTCTCCCGTCTGCTTTCTTATAAAGCTACTTTATTAGAGCTGCAGCAGACGAAAACAGGTATTTTTG
TTGCTTTCTTGTTGATTTCATATCTCTGTCTTTGTTTTTCTTTATTTCCTTTGTTCTTAGATCTTTTCGAGAGGTTCTGCGCTCTTCCAATTTGCTGGTGGAGATGGTTTTTCTGTCCATCAGCA
GATTCAATGTCGGAAGGCTCTGAAATCTTCTTGATTTCTCTTATCCCCTCATATGGTCTGAATCCTTCATAAAAGACTGCAGCAATTCTTCTTCTACTGTTCAAAGAGAAGGTTGTAGCAAA
GTACATTGCCTGTTCTACATATAGAGGAGAGAGATTCTCTCCATTAATGAATTTTGAAGAATTGAATATAGAGTTGAGATTTCAAGGTCTGATAGGTATTGGAGCGTACAGTAGGGGCTTGA
TGGAAAGCAGAGAAATGAAGTCGCCATTGTCTGGAAGAAAAAGGATTGTGGTGCCAAAAAAGCCTACTAAGCCTCAGGTAACGAATACAAGATCCTTATCTTTGGGCTACTTCAGTGTG
GAATCCTTCTTGTTGCTTGCCTGTCTCACAGCATCGTTGCTGTTTCTTCCTCTTATACTGCCGCCATTGCCGCCACCGCCGGTTATGCTGCTTCTTCTTCCTATAGGAATTCTGCTTGTTCTTT
TGATTCTGGCGTTTATGCATTCAGATATGAGGGATGTTTCCTCTTACTTATGAAGAGCAAAAGTCTGATTGAGATTGTTGTAGCTGTCTGTCAAATTAGTTGTGAAAAAAGAGTTTTTAAAA
ACTACTGTCTGCACATGTAATAAATTATTTTCGTTTAAAAAAGAAGT
>comp154169_c1_seq6
ACAACAATCTCAATCAGACTTTTTTAAACGAAAATAATTTATTACATGTGCAGACAGTAGTTTTTAAAAACTCTTTTTTCACAACTAATTTGACAGACAGCTACAACAATCTCAATCAGACT
TTTGCTCTTCATAAGTAAGAGGAAACATCCCTCATATCTGAATGCATAAACGCCAGAATCAAAAGAACAAGCAGAATTCCTATAGGAAGAAGAAGCAGCATAACCGGCGGTGGCGGCAAT

GGCGGCAGTATAAGAGGAAGAAACAGCAACGATGCTGTGAGACAGGCAAGCAACAAGAAGGATTCCACACTGAAGTAGCCCAAAGATAAGGATCTTGTATTCGTTACCTGAGGCTTAG
TAGGCTTTTTTGGCACCACAATCCTTTTTCTTCCAGACAATGGCGACTTCATTTCTCTGCTTTCCATCAAGCCCCTACTGTACGCTCCAATACCTATCAGACCTTGAAATCTCAACTCTATAT
TCAATTCTTCAAAATTCATTAATGGAGAGAATCTCTCTCCTCTATATGTAGAACAGGCAATGTACTTTGCTACAACCTTCTCTTTGAACAGTAGAAGAAGAATTGCTGCAGTCTTTTATGAA
GGATTCAGACCTGCTCAGAATCAAAGATTAAAAAGTGTTAGAAGTCCTAAATCAAATCAACAGAGTAGAAATTTTTGAAAGAAACCAAATTTGATCATATGAGGGGATGAGAGAAATGA
AGAAGATGATAGCTTCAACTCATTAAAGATTGAAATGGTTTCAGAGCTTGGCAGCCCATTGAGGGCTGTTGGATTCGGATTCCTTCACAAACCTCTCTCACAATATATATTTATCCTCTTTC
CGACATTGAATCTGCTGATGGACATAAAACA
>comp154172_c0_seq1
AAAAAAAAAAAAAAAAAAATTACGTAAGATAATCCCGTGAGACCGACCGTTTTAGTCCGACCGTACAATATACAAAACTCCAAATTTATCTTCCCATCTCAATCCTTCCCGACGATCGGCT
CTGCCCTTAATTTCCCCAAAAAAAAAAAATCCCTTCCCGACAACGTCGCGTGCGACAAAACCACCACACTCGATCTCCAGCCTCTACTCATACGTCTCCTACCATTTTCAACTCTTCATAA
CTAAATTCCGATATGTCCGCCGCAGCCGCCGCCGACGCCGTTGATCAATCTTACCCTCCCTTCCTCTCCGGCGCTGCCATGCCTCCTCCTCCACCGCCAGGCCATGAACCGCTCCACGTTC
CTCTGATTGACTTGGAAAATCTCGAACACAAACGGCTCGGCGACGTTTGCCGATCACTTGGCCTCTTCCGGCTCGTAAACCATGGCCTGCCGCGCGAGCTCTCGGACCGGGTTCAGGCT
GAGACGCGGCGGATTCTGGAGATGCCGTTTGAGCAAAAGCGAGCGGAGATGTCTGAACCGGACGGCGGCGGGCCGGTGGTTTATTTTTGGGGCACGCCTGCCGTGAGCTTGAGTCTTC
GGAGCGTCAACTGGGTCGAGGGGCTCCACGTATCGCTAGCCGGAATCCGCTCCGGGGACGGACTGGACTGCTCTGCTTCCCTCCGGTCTCTAATGGAAGATTACGGGAAACATATGGTG
CGCATTGTGAGAACCCTATTCGAATCAATGGCGCTGGATCTCAAACTGGATCCCAATCTTTTAGATTCGTGCATGGATCCATCTAACGGCATCTTTCGAATGTACCGTTACCCTTGCTTACCC
AAGGCTCAAAGCTGCTTAGGAATGGGAGCTCATACAGATAGCTCAGTCTTGACTGTGGTAGACCAGGATGATGTTGGTGGCTTGGAGATCCTCCATGATGGTTCATGGTTCCATATAATGC
CAGTTGCTAACACTCTCATCGTCAACACTGGTGATATGTTGCAGGCAATCAGCAACGACGAATATAAAAGTGTGGAACATAGAGTGCTAGCTAATGGAACCAAGGAGAGGATGTCTTTGT
GCTTCTTTGTTTTTCCTAAGGAGGATTTCAAGATCAGGAGCTCGAGTTACAGAGAGTTCACTTACAGTGAGTTCAAGGCTCAGGTGCAGGAAGACATTAAGGAAACTGGCGATAAAGTT
GGGTTGAAGAAATTCAGGCTGTAATGGATGAAAAAATCTCTGGATTTTGAATGAATTGAATGATTAAAGGCTTACATTTGTTTTCATTTGATTAATCATCTTTATTTTTGTGTTGCACTAATTT
GAGATTATGATTTGGTATGTTGGAGATGCTTTTATGATTTAATTGTTTAATGCTTCAATTAAGTTTTTGCTA
>comp154194_c0_seq1
AGTCTCCAATAGAGGAATCTCATAATGAAACTTGTTACATAGTTAATTTTGCAACTAGGCCAGGTCCCGTGATTTTTCCCACTCTGCAGCTATGTTGGTTGGGAACTTTTGTTACCAATCAA
GAGGTTTTGGGATCCAGCCTTGCGCAAGTGATTTGTGTCTAAACCCTAAACTCTTAATCCTAAACCCCCCAAAAAAAAAATACTGATGGTCGAACAAAGCATGAAAAGGTTGGCAGTATG
ATAATTATTCAAAGTACAGAGTTTAAATCCGCATTGCATCCTCCATTTAAACACAAATTGAAGCAATTGCATCCTTATTGTTACAGTTGTAAACTTTATGGAACAAATAAAGTATTCAAACAA
AGAGTTCACAGAATTCCATCACTCGACCATCAAAGGTATTCATGTATTTTACTCTTCAGCTGCAGCACTGGCTGCGCTGAGATCTACAGACAAATCAAGCTCCTTCCCGGTTATCTTTCTGT
ACATGGATATCACATCTTGAACGGTTGATTCAAAATGGGTTGTTGCATCATAGCTTGTTGAAATGAAGCAACTATCCTTCTGACTATTATTCACTGGCTCATATCTATCATATATATCAAAAAA
GGTTTCATCTACAGGTCCAAGCAGATCCATTCCAGGTTTCAATTCAACCTCAGGTCGGTCGGTCTCAGCTTCAGTTGAAACAAATGCAATATATTTTCCCTCTGGAGCTATGTTATGAGAAT
AAGAGCAGCAGAAAACGTACATGTCAGATTTGCGGCCCAATTGTTTCTGTGGAAGAATGATCTGCACTGAGTCTGAATTATTGGTATCTGTAATGGGATGACTCATTATGCAAATAGCACG
TGTTACTCTTCCAACTTTTTTCACCTTGTCAGGCAAGTAGGAAGGATCACAGACAACCTTTTTACACTTTGCAGTCTCTCCTTCAGATGTAACTCCATATGCTTTTCCATTTTGGTCAAACT
CAACCTTGCATTCTGGTTTGTTGAGCATATAGGTGCCACCATAAACAGCACTAAGACGAGCAAAACCCTGTGGTAGTTCTCCAAGCCCATATTGAGGGTAAATATAAGGAGATCCACCTTG
AAAGCGTGCAAGTGAATCTGCATATAGCTTCATTCTCTTTACTGTGTCAATGGCAGGCTTGTCCAGGTAATCATCATTCGTGTGAAGTGCCAAAGCATGCCCGATAAAGTCAACTGTGTTG
TCATCTAGCCCATATTTCGCAAAAACTTCTCTAGTTGTGACTTTGTTGAGGTCTAGTCCTTCATGAGATTTGGGATCATTCTCATCATAATCTTGAACATAAATGAACAGCTTTCGAGCACGG

CGTTTCTCAAAGAGCCCCATAAGAGTGGATTTTAGTGCTTCAACGTCTGTAGCAGGCACTTTATAAATCTTCCCCTTATTGTAAACAAAACTTCCATCAACAGCCTTGAAGTTAAGATATTT
CGTCACACTCGTGTGGATGAGCACACGGACTAGAGCACCGTTAGCCATCATAAACTTAGGGACCATGTCAACATTGTAATCCTTGGTTGAGCCCAGACTTTCAGGAGGTTTTTCATCACCT
CTAAATCGCTTCCAGAGCTGAACTAGATTAAGTGATGTTGATTCTCCACCATAGTAATCATTCCTATCCATGTGAAGAACCTCCTCCTCATCTCAATATGATTTTCCTCCCGGCATCCGAGCT
ATGGCGTCTCCCCGTGGTCCATGAAAGTTGAGCTCGCCCACCACAAAGAACGCCAAGGACCTCGTTTCTAAATGCATTTCTTTCTCCCATTCGGACTTCGACTCCCTTGACGCCATGAATC
GTCTCCTTCTGAAGGGCAACTGTGGCCATCATAACCTCCTGAAGCTGCCTGCAACAGCTCGT
>comp154196_c0_seq1
AAGAACAGGAACGAGACGGAGAAAAGGCTAATTCATTTCATGTAGTATTGAGTTACAATGTGTATTAAATAGAGATGAAACAGTAGCTGTAACTAATAGGACTCGGACACGTGTCTAGTA
CAATCAAGAAAGACTAATACGTGTTACTAATATCTTAACACGCCCCCTCAAACTATTGCATCGGACGATGTAACTAGTTTGCTTGAGAGAAGCTGAAACCGTGGAGCTGCGAGCGTCTTA
GTTAGTAGATTTGCAAGTTGGTCCGTAGTTGATATGTGATGAATTTGGATGGTGTTGTCTTTGATGTAATTTCGGATGAAATGGCAGTCAACCTCTATGTGTTTGGTGCGAGCGTGGAAGAC
GGGGTTATTGGCCAAAGCGATGGCGGATGTGTTGTCGCATAGAATGGGCGTAGCCGTGGATTGCGGGCTGTTGAGTTCTTGGAGAAGACGCCGTAGCCAAGTAACCTCGGCGGCGGCGG
ATGCAAGTGCCCGGTATTCTGCTTCGGTAGATGAACGAGCGACGGCGTTTTGTTTCTTGACGCTCCAAGATATGAGTGACGATCCTAGGAAAGTGCAGTAGCTGGTGGTAATTTTCGGTC
GGTCGAGTCTCCAGCCCAGTCGGAGTCCACATATGCACGTAGGTTGAGATTTGAGTTTGCAAACAGAGAAATACCAAAATGGAGGTACCTTGAATATACCGCAATAGTCTTTTAAGAGCA
TCAAAGTGGATGTTTAGAGGATTTTGCATATGTTGACAAACCTTGTTGACGGCGAATGAGATGTTGGGTCTTGTTAGCATCAGGTATTGAAGTGAGCCTGCAAGTTGACGATATAGCGTGG
GATTGGAGAAAGCACCGGAGTTGCTTTGTTTGGAGGGAGGTTTTAATTGGGACGGCATAGGAGCAGATTTGCAATTGGACATTCCAGCCCGGGAGAGAATCGTTTGGGCAAAGTGAGTT
TGATTTAGGTGTAGTCCATAAGAGGAGTGGACAGCTTGAAGACCCAAGAATTGAGATAGCTTCCCTAGATCCCGGATAGTGAATTTTGCTTTGAGATTTTGCATTAGATTATGTATAGATTT
GGTGCAATTTTCGGTAAGGAGAATATCATCGACGTAAACCAAGAAGTACATAGGCACGCCATTTTTATTGTAGATAAGAAGAGAAGGGTCGGACGTACTAAAGGAAAAGCCGAGCGACT
GTAAGTAACCGGATAGCGTGGAGTACCATTCGCGAGGGGATTGTTTAAGTCCATAAATAGCCTTTTTAAGTTTACAAACGTAGTTGGGATGAATGGTATCTTTAAATCCGGTAGGTTGTTTC
ATGTAGACGGTATGATTTAGACTACCGTGGAGAAAGGCATTAGAGACGTCGAGCTGGTGGATAGGCCATTGCTGATGAAGAGAGACTACAAGAAGGACCCGAACTGTAGGGATTTTGGC
CACAGGGCTAAAGGTTTCGGTGTAATCTAAACCATATTCTTGGTTGAATCCCTGAGCTACGAGTCTGGCTTTATAGCGGGAGATGGTACCGTCGGCAAGATATTTTGTGCGAAAGATCCAC
TTGGAACCAAGAGTATTTTGATCGGGTTGCGGAGGAACAAGCTCCCAGGTACCTTGAGATTGGAGGGCTTGAAATTCCTGAGACATAGCGTCACGCCAGTGTTTGTTTTTGACGGCTTCA
GCATAGGTAGTGGGATCAGACCTGGCTTGTTGATGAAAGAGATTGAAAATAGTTTTGGGTTTGAGGTTACCTGTTTTGAACCTAGTTTGCATGGGGTGAGTCAGATTTTGTGCTGCGGGC
AAGATAAGACGAGACGGACTTGAATTTCTAGAAACGGTGGATGGGAGAGAAGGAGTTTGGTTGGCAGGAGGAGATGGCACGGAGTAATGTGAAATAGAATTAGTGTTGTGCTTGTTATG
CGGTGATGTTGTTTGGGTATTTTAGAGAGCCGGTTTACCTGATAGGATGGATGTAGGAACGAGAGTGAGAGGTGGGATGAATTCAGGTGTTGAGGCTTCTTGAGTGATTTGAGATCCTGC
TTGATAGGGGAATATAGATTCATTAAAGATAATGTGCCGAGACGTGTAGATTTGCCTAGTTTTTGGATTAAAACATCTATAGCCTTTTTGAGACGAGGCGTAACCTATGAATATACACGGCGT
AGACATAGGTGAAAATTTGCTAGCGGAGTATGGTTTGAGCCAAGGGTAATAGAGGCAGCCGAATATTTTTAGATGATTGTAAGAGGGTGATTTTTGAAATAGCTTGTGGTAAGGGCTGTGA
TTGGCAAGGGATTGAGTGGGTAGCCTATTAATGAGATACACGGATGTGTGAAGAGCGTCATCCCAGAATGATTGAGGAAGATTGGCTTCAATAATAAGACTACGAACAGTTTCGAGGATG
TGTCTATGTTTTCGTTCGGCAACTCCGTTTTGAGAGGGAGTATAGGAGCAACTGTATTGATGGATTATTCCTTGTTGTTTGCAGAACGTTTGAAATGCATGATTTATGTATTCACCTCCACCG
TCGGATCGGAGCATTTTAATATTGGTGTTAAATTGCCGATTGATCATTTTAGAGAATTCGATGAATTTATGGATCACCTCAGATTTATGAATGATAGGGTATACCCAACTGTATTTGGAGAAAT
CATCGATAAATGAAACGTAGTATTTAAAGCCATTTATAGAAAAAAATAGGAGATGGACCCCAAACATCAGAATGAATCAGATCGAAGCAAGAACGACTAGAAGTGCAAGATCTATTAAAT
GGTAACCGGTGGCTTTTAGCCAACCGACAAGAGTTACAATGGGAATCATGTGCGGTGGTACATAAAGAAGAAATGTATCTAGACAAATGCTGAAGAGTGATGGCTGCCGGATGACCAAG
ACGTTTATGCCATAGTTGAGGCACGGCTTGAGTAGATAGAAGGGCTAGGTATTGGTGCGGAGTAGTAGACCGGATGGGATAGAGACCGTTAAGGCAAGGTCCCTTGAGTAGAAGACGAC

CCGTCGTCGAATCCGTGCGGAGTGGGTAGGACGCCTTTACTTGAATGTTGAATGGGAACCTGATACCCATTCCCGAGAGTGACTGGTTGTTGGCCGGCATACGGTTTGACAGATTGGATG
TTGGAAAGGTCAGAAGTGAGGTGAGTTGAAGCACCACTGTCAAGATACCAATCAACGTTGTTATTGGATTATGAAGGAGAGAATAGAGCTATTTTCTGTGATTGGGTATCCTCGGTATAGG
AAGTGTGGAGGCGGTACCAGCATTTTAAGGCTGAATGGCCAAGTTTCGAGCAGATTTGGCAAGTAACTGGATTCGGAGCATTATTCTTGTCGGGATGGCGAGAGTTGGGACGCGGAGAT
CTATTTGAATACGATGAACGTCCCCGATAATTGTTCTTACCTCTTCCACGACCGCGGGGCGCAAACAAAGCGACGGATGAGTCGGTGATTTGGAGATTTTGGAGATCTTTCATCGCTTCTT
GGGCAAGATTTTGTTCCTCACTACACAAAAGAGTGTAGAGGGTATCCATGCTAAGGGGTTGAAGATTGGTGCGGATGGCAGTTTTGAACGCTTGATATTGATTTGGGAGACCGTTTAAGG
TATAGAAAATGACTTCTTCCGAGGTAAGAGGAGAACCAGTCGCGGCTATGGCGTCAACCTTAGTTTTAACAGCCAGCAAGTATTGAGACATATTTAGATTTTTCATGGAGAGATAGTTAAG
CTCGTTGCGGAGTTGAATGGTGCGTGATCTTGATGAGGACTGGAGACGGTGAGAGGTGTCCCAAATATCGCTGCAGTGATCCAGGGATAAGACATAAGGAAGGATGCTAGGGGAGATGA
CAGAATAAAGAGCAGCCGCTAGGTTTTGGTCAAGAAGATTCCAAGATTGAGAGGAGAGGGAGGATGAGGAGGAAGAATCATTTGGAGAGGGGCAGCGGCAAGAGCCATCAAGAAAAT
CATCAAAACCATTTGCTCTAAAAATTTTTATAACTTGGGATCTCCACATTGGGTAGTTTTCGGCTGTGAGGGGGAGGGAGATGATGCTTTTGATATTACCTATGAGAAATTTTAATTGCGGT
GGATGAGAGGGTAAGACAGGGTAGCAGTTTCTTGAATTTCTTCCGGACCTCCGGTGGCGGACGAGACTGCCGGCACCTCCATGGAAGACGGGAAGAAGAAGAACGCAGAATGGAAA
AAGAAATCACGGACGAAGGCACTGAACCGCCTGCTC
>comp154196_c0_seq2
AAGAACAGGAACGAGACGGAGAAAAGGCTAATTCATTTCATGTAGTATTGAGTTACAATGTGTATTAAATAGAGATGAAACAGTAGCTGTAACTAATAGGACTCGGACACGTGTCTAGTA
CAATCAAGAAAGACTAATACGTGTTACTAATATCTTAACACGCCCCCTCAAACTATTGCATCGGACGATGTAACTAGTTTGCTTGAGAGAAGCTGAAACCGTGGAGCTGCGAGCGTCTTA
GTTAGTAGATTTGCAAGTTGGTCCGTAGTTGATATGTGATGAATTTGGATGGTGTTGTCTTTGATGTAATTTCGGATGAAATGGCAGTCAACCTCTATGTGTTTGGTGCGAGCGTGGAAGAC
GGGGTTATTGGCCAAAGCGATGGCGGATGTGTTGTCGCATAGAATGGGCGTAGCCGTGGATTGCGGGCTGTTGAGTTCTTGGAGAAGACGCCGTAGCCAAGTAACCTCGGCGGCGGCGG
ATGCAAGTGCCCGGTATTCTGCTTCGGTAGATGAACGAGCGACGGCGTTTTGTTTCTTGACGCTCCAAGATATGAGTGACGATCCTAGGAAAGTGCAGTAGCTGGTGGTAATTTTCGGTC
GGTCGAGTCTCCAGCCCAGTCGGAGTCCACATATGCACGTAGGTTGAGATTTGAGTTTGCAAACAGAGAAATACCAAAATGGAGGTACCTTGAATATACCGCAATAGTCTTTTAAGAGCA
TCAAAGTGGATGTTTAGAGGATTTTGCATATGTTGACAAACCTTGTTGACGGCGAATGAGATGTTGGGTCTTGTTAGCATCAGGTATTGAAGTGAGCCTGCAAGTTGACGATATAGCGTGG
GATTGGAGAAAGCACCGGAGTTGCTTTGTTTGGAGGGAGGTTTTAATTGGGACGGCATAGGAGCAGATTTGCAATTGGACATTCCAGCCCGGGAGAGAATCGTTTGGGCAAAGTGAGTT
TGATTTAGGTGTAGTCCATAAGAGGAGTGGACAGCTTGAAGACCCAAGAATTGAGATAGCTTCCCTAGATCCCGGATAGTGAATTTTGCTTTGAGATTTTGCATTAGATTATGTATAGATTT
GGTGCAATTTTCGGTAAGGAGAATATCATCGACGTAAACCAAGAAGTACATAGGCACGCCATTTTTATTGTAGATAAGAAGAGAAGGGTCGGACGTACTAAAGGAAAAGCCGAGCGACT
GTAAGTAACCGGATAGCGTGGAGTACCATTCGCGAGGGGATTGTTTAAGTCCATAAATAGCCTTTTTAAGTTTACAAACGTAGTTGGGATGAATGGTATCTTTAAATCCGGTAGGTTGTTTC
ATGTAGACGGTATGATTTAGACTACCGTGGAGAAAGGCATTAGAGACGTCGAGCTGGTGGATAGGCCATTGCTGATGAAGAGAGACTACAAGAAGGACCCGAACTGTAGGGATTTTGGC
CACAGGGCTAAAGGTTTCGGTGTAATCTAAACCATATTCTTGGTTGAATCCCTGAGCTACGAGTCTGGCTTTATAGCGGGAGATGGTACCGTCGGCAAGATATTTTGTGCGAAAGATCCAC
TTGGAACCAAGAGTATTTTGATCGGGTTGCGGAGGAACAAGCTCCCAGGTACCTTGAGATTGGAGGGCTTGAAATTCCTGAGACATAGCGTCACGCCAGTGTTTGTTTTTGACGGCTTCA
GCATAGGTAGTGGGATCAGACCTGGCTTGTTGATGAAAGAGATTGAAAATAGTTTTGGGTTTGAGGTTACCTGTTTTGAACCTAGTTTGCATGGGGTGAGTCAGATTTTGTGCTGCGGGC
AAGATAAGACGAGACGGACTTGAATTTCTAGAAACGGTGGATGGGAGAGAAGGAGTTTGGTTGGCAGGAGGAGATGGCACGGAGTAATGTGAAATAGAATTAGTGTTGTGCTTGTTATG
CGGTGATGTTGTTTGGGTATTTTAGAGAGCCGGTTTACCTGATAGGATGGATGTAGGAACGAGAGTGAGAGGTGGGATGAATTCAGGTGTTGAGGCTTCTTGAGTGATTTGAGATCCTGC
TTGATAGGGGAATATAGATTCATTAAAGATAATGTGCCGAGACGTGTAGATTTGCCTAGTTTTTGGATTAAAACATCTATAGCCTTTTTGAGACGAGGCGTAACCTATGAATATACACGGCGT
AGACATAGGTGAAAATTTGCTAGCGGAGTATGGTTTGAGCCAAGGGTAATAGAGGCAGCCGAATATTTTTAGATGATTGTAAGAGGGTGATTTTTGAAATAGCTTGTGGTAAGGGCTGTGA

TTGGCAAGGGATTGAGTGGGTAGCCTATTAATGAGATACACGGATGTGTGAAGAGCGTCATCCCAGAATGATTGAGGAAGATTGGCTTCAATAATAAGACTACGAACAGTTTCGAGGATG
TGTCTATGTTTTCGTTCGGCAACTCCGTTTTGAGAGGGAGTATAGGAGCAACTGTATTGATGGATTATTCCTTGTTGTTTGCAGAACGTTTGAAATGCATGATTTATGTATTCACCTCCACCG
TCGGATCGGAGCATTTTAATATTGGTGTTAAATTGCCGATTGATCATTTTAGAGAATTCGATGAATTTATGGATCACCTCAGATTTATGAATGATAGGGTATACCCAACTGTATTTGGAGAAAT
CATCGATAAATGAAACGTAGTATTTAAAGCCATTTATAGAAAAAAATAGGAGATGGACCCCAAACATCAGAATGAATCAGATCGAAGCAAGAACGACTAGAAGTGCAAGATCTATTAAAT
GGTAACCGGTGGCTTTTAGCCAACCGACAAGAGTTACAATGGGAATCATGTGCGGTGGTACATAAAGAAGAAATGTATCTAGACAAATGCTGAAGAGTGATGGCTGCCGGATGACCAAG
ACGTTTATGCCATAGTTGAGGCACGGCTTGAGTAGATAGAAGGGCTAGGTATTGGTGCGGAGTAGTAGACCGGATGGGATAGAGACCGTTAAGGCAAGGTCCCTTGAGTAGAAGACGAC
CCGTCGTCGAATCCTGTATGTGAAATTCATGAGGAAAGAACTTGACGATGCAGTTGTTATCAGAAGTTAGCCGGTGAATAGAAATATTAAAGGAGAGATTCGGAACGTGATAAAGATTGT
TCAGATTAAGCTTACCGTGCGGAGTGGGTAGGACGCCTTTACTTGAATGTTGAATGGGAACCTGATACCCATTCCCGAGAGTGACTGGTTGTTGGCCGGCATACGGTTTGACAGATTGGA
TGTTGGAAAGGTCAGAAGTGAGGTGAGTTGAAGCACCACTGTCAAGATACCAATCAACGTTGTTATTGGATTATGAAGGAGAGAATAGAGCTATTTTCTGTGATTGGGTATCCTCGGTATA
GGAAGTGTGGAGGCGGTACCAGCATTTTAAGGCTGAATGGCCAAGTTTCGAGCAGATTTGGCAAGTAACTGGATTCGGAGCATTATTCTTGTCGGGATGGCGAGAGTTGGGACGCGGAG
ATCTATTTGAATACGATGAACGTCCCCGATAATTGTTCTTACCTCTTCCACGACCGCGGGGCGCAAACAAAGCGACGGATGAGTCGGTGATTTGGAGATTTTGGAGATCTTTCATCGCTTC
TTGGGCAAGATTTTGTTCCTCACTACACAAAAGAGTGTAGAGGGTATCCATGCTAAGGGGTTGAAGATTGGTGCGGATGGCAGTTTTGAACGCTTGATATTGATTTGGGAGACCGTTTAA
GGTATAGAAAATGACTTCTTCCGAGGTAAGAGGAGAACCAGTCGCGGCTATGGCGTCAACCTTAGTTTTAACAGCCAGCAAGTATTGAGACATATTTAGATTTTTCATGGAGAGATAGTTA
AGCTCGTTGCGGAGTTGAATGGTGCGTGATCTTGATGAGGACTGGAGACGGTGAGAGGTGTCCCAAATATCGCTGCAGTGATCCAGGGATAAGACATAAGGAAGGATGCTAGGGGAGAT
GACAGAATAAAGAGCAGCCGCTAGGTTTTGGTCAAGAAGATTCCAAGATTGAGAGGAGAGGGAGGATGAGGAGGAAGAATCATTTGGAGAGGGGCAGCGGCAAGAGCCATCAAGAAA
ATCATCAAAACCATTTGCTCTAAAAATTTTTATAACTTGGGATCTCCACATTGGGTAGTTTTCGGCTGTGAGGGGGAGGGAGATGATGCTTTTGATATTACCTATGAGAAATTTTAATTGCGG
TGGGATGAGAGGGTAAGACAGGGTAGCAGTTTCTTGAATTTCTTCCGGACCTCCGGTGGCGGACGAGACTGCCGGCACCTCCATGGAAGACGGGAAGAAGAAGAACGCAGAATGGAA
AAAGAAATCACGGACGAAGGCACTGAACCGCCTGCTC
>comp154220_c1_seq15
TCACGACAGTTCCGAATTGCCCCCATCCCACTCTCAGCAATTCACGAACCGTTCTCTCTCCATATTTCGGAAGCCACCCAGCCTTCTTACGAACTAACGCACAAAACCACACGGAAAAAT
CCCAGCCCCATTCACGAATCGCACTCCTCTCCACTACTCCTCTCCTATTCGCACAGAAGGAAACCTCCAGCCCATTCGTTCTTCTCTCTACAGTTCCAAAATTCCTAAACCACCCCATCCTC
CCTGCAGAGCGAACTGCCTCCCCCTCTCTTAACTCCCGAACACCCTCTCTCACATCCCACTCTCAGCAATTCACAAACCGTCCAAGCTCTCTCTCACTCAGCGGTTCACAACCCAACGGC
TCCTCTCTCTATCTCACACAGAGAGAATCAAACTCCCACGAACTCCAGCCTCCTTCACTACAGTGCACGAAACCCCCAATCTCCTACAGTTCGCTAACTGCTCCCTCTCCCTGACTCCCTC
TAATTCACAAAAACCACCGCTCCCCCTCTAAGTTCAGTTCACAACTCCAGTCTCTCTCACTCTCTAATTCGCATACCTCCAGCCCGTGACTCTACCAAGTCGTGAAACCAGTCCAAAATCC
ACTGCTTTCTTACCAGCTCTGTCAAATCCCTCGTTGTTTCCTCTCGGCTCGCAAGAAGCTCCAACGCAGAATCCTGAAGGCCGCCTCTTCCTTCTGCATCCCGCCGCTTCTAGTTGCTCAG
GCCATTGACGCAGAACGTTTGACTGTAAAAAGGAGTGCGTAATATAGAGCAAATTCAGGTTAATGGCTTCATCAATCTCTCTCTCCGATGACATCCACATCTTCTACAATAAAATTTTTCAA
ATGTCCAGCCCAGATGATAATGTGGAAGAACCTTATCTCATCCTCAACTTCATTTACAATGTTGACGGGGACAGCATGGAGCATGATATTATTGCCACGTCAGAACATAACTTCACTGAATG
CAATCCATCTTTTCTTCGAGCTTTGATTGAAGAGAAGGAGCCTCTTCCATTGGATGAAATGATTGATCCACAAGATGCTGAGATTTCAAGAGAGGGAATCTCAAACTTCCTGGCTTTGCTG
GTAAACGATTGTGCTCGGGTTGAAGGGCTAAAGATATTGGAGGTTGACGTCATTGTAGATATCAGTCTCGAAGAAGAAGATTATGATTTTATGGGATTAGATGATGCGGACTTCCTTCACG
AGAGACGGATGAATTCGTTGCAGAAGAATGTTTATAAAAATTTGGCAAAGGCTGAGAAGGAGAATGAAATTTGTGCTATTTGCTTGCAGGAGATGAAGGTGGGGGAGGAAGTGGCTTGC
ACGCCTTGCGTTCACCGATTTCATCATACTTGTATTAGTAAGTGGTTCGAAAAATCTGGCTTTTGCCCATTATGTCGGTTCAAGATTGATCCATCTTGATGGAGGATTACTTTTGTTATCTTTA

TTTTGTGTTTTCATTTATCTAGTTTAAATTGTTCAATTGTGGAAAATTAAGAAACAGATGTTAACATAAATCTTTTTAATATATAAATTCATTGCAATTTTCTAGCTAAAAA

>comp154232_c0_seq9

ATATATATAGGCAATAGAGGGGAACTAAACAATCACTTAAAAGAGAATATAGAAGGTCAAGAGACCACTAATGAACAGCCCAACCAGCTGTCCAAAACAGCAGCCCAAAAGTCCATAAA

TCTGGCCAGAAACAGTGAAAAATTCAGCAGAAAAACTGCACTGAAAACAATGGAAAGACTCTTGGAATTCTCCCCAAAACTGCCACCAAACAGCCCCAAAAACTGTCCAAATTCAGTC

CAAATACTATTCATATTCCCATTTAAATTAAAAGAAATGAAAAGCCAAGAGGCACA

>comp154239_c1_seq5

TTTTTTAAAAAGAATAGTCATTTATTCAAATACTGCATATTTCAATAATAAATACTGATTGGTCAAAGGGAACGTGACATAAATTATACTGGTTCCCTTCAGCAGCATCAGCAAGAAAGCAA

ATAACTTAATATTGAAAACATATAGAGAGCATTGATAGGAATAGTAGCAGAAAGTAAGTAACTATGTTCCATATGGCAGGATCATCTTCAGCACGCAGAGCAAAAGACAAAGGTCCAGTCC

ATCCATCACACTACATATGTATATATCTATATAGGATAGAGGTATTAACAAAGTACTATGGTATTAACAAGTGAATCCTGCTATTATTGTTGCTCTGCTAATATTCCATGAAAGCTTTACCCCAC

CTCAGCCGTAGGCGGCCTTGTTTGCTTCTTGATCGGCTTCAGTGTTGTACTCCCTTGGAACATGGCTGATATCAAATTCGTCCAGCCCTCCGACCAGATCATTGACCCTTCGGCTAGGTTCA

AACAGGTTGCTGCTTCCGACTTTCCAGCTTCCATCAACCTGTTTACAGACAAGTTGTGAATCGCCCCTTGCGTGAATCTCTTTGTAGCCCATTTCAGTAGCTTTCTCAATTCCTACAATCAA

GCCCTCATATTCTGCAACATTATTGGTAGTAAAACCCAAAGGCTTGTTCACCCGAGCAACCACATCTCCCCTGGCATTGCGTATAACTGCTCCAGCGCCTCCTTTTCCAGGATTTCCTTTGC

AAGCACCATCAAACTCAACAGTGCAGGATTTGTTGGACATCGTTGAAAATAGCAATCCGTCAGACACAGAACTGAGGAGAGGAGAAATCAGAGAGACGCATTCTGATCTCGAACCGAT

GGGAATTTAAAGAGAAGGGCGGAGAGCGCTAGGGTACTGAAGTACATATCTCCAGATGAAGTCTGACCACCACGTGGTGTAGTGACTTAGAAATCG

>comp154270_c0_seq3

TTTTTTTTTTCTCTTTTTTTTTTTCAATAGGAAATAGATTTTTTCTTTTTAAATTTATTGGTTAGGTATTGGTCACAGAATAGGCAAGGCGCCGTAGTAGAAGTGCGGCGCTTTTTGGCATCTC

AGCCGTTGGATGCCGATCCGGCGGTCTTTTCGCTTTCCAAGCCGGCGCGATGGCGGTCGAGTACAGTCACCGGCGTGAGAGCAGAGTAGCCCGAATCACAGCCGTTCGATGCTGCCTCA

ATCCCGGCAGTCGTATCGTAGCTCTCCATCGCGGCGCAGCATTCTGCCAAAGACTCCTGATAATATTTGTAGAAATACCCTCAACAAAACAAGAAATAAAAAAAAACATATAGAATAAGAG

GAAAATGTAAATCTATGGTTATGCGCGCACTTTATTGAGATATTCACAGCAGAGCAGGGCAGTGCGGAAGGAAATGAAATGAGCAGGGAAGAGTTCGCGCGCGGCGGCGAGAAGCGCT

GCCGCAGCGATCACCGAAGGCTTGAACTCGAGCGATTTGACATCGTTTTGGGATTTGAAGAGGATCTGAGAGGCGTGGGTCTTGAGAGATTGGATGATGATTGGATTCGCCAGGGGAAA

GTAGGATGTGAAGAATCGGAGAAAGGAGAAGGGCGTGATCGATCGCATCCGCCATTCAAGCACGCCGAGCACGAGCAGTTCCATTCGCCGGATGGTCCGTGCATCGAAGATGAAGCCCT

CCTCCCTCTGCTCCGAACAATTTGGGGAAAATGTTGAATCGATCGGATCGAGATTGTTCGATAGAAGAGGTGAAGCTAGCTTCACGGTACCTGTAAATCAGCAGGGGAGAAACTGGTCTT

CTTCATCTTTGAGGCGAGGGAGACGCAGGAAATGGCGAGGAGTCGCGCGATCCATGGCTTCTCATTCGGGATCTCGCGTTTGGCGAGGAACCGATCGATATAGTTGACAGCAAGGTAGG

CTAGTGCTGGATCGAGATTATTGCTGAACTGGGCCTGGACGATAAGGGAGAGGGCATGGCAACGGGTGGAGAGCTGGTCCAGAGCAACCGCAAGCGAGCTCATGTGGTCCGATTCAGC

TGAGAAAAGCAACGAGGGCTCGTCGGAGTCTGTGCAGCCGATGAATAGTGCTTTCGAGAGTTGGATGTTTTGCTCATCGCTACCGGAGAACGGGTTCTCAAGGTCGAAGTCGAACTCCA

TTGCTAGTTTCTTACTCTTCAATCCGGCTGTGTGAGAAGAGGAAGAGAGAGAGAGAGA

>comp154281_c1_seq1

ACAAAAAAAAAATGTTTGGATGGGAATTGACGAGAAGTGAATAATCCATTAAAAGTTTTCATGTAAGATCGAGACTTTCCAGATGAAGAACTCGGATCCATAACTAATTTAAGTAAAGTAA

AATTAGTAGAAAGATTCATACAGAACTAGAAAAGAAAAAAAAAATCCTTTCAACACAATAAAAAAAAAACGAAAAGAACTTTCTAGGACCTGCCTGTATTTTCTCATTAGAACCGCTCCA

TCTAACTGCCCACGTCTACACGTGTCCATTTTTAATCTCCCGCCAGCGCACGTGTCTTCCCGCTCTTCAGCGACTCACTGTTCTAATCCCACAACCGCTGAACGAATTACAAATGATCCCCA

TTACTCACACCAGCAACTCGATCGTTTTCCTCTGATAAAATTTCCATCACCGAACTCCTCTTCTCGCTAACATGATGAACATCGTCGCCATCGCTTTCATCCTCTCTTCCATCGCCGCCGCTT

CTTTCTTCTCTTCTCCTCCCGTCCCCGGCCAGGACCACCACGTCCTCCGCGAAGGTCACCGCCTCATTGTCGTGGAGTTCGACCGCCAGATTCCACAATCCTCCTCCTTCGCACCAAGCG
CTAGCGAAGCTAACGATGAGCCCCTCGATGCCGCTTCCGTCCAGCCCAATGTCGGCCAATCCGTGTCTCAAACTCCAAAGGCAAAAATTTCTTCTGTCTTCACAGATGCTAAAAATAAGG
CTAGCCAGTTTGAAAAATCGACCGCCGATGCTCTTAAATCCGTGGCCGAGAGGCTCACTCATAAAGCCGCTCAAATCGAAGAATCTGTCTCCGACCGCCTTAAATCCGGCGCCGACGAG
GTCAAGCAAAAAGCCTCCAAAATCGAAGACTCTTCCTCCGCTGCTCTTAAAAACGCCGCCGAAAAAACCAAGCAAAAGGCCACCCAATTCCATGAATCCACCGGCAAAGCCGCGAAGA
ACGCTTCGGAGACCGCGGAAGCGGCCAGCAAGGGCGTCGCTGGCAATATTTCTAACATGGCGGCTGCGGCGATGAGGGAGATCCGGCGGAACCTGACCGAAATCCTTCAGCGAGCTTG
GGAGCTTGGCCAAGACGTGGGAGCGTTTGTGAGCGCGGGGGCGGCCGAGGCCGCGCGGTCGGCTGCCGCGGTGACCCACTTGCTAGGGTTTGCAACGGCGTATGGCACGTGTATGTGG
GTGACGTTCGCGTCAAATCACGTGCTGGCGTCTGCACTGCCACGGCAGCAACTTGGGATCGTGCAGAGCCGGATTTATCCGGTTTATTTCCGGTTTTTGGCTGTAGCCGTTGGTGTTGCGC
TCGCGGGTCATATTCTGAATGGGAAGGAGAGCGTGCAGCGGTATAATTTGATGGCGATGCTGGCGCTTGTGTTGCTGAATCTGATCTGGCTGGAGCCTAAAGCTACCAAGGCTATGTTTGA
GAGGATGAAGTTGGAGAAGGAAGAAGGAAGAGGGATGGATGTTGCTGATGTCTCTACTGAGCCAACCAACACGCCAGTTGCTGATGCTTTAGCCATAAGGAAGGAGGTGGCCAAGGGC
CGAATGACTGAGTTGAGGAAGAGACTAAAGAGTCTCAACAAATACTCGTCCTTTCTGAATGTGCTCACTCTAATGGGGCTAAGCTGGCATCTTGTCTACCTTGCTTAGATTTTGATGTCTT
CTAATTCTGAGTGTTGTAAATAGTTTATTTTTCTTCTTTTTAAGGCTTTGTTT
>comp154352_c0_seq1
TAAAGAACACAAACCATTCTTATAATTTATATAAAAATATATATAGCATCAAACTTATATTTATATAATCCAAATTCTGATCATTAATTTTTGTACAATTGGAAGTAAAATAACAAAATAATTCTT
CAGACTTTCTTCACCCAGTGTAGAGCCTTAGACGAGACGTAATGTTTGTTCTACAGATAGGACATTTCGACAGGTGATTCCCGCATTGTCTGCAGCATGTATGGCCACATCCGAAGGCCAA
ATCCTTCGCATTTGTCCAGCAAATCGGACATGCCTCGTTACGGTCATCAGCAATACTGGGATTTGAAGTGCTTGTTTCTCTCCTGAAAGATAATCGTTGTTTGTTTGCAGGTTGAGGAGGA
GGTCTAAACGTTACTTGTTTGGCCTTCCCAGTCGTACGCCCGAGAATGCCAAGTTCTTTGGTAGCTTTGTACTGGATGGGAACCTCCATTAGAGCAGCCAGTGCGAAAGCTGCTTCCTTCT
CTGCTGGGCTAGCATTTTTGGCCATGATCTTGGAGAAGTTAACGAACTGAAAATTGTCAAAGGCTCGAGCTGGAATCTTATCATCAAATTTTTGCATGTCTTCCCATGGTCCATCCCCAACT
CCTACTAATACAATTGACAATGGAACACAGCTGATAATTTCAAATTTGGAACAATTTTCCTGTAGCATTCGAGAACTTCCTCAAAGCCATGACAAGGAGAGTGATCGCCATGAAAGCTGA
ACACCTCCCTATCATGAGTTGTTGCATCGCCAAATCCAAAGCATGGGATGAGATTGTCTTCGTCAAATGGAGCTAAGGTCTTCCCTATTATGCTGATGGCTTCTTCATATGGATTTCGTTTAT
CACCAATTGCATGCAGGCTCTGGTTATTGAATGAGTATTTGCCTGCCCAATCATTGCTTTTTGTAAAATCAATTCCTAGGATCAAGTTTGATGACTCCAAGCCTGCTTCTCTTAGGGCTGCT
GTAACCTGGTCGAGAGAGTTATAGTTGTCAGGTATGTACGAGTACTTCCTAGTTATCATTCTCCTATTCGCTTCACTGTTTCTGCGACTCGTGCTCGGTTGATCTGTTATAACCCCTGTCGCA
GCCGACGCCGGCGATGGATCCCGGCGATCAACGTCGCTCAGATTCCGGCCACCCCGGCCAAAGAAACCTCCCCAAGAAAGTGCGAGGAAGATGTTCTTTAGCTTTGCTTTTGGCCCTCC
AATCGGACAAAGGAGAACCTTCTGCGTTTCTTCCACAAAGTGTTGTCTTTTTCACCTGCCACTTCAT
>comp154352_c0_seq2
TAAAGAACACAAACCATTCTTATAATTTATATAAAAATATATATAGCATCAAACTTATATTTATATAATCCAAATTCTGATCATTAATTTTTGTACAATTGGAAGTAAAATAACAAAATAATTCTT
CAGACTTTCTTCACCCAGTGTAGAGCCTTAGACGAGACGTAATGTTTGTTCTACAGATAGGACATTTCGACAGGTGATTCCCGCATTGTCTGCAGCATGTATGGCCACATCCGAAGGCCAA
ATCCTTCGCATTTGTCCAGCAAATCGGACATGCCTCGTTACGGTCATCAGCAATACTGGGATTTGAAGTGCTTGTTTCTCTCCTGAAAGATAATCGTTGTTTGTTTGCAGGTTGAGGAGGA
GGTCTAAACGTTACTTGTTTGGCCTTCCCAGTCGTACGCCCGAGAATGCCAAGTTCTTTGGTAGCTTTGTACTGGATGGGAACCTCCATTAGAGCAGCCAGTGCGAAAGCTGCTTCCTTCT
CTGCTGGGCTAGCATTTTTGGCCATGATCTTGGAGAAGTTAACGAACTGAAAATTGTCAAAGGCTCGAGCTGGAATCTTATCATCAAATTTTTGCATGTCTTCCCATGGTCCATCCCCAACT
CCTACTAATACAATTGACAATGGAACACAGCTTGCTATAACAATTGAGTTGATTGTGCTTTCCTCCTGTGGACTCAGATCCTTACCTTCTAAATCAACACTTCTGGTAACCTGCCCATCTGC
TATAATAACCAAAACGTGATACTGTCCTCCGCTGTTCTCGACGATGTGAACCGCAGCTTCTACTAGAGGTGCAAATGACGTCGGGCCTGATAATTTCAAATTTGGAACAATTTTCCTGTAG

CATTCGAGAACTTCCTCAAAGCCATGACAAGGAGAGTGATCGCCATGAAAGCTGAACACCTCCCTATCATGAGTTGTTGCATCGCCAAATCCAAAGCATGGGATGAGATTGTCTTCGTCA
AATGGAGCTAAGGTCTTCCCTATTATGCTGATGGCTTCTTCATATGGATTTCGTTTATCACCAATTGCATGCAGGCTCTGGTTATTGAATGAGTATTTGCCTGCCCAATCATTGCTTTTTGTAA
AATCAATTCCTAGGATCAAGTTTGATGACTCCAAGCCTGCTTCTCTTAGGGCTGCTGTAACCTGGTCGAGAGAGTTATAGTTGTCAGGTATGTACGAGTACTTCCTAGTTATCATTCTCCTAT
TCGCTTCACTGTTTCTGCGACTCGTGCTCGGTTGATCTGTTATAACCCCTGTCGCAGCCGACGCCGGCGATGGATCCCGGCGATCAACGTCGCTCAGATTCCGGCCACCCCGGCCAAAGA
AACCTCCCCAAGAAAGTGCGAGGAAGATGTTCTTTAGCTTTGCTTTTGGCCCTCCAATCGGACAAAGGAGAACCTTCTGCGTTTCTTCCACAAAGTGTTGTCTTTTTCACCTGCCACTTC
AT
>comp154401_c1_seq4
GCAACAAGAAAGGGGAAGAAGAAAAGAGAGAGAGAGAGGTAGAGGGAGAGGGCTTTTGCTACTCAATGTTTCCATTTTCTAGCAAATTTTCATCTAACACAAACTACTCTCTAACATAC
ACATTTTCACAAAACAAACACTTTACCTTTCACACTTATACACTTCTCCCAATCCTAAAATCATACTATACACTTTTCCTTATCAAAATATTTTTTAAACATTTCAATATTCAAAGCACCTTTC
TACACATTTCAAATTTCAACACATACATTTATCATTTCCAAAATAATACCTTTCATTTTCCAAAATCACAAAATTCACTTTTCAAACCAAACACACACTTTTCAAAATGCACATGCTTTATTT
CCAAAATCTACACCTCTCACCCTTCCAAAGTTCACACTTATACTTTTCAAAACATACACTTTTCAAAACATGTATTTCATCTTCCAAAATACTTCACCTTTCAAAATACTCCCAATCAAAGTA
AAGTACTTTTCTCCAACCTCTTTCTTTTCCCAAAGCATGAAGAGATGTCGTGTATGTATAGCATGAAGCACAACCCTGCATGACATCTCCCATCAAATATTTTCCACCACAATTCCACATATA
TCTACTTCCCAACTCAAAATTTTCACCAATTGTAGTTTAAAATAAAAAGAAGATTTGTTGCAAGTCTACTCTTTCCAAAGTTTAAGAAAACATCCCAACCAACATAAAGCAATATAATATTT
GGATTTATGCAAAACTCACATTTGAAGCGTATACGTGGATATTTTTCAACACAATTTTCAACCTTCACATCACATGCTATCATACATGTGGAGGAAACTTTCTGAACCCAAATTCATATATTT
CATTAAGCACGA
>comp154423_c0_seq1
TTATTGAGTAGGAGTTATAAAGGAGGTTATGAATATATTGGGATGAAAATAAAAACCCAGCTAATTCAATAGTAGATAATTCTTTAGAAAAAACAAAGTACAAAGTACAACAACAACTTATT
GTTGAATGTCATTATATGTAAAAGCAACCACAAAAAACAACAACGAAGAGTATAAGAAAAAATAAAATAAAAAATAAAAAATAAAAAAATAAAAATAAAAATAACAATACCGGTCATCTC
CCTTTTGTTTCTTTCCTTGGGAATATCTGCCAGACCACAGGCACAAACCACAGAACAAACAAACTCCCAAACCAAAAAAAATCCTTTCCAACTCCATTCTTAGCTAATCTAACAAAAATC
CAGCCAAAACAAACGCCATTATCAATGATCCAACCTTCCACATGCTTCTCCTCCTACCCTCAGAAGCTTCATTTCCAACATGAGCATGTTTACCATGTACCTTCGCCGGAGAATCCTCATCC
TGTTCCACCGGGGCCGGGGCCGGCGCCTCCACTTCACCAAGATTATACGCCTTGATCCCAATCTCAAACTTACAGCTCCCTCTGCTATAATCCTTGGTCGTCTCCTTCGCCAACCCATCAA
AAACACACGTCCCAGTCCCCTGTCCAAGCGCCTGAAAATACGAATTGAAGACATACGAAGCCTGCATTTCCAAATCCATTCCCGAGCAAGATGAACCGTAACTTAACGGCATACAATCAG
CACCATGACCGCAAGCGTAATCCATATTCGCACTCACTCTGCTTTCATTCTTCGCCTGCTTCGCCTCTTTGTTCAAAACGCACCATTTCTTCGCCAAGTAATTAACTTCCACCGCCGCGACA
GCCGCTTTTCCGGCCACTCCGCCGCCCGATAAGTCCACCGGAAACTTCGGCTTTCCGTCGGTTGAAAAAATGCCCCAGCTTCTTTCAAAAGGCCCCGGAAGTATGCTCTTTGCTTCTTCA
TCGAATAGCCCGAAGAGATAAACTTCTATATCCTCCTTAGGCCGCTGCGGCGTCCCGGTTTTTTTTGCTATCTTTTGCGTTATTCCTTTGTAGAATCTCTCGGCTATCGATACGTTGGCGTGTA
TATCGCCGTCGGTCGGCCAACCAACTTCGCCCACGATGATCGGAACGTCGCCGCAGCCCTCCGATTTCAGCGCGGCGAGCAGCGTGTCGTAATTCGCATCGAAGACGTTCGTGTAGATTA
AATCTCCATCAACGACTGGCTTCGCCTTTCCGTCGAAGAAGGCGAAGTCGGAGGGGAAATTGGCGTCGATATAAAGGCTGAAAAATGGGTAAATGTTGACGGTAAAAGGCGCGCCGGTT
TGATTATAGTATTTGACAATGTCGGACATAAGACCGGCGACATCGTCGCGCCATTTTCCGGCGGAGGGGACAGGATTATACCACGGCGAGTAGTAGATGTCGGCATTGATCGGAATCGTGG
CTTTGATTTCCTCGCCCAGTCCGGCTTCATCGAGAGCTTTTTGAATGTTTTTGAGGGCGGGCAAAGTTACATTTGTGAATCGATCTTTGTAGTCTTTGAGAAAAGGTTCGTTCCCGACGGC
CACGTATTTGATGTTGACCTTCGAATGAGTGCGATAATGGGTGACGTTAGCGTTCACCCAAGCTTTGGCGGTGGCGTAGTCGTTCATGGCGGCAAGGAGGAGATTGTTGACGGCGATCAT
GACCTGGATGTCGGTGTTGGAGAGAGTTTCCATGGTGTAGGGGTCGACGTCAAAAAGCTTGACTTTTTTGATGCCGTTGTCGTGGAGAAGGCGAGCGACGGTTTTGGGAGGGAGGCGG

TGGTAGGTGATGGTGCCCCAGTTGACGCCGAGAGAGGCGTTGCCGGAGAGAAAGAAGGTAGCCGCCGCGAGGAGGGCCGTCGCCCAAAAGACGACGACTTGGCGGGGGCTAGCCATT
GTTGGGTTTGTACGTTCGCTTATTTGGTGTCGAAGAGGAATTTGTTCTAATGTTTTATACATGTAAAGGGGGAGGGAGAAGGAATGGCTATACGGTTTCGACGGCGAGGGAGGAAGAGAG
AGAGAGGAGGTTTGTTGGAAGAACAGAGTATAATGTACGTTTATAGGATGGAAAATACTTGTGGAGAGGAGAGAATTGCTCTGAGGAGGGAGTTAGAGAGAAGGAACTCAGTGTTTTTT
TTGGACATTTAGGTCAATTAAAACAAACTCACACACAACAAATACGCAAGAGTCAAACTCAACATCTTTTAGTTGATAGCCAAAGCTTCAA
>comp154424_c0_seq1
TTATATATAACAAACAAATAGAGGCTCGCAAGAGTTCACCAATACACAAAATAGTAAAAACATAATCAGAGAGCCGCACAATTTGCCAGGAATTACGCCATTTTCCTCAGCCCGAGATCTC
TATGGACATCACGCCAACCTTCTTAACCTCCTCCTTAGGTACCGTGACGGTGAGAACGCCGTTCTCCATGGCGGCCCTCACGCCGTCCACCTTCGCATTTTCCGGTAAACGGAAGTGTCGT
TGGAAGTTGACACTACTGCGCTCCATGTGATGCCAGATTATGCCCTTCTCCTCTGTTTCCCGCTTCTTCTCCCCGCTGATCCAAAGCCACCGCCCTTGTTCCTCCACCTCCACCTTCACCTC
CTCCTTCTTCAGCCCTGGGAGGTCGGCCTTGAACACGTGCGCCTCTGACGTCTCCTTCCAGTCGAAACATACTTCGGCAAAATTTGAAATCTTGCCACATATACTAGATCGGGGATGGGA
GAGGCTGTTGGATCCGAAGTGAAACTCCCCCAACGAGTCGAAGACGTTGCCTCGGCGAATAATCGTCATTTCTCTCTGATCTC
>comp154424_c1_seq3
TGATTATGTTTTTACTATTTTGTGTATTGATTTGCTCTTGCGAAGTGCTTTTGTATGCATTCCTATCAAAATCTGAGACATGTTTTTCTTTGATATCTAAGAATCGAATAATCTACATATCTTAAC
CTGTGAAGTATTCAAATCATGTTGAATTAAATGCATAAATTCATGGCTTCGTGCGGTAAATTTTGGATCAACTAAACTGTTACATAAGCC
>comp154447_c0_seq1
ATTCTATTTATCCGACCGTTCCCCAATTATGAATCCCAGCTTTCACTTTTTCCCATCACAGAAAGAGAAACAGAGCGAGAGAGAAAGATGGCGTCTCCAATCATCTCGCTTCGTCTCGCAT
TTCTGGTTATCCTCCTGGCAGCGGCGCACGCTCGAAACACTCTACAGAAAGAATCCAGTCAGGATCCCGATCTTGTCACCGCCCTTCCCGGCCAACCGCCGGTGAAATTTCGGCACTACG
CCGGGTACATCGGCATTGGGGACCGACAAGCCAAGGCGCTCTTCTACTGGTTTTTTGAGGCCGAGCACCGCCCCAGTGAGAAGCCTCTCGTTCTCTGGCTCAACGGAGGACCAGGATGC
TCATCCGTCGCATACGGCGCGGCGCAGGAACTGGGGCCTTTTCTGGTACGGAGTAACGGCCCGAACATTACGCTGAATCCATTCGCCTGGAACAAAGTGGCCAATCTTCTATTTCTTGAA
GCTCCAGTTGGTGTCGGCTTCTCCTACACCAACCGATCATCGGATCTTCGCAAACTAGGTGATCGAGTCACCGCCGAGGACTCCTACTCCTTTCTTCTCTCCTGGTTCCGCCGCTTCCCCC
GCTTCAAGTCCCACGACTTCTACATCGCTGGGGAGAGCTATGCCGGACATTATGTTCCCCAGCTGGCGGAGAGAATATACGAAGGAAACAAAGCTGCAAAGCAGATCTCATTTATTAATC
TCAAGGGATTCATGATAGGGAACGCGGTTCTAAACGACGCGACGGACCAAATGGGGATGGTGGAGTACGCTTGGAGCCACGCCATTATCTCCGACGAGCTCCACGCCATCATTCTCAAG
GAGTGCGGCTCCTTCCAGCAGGATGGTGACCGGCCGGCCGGCCCGGGATGCTCTCCAGCCCTGCGAGCTTTTCTCCAAGCCTATTCCGACATCGACATCTACAGCATTTACACGCCGGTC
TGCCTCTCATCATTTGGCCGTTCTAGCCGCCGGAAGTCTAGACTTGTAGCTGCCCCTCTGCTCTTCTCTCAGCAAGACCTCTGGCCGGGAATGAGCAGAGTGGCCGCCGGCTATGATCCAT
GCACGGAGGACTATGTCGAGACATACTTCAACAGGGAAGATGTTCAGAGAGCTTTGCATGCCAATGTCACGCAACTCTCCTACCCATACTCCTCGTGCAGCGAGGTGATCGAGAAGTGG
AACGACTCGCCGGCCACGGTTCTGCCCGTCCTTCGCCGGCTATTGAACGCCGGCCTCCGCATCTGGGTCTACAGTGGCGACACAGACGGAAGGGTTCCGGTGACATCAACTCGTTACAG
CATCAACGAGATGCATCTAAAGGCGAAGCAGGACGAGAGGAAAGGGAAGAAGAAGGAATGGGGTGGGTGGAGAGCTTGGTATCATAAGGAGCAGGTTGCAGGATGGGTAGTGGAATA
TGAGGAAGGGCTTTCATTGGTGACAGTGAGGGGGGCCGGGCACCAAGTTCCAGTCTTTGCCGCTGCCCGTTCCCTTGCCCTCTTCTCTCACTTCCTCCATGGCCGGACGCTCCCGGCTGA
CCGCTTCGCCGGGGCCTAATTTGCTTTTGCTTTTGTGACTGACTTCTTTCCTCGTCTTCTTCTCTGTGCTTGCTCTGTAGTGAGGAGTTCTGCTCTGCAAGCTACACCAATTGTTTGGGTATT
TCATTTCACAGTTTTATTTTTTGCCATCTTTGATTTTATTTTATTTTTGTATCGATTGATTTGGTTGGTAAGACACCGAGTTGATGTTTGGAGTAGAATTTACGTTTAATGGAATTAATTGATTT
CTGATAATTCAGACGTGCTATTTAAGCTTCT
>comp154464_c0_seq1

TTATTCCAATGGCACTAATACAGTTGGAGGTGCTCATGGTGACACTGCACAGGATTTCCTGCCTTCTAGTTTCTGTCCAAAGAGAACATCTACCTGCCTGTTTCCTCCCCCTTTATGGAGGA
AAATCCACTATGGGCCATTTACCCAAATAAACCTCTTAATGCGTTTATTTTCAACTTTTTTTTGGATATGAATTTTCCTCTTATGAAACAAAGTGTTGGGTGTCCCCCATCCCTTCCCTCACC
CCCAACAGCCAGATAAGTCCCTGCTGTGAGGGCATTTCTGTTGCCTCTGAAATCTACAAATAAATATTGAAAGAAACAGCATCAAGTGTTCATGTTGAGTGATGGCACGAAGTGGGCCCC
AGAAACCTGTCAGGCAGAGCATTTCTGAGTCTGGACAGTCTCAAAGTGTCGTGGAGAATGTGGGGGGCTTGTTGCTGTGGTCACCTGTCCCTGGGCGCGGGGGCTGCGCCAGTCCCAC
TGGGCAGCAGGCTGCTGAGAAGCCGCCTACAATTTGTCCAGGAAGTTGTCCAGGGCCGGAATACCTTCCTTCAGGCCTTTGCGCTTGCGTGTCTCTGCCACCACCTGGCTGGGGCGACT
GGTGTTGTCGAAGGGATCCCCGGGCAGGATCTGCCAGTGGTCGAACACACACTGGGGAAAGGCCTGGCCGCCTGTGTTGGATCTCAGGTCAGCAGTAAAGCCAAAGGATTCATTGACA
GGCAAGTAAGCCTTCACAACAAACATGGGGGTACCAGCCACCTGGGACTCCTCAAAGACGTGGCCCCGCTTCCTGTTCAGGACACCATAAATGCCACCAACCACTTGTTCTGGACACTG
GATCTCCACAAGGTAGATGGGCTCCATGAGCCTGGGCTGGGCAGTCAGCACACTGGCATAAAGGCAGCGCCGTGCAGTAGGGATGATCTGGCCACCACCACGGTGGATGGCGTCGGCGT
GCAGTGTCACATCGTGGACATCAAATCGCACACCCCGCATGTTCTCCTCACACAGCGCCCCCTCTTTGGTGGCCCACTGGAAGCCAGCCACCACGCTGTCCTTGATCTCGTTGAGGTACT
GCACACCCTTGGTGATGTCAGTGAGGATGTTGGGGCCAGTGCCATCAGGGCCAAAGCACCAGATCTTGCGGGCCTCAGCCACGTCCCACTCGTACTTCTCAGCTAGGTAGCGGGCCCGC
TGCTTGAGTTCCTGGCGGGCAGACACCTCTCCCTTGTCGATGTCTTCAGCCAGGCCGTCAGGGAAAGGCCTTGCTTTCATGTATAGCCTGTTGTGCTTGTTGGGGGACTTAGACAAGCAG
AGCACATTTGACTCCTCACTGACAGTCTCACGGTAAGAGACAACTGGGTCAGATTTCTTGATAGGAATGCAGGCGTGGTCCTCCTCCAGGTCCTTCAGGCAGATCTCTAGGTGCAGCTCT
CCAGCCCCTGCGATGATGTGCTCCCCAGACTCCTCAATGATGCACTGCACCATGGGATCTGACTTGGCCAGCCGCTTCAGGCCCTCCACCAGCTTGGGCAGGTCAGCCGGGTTCTTGGCC
TCCACAGCGACCCTGACGACAGGGCTCACGCTAAACTTCATCACTCGCATGTTGTGGGCGTGCTCAAAGGTGGTGATGGTTCCTGTCTTCACCAGGAACTGGTCCACGCCCACCAAGCC
CACGATGTTCCCACAAGGCACGTCCTCAATGGGCTCCACGTAGCGGCCCATCATAAGGATTGTCCTCTGGATTGGCTTCAGGTATAGGTCCTCCTTCTTCCCAGGGGTGTAGTTGGGCCCC
ATGATCCTGACCTTCAGACCGGTAGACACCAGCCCTGAGAAGACCCGGCCGAAGGCATAGAACCGACCTTTGTCGGAGGTCGGCACCATTTTGGAAATGTACATCATAAGTGGGCCTTT
GGGGTCACAGCTTTTAATGCCCATGGCTGCCTCGTCGTCCGGAGGCCCCTCATACAGGAGCTCACAGCGGTACTTCTGGGCCGTCACGGGGGAGGGCAAGTGGATGGTGATCATCTGTA
GCAGCGCATCCCCGGCGGGCAGCCAGCGGCGCATCACAGCCTTCAGCAGTGGTTTGCCTTCTTTGTCCTTATCTTCACTGTCCAATTTGATGTCTAGTTTTTCAATCAGTTTTGCTGTCTCC
TCTTTCTTGAAATTCATGATGGCATCAAACACCTTGAAGATAGGGTCCAAGATGAGCTGGCAGAAGGTCCTCGGCAGCTTCTTGCCATCAGGGCTGGTGGCTGACTTGCTGAATTTACCA
TTGGCTGGATCAAAGTACCGGTCTCCCCATAGCTTTTTCATCATGTCCTCCACCTTCTTGGCCCGCTCAGCAGGCCCCAACTGGCCCTCGCCCTTGGCGGCAAACTTGGCAACGTACATCT
CTGCAAACTGCTTCAGGGTGAAGGCCCAACCGTGGAGACCAGACCCAAAGCCAACAGTACCAAGAACAGGATCAATCATGATGTTGCCCATGGGGCCACTCTCGCCCTCCCCATAGGTG
GAAATGATGACATTGACATTCTCCACAATGCGCTGGAAGGTCTGGTAGAGCTCCTCGGGCTCCAGCTGCAGCTCGAGCAGGGCCCGGTCCATCTTGTTCATCATCAGCACAGGCTTGATG
CGCTCAGCAATAGCCTGGCGCAGCACTGTCTCTGTCTGCACGCACACACCTGACACACAGTCCACCACCACCAAGGCACCATCGGTGACACGGAGAGCTGCTGTCACCTCTGAGGAGA
AGTCCACGTGCCCAGGTGAATCAATGAGGTTGATGAGGAAGCCAGAGCCATCTTTGCTCTGCTTGATGAAATTCAAGTCATTCTCTGAGAGCTCATAGAAGAGGGAAATGGCCGTTGATT
TGATAGTGATGCAACGTTCCTGCTCATCCTTCCGGGTGTCAGTGAAGCGGGTCTCCCCAGCCCGGGCGGAGGCGATGATACCAGCCTTGCACACCAATGAGTCCGTCAGAGTGGACTTG
CCATGGTCCACGTGGGCAATGACGGACATGTTTCGGATATTAGCCTTCTTGTCCATGATGGCCCGGATCTGGTCTACTGTGAAGTTCACCATGGTGACGGATGGCGGTGGATTCTACCGGG
CAGATCCGAGTAAGGTTAGTCGCGCCGAGGATGGC
>comp154610_c0_seq10
AAAAGGTGCAAACCGATTCATTCAATCTCGAGATAGAGCCCTAATTTGATAAATTCATTACTGAAAGAAATGATGATTCATAGAAGCCCTTCGCTAACCGAAAAACCCTAATCGGCTAATC
CCAAACAGAATAGTTTCCGCAATATGAAGCTAAAGAGACCCCCACTCCCTCAGAATTTCTCCACTACGTCGCCGTCCTCCGCTTCCACTCCGCTGATCATCTCCACCACCTCCTCCTCTAT
GCTCTCCGACTCCGAAGAACCTCGCCGATTCTTGAAGGCCGAAACCGCTCGGCCGCCGCCGCCCGATAGCAACGGCGAGAGGTGGTAGAGCGGCGGCGGAGGGCAGTAGTGCGCCGG

CGAGGGGTTGGCGCCATAGAGTGTGGTCCCCGCTGCCACGGCCGAAGCTGCTGCTGCTGCTGACGTGGCGTACCCTGGTGGGACCCAGATGCCTCCTAGGACTACAAGTTGCGGCGCCA
CCGCTGTTGTCGGCGGCGGTGGCGGCGGTTGCATTGGCCTTCTTGTGTGGAGCCTGTACTTCTGCGCATCGGTTAAGCAAAAGAGTCAAAAGGAGTCCGTTTGTAGTCAAAGCTTTCAG
GTTGTAGAAGAAAGTTCACTCCTCATTACTTATTTCTGTACAGAAAATTCCTATAATCATTTCTTATAGAAATTTAGATAAAAAATCCGTAAAGATATAATCTTTTTTGCAAATTATGTAAAAT
ATTTCTTTTTGAATTTATATCGCTGTTTGATTATTTCCTTGTGATTATAAAACTAAAAAAGAGTGCCGTTTTGTTGAGGAAGGGCCATTGGCTCCTCCCCTAAAACTCTATTTTGTGATATGAG
GTTCCTGTGCATTCATGATTAAAGAGCACTCAAAGTCTATAAATAAATAAATAATAAGAAAAGCATAATTAGTCAAGCTTTACACAATGTTGAGAGAGAGAGAGGACCTGCAGATGGCTTT
TCACCTCATCATTGGTAAGCCCATCAACGTTCATTAGCTCTCTGATTTGCTTTGGAGTGGCAACTTGAGACCCACCCAATATATGCAGGGCATTGACAAACCGCCGGTGCAACTCCGGTGA
CCAACATCTCCTCGCCTTCCTCTGAGTAGCCGCCGGTGCCGCAACGTCGCTGGAAACACTACAACTTCCTTCTCGCTTACCCTGTTTCTCTTCACCTTCATTACTCTTACTACAGCTCTCCT
TCTCCTCCATCTCCTTCTGAATAGAACGAGCCAAAGCAAGTTCAGAAATATCCTTTTTCTTCTGCTTTGAGAAAGGTTGAAATGCTCCTCCAATTCTTGGTTTAGCATCTAGTACCAGCTTA
GAGCTGAAAGTCACCAATGAATGCTCCTCTTCCCCTTGCTTTGCAGAGTCGGCCTCATCAGCAGCCGGGCTGCTCCATAGCTGTGCCGACACCATCCAGTTCGCCATCTCAGATTCTTCGA
CGCTCGTCTTCTTCAATGGGATGAATTCTTCGAGCACTGGCTGAGCTTCTTGGCTTGCTTGGTATGTCTCCAATTGCTTCCTGTATGCCTCCATGGCGTTGTTGAGTAGCTGCATGCAAAGT
GGGAGCTCACGCTTGAAAGCTTCAATCTTGAGACTCTCCTCCTCAAGACGAGCAAGAAAATCTTGCAGCTTGTGCATGTGATCTGCAATTGGATTCTGATTTTGGTTAATAGAGTAGCTAC
TGATTAGCTTGTAATCCAAACTAATTAGCTCTGAAGAAGAAGAAGAAGCCATAAGAATGAAATGAAGCAAATAAAATAGTTGTTTTGGTTATCGTCTCCTATGAATGATGATTATGATGATC
ATGGAGTTGGTGTGATGAATGAAAAGAGGAGCCAAATGGATGAGAACGCAGAGGAAATTGAAGAAAGGATCCCCTTCTTTTATGTGTGAGTGCGAGGAAGGGAATAAAGTGAAGTTTG
AAGAGAGGAAAAGGAAAAAGAAGTAATAAAAAGTA

>comp154610_c0_seq11

TCACTTTATTCCCTTCCTCGCACTCACACATAAAAGAAGGGGATCCCTTCTTCAATTTCCTCTGCGTTCTCATCCATTTGGCTCCTCTTTTCATTCATCACACCAACTCCATGATCATCATAAT
CATCATTCATAGGAGACGATAACCAAAACAACTATTTTATTTGCTTCATTTCATTCTTATGGCTTCTTCTTCTTCTTCAGAGCTAATTAGTTTGGATTACAAGCTAATCAGTAGCTACTCTATT
AACCAAAATCAGAATCCAATTGCAGATCACATGCACAAGCTGCAAGATTTTCTTGCTCGTCTTGAGGAGGAGAGTCTCAAGATTGAAGCTTTCAAGCGTGAGCTCCCACTTTGCATGCAG
CTACTCAACAACGCCATGGAGGCATACAGGAAGCAATTGGAGACATACCAAGCAAGCCAAGAAGCTCAGCCAGTGCTCGAAGAATTCATCCCATTGAAGAAGACGAGCGTCGAAGAAT
CTGAGATGGCGAACTGGATGGTGTCGGCACAGCTATGGAGCAGCCCGGCTGCTGATGAGGCCGACTCTGCAAAGCAAGGGGAAGAGGAGCATTCATTGGTGACTTTCAGCTCTAAGCT
GGTACTAGATGCTAAACCAAGAATTGGAGGAGCATTTCAACCTTTCTCAAAGCAGAAGAAAAAGGATATTTCTGAACTTGCTTTGGCTCGTTCTATTCAGAAGGAGATGGAGGAGAAGG
AGAGCTGTAGTAAGAGTAATGAAGGTGAAGAGAAACAGGGTAAGCGAGAAGGAAGTTGTAGTGTTTCTCTTCACCTTCATTACTCTTACTACAGCTCTCCTTCTCCTCCATCTCCTTCTG
AATAGAACGAGCCAAAGCAAGTTCAGAAATATCCTTTTTCTTCTGCTTTGAGAAAGAGTCGGCCTCATCAGCAGCCGGGCTGCTCCATAGCTGTGCCGACACCATCCAGTTCGCCATCTC
AGATTCTTCGACGCTCGTCTTCTTCAATGGGATGAATTCTTCGAGCACTGGCTGAGCTTCTTGGCTTGCTTGGTATGTCTCCAATTGCTTCCTGTATGCCTCCATGGCGTTGTTGAGTAGCT
GCATGCAAAGTGGGAGCTCACGCTTGAAAGCTTCAATCTTGAGACTCTCCTCCTCAAGACGAGCAAGAAAATCTTGCAGCTTGTGCATGTGATCTGCAATTGGATTCTGATTTTGGTTAAT
AGAGTAGCTACTGATTAGCTTGTAATCCAAACTAATTAGCTCTGAAGAAGAAGAAGAAGCCATAAGAATGAAATGAAGCAAATAAAATAGTTGTTTTGGTTATCGTCTCCTATGAATGATG
ATTATGATGATCATGGAGTTGGTGTGATGAATGAAAAGAGGAGCCAAATGGATGAGAACGCAGAGGAAATTGAAGAAAGGATCCCCTTCTTTTATGTGTGAGTGCGAGGAAGGGAATAA
AGTGAAGTTTGAAGAGAGGAAAAGGAAAAAGAAGTAATAAAAAGTA

>comp154610_c0_seq2

AAAAGGTGCAAACCGATTCATTCAATCTCGAGATAGAGCCCTAATTTGATAAATTCATTACTGAAAGAAATGATGATTCATAGAAGCCCTTCGCTAACCGAAAAACCCTAATCGGCTAATC
CCAAACAGAATAGTTTCCGCAATATGAAGCTAAAGAGACCCCCACTCCCTCAGAATTTCTCCACTACGTCGCCGTCCTCCGCTTCCACTCCGCTGATCATCTCCACCACCTCCTCCTCTAT

GCTCTCCGACTCCGAAGAACCTCGCCGATTCTTGAAGGCCGAAACCGCTCGGCCGCCGCCGCCCGATAGCAACGGCGAGAGGTGGTAGAGCGGCGGCGGAGGGCAGTAGTGCGCCGG
CGAGGGGTTGGCGCCATAGAGTGTGGTCCCCGCTGCCACGGCCGAAGCTGCTGCTGCTGCTGACGTGGCGTACCCTGGTGGGACCCAGATGCCTCCTAGGACTACAAGTTGCGGCGCCA
CCGCTGTTGTCGGCGGCGGTGGCGGCGGTTGCATTGGCCTTCTTGTGTGGAGCCTGTACTTCTGCAGATGGCTTTTCACCTCATCATTGGTAAGCCCATCAACGTTCATTAGCTCTCTGATT
TGCTTTGGAGTGGCAACTTGAGACCCACCCAATATATGCAGGGCATTGACAAACCGCCGGTGCAACTCCGGTGACCAACATCTCCTCGCCTTCCTCTGAGTAGCCGCCGGTGCCGCAAC
GTCGCTGGAAACACTACAACTTCCTTCTCGCTTACCCTGTTTCTCTTCACCTTCATTACTCTTACTACAGCTCTCCTTCTCCTCCATCTCCTTCTGAATAGAACGAGCCAAAGCAAGTTCAG
AAATATCCTTTTTCTTCTGCTTTGAGAAAGGTTGAAATGCTCCTCCAATTCTTGGTTTAGCATCTAGTACCAGCTTAGAGCTGAAAGTCACCAATGAATGCTCCTCTTCCCCTTGCTTTGCA
GAGTCGGCCTCATCAGCAGCCGGGCTGCTCCATAGCTGTGCCGACACCATCCAGTTCGCCATCTCAGATTCTTCGACGCTCGTCTTCTTCAATGGGATGAATTCTTCGAGCACTGGCTGAG
CTTCTTGGCTTGCTTGGTATGTCTCCAATTGCTTCCTGTATGCCTCCATGGCGTTGTTGAGTAGCTGCATGCAAAGTGGGAGCTCACGCTTGAAAGCTTCAATCTTGAGACTCTCCTCCTCA
AGACGAGCAAGAAAATCTTGCAGCTTGTGCATGTGATCTGCAATTGGATTCTGATTTTGGTTAATAGAGTAGCTACTGATTAGCTTGTAATCCAAACTAATTAGCTCTGAAGAAGAAGAAG
AAGCCATAAGAATGAAATGAAGCAAATAAAATAGTTGTTTTGGTTATCGTCTCCTATGAATGATGATTATGATGATCATGGAGTTGGTGTGATGAATGAAAAGAGGAGCCAAATGGATGAG
AACGCAGAGGAAATTGAAGAAAGGATCCCCTTCTTTTATGTGTGAGTGCGAGGAAGGGAATAAAGTGAAGTTTGAAGAGAGGAAAAGGAAAAAGAAGTAATAAAAAGTA
>comp154610_c0_seq5
TCACTTTATTCCCTTCCTCGCACTCACACATAAAAGAAGGGGATCCCTTCTTCAATTTCCTCTGCGTTCTCATCCATTTGGCTCCTCTTTTCATTCATCACACCAACTCCATGATCATCATAAT
CATCATTCATAGGAGACGATAACCAAAACAACTATTTTATTTGCTTCATTTCATTCTTATGGCTTCTTCTTCTTCTTCAGAGCTAATTAGTTTGGATTACAAGCTAATCAGTAGCTACTCTATT
AACCAAAATCAGAATCCAATTGCAGATCACATGCACAAGCTGCAAGATTTTCTTGCTCGTCTTGAGGAGGAGAGTCTCAAGATTGAAGCTTTCAAGCGTGAGCTCCCACTTTGCATGCAG
CTACTCAACAACGCCATGGAGGCATACAGGAAGCAATTGGAGACATACCAAGCAAGCCAAGAAGCTCAGCCAGTGCTCGAAGAATTCATCCCATTGAAGAAGACGAGCGTCGAAGAAT
CTGAGATGGCGAACTGGATGGTGTCGGCACAGCTATGGAGCAGCCCGGCTGCTGATGAGGCCGACTCTTTCTCAAAGCAGAAGAAAAAGGATATTTCTGAACTTGCTTTGGCTCGTTCTA
TTCAGAAGGAGATGGAGGAGAAGGAGAGCTGTAGTAAGAGTAATGAAGGTGAAGAGAAACAGGGTAAGCGAGAAGGAAGTTGTAGTGTTTCCAGCGACGTTGCGGCACCGGCGGCTA
CTCAGAGGAAGGCGAGGAGATGTTGGTCACCGGAGTTGCACCGGCGGTTTGTCAATGCCCTGCATATATTGGGTGGGTCTCAAGGTAATAAATTGAAGTACTATTGAACATAAATTTTTCA
ATAAAACTAATTATGTTTTATAAAAAATTTATCTTTTAATTAAAATGAAATTATGTGTAATATGGTGTCACTGTAACGTTGCTCTTTTCTATTAAAATGTTAATTCTTCAAATTTAAAGCAGATT
ATGAATCTGATCATTGGATTAACTACTCTTCCTCAATTGAGTTATCAATTCTGTCCACAGTTGCCACTCCAAAGCAAATCAGAGAGCTAATGAACGTTGATGGGCTTACCAATGATGAGGTG
AAAAGCCATCTGCAGAAGTACAGGCTCCACACAAGAAGGCCAATGCAACCGCCGCCACCGCCGCCGACAACAGCGGTGGCGCCGCAACTTGTAGTCCTAGGAGGCATCTGGGTCCCAC
CAGGGTACGCCACGTCAGCAGCAGCAGCAGCTTCGGCCGTGGCAGCGGGGACCACACTCTATGGCGCCAACCCCTCGCCGGCGCACTACTGCCCTCCGCCGCCGCTCTACCACCTCTCG
CCGTTGCTATCGGGCGGCGGCGGCCGAGCGGTTTCGGCCTTCAAGAATCGGCGAGGTTCTTCGGAGTCGGAGAGCATAGAGGAGGAGGTGGTGGAGATGATCAGCGGAGTGGAAGCGG
AGGACGGCGACGTAGTGGAGAAATTCTGAGGGAGTGGGGGTCTCTTTAGCTTCATATTGCGGAAACTATTCTGTTTGGGATTAGGGTTTTTCGGTTAGCGAAGGGCTTCTATGAATCATCA
TTTCTTTCAGTAATGAATTTATCAAATTAGGGCTCTATCTCGAGATTGAATGAATCGGTTTGCACCTTTTTTTTGGTGGGAAAAAA
>comp154614_c0_seq13
ATATATACATCCACGTTGAAGCAAGCTCTGAATTTGTCTTGCAAAGCAAAGCTGAGATTTAATTTGGTGAGCTCTGTACTGTTAGTTACAAACTCAGCCTTACTTGCTATTATATGTAGGCA
ACATCTTGGTTTTGGCACCATTCTCGTAGTTGTGGGCATGCACTGATTTCGACATTGTTGGGATTCAACATATCTGGAAGCAATTCATTATAGAGACGAAGTTGAGGACAGTTGCTTATTTT
TAAGAAATGGAGGAAGACTTGGACTTGCAGTCCTGTTAAAAGTTTCAGAGTTGAGCAGAAAGAAATCTCAAGTGAAGATAGATTCATTTCCCTACAGTTCAAAAAGTTAAGCTTATTGCA
TCTATCAATGGTCAACGATTGAAGGCCATTGGGAAACCGTCCAAGAAAAACGAGATTTGGACAGCTGCTTATATTCACGTCTTGCAGAGTGGTGACCCTTTCTAGCGCAAAAAGCTCTTT

CAGATGGGAGCAATCCCATATCCTTAGTTGCTTTAGTCTAATGAAATTTTTCCTCAATGAGATAGACTTCAAATGAACACAGGAGCAAAAAAGCGGGCCATTTATCTGTTTCAAGAATAGC
CTCATCGTAGTCCAAAACACATATGTTTTCAAAGAATGTATATTTTTCCTCAAGCCATAGCTTGATCTGCTCACTGCTCCTAACAAATAGTTCTTTGTGTGATTCCTTATTATTATAAGGTATG
TTATATCTTTGACACCAGTCTATGAGCTTTGGGCTGTTAAAAAGTTCTAGTTGAACTTGAAGCGTGAAGGGAAGTTGATCTGGTGGTGGGAGCAGAAGCTCGGGACACTCAAATATTCTT
AGGTTTCTAAGGGAGAAAAGTTGTTCCAGCCCAGGCAAGCACAAGATCTTGGGGCAGTTGCTTATGAGCAAGAAGTGAAGTTTGCATGGCAAATTGTGGTCTAGCGATAGTGAGAATTG
AGGACATTCGATTATTTCTAATTTTTCAAGGGATTCCATCATCTTTAATCCAGGCAGACATGTCAGAGCCGTGCAATGCCATATTTTCAACAGCCTCAGTGCAGGAAAATTTAGGTACGGAG
GAAAGAACATCAGACGTTGACATGAATTAAGATGCAATGTTTGCAGACTGCACAGAATCCATTCAGCTTTGAGGATGTCACTTTCATCAGACTCGAGTATATGCAAACTTTTTAAGGAGA
CGAGATTCTTTGAGCAATAATAATATATTAAATGGCTGGTCTCTCGTATGGTCAACTCCTCCATCTTAGATAATTCCTGAATAGTACTTTTGAATCCATATCTTTGGCACCATTTTTCTAATCGA
GGGCAATTGACAATCTCCAAAACACTAGGCATGGTTGAAAGCTTTTCTTCTAAAGGGAGTCTAAGTTTCGGACATAATTGTAGCTTCAATTCCTCAAGTGAACAAAGGTCTTGCAGGCCT
GTTATTGATGTCAACTTCCAACAATTTTGGATCCATAGAAACTGCAACTTGGAAGGAAGTCTCTCCTTTGCAGAGATGAAGAATTTATAACACTCTATTATTTCTAACTTTTCAAGTCTATTC
AAACTGTATAGCCCATGCAATGTTGTTATTTCAGGGCATAGTCTGACAACCAGTCTTCTAAGTGAGGATGATTGTACTATTTTTATATCTTTTATCTTAGCACAATTAATGATGGAAATACTTT
GAAGACA
>comp154655_c0_seq7
GAATTAATATTACTGCTATCACTTTAGGATATTTAGAATTGGTTGTAAGTTCCCCATGTCCAAATATGTCTGATATCCTAGCGTATCTTCTACTTAATACCGTGCATTTATAGAATTTCTTATTTT
ACATGTGAAATGTCATTGCAGAATCCAAGATTTCGGATGAGGTGGAATATTTAAACAAATCTAACTTTTTATGAGCCTTTTCCAGTTTAGCCTGAAGACTGAGCAAATGAGTTTTAGCTCC
AAGTCTCAAAAGTTAAGCTTCAAAATGTATAGTGTATGTATATATATTATCCCAACTATTTGAGTTTTTGGCATAATTTTCACTCATTTTAACTGTGGTATGCCAATTTGAATATCATTCAAAAC
TCCATAGCCAATATAAAGAATTAAGATTTGATATAATTTTATTGTTAAAAAAACTCTTTTGCTATTCTAC
>comp154663_c1_seq6
AAGGAAATCTCTAAACAGTCCCTAAATTCAAATTTCACTCTTAATTCCCCAAAAAATCATTCCTATTCGGCTCCCTTGAAGCTTGCTGATCATGCGACTCTTCCTGGAGGCGGAAGAGTTC
CACCTCAAATTACGTAAGCCAGTCCTGTCAGACCGCTAGACCGGAAGAGTTCCACCTCAATCAAATCACCAATCAAATCAACATAATTAGGAAAATAATCAACTCCCTATCAAATAAATAA
TGAGCCATCAAAGCTTTACAAAATAAAACTTATTTGTAATTGAAAGTAATTCCAAATCAACAAAAAAACCTTATTGGTATATCCTACTCTTCCATCATTAACTTTGTCATGAATCAATTTTTA
CTTGTTACAAAAACTTCAAAGTACCCACCGATGCTTAGCTCGATTGGTGGGCATGCTTGGTTGGGAACCCCCCATCCCGAGTTCGATTCCCTCGAGTGGCACTCGGCCTTTAAGTGGGGG
CTTCAAAGCGCCGGCTATGAGTTTGTGGCTTGGGTGGCCCATTAGGTCAGTCGGGGTTTACGGACTGCCATACACGCTCGATGAAGTCGGGTGAGGCAGCGGATGGCTCCTCGTCATAAA
AAAAAAAACCCTTCAAAGTTACATTAATTCAATATCATATTACTTTAGTGCACATTGCGAGCACCTTTGTGGATGGTGACATAAATCGGCCACTCTATCACTGCACCACACCCACCAGAAA
GAAAATCAAACATTTCAAGCTAGAAATAATACTTTTTTCTCCACTAATCTATTTACAATATTATTTTAAAAAAAGCTGATGCCAGATGTTCATATTAGGTGGTCTCATTTCATAAATCTAGTTT
ATTTGAATTTTTGGTAGCTACCCACCCCACCTCGCATCTAATGAAATTTTTAGTAAAATAAAGTGGTCGCCACATATCAATTTGAGTAGTGTCATAAGGCATAATTTGGAACAGTTTTTTTAC
TATTTTATAAAGCAGTTTTTAAGTATACAATTTTTAATTCAAAAATTTTTTTATTAAAAAATTACTTTACAAAATAGTAAAAAGACTGTCCCAAACGAAGCCTTAGGCTTTGACTTTTTTGCG
GCTTTATTTATAAAAAAACTTATAAAGCAGTTTTTATTAGTAAAAAATATCTTGAACTAGAAATTTTTGTATTAGAAAACTATTTTAGAAATCAGGCTTCGTCTGGGACGACTTTTTTCCTGC
TTCTTAAAGCGG
>comp154760_c0_seq5
AGATCAGCCTTTGCTGATCTGGAGGGATGCCCTCCTTGTCCTGGATTTTGGACTTAACGTTCTCGATGGTGTCCGAACTCTCTACGTCAAGGGTAATAGTCTTCCCTGTGAGAGTCTTGAT
GAAGATCTGCATCCCTCCCCGAAGACGAAGAACGAGGTGTAGGGTAGACTCCTTCTGGATGTTGTAGTCGGAGAGGGTTCGGCCGTCCTCAAGCTGTTTCCCGGCGAATATCAGCCTCT

GCTGGTCCGGTGGGATTCCCTCCTTGTCCTGGATTTTGGTCTTAACGTTCTCGATCGTGTCGGAACTCTCCACCTCAAGGGTGATTGTCTTGCCGGTGAGGGTCTTAACGAATATCTGCATC
TATACAACACCGAAGAGCCTCGAAATATTAGATCAGACAAGAAAAACACCACCACAAGAACGGGATCGAATGACATCAGAATAAATAAATGAATATTAATTGCTAACGTAAGCAACCATA
AAATAAGTAGTCAAAGAAAAATATCAGAACAAAATGGTTG
>comp154760_c0_seq6
AGATCAGCCTTTGCTGATCTGGAGGGATGCCCTCCTTGTCCTGGATTTTGGACTTAACGTTCTCGATGGTGTCCGAACTCTCTACGTCAAGGGTAATAGTCTTCCCTGTGAGAGTCTTGAT
GAAGATCTGCATCCCTCCCCGAAGACGAAGAACGAGGTGTAGGGTAGACTCCTTCTGGATGTTGTAGTCGGAGAGGGTTCGGCCGTCCTCAAGCTGTTTCCCGGCGAATATCAGCCTCT
GCTGGTCCGGTGGGATTCCCTCCTTGTCCTGGATTTTGGTCTTAACGTTCTCGATCGTGTCGGAACTCTCCACCTCAAGGGTGATTGTCTTGCCGGTGAGGGTCTTAACGAATATCTGCATC
TTGAGCGAAGATCGGAAACCTATCAATCCACGCGAGAAGATTGAGAATGACTATCGATGGCCGTCTTGTAGGTTTGTGATCCTTTTATAGCAGTATTAACCGACTCTCTTCCGTAATTATAC
GCAACCGGTACGATTA
>comp154760_c0_seq9
GATTGGTCATAACATACAAGTAAAAAGGAGCAGCTAAATAACTCCAGCAATGGTGACAGTACGTCCGATTCGACTAATTTGAAAAATAAATACGATTAGCAAAAAATGTAGTGGAACAAA
CTAAGAACTACATCGAAACAGAATATATTCAAATATCAAGACAGATGAGATTAGCCATGATTACCATCACAGTTCATATAGCATTTAAAAAGATTAAAGACGATATCAAAGTGCACAGAAA
CGAGGAACAAAAAAAAAAACGAGGACAATAATAGAAAACATGTGACAAATCCCCATTGTACTAGAACTTATCAAGGCCAGAGTACGATACGGCTAAAACGATAACCATTCCAAATCTATA
GATCATAGCGACGACACAAGAACGGAGCAGGACTGCCATTCACTGCCCACCGCGAAGGCGAAGAACGAGGTGAAGGGTTGATTCTTTCTGTATATTGTAGTCCGCGAGAGTGCGGCCGT
CCTCAAGCTGCTTACCAGCAAAGATGAGCCTTTGCTGATCCGGCGGAATCCCCTCTTTATCTTGAATCTTCGACTTCACATTCTCAATTGTATCCGAGCTCTCCACGTCCAGGGTGATCGTT
TTACCAGTAAGAGTCTTAACAAAAATCTGCATTCCACCACGAAGGCGGAGCACAAGGTGAAGGGTGGATTCCTTCTGGATATTGTAGTCAGATAGGGTTCGGCCATCCTCAAGCTGCTTA
CCGGCGAAGATTAGCCTTTGCTGGTCCGGAGCGATGCCTTCCTTGTCCTGGATCTTCGACTTCACGTTCTCAATGGTGTCGGAGCTCTCCACCTCGAGTGTAATGGTCTTGCCTGTGAGGG
TTTTGACAAAGATCTGCAT
>comp154796_c0_seq2
TGTCTTCCTTCTCCACTCCCTCGGCCATCCCCTTCTCACAACCTGAAGCACCTAAAAGCAAAGGGGAACTCGACGGCGGCGCCTCCTATTTCCGTCGAGCCACCCCTTCTTCCTCTTCCTT
GGCATTCCCACGATGGACTCGGCCGCCCGGCGCATCGGACTCACATTCGCCCTCTTTTCGCTCTACGCCGCTTCGGTGATCTCCACTCGTAGCGATAATGTAAAAGGAGTGGTATATGATG
CGCGTTCTTTCATTATTAACGGGAAGAGAGAGCTTCTCTTCTCTGGCTCCATTCATTACCCCCGAAGTACTCCGGAGATGTGGCCAAATATTATTGCCAACGCTAAGGCTGGTGGGCTTAAT
GTGATTCAAACTTATGTTTTCTGGAATGGACATGAACCCATTCAAGGGCAGTATAACTTTGAGGGAAGATATGATTTGGTGAAGTTCATTAAATTGGTCCAGCAAATGAGAATGTATGTAAC
ACTCAGGATTGGACCCTTCATTCAAGCCGAATGGAATCATGGTGGACTACCAGTCTGGCTAAGGGACATTGAGAACATTACCTTCCGGACTAACAACCCTCCCTTCAAGTACTACATGGC
GAAATTTGTGAGAAAGATTGTTGATATGATGAAAGAAGAAAAGCTATTTGCCTCACAGGGCGGGCCCATCATCTTAGCTCAAATTGAAAATGAGTACAACATGGTGCAAGATGCGTTCAA
GGAAGGAGGCAAGAAGTACATACGGTGGGCTTCTAACTTGGCCTTGAGCTCGGGGGCTGGAGTTCCATGGATGATGTGCAAGCAGCAAGATGCTCCTGGTCCAGTGATCAATGCCTGCA
ATGGAAGGAACTGTGGTGACACCTGGATTGGACCGAATCATCCAACTAAGCCTCTTGTCTGGTCTGAGAACTGGACAGCACAGTATCGTGTGTTTGGTGACCCACCATCACAAAGAACA
GTTGAGGATCTTTCCTACTCAGTTGCTCGATTCTTCTCCAAGAATGGCACTCTTGTTAACTACTATATGTATCATGGAGGGACCAACTTTGGCAGGACTGGTTCTGCTTTTGTGGCAACACG
GTACTATGATGAAGCACCTCTTGATGAATATGGTTTGCCGAAGCAGCCCAAATGGGGACACCTGAAGGACTTGCACCAAGCTTTGAAGCTAAGCAGGAAGGCAATGCTCTGGGGCACTT
ATAGTTTCCTGTCACATCGAAAAGACTTTGAGGCTAGAGTTTATGAGATTCCAAATAAGAAAGTGTGTGCCGCCTTCCTTACAAACACCCATGCTACTATAGATACAACGGTTAAATTTCGT
GGTGTAGACTATTTCCTACCTCACCGTTCCATCAGCATCCTCCCAGATTGTAGGACTGTGGTTTTTAATACTCAAAAGATAAATGCTCAACACAACTCGAGGAGCTTCACTCCAGTAGTTG

AGGTCAGGAAGAAGAGAAAATGGAAGATGTATAAAGATTATTTACCCAGATATCAAGATCAAGGAATTGTTCGTAACTATGAACCCTTGGAACTAATGAACATGACCAAGGATACCACTG
ATTATCTGTGGTATACCACAAGCTTCCACATTGAAGAAGAAGACTTGCCCAGAAGACATGACATTCGTCCTGTGCTCCAAGTGGCAAGCCTTGGCCATGTTGTGCATGCATTTGTCAATGG
GAAGTACATCGGAAATGCACATGGTAGCAAAATTGAGAAAAGCTTTGTTTTCCAAAAGCCCATGAAGCTGCATTCTGGAATCAACCACATCGCTATTCTTGCCTTGACAGTTGGTTTTCCT
GATAGCGGGCCTTATCTGGAACATAGAATGGCTGGAATTCACTCTGTGGCCATCCAAGGTCTTAACACTGGAACCCTGGATCTCACTAGCAATGGATGGGGACATCAGGTTGGTCTTTTGG
GAGAGAAGCTCCAAATTTATAGCCAGGAAGGTTCTTCTAAAGTTCAATGGACTCAACCTGTGGGCAACACAGCTCTTGTTTGGTATAAGAGATACTTCAGTACTCCACTAGGTAATGATCC
TGTTGTCCTGGACATGAAGACCATGGCCAAAGGTCTTGCTTGGGTTAATGGGGAATGTATTGGTAGATATTGGGTTTCCAACACCTCCCCTCTTGGCACAGCCACACAGACACTCTACCAC
ATACCTCGTGAATTCATGAAGCCAAAGGGTAACCTTCTGGTTGTGTTTGAGGAATCTGGTGGCAACCCCGATGGGATTCAAATATTGACTGTAAAGAGGGATGAGATCTGCACCTATGTCT
CTGAATACCATCGTTCATGGTCAAGGAGGAAAGGGAAGGTCGTCTCTATTGCGGAGCGTGTGAAGCCAGAGGCACAACTAAGCTGCCCCAACCAAAAGATCATCAAATCTATCACCTTT
GCCAGCTTCGGCAACCCCACTGGAGTTTGTGGGAACTTCACCGTTGGAAACTGCCATGCTCCACAAACCCAGTCCATTGTTGAGAAGACCTGTTTGGGTAGGAATTCATGTGTGATTTCG
GTGAAGGGAGAGGACTATTTAGCAGATGAAGATTGCCCAGGAACGACGGACACGCTTGCAGTTCAGGTCCTATGCACTCTGTAGTAGAGCCTCACAGTTTGAGGAGATTCTTTCTTTCGC
ACTAAAAATTTGGATTATTATTATTATTATTTTTTAAAGCCGAAGTGCACAAACTGAACTGTGATGACGATTGAAGCTTTTACTAGCAGCACTTGTATACTGGGAAATTGATAGGAGCTGCT
GCAATGCGCATCAAACAGATTTTCTTCAAACTATTCACAACACTTGATCTCTTTCCTCTATGGCTATTCTTAAAAAAATTATAAGAAAAAAAAA
>comp154796_c0_seq4
AATCCCCACCGCGAAGAAAGGGGAAGGAAATTTTCTCTTCTTCGATCGTCTCCTCTTCTCTTCTCTCTGGCATCCCTTTCTCGTCTACAATGGCTGCCGCCGGCCGGCGCGTCGGGCTACT
CCTTTCCCTCTTCGCCACCGTCGTTGCGGTATCTGCTCGCAATGGCGGAGTTAATAACAGAGGAGTGAGTTACGATTCTCGCTCGTTCATCATTAATGGAAAGAGAGAGCTCTTGTTCTCT
GGCTCCATTCATTATCCTCGAAGCACGCCAGATATGTGGCCTGGTATCATTGCCAATGCAAAGAAGGGCGGAATCAACCTGATCCAAACGTATGTTTTCTGGAATGGTCATGAACCAGTTC
AGGGACGGTATAATTTTGAGGGGAGGTATGACTTGGTGAAATTCATCAGGTTGATTCAAAAGGAGAAATTATATGTCACACTTAGGATTGGACCCTTCATTCAGGCTGAATGGAATCATGG
TGGATTTCCATATTGGTTAAGGGAGGTAAAGAACATCACCTTTCGAACCAACAACCCTCCATTCAAGTATCATATGGAGAAATTTGTGAGAAAAATTGTTGATATGATGAAGGAAGAGAA
GCTCTTTGCTTCTCAGGGAGGTCCTATCATCCTTGCTCAGATTGAAAATGAATACAACATGGTACAAGCGGCATTTGGGGAAGATGGCAAGAAGTATGTACAGTGGGCAGCTGACTTTGC
CTTGAGCCTTGGGATTGGAGTTCCATGGATGATGTGCAAGCAACAGGATGCTCCTGGACCAATAATCAATTCTTGCAATGGAAGAAACTGTGGTGATACCTGGAAGGGACCTAACCATCC
AACTAAGCCTCTTATCTGGGCTGAAAACTGGACTGCTCAGTACCGAGTTTTCGGCGACCCGCCGTCTCAAAGGTCAGCTGAAGATCTTGCATTCTCTGTTGCTCGTTTCTTCTCTAAGAAT
GGGACTCTTGCAAACTATTACATGTATCATGGAGGAACA
>comp154796_c0_seq5
GTATCATGGAGGAACAAACTTTGGAAGAACTGGTTCTGCTTTTGTCGCGACACGGTACTATGATGAAGCACCTCTTGATGAATATGGTTTACAGAAACAGCCAAAATGGGGACACCTGAA
GGACCTCCACCATGCTCTGAAACTTAGCAAGAAACCCCTACTTTGGGGTTCTTACAGTTTCAGAAAACTGGGGAAAGATGTTGAGGCTAGAGTCTATGACCTTCCTAGTAAGAAAGTGTG
TGCTGCTTTCCTCACAAACAAGAAGGCTGACCGAGATTACACAGTGAGCTTCCATGGCGTCAACTACTTCTTGCCACGCCATTCTATTAGCATTCTTCCAGACTGCAAGACTGAAGTCTTC
AATACTCAGAGGATTAATGCCCAGCACAACTCAAGGACCTTCATTCGATTTGTGGAGAGGAAGAAGAAAAAAAGATGGGAGATGTACCAGGATCATATCCCCAGATATCATGACAAAGG
AATTGTTCATAGCCCGGAGCCATTGGAGCTCATGAACATGACCAAGGATACCACTGATTACCTTTGGTATACCACCAGATTTAAGCTGCAGCAAGAAGACTTTCCATGGAGGAATGATATT
CATCCTGTGCTCCAAGTTGCAAGCCTTGGCCATGGAGTGCACGCATTTGCCAATGGCCAATACCTTGGAAACGCGCATGGTAGCAAGATTGAGAAGAGCTTTGTCTTACAAAAAGTCATG
AATCTGAAGCCTGGAAACAACCACATTACCATCCTAGCCATGACCGTTGGTTTCCCGGATAGTGGTCCTTACCTTGAACGCCGGCTTGCTGGAATTCGCTCGGTCGCCATCCAAGGCCTTA
ACACAGGTACCCTGGATCTCACTGCCAATGGTTGGGGCCATAAGGTTGGCTTTAGAGGAGAAAAGCTCCGGATCTACTCAAATGAAGGACTATCCAGAGTTCAATGGACCAAAGCTAAG

AGCAACTTACCCATTACTTGGTACAAGAGATACTTCGATACACCCCATGGAAACGATCCAGTCATCATCGACATGAGCACCATGGCCAAAGGTCTTGTCTGGGTCAATGGCGAAGGCATT
GGCCGATACTGGGTTTCCAATCTCTCCCCTCTTGGGACCCCAACTCAGACTGTCTATCATATCCCTCGCTCATTCTTGAACCAAACAAAGAACCTTCTTGTTGTGTTAGAAGAATCTGGAG
GAAACCCCGAGGGAATCCAAATTTTTACAGTTAGGAGAGACGACATATGTACTTACGTGTCTGAATATCATCCTGGTGTTACCAAATCTTGGTCGAGGAAGGAAGGCCAACTCAGGTCGG
TTGTTGAGGATGTTAAGCCAGAATCTCACCTCAAATGCCCAGACAATAAAGTCATCCAAGCCATCACCTTTGCTAGCTTTGGCAACCCCAGCGGCGTGTGTGGAAACTACACCATTGGAA
GCTGCCATGCTCCACAAACGAAGCTTGTTGTTGAGAAGGTCTGTTTGGGAAAAGCTTCTTGTGTGCTGCCAGTGAAAGGGGAGGTTTATGGTGCAGATGCAAACTGTCCAGAAACTACG
GCGACGCTTGCAGTTCAGGTCTTTTGTGGCCGTAAGTAGAGCTATATCTAAGGATTGAGATTACACATTTGTAGTAAAATTTGTTCATTTTTTTATTTTTTAAGGGGAAATGTAATAATCATG
GTGATTAATGAATCACTGTGTCGAGTTCTTATTGTAATATTTTGAACAATTCAAAGGCATGCCTTCATATTTTACTTTTATCAATTTTGCTAGAAAAAAAA
>comp154821_c0_seq1
ATCGTTCTCTTCTTTCGCTCGTTTTTTCGCCAAGTTCGATAGAGATAAAAAAATGATGAAGATGAACAGTGTAACACCAATATTGTCATCCCATTGTTCACCCGCAACCAAATAACAAAAAT
TTTACCTTCGGTTGGAGCTGCCGAGGGACATCATTGACGCGTTTCAAGCTGGGTTGGAGATATCCTAAAATTCGCTTCTCTATGCCAATAAATAACGAACACTCGTAAATCCTACCACAGT
CGGCAACCAGAATTTCGCTCCTCCTTTCGTTTGTAGAGAACCAATAACCTAATATTTTCTTCGTCGATGGCGCGTACGAAGCAAACTGCCCGGAAATCAACAGGAGGAAAGGCTCCTCGG
AAGCAACTTGCGACCAAGGCTGCAAGGAAATCGGCGCCGACTACCGGAGGAGTTAAGAAGCCTCATCGTTACCGTCCTGGAACCGTCGCTCTCCGTGAGATTCGCAAGTATCAGAAGA
CCACGGAACTGTTGATCCGCAAGCTGCCGTTTCAGAGGCTGGTGCGTGAGCTTGCTCAGGATTACAAGACGGATCTGCGATTTCAGAGCCATGCCGTGCTCGCTCTTCAGGAAGCCGCG
GAGGCGTATCTCGTCGGCCTTTTTGAGGATACGAATCTCTGCGCCATCCACGCGAGGCGCGTCACCATCATGCCCAAGGACATTCAGCTTGCT
>comp154821_c1_seq1
CTACACTATTAGACTTCATTACCAACTTTTAAGTCTTCACGAAAAAGAAAAATCTAAAAAATTAAAAAAGCAACAATAGAACTGCCATGGTTGATCAACTTAAGCACGCTCGCCCCTAATA
CGCCTAGCAAGCTGAATGTCCTTGGGCATGATGGTGACGCGCCTCGCGTGGATGGCGCAGAGATTCGTATCCTCAAAAAGGCCGACGAGATACGCCTCCGCGGCTTCCTGAAGAGCGAG
CACGGCATGGCTCTGAAATCGCAGATCCGTCTTGTAATCCTGAGCAAGCTCACGCACCAGCCTCTGAAACGGCAGCTTGCGGATCAACAGTTCCGTGGTCTTCTGATACTTGCGAATCTC
ACGGAGAGCGACGGTTCCAGGACGGTAACGATGAGGCTTCTTAACTCCTCCGGTAGTCGGCGCCGATTTCCTCGCAGCCTTGGTCGCAAGCTGCTTCCGAGGAGCCTTTCCTCCTGTTGA
TTTCCGGGCAGTCTGCTTCGTACGCGCCATCGACGATGAAAAGAATCGGTTAGGGTTTCTCTATACGCGGAAGCGAAAGAAGGAACGAAATTCTGGTTGCGAATGGTGGTAGAAATTATG
AGCGCTATTTATAGACGTAGAGAAGCGATGTGTTATGGGGTTGGGCCGGGTCGGTTCGCTTACGGCGTTCCAATTTTGAAGTTCTGATCGAACGGAATGGGATTCATTTGAGAAATTAGCG
ATCCGTGTGCTCTGTTCCTATTGGTTCAAATGCCATTGACATGGATTAAGAAACTTAATTTCTCTCCATTCCTTCTCTAATAATATGCGGAAGAGC
>comp154823_c0_seq1
AAAAAATTCCTCAACAAAGTTCACATGTCTCCTTATACCCTTCACTATATAAGTCCTAACTAAACTGAATCTGCCATCAGAAGCTTAATTACTTCCTGACCATGGACTGGTACTCATGGCTC
TTAAAATCTTCGAGGCTCGACCGGAATCTGGTCTACGAGTACTCTGTCCTTCTTTCCCAGAACGAGCTCGAGGAAGATGACATCGCCCACTTCGACCACGAGTTTCTCCTCAGCATGGGC
ATTGATGTCGCCAAGCATCGCCTCGAGATCCTCAAGCTTGCTAAGAGCCACCGCCGTTATCACCGCCCCGGCCGACCAGTCGCCGCTCTCCTGACAGTCATCCACGAAGCCTGCAACTGC
TTAACAAGGTATGTTCATGATCTAGTCCACCATACAAGCAAGGCAGTGGTGATTATGCCGAGGCCAGGAGGAGGGGGGCGACAGATGGTGAGCGGAGGGATCAGGAGGCTGGCTATGGT
GAAGAAAGGAAGGTTGCAGATCACTGATGTAGTCTGCAATGGAGTTAAGGCAGTTCAGCTCGGTCGAAGCCCGGCTTCGCAGAGCATGAGTCCTGTAGTCGGCAATGGAGTTAAAGCA
GTTCAGCTCTGTCGGAGCCCAGCTTCACAGAGCTTGAGCCCTGTAGTCGGCAATGGAGTTAAGGCAGTTCAGATCGGTCGGAGCCCGGCTTCACAGACCATGAGCCCAATGGCACTGCT
TGCTTCAAGGGAAGAAGGCCATGATGATGGCTTTACTTGGATTGGGTACAACGATACAGACCAAATGAGGTGGGACTCCATGTTTCAGGATCTGAAGCCTACCTGACTGGATGAGCTCTC
CTTTTGAAGATTTCAGAAGGTTCTTATCAGCTACAGAATCAAGATTTATTCGAAAGAACGAAGTTTTAACTAAATTTTGAATCAAAATTTGCATATAGTGATAATTAGTTTATGAGGAAAAA

GAATTGGTACCATCAGCACCACTGTTTCTACCACTAGCTCTGATCTGTTTTGGTGTCTCTGCTTTAGCTTTTTGGAATCCAGCTTTTGAGAGTGAGGTATGAATGGCTAGATTTACTTGAAG
GTGGAGTGTTTAGGCTTTTACTTTGCTTTTGTGGAAGAATGACTGTAAGAAATGCTAGTGGGAAATTATTATGCTAAAGGGGAGGTCCTATTTTTTTTTCTTCCATGGATTGATTAATTTGCT
TGATGGTTAAGTCAGGTAGATGAAAGTACTGTGGCATTCCACAAGATAAAAGAAGAGGTGATGGCCTGTCTCAGTCACAAATTAAAGTTTTAAGAATATTATGACAACTTTGGAGTTTCTA
TTCTGCAGCCAAAACAAAAAATTATACCTACCGGCATTGGAGACTTTGGCTAAGTTGGAAAGAGATTTGGTTTCATATATTATGGGCTAAAGCTAGCTTAGAAAGTAAGGCTTTTTTTGTTT
TATTCTGCAAGTTGTTCTTATTCTACAATAACTAAGGTATGATATTGATATCTTATTATGATGGC
>comp154823_c0_seq2
AAAAAATTCCTCAACAAAGTTCACATGTCTCCTTATACCCTTCACTATATAAGTCCTAACTAAACTGAATCTGCCATCAGAAGCTTAATTACTTCCTGACCATGGACTGGTACTCATGGCTC
TTAAAATCTTCGAGGCTCGACCGGAATCTGGTCTACGAGTACTCTGTCCTTCTTTCCCAGAACGAGCTCGAGGAAGATGACATCGCCCACTTCGACCACGAGTTTCTCCTCAGCATGGGC
ATTGATGTCGCCAAGCATCGCCTCGAGATCCTCAAGCTTGCTAAGAGCCACCGCCGTTATCACCGCCCCGGCCGACCAGTCGCCGCTCTCCTGACAGTCATCCACGAAGCCTGCAACTGC
TTAACAAGGTAACTATAACATTTCGACCGCATCTCTAAGCATAGGAGGAATCAGAGAGGGACTAAAGGTGACTAATTTCGCCTCATAGGTTTTTAAAAATTTTACATAATTGGTCATTATATA
TAATATGGAAATTTCATCTTTAAATTTTCGACCCTCCCTGTAAAATTTGGGGTAAATTATTTTGCTGTGATCCATGGCAGGTATGTTCATGATCTAGTCCACCATACAAGCAAGGCAGTGGTG
ATTATGCCGAGGCCAGGAGGAGGGGGGCGACAGATGGTGAGCGGAGGGATCAGGAGGCTGGCTATGGTGAAGAAAGGAAGGTTGCAGATCACTGATGTAGTCTGCAATGGAGTTAAGG
CAGTTCAGCTCGGTCGAAGCCCGGCTTCGCAGAGCATGAGTCCTGTAGTCGGCAATGGAGTTAAAGCAGTTCAGCTCTGTCGGAGCCCAGCTTCACAGAGCTTGAGCCCTGTAGTCGGC
AATGGAGTTAAGGCAGTTCAGATCGGTCGGAGCCCGGCTTCACAGACCATGAGCCCAATGGCACTGCTTGCTTCAAGGGAAGAAGGCCATGATGATGGCTTTACTTGGATTGGGTACAA
CGATACAGACCAAATGAGGTGGGACTCCATGTTTCAGGATCTGAAGCCTACCTGACTGGATGAGCTCTCCTTTTGAAGATTTCAGAAGGTTCTTATCAGCTACAGAATCAAGATTTATTCG
AAAGAACGAAGTTTTAACTAAATTTTGAATCAAAATTTGCATATAGTGATAATTAGTTTATGAGGAAAAAGAATTGGTACCATCAGCACCACTGTTTCTACCACTAGCTCTGATCTGTTTTG
GTGTCTCTGCTTTAGCTTTTTGGAATCCAGCTTTTGAGAGTGAGGTATGAATGGCTAGATTTACTTGAAGGTGGAGTGTTTAGGCTTTTACTTTGCTTTTGTGGAAGAATGACTGTAAGAAA
TGCTAGTGGGAAATTATTATGCTAAAGGGGAGGTCCTATTTTTTTTTCTTCCATGGATTGATTAATTTGCTTGATGGTTAAGTCAGGTAGATGAAAGTACTGTGGCATTCCACAAGATAAAA
GAAGAGGTGATGGCCTGTCTCAGTCACAAATTAAAGTTTTAAGAATATTATGACAACTTTGGAGTTTCTATTCTGCAGCCAAAACAAAAAATTATACCTACCGGCATTGGAGACTTTGGCT
AAGTTGGAAAGAGATTTGGTTTCATATATTATGGGCTAAAGCTAGCTTAGAAAGTAAGGCTTTTTTTGTTTTATTCTGCAAGTTGTTCTTATTCTACAATAACTAAGGTATGATATTGATATCT
TATTATGATGGC
>comp154839_c4_seq7
TCTCTCTCTCTCTCTCTTCCTCTTCTCACACAGCCGGATTGAAGAGTAAGAAACTAGCAATGGAGTTCGACTTCGACCTTGAGAACCCGTTCTCCGGTAGCGATGAGCAAAACATCCAAC
TCTCGAAAGCACTATTCATCGGCTGCACAGACTCCGACGAGCCCTCGTTGCTTTTCTCAGCTGAATCGGACCACATGAGCTCGCTTGCGGTTGCTCTGGACCAGCTCTCCACCCGTTGCC
ATGCCCTCTCCCTTATCGTCCAGGCCCAGTTCAGCAATAATCTCGATCCAGCACTAGCCTACCTTGCTGTCAACTATATCGATCGGTTCCTCGCCAAACGCGAGATCCCGAATGAGAAGCC
ATGGATCGCGCGACTCCTCGCCATTTCCTGCGTCTCCCTCGCCTCAAAGATGAAGAAGACCAGTTTCTCCCCTGCTGATTTACAGGTACCGTGAAGCTAGCTTCACCTCTTCTATCGAACA
ATCTCGATCCGATCGATTCAACATTTTCCCCAAATTGTTCGGAGCAGAGGGAGGAGGGCTTCATCTTCGATGCACGGACCATCCGGCGAATGGAACTGCTCGTGCTCGGCGTGCTTGAAT
GGCGGATGCGATCGATCACGCCCTTCTCCTTTCTCCGATTCTTCACATCCTACTTTCCCCTGGCGAATCCAATCATCATCCAATCTCTCAAGACCCACGCCTCTCAGATCCTCTTCAAATCCC
AAAACGATGTCAAATCGCTCGAGTTCAAGCCTTCGGTGATCGCTGCGGCAGCGCTTCTCGCCGCCGCGCGCGAACTCTTCCCTGCTCATTTCATTTCCTTCCGCACTGCCCTGCTCTGCTG
TGAATATCTCAATAAAGAGTCTTTGGCAGAATGCTGCGCCGCGATGGAGAGCTACGATACGACTGCCGGGATTGAGGCAGCATCGAACGGCTGTGATTCGGGCTACTCTGCTCTCACGCC
GGTGACTGTACTCGACCGCCATCGCGCCGGCTTGGAAAGCGAAAAGACCGCCGGATCGGCATCCAACGGCTGAGATGCCAAAAAGCGCCGCACTTCTACTACGGCGCCTTGCCTATTCT

GTGACCAATACCTAACCAATAAATTTAAAAAGAAAAAATCTATTTCCTATTGAAAAAAAAAAAGAGAAAAAAAAAGAAAGAGAAAAAAAAGAGAGGAAAATTTGAGATTCTTTTGCCG
TCGCCGCTGACCCTGAAGGTATAATCTCCAAAACTTAGATTTCCCATTCTCGTTGTTCGTGGCGGATCGGGGAACCACCTATACGCGCTCTCGTAGCACGGTGGGAGGGAGCCCGGTGCG
ATACCTACGACGAGACGGGTAATCACCTAAAACTCATATTCCCATTTCGATGTCTGCTGCGGACCGGGGAACCACCTATACG
>comp154839_c4_seq8
TCTCTCTCTCTCTCTCTTCCTCTTCTCACACAGCCGGATTGAAGAGTAAGAAACTAGCAATGGAGTTCGACTTCGACCTTGAGAACCCGTTCTCCGGTAGCGATGAGCAAAACATCCAAC
TCTCGAAAGCACTATTCATCGGCTGCACAGACTCCGACGAGCCCTCGTTGCTTTTCTCAGCTGAATCGGACCACATGAGCTCGCTTGCGGTTGCTCTGGACCAGCTCTCCACCCGTTGCC
ATGCCCTCTCCCTTATCGTCCAGGCCCAGTTCAGCAATAATCTCGATCCAGCACTAGCCTACCTTGCTGTCAACTATATCGATCGGTTCCTCGCCAAACGCGAGATCCCGAATGAGAAGCC
ATGGATCGCGCGACTCCTCGCCATTTCCTGCGTCTCCCTCGCCTCAAAGATGAAGAAGACCAGTTTCTCCCCTGCTGATTTACAGAGGGAGGAGGGCTTCATCTTCGATGCACGGACCAT
CCGGCGAATGGAACTGCTCGTGCTCGGCGTGCTTGAATGGCGGATGCGATCGATCACGCCCTTCTCCTTTCTCCGATTCTTCACATCCTACTTTCCCCTGGCGAATCCAATCATCATCCAAT
CTCTCAAGACCCACGCCTCTCAGATCCTCTTCAAATCCCAAAACGATGTCAAATCGCTCGAGTTCAAGCCTTCGGTGATCGCTGCGGCAGCGCTTCTCGCCGCCGCGCGCGAACTCTTCC
CTGCTCATTTCATTTCCTTCCGCACTGCCCTGCTCTGCTGTGAATATCTCAATAAAGAGTCTTTGGCAGAATGCTGCGCCGCGATGGAGAGCTACGATACGACTGCCGGGATTGAGGCAGC
ATCGAACGGCTGTGATTCGGGCTACTCTGCTCTCACGCCGGTGACTGTACTCGACCGCCATCGCGCCGGCTTGGAAAGCGAAAAGACCGCCGGATCGGCATCCAACGGCTGAGATGCCA
AAAAGCGCCGCACTTCTACTACGGCGCCTTGCCTATTCTGTGACCAATACCTAACCAATAAATTTAAAAAGAAAAAATCTATTTCCTATTGAAAAAAAAAAAGAGAAAAAAAAAGAAAG
AGAAAAAAAAGAGAGGAAAATTTGAGATTCTTTTGCCGTCGCCGCTGACCCTGAAGGTATAATCTCCAAAACTTAGATTTCCCATTCTCGTTGTTCGTGGCGGATCGGGGAACCACCTAT
ACGCGCTCTCGTAGCACGGTAGGAGGGAGTCCGGTGCGCCGCCTACGACGAAGCGGGTAATCACTTTAAATCCCATTTTCGACGTTTGCGGCGGACCGGAGAACCACCTATACG
>comp154856_c0_seq1
TTATTAGAAATGAAGTATGCTTACATTTTGCCACAATTAGGAGTGCAACTGAGTCAAGCCGACTTGCAAGCCAACTCAGACTTGACTCGATAATAGGCTCAACGAGACGAGCTTATCGAG
CTCGAACACAATTTCAAGCTCGATGGCTTATTGAGATCGAGCTCAAACAGGCCTCGGACTTGACTCATCGGCTGGACAAGCAGCTCGCGAAGGTGACTGGGCGGAAAAATGAAATATTT
GTTGTTGCGGCCAGGAGGACGAATCAAAGGTCAGGCGAGAAAACCAAAACGACAAGTGAGCTCAGGACGACGGATCTCGACAGCAGATGTGATGATGACTGATCTTGACGACGAACG
CGACAACAAGCTCGTGACAACGGATCTCGACGACATAGCTCAAGATGACAAAGCTCCACAACAAACGCGACGTTGATGAACCTCGGGACCATAGAGAACGCGACGACGAGCCAACTC
GGGACTAAGCTCAACGAGCTGCTCAACATGAGACGATTCGAGCACGAGCACGAATTTTGAGCTTG
>comp154859_c0_seq3
AAAAAAACTTCTGTGCATGGCTTCGAATCTGAACAGTAGTCTTCGATCCTATATGCTCTGCAAAAATGATTCATGGTGAGGAACAGAGGAAAGCTTGATATCCCAGAAAGCTTTCAAAGG
TGTCATGGTAGTTAAAGAGAGACGCTTATAAGGTGACGCAAGTCACCTTCTATTCTTCGCCAGGCTCGGCCATGAAGTTTCAGTGCTTCCAGAAATTTATTGTGCTCTTCAGCAGTCCATC
TTTCTCGTTGTTTCGTTATTATGTAAGGTTTCCTTACCTTGATAGCATGTTCAGAAATTGATGAATCAGCTTCTGCCATCGGGTGGAGACAATTTGCAGATGAGGCTTCTCCGTCCACACAC
ATTCTCTTGGAGAGGAATAAACAGTACTGTGTAATCCTCGCTGTTCCACCGAAGAACGCTGGAATAGGGTCTTCTCGCCGCCGATAGCCCTTCTCTAGTTTGCCGAAAAACCTCCGGCGA
AGTGAATTCGATTGAGAGAGAGAGGAGAGAGAGTGAGAGAGATGGGAGAGAGAGAGAGTGAGAGATAGATAGAGAGAGCAAGAGCAAGAG
>comp154859_c0_seq8
TCATCATCATCATCATCATCATCAACAACAACAGACGAAACAGCTGCAGCCATCAAATCTACAACTGAAATAAAACGAGAGAAGAAGATAAAACAGAGTGGGCAGAATCATCTCTAATCA
CCTCAACAAACAAATTGATAACAGTTGAGAGATTCTGTATCCTCTACTGACACCCAAGCTTTTCCCAATAGCTACTTCCTTCGATGTTGGCCACAGGTGTGCGCGGCCGTTCCTTCTCAAC
TACACATCTTTTGTAAGGCACAAAGCCTCTGTTAGAGTTTGCAGAAGATGGAATCAAACTGCTGAAAGCTGAGTTCTTGCTTGGCTTCAGCACTGGTGCTGGAGGAAATTTCTTCATTTG

AATACCAATCTCAGCTGCCTCAGAATTGCTATCAGAGGTAGTGCTGGAATCAGTTTGAGAACTTTCGTTATGTAAAAAGCTCTCATCTTTGGAAGGCATTGGACTTTGATCTGATGGATTCA
GAGCTCCTGAAGAGTGGAACGGCATAACTCCATACAAGTTCCACAAAGAAGGAAATGAAACCATATTAGGTTCCTGATATTCCGACTGATTCTTCTTACTCGAAGGCTGCTGCTGCAAGC
CAGCTACTACAACGGTCCTTCCAAACAATCTAAGACATGTTATGTTCTGTCCTGTAACTGGAGCCTCTTTAGCAGCAAAACAATCTTGAATGCTTAAGTCTAATTCCATAGGAGATATATCT
TGCAGGGTCAAGGAAGGAGAGGTATCCGATGATTTATTACATTTGTATTCAGCTGAGGTAATTTGTTCAACCAATATTTCCTCTACCGAGACTGTCTTATCAACAGATGAAACTGGTGACAT
ACAAGGCTTTAGGGTGTCAGCAGCAGTTGTTCCACTTGTATCAGATCCAAATGCAGACAACACCGATGTCGGCGATTTACTCTCCTGCTCGGAAACTGATTGATTTGCTATGGAAGGCCA
CAGCTGTGCATCTATAAGAGGGCCTTTCATGATGGATAGATGACCCAATTTACGGGGGTATGGATGATTTGGTTTCCTTTTGGGTCGTGGAGGAGGTATCTCCATAGCTTTGACAGTGCTTG
GATTATTGCTTGATGACTCCCGTGTCACCTTGGAAAAAAACTTCTGTGCATGGCTTCGAATCTGAACAGTAGTCTTCGATCCTATATGCTCTTCTATTCTTCGCCAGGCTCGGCCATGAAGT
TTCAGTGCTTCCAGAAATTTATTGTGCTCTTCAGCAGTCCATCTTTCTCGTTGTTTCGTTATTATGTAAGGTTTCCTTACCTTGATAGCATGTTCAGAAATTGATGAATCAGCTTCTGCCATCG
GGTGGAGACAATTTGCAGATGAGGCTTCTCCGTCCACACACATTCTCTTGGAGAGGAATAAACAGTACTGTGCAATCCTAACATCAATTACATAAAAATCAGATCTTTAAATAACATGACG
TACAGAAACCTAATAACTTGTGACATACATGCTATTAGCCCTCTTAAGAAACAAAGAAAGGACATTTGGCCTAAATAATATTCCACATAATACTGCCTAATTCAGATTTAACTTAAAAATATA
TATAGAATAGCTCCTCTTGGCATATATATACGGCTTATTGCCTTGTTCAGCAACAGAAAACCTGAATTGCGCATATAAATACAAGCAATACCATTCTTAGAAAGGAGTTAAAAACACAAGTT
ATTTAGACAGATTCTGTTCAATAACAATAATTACTGTATGACAGCCTAAAATTTGCCTACTATCATTTGTAACATAATTTTTTTTTTTCATCATTTCTGCAAGGAAGAATCTCTGTTCTTAGAA
TTAATAGGAAGCGGACTGTCTTTGCGAACGCCAAGAAATCGTCACCAGCCAAGATGGTGCCATGGAAACTTGGATGCTGAAAAAGAGTGAATTCTGTAACTTGTCAGTTCTAATCATGAC
CCCTGGTAGCCATATGTATGCTCTTGATTTGATATGAAAAACTAGGCATTATATATACCAAAATTTAAAATTAAATACGTTTAACGACTTGGAAAATTGTTGGCCATATCAATATTTAGTAAAA
TCCTACAAAATAGGGTAATAATTTTGT
>comp154860_c0_seq2
GAGAAATAATATCAAAATAAACACCATGTTTAATTTAAAAATTCATATTAATTATGTTTCTAATCAAACCCCAAGCAAAACTTATTTGCTTCCATCCAATCCCTTCATTCAGCACCTACGTACA
GACCCTTACAAAAAAATACCTTAACCTAACAGATTAAAATCCTATAATTATGAAATAAATATCTAATAGAAACTTATCAATAGATAATACAATTGATGCATAGCGACCTGTTAGTATTCAAAG
CAAAGGTTTTCAAATCCCATGTACTCTTGATGCCGAAGCTTCTGGATCATCCGCGAAATACCAACCCCACCTAAAGAAATAGCGCTTATAAAGATTGTAAAAGCCAGTATGAAGATGATGA
TAGAAGCTCTCCCCAGAATAAGTATGATCTTTCTCACAACGTGTTGGCCAATGAAAGCCGCAACTGTGGCCACGGCAGCGAAGTATAAAGCATACGGGACCGGGAAACGACGTAAAAGG
TAGTATTCGACGACGGACATCGAAGCAGAAAACGTCATAGCAAAGGTTGCCGTAGCACTTGACACCTGAGGTGGAATTCCTAACTCCAGGAAAAGAGGGCCGAGAACAAATCCTCCTC
CTAAGCCGAGCAATCCTCCAACTATGCCAGCAAATACACCAAATAAGCAGTATATTACAAGCTGATGAATCTTCCACTCTGTAACTACTTCTCCCTTTGATGCGATCACTCTTCTCCCTTTGT
ACAGACTAACAGCTTCATATAAACTGACACCAACAGCAACTGGAATCTGTAGAAAATTCAGCACCCAATACCAAACCGAACAAGTTCCTGTATGGTTCTGTATAAAGTTTACACAAGCAA
TGTCAATAGATTAAGAGTTGCAGATTAAAAATGGCAACGAAAGCTACCTGCAGGAATTGCTCACTTTTGAAATCTGTAGTGTTAGAAAGGATATCCAGACAAATAATAGAAGGCCGAGCT
CCTTCCAGGAGACATTGTCAAAGATTGGAAACTCCTCTTCAGAAGATGACTTAAGTTCCCTTTGAGCATTGTTACCTAGACCACCAGGTAGTGGCCTGTATTCTACTTCTTCACTCTCACTA
TTAGAGTCGAGGTTTTTCGCTGCATTCTTCTTCATCAATGTTTCCTTCTTCCATGTCTCAACACCCTTTATACATGCCTTGGTAGAGGTAACTAAGAAGAGGATGATCAGAAGAACTGTAAC
CATCCAATCAGGAAAAACTACATTGAAGATGACCCCGATGCTAATCCCAAGCATGAGCATAGGTTGGAAAAGCAAAGCAAGATCATAGTCAATGAGCGGTATATCTAGAGTAGGATGCCT
CAGCCTCAGATTATAGTAAACAGTCGAAGCGGCGGCACCTGTGATCATACATTTCGACATTGCAGTAGAAGATTTAGGATCAAACCCAATGATCAAAGCGAGCATGGGAACAAAAATCCC
TCCACCACCGACACCTCCCACGCTCCCTAATGCTGCTCCAAAGAATCCAATCACTGAACCAACTACGATTTGCCATCCGAATTTCAATGGCGGCCAAACATGCTGATACCGAGATCTATCA
GCTCGCTTCAATAAACCCAAAACCTTCATGAAATAGCTCGACTCATCCATGTCCTCGCTCGCCACGGCCGGAATGAGCTTGATTAAACCCCGATCAGCCGAAACCAAAGCTGCCATGACA
AAGATGAGAAGAATTGAGGAGATCAAACCCAGCCCTCTCCTCCATTTCCTCCCATTTCAGTTGATGAAGAACGATTAGGCTCAAGCAAGAAAGAGGAAAAAGCGCAGCGTTCCCTCCA

CAAAACAAAGAGGAGATTCTTCCAAACCAATTTAGAATCTGGATGTGAGAGAGCACGGGAGAGGTTG
>comp154895_c0_seq11
CCAGTTTGATGCAATGGTAGCATAGTTTTAAAATTTGACTCAGTGGATTGGGGTGAATATTCCAAATGATTTGGTTAGTTTGCTTCTTTTTAATTTTTTTTAATATTTTGCTCCTTTTCCTAATT
TAAATTAACAGCATACTCCTTTAAAAAAAAATCCCATCTCAAATAAGTAAGATTTACTTTGAGATTATACAATACAAATTTCAATGAATTACTCATATATATTACGCTCACCTCAAAATCAATA
ATCTTAAAACCCATCTAACTAATTTATGATCAAACGATCAAAATATCATGACCCAAAATTTCCTATGTTCAAATATAAGCAAATGAAAATGATAAAAAAAACTCACAGAACATATTGAAAGT
TCAAAACTCATTTCAGCACACTTTGCAAAGGCAAACTCTCTTCAAATGCCCTCTCCCTCGCCACTATTGCCTGCAAGATCAACCATAACATTTGAACACCATTGTAAAAAGTTATTAAAAT
AAGCTCATTAACCATACCTGTTGTTGCCAGTTAGTAAAAGTAGTTATTATTCCGAGAAGAAGGCTTTGAATCACAGCACCTGCTAATATACCAATCCACAGACCCTTACCTCCAATGTGGA
AGAAAAAGCCTAACAAAACTGCGATTGGGATTCCGACGAGATAAAATGCTCCGAGATTCACATAGGCTCCAAGGTGCTGCCACCCACATCCGCGAGCGATTCCAGAGAGCACTCCAAGA
CAGCCATCGGAGATAACTGAGATAGAAACAAGAGGGGTCATCTCCCTAACATAATCCACAACCTCCTTCTCGTCGCTATAGGCATGGCCAACTAGCCGGCGAAGGCTGAAGAGAGTTAA
ACTGACCAAAACTGCCTCTGTAATTGTGATTGATGTGGCCACACGTACAGCAAGATGAGCTTGCTCTGGATTCCCAGCTCCTAGTTCGTTTGACACGCGTGTGCTTGCAGCACAACCAAG
TCCATATGGTATGGTGTAGAGCAAGGAAATGCTTGTGAGGCATATAGAAAGAACAGAGGTTTCAAGCTTTGGATTTGGTAGGAGACCTGAGAGCAAGACAAGAAGCTCGAAGGACCACC
ATTCGAGGCATATCATTACTGCTGAGGGAATTGCTAGCCTCAAGAACTCATTAATGCCCCACAATGCCTCCATTGATAGCGGTGCACGAGTTGCATTGCAAGAATCTGAATATTTGATGTAA
AATGCAAGAATCAACACATTGATCCAATAGGAAATGCTTATAGAAAGTGCTGCTCCAACGTTGCCCAACCCTGTTTTGAACACCATGACCCAGCACAAAGGAATGTGGACGCAGAGGGT
CACAAATGAGCTCAGAAGCATAGGAAGAATTAAGCTCTGAGACTGAAGAAATTTCATAAGAGGTTGGGCAATAGCAAAGGCGAAGAGGCCTGGGATCATCCATACTGCATAAATGCCAG
CCTCTTGGGATATTAAGGGATCCTGGCCTATGAGTGAGAGAAGTTTCCCCATTGATACCCAAATGAGAGAAATTGGGAGGCAGACTAATACAAGTGAGAGTATGGCTCTGTATGTGTGAAT
TCCAAGCTTCTTGTATTGTTTTGCACCATATGCTTGGCCACAGAGAGTATCCAAGCCACTAGCCATTCCTAGAAGGACACTGAATCCAGTAACGCTGGAGAGTGAAGTGGCAATGGCGGA
GCTGGAGAGCGCGAGCTCACCGAGATGTCCGACCATCATCGACGAGGTGACCTGAACCAGGTACTGCGATAGCGTCACCCCCACCATCGGCCCGGCCAGGTAACCCAGCCTCCGCGCCT
CAGCTGTAAAATCACAGTACCATCCCCTCACCGTTCTCCCCTCCTCCCTCTCCAAAAGCCCTTCCTCCATTTCCGTAAGCTGTCCCTCTCCTCTTCGTTTCCCTTTTTCTTAGATGAC
>comp154895_c0_seq18
CCAGTTTGATGCAATGGTAGCATAGTTTTAAAATTTGACTCAGTGGATTGGGGTGAATATTCCAAATGATTTGGTTAGTTTGCTTCTTTTTAATTTTTTTTAATATTTTGCTCCTTTTCCTAATT
TAAATTAACAGCATACTCCTTTAAAAAAAAATCCCATCTCAAATAAGTAAGATTTACTTTGAGATTATACAATACAAATTTCAATGAATTACTCATATATATTACGCTCACCTCAAAATCAATA
ATCTTAAAACCCATCTAACTAATTTATGATCAAACGATCAAAATATCATGACCCAAAATTTCCTATGTTCAAATATAAGCAAATGAAAATGATAAAAAAAACTCACAGAACATATTGAAAGT
TCAAAACTCATTTCAGCACACTTTGCAAAGGCAAACTCTCTTCAAATGCCCTCTCCCTCGCCACTATTGCCTCCAATGTGGAAGAAAAAGCCTAACAAAACTGCGATTGGGATTCCGACG
AGATAAAATGCTCCGAGATTCACATAGGCTCCAAGGTGCTGCCACCCACATCCGCGAGCGATTCCAGAGAGCACTCCAAGACAGCCATCGGAGATAACTGAGATAGAAACAAGAGGGGT
CATCTCCCTAACATAATCCACAACCTCCTTCTCGTCGCTATAGGCATGGCCAACTAGCCGGCGAAGGCTGAAGAGAGTTAAACTGACCAAAACTGCCTCTGTAATTGTGATTGATGTGGCC
ACACGTACAGCAAGATGAGCTTGCTCTGGATTCCCAGCTCCTAGTTCGTTTGACACGCGTGTGCTTGCAGCACAACCAAGTCCATATGGTATGGTGTAGAGCAAGGAAATGCTTGTGAGG
CATATAGAAAGAACAGAGGTTTCAAGCTTTGGATTTGGTAGGAGACCTGAGAGCAAGACAAGAAGCTCGAAGGACCACCATTCGAGGCATATCATTACTGCTGAGGGAATTGCTAGCCT
CAAGAACTCATTAATGCCCCACAATGCCTCCATTGATAGCGGTGCACGAGTTGCATTGCAAGAATCTGAATATTTGATGTAAAATGCAAGAATCAACACATTGATCCAATAGGAAATGCTTA
TAGAAAGTGCTGCTCCAACGTTGCCCAACCCTGTTTTGAACACCATGACCCAGCACAAAGGAATGTGGACGCAGAGGGTCACAAATGAGCTCAGAAGCATAGGAAGAATTAAGCTCTG
AGACTGAAGAAATTTCATAAGAGGTTGGGCAATAGCAAAGGCGAAGAGGCCTGGGATCATCCATACTGCATAAATGCCAGCCTCTTGGGATATTAAGGGATCCTGGCCTATGAGTGAGAG
AAGTTTCCCCATTGATACCCAAATGAGAGAAATTGGGAGGCAGACTAATACAAGTGAGAGTATGGCTCTGTATGTGTGAATTCCAAGCTTCTTGTATTGTTTTGCACCATATGCTTGGCCAC

AGAGAGTATCCAAGCCACTAGCCATTCCTAGAAGGACACTGAATCCAGTAACGCTGGAGAGTGAAGTGGCAATGGCGGAGCTGGAGAGCGCGAGCTCACCGAGATGTCCGACCATCAT
CGACGAGGTGACCTGAACCAGGTACTGCGATAGCGTCACCCCCACCATCGGCCCGGCCAGGTAACCCAGCCTCCGCGCCTCAGCTGTAAAATCACAGTACCATCCCCTCACCGTTCTCCC
CTCCTCCCTCTCCAAAAGCCCTTCCTCCATTTCCGTAAGCTGTCCCTCTCCTCTTCGTTTCCCTTTTTCTTAGATGAC
>comp154895_c0_seq6
CCAGTTTGATGCAATGGTAGCATAGTTTTAAAATTTGACTCAGTGGATTGGGGTGAATATTCCAAATGATTTGGTTAGTTTGCTTCTTTTTAATTTTTTTTAATATTTTGCTCCTTTTCCTAATT
TAAATTAACAGCATACTCCTTTAAAAAAAAATCCCATCTCAAATAAGTAAGATTTACTTTGAGATTATACAATACAAATTTCAATGAATTACTCATATATATTACGCTCACCTCAAAATCAATA
ATCTTAAAACCCATCTAACTAATTTATGATCAAACGATCAAAATATCATGACCCAAAATTTCCTATGTTCAAATATAAGCAAATGAAAATGATAAAAAAAACTCACAGAACATATTGAAAGT
TCAAAACTCATTTCAGCACACTTTGCAAAGGCAAACTCTCTTCAAATGCCCTCTCCCTCGCCACTATTGCCTGTTGTTGCCAGTTAGTAAAAGTAGTTATTATTCCGAGAAGAAGGCTTTG
AATCACAGCACCTGCTAATATACCAATCCACAGACCCTTACCTCCAATGTGGAAGAAAAAGCCTAACAAAACTGCGATTGGGATTCCGACGAGATAAAATGCTCCGAGATTCACATAGGC
TCCAAGGTGCTGCCACCCACATCCGCGAGCGATTCCAGAGAGCACTCCAAGACAGCCATCGGAGATAACTGAGATAGAAACAAGAGGGGTCATCTCCCTAACATAATCCACAACCTCCT
TCTCGTCGCTATAGGCATGGCCAACTAGCCGGCGAAGGCTGAAGAGAGTTAAACTGACCAAAACTGCCTCTGTAATTGTGATTGATGTGGCCACACGTACAGCAAGATGAGCTTGCTCTG
GATTCCCAGCTCCTAGTTCGTTTGACACGCGTGTGCTTGCAGCACAACCAAGTCCATATGGTATGGTGTAGAGCAAGGAAATGCTTGTGAGGCATATAGAAAGAACAGAGGTTTCAAGCT
TTGGATTTGGTAGGAGACCTGAGAGCAAGACAAGAAGCTCGAAGGACCACCATTCGAGGCATATCATTACTGCTGAGGGAATTGCTAGCCTCAAGAACTCATTAATGCCCCACAATGCCT
CCATTGATAGCGGTGCACGAGTTGCATTGCAAGAATCTGAATATTTGATGTAAAATGCAAGAATCAACACATTGATCCAATAGGAAATGCTTATAGAAAGTGCTGCTCCAACGTTGCCCAA
CCCTGTTTTGAACACCATGACCCAGCACAAAGGAATGTGGACGCAGAGGGTCACAAATGAGCTCAGAAGCATAGGAAGAATTAAGCTCTGAGACTGAAGAAATTTCATAAGAGGTTGG
GCAATAGCAAAGGCGAAGAGGCCTGGGATCATCCATACTGCATAAATGCCAGCCTCTTGGGATATTAAGGGATCCTGGCCTATGAGTGAGAGAAGTTTCCCCATTGATACCCAAATGAGA
GAAATTGGGAGGCAGACTAATACAAGTGAGAGTATGGCTCTGTATGTGTGAATTCCAAGCTTCTTGTATTGTTTTGCACCATATGCTTGGCCACAGAGAGTATCCAAGCCACTAGCCATTC
CTAGAAGGACACTGAATCCAGTAACGCTGGAGAGTGAAGTGGCAATGGCGGAGCTGGAGAGCGCGAGCTCACCGAGATGTCCGACCATCATCGACGAGGTGACCTGAACCAGGTACTG
CGATAGCGTCACCCCCACCATCGGCCCGGCCAGGTAACCCAGCCTCCGCGCCTCAGCTGTAAAATCACAGTACCATCCCCTCACCGTTCTCCCCTCCTCCCTCTCCAAAAGCCCTTCCTCC
ATTTCCGTAAGCTGTCCCTCTCCTCTTCGTTTCCCTTTTTCTTAGATGAC
>comp154947_c0_seq26
GGAAGATAGCAGCGTTTTATAAAGTTGGTTGTGAATTTGTTGTGACTAATGTAACTTAGCTGCTTTGATTGTAAAAAGCAGCTTTTCATCACATAAATTCTTGAAGAATAGTAAGAAAGCAA
TAGTCAAATAAAATTGTAGTTGATATCTGAAAAACAAAGCAGTTAAAAATTGTCCCAAACAGACCCTTATAAGGGGTCATGGGAGAAATGTTGAAATTGCGTCGAAGTTTATATTGGATCT
GATAAAATTTATGAAACCATACAAGAAGCTAAATGTTATATCTCAACCACTCAACAAAAAAGATTGCCACACTGTATGTTTCATTAAGCCTAAAAAAAACTTAGCCTGATGCATTTGATAAA
GCCAGGAGAAAGTCTCTGTAGTTGTCATCATCAAGAACTCTCTCAACAATGGCTTCTTCAAGCTTCACCTTATACCTCATTTGAAATGCTTCCTTCACCTTGTTCATGCCGACCTCCACACT
GCCTATCATCACTCTTGTCAAAACCACCTTCTTGTCTACCTCAGCGACCAAACTCATATATATCATCTCAGAGTAGTACTCGTAAGGGGCACACATGCATTGTATGACAACTCTGAGGGAGT
CTTCAAAGTTCCCACTTCTATCTTCAAGTGCCTGAAAGTTTCTCATGAATAGTTAGTTATTTGATCACTTAGGGGCTCTAAAAAGTTATGTTCAATTGGCACTTATATGATCATTGTTTATAGT
TTTTTTTAGTTGGTGAAGTCACTTAAAATCGGCGGTCAGACGGTCAAATCGTTGGTTAATGTCGACGCCTGAAGAAAAACTGGAGGTATATATGTCCGACGATCAGTTTGCCGCCGATGTT
TTCAGCAGTTTATTATGAAAAACCGCCAGTAATTTCGGCAGTCAAATGACCGCCGGTAAATAGCGAATTTCCTGTAGTGTACTCAGAAGATTTGTCTATTAATATAGTAGTGATGTTAGCCA
CAAATTCCTCCCCATCTCCATCTAATATGATATCTAAGAGAATTTGTGCTTCAGTATATTCTGAGAGTGAATGGCCTGACATCATTCTTATAGTGGAAACCCCCTTCAGATAATCTTGGTTCA
AACCATTAAGCCATAGCATTTTTTACTTAGATTTTCTTCCCAACTACTTTTTTTTTCCCAACTACTTAATTATACTTTCAACACATTATATGAAGGCATAAATACTACTCAATCACCTCAATATA

ATCATTTCCATATATATGCCTGTAACAAGAGAATGCCAGCCTCAGCTGCGGTAAGCTTCTCTTGCTGAAGATCTCTATAATAGAAGATTCATCCATTCCTCCATCACTTTTCTTCAATGCCTCA
TTCAATCTCTTAGCATCACAATTGGCAATGTGTTGGCTGATCACTTCGTCATGGTGAGATCTATGTGAAGTTGCAAGAGCTGCAAGAATCTTTGGTCCCAGGTGTTCTGGCTCCACAGAAA
GGATATCTTTGTCCATATGGCTTCTGCACTTAGCAAAATAAGCCTGCTTGCTGGCAAAGAGATGGCTCGACTTTCTGCGCACATAGATCTCGACGATCGCCTTGTAGTCATCGGCACTTCCC
CGTTCTACGGCATCCTTCGCCACCACTGCGTCGCGCTCGGGCTGCTCCATCATCCATAGCCACAACCCAAAGTAAACTGGTTCACTTTCTGGATTGGCTTCCTTAATCCTAAGGAGTTCGA
CAAGTTCTTCGCCATAAATAGCTCGATAAAGCTCTTTAATCTGCCGCTTCTCATCTGAAGATTGTCCTGCCAAGACAAGAACAAGATGATTCGGCTGGTTCTGGTTCATAAAAATTTCTCTA
CATACTTTCTCATCATATCGGTCGACTGTGACTGAGGAGGAAGGAACCATGGTTGGGAGAGTTGAGGTGAAGCTTTGAGAACTAATGAGCATTTTCTGAAAGAAGTTGTGATTTTTCTGA
GGGCTTAGGAGCTTTATGTAGTGTGGAGGATTGTGCAAGTACTGTAACTTTAACTTGGGGTCTTTCAGCTTGAGTTGTTTCCAGCAATGAGAGAGTATTTGTG
>comp154947_c0_seq36
GGAAGATAGCAGCGTTTTATAAAGTTGGTTGTGAATTTGTTGTGACTAATGTAACTTAGCTGCTTTGATTGTAAAAAGCAGCTTTTCATCACATAAATTCTTGAAGAATAGTAAGAAAGCAA
TAGTCAAATAAAATTGTAGTTGATATCTGAAAAACAAAGCAGTTAAAAATTGTCCCAAACAGACCCTTATAAGGGGTCATGGGAGAAATGTTGAAATTGCGTCGAAGTTTATATTGGATCT
GATAAAATTTATGAAACCATACAAGAAGCTAAATGTTATATCTCAACCACTCAACAAAAAAGATTGCCACACTGTATGTTTCATTAAGCCTAAAAAAAACTTAGCCTGATGCATTTGATAAA
GCCAGGAGAAAGTCTCTGTAGTTGTCATCATCAAGAACTCTCTCAACAATGGCTTCTTCAAGCTTCACCTTATACCTCATTTGAAATGCTTCCTTCACCTTGTTCATGCCGACCTCCACACT
GCCTATCATCACTCTTGTCAAAACCACCTTCTTGTCTACCTCAGCGACCAAACTCATATATATCATCTCAGAGTAGTACTCGTAAGGGGCACACATGCATTGTATGACAACTCTGAGGGAGT
CTTCAAAGTTCCCACTTCTATCTTCAAGTGCCTGAAAGTTTCTCATGAATAGTTAGTTATTTGATCACTTAGGGGCTCTAAAAAGTTATGTTCAATTGGCACTTATATGATCATTGTTTATAGT
TTTTTTTAGTTGGTGAAGTGGCTATTCAAATGCTGGAAACCATTAAAGAAGTTATTTTTTTGTCATTAGTTTCATGTAGAAACTAAGCTATAGCTCATAATAAGGGTAAGCAAAGTTTGTATG
AAGCTCCTCATGACATAGAAAAAACTCTCATAGTAGTATATATGTAGAAGAGCTTCTCCAATGAAGTGAAAAGTAGATTAGGGACACTTGAACATGCACTTGGTACACAGTGAAGTGTCA
ATGATTGTGGCTGTTCAAAACTAAGTGATAATTAGTTCAGGTTATACCACTATTGGAAAATCATCAATTAGCGGCGGCTTTTTCGGACAGTTAAAGTAAGTTTTCTGGCGGTTTTGAAAATT
TCCGGCAGTCGGCCTGTATTTCAAAAACCGCCGGAATCAATTAAACCACGTAGAAAAGGAATTCCGACAGCCTTTTATCGGTGGTCAAACTTTCCGGCGGTTTTCCGGTGGCTTGACAGC
CGGAAAAAAAGTCACTTAAAATCGGCGGTCAGGCGATCAAATCGTCGGTAAATCTCGACGCCTGAAGAAAAACTGGCGGTATATATGTCCGACGATCAGTTTGCCGCCGATGTTTTCAGC
AGTTTATTATGAAAAACCGCCAGTAATTTCGGCAGTCAAATGACCGCCGGTAAATAGCGAATTTCCTGTAGTGTACTCAGAAGATTCAAATTCCTACCCATCTCCATCTAATATGATATCTAA
GAGAATTTGTGCTTCAGTATATTCTGAGAGTGAATGGCCTGACATCATTCTTATAGTGGAAACCCCCTTCAGATAATCTTGGTTCAAACCATTAAGCCATAGCATTTTTTACTTAGATTTTCT
ACCCAACTACTTAATTATACTTTCAACACATTATATGAAGGCATAAATACTACTCAATCACCTCAATATAATCATTTCCATATATATGCCTGTAACAAGAGAATGCCAGCCTCAGCTGCGGTAA
GCTTCTCTTGCTGAAGATCTCTATAATAGAAGATTCATCCATTCCTCCATCACTTTTCTTCAATGCCTCATTCAATCTCTTAGCATCACAATTGGCAATGTGTTGGCTGATCACTTCGTCATGG
TGAGATCTATGTGAAGTTGCAAGAGCTGCAAGAATCTTTGGTCCCAGGTGTTCTGGCTCCACAGAAAGGATATCTTTGTCCATATGGCTTCTGCACTTAGCAAAATAAGCCTGCTTGCTGG
CAAAGAGATGGCTCGACTTTCTGCGCACATAGATCTCGACGATCGCCTTGTAGTCATCGGCACTTCCCCGTTCTACGGCATCCTTCGCCACCACTGCGTCGCGCTCGGGCTGCTCCATCAT
CCATAGCCACAACCCAAAGTAAACTGGCTGCACATGAAAATCGCAAACTATTTAACAATCAAGTTGCATTTTTTATAATGTAACATGTAAAGTACTGTAACATAATTACTTCACTTTCTGGA
TTGGCTTCCTTAATCCTAAGAAGGAGTTCGACAAGTTCTTCGCCATGGATAGCTCGATAAAGCTTTTAATCTGCCGCCTCTCATCTGAAGATTGTGGAGACAAGACAAGAACAAGATGAT
TCGGCTGGTTCTGGTTCATAAAAATTTCTCTACATACTTTCTCATCATATCGGTCGACTGTGACTGAGGAGGAAGGAACCATGGTTGGGAGAGTTGAGGTGAAGCTTTGAGAACTAATGA
GCATTTTCTGAAAGAAGTTGTGATTTTTCTGAGGGCTTAGGAGCTTTATGTAGTGTGGAGGATTGTGCAAGTACTGTAACTTTAACTTGGGGTCTTTCAGCTTGAGTTGTTTCCAGCAATG
AGAGAGTATTTGTG
>comp154947_c1_seq1

AAATGCTCATTAGTTCTCAAAGCTTCACCTCAACTCTCCCAACCATGGTTCCTTCCTCCTCAGTCACAGTCGACCGATATGATGAGAAAGTATGTAGAGAAATTTTTATGAACCAGAACCA
GCCGAATCATCTTGTTCTTGTCTTGTCTCCACAATCTTCAGATGAGAGGCGGCAGATTAAAAAGCTTTATCGAGCTATCCATGGCGAAGAACTTGTCGAACTCCTTCTTAGGATTAAGGAA
GCCAATCCAGAAAGTGAACCAGTTTACTTTGGGTTGTGGCTATGGATGATGGAGCAGCCCGAGCGCGACGCAGTGGTGGCGAAGGATGCCGTAGAACGGGGAAGTGCCGATGACTACA
AGGCGATCGTCGAGATCTATGTGCGCAGAAAGTCGAGCCATCTCTTTGCCAGCAAGCAGGCTTATTTGCTAAGTGCAGAAGCCATATGGACAAAGATATCCTTTCTGTGGAGCCAGAAC
ACCTGGGACCAAAGGCACTTGAAGATAGAAGTGGGAACTTTGAAGACTCCCTCAGAGTTGTCATACAATGCATGTGTGCCCCTTACGAGTACTACTCTGAGATGATATATATGAGTTTGGT
CGCTGAGGTAGACAAGAAGGTGGTTTTGACAAGAGTGATGATAGGCAGTGTGGAGGTCGGCATGAACAAGGTGAAGGAAGCATTTCAAATGAGGTATAAGGTGAAGCTTGAAGAAGCC
ATTGTTGAGAGAGTTCTTGATGATGACAACTACAGAGACTTTCTCCTGGCTTTATCAAATGCATCAGGCTAAGTTTTTTTTAGGCTTAATGAAACATACAGTGTGGCAATCTTTTTTGTTGA
GTGGTTGAGATATAACATTTAGCTTCTTGTATGGTTTCATAAATTTTATCAGATCCAATATAAACTTCGACGCAATTTCAACATTTCTCCCATGACCCCTTATAAGGGTCTGTTTGGGACAATT
TTTAACTGCTTTGTTTTTCAGATATCAACTACAATTTTA
>comp155004_c0_seq2
CGGACCTAATAATTTTCCAAAGAAGAGGCAAATAGGTGTAAAAAACTTTTCATGGAAAGCAAAAACAATTTTACAACTTGTCACAGAAGCATAAATTCGAGGTTAGAGGAAGATTTCCCC
GTATCACCATTGATTTTTCTCAAATGCTGAAGGGGAACGGAAAACTGTTTCCAAATCATGGAAACATAACAACACAATAAGACTAAAAACACTATTTCCTACCTATATTTTTAGCAGCAATG
AAAGTTCGAACGAAACATTCCATAAACTAAAAAATAAAAATTGCATCTTGCACAAGACGAAAAGGCTTGGAAGAGAGTCAAATAACCAGCTGCTGAGATATCTGCCATGAATGAAGGAA
AACAGTTCTTGAGTCTCTGTTTGTTTTATGCAGACGCAGGCACTGGAGGTAAAGACCCAGAGCGGCCATGGGTGCAGCATTAGAGGCCATTTGATCAAGCCTGTGGTGTTCTATATCGCC
GCCTCGGCCAGAAGGGACCGCCACCCCTACCCCCTCTGTTCCCAAAATCAGGCCTGTAACTGTAGTCTCCTCCCCACCTTCCATAACCCCTCCCTCCACCATAATATCCACGACCCCTCAT
TCCATTACCGCGGTATCCAGCTCCTCTAACAGGTGCAAACCTTGCTTGGTTATTCACTCGTGGAGCATTGGTTCTTTTTTCCTCAACATAAGCTTTACGACCACCAATCAATACAGGTGAAG
CCTTAATTGCACTTTTAACGGCATCAGCCACCTCAAACTCCACAAAACCAAAGCAAAACCCATGTAACATATTGCGTCTAACTTGTATGCCATTGGGCTTGATTGGACCAAAGATTTTGAA
AACCTCTTCAAGTTGAGCACAGGTAGCATTCAAAGGCAGCCGTTTGATGTATATAGAATACCCTTCAGCTTCTGACTCCTGGGCAACGTTGGTTTCTGCAGCATAAGAACCTCGGGCAGG
AACTTTTGCTGCCCGTGCTAGTGCTGGTGCTGGTGTAGGAGCAGCAGGAGCTTTCTGTTCTGTATCAGAAGGTACTGCCTCGTTGGGAGCAGATTTGGCAACTGATACAGGTGCAGGTTT
TTCTTTCATCGCCATCACAATTGAAGCATAGGACACATTTGGCTTCCGGCCAGTTGAAGGATTTGAACCAGCCACAGTAGGCTGCAAAGCAGTTGGAACTTCATTGATCTCCTTCTCGACA
TGCTCTGCTGTATCATTTGAAGATTCTCCGCCATGTTCCGAAGGATCATAAACTTCTTCTTCAGTTATTTCATCCTTTTCCGGTTCTTCTTCACTTTGCTCAGAAGGATAATGCTCCTGGGGT
GTAACAGTACCATTCTCTGCAGAAAAAGGAACCCAGGTTTCATTCGCCAAACCCTGCCGCTCCTGTTGCTGGTCCTCTTCTTCAACAAATCGGAATATATCATTTAAAACATAGAAGCCTG
TCTCCTGGGTCGCAAGAAGGAAGCTTTGGGTGAAACCTCTCCTGACATCATCCTTCCCAAATAGGTAACCCGTGACAAGGACAAGGACCCCACCGTTAAGGGTTTCTTGTGCATCCACT
GTCTTTATCTGAGTTCGGTACTCGCTGTAGTCCATCGATAGAATCTTCTCGTTGATGTCCTGCAAGGAGGAGGTCGCGGTCATCTCGCCATTAGGTCCAGGACGTCCCAGCTTGCTACTTTC
CTTGTAGAATCGATGAAGCTGGTCCGGTGACTTATGCAGGACGAGGAAGAATTGCTGCACAAATGCATTTCCCACCAGTTGAGCATAGCGCACAGATCCCGCAGGAGTGGATTGTTGGG
AGGCCATCTCTCGTTGTTAATCTCGAAAAGGATGAAGAAACTCAAGAAATTGATCAAGCGAGTGCTTTTAATCCGTAAGGACGGGTGATCAGCAGCTGAGACCGAATGAAAAGCTCAAT
GACGCATAGCATCGGCATTCGATCGGATAAATAGCTGAAGCGGGGTCGCCTAAGACGAACTCTTCGGCGCTATTGTAGGGAGCACTCTGTGGTTTAGACTTTAGATCCTCGGATCTGGCAG
GAAAGAAAGGAGCCGAAGAGCAGACGGAGCCGAGTCCTCCGTTTC
>comp155008_c0_seq1
CAAGGCCCTGTAAACATATAAGGAAATTGACTTGAACAGTTAAAAAGGGTAGCAGATTTCAAATTTGTGATTACAAATTTTGATCAACCTACAGTTCACCGGATTTCTTTTACTTCACTTTC
CTTTCATCCAAACCTAGCCTAAAGAAACAACAATCATATCTGCTAACATAAATTTCTCCAATCACAACAGGACAATAACTTCTAAATGGTCTATTGATTAGATATCTTGTAGAAATATACATAT

AAAACCACCATGAAGCTAACTGATACAGAGTTTCATACCTTTCAGCATTCTGTTTAAATCCTGTTTCCTTGATCTTTGACAATGCTTTAGGTTGAATTTTAACAGAGAATATTTGATTCTAAG
CTAGAAGGCATTGAGTTTGCTTCATCGTGTCCTTTCATGGCTTCTAAACTCTGAAGTTAGATGTACAATCTATTTCTCAAAATTGCTTTGCCTTCACCTAGCATTCAACCTTGGCTGTTTTAG
AAATCTTCTTTCAAGTGCTGAAACTTTGGACTGTTCTTTCTAGCTGATCAAGGTTAGAATCTCTGATGTCTGAAACTGAGGCCATTCTTGTATACTTTGAAGCTAGGCACTGAGCTCACCG
TTTCCGGCAATGCTAAGCAATATTTTCCCCTCATATTCACCAGGTGATTTGACAACACCATGAAACTGGTCTTTGTTGGTGAGATGTCTGACCTCAGAGCCATTTGCAGCCTCTAAAATTCC
TTCAACACTCTTTCTCTTGAGATGTTCTTTAGCTTCCTGCAAGGCTTTGATTACTGTCTTATCTCCCGACATTTCAAGAACCAATACCTCATAATCTTCTACAGATGCCTCCCACCGCTCCAT
CTTGGCATTACAATCTGCTCTCCTTAGGCGAGCCTTGCTGTATTTTGGTCGTAAAATAAGAGCAGCATTGCAATCTTCAATGGCTTTTTGCCATTCACCAAGCTTAGACAGGCAAGCAGCT
CGATTGCTTAGTAGGATTGCATTCTGAGGGTCATGTTTAAGGCCATTGCCATAGGCAAGAGATGCCTCAGAAAATTTGGAGGCCTTGAAAAGATTATTACCCATTGACCGAGCTGATGCAA
CTGATCGAGCCTTTCGAGCAACAGCAGCAATTTCCCTGCTTCCAGGATCTATTTGTGCCGCCATTTGAGCTAATGAAACTGCATTTTCAAATTTTCCTTCAGCCATATCAACCTGTGCTCTT
ACCGAAAGATAATATGCGTTGCTCTGGGCGCCAAAGAACTTTGTTGAAGAAGTCTCAAATTTGGGCGCATCAATCATTGATTCCTCTGCATCTTCCAGCTTGCGGAGCATCAATAAGGCCT
CAGCTTGGTAGGCAAAGATCTGAGGAGATGAATCAGCACCAGCAGAAATTGCAGACTGACTCTCTTTTAGCAAAATATTCCAGTTCCTTGATTTCCTCGCTTCACTGCACTTACAAATGA
AAACCTGTATGTCCTTTGCATGCGAGATGTCATCATAGCTTGCTTCATTTTTCGCTAGTTTATAGTGTTTAGCTGCTCTTTCTGGTTCTCCCAATCTGAGATAGAGATTGGCA
>comp155061_c0_seq9
ATAGTATTTACATAAAGATTTAGAACAAAACATATAAATTACATGAAAACCCCAAAAAGAAAAGAATAATGAGATTTAGCCTAGATGAAATAATTTTACTAATATATGTCATTGATTACAAAT
GATGACATTCCCCATGATTGAGAAAGATGTAGACTTAAATTATGCAGAGCAAATTCTATGGTGACAGATTGAAGAGTCTTGTGGAATGGCAACTATTTTTAAGAGAGTTTAGAGTGAATGT
AAAGTTCTGCAGCTCCTGTACCATTACCACCATCACTTAGTCCAGGCGATAAGCATGACCCGGATCTTCTCGAACCTCGTGAGGTTGGTGATGAACTGGACGAGAGGCTTCTTGAAGTTG
TTGGAGTTGGAGCTCTTGGTGAAAGGTTTGTGAATTCTAGAGCCTGCGTTCTCAACTCCGCCGGATAATCTGCAACGCAACCAATCCTAGGGCCTGCACCAGTACACCATTTCAAGCTCA
CTTGATGCCCAAGCTGATAAGAACTCGATTCCATCTTCGATTTGATTCTCTCTTCAATAGCCTTCTCAGAAACATCCGCCATTGGACTCTGAAGCCCACCGGATAGAGTCCTTCTGTAAACT
TTGGCTTTGTTACCTTTGCCATCAACAATTTCAGAATCAATGGGGTCTTCTGAGGGTTCCTCGGTGAATGTTCTTGCAGGGATTTGAAGAGGTGTAGGTTTAGAGATGCGTGAGGTCTCAC
CCAGTTTCTTAGCTTGGGTTTCAGTGTTTTTGGTGTCATCATAATCGTCGCTGGACGATGAGCGTATCTCAACTGCGGTTAGATCAATTCCATTTTCTTTGAGGTAGTTCAATAATTGGTCAA
GGTTCTCATCAGATGGTCTGTAATGCCCACTGTATGCCCAAATAGCCTTGAGAACTCCATTCTCTGCAACGAATCTTCCAGCTGCTAGAGTGCTGCCTCCAGCGAGGAAGCTGGAATGGT
GAAACATTCCCTTCTTTTTCATGCCAGCATACAATTTCCTTGATGTGCACATGACGAAAATCCATTTTGCTCCTTCAGGTCCCTTCGTTGTATCAAGAGGTTCACCGGTTCTCTGGTGTACA
ATTTTTCCATCAACTGGTATATATTGATATTGTAGACGCTCTAGCTGAGGTGGAGCTCTGCCGCAAAT
>comp155065_c0_seq2
CTTGGAGCTACCCTTGCATTGGAGGATGCCAAGGCTGCATAGTAGGTGGTTCATTGATGCTTATGGGAAACAAGAAAATGTTAATCCCATATTGCTTGAGTTGGCCAAACTAGATTTCAAC
ATAGTACAGAGCATTTATATGACAGAGCTTAAAGAAATCTCTAATTGGTGGAGAAATCTTGGTGTTCTTTGTGATGAGCTTGATTTTATAAGAGACAGGCTAGTGGAGAACCATTTATGGGC
TTTAGGATTTACATTTCAACCTAAATTTTGGAGAAGCAAGAAAGCAATCACCAAGATTAATTCTTTGGTAACAACAATTGATGATGTTTATGATGTTTATGGCACTCTAGATGAGCTAGAGA
TCTTTACAAATGCCGTTGAAGAGTAAGGAAATACAAAAAATTAATTAATGTGCATACATAAGTTTTAATTAGCTTTGATTATCAAATATGTATAAATAATAATATTATTATAATCTAATGTAATT
AAATTTAAGTATTGAATTATCCTTGATTAAATTATTGCTAAATTAAATTGGAACTCCCAGTTGGAAAATGGATGCAGCTCAACAACTTCCAAATTGTATGAAGATATGCTTGATGGCACTTTT
CAATACAATGAATG
>comp155065_c0_seq3
CTTGGAGCTACCCTTGCATTGGAGGATGCCAAGGCTGCATAGTAGGTGGTTCATTGATGCTTATGGGAAACAAGAAAATGTTAATCCCATATTGCTTGAGTTGGCCAAACTAGATTTCAAC

ATAGTACAGAGCATTTATATGACAGAGCTTAAAGAAATCTCTAAGTATTTTGAATTTTTTTTTAATATACATGATCAATCCTTCTCATAATTCTTCTAAGAAATATTATTTCCTTTCACAGTTGG
TGGAGAAATCTTGGTGTTCTTTGTGATGAGCTTGATTTTATAAGAGACAGGCTAGTGGAGAACCATTTATGGGCTTTAGGATTTACATTTCAACCTAAATTTTGGAGAAGCAGGAAAGCAA
TCACCAAGATTAATTGTTTGGTAACAACAATTGATGATGTTTATGATGTTTATGGCACTCTAGATGAGCTAGAGATCTTTACAAATGCCGTTGAAGAGTAAGGAAATACAAAAAATTAATTA
ATGTGCATACATAAGTTTTAATTAGCTTTGATTATCAAATATGTATAAATAATAATATTATTATAATCTAATGTAATTAAATTTAAGTATTGAATTATCCTTGATTAAATTATTGCTAAATTAAATTG
GAACTCCCAGTTGGAAAATGGATGCAGCTCAACAACTTCCAAATTGTATGAAGATATGCTTGATGGCACTTTTCAATACAATGAATG
>comp155097_c0_seq4
TTTTTTATTTTTTATTTATTTTTTCAACCATTCTCTCCATGCTTTTAGAGAAAATAAAGCGAAGAGGAATGGAAGAAAATATAGAAGGAAGATTAGGAGGGAGAGAGGGAGGGAAGATAAA
TCAAATACAGAGATACATATAGTGGAATATGCACTGCTGCTGTGGGGGTTAAAAAAAATTGCACATTTATAGGTTTAACAAAGCTTCAGGACTGTCCGCAGTCTGAAGAATGATGCAGGGA
TCAATTTCTTCAGGTGTTTGAATGATTTCCTGAGGATCGATTCCTTCCTCCAGTTCCTGATCTTGATTCCACACATAGTTTGGACTTTGAGCCATGCGATAATATGGGTCTTTAAGGTTGATT
TTGCATGAAGAAGTCTGTAGTATTCCTCCTTGATCAGCACCAGGCCCAAACATTTTAGCTGACATGGTTAAATTAATTGTATAGCTTTGTGCACTGTGGGCAACTAGATTAAATCGTCCAGT
AATTTCTTGACCAGACAAGACATATATTGGCTGGGAAAGGAGACAACGCAGTTGATACCAATGCGTGGTAGGAGCACCAGGTGCAGTAGTTAGCCATCTTTGAACAGAACCAAAGAGAA
GATACGTGGCTGCGGTTGGGACTCCTGAGCAGAGACAACCTTCGGCTGGATTGGTTGGAATGGATTTGGAGACCTCCAAAACAGCAAAACGAGGAT
>comp155153_c1_seq4
ACATGTGCATTCATATGGAAAAGGAACCAAAGGGAATGCCGCTCTCAGATTTTTGTAAAGAACTCAAGTACTGTCATTTTTTTTAAGATTATCATGAAGAAATAGAAATAGGAGATGGAGG
AAGGAAGGAATAGGAGAAATGAAAGAAGAAGAAGAAGGAGAAAAAGGAGAAAGGAGGGGAAAGGGAAGAGTACTCACCTTGCAAGATGGGGAAGCTCGGGTATATCCAGGTAACGA
TCCTAGCTACTTCGGATCTGGTTCAGGCGTGTAAAGCCGAGAGCAGGAAAAGTCGGGTAGCTGCTGTCGAGCTCAGGTTCGGTCCGGAGAGGTCGGGGTCGGTCCGGGCGGCGAGAGC
AGGGAGATCGGTGGCGGCCATTGAGGTCGATCCTAGGGGTCGGACGACTGGTCCGAACGTGCGAAGCCGAAAGCGGAAGCAGGAAGGGGTTCCGCCTGGCTGCTGTAGATCGGACCG
GGGAAAGGCTTTGGCCTCGGCTAGGGCAAGGGGAGGTCGGCTTCAGGCTCTGCTCGAGAGTCGGAGCTCGGGATCGGTCCGCCTGGGGGCTTCTACCGAGAGTGAGAGAGGAGAAGG
AGCCGAGAGAGAGCCGATCCTCTTGCCCTAGAGAATCGAGAGCTTTTTTTTGAGATGATCCTGTTGTTTCGTCCTCTCGATCCTGCTTCCAAACGCTGAACCGGGCTCTGTCTCACTGCTC
CCCGTCGCTCGACCACTGTATTCTTCCCAAAATGAGCATATCACAAGCTCAAGCATCCCAGTAACGGTAGAGTTAGGATACGGGGAGATCAGAGCCATCGGTCGCATTTCTTGATTTAGGA
ATCAAATTCAAACTACTTAGAAAGACAAATGGGCCATAATATAATTAAATTAGGCTTATTTGAGTTAGGTTTGAATGCGAATTTGACTCAAATTGGCTCGATTAGAATCGGGCTAGGTTCGA
TCAATTTGGTCAAATCAAATTAAAGACGATTCGGATCAAATTGAACTCGTAGCAGTCAAAGCGAACTATTCGGGCTTGATTGAACAGGGTCGAGTCAAATTAAAATCAATTCAGATCGAA
TTCGAGGTCGGATTTGGCTCAACGAGGTTCAATCGAATTGATCGAATGCGCTCGACGATATTTGAGCAAAAAACGAGGTCAACATTCCTTTGTACACAACAGAGTGTACAGTTCATCAAG
GTCCAGTGGTTGTTGACTGGTGCAAATCGCTGTTTTGAATGCTTGATATGTCGCCGGCAAGCCGTTTAAGGTATGAAGTATGATATCCTCGGGATCTATCTTGCATCCAGCGGCTGCAATGG
CGTCTACTTTGGATTTTACAAGAAGTAGATATTGTGTCATCGAACTTTCGCCTTTAGTGAGAAATTGCAACTCATTCTTTAATTGCATTACTCGAGATCTTGTTGTAGCTTGAAGACGATGAT
TCAGCGTAGACCATATTTCTGTACAGTGCGTTAAGGATAATACATATGGTAGGATAGAAGGCGAAATTACCGACATTAACGCTGCCGCAAGATTTTGGTCCAGTAGCATCCATGTTTCATAC
GTTGATTTTGCGCTAGAATTTGAGTTTTCACCATTTGTATTACTTTGCGGAGGGCAAGAGCACGAACCGTCGAGGAAACCTTTGAATCCATTTGCAGCGAACACTTTTTCTACCTGTGATC
GCCATAACGGGAAGTTGTCGGCGGTGAGTGTGATGGAGACGAAATTCTTGATATTTCCGATCAAGAATTTTAGCTGGGGAGGAATCTGGACAGCGCTAATAGAGGATATGCTGTCGACAG
ACGATGTCGATCCTGCCATTGTGTCTTTCGTCGGAGAAGATGTCTGAGATTGAAAACCAAGAAAGCGGCGAATCAATCCTGCTCTGATACCATGTTACAAACAGAGAAGAGAAGTAGCA
AAGAGAGAAGACAGTTGGAGAATTCTGCTGTGACAAGCAGATTTCATTTTCATTACTTTTCTTGTATAATACACCTCTATTTATACACTTTGTATACTAGCTTATCCGTACAATACAGCTGGC
GTGGTAACTGAATTAGTTTCTTGATTTACATTATGCCTAATACCCTCCCTCAAGCTAATGTTGTCGGTAGAAGAGATTAGCTTGTCGGATAGTTGTTTGAACCGAATAGAGGAAAGTGATTT

TGTAAATAAATCCGCAATTTGATCTTTGCTTGAGATGTGATGAACATGTATTGTTTGCTCTTTAATGCAATTTCTGATAAAATGACAGTCTACCTCAATGTGTTTTGTGCGAGCATGATAAAC
TGGATTATTTGCTAGTGCTATGGCTGATGTATTGTCGCAATATAGAGAGGTAGGCATTTGTTGTTTGCAATCTAGTTCTTGTAGTAGTTGTCTTAACCATATAATTTCGGTGGCAGCCGATGCT
AGAGCACGATACTCCGCTTCTGTAGAGGATCTTGCAACTGTTTGCTGCTTTTTAGCACTCCAGGAAATAAGTGAATTTCCTAAGAAATTACAAAATCCTGTTGTTGATTTTCGGTCCAGAT
GATCTCCTGCCCAATCAGAATCAACGTACGAG
>comp155210_c1_seq5
GAATTTCAATATGCTAATAACTTTAATGATGTCAAAAATAATGATCAATCACCCAATACAAAAAAAACTAAATGAAAGTGGGAAAATAATCTTCTTAATATGAGGCTAAAAGTACATGTTTC
TATCTTTCTCATATGAATATAACTTACGGCTAAGATTAGAGCTAGCTTTGCAGAACTATTAATAGGTCTCATTAATAGTTTCCTTGTCTCAAAAAGTGCTTACATCTCAAAAGCCCAAAAATC
AGCACATTAACGAACCAAAAAAGAAGTGCAAGAAAAAAAATTAGGAAACAAGGAAGTTGGTGGCCCAAATAGGAGGTGCGTGGGCGGTGGGCCCAAGTGGGCTGTGGGCCCACCACC
AAGTTCACATCAACATTGACATCCATCACCCCAGAAATGAAAAGCAGAGAGCTTTCGGCCACCTGTTACTGCAGGATGGGAGAAGGAGACATCACCGGGTTCTGCCACGTGGTCCAACC
CCACCCTCCAATTGTAACTTGACACGTCATCATTGACCCTGTAGCTGACTACCACGTGGCAGATTTTTTTTTCTCTCTCCATCACTTTACTCTTTCTTTGTCTCTCTATTATTATTCAATTAAGT
AGCATCTACAATATGGGAGCACTTGATGTGGCAGAAGCTTTTTTTAGACCAATGAAGATTTGAGCTACCAAACCAACCCCTGCAATTTGAGCCCAGCTTTCATGCCTTTTTTTGAAAGTCC
TTGCATCTTGAAATATTTGCAGCAATAGAGAGATATATACTTTAGTGTCCTAGCTTTGTACGAAGATCATATACACGAACAAATAAAAAATAAGGTTGTATGTTCATCAGAGAGACCACAAT
TTTTACATGGAAACCCCTAAAAAGGGAGAAAAAGCATGGGGAATTGCCCAGTTTAAATGAATTCACTAGTGT
>comp155222_c0_seq2
TTGGAATTTTTAATTATTATTAATCAATTATTTTAATTAAATATTCAATTTCACTTAAAATTAAATTAAGCCTAAGTTTGCTGTCAAATAATTTTTGTTTTATTTTTCGTTTGTTAAGGGTTTGATT
CTGGTCAGTTAGAGGTCTTACTCATATTTTTTTGGAGTTAAAATGGATTTATTACATTTTCCAGAAGGTTTAATGACTTAATTAAGATTTAAATTTAGGAAAATTAATTAGGGGACTTAAAGA
TCTCTAAAACCAGTGAAAGTTTTTGTATGGGCTGAGAGTGTCACTAAGTTTAAGAGGTTAAAATTTCAAGTTCAAATGAGGTGTATCCTAATCACAATACATTTTCTAAGTTTTCTAAATAA
AGTCCTTAATTATTGTCCTTAACGGTGTCGCTAAATTTAATAGGCTTGAATCAAAGTTTTGACTAACCCTTGACTAAACATATAAGAGAAAATGCATAAAAACACAATCAAGTCCTTAAAAC
TAGGAGAGCCTAGGCTAGGTGGTGTGTGCACCCTAAGCATGCAATTAACAAGATAAAACTTGATTAATTAAGGGATTAACCAATGAACAATTAAAATAATTGCTAATTAATTAACCACCAC
TCCCCCATTAATTTTGTTCAAGTATGCTTAATCAAAGTTTAAAATTTCAAGGGATCTCTCAAGATTTAAAGATCTAAATAAGTAAAATTTTAAAATAAAT
>comp155230_c0_seq1
ACTAAAAAGTTGTTTTAGAAAGCTGTCCCCGGCTTTGTTTGGAAGAGCTTTTGACTACTTTTTCGTTGATTTTAATAAGTTTTTACGCTAAAAAGTTGATGTGAAAAATAATAAAAAAATTT
ATTACAAAAGAAGTATATGTATAAAACGGGACTTTGCAATTTGATAAAAATAGTTTTTGTTAAATTTTTTTATAATACTCTTAACTTTTTTTTTTTGGATTTTTTAATGAAAAATTAACTATCA
TCAAATTTCATGGTTTGGAGCCTTGCATTAATTCTTGTGCCAACGTGTCTTCAGTTCTTATACTTGCCCGCTAAGGAATTGAGGCACCAGTAGATTTTTTTTTTCTTCCCGTTCACTTGTATTA
TAAAGTGAACAAGACAGCTGCTTTCTTTGGTCAGAACTTGTCCTTACCATGCATATGTAAAAGAAGCGCGTCTATGGTTATCTACAATCATATCCAAGATATTACATATATAAAAGATTCACT
ATTAATTTAAAAATTAATCCATTAATATTTAAGTGTCTATTCTTACGAGTTAGTCTGCTTCTAACGTGTGACTTTTTATTCTGTGCAAATTCTGAATGAAGGAATCGTTCGATTTTTTCTTTGTT
TAGAGTAAAAACACAGTCAAAACATATTTGCTATAGTAACTATATTTTGACTGTAGACAGAGAGAAAATCAAACGGACAGAGAAAAAATCGGACGATTCTCTTAATTTTCTCCTAACCTGG
GGCTCTTTTTTCAGAGAAAAATCTTTCCACCGACACCTCTACAGCTAAAGCACGCCTTCATAAGTCTTTATCTTTAAATATGTCTTTATTATTATTATTACTGTTATTTTATGATTTGCATGAAT
ATATATCTATTTGGGAATGATGTGAGACTGTCAGTAACGAAGAAGGAAGCTCACCTGTTTCCCCACCTTGAATGGAGATCCATGATCAGCAACTGTTCTTCAGGCGTGATGTTCCCACGGC
GGACTGATAATGAGATAAAAAACTATTGGAGAACAAGAATCCAGAAGAAAGTTAAAGATGGTGAACCAGTCGATTGCTCGAGCCAAATAGTGAATGATGAAGGTAGCTCTAGCCATGCC
GGCGGCCTTGAGGAAGCCATGGCGGCACAGCCAAGCTATCCCATTTATTCAGATATTGCCAATCCTCACGAAGCTTATGTTCCAGCTGCCTACAGTGGAAGTATCAATACTAGCGAGACCT

ATTTGAGTGCTGAGGATTTCTGGGCCGTACAATCTCTGAATGGGTTATAGATCTGGAATAACACTCCATGATGCTTGACTGATGAAGGTTGGTAGCTTTGCTTAGGATCAATCTTGATGTAT
AAATGTAACTAAGGTTTCATTTGGGATGACTTTCT

>comp155230_c0_seq2

AGGAATCAGGACCGTACCTACTCCCTTTGATCTCCCTCGAGTTAAACCTTTTTTTTAGGACTAATGAAGATTTTTAAAAGCGAAATTCTTGCTTTTATTTTCTATAAATCTCTCAACAACCTC
AAGCCTCCTTGACATTCCCTAGTCTCCCTACTCTCTCTCTCTCTCTCTCGACCATGATGGGAGATCAAGAGGAGGCCATTAGTGTGGATAAGAGGATTAACAACAACGAGGAGTCTGAGG
TAGTGAGGAAGGGACCGTGGACCATGGAGGAGGACCTCATACTCATCAACTACATAGCCATTCATGGTGAGGGGGTTTGGAACAATCTTGCTCGATCGGCAGGTCTTAAGAGAACTGGA
AAGAGCTGCCGGCTTCGGTGGCTGAATTACCTCCGCCCAGACGTCCGCCGTGGGAACATCACGCCTGAAGAACAGTTGCTGATCATGGATCTCCATTCAAGGTGGGGAAACAGGTGGTC
CAAAATTGCGAGGCAACTCCCAGGTAGGACTGATAATGAGATAAAAAACTATTGGAGAACAAGAATCCAGAAGAAAGTTAAAGATGGTGAACCAGTCGATTGCTCGAGCCAAATAGTG
AATGATGAAGGTAGCTCTAGCCATGCCGGCGGCCTTGAGGAAGCCATGGCGGCACAGCCAAGCTATCCCATTTATTCAGATATTGCCAATCCTCACGAAGCTTATGTTCCAGCTGCCTACA
GTGGAAGTATCAATACTAGCGAGACCTATTTGAGTGCTGAGGATTTCTGGGCCGTACAATCTCTGAATGGGTTATAGATCTGGAATAACACTCCATGATGCTTGACTGATGAAGGTTGGTA
GCTTTGCTTAGGATCAATCTTGATGTATAAATGTAACTAAGGCTTTGTTTGGGAC

>comp155230_c0_seq3

AGGAATCAGGACCGTACCTACTCCCTTTGATCTCCCTCGAGTTAAACCTTTTTTTTAGGACTAATGAAGATTTTTAAAAGCGAAATTCTTGCTTTTATTTTCTATAAATCTCTCAACAACCTC
AAGCCTCCTTGACATTCCCTAGTCTCCCTACTCTCTCTCTCTCTCTCTCGACCATGATGGGAGATCAAGAGGAGGCCATTAGTGTGGATAAGAGGATTAACAACAACGAGGAGTCTGAGG
TAGTGAGGAAGGGACCGTGGACCATGGAGGAGGACCTCATACTCATCAACTACATAGCCATTCATGGTGAGGGGGTTTGGAACAATCTTGCTCGATCGGCAGGTATTCTTTACATGCATG
AATACATATATCTGCATGCATGCATGACGCATGCTCTCTCCCGATCTTTTCTTTTTTTCTTTCAATACCATTATGTTTGGTTTTGGTTGGTACAATTTGCATAATTGAGAAGGTCTTAAGAGAA
CTGGAAAGAGCTGCCGGCTTCGGTGGCTGAATTACCTCCGCCCAGACGTCCGCCGTGGGAACATCACGCCTGAAGAACAGTTGCTGATCATGGATCTCCATTCAAGGTGGGGAAACAGG
TGGTCCAAAATTGCGAGGCAACTCCCAGGTAGGACTGATAATGAGATAAAAAACTATTGGAGAACAAGAATCCAGAAGAAAGTTAAAGATGGTGAACCAGTCGATTGCTCGAGCCAAAT
AGTGAATGATGAAGGTAGCTCTAGCCATGCCGGCGGCCTTGAGGAAGCCATGGCGGCACAGCCAAGCTATCCCATTTATTCAGATATTGCCAATCCTCACGAAGCTTATGTTCCAGCTGCC
TACAGTGGAAGTATCAATACTAGCGAGACCTATTTGAGTGCTGAGGATTTCTGGGCCGTACAATCTCTGAATGGGTTATAGATCTGGAATAACACTCCATGATGCTTGACTGATGAAGGTTG
GTAGCTTTGCTTAGGATCAATCTTGATGTATAAATGTAACTAAGGTTTCATTTGGGATGACTTTCT

>comp155230_c0_seq5

AGGAATCAGGACCGTACCTACTCCCTTTGATCTCCCTCGAGTTAAACCTTTTTTTTAGGACTAATGAAGATTTTTAAAAGCGAAATTCTTGCTTTTATTTTCTATAAATCTCTCAACAACCTC
AAGCCTCCTTGACATTCCCTAGTCTCCCTACTCTCTCTCTCTCTCTCTCGACCATGATGGGAGATCAAGAGGAGGCCATTAGTGTGGATAAGAGGATTAACAACAACGAGGAGTCTGAGG
TAGTGAGGAAGGGACCGTGGACCATGGAGGAGGACCTCATACTCATCAACTACATAGCCATTCATGGTGAGGGGGTTTGGAACAATCTTGCTCGATCGGCAGGTCTTAAGAGAACTGGA
AAGAGCTGCCGGCTTCGGTGGCTGAATTACCTCCGCCCAGACGTCCGCCGTGGGAACATCACGCCTGAAGAACAGTTGCTGATCATGGATCTCCATTCAAGGTGGGGAAACAGGTGAGC
TTCCTTCTTCGTTACTGACAGTCTCACATCATTCCCAAATAGATATATATTCATGCAAATCATAAAATAACAGTAATAATAATAATAAAGACATATTTAAAGATAAAGACTTATGAAGGCGTGC
TTTAGCTGTAGAGGTGTCGGTGGAAAGATTTTTCTCTGAAAAAAGAGCCCCAGGTTAGGAGAAAATTAAGAGAATCGTCCGATTTTTTCTCTGTCCGTTTGATTTTCTCTCTGTCTACAGT
CAAAATATAGTTACTATAGCAAATATGTTTTGACTGTGTTTTTACTCTAAACAAAGAAAAAATCGAACGATTCCTTCATTCAGAATTTGCACAGAATAAAAAGTCACACGTTAGAAGCAGA
CTAACTCGTAAGAATAGACACTTAAATATTAATGGATTAATTTTTAAATTAATAGTGAATCTTTTATATATGTAATATCTTGGATATGATTGTAGATAACCATAGACGCGCTTCTTTTACATATGC
ATGGTAAGGACAAGTTCTGACCAAAGAAAGCAGCTGTCTTGTTCACTTTATAATACAAGTGAACGGGAAGAAAAAAAAAATCTACTGGTGCCTCAATTCCTTAGCGGGCAAGTATAAGA

ACTGAAGACACGTTGGCACAAGAATTAATGCAAGGCTCCAAACCATGAAATTTGATGATAGTTAATTTTTCATTAAAAAATCCAAAAAAAAAAAGTTAAGAGTATTATAAAAAAAATTTA
ACAAAAACTATTTTTATCAAATTGCAAAGTCCCGTTTTATACATATACTTCTTTTGTAATAAATTTTTTTATTATTTTTCACATCAACTTTTTAGCGTAAAAACTTATTAAAATCAACGAAAAA
GTAGTCAAAAGCTCTTCCAAACAAAGCCG
>comp155230_c0_seq7
TGAAAAATGAAATTTTCGGTTGGAATTAGGACTTATAAACTCAAATTTCTAATATATTTGTGTTTACTTGCTCTAGCATTTATATAGGTTTAATTGTCTTCATATTTCATTTTCATATTATATTAAT
TAGTATAAATATATATACTATTATTCATGGACCAATATAAGTGCTTAATAATCTCCTCAACATTTACCCAAAAAAAATAATCTCCTCAACAAACCACTAAAAGTTAAGTAGGCATACTTTCTCT
CCCTA
>comp155230_c0_seq8
AGGAATCAGGACCGTACCTACTCCCTTTGATCTCCCTCGAGTTAAACCTTTTTTTTAGGACTAATGAAGATTTTTAAAAGCGAAATTCTTGCTTTTATTTTCTATAAATCTCTCAACAACCTC
AAGCCTCCTTGACATTCCCTAGTCTCCCTACTCTCTCTCTCTCTCTCTCGACCATGATGGGAGATCAAGAGGAGGCCATTAGTGTGGATAAGAGGATTAACAACAACGAGGAGTCTGAGG
TAGTGAGGAAGGGACCGTGGACCATGGAGGAGGACCTCATACTCATCAACTACATAGCCATTCATGGTGAGGGGGTTTGGAACAATCTTGCTCGATCGGCAGGTATTCTTTACATGCATG
AATACATATATCTGCATGCATGCATGACGCATGCTCTCTCCCGATCTTTTCTTTTTTTCTTTCAATACCATTATGTTTGGTTTTGGTTGGTACAATTTGCATAATTGAGAAGGTCTTAAGAGAA
CTGGAAAGAGCTGCCGGCTTCGGTGGCTGAATTACCTCCGCCCAGACGTCCGCCGTGGGAACATCACGCCTGAAGAACAGTTGCTGATCATGGATCTCCATTCAAGGTGGGGAAACAGG
TGAGCTTCCTTCTTCGTTACTGACAGTCTCACATCATTCCCAAATAGATATATATTCATGCAAATCATAAAATAACAGTAATAATAATAATAAAGACATATTTAAAGATAAAGACTTATGAAGG
CGTGCTTTAGCTGTAGAGGTGTCGGTGGAAAGATTTTTCTCTGAAAAAAGAGCCCCAGGTTAGGAGAAAATTAAGAGAATCGTCCGATTTTTTCTCTGTCCGTTTGATTTTCTCTCTGTCT
ACAGTCAAAATATAGTTACTATAGCAAATATGTTTTGACTGTGTTTTTACTCTAAACAAAGAAAAAATCGAACGATTCCTTCATTCAGAATTTGCACAGAATAAAAAGTCACACGTTAGAA
GCAGACTAACTCGTAAGAATAGACACTTAAATATTAATGGATTAATTTTTAAATTAATAGTGAATCTTTTATATATGTAATATCTTGGATATGATTGTAGATAACCATAGACGCGCTTCTTTTAC
ATATGCATGGTAAGGACAAGTTCTGACCAAAGAAAGCAGCTGTCTTGTTCACTTTATAATACAAGTGAACGGGAAGAAAAAAAAAATCTACTGGTGCCTCAATTCCTTAGCGGGCAAGTA
TAAGAACTGAAGACACGTTGGCACAAGAATTAATGCAAGGCTCCAAACCATGAAATTTGATGATAGTTAATTTTTCATTAAAAAATCCAAAAAAAAAAAGTTAAGAGTATTATAAAAAAA
ATTTAACAAAAACTATTTTTATCAAATTGCAAAGTCCCGTTTTATACATATACTTCTTTTGTAATAAATTTTTTTATTATTTTTCACATCAACTTTTTAGCGTAAAAACTTATTAAAATCAACGA
AAAAGTAGTCAAAAGCTCTTCCAAACAAAGCCG
>comp155232_c0_seq12
CTGTACTTCGATTATTCTGTGCGTACATCAGACCATTTGGAGGTGAATCAGAATGTGGCACGTCACCCATTACACAGCGGTACACAGCAAAAGGTAACGTGACATGTTCTAATTCGTATCC
GAATGGCGTCCACATTCGCACAGAATAATTTCACCTATGCTACTCAAAGAAACCATAGCAGAAATCAAATAATATAAAAATATATGTTGTAAGGGACATATAGACATTTGACTTCTGTTTCAT
GTTAATGAATTACGAAAGATTTCTGCTTCAAAAACAGAATTATCCGTTTGGTAAGGGAAAGATTACCACATTTCTCTTAGTTCAAATGCTCGGCATGGAGCTTCCCATTGAATTACGAATCA
TCAGCCATTACAACTGTGTGGCATCACCTATCTGCCTGCCATTCTTTGTTGTTTCAGAATGTACATAGTTCCGGTCTTCTAGATGCTTCCCATTCGCCTGGTTCCTATAGTTCATTCAATAGCT
TGTGGGTCTCGTCCGATTTGCTTTCTTTCTCTTTAGCGACTAATGGGAGCAATGGCTGGTTTCCATTAGAACGGTCAGGCATCTCTGTCAATTTTGTATCAATAAAATATACCACCAAGTTTG
TAGTATACCATGCAACCACAACATCAACACTGTAATGCTTCCGTGAAGCTATTATAAGAAGACTTTGTATGACTGCCATCAACCATGCAAGCATTTTAATAAACCTTTTAGAACCATATTTAT
AGTATGTGAGAACAAAAACCAGTGTAAATATCATATGAGATGAAAAAATCAAATCGCCACAACCATAAAAGACTCCACGGACATTAATGAGAAATAGATCCAATATTCTTTCAGGAGGAG
CGAGTGTGGCATACTTTGAACCCTTGCGGCAGTGATAGTTTGGACCTGGAAGATGTGTAGAGTAGAATGTGATGATCCTCAAAGCCTGGCAAGTAACTAAAATTGCCAACACTCTACGCC
ATAGCAGAACTGTGTAGAAGCGTTTGCTATGATAAATGAAAGGATAGAATGTCCACTGCAAAACTTCAAATTACTTCGTACGTTGGAAAAGAAAGGAAAACAAAGGAATTAAAATAACT

GATGCATCATAAGCAGAAGGCAATAGTGACATGGTTAATACAAAGCATTCCCAACACGGAAAATCCTGGGAACTTCAACTAGGTTAGGAAGGCTCAATTCCTACAAATAAAGAAAGTGC
AAGGGTAAAATATGTTATCAT
>comp155232_c0_seq7
CTGTACTTCGATTATTCTGTGCGTACATCAGACCATTTGGAGGTGAATCAGAATGTGGCACGTCACCCATTACACAGCGGTACACAGCAAAAGGTAACGTGACATGTTCTAATTCGTATCC
GAATGGCGTCCACATTCGCACAGAATAATTTCACCTATGCTACTCAAAGAAACCATAGCAGAAATCAAATAATATAAAAATATATGTTGTAAGGGACATATAGACATTTGACTTCTGTTTCAT
GTTAATGAATTACGAAAGATTTCTGCTTCAAAAACAGAATTATCCGTTTGGTAAGGGAAAGATTACCACAACAGTAAGAAACTGCCGCCCATTTCTATTAGTTCAAATGCTCGGCATGGAG
CTTCCCATTGAATTACGAATCATCAGCCATTACAACTGTGTGGCATCACCTATCTGCCTGCCATTCTTTGTTGTTTCAGAATGTACATAGTTCCGGTCTTCTAGATGCTTCCCATTCGCCTGG
TTCCTATAGTTCATTCAATAGCTTGTGGGTCTCGTCCGATTTGCTTTCTTTCTCTTTAGCGACTAATGGGAGCAATGGCTGGTTTCCATTAGAACGGTCAGGCATCTCTGTCAATTTTGTATC
AATAAAATATACCACCAAGTTTGTAGTATACCATGCAACCACAACATCAACACTGTAATGCTTCCGTGAAGCTATTATAAGAAGACTTTGTATGACTGCCATCAACCATGCAAGCATTTTAA
TAAACCTTTTAGAACCATATTTATAGTATGTGAGAACAAAAACCAGTGTAAATATCATATGAGATGAAAAAATCAAATCGCCACAACCATAAAAGACTCCACGGACATTAATGAGAAATAG
ATCCAATATTCTTTCAGGAGGAGCGAGTGTGGCATACTTTGAACCCTTGCGGCAGTGATAGTTTGGACCTGGAAGATGTGTAGAGTAGAATGTGATGATCCTCAAAGCCTGGCAAGTCTG
CAGGAATAATCTTCATCAATATAAAGGAGAAAAAGATGACAGAGAAAAAGATTTCGCTGACATAGGCTTTATCTGGCCCTATCTCCGGAAGGAGACTGAAACCAAGATCATGTAGAATAG
GTCCAGGTCGATGCAAATAATGAATCCCGTGAGCAGCAACCCCATGAACATACTGAAAAAAGAGCCCCGCAAGCAGAAGCTTCCATTTCTCCATAAAAATTTGTAGTTCTACACAAGTCT
CCATTGCAATCTTCCTCCATAACTTCACAGCCTCTCGTACAATGTAAAGCGTCATATTGATCAGCAAAGGCATACAGTTCCATAGAAATCAGCTTCAAGCGAAGGATTCCTTCTGCATTGCC
TTCTTCATGATTTTCCTCTAGTAATGCCCGAAGCCAGGTTAGGTTCAACTTCTTCCGGTTCCTTCTATCGATGAAAAGCCTTCAAAGGAAAGTAAAAGAAAAAAAAAAGGGTATCGGAAG
AAAACGCGGGGATCGACAGAAA
>comp155232_c0_seq9
CTGTACTTCGATTATTCTGTGCGTACATCAGACCATTTGGAGGTGAATCAGAATGTGGCACGTCACCCATTACACAGCGGTACACAGCAAAAGGTAACGTGACATGTTCTAATTCGTATCC
GAATGGCGTCCACATTCGCACAGAATAATTTCACCTATGCTACTCAAAGAAACCATAGCAGAAATCAAATAATATAAAAATATATGTTGTAAGGGACATATAGACATTTGACTTCTGTTTCAT
GTTAATGAATTACGAAAGATTTCTGCTTCAAAAACAGAATTATCCGTTTGGTAAGGGAAAGATTACCACAACAGTAAGAAACTGCCGCCCATTTCTATTAGTTCAAATGCTCGGCATGGAG
CTTCCCATTGAATTACGAATCATCAGCCATTACAACTGTGTGGCATCACCTATCTGCCTGCCATTCTTTGTTGTTTCAGAATGTACATAGTTCCGGTCTTCTAGATGCTTCCCATTCGCCTGG
TTCCTATAGTTCATTCAATAGCTTGTGGGTCTCGTCCGATTTGCTTTCTTTCTCTTTAGCGACTAATGGGAGCAATGGCTGGTTTCCATTAGAACGGTCAGGCATCTCTGTCAATTTTGTATC
AATAAAATATACCACCAAGTTTGTAGTATACCATGCAACCACAACATCAACACTGTAATGCTTCCGTGAAGCTATTATAAGAAGACTTTGTATGACTGCCATCAACCATGCAAGCATTTTAA
TAAACCTTTTAGAACCATATTTATAGTATGTGAGAACAAAAACCAGTGTAAATATCATATGAGATGAAAAAATCAAATCGCCACAACCATAAAAGACTCCACGGACATTAATGAGAAATAG
ATCCAATATTCTTTCAGGAGGAGCGAGTGTGGCATACTTTGAACCCTTGCGGCAGTGATAGTTTGGACCTGGAAGATGTGTAGAGTAGAATGTGATGATCCTCAAAGCCTGGCAAGTAAC
TAAAATTGCCAACACTCTACGCCATAGCAGAACTGTGTAGAAGCGTTTGCTATGATAAATGAAAGGATAGAATGTCCACAATATAAAGGAGAAAAAGATGACAGAGAAAAAGATTTCGCT
GACATAGGCTTTATCTGGCCCTATCTCCGGAAGGAGACTGAAACCAAGATCATGTAGAATAGGTCCAGGTCGATGCAAATAATGAATCCCGTGAGCAGCAACCCCATGAACATACTGAAA
AAAGAGCCCCGCAAGCAGAAGCTTCCATTTCTCCATAAAAATTTGTAGTTCTACACAAGTCTCCATTGCAATCTTCCTCCATAACTTCACAGCCTCTCGTACAATGTAAAGCGTCATATTGA
TCAGCAAAGGCATACAGTTCCATAGAAATCAGCTTCAAGCGAAGGATTCCTTCTGCATTGCCTTCTTCATGATTTTCCTCTAGTAATGCCCGAAGCCAGGTTAGGTTCAACTTCTTCCGGTT
CCTTCTATCGATGAAAAGCCTTCAAAGGAAAGTAAAAGAAAAAAAAAAGGGTATCGGAAGAAAACGCGGGGATCGACAGAAA
>comp155255_c0_seq6

CAGCAAGACCGACCGCACCGAGTCCAAAGATTGCCACCTTCGATCCTTCTTCGACATTCGCCGTATTCCAAGCCGCTCCAACTCCGGTGGAGACGCCGCAGCTGAGAAGACACATTCTG
CTAAGCGGCGCATCAGCGGGGACTTTCACAACACACGCAGAGTCGATAACCGTGTACTCAGCGAAGGTCGACGTGTTCAAAAAGTGGTAAACAGGTCGTCGTTCTTCATCAGGTCCGAC
CCAAAAGAACCTAACCCGCCCGTCAGCCCTCATTACGCTCCGCAGCGGGTCCACCGCAAAACCCGAGCATATGTTAGTCTTATCCGACCGACACATGTCGCAGCACCCGCATTCCCCGGT
AAAAACCGGTACCACCCGGTCACCAGGTCTCAAATCCTCCACCCCCTCCCCAACACTTTCCACAACCCCCGCGGCCTCGTGGCCCAGAACACGAGGGAAGATCTGCTGCAGTTTGTTCT
CGCCCTTCCAAGCACTGAGGTCGGTGTGGCATATTGAGGTGAAGAGGATTCGAAGCCGGACTTCCAAACGCTGAGGAGGCTCCACCTCTATTTCCTCGATCTTAAAGGCCTCTCCGGGA
CCCCAAAGCACAGCCGCTTTGCATGTGATGACTTTGCCGGCCGTTTTACCACCGGCGTCTCCGTGCTCTCCGGCGTGGAAGCCATTGGAGTTCTCCGAGTTTAAGCTTCCATCTTCTCTCT
CTCTCTCTCTCCCTTCCTCTGTCTCTCCTATATATCCTTCTCTTTATT
>comp155318_c1_seq9
CAAGCTCATTTTTTTTTCTCCCTCACGTGACAAGAAGCTCTCATGAGTCTTGCTCTTGGGATAGACAAAAAAAAAAAAGAGGACTCCTTTTTTCTCTCAAGGAGAGCAACCGGCCCTACG
GCTGGAGCCTCTTGTTCTTCAGTTCCTCTCTGGCTCTTGGTTTTTCCTTGGACGTCCGCCTCCCGTCGCCCCTAGCAGCCGAACGCCCCAGGCTCCCGCTCCATCTCGGTTCCTCCCTTGC
AAGCCGATGCCAAGAGATGAACAAGCCGAAGCTACCTCGATCTCCTCCTCGCCCTCTCTCCTTTTCCCGTTTACGAAGCCCAGGAGAGAGCCAAAGTCGCCTCTCCCTAGCCGAGCCCG
ACATTGACGCAGGCCCTGTCGCCGAAGTTCCAATCTTGGCCGACCAGCCTTCTTCTATAGCAGCAGGTA
>comp155352_c0_seq1
TATTGTATGAATTTATAAGAGCTCAAGACATGAATTAATTATTTATTTAATTAATTCATGTCTTGAGCTCTTATAAATTCATACAATAATTATATTAAAATAATTAGAGTCCAAATTTTCTTCATTT
ATATGTAGAGTCTTACAAATCTATTTTCACAGGCCAATAAGCCAAAGATTAAATAAAAATCTCCACATCGCCGTCGAATCAGTCAAATTAGGAACCTGGAAAACGAGATTGCATGGCCATG
ATTAGCTCCACCCGTTACATTCCCTCCTCCCCCGCTCCTCAATGGCACCCATTCCTGAAACTCTGAGGTAGACAGAGAAAGAGAAACAGAACTCGCCAGAGGACTCATGGCCATGGCAG
TGGCCATAGCCCTCTTCTCCTTCTTGGTTCTTTTAGTCGCCCCCGTCGCAGTTGCAGATGAGGCCGAAGTCCTCCTCCTCCTCAAGGCCTCCCTCACCAACGCCAACGCCCTCTCCTCCTG
GTCTCCGGGAAATGGCGTCCCCTGCTCCCACGCCTCCTCGTGGGACGGCGTCACATGCGACGCCGGCGCCGTCGCTAGCCTCCGACTCGCCGGCCTTGGACTCTCAGGCAGCATTGACG
TCAACGCCCTCGCTCGCCTTCCTGGCCTCCGCTCCATCTCATTAGACGACAACTCATTCTCCGGCCCCATCCCACCCTTCTCCCGCCTCCACGCCCTCAAATCCATTTACATCTCCGGCAAC
TCCTTCTCCGGCCAAATCCCGGACGATTTTTTATCGCTGCGTCGCATCTCAAGAAGCTCTGGCTCTCAAACAACGCCTTTGAAGGCTCCATCCCGTCTTCTCTCTCCAAAGCCGTTAGCT
TGATGGAGCTCCACCTCGAGGGAAACCATTTCACCGGCGGAATCCCTAATTTACCAATGCCGACGCTAGCTTCCTTCAACGTTTCAAAGAACCAGCTTTCGGGTCCCATACCGTCGAGCC
TGAGGAAGTTCAACGCCAGCGCGTTTGCCGGGAACGTGGGGTTATGCGGGCCGCCGCTTGAGAACATCTGTGTAGATGCTGCAGCGGCAGCGGGAGCGTCTTCCGGGAGTTCAGCGGC
GGCAGTGGGATTAGTGTTGGTCATAATTCTGGCGTTGGGGGGAGTAATGGCTGTCGCCGCAAAACGTCGAAATAATTCTGGTTTCAGGACGCTCAGCCTCGAGCACGCGACGCCGGGCA
TTCATATGGCGGAAAGGAGGGAGAGGGAAGCACAGCAGCCTGAGGTTACCACCTCCGCTGCCGTCGCGGAGTCGACGGGAAAGGGGAAGAGGAGCAGCTCATCGGTAAAAGGAGGA
AGAGGGAGCGGGGAAAATGAGTTGGTGATGGTGAACGAAGCAAAGGGGACATTCGTATTGTCAAATTTGATGAAGGCTGCGGCGGAGGCGATGGGGAACGGAGGAATGGGATCAGCTT
ACAAAGCCGTGATGGCGAATGGTTTGACTTTGGTGGTGAAGAGGATGAACGAAATGAATCGGGTAGGGAAGGAGACGTTCGAGGTTGAGATGCGACGGCTCGGCAGACTCAGCCACCC
AAATGTACTCCCGCTGTTGGCCTACCTTTACCGGAAGGACAAGAAGCTTCTAATTTCCGAGTACATTCCCAAAGGCAGCCTCCTCTATATCCTCCATGGGGACCGAGGAAAAGACCACGC
GGCGTTGAATTGGCCGACGAGGTTGAAGATAATACGAGGAATAGCTCAAGGAATGGCGTTTCTGCACTCGGAGCTGAATTTCATCGTTATCCCCCATGGCAATCTTAAATCCGCCAACGTT
CTTCTCGGCCCCGACTTTGATCCTCTGCTCGTTGACTACGGATTCCTTCCTCTCGTCAATCCTTCGCATGCCTCGCAGACAATGTTTGCCTTCAAGTCGCCGGAGATTCTCGTCAACCGCCA
CTTGAGTCCAAAAGCCGATGTCTATTGCCTCGGCATCATCGTCCTCGAGCTCCTCTCTGGCAAGATTCCATCACTGTATCTCAACGACACCAAAGGCGGGACTGATCTCGAGCGCTGGACT
AATTCGGTCCTCGCGGAGAACATTCCGTGGAGTATTATTGATCCCTTCATCACGCAGGGGTTTAAGTCTGAGGTGTTTGAGATGGAGCAGATGGTGCGAGTTGGGGCGGAGTTGATAGAT

GCCGACATGGACCGGCGGCCGGATATGAAGGAAGCTGCGAGGAGGATTGAGGAGGTGGCGGAGAAGGCCGAGGGACGGAGAAAGGAGGACGGCAGCGCTTCGGCTGGCTTAAATGA
ATCGTTGATGAGGGAGGGGCAGACCCCCGCCGGATTAAGTGGAGATGGCGTCGGAGTGCGCCGAACGGCGAACACCCCAAAAAGGTCGAAGAGCATTGAGGATTATCTGTAGTATGTCA
TCTCTTGATTCTGCTTCAAAATTTGGTGGGTGGGGTTTTAAAGGTTCGAAGGATTGGAATATGTGGTATTTATTTTTTTTGGGGAAAATGTTTGGTATTTTCAATTTACTATTTATAGTATCAC
GATTTTTTTTCTTTAAATAGATTTTCTTTTTTAAATTTACTTTCAAAATTAGTATATTGTTATTTTTAATCTTTATAGGTTAGGCTTTATTTGA
>comp155352_c0_seq3
TATTGTATGAATTTATAAGAGCTCAAGACATGAATTAATTATTTATTTAATTAATTCATGTCTTGAGCTCTTATAAATTCATACAATAATTATATTAAAATAATTAGAGTCCAAATTTTCTTCATTT
ATATGTAGAGTCTTACAAATCTATTTTCACAGGCCAATAAGCCAAAGATTAAATAAAAATCTCCACATCGCCGTCGAATCAGTCAAATTAGGAACCTGGAAAACGAGATTGCATGGCCATG
ATTAGCTCCACCCGTTACATTCCCTCCTCCCCCGCTCCTCAATGGCACCCATTCCTGAAACTCTGAGGTAGACAGAGAAAGAGAAACAGAACTCGCCAGAGGACTCATGGCCATGGCAG
TGGCCATAGCCCTCTTCTCCTTCTTGGTTCTTTTAGTCGCCCCCGTCGCAGTTGCAGATGAGGCCGAAGTCCTCCTCCTCCTCAAGGCCTCCCTCACCAACGCCAACGCCCTCTCCTCCTG
GTCTCCGGGAAATGGCGTCCCCTGCTCCCACGCCTCCTCGTGGGACGGCGTCACATGCGACGCCGGCGCCGTCGCTAGCCTCCGACTCGCCGGCCTTGGACTCTCAGGCAGCATTGACG
TCAACGCCCTCGCTCGCCTTCCTGGCCTCCGCTCCATCTCATTAGACGACAACTCATTCTCCGGCCCCATCCCACCCTTCTCCCGCCTCCACGCCCTCAAATCCATTTACATCTCCGGCAAC
TCCTTCTCCGGCCAAATCCCGGACGATTTTTTTATCGCTGCGTCGCATCTCAAGAAGCTCTGGCTCTCAAACAACGCCTTTGAAGGCTCCATCCCGTCTTCTCTCTCCAAAGCCGTTAGCT
TGATGGAGCTCCACCTCGAGGGAAACCATTTCACCGGCGGAATCCCTAATTTACCAATGCCGACGCTAGCTTCCTTCAACGTTTCAAAGAACCAGCTTTCGGGTCCCATACCGTCGAGCC
TGAGGAAGTTCAACGCCAGCGCGTTTGCCGGGAACGTGGGGTTATGCGGGCCGCCGCTTGAGAACATCTGTGTAGATGCTGCAGCGGCAGCGGGAGCGTCTTCCGGGAGTTCAGCGGC
GGCAGTGGGATTAGTGTTGGTCATAATTCTGGCGTTGGGGGGAGTAATGGCTGTCGCCGCAAAACGTCGAAATAATTCTGGTTTCAGGACGCTCAGCCTCGAGCACGCGACGCCGGGCA
TTCATATGGCGGAAAGGAGGGAGAGGGAAGCACAGCAGCCTGAGGTTACCACCTCCGCTGCCGTCGCGGAGTCGACGGGAAAGGGGAAGAGGAGCAGCTCATCGGTAAAAGGAGGA
AGAGGGAGCGGGGAAAATGAGTTGGTGATGGTGAACGAAGCAAAGGGGACATTCGTATTGTCAAATTTGATGAAGGCTGCGGCGGAGGCGATGGGGAACGGAGGAATGGGATCAGCTT
ACAAAGCCGTGATGGCGAATGGTTTGACTTTGGTGGTGAAGAGGATGAACGAAATGAATCGGGTAGGGAAGGAGACGTTCGAGGTTGAGATGCGACGGCTCGGCAGACTCAGCCACCC
AAATGTACTCCCGCTGTTGGCCTACCTTTACCGGAAGGACAAGAAGCTTCTAATTTCCGAGTACATTCCCAAAGGCAGCCTCCTCTATATCCTCCATGGTAAATCAGATTGTTACTTGAAGT
AAAAATCATACCTTTAGCCTTAAGTACCCTTTTAGTTTTTCAAAGCAAGGATTTCTTTTCTTTTTCTTCTTACAGGGGACCGAGGAAAAGACCACGCGGCGTTGAATTGGCCGACGAGGTT
GAAGATAATACGAGGAATAGCTCAAGGAATGGCGTTTCTGCACTCGGAGCTGAATTTCATCGTTATCCCCCATGGCAATCTTAAATCCGCCAACGTTCTTCTCGGCCCCGACTTTGATCCTC
TGCTCGTTGACTACGGATTCCTTCCTCTCGTCAATCCTTCGCATGCCTCGCAGACAATGTTTGCCTTCAAGTCGCCGGAGATTCTCGTCAACCGCCACTTGAGTCCAAAAGCCGATGTCTA
TTGCCTCGGCATCATCGTCCTCGAGCTCCTCTCTGGCAAGATTCCATCACTGTATCTCAACGACACCAAAGGCGGGACTGATCTCGAGCGCTGGACTAATTCGGTCCTCGCGGAGAACAT
TCCGTGGAGTATTATTGATCCCTTCATCACGCAGGGGTTTAAGTCTGAGGTGTTTGAGATGGAGCAGATGGTGCGAGTTGGGGCGGAGTTGATAGATGCCGACATGGACCGGCGGCCGGA
TATGAAGGAAGCTGCGAGGAGGATTGAGGAGGTGGCGGAGAAGGCCGAGGGACGGAGAAAGGAGGACGGCAGCGCTTCGGCTGGCTTAAATGAATCGTTGATGAGGGAGGGGCAGA
CCCCCGCCGGATTAAGTGGAGATGGCGTCGGAGTGCGCCGAACGGCGAACACCCCAAAAAGGTCGAAGAGCATTGAGGATTATCTGTAGTATGTCATCTCTTGATTCTGCTTCAAAATTT
GGTGGGTGGGGTTTTAAAGGTTCGAAGGATTGGAATATGTGGTATTTATTTTTTTTGGGGAAAATGTTTGGTATTTTCAATTTACTATTTATAGTATCACGATTTTTTTTCTTTAAATAGATTTT
CTTTTTTAAATTTACTTTCAAAATTAGTATATTGTTATTTTTAATCTTTATAGGTTAGGCTTTATTTGA
>comp155354_c0_seq1
AGCTGGGTACCCTATACTTGTGTGGGACGATTTTCCCCGTGCGCACTCGGCACCTAATAATATTGACCCAACCCAAAAATCCAACTCGAAAATTTCGGTTTGGACCCAAAACAGGTCAGG
TTTAGGTTCAAAAAATCAACTCATTTAAAGAAATGAGTCAGGTTCGAGTTGACCCGGAAACTCTGAAATCACCCGGAAAAACATGTTTGCCCTCTCTGACAGCCCTTGTCGGCGTCTGCC

CGCATCGCCTACCAGAGTCGCCTGCCCCGGCTGCCTGTGTAGCATCAGTAGTGTTGTCTGGCCCGCGTGCTTGTTATCATGTTGCCGCCTTCCGACTGTCGCGCCTCTCTGCTGCTGGAGG
ACTGGACCACTAAGTTATGGGATTTGGCTCAACAAGCGCGTGAGTTATGGGAATGAGGTTTAAGACTTACGGGTCAATTCAATTCAGGTCCAAAAAACATACCTATTTTCAAAAACGGAT
TGGGTTAGGGTCAGCCCGAATAATACAAGACCAATTTTAGGTGGGTCAATACCACCCAATTTTGTCAGCTCTATAAATAAATTACAATGGATTTATTTGCAATGACCAACCCATATGCCGCTA
TTAAAAAAATGAAGAAAAAAAAAATTGCAATGACCAATCCTGCAATAAAATGAAATCATTTTGTATTTCTTTGAAAGCTTTTCCAATCATTTCTTCTCTCTCTCTCTCTCTTCATTTTTATCAG
AGCTTAATCGCAGCGGGCACAGACACTTCATCAAACACAATAGAATGGTCGATGTCTCTCTTACTAAACAATCCCGACAAATTAGAGAAGGCGAGGCAAGAGATCGATGACCAAATCGG
ACATGATCGCCTCCTCGAAGAATCAGATCTCATCAAACTTCCATACCTCCACTGCATCATCAACGAAACCTTCCGTCTGTATCCCGTCGCCCCTCTCCTCATCCCTCACGTAGCAAGAGAG
GACTGCGAGATCGGAGGCTACGCCGTGGACCGAGGAACTCTGCTCTTAGTAAATGTTCATAAAATCCATAGAGATCCTGCGATTTGGTCTGAACCGACCAAATTTCTCCCCGAAAGATTT
CAAAATGCGAAGGAAGAAGAAGGGTCGAAGATGACGATGATGCCATTTGGGATGGGGAGGAGGAGATGTCCGGGAGAAGGGCTTGCATTGAAGGAGATTGGATTAGTGTTGGGGATTC
TAATCCAATGCTTTGAGTGGAAGAGGATTGGAGCAGAAGAGGTGGACATGACGGAAGGGTCAGGACTTACAATGCCGAGAGCTAAGCATCTTGAAGCTCTGTGCAGGCCACATCAATAT
ATGATGAATGTGTTGTCTCAGATATGACTTTTTAGTTCAACTTTATGGTGAGATTAATAAGAATAAAAAGTATTTTGCTTGATTTGTTTTATTATGTTTGTGAGAAAAGGATTATGTAAGCTTT
TAGACTGTACTATGGAGAAATAAAATTTTAAAAATTATATACTTCATTTTGCAACTACCAATATGATCTGTTTTTTTTTTTTTTTTAATATATATTATATAGACATGTCATGTAATAACTCTAGCA
GCGGCCGGCAATCCAAGCTGCGCGGCCACCGCTCAAATTAACGGTGGTGGCCGCCACCGCCGCCCGGGCTCGGCCCCGACGGCAACCTCTCCATCCACGAAGCCATAATCTCTTTAGCC
TTCTCGTATACACTCTGCTCGACGGAAGGATCCTTGTTTCTCTCTCTAAGCTCTTCTGACATCGTTCGTATGGCAGTAGCATTCTGCGTGCTCGCCACGACGATGACGAAGACGACGAAAG
TGACGAAAAGTAGAGCTCGATACCTTCGGCAACTCATGCTTGACGCTTTCTTCGTGGAATTGGGGAAGAGGATTGATTATCAGACGAGAAACTGGAGAGTACTTATT
>comp155354_c0_seq3
CAGAACCCCTTCTTCCTCCTCCCGCCATGGACGCCATGAAGATCCTCACCAGCTTAGCTCTCTTCCTCTCCTTCCTCCTCGTAATCAAACTCTTCATAACCAACAAGAACAAGAAGAGGG
AGAAGAAGAAGCCGCCTCCAAGCCCTCCTAATTCGTTTCCCATCCTCGGCCACCTCCACCTTCTCCAGAAACCCCTCCACCAATCTCTTGCAAGTCTCTCCACCCAGCACGGCCCGATCC
TCCTCCTCCGCTTCGGAAACCATCCCGCCCTCATCGTCTCCTCCGCCTCCATCGCCAATGAATGCTTCACCACCAACGACATCGCACTCGCAGACCGCCCTCATTTCCCTTCCATGGCCGT
CATAACCTACAACCACACCAGCCTCCCCTTCGCCGCATACGGACCCCACTGGCGCGAAATGCGCCGAATCGCCACCGTCGAAGCTCTTTCGGGCCCCCGCCTCGCCTTCTTCTCCGATATT
CGGGCGGACGAGGCTCGATCCCTGGCCCGAAGCCTCTTCCATTCCTCGGGGAAAGTGGAGATCAGTTCAAGCTTGTTCGGGCTTGCAATGAATGTCATGATGAGGATTATAGCAGGGAA
GAGGTATTATGGGGAGAAAGGTGAAAAGGAGGAGGCGCATAATTTCAAGGACGTGATAGAGAAGATCTTCTCGCTTACCGGCGCGAACAACGTCGGAGACTTTTTGCCGGCGGCGATCG
GGTGGTTTGCACGTCGAGAAGTGGATAAGAAGCTGGCGAGGTATCACAAGTACAGGGATGAAATTGTTCAGTCACTTATAGAAGAGCACAAGAGGAGAAGAAGGGCAGAGGAGGAAG
AAGAAGAAAAAGAAGGGGGATTGAAGCAGAAGCAGAAGATTCATAAAACCATGATTGATGCTCTGCTCTCTTTGCAGGACGACCAGCCGGAGCAGTACACTGATGTCTTCATTAAATCG
CTTATTAATAGCTTAATCGCAGCGGGCACAGACACTTCATCAAACACAATAGAATGGTCGATGTCTCTCTTACTAAACAATCCCGACAAATTAGAGAAGGCGAGGCAAGAGATCGATGAC
CAAATCGGACATGATCGCCTCCTCGAAGAATCAGATCTCATCAAACTTCCATACCTCCACTGCATCATCAACGAAACCTTCCGTCTGTATCCCGTCGCCCCTCTCCTCATCCCTCACGTAGC
AAGAGAGGACTGCGAGATCGGAGGCTACGCCGTGGACCGAGGAACTCTGCTCTTAGTAAATGTTCATAAAATCCATAGAGATCCTGCGATTTGGTCTGAACCGACCAAATTTCTCCCCGA
AAGATTTCAAAATGCGAAGGAAGAAGAAGGGTCGAAGATGACGATGATGCCATTTGGGATGGGGAGGAGGAGATGTCCGGGAGAAGGGCTTGCATTGAAGGAGATTGGATTAGTGTTG
GGGATTCTAATCCAATGCTTTGAGTGGAAGAGGATTGGAGCAGAAGAGGTGGACATGACGGAAGGGTCAGGACTTACAATGCCGAGAGCTAAGCATCTTGAAGCTCTGTGCAGGCCAC
ATCAATATATGATGAATGTGTTGTCTCAGATATGACTTTTTAGTTCAACTTTATGGTGAGATTAATAAGAATAAAAAGTATTTTGCTTGATTTGTTTTATTATGTTTGTGAGAAAAGGATTATGT
AAGCTTTTAGACTGTACTATGGAGAAATAAAATTTTAAAAATTATATACTTCATTTTGCAACTACCAATATGATCTGTTTTTTTTTTTTTTTTAATATATATTATATAGACATGTCATGTAATAAC
TCTAGCAGCGGCCGGCAATCCAAGCTGCGCGGCCACCGCTCAAATTAACGGTGGTGGCCGCCACCGCCGCCCGGGCTCGGCCCCGACGGCAACCTCTCCATCCACGAAGCCATAATCTC

TTTAGCCTTCTCGTATACACTCTGCTCGACGGAAGGATCCTTGTTTCTCTCTCTAAGCTCTTCTGACATCGTTCGTATGGCAGTAGCATTCTGCGTGCTCGCCACGACGATGACGAAGACG
ACGAAAGTGACGAAAAGTAGAGCTCGATACCTTCGGCAACTCATGCTTGACGCTTTCTTCGTGGAATTGGGGAAGAGGATTGATTATCAGACGAGAAACTGGAGAGTACTTATT
>comp155387_c1_seq1
CTGTATGCTTTGGGTATTATATGGGATGCCCTTCGTGAAGCCTAACAGCACCCTAATAATAACCATTAATGGAGCCGGCACTATGATAGAGCTCATCTATATCATTATCTATGTCCTTTACTCG
GATGGGTCAAGACGGATCAAAGCCCTTTTACTTCTCTTCACAGACATTGCCTTTATTGCCGTCGTCGCTGCTATTGTTCTTTCCATCTTCAAATCCCATGAGGAACGAACACTAATTGTTGG
TATTCTATGTATTGTTTTCTGCATCATGATGTATGCGTCACCTCTCTCTATTATGAAAAGGGTGATAAAAACAAAAAGTGCAGAGTACATGCCTCTCTATCTCTCTATTGCTTACTTCTTTAAT
GGGCTATGCTGGACTACTTACTCGCTCATCCATCTTGATGTGAACC
>comp155388_c0_seq6
ACCCGAATAGCTAAAGGTTAATGTTGATGAATCTCTACTTTCTTCTTATACAACAGGTATTGCTGGAGTATTAAGGGATTGCAAAGGCAACTATTGGCTTTCGGTAGACCTTGCGTTCAATG
GGATATAGCATAATTGGAGCTTCTAGCAATTCATTCTTTGAAGGAGGTGATCAAGGATTGGATGCTCAAATACAGGTATCATAATTGAAGGTGAAAATTCGAATATCATTTCTTTTCTTCAAG
GTATTTGGAAAAAAAAGTTAAATACAAAGAAGAAGAACTTGGCTTTCTTAAGGAGTTTAATTATGTTATTTTCAATTGTATAGGTAGAGAAAGCAATAAGTTAGCAAATTTGTGTGCTAATTA
TGCTCTTTTTAGCAGCTTTAGTTGGGAAGAGTTTTCTTTCAATAAGGTTCCTCCTTTCTTTAATATTTTGAAGGAGGAATCCACTTTGTAATTGTTCTTATTTTTTTCTCTAGTTTTTAATATAT
GCAAGCTCCGGCTTTAGTCTCAGCATTGGAAGTGGATCAAAAAAGCTAAGTTGAATGCTCGGGTGGAAGTGTTTTGGTGGAGAGTAAGTAAAAATGCAATCCCTTCATTCAAATTCTTAT
GGTACAGAAGACTGATGGATAGTGCCAATTGTCTTAGAGGATGCAATGATATTGAAGATGTGGAGCATATTGTGTGCAACTGCTTGAAGTTAAAAGAGGTTGTTCATCATCTAAACCAATG
GGGGTTTGGAATTCCTTTATTTCTAAGCTTTGAAGATTGTATTAAATGGCTAGAATCTATTGCATTGAAGAAAGGTATGCTCGTTAATTTGTAGTGTAATGCAGTCTTTTTATCATGGAAAGC
TAGGAACAAAAGTATTCATGAAGGAGTGGAAGACATCCCTGCTTTTATTGCTTCCAATGCCATATGTTATACATCGGTTTCTCATAGTTTTAGCCATTTCTATTTGGGCCACTAGGATGTTAA
CCAGTCGATCAGACTGTCAAATACTTGGCATCCTCCTCC
>comp155396_c2_seq3
AGTGACAAATGATATGAAGCTTGGAACCAACAAAAAAGAGAGGGACAAATTTTATAAATGTGCATTGAAGCTGATGTTTTTCATTTTTTCTGGGGAAATTATTATGTGGGATATTGTACGA
AGGTTAATTGGGATGTGCCATATCATTAATTTATTATGTATTGTTGTGTATCGAGTGATGTGGCACGTTCTGATTGACATCCGGCTGGTCCGCTGACCGCGCGTAATAATTTTTCATTTTTCTA
ATGGTTTGTGTAGTCATAAAAGAATTTAGGAGATTTATATATATCACTCAAAGTATATCTAAATTTTATTATTTCTTTTAGTATCTCATTAATTCATGCAACTCATCTTCGTGTGTGTGGATTGGA
AGCTTTGATTATCAATTAACATATTTTGAATTTGAGTCTTACGTATGTGATGTGTGCATAGATTTATCTTAATTGGTCTAAATATAAAAAAAATGCATATCATTAATTCTTTTGATGAGAGATATT
AATCATAATTAATTAATTAGGATCATCATTGGACCCATTAATATCTTCTTATTGTGGGCTTATGTTCCCTTTATATATGGACATAAAACTTTGAGTCACCCCTTTCTAGGAAAAATTATTTTGTG
GACACCGAATGTCATCTGGAGACCAATTAG
>comp155409_c0_seq7
GGAGATGAGCAAAACAGAGGGGACGCTTCTTATTTGGGGAAGGAAACAGCTCAGTTTGTTTCAGTGAGATTTCAATTTTTCATCCTTTCCCTCAACGTGGGTCCCACTCCCACCTTAGTG
GGACCCACCAGGAGGAGGAACAGCCCTCCTCTGTTCTTCTGGTTCCTCATGACGACAGAGTTTTCTTCTCCCAAATTTTAGTTCTGATCTGTTTTTCCTCTTCTTCTCATAGACAATCAAA
AAGGGGACTTTTTGATCTCCAGATGGGGAAGAAATGGATTTCTGCAGTCAAAAAAATCTTCAGTTCTGAATCTACGGGGAACCATACCTTCGAGAAACCAGATAAGAAATTGCGCAAATC
AAAGCATTCAAAGAACTTTGTAGATCCTCTTGAAGATTCAAAGAATGGACAAAGCCAACGCGCTTATTCAGCGGCCGAGGCGGCCATGGCGGCAGCTCGGGCGGCGGCGGAGATGGCC
AGATTAACCACATCATTAAGAGCCTTCCATGAAAGACCAAGAAAGGAGGTTGCAGCAATCAAGATTCAGGCTGCCTTTAGAGGCTATTTGGTGAGAAGAGGAACAGGAGCTTTGAGAG
GATTGGTGAGGTTGAAGCTAATGGCCAACCGAAGGGCAGTCAAGCGCCAGACTATAAACACATTGCACAACATGCAAACCCTAGCAAGGGTTCAAGCACAGCTAAGGGAAAGAAAAGT

CAGATTGTCAGAAGAGAACCAGACTCTACACAAGCAGAGGCAACTCAAGCGTGAGAAAGAACTGGAGAAATTTAAGCTGGGAGAGGATTGGGTTGACAGTCCGCACTCAAAGGAACA
ATTTCAAGCTAAAATACAAAAGAAGCAAGAGGCTGCTCGAAGAAGAGAAAGAACACTAGCTTATGCCTACTCACATCAGTGGAGGAGCAATTCTGCCAGGTCCAAGAAACCAGCTCTC
ATAGATCCCACAAAGCAACAGTGGGGCTGGAGCTGGCTTGAGCGCTGGATGGCAATCAGGCCATGGGAGACCAACCAAAGCTTTATCACAGGCAGAGATTCATCCTCTAAAAAGAATGG
AGGCTTAAACAAAGCTCCCTCAGTTGCGTCAAATGATGATATCTGTGGCTTGTTTGGCTTTCAATCTGAACAACATAAGAGAAGGCACAGTCTTGGCAGTTCATTTTGTTTTGGAGACGTT
GAGAACAGAGGTAAGTCACGGTTCCAGGCGCCGCTAACTGGCAAGTCGAAGGCCAAGAAGCCATTGCCGTTAACTTCTGCTTCAAGGAGGCTTTCAGGCCCCCAAAGGATGGCAGGAT
ATGGGAAGCTATGGTAGTGAAAGTAAGTTGGTGATGTTTTTAAATTTCCATCAATGAATGTTAATGATGT
>comp155442_c0_seq1
CCAATCCTATAAGAGATCAAGCAAGCAAAACGACTAAGTATAAGCTGATATATCTTTCTACGATCAATTACTTCGAAGATCGAGAAAATGACAGAAATAGTAGCCACCAGCCTCCTCAGGT
TTATCTCTGAAAAACTGGCATCAAAAGCTATTGCAGAGTTTGCAATGCAGACCGGCATCGACAAAGAACTTCGAAAGCTTGAAAGGACTCTTTCAACCATACAAGATGTGCTTGAAGAT
GCCGAGGCTAGGCAGGTGAAGGAGAAAGCTCTTAGGAGCTGGCTAAGTCAGCTCAAGGAGTTGGCTTATGATGCCGATGATGTGCTTGATGAATTTTCCTTCAAAGAGGCAAAATTGAA
GAAAGCCAACATGAAGGAGAAGGTACGCAACTTACTTTACCTCCCTAAGAACATTGCTTTCCAGCGTAAGATTGCACTGAGGGTGAAGGAGATCAATGAGAGATTGGATGAGATTGCAG
AAGTAAGATCTAAGTTCCATCTCAAGGAAGGAGTAGTTGGAAGCTTTAAGCTAGAAAGAAGCATGACAAGGGAAGAAACAGGTTCCTTTATAAAGGAATCAGAAGTTTATGGAAGGGA
AGTAGATAAAGAGAAAATAGTAGAATTTCTCATGAATACTTCCGTTGGAGATGATATTGGCGTAATAGGTATTGTTGGCTTAGGAGGGCTTGGCAAGACAACACTTGCAGGATTAGTCTAC
AATGATGAAAGGGTTTGTGGGCATTTCCAGAAGAGGATGTGGGTTTGTGTATCTGAGGAGTTTGAACTTAAAAGGCTTGTTAGATTGATTATAGAATCAGCAACAGGAAGTGAATTTTCTC
CAATGAACATGGATGCAATGCAGAGATCCCTTATAAATCAACTCCAGGAGAAGAGATTTTTACTAGTTTTAGATGATGTGTGGAGTGAAAGTCATGAAAAATGGGATAGTCTCAGGGCTTT
GCTTACAGTTGGTGCAAAGGGAAGTAAAGTCATAGTAACCACAAGGAATGAGCATGTTGCATCCATAATGGGCACAATTTCTCCTCACCGGTTATCGAATTTGTCGTTCGATGATTGTTGG
TTGCTGTTTGAGAGAAGAGCTTTTGGCAATGAGGTTGAGAGGAATCCAGATTTCGTTGCGATCGGAAAGGAGATTGTGAAAAAGTGTGGGGGTGTTCCTTTGGCTGCAAAAGCGTTGGG
TGGCCTATTGAGATTCAAAAGAACTGAGAGTGAGTGGCTAGCTATCAAGGATAATGAGCTATGGAAACTTGCTTCTGATGAAAACTATGAGGTTTTACCTGCTCTGAAGTTGAGCTATGAT
CATCTATCTTCGTGTTTGAAACAGTGCTTCGCTTATTGCTCAATATTCCCCAAAGATTTTAATATAAATACTACCACATTGGTTAAGTTATGGGGTGCTGAAGGCTTCCTTGAGCCATCTGCA
GGGCATATTCACTGGGAGCAAATTGGTTTGCAATGTGTGGAAGAGCTGCAGGCAAGGTCACTCTTTCAAGTGAACATGATAGAGCAAGGAGGCGTCATAACACAGGTAAAAATGCATGA
TTTAGTACATGATCTCGCTCAATCTGTTGCAGGTATTGAATGTTCAATCGTGGATGATCTCACTAAAAGATATCCTTCACAAAATTCACGCTATGTTTCTTTCATTTGCAATCAAAAGCCACT
ATCTGCAACTTTAGAATTGCTACGTGATGCAAAGAAACTGCGAACGTTATATTTGGATTACAGAAGAAATGTTGACAGAGAGACAGAACAGAGCGTCCAGGAAGTTCTTCATGTTATATC
ATCTGACATTAATCTTCTAAGAGCTTTGCACTTACAAAGTTATCCCTTAAAAGCTCTGCCACATACCTTCAAGAAACTCAGGCATCTAAGGTATCTTGATCTTTCTAGCACTCCTCTTGAAA
TCTTTCCACAGTGCATCAACACGCTACATAGCCTGCAAACACTTAACCTCTGCAATTCTGAAATCAAATCAATACCAGACTCAATCGGTGACCTTCACAACCTCTGTACCTTGAAAATTAG
CCATTGCTTTAATCTTACATCATTGCCTGGTTCCATTGGCCGACTCAACTGCTTACGTGAATTAGACTGCTCTCGCACCAAAATAAACTCACTTCCTGAATCAGTTTGTACTCTCTCCAACC
TTCATACTCTGGGGCTTGAAGATTGTTTTTTCATCCATGAGCTTCCACAGAACTTGAGCAATATGAAAAGCCTTGTCAACCTCAACATACATGGATGCATTAATTTAACTTGCATGCCTATG
AATCTCAGGAAACTGAAGCAACTTCGAACACTTCCAATTTTCATTCCTGGAGGGAAGAACAACTGCACCCTCAAAGAGCTGGGTAGTTTGAAACTTGAAGGTATATTGCAGCTGAAAAA
TCTTCAGAATGTGAGGTGCGTTGAAGAAGCCAAGGCCGCGCAATTGAGTGAAAAGCATAATATCAGGTCACTGAAGTTATCTTGGTTGTGCAATTCTTACATAAATTCAGTGGAAGCCTT
CGATGAATATGACACCATTGTGGAAATTCTGCTTGCAGAAGATGTACTTGAAAGCCTCCAACCAAGCAAAAGCTTAGAAGAGTTAAAAATAGAAGGATATGTTGGAAGAGCTTTCCCTTC
TTGGATGAACTTATCACTTTCAAATCTTGTCCGTCTCACACTAACTTGTTGCGCTTGTGAAAAAATCCCAACACTTGGGCTGCTACCCCGCCTCCAAGTTCTTAACCTCGTAGAACTTCCT
CTAGTCAAGCATCTAGGTTCTGATTTCTATGGTGGCGCCAATGCATTTTCTTCGTTGCAGGAGCTGGAACTAAACTGCTTGACAGATTTGGAGGAATGGTGCAGTGCAGCAGGAGAGAAA

TTTCTCCCTCGCCTTCACACACTAATTCTGCTGGACTGCCCAAAGCTGAAAGCTTTGCCCTCAGTTTTCTCATCAGTTGAAAATCTCATTATGAATGTGGACGATAAGCTGCTACTGTCTTC
CCTGCAACATAGAGGATTTCCAAATCTGAAGCATTTAAAGATGAAAAACTATGATGATAAAGACCCGATCCCTGAAATAGTGACAGAGCGTGCCGAATGTCTCATTAGCTGGACGATGGA
AACAAAACCAGAACCCAAGGAAGGTGATCCTACAACTATGTCTACTTTCAACTTTGGCATGGTAACTTTGAGGTTTGAAGAGGGAAAGGGCACAAAGCAAAGTGGATCCATTCCTCTGT
TTCTAGGTAGATAGCGATGTTTTCTATAAATAATATAGCTATTTTATGTTATTTTTGCTTTAAGAAATGCGAAGTTATTCAGAAAGATCAAAGATAATTATGGTTCTTTTTTATTGTATTACATTC
AAGCAATCAAATTATGTAACATCTAAATGTCATCAAA
>comp155464_c1_seq3
AAAAAAAAAAAAAAAAAAACTTATAATAGATTGATCTAATGAATTATGTCGCCTGCACCCCCTCCCAATTATCACGATAACAAGAGAAGATTCAAACTGAATTAGAAACTCTAAAGTTTAA
AGTTCATCAGGCAAATCCAAGCTGAAGATTATTAGAGAAACTTTGATCTAGTTGTCTGTCTTTTAACCTGTAACCTCAGCTGTTGTTGACCATGGCAGAAAACAGCATCTGATTTATATCCT
CGTCAATATCCTTATCATCACCTGTCCTTGTTTTTAGTTGAAGCTTCCACTCATCAATGATCTTCTGAGGTAGTAGCCAAGTGGGAGCTAAGAAAGACTCGAACCCAATGCCTCTTAGTTGG
TAGCCTAAGCTC
>comp155485_c1_seq1
CTAATTGATAATAAATCACTTAATCTACATTCTCTTTTCTAATTTACAACCAACTAAATGAATCAATCAAATCCTAAAATATAATCCCTAAAATCAATCCTAAAATATTAATATTTAAACTCTAA
TTGAAATCTCTCTTTTGTCCTCTAACAAATCAAAAATGCAAACAATTTCAACTAAATCATCCTACATAGGATATATCCAAAACTACACAGCCTACACACACCTTAGACCGGTTTGATTAGGA
CACTACTTCTGGTCCTAGGTTAGGTTTACATTTCTCTTTTGCTATTCAGTATTTACTACTTATACATATTCTATAAATGCAATTCTTACATTTTCTGTCAGTTATATTTTCTTACTTGCATTTCTTT
TCCGAACGGCATCTACGCTTATATATATGTTTTCTTTTGTCTTACTTTGCGAGGTACATCCGGTGTTGCATTGTCAATGATCAAGCGGCGTCAATTGCTTATAAGCTTTTAAGTGGCAACGCC
ACATACAGTCAGATAACGATGATTGGTTCCCCTTTTTGGCCTCTCTGAATTCACCCTGGATGGTTTCACTTCCTCGGCTCAGGCAATCGGATGATTTTTTGATTAGTGCTACGCGCTGATTGT
AAACATGAAATTTTTGTTACCATCTAAGTCCAAACTATTTCATTCCCCTTTTATTTTGCTAGTTTTTTAGCATTTCCCTTTTGGACATACTTAGTTATATTTTTTATATTCTTTTTGTATACTGAG
CAATG
>comp155491_c0_seq10
TTTAACTAAAAAACATTTATTAAAGGCAAAATAAAAAATAGAAATTCGGAGCAAAAAATTAGAAAATAAATAAAAGAATGAGCTGCTTGCTGTTCTTTATCAAAGCAGGACAAACAAAAA
CAAAAAATAATTAGCAAGACAGCTAACTAGTCCAAAAGAGTCTTAAAAGCTCCTTGCCAGGGGTTGTTAGCCTGACATCTTATGCAGAATGCCGTTAAAAGATCCCAAATAAAGAAGAA
AAACTTTACAAGTCGCTGGCGATTGATTAAGCTTCACCAGCACCTTTACCTGCACTGATCGGCCCTCCGCTCTGTTGCGGATCTTGGCCCTTTAAATCTGCAGGAGCTTCCTCCGAATGGC
CACCAGCTTCCTGCTGAGTGTCTACGGGGCCACCAGAGCCTCCGTTGCCGCGGCCTCTGCGGCGGCCGGAACCCCCCCTGCCATATCCGCGCCTCCTGTTGCCTTCACCTCCATCCCCGG
AATCTACCATAGCAGGTCCATTTCCTCTGCCCTTGCCTCTGATGAGATTGCCACGGCCTCTACCTCGACGAGCAGGATAACTCTCTCTCTCGCTGTCATATTTAGCGTATAATTTCACCTGAT
CTTCAGATAAAGTAGATTGGTATCCAGCAGAAGACCTATCAAGTTCTTGCTTAGAGAGTGTAATCGCGATTACTGACACAAGCCTAGTAGTTTCCAGTGGAAGTAGGCCTTCCTCTAAAGG
CACCCATGAATCAGTAATGAATGTGGATCCTATTTTAGTATCTTGATGAAGACCAGCAACCCTTCTCTTAATTAGCTCCACAATCAAGACAGTCTTATTAATAGCTCTCCCCATTGCCTTGAT
GACAATTTTATTCGAATCTTTGTCCAGAAGCAAGTTAGTGGCATAAGTGATATAACCGCGCATCCTTCCCATAGAAGTTATACGAATTTCATTCTCCTTAGAAGGCGAAACTTCCCTGGGCT
TCAGCACCCTCTGATACCGATCCATCTTCTCTTCGCAAAACCTTGGCTGCGTAATAATTTTCGGCCCTGAATTCTGCTCCCGCAACGGCTTCACTTCATAATCCGCTTCGAAGCGAATTGGT
CTTCTTCTTCGGCAAGGGTTTGTTTCATTACGTGGCGAGAATTTGAGCAGTTTACGGTCAAGAATGATTTCTGCTCGATCGAGGCACGCAAAATCGTATTTTTTTAGCATATGATGTGCGG
CTCTGCGGGTTGCCATATAAAATTT
>comp155491_c0_seq13

GGCTTTATAAAGAAGAAAATCTTGCATGCAACCAATGATTTACTAAGCTTCGCCAGCAACTGCATTGATGGGACCATTTGACCTCGCATCGCGGCCCCTTCCTTGACGAATTTGCCGTCGG
CCACCTCCTCGACCTCGGCCCTGAACGGGAGCATGAGTAGCATCCTCAATGTAGACAGCATTGTCTTGCTGATAATTGGAACGACCACCGTAGCCTCCTCCTCTGCCCCAACCTCTGAAA
CCACGGCCTCTACCACGTCCCCAACCTCCCCTGCCAAATCCACGCTCTCTATTGTCCCAATTCCGTTCACCATAATCTACCATCATAGGTCCATTTTCTCTTCCCCTTCCTCTACCATGACCA
ACACGGCCTCTTCCTCGACCAGCAGGAGAAACCTCTCCCTCATAATCATATTCAGCAGATGGTTTCACCTGATCTGCCGGTAAAGGAGATTGATATCCAGCAGATAACTTATCCAGCTCCA
TTTTAGAGAGGGTTATGCTGATCATTGATACATGCCTTGTCGTTTCAAGTGGAAGTAAGCCTTCTTCTAATGGCTCCCATGTATCAGTTATGTCTAAAGACCCGACTGCAGTATTTTGATGAA
GACCAGCGACTCTTCTCTTAATTAGCTCCACAATCACAACAGTCTTATTAATAGCTCTCCCCATTGCCTTGATGACAATTTTATTCGAATCTTTGTCCAGAAGCAAGTTAGTGGCATAAGTG
ATATAACCGCGCATCCTTCCCATAGAAGTTATACGAATTTCATTCTCCTTAGAAGGCGAAACTTCCCTGGGCTTCAGCACCCTCTGATACCGATCCATCTTCTCTTCGCAAAACCTTGGCTG
CGTAATAATTTTCGGCCCTGAATTCTGCTCCCGCAACGGCTTCACTTCATAATCCGCTTCGAAGCGAATTGGTCTTCTTCTTCGGCAAGGGTTTGTTTCATTACGTGGCGAGAATTTGAGCA
GTTTACGGTCAAGAATGATTTCTGCTCGATCGAGGCACGCAAAATCGTATTTTTTTTAGCATATGATGTGCGGCTCTGCGGGTTGCCATATAAAATTT
>comp155491_c0_seq2
TTTAACTAAAAAACATTTATTAAAGGCAAAATAAAAAATAGAAATTCGGAGCAAAAAATTAGAAAATAAATAAAAGAATGAGCTGCTTGCTGTTCTTTATCAAAGCAGGACAAACAAAAA
CAAAAAATAATTAGCAAGACAGCTAACTAGTCCAAAAGAGTCTTAAAAGCTCCTTGCCAGGGGTTGTTAGCCTGACATCTTATGCAGAATGCCGTTAAAAGATCCCAAATAAAGAAGAA
AAACTTTACAAGTCGCTGGCGATTGATTAAGCTTCACCAGCACCTTTACCTGCACTGATCGGCCCTCCGCTCTGTTGCGGATCTTGGCCCTTTAAATCTGCAGGAGCTTCCTCCGAATGGC
CACCAGCTTCCTGCTGAGTGTCTACGGGGCCACCAGAGCCTCCGTTGCCGCGGCCTCTGCGGCGGCCGGAACCCCCCCTGCCATATCCGCGCCTCCTGTTGCCTTCACCTCCATCCCCGG
AATCTACCATAGCAGGTCCATTTCCTCTGCCCTTGCCTCTGATGAGATTGCCACGGCCTCTACCTCGACGAGCAGGATAACTCTCTCTCTCGCTGTCATATTTAGCGTATAATTTCACCTGAT
CTTCAGATAAAGTAGATTGGTATCCAGCAGAAGACCTATCAAGTTCTTGCTTAGAGAGTGTAATCGCGATTACTGACACAAGCCTAGTAGTTTCCAGTGGAAGTAGGCCTTCCTCTAAAGG
CACCCATGAATCAGTAATGAATGTGGATCCTATTTTAGTATCTTGATGAAGACCAGCAACCCTTCTCTTAATTAGCTCCACAATCAAGACAGTCTTATTAATAGCTCTCCCCATTGCCTTGAA
AACAATTTCATTTGACTCTTTTTCCTGAAGAAGGCTAGTGGCATAAGTGATGTAGCTGCGCATCCTTCCCTGAGTTGTTATGCGGATCTCATTATCGTTAATAGGTGTTTCCTCCCTGGGTTT
TTCCACCCTCCGGTACCGATCCATCTTTTCGTCCCGAGACCAAAAGCAGAATTGAGAAATTTTTAAAACGAAATCAACGCAGAAACTCAAATCCAAAAACATATCTACCGCCTATCCTTCC
TCAGTCCATCCAATATCTCGCACAAAAGACACACACACACAAAAATTGTTCCACGCGAAGACAGAGAAAAAAACGCCTAATTCCTAGCTTCCTCAGCCCAAGAAACCTCCCAAGCACA
GCAATTATCCATCAAGGTAAGCAAAAACAAAGAGAAATCACTGTGCCATAAATCGAAAGCAAACAGATCTTAACAAAAATCCAATCTCACAACCCATGACGCCAATCGAAGAAGAGCCC
TGACGAGAGAAAGACAAACCCGGAATCCAAAAATAATAATAATGAAAAAAAAAAAAACAAAATAGAACACCGTCGAAACCAAAGAACAAGCTAATTAGAAGATTTGGTTTCTTGGAAT
GAGAACAACGACGATTGAAGAAAAGGCAAAACAGAGACTCCATCCTAGCATTTCATACCTCGATCTGCTCTGTTCTCCTTTTTCCGCCCTTAATCCACTTCCCGCAACTTATCCGCTATGT
CGCTATATATAAACCCGCGCCGGAGGGAACTGCTCTCTAGGGTTCGTTAACCAGAAAGCGC
>comp155491_c0_seq21
TTTAACTAAAAAACATTTATTAAAGGCAAAATAAAAAATAGAAATTCGGAGCAAAAAATTAGAAAATAAATAAAAGAATGAGCTGCTTGCTGTTCTTTATCAAAGCAGGACAAACAAAAA
CAAAAAATAATTAGCAAGACAGCTAACTAGTCCAAAAGAGTCTTAAAAGCTCCTTGCCAGGGGTTGTTAGCCTGACATCTTATGCAGAATGCCGTTAAAAGATCCCAAATAAAGAAGAA
AAACTTTACAAGTCGCTGGCGATTGATTAAGCTTCACCAGCACCTTCACCTGCACTGATCGGCCCTCCGCCCTGTTGCGGATCTCGACCCTTTATATCTGCTGGAGCTTCCTGCTGATTGTC
TGAGGGGCCGCCAGAGCCTCCGTTGCCACGGCCTCTGCGGCGGCCGGAACCACCCCTGCCGTATCCACGCCTCCTGTTGCCTTCACCTCCATCCCCGGAATCTACCATAGCAGGTCCATT
TCCTCTGCTCTTGCCTCTGCTGAGATTGCCACGGCCTCTACCTCGACGAGCAGGATAACTCTCTCTCTCGCTGTCATATTTAGCGTATAATTTCACCTGATCTTCAGATAAAGTAGATTGGTA
TCCAGCAGAAGACCTATCAAGTTCTTGCTTAGAGAGTGTAATCGCGATTACTGACACAAGCCTAGTAGTTTCCAGTGGAAGTAGGCCTTCCTCTAAAGGCACCCATGAATCAGTAATGAA

TGTGGATCCTATTTTAGTATCTTGATGAAGACCAGCAACCCTTCTCTTAATTAGCTCCACAATCAAGACAGTCTTATTAATAGCTCTCCCCATTGCCTTGATGACAATTTTATTCGAATCTTTG
TCCAGAAGCAAGTTAGTGGCATAAGTGATATAACCGCGCATCCTTCCCATAGAAGTTATACGAATTTCATTCTCCTTAGAAGGCGAAACTTCCCTGGGCTTCAGCACCCTCTGATACCGATC
CATCTTCTCTTCGCAAAACCTTGGCTGCGTAATAATTTTCGGCCCTGAATTCTGCTCCCGCAACGGCTTCACTTCATAATCCGCTTCGAAGCGAATTGGTCTTCTTCTTCGGCAAGGGTTTG
TTTCATTACGTGGCGAGAATTTGAGCAGTTTACGGTCAAGAATGATTTCTGCTCGATCGAGGCACGCAAAATCGTATTTTTTTTAGCATATGATGTGCGGCTCTGCGGGTTGCCATATAAAA
TTT
>comp155491_c0_seq25
TTTAACTAAAAAACATTTATTAAAGGCAAAATAAAAAATAGAAATTCGGAGCAAAAAATTAGAAAATAAATAAAAGAATGAGCTGCTTGCTGTTCTTTATCAAAGCAGGACAAACAAAAA
CAAAAAATAATTAGCAAGACAGCTAACTAGTCCAAAAGAGTCTTAAAAGCTCCTTGCCAGGGGTTGTTAGCCTGACATCTTATGCAGAATGCCGTTAAAAGATCCCAAATAAAGAAGAA
AAACTTTACAAGTCGCTGGCGATTGATTAAGCTTCACCAGCACCTTCACCTGCACTGATCGGCCCTCCGCCCTGTTGCGGATCTCGACCCTTTATATCTGCTGGAGCTTCCTGCTGATTGTC
TGAGGGGCCGCCAGAGCCTCCGTTGCCACGGCCTCTGCGGCGGCCGGAACCACCCCTGCCGTATCCACGCCTCCTGTTGCCTTCACCTCCATCCCCGGAATCTACCATAGCAGGTCCATT
TCCTCTGCTCTTGCCTCTGCTGAGATTGCCACGGCCTCTACCTCGACGAGCAGGATAACTCTCTCTCTCGCTGTCATATTTAGCGTATAATTTCACCTGATCTTCAGATAAAGTAGATTGGTA
TCCAGCAGAAGACCTATCAAGTTCTTGCTTAGAGAGTGTAATCGCGATTACTGACACAAGCCTAGTAGTTTCCAGTGGAAGTAGGCCTTCCTCTAAAGGCACCCATGAATCAGTAATGAA
TGTGGATCCTATTTTAGTATCTTGATGAAGACCAGCAACCCTTCTCTTAATTAGCTCCACAATCAAGACAGTCTTATTAATAGCTCTCCCCATTGCCTTGAAAACAATTTCATTTGACTCTTT
TTCCTGAAGAAGGCTAGTGGCATAAGTGATGTAGCTGCGCATCCTTCCCTGAGTTGTTATGCGGATCTCATTATCGTTAATAGGTGTTTCCTCCCTGGGTTTTTCCACCCTCCGGTACCGATC
CATCTTTTCGTCCCGAGACCAAAAGCAGAATTGAGAAATTTTTAAAACGAAATCAACGCAGAAACTCAAATCCAAAAACATATCTACCGCCTATCCTTCCTCAGTCCATCCAATATCTCGC
ACAAAAGACACACACACACAAAAATTGTTCCACGCGAAGACAGAGAAAAAAACGCCTAATTCCTAGCTTCCTCAGCCCAAGAAACCTCCCAAGCACAGCAATTATCCATCAAGGTAAG
CAAAAACAAAGAGAAATCACTGTGCCATAAATCGAAAGCAAACAGATCTTAACAAAAATCCAATCTCACAACCCATGACGCCAATCGAAGAAGAGCCCTGACGAGAGAAAGACAAAC
CCGGAATCCAAAAATAATAATAATGAAAAAAAAAAAAACAAAATAGAACACCGTCGAAACCAAAGAACAAGCTAATTAGAAGATTTGGTTTCTTGGAATGAGAACAACGACGATTGAA
GAAAAGGCAAAACAGAGACTCCATCCTAGCATTTCATACCTCGATCTGCTCTGTTCTCCTTTTTCCGCCCTTAATCCACTTCCCGCAACTTATCCGCTATGTCGCTATATATAAACCCGCGCC
GGAGGGAACTGCTCTCTAGGGTTCGTTAACCAGAAAGCGC
>comp155491_c0_seq8
TTTAACTAAAAAACATTTATTAAAGGCAAAATAAAAAATAGAAATTCGGAGCAAAAAATTAGAAAATAAATAAAAGAATGAGCTGCTTGCTGTTCTTTATCAAAGCAGGACAAACAAAAA
CAAAAAATAATTAGCAAGACAGCTAACTAGTCCAAAAGAGTCTTAAAAGCTCCTTGCCAGGGGTTGTTAGCCTGACATCTTATGCAGAATGCCGTTAAAAGATCCCAAATAAAGAAGAA
AAACTTTACAAGTCGCTGGCGATTGATTAAGCTTCACCAGCACCTTCACCTGCACTGATCGGCCCTCCGCCCTGTTGCGGATCTCGACCCTTTATATCTGCTGGAGCTTCCTGCTGATTGTC
TGAGGGGCCGCCAGAGCCTCCGTTGCCACGGCCTCTGCGGCGGCCGGAACCACCCCTGCCGTATCCACGCCTCCTGTTGCCTTCACCTCCATCCCCGGAATCTACCATAGCAGGTCCATT
TCCTTGTACAGAGATGAAAATACATCGTTTCATATATTGTGCTAAATCCTATAATAGACATTTAGAGCATCAAATTAATAGCACCTCTGCCCTTGCCTCTGATGAGATTGCCACGGCCTCTAC
CTCGACGAGCAGGATAACTCTCTCTCTCGCTGTCATATTTAGCGTATAATTTCACCTGATCTTCAGATAAAGTAGATTGGTATCCAGCAGAAGACCTATCAAGTTCTTGCTTAGAGAGTGTA
ATCGCGATTACTGACACAAGCCTAGTAGTTTCCAGTGGAAGTAGGCCTTCCTCTAAAGGCACCCATGAATCAGTAATGAATGTGGATCCTATTTTAGTATCTTGATGAAGACCAGCAACCC
TTCTCTTAATTAGCTCCACAATCAAGACAGTCTTATTAATAGCTCTCCCCATTGCCTTGATGACAATTTTATTCGAATCTTTGTCCAGAAGCAAGTTAGTGGCATAAGTGATATAACCGCGCA
TCCTTCCCATAGAAGTTATACGAATTTCATTCTCCTTAGAAGGCGAAACTTCCCTGGGCTTCAGCACCCTCTGATACCGATCCATCTTCTCTTCGCAAAACACTGACACCAATGAGGTCGG
AAGGGAAGAAAATTAGGTATCCAATATGAGAAAATTTTATCCTTGCCCTGCTCCACCCTGTACCTTGGCTGCGTAATAATTTTCGGCCCTGAATTCTGCTCCCGCAACGGCTTCACTTCATA

ATCCGCTTCGAAGCGAATTGGTCTTCTTCTTCGGCAAGGGTTTGTTTCATTACGTGGCGAGAATTTGAGCAGTTTACGGTCAAGAATGATTTCTGCTCGATCGAGGCACGCAAAATCGTAT
TTTTTTTAGCATATGATGTGCGGCTCTGCGGGTTGCCATATAAAATTT
>comp155518_c0_seq13
AAACATAATGGTCGAATAGAAATTGTGATTTCAGTCTGGAAAACAAAACCAAAACCACTTTTTGAGAACCGAACCGGAACGGCCTGTTCAACCGTGAACCGGTCAGATGGCTGGTTCGA
TCCAACTTACAAAAGTTTGTCCGGTCTGATTTTCACAACATTGAGCAAAACAAAAAGAAAGTTGTTCCAAATAAAGCCTATATTACAAAATTAATTATTCAACAAAGAGAAATATAACCAA
GGTGTTGTGGAGACAAAGAAATAGAGCAACAAATGCAAGGACGCACCTTCAGAGATGAAGAAGCCTAATAGTATCACGTTCCCTCATTTGCATTCGCGAATCTGGAAAGTAAATCGCAA
GCACTTGAGGGCACATGTATTTCTTTCAGGTACCGAATAATTCATGAACCTGCAGGAGCCATACATCTTTGTTGGTTGATTCCAATGAGAGAACAGATTTGAGTTGTTCACACATAAGTAG
GAACGGGAAAAATATCATCTATCATGAGTAAATGCAATGCGTATGCTTATTAATTGTAGCATAAAACTAACGTAGAGTCATGACAAATCC
>comp155531_c0_seq1
GAATTTAAGGAAGTGGATATTGCCATTGTGAAGGCGACGAATCACGAAGAATGCCCACCAAAGGAAAGACACGTTAGAACTACATCTGTTGCTCGACCACGGGCTGACATTGCCTATTGC
ATATATGCACTTGCAAAGAGGCTAGCAAAGACACGAAATTGGGTGGTTGCAGTTAAAACATTGATAGTGATTCACAGAATTTTGCGAGAAGGTGACCCTACATTTAAAGAGGAGCTCTTG
AACTATTCGCACAAGGGACAAATTTTACAAATTTCCAATTTCAAGGATGACTCGAGTCCTTTTGCTTGGGATTGCTCTGCATGGGTTCGTACATATGCACTCTTTTTAGAAGAAAGACTTG
AATGCTTTGGAGTTTTAAAATATGACATCGAGGCAGAGCGTTTGATGAAAAATGCACAAGGTTCTGCCAAGGGACATACTAGAACTAGAACTTTATCTTGTCCAGATCTGCTAGAGCAAAT
GCCCGCCTTACAACAGCTATTGCATCGGCTTGTAGGATGCCAGCCAGAAGGATCAGCATCTGGAAACAATCTTATACAATATGCATTGGCATTGGTTATAAAGGAGAGTTTCAAAATTTATT
GTGCAGTTAATGACGGGATCATCAATCTTGTTGATATGTTCTTTGAGATGCCAAAATATGATGCCATTAAAGCGCTGGAGGTATATAAAAGAGCTGGTAAACAGGCAGAAAATCTTTCTGAT
TTCTATGACTTGTGTAAACATTTGGAACTCGCTAGAAATTTTCAGTTTCCGACTCTAAGACAGCCACCTCCATCATTCCAAGCTACAATGGAAGAGTACATACGTGAAGCTCCATGTATGA
GCAACATTTCAACTAAGAATTTGGTGAGTGAAAATAAATTTAACGAGCATATAAACGCTCACATAGATCACCATAATGTTTTTCTTTTTTGATATGATTGAGATGTTTACCAGAGTAATGAAA
TCAATCAAGTGCTACAAGTTTTATTATTAATTG
>comp155588_c0_seq2
TTTTTTGCGTAGAATAATTCCTTCATGTAAGAAGGGTGAGGATTAATATAAAAATGATACTATGTACGTGTCATTTTCAGGAGCATCATGTGCATGCCATGTTCACATTGCCACATATATATAA
CGCACAGTTTCAATCAATAATACTGACGCACCCATCCAGTAGTAGAAAGCACCCAGCCTGGCCTTCGATGAGCCACTTGGGAGTTCCCACAAATTTACATCAAATGGATCTCTGAATATCT
TCTTTCTTTTACATCACCACGTCATTAGTTATTAGTACACACACCCTGCTCATGAAAGTGACTGTTGAGCTTTGAACTTGGTGAGGGTTCTCCTGGTTAGGTGATGAGAACAGAGCAAGTT
GAATATGGACAGAGAATTCAGCAGAAGCCACTTTTTCTAATAGTACGTCTTGTTCAACCCCCATTCATAACAAAAGAGCTAAATAAACGAGTGCAACAATTTTTATTTGATCATCTTTTCTG
GTTCAGGATGTTGTTTCCTACAGCATTGACAGGAAGAAGAAAAGGGAAGAAAAGAACAAGTTGATAAAATAAACAGAGAAGAATAATAAAAAAAGAAGCCATGGATGGATGCTGGAGA
TCTACACATGGATTTCTAATCTTCGTTGCTATTTCTTTAGAGCAGATTGTTCTCGTCTTCTTGCCTGCAGTTTGGTGCTTGATTCAGAGCAGCGGAAGATTGAAGATAATTACCAATATGATT
CTTCAAAGCAGCCTCTTTCTGTGTAGGTAGAGCAGAAGTTGGCACATTTACTCAATAGCAAACAAAAAAGAGAGAGAGATTGAGGAAAAAAATGATAACAAAGAAGAAAAACCAGTGC
CCACCTTGCAATGGTTTGCAGAGAAACCACAGGAGAGTTCCAAGACATCATCCACAAGGTGATTCCTGCTGCTGCTGCTGATGCTACTGGTAGCCTCGGAGAAACAGAGCAAGGGAGA
ACTTGCAGTGTCATCTAGGAAGGAAGAGTGCTCCTGGAGTCTTCTTTGCTTCTGCAGGTTCTCTTCCTCAGCCTTTCTTCTCTTGCTGACCCCATTACGGGCAAATGCAGAGAGACCTAAA
TCTCGATCATTAGAACAATAGTAAAACTTGATGCAAGTTTCTTCTGTGCCATGTGGAGGACCAGAAGATGCATCGGAGAGCATTGACAAGTCCTCTTCTTCATCTTCATGAACGGAGACTT
CTCCTCCGCTAGGAGGAGTGGAGGTGGAAGGGCTGTCATGCGACTGGCAAAGGTACAAAGTCCAGCCAGATTGACATCCACTACTGCATTCAGAGAAGCCCCTCTCTCCCTTTACCACG
AACTCCTCCAAGTCCTCAACTTTCTCCATGACGAGCACTGCTTGACGAAATCAGAGTAAGGCGGGTTCTTCTTTCCCTCTGTGATTCAGGGAATGGTAGGAGAAGAAAAAGGGAGAAGA

AGAGGAGTTTGGAGCTTTGCGCGGAAAACAAAGAATTGTTTGGCACCCAAATCTCAGACCCATCTTGTCATTTACCCAATCCATCCCTGGAACAACACCATCTCGGTTTCCTCGCTGTGT
ATGAAATTAAAACTGCCAGTTGGGAATGGTGGGAAGGGAGAGGAAAATCCCCAAGTCTCTCTCATTTTCAGCCTTCACTGTGTACATAGAAGATAGAAAGAAAGAGATAGAAAGAGAAA
AGACTTCACATTTCACATGCAATACAGCGTGGCGAAGTGTACGCCCTACACCTTCATTATATCTCTGGAATCCCGTGAGGACTAAGAAGTTGAAG
>comp155610_c3_seq1
TTTTTTTTTTTTTGAAGAAGTGCATCAAATTGCATTAAGAACAAAACAAGACTTAATTACAGGTAATAAACGGATCACATTCCTTCTTGATTAAATTTAACAACAAGGAAGGAATGTTCTCA
AAATAAAAACTATCAAAAAAGAAACTACTCTCCAATGCCATAGAAGCACAACAATTAGCTATTTTTTACAACTTCTATAAGCATGGAAGAAAACAACTTTATTGAAATCATTTAAGAAACA
CAAACCTTTGTCGGCCATCTATCAACTTTCCACTTATTCTTCTTTAGAGGGTCTTGAATGAACTTGATAACATTGGTATTATCACTATCAATGATCAGCCCTTTACATTCAAACATACAGCTTC
TCAAAAACTCCTTCACCGAGAAAACTGCTTCTAGCTCTAATTGAGCAATATCCCAATGAGCTCCATTCTTCCCAAATGCACTTATAAACCTACCTTTGTGATCTCTAAAAACACCACCAAC
ACCAGCCAAATTGGAACTAAGCAAAGAGGCATCTACATTGATCTTAATTCAATCTTTCGGTGGAGGGCACCAAGCACTTTGAGACTCCCTAAGGAGATTAGTACCCCAGCTAGTATGATAT
GGACCAGATTTTGAAATGGCTGAGAACAAAGCATTAGACGAAACCATAGAGCATGGAAAAGCAGTCTTATTGTGCTTGACAACATTACAATTCTTCCAAGAGTGTTATACAATATTGCAGT
AAATATGTACAATCACAGATTTATTTTTTGCTATCTGTTTTAACCCCAGTAAACAGCAATTCAGAGAACTAAAAACAGGAATAGAAATACCCCATTCACCCATCTTCACGATGACATCAGTC
ATATATTTACAGTGTACCATTATATGTTCATATGTTTCACTCACTTGGCATCCTCTAGGACAAAGATCATTATCTGAAATTCTACGGTTCTTCAAAAAAAGATTGGTAGGAATCGCTGATTTTC
CCAACCTCCACCAGAAGATCTCCACCTTTTTCTTAAGTTTTAATTTTTGCAACCAGCTGAAGTAATCAGCATCCTCCAGGGTATACTTGTTGGACAACACTTGCTCATACACCAATGCAGA
AATTGTCTTTCCCGAGCACATCTTCATCAATTCTAGCTGATCCTCCTCCTCAAACTCAATATGAATTTGGCTAATCAAGAACACTAGATTGGGATGGAAGGCCCTTTGCAACTTGGTACAAT
TCCACACACTATTTCTAAGCAAAAACTGTTGTATGTACATTCCATCTAGAAAATCACAGTCAACATAAGTTGGCCAACGGTTGATACATCTATCAAGCAGCCATGTATCATTTAAAATATTAA
TCTTGTCACCCTTGCCCACTCTCCAACGAACAGCCATTTTCAAATATTTGCCACCATCCACCAAAATTTGCCAAGCTGTGGAAGTAATCTTGCTTTGGGTTCCATTCATCACATCATTACCA
TATCTAACTATCATTGCACGATGGAACAAAGATTGCGATTTCTGGATAAAATTCCAAGCCAACTTGGATCTTAAAGACCCAACTTTAAGGAGGGGAGAATGCAAACCCAACCCTCCCCAA
CTTCTTGGTTTACAAATATCACCCCAAGCAACATAGTGCATACCATTGGTCCCATCATTTTTATGCAAAATAAAACTTCTACAAAGTTTCTCCAACTCATGTAAAACCCTCTTGGGGACTATA
TAATGAGTAATTAAGAAATTAGGCATAGAAAGCAAAGAACTATCAATGAGAATCAGCTTACCTCCCAATGATAGGGACTTCTTACTCCAAGCATTGAGTTTATCCATAACATTACTAAGAAT
TTCTTGAAAATCAGCCATTTTAAGTCTATTAAGGGACATCTTCACTCCGAAATACTTCATCTCTTTAACAACTTTGAAGCCAAGAACCTTAGCTATTCTCTTCTTCATGGAGTAACTAACCA
CCTTGTCAAATATCATCTGAGATTTGCTTACATTGATTCATAGTCCAGTCCACTTACAAAAGTCCTCCACAATAGTCTTCAAAGCATTAGCTAAATCCACGGATGCATGAGAAAATATTAAC
ACATCGTCTACATAAAGGAGGTGTGTGATCATAGGTCCTCTAGGAGAAATTTGTATGCCAAGCTTCTGTCCCCTTTGCACAAGCGAGTTGGACAAGAGTTGAGAGCACAATATAAAAAGA
TACGGAGAGAGTGGACACCCTTGACGGAACCCACTATGAGAATTAATCGACTTAGAATTTCTTCCATTGATGATAATAGAGAATCTCACATCTACAACACATTCCATGAGAAGGTTAGAAA
AATCAGTAGGAAAACCATACCAAATCAAGATGTGCCGAAGAGATGCCCATCCCATACTATCATACGCTTGCTCCATATCAAGTTTTAAATCCATCAAGCCCTTCTTATTTTTAGAAATTCTGA
ACTTGTGAAAGATCTCCTGAGCCAAAAGGCAATGCTCTGCAATAGATCTTTCATGGATGAACGCCACTTGCTCCTCTGAAATCATCTTGGAGATAACCTTCTTTAATCTATTAACTAACATG
GTAGCCACAATCTTATAATTAGTTTGGCAAAGGCTAATAGGCCTAAAGTTAGAAGGAGTCATAGGGTTCTTAATCTTAGAGATCAAGACAATCAAAGTATTCTTCCACTCCCTATGCATATG
GCAGGATTTAAAGAAACAGCTAACTGCATTCCAAAGAGTCTCCCATACAATACTCCAATAGATCTTGTAAAAAGAAGAGGTGACTCCATCCAAGCCTGGTGACTTGTTGTTTCCTTGCTG
AAAAACCAAATTTTGCAACTCATTTACTGAAAATTCTGCATTGAGCACATCCATATCATCAGCTGCAATCTTCTGGTTTTAAAAAACTGTAGGCCAACCCGTCACTTTACAATCCCTGTGCT
TCCATTTCTTCTCAAAAAAATTAATGAAGACCTTCTCAATTATCTCATCCTCGTCATACAAATTGTTGAGCTCATCTTTAATTTGAATGATCCTATTTCCATTTCTTCTAGCCGAGGCATAATTA
TGAAACAATTTTGAGTTTGTATCACCT
>comp155617_c0_seq3

CAGAATAAATGTTCAATAAGTAAAAAAAAAAAAAATCTATAGATTTTATTTTTATAACCTGATATTTGCATATTTGATATATGATCAAATTGTTTTGAATTTGACGGAGAATAAAGTTCTCTAAA
TTGTAAGTGACATGAAGAAATAAAAGGTTTTATAATATCTACAGCGAAAATAGTCAAAATACTATGTAGGTGTTGATGAATTGAATGAGAAAAAAAAAAAAAGGAGAATAATAAGAAAACA
AGAATGAGATTATTATAGGTTTATCCCACCCTTTTATTTTATTTTATTAATCTATACTTGTATTAATCTGCTCCGCAGAGTCGCCTGCAAGCTCTTTTCACCTCCCCTTGTTCTGCTTCATCATTC
TTGCAATCCTGACGCGCAGCTTCTTCAGAGTTTTGAGATGGGTCGTCAACTTCATGAGCTATTATTTTAGCCATCCAAATGTCGATGTTTTTGTGGAGTTCTTCCAGGACCTCAGTGATTTC
AGAAGAATTTTCTTCTCCTCTATTTTCCAAGGATCGTTTAGCCATATCAAGCGCAATGGCGGCATTATAGGCTTCGAAAACAGCTTCCAGCGCTTTCACCCTCTCCAGCCCATTCTTCACCA
TCGCTACATGGCGAAGATAATCCCCCTTCATCTTCTTAAAGAAAGCCCTAGACTCCGTAGTCATCGCCTTAGCCGCCAGCCCTTCTTCGGAGAAAAGTACGCTGCGATCAATGAAATTGAC
AATATCCGCACAGGTTTTGGAGATCTCATCCGCAAGAAACTCCTGATATTCTCTGGCGGCTTCCCGCCGGGTAGGGCTGGTGGTCATATCGGCCGCTACGAAAGCGAGGTAGTGCCAGGT
AGTAAGTTGGCGATCGAAATAGAGGTAGCAGGCCAAGCTGTAGCAATTACGCTCCTTTTCAGAGAGATCTTCGTTCGGCATCACGATTTGAATGAGCTTCTTCATGTAGTTGAGCATGTAT
TCGTAGTTCTCGCTCTCCTCGGCGCATCGAGCGAGAAGGAAGAGCCCCTCCCTCGTCTCCTCCGGATCCTGCACTACCTCTGGCCTTGTCTCCTTGTCCATCCTCTGAGTGAATCCCTTCG
CTGTCGCCATAGAATGCTGGTGAGAGAATCTGTAGAAAAAACACAGCTAAATAGGGTTTTGGGTTCGAATTTTTGTATGAATTTGACTTAAATGATCTAAATGTCCCAAAAAAATATGTAG
AAGAAGGAGAAA
>comp155617_c1_seq2
TTTTTTTGGGACATTTAGATCATTTAAGTCAAATTCATACAAAAATTCGAAACCAAAACCCTATTTAGCTGTGTTTTTTCTACAGATTCTCTCACCAGCATTGTATGGCGACAGCGAAGGGA
TTCACTCAGAGGATGGACAAGGAGACAAGGCCAGAGGTAGTGCAGGATCCGGAGGAGACGAGGGAGGGGCTCTTCCTTCTCGCTCGATGCGCCGAGGAGAGCGAGAACTACGAATAC
ATGCTCAACTACATGAAGAAGCTCATTCAAATCGTGATGCCGAACGAAGATCTCTCTGAAAAGGAGCGTAATTGCTACAGCTTGGCCTGCTACCTCTATTTCGATCGCCAACTTACTACCT
GGCACTACCTCGCTTTCGTAGCGGCCGATATGACCACCAGCCCTACCCGGCGGGAAGCCGCCAGAGAATATCAGGAGTTTCTTGCGGATGAGATCTCCAAAACCTGTGCGGATATTGTCA
ATTTCATTGATCGCAGCGTACTTTTCTCCGAAGAAGGGCTGGCGGCTAAGGCGATGACTACGGAGTCTAGGGCTTTCTTTAAGAAGATGAAGGGGGATTATCTTCGCCATGTAGCGATGGT
GAAGAATGGGCTGGAGAGGGTGAAAGCGCTGGAAGCTGTTTTCGAAGCCTATAATGCCGCCATTATGATTTATAGATATGTTCCATCTGTAGGAAGAGGTATTGCAGTTGCCGGAGGCGA
ACCTAACTCGACTACAGTTAGCCCTCAGTTTGGCTCTGTTTCATGCTGAGATATGTGAATCACCCAAAATGGCTGTTTCTGTTGCAAGAGAGGCGCTTGATATGGCTAAACGATCCTTGGA
AAATAGAGGAGAAGAAAATTCTTCTGAAATCACTGAGGTCCTGGAAGAACTCCACAAAAACATCGACATTTGGATGGCTAAAATAATAGCTCATGAAGTTGACGACCCATCTCAAAACT
CTGAAGAAGCTGCGCGTCAGGATTGCAAGAATGATGAAGCAGAACAAGGGGAGGTGAAAAGAGCTTGCAGGCGACTCTGCGGAGCAGATTAATACAAGTATAGATTAATAAAATAAAAT
AAAAGGGTGGGATAAACCTATAATAATCTCATTCTTGTTTTCTTATTATTCTCCTTTTTTTTTCTTTGTTCTCATTCAATTCATCAACACCTACATAGTATTTTGACTATTTTCGCTGTAGATATT
ATAAAACCTTTTATTTCTTCAAAAA
>comp155625_c0_seq2
TTTCAGCTATTTTTCCTAAAGATATTATATAACAATCAATTTGATTTGTTAATGCAGTACTAATATAAGCTTTTCTTTCACTTGGTCAACAATGTCAGGCTTAAAGAGCAACACTAACAGCCA
AGCAACTTTATCATCAGCAAAGCCCCTCTCAAAGCGCTGGCAGTAGTGAAAAGTGCTTTTGGCATGAAATTCTCTTATTTTTATTGTGAATTACAATTTTGCAGAAGTTTCAAAACATCAA
CATTAAAGTAAGCCTCTGTCCAACATTTTGGTGTAGCCAACAGAGCTTCTATGACTCATCTGATGCTGAAGATATTTTATATTTCTCATATAGATAGTTTCTTCAATTTGTTGAGTGATGCCCC
TTAGGGTGTGGCAATCTTGTAATATTAGTATACATTATTTGATTCATTTCTATATGTTTTTTATTTGCTAAAAAGAATTATTTGTTGAACTCATTTGAAAGGAGCTTTATCATGTTTGAAATATAG
TAGGGATTTTTAAAAAAA
>comp155697_c1_seq2
AATGCTGTAAAAAAAAAAACGCTTAATCGAATTTTAATTTAGAACAAAAAAATATTAATCTCAGTTTTATTCCGTCCGAACGAGAAAAATCTATGTGACATAATAATGTAATAAAAAATATG

CACCCATTAGACAAAACTAAAAAGTTAAATGGGAATGTGTTATGAGGGGTAGAAATAGCCTTAATTTTCATGTGGGAAAACTTATAAGTAACCATAGGGTTGTCAATATTGGATCCGACCC
GAAAACCCGATCGAAACCTGATCCGAAAATTTTGAGTTTGGGTTGACCCGAATCCGAAACGGGTCGGGTTCGGGTTCCACAATTCGACCAGTTTCAAAAATCAGATCAGGTTGACCCTT
TGACCTAAAATTGACTCGTTTTTTAACTGAAAATTCATTTTTGACCCGGCAACCCGAGATTTACCTGTTTTTGACCTGAAAAGTGGAGGTTGACCCATTTTTGGGCCAGAAAATTAGCAGT
TTTTTGTTAGCACCGGGTCATTTGGGTCTGGTCGGGTTGACCCGGACCTGAAGTAAGTCAGCTTTGAGTTGACCCGAATAGTGTCGGGTCTATTTCGGATTCAACCCGAACCCGACCCGT
GACCCAA
>comp155751_c0_seq2
CAACATGTCATGGTCACCGCTGTTTTTAAAAAAAAGTCTCACTAATTATAAAGTTTTATTTCTTGTAATTAATTAAGAGCCAGCATTAACTTAACCTGTAAACAAGAGCTCTGTAACCTCTTT
TTAATAGATTCAGCTGATAAGGTAAACTGCTTGGGATGTCTTTCAGGTAATCGAAGTTGTTGCACCTCTGCCATTCACTCACTGTTCCCTTCCTGATTTGAAGGGCCTCTCTGGTGACATTT
TCGTTTGCCCAGAAATAACCAAGGTAATAAGCATAGCCTTTGCAGCGGAGTTCGGGAGCCTTCGGTGGACGGAAGAGCCGGCCAAAGCTGTCCTCATTCAGTGATCTCTTATAGTACTCT
CTGGCGATGTCTTTAGGCCTTGGGAAGTCCGTCGCACATTTGGGCTCCAATACGTCTGTCTCCGACAGCTCCCAACGGTATTTTTTAAAGGTATCGAGGCGAGCAGCGCACTCTGCAGTTT
TTGGATTTTCATGATCTTGTCCAGCACAATCCTTTTCAACGAGCTCTGCAAATTCATCAGAGATGATGCCCATTCCAAGGGCAAATGGAACTCGGGCTTTAGTATCAATCCTTTCACCCGTT
CTTGGATTTCCAATAATATAACCCTGAAGATTAATGAAGGGTTGTTCTTTCACATCATTTCCATCTGCAATAAATTGAGCAACGACGGGGACTAACATGCCGCCATAAGAATCTCCAGCAAC
ATAAAAGCCGTTTGAGCGAAAACGAGGGTGCTCTACTAACCACTTTTTTAGAAACCCATAAACTTGATTAGAAGAAGAAGAATCCCCTGTCATATAGGTTTGAGATACAGTTGGGACTGA
GAATCCAGTCCCAGCCGGGTAATCCAGAAAGATAATGCTAGATACCTGATCACACACAAATATACATATATATGCGCGCTAGCTAGTAATTGAAGAATTTTTTTTTCTTTCTTTCTTACTTAT
GGAAAAAAAAAGTTATTGATTTAGTGATCCAATCGCTTATTTATTTACCTTGGTCCAAGAGTAAGGATTATAAAGAAGAGTTGGCACACTCCCATCATAAGCATCTATTTGAAATTTAACAG
GACCAATTTCGAAGAAGAGAGCCGAGAAGCTTGAACAACCGGGGCCTCCACTAAGCCAGAGGAGGAGTGGGTCCTCGCTGGCGTTTCTCTCCGACTCAATGAAGTAGTAGAACAACTC
CACGCCATTAACTTCATCCACCGTCAAATACCCAGTTTCCATTTGGAAGGGAAGCTGCCCTTTGAAGCCTGGTAGTTGCTGTACATGATTTGGAGTTGAAGAGATAGATTTTTGTAATATCA
CGAGAAAACTAAGTAGAAGTAGTACAGGACTCCAACGAGCCATTGCCGGTTTGCCTTAATCCA
>comp155751_c0_seq3
TAGAGTTTATGGTCATACTATCAATTAAAAAATATTGAAAACTAATTTATGTCGCCACTGTGGAAAAGTACTTTTGAGTCTGCCACTGACTATAGGTTTATATCATTTTTGCAATGAATTACTA
CAAAGTAGAAAATAGTAGAAATAGAAAATGTTGAACTCGGATAATATTACATATAATCATAAAGATATTATAATGTAAAATTTTGGAGTACACCTCCCTTGAAATATATTTCAGAGAGGTTTC
TTTGAAGTCCACCTATCGAACATGGCGAAACATTGCTGAGGCTTGAACTCTGGCGCAGTGTGTCCGGCCGCCCTCACTGTCGCAAAAGTTAAATCGTTTGAATACAGAACTGTATACCCT
GCAACTTGGTCATCAACACTCCATGATCTCCATGGATCCACAACCGTTAAATTTAAGGATTTAATCCATAGCTTGGTGCCCACAAATGGGATCAACATGTCATGGTCACCGCTGTAAACAA
GAGCTCTGTAACCTCTTTTTAATAGATTCAGCTGATAAGGTAAACTGCTTGGGATGTCTTTCAGGTAATCGAAGTTGTTGCACCTCTGCCATTCACTCACTGTTCCCTTCCTGATTTGAAGG
GCCTCTCTGGTGACATTTTCGTTTGCCCAGAAATAACCAAGGTAATAAGCATAGCCTTTGCAGCGGAGTTCGGGAGCCTTCGGTGGACGGAAGAGCCGGCCAAAGCTGTCCTCATTCAGT
GATCTCTTATAGTACTCTCTGGCGATGTCTTTAGGCCTTGGGAAGTCCGTCGCACATTTGGGCTCCAATACGTCTGTCTCCGACAGCTCCCAACGGTATTTTTTAAAGGTATCGAGGCGAGC
AGCGCACTCTGCAGTTTTTGGATTTTCATGATCTTGTCCAGCACAATCCTTTTCAACGAGCTCTGCAAATTCATCAGAGATGATGCCCATTCCAAGGGCAAATGGAACTCGGGCTTTAGTA
TCAATCCTTTCACCCGTTCTTGGATTTCCAATAATATAACCCTGAAGATTAATGAAGGGTTGTTCTTTCACATCATTTCCATCTGCAATAAATTGAGCAACGACGGGGACTAACATGCCGCC
ATAAGAATCTCCAGCAACATAAAAGCCGTTTGAGCGAAAACGAGGGTGCTCTACTAACCACTGCAATATGCGAGATGTATATAAACAAGGATTGGAGCTAGCGCTCAGGAAATAAAAGTT
AAAAATGTATTTGATCTTCCAAAATATAGATATTTGTATGAGCTAGAGACAGGGCGAGCTGCATCAGACCTTTTTTAGAAACCCATAAACTTGATTAGAAGAAGAAGAATCCCCTGTCATAT
AGGTTTGAGATACAGTTGGGACTGAGAATCCAGTCCCAGCCGGGTAATCCAGAAAGATAATGCTAGATACCTTGGTCCAAGAGTAAGGATTATAAAGAAGAGTTGGCACACTCCCATCAT

AAGCATCTATTTGAAATTTAACAGGACCAATTTCGAAGAAGAGAGCCGAGAAGCTTGAACAACCGGGGCCTCCACTAAGCCAGAGGAGGAGTGGGTCCTCGCTGGCGTTTCTCTCCGA
CTCAATGAAGTAGTAGAACAACTCCACGCCATTAACTTCATCCACCGTCAAATACCCAGTTTCCATTTGGAAGGGAAGCTGCCCTTTGAAGCCTGGTAGTTGCTGTACATGATTTGGAGTT
GAAGAGATAGATTTTTGTAATATCACGAGAAAACTAAGTAGAAGTAGTACAGGACTCCAACGAGCCATTGCCGGTTTGCCTTAATCCA
>comp155758_c0_seq1
AGAATATTATCAAAAGTAAAATTCATACATTTTTCTGCAGAAAGAATGATAAACTTACAAGAATTCTCAGCTAATTACACATTCACACTGTCAGATTGTCTTCAAATATACAAAATCATATAA
TTTTAAAAAAGCAGAAATGATATACACACATGAATAAACAAACAGGACTGGCCACCAGCATATGGATCTACTGAATTCCCGAATTTCTGCTCCATGATAAGAACGAAGGATCTCCAAGTTT
CCTAGGCGTTACCAAATATAGCACAGTGAAGGCTGTTGCAAGAAAGAAGCACAATCCACCAACAGAGAGATATGCAATGCCAAGGAAATCATTTTTTCCTCCAAGCCAGGATGTGGTTG
AAAGCACAAGTTTTTTCTTACCACCAAAACTGTATGTGTTGTAGTTGTTTTCTAGGTTCACAGTAATGATGTCGTTCTCCTTGAGATCCATCTCTATTCTTCCATACAACTTTCTAAAGGTTG
GAAGGGCTGCAGTACGCATCCACACAATGAGATCCTCTTGCTTGCTCAAAGGTTTCTTAGAATCAAGCTTTCCACCACCTATCAGAGCTCCATTCTGGAAGTTTTTGGGATACACATCATA
TCCAAACTTGTGATCCCTGTCACTCTTCCAGGAAATATCCTTCTTGTTCACAATCAATGATTCACCATTGCCATGGGTGAAGCTGTATGTATCATTGAACAAACTCCATGCTATAAGCCCAC
AAGGAACAATAGGAGTTCCATCTACAGTGGTTTTTTCCGGGTCACAACCACTTGTCTCATAAGCTTTGCTAGCATCCCTCAACTGCTTATCATTTCGGCTCTTTACATACCTCCTGTGATTCT
GATAGAAGTTATCAAGCTGATAGTACACATATATGGGCTTGTCCATGTCCTTGGGTACCTTGAGCACCCGAGAACAAGCTTTGCTGATGGCTGGATTCTGAATGTACCCAACCTTATCATTA
ATCATGCTGGGTGGAACGCAGGCAGTTTCATACCGATCAACAATTTCAACAACATCGTTGGAAGCAAGCAGCGAAGCTAGGCCAATAGGAATGAAGATTATGCCAACCAAAGTGAACAA
TGAGATAACCCATAGTGGGGTGAGAATCGGCTTGCAGGCTGGAAGCTCCTGCTGAGTAAACCTCGAATATTTGGGTCTCTTGGAATTCCTCCGGTCGCCTGATCCCGAACTCGACCCCGC
CGCAGAAGCGCTGACATCGTCCATCATCGTAAGTACTGAACTACTGGCGGAAAGAAAAGCTTTGTATCTCCCTTTCGATAAATGTCTCTTCCCTTCTATCTCCCCTTTCTCGCCTCCTTACC
TATATAGCTCTCTCTCCCGAAAATTCTCTGTATGGGTACAAGGAAGAGAAGGGGAGACAAAGAGAAGCGCGAAGCGATCGAGAGAAGCGACCGCGGAAGAGGGAAAACCGTTAGCAC
CGATGACAAGCTGACCCTCACCGCGCAGACGCGCTTTTAAGAACGCGAGTCCGGTCAAGTTCCTCTAGAA
>comp155759_c0_seq7
AATATTAAAGTTTTAGATATATGTATGTATCCCCGAAAAAAGAGAGAGTGAGAGAGAGAGAAGAAAAAGCGAAAGAAGATTATTTTGGTTGCTTAGTTCACCATGCGTTGTCCCCCATCAT
TTGAAAACGTTAGCATCCGGATGGCGTGAGGCAATCAAGAACCTTATAATGCCTTCAGAACCTCTCAACTTTCCTACTCTCAAACACACAGATATAAATAGAACTTAGCACAGAGAGCTC
GGCCAAGTAGAAGAAAGAGAAAGCTAAAAGAGAAATGAAGCAAACGTCATATAGCAGCTTTCGCCGGATCAAGGCGGAGAAGCTCGGCCGTTCTGCTGTCTGTACTTGCTTTCTTATTA
CTGTCTTCTTCATCACGATAATTAAGCAAAGCTCTCATCCCTTACCGTTATCAAGTTTCCAGCTATCGGTGAGGACCAACTTGCCTTGTTCGAAGGTGAAGGAATTTCAATTAGGAAGCTTT
ACTAAGCTACGAGGCAATGAAAGTATAGTGTCAAAACAAGTCACTTATCCAAAGAACTCCATAGGAGAACAGATGCTACAAGGCAATGAAACTGAAACCTTATCACTAGATCTGGAAGA
AAATAATCAAGTTAATGTAAATATTAAGAGGATAGAAGTACATCAAATTGCAAATCAGAAGCAAGAGAAGGTTCACACCTTTGCAAGCAGATCTGAGGAGCAAAACTACATAATTTCAAG
CAGTGATCAAGAGAAGAATAAACTGAGTGGAAGTAAATACACATCACAAAAGAACTATCCACAGCATAACCACCCAATAACGAATAGTTCCCAAGTTCAAAAGCACAAAATCAGAAGTA
AACAAACTCTCTTGGAGTTCAATACATATAAAAGTGGCTAAGCTGAATCTCATTATATATATTAATGTTTTGTTTGAGACATCTTTCTTATTATTTTATGAAGCAGTTTTTTACTACAAAAATTT
TACATTAAAAAAAATTTGTGCTAAAAAATTGCTTCATAAAGCAGTAAGAAAGCTGTCCGAAACGAGGCGTAAGATTTGAACTTAACTGTAGCCTTACAATACATATGAGAGGAAGCCCAA
CTTTAACTAATTTGATTTCAGCATCTAAGCAGAACCTTTTATATTCAATATATGTAGGTGATATTGGTCAAGAAGGCAAGATAATCTGTGATTTTTCGGAAGCCAGATCTGATACCTGTTCCAT
GTTTGGTGATATAAGAGTGCATAGTAGCTCCTTCACCATCTTTGTAGCTATGTCCTCTCAATCTCCCTTTCCCTTGGAAAACTATACGCACACTATAAAACTTGTAATATAAACCATGCAGTT
CCTGCCATCATCTTCTCTACAGGTGGATTTCTAGGCAATTTCTTCCACGACTTCACTGATGTTCTGATCCCACTCTTTGTCACCTCTCGCCAATTCAATGGGAATGTTCAGTTCCTTGTAACT
GACTTCAATGATAAGTGGATCAATAAGTACAGACCAATTCTACAAGGCCTTTCCCGCTTTGAGATCATTAATATGGACATACAGAAGATTATCCATTGCTTCCCTTCCGTTCATGTTGGTCTA

AAAAGCCACAAAGTCCTTGGTATCTATCCTTCGAAGACTCCAAAAGGCTATACTATGTTTGACTTCAAAAATTTTCTCAGAGAAATATTTTCACTTAAAAGGGGTTCTATAGTTCAGAAGA
AATCAAGAAGAAAGCCAAGATTACTGATGATACTAAGAAAAGGATCAAGATCAATAATGAATGAAAAAGAAGTAATCGTGATGGCTCGAAGAATTGGTTATAAGGTGATCACTGCAAGA
CCAGAAGAGATGAAGGAATTGCCAAAGTTTGCTGATATTGTGAATTCATGTGATGTGATGATGGGAGTTCATGGGGCTGGGTTAGCTAATATGGTGTTCCTTCCAGAGAAATCAACAGTGA
TTCAAATAATTCCATTGGGAGGTTTAACATGGGCTTGTAGACATGACTATGGTGAGCCTGCTCCAGAAATGGGGATTAAGTATTTGGAATATGAGATTACAGATAATGAAAGTTCATTAATA
AAACAGTACCCAAGAGATCATGCAGTCTTCAAGGATCCTTTTTCAATCCATAGGCAAGGTTGGAATCAGTTATGGTCTATCTTTCTAGACAAACAGAAGGTCAATATTGATGTTGGGCGAT
TTAGGGGTTTGTTGTTGGAGGTTTATCATTCTATCAAACAATAGACAGTGGGTATTTCATCTATTTCTCATCATTATATTTTACATGATCAAAGTTGTAAAGAATAATTGCCTGCGTTCATATC
AAGGAATCTTGTTACATATGAAAGCTAATTTTGATAATTTTATCAAAGAAGAGCATTGATAATTTCTATAATAGGTTGTACGTGTCTTCCAGTCTCACCAATAGGAGAAACGTATCACTTGCA
AGTTGGAACACACTTAAAAAATAAATAAGCAGTTCATCGTCAAACCACCTAGGAAATTCTAGTATGAAGATTAGTATATAAATTTCGTGACGTTCAATTAA
>comp155772_c0_seq2
TTTTTGGAAAATAAAAACGCAAATTTAAATTTATTGATAGAGAGACATTTGTGCTCAGGTGAGCACAATCAAAGTTACATGACATAAAATATCATTTCTTATTGCACAATCCATTATTATTTAT
TCTATGTATTAGAAGACAGATACACTCCTGTTCTTCAGCACTTAGTTTGATAGCACACGAACCATCTCTACACCTGAACGTAATCAATTTCAATCTTGCCGGCATCCAAATTTGCGATAGTT
GAGAAAGCCTCTTGGGAGAGATCTATGGTGCCTCGGCAACCAGAAGGGCAATAATCGACAATCTTAACCACAACGCTTGCACCTGTACACGGGTGAAGATCACCAGCATTGGTTGGACC
CGTACAGGTCACTCGATAATTTCGTCCGCAGGCCGCACGATTATCCCATATTGTATCGCTCGCCGCAGCAATCAGCGTACCCATGTCGTCATACCCGTAACACGAAGAAGGTGTATATGGAG
GGGTATAGTAAGTTGCAGTTCCATCAATGGCAGAAGAGAGAGCAAAGTGGCAGAGAACAATGGTGAGGGCAAGAACAATATGTTTCTCCATTCTTCGATCTCTTAAATCTCTAATCGAAT
ACTAACAATATATCTCAACTATGATCGATGGAAGGTAAGGGGAAGAATTGGTCCTCTTTTATAGGAGAAGATCGATAGGCTAAACG
>comp155793_c1_seq1
TAACAATCACCAACAGAAAATGTAAATGGATAAAGTGCTGATTTTGTGCCAAAGCAAATAAAACTGAAGAATCTCTCCTAATTCTTATAAAATTGGACTACATGATTCAAAATTATTAAACG
CTTTAAATAGAATTTGATCCTTGCAAGTAACAACAATATGAATTATCTTCATGACTTGAAAAACATCATCATTCAGCAAATGTTCCAACAAATCCACCTTCCATACTCCAACAAAAAATTCA
ACAACTAAATGCTTGGAATGAGCGGCATTCGGAAAAATAGAAGCCAGGGAACCAATTCCAAGTTAGTCATCAAACCAAAAAGAGAAATTCCCAGCACGAATACCCAGCAAATGAAATTT
GCAGCTTCCCTTCTGATGTTACACGTTCTTTTTGAACAAAGCAGAAATCAATGTTTCTCCACATTAAGGGATCCCGACGGCGACGGAAGCGGCGGAGCCGGTCTCCCCCTTCGATTTGCG
TCGCCGGGATTGCTTCGGAACGGAGGGGGAATCCCGTGCCGGTGACTCGGATCCCGTGGTGGATACCTTGTAGCGACGGCGGCTGCGTCGGAAGCTTCCATCTGAAGGCTCCTTGGCCG
AGTAGTCGGCGGCAGCGGGGGACGAGCCTGCGGATTGTTGACGACGGGAGTGGCGGTTCTCGCGGTTAGAGGAATCCTTTTCGCGACTGGAAAGGGAGTCTGTGCCAAATGGAGAGTC
GGGTGCGGAAGAGCGCCGCCGATGCGGCCTCCTCACCGGCGACTGTGGAACGGGTTCTGCACTCTGAGATCTTGCGGGGTTTATCGATAACTCGCGCATTTCTTGAGTGGGTGCCTCTAG
GCCTTTCCCATTGTTGGCTGGCGTCAATTGATACTCTCCCATCCAGCTCGGTGCATTTGCAGAAGGTCCATCGCATCCAAAGTGAGCCAAATGTTTGACATCTGTAGGGTAGCCAATCTGC
ATCTCTTTCTCCTCTTCTTCATCAAATATTTGAGAAATGTATCTCAGGCCCTTTAACAGGCCTTTCATCTTATTGTTCATCATCAGCAAAGCAGATTATATGATGCTCCTCTCAGCTCCACCTC
AAATGTATATGAAAGTCACCAACCGATTCTCCTCTCGATCTGGCGCCTCCTCATTACCATAATCTCAAGATCAAATTTATGTGATTCAAATGATTCAGAACCTTACAGAAACGATTTTGGGA
GATGGGAGGCGGCAACAAAAGATGCTATCAAAAGATAACAATGCTACAGAGGCCGCCGAAGCCCCGCCTGGAAACAGCAATATCCAGAAAGGAGGGAGCCTGCTTCTTTCGGTTTCAG
AAGATTGATTATTTCTTCTTTTTTTTCTTTAAAGACTTTAACTTTTTTTAGCCAAGTTTCCTTTTTTTAATTTGTTAATTATTTTT
>comp155799_c0_seq5
TTCTTTGAATCCGAAAGTATTTATACAGAAGAAATATGTAACAGAGAGGCTATACAGCAGCTACACATACATCAATACGTCTATATGGAAACTTATTTCTAAATTTTGCTCGACGCTCCGACG
TCAAATAGAGATAAAGAATCATGAATGAAGATTTTTTGTCCCTAATTTTAGACGTCTTATCCTAATTTCTTTTTTGATCTTCGTCGTTTCTCATGTGACTCCATTGGTATATCATGTGGATCTAT

AATTTTTATCATTCGTTGCTTCTTTGCATTTTCTATTCTTTGATATCCTTCATGAAGCTTCCACTTCGAAATCTCCAGCTTGATCGACGCATCTGAAGCTTCCTTCAACTGCTTTCTCTTCACT
CCAGCAGATAAATGCCCACTTGGTTTTTGCTTCAAAGCTTGTTTTGACGAGCAGACAAGTTCAAAATCTGGTTTGTTGAGCTTGCTAATAGAATAATCTGTAATATTATTTTCAGAAGTTGC
AGCAATAGGTTTAGCCCATTCAGCAGCAACCAGCGCTTTCCAACCTTTAATGAGAGTCCTAACAAGTTTTTGAATCTGGCTACAGTTGTGTTTTCGTAAGGCATTCACAGTTCTTCCAATT
TCAGTTTCCTTCAGAGTTTCTACAGACAATTGCATCAATTGCAGACTTCTTAGTGATTCATACAAGAGTGAATCAGACTCATATAAGTGATTATTGGAAAGAATCTCCTTTATCTTCATCACC
TCTCCAACTATCTGATCATTCTCTCCGGGCTTGTCATAGTTTTTGGAGCCGTTTAACTCCTGCTTGGGATGTTCAACATCGCCATTATTGAGAGAAAGATGGCAATTGTTGATCTTCTCGAC
AATCAGATCCTTTCTATCATTAAATTCCTTTGGGTAATCCACCATCGCTATGAGAATCGCTTTGTCAATCAACTCAAAGATACCTGATTTCGCACATCTGAAAAATTTTCTCCAATAATCCAG
TGAATCTTTTGTCTCCGCCATTGCCACAAATTAAGACTGCTCCTTTCCTGCCGCTGCCCATATCCTGCACAAATCCACGCAGTCTGCAAGCAGCCTCGAATGCAGTCGCCCATCCATCGCC
TCCCTCAAGTATCCGCGCTCCTTGTTAAAATGCCGATGGAATGAAGCCTTTGCTTCTTCTTCTTCGACTGTACACACAAGAGAGCTCAAAATTTTTCTCATTTCTTCCATGTTCATTCACAA
TCCCGACCGAACTCCATCACAGGCCGCCGCCCCTTCCTCTCTGCCTGCATCCAACTTCATCGAACTCAAACAACAGCCCCTCGCCTATCTTCCACGCACACGAAATCGAACCTGTTCGAC
TCCTCCAACCCTTGAGCCGCTGCATCCCGCAAGCATCCGACTCCCTAGCCAAAGCCTGTCTGGCCTTCCTGTTTGAGGCCCGTCGATTCCAACCAGTTCCGTTCGTCTATACTCGCCGAGC
TCGTCACCCCATTCGACGACTCTTTCTTCCATCCTGCTGCAATCCTTCACCATCTCAATACCTAACCTCACGGCTATATACCACCTGTAATAGCCGCTCAAACCTTCCTCCATAGCCGCAGA
AATCCCACATAGAAACCCTAGCCTGCATCTCTTCCTTGCGCCAGATCCGGCTCCAATCCTCTGCCCCAGCTGGTATATGTTTTGTATTGC
>comp155819_c0_seq1
TAGAACTTATATTTTGGATATTAATTAAGATATAGATCTAATTGATTTTAATTTCAAGGACCACGATTTCATTTGCAAGCTTAAAATTCATACACATACCTTGTAGACATGATTGTCGTCAAAAT
CCTGTAAGGCATTAGAATTAGTGTTAGAATTTTCGAGGATGAGGATTTCATTTTCATAAACATACCTCGTCGAAAGTCGTCATTAGAA
>comp155828_c0_seq1
TGGAGGAGGAGGAGGAAGAGGAGGTGGAGGAGAATGAGGCAGTGGTGGAGTAGTAGATGGACCTCCAAGAGATGCTGGAAGTCCCTGGGACAAAAGATCTGCAGAACTTGGAGTAG
AAGTTTCAACACTAGTAAGGTTTGCTATATTTGATGATACCAAGGGTTGAAATGGTGGAGACGTTGCTCCATGACCTTCAGAAATAATTTCTTGCTGTAGAGGCGGTGATAAAGGTGGGTG
AGAAGTGAGAAAGGGAAACTCACCTTTCTTAACAATATTCGGAGAAGGTGTAGGAGGTATTGTTAAAAGGTCATTTATAGCACGTTTAACATTGATTGGAGGCAAGGGAGGTAGGTAAGA
TGCAAGTAATTCAAGAGCACTTTGTTTTTCCCTGAAAGGGGGTGTTGGAGGAGGAGGAGGTGGTGGTGATGGAGCTTGGAATATTCCTCCTCCATTTGGAAGTAAGAAAGAAGAGAGCA
GACCATTGGAACTGTTATGTATGAGATCTGGATAAGAGGCTGAATAGATTGATGAGCATGAAGGGTTTGGGGTTGGAGGTGGGGTTGCAATTGAGGGTGGAGGCGTTGAAGGTGGAGGT
GGAGGTGGGTGAGGGGTTGAAGGTGGTGGTGGAGGTGGGGAAGGAGGTGGAGGGATTGGGGGTGCTGAGCATGGCTGTGGCGATGGAGGAGATGCAGGAGCTATCCTGTTAGTTGGA
AACTCAGAAAGGAAGGGTGTGGGATGCGGGGAGAGAGGCGGTGAGAATGGTGCTGGTGCGGGTGACAATGTAGGAGGTTCCGTAACATGTACTGATGACTGAGGTGGGGAAGGTATGA
TATTAATTAGAGCAGTAGAAAGCAAAGGTGAGGTAATGAAAGGAGTAGCAGAATCAGGAGGTATAGAAGAAGCTAGTGGAAAAAGAGGAAGTGATGATGATGGTGGATTTAGAGGAGG
TGGAGATGGTGGAGTTATGCTTTTGGCTAGAGCAGTTGAGTGAAAAGTTGGAGGATAAGGAGGTGGTGGGGGAGGGTTTCGGATAGGTGAAGGGGAAATTGAAACATGCTCTTCCCTG
AATTTCAGAGTTGAAAGAGATAACTGTGGAGTTGGTGGTGGGGGTGGGCAAGGGGTTGTAGAAGTTATGCCAATGACGGGAGCAGCTGTAAGCAAGGATTCGGAATATGCAGGGACTTT
GTGAGGGAGTGGGTGCTGATATAACAATGATATTTCTCTCCCATTATGGGGTGACCAAGGTGGAAGAGGAGGTGGGCTTGGAGGTTTGTAAAGACCTGGGACTTGAGGAGCCATGCCTTT
GACTGGAGAAGAAGAATGGAAGGGTTGTGCTTGAGGGGTCGAAGGAGTAGCAGTAGTAGATGAAAGAGGTCCTAAATATAACGATGGAGTTTGTGCAGTTAGTAAAGTAGGAGAGTTAT
TTTCAGAAACGGCATCTGGTAAAGTAGGAGAAGAAATGTTTTCAAGTGGAAGGGTGCAAGTAGCATTTAAGTGATGTGTTTGTAATGACTGAGAAGAGGAATGGGGTGAACTCTGTGTA
GAGGTGGCAGAAGATGGGCAAGCCTTCGTGAAAGATGGTAGCAAAGGTAATATTACTGTTGATTCTGTAGCAACATTTGGTGACTCTGTGGATTCAACTTCTTGCAAAAATGTGGAGGTT
CGTAAAGAAGGTAGCAAGCTGTTAAGCATGAGTGAAGGAGATGGCAGGCGGATCACATTCGCATTAGAATATTTGTTGGCAGATTCCTTAGAAGATATTGTTTGTGATTCCTCATGTTTGG

CATGATTTTCAATCAATTTCTGTTGTTCCCCCTGTTGCACCGTTATAACTGAATCAGAAGGCATATCCACTGTGCAATTTGGTTCGTCTTGTCCAATAGTGAGAAGCTGTTCCTGAAGCTTTT
CTCTACAGGCACCAACCAATGAATTTTCACAAAGAACCTCAAGCAACTCCTTAGGCTGGGTGACAACATCATCTATTTGGAAATCACGAGCTTTGCTATTAATCTCATCAATTTCTGACCT
CCCGGAATGACTAGGCTCGCTACAACGGATTAAGTTTATTGCAGGAAAATCAGCACTAACATCCTTTTTGATATTTTCATCTTCCAAAGCACTTTCACCAAGCAAAAATGGAGTTTCCCAA
CTCTCAGTCATCTCACCTGAAGCAATTGGCATAGTGCCCTTCAAGCCCTGTCTCAAACTATGGGATTCTAAGGAGGTCATTCCTTGAACGTCTGAATTAAGAGTCTCCTCTGCGTCCTTTCT
GAAATCAGTCATGCTAGAGGTCCTAGAGTCAGGAACATTTTCATCAAAAACAGATCCCATTTCTATAAACTCCTTATCTAGAACCTTTTTAAAAACTTCATTTACTCCACAGTCCTCCAATT
TATCTGTAGACACAGTTTCTAACCTGCTACTTCCATCACCTACTACACTTGAATCAATTAAGCCTAAAGAATTGGCGTCTGTAAGGCACTCTGCTTCCTGATTCAAGTAACCAACAAACGG
TGAAATCATCCCTTCATTTACAAGAGAAACATTTTTTTTAACTTCTTGTATGAGGTCAAACATTTTAGGAGTTCTAAATATAGGACTACCATCATGAAATGATTGTTCCTCGGTGTCGTCATC
GTCAGCATGATAATTGGAAGGTTTGATTTCTAGGTTGTTCTTATCTTTATCCAGGGATGAAGTGATACAATTGATACTGCAATCTTTTCCAGAATCATCTAAGATAGAAATTTCTTGGGCAGA
ATCAGTCTTAAGGTTCCATGCTTTACCATATTCCAGTTTACGACTGAATAGACCTTCCCATTTTGAAATTTCAGATGGAGTGCTATCATTTAGAACAGTTCGAACTGTACGAATATCCGAATC
TCTAGATCCGTCTTGCAAATCAGCATTGCTAAAGATCTCTTCTGCTTCAAAAAATTCTTCCGCTTCCAATTCATCTTCATCTACCGCTGCAACTCTCCGGTGGTTGTCAGAATCCTTGTCATA
TTCTGAAAACAGTACCTGGTTCAGGCATCAATGTACATTCAACATCCTGAGTATCAGAGATAAACACTAGAATGAAAGTGAAACTAACAACCAGGAGTGGAT
>comp155852_c0_seq1
CTCTCCCAACAACCATGCAATCATATGCCTTAATTTCCTCCCATTTCTTCTGGCCTCCATCAAAACCCTCAGCTTTCTCCTCTCCGTCCCCCTCTTCGGCCTTCCCCCACCCCGCCGCCTGTC
TCCATGGCCAGTCAGGCGGCGCCATGGCTCCTCATCTTCCTCTACCTTGCCTACTCGACCATCACCGTCGAATCCGCCGGCGCCGAGGAAGGGGAAGCACTTCTCAGATTCAAAGCCACC
CTATCAATCGATCTCCCATCGTGGATCCCCGGCCCCGCACCCTGCACTCTAAACTCCACCACTTGGGAAGGCGTCATCTGCTTCAATGGCCGCGTTTGGGGGATTCAGCTCGAGAACAGA
AGCCTTTCCGGGACCCTAAATCTCCGACCTTTAATACCCCTACCTGGCCTCCGTACCATCAGCTTCATGGGCAATAAGCTCGAGGGACCTTTGCCTCCTGATCTTAAGCTCCTCGGCGCTCT
CAAGGCTGTTTATCTCTCCAGGAATAACTTCTCCGGCGAGATTCCGGCGGATGCGTTTGCGGGCATGAGATCCCTAAAGAAGCTTTTTTTATCGAATAATGGCTTTTTCGGTCCTGTTCCGA
GATCGCTTGTTGGCCTTGGTAAGCTGCTTGAGCTCCGTCTCGATCACAATAAGTTCGCCGGCGGTATACCGAGTTTCGGCCAGCGCCGCCTCACGATTGTTGACGTGTCTTTTAATCGTTT
GGAGGGGGCTATTCCAAATAGACTCAGTAGGATGGACCCCGCTTTATTTCAAGGAAACCCAAAGCTCTGCGGCGCCCCGCTCACCGTGATCTGCAACAACAAAATCCAACCAGCAAAAC
ATGGAGGAAGCTCCGGCTGGCGTCTTTCTCTCATAATCATTACTGCCTGCATCATTTCCTTCCTCCTCGTCCTCCTCCTCCTCTCAGCACTTCGCCGGCGCCAGAAAACCAGTAAGGTGATT
GGTCGCCCGTGCGCCACCACTGAAGACGACGGCGAAAAGCTGGAGCTGGGTACGGATTCAAAGCACCAACAGCCAGCCACCGCCACAGCAGCCGGACCGGTGGAAGGAAAAACTTC
CGGCGGACGAAAAAGCGAGTGCGGGAGGCTGGCTTTCGTTCAAGAAGGAAGGGAGAGATTCGAGCTCCACGACCTGCTTAGGGCATCGGCGGAGGTGCTCGGAAGCGGAAACTTTGG
TTCGTCTTTCAAGGCGGTGTTGTCGCCCGGTCCCACCGTCGTCGTCAAGAGGTTTAAGGAGATGAACGGAGTGGGGAGAGAAGACTTCAACGAGCACATGAGAAGGATAGGGAGGCTC
TCACATCCTAATCTCCTTCCATTGATCGCATATTACTATAAAAGAGAGGACAAATTGCTCATTACTGAATTCATTGCAAATGGAAGCTTGGCTCACATGCTCCATGGTAATCGTGGGTCAACT
CTGCCGCCGCTCGACTGGCCGACGAGGTTGAAGATAGTAAAGGGAGTAGCCCGTGGATTGGCCTACCTTTACGATGAGCTGCCGGTGCTAACCCTCCCCCACGGCCACCTCAAGTCCTC
CAACGTCCTCCTCAACGACACCTTCCAACCTCTCCTCACCGACTACGCTTTGGTCCCAGTTATGAACTCAGCCCACGCAACACAGGTCATGGTTGCCTACAAGTCTCCGGAAGTCGCCCA
ATTTGGCCTCCCGTCGAAGAAGAGCGATGTGTGGAGCCTTGGCATACTAATACTCGAGATCCTTACAGGCAAATTTCCGGCGAACTTCCTCCACAAGGGCTCCAGCGGCAGCACCGATCT
TGCCACCTGGGTGGGTTTAGTGATGGAAGAGGAATGGAACAGCGAGGTTTTCGATTGCGACATGCGGGACACTGAAAATGGCCGAGGTGAGATACAGAAGCTACTGAAGATTGGACTT
GACTGCTGCGAGGAGATTGTGGAGAATAGAATGGAGATGAGGGAAGCACTTGAAAGGATTGAAGAGTTGAAGGAGAGGGAGGATTGATTCAAAGAGCAGATCTTGGAATGAACTGGA
GAGCCTGGAAGTGAAGCTGGAAGTGTTTTATGATGCTGTGGGTGGAGGCCAATTCTACGACTTATAAAATCTTTTAATTTTGCATTGTTCTTTCTTCGTCATGGAGTTTTTTTGTGGCATTA
GATACAGTGCAGGTGATAAAAGTAAAGATGCCTTATTTCAACTTGTGCTTTTGCTCTTACATAAAATCTACATACATGATGAATTGTACTGCGTATGCTGCTATAAATAACACATT

>comp155972_c0_seq3
TTTTTGTAATATTCAAGTATTTAAGCAGCTAGACATTTATAACCCATTGCATCTCGCATGCAATGGATTTGAAAAACATGCATTTATATGCTTCATTCGACAAATCTACATTATCATTAGCCATA
AAAAAGACAGACTCTCAGGGAATAGTAGCATATAATGTTTGATTTTCGAATCTGCCCATCATTCCTCAACCAAGCCTATAGTACTTTTAAGTCTTGTTTTCTTAACCTCATGCTCACCTTTCC
AGAGTACGTTGATTCATAATAACTTCTTACAGGGAGAGGATGCTAGGTAACTTAGCTAAGTCTATCTAGGAGATGAGTTCTATACCCTGAAGCAGTAGTGCATCAGCCAAAATTTTGTTGG
CGGCTTCTGAAGGGTGGAAACTGTCCCAGAAAACATAGCCAGTAGCATTGGCACAAGTCCCCGGCGACCTCGCATTGCACAAAAGAGAAGTCTCAATGGTGCCTGTTCCACAACATGCC
TTTCGTGCCTCGAAGAAACCATTGTTCCCAGGGTTCTTGATGAGGTCTAACAAGGGATTGTACACATCAAATATGACAAGTTTGAGATCCGAGTGGCTTTTCTTAAGCGTTTCTGCAGCTG
CGCTCACCTTCTTGTTAAAGGCAACTGCATCATTGTTCAGGCGATCAACACAGTTATTGCTGCCACCACCAAACAAGGTTATTGCGGCGGGCAAGCAACCGATTGGCGGCAGCGATGTCA
CACCAATCCTCCGGGCTCCCAGGCCATACATGTTCTGAACAAACGAACCGAAGGAGAGGACGAGCAAGTTGGCGAACTGGTCAGGAGTGTAGGCTTCATTGATGAGGGGGTTGATGTA
GTAGTTCTGTACGTAATCGCTGCTCCCCGCACTGAGGATGTTGATGGCGCCGGAGAATATTCCGGCAGCTTTTGCTCTTCCACCTACAATATTCTCCACCTTGCTCTGGTACTCCTTATAGTT
CTCCAGCTGCTGGGTTAAGCTTATTGCATGATAAAGGTTTGCAGTCCTGTCCAGGTACCCGGAGGAAGCAGAGGCAAAGTTGGCACCGGTGAGGAGGTTCTTCCCAGTTGCTTCTCTGCT
GAGGTAAGCTGGCGGGTATGTGCTGAAACCCAGGTTTTCCACGGTGAAGTCAGTGGCTAGTTTGCCGTTGCAGAACCTGCCGGTGGGGGCGCCGCGGGTGGTGGTGAAGTCTCGGCCA
TAGGGAGGAAAGTTGGATTTGACGAGAGTGAGCAAGTAGTTATTGTTTCCTGTGTCCACAACCGAGTCGCCGAAGATCATCACGGCAGGCACCAGTCCTTGTCCCTCTGCTAAGCGCAC
CACTAAGCATGCTGCCGCCACCATTAGCAGAAGAGCAGAAGCAGCAGCCATGGCTAGTGGAAGAATGAAGAGTTTAGCTAGTGGTGTTTCATAGAAATATTGTTGCTGGATTTTATGGGA
TGAACTGATGAAGTGGGACTTTATTGTTTATGGAAGTGTGGTGGTTATGAGCATTTAAGAGTGGGAAGGGAAGCCCAGGGGCTGTGGCTTGAATTCGTGCCTTTCTTTATA
>comp155972_c1_seq1
GTTTGTAGTTGTATCACGTTGTTAGTACTTTTTCAAGGTAAACTTGGCTTTCCTATTGGGGGGATTACATAAAAAACACAAAAATCTCACCTATTTACAAAATGACTCATATGGATATGCATG
TCACAATCAACTCATTAAAAATTTGACAAGAGAAAAATATCTGTTGATGTTTTGTTGAGTATTTTAATATTAGGGTGAAATTTGACTGTCATATATATGCCCTCTTTATGATTTTGTTTGCAAA
ATAAGAAACTGAGGCATCAACACAAGTTAGGTAGTGCGCTATATTTATTGAGAATCAGTTGAATTAATTTCATGTCTATTGTCTAGTATCTATCAGTGGTCAAATTATTTACTGCTTCGCGGC
CTATATTTATTGTTGGTGTTGATCATGCTTTAGACACTTTGCCACATTTATAATATGTCAGTCTCACACTGAGTGGATACAGTGCAGTCCATTAACACAAAGGTGTCATTTTATGTCTGTCAA
AGAACAGAGATTGCATGTGGGCGTTGAAATGCCTTTGACACCTTTATGAAACCACTTTAATGGAGCAGAGCTATGAATGAAATATATCTTAGATTTCATGTGGGACGGCTTTCTTACCGTTT
TTTAATATAAATTTTAATTTTTGTACTTAAA
>comp155978_c1_seq1
CAAACAGAAATTGTGATTGTGATTTGAAAAATAAAATAATAAAAAGATTGTCCCAAACCAAACATAAATATAATCAACCCGAGATCTTATATTGACAACTCAACTATTATAACAACACCACC
TAGTAAAATGATTACAAAAGGATTACAAAAAAATTTATAGTTGGTAACTAACTTCTTCAATACAATTTACTCTCTTTGGTCTATAAAATAAAGTAATAAATTTTGCCTAGTAAAACAACAAA
AAAAAAGCTCACAGCAGAGCCATGAACAATAGAAAACAACTTTGTAAACAACCCCGGTTTCAGTTGAATTCCATCACATAGGAGGGAGAGTGTCACTTGCGATGGGACTGCACTGTTGT
GTTGGAGCTCTGCGGTGGAAAATCACCTTCAGTTAGTTGCATAGGTCTCTACATAGCAAGCGATTGTAGAACTTTTTGCGCTTGCAGAGCTTTTTTAATTTGTTGAACTTTTTTTGAAGGCT
GAGCATTTTTAAGTGGTTGATCATTCTGATCTTGCCGGAATGGAAGAATGTTGGGATATTGTTGTCCTTGTAATGACAAAGGGCGAAGATTCTGCAGGTCAGGGTCAGTAATGAAATCATA
TGGGATCTTCGAGTCATCCATCTTCCTAAATGTA
>comp155984_c1_seq3
ATGAAGCCTTAATTGTTTAAATATAAATTAAGATTTATTTATGGTCTATTTCGGACGGCTTCGATGTTTTATAAAGTCCATTGCGAAATTAAAGTAGGCAAAACATAGATATTTTAGTTGCACA
AAATTAGTTTTTTCTATTTTTTTTCCAAAAAAAAAAATTAACAGTAGAGTCTATTATAAGACTTATATAGTAAAAAAAAATTCCAAAAAAAAATAGTAAAACTATTTTGGGCTTTTGACCAAT

TATTCTAAAAAAAAAACCGAGCTATATTTAGATTCATCCCAACCTTTTTTTCTGCCCAAGCTAAAACAATGGGTGCCACATTTCATTACAACTCCCCTTATATTCCAAACAACAAACCCAAA
AACACCCAAAAATCCCACCTTTGAGCCCTTCGCTTCTTCCTCCCTCTCTCTCTCCCCGCACCATGCCCAATGAAGAAGACTTCAACGAATCCATGTCTCTCCACAACCGCCGCCACCCCA
CGCCGGCCGCCCTTGTGTCGGCGGCAAAATCCAAAGGCGGCGCCCTCCTTCTCTCCATCCTCCTCATGTTCATCATGATGCACATAGTCCTGTCCATCTTCTTCCTCTTCGTCGACATTGCC
GTCGTCATAATCCTCCTATGCATCGCCACCGTCACCATTGCCGCCGCCGCTTCTTCCAACAACCAGACATCGAAAGGCGCGACCTTTCGCGAGAAGAGATTCGCTACCTCCTCTATTTCAG
ATCCGCCGAATTGGTCTCCGACGAAATATAAATCTGGCTGTATTGAATGATCTTGAATTCATGAGTTTATGCACATCACAGATCTGCTTGATATGACTATAAGCATGTAAGATTTTAAGATCA
CTTGATCTAATTATTAATATCAAGAAGTTTTTAATTATGTTTGAAATGTTTAATTTGAGCTCGGAGAAGGGTGGGATCACTTGTGCTGTTGATGTGTTTAAGTCTTTAAAATGTTCATTTCGAC
TATAAGTTTATCAAATTCGGC
>comp155986_c0_seq3
ACAAACAAAAATAATTTCATACCAAAACACTAAGTAATAAATCACAACAAATCATGCTGTTAAAAAATTATAATTTTGATCTCAAGCGTACATCATGAAATGATAAAGGAGCAAATCTCTAT
AAGAAATGAAGAATTATTTATAGAATTTCTGAGACGACCACCATTTGATCTGCAACATATCAGACAAATCACTAAGAAGTGATTGATTTCCATCCAGAATCCAGCCCTCCAGTCTCACCGG
CGGCGGCTACTGCTACTCCGGCTGCTCGCGTCGTTCTCGGCGATCTCATCTGCGAGTTGCCTTGCAATACTGAAGCACGACAATGTCCGTGGCCTCCTCGGCTTCCCCCTCGAACTCCCTT
CGTCCTCCTTTACCTCCGCCTCCTTAACCTCCATCGCCGGAATTTCCGACCTCCACTTCTTCAACTTCTTCTCCAATGCCACCCGCTCCTTCTCCCCCACCCCGCCCCCCGACCCGCTCTTA
TCCAGCCCGGCCTCCCTCTGCTCCGGTGGCAAGATCAAAGCTTTGACCTTTTTCGCAGGCCTAGCCATAAGATCGTGGTAGGCGAAAGAAGATGCGCCGAGATCGGCGTAAATGGGGAG
ACTTCGGACATACGCGTCAATGAGAGCAACGCCGATGTTGAGAATACCTTGCGGGCGTAGAGAGGAGGGCCGGCGGATTTGCAGGGCGACGAAGTTGGTGGCAGGGGAGGGGCGGAG
AGTGCTGAGAATAGCGCGAGAAGTGCCGAGCAGAGTGTCGGATCCGGGACGATAGCGTGGGCGGGCGGCGTAGATGTCGACGTGGATGGCGGAGGTGTCAGAGCGGAGAGTGGAGTC
GTCGACGCGGAAGACGAACTTGTCGTTCCAGGTGGGCTCGGTGTGGCCAACGGCGTCGAGGCGCGTGCGGAGGCGCTGGTCGGAGCGGAGCCAAGCGACGGCGTAGGTGCGCATGGA
ACGGGACGAGGGGAAGAGGTCTTGAGCGGAGATGAGAGTGACTTCGAGGAGGTGGAAGTTGAAAGGGAACGGCGGCGGGGAAGGGAAGTACCCGCCGCCGCCCGTTAACATCGCTA
GGGGCGAGGGAGGGCTGGGGAAGAAGGCGGGGAGGGGAAGGAGAGGCGTTGATGGGAAGGCGGAAGGAGGGATGGTGGCGTGGTGCGGGGTTTGTAATAGGGGAGGCGGAGGAGC
TCTCTTATTTTGGTCGGAAACCGTTAGGTTCTAATGCTTGAAATATTTAGTAGGAGCTGTCATTCTCTGAATTTGAGAGAAATGGGGGTTTGGTGTAGGTGTAAATTAATTTAGTTGGTGATA
TTTAAAAAAAAAAATATTAGTAAATTTCAAGATTTAATTTG
>comp155987_c0_seq6
AGTTTCGTCCGGTGACAGCTGAGATGGGGTCAAACATAAAAGCCAATGGATCAGGATGAGCTACAATAATTAAAATTCTATTGACTTTTTAGTTGATAAATAAAAAATCTCAACCAAGAAA
TCAAGATGAGCTGCTAAATTAAATTTCTATTGACTCACCGAACAGATATTATAACGGTCAGCCAAATGGCCGAGAAAGATGGGTCAAAAAAGAAAATCTAATACCAAAAAGAGAGATCGT
TTCTTCCAAGTTTCGATCAAAGCGGCTCTGTGGCATAAACTTTCAAATAAATTAATAATTTTTAAATATGTTCGGTAGAATTTTTAAAAGGAGCATATTACTCTTTTAATTTAATCAAAAGAG
CTTAATGAATATTAAATTCAAGGAACAATTATTTGGTTCGGTGAACGTACGCTCCTCTATGTTCAGCCATCAACCTTTTAGCGCCACGTAGCACGAAGAAAAGTCGCCAACCCGACCCGTT
GTTTTTTGGGTTCTAGCGCATGCGTTTTTTTAATTCCTTGACGAGCCGATCAACATTCCATCAACTTTTGGATATATTTTAAATATCTAGCAAATACCGAGTTG
>comp155993_c1_seq13
AGGAGAAGGGTCTTCCCATTCGCATCTCTCCAAGGACTCGGACGTCTCCAGCTCTTCCCCCATGCTCCCGTCTCTCCCTCTGTTAGGAAAAAACAAGAGAAGCCGACGCTCCTCTCTCTT
GGTCTCTTCCTCAGTTCGCACAAACAAGAGGAGCCGAAACCCCTCCCATTTCCTCCTTCTCTCTGTTCGCGAATCCCAGGCGGAACCAAAGCTCCCTCATCGACTTGCCCTAGCAGCCGG
CTACCACTTGCAAGAGTAAAATATGCTAAATGGAAGGAAGAATGTCGCCAGATGGATACTCATGTTGGTTGTGGTACCATTATCCCAAGCCCCAATAATCACTGATGATGGAGAACCAATC
AAGTATTCTCTTGTTCTAATGCAAGCTGTGCCATCTGCTGACCTTCTTGATGATTTTGGAGCCAGTGAAGGAAGCCATGATGAAACCCAAATACATATAACAGACAAAAAAATTATTCAGT

GCAAACTAACTTCACATCAAATTGGTAGGTATTATTTGTTCAGCTCCAGTTACTGTTTTTGAATTATGTTATTTCTTTCATTAGGCAATTTATGACTTACTACCATAGCAGGCTCTTTTTTTGTC
ACCATTGAAATGCCCTTTTTAAATTAGAAAGTGTTTGGTATCCTTTTTTGAATGTGTAAGCTCCATCTTGTATATTTATTTGGGTTTCATCATCGCTC
>comp155993_c1_seq2
GCACTCCCACTCTCTCCATCTCTCTGTTTCTCTCGCTCGGATCCACTCAGACGGACAAGCCGAAGTCCTCCCTCGCTCCCTTCCCTGTTTCTCTCGCGACCTAGTTGAAGCTGGACCTTCC
CCGTTCGTCTCCAGGCATTCGCACCCTAGAGGCAGAACTCCGCCCCTATCCTCTATCTCGCACGGCAAGCGACGGGACTCCGCCTCCCTTTTCGAGCTTCCTCGTGAGCAGAGCCCGACG
AGCCCCTCTCACGTACGGCTTCATCTCCAGGTCCGAACCATACGACGACTCGAGTTCCTGCCTTGGCCCTCCTCCCTCGAACAGAGTAAAATATGCTAAATGGAAGGAAGAATGTCGCCA
GATGGATACTCATGTTGGTTGTGGTACCATTATCCCAAGCCCCAATAATCACTGATGATGGAGAACCAATCAAGTATTCTCTTGTTCTAATGCAAGCTGTGCCATCTGCTGACCTTCTTGATG
ATTTTGGAGCCAGTGAAGGAAGCCATGATGAAACCCAAATACATATAACAGACAAAAAAATTATTCAGTGCAAACTAACTTCACATCAAATTGGTAGGTATTATTTGTTCAGCTCCAGTTA
CTGTTTTTGAATTATGTTATTTCTTTCATTAGGCAATTTATGACTTACTACCATAGCAGGCTCTTTTTTTGTCACCATTGAAATGCCCTTTTTAAATTAGAAAGTGTTTGGTATCCTTTTTTGAA
TGTGTAAGCTCCATCTTGTATATTTATTTGGGTTTCATCATCGCTC
>comp156008_c1_seq2
AAAAAAAAAAAAGAATTGCCGAATTAATCAAAACCCTTTGATTTCTCTCCACCAATAAATTCTTCAATAATTTCCTTTGTAAGAAAATAACATTTGAGCTACTCAATATAAAGAGCTTCCTA
TCAAAGAACCGTTCAACCTTTCTTCTTCTTCTTCTTCTTCCTTCGATCTTCTTCTGATTTCGGGCCATGGGTTCGGGTCTCGGGCCGGGCCTTCTCTGTTTCGCTCTGTTCGCAATGGTGTC
GGAGGGGTGCAACATGTTCAACGGAAGCTGGATCGTGGATGAGTCGTACCCGCTTTACGATTCGGAAAACTGCCCGTTTATAAGGAAGGAGTTTGATTGTGTGAAGTATGGGAGGCCTG
ATAGGGAGTATTTGAGGTATAGATGGCAGCCGGATGGTGAGTGCGTGCTGCCGGCGTTTGATGGGATGGAGATGATGAGGTTGTGGAGGGGGAAGAAGGTTTTGTTCGTGGGGGATTCAT
TGAGCTTGAATATGTATAATTCGATGCTTTGCTTGTTGTATGCGGCTGCTCCGTATGGGAGGTTGAGCTATGTTGATAATGGAGTCCTATTTGAGGAATTCAATCTCACAGTATCATATTACAT
ATCACATTATCTTGTCGACATTGTTGAGGAGAAGATTGGAAGAGTCCTGAAGCTCAACTCTTTGCAGGCCAGCAAAGAGTGGCTCTCAGCTGATTTGCTCATCTTCAACTCTTGGCATTG
GTGGCCTCGCGCCGGACCGACACAACCGTGGGATTACATTCAAGATGGAGACCTAATAGTGAAGGACATGGACCGGACATTAGCGTTCTCTAAAGCTCTAACTACTTGGGCTAATTGGGT
TAGAGCCACCATTGACCCCTATGCTAAGAAGGTTTTCTATCAAGGAGTCTCTCCTTCCCATTACCATGGTAGTGATTGGGGTAAATCCTTCTCGAAGAGTTGCTCGGGAGAATCAGAGCCG
TTGGATGGATCAAAATACAGATATCAGCAATCACCACAAGAAGCCATAGTGAAAGAAATAATCCCGAGAGAAATAGTCACATTGCTCGACATCACATTTCTCTCAGAGCTGAGAAAGGAT
GCTCATCCTTCCAAATACAGTGGAATCAGAACCAGAAATGACTGTAGCCATTGGTGCCTTGCTGGCTTGCCTGATACTTGGAATCTGCTTTTGTATGCGTTTCTTATACAGTAATTTTATTCT
GATTTTTGCTTTCAGAAGAAAAAAATAAAAGAGAGAGAGAGAGAGAGAGAATAA
>comp156008_c1_seq6
AAAAAAAAAAAAGAATTGCCGAATTAATCAAAACCCTTTGATTTCTCTCCACCAATAAATTCTTCAATAATTTCCTTTGTAAGAAAATAACATTTGAGCTACTCAATATAAAGAGCTTCCTA
TCAAAGAACCGTTCAACCTTTCTTCTTCTTCTTCTTCTTCCTTCGATCTTCTTCTGATTTCGGGCCATGGGTTCGGGTCTCGGGCCGGGCCTTCTCTGTTTCGCTCTGTTCGCAATGGTGTC
GGAGGGGTGCAACATGTTCAACGGAAGCTGGATCGTGGATGAGTCGTACCCGCTTTACGATTCGGAAAACTGCCCGTTTATAAGGAAGGAGTTTGATTGTGTGAAGTATGGGAGGCCTG
ATAGGGAGTATTTGAGGTATAGATGGCAGCCGGATGGTGAGTGCGTGCTGCCGGCGTTTGATGGGATGGAGATGATGAGGTTGTGGAGGGGGAAGAAGGTTTTGTTCGTGGGGGATTCAT
TGAGCTTGAATATGTATAATTCGATGCTTTGCTTGTTGTATGCGGCTGCTCCGTATGGGAGGTTGAGCTATGTTGATAATGGAGTCCTATTTGAGGCCAGCAAAGAGTGGCTCTCAGCTGAT
TTGCTCATCTTCAACTCTTGGCATTGGTGGCCTCGCGCCGGACCGACACAACCGTGGGATTACATTCAAGATGGAGACCTAATAGTGAAGGACATGGACCGGACATTAGCGTTCTCTAAA
GCTCTAACTACTTGGGCTAATTGGGTTAGAGCCACCATTGACCCCTATGCTAAGAAGGTTTTCTATCAAGGAGTCTCTCCTTCCCATTACCATGGTAGTGATTGGGGTAAATCCTTCTCGAA
GAGTTGCTCGGGAGAATCAGAGCCGTTGGATGGATCAAAATACAGATATCAGCAATCACCACAAGAAGCCATAGTGAAAGAAATAATCCCGAGAGAAATAGTCACATTGCTCGACATCA

CATTTCTCTCAGAGCTGAGAAAGGATGCTCATCCTTCCAAATACAGTGGAATCAGAACCAGAAATGACTGTAGCCATTGGTGCCTTGCTGGCTTGCCTGATACTTGGAATCTGCTTTTGTA
TGCGTTTCTTATACAGTAATTTTATTCTGATTTTTGCTTTCAGAAGAAAAAAATAAAAGAGAGAGAGAGAGAGAGAATAA
>comp156046_c0_seq2
CCTCGCCGCTTGGTTGTCGCCACCAAAATTCGAAAGGTACCGTCTGAACCATTTATTTCCAGGGGCATTTACCCTTTATACGGGGGTTTACCCTTAATATCCCGCACCCTCCTTTACTCTCC
CACCTTCCGTCTCGCCATGGCCGCCGAAGATACCTTCTCCTCGACGTCGGCTGCATTATGTTGTATCTCTCTCCCTCCTCCCCCCTCCCTGCGCCGCCTATGGAGCACCTTTTCGGAGCCGA
CTCGTCCGATCGTTTCAACCCGCCGGGGCCAACCTTTTCTGAGCCAACCCGCCTATGGAGCACCTTGGGCTACGCTTCAGGGTGGCCTTGTCGGCGAAGCGGAGGCCACTTCGTCACAA
ATCGTTGGGAAAGGTCTGGACCCCGCTGAAGCAGTTGCTTGGGAGCTTTTCACGCCGCTTCACCGCGTACTCCTCGTCGCCATCATTGCCATTGCGTCTGCGGAGTCAAGGCGGTCCCAG
AGAATCTCGCAGCTTCAGCGAAATGTGGAAATTAGGGATGAGGCGCTCAAAAACATCCAAGCAAAACTTGATAATTTGTGCGAGCAAATAAAGTTTATCAAAGACCAGTTTCCTACACCA
GTCTGCCGCTTTCTGCAGGATACTGAGCACTTTATAATTGGAGAAATGACAGAGGCAAGTGAAGATAAATTCTCTCATCTTGGTACTCGAGCATTGCACCTCAATCCTGATAGTGGTATTGT
CCCTCTTTGTAGTAGCCAATCAATTAAAACAAAGGATGTTTACATGAATGAGATGAGCAAAGGAAGATTGTCCGTGAACAATCACGAAATCGCATCGGAGCTAGAGGAGCGCAGAATGTC
CGATCTGTCAGATTTTTGTTGCAGTGTTGCGTCCTCTGCAGATTTTCAGCCTGTAGAGCTGAGCACATTAGCAGCAGAGCAGGAGCTTTACAACCTTCGAAATGAATGTGAAGAGAAGGA
TGCCAACATCAAGGAACTTACTGCTACTGCCCATGCTTCCAGTGTTGCTTACTCTAAGAGGATTGCAGAACTGGAAGAGGTCATAAGAAGGAAAAACATGATAACCACAAAGCTGAGGA
AGGATATGTTATTTTTAGAGCAACAGATTAGCAAATTATTAAGGCTTCGAAGACAATCCTCGCCTTCTTCTTCTTCTTCTGTCATTTCAACCACCAAACAACTTCCACGCATGACGGAAAAT
ATTCTCTATGACATGAGCAGCACCAGCTCCTCTTCTTCGGACCGTGATTCTCCTGACAGGGATAGAGTGAATCCGGTTCTTCTGCGAAGTATGGAACAGAGCAGGCAAGTCTCAGCAATG
CCTCTGAGTTCTTTTGTCTCAAAAGAACAGCGGCCAATCAGTCCTCTCAAGGAAAATCGTATGAGCAGGAAAGTTGAAGCAAATACTACAGTGAGGCAAGTCAATTTGAAGCAAATCCT
GTAGTGAGGCAAAGGAAATTAGTTTCTCCAAGTATGGATAATAAGAAGTCTCGGAGAAAAAGCTTGAAGGAAGATAAGAACATGAAGACTTCTCAGAAGAAAACAGATTGAGGTGCGG
TTTGATCCAATTTTTTCTGGATGGTAATATTATTGTTATTTTGATGATTTCAGATTTTGGTAGCAATTTTATTTATTAAATATATCATGGTTGGTTCAATGAGATGTAGCTTAAAGAATTAATGAT
TTTTTTTAATTTATTAATATGATGATACCCTTTTCCCATGCCAGTGAATTTGGCAGAGGATTTTTGATGTATTGACATTTG
>comp156046_c0_seq4
ACTTTTCAATGTCAATCTCCGCAAATGTCACTACATCAAAAATCCTCTGCCAAATTCACTGGCATGGGAAAAGGGTATCATCATATTAATAAATTAAAAAAAAATCATTAATTCTTTAAGCTA
CATCTCATTGAACCAACCATGATATATTTAATAAATAAAATTGCTACCAAAATCTGAAATCATCAAAATAACAATAATATTACAATCCAGAAAAAATTGGATCAAACCGCACCTCAATCTGTT
TTCTTCTGAGAAGTCTTCATGTTCTTATCTTCCTTCAAGCTTTTTCTCCGAGACTTCTTATTATCCATACTTGGAGAAACTAATTTCCTTTGCCTCACTACAGGATTTGCTTCAAATTGACTTT
GCCTCACTGTAGTATTTGCTTCAACTTTCCTGCTCATACGATTTTCCTTGAGAGGACTGATTGGCCGCTGTTCTTTTGAGACAAAAGAACTCAGAGGCATTGCTGAGACTTGCCTGCTCTG
TTCCATACTTCGCAGAAGAACCGGATTCACTCTATCCCTGTCAGGAGAATCACGGTCCGAAGAAGAGGAGCTGGTGCTGCTCATGTCATAGAGAATATTTTCCGTCATGCGTGGAAGTTG
TTTGGTGGTTGAAATGACAGAAGAAGAAGAAGAAGGCGAGGATTGTCTTCGAAGCCTTAATAATTTGCTAATCTGTTGCTCTAAAAATAACATATCCTTCCTCAGCTTTGTGGTTATCATG
TTTTTCCTTCTTATGACCTCTTCCAGTTCTGCAATCCTCTTAGAGTAAGCAACACTGGAAGCATGGGCAGTAGCAGTAAGTTCCTTGATGTTGGCATCCTTCTCTTCACATTCATTTCGAAG
GTTGTAAAGCTCCTGCTCTGCTGCTAATGTGCTCAGCTCTACAGGCTGGAAATCTGCAGAGGACGCAACACTGCAACAAAAATCTGACAGATCGGACATTCTGCGCTCCTCTAGCTCCGA
TGCGATTTCGTGATTGTTCACGGACAATCTTCCTTTGCTCATCTCATTCATGTAAACATCCTTTGTTTTAATTGATTGGCTACTACAAAGAGGGACAATACCACTATCAGGATTGAGGTGCAA
TGCTCGAGTACCAAGATGAGAGAATTTATCTTCACTTGCCTCTGTCATTTCTCCAATTATAAAGTGCTCAGTATCCTGCAGAAAGCGGCAGACTGGTGTAGGAAACTGGTCTTTGATAAAC
TTTATTTGCTCGCACAAATTATCAAGTTTTGCTTGGATGTTTTTGAGCGCCTCATCCCTAATTTCCACATTTCGCTGAAGCTGCGAGATTCTCTGGGACCGCCTTGACTCCGCAGACGCAAT
GGCAATGATGGAGACGAGGAGTACGCGGTGAAGCGGCGTGAAAAGCTCCCAAGCAACTGCTTCAGCGGGGTCCAGATCTTTCCCAACAATTTGTGACGAAGTGGCCTCCGCTGAAGCA

GTTGCTTGGGAGCTTTTCACGCCGCTTCACCGCGTACTCCTCGTCGCCATCATTGCCATTGCGTCTGCGGAGTCAAGGCGGTCCCAGAGAATCTCGCAGCTTCAGCGAAATGTGGAAATT
AGGGATGAGGCGCTCAAAAACATCCAAGCAAAACTTGATAATTTGTGCGAGCAAATAAAGTTTATCAAAGACCAGTTTCCTACACCAGTCTGCCGCTTTCTGCAGGATACTGAGCACTTT
ATAATTGGAGAAATGACAGAGGCAAGTGAAGATAAATTCTCTCATCTTGGTACTCGAGCATTGCACCTCAATCCTGATAGTGGTATTGTCCCTCTTTGTAGTAGCCAATCAATTAAAACAA
AGGATGTTTACATGAATGAGATGAGCAAAGGAAGATTGTCCGTGAACAATCACGAAATCGCATCGGAGCTAGAGGAGCGCAGAATGTCCGATCTGTCAGATTTTTGTTGCAGTGTTGCGT
CCTCTGCAGATTTTCAGCCTGTAGAGCTGAGCACATTAGCAGCAGAGCAGGAGCTTTACAACCTTCGAAATGAATGTGAAGAGAAGGATGCCAACATCAAGGAACTTACTGCTACTGCC
CATGCTTCCAGTGTTGCTTACTCTAAGAGGATTGCAGAACTGGAAGAGGTCATAAGAAGGAAAAACATGATAACCACAAAGCTGAGGAAGGATATGTTATTTTTAGAGCAACAGATTAGC
AAATTATTAAGGCTTCGAAGACAATCCTCGCCTTCTTCTTCTTCTTCTGTCATTTCAACCACCAAACAACTTCCACGCATGACGGAAAATATTCTCTATGACATGAGCAGCACCAGCTCCTC
TTCTTCGGACCGTGATTCTCCTGACAGGGATAGAGTGAATCCGGTTCTTCTGCGAAGTATGGAACAGAGCAGGCAAGTCTCAGCAATGCCTCTGAGTTCTTTTGTCTCAAAAGAACAGCG
GCCAATCAGTCCTCTCAAGGAAAATCGTATGAGCAGGAAAGTTGAAGCAAATACTACAGTGAGGCAAGTCAATTTGAAGCAAATCCTGTAGTGAGGCAAAGGAAATTAGTTTCTCCAAG
TATGGATAATAAGAAGTCTCGGAGAAAAAGCTTGAAGGAAGATAAGAACATGAAGACTTCTCAGAAGAAAACAGATTGAGGTGCGGTTTGATCCAATTTTTTCTGGATGGTAATATTATT
GTTATTTTGATGATTTCAGATTTTGGTAGCAATTTTATTTATTAAATATATCATGGTTGGTTCAATGAGATGTAGCTTAAAGAATTAATGATTTTTTTTAATTTATTAATATGATGATACCCTTTTC
CCATGCCAGTGAATTTGGCAGAGGATTTTTGATGTATTGACATTTG
>comp156059_c0_seq1
TAAACCCAAAAAAAAAAAAATCATAACAGCATAGCACATTACAAATATTTCAAAACGAAGAAAAAAAGAAAAACTTATTTTGCTTTTTGCAAATTCCAGAAAAAACTAAAACAAGAATT
CCATTGATTTTTTTCATCCAACAAAAAGTGTTATTTATTGCATGTTTTCCCCACCAAAAAATAATCAAAAAAGCTACCCGAAACAAACTTTATTATTTAAAGAAAACTAAATTTTGTAGTAA
CGGCCATCCTATTCAAATCTGAGATTCAAAAGGAGGCTAATACTCGAAATCTGGGACAGGAATTTACAATTCTTTTCAATGAGAATCTATATATAAAAGAAGAAAAGGAATGCTAACTTGC
GATATGCGGTGAGGAGAGTTTTTCTGGAGAAGAATGAGCTGAGCCTCCTCTGTCTTCTCCATGGCTCTGATCATTTATTCTACTAGTTGGATTTACATCACCAGGCCTCTCATCATTTAGTCT
CTCTGCTGTATCCATACGAACGGAGAGATTAGAAGATTGCTTGCTGCATTTTATCCAACTTCCTGCTGTGTGTTTTTTATCACCTCTGTCATTATTGTAACCAACCCTGTGATTTCTTTTGCTT
GAATGTCGATCTCTAAAGCAAGCTCTAGGCTTTGTGGGGTTGGTTCCAGTACCAGTCCAATAACTTGGTGGTCCATTTCCATATCTATCACAAACCTTAGATGACCTGATAGGAACTGGCT
GCAGAGGAGTAACTGGAACCAAGTGAGGGCCATCATAGCCCATGACCTGAGTTAAAAAGTTGATGTTAGGTCTTCTAACAAGACCATCCAATGGATAACGATCCTGTAAATATACAGGAG
GAATCATAACAGGAGGAGAATAGACAAACGGTACATGATTTGAGGTGTTCTGACAGACCCTTCCATATTGTAAATTTTGCCAATGGCTATCAAAATCACTATTTAACATATCCTTTACGTGC
ACTTCAGTACACTCCAGAGCTGCAGCACTTGTGGTACTTGGAGATCTGTAATTTTGTTCTCCATCAACAAGGTTTATTGAACCATCAGAATTTGCCCCATCGGCTGGAAAATTGTACACAG
GAAACATGGCTACTGGACCTGCAGGATAAAAGGACAGTGGAGCTACATCTGAATTATCCACCGCATTTTGCAACGAACTCCCAACAATCGCGGGTTCTAATGGAGTTGATGTATCTGCTG
CACTTATTTGTGTGGTTAAGTGCTGCATTTCATCAGAGTGAATATGCAAACCTTCGTTTTCAGTTTCAGAACCATCGATGGACCCAGCATCTTGATCATACCATGCGTCATTCTCTCCATCCT
CGGGGCTAGTCTGGCCAGAATTCTCATTGACAGGATCTGCTTCTACTGAGTTTGAGTTTTGGGTAGCATTAGTAGAATAATAGGGCAGCTCTGATGAACCGAAACCTCCAGAAAACTGCAT
AGAATATGCCCAGGAAGGGCCAATCAACGTAACAATGGATGGAACAATTCCAGTCAAATTTCTCTGAGCATAACCCATTGAAGCTAGGCCAGGAGACGAAGGAAGAGGTAAATGACAAG
CGAAATACATGGTTGGACCAGGCCAATTGAATTATGATTCACTGAAGATTCCATTAGATTAGCCAGATCCTGCGGTTCCTGTTGTTGTAACTTCAATGTCTCAGTATCAGAACCAAGCCCTG
CACTCATGGCTGTAAAACTAAAATCATCTTGATAACCATTTGAGATTATGCTTGAATCAGTTGGACCTTTAAGGCTATGATGAGATGAGCTTTGCCACAGAAGCTTAGAAGCATAACCACCT
GAAGCACGTCCCCCTCCAACCTGACTCTTGGGACTAGTGATGCCTCTAGAATTATTAGGACCCTCATTTGGGCAATTGATTGAAGGATGCAAGGAGCAAATACTAGAGATAAGGACATCA
GGCCGATTGCTGCCACTTCCATGCCTTTCCCAGGTGTTCCTAAAAAACCAATTGACTTCTGCAATCAGATTCTCGTTTGGACAATCAAGTAAATTGGCCAGCTTTTTGGCTCCAAATGCAA
ATGCATAGCGTATCCTGTAGAAGTTTCCTTTATTGACACTTCGCCCAAGATTATTGCTTGTACGTAAAGGATCAATAACATTGAAATGCTTGGATGCAAATCTTCGACCTTGATTTTCTTGAC

CACCAAGAGAAACAGCATAAATTGTACTACAAGCCTCAAGAAAGGCTTCATCAAATAACAACTTACCACCATCATTTCGAGGAAGCTCCGCTGTTATATCTGGAATTGAACTGATTGGAA
CTGGGCCCCAAAGGCTGACACAGAAGTTATTCCAATCAAAATTACTGAAAAATTGCAGGAACCGATACAATACCTAATAATACAGCGCAGTCCAGGTCAATGTGCAATATGTTTCAAGTA
ACAAAAGATAATATAAAGAAAAAACATTACCTCAAATGGTCCAGCAAAGGTATTATTGAAAATATGAAATATGTAAAGAACAAGGATCTCTAAAGCATATGTCGATATAAGCCCATGAGGA
GCACCAAGTATCCGGCTCTCATAGTAACTCCAGGCTTTGATCAATATTATGCTCCTCTTCAAAAGATGGTTCTGGTTTATAAGATGATCAACCTCTTCAAGAAAACATAGAGTGCACAACCC
ACCAACCTGACCGAAAGATATATCTACAATTATATTTTCAACAAGACACTTTATTACTTTTACTTCTGCATGAATGTACTTCACTTCCTTCACACGAAATTCAGCATCCTCAAGCTCTTTTTC
ATTCTCTAGCACATTGAGAACCGCATTGGCCCAAGTATTTTTCGTGTATTCACTGTTACTAAAAATAGTCAAGTCAATGTCTCCATCCGGTAAGTAGGTCTTGAGGGGTACTGAACCAAACA
TGAAAACCTCACAAGAGAAGCACTTCTTGATAAGCGTCCGGACATAGTTGGCCACGGCATTCCGGAGCTCCTCAGATGGCCGGTTGGGCTGAATACAATCTATTAGTTCAGCAGTTCTCT
CCTCAACCTTGGACCATCTCTCTGCATCAAGCAGCCTAATCACATTGTCCGTTTCATTGGGCAGAAGGCCATTTAGCAGCAGCTCACCTGGATGTGACAAGGCTCCAAGGTCGCCCATATA
AGGCCACTAAAGATTACTAAAAGCAGTGGCTTTAATGGCGATACTGTTGCCGTTTTAATGACGAAGAAGTAGAAGAGGATAAAAGGACCACGAGCACTAGCCAAAGCTGCAATGGCGAT
ACTGCAGATGGACGAGCAACCAAAGGAAGTCGTGGTGTATGCCAAATTCTAACAGCTTGCCCCAAAGCGAAACAGAGGAGGAAGATACCAAAAGCAAACCCACACAATCAGAACCAA
ATTCCAAACACACCGAGGCGATCAAAAGTCCCCGTCTTCCCACCGCGCATACTATACGCCCGGAACCAATCCTCCCAATGGCGGAATTCCTCCCATAAACTCACACACAGGCAAGCAGA
GTCCCTCGAACAACTAGTTACAACGAACAAAACCCTACATAAAAGAAGAACCGCGCCTTTTCAATGGGGCTGCGAATTTGCCGGCAAATTGAAACTT
>comp156059_c0_seq3
TAAACCCAAAAAAAAAAAAATCATAACAGCATAGCACATTACAAATATTTCAAAACGAAGAAAAAAAGAAAAACTTATTTTGCTTTTTGCAAATTCCAGAAAAAACTAAAACAAGAATT
CCATTGATTTTTTTCATCCAACAAAAAGTGTTATTTATTGCATGTTTTCCCCACCAAAAAATAATCAAAAAAGCTACCCGAAACAAACTTTATTATTTAAAGAAAACTAAATTTTGTAGTAA
CGGCCATCCTATTCAAATCTGAGATTCAAAAGGAGGCTAATACTCGAAATCTGGGACAGGAATTTACAATTCTTTTCAATGAGAATCTATATATAAAAGAAGAAAAGGAATGCTAACTTGC
GATATGCGGTGAGGAGAGTTTTTCTGGAGAAGAATGAGCTGAGCCTCCTCTGTCTTCTCCATGGCTCTGATCATTTATTCTACTAGTTGGATTTACATCACCAGGCCTCTCATCATTTAGTCT
CTCTGCTGTATCCATACGAACGGAGAGATTAGAAGATTGCTTGCTGCATTTTATCCAACTTCCTGCTGTGTGTTTTTTATCACCTCTGTCATTATTGTAACCAACCCTGTGATTTCTTTTGCTT
GAATGTCGATCTCTAAAGCAAGCTCTAGGCTTTGTGGGGTTGGTTCCAGTACCAGTCCAATAACTTGGTGGTCCATTTCCATATCTATCACAAACCTTAGATGACCTGATAGGAACTGGCT
GCAGAGGAGTAACTGGAACCAAGTGAGGGCCATCATAGCCCATGACCTGAGTTAAAAAGTTGATGTTAGGTCTTCTAACAAGACCATCCAATGGATAACGATCCTGTAAATATACAGGAG
GAATCATAACAGGAGGAGAATAGACAAACGGTACATGATTTGAGGTGTTCTGACAGACCCTTCCATATTGTAAATTTTGCCAATGGCTATCAAAATCACTATTTAACATATCCTTTACGTGC
ACTTCAGTACACTCCAGAGCTGCAGCACTTGTGGTACTTGGAGATCTGTAATTTTGTTCTCCATCAACAAGGTTTATTGAACCATCAGAATTTGCCCCATCGGCTGGAAAATTGTACACAG
GAAACATGGCTACTGGACCTGCAGGATAAAAGGACAGTGGAGCTACATCTGAATTATCCACCGCATTTTGCAACGAACTCCCAACAATCGCGGGTTCTAATGGAGTTGATGTATCTGCTG
CACTTATTTGTGTGGTTAAGTGCTGCATTTCATCAGAGTGAATATGCAAACCTTCGTTTTCAGTTTCAGAACCATCGATGGACCCAGCATCTTGATCATACCATGCGTCATTCTCTCCATCCT
CGGGGCTAGTCTGGCCAGAATTCTCATTGACAGGATCTGCTTCTACTGAGTTTGAGTTTTGGGTAGCATTAGTAGAATAATAGGGCAGCTCTGATGAACCGAAACCTCCAGAAAACTGCAT
AGAATATGCCCAGGAAGGGCCAATCAACGTAACAATGGATGGAACAATTCCAGTCAAATTTCTCTGAGCATAACCCATTGAAGCTAGGCCAGGAGACGAAGGAAGAGGTAAATGACAAG
CGAAATACATGGTTGGACCAGGCCAATTGAATTATGATTCACTGAAGATTCCATTAGATTAGCCAGATCCTGCGGTTCCTGTTGTTGTAACTTCAATGTCTCAGTATCAGAACCAAGCCCTG
CACTCATGGCTGTAAAACTAAAATCATCTTGATAACCATTTGAGATTATGCTTGAATCAGTTGGACCTTTAAGGCTATGATGAGATGAGCTTTGCCACAGAAGCTTAGAAGCATAACCACCT
GAAGCACGTCCCCCTCCAACCTGACTCTTGGGACTAGTGATGCCTCTAGAATTATTAGGACCCTCATTTGGGCAATTGATTGAAGGATGCAAGGAGCAAATACTAGAGATAAGGACATCA
GGCCGATTGCTGCCACTTCCATGCCTTTCCCAGGTGTTCCTAAAAAACCAATTGACTTCTGCAATCAGATTCTCGTTTGGACAATCAAGTAAATTGGCCAGCTTTTTGGCTCCAAATGCAA
ATGCATAGCGTATCCTGTAGAAGTTTCCTTTATTGACACTTCGCCCAAGATTATTGCTTGTACGTAAAGGATCAATAACATTGAAATGCTTGGATGCAAATCTTCGACCTTGATTTTCTTGAC

CACCAAGAGAAACAGCATAAATTGTACTACAAGCCTCAAGAAAGGCTTCATCAAATAACAACTTACCACCATCATTTCGAGGAAGCTCCGCTGTTATATCTGGAATTGAACTGATTGGAA
CTGGGCCCCAAAGGCTGACACAGAAGTTATTCCAATCAAAATTACTGAAAAATTGCAGGAACCGATACAATACCTCAAATGGTCCAGCAAAGGTATTATTGAAAATATGAAATATGTAAA
GAACAAGGATCTCTAAAGCATATGTCGATATAAGCCCATGAGGAGCACCAAGTATCCGGCTCTCATAGTAACTCCAGGCTTTGATCAATATTATGCTCCTCTTCAAAAGATGGTTCTGGTTT
ATAAGATGATCAACCTCTTCAAGAAAACATAGAGTGCACAACCCACCAACCTGACCGAAAGATATATCTACAATTATATTTTCAACAAGACACTTTATTACTTTTACTTCTGCATGAATGTA
CTTCACTTCCTTCACACGAAATTCAGCATCCTCAAGCTCTTTTTCATTCTCTAGCACATTGAGAACCGCATTGGCCCAAGTATTTTTCGTGTATTCACTGTTACTAAAAATAGTCAAGTCAAT
GTCTCCATCCGGTAAGTAGGTCTTGAGGGGTACTGAACCAAACATGAAAACCTCACAAGAGAAGCACTTCTTGATAAGCGTCCGGACATAGTTGGCCACGGCATTCCGGAGCTCCTCAG
ATGGCCGGTTGGGCTGAATACAATCTATTAGTTCAGCAGTTCTCTCCTCAACCTTGGACCATCTCTCTGCATCAAGCAGCCTAATCACATTGTCCGTTTCATTGGGCAGAAGGCCATTTAGC
AGCAGCTCACCTGGATGTGACAAGGCTCCAAGGTCGCCCATATAAGGCCACTAAAGATTACTAAAAGCAGTGGCTTTAATGGCGATACTGTTGCCGTTTTAATGACGAAGAAGTAGAAGA
GGATAAAAGGACCACGAGCACTAGCCAAAGCTGCAATGGCGATACTGCAGATGGACGAGCAACCAAAGGAAGTCGTGGTGTATGCCAAATTCTAACAGCTTGCCCCAAAGCGAAACAG
AGGAGGAAGATACCAAAAGCAAACCCACACAATCAGAACCAAATTCCAAACACACCGAGGCGATCAAAAGTCCCCGTCTTCCCACCGCGCATACTATACGCCCGGAACCAATCCTCCCA
ATGGCGGAATTCCTCCCATAAACTCACACACAGGCAAGCAGAGTCCCTCGAACAACTAGTTACAACGAACAAAACCCTACATAAAAGAAGAACCGCGCCTTTTCAATGGGGCTGCGAAT
TTGCCGGCAAATTGAAACTT
>comp156063_c0_seq1
GCCTCAAATTTTTTTATGAACAGAAAAAAAGGAAGAAATTCCATAGGGTATCAAAATGAGGTATCACAGTTAAATCAGAGTACAAAAACAAGGAAAAAACTTCAAATCCTCTGTAATTG
TGATGGTCCTTATCACTAATTTCTACCTATAAAAAAATATAAAAATAAGCAATGAATGAATTTGAAGAAGCAAGAGGGAAAAGATCGAGTGCATCAGGAAGAAGGTTATGTGTTTAGGAG
AGAGAATTTGTTTATGCAATCTCCTTTAGGTCTCTATCCAAGAAAGAATCCAAAGTAACCTTCGCATCAGATTTCTCATCATCTGCTGTAGTTTTAGTTTTCTGGGGGAATGATGCCTGCTTT
AAGAGTTGTTTTGTTGCTGCTGCTGAGTCCTGAGAAGCTCTCAAATTAAGATATGTGTAGAAAGACATTCCAGAGAGAGCAACAATGGCGCCGCACAGGCTTACAATTCCTGGATCAGAA
TTGAAGAAGAGGTATCCACCAAGCATAATCACACAAGTCTTGAACTGGCCTAACACAACATGAGAGGTCGCAGATGTTGCACCTAGAGCAAGAGCCCCAGACCATTGAAGGAGAAACC
CAAAAACAGCAGATATTAAGATAGCCGTTAAGTTGTTGGAATCCCAATTGAAAGAGAGCAACCCAGGCGGGTCTAGCAATGGCATCAACGCCAAGAAGAAGAAGATAGTTATTGGAGTA
GTTTTCCACATCAACGAAAGAGCCGTCCAGTTGCCAGTTTGCTGCATGTTAGACCATAGAATTTTATTAACAGCACTTGGGACAATCCAGGCCAATGATATGCTAGCACCAAACAAATTGA
ACTCTAGATCAGTGACAGTTGCAACAGCCACACCTATAGAAACCACACCAAGTGCAAGAACCTTGTAGAGAGTAACCTTTTTACTGAAAAAAAGGAATTCAGCAAGAGCAATTGTTGGT
GTAACGGATATCTTGGCCATCTGATAGAACCCAACACTGTTGTGCTTCAAACTTATATTAGAAAGTCCTGTTGAAAACGACATAACAGCACCCAAGAGAAATAAAGAGGAGAATGGTGTG
GATTTTGAAGGAGGGGATACAGGAAGCAATGAGAATAATCTAAAGACTGCCATCAGAAACCATGCAGTCACATAGTGTATCAAAGATAGTGCGACCGGGAAATTGAAACCGATTTTGCCC
ATCACCAATTTATTTGCCATGATTATTCCAACAGAGATCATGAAATTAAAAGACATTGCCACAAAAGGACCACAATATGTCTGTTGTTTCCGTCTTGCTCCCTCTGAAGTATTGAAATCATTA
TACAAAGAGCTGCGAAATTCTTCAAGCGAAAGACCTAGTTCCCCTGCATCGCTATCCTTCCTTTTGATGAACCTCCTCGCGTTCTTCCCCAAAACAGAATCAAGCAGACTCATTTTGCCGA
AATCCAGGATATATCAGAGCATAGCTTTGAGCCTTTGAATCAAAGGAATCAAGACCAACCTTTCCTTAATCGTCCAATCACTTTATACGTTTTTTAAGCTACGATTTCCAAGGAAGAAAGA
GAAATGGAGATATGCGAATAGAGAGCGACAGAGAGAAATCAGGGGCAGTCCTGCAGAATGAACGAAGACGGGGAGGAGCTGCGTTGT
>comp156071_c0_seq9
TCAACATTTCAAACAATACTCAATATGAAGGAATGAAAAATGACAATTCATAGAACAAACACAAATAGTTAGGATAATGATGATGATTTTGGGAAAAACGAATAATAAGATCTACAATAAA
CAAAAGTTCAAATAATTTTGTCCACACTGCGAAGATGCAGAGTTAGATCCTCCAAAGCTGCTGAGAAGAAAACTCATTCCTTCACTTTGCCAAACTTACTTCTATCCAGGACAATGCAAT
CATTGTGATAGATGTCATAACGACTAAGCCAACCTCCAGTGCCATGCCCACCAAACACCAAGAGATAGTGTCCCCCAGAAGTTATTGTGTGGCCACAGCGAGGAGAGGGTGATTGCCCA

GGAAGCTTTAACTGAGTCCAACCTGGTGTCTCATTCTCTTCCACAAGAAAGGTATAAGTATCTCATCAGAATCATAAAA
>comp156074_c0_seq6
GAAGTTAAACTGGGACTAAGTCAAAGAGGACCTAGTTTTGATTGAAATCTTCTATGTAGGTTTTGATAGGGTTTCAAGGTTAATAAGCATCATGCCCCATGCTGGCTTTCTCCTAACAGTTG
ATGCAGGACACAACAAAGCAAAAGTTGTAAATCATACTCATTATTTTATTATAATTCTAGTCATCGAAAAAGACATCAAATTTAAAACATCTTAAACACAAACTAATACCATAAGCCAAAA
AAAAAAACTATCAAAATTTGTATTTCCCAAATAATTTACAGAGTCAAAAAAACAAAAAAAAAAAAACTATAAACGGCACCACACACATTATATCGGCAACATTGAAGGTGGATGAGACAT
TGTAATCTCCTGGAAGATTAATTTGATAGGCATTGTAATTATTTTTAATGACCATGAAAGGCTGATATTTATGTTTAAGTTTCCCAAAAGAACCATGAGGAAATCGATCTTTCCTTAGCTTGA
TCCACACGAGGTCACCTTCTTTGTAAGTTATATAGCTATTTGGGACTTATTGGCGTGGGTTTGACACTTGGTATTCTGTTTCTGGATCAGTTCTTGGACTTGTTGGTGCAGTTTCTGAATTTC
AAAACATCGTTGTGCTCCTTCAGGACTGAAATTGGTGGCCGGCACTTTTGGAATAAGATCAAGTAGTGTTTTAGGATTACTGCCGTATACAATTTCAGAAGGACTTTTAGTTGTAGCTTGA
GTGGTGGATCTATTGAAGGTGAATTCGACTTGAGCCAAAATTTGATCTCATTGTCGAATGTTTTTGCCTATGAAACAACATAGAAGATTAACCAAACTTTGATTGATAGTCTCCATTTGTCC
GTCCGTTTGTGGATGGACGGTGGAACTAAACTAAACTGAAGTTTAGTCTCTAGCTTACCCCAAAGAGTCTTCCAAAAAATGACTCAAAAATTTTACAGCAAGATTTGAAGTATTCATCTTG
GAGATTGCGTACAAGCAAACGATCTCTTTAAAATATAAATCGGCCACATGGGCATCATCAAGAGTCTTATTG
>comp156101_c0_seq1
CTCCCTCCGTTCCTTTCAATGGCTCAAATGCCGCTCTTTTCCCTCGTCGTTCTCACAGACATTTCGGCTGGTGAGTGGTTCAGCTGTTATCGATCTCTAATTTTAGAACCGGAATCTCTCGC
TCTCTCTCTCCCACCTTTCTTTTCTCCGGCTTTTAACTAATGGTCGGCTCGATTTGCACCACAGAGGATTTCATCTGTGGCGGCAATTTTTCTGTTATGCTGGATCGATATATATCTCTTTCTC
TCTGCTTGTAGATGTTGATTTTCTCGTAAAAATTAGTCGTCCAGATTGCGGGTTTCCGTAGTTCAGATTTTTTTGAAGAAAGTTAGCGGTTTGAGCTTTCCAATTCCAATATTCTGCTGAATA
CGAATTGATGCGTAGTGTCCTTATGATGCATTTGTGGTAGGAAACTGACTTCCCAGAAAATAAAGGTGGAAACTTTTTCTCCTTCAAATGCAACGCTCATTCAACTGTGATGGGTTTTTTG
TTCTGTAACTAGATAGTTTTGGCAGAATGGATTCAAGGAAGGGAGAGGATAGTAATAACGTTGGAGTAGTTGGTGCTGAGGCTATGGAAAGATCAATGTCTCAGTTGAAAGGACATGGGA
GCTTCAAAAATAGGGATATTTTATTTAGGGCTGATCAGATTGATCTCAAAAGTCTAGATATTCAGTTGGAGAAAAAACTTTCCAAGATTTGGCTGAATGATGTGCTTGGCTCTAGAAGATCC
AAGAAGGAATGGGAAATTGATCTTTCTAAGCTTGAAATAAGGTATCTTGTAGCTCAGGGGACACATGGTACTGTGTTTCGAGGCATTTATGATGGTCAAGATGTTGCAGTGAAACTATTGG
ACTGGGGAGCAGATGAACATGCAACAGATACTGTTACTGCTGCTCTTCGATCATCATTTCAACAAGAAGTTGCTATGTGGCACAAACTTGACAACCTACATGTGACAAAGTTTGTTGGAG
CATCGATGGGAACCGCTGACCTGAAAATCCCAAGCAGTAACTCACTTCCAGCACAAGCATGCTGTGTTGTTGTGGAATACGTTCCTGGAGGCACATTGAAACAATATCTTATCAGACACA
GTCGAAGGAAGCTGCCTTTCAAGGTTGTGATTCAGCTCGCATTAGATCTTGCTCATGGCCTGAGTTATCTTCACTCAAAAAAGATCGTCCATCGAGATGTGAAAACTGAAAATATGTTGCT
GACTGCCAATAGAACTGTAAAGATTGCTGACTTCGGTGTTGCTCGTGTTGAAGCTCAGAATCCCAAGGATATGACAGGTGAAACTGGGACTCTTCAATATATGGCTCCAGAGGTGATCTTT
CATAAAATTGTTTTATTGTTCAAGCTGAAATATGTTATTTTGGCTTGAAAATGGAAGTTTTCTGAGTGATTTTTTGACAGAGTCTTAAGCTTTTGTAGGTTCTTGAAGGAAAGCCTTATAATA
GAAAGTGTGATGTCTATAGCTTTGGTATTTGTTTGTGGGAGATATATGTTTGCGACATGCCATACTATAAGCTTACTTTCAGTGAAGTTTCTTACGCTGTAGTTCACAAGGATTTGCGACCAA
AAATACCACGATGTTGCCCAAATTCATTGGGAAATATTATGAAAAGATGTTGGGAATCAAATCCAGACAAAAGGCCAGAGATGGACGAGATTGTCAAACTTTTAGAGGCTATTGACACAA
ACAAAGGGGGTGGCATGATCTCTGAAGACCAGTTTCAAGGTTGCTTTTGCTTTGCAGCTCGTGGCCCTTAGAAATTTTAATATGTTTTTAAAAGCTGAAAGAGTAATTAGGATGTGCATTA
TTTATATCCATTGTAGTTTCTGAAGTTTAATCTTATATTTATTGATCATTTATTTGCCCCCTTTTGTTATTGAATCATATTCTGCTTATTTGGGGGCTCAAAATGATTTTTGTTTTTTCTAATGTTA
GAGATTGTTTGTAACTGATTTGTTGAAAGTAATATTCATTGATTTA
>comp156101_c0_seq3
CTCCCTCCGTTCCTTTCAATGGCTCAAATGCCGCTCTTTTCCCTCGTCGTTCTCACAGACATTTCGGCTGGTGAGTGGTTCAGCTGTTATCGATCTCTAATTTTAGAACCGGAATCTCTCGC

TCTCTCTCTCCCACCTTTCTTTTCTCCGGCTTTTAACTAATGGTCGGCTCGATTTGCACCACAGAGGATTTCATCTGTGGCGGCAATTTTTCTGTTATGCTGGATCGATATATATCTCTTTCTC
TCTGCTTGTAGATGTTGATTTTCTCGTAAAAATTAGTCGTCCAGATTGCGGGTTTCCGTAGTTCAGATTTTTTTGAAGAAAGTTAGCGGTTTGAGCTTTCCAATTCCAATATTCTGCTGAATA
CGAATTGATGCGTAGTGTCCTTATGATGCATTTGTGGTAGGAAACTGACTTCCCAGAAAATAAAGGTGGAAACTTTTTCTCCTTCAAATGCAACGCTCATTCAACTGTGATGGGTTTTTTG
TTCTGTAACTAGATAGTTTTGGCAGAATGGATTCAAGGAAGGGAGAGGATAGTAATAACGTTGGAGTAGTTGGTGCTGAGGCTATGGAAAGATCAATGTCTCAGTTGAAAGGACATGGGA
GCTTCAAAAATAGGGATATTTTATTTAGGGCTGATCAGATTGATCTCAAAAGTCTAGATATTCAGTTGGAGAAAAAACTTTCCAAGATTTGGCTGAATGATGTGCTTGGCTCTAGAAGATCC
AAGAAGGAATGGGAAATTGATCTTTCTAAGCTTGAAATAAGGTATCTTGTAGCTCAGGGGACACATGGTACTGTGTTTCGAGGCATTTATGATGGTCAAGATGTTGCAGTGAAACTATTGG
ACTGGGGAGCAGATGAACATGCAACAGATACTGTTACTGCTGCTCTTCGATCATCATTTCAACAAGAAGTTGCTATGTGGCACAAACTTGACAACCTACATGTGACAAAGTTTGTTGGAG
CATCGATGGGAACCGCTGACCTGAAAATCCCAAGCAGTAACTCACTTCCAGCACAAGCATGCTGTGTTGTTGTGGAATACGTTCCTGGAGGCACATTGAAACAATATCTTATCAGACACA
GTCGAAGGAAGCTGCCTTTCAAGGTTGTGATTCAGCTCGCATTAGATCTTGCTCATGGCCTGAGTTATCTTCACTCAAAAAAGATCGTCCATCGAGATGTGAAAACTGAAAATATGTTGCT
GACTGCCAATAGAACTGTAAAGATTGCTGACTTCGGTGTTGCTCGTGTTGAAGCTCAGAATCCCAAGGATATGACAGGTGAAACTGGGACTCTTCAATATATGGCTCCAGAGGTTCTTGA
AGGAAAGCCTTATAATAGAAAGTGTGATGTCTATAGCTTTGGTATTTGTTTGTGGGAGATATATGTTTGCGACATGCCATACTATAAGCTTACTTTCAGTGAAGTTTCTTACGCTGTAGTTCA
CAAGGATTTGCGACCAAAAATACCACGATGTTGCCCAAATTCATTGGGAAATATTATGAAAAGATGTTGGGAATCAAATCCAGACAAAAGGCCAGAGATGGACGAGATTGTCAAACTTTT
AGAGGCTATTGACACAAACAAAGGGGGTGGCATGATCTCTGAAGACCAGTTTCAAGGTTGCTTTTGCTTTGCAGCTCGTGGCCCTTAGAAATTTTAATATGTTTTTAAAAGCTGAAAGAG
TAATTAGGATGTGCATTATTTATATCCATTGTAGTTTCTGAAGTTTAATCTTATATTTATTGATCATTTATTTGCCCCCTTTTGTTATTGAATCATATTCTGCTTATTTGGGGGCTCAAAATGATTT
TTGTTTTTTCTAATGTTAGAGATTGTTTGTAACTGATTTGTTGAAAGTAATATTCATTGATTTA
>comp156111_c0_seq5
ACGCACTCTATCTACTCAAATTTTATAGCACATAACATCTTAGTTTACAATCAATTTCATATAATACATTATTATGTCGCTTGCTCCTATAAAACAAAGAACAAAAAAAAAACAAAGAGGAA
GAATTACAATCAAATTCCTTATCAACTATCAAGTATAACAAAGCAAATAAATATAAACTAAATGAATAAAATGATAAAAAGGTTCACATTTTGTCACAACTTCAAGCAAAGAAATGCATTTA
AAAATAATGAAAATCATCGTCGTCATCATCATAGAAAAAATCAGGCAGCTCCGATTCTGTCCTGCCTTCCGTCCGTACTTGCCTCATCCATCTTCGAAAAGAATAGTTATGCAGCTTCATAA
ATGCTTAATCAATTAAAAAAAGAGAAATGTTACAAACATATATATCGCAAATTTATTGAAACAAGACTATGTTTATTCACTTTATTGTGCCTTTAGGCTTCCTTTGGGACGGCTTTTCTTCTTC
TTTCTTAAAACATCTTTTTATTATAAAAATTTTTAAGTTTTAACCTAAAAAATTGTTTTAAGAAGC
>comp156111_c0_seq6
ACGCACTCTATCTACTCAAATTTTATAGCACATAACATCTTAGTTTACAATCAATTTCATATAATACATTATTATGTCGCTTGCTCCTATAAAACAAAGAACAAAAAAAAAACAAAGAGGAA
GAATTACAATCAAATTCCTTATCAACTATCAAGTATAACAAAGCAAATAAATATAAACTAAATGAATAAAATGATAAAAAGGTTCACATTTTGTCACAACTTCAAGCAAAGAAATGCATTTA
AAAATAATGAAAATCATCGTCGTCATCATCATAGAAAAAATCAGGCAGCTCCGATTCTGTCCTGCCTTCCGTCCGTACTTGCCTCATCCATCTTCGAAAAGAATAGTTATGCAGCTTCATAA
ATGATTTACGCGGAGACTCATCATTAGGTACATCATCCCGATCAATGAACTTCATTAGTCCATATACAGTCACAAATAGCACCGCGAGGATGTTTGATCTGAAGAAATTAGCCACATCCATG
TTCTTCATCTTCTTCTTCTTCTTCTTTCTAGCTTTTCTATTCTC
>comp156111_c0_seq7
AATGCTCTAAATCCGGATGTCGGAAACCTTAAGGGGCCTAAATCCTGAAAACTGTTTTGTAACATTCATGATAACTCTTGTTAAATCTCATGTGATTGTTTGGCCATATAGTGGATTTAACGA
GCAGACTCCTCGCAAGCCGTTGGATTTCGTAAATTTGGCAAAATACAAGATTTCAAAAATCCAGGATTTAGCACGAAAAGATGAAGTTTTTTGGTATATGGCGGCCTTCATTTGCCGTGAG
ATCTGGCTTTGCATTTTAGGCCATTTGGCTGAATTTTGCTCAAAACCGCCTCAAATCCGGCCTAAATCCTGCTACCAAACAGCCCCTAAAGACCCTCCTAATTCTTAAAAGATGAAAATGTT

TTATGGCCTTACATTTACGCGGAGACTCATCATTAGGTACATCATCCCGATCAATGAACTTCATTAGTCCATATACAGTCACAAATAGCACCGCGAGGATGTTTGATCTGAAGAAATTAGCC
ACATCCATGTTCTTCATCTTCTTCTTCTTCTTCTTTCTAGCTTTTCTATTCTC
>comp156168_c0_seq1
AATTATATATTTTTATAAATAATTAATGAGATAAAAATGCAATATATAAAACTCATTAAAATAAATAATATTAAAATATTGTCACACAATTTTTAATTTTTTTTCCTCACAAAATAAAGGCCTATC
TTGAATTATCTAACCCGTGAGCAGAGAGGAGCACCGGCTGCGATAGAATTTTTAAGCCTCTGCATCTCCTTTCTCTTTCTCCTCGCATCGGCAGCTGCCAATCTCCGGCAAGCCGTCGCGT
AAACCTCCTCCTGCCTCCGCCGCATCTCGTCAGCGGCTGCGGCAGCTGCGGCAGAAGCCGAAGCTAGATCTTCTGCACTCATGTTCAAATCCGGAACTACGAACTCCCTTCCGCCGCCGA
TGCCTTTCCACTCCGGCCAAAAACGAAGCTCTCTCTGTTTATAATCTGAAATCCCTGATTTGAAATAACCCTGGATCAAATTTTGGCAGGATTCACGCGATTCTACATCTACCGGAGCTTTC
AATGGAGAAGCCAGATCATCATCCTTCTCCTTGCAAAGAGAGAACGAAATCGGCAGCGCATTAGAGCTCGAAACAGAAATAGGTTCTCCAGCATAAGAAACAGGAACAGTAGCTGAAG
CGTGAACGGAACGCTTCCTCTTCCCATTCCACCTCGGGAACCCGAAATCCAAACCCATTGTTTTTGAACTCCGGCGATCAAACCGACGATCGGAAACGGAATCTTGCCGGTGAAGAAGA
TAGATGATTATATCTGCTACCTCTCGCTCCAAAATCGTGGGCTCACCGTCTCCCGATTCGGATTCTTCTTTCTCTTTACAATTTTTCCCATTTGATTCCAACTGAAAAAGGTAGGGCGGCGAC
ATTGAAAGGAAGGAAGAAAGGAAGAGAAGCAAGAAAAGAAAGAAGAGAAGAAATAATTTTCATCAGCATATAAAAGCCAATTAGTAAATGCTCTGCTACGAGTGGGAAGAATAATCTT
ATAAAAACTTGATAACTTCGTATATGTATTTAAAATTATTAAGGAATTTGATCAAAAA
>comp156190_c0_seq1
AAAAAGAAACTACAATGGATTTGCTAAATCTTTACTCTTTTTGTTATTTTTTAAGCTCTAGCATAGTTTATTTTCAAGTGATCAATATACATCGTAAGGGTATCTCCAATCGAAAAACTTATTT
TTCATTCCAAAATACAATAATGCTCCAAAATGATGTAATTCATCTCTAAGTGAGCTCTAAAATATAGTAGCGCTCCAAAGTGACCCGGTTTAGTTTTAATTGTTAGTTTTGTTAGTACGACGA
GGTTGATTAGATTAAAAAACAATAAAGACCAAACAAAAGAGAATCCGAACATGTTATATATAACATGATTATATGACATGATTTTGATATGTAGCTCTTCTTGATTTGTTGGTTGGTTGGGAG
TTTGACTCCCAACTAAGAGGTGTTGGATTCGAGTCTTGCATAAATATTGTGTGCGTTTGGGCATTGCTCCTAATTTTCCCAAAAAAAAACATGATTTTGATAGTTAAACCTTTAGTACTTTAA
TTATTGGCCAGTTCAGACACGACAAAATGTTGAGCTATTAATTAAGAAGTTGAAAACAATATAAATGTGAAATTATTAGTTAAAAAGTTTGCCATATTCCTTGGAAAGCTCAGAGAACATTT
ATACAAAATTATTTGAGTCTTATGAAATCTTTAGCTCCCGATGCTACATGTAATAGTTAAGCAAACCAAGCGCATGCTATCTCTCACTTGATTTATTACATTGTAACCATGATAATTAGCAACT
TTTGTACAAATGCAAGCGCGACATTTATGCATTACTAACCAGCACAATCATATAAATATATATATTCCTGAAGCTGATCAAAGCCATTTACCTCATTCATCAATAGTTCTTACATATAAGCATG
ATGGTTTCTTGATATCTAACTTAATTAATCACCATTAAGAGATTGGTCAGATTGGATTGACCAGAAATCCTCCACACTCCAAAAGTTCTCTCCCAATTCACCAGTGTTGTATGAAGGAGGAT
AACCTTCAGGATTTGTATTTGGGTGAACGGGATAACTTTGCTGAGCTATTCCTTCTTCAATGCTACTTGTATGGCTTGTGCTGGCCTCATCAATGATCATTTGGCTCGAGTAATCGGCTGCAT
CGCCATTCTTAACCTTCTTCTGAATTCTAGTCCTCCAGTAGTTCTTGATCTCATTATCAGTCCTCCCTGGAAGTTGCCTTGCAATTTTTGACCACCTGTTTCCCCATCTGGCATGCAGCTCCA
TGATCAGCAGCTGCTCCTCCGGCGTTATGTTCCCCCTCCTCACGTCTGGCCTCAGATAGTTCAGCCATCGAAGCCGGCAGCTCTTCCCTGTTCTCTTCAAACCTTCTCATAAAACCCACCA
ATATATATATCACCTTAATCAATCAAATCATTCTCAAACTCAAAAAATAAAAATCCTTCAAGAAATATTTGCATCTCAATCACCTGCAGATCTGGCAAGATTATTCCATACTCCCTCTCCATGG
TTGGCTATATAGTTTATGAGGATGAGGTCCTCTTCCATAGTCCATGGCCCCTTCCTCACCTCAGCCTCCTGGTTTCCGTTTATCCTCTTATCCATGTTGCTCGATTCTTCCACCATGGCAGAGT
CGGCGGAAGAGAGAGAAAGTTAGGTTTGTAGTATGGTATGATATGAGAGAGGAAAGAGTGTGTGGAAGAGAGAGAGGGGGGTGGTGAGGACGGCAGGAAGTTGTTGGTGAGGTCTTT
ATAAAGAAGAATAACAAGTACCTTGTCTTCATTAGGCTTAATTAACGATGTTTAATTATTGCATATATTGATGTTTAACGTCATAGAAATTGATCAAGGTTCATTATATATAGCTCTAGGGGAA
CGTGTAACGATCTTGAACGCTTCTTAAGAAACGCAAGCTTGCATGGAAACTATTGTTGTAGTCCGCTGGAACCAGTTAGCCACGAGGAATAATATCTTTTTAATCCCTCCAGAAAAGTCTA
GACGGTGACAGGCTTTCCGTCTATGCTTTACAATTTAGACGTGCTGGTTCATCGTAACGCAGCTTTTGAGGCAAATATACTGTTTTTTATTTTCATAAACTCAAATGAAATTTATGTAATTTA
CCCACAGAGATTTATGGTGGTTAGTTGATTAGCTTGATTAAGAATCCCCATTCCGAATTTGAATCCCCCTATTACAAACTCATTTTTTCAGTAGGGCAAAGCAAGTGTGGGCCCAAAGACG

GAAGGTGAGCTCGCTAGGTTCAGCTGACGGATTATTTATGCCGTCAAATACTTTCTGATACCCGTCAAAAAATTAGCTTGATCGGGCACGAA
>comp156190_c0_seq10
ATTAATTAACTAACATTAATTAATTAATTTAAATAAACTAATTAAGTTATTTAAATTAACAATCAACTAATTTAAATTCGATAATTAACTAATATTATTTTCACTAGAAACTAGTATTTTCGTCAG
AAAAATCCATCAGAAAAGGGCGTTCTCTTGTGGTGGGAAAAGCTCTAGCATTTCTACCTTTCAAAATGTTTTTAGTTTAGCCTCTAACTTAGAGAAAATTATTATCATTTTTTTAATTCTATT
TTTAACAAAGATGATCTTGTCTACACTATTGATTTATAAGTGGGTCCTCTTCTCTTCACTTACCTAGACTTTAATAGCATAATTTTTAATTTTTTAGAAGTTGCTTTGTGGGCACCACAACATC
AACGTTGTCGAGGCCCCAATTTTGAGCTCTAAAGAAAATGGAGAAATGGAGGAATACAATGTGTTCTTCACCTCAGTGCTGAGGCACATATGAAGATTCTCTCGGAGCATAGCGGAGCTG
AGGAATTGAGTGCCAAAACACTAGGCCCAAAACTTCATTTTTATAATAAAAATATCTTGTTGAGGATTCAAAGAGTAGAGAAAGGGAGAGAGGGAGGTGAAGAAAGAGAAGAGAGAGA
GAGAGAGAGTTCATTAGATTGATTTCTGA
>comp156190_c0_seq11
TTGATATTAAAACTCTATTTTAAGAAGTAATAAAAAGGTAAACTTAAATCATTTCGTAGATGAATAATTTTAACGAAAATCTTTTATAATTATCTGTATGATTATCATGACCACACCTGACATA
AAATTAATTATTGAATTGATTAATAGAAAAAAAATAGATAGAGAGTTATACTTCTACACCAGGCAAACTTTATAAATATTCCATTTTTTAGCTTCTGTTATCCACTTTCATAGTTTCTTCTATGA
CAGCTTAAAGAGGATGCAGG
>comp156190_c0_seq2
AAAAAGAAACTACAATGGATTTGCTAAATCTTTACTCTTTTTGTTATTTTTTAAGCTCTAGCATAGTTTATTTTCAAGTGATCAATATACATCGTAAGGGTATCTCCAATCGAAAAACTTATTT
TTCATTCCAAAATACAATAATGCTCCAAAATGATGTAATTCATCTCTAAGTGAGCTCTAAAATATAGTAGCGCTCCAAAGTGACCCGGTTTAGTTTTAATTGTTAGTTTTGTTAGTACGACGA
GGTTGATTAGATTAAAAAACAATAAAGACCAAACAAAAGAGAATCCGAACATGTTATATATAACATGATTATATGACATGATTTTGATATGTAGCTCTTCTTGATTTGTTGGTTGGTTGGGAG
TTTGACTCCCAACTAAGAGGTGTTGGATTCGAGTCTTGCATAAATATTGTGTGCGTTTGGGCATTGCTCCTAATTTTCCCAAAAAAAAACATGATTTTGATAGTTAAACCTTTAGTACTTTAA
TTATTGGCCAGTTCAGACACGACAAAATGTTGAGCTATTAATTAAGAAGTTGAAAACAATATAAATGTGAAATTATTAGTTAAAAAGTTTGCCATATTCCTTGGAAAGCTCAGAGAACATTT
ATACAAAATTATTTGAGTCTTATGAAATCTTTAGCTCCCGATGCTACATGTAATAGTTAAGCAAACCAAGCGCATGCTATCTCTCACTTGATTTATTACATTGTAACCATGATAATTAGCAACT
TTTGTACAAATGCAAGCGCGACATTTATGCATTACTAACCAGCACAATCATATAAATATATATATTCCTGAAGCTGATCAAAGCCATTTACCTCATTCATCAATAGTTCTTACATATAAGCATG
ATGGTTTCTTGATATCTAACTTAATTAATCACCATTAAGAGATTGGTCAGATTGGATTGACCAGAAATCCTCCACACTCCAAAAGTTCTCTCCCAATTCACCAGTGTTGTATGAAGGAGGAT
AACCTTCAGGATTTGTATTTGGGTGAACGGGATAACTTTGCTGAGCTATTCCTTCTTCAATGCTACTTGTATGGCTTGTGCTGGCCTCATCAATGATCATTTGGCTCGAGTAATCGGCTGCAT
CGCCATTCTTAACCTTCTTCTGAATTCTAGTCCTCCAGTAGTTCTTGATCTCATTATCAGTCCTCCCTGGAAGTTGCCTTGCAATTTTTGACCACCTCGTGGGAGAAAGAAATGAATTTTAA
AGTGTAATTAGATCATAAAGTCGAAGAGGGTGGAATTAATTAATTCCAATATGTTAGTATATACTTTTACATTGATAATTCTTTCTAAGCTGTAAGACAGTTCTTCTATTCTATAAATTTATTAT
TTTATAATCATCTTGATTGTTTTCTTGCAAAGTAAAAGCAAAATAATTACATTAATCTAATAAGATTTCATAAGCAAAAATATTGAAATCCCTAGTTAAACTTTCAATAACAACAAATCTTTAT
CTCACTATGTGGGGTCGGCTACATGA
>comp156190_c0_seq9
AAAAAGAAACTACAATGGATTTGCTAAATCTTTACTCTTTTTGTTATTTTTTAAGCTCTAGCATAGTTTATTTTCAAGTGATCAATATACATCGTAAGGGTATCTCCAATCGAAAAACTTATTT
TTCATTCCAAAATACAATAATGCTCCAAAATGATGTAATTCATCTCTAAGTGAGCTCTAAAATATAGTAGCGCTCCAAAGTGACCCGGTTTAGTTTTAATTGTTAGTTTTGTTAGTACGACGA
GGTTGATTAGATTAAAAAACAATAAAGACCAAACAAAAGAGAATCCGAACATGTTATATATAACATGATTATATGACATGATTTTGATATGTAGCTCTTCTTGATTTGTTGGTTGGTTGGGAG
TTTGACTCCCAACTAAGAGGTGTTGGATTCGAGTCTTGCATAAATATTGTGTGCGTTTGGGCATTGCTCCTAATTTTCCCAAAAAAAAACATGATTTTGATAGTTAAACCTTTAGTACTTTAA

TTATTGGCCAGTTCAGACACGACAAAATGTTGAGCTATTAATTAAGAAGTTGAAAACAATATAAATGTGAAATTATTAGTTAAAAAGTTTGCCATATTCCTTGGAAAGCTCAGAGAACATTT
ATACAAAATTATTTGAGTCTTATGAAATCTTTAGCTCCCGATGCTACATGTAATAGTTAAGCAAACCAAGCGCATGCTATCTCTCACTTGATTTATTACATTGTAACCATGATAATTAGCAACT
TTTGTACAAATGCAAGCGCGACATTTATGCATTACTAACCAGCACAATCATATAAATATATATATTCCTGAAGCTGATCAAAGCCATTTACCTCATTCATCAATAGTTCTTACATATAAGCATG
ATGGTTTCTTGATATCTAACTTAATTAATCACCATTAAGAGATTGGTCAGATTGGATTGACCAGAAATCCTCCACACTCCAAAAGTTCTCTCCCAATTCACCAGTGTTGTATGAAGGAGGAT
AACCTTCAGGATTTGTATTTGGGTGAACGGGATAACTTTGCTGAGCTATTCCTTCTTCAATGCTACTTGTATGGCTTGTGCTGGCCTCATCAATGATCATTTGGCTCGAGTAATCGGCTGCAT
CGCCATTCTTAACCTTCTTCTGAATTCTAGTCCTCCAGTAGTTCTTGATCTCATTATCAGTCCTCCCTGGAAGTTGCCTTGCAATTTTTGACCACCTGTTTCCCCATCTGGCATGCAGCTCCA
TGATCAGCAGCTGCTCCTCCGGCGTTATGTTCCCCCTCCTCACGTCTGGCCTCAGATAGTTCAGCCATCGAAGCCGGCAGCTCTTCCCTGTTCTCTTCAAACCTGCAGATCTGGCAAGATT
ATTCCATACTCCCTCTCCATGGTTGGCTATATAGTTTATGAGGATGAGGTCCTCTTCCATAGTCCATGGCCCCTTCCTCACCTCAGCCTCCTGGTTTCCGTTTATCCTCTTATCCATGTTGCTC
GATTCTTCCACCATGGCAGAGTCGGCGGAAGAGAGAGAAAGTTAGGTTTGTAGTATGGTATGATATGAGAGAGGAAAGAGTGTGTGGAAGAGAGAGAGGGGGGTGGTGAGGACGGCA
GGAAGTTGTTGGTGAGGTCTTTATAAAGAAGAATAACAAGTACCTTGTCTTCATTAGGCTTAATTAACGATGTTTAATTATTGCATATATTGATGTTTAACGTCATAGAAATTGATCAAGGTT
CATTATATATAGCTCTAGGGGAACGTGTAACGATCTTGAACGCTTCTTAAGAAACGCAAGCTTGCATGGAAACTATTGTTGTAGTCCGCTGGAACCAGTTAGCCACGAGGAATAATATCTTT
TTAATCCCTCCAGAAAAGTCTAGACGGTGACAGGCTTTCCGTCTATGCTTTACAATTTAGACGTGCTGGTTCATCGTAACGCAGCTTTTGAGGCAAATATACTGTTTTTTATTTTCATAAACT
CAAATGAAATTTATGTAATTTACCCACAGAGATTTATGGTGGTTAGTTGATTAGCTTGATTAAGAATCCCCATTCCGAATTTGAATCCCCCTATTACAAACTCATTTTTTCAGTAGGGCAAAG
CAAGTGTGGGCCCAAAGACGGAAGGTGAGCTCGCTAGGTTCAGCTGACGGATTATTTATGCCGTCAAATACTTTCTGATACCCGTCAAAAAATTAGCTTGATCGGGCACGAA
>comp156207_c0_seq3
AATGAAAAATATTTGATCTAACAGCAAGGCATGTTTGAAAGCAAAAATAAGTTCAAAATATACTTAATTATAGTTATTTGCTTTCACATTAAGAAAAAAGAAATCTTTAAAAGCAAGCCAG
ATATGTTCTCAGCTTTAGCTAACAAATGGCACAGATGCATCATGCGGATAAGTTTAATGGCGTCCCATACGGGCTCTTTCTGGGGCATAAGGTCAGTGGAGCTTTAATTAATTGCTTTAATTG
TTCTTTTTTATGTATTGTGTTAATTTATGTGAACACAGGAATTGGAGGAGGTTGGAGGGAAGGAGGAATTGGAGAAGGAGATTAAGAGAAGGATTAAGGTTTTTAATAGCAGCTCCAATG
CTAGCTAGCTGAACTTCTGGTTTTATTGTTTTGATTTTAATATTTTCTTAGGTTTTGTTTGGGACGGTTTTTT
>comp156209_c2_seq1
TTTGGAAAAATTCAGATTTCAATTCAAAATTCAGATTTCAATCTATGAACAATTTGATAGAAGTCAAATCCATATTCATTTTTTACATGATTTGCTATATAATTTGATGCATAAAAAAAAAAGT
TTTTTTTCTGAATTATACATAGTAAACCATGCCTACTTTAAGCAACAAAGAGTGAGGCACACTTAACTGAAGATCAGGAGCTCTGCAATTCTTGCTAAGGAAAGCATAAACAATATCTATG
GCCAATTTCTTTATCAAGGAAGATGAACTCCTTGCCTTAGCGTAAGAGTGTCCAGTAATGTATACCACACCAGATTCTTTAGCCTTAAGGATCTCCATAGATTGCTCACTTCTAACAAAACC
CTCTACCAGACCAAACTCTCCAGGTTTTACTAAACCTGCATTCTCTTCTGATTTCAGAACTTCTTCCATCTCCACAAATGAGATAATTTTAGACACAAGCCTGTTCTCAAAGTCATAGTTTT
CTTGGCGCAAATCTTTGTATCCATATCTCACAATGCATCTGAACATTAGATGTTCCTTCGGCCCAATCCAGCCGACGAGAAATCTTTCACACTCAGGGATGAAGGGAACCTGAACTGATTT
AATGCACACAAAGATTAGAACTTGATGAAAGGCTGGAAGGCTCATCGCAAAATGTCCAAAAACAGCAGGAACACCGGTCACCAAGTCAGTATAAACCAGTCCAATGCCCGGCACCCGA
ACAAGTCCAAGGTTAGGACCTAAAGCTAATATCTTCTGAACTGAGACCTTATTCTGTAAATCAAATTCATGCTTAAGCAGTGTACCATAGTTCCAGATGTACATCACACACATAAAGATTAT
AGAAAGTGTAAGAGGTATCCAACCCCCTTCAGGGATCTTGATGAAGGAAGCTGAGATGTATATAAGCTCTATAGATCCAAAGAATATAAGAAAAATGATAGCTGAAATAATCTTCTGTTTC
CAGACAATTATTATCACCAGAGACATCAACCAAGTTGTTACGAAGATCACAATTGTGACAGCAAGCCCATATGCATGTCCAATCATATTTGTATTTCTAAGTCCAATTGTTACAGCCAAACA
AAGGCACATTAGAATCCAATTGACCTCTGGGATGTATATTTGACCATATATGTGATTTGAAGTATGAATAATTTTCACTAGAGGGAAGCAATTCAATGCACAGCACTGGCTTATAATAGAAA
AGGTTGCTGAAATCACAGCCTGACTTCCAACTACTGCAGCGCATGTTGCTATTATGAATACAGGCCAAAATACAGTTTCTGGTATAGCTTTATAGAAGCTTCTCTGTATATCATCGTGGTGC

TTAGAAAGAAATGCAGCCTCACCCATGTAAGCAAGAACGAGGCAAGGATAGACCAGAAAGGAGAAAGCAATCTTAATTGAAACTGGAGAGAAATGACCCAAATTGGCAAACATGGTCT
CTGCACCTGTAATGCATAGGACCACACCCCCCAAAGAAACCCAACCTTCTACCCCAGTACTGCTGAGAAATTTGTACATGTACACTGGTGAGATGGCATGGAAGATACTTGGATTCCATTT
AAATATGTTATATATACCAATAACACTAATGCACAGAAGCCAAGCAATTACAATTGGAGCAAATATGAAACCAACTCGATGTGTTCCAAAGCGTTGAAGTGAGAAGAGTGCCACCAGGAT
CAAACATGATAGTGCCACTATATAATTTTCATGAAGATGAGTTATTTTCACTCCGAGTCCAGAAACAGCAGAAAGAACTGAGATTGTGGGAGTGAGCACCCCATCACCAATCGACATGCA
AGTTCCAAGCAAAACAACAATTAGAAGCCCATGTTTTAATCTCGGGTACCTACTGAAAAAATCATTAAGCATAACACTCTGCCAAGTGTCTGTAGATCCTTCAACATCATAAGTTGACAAA
TTCTCATCAATAGCTTGTTGGTTAGGCAAAATTGACAATCTGGCATGCCGACATAGCAGAGAGTATAGTGCAAATGTACCGCCTTCACCATTATCATCAGCTGATAAGACAAAGTAAATGTA
TTTGAACAGTGGAATGAGAGTGAGAGTCCAAAATATGAAAGAAAAAACCCCAAAAACTTCCTCATCACTCTCGTGCAGGCTCAATTTCCCTGAGAAAGTAGTCTTATAAACATACAAAG
GTGAAGTGCTAAGATCACCATACACCACACCCAGACTTTGGTAAGCAAGTATGAGTACATCCTTGTAAGAAGAATTCTTCGGATTGGAGGGTTCTGGGCCTTCCTCCTGTGCAAATGCTG
GTGGTTTTGAAGAGCTCATGAGGAAGAGGGAAGCGGCTGAAGCAGTGATGAAGGAGGGAGGAAGGTTAAGT
>comp156217_c1_seq1
ATTATCTGAAAACATTATGGGCAATGTGAAGGAATCGGAGAATGGTGAGGCATATTTTTACATTTAATGGCTGATCATTCATTAATTATTTAAATTATTATTCATTTTATATTTAATTGTTCTACT
AATAATTTAATTAATTATTTAAGTGTCAGTAAGTTAGTTTTAATAATAAATTATACTACTAATTATCTCAGTAATCACCTAATTAGTTTTAATTATAAGCGATAACTACAAAAGTTGGTTGCTCCT
TAAAATGTTAAGCCGAAAGAAAAAAAGTAGAGGAAGAGGAGATAGAGAGCAAGAGACCCTACTTCTCACTCAAGTCAAAGCAGTTAGAATATTCTTTGAGATCAAAGAGAGATGAGGC
CAAGAAAACTTTCACTATAATAAAAACAAAGGCATGGAATATCCGGCTGTAGATATGACTAAGTTTTATCAATGAGACAAAAAAATCATAAGGAGAACCTTTCAATCTGAAATTGTAAGCA
AATCAATATATAATTATAGATTGAGATAGTGGTTAAAATTTCCTTTATGAGACCCTACATGCTAAATTAGGTTTACATACTAGAACAAACCAAGGCCTCCTTTATTCCTTAAAGACTTATGAAT
AAAGATTCTAATTTTATGGTTGATAGAAAATGTGATGTAAATTTTGTCATAACAAGGCAATATACTGGTGATGTTACATGTGCTATAATTCCTTTAGATGTGTGTCATGTAATCCTATAGAATA
CATTTTATTGAATAAATATAATTTGATTATAAAAGATAGCAAACTTATAATTTTTAAAAAATAGTAATAATTATGTGGAGCATAAGGCAATTGCATAGGTGAAGGACATAAAAGAAGCACAAT
AAGCATGATAGAAGAAGATTGTTTTCAATGATTAGAGCCTTTTCAATAGTATTTAACTAAATAAGAGTGTCAAATCTAAAGCATAAAAGGAGGAGGAGGCCAAGTGATATAGTGCATCATA
AACCTATCATAAGGTCAATGACAAAGAATTAGTGGCAATAATCAACCAAGGTGTCTTCATATGATTCCTAAATTTGTGGGGAGCCACACCAAGACTTATGCAATACATTTCCAACATCAAG
GGATAACCAAGTGTTGCTACAAATCATTTTCCACTACTCAAGAAGCCAAACAATGAACACTTCTCAAAATAAGTGTCTACCTTGACATCACCAAGAAAGTGCCAAACCACCTCGGCATTC
AAGGAACCACACCAACTTAAAGAGTTTTTTATCATAAGGCACAACCTTGTTTATCAAAGATGAATAACAAATTATAGTGTAAAGGTCCTCATAGAGTCCTAGCGCATAACAATGGTGCCG
CATGTGCTATTGTAAACATGGAATGATAAAACAATATGAAAAAAAAGTAAAAAAAGTAACTAATACTGAAATACACTCTAGAAAATTTTCAAAAACAAAATGTAGTTTAGAAAACTGATTT
GTTTGTTAAGTAGCAAATTTTGGGTTTTAGGCTTCACCTATTAATGAGAAGGCCTTCAACTACGATTTTAGCTAAAGGAATCATCTAAAAAAAACATAAAAAATCATAGAGAACCTTAACTT
TATTGCTCAAAAGACTAAAGTTATGGGATGAGACAACTTGCCTAATATGGACCTCAATAGTGAACAAAGGTGCAATGGAAGGTTAAAGCTGAATGCAAGCTTGCCATTCCATTAAGGTGT
CCTTTGATTCCCCCTTATTACTTATTTGAAGAATTTTGTTATCAACATATTTTTATTATAATTCATAATAAAAGAGAAAATATGTGCTACACAATAAGATGATGAAGAGCTCGATGACAATTTG
TCTATTTTCACATCTTATTCTTTAAGTTCAAATGAGTTTCAAAGTAAGGAGACCTAGTATCAACCACAATAACATCAAGCCTTTATCCC
>comp156256_c1_seq3
AGGATGCTTTTTTTTTTTTTTTAAATCAATAACAATTTTCAGAAAATCTATGAACTTAACAATCTTCCTATGCTGTTCTTATAATTACACCTTCCAGGCTTGCAAGATTTACCTTAAAAATATAT
AAATATATATACAAATTAAACCAAAATAAATAAACAGATTCATGAATATGAATAGAGAGGGGAAGAGAAGTATATGAAGAAGGCTATAAGTGAAAGGAGGAGCTACAGCAAGTCTAAGCC
AGGAGCCAGAAGTTCTTGTGTGTCTTTGTGTTGAGAAGATAACCTGCATTCTGACCTTTCTTCTGGACTCCAACGCAGAGGCACTTGCAGTGCTCTATCAAGAACTCTGCAGTGTCCAAC
CCTCTCCCACTCATGCTTCCACTGAGCTTGCATCAGTTGGAGATCACGAGGTTCCAGCTTCTTCATGATTTCGTCCGGCATGTATCCCCAATCCCAACAACACCGCTTTGATCCTCCGCTCA

CATCGGCGATTCTTCCATGTCATCATTTTCATCAGAGAAATTACTCTTCGAAGCCATGGATATATATGCGAGTCAAATTATAAGCAGGAAAATCATCCAAATAAGCAACGATGTAG
>comp156256_c1_seq5
AGGATGCTTTTTTTTTTTTTTTAAATCAATAACAATTTTCAGAAAATCTATGAACTTAACAATCTTCCTATGCTGTTCTTATAATTACACCTTCCAGGCTTGCAAGATTTACCTTAAAAATATAT
AAATATATATACAAATTAAACCAAAATAAATAAACAGATTCATGAATATGAATAGAGAGGGGAAGAGAAGTATATGAAGAAGGCTATAAGTGAAAGGAGGAGCTACAGCAAGTCTAAGCC
AGGAGCCAGAAGTTCTTGTGTGTCTTTGTGTTGAGAAGATAACCTGCATTCTGACCTTTCTTCTGGACTCCAACGCAGAGGCACTTGCAGTGCTCTATCAAGAACTCTGCAGTGTCCAAC
CCTCTCCCACTCATGCTTCCACTGAGCTTGCATCAGTTGGAGATCACGAGGTTCCAGCTTCTTCATGATTTCGTCCGGCATGTATCCCCTACAAAACATCAAAAGACAAGGAGATGATTAG
TTGAATAAATGAACAACGAACAAATATATTGAACCTCAAGAACTCAATGGTCATGCCAATCCCAACAACACCGCTTTGATCCTCCGCTCACATCGGCGATTCTTCCATGTCATCATTTTCAT
CAGAGAAATTACTCTTCGAAGCCATGGATATATATGCGAGTCAAATTATAAGCAGGAAAATCATCCAAATAAGCAACGATGTAG
>comp156256_c1_seq6
AGGATGCTTTTTTTTTTTTTTTAAATCAATAACAATTTTCAGAAAATCTATGAACTTAACAATCTTCCTATGCTGTTCTTATAATTACACCTTCCAGGCTTGCAAGATTTACCTTAAAAATATAT
AAATATATATACAAATTAAACCAAAATAAATAAACAGATTCATGAATATGAATAGAGAGGGGAAGAGAAGTATATGAAGAAGGCTATAAGTGAAAGGAGGAGCTACAGCAAGTCTAAGCC
AGGAGCCAGAAGTTCTTGTGTGTCTTTGTGTTGAGAAGATAACCTGCATTCTGACCTTTCTTCTGGACTCCAACGCAGAGGCACTTGCAGTGCTCTATCAAGAACTCTGCAGTGTCCAAC
CCTCTCCCACTCATGCTTCCACTGAGCTTGCATCCGAGAAATGAAGAACCCGAACGGCGCTGACCAATAACAAGCAAATCGACTCCAAGTACCTTCGTCTGCGACAGAATCACCGCCGC
CTTTTCCCTTCCCTCCATCTCCACCTTCTCTACCTGAACCTTAACCTTCGGCTGCGCCGCTTCGCACGCCGCTCTCATCGCCTCCAAGAACTCGTACTCCCCCCCATCCCCCGACCCAGCTG
ACACTACCGATCCAGACGACGACGACGAAGACGACGAAGACGAAGATGACGTCGACGTTAGAGCCAACTGCTGATGGTGCTGCTGGTTTCCAGCACTTCCTGACGACATCGGCCGGCG
GAGGAAAGCCGATAAGCCGCCACCGCCGCGTCGACTAGGGTTTGACGTCTCGACGTGGAGGAGAATGAGGTCATCTTGTTCGAGAATGGCGTGGGAAAGGGCCCATTGAAGGGCGACG
GAAGATTCGCGGCTGGCGTCGGCGATGACCATGACCCGACGCTTGGTTGGAGGGAGAGGGCCGCCGCGATCGGCGGAATCCATGGCGACCGACGGCGCCGTGTCGTCGCCGGCGCTGA
GCTTTTTTTCCTAACGCTATTACAGGTGGCACTAAGAGGGCATTTTTGTTAAGAGAAAGGTAGAATAGTGGTTGTGGGTTTCTGGAGCGGAACAAA
>comp156315_c0_seq1
CTCTTTCACCTCTTTCTTCAGGCCTTCCGGCCAAAAAAGCTCGGAGAAAGACTAGCTAGGCGGGGAGAAGAGGAAGATAATAATAAAACTACGTGCGTGTCTTTCCATAAAAACTTCCAT
TACCATCTCTCCCGCCATGGCAGCCTTCTCCGACGAAGAACAGCGGAAGCGCCGCTACAGGCGGATCGCCGGCTCCATCGCTTCTCTCATTCTCCTCCTCGCCGTCGTCGGCACCGCCTG
CACGCTCTACAGCCCAGAGAAAGACACTCCTTCCTCCCACATCGTCACATCCGCCACTGCCGGTTCCCCCGCCACGCAACTGCATTCCACCGCCAAGTCCATTCAGGTAATGTGCGAATC
CACCGATTACAAACAAACCTGCGTCTCCAGCCTCAACAAAGTCGTGAAGAAGAACACCACCGACCCCAAAGAACTCGTCAAGGCCGCCGTCGCCGTTGTTCTCGACGAGATCAGCACG
GCCTTCCGCCGCACCCGCCTCCTAGCCGACAAGGACCCGAGAGTTCGTGGCGCCGTCGAAGACTGCCGGCAGCTGTTCGAGGACTCCAAGGACGAGATCAAACGTTCGCTCGACGACA
TCGTCTCCACCGGCATTGAACATATCCCCAGGAAGTCGCGCGACATCAGAACATGGCTGAGCTCTGTCATGTCGTATCAACAAACCTGCATTGACGGTTTCCCTGACGGTGAGCTTAAGG
TGAAGCTGAAAAAGACGTTGAAGACAGCAAGGGAGCTAACGAGCAACTCTCTTGCAATTGTTAAGGAGCTATCGAGCTTTCTTGAAATGCTGGAGGTTCCGGGATTGACCGTTAGTGGA
AGGAAGCTGCTCGCTGAGAAAGAAGAACTGCACCAATTTGACGATGAGGGCATTCCTTCGTGGGTTCATGAGGATGAGAGAAGGGTTCTCAGGCAGGTCAATACGAATCTAAAGCCAA
ATGTTACGGTGGCTAAGGATGGGACCGGCGATTACACGACGATTTCAATGGCGTTGGCGAAGATTCCGAACGAGTACACTGGAAGGTATGTGATCTATGTTAAAGAGGGAGTGTACGAGG
AGACAGTAAATGTCACGAAGAAAATGCCGAACTTGACCGTTTACGGCGATGGAAGTGCGAAGACGATCATCACTGGAGAGAAGAATTTCGTCGATGGCGTCAGAACTTTCATGACTGCT
ACGTTTGTGGTCTCCGGCGACAACTTCATGGGCATTGGCTTTGGCGTCCGCAACACCGCCGGAGCCATCAAGCACCAAGCCGTGGCAATCCGGGTCCAATCCGATCGTTCCATCTTCTTC
GAATGCCGCTTCGAAGGCTACCAAGACACGCTCTACGCCCAAGCCAAGCGACAATTCTACCGTAGCTGCGTCATCGCCGGCACCGTCGATTTCATCTTCGGCGACTCCGCCTCTGTCTTC

CAAAACTGCCTCATGGTCATCCGTCGCCCCCTCGACAACCAACAGAACATCGTCCTCGCCCACGGCCGCGTCGACCGCCACGAAACCACCGGCTTTGTCCTCCACAAATGCAAGATCAT
CGGCGACGACAAGCTGTTGCCTTTCAAGAACAAGATCAGGAGCTACCTCGGCAGGCCGTGGAAGGAGTACGCGCGGCATGTGATCATGGAGACGGAGATCAGCGATGTTATACATCCCG
ATGGGTATTTGCCATGGGAGGGGGATTTCGCGCTCAATACTCTATTTTACGGGGAGTATAACAACACAGGGCCAGGGGCGAAGTTTGATGGGAGGGTGAAGTGGAAGGGCGTGAGGAAA
CTGAAGAGGTCGGCGGCGAGGTTCACGGTGGCGGACTTCATACAGGGCACTGAGTGGATAAATAATGAGAGAGGGGGAGTCACCATTCCTGTGCGTTATGGCTTGTATAACTGAAGGGA
ATTGCAAAGCTGTAGCGTTTGATTTTGCTGTTAATTCGTTTGGGAGGGGTTTGATTTCTGTTTATTAATTTGTGTTTGATCGACCGTTAGAAAAGATACAAATTAATGAAGGGAAAAAAGAA
AAAGCTGTTGCTTGCAAAGCCATTTG
>comp156316_c0_seq1
AATAAATAAATAAATATTGTGAACCTTTTCTCTCGCTCCTTCCGCCTTCCATGGCCGTTTAACCAATCTTTCAACCGCGATTTTAATTCGTTTGATGCCCCAGATCCCAAAATGTTGATAGAA
ACCCGGTAAAACTCTGCAAATCCCATGGATTCTGATTCGCTTCTCCTTATATTGCCTTCACTCGTAGCTGTACGCATGAACGTGTCCTCAGAGAGGAACAGGGAACGAACTCGTTGAGAA
AATTCTCGAATGTCAAGAATCGGATGAATCAAAATTTTCCAAAAATTTCGTAATGCGTTCGTTCAATTCCCCTATCACTGCAATCGACGCTGCAAAGTATTTTCACAGCCCCGTGCACAAA
GCCGTGCTCACTCGCGATCATGCTGCCCTAAAGCAAATCCTTGCCGCTATTCCCCGCATCTCCCCGCCTTCTCAAAGCCTAACCGAGGCCGAATCCATCGCCGAGGAAGACAAGGCCAAC
GCCATTTCAGCTGTCATTGATCGCCGCGATGTCTACGAGCGTGAAACCCCTCTCCACCTCGCCATCAAACTCGGCGATGCCACCGCCGTTGAGATGCTCATGGCCGCCGGAGCGGATTCA
AGCCTCCAAAACATGCAGGGATGGAGCCCTCTTCAAGAAGCCATCTGTGCTCGAGAGGAACAATCGCAAAAATCATTGTTCAGCATTACCAGGCCTTAGCCTGGGTCAAGTGGTGTCAC
CGAATGCCACGCATTGTGGCTGCCATGAATCGGATGAGAGATTTCTATATGGAGATCAATTTCCATTTCGAAAGCTCCGTGATCCCTTTCATATCAAGAATTGCCCCATCTGATACCTACAA
GATATGGAAGCGGGGATCGAATCTGAGAGCAGATATGACGCTTTCCGGCTTCGATGGTTTGAAGATTAAGCGAGCCGAGCAGAGCGTTTTGTTTTTGGGAGCAGGTTCGGTTGATGGGAA
GATAAAGCCTGCTAGGTTGTACGTAATATCGCATAAGGATAAGGAGGTTCTTGATGCTTTGGATGGAGCAGGGGACTTGGTTTCTGATGCTGACTTACAACAGGAGGTTGCAGCTCTGTTT
AGAACCAATGTTCTTCGACCAGGGCTTGATGTTACCCGTACGGTGCTAGTTCCACAGATGAATTGGAGAAAGCAGCAAAGGTTGGAGATGGTTGGGCCATGGAAAGCTAAGGTCTACAA
CATGCATCATGTAGTTGTTAGTATAAGGTCACGGCGGGTTCCTGGTGCCATGACTGATGACGAGCTATTCGCTGTTAAGAATGATGGAGAGGATGTTGGGTTTGGTGATGAGCTTACTGAG
GGAGAGAGGAACAAATTGGAGGCAGCTTTAAAGATGGAAACTTCAGATTCTGGCTATTTCACTCAGCAAAATGATCAGGGCCCGGTTGTTGATATGACTCCTACTCGTAATGGGGGATGT
AGTGATGTAAATAAAGAGCGGAATGGATGGTTTGGAAATTGGAATAATGGTAAAGCTCCCAAGCATGGCTTGGAGAAGAAGGATTTAGTTTCAAGGCAATCTCTTTGTGGTGAAGAGAA
GGTAAATGATCTCCCGAAGGGTTCATCAAAGATGCCATATAAGCAAGAAAAAAATTCTATTGATAGGAGTAGAGATGAGTCCAAAAAAATTGCTGCAAGTTTGGAGAATGGTCATCAACT
CAAGGAGAGTGTAAGAGAGAATGAATATAGCAAGGGTTTAAAACCAGTTCTATGGCTTTCTCCTAATTTTCCATTGCAAACAGAGGAGCTTCTCCCTTTGCTGGATGTTCTTGCAAACAA
GGTTAAGGCTGTACGTCGTTTGAGGGAACTGCTTACCACTAAGCTTCCAAGTGGCACGTTTCCTGTGAAGGTTGCCATTCCAGTGATCCCAACAGTTAGGGTTGTGATCAGTTTCACAAA
GTTTGAAGAACTACAATCGACAACTTCTGAAGTATTCCGCACACCACTTTCAACCCCTAAGAGGGAATCCCCTGTAATAAATTCTGCTAATTGGGCTCAATGGTTAATTTCTTCCTACTCTA
CTGATAACTTCCAACCTAAAGCATCACCAGAACATAGCAACTCTTCCATGGATTTGCAGGATCCCTTCATTATTCCTCCTAATTATGAGTGGACAACCTCTGAAGCAAAGAAGAAGAAGAA
GAAGAACCAAGAGGCTAATGGGAAACCAAAGAAGCGGCAGGGTTAGTACATATGTTGACTTCTCAACAATTGTTACTTTCATGATTTGTTAATACAACTGAGATTACAGAATTATTAAGTG
AAATACATAAGTATTTGGAAGGTGAATGAAGTCTGCATAGTAAAAATCTTGCTTGCGAGTTATGTAATTTGTGATTTTTTTATAATATGAAAATTGCAATAGCACTTGCAAGCTTTAACTTG
CTCTCTATGTTGAACCGATACACCTATTTATGAAGG
>comp156318_c0_seq3
GAAATTATGTATTTTTCCTAAGTTTAGGCGGAAAATTCTGAATTTTTCAGTCGATTTCTGACGAAAATATTTTTAATGATAAATTGAGCGTCGACAAAAAAGTTTTACGACGGCTGTCCGAC
GGAAACTCAAAGGGAATAACTAAAAAAAGATTTAAAAATTTTAATTTTGGCATCTGTCCGTCAAACGTAAGTCCGCCAACCATGCCGGCCCTTAGTAGCGCCCGCCCATGTGGCTCGCA

CCAGCCCTAGTGCGCCCGCGCACAGCACCCGCCCGCATGGCCTGTATCAGCCCGCGGTGCGCCCAGCGCCCACCCGCATAGCCAGCGCAGGCGCCGCCGAAAAAAGTACATGAAAATTT
GTCGGAAATATTTTAATGTAACCCGTCTAAAATCCACCAAAAGATCTGTAGATAATAAACACTTTTCTTGTAGTGCTCGTAATTTTCTTGGTCGTTAGTGAAGAATAAACCAAGTTCATAAG
GAGCAAAAATACCTTGAAAAAGCCAGCATTTTTGCATGCCATGCCGATTTCAGTGACCACCTTGTGGCGATGAGGGCCATTCAGACCTGAAAGATCAATCACAGGGATGGCTTCACCTGA
GTTTTCGACGCCATCGAGCTCTGGTCGGTCACCGGCCGGTCGTATGTAGTTTGAGGGAATGAAGGGAGAGTCAGAGGCAAAGTCGGCAAGGAGAAACTTGAAGGAAGACGATGCCGCC
ATTGTTGACCTACAAAGAGAAACTAAGTTTTGAATATCTCTCTCTTTATAAATTCTATCTTTGCTTGCTCTGAAGGAGGAAGGAAGCAGGTGCCACATTTATATACACGAATGGCGAGTCTT
TAGTTGCGGCTAGATCTGTTCTTTTGCGTTTAGATGTAACAACGGGGTGATGTCATGTGC
>comp156329_c0_seq4
CCAATCCTGTCTATAACAAGACTAGTAAAGAAGGAGATGGAGAAGAAGGAAATAGTTGAGGAGGAATATGCGCAAGAGGTTCCAGATCTTCAACCTATGGAGGAAGATGTAATAGAGGA
GATTGAGATGATCTTGCAGTATAGATTTAGGAATAAAATGCTTCTTGCTCAGGCATTCACCCAGTCATCTTTTAACAACCCTTTCAAAAAGGCTGGGAAATCTTATGAACGATTGGAGTTCA
TGGGTGATTCGGTGCTTGGCCTCTTAATGGCTAGAGAGGTATTTTCTTCTTATGAAGATTTGCCTCCGGGACAACTTACACTTCTAAGGGCTGCTAATGTTGATACTGAGAGGCTAGCTCGA
ATAGCCATTAAATCTAATTTTCATCGATTTCTACGTCATAATGCTCCTCAGCTTGAATGGCAGATTAAGGAATTCATGGAGGGCATAAATCAATATCCTATACATTCAAATGGTCTCCTTGATC
CACCAAAAGCCCTTGCAAACATCGTTGAATCTGTCATTGGAGCCATTTTCATCGACAGCAATAGTTCTTTAGAGATTGTCTGGAAGACATTCAAGAAGTTAGCAGATCCACTGATCAGCCT
CGATACACTAGGAAAACACCCTGTGAGCGAGCTTTATGAACTATGTCAGAAGAACAAAATGAATGTAAATTTCGAGAAAGATACATGGACGAAGAGCATGACGGTCAAAGTTTTTATCAA
CGGAAACTTGTATGGAAGTGGCACTTACGGTCATAAGAAGGAGATCGCTCAAAATCGGGCTGCAAAAGCTGCTTTGGATAGACTCAGGATGATACTTCAGAAAGCACAAATTGGTTCAT
GTTATTCTACATCAAACTAGTTAAGGCTATTGGCATGGTCAGATCCACCTTAAGATCGGGGGACGAAGCGTATAACGATGAACAAATATGGGTGGAGAGGCAGCGCCCTCAATACAAATTC
TGGTTGTAAAGATATTTATGAAAAAAAAATTACATTTTTACTTATAAGTTTGACCCCCACTGAAAATTAAGGCTGGATCCGCACCTGACTATTGAAATATTCATACACTTATTTTTGAAGATG
AAACAAAAGAAATGTAGATATGTCATTGATCCCTCCTCACATAACATGTACATATGTTATGAAGCTTATTCAAACAAATAAAGATTCAGAGGGTTTTTATGATGATATTGTGTTTGTTATTGA
AAATTTTCATTTTATATTCTGACATAAAGTCATGCTGTTTTTTCTTTTCATTATAATATTTT
>comp156382_c0_seq6
AACTTTCAAGCCATGCAGCAGCTGCTCCTGTCTTTCCTAATTCCACCTGCGCATACGAATCTATGTTCTTTTTCTGATTTTACAAGAACAAGGAAGCTCAAGAAATTGCCACGGATTCCCTT
TTTCCAAGAGCAGTGCCTATCCATAATCAAGAAGTACTGGTCTTCCTTCCCTGAAAAGCTTTACTCTCCCATCTTCATCGCCCCAAGCTAAGAAGAGATGAGAAATGCTATGAGATTTCTC
TGTGAAGATTGTCAAAGGCCTACCGCAGCTGATGAGCTCCAAGTATTGGATTCTCATGGAGTAACTATTATTCAAATTGTTGGGGTTGCAGCTTTAGTAGTGGAGCTGCACCACTTAGGCA
TCACCTCTCATGGATTTCTAGCTTTCTATGCTATTCCTTATGTTCCACAAACTATTGAACAAGACAGAGAGGCTACAACATCAAGGCCTGAAGGGATCACACCACCATGCATGCCTGAAGG
GGTCCAGTTTGAGTTGCTAACACTGCCAGTAGATAAAAGGGCTATTGGGGACGGAGATGGCTTTACTGCCTATGTCAATATAACAGAGCCAAGGGAATTGAGGCTCGTTCCTCAAAAAGT
TATAGATGCAACCAAATTGATGGAAAATGCTCGTGATGCGAAGGACTTTAAACTAGTAAAACTAATTAAGAAAAGCGTTATTGAATTTGGATATAGGTAAATCATATGCGTAATCACACTAA
AAATGTAAAATGTTTCCAACTAACATATCTTTTCCTGACAGATTCATAAACCTTCCAAATCAGCAAGAGGTTCTCGCACGTAAATACAGAATCAGATTAAGGGGAGTTGATGCACCTGAGA
TTGGTATGAAATATGGAGATGAAGCTAAACAGGTGCTAGTGAACATAATACAAGGGAAGTGCCTCAAGATTCATGTCTATAATGAAGATAGATATGGTCGAAGTGTTGGAGATGTTTACTG
TAATGACATCTTTGTACAAGAACAATTGTTGAAAAGAGGTTGTGTATGGCACTATTTGGCTTTTGATCCTCGTCCAGAATTTGCAAAGTGGCAAAAGGAAGCACGCGATACTCGTAGAGG
CTTGTGGGCTTCACCAGATCCTGAGAAACCTTGGGAATGGAGGAGAAATAATCCACGAGAACACAGTAGAGAGAAGCCACTATTTTGTTCAGAGATTCTGATTCAATAGAAAATACTAGG
ATAAAAATTACTAGTGTATGTTTATAGAGTATTTGTACAAAATAATTTGCTTGTAGGTTTAGACATG
>comp156382_c0_seq9

AACTTTCAAGCCATGCAGCAGCTGCTCCTGTCTTTCCTAATTCCACCTGCGCATACGAATCTATGTTCTTTTTCTGATTTTACAAGAACAAGGAAGCTCAAGAAATTGCCACGGATTCCCTT
TTTCCAAGAGCAGTGCCTATCCATAATCAAGAAGTACTGGTCTTCCTTCCCTGAAAAGCTTTACTCTCCCATCTTCATCGCCCCAAGCTAAGAAGAGATGAGAAATGCTATGAGATTTCTC
TGTGAAGATTGTCAAAGGCCTACCGCAGCTGATGAGCTCCAAGTATTGGATTCTCATGGAGTAACTATTATTCAAATTGTTGGGGTTGCAGCTTTAGTAGTGGAGCTGCACCACTTAGGCA
TCACCTCTCATGTATGACAATTTTATATGTTAGGAATTTGATTGGCTTTTCTTATATAAATTGTGTAACATGTCATTATTTATACCTACAGGGATTTCTAGCTTGGTATGCCATTCCTTATGTTCC
ACAAACTATTGAAGAAGACATAGAAGCTACAACAACAAGGCCTGAAGGGATCACACCACCATGCATGCCTGAAGGGGTCCAGTTTGAGTTGCTAACACTGCCAGTAGATAAAAGGGCTA
TTGGGGACGGAGATGGCTTTACTGCCTATGTCAATATAACAGAGCCAAGGGAATTGAGGCTCGTTCCTCAAAAAGTTATAGATGCAACCAAATTGATGGAAAATGCTCGTGATGCGAAGG
ACTTTAAACTAGTAAAACTAATTAAGAAAAGCGTTATTGAATTTGGATATAGGTAAATCATATGCGTAATCACACTAAAAATGTAAAATGTTTCCAACTAACATATCTTTTCCTGACAGATTC
ATAAACCTTCCAAATCAGCAAGAGGTTCTCGCACGTAAATACAGAATCAGATTAAGGGGAGTTGATGCACCTGAGATTGGTATGAAATATGGAGATGAAGCTAAACAGGTGCTAGTGAAC
ATAATACAAGGGAAGTGCCTCAAGATTCATGTCTATAATGAAGATAGATATGGTCGAAGTGTTGGAGATGTTTACTGTAATGACATCTTTGTACAAGAACAATTGTTGAAAAGAGGTTGTG
TATGGCACTATTTGGCTTTTGATCCTCGTCCAGAATTTGCAAAGTGGCAAAAGGAAGCACGCGATACTCGTAGAGGCTTGTGGGCTTCACCAGATCCTGAGAAACCTTGGGAATGGAGGA
GAAATAATCCACGAGAACACAGTAGAGAGAAGCCACTATTTTGTTCAGAGATTCTGATTCAATAGAAAATACTAGGATAAAAATTACTAGTGTATGTTTATAGAGTATTTGTACAAAATAAT
TTGCTTGTAGGTTTAGACATG
>comp156405_c0_seq1
TGGATATTTAGGTCAGTTAAGATAAACTCACACCTAATAAATTTCATCATTTTGTAAAAAGAAAACTCACACCATCAGCACAGAAATAAAAGCAAGAGCTTGATAAGATTCCACATATAAC
ATGCATTAAATTTTAAATAAAGATAATTTATGTACAAGATAATCGCATATCATCTTCGACGATGAAAGCACAGAAATTAGTATGTAATCGACCAGACCAAACTCAGCATTCAAACGCCAAAT
TTAACTCGAAGCTTTACCCTCACTGCTTCCTGCTCTGGAGGACGCCCAAAAGGGCACGGCAGGAACTCGCAGAAAAGCCTTCACTCCCAACGGGTTATTCTTGCTCGTTAGGCAAGTTC
CGGCCCGAAGATCGCAAACGGGGAAATCAAGAGGGCAGCAGCTGCTCTGGTCGTCACAACAGGTGGCGGCTTCGAGGGGGCAGCAGCCCCAAGCAAGGCAAAGCCGGCCGTACTCAT
ATACACAGCAGCAAGTGGTGCTTGCTGGGCAGGAGTAATAGTTATCGCAAAGCTTAGGAGGCTTTACAGGAGCGGGAGGGCTTGGGGTTGGTTTTGGTGGGTTTGGACCAGTTTTGATA
GGATAGGAGGCCTGCATTGCAATTCCGCATTTGCCGCTGGACGAATTCACATTACGCTCCATCCTTATGAAGCCTTCCTCTCCCCAGTCTGCGCCCCAGGAGTTCTTCACGACCCAGTAAT
CCTTGCCGTGCTCAGTCCCGTAGCCTACTGCAAGAACACCGTGATCTAGTTCGGTCCGGCAACTTCCAGTGAAAATGCCCGATTGATACAGCTGGAATGCCCTGCCAGCAGCTTCTATGG
CGACACTTACAGGCTGATTTGCAACTGCCTTCTGCAAAGCCTTTTCATTGTTTACAGGCACATCTTCATATGAATCAATAGACACGACCCTAGCATTTCTCCTGTAAGAATCACATTTGCCA
TTACGGCCGCTGTAAGGATAATCCTCCTCAGAGTCAATGCCACCGTTCCTAATGATGAATTCGAAGGCATCATCCATAAGGCCGCCGTTGCATCCCTGATTGCTGCTCGTGTCGCAATCCAC
CAACTCTTGCTCTGATAGCGAAATGAGGTCTCCGGTCACAATCTGATTAATCCCTTCCACGGCCGCAATGCTCGAGAAAGCCCAGCAGCTCCCGCAAGTGCCCTGGTCCTTGACAGGGGC
AACGGCTCCCTTGGCCCTCCAATCAACAGAATCGGGCAGGGCCTCACCAAAAGCGACGTGGTAACGGTCACTAGGATTCCGACGGTGTAGATGTTGCCGTGTACCAAGGTAGGATGCGC
GGTACTCTTCATTGGTGAGATCGGCGAAGCGGTTGAGGCCGAGGCGGAAGGAGTGGACGCCGGCATCGGCAGCGGCATTGTGCTCGTCTATGTAGCGGAGATTCTCCTTGAAGACTTCG
AATCGCCGCTCCTTCTCGTCGAGCGCGTTATAGTTCCGGCCATGCTGCTCCATCCACCTCTCGTAGATCTCCCGCATCTCTGCCTCGCCCCTATCGAGTCCCCGCATGCCATGACGTTCAAC
GTAGCTTATGATCGACATATCTGGTCCATTGCCGCCGGCACCCGGGGCAGCCGCTACGCCGTTGAGGAGGAGGAGGAGGAGGAGAGA
>comp156445_c0_seq12
TTTGTCTACAAAAAGTTATAATTGCCATTTGAAAGGCAAAACAATAAGAAAGATATCCCAAATTTATGACATAGATAATGGATGACAAATTTATGACATTTTAATGCTCTCCAAAGTAGGTTA
TCTCAATAATATTTGTCTAGATTACAAAAGAGTGAGAGGAGATTCCTCTTAAATTAGGGCCTCTAAAAAGAGAGGATAATGGAGAACCACATAAAGAGAAGGGATGACAAATTTTCTATCT
TCAACTAATGATGTATTTCTGCCTCTTTTATCTCTCTTCGAAATCCACACCTTCAAAAAAATATTTTCCAATTTTTTTTCAAAGAAAATTAAGAGTCTCTAACCAGAAAAAAAATATTAAATT

TAAGATGCATCGCTTAACCCATCCAAAACTATAAATCTTCTCAAAATTCTTCACCTTTATCGAAGAAAGAAAATGAATTTCCCTGATATGGAAGATCTAATACCAGGCCTTCCAGAAGAAAT
AGGCTTGGAGTGCCTTATTCGAGCCCCCCACAAAACCCTCCATCTTCTTCAACAAGTCTGCCGGAGGAAACAAGCAACTGAATCCCCCTTCTTTCACCACCTCCGTCACTCTGCTGGTTC
CTCCCACCATATCATCATCTTCATAGAGTCCCACAGCGCAGCCAGACGCTCCCTCAGGTTCTTTTCCTCCCACCCCTACTGCCGTCTCGCTACCCTCAACCAGTCCACTGGTGAGTGGGCT
CCCCTCCCACCAATCCCTTTCTACGTACGCCACCCCTTTTTCTTTTGTATCGTTGCCTCCGGCCTCCACCTCGTCGTCATTGGCAGCTTGTATCGAGACTCCTTAAGTTGCAATGATAGCGTA
TATGTCTATAGCTTCCTCTCCAACACTTGGCATCTCGGTCAGCCCTTGCCTGGCCCCACCCGCTTCTTATTCGCTTGCGCCGGCAGCCCAGACAACCAAGTTTTCATCGCTGGGGGCCACG
ATGAGCGAAAGAATGCCCTGAAATCGGCGATGGTTTATGATATTGCCAAAGACAACTGGACAATGTTGCCGGAGATGGCGAGGGAACGTGAGGACTGCACGGGAGTTTTTGCAGCTGGA
TTGTTCCAGGTAATTGGTGGTTCGATGCAGAAGAGATTAGAGTGGAGTAGAGAGGCATTTGATGTAGGAGAATGGCGATGGCGAGCAATGGAGGAGGATGCGGTGCCGGAGGAAGAGT
TTTTGAGGACCTGCGTAATGGGAGGACTTGGGCGAATTTATAAATGTGGCGTAGGAGGTAAGATGTTGCTGGTCAAGGAAGGAGAGGATTGGAGGGAGGTGGCAGAGTTGCCAGAGGA
CGTCAGAGGTTCTCCAATAATGGTATCTTGGGCTGAAAGATTGATGGTGGCGAGGGTGGAGTCTAGTGGCTGGCCTTCGGCTATGTTTGTATTGGAGATGAAGAAAGGGGTGGCCATTGG
GTGGCGAAGGGTGGCGTTTCCAAAGAAATATCATGGATATATTTATACTGCTTGTGTTGATATCTAAAGCAGGCTACATTAGCTCTGGCGTGCTTAATGATCGAAGATAAGCGTATATAAAAT
CTAGTATTCTGATTTAGCTATATCAGAGAAACTGTTGTGTTATTGTAGTTCGAATTATGCATTTTGATTTTGTTTTGAGGATATAAGAACTATTCTTCAGTGTTTCCAAGAGGAATTTAGGCTT
>comp156488_c0_seq1
GGGATTTGTTGAGTTTACAGACGTGATTTGGAAGCTGAGGATCTTCAAACCCCTGTGGTTGACGCATGTAGATTGCTTCTGGTAGATCGCCGTGAAGGAATGCATTAGTAACGTCGAGTTG
TAGGATGTTCCATTGACGATTGACTGCAAGAGTTAACAGCATCCGAATTGTGGTCATTTTAGCCACAGGGCTAAATGTTTCCGTGTAGTTCTCGCCGAATTTTTGTTCATATCCTAGAGCAA
CGAGACGTGCTTTGTGACGATCAATTTGGCCGGATGGCAAAAGTTTGGTCTTGTAAGTCCATTTACATCCGAGAATGCGAGTGTTGGGAGGCGGTGAGACAAGGGTCCATGTGTGTTGCT
GTTGAAGGGCACAGTATTCAGTCTGCATAGCTTGTTGCCAGTGAGCATGAGGCGCGGCTTGCTTGTAGGTAGTGGGAGTAGGAGGAGCGGTTCTTGGGAAATCTTTTATTTCGATTCTTGT
TCTGGTTCACGGACGAGGCATAGAGAGCAGAGGCCGAGTTGTCAATAGATTGCTGTTTATGCAAGTCTTGATTAATGTGAATCTCCTCGCTGCAAAGCATGGAGTATAGGTTGTCAAGATC
GGCTGGAAGAGCAGAGGTG
>comp156510_c0_seq2
TCAATGCAAGCTGGCTTGATTCAATCATAATCACTTGCGTCCACGGTTAAAAAAAATAATTGTACATAAACATATCTTCACACAATCTCGCCAGGAAAATATGGCGTGGTGTTGTTTTTATTA
ATCTTATTGAATTCAATCCTTATCACCGCTGCTCTATATACCAAAAGCAAGACAAATAGATATATATAGGAAAACAAGAACAAAGGATATTTAGAGATCGAAGAGAAACTGAATACTACACC
CACCATCTAAAATGCCTCAAACCCAGATTGATCCATAGAGCTCCAATCTCCAACCACAAGTTCTTGAAAATGATGATTAGATCCAATTTCTTGCTGTACCCGCGTAGACGCCACGGCGAGA
TTCTGCTCCTGCGCCGGCCACGGCATATTCGGATAGACCGAACCCCAATTCATGGAAAACACATCTCCCCCGTTCATCTCCGGCAACATCGAAGATGAGTACTCCAAGCGAGGAAACACA
ACAGACTCCTCCATCCCACAATTGCTCGCCATAAATCCCAGATTGCCTTCATCTGCTTGACTAACAGTCTGGCAGTTCATATCAGTTGATGGTGTTGACGACGATGACAACTCGGCTGTCA
CGGATCCAATAAATCCAAATGAATCGTCAATCTCTACAGGCAATTTATCAGGCACCTGGTTTAAGTACCTAGCATACATAACTGCCATATCTATATTCGAACCGCCGGAATCCGAACTCGGG
TAATAAGTGTCGAACATGTCATCGAGTGCTTGGTCAGGGCGAAGCCCGGAGTCCAGTACTCTGGTGGTATCAATCCGTAGGCTGCTGCTTGATGATGTGGAGGAGGTTATTGAATCGGAG
GAAAGTCGAGCCGATTTACCACGGCGGTTTTTGCGGCAACCGCCTCCGACGGGGACGTTGCGGAGTGAGCCGCCTTTGGTCCAGTACCTTCGACAGCCCTTGCAGAAGTACCGGGGCT
GGGTGAGACTGTAGTTGTTGTAATAGCAGAATTTGGTGTTTGAGGAATCACAGCGAGGGCAATTCGGGGCTATCTCTAGTGACGGTTTCCATCTCCGGTGATCTACCGGCGCCGATCTCGT
CGCGTAGGATAACATACGGTGATCTTCGGGTGAGAACATCGCGAGGTGGAAAGAGGGAGAGAG
>comp156524_c0_seq3
ACCAGCAACTGGCTAGCCAAAACTGATCTTAGCCCCCAGACATCCCAAAGAACCACATGAAGCCAAGAACTCACAACATGAGCACCAAAGTTACATTCATCTCTAGCAGAAATAGCAGA

CGAACTTAAATGCTGAGATCTATGGAGGACAACCTTGTAGTAGCATAAGAAGGCGTATCCACAAGAAACGAGTCCTCATTCTCTCCTGAAGGCAGTTGTAGAGTGATAGCTCCTAAAGGG
TTTGAAGAAGAGAAAAACCCCATAAACTCTAATACCTTGTAATCAAAGATGCTCAAAAGGAACCTCACAAGAAATCTTCTTCTCC
>comp156530_c0_seq4
ATCTTCATTTTTGCCCCCAAAAAAATGAGTTTCTGATAATAGTATGACATCAACATTAACATTGCACTGCTCACAGCCTTAAAAAAAAATTATTGTTAGCGTTCAGATGTAGGCTCACAGTA
GCTGTAACGAACATTTGCAATTCAGTTGGAGATGCCCTAAACGCATCAAAGTGGATAAAAGTTGTCAGTATATAAGATTCAAATGCAAATTATATTATAATAAATTTTAAAATTTATGATATA
ACAAAAATCATAAATTTTAAACCTATCCTCTTTGTAGTAGTCTGATTGGAATCAGCAATTGGGAAATAATCCAAGAAAAATGCAAAGAATATTGATTATGCCTTCATGGGATGTGATTGTAG
GTGCGCATGACCGATTATGGGTTATTTACATTAAAAATAATAATAATAATGCATTACTTCCTATCTTGTCCTGTTCAATTTTCATTAAGCAGAGAAGTTGCCAAAACAGTTGAACTATTTGCA
CTGAGATAAATTCATCTTTCTTTCACCAAAAATCCTGTCAGAGTTTATACATAGTATGGCCGCTTGTGATAATGATCAGGAGTAAGATTTCCCCACTGCCTAATGAACCGAGGATCTGGAGA
CATCTCAGGCTGATCAAAATCCATCGGTGGATCGCTTGTGCACCTGCGTTTTACCTTTCTGTCCATTTTCTCAAGCAGAATACCATCCCTACAACCCTTGCTCATGCAAATACTCCTAACTTC
TTTTTGCGTCTTTGTTTCGCGTTTAGAAGCTTCAGCAGGAGTTTTATTATGGAACTGCAGTAAATACTGTTCATTAAGGTTCATGCAGGTCTCCAGAGCTTTTACTGCTTCAGCCATGGTGG
GTCTCCCCTTGGGATTCTCGCTGAGGCACTGGTAGGCCAAGTTGCCGGCTATCTGTGCAGCTTTGTGAGAGTATTGACCTGCCATTCTGGGATCAATAATCCTCAGCAGCTTATGGTGGCG
GATGAGAAGGGGTCGAGCCCACTCGACGAGGTTGTGCTCGCGTCCGGGCCTGCTGGTGTCAAGTGCTTTACCGCCGGCGAGCATTTCTAGGAGTACAACACCAAAGCCATAGACGTCAC
TTCTTGCAGTTAGATGGCCAGTCATCACATACTCTGGAGCTGCATATCCATAGGTACCCATTACTCTGGTAGAAACATGTGTTTTGTCACCTATTGGTCCCTGCTTCGCAAGGCCAAAGTCC
GAAAGCTTGGCATTGTAATCCGCGTCAAGCAAGATGTTAGAGGTCTTGAAGTCACGATAGATTATGGGTCTTTTAGCTCCATGAAGAAAAGCAAGCCCCTTTGCAACATCCAAAGCAATT
TTTATCCTAGTGTCCCACGCAATTGTTTGAGGTACCCGTTGGAAAAGATGCCTCTCTAGGCTACCCAAAGCCATATACTCGTAAACCAATATCCGGTGATCATCTTGACAACAGTATCCAAT
GAGTTTGACAAGATTAGGATGGCTCAATTGCCCGAGGTAATTAACTTCAGCCAGCCACTCCCTATCACCTTGTAGACCATTTCGGTTGAGATCCTTAACAGCGACAAACATGGGCTTGAG
ACCTGGCCGAATGTTCTCATCTATGACTCCCTTATAAACAAGCCCAAAGCCACCCTCTCCTAGGATTAGATCTTCTCGGAAGTTCCTGGTTGATAATCTCAACTCATCATATGTAAATATGAC
AACATTTCCATAACTAGTTATTTGTTGAAAGTCTTCCACATCTCCATGCATCACTGGCTTGCTCTCCACATAGCCAGTTCTGTCTGCATCCTCATTTGGAGAAAGTGATAGTGCAATTGAAG
CTTTCACTGATGATATGGCCGCCTTGGTATATTTTGGTGCAGTAAAATCAATTTCTGATGGTACGACAGCTTTGTTCTTTGATGGCCTGGGAGCTTTACTCCTTGACGGTGCAACTGCAAAT
CTATTTGATGGAATAACTGGGTTCCTTCTAGCAAGAGCCCTAGGATCCTTGGAAGAGAGGCAGTTCCCCATCTGTGCAGGTCGTCTCACCACTCGCGGTGGACGAAAATGGTCAATCGCT
TCGGCCCATCGCCGATCTTCTTACCTTGCTTCTTAATCTTCGTCACTAGCGAAGATTCCGTCGATCAACGATTGTTCCTGCGAGATCGCCGGACGACTACGTTTCGTTTCGCGTCTCTG
>comp156576_c0_seq1
CGAACGAATCCCTTTGATTTTTTATTGCTTTTTTTTTAATTAAAAAAAAATCATTCCCTCACTTTCAAAGCTCTATGAATTCTAAGAACTCATATGATATAATTATTATATACAACTAAGTGCAAA
AAATTAAGCTTTCCCAAACATTAACTACCTTTAAAGAAGTGATTAACCATTCAGTAATACACACTTAATTAACCACACTGGTTCTCCTTCACGGCAACAGAACTTTTGCAGTGCGCAATAG
CAGCTTGAATGGCATTCTGTAACTCCTCCATAGTGCTTTCATCAGAAGAGGAAAACGTACTCGACGGGGCCACAAGAAGCCCGCTGTTCGCCGGTGACGTCCGCATCGACGCGGGAGCA
GAGAAATCTCCCCGCCACCGCCGCCGCCAGCCACCTTCCCGTGGATTGCAGTCGCGGGAGAAGGAGTAAGGACTTCGCCGCAATTCTCGCTTGTCCTTCATGGGCTTGAAGAAGAGAAG
CGGCTCTATCAAGCTCGCATATTTCTTCCATAATCTGCCCAACACCTTCGTCTTCTTCTTTCTCTGTTTTTCTGCTTCTCTGTCCTCGCTTCCTCTTAGCCACTTCCGGAGAGCGAAGAAGGT
GGAGAAAGGTTTCGCCTTTGTGCGTTCCTTGAATTCTCCGTGGTTGGAGGAGGCGTCGGCGCCGCCTTCAATGACTCTGTTTGTGTTTGGTTTGTTCTCCGTGTTTTTGCAGAGGTTGTGT
TCTGTTTCGATTGAGGCGTTGGAGGCGCGGTGTGAGAGGAAGTGAAGGGGGAGGAGGTGGCCGTGGAAGAAGATTTCGTCGGCCGGGGAGAGGTCGAAGTCGAGGAGAGGGGCGGC
GGGTTCTTTGAGCTTGAGGGAGGCAGTGGTGTTGGAAGAGAGAGGGGTGGGAGGGTGGAGAGAGATGGTGAAGGAGAATTCATGGGATGGGGAAGAGTTTAAAGGAGGAGGAGGTG
ATGAAGGTGGTGATTCCGGCGGTGGCGGAGGCTCCCGCGCCGAGGCTGGTGGTTTGTGATGTTTCTCTGTTTCCATGGTTTGGTTTCTGATGCTGGATTTGAGGAGAGAGGGAGAATGCA

GGGAAGTTGATAAGGGAAGTCGAGAGATAAGGAGGAAGTGAAGGGAGAGAAAAGAGGAGGAGATGGAGGGGGTGAGGAGAGATTCATGGGCCATATGAGCTTAGTTACATGTCTCTTT
GT
>comp156588_c0_seq3
TATCATAGTCAGACAATTGATTGAACTTCATTATGATCTTTCGAGATCATATGTTGTTTCCCCTTGAGTCTGACTATAATAATTTAGATAAATTTAAAATGATAATAATTCTGACAAAAATTATA
ATATGCAAGTAAATCTCCCATATTTATTTTTATGAAAAAAAATGGTTGAAAAAGAGGCAGAAATAGAATAAGTATATGACATACACCACACGAGAAAGGAAAGAAACGAAGAATTGCAGG
CCTTACAAGTCATTCAATCACCGCTGGCTACTGCTGCTCCAGCTACTCGTGCTACTGTTATCATCTTCTTCATTCTCACCCGCATATTGCGCAGCAAGATTAAAGCAAGACATCGCCCTAGG
CCGCCTCGGCTTTGCCCTTCTGGACCCCCCTTCATCATCAGTTGCCGCCGGCGATTGCCCCCCTTTCTCCCATCCCATCTGATCCTCCACGGCCCAAATCTCCGATCTCCACTTCTTCAGCT
TCTTTTCCAAAACAACTCGCTCCTTCTCCCCCGACCCGCCCACCGACCCGTTTCTATCCAGAGCCGCAGGCTCAGGCTGAGGTTGCGGAAGCCGTGGCTGAGCGGCGACGAATCCCTTT
AACCCATCCTTCTTCCTCGGCTTGGCGGACATGGCGGCTGCCATTAGATCGTGGTAGGCGAAAGCGGAGGTGCGGAGATTGGCGTAGAGCGGGAGGCTACAAACGTAGGGATCGGCGAT
GCCGATGCCGATGTTGAGGATACCCTGCGGACGGAGGGAAGTTGGGCGGCGGACTTGGAGGGCGAAGAATCGGGTGGAAGGGGAGAGGTGAAGGGTACTGAGAATGGCGCGGGTGGT
GCCGAGGAGAGTATCGGGGCCGGGATGGAAACGAGGGCGGGCAGCGTAGATGTTGATTTGGATGGCGGATGTGTCGGAACAGAGAGTGGAATCGTCGATGCGGAAGAGGAATTTGTCG
TTCCAGGTGGGTGACGTGTGACCGATGGAGTCGAGACGGGTGCGGAGGCGGTGGGAGGGGTGGAGCCAAGTCACGGCGTAGGTGTGGAGGGAGCGGGAAGAGGGGAAGAGGTCTTG
GGCGGATATTAGGGTTAGCTCGAGGAAGTTGAAGGTGGACGGAGGCGCCGTTGGGGAGAGGAGGAAGTCGCCGCCGCTGGTGCCACTGACATCCGGCGACATTTTTAGGAGGAGGTGG
TGGGAATTTGGGGTTGGAGGAACGAGGAAGGACGGAGATCGTGTAGGTTAAAGAAGGGGGAAGAGAGGAAGGTGCGGTGGAAGGGAAAGCGGAAGAGGCGCCAGGGAGGGCGGGA
CGCGCGGTTAATTTAAAGGAGAGGGAATAATTTGCTTAATTTAGTATGATTTTGTTAATATGTTAGGAATATTTAGGCTAATCTGGGAAATAAAAAAATAGTAAGAAAATATTGT
>comp156601_c0_seq1
CACCGCCGCCTCCGCCGCCTTAGAAGCCGAAGCAGATGGTGTTGCTCTCACACAAAAAGAGATCTTTGCTTCAAATATTTCTTCCTCTGCTTCTTCCATCGAAGCCGATGACCATATCTCC
TTCTCGCCGGCACCACAACCGGCAGTATCATCTAATGAGCTGATACAACTAGAAGACTCTTCTCCACCTGTAAGAGCGAGGAGAAGAGATGTTCATCTAGAAAGACTTGAATTTAACCTT
GCAAAAGCTCGAGCTTCCATCACATTTGCTGCACAAAATCCAAATAAAACTTCTGCAGAAGATAAAGATTATGTCCCAATCGGCCCAGTTTACAGGAACCCCTATGCATTTCACAGGAGCT
ATCTTGAAATGGAGAGGCTCTTTAAGGTCTATGTTTATGAAGAAGGCGAACCGCCATTATTCCATGATGGTCCCTGCAGAAGTATCTATTCAATGGAAGGAAGGTTCATTTATAACATGGAG
ATGGGAAATAGATTTAGAACCAGGAATCCTGAACAAGCACACGTTCATTTTCTTCCATTCAGTGTAGTCAAAATGGTGAAGTTCATCTACCAGCCAAAGACATTTGATATAAGCCCCATTA
AAAGAACTATTGTGGATTACATAATGTTATATCTTCAAGATATCCCTATTGGAATAGAAGCCTGGGAGCTGATCATTTCATGCTCTCTTGCCATGATTGGGGGCCACATGCTTCCAAGTCTCT
CCCTTACCTCTACTCCAACTCCATCAGAGTCCTCTGCAATGCCAACACTTCAGAAGGCTTCAAACCCCTCAAAGATGTCTCCCTCCCAGAAATCAATCTTAAGACCGGGGTCACCGCCGG
CTTCATCGGGGGCATACCGGTCTCTCGCCGCTCAATTCTCGCCTTTTTCGCCGGCGGCATGCACGGTCCGATCCGGCCAATTCTCCTCAAACACTGGAAGGACAAACATGACAATGACAT
GCAGATCCATGAATATTTGCCGCAAGGTGTGGACTACTATAAGATGATGAAGAGTAGTAAGTTCTGCATTTGCCCAAGTGGCTATGAGGTAGCAAGTCCAAGAATAGTGGAGGCAATCTAT
CTTGAATGTGTGCCGGTTATTATATCAGATCACTATGTTCTTCCCTTCAGTGATGTGTTGAATTGGAAGTCTTTCTCGGTTCAAATTTCGGTTGATCAGATACCTGAATTGAAGAACATATTG
GCGAGGATATCGAAGGGGCAGTATATAAGGATGTGGAGAAGGGTGAAGATGGTGCAGAAGCATTTTGTGGTGAATAGTCCGCCTGAGAGATTTGATGTGTTTCATATGATTTTGCATTCTA
TTTGGCTTAGGAGATTGAATTTTCGTGTTCAGGATTTGGATTGATATTTATTTGTTGGGGGAAATAAAGAAC
>comp156630_c0_seq2
AATTTTAAAAAAAATAAAATAAAATATAAATAATTAAAAACTTCTTTATCAAAAGCTTAATGAATTTACAAAGCCAAGAAAATTTGGATACTATAGCTTGATTTCATAACCTGATTCTCTTAT
GAAAGTGAGAACACCCATCAAATGTTTCATAAAGCTACTACATATGAGAAAGTTGATTGTAAAAAAGCAGTTTCTTGCAAAAAAAATAGAAAACAATTAGAAAAGTATAAAAAAATAGTAG

CCGAACAAAATATAATTGTGATTGTGGTTTGAAAAGCAAAGCTATGAGAAAGAGACCCTAAAATCACTTTGTTCCACATACAAAAAATATATATAAACAACATAAATTTATATGAGAGCAA
AAATTTTATGGCACTCAACCTAACAAAAGCTCATTAGAACAACATTAATACAATGTTACAATAGAAGCCTTACATATAATGTTAAAAGATGCAAAACATATAATTTTAGTCAACTTTAACTAC
CCATCCTTTTGGGAAGGCGCTAGATAAAGACTTATAGAAGAAAGAAAATGAGAGACTCGGCCGGTTGACTCGAATGAAAAAAGAAAAAAAAAAATGTGAGCGCTTTCGTCTTCAATGA
GGAATAAGGGACGGAGCTTGAAGAAGCGAGCCTAAGCTATTACATTGCTTTCTATGGCTGATTAGTCTTAGTCAAGCGCTAGCGCCCCTCTTAAGTAAGGTAAGGCTATTCTACTATCAAT
GAATTTTTCGTTGGAATGACGAGACTGATTCCCCAGCGCTGAAATGCAGCAATAATGGAACGCATCGGCCGGGCACAGCTACGCATGGAAGACGGTCAACTTTACTAAACTGAACAAAC
TAGCACCTTTTTTTGGTTTGAAATGCACCCCAAAATAGTCAGGCGTGGGGAATATGGTATCCACCCTGACAGTTTGAGGGAAGAGACGACTGTCTTGATGATATCATCTCTTCTATCTCCCC
TTCTTGAATGGCAGAGCGCGAACCCTCTTCGTGCAACTCTTCCCGGGAAGTGAATTTGCTTCTTCTCCTTTTTCAGAAAGACTGAGTGAGTGGTCCTCTTCAAGCCACCACTTGTCCTCC
AGATCGATTTCCATCAATGTGAGATACTTTAGAAGAAAGAACATGGAAAAGAGCTACAAGCAAAACTCAGACCACATATTAAGAGACAAACTAATTGAAACCAATAAAACCAAGAAACA
AGACATTAATAAGATCTAATTTATCATTCCTTAAACTTAAAGCTAGGTTAAGTTAGAAGGGCAAAGTATTTTGAATGTACAAGAGAAATCAAATGAAATAAAACTTTAAATAGAGTGCATAA
CAAAAAGAATCATATTGAGAGCTAAGAGAGCCGTAACACATGAGACCAA
>comp156659_c0_seq7
AAGAGCAAGGAAATAGGGGCGAGCGAAGTGGTACAGTGGAGGAGAAAATGGCATGGCTTCTTCCTTTGATCCATATCTTCCTCTACTACCTCATTGCAAGAGCTCCAGTCTCCAGTGGCT
GGGGAATAGAGGGCCATGTCATGGTCTGTAAGATAGCACAGGAACATTTCACTAACTCAACAATGAATGCGGTGTTAGAGTTGGCTGGGGGAGAATTAGCACCGTTGTGCCCTTGGGCTG
ATGATGTTAGAAAACAGTACCGTTGGTCAAGGACTCTCCACTTTGCTGATAGCGCTGGTGACTGCACTTTCCAGTATTCAAGGGATTGTCACAATACCAAAGGAGAGATAAACATGTGCA
GTTGGAGCAATCAACAATTATACCAGTCAGCTCCAGAAACGCAAAGATTCATCAAATAATTATAACTTAACAGAAAGCTTATTGTTCCTTTCCCATTTTGTGGGAGATATCCACCAACCTTT
ACATGCTGGTTCTGAGCTTGATTTAGGAGGTAACACCATCAAAGTGGAGTGGTATGGAAAGAATACCAATCTTCACAAGGTCTGGGATGAGGATATAGTAGAGAAAGCCAAGCAGAAATT
CTATGATGATGATATTGATACAATGGTTGAATCCATTCAAATTAACAATAGAGTAAGTTCATTGTTTTATGAATCTTTGATTGTTCATCATGAAAGTTTTCTGGATATTTATGTACTTGTCAGTT
GTTTTCTTCTGAATTGCAAGCTATCTACAGGACATTTGGTCAGATGAGATTGATCAATGGATGAATTGTTCTACCAAAAACTTTACTTGTGGAAGCATCTATGCTTCCGAGAGTGCCCGCCT
GGCCTGCGACTATGCTTATAAGGACGCCAAGCAAGGTTCAGATCTTGGAGATGAATATTTCATCTCTCGTCTTCCAGTAGTGGAGAAGAGAATAGCACAAGGTGGAATTCGCTTGGCTGA
GATACTCAACAAAATTTTTGATGATGATTCTGGTCAGAAAGATCAGAAGCTGCTCAAAGAGAGAAGAATTGGTGGTGTTTATCAAGCTTAATCTGCCGCATGCCATGAAAAACTGCTTATT
TGGAAAGAAGTTTGAGCTTTAAGTAGTTTTTTTTTCCTAGACAATGAAGCTTGGTTTGGATAGATTGAAGTGAAGAAAATTAAATAAAATTCTAGTGATTATGGACAGATTTTGAACCTATA
CCTATCGATTAGAATTTTTA
>comp156682_c0_seq5
GGCAAGAAAAATCTTGTTTGATTTTTTATAAGTAAATTTATAGTATTCACAATAATTAAAAAAATGTTTTTTTTATATCAAATCTATAGTAACTTTTAAAAACAATTGCAAATCATCCCAAAAA
ATCCTAAACATGAAGAAAGAATACTAACCTTTCAAACTTGATAACTTGGCCAAGTATTATTGTCGAAAGAACCGTGGAGCAAACGGTCTGAATGGGATTAAACATAGAGACCATTACTGG
CCCCTTCCTTAGCACAGCCCAAGTTTGGAATGCAACACATAGTGCAATCAACATTCCTCCCAGGAAGACAAAGGAGATGATGGCTTGGATACTGATAGTTGGCTGACCAAAATCCAGCTT
CCCTTCCATGATAAATTGCACAATTGTAGTAAGTACTGAGGCCAGCAGGGATGTTACACAGCACAAGGACAAGGGAGCCGGGAACTCCATCATTGTTGCAGCCTGCAAAACCGTGCTAC
AGGATAGAACGAGAACTGCTCCCAGCAGACTGCAGCAACCAATGATCCAGTCCCTGTACACATTAACTGGCGACGGATTAGGCAAGACAGAAGAGCGTCCAAAATCCGTTGGATCTGTA
GGACTTTGCAAGATGCTCAAGGCTACTGCTCCAGTCAAGCAAACCAATGTCCCTAGGATCTTTGTTCTAGTGTAGTTGCACTTAAAGTCTAGTTTTTCAAGCCTACAATGGAAATCAATCT
CATGTATTACTGGTCCATTGCAATCCCAATA
>comp156725_c0_seq12

TTTCAATAAATTGAGATGGTTTTCTCCATCAGTAACCGCGTCCTCTTCCGCTTCTTTTTTTTTTTTTAATACTGGAACTAAACATGCAAATGTCCATCAAGAAGACAACAAACACGATTAAC
CAACAAAGCAAGCAAGATCAAACCATGCATGCAACCGCTTATCTACTTGTAATGAGCGTACAGGTCGAAGTTATCCTCCAGCTTTTGAGAAGAACCTGTCACCAGATCTATCAAACCATG
CATCCAATCGGCTACCTCTTTCTTTTGGTTAGGGGTTGTGATCTCTGCCTTTCTAATTTCTAGTATTGTTGAGTGTTCTTTCTTGATTCTTTACACTGAGTTCTGTATGATCTTTACGGCTTATG
TTTTTTTTTTTTTTTTATTTGAATGGAATGGAAGATAAGAATTAAAATAGATAGAATTCATTGAGATTATTGAGATTTTTATGGAGGAAATAGTGGATGGATTGGGATGGTTTTATTTTTTTAA
CCTTCAAATGATAATGGGTCAATCACTGATCACAGAAAAGTACGGGCTCCTAGGATTTTATCTCATGAAGAAAAATAAAAATGCACAAAAGATAGAGACCAATGGAATGAATAGAAAGAT
GAGAAATAGTGGAAAACTGGAAGAAATTGAGGAAACAAAT
>comp156725_c1_seq2
TGTCACACGATCTCGCTGGATCTGCTAAACTCCAAGATCATCACAGTGGCCGCATATAGATTCCCATCTTCCACACTTTTCCTAAGCAATATCTAAAAAAACCTAAAGCACTATAGCTGGAA
AAAAATCAAAAATAAAGAAGTGCAGCGGAAAGCCGGAAACCCTCGATCTCCTCTTCTCTTCTTCCTACTTCACTCTTCAACCATCTTCTCTGCTTCGCTGATTTGAATGCTGATCTGCTGG
CCAGTAATGAGCATAAGAAAGCAGGCTTCGAAGCTCCCAGGAGGGGCATCCCTTCTTCATCTTCAACTTCATTATCACTATCTGAGCAGGATGAATGGTCGCCGGGACCGCCTCCTCCGT
CGCATTTACAGTTGTTAGAAGCTTGCGCAGGCGAGTTGCAGTGCCAGCTCAATCCATCTCGTGTAAAGAATCAAGAAAGCAC
>comp156735_c0_seq7
TTAATTGAAGAGTTTAATGCAATTAATAGTAATAGAATTATTTGCAACAATAATCTACAGGTCTTATAGCAGTACTTCAGTGACAATATTTTGTTAGTTAAAGTCTACAAATGCAAACAAAAC
TGAACTGAATGCATTACCAAAGTAAATAACAAAGCAAGGTACATATTAAATCTAACTCACCCTGTCTAACATTTCACTGTAGACTATTGCCCGTTTATATGTTAAATAAACCTCTATTTTAAG
AAGCTCAAATGCTCACATTGGGTTTGATTATTCTGATTTCTTCTCCATTGTTTCCTCAATGCCTTCAGCAGATGGTGGTAGACCACCATGCTTCTTACTTCTTCCCTGGCAATAACAAGAATG
ATGAAAATCCAACATTTGTGAACTTGTGATCAGCAGTGGAATTAGGTTTTAGAACTATATCAAAGCTATTATACTGTATGAGAATTTCTTCTAAATTTAAGCTGTGACTTGGGGTGTACACG
AGTTGAGTAATGACGCTATCGAGCTCGACTCGTCAGTCAAAACACCAGCTCGAACTCGAGCTCATCCTATTATAAATGAGCTCGAACCAGCTCACAAGCCTTAGGCGTAGCTGGCATTAA
ATGCTGCAGGATACATTAAATGATATAGCATAACTTTAATATTTAATATGTTGTTATGCTCGGTTCATTCTCTAGCCCACCCGATTCCCGAAGTCTGACTTCAGCACATTTTAGGCGGGATTAT
AAAAAAATTATTTCATTTTAAGAATGATTTAGGCGGGATTAAAAATATATATTGATTTTTAATAATATTTAATTTAATAAATAAAAAAAAAATATTCAGACTAGCTTGCGAGCCGAGTCTCAT
CAAGCTCGAACTCAAGCTCGTTTAATAAACGAGCTTGAGTTTGAGCTCAAGCTCATCTCGTGAGCCCATAAATGAGTAAAGATCGAGCTAGCTCACGAGCCGGCACCGTTTTCACTTACA
CCCTAGCTGTGACCCCTCTGCCACTTTTGACTCCTAGAATACTCGAGAAATAGTTCTAGTCATAGAAAAGATTAC
>comp156769_c2_seq1
CTAAGCGGCGCTTCCATGAGGTGAGCTTGCTCCCTTCATCTTCAATCCTCTTCTTTTGTTTTAATTGAACGCCCATGAGCCAATAATTTGGATTGGGATTTGTTAGGAGTAAAGGATATGCTT
GGAGAGTTTTTTTTTTTTTTTACTTATATTACCATTTTTATTTGTTGCATTTTTTCAGACTACTTGCCTTTTGATGGTTTGATTGTTCTAATAACTTTTTTTTCTATGGTTTTGTTAATAACTGGTC
TGCAAAGAGCTAAAGAGCCAGGTTATATGATTTAAGAGCTCTGAATTTGGTTATTTTCCATTTATCTTAGGCTGGACAATTTTCTTAATGCTTTCTAAAACATTTTTTAATAACACAAATTTAA
TCTGAAAAAATTTTGTACTGAAAAAGGCGTTTCAGAAAGTAGACAGAAAGCTGTCTCAATTAAGACCTAATTTAGGCTCCGAAAAGTTTTGGATAAACATAGAAATAGTGTAGAAAAAAT
TATTTTTATTTAATTATTGTGAGTATTATTTTCTTTTTTTTAAATAATAATAATATTTTTTCTTATAGCTATCAGCTTTAGTAGAATAAAAAACTTTTTTCTCATGAAAAAACAGAAAAAGGGAA
ACCTCAGCCAAGAACC
>comp156769_c3_seq2
TTTTTTTTTTATAAAATAAAAGCACTTTCTTATTAATTTAATTTATGAAGCAACTTTTAGTACAAACTTTTTTAATTTAAAAACTGCTTTAAAAACAGTAATACAATCATTCATTCGCCGAGAA
TTCTGAAGGAGACTAAGGGCATTATTTGCTGTAAAAGTCTAATATGCTGGTGCTTCTGCGCTTCGTCTTCGCATACGAGCTCCCCAAGAGAGATTTTCTTCTGAATTCTTCATTGTGCTCCT

CCTGCTTAGCTCCACAAGCTGATCATGAAAACGTTTCTGCAACCAAGATTCCTTCTCCATTTCCTAAATCAAGAGGAAACCATACGAACAGCCGCATTAGGAACTAATTTTCTAAAAAATT
TTGGACAAAATGATTGAATGAATGAGAAACTTCGTTCAGTGAGGAATAAATGCAGAACCAGAATAACAGGAAGGAGGCTATTTCATTTAATTTCATTGATCAGAACCGCCCCTGTTTTCTA
TCAGAGAAAACCAAACAGTTTTTCGGTTCTGTCAGATGGAGAAAAGTCAATGGAGTTACTGGACAATTTTTTTTTCTTTTAAATGTATATATATGTGTGGTGTGCATAATTGCATGTCTATTT
TCCATGTGGCAAGAGAAAGAGAGAGAGAGAGGGATCTATGAATTAATAAATATATTTAAGTTTTTTTGGGACAATTTTTATAATGTTTTTTAAAGCAATATTTTAGTTAAAATTTTTAAATTC
AAAATTATTTTATTCTAAAATAGTTGTTTTAAAAAGTAAGAAGT
>comp156769_c3_seq3
AAAAAGCCGTCCCAAACGAAACCTTAATTAGCAGCAATGCTAAAGAAATTTACATCACTTAAGCTTCTAATTGATCTCACACTTGAGGCATTTGCCACTTTCCACTTTCTTTGAAGCAACT
GATCTTGTAGAGAGTAAGAAATTGGTGAGATCTATAGATACAGCCTTACTGCTGAATCCATAAATCTGATGAAGCTTCAGGCATGATTGAAGAAGCCTAATCGGCTAAAGATTCCGCCCTT
CGCTTTCGCATGCGAGCTCCCCAAGAGAGATTTTCTTCTGAATTCTTCATTGTGCTCCTCCTGCTTAGCTCCACAAGCTGATCATGAAAACGTTTCTGCAACCAAGATTCCTTCTCCATTTC
CTCATGGAAGCGCCGCTTAGCGGTGGAGAAGGCCAGGTGGAGATCGCTGCGAGCAAGGTTGCGATCATAGATGGCACGGTGACAAGGATCTAAGAGGGTGTCATAGGCCTCCTTTACCT
CGATAAACCGCTGAGTATACTCCTCCGTGCGATCCGGGGGTGAGACGTCAGGATGGTACTTCCTCGCGAGGTGCTTGTAGGCGCGCTTGATATCGTCGGTGCTCCCACTCGCCGGAATCC
CTAGAAGCTCGTAGAAGCTACCCTTCGTCTCCGGAAGCTCGACAACGGCCCCGAAATCCCTCATGGCCGCCCGCGGGCCAGAGATTAAGCGCGACTTGCGCGGTAGAGCCGTCCAGCTG
CGTCCCCGCACGGCCGCCGGCATCCAACTTTGGTAACAGCCTCCGGCGGCGGGCGCGAGGCTGGTGGTTCTTAGAAAAGGCGAAATGGAGCTATTTCTGCAATTCGGAACGGCGGATTG
AGACTTGATCATTTCTCTCGCTTGCTCTCT
>comp156769_c3_seq5
TTTTTTTTTTATAAAATAAAAGCACTTTCTTATTAATTTAATTTATGAAGCAACTTTTAGTACAAACTTTTTTAATTTAAAAACTGCTTTAAAAACAGTAATACAATCATTCATTCGCCGAGAA
TTCTGAAGGAGACTAAGGGCATTATTTGCTGTAAAAGTCTAATATGCTGGTGCTTCTGCGCTTCGTCTTCGCATACGAGCTCCCCAAGAGAGATTTTCTTCTGAATTCTTCATTGTGCTCCT
CCTGCTTAGCTCCACAAGCTGATCATGAAAACGTTTCTGCAACCAAGATTCCTTCTCCATTTCCTCATGGAAGCGCCGCTTAGCGGTGGAGAAGGCCAGGTGGAGATCGCTGCGAGCAA
GGTTGCGATCATAGATGGCACGGTGACAAGGATCTAAGAGGGTGTCATAGGCCTCCTTTACCTCGATAAACCGCTGAGTATACTCCTCCGTGCGATCCGGGGGTGAGACGTCAGGATGGT
ACTTCCTCGCGAGGTGCTTGTAGGCGCGCTTGATATCGTCGGTGCTCCCACTCGCCGGAATCCCTAGAAGCTCGTAGAAGCTACCCTTCGTCTCCGGAAGCTCGACAACGGCCCCGAAAT
CCCTCATGGCAGCCCGCGGGCCAGAGGTTAAGCGCGACTTGCCCGGTAGAGCCGTCCAGCTGCGTCCCCGCACGGCCGCCGGCATCCAACTTTGGTAACAGCCTCCGGCGGCGGGCGC
GAGGCTGGTGGTTCTTAGAAAAGGCGAAATGGAGCTATTTCTGCAATTCGGAACGGCGGATTGAGACTTGATCATTTCTCTCGCTTGCTCTCT
>comp156769_c3_seq6
AAAATAATACTCACAATAATTAAATAAAAATAATTTTTTCTACACTATTTCTATGTTTATCCAAAACTTTTTGGAGCCTAATTAGGTCTAGTTTGAGACAGCTTTCTTTCTGTTTTCTGAAACG
TCTTTTTAGTACATAATTTTTTCAGATTAAATTTGTGTTATTAAAAAATGTTTTAGAAAGCATTAAGAAAATTGTCCAGCCTAAGATAAATGGAAAATAACCAAATTCAGAGCTCTTAAATCA
TATAACCTGGCTCTTTAGCTCTTTGCAGACCAGTTATTAACAAAACCATAGAAAAAAAAGTTATTAGAACAATCAAACCATCAAAAGGCAAGTAGTCTGAAAAAATGCAACAAATAAAAA
TGGTAATATAAGTAAAAAAAAAAAAAAACTCTCCAAGCATATCCTTTACTCCTAACAAATCCCAATCCAAATTATTGGCTCATGGGCGTTCAATTAAAACAAAAGAAGAGGATTGAAGATGA
AGGGAGCAAGCTCACCTCATGGAAGCGCCGCTTAGCGGTGGAGAAGGCCAGGTGGAGATCGCTGCGAGCAAGGTTGCGATCATAGATGGCACGGTGACAAGGATCTAAGAGGGTGTCA
TAGGCCTCCTTTACCTCGATAAACCGCTGAGTATACTCCTCCGTGCGATCCGGGGGTGAGACGTCAGGATGGTACTTCCTCGCGAGGTGCTTGTAGGCGCGCTTGATATCGTCGGTGCTCC
CACTCGCCGGAATCCCTAGAAGCTCGTAGAAGCTACCCTTCGTCTCCGGAAGCTCGACAACGGCCCCGAAATCCCTCATGGCCGCCCGCGGGCCAGAGATTAAGCGCGACTTGCGCGGT
AGAGCCGTCCAGCTGCGTCCCCGCACGGCCGCCGGCATCCAACTTTGGTAACAGCCTCCGGCGGCGGGCGCGAGGCTGGTGGTTCTTAGAAAAGGCGAAATGGAGCTATTTCTGCAATT

CGGAACGGCGGATTGAGACTTGATCATTTCTCTCGCTTGCTCTCT
>comp156771_c1_seq2
AAATCTGCTGCGACAACAAAGTCAATGTTGAGCACTTCATTTCTGATTGCGTCGCCCTCGAAATAAGCTCTGTTGTCTGTCCTGCCATTTTCTCTTAGATCACCGCCGTGCTTTTTGAACTC
TCTTGCATGGTGGGTGCGGGTGAAGTTTGGACTCACAACGGTTGCACCGCTGGCATCCCCAATCGATTGATAAGAGACCATATAGAACCCCCAAAACAACAAATCATAGAATATAAGCTC
CATCTTTTATGAGCTGGAGATATGGTGTTGCTTTCACAATCATTCTTCTTTTCAAAG
>comp156809_c0_seq3
TATATATATATATATTATTTTCTACATAATAACTTCCTAAATTATTATGTAGAAAATAATATATATATATAAATATAAGCAGCACCGTTATCATTTCCCTGTAGATAAAACTCACATAATGAGCTGG
ACCTTTCACAGTGCAGCAGATTTCAACCTATGCACCGGCTATATCTGACAAACTAATAAATACTACCAACATTACAAAGGGATGGGATTGAATGCGGAAGCCACATCGTTTTCTTTTGTCTC
AAATAAATAACGTAAGGATGAATGAGTTTCCTCGGCGAAGCACAAACGCGCACATTTGTCCAACCCGATCTCATCTCGTACCCGAACCATACAAAGAATTTGCCCCCACATGCCAACTGA
GGAAAGAGAAGGGCGTAAGGGGACCGTTCGGCGTGGCGCACACGCCGTCCCTCACCGGCCTGGTTGTCGCCTCCGATGTGACCCGATGGGTCGGCAGCTAGCCAGCATCGACCTGCCC
GCCCCTTCGGGCCAAAATTCCCGGGCGATCGGGCCTTCCCTTTCCGCACGCGCCGTGGCCGCCTCGGTGTCTTCCAAATCCTAACCTCCTTGTCGGCGATTGCCTTCGTCAATTCCATCCT
GCGTTTTCCCAAGAACTATTTCGACATGACATATGTATATAAATAGATACATAAAAAACCAGCTTTTAGAGAGAGAGAGGGGGGGGGAATCAAATTAAAGGAACAAAATGATCATGAAAATT
TAAGAAAGATGGCGAGGATGACGCCGCACTTGCGAGATGCTAGGTTTCTGCTCCAAACCACGTCAGGTCGAGTCTTTGTCCTTTCTCTTACAGAAATGCGCTGCATCTATGAATGATACG
AAGAATATTGGCTCAAGCAAACCATGTAATATGAAAATATAACAGAAAAGAAAGAAATAGCTAAGAGATAAATAAAACACTTGCGAGGTTAAACCATGTAATGCCTGTGGTAAAGGAAGG
AGAAGAATAGATGCTATTTTTTTGCCATGATAGGATATTAAAAAAAAAAACGAAAGAAGAAAGTTTTGATAGCAGAGGAAAGACAGCTATGGCGTGTGAAAGCACAGAGAAGAGCGGAT
AAGCGGTGTTATCTTGCGCAAGACCACGCAGCCAGGCCATAAGCAGCGCATCGGCGTGCAGACCTATCCATTGATCAGTACTCGATCTTCTCTTTCTTCTCTTCTGCTGCAAAGAAGAAG
CTCCTCTAATTGATAGGCCAGTACAGCGATGCGGTGTAATATGGAAACGTTGCGCGGTCTTGAGCAAAACACCGGCCACTATGCACCCACAACCTTCACTCTCACATGACCCAGCTTATGA
GGAAGACAACACAGGAAATAGCTAGGCTGTAAGAGACATATATAGGAAGAGGTATGGCAATGAATTTGTGAAACAACGGAGATGGCTGTGTTGAAGGAAAGGGGACTTCAAATTGGTG
AAAGAGGCAGGCAAAAAGACTTTGAAATAAATTTGTGAAGGAGAAGGGAAGTGAGAGAAGAGGCTGAAGAGAGGGGTCTTGAATGTGGAGGAGAGATGGTTAGCCAACTAAAGGTA
AAAGGTGTGACTTTCTTGGAGGAAGAGAGTATTGATGAAGTGATACATTTATAGAAGGGGCAGCTGCAACCGTAGCAGAGATCACAGGGACGGGGTTGAGAGGCTGAATGGTAAATCGT
GTTTTGTGGGTACCATGGCATTGAAGGGTTTTGACCCTGTTATTTGGCCTTCCGTTTCAATAAACGATATGTAGTGCTTAAGCTCCGCGTGTTAATTTGTTAGCTTCATTAAAACCCTCTGAT
TAAGCACACCGTTTAAATCACTGAGTATCTATCTCAAAGCATTC
>comp156821_c0_seq2
TGCATCTATATTATTGTACACCTTAAATGCATCAAACCAAACAGTCTCTAAGCCATTTGACACAATAACATCACATTAATCGTTAAAAGAAAAAAAATCATATTTTCTTAGCTCACATTTCTC
ATTCTTGCTTGAACAGTTTTGTACGCCCTAAAAAACTGTCATATGCAAAAGGCGTGGGTAATAAATAGAATCCGAATAAAGTGGGATTGTTAGAGAGAAATTAGGGTTCGAGATGTTCCCG
AGTGCGACGTGGGCAGCTTGTTGCAGGCTGCAACGTAAGTTTAGCCAACTCAAATCGTTACGACCGTTAGGGTTCAGAGCATGGTGGGTCCGGAAAGAGCTTTGCTCCATTTTTCTCCTT
CTCCCCGGAGAAAGCAGTGATTCCAAAGTGAGATAGGGAGAGCCCTCTCAACGGCTCCTCCCATCTCCTTCTTCTACTCTATTGGAAGGAGAACACACCAGCAATGGAGAATGAAGAGA
ACAATGCTGAGTCAGGGTTGGAGATTGTAGAGGAACCTGCTGCAATCAAATACTATGGCTGGAAAACAATGCCTTATGTCATAGGTAAGTCAATAAGTTAGCTGAGGAAGAATAAAATAA
AATATATATGCAACTTTCAAAATATTTTGAACGTAAATGCCATGCTCAGGATGTATATAACAAAATTTTCCCAAAATTATGAGGAAATGAGTGCTCCCTTTCCATGGATTTGTTAGAATTGCT
GAAATTTATGAGGAGTCTCTAGTGAAATCTAATCTTATACGTACACATGAAGATTTTCTGCATCATTCCTCTTTGATTGGTATGAAATTAATATTTTTTTAAAGTTAAACAGGCAATGAAACAT
TTGAGAAGCTTGGGGGCATTGCCACAGCATCAAATATGCTTGTCTATCTCACCACTGTCTTCCACATCAAAAGCATCCATGCTGCAACGATCCTTAATGTTTTTAATGGAACTACAAGCCTT

GCTACTGTTCTTGGGGCCTTCATCTCGGACGCCTTCTTAGGACGCTACAAAACCTTGGGTTTTGCTTCTGTAGTTACACTTTTGGGTATGTTTGTCATCATGATCACTGCTGCAATCCCAGC
TCTGCATCCACCTTCCTGTGTACAAGGAACAACTTGTTCAGGTCCTACAACAATGCAGCTTGCTATGCTTGCTCTCAGCTTTCTATTTCTAGTGATCGGAGCGGGCGGTATCAGGCCCTGC
AATCTTGCCTTCGGTGCTGACCAGTTTAATCCAAAAACTGAATCTGGAAAGAAAGGAATTAGCAGCTTCTTCAACTGGTATTATTTTACCTTCACCATTGCAGTCATGATCTCCTCGACATT
CATAATCTATATTCAGAGCAATGTGAGCTGGACACTGGGCTTGGCTATCCCTGCCATCATGATGCTCATCTCTTGCATCTTCTTCTTCCTTGGCACGAGAATTTATGTCAAGGTGAAGCCTG
AAGGGAGCCCTTTTATTAGCTTTGCTCAGGTTCTAGTGGCAGCACTCAGGAAGCGGCGTTGGCAATTATCAGATGATCCAAAGCAGTCTCTCTTCAGTCCTCCTCATTTCAGCTCTCTCAT
CTCCAAGCTTCCACATACTAATCAGTTCAGATGGCTTGACAAGGCTGCAATCATAACAAACTCAGATGAAATCAAAATAGACTCAGCTGCGGCGAATCAATGGCGATTATGTAGCATCCAG
CAGATTGAAGAGGTGAAATGCCTCCTAAGAATAGCTCCAATTTGGTCCACAGGCATCCTATACTACATCGGCATGGCACAGCAATCAACATATGTTGTTCTCCAAGCAATGCAATCTAACC
GTCACATCGGAAACAAAAATTTTGAGATTCCGGCCGGCTCCTTCTCCATCTTTTCCCTTCTCGCTCTCTCCATATGGTTACCGATCTACGATAGCATTGTCATCCCATGGCTAAGAAAAATC
ACCAAAAAGGAAGAAGGCATCACACTTCTACAAAGAATGGGAGTTGGAATGGTGATTTCCATAATGGCAATGATTGTTTCAGGAATGGTTGAAGAGAGGAGGAGAAACACTGCTCTTCA
TCAACCAACTATTGGAATCTCAGTAAGTGGTGGAGCCATTTCATCCTTCTCTAGCCTTTGGTTGGTTCCTCAGCTCATGCTTTCAGGCCTGGCTGAGGCATTCAACACAATTGGGCAAATG
CAATTCTACTACAATGAGTTCCCTGAACACATGAGGAGTGTTGCAGGTTGTTTGTTCTTCTGCAACGTTGCACTTTCAAATTACTTGAGTGGATTTCTGGTGACGATTATAAATCGAGCTGG
TGGCGACGATGGGAGTTGGCTGGCTGGGGATCTTAACAAAGGGAGGTTGGAGAATTTCTACTATCTAATTGCTGGGATTGGAATTGTGAACTACATGTACTTCCTTATTGGTTCCAGGTGG
TACAAATACAAAGGATCTGAGATTTCCCAGAAAGTAAGTAGCTCTGAGTGTCAACCAATTTAACACATTGATTTAAGCTAAACAGGTGGATTTCATTTTGGTTTTTTAATGGAATTTCGAG
ACTACATTTATCTATATGATATGCAATCATTTCAGTTGCATACTAAACAGTTTTGGAGTATTATTTCATCATAGTCATGTGGAAAAGATATTGATATGGGAAGAACTGATCTGTGTTACATTATT
ATACCTTATAATTATTAATTGGATATTACACCTCTCA
>comp156821_c0_seq3
TGCATCTATATTATTGTACACCTTAAATGCATCAAACCAAACAGTCTCTAAGCCATTTGACACAATAACATCACATTAATCGTTAAAAGAAAAAAAATCATATTTTCTTAGCTCACATTTCTC
ATTCTTGCTTGAACAGTTTTGTACGCCCTAAAAAACTGTCATATGCAAAAGGCGTGGGTAATAAATAGAATCCGAATAAAGTGGGATTGTTAGAGAGAAATTAGGGTTCGAGATGTTCCCG
AGTGCGACGTGGGCAGCTTGTTGCAGGCTGCAACGTAAGTTTAGCCAACTCAAATCGTTACGACCGTTAGGGTTCAGAGCATGGTGGGTCCGGAAAGAGCTTTGCTCCATTTTTCTCCTT
CTCCCCGGAGAAAGCAGTGATTCCAAAGTGAGATAGGGAGAGCCCTCTCAACGGCTCCTCCCATCTCCTTCTTCTACTCTATTGGAAGGAGAACACACCAGCAATGGAGAATGAAGAGA
ACAATGCTGAGTCAGGGTTGGAGATTGTAGAGGAACCTGCTGCAATCAAATACTATGGCTGGAAAACAATGCCTTATGTCATAGGCAATGAAACATTTGAGAAGCTTGGGGGCATTGCCA
CAGCATCAAATATGCTTGTCTATCTCACCACTGTCTTCCACATCAAAAGCATCCATGCTGCAACGATCCTTAATGTTTTTAATGGAACTACAAGCCTTGCTACTGTTCTTGGGGCCTTCATCT
CGGACGCCTTCTTAGGACGCTACAAAACCTTGGGTTTTGCTTCTGTAGTTACACTTTTGGGTATGTTTGTCATCATGATCACTGCTGCAATCCCAGCTCTGCATCCACCTTCCTGTGTACAA
GGAACAACTTGTTCAGGTCCTACAACAATGCAGCTTGCTATGCTTGCTCTCAGCTTTCTATTTCTAGTGATCGGAGCGGGCGGTATCAGGCCCTGCAATCTTGCCTTCGGTGCTGACCAGT
TTAATCCAAAAACTGAATCTGGAAAGAAAGGAATTAGCAGCTTCTTCAACTGGTATTATTTTACCTTCACCATTGCAGTCATGATCTCCTCGACATTCATAATCTATATTCAGAGCAATGTGA
GCTGGACACTGGGCTTGGCTATCCCTGCCATCATGATGCTCATCTCTTGCATCTTCTTCTTCCTTGGCACGAGAATTTATGTCAAGGTGAAGCCTGAAGGGAGCCCTTTTATTAGCTTTGCT
CAGGTTCTAGTGGCAGCACTCAGGAAGCGGCGTTGGCAATTATCAGATGATCCAAAGCAGTCTCTCTTCAGTCCTCCTCATTTCAGCTCTCTCATCTCCAAGCTTCCACATACTAATCAGT
TCAGATGGCTTGACAAGGCTGCAATCATAACAAACTCAGATGAAATCAAAATAGACTCAGCTGCGGCGAATCAATGGCGATTATGTAGCATCCAGCAGATTGAAGAGGTGAAATGCCTCC
TAAGAATAGCTCCAATTTGGTCCACAGGCATCCTATACTACATCGGCATGGCACAGCAATCAACATATGTTGTTCTCCAAGCAATGCAATCTAACCGTCACATCGGAAACAAAAATTTTGA
GATTCCGGCCGGCTCCTTCTCCATCTTTTCCCTTCTCGCTCTCTCCATATGGTTACCGATCTACGATAGCATTGTCATCCCATGGCTAAGAAAAATCACCAAAAAGGAAGAAGGCATCACAC
TTCTACAAAGAATGGGAGTTGGAATGGTGATTTCCATAATGGCAATGATTGTTTCAGGAATGGTTGAAGAGAGGAGGAGAAACACTGCTCTTCATCAACCAACTATTGGAATCTCAGTTG

TTTGTTCTTCTGCAACGTGGCGCTTTCAAATTACTTGAGTGGATTTCTGGTGACGATTATAAATCGAGCTGGTGGCGACGATGGGAGTTGGCTGGCTGGGGATCTTAACAAAGGGAGGTT
GGAGAATTTCTACTATCTAATTGCTGGGATTGGAATTGTGAACTACATGTACTTCCTTATTGGTTCCAGGTGGTACAAATACAAAGGATCTGAGATTTCCCAGAAAGTAAGTAGCTCTGAGT
GTCAACCAATTTAACACATTGATTTAAGCTAAACAGGTGGATTTCATTTTGGTTTTTAATGGAATTTCGAGACTACATTTATCTATATGATATGCAATCATTTCAGTTGCATACTAAACAGTT
TTGGAGTATTATTTCATCATAGTCATGTGGAAAAGATATTGATATGGGAAGAACTGATCTGTGTTACATTATTATACCTTATAATTATTAATTGGATATTACACCTCTCA
>comp156821_c0_seq6
TGCATCTATATTATTGTACACCTTAAATGCATCAAACCAAACAGTCTCTAAGCCATTTGACACAATAACATCACATTAATCGTTAAAAGAAAAAAAATCATATTTTCTTAGCTCACATTTCTC
ATTCTTGCTTGAACAGTTTTGTACGCCCTAAAAAACTGTCATATGCAAAAGGCGTGGGTAATAAATAGAATCCGAATAAAGTGGGATTGTTAGAGAGAAATTAGGGTTCGAGATGTTCCCG
AGTGCGACGTGGGCAGCTTGTTGCAGGCTGCAACGTAAGTTTAGCCAACTCAAATCGTTACGACCGTTAGGGTTCAGAGCATGGTGGGTCCGGAAAGAGCTTTGCTCCATTTTTCTCCTT
CTCCCCGGAGAAAGCAGTGATTCCAAAGTGAGATAGGGAGAGCCCTCTCAACGGCTCCTCCCATCTCCTTCTTCTACTCTATTGGAAGGAGAACACACCAGCAATGGAGAATGAAGAGA
ACAATGCTGAGTCAGGGTTGGAGATTGTAGAGGAACCTGCTGCAATCAAATACTATGGCTGGAAAACAATGCCTTATGTCATAGGCAATGAAACATTTGAGAAGCTTGGGGGCATTGCCA
CAGCATCAAATATGCTTGTCTATCTCACCACTGTCTTCCACATCAAAAGCATCCATGCTGCAACGATCCTTAATGTTTTTAATGGAACTACAAGCCTTGCTACTGTTCTTGGGGCCTTCATCT
CGGACGCCTTCTTAGGACGCTACAAAACCTTGGGTTTTGCTTCTGTAGTTACACTTTTGGGTATGTTTGTCATCATGATCACTGCTGCAATCCCAGCTCTGCATCCACCTTCCTGTGTACAA
GGAACAACTTGTTCAGGTCCTACAACAATGCAGCTTGCTATGCTTGCTCTCAGCTTTCTATTTCTAGTGATCGGAGCGGGCGGTATCAGGCCCTGCAATCTTGCCTTCGGTGCTGACCAGT
TTAATCCAAAAACTGAATCTGGAAAGAAAGGAATTAGCAGCTTCTTCAACTGGTATTATTTTACCTTCACCATTGCAGTCATGATCTCCTCGACATTCATAATCTATATTCAGAGCAATGTGA
GCTGGACACTGGGCTTGGCTATCCCTGCCATCATGATGCTCATCTCTTGCATCTTCTTCTTCCTTGGCACGAGAATTTATGTCAAGGTGAAGCCTGAAGGGAGCCCTTTTATTAGCTTTGCT
CAGGTTCTAGTGGCAGCACTCAGGAAGCGGCGTTGGCAATTATCAGATGATCCAAAGCAGTCTCTCTTCAGTCCTCCTCATTTCAGCTCTCTCATCTCCAAGCTTCCACATACTAATCAGT
TCAGATGGCTTGACAAGGCTGCAATCATAACAAACTCAGATGAAATCAAAATAGACTCAGCTGCGGCGAATCAATGGCGATTATGTAGCATCCAGCAGATTGAAGAGGTGAAATGCCTCC
TAAGAATAGCTCCAATTTGGTCCACAGGCATCCTATACTACATCGGCATGGCACAGCAATCAACATATGTTGTTCTCCAAGCAATGCAATCTAACCGTCACATCGGAAACAAAAATTTTGA
GATTCCGGCCGGCTCCTTCTCCATCTTTTCCCTTCTCGCTCTCTCCATATGGTTACCGATCTACGATAGCATTGTCATCCCATGGCTAAGAAAAATCACCAAAAAGGAAGAAGGCATCACAC
TTCTACAAAGAATGGGAGTTGGAATGGTGATTTCCATAATGGCAATGATTGTTTCAGGAATGGTTGAAGAGAGGAGGAGAAACACTGCTCTTCATCAACCAACTATTGGAATCTCAGTAA
GTGGTGGAGCCATTTCATCCTTCTCTAGCCTTTGGTTGGTTCCTCAGCTCATGCTTTCAGGCCTGGCTGAGGCATTCAACACAATTGGGCAAATGCAATTCTACTACAATGAGTTCCCTGA
ACACATGAGGAGTGTTGCAGGTTGTTTGTTCTTCTGCAACGTTGCACTTTCAAATTACTTGAGTGGATTTCTGGTGACGATTATAAATCGAGCTGGTGGCGACGATGGGAGTTGGCTGGCT
GGGGATCTTAACAAAGGGAGGTTGGAGAATTTCTACTATCTAATTGCTGGGATTGGAATTGTGAACTACATGTACTTCCTTATTGGTTCCAGGTGGTACAAATACAAAGGATCTGAGATTTC
CCAGAAAGTAAGTAGCTCTGAGTGTCAACCAATTTAACACATTGATTTAAGCTAAACAGGTGGATTTCATTTTGGTTTTTAATGGAATTTCGAGACTACATTTATCTATATGATATGCAATC
ATTTCAGTTGCATACTAAACAGTTTTGGAGTATTATTTCATCATAGTCATGTGGAAAAGATATTGATATGGGAAGAACTGATCTGTGTTACATTATTATACCTTATAATTATTAATTGGATATTA
CACCTCTCA
>comp156825_c1_seq2
CAATATCTCATTTGTTGCCCTAGCCGACCATATTCGACTCCTTCACAACCGCAACCTTCATCTTCGTCGGAGATCATCCACGCTGAATCAGCTAGCCTTGATGCCGAAGCCTCCCATGTCAA
CGCCGAATCTTGCTATCTCGATGTCTAATCCTGTTATCCCGATGACAAATCCACCCATCTTGATGCAAAATCCAGCCGTCCTGATGCCTAATCTAGCCATCTCGATGCAAAATGAACCATCGC
TAACCATTTGTTGGTGATCTTAGAAGAACCAAATCCCTTCTTAAAAGAGGAAAATGTGGGAAGAAGAATAAGTCTGGACTCATCTTATTAGACTATTGCTCCAAATTTGAGAAACAAAT
>comp156863_c0_seq1

CATATATTTCTTCAAAATTCACAACATCAATAAAATTGCAGTTACATAATTTAGAAAATGCTACTAGAAGGACCTCAATAATGTTAAATCTTTGTATAATCCTATTTTTAAATTTGTCTCAGCT
CAAAGCCTTATAATTTATTTCAAATAAATCAGGATGAAGACTAATACCAACTGCATTTGTCCTCTGAGAACTTTACAGGCCTATTACTGTTGGAATCAATGGCAAGATTACATATAATATGCC
TGTGACTCTTCACCCTTACCAAGAACCCTACAAGATATCCTTGGCTAGCCATGTCCAAGTTTAGTGTCATTGGAGCTTTAATAGAAGTTCTTGACAGATCTAAGCCCAGGCTAGCTCCATAA
AGAGGAACCTGGTCACCCATCAGAATGATAGACTTAACTTGATGGTTTTTTCTGGGCAGATAATACTCCTCCACCTGATTTACAGCAACCCGTATTTTGGAATAGAAGAGACTAACATGGG
TAGAGGTAACATGAACGCCAAAATATGGAGCAGGATTGAACACGTTGATCTTCACAGTACAATTAAGACCAAGCATTTTTGTTGGCACTCCAGTACGATCTTGTCCAATTCCAGCATGAA
AGTAATTCACAACCAAGCTCTCCACTGCCACCTCAGGTGCATATTGCCTGCTTACCCCCCATATGATGAAGCCAGCAACAGTACATATCAAAATAAAAGCAACTAAACCAAAAAACATCTT
GAATACAATTGAATGCCTTTCAACATCACCATAATTGAGCTCCTCGTCAATGGTTGAATAATCAAACCATCCTTTTTCATTCAGCCTCCTCCTCTCCTGCGGCCCCGTCGTAGATAAATTAAA
GGACTCAGAAATCAGGCTGGCCGATGAAAACCTTGCGGGAGAATCTGAAGGGCTTTTGTAGACTGGAGTGGTTCTTGACGAGGACTTATTTCCATCATAAGAATCCCCAGATGGGCTCTG
CACATAGTAAGCTGGAAGTATATTTGCGAATCGATATGGCGAGGAATCATTTAGGGTTGAGACGTCTGAATCAGAAATAGTTCTCATCATCTTGACCTAAGGGAGCAGTAGAAAGACGAA
AAATCTCGTCTTGCAGAAGGGGTGATTTGCTTTAAAAGAGGAAAAATCTCAAAGGACAAAATTTTCAAGGGAAAACCAGTACATGAAAGAATCAGAACAATG
>comp156883_c0_seq2
AGATATGTAGGAATACAAAGCTAGGTAGAGACAGATGGAATCTGAGAAGATAATTGCTTAAGGCCTGCATTATGGAGAAAGATACATAAAGAATTATTATTTTTTAAAAAAAAAATTGAACG
ATATATGTGATGTTCAGAGGTAGACATGTGGACTAAAGCAGATGCCTTAAACACTAACATTTAAAGTGGTGGCATCGTTTCTTCTATGCGAAATGAAACAGAAAGAGCGACATTGGGGTC
AATTATATCTAGCTTTTTCTTTAACGAGAATTTTTCCACTTCTTTGGTGTGATGAGTAGAAGAAGTATAATTTTGATAGTTCTTATTAATGCGTACAAGAAAATGTAGAAAATTCGTTTCTTCT
CTTCCTTTTCCTTCTTTTAGATCATCTCCAACAGAACTCCATACCATTATATGTCATCCGCCATTTACTCCATTAGAACTCCAAATCGCACGCCAAATTGTCGTCGGACTGTTCACCTGTGCC
GAAAATATTCGCTGCGAGATTTGTCAAATAAGCGCGCTAAAGAAAGAAAAGCGCGCCAAAGATGATTACCATCGCAGGAAGAGCATGAAAAAAAGAAGAAATGATGTTCTGTTGGAAA
AGAATTATTGCATTTTAAAGTTTTACTATATTTTGGAATGAAAAATGACTTTTTGGGTTGGAGATGCCCTTAAAACTTAATGGAGGCAATTGCTTCTTTGGATTCTTCTTGTTTACGGCTTCT
TCGCTACAAAACAAAAATGGGCTGCCGAAGTAGAAGCAATCGAATTATCCCCACCGTAATATAAGGAAAGCATCTTCAAGTGCTCATGTTGCTGTTTAAACTTGAAAGCATTAAAATAATA
ATATATT
>comp156900_c0_seq1
CTCCCCCTCTCTCTCTCTCTCTCTTATGCCTCCGAATTCAACACGAGGAATTTTGGGAACGGCCTTCCCTGTTTGCCATAATTTTATAATTTCTTCTAAGAGAAAATATCAAAATAACAGATT
TTTTTGTAAACTTTCGTATTTTTACACATTGAATACAATAATAAAAAAGCGGGTATGCACATAGATACACTGATACTGTCACTTTTTGCTAATTAAATTAATTTACGAACTAAAGCTCTTAATT
TT
>comp156907_c1_seq5
AAAATTATATATAAACTTAAATCATATCAACTTTTAGTCAGGAGAATGAATAAAAATATTCTCCAAACAGGTGTAAGAAGCTCTCCCATCTAAGAAAATGGCTCCACAAGAACTTTTGTATA
ATTTTTTTGAGGAGCTTAACAGTATTCTCCTCCCCTAAAAGCAAAACCACTACTTATTATTTCATATAGGATGCAATACAGAGAGCTTCATCCTATATTATCAGCACTCTACCTTGTGTTAGTA
AGCTCAAGAACATATACATTAGTTTGATTTAAACTACATTTGGGACAAATTTCTTACTACTTCTTGAAGCAGTTTTTTAGTATTGTATTAAAAATCTGCTTTAAGACGCAGTAAGAAAGTCAT
CCCAAATG
>comp156910_c0_seq1
AGCTCGAGGAGAAGGAGGAGAGCAAATTGCTTAAGTTCCTAGGCTTCATGTGGAATCCCCTGTCATGGGTCATGGAGAGCGCTGCTTTGATGGCCATTTTCCTAGCCAATGGCGGTGGAA
AGCCGCCGGACTGGCAGGACTTCGTCGGCATCGTCGTTCTGCTCATCATCAACTCCACCATCAGCTTCATCGAGGAGAATAATGCTGGTAATGTCGGAATTTGGAAATGTTTAATCAACAG

GTAATTAGAGCCGCATTTTGATTAAATTATGCATCGACAGGTAACGCTGCGGCCGCGCTGATGGCTGGACTCGCCCCGAAGACCAAAGTCCTCCGCGACGGCCGGTGGTCGGAGCAGGA
CGCCGCCATTCTCGTTCCCGGCGACATCATCAGCATTAAGCTCGGGGATATCATCCCCGCCGATGCTCGTCTTCTAGAAGGAGACCCACTTAAGATCGACCAATCCGCCCTCACCGGCGAA
TCTCTTCCAGTCAATAAGCACCCCGGCGACGAAGTCTTCTCCGGCTCCACCTGCAAACAGGGCGAGATCGAAGCCATCGTAATTGCCACCGGCGTCCACACCTTCTTCGGCAAGGCGGC
CCACCTCGTCGACAGCACCAACAACGTCGGACATTTCCAAAAGGTTCTCGCTGCCATTGGCAACTTCTGCATCTGTTCCATCGCCATTGGCATGCTCGTAGAGATAATCGTCATGTACCCT
GTCCAGCACCGGCTGTACCGCGACGGCATTGATAATCTCCTCGTTCTCCTCATCGGCGGAATCCCCATCGCCATGCCCACCGTGCTTTCAGTCACCATGGCGATCGGGTCCCACCGGTTGT
CGCAACAAGGTGCCATTACCAAGCGGATGACCGCCATTGAGGAAATGGCCGGCATGGATGTGCTCTGTTCAGATAAAACTGGCACCTTGACGCTTAACAAGCTAACCGTTGACCGCAGT
CTGATAGAACTTTTTACCAAGGATGTTGACAAAGACGGTGTTTTGCTGTACGCCGCACGAGCATCCCGTGTCGAGAATCAGGACGCCATTGACGCCTGTATCGTCGGCATGCTCGCCGAC
CCTAAAGAGGCACGCGCCGGGATAAAGGAGGTTCATTTCTTACCCTTCAACCCTGTTGAGAAGCGTACCGCCATTACCTATATCGACGAACAGGGGAAGTGGCATCGGGCCAGTAAAGG
CGCGCCGGAGCAGATCATCGAGCTATGTGGCCTTAAAGAGGACGCTAGTCGGAAAGTACATGCATTAATCGACAAATTCGCTGACCGCGGTCTGAGATCCCTCGGCGTGGCGCGTCAGG
AGATTCCTGAAAAGACGAAGGACAGCGCCGGTGATCCATGGGAGTTCCTCGGTCTGCTCCCAGTCTTCGACCCGCCGCGGCATGACAGTGCTGAAACCATTCTCCGGGCTCTCAACCTC
GGCGTCAACGTCAAGATGATCACCGGTGACCAGCTCGCGATCGGGAAGGAGACCGGGAGGCGACTCGGGATGGGAACCAACATGTACCCTTCATCAACTCTGCTCGGCGATAAATCAA
CAGAGTTAACCGGAATCCCAATCGACGAACTCATTGAAAAAGCCGATGGATTCGCCGGAGTTTTCCCGGAGCACAAATACGAGATTGTGCGGAGGCTTCAAGAACGAAAACACATATGC
GGAATGACCGGCGACGGCGTCAACGACGCGCCGGCGCTGAAGAAGGCGGACATCGGTATTGCGGTGGCCGACGCCACCGACGCGGCGAGGGGCGCATCTGACATAGTATTAACAGAGC
CTGGGTTGAGCGTGATAATAAGCGCAGTGTTGACAAGTCGCGCCATTTTCCAGAGAATGAAGAACTACACCATCTACGCCGTGTCCATCACCATTCGAATCGTTCTAGGATTCATGCTCAT
CGCTTTAATCTGGCGCTTCGATTTTTCCCCCTTCATGGTTCTCATCATCGCCATCCTCAACGATGGAACCATCATGACCATCTCAAAGGATCGCGTGAAGCCATCCCCTGTTCCGGATTCAT
GGAAGCTGAAGGAGATTTTCGCTACGGGTGTCATCCTCGGCGCGTACATGGCCCTCATCACCGTCATCTTCTTCTACCTCGCCCACGATACCGATTTCTTCACCGAGAAATTCGGGGTTCA
TCCGATTAAAGAAAAAGCCGACGAGCTCACCGCGGCGCTTTACCTGCAAGTAAGCATAATCAGTCAAGCCCTTATCTTTGTCACAAGGTCGAGGAGCTGGTCCTTCGTTGAACGGCCTG
GGATGCTGCTTGTCACCGCCTTCCTCGCAGCGCAGTTAGTTGCGACGGTGATCGCCGTGTACGCCTCCTGGAGCTTCGCCAGAATAGAAGGAATCGGTTGGGGATGGGCCGGCGTGATTT
GGATCTTCAGCTGCATCACTTACTTTCCTCTCGACGTACTCAAGTTCACCACCCGCCACACTTTAAGCGGCAAGGCGTGGGATAATTTGTTACAGAACAAAACTGCGTTTACGAACAAGA
AGGATTACGGGAAAGGCGAGCGCGAGGCACAGTGGGCCGCAGCGCAACGCACGCTTCATGGACTTCAGACCGTCGAGACCTCCGACCTTTTCCATGATAATAACAGCTATCACGAGCTC
TCCGAAATCGCCGAGCAGGCTAAGCGCCGTGCAGAAGTAGCACGGCTGAGAGAGCTGCACACGCTCAAGGGACACATGGAGTCCGTGGTGAAGCTCAAAGGTTTGGACATTGAGACG
ATTCAGCAGCATTATACCGTCTAATAAGGAGAAGCAACCGCCATTGATGAGCAAAAAAAGAGCAAATCATTTGGTGCCTTAAACTTGAAGGTTCTGGGCCTCAAGTTGGAGAAATTATTA
CCTGCGGAGTTTTGAACGTTCGGTCGGATGCAAAAAAATGAGAAATTTATTCTCAAAATCTCATTTTTTTGAACGTCTAAATGAATTACTTTTTTAATAGGAGTTTTGAGCGTATTTGTACT
AAAACAATTATGTTGATATTTACTATACTAAATCTAGAATATTTATTATTTTTTAAAAAAA
>comp156910_c0_seq3
AGCTCGAGGAGAAGGAGGAGAGCAAATTGCTTAAGTTCCTAGGCTTCATGTGGAATCCCCTGTCATGGGTCATGGAGAGCGCTGCTTTGATGGCCATTTTCCTAGCCAATGGCGGTGGAA
AGCCGCCGGACTGGCAGGACTTCGTCGGCATCGTCGTTCTGCTCATCATCAACTCCACCATCAGCTTCATCGAGGAGAATAATGCTGGTAATGTCGGAATTTGGAAATGTTTAATCAACAG
GTAATTAGAGCCGCATTTTGATTAAATTATGCATCGACAGGTAACGCTGCGGCCGCGCTGATGGCTGGACTCGCCCCGAAGACCAAAGTCCTCCGCGACGGCCGGTGGTCGGAGCAGGA
CGCCGCCATTCTCGTTCCCGGCGACATCATCAGCATTAAGCTCGGGGATATCATCCCCGCCGATGCTCGTCTTCTAGAAGGAGACCCACTTAAGATCGACCAATCCGCCCTCACCGGCGAA
TCTCTTCCAGTCAATAAGCACCCCGGCGACGAAGTCTTCTCCGGCTCCACCTGCAAACAGGGCGAGATCGAAGCCATCGTAATTGCCACCGGCGTCCACACCTTCTTCGGCAAGGCGGC
CCACCTCGTCGACAGCACCAACAACGTCGGACATTTCCAAAAGGTTCTCGCTGCCATTGGCAACTTCTGCATCTGTTCCATCGCCATTGGCATGCTCGTAGAGATAATCGTCATGTACCCT

GTCCAGCACCGGCTGTACCGCGACGGCATTGATAATCTCCTCGTTCTCCTCATCGGCGGAATCCCCATCGCCATGCCCACCGTGCTTTCAGTCACCATGGCGATCGGGTCCCACCGGTTGT
CGCAACAAGGTGCCATTACCAAGCGGATGACCGCCATTGAGGAAATGGCCGGCATGGATGTGCTCTGTTCAGATAAAACTGGCACCTTGACGCTTAACAAGCTAACCGTTGACCGCAGT
CTGATAGAACTTTTTACCAAGGATGTTGACAAAGACGGTGTTTTGCTGTACGCCGCACGAGCATCCCGTGTCGAGAATCAGGACGCCATTGACGCCTGTATCGTCGGCATGCTCGCCGAC
CCTAAAGAGGCACGCGCCGGGATAAAGGAGGTTCATTTCTTACCCTTCAACCCTGTTGAGAAGCGTACCGCCATTACCTATATCGACGAACAGGGGAAGTGGCATCGGGCCAGTAAAGG
CGCGCCGGAGCAGATCATCGAGCTATGTGGCCTTAAAGAGGACGCTAGTCGGAAAGTACATGCATTAATCGACAAATTCGCTGACCGCGGTCTGAGATCCCTCGGCGTGGCGCGTCAGG
AGATTCCTGAAAAGACGAAGGACAGCGCCGGTGATCCATGGGAGTTCCTCGGTCTGCTCCCAGTCTTCGACCCGCCGCGGCATGACAGTGCTGAAACCATTCTCCGGGCTCTCAACCTC
GGCGTCAACGTCAAGATGATCACCGGTGACCAGCTCGCGATCGGGAAGGAGACCGGGAGGCGACTCGGGATGGGAACCAACATGTACCCTTCATCAACTCTGCTCGGCGATAAATCAA
CAGAGTTAACCGGAATCCCAATCGACGAACTCATTGAAAAAGCCGATGGATTCGCCGGAGTTTTCCCGGAGCACAAATACGAGATTGTGCGGAGGCTTCAAGAACGAAAACACATATGC
GGAATGACCGGCGACGGCGTCAACGACGCGCCGGCGCTGAAGAAGGCGGACATCGGTATTGCGGTGGCCGACGCCACCGACGCGGCGAGGGGCGCATCTGACATAGTATTAACAGAGC
CTGGGTTGAGCGTGATAATAAGCGCAGTGTTGACAAGTCGCGCCATTTTCCAGAGAATGAAGAACTACACCATCTACGCCGTGTCCATCACCATTCGAATCGTTCTAGGATTCATGCTCAT
CGCTTTAATCTGGCGCTTCGATTTTTCCCCCTTCATGGTTCTCATCATCGCCATCCTCAACGATGGAACCATCATGACCATCTCAAAGGATCGCGTGAAGCCATCCCCTGTTCCGGATTCAT
GGAAGCTGAAGGAGATTTTCGCTACGGGTGTCATCCTCGGCGCGTACATGGCCCTCATCACCGTCATCTTCTTCTACCTCGCCCACGATACCGATTTCTTCACCGTCAGTATTATTATTTACC
CTTGTTACCCGCAATTCATCAAACACTTCTTATTACATAAAATTCAATCTTTTTGCAGGAGAAATTCGGGGTTCATCCGATTAAAGAAAAAGCCGACGAGCTCACCGCGGCGCTTTACCTG
CAAGTAAGCATAATCAGTCAAGCCCTTATCTTTGTCACAAGGTCGAGGAGCTGGTCCTTCGTTGAACGGCCTGGGATGCTGCTTGTCACCGCCTTCCTCGCAGCGCAGTTAGTTGCGACG
GTGATCGCCGTGTACGCCTCCTGGAGCTTCGCCAGAATAGAAGGAATCGGTTGGGGATGGGCCGGCGTGATTTGGATCTTCAGCTGCATCACTTACTTTCCTCTCGACGTACTCAAGTTCA
CCACCCGCCACACTTTAAGCGGCAAGGCGTGGGATAATTTGTTACAGAACAAAACTGCGTTTACGAACAAGAAGGATTACGGGAAAGGCGAGCGCGAGGCACAGTGGGCCGCAGCGC
AACGCACGCTTCATGGACTTCAGACCGTCGAGACCTCCGACCTTTTCCATGATAATAACAGCTATCACGAGCTCTCCGAAATCGCCGAGCAGGCTAAGCGCCGTGCAGAAGTAGCACGG
CTGAGAGAGCTGCACACGCTCAAGGGACACATGGAGTCCGTGGTGAAGCTCAAAGGTTTGGACATTGAGACGATTCAGCAGCATTATACCGTCTAATAAGGAGAAGCAACCGCCATTGA
TGAGCAAAAAAAGAGCAAATCATTTGGTGCCTTAAACTTGAAGGTTCTGGGCCTCAAGTTGGAGAAATTATTACCTGCGGAGTTTTGAACGTTCGGTCGGATGCAAAAAAATGAGAAAT
TTATTCTCAAAATCTCATTTTTTTGAACGTCTAAATGAATTACTTTTTTAATAGGAGTTTTGAGCGTATTTGTACTAAAACAATTATGTTGATATTTACTATACTAAATCTAGAATATTTATTATT
TTTTAAAAAAA
>comp156929_c0_seq18
TAATCCAATATCTAATGAGCTGGTAGTCTGAGAGTAACAGGAGAGAGAAGGTGAGCAGGCCCATTTATTTATCGAGATCTTGCTTCCATGTGGGATTCGAGCGACAGCCACAGCAGAGCG
ACAGATTTAGAGAGAGAATTCAGCCAGCGAATGCTGAGACGATAACTGAGGAGATCGAGAGGACGGTGCGGGCAAAAACAGGAAGCGAATCCGGCCTGCCCTTTACGATTCGGATCTC
GGATTCTCCGCCGATCCGCTCGTTCGGATCCGTAAAGGCAGGATCCCGCCTGCTCTATTTAAAGCCCGCACGATTCAACCTATCCTTCTCGTGTCTTCAAGTTTGATATTTTCGTCCGCGAT
TTCATCGTATATACAATTTGATCATCGACGTAATGTCTTCAGAAAGAGGCGGAGCCGCCGGTGAGGGGCAACGGGAATTGCACGTTCTCGCCGTCGATGACAGCTCCGTCGACCGCGCCC
TCGTCGCTCACCTTCTGCGTAGCTCTAAATTCCTGACTACGGTGGATAGCGGGAAGCGTGCGCTGGATCTGCTGGGAATGGAGAGGAATGTGAGCATGATAATTACGGACTACTGGATGC
CGGAGATGACCGGACTGGAGCTGCTGAGAAAGATTAAGGGTTCGTCGGAGTTAAAGGCGATTCCGGTGGTGATTATGTCGTCCGAAAACGTCCCCAACCGTATACGCACGTACGATCATT
ATTTTGTTTTTAACATTTTTTTTATTTGAAATGGTAAATGTGTAATATAGTTATTTTCGTGGTGATTTAGGTGTTTGGATGAGGGCGCTGAGGATTTTCTTCTGAAGCCGCTGCAGCCGTCGG
ATGTGTCACGGTTATGCAGCCGCGTGCTGAGATGATTCTGGCCGTCTGATTTTGTTACTGAGCTTTTCCTATCGGCTGGGTTTCGTTTTGTTTTTCTGGGTTTTACTTTGCTAGCATTGTGAA
AATTTTAGGAATTTACTGGTAAAGAAAGAAGTTAGTTACCTGATATTTTGCCTTTTTATATCGAATAAATG

>comp157049_c0_seq2
TGGGTAAAATACTGTCGTTTATTTGCAATTTGGCATTCCACATTTGGGACCAAATAATATTTGTACTATGAGAAAACGTTGCATAAAACATTGAAGAAGACTGGGGAATCTTTAATTGCTGT
AATTAAGAGGAATCAAAAACAAAAACAAAATAGAGTAACGACGAGGTTGCGAGCCACTCTTAGAGATGGATACGCAGAAATTATTTTGGCGTCGTTGAAGTAAAAATAGCGAGACGAAC
GCAAATATGCCAGGTTTTGTTGCACACAGTGCGTCTCGACATCTCTTCAAGGACCTGCTTGTCACTGGCCCGCACTATCGCTGATGTGCTCTGCACGCCAAAGTCGGACGGGGAGACCGT
GCTTGGGAACAGGGAATGGACTATACTCCACCGAATTGCGAGACCCAACCACCTCGAATCTGTATTGAGTCACCAAATGGTGGATAAAGGAAAACATCTCAAGCTTGGCGATCTCGTTCC
CAGGGCAAGAGTGAACGCCATTTCCGAAGGGCATGAAGGTGTTGGGCTTAGGTGAGACCTCAAATCTAGAAGAATCAAATTTCTCCGGGTCTGAGTAAAATTCCGAATTGTGATGGATAT
TCCTGAATAAAGGCATCACCTTCCATCCCTTGGGGATAAGATACCCTTTATACTCCACATCGGCCACAGCCTCCCTAAACGTGAAAGAGATGATGCTTGCCATCCTTAAGCTCTCCATTATA
ACCCTATGAGTGAGAGGCATGCTCCTGGTTTGGGCCCACGTCAATGGCTGTCTTCCACATCCATTCGACTCATAAATCGCCATCTGTTCGGCCTTTACTTCCATGAGAAGTTTTGGGTCGTC
GTTGAGATACTTGACGATCCACGTAAGAACGCTGGCCGTTGTGTCCTGCGCCGCGAAGAGTACGCCGATAATGTTGTCGGAGATCTGTTCATCACTCAATTGTTCTCCTTTCTCATCTCTC
CACTCTACAAAACATGACAATAGATCCTTCTCCACCGCTGCTTCTTTCTCTCTCCTCTCCTTCATGATCTCGTTAAGTATCCCCCGAAGCCTCTTCCTCGCCTTAATGGCGTTGCTGAACAG
AGTTCCGGGAAAGTTGGTGGGGAAAGAATTGTATCCCTTGTCAACGATGAAGTAATTTTTTTCCAGCGCTTTCTTGCGTTGCTCGCTCAAATGGCTGCCGAAGATGACGAGGATGCCGAC
GTCGAAGGAGAGCTTCTTCATAACTTTGAAGGTGCTGAGGACATTGCCATCCCAGGAGCGGAGAATGGCAGCTGCGGTGCCTTCAATTTGATGGACAAGACGGCGGAGCGACTCCGGC
GAGAGCGAGGCCTGCACGAGCTTCCGTATGCGGGCATGGTAATCATCCTGATGGAAGAAGAGCGCCCAAGGGCCGATCATCTGCTCCTTGCTCCGTGGGTAGGTGGGCTTGAAGAGATG
AGCCTCGCTCACCAACACCATCCTCGCCGCTTCTGGGCTTGCCAGCATCACGCACGGGTAACCTAGCACATGCGTCTTAAAGATCTCACCATACTTTTTAAGCCTGGAGGCGAAGAAGAC
ATTTGGGTCCTGTGAGTAAAGCTGGAACGTCTCGCCGATGTAGGGCAAGCCCATGGAGCCTGGTGGCAGGCGCAGCTTCCTCATTTGCTTGCCATGTTTCGTAATACGGAGACAGGAGA
AAAGGGCAAGGGCGAGGGACGTGATTATTATTAAACTGCAAGAAGTTTCCATTTGCAACTCTATTCTTAATCTTCTCTATTGCTAGAGGAAATGCTGTTGGAGACAGAGGAATGAGGCCG
CTCGGCGAGAGGAGAGGTGGGGGAGCTTTTATATATAGAAGAACAGAGCCCGTTACACACGCGTCTATCTCACAGAATCGCACTGTATTTATAGAATTGCGCATCAGAAACGGATGGAAG
AAGTCATAAATTTTTCATGAATTTAAAGGTGAATAAACACAAATCACCATTAAAATTGGAATCCCAATACAACTTAATTGAAATCCGCCCATGATTC
>comp157061_c1_seq11
TTGCCGAGCAAAATCGACTAATACCTAGTAATACCGAGCAATACCTAGCAAAATTGAGAAATATTGAGCCATCCTGGGTCCGTTCCGAATCAGATTATCGGGCCGCGTGATAAATAATGAG
AACGCGTGGCGCCATCTGACCGGACCAAATAAAAGTTCAGTAAAAAGACATTTCTTTCAAAATTTTCTATAAAGATTCCTTGTAGAATCTCTGTCATACACGCAAATACCAACCTGCCTCA
ACTAGCTAGAGAACTCTTCCAAAAGCTTATACCTCCTCACCTTTCCTCTAAACACCTGTCGTCCCCTCCCAGAACCAGGCGACAGCTCCTCCCCGCTGCCCCGGTCGCCGGAATCCGCCG
GTGGCTGCAGCCGCCTGTCACCCAATCTCTGCTTCTTCACCGGTTTATTCAGAAGCTGTGGCGAGAACGCCCCACGAGCGAGCTTCAATCCCGAGCTTGTGCCACCTACCGGAGAAGAG
GAAGCTGAAATAATTCGATCAACCGTCGTCTGTTCTACCAAAAGTAACCTGCTTCCTGCTTCCTTCCTCAGGCACCAGTTACAGGCTCGATAGACCTCTGCTCTCGGGTATAGATCGCTGC
AATATCTAATATATCAATCAAATATTCATCAATCAGGTGGGGATTAAGATGGAGGAGTTGAAGACCAAGAAAAAATTGAATTGGATCATAGCCTTCATAGGGATTGAAGCAAATTTGAGAG
CTTGAATTAGACTATGTTAATTGTT
>comp157061_c1_seq2
AAATTAATTAATTTAATTATGTAAAAGAGATAATAATTAACCGGCAGTTCTTAAAAGAAGTTCGAACCTGTTTTTTTCTGGTACAACGCAAAACCGATTTTGAAAATAGTTTAGGCCGACTA
CTCAATCAGTTCTAATTCGGCTTTCAAAATAAAGGTAGTAATTCGCGAACAAATCTCTACAAAAGATAAAAACTTAACTAAATACAATGATCAATTGATCATATAGAATCCCATAGTAAAAA
GACATTTCTTTCCAAAATTTGCTATAAAGATTCCTTGTAGAATCTCTGTCATACACGCAAATACCAACCTGCCTCAACTAGCTAGAGAACTCTTCCAAAAGCTTATACCTCCTCACCTTTCC
TCTAAACACCTGTCGTCCCCTCCCAGAACCAGGCGACAGCTCCTCCCCGCTGCCCCGGTCGCCGGAATCCGCCGGTGGCTGCAGCCGCCTGTCACCCAATCTCTGCTTCTTCACCGGTTT

ATTCAGAAGCTGTGGCGAGAACGCCCCACGAGCGAGCTTCAATCCCGAGCTTGTGCCACCTACCGGAGAAGAGGAAGCTGAAATAATTCGATCAACCGTCGTCTGTTCTACCAAAAGTA
ACCTGCTTCCTGCTTCCTTCCTCAGGCACCAGTTACAGGCTCGATAGACCTCTGCTCTCGGGTATAGATCGCTGCAATATCACATACTTGTGTTGGGAGCGAAAGGCGCAGAGTTTGCACC
GAAAGAGCTCCCGTGATAGCCCCCGATCTCCGCACATGCAACAAACCACCGACGGCGACGACGGAGACGACGGGGATTGTAGTGCGCCACCACCTTTGCTACCCATTCTCTCTTCTTTCC
CCTCTCTACTGTATCTCAGGCTGTTTCGACTTTATTATGTCGCCCACAAACGTGGAAAGAAAACGATGGGGGAGGTGGAAGATGAGCTTCATTCGATCAGTATGTGGCAGCCATTGTAGTT
CGATTTTGTTGAGTAGTGGTTGAGAAACAAGTCAACAAATACTGCAGCTTTTGTGGCCTTTATACTTTATAGGTTTATTTATGGAGAAATTATTTTATGTAGGCATCGGATTGTTCCGAAGTG
AATCAGAGCGTACCACATCATCCAGTACACAGCTGCGGTACTCAGCAAAGGGATGTCGTGGTGTGCTCTGATTTGACTTC
>comp157061_c1_seq6
TTGCCGAGCAAAATCGACTAATACCTAGTAATACCGAGCAATACCTAGCAAAATTGAGAAATATTGAGCCATCCTGGGTCCGTTCCGAATCAGATTATCGGGCCGCGTGATAAATAATGAG
AACGCGTGGCGCCATCTGACCGGACCAAATAAAAGTTCAGTAAAAAGACATTTCTTTCAAAATTTTCTATAAAGATTCCTTGTAGAATCTCTGTCATACACGCAAATACCAACCTGCCTCA
ACTAGCTAGAGAACTCTTCCAAAAGCTTATACCTCCTCACCTTTCCTCTAAACACCTGTCGTCCCCTCCCAGAACCAGGCGACAGCTCCTCCCCGCTGCCCCGGTCGCCGGAATCCGCCG
GTGGCTGCAGCCGCCTGTCACCCAATCTCTGCTTCTTCACCGGTTTATTCAGAAGCTGTGGCGAGAACGCCCCACGAGCGAGCTTCAATCCCGAGCTTGTGCCACCTACCGGAGAAGAG
GAAGCTGAAATAATTCGATCAACCGTCGTCTGTTCTACCAAAAGTAACCTGCTTCCTGCTTCCTTCCTCAGGCACCAGTTACAGGCTCGATAGACCTCTGCTCTCGGGTATAGATCGCTGC
AATATCACATACTTGTGTTGGGAGCGAAAGGCGCAGAGTTTGCACCGAAAGAGCTCCCGTGATAGCCCCCGATCTCCGCACATGCAACAAACCACCGACGGCGACGACGGAGACGACG
GGGATTGTAGTGCGCCACCACCTTTGCTACCCATTCTCTCTTCTTTCCCCTCTCTACTGTATCTCAGGCTGTTTCGACTTTATTATGTCGCCCACAAACGTGGAAAGAAAACGATGGGGGA
GGTGGAAGATGAGCTTCATTCGATCAGTATGTGGCAGCCATTGTAGTTCGATTTTGTTGAGTAGTGGTTGAGAAACAAGTCAACAAATACTGCAGCTTTTCTTGCCTTTATACTTTATAAGT
TTATTTATGGAGATATTATTCTATGTATGTATCGGATTGTTCGGAAGTGAATCAGTGCGTATCACATCACTCAGTACATAGCTGCGATATCCAACAAAGGGATGACGTGGTGTGCTCTGATTT
GCCTTTGTACGAC
>comp157077_c1_seq2
ATCTAGCATTCCTAGATAGTATTATGAAATGTGAATATCTTTTTATGATGTGAGGAAAATTTGCTGTTTCTTTTGATTAAGATGCAAAGACATGGGACAATTAATAGTGGCAAACAGACATAC
ATTTACATTCATCCAGATGATAAGCAAATATTGTGTAACTATATGATGATGTTGTTCAACAAAGAGATCATCATCGTTATAATCACCCAACAATTTGCAGCCGGTTTAAAGATCCTTCTTCTCT
ATTCATTTCTATCTAGCATTGTATGTAGTGCAAACTTTAGTTTATGCAAGTTATTATTTAACACAGAAGGCAAAATTAGCCTTGATCTACTAATCTACTAGTTCTATTTCAATGACTTATGAGCC
AAATAACTGTTATCAGCAGAAACAAAATCACACAACACAAATACAATGAACATGACGATTTATGTGGTTTGGCGAAAATGCCTACATCCACAAGGAGGGGATGATAATTTTACTATATTGG
AGAACAATAAGGATTACAAATTGGCTCTCTTTTATCTCATGCATACATTTTAATGGCCCTCAAGCCCAATTTAAAAATTTGAAATAAACACAGTAAGGAAAGCATAATCAATAAGAAAATAA
GAAATCTAATCATGCCATAATTAGATATCTTAATCAATACATAAATCAAATCTCCTAATTAATACAATAATTAGATCCCCTAATTAATGCATTGATAGGAAACACTTAAAGATCTCAAGTATTTC
CTGTCAATCTTCGGA
>comp157078_c0_seq1
TGAGACAAGTTATTTGCTCACTTCACTTTAATTTTTTTTCTCTTATTCAATTATTTATCATTATTACAACCATTTGAGCTCCACCCTCTCTATGAACAGAAACTACCATGCACAAGATTTTTTTT
TTTTTCAAGTGATTATTTCTCCTTGTAAAGAGAATACATCAGATAAGTAAAACAAGTGACATAATAATATACATTATATAAATGCTTCTGCCTTCCAATGATATCTTAAATTATGTACAAGATAT
TCTTTTAATCATCCTTAAATATGGAAGCGATACTTCAAACAGCAGAGATGAAGAAGGTATTGAGATTTGAGACGTTTAATGGCCACATTCAGCAACACGTCTCTTCTATTCATTAGTAAACC
CAAGAAGGAATCGCAGGAATGCGCCGCGAAGCCTTCACCCCCAGCGGGTTGTTCTTGGCCATAAGGCAAGTTCCAGAATGAACATCACACAGAGGGTAATCTGGAGGGCAGCAGCTGT
TGTGATCTTCACAGCAGGTAGCGCCATCAACGGGACAACATCCCCATGCAAAGCAGAGCTTGCCATACTCATATACACAGCAGCATGTGGTGCTAGCAGGACAGGAGTAATATTTATCACA

TTCTGTGGGGGGTTTTACGGGAGCAGGTGGTCTTGGTCTTGGGCTTGGGCTGGGTTTTGGCGGGTTTGAACTATGTTTGACTGGATAAGAAGCTTGCATCGCAATTCCGCATTTGCCGCTT
GCTGCCTTCACATTGCGCTCTATCCTTACAAAGCCTTCCTCTCCCCAGTCTTCACCCCAGGAGTTTTTAACAATCCAGTAGTCTCTTCCATTTGCAGTGCCATAGCCAACAGCGAGAACGC
CGTGGTCTAACTCGGTCCCACAGTTGGCGCTGAATATGCCCGATTCATACAACTGGAAAGCCCTTCCAGCGGCTTCTATGGCGACGCTGACGGGCTGATTTGCAATCGCCTTCTGTAGAGC
CTTCTCGTCGTTTACCGGAACATCTTCATATGAATCAATAGAAACAACCCGAGCATTTTTTCTGTAAGCATCGCATTTGCCACCATGGCCTTTATAAGGATAGTCTTCCTCCGAGTCAATGCC
CCCATTATTGATGATGAATTGAAAGGCGTCGTCCATAAGACCACCATTACATCCTTGGTTTCTGGCGGTATCACAATCCACTAGCTCTTGCTCTGATAGCGATACGAGCTTGCCAGTCACAA
TCTGGTTGATCCCTTCCACGGCAGCAATGCTCGAAAAAGCCCAGCAACTCCCGCAGGTCCCCTGATCCTTGACAGGGACAACGGCGCCCTTTGCCCTCCAGTCGACGGAAACAGGGAG
ACCCTCGCCAGCAGCGAGGCGGTAGCGATCGCTGGGGTACTGGGGGTGAGGGGAGCGACGGAAGCCAAGATATAAAGAACGGTACTCTTCGTTGGTGAGATCGGCGAATCTGTTGAGA
CCGAGGCGGAAGGAATGAACGCCGGCGTTATGCTCGTCGATGTAGAGGAGATTGTCCTTGAAGATCTCGAAACGCCTCTCCTTCTCGTCAAGTTCATTGTAGATCCGGCCATGCCTCGCC
ATCCAGTTATCGTAGAGCTCCCGCACATCTTCTTCGCCCCGCTCGAGCTCCCGTGTTCCATGGCGCTCCCCGTAGGTTATGATCGACATATCCGGCCCATTGCCACCCGCGATCGCCACTGC
GATGGAAAGAGAAAGTAGGAGAGCAAGCGAAGTAATGGAAACTGAGGCCATTGTAGAGAGAAAGACAGCTTAAATTGCGGGCGGGGCTTCTTGGATTTCGCCTTCTTCCATCAGAGCT
CTCCTGGAAGGTTATTTATAGGTATAGAGGAAGAAGGGGCGCAAGCTCGGCAAAAG
>comp157186_c0_seq1
TTTTTTTTTTTTTAATATATAAAATTTTTTTATATAACGCGATACTAACACAAATAAAAATGATTCTTAAAGCTTATTTCATATCTTTGTGATTGAACTGCATAGTCAGAATCCTTACAATCTCCA
TGTAAAAAATTGATAAGCACCCTTTTATCCACTTACAAACAATTCCACACACAATACCCTTTAACGTACACACTTAACCACTTAGAAATAGAACCTTATTGCGAGGAGTTTCACCATTTCAA
CTTAGCAGGAAAATACTTCTCAATGAGAGAAAGGTAGTATGGTTTCACCTTCTCCACATCAATCCTAACCTTGCTTTTACTATAGAGGTCATATTTGTTGAATACATGCAGCCATTTCAGGTT
CTCTTTGTCTTCCTCATTCATCAAGTATGAGTAGGCTCCAAATCTATGGAGTGGATAGAAGGAATGGTATCTGATGATGAAGAGAGCAGCTGAAGGCAAAGTAGTCTTGTTCTCCTTAGCC
ACCAGATACATGTAATCATCATGACCCCATGACATTAGCACATTCTCAAGGCCACACCCTTCAGAATAAACACCATACTTGGTATTGAACTTGGGATTGTGGTAGTCTGAGTTCCCTTCAAA
AAACTGAAATAATTGCAATGTAATTCTTTGTGAGCAATATATAGATCTTTTAATCATTTCTACAGAACATTTATAAGGCATAAATGTTTTATAGGACCTTGTGGTGCACATTAGATTCATCAAA
AGCACAGCCCACAGGAAATGTGTCACCAACAACAGCCCATTGAGGAAGCTCACCAAAACTAGGATGAAGAAGAACCTTTCCAAGATCATGAATAAGGCCAGTCAAGTGAAGCCAATCC
TCGTTTGGATAATCCTTCCTGATAGCTTCTGCAGTCTGCAACAAATGCTCAATCTGGGGCTCATCAAGATCAGGATCACTGTCATCGACGAATTCATTAAGCAATTCAATGCTTTCCCATAT
GCTCATCTCCACTCTATTTAGCTTCCCATACTCTTCTCTCATCCTCTTCACAAAAGCATAAGATTGATGAAGATGATTAGTGCGGTAGAATTCTTCGACCGTCTTTTTCCTCTCAGATTCAGC
ATTGTAATCCCTGAAAGTCTGGCCAAAGGCGTTTGTTTCTGGAACAGAGAAACCGCCATCTAAATCCAGTTCGTTTGTATCAGGGATTACAATGCTGAATCTGAGAGGAAAAAGACGGTC
GAAGAATTCTACCGCACTAATCATCTTCATCAATCTTATGCTTTTGTGAAGAGGATGAGAGAAGAGTATGGGAAGCTAAATAGAGTGGAGATGAGCATATGGGAAAGCATTGAATTGCTTA
ATGAATTCGTCGATGACAGTGATCCTGATCTTGATGAGCCCCAGATTGAGCATTTGTTGCAGACTGCAGAAGCTATCAGGAAGGATTATCCAAACGAGGATTGGCTTCACTTGACTGGCCT
TATTCATGATCTTGGAAAGGTTCTTCTTCATCCTAGTTTTGGTGAGCTTCCTCAATGGGCTGTTGTTGGTGGCTAAGGAGAACAAGACTACTTTGCCTTCAGCTGCTCTCTTCATCATCAGA
TACCATTCCTTCTATCCACTCCATAGATTTGGAGCCTACTCATACTTGATGAATGAGGAAGACAAAGAGAACCTGAAATGGCTGCATGTATTCAACAAATATGACCTCTATAGTAAAAGCAA
GGTTAGGATTGATGTGGAGAAGGTGAAACCATACTACCTTTCTCTCATTGAGAAGTATTTTCCTGCTAAGTTGAAATGGTGAAACTCCTCGCAATAAGGTTCTATTTCTAAGTGGTTAAGTG
TGTACGTTAAAGGGTATTGTGTGTGGAATTGTTTGTAAGTGGATAAAAGGGTGCTTATCAATTTTTTACATGGAGATTGTAAGGATTCTGACTATGCAGTTCAATCACAAAGATATGAAATA
AGCTTTAAGAATCATTTTTATTTGTGTTAGTATCGCGTTATA
>comp157186_c0_seq18
TTTTTTTTTTTTTAATATATAAAATTTTTTTATATAACGCGATACTAACACAAATAAAAATGATTCTTAAAGCTTATTTCATATCTTTGTGATTGAACTGCATAGTCAGAATCCTTACAATCTCCA

TGTAAAAAATTGATAAGCACCCTTTTATCCACTTACAAACAATTCCACACACAATACCCTTTAACGTACACACTTAACCACTTAGAAATAGAACCTTATTGCGAGGAGTTTCACCATTTCAA
CTTAGCAGGAAAATACTTCTCAATGAGAGAAAGGTAGTATGGTTTCACCTTCTCCACATCAATCCTAACCTTGCTTTTACTATAGAGGTCATATTTGTTGAATACATGCAGCCATTTCAGGTT
CTCTTTGTCTTCCTCATTCATCAAGTATGAGTAGGCTCCAAATCTATGGAGTGGATAGAAGGAATGGTATCTGATGATGAAGAGAGCAGCTGAAGGCAAAGTAGTCTTGTTCTCCTTAGCC
ACCTATGATTAATCATTTATTAGAGAAACCTGAAGGAAGAAGTCTGACTATATATAATATCAATGTATTAACTAATTTTGGTGTCTGCAAAGTTTACGGATATTTGATTTTAATTGAATGCCATA
TAATAGAGTTGCATTTTGGTATTTTACTTCAATTGGATGTTCCTATAATTATAAATACATTTCAAAAGACTATTTGATAAGAACTTGT
>comp157282_c0_seq4
GCTGGAATAAGTGGACAGCAAGCAGAATTCCAGGTTTGGGAGGTAGAAGACTTCACGTTGGATGGAGGACGGGAGGATTTAATGGAGGAAATCTAAACAGACTAGTGGGAATAGATGG
ACAGCAGGTTGGTCTAGTAAAACAGTAGCATTGAAGCTTCACATCACTGGAAAGACACTTAATAGTTGCTGGAATTCTCTGGCTGCAGCCTTAGAAGGCAGAAAACTCAACGTCAGATG
GAGTTTGGGGAGAAATTTGTGGAAGGAAATTGATATACGGGAATTGGGAAGTTTCAGCCAGCCGGGGGATGAAGGCCTGACGTGATCTCCAATTTAATTCAATGGGGTTGGAAAGATTTA
ATGAGGTGAATGCATCAAGGGGAATTTCGACCAGGTCATGAAGATGGGTTTTAAATTCTTTTCAAGTGGGAATAGAGGATTAGGAAATAAATCCAGGAGATTTATTAAAGGGCTAGCAGTT
TAAAGATGGAGGAGATTGGGGAGGTGATTTTGGGTGCTTAATTTAAGGCTTTAAAATTTAGGAAAGTGTTTAAACATATCCTATTTG
>comp157318_c0_seq4
CCCTTAACTTTTTTCATCCAGATTTAAGACCTGGCAACGAAATGTAGACATAAAAAGACTTTTGTCTCAGGCAAATAAATATAAAATTTCCCTAAACTTAACAAACTCAAACAATGTTCTTG
GATAAATGCATGACATCATACACCACAAAAAAAAAAAAAAAAAAAAGTTTACCATGATTTTCACCTTCAAGACAATGTGCCTTCTTGCATCATTCCCAAGGCGAGTGCAACGTTTTCATAA
GCACTTTCCAGAATGCATCTACTTTATATTAAGGTGTACTAGAAGAATCATTCGAGCTAATAGGAATAGAATAATTGTATTTTCTGAACACTCTTTCCTGATTTTTTTTCCACCAATCTTCTGC
CTCCTGCTCGGAAAATCTTCGCTTGCTGAATTTGACTCTTTTGAAGAGCTTAGTGCCATCTAGTAACTGCAAAAGAGTCGCATATATACCAGGCTCAAATTGCTCCATCAATTCAACTTCTC
CCTTGTTGTTAATTATTCTTTTAGAAGCTCGAGGTGACCTTGAGCTACTCTCCATGCTTTGGCGATTTGAGTATTAGGAGACTCTATGCTTGGTGTGGCACTTGAAGGCAGCTCTGAAGCTC
GGGTTTTGTTATTTTCTAGAAACAATTCTGCTGAAATAATCATTGATTTTATGTTGTCACCTAGGTCATTAGGGACTTTCTGAGCTACATCCTTCATCTGGGCTTCAATTGTCCTGATAAGCTC
AATGGCAGCATTAAACTTAGCAGATTCTTGTGCAGCTACAAAAACTTCTTTAGCTTCATTTGAAACATTATGTGCAACTGCATCTTGTATTTCTAGTTTTTGTTTAAGACTTTTGATCTCCCC
TTGCAGCTTTAGGACTTCTCTATCCCAGTGCTCATTAGTCCTCTTTATAGTCTCCATTAAGTTTCCAGAAAAATCAGGAGTTGTAGATAGTGAAGGGTTTGGCTTCCATGAGTATGGAGTTA
CAAACTTAGAATTTGCAGCAGGCAATGGTGGAGGTGCTGTCGGTGTCATGATTGGCCTTGAAGTCGTTTGAAGTAAGCTCAATGAGCTTGGGAAGGCAATATCTTTTAGCTGTGAACGAG
ATGAAACTTGAGAAATTCGGTTTGAAGTTCGTTGATTCTTACTATATCTAATATCATTATATCTTCCCAACTCCTTAGGAACAAGTAAAAGCCTTGAATATCTAATTTTTCCCTTCTCAAACAT
TTCCCTAGTATCAATAGATTGACGAGAAATCACATTATTTCTACCAACAACCAAATGCATACCAGTTTCCGCAGCTTTAAGTTTCGAATAGCATGAATCACATACCCGATGAGGTTTGCCTG
GAGTTGGTGCCAATACAGCTTTCAAAACTTTTCTGGAACTGCAAGCATGACAATATACTAGACCACAATTATAGCAATTATGTCTTTTTCTAGTAAATCCAAATGCTTGTCGACAGCCGGTG
CAGACCGATTGGTCAGCACTAGATACCCACTTATGCATGCAAATACATGCTGTGAAATTAGAGCCACAAGAGATACTCTTAACATGCCTGTCTTTCAAAGCTTCAACAAGAATGGGTGTCT
TTCTGTCATCAGTATCTCCATGTCCTAGTCTTCCATTAGCACCTCTTCCCCATGAATAAACTTCACTTCGTGAAGTTAATACCACAACATGATGGGATCCACAAGCAATCTCTTCAACTAACT
CACCTACTAATCTATCTTGCACTAAACAGGGAACTTTACCATCAAATTGTGGATTTCCGAGCTCGCCATACATGGAATGTCCCATGGTGAAAACATGCCCAGAAGTTGAGAGGGCAATGGT
AATATTATGTCCGCAAGCCAACTGATGAAAGTTGTAATCAATAAGTGATGCAACACAAGTTGGAACCAATCTTGCTTCCTTATCACCATGGCCTAGACGGTATCCATCGCCATCGCCCCATG
TAAAGAGTTTACGTGATACAACATTCGTACCAGCATGTCCCATTACTTCTACAATTGCGGCCGTATGCCACACTCCGCATGCAATCCTAATGGTTCGTAAACCACTCAAAGATTCGATTTCC
CTGGGATAAGCAACACTCTCTCGATCACCATGGCCCAAAACACCAAATGAACCATCACCAAATGAATATATTTTTCCATTTGAAGTAGCCAGTACAGTATGCCATGTGCCACATGCAACTA
AAGACACTTGAAATCCTTCCAAAGTTCCAGAAACTCTCTTAGGCATCCAATGGCTGGCCTCAATACCATGGCCAAGAAGTCCAGTATTATAAGCTCCATCACCCCAAGTAAACAAGTCAC

CTGCTGTAGTTATAGCACAAGTATGAAACTCGCCACATGCAACAGAATCCACATTGGATAACGCTAATGATTCAACTAAACGAGGTCGACTAATATCAATATCAGTTCCATGCCCAAGTCTA
CCACCGCATTCTTCACCCCATGTAAATACTT
>comp157318_c0_seq5
CCCTTAACTTTTTTCATCCAGATTTAAGACCTGGCAACGAAATGTAGACATAAAAAGACTTTTGTCTCAGGCAAATAAATATAAAATTTCCCTAAACTTAACAAACTCAAACAATGTTCTTG
GATAAATGCATGACATCATACACCACAAAAAAAAAAAAAAAAAAAAGTTTACCATGATTTTCACCTTCAAGACAATGTGCCTTCTTGCATCATTCCCAAGGCGAGTGCAACGTTTTCATAA
GCACTTTCCAGAATGCATCTACTTTATATTAAGGTGTACTAGAAGAATCATTCGAGCTAATAGGAATAGAATAATTGTATTTTCTGAACACTCTTTCCTGATTTTTTTTCCACCAATCTTCTGC
CTCCTGCTCGGAAAATCTTCGCTTGCTGAATTTGACTCTTTTGAAGAGCTTAGTGCCATCTAGTAACTGCAAAAGAGTCGCATATATACCAGGCTCAAATTGCTCCATCAATTCAACTTCTC
CCTTGTTGTTAATTATTCTTTTAGAAGCTCGAGGTGACCTTGAGCTACTCTCCATGCTTTGGCGATCTGCAGTAACTCTGATATCATTAATGACCGATTTAATATTATTTTGAGCATTGTCAAC
GGTAGAGTTTTCAAAGCCATTTTTCTGATGTACTTGAGTATTAGGAGACTCTATGCTTGGTGTGGCACTTGAAGGCAGCTCTGAAGCTCGGGTTTTGTTATTTTCTAGAAACAATTCTGCTG
AAATAATCATTGATTTTATGTTGTCACCTAGGTCATTAGGGACTTTCTGAGCTACATCCTTCATCTGGGCTTCAATTGTCCTGATAAGCTCAATGGCAGCATTAAACTTAGCAGATTCTTGTG
CAGCTACAAAAACTTCTTTAGCTTCATTTGAAACATTATGTGCAACTGCATCTTGTATTTCTAGTTTTTGTTTAAGACTTTTGATCTCCCCTTGCAGCTTTAGGACTTCTCTATCCCAGTGCT
CATTAGTCCTCTTTATAGTCTCCATTAAGTTTCCAGAAAAATCAGGAGTTGTAGATAGTGAAGGGTTTGGCTTCCATGAGTATGGAGTTACAAACTTAGAATTTGCAGCAGGCAATGGTGG
AGGTGCTGTCGGTGTCATGATTGGCCTTGAAGTCGTTTGAAGTAAGCTCAATGAGCTTGGGAAGGCAATATCTTTTAGCTGTGAACGAGATGAAACTTGAGAAATTCGGTTTGAAGTTCG
TTGATTCTTACTATATCTAATATCATTATATCTTCCCAACTCCTTAGGAACAAGTAAAAGCCTTGAATATCTAATTTTTCCCTTCTCAAACATTTCCCTAGTATCAATAGATTGACGAGAAATCA
CATTATTTCTACCAACAACCAAATGCATACCAGTTTCCGCAGCTTTAAGTTTCGAATAGCATGAATCACATACCCGATGAGGTTTGCCTGGAGTTGGTGCCAATACAGCTTTCAAAACTTTT
CTGGAACTGCAAGCATGACAATATACTAGACCACAATTATAGCAATTATGTCTTTTTCTAGTAAATCCAAATGCTTGTCGACAGCCGGTGCAGACCGATTGGTCAGCACTAGATACCCACTT
ATGCATGCAAATACATGCTGTGAAATTAGAGCCACAAGAGATACTCTTAACATGCCTGTCTTTCAAAGCTTCAACAAGAATGGGTGTCTTTCTGTCATCAGTATCTCCATGTCCTAGTCTTC
CATTAGCACCTCTTCCCCATGAATAAACTTCACTTCGTGAAGTTAATACCACAACATGATGGGATCCACAAGCAATCTCTTCAACTAACTCACCTACTAATCTATCTTGCACTAAACAGGGA
ACTTTACCATCAAATTGTGGATTTCCGAGCTCGCCATACATGGAATGTCCCATGGTGAAAACATGCCCAGAAGTTGAGAGGGCAATGGTAATATTATGTCCGCAAGCCAACTGATGAAAGT
TGTAATCAATAAGTGATGCAACACAAGTTGGAACCAATCTTGCTTCCTTATCACCATGGCCTAGACGGTATCCATCGCCATCGCCCCATGTAAAGAGTTTACGTGATACAACATTCGTACCA
GCATGTCCCATTACTTCTACAATTGCGGCCGTATGCCACACTCCGCATGCAATCCTAATGGTTCGTAAACCACTCAAAGATTCGATTTCCCTGGGATAAGCAACACTCTCTCGATCACCATG
GCCCAAAACACCAAATGAACCATCACCAAATGAATATATTTTTCCATTTGAAGTAGCCAGTACAGTATGCCATGTGCCACATGCAACTAAAGACACTTGAAATCCTTCCAAAGTTCCAGAA
ACTCTCTTAGGCATCCAATGGCTGGCCTCAATACCATGGCCAAGAAGTCCAGTATTATAAGCTCCATCACCCCAAGTAAACAAGTCACCTGCTGTAGTTATAGCACAAGTATGAAACTCGC
CACATGCAACAGAATCCACATTGGATAACGCTAATGATTCAACTAAACGAGGTCGACTAATATCAATATCAGTTCCATGCCCAAGTCTACCACCGCATTCTTCACCCCATGTAAATACTT
>comp157318_c0_seq6
CCCTTAACTTTTTTCATCCAGATTTAAGACCTGGCAACGAAATGTAGACATAAAAAGACTTTTGTCTCAGGCAAATAAATATAAAATTTCCCTAAACTTAACAAACTCAAACAATGTTCTTG
GATAAATGCATGACATCATACACCACAAAAAAAAAAAAAAAAAAAAGTTTACCATGATTTTCACCTTCAAGACAATGTGCCTTCTTGCATCATTCCCAAGGCGAGTGCAACGTTTTCATAA
GCACTTTCCAGAATGCATCTACTTTATATTAAGGTGTACTAGAAGAATCATTCGAGCTAATAGGAATAGAATAATTGTATTTTCTGAACACTCTTTCCTGATTTTTTTTCCACCAATCTTCTGC
CTCCTGCTCGGAAAATCTTCGCTTGCTGAATTTGACTCTTTTGAAGAGCTTAGTGCCATCTAGTAACTGCAAAAGAGTCGCATATATACCAGGCTCAAATTGCTCCATCAATTCAACTTCTC
CCTTGTTGTTAATTATTCTTTTAGAAGCTCGAGGTGACCTTGAGCTACTCTCCATGCTTTGGCGATCTGGGCTTCAATTGTCCTGATAAGCTCAATGGCAGCATTAAACTTAGCAGATTCTTG
TGCAGCTACAAAAACTTCTTTAGCTTCATTTGAAACATTATGTGCAACTGCATCTTGTATTTCTAGTTTTTGTTTAAGACTTTTGATCTCCCCTTGCAGCTTTAGGACTTCTCTATCCCAGTG

CTCATTAGTCCTCTTTATAGTCTCCATTAAGTTTCCAGAAAAATCAGGAGTTGTAGATAGTGAAGGGTTTGGCTTCCATGAGTATGGAGTTACAAACTTAGAATTTGCAGCAGGCAATGGT
GGAGGTGCTGTCGGTGTCATGATTGGCCTTGAAGTCGTTTGAAGTAAGCTCAATGAGCTTGGGAAGGCAATATCTTTTAGCTGTGAACGAGATGAAACTTGAGAAATTCGGTTTGAAGTT
CGTTGATTCTTACTATATCTAATATCATTATATCTTCCCAACTCCTTAGGAACAAGTAAAAGCCTTGAATATCTAATTTTTCCCTTCTCAAACATTTCCCTAGTATCAATAGATTGACGAGAAAT
CACATTATTTCTACCAACAACCAAATGCATACCAGTTTCCGCAGCTTTAAGTTTCGAATAGCATGAATCACATACCCGATGAGGTTTGCCTGGAGTTGGTGCCAATACAGCTTTCAAAACTT
TTCTGGAACTGCAAGCATGACAATATACTAGACCACAATTATAGCAATTATGTCTTTTTCTAGTAAATCCAAATGCTTGTCGACAGCCGGTGCAGACCGATTGGTCAGCACTAGATACCCAC
TTATGCATGCAAATACATGCTGTGAAATTAGAGCCACAAGAGATACTCTTAACATGCCTGTCTTTCAAAGCTTCAACAAGAATGGGTGTCTTTCTGTCATCAGTATCTCCATGTCCTAGTCT
TCCATTAGCACCTCTTCCCCATGAATAAACTTCACTTCGTGAAGTTAATACCACAACATGATGGGATCCACAAGCAATCTCTTCAACTAACTCACCTACTAATCTATCTTGCACTAAACAGG
GAACTTTACCATCAAATTGTGGATTTCCGAGCTCGCCATACATGGAATGTCCCATGGTGAAAACATGCCCAGAAGTTGAGAGGGCAATGGTAATATTATGTCCGCAAGCCAACTGATGAAA
GTTGTAATCAATAAGTGATGCAACACAAGTTGGAACCAATCTTGCTTCCTTATCACCATGGCCTAGACGGTATCCATCGCCATCGCCCCATGTAAAGAGTTTACGTGATACAACATTCGTAC
CAGCATGTCCCATTACTTCTACAATTGCGGCCGTATGCCACACTCCGCATGCAATCCTAATGGTTCGTAAACCACTCAAAGATTCGATTTCCCTGGGATAAGCAACACTCTCTCGATCACCA
TGGCCCAAAACACCAAATGAACCATCACCAAATGAATATATTTTTCCATTTGAAGTAGCCAGTACAGTATGCCATGTGCCACATGCAACTAAAGACACTTGAAATCCTTCCAAAGTTCCAG
AAACTCTCTTAGGCATCCAATGGCTGGCCTCAATACCATGGCCAAGAAGTCCAGTATTATAAGCTCCATCACCCCAAGTAAACAAGTCACCTGCTGTAGTTATAGCACAAGTATGAAACTC
GCCACATGCAACAGAATCCACATTGGATAACGCTAATGATTCAACTAAACGAGGTCGACTAATATCAATATCAGTTCCATGCCCAAGTCTACCACCGCATTCTTCACCCCATGTAAATACTT
>comp157331_c0_seq6
AAGTTGCTGATTTTCTTGCTCGCATGGATCCCTCCAGAATCAAGCTTTTCGAACTGGACGAGATGGATGAAATTATGAACGGATGTGTTGGTGCGGGCGCATTTGCCGAAGCATTGCATAA
GGATATCGAAGGGATTGATTCCACCAATCTGGCAGCTTCCATCACCAATCGCAATGCTGGAGTATTGACTCTGGATGATAGCTCCACCACATTGAAGAATAATTTGCAATTCACTAAACATC
TAACTCAGTTGGAGCAAGTTAAAATTTTTCCTTCTCGCTATCAAAATCAGCATATTGATTCTCAATTACAATGTGATCATAGACTACTACAGCATGGAAATACACATTGTGAATGGCAAAAA
GGGCATTCAGATAATTTTTCTCGGCAAAATGTTGATAATAACCCATCCCAAGACATACATCAGCATTCCAAGGTACAAAAGCAGAAAGTCCAGCAGTTCAATAATCAACAACCTCCCAATA
CTGACCTTTCAAATAATTCCTTGAACTGGAAGATACCGAAAAATTCCCTGGAGTTGAATATGTTAGTTCCACAATTACAGCCTCTCCTTGATAAAGACAGGTCAATGCTGCTTCGGGATTT
GTATATTAAATTATTGAATAATGAAATTAGTAAGGAGAACTACTTAAAAGTTTGCAATAAAGTTGTTGCGGACCGAATGATTAGAAGAGAGGCTGCAGTCCATCAAGCGCAGATGCAGAAC
CCAGTGCAGTCACAATCTCAAAGTCTCTATCTAATTCGTGGAACTAGCATAAAATTTCCTCCAAAGAAACGTCCCATTGGTAAGAAAAAGACGCTTGAAACTTCAGGGAACTCCCAACCC
CAAGCAAGGTGAACAGTTCCATTGATCATTTTTTAGTTATCTTGATTTCAACAAAAACTTACTAAATATTTACTTTATGTGATTATCTGATTCCAAGTGATGTTTAATAGCAAAAAGCAGAAG
ACTAGTGGAGCTTTCCTGGATCAAAGCATTGAACAGCTTAATGATGTGACTGCTGTAAGTGGAGTCAATCTAAGGGCAGAAGGGGAGCAACTACTTGTTGCTCGGACAGAAGGAACACA
AGCTTCAGAGGCAATTCAAAGAGTTGTACAGGAGGAAGAAGAAACATCAATTCTGCAGAAGGGACCTCTACTGAAAAAACTGACGCAAATCATTTCCAGATGTGGTTTAGGAGGTATAA
GTAAAGACGTGGAGTATTGTCTATCTATGTGTGTAGAGGAACGTTTGCGGAGTTTCATAGGCAAATTGATAAGATTATCTAAGCAGCACCAGATTTTGTCTATGAATCAAAAAGCCAAGAA
AGAACGGGAGAAAAAACAAGCTGAGGAATCAGAAAAGTTTTGTAAGCTAGACAATGCTTTAGCTGATGCTGAGAAAGATGCAGAAAAGGACAAAGATAACAGACCTTCTAAGAAACAT
AAGTTTCACCGGGAGGAAGACGATAAGATGCGAACGACAAATGCGGCTGCTCGAGCTGCCATTGGTGCAGATGATACGCTTTCTAAGTGGCAGCTGATGGCTGAGCAAGCTCGTCATAA
GCGTGAAAGCAAACTGGGTGTATCTTCCAGTCACCAACAAGGTACTAGTATAGATGAGAACCACTCCCTCAACTTCAGAGAAAATCTGAGAAATCTAGAGGAAGATGGTGGCATGAGCA
GACATGGCCGAAGCATAGGAAACGTGTCTAATCCTAAAATTGATCATACAATTCTTATTAAGGATGTCATTGCAGCTCTTGAGAGGGAACCAATGATGTCGAGATCAACCCTTCTGTGTCG
TCTCTATGAAAGAAGGTCTAGCGGTTATGGTGCCGGATGATCTGCAGTTAATGAGCTTCCATTTAGCAGGTACACAATTTCAACTTTGATTGGTTAACTTCGGGGTAAATATTTCAACTTTC
TTGATGAAATTTGAAAAGAATTTCGATATTACCCATCAAGCATTTATTATGATTATTCTTTAATTTGATATGTATATAAATTTTTTTATTGATT

>comp157334_c0_seq4

GGGCTAAGCCTCATCATTTTCAACAGAAATAGAGGAAGATTGCATTAAAAGTAGAGGAAAACCACTGCCTTATGCAGAAGTAAAACAATTTCAAGGGTAGAAGTGATAGAGCTGGATCC
AGCTGAAACAAAAGAGAAAAAAACAAATGAGCACAACTGTGTTGCTATTTGCTAATGAAATGTCAAAGAACTGACGACAAAGAAGTTCTTTTTCCACTGGAACATGCCCGTTCTGGGC
AAAAGATCATTCGCTGATCTCCTCCGCTTCCTGCTCGCTGCTTCCGTCCTCATCCCTTGCTTCCTCACCGGCCTTATTAGCGGCCTTGGCGGTCGCCGCATTGCTGTTGGCGCTGCGCTCTG
TTCTTGCAGCCGTGATAAGATCACTCGAATTCTGCGTCTTGCTCAGCAAGATCTCAATCTTGGCTTTGCGAACAAGACGGCCGTTGGCTTCGTCCTTGGTGCCAACAGTAGAGGTCATGAT
CTTCTTCTCAGTAGCCAGGCCGTTGTTCTTGAGTATCTCGGCGACGGTGACGACTGTGCCGATGGCCATGCCGAGGGCAGAAAGTTCGACCTCGTTGTGGCGCTCCATAAACCTCTTGGC
GAGATTTACGTAAAAGTAAACAGGCTTTTTATTGGTGGAGACCTGAATCCGATTCCCCTTCTGTAGACCGACAACCGCCGCGGGATCTTTGATCGCCACCGCCGCCATCATCGTCTCCATC
TCTAACTCGATCTGGCTTACCTGTCTGTACTCTCTCTTAATTTCTCTCTCTGATCTCTCTTTGGCTCTGGCTCGCGCTCGAAGGA

>comp157357_c0_seq11

AAAAAATAAAAAAAAAGTGAAAAAAAAATTAAGAAAATTGATGACCAAATAAAAATGTGTCACTTTTATTATAACTCCACTAAGAAATCCCATTCAATAGATGCTATGAATAATACTCAGA
AAACAAAGATCTATGCTCTATAGCCCTATTGTATTATGCATGCATCATCATCATCATTCCCTTGATAGCTCAACATGCAAAGAATTAAAAAAATAAATTTGTTCTAACAACTTAAAAAAAAT
AAAAAAATAAAGTCTTCAGTTTTTATTGTTCCTTTTCACTATCATCACCTTTATCAAATGCTCTTGATGGTCCAGCCTCAGGATCAACACCCTTTACATACTTCCCACGCCGGCGCCGATCA
AAAGCCTGCCATCCCTCAGCCTGACGAAGATAATCATTGGTGATTTGTGTGCCATAAATACTAAGAACTGATAGTTTTTGGCAAAATTTGAACAAAGTTGAAGGAAGCGTTTTGAGGTTAT
TGAGACGCAGATGCAAAACCTTCAGATTTCGTAATTGTCCAAATGTTTCTGGTAATTCGGTCAAAATATTAGCTGATAAATCTACCTCATTAAGCAAGGAACAATTTCCAATGCTAGCAGGC
ACCGAGCTTATCCTATTGCTTCGTGCTTTAAGAATCTGAAGTCGATTAAGACATCCTAATTCATCTGGTAAGCATGTTATCTTGTTCATTGAAATATCGAGTTCACTCAGAGAGGTCAATTCA
CCCAAAGAAGGCGACAAAGTAGTTAATCTGTTTGTGTTTAAAGATAGTACTGTTAAAGACTTCAACTGTGAAAGGCCATCCCAGCTAAACTTCTCATCGCATAAATGGTTATCATTCAGGT
ACAGATTCAAGGACTTCAAAATACCAATTTTATTCGGCAATTCTTCAATAGAATTGGAGCTGAATTCCATCTTTATTAAAAACGGTGCACAATCCCAGACATCATCAGGCAAAGCCTCTATG
TGCTGATCCAGAGTAGCTATCATCATTAGTTTGGAACCATCAGTTATCTGCACTGACCTCAAACTCACTACATCTGCATGAATTTTGCCTTTAAATATGAGTTTCTGACAGCGAGTGAGGAC
GTCGGTAAGGGAGTGGAGGCGGGATTTAAGATCCAGAATGGTAGATTCAGGCGAAAGGGTAACTGAGATCTTGCGGCCGCTGAACTTTACGTGGACGGTGATGGGATCATCAGCTCGAA
TGAAGGAGGCCGCGGCTTTGGCTTTTTCACTTTCCTCTATCCTCTCCATGGCCGCTGCTGCAGTTAAACGGAATAGCTTAATCGCGGAAACTGTATCTCCCTGTTGCAGATAATCCTTTGGG
AAAGAACGGAGCATATTTACGGGATGTTGGCGGCAGGATTTTCGCCGTATCCACGCCGGCATTTTGGGTGCCAGGAGGGGGAACA

>comp157357_c0_seq13

AAAAAATAAAAAAAAAGTGAAAAAAAAATTAAGAAAATTGATGACCAAATAAAAATGTGTCACTTTTATTATAACTCCACTAAGAAATCCCATTCAATAGATGCTATGAATAATACTCAGA
AAACAAAGATCTATGCTCTATAGCCCTATTGTATTATGCATGCATCATCATCATCATTCCCTTGATAGCTCAACATGCAAAGAATTAAAAAAATAAATTTGTTCTAACAACTTAAAAAAAAT
AAAAAAATAAAGTCTTCAGTTTTTATTGTTCCTTTTCACTATCATCACCTTTATCAAATGCTCTTGATGGTCCAGCCTCAGGATCAACACCCTTTACATACTTCCCACGCCGGCGCCGATCA
AAAGCCTGCCATCCCTCAGCCTGACGAAGATAATCATTGGTGATTTGTGTGCCATAAATACTAAGAACTGATAGTTTTTGGCAAAATTTGAACAAAGTTGAAGGAAGCGTTTTGAGGTTAT
TGAGACGCAGATGCAAAACCTTCAGATTTCGTAATTGTCCAAATGTTTCTGGTAATTCGGTCAAAATATTAGCTGATAAATCTACCTCATTAAGCAAGGAACAATTTCCAATGCTAGCAGGC
ACCGAGCTTATCCTATTGCTTCGTGCTTTAAGAATCTGAAGTCGATTAAGACATCCTAATTCATCTGGTAAGCATGTTATCTTGTTCATTGAAATATCGAGTTCACTCAGAGAGGTCAATTCA
CCCAAAGAAGGCGACAAAGTAGTTAATCTGTTTGTGTTTAAAGATAGTACTGTTAAAGACTTCAACTGTGAAAGGCCATCCCAGCTAAACTTCTCATCGCATAAATGGTTATCATTCAGGT
ACAGCTTCTACACAATTAAGGCACAAAAGGCTCCATATTTCACATTTAGTAAGGCTAAAGCTCCAAAGCTAATCATATTTTCAAAGGGAATGTTTCAACCAGATTAAAAAAACAACATAAA
ATAATGTATTATAAGAAACATACATTCAAGGACTTCAAAATACCAATTTTATTCGGCAATTCTTCAATAGAATTGGAGCTGAATTCCATCTTTATTAAAAACGGTGCACAATCCCAGACATCA

TCAGGCAAAGCCTCTATGTGCTGATCCAGAGTAGCTATCATCATTAGTTTGGAACCATCAGTTATCTGCACTGACCTCAAACTCACTACATCTGCATGAATTTTGCTTTTGTCTAAAAGATG
ATCAATATAAAGTGCTTTCTGATAAAATTCTGAAACAGAATTTAAATTAAGCAAACTGTTTCTACAAGCTCTATGTCCGCCGCCTCACTGATGTCAACTATCGACTTAGGTTCTTGCTTATTT
GGCTTCCATCTTGCTTATGAAACCCTTTAAATATGAGTTTCTGACAGCGAGTGAGGACGTCGGTAAGGGAGTGGAGGCGGGATTTAAGATCCAGAATGGTAGATTCAGGCGAAAGGGTAA
CTGAGATCTTGCGGCCGCTGAACTTTACGTGGACGGTGATGGGATCATCAGCTCGAATGAAGGAGGCCGCGGCTTTGGCTTTTTCACTTTCCTCTATCCTCTCCATGGCCGCTGCTGCAGT
TAAACGGAATAGCTTAATCGCGGAAACTGTATCTCCCTGTTGCAGATAATCCTTTGGGAAAGAACGGAGCATATTTACGGGATGTTGGCGGCAGGATTTTCGCCGTATCCACGCCGGCATT
TTGGGTGCCAGGAGGGGGAACA
>comp157357_c0_seq8
AAAAAATAAAAAAAAAGTGAAAAAAAAATTAAGAAAATTGATGACCAAATAAAAATGTGTCACTTTTATTATAACTCCACTAAGAAATCCCATTCAATAGATGCTATGAATAATACTCAGA
AAACAAAGATCTATGCTCTATAGCCCTATTGTATTATGCATGCATCATCATCATCATTCCCTTGATAGCTCAACATGCAAAGAATTAAAAAAATAAATTTGTTCTAACAACTTAAAAAAAAT
AAAAAAATAAAGTCTTCAGTTTTTATTGTTCCTTTTCACTATCATCACCTTTATCAAATGCTCTTGATGGTCCAGCCTCAGGATCAACACCCTTTACATACTTCCCACGCCGGCGCCGATCA
AAAGCCTGCCATCCCTCAGCCTGACGAAGATAATCATTGGTGATTTGTGTGCCATAAATACTAAGAACTGATAGTTTTTGGCAAAATTTGAACAAAGTTGAAGGAAGCGTTTTGAGGTTAT
TGAGACGCAGATGCAAAACCTTCAGATTTCGTAATTGTCCAAATGTTTCTGGTAATTCGGTCAAAATATTAGCTGATAAATCTACCTCATTAAGCAAGGAACAATTTCCAATGCTAGCAGGC
ACCGAGCTTATCCTATTGCTTCGTGCTTTAAGAATCTGAAGTCGATTAAGACATCCTAATTCATCTGGTAAGCATGTTATCTTGTTCATTGAAATATCGAGTTCACTCAGAGAGGTCAATTCA
CCCAAAGAAGGCGACAAAGTAGTTAATCTGTTTGTGTTTAAAGATAGTACTGTTAAAGACTTCAACTGTGAAAGGCCATCCCAGCTAAACTTCTCATCGCATAAATGGTTATCATTCAGGT
ACAGCTTCTACACAATTAAGGCACAAAAGGCTCCATATTTCACATTTAGTAAGGCTAAAGCTCCAAAGCTAATCATATTTTCAAAGGGAATGTTTCAACCAGATTAAAAAAACAACATAAA
ATAATGTATTATAAGAAACATACATTCAAGGACTTCAAAATACCAATTTTATTCGGCAATTCTTCAATAGAATTGGAGCTGAATTCCATCTTTATTAAAAACGGTGCACAATCCCAGACATCA
TCAGGCAAAGCCTCTATGTGCTGATCCAGAGTAGCTATCATCATTAGTTTGGAACCATCAGTTATCTGCACTGACCTCAAACTCACTACATCTGCATGAATTTTGCCTTTAAATATGAGTTTC
TGACAGCGAGTGAGGACGTCGGTAAGGGAGTGGAGGCGGGATTTAAGATCCAGAATGGTAGATTCAGGCGAAAGGGTAACTGAGATCTTGCGGCCGCTGAACTTTACGTGGACGGTGA
TGGGATCATCAGCTCGAATGAAGGAGGCCGCGGCTTTGGCTTTTTCACTTTCCTCTATCCTCTCCATGGCCGCTGCTGCAGTTAAACGGAATAGCTTAATCGCGGAAACTGTATCTCCCTG
TTGCAGATAATCCTTTGGGAAAGAACGGAGCATATTTACGGGATGTTGGCGGCAGGATTTTCGCCGTATCCACGCCGGCATTTTGGGTGCCAGGAGGGGGAACA
>comp157383_c0_seq1
TGTCTAATAATTATCAAAATAGGAACGACTTCGTTGCGGTTTGGGTTTCTGCATGAGAGATCGGGAGGGGAATGCGGGAGGAAAGAAAGAGCGCGAGCAACGGTGCGATAGCCAGCGAT
GAGTTTTCTCAAGGCTTAAAATCTCTGATTGATTCCTTCCCCAACCCTGCATTCCAGTTTCTCAAAATTTTCGCTGGTAATCCTCTTCCAGCCGATCATTCACACAGAGCTACGGAACCCTA
GCTGTAACCCTAGCATGTCTCTTGTTTCGGACCTACACGTCCTCCGCAATGTCTCTGACTCCGCAGGGTTCGTTGCCGTACGCGGGGAATCCGAGGCGGATGCCGTTGCTCCGCCTCATTC
AGCAGCTACCGCGATTCCCTTACCCGAGCCAGCAGTTCTTCCTCTGAATCGGCGAGAACACGCGCAAGACTCTGTTCCTTTTCTTGCCGGCAGCAATGCCTCCCATGGTCCCGCTTCCGA
AAGGGAAAATAGGCCTGTATGGGGAAGGGCGCCGAATGGAGCCATTGATACTGCTCCGCTAGTTTTGGGAGGCTCCTCTTCTTGGCCCGCTTTATCGGAATCTGCCAAAGCCTCTACCAG
ACCTTCATTCGATGAATTGGCCCCCTCCCTGGGCTCTAACTCTCTTGGCGGTGGAAATAATGGCGATGATGTTGGAACCAGTGGTAGATTTTCCAGTAATGGTGATCCCTCATCATCTTCTT
CAACATCAAGTTCAACGGAATTATCTCAGAACATGACAGATAAGTTGCCTGATAAGGGCAATAACAGCAATTGGGACCATCGTCCTCGTGAATCCCATGGCTGGGTGGATCAAGGGGATC
GACGGAGGAGGCATGGTGGCAGCAACCGAAGGGGAAGCTATGGAGGCAGGCGTGATGAGGACCGTGTTTTCCGTGATTGGAGTTCTTATAGGCCTCTGAGTGTTAGAGATTCCCACTTG
CAGCGAAATCACCCGAGCCATGCAATCATGCGACCTCATATGCCTGCTATGGTGCCATTGTATGGCCCTCCTCCGCTTATCCAACATTTTCCTGGCCCCATGGCCTACCCTGATATTCCACCT
CCAATGTATTTTTTCCCAACTCACCCTCATCCCGAACCTTTCGGAGGGTACCCATTTGTTCCCAACCCAGCACCTCCTGCAATACAAGAACCTATGTTTTTTCCATCTGCGGAACAAAAAC

GCACTTTGATATTGAAGCAAATTGAGTATTACTTCAGTTCAGAGAACTTGTGCAGTGATTTATTCCTGAGATCGAACATGGATAACAATGGGTGGGTTCCAATCGATTTGATTGCTAAATTC
AATAGAATTAAGCTATTAACAGATAGCATACCATTTATATTGGATGCAATCAGGCCCTCAGATGTTTTAGAAGTGGAGGGCAACAAAGTCAGAAAACGTGACGGATGGCAGATTTGGTTAT
TTAACCCACGACAAAATAAGGATAACACGGAATCTGAACCTAATTCTCAGACTACCACAAAACAAGATTCTCTTGTTTCTCAGATGGAGAATATTGCGCTCAACGAAGGAAATACTCGGA
CCAATTGAAATCTTTATTTGGGTCAGGCGACTTTCGACTGTAGCCTTTAAGGTAACAAAAAAAAAAAAAACTGTATAGTTTTTCCCCTTATTATTATTATTTGGAGTCCTTTTTGGGTGCTG
CTGCTGCCTCCTCCTCCTCCTCTCATGATCATTTGTGAACTAACTAACCCTCTCCATCGTCTTTGGTCTTTCTTATTTCTCTTTCTCGAGTTGCAGAAACTTCCATTCTTTTGTGAGTAGCGG
AGTCGGAGAAGTGATTCAATATCATTCCTCCCTCGCTCTCTAATCCTCCTACTTACAAAATTACTCGTCTCTTTTCTTCCTGAAGAACTTGCATTATATTACTTAAAATGTATTTTTTTTTTTT
AATTTCTGCTGGATCTGCTTTCACATTGAGCTTTCTTCATGTTCATAATATCATATGAATCCTTTATCTATTTTATTATTTATTAATTTATTTGTTTATTTATTCTGATATTTCTAACTCATACATGT
>comp157421_c1_seq4
AATTATATATTTTTCCCCAGAAAATATGATTAGTTAGAATATGTGCTTTATTACACAAAGGAAATAATCACCTCGGATTACAGTACAAATAAGAAAAAAAATGCACACAATCACACCAAACA
AGTACAGATTGGGCCATGAAAGCTTACAAGCTAAAACACCAAGCTTCTTCACCCTTCTTATTGACCAAACTACATAAACAATAGTATAATTCTTGAGGCCAGAAAATAAAGCATCTGCCTC
AGTACAAGCCACCGCCAGTCTCGTGCGCAGTCCGGTTTGTTTCAGTGCCGGCAAGTGGCCCATCAGTCCTGTGCAATCCCCCGGCCCCACTAGTCGGCTCAACTAAACCGATATGGTGTC
GAACTCCTGTGTGATGCTCACCCAAGCCAGTTTGAGGTACGACACCATTGTGATGCCCATCCAAGCCGGTTCCGGTTTGGTGGCCGACTCCTAAGTGCTGTGCTTCTCCTTTTAGTATGGC
ATTCTCCTTGTACGCCTGCTGCTTCATCAGCTCAGCTTGACGAATCTTCTCCTCCTTCCTTTCCTCCACCATTTCCTTTTGCAGTGGATCCCTTGTCTTCATTTTCTCGATCTTCGATGGAAG
AGTTCGCGATTATCGACGACGAACAGGAGAGGAGAGAGAGAGAGAGAGAAACGGTTGTTCTATATTCTCTCCATCTATTGCTCTTCTCGCCCTGTTTCTGCGGTCTAGACGGGAGAACAA
GAAAGATATTGCTAGTTGTCCATTCAAAGGGATTCCAATTTATGGGCATTTTATTAAGCTCGCCTCGGGGCTCGTATTTCTTGTTCGGGAAAACGCGAGGACGGTTTATTTATCGCCAATCTT
TTATCGCCC
>comp157449_c0_seq1
TATGCATCATATTCATATTACATAGTATATACAATTTCTTCAATGGAGTCTTCAATATTTTGTAAATTTTACCCCATGGAATCTTCCTTCTCTAAAACGACAATGAATCTTTAATTTTGTTTCTTC
CTCCGTTAACAAATACATATAAAGATTAAGATCCATGGCGCAGCAATTACCCAAGTTGACTAACGAGACGAACTGCAGTGCTTAGTCTTGCCTAAACCTCGACTGAATCATTAGCATTCAC
TATGTTAACGACTTCCACCTCGGGAACCTTGGTCGCCGCCGGCAAGAATTTCTTGATGGCAGCGGCCGCCCAGTCCCAGGCGTTGAGTAGCACGAATACGGCGATTCCGCCGATGAGCCC
GTACGCAATGGAGTATGTAAGTGGCATCAAGATTAGAGTTATGAAAGCTGGAATTCCCTGTCTCATATCCGCCCATTCGATCTCTGTCACCGCCTTCATCATCATCACCCCAACAACTATCA
GCGCCGGCCCCACCGCCCATGGAGGTATTGACGCCAGCAATGGCGTGAAGAACAGCGAAAGGATAAAGTACGCCGCCGCCGTCAGCGCCGTTAGCCCTGTTCTTCCTCCTTCCCGGATA
CCTGTTGACGATTCTATAAACGCCGTTACCGGCGACGTCCCAAGTAGCGAGCCGGCGACAATAGCTGTAGCATCGGACATGAAGGCGAAGTATTGCCCTTCAAATTCCCCGTTCTCTTCG
ACGAATCCTGCGAAGCGCGCCATGGAATAAAGAGTGCCGGTGGTGTCCAGAATGTCGACGTAGAGAAAAGTGATGACTGCTTCCCAGAAACGGCCGCGGGCGATGTCGGAGAAGCTGA
GGGCGCCAGCGGTGGAACTTATTGAATGAAAGTCGACGACTTTTTTGAAATAAGCATAGGATTCGTTGCCGGCGAGAGTGTCAGGGAAGGCAGTGACGGCGGTGCCGCGGAACCAGGA
GATGGCGGTGACGAAGACGATACCGTAGATTATGCCGCCGTTGATATTTCGGATGAGGCAGTAGGAGATGATGAGGAAGCCGGCGGCCGCGAGCCAGAAAGTGGGGCTCTGCATGCGAC
CGTGGAGGCAGAGGAGCTCGCCGGAGACGGTGGCGCCGGGGATGAGGGAAAGGGTGCCGTTTGGAAGGGTTTGGACAGGGGCTAGGGAGGCGCGGGAAGAACGCGGGCAAGCGCC
AAGGGTGAGGAGAGTAGATGGGCTGAAGCCGACAAGCCCTATGCCCTGATTGTTCTGCAGGCCGATAAACGCGAGAAATAAACCGATTCCGACTGAAGACGATGACCTAACTGGGCGG
GGAATGAGACGGGCCAGACGAGAGCGGAAGCCGAGAAGAGAAAGGAGAAGGAATAAAACGCCCTCGATGAACACGGCGGCAAGGGCAGAGCGGTAAGGCACGCTGCCGGAACCGT
GGAAACCGACAATGGAATAAGCAAAATAAGCGTTTGTTCCCATTCCAGGGGCGAGTGCGATGGGGAGATTGGCGAAGAAACCCATTAGAACAGATCCTATTACGGATGATGCGGCGGTG
GCGACGATGAGGTCGCGGCGAGCGCGGGCAACGCAATCAGCGTAGCCGGGATCGATAGCTGGTGGGAATTTGCAGGACGGAGAAGGATGGAGGCAATCGGAGACGGAGCAATTCGCG

CCAGAGTCGGAGACGATGGAGGCGTTGACGGCGAGGATGTAGGCCATGGTTAAGAATGTGGCCGTACCGGCACGGAGCTCAGTCGAAAAGGTGGTGCCGCGTTCTGAGAGCTTGAAGC
GCCGTCCAAACCAGGAAGACGCCACCGCCGCGTTCAGATTGCGGAGGCGGGAGGGCGGGGGCGGTGGATGCATGGTTGGCCGCCTGCCGGCGAGCCGAGGAAACGGGAGCAGAGAG
TTTGCTGAGGGGCGAGATTTATGGGGTGGATTGGCTATAAGACATTGGGGGTTTTAGTTCAGAGGTTATCTGCATCTTATGTAAATGTTCGTTTGTGGGCGGTTGTGGAATTATGGTGATAA
GAAAAGTTCATTTCTAGAGGGGAAAAA
>comp157450_c1_seq12
TATTTCGTAAGTACATGTTATCATTTTGCAGCTCCATTTCCTGCAACAAAATCAGTTTCAAGTGTTTATATATAATCTATTTTCTTGGAGCTAAATTTGCTTGCTGCTTTTCCAGCTGCAAAAA
TAAGCAGATATATAGAAAGCAGAAAGGAGAGTTTGGTGGATTAATAGGAACAAGCTTCTTGTTTTTACCTTCTTCTGCATGTACTCGATCTCATTATACAGCAACTCATTCTTTTTAGATCTT
ATTTTGCTGATGCCTTTCTCCAGTCTAGTTTCTAGTTGTTTGAGGTCCCTTAAGTTCATTGTGCTGAGAGCCTCACCCATCAAATTCCTATAGGCATTTAAGGATTCTGTAACATATGGAATTT
ATAAACGGAGAATAGTGAAGTGGACTGTATGTTAATTTATTA
>comp157450_c1_seq2
TGGAATTGCTAAGACCAAATTTTTGTTATAAAATTATGTTTATTTGTAACAATACAAAGCCATCACAAGCTATAAAACAAAGAGTTGGCAATATATATACAACAAATTAAGCTCCTGGACAA
AAAGTCTATTCTTGACAATTCATGCATATTTATGGTTTATTTCCATGAAGAAAACCTGATCTCCAGTACCTGACATCTCTTCCACTAAGGTATAGGCTGCCTGTTGCTAGGAAGAAGATTTAC
TTGCAGAAAGCTTCGAGAATCAAATGGAGGCATAACTTCACAATCATTTCTTGTTGAAGGCAGCATGTTCATCTGATGCTGCTGCACTCTCTCATTCTCAGCAATCTGAATAATTTATAAGT
TTCAGACAGAAAAGTAGTGATAAAAATTCAATCTTTCAGTGCATTAATGCAGAAAAATAAGGAAGTTTCCATTTATTTCTATGTATGATTAAATTTACAGAAAAGTAGACCTTATTTC
>comp157450_c1_seq4
CCTTCATCCTTCTCTTATCAGCTACTGCTCATATGAACTATTGAAAAACCATCAAATAAGATTATGATACTCCTACATATAATATCTTATAAAGTCTCAACATTGAACAAGCCTATGACACCAG
TTCCATAAAATAATATAGAGAAAAGTCTATTGCACAACCATCACCAACTCTTAAGCTTTGCATCCAGACTTTCCTTACAAAACATACCAAATATATTTTCTTCTGATACTTGAGAAACTCAGC
TAGCATCTCCAAGCTATCATTGCAGCAATTCTCCATTACCCAAGTTGCAGAGCTGTTTGTTGCTGGTGAGCATAATGATCATTGGGATCCAACAGATTAACTTGCAGAAAGCTTCTGGAATC
AAATGGAGGCATAACTTCATATTCTGCGCTTGTTGATGGCAATATATTCATATGCTGATGCTGCTGTGCTCTTTCATTATCAGATATCTTCAAAATATATTCACAGTTATTTTTAGAAAAAAAT
TATATAAAGTTGATATTTTTCAATAACATACAGTATATCACAGACAAATGTGTTGATTTTGTGAGAGTAAGAGATATTTTATTAATTAATTTATTTATTGAATAGTTACTACATTGAAAATAATTT
ATGGTGTGAGAATTTAAAGACTGAGGTTTTATTTGGGACGGCTT
>comp157450_c1_seq7
CCTTCATCCTTCTCTTATCAGCTACTGCTCATATGAACTATTGAAAAACCATCAAATAAGATTATGATACTCCTACATATAATATCTTATAAAGTCTCAACATTGAACAAGCCTATGACACCAG
TTCCATAAAATAATATAGAGAAAAGTCTATTGCACAACCATCACCAACTCTTAAGCTTTGCATCCAGACTTTCCTTACAAAACATACCAAATATATTTTCTTCTGATACTTGAGAAACTCAGC
TAGCATCTCCAAGCTATCATTGCAGCAATTCTCCATTACCCAAGTTGCAGAGCTGTTTGTTGCTGGTGAGCATAATGATCATTGGGATCCAACAGATTAACTTGCAGAAAGCTTCTGGAATC
AAATGGAGGCATAACTTCATATTCTGCGCTTGTTGATGGCAATATATTCATATGCTGATGCTGCTGTGCTCTTTCATTATCAGATATCTTATTACGCAGGTACATGTTGTCAGTTTGCAGGTCC
ATTTCTCTTTTCTGCATGTAGTCAATCTCAGCGTGCAACAGTTCATTCTTTTTAGATCTTATTTTGTTGATGCCTTTCTCCAATCTTGTTTCCAGTTGTTTCAGGTCCCTCAAGCTCATGGTGG
TGAGAGCATCACCCAGCAAATTCCTGTTGGAATTTTGTAAGTTGGTGATTTGCTGACGTAGTTTTGTGGCCTCTTGTTGGTAATACTTGTTCAGAAATAGAGAAATGTCAGAGATAGAGAT
CATCAATTTGACAAACATATATAATATTTAAATGGAATAGTGATATATATTTGATAAAATCCTATTATATAAGAAAATCAACATAATTGTAGACTGAAGGCTAGCTAAGTTACTGATGACCAAA
TCTGGTTTCATGATCATGAAATATCCAGACAAAAGTTGAGCAATGATCGATTGGCAATTTCAAAAACTTTCATTTGGCCTAAATGTTTATTCAATTGCTAGTTATTTGCTAAATAAAATAGAT
TAATGACAGTTCATTTAATGCATGTTCTCTACTAAAAGTATCATAAGGTTGTATAAATTAGCTCCTTGATAGGGTGTGTATGTTTTGATTAAATCCAGTTTTGGTAATTTTATAATAAAAAAAA

TTAACTTAAAAGGCATTATTATATTTTAAGGTTTCTACTTCATTCGATTTCTATCAGTATTGATCCATTTTTAGACTTCGTTTGGGACGGTTTTCTTACCACATCTTAAATCAGCTTTTTAGTGT
AAAAATTTTAAGTTTGTACTAAAAGCTGCTTTAAGAAGTACTAAGAAAGCCGTATCAAACGTAGCCTAAAATGATGATATGTCTATCTAGATAGCTAGACTCGGAGCTTCATTTGTAATTTT
TTGTACTAAAATTCCGTTTTATGAAGCAATAAAAAA
>comp157487_c0_seq7
TTGTTCATATCAAATATAATGAGAAAACAACGACTAATAAAAGAATGATACACCTATTTCTTTAAATAAGTTCGGACGCGATTATGAGCGATTTTTTTTTTAAGGTTTAAAAAGAGTGAGTTT
GAATAAATATTTGCATGCATAAATCTCATTTTGACCGAATCAATATATGAAATTTCTGCACAAATTAACTATAATCATTATGTCAAAAATCATGTTAAATTTTTTTATGCTATTTTTTTTTTACCG
AAAATATATTTATAATAATTTCATTTAGATAAGATAATAAAATTAACAGTACATTTGCATTGTAGTGCCTTGACAGACAAATGGAAATTGACTCATGAGATGTATACGCCAATATCACTTTTTT
TTAACTAAGAAACAATACTAAGTATATCGTTATGCACAAATTTCCTCAATCGATCATGAATGATATACATAAGATGCTATATAAATGATACTAAACATGCTTCCTAAACATCTTTCTTGAAGTT
ATAATACATGCGAACGTCACTTAGGCACCATGAAAGAATTATGCATTTGATCACATGATTTGATTGTGGCTCATAGTAGTGGTGCAACATATAGGCAAGATTCTTATGGAAATAGATCACCA
ACCATGACAAGAATCTCAATCCTTTCCCAATAACAATATTAAAGAATAAGCTTAACATGATTTTTCTTAGCTCTCCCAAACTATACTCACGTTTATACATGTACACAAAAACTAGCATTTGGA
GAAAAATCATTCGATAGATCCAAGAAAGAAAAATAAATTTATTTCTCCCTTAAGTATTTTTTATTCTCCACAAACCAAATACCTTTATTTTGAACTTATAAATCTACTCATTTAAATTTAGAAT
ATGAAGCTACTTATTCTCATCACCAAGCTTCGTATCTATGATCGTATACAAATATATCATCG
>comp157487_c1_seq1
TTTTTGTCCTTAGATGTCACTAATGTTATAGGGGAACGAAAGGAGGTGTTTTTCTAGCCCTTCTAGACATCGTGCAACGCTATATAAACTATATTTTTTTATATTGACTTTAGAAGCGAACAA
TTTAGAAGAAAATAAAGATATTCATGATATACAAATAACATTACCCGAAGCCATTTTCGTTCATTGTTTCATTTTCAATGTGTATATGTTAGATGCAAAGTAAAAGAATGTGGTATATTTACAA
TAACAATAAGCATGCAATTTGTCAGATTCATTTCTTTATTGACAACACATATACATACATCACCAAAGTATAAAACTGTACGAATTCGATACAACCATAAACTATATATAATGACCATGGTACA
AAGCATATTCTCATTGTAATGATGCATGCTTGTATTAAATATATTAATATCATCATTCAAATGTATATATCAACACCCCAACAACTCTACATAAATCTTTGGCAATTAGATGTACAACAACAATT
AAAACAAGAATAAGTGCGTCCGACTAGGTTCATGAAATTAATCAGCCACCTTAAGGTTTGGAGGGTTAACAAAGTTGCTAGTTTTATTGATTCAAGATAACAAGAAGAGAGATTCACCTA
GTCGATCACTATTTATTTCTTAAATGAAGATAATTAAAATAATAATAATATTAATAATAACGAGATTATCTTAATATATAACTACTCCATTCTCCCAAGAAAATTTAGTTCAAACATGCTTAGGA
GAAAATTCAATTCAAAATTTCCTTACAGGAAAACCACATCCATAATGTTCTTACAAGAAGGCTAATTGGAACGAGATAGTCATTTCAATCTATTATTTAATATAACATTAAATTCAAGAAAG
GAGAATAGAAGAAGGTTTGACAAAGACTAATGTTGTAGTATTTCCTTTTTTTTTTTTGATGACTA
>comp157561_c1_seq1
AGCTTCTAAATACCATTTGAACGCATAACCATTTTTTTAATTGATAAAACATCTCCATCTCCCAAATTCTGAAAAATAATTAAAAAAAAAATCAACAATTTCAATAAATAGAAAGTTTTTGTT
GATAACAATTCAAAGATAGCCATAATCTTTTTAAGTATCCTTCTCCCAACAAAGCATCCCAACATTCTTCATTGATCAAGAAAGGCAATTCACAATCAAGCAAGCAAACAAACTTCAGAAA
AATGTAGAACAACAACGTAGTGGACTAGAAATCTTCTTCATCTTATTGACTTTTCGTTACTTTTTGATCTGTAGTAGCTTAAGAGTAAAACTTGTAAGTCTTTGCCTGGAGTATGATCTGCC
TTAAACCTCCGATTGGTGAGAAGACCATCAACAAAACTCCCAGCATAATACAAATCCAGTTGATTATCCATGACAAACTGAACTTCTTAGGCTTGTAGATAGCAAGCCACATGACACAGG
GAAGAAAGTATGTTGTTGGGGAAAAAGCAAAGCCTCCGAAGAATCCAAGGAGGGCACCGAAGAAAGGAAAGGTTATACCAATAAACATTGTGACAGCAACATAAACACTTCGAGCAAT
CAAGCGAAGAGGAAGGCCTGGAGGGAAAAAGAGTTTCTTAACTAGCACAGTTTCGATCATGTCGAACACAGGCATGGCATAAATCTGATAGCTCCCAATAACATGGATCACAACGAACA
TATTAGCTGCAGCAATCAGCCACTTGGGCTTTTCAAGGGTGATAAGAATATTATCATCAACATTATTTCCAAAAACATAATACCCAACAAAAGCGACTGGTAAGTAGCAGATTGCAACTATA
ATGTATGCAACAATGACTCCCTTCCACATAGGCTTCTTGGATGGCTTATCAGGCGAGGAAGGGATTGTTGCTTGGATCTCTAGAACAACATTGTGGCCTGCATAGGCAAAAGCAACATCTC
CTAGAGCATTAAAGAAATTGAAAACGTTATCAGCGTTGTTCTTTCCAGTTAAGTCATAGTGCACTTCTGGCTGCTTCCCTTTCGCTCCAGATGCAGCCCAAGCAATGGTCGAATAGCTGAG

AGACATGACAGCAGCAGCCAAGGAAACGCCAGAGATTGAGTTGAAGTTGGGGAGCTGGGCAAGAACAAAGTGAATAGAAGCAAAGATCATGATAAAGTAAGACAGTCTAATGTTACTG
CAGTCTGGGCAAACAAGGTCATGGAACTTCTTGAGAGATTTGCCCCCTGTGACCATGTACACAATGTCGACCCCAACTTCGACTATAAGCTGTTGGGGAACAACAATCCAGAGCCCAAG
TTTGTCGCCGAAAGCGTGCTGCCCAAGCTCATGGTAACGGTCAAAACGTTTCCCTGGCGCCCTATCTGGCATTTCGTGCATCTCCACCATTTGCCACAAGGTGTAAAGGGTGATGATCCAT
GACAGAATCATGACAGTGATTCCAGGACCCCATCCAAGATGCGACATCGCGTAGGGCAAGCTGAGAACACCAGCACCGACCATGGCCGTCACATTGTGGAAAGCCGAGTACCACCATTT
AGCATTCCTGGACGAAGTAATCGGAAGCCAGTCATCCAGCACCTTCGCCTTCTGTACCTGCGTCTCCTTATCCTCAAGCTCCATTTCAACCTCGATTGAATCCGATAGCAACGAACTGAAC
GGGGAAGGAGGAAGGACAATCTTGAATGAACAGTAAACGAGAAAGCTCGATCGATGAAAAAGAAGAAGAAGAATATCTGCCAATGAGGCGAGGAGTCTCTTTCCTTCTTCTCCTCCAC
TAACCCATTGCCATCAGGCCTCCGGTCTTGCTTATGTAGCGGTGGAGATTACCATTTTTAGAATCACAGCTCGCAAAAACCTAGACG
>comp157561_c1_seq10
AAGCCCAAGTGGCTGATTGCTGCAGCTAATATGTTCGTTGTGATCCATGTTATTGGGAGCTATCAGATTTATGCCATGCCTGTATTCGATATGATCGAGACGGTTTTGGTAAAGAAACTCGAT
CTCCCACCGGGCTTAGCCCTCCGCTTGATTGCAAGGAGTCTATATGTTGCCTTTACAATGATTGTTGCAATCTGCATCCCTTTCTTTGGTGGCTTGCTTGGTTTCTTTGGAGGATTTGTATTT
GCCCCAACCACATATTTTCTCCCTTGCATCATGTGGCTTGCTATCAAAAAGCCTAAAAGGTTTGGCTTGTCCTGGTCCATCAACTGGATCTGCATCATTCTGGGAACAATGCTGATGATTCT
TGCACCAATTGGAGGGCTCAGGTCAATCATCCTCAGTGCCAAGAGCTACAAGTTCTTCTCATGAACTTCACTTGATGGAAATGATAGCAAGGTTCTTTCTCTTAAGGGCCTCGTGCTTGCC
TACCCAGCATGTCTATAATTACTAATATGCTATATGAAGTTGTCTCTTAATCTTCATTGTGTAGGAAAAAGATTTCATTGCTACTTAAGGTGGATAACCTAATTATGTTTTCTCAAGTATTTGTG
CTCCTAAGACCATTTTTTTTATACCATGATTATGATCGGGTCGTACAGACTCACTGAAGCAAATGTATGTTGGTATAAATTTTACTTTTATTCCTGCTGCAAGTATGGCTGTGTTATGTGTATG
TGATTTCCTACTACAATTTAATTCAGCCATACTTGCAGCAGGAATAAAGATAAAATTTATACCAACATACATTTGCTTCAGTGAGTCTGTACGACCCTATCATAATCATGGTATAAAAAAATG
GTCTTAGGAGCACAAATACTTGAGAAAACATAATTAGGTTATCCACCTTAAGTAGCAACGAAATCTTTTTTTCCTACACAATGAAGATTAAGAGACAACTTCATATAGCATATTAGTAGTTAT
AGACATGCTGGGTAGGCAAGCACGAGGCCCTTAAGAGAAAGAACCTTGCTATCATTTCCATCAAGTGAAGTTCATGAGAAGAACTTGTAGCTCTTGGCACTGAGGATGATTGACCTGAG
CCCTCCAATTGGTGCAAGAATCATCAGCATTGTTCCCAGAATGATGCAGATCCAGTTGATGGACCAGGACAAGCCAAACCTTTTAGGCTTTTTGATAGCAAGCCACATGATGCAAGGGAG
AAAATATGTGGTTGGGGCAAATACAAATCCTCCAAAGAAACCAAGCAAGCCACCAAAGAAAGGGATGCAGATTGCAACAATCATTGTAAAGGCAACATATAGACTCCTTGCAATCAAGC
GGAGGGCTAAGCCCGGTGGGAGATCGAGTTTCTTTACCAAAACCGTCTCGATCATATCGAATACAGGCATGGCATAAATCTGATAGCTTCCAATGACATGAACAACAACAAACATATTAGC
CATGGCTATCAACCAGCGAGGTTTCACTAGACTGAGAAGTATGTTATCCTCAACTGAGTTGCCGAATGCCCAGTATCCAACTAGAGCTACAGGGAAGTAGCAGAGAGCCACTACGATATA
GGCAACAACAACACCTTTCCACATTGGTTTCTTTGAAGGCTTTTCTGGAGTAGAAGGAATGGTTGCTTGGATTTCAAGTACCACGTTATGACCTGCATAGGCAAAGGCCACGTCGCCTAA
TGCACTGAAGAAGTTGAATACAGTCCCAGCAGTGCTGTGAGCTTTGTATCCATATTCAACCCCTTCTTGTTTCCCCTTGTGAGCTGATGCTCCCCATGCAATGGTGGAGTAGCTTAGGGAC
ATAACAGCAGCGGCAAGAGAGACACCAGAGATGGAGTTGAAGTTAGGTAGTTGGGAGAGGACAAAGTGAACGGAGGCGAAAATCATGATGAAGTAGGAAAGCTTGATGTTCTTACAAT
CAAGACATACAGTATCATGAAATTTTTTGAGAGATTTGCCTCCAGTTACCATATAAACAATACAAACACCAACTTCACAAATGAGCTGCTGAGGAACTACTATCCATAATCCAAGTTTTTCT
CCAAAAGCATATTGACCAAGCTCATGGTATCTATCAAATCTTTTTCCAGGCACCATCTCATGCATCTCAACCATCTGCCATAATGTGAATAGAGTGATGACCCAAGACAGCACTAAAACTGC
AACCCCAGGACCCCATCCAAGCTCAGACATGGCATAAGGCAAGCTGAGCACTCCAGCTCCAACCATAGCCGTGACATTGTGGAAGGCAGAGTACCACCATTTTGCATTTCTTGAAGATGT
GATTGGAAGCCAATCATCAATAGCTCTTTCTCTATCAGACTTCGTTAACTCGGTAGTACTGTTGTCGACATCTTTCTCAGAAGGAGCAACTGCTTTCATGGCGGCAGAGAGACTTTGTGAA
TGGGATGCAGTCAGAACTCTATGTCAGAAAGAGAGCTCGCAAGTAAGAAGGGGAAGAAATTATCGTGGACTGAGGAGCTTTGAGAGAGAGAGTGAGAGAGTAAAATGGATGTTGCAG
ACACAAGAAGGTATGCGGGAAGGTAGGAGTTATATATAGAGTATAGAGAGAAGAGTCCTGAGTTTCAGTTAATTTGGACTTTTAAGACACGCTGTAATGACCCATACTCCGGCCACTCCG
>comp157561_c1_seq2

AAGCCCAAGTGGCTGATTGCTGCAGCTAATATGTTCGTTGTGATCCATGTTATTGGGAGCTATCAGATTTATGCCATGCCTGTATTCGATATGATCGAGACGGTTTTGGTAAAGAAACTCGAT
CTCCCACCGGGCTTAGCCCTCCGCTTGATTGCAAGGAGTCTATATGTTGCCTTTACAATGATTGTTGCAATCTGCATCCCTTTCTTTGGTGGCTTGCTTGGTTTCTTTGGAGGATTTGTATTT
GCCCCAACCACATATTTTCTCCCTTGCATCATGTGGCTTGCTATCAAAAAGCCTAAAAGGTTTGGCTTGTCCTGGTCCATCAACTGGATCTGCATCATTCTGGGAACAATGCTGATGATTCT
TGCACCAATTGGAGGGCTCAGGTCAATCATCCTCAGTGCCAAGAGCTACAAGTTCTTCTCATGAACTTCACTTGATGGAAATGATAGCAAGGTTCTTTCTCTTAAGGGCCTCGTGCTTGCC
TACCCAGCATGTCTATAATTACTAATATGCTATATGAAGTTGTCTCTTAATCTTCATTGTGTAGGAAAAAGATTTCATTGCTACTTAAGGTGGATAACCTAATTATGTTTTCTCAAGTATTTGTG
CTCCTAAGACCATTTTTTTTATACCATGATTATGATCGGGTCGTACAGACTCACTGAAGCAAATGTATGTTGGTATAAATTTTACTTTTATTCCTGCTGCAAGTATGGCTGTGTTATGTGTATG
TGATTTCCTACTACAATTTAATTCATTTTTATTTGTGTGTATTGCATGTTCTTCGATCAGATTCTGTATTTTTATCTGTTTGTAGCACTTGTAGTCAGCGACGGAGTCAGAAATTTTATTTAGAG
AGAGGGCCTAAATTAAAATAGTCATGTTTGCCATCTCATATAAAATGTTAGAATGCATTAAAATTGGTGTGGATATGTGACATAAACTTGTTGAGGATCATTATTAAGGCTTCATTTAGAACA
GTGTTCTTATTGTTTTGAAAAAAA
>comp157561_c1_seq4
AAGCCCAAGTGGCTGATTGCTGCAGCTAATATGTTCGTTGTGATCCATGTTATTGGGAGCTATCAGATTTATGCCATGCCTGTATTCGATATGATCGAGACGGTTTTGGTAAAGAAACTCGAT
CTCCCACCGGGCTTAGCCCTCCGCTTGATTGCAAGGAGTCTATATGTTGCCTTTACAATGATTGTTGCAATCTGCATCCCTTTCTTTGGTGGCTTGCTTGGTTTCTTTGGAGGATTTGTATTT
GCCCCAACCACATATTTTCTCCCTTGCATCATGTGGCTTGCTATCAAAAAGCCTAAAAGGTTTGGCTTGTCCTGGTCCATCAACTGGATCTGCATCATTCTGGGAACAATGCTGATGATTCT
TGCACCAATTGGAGGGCTCAGGTCAATCATCCTCAGTGCCAAGAGCTACAAGTTCTTCTCATGAACTTCACTTGATGGAAATGATAGCAAGGTTCTTTCTCTTAAGGGCCTCGTGCTTGCC
TACCCAGCATGTCTATAATTACTAATATGCTATATGAAGTTGTCTCTTAATCTTCATTGTGTAGGAAAAAGATTTCATTGCTACTTAAGGTGGATAACCTAATTATGTTTTCTCAAGTATTTGTG
CTCCTAAGACCATTTTTTTTATACCATGATTATGATCGGGTCGTACAGACTCACTGAAGCAAATGTATGTTGGTATAAATTTTACTTTTATTCCTGCTGCAAGTATGGCTGTGTTATGTGTATG
TGATTTCCTACTACAATTTAATTCAGCCATACTTGCAGCAGGAATAAAGATAAAATTTATACCAACATACATTTGCTTCAGTGAGTCTGTACGACCCTATCATAATCATGGTATAAAAAAATG
GTCTTAGGAGCACAAATACTTGAGAAAACATAATTAGGTTATCCACCTTAAGTAGCAACGAAATCTTTTTTTCCTACACAATGAAGATTAAGAGACAACTTCATATAGCATATTAGTAGTTAT
AGACATGCTGGGTAGGCAAGCACGAGGCCCTTAAGAGAAAGAACCTTGCTATCATTTCCATCAAGTGAAGTTCATGAGAAGAACTTGTAGCTCTTGGCACTGAGGATGATTGACCTGAG
CCCTCCAATTGGTGCAAGAATCATCAGCATTGTTCCCAGAATGATGCAGATCCAGTTGATGGACCAGGACAAGCCAAACCTTTTAGGCTTTTTGATAGCAAGCCACATGATGCAAGGGAG
AAAATATGTGGTTGGGGCAAATACAAATCCTCCAAAGAAACCAAGCAAGCCACCAAAGAAAGGGATGCAGATTGCAACAATCATTGTAAAGGCAACATATAGACTCCTTGCAATCAAGC
GGAGGGCTAAGCCCGGTGGGAGATCGAGTTTCTTTACCAAAACCGTCTCGATCATATCGAATACAGGCATGGCATAAATCTGATAGCTCCCAATAACATGGATCACAACGAACATATTAGC
TGCAGCAATCAGCCACTTGGGCTTTTCAAGGGTGATAAGAATATTATCATCAACATTATTTCCAAAAACATAATACCCAACAAAAGCGACTGGTAAGTAGCAGATTGCAACTATAATGTATG
CAACAATGACTCCCTTCCACATAGGCTTCTTGGATGGCTTATCAGGCGAGGAAGGGATTGTTGCTTGGATCTCTAGAACAACATTGTGGCCTGCATAGGCAAAAGCAACATCTCCTAGAG
CATTAAAGAAATTGAAAACGTTATCAGCGTTGTTCTTTCCAGTTAAGTCATAGTGCACTTCTGGCTGCTTCCCTTTCGCTCCAGATGCAGCCCAAGCAATGGTCGAATAGCTGAGAGACAT
GACAGCAGCAGCCAAGGAAACGCCAGAGATTGAGTTGAAGTTGGGGAGCTGGGCAAGAACAAAGTGAATAGAAGCAAAGATCATGATAAAGTAAGACAGTCTAATGTTACTGCAGTCT
GGGCAAACAAGGTCATGGAACTTCTTGAGAGATTTGCCCCCTGTGACCATGTACACAATGTCGACCCCAACTTCGACTATAAGCTGTTGGGGAACAACAATCCAGAGCCCAAGTTTGTC
GCCGAAAGCGTGCTGCCCAAGCTCATGGTAACGGTCAAAACGTTTCCCTGGCGCCCTATCTGGCATTTCGTGCATCTCCACCATTTGCCACAAGGTGTAAAGGGTGATGATCCATGACAG
AATCATGACAGTGATTCCAGGACCCCATCCAAGATGCGACATCGCGTAGGGCAAGCTGAGAACACCAGCACCGACCATGGCCGTCACATTGTGGAAAGCCGAGTACCACCATTTAGCAT
TCCTGGACGAAGTAATCGGAAGCCAGTCATCCAGCACCTTCGCCTTCTGTACCTGCGTCTCCTTATCCTCAAGCTCCATTTCAACCTCGATTGAATCCGATAGCAACGAACTGAACGGGG
AAGGAGGAAGGACAATCTTGAATGAACAGTAAACGAGAAAGCTCGATCGATGAAAAAGAAGAAGAAGAATATCTGCCAATGAGGCGAGGAGTCTCTTTCCTTCTTCTCCTCCACTAACC

CATTGCCATCAGGCCTCCGGTCTTGCTTATGTAGCGGTGGAGATTACCATTTTTAGAATCACAGCTCGCAAAAACCTAGACG

>comp157617_c0_seq2

GTATTAAAACGATGCTTGAAGCTAAAAGCGTCTAATACCAAAATAATAATATACAGCATTAAATAAAGACTTCAATTATATACATAAAACAACCATATGCTCGCAATCTTACTGCATTCATAC
AAATCATATGTATTTACATCGGTAGAATGTCAGATCAAGCTTTTTGGATCACATCCCAATAATTTTTATTTTTATTTTATACATCTGCTGCAGTGCAAATCACCAAGAAAATTAAAATGCAATA
TACATCTAGCTTTCATTATACAAGCAACATCAGTGTGAAATTGGTATCCTCTCCATGAACAAATAAAACTAAGTTAGACAGACCAGAATGTTCACAAATAGTTTCCAAATAAGACTGGAAA
TGTATGTATAATGAGTGGAAGAAATCAGTTCACTCAAATCAAACTTGAATTGCCTTGTGATGGAGCATTTTGTGAGTACTCGGTGAATGGAACAGCAAAAGGATTTGTTTGACCTGCTTGC
AAGTGATGATTCTCCGTTTGATAGCCATATGGCACCATCATCATCATTTGTTGTTGTTGGTAGCGCTGAGACATTTGTTGCTGCTGATAATACAGTTGTTGTTGGTGGTGGTAGTGATGGTGA
TGATAATATTGTTGTTGTTGTTGTTGTGTCATTAGCGTCATTTGAACATCGGTAGGAGGTGTAAGGCCGCTCGGCATAGTGAATGGATTACTCGGCTCGAACGAATTGACATTCGAACAACC
AATGGAGTTGTCTCCAGCAATTTGTTGTCTTCTTGATGCATCATCATACAAGCTATCTAGTAACAGCTTATCAAAACCTCCTGCCAGTTTGCTTTCCACAACATTGCTGATATTGTTACTCGG
GGTGGACACCAGTGCTAACTCCCATCCTGAGCCGCTACTGAGTAAATCTTTAAGTGTTGCTGTCCTTAATCCTTCCTCTGGCTGGATGATTGAAAGAGCCAATGCATTTTTCTGCTCAATGT
CTGAAGCAATGGATCTTATCTCTTCAATACCCAGAAGATCTGTTGTAATCTCCAAGGGGGGTTCGACCTCAGTGATTCTTTCTAATGGTTCTACAGCCTCAACAAGTTCTTTTACCTCATCT
ACTGAAAGCTCTGTATTCTCTGTTGTATCCGCATGTTCTTCTTTTTCGTAGGTTAAGAGGTTTGTCTCTTGATGCTCCAAATTCTTAGTTGAAATGTTGCTCATACATGGAGCTTCACGTATG
TACTCTTCCATTGTAGCTTGGAATGATGGAGGTGGCTGTCTTAGAGTCGGAAACTGAAAATTTCTAGCGAGTTCCAAATGTTTACACAAGTCATAGAAATCAGAAAGATTTTCTGCCTGTT
TACCAGCTCTTTTATATACCTCCAGCGCTTTAATGGCATCATATTTTGGCATCTCAAAGAACTGCAAAAATTTTGAAGTACATATCAACAAGATTGATGATCCCGTCATTAACTGCACAATAA
ATTTTGAAACTCTCCTTTATAACCAATGCCAATGCATATTGTATAAGATTGTTTCCAGATGCTGATCCTTCTGGCTGGCATCCTACAAGCCGATGCAATAGCTGTTGTAAGGCGGGCATTTGC
TCTAGCAGATCTGGACAAGATAAAGTTCTAGTTCTAGTATGTCCCTTGGCAGAACCTTGTGCATTTTTCATCAAACGCTCTGCCTCGATGTCATATTTTAAAACTCCAAAGCATTCAAGTCT
TTCTTCTAAAAAGAGTGCATATGTACGAACCCATGCAGAGCAATCCCAAGCAAAAGGACTCGAGTCATCCTTGAAATTGGAAATTTGTAAAATTTGTCCCTTGTGCGAATAGTTCAAGAG
CTCCTCTTTAAATGTAGGGTCACCTTCTCGCAAAATTCTGTGAATCACTATCAATGTTTTAACTGCAACCACCCAATTTCGTGTCTTTGCTAGCCTCTTTGCAAGTGCATATATGCAATAGGC
AATGTCAGCCCGTGGTCGAGCAACAGATGTAGCTATAAATATTTTTCTAACGTGTCTTTCCTTTGGTGGGCATTCTTCGTGATTCGTCGCCTTCACAATGGCAATATCCACTTCCTTAAATTC
ACTACTGACTTTTGCCAGGCCAACCATTGTAGAATCCTTAAGGGCGCCATAAGCCCTCCTCCATGTTCCCATTTCCTCTTCTCCCCCTATATATTCCTCTATCTATCAATCCACTGCGAATAGT
TCTCTCAATCCTCGAGAGGTGAGAACATAGAGAGAGAAAGGATGAGGAGAAGGAGATCGCGCTCCCCTTCTCTCCCTCTCTATACAGCCGCGAGTTTCGTTTCTCCTCAACCACCTTCTC
CTACGTCCGTTCCCTCTTTGCTGTCAGGAAAATGCCGGTTGAATAGTTATTTGCGCAGAGAGCGAAGCCTTCC

>comp157629_c0_seq4

CAAATTTAACATGCTGTATATATTGCAATTAAACAAAATATCCAAAATTTTCAATGAAATCAATATTACCTCTGCCCTAACTTAACAAATGCTGAGCTAGGTATAAGTCTGAGTCGGACAGGG
CTAGGGTTCCTGATAGCGCGATATGATTGACCTTCCTGCGGCGGCGGCGAGGGCGACGGCGGAGGCGGAGGCGGAGGCTTTACCCGACGATGTATTATTGCTTGATTTCTCCAGCGACCC
AAACTGTAACCTGGATGCCCCTACGCCGCAGCTCTTCCAGGATATCTTCGACGCAGCCATTGACGACCCCGGTTCGGCCGTGGATGGGGAGGAAGAAGAGCTGGAATGGCTGGCGAACA
AGGACGCGTTCCCCCCTTTAGAAACCTCTTTCGACCCAGTTTCACGGCTGCCTTTCCGGCCGCCTTCGAGGGCACGGAGTGGCGGCCGGCGTCGGAGACGCAAGGCGCTGCCGACCTTT
CCGCCGTCGACGGCACCGGAGAAGAAGCAGTGCCGGCACTGCAAAGCGGAGGAAACGCCACAATGGAGGTTGGGGCCGGAAGGACCGAAGACTCTCTGTAACGCGTGCGGAGTGCG
GTATAACTCCGGCCGGCTTGTGCCGGAGTACCGGCCTGCCAACAGCCCCACTTTCTCCGCCGCTATACATTCCAATTCCCACCGCCGGGTTATGGAAATACGCCGCCGGAAGTACGGCGG
TCGTCGAGGACGGCCGAGGGTCCGGTTGGTTTCCGAAAGTTCCTAATTTGGTTGGAATTTCGCTTATTTTTAGGTAGGGATTTGTAGAAGAAGAAAATATTTGTTTGTGCTCATTTTTGTTT
TTAATTTCCTTTGAATTTTTATTTTGATTGGCTGGAATGTGTGGTGGGAGGTGATCTAAAACATTGGTTTTGGAATTAAATTTTTTTATTTTATTATGTCATTTATTTATATATCTAGTTTTGAGA

>comp157675_c0_seq1
ACGTAATAATTGTTCCTTAGAGATGCTTATTATTCTCTCTAAAACTGTGTAATTAATGATTGCTTTTAATGCCAACTGAGTAATTAAGGTTCAAGTTTCTTCTCTTTGTGAGCTCTTCTCCCTG
CTCAGCGCCATGAAGAAAGCATCAACGGAAGAAGTATGGTGAGGAGAAGTGGTCGTCGGTTCTGGAAGACAAGGAATCGGATGTTTACGACCAGGTTTTGAAGGCTGATCTAGCGGCA
GAGCAGGTCGATGTTTTCATTGTTGATGCTTCGTCTGTTGAACACGAGCAGTTTTTTAACTCATTTGAGGATGAACAGCTTCAAGTACCTTCAATGGAGTCCGATCGTTTAGCTAATGGTG
ACGAGCTGGTGTCGGGTGAGCCAGTTCCGTTCGGTTTTGCGTTCGCTTTGGAACGGGATTCTTCTTCGTTCGTTCGTCATTGCCTCGACCTTTCCGTCTGCGAATTCGAGTTCGAATGTAT
CTCGTTCTTGCAGGCTGGGGAGATCGAACCACAACCTCCGGCGAGTAGCGTCTCGAGCGGCTCCGATCAGGCCCAGGCAAGTCCGACTTCTGCTATGGAACCAGGCGTTTCTGGATCAG
GGAAGGTTCAAACTGCAGCTTTGGTTTTCTTGCAAATCAATGCTAGTCTGACTGCTGGTTTTGCAGTCAATATGAGCAATGGTGGATCAGAAATTGCGAAGGACTGTAGGTATTTGAATGA
TCCTATGGATTCTACTGTTTGTTCTGAGATTGATCGCATGCATGTAAATACATTAGATGAAGCCATTCATGTTATTTGTAAATCTGCAAATTACAAATCAGGTGTTTGTGCAAATGCCTCTACC
ATAACTGCTTGCTTGAATGGCCAAACTACTTATTTTCAACAATCCTCCAAGCATCCAAATGGTGCTAAACCTGCCCATGACCTATCCTATGTATGGAATAGGTTGGACGTAATCAAAATTGA
TGATGTTCCTAAGGCGGCAGTTATGTTAGATGATGGAAGTTCTGTGACTTTGAACATGGAGTCCGTCCATGCCAACTTAGTTAGGCTTGAAAGATGGCTAGTGTGCAAGCTTATTGGGTGA
AGGTTACCTTACTTTGTGCTAAACAACGAATTAAAGTTTGAATGGTCAATATTTGAAGATTTTCAGCTCCTTTCCATTTCCCTGGATTCTTTCATTTGCCTCTTTGAAAGTGCTGGGCTTTGG
ATGCTGTCCTTATGGGTAGACTGTGGGTTATTGGTGGGTATACACAACTTCTCTGCCCTGTTTTGTTTGTTGATGTTGTTTTAGGAAAGGTTTTTTTGCATTCAGATCTACTTGCCAATTTCG
CTTCATTCTTTCAACATAGATTGAACTATGTGAGACATAAGCCACCCATTGCCTTTCAAGGTAAATCCTCTCTTATTTTTATTGATGTTGAGATTTTTGATGTGGTGAACTCCTTTTTCCTATA
TCTTGCTTGGTAAATTTTTCTTGGTTGCCCAAGCTTGGAGATTATTCATACTTTTATTTCACACTTGCCTCTAATGTATTGGGACAACACCAATCTTGTTCTGATAGCTTTTTTCTTTGGACCA
CCTCTATGAGTTGATGCTCAAACAAACTCCTAGGGGAGAAATGCTTTTGCTCGTATTTGCGTGCAGATGGATTTGGTGCCAAGCTTCCTCCAGGAGTTTGGATCTCTGGGCTCCATGGCAA
GTTTTTTTAAAGGGCGGAATATAAGGATATCACTCACCTTTGCTTCCAATGTGGCTTTGTTCGACACAACATAAGTAATTGTCCCAATGTTCCTCAATCCTCCAAGCTGCCCACACAACAAC
CTCAATGTAGATCGAATCCTCCATGTGTTTTGTGCAAGATTAGCAGCACCCCTCTTCTAGTGACCCTCTTGTCACTAATAGTTGCCCTCCAATGGCGATTAATGACCCAAATTCATCTTCTAT
GATCCAGCCAACTCTCTATCCACTCCGGTTGGAAATTGCGCACTCTCCTTCTACTATCAAGCATAGTGCAAAAGAAGGGAAAATTTCAGAAACCTATGGGCCTTGGAGTCTTGTTACAAA
AAATCTAGGAGACATAACAAGAAGAATTCCAATGTAGGTATTGGAGATCAGAAGTCTTTTCTCCCTTCCTTGCAAATCTCAAGTGGGGAAGTTAATGTTCCAACCAAGCAAGTTGTTTCAT
CTAGGGATGGTGATATCTCCAACATACCCTTCAAGTTTTCCACTGAGGCTTTCCTCATGCATCCAAGAAGATCTGACCCAAGGTATGATAGAGTCTATAAGACTCGTTTTGCTAAGGATCTT
CATCGTTTGGGGGCCAATTACTGATAATCCTAGGAAGTGGAAGGAGGGTGGCGGGGACGACTCTCGTTAAGGTGTTCAATATTGGGGGTCTCTACATGGTCTTCTTGTAAGGGGCGCCTC
AATTCTCTTGGTATTCCCCCTTTTGGAGTGGACCTTACCCTATTTGTTTGATACTTGCTAGGAGCTCTGGTGTGTCCTCACTTTGACAACAAAACAATGATTTTTTGTCCCTTTTTTGTGAAG
TATAGGCATATTTGGGAAGTTTTAGCTTGTTCTATGCTTCTCACTTTTTTATTTTGTTTTATCTTGGGAGGTTTCTTTTTTATGCTTCTTCCTAGATTATACTCATTGTATACTTTTTG
>comp157692_c0_seq3
ATTTATTTTATTTTAGCATTATTTAATTTAATTTAACAAAATGAATAATTTTGAATATTACTAATTAATTGTCATTTTCTAAAAAAAATTTAATTTTGTCAGAAATCCATCGGAAAAGCTAGCATA
AAATGCGTCAGAGATACCTTCAAAAAATTCGTTGGAATTTTGCCGGAAAATTTGTCTGAAATATTTTGCCCGAAATTCGTCGGAAAATTTGTCGGAATTTCTGAAGGATTTATTTCTGCCGG
AAATCAATGTTTTTCTTGTAGTGTTTGGATAACTATTATGCACTTAATAACCCGTTTAGTATAATAATTTATTATACTGCACTTTGATTATATCGTACTTTGAAAATTATTGAAAATGTTAAGAA
AGGAAATATTAAAAACTTAAGATTGGTGGTTTTTGCTTGCAGGCCTTTTGTAAATCACTATGGCTATGCTTTTTGTTTTTAGGATCTGAGAAAATGAATGAACATGTTGACTAGCAGTTCAG
AATCTAATCTATATTTATAGTTATTTCTTTATTTGTATTTTTAATACGGTTATAGTAATTAATTTTTAGATGTTTTACTTTGTTCTCCATTCAGTAGCTCGGCACTATAGTGTAGATTTTATATATAC
ATTTTGATGAAATAAAAGAGATTGTATAAACGAGTTTATTTTCATTACACTGGATAATTTTAGTAGCTATGTATTCAATATTTATTTAAATGTTTAAATTAGATGAAGAAATTTGAAGCATTTTT
CAAAACTAGAACATGGGATAAGCATGTCGCAGCCTAATAAGATAGGACCTGTTCTTTTGCATTGGGTTGAAAATGTATCATAAGATAGAGCTATATGAATTAATCTATAAGTCAAAAAGTTA

AGATAGTTTTGAGTCTATTTGACTGTTCATTATTATTTATTATCATGTGTATATATATTTAGTTGCATATTTTGTGTGATTATGTATCCGACGAACCCAAATATGATAGATGGATAGAGTGCTAGT
CGGACAACTCTTATATATAGTACTGCTCAGATAGTGAGTTTTAATAAAATTTCTCAGTTTGCTTGTCTGATAACACAAATGATGGGTGAGGCATAGAGTAGGATTCCTGCTATTAGATCTAAT
GATGTAGATAAACACTTTCATATATTTTTGAGATTGAAGCCTTTAAATTTGAGGGTGTTATTGAGGACTAGTTGAGGAGGCTTGAGCATATATTTGACAATATTTAGTTTCCTCCTTAAAGGA
AAGTATAATTGGAAATTATTCTGTTAGATGGTAAGGCAGGGCACTGGTCGATTGACCAGTAGAAGAAGAAGTTTTTAAGGGAAATTGAATTATCTGATTAGGTGGTAGGAGTTTATTAATGT
TTTTTAATCCTAGTTTAAACAAGCATCTGCACGTTAGTAGATGTAGGAGACATTTCTAAGATTGGTTCAAGGGAGTAAGATTGTGATATAACATGAGACGGAGTTAACTATTTTGGTTATATA
CTCCGTAGTTGGTTAGAACTCTCGTAGATAAGTGTTACATATTTCTAAGAAGACTTTAAGATAGTTTAAGATAGACACTAGTATCATTTCATATTTCTAATAATGTCACTAATAAGTTACTTGA
CAAAATTTGAAGTTAGTCTTCTCCTAGTAACATTTTTTTTACTTTGAGGATTATTAAAGGCTTTATTCTTATTGATGAGATGTTAAAAGTTAAAGGCTTGTATATTACGTCTAAATACCATTTTT
TTTCATTTTGATACTATTAATCGTGTTGTTTTTAATGGCCCTATTACTGCTAGGGTTTGGTATATTTTGGAGAATACTTTTGAGTTAAATATTAGTAGCACTAAGAGTAGTTGTTTTAGTTTTTTA
TTTGACTGGTTTTGCAATATGCCCAAAGGTCATATTCTCACGATTATTCTTATGATCATTTTTTTGGTTTTTGTTGCGTCCTGGAATATTCGC
>comp157705_c1_seq1
GTTACCTTCCATCCATTGATACTAATTAAAAAATTATGTCAATGTACGATTTTATAAAAATGACTATATACAAATGAAATTACATTATAAAATTTAGAACAAAGACATCTGAAAATAATTTATA
AAACGCTGCAACTCCTTATCTAGCAACAAACAACCAATAAAGTCGTTTAAAGGAGAAACCAAACAAAATATACACTATCATAACCTTCAATCTTCTCATTTAAATCTCGGTCACTATCACA
ATCCCTCCCTCATCATTGTTGTTCTCAAACTGAATGCACTTATACCACATGGCGCATAGTATGTAAAGCACTAAATCCACAGCGGTCAAAGCTGCAAGGAGGAAGAAGAACCGGTCTAGG
CGACCGGAATTGAGGTTACTCGGAATCCATCCAGGTCTCGCTCCGCTTGCGGTCGCAGCCATTACAACACTAACAAGCAAGATGCTCACGTAGTTGCCGAGTGAGATGGAAGCCATGTTG
AGGGAGCTCCCAAAGCTCTTGATGCCATCAGGTGCTTGCCCATTGAAAAAATTTAGCTGCCCAACATACATAAACACCTCTGAAGCTCCAATGAGGGCATACTGTGGAATTTGCCAAGTG
ATTGGGAGTGAGCTCTCCTTATCTGGAGAAGCAACTTTCTTTAGCCTAGCTAGTTCAACAACACCAGCAGCAATCATTGCCAGCATAGCGATTAAAAGGCCAACACCCATCCGCTGGAGT
TCGGATAACCCATCAGGATTCTTCGTAAACTTCGCTAGCACTGGCATAAGAATCCGACGATAGATAAAGATGAAAAATATAACACTAATAATGTCAAAGACCGACATGCTGGCAGGAGGGA
TGGTGAAGGAACCAAACTGTGTACTCATCATGGCGCCTTGCTCGACAAAGAGGGAGGCCATCTGGGTGAAGACAACTGAGTAAATTATGGTGCACAGCCAGATGGGAAGCATCTTTAGC
ACACATTTAACCTCCTCAACCTGAGTCACGCTGCACAGCTTCCATGGACTCTTGGCCTCAGGCCGCACAGAGTGTACATCAGTTGTCATTGTTGCAGCTTTGTCCAAGAACCGAAACCCA
TTGCTGTGGGAGATCTTTCTACTCCCTGCAATGGCAGATTCCTTTCCTTCCACTTCATAAAGCAAGGCGTCATTCTCAGGAACACTAACATTCCACTTTCGAGCCGCAGCAACAAAGACTT
GTGCAATGCGAGTCAAGGGGTTGCCTGATGCCTTGAAATGCCTGTATCTTGGAGTTCCAAGTAAGAAAAGAAGAAAAGCAATAACAGCTGCACCAGTTGATGTCCAAAATCCAATAACC
CACTGTCCAGAGTCCTCATAGTACACTAAGATTGTGTTGGAGAAGAGAGAACCTGCATTAAGAGCAACATAGAAGTAACTGAAAAAGGCTGCCTTTGAATCACTTTCTGAAGGGTTCATC
TCATCAAACTGGTCTGATCCAAATGTAGCTATTGTGGGCTGATATCCCCCATTGCCAAATGCAATTAGATATATTGATATATAAAAGATAGCAGTTCCAAGAGCTGAAACTGGTTCGCATTCC
AGTTTCCCATCACCACATCCAGAAGGATTCACCAATAAAATCCAAGACGACAAAGATAAAAGGGCTAGTCCCATGATGAAGATTAACTGAAATATAGCGCAGGTTAAGTAACGGCCCCAG
TAGGAATCACTCAAGAATGCTCCGACGAGTGAAAATATGTACACTGTGCCAGTCCATTTGCTGACATTATTTGCTGCGTCTGCATTCTCTTGTTGCATGACTCTTGTTAGGAAGAGAACAA
GATTAACCCCAAGTCCAAAAAAATGCAAATGTGACAAGACCATAGTTCACAGTGCACATGTTCTGGATATTCTCTTTTTTCCATTTTATGAGAACAATTTTGGAACACACAAGAAAAAGACC
ATCACAGAAGAAATTAAAGTTCGAAGTTCACTCAAGCAGGTTTAGATGCATTCATTGGCTGTTAATATAGTCAACCTAGCAAAAGAATTGCAGATTTCCATCTTCCAGTTTTTGATTTGAGT
GCAGGTTTCCCCATCCTGTCCAACGTGCCATCCTCAGTGATGGGAGAGATTTCCCTTTCATCCACACCAGCCGTGTTTTCAACCACATCTTCCTTCTTCTTTAGAGGAACGCCATTGATTTC
TAGAGCTTCCATTACCTCTTAAGTAGTACGTTGCCGACGGATTCCGGTGAAGGTGAAGAAAGCGAAGTGATGAAGGATATGAAATCAGTCCCAGAATCTAGAACGAGATCCAAAGTCCCT
TGCAATTTGAAGAACTCCTGTAAACCTATTGTCATTTATGTCGCCGACTGCTCTATGCGTTCCACAACAACACAAAATGCCCCCTTCTCCAATTCGCACGAGACG
>comp157766_c0_seq3

AAATGGGACTCTCTCTTTATTCTTGGTTGATTAATCTTCAAAAGATTTTACCAAATTCTAGAATGAATCATTTGTTCACGGAATATTCAAATTTATTTTTCAGTTTGAACTTCAATAAGAATTG
ATTGACAAAAACTCTTCAATTTTTCACCTGCCAACATTTTTTCACGTAAAGCTTCTCCATTCACAGTACATAAACACAGAGAACAGTCCATACAAAGGGTACATATACACGTCCTTCCAAG
AGCGCGCCACTTACATCTCACACGGCAACACGGCCCGCCACGCCAAGCTTTACGGCGACCTCAAACTAGCTAAGATCTGCGGCACCATCGCCGCAGACGAGAAGCGGCACGAGGCGGC
CTATTCGAAGATCGTCGACAAGCTCTTCGAGCTCGACCCCGATGGAGCCGTCATCGCCTTCGCCGATATGATGAAGAACAAGATCACCATGCCGGCTCACCTCATGTTCGACGGCCGCGA
CCACCGCCTCTTCCACCATTTCAGCGCGGTTGCTCAGCGCCTGGGAGTTTACACGGCTAAGGACTACGCAGATATTACGGAGTTTTTGGTTGGGAGGTGGAAGGTGGAGAGCCTCGTGG
GTCTATCTGATGAGGGGAGGAAGGCGCAGGATTTTGTGTGTAGGTTGGCGCCCAGGTACAGGAAGCTAGAGGAGAGAGCTCAAGGACGGGCTAAACAGGGCTTCAGTACTGTGCGTTT
CAGTTGGATTTTCGACAGAGAAGTACAGGTATGAGCTGTGGAAATTTCCTGTGCTGTTCAAGATAGGAAATTTCTTATAAATCGGTTGTTATATAATATTATGTTAATTGATGAAGATTAAAG
ATGTTGTGACTTCTTGAGACTCTACTTTCATAGTATTTAAATACAGAATATGTAATATGACTGGTGTATATTGCGCTTCATTATAATTAAGATTTTGTTTTAGATGGATTAAAA
>comp157771_c0_seq1
CCAAATCAAACCTAAATTATCAGCAAGCAGAGTTCACATAATCTGCCTCCATAATATTTGACAAAATTAACCCAAAAACTTCAAACAGAGCAGCCAAAAAGTACCAGCAAATTCCAGAAA
TTAGAAAACTTTAAGAATCAAAGTACACAATACAAAATAGGTTCAAAGGAGGAAATATTTCACATATAAGTGATAAAACCATCATGATTTTCAACTAAGAGATGAAAAGAGACAAACCTA
GCAACCATCCTCAGTTCATCAGTGTTCAGACTTTATAGAGCTAACTTCCATCTGCTTCAGTCACTTATCCAAGCTGATTAAGCTGTGCTACAGAACTGCTGTAAATTGATCGAGCAACAGA
ATTCCATCTGAAATTATATTATATACTGTCATCCATCATCTGAATCTCCTTAAAATGGTTCACAACTTCAATCATTGTTGGCCTTTTGGCTGGCTCATCAGTGGTACACATCTTGGCAACCCTC
AGAACACACATCATTTGCTCTCTCCACAAGTTGATAGGTGAAAGGGATGGATCAAATACCTCATCAACCAACCCAAAGCCCTCCATCCACCTCACCCATTCCACCAGATTACCTCCCCCCT
TCTCTTCTTCCTGGCCAGTAGGAGTTCTTCCAGTCAACAACTCCAACAATACAACCCCAAAGCTGTAAACATCACCCTTTGCTGTTGCCTTCATTGTTAAGCCGTACTCTGGTGGGATATA
GCCAAAGGTCCCAGCAAGGTCAGTAGTTACATGAGTCTCACAAGCACTGATAATACGAGCAAGACCAAAGTCCGCAACCCTGGGCTCGAAGTATTGGTCAAGAAGGATGTTACTTGATT
TCATGTCCCTGTGAATAATGTGAGGGACAAAGCCATGATGCAGGAAAGATAGCCCCCGGGCAGCGCCTAAGCAGATCTTTAGCCGAATTGACCAGCTAAGTGTTCCATCAGCATCAGCTT
GTTTCCTAAGCCATTGCTCAAGACTCCCATTTTCCATGTACTCGTAGATTAGAAACCTCTCTTCACCAAAGACACAGTAGCCAAGCAAGGGAACAAGATTCCTGTGCTTCACCTTTCCAAT
AGTCTCCATTTCAGCCAGGAACTCCCGGTCACCCTGAAAATGTCTCCCACTATTGAGCCTTTTAATTGCAACAACCTGGTTTTCAGGTAAAGACGCCTTATAGACCGTGCCGAATCCACCA
TCCCCAATAATATGAGACTTGCTAAACTCATTTGTTGCTTTCATGATATCAGCAAGAGTGATGCGAAACAAAGCATGCTCAAAAGTGGCAATGTTGATGCTGACTGGCTCCTTTAGTCTTTT
GCTCAAAAGCTCCTCAGAGGATGCTGGCTCAATAGCTGATGACTTGGTCTTATTTGCTGGAGCGAGGGCTAAATTTTTCTGGTAATGTCTCCTCCATTTGATAATAATGAAAAAAATTAACA
CAGAAATAAATGCGACAGAAATAATAATACCCCAAACTAAAGCTCGACCAAGCAATCGTGCAGAAGGGTATGCAGCCAATGGCTTTGAGATAATGTGACTCCCTACACATGCCTGGGACA
TCGTGCAGCTCTCTGGTGCAAAACTGTCAAATTTATTGCCAGAAAAATTCACAAATGTGAGCCCAACTATGCTGCAAATCTTACAAGGGATGGCATCCTGGAGATTGTTGTTAGAGACATC
AAGATAAGTGAGAGAATTTAGACTGGACAATAAAGATGGAAGACTTCCAGTGAAATTATTGTTATGGAGGTCGAGAATAGAGAGAGAAGTTAGGTTTGAAATAGACTCTGATATAGCACC
AGAGAATAAATTATTGCTGGCATTGAATATAATAAGTGAGCTAACTCCACCATCAATGTCTATGAGAAACGATATAGACCCAGAGAGAAAATTTAGGCTAGTGTCCAAATGCGTAAGGCTC
TTAAGAACAAAAAGAGATTCAGGAAGAGGGCCAGTAAGTCGATTACTTGATAAGTTAAGCTTCACTAAACTTGGCATCATCAAGCCAAGATTATCAGGAATTGGACCCTCTAGATGGTTG
TTTGACAAAAGTAAGCCCTGAAGGTTCTTTAAAGAGAACTGTGCAGACAATAATGTACCTGAAAGAGAATTGAAAGAAAGATCAAGGACAGCCAGGTTCAGGAGGTCATTAAGCTCAG
GAGGGATGATGCCATTAAGCTTGTTTCCTTGCAACATTAGCTCTTTTACTTCCACACAGTTCTTGATTGTTTTTGGAATCTGACCTTTAAGATTGTTATATGATAGATCAAGCACACCATAAT
GCTGATTGAATTCAGAATCCGGGTGCTGCGCCAGCTGAAACCCCGCACAGATTTCATCAGGTATTTCACCAGATAATAGATTGTTGGAAAAAACTAGATCACCTAGAAGCTTCAACTGAG
ATATGACCTTAGGAATAGACCCAGACAATCTGTTTGATCCAATGTCAAGTTCTACCAGGTTAGTACAGTTGAAGAGATCTAATGGGATCCCCCCAGAGAGTTTATTCCCATGAAGTGACAA
ATTAGTTAAGTTGCTTAATTTTCCAATAGAGTTTGGAATGACTCCTTCAAAATAATTATTGTCAAGTTGCAACCTCCGAAGAAGAGATAAACCTCCATTGCTATCTGGGAGCTTACCGCTAA

GTTGATTGTCACAGAGATAGATTTCAACTATGGTTTCAGACTGCAACAATTGTGAGGGAAGTAAGCCAGAGAAGTTGTTGTGCGAAAGCTCTAGGGTAACTAAAGGCAACTCGGCTAAAT
AACCTGGAACCTGTCCGAAGAACTTGTTCCCGTTTAAGATTAAATCAGTGAGGTTTGAGCATCCTCTGAAGGTTTCATGAATGCTTCCTGTGAGTTCATTGTCAGCAAGAGAAAGATGAG
AGAGTGACTCAGCCTCACAAATATCAGCGGGGATCTCCCCTGACAGATGGTTTGCATCAGCAGTGAATGAGGTCAAGTACTGTAGCCTTAAAGAAGGCAGAGTCCCACTGAAGTTGTTC
TTCTCCAAACTGATGAATCGTGCCATGCTCCAATTTGACAACCAGCCAGGAATTGGTCCAAACAAATGGTTATCATCGACAAGGAATGAGTTGATGGATTCAAGCGCAAAGAGACTATCT
GGCAATGGACCATAGAAGTGATTGAAGGAGAGATCCAATATCATCAGATTCTTCAGATTTCCCAACTCCTCGGGTATCTTCCCAGTGAGTCCAGCATGGGATGCAACGAGGTAATTAAGAT
TTGTCAGTTCCCCAATACCTGGAGGAAGCTCCCCTTCAAAGTTGTTGTCAGATATGTCCAGGTCTGTTATTTTCTTGAGTTTGGAGATCTCACTGGGAATTTTCCCAGTGAACTTGCAGCT
AGGGATTGAAAAGATTTCGAGCTGTTCAAGATTTCCAATTTCCACTGGAATACTCCCATTGAAGCCATTAGGACCCAACCATAAAGATATTAGACTTGACAACTTCCCAATATCTTCCGTTA
GAGGTCCTGTAAAGCTGTTAGAAGAG
>comp157801_c0_seq1
GATAGAATCAAGAACTGAATTTTACTCTTTTAATTACCTATACGAGGGGTTATTAGGGGAAATAAGACTAGCTTTTAACATAAAAAACGCCCAAATTACACAGCATTTCAGCAGAGATCGAT
ACATATAGACATATATGGAAGATACATGCATGCTTAAGAAAAAATATTTATGAAAACAAAATTATGTCAAAAGATATAGATTATAATACGATGAGCTTGAAAATTTTTAAATTTTTTTTGAGA
TTATTAATGATAGTAAATGAAGAATTTGTTTTACACAAATCTTAATAGAAATTTAATAATTTTCAAGCTCATGCATATATAAGTACATGTGGACATAATTTTTCTTCATATTTATGTATGTATGTA
TATATATGTATACATACATGATACCAGTTAAACGAGATCCTGATCAACCTCACCAATCTCAGAGTGTCCAGCCGTAGAGCCCGTCGCCGGCGATCACCGGCGTCTCAAACACCACCGACTC
CATCAAAACCTCACCGTAATCTTCCACACTTTCAAACCTCTCACCATACAACTCAAAACGATCACCATCTCCATAACCACTTTCTGCCTTGAAATCCGGCGGCGGAAAGTCAAAGATTCCA
GCTTGGTCTAACCCGCCGCCTGCGAGCAGATCAAGAGGGCTGTAGCTGTCGCAAACGCGGCCCACCGTGGCCGCCGGAGGATGGCCTGCCAGTAAGTCGGCGATGTGAGAGTCTCTGC
CGGTGAGTTCCTGTACGAGAGCTCGGAAATCAGCAACAGTAGTTGTGACTCTCACGGGATTGGAGAAGTGAACCACTTTAATTTTCTGCTGATTCATCTTCTTCTTCTTCGTCGGCTCGAT
CTTAGTTTGCTTTAAGAGATTTTGATGAGCTGGTCTTGAGGTGTGATCCATGGCGGCGATAAGTTGGAGGTGGCGATAATTAGGGGTAAAGGTGATGTTTTTATATGGAAAGATCTTTCCTT
TTTGTAGGATTGGAAAGGAGAAAGAGTTTGGTTTCCAGGAAGGCAGAGAGAGATGTGGAAGAAAGAGGGGATAATTAATAACAGTGGCTTTTTTTAAGGGAGAATAATTCTAG
>comp157827_c0_seq1
AGATTTGAATTTTATTGATGTTAGCTTGACAATCAATCATACAACTTGTATAGAAATTCATAAAAAGAAGTATAGTTTATTTTATTTATGCAGAATCTCTTAATTCATTGGTGTCCTTACAAAA
CGCGCTATTTATGATCACCAATGAATAAGAGTAAGAAAGTTACAAAAACTTGGAGATTGCTTTTGTACAGATAATATTTTTTTGGTCATCCAACCAGAACCAGAAAATTTAACAAATAACG
GTCGACAAAATTTGAATCCAATTCCGATAAAGATGTACACTGTACTGAAAGCAACTGTCGCCATGCCGTCATTTTATTTAGGTCGTAAATATAGGACCTAATTACTGGCTGTAAATGCAATG
TCAAGAAAGGGAAGAACACCAAACTCTAAAACAGTTTGTGAGCTTCTAGAGATCATAACCAAGACGCAACAGGGTAGTTGAAGTCGAAGGAAATCACTTCACTAATGTGAGCTGGAAT
GTCTGAAGAGTCGACGAGTCTATCTTCGTTGTTCCTCAACGCCATCTGCGACTGTGCTGAGACCTCACCCAATTCTTGCTCAAAATCTTTGCCATCTGCCCCTTTAACATGTCCCAATTGCT
CCGTATGCTATCTCTTCATCATTAGCAAAGCTAGGCATTGGCATCACATCACTAAACTCTTCGATCTTCGCAAATCTACCTCGAACTCGAGGCCTGCTATCAGCTAGGATCTTCCTACATGTG
TACTTGATCGTTTTGCTAAAATTCCTCTCCTTCTTCTTCCTAAGATACCTATCTAACTTCTTCCTCCTTTCCTCCGGTGATGCTCGACTAACTTTATTGTTTGAGTCATCCATTCTGGGGATATC
GGAAGGCATTTGTAATGGAGCAATACTGGTGCTACACATTGGCCTTACATTTTGGTTATCTCTCAGTACTTGTAAGTTTCCGGGGCTGTAGAGTCTCGGCATAGGTTCATATGTGTAGATTC
CTCTTAGATTGCAGGCTTCTGCAGCCTCCATCGGCCTCACGGGCATGCCAACACTAGTATTTTTCCCCATCCCATCATCTGCTACAAATGTCGGCATTCTCATTCTTTCAGCATAAAGCTGCC
CTCCAATTCCACCATTAGCATCCATGAATGAGCTGCGCTGCATTGCTGATGGCTCCATGCCCATATATCCAGGCAAGCCCAAATGACCCGAAGGAAGCTGCTGGTTCACTACTGAGTTTGG
CGGGTTAGATGTGAAGGGAGGATTACAAATCATTGGATTGGGCTGATTCATAGGATGGATTGGAGCCATTGATGCAGCTGGAAGTGATGGAAAGCTATTAGGTTCCGGTTCAGGTGGAAG
TTGAGGATTGATAAGAGAGGATATTAGTTCTGAGTTGAAGGAATTGTCATAGCCGGGCAAGTTGGCGATGTTCGTATCAGGTTCAGGGAAGGTTATTGTGACGGGGACGTCCGAAGAACA

AACACCATTGTTGGTTGAGGATAAGACTCTTGGTGAGTAGCCAGCTTCTCGAAACAAGTTATCTTCGTAATCAAAGTTGGGCATTTTGTCTTTTACAGAACTGGATAAGCACTCATCAAGG
GATGGATGCACCGGCGATCGAATCAAATCCTCCAGCATTTTGTCAATACAAAGCGATTCCGAAGAGGGAGTTCTAATTCCTTTTCCATTATCAGGGGAATGCAGAAGAAGAAGAAATTA
AGGAACGGAAAAAATATTGGTTGATGCATGCAATGATGAGGCTGTAGATGTTTATAGGGGCGGCGAGCGAAGAAAACAGACAATTTATGCCCAGCTTTAGAATGTGCCTATTTGCCTCCG
GACTGTAAGGGAAGCGACCAGTTTTAATCACGGGCCCTTTTGGACCCACCAATAATTTTTGGCCACGTCAGCTATTAGTTATGTGGAATGCGAATGTGGCACAATTGAAACTTAAAATGTA
ACAAAAAAACCAGTAATTAGTTGAGAGGCTAGCTGTAGTAAACGGATTGCTCAATCTA
>comp157827_c0_seq10
CTGTTCCTGATCTGCAAAAAAGATTTTGAATTCTCTCTTCGTGTTCGTTTCGTCTTGACAAAATGCTGGAGGAATTGATTCAATCGCCGGTGCATCCATCCCTTGATGAGTGCTTATCCAGT
TCTTTAACTGACCAAATCCTCAACTTTGATGATGAAGATAACTTGTTTCGAGAAGCTGGACACTCAACAAGAAGCTTATCGCTAACCAACAATGTTGTTTGTTCTTCGGACGTCCCCGCCA
CAGTAACCTTCCCAGCAACTGACACCACCACCACCGACTTGCCCTGCTATGACAATTCCTTCGACTCGGATCTAATATCCGTTCTTCTCAATCCTCAGCCTCCACCTGAACCGGAACCTGA
TATCGTTCCATCACTTCCAGCTGTATCAATGGCTCCAATCCATCCTATGGATCAGCTCGATCCAATGATTTGTAATCCCTCCTTCACATCTTACTCGCCAAACTCAGTAGTGAGCCAGCTTCC
TTTGGATCATGTTGGCTTGCCTGAATATATGGGCATGGAGCCATCAGCAATGCAGCTCTGCTCATTCATGGATGCTAATGGTGGAATTGGAGGGGAGCTTTATGCTGAAGGAATGGGAATG
CCGATATGTGTAGCAGATGATGAGGTGGGGATATATGCCAGCGTTGGCATGCCAATGACGCCAATGGAGGCTGCAGAAGCCTACGGTCTAGGAGGAATCTACACATATGAAGCTATGTCG
GGCGTCTACAGCCCCAGAAACTTACAAGTACTACGAGATAGCACCAAGATTGCTCCATTACAAGGGACTTTCGATATCACAACAATGGATGACTCAAACCATAAAGTTGGCCGGCTGTTC
GTTGCGATTCGACTTGGCCTGCTTGGCGTGACGCCTCCTTCGACGACGTCTCCTCGCCGTTCTTCTGCAGGATTGGAAGGAAAAAAGACGACGGGAGCTTGCTCGTAGCGAAGGTAGCG
ATCTTTCTTCCTGCGCGATTCTTCGGCCAAATCCCGAAAATCCTGTTTGACAGGATATAGCTTAAATCCTGCTCTAATCCAGCATAAATCCTGTATC
>comp157827_c0_seq11
AGATTTGAATTTTATTGATGTTAGCTTGACAATCAATCATACAACTTGTATAGAAATTCATAAAAAGAAGTATAGTTTATTTTATTTATGCAGAATCTCTTAATTCATTGGTGTCCTTACAAAA
CGCGCTATTTATGATCACCAATGAATAAGAGTAAGAAAGTTACAAAAACTTGGAGATTGCTTTTGTACAGATAATATTTTTTTGGTCATCCAACCAGAACCAGAAAATTTAACAAATAACG
GTCGACAAAATTTGAATCCAATTCCGATAAAGATGTACACTGTACTGAAAGCAACTGTCGCCATGCCGTCATTTTATTTAGGTCGTAAATATAGGACCTAATTACTGGCTGTAAATGCAATG
TCAAGAAAGGGAAGAACACCAAACTCTAAAACAGTTTGTGAGCTTCTAGAGATCATAACCAAGACGCAACAGGGTAGTTGAAGTCGAAGGAAATCACTTCACTAATGTGAGCTGGAAT
GTCTGAAGAGTCGACGAGTCTATCTTCGTTGTTCCTCAACGCCATCTGCGACTGTGCTGAGACCTCACCCAATTCTTGCTCAAAATCTTTGCCATCTGCCCCTTTAACATGTCCCAATTGCT
CCGTATGCTATCTCTTCATCATTAGCAAAGCTAGGCATTGGCATCACATCACTAAACTCTTCGATCTTCGCAAATCTACCTCGAACTCGAGGCCTGCTATCAGCTAGGATCTTCCTACATGTG
TACTTGATCGTTTTGCTAAAATTCCTCGCCTTTTTCTTGCTCACATACCTATCTACCTTCTTCTTCCTCTCCTCCGGTGATGCTCGGCCAACTTTATGGTTTGAGTCATCCATTGTTGTGATATC
GAAAGTCCCTTGTAATGGAGCAATCTTGGTGCTATCTCGTAGTACTTGTAAGTTTCTGGGGCTGTAGACGCCCGACATAGCTTCATATGTGTAGATTCCTCCTAGACCGTAGGCTTCTGCAG
CCTCCATTGGCGTCATTGGCATGCCAACGCTGGCATATATCCCCACCTCATCATCTGCTACACATATCGGCATTCCCATTCCTTCAGCATAAAGCTCCCCTCCAATTCCACCATTAGCATCCAT
GAATGAGCTGCGCTGCATTGCTGATGGCTCCATGCCCATATATTCAGGCAAGCCAACATGATCCAAAGGAAGCTGGCTCACTACTGAGTTTGGCGAGTAAGATGTGAAGGAGGGATTACA
AATCATTGGATCGAGCTGATCCATAGGATGGATTGGAGCCATTGATACAGCTGGAAGTGATGGAACGATATCAGGTTCCGGTTCAGGTGGAGGCTGAGGATTGAGAAGAACGGATATTAG
ATCCGAGTCGAAGGAATTGTCATAGCAGGGCAAGTCGGTGGTGGTGGTGTCAGTTGCTGGGAAGGTTACTGTGGCGGGGACGTCCGAAGAACAAACAACATTGTTGGTTAGCGATAAG
CTTCTTGTTGAGTGTCCAGCTTCTCGAAACAAGTTATCTTCATCATCAAAGTTGAGGATTTGGTCAGTTAAAGAACTGGATAAGCACTCATCAAGGGATGGATGCACCGGCGATTGAATCA
ATTCCTCCAGCATTTTGTCAAGACGAAACGAACACGAAGAGAGAATTCAAAATCTTTTTTGCAGATCAGGAACAGATAAAATATTGGTTGATGGATGCAATGATGAGGCT
>comp157827_c0_seq13

AGATTTGAATTTTATTGATGTTAGCTTGACAATCAATCATACAACTTGTATAGAAATTCATAAAAAGAAGTATAGTTTATTTTATTTATGCAGAATCTCTTAATTCATTGGTGTCCTTACAAAA
CGCGCTATTTATGATCACCAATGAATAAGAGTAAGAAAGTTACAAAAACTTGGAGATTGCTTTTGTACAGATAATATTTTTTTGGTCATCCAACCAGAACCAGAAAATTTAACAAATAACG
GTCGACAAAATTTGAATCCAATTCCGATAAAGATGTACACTGTACTGAAAGCAACTGTCGCCATGCCGTCATTTTATTTAGGTCGTAAATATAGGACCTAATTACTGGCTGTAAATGCAATG
TCAAGAAAGGGAAGAACACCAAACTCTAAAACAGTTTGTGAGCTTCTAGAGATCATAACCAAGACGCAACAGGGTAGTTGAAGTCGAAGGAAATCACTTCACTAATGTGAGCTGGAAT
GTCTGAAGAGTCGACGAGTCTATCTTCGTTGTTCCTCAACGCCATCTGCGACTGCAATTCAGCAAAGAAAATATTCAGAAATCTGCCACTTCAGAATTGCATTCATGTTGATAACAAAACG
TTTTTTATATGCAGAATCTTTTGATGTTTCGCATTGACATGCTTTACTAAGAAATGTATTCTTATTCAAGACGGCAATTTATTGACTGAGGAAGGAAAGACGAGGATTATGTCTTTCTGTTTT
GTTCTATTGATCATTCTATCAAGGTAGTTTTCTTAGTACAGTGTCATATCAGATTGTCTCCCAAATCTATACAGATTTGA
>comp157827_c0_seq5
AGATTTGAATTTTATTGATGTTAGCTTGACAATCAATCATACAACTTGTATAGAAATTCATAAAAAGAAGTATAGTTTATTTTATTTATGCAGAATCTCTTAATTCATTGGTGTCCTTACAAAA
CGCGCTATTTATGATCACCAATGAATAAGAGTAAGAAAGTTACAAAAACTTGGAGATTGCTTTTGTACAGATAATATTTTTTTGGTCATCCAACCAGAACCAGAAAATTTAACAAATAACG
GTCGACAAAATTTGAATCCAATTCCGATAAAGATGTACACTGTACTGAAAGCAACAATCTCCATGCCGTCATTTTATTTGAGTAAATATACGACCTGGTTAATGGCTGTAAATGCAATGTTA
AGATAGAGAAAGAACACCTAAATCTAAAACTGGTTCTAGAGATCATATCAAAGACTCAACAGGGAAGTTGAAGCCGAAGGAATTCACTCCAGTAATATAAGACAGTATTTCTGAAGAGTC
GAGGGGCATATCTTCATCGTTCCTCGACGACGTCGCTTCATCATCATATCCACCGTTAGCAGAGCTAGGCCTTTGCACCACCTCACTGAACTCATCGTTCTTCGCAAATCTACCTCGAACTC
GAGGCCTGCTATCAGCTAGGGTCTTCCTACATGCGTACTTGATCGTTTTGCTAAAATTCCTCTCCTTCTTCTTCCTAAGATACCTATCTAACTTCTTCCTCCTTTCCTCCGGTGATGCTCGACT
AACTTTATTGTTTGAGTCATCCATTCTGGGGATATCGGAAGGCATTTGTAATGGAGCAATACTGGTGCTACACATTGGCCTTACATTTTGGTTATCTCTCAGTACTTGTAAGTTTCCGGGGCT
GTAGAGTCTCGGCATAGGTTCATATGTGTAGATTCCTCTTAGATTGCAGGCTTCTGCAGCCTCCATCGGCCTCACGGGCATGCCAACACTAGTATTTTTCCCCATCCCATCATCTGCTACAAA
TGTCGGCATTCTCATTCTTTCAGCATAAAGCTGCCCTCCAATTCCACCATTAGCATCCATGAATGAGCTGCGCTGCATTGCTGATGGCTCCATGCCCATATATCCAGGCAAGCCCAAATGAC
CCGAAGGAAGCTGCTGGTTCACTACTGAGTTTGGCGGGTTAGATGTGAAGGGAGGATTACAAATCATTGGATTGGGCTGATTCATAGGATGGATTGGAGCCATTGATGCAGCTGGAAGTG
ATGGAAAGCTATTAGGTTCCGGTTCAGGTGGAAGTTGAGGATTGATAAGAGAGGATATTAGTTCTGAGTTGAAGGAATTGTCATAGCCGGGCAAGTTGGCGATGTTCGTATCAGGTTCAG
GGAAGGTTATTGTGACGGGGACGTCCGAAGAACAAACACCATTGTTGGTTGAGGATAAGACTCTTGGTGAGTAGCCAGCTTCTCGAAACAAGTTATCTTCGTAATCAAAGTTGGGCATTT
TGTCTTTTACAGAACTGGATAAGCACTCATCAAGGGATGGATGCACCGGCGATCGAATCAAATCCTCCAGCATTTTGTCAATACAAAGCGATTCCGAAGAGGGAGTTCTAATTCCTTTTTC
CATTATCAGGGGAATGCAGAAGAAGAAGAAATTAAGGAACGGAAAAAATATTGGTTGATGCATGCAATGATGAGGCTGTAGATGTTTATAGGGGCGGCGAGCGAAGAAAACAGACAATT
TATGCCCAGCTTTAGAATGTGCCTATTTGCCTCCGGACTGTAAGGGAAGCGACCAGTTTTAATCACGGGCCCTTTTGGACCCACCAATAATTTTTGGCCACGTCAGCTATTAGTTATGTGGA
ATGCGAATGTGGCACAATTGAAACTTAAAATGTAACAAAAAAACCAGTAATTAGTTGAGAGGCTAGCTGTAGTAAACGGATTGCTCAATCTA
>comp157827_c0_seq8
CTGTTCCTGATCTGCAAAAAAGATTTTGAATTCTCTCTTCGTGTTCGTTTCGTCTTGACAAAATGCTGGAGGAATTGATTCAATCGCCGGTGCATCCATCCCTTGATGAGTGCTTATCCAGT
TCTTTAACTGACCAAATCCTCAACTTTGATGATGAAGATAACTTGTTTCGAGAAGCTGGACACTCAACAAGAAGCTTATCGCTAACCAACAATGTTGTTTGTTCTTCGGACGTCCCCGCCA
CAGTAACCTTCCCAGCAACTGACACCACCACCACCGACTTGCCCTGCTATGACAATTCCTTCGACTCGGATCTAATATCCGTTCTTCTCAATCCTCAGCCTCCACCTGAACCGGAACCTGA
TATCGTTCCATCACTTCCAGCTGTATCAATGGCTCCAATCCATCCTATGGATCAGCTCGATCCAATGATTTGTAATCCCTCCTTCACATCTTACTCGCCAAACTCAGTAGTGAGCCAGCTTCC
TTTGGATCATGTTGGCTTGCCTGAATATATGGGCATGGAGCCATCAGCAATGCAGCTCTGCTCATTCATGGATGCTAATGGTGGAATTGGAGGGGAGCTTTATGCTGAAGGAATGGGAATG
CCGATATGTGTAGCAGATGATGAGGTGGGGATATATGCCAGCGTTGGCATGCCAATGACGCCAATGGAGGCTGCAGAAGCCTACGGTCTAGGAGGAATCTACACATATGAAGCTATGTCG

GGCGTCTACAGCCCCAGAAACTTACAAGTACTACGAGATAGCACCAAGATTGCTCCATTACAAGGGACTTTCGATATCACAACAATGGATGACTCAAACCATAAAGTTGGCCGAGCATCA
CCGGAGGAGAGGAAGAAGAAGGTAGATAGGTATGTGAGCAAGAAAAAGGCGAGGAATTTTAGCAAAACGATCAAGTACGCATGTAGGAAGACCCTAGCTGATAGCAGGCCTCGAGTTC
GAGGTAGATTTGCGAAGAACGATGAGTTCAGTGAGGTGGTGCAAAGGCCTAGCTCTGCTAACGGTGGATATGATGATGAAGCGACGTCGTCGAGGAACGATGAAGATATGCCCCTCGAC
TCTTCAGAAATACTGTCTTATATTACTGGAGTGAATTCCTTCGGCTTCAACTTCCCTGTTGAGTCTTTGATATGATCTCTAGAACCAGTTTTAGATTTAGGTGTTCTTTCTCTATCTTAACATT
GCATTTACAGCCATTAACCAGGTCGTATATTTACTCAAATAAAATGACGGCATGGAGATTGTTGCTTTCAGTACAGTGTACATCTTTATCGGAATTGGATTCAAATTTTGTCGACCGTTATTT
GTTAAATTTTCTGGTTCTGGTTGGATGACCAAAAAAATATTATCTGTACAAAAGCAATCTCCAAGTTTTTGTAACTTTCTTACTCTTATTCATTGGTGATCATAAATAGCGCGTTTTGTAAGG
ACACCAATGAATTAAGAGATTCTGCATAAATAAAATAAACTACACTTCTTTTTATGAATTTCTATACAAGTTGTATGATTGATTGTCAAGCTAACATCAATAGAATTCATTTTTTTTTCTGGGA
TTAAACCTGTAATGGTTGTTTTATTGAAAGTTTCTGCTTGATATCGAAGAAGATTGGGATAGTCTCAAATAGCAGGGTGATAATTGTTGCGA
>comp157827_c0_seq9
GCTCATTCATGGATGCTAATGGTGGAATTGGAGGGCAGCTTTATGCTGAAAGAATGAGAATGCCGACATTTGTAGCAGATGATGGGATGGGGAAAAATACTAGTGTTGGCATGCCCGTGA
GGCCGATGGAGGCTGCAGAAGCCTGCAATCTAAGAGGAATCTACACATATGAACCTATGCCGAGACTCTACAGCCCCGGAAACTTACAAGTACTGAGAGATAACCAAAATGTAAGGCCA
ATGTGTAGCACCAGTATTGCTCCATTACAAATGCCTTCCGATATCCCCAGAATGGATGACTCAAACAATAAAGTTAGTCGAGCATCACCGGAGGAAAGGAGGAAGAAGTTAGATAGGTAT
CTTAGGAAGAAGAAGGAGAGGAATTTTAGCAAAACGATCAAGTACACATGTAGGAAGATCCTAGCTGATAGCAGGCCTCGAGTTCGAGGTAGATTTGCGAAGATCGAAGAGTTTAGTGA
TGTGATGCCAATGCCTAGCTTTGCTAATGATGAAGAGATAGCATACGGAGCAATTGGGACATGTTAAAGGGGCAGATGGCAAAGATTTTGAGCAAGAATTGGGTGAGGTCTCAGCACAGT
CGCAGATGGCGTTGAGGAACAACGAAGATAGACTCGTCGACTCTTCAGACATTCCAGCTCACATTAGTGAAGTGATTTCCTTCGACTTCAACTACCCTGTTGCGTCTTGGTTATGATCTCT
AGAAGCTCACAAACTGTTTTAGAGTTTGGTGTTCTTCCCTTTCTTGACATTGCATTTACAGCCAGTAATTAGGTCCTATATTTACGACCTAAATAAAATGACGGCATGGCGACAGTTGCTTT
CAGTACAGTGTACATCTTTAACGAAATTGGATTCAAATTTTCTCGTCGGTTATTTGTTAAATTTTCTGGTTCTGGTTGCATGGCCAACAAATATAATCTCTCACTGGTGCTTGTCAAGTGGTT
TTCGATCGTAGCACCTCTATTTGAAAAAAAAATGGAGATGACTGATCTCCTTAACTTCTCCCTCCAAGCCATTACTATTGTTACTTTATAAAGATCTTCAATTTGATGTTCTTCCCTCTTTGGC
GGCTGAAATGTTGAGCAGATTTCTCCTCCTTGCTAGCCCAAATCTGCAGTTATTCTTCTTGTTTGGTGGCTGAAACATATAGCTATTCTTCCTTATTATTGCTGGCAAAATGTAAAGCTGTTT
TCCCTCTATTGTTGGCTAAATCTGCATCAAATCCTCCCTCTATTGCTGGCCGAAATCTGCTATAGGA
>comp157865_c0_seq1
TCGGAAACACAAAATATAATACAATAAAATAAAATATATTTGCATTCCCTCCAATAAAATGCAAATGATGACAACATATACCATTTTCATACCAAAACGGTCATATTTCAACAATCATCAGAT
TGAAGAAGCATTAATTCTCCAACGAAGACAAAAATATGCTAATAATCTATAAATCAGAGCCATAGCTGCAAGAATCCATATTTCATTCCATTGACTGTACAGATTTATAGTGTCAAATGATGG
TGAACTTTGAAGACTTCGGCATCCTCCTTTGCTTTGGCAATCATAGAGGAGATTTCCTGAATACTGTACTTTCAGAAGAAGTCTGAATCCATGATGCATGAAAGATACCTGCTTCATCCACC
TCATGAATCTGGGTAGGTGCTGAACATAATAGCCTCCAGTGAGCAGAAACAACATTAGCACCAAAGAGGCCATAAATCCTGCTCTTTTTACATTTAGAGTAGCTGCTCCGAATAGCTCTCC
TGTTCCCTGGCTTGTGACAACAATTAACAATATAGCAAATAGCGTCAGGAAGAAATGAGATGCTGATCTTCTCAAATCCACCATGAAGTACAAAATGAGAAGAAAAAATGGTAGGATAGAA
AATATGGGCTATCATGTCGCAGAGAGTGCTGGAAACATAGTACACACTTAGTCTATACATATCTGCTTTTTTCTCCTTCACCAAATAAGCCTTCTCAAATGGAAATACATATACTGCTCCAAA
TAAAGCAGAAGATGTCCAAAATATGCATAAGTAGAACATTAGCCCAACCTGATCTCTGAGTTGCGCCTCACTTCCTATTTTAGATTTCCACCAGAGAAGGCCAAGAAGAACTGCAACTCC
AACAGTCTGTAAAAGTCTCAACTTGTCCAAATAGTCCATGCTTCTTTTCCTGAATGTTCTCTTGGATATTATCAAGAACTGCTCAATCCATCCTATTGTCCAATCCTTCTTTACTAAAATTGC
CAAACTCAGCTGCTTCGGTATGGTGCTGTTCTGATGACTCTCCTCTCTCTCGTTGGTTTCTATATCTGATTTATACTTTAATTGTAGAAACTTAATGACTTCCGTCTCAAAATCTTGTGGATTC
AGCGATTTTCTAAGATCTTCGGGTATCTTGATATCGTTAACTTGACCAGTTGCAAGATCTAATAAGAACTCTGCAGGGTTCATGGCAATCTCAGGTACAAATCCTAGTGAAGAGAAGTACC

ACACAGAGTCACATGCCTTGCCATGGTATATAGGGTATCCATCTGATATAAGCAAAAGTTTGTCGAACATGTAAAATATCCGGCTTGAAGGCTGATGAATGGTAGTAATGATTGTTCTACCA
GCTTTGGCAAGGCTCTGAAGAACTAGAAGGAGCTTATTTGCAGAAGCGGAATCAAGGCCTGAGGTTGGTTCATCTAAGAATAAGAGGGAGGGATCAACAAGGATTTCATACCCAATGCT
AGTTCGTTTTCTCTCTCCACCTGATATTCCTCTAACAAATGCTCCCCCAACTTTAGTATGACGACACCTCTCCAAGCTAAGTTGCTTGATGATGGCATCTGCCTTGGAGTACTTGTGCTGCC
TTGTCATGTTCTTTGGGAGCCTCAAGAAAGCAGCAAATACTAGGGTTTCCTCCACTGTTAGCTGGGGAAAGAGCACGTCATCTTGTGTTACAAATCCAATCCTGCGCTTGAGAGCAAGAG
TGTAGGGAGTATCGTTGTAAGTGACAGCGCCGGAGACATCACCGCCGGCATCCAAACGGCCGGCGAGGATTTTGAGAAGAGTTGTCTTGCCGCTGCCCGAGGGGCCCATTAGGGCCAG
GATCTCGCCGGGGCCCACGCCTCCAGATATGCCCTTC
>comp157939_c0_seq3
CGGCGGCCGCTACCACCTATTGCTGGCCACCACCAATGCCAACCGCCACCGATGGCCACTTCCACCACTAGCCAGGGCGGCCGCTACCTCCACCTCCTCCTCCTCCACCAGCCGCTAACA
TTTTTGAATTTTACATTTTACACTGAATTCCTTTTACACTTTTTTTTTTTTACACTTTTTTTTAGCTAACCAAATAGTGCCTTACATAAGTTGCTTTTATCTTACCTCCCCCACTAGAATGCTCC
AAAGCTAAAGGAGGTAAGAAAAAAATCTAATAAAGCATTTTGAGTTTTGAGACTCTTATGTTAAATTATGGAGCATAATTTTGACCTATGAAAAAAATATGCTGCGTTGACTCGGTCCTTG
GTCCACCTTCAAGGTAAGGTTATCGTGAAAGTTATAAGAGGGAGGGGAACAGCTTTTTGTGGGGCATAGCTGTGAAAGAGACCAATATTTATGGGTTACATTATTATGGGCATTTTGGAGA
GTTAAATATTTATAGGTTACTTTATTATGGGCATCTATTTTTTTCTTTATTAAGTGGGGGCTTCATCTTTATATTGCCTTTGTAATTCTCCATAATCAGAATTATAGTGAAATCTGCAATAGAAGT
GAAATTTATATCATCAACAACAACAGGATGTTGCCATCAATTATGTGAATCCATATAAAATTTTGTATTTTCGTGCATTGATATTCTTTTTTATTTCTTAGTGTTTTAATAAGGCTTCAAATTGC
TAGACAATTGTACATATTTTATTTGGTGTAAATTACTAAAATGTTGTAGCAACGCAGAACATCAAGCAAGTTCTTTCCAGATTTGGAAGCAACATAATATTCTCTAATGGAATGAAAGACCC
ATGGAGCAGAGGAGGGGTACTTGAGAACCTGTCATCATCCATTGTTGCTCTTGTCACGGCAAAAGGTGCTCATCATGTAGATTTCAGATCTGCTACAGAAGAAGATCCAACTTGGCTTATA
GAACAAAGGACGCTAGAAGTGGAAATAATTAGAAGTTGGATAGATAAGTACAGAGAAGACGAAATGATATGAAGAAATAACTGCAAACAATTTGATGACCGGGGGATGTATTGTAAAAC
AATAAGTCCCCACAAGGTGGCTCATTACATTCATCACAATTCCAATAATTTGTAATTTGTTACCAAGTCAATTATTTTTATCTTCAATATTATGCTCAAAGAGTTTATTCTCTTTCTATGCCACC
ATATTATATGAATTTTAATTTATTTG
>comp157971_c1_seq1
TTAGCAAGAAAACTTCTTTGAAAAAGTAGCTGTAACAGAAAATGGATTTAACAGGCAACATAAGGCAGTGGGATTTCAGCATTTCAATGCACTAGTAATGTTTAAAACCACAGAATCGGC
TTCATACAAGTTGAAGACATGAGCATAGAAATATCAATAATACAAATTTATGTGCATTTGCTTATAAAAGTTGAATTATTGGACTTCTTACTATCTATACATGTTGTAAAAATTTATTGTCATGC
AACTGGAACTTTGCAGCAAATATTGAGGACTTCTTCCAAAGGTGCATGGTCACCTGTTCCTTCATCTTTCCTAGCATACAACAATTCTTTTCCCTTGAATACAAGCATCCCACCCTGCTGCA
GAACACTGTCTTATCGTCCGGTGTCGCTGAAATAGTGTAGTTAGCTGCAGCTTTTCGAATTGAACTGAATCTTGAGAAAACTTTGGTACTGGCTGGATTAAAGAAGGTACGGCCTAAACC
ATGATACAGGCCCAAAACATCGTAAGCCTTCCGATCTGGGTCTGCATAAAGATAATCCAACGGAAATGGTAGCCGTTGGGCAAGAATACGAGCGTTGTCAGGGCTACCGACACCAATAGC
AATCAATTTGACTCCGGCAGCATCAAACCTTGGTATCAATTCCTTTAGTTCAGAAGCAAGTTCCCAACAACAAATACATCCGAAATGTCTCAGAAGTGCAACCACAGCTATTCCCTCCTTC
TGATCCCATAAATCCTTAAGGAACGCCGGCTTCCCGGTAGAGGAGGTAAGAACCTGGACATTCCCAAGAGCATCCACGGCGGTTTCGTCGAATCCGGCGGTGGAAAGGTTCGACGAAGA
GGAAGAAGAAGAAGAAGAAAAAGCACTAGCGATGAAAGAGAAAGAAGTACCCATCGCCACGTCGGTCCTTTTGGCCGCCAACAGAAAGCACGTCGATGAAGAAACGGGAAGGGAGA
GAGAGAAGAAAGAGAGTGGTTGAGGAGAAGCAATGAGACTGCGGGGCGGAGCCAAGACGGAGTTCCAAGGAGCGAGCGCCATATTTCTGCATGTCCTTGCTTGGAGAAATTAATTATT
AAGTACGGTCACGGAACCTTGGAAAA
>comp157978_c0_seq2
CTTCAACTCCCCCTTCAATCAATCTTCACTTCACACTTGTCACTAAGCTTCCTTCTTCGGGGCTTCTAAGAACGAGAATGACGGCAATATTCCTCGCAGATCCATTTCTCCAGATATCCTCA

ACGGCGAACTCTGAGATAGATAGAGAAAGAAGACTAATAAGAAGAATAAGAAGAAGAAGAAATGGAGAGCCCACCAGAAAGCGCTAGGTCGAGCCCGGAGGCGGAGGTGGGAATGC
GAGTGGAAGATCTATGGGAAATCCAGGAGCCGCAACTCAGCCCCTCTGAGAAGCTCAATTCTTGCTTTGAGAGTATTTTCGTGACCTCTTTCCCACAAGCCCCTTCATCTCAAGTCATAG
AGATACCTTCAGATTCCAGCTTGGCTGATGCTGTCAAAATTCTATCTAAGAATAAGATTATGAGCGCTCCTGTGAGAAATTTAAAAGCTCCGAAAAATGCTAGTTGGATTGATAGATACATT
GGAATCGTAGAATTTGCTGGGATTACTGTCTGGCTTCTGCATCAGTCAGAATTGGCAGCCAGTGAGAAAGGTGCTGGCTCTCCTATAAAAGGGCCTATCACAGAAGGAATCGCGGCCAGG
CTTAAAAGTGTTAAAGTGAAGAGTAAAGATGATGGAATAACAACTTCTGTTCTTGATGCAACTGATTCTATGGGTGGAACATTCCTTGAAACTCTGATAACTTCAGACTTCTATAAGAAGA
CAAAGGTTCAAGACATCTCAGGATCATTCCGCTGGGCACCCTTCCTCGCATTGCAGAAATCTGACTCTTTTCTAACTATGCTTCTGCTGCTCTCGAAGTACAGGATGAAGAGCCTTCCTGT
AGTTGTTCTTGGCGAAGAGAAGATTGATAATATAATCACACGATCTGCTGTTGTACACATGCTTGCAGAATGCGTTGGGCTCCATTGGTTTGAGAACTGGGGAAAAATGAAGCTTCATGA
GCTTGGCCTGCCAAGTAACAGAAATAAAATTATTAAGGTTAGCGAAGATGACTCCATTATAAAAGCCTTCCAACTGATGAGGAAAAAAAGAATTGGTGGGGTACCTGTTGTTGATAAAAG
TGGGAACAAGGCAATTGGAAATATAAGCATCAGAGATGTTCAGTACCTTCTTACAGCTCCAGAAATGTATAAGGGTTACAGGTCGATAACAGCCAAAGACTTCATAAGGTCAATTAAGAG
CTATCTGAACACGCATGATGATGCTTCACCGAGATTTCTTGGCGTACTAACTTGCCATAGTGAAAATACAATCAAAGACATAATTCTAAAGCTGGATGCAGAGAAAATGCAACGGATCTAT
GTTGTTGATAAGGATGAAAACTTGGAAGGAGTGATTACACTTAGAGATATAATATCGAGGCTGGTTCATGAGCCAAATGGCTACTTTGGTGATTTCTTTGATGGTGTTGTTCCTTTACCTCA
GAACAGCAGGGTTTAAAGATTTAACTACTAGTAACTGTTTTCAATTATCATTTCTCTTTTAGCTTCTTGTTTCCCCTGTATTTGGTTTTCTTAGTATCTTTTATACTTTGTTTTTTTTTTTT
>comp158030_c0_seq11
CCCCTCTGAGATTTTACCTTTCAGCCCTTTTCCTTCGCGCCGCGCTACGTCTTTTCTCAAGCTCTTTCGTATGTCTCCCTGGGCTCTTCCTCGAGCGCTCCACATCACTGATATTGCCTATTA
GGGTTTCTCTTCTGTGGCATTTTGGCGATCGGGCTCTACGTAGTGTTCGATCTCGATTCGCTATTCTGAGATCTCTTCTATTCACTACAGGAATCGGCTCCAGGAAAATGTGTACTTAATGGA
CTGCACCCCTAACTGCAGTGATAAGAAGACTCTAAACAAGTGGTTCTTCATTGACAAAATGGTTGGGTAGTTGATCACCAACACGCTAAAGAATAAAATAACAAGTTCCTTTACTTTTGG
AATATTGGACATCACAAAGGATCTGAAGCAGAGCCACAATTCTCCAGTTCTCACTGATCAAGTGCGTGCAAGTCAGTCCAGGTTGGGAATGTCTTCTAGTTTGTCACCTTACTCACCACC
ATCTGCAACGTTTTCTTCTACTTCCTACTTAACAATTCCAAGGAGAAAGCCTATTAATGACTGCCTCGATGCCATGAAATCGTCTTCTCCTCCTTGCAAAAAAGTTAGTAAAGATATCAGCT
TTGATGCTGCTTTTGAGGAAGCTGATGCAGTTTATCGAAATTGGATGAGCAAGTATCCTTCAGCTCTGACATCGTTTGAACAGATCACAAACTATGCTAAGGATAAGAAGATAGCTCTCTT
CATGGACTATGATGGAACTCTTTCCCCTATTGTGGACAATCCTGATCTTGCATTCATGTCGAGTGCTATGCGCACTGCAGTTAAACATGTCGCCAAGTATTTTCCTACTGCCATTATTAGTGG
GAGAAGTCGTGATAAGGTTTACGAATTTGTTGGGTTGAAAGAACTGTACTATGTTGGCAGTCATGGTCTGGACTTGGTTGGCCCAGTCAAATATTCTGAATCTATCGCTACAAATCACAAT
TGCATTAGTCCTACTGATGAGCTGGTCAGCACTGCAAACTTGTTTCAAACTGCAAGAGAGTTTTTACCCATGATTGATGAGGTTTTCAGATCTCTCATCGAGAAAACAAAGGATATTAAAG
GCTCAAAAATTGAGAACAATAAGTTCTGCCTTTCTGTGCATTACCGTCTAGTAGACGAGAATAGTTGGACTGTGATTGCTCAGTGTGTTCATGACATTCTCAAGAACTATCCTCGTCTGGA
GCAAACTTATGGGATGAAGGTAAATAACACTCACAATTTTTGCATATTTGATCATTATTTTGCTATGTATTGCTCTGTATTGATAATGCTATAATTCTAAGACAGAACTATATGAAGCCAGAAT
ACTAACCTTACAGATTGCATTAAAATTAAATGAGCTACACTAACCTAAGAATCTCCCAAATGGCTAAGGTGCTATGTTTTGTATTGCATATGCTTCATGGCTTTGCAAATTGTAATATTTTATT
TAATAGTTGTTGTGGTGCAGGTTTTAGAAATTCGCCCAATAATTAACTGGAATAAAGGGGATGCTGTAGAATTCATGCTTGAATCTTTAGGACTAAATGATCGCGGGGCCATGCTTCCTATAT
ATATTGGAGATGATCGTACTGATGAGGATGCATTCAAGGTCTTAAGAGAAAGTAACAGGGGTTATGGTATTTTGGTTTCTTCAGTTCCTAAAGAAACTTATGCCCATTTCTCATTGAAAGAT
CCATCTGAGGTCATGGTGTTTCTCAGATCTCTTGTGAGATGGAAGAAGTCTCAAATAGCAGTAAATAGATCTTCGTGATCTTTTTCCAGTATATCTATTATCCAGTAAAACAATAAAGGATCC
AGGCTTTTATTTTCTATCCTTAAGCTCTTTAAATAAGTTTCTTTTTGCCGGTTTTGTGCGGAAAGCAAGCTATGTTTAATGTAAGATCTGATTGTATGCTACATATAAAACTCGATCCAATTGA
GTTTTATATGTAGCATACAATCAGATCTTACATTAAACATAGCTTGCTTTCCGCACAAAACCGGCAAAAAGAAACTTATTTAAAGAGCTTAAGGATAGAAAATAAAAGCCTGGATCCTTTAT
TGTTTTACTGGATAATAGATATACTGGAAAAAGATCACGAAGATCTATTTACTGCTATTTGAGACTTCTTCCATCTCACAAGAGATCTGAGAAACACCATGACCTCAGATGGATCTTTCAAT

GAGAAATGGGCATAAGTTTCTTTAGGAACTGAAGAAACCAAAATACCATAACCCCTGTTACTTTCTCTTAAGACCTTGAATGCATCCTCATCAGTACGATCATCTCCAATATATATAGGAAG
CATGGCCCCGCGATCATTTAGTCCTAAAGATTCAAGCATGAATTCTACAGCATCCCCTTTATTCCAGTTAATTATTGGGCGAATTTCTAAAACCTTCATCCCATAAGTTTGCTCCAGACGAGG
ATAGTTCTTGAGAATGTCATGAACACACTGAGCAATCACAGTCCAACTATTCTCGTCTACTAGACGGTAATGCACAGAAAGGCAGAACTTATTGTTCTCAATTTTTGAGCCTTTAATATCCT
TTGTTTTCTCGATGAGAGATCTGAAAACCTCATCAATCATGGGTAAAAACTCTCTTGCAGTTTGAAACAAGTTTGCAGTGCTGACCAGCTCATCAGTAGGACTAATGCAATTGTGATTTGT
AGCGATAGATTCAGAATATTTGACTGGGCCAACCAAGTCCAGACCATGACTGCCAACATAGTACAGTTCTTTCAACCCAACAAATTCGTAAACCTTATCACGACTTCTCCCACTAATAATG
GCAGTAGGAAAATACTTGGCGACATGTTTAACTGCAGTGCGCATAGCACTCGACATGAATGCAAGATCAGGATTGTCCACAATAGGGGAAAGAGTTCCATCATAGTCCATGAAGAGAGCT
ATCTTCTTATCCTTAGCATAGTTTGTGATCTGTTCAAACGATGTCAGAGCTGAAGGATACTTGCTCATCCAATTTCGATAAACTGCATCAGCTTCCTCAAAAGCAGCATCAAAGCTGATATC
TTTACTAACTTTTTTGCAAGGAGGAGAAGACGATTTCATGGCATCGAGGCAGTCATTAATAGGCTTTCTCCTTGGAATTGTTAAGTAGGAAGTAGAAGAAAACGTTGCAGATGGTGGTGA
GTAAGGTGACAAACTAGAAGACATTCCCAACCTGGACTGACTTGCACGCACTTGATCAGTGAGAACTGGAGAATTGTGGCTCTGCTTCAGATCCTTTGTGATGTCCAATATTCCAAAAGT
AAAGGAACTTGTTATTTTATTCTTTAGCGTGTTGGTGATCAACTACCCAACCATTTTGTCAATGAAGAACCACTTGTTTAGAGTCTTCTTATCACTGCAGTTAGGGGTGCAGTCCATTAAGT
ACACATTTTCCTGTAAACTGGAGCCGATTCCTGTAGTGAATAGAAGAGATCTCAGAATAGCGAATCGAGATCGAACACTACGTAGAGCCCGATCGCCAAAATGCCACAGAAGAGAAACC
CTAATAGGCAATATCAGTGATGTGGAGCGCTCGAGGAAGAGCCCAGGGAGACATACGAAAGAGCTTGAGAAAAGACGTAGCGCGGCGCGAAAGGGCTGAAAGGTAAAATCTCAGAGG
GGAGGGAGAGCGAGTGGGAGAGAGAATGAAAACAAAAGAAAGGGCCAACGGTATTTATAGAGCTCCGCCGTGACGGATAGGATCCGCTTGTTGCAACTAGCTAGTCTAA
>comp158030_c0_seq18
CCCCTCTGAGATTTTACCTTTCAGCCCTTTTCCTTCGCGCCGCGCTACGTCTTTTCTCAAGCTCTTTCGTATGTCTCCCTGGGCTCTTCCTCGAGCGCTCCACATCACTGATATTGCCTATTA
GGGTTTCTCTTCTGTGGCATTTTGGCGATCGGGCTCTACGTAGTGTTCGATCTCGATTCGCTATTCTGAGATCTCTTCTATTCACTACAGGAATCGGCTCCAGTGATAAGAAGACTCTAAAC
AAGTGGTTCTTCATTGACAAAATGGTTGGGTAGTTGATCACCAACACGCTAAAGAATAAAATAACAAGTTCCTTTACTTTTGGAATATTGGACATCACAAAGGATCTGAAGCAGAGCCAC
AATTCTCCAGTTCTCACTGATCAAGTGCGTGCAAGTCAGTCCAGGTTGGGAATGTCTTCTAGTTTGTCACCTTACTCACCACCATCTGCAACGTTTTCTTCTACTTCCTACTTAACAATTCC
AAGGAGAAAGCCTATTAATGACTGCCTCGATGCCATGAAATCGTCTTCTCCTCCTTGCAAAAAAGTTAGTAAAGATATCAGCTTTGATGCTGCTTTTGAGGAAGCTGATGCAGTTTATCGA
AATTGGATGAGCAAGTATCCTTCAGCTCTGACATCGTTTGAACAGATCACAAACTATGCTAAGGATAAGAAGATAGCTCTCTTCATGGACTATGATGGAACTCTTTCCCCTATTGTGGACAA
TCCTGATCTTGCATTCATGTCGAGTGCTATGCGCACTGCAGTTAAACATGTCGCCAAGTATTTTCCTACTGCCATTATTAGTGGGAGAAGTCGTGATAAGGTTTACGAATTTGTTGGGTTGA
AAGAACTGTACTATGTTGGCAGTCATGGTCTGGACTTGGTTGGCCCAGTCAAATATTCTGAATCTATCGCTACAAATCACAATTGCATTAGTCCTACTGATGAGCTGGTCAGCACTGCAAA
CTTGTTTCAAACTGCAAGAGAGTTTTTACCCATGATTGATGAGGTTTTCAGATCTCTCATCGAGAAAACAAAGGATATTAAAGGCTCAAAAATTGAGAACAATAAGTTCTGCCTTTCTGTG
CATTACCGTCTAGTAGACGAGAATAGTTGGACTGTGATTGCTCAGTGTGTTCATGACATTCTCAAGAACTATCCTCGTCTGGAGCAAACTTATGGGATGAAGGTAAATAACACTCACAATT
TTTGCATATTTGATCATTATTTTGCTATGTATTGCTCTGTATTGATAATGCTATAATTCTAAGACAGAACTATATGAAGCCAGAATACTAACCTTACAGATTGCATTAAAATTAAATGAGCTACA
CTAACCTAAGAATCTCCCAAATGGCTAAGGTTTTAGAAATTCGCCCAATAATTAACTGGAATAAAGGGGATGCTGTAGAATTCATGCTTGAATCTTTAGGACTAAATGATCGCGGGGCCAT
GCTTCCTATATATATTGGAGATGATCGTACTGATGAGGATGCATTCAAGGTCTTAAGAGAAAGTAACAGGGGTTATGGTATTTTGGTTTCTTCAGTTCCTAAAGAAACTTATGCCCATTTCTC
ATTGAAAGATCCATCTGAGGTCATGGTGTTTCTCAGATCTCTTGTGAGATGGAAGAAGTCTCAAATAGCAGTAAATAGATCTTCGTGATCTTTTTCCAGTATATCTATTATCCAGTAAAACAA
TAAAGGATCCAGGCTTTTATTTTCTATCCTTAAGCTCTTTAAATAAGTTTCTTTTTGCCGGTTTTGTGCGGAAAGCAAGCTATGTTTAATGTAAGATCTGATTGTATGCTACATATAAAACTCG
ATCCAATTGAGTTTTATATGTAGCATACAATCAGATCTTACATTAAACATAGCTTGCTTTCCGCACAAAACCGGCAAAAAGAAACTTATTTAAAGAGCTTAAGGATAGAAAATAAAAGCCT
GGATCCTTTATTGTTTTACTGGATAATAGATATACTGGAAAAAGATCACGAAGATCTATTTACTGCTATTTGAGACTTCTTCCATCTCACAAGAGATCTGAGAAACACCATGACCTCAGATG

GATCTTTCAATGAGAAATGGGCATAAGTTTCTTTAGGAACTGAAGAAACCAAAATACCATAACCCCTGTTACTTTCTCTTAAGACCTTGAATGCATCCTCATCAGTACGATCATCTCCAATA
TATATAGGAAGCATGGCCCCGCGATCATTTAGTCCTAAAGATTCAAGCATGAATTCTACAGCATCCCCTTTATTCCAGTTAATTATTGGGCGAATTTCTAAAACCTGCACCACAACAACTATT
AAATAAAATATTACAATTTGCAAAGCCATGAAGCATATGCAATACAAAACATAGCACCTTAGCCATTTGGGAGATTCTTAGGTTAGTGTAGCTCATTTAATTTTAATGCAATCTGTAAGGTTA
GTATTCTGGCTTCATATAGTTCTGTCTTAGAATTATAGCATTATCAATACAGAGCAATACATAGCAAAATAATGATCAAATATGCAAAAATTGTGAGTGTTATTTACCTTCATCCCATAAGTTT
GCTCCAGACGAGGATAGTTCTTGAGAATGTCATGAACACACTGAGCAATCACAGTCCAACTATTCTCGTCTACTAGACGGTAATGCACAGAAAGGCAGAACTTATTGTTCTCAATTTTTGA
GCCTTTAATATCCTTTGTTTTCTCGATGAGAGATCTGAAAACCTCATCAATCATGGGTAAAAACTCTCTTGCAGTTTGAAACAAGTTTGCAGTGCTG
>comp158030_c0_seq24
CCCCTCTGAGATTTTACCTTTCAGCCCTTTTCCTTCGCGCCGCGCTACGTCTTTTCTCAAGCTCTTTCGTATGTCTCCCTGGGCTCTTCCTCGAGCGCTCCACATCACTGATATTGCCTATTA
GGGTTTCTCTTCTGTGGCATTTTGGCGATCGGGCTCTACGTAGTGTTCGATCTCGATTCGCTATTCTGAGATCTCTTCTATTCACTACAGGAATCGGCTCCAGGAAAATGTGTACTTAATGGA
CTGCACCCCTAACTGCAGTGATAAGAAGACTCTAAACAAGTGGTTCTTCATTGACAAAATGGTTGGGTAGTTGATCACCAACACGCTAAAGAATAAAATAACAAGTTCCTTTACTTTTGG
AATATTGGACATCACAAAGGATCTGAAGCAGAGCCACAATTCTCCAGTTCTCACTGATCAAGTGCGTGCAAGTCAGTCCAGGTTGGGAATGTCTTCTAGTTTGTCACCTTACTCACCACC
ATCTGCAACGTTTTCTTCTACTTCCTACTTAACAATTCCAAGGAGAAAGCCTATTAATGACTGCCTCGATGCCATGAAATCGTCTTCTCCTCCTTGCAAAAAAGTTAGTAAAGATATCAGCT
TTGATGCTGCTTTTGAGGAAGCTGATGCAGTTTATCGAAATTGGATGAGCAAGTATCCTTCAGCTCTGACATCGTTTGAACAGATCACAAACTATGCTAAGGATAAGAAGATAGCTCTCTT
CATGGACTATGATGGAACTCTTTCCCCTATTGTGGACAATCCTGATCTTGCATTCATGTCGAGTGCTATGCGCACTGCAGTTAAACATGTCGCCAAGTATTTTCCTACTGCCATTATTAGTGG
GAGAAGTCGTGATAAGGTTTACGAATTTGTTGGGTTGAAAGAACTGTACTATGTTGGCAGTCATGGTCTGGACTTGGTTGGCCCAGTCAAATATTCTGAATCTATCGCTACAAATCACAAT
TGCATTAGTCCTACTGATGAGCTGGTCAGCACTGCAAACTTGTTTCAAACTGCAAGAGAGTTTTTACCCATGATTGATGAGGTTTTCAGATCTCTCATCGAGAAAACAAAGGATATTAAAG
GCTCAAAAATTGAGAACAATAAGTTCTGCCTTTCTGTGCATTACCGTCTAGTAGACGAGAATAGTTGGACTGTGATTGCTCAGTGTGTTCATGACATTCTCAAGAACTATCCTCGTCTGGA
GCAAACTTATGGGATGAAGGTTTTAGAAATTCGCCCAATAATTAACTGGAATAAAGGGGATGCTGTAGAATTCATGCTTGAATCTTTAGGACTAAATGATCGCGGGGCCATGCTTCCTATAT
ATATTGGAGATGATCGTACTGATGAGGATGCATTCAAGGTCTTAAGAGAAAGTAACAGGGGTTATGGTATTTTGGTTTCTTCAGTTCCTAAAGAAACTTATGCCCATTTCTCATTGAAAGAT
CCATCTGAGGTCATGGTGTTTCTCAGATCTCTTGTGAGATGGAAGAAGTCTCAAATAGCAGTAAATAGATCTTCGTGATCTTTTTCCAGTATATCTATTATCCAGTAAAACAATAAAGGATCC
AGGCTTTTATTTTCTATCCTTAAGCTCTTTAAATAAGTTTCTTTTTGCCGGTTTTGTGCGGAAAGCAAGCTATGTTTAATGTAAGATCTGATTGTATGCTACATATAAAACTCGATCCAATTGA
GTTTTATATGTAGCATACAATCAGATCTTACATTAAACATAGCTTGCTTTCCGCACAAAACCGGCAAAAAGAAACTTATTTAAAGAGCTTAAGGATAGAAAATAAAAGCCTGGATCCTTTAT
TGTTTTACTGGATAATAGATATACTGGAAAAAGATCACGAAGATCTATTTACTGCTATTTGAGACTTCTTCCATCTCACAAGAGATCTGAGAAACACCATGACCTCAGATGGATCTTTCAAT
GAGAAATGGGCATAAGTTTCTTTAGGAACTGAAGAAACCAAAATACCATAACCCCTGTTACTTTCTCTTAAGACCTTGAATGCATCCTCATCAGTACGATCATCTCCAATATATATAGGAAG
CATGGCCCCGCGATCATTTAGTCCTAAAGATTCAAGCATGAATTCTACAGCATCCCCTTTATTCCAGTTAATTATTGGGCGAATTTCTAAAACCTTCATCCCATAAGTTTGCTCCAGACGAGG
ATAGTTCTTGAGAATGTCATGAACACACTGAGCAATCACAGTCCAACTATTCTCGTCTACTAGACGGTAATGCACAGAAAGGCAGAACTTATTGTTCTCAATTTTTGAGCCTTTAATATCCT
TTGTTTTCTCGATGAGAGATCTGAAAACCTCATCAATCATGGGTAAAAACTCTCTTGCAGTTTGAAACAAGTTTGCAGTGCTGACCAGCTCATCAGTAGGACTAATGCAATTGTGATTTGT
AGCGATAGATTCAGAATATTTGACTGGGCCAACCAAGTCCAGACCATGACTGCCAACATAGTACAGTTCTTTCAACCCAACAAATTCGTAAACCTTATCACGACTTCTCCCACTAATAATG
GCAGTAGGAAAATACTTGGCGACATGTTTAACTGCAGTGCGCATAGCACTCGACATGAATGCAAGATCAGGATTGTCCACAATAGGGGAAAGAGTTCCATCATAGTCCATGAAGAGAGCT
ATCTTCTTATCCTTAGCATAGTTTGTGATCTGTTCAAACGATGTCAGAGCTGAAGGATACTTGCTCATCCAATTTCGATAAACTGCATCAGCTTCCTCAAAAGCAGCATCAAAGCTGATATC
TTTACTAACTTTTTTGCAAGGAGGAGAAGACGATTTCATGGCATCGAGGCAGTCATTAATAGGCTTTCTCCTTGGAATTGTTAAGTAGGAAGTAGAAGAAAACGTTGCAGATGGTGGTGA

GTAAGGTGACAAACTAGAAGACATTCCCAACCTGGACTGACTTGCACGCACTTGATCAGTGAGAACTGGAGAATTGTGGCTCTGCTTCAGATCCTTTGTGATGTCCAATATTCCAAAAGT
AAAGGAACTTGTTATTTTATTCTTTAGCGTGTTGGTGATCAACTACCCAACCATTTTGTCAATGAAGAACCACTTGTTTAGAGTCTTCTTATCACTGCAGTTAGGGGTGCAGTCCATTAAGT
ACACATTTTCCTGTAAACTGGAGCCGATTCCTGTAGTGAATAGAAGAGATCTCAGAATAGCGAATCGAGATCGAACACTACGTAGAGCCCGATCGCCAAAATGCCACAGAAGAGAAACC
CTAATAGGCAATATCAGTGATGTGGAGCGCTCGAGGAAGAGCCCAGGGAGACATACGAAAGAGCTTGAGAAAAGACGTAGCGCGGCGCGAAAGGGCTGAAAGGTAAAATCTCAGAGG
GGAGGGAGAGCGAGTGGGAGAGAGAATGAAAACAAAAGAAAGGGCCAACGGTATTTATAGAGCTCCGCCGTGACGGATAGGATCCGCTTGTTGCAACTAGCTAGTCTAA
>comp158030_c0_seq41
CCCCTCTGAGATTTTACCTTTCAGCCCTTTTCCTTCGCGCCGCGCTACGTCTTTTCTCAAGCTCTTTCGTATGTCTCCCTGGGCTCTTCCTCGAGCGCTCCACATCACTGATATTGCCTATTA
GGGTTTCTCTTCTGTGGCATTTTGGCGATCGGGCTCTACGTAGTGTTCGATCTCGATTCGCTATTCTGAGATCTCTTCTATTCACTACAGGAATCGGCTCCAGTGATAAGAAGACTCTAAAC
AAGTGGTTCTTCATTGACAAAATGGTTGGGTAGTTGATCACCAACACGCTAAAGAATAAAATAACAAGTTCCTTTACTTTTGGAATATTGGACATCACAAAGGATCTGAAGCAGAGCCAC
AATTCTCCAGTTCTCACTGATCAAGTGCGTGCAAGTCAGTCCAGGTTGGGAATGTCTTCTAGTTTGTCACCTTACTCACCACCATCTGCAACGTTTTCTTCTACTTCCTACTTAACAATTCC
AAGGAGAAAGCCTATTAATGACTGCCTCGATGCCATGAAATCGTCTTCTCCTCCTTGCAAAAAAGTTAGTAAAGATATCAGCTTTGATGCTGCTTTTGAGGAAGCTGATGCAGTTTATCGA
AATTGGATGAGCAAGTATCCTTCAGCTCTGACATCGTTTGAACAGATCACAAACTATGCTAAGGATAAGAAGATAGCTCTCTTCATGGACTATGATGGAACTCTTTCCCCTATTGTGGACAA
TCCTGATCTTGCATTCATGTCGAGTGCTATGCGCACTGCAGTTAAACATGTCGCCAAGTATTTTCCTACTGCCATTATTAGTGGGAGAAGTCGTGATAAGGTTTACGAATTTGTTGGGTTGA
AAGAACTGTACTATGTTGGCAGTCATGGTCTGGACTTGGTTGGCCCAGTCAAATATTCTGAATCTATCGCTACAAATCACAATTGCATTAGTCCTACTGATGAGCTGGTGAATCACCATTAA
TAAAATGCTAAATATTTGCTTTGTTTTCTTAAGACCACCACAACATTAAAAAGAAGAACAAGATCAGACACAACCAATAAATTTAGATATCTATTTCTATAGTTTGCTTCGGTTGTAAATATA
TTGTCTTAAGTATTTTCTGCAGGTCAGCACTGCAAACTTGTTTCAAACTGCAAGAGAGTTTTTACCCATGATTGATGAGGTTTTCAGATCTCTCATCGAGAAAACAAAGGATATTAAAGGC
TCAAAAATTGAGAACAATAAGTTCTGCCTTTCTGTGCATTACCGTCTAGTAGACGAGAATAGTTGGACTGTGATTGCTCAGTGTGTTCATGACATTCTCAAGAACTATCCTCGTCTGGAGC
AAACTTATGGGATGAAGGTAAATAACACTCACAATTTTTGCATATTTGATCATTATTTTGCTATGTATTGCTCTGTATTGATAATGCTATAATTCTAAGACAGAACTATATGAAGCCAGAATACT
AACCTTACAGATTGCATTAAAATTAAATGAGCTACACTAACCTAAGAATCTCCCAAATGGCTAAGGTGCTATGTTTTGTATTGCATATGCTTCATGGCTTTGCAAATTGTAATATTTTATTTAA
TAGTTGTTGTGGTGCAGGTTTTAGAAATTCGCCCAATAATTAACTGGAATAAAGGGGATGCTGTAGAATTCATGCTTGAATCTTTAGGACTAAATGATCGCGGGGCCATGCTTCCTATATATA
TTGGAGATGATCGTACTGATGAGGATGCATTCAAGGTCTTAAGAGAAAGTAACAGGGGTTATGGTATTTTGGTTTCTTCAGTTCCTAAAGAAACTTATGCCCATTTCTCATTGAAAGATCCA
TCTGAGGTCATGGTGTTTCTCAGATCTCTTGTGAGATGGAAGAAGTCTCAAATAGCAGTAAATAGATCTTCGTGATCTTTTTCCAGTATATCTATTATCCAGTAAAACAATAAAGGATCCAG
GCTTTTATTTTCTATCCTTAAGCTCTTTAAATAAGTTTCTTTTTGCCGGTTTTGTGCGGAAAGCAAGCTATGTTTAATGTAAGATCTGATTGTATGCTACATATAAAACTCGATCCAATTGAGT
TTTATATGTAGCATACAATCAGATCTTACATTAAACATAGCTTGCTTTCCGCACAAAACCGGCAAAAAGAAACTTATTTAAAGAGCTTAAGGATAGAAAATAAAAGCCTGGATCCTTTATTG
TTTTACTGGATAATAGATATACTGGAAAAAGATCACGAAGATCTATTTACTGCTATTTGAGACTTCTTCCATCTCACAAGAGATCTGAGAAACACCATGACCTCAGATGGATCTTTCAATGA
GAAATGGGCATAAGTTTCTTTAGGAACTGAAGAAACCAAAATACCATAACCCCTGTTACTTTCTCTTAAGACCTTGAATGCATCCTCATCAGTACGATCATCTCCAATATATATAGGAAGCA
TGGCCCCGCGATCATTTAGTCCTAAAGATTCAAGCATGAATTCTACAGCATCCCCTTTATTCCAGTTAATTATTGGGCGAATTTCTAAAACCTTCATCCCATAAGTTTGCTCCAGACGAGGAT
AGTTCTTGAGAATGTCATGAACACACTGAGCAATCACAGTCCAACTATTCTCGTCTACTAGACGGTAATGCACAGAAAGGCAGAACTTATTGTTCTCAATTTTTGAGCCTTTAATATCCTTT
GTTTTCTCGATGAGAGATCTGAAAACCTCATCAATCATGGGTAAAAACTCTCTTGCAGTTTGAAACAAGTTTGCAGTGCTGACCAGCTCATCAGTAGGACTAATGCAATTGTGATTTGTAG
CGATAGATTCAGAATATTTGACTGGGCCAACCAAGTCCAGACCATGACTGCCAACATAGTACAGTTCTTTCAACCCAACAAATTCGTAAACCTTATCACGACTTCTCCCACTAATAATGGC
AGTAGGAAAATACTTGGCGACATGTTTAACTGCAGTGCGCATAGCACTCGACATGAATGCAAGATCAGGATTGTCCACAATAGGGGAAAGAGTTCCATCATAGTCCATGAAGAGAGCTAT

CTTCTTATCCTTAGCATAGTTTGTGATCTGTTCAAACGATGTCAGAGCTGAAGGATACTTGCTCATCCAATTTCGATAAACTGCATCAGCTTCCTCAAAAGCAGCATCAAAGCTGATATCTTT
ACTAACTTTTTTGCAAGGAGGAGAAGACGATTTCATGGCATCGAGGCAGTCATTAATAGGCTTTCTCCTTGGAATTGTTAAGTAGGAAGTAGAAGAAAACGTTGCAGATGGTGGTGAGTA
AGGTGACAAACTAGAAGACATTCCCAACCTGGACTGACTTGCACGCACTTGATCAGTGAGAACTGGAGAATTGTGGCTCTGCTTCAGATCCTTTGTGATGTCCAATATTCCAAAAGTAAA
GGAACTTGTTATTTTATTCTTTAGCGTGTTGGTGATCAACTACCCAACCATTTTGTCAATGAAGAACCACTTGTTTAGAGTCTTCTTATCACTGCAGTTAGGGGTGCAGTCCATTAAGTACA
CATCTTCCTGTAAACTGGAGCCGATTTCTGTAGTGAATAGAAGAGATCTCAGAATAGCGAATCGAGATCGAACACTACGTAGAGCCCGATCGCCAAAATGCCACAGAAGAGAAACCCTAA
TAGGCAATATCAGTGATGTGGAGCGCTCGAGGAAGAGCCCAGGGAGACATACGAAAGAGCTTGAGAAAAGACGTAGCGCGGCGCGAA
>comp158030_c0_seq48
CCCCTCTGAGATTTTACCTTTCAGCCCTTTTCCTTCGCGCCGCGCTACGTCTTTTCTCAAGCTCTTTCGTATGTCTCCCTGGGCTCTTCCTCGAGCGCTCCACATCACTGATATTGCCTATTA
GGGTTTCTCTTCTGTGGCATTTTGGCGATCGGGCTCTACGTAGTGTTCGATCTCGATTCGCTATTCTGAGATCTCTTCTATTCACTACAGGAATCGGCTCCAGTGATAAGAAGACTCTAAAC
AAGTGGTTCTTCATTGACAAAATGGTTGGGTAGTTGATCACCAACACGCTAAAGAATAAAATAACAAGTTCCTTTACTTTTGGAATATTGGACATCACAAAGGATCTGAAGCAGAGCCAC
AATTCTCCAGTTCTCACTGATCAAGTGCGTGCAAGTCAGTCCAGGTTGGGAATGTCTTCTAGTTTGTCACCTTACTCACCACCATCTGCAACGTTTTCTTCTACTTCCTACTTAACAATTCC
AAGGAGAAAGCCTATTAATGACTGCCTCGATGCCATGAAATCGTCTTCTCCTCCTTGCAAAAAAGTTAGTAAAGATATCAGCTTTGATGCTGCTTTTGAGGAAGCTGATGCAGTTTATCGA
AATTGGATGAGCAAGTATCCTTCAGCTCTGACATCGTTTGAACAGATCACAAACTATGCTAAGGATAAGAAGATAGCTCTCTTCATGGACTATGATGGAACTCTTTCCCCTATTGTGGACAA
TCCTGATCTTGCATTCATGTCGAGTGCTATGCGCACTGCAGTTAAACATGTCGCCAAGTATTTTCCTACTGCCATTATTAGTGGGAGAAGTCGTGATAAGGTTTACGAATTTGTTGGGTTGA
AAGAACTGTACTATGTTGGCAGTCATGGTCTGGACTTGGTTGGCCCAGTCAAATATTCTGAATCTATCGCTACAAATCACAATTGCATTAGTCCTACTGATGAGCTGGTCAGCACTGCAAA
CTTGTTTCAAACTGCAAGAGAGTTTTTACCCATGATTGATGAGGTTTTCAGATCTCTCATCGAGAAAACAAAGGATATTAAAGGCTCAAAAATTGAGAACAATAAGTTCTGCCTTTCTGTG
CATTACCGTCTAGTAGACGAGAATAGTTGGACTGTGATTGCTCAGTGTGTTCATGACATTCTCAAGAACTATCCTCGTCTGGAGCAAACTTATGGGATGAAGGTAAATAACACTCACAATT
TTTGCATATTTGATCATTATTTTGCTATGTATTGCTCTGTATTGATAATGCTATAATTCTAAGACAGAACTATATGAAGCCAGAATACTAACCTTACAGATTGCATTAAAATTAAATGAGCTACA
CTAACCTAAGAATCTCCCAAATGGCTAAGGTGCTATGTTTTGTATTGCATATGCTTCATGGCTTTGCAAATTGTAATATTTTATTTAATAGTTGTTGTGGTGCAGGTTTTAGAAATTCGCCCAA
TAATTAACTGGAATAAAGGGGATGCTGTAGAATTCATGCTTGAATCTTTAGGACTAAATGATCGCGGGGCCATGCTTCCTATATATATTGGAGATGATCGTACTGATGAGGATGCATTCAAGG
TCTTAAGAGAAAGTAACAGGGGTTATGGTATTTTGGTTTCTTCAGTTCCTAAAGAAACTTATGCCCATTTCTCATTGAAAGATCCATCTGAGGTCATGGTGTTTCTCAGATCTCTTGTGAGA
TGGAAGAAGTCTCAAATAGCAGTAAATAGATCTTCGTGATCTTTTTCCAGTATATCTATTATCCAGTAAAACAATAAAGGATCCAGGCTTTTATTTTCTATCCTTAAGCTCTTTAAATAAGTTT
CTTTTTGCCGGTTTTGTGCGGAAAGCAAGCTATGTTTAATGTAAGATCTGATTGTATGCTACATATAAAACTCGATCCAATTGAGTTTTATATGTAGCATACAATCAGATCTTACATTAAACAT
AGCTTGCTTTCCGCACAAAACCGGCAAAAAGAAACTTATTTAAAGAGCTTAAGGATAGAAAATAAAAGCCTGGATCCTTTATTGTTTTACTGGATAATAGATATACTGGAAAAAGATCACG
AAGATCTATTTACTGCTATTTGAGACTTCTTCCATCTCACAAGAGATCTGAGAAACACCATGACCTCAGATGGATCTTTCAATGAGAAATGGGCATAAGTTTCTTTAGGAACTGAAGAAAC
CAAAATACCATAACCCCTGTTACTTTCTCTTAAGACCTTGAATGCATCCTCATCAGTACGATCATCTCCAATATATATAGGAAGCATGGCCCCGCGATCATTTAGTCCTAAAGATTCAAGCAT
GAATTCTACAGCATCCCCTTTATTCCAGTTAATTATTGGGCGAATTTCTAAAACCTGCACCACAACAACTATTAAATAAAATATTACAATTTGCAAAGCCATGAAGCATATGCAATACAAAAC
ATAGCACCTTAGCCATTTGGGAGATTCTTAGGTTAGTGTAGCTCATTTAATTTTAATGCAATCTGTAAGGTTAGTATTCTGGCTTCATATAGTTCTGTCTTAGAATTATAGCATTATCAATACAG
AGCAATACATAGCAAAATAATGATCAAATATGCAAAAATTGTGAGTGTTATTTACCTTCATCCCATAAGTTTGCTCCAGACGAGGATAGTTCTTGAGAATGTCATGAACACACTGAGCAATC
ACAGTCCAACTATTCTCGTCTACTAGACGGTAATGCACAGAAAGGCAGAACTTATTGTTCTCAATTTTTGAGCCTTTAATATCCTTTGTTTTCTCGATGAGAGATCTGAAAACCTCATCAAT
CATGGGTAAAAACTCTCTTGCAGTTTGAAACAAGTTTGCAGTGCTG

>comp158030_c0_seq49
CCCCTCTGAGATTTTACCTTTCAGCCCTTTTCCTTCGCGCCGCGCTACGTCTTTTCTCAAGCTCTTTCGTATGTCTCCCTGGGCTCTTCCTCGAGCGCTCCACATCACTGATATTGCCTATTA
GGGTTTCTCTTCTGTGGCATTTTGGCGATCGGGCTCTACGTAGTGTTCGATCTCGATTCGCTATTCTGAGATCTCTTCTATTCACTACAGGAATCGGCTCCAGGAAAATGTGTACTTAATGGA
CTGCACCCCTAACTGCAGTGATAAGAAGACTCTAAACAAGTGGTTCTTCATTGACAAAATGGTTGGGTAGTTGATCACCAACACGCTAAAGAATAAAATAACAAGTTCCTTTACTTTTGG
AATATTGGACATCACAAAGGATCTGAAGCAGAGCCACAATTCTCCAGTTCTCACTGATCAAGTGCGTGCAAGTCAGTCCAGGTTGGGAATGTCTTCTAGTTTGTCACCTTACTCACCACC
ATCTGCAACGTTTTCTTCTACTTCCTACTTAACAATTCCAAGGAGAAAGCCTATTAATGACTGCCTCGATGCCATGAAATCGTCTTCTCCTCCTTGCAAAAAAGTTAGTAAAGATATCAGCT
TTGATGCTGCTTTTGAGGAAGCTGATGCAGTTTATCGAAATTGGATGAGCAAGTATCCTTCAGCTCTGACATCGTTTGAACAGATCACAAACTATGCTAAGGATAAGAAGATAGCTCTCTT
CATGGACTATGATGGAACTCTTTCCCCTATTGTGGACAATCCTGATCTTGCATTCATGTCGAGTGCTATGCGCACTGCAGTTAAACATGTCGCCAAGTATTTTCCTACTGCCATTATTAGTGG
GAGAAGTCGTGATAAGGTTTACGAATTTGTTGGGTTGAAAGAACTGTACTATGTTGGCAGTCATGGTCTGGACTTGGTTGGCCCAGTCAAATATTCTGAATCTATCGCTACAAATCACAAT
TGCATTAGTCCTACTGATGAGCTGGTGAATCACCATTAATAAAATGCTAAATATTTGCTTTGTTTTCTTAAGACCACCACAACATTAAAAAGAAGAACAAGATCAGACACAACCAATAAAT
TTAGATATCTATTTCTATAGTTTGCTTCGGTTGTAAATATATTGTCTTAAGTATTTTCTGCAGGTCAGCACTGCAAACTTGTTTCAAACTGCAAGAGAGTTTTTACCCATGATTGATGAGGTTT
TCAGATCTCTCATCGAGAAAACAAAGGATATTAAAGGCTCAAAAATTGAGAACAATAAGTTCTGCCTTTCTGTGCATTACCGTCTAGTAGACGAGAATAGTTGGACTGTGATTGCTCAGTG
TGTTCATGACATTCTCAAGAACTATCCTCGTCTGGAGCAAACTTATGGGATGAAGGTAAATAACACTCACAATTTTTGCATATTTGATCATTATTTTGCTATGTATTGCTCTGTATTGATAATG
CTATAATTCTAAGACAGAACTATATGAAGCCAGAATACTAACCTTACAGATTGCATTAAAATTAAATGAGCTACACTAACCTAAGAATCTCCCAAATGGCTAAGGTTTTAGAAATTCGCCCA
ATAATTAACTGGAATAAAGGGGATGCTGTAGAATTCATGCTTGAATCTTTAGGACTAAATGATCGCGGGGCCATGCTTCCTATATATATTGGAGATGATCGTACTGATGAGGATGCATTCAAG
GTCTTAAGAGAAAGTAACAGGGGTTATGGTATTTTGGTTTCTTCAGTTCCTAAAGAAACTTATGCCCATTTCTCATTGAAAGATCCATCTGAGGTCATGGTGTTTCTCAGATCTCTTGTGAG
ATGGAAGAAGTCTCAAATAGCAGTAAATAGATCTTCGTGATCTTTTTCCAGTATATCTATTATCCAGTAAAACAATAAAGGATCCAGGCTTTTATTTTCTATCCTTAAGCTCTTTAAATAAGTT
TCTTTTTGCCGGTTTTGTGCGGAAAGCAAGCTATGTTTAATGTAAGATCTGATTGTATGCTACATATAAAACTCGATCCAATTGAGTTTTATATGTAGCATACAATCAGATCTTACATTAAACA
TAGCTTGCTTTCCGCACAAAACCGGCAAAAAGAAACTTATTTAAAGAGCTTAAGGATAGAAAATAAAAGCCTGGATCCTTTATTGTTTTACTGGATAATAGATATACTGGAAAAAGATCAC
GAAGATCTATTTACTGCTATTTGAGACTTCTTCCATCTCACAAGAGATCTGAGAAACACCATGACCTCAGATGGATCTTTCAATGAGAAATGGGCATAAGTTTCTTTAGGAACTGAAGAAA
CCAAAATACCATAACCCCTGTTACTTTCTCTTAAGACCTTGAATGCATCCTCATCAGTACGATCATCTCCAATATATATAGGAAGCATGGCCCCGCGATCATTTAGTCCTAAAGATTCAAGCA
TGAATTCTACAGCATCCCCTTTATTCCAGTTAATTATTGGGCGAATTTCTAAAACCTTCATCCCATAAGTTTGCTCCAGACGAGGATAGTTCTTGAGAATGTCATGAACACACTGAGCAATC
ACAGTCCAACTATTCTCGTCTACTAGACGGTAATGCACAGAAAGGCAGAACTTATTGTTCTCAATTTTTGAGCCTTTAATATCCTTTGTTTTCTCGATGAGAGATCTGAAAACCTCATCAAT
CATGGGTAAAAACTCTCTTGCAGTTTGAAACAAGTTTGCAGTGCTGACCAGCTCATCAGTAGGACTAATGCAATTGTGATTTGTAGCGATAGATTCAGAATATTTGACTGGGCCAACCAAG
TCCAGACCATGACTGCCAACATAGTACAGTTCTTTCAACCCAACAAATTCGTAAACCTTATCACGACTTCTCCCACTAATAATGGCAGTAGGAAAATACTTGGCGACATGTTTAACTGCAG
TGCGCATAGCACTCGACATGAATGCAAGATCAGGATTGTCCACAATAGGGGAAAGAGTTCCATCATAGTCCATGAAGAGAGCTATCTTCTTATCCTTAGCATAGTTTGTGATCTGTTCAAAC
GATGTCAGAGCTGAAGGATACTTGCTCATCCAATTTCGATAAACTGCATCAGCTTCCTCAAAAGCAGCATCAAAGCTGATATCTTTACTAACTTTTTTGCAAGGAGGAGAAGACGATTTCA
TGGCATCGAGGCAGTCATTAATAGGCTTTCTCCTTGGAATTGTTAAGTAGGAAGTAGAAGAAAACGTTGCAGATGGTGGTGAGTAAGGTGACAAACTAGAAGACATTCCCAACCTGGAC
TGACTTGCACGCACTTGATCAGTGAGAACTGGAGAATTGTGGCTCTGCTTCAGATCCTTTGTGATGTCCAATATTCCAAAAGTAAAGGAACTTGTTATTTTATTCTTTAGCGTGTTGGTGAT
CAACTACCCAACCATTTTGTCAATGAAGAACCACTTGTTTAGAGTCTTCTTATCACTGCAGTTAGGGGTGCAGTCCATTAAGTACACATCTTCCTGTAAACTGGAGCCGATTTCTGTAGTG
AATAGAAGAGATCTCAGAATAGCGAATCGAGATCGAACACTACGTAGAGCCCGATCGCCAAAATGCCACAGAAGAGAAACCCTAATAGGCAATATCAGTGATGTGGAGCGCTCGAGGAA

GAGCCCAGGGAGACATACGAAAGAGCTTGAGAAAAGACGTAGCGCGGCGCGAA
>comp158030_c0_seq6
CCCCTCTGAGATTTTACCTTTCAGCCCTTTTCCTTCGCGCCGCGCTACGTCTTTTCTCAAGCTCTTTCGTATGTCTCCCTGGGCTCTTCCTCGAGCGCTCCACATCACTGATATTGCCTATTA
GGGTTTCTCTTCTGTGGCATTTTGGCGATCGGGCTCTACGTAGTGTTCGATCTCGATTCGCTATTCTGAGATCTCTTCTATTCACTACAGGAATCGGCTCCAGTGATAAGAAGACTCTAAAC
AAGTGGTTCTTCATTGACAAAATGGTTGGGTAGTTGATCACCAACACGCTAAAGAATAAAATAACAAGTTCCTTTACTTTTGGAATATTGGACATCACAAAGGATCTGAAGCAGAGCCAC
AATTCTCCAGTTCTCACTGATCAAGTGCGTGCAAGTCAGTCCAGGTTGGGAATGTCTTCTAGTTTGTCACCTTACTCACCACCATCTGCAACGTTTTCTTCTACTTCCTACTTAACAATTCC
AAGGAGAAAGCCTATTAATGACTGCCTCGATGCCATGAAATCGTCTTCTCCTCCTTGCAAAAAAGTTAGTAAAGATATCAGCTTTGATGCTGCTTTTGAGGAAGCTGATGCAGTTTATCGA
AATTGGATGAGCAAGTATCCTTCAGCTCTGACATCGTTTGAACAGATCACAAACTATGCTAAGGATAAGAAGATAGCTCTCTTCATGGACTATGATGGAACTCTTTCCCCTATTGTGGACAA
TCCTGATCTTGCATTCATGTCGAGTGCTATGCGCACTGCAGTTAAACATGTCGCCAAGTATTTTCCTACTGCCATTATTAGTGGGAGAAGTCGTGATAAGGTTTACGAATTTGTTGGGTTGA
AAGAACTGTACTATGTTGGCAGTCATGGTCTGGACTTGGTTGGCCCAGTCAAATATTCTGAATCTATCGCTACAAATCACAATTGCATTAGTCCTACTGATGAGCTGGTGAATCACCATTAA
TAAAATGCTAAATATTTGCTTTGTTTTCTTAAGACCACCACAACATTAAAAAGAAGAACAAGATCAGACACAACCAATAAATTTAGATATCTATTTCTATAGTTTGCTTCGGTTGTAAATATA
TTGTCTTAAGTATTTTCTGCAGGTCAGCACTGCAAACTTGTTTCAAACTGCAAGAGAGTTTTTACCCATGATTGATGAGGTTTTCAGATCTCTCATCGAGAAAACAAAGGATATTAAAGGC
TCAAAAATTGAGAACAATAAGTTCTGCCTTTCTGTGCATTACCGTCTAGTAGACGAGAATAGTTGGACTGTGATTGCTCAGTGTGTTCATGACATTCTCAAGAACTATCCTCGTCTGGAGC
AAACTTATGGGATGAAGGTTTTAGAAATTCGCCCAATAATTAACTGGAATAAAGGGGATGCTGTAGAATTCATGCTTGAATCTTTAGGACTAAATGATCGCGGGGCCATGCTTCCTATATATA
TTGGAGATGATCGTACTGATGAGGATGCATTCAAGGTCTTAAGAGAAAGTAACAGGGGTTATGGTATTTTGGTTTCTTCAGTTCCTAAAGAAACTTATGCCCATTTCTCATTGAAAGATCCA
TCTGAGGTCATGGTGTTTCTCAGATCTCTTGTGAGATGGAAGAAGTCTCAAATAGCAGTAAATAGATCTTCGTGATCTTTTTCCAGTATATCTATTATCCAGTAAAACAATAAAGGATCCAG
GCTTTTATTTTCTATCCTTAAGCTCTTTAAATAAGTTTCTTTTTGCCGGTTTTGTGCGGAAAGCAAGCTATGTTTAATGTAAGATCTGATTGTATGCTACATATAAAACTCGATCCAATTGAGT
TTTATATGTAGCATACAATCAGATCTTACATTAAACATAGCTTGCTTTCCGCACAAAACCGGCAAAAAGAAACTTATTTAAAGAGCTTAAGGATAGAAAATAAAAGCCTGGATCCTTTATTG
TTTTACTGGATAATAGATATACTGGAAAAAGATCACGAAGATCTATTTACTGCTATTTGAGACTTCTTCCATCTCACAAGAGATCTGAGAAACACCATGACCTCAGATGGATCTTTCAATGA
GAAATGGGCATAAGTTTCTTTAGGAACTGAAGAAACCAAAATACCATAACCCCTGTTACTTTCTCTTAAGACCTTGAATGCATCCTCATCAGTACGATCATCTCCAATATATATAGGAAGCA
TGGCCCCGCGATCATTTAGTCCTAAAGATTCAAGCATGAATTCTACAGCATCCCCTTTATTCCAGTTAATTATTGGGCGAATTTCTAAAACCTTCATCCCATAAGTTTGCTCCAGACGAGGAT
AGTTCTTGAGAATGTCATGAACACACTGAGCAATCACAGTCCAACTATTCTCGTCTACTAGACGGTAATGCACAGAAAGGCAGAACTTATTGTTCTCAATTTTTGAGCCTTTAATATCCTTT
GTTTTCTCGATGAGAGATCTGAAAACCTCATCAATCATGGGTAAAAACTCTCTTGCAGTTTGAAACAAGTTTGCAGTGCTGACCAGCTCATCAGTAGGACTAATGCAATTGTGATTTGTAG
CGATAGATTCAGAATATTTGACTGGGCCAACCAAGTCCAGACCATGACTGCCAACATAGTACAGTTCTTTCAACCCAACAAATTCGTAAACCTTATCACGACTTCTCCCACTAATAATGGC
AGTAGGAAAATACTTGGCGACATGTTTAACTGCAGTGCGCATAGCACTCGACATGAATGCAAGATCAGGATTGTCCACAATAGGGGAAAGAGTTCCATCATAGTCCATGAAGAGAGCTAT
CTTCTTATCCTTAGCATAGTTTGTGATCTGTTCAAAAGATGTCAGAGCTG
>comp158030_c0_seq69
CCCCTCTGAGATTTTACCTTTCAGCCCTTTTCCTTCGCGCCGCGCTACGTCTTTTCTCAAGCTCTTTCGTATGTCTCCCTGGGCTCTTCCTCGAGCGCTCCACATCACTGATATTGCCTATTA
GGGTTTCTCTTCTGTGGCATTTTGGCGATCGGGCTCTACGTAGTGTTCGATCTCGATTCGCTATTCTGAGATCTCTTCTATTCACTACAGGAATCGGCTCCAGGAAAATGTGTACTTAATGGA
CTGCACCCCTAACTGCAGTGATAAGAAGACTCTAAACAAGTGGTTCTTCATTGACAAAATGGTTGGGTAGTTGATCACCAACACGCTAAAGAATAAAATAACAAGTTCCTTTACTTTTGG
AATATTGGACATCACAAAGGATCTGAAGCAGAGCCACAATTCTCCAGTTCTCACTGATCAAGTGCGTGCAAGTCAGTCCAGGTTGGGAATGTCTTCTAGTTTGTCACCTTACTCACCACC

ATCTGCAACGTTTTCTTCTACTTCCTACTTAACAATTCCAAGGAGAAAGCCTATTAATGACTGCCTCGATGCCATGAAATCGTCTTCTCCTCCTTGCAAAAAAGTTAGTAAAGATATCAGCT
TTGATGCTGCTTTTGAGGAAGCTGATGCAGTTTATCGAAATTGGATGAGCAAGTATCCTTCAGCTCTGACATCGTTTGAACAGATCACAAACTATGCTAAGGATAAGAAGATAGCTCTCTT
CATGGACTATGATGGAACTCTTTCCCCTATTGTGGACAATCCTGATCTTGCATTCATGTCGAGTGCTATGCGCACTGCAGTTAAACATGTCGCCAAGTATTTTCCTACTGCCATTATTAGTGG
GAGAAGTCGTGATAAGGTTTACGAATTTGTTGGGTTGAAAGAACTGTACTATGTTGGCAGTCATGGTCTGGACTTGGTTGGCCCAGTCAAATATTCTGAATCTATCGCTACAAATCACAAT
TGCATTAGTCCTACTGATGAGCTGGTCAGCACTGCAAACTTGTTTCAAACTGCAAGAGAGTTTTTACCCATGATTGATGAGGTTTTCAGATCTCTCATCGAGAAAACAAAGGATATTAAAG
GCTCAAAAATTGAGAACAATAAGTTCTGCCTTTCTGTGCATTACCGTCTAGTAGACGAGAATAGTTGGACTGTGATTGCTCAGTGTGTTCATGACATTCTCAAGAACTATCCTCGTCTGGA
GCAAACTTATGGGATGAAGGTAAATAACACTCACAATTTTTGCATATTTGATCATTATTTTGCTATGTATTGCTCTGTATTGATAATGCTATAATTCTAAGACAGAACTATATGAAGCCAGAAT
ACTAACCTTACAGATTGCATTAAAATTAAATGAGCTACACTAACCTAAGAATCTCCCAAATGGCTAAGGTTTTAGAAATTCGCCCAATAATTAACTGGAATAAAGGGGATGCTGTAGAATTC
ATGCTTGAATCTTTAGGACTAAATGATCGCGGGGCCATGCTTCCTATATATATTGGAGATGATCGTACTGATGAGGATGCATTCAAGGTCTTAAGAGAAAGTAACAGGGGTTATGGTATTTT
GGTTTCTTCAGTTCCTAAAGAAACTTATGCCCATTTCTCATTGAAAGATCCATCTGAGGTCATGGTGTTTCTCAGATCTCTTGTGAGATGGAAGAAGTCTCAAATAGCAGTAAATAGATCTT
CGTGATCTTTTTCCAGTATATCTATTATCCAGTAAAACAATAAAGGATCCAGGCTTTTATTTTCTATCCTTAAGCTCTTTAAATAAGTTTCTTTTTGCCGGTTTTGTGCGGAAAGCAAGCTATG
TTTAATGTAAGATCTGATTGTATGCTACATATAAAACTCGATCCAATTGAGTTTTATATGTAGCATACAATCAGATCTTACATTAAACATAGCTTGCTTTCCGCACAAAACCGGCAAAAAGAA
ACTTATTTAAAGAGCTTAAGGATAGAAAATAAAAGCCTGGATCCTTTATTGTTTTACTGGATAATAGATATACTGGAAAAAGATCACGAAGATCTATTTACTGCTATTTGAGACTTCTTCCAT
CTCACAAGAGATCTGAGAAACACCATGACCTCAGATGGATCTTTCAATGAGAAATGGGCATAAGTTTCTTTAGGAACTGAAGAAACCAAAATACCATAACCCCTGTTACTTTCTCTTAAG
ACCTTGAATGCATCCTCATCAGTACGATCATCTCCAATATATATAGGAAGCATGGCCCCGCGATCATTTAGTCCTAAAGATTCAAGCATGAATTCTACAGCATCCCCTTTATTCCAGTTAATTA
TTGGGCGAATTTCTAAAACCTTCATCCCATAAGTTTGCTCCAGACGAGGATAGTTCTTGAGAATGTCATGAACACACTGAGCAATCACAGTCCAACTATTCTCGTCTACTAGACGGTAATG
CACAGAAAGGCAGAACTTATTGTTCTCAATTTTTGAGCCTTTAATATCCTTTGTTTTCTCGATGAGAGATCTGAAAACCTCATCAATCATGGGTAAAAACTCTCTTGCAGTTTGAAACAAG
TTTGCAGTGCTGACCAGCTCATCAGTAGGACTAATGCAATTGTGATTTGTAGCGATAGATTCAGAATATTTGACTGGGCCAACCAAGTCCAGACCATGACTGCCAACATAGTACAGTTCTT
TCAACCCAACAAATTCGTAAACCTTATCACGACTTCTCCCACTAATAATGGCAGTAGGAAAATACTTGGCGACATGTTTAACTGCAGTGCGCATAGCACTCGACATGAATGCAAGATCAGG
ATTGTCCACAATAGGGGAAAGAGTTCCATCATAGTCCATGAAGAGAGCTATCTTCTTATCCTTAGCATAGTTTGTGATCTGTTCAAACGATGTCAGAGCTGAAGGATACTTGCTCATCCAAT
TTCGATAAACTGCATCAGCTTCCTCAAAAGCAGCATCAAAGCTGATATCTTTACTAACTTTTTTGCAAGGAGGAGAAGACGATTTCATGGCATCGAGGCAGTCATTAATAGGCTTTCTCCT
TGGAATTGTTAAGTAGGAAGTAGAAGAAAACGTTGCAGATGGTGGTGAGTAAGGTGACAAACTAGAAGACATTCCCAACCTGGACTGACTTGCACGCACTTGATCAGTGAGAACTGGA
GAATTGTGGCTCTGCTTCAGATCCTTTGTGATGTCCAATATTCCAAAAGTAAAGGAACTTGTTATTTTATTCTTTAGCGTGTTGGTGATCAACTACCCAACCATTTTGTCAATGAAGAACCA
CTTGTTTAGAGTCTTCTTATCACTGCAGTTAGGGGTGCAGTCCATTAAGTACACATTTTCCTGTAAACTGGAGCCGATTCCTGTAGTGAATAGAAGAGATCTCAGAATAGCGAATCGAGAT
CGAACACTACGTAGAGCCCGATCGCCAAAATGCCACAGAAGAGAAACCCTAATAGGCAATATCAGTGATGTGGAGCGCTCGAGGAAGAGCCCAGGGAGACATACGAAAGAGCTTGAGA
AAAGACGTAGCGCGGCGCGAAAGGGCTGAAAGGTAAAATCTCAGAGGGGAGGGAGAGCGAGTGGGAGAGAGAATGAAAACAAAAGAAAGGGCCAACGGTATTTATAGAGCTCCGCC
GTGACGGATAGGATCCGCTTGTTGCAACTAGCTAGTCTAA
>comp158085_c0_seq7
GAGAGAGAGAGAGAGAGAGTCCTGTTTTGCAATCAGGGATTGAAAATTTACAATCTACTTCATATATTTTTTCCATATTATTAACAAATTCAATGACAGGATTGAAACATGCATAATTTGCA
AATAGACTGTTCAAATCAAGAAAAAAACAAATTACAAGAGAACTCATAAGATTCCAATTAACCCATTCATACTTCATTAAAATGACTCACAAAAAAAAAAGAGAAAAATGCTCCACTTTTA
ACATTCTGATTAAGATTTCGTTTGAGATAACTTTCTTATTGCTTTCTGAAACAGAATTTAATATCAAAATTTTTAATTCAAAAAATTTTTATACTAAAAAAATTACTTTATAAAGCAGTAAGAA

AGCTGTCCCAAATGAAGCCTTAATTTAGATCTAGAGTTCTCAAGAACTCGTCTTCTGTATT

>comp158085_c2_seq1

AAGATTTAAATGAAAAATTAACTTCAAACAAAACATATTTAATAAAAATTCAGGTAGCCCATACAACGTAGCAGGATAAACACTTGTTTGTTTTTAGCCCTAATGAAAAGGGCTCACAATG
ATGAATATTTTTTTCTTTGAAATTCAGACCCGAAACCTTTCATCCCCAAGGCTCCAAATGCCCGTGCTTAAAAATTAAAATTGCTGAAACCCCCGACAACGTAGAACCGAAAGTCGCGAA
GGGTTCGACCAGCAGTGTGTAAAACAGAAGACGAGTTCTTGAGAATTGTGGATCTAAGTTAAGGTTTCGTTTGGGACAGCTTTCTTACAGCTCCGTAAAACAGCTTTCAAGTAAAAAAAT
AATGAGAGAACAAACGTAAACCGGGGAAGGAATGAGATTTAAGATCCTCTAGGATTTAGATGATAAAAAAGCAAGGGCTCGTTTGTTTCCACGTTATTTCTCCTTTTTTCCGTTTGAAACT
GGAAAACGCAGTCGTGGAAGATGTTTGGATGTGTAGCAGACTTTTTTAAAAACTCCGTTTTTGTATTGTTTTGAGAAGCATTATTTATTAGCTTCTCCGTTTTAAAAAGAACCCTTTCTCCT
ATTGGCGTTTTTTATACCGGAACGGGAAAAAAACCAAACGAAGCTTTAAGATAGCATCAAAATAGTTAGAAAATTAATTAAAAAGATTATTGTTATTAGTACATAGTAATTAGCAATATGATT
ATGATTAGAAAAATAAGAGAATGACAATTAACGTGAAAGAAGTTTTATGGGCTTAATTATTTGTTTTCTTTTAGTTTATTTGTTATTGAAAGAGAGGAGGGGTTCTAGTGAGAGCTGGTTCT
GAAGCTGGATCGTACCGGCCACCAGACCGGTTCACGGTCGAACCGGTTGAACCGGCCGGTCCGG

>comp158087_c0_seq18

AGACAGCAGAAACTGGTCTAATTTACTCAATAGAAAAGAAAATGGCAAGTCAAAGTTACAACTTGAGCTTGATGACTATATCTATCTTAGTCTACCCTCTAGTTATGCAACAAACAAAAG
ACCTGAGGTTTTGCATTCTGTCTGCAGTCCAAACACTCATTGGCAGGCTTAGTAGCGCAATGAAGATTTCATGCTCGAGGATCATGTTCTTATTCACTAGTAGAATTCATGGTGCGAGATTG
CCAGTAACCTAGAAAAATGCTGTGGTAAGCTGACAATTTTAAACATTATTGCACTTAAAAAAAAAGAAATTAATACTTGGATCAACTAAGATTTACAAGCCAAGGAAACGGTTCTGTCCA
GGTGGCTCCTGGAGGAAGGAGATCCTTACATTGATGAGCTAGAATATGACTAAAAATGGCACCAAGCACGTCGCAAAACAAAATCAGCTCATTTGCTTGTAGTAAAATCAAATTACTGAA
GCAGAGTTCTCCGCTCCACCGTCTCCACCCCCATCAACCACATCATCTTCATCTTCATCTTTATCTTTGGGTACAGGCTGAGAATAAGCAGATTGAAGTAGCAGGTGGATTTCTTCCATCTT
TCCAGACATCTCATCCTCTCTATCCATCTCAACGAGGGCCTGTGCAGAGAAGTTTTTGAATATTGGGAGGAAGCGTTTCCTGCAGTACTCATGGAAAAGGAGGGTGAATATCACAAGAGG
GACGACTAGACCTGATGCAACCGTTGATTTCTTTATTCCGAATACTCCGAGGGCTATTATCTGTGCAAGGACAAGGGAGAATATTGTGGAGTTGTGCACAACGGGCCATAGTTGTCCCCCA
CTCTCATATTTTGTACGATAAACATTAAGCATCTGGTTGCGGTATATCAAGTAGCCAAGCAAGAAATACACCATCAAGAATGGCAATATCAATGGTGCCAAAATAGAACATGTGAATCCGAG
AAGCCCAAAAAGCAAGACTCTCGGAACTTCAGTGTGATATGGGAATGTTGGCACCATATTTGGATCATCTAACTTTTTTCTGAATACATACTTTTGTAGCAGATTATAAGTAAGGGGAAAA
CATTGTACTAGTTCAGCACATAAACTTGTCCAGCCTGAAGTCAAGACATAAGTAATAAAAAAGGTAGCCTGTCCTGGTAAAGTTCTAGCAAGAACAAAAGGGATGTCTTTTGGATGGGAA
ATGAACATTAGCTGATGGATAACAGATGCAGATAGAACGTTGACAAAGAAAACATTCCAAATCGTGAAGTATAATACTTTGCAACATGCACTCTTTTTTCTGCCACTGCGGGAGATAGCTC
CTTCCACAGTAGAAAATAGCATCATTGTTGGTGGTACAGCATAGAAAAATAGAAGCAAAATGACACTGGGAAGATAGCCAGTTACCAGTTGGATCATGTATTTCTTTGTTAGTATACCTCTT
AGAAATGGGAGTTTTTGCTGTAATGTCTCCAACTGTGTAAGACCTTGCACAAATGTAACTGGGATGAGAAACAGAAACAAAAATAGAATAGTAGCCACCACTATTGATATTCGGCGAATC
CAAAGTTGCCTGTACGGTATTGAAAGGTTTGACCAGTATAAGTCATCAGGTTCCGGAGCCAAATCAGTAACCCATAACATAGGGTTTGATGATTGAAGAACTCGAGAAGCCACTACTGCA
GCATACCGTGTCTTAAAAAATACCAATGCAGCTGAACAATCCTCTTCCTTTTTCCCGGAATCTGAACGGCTAAGTGGAGTTCTTCTCTCCTCAACGTCAGAGGCATTGTCATACAGATCAT
AAGATCTAGAAGTTCCACCACAAAGACCACATCGATATGAAGCAGGTCTTCTTTTAAGATCAAAACTTGCAGCCCTTAGGCGTACAAACTTCGTACACGCCTTCTCCGCATTGGTCATAAT
TTTGTGAAATGCTCCACTCCGATGTATTCTTTGATGTGATAAGTAACTCATTCCATGATAAGTTATGAAAAAATTTTTAATGACATCATCAAGTGGCATGTCTCTTAATCTCGGTATTGACCG
AACAAGAATACTGAAATGGCTAAGTTTAGGAGGACGTGCTGTAACATGATCCAGTCTTAGTTTGGAAATTCTTTGATATTCGAAGTAAAGAAGGGTGCAAGCTGAGAAAGAGATAATATG
CAGGGCAAGGCAATGAACCCACAACCTGAAAACAATCATCCTTAAAAAGACAACAGCATCAAGGCCGACGGATGCCAATATCTCATTTTCGGTTGGTCCCCATGCCTTGAGTATCCAACC
TGGGAAAGGCAAAAAATTTTCAATATTGAAGGAATCATTTGGCTTGTTCCGCTCCTGGGTGAGACTCCGCCCAAAATACACGTACAAATTTGAAGGCTGTTTCCTGATCACCGAATAGATC

GAGATAAAGAACACACAGAGGCCGATGTTTATGCCTGCGGAGGTCAGCAGAGCGTCAAGCTTCATCTCAGCCTACCGATATCTGACCCAATGAGCATTTCTCCTGCTTACAGCGCACACC
GCCTAAAATGGGCAAAAACTGCCGATCGATCTTCGCATAGGGTTAGGGCGAATATCCGAGAGGAACAGAGCTGGGCATTTAGACAAATTCTTGAGATAATAAGAACCCTGTTAAGACCAG
AGTAGATCGATGTAAGAAAACCTAGATAAAGAAGGACATTTCGAGTCGGCCATGGGAGAAATGGATGAGGAAACCAAGGAAAGAGCGCGCCAGTTTGGGGTTTGGGAGGAAGATGTAG
GGGCCACAATGTCATGATACTGCACGCGTGCCTTTCGCCTATTGATTGAACC
>comp158119_c0_seq1
TTTTTTTAATTATTAAGACTTCTATTTCTAAAAACAAATCCATTATACACAAAGATACCCATACGTCTCATTTCTTTCTTTTCTTTTTTTTTTTGTTTTTGTTTTTCTAAATAATACATATGCTTA
TGCTATCGCTATTAAGGCTTTGTTTGGACGACTTTACTGTTTCTTAAAGAAATTTAAGTGAAAATTTACTTTAAGAAGCCCTTAAAAAGCCATCACAAAGGAAACCTAGCTAAGTTTAGGA
GCAGAAAGTCCAAATCTGGCTAGCAAGTACTTGCACACTGCACACACATTTCCAGTA
>comp158162_c1_seq4
CAAGGCTAATTTTTAATTTATGTATGTAGTAGAGTCAAATTACAAATAAGTATGCATGATCCAGCTACTAAGAGAGGGTATAAAAGTAGATACAGATCGAATTGCACATAGGGAGATATTGA
GTATTTATTTTAAAAAGTCTCCAGGTGTACTCAAAGAATATTAACTGAATAATTACTATGAGTGGTTGATTTCAAGAATCAAATGCTGGGTAAACTATATATTTAAATGAATTAGGAGAGAAA
AAAGTAACTAAGCTCTATTACTCTGTGGCTAAACAAAAAAAAGTGGAAGATTTTGGTCTTTATTTCCTTTTTCTGTCATCCCATCTCCAGCTGTACCACGGTTTGTTAATTTTCTTTTCTTGG
CCTTTCCTTATCACCCAAGAATGACGAAATCTATACATGCTTGTGCAATCAGTTCCATGAGTGTTGCAATGATCTGGATTTTTACCTGAAGAATCAGTTTTTTCTGCAATGGCTTTGTTGTGC
CTTTTATGCTGTGTTTAGTCTCAGAAAATCGAGAATCTTCTTATAGACTTCTTTCTATCTTTGATTGAGTCATTCAAGGATGAATCAGTGTAGTTTTTTGCACAAGCAACCAGGAATAGGATT
TGCGTAAAAATTTTCTTCTAGCTTTGATGTGGGTCCAGCTTGCCTTTCGTATGGTCCTCCAAGTCTTCTAGCATGGTGCCTTCTAACTTGATCTTCAAATTTCTCCCATGTTATGGTGCCCTT
GTCCAATTTATCTATCAATCCCACTATCTTCCGCCTGTCAGGATTTATGGTTTTAAGGAATCCTTTAAATCTTCTGTGGCCTTTCACCGTTTTCGCCATATTACCATCATAGGTATACACAAAA
ACATCACTGTTTAGGGCTACAATGTAGTCGATTGCAGCAAGCCGGTTATGGTATGGTTTGAATGGTCTCAACTCTTCTTCTGTTGCTAAGCTTTTATGTGTATGAATGTTGGGATACTCTTTC
TTTAAAGAAGCCATGCTGTTGCGTCCAAAAGTTTCTCCAGCAACTATGTAGATGTTGGTTGTTGATGGATATCCCATGGCTTTTAAGAATAAAGCAGCCTCTCGCGGTGTCAATGGACACC
CTCCTTGAATTCTCCTCTCTGCACCATTTATACTCTTTTCCTTCCAATGCTTCACTCGGTATCTCATTAATTGAAGAACTTCTGCCTCCTCCTTTGTAAGGTTGTGACTGCAACCAGTGAATG
CAAGCATGTCCTTCTCATATCTCAAATGCAGTGCAATGTAGTGATTGCTTCCATTTCTTAGCCTATCAACCAGTTCCTTCCCAAGCTCTTCAATCTGTGGTGTGTATCTCAGTGCCAGATAGT
TAACCCGGCATCGGAGCCTCTGGATCGACGGAGGAAGTCCATTGTTTGCAATTCGAGAATCGGTCTTTATGAATACCACCACCTTCCGTTTCCTTAAAATTTGGCAAAGTCTCTATAGTAC
GGGCTCTTGGAATAGGAGATTGGCTCCTTAATGAGTGGTTTGATTCCAGCATATTTGTGTGGTAGAGATTCTACAATCTCAATATCCTCTCTCAATGCATTCACAAAGTGCTTCCAGTCAAA
AATATCTTTGAATCCACTTGAATCTTTCCAGAAAGAATCATGATCTAAATGGGGAAGAACAAGTGTTGCATCCATTAACTTAGCCGCTGCCACCATATCACTTATTCCTATTCTCATTTGATT
CAGTCCGCCATTGGCATGAACTATGAGATAGCCATTCGAGGTTGAAGTTGCAGGTCTTATTTTATCTTTTGGTCTGGAGATGCATTGCTCGTAGCCTTCGTTATAAGGTTTTCTCCATATTTC
TGGGACCGGAGCGTGACTCTTTCGTATGGCCGTTAGCTTGTCCATCTCTACATTGAACGCAAGGCCGCCGCCGTCGCCGGCGATCGGATGGTGGTGCAGAACGCCGTCACCGGTCGTAAT
TCTGCTCATCTGAAAGACGCTCGCAAAGCCAAATAGCATTAACACGGCGAGCATGAGCACAAGCGCTGCCCCCCTCCTCCCTCCCCTTCCCCTCCTCCATTCCGCCGCAGGCGGAGTCTC
CTCTCCGTCATCATCGGAATATGCTCGTTCGATGTCGCCGGCCATGTTGTCCTGTACATGGCGGCGCGTCGGTCGGCAGAGCCTCGGGCTTTGCGGCAGACTTGTGCTGGTTTTCGGCCCG
ACGGCAACTGCCATTGCCCGCCCGGTCGGATTGAATGAGACTATTTATATTAACATAGAGAGAAGTGATCGAATGAGAGAAACGGATCGAACGAAAGAGAAGAAGAGGCCGGGAACCG
CTTCTCCCTCTCCTTAATAACGAAAATTCGGCCTTCAATTTCTGATTTGGTTCTATTTTTGTGATAA
>comp158162_c1_seq5
CAAGGCTAATTTTTAATTTATGTATGTAGTAGAGTCAAATTACAAATAAGTATGCATGATCCAGCTACTAAGAGAGGGTATAAAAGTAGATACAGATCGAATTGCACATAGGGAGATATTGA

GTATTTATTTAAAAAGTCTCCAGGTGTACTCAAAGAATATTAACTGAATAATTACTATGAGTGGTTGATTTCAAGAATCAAATGCTGGGTAAACTATATATTTAAATGAATTAGGAGAGAAA
AAAGTAACTAAGCTCTATTACTCTGTGGCTAAACAAAAAAAAGTGGAAGATTTTGGTCTTTATTTCCTTTTTCTGTCATCCCATCTCCAGCTGTACCACGGTTTGTTAATTTTCTTTTCTTGG
CCTTTCCTTATCACCCAAGAATGACGAAATCTATACATGCTTGTGCAATCAGTTCCATGAGTGTTGCAATGATCTGGATTTTTACCTGAAGAATCAGTTTTTTCTGCAATGGCTTTGTTGTGC
CTTTTATGCTGTGTTTAGTCTCAGAAAATCGAGAATCTTCTTATAGACTTCTTTCTATCTTTGAATCAGTGTAGTTTTTTGCACAAGCAACCAGGAATAGGATTTGCGTAAAAATTTTCTTCT
AGCTTTGATGTGGGTCCAGCTTGCCTTTCGTATGGTCCTCCAAGTCTTCTAGCATGGTGCCTTCTAACTTGATCTTCAAATTTCTCCCATGTTATGGTGCCCTTGTCCAATTTATCTATCAATC
CCACTATCTTCCGCCTGTCAGGATTTATGGTTTTAAGGAATCCTTTAAATCTTCTGTGGCCTTTCACCGTTTTCGCCATATTACCATCATAGGTATACACAAAAACATCACTGTTTAGGGCTA
CAATGTAGTCGATTGCAGCAAGCCGGTTATGGTATGGTTTGAATGGTCTCAACTCTTCTTCTGTTGCTAAGCTTTTATGTGTATGAATGTTGGGATACTCTTTCTTTAAAGAAGCCATGCTGT
TGCGTCCAAAAGTTTCTCCAGCAACTATGTAGATGTTGGTTGTTGATGGATATCCCATGGCTTTTAAGAATAAAGCAGCCTCTCGCGGTGTCAATGGACACCCTCCTTGAATTCTCCTCTCT
GCACCATTTATACTCTTTTCCTTCCAATGCTTCACTCGGTATCTCATTAATTGAAGAACTTCTGCCTCCTCCTTTGTAAGGTTGTGACTGTCTATCCTTTTAAAGGGACGGGAAGGTACCTCA
AATGCAGTGCAATGTAGTGATTGCTTCCATTTCTTAGCCTATCAACCAGTTCCTTCCCAAGCTCTTCAATCTGTGGTGTGTATCTCAGTGCCAGATAGTTAACCCGGCATCGGAGCCTCTGG
ATCGACGGAGGAAGTCCATTGTTTGCAATTCGAGAATCGGTCTTTATGAATACCACCACCTTCCGTTTCCTTAAAATTTTGGCAAAGTCTCTATAGTACGGGCTCTTGGAATAGGAGATTGG
CTCCTTAATGAGTGGTTTGATTCCAGCATATTTGTGTGGTAGAGATTCTACAATCTCAATATCCTCTCTCAATGCATTCACAAAGTGCTTCCAGTCAAAAATATCTTTGAATCCACTTGAATC
TTTCCAGAAAGAATCATGATCTAAATGGGGAAGAACAAGTGTTGCATCCATTAACTTAGCCGCTGCCACCATATCACTTATTCCTATTCTCATTTGATTCAGTCCGCCATTGGCATGAACTAT
GAGATAGCCATTCGAGGTTGAAGTTGCAGGTCTTATTTTATCTTTTGGTCTGGAGATGCATTGCTCGTAGCCTTCGTTATAAGGTTTTCTCCATATTTCTGGGACCGGAGCGTGACTCTTTCG
TATGGCCGTTAGCTTGTCCATCTCTACATTGAACGCAAGGCCGCCGCCGTCGCCGGCGATCGGATGGTGGTGCAGAACGCCGTCACCGGTCGTAATTCTGCTCATCTGAAAGACGCTCGC
AAAGCCAAATAGCATTAACACGGCGAGCATGAGCACAAGCGCTGCCCCCCTCCTCCCTCCCCTTCCCCTCCTCCATTCCGCCGCAGGCGGAGTCTCCTCTCCGTCATCATCGGAATATGCT
CGTTCGATGTCGCCGGCCATGTTGTCCTGTACATGGCGGCGCGTCGGTCGGCAGAGCCTCGGGCTTTGCGGCAGACTTGTGCTGGTTTTCGGCCCGACGGCAACTGCCATTGCCCGCCCG
GTCGGATTGAATGAGACTATTTATATTAACATAGAGAGAAGTGATCGAATGAGAGAAACGGATCGAACGAAAGAGAAGAAGAGGCCGGGAACCGCTTCTCCCTCTCCTTAATAACGAAA
ATTCGGCCTTCAATTTCTGATTTGGTTCTATTTTTGTGATAA
>comp158171_c1_seq2
TCACATCTCTATTACCTTAATGTAGATTGAGAGAATTTTCCACTAAAGTTCATTCATCCGGCCATATTATAATATCATACAAGGTTTTTCCTCCAACTATTGGATAAATAATGACCTGGATCCAT
GATATGACCTTCAACTCTCTTTATATTTTAAGCTTATAAACTTCCAACTACAATGTTAAATCCACTCATAACCCCTTTTAATAGGGTCCATTCACTACATAAAAGTTTTCTTTTTGCGGCACAC
AAATCCACCGCAAAATAATGAAATCTGTCATATACAATTAGTGGTAAATTTGCAACGAAATATAATATGACGCAAAAAGTGGCATCGTAAAATTATTTGCGGAAAGAAAAATATGCTGCAA
ATATTTTTATGATGAATTTGCCACATATTTATGGTGAAATTTTAGCCAAAAAAATCAAAAAAAAAAATCATAAATTTCGCCTAGCCGGGTATGCTGCCAGCACCTGACTAGACTGCCTGCTT
CATCTCCAGCCAGTCTGACTGGCTTGCCTGCACCTGTTCGCTTCCTGCATCTGACCGGACTATCCGAGATTCGAACCTTGCTCCAACAAAAACCATTGCCCCTCTCCACTCGCCAGAGGA
GACAAAATAGCTTAAATAGCTTTTTTTTATTTATAGTATAACTCATCTTGCAACAGATTTGTGGCAACAAATTCCGCTACAACTGCTCAAAAAATAGCAAAAAAATTTGTCGCAAATTTGTC
TTCATTTTTGACAGAAAACAATCCGTTGTAAAGTCCGTCGCAAATCCACATCAAGCTGCTAACTTTCCAGTAGTGCTTGAGATATTTAAAATGAAAAACTCTACCACATGGCTAATAGAGT
TTCCCATGGGGAAAGATTTCAACCTACTGATGTTAGACTAATTTAGGCTTCATTTG
>comp158173_c0_seq2
TTGTCATGCAATTTGGCTTCGAAATTTATTGAAGGAGCTAAGACTGCCGCAAGAAGAGCCAACAAAGATTTGTGTCGATAACAAGTCAGCTATTGCCTTGGCCAAGAATCCTGTTTTTCAT
AATCGAAGTAAACATATCGACACACGCTATCACTTCATCAGAGACTGCATCACACGAAAGGATGTGCAAATAGAGTATGTGAAGACTTATGATCAAGTAGCTGATATATTCACCAAGCCAC

TCAAGCATGAAGATTTTACAAGGATAAGAAGATTGCTCGGGATAACAAATCAAGTTTAAGAGGGGGTGTTGAAAATTAAACTTGATTAATTGGGCCTTTGATTGAGTGGGCTTTCTAATTG
AGCCCATAGCCTAGAAAAGTCTTTAGAAAACTCTAGAATTCTACTTTATGGTGAATGTTTTAGAGGATTCTAGAAATATATTCTAAAAAGAGTTGTTTAGAGTATTCTAGAATATGCAATTAA
TGACTTTTGTAAAAGAAGATAGGAGAATAATCTAGAGAGTCAATATTAGCCATTGGATTAAAATGTGTTGGCACAATCCTAAGGGGGAAGTCACTAGTATAAATAGGTGTGGAGTCTTTGT
GTTTATGTAAGAAATATAATCAAGAGTTCAAGAGTGAAATAAAAGTGAGTTTTATAAGTGTTCCTCTTCTCTTCTAAAATTCTTCCTCTTTCTATAGCCTCTTATTTTCCAACAAATTGGTATC
AGAGCCGAAGTTTCTTCCTAAAGAGATGGCGAGCAATGGTATGATCCCTTTCCAAGTCCCAACGCTCAACAATAGCAACTATGACAATTGGAGCATCAAAATGAAGGCCCTTCTTGGAGC
GCAAGATGTATGGGAGATGATAGAGAAAGGCTACAATGAGCCACAAGATGAGTCAAGTTTATCCCAATCTCAAAAGGATAGTCTAAGAGATTCAAGGAAGAGAGACAAGAAGGCTCTCT
ATCTAATCTTTCAAGGATTAGATGATGACTCTTTTGAGAAGATTTCCGATGCGAAGTCTGCCAAAGAAGCATGGGAGAAGCTTCAGACCTCTTACAAAGGAGC
>comp158194_c0_seq4
CTATAGGCATGCCGTTTGAAGTGGCCGAGGAAGTTCTTGTGATAAAACATTACTGGGATGACTGGTTATATTGCACGTAAAAAAAGACAACGCTCCAGTTCACAAAAATATGCATGTTAAA
TGATGATCATAATACATTCAAACATATGAATGTTCCATAATATTAACTACACTCTATAGGCATGCGACTCTTTAAGTCCCTAAATGAAGATGGATTGAACAGTAAAAACAATTTGTATTATCTT
CATTCAGGCCTTAATGGAAATACAGCGTACAATCCAAATATTCAATAATAAAAGCCAGGTCTAGGCAGAGAATTGCGTCCCCCTTGTGAGTTTAAGGTCACGTACTGAAGTTATGAAAGGG
AACCTCTCCTTTTAGTAAGGGTACAATTGCATAGATTAACGACCATTCTAGAGCCCATATTGATATGATCCTCATTCTCGGCTATGCCCTTTTTTCTTATAGGTTTATGGAAGGATGCAAGTCT
ACATATATCTGCTTCATGATTAATTCCTCAGAGAAGATCATCAGATGAATAATGCAAATTCAACTAGCCAAAACAAATATGAATAGTCTTGCAGAAATTTGCAGAAAAAAGCATCATAATGT
AGAAGGACGAATTTTGAACATTTTATTTAACTGAATCCTTACCAGTTACTCATTCTTGAATGATTGTCATAAGATTCGAAAATGGCATCCTAGCAGTCCACGGCCATGCCAGTGTCTAGGTG
TTATAGTGAAATTCATTAGAATGCCCTGCCTCATTATCAGTACAGAAACAGGATGGCCCTCATTTGACTGTGCTTCTGAGGCAAGCCTTACCATCAATGCATCACCAATTTCAACATTCCCA
AACTTTATTATCTGGTCACCAAGCTGTAAACCATCTTCTGCTGCTGGAGAAGCATCGCTTATTTCATCAATCACAGCAAAAGGAATCCTTGCAATCGAGTCCACAACCATGGGAGAAGTTT
GAGATGCTCTATCATGAACAGAAGCTGATGGATCTGAATGTGGTGGACGTTGTAGATCGTTTCTAGCTAACTTGGCTGAGTGCAGAACTTGCAAATTTTCATCTATTTTTGCAGTGATAGCC
TTGTGATCATTCCGAAGCGCTGATACAAAGTAAAACAAAAAACAAAAAATGGCCAACAGGTTTTTAGGGATAAAAACTAAGCATAAAATAACAGCATTCTAACAAAAAGAACCAAATTT
CACCTCCAAGCTAAAACATAAAACAATATATCATTCAAACACAAGGAATCAAACTTCACTTGGCTCTCATTATTTCAACAACAAAAAAAAATCATCAATGGCAAAAGGTTTACAAAGAAA
AGCATAAAAGGAAAATATGACAACTAAACAGGTAAGCAATTTGCTCGTATGCAAATCGTATGGCGAGATGATTAAAATATATATATCGACAGAAGGACTAATAATATGAAAGTAAGTACTAT
AGATAGCCTTCAGATGTTACTTTCAATTAAATAGCCACAAAAAAACACCAGATGTTCCGATATTCAAACAAATTTCATGTCTTACAATCTCGATATCCAAACTCAAAAACAAAATGTTCCTG
ATGTCCCCCAATCTAACCCGTCTACCTCTCATATAGGGGTGTCAAAATGGATTTTTAGGTCAAGCTCGGGTCCAAGCTTCAATTGACCCAACACGACTAGGAAAATGGGTCAGAAATCCA
AACCCAACCCGACCCGACCCAACTCGCTAACCCATAAGTAGCCATGCCCGAGCGATGCATGTTTTTAGGTTGGGTCAGAAATAAGTTGATTTCGGGTGAAAAAAAAGTTGATTTCGGGTT
TTTAGGTTGGGGTCAAAAAAAACTTGATTTCGAGTTTTTAGGTTGACACAAATTGAACCCGAAATTTTAAATGGGTCAGAAAATGAAATATAAACCCGACCCATTTGCAAATGGGTTAATC
CAAACCTAGAATTTTTTGGGTTTGGTCTAGGTCGTGTTTCGAGTCGGGTCAAATATTGACATCCCTACTCTCATACATCAAAATCTACAAGAGCCAAAAAATGACAAGAAGGCAACAAAC
TAGCTCACTAAATCTCAATGACCCTGCCTCCCATTATTTGGAACATCAAAGGATTCAATAGCACTGATAAAGTCCTCAGTTGTAAGGAGCTTACCCACACTTTTGCTCTAAACATGCCCTGC
ATTCTAAAGGCAAAGATAAACGGTACAGTGCTGTCATACCCTTGGTTTTCTTCCTCACATAATATTTTTGTTAATGAAGAAAGCTGCCACAATTTTTATTTTTCTAAAAAATAGAAGAATTTA
GATCAAAATGGAACTCCTCTAAAATCTCTTTCACCTCCTCTTTCTTCTCAAGCCAAATGATACATGGGACCATCACTGTTGGGTCCCACCCCTCTTTTCAAACATCTATAGTTTATGCTTCTA
ATTCTTTGGATGAAAGGCTCCTTTCGGATCAAATCAAAGCCATCGTTGATAGTTCTTCTCAGCCTTGAGCAATCTTTGGTGACTTCAATTGCTATAAGTATCATTTTGAAAAAAGCAGGTGG
CAATCCTATTCATAAGAGACCTATTCATAACAGCAAGCTCTGGGATTTCGAGTCTCTTATATATGATATCAGTAAAAAAAAAATATGGCATTTGTTATTTTTTTTACTTGGTTTAATCAAAGATC
AAATTATCCTATACATATCAAGTTGGATAAATTTTTTTATTAATCAGATACAACCCTAGATGTTATAAAATTTTGTCACTGGGACGGTAAGAGAAGCAATTCTCACTCTTATTATAGGATCGAC

AACCCTAGACGTTATATCATTCCCTCCTCATTCTGCAATCCAATCCAATCGCCTTATTTCAAAAAACCACATGTTATTTAAAAACTATTGGACTAAGAATGCCAGCTATTGGGAAATTCATCA
GGGTTTTCTCTCAAAACCCCATTGGAAATCCTATCTCATCACTCTACCAAAAGCTTCAAAATCTTAAAACCCTCATTAACTAAAGCCTGGGCTTCCTCCACCTACCTAGCCACCACCTCTAT
GACTGACCTCCAACAACAACAAACTTTATATCTTTAACAACTGGATCATGGTCCTTTAAACACCTTTCTAAACTATCTTTGAAGAATGTTAACAGACTACTTGTCAAATGCTCCTCCTCTTG
GGCTTCTTTGATCATCCAAAGGTCCAAAGCAAAATGGCTTTCTCATGGTGAAAATGATCTCAAGTTTCTAAATGCCAAGATGAAAATAAGACAAAACTACAACAGAGTTAAAAAGCCATC
ATCGCCTGTTGGAACTCCATACTCAACACGGGACTCTATTAATGTTATTATCTTTCACTTTCAAAAACTTTTCAATACCCCAACTAGCCCTTGGCTGACTTAACTCTCATTCCACTAGGCTCA
ATAAATCCCCCCCCCACCTCATTGCTTCTCTTACTTCCCCAGTGCAGATGATGAAATCCAATATATCATTCTCTCTAATCCTACCTCTTCAGTCCTTGGTCTTGATTGATACACATTTGAATTCT
ATAAAAGTGACTGGAATATTATTGGACCTCAAGTTTACAAGGCTGTTAAATTCTTTTTTCAACATTGCTATTTATCAATGAGAGACAAAGCCACAGCTATAATTTTGATTCTCAAAACCAATC
ATGCTAATAATATTTCTAACTTCATGCCTATTTCTATTTGCAATATATTTTATAAAGTGATTGCAAAAATT
>comp158194_c0_seq5
AAAAAAATAACAAATGCCATATTTTTTTTTACTGATATCATATATAAGAGACTCGAAATCCCAGAGCTTGCTGTTATGAATAGGTCTCTTATGAATAGGATTGCCACCTGCTTTTTTCAAAAT
GATACTTATAGCAATTGAAGTCACCAAAGATTGCTCAAGGCTGAGAAGAACTATCAACGATGGCTTTGATTTGATCCGAAAGGAGCCTTTCATCCAAAGAATTAGAAGCATAAACTATAGA
TGTTTGAAAAGAGGGGTGGGACCCAACAGTGATGGTCCCATGTATCATTTGGCTTGAGAAGAAAGAGGAGGTGAAAGAGATTTTAGAGGAGTTCCATTTTGATCTAAATTCTTCTATTTT
TTAGAAAAATAAAAATTGTGGCAGCTTTCTTCATTAACAAAAATATTATGTGAGGAAGAAAACCAAGGGTATGACAGCACTGTACCGTTTATCTTTGCCTTTAGAATGCAGGGCATGTTTA
GAGCAAAAGTGTGGGTAAGCTCCTTACAACTGAGGACTTTATCAGTGCTATTGAATCCTTTGATGTTCCAAATAATGGGAGGCAGGGTCATTGAGATTTAGTGAGCTAGTTTGTTGCCTTC
TTGTCATTTTTTGGCTCTTGTAGATTTTGATGTATGAGAGTAGGGATGTCAATATTTGACCCGACTCGAAACACGACCTAGACCAAACCCAAAAAATTCTAGGTTTGGATTAACCCATTTTG
CAAATGGGTCGGGTTTATATTTCATTTTCTGACCCATTTAAAATTTCGGGTTCAATTTGTGTCAACCTAAAAACTCGAAATCAAGTTTTTTTTGACCCCAACCTAAAAACCCGAAATCAACT
TTTTTTTTCACCCGAAATCAACTTATTTCTGACCCAACCTAAAAACATGCATCGCTCGGGCATGGCTACTTATGGGTTAGCGAGTTGGGTCGGGTCGGGTTGGGTTTGGATTTCTGACCCAT
TTTCCTAGTCGTGTTGGGTCAATTGAAGCTTGGACCCGAGCTTGACCTAAAAATCCATTTTGACACCCCTATATGAGAGGTAGACGGGTTAGATTGGGGGACATCAGGAACATTTTGTTTT
TGAGTTTGGATATCGAGATTGTAAGACATGAAATTTGTTTGAATATCGGAACATCTGGTGTTTTTTTGTGGCTATTTAATTGAAAGTAACATCTGAAGGCTATCTATAGTACTTACTTTCATAT
TATTAGTCCTTCTGTCGATATATATATTTTAATCATCTCGCCATACGATTTGCATACGAGCAAATTGCTTACCTGTTTAGTTGTCATATTTTCCTTTTATGCTTTTCTTTGTAAACCTTTTGCCATT
GATGATTTTTTTTTGTTGTTGAAATAATGAGAGCCAAGTGAAGTTTGATTCCTTGTGTTTGAATGATATATTGTTTTATGTTTTAGCTTGGAGGTGAAATTTGGTTCTTTTTGTTAGAATGCTG
TTATTTTATGCTTAGTTTTTATCCCTAAAAACCTGTTGGCCATTTTTTGTTTTTTGTTTTACTTTGTATCAGCGCTTCGGAATGATCACAAGGCTATCACTGCAAAAATAGATGAAAATTTGCA
AGTTCTGCACTCAGCCAAGTTAGCTAGAAACGATCTACAACGTCCACCACATTCAGATCCATCAGCTTCTGTTCATGATAGAGCATCTCAAACTTCTCCCATGGTTGTGGACTCGATTGCA
AGGATTCCTTTTGCTGTGATTGATGAAATAAGCGATGCTTCTCCAGCAGCAGAAGATGGTTTACAGCTTGGTGACCAGATAATAAAGTTTGGGAATGTTGAAATTGGTGATGCATTGATGG
TAAGGCTTGCCTCAGAAGCACAGTCAAATGAGGGCCATCCTGTTTCTGTACTGATAATGAGGCAGGGCATTCTAATGAATTTCACTATAACACCTAGACACTGGCATGGCCGTGGACTGCT
AGGATGCCATTTTCGAATCTTATGACAATCATTCAAGAATGAGTAACTGATTCTTAAAACAGTGAACTGGAAATTGGGGAAGAAATTACGAAGTGAACGTAAGCATAATCTCCTGATGCAT
GAATGGAACTTTCACTGACTTCCAAATTAGAAAGGAAAGGGAACCAGCAGGTTCTGCAGCAGTTGCATTAATTACGTCTCCAACAACTCTTCTTGCCTTGGCCCTGGTCCTCCAAACCTG
CCCTTCAGTAATGGATCTACAATAAAATGCTGACATCCCCATTTTCAGTATCTGTAGTTGCTGCTTTAAGGGAATGTGTCTATTCACTTGTTACCAAAAGGCAAAATATAACCTTTAAATCTT
CGTACTTCTGGAAGTGCTGAAATCCATTTGTGATCTGTTGCAACAAATTTAAGATACTTGCCAATCAGTAATCGTGGAATAATGGAACTCTATTTATTTTCTCTGCATGCAATAGCTTGTATC
GTAAATGACTAGTCATTCTCTATGACTTTTTTTTGCCAGGTTAAAAGTCGTTGCATGTGGTGTCCCTAGCGATAAGAAATGTTTTACTGGTAAAGTTACGGCTGGTTTGGACCACTTGTGCT
TGCTGCTGATGCTTTTTTCTATATATATATTTTTTAGGAGATGCTTTAGGATGCTTTCTTTACCTTTAACTCCAGTATAAAAGTAGCAAAAAAAAAAAAGGCTTTAATCAG

>comp158236_c0_seq4
TAAACTGGCTTGCTCTATTTAGCTCTGGTTGCTTGAGCTTCTTAGCCCTTTATTGATTCTGGAAGATAGCAAGAATTTGCAGAGGTTTAGTGCATTCGAAGAATGAAACAGTTGTATCTGAG
GAGCAGAAGCCTCAAGCGTCTCTTCTCCTCCCTCTCTCCCTCAGGCATCTCCAAGAAGGGCTCTACTGAAACTTCCATTGATTCCGGCAAGGCCGAGCCTGCAGTGAAACCAGCACCGA
AGCCCACATGGAGGTGTTTCTCCTATGAGGAGATTCACAAAGCTACTAATGGATTTCATCAAGATAACTTAGTTGGAACTGGAGGGTATGCAGAAGTTTACCGGGGAGAACTCGAGAACG
GCCAAGTAATTGCAGTGAAGCGGCTGACAAGAGCAGGCAATGATGAACAGAAGGAGAAGGAGTTCTTAACAGAGCTGGGAACTGTTGGTCATGTCAGGCATCCCAATATAACTGCATTG
TTGGGCTGCTGCATTGATCAGGACCTGCATCTCATCTTCGAGTTCTCTTCTCGTGGCTCCGTTTCTTCCAATCTCCACAGTACAAATTCTCCTCCAATGGAATGGAAACTCCGGTACAAGAT
TGCTCTCGGAACTGCTCGAGGCCTCCATTATCTGCACAAGGGATGCCAGAGGAGGATCATTCATAGGGACATCAAAACATCAAACGTTTTGCTGTCAGATAGCTTTGAGCCTCAGATTTC
AGACTTTGGGCTAGCAAAATGGCTTCCAACAGAGTGGAACCACCGTGCCGTCGCACCGATCGAGGGCACCTTTGGATGCTTAGCGCCTGAGTACTTCACACATGGGATTGTGGATGAGA
AGACTGATGTATTTGCATTTGGAGTTTTCATATTGGAGATGATATCAGGGAGAAAACCAGTGGATGGATCCCACAAAAGCCTGCTTAGCTGGGTATTTTGTCATCTGAATTTTTAATTATATC
TCTAATGACATCATTAGTTATATAATCTATTTTGGCTTTGATGTACAAATTGTCACAGGCAAGACCATATTTAAATGATGGAAAATTAGAAATGTTGGTAGATGCAAGATTAGGAGAGGATTA
TGATATTAATCAATTGAAGAGACTCACCTTCATAGCTTCCTTATGCGTCAGGGCAAATGCAGCATGGCGACCTTCCATGACAGAGGTGTTGGATCTCATTGAGCATGGCGATATGTCGTCGG
AGCGGTGGAAGATGCCAAAGTCGGAGGAGGAGGAAGAAGAGGATGAAGAGAATGAAGAAGAGGAGGAGAATGCAGAGGGGAGCGCGTAGGCCGGAATGGTAGAAAAATAGGTACA
AATGAATTAGGTTGCTCTCTGTGTGGATTTTAGGTAGGATGAATGTGGGTTTTTTCGGCCCTTTGTGGCACATTGTGTGTGTTCAGATTTAGGCCTTTTGTTGAAGATTGGTGTTCTGCTTCT
GTGAACTGTTGGTGTGTTTCTTTCTTTTTTTTGTTTTCTTGGAAATGTTTTCGTCGTAGATTCTTTCAACCATTCAACTTGTTGTTGGATGAAAGAATACTTTCTATATGTACTTTTTTGTTTTT
GATATAAAGTTAGATATTGTTAAACTTTTTTTTCTTATATATTGGAAGATTCCCTCAAAAAA
>comp158263_c0_seq8
AAAAAAAGCTATTATTTCTCTATCAGAAATGGGTTATGAAGTTGTTTGTTATATTTGCATTTGAATAACTACATCAAAAGTCGCTTTTTTTTTAATGGCACGCAATAAATACTATCCTTTATGC
ATGAGAAAAGGAACAAAATAGTCCCTCGACTCTTTGTGAGGGATTAAAATGGTCTTGAACTCATTTGAGGGACCAATTTGGTCCCTCAAATGAGTTCAAGACCATTTTAATCCCTCACAA
AGAGTCGAGGGACTATTTTGTTCCTTTTCTCATGCATAAAGGATAGTATTAATTGTGTGCCATTTAAAAAAAAAGCTACTTTTGATGTATTTATTCAAATGCAAATATAACAA
>comp158302_c1_seq7
GGTATACTCACATATATGCCTTTCTCCCCTGTACTAGAAATAGGAATAACTAACTAAAGTGCTTTATGCACGGCAGCAACATAAAAACAGAAAGTTAAGTAGTGGAGAAAATTCTACTGCA
GTCTAAAATAAAAGTAATAATACTGTAGTAATATTATAAGATAACGTGTTCCCTTAGCTGTAGATGATCCTCATCACATTCCCAACCTCCTCGCCCTCTCCACCTCCTCCTCATTCCCTCCTCC
CAATATAACCCCACACACCGCCGCACACGCCGCTAGCTTCCCCTCCCTCTCCTCCTCTTCCACAGCCGCGACGGCCGCAGCCGTTTTCGAGATCTCCATCAACACCTTCAGCAGTCTTGTG
GCGGCCTCCGCATCACCGGCCGGCGTTCGAGCCACCATCTCGTATCCCAGAGCCAGAAGCGCGTCTCCAGTCATAAGCTCTGTTCCGAGGGAGAATTCGGTGAGGGGATGGTGGCTGAT
GACGGCGTTGTGGGCGTGTCTGACGGCGTGGATGAGTTGGAGGGCGCAGGCAGCGTTAATGGCAAGGTTAGGGTCTCCGCCGACGAGTTCGGAGGCGGCGAGGCAGAGGGAAGGGGC
GATGGTCGGGGGAGTGGAGGAGGAGAGGAGGCGGTGGATTGGGGTCTTGACGGAGGGGTGGAGAGTGGTGGGGAGGCTGCGGAGGAGATGGGAAGATATATCGGCGTTGGCGGTGGC
GAAATAGGATTGATCGGAGGCGGTGGTGGAAAGGGTTAGGTTGGGGGCCTTGGGGCGGCTGAGCCGGCGAGGCAGGATTGCCGAAATTTGAGCTGGTTTAAAGGAGCAGGGGATTGAG
ACAAAGGCTGCTGCCATTTTTAAGAGAGAGAGAGAGATAGAGAGAGAGTGCGGTGTGGTTCTGTACTTTGTAGGGGTGGGGGAAAAGCAAGGAGCTTTATAGTAAGGTTGCGGGAGAAAT
GCGACCACCGCTGTGCCGTGAACCAGACTCAACCCTTAAGTCTTCTTTGACGCATTTTAAACGATAGAGGAGG
>comp158306_c0_seq4
AATACGGAGATTTATATAATGAGGTCGGAGCTTAAATGTCATAGTGCAATTTCTTTCATTTTAATAACCCAATTAGGTAGAGTACCCATATCCAATAATAACAAAGACTTATAACCACAAATT

CAAAGATGACGATAATAACAAACTTACATAAAGTTCTTCACAGAGCTTATTCGAACTGATACTGAAACTTCTAAATGACCAATCTGTTTCAGGCCAAGTCGTATATCCCTTCAATAAACTTT
GCCAACGTGGTGTTAACCACCGTCGCTCCGCCGTCGATAACGAGGTTATGTCCGCTTATAAAGGCAGAGTCGTCGGAGGCCAAGAACAGCACTGCCGTCGCCACATCATCTGCCCTCAG
CACCGCCCCTTTGAGTATCGCCCCCGTGCACAAAAGCTTCTCAACCTTTGCCACGTCCGCACCAGGCACTCCGCAGACCAGAGGCGTTGCCACCAAATACGGGGACACACAATTCACCC
TCACTCCTTTCCCACCAAGCTCCGCCGCCGCCTCCTTCAAAAGCCCCAAAATCGCGTGCTTTGAGGTCATGTAGGCCACCGGCGCGAACCCGCCTTGTATTCCTGAGACGCTCGCATGGC
AGATGATGGACCCCCGAGTCCCTGCCGCCACCATTGCCCGACCGGCGTGTTTGATCGCCAGCGCGGTGCCGCGGACGTGGACGGCGAGAGTGTGATCTAGGTCGGCGGGGTCGAGGTC
GAGAACACCGGTAATGGGGCCGGCTATGCCGGCGTTACTGAACATGATGTCGAGGCGGCCGTAGGTGGTAATGGCGTGGTCAACGGCGGCTGCGAGGTGGAGTTCGTTGCGCACGTCGC
AGTGGATGAAGGAGCAGCGGCCGTGAGGTGAGATTGAGGCGGCGACGAGGTGGCCGAGGGAATCGTTTATGTCAGCGATGATGACTGAAGCGCCGTGGGCAGCGAAGAGGCGGGTGG
TGGCTTCGCCTATGCCGCTGGCTCCGCCCGTGATGATGGCTACTTTCCCCTCGAGCTTCAGCTTGTTAGACGCCATGCGCTCTCTCTCTCTCCCTCTCTCTCTCTCTCTCTCTC

>comp158306_c1_seq1

ATTGAGTTTTATATTGAAGACTATTGGTGACCAAGTATTTGTCCAGTTGCTCTTATTGACAAGCAATAACCCTTGACTGGCTAATTTTTTCCGGCCTTTCCGTTGGCTAAAATAAATTTATCGT
ATAGCACATACTGATCCCAACTTTGCATAAAAACCAAACGAAACTCTCCCCATTGATGACTTCAATTTCACACATGGCTTCAAACAATCTGAGGCTGGAGGGGAAAGTAGCCATCATCAC
CGGCGGAGCCAGCGGCATAGGCGAAACCACCACGCGCCTCTTTTCCGCTCACGGCGCCTCAGTCATCATCGCCGACATAAACGATTCCCTCGGCCACCTCGTCGCCGCCTCCATCTCACC
TCCCGGCCGCTGCTCCTTCATCCACTGCGACGTCCGCAACGAACTCCACCTCGCCGCCGCCGTCGACCACGCCATCACCACCTACGGCCGCCTCGACATCATGTTCAGTAACGCCGGCAT
AGCCGGCCCCATTACCGGTGTTCTCGACCTCGACCCCGCCGACCTAGATCACACTCTCGCAGTCCACGTCCGCGGCACCGCGCTGGCGATCAAACACGCTGGTCGGGCAATGGTGGCGG
CAGGGACTCGGGGGTCCATCATCTGCACGGCAAGCGTCGCCGGAATACACGGCGGGTTCACGCCGGTGGCCTACATGACTGCAAAGCACGCGGTTTTGGGGCTTGCGAAGGCGGCGGC
TGCGGATCTTGGTGGGAAAGGGGTGAGGGTGAATTGTGTGTCTCCGTATTTGCTGGCGACGCCTCTGGCCTGCGGTGCGTATGGGATGGACGCGGCGAAGTTGGAGGAGACTTCGAGCG
CGGGGGCGATTCTGAAAGGTACGGTGCTGAGGACAGATGACGTGGCTACGGCGGTGATGTTCTTGGCCTCCGACGACTCAGCTTTTATAAGCGGACATAACCTCGTCATCGACGGCGGA
GTGACGGTGGTTAACACCACGTTACCAAAGTTTGTCGAAGGGAATTTCCACTTGGCCTGAAACAGATAGTTAATTTAGAAGTTTGAATAAAAGTTCGAATAAGTTCTGTGCAGAACTTTA
TGTAAGTTTGTTTTCGTCGTCATCTTTGAATTTGGGATTGTAAGTCTTTGTTATTATTGGTTGGGTACTCTACGAAATTGGGCTATTAGAATGAAAGAAATTGCACTGTCACATTTCAGCTCC
GAGCTCATTAACGATCCTATGTAGTACGTAAACATAATTTTTTTTTTTTTTGAAAAAATGATGTTGAGTATAAGGTTTAGTTAG

>comp158306_c1_seq2

ACTCTATATAACAACAAAGCTCTCGCCATTCCACAACAAGAGAGAGGGAGAGAAATATCAAATTAATGGCGTCTAACAAGCCGAAGCTTGAGGGGAAAGTAGCCATTATCACAGGCGGA
GCTAGCGGCATAGGCGAAACCACCACCCGCCTCTTCGCTGCCCACGGCGCCTCAGTCATCATCGCTGACATAAACGATTCCCTCGGCCACCTCGTCGCCGCCTCAATCTCAGCTCCCGGC
CGCTGCTCCTTCATCCACTGCGACGTCCGAAACGAACTCCACCTTGCGGCCGCCGTTGACCACGCCATTACCACCTACGGCCGCCTTGACATCTTGTTCAGCAACGCCGGTGTGGCCGGC
CCCCCTACCGGAGTCCTCGACCTCGACCCCGCCGACCTAGATCACACTCTCGCAGTCCACGTCCGCGGCACCGCGCTGGCGATCAAACACGCTGGGCAGGCGATGGCGGCGGCAGGGA
CTCGGGGGTCCATCATCTGCACGGCAAGCGTCGCCGGAATACACGGCGGGTTCACGCCGGTGGCCTACATGACTGCAAAGCACGCGGTTTTGGGGCTTGCGAAGGCGGCGGCTGCGGA
TCTTGGTGGGAAAGGGGTGAGGGTGAATTGTGTGTCTCCGTATTTGCTGGCGACGCCTCTGGCCTGCGGTGCGTATGGGATGGACGCGGCGAAGTTGGAGGAGACTTCGAGCGCGGGG
GCGATTCTGAAAGGTACGGTGCTGAGGACAGATGACGTGGCTACGGCGGTGATGTTCTTGGCCTCCGACGACTCAGCTTTTATAAGCGGACATAACCTCGTCATCGACGGCGGAGTGAC
GGTGGTTAACACCACGTTACCAAAGTTTGTCGAAGGGAATTTCCACTTGGCCTGAAACAGATAGTTAATTTAGAAGTTTGAATAAAAGTTCGAATAAGGTTTGTTTTCGTCGTCATCTTTG
AATTTGGGATTGTAAGTCTTTGTTATTATTGGTTGGGTACTCTACGAAATTGGGCTATTAGAATGAAAGAAATTGCACTGTCACATTTCAGCTCCGAGCTCATTAACGATCCTATGTAGTACG
TAAACATAATTTTTTTTTTTTTTGAAAAAATGATGTTGAGTATAAGGTTTAGTTAG

>comp158332_c1_seq1
CAAATTCTCGTTTAAATCTAATAGAGAAAAAAATTAACTCAATCTTCCTTCCAATGAAATTCAAATTGGGAGAGAGTCAATAATGAATATACAATCCAACAACATATGGAAAATAGATAATC
TCTGTCATCAAATATGCACATTAAAGGAACAATCAAACAAATTCATTCATTATTGCCTTGAAATGAATTAATTACTTTACTTCTAATTCTTCTTCTCAGCAACAGTATCTTCAGAAGCCTTCT
CTTCAAATGGGGCTACCACCACCTCCTCTTTGACATCCTCTCCACCTGCGCTCGCTTTTTCATCTTGAGATCCGGATCCTTCAGCAACGAGCTTTTTCGCCTCTCCATCACTTACAATCACA
ACCTCCTTCTTTACCACTGAAGAATCTTCAACTTCCGAGATAGAATCAGCCAGCGAGTGATCATCATCCTTGTTCAGAACATTTCCAGCAGCAATAGCTTCCTCGTCCTTCCTCGACGAGA
AATAGCGGTCCGGCGAGTCTTTGGAGATGAGAGGAGCCGCCACCACCTCTTTCGTCTCAGAAGCAGCAGAATCAACCGGCGGCAACGAGGTATTCGACGGTAAAGGATTGTTTAATTGA
AGGGATTTGGCGGCGACTGTGTTGCCGACGGCGACCTCGTCCTTGGAACCACCGCACCCCATGGTTTGAGGAAGAGAGAGATCGGCGGGGAATTGAAGAAGGATTTGGGGAATTTAAG
GGTTTTGGAAGGGATATAGATTGGTTTTGTTTATAGGTTTTGGCAATGATCTGTTTATGGGGAGAATGGGAGGATGGCTTTGGATTTG
>comp158385_c1_seq5
AAGGCTGGCTAACCCAGGTCGGTCGAGCTGAATATGACCGCCGATTAGAAATCTGGAAACAAATTGGCGCTCCGCAAGTGCGGCCACCACTCTCCGACGAAGTAGAATCTTCCATCTCC
GGCCTTCTCCTTACAAGAAACTCTTTTCAACCGGAATCCGGCATTGGCGAAGGAAAGAGGGTGGCCACTGGCGCATTACCTGTAATTGCCTTAGCCATCAAATCGGGCTCGTCGGCGCTG
CCGCCAACAACCCTTTCTTGCACTGACAGAACTCTTCCGCCTCGTTACATGAGTCCGAATTGCTGGAGAAATGCTTCGCCTCCGCGG
>comp158391_c0_seq1
ATCATCGCCTTCTTCTCCAGCCTCGACGTCCTCGCGAGTGTGTCCTCCATTAGCACCCTCTTCATCTTCATGCTCATGGCCGTGGCGTTGCTCGTCAGAAGATACTATGTGAGAGAAGTCAC
TCCAAAATCCAATCTTTTGAAGCTCAATTGCTTGCTTTTCTTGATCATCGGTTCATCTGTTGGGATATCAGCGTACTGGAACTCAGGGGCGAAGGGATGGATTGGTTATGCAATTGCTGTGC
CGCTCTGGTTTTTGGGCACGCTTGGGTTGACGGTGGTGATTCCTCAGCAGCGGACGCCTAAAGTGTGGGGAGTTCCGCTGGTGCCTTGGCTGCCATCGGCCTCCATAGTGACTAACATTT
TTCTCATGGGATCTCTTGGATACCAGGCATTTGTGAGGTTTGGAATCTGCACTGTGGTAATGCTGGCCTATTATGTGCTTATTGGTGTCCATGCGACTTATGATGTTGCTCATGAGGAGGAA
GATGATAAGTTGGAAGGCTTGAAGGTGGAAACTGGCAGTCGTGGAGCTTAGATTATAAGATATCAGGTACTTTTCCGTTAAAAAAATATAAATTTATAAAAAGAAGATCAAAGAAATTTTA
TCAGATATTCAGATTATTGATTTTTATTTTTTTCCTCACAAGAGGGAGGCTGTGCAGCAGAGTTCCAACTCTCAGCTGTGCTGCTATAAGTAATGAAGCTCGAACTGAGCTGAAGATCTCCA
TAGAAACAAAGCCTGCTCTATAATTTGTTCTTCTTGACTTTTCCTTTTATTTTATTTCAAAGGTTTGAGAAGCTATGTATCTGTTATGGAGAGCTTTAGTTCTTTGTTCCTTGTTTTATGGCTG
CTTGATATCTTGCAATTGTAATGTAAAGTCTCCCTGCTAAAAAGTTGTCAGAATCTGGCTTAAATTTTATGATCTGAGAATCTGTGATCATGTTTGGAAACTTTTATGTTTTTTAACATAGAA
AGCG
>comp158429_c0_seq9
TTACTGTACTGTATTAACAGAAAGAGAAATAAAGAATACTTTTCCTTTCACTGTTTACACGTCTTCTTCTTCTTTCCAATCTGTTCTTGCGGGAAACTTAAATCCAAAGTTTCTACAAATATC
ACATATAACATCTTTAAAAATATAAAAAAAGGGTAAACTCTATTTTGTGGTAAAACAAAGAACAAGGCCTAAAATTCATTTAATTAAGTTCACACTGAAAAAGCAATCCCACTTCATTTTCA
TAGTTTCAATCAAAGAACAATTTTAACATTAAACATGTAATTCATTTTGCGTGTAACCATTTGAATCCGTGTATGGGGTAGTTGTTACGAACAATTACCTGTTGCACGACACGAATATCCAA
CTGATTACTGTCATTGCTTATGCAAATAGGGATCCACAATGACTTGGGGAATGAAATCTGGTTTCAAGTACCGTGAAAGCAAATCCGCATATAATTCATAACGAGCAGTCATTCGAAAGCC
CCATTTATCCTTTAACTGTCTGCAAAGATTCAAGTCCATGTCAGAGACTAGCAAACCATCTCTATATCTTGACAGAGATGGAGTGCAAGATGCATCTGACATGGACTTGAATCTTTGCAGA
CAGTTAAAGGATAAATGGGGCTTTCGAATGACTGCTCGTTATGAATTATATGCGGATTTGCTTTCACGGTACTTGAAACCAGATTTCATTCCCCAAGTCATTGTGGATCCCTATTTGCATAAG
CAATGACAGTAATCAGTTGGATATTCGTGTCGTGCAACAGGTAATTGTTCGTAACAACTACCCCATACACGGATTCAAATGGTTACACGCAAAATGAATTACATGTTTAATGTTAAAATTGT
TCTTTGATTGAAACTATGAAAATGAAGTGGGATTGCTTTTTCAGTGTGAACTTAATTAAATGAATTTTAGGCCTTGTTCTTTGTTTTACCACAAAATAGAGTTTACCCTTTTTTTATATTTTTA

AAGATGTTATATGTGATATTTGTAGAAACTTTGGATTTAAGTTTCCCGCAAGAACAGATTGGAAAGAAGAAGAAGACGTGTAAACAGTGAAAGGAAAAGTATTCTTTATTTCTCTTTCTGA
TAATACAGTACATTAACCTATTTATAGAACGTATATGTAATGGCAATATATAAGGTAATATACATGGCAACATATTCTAATGTCAAGTCTTATTTGTTGTTACTAATAATATTCCATGCTTCTTCG
GTCATCGCATTCGCAAGTAGCCGAGCATCATTAGTATAAAAATGACAATTAGAGCAAAGGCAGCCATGTTGAGCTATCGATTAGAAGTAGGCAACTGTTCTCTTGAACTTCACAAGTTTCA
CCATTTGAAGTTCTTTGACTTCCTTTCTAAACATTCCTAGCTTTGAACTAGCTGCTGAACATTCCTTGGGAATGAGGCTTGCAGATTTCCTTGGTATAGATCCTTCGATGCAATAACATTGAA
AGCATCTGTAGGATGGTAAGGATCCCAGAACACATACTCTGACCGGTTTTGGCATGGAGTAAAAACTGGTAAACAGAGGATCTGACCTTTCGATTTGCCGCCACCACAGCAGGCTGTGTT
TTGGTTCTTGAAGCCATAGTTGGCATAGTTTTGGATGACATTGATGCCAAAGGAGAATGCATCCCAGTAGAGGAATGTTGATTCCTTGAGCTGTGCATTGTACTGATCAACCATCTCCTTAA
GCTTTCTGTTATAGTGAGATACCAATAGATTGACTGATTCAACACACGCACCTGTATCACTTTCCTGCGCTGCCAACTGGTTCGGTAGGCAGCCGGTAGGACTGATATTGACTATCAAGAAT
TTCCTCACACCCAAATCATACAAATACTTGAGTTGTCTTCCAAATTGTTGAATGAGTAGATTGGTGAAAGCTTCAGGGGTGTATTCGTAATGTATTTTTGAAAGTGGTAAGAGATAGTTATT
GATATAGTCATTGCTGCCTTGAGCTACAAAGAAGAGAGATCTTCTTATGTGGTCTTGGGTCTTGTCAAGAAGCATTGTGCTTAGTTCTTCTATTGTTGCATTGAAGTTTTGTAGCTGTTCATC
CATGGGGATGCGCATTCCAAAATTTTCTCCAGTGTCATTGAGAATTCCAGATGCTGCAGAGGCATAGTTAACTCCTTGAAGTATTGCAATGCCATGTGTGTCTGGATTGTTGAATGCTGGAA
TCAGAGGAAGTCCCAAAAGATAACCCAAATGATCAACGAGTGTTCCACCATTGCAAAACCTTCC
>comp158429_c1_seq2
AGGCTTGATGTTATTGTTGTTGTTTATGAATTCATATCATAATTTAATATATTGCCATAAAACTCAACATGAGAGTTAAAAAAAAAAAAAATTAAATTGACTTGCATGATATACTCTATGTCCAT
AAGGGCACTTCCATCCATATATTCATGCAACCCCGCACGTGTGTAGCACACATGCTCGACAGCACTAAAAAATTAAAGAGATTTTTCCCTCGTAGGTTATCTTCTGGGGCTTCCTCTGATTC
CACCATTCAACAATCCAGACACACATGGCATTGCAATACTTCAAGGAGTTAACTATGGCTCTTCAGCATCTGGAATTCTCAATGACACTGGAGAATTATTTGGAAAGCGCATCCCCATGGA
TGAACAGCTACAAAACTTCAATGCAACAATACAAGAACTAAGCACAATGCTTCTTGACAAGACCCAAGACCACATAAGAAGATCTCTCTTCCTACTAGCTCCAGGCAGCAATGACTATAT
CAATAACTATCTCTTACCACTTTCCAAAAGACGTTCCAAATACACCCCTGAAGCTTTCGCCAATCTACTCATTCAACAATTTGGAAGACAACTCAAGGATTTGTATGATTTGGGCGCGAGG
AAATTCTTGATAGCCAATATCAGTCCTATCGGTTGCATACCGAACCAGTTGACAGCGCAGGACAGTGATACAGGTGCGTGTGTTGAATCAGTCAATCTAATGGCATCTCACTATAACAGAA
AGCTTAAGGAGATGGTTGATCAGTACAATGCACAGCTCAAGGAATCAACATTCCTCTACTGGGATGCATTCTCCTTTGGCATCAATGTCATCCAAAACTATGCCAACTATGGCTTCAAGAA
CCAAAACACAGCCTGCTGTGGTGGCGGCAAATCGAAAGGTCAGATCCTCTGTTTACCAGTTTTTACTCCATGCCAAAACCGGTCAGAGTATGTGTTCTGGGATCCTTACCATCCTACAGAT
GCTTTCAATGTTATTGCATCGAAGGATCTATACCAAGGAAATCTGCAAGCCTCATTCCCAAGGAATGTTCAGCAGCTAGTTCAAAGCTAGGAATGTGTTAGACCCGACGGGACCGGCTTAC
GCGAAGTTCGGCGGAAGTCTCCGCCTCTTGTACGCAGGAGCCGACCGCTCGGTCTTCACAAAATCAATTTGGAAGGATTTCTTAGGGAGTTAGAAGGGAAATTTGAATGTACGAACTGT
TTATAGATTTCCTTTTGATTTGTACGCATTAAATAGTTTGTTTTCCTAG
>comp158429_c1_seq8
ACATTTCTCTCCTCGACAATGAAAGCACACAACTCCACCTAATGTCAACGTACTTTTTCATTAATCAAAGAAATAAATGACCTTAAAACTTAACCCAGAAAGAGTTCTTCCCTCTCATTCA
GCTTCTAAAATTGAAGAAGACAGAACGTGAAAAAAAAAAAAAAGGTGTCTTCCTCTTCCAGATTCTGTCATTTAATAAACAAGTGTTATCAGGATAAAACATGCAATTTACTGAAGTCATTT
ATTCCTATGCAGCAGAAATATGGGAATTAAGCTTTATATAAGGCTTGTTGCTATTGGTCTTTGAGTAGCCATGGCTTCTCTGTTCTTCTTCTTCTGTCAGCTTCTTTTGAGTTTGGGAAGGAG
TTTCGTTGCCGGAGCAGATCAGGTTCCGGCCATGTTCGTGTTTGGCGATTCATTAATCGACCCAGGAAATAACAATCAGCTATTAACACTGGCCAAGGCTAACTTTAGACCGCATGGTATA
GATTTTCAAGAGGGGACGACTGGAAGGTTTTGCAATGGTGGAACAGTCGCTGATCATTTGGGTTATCTTCTGGGGCTTCCTCTGATTCCACCATTCAACAATCCAGACACACATGGCATTG
CAATACTTCAAGGAGTTAACTATGGCTCTTCAGCATCTGGAATTCTCAATGACACTGGAGAATTATTTGGAAAGCGCATCCCCATGGATGAACAGCTACAAAACTTCAATGCAACAATACA
AGAACTAAGCACAATGCTTCTTGACAAGACCCAAGACCACATAAGAAGATCTCTCTTCTTTGTAGCTCAAGGCAGCAATGACTATATCAATAACTATCTCTTACCACTTTCAAAAATACATT

ACGAATACACCCCTGAAGCTTTCACCAATCTACTCATTCAACAATTTGGAAGACAACTCAAGTATTTGTATGATTTGGGTGTGAGGAAATTCTTGATAGTCAATATCAGTCCTACCGGCTGC
CTACCGAACCAGTTGGCAGCGCAGGAAAGTGATACAGGTGCGTGTGTTGAATCAGTCAATCTATTGGTATCTCACTATAACAGAAAGCTTAAGGAGATGGTTGATCAGTACAATGCACAG
CTCAAGGAATCAACATTCCTCTACTGGGATGCATTCTCCTTTGGCATCAATGTCATCCAAAACTATGCCAACTATGGCTTCAAGAACCAAAACACAGCCTGCTGTGGTGGCGGCAAATCG
AAAGGTCAGATCCTCTGTTTACCAGTTTTTACTCCATGCCAAAACCGGTCAGAGTATGTGTTCTGGGATCCTTACCATCCTACAGATGCTTTCAATGTTATTGCATCGAAGGATCTATACCA
AGGAAATCTGCAAGCCTCATTCCCAAGGAATGTTCAGCAGCTAGTTCAAAGCTAGGAATGTTTAGAAAGGAAGTCAAAGAACTTCAAATAGTGAAACTTGTGAAGTTCAAGAGAACAG
TTGCATACTTCTAATCGATAGCTCAACATGGCTGCCTTTGCTCTAATTGTCATTTTTATACTAATGATGCTCGGCTACTTGCGAATGCGATGACCGAAGAAGCATGGAATATTATTAGTAACA
ACAAATAAGACTTGACATTAGAATATGTTGCCATGTATATTACCTTATATATTGCCATTACATATACGTTCTATAAATAGGTTACTGTACTGTATTATCAGAAAG
>comp158462_c0_seq13
CCTTAAGAGAGCACACTTTGTTTGCCAATCAGAAGAATTGTTCTTTCGTTCAACCCAAAGTGGAGTATTTAGGGCATGTGGTTTCACAAGAGGGACTGGCAGCTGACCAGAGTAAGATTG
AGGCCATGCTCAGATGACTTGTACCCAAGACTTTAAAGGAGCTAAGAGGATTCTTATGACTCACAGGCTACTACCATATATTTGTGAAATGATACGGTTCTATAGCGAGGCCATTGACAAA
ACATCTCAAGAAGGATAACTTTAAGTGGGGATAGTCATCCACCCAAGCTTTTGAGGAGTTAAAGGCTATGACCAGTGTGTCAATATTAGCTCTTCCATATTTCAACCAGCCCTTTCTAGTTG
AGATAGATGCCTCAGGTCTTGGGTTGGGAGCAATACTCATGCAGAAACAAAGATA
>comp158469_c0_seq1
TTGAATCACAATACATAACTTATATTTCCATTACTAAGAATCATTAGAAGAAAAAATAAATAACAACATAAAATATTACATTGTCAAAAATGTGGCAAAAGAAACCAAAATGTTATAAAGCA
TAAATCAGCAATATTATGGAAAATCGTGCAAACAATAGGGAAATAATGAAATGTGTCACTTGTACTCAGGAGTAGTGGCGATATTGTTTGGGGGATCAGATACATACATTAGGCTCTGCAAT
CCCGGTTTTAATTCCCTGTTATAGAACTCCTCATACTCTCCTCTGTCACCTGCTTTCTTCTTCACCTCCACCATCACAAGTGAAGAAGTCAACTCAAATATCTCTGCGGCTATTGTCAATGGC
CCCTTCTCTCCCTCCCTAGTCCCTTCCAAGCTCACCTGGAAGTCTTTCTTCCTCACTGTGAAGCTCACCAACTTTGCAATGTCCTCCAATTTTGATATAATCATAGGCACGGGCTCACCAGA
TACAAACCTTGCCTCCTCCCCCCTCTCTTCAAACAATCCAGAAAGATTAAAACCTTGCGAGAAGGATATGATGTCGAAAGCATTCAGGCTGGCTGGTCTGGGAAGCCCCATTATCAGCCC
CTTAGAGATGTACATTGTGGCAGGGCAGGAGGCCGCGGAGCAATCGGAATCGCAGCCAGAATCCGACGTCTCATCATGCTTCTCTGATGACGGAGTCTCCGGGGCATTTGTAGGAGGCG
ACTCATCGATTTCATCGATGCTGTGGAGCTTGTCATTCTCGATGTAGAATCGAATATGGCGAAACCCTTTTTTGAACCACAGATTCTCCATGATTTCAGGAATGGTGATCCGCGTCTGGGGA
TTCGTGTCCAGAAGGCGAAAGAGAAGCTTGCTGAGATCGGGGGAGAACCAGCTAGGGCATCGAAATGCCCCTTTATAGATTTTGCGGTACATCGCCAAGACGTTCTGATCGTGGAAAGG
GAGATAGCCAGCCATAAGCACGAAGAGAATGATCCCAGATGACCAGATATCAACCCTAGCCGCGTCGTACCCTTTCCTGGAGAGCACCTCCGGCGCGACGTACGCCGGAGTACCGCAAA
AGGTGTGGAAAAGCCCATCCTGCCGAATCTGATCGGAGACCGCGCTGAGCCCAAAATCAGAGACTTTGAGATCCCCCTTTTCGTCAAGTAGTAGGTTCTCCGGCTTGAGATCCCGATGG
AAAACGCCGCGGGCGTGGCAGAAACAAACGGCCGATATCAATTGCTGAAAGTATTGGCGGGCCGCGTCCTCCTTTAGCCGGCCTTTAGCGACTTTGCTGAAGAGCTCCCCTCCGCGGAC
GTACTCCATCACAAAGTAAATCTTGGTCTTGGTGGCCATGACCTCGAAGAGATGGACAATGTTGGGGTGGCGAACACGGCGGAGGATTCCGATCTCGCGCTTGATGTGAGCGGCAAGGC
CGCTCTTTAAGATGCGCTCCTTGTCAAGGACCTTAATGGCGACAGATTCGTCGGTCTTCAAGTTTTTCGCGTAGAAAACCTTGGCGAACGTGCCTTGGCCAAGGAGTTTCCCCAGTTCGT
AACGGCCAAGGATGACAGCGCTCTGCCTCTCCTTCTTAGGCTTCAGCGACGGCTCCACCGCCATGGGAGCCATAGCTCCTCGAAAGAGCAAAACTTTGTTCTCCTACGTTTATAGAAGGG
AGGGGAGGGTATAGTGGCCGTCTCCAGAGGTGAAAATAGAGGCGACACGGTAGGTTGGAATGGGCGAATTGG
>comp158513_c1_seq1
CTCTCTCTCTCTCTCTCTCTCTACGTCCCCCAACAATGCACTTACTCCACCTTTCACAGGCCATCTTCATTGAAAGAGAAGTCTGGCTCATCCGGCTTCATGATCAGCTTCGGCATCTTCAC
ACTGAGGGAAACAGTATCGTCCCGCTTAAGCTTTTGATTTGCTCCTCTCTTCGTGTTCTTTGCAAAGTTGGCATTGCCTCCAAAAGCACTGTCACCAACATCCGCATATTCAGCGAGCAAT

GGAGCATCGGAATCCTCATTAACCGGTATCGTTTCAGCCATGGCTGGTGAGTAGTAGAGATTCTCTTGCTTAGCCAGTTCCATTCCTAGCTTCTTCTTCTTGTATCTTCTCCATGCAGTTTGG
ATGAAGCAGGCTCCCCATGTCCTCCATTGATGGGAATAGAACCTGAAAGCATGCTGTAGCTTCTTGTTGTGGAGACGTTTGAACTGATTTGCAACAAATTTTAGGTCTTCAGCTCGAAGC
GCAAATGCCTCTACTTCGCTAAGGGCTCTAACAGTTCGGGTGGAGAGTGGTAGATTGACGCTTGGATTTGGCATGAGACCCCATGTGAGAAGCTCTTCACCGCAGAAATCTCCGGGTCTT
AGGGTTATGGAGTTGAAAAATCCAGTTCTTCCGCCGTCTGTAGTTGAGCTCTCGAGCTGACCGCGGATAATAAAGAGCATCTCGTCCACTGGGTCACCTTCCCGGACAATGTAAGTATCTT
TGGTGCTCAGGGATGAGACTAGTCGTTCGCATATTGCATCTAGTAGTTGGTCATCCATTTGGGAGAAAAATGGAACCCTACGAACTAGAGCAAGACAAAGATGTCTTCTAATTTCACGAC
GAAGGTCCAAAGGTAAGGAGACCAAAATTGAGTCTTCATCAACACCTCTTGTAGCAAGCCATTTGTACTGAACAAACCTCCTAACCCGTTCTTGCAAATCTGGTGGTAGTTGACGATGTC
TCATCCATTCCTCAATGTCTCTTCTTTTTAATCTCCATTCTTCAAGCCTCACAGTAATGGATTGCAAATAGGTCTGCATATTTCCTATCAAATGTGAAAACAGAACAAGGCCCATTATACATAT
TAGAATGGCAAATAAAGTTTCCCCAATGTAAGTGCTTGTAGCCAAATTCTGTCCATACGAGCTCAAATTTTGCAAGCCCCACCAGAGACAATACAGGTACTTTGCTATGAAGGTTGCTCCA
ACAACTTCATTATTCAATGCATCAGAGAACATGCCAAAATTGAAATTAACATTACTACTAGTTGCATCACAATTGCTTATCACTTTAGTAGCATTGATCCAGGCTTTTCGCTCAGGTAGATTA
AGACTATTACAGTCCAGAAAACTTGGATTGCACAGTGGCATCTTCATGGTGCCATTCTCTTTCATGCATTCTGTCAACCAGCAAGTATATTGGCGTTCCATTGATAAAAGATACCATAAAGC
ACCTAAAACATGGCTCGCAAGCATATATAGGAGAAGATTGTACGCAGCTCCTGCCCATGCAGTTTTCGTAACAACTCCGGTGGCTTTGACGATCCGTGAATTAAGAGGAAATATAAGAAA
CAACCTTGGAATGTACTGAATGAAGACAATCATTGACAGTGTGTTGTTGGTGTGATTGGCTGATGAGCTACTCACTGCCGGTATTACAAACCAGATTAGTATCTGAGGAATCGGAAGCATG
GCAGCAAGATCGATGAAAAAATCCCCTTTAAGGTATCTCATGGCGATCTGATGGGGGTCCTTCACAAGATCACCCCTCCCAAAAACCCTGGAGCTAGGCGCAACAAAGGCCGTGCGAAA
CTTCAGAAGCATGTGAGCGAGATAAAACAAGTCAGCTACCGTGCGAAAGAAGGTAACAATAATGCCGATGCCCTGATCAATCCGCATGCAGGCGGGACCGCCGATGAACATTAGATAGA
AGTAGAGGGGATCGAGGAAGAGTGCGACGAGGCAGGAGATGAGGAAGATACGGTTCCATTTGAGGACGACATCGCCACCAGGATCGAGGATACGGCGAGGCCGGTGCCACTTTTTGCG
GAGGAGATTCGGTGTTCGATAACTCCCCTGCCTGAAGGGGAGGAATTTCGACGCCGGCGAGGGAAAGCCGAAACCGAGAGCGTTCATCTTCCATGAGGAGGTGAACGGGAAGGAGGA
GGAGGACGCGGACATTGAGGCGGGCCAGAAATTATGTCACCTCCATGTTTCTGACGGGTTTGAGGGAAGCCGGTAGTGGCCGAGCGTTTCTCGGAGGCGACATTGTTGGGGCAATGCAG
AGGTGATTAGAGAAACAGCGCCGGTTTATTGAAATCTTTCTTTTTTTATATCCCGCCAACAACCGTTTCCTTT
>comp158532_c1_seq19
TAATTGTAAAAACTGCTATAATATTCTCAAATTAATAAGAGCTTTCGTTGTTCTTAAAAAAAAATATGACTCATCATAACCCTCTATAAAAAAACAAACAAACAAACAACAAACAAAACAAA
TTTCTCAACAAGGAGACGCACAGATCTGAAAAAGAGCACATTGGAGATGAACACCTTACCGATTATGAGGAAAAATATAAGTTGTGTTGCCAGCATTGTAAAGAGGAATAAGAAACTCA
TGCTCTGGACGAAGTAGAAGAAATGTGACCTGCAATTCCGTTACTTCCATAAAAAAAAATAAAAATAGCCTTTCATTCTTTCCCAAAGGGCCTAGCTTATTTGATCCAACATTTGTAGAAAG
AAGAAAGACCAAGAAGGTAAGAACGCAGATAATC
>comp158640_c0_seq2
GCAACAACGATTTGGTAGCCAAGGCGCAAAAAAAGCAAGTTTCCTTCATCTTCCAAAACATTTATCCCTATATTTATACCATCCTCTCCGACGATCCATCGATCGATATATTTAACGATGGAT
CAATCTATTTCCTCTCTACATATGAGGAAGCTTTGCCCAAATCTAGAGCGAGAAGATGCCCTTGACACCGTCCTCGAGGTTCCTTTACCGGAGGAGATGTTTCCCAACCGGAAGGGAGGA
TCGTCGTCGTCATCGACCGACCAAGACAATCACTACGCTTGGCTCAAGTCGCAAGCTTTCAACAAAGCCACTGACAGCCCGCCGCTGATGCTTTCAGGCCGGACCGCCGAGCTGCAGCT
CCTCCTCAGCGTCATCGGCTCGCCGCTCATCCCTATGCCCGTGCCGTTCGACACCGCATTCAGTAACCGCTCCGTCTGTGAATCCTCCATTGGCCTAGACCCGCGCTCAACGGCAAATCTC
TTCGTCGACGCAGTCTGCATCGGCGAAAAAATCATAAACGGCGAAGAATGCTTCATTCTAAAGCTCGAAGTCAGCCCCAATGTCCTCAAATCCCGCAGCGCATCCAGCTTCGACATTATC
CGCCACACCGTTTGGGGCTACTTTAGCCAACGCACTGGTCTCCTTATGCAGCTGGAAGACTCTCATCTCCTCCGTATGAAATCTGGCTGCAGTCGCATCTCCGCCTCCACCCAGATCAGCT
GCAAAAGCATCAACAATGATTCTTCCATATTCTGGGAAACTTTTATGGAATCCATCATTGACGATTATCGCTACATTGATGGCATCAATATCGCTCACAATGGCCGAACCGTCGTCACTCTGT

TCAGATACGGTGAGGGATCGATTAATCATAAGCGCCGGTTGGAGGAGTGTTGGACGATTGAGGAGGCCGATTTCAATCTGCATGGCCTTGCGATGGATTCTTTTCTTCCACCGTCAGATCT
TAAGGTCGATTGCGACTAGAGTTTGTGAGTTTTTTCCCCTTTTCGATTAGTTGATTTTTTTGTTATGATGTACTTTTAATTGCTCTTATTATTTTGTTTCATTTTAAGTAATTAGGAAATAGTGA
AGAGGGAGGAACTGTAAAGATGGAGAGCAGGGTTTCCATTGTTTTTATTCGTTTTCATTTAACACCAAGAAAGGATTTGTAAGTTAAATCCAGAGTTTGGAGAAATTAGTGAGAAAGTGT
AACTCATTATGTGTTGAATTGTTATTGCGATGCAAATCATTCATATCAAAAGAGTATAAAGTAGTACAAATTAAAAGCATGTGTCTAGAAAAGATAGAATTACGAGGC
>comp158656_c1_seq12
TGGTAGCCGGTGGCGGGTAGTCCACGGGATGACTAGTGAGCATCCAACTAGCGTGAACAAGATGCGATTTAAGTAATCCTCCACGGAAGAATCCACGTCAAAGTTTACGTCGAAGAAAG
GGGATTCAAGGACGTTGGTTCGTCCTTCAAATCAGCAATCCCCTTAGCCTCAATAGTCTGGACAGCAACCTGGAATAGCTATGGAATTCTGGACGGCAACGGAAACCTTTGGGTTGAGAG
ATAATGTGTAGATATGCCCGCAGAAATCTGTGGACAGCAATGGGGCCTTCCTGGAGCAGCAGTTTTGCAAAGGTATTGGGCTAGAATTCAATGATGAAGACGTCTCAAACTCTGGACAAC
AGGATCACTCCAGAAGGAATTCTGTTTCAGCCATCACGGGGAAGATCTGGACGGGCTAACAAGTGGAGGAAGGGGCAAGGCTTTGGGCCAGATTTGGGCTCCGTAGTTAGGAAGCCAA
GGAGGGAAATTCTGAAATGTATTTCAGATTTGATGAATTAGGAATTAGGAGGGAGTTCGAATTTCAAACCCCAAGTAATTAAGGAGAGTAAAGGGGTGGCTGATTTGTGGAGTTATTAAG
GTGATT
>comp158679_c0_seq3
CGAGCATATTTGTGAGATATATCATCTCTTTGATGGTACTATAACAAGATAATTTTCACATCTCTTCAATCATGTAGTTGACTACCATATCCTTATCATTGAGTTCTTTGAAACCTTCATTTCAG
GTTCCTTTACATCTCTGATATTGTAAACGATTTCACCTCTCCTTCTGATGCCCTTTCGAACCAATTGGGCCCAACAAAGAAAATCACCCTCATTCAATCAGATTGTGATGATTTTAATTGAGG
GAGAGGGGAGAGCTTTGAATTGATTGATGTTCGAATGAAGGTAGACAAGTTGAAGTAACTTGGTTATACTGGGCAGCATAAGGAGTAAAGATAGAACCGCGACAGAAGGTTTGTTCAGC
GCCTCGAACAGGAAGGCCAGCCCGTTCTGATTTGCTTCAAAACCGGTATTACATAGAACTAGCTCGGAATTGTTCAAAAGAACAGTAAAACCGGTCCCGTTTTCATATTATTGGCCGGTTC
ATCTGCGATATATTTTGAACAATTCACTGAAGATAGGACGGCTCAAGTAAGCTTATACAAAACCGAAGAAATAAAAAACCCTACCATTCATTCGACGGATTGTATCTATTGTGTCTTCCCCA
ATTCTCTTACTAGTTCGGGAAGCCAACCGAGGAGGAAGCAAAAAAATAGTTTTTGGGACAATTTTCTAAATGCTTTTAAAAACAATTTTTAGTAAAAAAAAAATTAATTT
>comp158680_c1_seq1
GACTTATTTATATTCCTGATGAGATCTGTGTATTGCCCACAGATAAATTTGAAGTCAGCCATGATCAAAATTGAAATACAGTTCTTCAATACAATACCACATTTATATAATAACATTATCCTGTA
CAATCAGTAAGCATTCAAATAGAAATTCATGCACATACTTTTTCTTACAATAAGACTGCATTGCTTTATATTTCAGAACAAATAGCTTCCAATTGTGCTCAAATATCTTAACCTTCTGATGCA
AAACCTGACGAGAGCTTAAATTTGGAGAGATTGCTATGACATATAGGAGCGCTGATTTACATTTATCGGCAATCATCTCCTATTAGTAGATCTCTCTTTTGCCAACCAAGCGCACAATTGCA
TGAATGCGAGAGACTGCGTGAAGAAAAAGTTCTTCACCTTTTTCCGCTCTGTCATCTTTGATTCGAAGAGAACGACGACGACAAAGCTGAAGGGATAAATAAAGCCAGAGAAAGCACA
ACGGGAAGACACACAAGACAAACATCAAGGACAAACAGACAAAAGAAAAGAAGAAATATAAAAAGGAAATAGGGAGATCGACAAATACGGGAATAAGCAATTAGCAAAGTTTTTTTT
TTTTTTTT
>comp158685_c0_seq1
GGGCAATGTGAGGATTTGAGAAATTGAGATACTTGTCAAATCTGTGAAAGCTCTCACCACAGGTCTCAACAGCACCATTTACATTGCCCTTAAGATCTATTAACCATAAAGGTGTTAAATC
CTTCTGAACAACAATATCATCATCTAGGAACAAGATTTTGTTCAGCTTAGGATAAACTTGTGGTAGATAAAACCTCAAGTGATTAAGCATTGAAAGGTATTTTGGATTTCGGTATTTCAAAT
TGTCAGGACCAGATGAAAGGGTTGTGGGATGAGCAGCCATGAAGTAGTATTCTTTCATGGGTACAGATTCAAGCTGACGAAGGACTGGGCAATATGAAGAATTCAACCACTTGAAATCAT
CAACGTTCTCCACATGGATGGTTGCATCTCCTGGTGGATTCAACAGAAACCACATGTTCATGGCTCCGAAGTTTAGTTTATCAGTAACTAGATGAAAGACATGTTTCGCTGGCTCCTTAGC
ATTCATTATGGTTGAGTTCACTACAACTGAGGCAGCCAATACATTGTCAGAGAAGAGTGCATAATGGTAGAGATTTGGGTTCTCCAAATTTTCACTTTTAGGAAATTTTCTTTGCTCTGGAG

GGAGAAGGTAATAGTCTATGCTCAGACGCATTGATAAACAATGAATGGCATTTGGAATTGTTTTTGCTGCCAATTGACTAAGGAATGTACTTTGCTTCTTCAAGCCTCTAACCCGCTCATCC
GCAGATTGAAGCATCCCTCTTAGATTCCGAATAAATATCTTGCAATCATAAAGCTGACCTTTTGCTTTTAACAAGACTTGATTCATTGCTCTGATTGTCTCCGGTGCACTTTGTGGGAGATC
GGCATCAGCATTAGCCTTTCCTAGGACGCGCTGACTTTCCTTGAGACGATTCAATAACTCTTCATAAATACCAATCTTACTCTTTGACTTAGCAAGGGCCATATAAACCTTTGACATAGTCAT
TTGATCTCGCATGAACTTTACTTTCGCATCAGCATGTTCATTATCATATTCTCGCCTCCATAGATTATATTTACCAAGTACTGCAGAGTCCACAGCTATTGAGTGCTCGATGGCAGCATTTTCG
AGTTGAACAAATACTTCATCATCCACTTTGTTAGAAACATTTGCAAAGCTGCTTTGATAATTGGTCAAGGTGGTCAGAGTTCCAAACACCACCGGGACAAACAGCGGCGCCAGAAGCGA
AAGAACGAGGAATTGTACGATTAGAGCGCGAAAGCCTCGGAAGCTCGGGTACAAACCGCCGCTCGAGAAAGAACGTGTCAAAGGAACCATGGCAGGCAATCGGAGACCGGATCAACG
GTGGGCTGGGATCGAAGTAATGGTGCTCCGGACCCTTCGGTGACCGCACATAAAATGCGGAGGCCCTTCTTCTTGAATCGGAAATCAGGGGAGTTGCGGTTGCTTTTT
>comp158685_c0_seq2
GGGCAATGTGAGGATTTGAGAAATTGAGATACTTGTCAAATCTGTGAAAGCTCTCACCACAGGTCTCAACAGCACCATTTACATTGCCCTTAAGATCTATTAACCATAAAGGTGTTAAATC
CTTCTGAACAACAATATCATCATCTAGGAACAAGATTTTGTTCAGCTTAGGATAAACTTGTGGTAGATAAAACCTCAAGTGATTAAGCATTGAAAGGTATTTTGGATTTCGGTATTTCAAAT
TGTCAGGACCAGATGAAAGGGTTGTGGGATGAGCAGCCATGAAGTAGTATTCTTTCATGGGTACAGATTCAAGCTGACGAAGGACTGGGCAATATGAAGAATTCAACCACTTGAAATCAT
CAACGTTCTCCACATGGATGGTTGCATCTCCTGGTGGATTCAACAGAAACCACATGTTCATGGCTCCGAAGTTTAGTTTATCAGTAACTAGATGAAAGACATGTTTCGCTGGCTCCTTAGC
ATTCATTATGGTTGAGTTCACTACAACTGAGGCAGCCAATACATTGTCAGAGAAGAGTGCATAATGGTAGAGATTTGGGTTCTCCAAATTTTCACTTTTAGGAAATTTTCTTTGCTCTGGAG
GGAGAAGGTAATAGTCTATGCTCAGACGCATTGATAAACAATGAATGGCATTTGGAATTGTTTTTGCTGCCAATTGACTAAGGAATGTACTTTGCTTCTTCAAGCCTCTAACCCGCTCATCC
GCAGATTGAAGCATCCCTCTTAGATTCCGAATAAATATCTTGCAATCATAAAGCTGACCTTTTGCTTTTAACAAGACTTGATTCATTGCTCTGATTGTCTCCGGTGCACTGAAAACATAATTT
TGTATTATAATAACTACACATCACATTAGTTAAATAGAAAGGAGGAAACCTTTGTGGGAGATCGGCATCAGCATTAGCCTTTCCTAGGACGCGCTGACTTTCCTTGAGACGATTCAATAACT
CTTCATAAATACCAATCTTACTCTTTGACTTAGCAAGGGCCATATAAACCTTTGACATAGTCATTTGATCTCGCATGAACTTTACTTTCGCATCAGCATGTTCATTATCATATTCTCGCCTCCAT
AGATTATATTTACCAAGTACTGCAGAGTCCACAGCTATTGAGTGCTCGATGGCAGCATTTTCGAGTTGAACAAATACTTCATCATCCACTTTGTTAGAAACATTTGCAAAGCTGCTTTGATA
ATTGGTCAAGGTGGTCAGAGTTCCAAACACCACCGGGACAAACAGCGGCGCCAGAAGCGAAAGAACGAGGAATTGTACGATTAGAGCGCGAAAGCCTCGGAAGCTCGGGTACAAACC
GCCGCTCGAGAAAGAACGTGTCAAAGGAACCATGGCAGGCAATCGGAGACCGGATCAACGGTGGGCTGGGATCGAAGTAATGGTGCTCCGGACCCTTCGGTGACCGCACATAAAATGC
GGAGGCCCTTCTTCTTGAATCGGAAATCAGGGGAGTTGCGGTTGCTTTTT
>comp158685_c2_seq1
TCTTTGGATTCCTTAGGTCTCCGATTGCCTGCCATGGTTCCTTTGACACGTTCTTTCTCGAGCGGCGGTTTGTACCCGAGCTTCCGAGGCTTTCGCGCTCTAATCGTACAATTCCTCGTTCT
TTCGCTTCTGGCGCCGCTGTTTGTCCCGGTGGTGTTTGGAACTCTCACCACCTTGACCAATTCTATAGAATAACGAGGTTCTTAGGTCGTTTGGGATGCAATTTCTGCATTTATTTTGGAAA
TTTTGATTTTAACTGGATGGCGGAAGGCGCTTCTTGGTTTGGGA
>comp158704_c1_seq1
TTGGCATAGCTCTTCGCACGGCACACGTAGCCCTCCATTCCGCTCTTACCCAAGCAACGCAAGGGAAGGCCACATCTTTCCATCGCTGGAAGCCACAAGTCCACACAAGCAGGAGAGAG
AAGCAGCTGAGGGAACTCATCGAATCTGACGGTAAAGAGGGTTTTTCTCGCGGATTGCGGCGTCGATATCTATGTTATACGCTATTCCGGCGGCCGAAGGCGGCGGTAGCGAGTTCAGAG
GACAAGCAGGATTTTCTGCGGACTCTTTTCATCGTCATCATCATCATCAGCAGCAGCAGCAGCAGCAGAAGGTTGGCCAGTGCCTCTCGCCGGCAATCCATGTCAAGGAGGCTCATATCC
CCTGTGCATGAAGTGGACCAAAAAAATATCGCATTAAAAAAAAAAAAATGGGCATTTATAATTCATGCATGATGTGATCTAAAAAATGAACCATATATTATTTTGGGTGCAGCATGCATTAATT
ATTTGAAGTGAGATGCCGTTTTTATTTTAATGATGGTAGGTAGACTAGTAGTTTTTTATTTCCTCAAAACCTAATGTAGGTTTATGGATCAATTTCATATGTTATTTTGTATTAATTTATATTAAT

GAATTCATATTTATTAATATAAAATAATATGAAATAATATATTAAGTCGAATAATATATACTCTCCTTGAATGAGAGGAATTATTAGGTGGTGATTTGCACGGTTGTGCAAAGTTGAATTTAGGG
TCTTGGATTTAATGAACTCTTATGTATATGTATTCTTATGGAGTGTTGGTGGCTCATGAGTGTGAGGTAATAGAAATATTTATATGCATTGAATCTATACATTTCTATGATAAATATAATAATTT
ACTCATAATATCCACTTGGTGACTC
>comp158731_c0_seq11
CTAAATTAGGCAAAGAAATTGAGAAGTAAGAAAATATTTTGACGGACATCCACTGGAGAAATCATTTGCCCTAATGCGTCATTGCTCGCGCCATGACGTAGATGGTGCGTGCCAACTTGA
ACATGTGTGCCACGCCGAGCCATGCGACATGGCGCATGCCATTTTGTGCCATTGACAAGTATGAAGGGCTCCCATCCCTTGGCTCATAGCTTTGGTCATTTTGGATCTGGTACTTCACACA
ACTAGGAATATATATTGAGCACAAATGATTAATAGTAAGTGCATATGAGTTTGATAGTCTTTGGATGAAAACATGATTACTAATTGGGTATTCTGTTTTGGCTTTAGAATTAGGATCATAATAG
GGACACTCTTGTGAACCTTTGTGGATGTTCGTTGACAATATTTTGTATAGGCTACAGTGCTTGATTATGATTTGGGATTACCTGAGAACAATTGATTATGATTATGATTTGCATGCTTAGAAA
GCTGGGTAGTGCCATAATGGTAAGTTTTTTTTTTTTTAATTCTAAGTGTTTCCAGTTCCACTCCGTATAAAAATAAAAATAAAACATTAGTTTTCAAATTGTTAGAAAAAGATAGTGATAAAC
GAGAGAATTACAAGAAGCACAGACAAGC
>comp158743_c0_seq8
TAAGAAGGAAAATTGGTTTAGTCCTGAGGGAAGTTCACAGAGGCAGCAAGGGAATTTAGTGGGAAGGTTTCATCTACTCGAGTTTAAGAGTTCTAAATGTGTTTTGGCTCTAATCAAGTA
AAGGCATGCACCTTAGTGGCACCTTGTACAACTGCTAATATTTTTTAGGTAGCTTTCAGGAAGTAGTTTTATATCATTTAGACTTCATTTGAGACAACTTTTTTAATTCTTTTTAAATCAGCTT
TTTAGTAAAAAAGAAAAAAAATTGTACTAAAAAATTGATTTGAGAAGCAATAAAAAGACCGTCCCAAAGGAAGCCATAGTTGATATCTATTATCCTTTCTTTCATCATAAATAGTGCATCAT
CTTTCAATTGAGTTGCATTCAGTGGGCATTCTCTAATGCACAAGTCCAAATCTTTATATATTTTTGCTCTTTTCTCTCAATTTCCCTCTCCCCTATCTTGTCTTGGGGGCATTTCATTGTTCTTC
TCAGTTATGTCTAATAATAGATGTGTTATGAAAGATTTTGTTGATATGATTAAGGAATGTGATTTATCATATAAGAAATTTCATGCATCTAATCTTACTTGGCTGAATGCTCACTGGAATCCTTT
TCCTCCGGCTTGATCAATCCCTTTGAATCTTTGCTCACAATTCAACTCAATTGAACTCCTCCAGATCATTC
>comp158759_c1_seq2
CTTCTCTCCCTTGTTCTGCCGATTCTTTTTTCCCCGGTCCTAGAAGTCAAAGTGTTGTGATTTTGCACTGCATTTAATCGAATTCGAGAATTTGTGCTACGTGCTGCGATATGAACTTGCGA
AGAGGTCTAACTCCTTTTCTCCGTGGCTTTTTTGAACTTTATTACGGAGATTTGAGATGCAAGTCCACCTCCTTGTGCGAATTCGAGCGTTACGGATTCTCAGAGACCTGCAAAAATGTCC
CTGAATTCTCTAGCCCATTTGGGGAACACACGAATGACTTGATTAAATTTGCTACAAAAAAAGTTTGCCCTGCCGTTGTCTATGTCTCTCATGAAAAGGGAATTGGAGGATCTGGGATCAT
CATAGATTCCGATGGCACAATCTTGACCCGCGCTTGTACTCGTTTTTCTATGGATTCTAGAAGCAAGATTAATGTCAGTCTACATGATGGAAGAGAATTTTCAGGAACTATTCTGGAGTCCA
ATGAGGATCATCATCTTACTCTTTTAAAGATACAATCAGAATCTCCCTTACCAACAACAACGTTGGATTATTCGAAAGATTATAAGGGAAGAGAATTCATTTCTTTTGAACGTCTATTGAGA
AGAACAACTTATCCAAGACTTTCAAGTCAAAATGAATTAGATAATGAGATTATTATTAGTCCACATGATGGAAGAGAATTTTCAGAAACTTTTCTGGAGCCCAATGAGGGCAACTTAATGC
TGTCAAATGAAGAACTCATGTCTAAAATTGATATTGACAATTTTCATTTGGGTAGTTGTGGAGGGCCAATTGTGTCTCTTGATGGTGATGTTGTTGGAATTTGCACTAAAACTAATATGCTTT
GGCCTTGCGTTAATGGTAATTATTGTGGAACTATTAATGCAATGGACATTGAGGAAGCCTTATGCGAAGTAGGATACATAACAAAATGATCGTTGGAATTTAAAACAGTTGAAGATGTTTAA
AGTTATTCTGCACGACGTGAGCATCATGAGAAGGTTGAATGGTTCTCTACATTTTAAGATTGTTCTTTTTAATTTATTACTAAATTGTTTGTGATTTACAATCTTTATGGCACATGATTAA
>comp158777_c0_seq1
GTAGTATAGAAGAAAAACACTCTCATATTAGACAATCCGAACTGAGAATGGATCAATCAAACATTTCCGAGAAGAGAAAAAAATCAATGCAGAACTAACGGAATTAACACGACATTCCGA
TTCCTCGTCCGGCACGACAAAATCGCTAACAAAAAAATAGTAACCAGTCAAACTAAAGAACTCATAAATAACAGCTCGAAATAAATCCCTTTTAAATCCTTCGCCATGAAACCTTCCGCC
TCACCTTCCAACATGGCCGGAATTTCACTTCCCACTTCTTACCGGCCATCCATTCCCACCCTCCTTCTCCTCCTTCATCTCGTCTTTCTCTTCTTCCCTACCGCCATCCTCTCCATCGGTATAA

ACTATGGAACCTTAGGCAACAACCTCCCGCCTCCAGCTGAAGTCGCATCGTTCATTAAATCTCGGACAACGATCGACCGGGTGAAGATCTTCGACTGCAATCCGGACATCCTCCGGGCCT
TCGCCGGCTCCGGCATCCCCCTCGTTATCACCGTCCCCAATGGCGACATCCCCTCCCTCTCTCGCCTCCCCAGCGCCACCGCCTGGGTTGCCGCCAACGTTTCCCCCTTCTATCCCGCCAC
GAACATCTCCCTTATCGCGGTCGGAAACGAGATCTTGGCCACCGGCGACCGAAACCTAATCGTTCACCTCGTCCCTGCAATGCGATCCCTCTTTTCCGCCCTAACAGCAGCCGGATTCCCC
TCTATTCGCGTCTCGACACCTCACTCCCTCGGCATCCTCGCCGCCTCCCAACCCCCTTCCGCCGGCCGCTTTCGCTCTGTTTACGACCGCGCCATCTTCTCCCCGATGCTCGATTTCCACCG
CCGCACAAAAACACCTTTCCTCTTCAATCCTTATCCCTACTTCGGCTACACAGATGAGACTCTTAATTACGCGCTTTTCAAGCCGAATCAGGGAGTATACGATCCGGCAACGCAGATTACTT
ATACGAATATGTTCGATGCACAAATGGATGCAGTGTTCTCCGCCATGAATCAGTTAGGGTACGGCGATGTGGAGATTGCGATCGGGGAGACCGGATGGCCGACTGCGGCTGAACCAGGGC
AGGCAGGGGTCGATCCCGCTGCCGCTGCGTCTTATGTTACGAATCTGATCCAGCATGTGAATTCCGGGAAAGGGACGCCGTTGATTCCGAATCGCAAATTTGAGACTTATATATTCGCGCT
TTTCAACGAGAATTTGAAGCCTGGGCCGATTGCCGAACGAAATTGGGGCCTCTTCCAGCCTGATTTACACCGGTGTACGACTCCGGCGTTCTCCTCGGCGGAGGCAAGGTCATGGGAG
GCCGAGCTGCCAAGTGGTGCGTTGCGAAGCCAGGAACGAGCGTGGCGGCCCTACAGGCGAGCGTCAACTACGCCTGTAGCGTGGTGGGTGTAAACTGCCGGCCCATTCAAGAAGGCG
GGGCGTGTTTTAAGCCCAACACAGTCCAAGCCCATGCAAGCTATGCCATGAACGCCTACTACCA
>comp158795_c0_seq13
ATTTTCTAAATCCAAATTTAGAGGATTTGAAAGTTAAATCTATTCGGATACCAAACAACCCCCTTACTTTTCTACCCATTTAAAAGATCTCTTTATTTTTTCTTAACCCTAGCAGTCAAGAGC
TGTTGATTTCGCCCTTCGCGCCCCATCAAATTCCAGAACTTCTGTGATACAAACCTCGATATGAACTTGAGAGGAGCTGTAACGCCCTTTGCCCTTGGCTTGTTTAAACATAAAGGAACAT
GGAGACGCACGTCCGGATCCTGGTGCAAATTCGAGCCTTACGGATTCCCAGAGACCAGCAAAAAGGAAGAAGACAAACCCAGTAAAATATATCGGCGATTTGGGCCACGCTCGGATTCC
TTCGTTAAATTTTCAACGGCGAATGTTTGCCCTGCTGTTGTCATTGTTTCTGCTGAAAAGAGAAATGGAGGGTCTGGAATCATAATAGATTCCAACGGCACAGTCTTGACCCATGCTCGTA
ACATTTTTTCACCGGACTTCAATAGTAAGATTAATGTCAGTCTACATGATAGAAGAAAATTTTCAGGAACTATTCTCGAGGTTAATGAGGATCATGAGCTTGCTCTTTTGAAAATACAGTCA
GAAATTCCCTTACCGACAACAATGTTTGGATCTTTGGGAAATTACAAGAGAAGCGAATTGATCTCTGTTGAACGTCTATTTAGAGAAAAGGATTACCCAAGAATTTCGAGTCCAAATGATG
TAGGTGAAGAGGCCAAGTTGATGTTGTCAGGTGAAGAAATCATACCTAGATGGAAGTACGAACAATTTTATTTGGGTAATTGTGGAGGACCAATTGTGACTCTTGATGGTGAACTTGTTG
GAATTAGCACTAAAATTCGTTTGCTTAAATCTCTACATAATGATGCGAGCTTTGGAACTATTGATGCAATGGGAATTGAGGAAGCCTTAAGCAACGTGGGATACACAAGAAAAAAATGATAG
GAACAATTACTTTTCTCTTGCTGCAGATTCTTGGGGTGATTAATAAATGAAGGTGGATAAAGCTCTTGAAGGGATCTTATATTCTAACTATATTTGTTGTATGATATGGCCAACATGAGAAGG
TTGAGTGGTCCTTTACTTCTTGAAATTTTCTTTCATATTTTATTACTATATAAGTTGAAATATGATGCTTTTTATTATTCCTCTATAGAGATCTTAAGACTTTAATTATGAGAGATATGATTATGAT
TTTATATTTAAGTTTGTGCAGCAATTTCTTTGTGTTGATATTAAGAATTTAATTACATGTCGTGATTACAATTATTGCCTTATATTTCGTGATAATT
>comp158795_c0_seq27
ATTTTCTAAATCCAAATTTAGAGGATTTGAAAGTTAAATCTATTCGGATACCAAACAACCCCCTTACTTTTCTACCCATTTAAAAGATCTCTTTATTTTTTCTTAACCCTAGCAGTCAAGAGC
TGTTGATTTCGCCCTTCGCGCCCCATCAAATTCCAGAACTTCTGTGATACAAACCTCGATATGAACTTGAGAGGAGCTGTAACGCCCTTTGCCCTTGGCTTGTTTAAACATAAAGGAACAT
GGAGACGCACGTCCGGATCCTGGTGCAAATTCGAGCCTTACGGATTCCCAGAGACAAGCAAAAGTTCGATAAGATATTCCTGTCAAAAGCTCTCTTTTTCTCCTTATTGTCTATCTCTCTTT
TTAATTCCAATTGGGTCTGTAGAGGAGGAAGACAAAGCCACCAAAATTTTTCAGCGATATGGGGAGATGGAGGGTCCGGAATCATCATAGATTCCGATGGCACAGTCTTGACCCAAGCTC
GTAACGTTTTTTCACCGGTTTTCAGAGGCAAGGTTAATGTCAGCCTACATGATAGAAGAAAATTTTCAGGAAATATTCTCAAGGTTAATAAGTATCATGATCTTGCTCTTTTGAAGATACAA
TCAGAAATTCCCTTACCGACAATAATGTTTGGATCTTTGGGAAATTACAAGAGAAGCGAATTGATCTCTGTTGAACGTCTATTTAGAGAAAAGGATTACCCAAGAATTTCGAGTCCAAATG
ATGTAGGTGAAGAGGCCAAGTTGATGTTGTCAGGTGAAGAAATCATACCTAGATGGAAGTACGAACAATTTTATTTGGGTAATTGTGGAGGACCAATTGTGACTCTTGATGGTGAACTTG
TTGGAATTAGCACTAAAATTCGTTTGCTTAAATCTCTACATAATGATGCGAGCTTTGGAACTATTGATGCAATGGGAATTGAGGAAGCCTTAAGCAACGTGGGATACACAAGAAAAAAATGA

TAGGAACAATTACTTTTCTCTTGCTGCAGATTCTTGGGGTGATTAATAAATGAAGGTGGATAAAGCTCTTGAAGGGATCTTATATTCTAACTATATTTGTTGTATGATATGGCCAACATGAGA
AGGTTGAGTGGTCCTTTACTTCTTGAAATTTTCTTTCATATTTTATTACTATATAAGTTGAAATATGATGCTTTTTATTATTCCTCTATAGAGATCTTAAGAGGAATAATAAAAAGCATCATATTT
CAACTTATATAGTAATAAAATATGAAAGAAAATTTCAAGAAGTAAAGGACCACTCAACCTTCTCATGTTGGCCATATCATACAACAAATATAGTTAGAATATAAGATCCCTTCAAGAGCTTTA
TCCACCTTCATTTATTAATCACCCCAAGAATCTGCAGCAAGAGAAAAGTAATTGTTCCTATCATTTTTTCTTGTGTATCCCACGTTGCTTAAGGCTTCCTCAATTCCCATTGCATCAATAGTT
CCAAAGCTCGCATCATTATGTAGAGATTTAAGCAAACGAATTTTAGTGCTAATTCCAACAAGTTCACCATCAAGAGTCACAATTGGTCCTCCACAATTACCCAAATAAAATTGTTCGTACTT
CCATCTAGGTATGATTTCTTCACCTGACAACATCAACTTGGCCTCTTCACCTACATCATTTGGACTCGAAATTCTTGGGTAATCCTTTTCTCTAAATAGACGTTCAACAGAGATCAATTCGCT
TCTCTTGTAATTTCCCAAAGATCCAAACATTATTGTCGGTAAGGGAATTTCTGATTGTATCTTCAAAAGAGCAAGATCATGATACTTATTAACCTTGAGAATATTTCCTGAAAATTTTCTTCTA
TCATGTAGGCTGACATTAACCTTGCCTCTGAAAACCGGTGAAAAAACGTTACGAGCTTGGGTCAAGACTGTGCCATCGGAATCTATGATGATTCCGGACCCTCCATCTCCCTTTTCAGGAG
AGACTGTGACAACAGCAGGGCAAACATTCTCCGTTGAAAATTTAATGAAGGAATCCGAGCTTGCCCCATATCGCTGAAAAATTTTGGTGGCTTTGTCTTCCTCCTCTACAGACCCAATTG
GAATTAAAAAGAGAGATAGACAATAAGGAGAAAAAGAGAGCTTTTGACAGGAATATCTTATCGAACTTTTGCTTGTCTCTGGGAATCCGTAAGGCTCG
>comp158795_c0_seq30
ATTTTCTAAATCCAAATTTAGAGGATTTGAAAGTTAAATCTATTCGGATACCAAACAACCCCCTTACTTTTCTACCCATTTAAAAGATCTCTTTATTTTTTCTTACCGCAGCACTTCTGTGATA
CAAATTGCGATATGAACTTGAAAAGAATTGTAACACCCTTTGCCCTTGGCTTGTTTAAACATAAAGGAACATGGAGACGCACGTCCGGATCCTGGTGCAAATTCGAGCCTTACGGATTCC
CAGAGACAAGCAAAAAGGAGGAAGACAAAGCCACCAAAATTTTTCAGCGATATGGGGCAAGCTCGGATTCCTTCATTAAATTTTCAACGGAGAATGTTTGCCCTGCTGTTGTCACAGTC
TCTCCTGAAAAGGGAGATGGAGGGTCCGGAATCATCATAGATTCCGATGGCACAGTCTTGACCCAAGCTCGTAACGTTTTTTCACCGGTTTTCAGAGGCAAGGTTAATGTCAGCCTACAT
GATAGAAGAAAATTTTCAGGAAATATTCTCAAGGTTAATAAGTATCATGATCTTGCTCTTTTGAAGATACAATCAGAAATTCCCTTACCGACAATAATGTTTGGATCTTTGGGAAATTACAA
GAGAAGCGAATTGATCTCTGTTGAACGTCTATTTAGAGAAAAGGATTACCCAAGAATTTCGAGTCCAAATGATGTAGGTGAAGAGGCCAAGTTGATGTTGTCAGGTGAAGAAATCATACC
TAGATGGAAGTACGAACAATTTTATTTGGGTAATTGTGGAGGACCAATTGTGACTCTTGATGGTGAACTTGTTGGAATTAGCACTAAAATTCGTTTGCTTAAATCTCTACATAATGATGCGA
GCTTTGGAACTATTGATGCAATGGGAATTGAGGAAGCCTTAAGCAACGTGGGATACACAAGAAAAAATGATAGGAACAATTACTTTTCTCTTGCTGCAGATTCTTGGGGTGATTAATAAAT
GAAGGTGGATAAAGCTCTTGAAGGGATCTTATATTCTAACTATATTTGTTGTATGATATGGCCAACATGAGAAGGTTGAGTGGTCCTTTACTTCTTGAAATTTTCTTTCATATTTTATTACTATA
TAAGTTGAAATATGATGCTTTTTATTATTCCTCTATAGAGATCTTAAGAGGAATAATAAAAAGCATCATATTTCAACTTATATAGTAATAAAATATGAAAGAAAATTTCAAGAAGTAAAGGAC
CACTCAACCTTCTCATGTTGGCCATATCATACAACAAATATAGTTAGAATATAAGATCCCTTCAAGAGCTTTATCCACCTTCATTTATTAATCACCCCAAGAATCTGCAGCAAGAGAAAAGT
AATTGTTCCTATCATTTTTTCTTGTGTATCCCACGTTGCTTAAGGCTTCCTCAATTCCCATTGCATCAATAGTTCCAAAGCTCGCATCATTATGTAGAGATTTAAGCAAACGAATTTTAGTGCT
AATTCCAACAAGTTCACCATCAAGAGTCACAATTGGTCCTCCACAATTACCCAAATAAAATTGTTCGTACTTCCATCTAGGTATGATTTCTTCACCTGACAACATCAACTTGGCCTCTTCAC
CTACATCATTTGGACTCGAAATTCTTGGGTAATCCTTTTCTCTAAATAGACGTTCAACAGAGATCAATTCGCTTCTCTTGTAATTTCCCAAAGATCCAAACATTATTGTCGGTAAGGGAATTT
CTGATTGTATCTTCAAAAGAGCAAGATCATGATACTTATTAACCTTGAGAATATTTCCTGAAAATTTTCTTCTATCATGTAGGCTGACATTAACCTTGCCTCTGAAAACCGGTGAAAAAACG
TTACGAGCTTGGGTCAAGACTGTGCCATCGGAATCTATGATGATTCCGGACCCTCCATCTCCCCATATCGCTGAAAAATTTTGGTGGCTTTGTCTTCCTCCTCTACAGACCCAATTGGAATT
AAAAAGAGAGATAGACAATAAGGAGAAAAAGAGAGCTTTTGACAGGAATATCTTATCGAACTTTTGCTTGTCTCTGGGAATCCGTAAGGCTCG
>comp158830_c1_seq4
GATGCTGTTGTAATGTATGATCACGACACTCAGAGGCCAAGAGGCTTTGGTTTTATAACTTTCACTTCAGAAGATGTTGTTGACAGAGTTTTGCAGAATTCCTTTCATGAACTGAATGGAA
AGATGGTGGAGGTCAAAAGAGCTATCCCAAAAGACATCTGCCAAGGACCTACTAACTATGGCTTGAATAGATTCAACAGTTCTCTCAATCTATATGGTCAAGTGAACAATCCAAGCTCTCT

CGGTAGCTTTCGGCCAGCGATCGGCGCTCGGAACGGGTCCCCTTTCACTCCTGGTTATGAAATTGGGTTGAGTTTTGAGCATGGAGTGAGCCCAAGCTATGGAGAAAACTTAAATTTAAG
TAGCAATATTGGTTATGGACAAGCCTTGAGCCCATATTATAGTGGGAATTTGACTAGATTTGGTGGCTCTATTGGTTACAGTGCAGGTTGTGGTAATCCTGGTTCATCTTTTGATTCAAGAGC
TCTTAATTTATGGGACAATGGTGACTATAATATTATCCCTGCAAGTGCAAATTCCTTCGCAGCCTCTGGAATAGGGAGCATTAATGGTTTCGACAATAATATTATCAGAAATCTGGATGATAC
TTTTGGTTTTTCTAGTGATAATTATCCAAGGCGTTATGACATTGGCTCTCTTAGTGCTTCTTTCACAGCTAGAAGAAACTATATGGATTTATATGGAAGTGGTTCAATTTATGGAGATCTTTCT
TGGCACTCAGGGTTGAATGAACTTGATGAATCTAGTTTATTTGGTTATGGGCTTGGCGCCACAGCTACAGATGTAATTTCCAGTGGTTCAATAGATTACATAAGTGATTATGATGTTACAAAT
AGGCGTATAAATAGAGAGGGATGAGCGTTCATTTTTTCACAATGATCGGGACATCAGATCGTGGAGAGTCTATCAGAGACATGACCGTCAATCTATGACAACGATTGAGTCACTGTATTTC
TGAGTTGTTTTATATAATTTTTTATTATGATATCATATTGTATTTATTTTATTAGTTTGCTTTATTATTTTCCCGTTGTACCATTTTATTA
>comp158918_c1_seq7
GAACTTTGTATACGCTATTAAACTATTAAGTTCAAATCATATTGCAATCACCATGTTTCAACGAAGCAGCTTGAGACGACTTTTCTCTTGTCTAACCTCTCCTACTTCTTCTCAAACCAGAA
AAAGAACTAATCACATTACTCCTCTTTATTTCCTCACCATTTACTGAATCAATGTTCCACCTTCAACCACATAAGCCAAGCTCATACCTTTATGCTCTCCCGAGGCCTCGATCAAGACAAA
CTCCTCCTCAGCAAGTTCATCCATGTTTGTTCTTCGTTAGGCTTCGAAGACTACGCCTTTACGCTTTTTAAACTCGAACAAAGTCCTGATCTTCACCTCTATAACACCATGATCAGAAGTCT
CTCTCAATCGGATTCAGCTGGAGAGGCTATTAATTTGTTTAACAATATTCAATTTGCTGGCTTTCGACCAAACACTTATTCATTTCCTTTTGTGCTGAAAGCTGCCGCACATTTATCCAACGT
TGAAGTTGGCAGAGAGGTTCACGGACAGGTTTTTCGACTTGGTTTTGAATACGACATTCATGTCTCAACAGGTCTAGTTCATATGTA
>comp158921_c0_seq5
TTATATTACCAAACATGCCATGTACTGAGGCTATCTTAAAGCCTATATATACTAGACTCTGTACCGAAGGCTAAATAAGAAAATACATTACTTTACTTTCTTCTCCACTTTCGGCTCTTTGCTT
TGTCCTCTGTCTTCTTGAGATCTTCTTGTTCCTGGGCAAGAACTGGATCAATATCCAATAGTTCTTCAATATTGCTATAGTAGAGGTTGGACAGCTTCAAGAACTGATGATCAACTCTCATTC
TCTTCCATTAGCATCAGTAAGTAGGCTTCTAGAGCGGACTGCAGCTCCCGGATGAATAAGGATTTGTTCAATAAATTTTGTTTTTTGATCTCCTCTTCTATAAATGCTTGTGACGGCATGAGA
ACCAACTATTTACCTTAGAGTAAGAAAGAGTAATTGTTTGCAGCTCCATGTATTGTTTTTCGGTCATATTGCCGAGGTCGACCCAATAGCAAATGACAAGCATCCATTGGTAACATTGCACA
AAACTTGATCTCCAAACTGGCCAATTGAGAAATTCAAAAAATACTTTTTACAAACCTGTACTTCTTTATTTTTTTGAATCCATGATAAGGTGTAAGGATGTAGATGGTCCGTAGTGTCCAAT
TGAAGCTTATCAACCATAGTGGCAGCGACGACATTTTTGCAACTTCCGAAATCAATAATAACTTGGCAAATCCTCCTTTTCACCGTACATCTTGTTTTGAAAATATTGTGATGGGGCCAAAA
ATATGGGGTATTTACTTTTGACTGATGCCAG
>comp158940_c0_seq2
TCCGGTGTCCGGACCTAATAATTTCCCTAAAACAAGAGAGCTTAATAAAAGAATAAAAGCTTTTGGTCAGATGTTAGTAAAAAAATCTTCAGGTTTTGCGGTTACGAAGATTTGGAGATGT
CAGAAGGCCGGAAAGCAATTTGCATGAGTTCCAGAGTTGTTATTCTCAGTTCATAAGTGATATGTTGAACAATATGGGCCAGTACATTAACATCTTGCATTGCTCTATGCGCAGGACCCTCC
ATAGGAATCCTGTAATGTTCACGGAGTGCATTTAGTGAGAAAGAGCCAAGCTTTGAACCATCTGACTTTCTCAACTGGCGTGCAAGATAAAGTGTATCTAGGAAGTACCAATCTTCAGGA
ATTTCCATAGAGCAACGTTTGAATTCCCTAATGAGGAAAGGCACATCAAATCTCCGTCCATTATGAGCAATCCACAGGACTGGTCTGCTACTCCTTTGACGGCTACTTACAAATTGCAGTA
GGATTGGAATAAGTTCCTCAAAACTTGGAATCCCAGGCCTGCTAACCATATCCGAGCTAATGCCATGAACATCGGTATTTGGTACAGCTCTTCCAGGATTTATAAGTGTTTGAAATGTACTG
TTCTTACCCCCGATTAGATCTCGAATTGCAATTTCAATTATCCTTCCCCGTTCTCTACTAAGACCAGTGGTTTCAGTGTCAAATACTAAAATTGTAGCAGGTCTGTCATATGCTTTGTCTTCTG
AAACTTTCTGGATACTGCAATACTCATCAGACTTGTATTGATGAAAATCTGACTTTGTTTTACTAATATTCTTCAAGATTTCAGCAGCACCACCAGGAACCCTAAGGTTGATGTCAAAAGCA
TCACTGTTGTGTGACCACTGACTCCTTCGTTCTACTTTTGATGCTAAAGTCCGTAGACAGCATATCCTAGGTTCCAATAGTTTTCCAGGGAAGAACTCAGCGTGGGATTTGAGAATAGTGA
GAGTACAACTGCTCCTGACGCAGATCCTACATTTCCTTCAAGGCAGCGAATAAATTGTTGGCATTCTTTGCCGAATAAAAAATATCTATGTTGCCCCCCATCTAAGAAAACATGGCCTTAAA

TTTTGGTACCTATGTGTATGAGAGATGTACTCTAGGAATTATATCTGCATTAGACAATGTAAAGTAGTATGAGGTTATGAAATATAAAAAAATATTTATAGTTTAAATTAAACTTAAATGAAAT
TAGGACTCACCAATAAA
>comp158944_c0_seq3
GGCAATGAGAACAATCCTGCTCTCACCATCACCAACACTCATAATTCTTGCATAGCAGTTGGGCATGCAAGAGTGATTAATCAAGCGTGCTATGTTTCCTCTGTTTGTAGCATCGATAACTA
CTTCATCACTGATCTTAAAAAGATAACAATCTTTTCCTTCCAAACGGTACCGGGTCTCCCTCAAGTCAGCAACGCTACGTCTGACCTGCTCACCCCGATATTCCAGTATCATTTCTCCTTCT
TGAATCTTTCTACAAGCAAACAGGCCCCATCCATGAATTCCAGATCTACCAAAACATACTCTACTTCTCTCAGTCGACTGCAAATAACGAAGGTGCTCCCTGAATGTTGAAAACGATTTCT
TCACATCCTTAGATGCATTCAAGCATTCAATTTTATCTAAAGAATGATGCAGTGAACCTCTCACTTGGTGCGGGATGCCAAATTCTCTGTTTTGCTGATTTTGCATCTTGTTATAAGCTATGC
ATCTTGCAGCATCTGAGGGGCTAGACTGATTGTTGGGTAACACTAAATTGAGGGGAGCATCTTTCTTAATTAACCGTGAGCCAGATTTCTTATCATTATTCTTGAGCAACTTTTTGCTAGAG
AAGATACCAGAAGGCGTGTGTATAATCAAAACAGTGTCTGGGTTAGGTGCCCTGTGGAATGAACAAAATGATAGCTTTTTTGTTGTCTGTATTCCATTCTTCTCAAAGCAATGTAACTCCAT
GCGATATCCAGCTCTTGATGCACACATTGCATGATAATAGGTTGAACACTTACAACATTGTGCACATGAACCATGCTTTTGCTTACATATTACACATATCTTCATAAAAGACTGTGGTGGTAT
ATTCAATATTCCCACAGCAGGCTCCATTGTTATGTCGTTAGCAAAAGAAACAGTGGGCTGAAACCAGGCACATGTAACATGAACCCACAAACAATCAACATCAGTTGGCTTCAAAGCCCC
ACCTTTTACTGGGCAAAGGCAGCATTCCTTGTTTTGTTGAGGTGTTTCACATGCTGTGCAAACCCAGGATGTGAAATCTCGTTCACCTCTCACCCCATAGCATTCCTGGTGGACAGCTACC
TGACATCTGATGCAAATGATGATTTTATTATAATCCCAGTCCTCAATCCATCTGCAAATTGCACACCTTTCAGTTGTCCATTTAGCATAGACTGGTTCATAAGCCTCTTGCAACAAAGTAACT
AGTTTTTGTTTTCTCTCTTTAAGAGACGGTTGTTTAGCGTGATTGGCTGGTATTGTATTGTTAAGGTGATATCCAATCCATTCCCCGAGTGGCCTCATGGTACTCTTGATTTTGACGCTGATC
TTCCATTTTTTACTTCTACAACCTGTATGACGCTCCCATTCACTAAGTTTGGCTTTTTTCCCGTTGCAAGTGTGGCAATGACATATCACCACATGTTGCTCAGGTAAGTATGTCCCCTCCACA
CCACAACAGCAAACAAGAATCTTCTGGGGTGCAGAGACTTGCAAATTTCTAACTTGTTCTTTTTTCTTCTCTGTATCTGGTAAATCAAACTTATTCTGCAACTCGCACTCAAGAGATGAAT
AATCACCATCTTCCAGACTCTGCCTGGTGTGGGCTTTTTTCAAAAGCGAGCCTTCTAAATCACCCTTCACTTTCTTTATGCTTGCAATTGAAAGGCAAGCCACATAATTGTCACAATGAAG
GCCTGGATTGTTCACGTCCTGTAAGCATTCCTGATCATGGTTGGAGCCTGTCGCTTCTTGAAACTCACCATAGAAAGGATCACGAGGATATGTGTGCAGCCTGGACATGTCCATCCCATCC
ACCTCCATTCCCATAAAACCATGCTCAGCCAGGAAAGCCTCCTCTATTGCCAACCTTAACTCAGAGGGCTTACTGTTATATAATTCTGTCTGCCCCTGGAACCTGTCTAGATTGTCTATAAAT
GGAAAAACCATCTCTTGCTTCACCAAAGCACACTTGCGCATGACTTCTCCACTTCCAGAATACCCAAAGAGCATCACACAAATGGATCCAACGTTAAGGGATTTAGAAACTGGCTCGGG
AGCTTGTGGCGTTAGGTTTACGACGATAGCTGGCCAAGCAGGATATGTGTTCCCTGGCTTAGCCCAGACCACATCCCCCACAGCAAACTTCATGGAAGAGTTTAGATATTCTCTCTGTGAC
ATTTTATCAGCCGATATATTCCCCAAATTTCTCACCGATTCATCTAGATTCGGCAACGCCGGGCAAACCATCACCTCCTCTCCCTCCGACGACTCATGAAGAAACGTCAAAGTGCTCGGTG
AAAGAGATTTCTTCATTGATTTTATATTCCAGCACGCTCTGTAACAATCCTGTTTTCCTAATAATTCGAATGAATTCTTCGACTCAGTTTCAGTCACATTAACTTCTCCGAAATCGGAATCAAT
AACCCCAGACTCGAACTTCTCCTTCCTCCAGGTTTCAATGGTGAACGAATCACCGAACCGCGAAGGAAGCACCTGCGCACGTCCCCGCTTTGTTCTCACAGCCAGCGACCGCCGCCGCC
GCGGCTGGCCCCAGCTGGCTATCGACCCGGATACGACCTCGCCGGAGCTCGTAGACGGCCCCAAGTCATTACATGCGGCGTACGGAATCCCGGCGAAACTGCCCAAATCCCCAAGCATC
TCCAATAAGAAGTAATCCCCCTTGCTGCTCTTCCACCGTTTCGCGCTACGCCCAACGCCAACCACCCCGCCTCCTTCGTCCGAATCCGCCCTGCAACGCTTGAGGCTCGGCATTTGCGCCC
TGTGCGAACGCTTTATGATCATCTCCGCAAATACCGATGTAAATTCATAACCCTAGACTCCGGCGTGCATTGGAGGCGCAACCGACACAAACAGAGGATTCGATTCAAAATATCCCAATCG
GCAATCCCACACTTGAATCGACTAGTTTGCAGAGATGCAATAGGAATGAGAACCAGAAGAAGAATACCCTAATTCAGGGAGAAAAAACGACGGATCTCCGGCTTCACCCAGACCTCCAA
AACCAGCGCGGTATGAAATATTTCCTATTTCATCACGTAATAAATAAAAAAGAAGAAA
>comp158945_c0_seq6
AAAATAAGATAAAAGAGAAATATATATATGAGCATAGACGCCATTCAGAAAATAAATGCAAGTAAGTAAAAAATATAACTAACAGGAAATTTAGGAATTGCATTTATAGAACATGTGCACG

CAGTAAACACTGAACAACAAAAGAAAGACATTGGCCTAACCTAAAGTTAGTAGTCGTGTCCTAGCAAACCAATCTAAGTTTGTGTAGGTTAGGTAGTTTTGGATGGGTTTATGTAGGGAA
GTTTAATTATAATTGCTTTCATTTGATTTGTTAAAAGGCAAAAGAAAAATTTTAGTTTAAGTTTAAATATTAATGACTTAAGATTTATTTTAAGAATTTTATTTTGTGTTTAGTTGATTTTATTTT
AATTGGTTGTAAATTAATAAAGTAAAAAGATTTTAAATAATTTATAATCAATTAAAGGATTAAATTAATTAAATTTTCAATCAACTGGTTCATGGCATTGTGC
>comp158970_c0_seq1
CGCTCGTCGGGGCCCATGCGGGTTATATGATTGCACTTGCCCGCTTTTGTGTACATATGTGCATACATATTATAGTGCATGTGCGGGGGCATTCTTAAAATATTTGTAAACGCCTTCTATCCGC
TTGCATAGATGTTGTGGATCCGGTGGCGTGGCGTCGTGCTCAGTGGCCCGTGGTTCTTACTACTGATTCCCTGCTTTTGCTTACATGTGAATCGTAACATTGTCACCTGATTCCCACAAAAT
ATCTCATTGTTCTGGTGTAAACTCCCTTTTCTCATTCACTGGGAGGTCTTTCTAAGGCACGTTTAAATGATTGCATTCTTACTGATGCGTGTATCCTTTTCAAGATCTACAGTATTGTGGATTT
CTATAAGATGTAGCCTCCATGGTTAAGTAGTTGGCCTTTGAGATAAGTTGAGGTATTTAGGTGCTCTTGTCGTTCCTGACTTCTTCAATAAGAACAATAGTCTGATCGTCATAAAGATATTGA
AGGAATTTATATTTTATTGAAGGAACTTTGGAACACCAAGAAGTTTAGTTTGAATTTGCTCAAATATCTCTTCCATTCTTACATGTGCCATTCTCATACAAGGGTTCTCTTCGAGGAGTAAAC
CGATGCTCGTATTTGTCATATGCTCTTGTATACTTTTGTGTCCACATGGGTCTTCCACAATGTACCCACTGTTGTTGTCCACAAATGGATCATGTCTATTTACAGGGACCGGTCATATTCAGTA
CGATCTCTTGAAAGTGTTCGTAGAAGAGTCATTATTCCTGTATCATATCCACCTTTCACCAATTTCAAGTGATTTTTATGCACTTGTTGGCGGTAGTGAGTTGTATACAGCCTGTTGGTGTTG
ATTGCAAAGCATCACAGCCTTTAGTATCTCTTGTCTTCACGTGATTTATTGAATGCGATAGGTTTTCCCTATTAATTTTAGGTGCTTCTATTTCAATCTACAAGAGAACTGTCATTTGTATAAC
AAATCATCTTTTCATCAATTTCCAGTGTTGTTATCTCAATCTACAGGCTAACTACTATTTGTATTGAGGCTCTCATCGATGTAAGTAGGAGACACCTGTGTATTCTTCAGTCAATTGATGTTGC
TTACAAGTACATTGTATTTGACTACATAGGGACTATTGTAGGAGTTTATTGTATCTGCATCATGTCCACCTTCATCATTCACAGGTGATTTGGTGTATTTGTTTGCAGTGATGGGCCTTCTACT
ATCACTTGCCACTGTAGCAATCTCAAACTATAAGCATTGTTGCTTGTGTTAGGGATTTCGTTGATGCATGTGGGAGACATCCATGCATTCAGTAGTTGGTTGTTGTTTGCAAGTACAATGCA
TTCAACCTCATAAGGACTATTATAGGAGTCCACTGCATCTATCAACATCATTTGTTATGTACCGTATATTCAGGTATTGATGATTGATCCCCGTTTGTAGCTACTCATATTAATAATCATTCATT
GCATGTATGCCTTGGGGTCTTTATTCATTAATTGTCAATGTCGCCCTCTCAATATAAATGCTAACTGTTGTTTGCATGATGGGTCTTTGATCGTTGCATGTGAGTAGTACTTGTGTATTTTGAA
GTTGACTGATATTGATTATAAGTCAGTTGCACTTGAAAGTTTTCCCACTGCGAGTCCCCTGCTTATGGTTTGATATTTGTTTGAATAATCAAAATTATCATTTATTTTTTTGTATTTTGAATATT
ATTGTGCATTTTAGGGTTATGTTCCCTGCAGTTTAGTTCAAGCCCCCTGTTATACAAGTCTCATGTGCATCTGTTCAGGTTAGGTGCATTTATGTTCAGGTACAGATACTATGTATTACTACTC
AAGTTAATCCTTCTTTTTTGTTCAGATCTTGTATACTTCAATGTAAATTTCAACAACATCCCGACATATCCCTCGTTTGAGGGAGATATGGGGACTGAGCCATCCCACTACGCTACTAGAGC
GTGTATGGTAG
>comp158970_c0_seq2
CGCTCGTCGGGGCCCATGCGGGTTATATGATTGCACTTGCCCGCTTTTGTGTACATATGTGCATACATATTATAGTGCATGTGCGGGGGCATTCTTAAAATATTTGTAAACGCCTTCTATCCGC
TTGCATAGATGTTGTGGATCCGGTGGCGTGGCGTCGTGCTCAGTGGCCCGTGGTTCTTACTACTGATTCCCTGCTTTTGCTTACATGTGAATCGTAACATTGTCACCTGATTCCCACAAAAT
ATCTCATTGTTCTGGTGTAAACTCCCTTTTCTCATTCACTGGGAGGTCTTTCTAAGGCACGTTTAAATGATTGCATTCTTACTGATGCGTGTATCCTTTTCAAGATCTACAGTATTGTGGATTT
CTATAAGATGTAGCCTCCATGGTTAAGTAGTTGGCCTTTGAGATAAGTTGAGGTATTTAGGTGCTCTTGTCGTTCCTGACTTCTTCAATAAGAACAATAGTCTGATCGTCATAAAGATATTGA
AGGAATTTATATTTTATTGAAGGAACTTTGGAACACCAAGAAGTTTAGTTTGAATTTGCTCAAATATCTCTTCCATTCTTACATGTGCCATTCTCATACAAGGGTTCTCTTCGAGGAGTAAAC
CGATGCTCGTATTTGTCATATGCTCTTGTATACTTTTGTGTCCACATGGGTCTTCCACAATGTACCCACTGTTGTTGTCCACAAATGGATCATGTCTATTTACAGGGACCGGTCATATTCAGTA
CGATCTCTTGAAAGTGTTCGTAGAAGAGTCATTATTCCTGTATCATATCCACCTTTCACCAATCTTAGATGGATTGATGATAGAATTTGATGCAGGGACAGATCTTTGATTACTACATATGGAT
AATACTCATACACTTTGCAATTGTCTGTTGTTGCTTACAAGCATAATACATTGACTTTATAAGGATTGTTGTAGGAGTCTGTTGTATTCATCAGTATCATCCACCATGTATTTTGTCTCATGTGT
TCATAAGTTTTGGTAATTAAGATTTGTTTATGATTGTTCAAATACTGTGAATTGATTACTGTTCATTGCTTGTATACATCAAGGTCCTGTTCGCCACA

>comp159055_c0_seq4
ATCTTAGCTCTATTTTTCCATGACCACTTTCACTCAAAATTTTGATTTGACACACTCTTCACTGCTACATATCTTATTAAATGGCTCCCTTCAAAAACACTACATAATACATCCCTCTATGAGC
ATTTATATCACCATCCTCTAGACTACAGTCACTTTAAAACCTTTGGTTGCCTATGTTATCCTTGGTTCCCAACCAAACTTACAAATAAACTTCAACCTCATTCCAGCCCGTGTATCTTTCTTG
GTTATGTTGCTCATTCAAAAGGCTATATATGCCTTGATTCCACTACGGGTCATTTGCATATTTTCCTACATGTCCAATTTCATAAATCCATTCATTCTCCTTGGTTAATTTCTCTTTACTATTTGG
TTAATTTCTCTTTGTATTTGGTTAACTGGGCAAAAGAACTTAGATAATTGAGTAAATGTGGAGACAAACATGTCCAATGAAGAGCAGGAAAAAGCAACAAGATTGCTGATTGCAAGCAGT
ATGTACAAGCGCACAATTGGATTAAAATCAAAAAATAAAAGCAATATGCACACGTTTACAAGGTAGTTAATACCAAACAAAAAACAGTATGTTAAAAGAGCTAAGGAAAAATAAAATGGA
TTTGAAAAATAGCATGCATTTGAAAGATTTCAAAATAGTTCGGCGAGCTTATATGAAATGCGATGGGGAGTTCAAGCCAACTGGTGATTTGATGGTTTTGGCGATCCACAAAGACGGATG
AGGGGTTGTGTTCGGCGAGAATGAGATGAACGAGAACGTCAAAGAGTTAGAAAGCGAAAGAAGTGAGAAAGTGATGAGTTCGATTTTAAATGGAGATGTGTTCGGCAAGGTACCTTTT
CAGTGCGGCGATCGGATTTGGAACGGTCGGGATGGAGAAGTAACCGATCGGGTGTAAATGCATATGCTTTGGTGCAAGTGAAACGGCATGTTGCGGCAGGCGGAAGTGGAATGCTCGGG
TGTGATAGCTTTCGAAACGGTCCGGAGGAGATGAAGTCGATCGCCTGGAAGATGATCCGTTCGGCTAGGGTTATTGTAACCTTGCGGGAATGCAAAGACGAACGGACGCCCGGAGTGAT
AGCATCGGAATTCGTTTGGCTATAAGATGTACGCTTTCGGCTAGGGTTAC
>comp159070_c0_seq10
ACAAATAGGTACTGAACAAATATCCGTAGAAGAGATTTAACATAACAAACTATAAAAGACTAATGAAATAGTTCTAAAATAAAAAATCACTCAAAGAACGAAAAGATTTTAAATTATAATC
TCATTAGTAGCCGAATACATCATTCATTTCTTCTTGCCCGACTTGGCAACATCTCCAGCTTTGGTCTTGTTCACACCACGGATTTTCTTAGCTCTGTTCTTTCTTTCTTTCATCTGCTTCCTTG
ATTTCTCCACCTTTGTGGCCAATCCATTCTGCCAATTATACACAATCAGCACACAGGTTTGTATTGAGAAGCAACAAATTTAAAATGTTTTAAGCAAGCATGAGAGAAATATTTGAAATGAT
GATGAGGAAAGTGCCAAGTAAAATATGTACAATCCATGTTAGTGCCAAGAAATATCATTTTAAATGTCTGCATAGCTAAATAATATGGTTTCTACAAGTATAAATGATAAAAGATGAAGATCT
AATTTTTAGATTTTAAGATTACTTTCTAATCTGAAGGATGGGTATCATTGATGATTAAGGGTCCACTTCATGATTAATTGTTCTGTATGGATACCAAACGACGTCTGGAGATCAATCAAAATG
TGCTACGTCACCCGTGCGTTTGATACCGCGGCTCAGTACAGCTGACATGGCGCATTCTGATTGGCCACCGGATGGCGTTCAACGTCTGGTCTCCGCACAGAATATTTCTCCCACTTAAAAT
GGTCCTCTACTTGTAGAACTGCCGTGGGTGTTATTAACTACCATGATAACATGCCCAATTTTAAGTTTAAAAGTTGAATGAGAAAATTATAGCTGAATAGAATTCTGCCAACTATCTGATAG
CTAAAATTGAAGATCGAGTTCTGATCCTTAACATCGTACAGCTTCGCTAGCTTCTCTTTCAACTCAACCTTTAGACAAGCAAGCAACAACAATCAAAATTTTAACAAAGAATGAAACCGA
AACGATCGAAAAACTCAAATTAATAGCTAGATCTCAGCGTA
>comp159070_c0_seq9
CCCGGATGAAGAACATCGATCACCTTAAAACACAATACAAATTAACTCAAAAAAAGATTTAAAATCAAAGGCTCGCTCTCAGAAAATAGACAACTGAGGTTTTAGGATTAACTCACGAAC
TGCTTGCGCGAGAGAAGGCGATTAGTCATGAACTTTCGGGTCCTGATGGTAACTGCTTTGGAATCTGCCATTGTTTTTCCTCCCTCCGATCAGGAAAAGCTCGCGCCGCCTGTTCAGGTG
GATGTGTGATTTTACTCTTAGAGACGGAGTGTGAAACGAGTGTATATGTGAAAAGTTTGGAGTGGCTTTGGTCGCATAAAATGTCGGTCTTCTGATGACCTGGTAAATACGAGAGCAGATG
TATTAGATTTGATCTATATTAATGCGGAGGTAGACCTAAAAAAAAACTTGGTTAGAAAATATT
>comp159092_c0_seq3
TAGGCAAGCCTTTCTTTGTATCTTAGAAGAAAACAGCAAATGGCACGTTAGGCATGTTAGGAATGTAAACGTGATGCGCTTTCATTGATTCGTGCACATAAACTGTCGGTTTTTAATCAGG
CAATTGGACAAACTTTAATAGGCGGAGGATAACGGAGGAAGAGACATTAACGTGGATTCCTCCAGCTGCCAGCCTTCCTAAACAAAACCATTGACCTTACAAGCTGCCCGGACATTCCC
ACATCGCTCGCCGGCGGCCAGCCCACCCCTCCTCTATATCTCTGCCCCTCTTACCCCCGCGCGCCGACGAATTATCTATATTGTATACCACCCCTTTCGCCCTTCCATAGCCAAAACAGATTA
GACAGAAAAACGCTTAGAAAAGGCAGCAGCGACGCCCGGTGGGCGCGCGTCGCAATGACGTCGTCAACGAGGCAATGGTGGAGGCGAGCAACTGCGGCAATGAAGGACAAGCGGAG

CGTGTACCTGAGCCGAGTTGCGGGCGGTGGGCATCGAGCTGAGATGGAGGCTGCCGTGATTCACGCCACCAACCACGATGAGCGCTCCATCGATTACAAGAACGCGGCGAGGGTGTTC
GCCTGGGCGCGCACCTCGCCCGCCTGCCTTGCCCCTCTTCTGCGAGCGCTTACGCGCCGTGCGACCCGGACGCGCTCGTGGGCTGTGGCGCTCAAAACCCTGCTGGTCACTCACGGCGT
CCTCCTCTGCTCCGCTGCCGCCCCGTGCGTCGGCCGCCTCCCATTCGACTTCTCCGACTTTCGTGATCAATCCGCTTGCCCCTCGGTAGCCTCCTACGGCTTCTCCGCCTTCGTTCGTGCCT
ACTTCCAATTCCTCGATTACCGCTCCGTCCTCTCCTCATCTAGTTCCCAATTTGATAACCAAGACAACAACAACTCCTCCTCCGTCAATTCAATCGAAGGTGATCTCGAGATGTTGGCGAA
GATGCAAGATCTGCTCGAGCTGTTAATGCAGATCCGGCCATATGCTGACGGAATGGAGGTGGGGCTCATCCTTGAAGCGATGAATTGTGTCATCATTGAGATCTTTGAGGTATATAGCTGCA
TTTGTAGAGGCATCGCCAGATTCCTCATCGATGTGCTCGGCTCTGATGTTTCCAACTCCTCTGCCTCCCACACCGCCGTCGCATCCTCAAAGGCGAAGATGGCCGTGCCTTCCGAGGAGC
GGAAGCGACTGGGTATAGCAGGGATGAGGAATCTGGCGATGCCTCTACAAATGCAGCTATATACCTCAAAGATCTCAATGATGACACAATTCATCGCTTCAAGGATGAGCCCCACCTCCAT
TCCGTCAGCATATGGCCGGATCTGCATTAACAGCTCGAGCAGATCTTGCATCTTCGCCAACATCTCGAGATCACCTTCGATTGAATTGACGGAGGAGGAGTTGTTGTTGTCTTGGTTATCA
AATTGGGAACTAGATGAGGAGAGGACGGAGCGGTAATCGAGGAATTGGAAGTAGGCACGAACGAAGGCGGAGAAGCCGTAGGAGGCTACCGAGGGGCACGCGGATTGATCACGAAAG
TCGGAGAAGTCGAATGGGAGGCGGCCGACGCACGGGGCGGCAGCGGAGCAGAGGAGGACGCCGTGAGTGACCAGCAGGGTTTTGAGCGCCACAGCCCACGAGCGCGTCCGGGTCGC
ACGGCGCGTAAGCGCTCGCAGAAGAGGGGCAAGGCAGGCGGGCGAGGTGCGCGCCCAGGCGAACACCCTCGCCGCGTTCTTGTAATCGATGGAGCGCTCATCGTGGTTGGTGGCGTG
AATCACGGCAGCCTCCATCTCAGCTCGATGCCCACCGCCCGCAACTCGGCTCAGGTACACGCTCCGCTTGTCCTTCATTGCCGCAGTTGCTCGCCTCCACCATTGCCTCGTTGACGACGT
CATTGCGACGCGCGCCCACCGGGCGTCGCTGCTGCCTTTTCTAAGCGTTTTTCTGTCTAATCTGTTTTGGTTATGGAAGGGCGAAAGGGGTGGTATACAATATAGATAATTCGTCGGCGCG
CGGGGGTAAGAGGGGCAGAGATATAGAGGAGGGGTGGGCTGGCCGCCGGCGAGCGATGTGGGAATGTCCGGGCAGCTTGTAAGGTCAATGGTTTTGTTTAGGAAGGCTGGCAGCTGGA
GGAATCCACGTTAATGTCTCTTCCTCCGTTATCCTCCGCCTATTAAAGTTTGTCCAATTGCCTGATTAAAAACCGACAGTTTATGTGCACGAATCAATGAAAGCGCATCACGTTTACATTCCT
AACATGCCTAACGTGCCATTTGCTGTTTTCTTCTAAGATACAAAGAAAGGCTTGCCTAGGGCTCCGTCGCTTCCGTTTGATGGGCG
>comp159094_c0_seq9
ATTTTGTTAAAATTTTAATCAAAAGTGAAGGTCTTCAACATCTACAATGCATTGTGGGTGATTCTGGTTCGGATGGGTCTTATGCTAAGATGCCTCTTCTCCCAGCAAAACTTTGGCTGGCC
TGGATAATATTTTTCTATGCTGTTGAACTTGATTCTGCGGCAGGACTACGTGTTTTCATTTCTTCTAGATTTTGGTGCCTCTTGGGTTTTTCATGAAGAGAAGTATAATGTTGCTATTATCTATA
GGTCAGGTTCGCTTTTTTTTTTTTCTGCTGTTCCTATATGAGGATATGGTTTTGGAGGTGATTTTTGTAACCCTCACTCCTTTAGGCTGTCTTTTCATGCCTGAGTCTCCTAACTTAGATCAAT
CCCTAAACATCAGTGATGGGGACATCAAATTTCGACACGTGTACCATCGCCG
>comp159122_c0_seq1
AAAAAGGAAGGGAAAATAAGAAAAAAAATATAAAAATCAAAGCTAATGAGAAATAAAATAAAATAAAATTTACATAGGGTTATGGAATATAATAATCACTTTTAAATTCTCAATAAATTCAT
AAGACCATCCAGCACTGTAGTATTCTATGCCTTACATGTCTTATATTCTTATTCATAGAAGCGAGGATTGTATCTACATGCGCCGCCAATAGGATCCGACGGCAACACCAGGAATCAAGACG
TGGGGTTTCGCAATCGGAACAACTCCTCTGCATTGGCGCCATCGTCTCCGTCGTCAAAGTTAAGTGCGTAGCTGATAGGGTCGTACCTGAAGTCTCCAAATTGGCGGCCTGATGATTTCCC
GATCAGCCTCCGGCAACATTCCTTCATCTCCGGGAGCTCCATAGCCTTTGCACGGATCCACGACGGCGTTGATCGAATCACCGATGCCTCCACCACCGGCGGATCTTCCGAATTGGCAAC
GCGACTGCAACAAGGGAACCTGATTATCGGCCGGGTTTTATGGCGCGGCGATGGAGAAGGTGATGCGCCAATGTTCTGGTCGCGGCATTCCATTTCCAGTGAAGAATTACTCTAGGGTTA
GGGTTTTGGAAGAACGGAGCAAGGTGATGAAGGCGGAGCAGTCCATTTATAGCTTCGGAGGGAAAGGGGTGGATAACGACGACGCGGCCGAAGAAAGAAAGACCGTTTCGGTTTCGG
TTTCGGTGGAAAAAACCTTCGCGTCGAAGGCATTAACGTTATTTTTCATGGGAGTAGTTCACGATTTTCGCGGTCATTGGAAGAATTTTG
>comp159139_c0_seq5
ATAGTCTATAAAAGTTTTTTGTTGAATCTATTTTGAAATAAATATATTATAAATTTATTTTTTCTTAATTATTACACTTATTTTAAAATAAATTTAATGACCTTACCTACAATCAAATTAAAAATTT

GATCATATATCCTGAATATAATTTCTTTGCCACTGCCTACTCTTCTTTTTTGCATGCATGCAAAAGCTTAGAGTCTCCGAAACCATGACGAACGCACCGCTGATCCTCACCTTAGATTGTGAC
A
>comp159218_c1_seq3
TTTGATTAATGCATTTTTCCCCTTCTTACAGATGCAAACTTTGATAAGCCGTGCACAGATGGATTCATTAATATCATATTATAATCCGCTGGTCTATTATTGTTATATATGACAATTCCCATGTTC
AAATTATTACAAATACAAACCAAACCACACAATATACCCGTCTTTTATCTCTCGCCACCCATCGACAATATAAAGGTCGAGCAACGGAGAGTAATGGTTCATTGATTCTGGCTTCTCAGTGC
AAACTGCTCATAGCAGAAGCCTTGAAGTTCTTCGTTGAGAGCCTGGGAAGTACAAATAAACCAACAATACCGCAAACGAAGGCAAACACTGAAGCAACGGCAAATGCAGGCATGTTCC
CCTTCTTGAAGATCCCATCCCATGGACCAGCTCCAAGTGCAATTACCACCTGTGGTATAACAATAGATATATTAAGTACTCCTGTGCACAGTCCTTGACCACCTCCTTCGTTAGCAGCTAAT
TGTGCCGTGACTGCAAATGGCACGCTGTACAGAACTGCTAAAGGAGCGCCCAGAACCGCGAAGACGACCAAGGCAACAGCCTTGACGCTACCGTCTGGAGAGATGATAGCATCATGAA
TGCTGCCATGGTAGCTGTGCACAGACCAGCTGCTGATGATGGCGGTGGCGGCCATGCATATAAACATGCAGAAGTTGCACATAACCCACACTATTCTTGTTGTGAATTTTCGACACAATGG
CTCTATCAAAATTGAGGTCACTCCAAGAACCACAGAGTTTATGAGCAAGCCTAATGCGCCTGCTCTGACTCCATTATCATAAGCTTTCATCTGAGCTGGAGTTCCCTTTGGATCTCCATGGA
ATATTTCTCGGCCCATCCAATCTGTATCGTAGAGGTTGAATGGAAACCATGACAGCCAGGTGAGGGAAGTGACTAGAAGAATATAAGGCATTTTTGGTGGAAGATTCTTGATAGTTCTGAA
GATTTCCATTATGCCAACCCTTTCTTCTACTGTAGATGGAGCTACTTCTTCTCTACCAGCAATCGAAGTCACGAGTACACTTAGCAGAAGAAAGACCACAGCAACTAAAAATGCTCCTTTC
AAATTTGCGCAAGCTTCACAGCAGGCTCTGTTAGAAAGGAACGGAAACCACTCGTGCCACTTTCCACTGGCACCAGAAGAATAGCCTAAGATGTTCCCGATGGCCATCCATGAGGCAAA
TATTGCATTTGCTTTACTGCATCCATGAGTCCCTGTAACAGACAGATCAGCCATGAGGGCTCGAGTTGGACCCTGAACTGTATTATTTGATAGATCGAGCAGCCAAAATCCGGCGACGTAG
ACTATTGCAGCCCTCCACCGTGGGCCGTGGTAAATGCTGCAATCTTCCTTGGTATCGCCTAACTTGTATCCAATGTCAGAAGAGAAGGCAATTACAATCACTGCAAAACAAATAAAGATGC
AACCGACGAGTATGAATGGTCTTCTCCTTCCAAATCTTGAACGGCATCGATCGCTCCATAAACCAACGCATGGTTGAACAACAAAGCCAGCTACAGGGCCGCATAGCCATATCAAAGAAG
ATAAAGTATGAGACAGTCCCAGCGTCTGGACGTAGGGAGTGAGCAGCGATAGCTGAAGCGCCCATCCGTACTGTACTCCCCCAGCCACCATGCAGGAGAAGATCAGCCTCACTAGGCTT
ATCTGTTTTCCCACCGCCGCCGCCGCCGCCGCCTGCTTCGCCAGTCTTCCTCGAACGATTCCGTCCACGATGCATGTGGCGCTCACAGTACTTCTGGTTGGGAACCACGTCACGCGAACA
CCGCCATTTCTTTCCATCCGTCCTTCTACACCTCCCCGGCTCCGGCTCCAGACTGTTCCTGTAGTCCAAACACAGATCTCCGCATCCCAAAACGCAGTTGGCAGCGGCGACGCTTTTCCAT
ATGGGGAGGACAAGGTGCAGCGGGACGGGGAGGCCACCCGCCATGTACTTGTAGATGAGGGCTTGATGCTCCAGCTCTTGCATTTGCATGAATGTAAAAATCGGTTTTTTGGGGGGGCT
GGAAACGGAACTCCCATTGAGACCTAGTTGAAGGGCGAGGGTTAGAGGGGAGGGAGACGCCATGGCCGCGGCTTCTTGTGACGTCGGATATGAAGAGAGACGGGGGATCTTGGAAGG
AGGAAGAAAAGGGGGAGTGTTGCTGTGGAAAGATGTGTGTTCTTTGGTTGCCATTGTGGTTCTAATGGCACGAGAGCTGATGAAATATGAGTTGTGTGCCCCTCGGCTTGGTCATTTGAG
ATGCAGGGCGGAAGCTGCTGCGTTCAGATCGAGGGAATGATAACAGAAGAGCTTCAAAGTTTTATTGGGCCTGTGCTCTATGAAATATGAGTTGTGTGCCCC
>comp159231_c0_seq1
CCGACCCCCATATAGTGGGATAAATGCTTGTTATTGTTGTTGTTGTTAATAAAAAAAAAAAAACTATTATTATTTTTAATACCTTCTAATTTTAAATATTTTTCACCTTATATTATTTAGCATCTTA
TTTTAATACAAAATGTATCACGATATTAATACAATTTTGTTTGCAATACACAATGTGAACGAATGGATATGAGTAGATCGTTCTAAACAAACAAAAACAAAAAACAAAAACAAAAACAAAT
GCCTTCCGGCTGAAACGTCAATCAATCGGTCATCTTCAAATGTAGAAACCAAAAAGCTAAAGAAAATAGAACTAAAGAAAAGCCAATGGATGGATGCATGGATTAGCAAGGCAATCCGA
TCTTGCAGTTAAGGGTCCCAGAGTAGCGTGTGAGCCTAGTTACGAACGTCCCTGGCTTGGCCCTCATCATATCATGCCTCGTGTATCTGCTAATAAATCGTTGCCCTGACTCAACGAAGAA
CGCTCCATTTATCATCAGATCACCCTGCGACTTCCACGTCCACTGGCTCCAGATAGCCTCCGGTGCATAATCCCTTTTCGTCACCTGCTTGGCGTTGATATCCGGCGGAGCAATGTATCGAT
TCCCCTGACTGATAACAGTGGGATGCTGGCTTCCCCCGACGGCGTACATTTGCCAATGTGTGTAGTCATTGTTGACGACATGGACAAAGCCCCACCGGCAACGCGGCATCCGTTGCACGA
GTCCACGCCCGAAGTGGTTGAAGGCAACCGTTACCTGCATAAGGGCATCCCCGGCGAAGGAGTCGCTGGCGCCAAAGAGCATTACATCGTCATGATTTGTCATGTGACAATTGGAAATG

GTGATGGCGGTGGATCCCATGATGGCGTCTATTAGGCCATCAGCACAGTTGGACATGGATATGTGGTCAATCCATATGTTGGAGGCACCGTAGATGGAGATGCCATCACCATCGCTGCGAG
TGCGAATGCCATAATGTGAAGGGGAGTCGCGGATCATGCCGCCGTTGCCAGGGCTGAGATGGTGGATGTGGAGGTTGTGGATGATGATGTGGTGGACGAATTGGATGGTGATTTGGGCGC
CATAGGCGATGTGGATGTTGGCGCCACGACCGTCGATGGTTTTGTAGCTATTTACTATTAGCTCCTCGTTGAGGCGGATCACCATGTCACGGGCGAACACTATCCACAATGGCTCAGCCTA
AGTATATATCATGCGAAAGTTGTGAACCTGAATAACGGCATGGCGAAGTGTCCCCGGCTTGGGGTTGACAAGATCATTGTCGGACGCATCTGTCACTACATAGAAGCGACCTTTGACACC
ACCCATGGCATTGCGGCCAAAGCCCTTAGCACAAAAGGCCAATCTCTTGCGGTAACTGGCCCAATTAGATTGGCAGCGCCAGCATCGATCAATGGGGTTTGTGGCCCAGCATGGATCTGT
AGGACTTCGCTTCCTTGATATTAGGGATCTGTGTGTATTGTTCTTCTCCAATGCCATGAACACATCTCTATTGAAGTCGTTGGTGGCCCCATGTGGGTCGGGGTTATAAGACTCACGGACCT
TAGCGCGGGAAGCCTCAGCCCGCTTCTGCCAGAAATCGTCGAAATCACCAATGTGCGAGCGAACGGGCGCGGTGAGGATGAGGGCCGAGAAGAGGATGAGGAAGCAAGGCCTGAACT
GTACGCACTCCATTCTTCTTCTTCTTCTTCTTCTCGCTTTTTTTCTTCTGTTTACAGTGCGTGTTTTCTTCTGTAATCGTGTTACTTTTTTTTTTTTTAAATCGGAAAGCAAAAAAGATTACAG
ATGGTCGGAGAGAGTCTGGAAAAAAAAGGTTTTGGAAGCAAAGCAGGATAACCAGACCCGACCGCTGGCCGAGCGAACCTCGAAGATCGCTAGAATGTGAGGAACATTGGGTTGGTGG
GGTGGGTATGTTTTTAGGGCTTCGGCTAGGGAATGGAGCTATATAGGATTGTTATGGAATCCAGTAGGAATAAACAGAGGAAAAGAAGGATCGGGCCGACAAGAGTGGCCAGCCATTAGC
AATTCCGCATTTAGGGACGGGAGATTTGGCTGCGCATATTAGTTTATCTGGCTCTGCGTATTTGCATATCTGGCGGTTATAGATCTCCTATATTGTTAATATTTCCTTGATATACTTCTCTTAGC
ACGGAAAGGTGAAGGATGAGGAGAGAAAATAGAGAGGGTGGTTGTTAAGGGCGATTGGATCGTTTATGAAATCGGGAAATT
>comp159231_c0_seq10
TCAATAATTGTTGAACAACTTCAGCATCTATTCAAATGCCCTTATTCTCAATCATCAAAGAATTTCCAAACATTCATTCAATGTACCTATATATAATATTCATGATACAGTGCTTCTCCATTCCA
ATTACTACAACTTTAAGAAAGTACTCAACATATAATTTTAAAAATAATTAAAAAAAAAAAAACACAGTCACACAATATGAGAATCACATATGATATATCCCATAAAAGAAGCACTTTTATGAA
GAAAAAATAAAGAACATGAAATAAATTCCATATACTTTCCCAACACCAAAGGAGTGAAGCAATCAGCAGCTCAACACTGCCTTCCAACTCTACAACGCAAAGTTCCTGCAAACCTCGTC
AATCTCTTCACAAAAGTGCCAGGCTTGGCTTTTATCATGTCCTGCTTCGTATATTTCCTTATGAACTCCTTCTTCCCCGACTGAACAAAAAACGCCCCATTTAACATCAAATCCCCTTCTGAT
CTCCAATGCCAATTCCTCCATTCCCATTCCCCAACATAATCCCTATGCGTCACCTCCTTCGCCGCCCGATTTGGTGGCGCGATATACCTATTTCCTTGACTCACTATCGTCGGATGACTACTC
CCACCAACCGCATACATCAACCAGTGCGTGTAATCGTTATTCACCACATGAACAAACCCCCAACGACACCTCGGCATTCTCTGCACCAATCCCCTTCCAAAATGGTTAAAAGCTACAGTTA
CCTGCATCAGAGCATCCTCCGAATACGAATCGCTCGCACCGAACAGAAACACATCGTCGTGGTTCGTCATGTGGCAGTTGGACACAGTGATTGCCGTCGACCCCATGATCGCGTCGATCA
GCCCGTCCGCGCAGTTGGACATTGAAATGTGATCGATCCAGATGTTCGAAGAACCGTAGATCGAAATCGCATCGCCGTCGCTGCGGGTGCGGAAGCCGTAATGGTTGACGGAATCGCGA
ATCATGCCGCCGTTGCCGGGTTTGATGTCGTGGATGTGGAGGTTGTGGAGGATGATGTGGTGAACGAATTGGATGGTGATTTGGGCGCCATAGGCGATGTGGATGTTGGCGCCACGACCG
TCGATGGTTTTGTAGCTATTTACTATTAGCTCCTCGTTGAGGCGGATCACCATGTCACGGGCGAACACTATCCACAATGGCTCAGCCTGAATAACGGCATGGCGAAGTGTCCCCGGCTTGG
GGTTGACAAGATCATTGTCGGACGCATCTGTCACTACATAGAAGCGACCTTTGACACCACCCATGGCATTGCGGCCAAAGCCCTTAGCACAAAAGGCCAATCTCTTGCGGTAACTGGCCC
AATTAGATTGGCAGCGCCAGCATCGATCAATGGGGTTTGTGGCCCAGCATGGATCTGTAGGACTTCGCTTCCTTGATATTAGGGATCTGTGTGTATTGTTCTTCTCCAATGCCATGAACACA
TCTCTATTGAAGTCGTTGGTGGCCCCATGTGGGTCGGGGTTATAAGACTCACGGACCTTAGCGCGGGAAGCCTCAGCCCGCTTCTGCCAGAAATCGTCGAAATCACCAATGTGCGAGCGA
ACGGGCGCGGTGAGGATGAGGGCCGAGAAGAGGATGAGGAAGCAAGGCCTGAACTGTACGCACTCCATTCTTCTTCTTCTTCTTCTTCTCGCTTTTTTTCTTCTGTTTACAGTGCGTGTT
TTCTTCTGTAATCGTGTTACTTTTTTTTTTTTTAAATCGGAAAGCAAAAAAGATTACAGATGGTCGGAGAGAGTCTGGAAAAAAAAGGTTTTGGAAGCAAAGCAGGATAACCAGACCCGAC
CGCTGGCCGAGCGAACCTCGAAGATCGCTAGAATGTGAGGAACATTGGGTTGGTGGGGTGGGTATGTTTTTAGGGCTTCGGCTAGGGAATGGAGCTATATAGGATTGTTATGGAATCCAGT
AGGAATAAACAGAGGAAAAGAAGGATCGGGCCGACAAGAGTGGCCAGCCATTAGCAATTCCGCATTTAGGGACGGGAGATTTGGCTGCGCATATTAGTTTATCTGGCTCTGCGTATTTGC
ATATCTGGCGGTTATAGATCTCCTATATTGTTAATATTTCCTTGATATACTTCTCTTAGCACGGAAAGGTGAAGGATGAGGAGAGAAAATAGAGAGGGTGGTTGTTAAGGGCGATTGGATCG

TTTATGAAATCGGGAAATT
>comp159231_c0_seq13
CCGACCCCCATATAGTGGGATAAATGCTTGTTATTGTTGTTGTTGTTAATAAAAAAAAAAAACTATTATTATTTTTAATACCTTCTAATTTTAAATATTTTTCACCTTATATTATTTAGCATCTTA
TTTTAATACAAAATGTATCACGATATTAATACAATTTTGTTTGCAATACACAATGTGAACGAATGGATATGAGTAGATCGTTCTAAACAAACAAAAACAAAAAACAAAAACAAAAACAAAT
GCCTTCCGGCTGAAACGTCAATCAATCGGTCATCTTCAAATGTAGAAACCAAAAAGCTAAAGAAAATAGAACTAAAGAAAAGCCAATGGATGGATGCATGGATTAGCAAGGCAATCCGA
TCTTGCAGTTAAGGGTCCCAGAGTAGCGTGTGAGCCTAGTTACGAACGTCCCTGGCTTGGCCCTCATCATATCATGCCTCGTGTATCTGCTAATAAATCGTTGCCCTGACTCAACGAAGAA
CGCTCCATTTATCATCAGATCACCCTGCGACTTCCACGTCCACTGGCTCCAGATAGCCTCCGGTGCATAATCCCTTTTCGTCACCTGCTTGGCGTTGATATCCGGCGGAGCAATGTATCGAT
TCCCCTGACTGATAACAGTGGGATGCTGGCTTCCCCCGACGGCGTACATTTGCCAATGTGTGTAGTCATTGTTGACGACATGGACAAAGCCCCACCGGCAACGCGGCATCCGTTGCACGA
GTCCACGCCCGAAGTGGTTGAAGGCAACCGTTACCTGCATAAGGGCATCCCCGGCGAAGGAGTCGCTGGCGCCAAAGAGCATTACATCGTCATGATTTGTCATGTGACAATTGGAAATG
GTGATGGCGGTGGATCCCATGATGGCGTCTATTAGGCCATCAGCACAGTTGGACATGGATATGTGGTCAATCCATATGTTGGAGGCACCGTAGATGGAGATGCCATCACCATCGCTGCGAG
TGCGAATGCCATAATGTGAAGGGGAGTCGCGGATCATGCCGCCGTTGCCAGGGCTGAGATGGTGGATGTGGAGGTTGTGGATGATGATGTGGTGGACGAATTGGATGGTGATTTGGGCGC
CATAGGCGATGTGGATGTTGGCGCCACGACCGTCGATGGTTTTGTAGCTATTTACTATTAGCTCCTCGTTGAGGCGGATCACCATGTCACGGGCGAACACTATCCACAATGGCTCAGCCTA
AGTATATATCATGCGAAAGTTGTGAACCTGAATAACGGCATGGCGAAGTGTCCCCGGCTTGGGGTTGACAAGATCATTGTCGGACGCATCTGTCACTACATAGAAGCGACCTTTGACACC
ACCCATGGCATTGCGGCCAAAGCCCTTAGCACAAAAGGCCAATCTCTTGCGGTAACTGGCCCAATTAGATTGGCAGCGCCAGCATCGATCAATGGGGTTTGTGGCCCAGCATGGATCTGT
AGGACTTCGCTTCCTTGATATTAGGGATCTGTGTGTATTGTTCTTCTCCAATGCCCTTAGCGCGGGAAGCCTCAGCCCGCTTCTGCCAGAAATCGTCGAAATCACCAATGTGCGAGCGAAC
GGGCGCGGTGAGGATGAGGGCCGAGAAGAGGATGAGGAAGCAAGGCCTGAACTGTACGCACTCCATTCTTCTTCTTCTTCTTCTTCTCGCTTTTTTTCTTCTGTTTACAGTGCGTGTTTTC
TTCTGTAATCGTGTTACTTTTTTTTTTTTTAAATCGGAAAGCAAAAAAGATTACAGATGGTCGGAGAGAGTCTGGAAAAAAAGGTTTTGGAAGCAAAGCAGGATAACCAGACCCGACCG
CTGGCCGAGCGAACCTCGAAGATCGCTAGAATGTGAGGAACATTGGGTTGGTGGGGTGGGTATGTTTTTAGGGCTTCGGCTAGGGAATGGAGCTATATAGGATTGTTATGGAATCCAGTA
GGAATAAACAGAGGAAAAGAAGGATCGGGCCGACAAGAGTGGCCAGCCATTAGCAATTCCGCATTTAGGGACGGGAGATTTGGCTGCGCATATTAGTTTATCTGGCTCTGCGTATTTGCA
TATCTGGCGGTTATAGATCTCCTATATTGTTAATATTTCCTTGATATACTTCTCTTAGCACGGAAAGGTGAAGGATGAGGAGAGAAAATAGAGAGGGTGGTTGTTAAGGGCGATTGGATCGT
TTATGAAATCGGGAAATT
>comp159231_c0_seq15
CCGACCCCCATATAGTGGGATAAATGCTTGTTATTGTTGTTGTTGTTAATAAAAAAAAAAAACTATTATTATTTTTAATACCTTCTAATTTTAAATATTTTTCACCTTATATTATTTAGCATCTTA
TTTTAATACAAAATGTATCACGATATTAATACAATTTTGTTTGCAATACACAATGTGAACGAATGGATATGAGTAGATCGTTCTAAACAAACAAAAACAAAAAACAAAAACAAAAACAAAT
GCCTTCCGGCTGAAACGTCAATCAATCGGTCATCTTCAAATGTAGAAACCAAAAAGCTAAAGAAAATAGAACTAAAGAAAAGCCAATGGATGGATGCATGGATTAGCAAGGCAATCCGA
TCTTGCAGTTAAGGGTCCCAGAGTAGCGTGTGAGCCTAGTTACGAACGTCCCTGGCTTGGCCCTCATCATATCATGCCTCGTGTATCTGCTAATAAATCGTTGCCCTGACTCAACGAAGAA
CGCTCCATTTATCATCAGATCACCCTGCGACTTCCACGTCCACTGGCTCCAGATAGCCTCCGGTGCATAATCCCTTTTCGTCACCTGCTTGGCGTTGATATCCGGCGGAGCAATGTATCGAT
TCCCCTGACTGATAACAGTGGGATGCTGGCTTCCCCCGACGGCGTACATTTGCCAATGTGTGTAGTCATTGTTGACGACATGGACAAAGCCCCACCGGCAACGCGGCATCCGTTGCACGA
GTCCACGCCCGAAGTGGTTGAAGGCAACCGTTACCTGCATAAGGGCATCCCCGGCGAAGGAGTCGCTGGCGCCAAAGAGCATTACATCGTCATGATTTGTCATGTGACAATTGGAAATG
GTGATGGCGGTGGATCCCATGATGGCGTCTATTAGGCCATCAGCACAGTTGGACATGGATATGTGGTCAATCCATATGTTGGAGGCACCGTAGATGGAGATGCCATCACCATCGCTGCGAG
TGCGAATGCCATAATGTGAAGGGGAGTCGCGGATCATGCCGCCGTTGCCAGGGCTGAGATGGTGGATGTGGAGGTTGTGGATGATGATGTGGTGGACGAATTGGATGGTGATTTGGGCGC

CATAGGCGATGTGGATGTTGGCGCCACGACCGTCGATGGTTTTGTAGCTATTTACTATTAGCTCCTCGTTGAGGCGGATCACCATGTCACGGGCGAACACTATCCACAATGGCTCAGCCTG
AATAACGGCATGGCGAAGTGTCCCCGGCTTGGGGTTGACAAGATCATTGTCGGACGCATCTGTCACTACATAGAAGCGACCTTTGACACCACCCATGGCATTGCGGCCAAAGCCCTTAGC
ACAAAAGGCCAATCTCTTGCGGTAACTGGCCCAATTAGATTGGCAGCGCCAGCATCGATCAATGGGGTTTGTGGCCCAGCATGGATCTGTAGGACTTCGCTTCCTTGATATTAGGGATCTG
TGTGTATTGTTCTTCTCCAATGCCATGAACACATCTCTATTGAAGTCGTTGGTGGCCCCATGTGGGTCGGGGTTATAAGACTCACGGACCTTAGCGCGGGAAGCCTCAGCCCGCTTCTGCC
AGAAATCGTCGAAATCACCAATGTGCGAGCGAACGGGCGCGGTGAGGATGAGGGCCGAGAAGAGGATGAGGAAGCAAGGCCTGAACTGTACGCACTCCATTCTTCTTCTTCTTCTTCTT
CTCGCTTTTTTTCTTCTGTTTACAGTGCGTGTTTTCTTCTGTAATCGTGTTACTTTTTTTTTTTTTAAATCGGAAAGCAAAAAAGATTACAGATGGTCGGAGAGAGTCTGGAAAAAAAGGTT
TTGGAAGCAAAGCAGGATAACCAGACCCGACCGCTGGCCGAGCGAACCTCGAAGATCGCTAGAATGTGAGGAACATTGGGTTGGTGGGGTGGGTATGTTTTTAGGGCTTCGGCTAGGG
AATGGAGCTATATAGGATTGTTATGGAATCCAGTAGGAATAAACAGAGGAAAAGAAGGATCGGGCCGACAAGAGTGGCCAGCCATTAGCAATTCCGCATTTAGGGACGGGAGATTTGGCT
GCGCATATTAGTTTATCTGGCTCTGCGTATTTGCATATCTGGCGGTTATAGATCTCCTATATTGTTAATATTTCCTTGATATACTTCTCTTAGCACGGAAAGGTGAAGGATGAGGAGAGAAAAT
AGAGAGGGTGGTTGTTAAGGGCGATTGGATCGTTTATGAAATCGGGAAATT
>comp159231_c0_seq17
TCAATAATTGTTGAACAACTTCAGCATCTATTCAAATGCCCTTATTCTCAATCATCAAAGAATTTCCAAACATTCATTCAATGTACCTATATATAATATTCATGATACAGTGCTTCTCCATTCCA
ATTACTACAACTTTAAGAAAGTACTCAACATATAATTTTAAAAATAATTAAAAAAAAAAAACACAGTCACACAATATGAGAATCACATATGATATATCCCATAAAAGAAGCACTTTTATGAA
GAAAAAATAAAGAACATGAAATAAATTCCATATACTTTCCCAACACCAAAGGAGTGAAGCAATCAGCAGCTCAACACTGCCTTCCAACTCTACAACGCAAAGTTCCTGCAAACCTCGTC
AATCTCTTCACAAAAGTGCCAGGCTTGGCTTTTATCATGTCCTGCTTCGTATATTTCCTTATGAACTCCTTCTTCCCCGACTGAACAAAAAACGCCCCATTTAACATCAAATCCCCTTCTGAT
CTCCAATGCCAATTCCTCCATTCCCATTCCCCAACATAATCCCTATGCGTCACCTCCTTCGCCGCCCGATTTGGTGGCGCGATATACCTATTTCCTTGACTCACTATCGTCGGATGACTACTC
CCACCAACCGCATACATCAACCAGTGCGTGTAATCGTTATTCACCACATGAACAAACCCCCAACGACACCTCGGCATTCTCTGCACCAATCCCCTTCCAAAATGGTTAAAAGCTACAGTTA
CCTGCATCAGAGCATCCTCCGAATACGAATCGCTCGCACCGAACAGAAACACATCGTCGTGGTTCGTCATGTGGCAGTTGGACACAGTGATTGCCGTCGACCCCATGATCGCGTCGATCA
GCCCGTCCGCGCAGTTGGACATTGAAATGTGATCGATCCAGATGTTCGAAGAACCGTAGATCGAAATCGCATCGCCGTCGCTGCGGGTGCGGAAGCCGTAATGGTTGACGGAATCGCGA
ATCATGCCGCCGTTGCCGGGTTTGATGTCGTGGATGTGGAGGTTGTGGAGGATGATGTGGTGAACGAATTGGATGGTGATTTGGGCGCCATAGGCGATGTGGATGTTGGCGCCACGACCG
TCGATGGTTTTGTAGCTATTTACTATTAGCTCCTCGTTGAGGCGGATCACCATGTCACGGGCGAACACTATCCACAATGGCTCAGCCTGAATAACGGCATGGCGAAGTGTCCCCGGCTTGG
GGTTGACAAGATCATTGTCGGACGCATCTGTCACTACATAGAAGCGACCTTTGACACCACCCATGGCATTGCGGCCAAAGCCCTTAGCACAAAAGGCCAATCTCTTGCGGTAACTGGCCC
AATTAGATTGGCAGCGCCAGCATCGATCAATGGGGTTTGTGGCCCAGCATGGATCTGTAGGACTTCGCTTCCTTGATATTAGGGATCTGTGTGTATTGTTCTTCTCCAATGCCCTTAGCGCG
GGAAGCCTCAGCCCGCTTCTGCCAGAAATCGTCGAAATCACCAATGTGCGAGCGAACGGGCGCGGTGAGGATGAGGGCCGAGAAGAGGATGAGGAAGCAAGGCCTGAACTGTACGCA
CTCCATTCTTCTTCTTCTTCTTCTTCTCGCTTTTTTTCTTCTGTTTACAGTGCGTGTTTTCTTCTGTAATCGTGTTACTTTTTTTTTTTTTAAATCGGAAAGCAAAAAAGATTACAGATGGTCG
GAGAGAGTCTGGAAAAAAAGGTTTTGGAAGCAAAGCAGGATAACCAGACCCGACCGCTGGCCGAGCGAACCTCGAAGATCGCTAGAATGTGAGGAACATTGGGTTGGTGGGGTGGGT
ATGTTTTTAGGGCTTCGGCTAGGGAATGGAGCTATATAGGATTGTTATGGAATCCAGTAGGAATAAACAGAGGAAAAGAAGGATCGGGCCGACAAGAGTGGCCAGCCATTAGCAATTCCG
CATTTAGGGACGGGAGATTTGGCTGCGCATATTAGTTTATCTGGCTCTGCGTATTTGCATATCTGGCGGTTATAGATCTCCTATATTGTTAATATTTCCTTGATATACTTCTCTTAGCACGGAAA
GGTGAAGGATGAGGAGAGAAAATAGAGAGGGTGGTTGTTAAGGGCGATTGGATCGTTTATGAAATCGGGAAATT
>comp159231_c0_seq2
TCAATAATTGTTGAACAACTTCAGCATCTATTCAAATGCCCTTATTCTCAATCATCAAAGAATTTCCAAACATTCATTCAATGTACCTATATATAATATTCATGATACAGTGCTTCTCCATTCCA

ATTACTACAACTTTAAGAAAGTACTCAACATATAATTTTAAAAATAATTAAAAAAAAAAAAACACAGTCACACAATATGAGAATCACATATGATATATCCCATAAAAGAAGCACTTTTATGAA
GAAAAAATAAAGAACATGAAATAAATTCCATATACTTTCCCAACACCAAAGGAGTGAAGCAATCAGCAGCTCAACACTGCCTTCCAACTCTACAACGCAAAGTTCCTGCAAACCTCGTC
AATCTCTTCACAAAAGTGCCAGGCTTGGCTTTTATCATGTCCTGCTTCGTATATTTCCTTATGAACTCCTTCTTCCCCGACTGAACAAAAAACGCCCCATTTAACATCAAATCCCCTTCTGAT
CTCCAATGCCAATTCCTCCATTCCCATTCCCCAACATAATCCCTATGCGTCACCTCCTTCGCCGCCCGATTTGGTGGCGCGATATACCTATTTCCTTGACTCACTATCGTCGGATGACTACTC
CCACCAACCGCATACATCAACCAGTGCGTGTAATCGTTATTCACCACATGAACAAACCCCCAACGACACCTCGGCATTCTCTGCACCAATCCCCTTCCAAAATGGTTAAAAGCTACAGTTA
CCTGCATCAGAGCATCCTCCGAATACGAATCGCTCGCACCGAACAGAAACACATCGTCGTGGTTCGTCATGTGGCAGTTGGACACAGTGATTGCCGTCGACCCCATGATCGCGTCGATCA
GCCCGTCCGCGCAGTTGGACATTGAAATGTGATCGATCCAGATGTTCGAAGAACCGTAGATCGAAATCGCATCGCCGTCGCTGCGGGTGCGGAAGCCGTAATGGTTGACGGAATCGCGA
ATCATGCCGCCGTTGCCGGGTTTGATGTCGTGGATGTGGAGGTTGTGGAGGATGATGTGGTGAACGAATTGGATGGTGATTTGGGCGCCATAGGCGATGTGGATGTTGGCGCCACGACCG
TCGATGGTTTTGTAGCTATTTACTATTAGCTCCTCGTTGAGGCGGATCACCATGTCACGGGCGAACACTATCCACAATGGCTCAGCCTAAGTATATATCATGCGAAAGTTGTGAACCTGAAT
AACGGCATGGCGAAGTGTCCCCGGCTTGGGGTTGACAAGATCATTGTCGGACGCATCTGTCACTACATAGAAGCGACCTTTGACACCACCCATGGCATTGCGGCCAAAGCCCTTAGCAC
AAAAGGCCAATCTCTTGCGGTAACTGGCCCAATTAGATTGGCAGCGCCAGCATCGATCAATGGGGTTTGTGGCCCAGCATGGATCTGTAGGACTTCGCTTCCTTGATATTAGGGATCTGTG
TGTATTGTTCTTCTCCAATGCCCTTAGCGCGGGAAGCCTCAGCCCGCTTCTGCCAGAAATCGTCGAAATCACCAATGTGCGAGCGAACGGGCGCGGTGAGGATGAGGGCCGAGAAGAGG
ATGAGGAAGCAAGGCCTGAACTGTACGCACTCCATTCTTCTTCTTCTTCTTCTTCTCGCTTTTTTTCTTCTGTTTACAGTGCGTGTTTTCTTCTGTAATCGTGTTACTTTTTTTTTTTTTAAAT
CGGAAAGCAAAAAAGATTACAGATGGTCGGAGAGAGTCTGGAAAAAAAGGTTTTGGAAGCAAAGCAGGATAACCAGACCCGACCGCTGGCCGAGCGAACCTCGAAGATCGCTAGAAT
GTGAGGAACATTGGGTTGGTGGGGTGGGTATGTTTTTAGGGCTTCGGCTAGGGAATGGAGCTATATAGGATTGTTATGGAATCCAGTAGGAATAAACAGAGGAAAAGAAGGATCGGGCCG
ACAAGAGTGGCCAGCCATTAGCAATTCCGCATTTAGGGACGGGAGATTTGGCTGCGCATATTAGTTTATCTGGCTCTGCGTATTTGCATATCTGGCGGTTATAGATCTCCTATATTGTTAATAT
TTCCTTGATATACTTCTCTTAGCACGGAAAGGTGAAGGATGAGGAGAGAAAATAGAGAGGGTGGTTGTTAAGGGCGATTGGATCGTTTATGAAATCGGGAAATT
>comp159231_c0_seq5
TCAATAATTGTTGAACAACTTCAGCATCTATTCAAATGCCCTTATTCTCAATCATCAAAGAATTTCCAAACATTCATTCAATGTACCTATATATAATATTCATGATACAGTGCTTCTCCATTCCA
ATTACTACAACTTTAAGAAAGTACTCAACATATAATTTTAAAAATAATTAAAAAAAAAAAAACACAGTCACACAATATGAGAATCACATATGATATATCCCATAAAAGAAGCACTTTTATGAA
GAAAAAATAAAGAACATGAAATAAATTCCATATACTTTCCCAACACCAAAGGAGTGAAGCAATCAGCAGCTCAACACTGCCTTCCAACTCTACAACGCAAAGTTCCTGCAAACCTCGTC
AATCTCTTCACAAAAGTGCCAGGCTTGGCTTTTATCATGTCCTGCTTCGTATATTTCCTTATGAACTCCTTCTTCCCCGACTGAACAAAAAACGCCCCATTTAACATCAAATCCCCTTCTGAT
CTCCAATGCCAATTCCTCCATTCCCATTCCCCAACATAATCCCTATGCGTCACCTCCTTCGCCGCCCGATTTGGTGGCGCGATATACCTATTTCCTTGACTCACTATCGTCGGATGACTACTC
CCACCAACCGCATACATCAACCAGTGCGTGTAATCGTTATTCACCACATGAACAAACCCCCAACGACACCTCGGCATTCTCTGCACCAATCCCCTTCCAAAATGGTTAAAAGCTACAGTTA
CCTGCATCAGAGCATCCTCCGAATACGAATCGCTCGCACCGAACAGAAACACATCGTCGTGGTTCGTCATGTGGCAGTTGGACACAGTGATTGCCGTCGACCCCATGATCGCGTCGATCA
GCCCGTCCGCGCAGTTGGACATTGAAATGTGATCGATCCAGATGTTCGAAGAACCGTAGATCGAAATCGCATCGCCGTCGCTGCGGGTGCGGAAGCCGTAATGGTTGACGGAATCGCGA
ATCATGCCGCCGTTGCCGGGTTTGATGTCGTGGATGTGGAGGTTGTGGAGGATGATGTGGTGAACGAATTGGATGGTGATTTGGGCGCCATAGGCGATGTGGATGTTGGCGCCACGACCG
TCGATGGTTTTGTAGCTATTTACTATTAGCTCCTCGTTGAGGCGGATCACCATGTCACGGGCGAACACTATCCACAATGGCTCAGCCTAAGTATATATCATGCGAAAGTTGTGAACCTGAAT
AACGGCATGGCGAAGTGTCCCCGGCTTGGGGTTGACAAGATCATTGTCGGACGCATCTGTCACTACATAGAAGCGACCTTTGACACCACCCATGGCATTGCGGCCAAAGCCCTTAGCAC
AAAAGGCCAATCTCTTGCGGTAACTGGCCCAATTAGATTGGCAGCGCCAGCATCGATCAATGGGGTTTGTGGCCCAGCATGGATCTGTAGGACTTCGCTTCCTTGATATTAGGGATCTGTG
TGTATTGTTCTTCTCCAATGCCATGAACACATCTCTATTGAAGTCGTTGGTGGCCCCATGTGGGTCGGGGTTATAAGACTCACGGACCTTAGCGCGGGAAGCCTCAGCCCGCTTCTGCCAG

AAATCGTCGAAATCACCAATGTGCGAGCGAACGGGCGCGGTGAGGATGAGGGCCGAGAAGAGGATGAGGAAGCAAGGCCTGAACTGTACGCACTCCATTCTTCTTCTTCTTCTTCTTCT
CGCTTTTTTTCTTCTGTTTACAGTGCGTGTTTTCTTCTGTAATCGTGTTACTTTTTTTTTTTTTAAATCGGAAAGCAAAAAAGATTACAGATGGTCGGAGAGAGTCTGGAAAAAAAGGTTTT
GGAAGCAAAGCAGGATAACCAGACCCGACCGCTGGCCGAGCGAACCTCGAAGATCGCTAGAATGTGAGGAACATTGGGTTGGTGGGGTGGGTATGTTTTTAGGGCTTCGGCTAGGGAA
TGGAGCTATATAGGATTGTTATGGAATCCAGTAGGAATAAACAGAGGAAAAGAAGGATCGGGCCGACAAGAGTGGCCAGCCATTAGCAATTCCGCATTTAGGGACGGGAGATTTGGCTG
CGCATATTAGTTTATCTGGCTCTGCGTATTTGCATATCTGGCGGTTATAGATCTCCTATATTGTTAATATTTCCTTGATATACTTCTCTTAGCACGGAAAGGTGAAGGATGAGGAGAGAAAATA
GAGAGGGTGGTTGTTAAGGGCGATTGGATCGTTTATGAAATCGGGAAATT

>comp159231_c0_seq8

CCGACCCCCATATAGTGGGATAAATGCTTGTTATTGTTGTTGTTGTTAATAAAAAAAAAAAACTATTATTATTTTTAATACCTTCTAATTTTAAATATTTTTCACCTTATATTATTTAGCATCTTA
TTTTAATACAAAATGTATCACGATATTAATACAATTTTGTTTGCAATACACAATGTGAACGAATGGATATGAGTAGATCGTTCTAAACAAACAAAAACAAAAAACAAAAACAAAAACAAAT
GCCTTCCGGCTGAAACGTCAATCAATCGGTCATCTTCAAATGTAGAAACCAAAAAGCTAAAGAAAATAGAACTAAAGAAAAGCCAATGGATGGATGCATGGATTAGCAAGGCAATCCGA
TCTTGCAGTTAAGGGTCCCAGAGTAGCGTGTGAGCCTAGTTACGAACGTCCCTGGCTTGGCCCTCATCATATCATGCCTCGTGTATCTGCTAATAAATCGTTGCCCTGACTCAACGAAGAA
CGCTCCATTTATCATCAGATCACCCTGCGACTTCCACGTCCACTGGCTCCAGATAGCCTCCGGTGCATAATCCCTTTTCGTCACCTGCTTGGCGTTGATATCCGGCGGAGCAATGTATCGAT
TCCCCTGACTGATAACAGTGGGATGCTGGCTTCCCCCGACGGCGTACATTTGCCAATGTGTGTAGTCATTGTTGACGACATGGACAAAGCCCCACCGGCAACGCGGCATCCGTTGCACGA
GTCCACGCCCGAAGTGGTTGAAGGCAACCGTTACCTGCATAAGGGCATCCCCGGCGAAGGAGTCGCTGGCGCCAAAGAGCATTACATCGTCATGATTTGTCATGTGACAATTGGAAATG
GTGATGGCGGTGGATCCCATGATGGCGTCTATTAGGCCATCAGCACAGTTGGACATGGATATGTGGTCAATCCATATGTTGGAGGCACCGTAGATGGAGATGCCATCACCATCGCTGCGAG
TGCGAATGCCATAATGTGAAGGGGAGTCGCGGATCATGCCGCCGTTGCCAGGGCTGAGATGGTGGATGTGGAGGTTGTGGATGATGATGTGGTGGACGAATTGGATGGTGATTTGGGCGC
CATAGGCGATGTGGATGTTGGCGCCACGACCGTCGATGGTTTTGTAGCTATTTACTATTAGCTCCTCGTTGAGGCGGATCACCATGTCACGGGCGAACACTATCCACAATGGCTCAGCCTG
AATAACGGCATGGCGAAGTGTCCCCGGCTTGGGGTTGACAAGATCATTGTCGGACGCATCTGTCACTACATAGAAGCGACCTTTGACACCACCCATGGCATTGCGGCCAAAGCCCTTAGC
ACAAAAGGCCAATCTCTTGCGGTAACTGGCCCAATTAGATTGGCAGCGCCAGCATCGATCAATGGGGTTTGTGGCCCAGCATGGATCTGTAGGACTTCGCTTCCTTGATATTAGGGATCTG
TGTGTATTGTTCTTCTCCAATGCCCTTAGCGCGGGAAGCCTCAGCCCGCTTCTGCCAGAAATCGTCGAAATCACCAATGTGCGAGCGAACGGGCGCGGTGAGGATGAGGGCCGAGAAGA
GGATGAGGAAGCAAGGCCTGAACTGTACGCACTCCATTCTTCTTCTTCTTCTTCTTCTCGCTTTTTTTCTTCTGTTTACAGTGCGTGTTTTCTTCTGTAATCGTGTTACTTTTTTTTTTTTTAA
ATCGGAAAGCAAAAAAGATTACAGATGGTCGGAGAGAGTCTGGAAAAAAAGGTTTTGGAAGCAAAGCAGGATAACCAGACCCGACCGCTGGCCGAGCGAACCTCGAAGATCGCTAGA
ATGTGAGGAACATTGGGTTGGTGGGGTGGGTATGTTTTTAGGGCTTCGGCTAGGGAATGGAGCTATATAGGATTGTTATGGAATCCAGTAGGAATAAACAGAGGAAAAGAAGGATCGGGC
CGACAAGAGTGGCCAGCCATTAGCAATTCCGCATTTAGGGACGGGAGATTTGGCTGCGCATATTAGTTTATCTGGCTCTGCGTATTTGCATATCTGGCGGTTATAGATCTCCTATATTGTTAA
TATTTCCTTGATATACTTCTCTTAGCACGGAAAGGTGAAGGATGAGGAGAGAAAATAGAGAGGGTGGTTGTTAAGGGCGATTGGATCGTTTATGAAATCGGGAAATT

>comp159231_c3_seq2

AAAACCCACCAACGGGCCTACGAGTTAAATAAAAAAGGAAATACAAGATCCTTAAAAGGATCTCCAGCTCATAATTGCTCTAACGAGCTACAATAGAAAATAAAAAAGATCCTAAGAGT
CGGAGTTCCAAACTAAGCACAATTACAAGGCAACATAAGAACATTCTAAAAATCCAAAAAAGAAGGAAAAAAATTGAACATGTTCAGCACCGCCTTCTCCACCAAAATAGAATCCTGCA
CAAAATGAGAACTGATCTCCAAAAACCTGAAAGACAGGCAGTTCAGGCCTTGCTTCCTCATCCTCTTCTCGGCCCTCATCCTCACCGCGCCCGTTCGCTCGCACATTGGTGATTTCGACG
ATTTCTGGCAGAAGCGGGCTGAGGCTTCCCGCGCTAAGGTCCGTGAGTCTTATAACCCCGACCCACATGGGGCCACCAACGACTTCAATAGAGATGTGTTCATGGCATTGGAGAAGAAC
AATACACACAGATCCCTAATATCAAGGAAGCGAAGTCCTACAGATCCATGCTGGGCCACAAACCCCATTGATCGATGCTGGCGCTGCCAATCTAATTGGGCCAGTTACCGCAAGAGATTG

GCCTTTTGTGCTAAGGGCTTTGGCCGCAATGCCATGGGTGGTGTCAAAGGTCGCTTCTATGTAGTGACAGATGCGTCCGACAATGATCTTGTCAACCCCAAGCCGGGGACACTTCGCCAT
GCCGTTATTCAGGCTGAGCCATTGTGGATAGTGTTCGCCCGTGACATGGTGATCCGCCTCAACGAGGAGCTAATAGTAAATAGCTACAAAACCATCGACGGTCGTGGCGCCAACATCCAC
ATCGCCTATGGCGCCCAAATCACCATCCAATTC
>comp159250_c8_seq4
AATAATTAATAATTATTATAATTAATTAACAAACAAAAAAGTCTCTCTTTTTTTTTTTTAATTTTGGCTGAAGAAGCCAAAGGGAGAAAGTTACCTATACAGCAAGCGGACTCCCGTTCTTC
CCCCCGATCAAAATGTTGCAACTTCCGATTTCGACAGGAAGGGAAAGCCGTGGAAGGAGCGGGGTCGACCGGCTAGGGAGGGGGAAGACCGTCGGCTTGGACAGGAGGTAGGGAGAT
CTATCTCTTTCTCTCTTTCTCTCCCTTTCGGGAGTTGGCCGAAGGAGGAGAAGGCACGGCACAGGTGTTCGGAGGAGGCGACGAGAGCTCGGCGGGCTTCAGTCGCCGGAGTAGGGATT
TGCGAACGGGAGAGGGGAGATCGG
>comp159426_c0_seq1
GGACGAAATAAAAGTTCCTAGGAGAAGATAGGCTGTCACCAAAGAATTCTATAGTAATATGGTCTTATTATTAAATAATAATAATAATCATATATATATATATATATATACTACGGCATCATTCAC
AAGAACTTGAAAGAATAATGAAGGAGAAACCTCAAACAGAGAACACCCCCAAATCTGCGATGTGAAAGCCTACCACACGGAGACCAGTCAAATCGGAGAGGATTCTTACGTATTTTTG
AGCACTCAATTGGATAACATTTGCAGGGTCTGTTTCACATCTGAACGGCTGCATGTGAAAGAACTCTCGAAGAACTTGTCGGACCTCCGGTATGTTAGTGGAAGCTCCGGCGAGCACGA
CCTCATCAACGGCTTCTTTCTCGATTCCGGCTTCCGTTAAGCACCTCATGATTCCGCTGCGCAGAAGGTTGAGATTCATCTCCACCAACGATGCACGACTGAGCGTTTCTATCACCTCCCT
GCCTTGTAGATCAAACTTAAGCTCCACGGAATCCGAGCCCAAACAAAGGATGTTCTTGGCGCGCTCGCATTCCGTTCGGATGCCAATGAGAGCCTTGACGTCACTGGTGAAATCGAATTC
GAGCTTCGACGAAAGCTCTGAAAGCATGACGTGGAAAAGTGGGCAGCCCAAGTCGGAAACGGCGGCCTTTACAATTAGATCTAAATCTTTGGTGATCAGAGAGATTTCAAGAATGCGAT
CACCGAGTTCGAATACGAGGACGGTTTTTGGTTCGGGTCTGAGAGAGTCGTAATAGGCGGCGGCAAGAACCGGTCTTTCGATGAAAAACAGAGGGTGAGCTCCGGCAGCGGCGGCAGC
GTCAGAGACTTGTGATTGATACACTGGGGTGTTGCTGCTGGCGGGGAGGATGAAAATGATATTATCGACTCCGCAGCCGGTGATGCTTTCCATGTCGGCTTTCTTTTCCTTGAAATGTACC
GTGCTGACTTGGGCGGGAGTAAGGAGCTTTTGCTCGTTATCGGCTTGGGTCGTCACGTAGTAGCGAGTGCCTTCGGGGGAGACATAACGCCTGAAAGGGTACAGGCGGCGGAGAAACC
AGTCTTTTCCGGCGGCGGAGTGCAGGTGGACGACGCAGTTATCGGGACAGCGGTCCATGGCGGGTTTCTTGTTCTCCTCTTGCCTTCTGAGCTTCTTGGCTTCAGGAAAGACGAGGATTA
TATTATAGCGAGGCTTCTTCTTGGCTTCTCATTTTTTAAGTGGGAGCGTTGTTTTAGCGAGGGAATGCAACTTAAGGTTAATTTTGGAGCCTTTTCTATGGTTTTTAAATAGAAT
>comp159470_c1_seq3
GGCCTTTGATTATGTCCACATAAGCCGACAAGCCTTAAAATATTCCTTCACAAATCAGAGCCTCTAACTTCATTTCAATTATTTAAAATTTCTCTGTTCATCAATCATTCTCTTACATTAAAAA
TTGATATCAAAACTCAGCAAATGATAAAATATGTTTGAAAGCAGAAATCTTTCATTTTTCTATGAAATAAAGTACAGAAACTTTTTCAATAACTCCACATTTATTAGCACTCCAGAATGGCTC
ATTTCCATTTATTCTGACTCTGCTCAACTAAAATTTGCAGACCTTAGCAGGATTCCTGAACACAGACGACCTTGCCACACCTAATCGTTGGCCTTAAAATGAACCAGCAACACCTGCAACC
TTCAATGAAACAAGCAGATAAAAGTAAGCTTTGTAAGCCCACTATAAAAGATTTTTACTCCATTTCATTATCTTCCAAAACAAAGAAAAATTTGAAACTATCATAACCGTTGGATTATCGAT
GAAATTAGTGCAATATATGAGTGATCAATCAAAGTATTTATTACAAAAATTTCAAAATAAATTCAATAATTTAAAGATTTTGCAATCAGAATATTCTTATCCAAAAATTTTCTGTAAGGAAAC
ATACCTTGGGGGTGAACAAGGTTCTTATGTTCCTTAACGTGAGCCAGAGCTCCTGTGCTTGTTGTGATGAAGAGTAGAAGAGCCATTAGCCAGAGGAAAAGCGTAAGTAGATTTCTCCCC
TTCATTCTTCTTTATTCTGTCAGACCAGAAAACAAGAGAAAGAGATTTTAGGCTGCTGGGTAGGAGATGAGCCGCCGCAGCCTATTTAAACTGGTAGGACTTGAAAGAAGAATTTTTGGA
TGAGTTTCCGTATTCAAATTTTCTTTGAATTTTATAAAGATGTTAGATCTTCGCAGTATCTGTTTTAATAAAAAAGGCAGCAGACCTTCGTGGTCCATTACAGCTTCAAGGAAAGACACCAA
TGACAGGTCAAAGACAAACAGGTTGGATATTTGACATTAATGACTTTGTCGGTCAAGTTTTGCTGCTAGTAGAATAGCACCACTGGAATGTGACCAGAGTAAAAAAAGGCTAATAATGCA
GCCACAATTTGGGAGGGTGGTGAATAATGACTTCTAATTAGGGGGTGCAAGATAAATTAAATGAGTTGTTAAGTGGTTCACTCGAGGATTTTAGAATCAGATCTGAAGATTCAAAGGTTCA

ACTAAGATTTAGGCTTTGCTTGGGACTGCTTTTT
>comp159479_c0_seq2
TACCGACTGCGGCTCCGGTGATCAAGGAGCGAACAAACTCGGCGTCAGATTCAGCCGACTTGTATAGATTAGCAAATCCCGGGCGAATTTGCAGATGAGCGTCTTTGGCGGCCCCACCTT
GGTGGGACCCAAGGAAGTCTTGCTGGCAGATTGCAATAGGGTCTGTTCAATGGAAAGATTGACACCTGGTAGAAATTTACTGTGAGTCTTTGTTGGATTCCCATTTGAAACTTATCAACA
AGTTGCAAGAGGTGCAAATCTGCTTGAGCAAAAATCTTCT
>comp159484_c2_seq1
AACTACCACACCAGCACTGTAAATAGACAAGTCATCCATTCAATATTAACATGAATTTTGTAATAACAACATATATCATTCTATACAGATCTTTTGAAATTAAGTCTCTCTCATTCTGCACCAA
TAAAACAAGACCAATCTCTCTCTCTCTCATTCTGCAGCAAGAACACAGAAGACCAACTTCACAGTTCTTATTCAAGCCTCCCTATAGAAATATAGTTATCTCAGGTGCTGAACGCATACAA
ATTGGTATCTTTTAGCATAAAACAGATGCTATAATTTACAAAATTTTCACATTTAAGAGACAAAAATTTACATATGACGCCTATCAAGGCATAGGAGAAGAATAGAACAGAAATAGCTGAAA
GATATTACATACTAAGAAAGAAGGATTGATTATATCCTCAACTGGAAGATCCTACGAGGAGCCGTTAGAGGGAATAGTATCATTGGGTGGTCCTTGCAAATTGAAAAGGTGTAAAGAAGCT
ACCAGATTCAATGATCAAAAAACTACCAAATGGATATTTTTGTCTGTTCCATGGTAAGATGAAA
>comp159508_c0_seq4
CCTTTTCATTCATCCCTCTCTTAAGTTTCTTCATCAACCAAGCTTCTCTCTGATCCTATCATCCTCTAATCGACTTCCAAACCCTTAAGAAGTTTTTATTAATTGCTTACACGAATAATTTTTCG
ATTAGGGCTAACTCAATTTTCAAATGTCAATTTCGAAAGATGAGGAAGTGACTAGTGAGAGGCTGGAAGAGCTTGTCGACTCCTCAACCTCGTCTAAGCCGCTATTGGAAAAGGATGATT
CAGGTGAAGCGGTTATGCAAGGGACAATGGCGGATAAAAATGAGCGGCGAATAGAGCTTTCTATTTCTTTATCTAGAAAGGAGATCGAAGAGGACTTCCTTAAAATGACGGGGAGAAAG
CCTAATTTTAAACCTAGAATGATACCAAAAATCATGCGAAAGCAACGTGATGCTCTATTCCCTGGACTTGGGCTGCCAGAAATCACAAAAGATATGCTTGAAGAAGATGATTGAGATGATT
CTTTATTCTACTTTTAGATAATGCCACCTGAAAATTTATATCAACAAGAACAAGATATGATCTTCTTGGTAGAAGGAGCAACAGAGGACATGTCATTCAACATTTACAGAATGGAAGTGATT
ACAAGTTGCAAGGCAGTTATCACTATTATTTAGCTTTTGTTTGAATGACATGTCCTCTGTTGCTCCTTCTACCAAGAAGATCATATCTTGTTCTTATTGATATAAATTTTCAGGTGGCAATATC
TAAAAGTAGAATAAAGAATCATCTCAATCATCTTCTTCAAGCATATCTTTTGTGATTTCTGGCAGCCCAAGTCCAGGGAATAGAGCATCACGTTGCTTTCGCATGATTTTTGGTATCATTCTA
GGTTTAAAATTAGGTTTTCTCCCCGTGATTTTAAGTAAGTCCTCTTCGATCTCCTTTCTAGATAAAGAAATAGAAAACACCTTTTGTCGATCATTTTTATCGTCCACTGTCCCTTGCATAACC
GCTTTACCTGGATCGTCATTTCCCAATAGTGGCTCAGACGACCCTGAGGAGTCAGACGACGTTGAGGAGTCCGGAAGCTCTTCTAGCCTCTCACTGGTTTCTTCCTTATCTTCTGAAATTG
GCATTTGAAAAATGAATGGGC
>comp159508_c0_seq8
CGGTACCGAGCGGAACCGAACAGTATCGGGGTAACGTGGTGCATTCTAATTATTCTTTGGATGACGTTTGGTGTCCGCACAAATTAGACTGTTAGAACTTCATGCTGATGGTAAACTAAAA
AATACCAATATAAATTCCAATAGTCTTATTAAGAAATTTAAGCATGAATATGACAAACTTGAAAATTATTCAAACAAAAGCTAAATAATAGTGATAACTGCCTTGCAACTTGTAATCACTTCC
ATTCTGTAAATGTTGAATGACATGTCCTCTGTTGCTCCTTCTACCAAGAAGATCATATCTTGTTCTTGTTGATATAAATTTTCAGGTGGCATTATCTAAAAGTAGAATAAAGAATCATCTCAAT
CATCTTCTTCAAGCATATCTTTTGTGATTTCTGGCAGCCCAAGTCCAGGGAATAGAGCATCACGTTGCTTTCGCATGATTTTTGGTATCATTCTAGGTTTAAAATTAGGTTTTCTCCCCGTGA
TTTTAAGTAAGTCCTCTTCGATCTCCTTTCTAGATAAAGAAATAGAAAACACCTTTTGTCGATCATTTTTATCGTCCACTGTCCCTTGCATAACCGCTTTACCTGGATCGTCATTTCCCAATA
GTGGCTCAGACGACCCTGAGGAGTCAGACGACGTTGAGGAGTCCGGAAGCTCTTCTAGCCTCTCACTGGTTTCTTCCTTATCTTCTGAAATTGGCATTTGAAAAATGAATGGGC
>comp159519_c0_seq23
TCCAGAATGCCGCTGGGATGATTTAAGTTTTGTGAACGTGCAAGATCAATCAATTTACATAAAATCTATTACATGTTTTCTTTTTTCGTGGAAATAATGTAAATTATGTGAATCCTAAGTCAG

GCTCCTCAACACTCATCTTATAAAAATTTTAGAAATTTGTTTTTTCTTTACCTGCTACAAGGGGTAAAAAGTTCTTTAATTTAGTTAATCTATCTTTTATTTCCTTTCTCATATTTGATACTGTA
GGATTTGTTTCTTGCTGCAGTTGAATCAAAATCTGTGTACGTTGGAAATCTTCCTGCAAGCATATCCACTTCTGACCTTGAGCAAGAGTTCAGGAACTTTGGTAGAATCAAACCTGACAGA
GTTACTATCAGAAGCCGGAAGGTAACTTGCGTGGAACTTTATTTGCAAGTATATTGTTGTAGGCAAACCATGATCTTTTAAGTTTCTTTTTTGATAGTTGAGTAGCATTTGATATGTTTCACT
TATATTTCATCCACCATTTGATCTGTTTCACTTTTTTGAAAGTTGAGTAGCCCTCCATCCCTTTCCGTCTTTGTCGTAAGTAAATTTAAGCTCACAAGAGGTGTAAGGATTTTCTAACTTTCT
GAAACCACTGCTCTGCCTTATTTG
>comp159529_c1_seq2
GAGTTTTAGCTATCAATTAAGAAATTTAAATTTAATCTAACGTATGTAATTTTATCTTAATTATCCAGTCTCCTCCAAAATAAAAAATCATTAATCCCAATAATTTCTCATATTCCACACATTAC
CATTACCTTATAATCATAACAAAAACCCTAAACATAACAAATCTGATTCACATTACAACGAACATTGACGCATCAGAATTCATATCATCTATCGATAAACAGACAAAAATCAAAACTTACGT
GATTAAAAAACATACGAAATTATCATTCCGTAAGAATCAAGTGGCCAATCCCAAAGAGTACCATGCAATTACAGAACATATTATTATCAGCTTCAAATAATCAAATAGCAACCAAACAACC
CATCTCAAAGAAATTCCGGCGACGACCTAACCGCCGAATCCATAAATGGTACGGCCAAGGCGCTTGAGGGCGTAGACAACATCCATAGCAGTGACGGTCTTGCGATGAGCGTGTTCGGT
GTAGGTGACCGCATCACGAATAACAGTTTCGAGAAAGATCTTGAGGACGCCTCTAGTTTCCTCGTAAATCAAGCCGCTGATCCGCTTAACTCCGCCGCGCCGAGCAAGTCGCCGGATCGC
CGGCTTCGTGATGCCCTGAATGTTGTCACGGAGAACTTTGCGGTGGCGCTTTGCCCCGCCTTTTCCCAAACCCTTGCCTCCTTTTCCTCTTCCAGACATTTCTCTCTTCTTTAGTTTTGCGA
AAAAA
>comp159616_c1_seq5
TATTTAATTAAAATAAATAATTAATAATAATTAAAAATTCCAAAAATTTCCCAAACACTTGGGAATCTCTCCTTAGGTCATTCCTGACCTTTGGAGTTGATTTCAAGGAGTTGGGACTTCATT
TGGAACAAAATCCCAAAAGGGGCCTCTAATTAAACAATTTTTAAAATCCATTAAATAGTCAAAACTTTGAATATTCATAACTAATTCAACAAAAATCCAAATTGACTAAACTCAATTGCATT
CAACTCATAATAAGGCCTAATATTTGTTAAAAATGTTAAACTCCAGAAATCTGTAATATTTCAAAAAGTCAACTCTTTGGTCAACCATAGGTCAAAACTAGTTCAAAACTTCAAACCTTTGT
AATAAAATATAGAAAAATCCGATTTCGACGTACTTACTGTCTAACTGATCGTAGTAAGATCTAGAATTTTAATAAAATGCTAGCTGTCCAGAATTTGACCTTAATTAAAAAGTCAATTTTTGA
CCCTTTAATTAATAATTAAAATATTCCAAAATTCATAAAAACTTGAAATTCATTTTAAAACTC
>comp159631_c0_seq3
ATCTGTCCACCGCCTACACCATCATCTTCGAGCAAATCGGACCCATCCATCCTCAGGCTCTCAAAATGAGCAAAGGCAAATTCCGACTCATCGAATCGAATCAGAATGCTTGATAAGTCAG
CCCCCTGATTCGATTCGATGAGTCGGAATTTGCCTTTGCTCATTTTGAGAGCCTGAGGATGGATGGGTCCGATTTGCTCGAAGATGATGGTGTAGGCGGTGGACAGATCACGCCGAATTCG
ATCTTGGAAGACCCCTTGCAGTACAAGGGGAGCAATGGGGGATCGAGTGCCGTTGATGGTGGTAGGGGTGCCGCGGGAAGAAGCGATGCGGCGAAGAGAAAGAAGAGTGGTGGCGGG
GCCGGGGGCGGTGGTGGCGGCGGTGGTCGAAGTTTCTTTCCAGGAATAGTGCTCTCTCTCAGTAACAGGAGGAAGGGGGCTCCTCATAGGTCTCCCCTATCTTAAAATAGTTAAGGGAG
CATCAGCTTGATGAAGCTAGTTTAGGTACTAGTGTTTGCATGTTAATTTGTCTTTAATGGTATCAAATTACTGTAGATTGTCATGCCCGGAATACCCTTGCTAACTCAGTTATGGGGGTATTAT
GGGCATAAATGTTATGGTATTTGGCTGTTGGCGGTTTGGAAGGGGGAATGGGCATTTCCATTGCCTGCTAGGTGGTTGCAGCAGGTCATATTTGAGACTGCAGTGGCTGCCAATTTTGTATT
TAGGTGATGGAATATTGCATACTTCTACATGATCAATATATATATAATATTTATGTAAAAAAAAAA
>comp159684_c1_seq10
TTTGGGAAAAAGGAATAATATGGAGTCTTTTTTAAGGCTAGTAACATTATTCCATTCAAGAGTAAGAGAATAATAAGAGGAAGAAGAATTCTCTCTCTGTGATCTCCTGATCATTATCAGAT
ATCAAATAAACTAACGGTCATCATATTTAAATCTCACAGATGGCTACTATCTATGCCTTCTATCATCTATGACTTCTATTAGATTTGAATGAAAGAAAAAGAAAAAAGAATACACACACACA
CATTAAAAATTCTAATCGATAGGTATAGGTTCAAAATCTGTCCATAATCACTAGAATTTTATTTAATTTTCTTCACTTCAATCTATCCAAACCAGGCTTCATTGTCTCAGGAAAAAAAACTAC

TTAAAGCTCAAATTTCTTTCCAAATAAGCAGTTTTTCATGGCATGAGGCAGATTAAGCTTGATAAACACCACCAATTCTTCTCTCTTTGAGCAGCTTCTGATCTTTCTGACCAGAATCATCA
TCAAATATTTTGTTCAGTATCTCAGCCAAGCGAATTCCACCTTGTGCTATTCTCTTCTCCACTACTGGAAGACGAGATATGAAATATTCATCTCCAAGATCTGAACCTTGCTTGGCGTCCTTA
TAAGCATAGTCGCAGGCCAGGCGGGCACTCTCGGAAGCATAGCTGTAATTAGTTAACTCTTTTGAATACTGATATCTCATTGAATTAATAGGCAGGCCCTTGTGTGAAAGATTTCATGGGA
AACATTATATTTTTTTTTATCTTACTGCTATCTGAACATTCTTAATTTTGCTAAAGACAAAGGAAGTGACATAGAGAGCACAATGAAGTAGAGGAAAAACATAGAGTTCATTAACTAATTGA
TTCAGAGAGTTTCTAGAAATTGAAAAAGATTGAAAAGAAATCTTTCAAATTATTCAATCAAAAGAAACCACCAATATACAGAGGCACTACAAACGTTTTAAAAGAAATCCTTAACAACAA
CAAATAAGAAAATTGTAATCACATCGCTGAATCATTTAAAACAGTCAAAATGGCATCTAACTTGAACAATAAATCATTCAAATATCAATTTGGCCACCTAAATGATGGATTTCACCCAAAAT
CACCCAGGTGGAGACTGTTCAGGATGTTGCTTCCCCTCAAAGAAGTTGTATCATACGTCAAAGTTAAGCTTTATTTAAGGTTTTGGCTGAATTAAGGCTTGGGGGCCTTTGGGTGGTCTTT
GAGCATAAACTCCTCTTTTTGTGTTGCATGGAATGATAGTATTAGAAAAGAAGTATGAAATTCAAATGAAAATTTTATAAAGTTAAGTAGAATGGGAATTGACTTCAGTAATCTTACTTCAA
ATCAAGTCAACTCACATTACAAATACATGAGTAGAACACTCACGTAAAGCATAGATGAACAATGAAAGAAACTTGCTTCTCAAGTTCATTTTGACTAGCACAATAACTTCATTTTAGTTAAT
ACCATTTTAATATTTTATTGATATTTATATATCATAATACATCATAAATTAGAAAGAAAAGTGTCATGTAAGTTTGGGGTTAGTTACATGCCAGGAAGAGGGAATTGAGAAGTGTAGTTCATTC
TACCCTTGCGAGAAATGTGCATCCCTCCATCTCCATCTCTGTGTGTGTGTGTGTATATATTTTATACACTTATATAATCAAATGATAATTTGAGTTAGTTTATTTTTCCTGATTCATATTTATAAAT
ATTTTGTAATGAGTTAAATATAACTTATTTTTCTGTGTTGAATAATTTCTTCAGCCAGCTCAAAATAAAGCTAGTCCGAGAGTCAAAACAGGTGAGTTATGCCTTGACAATTAAAAGCCAAC
TCAATCTAATTATGATTTAAATTGAAAATCATTCATTTCCAGTTAAGATTGCTCAATTTGAACAGTAACTCAATTTCTTGTCATCCTACTTTTTCATTAAACATAAACCTCCGATCAAGATGCC
AGGCTTAGAATAAGAAGAACTGCCAATTAACTGCTCAAATTAAAAATGGAAATCTTACATGCTTCCACAAGTAAAGTTTTTGGTAGAACAATTCATCCATTGATCAATCTCATCTGACCAA
ATGTCTCTATTGTTAATTTGAATGGATTCAACCATTGTATCAATATCATCATCATAGAATTTCTGCTTGGCTTTCTCTACTATATCCTCATCCCAGACCTTGTGAAGATTGGTATTCTTTCCATAC
CACTCCACTTTGATGGTGTTACCTCCTAAATCAAGCTCAGAACCAGCATGTAAAGGTTGGTGGATATCTCCCACAAAATGGGAAAGGAACAATAAGCTTTCTGTTAAGTTATCTAAAACAA
ATTTGATGAATGATTGTTAGGACTATAACAATATAGTGTTAGGCACTGAATTATCCAATTAATAAAATAGCAACTTTATGAAAGATAATTGTACAATTATTTGATGAATCTTTGCGTTTCTGGA
GCTGACTGGTATAATTGTTGATTGCTCCAACTGCGCACATGTTTATCTCTCCTTTGGTGTTGTGACAATCCCTTGAATACTGGAAAGTGCAGTCACCAGCGCTATCAGCAAAGTGGAGAGT
CCTTGACCAACGGTACTGTTTTCTAACATCATCAGCCCAAGGGCACAACGGTGCTAATTCTCCCCTAGCCAACTCGATCACCGCATTCATTGTTGAGTTAGTGAAATGTTCCTGTGCTATCT
TACAGACCATGACATGGCCCTCTATTCCCCAGCCACTGGAGACTGGAGCTCTTGCAATGAGGTAGTAGAGGAAGATATGGATCAAAGGAAGAAGCCATGCCATTTTCTCCTCCACTGTAC
CACTTCGCTCGCACCTATTTCCTTGCCCTTTATCAGCTTCTGTAAAGCTTTAGAGCACACGGAGATATTATTCTGT

>comp159684_c1_seq12

TTTGGGAAAAAGGAATAATATGGAGTCTTTTTTAAGGCTAGTAACATTATTCCATTCAAGAGTAAGAGAATAATAAGAGGAAGAAGAATTCTCTCTCTGTGATCTCCTGATCATTATCAGAT
ATCAAATAAACTAACGGTCATCATATTTAAATCTCACAGATGGCTACTATCTATGCCTTCTATCATCTATGACTTCTATTAGATTTGAATGAAAGAAAAAGAAAAAAGAATACACACACACA
CATTAAAAATTCTAATCGATAGGTATAGGTTCAAAATCTGTCCATAATCACTAGAATTTTATTTAATTTTCTTCACTTCAATCTATCCAAACCAGGCTTCATTGTCTCAGGAAAAAAAACTAC
TTAAAGCTCAAATTTCTTTCCAAATAAGCAGTTTTTCATGGCATGAGGCAGATTAAGCTTGATAAACACCACCAATTCTTCTCTCTTTGAGCAGCTTCTGATCTTTCTGACCAGAATCATCA
TCAAATATTTTGTTCAGTATCTCAGCCAAGCGAATTCCACCTTGTGCTATTCTCTTCTCCACTACTGGAAGACGAGATATGAAATATTCATCTCCAAGATCTGAACCTTGCTTGGCGTCCTTA
TAAGCATAGTCGCAGGCCAGGCGGGCACTCTCGGAAGCATAGATGCTTCCACAAGTAAAGTTTTTGGTAGAACAATTCATCCATTGATCAATCTCATCTGACCAAATGTCTCTATTGTTAAT
TTGAATGGATTCAACCATTGTATCAATATCATCATCATAGAATTTCTGCTTGGCTTTCTCTACTATATCCTCATCCCAGACCTTGTGAAGATTGGTATTCTTTCCATACCACTCCACTTTGATGG
TGTTACCTCCTAAATCAAGCTCAGAACCAGCATGTAAAGGTTGGTGGATATCTCCCACAAAATGGGAAAGGAACAATAAGCTTTCTGTTAAGTTATAATTATTTGATGAATCTTTGCGTTTC
TGGAGCTGACTGGTATAATTGTTGATTGCTCCAACTGCGCACATGTTTATCTCTCCTTTGGTGTTGTGACAATCCCTTGAATACTGGAAAGTGCAGTCACCAGCGCTATCAGCAAAGTGGA

GAGTCCTTGACCAACGGTACTGTTTTCTAACATCATCAGCCCAAGGGCACAACGGTGCTAATTCTCCCCTAGCCAACTCGATCACCGCATTCATTGTTGAGTTAGTGAAATGTTCCTGTGC
TATCTTACAGACCATGACATGGCCCTCTATTCCCCAGCCACTGGAGACTGGAGCTCTTGCAATGAGGTAGTAGAGGAAGATATGGATCAAAGGAAGAAGCCATGCCATTTTCTCCTCCACT
GTACCACTTCGCTCGCACCTATTTCCTTGCCCTTTATCAGCTTCTGTAAAGCTTTAGAGCACACGGAGATATTATTCTGT
>comp159684_c1_seq13
TTTGGGAAAAAGGAATAATATGGAGTCTTTTTTAAGGCTAGTAACATTATTCCATTCAAGAGTAAGAGAATAATAAGAGGAAGAAGAATTCTCTCTCTGTGATCTCCTGATCATTATCAGAT
ATCAAATAAACTAACGGTCATCATATTTAAATCTCACAGATGGCTACTATCTATGCCTTCTATCATCTATGACTTCTATTAGATTTGAATGAAAGAAAAAGAAAAAAGAATACACACACACA
CATTAAAAATTCTAATCGATAGGTATAGGTTCAAAATCTGTCCATAATCACTAGAATTTTATTTAATTTTCTTCACTTCAATCTATCCAAACCAGGCTTCATTGTCTCAGGAAAAAAAACTAC
TTAAAGCTCAAATTTCTTTCCAAATAAGCAGTTTTTCATGGCATGAGGCAGATTAAGCTTGATAAACACCACCAATTCTTCTCTCTTTGAGCAGCTTCTGATCTTTCTGACCAGAATCATCA
TCAAATATTTTGTTCAGTATCTCAGCCAAGCGAATTCCACCTTGTGCTATTCTCTTCTCCACTACTGGAAGACGAGATATGAAATATTCATCTCCAAGATCTGAACCTTGCTTGGCGTCCTTA
TAAGCATAGTCGCAGGCCAGGCGGGCACTCTCGGAAGCATAGATGCTTCCACAAGTAAAGTTTTTGGTAGAACAATTCATCCATTGATCAATCTCATCTGACCAAATGTCTCTATTGTTAAT
TTGAATGGATTCAACCATTGTATCAATATCATCATCATAGAATTTCTGCTTGGCTTTCTCTACTATATCCTCATCCCAGACCTTGTGAAGATTGGTATTCTTTCCATACCACTCCACTTTGATGG
TGTTACCTCCTAAATCAAGCTCAGAACCAGCATGTAAAGGTTGGTGGATATCTCCCACAAAATGGGAAAGGAACAATAAGCTTTCTGTTAAGTTATCTAAAACAAATTTGATGAATGATTG
TTAGGACTATAACAATATAGTGTTAGGCACTGAATTATCCAATTAATAAAATAGCAACTTTATGAAAGATAATTGTACAATTATTTGATGAATCTTTGCGTTTCTGGAGCTGACTGGTATAATT
GTTGATTGCTCCAACTGCGCACATGTTTATCTCTCCTTTGGTGTTGTGACAATCCCTTGAATACTGGAAAGTGCAGTCACCAGCGCTATCAGCAAAGTGGAGAGTCCTTGACCAACGGTA
CTGTTTTCTAACATCATCAGCCCAAGGGCACAACGGTGCTAATTCTCCCCTAGCCAACTCGATCACCGCATTCATTGTTGAGTTAGTGAAATGTTCCTGTGCTATCTTACAGACCATGACAT
GGCCCTCTATTCCCCAGCCACTGGAGACTGGAGCTCTTGCAATGAGGTAGTAGAGGAAGATATGGATCAAAGGAAGAAGCCATGCCATTTTCTCCTCCACTGTACCACTTCGCTCGCACC
TATTTCCTTGCCCTTTATCAGCTTCTGTAAAGCTTTAGAGCACACGGAGATATTATTCTGT
>comp159700_c1_seq1
TTTTTTTTTTTTTTTTTTGTACAAAGCCGTAAAAATTTTAACCAAAATAATATAAAAAGAATGAAAATGATATCATTAAATAAAATATATAAACTATTTTTTATGTTTAATTTTGATACTTCCCC
ACCCTCTTTTTCTTTGCAGCATATACGCAAAAGAAATCAATAAATTTTCTCTTAACTTTCTGACCTTTCTGTAATTCCGTTACAAAGAAACAGCAGAGATATCAGCTCCGCCAACTAGCCAT
CGCTCATAACAAAAAAGTTGATGGCGACCTCAGACATATTTCAACGGTCAGTAGCAGTTAGTAGCCCGGATAAATTGGTGGTGGCGGAGATGCGTAGCTATATCCACCGACGGGTGGGAG
AATAATATCAGGGAATTCCGGAGGCGACAAAGCTGGCGGTGGTGGTGAGATGATTGGCTGCGGTGGAGGCGATGCTAATGGTGGACGTGGCGGGGAGTAGACATATTGAGGAGGTGAGC
GACTTGGTGGTGGCGGAGGAGAGTGGACTGGTGCTGGTGGAGGTGAGTATACCGGTTGGGGTGGAAAGTGAATCGGAGGTGGTGGGGAGTGGACAACTGATGGGGGTGGGGGTGAGT
ACACCGGTGGGGGTGGTGGGGGTGGTGAGTACACTGGAGGTGGCGGGGAGTTGACAACAGCTGGCGGTGTTAGTGAATGCACCGGAGGAGGCGGGGATTTGACCGTCGGCGGCGGTG
GAGGAGAATGCACTGGTTGCGGAGGAGATGAGAACAAAGGGGGAGGTGTAGGTGGCGGTGAACTGGGTGGAGTCGGCGGCATGGAGATTGGTGGAGATGATATCTCCTCCGTAGGCAA
AGGAGATGGAGCATCATCCACATGCGGCGGCGAGCTACTAACAGGCTTTGGTGCAGGTGTCGGTGATTGCGATGGAGATGGGGTTGGAGATGGGGCAGTCACCGGACTCGAGCTGGGC
TCTGGCGAAGGTGTAGGTTTCTCCGGCGACAAAGTGGGGCTATCAGCCACGGGAGCGGGAGAAGGATTTACTGAAACCGGAGCTGGCGTTAGGTTCGCCGGAGAAGGCTTTGGCGTAT
CCGGCAAGGGAGCAATGGGCTTCGGGGTGGGTTTTGTACTGGAACCACCAGCGCACTTAGACTTGCTGCAGTCGACGGGCTTTCTTACTAAATGATCGCATTCCTTAGCCGTCTTCTGCC
CAGGCCGATCGGCAATACAGTTGCCTTTGTCGTCAAAAACTACGCCTGGCCTTGAGATAGCTGAGCACTCAGGGGCATTGCCCTTGAAGTAATTAAAGGAGTAACTAAAATTCGTAAGTT
TGGGCAGACGGCAAACCTCTGCGGGAACAACACCACTAAGTAAATTGCGACCCAGATTAATCTCTTCGACGCTGCTGAGGCCTGCGAAAGTCTTGGGCACAGAGCCAGTGAAGAAATT
ACCGCTGGCATCGAAAACAGTGAGACTAGCAAGCTTACCGATCTCCGGCGGTAGGCAGCCTGAGAGTTCGTTCCCAAGGAGGAGGATTTCATTAAGTTTTCCAGACATATTGCCTACACT

CCTTGGGATGCACCCGATGAAATGGTTGTTGGCGAGGATGATAGCGGAAGCGGGAGAGCTGCCGAGGTTTTCCGGGATGGGAGAGTGGAAGCGGTTGTCGTTGAGAAATACCGCATCG
AGGGCCTTATCAAAGAGCTCGGCGGGGAGATGCCCCTCGAAGTCGTTGTACCGGAGATCAAGATACTTGAGAGCGGGCATGGAGAGAACAGCGGAGGGGAAAGGACCGACGAACCGG
TTGTTGCTGGCGTCCAACTCGTGGAGGAGGGAGAGGCGGGATAAGCTCTTGGGAATGATGCCGCAGAAGCGATTGGAGTTTATGTGGATAAGTGCGACGTCGGTGAGGAGGCCAAGCT
CGGCGGGGAGGTAGCCAGCTAGGTCGGCGCCATTGAGGTCGACGCCAGCGACGACGTCGACGAAGGGGTCATCGAGGGCGGGGGCGCAGAAGACGCCGGTGTAGTTGCAGACATCGG
GTCCAACCCAATTGCCTGTTGTGTTGAAGGGGTCAGAATATAAGGCTTGTTTCCAGGCGTGAAGGGCGACGTAGGCGTGGCGGAGGCGAGTATTTTTTATGTGGACGTCGATCTTGACGT
TGAACTCAAAGTCTTCAGGGAGGTCTCCGTTTTCCGGGAGGTTATGAAGCTGGCGGCGGGCGATGGTGGCGGGTTCGAAGTTGGGAATTGAGGAGGCAACGGCGAGAAGAATACAGAA
GAGGAGGAGGAGGAGAGGAAGGTGGAGGGATGGGAAGCAGTCCAAGGCCTGCATCGCGCATGAACCCTCGGAGGGGCCTTAGAGAGAATGGAGGATAACGAAAGAAGAGTAGGGGA
GGTTATAGTGGGGAATGGGGAAACAGAAGCCATAGGCGGGC
>comp159705_c0_seq17
CTATATCAATCAACCATTCTATTTTTATCATACAACCAATTTTATCCAGTCATATCATTTAATCATTCTCGCTAGTATATTAGTTATTCAATTATAAGGCAGTGTTAGTATCTGTATTCCGTAACAA
TAAGCAGTAACAGAAATTAGTGTCCAAAAGGTCAATGTCCGTACAGTTCCAATTTGAATGTGTTTTGCGGAGAAAATTAGGAGAGAGGGGTCGGAGCGACTGAATCCGAAACCACGTGC
GAGGAGAAAGGTGGTAAATTTCTGAAACCACTGACGTGGAGCTTGTTTGAGTCCGTATAAGGATTTTTGTAGTTTGCAGACGTGGTTTGGAAATTGAGGATCTTCAAAACCTTGTGGTTG
ACGCATGTAGATAGCGTCGGGAAGATCTCCGTGTAGGAAGGCATTTGTGACATCGAGTTGAAGAATGTTCCAGTTGCGGTTGACGGCGAGTGTAAGAAGCATTCGGATAGTCGTCATTTT
GGCCACCGGGCTAAATGTTTCCGTATAATTTTCACCAAATTTTTGTTCATATCCTAATGCGACAAGCCGTGCTTTGTGGCGGTCTAGCTGGCCGGAGGGAAGAAGTTTAGTCTTATACGTCC
ATTTGCAGCCCAGAATGCGAGTATTGGGCGGTGGTGCGACTAAAGACCAGGTGTTTTGATGTAGAAGAGCTCGGAATTCTGTCTCCATTGCCTTTTGCCAGTGAGGTTGAGTCGAAGCTT
GTTTGAACGTGGTTGGAGTAGTCGGTTTTGTGACCTAAACAAGAAAATAGAAAGCAAGAAGAAAGCAAACTGACAAAAAAAAAATTACCCCACCAGAAAAAAATAGTATAGCAGGTTTC
TATTGTACCAAACACTAGTAACGTCTTGTATAAGCTAGTGTTTTGTGCATTCCTTTGCAAGTTTGACAAACAGAGCAGCAATCCATGAATCTCTGTTGAAGTTTTCACCATAAGAAAAGAG
TTTAAAGTAGAACTAAGTTTTTGTTAGGCGAGACTACCCAAGTGCAAGTCCTATATTAGCTGTTCCTTGATCGAGATTACCCCTTAATAAAAATTTGTCTGATTAAGAAATCTACAATGGTTT
GATGTTTCAAACATTTATTGTAGATGTTGGTGAAGTCTGGATCAGCTTAATAAAGCTCCTTGAGGAATGCGCAGCCCGTAGTTCTTACTGGTTTGTAAAGCCACCATCAAGGAAGAGCAGT
GACTTAAAGCATCGGACACTCTATTTGTTTTCAGTGTTTAAACACAAAATTGAATTTCTACAAGAATAAAATCCATCTCGTTTGTATCCTGCTACATGTTTTATGGGTCTGTAAGCAATGCGA
GGTCTGATAATTACATAATAGCAAAAAATCCTGATGATGAAGGTAGTGCTCCCACTGCTTGAGCGTGCGAACGATTGCAAATAATTCCAATTTATAAACAGTTCACCTCTGTTGTAGCAAAC
CCAATTTCTCATTGAAGAACTTAGCTGTTCGATCTTCTTGTGATAAGACAATGCTGATGCCGATAGCTGAAATGTCCGTATCAATCAGGGAAGGCTTGTCAAAAATTAATGCAGTCATGAA
AAAAAGTGAAGCACATGTTGAAGTCACAAACTTACGTGTTGCAAATTTGAAATTTATGTTCTTACTTAGGTGTAGCAAATTTGAAACTTAGGGTGTCATGCGCACTACCTAGCATGAAAA
AGTGAAACACGTGTTGAAGTCACAAACTAACATAATATCTTATATTGTTGAACCACATCTTCCAATATAAGTGACCTTTTCAGCACTCGGAACATGAGCATCTACATGCATTCATCAATGGA
TCATCTACCTTAAAATATGACATATATCAACTCTCATAGAAAATATCCCTTGGGGTATTTTGATAATCTCACTAAAGCTTCCAATACAATAGAGAATAACCTACTGATAGTCTCCATTCAATGC
TGAAATTGCTCAAATGTCCATCTTATTAATTCATTATGCAATATAATATATAAGGTGGTTGCAAAAATACTTGTCTCACTTGTTGACTGTGATGACTCCCCGACCGAT
>comp159720_c0_seq1
TTTTTCTTAAAAAATTCATATTAGAAGAATTTGAAAACAAGTTTATATACAAGAGACTAGAGAGAGAGAGAGATTCCTACATTAATTAAATCATGGTACAATAGCTGGTCTAACTAACCTTG
CACTTAATTCAATAATTAAAATTCATTAATCTAATCAGCAAAACATACTCCCACCAAACAACCTCTTAAGATAAATCACTAAACCCTAACCCTAATTTAAAAAAAAGAAAAGAAAAGAAAA
GAAGAAGTAATCTATGTGGTGATAGTACAGTTAATGGTTTCAACCTTGGGCACATCAACAGGGAACTTATCAATCTCCTCCAGCCATGGATAGCTCCCCTTTGGATTTATATCTCTCAGCGC
CTTCCAAACCACACTGTTGCACTTAAATCCAGAACCAAATCCGATTTGCCAGACCCTATCCCCTTTCTTAACCCTGTTCTTCGCCTCCGAATACCCTAATTCATACCACAACGAGCTGCTCG

AAGTGTTGCCGAATCTGTACAGAGTCATCCTCGACGGCTCCATCTGCCAGCTTCCAAGATCCAATCTTTTCTCCAGTTCGTCTAATATCGCCCTTCCTCCGGCGTGGATGCAGAAGTGCTC
GAAGGCTTTTTGAAATCCGGCACATAGGGTTTCGAAACTGATGAGTGTGGGCCGAAGCATTTGGTTGAGATTAGGGAAAAGGCGAAGCGGAGTTGTTCGGACAAGGGGAGGACGCGG
GGGCCGAGGGCGGTGATGTTGGCCTTCAGGGCGCCGCCGGCGACGGACATTAGGTCTTTGGAGAGGGAAACGCCGACTTTGCCCTGATCGTCTTCTTCCTGAGAGACGCAGAGATAGG
AGGAGTCGTCGGAGCCTCTGTGAGTGCGGATCAGGTCGGATAGAGAGTACTTGGCGCGGTGGAGATCGGAGCGGCGGTTGGAGAGGAGGACGGCGGCGGCGCCCATACGGAAGAGGC
AGTTGGTGACGAGCATAGGACGGTTGTTGCCGTAGTAGCCATGGAGGGTTAGGTTTTCGGTGCTGACTATGAGGGCGTAGCTGTTCGGCCTCACCTTGAGAAGGTGGCGAGCGAGATCA
ATGGAGATAACACCAGCGCTGCATCCCATCCCACTAAGATTAAAGCTCGCCACATCCTCCCTCATCTTATACTTGTTCACAATCATGGCCGACAGAGAAGGCGTCGGCCCGAACAAGCTAC
AGTTAACCACCAAAATACCGATCTCCTTCCTCACATCAACTCCGGTCTTCTCCAACAGTCGATCCACAGCACCGAATATCATCATCTCCGCCTCCTTCCTGGCCTCCGCTATGAACAAGGC
CGGCGGTATTCTCGCCACCGCCTCCGGGAAGTAAGTGGAGTGGCCGACGCCGGACCTCTCCAAAATCCGTTTTTGGAAGGCCACATTGTCTTTGGTGAAGGTTCCGAGAACTATGCTGC
GTTCGACGTAGGCTTGGTGAGTTTGGCTGCACGATTCGGTGGCTTTGTGGCAGGCGAAGTCGATGAGGAGGATTGTGTTGTGGCGGCTCAGTAGGAGCTGGAAGAGGAGAAAGAGGAG
GACGACGACGACCAAGAGGAGGAGGACCAGAGTGAAGAAAAAGCTCATTTTGCAGGTACTAATCAATAGTTGATCTTGTTATGAATCAAAGTTATTG
>comp159725_c1_seq1
AATCTCTCTTTTTTTTTTTTTAATGTAGGGGTGATGCTCTTGAAGTAAATACATGGTTGAGACGTCATACAAAATTGAGTATGGCTCGTGATTGGCATGTACGCAATCTCAAAACAAGTCAA
AGTATGATTCGGGCTTCTAGTATTTCTGCACTCATGGACAAAACCACACGTAAACTTTCAAAGATACCTGATGAAAAAAAAGTTGAATTAGATCTCCACTGTAATGATACTACTTTTATTTT
GAATGAGGACAACAGAAAGTTCCCTAAAGTTAATGATGAGAATGCAGATTACATCATAGATGGCTTAACTCCTAATTGGGCCGATTTAGATGTTAATATGCATGTGAACAATGTCAAGTACA
TTCTGTGGATTTTTGAGGTAAAATAAATGATTAGCTTTGTTAAAATTTGTTATCATTTTACCCCTCTTCGTTCTATTTTAAGAAAAATAAATAAATAAATAA
>comp159725_c1_seq15
ATGCTTTCTTGTCTTAATTTTGGCTTTATGAATTCTTATTAAACATTCATTTTTATTCTAAGTGGCCACATTAGATCTTAATTTTTATTATTAACAAATATTTTTGCATAACATAGTTATTTTTTGC
TAATTTCTTGAATATATAAGTGGTGTAATAGAACTACCTTTGCACATTTGTAGTTCATGCCATATCTTGCTCACATATGCAGCTGTCGGTGCAACATTGCTCATTTGAATTGGTTAACAAGAA
AATTGAAGGCCTTACTATTTTCTTTCAACTTGGATTTGGCAGGCCATTCCAATTTCTATCTGGGAGACTCATGCGATGTCCAGCATGGTCCTTGAGTTTAGGAAGGAGTGTAAGATGGGCG
ACGTATTGCAATCCCTCGTTGTTGTCTCCTATGTAAGCTCCAATTCCTCATCTGAATTTAAGATTGAGTGTAGGCATCTGCTCCAGCTTAAAAGTGGACCTCCGCTTGTGCAAGGACTAACT
ACTTGGATACAGAAGGTTTGAAAACTAGATGACTTCTTGAGCAACATCCCTAACACAAAGTTTCAACCACTTGAAAGATAAATGTAATTTCCATAATAAAATAATGACAGATAAGTTCTTG
TCCCTACTTAAAAGCTCAAACGATGGCCTTCGCCTCGGCTTCTAGCGAATAATTATAATAAATTGAAGGTTTGGTGTTCTTGTGGTATCCTTGTCTATAGTGCTTCAATTAATATATTACGAGT
TAGTAGTTGTTGCTTATATGGAGGAATTGTAAGCCTTGTATTCCCATGTATATCTTTCGGTCAAATCTGGCTCTTATGTTTCAGAAAAAACTTTGTTTACATATGATATAAGTGTATTAAACTTT
ATAATCTATATATTATATTATCTTGGTTGCAGCATGAAACGCTCTACTATGATGTTACCGATTTGGATTTT
>comp159725_c1_seq18
TCTCTCTCTCTCTCACACTCTCTTCGTTTGAAGAAAGCCGGCATTTGATAACGGCTTCTTTGAGGTGTGAAAGTAGCACAATCATGACTGCTTCAACTGCTTTCTCAACCATCTTCTCAATT
CTATCATCGTCACACCCAATTTCATCCTCTTGGACTGGTTCGTTTGATGTTTCATTTGCTTTTACCAGTGGAGTGAAAGCTAAATTTCCATGTTCTCTTAAGGCTTTTCAAATCAAAGGATAT
GTCAAAAAGGCCAATACTGTTACTTTTAGCTTTAAGAGTGACTTAGCAAATGTTGAGGAGCCTAGGAGGCCTGACCTGTTCGATGATTCATTTAGTCATGAACAGATAATTGATAATATTTA
CATGCAGAGAAACTCTATCAAAGTATATGAGATTGATGCTACTGGAAAAGCATCGCTTGAGGCAATAATGAACCTTTTACAGGAAACAAATGTCAACCATTTCAAAATTCTTGGGTTTTTG
GATGATTCTATTCGTTCATACCCAAAGATACATGGCGGAAACGATATATTTGTCATACTAAAAATGCATGTTCAAGTGGAACATTATCCTTCATGGGGTGATGCTCTTGAAGTTAATACATGG
TTAAGACATCATAACAAATTGAGTATGGCTCGTGATTGGCATCTATGCAATCACAAAACAGGTCAAAGTATGATTCGAGCTACGAGTATTTGGGCACTTATGGACAAAACAACACGTAAAC

TTTCAAAGATACCTGATGAAAAAAAAGTTGAATTAGATCTCCACTGTAATGATACTACTTTTATTTTGAATGAGGACAACAGAAAGTTCCCTAAAGTTAATGATGAGAATGCAGATTACATC
ATAGATGGCTTAACTCCTAATTGGGCCGATTTAGATGTTAATATGCATGTGAACAATGTCAAGTACATTCTGTGGATTTTTGAGGCCATTCCAATTTCTATCTGGGAGACTCATGCGATGTCC
AGCATGGTCCTTGAGTTTAGGAAGGAGTGTAAGATGGGCGACGTATTGCAATCCCTCGTTGTTGTCTCCTATGTAAGCTCCAATTCCTCATCTGAATTTAAGATTGAGTGTAGGCATCTGCT
CCAGCTTAAAAGTGGACCTCCGCTTGTGCAAGGACTAACTACTTGGATACAGAAGGTTTGAAAACTAGATGACTTCTTGAGCAACATCCCTAACACAAAGTTTCAACCACTTGAAAGATA
AATGTAATTTCCATAATAAAATAATGACAGATAAGTTCTTGTCCCTACTTAAAAGCTCAAACGATGGCCTTCGCCTCGGCTTCTAGCGAATAATTATAATAAATTGAAGGTTTGGTGTTCTTG
TGGTATCCTTGTCTATAGTGCTTCAATTAATATATTACGAGTTAGTAGTTGTTGCTTATATGGAGGAATTGTAAGCCTTGTATTCCCATGTATATCTTTCGGTCAAATCTGGCTCTTATGTTTCA
GAAAAAACTTTGTTTACATATGATATAAGTGTATTAAACTTTATAATCTATATATTATATTATCTTGGTTGCAGCATGAAACGCTCTACTATGATGTTACCGATTTGGATTTT
>comp159725_c1_seq2
TCTCTCTCTCTCTCACACTCTCTTCGTTTGAAGAAAGCCGGCATTTGATAACGGCTTCTTTGAGGTGTGAAAGTAGCACAATCATGACTGCTTCAACTGCTTTCTCAACCATCTTCTCAATT
CTATCATCGTCACACCCAATTTCATCCTCTTGGACTGGTTCGTTTGATGTTTCATTTGCTTTTACCAGTGGAGTGAAAGCTAAATTTCCATGTTCTCTTAAGGCTTTTCAAATCAAAGGATAT
GTCAAAAAGGCCAATACTGTTACTTTTAGCTTTAAGAGTGACTTAGCAAATGTTGAGGAGCCTAGGAGGCCTGACCTGTTCGATGATTCATTTAGTCATGAACAGATAATTGATAATATTTA
CATGCAGAGAAACTCTATCAAAGTATATGAGATTGATGCTACTGGAAAAGCATCGCTTGAGGCAATAATGAACCTTTTACAGGAAACAAATGTCAACCATTTCAAAATTCTTGGGTTTTTG
GATGATTCTATTCGTTCATACCCAAAGATACATGGCGGAAACGATATATTTGTCATACTAAAAATGCATGTTCAAGTGGAACATTATCCTTCATGGGGTGATGCTCTTGAAGTTAATACATGG
TTAAGACATCATAACAAATTGAGTATGGCTCGTGATTGGCATCTATGCAATCACAAAACAGGTCAAAGTATGATTCGAGCTACGAGGTTAGCCGTTCACGTGTGTCAGAACTTTCCACTAC
ATGCCAAATGAAATATATCAATTTTTAATGTGATTATGCTAATTATGGTTTCTTTAGTATTTGGGCACTTATGGACAAAACAACACGTAAACTTTCAAAGATACCTGATGAAAAAAAAGTTGA
ATTAGATCTCCACTGTAATGATACTACTTTTATTTTGAATGAGGACAACAGAAAGTTCCCTAAAGTTAATGATGAGAATGCAGATTACATCATAGATGGCTTAACTCCTAATTGGGCCGATTT
AGATGTTAATATGCATGTGAACAATGTCAAGTACATTCTGTGGATTTTTGAGGCCATTCCAATTTCTATCTGGGAGACTCATGCGATGTCCAGCATGGTCCTTGAGTTTAGGAAGGAGTGTA
AGATGGGCGACGTATTGCAATCCCTCGTTGTTGTCTCCTATGTAAGCTCCAATTCCTCATCTGAATTTAAGATTGAGTGTAGGCATCTGCTCCAGCTTAAAAGTGGACCTCCGCTTGTGCAA
GGACTAACTACTTGGATACAGAAGGTTTGAAAACTAGATGACTTCTTGAGCAACATCCCTAACACAAAGTTTCAACCACTTGAAAGATAAATGTAATTTCCATAATAAAATAATGACAGAT
AAGTTCTTGTCCCTACTTAAAAGCTCAAACGATGGCCTTCGCCTCGGCTTCTAGCGAATAATTATAATAAATTGAAGGTTTGGTGTTCTTGTGGTATCCTTGTCTATAGTGCTTCAATTAATAT
ATTACGAGTTAGTAGTTGTTGCTTATATGGAGGAATTGTAAGCCTTGTATTCCCATGTATATCTTTCGGTCAAATCTGGCTCTTATGTTTCAGAAAAAACTTTGTTTACATATGATATAAGTGT
ATTAAACTTTATAATCTATATATTATATTATCTTGGTTGCAGCATGAAACGCTCTACTATGATGTTACCGATTTGGATTTT
>comp159725_c1_seq9
TAAAATGCATATTCATGTGGAACATTATCCTTCATGGGGTGATGCTCTTGAAGTAAATACATGGTTGAGACGTCATACAAAATTGAGTATGGCTCGTGATTGGCATGTACGCAATCTCAAAA
CAAGTCAAAGTATGATTCGGGCTTCTAGGTTAGTCATTCATACATGTCAGAACTTTCTCCTTCAAGCCAAACGAAATATATCGCTTGTTAAACTGATTATGCTAATTCTGATTTCTTCAGTAT
TTCTGCACTCATGGACAAAACCACACGTAAACTTTCAAAGATACCTTTTGTAGTTCATGCCATAGCTTGCTCACATATGCGGTTGTCAGAACAACATTGCTTATTTGAATTCTTTCAACTTG
GATTTGGCAGAGCATTCCAATGTCTATGTTGGAGACACATCAGATGTCCAGCATGGTTCTTGAGTTTAAAAAGGAGTGTAGAATGGGCGATGTATTGCAATCCCTCGCTGCTGTCTCCTCT
GTAAGCTCCAATTCCTCATCTGAATTTAAAATTGTGTGTAGGCATCTGCTCCAGCTCAAAAGTGGGCCTGCTCTTGTGGAAGGACTAACAACTTGGGTACAAAAGGTTTGAAAACCAGAT
GATTACTTGAGCAACATCCCTAAATCAAATGTTCAGACACTTGAAAGATAAATATAATATCCATAATAAAATAAAGACAGCTAATTTCTTGTCCCTATTTTAAAGCTCAAATGATGGTCTTTG
CCTCGGCCTCTAGCGAGCAATTACAATAAATTGGAGGTTTGGTGTTCTTGTGGTATCCTTGTCTATAGTGCTTCAATTAATATATTACGAGTTAGTAGTTGTTGCTTATATGGAGGAATTGTAA
GCCTTGTATTCCCATGTATATCTTTCGGTCAAATCTGGCTCTTATGTTTCAGAAAAAACTTTGTTTACATATGATATAAGTGTATTAAACTTTATAATCTATATATTATATTATCTTGGTTGCAGC

ATGAAACGCTCTACTATGATGTTACCGATTTGGATTTT
>comp159725_c2_seq2
TTTTTTTATTTTTATTTTTTTAATTTTTTTTTAATAAAAAGCCCCGTTGAAATTTATATAAACCATGAATTGCAAAAGAAATAAAGCAAGCCTTAACAAAACTATTAGGCTACAAAAAGGACC
TTCTAACAATTAGTTCAGGTAAATGGGCTAAAGATGAAAAACAAATCAACTGACTACATTGACATGATCATTACAAAATGGAAGGGATTATAACCCCAATATATAGAACATGTGCAGCAAA
TTCAAGAAGATTGATAACGACATGCTTGATCATGCGAGCTTTTTGACTAACCATTTCATTAACAAAATACAATTAATCAATAATCAATGAGGAAAAAAACAGCATTGACAAAATTGTGAAG
AACCGGTCCATGAATTTCAGAGTAGACCAAAGAATTTATAGAGTATCGAAAAGGACAAGATCTAGCTAATAGAATATAAAAGATTGGTTCGAAATACTAGATTCAACCGAGGCTAGCCTTA
ACCAAATCACTTGATCATGGCTGAGTAGACCATGGTAAAACCACCTAATGGTCAAAGGGAAAAGTAAGAGTAAAAGAAAAAGGCATATTTCAATGATCAGATAGTAAATTATTCCACGAA
GGAATCAATATCAATAAAATCTCTAAGCCAAGCTTAGTATCTCTCTTGTTTATGTTTCATTTTTACTGACTCTTTCCCACAATCTCAACAAAACTTGAAATTGTGAAATATTTGATCAACCAG
CCTATAACTAGACTTGTATCGGTATAAAATTTAGTCGAATAACTTGGAGCTAATCTCATACATCCCAAGCAATCCGCGAAGCTTTAGCTAATCAGTGGTTATCGCCTCCACAGAAGTTAGAA
GTGAGATATGCTTGATGACTAGTCTTACATATCAAGTTTCATTCAATTAAAGGCTAATCTCGAACATTATATACGGCGAAATCCCCTTATTACCAAAAAAATCTGAGATTTCAATTGAATAGC
TGTAAACAACGATACGAACTAATCAATCCAAGAAAACAGTGCAGAATATTAAATGCCGATAACGAAAGAACCATTCTGGAAAAGAACGTTTGCCGAAAAAGATGAAAGATGTAATGTAG
GACGCTGGTTTGTATTGGACTCTTACAAGATCTTACCCGTTATCAAATGCCGGCTTTCTTCAAACGAAGAGAG
>comp159755_c0_seq2
CTCATTCAAGTTATTTATAGTATTTCAAAATTTATGTTTATTTTTCTCTTTTCAAGGACTATCAAGATATTTGTTCAATAAACCCTAGCTCCTTGCCTAGTTGGTCGAGGAGGAAGAAAAAAC
CTATAACCCTCCATATAACTCTTTATTAATGATCATATGGCTACTACTCATTTTATAACATTGAAGATTTAATGCATTATCAAGGAAGTTTCGAACATCGAGATTTGCTTATGAATTTATCTACA
GGTGGGATTTCTACTACAACTACTACTTTCATTGAGTTCGTTGTTTCTATTGCACCTGAGTACGATGATGCAGAGCATATTATTTTAGATGAACTTGTACTTGGAAAAAATGCTGGGCACAAAT
TCTTGATCAAATGGACTATCGCCTCTATCAGATGATACGTAGATTATAAAAGTAGAATTGCTAAAGCTTGTTGCTGATCTCTTGGTTGATTATACTATTGCTAACTCTCTACAGCAGAGTTCCT
TGCGGTTAATGGGAGTTAACCCAATATCTTTTTTGTATGATGATTGTTCATCCAAATTAGTACACGGAATTCTACTGTAGAGAGTTAGCAATAGTATAATCAACCAAGAGATCAGCAACAAG
CTTTAGCAATTCTACTTTTATAATCTACGTATCATCTGATAGAGGCGATAGTCCATTTGATCAAGAATTTGTGCCCAGCATTTTTCCAAGTACAAGTTCATCTAAAATAATATGCTCTGCATCA
TCGTACTCAGGTGCAATAGAAACAACGAACTCAATGAAAGTAGTAGTTGTAGTAGAAATCCCACCTGTAGATAAATTCATAAGCAAATCTCGATGTTCGAAACTTCCTTGATAATGCATTA
AATCTTCAATGTTATAAAATGAGTAGTAGCCATATGATCATTAATAAAGAGTTATATGGAGGGTTATAGGTTTTTTCTTCCTCCTCGACCAACTAGGCAAGGAGCTAGGGTTTATTGAACAAA
TATCTTGATAGTCCTTGAAAAGAGAAAAATAAACATAAATTTTGAAATACTATAAATAACTTGAATGAGGAAGAGGGAATAGGAATCAAACTCTAAGTACACAACTAAGAGCACTCCGCAT
ACGCAACTCGGATTAATGAAGGAAAAAGCTACTAACAACTAATGGTCTCATCAAATGATTTATTCATCAATAATCCAAAAGCAATTAGTCATATTTATAAAAGAAAGCAAACCCTAAAAAA
GGATGAAATTATTCTAATACTTTAAGATTTAATCTAATAATAAAAATTGAAAAGAAATACTACAAAAATAAAAACACACAAAGACACATATTAAGTTTACAAAAATTTTTAATTAAGTATTAA
AA
>comp159764_c1_seq4
CCTACCTACTTTTCAAATGTGCTTATTTTAGCACATGCAACCTAACGTCCTCGTCGGCTCCTCCCTTCCCCTCCACTACTTCTCACACAATCGGTCCTTTTTCTCCTCAAATCCCTAATTTCC
ACACCTTCGCACGTTCTAACTACCTTTACCCTACCAAAAATTCAACAATTTTCGTCATTTTAGTAAATTTCAATGGGCCAAATTTAAATCTTTTATTCTCGCCGT
>comp159767_c1_seq1
GCTCATTAGTTTAGTTTCCAATCATAGTACAATAACAAATGATGTGCACGTATACTATAATGATTTTCATCATTTTTTTTACAGAAATCAAATTTCTTATCTGCCAAAAATTTGAAAAGCAACC
CTGAAAGTTGTTATCAAAAAGGATATAGAATGCATGCCATTTACATCACGAAGAGTTGCTGAATACTAACATTGTTTGATCAGACAAAAAATGAGTTACTCACAGAGGAATTTATGCCATA

AATCAGGCAAAATCATCAATCCAATCCCCATAGATGCGACTAATTTTTTTCAAAGTAAGAGTTCTAATTTTTGGCTGAGATACAGCAGCTTCTCTGGACTCTCTAGGCTCTGCCGTTGTCTT
AGATAAAATTTCATCAGTTATGTTAATAGCACATTTGGGACGAGCTGCAACAACATTCTTCTCAACCAATTCATCATTATCCCCTCCGAGCTTCCTTAATTCTGAATGTCCATGAGCATAACT
GCATTGATCACCATATGAGCAACTACCAGAAGTCGACCACTTATGACAAATCTTTGTCTTCCAACACATAGGTTTCTGACTGGGTAAGGAGCTATCCTGAAGCGAACCTGGCTGTACATGA
CTAAAACTGCACTTGTCACCATAAGGACAGAGCTCACCATGATAAAACTTCCAGCAAACTTTGTTCCTATGCAGCTTTTGCTTCTCCCTTGCATCAAAAAGTTCTGAGGAAACAAGGCTC
TCACATCCACATTTCCAGCAGGCCTGCGGAGTTCTTCAATGCTGTGAGCGAAGTTGCAGCTGCCACCTTTGGAACAGGAACCAGACATGAATTTCACACACATACGGGTCTTGAAATATA
CTGATTGAGGTGTACTGGAGCCGTGGTATGTTAGATTCGGGAAAAAAGGTGGAATTTGAGGACAATTCCCGATTTCTTCTGAGATTCTCTGTTCCGGGGTGATCGAAAAGTCGAATGGGC
CAGGGAAAGCAAAGGGATGATCCATGGTTGAACAGAATATTTTTAATAGCACAGGAGAAAGGCAAAGAGCTACAATGGGCTTTCAAGATAAATGAAGGCGATATATCAACAAAGCATGG
AAATTACTAATGTTCACAAAAGAAAAAGTAATATATATGAGAAATGCAGGAAAGCACCAACAGTGATATATGAAAGACAATCAAACTCAGAATCAAGATTTAGGTTCATCATCTAAAGAC
GAACCTCCGCGTAAGAGATACGAGGATTCAATTATAGAATGAGGAAAGTCGTCTATATCAGAATTAGATAGGCCCGGGACGAATTCAATCGAAAATGGCGGTGAATAAGAGAAACCCTAG
AATCGACGAGAGAGATCAACGAGGGTTTTGATTTCGAAAACCCTAAAAATTGAAGAAATGCCTTAAGCCTTAAGGAGTATAATAATAAAGGAAATTTTAGGATTTAATCCATCCGAAGGTT
TCCGTTAATAGAATCTTTGTTGCTTAGCACGTCTGCCCCGCCTCTGCCCGCGAGATTTCAGCTTCTCCGCGCGTTAACCGTACGGAATAGAACCGGCCTTATGGCAGCGCGGGTGGGGGCC
CACG
>comp159767_c1_seq4
GCTCATTAGTTTAGTTTCCAATCATAGTACAATAACAAATGATGTGCACGTATACTATAATGATTTTCATCATTTTTTTTACAGAAATCAAATTTCTTATCTGCCAAAAATTTGAAAAGCAACC
CTGAAAGTTGTTATCAAAAAGGATATAGAATGCATGCCATTTACATCACGAAGAGTTGCTGAATACTAACATTGTTTGATCAGACAAAAAATGAGTTACTCACAGAGGAATTTATGCCATA
AATCAGGCAAAATCATCAATCCAATCCCCATAGATGCGACTAATTTTTTTCAAAGTAAGAGTTCTAATTTTTGGCTGAGATACAGCAGCTTCTCTGGACTCTCTAGGCTCTGCCGTTGTCTT
AGATAAAATTTCATCAGTTATGTTAATAGCACATTTGGGACGAGCTGCAACAACATTCTTCTCAACCAATTCATCATTATCCCCTCCGAGCTTCCTTAATTCTGAATGTCCATGAGCATAACT
GCATTGATCACCATATGAGCAACTACCAGAAGTCGACCACTTATGACAAATCTTTGTCTTCCAACACATAGGTTTCTGACTGGGTAAGGAGCTATCCTGAAGCGAACCTGGCTGTACATGA
CTAAAACTGCACTTGTCACCATAAGGACAGAGCTCACCATGATAAAACTTCCAGCAAACTTTGTTCCTATGCAGCTTTTGCTTCTCCCTTGCATCAAAAAGTTCAGAAATTATGGGATTTT
GTTCATTCCTTCTACTGCCGTCTACCTGAGGAAACAAGGCTCTCACATCCACATTTCCAGCAGGCCTGCGGAGTTCTTCAATGCTGTGAGCGAAGTTGCAGCTGCCACCTTTGGAACAGG
AACCAGACATGAATTTCACACACATACGGGTCTTGAAATATACTGATTGAGGTGTACTGGAGCCGTGGTATGTTAGATTCGGGAAAAAAGGTGGAATTTGAGGACAATTCCCGATTTCTTC
TGAGATTCTCTGTTCCGGGGTGATCGAAAAGTCGAATGGGCCAGGGAAAGCAAAGGGATGATCCATGGTTGAACAGAATATTTTTAATAGCACAGGAGAAAGGCAAAGAGCTACAATGG
GCTTTCAAGATAAATGAAGGCGATATATCAACAAAGCATGGAAATTACTAATGTTCACAAAAGAAAAAGTAATATATATGAGAAATGCAGGAAAGCACCAACAGTGATATATGAAAGACA
ATCAAACTCAGAATCAAGATTTAGGTTCATCATCTAAAGACGAACCTCCGCGTAAGAGATACGAGGATTCAATTATAGAATGAGGAAAGTCGTCTATATCAGAATTAGATAGGCCCGGGAC
GAATTCAATCGAAAATGGCGGTGAATAAGAGAAACCCTAGAATCGACGAGAGAGATCAACGAGGGTTTTGATTTCGAAAACCCTAAAAATTGAAGAAATGCCTTAAGCCTTAAGGAGTA
TAATAATAAAGGAAATTTTAGGATTTAATCCATCCGAAGGTTTCCGTTAATAGAATCTTTGTTGCTTAGCACGTCTGCCCCGCCTCTGCCCGCGAGATTTCAGCTTCTCCGCGCGTTAACCG
TACGGAATAGAACCGGCCTTATGGCAGCGCGGGTGGGGGCCCACG
>comp159784_c2_seq18
AAATCCAAAGGCATAGTCCATGAACCATACCTGTAAGAAAAATAAAATGAAACAAACTTCCAAACATAATCAGAAAAGCACATTCCCCAAAAAGAAAAAAATACACCACCAAAACAGA
AGCTGATCAATAACCAGATGACCAGCAAACACAAATAACCAGCCAGAGAACAACCAGATAAGTCCACCACCAAAAAAAAAAAACCCAAAAACACTTGCTAAAAAAACCCATACCATAA
GGCCAAAGCACTAGAGGAACAAAACATTGCCCCAACACCAATAGCAGAACTAATCCAAAGACTGATTAGAATGAGCATAGATCTAGTAGTTCTGCCAACAATTTTTTTTTTCTGTTAAAA

CCCACGGAGGGGTGTACACGTTTATAATAAAAAACGATTACAAAATCATTAAGGGAGACCATTAGCTCGCTACTTCTCAAACGAGCTATAGAATAAAACAAAAGAAAACCTAAGAGCTGA
TTTTTTCCACTAAGCCAGATTAAAAAAAGACCTAATAACATGCTAAAGATGCTAAAGCCAAGCAACAAAAAAAGAAAGATCAAGATATGAACTTTAGGCACAATCTTTTTGCAAAACCTT
TTGAAGACAGCCATGAGCCAATCCACTTGAAAATAATAGAAGCTACAACACACAAAATTATAGCACAAAAGAGCATATATGTAATATAATTAATAGCCAAAATCTAAATCAAGAAAAAAGT
AAATATAAAAAGTACTGTAAGTAAAAGGAAGCTGCCAGGAAATAGAACCACCAATAGGATAAAGACTCATAGAGCTCAGGTAAGAATTAAATCATGAAACATCACCCAAGTAATCCACAC
GCCACTCAATCCTACCAACAAGAGTAAGCATAAACACCAAATCATAACAGGACACAGCAGAACAACACAAAACCTCCAAGTTAACCCCAACCGTTAGGCCAAGAAATAAAGATAAAGG
CAACCCAGCCAAAAAACCCAACACTAGAACAGAAACATGGAATCCCCAAAGCCCCAAATGCTCATATATATATATATATATATTGTTTAACACACATGATTGAAATAATTTCAACTACAAAA
CAAGAACACAAGACTACAGGCTCTGAAACTATAATCTTCATCTTCATCAGCAACGTCATATATAATAATTCCAAGAACTCTCGACCGTTAGCTTTGGAAATTCATGCTGTTTCTCCATGTTG
AAAGATGCAGAAACCTTGATCAGGTAGTGTTGGCTAAAAGTAGCGGTGAAGCGAGGCAAATTGGTCGCTGATCAAAGCACGATCAGCCTCGTGCTATTGCTATAGTTGGTCCCAGTGAG
CGATAGACTCCTGGATGTGTGTTTCCAAGCACATCTCTAAGCAGTCAAATTGCTTTATCATAGTATCATTAAACGATGTTGATATGTAGGTGCTGGTGTTGGTTTTGGTACCTCTGGGACTGG
TAGGGCTCCTCTCGAGATCATGCTCCTCCTTTGCATGCTAGGCCTCGTGGCGTCTGTCACCTAAGAACAGGTGGAAGGAATTATCCGTGTAGTATGAAGGGAAGAGGTCGTTGGGTACAT
GAGCTGATATTTTGTATCGAAGACATAAGCAATAATGTGTCATAGAGCTAGATTTTGCCTACGTCGGACTCCAATATTTCTAATAGATGTCAGATTGTCAATATAACAGATAATCATATCCGTT
GCATTGAATTTTGTGTCAGACATAATAAAGTAACTATAAACAACAAAAGGGTGATGTTTATCCTATCTCCAGCCTTAAGGTAAAATAAAAGTTCTAAGAATATGTTGATCTTGCTGGATGAT
GCATGCATTCTGGGTAGGAGTGTTCACTAGAGGTAGGTGATGACTGATAGGTGCATTTCCAATGGAAAGATTTATTTAGGTCCAGTCATAGTCTCCCCTAGAGCTAACACTAAGCAGTGCT
ATACCTGTGATAGAAAGATGTAAGATTTCATCAAGGTCTTGATTACTAATTTATACTGGACGCCTACAGACCTAAGATTGCATTATTGTTTTTTCAGCATTAATTTTTAGACTTGAATGCAATA
AATGGAGTTTGATAACTACCACTTACGAGATTTTTTGGTCAGTTCTATAGAAATATTATTTGCATATATTATATGTTATTATCCTTGGAAATAATATCATCATCCCTTCCTTTATCACTTTTATTAT
GTAATGTGTGCGGTGTACCACATCCTCCATCTACATTAAAGATGGGAATACTTTCTTTTGTTATTTGTTCAACCTCCAAAATACTTGGTTTTCCTTCCTCAACTCCCACGCACAGCATATGAA
AAGCCCTAAACAACAGACTACAAGAGACGATCTACAGATCTACATAAGATTGTAGTTAAGAAACTTACAAATAGAGCCCTTAAAATCTCAAATTTAACAAGCTTCTTCTTATTCAATTATCT
TAAGAAAGCACGTTATAATCAGGGCGCTTTAAAGTGAGATAGGGTTCGAAGAAATTGACA
>comp159794_c0_seq12
AAATTCATATAATATGGTGGCATAGAAAGAGAATAAACTCTTTGAGCATAATATTGAAGATAAAAATAATTGACTTGGTAACAAATTACAAATTATTGGAATTGTGATGAATGTAATGAGCCA
CCTTGTGGGGACTTATTGTTTTACAATACATCCCCCGGTCATCAAATTGTTTGCAGTTATTTCTTCATATCATTTCGTCTTCTCTGTACTTATCTATCCAACTTCTAATTATTTCCACTTCTAGC
GTCCTTTGTTCTATAAGCCAAGTTGGATCTTCTTCTGTAGCAGATCTGAAATCTACATGATGAGCACCTTTTGCCGTGACAAGAGCAACAATGGATGATGACAGGTTCTCAAGTACCCCTC
CTCTGCTCCATGGGTCTTTCATTCCATTAGAGAATATTATGTTGCTTCCAAATCTGGAAAGAACTTGCTTGATGTTATGGCCTCCATACTCTGTAGTAATCCAATGGGGCCGAGGTCTTACAC
CATACGTTTTCTTGCATTGGTCTCCAAATTCTTTATAATCATATGCTGATGGTGGGAACATGCTTTTGTTAGAGCAAGTCATTGGCATGACCATTTCTGTGCAAGCCTGCCAATCCCACCCAT
CAAGTCCATGAACATCAGCCTCATATCCTATATTAAAACAAGATTGACTGCCTGAATAGTTGTAATACAAGCTTGCAGCTGAAAACACTTTGGTGAGTATGTCAACCTCTGGTGGGAATCC
ATCTATGATTCTGCACATCTCCTGAACTGGATTGGCTGGCAATGGTTTCATGAAATTTGCTTTTGTTGGATAATCAACCATTGCCGTATATACAAATGCTTCCCATAGCCAATCTCGTGCTTGA
TATACTGAATGAAGATTTAATAATTAGAAGAAATCTCTGAATTGAATCGATAGATGAAAATGCCAAAAAATCAACTTTCCCTCTCTTGTGGCTCTCCTCTTCTTCCCCCTTTCCCTCTCTTCT
TCCACGACAAGCCGCGCTCAAATGGGGGAGGTGGCGGCACTGAAGCAACTTAATGCTTACGATTTAGAGTAATAAATATACATAAATATTGTTAGTAGCCTAGCGTTGCAACGCTGCATGC
TGAATCCAGAATTACTGTAAAATAGAAACTCAAACATCGCAGCATCAATTTCTAGCGCCGCTGTGGTGCTTTAAGCATAGCACCTCATCGTTACAAAACACAGTGCTAAAGCATTATTATTA
TATTTTTTATTTTAAAATATTTTTCATTTCTTCGGGTTGTTAGAATTAATGACTTGAAAACACACGTGGTAAGAGTGCGAGTTGTTGCTCTTTACAATTTTTAAATTCTGCACCTAATGTTGAA
ATTATGAAAATTTATTTTGCTTTAAAAATTTGTTCATTTAAGAATTGGTCAACTTGTGTCAAATAAATCATGTCTCAGGACAAATTTTAAAAAATAGTATGATAAATAATTTAATTATTACAAG

TATATATATACAAAAAAAATTTATGTAGTTGCTCG

>comp159794_c0_seq16

AAATTCATATAATATGGTGGCATAGAAAGAGAATAAACTCTTTGAGCATAATATTGAAGATAAAAATAATTGACTTGGTAACAAATTACAAATTATTGGAATTGTGATGAATGTAATGAGCCA
CCTTGTGGGGACTTATTGTTTTACAATACATCCCCCGGTCATCAAATTGTTTGCAGTTATTTCTTCATATCATTTCGTCTTCTCTGTACTTATCTATCCAACTTCTAATTATTTCCACTTCTAGC
GTCCTTTGTTCTATAAGCCAAGTTGGATCTTCTTCTGTAGCAGATCTGAAATCTACATGATGAGCACCTTTTGCCGTGACAAGAGCAACAATGGATGATGACAGGTTCTCAAGTACCCTGA
ATTCAAATTAATGGCATTAAAAATTCAAGGACATTTGATAGAATCTGATTGATCTCTTTTAAATAGCAATTTTGTAGTTGAAAAATATAATGTTAAATGAACACAATAAGATATTCAATCCTCA
CCCTCCTCTGCTCCAT

>comp159794_c0_seq7

AAATTCATATAATATGGTGGCATAGAAAGAGAATAAACTCTTTGAGCATAATATTGAAGATAAAAATAATTGACTTGGTAACAAATTACAAATTATTGGAATTGTGATGAATGTAATGAGCCA
CCTTGTGGGGACTTATTGTTTTACAATACATCCCCCGGTCATCAAATTGTTTGCAGTTATTTCTTCATATCATTTCGTCTTCTCTGTACTTATCTATCCAACTTCTAATTATTTCCACTTCTAGC
GTCCTTTGTTCTATAAGCCAAGTTGGATCTTCTTCTGTAGCAGATCTGAAATCTACATGATGAGCACCTTTTGCCGTGACAAGAGCAACAATGGATGATGACAGGTTCTCAAGTACCCCTC
CTCTGCTCCATGGGTCTTTCATTCCATTAGAGAATATTATGTTGCTTCCAAATCTGGAAAGAACTTGCTTGATGTTATGGCCTCCATACTCTGTAGTAATCCAATGGGGCCGAGGTCTTACAC
CATACGTTTTCTTGCATTGGTCTCCAAATTCTTTATAATCATATGCTGATGGTGGGAACATGCTTTTGTTAGAGCAAGTCATTGGCATGACCATTTCTGTGCAAGCCTAAATGAAAAACAAA
GCAAAAGGAATGTAATAAAATCCAAGAAAGTTATTCTAAAGAAAATAAGCATTACATGTGATCATTTAGGACTAAAGAATTCCTTCTTTCTAATTATGGCAAGCATGTACATGAAGATTTAT
GCAGATGAACACAGAAGATATTGAAATTCATCAGTATTAAGATTTAAATCAAGACAATTACTAATATGTCCTAAGAAATTAATTGCTCCAGCTCAATTTTAATCTTTCTTAGGGGCCCATTCT
GTGAAAGATTCTAAGTCGAAAACCATTTGAATCAAAACTCCCTCTACTATTAAAGGAAAACCTGCTATTATCCGATTGTGTGCTGTTGA

>comp159796_c0_seq1

CTACAAAATAAGCTAGACCACAAACTTTAGCAGGTTGATACAGGAACTGTCAACAATCCAATTAACAAAGATTATTGAACAACAGAATAATTTAAAAAACAGATAATCATTTCAGATATCC
ATAGAACATTGACTGCAATATCCTTATTATTTATAAAAATAGATTTTATAAGAGAATGAGGGGTTAATCTAATGTGACAGTTTCTCCTGTTTTCAACGCTTACCACAGCTTATCGTGTAGAAC
AATCACAGGCAAGCAAAACCAACTTCCCCGATCCAACGGCTGGTGATCCTCATCAGCTCCGGTGGACTCCCCGCCCTCGCCTCTCTCCTTTATCTCCTTGAACATATATATTATACTAATAC
TGATCATAATCGATCAGTATCAATCACAACAGTAATCTTCAGCATAATTTTTGTGAAGTCACAAAAAAAGCTTACGAAATTAAAACCTTTGTGTATATCCTCCCCTGTTATATGGATAGCCCC
CACCTCCCGTCGCCGGTAGCTTCCCCTGCATCCCATCCCTAGCGTACTGCTGCCACATCAGCTGCTCCTCCACAAACAACCTCTGCTTCCTCTCCAGCTCCGACATCTGGACCCACGGAGG
CGGCGCTACTGCCGCCGAAGCCTCAAAAGGGTCACCTGACCCGCTTGCTCTGACGCCGCCGCTTGCCGGGGCCGGCAATGCCAGCATCCCCTGCGCCGCCGGTGCCATCACAGCCATAT
TGCTGGCGTTTCCTCCAACTCTAGCATCCGTAGCGGTGCCCTGGCTGTACATTCCCTCGAGCATCAGCATGTCGAATCCGCTGGCTGACGCCGGCTTCTGCTCGTGGAAGGTGCTCGCGG
ATTCGACGAGTGCCATTTCCCAATCGGCCGTTTCCTTGAACGGCTCCCATGGCTGCGCCGGAGGCGGCTCCGCCGGAATGCCGTCGAACAGGGCGAGAGCGAGGCTGTCCGGGTTACGT
TCGCTGGTCGCCGCGTCTTCCTGAATATGGAGCAAGTCTGCCAGCTTCTCCTCCTCTGTAGTCGACGCAGGCGCCGCTTCCGGGGCTGCTGCCAACTCTACCAACTCATTTCCAATCTCCT
CCACCTCCTCCGGCGGCTCCTCAGGCGGTGGCGCCTGAATGGACTTGACGTCCTTGACGGCTTGAAAAGCGGCATCCTGTTCTTGCGTGGCAGGGCGTTGAGAAAGCTGGCGGTCCGG
GAGCGAGGAACGTTCTCGGATGAATTCTTCCATAACTTTAAGGTTGTTCTCGGTGATGTGCTCGATTTCAGGGTAATCGGAGGGGCGGGCGATGCCGGCGGCGCGTGACCAGGAGTGGA
AGGAGTCGAGCTCCTCGAATTGCTTGGAGAGCTTGGAGAAGAGATCGTGAACGCGGACGCAGTCCGTAACGAGAAGCTCCGTGAAGCGGTCTACGAAAGCGGAGGTGATCTCCGTCAT
GTCCAAGTATATTTGGAAGCTCTCTTTGATGATTGGGTACAATGCCATTGCTACGATCCTGTCGTCCTTGGCAACCCCCGTTGGACGGCAGGCAAGCAATCTGTCTAGCAACATCTGGAGA
TGATGCGCCTTCACCAACAATCGATCCGTCTTCATCTCTCTCACCGGCGTCAATCTCGCCGGAGTCACCGCTCCCTCCCCCTCCTCCCCCTCTTCATCCCTCCCCATCCTCCTCCATCCCGA

CCACCCCACCGTCGCGGACAACAACGTCGCCGATCCTCCCCTGTACCTCCTCCCAAACATCTTGTATTCCAACCTCTCATCAAGGTACCTCGCATAAGTCCTCACAAAGGCCGAAAAATC
CCACGCATCAGACCTCGATCCCGCGTCCCTAAAATCCGCCATATTGAGCATCCGCGTGCCGCGCCGCGTCCCGAAGAAAACCTCCCGCTCGTAGGCGGGATCACCGTCGGCGAGCAGGC
GGTGGATGAGCACAAGGGCCTTGAGCGCCACAGCCCAGCTCCGGGTTTTGCCGAGCCGGCGGGAGAGGAGAGCCACGCAGGCGGCGATGTTGGTGCGGGAGTAGGATGTCAGGGAAA
GTATCTCCCGCACATGCTTCTCCTCGGCGGGGTACTCGTCGTGGCGGGTTGCTTTCACGATGGCAACCTCGAGCTCCGCCAGCGTGGTGCTGCTGCTCACTTTTGCGAGGCCTATGCTCGT
TTGATCTTTTACTGCGCCCAGCGCTTTTCGTATCTTGCTTGGTGCCATTTTTTCCTTTGTTGGGTTGTTCTGATCATGTAGTTTGGAGATATTGAAGAAGAGAGGAGGAAGGGGAGATGAG
AGAGAGGAGATAGATCAATGGTGCTCTCTCTCTCGCTTTGGCTCTCCTCTCTTGTTCTGTTTGTTGATTTCTTGGCGATTTTGATTTCCTGTTTTTGGTAATGGTTTCTCGTGTTCTGTTTTCT
CTTTGCGGATGAAAGGGCAGGCTGCAACGGATG
>comp159806_c2_seq6
AATTTTTTAGAAAATATCTAATCAAAAAAATATTTTATAAAATCCTCAAATTGACAAGTTCCATGGGTCTAAGCTTAAATTCTGGTTTGTATTTATATTCATACTATTTATGTATGCGAGAAATG
CTTCGTTCTCAAAGCCATAAAATTGAAGTATTTTAATCATTATAAAGTTGCAGCATCTCAAATATATAAAAAACAAGATTAAAAGACATAAGAAAATCAGATATTAACATAGAGGGCTTTCT
TGATTCAGACCCGAGACAATCGAAGAAATGAGCTGAATATTGAAGGAAGAACTTAAAAAAGAAAACCAACCACAGAAAATACCATACATCCTGGCAAGTGAGCGACTGCGGCTGTCAG
AAGCGCACCGGAGATGTATTGTGCACGGCTGCTGTGAAGGCAGAATCGTACAGAAGCAGAAACGGTGCGTAATGCTCTTCCTCAGGGAGATTGGGTGTGCGACTTTGCAGATGAATAAG
ACAAGAGCGAAGAAGCGGCAAGTAGCGTGGAGCGGTGTAATCTGCGCTA
>comp159808_c0_seq3
GAATGAATTTGAAATAGCAACATGTACTTCATGGCTCTTCTTCACATTTGCAACAGTGTCAATTGTCACCTCAGGCTCCAAGTCCTGCATTGCATATAACACATGAAATAAATTTTACTGAA
TCTGTAATGAAATTGGAGTGTTTGGGGTTTAAAGCTAAGAATCTGATACATATTGGTTAAGAGTAGGATGAAGCCAGCCTGTGGTTATGGCTTTGATGGTTGCGATCGCCTCTAGGCCCCC
AGAAGCTCCCAGGCAATGTCCAATCAGAGACTTTGTAGCATTCATTTTAATTTCTGAAGTCTCCTTGAAAACTTTCTTAATAGCATTTACCTCAGCTAAATCACCAGCAAGGGTTGATGTTG
CATGAGCATTGATATAATTTACCTCCTCAGCTGAAACTCCGGCATCCTCTAAACTCTTTTCTATACAAGAAGAAACTCCGAGCCCATCGGAGCGGGGATTGGTCATGTGATGAGCATCACA
ATTTATAGCTCCTCCCAGATACTCTGCAATTATAACTGCGCCCCTTCTTCTTGCATGCTCCAAGCTTTCCATGACCTTTGATACAGAAAAATACAAAGATCACACCAAATTAGTTAGATAATT
AACTCTTTCAGATGCTATATGGTTCACTCTGGTTTGAAGTGTGTTCAAATTTTGCTTACGGTGTGTTGATGTCAGTTTTCAAGCATTTTCTAGTTTAACAATGGAGAAATTATTAGTTGCGGA
AACCGGGCACAGAGAATCA
>comp159810_c2_seq2
AGTTTTAAATTGAGTTTGTCTCCAAATTTCTTTTAATTCAACTTTTCACATATCTCCAAATGAAGCATATATTTAGATCCAATGGTCAAGATGTATGCAACAAAAGAGATATATCTTAATGCTT
TTCCAGGAAAAGATATCAAGAGGACAATGGTTCACTTTTGCTTTTTCAGAGAAAGAAATTGCTCAATTAGATCTCAGCCGTCTAATGGAGAGAGCTCCTCATACATGAACTCTTGGAGGG
AATGAGGATACGGGGGACATGGACAGAGAAATCTCTTGGTCTTCCGAGGACTTCAGCCGGCCCTAGCTCCTCATCCCCCAGGCCTTTCCTTCCTCTGAGATGGCTCTCATAAGCAAGCAA
CAGGGGAGAGCTCCTCGGCCCTCATGACAAGACACGATTTCTCACACTAAGAGGCAAAGAAATGTTCTCTTACCATCTCGACCGCACGCCGGCAACCCCGATGTCGACCTCCGAGCTCA
TGGTCCACTATCGTTGGTGTCCAGTTCTTGTCGTCGACCCCAGCCGCCTCACTCGCCGCCGCCGACAGTTCTCTCGAGCCATTCCTCCTTCCAACTCTTCCGCCATTCTCTTGGCCACGAC
ATGGGGTCCGTCGTCAATGCCGACCAGCTGACCCAACCCAGCGGCGTTGCCCCCAACCTGTCGCCGGCCGTTAATAAGCCGCCGGTCGTTTGACCACCAGCTATTCTCCTCCCAGCCACT
GGCGCAAACCCCCTTGCTCGGTCCGACGCCAAACCTGGCCTCCGACGTCATTGACCACGATCTCGATTCTATGAGGTCCATGGGAAGGCGTTAGAAAATGTTGAGTTCGAGATTCAGACC
ACCTCGCTGTCTTTGAAAATTTCACTCATTCGTTGGGACTCAAGTCCAAGTAGAAATGTAAGTCAAATTATATGTAAATCCATACTTGATGTTTGATACTATGGATCATAACCACTTTCAGCT
AAAAAAAAAAGGTTGATAGAAGGTGCATTCTGTAATTTCCCATGTTTTAGGTGACGTCGTTTGAGACTTTCAATGTTCGTCGGTTAGTGATGAGAGTTTATATCCTCTCATTTGCTGGTCAT

TTAATGAATATTGCTATGGAAA

>comp159812_c0_seq1

GCGAGCAAGCCTGAACTGTTCAAATTCTTTCAACATTGCAACTTGCAAGAGATTATTCTTTATTTATATTTAATAGTTTAATTCGTACATAAAGAAAAATCTCCCTAATTTTAACCAAGAAAA
AAAATCATATAAAAATATTCTAACGTAATTTATTTCACAATTCACTTGAACTTATATGCTTAATCTCAACATAAATTCGTTCTCCCTTAATTAAGCCCATAAGCCGCCGCCGGCAGCCTCGGCG
TCGCCATCACCGCTAGCGGCACAGCCTTCGGCAACACCAACCCCGGCCCTTCCTCCATGTCCAGCTCCCCAACCCCTTCCGGCAAACTCCAATCAAAGGCATGCACCAACGTTCCCAAAT
AATACTGCACCATCACCATCCCCGCTAGCTTCCCCGCGCAAATCCTCCGCCCGGCTCCGAACGGTATGAGCTCGAAATCATTTCCCATCGGATCGATCCTCGCCATCTTCCCTTGCAGAAA
CCTCTCCGGGTCGAACACCAGCGGGTTCTCCCACACGTCCGGGTCCCGCCCAATGGCCCATATGTTGACGAGCAGCCAAGTCTTGCCGGGAATGTGGTAGCCTTCCACCTCGCAGGGAT
CGGCGGCGTAGTGCGGTATGCTGAGCGGCGTTGGAGGGTGCTTTCGGAGAGCTTCCTTGCATATGGCTTGGAGGTAGGGGAGGTTTGGTATGTCGGATTCGTCGAGAAGGCGGTGGCGG
CCGACGACGCGGTCGGTTTCTTCTTGTGCTCGTCGGAGGATTGATGGGTTTTTAAGCATTTCCGCCATCGCCCACTCTATGACTATGGCGGAGGTGTCCGTCCCCGCCACGAATAGGTCAG
AGATGAAGCCCTTAATATTATCTTCACAAAGCCCTTCTCCATCCTCACCTTTAACCTCACTAGCCAAAACAAGATCCAGCAGATCCGGGTTCCCCCGCCTCTCCCCTGCAGTCTCAGCGTG
CTCCACCAGCAACTTACGCACCATCCTATCAAACCTCCGATTCAATCCCAGCATCTTCTTCCTCACTCCCATCGGATCCAACCACCCGATCGCCGGCACCAAATCCTCCATATTCGAAGCCC
CCTGCCCCACCAACAGCTCCTTAATCATCTCCTTAAACTCCTTCGCCTCCTCCCCGTCCTCGTGAAACACTCTTTTGCTCATCGCGATCCTCCCAATCACATTCGCCATGGCACAAACCAAC
AAATTTGGTAGCAGAACCGGCTTTTCCGAATCGCTGTGTTTCTTCAAGAATTGAATCATGGACAAGGCCTCGTCGCGCCGCACGCCGGCCCAGCGAGACATGGCCTTGGAGCCGAGGAG
ATGGAGGCTGGAGACTTTGCGGAGGAGCTTCCACTTGGGGCCGTAATCGGCGAAGACCATGTTCTGGCCGTTGTAACTGATTTCCTTGGAGATGACGCCGGAGGGGCGGTCGGAGAAGT
GGGAGTCGAAGGTTTTGAGAAAGGTTCGAGCGGCGGCGGCGGAGGAGGCGACGACGACGCTGCGGATGCCCATCTTGAGGAACATGATGGGGCCGTATCGGCGAGCAAGGAGGGCGA
GGCCGGAGTGGGGCATGGGGCCGATGAAGGAGAGGGCGCCGATGATGGGGAGGTTTGGGGGGCCGGGTGGGAGGGGTAAGCGGCTGCAGTAGCGGCGGCGGAGGAGGAGGTGGAGA
GAAAGGCAGAGGAGGAGTGACGCGATGAGGAAGAGAGACATAGTTGAGTTTGTTAGCTTCAAGAGCCTTTCACATGCGAGTGCTTATATAGCAATGGGAATTATGTTAAGGGGGGTTTG
GAAAAAAAAACATTCGGTACGTACACAAAATATATTTGCATAT

>comp159863_c0_seq3

TTTGATAGAAGAAATTGCAAAGCTCCCATAACTAATCACAACAAAGCAATGCAACATAAGTTAATTCCTGCTGCTACTTGGCCAATCTCACAGTTCTTACATTCGTTAAGCCAACCCTAAA
CGGCTAAACTTGAATGGCATAGCTATTATAAAAAACTGATCATAGATAGCATCATAGCAGTAACAATGGAATTGAAAAAATCTTACACCAAAAATAAACTGATTCACATAAATCATATTTAAT
TAGGGAGATTTATCCCATCCATGAATTAATTACAGGAAGCAAAAAGAATTAACTGCTCTTCTGGGCTTTCTTTGCAGGAGGTAAAGTCCCAAACACAATATCCCACAATGAAGATGTGATC
CCAAAACCCTTGGTTTGAATTTTGAAGTGATGGTTTAGATGATAACGCTTGAGATGTCTAGCTGGTTCTATGGATGGTGATCCATGGTGGAGGTAGTAGTGTGTGCAATCATAGATCACATA
ACCGAGCAAACCACCAGCAAATATGGCAGGAGCAGAAGCTGGGGTCGATAAGAGTCTAATTAAACCCCATAACAGTAACTTTTTGTCTTCACGTGGAAAAGGAATCGATGCAATGTGTA
CTCGAGCAATGTCCATACCAGTACTCCAGTCATAATCAGCAAAGCAGCCCGAAATGGCAACATTCCCATGCGAATTGAAATCAATGCAGACCAGCAAACAACTGGAAGCCATACAATTGG
GATAACCCACCATAATGTTCGCGTCAAGAACTCCATAAAGTTATTTTCAAAAAAACGTGGTCCTTCCTTGCTCACAATAGGCTGATGAACCCATTCTTGGTAAGCTTCCTCAAGTTGACCG
ACCTGACAAACTAAAGGCTTGTTTAAGTCCACTTTGAACTCCTTTGCAACCATTGTGATTCCCAGTGTGCTCAAGAAAAAGGAAAGAAAAAAAACTGCTAACTGATCGGCAGCCCCCCC
TGCGACTGCAAAATCGCGAACCCTTCCAAGGGTTTTCCTCTTTCTGTTCTTCCTCTCCTTCACAAAAGGCGGCAAAGATGAGGAAGTATAGCCACAGATCGGAAACCAT

>comp159863_c0_seq7

GAAGTCCAATTTAAAGGGTACAATGAGCAATACATAATAAATAAGAAATTTAATATAAAAAATATTATACTCCTACCTCAACATATTATGACAAACCATTTTTATGAATACATATCATGTAAAA
CATAACTGAAAACGATACAATCTTAAACCAAAAGTAAACAATTCACATAAACTAGGTTTAATGAGGGACAATTATCCCAACCTTCAAAGAATCACAACCATTATAAATCTGCAATGAAAAA

GAATCAACTACTCTTCTGAATTTTCAAAGCAGGTGGCAAAGTCCCAAACACAAAATCCCACAGTGAAGAAGTGATCCCAAAACCTTTATTCTGAATTCTGAAGTGATGACTAACATGATA
ACGCTTGAGATACCGAGCTGGCTGGTTGGATGGTGATCCATGGTGAAGATAGTAATGAGTGCAATCATAGATCACATAGCCCAATAAGCCACCAGCAAATATAGCAGGAGTAGCAACAGG
GGTTGATAGAAGCCTAATTAAACCCCAAAATGGAATGCATATAATAGCAGCTGCAGCAGGGGGAAAAACAAGGCGCAGGCTATCCATTGGGTGCTTATGATGACATCCATGAAGCAGATA
ATGCATTGTATTTGACCAATAATTTTTTGTACTCATGTGGAAAATGAATCTATGCAAAGAGTACTCCACTAATGTCCATATTAACATTCCAACCATGACTAGTAGAGGAGCTTGAGTAATTGA
TATTCCCATGCGAATAGCCATTACCAAACAGCAGCTCACTACTGGAAGCCATACAAGCGGAATGATCCACCATTTTGTGCGTGTCAAAAACTCCATGATATCATTTTCAAAAAGCCGTGGA
CCTTCCTTGCTGACAATAGGCTGATGAACCCATTCTTGGTAACCTTCCCCGAGATGTCCAACCTGAAAAACAAGAGGCTTGTTCAAGTCCACATTAAACTTCTCTGCAACCATAGTGATTC
GTGTGCGATTCATAATTTAGTCGAGAGGCGCTCCAACGAAGAAATCATCTTTCTACATCCAAAACACCAATCCAGCAGAAAAATCAACAATGCGGGCTCCCGAAACCAAATCCAACCCCC
AAAACAGTGAAAAAGCTTCAAAATCTCCAGACATAATGAAGAAACCAGCAAAAGAGAGATGCTTGTGCGCTCACCCTAATGAGATCTGGCGATGCGCTTCTGTTCTCCTCTTCTTCCCG
ACAAGCAAAGAAGAAGAGGAAAGAGAGGC
>comp159873_c3_seq1
TCGCAAAATAAGATAAAAGAGAAAGAGATATATGAGCGTATATGCCATTCAGAAAGGAAATACAGGTAAATAACAAAAGTATAACTAACAGAAAATTAAATATAACTAACAAGAAATTTAA
TAATTGCATTTATAGAACATGTATATGTAACAAGCATTGAATAACAAGAGAAAATGTGAGCCTAACCTAGAGCTAAAAGTCTTATTCTAATCAAACAAGTCTAAGGTATGTGTAACAGGTTG
TATGGTTTTAGATAGGTCCTATGTAAGATAGTTTAATTATAGTTACTTTCATTTGATTTGTTGAAGGGCAGAAGGAGGATTTTGATTTAAGTCTAAATATTAGTACTTTTAGGATTGATTTTAG
AAATTATAGTTTAGGATTTGATTGATTATTTTATTGGTTGTAAATTAAAAGAAAAGGTGTATTAGATGATTTATTATCGATTCAGGGATCAAATTATTTAAGTTTGTTATCAACTGGTTCGTGGC
GTTGTG
>comp159897_c1_seq9
TAATTTTATTAATTTTTTATAATATTATTATTTATAATTATGTCAAGCATGGACCTATTCTTCCGATAACCTTATGATATCACTATTTTTTTCCGAAAATTGGATGGCGGAAAACAAGAGGACCTT
GCACGAATTTCCAAAAATCAATAGATATCCAAGCAGGTTAGGCGAAGCCTTCATGTTGGCGGAGCTGTCAAGCTCCAGCGACGCCATG
>comp159902_c0_seq4
TATGCTCCACTGTCTTCAAGCTAAATATTGTGTTGTATCAGGCGGCTGCCACTAGATGCATTACAAGTTTCCAAGCTTCCAAGCCACCCAAAATTGTTGTTGCATTCCGAGTTTCTGAGGTT
GAAAGACGCCAAAAGTGGTTCCTTTCCAAGTTTCCTAGCTTCCAAGCTGCAACAAGTTGTCGATCCATTATAAGTTTCCTAACTTCCGAACCTCAACAAGTTGTTGATGCAATCCAAGTTC
CTAGCTTTCGAGCCGCAACAAGTTGTCGATGCAATCCACGTTTTTGGGCACAAATTAAAAAGTAATGAACGAACTACTAATAAACATATGTAACAGTGCAAATAAATTAACCATACAATAT
AAAGAAGGAAATAAATAAAACTTAACTTAGTTTCCTTGATGATGGGCAAAAAAACAAAATCCAAGCATAATTGTATGCTTCATTGTCTTCAAGCTAAACATTGTTTTGTATTGGGCCCCCA
CTAGTGGGATTCCGAGTTTCCGAACCACCACAAGTTATTTATGCATTCCAAGTTATCGAGCTTCCGAGACGCCAAAAGTTGTCGATGCATTCCGAGTTCCCTAGCTTCCAAGCCGCAACAA
GTTGTTGATTCATTCCAAATTTCCTTGCATCCAGCTGCAACAAGTTGTCGAGCTTCTGAGCCATCTCAAGTTGGACCAACAATAGAAAATCCAGTGCTGAAATGTATGCTTATAAGAAAGT
GACAATCCTTAATGTTAATTTACACATTCTTACAAAATACCATGAAAAAAAGTTCAGCCCCTTCATAAAACTTAAAAAAACATTAAACAATTGCAAAAATTACAATGTACGTTGATCCGAA
GAAACCTCCCCTTATGCAAAAAGATCAAAGAGCTAAGACCAGAAGTACAATAGCCGCTGAAAAAACCATATAGATCACACAATGTATACAACCATCATACAAAATTAGATAAAGTAAAGA
ATATTCTGTTAGGATAATAAAAAAAAAGTTAGAAATCATTCATGCTTTGGAATATGGATCATCAATTTTTAATAATATGTTAATATAGAAACAATAATAAAGCAGCCATTTCCAACCAAACATTC
TTCCGTAAGCGATTACCGCTATCGCTCGAACGTATTGAGGGCTCAAATAG
>comp159904_c1_seq11
CCTTTTTCAGCCAATGTTCGCCTCCCTGATCAGAGACAACTCATTTGAACCTACAATGCCTCCGGGTGATCATTATTCTTCCTCCATTCTCAGAGATTAACCAAAATCAATGTTTTCTAAATC

CAAATTTGAAGAATGAATGCTTCATCTTCCTTTTTGAGGTCCGATTAACCAAATCCCAGACGCAATTTCCAATCCCTCCGCAAGGCAATCATCCCGTCGACAGCGACGAATGGGAAATGA
GGACAAAAATTCCTCCAGCTGTGGCTTGCTCATTCTCTACAACGCCTTCTTCCGCCGCCGTGCTGGATCAGCCCCCGTCTCTCCTCGCCACCCCTCTCCTTCCCCTATTGTCGCCAATGCCT
CCCTTTCTGGTGGAAGCAGCCATAGTACCAAGCGGCGGCGTCCTGGCCCCGACCAATCCCCTGCCGCCGTCACACCAAAGATCCTAGTCTCCCCCAAGCCTGCCGCGGCCGCGTGTCCC
CCAGATCAAACCAATCGGCCATCCGCTGGTCGCGGCGTGCGGCCACAAACCACCAGCAGCATATCGGCAGATCTCGACAGTATGATCTACGATCACCAACGCGCCAAGGGGAGCAATAC
CCTTGTCCGCGCTTCCTCTGGTAACGTCATGCTCTACAGCAATTTGGGAAACCTCCGAGCTCCCGGCGCCGTCACTCCGAACCGCGATGTCCTCGACTATCTCCCCAAGACGGCAAAAGA
GATGGAATCGGAGGTGAGGCGTAGAAGTAGCTCTGAGTATGAGGCTGAGATTCCGGTGTCCATGTGCCGGGCGCTTTCAAGAAGACTTGATCCTGAAGAGCTGAAGGAGTTGGGGAAC
GAGGAGTATAAGAACGGGAGGTATGCGGAGGCCGTGGCGTTCTACGATAGAGCGATAGTGATTGATCCAGAAAATGCTTCTTACTGGAGCAACAAAGCGGCAGCGCTAATCGGCCTCGG
CCGTCTCCTTGAAGCTTTGCAGGAGTGCAGACAGGCTATCAGGATTGATCCTTGTTATTTAAGAGCGCATCAGCGGCTTGCCAATCTCTATCTCAGATTGGGTGAACCAGAGAGAGCAGC
CAATCACTATAAGCTAGCAAAGAGTGAAGCCAGTCATGAAGACATCTCTAGAGCTAAGGCTGTGCAAGTTTTCATTGGCAAGTGCAGTGAAGCAAGGAAACAGAGGGACTGGAATGCT
GTATTAAAAGATAGTCAGTCTGCCATTTCCACCGGTGCTGATTCAGCTCCACAGGTTTATCTCCAGTTTCTTTAAATATTTATTCCAAGCGGAGGCCTTTCTGATGCTCCTCAGACAGGAAG
ATGCGGAGGAAACCATGATTGATGCACCCAAATTCGAAACTGATGCTGCTACACAGTTCTTTGGCGCCCTTAGCAATGCATATCACCTTTCAATAAGAGCACAGGTTGATATGGCTACAGG
AAGATTTGAGAGTGCTGTTTCATTAGCTCAAATGGCTGCACGGATTGATCCTGGCAGCAGGGAGATTGCTGCTATTGCTCGAAAGGCTCGGTCAGTGGCATCAGCTCGCTCAATGGGTAA
TGATCTCTTCAATCCTACTATGCAACCGAGCTGCTTGCCGGTCGAAACTTGGGCAATGGGAAAAGGCCATTGAAGATTGCAATGTTGTTCTTAATTTGCGGCCCAGGTACAGCAAGGCTC
GGCTAAGGAGGGCTGACTGCAATGTCAAGTTGGATCGGTGGGAAGATTCTATAAAAGATTATGAAGTATTGGCTCTTGAAATGCCGGGAGATGAAACAGTAAACAAAGCCTTGCAGGAT
GCTAAAGCGCATGTCAGGCGAAGACAAAGTGAAGGCCTTAAACAGATGGCAGATGACTCTGCCGTTCATATCACTACAAAAGACCAGTTTCGTAGTGTTATCACATCATGTGGTACGTGT
CATTAGCATTAGAGATTCTTAAAAAAATTAAGGAATGCTACATTACTATGTGGCATCTGGCCAAAGCATATGGAGATTGGCGATTTGTTACATTTAAAACGTGCTTGCATAAGAGACACATT
TCCTGTGCCCATATGCCTTACTGATTATCATTCCATCTACCATGTAATATGTGAAGAAAATGCAATTTTTGTGTGCTACATGTTAATGTTATGCACGTAGCTAACCCCAAGTAGTATTTGTATA
AGGCTAATGATGACGATATATGATGCATTTGATAATATTCCCCTCTTTTAATTTCTTGCTTCATGTAATGGATTTTGATAATTGTTAGGTTGGCATGACATTGAGACCACTCTTCATATCATCCA
CTGTTTAGGAAAAAAAGATCAAAATGATTCAGACTATTTATAGGAGGTAGTTTTGATTTGAAGCAATTTAATGTTCTAATTTAATTCCTCGACTCTTTAAACTAGATTCAAAATAACCCATTG
ACATCTCAAAATTGATCCAAAATAATTCAATAGTAAGACTATTTTGGATCATTTTTAATTAGTTAAAAGAATAAATTGAACACATTAGATCGTTGGAACTAAAGTGATATCATACAAATTGTG
CAAATTCCACTTTAGCCTTTTTCTCTATTGTTTTGATTCACATTTTAATGAGAATGTTAAGTTATTATGCAACTGCTAAAATCTGTAAATTACTTAAGTTCTTAATACATAGTTCATGGCATCTA
TGTTTGGCAGGACTGTCGCTGGCACTATTCTATGACCAATCAGATGAAGATTCAAAAGGAATATTGCATCATCTAGAGCAGCTCTGCAAGCAACACACGTCTGTGACTTTTCTCAAGTTTG
ATATCCATACAGGTGAACATGAAGGAACAAGCTAGCATTGTAAGTTCAGAGAATGTGAGTTCAGTGCCTATTTTCAAATTATACAAGAATGGCTCCAACATAGAAGACGTTCCAGGTTCTG
ACATAGAACAGCTAGAGAGGACTCTCCAAATTTTCAGCCGTTGTTGAAATGAGAGGCTATAAAGAAGCATTAAAATTTTGAAATATCCATGCAATCAAAATAAAGGATTGTACATCTTATTT
CAAAGGCTTGAAGCCATAGACGAAAAAAAAAAGAAGATAATGCAAAATTAATTTTCTGGAAGCTCAGAATTTTTTTTCCTTGTTTAAGATTTGACTAAGCCCATTACTTGGTATTAACTAT
GAAATTGAGGACATCAAAAGAATGTTGAAGGGACACATTTAGTAAATGTCATAACTGTACAAAAATTACCATTTACGCTGCAAAATTAAAGGACGTATGTTCCAAATAAAAATATCTTATTT
TTTTTAGGAAA
>comp159904_c1_seq12
TTTTGTTATTCTTCAAACCATTTGATTAATGTATTGGCCAAGGAAATAGTGTATTTTTTTCTTCCTATCCAGCTATGAACTTACACAAAAATAAAAATAAAAAAATTAGATTGGGTGAACCAG
AGAGAGCAGCCAATCACTATAAGCTAGCAAAGAGTGAAGCCAGTCATGAAGACATCTCTAGAGCTAAGGCTGTGCAAGTTTTCATTGGCAAGTGCAGTGAAGCAAGGAAACAGAGGGA
CTGGAATGCTGTATTAAAAGATAGTCAGTCTGCCATTTCCACCGGTGCTGATTCAGCTCCACAGGTATTTGCCTACCAAGCGGAGGCCTTTCTGATGCTCCTCAGACAGGAAGATGCGGA

GGAAACCATGATTGATGCACCCAAATTCGAAACTGATGCTGCTACACAGTTCTTTGGCGCCCTTAGCAATGCATATCACCTTTCAATAAGAGCACAGGTTGATATGGCTACAGGAAGATTT
GAGAGTGCTGTTTCATTAGCTCAAATGGCTGCACGGATTGATCCTGGCAGCAGGGAGATTGCTGCTATTGCTCGAAAGGCTCGGTCAGTGGCATCAGCTCGCTCAATGGGTAATGATCTCT
TCAATCCTACTATGCAAC
>comp159904_c1_seq43
CCTTTTTCAGCCAATGTTCGCCTCCCTGATCAGAGACAACTCATTTGAACCTACAATGCCTCCGGGTGATCATTATTCTTCCTCCATTCTCAGAGATTAACCAAAATCAATGTTTTCTAAATC
CAAATTTGAAGAATGAATGCTTCATCTTCCTTTTTGAGGTCCGATTAACCAAATCCCAGACGCAATTTCCAATCCCTCCGCAAGGCAATCATCCCGTCGACAGCGACGAATGGGAAATGA
GGACAAAAATTCCTCCAGCTGTGGCTTGCTCATTCTCTACAACGCCTTCTTCCGCCGCCGTGCTGGATCAGCCCCCGTCTCTCCTCGCCACCCCTCTCCTTCCCCTATTGTCGCCAATGCCT
CCCTTTCTGGTGGAAGCAGCCATAGTACCAAGCGGCGGCGTCCTGGCCCCGACCAATCCCCTGCCGCCGTCACACCAAAGATCCTAGTCTCCCCCAAGCCTGCCGCGGCCGCGTGTCCC
CCAGATCAAACCAATCGGCCATCCGCTGGTCGCGGCGTGCGGCCACAAACCACCAGCAGCATATCGGCAGATCTCGACAGTATGATCTACGATCACCAACGCGCCAAGGGGAGCAATAC
CCTTGTCCGCGCTTCCTCTGGTAACGTCATGCTCTACAGCAATTTGGGAAACCTCCGAGCTCCCGGCGCCGTCACTCCGAACCGCGATGTCCTCGACTATCTCCCCAAGACGGCAAAAGA
GATGGAATCGGAGGTGAGGCGTAGAAGTAGCTCTGAGTATGAGGCTGAGATTCCGGTGTCCATGTGCCGGGCGCTTTCAAGAAGACTTGATCCTGAAGAGCTGAAGGAGTTGGGGAAC
GAGGAGTATAAGAACGGGAGGTATGCGGAGGCCGTGGCGTTCTACGATAGAGCGATAGTGATTGATCCAGAAAATGCTTCTTACTGGAGCAACAAAGCGGCAGCGCTAATCGGCCTCGG
CCGTCTCCTTGAAGCTTTGCAGGAGTGCAGACAGGCTATCAGGATTGATCCTTGTTATTTAAGAGCGCATCAGCGGCTTGCCAATCTCTATCTCAGATTGGGTGAACCAGAGAGAGCAGC
CAATCACTATAAGCTAGCAAAGAGTGAAGCCAGTCATGAAGACATCTCTAGAGCTAAGGCTGTGCAAGTTTTCATTGGCAAGTGCAGTGAAGCAAGGAAACAGAGGGACTGGAATGCT
GTATTAAAAGATAGTCAGTCTGCCATTTCCACCGGTGCTGATTCAGCTCCACAGGTTTATCTCCAGTTTCTTTAAATATTTATTCCAAGCGGAGGCCTTTCTGATGCTCCTCAGACAGGAAG
ATGCGGAGGAAACCATGATTGATGCACCCAAATTCGAAACTGATGCTGCTACACAGTTCTTTGGCGCCCTTAGCAATGCATATCACCTTTCAATAAGAGCACAGGTTGATATGGCTACAGG
AAGATTTGAGAGTGCTGTTTCATTAGCTCAAATGGCTGCACGGATTGATCCTGGCAGCAGGGAGATTGCTGCTATTGCTCGAAAGGCTCGGTCAGTGGCATCAGCTCGCTCAATGGGTAA
TGATCTCTTCAATCCTACTATGCAACCGAGCTGCTTGCCGGTCGAAACTTGGGCAATGGGAAAAGGCCATTGAAGATTGCAATGTTGTTCTTAATTTGCGGCCCAGGTACAGCAAGGCTC
GGCTAAGGAGGGCTGACTGCAATGTCAAGGTGAGCAGAACCTTTTATCCTTTGTCAGATGAATTTCTTTATCTGGAAGCTAAAGGTTGTCACATCTTGAATCTTGTATTAGTTGGATCGGT
GGGAAGATTCTATAAAAGATTATGAAGTATTGGCTCTTGAAATGCCGGGAGATGAAACAGTAAACAAAGCCTTGCAGGATGCTAAAGCGCATGTCAGGCGAAGACAAAGTGAAGGCCTT
AAACAGATGGCAGATGACTCTGCCGTTCATATCACTACAAAAGACCAGTTTCGTAGTGTTATCACATCATGTGGTACGTGTCATTAGCATTAGAGATTCTTAAAAAAATTAAGGAATGCTAC
ATTACTATGTGGCATCTGGCCAAAGCATATGGAGATTGGCGATTTGTTACATTTAAAACGTGCTTGCATAAGAGACACATTTCCTGTGCCCATATGCCTTACTGATTATCATTCCATCTACCAT
GTAATATGTGAAGAAAATGCAATTTTTGTGTGCTACATGTTAATGTTATGCACGTAGCTAACCCCAAGTAGTATTTGTATAAGGCTAATGATGACGATATATGATGCATTTGATAATATTCCCC
TCTTTTAATTTCTTGCTTCATGTAATGGATTTTGATAATTGTTAGGTTGGCATGACATTGAGACCACTCTTCATATCATCCACTGTTTAGGAAAAAAAGATCAAAATGATTCAGACTATTTATA
GGAGGTAGTTTTGATTTGAAGCAATTTAATGTTCTAATTTAATTCCTCGACTCTTTAAACTAGATTCAAAATAACCCATTGACATCTCAAAATTGATCCAAAATAATTCAATAGTAAGACTAT
TTTGGATCATTTTTAATTAGTTAAAAGAATAAATTGAACACATTAGATCGTTGGAACTAAAGTGATATCATACAAATTGTGCAAATTCCACTTTAGCCTTTTTCTCTATTGTTTTGATTCACAT
TTTAATGAGAATGTTAAGTTATTATGCAACTGCTAAAATCTGTAAATTACTTAAGTTCTTAATACATAGTTCATGGCATCTATGTTTGGCAGGACTGTCGCTGGCACTATTCTATGACCAATCA
GATGAAGATTCAAAAGGAATATTGCATCATCTAGAGCAGCTCTGCAAGCAACACACGTCTGTGACTTTTCTCAAGTTTGATATCCATACAGGTGAACATGAAGGAACAAGCTAGCATTGT
AAGTTCAGAGAATGTGAGTTCAGTGCCTATTTTCAAATTATACAAGAATGGCTCCAACATAGAAGACGTTCCAGGTTCTGACATAGAACAGCTAGAGAGGACTCTCCAAATTTTCAGCCG
TTGTTGAAATGAGAGGCTATAAAGAAGCATTAAAATTTTGAAATATCCATGCAATCAAAATAAAGGATTGTACATCTTATTTCAAAGGCTTGAAGCCATAGACGAAAAAAAAAAGAAGATA
ATGCAAAATTAATTTTCTGGAAGCTCAGAATTTTTTTTCCTTGTTTAAGATTTGACTAAGCCCATTACTTGGTATTAACTATGAAATTGAGGACATCAAAAGAATGTTGAAGGGACACATT

TAGTAAATGTCATAACTGTACAAAAATTACCATTTACGCTGCAAAATTAAAGGACGTATGTTCCAAATAAAAATATCTTATTTTTTTTAGGAAA
>comp159919_c1_seq4
TTTTACTAGAAAGCTGCTTCTTACAATATTAATAAAAATACTAACAACAGCTTTACAAACACCTTTACAAAAGTTGTGGTCGTCTCTAATAGACCCTAAATTACTTGATAATATAATTTGTTA
CTTTCATCTAAATAGTTATAAGAGCTATTGCATCTGATATAAAATTTAAATTCATGTATTCTATTATTTGTAACATTTCACAATGTTACAAATAACAAAAATGAAAATTCATTTGATCCCAAATT
TATTTTTATTTTTACAAAAAATTTAAATACTAATGATTTTTTTCTTTTACTTTACTTATACTAGACATGGCATATGCATGGTTCAATCAACACCGAGACCATATAAAACTCCAGCACATCTCTGT
ACAGATCCATTTGCTAAACAAGATTTCAACTTTGTGAACGTGGCAGCAGTGAGCAAACTATCTGATCCAGCCTGGTGACAGGTTCCCATCCTCTTCACCCCAAGTAGCCCTGCGAGCTTG
TTCAGCCCGCCATAGAAGTTAGAGCAGATCTTCGTCAAATGCTTGATGTCATAGATGTTTGGAAAATACAGCCGCAGAGAATGGAAGAAATCCTCCTGAGAGTCCGGCAGATTGCGGCAA
GTTAACATTTTGAGAAGATAACCGAAGTCATAGCAGCCATGGAAAGTGATCCATTGGACGGTGTCATTCAGCACGACGCCAGAAGCGATCATGAGTTCGGCAAAGCGATTGGCAGCGAC
GCCGAGGCAGTGATTCTTTTCGAAGTTTATTCCACTACGGCGGAGAAGCTCGATGGAGTCAGGGGCGAAGAAGTCGCTTGCAGCATTGAATTCGCGAAAGTTGAACTGCCAAATGCAAG
GGCGAGGGGGAGAGCCGAAGGAAGGGAGGTGGCCTGAAGAGTCAAAGAAGGTGAGGCCGAGTTGGATGAGTTTGAGGATGTTGACGTTGAGTTGGAGAGTCTGGTAATTGAATTTGG
TTATTGAATTCGGAGAAGTGACGACGGGGAGAATTGCAACGCCAGGGAATTCGGTGTCCATGGAGACGAAGGGGTAGTCGGCGACAATAGAGCTGATGAGAAAGAATTCCTCCTCCAG
ATTAAAATTCCATACCTCGCGAATCTCAACAGAGTCGACGGCATCGACGGCAGCGGCATAGTCACCATAAAGCATCTGAGGCGCCGAAGTCATGTTCAAATAGTTGTGAAGGGAGAAGG
AATTGTTTTTTTGAAGGAATTGTTTAGCAATAGGGTTTAAAAAGAAAACTTTAAATCCTATAAATAGTTATTCTCCTCTGTTGACCCGTAATTCCTGCAATT
>comp159946_c0_seq1
TGTCTCCATTTTGCCCAGCGGCGAAATTCATTCGCCATTTCGCGGATAAATTTACGAAGTATTACCAGCTAGCTCGCGTCTCGCGCTCTTTCGCTCTATAAATACGGAATTCTCTGTGAGCG
GATCTGCGGACTATCCTAGTTGAGACGTGGTGATGGGGAACGCGAGCGATAAGGAGGAGATAGATAATGGCAGCGACGACTCGAATGAGAGATCGGACGGCGAAGCTAATCACCGGAG
CGGCGGTGCACGCCGCTCGCGCTCGCTGGAGCTGATCCCGGGCTCACCGCCGGACAGGTCCAATCGGTCCCTATCGCCACTCTTGTTTGTTCCTCAGGTTCCTGTAGCTCCCTTGAATAG
GCCTTCTGATGTCCCTCCAGTTTTCAGTCAATACTGGATGAATGATCCCTACGTTGTTTCAGATACTCCCCGTGAAAAGGAAATTCCGGTGTTAATAACGTGGAACTATGGTGGAAACAGT
GTTTTAGTGGAAGGATCATGGGATGACTGGTCTTCTAGGTACAAGCTTTTTAATTAATAGAGCTCTATGGAAATCTCATCGCTTCATTATCATTTATTTAAGTTACATTATGTAGCTATTCCTGT
TTCTAGGAACTTAAAAGGTTGTCAAAACATATGAGCATAGTGTATATGCAGGAAAACTCTCCAGAGAACTG
>comp159946_c0_seq2
TGTCTCCATTTTGCCCAGCGGCGAAATTCATTCGCCATTTCGCGGATAAATTTACGAAGTATTACCAGCTAGCTCGCGTCTCGCGCTCTTTCGCTCTATAAATACGGAATTCTCTGTGAGCG
GATCTGCGGACTATCCTAGTTGAGACGTGGTGATGGGGAACGCGAGCGATAAGGAGGAGATAGATAATGGCAGCGACGACTCGAATGAGAGATCGGACGGCGAAGCTAATCACCGGAG
CGGCGGTGCACGCCGCTCGCGCTCGCTGGAGCTGATCCCGGGCTCACCGCCGGACAGGTCCAATCGGTCCCTATCGCCACTCTTGTTTGTTCCTCAGATGAAGGATTCAAATACCATTCC
TGAGATTTATATTCAAGAAAATGAGAAAGTTCACATTGTAGATAATGTGGTAGAGGCATCTGATGATACATGTAATGATGATTCAGTCGTAATTAAGAAGCGCAGAAGGATAACTTCCAAG
AATTT
>comp159949_c0_seq1
AGGTCCTCGCCAAGCAAATTCCTAGAGCACTCGCAGATGAAATAATGAGATAAAGAGTATTATTTCTCTCTTGTATCTAGCAATATCGAATACCTGCCTAGAGAATTTAAAAATGTAGGTAA
AGTGATCAGAGTGGCAAATGTTTCACCTCTGAGATCTTTGTAAGGCCTCTACTCGGGCTTTAAGTTTCAAGTACTCCTGCTGACTACTCTGGGTCTCTCTTGATATTATATTTGTTTCTGGGC
CTTCATAATTGCATTTTTGGTACCGTTCCAAAGTCTTCAACATACTTGAGCTGCTGCAGAACTCGTAAAGCTTTCCGCGGTTGGAGAAGATGATGAGAGCGACCTCCGCATCGCAGAGGA
TGGAGAGCTCATAGGCCTTTTTGAGGAGGCCGTTACGGCGCTTGGCAAAGGTTACTTGACGGTTAATCTTGTTCTCAATCCGCTTCAGCTCCACTCTCCCTCTCCCCATCTCTTACTCTCGT

CCGGTTCTCTCACTTTTCAAAGCCGCCGACGATCGATGGAGAGGGAGGAGTAGAGACGAGAGGCAAAAACGGGGCTTGCTTCCGGCGAGTGGTAGGGATGAGGCCTTCCCTTTTGCTAT
TTATAGGACGAGAAAGCGAAATATGAAGAGGGAGAGGGCCCTCTCCCTTCGTTACCCCAAGTCTCCCCATTTCCCTTAGCTTTTTCACCTTTTGATCTGTCGTCGTCTTGTGGGCGGTGAA
AGAACTTAGAAATCTAAATAG
>comp159949_c0_seq5
TTTTTTTTGAAAGCAAAACCTATCCAACAAAGGATATGATTGCTAAATATTTGTCTTCTATTACAAAGTACAAGCACAGCTGTTTTATAGATAAGAATTCCAAATAATTATGACCGGGATGAC
TTCAAATAGAATAGACATCTTGTACAGACATGGCCACAATTGACACTAAAAAAAAAGTTTTGAGGATACAGAACATAATAAGGTAGCAAACAAGAAATTTTTAGCCTTTCATACGAGCCA
ACCAGGCGGCATGTAATTACTGACGTTTGGAGCAGTCGTTGGTGCCATTGTTATATCAGAATGATACCTGTTTAAAATAAAAATAACAAAAGGAGAAAAATAATGTAGTCATATTTAGAAA
AATTAGAATCTCTGTTCTTTACGTTTAAGAAATGATGATAGTGTTGTGCAAGCAACCTGCAATACTAAGTCTAAGTGAACATAAAAATCGACAGTATTCATGTATAAATATTGACAGAGTTG
GTGTTAAGAGTATTTCTATTTTTATTTGAGCTACCATTCGTTAATAACTACCGATAATGTCCAAGAAAGGAGAAATTATTAGGTGCAGAGTTCGGACACAGAGATACGTTCGGACATCCACT
TAAAACGTGAGACGTGGAGCGCGCTTTGATTGTACTTTTTTGTGTCATTTATTTGTGTTTTACCTACACTTTAGTTGTGCTTTGACTGCTCTTTTGTTACAGTTTTTTACTGCGCTTTGGTTG
TGCTTTTGATGTGTTTTGCTTAAAGTTCCACGGACACATTACATATATGAAGAAATAAGTCGAATTGAAAATCCATGTGTCCACTCTACATGTCAACATTTTATTGGAGTCCGGACATATATC
TATATCCGTACTCCTCACCTAATAATTTATCCTGCTTCTGAGCTTGTTCTTAATTGACTGAATCCAACAGCATTTAAATGACAGAATAACAAGTTTACATATTGAACTACGCATGATCAAAAC
TGCAACAGGACTAAAGATTATCTGGAAAAAAAGAGGAACAAATGCAACAGCACAAGTTTGAAGCTTACTCAGAGGTAAATTTTATACTAAC
>comp159954_c3_seq19
GTATCAATCTGTGACATATGTGAGAAGCTTCAGTTACCATTTGATAAAATGTTTTTTCTAGTTTATCTGATTAAATAATACGCAGCCAAACAAAAACAATTTAATTTCTTACTCCAGTCTCCT
CCTTCACCTCTCGAACAGCACCATTAAATATTTCTTCATTCTCAAGGATAAAACCTGTAGGTAATTTCCAAACATCTTCAAATGAATAATGATGCTTCTCTTGCACTACAAGAACCTCATTTC
TGTGATTGAGAACAAAACCACCAACTCCTACTTGATGTGATGCATTAGCAGGAAGCATGCAAGGCCCTTCAGGAATCCAATATGTTAGCATCAAATATGTTCGCTCAGCATGATGGTACTT
GAAGCCCTCCTTAACAGCAACTGGAACAAACTCAGCTTGATCCAGTGGCAGTCTAAGCCAGACCCCCTTCTTACCCTGCAAATCATTCAATAGAAAAGTTTATTCTGAATTATGGAAGCT
CATTTAAGGATAGATAACACGAATACACGAAGGCCACTATATGTGAAACCTAACTGCATTCTAAGGCTT
>comp159954_c3_seq22
CATTTCATGTGAACATTACTACTTGAAATCCTTTGTAACATGGACAATATCTACAAAAGAGCTCTTTTGATTTCCAAATATCATTATTTCCATAGAATTTGTAATTATCATATTGATATACATGT
TCCTCTCAATATAATTCTATAGCAGCAGCACTTATGCCACATCTAAGACACTTGGCAGCTATAGTCCTCAGGCTCAACTACATTATAATACAAGGTAGAAGATCTAGAATCAAACTTTGAAAT
CACCTGATGAGCCGTTAATCCACAATAACACTTTCCGAGCCGCGCAATGCAGATGTCGATAATCTTCTTGAACATGTTGTCTTCTTGAATGCACGGTTGCCTCACAAACTCAGCCAGAGGC
ATCCACTTGGCTGCTTGTATTTCGAGTTCATCTACAACAATTTGAGTTGATAAGGGTTTTAGCATGCAGATGAAGAACAGATCAGACTTCTCAAATGCAACATGGTGAACATGCCTTTGTTT
TAAAAATTGATGTGATTAGGCATTTGTTTCTTTGGACAGTGTTAGACTGGTTCATTGGACGGGTCAAACCGGTCCCTACGGGACCGACTCAAGCGGAATAGAGGTGGAACTTCCGCCTCT
CCTGTACGCGCTGAATGCACACTTTTCTTCCCTAAAAGCAATTACTTCCAAGAATTCTGTATCAATTCCAGTCTCCTCCTTCACCTCTCGAACAGCACCATTAAATATTTCTTCATTCTCAAG
GATAAAACCTGTAGGTAATTTCCAAACATCTTCAAATGAATAATGATGCTTCTCTTGCACTACAAGAACCTCATTTCTGTGATTGAGAACAAAACCACCAACTCCTACTTGATGTGATGCAT
TAGCAGGAAGCATGCAAGGCCCTTCAGGAATCCAATATGTTAGCATCAAATATGTTCGCTCAGCATGATGGTACTTGAAGCCCTCCTTAACAGCAACTGGAACAAACTCAGCTTGATCCA
GTGGCAGTCTAAGCCAGACCCCCTTCTTACCCTGCAGCTTCCAGTGGAAAAGAGATGATTGAAGGATAGTAGCAAATGCATCTGCTGTTTGTGGAAGGCATTCTGGTCTAATTATGACTCC
TCCATACTCATCTTCATCAGCATCAAGCATGTAACTTTCTCTTGATGATATCCATGGATAAGCATCATAGCTTCCATTTATAACTAATTTTTCTTTCCCCCATATATTTTTAGAAGTCAAAGCTA
GTATTTTCATTAGGGGTTTTGGCAATCAGGTAGGAAGACTTAACTGAAACATCT

>comp159954_c3_seq9
CATTTCATGTGAACATTACTACTTGAAATCCTTTGTAACATGGACAATATCTACAAAAGAGCTCTTTTGATTTCCAAATATCATTATTTCCATAGAATTTGTAATTATCATATTGATATACATGT
TCCTCTCAATATAATTCTATAGCAGCAGCACTTATGCCACATCTAAGACACTTGGCAGCTATAGTCCTCAGGCTCAACTACATTATAATACAAGGTAGAAGATCTAGAATCAAACTTTGAAAT
CACCTGATGAGCCGTTAATCCACAATAACACTTTCCGAGCCGCGCAATGCAGATGTCGATAATCTTCTTGAACATGTTGTCTTCTTGAATGCACGGTTGCCTCACAAACTCAGCCAGAGGC
ATCCACTTGGCTGCTTGTATTTCGAGTTCATCTACAACAATTTGAGTTGATAAGGGTTTTAGCATGCAGATGAAGAACAGATCAGACTTCTCAAATGCAACATGGTGAACATGCCTAAAAG
CAATTACTTCCAAGAATTCTGTATCAATTCCAGTCTCCTCCTTCACCTCTCGAACAGCACCATTAAATATTTCTTCATTCTCAAGGATAAAACCTGTAGGTAATTTCCAAACATCTTCAAATG
AATAATGATGCTTCTCTTGCACTACAAGAACCTCATTTCTGTGATTGAGAACAAAACCACCAACTCCTACTTGATGTGATGCATTAGCAGGAAGCATGCAAGGCCCTTCAGGAATCCAATA
TGTTAGCATCAAATATGTTCGCTCAGCATGATGGTACTTGAAGCCCTCCTTAACAGCAACTGGAACAAACTCAGCTTGATCCAGTGGCAGTCTAAGCCAGACCCCCTTCTTACCCTGCAGC
TTCCAGTGGAAAAGAGATGATTGAAGGATAGTAGCAAATGCATCTGCTGTTTGTGGAAGGCATTCTGGTCTAATTATGACTCCTCCATACTCATCTTCATCAGCATCAAGCATGTAACTTTC
TCTTGATGATATCCATGGATAAGCATCATAGCTTCCATTTATAACTAATTTTTCTTTCCCCCATATATTTTTAGAAGTCAAAGCTAGTATTTTCATTAGGGGTTTTGGCAATCAGGTAGGAAGA
CTTAACTGAAACATCT
>comp159968_c0_seq1
CCAACCTCTCCCGTGCTCTCTCACATCCAGATTCTAAATTGGTTTGGAAGAATCTCCTCTTTGTTTTGTGGAGGGAACGCTGCGCTTTTTCCTCTTTCTTGCTTGAGCCTAATCGTTCTTCAT
CAACTGAAATGGGAGGAAAATGGAGGAGAGGGCTGGGTTTGATCTCCTCAATTCTTCTCATCTTTGTCATGGCAGCTTTGGTTTCGGCTGATCGGGGTTTAATCAAGCTCATTCCGGCCGT
GGCGAGCGAGGACATGGATGAGTCGAGCTATTTCATGAAGGTTTTGGGTTTATTGAAGCGAGCTGATAGATCTCGGTATCAGCATGTTTGGCCGCCATTGAAATTCGGATGGCAAATCGTA
GTTGGTTCAGTGATTGGATTCTTTGGAGCAGCATTAGGGAGCGTGGGAGGTGTCGGTGGTGGAGGGATTTTTGTTCCCATGCTCGCTTTGATCATTGGGTTTGATCCTAAATCTTCTACTGC
AATGTCGAAATGTATGATCACAGGTGCCGCCGCTTCGACTGTTTACTATAATCTGAGGCTGAGGCATCCTACTCTAGATATACCGCTCATTGACTATGATCTTGCTTTGCTTTTCCAACCTAT
GCTCATGCTTGGGATTAGCATCGGGGTCATCTTCAATGTAGTTTTTCCTGATTGGATGGTTACAGTTCTTCTGATCATCCTCTTCTTAGTTACCTCTACCAAGGCATGTATAAAGGGTGTTGA
GACATGGAAGAAAGAAACATTGATGAAGAAGAATGCAGCGAAAAACCTCGACTCTAATAGTGAGAGTGAAGAAGTAGAATACAGGCCACTACCTGGTGGTCTAGGTAACAATGCTCAA
AGGGAACTTAAGTCATCTTCTGAAGAGGAGTTTCCAATCTTTGACAATGTCTCCTGGAAGGAGCTCGGCCTTCTATTATTTGTCTGGATATCCTTTCTAACACTTCAGATTTCAAAAAACCA
TACAGGAACTTGTTCGGTTTGGTATTGGGTGCTGAATTTTCTACAGATTCCAGTTGCTGTTGGTGTCAGTTTATATGAAGCTGTTAGTCTGTACAAAGGGAGAAGAGTGATCGCATCAAAG
GGAGAAGTAGTTACAGAGTGGAAGATTCATCAGCTTGTAATATACTGCTTATTTGGTGTATTTGCTGGCATAGTTGGAGGATTGCTCGGCTTAGGAGGAGGATTTGTTCTCGGCCCTCTTTT
CCTGGAGTTAGGAATTCCACCTCAGGTGTCAAGTGCTACGGCAACCTTTGCTATGACGTTTTCTGCTTCGATGTCCGTCGTCGAATACTACCTTTTACGTCGTTTCCCGGTCCCGTATGCTT
TATACTTCGCTGCCGTGGCCACAGTTGCGGCTTTCATTGGCCAACACGTTGTGAGAAAGATCATACTTATTCTGGGGAGAGCTTCTATCATCATCTTCATACTGGCTTTTACAATCTTTATAA
GCGCTATTTCTTTAGGCGGGGTTGGTATTTCGCGGATGATCCAGAAGCTTCGGCATCAAGAGTACATGGGATTTGAAAACCTTTGCTTTGAATACTAACAGGTCGCTATGCATCAATTGTAT
TATCTATTGATAAGTTTCTATTAGATATTTATTTCATAATTATAGGATTTTAATCTGTTAGGTTAAGGTATTTTTTTGTAAGGGTCTGTACGTAGGTGCTGAATGAAGGGATTGGATGGAAGCAA
ATAAGTTTTGCTTGGAGTTTGATTAGAAACATAATTAATATGAATTTTTAAATTAAACATGGTGTTTATTTTGATATTATTTCTCAGTGATAGTTTATAAAAAAAATGTTTCAATT
>comp159979_c0_seq2
CCATTCTCTTCTTCTTTCCTCTCATTTTCTCCTCTTATTCTTCATTCTCTCTCTACTTAAGGGTTCTTGCAATTTGATTCCATCCTTCTTCGCCTGTATCGCATTCTCTTTGTCTGTTTTCGAACC
CGCAAATGGATTCTTCGTTGATTGATTGAGGGAATATTTCAACACTTGATATTTGTCAGATATTGCATATTTCTGTGAAGATTAGAACTGATCAAGGCCATAGAAATGAAAATAAGTTTCGGC
TTGTGATTTGGTTGTGCATTGGAACAATTCATCAGTTTACATCCGAGGAGAATTTTAAGTTTGATTTTTTTGGTGATTCTGTGCTCCTGCAACGTTTGACCATGTCCTGCTGTGGAGGCACA

GAAGAGGAGAGCTATGGTCCACCAGCTAACCAGTATACAGCTCCACCTAACCGCCCAACTCCTGGCCACGATAGGGAACCAAGAGGCCCCAATGCTGCCAGGAGTGGAGCTCCACAAG
AGATCCTTCCTATTGAGATACCTTCCATATCCTTGGCAGAGCTGAAGAAATTAACCAGTGATTTTGGACAAAAAGCTCTTATTGGTGAAGGTTCTTATGGAAAGGTTTTCCATGCCATTTTA
AGCACAGGAGAATCTGCTGCAATAAAGAAAATAGATCCCAATGCATCTCAAGAGAGCGATTCAGAATTTGCTGCTCAGTTAGCAATGGTTTCCAGGTTGAAGCATGAATGCTTTGTCCAG
TTATTGGGTTACTGTTTGGAAGCAAATAATAGAATACTGGTCTACCAGTTTGCTACAAAAGGATCCTTACATGATGTACTGCATGGTAGGAAAGGGGTGCAAGATGCTGAACCTGGCCCTG
TACTTAGCTGGATTCAGCGAGTGAAAATCGCCTATGGAGCAGCTAGAGGTCTCGAGTACCTACATGAAAAAGTCCAGCCTTCTATAGTCCATCGTGATGTCAGATCCAGCAATGTTCTTCT
CTTCAATGGCTTTGAATCCAAATTGGCGGACTTCACATTAACAAACCAGTCTCCTGATACTGCTGCTCGTTTGCATTCAACTCGTGTTTTAGGGACTTTTGGCTACCATGCTCCAGAGTATG
CAATGACAGGGCAGCTGACACAGAAGAGTGATGTTTATAGCTTTGGT
>comp159987_c0_seq13
GCACTTGATATATTTGACTCAACTACCCACAATACCCTCTAAACAAAGTCCCAAAATAATAGAAGAGAAACAGCCTCCCAAAATCCAAAACTAAAACAAATCCTTTTCTTTTGCATATCTC
CCAAAATTCATCTTTTAGAAACAAATGTGAATCAATAGAAAGAAAATCTGTACACTAAACATTAACTTGCTCTAAAAAGATATTAAAGGGGCTCACTACTCAAAGCAAATCATAACATTAG
AAGTTCCAAAATGCCTTACGAAAACTCCTAAATGAAATACTACACAAAACTTCACATAAAGCTTCTACCAAAATCTTTTGACACAAAAGACAATCAAGTGCTTCTTATATTTGGTGTGGGG
TAAAACATAAAGCTTTCCAACAATAAAGTTGTATAATACCCCTCGTTGTAAAGCCTTCACGAAACTCATCGTACAAAACCTTTTAAAACAAAACCAA
>comp159987_c0_seq15
CAAATTGAAGTTTTGGCTGCATGAGTGTGTTGGTTCGAAACAAGCAGAAAAAAAAAAAAATTCTGTAGAAGAACTTATCTTCCTATTGACACAAATCCTGTTGCTTTGGAGGAAGGTTGGT
CGACTAGAGCAGAGATTCCAACGACTAGACTTGCATTGGGCTCGGCTGGGAAGTCGGAGCCGGGTCATCGGGTTCGGTCCAAACAGAGGCGAGTGTAGGGCTTCGGTCGGAGAGGGTC
GGGGCCAGATCGTCGGGTTCGGTCTTGAAGGCGAAGTCGGCTGGGAGACGGAGGTCGGTCTCAGCATTGGAGTCGAATGGGAGGGATTCGCCTTGGCTGTGCGAAACCGAGAAGGATA
GAGCCGGGGTTTCGTCGCCTGACCTCCGTGAACCAAGGGAGAGAAGCTGGGGTTCCGACCTCCAGTGTCATACGAATCACAAGGAGACAGAGATCTCTGCTAACGGGGGAGACGGAC
AGAGCTTCGGCTCCTTAGGATTTTGAACGGGAGAGAAAAGGGAGACCGGGCATGAGGCTTCGGCTCTTCTCCGACCCAAAGAAGACGGAGAGATAGGGAGGTAGAGGCCGGCGTGCC
AGCAGAGAGATCGGGAGGGAGCTTCCGGTTGTGCGAATGTAGAGAGTCTCGGGAGAGAGGGAGAGCGGCCTGAGTGATCCCACTGGGCGTGAATGAGAGAGGTTCGGCCTTAGTTAG
GGCATCATAGGGAGAGCGGCGTGAGTGATCCGAGCCGTCGGATCAAATTATTGATCCAACGGTGATTCAGATTGGCTTCGAAAATCAATCGAATTTTGATTATGGATTAGCTAAAAGATCG
GAAGAG
>comp160017_c0_seq1
AACCGCATGAACCTTTCTCTCAGTCCAGATGGCTTCCTCCTTAAAATCTAGGACTTGTGGCTGTTAAGGAGGTACCGATAACTGCTAACAACCTCTTTTTTTCCGCATATCTCGCAGATGAT
CTATATATGTTCTTGAAATAATCGATCTATCTCTTTTCTCAATCTTCTCGCTCGTCTCCCTTTCACTTCCTTTGATTCATTGCTAACTCCATCGATTGACGCCGAATTGGGAAGAAAGAAGGTA
AATTCATCGATAATTAGCAATTATGTCGCTGGAAATCACGCCGGAAGACTTCTATAAGAATGTGACGGCGAAGCAGGAAAGAGTGTTCTGCTTCCATGCGATTATGACTACCTCGACGGGC
ATATGGCTCGCAGACAACCCTCTCTTGTTCTCCCTTCCTCTCCTTCTTTTTCAGATCGTTGTCGTCTTCATCTTCACACGCATCAGCCACTCCATCCTCCGGCGCCTCTCCATTCCTATCGTC
ATCTCGCAGATCATAGCGGGGATTCTACTGGGCCCATCCTGTATGGGACGGATCAAACCCATCGAAAGGATTCTTTTCTCCCCTCGCAGCTGGGAACAGCTAAACACCATCTCGCTTCTCT
CCTTCATGCTCTTCCTCTTCCTCATCGGCCTGAAAACGGACCTCAGCATGATCCCAAAAGCAGGCAGAAAAGCCTTCGCCATCGCCATCTGCACATCCTTCTTCCCATTCGCGATCATCGT
CCTCACCGGCTTCCTCCTGAAATCCAGCATCCCCTTACGCTTCAAACAGAGATTCCTCATCACCAACTTAGCGTCCTCCTGGAGCATGACTTCCTACACCGTCGTCTCATGCGTCCTCGCC
GATCTCAATCTCCTCACATCAAAACTCGGCCGCCTCGCAATGTCCGCCACTCTAATCACCGAGTTCACCAATCTCTTCATCACCGCCGTAATCGGCTCTTACTTGCTGGGAAGCAAGGGCG
ATTCTGCCCTAATGGGAGTGACATCAATGGCTGCTTTTCTAGGGTTTATGGCGTTTCTTGTGTTTGTGGCGAGGCCATTGACGATTTTGGCGATAAGAAAAACAACGCCGGAAGGTGGAAT

GCTCGATGAGGCGTGCTTTGTTGGGGTGATTCTCCTTGCTTTCGTTTGTGGGTTTGTGAGTGAGCTGATTGGGTATCATGCGACGATGGGGCCGTTTATGTTGGGATTGATGTTGCCGGGA
GGGGCGCCATTGGGGGTGACGATGGTGGAGAAATTGGAGAGACTGGTGGTGGGGGTATTCTTGCCGGTGTTCTTGGCGTCGGCGGGGCTGAGGTTGGATTTGTGGACATTGGGGGATAG
GGTGGAGGAATGGATGGTTTTGTTCTTCTATTTGATGATTGGGGTTTCAGCGAAGTTTGTCGGTGGGGTGGTGCCGTGCTTCTATTGCAAGATGCCGATGAGGGATTGTCTTGCCATTGGG
TTGATGATGATTAGCAAAGGGATTTATGAGATCGATTCGGCTTGCAGGTGGCAGGATAACAGGATTGTTGACCGGCAGCTATTCACAGTCCTAGTCTTAGCTATCGTAGTTGTGGGTGGCG
GCTCAGCGCCACTAGTCAAACACATCTACCGTCCCGAAGACCGCTTCGTAGCTTACAAGCGCCGCGCCATCCAATTCCTCAAGAGCAACAGCGAGCTCCGCATCCTCTCCTGCATCCACG
GCCAGGAACACGTTAACGGTATGCTCACGCTCCTCGACAGCGCCCACCCTACCGCTCTAAGCCCCGTCTGCCTCTACGCTCTCCACCTAAAACCTCTCGCTGGCCGCGCCGCCACACTCC
TCGCCCCTTATACCCGCCACCGCACCCCCTTCGATGCCTCCGCCGCCTCTGACACCGACCACATCTTCAACGCCTTCCTCCAAGCCGAGCAGCTCTACACTGGCTGCTCCATTCTCCCCTA
CGTCTGCATCTCTCCTTACGCCAGCATGCATGAGGATATCTGCTCCCTCGCTCTTGACAAGAAGGTCTTCCTCATCATCGTCCCCTTTCACAAGCACCTCGCCATCGACGGCTCCGTCTCCG
CCCCCATACCCGCCATCCAAGCAGTCAATTCGGCCGTCCTTCGCTTCGCACCCTGTTCCGTCGCAGTCCTCGTCAACCACGGCCTCGCAGCAGAATCGTCCGCCTTCCGCGCCGGCGGCA
TTCCGCACGCCGCCGTATACTTCTTCGGCGGACCGGACGACCGCGAAGCACTCGCTTTAGGGCTCCGCATGGCCGAAGACTCCTCCGTCGGGCTCACCGTGGTGAGGTTTCGACTACCG
AATGCCAGTATGGAGGGAACAGAGGAAAGACTGGATGAAAGAATGATGGAGGATGTGAGGATAAGGAAATCGGATGGAAGCAGAGTGGTTTACAGGGAGGAGGTGGTGAAAGACGGG
GAGGGGACGGTGGCGGTGATTAGGGAAATAAGCGTGAGGTTTAGTTTGTTGTTAGTTGGTCGGGGCGATGGGAAGGAGACGGAGCTGACGTCGGGATTGTCGATTTGGAGTGAGTTTGC
GGAGCTGGGGACTATCGGGGATTTGTTGGCCTCGACGGATTTTGGAGGTAGGGTTTCGACTCTGGTGGTTCAGCAGCAGAGGAGGGTGGCGGGGAATGGGCAACCGGAGAGTGTGAGG
ATGGGTAAAGCGGTTAAGAACGAAGTTGATGAAGAAGATGATTGATTTGGGAGACAAATTGAAGATTTTTGAAGAAGGAATCGACGGTTTAGTGAATCTGAATGTGATTATTAGAGTTGA
ATACTGATAGTATAGTTTTTTATTTTCTTCAATTAGTGGCGACGAGCTCTTTCGGTGAACGAATTTCAATGAGCTAAGCGTCGTTCGGTTGTTTATTATTATTGTTTTATTATTTTGCAGTATTT
TCTTGAGAGAAATGCTATTTTTCATTATCTTAAATTTATATATTTGTGTGGTGACTGGCAAGCACATTAAGGCTTC
>comp160038_c0_seq4
GCGCTAACTCTTCCTCTCTCTCGCCGAGGACCGCGCGAAAGGGGAAAAACAATTTTTTTTTATTATTTAAAATCTCAGAACGTTTTTTTGATCAGCAGGGAGTAGGGAGAGCTTATAAACC
CTAACCCGACTTAACAATTTGGTCGTCGCCTGCTTCTTTCGGCGGCAATGGCGACCACTAACGTGCCTGCTCCGCAGTCTCCTTCCTACATCTCGACCCCACAGCCCTCGCCGGGACCAC
TTCAGGCCGCGGCGTTCAGCAATGCTTCTCTTTACGTCGGCGATCTCGACAAATCTGTTACGGAGGAGCAGCTCTTCGAACTCTTCAACCAGGTTTCCCCCGTCGTCTCAGTGAGGGTTT
GCAGGGATATGATCAAGCGGGTGTCGCTTGGTTACGGTTACGTCAATTATCGCACACCGCAGGAAGCTAGCCGTGCCATGGAAGCTTTGAATTTTACTCTATTAAGTGGAAAAGCTATAAG
GGTTATGCTCTCGCAACGCAATCCTAGCGTTAGGAAAAGTGGATATGCTAATATATTCATAAAGAATCTTGAAACAACAGTTGATAACAAGTCTTTGCATGATGTTTTTGTGTCCTTTGGTC
CTGTTCTTTCATGCAAGGTAGCTGTAGATAGCAATGGCCAGTCGAAAGGGTATGGCTTTGTACAGTTTGAGAATGAGGATTCAGCTCAACAAGCTATCATAAAGCTTAATGGTATGCTTATC
AATGAAAGAAAAGTGTATGTTGGCCTCTTTATACGTCGACAGGACAGAGATAGAGAAAAAGGATCTCCTAAATTTACTAATGTATATGTAAAGAACTTTCCTGAAACATTTAATGAAGAGG
ACTTGAGAAAGGAATTTAGTGAGTTTGGTGATACAGATAGTGTTATAATTATGAGAGATGCTGATGGAGTTTCAAGAGGTTTTGGTTTTGTTAATTTCACGCAACCAGATGCTGCTGCTGCT
TCAGTTGAAAACCTTAATGGAAAAACTATAAATGATAAGGTTTTATATGTTGGGAGGGCCCAGAAAAAGGTTGATAGAGAGGCTGAGTTGAAGCGAATTTTGAACAGGAGAGAAATGGA
CGATTGGAGAAGCTTAAGGGTGCTAATTTATATCTGAAGAATCTTGATGACACAGTGAACGACAAAATACTTCATGAGCTTTTTGCTCCGTTTGGCAAAATTATCTCCAGCAAAGTCATGC
TTGATTCACTTGGGCAAAGCAAGGGTACTGGTTTTGTGCAGTTCTCCTCGACTGAGGAAGCTAATAAGGCTGTAAATGAGATGAATGGGAAGATGATTGGGAGGAAGCCATTATACGTTG
CAGTAGCTCAGCGGAAAGAAGAAAGAAGGGCTAAACTGCAGGCTCATTTTGCTCAGCTTAATGCTCCAGGGGCGATGAATCCTCCAATGCCTGCTTTGCCTGGTTTTCACCCTGGTCCAC
CTAGGCTTCTTCCGCAGCAACTTTACTTTGGCCAGGGAGGGGCTGGACTGGTGACACCACAACCTGCTGGCTTTGGCTTTCAGCAGCAAATTATTCCTGGAATGCGCCCAGGTGTTGCCA
CAAATTTCATGATGCCTTATCATCTTCAAAGACATGGACAACCTGGGCAGCGGATGGGTTTTAGGAGAAGTGGAATTTCACAGCAGATGCAGCAGCAGTTCAACCATAGGAATGCTAATC

AAGGCTTCAGATACATGCAAAACACTCGTAATGGTGTTGATCCATTGGTGCCTCAGGGCCTCATGGCTCCAGTGATGCCTTTGTTAATTGAAGCAAACAACGTCTACACTCCTCCCGCCGA
TGCAGTTCGAAGCCCGATTCCAATATCGGCGCTTGCATCTGCTTTAGCTTCCGCATCGCCCGATCAACAGCGAGTTATGCTAGGCGAGCAATTATACCCTCTTGTCGAACAAATCGAACTG
GATCATGCAGGGAAGGTGACTGGGATGCTACTGGAGATGGATCAAACAGAGGTTCTTCATCTTATAGAATCGCCGGATGCGCTAAGGAAGAAGGTTGGAGAGGCCATGGAAGTTCTTCG
ATTAGCAAATGCAACTGGTTCAGATGCTGTGGATCAGCTTGGTTCAATTGGACTTAATGACTGAATGCATTGATGTGATTAGTTTGATTTTGACCATTTATTAGGTTTTTTGAGATTAAGTGA
TTGGCACCAATTTTTGTAGTGTGTTTGGTTAGAAATGCTGAGTAAGAAGAGTTTATTACACGGATGGATTTAGGTTTTTTTTTAATAAAATTTTTAATGGTGTGTTGAATGTATGGATCAAA
AAA
>comp160065_c0_seq8
ATCAATCACGAAAAATCAACATAAAATCCAAAAGCACAAGAAAAAGAAAGTAAATTTTTCATTGCTCGCCAAGAGCAACTCCATGCCTCCATACAAGCTCGTGATTCCTTAATTACATTCA
ACAAATATTACATAGTAAAATATTACTTTTCTCAAGTAAAAATAATTGCAATTAAACTACCTACCCAAAAAACTGTGAAACTATCCTGAAACTATACAATTAAAAATTTGAAATGGCTGATAA
AATGGTCTACACAGAGAACATCAAATCAAAGAGAGCCAAAAATATAAGCTCTGTTGAGATTGAATTACTTCATCTACAAAATTCCTCAATGATCACCAAATTTCCACCATCTTGCATCGGT
GAAAAAGTTTGTGCTTGGCTTTCCCAGCTGCATAAGCAGCCTTCTCATCTCCTCAGAGCTTTTCAATGTTGAGCAAGCAGAGAAAAACAAGCAGTTCTATCAGAACAACTTAAGAGCTGT
TTTCAGCTTGTTCTGCTTCCACTTTGGTAGCTTGTAGAAAGCACTTTTGCTCATATTGAACTTCGCTTTAAACTCAATAGAAGATAGATATGCTTCTCGTTTGGTTACATCGATATCTGTAAC
AGGGTCTGTGGATGATGTTATTAAGCATTCATAAGGGTAAGTAGGAAGATCTTCTTCATCATTGACTTCCTCTTTTGGATCTTCGCTTATGGTGAGATTTCCAATTCGGCCACTCAATGCGCT
TCCATTGTCTGTGGACTCTGCTTTAGTAGTTCTAACTGGAAGCCTCACTCTAGATAATGGTTGCCCAGATGATTGTGCTGCCGATTCAAAAGAAGAAGTTAGTGCAGCTATTGCTGCAGAA
CGAGGAGCTGTCTTTGTATCTGATAGTGAAGATTTTGGATAAATTTTTCCAACAACTGGAGGAGTTGAGAAATTTCTGGAGTTCAGATTCTCAAACGTAGAAGCTATTGCATTGAAGGCAG
GAGATCTTCCTCTTGGACGATGTCTGTCTGCGGTGCTGGAAACACTTCGGGAACGTGGAGATTTGTCCGTTAAAATTGACCTTCCACTGTACGTTGGTAAAAGCCGCTTGGATTTAGTTAT
TACTGGAGTGACTCCATGTTTTACTATGGCAAGCTTCCTTTGATATGAATTACCATGCACTGCTGACTTGGAAGAGTCCCAACTGAAAAAGCGAGTGAAATATTGAGGTTCACTTCCCTCT
GTAACAATAAATATTGGAGTTTCTCGATATAACTTCTCCATAAGGAAATCACGTTCAAGAAATTTCTTTCCTATGTTTAAAGCCTCCAATTTACTCTTGGAATCCACCTGCTGCCCAACCCAG
ACGAAGATGTCTGATTGACAGTCCAAAATGTAGATATCTTCTGTCATCAAATCATCTTGACTAAAGTTGAAAACTTCCTTGACCTTCAATGTGCCTTGAGAATAGGAGCAAAAGAAAAGG
TGAGGATCACTTGCGGGTTGTTTTGCAATTTTTTCATTGGGGTGTTCTCTTTTACCCCCAAGTAAATCCCAAAATTGCTCCGTTTCTGCTCCTTCTTTCAGAGGCTTGTACTGCAAATTTGG
CTTTATCAGATCTAGTTGCCTTTCCAGAAGTTCATGATCCTCTGAGGTAGTAAGGTTTCCAGCCCATGTGTAAAGGGCATTACAAGCCTGGAGTATGAAACAGTATGATGAATTCAAAGAA
GATGCCACCGCATCAACTTGGATGGCTTGCATAATATCTGGTCCCAAACCTTGAACGCGAAAAAGAGCAGTTCCATCTTCTTTATACGAATCATCAACAATGTTTCCCTCTGAAATGAATTT
CTTATACCCAGAGCTATAGCCACCCTTCAACACTATAAATCTCTGGAAGATGGCACAGAATTGGAGAGGTTCCCTTCCTTCAAATATATGAACCTGAACTGCCCGAGACTTACAAGATAGA
GCCATCTTATAAGCTATTGAAATAGCATCAGCCCTTTCTTCCTCAACGCTCTTCTTCCCAAACCATGTGCCAATAAGATACTCCTCACCTTCTTCTCCAGGAGATGTATAATGAAAAACATAA
CAATCTCCAGTATAAAGCTTTGACTGCTCTGAAGATGAAAGAAGGGCCTTTTCATTGCCATCTATACGCCAAACCTGTAAAACGCCTGAGCTATCAATGCATGGTTCAGGCTTCTCTTTTAC
AGGATCAGATTTCATAAGGCCCTTTACATCAAGACCTTGAAGCTTCAAGAGAGCTGCAACTTTGCCACGGCCATCTTCCGATGCAGCCACATTAGATGTCTCAGGCCATGAATCAAACTTA
GATTGAAACATCACAGTTTCAAACCCCTCAATTATCCGAATCAAATGAGTTTTTTGTCGATCTGGATCTTGAACTAATCCTTCTGCAACAACACTTGCGCTTTTCCTATCATCGAGGGATGT
ATATCTGCCCATCCACACAAAAACTTCAACTCCACAATCGATCATGTAACACTTGTTTGACTCCAATAATTCTCTAGTCAACAAATCAGTTTCAATGGTTGTTGTCTTCCCTTTGTGAACAC
ATAATAGCTTTGCTGAAAAGGCCTCTTCAACATTTTCAGCCACTGAGGTTGCTCTTTTATGAAGAGGCGCAAAGCCACCAAAAAGAGACCAGAACTCTCCAGCATCAACATCAGCCATGA
GCTTTCCATCATCAACAACTGCGACCTCACACTTCCCATCATGGTGTGTTTCCCTAATGTACTGCACAACTTCTAGAGCTTTAGCCCTCTCTTGAATGGATGAATTGGACCCATTGAATTGA
AAAACCTTAGACTTTGTGTCCAAAATGAATACA

>comp160065_c0_seq9
ATCAATCACGAAAAATCAACATAAAATCCAAAAGCACAAGAAAAAGAAAGTAAATTTTTCATTGCTCGCCAAGAGCAACTCCATGCCTCCATACAAGCTCGTGATTCCTTAATTACATTCA
ACAAATATTACATAGTAAAATATTACTTTTCTCAAGTAAAAATAATTGCAATTAAACTACCTACCCAAAAAACTGTGAAACTATCCTGAAACTATACAATTAAAAATTTGAAATGGCTGATAA
AATGGTCTACACAGAGAACATCAAATCAAAGAGAGCCAAAAATATAAGCTCTGTTGAGATTGAATTACTTCATCTACAAAATTCCTCAATGATCACCAAATTTCCACCATCTTGCATCGGT
GAAAAAGTTTGTGCTTGGCTTTCCCAGCTGCATAAGCAGCCTTCTCATCTCCTCAGAGCTTTTCAATGTTGAGCAAGCAGAGAAAAACAAGCAGTTCTATCAGAACAACTTAAGAGCTGT
TTTCAGCTTGTTCTGCTTCCACTTTGGTAGCTTGTAGAAAGCACTTTTGCTCATATTGAACTTCGCTTTAAACTCAATAGAAGATAGATATGCTTCTCGTTTGGTTACATCGATATCTGTAAC
AGGGTCTGTGGATGATGTTATTAAGCATTCATAAGGGTAAGTAGGAAGATCTTCTTCATCATTGACTTCCTCTTTTGGATCTTCGCTTATGGTGAGATTTCCAATTCGGCCACTCAATGCGCT
TCCATTGTCTGTGGACTCTGCTTTAGTAGTTCTAACTGGAAGCCTCACTCTAGATAATGGTTGCCCAGATGATTGTGCTGCCGATTCAAAAGAAGAAGTTAGTGCAGCTATTGCTGCAGAA
CGAGGAGCTGTCTTTGTATCTGATAGTGAAGATTTTGGATAAATTTTTCCAACAACTGGAGGAGTTGAGAAATTTCTGGAGTTCAGATTCTCAAACGTAGAAGCTATTGCATTGAAGGCAG
GAGATCTTCCTCTTGGACGATGTCTGTCTGCGGTGCTGGAAACACTTCGGGAACGTGGAGATTTGTCCGTTAAAATTGACCTTCCACTGTACGTTGGTAAAAGCCGCTTGGATTTAGTTAT
TACTGGAGTGACTCCATGTTTTACTATGGCAAGCTTCCTTTGATATGAATTACCATGCACTGCTGACTTGGAAGAGTCCCAACTGAAAAAGCGAGTGAAATATTGAGGTTCACTTCCCTCT
GTAACAATAAATATTGGAGTTTCTCGATATAACTTCTCCATAAGGAAATCACGTTCAAGAAATTTCTTTCCTATGTTTAAAGCCTCCAATTTACTCTTGGAATCCACCTGCTGCCCAACCCAG
ACGAAGATGTCTGATTGACAGTCCAAAATGTAGATATCTTCTGTCATCAAATCATCTTGACTAAAGTTGAAAACTTCCTTGACCTAAAAAATATTATTGCTATGGTTAAAAGTTAAAATCTT
AAAACGAGTTAGTAGTTGAAAATCTACCAAAGTAATTTATGTAATTAAAGCACTAATTCGGAATTTATATTGATATGTTGTGATGAAGATTCATATCAAGATGTCAAATTTGTAGAGCTCCCA
CAGAACAAACCTTCAATGTGCCTTGAGAATAGGAGCAAAAGAAAAGGTGAGGATCACTTGCGGGTTGTTTTGCAATTTTTTCATTGGGGTGTTCTCTTTTACCCCCAAGTAAATCCCAAA
ATTGCTCCGTTTCTGCTCCTTCTTTCAGAGGCTTGTACTGCAAATTTGGCTTTATCAGATCTAGTTGCCTTTCCAGAAGTTCATGATCCTCTGAGGTAGTAAGGTTTCCAGCCCATGTGTAA
AGGGCATTACAAGCCTGGAGTATGAAACAGTATGATGAATTCAAAGAAGATGCCACCGCATCAACTTGGATGGCTTGCATAATATCTGGTCCCAAACCTTGAACGCGAAAAAGAGCAGTT
CCATCTTCTTTATACGAATCATCAACAATGTTTCCCTCTGAAATGAATTTCTTATACCCAGAGCTATAGCCACCCTTCAACACTATAAATCTCTGGAAGATGGCACAGAATTGGAGAGGTTC
CCTTCCTTCAAATATATGAACCTGAACTGCCCGAGACTTACAAGATAGAGCCATCTTATAAGCTATTGAAATAGCATCAGCCCTTTCTTCCTCAACGCTCTTCTTCCCAAACCATGTGCCAA
TAAGATACTCCTCACCTTCTTCTCCAGGAGATGTATAATGAAAAACATAACAATCTCCAGTATAAAGCTTTGACTGCTCTGAAGATGAAAGAAGGGCCTTTTCATTGCCATCTATACGCCAA
ACCTGTAAAACGCCTGAGCTATCAATGCATGGTTCAGGCTTCTCTTTTACAGGATCAGATTTCATAAGGCCCTTTACATCAAGACCTTGAAGCTTCAAGAGAGCTGCAACTTTGCCACGGC
CATCTTCCGATGCAGCCACATTAGATGTCTCAGGCCATGAATCAAACTTAGATTGAAACATCACAGTTTCAAACCCCTCAATTATCCGAATCAAATGAGTTTTTTGTCGATCTGGATCTTGA
ACTAATCCTTCTGCAACAACACTTGCGCTTTTCCTATCATCGAGGGATGTATATCTGCCCATCCACACAAAAACTTCAACTCCACAATCGATCATGTAACACTTGTTTGACTCCAATAATTCT
CTAGTCAACAAATCAGTTTCAATGGTTGTTGTCTTCCCTTTGTGAACACATAATAGCTTTGCTGAAAAGGCCTCTTCAACATTTTCAGCCACTGAGGTTGCTCTTTTATGAAGAGGCGCAA
AGCCACCAAAAAGAGACCAGAACTCTCCAGCATCAACATCAGCCATGAGCTTTCCATCATCAACAACTGCGACCTCACACTTCCCATCATGGTGTGTTTCCCTAATGTACTGCACAACTT
CTAGAGCTTTAGCCCTCTCTTGAATGGATGAATTGGACCCATTGAATTGAAAAACCTTAGACTTTGTGTCCAAAATGAATACA
>comp160069_c0_seq1
CTACTATATTTCAAACATGATAAAGCTCCTTTCAAATGAGTTCAACAAATAATTCTTTTTAGCAAATAAAAAACATATAGAAATGAATCAAATAATGTATACTAATATTACAAGATTGCCACA
CCCTAAGGGGCATCACTCAACAAATTGAAGAAACTATCTATATGAGAAATATAAAATATCTTCAGCATCAGATGAGTCATAGAAGCTCTGTTGGCTACACCAAAATGTTGGACAGAGGCTT
ACTTTAATGTTGATGTTTTGAAACTTCTGCAAAATTGTAATTCACAATAAAAATAAGAGAATTTCATGCCAAAAGCACTTTTCACTACTGCCAGCGCTTTGAGAGGGGCTTTGCTGATGAT
AAAGTTGCTTGGCTGTTAGTGTTGCTCTTTAAGCTTGGAGATTTCAAGCCATCTTTAGAATTTATAGGCTTCAAAGCACCTTTTGTAGCAAGAGAGGTTGGAGATTTCAAAGAATCCTTCA

ATTTTGAATGGATCACAGTTTCCTGTGCAATCTTTGGTTGCAATGGCTTAGCTACACTTGATTTAACTCTGGACATCTTTTCTTTTGTACCATTACCATTGATTAGCCTGTTCTGCTCAGGGA
GGTGAGAGAACTCAATAGCACTAACTAACCCACTGGTTTCCAAAAGACAGCCATTATATGCATCACTTAATCCTTCTTCTGAATCTACACCATTGACTGCCACAATCTGAAAATCTGCCGG
ACAACTCAAACCAGGTTCTTCACCAGAATGCTTTGAGTGACACAAGCTTTCCCTCTTGTGCTTGTCATCCACAGAGTGCTGCGAACTTATTTCAGAATGTCTGTCGCTGTTGTCTGAGTTA
TCAGAATCTAAAGAAGCTTCATTATTAGATCTGACGATCCTTTGAACTGATAATCCCAGGCCTTGTTGGGAAGTCAAATCTTTTGAGGACATTTCAGGAGAGAAGGATGAGTGCCTCAAA
ACAAAATTACCATGTTTTACATGGTTTACACCAGAAGAGAATGTTCCGGTTTCCCTGAGCATTTGCAGCTTTTCAATCTCCTTATCTTTCCTTGCATTGGCTTCTTTAAGCAATGCTACCTGT
TCAGTCAAATTCTTGACATCTTTGCTCTCTTTGTTACTACAAGCAGCACCTAACTCTACTCCAGACACTCTTTCAGCAAATTTCAAGGCACTTAAACTCTCTGAATATGACTTTACATCTGG
ATTTATCTGCACAAACATAAGTGTTTTTGCATGGCCACCCAAGGAGCTTTGTAAGACTTGAGTGAGCTTGCTATTTCTGTATGGAATATGAACACTTTTCTGGGACAGAGCAAATATGACGT
CGCCTAGAGCCGACAAGGATTTGTTAATATGCTGAGTTTCCTTGAGCCTATCTCCAGTCACTTCAGAGCGTTCAACTCTCTCACTCCCAGCAAGGTCTATGAGATGAAGGGATCCAAACA
GAGTTGTTCCAGCCTTCAAATCACTACCTCGGACATGAACGGTTACAACACTATGTGAACGGCTGCTACTTTGATTTAGAGCAGTGGCACTTATAGCCCTGTTAGCTTGTCCAATCTGCAT
CAGTTTCAGCACATCAGAGGTTGAATCAACGGAGTGCATGCTTGCATCAGGGATAGCAAGTCCACTAGCTTGAGATCTACTCACAATCCCATCTCTTTTGTGAAGCATCATTGGAAAGTAG
ATCACGAACTTGTTCATTATATATCTCAATCATCTGCACACCAACTTCATATTCTACAGTACTTCTACTTCGAGATATATGAAAAAGATCATTCAAAGCTCGATAATTGACTCCCCATTCTTCT
TTCGATGGTGAATCGGGCCCATTCATTGTGTATGTTTTGCCTGATCCAGTTTGTCCATATGCAAAGATACAGACATTGTATCCATCAAGAACTGATCGCACTAAAGGCTGAATATCTGAGAA
CACTTCCTCTTGGGTAGCAGCTGGACCAAAGACCCTATCGAATTTGAACATTTTTTGTCCATCCTTTCCTTGTTTGGATGGATTTACAATAGCAAGCTCGCCATTCTCACCGATATTGTCGAT
GACTGTAGACTTCGTATCTTCTCCAGAAAGTAAAGGCCTTATCCGACAATAGACTCGGATATTACCTTTCAATTCTTGAACCTCATTGTACAATTTTATATTCTCTAAGAAAATAGTGTGATA
ATTTTCAGCAGCCTTTGTTAGTACTTTCAACTTTATCCCAAACTTTGCAATTTCTTCTCTCCATCTTTCTTGCACATCTATAACTTCTTCTTTTATTGATTGAGAAGACGTCCTCAAGACCTGC
AACGATTGCAATTCTAAACACATTAAATTGTTGAAGATGTGCTCCTTCTGGTTCCAGTTTTGACACTTTGATTCTGAATAAACCTCCAGTTCCTTTGACCTCCTCCTTGACTCTTCTAAAAG
GGCTTCAACCTCCTTTATTTTCTTTTCTAGCTCAAACTTAGTTCGATTGGCTTCAGTTTCTAGGCTCTGATATTTTTGTTCATATGAATTCTTGGTCGATTCTATAGCCAGCTTAAGTTCTGAA
ATCATGCTATCACTATTTTCTTTCTCCTTCATCAGCCTAAATACTTCTTCCTCCCCGAGTCTCTTTTGCTCCACAAATTTTTTCTTATCTGCCTGTAGGAGCTGGACCTGGTTTGCAGTAATCC
GTGTTTCTTCATTCGTTCCCTTAGCGAGTGTCTCAAGCACTTTGATCCTTGATAGATACTTCTCCTCACGCGCTTTCATCATATTACTTTGATGTATGATATGCCCAGCTTGAGTTGTAACACG
CCTTTCGATTTCTTGCAAAACCTTTTTCAACAAGCACGCCACACGCTGATGTAATTCCCCATTTTTTTTCTCAATGCTTTCATCAAGCATCTCATTGGCAACACTCAAAAGTAATTGTGTTG
GAGCAATATCTAAAGTGTTCAGTTTCAAGATTTCGGAAATCTTAGCAGAAGGAAGTTCAGAAAACTGCCCACGTTTTAGCTGGAAAACCTCATGAAGTTTATTTCCAGCATATTGAGATAA
AGGACTAGTTGTATCTGGGGAAACAAGACTTCTCGAGGCATGTTGGAACTTCCTTTCAGCAAAACTTGATTTTTTATCATCTCCGAAGACTGGAGAACTCTGCCCACCATGAATGCCATCA
GTTAATAAACCATCCAGGCTTTCCCTATGCTTCCACTTCCGAATTTGCAGGCTCTTGCACCTAGTTAGTGGGCTCAAAACCTTGTCTTTCCTAACTGTGGAATCTATGTGGCTCCGAATGGC
GAGAAGGCATTCCAAAACTGCAGAAAAATTTCCCTTCTCCAAATCTGAGACTTGAAAGCTGGGCAACCCCAAGTTATTGACGACAGAGAGGAACAGCTTCAAAATTTCTGGGCGCCTCT
CAGAAGGAACATAATCAGCGTATCTAATCTCGTCCACAGCACCAGCATTCAACATCCTAAGAATTCCATTAAGAACAGAGCCATCAAACAGCTGCACTCTGAGCTCCCATTCCGTGGCCT
CCTCCGGCAATTTCAGCTCCGGGAATAAACTATTCAGCCATGCAACTACTTCCGCTCGCATCTTTGCATCGTCTGCTTCAAAATTGAGGCTTCCCATGTAGTCTCCGTCTAGGGTTTCCTCC
AGGCCAGACGAACAATGCAAGCAGAAATTGCGAAATCGGATTCGGCAATCGAATTAAGCCATGGATCAAAACCCTAAAAAAGACGAGAGGGAAGAGTTTCTTGATTGAACGCTGGGGG
CCAAAACGCGGGAGGGTTCTTCCGTTGTAGAGGAGCTCAAAGTAGGAGAAGAGGCGGAGAAGAGGGAAATGCGGAACCAGTCTGTTTCCGCTAAATTCCATGTCAATTTGTTCGCTTCT
CAACGGCCAGGAAAGATGCCGGGAATTCAAAAGTGGTTTCTTGATTGA
>comp160089_c0_seq2
CAATCCTTCATCGGTCTCCACCCCCAACAAAAATGGTAAACAAAGCTGCCATCGGCGGCTTCTCCGCCATCCTGGTCGTCGCCGCCGTCATCAGCGTCATCGCCATCTCCTCCCGATCCTT

CAACAAGCCTACTGCTTCCAGCCAACTCGCCACCACCGTTAAGTCCTCTGTCTCCGTTCTCTGCGCACCGACATCCTACAAAGATGCATGTGAGCGCACCCTCACCGGCGCCATCAACGA
CACATCTTCCGCCGGCGATATTATCCGCGCCGCCGTTAAGGTGATCACAACTCACGTCGAAGCTGCGAATGAAAAAGCTAACGAGCTTCATAAGCTGGCTAAGGGCCCCATTGACAACGA
CGGCTTCGAATTGTGTAAGGAGCTTCTGGTGGATGCCGGCGATCGGCTCGCCGCCGTGCTCGCCGAAGCTGATAATGGACATCGGCTCGATGATTTTCAGGCCTGGCTCAGCATGGTCGT
CTCCTACCGCACCAACTGCCTAGCCAGCATCGAGTCGGAAGAGCTCAACGCCGAGATGGCCACCGCCCTCCGCAACGCCACCGAGCTCACCGACAACGCCATCGCCATTATTACGGCCG
TTTCCGACCTCACAAAAGATCTCAACCTCGACTTGAATGCCACCGTCGCCGCATTAACCGGGCGCCGCCTCTTGAACTATCCCGAGTGGGTTCCGGCGGCCGACAGAAAGCTTCTGGCT
GCCAGATGGAACGATCAGAAGCTGCCACCCAACGCCGTGGTTGCGAAGGATGGCAGTGGACAATTCAAGACAATCAACGACGCGCTCAATGCCATGCCGGAGACTTACAGAGGGCGCT
ACGTGATATATGTGAAGGCTGGTGTGTACAATGAGAAGGTTACTGTAGATAAGACTAAGCCCAACTTACTTATTTACGGGGATGGGCCGCGAAAGACTCTGGTGACGGGCCGGCTGAATT
ATGCAGAGGGGGTTGGGACCTTCAAGACCGCCACTTTCTCGGTATTGGCCCCCGGCTTCATCGCCAAATCAATCGGCTTCCAGAACAACGCCGGCCCGAAAGGCCACCAAGCCGTCGCC
CTCCGCCTAAACGCCGACGCCGCCGTCCTCTACAACTGCCGCATCGACGGCTTCCAAGACTCCTTCTATTCCCAATCCGGCCGCCACTTCGTCCGCAACTGCGTCATTTCCGGCACCATCG
ACTTCATCTTCGGCGACTCCCAAGTCGTCATCCAAAACTCCCTCATCATCGTCCGCCGCCCAATGGACAATCAACAGAACACCGTCACCGCACACGGCAAAGACACCCCCCACGAACGC
ACCGCTCTCGTCATCCAAAACTGCCGAATCGTCCCCGACCAATTCCTCTTCCCCGACCGCTTCAAAATCGCTAGCTATCTCGGCCGCCCATGGAAGCAATTCTCCACCACTATTGTCATGG
AATCCACTATCGGGGATCTTATTAGACCTCTGGGGTGGATGCCGTGGAACGGAGATTTTGCCCTAGACACGCTTTATTATGCCGAGTATAACAACAGGGGACCGGGCTCGGTTACCAGTGG
CAGGGTTAATTGGAAAGGATTTCATGTGATTAACAGGAAGGAGGCGGTGAGGTGGACTGCAGGCCCGCTGCTGGCCGGCGTGAACTGGATTCCGTTCGCCGGCGTGCCCCATGCGCTTA
CCCTCATCAAGTGATCGGGTCGGGAGAGGCGATGATGGAGAAAGAACAGAGCGATTAATTAATGTTCGATTACTTGTATACCATATTACGGATTTTCACATCTGCTTGACTGCTTCGATATG
ATTTTATTTTGGTTTCTGACATTCTGATTATTGTCACCTTATATTTATGATTTCGCAGAAATTTTAATTAAAATTGTAGAATTTAGTTGTTTTAAGATCATTTAATTCACTTTTAT
>comp160089_c1_seq2
TTTTATTAGTATTTTTGGATTTAAAAAATTGTTTTGATAAAAAAATTATCTTATTTAGTATCTTCAAAAATAAATAAAATATTAGAAAACCAAATGAATTCCTTGCTACAATTATTCAAAATATA
ATATGATTATCGCAGCCGTGCAAGCACCCGGATTCATAGCCCGGTCGATGGGATTCACGAACACAGCCGGACCAAAGGGTCACCAAGCTGTAGCTCTCCGGCTAAACGCCGACGCCGCA
GTGATCTACAACTGCAGAATCGATGGTTTCCAAGATTCCTTCTACTCCCAG
>comp160091_c1_seq1
AGGAGGCAAATTAAAAGAGATTTGAGAAAAAAGTCTTGTGAAGATCGAGATGGAAGCAAGCAGAGAAAGGATGGAAGGAGATGAGGTAAAGATGAGGAGGCTTCACATAGTTTACTTC
TTAAGCAGGAAAGGGAAGATCGAGCAGCCTCACCTCATCACAGCTCACCACCTCAATAACAATGGTGTTCACCTTTGAGAGAGCAACAAGCAAGGGAAAGAAGAGCTCCTGAGGTGAT
GAAAGATCAATTTTAAATGGCAACGGTAGAGGAAAAGTAGCAGCAGCTGGGAAGAAAAGTAAGGATCGGCCAGCAGATCGTACAACTGGAAGACTAAAAGACAAGCAATTACTGTAGA
AGGAAACTATGTTAGAATGTCTTGGAATAAGGATGAGTTCGAATATGCTTATATTTGTTTTGAGATTAATGTTATATTCGAAGAGTTTGTATTGCATTTATTTAAGTTTTTTATAGTAGTGTAGC
AATTGTTATGATTAATGGGTAGTTTGTTTGTAATTGAATAATTAGTGTAACTACATTGTAATTTGCTTGAAACTACTGTAAACAAAACTTCAATGGCTCGCAGGGGGGCCAAAATAGAATTT
CCCTGCATGTTCTCCTCTTAT
>comp160152_c0_seq1
AGTCGTCCCAAACGAAGCCTGAATCTCATGGAACTACCCCACCTTATCTTCATCTGCCGTGCTCATGCTCTCCAAAGCCATGGCTGCACTTTCCACATGCCTGAGAAGGATCTCATAAATC
TTCCTTCTCACCCTTTCACACGCTTCGGCATCATCATCCTCCAATAGAAAGCTTTCTTTCGAGCGTTCGACAGCCCAAGATTTATCCTTGGCACTGATTGTGGCCAACCAGTCATCAACTCG
CTTCAGTTGACACAGTAAAGATGCAATCTGAGACTCTTTTGCTTTACAACTTCTGTTGCTATGGATAACATCTAAGAATTTCTCAATGTATCTTAGAAACCATCTGTTGCATTCAGATTGCAG
TGCCTTTGCCAAATCAACAGTCATTATATTGCAGTTTAATGTCGTTGGAAGAGCACTTACAGATGACAACTCAGTGCTCTTCTTTTGCCTGCTAATGGAGGTATGTGGCTTTCTGTCAGAAG

ATGAGGATTCTATAGGATCTACAGTGGTGTTTGTTATCCTTGGAGATTTTGAGACTTCTGCTTTCAGTGCTGCTTTGATGCATGAAGCTGCGCATTTTCTTCTTTCTGATGCATCTTTTGCTG
CCTGTATAGTGGAAGTTGATATAGACTGAGAAAAGCTGGAATTTGTTTTAGCCAATGACTGAGTAGATAAGGCGGCTCTGGAAAGGTCTTCATGGAAGTCCAAGAATTTGTCTACTACCTT
CTGGGGATTACGGTCCTTGTTGGCTTGTAGCTCTGAGTATGTGCTGACAAGAGAAGATGGAACAGAAAAAAAGCAGCAAAGTTGGAAAGTATTAGGCATTTTTTTGATTGAAATAAATAC
CCAAGAATTTCTCGGATTGAGGTAAAAAGAAAGGATCCACTCACAGTACTGGAACTTCGCGTTTTGAGGATAGGCTTGTCCTCGCGCTTCCTATCCTTCATGAACTCATCAGGTTCCCAGA
TCTTCCTCACCGTCTTTGGCCTTGGAATCATCGACACAAAAGAATGCGAGGAAGAGTTCGAGATGCTAGTAATAACTGAAGACTTATCAGACTCGTCACTCTCCTCATCAATATGTGAAAC
ACCTATCTTCATCGGCTGTCTCACCACAGAGAAAACCCTGTCTATTTCCTTCCTCTTTGTCGGCGAACTCGGACAAGAATTAGAATGCAGCATCCCATGCCCAACCTTCCGCCCATCCATCT
TCCAAGTGTTCGCAGACCGCGGCTGCCGCTTCTTCCTCTCTGGCGTGGCTGGGCAAATCGTCGAACATGTTCCGGACGACGTGGCGTGTATGGCCGCGGTGTGCTGAGGGAAGGGACGC
CGGCCTGGGATGAGACGTAGGCCGTGGAGGATGGGAACAGGAGACGAGGCTTCGAGACGGCGGACGAGGATGAGCTGTCCGAGCTGGAGATTGTCGCTGAGGATGAGATCGTCGTGG
CCCGGCGGTAGGGAGACGTAGGTGGAATGCGAAGAGTCGGAGACCTGGAGATAAAACCCTCGGTCAGGGAAAAGATCGGAGTCGGCCAGGGCAGGGACGATGCCGGTCACTTGAAGG
ATGGATGAACCGTCGTCCATGTCGTCGTCGGAGAGATAATCGTCGGCGAGGAATCCGGAGTCGGAGGAAGATGACGACTTCATGTCGCGAATGAGCTTGAGGAGGACGCCGGAGCTCA
AGTTCGCCATTGCTGGGGCTTGGGCGTGAGGCCTAACAGGCAGAAGGAAATAGAAAAGCTTTCGCCTCTAGCAGTTAATCAATGGTTCGGCTTCGATTCCTGGCTGCAGGGAAGGCTATA
CGGGTTGGGAAGGGCGGGCGGCGGGGAAAGGAAAAACGTTGGTGAGAAACGATAGATCACCATCCGTTACTGTCGAGATTCGTGTTTTTGATGCGATGGAAGTTGATCCATTGTTTCTT
GCAGCTAAGAATTCGAATGGGATTTCTGTCCAAGTTGGGGGGTCCAAACGGAAATAACTGCCGATATTCTCTTGTGGGAAAGGTCAAGACG

>comp160152_c0_seq7

AAAGGACTATGGTATGAGAAAAGTCTGGAAATTGCTTTTAAAGTGCCAACCAGCACCAAGAATGATATAGAAATCAGATTTTGCATTTCCACCAGGAGATTCACCATGATTTCAAAGAGA
AATTGCAAGTCATTAGACCTATTACAAGCATTTTAATTAGGTTACCCATATCACCAATGAACGATCCTAGTGTCCCTTGATTTCTAGTTAATCCCTGATGTAAGGGATTATGGAATCATTCTAT
TATGCCTGGAATTCACTAAATTAAGCAAGTTGATTTTGTCTGTTCAATCAATATCACCATATACAGCACATGAAGTTTCACAACTTTCTTTTTCATGTAAGTATTCATGCTATAAATCTATGAA
GGACAAATATGAACCTTGAAATGTATCTCACCTCAGACATCCAAGTAACCTCTCCAATGCACATGCCTCCTGCAAGGAATCCAAAGTAGCATAGAGAGCGCTATCCCTTCTTCTCAAGACT
TCCTTCCCATACTTTACTAAGTTAGATGGAAGCGATCCCCACAACACATTGCTGTCTGGCCACTTCATAACATCATATGTACTTAATGGAATTGTTGTAGAAGGATTCTTTGTGGACGCCTTG
CTTGCAGTGAGAATCTTCCTTTTGGCAGAATTACTGAGAAGTACCTTTAATTCTTCTCCATCTCTTTTTCCAGCCCATGATTCCTCCAAACTTTCACCTTTGGATATGCAGGAACTATAATGG
GCAAAATTACCAGTACTGGAACTTCGCGTTTTGAGGATAGGCTTGTCCTCGCGCTTCCTATCCTTCATGAACTCATCAGGTTCCCAGATCTTCCTCACCGTCTTTGGCCTTGGAATCATCGA
CACAAAAGAATGCGAGGAAGAGTTCGAGATGCTAGTAATAACTGAAGACTTATCAGACTCGTCACTCTCCTCATCAATATGTGAAACACCTATCTTCATCGGCTGTCTCACCACAGAGAA
AACCCTGTCTATTTCCTTCCTCTTTGTCGGCGAACTCGGACAAGAATTAGAATGCAGCATCCCATGCCCAACCTTCCGCCCATCCATCTTCCAAGTGTTCGCAGACCGCGGCTGCCGCTTC
TTCCTCTCTGGCGTGGCTGGGCAAATCGTCGAACATGTTCCGGACGACGTGGCGTGTATGGCCGCGGTGTGCTGAGGGAAGGGACGCCGGCCTGGGATGAGACGTAGGCCGTGGAGGA
TGGGAACAGGAGACGAGGCTTCGAGACGGCGGACGAGGATGAGCTGTCCGAGCTGGAGATTGTCGCTGAGGATGAGATCGTCGTGGCCCGGCGGTAGGGAGACGTAGGTGGAATGCG
AAGAGTCGGAGACCTGGAGATAAAACCCTCGGTCAGGGAAAAGATCGGAGTCGGCCAGGGCAGGGACGATGCCGGTCACTTGAAGGATGGATGAACCGTCGTCCATGTCGTCGTCGGA
GAGATAATCGTCGGCGAGGAATCCGGAGTCGGAGGAAGATGACGACTTCATGTCGCGAATGAGCTTGAGGAGGACGCCGGAGCTCAAGTTCGCCATTGCTGGGGCTTGGGCGTGAGGC
CTAACAGGCAGAAGGAAATAGAAAAGCTTTCGCCTCTAGCAGTTAATCAATGGTTCGGCTTCGATTCCTGGCTGCAGGGAAGGCTATACGGGTTGGGAAGGGCGGGCGGCGGGGAAAG
GAAAAACGTTGGTGAGAAACGATAGATCACCATCCGTTACTGTCGAGATTCGTGTTTTTGATGCGATGGAAGTTGATCCATTGTTTCTTGCAGCTAAGAATTCGAATGGGATTTCTGTCCA
AGTTGGGGGGTCCAAACGGAAATAACTGCCGATATTCTCTTGTGGGAAAGGTCAAGACG

>comp160244_c0_seq2

CTCCAAAAACAATTCATGAGAATCTGTATTCGGTATTATGTAAAAGATATTTCCCTCTAGAAGACAGTTACATTCAAACCGTTTTGCATTTGAACTATCACGTCCTTTAACAGAATTTTTGTA
ATATAATTTCTCGACAACAAAAACCTGAAGGTTGATAGTTTTTGTAAATTCAAGCTCCTTTAGGTGCAAATATAGGTCAGGATGCATCTTATGTAGGTACCGTTGAGCATAGGGAAGAATCC
AATTAATTAAAGAAGCTTCATAATTGTTGTCTGCAATACCATATGAAATGGCTTCACGAGATATGACCTCTGCAAGTGCACGAATACCTATGTTTTGCATAAATATGGAAACCTTTCCACAA
AGCATTTCCCTCTCATTGGTAGTAAGTTCACCAAATTGTAAAATATGAACATCATTGAGATCCTTAAATTGCTCCATTCTTTCGTCATCATCAGTCCAACATACGGTACCGAATGTTGGATTA
AGGGAAACCCATGTATCCTGCATCGTTGGAAGCACTGTGTTTTCTGATTTTAAAAGAAAATATTTTACGTCATTTATTTCATCACAACTGAATAATCCAGATTTCAAATCATCGGCCCACTTG
AGAAATACCCGAAAAACTTCATGAGCAACCTCTGAAGGTAATGCAACATATGATAAATGACGCAGTATTGTAAGGTAAGCACGGAACGAAGGTGCTTCAGGTATACCACAGATAGTCACA
AGAAAATTATAAAGATCTGGATATAGAATGGACAGTGTCAAGCATGTTGGAAGCTTTGTTGCACTTGCTGGGTCATGCGAATGGGTTAATTCCCTCAATCTTCCCACACAACCAGTTGCAT
CATGCCAGCATAATTTATCTGGAAGCATAAATGTACCAGACACAACATCCTTGTTTTCAGATTTACATACTGATGGAACGAATATGACAATATTTGATTTCACATCGGTGATCTTTTCCTTTGA
TTTAGCCAATTCTTCTTGAACAAAAGTGTAAATTTGATTCATCTGAAATATGCTGGAAGAAGTCTTGGAGGTTCTCCAGAGCTGTAGCACTCTGATAGCATCATCAAGTGTAACTTGAACC
TTTAATCCAATATCCTTTACAAATCTTTTGCTCGAAGCCTTTGGAACAGCATATGGAGCAGCAGTTCCCAGAATAGATTGAACTGCCTTGCAATTATAGAAAAGATCTTCAGGGCAGTGAA
GTTCATCATCAATGCTAGAAGCTACCCATTTAAATTTACGTATACATCTCATGAATGAGGACTCAAAGTTCTTCAATTGCACTCCACATTGAAAAGGATTATGACATGTAGATTTCAAGATAT
AAAAATCATCCCACATTTTATCAAAAACTTCCAGCAGATATTTAAACTTATCCAAATGTTTCTTTGTTGAAAGTGCAGATAGTAAATTGATTAACTCAGGCGACTCCCAATCTTCAATGATTG
TTTCACCATCAATAGCATCACCATAAACTGGCATGCTTTTAGAATCCATGTCTAAAACCTCTTTTAAACTTTTTTCTACTCGTCTTACTTGCAAAAAATCAGTGATACCTAATTCCATTAAAA
ATTCTCTCCACTTGTTCAAACCATTAGATAATGACTGGGTGCTGGGATGTTGCAAGTAGCAGTTGTCTAATTCATTCCACTCAATATCGATATCATCTATCAATTCTTTCATGTCACAAGGATT
GCCAAATTCTTTGCTGAAATGGATCGGCTTTTCAGTAGGGCATATGTAACCATGATTTGTTGATATAATTGACCGTCTCTGTAGCTCAGAGATGATGTCTCTTCTTTCTGTATCACAACTGGA
GCAGGCAGATTGAAGATGTGTCATTATATAAAAGAGGTACTCAGTCATCAAGTCTGCGGACACACCGGAAATTCTTCCATAAGAAATAGATGGCAAAATATGATTTCTGATAACATCATGT
GAAGACAATTGCCGCACACCGATTTTCAACAGGATTCGGATAATATTGCTCACTACATTCTCCTCAAAATTGCTACTCTCTGAAAATTTTGTAGAAAATAGGAGGGGATTTACAATCCTCAG
GTTGGAATAAAGACCAGGAAGTTGCTTTAGAACATTTCCACTTTCATGTTTCAAAGTGGAAACATCAAAGGGCAACCAAACTGGCCCTTCTGCTACTGAGACGTATGAATCATCAGATAG
TGGAATAAATGAAAGGCCTTTTAATTTTTTGACAAGGCTGAATTCCATTTCAGTATTTGCTGATGCTAAATTCTTGGAAGCATTAGATGAAATGGAAATACAAAGAGCATTAAGCCAACAA
GACAACCACTCTATGTTCAATAAACTAGTCCCACCTGAACGACATGCAGAGGATTGGAGTTCAACTAAAAATTCAGGTCCATATTCTTGAATACCCAATTCTTTAGCCAATGAATCATGCA
AGAATATGTCCCTATGCAAGTAACTAAGACCTAAATGCTGTTGAAGCAAACTATTCGACAAAAAGTTTCGAAGCTGCTCATCACAATTTCGAAGTACTCTACAAGGAGGAACCCACTCAA
CACTAGATCCATCCAAAAGTAAGCATTTTGACTTGCGAAGTTCTTTTGAAATCATTCGTGGAAGATCTGAGAAAAAACCATGAACTTCACCCATCAAAGGAACAAAACTCATGAAAGTTG
TAACTGCTTTCCCCAGATTCTTCTGAAAGCAAGGAAGTGAGCAGAATGACTCCTCTGCAGAGACAAAAAGTTCTGGTATCCTTGATAACAAGTACTGGTTCCAAGCACTATTTCCATCAA
CTTCTTCCCTAGAAGAAGGCAATATAAAATCACCTTGCAGAATGAATTTAAGGCCATATTTTCTTAAAGGAAGAAAAGCAAAAACATGTTGTTGATCAAGCTGCGCTTTATATTTTGAAATT
TCTGACTCTTCAAGTGGGAAAGCTAATGTGATCTTTGTAGTTTGAACCTCAGGACGGATCAAAGTTGGTTTTAGATTTTGGTTAACCAACAACCAATTCATTTTATTCTTTCCAATAGAAAC
CGTAGCTATGCCATTTTCATCACTTCCCCTTCTGAAGACAACTAACTCATTGGATATCATATTCTTATATCTGATACACTTAAGATGCCGAAGAAAAAGTAACAAGTGAGGATGAATATCTGA
AAACATTGAGGGAATCGGACTCATACCCTTGCCTCTTTTAAGCTCTGATTTGAATGGAAGCACAATGCAACTGTTCCAGAAGGTGGCATGTGGTTGGTCAGAATCACATATCACTTGCCTT
CTAAAAAAGTCAATATCGCATGGGGGTATTATAGTTGGTAGGACAAAACCAATCTGGCCATCATTAATATCAAACTTGACATGAAAACCATTGGAGTGAATTTCTGGAGCATCTGTTACCCG
GAAAACTGATTTGAAGCCAATACCCTTCTGCCCAATGTAGCCACCACTTGATCCCTTCTTTGTTGAATTTCCAACATCACAAAGTGCCCTCATATTCTGAGCAGAAAACCCCACCTCATTG
TTTAAAACAATAACTCCATTTTCCTGAAGAATGAAGACCAGCGTAGGCTCCACGTTTTCTGGATAGCTATTATCATCAGCATTTTGTATCAGCTCTAGGATAAAATGTGAATCCTGTGAATAC
AACTCTTGTGAAAGACAATTGAGTGCTCTTCCTAAACGAGCATGTTGCTTCTTTAGTAAGTCAGTATCTGCAGTATCTAGATCCAAACCAAATTCCTCCCTTCGAATTTGTTCAATAAGAGC

GGCTGCATCCCGGAATTGAAAATCTTTATTTTCGAGCTGTGTAGAGGTATACATCAACTCAGTGCTCAATTCCTTGCAAAAACTTGCACAAACTGCTGCTTGCGGTAGACCTGACATTGGA
AGTGGTGTTTCTATATTCTTTTGTGCTTCCCCGTCTGAAGACAATATTGTTTCAAAGACATCAGGCAGATAATTACTAAGTTCAGGAATGGCCCATTCTTCAGACCCCCTTCCTTGAGTTCC
AAGTGGTTCAGACATGCGAGCAGGAACTTTTGAAGAAAAATCCTTGTAATCATTGATCCATTCCATTATTCCAAGGGACAATCCAATTTCATGAAGCATTATGCGCTCATGGACATGTCTGC
ATTCATTTAGAATGATTGTGACAGCATTTTTGGAGGAAAACTGCATGCCTGATACCAAAATGTCTGCTGCAAAACTATGAAATTCTGAAGGTAAATGGCCAAGGCAATCGAGGATAAATAC
AGATATGTACGAATAGGCTTTGCTTATCTGTGAGATATCACTACTACCTCCAATTTCTGACATTTTGTTAGGATTCTTGGGCAAAATGCTCCAGTCATTTTTTGTAACACTTTTACTTTGTTGT
GTAAAGAAAGATCTCTGAAGTGGAATAGTTTGGCTGGAGTTCGCAGAAGCTTCTATATGATCAGCAAATTTTTTTGTTACAACAACAACAGCTTGTTGGAAATAACATTTTAACAGTGAAA
CAGGAGCTTTGCATGAACCCCCATAGATCTGAAGCAAAGACAACAACTTCAACGCTGCTTGAAATGATGAACCTCGAATTGAAGCTTCCAAAAATTCATCAGGAGTGGCTAAATGATCAA
TTCTGATGATCCTTCCATCACTTGTTATAATACATGAAAATTCCTCAATAGACCCTTGATTAAGTAACCATTTCCAAAGAGGACCAAGTGTTGGAGCAAACACATGATCCCAATGTGACCAA
GATTGCAGCTCTGATAGCATGGGTGCCTTGAGTAAGCACTCAATTGCATCTTTTGCAGATACAGGTCCAAGTGAACCAAATGGTATACCCCTTTCTGTAACCACACCAGATGATTCTTCAA
ACTTTTCACTTTGGGAAAATAAACTTCCCCTGCAGAAGTTCCCCAACAAGGTAGCAGAAAAAACTGCCGAGGAGGCAATAAATTTTTCTTTGTAGTTAATTAAATCAGAAAGCATCTCGT
CAAAGTCATTTATTGTTACCATTAAACCAATTAATGGAAATTGTTTTCTGAGAAGTATGAAAATATCCTCTTGACTTATAACAATCTCCTTGTCCATGTTTCCCACTGCTTGAAGTAGTAAGC
CTAAAAACTGCTTTTGATGCATAGAAATTTCACAGACAGACTTTT
>comp160254_c3_seq6
CAATTCGAGGATGAGTCCAATTAAATTTCTAAGCAGATTGAATCCTCTCTCAAATTAGGAAATCAAATCAACGGCTTTGATTTCAATTCTGCCCTTTCCATGTTGGTTCAGCCATGCGATTT
GCTTATTTGGTGGAGTATATAGGAGGCTTTAGCAAAATTCAGTGGGCAGAGGATCATTTGATATATGGAAGAAGTAGGAGAGCTACTTATTTGAAGGGATCAATTCAAAAGAAGGGAGGAT
CTCTCTCACACTCACTCGTCTCTCATGTTTGAACAAAACCGAATGCATGAAGCCCCCTCCTTTCTCATCTCTCTTGTTTGCACGCCCTAGCCATAGTCCCCCTCCTCTCTCCCCCTATTTCTT
TGGTTCACACACCCCAACGAAGTCAACCCCCGACCCCTTCGTGTTTGATCCGTCCAGAGAAGCTACACGCCCCAGCTGAGCCAGACCTTTGACGCCTAACCCTCTCTCGCACAGGTAAC
TTCATCTCGTTCTTCTTTCCCCTTCTTTCTTCTTATTCTCCTCGTTTCTTCTTCTTCTTTCTTCATCTTCTCTTTTTTTCTTATTCTTCCTTCTTTTCCTCTTCTTTCTTCTTCCCTTTTTAGTAATT
TTCCTTCTTCTTCTTCTTCTTCTTCTACTTCTTCTTCCTGGAAAGGGTCCAAAGACTTCTTGCTCAGGAGGAGGGGAATAAGTAGCTTGTCCTCATCACCGCAGTCCATGGAGAACTGATAT
TCCTTCCTCTCATCACCTTCTCCAAATTCACCTTCGTCATTGTCTGAAAGTTGGCTTCCGGCCTCGATGGTTTCCTCAGTTTTCTTGCAGAGAGTCTCGAGCACACTTTCTCCTGCCTCGAG
CCTGCAGGACTCTCGCGTCATCATTCTGCCAATTTCCGAGAGGCAGAGGCCGTAATCCACCGCTTTCTTTAGGAATATAGTGCAAAGTATGAGGATTCTGATCGCTGGTTCTCGAATCGAA
GGCAGTTCCGTGCGGAGGAGCTCGGCATCTTTGAAAGGATCGAGCTTTGAAACATATTCTATGACCTCGTCCGAGAATGGCACGGCGGCCTGCGGCCAATGGAGCCACTCGAAATAAGG
GTCGTCGAGCAGCTCCGGCAAGCAGAGACCGTGGTCGATGGGCACGAGCTCTGTGGTGAGATCTCCGCCTTCAAAGCTTCGGTTATTATCAATTCTATGTTTTTTTACTAAAATGTTACCG
GCGTGGCGGTCGGTATTGAGCAGCCTGATATCAAGAATGGCTATCCGATGGACGGAGGAGACTGAGAACCTCGATGGGCCGACGTCGCCAGCGTCACAGTCATGAGGAACAAAGCGTT
GGATGGAGGCGACGCTTTGCGCCGGAGGGAAAGAATGGTGGGAGATTCGGATGAGGGCAGTGGGGGGGACTCCGGCGAAGGCGCCGTGGTCGAGCAGGTAGGCGGCGGCCTCACGG
GCGCCGGTCTCGCAGGCACGAATGCAGGAGCTGGGGAAGGCCATCTGTTCGTTGTCGTCGTCGATGACGACGGGTTTCACAACGGCAACGCAGTCGCCGGCCTGGTCGTCGAGGAAGT
AGGCGCTGCCTAGGCCAGTCTCCGTGGGGACAGGGCAGGCACCAAAGGTCATGGTAGCGGCAGCCTCGGCGACGAGGGCGCGGATGCCAGGGGCGCTGCGACCGCCGATGATCACCA
CACTTGCGACGTCGTCGTCGGTGGAAGCAGAGGAGAGTAAACATGGAGTGGAGCGGCTGCGTTGGAACAGGGAACTGGGAGGGAAGGAGAGAAGGTCAGGGTGGTCGAAGGAACAA
AAGTGATAGCGTTTGAAACCGCTGCCGCTGCCTCCGCCTGGAAGGTGGTGGCATCCGGCGGCAATAAGAGCCATGGTCGGAGCTCTGTTCCACGGTCCGGTAGTCATTCATGCGAAGGC
CTCGCCTTCTGATTTCTCCCTTCTTTTCTTTACGATTTCTGATCAAAAAGAAGGATTTTTCTTCTAATCTCTCTCTCTCGCTCTTGCTAATCCAATTATTTCCTTTGAACCATTGAGGACGATC
GATCATCGAACAAGGAAATTAAGGCCAAC

>comp160316_c2_seq1
ACTTCAACGATCTGCTCTTTCCCTCAAACGTTCCGAAACATCCAAAGAAGGCCAACTCCAGTCGTTCGCACGAACCTCTCCAGTCCGTCGCCTGCTATTCGCACAATCCAGTCCATCTCC
CTCAGCCATTCGAAATCCAAGGCCGAACTCCCTCAGTTTCCCATCTCTCGCAAAATCGAAGCCCCAAGGAGATCCGACATTGGCTCTAGCCACTGGATCGAAGTCTTAGTTCCACCGACT
TCTTCTTGTATATTGGTAAAAATCTTCTTCTTCTTGCTTGCTTAAGGAATACATCAATCCTTTATTCTCTTGAG
>comp160397_c0_seq1
TCACTAAGGTCCGCCCCATCTAGAATCACTAGATCTAAACTTCCTTCTCTTCCCAAAAGAATGCATAACTAAATGTTTCTCCACTAAATATGTACCTGCTACTAGCCCATTGCTACTGAGTTG
CTGATATTTTATTCTCAATCAAATACGTTCTCTCCACTAGCTTAGAGAAATAAAAATATGAAATAGAACCATCAGTCATCTTAAAATATCTCAGGATAAGTGGCCGAAGCGTCAATCTGGAC
ACATACCCTCGCATAGGTTAAACGGGATTTGTTCGCAGTAAGATCGTCTACAGCGAGGGGGATACCAATTTTGGTAGCAATTTTTTATATTCCTATCGGGGTCCAACAGCAGAGAGGTAGG
TCAAGGATTTTGATCCAGATTGGGACCGAGGTGAACTCCTCCTTTTTGGGGGTGAACAAAGGCGACCACTTTTGTAGAACAAAAGGTTTTCCCAAGATAAACCAGACACCCTTATTCCAT
ACATATTCAAAGTCCTCAATTGAAGAAAATTTAAAGAGAAAGAAGCCATCACTCAAGGAAATCATTTTGAGACTACCTTTAAGCTTCCATACCTTGTTTACTGCAGTAAGGAGGGAACCAT
AGTAAGGCCTCTTACCAAGGGAGTATCCTACCAAAGCAAGGCTCCATTCCGAGGAAGCTTCGACCACATCCTCGTGAGCGAACGGGATGATGTCCTCCGGAGTTGGGAAAGCAGCGATA
TCGAACTTGCTCTGGTTCGGCCTGTCGGCCAGGATACGCGTCCAGGGGAGCGGGTTTTGCGAAGAAGCAAGCGGCGAAACCTGGTGCATATTAGGCAATGGCGGGCTGGAAGGGCGAA
GCGGCGGAAAAACGGAGTTTGAAAGGGAGGGATCCATAGATCCCTCCGCCAACAGGTCGGCGAAGCACGAGAGACGGCGCGGCGCAGAAAAAACCCTAGCAGAGCTTTTTCCTTTCT
TTA
>comp160397_c0_seq3
TTTTCATAGAACATGCATATAACAAATTGGATAAGAGCAGATGCATACGTACAGTAAGGAAAAAAGTATTACATTGTCTTCTTACTCAAAAGAAAATATGATATAACAATGAAGGTTGAGGC
CATCCAATACAGAGAAAATATAGCATAATGTTTCTTCTTCGACCTATATGGAATGGCATCTGTACCTTGCATGTAGTGATGTGGCGTCCTAAGAGGTCTATCCCTCAACTCGTGCAACTCTGC
ATGATGCGCAACTGCCTCCTCATATGTAGATGGAATGACCGTATGAATGTAGTCATATATATGTGGGCCAAGATGGAATGTTAAGCGGTGTCAGCTGGCACAAGAATCTTGAGATGACAGT
GCTCTTTGTAAGATAATGGGCTCGCAACCATGCTATGTGGTTGCACAAATGATGGGGTGCCATTGAAGTAGGTGTACTAGGTATGTGTAGCTTATGTATAATGATCATCTTCGATATGCATAT
GTCTTCACATATCATGAATCAAGTCCTCCATATGGTCGATAGCTGGTTGGATCACTCCTAAGTCATGTCTACCAAATATGGCTGCAATGTGGTCTCTGAATTGAGAAAGTAATTACGTTGCA
CATATAAAGCTTTGTTGGTTAGAGATGTATGGCTACAAATTTTTACGTACGAACACACTGAATTGAAAATTTTAGGTTTTTATGACTAACTATACGACGCTCCAAGGGTGTACATTGGTAGTA
AGTGATTGGAGGCTCAACAGGTGGTTTCGGCATGTACAATGTTTCCCATGAATGAAAACACTACAAGTAAGGATGAATGAGTCGTTCATTATCAAGATCTACAGCTATACCTTGCAGAATA
CAAAACTGATTGTCCTCCCCATGCCAATGATTATGATAGTATATTATCCAATCTATGTAAAACTTCCGGTGACCATCTGAACGGATGAAGCTAGGATATCCAGTGGAAGTATATCAATTGAAG
GGACATACCCAAATTGTATTATCACTTTATCAGGAAAGTTCTACTCAACTCTCGTCCAGCTAATCAAAGGCACATGTGCCATCCAGATCTGCTCTCCTATGAGGCATATTTCTGGAAGTTGT
GCACAAACATCTAGAGTGTATGGCTCCCAAATAATCTATACAAATTAAAATATTTGTGTAAGTGTTTGATATCGTGAATTTTTAGTTAACAATATAAGAAAATTGCTTAAATTTAAGGACTTG
ACTATCCAAAAGTCCATCTAGCTCATCTTTATAAGTACTAATGGTTGTTACGAGAAGCTATCTCGCTTTAGCTTCATTCCACCTGTAAGTAAACATGAGAAAATCAGTAGTGCCAAATATATA
AAAATTAGACATTCCAAACACTTACTTTTTTTTTCCTTTTTTTACCTTAATTGATGCTAAGATCTTCAAGAGGGACATGTTGTGGAACATGGAGTGTCGGTCGGCCAACGAGAATGTGCTCC
CAACACTATATTTGTAGTAAGTTGATGCACCCAGCAATGTTAGCCCAATTGGGCTTAGTAGCAAGAGCGAACTCTCTATAGTGGTGACCTAAAATTACTCCACTAAGGGAGTAAGTGATGA
ATAATCCCAAATCTTCTAGCAAGTAGATTCCTCAAGTGTATCTCAAATCTCAAGGTATCATGAAGCAGGTAGCATGCATCCTAGCAAGAACAAAATGAATGCTCTAATGTGCCTATAGACTT
CCATCTCACCTAAATCAATGTAAGAATCCTACCTAAATGTTTATTGAAGCCAGCGAAGTGGTAGGAAAGTCTATGCATGTGGCTGACCCATCTTGAACTTATAGCTGATATGACGGTTTGTT
GAGTCATAGTAAACAACATCAGGCTTGGGATAGTACCCTAGAAGATCATAACAACAATCTGGCCAACTATGCCAACATCTTCCTGGACTTTCTACATTGTGGCCAACAAATGTGGGGCCAT

CAATTTGAACACCAAGGATAATATCCACATCCTACAAAGTGATCGTCATCTCACCAACAGGGAAGTGGAATGTGCTGGTCTAAGGTGACCAACACTCGACCAAAGTAGTAACTAGGTTAT
GATCCATCCTGATATATCGTAGCTGACTGATACAAACCAAGCCCATCATTTACAATGTAGCCACGAAATGAGGGCAATTGATCAGTCATTCATGCATCATGAGCAAGTATGATGTAACTTCT
AGAACTATTCCATAAATAGCAAAAAAATTAAAAAAAATTCAAATCATAACATCATACTGAAAAAATATTATATTAACCTCGTCTAAATGTGTAGCTCTATGCATACCTCGTAATGATGCAATA
CTGGGGTGCATATCTACCAAGGGAGATGATTGCACTTCTTCTTCATCCTTTTGTGGATGTCGAGATCGACATCTACAGGCCTTATCTTGGTTGCCTTCCATGAGTCCTCCATAACATAAAATG
GAAAAAAATGATTAGAACATTTAATTCTAATAAAGAGTAAACAAAAATATTTGCAAAACTTTTACATGAATTATGAAATGAATAAAATAGGAAAAAATATGTGCTACTCAACATTAAAAGTA
GTGGAGACTCATCAACCATAATTGGGAGAAAAATTTCTATCTCAAATATTCTATTTAATATAAATTTTCCATGTCTTCTAAAAAATTAGTAGAGTATTACCTAGTGGGACATGTGCTTCTGTTG
TGACCACTGACATGGAAAACACTACAAACTCTACCACTCTGGCTCCTACTTTCATCAATCGTATTCCAATATTGATGTATTCAAGGCCTTCCTGGTCACCTCCTATAGTTTGGTGTTTGGAG
AGGGTATAACTCGAAGGCAGGATCATGAGAAACATAATATTATCTATCTAGAATTACATGAATGATCTGAGTATGTTAATCTTAAGTTTTTCAAACTATAATACTCTAAGACATATTGCTAATG
ACTTTGCCTAAGTGTGGAAACACAAGCAGTCACATGTGCACAAGGCATGTGGTAAATAATTGTTTTGCCACAACAGTATATAAAGATATCGTCAGGGCCCAAGTCAACTCTATAGAATCTT
CAAGTCATATCGAATACCTGAAATTTGTTCATTGTAATCCTAGTTGGCTCGGCTAGTCTCATTGCCTCCATTTCTAATTTATGAACTTTAGCATGTGCTTTAGGGGCCAAGTGTCCCTCATATA
TATGTTCACTGTTATTTTTTTTTGTCATTTCAACAAATGAAAATGGCTACAATGTTGTAATAAACTAATGTAATTAATGCAATGTCACACTTCAGTGCTCTAAGAACCTGTGTCTTAGGCTGG
TGTAAATTTAACCTTGTCGGAACTTATAAGGGATAGTTTTTACCTCCAATCTTTGAACTTTAGCTTGCGCTTTAGGGGCCAAGTGCCCCTCATACCAATGTCCATACAAATTTTTTATGCCAT
TTCAACAAATGAACATGGCCTCGTGCTCTCTTCAGTACTCCATTGAAGAACTTTAAAACATTAGTAGTCATCCCTTCATATCTATACCCACCATCATGCATTAAAACCCACTTCTTCACATCC
CTATAACCTAATCATTGATGTAGTTTGGGTTTTATGTTGATGATATCAACCATGCGTTTGTTGAACAAGAATGGATCATCTATGTACTAGCCACATACATCTTCTGGAAGATGTAGTCAACATC
TAAAATCCCCAATTAAGATTTTTTTTTTTTTTGAAATTCTCACACAAGTGCCGCAAAAAATGATTGTTCATGGTATTGGGGAATATTTCATTACACCCACATACCTGATCGACATGCTGATCTG
GGATGATACATAGATTTAATCTAGTCCCAATGGATGGGAGTAGCTCATTCAGGGATGTAAGAAACCATATATAGGATTCAACACACTCTGACCAAATTATAGCAAATACCAACATGAAAAAG
TTGTTACTGGCATCCAGA
>comp160422_c0_seq1
CGCTTCCTCAACCGATTACCTCACGTAACCCTAAACCCATCAAGCTCTCGCTTTCTCCTTCTCATCTCACATCCGACGATGACGGTGGCAAATGTGACTAAAGCCTGGAGCGGGATCGGTA
CTTGGGCTCTCGAAGCTGAACGAGCCGAGGCAGAGGAGAATGAACGCGCTGCTGCGGACTCCTCTACCGCTCGCCCAACTTTCCTCGCCGATGAGCCAATGCAGGGCTTTCCAAGCCTC
AAGGAAGGAGCAAGAAAACCTAAGAAGAAGAAAGGCGTCTCTCTTACGCTTTCCGAGTTCACCTCCGGCACCTACGTCGGCCCTGGAGGTGCTCTTCGCAAGCCCACGTTCGATTCCA
ATGGTCTCACAGTCGAAGAGATGGCCCGCCTTCCTACTGGTCCCCGTCAACGCACGGCTGATGAGATAGATCATTTACGCCTCGGTGGTGGCTTTCGTTCTAATGGTTATGGCGGCGGCGC
TCCTCCTCGCCACCTGAGTGAGGAATTTACCAAGGACTCAGATCGCGATCGTCCCTCGAGGGCTGATGAGGTAGATAACTGGTCTCATTTTAAGAAGCCGTTTTCCATGGATTCCGGCCG
GCAAGATCGTTACGGCTCCCTGGGCACATCCAAGGCGGACGAGGTTGATAATTGGTCTTTAGGAAAGAAATCTGTTGCTCCTCCATCTAGGCACTCTGGCTTTGGATCTGGATTTCGGGA
CTCTTTCGGGTCGGGGATGCATTCTGATCGTTGGGATAAAGGAAGCGTGCCTTTTCCGAGGAATGGGGAGAGAGAAAGGCCGAGATTTGTACTCGATCCTCCAAAAACAGATTCTGAATC
ACAGCCAGAGCCTACGCGTAGCAGGCCGAGTCCTTTTGCTGCTGCAAGGCCAAGGGAGGAGGTGTTGGCTGAGAAGGGGGTGGATTGGAAGAAGATGGAAATCGATATAGAAATGAAG
AAGACTAGTGGCTTCACAGACTCTTATTCAAGCAGGACATCCAGTGCGCAGTCAAGCAGGCCTGGGAGTTCGGGCTCACAGGCTTCATCAGAAGGAGGGGTGCCAAAGCATCGGCCTA
AGGTTAATCCTTTCGGAGATGCAAAACCTCGGGAGGTTCTTTTGGAAGAGAAGGGAATGGATTGGAGGAAGATTGATCAGGAACTGGAGCATCGTGGGATTGAAAGGTCTGAGACTGA
AGAAGAAAAGAAACTGAAAGAAGACATTGATAATCTGAAGAAGGAACTTGCTAAGGAAACTGAGGATAATGTGAATGGTGATTCCTCGCATCTTCCTACTGAACAATACGATAGCTTGC
TTGAGCAAATAAGGAAGATGGAGAAAGAGCTGGAAAATCTAATACGCGAATTAGATGATAAGGTGCGGTTTGGTCAGAGAGCCTTTGCTAATGGCAGGCCTGGTTCTGGATCTCGAAGA
ACTCCTTTCTCTGCAGAGAAGCCATCAGTGCAGACTGGCTTTTCAGAAGATTCAAGGCAGACCGAGTTTGTAGACAGGCCTCGATCTAGAGGGACGGGTGATTCATGGTACAGACCCTT

CATTGAAAAACGGGGATTCCAATCAAACAGGGATTCAACCCTTCACGCCAACAGGAACATGGATAGGCCAAAATCCGGGGAAAGATGGTGAAGGAGCTTATATTTCTTCTGGGGCCATT
AATATTTTTTATGCTGAATCTGAAGGCTCTGCTCTTTTCTCTTTTTTTCTTTTTTTTTTTAGAATGTTGAGCG
>comp160462_c0_seq3
GCGGATGCCGAACGTCTTCCAGAGCTCAATGAAAATGCATCACGTCACCTCCACTTGGTACCGCGGCTCGGTACTCGATGACGTGGCTGTACCTGATTCACCTCTGGACGATAAGCTATGT
CCGATGTCCGCCCAGAATAATCTCTCGTTGGAGAGGGGAACTAAAGGGATTAAATTTAGGTTTATACACATTAATCAAATCCAAACATCTCAACAGTGCACCTCTTCATATCATCATTATCTT
GCACTTTCACCAACATAAAAAAACTCTCGATGTAATTATTTTTCCTAGAATATGAGATCAAACCAATACCCTCACACTGTTTTGCTGGACCAAGAATTTCTACAATTTATGTTTCAAATAAAT
CAAAATTATGATATGATACTTTCTATAATCCATCGCAAAAACAACCTGCAAACAAAAACAAGAACATACCTAACCATTTCTCTCTGCTCTAATCAAATTCTCCCTCAGGTTGAAGTTCTGAG
CTGCATTCGGCATCCAAATATGGTCCTGCTTCTCGGCGCCTGCCCTGAATACGGCTGCCTCGTCTACGAATACATGGCCAACGGAAGCTTAGACGACCGCCTCTTCCGCCGGAACAACAC
CCCTCCCATCCCCTGGCAACACCGATTCCGCATCGCCGCCGAGATCGCCACCGGCCTCCTCTTCCTCCACCAAACCAAACCCGAACCCCTCGTCCACCGTGACCTAAAACCCGGCAACAT
CCTCCTCGATCGCAACTTCGTAAGCAAAATCAGCGACGTCGGCCTCGCCCGCTTAGTCCCCCCTTCCGTCGCCAACACCGTCACCCAATACCGAATGACTTCCACCGCCGGCACCTTCTG
CTACATCGATCCCGAGTACCAGCAAACCGGAATGCTCGGCGTCAAATCCGACATCTACTCCCTCGGAATCATGCTTCTTCAGATCATCACCGCCAAACCCGCCATGGGACTCACCCATCAT
GTCAGCCGAGCCTTGGAGAAGGGGACTTTCGAGGAGCTCTTGGATCCGAGCATCGCGGATTGGCCGGTGGAGGAGACGGTGAAGTTTGCAGAAATGGCTTTGAAATGCTCGGAGTTGA
GGAGGAAGGATCGGCCGGATCTCGGGACTGTCGTGCTTCCCGAGCTGGAGAGGTTGAGATCGCTTGGGGAAGATAATATGCACAACTGTGTGATGCTGAGAAGCGCGAATAGCTCGCCT
CTGAACAGTCAGGTTTTCTTGCAGGATGTGCTGAGTGATCCCAATATGATTCAGTCTGGATATTATGAGAGTTCCACCAAGACCAATTCTGGTTTGTCTGGAGAAAGAAGATCAAATTTGA
GTTGAGAGATAGCAACAGGTGATTGTTTTCAGTTTATTTATTCCTTTGTTTTTTTTGCTTTGTCTTGCAACTTTTTGAATGGTGATGGATTGGCCTCAGAATGATTTGGCTTAAGAGTTTAAA
TTTTTTTTTTCTTGTTTGTTATATTAGTAGTAGCAAGCTTTTACATTCATGATTGTATGAATTTCATGTAAATAATCTATTATG
>comp160462_c0_seq4
AAAAAAAAAAAAAATCTCATTTTTGAAGGCAGACAAATTAAGTTTGGAAGAGTGTCCATTATTTTTGTTTGAAAAGAATAATTATTTGTCCATCTATTCAAGTGATGCTAATACAGAATTTT
TTTTCTTTGGCCATCCTTTTTGCAGGTCACCATTCACAAGGGGAAACCGAGTGAAATCCAATGCAGATCTGTTATATAATGATACTGACATATCTTTTGTGAGCTCAGGGAGGCCGAGCATT
GATAGATCATTTCAACCAAGGCATTCCAATGCTTCAGATGGCCATGATCATAGTTTTGAGTCAGCTCGCACACCAATTCGCTCCGTTGATTCATACTCGTTCGGGAATGAGTTCTCTTCTGG
TTCCGGGGCCAGTAGTTCAAACTCTTGGTCATCTCAAACCATGGATGATGTAGAGACTGAGATGAAGAGACTGAGGCTGGAGCTCAGGCAAACTATGGACATGTACAATGCTGCCTGCA
AAGAAGCACTTATCTCAAAGCAGAAGGCTCTTGAGCTCCACCGATGGAAGATGGAAGAAGAGCATAAGCTGGAAGAAGCCCGGCTTGCCGAGGAATCGGCCATGGCGCTTGTAGAGAG
AGAGAAGGCAAAGTGCAAAGCAGCCCTGGAAGCTGCTCAGGCGCAGCAGAGGATAGCAGAGTTGGAAGCTCAGAAGAGAAGATATGCAGAGATGCAGGCCTTAAGAGAAGCAGAGG
AGAGGAAGGCAATGGATACTTTTTCTCCTACTGTTAGGTACCGAAGATACACGATAGAGGAGATTGAAAAGGCTACAAACTACTTTGCTGAGAATCTTAAGATTGGTGAGGGTGGTTATG
GTCCTGTGTATAAATGTTTTTTAGATCACACACCTGTTGCTGTTAAGGTACTCAGGCCCGATGCTGCTCAAGGAAGAACACAGTTTCAACAGGAGGTACAAAACTTTACTCGTCTCTTTTA
ATTTGCTTGCTTCTTTTTCATACTTCATGAACAAGCCTGCAACTTTTCCTCTTTTTTACTTTGAAAGGACTTGAATAGTGGGTAATTCAAACTGCCAGAACTTGGGAAATGATTCAGTTATG
AAAAGTATTTAAATATAAAAAATTATGATACTTGGTCTCTTCTTTCATCTGTTGCAGGAAAAAAAAATTGGCACTAT
>comp160462_c0_seq5
TCGATCAGCGGCAGAGAAACGAAGCGAGAAACCTATCCGACAATGAAGGGGAACCTCAGATGGCGAAGATCGAGAGAAAGAAGCTGGATAACTACCATAAAGACTTCATTGCTCCTTC
TTCCTCTTCGTCCCTCTTCTTCGTCTCTTGGAAAAGGCAAGGATACAGATTATTTACAGGAAATAATCAAGGAAGAGGAGAAAGATGAAGGATCACGACGCCATCGCCGCATATCCGTTG
GTAGCTGTGGCCATTAGCAAGGATAAAAGCAGCCAGAATGCTCTGAAATGGGCGGTTGATAACCTCGTCGCCAAGGGCCAGACTCTCACCTTAGTCCATGTTAACATTCAGGCTTCAGCT

TCACAGCAAAACGATCCATCCGATGGCTACAAAGGCGCTGTGGATCCTCAAATGAAGGAACTATTCCTCCCATTTCGTTGTTTCTGCACGAGGAAAGATATACATTGCAAGGATGTGGTG
CTTGAAGACACAGATGTGGCAAAGGCAATTGTAGAATTTGTGTCTCAAGCTGCAATTGAGAAGCTTGTTGTTGGAACTCCATCTAAAGGAGGATTTATTAGATTTAAGAGCGCAGATGTC
GCATCACACGTCTCTAAGGGAGCACCAGACTTCTGCACAGTTTATGTTATCTCTAAGGGAAAATTGCTTTCAACTAGAAATGCTGTGAGACCAGCTCCGGTGATGTCTCCACTGCGAGCT
CAGATACAGAGCCAGGCCAGCTTCAAGCCTGATACTGGAGAGCCACACCATTTGTTGGGAACAAAAGGTGAAATTACATCTAGAGCTCACAGCCCTCACAACTTGCATGATGAATCAAT
CAAGTCACCATTCACAAGGGGAAACCGAGTGAAATCCAATGCAGATCTGTTATATAATGATACTGACATATCTTTTGTGAGCTCAGGGAGGCCGAGCATTGATAGATCATTTCAACCAAGG
CATTCCAATGCTTCAGATGGCCATGATCATAGTTTTGAGTCAGCTCGCACACCAATTCGCTCCGTTGATTCATACTCGTTCGGGAATGAGTTCTCTTCTGGTTCCGGGGCCAGTAGTTCAAA
CTCTTGGTCATCTCAAACCATGGATGATGTAGAGACTGAGATGAAGAGACTGAGGCTGGAGCTCAGGCAAACTATGGACATGTACAATGCTGCCTGCAAAGAAGCACTTATCTCAAAGC
AGAAGGCTCTTGAGCTCCACCGATGGAAGATGGAAGAAGAGCATAAGCTGGAAGAAGCCCGGCTTGCCGAGGAATCGGCCATGGCGCTTGTAGAGAGAGAGAAGGCAAAGTGCAAAG
CAGCCCTGGAAGCTGCTCAGGCGCAGCAGAGGATAGCAGAGTTGGAAGCTCAGAAGAGAAGATATGCAGAGATGCAGGCCTTAAGAGAAGCAGAGGAGAGGAAGGCAATGGATACTT
TTTCTCCTACTGTTAGGTACCGAAGATACACGATAGAGGAGATTGAAAAGGCTACAAACTACTTTGCTGAGAATCTTAAGATTGGTGAGGGTGGTTATGGTCCTGTGTATAAATGTTTTTTA
GATCACACACCTGTTGCTGTTAAGGTACTCAGGCCCGATGCTGCTCAAGGAAGAACACAGTTTCAACAGGAGGTTGAAGTTCTGAGCTGCATTCGGCATCCAAATATGGTCCTGCTTCTC
GGCGCCTGCCCTGAATACGGCTGCCTCGTCTACGAATACATGGCCAACGGAAGCTTAGACGACCGCCTCTTCCGCCGGAACAACACCCCTCCCATCCCCTGGCAACACCGATTCCGCATC
GCCGCCGAGATCGCCACCGGCCTCCTCTTCCTCCACCAAACCAAACCCGAACCCCTCGTCCACCGTGACCTAAAACCCGGCAACATCCTCCTCGATCGCAACTTCGTAAGCAAAATCAG
CGACGTCGGCCTCGCCCGCTTAGTCCCCCCTTCCGTCGCCAACACCGTCACCCAATACCGAATGACTTCCACCGCCGGCACCTTCTGCTACATCGATCCCGAGTACCAGCAAACCGGAAT
GCTCGGCGTCAAATCCGACATCTACTCCCTCGGAATCATGCTTCTTCAGATCATCACCGCCAAACCCGCCATGGGACTCACCCATCATGTCAGCCGAGCCTTGGAGAAGGGGACTTTCGA
GGAGCTCTTGGATCCGAGCATCGCGGATTGGCCGGTGGAGGAGACGGTGAAGTTTGCAGAAATGGCTTTGAAATGCTCGGAGTTGAGGAGGAAGGATCGGCCGGATCTCGGGACTGTC
GTGCTTCCCGAGCTGGAGAGGTTGAGATCGCTTGGGGAAGATAATATGCACAACTGTGTGATGCTGAGAAGCGCGAATAGCTCGCCTCTGAACAGTCAGGTTTTCTTGCAGGATGTGCTG
AGTGATCCCAATATGATTCAGTCTGGATATTATGAGAGTTCCACCAAGACCAATTCTGGTTTGTCTGGAGAAAGAAGATCAAATTTGAGTTGAGAGATAGCAACAGGTGATTGTTTTCAGT
TTATTTATTCCTTTGTTTTTTTTGCTTTGTCTTGCAACTTTTTGAATGGTGATGGATTGGCCTCAGAATGATTTGGCTTAAGAGTTTAAATTTTTTTTTTCTTGTTTGTTATATTAGTAGTAGCA
AGCTTTTACATTCATGATTGTATGAATTTCATGTAAATAATCTATTATG
>comp160501_c0_seq1
AATCACAAAGCAAAATAAAAACAAATCTGATAAATAACCCAAAAACTGTGGTAGCTATGCTACACATCTCTGTTGCAAGCCATTATTCTACACACAGAGGCTAAATAACATGATTGATAGC
TTAATAAGCACAATAGTATGGGTAAGCTATGAATCCAGTTGGACGTTGATAAGCTCAAGTTGGACGTTGATATGCTCCAGTTGAAGGTGGATATGCTCGATATGCTCCAGTTCGAGGTTGAT
AAGCTCTAGTTGCAGGTGGATACGCTCGATATGCTCTAGTTGGAGCTGGATATGCTATAGTTGGAGGTTGATAAGCTAGAGGAATTCCATAAGTCTCGTATCCACTAAAATG
>comp160521_c0_seq38
TGCAGTGACAAATATTCCATAAAAAATTCTACTCACAGTTTCAGTTTTAGCCAAAATAAACTTTGCATAGATATAACTTGTCATCCACTAAATCAATCACAGGATTTAGAACTAAAAATGAG
AGGAAACAACAGTAGAAGAAGAAGTTTAAGGGGAAGAAGGAAGAGAAGGGGATATACCTGCTGTAAAGAAAGAAGTGAAAGCCTAGCTAAAGCAAACCGTGCACAAGCTTGTGCTGC
CTTGGCTCGGCCAGGTGGGAGAGGCGGAACAGGGAGTTCGGGTTCGTCTGAGATTCGCGAAGCAGCGAGAGAAAGGGGGTCGGTCCCGAGGGTTTCGCACACCTGGAGGTCGGACGG
GAGCAAGTCCACCGCCTCGGCTAGGGCAGCATTGGGTTCGGCCAAGGGAGGAGTGGCAGCGGCTGGGTCGTACGAATGGAGAAATCAGGGAGGCGACGGGCTCGGTCCAGACGTGCG
ACATCGGGAGCTCGTCGGGTTAGAGGGGCTCCGCTCGGGTCAGCCACACGAGAGAGGGAAGGAGCTGGAGTCGAGCTCTGCGAACAAGGAGGAGGAGATGGACCAAAGGAGGGGCT
TCAGCCTCTAGGAACCTGCGAACCAGCCGGAGATGGAGAGAGCTAGAGCCTCTCTTCTTTTTTGTGTGCCGCTTCCTCTTGTCTCCTCTCCACCGAGCCCGAGCGAGAGAGGGAGGCTT

CGGCCTCAAATTAGGGCACGAGAGGGAGAGAAGGAGATCAGAGCCATCCGATGAAATTCCTTGATCCAACGGTAGGATTCAAACCGGTTTGGAAATTCAATCAAATTTTGATTAGGAC
CTAACTAAACCCAAGGGGCTGAAATCAGGTTCAAGATTGAGTCAGGTCTGGTTCGATCCGAGTCAACTCGAGCTCACCTCGACTCGACCAAAACTAATTCAATAAAATAGGATAAAATAA
ATCGAACTCAAATTAATATTAATTCGAACCGAATCGGGCTCGACGAGGCTCGATCAACACGATCAAATACGTTCGAGGGAAAAGCTAAGCTAAAAACGAGGTCAACAACAACAAACCAT
GGAAACAAAGGCCCGAAAACAAAAAGCAGCTAGATAGAATTATATAGTGAAGCAACAATACAACTCTCCAACATTTCGGTAGAAACAGAAAATCAAAGACAGTAAGCATCTCGACAAA
GGCATGAGTGAGTCCATGGACACACACACCCAGTATAGATATCAAACCAAAATGTTGCCCAGTTTAGTCTAGCAAAAATATAAGATGATTTATCATTATAGACTCCAAAATGAAATAAAAA
ATACTACAATTAAAGGCAATAGCAGAACAACTGCAAAAAAAAACTCTAGCATCAAAGTGCTAATGAAATGGACGGAACTAGAAACCCCAATTGGCACCACCATCAAGGATCCAAAGAAG
CCTTCATTTAAAATACACTCATT
>comp160634_c1_seq3
AAATGGCACATTCCTTATTCTTTTTTCAGTATCAATGCATCCCTACACGAAATTGCGCATTCCCTTTGTAAAAAATTTCATGTGCTTGCAATGAAATGTCTATACATGCGAATGTTTTACTCGC
CGGATAGAAAATATCCCAAGGTGAGTTTCTCTTAACCTTATTCCCAGTTTTTTTTTACTTGCCTCAGCCTTTCACCGATGGCCCCTCTATCCTCGATTTCTTCTCTCAGAACATCATATTTTTT
TTTCTTCCATATAGATATTTCTTATTCATATTTTGTACCTACCAAAATCTTAATATTGTTCCTTTTATTTTTTTTTGTTTGAACTCATATACTACTACAGTTTTATCTCTCAAGATGCTTCTTTAGA
CTGTGCAACAACCCTTCGCTCTATTTGAAGTTTTTTTTTCATTTGATTTCTCTTATACATTCAAAATACTTCGCCCTTCTAACTTAACCTGGCTTTAAGTTTAAAGAATGATAAATTAGATCTT
ATTAATGTCTTGTTTCTTGGTTTTATTGGTTTCAATTAGTTTGTCTCTTAATATGTGGTCTGAGTTTTGCTTGTAGCTCTTTTCCATGTTCTTTCTTCTAAAGTATCTCACATTGATGGAAATCG
ATCTGGAGGACAAGTGGTGGCTTGAAGAGGACCACTCACTCAGTCTTTCTGAAAAAGGAGAAGAAGCAAATTCACTTCCCGGGAAGAGTTGCACGAAGAGGGTTCGCGCTCTGCCATT
CAAGAAGGGGAGATAGAAGAGATGATATCATCAAGACAGTCGTCTCTTCCCTCAAACTGTCAGGGTGGATACCATATTCCCCACGCCTGACTATTTTGGGGTGCATTTCAAACCAAAAAA
AGGTGCTAGTTTGTTCAGTTTAGTAAAGTTGACCGTCTTCCATGCGTAGCTGTGCCCGGCCGATGCGTTCCATTATTGCTGCATTTCAGCGCTGGGGAATCAGTCTCGTCATTCCAACGAA
AAATTCATTGATAGTAGAATAGCCTTACCTTACTTAAGAGGGGCGCTAGCGCTTGACTAAGACTAATCAGCCATAGAAAGCAATGTAATAGCTTAGGCTCGCTTCTTCAAGCTCCGTCCCTT
ATTCCTCATTGAAGACGAAAGCGCTCACATTTTTTTTTTTCTTTTTTCATTCGAGTCAACCGGCCGAGTCTCTCATTTTCTTTCTTCTATAAGTCTTTATCTAGCGCCTTCCCAAAAAGATAG
CCAACCAATAATTATCCGCGCCTGCCCGAGTGCTCAAAATCTTTTCCTCCCGGGGCTGCTTGCTTGGTTGAGTGATCGCCAGTGGCAGCCACGCTCATTCTAGATTATCTCTCTGGTTGGG
CATCCCAGGGCACATAAAGTTCCTATAGAACCATTAGAATTAGTTGGTGAGGACATAAGGAATTGAACAACAATTGCTAAATACTTGTACGGCTCCAGTGAGACTTCCAGGTACGCAACTT
CATCAAGGACTTATACAGGTTGGTGATTTGGATCATTAGCAGAACTCCTGGGCTTAGGCGGCATTCCAGATTAGGATTAGAAGAACACCCTGGGCCCACTACCACTTCATCTAGCAAAGTC
CAATCCCTGGGCAGGGATTCAGGTAATTCCATTAGAAAAGAAAAGAGAAAGAAAGAAGAGAAGAAGAAAACTAAGGCTGCTCGTCCGGGCAACCCTGTTTACCGGGGTTGTTCCCACA
ATCATCTTTCTTGTACCTCGATAACCAAATCCCACATGTACTATCACGAGTTACTGGCTTTCACAAGAACAAGAAAGAAAGGGAAAGCAACCGGAGATGCTGGTTCAATTCCAGCTTGTG
AATCAAAGAAAACAGAGCATCAATTTCCAGGTCTATCTATTCTACGTTATTTTACCGATGCGAGTATCCGAATCCTTCTATTACATGCCAATCCCAGAGACTCGAGGGGACTCAGGCTATTC
CTGTTCTCCGTGCAGCAGCTATTCATTCTACTCGGGAAAACTTTTGCGGGGACAGTCCTATTGTTCTGTGCCGGTGAATTCCTTCTT
>comp160634_c1_seq8
GGAAGTGGCAATAAAGGTTCTCCCATCCATTTTCACGTTGGTCCAAATCGATGTCTGGGAAGAGATGGTCACGGCATAAATCTACTTCAACAATTTTATATCCTAATAATAGTGGCTTCTTC
TCTTATTGGATAGTTAATCCATCAACCCATCAGTTCGTTCAAACACTCCTTCGATTTTCATCTCTTTTCCATGTTCTTTCTTCTAAAGTATCTCACATTGATGGAAATCGATCTGGAGGACAA
GTGGTGGCTTGAAGAGGACCACTCACTCAGTCTTTCTGAAAAAGGAGAAGAAGCAAATTCACTTCCCGGGAAGAGTTGCACGAAGAGGGTTCGCGCTCTGCCATTCAAGAAGGGGAG
ATAGAAGAGATGATATCATCAAGACAGTCGTCTCTTCCCTCAAACTGTCAGGGTGGATACCATATTCCCCACGCCTGACTATTTTGGGGTGCATTTCAAACCAAAAAAAGGTGCTAGTTTG
TTCAGTTTAGTAAAGTTGACCGTCTTCCATGCGTAGCTGTGCCCGGCCGATGCGTTCCATTATTGCTGCATTTCAGCGCTGGGGAATCAGTCTCGTCATTCCAACGAAAAATTCATTGATAG

TAGAATAGCCTTACCTTACTTAAGAGGGGCGCTAGCGCTTGACTAAGACTAATCAGCCATAGAAAGCAATGTAATAGCTTAGGCTCGCTTCTTCAAGCTCCGTCCCTTATTCCTCATTGAAG
ACGAAAGCGCTCACATTTTTTTTTTTCTTTTTTCATTCGAGTCAACCGGCCGAGTCTCTCATTTTCTTTCTTCTATAAGTCTTTATCTAGCGCCTTCCCAAAAAGATAGCCAACCAATAATTAT
CCGCGCCTGCCCGAGTGCTCAAAATCTTTTCCTCCCGGGGCTGCTTGCTTGGTTGAGTGATCGCCAGTGGCAGCCCTCTTCCTAAAGAGAAAGAGTCCTTTAGGGCAGGTCTACTGCTCG
AAAAA
>comp160654_c0_seq2
TCAGTATACTAGCTGACTTTATTAGGAGCTCAGCAACGTAAGATTAAGATCCTAAATGGACGGGAACGGGCGGCGATTTCTCGCGCAGAGGAGTACGGGTGCTCTGAGGTGGCCGGATCC
ACGAGAACTTCGGGCGGCGGACCCACGAATCTGTTGCTTTACTGCTCTCGTGTTCTCATATCTGGAACTGACAACCCTCGACGTAATCCCTCGCCTACGGCCGCTTAGGGCGACGAAAGG
ATCAAGGACCGGTGCAAGAAGAGGTGGCGGAGAGAGTTCGATACGTTGCTGAATTCGATCTCATTGGCCTTTAGAGAATACTTCCGTAATCCGATCATAATGGCCGATGATAAGCAGAAG
GCCGACCATGAAGACGAGTTTTTTACTGAGTACGGTGAAGCAAGCCAGTATGAGATAATGGAGGTTATTGGAAGGGGAAGCTATGGTATAGTTGGGTCGGCAGTGGACACTAAAACTGG
GGAGAAGGTCGCCATCAAGAAGATCAATGATGTCTTTGAGCATGTTTCAGATGCAACTCGAATTCTTAGAGAGATCAAGCTTCTCAGGCTGCTTCGGCATCCTGACATTGTAGAAATTAA
GCACATAATGCTACCTCCATCTCGTAGGGAATTTAGAGATATTTATGTTGTTTTTGAGCTAATGGAATCAGATCTTCACCATGTTATAAGGGAAAATGATGACCTTACTCCTGAGCATCACCA
ATTTTTCCTGTATCAGCTTCTTCGTTCATTGAAGTACATCCATTCAGCTAATGTATTTCATCGGGATTTGAAGCCCAAAAACATCCTTGCCAATTCAGACTGCAAGCTAAAAATCTGTGACTT
TGGACTTGCTCGGGTGTCCTTTAATGACGCTCCATCGCCTATTTTTTGGACTGATTATGTGGCAACAAGATGGTATCGTGCACCAGAATTATGCGGTTCCTTTTTCTCCAAATACACCCCCG
CTATTGATATGTGGAGTATAGGGTGCATCTTTGCTGAAATGATAACAGGAAAGCCACTGTTTCCTGGGAAGAATGTGGTTCATCAGTTGGATCTCATGACTGATCTTCTAGGCACTCCTACT
CCTGAAACCATCACAAAGATACGAAATGAAAAGGCTAGAAGATATCTAAGTAGCATGCGTAAAAAACATCCACTTCCCTTCTCAGAGAAATTCCCTAACGTTGATCCCTTGGCTCTTCATC
TACTCGAACGTCTGCTTGCTTTTGATCCTCAAGATCGTCCCACTGCTGAGGAGGCACTAGCTGAACCATATTTCGATGGATTGGCAAATGTAGAGCATGAACCTTCAACGCAACCAATCTC
AAAACTTGAGTTTGAATTTGAAAGGAAGAAATTGAATAAAGATGATGTAAGAGAATTGATT
>comp160660_c0_seq3
ATATTAAAGTCAAATATGTTAACAAAAGAAAATTTAATTTGGGAATAACATAATAATGGTTTATTCATGTTCGAATGCATCTATCTTAAAAAAGATTTTACAAGATTTTACACCAAGACAGAT
GCAAAAATCGTACCTTGCAAGTTATCTTAGTGTGTGATGAGGAGGAGGTGGACAAGGCAGAGGTAGCGAGGGAGGAAGAAGAGCCTATACACTTGGAGGTGGCTGAAGTTTCATTGAA
CTCTGTTGTAGAATTCACATTGAATCACACTATGAAGGTGAGAGGCTAGATATGAGAAGGGGCGGTGGCAATCCTGATGGACAAATTGGGGCATAAACTGATAGATACGAGATGTTTTGG
AGTGATGATGGGCATGGGGAAGGTGGAGATAAAGCAGAGGGATATGTCAAGGTTTGGGGGTAGAATTGCAGGGGATTCACATCAAGGAGGATTTTCTCCGATTGAAGTTTGAGTACTTAT
TTGATTCTAGGAATGAAATGGCTACAAATATTGGGGGAAACTAGGGTAAACTGCAAGGCATTATCTATGGAGATCCAGGAGGAGGGGAGAAAAGTGATAATTAAGGGAGACCCCAACTTA
TCCAAAGCCATGGTTTCTTTGAAAATCCTGGTTAGTGCGCCACAAGGGAAAGGAGAGGGATTCTTGGTAGAGCTCCAGAGTTTAGATGGAGGAGAGACAAAGGATGTAGAGGACATTTC
AAACATGGTCCAGCCACTTCTTCAGCAATTTAGGGAGGTATTTTTGCTTCCCCAAGGATTGTCACCCCCTCGAGAGCATGAGCACTATTTCATTATGAAGGAAGGAATCTCTCCAATTAGTA
TCCTTCCATACCGCTACTCTCGGGCCCAGAAGGATGCGATT
>comp160709_c0_seq4
GCGACGAAACGTCGAAACCTAGATCTCGGGCACGTCTTGATTTCCTACCACGTCGGAAGGCCATTTCCCGACGAACTCGCCGGAAGAGGATCAAAGGCTTCGTTCATTTCCTCCTCTGAT
ATTCGACCCTTTTCTTCGCATTTCGCGCAGTGGCTTCTGCATTCGGAATCAGCCATTTTTTCCATTATAGAGGAAAGGTTATTCGGGGTCTTCCCGAGATTGGATTGCATAGGATTTTGATCG
GAAGAACATATTGCAGCTATTGATGAGTTGATAAAGGCTGATCCTCTCTGAGAGCTCGTTCCGATGTATGATGGCTATGAGAAGTGATGTTCTTTTAGCCGCGTGCGCGGTTTTTTTCCTAG
TATTTGCCGTACCGAGGGGTTCGAGAGGAAGGCGAGCGGGCGATGGATTGTTCTTTGGATCCGAGGATACGATGGTAACGGATTTGTCAGGTACGGGAGAAGAAATGGTAAGAATTGTTT

TGGTATTATTATTGATATTTGATATAAGAACTTGATCTTTTTCTTCTTGCGCGTTCTTTCTTTTGGCGTAGAAATTTTCTCATGTGAATAGTAATGATATGAAATGCTTCGGTGTTAATTGCAATA
TGATTTTGTTTGATTGAGTATT
>comp160713_c0_seq12
AAGCCTTAGTGATTATTTTCTCATTACTTAAAAAAAAAAAATTGTTCTATGAAGCTGCTTTCTGCAACAGCCTTAAAAATCACTTTATCTATAGCAGCTTTTCAAAATTATCCCAAATGAAGCA
GTAATCACAACAAAACTAATAACAAATAAAACCCAATATTTGAGCATATTACACATACATATTATATACATCTTAGAAATTACAAAAACATAGAGCTTAATATCTCTACATGTCAAAAGCCATT
ATATCTGCCTGAAAAATGCCATCATAGAATCATATACTACTTGCTTTGCAGAGACTCCCATTATGAAATCAGCATGAGCAAAGTTCTGTACATAATGAAGAACTAATTGATCAGTTTCATGCA
GCTTTAGATCCTGAAGCAAATGCTCCACATCTTCTGTATCAGACAGAGAGTCTCTCCCACCATAGCTTAGGTAAAGTGGCAGACTGTTGGGAATATTTGATAAATTGTATACAGGAGGTGT
GCTCTGACCATATCTTTGTATGTTATTAACACCATAGTTATATTTTGTTAGGGCTCCATCCCTGAATGTTTGTGCTAAATGTACCATATTTTTGGTTGATGTTGGCTGTGGTTCATACTGGAGG
AATAATTGAACTGTTGAGCCATTAAGACAACAGTTGTTACCTGTAAATGCTGTCAGGAGATCATAGCAGTTCACATCTGGATAACTACAGAGTATTTTCAAATATTTGGATGCTTCCAGCCT
GCAATTGAAGCTCACAGCTTAGTAGAGAATGAAGTAGAAGAAAAATGGTGTGTTTGGATTCATATTTTATTATGTTTGTTTCTTATTTCTTACCCTTTGGGATTGAACTCTGCAAGTCCTAGC
AAATTGCTCAAGATCTCACCCACAAAGAGTCTGGCAGCCAGAATGCCTAATTTTGTTTTCATGTGATCAAGGTAGGCTATTGGACACAAAAGTGCTGCTGATCTTAGCTTGTCCACCAGCC
TTCCTTCTGAGAAAGATGCCAAAGCAATGAGCGTTCCCTGCAATTATTTACAATCAGAAGCTTTGCACTATGTACAAAACTATAAATTTGGCTAAGTAAAGCTGATGAAGACAAAGAAGA
AGAAGAAGAAGTTTCTTACACACCAAGGAGTGCCCAACATAGTGCAGCTTCTGCTTTGTCTGTTTGGCAACCAAGTCTACCATAGCTGGGAAATCATTCTCCACTAGTTCATCCCATGAC
CATGCCCAATAAGCCTATGAAGGATCAAATTAAGGCCAAAATTTTCATAAAAATATTAACATATGCACATATTAAGACTTCGTTTGGGACAGTTTTATTATTTTTCTTTCTTTTCGAATTGTAA
CTATAATTTCTATTTAGTTATTATTTTTCACCGTTTTTTTACTCACAATAGTTTAATTTATAATCAACTTTATAGAAAGCTGCAGTTATTCCAAACGGATGTTAAAAGCATGCATACTTCAAATAT
TTTATTATTATTGCACACAATTTTCATATGTAATATTATTTCTTTTTTTGAAGAATAAAAATATGTCATTACTTAGTTTTATGTTGCTTATTATGGTGTAGTTAATAGATATGTTGGAATGAGCACA
GAGGGTTTGATATGAAAATATTGAAATGTTGCTGGACTTTAATCTTAATTATCCCAAAAGATTAATGAAGGATTAGAAGAGTTAAGTTAATCATGAGGGATTAGCCTAGTAATGGAAGTAGT
TTAACTTTAAAGAGATCGTCATATATATATGTAAGGAAAAATGATTCAGGGTGGTAAATGAAAATTACACACCCGATCGGATGGCGTAAGAGACACGTGGCGATTGCTGGAGCGAGTGCCT
CTAATATTACCGATCCACACATCAAATCCGTTATCGGCTAGCACAAAGGCCAGGGATTGCTCCGGAGAGTTCAATAGCCAGGTCATCCCATCCATAAGAACACCGTGTTGCAGCAATACTG
GCTGCCTTGCCCCTCCAATAACTCCATTGCGCCCCACAGGTATCCTCTGCATGCCCAGTATGTAACCATCCTCAGTTGTCACCTATCTCCATAAATTTATCTAAATTAATCCACTTTTCTTCAT
AAAATCAACATAAATATGATCAGATTAATCGTTCATAATCAATATAAC
>comp160713_c0_seq16
AAGCCTTAGTGATTATTTTCTCATTACTTAAAAAAAAAAAATTGTTCTATGAAGCTGCTTTCTGCAACAGCCTTAAAAATCACTTTATCTATAGCAGCTTTTCAAAATTATCCCAAATGAAGCA
GTAATCACAACAAAACTAATAACAAATAAAACCCAATATTTGAGCATATTACACATACATATTATATACATCTTAGAAATTACAAAAACATAGAGCTTAATATCTCTACATGTCAAAAGCCATT
ATATCTGCCTGAAAAATGCCATCATAGAATCATATACTACTTGCTTTGCAGAGACTCCCATTATGAAATCAGCATGAGCAAAGTTCTGTACATAATGAAGAACTAATTGATCAGTTTCATGCA
GCTTTAGATCCTGAAGCAAATGCTCCACATCTTCTGTATCAGACAGAGAGTCTCTCCCACCATAGCTTAGGTAAAGTGGCAGACTGTTGGGAATATTTGATAAATTGTATACAGGAGGTGT
GCTCTGACCATATCTTTGTATGTTATTAACACCATAGTTATATTTTGTTAGGGCTCCATCCCTGAATGTTTGTGCTAAATGTACCATATTTTTGGTTGATGTTGGCTGTGGTTCATACTGGAGG
AATAATTGAACTGTTGAGCCATTAAGACAACAGTTGTTACCTGTAAATGCTGTCAGGAGATCATAGCAGTTCACATCTGGATAACTACAGAGTATTTTCAAATATTTGGATGCTTCCAGCCT
GCAATTGAAGCTCACAGCTTAGTAGAGAATGAAGTAGAAGAAAAATGGTGTGTTTGGATTCATATTTTATTATGTTTGTTTCTTATTTCTTACCCTTTGGGATTGAACTCTGCAAGTCCTAGC
AAATTGCTCAAGATCTCACCCACAAAGAGTCTGGCAGCCAGAATGCCTAATTTTGTTTTCATGTGATCAAGGTAGGCTATTGGACACAAAAGTGCTGCTGATCTTAGCTTGTCCACCAGCC
TTCCTTCTGAGAAAGATGCCAAAGCAATGAGCGTTCCCTGCAATTATTTACAATCAGAAGCTTTGCACTATGTACAAAACTATAAATTTGGCTAAGTAAAGCTGATGAAGACAAAGAAGA

AGAAGAAGAAGTTTCTTACACACCAAGGAGTGCCCAACATAGTGCAGCTTCTGCTTTGTCTGTTTGGCAACCAAGTCTACCATAGCTGGGAAATCATTCTCCACTAGTTCATCCCATGAC
CATGCCCAATAAGCCCGATCGGATGGCGTAAGAGACACGTGGCGATTGCTGGAGCGAGTGCCTCTAATATTACCGATCCACACATCAAATCCGTTATCGGCTAGCACAAAGGCCAGGGAT
TGCTCCGGAGAGTTCAATAGCCAGGTCATCCCATCCATAAGAACACCGTGTTGCAGCAATACTGGCTGCCTTGCCCCTCCAATAACTCCATTGCGCCCCACAGGTATCCTCTGCATGCCCA
GTATGTAACCATCCTCAGTTGTCACCTATCTCCATAAATTTATCTAAATTAATCCACTTTTCTTCATAAAATCAACATAAATATGATCAGATTAATCGTTCATAATCAATATAAC
>comp160713_c0_seq2
AAGCCTTAGTGATTATTTTCTCATTACTTAAAAAAAAAAATTGTTCTATGAAGCTGCTTTCTGCAACAGCCTTAAAAATCACTTTATCTATAGCAGCTTTTCAAAATTATCCCAAATGAAGCA
GTAATCACAACAAAACTAATAACAAATAAAACCCAATATTTGAGCATATTACACATACATATTATATACATCTTAGAAATTACAAAAACATAGAGCTTAATATCTCTACATGTCAAAAGCCATT
ATATCTGCCTGAAAAATGCCATCATAGAATCATATACTACTTGCTTTGCAGAGACTCCCATTATGAAATCAGCATGAGCAAAGTTCTGTACATAATGAAGAACTAATTGATCAGTTTCATGCA
GCTTTAGATCCTGAAGCAAATGCTCCACATCTTCTGTATCAGACAGAGAGTCTCTCCCACCATAGCTTAGGTAAAGTGGCAGACTGTTGGGAATATTTGATAAATTGTATACAGGAGGTGT
GCTCTGACCATATCTTTGTATGTTATTAACACCATAGTTATATTTTGTTAGGGCTCCATCCCTGAATGCTGCAAATTCATCCAGAAAAAATTGAAATCTGTAAATTATATTTGAAAATTTGATG
AAAACTTGATATGAATTTAGCTTATAAATGATTAAATATTTGATATTTTTATATGGACGGTGAAAGTTCTATAAGAAGGTGGTGAGACCTGCAATGTTTTATGGCGTAGACTGTCGGCCCTCA
AAAGAAAAGCATAAAATTATGTCAAGTGTTGAAGAGAAGAGGATGCTCAGGTGGATTAGTGGCTTTACACTTAGAGACGGAATGCCAAACGAGTATATACGTGAAAATGTTGAAGTAGG
CTTAGATTTATGGTTTGGTCACACAAATTGTCAGCCTTCTGATGACTCAGTTAGTAAAGTGGATGTATTAGGTCTGATTTATATTAAGAAAGATAGAGGTAGACTTAAAAAGATTTGGTTAA
AGAATATTAGAAATGACTGTTCTTTATTAGTAAAAGAGAATTTGACTTGTAACAGAACACAATGGAGACCCAACGTAGTGGGATAGAGGCTTGTTGTTGTTGTTAGACCCTATTATAGGGT
AAGCTTGTTTATTTCATGTAGTTACTAAAAGGCTTATCGAAAATTTAAGTGAGCCGAAGGTCTCCCTCAATCACCCCCTTGTTTCACAATAGAATACGAGTGGGTTTACAAGTAACTCATCC
AAGAACATTCATGCGAATGATCAACATTTACATGTCTATGACATATCAAGCATTTGGGTTACTTGCAAGTTATTTCAAGTTTAAGTTTTTATATGATAGTTAAAACATCTGTGTTTTATTTGGA
TATGTTTTATGTTATAAATAGTTCAATGCCTTTTTAGTATTGTTTTTAATTGAATCGTTGAGGTTTGTTATTTGTGATCTATTAAACGTAAATGAACTGCCTGTGAGCTGCTCCTGATTTTCTGA
AATTATACCCTTGCTGGTCTGATTTAGTATATATTTCTGGGACTATAATCCATTCATTTGTTATACCGGTGCAGAGATCTTGGAGCAATTGCGGGAGTTGTATATGTTCTATTTTGCAACCATTG
CAATTTCGGTGACCCCAACGGCAGGATTTTATGGCT
>comp160713_c0_seq21
AAGCCTTAGTGATTATTTTCTCATTACTTAAAAAAAAAAATTGTTCTATGAAGCTGCTTTCTGCAACAGCCTTAAAAATCACTTTATCTATAGCAGCTTTTCAAAATTATCCCAAATGAAGCA
GTAATCACAACAAAACTAATAACAAATAAAACCCAATATTTGAGCATATTACACATACATATTATATACATCTTAGAAATTACAAAAACATAGAGCTTAATATCTCTACATGTCAAAAGCCATT
ATATCTGCCTGAAAAATGCCATCATAGAATCATATACTACTTGCTTTGCAGAGACTCCCATTATGAAATCAGCATGAGCAAAGTTCTGTACATAATGAAGAACTAATTGATCAGTTTCATGCA
GCTTTAGATCCTGAAGCAAATGCTCCACATCTTCTGTATCAGACAGAGAGTCTCTCCCACCATAGCTTAGGTAAAGTGGCAGACTGTTGGGAATATTTGATAAATTGTATACAGGAGGTGT
GCTCTGACCATATCTTTGTATGTTATTAACACCATAGTTATATTTTGTTAGGGCTCCATCCCTGAATGTTTGTGCTAAATGTACCATATTTTTGGTTGATGTTGGCTGTGGTTCATACTGGAGG
AATAATTGAACTGTTGAGCCATTAAGACAACAGTTGTTACCTGTAAATGCTGTCAGGAGATCATAGCAGTTCACATCTGGATAACTACAGAGTATTTTCAAATATTTGGATGCTTCCAGCCC
TTTGGGATTGAACTCTGCAAGTCCTAGCAAATTGCTCAAGATCTCACCCACAAAGAGTCTGGCAGCCAGAATGCCTAATTTTGTTTTCATGTGATCAAGGTAGGCTATTGGACACAAAAG
TGCTGCTGATCTTAGCTTGTCCACCAGCCTTCCTTCTGAGAAAGATGCCAAAGCAATGAGCGTTCCCTGCAATTATTTACAATCAGAAGCTTTGCACTATGTACAAAACTATAAATTTGGCT
AAGTAAAGCTGATGAAGACAAAGAAGAAGAAGAAGAAGTTTCTTACACACCAAGGAGTGCCCAACATAGTGCAGCTTCTGCTTTGTCTGTTTGGCAACCAAGTCTACCATAGCTGGGA
AATCATTCTCCACTAGTTCATCCCATGACCATGCCCAATAAGCCTATGAAGGATCAAATTAAGGCCAAAATTTTCATAAAAAATATTAACATATGCACATATTAAGACTTCGTTTGGGACAGTT
TTATTATTTTTCTTTCTTTTCGAATTGTAACTATAATTTCTATTTAGTTATTATTTTTCACCGTTTTTTTACTCACAATAGTTTAATTTATAATCAACTTTATAGAAAGCTGCAGTTATTCCAAACG

GATGTTAAAAGCATGCATACTTCAAATATTTTATTATTATTGCACACAATTTTCATATGTAATATTATTTCTTTTTTTGAAGAATAAAAATATGTCATTACTTAGTTTTATGTTGCTTATTATGGTG
TAGTTAATAGATATGTTGGAATGAGCACAGAGGGTTTGATATGAAAATATTGAAATGTTGCTGGACTTTAATCTTAATTATCCCAAAAGATTAATGAAGGATTAGAAGAGTTAAGTTAATCAT
GAGGGATTAGCCTAGTAATGGAAGTAGTTTAACTTTAAAGAGATCGTCATATATATATGTAAGGAAAAATGATTCAGGGTGGTAAATGAAAATTACACACCCGATCGGATGGCGTAAGAGA
CACGTGGCGATTGCTGGAGCGAGTGCCTCTAATATTACCGATCCACACATCAAATCCGTTATCGGCTAGCACAAAGGCCAGGGATTGCTCCGGAGAGTTCAATAGCCAGGTCATCCCATCC
ATAAGAACACCGTGTTGCAGCAATACTGGCTGCCTTGCCCCTCCAATAACTCCATTGCGCCCCACAGGTATCCTCTGCATGCCCAGTATGTAACCATCCTCAGTTGTCACCTATCTCCATAA
ATTTATCTAAATTAATCCACTTTTCTTCATAAAATCAACATAAATATGATCAGATTAATCGTTCATAATCAATATAACCTCAAACTCCTGGCATTTATACCCATATGGGCGCACCACAGCCTCG
CAGATTCCTACTTCTTCAGGCGAAGGAGAGAATCCCCCCTCGCCAAGGAAGAACAAGAAGAAGATGATGATGAAAGGAAGCTG
>comp160713_c0_seq35
AAGCCTTAGTGATTATTTTCTCATTACTTAAAAAAAAAAAATTGTTCTATGAAGCTGCTTTCTGCAACAGCCTTAAAAATCACTTTATCTATAGCAGCTTTTCAAAATTATCCCAAATGAAGCA
GTAATCACAACAAAACTAATAACAAATAAAACCCAATATTTGAGCATATTACACATACATATTATATACATCTTAGAAATTACAAAAACATAGAGCTTAATATCTCTACATGTCAAAAGCCATT
ATATCTGCCTGAAAAATGCCATCATAGAATCATATACTACTTGCTTTGCAGAGACTCCCATTATGAAATCAGCATGAGCAAAGTTCTGTACATAATGAAGAACTAATTGATCAGTTTCATGCA
GCTTTAGATCCTGAAGCAAATGCTCCACATCTTCTGTATCAGACAGAGAGTCTCTCCCACCATAGCTTAGGTAAAGTGGCAGACTGTTGGGAATATTTGATAAATTGTATACAGGAGGTGT
GCTCTGACCATATCTTTGTATGTTATTAACACCATAGTTATATTTTGTTAGGGCTCCATCCCTGAATGTTTGTGCTAAATGTACCATATTTTTGGTTGATGTTGGCTGTGGTTCATACTGGAGG
AATAATTGAACTGTTGAGCCATTAAGACAACAGTTGTTACCTGTAAATGCTGTCAGGAGATCATAGCAGTTCACATCTGGATAACTACAGAGTATTTTCAAATATTTGGATGCTTCCAGCCC
TTTGGGATTGAACTCTGCAAGTCCTAGCAAATTGCTCAAGATCTCACCCACAAAGAGTCTGGCAGCCAGAATGCCTAATTTTGTTTTCATGTGATCAAGGTAGGCTATTGGACACAAAAG
TGCTGCTGATCTTAGCTTGTCCACCAGCCTTCCTTCTGAGAAAGATGCCAAAGCAATGAGCGTTCCCAAGGAGTGCCCAACATAGTGCAGCTTCTGCTTTGTCTGTTTGGCAACCAAGTC
TACCATAGCTGGGAAATCATTCTCCACTAGTTCATCCCATGACCATGCCCAATAAGCCCGATCGGATGGCGTAAGAGACACGTGGCGATTGCTGGAGCGAGTGCCTCTAATATTACCGATC
CACACATCAAATCCGTTATCGGCTAGCACAAAGGCCAGGGATTGCTCCGGAGAGTTCAATAGCCAGGTCATCCCATCCATAAGAACACCGTGTTGCAGCAATACTGGCTGCCTTGCCCCT
CCAATAACTCCATTGCGCCCCACAGGTATCCTCTGCATGCCCAGTATGTAACCATCCTCAGTTGTCACCTATCTCCATAAATTTATCTAAATTAATCCACTTTTCTTCATAAAATCAACATAAA
TATGATCAGATTAATCGTTCATAATCAATATAACCTCAAACTCCTGGCATTTATACCCATATGGGCGCACCACAGCCTCGCAGATTCCTACTTCTTCAGGCGAAGGAGAGAATCCCCCCTCG
CCAAGGAAGAACAAGAAGAAGATGATGATGAAAGGAAGCTG
>comp160713_c0_seq40
AAGCCTTAGTGATTATTTTCTCATTACTTAAAAAAAAAAAATTGTTCTATGAAGCTGCTTTCTGCAACAGCCTTAAAAATCACTTTATCTATAGCAGCTTTTCAAAATTATCCCAAATGAAGCA
GTAATCACAACAAAACTAATAACAAATAAAACCCAATATTTGAGCATATTACACATACATATTATATACATCTTAGAAATTACAAAAACATAGAGCTTAATATCTCTACATGTCAAAAGCCATT
ATATCTGCCTGAAAAATGCCATCATAGAATCATATACTACTTGCTTTGCAGAGACTCCCATTATGAAATCAGCATGAGCAAAGTTCTGTACATAATGAAGAACTAATTGATCAGTTTCATGCA
GCTTTAGATCCTGAAGCAAATGCTCCACATCTTCTGTATCAGACAGAGAGTCTCTCCCACCATAGCTTAGGTAAAGTGGCAGACTGTTGGGAATATTTGATAAATTGTATACAGGAGGTGT
GCTCTGACCATATCTTTGTATGTTATTAACACCATAGTTATATTTTGTTAGGGCTCCATCCCTGAATGTTTGTGCTAAATGTACCATATTTTTGGTTGATGTTGGCTGTGGTTCATACTGGAGG
AATAATTGAACTGTTGAGCCATTAAGACAACAGTTGTTACCTGTAAATGCTGTCAGGAGATCATAGCAGTTCACATCTGGATAACTACAGAGTATTTTCAAATATTTGGATGCTTCCAGCCC
TTTGGGATTGAACTCTGCAAGTCCTAGCAAATTGCTCAAGATCTCACCCACAAAGAGTCTGGCAGCCAGAATGCCTAATTTTGTTTTCATGTGATCAAGGTAGGCTATTGGACACAAAAG
TGCTGCTGATCTTAGCTTGTCCACCAGCCTTCCTTCTGAGAAAGATGCCAAAGCAATGAGCGTTCCCTGCAATTATTTACAATCAGAAGCTTTGCACTATGTACAAAACTATAAATTTGGCT
AAGTAAAGCTGATGAAGACAAAGAAGAAGAAGAAGAAGTTTCTTACACACCAAGGAGTGCCCAACATAGTGCAGCTTCTGCTTTGTCTGTTTGGCAACCAAGTCTACCATAGCTGGGA

AATCATTCTCCACTAGTTCATCCCATGACCATGCCCAATAAGCCCGATCGGATGGCGTAAGAGACACGTGGCGATTGCTGGAGCGAGTGCCTCTAATATTACCGATCCACACATCAAATCC
GTTATCGGCTAGCACAAAGGCCAGGGATTGCTCCGGAGAGTTCAATAGCCAGGTCATCCCATCCATAAGAACACCGTGTTGCAGCAATACTGGCTGCCTTGCCCCTCCAATAACTCCATTG
CGCCCCACAGGTATCCTCTGCATGCCCAGTATGTAACCATCCTCAGTTGTCACCTATCTCCATAAATTTATCTAAATTAATCCACTTTTCTTCATAAAATCAACATAAATATGATCAGATTAAT
CGTTCATAATCAATATAACCTCAAACTCCTGGCATTTATACCCATATGGGCGCACCACAGCCTCGCAGATTCCTACTTCTTCAGGCGAAGGAGAGAATCCCCCCTCGCCAAGGAAGAACAA
GAAGAAGATGATGATGAAAGGAAGCTG
>comp160732_c0_seq14
ACAGTAAATGCTTGTCTTTTAGTCTTCCATCTATACGCTCTGTTGGCAGATCCCAACTTTTCTTCCCAATTGCTGCAACTTTTCCCTACTTTATCTCTATCTTTCTTCGCATCAGAAGCTCTTC
TTTCCCTTGTTGCTCTCTAAGTACTGCTACGGTTATTCTTTCTACTACCACTCTGATTTTGATTGCCAGCTCTCATAATAAAGTCCTTCGTCCCCACCATTCTACTTCTCAGAAAATCTTTCAG
AACGAATGGTGCCCAGCTGCGACCATTATCATCACTCGAAGGTGAACACTATTGTTGTTGAGGTGGTGAACTGTGATGAGGTGAGGCTGCTCGA
>comp160732_c0_seq8
CTTGCAGGGAAATTCTATCTTGGCCCCCCTGCGAGCCATTGAAGTTTTATTTATAGAAGTTTCAAACCAAATACAAAGTAGTTACACACTAAATATTCAATTACAAGCAATATACCCACTAA
TCATACAATTGCCTACACTAATATTGAAAATTTAGATTAAAATATCATCATACAAATTGTATCAGGATGAGTTTAAATACTGCTAAATTTATCTTGCAATTAGTGTAGTGCAAATTCTTCAAATA
TAACTCATCATGATACCATTTGTATTAAGATATTCTAACCAAGTTTTCTTCTACAATAATTGCTTAAATGAAAATTGCTTGTCTTTTATTCTTCTAGTTCACCTTGCTGCTGCTATTTTTCCTCC
ACTATTTCCATTTAATATTGATCTTTCATCACCTCAAACAGAAAAATTACCTAAGTACTGTTGCTGCTATTCTTTCTACTATCACTCTGATTTTGATTGCTCATAACTCTCATAATAGAGTCCTT
CGTCCCCACCGTTCTACAACTCAGAAAATCTCTCAAAACAAATGGTGCCCACCTGCGAGCTTTATTCTCACCACTTTCCTTACTTTTACCTTCAGTAACCTGCCTCCCATTCTCCACAGGA
ACTATTTCTTCACAGCTGAGTTTCTCTACTCTTTTCATCTCCTCCTTTGAAGAATCTATCTTGAAAACTGCAGTTTTCCATTGGGCTTCTTCAGAGCATGAATTCAATGTTTCTATTGGCGGC
TGTTCCTCTAATTCTGTAGGAGGCTTTAGAGAAGCAATTTTTTCTCCTTGCAATATTTCACCTGGCATTCTTAAAGTTGTAGGAGCAGCAAAGGACTTTGCTTCTTCCTTCAACTTTTTAAA
GGTTTTGATGGGAGAGAGGATAGATCCTTTTAATACATACTCACAGTAGCAGGTGGGCATGATGAGGTCATCGTCGGTGAGGTCCTGCCATATATAACCGGTTTTGTACTTCCTCTTGTATG
ACCAAGTAAAGGATTCCGGCATTTCTTTTCCCCTGAGCTCAGATAGCCATCTCTTAAAATCTCGAAGGTAAACACCATTGTTGCTGAGGTGGTGAACTGTGATGAGGTGAGGCTGCTCGA
>comp160772_c0_seq6
TAATTCCTCCGTTAACCTACGCGGCTTCCTAACGGCTAGTTCAAGTAGCCGTTACGAGCGTGGTCAACTAATTTTAAACTCCAGCCCCTGCCGTTCTTCTCTCCTCGCCACTCACTCCTCTC
ATCCACAACTGTCATTCCCTTCTTTCCTCCTCGTTATCAACCGTCCTCCCCACCTCCCCTCCCTCATTTCCAATCGAATCTCCATCTCCTTCCCCACCCCCTCGCCGTCTCCTCCATGGACGC
GGCTGAACTCAAACGCGTCTTCCAACTATTCGACCGCAACGGGGATGGCCGAATCACCAAAAAAGAACTCAGCGACTCTCTCCACAACCTCAGGATCCACATCCCGGACGACGAGCTC
CAGTCCATGATCGACAATATCGATGTCAATGGCGACGGTTGCATTGATATCGACGAGTTCGGGGTGCTCTATCAGAACATAATGGACGAGCAACGCGGCGACGAAGACGAAGAGGAAGA
CATGAGAGAGGCTTTCAATGTCTTCGATCAGAATGGCGATGGTTTCATCACTGTGGATGAGCTAAGGTCTGTTCTCTCCTCGCTTGGGTTGAAACAGGGGAGAACCGTGGAGGATTGCCG
GAAAATGATCAAGAAGGTTGATGTGGATGGAGATGGGAAGGTTAATTATAAGGAGTTTAAGCAGATGATGAAAGGAGGAGGCTTCGCCGCCTTGACGTGATCAGATTTGTATTGGCTTTG
GCATTGGTGAGGGCCTGAGAGGGGCGATGAGCTGATGCCCTAGGTTAGTGAAGAAAAAAAGAAAAAAAATGGTTTGTTTTGGATTTTTGTGAAGAATAACTGAATCTGGAAACTTGGAG
ATTTCACAAAAAAAAGGCCAGTTGACCCCTTACAGGCTTATTATACACATCATTTATTATAGGCAGTTGTGCAATGAAGGCTTCCATGTTCTCGCAGCCAAACATACAATGCAGATCGCTTG
CAGACTCGCATGTTCATGAATATTGTAATCATAAAGAAAGTTGAAATGTTTAGTATTTCTAAATAAAGAAGGTTGAGTATTGAGGTATTTTTTGTTCGTGAGATGAGGTATAAACCCAAGGC
TTTGAGGCGTAAGTCTTAAAATTAGAAATTTGCATAAAGTCAAAATGAGGATATCATTTGCTAGGACTTCTTTCATAAATATAATTTTGTTGATTTAGAATAATTCATTTTTTTCTG
>comp160776_c1_seq34

CGACGGGAGCTCGCTCGCTGCAAAGAAGAATGGGGATCAACTCCATAGAGCTTGCTGGCCCGCTGAAAGCTCTTCCTCCAGTGGACTTTTGTTGTGCTTATGGTTCCTCTCTCCTTTCCA
ACAACACAGACAAGTCTTCTATGGTGGACTATATCATAGGGGTAGCAGACCCAGAAAAATGGCATGCAGAAAATTTTGAAAGGAACAAGATCCATTACTCGAGGTGGATGTCAATTCTAG
GGCCAAAAATGATCACTTGTGTTGCTGATAAAATTGGGGTTGGGGTTCACTTCAATCCATTTGTGGAATGGAGAGACAAGATAATAAAATATGGTGTTGTGAGGATGCAGGATTTGGCACT
GGATATATTGACTTGGGATAGATTCTATCTTAGTGGTCGTTTGCAGAAACCTGTACACGTTCTTGTTGATAATTGGGACATAAGAAAGGTCAACTTAGTCAATTTGAAAGCTGCCACTTGTG
CTGCGCTTCTTCTTCTGCCTCCTGTTTTCACTGAGGAAGATCTTTATGTCAAAATTTGTAGCCTTTCTTACATGGGTGATCTACGCATGCAATTTGCAGAGGACAAAGATAAGGTTAAGAAG
ATTGTCCAAGGGAGTTTCAGAACATTCCAAGTAATGTACAAGCCTCTTTTAAAGGAGTATGCAGCTGAAGGGTTACTGGAATTCGGATATTATCCTCATTCCACATTTCAACAGGACTGCG
GAGTGTCTGCCACAACATCACTCTTCTCTTCTCTTCCTTCTGCAGTACATCGCAGGATGTTTTGGGGAAAGCACAGGCCTGTCATGACTGGTGAAATACCATCAGAATTTTTCATTACCTCT
AGAGAGCAGGCTTCTAGCTGTGTTCTCAAAGCACTGCGGCGTATAGTTCTGGTTTCAAGTGCGAGACAAGCAGTTTCTGGATTGCTTGCAACTGGAGGAGTTAATGCTGCTCGCTATCTC
ATGAGGAAGATGTCCAAAGCATGGAAATCCCGGACTGCATGATTCCTTCAGAAATGGAGCTGTTTATCAAGTGCACTTATTCGCGGATTGATCGACCAAGAAAGCATTTTGGTTGAAAGG
TTCTTCTGCGTATTTTAAGGGAGGGCAGGCGATCACCATTCAAAGAGAGTTCATCCAATCCAATTCGTAATTTTGATATACCGATGAAAGAGAACGTACACATTTTCCTATCAAATACTCAA
TATTTTTACCATAATCTTTTAAACCCCTCTCTATTTTAAGATGTAGAATAAGTGTATTGCTTAATATTAGCAAACCTCTAACAAAATTACAGATTCACTACAGGACCCACCTGCACGAAACTC
ACTTCTTGCCTATCCTTGATCATATTAATTATCCTGCTTAAGTTTTCGTTACTTTTAAGGTTTGACCGTCAGTTTATTTGTGCAATGATTAGTAATTGTTTCTGCATCTTTATGTTGTATCGATTG
CGTCCGCATTGTTCGCACATTTTCTCCATAAAGGATTGTTTATTTCTTCTTTCTATGGAGCGATCCATATGTTTTTATTGTATTATTTGAAATGGAATGATCTCTTTCATGATAAATTGGCACTT
TGTGTTCAGTTTAAAAAA
>comp160796_c0_seq18
AAATCTATAAGTTTAAAAGATCTATTTAAAACGGTATCCTCCAATTGAGGAACTACCGACAATATAATCATTAAAACAATTTTTGTCCAGTTAATTGCATCATTCCTTTCTTAGCCATGGCCG
CTTGATTTGGTCTTTCACATGTGATTATATTGCAAATTTATCACCTTGATCTTTGGTTTTGATTGTTTTGTACAATCGTCCAATCTTTGCAGAGACCTCTAATAACAGTAAAGCTTGCCCGTAG
GAGCAGTTTGAGGAAGAGTGGAGCTCCATCTAACACTAATTCATAATTTTCAGAGATGGCCTCTTCCCCTCAGACCGCACCACCATCACATACCAACTCCTCATCTTCAAATAACACACAA
ACCAATCCATCACCACCATCTCCAACCCCTCCGGCCGCCTCGGCACCACCGCCATCATCCTCACCACCACCACCAGCCCCTCCATCCCTAAGCCCTCCGACCACCTCAGCACCACCTCCA
TCATCACCAGGCAGATCTTCTCCACCCTCACCTCCCGTGCCTCCACCGTCACAACCCCCTCCAACGCCGCCATCATCGTCCAAGTCGCCGCCTTCTTCCGGCAATTCCGCCCCACCTCCCC
CGCCAAAACGGCCGGCGAAGCCCTCGCCTTCTACTCCAACCCCTACATCTGATGATAAAAATGCTAATTCAAGAAAATCATCAGGTGGATCTGATTTCAATTTGCCTCTGGTGGCTGGAGT
TGCTGCTGGCATTGCTCTATTTATTCTGATTATTGGCATTACAGCATGTTTCTGTTGTGGGAAGAAAAAGAAGAAGAGGAATCATAACGCAATGCACTACTACTCAGATTCTGATGTTAAAG
GAAGTGATTACTACAGCAGTGGAGCAGGCCCTCTTCCGAAATGGCATAAAGGAGGACCAAATGCTGCCATCAATATGCCAATTTCTCAAGGAGTGGCTTGGCATGCACCTCCTCCCCATT
CCATGAACAGTGAAGCGAGCATGGAATACTCAGGACCACATGGACCAGCCTTGCCTCCGCCCTCTCCCGCCCTTGCCCTCGGCTTCATTAAGAGCACATTCAGTTATGAACAATTGGCAT
CAGCAACAAATGGATTCTCTCATGAAAATCTAATAGGACAAGGTGGCTTTGGTTATGTGTACAAGGGAGTTTTGCCAAACGGGAAAGAAGTTGCTGTGAAACAGCTCAAGGCAGGTAGC
GGGCAAGGAGAGAGGGAGTTCCATGCAGAGGTTGAGATTATTAGCAGAGTTCACCATCGCCATCTGGTCTCTCTTGTTGGTTACTGCATTGCTGGCTCTCAACGATTGCTTGTTTATGACT
TTGTTCCTAACAAGACTCTTGAGTTCCACCTGCATGGTAAGGTTGCCTGCATTTTCATTCATGTCTACTTAGGGCATCTCCAACTGAACTCC
>comp160796_c0_seq2
AAGGTTTACTAATTCTTTTGTAGCTATTCAATATTAATTTCATCTACCTCTTTTAATATCACTAGCCTAATTATAACATCTTCTCATAATTAAAATGAAACTGAAGGATTGATGATACCATCTTAT
CATAAAACACTGATGAGTCTAATTTCTTTATATTTTAACCATCATTATCAAACTTCTGTTAAATTTGTTCTATTTTTAAGTTTTTGAATCTCCTTGTCGTATTCTTTCTGTGGTTGCTGAACTGT
ATTAAAGTTACAGAATGATGAAGTTCGTCTCATTGCAGGAAAGAACCAGCCAACAATGAACTGGCCTACCAGAATGAAGATTGCTTTAGGATCAGCGAAAGGCCTTGCCTACTTGCATGA

AGATTGTCATCCTCGCATCATCCATCGAGACATCAAAGCTTCAAATATTTTACTGGATTATAGATTTGAGGCGAAGGTTGCGGACTTCGGACTGGCTAAGCTATCTTCAGACAACTACACCC
ACATCTCCACTCGTGTCATGGGAACGTTTGGGTATCTAGCTCCTGAATATGCATCAAGTGGCAAGCTTTCTGAGAAGTCTGATGTCTTCTCATTCGGTGTGATGCTTCTGGAGCTTATAACA
GGAAAGAGGCCAGTTCACAGCAATCCTATGGAGGATAGCTTGGTTGATTGGGCAAGGCCTCTATTGACACAGGCATTAACTGATGAAAACTATGAGGAGCTGGAGGTGCTGGTTGATCC
AAGACTGGAGGGAAATTACAGCAAAATGGAGATGGCGCGGATGGTAACATGCGCTGCTACTAGTGTTCGCCATTCTGCTAAAATGCGTCCAAAGATGAGCCAGATTGTAAGAGCACTGG
AAGGTGACTCATCTCTTGAGGACTTAACTGAGGGCGTGAGGCCTGGGCAAAGCATGCAGTTTACTTCTAGTTCAGAGCATGAATCAGGCTCCTACGCCTCTAAGATGAGGCGTTTCAGAA
AGGCTGTATTTAACAGCAATGATTTGAGCCAAGAGTACAGTGGACAGACTAGTGACTATGGCCGCAATCCCTCATCATCAAGCAGTTCTGAAGAGATCCGCACAGACTGGACTAGAGAA
GTGAAAACATAATATAACAGCAAACAAAAATCACTTAATCTATCTACTGGTGTTCTGTTGGAGGGGTAGGGACTTGGTTTATGTGCTATACTTTATATATATATTCTGAGCAAGAATGGCTTT
TGTGATTCAAAAAGACTGGAGACTTCTGATATTCTCTTAGTTGCCATCAAGTTCATATTTAGAATTTTTCATGATCAAATTATTGTTAAGAGAAAGTCTCTCATTTTCCTTAAATTTAATGGTT
ATTATGAGCTCTTATTGAACTATTATGATGATGAGAATTTATGTTTTATGTTTAAAAGGGAGAGGGGAAGCTCCACCAAATGCACATACATCATATTCC
>comp160796_c0_seq7
TGCATATATTTTTTCTCAACGGAATTTTTTACTTCAAAAAAATTCGTTGGTGAACAATGGCATCACACTATTCACTTTGTTACATTACCATGTGAATTGGAATTTGGTTAAAGCTGTCGGTGG
ACTGCATACGCATGTGCTCCCTTGGAGAAGAAATTATTATGTGCAGAGGCCGGACACAGAAGGCTGTCTGGAGACCAATTAGCATGCGAGCCACAATACCGGTACCAAGCGAAGGGGTG
ACATGTCCCATTCTGATTAGTCTCCGGACAACGTTCAACGTCCGCGTCTGATGTTTGATTGATTTTTTAACCAGACATAGTAAATACAAAAAGGGTCAAATATTGCAGGAAGTGATTACTAC
AGCAGTGGAGCAGGCCCTCTTCCGAAATGGCATAAAGGAGGACCAAATGCTGCCATCAATATGCCAATTTCTCAAGGAGTGGCTTGGCATGCACCTCCTCCCCATTCCATGAACAGTGAA
GCGAGCATGGAATACTCAGGACCACATGGACCAGCCTTGCCTCCGCCCTCTCCCGCCCTTGCCCTCGGCTTCATTAAGAGCACATTCAGTTATGAACAATTGGCATCAGCAACAAATGGA
TTCTCTCATGAAAATCTAATAGGACAAGGTGGCTTTGGTTATGTGTACAAGGGAGTTTTGCCAAACGGGAAAGAAGTTGCTGTGAAACAGCTCAAGGCAGGTAGCGGGCAAGGAGAGA
GGGAGTTCCATGCAGAGGTTGAGATTATTAGCAGAGTTCACCATCGCCATCTGGTCTCTCTTGTTGGTTACTGCATTGCTGGCTCTCAACGATTGCTTGTTTATGACTTTGTTCCTAACAAG
ACTCTTGAGTTCCACCTCCATGGAAAGGACCAACCAACAATGGATTGGCCTACCAGAATGAGAATTGCTTTAGGATCAGCTAAAGGCCTTGCCTACTTGCATGAAGATTGTCATCCTCGCA
TCATCCATCGAGACATCAAAGCTTCAAATATTTTACTGGATTATAGATTTGAGGCGAAGGTTGCGGACTTCGGACTGGCTAAGCTATCTTCAGACAACTACACCCACATCTCCACTCGTGTC
ATGGGAACGTTTGGGTATCTAGCTCCTGAATATGCATCAAGTGGCAAGCTTTCTGAGAAGTCTGATGTCTTCTCATTCGGTGTGATGCTTCTGGAGCTTATAACAGGAAAGAGGCCAGTTC
ACAGCAATCCTATGGAGGATAGCTTGGTTGATTGGGCAAGGCCTCTATTGACACAGGCATTAACTGATGAAAACTATGAGGAGCTGGAGGTGCTGGTTGATCCAAGACTGGAGGGAAATT
ACAGCAAAATGGAGATGGCGCGGATGGTAACATGCGCTGCTACTAGTGTTCGCCATTCTGCTAAAATGCGTCCAAAGATGAGCCAGATTGTAAGAGCACTGGAAGGTGACTCATCTCTTG
AGGACTTAACTGAGGGCGTGAGGCCTGGGCAAAGCATGCAGTTTACTTCTAGTTCAGAGCATGAATCAGGCTCCTACGCCTCTAAGATGAGGCGTTTCAGAAAGGCTGTATTTAACAGCA
ATGATTTGAGCCAAGAGTACAGTGGACAGACTAGTGACTATGGCCGCAATCCCTCATCATCAAGCAGTTCTGAAGAGATCCGCACAGACTGGACTAGAGAAGTGAAAACATAATATAACA
GCAAACAAAAATCACTTAATCTATCTACTGGTGTTCTGTTGGAGGGGTAGGGACTTGGTTTATGTGCTATACTTTATATATATATTCTGAGCAAGAATGGCTTTTGTGATTCAAAAAGACTGG
AGACTTCTGATATTCTCTTAGTTGCCATCAAGTTCATATTTAGAATTTTTCATGATCAAATTATTGTTAAGAGAAAGTCTCTCATTTTCCTTAAATTTAATGGTTATTATGAGCTCTTATTGAAC
TATTATGATGATGAGAATTTATGTTTTATGTTTAAAAGGGAGAGGGGAAGCTCCACCAAATGCACATACATCATATTCC
>comp160796_c0_seq9
AAGGTTTACTAATTCTTTTGTAGCTATTCAATATTAATTTCATCTACCTCTTTTAATATCACTAGCCTAATTATAACATCTTCTCATAATTAAAATGAAACTGAAGGATTGATGATACCATCTTAT
CATAAAACACTGATGAGTCTAATTTCTTTATATTTTAACCATCATTATCAAACTTCTGTTAAATTTGTTCTATTTTTAAGTTTTTGAATCTCCTTGTCGTATTCTTTCTGTGGTTGCTGAACTGT
ATTAAAGTTACAGAATGATGAAGTTCGTCTCATTGCAGGAAAGAACCAGCCAACAATGAACTGGCCTACCAGAATGAAGATTGCTTTAGGATCAGCGAAAGGCCTTGCCTACTTGCATGA

AGATTGTCATCCTCGCATTATTCATCGCGACATCAAAGCTGCAAACATTCTTCTGGATGATAAATTCGAAGCTATGGTTGCAGACTTTGGACTGGCCAAGTTAACTTCGGACAACTACACC
CATGTCTCTACTCGAGTCATGGGCACATTTGGATATCTGGCTCCTGAATATGCATCCAGTGGCAAGCTTACGGAGAAATCGGATGTTTTCTCGTTTGGTGTAATGCTTCTGGAGCTGATTAC
TGGAAAGAAACCAGTAGACAGTGATGCAATGGAGGATAGCTTGGTTGACTGGGCAAGGCCTTTATTAGCAGAAACATTATCTGAGGAGAACTATGATGAACTAGCTGATCCAAGATTGGA
AGGAAATTTTAGCAATCTGGAGATGACACGGATGGTAGCATGTGCTGCTGCTAGTGTTCGGCACTCGGCGAAAAGACGCCCCAAGATGAGCCAGATTGCTCGAGCACTGGAAGGTGATT
CATCCCTGGATGACTTGAATGAGGGGGTCAAGCCAGGACAAAGTATGCTCTTCAGTTCGAGTTCAGACCATGACTCGGGCTCCTATGCCTCTAACATCAGTCGTTACAGAAAGGTTGCAT
TTGGAAGTAATGAAATCAGTCAAGAGTACAGCGTACGAACCAGTGACTATGGGGTTAACAATTCAACATCAAGCAGTTCTGGCGAAGTGAAGTCAGTCAGACCTCGTTCTAACAAAGGT
GGTCCAAAGCAGTGAAAATAAGCAAAAGAAAAATAATCAAGATCATGGTTTAGATTTTGACTGATTCTTATACATTAAAAACTTATAATTTATAACAGATTCAATTCTTTCAATGTGAAGG
>comp160804_c0_seq3
CCTCTCCAACTCACCCGCTATAAACCCGGCTCCCTGATCCTTCCTTCTTCAATTCAGCCCTCAAGAGCTGCTTCTGGTTCGGTGGTGAAAGAGCCAGGAGAGTGAGAAAGATATCGGAAG
ATAGAAAAAGGAAATCGGCCTAAGATATTGCTGATGGCGGTGGCCGCGATGGCTGCGAAGACTCCGGCGGCAAGCCCTGCCGCGCTGCCACTGTCGGACTTGGTGGCGAGAGACACGA
TCATTGCCTGGTACAGAGGCGAGTTTGCAGCTGCGAACGCAATTATTGATACGCTCTGCTCCCATATCGTCCAGATAACCGGCGTTCCTTCCGAGTATGAGGCCGTGTTCGCTGCGATCCA
CCGCCGGCGCTTGAACTGGATCCCCGTCCTTCATATGCAGCGATATTTCTCCATTGCAGACGTATCAAACGAACTCCGCCGCGTCGCCGGCAATCGCAAGCCCCTGAATTCCACCGCTGTC
AGCGAAGCTCAGAGTGATGCCGCTGTGACGCCGATTAATAGGGATGTGCCAGGCACTGGATCAGATGTCACCGCTGTTGAAGAGATCAGAAGGTCCCTCGACGAGGAGCGGTCTTCAG
GAAGAATATCGGAGGGCACCCGAGAGGAACATTCCACTGCTTATTCATCAGATTGTAATATTGCAACGGAAGGCAAATCTGTTGATGCCGAGTTTGCCGATGACGGAGGATCTCATCAAG
CGTTGACAGAAAATGATCAAATATGCACGCATCATGAGGAATGTGTGACGCGTCCTGAGAAGATCAAGTTTTTTAAGGGTTTTGTTGCAAAGGAGCCTGTGAAAGGGCATATGGTCAATG
TGGTGAAAGGGCTGAAGCTTTATGAAGACGTGTTCACAAACTTTGAACTACACAAGCTTACTGAATATATAAATGAACTCCGTCTTGCGGGCCGAAGGGGAGAACTATCAGGTGAAACAT
TTATTTTCTTCAATAAGCAGATGAAAGGCAACAAGAGAGAGATTATTCAACTTGGCGTTCCATTATTTCAACCAACAAATGAAGAGACAGCAAGCAGCATTGAACCTATTCCTCAAATTCT
GCTATCAGTCATAGATCATCTGACGCAGTGGCACTTAATACCAGAAATTAAAAGGCCTAATAGTTGTCTTATACATTTCTTCGATGAGGATGAGCACTCGCAACCGTACTTCAAACCTCCGC
ATTTAGAAAATCCCATCTCTACACTTTTTCTTTCTGAGACAACCATAGCTTTTGGCCGGGTGCTCTCCAGTGATCAAAATGGAGATTATAAAGGCTCCCTTACACTCGCACTCTCCGAAGG
GTCACTCCTGGTGATGCGTGGTAACAGTGCAGACATGGCAAGGCATGTCATATGTCCGTCTCCTAACAAAAGGGTAAGCGTTACCTTCCTGAAAGTCCGATCTGCTACAAACCAAATCGA
ACTACCATCTCCCGCCTCCCAGCAATCTAAAGCCATTACTCTATGGCAACCCCCACAACCAGTCGAAGTGCCTGAAACAGGGCCTATTGCTTATGGACCACAAGCCATGGTGCCACCATG
GGGGCTTTCTCTTCGAGCTCCTGTGTTCATGTTAGCGCCGACGGCGACGAGGCCAATGGTCATGAACCAAGGGAGGAGAGGTGGACGCGGCGGCACCGGAGTGTTCTTGCCGTGGAAT
GTGGGTCCAAAGAGGTACACCAGGCATCTTCCTCCTCGGTTTCAAAGAAGGAGACCACTAGAAGCACAAGTGGAGAAACAAAAGTAAGCCTCTTCTTTGCTGTTTTCTCAGTTTGCTAA
AAAGAAATACTGTAGAGATTAATATTCAGGAGGAAAAGGCTTGTTTTAGTAGTCTTTCTTGTTTCGACTGGTTACTGCATCGATTTTTGCTTAGTTTGGGTGATTTTTTTTTTTTTTTGGTTG
TGTT
>comp160824_c1_seq9
CGCCTCCTATAAGTTCCCTCCCTCCCTCCCTTCCCTCTCCACACCTCAAACCAAACCAAGCCAATTCTCAACAATGGCGCCATCACTCTCCCTCCTCTTCTCCTTCCTCCTCCTCGTTGCAG
CGGCCTCCGCCGATGACTACGGCTGGGAAAGCGCCCACGCAACCTTCTATGGCGGCGGTGATGCCTCCGGCACAATGGGAGGCGCCTGTGGTTATGGAAATCTGTACAGCCAGGGGTAT
GGCACAAGCACGGCGGCACTAAGCACCGCCCTATTCAACAACGGCATGAGCTGTGGATCTTGCTACGAAATGCGGTGCGATGATGATCCTAGATGGTGCCTAGCCGGCTCTATAATCGTC
ACGGCCACCAACTTTTGCCCGCCCAACAACGCTCTGCCCAACGACAACGGCGGGTGGTGCAACCCTCCCCTCCAGCATTTCGATCTCGCCGAGCCCGCCTTCCTCCAAATCGCTCAATAC
CGTGCTGGCATTGTCCCCGTTGCCTTCCGCAGAGTCTCCTGTGTGAGGAAAGGAGGGATTCGGTTCACAATCAACGGACACTCCTACTTCAATCTGGTGCTAATCACAAATGTGGCTGGC

GCTGGTGACGTGCACGCGGTGGCTATTAAAGGATCGAACACCGGATGGCAAACAATGTCGCGAAATTGGGGTCAGAATTGGCAGAGCAACTCGTTTCTCAACGGGCAGAGCCTGTCCTT
CCAAGTGACCACCAGCGACGGCCGCACCACTACTAGCTACGATGTTGCGCCCGCCGGATGGCAGTTCGGGCAGACCTTTGAGGGTAGCCAGTTCTGAACGTTACGGCGGAATAATTAAG
AGTTGTGAGCCAGATTTGGAGCTGTGGTTGCTTCATATTGTTTAGCACCCGCTGTGGCCTTTCTCATTATAGTTAGGTATAATAGTTAACTAGAAATATTTATTAAGAATTTGTGCAGGTTAA
ATGGGGTGTTATGAGCTTGATTTGTGTCAGAAAGGGGCCATGGGAAAGGAGTTTCTAGATTTGTCATGTTGTTAGAGTAGGCTCTGAGAAAGGAGATCTGGGCCTCACATGGGTGATATA
AATTAGTGGTTCTATTGATAAAGAAATTATTGGTTGTTAGTCTTTACGATGCTTCACTTGATTGTTTGCGGCGCTTTTTATTAATGTGGGTTGTTGACATCTTCACTCTTCTTGTGTAAGATCA
GGCTTCGCATGGATTTTAAGTTTTACATTATTTAACCTTTAAAAAACAAAATTACCTCAAAAAAAA
>comp160866_c0_seq1
AAAAGTATACAATGAGTATAATCTAGGAAGAAGCATAAAAAAGAAACCTCCCAAGATAAAACAAAATAAAAAAGTGAGAAGCATAGAACAAGCTAAAACTTCCCAAATATGCCTATACTT
CACAAAAAAAGGGACAAAAAATCATTGTTTTGTTGTCAAAGTGAGGACACACCAGAGCTCCTAGCAAGTATCAAACAAATAGGGTAAGGTCCACTCCAAAAGGGGGAATACCAAGAGA
ATTGAGGCGCCCCTTACAAGAAGACCATGTAGAGACCCCCAATATTGAACACCTTAACGAGAGTCGTCCCCGCCACCCTCCTTCCACTTCCTAGGATTATCAGTAATTGGCCCCCAAACGA
TGAAGATCCTTAGCAAAACGAGTCTTATAGACTCTATCATACCTTGGGTCAGATCTTCTTGGATGCATGAGGAAAGCCTCAGTGGAAAACTTGAAGGGTATGTTGGAGATATCACCATCCC
TAGATGAAACAACTTGCTTGGTTGGAACATTAACTTCCCCACTTGAGATTTGCAAGGAAGGGAGAAAAGACTTCTGATCTCCAATACCTACATTGGAATTCTTCTTGTTATGTCTCCTAGA
TTTTTTGTAACAAGACTCCAAGGCCCATAGGTTTCTGAAATTTTCCCTTCTTTTGCACTATGCTTGATAGTAGAAGGAGAGTGCGCAATTTCCAACCGGAGTGGATAGAGAGTTGGCTGGA
TCATAGAAGATGAATTTGGGTCATTAATCGCCATTGGAGGGCAACTATTAGTGACAAGAGGGTCACTAGAAGAGGGGTGCTGCTAATCTTGCACAAAACACATGGAGGATTCGATCTACA
TTGAGGTTGTTGTGTGGGCAGCTTGGAGGATTGAGGAACATTGGGACAATTACTTATGTTGTGTCGAACAAAGCCACATTGGAAGCAAAGGTGAGTGATATCCTTATATTCCGCCCTTTAA
AAAAACTTGCCATGGAGCCCAGAGATCCAAACTCCTGGAGGAAGCTTGGCACCAAATCCATCTGCACGCAAATACGAGCAAAAGCATTTCTCCCCTAGGAGTTTGTTTGAGCATCAACT
CATAGAGGTGGTCCAAAGAAAAAAGCTATCAGAACAAGATTGGTGTTGTCCCAATACATTAGAGGCAAGTGTGAAATAAAAGTATGAATAATCTCCAAGCTTGGGCAACCAAGAAAAAT
TTACCAAGCAAGATATAGGAAAAAGGAGTTCACCACATCAAAAATCTCAACATCAATAAAAATAAGAGAGGATTTACCTTGAAAGGCAATGGGTGGCTTATGTCTCACATAGTTCAATCTA
TGTTGAAAGAATGAAGCGAAATTGGCAAGTAGATCAATAACCCACAGTCTACCCATAAGGACAGCATCCAAAGCCTCAGCACTTTCAAAGAGGCAAATGAAAGAATCCAGGGAAATGG
AAAGGAGCTGAAAATCTTCAAATATTGACCATTCAAACTTTAATTCGTTGTTTAGCACAAAGTAAGGTAACCTTCACCCAATAAGCTTGCACACTAGCCATCTTTCAAGCCTAACTAAGTT
GGCATGGACGGACTCCATGTTCAAAGTCACAGAACTTCCATCATCTAACATAACTGCCGCCTTAGGAACATCATCAATTTTGATTACGTCCAACCTATTCCATACATAGGATAGGTCATGGG
CAGGTTTAGCACCATTTGGATGCTTGGAGGATTGTTGAAAATAAGTAGTTTGGCCATTCAAGCAAGCAGTTATGGTAGAGGCATTTGCACAAACACCTGATTTGTAATTTGCAGATTTACA
AATAACATGAATGGCTTCATCTAATGTATTTACATGCATGCGATCAATCTCAGAACAAACAGTAGAATCCATAGGATCATTCAAATACCTACAGTCCTTCGCAATTTCTGATCCACCATTGCT
CATATTGACTGCAAAACCAGCAGTCAGACTAGCATTGATTTGCAAGAAAACCAAAGCTGCAGTTTGAACCTTCCCTGATCCAGAAACGCCTGGTTCCATAGCAGAAGTCGGACTTGCCT
GGGCCTGATCGGAGCCGCTCGAGACGCTACTCGCCGGAGGTTGTGGTTCGATCTCCCCAGCCTGCAAGAACGAGATACATTCGAACTCGAATTCGCAGACGGAAAGGTCGAGGCAATG
ACGAACGAACGAAGAAGAATCCCGTTCCAAAGCGAACGCAAAACCGAACGGAACTGGCTCACCCGACACCAGCTCGTCACCATTAGCTAAACGATCGGACTCCATTGAAGGTACTTGA
AGCTGTTCATCCTCAAATGAGTTAAAAAACTGCTCGTGTTCAACAGACGAAGCATCAACAATGAAAACATCGACCTGCTCTGCCGCTAGATCAGCCTTCAAAACCTGGTCGTAAACATCC
GATTCCTTGTCTTCCAGAACCGACGACCACTTCTCCTCACCATACTTCTTCCGTTGATGCTTTCTTCATGGCGCTGAGCAGGGAGAAGAGCTCACAAAGAGAAGAAACTTGAACCTTAAT
TACTCAGTT
>comp160902_c0_seq7
GCGGTCACCATTAATGATAATTTTGATTATAATGTACTATAATAGGTGGTGCATCAAGACTAAGCTAGTGATGATCAACGAGCAATAACTGAAAATGAAAATTTGGAGAAGGATTATTTTCAT

TTGGTTGGATCCAACAAAAACATGCATTAATATTTGTACTTTTTTATGAGTCAGCTCATTTCCTTGAGTAATGGATTTTGAGATAGGGGTGTCAATTCAGGTCAATGGGTCGGGTTGTGGTC
GAACCCAACTTGACTTGACCATAAACGGGTTGACACTAACCCGACCCGTTTTTGCTAATGGGTCAAGATTGTCAACCCAGACCCGTCTCGTGCGGGTTATCAGGTTGACCCGACCCTAGC
CGCGATGGCTAGAAATCACTGGAGCCATCTATGGCAGGACGAATAGTGGCATCTTTACTATTGGACGGATGCACTCAGCAGCAGCAGTTCGGACAGTCGGACTCGACCAGATGTCGGCG
GCAGCTCGGACTCAACACAGATGTGATGGAAAACAAGACACATACACATATGCGGGTTATCGGGTTACGCGCTGAAACCTAACCTGACACGAGTGATCGATGCAATGACTCACGACGCT
AAATCAAGTGACACAACGACTCACGCTGACGCGTGACTGGCAATAAACGGGTCAACCCGGGTTGGTTTTGGGTTTTCGGGTCGACCCAATTTTACAAATGGGTTAGAATTACAGACCAG
AACCTGAT
>comp160916_c0_seq5
CGACTTTATTTCCAAAAAAATTTAAAATATTTCAAGAAAATTTAGGCACCCAATTTCTCAAATAACATCCCATTTTAGCCAAAACTAAAAATTTTTCACTACAATTAGCAGAATTCCAGACG
ATAAGTTCATGAATAAAATGAAAAATAATTTAAAAGAATAAAAATACTAGCAAATTCAATTTCTAGAACAGCATAGGTCATACCATCACGCAACTGGGCTTGTTGTTCCATAGTTTGTAAGC
GCAGCCTAAGTTCTGCATTCTCTGCAGCCAAACCGGTTGTGTCTCTCTGGAATAAAGTAAGTTGCGCAGATAGTGTTGTAGCTTCAGTCTGAAGAGTTTGAACTCTCCTCTCAAGATCAG
AAATATAGCGTGCCTTTCTTTCTTTAGAGCGAGCCGCAGACTGTCGATTAGCTAAAATCCTTCTTGCCCTCTTTGGATCAATGGCCGTGAGCTCCGCTAGCTGCTCCGCCGTCATGGCCTTC
TTCGCCTCCAGAATCTCCCCAAACACTACCTCGCCCTTCCTGGCCGCCATCGATGAAACTGACGAACCGTCCAACGAGTTGCTGTGCCGATGCTTCGCCCTCGAGGTCTCCGCAGACTCG
CCATCTCCTGCATTGCCCGTCCTCGGCTCCTGGTCCCCACCTGAGCTCTCCGCCACCCGATCTTGACCCACGCCGTCCTCCGACCCCGATCCGCTTCCCTCCAACTTGCATCCGATCTTCTC
AATGTCGATATAAGTAGAAAAAAGGTCTTCCTCGGACCCGATCTCTTCGAAGCTACCCGTCTCCATAGGATCCCCGGAGCTCAGGCCGAGGTCTTCAGGGATCCGCAGAAAAAACTCGGA
TCTAGCACGTCTATGGAGCGGGCTGCAATTTGGAACCAACGAAGTGCCCGTAGCTCCAGTAGCCACCGCCGGTGGCAACACCGTCGACAATGCATTGCTCCCCACCTGCCGGGAGAAAC
TGGGGTTTAGGCCAGTGTTAGTGCTCAAGGTTTGAAATGGATCTATGGGATTCGTTTGCATTTCTTAACCTCACCCGTCGTTTCATAAGACAGAGAGCGAAGTCGGCGGGGAGGGACGGA
AGGATCCCAACGGAACGAAGAAGCTGAAAGTCCTCCTTTTATCCTGGTTCTTCCTGCGAGCCAGAACCGCACCGTCGCATGTTGACGCCTATACAGGGAGAAACAGAATCCGATTGCGA
CGTTTGAGCCTTAAACGCGCGCAGATCTAGCGGATTGATTTCTTTAATAACGTAACTTCT
>comp160945_c0_seq3
AAATTTAAGTAAATTTTAGCTATAAATTTAATTAAAAAAAGGTATGATTTATTCATTGAAATCAATAGTTATAATGAGGTAAAAAATAGAATAAAATAAAAGAATTATAATGAGGTTGAGGGG
AAAATTTATTATCATTATATAATTACAAGAATAATGTACTGTATATTTATCAATAGCATTTTCTCAACTAAAATTCCAACGGACATAAGAAGATAATAAAGTTAAATATTAAAGCCAAAAATTA
AAAAAGTACTCGTTTGAATCAAACGGCCCAAAGCTAATTTCACCAGAAATCCCACAACCCTTACCCAAACGACCGGGAGCACTCTCTCTATCTCACTCTCGAGAGAGTTAGCTTTCAACT
ATACACGCAGTATATAATTTTATAACTGCAAACTTCTATTGGACCGGATAGACTTCGGGCCCGATGACCAATCAGCCATTGAACCAGATCAATCATCCAAAGTACCTTAATACCCCCACTTG
GGTTACAAGCTCACGTTACCGAGCTTAGCGACTATTCAACTCAAATGAGAAGTCTATATCCTATAATATTTTCAAATAATTCAGCGATGTG
>comp160945_c1_seq3
TCTACAGCAAGTAAGCGATAAAAGATATTTTATTATGCTAAGTTTCCACCTTTTCCTGCATATTAATCCCACGTCGCTGAATTACTTTTAAATATTATAGACTATAGACTTCTCATTAAAAGTC
GTTGAGCTCGGTAACGTGAGCTTGTAACCCAAGTGGGGGTATGAAGGTAATTAGGATGATTGATCTGGTTTAATGGCTGATTGGTCATCGGGCCCGAAGTCTATCCGGTCCAATAGAAGTT
TGCAGTTATACAATTATATACTACGTATATAGTTGAAAGTTAACCTCTCGAGAGTGAGATAGAGAGAGCGCTCCCGGTCGTTTGAGTAAGGGTTGTGGGATTTCTGGTGAAATTAGCTTTGG
GCCGTTTGATTCAAACGAGTACCTTTTTTATTTTTGGCCTTAATATTTAACTTTATTTT
>comp160999_c0_seq15
ATATTATAAATGCTAAGGTGGTGGCATGATATTATCTTACAAATTAAGATGTGTTTCAGCTTAATAATTGCAAATAGATGAAGCAAAACCCCAATTCAAAGTAGATGCATCTTCAGTTGCGTT

ACAGATTTATTGCAGAAATCTTTAATAAGAATACACAGACTAAAAAACATTTAAGGTGGTGGTAAGTAGTGAGCATCATGTGGAACCTGATATGGAGGATACATCACGTTGGTAAGTGCAG
AAGTCTCCATTGCTGATCCAATTGTTTTTGTCTCAGGATCATAAGTTGACAGCCAGTTTATCAATGGATCAAACTCAGGGGTAGACAAATTGATGATCAAATTTTTCACCAATATTAAAACA
GTTTTTGCAGTGATTGGATCCATCTCCTTAAGCTTTTGCAAATACATTCCCATTGTGCTATTGATATCAGGGTCAATCAAAGTACCAATGAAGTTGTAAACCTTAGAAAACTCTGGAATGGC
AGCATGAAATGATTGTCGACTCCCTTGCTCTGGTGCTTCAGAAGTAACCTGTGATGTGCTTGAACTGCCAGTCCAACTAACATTGTGGTTATAGATATTTGTCAAGCCCAGTGTTCCATTAT
CACATTCAACTCCACAGAGGGCATTGTAGTAATTTGAAGAGGACATTGCATCATTGGAGGTGTAATCCCTCAGGAAAGATGGATCATCCCAACTGGAAGACGAAACAATAAGACCAGTG
GATGCAGATGGGCAGATCATAGATGACTGCAGCTGCAGAGTTGTTGGATCTGCAGAAGAAAATTGTTGGTCGGTCTCCATATATTATCATTTTAATATAACTTGTAAGAAGTCCTAATCAAA
ATAAAGGAAAGAGAAAACCATCATCAGGTTTCTTTGGATATGGATGAGTAGAATTTCTTTTTGGACGGGCCGGGGGTACATGTGCCATTATCCCATTCTTCTGAACCTTCAAGAAGTATTTT
TGAGCATGACTGCGGATCTGTATCACTGTCTTTGTGCCAACAAAATCTTCAATTTTCTTCCATTCTCGACCAAACAAAAGTAGTGCGTCGAGGAAGCGCTCATGCTCCTCATCCGACCACC
TATCTCTGGACTTGGTAATGGTATAGGGCTTCCGCAGCTTCTTGATCGTTCCATCTGCGTCCATAGATAAGAGGTGTGGTGAGATCCAAGTCAAGAAGAAAACAGAAGCATATCATCTGCT
TGGAACAAAAGTAAAGGTGAAACGGAGAACAAGAATGGAAAGGAGGGAGA
>comp161031_c0_seq3
CAGGCGTCTAAATATCTAAATAAACATATAATCCATAAAAGGTACATAGCTATCAAACAAGTGACAAAAGGAATTAGCAAACAATTAATAAAAGTTTACAAACTAATCTAACCAATCAAAG
AGATTACCTTAAGAACATAGTTATAAAAAATTGAGAATAACATAGAAGTCCTTTACCATGACTACTAATGACTTTCGAGATGAATTAGCAGAAAACCCTTCACGAAATACTGAGTAGGTATC
TACTACACTTGAACAGTGCTGAAGCCAAAGACAATAAGCTTAAATACATAGTACTGCAGTCATGAAGCAGTTACAATATCAATATCAGTAGCACGCACATATCTCATGAACATCACTGAAG
TTCTTCATATTCTAATGAAACTGCTAAAAAGCTAATATTAAAATTCATTTAATTAAGAGGAAAGTAGTAGGCTAATAGTGTTACTTATTTCCCTGCAAGTTTGGCTGGATGGGCTGCACACGA
AAGGTCAATGGCATCTGAGCTGCATACTCCCTATCTTTCTGATGATAGCCGATGTCCAGTTCTCTCATGCTCCCTTCCATTGCTAGTTGCTGATGATGCTGTGAAATTGAAAACAGCGATTT
CCAGAAATAAGAGGCAAAATGAAACTTACAAGAACTATCTAGGAAAGATGAGCTCAATTGCAATATGAACAACAAATTAAAAATTAAAAAAGAAATATAAGTCTTGTGGCAGGATTGTTT
CAAGGTTTAAAACACAATTAATTATTGAGGCAATTTTTGTTCGAGCCTCAAAATTAAAAAATTATATAAAGTGAAAATAAAAGTTAAAAGTTATTTTTATATAACAGCCAAATAATATGTTAA
ATGATGGTATATTAACTTTCTATGGAACTATGAAGTA
>comp161059_c0_seq12
GGTACATTGGAGCTTAATGATCGGAAACTGAACGCGACCGTCTGCTTACAAGCTTTTGAGTGGCAGCGCCACATACAGTCAGAACAAAAGAAAATCATCGGATCGATTAGTTGTGTATTC
TTGGGACTGTTTTGCGGGAATTAGAAACCCGCTCATATTGAGCGGGTTACAAATTTGCTCGACGAAAATTCCTTGGCGCGGTCGTTACTATTCGGCTTCATTGCTTCGCACGTGAAGACCC
CTACCAATTGCTCAAGTAAAGGATCTCACTTATGGCAGCTATAGTTTCATATAAAAAGGCACAATTATTTTATCATTGTAAATATTATTAACATCTGATTCATCTTTTTTTTTCTTATATAGATAT
ATTAACATTTAACTAA
>comp161068_c1_seq2
TCATTGTCCATATCCAAAAGAACTAAGGCAGCCAAAATCATAAAAAAAAACCCAAAAATAAGGCCCAATAAATTATTTCTTTCTCGACTTTCGTTTACGATTTCCGACAATATTATCCAACGA
ACCAATTTCCAAATCACCATCAAAAGTATCCTCGCCTTCAGACAGCAAATCATCGTGTAACCAATCACATTGCCACCGGGTATTGTTCCTAGCTAGATGGGCTTGTAAGTTAGCGTTTAAAA
TAACCGAAATAGGCACTTCCACCAGCAAATACACACCAACCCCTCAAGGTATGATCGCGGCAGTGAGTGGGCGTCTTGGAGGTCCGGCGGGAACTGAAGAAGTTGAAGGATCTATCACC
TTCTGCACAACAGCTTTGTCACTGGAGTTAGCAGATGGAACATGTGAGGGCGGCCACGGGAGCGGGAGGGCGGCGGTGACGTCGTCTAGGCCGGCGAAGAAGGGATCATTAAGAAGCT
CGGCGGCGGAGGGGCGGGAGCGGGGAGTGCCGAGGCAGCGTTCAATGAATGTCTGCACTTCGGGGTCACGGACCTTGCTGAGGGAGGCGGGACGAACGCCGGCGGTGACCTTGCGGT
AGATTTTAGGTACAGAGTCGCACTCGCTGTAGGGGATTTCTTTCGTCATCATCTCAAGGACGCACATGCCGAAGGAGTAAATATCTACTTGCTCCGTGTAGTCCTCCTCGTAGAGCTCCGG

CGCCATGAACTCCGGCGTTCCGAGGATCGAGTGGGCTAAGTGGCTCTGGCCAACGATGGCGGCAAGACCCAGGTCGCCGATCTTCACCTTGCCGGTGTTCCCATTCATGAATACATTGCT
GCAGTTGAGATCGCGGTGAATGATGCACGGATCGTGCTTATGGAGATAATCCAGACCAACGAGGATCTGGCGACTCCACTTTTTCATGGCCTTCAGAGAGACGTGACAATGCTTTCTTCG
GTATTCTCTCAGATTACCTGAGGTGCATACCTCTGTTATAAAGTTCAGGGTGCTGGTCTCGTCGTCGGTCCACACGTTATACAGCACAATAATGTTCTCGTGACGCAGAGACCGGAGAAGA
CGAACCTCCGAGAAAAGTCTCTTAAGCATCTGCGAGTCCTCTTTGAAGCTCTGCAGCCTCACTTGGTTCCAGGCTACCTCAATACCCTCTTCCTGGTCGAACCCTCTGTAAACCTTCTTGG
TGGCGCCGATACCGAGGAGATCTGCATATCTCCCATACCTTCCCGTTGGATCCATCTCCACAAACGGCTCTGCATCCTTGTCCGACGGATCCGGCCTTACGCACGGCATCCTCGCTCTAAG
CGCAACCAAATCCAAAACACTTGCTAAATCTCCGGCACCCGGCAATCAGGAGAGCCGCAGTCGGAGAAACCCTGCAGCGATCAAATAAACACGGAGGCGGCACAGATCACAAGGAAAT
ATGAAGTTCTTGATTTCCTCAGGTGGCGATTACTACTTGCCATCGGAAACCTAAAAGGAACGTAAAATCAATGGAAACCCTAGGGTTTCTGCCTTTGATTCGAAAAATACCGAGAAGGAT
CGAAAAGAGAAAAAGACGAGAGAAAAAGAATCAAATCAACTAGCGGAAGATGCTTGAGCTATTCCCTCTTCCGAATCCAAGGAAGAAGAACATCAAGCGGCTGCGAAAACTTTGCGG
AGAAAGCTTGCGAAGAATTCTGGATAGAAAAAGAAGGTTGTGATAGCCTAGCTGTATTTATAGGAAGGAATAACTTT
>comp161130_c0_seq3
TTTTTAATCCATCTAAAACAAAATCTTAATTATAATGAAGCGCAATATACACCAGTCATATTACATATTCTGTATTTAAATACTATGAAAGTAGAGTCTCAAGAAGTCACAACATCTTTAATCT
TCATCAATTAACATAATATTATATAACAACCGATTTATAAGAAATTTCCTATCTTGAACAGCACAGGAAATTTCCACAGCTCATACCTGTACTTCTCTGTCGAAAATCCAACTGAAACGCACA
GTACTGAAGCCCTGTTTAGCCCGTCCTTGTGCTCTCTCCTCTAGCTTCCTGTACCTGGGCGCCAACCTACACACAAAATCCTGCGCCTTCCGCCCCTCATCAGATAGACCCACGAGGCTCT
CCACCTTCCACCTCCCAACCAAAAACTCCGTAATATCTGCGTAGTCCTTAGCCGTGTAAACTCCCAGGCGCTGAGCAACCGCGCTGAAATGGTGGAAGAGGCGGTGGTCGCGGCCGTCG
AACATGAGGTGAGCCGGCATGGTGATCTTGTTCTTCATCATATCGGCGAAGGCGATGACGGCTCCATCGGGGTCGAGCTCGAAGAGCTTGTCGACGATCTTCGAATAGGCCGCCTCGTGC
CGCTTCTCGTCTGCGGCGATGGTGCCGCAGATCTTAGCTAGTTTGAGGTCGCCGTAAAGCTTGGCGTGGCGGGCCGTGTTGCCGTGTGAGATGTAAGTCGCGCGCTCTTGGAAGGACGT
GTATATGTACCCTTTGTATGGACTGTTCTCTGTGTTTATGTACATCCCACAACCAATTAGATACTGTATCGTCTTCTCGATCTGCCTCATATCCACTCTTCCACAAAGATAAAGATACTGCTTA
AGCAAGTCCCCATGCCTGTTCTCTTCTGCAGTCCATCCTCTCGTCCAAATAGCCCAGGATGTGAGGCTTGCCCCTGTTTCATCCCTCACACCGTCAAAGGTGTTCAGCATTGTCTGGTAAG
TTGGAAGAGCTTCCTCTGTGATCAAGTCCCCAACTAAGCATACGTAGTAGTCATCAGGGATCTCTTTAGCCCTCTTCCTCAGTTCCTCCACTTGCTCATAGAACTCACTCGATGCAGGAGA
AGGTAAGAAGTCCTGAGGTTGCCATGAGCTCTCCACTGGTTTCAGATGAGTCAGTATGTTCTGCTCTGCCCACTTATCTAAGGACTTGAATATCTCCATCTTTTCTGATGGCATGGAATGAA
TTATTTGATTTTCTACTTCATGTGGAGGTCTGAAAGGCTTCTTATGAGTGCTTACTTCGGTATAAGTTGTGGAGGTCATGGAAACCTTTGGGGAACTTCCAGTAGCTTTGGTGGAGTTAGCA
GATAAGCGTAGCGTTTTTTGGAAATAGTTCCGTGGAGAAGAGAGACTAGACAAAGCCATTTCTCGAGAGAGAAAGAGGGGAGAGAAACCCTAAACGAGCAGAGAGAGGATGGAGGCC
GCGGGTTTGCTTCATGTTCGGAACTCTCTGCCCCTCTTTATACTGTAGGAGACTAAGGCAGTAACCTGTTGTTAGAATCGCCCACCGAGTCCTGTTCAACGCTTAAGTTTTATTTAATCTAA
GCAGCTGGGTGATGTAAT
>comp161132_c0_seq13
CGAAATATCCAACAGAAATATTTCCGGTGAATATATACTACGTCGAAAATATAAAAAAAATGAAGAAAAAAAAAAAAAACCGTATGCAATGAACATGTTCTTCATCAAATTTTGTGAAACT
GTATGTGTACATTATGAGATACTACACTGCAAAAAAAAATTACACAAACTCAAGGCATGTACGCACTTAATGTGGTGGACCAATACAAATTTTTCGAGTAAAAGCGGGCGACGTTTTCATT
ATGCTTGAACTTTGCTTGACTGTTAAGATTGCTCCCAAAATTGATTGGTTCACCCATAAAAATTGGTCCAAATAAATTTTTATGTGTGCACAGATTCCTGGTGAATATTTTGATATGATCTGT
TTGAAGTGCAGCACTGAAAGCAGGAAGCAACTCTAAACGCAAAGAAGTTTGAATTCGTTGAAGCTTCCAATCGTGAACTGATGGGCGAAAACCTGGAGTCCCTCTCATTGGAAGATCT
GAATAAGTTAGAAGGTCAGCTTGAACAAGACCTAGGCAACATTAGAGGAATAAAGATTCGCTTGCTATCAGGGCAGGTAACACACCTTAAAGAAAAGACGAGGGTGTTGTCAGAGGAA
AATGAATTGCTACAGAATCAATGCAAGGAGACCCAGCTAATCTTAGATTCCACCCGGTTGGTGGCTCTCGAAGACAACAATAATCAGCACAAGGATGTAGAAACCGACCTGTACATTGGA

TGTCCGGGAAAAAAAAAGATCTAATAATGCCAGAGAGGGTGACCATCTACAGTCTCAAATATTCAATAACAAAGGTTTTCTTCTTAAAATAACTCCACCAATTCTCTATAATAATTGGATGT
CCGGACAGAATAATAGATGATGTAGTTATGTTTACATAATCTTCTATTCATCGAAGTTGCATAAAGCTGGTTTATTTTGTTTAGCCTGAATCGTCTACGAACTTAACTTGACGATAAACGTTA
AAGGCATTTTATACATTGTACTTTGCTTTCTGCTGGCACAGTCTAGACAGAATTGAGTTTCTGGAGATGATAATTAATGTATATATACAGAGAACTGGTCTTGTTGTGTGTGCTTCAGATATG
AAAAGAAATGGTCTTTGCCTCTGATAAAAAAA
>comp161132_c1_seq5
GCTGGGTCTCCTTGCATTGATTCTGTAGCAATTCATTTTCCTCTGACAACACCCTCGTCTTCTCTTCAAGTTGTGTTACCTGCCGGGATAGCAACCGAGACTTTATTCCTCTAATATTGACTA
GGCCTTGCTCAAACTGACACTCGAACTTGTTCAGATCTTCCAATGAGCAGGATTCCAGGTTTTCGCCCATCAGCTTACGATTGGAAGCTTCAAGGAAGTCAATCTTCATCGCATTTAGAGC
TGCTTCCTGCTTCCAACGCTGAATATTTTGCTCAGTTGCACTGCTAGTGCTGCTCAAGTCTACCAGGTTCATTTTATATCGCTCGATGACATTTTGCATGCTAAGACATTTTATAGAAAAATG
AAAAAAATTAGATTATACTCTGCCTGTTGCATAAATAATCAGCACGCGCACACCCTCTTTACAGTGAATTGACAAGCACAGTGTCTCTTTAGAGATGTGAGTGCGTGCGCCAGTTGGGGA
GTGAATTTAATCAGAAGGCCAAAAAAATTTAATAATATTGAAAGATATTTAATCAGAAATATAAGACACTTGTATAATAACTGCAAAGCAATTATTTTGCAGGAAAAAATGACACTCAAAGC
TCATTATTAGTCCCATGAAAAA
>comp161170_c0_seq13
ATGATTAAAAAAATTAAAAAATTTAAGCTCAAAATTGTTCTACTGTAATACAGATTATAAAACTTGCACAAAAAAGATAAGCTTCCTATCATTAAAAAATTTCACTGCTCCGGGAGAGAAAT
ATATACATAAAAGCTGCTTCTTTGATTAACCAATATCAACTAATTGAGATAAAGATCAAATTGATTTCTCTCCTCTGCTTCGGTCGATCAATCTCTTCAAAAAGAAGCCAAATTTTGTGATGA
ATTCGGAGAAACTAAGCTCGAGATATAGCACTCCTGCGTTGAAACATCGACGCCGGCGTTGATCAAAAAGGCCCAAACGAATTCTGCAAATCTTCCAGGTTCAGATCCGAGTACCAATCG
TATTCCTCCTTTATCGCCGGCGCAGCTGGCGGAACTTGCGCCGCGCCTGAGCTGCGATTCATATCCACTGCTTCGTGACCCAAATCATCCAAATCCGGCAACTCGATGTCAGATTCCGGCG
TCCTGAAACTGTCCTCCGTCAAGTCCGTCTCCTCCTTCGGTATCCTCTCCCACCCTTTCTTCTTATTATACAATCGGCACAGCACCCAATCATCCAACTGCAACAAAAGTTCCATCTTTGAT
CAGTTCTTTACCGTTTTCATTTCCCAATTTATAAAAAAATAATAATAAAGCAAAACTTTGGATGATTGATTATTACCCGCAAGCTCCCATTTCTTCTCGCGCTGCGATTGGCATCAGCGAGGC
GATACTCATGCATGATCCAATCCGTCTTTGCGCCTTTCGGCGGCTTCCCGTCGTAGAAAACCAGGGCTTTCTTTATACCAATCGGCCGGGAATATCCCGCCGGCGTCACCGGCTTGTCCGC
CCCTGTTGCTTTCCAGTACCCTCGCCCGGCGGCGCGGTTCGGCCGTGATCCGTTGGGGTACTTCCGATCTCGCGGCGTGAAGAAGTACCATTCCCGCGTTCCGAACAACGCCTTATCTGC
TCCCAAAAAATCCAGTTCAGCGAGGAAAATGATATAACCATAATAAAGCGAATGAAGACAAGCTGTTCGACGAAAGTACCAGGTAAGTCCCAAGGGTTATATTTGTACAAATCGAGCTCG
GCGATGATGGGAACGGGGAGAAGCTGATGAGAAGCTCTCCGGCAGAGGTAGTGGACCAGAAGCTCTTCGTCGGTGGGGTGGAACCGGAATCCCGGCGGCAAGTTGAGCTCCGCCTCC
GCGTCTCCGATCCTTCTTCCCATATCTCTTTCTATCTATTCCTCGCTCTCCGCCGGGTTCTTTGGCGAGGTATAAATACTTGGATATTTCGGCGCGGACTAAGCGTCGATTGCGTCGGTCGAC
ACGTGGCGGGTGGGTGCTGGCCGGTTTGGAATCCT
>comp161261_c1_seq1
ATTTTATAATGCAACACTCTCTATCTAAGAAATAAAATGATTGCATTATGATATAACTTCAAAAATTTATGGGCTATGATTTATCACTAAATCCATTATTTTTAGAATCTTTCACATTTATCAATA
TGAAATAAATAAACATTCGAGTACTATTATTTATAACAACATGACCTCAAATTTAGGCTTCATTTGGTATGGCTGCTTCACTGTATCTTCTTAAAGCAGCTTTTTAGAAAGCTGTTTTAAGAA
GCAG
>comp161264_c2_seq1
TTTGAAAAAAGAAGCTTAAAATAATATGATTAACAATAAGCATAGGTACACTCAATAAGAAAAAAAGACTAAACTATTGTGCACATTCAAACAAGTTTCTAAACACAATAAATAGACATTT
TTTTTAAAAAAAGGAGACAACCTATATAAGAGTTTATGCTACTACACAAAGATTCACAAAAGCACGCTCGTAAATTAAAAAAATACATATTGAGGAAAATTTACCAAGTATTCAATCCCAT

TCAATTCTTCTCACCTTCATCATCACCATCATCATCACCATCACCATTGTCATCCTCAGCATCTTCATGATGACCATCACCATGGTCATTCTCAGCATCTTCATCATCACCATCACCATCTCCA
TGGTCATCCTCAGCATCTTCAGCATCTGTCATATTATTAATCACTTCTTCAA
>comp161275_c0_seq1
CTGGGTCATTCTCGGGCTTTTGGGTCAACCCGAACCTTACCTAATTTTTGAAATGGGTCAGTTTGTTGAACCCGAACCCGAACCTGGCGGTTTGGGTTCTCGTGCACGATTTGAGAAATC
GTTAGTGTGGGTATGGGTAGGGAGGTGCACGACTTCCAGTATGGGAGATTTCTTTTGTATAAAATAAGTCAACAATTATCTTTGTGGCCCTCATCACCTCCATTATGTTCATCAGTATCCTCT
ACTATTGTAACTTCACCGATTTCCTCAAAATGTATATAGCACTTTGAGATTTAACCTAACTCGATGAAAAGATCTGATACATCACCAGACGTAATCAATGAAATTTTGCTATGATACAACAAT
GATTCAGATAAAGTTATTCCTTAAAATAAACTCTATTGATTTATGATATGCTATACGTGGGAAACAATTGTATTCAAATTCTGCCCAACATTAATTTTTGGAACGTCATGTTTTTTTCTCATGA
ATAAAGTTGTAAGAAATGCAAACTTTATGAATCCAAAAAACTTTCACAAGAAAATACTTTTAAAAGTTGTTACATAGCAGCAACATTCTATGACTTGGCTTCATTATACCTACCATCTATTTC
TCAGGCCATTGATATCTCCTTCCAATATATTCCGCAGACTTTTTCCTTGGCTTCCTTATCAACTTTGCAACATGTGGGGGTCATCCATGGTATCCTGTATGGAAACTTGATTGTTCATTGGCGA
TTTATATGTGTTCTTTAGTGTACAGTACTGCATATTTTCTTCTCCAAGAGATCTTAGCCTATTGAGCTCTGGTAGTACCTCTGTGGCAAGGTCTGGCCGGTCCTTCCATAGGAGCGCTGCACA
CTTCAGTGCTATCTCAGCAAATTTCATGGTTTCCTCAATTGGCCAGTCTGTAACTGTTGCATCCAGTAACTCCAGGAAGGTCTCGTTCTCAATTGCACCACTAACATGGTTTGCTAACCCCA
TAGCTGGTTTTGCAGTGATGATCTGGAGCAGAATGATTCCAAGAGAGTAGATGTCAGATTTGACTCCCAACATGCCTGTCTGCTGGTATTCGGGATCTATATAGCAGAAAGTTCCTGCAGC
AGAAGTCATCCGGTATTGTGTGACCCTGTTAGCAACTGAAGGAGGAATCAGGCGAGCAAGCCCTACATCACCAATTTTGCTCACATAGTTTTGATCAAGGAGGATGTTGCTCGGCTTCAA
GTCTCGGTGTACCAATGGCTCAGGCTTTGTTTGATGCAAGAAAAGTAGGCCTGTGGCAATCTCTGCAGCAATGCGGAATCGGTGTTGCCAGGGG
>comp161275_c0_seq3
CTGGGTCATTCTCGGGCTTTTGGGTCAACCCGAACCTTACCTAATTTTTGAAATGGGTCAGTTTGTTGAACCCGAACCCGAACCTGGCGGTTTGGGTTCTCGTGCACGATTTGAGAAATC
GTTAGTGTGGGTATGGGTAGGGAGGTGCACGACTTCCAGTATGGGAGATTTCTTTTGTATAAAATAAGTCAACAATTATCTTTGTGGCCCTCATCACCTCCATTATGTTCATCAGTATCCTCT
ACTATTGTAACTTCACCGATTTCCTCAAAATGTATATGCACTTCTATGCCTTCTTTGTCCTAACCTCTATGGATGGCTTGATCCTCCTTTCTTTACTTTGACATTTCTCTTCCTCTAGATGCGCT
ATTGAAGATCCTCCTCTTACTCAATTTTTTTGTCATTGGTGTCCTCTCCATTTTCACGCATCGCCATCATTATGCACTAATCCTACATTATCTCCCTTGACATCGTTGTCATTATTTGGCTCGCC
AAGCTCTAAAAGTGGACTGCCTAGACCCTTCTTGCTCTCATTGACTACAATCTTGGCAATCCTATGTTTCACCACTTATAGGCTCAAGAAGATGGTATATTGTCAACTGAATCTCTCCTATC
GACTAGATCAATTATGAGCTTTTTTTTTTCAAATGCAAGTTGAGAGCTGCTGCCTCAAGTTTGCAGGGGAATATTACCATATCTATATTATCACATGTTCTAAATATTTATAGAAGGAATATGC
AATTGTTACTATTATAATATATTTTCATTATTAAGGATATTCTTATTTATTGTGTTTCTTTATATTTCTATATTCTCTTGTTTATATATACACATACTTGCACTATCATAATCAATCAAGTAAAAGATA
TAAACCCTATGATGTTTTCTATCACGCTGAAGGAATGCTACTACTTCCATCCATGGCATTCACCCAATCAGTTCACACAAGGTAGAAGATTTGATCTTATCCAATCCATTTAGAGATGATTTA
TCTAGTCCAGGCCTCATATGAACTCGCATGATTTTGATGTCATTGGAATTGTTATGGTGAGAAGATTGTTTTAACTTTAAGTTTGCACCATTGGAGTATGTTTGATCTCAAAAATAAATTTTTT
AAGTAGTAGTTTAAATTTCTTTTAGCACTTTGAGATTTAACCTAACTCGATGAAAAGATCTGATACATCACCAGACGTAATCAATGAAATTTTGCTATGATACAACAATGATTCAGATAAAG
TTATTCCTTAAAATAAACTCTATTGATTTATGATATGCTATACGTGGGAAACAATTGTATTCAAATTCTGCCCAACATTAATTTTTGGAACGTCATGTTTTTTTCTCATGAATAAAGTTGTAAG
AAATGCAAACTTTATGAATCCAAAAAACTTTCACAAGAAAATACTTTTAAAAGTTGTTACATAGCAGCAACATTCTATGACTTGGCTTCATTATACCTACCATCTATTTCTCAGGCCATTGAT
ATCTCCTTCCAATATATTCCGCAGACTTTTTCCTTGGCTTCCTTATCAACTTTGCAACATGTGGGGGTCATCCATGGTATCCTGTATGGAAACTTGATTGTTCATTGGCGATTTATATGTGTTC
TTTAGTGTACAGTACTGCATATTTTCTTCTCCAAGAGATCTTAGCCTATTGAGCTCTGGTAGTACCTCTGTGGCAAGGTCTGGCCGGTCCTTCCATAGGAGCGCTGCACACTTCAGTGCTAT
CTCAGCAAATTTCATGGTTTCCTCAATTGGCCAGTCTGTAACTGTTGCATCCAGTAACTCCAGGAAGGTCTCGTTCTCAATTGCACCACTAACATGGTTTGCTAACCCCATAGCTGGTTTTG
CAGTGATGATCTGGAGCAGAATGATTCCAAGAGAGTAGATGTCAGATTTGACTCCCAACATGCCTGTCTGCTGGTATTCGGGATCTATATAGCAGAAAGTTCCTGCAGCAGAAGTCATCCG

GTATTGTGTGACCCTGTTAGCAACTGAAGGAGGAATCAGGCGAGCAAGCCCTACATCACCAATTTTGCTCACATAGTTTTGATCAAGGAGGATGTTGCTCGGCTTCAAGTCTCGGTGTAC
CAATGGCTCAGGCTTTGTTTGATGCAAGAAAAGTAGGCCTGTGGCAATCTCTGCAGCAATGCGGAATCGGTGTTGCCAGGGG
>comp161297_c0_seq1
AAAAATTCTCAGCGCTCTTAATTCCACATAACTTTTATGTTCAGTAGGTCGCTATTGTTGACAGACAAACATATATAGAAAAAAAATGAAAATCTATGAAGTTTTAAGAGGAATTCTCTCTG
CCTTCTGGCACCAATTTATTTGGATTGTAGCTGTACCCTAGAGATCCTTTTGAACAAAAGATGATGACAACAACAGTATTCTTATAGTTTAACTACACTAAAACTTACCAATTCTTACAAATC
CTTTAGATTCACACAACAAGCAGCTTGGAAAGCAAGCCCTCCACTGTTAATTTTCACCTTCAACTTCTCCAGTATAAAATATGCCAAATCAGCTTACAATGTAACTGGACCACTAGCACCA
GCACATCTCTGCTCATCATAAAGAAAACCCAGACTTCCTTTGGCTGTGATGCTTGTGCAATGGTGGGGTAATGCATGAGAATATGTAAGGCTTCTTTCGTCTCTGCCCGAATAAGAAAGTT
CTGAGTGGAACCTTAGACCTTGTAGAACAGTGTCTGAAGTTTTGGTATTGTTGATACCCATTTGAAACTGAGAGATCGAAGCTCTCCCTCAGCTTGGAGAGAGACTTGGCAACCTGAGAC
TGAAGAGCCTTCACATGGTCCAGACCGGTTTGCAACTCCATTGCAACTTTGTTGTTCTCTTGTTGCATGTTGAGCAGTTCACCCGAACTTTGCTGCTTGGTATGGTGTGATCTTGGAATGC
TCTCCCTCGGTATCGATTGAGGCTTTGGAAATCTCTTCCTGGATGTTGCAGAGGGATGTAAACCGGTATTGAAGCTCTCCCTTCAGCAAGTCGTTTTGTTCTAACCAAACTTGGAGCTCTG
TATGGAGCTCCCTTAGCTTCTTATCGAGTGGCCAGGTGTCCTGTTTTGATGAATGCTTTGGGGATGCAGGAGCTGATCCACCTTCCTTGGGATCCTTCGTCTTATCCAGGCCAGCAGCTTCC
AACTCCTTGAATGATGCTGCAAACTTCTGTATTTGATGATATGAGGTGCTGAATCTTAGCCAGAAGTTGAGGTTCTCATCTAGAAGAGTGTCTAGTTCAGCTCTGAACTTTTCCTCAATCGG
TGAAGTTGAGTGCTGCTGATCTAAATGGCATAATTTGATCTGCTCTTCAGCTTCAATGATCTCGATAGGAGATTCTGGTTTTACTGCGAGCATTTCAATGGAGCTTCCATGCTCTTCTATGTT
CTGTATATGAGTTTCTTTATAAGTGCTTGTTGCAACCTTGGAAGAAGTGTGATAGAGCAATTTACTCCTCAAAGCTCTGATCTCTTGGTCCTTGACAGCATTCACACTCTTCAGCTCCTGAA
TCTGAGCCATCATCTCAAAATGGTTCTCTTGGTTCTTCTTCTCCATCTCGGCTAGTTTTTTCTTTGTGTCTTTGTAGTTGTGGAGAATTGATGAATATCCACACAGCAGCAATTTCTCTCTGC
CTTCTGGCCCACTATTCAATAGATGCTGCAAGTCTGGAATATTATCATTGTCATCAGGCTCCAAATCCTTCAATAAAGATGAAAACATTGCACTGAGATGTTCTCCCTTTACCATCGATGAAC
TATTAAACAACTCAGTCTGATCCTTTGTATCTTCAGCGTTGGGCTCAATCTGCTGCTTCACAAGAACCTCATTCTCCTCAGCTGTTGCATCACTTCTCTCATTTTTGACATCCTCTTTTATTTC
TTTGTCCTGCTCCAAGTTTTCAATATTTGAAGAAGCTAAACTTCTATCCTTCTCCTTAGGGCTGGTAGATTGAAGTTTTTCTGATATATCATTAAGACTATTGCAGGCCTCTATAAAATGAGTT
TGAAAGATCCCTTTCTCATGATGCACGATTTTCTCAAGATCCTGAACTCCATGCAACTCATCTTCTGCCTGCCTCAGCCTCACATTTGAGCTGTTTGAGTCAATTATCAAATTAACTTTTTCT
TCTTCCAGGCATCGGAGGTGCTTCTCAAGCATGTCAGTCTCTGACCTTAGTGTGTGTATTTGCGCTGTTTGCGATGAGACTGCTACCTCCAAACTAATGACTTTCTCCACAAGTTCATCAAT
TTTCTCAGCAAGGTTCACAACTGAAGTGTCAGAGTCCATCTGGAAGTGCTGTTTCACCCGTGCACATACAGAGAACAGGTCTAGCCGCTCATTTTTTAGGATAAAATCTTCTCCCTCTTCA
TTACTTCTTTCTTGGTTAAAGGTTTCCTCTATTTCTGGCGAGCATTTTTCACCCTTGAGAAACTTCAGCCTGTCTTTGACTTCCCTGATTCTTTTATATTCTATCTCTGCCTCTTCACCTGATTT
CTTCTGTTGCTTCTGTAAGCTGACTAGGCTTTCTTCGCATGATTTGATTGCTGTGGCTGCCATCAAGGCCCTGGCTTCATTATCTTCAATGATAGAACTTGCACTGTACTCATCCTGAAAGGT
GCAGACCTCGTCTTGTAAATCAGATATCTTGCTCTCAATCTCCCAGAACTTTGCAAGGCTACTTTCATAGGAACTCTTGACAAATTCTTTCTCAGTCTGGAGAACAAGGATTTCTTTCTGA
AGCTTGTCTATTTCATTTTGCGCATTCTCCTTGGTGATATGAGAACAAACTTTCTTATGGTGCTTCTTTAAGGCAGTCAACGCATCTCTGCTTTTGTTTCTGAAATTGATCTGTGGCACCTCC
CCGGGTAGGAGCTTGCGGAACTTACTGATATCTACGCCTGTGAAAGCCGTTGGGGAACCATCTTCATCGTCATCTTGCATTGCAAACTGGACTTGATCAGGGAAAGCTGACGCAATGGTG
TGGTTGGCCTTATGCAGTTCTCCAGATATGTGATCATATCGGTCGGCCAAAGCTTTATAAGCCTTGTAAGTCTCTTCAACAAATTTTATTAGTTCTGGTCTTCTCTGGTAGTACATCTCTGCCT
TTTTAGCGAAGGAGTCAGCATCCACTTCTATAAGCATGATCATGGCTTTCACCTTCTCCTCCATTTCTTGGAGGTTGCTATCAAGCCACTTAGATTGCTTGGTCCGGATGTTACTCGCCCAC
CACCAAGAATAAGCGTTGCTTGCTGCTCGCTGCAGCATTTCTCCCTCCCCTCTCTCTATCTCTCTTTCTCTCTTCTCCAGCTCTGCAGACTTCAATGTGGCGCAAAGCACGAAGGATCGTAT
GGAAAAATTCGAATGAGGAGAAGGGAGAGAAGGAGAAGAGGAAGAAGATAAAATAGAGAGGAATAGGTTTGCTTGTCTGGGCAAGGAAAATGTGAGGCATGGAGGGAGACAAACAG
ATGTCCTCGGCTTCTCCGGGACGGAAGGGGGAACCTGAGGCGGAGCGGTTGATGC

>comp161305_c0_seq1

GTGAAGAAGGGAGAAGCCAATAAGCGGTGGATCGATAGCAATGGCGGCCGAAAACCAGCCCGGCTCCAACAACAACAACAAAGGCAAAAAGAGAAAATTTTTGCCTCACGGTAAAC
CCGTTAAGAAGGGGTTGTATCCGCTTCGCCCAGGAGTGCAGGGTTTCTTCATCACTTGTGATGGCGACAGGGAGCGACAAGCCACAAACGAAGCCTTGAATCTGCTTCAATCGCGGACT
CCTGCTACTATCCGGGATCAGTGCCGTCAGGTTTTATTCAGTATTCAATATTCGGAATCTGTGCTGTGCCGGTTATTCTGGAATCAGAAATTTTTTATTTTGAGTTTATCTATTTTACTCTGCA
AATGCCTCCCGCTTCTTCTCAGCGATCGTTGACAACCCCAAGTATTTCGGTGGCCTCAACAGATCGAGATAAGCCACACAGGCCTGGGCCTTCTTTTGAGTCAGCTCCCAATTGGGAGAA
GAGATTCTGCTCTCAAATGAAATGGAGAGACTTCTGTGAAAAGAAGAGAGTGGTTCATCTTTTCCCGAAAGTGCTAGATTGGGATGACGCGGCGGGAAAAGAGGCCTTCGACAATGCTA
AAGCTCGTTATTGGGCACAAATTAATAATCTCCCTTCGGAAATTCCATCGCCGGATCCAGATATGTACGTCCAAGAGGTTGATTATGACGCTTATATTGACCCTTCGCTGCTAGAAGATTTGT
ACGAACTGCCGCCGCCTCCTCCAGAGACTCCAGACCCGAGTTCCATTAAAATTAGTGAAATTATACCAACTGGATGGGATGTGGAGGATCCTCCAATCAACAACATTCCAAAATATATTTT
TATCAAACCATCCACAGCTGATGACATAGTTGATAATTCTGCTCCAACTATACCTACAGGATGTGCTGATGGTGACAAAATTGACATTACTGCTGCTTCAATTACCATCATTCCTACAGGTTG
GGGTGATGAGGAAAATGTTGAGCCCAACAATGCTGGAAACAAGAATGTGGAAAATGTTCTTGGAAATACAGAGAATCATAGTACTTACTGGCAACAAGATAAGAGAAACGATACTATGT
TTTCTAGTGAAAAACTCAGTTCCTTTGAACAAAGGATCGACTCCCGAGATCGAAGATATCGAAGGAGTAGCAGAGATCAGTTCTTCGATTTAAATCCTGCAAATCTAAGGTGTCATGAGC
AAGGTTATCATAAGGAGAGATGGACAAAGTACATCAAAGAGCAGTATTTTTGGCGTCAAAAACACTCTTTGGCTAGAGGGCTCTCTTGTACACCTGAGAATAATTAGCTAAACACAGACG
AGACCTGAAATTCTTTAGAAAAAAGAAAACTTTGGAGGTTCTTTACTTGATAAACTTCTTTGTTAGCAGGAATAGGTATAAGAACTTGCAAGGAAGGCAAAGTTCAGTTTTCACAGATTT
CTTTTATTACTAACCTTCTTATGAGCTAGTATGAGCTTGGTAACATTTGCATAGGCTTTTCGTGGGATAACGGTATTTTGCCGTAGAAATTCTTTGTAATGTTTGATATTTTGTGCTTGGCTAAT
TTGTTTCTGTTCTTTCAAGTGTATGACATTCTATTATTTGTATTAATTTTTGTTTGCTGACATAATGTG

>comp161305_c0_seq5

GTGAAGAAGGGAGAAGCCAATAAGCGGTGGATCGATAGCAATGGCGGCCGAAAACCAGCCCGGCTCCAACAACAACAACAAAGGCAAAAAGAGAAAATTTTTGCCTCACGGTAAAC
CCGTTAAGAAGGGGTTGTATCCGCTTCGCCCAGGAGTGCAGGGTTTCTTCATCACTTGTGATGGCGACAGGGAGCGACAAGCCACAAACGAAGCCTTGAATCTGCTTCAATCGCGGACT
CCTGCTACTATCCGGGATCAGTGCCGTCAGGTTTTATTCAGTATTCAATATTCGGAATCTGTGCTGTGCCGGTTATTCTGGAATCAGAAATTTTTTATTTTGAGTTTATCTATTTTACTCTGCA
AATGCCTCCCGCTTCTTCTCAGCGATCGTTGACAACCCCAAGTATTTCGGTGGCCTCAACAGATCGAGATAAGCCACACAGGCCTGCGTTGGAGGTTATTGGAACATGGGCAATAGGCGG
TGGAAGAAAAGGGCGCCGGGTTGGCAGCGAAGGGACGCAGAGTTCCACACAATGCTTCGGATGCTCGATCGACAGTCGGCGTCTCCTGGGCCTTCTTTTGAGTCAGCTCCCAATTGGG
AGAAGAGATTCTGCTCTCAAATGAAATGGAGAGACTTCTGTGAAAAGAAGAGAGTGGTTCATCTTTTCCCGAAAGTGCTAGATTGGGATGACGCGGCGGGAAAAGAGGCCTTCGACAA
TGCTAAAGCTCGTTATTGGGCACAAATTAATAATCTCCCTTCGGAAATTCCATCGCCGGATCCAGATATGTACGTCCAAGAGGTTGATTATGACGCTTATATTGACCCTTCGCTGCTAGAAGA
TTTGTACGAACTGCCGCCGCCTCCTCCAGAGACTCCAGACCCGAGTTCCATTAAAATTAGTGAAATTATACCAACTGGATGGGATGTGGAGGATCCTCCAATCAACAACATTCCAAAATAT
ATTTTTATCAAACCATCCACAGCTGATGACATAGTTGATAATTCTGCTCCAACTATACCTACAGGATGTGCTGATGGTGACAAAATTGACATTACTGCTGCTTCAATTACCATCATTCCTACA
GGTTGGGGTGATGAGGAAAATGTTGAGCCCAACAATGCTGGAAACAAGAATGTGGAAAATGTTCTTGGAAATACAGAGAATCATAGTACTTACTGGCAACAAGATAAGAGAAACGATAC
TATGTTTTCTAGTGAAAAACTCAGTTCCTTTGAACAAAGGATCGACTCCCGAGATCGAAGATATCGAAGGAGTAGCAGAGATCAGTTCTTCGATTTAAATCCTGCAAATCTAAGGTGTCAT
GAGCAAGGTTATCATAAGGAGAGATGGACAAAGTACATCAAAGAGCAGTATTTTTGGCGTCAAAAACACTCTTTGGCTAGAGGGCTCTCTTGTACACCTGAGAATAATTAGCTAAACACA
GACGAGACCTGAAATTCTTTAGAAAAAAGAAAACTTTGGAGGTTCTTTACTTGATAAACTTCTTTGTTAGCAGGAATAGGTATAAGAACTTGCAAGGAAGGCAAAGTTCAGTTTTCACAG
ATTTCTTTTATTACTAACCTTCTTATGAGCTAGTATGAGCTTGGTAACATTTGCATAGGCTTTTCGTGGGATAACGGTATTTTGCCGTAGAAATTCTTTGTAATGTTTGATATTTTGTGCTTGGC
TAATTTGTTTCTGTTCTTTCAAGTGTATGACATTCTATTATTTGTATTAATTTTTGTTTGCTGACATAATGTG

>comp161305_c1_seq4
TTTTTGCCAGCAAACAAAAATTAATACAAATAATAGAATGTCATACACTTGAAAGAACAGAAACAAATTAGTCATGCACAAAATATCAAACATTACAAAGAATTTCTACGGCAAAATACCG
TTATCCCACGAAAAGCCTATGCAAATGTTACCAAGCTCATACTAGCTCATAAGAAGGTTAGTAATAAAAGAAATCTGTGAAAACTGAACTTTGCCTTCCTTGCAAGTTCTTATACCTATTCC
TGCTAACAAAGAAGTTTATCAAGTAAAGAACCTCCAAAGTTTTCTTTTTTCTAAAGAATTTCAGGTCTCGTCTGTGTTTAGCTAATTATTCTCAGGTGTACAAGAGAGCCCTCTAGCCAAA
GAGTGTTTTTGACGCCAAAAATACTGCTCTTTGATGTACTTTGTCCATCTCTCCTTATGATAACCTTGCTCATGACACCTTAGATTTGCAGGATTTAAATCGAAGAACTGATCTCTGCTACTC
CTTCGATATCTTCGATCTCGGGAGTCGATCCTTTGTTCAAAGGAACTGAGTTTTTCACTAGAAAACATAGTATCGTTTCTCTTATCTTGTTGCCAGTAAGTACTATGATTCTCTGTATTTCCA
AGAACATTTTCCACATTCTTGTTTCCAGCATTGTTGGGCTCAACATTTTCCTCATCACCCCAACCTGTAGGAATGATGGTAATTGAAGCAGCAGTAATGTCAATTTTGTCACCATCAGCACA
TCCTGTAGGTATAGTTGGAGCAGAATTATCAACTATGTCATCAGCTGTGGATGGTTTGATAAAAATATATTTTGGAATGTTGTTGATTGGAGGATCCTCCACATCCCATCCAGTTGGTATAAT
TTCACTAATTTTAATGGAACTCGGGTCTGGAGTCTCTGGAGGAGGCGGCGGCAGTTCGTACAAATCTTCTAGCAGCGAAGGGTCAATATAAGCGTCATAATCAACCTCTTGGACGTACATA
TCTGGATCCGGCGATGGAATTTCCGAAGGGAGATTATTAATTTGTGCCCAATAACGAGCTTTAGCATTGTCGAAGGCCTCTTTTCCCGCCGCGTCATCCCAATCTAGCACTTTCGGGAAAA
GATGAACCACTCTCTTCTTTTCACAGAAGTCTCTCCATTTCATTTGAGAGCAGAATCTCTTCTCCCAATTGGGAGCTGACTCAAAAGAAGGCCCAGGAGACGCCGACTGTCGATCGAGCA
TCCGAAGCATTGTGTGGAACTCTGCGTCCCTTCGCTGCCAACCCGGCGCCCTTTTCTTCCACCGCCTATTGCCCATGTTCCAATAACCTCCAACGCTGCGCAGAACAAACAGATTGAAAG
GAAAAAAAATTACAGAAATTACTCCTTCCTTCCGCTTTAAGTACACAGAACGGAAGAGAAGTCGGCGAAGAAAACGTCGGACCAGAGGATTGTGTCTTTGCATGCGAGAGGAGGAAAG
GATTTCGCGATGATGCTCTAAATCAAGCGGCGGCCACGATAATTTATAGGTGAGGAGGAATATTGGATTTCCTTTAATATTTACTGAATATTCGAAATCTGAACAAAAATAAAGATAGATTCG
CTTTTATTCTATTTTCTGTTATTAATTTGCTTTAAATAGAATATAATTAATTTTGCTTAAATAACTTCTATTCAAT
>comp161322_c1_seq2
AATGTGATCGTCATGAATCTACTACCACAATTGAGGAGCATTTCGAAGATAAAAGAGCCGGGGGAAGAGTTTACCCAAAGACGAGAGCTTACGAAGCTTCCCGTTTGATCATCAAGGTAC
TGGATGAAGAACAACTAACAGTGCCAAAGCTGGGAGACATTCAGGAAGGTGATCCTTGACTTCTTGGACAGCAGGGCAGCAAGAAGATAGCCACTAAATCGACTGGACAACAACTGGG
GATGCGTAGGCAGCAACAATTGTTGGTGGAAAACCCCTTTTGGTCTTCCAAATACTCCCAACAGCAGTTTGACAGCAAATTAACACAAGGGGACAGCAGTTTGTCCTGTCAAGTGAGGC
TTATGCAGCCATCCGGGCTTACTGGAGAAGGCAGAACAGTACTCTTAGGCTGCTGTAATTCACGGCCACATCCAGGAATTATATTGACATAAAATCAGATTTCTGGAAATGAAGGGAGGA
GTTGGGTGGAGATCTCAAGCCAATTCCAGGGAAGTACTCGGGTAGGAGAAAGGACTTGAGGAAAATTTCAGTTTTTCTGAAACTGAATTTTAGTGAGGAGAAATGGGGGAAGCTGATTT
GAAAATCCAATTAATTTCCAAAAGGAAGGGCCAACGGTTAGTGGGGGATTAGGGGTTTAATGGGAGGCAATTAATGTAATTGAAAAGAGAATTAATGGGCAAGGGTGGCCGGCCATAGA
GCAAGGAGAGAGAGGCGTGGAATTTAAAAGGAGGGGCCCACGTTTGAATAATTAATTCCTAATCAAATCTGAAATTATAATTTTAGATTTGATTGAGAGAATTATTTATCCAAACGTGGGC
CCCACTTTCTAAATTCCAAAGCCTATCTCCTTACTATATGGCCGGCCACCCTTGTCCCTTAATTCTCTCTCTAATTGCCACCTAATCATCTCCTGATATTTCTTTTATTCCTCTTTCCCTCTAAA
CCGAATTTGGATTTCAAAACCTGAACTTGAAATTCAAATCCGTTATTTTCCTCCTATTCAAAACTGAATTGAGAATTCAGTTTTTTCTCTTCACACAAAACAGCCACCACTTCTCTCTTCTC
TCCCTCCCAACCGAGATCCCACATCTCCTTTAAACCCACGGCTCAAATCCTCTCCCCTCTCCAAGCCGCCGCCCCTCTCTCCCTCCCATTGGAGATCCTCTTGCGGCGATCACCCGTTCTC
ACTTGCAAGCCGAGTGGAACTTCGGGTTTGAGTACGACAACCAAGGAAAAGTTGACATCCTCAAATCACCCTTCTTCAACGTAGGCTTTGATTTAGACGACACCGTCGAAGAATATTTG
GATCGGATTCTACCAACACTAGTGGATTCCATCGACGACCAATTCTCCGACTACACGTGGATCATCGAAGGTCATCCCACCGTGCCACCTCCACCTCTGAGTT
>comp161332_c1_seq3
GGGAAATTTTTCGCTCGCAAATAGCAGAAGCCTCAACCATCAGAAAGCCGTGGGGGAAGAAAGAGAAATCCCGTAACTCCTACGCGCGAGGAGGGGCGCTAATTCGAGTAGGGGAAGT
TTCCCACTCAAAAATAGCGGAAGCCTCAGCCGCTAGAGAGCCAGAGGGAGAAAAAAAGAGAAGTTGCAAATTTTCTAAAGGCCTAGGAGGCTTATTTATAGGTTTTGAAGAGAATAGGA

GGGAGTTATTGACATTTTCGAATTTCCGTATTTTCTAAATTTTTTCGAATTTTTTAAAATTTTTTGAGATTTTTTTTGGAATTTTTAGGACCCAAAAAAACCAAAAAAGACATCTTTTCGCGT
TTGTGGGGCCCACAAAGGCGGTGGGTCGAACCACCTCTTCTCTCTCCTCAAAACCGCGCGGACAGCTCACGGATCCACGAGCATTTCAAGCGGATCGAGACAGCAGCTCCCGCGGCTG
CAGCGGCGGCAAGCATTTCTCGCGAGCATTTCACGCGGAAGCGGTCCGCGGAGCAATTCGTGGAACAGTCATCGGAACCAGTCCGTGGAAGCGGTCCGCGCCGCGGACCGTGTAGGCC
TCTGCGCGGGAGTGTCCGGCGGCCGTTGTTCCGCGCCGCCGGCACCGGCGTCGAAGGCCGGCCGGTTCGGCCGCCCTGTCCGTTTTGGACCTGTTTTTTTTCCCAGAAATAATCAAATTC
AATATTTTTGGGGTCCCCAGACACGTTTTTTGACGTTTCGAACTCAATTTTTCAACTTTCAAGGCTCAACGAAGCCTCTATCATTAAGTTTCGAGAAACACTTAATTTTCAGAATGCAATTT
TTGGCGTTTTGACTGATACAAATTTTCGAAGCATATATCTTCGGACTCCTGGAGCTTCGATGTACGAAAATAGGAGGGCAACTGATACGGCATCACAAAATTATCTCGATCGGGCAAGAAT
CTCGGGGCAAAAGCTAGTTGGAAACTTCA
>comp161332_c1_seq4
GCCCGCTTAAATCTCGCCGGCACCTTCAACACCAAGTCATTAGGAAAATGAAATTTTTTTTCTGAAAGAGATGAAAGAGTCTTCGGTAATGGTGGAGATGACATCGTCGGCTTTTGTCGC
CTTAGAAGTCTTCTTCGAACCTTCTGAACGGGCAGTAGGACGATCTTCTACAACAGAAACATCTTTTGTAGAGATGCGATCACCCATTTTCAATTGCTTAAGGAAGGAGGAAGAGGAAGA
AATCTCGTAGGCTTCTGCACGCGAAGAGGGGCACTAATTCGAGCAGGAAAAAAATTTCAACTCGCAAAATAGCGGAAGCCTCAATCGTCAATAAGCCGGGGGAAGAGGAAAAAAAATTCC
GTAGGCTTCTGCATGCGAGGAGGGGCGCTAAT
>comp161344_c3_seq1
TTAAATAAAATCTAAAAATTTTAAAATTCATCTTAATTAATTTTTTAAAAAAATTTTGGGAACCAAACTTTATTCTTTCAAACATATTTGTAATCATAATTTATTTAATTAACATTGATATTCAATG
CTCAAATTTATTTTCAACTAAAAAAACGATAGGCTTGCATAAAAAAAGCATAAAAAAATCATACGATGCCCCCTTTGCATGCCCACGTTAAGGGTCGGGGCGGGTGCGAAAACCTCCCGGT
AGAGATAAGGTCAAACAACACCTTATTCGCGGCATCAGTATAGTGAACTCCGTCCCACACCACCTTTTTTGATGGCTCGGGGCACGATCTTCCAAGCAAAACCTCACTGCCGTTCACCGT
TGCCGTGCCCCCACACCGGATGTTGTAGTCGAAATTGTACTTGCCACCACCGTACCCGCAGCATACCCTCAGAGGGTCCTCAAACCCTTGCTAAAAGAGGCACCCACCTCAGAAACTTCC
TGGCCCGCATTGATTGGACCCCACTTAAGAGGCATTGCCGCTGGAGGGGAATCGAGCACGGGTTACTCGCCCACCAATCAAGCTAATCATCGGTGGGTTAATCTAGTTTTTGATCCTCAAA
TAGATACATAGGATGAATAAAATGACAAGTCTAGTGCTAGCTCAGATCACACATGCTACATGCATCATTAAACATTCAATATAAAATGACCAAGGGATTTGATGTTGTTGCTAGTTTTAATTG
TAGCTAACCACAAGAACAAAAGAGCATTTCCATTAAAATGATCCTTCTTGGATGTTAAGAACAAATTATATATATGGCAGAGGGTAATTATGAGATGGCAAAGTACCTGCAAAATGTCAAA
AACCTTTT
>comp161365_c0_seq3
AAAAAATACTATAATTAATAACAATATATGGACACTTCATATATCCTCATCACATCCATAACAATGTTCTTTTTTACATATATTTGCCATGGCCAAACCCAAACCAACCCATTCATCTCATCAAC
ATACCAACTCCTGAAAAAGAATAATAATAAAAAATTATGCGTTTTTAATTATTATTTTATTCGATACGACTTAATATTAATAATTAAAAACTATCAATATTAAATAATATCAACAATTTTCTGACT
CTAAACGGGAAAAGTACCAAGCAATCAAAAATTATCTGATCAGCAATTAAGATAAAATTCTTAAAAATTTTAATTTTTTTTTTAAATGAATATAGTATAAAAATTAATATTCATTTTAGAATAA
ATTTAAAAATAATAAATTTATCTTAATTAAAATTTGATCACTTCCTCTTGGCCATCTGAAACGTCCTCTTCAAGAACCGGTTAGCCACCTCGTCGTCCCTGTGACAGATCACACGCAAAATG
GTATTGGGATCCGATCTGTTCCACTTCCCCGTCGTCGGCGGCATGGGCACCTTATGCGCTCCGATCCCACTCGCCTCGCACGCCACCGTCGTAAACTCCTCCAGCAATTTCTCAGTAGGCA
AAGCCATGAGGGCCACGATCCCCTCCGCCACCTCCATCTCTTTCTCCACTACCTCCTCTGCCACCAACCCCTCACCCGATGTGCAGAAAACTCGCCGCAAACTCTCCAAATCCTCCGCGA
CCATCTCGCTGTCACCCCGCCCGAAGGCTCTCTCGCTTCCGCCGGCCAACAACACCATCAAAAAGGCTTCTAGTGCCGCCTTCATCACCTCCCGCACCGCCACCGGCTGAGCCCTGTCGG
TTAAAATTGTGACGATGAAGTTTAGGTTCTGTTTTAGCAGGCGGAGGGAAGGGCGGATTCGGGCGGCGGCAACGCCGCCGGCGTAGAGGCCTTGGTAGAAGGCTTGGTGGGAGTCGAC
GAAGATGAGGCGATAGGCGGCCACTTCGGAGACGTGGAAGATGGCGGATTGAAGGGAGGAGCGGGCAAGGTCGAAGCAGGTGGGGGCGATGCGGCGGCGGGAGGTGGAACCGGGGC

CGGCGAGGCGGGGGTGAGGGGAGCGGGAAGAGTTTCGGCCAAAGAAAGAGAGGGATTTGTCGATGGAGTGGAGGACGGCGAGGACGTAGTGGAGGGAGTTGAGGCGGATGTAGAAG
CGTTGGGTGGCGCGGCTGGTGGAAGGGCGGGGATGGTGGCTTTCGGCCGGAGTGGGTTTCTTTCTGGTTTTGGGATGGACTAATCCGATCAAGGCCTGAGCTTTCTTCCATAGAGTGGTG
AATCTTGAGTCCTGGTTGCATCTGGTTAGCGGTGGGAGGTTGGGGATGTAGCTCTGCTTCGTCCCGCAGGAAGCAACGAAGGAAGTGTAATCCTGGAAGAGGTTTTCCAGGCCATCGGC
GAGTTCCTGAACCAGCTCTTCCCTTGCAGCCACCGGTATTTCAAAGAACTCATCCACTGTTACTTTAGCAAGCTTCATCAGATCAACTGCAGACTGTGCGTAGGGCTCTGATTTGGATTTT
CGGTTCCAGGTCTCTGTTTCCTTTGCTCTGCTGAAGCACTCCCTGCCAATTCTCAGCCTTCCCGTTATCCAATTCTTCATCAGGCTAAGGATAATGGAGTCCACATCGTAGAGCACCATCTC
CCTCACGATAGCCTTACCTCCATCATCGCAGTTGGCTGAGTCCTCCACCACCATCTGTACCAGCGTCTTCTCCAGTTTTCCGGCGGTCTGAAGAACCCGAACAGTCTCATTTTTCAGTCCA
GGGATTCTAGCCAAGTACTGCTTCAACACAATTCCGTAGCAGCTATGGAGTGTCGCCACAGCCACGGCCATCGAACCAGGATGCCATCTCTGGAGCACCGGACTGTAAGTCTCCTTCTCG
AGGGCCACCAAGGTCTCTGTGTCTTTGGCTAGATGCACTAGAGTCTCGCTAGGTTCTTCGTCTACCTCCACCACCATGCTGTCCACGTTCCCGTTCCCGTTCTCTAGTATCTTAGTGAATGC
GCTTTTGAGGGAAGACTTTATGTAGCGTTCGATTGGGGACACCGCAGAATTGATGACCTCGCCATTGCCGTCTTCATCGGCTGAGTTTCGTTGGTCGTCGTTCATGATTTTGGCTGTGGTG
AGAGCCAGCGAGAGAACCTTCTCCATGAGCGCAACGGAACATTTCCCGCGGAATACAGTGTGGTAATCAAGCAGCTTCCTTTCGGCCCACGCTAGCATCGACGACATCACCGGAGAAAG
GACTTTGAGGAATTCTGCCTCTTTTTTGGCTTCGGTGGCGATTTCGCCGAGGATGATCAGCGACGCGCCGAGCAGGTCCTGCTCGATCTGTCCTGTCTTTACGTACTGCTCGAACATCGTC
CAGGCCAGGCAGACATTGTGTGTCATCTTGTTGATGCCAAGCGTAATCCATGTCTTCTTCATCAGCTCGATCAGCTCGTCGACCTCCTCGAGCACCACGGTTTCGTCGCGGAGGTCGAAG
ATGGATCGGAGGAGGGAAATATAAAGGTGGATGTTAAGGGGGAAGCCGTCGGCCCAATGGCAGGCCTCAGCGGGGGATCCATTGGGATTCCGCCACGCGAGGGAGACGACTGCATTGC
AGAGAGCTTTAAGGGATTCGGAATTCTTGCTGTTGTCGATGGGTTTGAGCTCGCCGGAGCGAATTACCTCACGGAGGCGCAGTGCCGATGAGTTGAGGCGATCAAGGGGGATGGAAGGG
TGATGGAGCAGGCCGGCCTCGAGGATCTTCAGCTGCCGCCGTTGCCACTGATGGTATTCCTGCGCGTCGCTGAATTCCGATGGTTTTAGATGCCTCAAAAGCTCCAATGGGAGGATGATG
GTCTCCGCTCTCTTTGCGGCCTGGCCGACGAGCGTGCGCATTATGGTTTTCCGGAGGCGGTTGTCGCTCTGCTCGGTGACCCCCATTTGCTGCCGCATGATCTCCGCGGAGGTCATGGGG
CGCTGCTTGAGCCGGCCGGCACCGGGGGATGAGGCTCCGGCGGCCCCGCCTACCGATACGACGGGTGATGAGGAGTGTGCGCTCATGACGCGCATCGGTGGCGAGCCGCGACGGGTGC
GGAGGCCGAGTGCGCGCTTGATCCTGCTGTTGACGACCATAATGCCGGTGCCGGAGGTAGCCGCGGTGTTACCTCCTTCCGGGGATGGCGGGTGGTGGTTTGAAGGGTAGAATGAGAGG
GGGTTTCTACCGCCGAAGCCGGGTGAGGAGCGGCATGAGGCGAAGAAAATCTCGTAAGCCGTTTCGCGGAGTTCGCCGCGATCGACGGCGTCGATTCTTCCGAATGGCCATTCGACATC
GGCATCGCCGGCTTCGGAGTCGTGCCCCACGGAAATGGTGAAGTCGGAGAAGGAGGAAGCGGAGGGATGATGGTGAGCCATACGCGCGGCAGCCGCGGCGGCGCGGCGTAGGAGGGT
GGTCA
>comp161365_c0_seq6
AAAAAATACTATAATTAATAACAATATATGGACACTTCATATATCCTCATCACATCCATAACAATGTTCTTTTTTACATATATTTGCCATGGCCAAACCCAAACCAACCCATTCATCTCATCAAC
ATACCAACTCCTGAAAAAGAATAATAATAAAAAATTATGCGTTTTTAATTATTATTTTATTCGATACGACTTAATATTAATAATTAAAAACTATCAATATTAAATAATATCAACAATTTTCTGACT
CTAAACGGGAAAAGTACCAAGCAATCAAAAATTATCTGATCAGCAATTAAGATAAAATTCTTAAAAATTTTAATTTTTTTTTTAAATGAATATAGTATAAAAATTAATATTCATTTTAGAATAA
ATTTAAAAATAATAAATTTATCTTAATTAAAATTTGATCACTTCCTCTTGGCCATCTGAAACGTCCTCTTCAAGAACCGGTTAGCCACCTCGTCGTCCCTGTGACAGATCACACGCAAAATG
GTATTGGGATCCGATCTGTTCCACTTCCCCGTCGTCGGCGGCATGGGCACCTTATGCGCTCCGATCCCACTCGCCTCGCACGCCACCGTCGTAAACTCCTCCAGCAATTTCTCAGTAGGCA
AAGCCATGAGGGCCACGATCCCCTCCGCCACCTCCATCTCTTTCTCCACTACCTCCTCTGCCACCAACCCCTCACCCGATGTGCAGAAAACTCGCCGCAAACTCTCCAAATCCTCCGCGA
CCATCTCGCTGTCACCCCGCCCGAAGGCTCTCTCGCTTCCGCCGGCCAACAACACCATCAAAAAGGCTTCTAGTGCCGCCTTCATCACCTCCCGCACCGCCACCGGCTGAGCCCTGTCGG
TTAAAATTGTGACGATGAAGTTTAGGTTCTGTTTTAGCAGGCGGAGGGAAGGGCGGATTCGGGCGGCGGCAACGCCGCCGGCGTAGAGGCCTTGGTAGAAGGCTTGGTGGGAGTCGAC
GAAGATGAGGCGATAGGCGGCCACTTCGGAGACGTGGAAGATGGCGGATTGAAGGGAGGAGCGGGCAAGGTCGAAGCAGGTGGGGGCGATGCGGCGGCGGGAGGTGGAACCGGGGC

CGGCGAGGCGGGGGTGAGGGGAGCGGGAAGAGTTTCGGCCAAAGAAAGAGAGGGATTTGTCGATGGAGTGGAGGACGGCGAGGACGTAGTGGAGGGAGTTGAGGCGGATGTAGAAG
CGTTGGGTGGCGCGGCTGGTGGAAGGGCGGGGATGGTGGCTTTCGGCCGGAGTGGGTTTCTTTCTGGTTTTGGGATGGACTAATCCGATCAAGGCCTGAGCTTTCTTCCATAGAGTGGTG
AATCTTGAGTCCTGGTTGCATCTGGTTAGCGGTGGGAGGTTGGGGATGTAGCTCTGCTTCGTCCCGCAGGAAGCAACGAAGGAAGTGTAATCCTGGAAGAGGTTTTCCAGGCCATCGGC
GAGTTCCTGAACCAGCTCTTCCCTTGCAGCCACCGGTATTTCAAAGAACTCATCCACTGTTACTTTAGCAAGCTTCATCAGATCAACTGCAGACTGTGCGTAGGGCTCTGATTTGGATTTT
CGGTTCCAGGTCTGTTCAATATAATAAATCTTTGCCGTCAGTTAATTCACACGAGATTTCTCTATAACACCCATGATTTGACGTACTTCTTCATGAAAATGCTAAAAAAATCATTTGATGACA
AAATCAACTAACAAATTTGAAACAATATAGTTCATTCCAACAGAAATTGATGTTTTTAAAATTTGAGACATCGAATATTTAGGGGGAGATAGTTTCGCTCTGCTAGGGTTTTGGCGGGGTTT
TGCTTCGCCCCGCCGTTTTCTTCTCCGTTTGGGGAGGGCCTCCTCCCCTCCCCTCCGTGCATCCGATGGCGTCTTCCTTCTACACTTCGGACTTTCCTCCGCTTCCTGGCTCCGGCTCATCT
ACGCTCTCCGCTCCTCCCGGCCTCGACTTCATCGGTAATCTTACCAAACA
>comp161365_c0_seq8
AAAAAATACTATAATTAATAACAATATATGGACACTTCATATATCCTCATCACATCCATAACAATGTTCTTTTTTACATATATTTGCCATGGCCAAACCCAAACCAACCCATTCATCTCATCAAC
ATACCAACTCCTGAAAAAGAATAATAATAAAAAATTATGCGTTTTTAATTATTATTTTATTCGATACGACTTAATATTAATAATTAAAAACTATCAATATTAAATAATATCAACAATTTTCTGACT
CTAAACGGGAAAAGTACCAAGCAATCAAAAATTATCTGATCAGCAATTAAGATAAAATTCTTAAAAATTTTAATTTTTTTTTTAAATGAATATAGTATAAAAATTAATATTCATTTTAGAATAA
ATTTAAAAATAATAAATTTATCTTAATTAAAATTTGATCACTTCCTCTTGGCCATCTGAAACGTCCTCTTCAAGAACCGGTTAGCCACCTCGTCGTCCCTGTGACAGATCACACGCAAAATG
GTATTGGGATCCGATCTGTTCCACTTCCCCGTCGTCGGCGGCATGGGCACCTTATGCGCTCCGATCCCACTCGCCTCGCACGCCACCGTCGTAAACTCCTCCAGCAATTTCTCAGTAGGCA
AAGCCATGAGGGCCACGATCCCCTCCGCCACCTCCATCTCTTTCTCCACTACCTCCTCTGCCACCAACCCCTCACCCGATGTGCAGAAAACTCGCCGCAAACTCTCCAAATCCTCCGCGA
CCATCTCGCTGTCACCCCGCCCGAAGGCTCTCTCGCTTCCGCCGGCCAACAACACCATCAAAAAGGCTTCTAGTGCCGCCTTCATCACCTCCCGCACCGCCACCGGCTGAGCCCTGTCGG
TTAAAATTGTGACGATGAAGTTTAGGTTCTGTTTTAGCAGGCGGAGGGAAGGGCGGATTCGGGCGGCGGCAACGCCGCCGGCGTAGAGGCCTTGGTAGAAGGCTTGGTGGGAGTCGAC
GAAGATGAGGCGATAGGCGGCCACTTCGGAGACGTGGAAGATGGCGGATTGAAGGGAGGAGCGGGCAAGGTCGAAGCAGGTGGGGGCGATGCGGCGGCGGGAGGTGGAACCGGGGC
CGGCGAGGCGGGGGTGAGGGGAGCGGGAAGAGTTTCGGCCAAAGAAAGAGAGGGATTTGTCGATGGAGTGGAGGACGGCGAGGACGTAGTGGAGGGAGTTGAGGCGGATGTAGAAG
CGTTGGGTGGCGCGGCTGGTGGAAGGGCGGGGATGGTGGCTTTCGGCCGGAGTGGGTTTCTTTCTGGTTTTGGGATGGACTAATCCGATCAAGGCCTGAGCTTTCTTCCATAGAGTGGTG
AATCTTGAGTCCTGGTTGCATCTGGTTAGCGGTGGGAGGTTGGGGATGTAGCTCTGCTTCGTCCCTGCAACCAAGAGCGTCAAGTAACGAAGAAAACAACTAGCTGGAAATTTAGTGAA
TAAATAACATCAGTTGCTTACCGCAGGAAGCAACGAAGGAAGTGTAATCCTGGAAGAGGTTTTCCAGGCCATCGGCGAGTTCCTGAACCAGCTCTTCCCTTGCAGCCACCGGTATTTCAA
AGAACTCATCCACTGTTACTTTAGCAAGCTTCATCAGATCAACTGCAGACTGTGCGTAGGGCTCTGATTTGGATTTTCGGTTCCAGGTCTCTGTTTCCTTTGCTCTGCTGAAGCACTCCCT
GCCAATTCTCAGCCTTCCCGTTATCCAATTCTTCATCAGGCTAAGGATAATGGAGTCCACATCGTAGAGCACCATCTCCCTCACGATAGCCTTACCTCCATCATCGCAGTTGGCTGAGTCCT
CCACCACCATCTGTACCAGCGTCTTCTCCAGTTTTCCGGCGGTCTGAAGAACCCGAACAGTCTCATTTTTCAGTCCAGGGATTCTAGCCAAGTACTGCTTCAACACAATTCCGTAGCAGCT
ATGGAGTGTCGCCACAGCCACGGCCATCGAACCAGGATGCCATCTCTGGAGCACCGGACTGTAAGTCTCCTTCTCGAGGGCCACCAAGGTCTCTGTGTCTTTGGCTAGATGCACTAGAGT
CTCGCTAGGTTCTTCGTCTACCTCCACCACCATGCTGTCCACGTTCCCGTTCCCGTTCTCTAGTATCTTAGTGAATGCGCTTTTGAGGGAAGACTTTATGTAGCGTTCGATTGGGGACACCG
CAGAATTGATGACCTCGCCATTGCCGTCTTCATCGGCTGAGTTTCGTTGGTCGTCGTTCATGATTTTGGCTGTGGTGAGAGCCAGCGAGAGAACCTTCTCCATGAGCGCAACGGAACATT
TCCCGCGGAATACAGTGTGGTAATCAAGCAGCTTCCTTTCGGCCCACGCTAGCATCGACGACATCACCGGAGAAAGGACTTTGAGGAATTCTGCCTCTTTTTTGGCTTCGGTGGCGATTT
CGCCGAGGATGATCAGCGACGCGCCGAGCAGGTCCTGCTCGATCTGTCCTGTCTTTACGTACTGCTCGAACATCGTCCAGGCCAGGCAGACATTGTGTGTCATCTTGTTGATGCCAAGCG
TAATCCATGTCTTCTTCATCAGCTCGATCAGCTCGTCGACCTCCTCGAGCACCACGGTTTCGTCGCGGAGGTCGAAGATGGATCGGAGGAGGGAAATATAAAGGTGGATGTTAAGGGGGA

AGCCGTCGGCCCAATGGCAGGCCTCAGCGGGGGATCCATTGGGATTCCGCCACGCGAGGGAGACGACTGCATTGCAGAGAGCTTTAAGGGATTCGGAATTCTTGCTGTTGTCGATGGGT
TTGAGCTCGCCGGAGCGAATTACCTCACGGAGGCGCAGTGCCGATGAGTTGAGGCGATCAAGGGGGATGGAAGGGTGATGGAGCAGGCCGGCCTCGAGGATCTTCAGCTGCCGCCGTT
GCCACTGATGGTATTCCTGCGCGTCGCTGAATTCCGATGGTTTTAGATGCCTCAAAAGCTCCAATGGGAGGATGATGGTCTCCGCTCTCTTTGCGGCCTGGCCGACGAGCGTGCGCATTAT
GGTTTTCCGGAGGCGGTTGTCGCTCTGCTCGGTGACCCCCATTTGCTGCCGCATGATCTCCGCGGAGGTCATGGGGCGCTGCTTGAGCCGGCCGGCACCGGGGGATGAGGCTCCGGCGG
CCCCGCCTACCGATACGACGGGTGATGAGGAGTGTGCGCTCATGACGCGCATCGGTGGCGAGCCGCGACGGGTGCGGAGGCCGAGTGCGCGCTTGATCCTGCTGTTGACGACCATAATG
CCGGTGCCGGAGGTAGCCGCGGTGTTACCTCCTTCCGGGGATGGCGGGTGGTGGTTTGAAGGGTAGAATGAGAGGGGGGTTTCTACCGCCGAAGCCGGGTGAGGAGCGGCATGAGGCGA
AGAAAATCTCGTAAGCCGTTTCGCGGAGTTCGCCGCGATCGACGGCGTCGATTCTTCCGAATGGCCATTCGACATCGGCATCGCCGGCTTCGGAGTCGTGCCCCACGGAAATGGTGAAGT
CGGAGAAGGAGGAAGCGGAGGGATGATGGTGAGCCATACGCGCGGCAGCCGCGGCGGCGCGGCGTAGGAGGGTGGTCA
>comp161371_c0_seq7
TTTTTTTTTGTGTGTTTATATTCTATAGAGGGTGCTCGAAAAATTAGGGGAGAGGTAAATCTATAATCGAAAATAGAAAAAGTTCTTCACTTACAAGATACAAACCTTCTCGTGTTTATCCT
TAATAAATATATTCTCTTTAGTTATAAAATCATCATATCAGAGCTCTATCAATCTGAGAACTTCATTGGAGAATGTATTGGTGGAATTCTATTTTATAAAGTAAATCAACATTGTTAAAATTATT
ATTATACTTTTTTTTTAATTCATTCACAAG
>comp161375_c0_seq1
TAATTAATCTTTCAAAAGCAATGCAAGCCTACGTTCTTCAAAGGATATCTCCGTCTCCTGTGGCTCCATTTTCAGTGCTGTAGCCTCTTGGGCAAAATCAGGTGGGTCAAGCTTTGTTGTCA
TGCTTTGGCGTAGACACCGTTCTGGGTGAATTCAGAGCTGAGACTGCTGAAGAACCCATAGGTATCATGTTTTCCTCTTCGGTGTTGCTTATACGTTTATATGGGGATGGTAACTGTATGCT
GTGGGGACGGCTTAAGGGCGTGAAGGGCCTACGAGGAGTCTCTACGTCAGAAGCGTCAGGATCAACAGATTGAAATTCTGGGTCTCCCGTGCCTCCAGAAAGAGAATCAGGCGTCTTT
GCAGACGAAGGAGACTTGAATGAAGGAAGTGAATCTAATCTTGTAGGCTGCAATGTGGCTCCCCCGACCGATAGCCTTCTGGTTGGTCCTAAAGGTAAAGTTCCAGCAAATTTCTTGGCC
GACTGGGACTTCGAAAGAGTTGGTTTTGATCCAAACATTACTTCCTTCTCGGCTACAAGCTGCCCTTGAAGCCTTCTCTGGTCCCTCAGTCTCTTTCTTTCATGTTCTTTCTCTTGCCTCAG
GATGGTGTGCTCTTCCAGCATGGACAAAAGGCTGACACCATCATATTTGAATTCGGTCCCCCTCTCGTTCTCCCATGACATAATTTTGGTGGTCAAAGTCTCCACAATCGCTGGAATTTTGC
TGACAACTGCACGGGCTTTTTCAGCGCGCTTGAGCATAAGGTGAGTTCCTTTTCCGGCGTTATAACGATTTTCATCCCTGTTATAATCTTCAAGCCAGGACTCTTCCTCACAAGCAGCAAA
CCATTTCTCAACCCTTTCAAGAATCTCCTTCCTGCTGAAGGCTTCCTCTTTCAGAATTGAAATTTGAGCCTCGATCTGTTCCAAAACTAGGGAGGAATCAAGAGATTCAGCAAAACAGTCA
TTTTCTGCAACAAGATGTGTGCGTCGGCACAGCTCATCCAGCTCAATCTTCTTTTTCAAAATCAAGTCCTTCAACTTGCTCGCTTTCAGCGCTTCGAGTCGTGTAACTTCGGCTTTCACAT
AATTGATGAATCCCATTGAAAGCGACTTGGGTTCTGTTATTTCATGCTCTGAAGCAGCAATATTACATGTTAAATTCTGAAACTGCTGCTGCTCTTCAGTTGGAGTATCCATCAGATACCAA
AGCTCCAACATTGCGGTTGCCAGATCTTGAAGCATGCGCATTCGTTGTAACTTAACTTCCCGCAGTCTCTGCGTGGTACTTATGAGCCTCTCAATTGTATCGCTATTGACATTTTTTGACTCA
TCGGGTCCAACTAAACTAGGATGAACTTCACTGGCTGTCTGTTTGAAATCTACATCTAGCACTGAACATAAAGAGTTCAACGTATTCAAGTGGTGCAGCACCTTCCTTAGGCGGTCATTCT
TCTCTTTATGGAGTGCATCTAAGCGTCTGTGAAGATCTTGTAGATTTCTTATGGACAAATCAGATTCATCAAGAACTATCTCAAGGCCAGGTTCAGGATTAATCTCGGTTGAGACGCACAGT
ATTTGCTCCCTTACTTTACGAAATTGACTCAATCTTTCATTCTTTCTTCTTCGCAAATCTTCTAGTTGCTGAATGATGACCTTATGCTCTTCTTTCAAGCTTCTGTCACCCTGTCTAAAATGAA
CTGGCCTCTCACCCATCGCAGAGCATAAAGTTGCAATCTCTGCTTCAGAATCTGCAATAGTTTGCCGAAGCTGAGCTCTGTGCCTGTTTGCTTGGTCAACCTTTCTTCTATATAAATCTAGG
CACTCTTTCTCTAGTTGGAGTAACATCACATCCTTTTCAACATCTGATTCTCCAATATCATCCCATATAGTCTGAAGTTCATCTAAAAGAGAACTGCAATCTATATCATTTTGCAGAAGAGGA
CCACGTTTACCTTTCTCCATCATGGAACTAACCGAAATCGGCGGCCCCTGCAAGGGGTATTGAGTAATAACTGCTGCTCGTCCTTGACGTTGAACACTTAGAGGAGGAGAAGAAGAAGA
AAGAGGAAAAGGAGAACAAGAAGGAGAAGGACGA

>comp161375_c1_seq1
AGAAGGCTATCGGTCGGGGGAGCCACACTGCAGCCTACAAGATTAGATTCACTTCCTTCATTCAAGTCTCCTTCGTCTGCAAAGACGCCTGATTCTCTTTCTGGAGGTTTGGTCCTTGTTG
ACTGAAATGCTTTTCTCTTTGCTTCAACCATATTTACTATATAATGTTATATTATTAGTAGTATGATTTCCACGTATTTTTAATTACTATTTATATCATATTTTAGTGATATTAATCTAGATATCATAC
AACCTAATGACTTAGAAATGTCAAATGTATTTTTCTTTGGAGTAT
>comp161439_c0_seq16
TCACTCAAGTTATATATTTGCACTGGTATATCTAAGAAGTAAGAAATAGCTTTAAGAGCATCATCCTCTGGTCATTAATTTAGGAGGAATATATCTTTTTATTCATTAATATCAGAATGTTTATA
AAGTGAATATGCCGATTCCACCCCCTAATTTTGCATCTAGAAACTTCTAAAAAAAGTAATATTTAGTTATTTAATTAATGTTTAATCATATATGATACAAACTGCCAGGGCCTCCTAATTTTTG
CCTAAAAACTTATTGTTGATGAGAATACATGGATGCAGTGTAACATTTCTTTCTTTTCATGGAAGAATAGTGAAAACATGAATAGATTTTAAATTCCTGCCACCATGACATCTTGGCTAACTT
CATGGGGATGCCTTCATCTTCTTTGGAAGTTCAGAGCTTTAATTGAAAATCCGAACAAGAAGGCAAAGAGTGTTGGAAACCCAACAATTGCAGCTGCAACTACTCCTAAGAAATCATGTC
TGAATCCAAAATAGTTTCTCACAAAATGAGAGACAACCTCACCAGTGTCAAGCTTGTCTTCTATATCTCCAAACTGTGAAGCTACCAATCCATAAAGTGTCCATGAAACTGGGCATGCCCA
GAAATACCATCTCCACCATACCGGAACCCTCTGTTGAAAATAATCTTTTTAGTTCCATCCACATTAAATTAAACAAAACTATCTGCAAATTACAACTTACCGGTCGTGGAATTATCATACCCG
AGAAAAGATTCCATAATAAATAAAACCATGTAGATGCTATCGTACCCACGTTTAAATTGGGTGTGAAGCCCACACACATCATTCCATAGTATGTGAAGTATAGGAACGTGAAATACATAATG
AACAAATACCAAAAGAACTTGGCAGTTGTCCATTCAAATCCAATCATTGAATAGACTATGACCCCATAAACGATAGCCTGACCCAATACATATGGGATCTCAATTGCGACCTGTCCAAATG
CATATGGTAAACTTGAGTACATTCCTGCTGCTCTTTCCCTATAAAAGACAGTTCTCTCGACAGACACCACAGGTTGAACTGAAGAAGCATTCGAAATTCCAAGAAACAAAACAGAAGCAT
ACATTGAACCCATTGCATTAAACAAATCCTGTTGTTTGCTCTTTTTGCCACCTAGATCCCAAAAAATTGTTCCAAATATCAAGGCTATGATCAATGTGAAGAAAAACCGCACTGCTGTGTAT
GGTGGATTTCTCCAGTATGACAAATGCTGTTTCCAAAGGCTAGCCATGCATTGAGAAACGAAGGTCTGCGAGTACTGTGTTGGGAAGAAAAGATCATTTGAACCAGGTGGAGGTATGCTC
AACTCCTTGATCAAAGCCTTATTCCTCTGAAACAATTCAGAATTCTTGTAGACATCAGTGAAATCAACCCCTAGTATTTCCTCTTGTGCTATTGTTGTCACTTCTAACATCCATGTTGCTGGG
TTATAGCTATCCTTTATTTTGCTTACTCCTTCAATTCCCTCAAAGTAACTAATCAGTTGACTAGAATGGTGACCCAATGGTCCTACATAGATCTCTTTTCCACCACGTTTCATTAGGAAGAGC
TCATCAAATGCTTCAAATATGTCTATGCTAGGTTGATGGATTGTGCACACTACAGTTCTGCCAGTGTCTACAGTATTCCTTACTGTTCTCATGACAATAGCAGCAGCTCTGGCATCAAGTCC
AGATGTAGGCTCATCCATGAATATAATAGCTGGATTAGCAACGAGCTCAACTGCAATGGTCAATCTCTTGCGTTGCTCTGTTGATAGTCCATTTACTCCTGGCAATCCCACCAATGCTCCCC
TCAATGGAGTAAGTTCCACAAGCTCCATTATCTCTTCTATAAACATCTTTCCTTTTTCAGAACCAACCTCAGCAGGCAATCGAAGCCATGCTGAATAAACCAAAGACTCATAAACTGTCAC
ATAAGGGGAATTGATATCATTTTGTTCACAATATCCTGAAACTCGAGCAAAGGTTTCCTGTTTCTTAGGATAACCATTTATCATGATGCTTCCTTCTATATATCCTCCAGTTTTTCTTCCGGCC
AATACATCCATCAAAGTTGTCTTACCAGCCCCACTAACACCCATCAGCGCTGTGAGCACACCTGGTCTGAAAGATCCACTTACACCTTTTAATAGCTCCAAACGGTCTTCTTGAATCCCTT
TGGCCTTCATTTCCTCTGGCATGTCCACAGAGTATCTTATATTGTCAAATGTCATGCTGAGTGGAGTAAAAGGAAGAACCATTCCTTTCTTGTTCAATTTGGATTCTGCAATGGCTTCTACAT
TTTTTGTTCCAATCCTGTTCTCATTCCCTTCAGAGCTCTTTGCTGTTGATGATATTTCAGTTATCTCTCCAGTAATATTTGCTTGTTTCTCCTTCAACGTCTCCTCAGATATGATTGGCTGGGAT
TTTCCAAATGGTTTTACAAAGGTAAGAGCCACCGTGAAAAGAAAATTAAAAAGGAATATGAAGCCAATCAATGCTCCAAAACCTATCCAATACCATTTTGCTTCTGTAAAGGTTCCACGA
GACTTCAAGATGGTCACCCCTAATGATTCTGTTGACCCAGGAAGAATATGTTTCCAGCTTTTCCCAAGGAACTCATTTGTTGCTAGAGCATTCAAGGAGTACATCATAGGTGAGATCCAGT
AGCCCCATATCCACCATTTCTTCATGTCATCGTGTGATAGAACAAATCCAGCCAATGCTACGACCACAAGCAGTGCAAATGATCCTAAAGAATTAGCAACAACCATGTCTCTGCTCAATCC
AGCAATTAAACGAAAGAACGCAGACATTGCCTGTTGGAGTGCTAAAAACAGCAAATACTGTTTAAAAAACCTTCCAACATTAGGATCATAACCTATAACATAATAGCTGACAAACACCCA
GGTACCAACTTCAACTAGAGCTATGGGAATCTTCAGAATCCACGAAGGTAATGTATATGCCCATGCAGGATAGAAGAGGTAGTCTCTTGCTTGAGGAAGACTGGGAGTTTCCAAATAGTC
AATGGAATCTCTGTGAGCCCATTAAACACGATCGTCTGTATAGAGAAGAACAATGAACCCACAAAAATTCCACCATCCAAGACTGAATCACGGTGCATATTTGTCCTTAGGAACAGCGTC

ATTGCAATAGATGCTAAAAGCAGTAGCTGAATTGCTCTGAAAACATATGCAAACCAGTTGCGCTTCATCAGCAATAATTCTCTTGAGATGCAAGCTTTTAGCAGTTCCTTTTTGCTCACACC
ATATTTTGAAGTTGTTAAAGCTGCAGGATGTCCCTTTCTCGCGTCGAATGGGACAGCAAGCTCCTTCGTTAATTTGTCACCGATATGGAATGATTGGAATGCTTCTGAGAATTCTCTTGCAG
TTACATATCTGTAAGGTTCTTCGTCACGTGCCCAATACTGCTGCTGATCTTTCCTTGATGTAACTTCCTGCAAGAAATCCGCAACCCCTTTTCTCTCCGGGCATTTGAAGCCCATCAATTCA
AAGAATTCAAGGACATTCTCACGAGGGCCATGGTACACAATTTGAGCATCCGAGAGAAGAATTATATCATCAAGAGGTCAAATGTTTCTGGTGCTGGCTGGAGAAGGGAAATAACAGCG
GTTCCATCAAGTATATGTATATATTGTCTCAATGAGCTGACAATTTGGTAAGTTGTGGAGGTGTCAAGTCCTGTGGATATCTCATCCATGAACAAAGCACTTGCTGGTCCAACAATCATCTC
ACCTGTTGTAACTCTTTTCTTCTGTCCTCCAGAAATACCTCTAATCATCTCATCTCCAATCATTGTGTCAGCACAGATGTCCAGCCCTAAAATCTTTAGTACATAATCAGTTACTAGACCCGT
CTCTTGTCCTTCCATTGAACTTGCCTTCATAAAGACATCAACGTCAGGGTCTGGCTTTATATTTGCTGCTTTCTCTCTCCTTGAAAGCTCTGCCAACATATCATAACGTTTACCAACTCCTTG
ACACCTTGCAGAGAATGATAGTGTTTCTCGAACCGTCATTTCTCCGATATGGACATCATACTGGCTGATATAGGCAGCATTTCTTTGAGGTACAAACTTATTCATTTCTTGACCATTGTAAGT
CACTCTTCCTGAAACCTTCACATCAGAGTCCAATTTTCCAGCTAGAGCCAGCAGTAAAGTGGTCTTCCCTGAACCAGGAGGTCCTAAGAGTAGAGTCATCCTTCTAGGCTTTATGAGTCC
ACTAACATTATGAAGTAAGGTTAATGGTTTCTTCCTGCTTGGTAGAATGTGAAGGTTGTTGGCAAAAGCCTCAAGCACGTTTATTACAGAATTGAGCAGCGTAGGCAATCCTCTGTTGCCC
ACATATGCTTCAGCTTTAACATTAAGGTTCTCATACCGTACTTCGATGGTTGGCAATTCAATCCCAACATTATCAATCCTGTTTCTGATTCTCAGCAAGAACTTCTCATTATCATCATCTGCCA
CTCTCACCAACTTCTCCAACAACTCCCTCCTCTCCAGAAACCCGAGACTCTCTATATCAACTTCCTTCTTACCTCCTTCAGCCAAGTTCAGAATGCCTTTCCTCATTCTATTGTAAGTCGGC
AGCCTCTCGATGGCTGCCCATGTTAGATCCTCCTCGTCGTCTTCACTGTGCCTGGACCTTGAGAAGACCTGATCTCCATTCCATATGGAAGAGCGGCGGGACCTTGAGCTTGTGGCACGC
AGAACAGACCCAGCTTCCATTTCAATGAACACCTTTCCTTCTCTCTTGAATGCAATGGACAAGAAGTAAAAGATACCAAGAAAATGAGATTGTTTAGTATGGTAAGAAGAACGATTCTGA
TGAAGTTTGAAAAAAAATATTTTGGAATGGAAGGGTTTTAAAAAGGTTCAAGCATAGCGGAAACCATTATGAAGGACAACTGCTTTACAGTGAAAGGTTTACCCTTC
>comp161439_c0_seq17
TCTCCAGTAATATTTGCTTGTTTCTCCTTCAACGTCTCCTCAGATATGATTGGCTGGGATTTTCCAAATGGTTTGAGATAAGAAAGAGCAACGGTGAAAAGAAAATTGAATACAAACATGT
ATCCAAGCAATGCACCAATACCAATCCAGTACCATTTTGCTTGTGTAAAGACTCCACGTGACTTCAATACTTGTACCCCTAGGCTTTCTTTTTGTTCCGGCAAAATATGACTCCAAGCGTGG
CCAAGGAACTCGTTTGTTGAAATAGCATTCTGGGCATACATAAGAGGTGAGATCCAGTAGCCCCATATCCACCATTTCTTGACATTATCATGTGATAGAATGAATCCTCCCAAAATAATCAT
CACCAACAATGCAAATGATCCAAAAACATTGGCAACAATTAAGTTTCTCCCCAGTGCAGCAATGAAACGAAATAAAGCTGAGGCCATTTGGTTGACAGCTAGTAGTAGAAAAAATTGTTT
AAATAACCTTCCAACATTAGGATCATATCCTATGACATAATAGGAGAGAAATACCCAGCCACCAACTTCCATAACAGTTATAGGAATCTTGAGTATCCATGTTGGAATTGCATAAGCCCATG
CAGGATAAAAGAGATAATCTCTTTGTTTGAAGAAGACAGGGAGCTTCAAAATTGTCAATGCAAGCTCAGAGAAACCATTGAACATTATCATGATAATTGAGAAGAAGAGTGAGCCCATAT
AAATTCCTCCATCAGGGGAGATATTATCTCTATGCATATTAGTCCTTAAAAAGAGTGTCATTGTTATGGTTGCCATCACCATGAGCTGAAATCCTCTAAAACCATAGACAAATGAATTCCTTT
TCATGAGCAAATATTCTCTTGAAAAACAAGCTTTAAGAAGTTCTTTGGTGCTAACACCATATTTTGAAATTGTAAGAGCTGCAGGGTGTTCCTTGCTCTTATCATATGGGACAGAAAGCTCA
TTCATTAGTTTCTCTCCAACATGAAAGGATTGGAATGCTTCTGCAAATTCCCTTACAGGCACAAATCTGTAAGGTTCATTGTGACGTTGCCAGTATTGTTGCTGATCTTTCTTAGATGTTACT
TCTTGTAAGAAATCAGCAACACCTTTTCTCTCTGGGCATTTAAAGCCTAGAGACTCAAAGAACTCTAGCACATGCTCGCGGGGACCGTGATACACAACTTGACCATCTGATATAAGAATTA
TATCATCAAAGAGGTCAAATGTTTCTGGTGCTGGCTGGAGAAGGGAAATAACAGCGGTTCCATCAAGTATATGTATATATTGTCTCAATGAGCTGACAATTTGGTAAGTTGTGGAGGTGTC
AAGTCCTGTGGATATCTCATCCATGAACAAAGCACTTGCTGGTCCAACAATCATCTCACCTGTTGTAACTCTTTTCTTCTGTCCTCCAGAAATACCTCTAATCATCTCATCTCCAATCATTGT
GTCAGCACAGATGTCCAGCCCTAAAATCTTTAGTACATAATCAGTTACTAGACCCGTCTCTTGTCCTTCCATTGAACTTGCCTTCATAAAGACATCAACGTCAGGGTCTGGCTTTATATTTG
CTGCTTTCTCTCTCCTTGAAAGCTCTGCCAACATATCATAACGTTTACCAACTCCTTGACACCTTGCAGAGAATGATAGTGTTTCTCGAACCGTCATTTCTCCGATATGGACATCATACTGG
CTGATATAGGCAGCATTTCTTTGAGGTACAAACTTATTCATTTCTTGACCATTGTAAGTCACTCTTCCTGAAACCTTCACATCAGAGTCCAATTTTCCAGCTAGAGCCAGCAGTAAAGTGGT

CTTCCCTGAACCAGGAGGTCCTAAGAGTAGAGTCATCCTTCTAGGCTTTATGAGTCCACTAACATTATGAAGTAAGGTTAATGGTTTCTTCCTGCTTGGTAGAATGTGAAGGTTGTTGGCA
AAAGCCTCAAGCACGTTTATTACAGAATTGAGCAGCGTAGGCAATCCTCTGTTGCCCACATATGCTTCAGCTTTAACATTAAGGTTCTCATACCGTACTTCGATGGTTGGCAATTCAATCCC
AACATTATCAATCCTGTTTCTGATTCTCAGCAAGAACTTCTCATTATCATCATCTGCCACTCTCACCAACTTCTCCAACAACTCCCTCCTCTCCAGAAACCCGAGACTCTCTATATCAACTTC
CTTCTTACCTCCTTCAGCCAAGTTCAGAATGCCTTTCCTCATTCTATTGTAAGTCGGCAGCCTCTCGATGGCTGCCCATGTTAGATCCTCCTCGTCGTCTTCACTGTGCCTGGACCTTGAGA
AGACCTGATCTCCATTCCATATGGAAGAGCGGCGGGACCTTGAGCTTGTGGCACGCAGAACAGACCCAGCTTCCATTTCAATGAACACCTTTCCTTCTCTCTTGAATGCAATGGACAAGA
AGTAAAAGATACCAAGAAAATGAGATTGTTTAGTATGGTAAGAAGAACGATTCTGATGAAGTTTGAAAAAAAATATTTTGGAATGGAAGGGTTTTAAAAAGGTTCAAGCATAGCGGAAA
CCATTATGAAGGACAACTGCTTTACAGTGAAAGGTTTACCCTTC
>comp161439_c0_seq27
TCACTCAAGTTATATATTTGCACTGGTATATCTAAGAAGTAAGAAATAGCTTTAAGAGCATCATCCTCTGGTCATTAATTTAGGAGGAATATATCTTTTTATTCATTAATATCAGAATGTTTATA
AAGTGAATATGCCGATTCCACCCCCTAATTTTGCATCTAGAAACTTCTAAAAAAAGTAATATTTAGTTATTTAATTAATGTTTAATCATATATGATACAAACTGCCAGGGCCTCCTAATTTTTG
CCTAAAAACTTATTGTTGATGAGAATACATGGATGCAGTGTAACATTTCTTTCTTTTCATGGAAGAATAGTGAAAACATGAATAGATTTTAAATTCCTGCCACCATGACATCTTGGCTAACTT
CATGGGGATGCCTTCATCTTCTTTGGAAGTTCAGAGCTTTAATTGAAAATCCGAACAAGAAGGCAAAGAGTGTTGGAAACCCAACAATTGCAGCTGCAACTACTCCTAAGAAATCATGTC
TGAATCCAAAATAGTTTCTCACAAAATGAGAGACAACCTCACCAGTGTCAAGCTTGTCTTCTATATCTCCAAACTGTGAAGCTACCAATCCATAAAGTGTCCATGAAACTGGGCATGCCCA
GAAATACCATCTCCACCATACCGGAACCCTCTGTTGAAAATAATCTTTTTAGTTCCATCCACATTAAATTAAACAAAACTATCTGCAAATTACAACTTACCGGTCGTGGAATTATCATACCCG
AGAAAAGATTCCATAATAAATAAAACCATGTAGATGCTATCGTACCCACGTTTAAATTGGGTGTGAAGCCCACACACATCATTCCATAGTATGTGAAGTATAGGAACGTGAAATACATAATG
AACAAATACCAAAAGAACTTGGCAGTTGTCCATTCAAATCCAATCATTGAATAGACTATGACCCCATAAACGATAGCCTGACCCAATACATATGGGATCTCAATTGCGACCTGTCCAAATG
CATATGGTAAACTTGAGTACATTCCTGCTGCTCTTTCCCTATAAAAGACAGTTCTCTCGACAGACACCACAGGTTGAACTGAAGAAGCATTCGAAATTCCAAGAAACAAAACAGAAGCAT
ACATTGAACCCATTGCATTAAACAAATCCTGTTGTTTGCTCTTTTTGCCACCTAGATCCCAAAAAATTGTTCCAAATATCAAGGCTATGATCAATGTGAAGAAAAACCGCACTGCTGTGTAT
GGTGGATTTCTCCAGTATGACAAATGCTGTTTCCAAAGGCTAGCCATGCATTGAGAAACGAAGGTCTGCGAGTACTGTGTTGGGAAGAAAAGATCATTTGAACCAGGTGGAGGTATGCTC
AACTCCTTGATCAAAGCCTTATTCCTCTGAAACAATTCAGAATTCTTGTAGACATCAGTGAAATCAACCCCTAGTATTTCCTCTTGTGCTATTGTTGTCACTTCTAACATCCATGTTGCTGGG
TTATAGCTATCCTTTATTTTGCTTACTCCTTCAATTCCCTCAAAGTAACTAATCAGTTGACTAGAATGGTGACCCAATGGTCCTACATAGATCTCTTTTCCACCACGTTTCATTAGGAAGAGC
TCATCAAATGCTTCAAATATGTCTATGCTAGGTTGATGGATTGTGCACACTACAGTTCTGCCAGTGTCTACAGTATTCCTTACTGTTCTCATGACAATAGCAGCAGCTCTGGCATCAAGTCC
AGATGTAGGCTCATCCATGAATATAATAGCTGGATTAGCAACGAGCTCAACTGCAATGGTCAATCTCTTGCGTTGCTCTGTTGATAGTCCATTTACTCCTGGCAATCCCACCAATGCTCCCC
TCAATGGAGTAAGTTCCACAAGCTCCATTATCTCTTCTATAAACATCTTTCCTTTTTCAGAACCAACCTCAGCAGGCAATCGAAGCCATGCTGAATAAACCAAAGACTCATAAACTGTCAC
ATAAGGGGAATTGATATCATTTTGTTCACAATATCCTGAAACTCGAGCAAAGGTTTCCTGTTTCTTAGGATAACCATTTATCATGATGCTTCCTTCTATATATCCTCCAGTTTTTCTTCCGGCC
AATACATCCATCAAAGTTGTCTTACCAGCCCCACTAACACCCATCAGCGCTGTGAGCACACCTGGTCTGAAAGATCCACTTACACCTTTTAATAGCTCCAAACGGTCTTCTTGAATCCCTT
TGGCCTTCATTTCCTCTGGCATGTCCACAGAGTATCTTATATTGTCAAATGTCATGCTGAGTGGAGTAAAAGGAAGAACCATTCCTTTCTTGTTCAATTTGGATTCTGCAATGGCTTCTACAT
TTTTTGTTCCAATCCTGTTCTCATTCCCTTCAGAGCTCTTTGCTGTTGATGATATTTCAGTTATCTCTCCAGTAATATTTGCTTGTTTCTCCTTCAACGTCTCCTCAGATATGATTGGCTGGGAT
TTTCCAAATGCTACAATGATATGGAGGAATGGTTCATCTACTTTAATTTACATAATGTTAATATTGTATGGTACGTTTATGTGGATACTTAGTTGGAATGATACCTACGTTTTACAAAGGTAAG
AGCCACCGTGAAAAGAAAATTAAAAAGGAATATGAAGCCAATCAATGCTCCAAAACCTATCCAATACCATTTTGCTTCTGTAAAGGTTCCACGAGACTTCAAGATGGTCACCCCTAATGA
TTCTGTTGACCCAGGAAGAATATGTTTCCAGCTTTTCCCAAGGAACTCATTTGTTGCTAGAGCATTCAAGGAGTACATCATAGGTGAGATCCAGTAGCCCCATATCCACCATTTCTTCATGT

CATCGTGTGATAGAACAAATCCAGCCAATGCTACGACCACAAGCAGTGCAAATGATCCTAAAGAATTAGCAACAACCATGTCTCTGCTCAATCCAGCAATTAAACGAAAGAACGCAGAC
ATTGCCTGTTGGAGTGCTAAAAACAGCAAATACTGTTTAAAAAACCTTCCAACATTAGGATCATAACCTATAACATAATAGCTGACAAACACCCAGGTACCAACTTCAACTAGAGCTATGG
GAATCTTCAGAATCCACGAAGGTAATGTATATGCCCATGCAGGATAGAAGAGGTAGTCTCTTGCTTGAGGAAGACTGGGAGTTTCCAAATAGTCAATGGAATCTCTGTGAGCCCATTAAAC
ACGATCGTCTGTATAGAGAAGAACAATGAACCCACAAAAATTCCACCATCCAAGACTGAATCACGGTGCATATTTGTCCTTAGGAACAGCGTCATTGCAATAGATGCTAAAAGCAGTAGC
TGAATTGCTCTGAAAACATATGCAAACCAGTTGCGCTTCATCAGCAATAATTCTCTTGAGATGCAAGCTTTTAGCAGTTCCTTTTTGCTCACACCATATTTTGAAGTTGTTAAAGCTGCAGG
ATGTCCCTTTCTCGCGTCGAATGGGACAGCAAGCTCCTTCGTTAATTTGTCACCGATATGGAATGATTGGAATGCTTCTGAGAATTCTCTTGCAGTTACATATCTGTAAGGTTCTTCGTCAC
GTGCCCAATACTGCTGCTGATCTTTCCTTGATGTAACCTGCATGCCAATAGGCAATTTGCTATTTTTTTACCTTTATATATATTTCAAGATCAATTGAGAGCATTGAGGAAGGCGAACAACGG
ATAATTCGAAATTTTATTCTATCGTGGAACAGATCACATTGATGCACCCAAAGCATTGAATATATCAAAGAAGAGCCATTCGTGCGTTGCTCAATTGAAAATGACGATGAAAAATAATGGTT
GGCAAGCACGATACTCCATAAAATTATCCTCATGAATTCAAATCATCAAATGGATTGAGCCGATGTGATGTATTCGGGGCATCAAATTTACATAAGAGGGGATCCCTCACAATGGAAGATCT
AAATTAACAAACTACAGATGGAACAATATGAGATGTTGACCAAATGTCTTGCATTTCGAAAATCCTGCTGCATATAGATACTGCAAGGTTTACACTACTAAAATCCTGCTGCAGCAGTTGC
CCTATCCTAAATCTGTCAGTTCAGTGAAAATAAAAAATCTCTGTTAAGGTAAAATCATCTCCACTTTCTATTCAATTAGGGCATCTACAACCTAAAAATTGATTTTTCATTTCAAAATACAGT
AACATTCATATCAAATGTTAATTCATCTCCAACCAAACTCTACTTCAACCCTATTTTTATTCTAAAAAATAAGTCTCTATTAACTTCTAAAAAATGTGCTGTCAATATTGACATTTATTTATTCA
CGGAGGAGAAAAATGAAAATAATGTGTTTGCGTTTGGGGTTTGGGGTTTGGAATTTGGGTGGGAGGCTTGCGGCAGCTGTAAAGGCCTGTTTGCAGTTCGATTGAAGATGAACTTATATA
AGCAAAATACACTTGAACTCCCTCTGACCCTTCTCCCTCTCTGTTTGATTCCTACCAAACCTCCATCTCCCTTATTCTCCTCTTAGTCCTAACGCAACCAGCACGAACTCCTCTCCACTCAC
CCTACTC
>comp161439_c0_seq28
TCACTCAAGTTATATATTTGCACTGGTATATCTAAGAAGTAAGAAATAGCTTTAAGAGCATCATCCTCTGGTCATTAATTTAGGAGGAATATATCTTTTTATTCATTAATATCAGAATGTTTATA
AAGTGAATATGCCGATTCCACCCCCTAATTTTGCATCTAGAAACTTCTAAAAAAAGTAATATTTAGTTATTTAATTAATGTTTAATCATATATGATACAAACTGCCAGGGCCTCCTAATTTTTG
CCTAAAAACTTATTGTTGATGAGAATACATGGATGCAGTGTAACATTTCTTTCTTTTCATGGAAGAATAGTGAAAACATGAATAGATTTTAAATTCCTGCCACCATGACATCTTGGCTAACTT
CATGGGGATGCCTTCATCTTCTTTGGAAGTTCAGAGCTTTAATTGAAAATCCGAACAAGAAGGCAAAGAGTGTTGGAAACCCAACAATTGCAGCTGCAACTACTCCTAAGAAATCATGTC
TGAATCCAAAATAGTTTCTCACAAAATGAGAGACAACCTCACCAGTGTCAAGCTTGTCTTCTATATCTCCAAACTGTGAAGCTACCAATCCATAAAGTGTCCATGAAACTGGGCATGCCCA
GAAATACCATCTCCACCATACCGGAACCCTCTGTTGAAAATAATCTTTTTAGTTCCATCCACATTAAATTAAACAAAACTATCTGCAAATTACAACTTACCGGTCGTGGAATTATCATACCCG
AGAAAAGATTCCATAATAAATAAAACCATGTAGATGCTATCGTACCCACGTTTAAATTGGGTGTGAAGCCCACACACATCATTCCATAGTATGTGAAGTATAGGAACGTGAAATACATAATG
AACAAATACCAAAAGAACTTGGCAGTTGTCCATTCAAATCCAATCATTGAATAGACTATGACCCCATAAACGATAGCCTGACCCAATACATATGGGATCTCAATTGCGACCTGTCCAAATG
CATATGGTAAACTTGAGTACATTCCTGCTGCTCTTTCCCTATAAAAGACTGTTCGCTCAACTGACACCACTGGTTGAACAGAGGAGGAATTTGACACCCCGATGAATAAAACAGCAGTATA
CATTGAGCCCATTGCATTGAATAAGTCTTGTTGTTTTCTCCTATTGTAAGCCATTCGTAATTATAATGCATAAGTTCATGATTGATATGTGAGAGAATGGAAATAGGATACCAGTATTTTGATG
CAATTGAATAAATATTAACTTACATTTTACTACCAAGATCCCAGAAAATTGTTCCAAAGATCAAGGCTATGCCCGATGTGAATAAATACCTCATTGCAGTGTATGGAAGATTTCTCCAGTATG
AAAGGTGTTGTTTCCATAAGCAAATCATACATTGTGAAACGAAAGATTGTGAGTACTTGGTTGCGAAGTAAAGATCACTTGATTCAGGAGGAGGTATGCTCAGCTCCGTGATCAAAGACT
TGTTCTTTCTGCATTAACATGAAATTTTGTGTAAGATTACAGTAGATCCTTTCTCAAAGCAAAACATAGATGTGAAAGACTACGAATTCAACTCACTGAAATAGCGCAGAGTTCT
>comp161439_c0_seq29
TTAATTTCATATCTTAGACACAATTGAAAATCATATAATCTACAAAAAATAATTACAAATAAAAATAACTTTGCCTTAGCATTTCCCTAAAATCATAAAGAAATGAAGAAATACATCAAGGTC

ACAAGACCGTTCTTCTCACAGAACACATAACAAACAAATTTTGCTTCAATCATGACTAGTAGAACTTCTTGGTTTACTTCATCAAATCCTTCATCTTCTTTGAAAGTTCAATGCTTTAATGG
CAAACCCAAATAGGAAGGCAAAGAGTGCTGCGAATCCAACAACTACAACAGCAACCACTCCCAAGAAATCATGTTTGAATCCAAAGTAGCTTCTTATAAATTGAGAGATGACAACATCA
CTATTTTCAAATGTCTCATGTAAATCTCCAAATTGTGAAGCTACCAATCCATATAGTGTCCATGCAACAGGACATGCCCATGAATACCATCTCCACCAGACAGGCATCCTTGGTCGTGGAAT
AATAAAGCCAGAGAAGAGGTTCCACAATCCATAAAATGCTGAAGATACTATGGAAGCGATGTTATAATTGGGTGTTAAGCCTACGGCCATCATGCCATAGTATGTGAAGTATAGGAATGTG
AAGAACATGAAGAACAAGTACCAAAAGAATTTGGCAGCTGTCCATTCAAACCCAATCATTGAATAAACTATGATCCCATATATGATTGCTTGGATAAAGACATAAGGGATTTCAATTCCAA
CCTGTCCAAATGCATAAGGCAAAGCTGAGTACATTCCTGCTGCTCTTTCCCTATAAAAGACTGTTCGCTCAACTGACACCACTGGTTGAACAGAGGAGGAATTTGACACCCCGATGAATA
AAACAGCAGTATACATTGAGCCCATTGCATTGAATAAGTCTTGTTGTTTTCTCCTATTGTAAGCCATTCGTAATTATAATGCATAAGTTCATGATTGATATGTGAGAGAATGGAAATAGGATA
CCAGTATTTTGATGCAATTGAATAAATATTAACTTACATTTTACTACCAAGATCCCAGAAAATTGTTCCAAAGATCAAGGCTATGCCCGATGTGAATAAATACCTCATTGCAGTGTATGGAAG
ATTTCTCCAGTATGAAAGGTGTTGTTTCCATAAGCAAATCATACATTGTGAAACGAAAGATTGTGAGTACTTGGTTGCGAAGTAAAGATCACTTGATTCAGGAGGAGGTATGCTCAGCTCC
GTGATCAAAGACTTGTTCTTTCTGCATTAACATGAAATTTTGTGTAAGATTACAGTAGATCCTTTCTCAAAGCAAAACATAGATGTGAAAGACTACGAATTCAACTCACTGAAATAGCGCA
GAGTTCT
>comp161439_c0_seq30
TCACTCAAGTTATATATTTGCACTGGTATATCTAAGAAGTAAGAAATAGCTTTAAGAGCATCATCCTCTGGTCATTAATTTAGGAGGAATATATCTTTTTATTCATTAATATCAGAATGTTTATA
AAGTGAATATGCCGATTCCACCCCCTAATTTTGCATCTAGAAACTTCTAAAAAAAGTAATATTTAGTTATTTAATTAATGTTTAATCATATATGATACAAACTGCCAGGGCCTCCTAATTTTTG
CCTAAAAACTTATTGTTGATGAGAATACATGGATGCAGTGTAACATTTCTTTCTTTTCATGGAAGAATAGTGAAAACATGAATAGATTTTAAATTCCTGCCACCATGACATCTTGGCTAACTT
CATGGGGATGCCTTCATCTTCTTTGGAAGTTCAGAGCTTTAATTGAAAATCCGAACAAGAAGGCAAAGAGTGTTGGAAACCCAACAATTGCAGCTGCAACTACTCCTAAGAAATCATGTC
TGAATCCAAAATAGTTTCTCACAAAATGAGAGACAACCTCACCAGTGTCAAGCTTGTCTTCTATATCTCCAAACTGTGAAGCTACCAATCCATAAAGTGTCCATGAAACTGGGCATGCCCA
GAAATACCATCTCCACCATACCGGAACCCTCTGTTGAAAATAATCTTTTTAGTTCCATCCACATTAAATTAAACAAAACTATCTGCAAATTACAACTTACCGGTCGTGGAATTATCATACCCG
AGAAAAGATTCCATAATAAATAAAACCATGTAGATGCTATCGTACCCACGTTTAAATTGGGTGTGAAGCCCACACACATCATTCCATAGTATGTGAAGTATAGGAACGTGAAATACATAATG
AACAAATACCAAAAGAACTTGGCAGTTGTCCATTCAAATCCAATCATTGAATAGACTATGACCCCATAAACGATAGCCTGACCCAATACATATGGGATCTCAATTGCGACCTGTCCAAATG
CATATGGTAAACTTGAGTACATTCCTGCTGCTCTTTCCCTATAAAAGACAGTTCTCTCGACAGACACCACAGGTTGAACTGAAGAAGCATTCGAAATTCCAAGAAACAAAACAGAAGCAT
ACATTGAACCCATTGCATTAAACAAATCCTGTTGTTTGCTCTTTTTGCCACCTAGATCCCAAAAAATTGTTCCAAATATCAAGGCTATGATCAATGTGAAGAAAAACCGCACTGCTGTGTAT
GGTGGATTTCTCCAGTATGACAAATGCTGTTTCCAAAGGCTAGCCATGCATTGAGAAACGAAGGTCTGCGAGTACTGTGTTGGGAAGAAAAGATCATTTGAACCAGGTGGAGGTATGCTC
AACTCCTTGATCAAAGCCTTATTCCTCTGAAACAATTCAGAATTCTTGTAGACATCAGTGAAATCAACCCCTAGTATTTCCTCTTGTGCTATTGTTGTCACTTCTAACATCCATGTTGCTGGG
TTATAGCTATCCTTTATTTTGCTTACTCCTTCAATTCCCTCAAAGTAACTAATCAGTTGACTAGAATGGTGACCCAATGGTCCTACATAGATCTCTTTTCCACCACGTTTCATTAGGAAGAGC
TCATCAAATGCTTCAAATATGTCTATGCTAGGTTGATGGATTGTGCACACTACAGTTCTGCCAGTGTCTACAGTATTCCTTACTGTTCTCATGACAATAGCAGCAGCTCTGGCATCAAGTCC
AGATGTAGGCTCATCCATGAATATAATAGCTGGATTAGCAACGAGCTCAACTGCAATGGTCAATCTCTTGCGTTGCTCTGTTGATAGTCCATTTACTCCTGGCAATCCCACCAATGCTCCCC
TCAATGGAGTAAGTTCCACAAGCTCCATTATCTCTTCTATAAACATCTTTCCTTTTTCAGAACCAACCTCAGCAGGCAATCGAAGCCATGCTGAATAAACCAAAGACTCATAAACTGTCAC
ATAAGGGGAATTGATATCATTTTGTTCACAATATCCTGAAACTCGAGCAAAGGTTTCCTGTTTCTTAGGATAACCATTTATCATGATGCTTCCTTCTATATATCCTCCAGTTTTTCTTCCGGCC
AATACATCCATCAAAGTTGTCTTACCAGCCCCACTAACACCCATCAGCGCTGTGAGCACACCTGGTCTGAAAGATCCACTTACACCTTTTAATAGCTCCAAACGGTCTTCTTGAATCCCTT
TGGCCTTCATTTCCTCTGGCATGTCCACAGAGTATCTTATATTGTCAAATGTCATGCTGAGTGGAGTAAAAGGAAGAACCATTCCTTTCTTGTTCAATTTGGATTCTGCAATGGCTTCTACAT

TTTTTGTTCCAATCCTGTTCTCATTCCCTTCAGAGCTCTTTGCTGTTGATGATATTTCAGTTATCTCTCCAGTAATATTTGCTTGTTTCTCCTTCAACGTCTCCTCAGATATGATTGGCTGGGAT
TTTCCAAATGGTTTTACAAAGGTAAGAGCCACCGTGAAAAGAAAATTAAAAAGGAATATGAAGCCAATCAATGCTCCAAAACCTATCCAATACCATTTTGCTTCTGTAAAGGTTCCACGA
GACTTCAAGATGGTCACCCCTAATGATTCTGTTGACCCAGGAAGAATATGTTTCCAGCTTTTCCCAAGGAACTCATTTGTTGCTAGAGCATTCAAGGAGTACATCATAGGTGAGATCCAGT
AGCCCCATATCCACCATTTCTTCATGTCATCGTGTGATAGAACAAATCCAGCCAATGCTACGACCACAAGCAGTGCAAATGATCCTAAAGAATTAGCAACAACCATGTCTCTGCTCAATCC
AGCAATTAAACGAAAGAACGCAGACATTGCCTGTTGGAGTGCTAAAAACAGCAAATACTGTTTAAAAAACCTTCCAACATTAGGATCATAACCTATAACATAATAGCTGACAAACACCCA
GGTACCAACTTCAACTAGAGCTATGGGAATCTTCAGAATCCACGAAGGTAATGTATATGCCCATGCAGGATAGAAGAGGTAGTCTCTTGCTTGAGGAAGACTGGGAGTTTCCAAATAGTC
AATGGAATCTCTGTGAGCCCATTAAACACGATCGTCTGTATAGAGAAGAACAATGAACCCACAAAAATTCCACCATCCAAGACTGAATCACGGTGCATATTTGTCCTTAGGAACAGCGTC
ATTGCAATAGATGCTAAAAGCAGTAGCTGAATTGCTCTGAAAACATATGCAAACCAGTTGCGCTTCATCAGCAATAATTCTCTTGAGATGCAAGCTTTTAGCAGTTCCTTTTTGCTCACACC
ATATTTTGAAGTTGTTAAAGCTGCAGGATGTCCCTTTCTCGCGTCGAATGGGACAGCAAGCTCCTTCGTTAATTTGTCACCGATATGGAATGATTGGAATGCTTCTGAGAATTCTCTTGCAG
TTACATATCTGTAAGGTTCTTCGTCACGTGCCCAATACTGCTGCTGATCTTTCCTTGATGTAACCTGCATGCCAATAGGCAATTTGCTATTTTTTTACCTTTATATATATATTTCAAGATCAATTGA
GAGCATTGAGGAAGGCGAACAACGGATAATTCGAAATTTTATTCTATCGTGGAACAGATCACATTGATGCACCCAAAGCATTGAATATATCAAAGAAGAGCCATTCGTGCGTTGCTCAATT
GAAAATGACGATGAAAAATAATGGTTGGCAAGCACGATACTCCATAAAATTATCCTCATGAATTCAAATCATCAAATGGATTGAGCCGATGTGATGTATTCGGGGCATCAAATTTACATAAG
AGGGGATCCCTCACAATGGAAGATCTAAATTAACAAACTACAGATGGAACAATATGAGATGTTGACCAAATGTCTTGCATTTCGAAAATCCTGCTGCATATAGATACTGCAAGGTTTACAC
TACTAAAATCCTGCTGCAGCAGTTGCCCTATCCTAAATCTGTCAGTTCAGTGAAAATAAAAAATCTCTGTTAAGGTAAAATCATCTCCACTTTCTATTCAATTAGGGCATCTACAACCTAAA
AATTGATTTTTCATTTCAAAATACAGTAACATTCATATCAAATGTTAATTCATCTCCAACCAAACTCTACTTCAACCCTATTTTTATTCTAAAAAATAAGTCTCTATTAACTTCTAAAAAATGT
GCTGTCAATATTGACATTTATTTATTCACGGAGGAGAAAAATGAAAATAATGTGTTTGCGTTTGGGGTTTGGGGTTTGGAATTTGGGTGGGAGGCTTGCGGCAGCTGTAAAGGCCTGTTTG
CAGTTCGATTGAAGATGAACTTATATAAGCAAAATACACTTGAACTCCCTCTGACCCTTCTCCCTCTCTGTTTGATTCCTACCAAACCTCCATCTCCCTTATTCTCCTCTTAGTCCTAACGCA
ACCAGCACGAACTCCTCTCCACTCACCCTACTC
>comp161439_c0_seq33
TCACTCAAGTTATATATTTGCACTGGTATATCTAAGAAGTAAGAAATAGCTTTAAGAGCATCATCCTCTGGTCATTAATTTAGGAGGAATATATCTTTTTATTCATTAATATCAGAATGTTTATA
AAGTGAATATGCCGATTCCACCCCCTAATTTTGCATCTAGAAACTTCTAAAAAAAGTAATATTTAGTTATTTAATTAATGTTTAATCATATATGATACAAACTGCCAGGGCCTCCTAATTTTTG
CCTAAAAACTTATTGTTGATGAGAATACATGGATGCAGTGTAACATTTCTTTCTTTTCATGGAAGAATAGTGAAAACATGAATAGATTTTAAATTCCTGCCACCATGACATCTTGGCTAACTT
CATGGGGATGCCTTCATCTTCTTTGGAAGTTCAGAGCTTTAATTGAAAATCCGAACAAGAAGGCAAAGAGTGTTGGAAACCCAACAATTGCAGCTGCAACTACTCCTAAGAAATCATGTC
TGAATCCAAAATAGTTTCTCACAAAATGAGAGACAACCTCACCAGTGTCAAGCTTGTCTTCTATATCTCCAAACTGTGAAGCTACCAATCCATAAAGTGTCCATGAAACTGGGCATGCCCA
GAAATACCATCTCCACCATACCGGAACCCTCGGTCGTGGAATTATCATACCCGAGAAAAGATTCCATAATAAATAAAACCATGTAGATGCTATCGTACCCACGTTTAAATTGGGTGTGAAGC
CCACACACATCATTCCATAGTATGTGAAGTATAGGAACGTGAAATACATAATGAACAAATACCAAAAGAACTTGGCAGTTGTCCATTCAAATCCAATCATTGAATAGACTATGACCCCATAA
ACGATAGCCTGACCCAATACATATGGGATCTCAATTGCGACCTGTCCAAATGCATATGGTAAACTTGAGTACATTCCTGCTGCTCTTTCCCTATAAAAGACTGTTCGCTCAACTGACACCAC
TGGTTGAACAGAGGAGGAATTTGACACCCCGATGAATAAAACAGCAGTATACATTGAGCCCATTGCATTGAATAAGTCTTGTTGTTTTCTCCTATTGTAAGCCATTCGTAATTATAATGCAT
AAGTTCATGATTGATATGTGAGAGAATGGAAATAGGATACCAGTATTTTGATGCAATTGAATAAATATTAACTTACATTTTACTACCAAGATCCCAGAAAATTGTTCCAAAGATCAAGGCTAT
GCCCGATGTGAATAAATACCTCATTGCAGTGTATGGAAGATTTCTCCAGTATGAAAGGTGTTGTTTCCATAAGCAAATCATACATTGTGAAACGAAAGATTGTGAGTACTTGGTTGCGAAG
TAAAGATCACTTGATTCAGGAGGAGGTATGCTCAGCTCCGTGATCAAAGACTTGTTCTTTCTGCATTAACATGAAATTTTGTGTAAGATTACAGTAGATCCTTTCTCAAAGCAAAACATAGA

TGTGAAAGACTACGAATTCAACTCACTGAAATAGCGCAGAGTTCT
>comp161439_c0_seq4
TCACTCAAGTTATATATTTGCACTGGTATATCTAAGAAGTAAGAAATAGCTTTAAGAGCATCATCCTCTGGTCATTAATTTAGGAGGAATATATCTTTTTATTCATTAATATCAGAATGTTTATA
AAGTGAATATGCCGATTCCACCCCCTAATTTTGCATCTAGAAACTTCTAAAAAAAGTAATATTTAGTTATTTAATTAATGTTTAATCATATATGATACAAACTGCCAGGGCCTCCTAATTTTTG
CCTAAAAACTTATTGTTGATGAGAATACATGGATGCAGTGTAACATTTCTTTCTTTTCATGGAAGAATAGTGAAAACATGAATAGATTTTAAATTCCTGCCACCATGACATCTTGGCTAACTT
CATGGGGATGCCTTCATCTTCTTTGGAAGTTCAGAGCTTTAATTGAAAATCCGAACAAGAAGGCAAAGAGTGTTGGAAACCCAACAATTGCAGCTGCAACTACTCCTAAGAAATCATGTC
TGAATCCAAAATAGTTTCTCACAAAATGAGAGACAACCTCACCAGTGTCAAGCTTGTCTTCTATATCTCCAAACTGTGAAGCTACCAATCCATAAAGTGTCCATGAAACTGGGCATGCCCA
GAAATACCATCTCCACCATACCGGAACCCTCGGTCGTGGAATTATCATACCCGAGAAAAGATTCCATAATAAATAAAACCATGTAGATGCTATCGTACCCACGTTTAAATTGGGTGTGAAGC
CCACACACATCATTCCATAGTATGTGAAGTATAGGAACGTGAAATACATAATGAACAAATACCAAAAGAACTTGGCAGTTGTCCATTCAAATCCAATCATTGAATAGACTATGACCCCATAA
ACGATAGCCTGACCCAATACATATGGGATCTCAATTGCGACCTGTCCAAATGCATATGGTAAACTTGAGTACATTCCTGCTGCTCTTTCCCTATAAAAGACAGTTCTCTCGACAGACACCAC
AGGTTGAACTGAAGAAGCATTCGAAATTCCAAGAAACAAAACAGAAGCATACATTGAACCCATTGCATTAAACAAATCCTGTTGTTTGCTCTTTTTGCCACCTAGATCCCAAAAAATTGT
TCCAAATATCAAGGCTATGATCAATGTGAAGAAAAACCGCACTGCTGTGTATGGTGGATTTCTCCAGTATGACAAATGCTGTTTCCAAAGGCTAGCCATGCATTGAGAAACGAAGGTCTG
CGAGTACTGTGTTGGGAAGAAAAGATCATTTGAACCAGGTGGAGGTATGCTCAACTCCTTGATCAAAGCCTTATTCCTCTGAAACAATTCAGAATTCTTGTAGACATCAGTGAAATCAAC
CCCTAGTATTTCCTCTTGTGCTATTGTTGTCACTTCTAACATCCATGTTGCTGGGTTATAGCTATCCTTTATTTTGCTTACTCCTTCAATTCCCTCAAAGTAACTAATCAGTTGACTAGAATGGT
GACCCAATGGTCCTACATAGATCTCTTTTCCACCACGTTTCATTAGGAAGAGCTCATCAAATGCTTCAAATATGTCTATGCTAGGTTGATGGATTGTGCACACTACAGTTCTGCCAGTGTCT
ACAGTATTCCTTACTGTTCTCATGACAATAGCAGCAGCTCTGGCATCAAGTCCAGATGTAGGCTCATCCATGAATATAATAGCTGGATTAGCAACGAGCTCAACTGCAATGGTCAATCTCTT
GCGTTGCTCTGTTGATAGTCCATTTACTCCTGGCAATCCCACCAATGCTCCCCTCAATGGAGTAAGTTCCACAAGCTCCATTATCTCTTCTATAAACATCTTTCCTTTTTCAGAACCAACCTC
AGCAGGCAATCGAAGCCATGCTGAATAAACCAAAGACTCATAAACTGTCACATAAGGGGAATTGATATCATTTTGTTCACAATATCCTGAAACTCGAGCAAAGGTTTCCTGTTTCTTAGGA
TAACCATTTATCATGATGCTTCCTTCTATATATCCTCCAGTTTTTCTTCCGGCCAATACATCCATCAAAGTTGTCTTACCAGCCCCACTAACACCCATCAGCGCTGTGAGCACACCTGGTCTG
AAAGATCCACTTACACCTTTTAATAGCTCCAAACGGTCTTCTTGAATCCCTTTGGCCTTCATTTCCTCTGGCATGTCCACAGAGTATCTTATATTGTCAAATGTCATGCTGAGTGGAGTAAA
AGGAAGAACCATTCCTTTCTTGTTCAATTTGGATTCTGCAATGGCTTCTACATTTTTTGTTCCAATCCTGTTCTCATTCCCTTCAGAGCTCTTTGCTGTTGATGATATTTCAGTTATCTCTCCA
GTAATATTTGCTTGTTTCTCCTTCAACGTCTCCTCAGATATGATTGGCTGGGATTTTCCAAATGCTACAATGATATGGAGGAATGGTTCATCTACTTTAATTTACATAATGTTAATATTGTATGG
TACGTTTATGTGGATACTTAGTTGGAATGATACCTACGTTTTACAAAGGTAAGAGCCACCGTGAAAAGAAAATTAAAAAGGAATATGAAGCCAATCAATGCTCCAAAACCTATCCAATACC
ATTTTGCTTCTGTAAAGGTTCCACGAGACTTCAAGATGGTCACCCCTAATGATTCTGTTGACCCAGGAAGAATATGTTTCCAGCTTTTCCCAAGGAACTCATTTGTTGCTAGAGCATTCAA
GGAGTACATCATAGGTGAGATCCAGTAGCCCCATATCCACCATTTCTTCATGTCATCGTGTGATAGAACAAATCCAGCCAATGCTACGACCACAAGCAGTGCAAATGATCCTAAAGAATTA
GCAACAACCATGTCTCTGCTCAATCCAGCAATTAAACGAAAGAACGCAGACATTGCCTGTTGGAGTGCTAAAAACAGCAAATACTGTTTAAAAAACCTGCATTTTTTTTTTATTTTGTTAA
ATGCTGTAACACATTCGTCCTTCTAGGAAGGCCAACTAAAGCATATATCGTAATGCATCATCACCTTCCAACATTAGGATCAT
>comp161439_c0_seq40
TCACTCAAGTTATATATTTGCACTGGTATATCTAAGAAGTAAGAAATAGCTTTAAGAGCATCATCCTCTGGTCATTAATTTAGGAGGAATATATCTTTTTATTCATTAATATCAGAATGTTTATA
AAGTGAATATGCCGATTCCACCCCCTAATTTTGCATCTAGAAACTTCTAAAAAAAGTAATATTTAGTTATTTAATTAATGTTTAATCATATATGATACAAACTGCCAGGGCCTCCTAATTTTTG
CCTAAAAACTTATTGTTGATGAGAATACATGGATGCAGTGTAACATTTCTTTCTTTTCATGGAAGAATAGTGAAAACATGAATAGATTTTAAATTCCTGCCACCATGACATCTTGGCTAACTT

CATGGGGATGCCTTCATCTTCTTTGGAAGTTCAGAGCTTTAATTGAAAATCCGAACAAGAAGGCAAAGAGTGTTGGAAACCCAACAATTGCAGCTGCAACTACTCCTAAGAAATCATGTC
TGAATCCAAAATAGTTTCTCACAAAATGAGAGACAACCTCACCAGTGTCAAGCTTGTCTTCTATATCTCCAAACTGTGAAGCTACCAATCCATAAAGTGTCCATGAAACTGGGCATGCCCA
GAAATACCATCTCCACCATACCGGAACCCTCTGTTGAAAATAATCTTTTTAGTTCCATCCACATTAAATTAAACAAAACTATCTGCAAATTACAACTTACCGGTCGTGGAATTATCATACCCG
AGAAAAGATTCCATAATAAATAAAACCATGTAGATGCTATCGTACCCACGTTTAAATTGGGTGTGAAGCCCACACACATCATTCCATAGTATGTGAAGTATAGGAACGTGAAATACATAATG
AACAAATACCAAAAGAACTTGGCAGTTGTCCATTCAAATCCAATCATTGAATAGACTATGACCCCATAAACGATAGCCTGACCCAATACATATGGGATCTCAATTGCGACCTGTCCAAATG
CATATGGTAAACTTGAGTACATTCCTGCTGCTCTTTCCCTATAAAAGACAGTTCTCTCGACAGACACCACAGGTTGAACTGAAGAAGCATTCGAAATTCCAAGAAACAAAACAGAAGCAT
ACATTGAACCCATTGCATTAAACAAATCCTGTTGTTTGCTCTTTTTGCCACCTAGATCCCAAAAAAATTGTTCCAAATATCAAGGCTATGATCAATGTGAAGAAAAACCGCACTGCTGTGTAT
GGTGGATTTCTCCAGTATGACAAATGCTGTTTCCAAAGGCTAGCCATGCATTGAGAAACGAAGGTCTGCGAGTACTGTGTTGGGAAGAAAAGATCATTTGAACCAGGTGGAGGTATGCTC
AACTCCTTGATCAAAGCCTTATTCCTCTGAAACAATTCAGAATTCTTGTAGACATCAGTGAAATCAACCCCTAGTATTTCCTCTTGTGCTATTGTTGTCACTTCTAACATCCATGTTGCTGGG
TTATAGCTATCCTTTATTTTGCTTACTCCTTCAATTCCCTCAAAGTAACTAATCAGTTGACTAGAATGGTGACCCAATGGTCCTACATAGATCTCTTTTCCACCACGTTTCATTAGGAAGAGC
TCATCAAATGCTTCAAATATGTCTATGCTAGGTTGATGGATTGTGCACACTACAGTTCTGCCAGTGTCTACAGTATTCCTTACTGTTCTCATGACAATAGCAGCAGCTCTGGCATCAAGTCC
AGATGTAGGCTCATCCATGAATATAATAGCTGGATTAGCAACGAGCTCAACTGCAATGGTCAATCTCTTGCGTTGCTCTGTTGATAGTCCATTTACTCCTGGCAATCCCACCAATGCTCCCC
TCAATGGAGTAAGTTCCACAAGCTCCATTATCTCTTCTATAAACATCTTTCCTTTTTCAGAACCAACCTCAGCAGGCAATCGAAGCCATGCTGAATAAACCAAAGACTCATAAACTGTCAC
ATAAGGGGAATTGATATCATTTTGTTCACAATATCCTGAAACTCGAGCAAAGGTTTCCTGTTTCTTAGGATAACCATTTATCATGATGCTTCCTTCTATATATCCTCCAGTTTTTCTTCCGGCC
AATACATCCATCAAAGTTGTCTTACCAGCCCCACTAACACCCATCAGCGCTGTGAGCACACCTGGTCTGAAAGATCCACTTACACCTTTTAATAGCTCCAAACGGTCTTCTTGAATCCCTT
TGGCCTTCATTTCCTCTGGCATGTCCACAGAGTATCTTATATTGTCAAATGTCATGCTGAGTGGAGTAAAAGGAAGAACCATTCCTTTCTTGTTCAATTTGGATTCTGCAATGGCTTCTACAT
TTTTTGTTCCAATCCTGTTCTCATTCCCTTCAGAGCTCTTTGCTGTTGATGATATTTCAGTTATCTCTCCAGTAATATTTGCTTGTTTCTCCTTCAACGTCTCCTCAGATATGATTGGCTGGGAT
TTTCCAAATGCTACAATGATATGGAGGAATGGTTCATCTACTTTAATTTACATAATGTTAATATTGTATGGTACGTTTATGTGGATACTTAGTTGGAATGATACCTACGTTTTACAAAGGTAAG
AGCCACCGTGAAAAGAAAATTAAAAAGGAATATGAAGCCAATCAATGCTCCAAAACCTATCCAATACCATTTTGCTTCTGTAAAGGTTCCACGAGACTTCAAGATGGTCACCCCTAATGA
TTCTGTTGACCCAGGAAGAATATGTTTCCAGCTTTTCCCAAGGAACTCATTTGTTGCTAGAGCATTCAAGGAGTACATCATAGGTGAGATCCAGTAGCCCCATATCCACCATTTCTTCATGT
CATCGTGTGATAGAACAAATCCAGCCAATGCTACGACCACAAGCAGTGCAAATGATCCTAAAGAATTAGCAACAACCATGTCTCTGCTCAATCCAGCAATTAAACGAAAGAACGCAGAC
ATTGCCTGTTGGAGTGCTAAAAACAGCAAATACTGTTTAAAAAAACCTTCCAACATTAGGATCATAACCTATAACATAATAGCTGACAAACACCCAGGTACCAACTTCAACTAGAGCTATGG
GAATCTTCAGAATCCACGAAGGTAATGTATATGCCCATGCAGGATAGAAGAGGTAGTCTCTTGCTTGAGGAAGACTGGGAGTTTCCAAATAGTCAATGGAATCTCTGTGAGCCCATTAAAC
ACGATCGTCTGTATAGAGAAGAACAATGAACCCACAAAAATTCCACCATCCAAGACTGAATCACGGTGCATATTTGTCCTTAGGAACAGCGTCATTGCAATAGATGCTAAAAGCAGTAGC
TGAATTGCTCTGAAAACATATGCAAACCAGTTGCGCTTCATCAGCAATAATTCTCTTGAGATGCAAGCTTTTAGCAGTTCCTTTTTGCTCACACCATATTTTGAAGTTGTTAAAGCTGCAGG
ATGTCCCTTTCTCGCGTCGAATGGGACAGCAAGCTCCTTCGTTAATTTGTCACCGATATGGAATGATTGGAATGCTTCTGAGAATTCTCTTGCAGTTACATATCTGTAAGGTTCTTCGTCAC
GTGCCCAATACTGCTGCTGATCTTTCCTTGATGTAACTTCCTGCAAGAAATCCGCAACCCCTTTTCTCTCCGGGCATTTGAAGCCCATCAATTCAAAGAATTCAAGGACATTCTCACGAGG
GCCATGGTACACAATTTGAGCATCCGAGAGAAGAATTATATCATCAAGAGGTCAAATGTTTCTGGTGCTGGCTGGAGAAGGGAAATAACAGCGGTTCCATCAAGTATATGTATATATTGTCT
CAATGAGCTGACAATTTGGTAAGTTGTGGAGGTGTCAAGTCCTGTGGATATCTCATCCATGAACAAAGCACTTGCTGGTCCAACAATCATCTCACCTGTTGTAACTCTTTTCTTCTGTCCTC
CAGAAATACCTCTAATCATCTCATCTCCAATCATTGTGTCAGCACAGATGTCCAGCCCTAAAATCTTTAGTACATAATCAGTTACTAGACCCGTCTCTTGTCCTTCCATTGAACTTGCCTTCA
TAAAGACATCAACGTCAGGGTCTGGCTTTATATTTGCTGCTTTCTCTCTCCTTGAAAGCTCTGCCAACATATCATAACGTTTACCAACTCCTTGACACCTTGCAGAGAATGATAGTGTTTCT

CGAACCGTCATTTCTCCGATATGGACATCATACTGGCTGATATAGGCAGCATTTCTTTGAGGTACAAACTTATTCATTTCTTGACCATTGTAAGTCACTCTTCCTGAAACCTTCACATCAGAG
TCCAATTTTCCAGCTAGAGCCAGCAGTAAAGTGGTCTTCCCTGAACCAGGAGGTCCTAAGAGTAGAGTCATCCTTCTAGGCTTTATGAGTCCACTAACATTATGAAGTAAGGTTAATGGTT
TCTTCCTGCTTGGTAGAATGTGAAGGTTGTTGGCAAAAGCCTCAAGCACGTTTATTACAGAATTGAGCAGCGTAGGCAATCCTCTGTTGCCCACATATGCTTCAGCTTTAACATTAAGGTT
CTCATACCGTACTTCGATGGTTGGCAATTCAATCCCAACATTATCAATCCTGTTTCTGATTCTCAGCAAGAACTTCTCATTATCATCATCTGCCACTCTCACCAACTTCTCCAACAACTCCCT
CCTCTCCAGAAACCCGAGACTCTCTATATCAACTTCCTTCTTACCTCCTTCAGCCAAGTTCAGAATGCCTTTCCTCATTCTATTGTAAGTCGGCAGCCTCTCGATGGCTGCCCATGTTAGAT
CCTCCTCGTCGTCTTCACTGTGCCTGGACCTTGAGAAGACCTGATCTCCATTCCATATGGAAGAGCGGCGGGACCTTGAGCTTGTGGCACGCAGAACAGACCCAGCTTCCATTTCAATGA
ACACCTTTCCTTCTCTCTTGAATGCAATGGACAAGAAGTAAAAGATACCAAGAAAATGAGATTGTTTAGTATGGTAAGAAGAACGATTCTGATGAAGTTTGAAAAAAAATATTTTGGAAT
GGAAGGGTTTTAAAAAGGTTCAAGCATAGCGGAAACCATTATGAAGGACAACTGCTTTACAGTGAAAGGTTTACCCTTC
>comp161447_c0_seq1
AGCTTGTAACCCAAGTGGGGGTATTAAGGTACTCCGGATGGTTGATCCGGTGGGCCCACCGGCTAATTTTCATTGCGCTGAGGTGCCCGACCCGATACAAGTTTGCAGGAAAATGCTGTG
GGTTTGCAGATAAGTAACTGCGTTTATAGGCAGGCGAAAAGCCTGGGTTTAACGGCGGACGTGAGAGAGAGAAGAGTGCTCCCCGTCGTTGGGTTAATGGTATTGGTATGGGTAGCTGG
GCCTTTGGTTTAGTGAAATTAGCCTTGGTCGTTTAATTCAGACGGGTACCTTTTATGTTTTTACCTTAATATTAACTTTATTCTTTTCTTCTGT
>comp161448_c0_seq5
TAAAATTTTTTGCTGCCACAAAGACTATAAATAAGAAAAATTATTTTGTGCGGGCGTCGTACAAAGGCAAATCAGAGCACACCACGTCATCCCTTTGTTGGATATCGCAGCTATGTACTGA
GTGATGTGATACGCACTGATTCACTTCCGAACAATCCGATACCTACATAGAATAATATCTCCATAAATAAACTTATAAAGTATAAAGGCAAGAAAAGCTGCAGTATTTGTTGACTTGTTTCTC
AACCACTACTCAACAAAATCGAACTACAATGGCTGCCACATACTGATCGAATGAAGCTCATCTTCCACCTCCCCCATCGTTTTCTTTCCACGTTTGTGGGCGACATAATAAAGTTGACACA
GCCTGAGATACAGGAAAGCAAATAGAGAGGGGAAAGAAGAGAGAATGGGTAGCAAAGGTGGTGGCGCACTACAATCCCCGTCGTCTCCGTCGTCGCCGTCGGTGGTTTGTTGCATGTG
CGGAGATCGGGGGCTATCACGGGAGCTCTTTCGGTGCAAACTCTGCGCCTTTCGCTCCCAACACAAGTATGTGGTAAGAAACATGAAAGGATGTTGTAAAGATTATAAATTTCTTCTGTTT
TTGAAAAAAAAAAAAAAAAACTGATAACAATTAACATAGTCTAATTCAAGCTCTCAAATTTGCTTCAATCCCTATGAAGGCTATGATCCAATTCAATTTTTTCTTGGTCTTCAACTCCTCCA
TCTTAATCCCCACCTGATTGATGAATATTTGATTGATATATTAGATATTGCAGCGATCTATACCCGAGAGCAGAGGTCTATCGAGCCTGTAACTGGTGCCTGAGGAAGGAAGCAGGAAGCAG
GTTACTTTTGGTAGAACAGACGACGGTTGATCGAATTATTTCAGCTTCCTCTTCTCCGGTAGGTGGCACAAGCTCGGGATTGAAGCTCGCTCGTGGGGCGTTCTCGCCACAGCTTCTGAAT
AAACCGGTGAAGAAGCAGAGATTGGGTGACAGGCGGCTGCAGCCACCGGCGGATTCCGGCGACCGGGGCAGCGGGGAGGAGCTGTCGCCTGGTTCTGGGAGGGGACGACAGGTGTTT
AGAGGAAAGGTGAGGAGGTATAAGCTTTTGGAAGAGTTCTCTAGCTAGTTGAGGCAGGTTGGTATTTGCGTGTATGACAGAGATTCTACAAGGAATCTTTATAGCAAATTTTGGAAAGAA
ATGTCTTTTTACTATGGGATTCTATATGATCAATTGATCATTGTATTTAGTTAAGTTTTTATCTTTTGTAGAGATTTGTTCGCGAATTACTACCTTTATTTTGAAAACCGAATTAGAACTGATTG
GCTAG
>comp161536_c1_seq1
GGTTCGGTTGACCGGATGCAAGAAGCATCCGGTCGTAGGAAAAAAAAAGGCGGTACCGAGGTGTACCGAGTTTTTCAAAAAACTCGGTACACCACGGTACCGAGTTTTTTTGCCTACG
ACCGGATGCTTCTTGCATCTGGTCAATCGAACCAAATAAAAGTTCCTCCCCTACACAATAATGAATACAGATTACACAAACCCAGCAACTTCATAATTTCTGAATTAAAAAAAAAATCCCA
TCTGCAAATCCCTTCACTAATCTCCTCCGCTTCCACCCTCTGTTTTTGGCCTTTTTGCTTCCGG
>comp161576_c1_seq16
AAAATTCACTTTGAAGAGTGAGAAATTGTACAGTTTTTTCACTATTGCAATATTATACAGCCTTCAGACTTTCATACACAGAGCATAACCACCGTTAAGTAACAATTTACAAAGGTGAAA

AGAAAAAGACTAAGTTGGACTACAACAACGTGATGTTGGACCTACTGCGTCTTAGTGTTGGGTTCTCTCTCTCACAACGGGTGGTCCATAGTGCCTTAGGCCTTCTCCAACCAAACTCCA
AACGTATGAATGATGTCGCTTGCCAGCTCCAGCCAAACTGTAGCCAGCTCCAGCCAAACTGTAAACCTTTCGGTATTTGGTTGCGGGTGA
>comp161633_c0_seq2
ATAATTAATGAATTATTATTCAGATAAGTAATCGATTACAACTCAACTCGATCCTTAACTAGAAGCCAAGAAGCCAAGAAGCCAATAGAAGCTTCTAGCATTATCTCGATTTTTCTTTGAACA
AAATGCCCAGCCCCGTCATCCGCCCCTATATATTCCTCCCTTGATCCCTAACACTTAACATATTTTCAAATCTCAAGAAAAAGTTGAGCAAGAACAAGAAGACAATGGATGGTCAGTTTTT
CGGAATGCGGAGCCAAATTTTCCCGTCGCTGCCGATCGTGCCAGAAGACGTGCCGGGAGAACGGGAGAAACTGGCGAAGATCTGTCTTCGCGAGATGAGGGAGAGTGTGCCGACGCTG
GCAAACGTCAAGGAGATGCCGTCGGCCTACGAATTCGTAGTCGATATGCCTGGGGTCAAATCTG
>comp161644_c0_seq1
CAAACAAAGCCGAAGTCGAGCAATTATAAACATCGATACAGTCAATCAAACAAACTCTGCTAGCGTTCCAAGCACAAATTTTCTCTTGAACTCAGCTGGTGCTAAGGTAAAGTTTCCTTA
ATTCTTAACAAACAAAAAATAGAATAGAATAAAATGTACAAAATAGCCTCTTACAGTAAGCTATTTCTGGTTTATAAATAAAAGAAATTAATATGTAGTTATAATAAGCCAATGAACTTTATG
AAGCAAAATATAGTACCCATGGAGAAGGTGATGATTATAATCAACTGGGTAGTCTGGTGAGACACCTCTTCAGCCTCTTGCGCTTGGACTTTTCTAGAAGAATCAATAAGCCGGTCTTCTG
ATTTGATAAACCAATGGGGTACCTGATATTATAATAGCTTGCGGGCTTTAGTTAACTCTGATAATACGCTACAAGGTAGAGACAAAAAAACAAACACCCAATGTGCATACTTGATCCTTGGT
GATTTCTTCCTCCAAGAGAAAAATGATGCCACCCACTTAACCACCGACATTATAGAGAAACTCCTTATTAAAGAAGGCTTCTTTACAATCCTCGTCTCTGGCACCACCTCCTTAGACAACT
GCTGAGCAATCTCCTTAAGCAGAATCCTCTGTGCCTTATCCGTCCCCATCGTCATGATAACCTGCTTCATTGACTCCCTGTCTGCCTCCAACGCTTGCAGCCTCATATAGAGCTTCTTAACCT
CTCCTTCATCCACAGTTCTATTACCCGCATCCCTCCTGCTTAAAAAGTCCCTCGCCGTGCTCATATCAGCCCTATCATACATCCCATCCACGTCGTGCACGGCATCAATAGTGCACACTCGAT
CACTTATGTCATCCCTCACACCGGTACATGCATCAGAGGCGCAATCTGCCATGGATGGTAACTCAGTGCTTCCAAACTCCTGTCGGGATGAAGCAGAACCCAAACTGTCAATGGAAAGCC
GGCGCATAGCCCGCTGTGCCCAGCTGGGTGAATGGCTATTTTCGATTACACCCTTTTCCATGATGATACTAGGCATGCGCTGAATGGGACTACGGGGTTTGTCACCGGCGGCATACTTGAG
CTCCGGGTCCACAACATTTGTCCTGAAATCGGCCAATTCACAGCGGAGAGAGGAATAGGCATTGCCGCCGTCCCCAGCGGCTGTTGAGGTGGTCGCAGCGGTGACGGCGTTGGAGGAAT
TGGATTCAATAGTGGCAGGGTCGATGCCATAGGAGAGGAGACAGTGACGGTAAGCCTGGAGCTCGCAGGACAAGGATTGCACAGCTTCTTCTCGCTTGAAAAGAAGATCTTCAAGGAG
AGCAATCTCTTGCTGATCATGAGACATTTTCTCCTCGGATAGGCGCTTGAACTGACGTGCGTCCATCTGGGCCTCTGCTTTATCTCGTTGGAGGCGGAGAATCATCGACATGGCCTCGGAT
GCGGCAGTGGAAGCTGCAGCCCGCTCCTTCTCGAGCTCGGTACAGAGCACCTGCATGGATTGCTGCTGGTTTGCGAGGGCTTCCCGAAGAGCAGCCGCTTCTATCTCGATCTCGACGCG
CGCCACATCCTCCGGACGCCCGCACTGAGCGTCCTCCTCCATTTGCTCAAGCTTGCGCTTCAAGGCTCTGGGCAAGGGGGAAGAAGGTTGAGAACCAGAACCGCAGTAAGGGCAAGAG
CAAGAGCAATTGCATGGCTGAAAATTAACGCTACCCCAGCCGCCGCTGCTGATCTCCACATCCATAAACGTGGTGGAGGCAGCGACAGCAGCATTCAAGAAAGTATCGGCTCCGTCGGG
CTTGATTGTATCTTTCCTACGTCGGCGAATGCAAGTCGTTAAAGACAGCTTTCGCCTCTCTCTAGCTATGCTGAGACGGAATTAGCGAAAGGAACAAGGTAAACAATAGAATGATAAGAA
AGAGAAATGCCAGCGATATCCGTGGAATCGTAATTCGAAGCTGCATGGAACGAGGGAGGGAGGAGGATTTGAAACCCTAGGAGGTCCTTTTCTTTTCAGTTTTTCATTCTTTTGGACCTC
GCTGTCGTTTGTTGCTGTTTTTCTCTCTCTAACTGAAATTTTCTGCTTCGATTTGCTAATTTAAGAGAATAATAATACGTCGAACAAA
>comp161798_c0_seq2
CCGCCACTATCTACCTTATGTTATGTTCATTATCTTTAGTTTCTCTATTACTCTTATTCTCTAGCTTTTGTCCTTTTTCAGTTACTTAAGTATGCTATAACTTTTAGTATGTGGGACCTAATATTGT
AATAGAAGGAAATACATAGAAGTATAAGCAGGGAAAGAACAAAGGGGAGGCACCTTTTTGTTTTTGGAGAAAGAATTTCTAACATTTAGTGGAATCCCTGTTTTATAAGCTGCTTCCCTGT
ACTGTTTTTTCTTGTATAAGTCCACTTAAACAAAATTAGTATGGATTACATGTTTGACAATATATTTTGAACTCTGTAAGAGAAGTTATAAAATTTGTGGCAAATATCGTCACCCAGAAAGAA
GGCATCAGCTGGAATAGCCCACTCTATGTTTTTGCTTTGCTATGCATGATGTCCCGTTGCAGGGATGCTTTAGTGCTGGATCATCTGCAAGTGGATGCTGCCCAAAGATAATCTCACAGCTT

TTGTCTTCAAAATTAGGAACCATGTTGGTGGGCATCCCAAGTGAGGTGTTGATGAACTTTTGAGAAGGCAACTTGCAGAGATTAGTGCACATTCCTACACAATTTGTGGCCTCCAAAAAC
CTGCATTTAGGAATGTAGACCACATTCTTCTCCCTCTTCCCTTGGAAATCCGACTCCCTGACCTCACAGGGACCTACGAGCCAGGAGAAAAACACTGTTGTGAAGATTGCGAAGAATTCT
CTCGAAAACTTTGATGGTGGTAGAAGAGCCTTCAACAGTAGACTCTTCCTCGACCACTTTCGGAATTCCACCTGGCAGCCTACTGATGCCAGAGTTCTGCCCTAACGTCAACTGATGCCA
GAGTTATGCCCAGTTGTTGACTGACATTGGAGTTCTGCCCGACTTTCACCCGTCGT
>comp161798_c0_seq5
TGTATTCTTTTTTACTTCAAATCAAACATCAACATTTTCAAAAATTTCTGTATGTGAAATACGTTACACTCCTAATGTAGCAAACCAAATGATCCCTTTGTCAAATCAGAACCTCAATATAAT
CTTTAGAAATAGTTTTGATAAATTTCAAAACAGGTCATTGTTGTTCATATTTGGAGCTTTTGTAATATTGCTTCACGCTACCAATTGCCAACATTCAATGCTTGTTTTTCAAATTTGTCCACCA
ATCTTCTTGTAAGGAAGATGACCAATCTTGCAGCCTCCTAAAGTCTGCCATTTGCTAACTTCGACATCCACCAACAACATACATCTAATGTTAGCCAACCTTCGATGTCCAAAGTCCATATG
TTAGATCGCTCAACCTCTTTTCATGTAACGAGAGCAATTAATTGAACTCTTGTTATTGTAATAGATAACATTGATGCTGCTTTCAATTGCCTTTTTGTTTTTGGAGAAAGAATTTCTAACATTT
AGTGGAATCCCTGTTTTATAAGCTGCTTCCCTGTACTGTTTTTTCTTGTATAAGTCCACTTAAACAAAATTAGTATGGATTACATGTTTGACAATATATTTTGAACTCTGTAAGAGAAGTTATA
AAATTTGTGGCAAATATCGTCACCCAGAAAGAAGGCATCAGCTGGAATAGCCCACTCTATGTTTTTGCTTTGCTATGCATGATGTCCCGTTGCAGGGATGCTTTAGTGCTGGATCATCTGCA
AGTGGATGCTGCCCAAAGATAATCTCACAGCTTTTGTCTTCAAAATTAGGAACCATGTTGGTGGGCATCCCAAGTGAGGTGTTGATGAACTTTTGAGAAGGCAACTTGCAGAGATTAGTG
CACATTCCTACACAATTTGTGGCCTCCAAAAACCTGCATTTAGGAATGTAGACCACATTCTTCTCCCTCTTCCCTTGGAAATCCGACTCCCTGACCTCACAGGGACCTACGAGCCAGGAG
AAAAACACTGTTGTGAAGATTGCGAAGAATTCTCTCGAAAACTTTGATGGTGGTAGAAGAGCCTTCAACTGTGATGGCTGCAATGCTGCTTGAGAGATGATGGATGGCTAGGCGGCCAA
ACCAGTCGTCTTTATATACTGTCCTCTCCTCCATCGCCGGCGTTGATTTTGTTTCGGGGATTCTGTAATTGGCAATTTCAGGGCTATTCGGGTCTGTAATCGCTGCCATCGTCGACAGCTTCT
TTCTCGAACACCGCATCGACATGGAGCACCAGGTTGACTGACGGCAAGTTGGGAGAGGGATTAAGCCTCCAAGCTGAACAATAAGACCCCTTTGCATGATTTCTGCGTATGAGGAAGAC
GAAGTGAATGTTGTAGCGTTGAGCCT
>comp161812_c1_seq1
CAAATCTCCTGCTTTATTGTAGATGCTCGCATCCATTGTAGTTCTTTGGCATTTATTCTTAGGTTGTATGTAATATTCGTACTGGAGCGCCAGTAGAAGCTCGTGTGAGCTTGTATCTGTCGTA
TTCTTTATCCACTTTGTTGTAGTATCTGGCTTACGTAGTACATCCGTCACTGCTGTATGTAGGTAGATAGATGTTTTC
>comp161816_c1_seq2
TCTTGCCACAAACAGAATTGAAAGCCTGGATCCAGGCTTGCTTCGGCCAGGACGCATTGATAGAAAAATTGAGTTTACTCTTCCTAATGTCAAAAGTCGGCGAAATATTTTTGAGATACAC
ACTTCCAGGATGACACTAGCTGATGATGTCAATCTTGAAGAATTCGTGATCACAAAGGATGAATTTTCTGGAGCTGATATAAAGGCCATATGCACTGAAGCTGGTTTGTTTGCTTTGCGTG
AACGCAGAATGAAGGTAACTCATGAAGACTTTAAGAAGGCAAAGGAAAAAGTCAAGGTTAAGAAGAAGGGAGTGCCGGAGGGACTTTATTTGTGATAATGACGAGTGTTGTAATGTGA
AATGATATCCAGTACTAATCGAACAGGATTCAACTAACCAACATGCCCAGTTAAAAACAAGTTTTAGGCAAGAAAAGGGATGTTGTGCTGTCAAGCGAAGCATTACTTCTAAAGTACATT
ACCTCCTTCAATGCTCTAATTTGAAATTCAGTTTCATAACTGCTACATTTCAATTTCTGCTTGTTGTGGTTTTACACTTTGGGTCAAGAAAAAAATACAAATTGCTCATGTCACACCACAATTC
TTTCTACATTTGACTTTGCTTTTTCTTCATAATTTTTTAAGACACCAATTTCTTTGTTCTGTCCTGCACATATCACTCACTTGCTGTCTCTATGGGGTTTCATTCACAATTATAGATAATTTTATT
GCAAAGTAGCATGAGAAAGATGGCGATTTTACTTTGATTTAGCATAGTGTAACTCTGGTGCAGATTTCATTTTGAATAA
>comp161823_c4_seq6
ATCGAGGATCAAGTCCAGGAAGCTCGTGATTATTTCTACGACGTAGAGGTGAAGGCCTTAGAAGAGGAGCTTATGCAGGATGGTTTCATCAAAGGCTTTATGAAGGGTGTTCGTGCTGTG
AAACGCAGAAATGGAGGGGACATCGAGGGGTTGACCCCAAGCCAAGCTTCTGGTGATCCTTCTCCAGATTCCGACGACGAAGAGTTGGAGAGTGAAAGGCAAAGGATCTTTGCGTTGG

AAGAGGACGACGAGGATGATCTTGAGATTCTGTAAAATTATTTTGATGCTTTCCGTGTCACTTTTATTTTTGTTACGATGATTGTTCGTGTGTTGCTGCTGCTTGCGTGTTGCTGTCGCTCGC
GTGTTGCTATTTTTGGATGTTGTACTTGATGTTCGTGGGTTTATATAAAAAAGACAATATTCTTTCTTATATATGTTACCTCCTGTGTTGAGGAAGTCTTTCTAATTTGCATGAATATGAGAGAT
ATCCGCTTTGGGATACTCTGACCTATCCGAGGTTGTTAATCAAAGCGTTTGCTTGATGATTTTAACCTGTGTGGAAGAGGTCGGACCGGGTCCCCCTGGGATTGGTCGTTTACTGATTGAG
ATACAACATAGGTTGTGTGTTTCTCGAATCATAAAAGGAAGCTCAGAACAGAAGAGACGATGCAATGATAAAGTAAACATCACATTACTGTATTTAAGAAGATAGTATAATAATTTCCTAAT
ATAACTTAGGAAATTGAAAAAGAGTTCTCCCACAATCAACACACAAGGTGTGTATGAAAAGTCAGCATAAAACTTGAAAGATACAAAGTATAACCACTTAAGGGGACATAACACGAGAC
AAATACTACCAACAACATAACATGAGATAAATAGTATTATCAACTTCTACTGATAGTACTTATGAAGGTTGCCAACATTCCAAGTTCGGAGGAGCATTTTTCCACTAGCATCCTTCAGTTTG
AAGGTTCCTGGCTTGACCATAATTGCTACCAAATAAGGACCCTCCCAGAGGGGGGAGAGTTTGTTACGTTGAGGACCTTTTCCTGCTGCCTCGATGCTACGTAGAACCAAGTCTCCAATT
GAAATATGTCGAGGTTTCATTCTTCGATTATAATAACGGACAACTCTTTGATGATATGCAGCAACTTTAAGCATTGACGCATCTCTTACTTCCGGAAGGAGATCTAAATTATTTCGGAGCTTC
TCCTTATTACTGTTGAAGTCAAAATCTTGGACTCTAGGACTTGGAATTCCAATATCGATGGGAAGTACTGCTTCTGTGCCAAAACATAGATTATACGGAGTTTCTCCTGTTGGAGTTCTGGT
GGTGGTACGGTAAGGCCAGAGGACATTCAGGAGTTCATCAAC
>comp161860_c0_seq5
GCCGAAGATGAAGTCGACGGTGCCTGAGATGCGGCAGTCGCGGTAGAATTGGCGGAGAGAGTGGGCGTAGAGGGTGTCTTGGTAGCCGCGGATGCCGCAGCGGTAGAAGACGGAGAG
GTCGGAGTCAGATCGGAGAGCTACGGCTTGGTGTTTGGATGGTCCGGCGGTGTTCTCGAACGTTATATCGCGGGCAATGAAGCCTTTTCCGGCGACGGCTGTTGTGCAAGTAAGTGAATG
ATTAATTTAGTACAATTTTCTTATTATTTTAATTTTTAAATAACCACTATAATTTTATTTCACTATTATTTTTTCATTATTTTTTAAAATTTATATATCATATAAATTTTAAAAAATAATGAAAAAATA
ATAGTGAAATAAAATTATAGTGGTTATTTAAAAATTAAAATAATAAGAAAATTGTACTAAATTAATCATTCACTTACTTGCACAACAGCCGTCGCCGGAAAAGGCTTCATTGCCCGCGATATA
ACGTTCGAGAACACCGCCGGACCATCCAAACACCAAGCCGTAGCTCTCCGATCTGACTCCGACCTCTCCGTCTTCTACCGCTGCGGCATCCGCGGCTACCAAGACACCCTCTACGCCCAC
TCTCTCCGCCAATTCTACCGCGACTGCACCATCTCCGGCACCATAGATTTCATCTTCGGCGACGCCAAAGCCATCTTCCAAAACTGCCAAATCTTCGTTCGCAAACCGCTCGAAAACCAG
CAAAACATCGTCACCGCACAAGGCCGCAAGTCCCGTCAATCCGCCACCGCAATCATCCTCCACAACTGCACCATCACCGCCGACCCCTCTTTCCCCCCCGCCTTCCACGCCAAGAACCCT
ACCTACCTCGGCCGGCCGTGGAAGGAGTTTTCAAGAACGTTTGTCATCCAGTCCTTTCTCGATACGCTCGTGCATCCTAATGGGTGGCTGCCATGGCTCGGGGATTTTGGGCTGAGGACG
TGCTTTTATACAGAGGTGGAGAACAGGGGACCGGGGGCGGATAAGAGCCTCAGGGTTAAGTGGAAGGGGGTGAAGAATATTAGCTATGCGCATGCGCAGAAGTTTACAGTTGAGCGTTT
TCTGTGGGGAAATGACTGGCTCCCTTCGACCCAGGTGCCATATATTCCGGTATTGTTGCCGCAGTCGTTGCCTGAAAGGATTCACTGATGAGAGATTTCTTCTTGTTGTCTTCATATAAACA
TATCCTTGTCTTCCATAAAAGAGATGAAAGTTAGTATCTTTATGATGCATACGTTGTTTTGTCTTAAAAATTTCATTTTTTTTCCTTCGGTGTTCCGATGCCTGTAAAAGG
>comp161913_c0_seq1
TCCATCACAAGATGAACTGAATTCCTGTCCTCATATGAGCCCTTGAATTCTACAATATTTGATTGACCTGTTAAGTGTTGCAAGATTTGCATCTCCCTCTTGATATCCTCCTTATCATTTTTAT
GAACAAGTTTGCGCTTTGAGATCGACTTGCAGGCGTAAGAATGGCCTGTGGCAATCTCCTTGCAGAAATAGGTGACGCCGAATTGGCCGCGGCCAAGCTCCTTGCCGAGTGAATAATGG
CTGCGAACATCCTCAAATGGCTTGCCAAGGATTGTGTTATTGGTTGGGGCAGGCTTGGGTGGAGATGCAATCGGTGGGTTTGGCTTAGCCGGAGATGGTGGAGCATAATGGGTTTCCGGC
GATGGACGTGAGGGTTTCACCGGTTGTACCGTCGCGGGAGGCTGGTGGGAATGGCCATTGGCTTCCAGCCCTGTTCTTTTCCCTTTTCCGCAGCAAGATCCCATCTTTCTTCTTCCACAGT
CGATACTTTTGATTTTGATATTGGATTCAACTTCTTTCTAGGAAACCTTGATGATCCAGATCTTTACAAAATCTTGCAAGTTAAGAAGATATCAAAATGGCCAAAATCATTTTTTTCTTTTTTT
TCCTTTCAGTGGAAGAATGATGGGAAGGAACCGCGTTATCCAAATTAAAAAAATAATGACAGGAACGATTCGGAATCTGATAAAATGTACAAAGGAGGAAGAAATGGAAAGGAAAGCCT
GTTAGCCGCCATATTGCGTTAACAGATCAGTGGCAGAAGGAAGTTAGGCTCTTTCCAACGAAAAAATGATTCACGGCCATTATTTTATTTAAATTCCTAAATTTAGGATAGGAATGTGGGCC
GAACGACGAAGTCT

>comp161913_c0_seq2
TCCATCACAAGATGAACTGAATTCCTGTCCTCATATGAGCCCTTGAATTCTACAATATTTGATTGACCTGTTAAGTGTTGCAAGATTTGCATCTCCCTCTTGATATCCTCCTTATCATTTTTAT
GAACAAGTTTGCGCTTTGAGATCGACTTGCAGGCGTAAGAATGGCCTGTGGCAATCTCCTTGCAGAAATAGGTGACGCCGAATTGGCCGCGGCCAAGCTCCTTGCCGAGTGAATAATGG
CTGCGAACATCCTCAAATGGCTTGCCAAGGATTGTGTTATTGGTTGGGGCAGGCTTGGGTGGAGATGCAATCGGTGGGTTTGGCTTAGCCGGAGATGGTGGAGCATAATGGGTTTCCGGC
GATGGACGTGAGGGTTTCACCGGTTGTACCGTCGCGGGAGGCTGGTGGGAATGGCCATTGGCTTCCAGCCCTGTTCTTTTCCCTTTTCCGCAGCAAGATCCCATCTTTCTTCTTCCACAGT
CGATACTTTTGATTTTGATATTGGATTCAACTTCTTTCTAGGAAACCTTGATGATCCAGATCTTTACAAAATCTGGAGTTTTAAACGCAGGGAAAAGAGCATGTTTAGGGTTTGAACTTCGA
ATAAAAACTCAAGCCAGATAAGAAATCCCGTGAAGTTTTCTAAAAGACAGCCGAGAAAAGAGACACCTTGCAAGTTAACATAAGATCAAAATGGAGAGAAACAATGAAACGGAACAGG
AGAAAAACGAGAAAATTTTGAATTTGCTTCAAACGCTCTCCAGAAATTCCACGGCAAGAAAAAGTAGATGAGGAGAGGTCTGGTTTTTCAGCTTTTGAATACCAATTAACCCCAAGCAA
AGAAAAGAAATCGCGTGAAGCTTTCTCAAGATACAAAAAACCAAGAAAAGGCGACACCTTGCAAGTTAAGAAGATATCAAAATGGCCAAAATCATTTTTTTCTTTTTTTTCCTTTCAGTG
GAAGAATGATGGGAAGGAACCGCGTTATCCAAATTAAAAAAATAATGACAGGAACGATTCGGAATCTGATAAAATGTACAAAGGAGGAAGAAATGGAAAGGAAAGCCTGTTAGCCGCC
ATATTGCGTTAACAGATCAGTGGCAGAAGGAAGTTAGGCTCTTTCCAACGAAAAAATGATTCACGGCCATTATTTTATTTAAATTCCTAAATTTAGGATAGGAATGTGGGCCGAACGACGA
AGTCT
>comp161924_c1_seq7
ATGATGACACTATAAAGGACGCATGGAACGCAGGAAGCTTTAGTGTTCTGGCCTTGTGGTCATCATCTCCTAGTCTATTCCATACGCAGTGTGGACTCCACTGAGAAGAAGCAGCAAGAG
CTGAAGAGCAAATAAAGAAGAAGAGAAAGCGGGCCGAAGTAGTATCTCCTTCCGGCCGGAATTTCTGGCTTCTCTTTGATTACCGGAGCAGAGGAGGAGGAGCAATTGCATAATTATGG
TAACAGTATGAAGTGTGATGCAGAAATGTCAGTACCCTAATCGACAGACAGCTGGTTTGGTCCCTTTCCAATTTCCTTCTGGCGGGCCAAGTTCACAAATCTGAACCTTTTTTCACTTTTT
GCCTTTTTTTTACCTCCAGTCTCCTCCACTTCCATTGAGCAGAGCAGTAAGAGAGGCTCCTGTCTTCCATTTGTCTTTTCCCCTCCAAGAATATAGCTTTATGATTTCGCTTTTATTCTTCCA
CTTTCTTTTCATTCTCCATCATTGTATCCCCTTTTTCTCCAAGTTTATTGTCTGAGAGTGTTTTTATTCTTCTATCTGCAAGTGATTTTTTGCCCAATCTCTCAACTTTTGGAGCAATGAAACT
GAGTTTTGACCTGGAAGTTGATGTCAATGGTGAGGAGATCTTTTTTGTTAACAAGGAAAAACTTTGTTCATTCTCTGGCAAGTTAAACAAATTATTAAGCAAAGCTCCAGTGACATCGGTT
ACAAAGCCTCTGAAAGTGATTTTCAATGATTTACCAGGAGGGGCAGAGGCCTTTGAACTCTTGATAAGGTTCTGCTATAACAATGGCAGAATCCAAATTACTCCAAACAACACTTGCCTC
CTCTACTTCATAGCTCAATCCATGGAAGTGACTGAAGAAGAAGTTCATTCATCTGACAGTTTAATCAAACAAACTGAGAAAGCTGTTGAGAGTATCTCATATTGGTCTTTGCCTGAAGTCT
TGATATCTCTAAAACAATGCCAAAATTATTTTCAATTATCGGAATCTTCAGGCATCCTTGACAAAGTAATTGATTCTCTTGTAGCTAGAATTACCACAGCAAGTGATACCAGCCCTTCAGCTT
CTTCACCTGATAGCTCTTTCTTCCGTATCTCGTTCGATACAAAGAGTAACATTAGCACGAAGAACAGTCATCGTCGAGCTTGGTGGTTTGATGATCTTGTCATATTAAACATTGAAATGATT
GATAGGGTTATTGGAAGGATGATTTCTCTAAAACTAGAGCATAATAAAATCAGCAAATTCCTCTTTTACTATCTGAAGTGCAAGCTCTCCTCAGCTCCTGATGAGAAGAGGATGGTGACCG
AGACTGTCATCGACCTACTCTACTCGCTCGATAAAAACTCGGTATCATGCAAAGGCTTGTTTGATATTCTTCGCATCTCTTCATCTCAAAATCTCAGTGGTAGCTGCAGAGAAAGATTGGA
GAATATGATTGGTAGCCAGATCGATCAAGCTACTTTGGACAGTCTATTGATTTCCTCTCCTTCTAGAGTTTCCCAAGGCATGCGAAGAATGTCATCGATGCTTATTTTATCAAAACCGTTGTC
GGAGAGGCTGGCATCCATGGCGTTGCGCCGCGCGATGGTGTAAACTTGGCAGCACTCTGTTTCGTAGCCAGAGGTGATCATGGCGCTGGCAATGTGGCGGAGACGGGTGGTGCTTTGGT
GATCAGGGTGAGGGGATGTCAGGTCAGCGGAGGGGGAATCAGGAGTCGCGGAATGATCGGAGAGATTGGGTTGGAGAACGCAGCGATCGGGCTCGTGTACGAGACTGAATGACGGGC
GGCGTTTAGGCTTGGATGCGCCGGAGGTGGAATCCGAAGGGGGTTTCCTCGACTTGGAGGAAGATTCGTTGACAAGAATGGAATGAAACTCGTCTTCTAAGAATGCCATGGCGTGGTGG
ACGACGGCGCCGATTTCGTTCATGGCTTGGTAGTACTGGGGTTCCGAGCCGGAGAATTCAGAGAGGGCGGCGCTGAGTTTAGAGGCGCGGTCGACGGAATCGAGAAACGAGGCAACTT
CATGGGAATCCGAAGAAGACCATCGATCGCCGCCGCAATCGTACTTGGCGATCTCTTTCTCGAGCAATGCGCCGAAGCGGGAAATGGCGATATCAGGAATCTCCGGAATGGTCTCCGGCG

AATTGTGGAGGATTGCGAGGAACTGGTCGATGTCATCGGAGAGGGAAAGGAGATCGTAGTGGTCGGGGATTTCTCCGGCCGTTGTGGCTGTCTCGTCGTTGTTGTCCTGCTTCTCATTAG
CGGGTGAATCTTCTTTCTCTTCTCGTTCCTCATTATCGTCTTTTTCTACCTTGTTTGCGTCGGCGACTTCAGCGACGTCCTCGGAGATGGGATGAAAGTGGAGAGAGCTGAGGGAGAGGTT
GCGATCGGAGCCGCGGAGTTTCTCCTCGCGAGAGGAGATGAAGCTAGAGGATTTCTGGGGAATGTGAGAATGGGTTCGTTCCATTGTTTTTGTTCTTCTTTGCTGCTATCCAAAAGAAGA
GAGGACAGATAAAGGAAATGGGCATGAAGGGAAGGGAGGGGGAGACTGAGGAAGATGAGGAGGGGGAGGAGAGAAGGGAAGAGAAGAGGTTGAAACGGTATTGGGGCTTTGCATAC
GACGAGAGGTTGGGGGAGTAGAAGCCTGCACGATGCGCGCTGGTCGATTAGATAATGGCCAGCCCGGTTTTTAAA
>comp161932_c0_seq2
AACGTCTTCTCCTGCTATTTCTCCCTCTGTAACAAACCGTCGCATCTCTATAAATAGACAAGCCATGCAGGTTAAATTATTTCGGCTCCGCACGGCCTCTATCGAGTATCTCTTCCAGTCTTC
AGTTTCAGTTGAAAATTATTATTGTTCGTTTCTCAAGAGAGTTTTTTGATAGAGGCAAGAGTAGTACTTGACATGGATGAAATGGATGGAAATATGGCCATTTTTTATGATTGGGCGGCTTT
CAATCCTAGCCCAATAAGGCAATAACCTATGAGTTTTTCAACGAGGATTTTGTTTCAAGGCCTATTCTTGATTTCGGAGAAGATGATAGTCAGAGTTCTTCTATCACAATGGACTTTGAGAA
GAAGAAACCACCTATTAGCTTCGAAGAGGGGGGAAAGAAAATGGGTTTGGACTGCACGAGTGACGATTCTTCAGAATTAATAGGATTTAATGTTCAGAAATCATGCTTGAAAGGGGTTCA
TAATGAGAAAATGGCAGAAATGGCTCGTTTAAACATGTTAAAAGCTTTTGTGAGTCAAAGTGATCAAGAGAATATCATGTTTGAGGTTAATTCTAGTCAAAACGAGTCAAAGAATCTTATA
TCATCATTGCCAGAGGAATCTTCTTCATATATGTTGCTTTCGCCAGGCGTTAATCTTTCATCAATGGAGTATCTAGATAATTTTTCAGTTTCTCTGGCCTTACCAGACAATAATGCATCCCTTC
AAGAAAATTTCGAGCTTTTGGAGCCGTTTGGAAGAAAAGAGAAAACTCATGATATTGCTGCTAATCAAAAAGCATCCCATTTCTTGGATGTGAATGGAAGTCCCCCACCTCCACCCCCTG
ATGACCATCAAAATGCAGAAAAAGGCGTCAACGTAGAGTTCTCATCCTTTCCTGAAAGTGACCCAACAAAGGATGGTTATAACTGGCGGAAGTATGGCCAGAAACAGTTGAAGGGCTGT
GAGTTTCCTTGTAGTTATCATAAGTGTACCCACCCAAACTGTCCTGTTAGAAAGAAGATTGAACGATCTCTTCAGGGCCATATCACTGAAATAATCTATAAGGGCTCTCACAACCACCCTA
AGCCTCCTCCAAATCATCGGTCCAGTTTCTCATCTTCAAAACAACTTCATGGTCCACAATCAGTCAGTGCAGATCATCCTGGTTCGGTGGCCAATTTTAAAGGAAATCCCTCATTGGCAAG
TTCTTATGATGGATTTAAAGGACCCGAACGGAGTGATGGTTTCGGAGGTACTTCTTCAGCTTCTTTTACTGAGTTTAATGACCCTTCTAATGTTTTGAAGAGCTCTCAATTAGAATTGGAGT
CGATGGAATCCTCATCATGTGTAATATCTATTGACAAGGAAGATAATGAGGCAACTCATAGCAGCTTATCCGCTGATTATAATATTGACCAAGAGGAGATGGAATCGAAAAGAAGGAAGCA
GGAACTCTGCTCTATTGGCAGGAATACTACTTCAAGGGCCATGCGTGATCGTCGGATTGTGGTGCAGATTCCTAGTGATTTAGATATCCCCTATGATGGGTACCGATGGCGTAAGTATGGGC
AGAAGGTTGTCAAAGGAAATCCAAATCCAAGAAGCTACTATAAATGTACAAACCGTGGATGCTCCGTCCGCAAACATGTGGAGCGAGCTTCAGATGATCTAAATTCAGTAATCTGTACAT
ATGAAGGCAAGCATAACCATGATGTCCCTTCTGCAAGAAATATCAACCTCGCAAGATATTTTCCATCCAGCCTTGCTGAAACCGCAGCAATGCAGCTTCCTGGTCTTTACACAAGGCCAG
AATTAGCTCAAGACAGCTTCATGAGTCTTGAAAACTGTGCTCCAATATGTACATATGGCCTTATTGGTAGAGGAAAGCAACTTGAACCAACAACTAGTTTTTCTTTCGGGTTCGACCAATC
AAATTTGGCGAGCCTTTGCCCATCGGGTTCAGACTTAATGAGGCGATTCAAGATGCCATTTTTTCCTCCAATACATCAAAACATGGATCGGCAGGTTAGCAGTGGAAATATTATACCAATGA
GAGAACCGAAGAACGAACCGATGTAAGAGTCTGATGTGCCGCCAATTGCCAATACAGATGCAGGTTATCAGCAATAGTGGAGTCGGTTAGTTTTTTTTGCTTTTGTTTTTATGACTGTGTG
CCTGAGATCATACTCCTCACACACACACACACTCATATATATATAAGGCTTGATTTGCTAATGATATAATTTTGTCAGCAAGCTTTGTGGCTGTAAGAAGTTTTCTAAGTTATTTTGATTAGCA
AATTGGCGTGTTTTGCCAAAGAGATGCTTCTAAGAAAATAAATTTGTTATTAAA
>comp161933_c0_seq1
GAGAGAGAGAGAGAGAGATGGCAACGCTCAGAGTTCCATTGCATGTTCCTTCTGCAGTAGAAGATGCAAAGAATCTCTGGATGGCCTGCGAAGGAAGCATTATACTGTTGGATGTTCAA
TCATGTCCAGAGAGGAGCAATCATAGCTAATATGGCTATGTGGAAATGGTTAGACTATAGAGCAATCGTCGAGACGGCGTGTATCAACTCAACAAAAGAACTTTTAGAGGTCAAGCAAGC
CTACCATGATCTTTTCAAGCGTTCGTTGGAGGAAGATGTTGCAAAAATGACTAATGGCGATCTTCGAAAGCTCCTTCGATCGGCACTCAACAAAGAGAAAATTGACGATAATACACTTGC
TCGAATTATTGTCACTCGAGCGGAGAAAGATTTACAGGAGATCAAAGATATGTATGAAGAGAG

>comp161933_c0_seq15
GAGAGAGAGAGAGAGAGATGGCAACGCTCAGAGTTCCATTGCAAGTTCCTTCTGCAAAAGAAGATGCAATGAATCTCTGGATGGCCTGCCAAGGAAGGGAGGCAGATGAGAAAACATT
GATCGATATTTTAGCGCATAGAGATGAAGCACAGAGGCTACAAATCATCCAATCTTATGAAGATCATTACAACGGAAATCTCATTGATAGACTTGAATATGAGCTATCGGGAGAATTTAAGG
AAGCATTATACTGTTGGATGTTCAATCATGTCCAGAGAGGAGCAATCATAGCTAATATGGCTATGTGGAAATGGTTAGACTATAGAGCAATCGTCGAGACGGCGTGTATCAACTCAACAAA
AGAACTTTTAGAGGTCAAGCAAGCCTACCATGATCTTTTCAAGCGTTCGTTGGAGGAAGATGTTGCAAAAATGACTAATGGCGATCTTCGAAAGCTGTTGGTTGGTTTAGTGAGCACATA
TAGATATGATGGCAAGGAAATTGTCAAGAGCCTTGCATTATATGAAGCAAACATTCTCCATGATGTTATCAGAAAGAAACTCTTCAATCATGATGAGGTTATAAGAATCTTTACCACAAGGA
GTAAAGCTCAACTCATTGCAACTTTCAATAAATACAAGGATGAGTTTGGAATATCAATCTTAAAGGACTTGTCAAGTGGGAGTCCAGATCTCTTTCCTTCGGTGCTTAAAATTATCATTCGT
TCCATCATTTCTCCTCATAAGTACTTTCAGAAGCTCCTTCGATTGGCGCTGAACGGAGAGGTAACCGACGAAAATGTGCTTGCTCGAATTATTGTCACTCGAGCGGAGAAAGATTTACAGG
AGATCAAAGATATGTATGAAGAGAGAGGAAAAATGAGTCTGATAGCTGCCATAAACAACAAAACTTCAGGGCACTTCAAGAACTTCATCTTGGAATTGATTGGGAATTGAAGATGAAATA
CTTCAGAAACTACTTCTATGTTTGTCGTAATAAGAAAAAACTCACAACTATTGGATATATATTTTTGAAATTACTGTTGATGACAGTTTGTATTTCAGTAGGCTTCGTTCGGAACAACTTTCT
TACTATTCTTTAAGATTGTTTTTTAGTACAAAAAATTTAAGTTCAAAAAAATTTTGTACTAAA
>comp161933_c0_seq16
GAGAGAGAGAGAGAGAGATGGCAACGCTCAGAGTTCCATTGCAAGTTCCTTCTGCAAAAGAAGATGCAATGAATCTCTGGATGGCCTGCCAAGGAAGGGAGGCAGATGAGAAAACATT
GATCGATATTTTAGCGCATAGAGATGAAGCACAGAGGCTACAAATCATCCAATCTTATGAAGATCTTTACCACGAAAATCTCATTAAGAAACTTGAAAATGAGCTCTCAGGAGAACTGAAG
AAAGCATTATGCTCTTGGATGCTCAATCCTGCCCAGAGAGGAGCAATCATAGCTAATAAGGCTCTGAAGGAAGGGTTAGACTATAGAGTAATCGTCGAGACGGCGTGTATCAACTCAACA
AAAGAACTTTTGGCGGTCAAGCAAGCCTACCATGATCTTTTCAAGCGTTCGTTGGAGGAAGATGTTACAAAAACGACTAATGGCGATCTTCGAAAGCTGTTGGTTGGTTTAGTGAGCACA
TATAGATATGATGGCAAGGAAATTGTCAAGACCCTTGCATTTTATGAAGCAAACATTCTCCATGATGTTATAAGAAAGAAACCCTTCATTCATGATGAGTTTATAAGAATCTTTACCACAAG
GAGTAAAGCTCAACTCATTGCAACTTTCAATATATTCAAGGATCATCATGGAATATCAATATCAAAGGCCTTGTCATTTGGGAGACCAGATCTCTTTACTTCGGTGCTTAAAATTGTCATTCT
TTCCTTCATTTCTCCTCATAAGTACTTTGAGAAGCTCCTTCGATCGGCACTCAACAAAGAGAAAATTGACGATAATACACTTGCTCGAATTATTGTCACTCGAGCGGAGAAAGATTTACAG
GAGATCAAAGATATGTATGAAGAGAGTGGGAAAATGAGTCTGATAGCTGCCATAGACAACAAGACTTCAGGGCACTTCAAGAACTTCATCTTGGAATTGATTGGAAATTGAAGATGAAA
CTCTTCAGAAACTACTTATATGTTTGTTGTAATAAGAAAGAACTCACAACTATTTGATATATATTTTTTATATTACTGTTGATGACATTTTGGATTTATTCATACGACGTAGATTCAGTAGAGTT
GGAGATAGAAAAAAGCTAGACTGCAACCCGTCTTTTTGATGAAAAAGCATTTTTCCGGATCCGAGAAAAACAAAACTTTCATGCCACCCATGGGAAAAAGTCATAAACCGTCTTTCCCTT
AGTGAAGTCACATTTCAATCTGGAAATTGTCAATCAACATGGGGTCAAAGTCATAAAA
>comp161933_c0_seq3
GAGAGAGAGAGAGAGAGATGGCAACGCTCAGAGTTCCATTGCAAGTTCCTTCTGCAAAAGAAGATGCAATGAATCTCTGGATGGCCTGCCAAGGAAGGGAGGCAGATGAGAAAACATT
GATCGATATTTTAGCGCATAGAGATGAAGCACAGAGGCTACAAATCATCCAATCTTATGAAGATCTTTACCACGAAAATCTCATTAAGAAACTTGAAAATGAGCTCTCAGGAGAACTGAAG
GTACCTGATGGTGTTGAACATATTCAACAATATCACTATAGGAAATTCGCAAATAACCGGCGGGACTTTCCAGCTGTTTTAAAAATTTCAGGCGGTTTTAAAAATTCACTTATCACCGGCGG
CCAAACCGCCAGTCATATGCGACGCCCAAACAAAAAATCGGCAGCATATTTGGTAGTCATTTTACTGCCAGCATTTCCGGCGGTTTTTTATTAAGAACTGCAAGTTTTAATCGGCGGTCAC
ACAGCCGGTTTAACCTCCAGTAGTTCCGGCGGTTCTTTGACTGCAGGTAATTGATGATTTCCTGTAGTGCATATAAAGAGCCTAACATTTTCAAATTTGTTTTTATTGCAGAAAGCATTATGC
TCTTGGATGCTCAATCCTGCCCAGAGAGGAGCAATCATAGCTAATAAGGCTCTGAAGGAAGGGTTAGACTATAGAGTAATCGTCGAGACGGCGTGTATCAACTCAACAAAAGAACTTTTG
GCGGTCAAGCAAGCCTACCATGATCTTTTCAAGCGTTCGTTGGAGGAAGATGTTACAAAAACGACTAATGGCGATCTTCGAAAGCTGTTGGTTGGTTTAGTGAGCACATATAGATATGATG

GCAAGGAAATTGTCAAGACCCTTGCATTTTATGAAGCAAACATTCTCCATGATGTTATAAGAAAGAAACCCTTCATTCATGATGAGTTTATAAGAATCTTTACCACAAGGAGTAAAGCTCA
ACTCATTGCAACTTTCAATATATTCAAGGATCATCATGGAATATCAATATCAAAGGCCTTGTCATTTGGGAGACCAGATCTCTTTACTTCGGTGCTTAAAATTGTCATTCTTTCCTTCATTTCT
CCTCATAAGTACTTTGAGAAGCTCCTTCGATCGGCACTCAACAAAGAGAAAATTGACGATAATACACTTGCTCGAATTATTGTCACTCGAGCGGAGAAAGATTTACAGGAGATCAAAGAT
ATGTATGAAGAGAGTGGGAAAATGAGTCTGATAGCTGCCATAGACAACAAGACTTCAGGGCACTTCAAGAACTTCATCTTGGAATTGATTGGAAATTGAAGATGAAACTCTTCAGAAACT
ACTTATATGTTTGTTGTAATAAGAAAGAACTCACAACTATTTGATATATATTTTTTATATTACTGTTGATGACATTTTGGATTTATTCATACGACGTAGATTCAGTAGAGTTGGAGATAGAAAA
AAGCTAGACTGCAACCCGTCTTTTTGATGAAAAAGCATTTTTCCGGATCCGAGAAAAACAAAACTTTCATGCCACCCATGGGAAAAAGTCATAAACCGTCTTTCCCTTAGTGAAGTCACA
TTTCAATCTGGAAATTGTCAATCAACATGGGGTCAAAGTCATAAAA
>comp161933_c0_seq5
GAGAGAGAGAGAGAGAGAGATGGCAACGCTCAGAGTTCCATTGCAAGTTCCTTCTGCAAAAGAAGATGCAATGAATCTCTGGATGGCCTGCCAAGGAAGGGAGGCAGATGAGAAAACATT
GATCGATATTTTAGCGCATAGAGATGAAGCACAGAGGCTACAAATCATCCAATCTTATGAAGATCATTACAACGGAAATCTCATTGATAGACTTGAATATGAGCTATCGGGAGAATTTAAGG
AAGCATTATACTGTTGGATGTTCAATCATGTCCAGAGAGGAGCAATCATAGCTAATATGGCTATGTGGAAATGGTTAGACTATAGAGCAATCGTCGAGACGGCGTGTATCAACTCAACAAA
AGAACTTTTAGAGGTCAAGCAAGCCTACCATGATCTTTTCAAGCGTTCGTTGGAGGAAGATGTTGCAAAAATGACTAATGGCGATCTTCGAAAGCTGTTGGTTGGTTTAGTGAGCACATA
TAGATATGATGGCAAGGAAATTGTCAAGAGCCTTGCATTATATGAAGCAAACATTCTCCATGATGTTATCAGAAAGAAACTCTTCAATCATGATGAGGTTATAAGAATCTTTACCACAAGGA
GTAAAGCTCAACTCATTGCAACTTTCAATAAATACAAGGATGAGTTTGGAATATCAATCTTAAAGGACTTGTCAAGTGGGAGTCCAGATCTCTTTCCTTCGGTGCTTAAAATTATCATTCGT
TCCATCATTTCTCCTCATAAGTACTTTCAGAAGGTAAGTAACTTGGATCGAAATTACGTACAAAATTCATAGCGCACTAGTTGGAAATAAATTTGAAAAATCTGATAGGTAATATTCAAAGG
CTCACCTCTCGTAAAGAAAAGATTAGGAACAAAATCATATATATATAAAATGTTCAGAATATCCTTATAACAAAAGGTTGATCTCATATAGAATTACTTAGAAGATGAAATGTAAACATTCTC
TTTCATATATTTTATGTTAAGGGGTTCTTCTAAAAAATTTTGAAGGTGTTGTAGAATTTGAAAATATATTAGTTCATAGCTACAAAAATGTTGCTACAAATGTCTATTATCTTTCTTATTAGAAC
ATCTTGCTAGAATTTTATCCTTGGTGATATATCTTCGGGTGAAAAATTTATGAACCTAACTTATGAAGAAAACTTTTGGACCAATCGCAGCTCCTTCGAATTAAGAGCAATGAAAATTGCAG
CTATCGCTCTATAAAATTAGGAAAAACCTTTGGAAATTACAATCAACACACCATTAATTCCAAAAATCTTTTCAATTGTTGTCCTTCACAATTCAAACAGAAATATTTCAAGAAACCAGTCA
GCCAGTAGTATGTCAGCTGCAGCTATTTGCACATACGCGAGTTTTACAGGTCAACAACTACTATTCATCGTCCCATGTCCATTTGGTTGCCTCGTCATCATCTACTAATACATACCGCCCTGA
CATCACCTTAATGGGCATCGTTGA
>comp161933_c0_seq9
GAGAGAGAGAGAGAGAGAGATGGCAACGCTCAGAGTTCCATTGCAAGTTCCTTCTGCAAAAGAAGATGCAATGAATCTCTGGATGGCCTGCCAAGGAAGGGAGGCAGATGAGAAAACATT
GATCGATATTTTAGCGCATAGAGATGAAGCACAGAGGCTACAAATCATCCAATCTTATGAAGATCATTACAACGGAAATCTCATTGATAGACTTGAATATGAGCTATCGGGAGAATTTAAGG
AAGCATTATACTGTTGGATGTTCAATCATGTCCAGAGAGGAGCAATCATAGCTAATATGGCTATGTGGAAATGGTTAGACTATAGAGCAATCGTCGAGACGGCGTGTATCAACTCAACAAA
AGAACTTTTAGAGGTCAAGCAAGCCTACCATGATCTTTTCAAGCGTTCGTTGGAGGAAGATGTTGCAAAAATGACTAATGGCGATCTTCGAAAGCTGTTGGTTGGTTTAGTGAGCACATA
TAGATATGATGGCAAGGAAATTGTCAAGAGCCTTGCATTATATGAAGCAAACATTCTCCATGATGTTATCAGAAAGAAACTCTTCAATCATGATGAGGTTATAAGAATCTTTACCACAAGGA
GTAAAGCTCAACTCATTGCAACTTTCAATAAATACAAGGATGAGTTTGGAATATCAATCTTAAAGGACTTGTCAAGTGGGAGTCCAGATCTCTTTCCTTCGGTGCTTAAAATTATCATTCGT
TCCATCATTTCTCCTCATAAGTACTTTCAGAAGCTCCTTCGAATTAAGAGCAATGAAAATTGCAGCTATCGCTCTATAAAATTAGGAAAAACCTTTGGAAATTACAATCAACACACCATTAA
TTCCAAAAATCTTTTCAATTGTTGTCCTTCACAATTCAAACAGAAATATTTCAAGAAACCAGTCAGCCAGTAGTATGTCAGCTGCAGCTATTTGCACATACGCGAGTTTTACAGGTCAACA
ACTACTATTCATCGTCCCATGTCCATTTGGTTGCCTCGTCATCATCTACTAATACATACCGCCCTGACATCACCTTAATGGGCATCGTTGA

>comp161938_c1_seq1
ACCGTTCTATATAAAATTAATATAGAAAAAGAAATATCTAAATATTTCTTCTATACATATTGAGCTATTCCTTTGTACTTAATAACCTAATAAAAATGTCATTTCTTTAATGAGTTAACCATCGA
AAAATAGAATGGTAAAGGGTCACATCCTGGCTCCAAGATGTTATTGTAATTAGATCGATCAAGAAAATTAAAGGACTCGAACAAATCTCCATTGTTAATCGCAGCCTCAACATTATTGCAAT
CCTGATTGCTAGTGGAAGCTCTTTCCACCATCTGTTCTGACTCGCCAGTCTTTGCAAACCTTCCTTTAACTCGAGGTCTGCTGTCTGCAAGTGTTTTCCGGCTTGCATACTTGATTTTCTTG
CTAAAATCTCTTCTTTTTTTCTTATTTTTGTATCTTTCGAT
>comp161940_c2_seq11
GCTATCAAAAACTTTTTCAGAAACCATTGCAATTTAGTTTGATTCAATACATTTCCACATCAATGGAAATTTCAGTCACAATGACCTATTAACCCTATGAACTTCCCATCAATGGAAATTTTC
AGTATGCTAGCTGGTACTAATAAGCAATTTATTAGACCAAAGTAACAATCCCAGTGAAGCTTTTAGTCTTTTGCTAGGAGAAACAATGAAGAATACACAAACAGGAATCATACCTACAGGT
TATCATCTTGTTTTACATCTTTGAAATGACCTTCTCATTTCACTATCATACTAGACTTTTATAAGTTTCTAATGCACTATTTGTCACCCAGCAGAGTGAGCAGAAAATCCCTGTAATAACCAG
AGGTATCTCCAACAACATCATCATGAAGTGTTCTGTTGTATCTGTTCTTATACTCCTCTTTGATCAGCTTCATATCGATTTCTGCTCGAGTAACAATAGCTCTTGTAAGAGAATCCTCATCTGT
TCCCAATCCAATGATTGAGGTATTCACAACCTGGGCGAAGTGCTTCTCTGGCGACTGTAAACAACAGATAGCAACTTTTATGATTGATGAGAGCTTCTTGTCATCACCACTGTTGCACTCT
ATGTCCTCAATTATGGATATTCCATATTCTTGTTTATAATGCTGGAAAATAGCATTAAGGTGAGCCTTGCTCCTTGTGCTGAGTACACGAATAACCTCATGATAAGGCCTCTGCTCTCTTATAG
CACAGAAGAGTGAGTTTGCTTCAGATTTCACCAGTTGCTCATCCACATCCTCCCCTTCATACCTATAGGAGCTCACAAGACGCAACAAAAACTGCAGAAAATATATCTTTCTCAGTGGTAA
AGCACATACAGGTTATATATTAAATATCGCCATATCATCATCATATTCAGTAGCCTTATCCTAACTATTTAAAATTGGCTTTATTATCACTATCTTATAGAGGAAAAAGTAAAAATAGTCCGTTC
AGTATTTATGTGTGAGCATTTTAATTCCAAACTACTTAATATGCCATATTTAGACTTCGTTTGGCACGGCTGCTTCTTAAAGCAGCTTTTTATAATAAAAAATAAAATTTTTTAATTAAAAAAT
TACTTCAAAAAGAAGTAAGAAAGCCATCGCAAACGAAAACATAGTCTCATCCTTAAAGCTGAAGCTTTGGTTTTATTACGAGCGATATTATTTTTTATTAGGATGAGCTTATTTGTTTGAATA
TCAATTCTTCTATTCCTAACCAAAATTTTATCAGAATATTCAGCTGAAGAGAAGAGTCTATCAGGATCTTCGCTTTATTCCAGTGTTCAACTGTAAGTTCTTTCATGCTTAGAGGAAGAAAA
TGTTGAATTAGGCGCACGCTTCTTAATGCTTTCTTTTTTACTACCTATGTGAACAGTTTAAATTCTCTTTTCATTACATAATTTACTTTACACATCTCAGTAAGTAAAAGCAGAAAACATGAA
GTACTGACCGCTGTTACCCTTCACAGACTTGGAGTTGAAGCTTAGTATTGTTGTGCTTGTTACATTAACTTTGTATGGTAAGGTTCTTTCAAGTGAGTGAATTGATGTATGACATCAATAGG
ATTCTTGTTAAGAGGAGCTCCACAAGATTTGCATTAGCTCTTTGAGTCATTTTAAAATAATTATCCCTATCAACCACAACTATCATTGT
>comp161940_c2_seq2
GCTATCAAAAACTTTTTCAGAAACCATTGCAATTTAGTTTGATTCAATACATTTCCACATCAATGGAAATTTCAGTCACAATGACCTATTAACCCTATGAACTTCCCATCAATGGAAATTTTC
AGTATGCTAGCTGGTACTAATAAGCAATTTATTAGACCAAAGTAACAATCCCAGTGAAGCTTTTAGTCTTTTGCTAGGAGAAACAATGAAGAATACACAAACAGGAATCATACCTACAGGT
TATCATCTTGTTTTACATCTTTGAAATGACCTTCTCATTTCACTATCATACTAGACTTTTATAAGTTTCTAATGCACTATTTGTCACCCAGCAGAGTGAGCAGAAAATCCCTGTAATAACCAG
AGGTATCTCCAACAACATCATCATGAAGTGTTCTGTTGTATCTGTTCTTATACTCCTCTTTGATCAGCTTCATATCGATTTCTGCTCGAGTAACAATAGCTCTTGTAAGAGAATCCTCATCTGT
TCCCAATCCAATGATTGAGGTATTCACAACCTGGGCGAAGTGCTTCTCTGGCGACTGTAAACAACAGATAGCAACTTTTATGATTGATGAGAGCTTCTTGTCATCACCACTGTTGCACTCT
ATGTCCTATCCATCCCAGAGTTTGCTAATACATTAGTGACTGGTTTTTAAGCTAAAACTCCACAGTTAGAAGACAAACATACTATTCGAATAGGTCGCTACTGCTTAAACAGTATAAATGAC
GCTTAGATGAAAAATGAGCATGTATTTCCTCATGCTAAATTATGTTAACTTGTATAGAAACTACACAAACACAGAGAGAAGATGACACTATGAAATATTACAACTTCAGGAACCTGTCTGT
GGGTGATTTATACAATATTCCATGTATCATGCAGAGGTTGATTTAGTTCTTTATATTGTGAACTGGAGGAAACTCACTCATTGATCTAGTTAGGCAATGAAAGAACAAAAAATTTAGAGAAA
ACGAAAAGATTTATCCCAAACTTAAGGGGGAATTAAACTTTTACAACATTCAAAATTACAAGAGGACTTCCATGGAAATACTTCTAGGGGGGAGTTATAAATGTGTGGTTTCAAGTGGTAAGC
TGATAGGAAATATATGCACTTTTCACAAAATCAGCAGATGAAATATGAGAATTGCTGAATTAAGAAGAACACCCAAAAAACAAGATACAATTGAATCTTCAACTGGAGAGGAAGATTCGA

CTCTCCCAAACCCTAGCCGTCCACTAGAGTCCCTCCTTCTCCTTAGGAAAAAACAAAACTCAGCAAAATGAACGATAGCCCAAACCCTACGAAACAATCCTACTAAAACATACAAAAATT
CTCTCCGATTCCAAAACAACCCCTCATTTTGAAATTTCCCTCCTAATCTCTCTTTAATTTTTAATTAATTTGCCTGCTGGACCCCACCCCCAAAGGGAGCGTCATTGACCGAAGCGGAACAG
CCTCTGGTAAACCAAGGCCGGTCTAGGCTAGACCAGCTAGACCGATCCAAGGACTAGTCTAACATAAGGCCAAAAAACCAACTTGATCGTTTCATCATTAATTACCACATTACTGATGTTA
TCTTGATTAAGCATGGTAATGGCATATTAAAGAGGGAAATATTTCAATTGTCAGTTTAATAAATATGGAATAACTATTATTGAATGACTTATGGATAGAAATTGCAGCGTCTGGACTAATGAAT
TGCTTCCACAGGGATCTATAAATGTTCCAGCTTAAATTTTACTGCAAATGGCATATTGCGTACCTCAATTATGGATATTCCATATTCTTGTTTATAATGCTGGAAAATAGCATTAAGGTGAGCC
TTGCTCCTTGTGCTGAGTACACGAATAACCTCATGATAAGGCCTCTGCTCTCTTATAGCACAGAAGAGTGAGTTTGCTTCAGATTTCACCAGTTGCTCATCCACATCCTCCCCTTCATACCT
ATAGGAGCTCACAAGACGCAACAAAAACTGCAGAAAATATATCTTTCTCAGTGGTAAAGCACATACAGGTTATATATTAAATATCGCCATATCATCATCATATTCAGTAGCCTTATCCTAACT
ATTTAAAATTGGCTTTATTATCACTATCTTATAGAGGAAAAAGTAAAAATAGTCCGTTCAGTATTTATGTGTGAGCATTTTAATTCCAAACTACTTAATATGCCATATTTAGACTTCGTTTGGC
ACGGCTGCTTCTTAAAGCAGCTTTTTATAATAAAAAATAAAATTTTTTAATTAAAAAATTACTTCAAAAAGAAGTAAGAAAGCCATCGCAAACGAAAACATAGTCTCATCCTTAAAGCTGA
AGCTTTGGTTTTATTACGAGCGATATTATTTTTTATTAGGATGAGCTTATTTGTTTGAATATCAATTCTTCTATTCCTAACCAAAATTTTATCAGAATATTCAGCTGAAGAGAAGAGTCTATCAG
GATCTTCGCTTTATTCCAGTGTTCAACTGTAAGTTCTTTCATGCTTAGAGGAAGAAAATGTTGAATTAGGCGCACGCTTCTTAATGCTTTCTTTTTTACTACCTATGTGAACAGTTTAAATTC
TCTTTTCATTACATAATTTACTTTACACATCTCAGTAAGTAAAAGCAGAAAACATGAAGTACTGACCGCTGTTACCCTTCACAGACTTGGAGTTGAAGCTTAGTATTGTTGTGCTTGTTACA
TTAACTTTGTATGGTAAGGTTCTTTCAAGTGAGTGAATTGATGTATGACATCAATAGGATTCTTGTTAAGAGGAGCTCCACAAGATTTGCATTAGCTCTTTGAGTCATTTTAAAATAATTATC
CCTATCAACCACAACTATCATTGT
>comp161940_c2_seq9
GCTATCAAAAACTTTTTCAGAAACCATTGCAATTTAGTTTGATTCAATACATTTCCACATCAATGGAAATTTCAGTCACAATGACCTATTAACCCTATGAACTTCCCATCAATGGAAATTTTC
AGTATGCTAGCTGGTACTAATAAGCAATTTATTAGACCAAAGTAACAATCCCAGTGAAGCTTTTAGTCTTTTGCTAGGAGAAACAATGAAGAATACACAAACAGGAATCATACCTACAGGT
TATCATCTTGTTTTACATCTTTGAAATGACCTTCTCATTTCACTATCATACTAGACTTTTATAAGTTTCTAATGCACTATTTGTCACCCAGCAGAGTGAGCAGAAAATCCCTGTAATAACCAG
AGGTATCTCCAACAACATCATCATGAAGTGTTCTGTTGTATCTGTTCTTATACTCCTCTTTGATCAGCTTCATATCGATTTCTGCTCGAGTAACAATAGCTCTTGTAAGAGAATCCTCATCTGT
TCCCAATCCAATGATTGAGGTATTCACAACCTGGGCGAAGTGCTTCTCTGGCGACTGTAAACAACAGATAGCAACTTTTATGATTGATGAGAGCTTCTTGTCATCACCACTGTTGCACTCT
ATGTCCTATCCATCCCAGAGTTTGCTAATACATTAGTGACTGGTTTTTAAGCTAAAACTCCACAGTTAGAAGACAAACATACTATTCGAATAGGTCGCTACTGCTTAAACAGTATAAATGAC
GCTTAGATGAAAAATGAGCATGTATTTCCTCATGCTAAATTATGTTAACTTGTATAGAAACTACACAAACACAGAGAGAAGATGACACTATGAAATATTACAACTTCAGGAACCTGTCTGT
GGGTGATTTATACAATATTCCATGTATCATGCAGAGGTTGATTTAGTTCTTTATATTGTGAACTGGAGGAAACTCACTCATTGATCTAGTTAGGCAATGAAAGAACAAAAAATTTAGAGAAA
ACGAAAAGATTTATCCCAAACTTAAGGGGAATTAAACTTTTACAACATTCAAAATTACAAGAGGACTTCCATGGAAATACTTCTAGGGGGAGTTATAAATGTGTGGTTTCAAGTGGTAAGC
TGATAGGAAATATATGCACTTTTCACAAAATCAGCAGATGAAATATGAGAATTGCTGAATTAAGAAGAACACCCAAAAAACAAGATACAATTGAATCTTCAACTGGAGAGGAAGATTCGA
CTCTCCCAAACCCTAGCCGTCCACTAGAGTCCCTCCTTCTCCTTAGGAAAAAACAAAACTCAGCAAAATGAACGATAGCCCAAACCCTACGAAACAATCCTACTAAAACATACAAAAATT
CTCTCCGATTCCAAAACAACCCCTCATTTTGAAATTTCCCTCCTAATCTCTCTTTAATTTTTAATTAATTTGCCTGCTGGACCCCACCCCCAAAGGGAGCGTCATTGACCGAAGCGGAACAG
CCTCTGGTAAACCAAGGCCGGTCTAGGCTAGACCAGCTAGACCGATCCAAGGACTAGTCTAACATAAGGCCAAAAAACCAACTTGATCGTTTCATCATTTGTCTTGATTAAGCATGGTAAT
GGCATATTAAAGAGGGAAATATTTCAATTGTCAGTTTAATAAATATGGAATAACTATTATTGAATGACTTATGGATAGAAATTGCAGCGTCTGGACTAATGAATTGCTTCCACAGGGATCTAT
AAATGTTCCAGCTTAAATTTTACTGCAAATGGCATATTGCGTACCTCAATTATGGATATTCCATATTCTTGTTTATAATGCTGGAAAATAGCATTAAGGTGAGCCTTGCTCCTTGTGCTGAGTA
CACGAATAACCTCATGATAAGGCCTCTGCTCTCTTATAGCACAGAAGAGTGAGTTTGCTTCAGATTTCACCAGTTGCTCATCCACATCCTCCCCTTCATACCTATAGGAGCTCACAAGACGC

AACAAAAACTGCAGAAAATATATCTTTCTCAGTGGTAAAGCACATACAGGTTATATATTAAATATCGCCATATCATCATCATATTCAGTAGCCTTATCCTAACTATTTAAAATTGGCTTTATTAT
CACTATCTTATAGAGGAAAAAGTAAAAATAGTCCGTTCAGTATTTATGTGTGAGCATTTTAATTCCAAACTACTTAATATGCCATATTTAGACTTCGTTTGGCACGGCTGCTTCTTAAAGCAG
CTTTTTATAATAAAAAATAAAATTTTTTAATTAAAAAATTACTTCAAAAAGAAGTAAGAAAGCCATCGCAAACGAAAACATAGTCTCATCCTTAAAGCTGAAGCTTTGGTTTTATTACGAGC
GATATTATTTTTTATTAGGATGAGCTTATTTGTTTGAATATCAATTCTTCTATTCCTAACCAAAATTTTATCAGAATATTCAGCTGAAGAGAAGAGTCTATCAGGATCTTCGCTTTATTCCAGTG
TTCAACTGTAAGTTCTTTCATGCTTAGAGGAAGAAAATGTTGAATTAGGCGCACGCTTCTTAATGCTTTCTTTTTTACTACCTATGTGAACAGTTTAAATTCTCTTTTCATTACATAATTTACT
TTACACATCTCAGTAAGTAAAAGCAGAAAACATGAAGTACTGACCGCTGTTACCCTTCACAGACTTGGAGTTGAAGCTTAGTATTGTTGTGCTTGTTACATTAACTTTGTATGGTAAGGTT
CTTTCAAGTGAGTGAATTGATGTATGACATCAATAGGATTCTTGTTAAGAGGAGCTCCACAAGATTTGCATTAGCTCTTTGAGTCATTTTAAAATAATTATCCCTATCAACCACAACTATCAT
TGT
>comp161963_c0_seq1
TCCTCTTCTTTCTTTAAAGCTATTTTAAAAACTACAATGTACTATAGCTACTGTGATATATGTGCAGGACTATAAATGCACTCGTGTGTGTATTTCTTCTTTTTTAGGGAAAACGTATTTCAAA
ATTTCAATGTAATCCTTCTATGTAATGGCATTCATGAGCTGTAATCCTCAAGATGATTATAAAGACATCACGTTTTGTTTATGAAATGTCAGTGGAGTGTTATATTTTCACTTGATTTTGTCCCT
ATGAAGGGCAATCTTTAAATCACTAATATTGCAGCTTTATTTCCCAGTTTTTCTCGACTAAACCTCTTGCCTGCGATCCTTCTAGCTTGCCTAAATATCCTCCAAGTAAGGAATATGATGTTA
AACTTCGAGATGTGGAAGCAAGGAGGCAAAAAGCTGCAGCTCTTAAAGGCAAGCCATTTGATTCTGGAAGGAAAGGCCCATTGGATAATAAACCTATGACCGGGCAAGAAGCCAACGG
AGACCCAAAGAAGTGGCGTGAGCAATCAAATACCAAAAGTACGAGTGAGAAGTACAGTCCTTTAGAAGAAGTTGGTTCAGGGTTCCCCATTGATCCTCCCAGAGGAGCTCCACATGGCT
TGTCTCATTCCAACACTACATTGCACCCCAGTGCATTTGGGAAGAACGATGATATCAGGACGATTCCTAACCAAGCATCTAGTACTATGTGGGCACCCAACGTTCCACGATTACGATCTCA
GAGATCATATATGCCTCAGAATAGTGGTGGCATGGACTTTGCCAATTCAAAGAGCAATATGAGCTCCACGTATAATAGGCTTGATGTTGCTGAGTCATCTGAGAAGCAGCATGTACTAGATA
GACCTTCTTCTACCTATAAGGGAGATGACAAGGTTGGAGCCAAGGAAGCTGTCATGAGTTACGGTGGAAAAAACAAAAGAATTCACTACTCAGGGCCATTAATGCCCCCAGGTGGTAAC
ATAGAAGAACTGCTTAAAGAGCATGAGAGGCAAATCCAGCAGGCTGTGCGCAAAGCTCGTTTAGACAAGGTAAAAACAAAGAAAAATTTCTAACAAGAGCCTGAAGATTATCTCCTCC
AATGTAAATGTCCCTTTTCCCACAGAATTTACTATTAATATAGCAGTATATTTGCCTCTTCTGTCTAATTTCCGAGATTCTTGGGAAGTTGCTTCTAGTGAATTAGAATCCATCTATGTACTGCT
TGCACAGCTAGAGCTACCCATCTGTAAAAGTTTATTTATGCTGCTAAATGCACATTTAGTGTGCAAGTCTGAGTTATATTCTTAGTAGAAAGTTTCATTTGTCAGTTGATTAAAATTTATTGTT
TTGTTAACATTGCTGTTTTATTTTCAGTATCTTTCCATGACTGTTTTAGCCC
>comp161963_c0_seq13
TGAATATATGGATCATGATCTTGCAGGACTTGTTGCAACACCTGGAATCAAGTTCACCGAGCCGCAGGTTAAGTGCTTTATGCAACAATTACTTCGTGGCCTCGAACATTGCCATAAACGC
AAAATTTTACATCGCGACATCAAGGGATCTAACCTTCTGGTTGACAGCAATGGAATCCTCAGAATAGCAGACTTTGGCCTGGCTACGATTTTCGATCCTGATCAGAACCAGTCCTTAACAA
GTCGTGTGGTGACTCTATGGTACCGGCCTCCTGAGCTTTTGCTTGGTGCTACTGAATATGGTGTTGCTGTTGATTTATGGAGTGCTGGTTGCATCCTTGCAGAATTGCTTGCTGGGAAACCT
ATCCTGCCAGGAAGAACAGAGGTTGAACAACTACATAAAATCTTCAAACTTTGTGGATCCCCACCAGAAGACTTCTGGAAAAAATCTAAGTTGTCGCAGTCTACTATTTTTAAACCTCAT
CACCCCTACCCTCGGCGTATTGCAGAGACATTCAGGGAGTTTCCTCCTTCAACATTGTCTCTTCTTGATACCCTTCTCGCAATTCAACCAGAGGATCGAGGAACAGCATCTTTGGCTCTGC
ACAGGGAATTTTTCTCGACTAAACCTCTTGCCTGCGATCCTTCTAGCTTGCCTAAATATCCTCCAAGTAAGGAATATGATGTTAAACTTCGAGATGTGGAAGCAAGGAGGCAAAAAGCTGC
AGCTCTTAAAGGCAAGCCATTTGATTCTGGAAGGAAAGGCCCATTGGATAATAAACCTATGACCGGGCAAGAAGCCAACGGAGACCCAAAGAAGTGGCGTGAGCAATCAAATACCAAA
AGTACGAGTGAGAAGTACAGTCCTTTAGAAGAAGTTGGTTCAGGGTTCCCCATTGATCCTCCCAGAGGAGCTCCACATGGCTTGTCTCATTCCAACACTACATTGCACCCCAGTGCATTT
GGGAAGAACGATGATATCAGGACGATTCCTAACCAAGCATCTAGTACTATGTGGGCACCCAACGTTCCACGATTACGATCTCAGAGATCATATATGCCTCAGAATAGTGGTGGCATGGACT

TTGCCAATTCAAAGAGCAATATGAGCTCCACGTATAATAGGCTTGATGTTGCTGAGTCATCTGAGAAGCAGCATGTACTAGATAGACCTTCTTCTACCTATAAGGGAGATGACAAGGTTGG
AGCCAAGGAAGCTGTCATGAGTTACGGTGGAAAAAACAAAAGAATTCACTACTCAGGGCCATTAATGCCCCCAGGTGGTAACATAGAAGAACTGCTTAAAGAGCATGAGAGGCAAATC
CAGCAGGCTGTGCGCAAAGCTCGTTTAGACAAGGTAAAAACAAAGAAAAATTTCTAACAAGAGCCTGAAGATTATCTCCTCCAATGTAAATGTCCCTTTTCCCACAGAATTTACTATTAAT
ATAGCAGTATATTTGCCTCTTCTGTCTAATTTCCGAGATTCTTGGGAAGTTGCTTCTAGTGAATTAGAATCCATCTATGTACTGCTTGCACAGCTAGAGCTACCCATCTGTAAAAGTTTATTTA
TGCTGCTAAATGCACATTTAGTGTGCAAGTCTGAGTTATATTCTTAGTAGAAAGTTTCATTTGTCAGTTGATTAAAATTTATTGTTTTGTTAACATTGCTGTTTTATTTTCAGTATCTTTCCATG
ACTGTTTTAGCCC
>comp162027_c0_seq7
GGCTCCCCTCCTGGCACCCAAAATGCCGGCGTGGATACGGCGAAAATCCTGCCGCCAACATCCCGTAAATATGCTCCGTTCTTTCCCAAAGGATTATCTGCAACAGGGAGATACAGTTTC
CGCGATTAAGCTATTCCGTTTAACTGCAGCAGCGGCCATGGAGAGGATAGAGGAAAGTGAAAAAGCCAAAGCCGCGGCCTCCTTCATTCGAGCTGATGATCCCATCACCGTCCACGTAA
AGTTCAGCGGCCGCAAGATCTCAGTTACCCTTTCGCCTGAATCTACCATTCTGGATCTTAAATCCCGCCTCCACTCCCTTACCGACGTCCTCACTCGCTGTCAGAAACTCATATTTAAAGGG
TTTCATAAGCAAGATGGAAGCCAAATAAGCAAGAACCTAAGTCGATAGTTGACATCAGTGAGGCGGCGGACATAGAGCTTGTAGAAACAGTTTGCTTAATTTAAATTCTGTTTCAGAATT
TTATCAGAAAGCACTTTATATTGATCATCTTTTAGACAAAAGCAAAATTCATGCAGATGTAGTGAGTTTGAGGTCAGTGCAGATAACTGATGGTTCCAAACTAATGATGATAGCTACTCTGG
ATCAGCACATAGAGGATGAACCAAGTACAAAAGATGGAGAAGACTATTTAAGTATTGGCGGTTCTTTTACCAGAACTCAAAAAGTTGCCATTGCAAGGGCTATGACAATATCTCGACGTA
AACGTTGGAAGAAAAGTGGTGTTGCTAATTGGTCGGGGAGGGCCTTGAAGGCTTTGCCTGATGATGTCTGGGATTGTGCACCGTTTTTAATAAAGATGGAATTCAGCTCCAATTCTATTGA
AGAATTGCCGAATAAAATTGGTATTTTGAAGTCCTTGAATGTATGTTTCTTATAATACATTATTTTATGTTGTTTTTTTAATCTGGTTGAAACATTCCCTTTGAAAATATGATTAGCTTTGGAGC
TTTAGCCTTACTAAATGTGAAATATGGAGCCTTTTGTGCCTTAATTGTGTAGAAG
>comp162029_c0_seq1
TCATAACTGAATAGAAGTCATATAATCATCAACCAAGCATGGGTTGTAGTATAAGTTAAAAATAATCAAGAAATCTCTAAGACCAAAGCCAACCATCCTACACTAAAAAGTATTAAGGAATT
TATAGAATGATACATATTTCAACTTACAATAAACATCATATAGCCAATTTCATCAACCAGTGAGAGTTGATAAGGGCATGTCATATGTGAGGGATCATGCGAAAGAAGTATTATTTGCTAAGA
GAGCATAACTTCAATAATTAATGGGTTGATGGCTTGCTGAACAGTGAACATAAACATTAATAACAACAACTCAATATAACAACATAACTTCCAAATACAAATATAACAGCATAACATATCCT
CTGAATATAGAAAAACATAATTGGAACCAGATATAACTGAATAGAAGTCGCATAACCATCAAACAAGCATGGGTTGTTGAACAGCAACTCAGCATAACAGCTAAGGGTGTCAATAATTTAC
ACGATACAATCTCTAATAACAGCATAACGCCATAACACCATAATAGCATTAACTTAACAAAAAACCAGCACAAACTAAATAGAAAACCCAGCACATCGAGCTCGACTTGGCTCGTTTGCA
CATCTACTGCGATTGATTAAAAACCAACAAAGACTGAACAAAAAAACCAGCACAAACAAAAAACTACTCTAAAACCAGATTTAATTAAACAGCATTTCATCAATTTTTCGGCCAAAACA
ACACCAAACCAGAAAGTATGTCAATCCAGAAATGCATGCCAAACTTTACCAGATTTTCAAAAAAACCTGCTATAATATTCGAGATAACAACAAATCAGCAAAACAACAACAAATCGGAC
AAACCCTAAACCCGAAAATTTCTTACGAACTTCAGAATAATTTGATCAAACAGCAAGATAGCCCTCAACTCTGCTCATAGATCAAGCTTCTAAAAGAAGAAAAAAGAAAAAAAGATAAG
AGAAACAAATAGCGAGCAATGTAAGTGTTAGTGTTGAGCCAAACGGGATGTACACGATGAACACCACGGACCAACAATATGCCAGAGAAGGAGAAGAGAGGAAACCGTTGACAGAGC
CAGAGATCTGAGGAGATTCGAAGAGAAGAAACGGAAACCCTAAACGAGACAGCGAAGGAGAGGAGAGGATGAAGCGGACGCGAAATGAATATGTGCGAATGCGGAGCCCTAATTTAA
TCCTCGTCGCGTGAAATGAAAAATACGCAGGTCAAAACGGGTCCGATTTCGGGTTGTCAGGTTAACCCAGACCTGACCCGAAAGTTGAACAAGGTCATTTTACTGAACCCGAACCCGAC
CCGTTTTGA
>comp162056_c0_seq2
TTAATGCCTCCCATAGTCCCATTTCTATCCCGATTCGTATTCCCTCCTACAATTCCTCTTCGTCTTCTTCTTCTTCTCCTTCTTTTTCATCTGCTTCTAGGGTTTATCTGACTGACGAGTTAGCC

AAAGCAGCCATGGAGCGGAAGAAGTATCCGATCCGCGCGGAGGAGTACGAGCTTTACGAGATGGTTGGACAGGGGGCAAGCGCCGTCGTGCAGCGGGCCTTATGTGTTCCTCTCAACG
AGATCGTGGCTATCAAGATTCTTGACTTTGAGCGCCACGGAAGCGATCTGAGCAACATCTCGCGGGAGGCACAGACAATGATTTTGATAGACCACCCCAATGTCCTGAACGCGCACTGCT
CCTTCGTGAAGGACCACACCTTGTGGGTAGTCATGCCGTACATGGCAGGAGGGTCCTGCCTCCATATCATGAAGTCAGCATTTCCAGATGGTTTCGAGGAATCGATTATTGCCACGATTTT
GAAGGAGGCGCTGAAAGGATTGGATTATCTTCATCATCATGGCCATATCCATCGTGATGTGAAAGCTGGCAACATCCTAGTTGATGCTAAAGGTGGAATTAAACTCGGAGATTTTGGGGTT
TCTGCTTGCCTTTTTGATTCAGGTGATAGGCAACGTTCAAGAAATACATTTGTTGGGACACCTTGCTGGATGGCACCTGAGGTGATGGAACAATTGCATGGCTATGATTTCAAGGCAGACA
TATGGTCTTTTGGAATAACTGCTTTGGAACTTGCTCATGGTCATGCTCCTTTCTCAAAATACCCTCCAATGAAGGTTCTATTGATGACTTTGCAAAATGCACCCCCGGGCCTTGACTATGAA
AGTGACAAGAAGTTTTCAAGGAACTTCAAGCATATGATTGCATCCTGCTTGGTTAAAGATCCTTCAAAGAGGCCTACTGCTCATAAGTTGTTAAAACATGCATTCTTCAAGCAAGCTCGAT
CACAGGATTATTTGCACGAAAAATTTTGGAGGGTTTGGCTGCACTTGGCGATCGATACCAAGCGCTAAAGGAGAAGGAAGAGAGCTTGCTTGCACAAAAGAAAATGCCTGATGGAAA
GAAAGAGGAGATCTCGCAGAATGAATATAAAAGAGGAATCAGTGGGTGGAACTTCGATATCGAGGATTTGAAGGCCCAAGCATCATTGATCACATGTAATGAAGATGCAGGAAAAGATC
AAGGAGTCTCAAATTCCTTTTTTGAGTGTGACAATTTGCAAGATAGAATGTCAGATAGGCCCTATTCCAGAAATTGTTCATTCAAGGAACATACAGACATTCAAAGCGATGGGTTGCAGG
ACAAATCATGTACTGTTTTACCGCCAGATGAAACATCATGTTCTCAAAGAAACCGATCTGATGGATCTGATAACGAGCTGAAGAATATGGGGGCAAGCGATCAACAAGTTGTAATGCAGC
TCCAAAAGACTGAGATGGATAATCTTACTAAAAATTGTGAAAAAGGGTTAAATGGACGGTCTGTTTTCTCTGAGGGTGAAAAGAGGATTTCAATCGCTTCAGAAATTGATGAAAAAAATG
TGAATGTTTCTCCTACTTATAGTCCCGAGGAGCCAAGGACTTTTACGAATTGCCCAATCGATGTTCCTTCATTATCCAGAGGAGCAAGCCTGAAGCAACAAAATCAGCAGCAAAACAACA
TGAATTCCAATGGAGAGGTTTCCCAGACATCAAATGAATCACCTGCTGAGATTGTGCCAAAAGGGTCTAAACCAATGGCTCCAAATGGTGATGACATTGATGACAAGTCAAAGCCTCCTG
TTGTTCAACAAAAAGGGCGTTTCAAGGTGACCTCTGAAAGCATTGATTTTGATAAGCCTTCTTCCATTGGTCTACAAAAGAGTCATAGCATTCAGGTGATTTCTCAACTTCAACCGCAAA
GCCTTTCACCGCTGGCTGATTCTTCTCCAAGCTATGCGAACTCATTCTTTCAACAGTTGCAGCTTATATTGCAGAGTAATATTGTTCAAAGGGATCATATTCTTAGCATCATGAAGCAGATAA
GCACCGGTGATCCATCAGGTATGGATTTTTCATGTCCTTCTTCATTTTGAATATATTCAATAAGTATTTCCCGAATGCAGCTAACCGCTCAGTTGACGGAGCCTCTTTACCTTCACCTACTAC
AGCACCTGAAAAGTCATTGGTAAGTTTTGTTTCACAGTTCTTTGGCAGCCTGACAGTAGCATTATTTTCTCTACATTCTGAGTTGGATACAACTATAAAGGAACAAACATTCAATATTCATA
CTTGTTTTCATGAGCTTATTTATTTAGCAAAAACATAATGCTTAACATTAAAGTATTTTTCTTATGTATAGTACACTCTACAATGGAAAGCCAATATGTTACACACTTTTTTATGACAAGTTAA
AACATCTTGCATACATGCTATACGCATCGTAGTTTACTGCTATCAAATGTACAAGGTCTAAAATATGACTATATTGTGAAGAAAATAAATTTTGTATAAATAGATTAAATAAATATTGCTTGTTA
ATGAGCTCCTAAATGGAAAATAGTATAAT
>comp162110_c3_seq3
TCATGCAATAAGTGTCCCACCATGTTATGATCACATAATCACACAGTGCTCATGCAATCATACGTCAATCACATAATCATGCAATCAAGCAGTCGCATTATCAAATCACAATATTATATAATTA
GATAAACATCACATAACCATGCTACATTCAACTAATCATATAACAATAATTTAGTCATATCTCAATCATGCAATCACATAGTATCCACACCACCGTATGATAAATATGCAATAATCATCTAATCA
CATCGAATCACATGCTAACACACAAGTGATCATGCAATAATCACACAACAATCATAAAACTATGCAATGATCACACGATCACACGATAATCCAACTATCATCCAATCATGCAATGGTCATAT
GATCATGCAACCAACCTAAATATCCAACAATCACTCAATCATACAACAAGCGATCAAGTATATAATGATTATCCCATAATCACAAAATTATGTAATGACCGCGCACTCATTCAATCAACCACA
TAGGCATGCAATGCTCACCCAATCATGCAAGCATGGTGGGACAAAAGAGAGAAAGAAACCCAACGTTCGCCTAACCAAATAGAAATGGAGAACATGCACAACTCGATACTCACCTAACC
AGATCGAAACAGTGAGAACAAGCTAAAAGAAACCCAACACTCACCAAACCAACCGAAAACAGTTAGAAAGAGTGAGGAAAACAACTCAACAATCACTAGTGAAGTGAACAACCGCT
C
>comp162111_c0_seq1
CCAAAAAAAAAAAAAAAACAATATGGCATTAAAACAATGAGCCGCGGCGCTATATATCGGCAAATTATTCTGTGCGAATGCCGAAAATCATTCATAGACCAACTATAAAGCGCCATGTCAC

CCCCGTTCGGTACGGCAGCTCGATACCTCGTTCGGTACCATGCTCAATACTGCTGACGTTGCACATTCTGATTGGCCTCCGAACGATAAACTAAGTTGGGCGTCCGAACATAATAATTTCTC
GCTATACACCACACATACTTTCAACTCCATATAGACATATAAATAATTATTATTCTACGTGAACACTAAAACTGGTTCCCCTCAAAAATCTGACCAAAACGCCAGTTCCTCGGCACCACATT
CAAAGACTGCACCGTCCTCCCATCACTCGTCGTCACCGAAAACGAAACACTCCTTCCCACCAACTTCTCCGACGTCTGCCAAACCTGCCCCCAATTCCTGATCATCGCCGTCCATCCACC
CCCGTCCGCCTTCACGGAAACAGCCGTCACATCCCCATCCCCAGCGACATTGAACACAACCACAAATGGCGTCCACTGATTCCCTTCATTTGGAACTTTATCCCGCCCTTTCTGTTGCAGG
TGACGCGGCGGAATTGGACGGGAACTATGCCGGCGTGATAGTCCTTTGTGATCTTGACGAACATCGGCATGGAGAGATCGAAGTGTTTTAAGGGTGGGTTGCACCAATTTTCGTAGGGTT
TGGAGTAGTTGGGAGGGCAGAAGTTGGTGGCGGTGACGATGATGGAGCCGGGAGCGCACCACTGGGGGTCGTTGAAGCATTGGATTTCGTAGCAGGCGCCGCAGGTGGCGCCGCCGTT
GAACAGGGCGGTGCTTAGAGCGGTTGTTTTGAGGCCGTAGATTTGCAAGAATAAATCGCTGTATCCGCAAGCTCCACCTATGGTTTCACCGCCGCCCATATCGCCATAAAAGGTCGCATGG
GCTGTATCCCATCCTCCATTGTCGGCACCATTAAGCCGAACAACCGAGAGAAAGAGGAAGGAAAGACAAATCCAGAAGAGATTCATGGCTGGCTTTCTCTCAAGAATTTGCGAGAGGAG
CTGTTCTTT
>comp162111_c1_seq1
ACAAGAACAGCTCCTTCCTCGCTAATTCTTGAGAGAAAGCAAGCAAGCCATGAATCTCTTCTGGATTTGTCTTTCCTTCCTCTTTCTCTCGGTTGTCGAGCTTGTTGTTGCCGACAATGGA
GGATGGGATACAGCCCATGCGACCTTTTATGGCGATATGGGCGGCGGTGAAACCATAGGTGGAGCTTGCGGATACAGCGATTTATTCTTGCAAATCTACGGCCTCAAAACAACCGCTCTAA
GCACCGCCCTGTTCAACGGCGGCGCCACCTGCGGCGCCTGCTACGAAATCCAATGCTTCAACGACCCCCAGTGGTGCGCTCCCGGCTCCATCATCGTCACCGCCACCAACTTCTGCCCTC
CCAACTACTCCAAACCCACCGGAAATTGGTGCAACCCACCCTTAAAACACTTCGATCTCTCCATGCCGATGTTCGTCAAGATCACAAGGGACTATCACGCCGGCATAGTTCCCGTCCAAT
TCCGCCGCGTCACCTGCAACAGAAAGGGCGGGATAAAGTTCCAAATGAAAGGGAATCAGTGGACGCCATTTGTGGTTGTGTTTAATGTCGCTGGGGATGGGGATGTGACGGCTGTTTCC
GTGAAGGCGGACGGGGGTGGATGGACGGCGATGATCAGGAATTGGGGGCAGGTTTGGCAGACGTCGGAGAAGCTGGTGGGAAGGAGTGTTTCGTTTTCGGTGACGACGAGTGATGGG
AGGACGGTGCAGTCTTGGAATGTGGTGCCGAGGAACTGGCGTTTTGGTCAGATTTTTGAGGGGAACCAGTTTTAGTGTTCATGCAGAGTAATTATCAATTATATCTTTATATAGAGATGAA
AGTTTGTGTGATGTATAGCGCTTGACGCCGCGGCTTATTGTTTCAATGGCAGTTTGTTTTTACGTGGTCTGTATTGGGAGAGATTATTCTATGCAGATATCGGACGGACACTCAGGTATTGA
GAGTTATCGAGTACCGAGTACCGTTCGGTATCGGACGTGACGCATTTT
>comp162134_c0_seq1
AAGGTCCTGGAGGAGAAGCGTAAGAGCACGAAGGTTCAAGGCTGTGAGGTCTGCTCGACATTAGTGGGAAAGCTGGATGGTGAAGAGGAGATGACTGGCCTTGCACAAAAGCTTGAG
GATGTGGTGGCAATCCAGGATAGTACTGTGGATCCTGCAAAGACAGATGGTCTAGTTCTACCAGAAACCATTCAGAAATCGTCAGAAGAATTTTCTGAGAACAAAAAATGCATGATATGT
TTCTTTGGTAATCATAAAAATCACAGTGATAAAAAATCAAAGGAAGAAATTCTTTTTTTATGTGAGGATTGCAAGTACCAGATAGCAACTTTTTATGTCCTGCATGTTCAGAAATGGCATGA
GCTCCTCAGTCCAAGAAGCAAAAGGAAGATAAACCCCTTTGAAGGTAACAAAGAGCAGTCTCCATCATGCGTTTCTTCCTGCAAAACTCGAATAAGAAGATCAAAAAAATCTAGAAAA
AAAACAGGAAAAAAAAGTCATCAGCAAGTCCTTTGGGAAAGGTATGAGAATGGTTATCCTACAGTGGGACCATTGGGTGAGCCAAGTGGGAAATTTGATTATATAGTAGAGTATGCGACT
GAAGAAGGAAAAAGAAGAGTACTATTAGACACATCTTATGATCAGATCATTCCCACAGGATGGGATGTAATTAATTAACTAAATGTCACTATATAGCTTAATTGTTTCTTTTAGTCATGATGT
TAGTCTAATCTTATGTACTGAATAATCATGTTGTTAGTCTAATTTTATGCTTAATTGCTGCTTTTAAAAGTCCCCTTTCATTGAAACATTGTTTTGTACTTAGTTTGAATTTTAGTTTATTTTCAC
TTGTGGAGTTTCTGTGTGAAAAACAATTTATGTTTCGGTTATGTAATTCTCTA
>comp162134_c0_seq22
CTCTTCAATCAGGAGCGAAAACGAATTTTGGACAGGAGTGACGGTATTCTTAATTCTTCCATGAATTTTTATTCATCTTTGCATCTTATATATTGTGGTTGTATGTATGTATGTAGATGTCTTA
GATGGAGCATAAGCCAGAAAAAAAATGACTGATTATGAATTTTGGACTTGAGTTTAATTACGCCGACGCAATTTTTGCTGGTAACTCCGCCCGTAATTACCGGTGGAAAAATGCGCTGAA

AACCTTTAAAATATCGGTGATTCAGCGATGGAAATTGCGTAGGATATTTCGCAGTTAAGCTACCGGCGGGTAATTTAATATATTTTTTAAAATTATTAAAATGAAATAAATAGCCAGGATATTT
TCAAATGATTAAAATGATCACGGGGATGGAAACTCAAGCCACAATTATAATTGTAGTATGGATATAGAGATAGATATGATTAGCATGATGAAAAGTTCTAACAACTTTGTAGGCCATGCATTT
ATCTGTCATAAATCAATTAAAAAAAAGTTGTTCGTTTGTTGTAATAAATAATAATACATTCATATCCATCAATGAAAGCCAAATAAAAGAATTTTGCCTGATCTCGACCAAACAAAACATTG
AATGTCCATCACTCATCATGTGTGGCAATCTCGATCGGCAATATATAGAAAAAAATGGCAGATCAAAAGCCCAAAAAAACCTTCCACCTTCCTTCGATTTTTATTCATCTTATTGCTGTTTTA
TATGTACATATAATGTTGATTTATATATGCAGATGTCTGAGAAGGAGAATAAGCCACGAAAAGTCTATGAATGCTGGAAGGAAAAGCATAAGAACACCAAAGAGCAGGTTCCATCATGCGC
TTCTTCCTGCGAAACTGGAAGAAGAAGGTCAAACAAATCTAGAAAAAAAACTGGGAAGAAAAGTCATCAGCAAGCCCTTTGGGAAAGGTACGTGAATGGTGATCCTACAGTAGGACCA
TTGGGCGAGCCAAGTGGGAAATTCGATTATATAGTCGATTATGCGCCTTCAGAAGGAAAAACAAGATTATCATTAGACGACATCATTCCCACAGGATGGGATTGAATTAACTAAATCACAA
GAGCTTAATTGCGCGCTGGTTTTAATCATGTTCTTAGTCTAATTTTATGCTTAATTGCTGCTTTTAAAAGTCCCCTTTCATTGAAACATTGTTTTGTACTTAGTTTGAATTTTAGTTTATTTTCA
CTTGTGGAGTTTCTGTGTGAAAAACAATTTATGTTTCGGTTATGTAATTCTCTA
>comp162166_c1_seq4
CTTTTTCAGGTGTTTTATAAATTCCTTCAATCAAATAAATTCAAAACACATAAAATATGAAAATTTTAAATTCCACAATGGTTTGTTTTTTTCATTTCCATTTTTCAAATCAAATTCTTAACAA
CTAAAACACAATAACATGCACTAGTTATTTCACGCCTTAAAGGCTAAAACACATCACAACAATCAACTACTGTTCAATTACAAAATATTTACGCGCAAAGGCTAAAAAAAATAATAAACAA
TAATTAACATATAATATTAAAGGGCTTCTCCATTGTTATTGAATAGAGATCATTTGCGGCAAATTAGAATGCGCCAGGTCAACGAATACACAACACCAATATGACGTGGCACATACTGATTTG
CCTCTAGGCGTCATCCAATGACTGCATGGAATAATTTTTCAGAACTAAGTGGATACATATCTTAGAATAAAAATGCCGTTCCTTTCCTCATAGTAGCCAGCGCGCTCCTTCTCAC
>comp162305_c0_seq4
TTTGCTCAACTCCCCATTTCGGCCCAAAGAAATAGGAAAGACAACTCCGGTGGTTGCTTGGGTGCTTGAGAGGAGGTAGACAGCTACCACGAGCCTTCGAGATGTAGCGGGACATCAAT
ATGGACTTTAGAGGCAGCGGGACAACAATAGGAGGTTGCCATGATTGCTGGACAGCAGCTTTGGTACTTGGAAAATCCTGGACAACAGATTAAATACAAGAGTCTAAACAGCAGCTTTA
GGTTTTTAGAAGTCTTGGACAGCAGCTCTTCTTGTTGGAAATCCCTGGAC
>comp162324_c2_seq12
GTCGAACCAAATTCAAGGCAACTCCAAACAAAGTATTTTTTTGTCATTATTGACCCATTTTCTTGTAGAGAAGAGTCGAGCAAAATTAATGCAACACCAGCAGTTACTTATGGGTGTCAAT
TTTGGGTCACGGGTCGGGTTCGGGTTGAATCCGAAATAGACCCGACACTATTCGGGTCAACTCAAAGCTGACTTACTTCAGGTCCGGGTCAACCCGACCAGACCCAAATGACCCGGTGC
TAACAAAAAACTGCTAATTTTCTGGCCCAAAAATGGGTCAACCTCCACTTTTCAGGTCAAAAACAGGTAAATCTCGGGTTGCCGGGTCAAAAATGAATTTTCAGTTAAAAAACGAGTCA
ATTTTAGGTCAAAGGGTCAACCTGATCTGATTTTTGAAACTGGTCGAATTGTGGAACCCGAACCCGACCCGTTTC
>comp162329_c1_seq3
ACATTTTTTTACCAATGAAAAAGGATTCGACGAGAAGAAATAGATAAGCAATGGAAATATTGCAATCAGTATAATAGATGAATAAGTCTTATTATAATTTTTTTTTTTTTTGGGAATTTTCTTA
TTATATTGTTAGGATAGTTATTTTAAAATATTTATCAGTACAGACGACATTGAAACATTAAGCGTTTTTGCCGTGGATAGAAACTGTCATCTCAGCAGCCTTCCATCATCCTCTTCACAAATTC
ATCATAGCTGATTTGCCCATCTCCATCCATGTCTATCTGACTTATCATTTCGTTCAACTCCTCATCACTCAGTTTCTGTCCAAGGTTTGCCATCACTTGACGGAGCTCTGCTGCAGAAATGAA
GCCATCCTGATCTTTATCAAAGATGTTGAAAGCTTCCCTAAGTTCCTTCTCAGTTTCTTTTGTTTTGCTCGTCATAAGATTCAGAAACTCTGGGAAATCAATGGCGTTATTTTTATCAGCATC
AAACTCACTGATCGTATTCTTCAATTGCAGTTCAGTAGGATTTAGGCCCAATGATCTCATGACTGTACCAAGCTCTTCGGAAGTAATGAAGCCATCGCCATTCTTATCGAAGAGGTTGAAA
ACCTCCTTGAGGTGTGCTATCTCTGCACTCGTCAGCCTCTCCATCTTTTGTTTGAGCTAGAAGAAAAATAAAAAGAAGACAAAGTTATACTCTTCTCCCCTTTCTTTCTATATCTATTCCCCT
TCCTTGATTGGATTAGTGAAGGAGAGGATGAGTTGAGAGAGCTT

>comp162329_c2_seq1
AAGCTCTCTCAACTCATCCTCTCCTTCACTAATCCAATCAAGGAAGGGGAATAGATATAGAAAGAAAGGGGAGAAGAGTATAACTTTGTCTTCTTTTTATTTTTCTTCTAGCTCAAACAAA
AGATGGAGAGGCTGACGAGTGCAGAGATAGCACACCTCAAGGAGGTTTTCAACCTCTTCGATAAGAATGGCGATGGCTTCATTACTTCCGAAGAGCTTGGTACAGTCATGAGATCATTGG
GCCTAAATCCTACTGAACTGCAATTGAAGAATACGATCAGTGAGTTTGATGCTGATAAAAATAACGCCATTGATTTCCCAGAGTTTCTGAATCTTATGACGAGCAAAACAAATTAACTGAG
AAGGGACTTAGGGAAGCTTTCAACATGTTTGATAAAGATTAGGATGGCTTCATTTCTGCAGTGGAGGAAAGCACTCAGCTTCTACTCTCCATTAAAGCACAAATAAAATGGAGTTTTTAA
AGCTATCTGAATTGTGGGACATTTGCAGCTCGGTCAAGTGATGGCAAATCTTGGGCAGAAACTGATTGATGAGGATTTGAACGAAATGATAAGTCAGATAGACTTGGATGGAGATGGGCA
GGTCATCTTTGATGAATTTGTGGTGAAGATGATGGAAGGTCACTGAGATTATAGTTTCTATCCATGACAAAAAGGCTTAATTAATGTTTCAAATGTCATCTGTACTGAAAAATATTTAAAAT
AACTATCCTAACAATATAATAAGAAAATTCCCAAAAAAAAAAAAAATT
>comp162372_c1_seq1
CATTGTTTGATTTATCCTTAGACTAGAGCAATTAAAAAAGGGTTAAATCCTACGCACTAGTCAGCCACATGCGCAAGAAATGAAAAGGGAAATCACGTAATTTTCAGTACAGGATCTGAAT
TTTAAAGTCAAAATCCCAGCACTTGGTGCACAGAGCATTAACAAGCTCAACAAATAAGAAAATTTAGAATATTTGCAAATTCCAATTACTTCATAAAGAATGCAAATATGGTATTTTGTCTG
CTTTTAAAGTCAATTTTGTAAACTCGTAAATGTTGACTATTGATATTCTCACCGAATAAAAAAAAAGTTTCTTGTCAGTCCCCAAAGTTCTTACTAATTTTGATTAATGGGTGAACTAGTGG
AAAACAAAATGCTGGGTGTGAACAGCTTTTATTCTGTGTGGACACCGGACGGTTCTCCAGTCAGGTTAAGCGCTCGGTACCGAGCGGTACCGAGGGCGGGTACCGAGCACATTACATTT
TTGGGGTGACGTGGCACAATTTTATTGGTCTCCAGACGCCCTCCGGACTCCACACAGAATAATAAATCGTGAACTAGTGGATCACAAAATGCAAAGTTATTTTCAATATTACTATATTTGCA
CGACAACTCTAGGATATGCAGGACCTAGGAAAATTGAAGCATTATTAATAGTTTTTGCTCATGTCAGCTTTGATAACAAGCAGTCATATTTGCTTTTGACATATCAATTTTCATAACATCATA
GCACGAAAACACCATGACCCAAATAGACTGGCGTCTACAATAAAAAATAATAAA
>comp162397_c1_seq11
TTTTCTTCCTCTAAATGCGGCTTCGTTCTCCTTTGGAATCCTAGCTTTAATATGAGTTCCATGTATCGCACCAATACAATCCTAAAGTTGACACAAAGTATATTTAGGATACCGTAATAAAAG
AGAGAATTTAATTGAATAAAGATTGATTATACCTTGAAAAATGGCATGAACATATTACTTGATTTAATCTTATATGGAGTTTCAGGAAGTGGAGGCTTGATATACTCATGGCGTAACTCCCCT
ACAACATGTAATGCAGCTTTGAAGTACCTACTAGCAATTTCACCAGAGCGATGAAAGTTAAATCGGATGACTCGGTTACGAACACTATGCCCGAGTGTATGTAATAACATAGCAACTTGTT
CCTCGATTCTAACATTCAGTGTGTCTAACAACAATCCTCGTGTACGCATTAAATCACATAATTTGAAAAAAGGCGTTCGCCTTATGCGTACCATATCAACACAGTCATAATCACGTCCATTAT
ATAACCTAGAAAGATATGTGTCACGTTGTATATTCCGTTGAGGATCATATTGATGAGGACATCAGATTTCGACATGTGTA
>comp162408_c0_seq3
TTTTTGAAATATAACCAAATGTGTTATTTATAGCAGCATACGCAGTACAATTCATCATGTATGTAGATTTTATGTAAGAGCAAAAGCACAAGTTGAAATAAGGCATCTTTACTTTTATCACCT
GCACTGTATCTAATGCCACAAAAAAACTCCATGACGAAGAAAGAACAATGCAAAATTAAAAGATTTTATAAGTCGTAGAATTGGCCTCCACCCACAGCATCATAAAACACTTCCAGCTTC
ACTTCCAGGCTCTCCAGTTCATTCCAAGATCTGCTCTTTGAATCAATCCTCCCTCTCCTTCAACTCTTCAATCCTTTCAAGTGCTTCCCTCATCTCCATTCTATTCTCCACAATCTCCTCGCA
GCAGTCAAGTCCAATCTTCAGTAGCTTCTGTATCTCACCTCGGCCATTTTCAGTGTCCCGCATGTCGCAATCGAAAACCTCGCTGTTCCATTCCTCTTCCATCACTAAACCCACCCAGGTG
GCAAGATCGGTGCTGCCGCTGGAGCCCTTGTGGAGGAAGTTCGCCGGAAATTTGCCTGTAAGGATCTCGAGTATTAGTATGCCAAGGCTCCACACATCACTCTTCTTCGACGGGAGGCCA
AATTGGGCGACTTCCGGAGACTTGTAGGCAACCATGACCTGTGTTGCGTGGGCTGAGTTCATAACTGGGACCAAAGCGTAGTCGGTGAGGAGAGGTTGGAAGGTGTCGTTGAGGAGGA
CGTTGGAGGACTTGAGGTGGCCGTGGGGGAGGGTTAGCACCGGCAGCTCATCGTAAAGGTAGGCCAATCCACGGGCTACTCCCTTTACTATCTTCAACCTCGTCGGCCAGTCGAGCGGT
GGCAGAGTTGACCCACGATTACCTTGAAATAAAGCGGGGTCCATCCTACTGAGTCTATTTGGAATAGCCCCCTCCAAACGATTAAAAGACACGTCAACAATCGTGAGGCGGCGCTGGCC

GAAACTCGGTATACCGCCGGCGAACTTATTGTGATCGAGACGGAGCTCAAGCAGCTTACCAAGGCCAACAAGCGATCTCGGAACAGGACCGAAAAAGCCATTATTCGATAAAAAAAGCT
TCTTTAGGGATCTCATGCCCGCAAACGCATCCGCCGGAATCTCGCCGGAGAAGTTATTCCTGGAGAGATAAACAGCCTTGAGAGCGCCGAGGAGCTTAAGATCAGGAGGCAAAGGTCCC
TCGAGCTTATTGCCCATGAAGCTGATGGTACGGAGGCCAGGTAGGGGTATTAAAGGTCGGAGATTTAGGGTCCCGGAAAGGCTTCTGTTCTCGAGCTGAATCCCCCAAACGCGGCCATTG
AAGCAGATGACGCCTTCCCAAGTGGTGGAGTTTAGAGTGCAGGGTGCGGGGCCGGGGATCCACGATGGGAGATCGATTGATAGGGTGGCTTTGAATCTGAGAAGTGCTTCCCCTTCCTC
GGCGCCGGCGGATTCGACGGTGATGGTCGAGTAGGCAAGGTAGAGGAAGATGAGGAGCCATGGCGCCGCCTGACTGGCCATGGAGACAGGCGGCGGGGTGGGGGAAGGCCGAAGAG
GGGGACGGAGAGGAGAAAGCTGAGGGTTTTGATGGAGGCCAGAAGAAATGGGAGGAAATTAAGGCATATGATTG
>comp162437_c0_seq1
GGTGTTCGCACAGAATAATTTCTGCTTACATAAAAATTTTAAAGATGTTTCACATGATTAGATTAATTCAAATATAGCGTGACTTCTCAATACAAAATCTAACGGTCTTCAATCTTCAAATCA
AATTAAACATCCTCCTAATAAAAACAGAGGTGCATCTAATAATTTACAAATCTCAACCTTCCCCTACATAAGTACAAAATTTAAGAAAAGTATGCTGCACACTACCAATCAAGCTCATCAAC
CCCCCTTCTCCTTCTTCCCTAAATTCCCTCATTGTAAGAATGGCCAATATCAGGCGGGCTTATCGCAATCCACAACAAGGAGATGGTAATGGCAATCAACCCCGACCACACATAAATAATGG
TCGGAGTCTTCCCCCTTCTTCCCATCAGCCCCTTACAGAAAGGATACATATGAGCCAAAACCCAGAAGCTAAAGAACAATCCGCCAATCAATTTGCTCCACTGCGGAATAACGCTGTAGA
GCGTCCTCGATACGCCGATGAAGATAGCAATGATGTTGATAGCGAGGATAGTGAGCGGCGGGACGAAGAGAGCGGTCCACTTGACTATATAGAGGTCGGCGAAGAGATCTTCGTCGTCCT
CGTTAGCAGATTTGGAGGTGAGAGTGAAAGAGATGTCGATGCCGGCAATGACTTTGAGAAGGCCTTGGAGGACGGCGGCGAGGTGGGCGGAGGTGCCTCCGATGACCCAGAATTGTTC
GTTCCGCCAGTATTCTTCGAGGGAGATGCCGGACCATTTGACTTCGAGGATGGAGAGGAGAGAGAGGGTGAGGGTGATGAGGAGGAGGTAGATGAGGAAGGTGGTGTTGAGGGATTGG
ACGACGAATTCGCCGGTGAAGAGGGAGACAGCGGGAAGAAAGCAGTAGATCACGAGGAAGATGGAAGTAAAGGGGTAGATTCCGACGTTGATGTAGGCGACGCGTTGGAGGAATTTC
AGGCGGGAGCTCGCGAGCAGAGCATTGTTCTTGGAGAAGAAGATCTCCACGGACCCTGTAGCCCACCGGAGCACCTGATGCAGGCGGTCTGTCAAGTTAATTGGTGCCGTCCCTCTAAA
CGCGTCGCGCTTAGGAATGCAATACACTGACCTCCATCCCCGATTATGCATTCTGTATCCCGTCACCACGTCCTCCGTCACCGACCCGTAGATCCACCCGATCCTATCCCCCATTCCGTCTT
ATCCTCATACCAGCAAGAAATGACAGATACAGCCTCCGCGACTGTCAAAGCATCCAACGGCGGCCGAGGAGCAAGCAACGCACCAGGCGGCCTTCCATTCTTCACAGACGGATGGTCTG
CCAATGGCCGCCCTTGAAACTCCGCAAATGCAATCGAATCCAAAAAAATACCGGAGTTGCCAAATCTTTCTGGCATGTCAAGATCTGGATGCTCGTTTAGCGGCTGAGACTCATCATCTCC
CTCCCCTCTCTTTCGCACAACCTCTGCTTTCCTCTTGTGTTGCCCGTAAAGCCCCAAATACTCGTTCGCACGCGGTGGACAGAAACCGTAGAGCGCGTATCGCCGGAATAAGCATCCAGTT
CCGACATACATTGGACCCTGTAATCCATCCAATGCGCGCATGTTCCCGTCGAAGAAAACTGTGTTGTGGTTCGCATAACGGTCGGACGGATCGATTCCCTCGAATCGTTGGGGATATTGAA
TGTAGCAAATTCGGTCGCCGCCTCGATCCATCATGTAACACATACCTTCACGAATGGCGAGCGAATTGTATATGTAATGATCACAGTCGAAGTTGAGGATGAAAGGGCCGTTGGAGAGGAT
GGCAGATGCGCGCACAAGAGCGTTCATTGCGCCGGCTTTTTTATTATGGTCGTAGCCTTGGCGTTTCTCTCGGGAGACGTAGACGAGCATTGGAACCCGAATGTCGACGGAGGTGAATTC
GATGAACGGATGGTCCCCTGTGTTGCCGTAGACAGGGCTGTGGTCAGGTGCTTTGATCATGATTTGAAGAATGCCCGCATGGTCTTTGTTGGAGTGGTCAGGGGAAGGGGATTTCCAGGT
ACCGGGCCAGTGGGTACCGTCGGCCATCCATGTCGCTATTGGAATGTTGGATTTCGCTGCGGCGGGATCTTCTCCATTTTCTTTCGCGAGGTTTACGGCTTTCTTCTGTTCTTTGGAGTTTA
GGGTTTTGGAGCGCTTTCGGATGGATTCAGGAAGGCCGTTGATCCGGACCTTGAATTCGTCGTATTCGCGCTTGATCCAGCGCCTATCCTTCACGAAATCTGGTTGCTTTTTGTTTTTGGTT
GGATCCAGTTTGAGGTTGAAATAGGCCTCAGGGTTGCGAGGCTCGACACAGTGTTTGCGGCAGAAGGGCACCCAGATTTCGGCGAAGGAGGCGGCTTCCGCCATGGCTTCAAAGGTGA
GGAGGGCGGCGCCGTCGTCGGAGACATAGATGGCGAGTTTATCAACAGGGTACTCTGTGGCGAGGACGGAGAGGATGGTGTTGGCAGTGACGAGAGGGGGCTCTTTCTCCGGATCGGC
GGTGGAGATGAACACATCGATTCCAGGGAGGTCGGAGAGGCCCATTGGGTTCGACGGCGAGGGAGACTCGAATTTCTCCTGGAGTGCGGCGAGGTCGGCTGCGCGGTTGATGGGGTTG
ATCTTTGTGAGGATGTCGAGGAGCCAGGAGAAAGCAAACCAGATCTCGCAGGCGGTGGATAATCCCCATAGCCATAAAGCATCTGTGTTTGCATTCGTTATTCTCCAAATTAGGAAGCCG
ATTAGACAAACCAGACGGCCCAACACCAACAACCTGTATGGGCTTAGAATTACGGCTGGTACTCGGACTTTTCTTGTGAGGGGCTTCCATGGCTTATCCATGAAATCCGACATATTCATGC

CGCCACCGGCATCATCATAGCCGCCGTCCGGCGACCAAAAGGCGTTGCCAATGCCGTACGTTCCCTTTGACTCAAAAAGCCACCTGTTGTGATCAAAATCGCCGGTTTGGCTCCGGAGA
AGCATCGACTTATTCTGACTCTTCAATAAAGATAAACGTCGGTCCATCTTGGCAGCCGAATCTCCTCCTCTCCCGCCGTCGTCATGCTTTCTAGCACCACCGCCACGCTGATTCGTTCGCTT
GGCTGCATTGGTAGGCGGTGGAGGAGGGAGGTCTTCAGACTTGGTGCTGGAGCTGCCGACGGCGGTGTAGGGCTGGTTGTCAGGGGTGGGGGGCATGAGTACGGTGTAGCTCATGAAG
TCATTGGGCCCGCCGGAGGACGTGGAGGTGAGCTCCGGACCGCCGGATTCGATGTCGCTGCGGGAGGAGCTCAACATGCGGCCGCTGGAGGTCCGGCGAGTGGATTTTACCCCCGATTT
CTTTGGTGATGATGACGACATGTCGATTTCTTTTGATTTTTTTTCTCGGGCCGTAGTATTTA
>comp162444_c0_seq1
GACGGAGGCGCTCTCCTCACCTTCGAGGCTATGGCGGAGGCGGCGAGCTTCGCAAACATATGGGTACCGTTCTGCCGCAAACACGACATCGAGCCGAGAAATCCAGATAGCTACTTCAA
CATTAAGGGGGACCCCACGAAGAACAAGTGGCGGACGGATTTCGTTAAGGATAGGAGGAAGGTGAAGAGAGAGTATGATGAGTTTAAGGTACGGATTAACGGGTTGCCGGATTCTATTC
GCCGACGATCGGATGCGTTTAATGCAAGGGAGGAGATGAAGATGATGAAGCATCTAAGGGAGGCCAGGGGTGACTCGGTGGAGCCCATGAAGCTTCCGAAGGCCACATGGATGGCTGA
TGGAACGCATTGGCCTGGGACTTGGACTACAGCCGCTCCCGATCATACTAGAACTGATCATGCCGGCATTCTTCAGGTGATGCTCAAGCCTCCATCAAACGAGCCCCTTTTTGGCCTCGCC
GAAGAAGGAAACCAGATGCTCGACCACAGCGATACCGACATCCGCCTCCCCATGCTCGTGTACGTCTCACGCGAGAAACGACCAGGCTACGATCACAATAAGAAAGCCGGCGCCATGA
ATGCTCTCGTCCGCGCCTCCGCCGTCATGTCCAACGCCCCCTTCATCCTCAACCTTGACTGCGATCATTACATCTACAACTCCCTTGCTGTTCGTGAAGGCATGTGCTTCATGATGGATCGT
GGCGGCGAAGATATTTGCTACATTCAATTCCCCCAACGCTTCGAGAACATCGATCCCTCCGACCGCTACGCCAATCACAACACCGTCTTCTTCGACGGCAACATGCGAGCCTTGGATGGC
ATCCAAGGTCCCGTTTATGTTGGGACAGGATGCCTCTTTCGCCGCTTCGCACTTTATGGCTTTGATCCACCTTCGATTACTGATTACTCTCGGCGGGAGATTCAGTCCCCTTTGGATCTGGA
TGATGTTGAGATGCAGAAGAGGCCACTCACCGCAACGGATTTTGATGATGAGCTCGATCCCAATCTTCTTTCTAAGAGATTTGGAAATTCTGCCGTGCTCGCGGAATCTATTCCTGTTTCG
GAGTTTCAGGGACGACCTCTTGCCGATCATCCGGCTATCAAGTATGGAAGGCCTCCCGGCGCCCTTGTTGTTCCCCGTGTGCCACTCGATTCTGCTACGGTCGCAGAAGCAATCTCCGTCA
TATCTTGCTGGTATGAAGACAAGACCGAGTGGGGAGACCGCGTGGGCTGGATCTACGGCTCGGTGACAGAAGACGTGGTCACCGGTTATCGTATGCACAACCGCGGCTGGCACTCCGTC
TACTGCGTCACCAAACGCGACGCATTTCGCGGATCTGCTCCCATCAACCTCACTGACCGCCTCCACCAAGTACTGCGATGGGCCACCGGCTCCGTCGAGATATTTTTCTCACGCAACAAC
GCCCTAATCGCTACCCGCCGCCTCAAGTTCCTCCAGCGCATCGCATACCTAAACGTCGGCATCTACCCTTTCACCTCCGCCTTTCTCCTCGTCTACTGCTTTCTCCCCGCCCTATCCCTGTTC
TCCGGCCAATTTATCGTCCAAACCCTTAACGTCGCATTCCTCATCTACCTTCTTACTATCACCCTCACCCTCATCGGCCTTGCCATCCTGGAAGTCCGATGGTCCGGCGTAGGGCTTGAGGA
GTGGTGGCGTAATGAGCAGTTCTGGCTCATATCTGGAACTTCCGCGCACCTCTATGCTGTTGTGCAGGGGATTCTTAAAGTCGTGGCTGGGATTGAGATTTCCTTCAAGCTTACATCCAAG
TCGGCAGCGGAGGACAATGAAGATATATACGCTGATCTTTACGTCGTCAAGTGGACCTCGCTGATGATCCCGCCAATTACAATTATGATGGTAAATCTCATCTCCATCGCCTTTGCTTTTGC
GAGAACTATTTACAGCGAAATCCCTCAATGGAGTAAGCTTATGGGCGGGGCGTTTTTTAGTTTCTGGGTTTTGGCGCATCTGTACCCATTTGCGAAGGGGCTAATGGGGAGAAGGGGGAA
GACACCTACTATTGTGTTTGTGTGGTCGGGTCTTATTGCTATCACATTATCTTTGCTTTGGATTTCCATAAGCCCACCCAAGTCCTCCGGCACAGTGGCAGGCGGCGGCGGCAATGGCGGT
GGATTCCAGTTCCCATGATCAGTGGAATTTTGGTTTTATTCTTTGATATTTCTCTTCTTGTTTGATCTGAAAGATGGTAAGGAAGAGTTTGATTTGTTATTATTTTTGTTTAAATTTTTAATTCA
TTTTAAGATTAGGGGATTAGAGTGCATTTTTTTTTTAATCGGAGGAACTATTAGATTGCAGATTCAGGTTTTAGCTTATAGATTGTACACACAATCAGAGAATAATGGATTAAAATTTATGGT
TTTTCTTTTTGTGTGTACTATTTTTTTGGTGCTATATGTATATATCAGCTTAAACACTGAGGGCCCCTTTAGTTGAATTTGTCCTAACCACATTTACTGAATTTTATTTAATGACCA
>comp162460_c0_seq1
CAATACTTGTTGAAACTTGTTATCAGACCTAAACTTATATCCCAGCGGCTAACAAAGAGGAGGGTAAATTGGTAATTAAAAAATTTCCCTCTTTAATTTTTAAACCAATAAAAAACTTTAAA
AACTTTTTGTAAAAAGATTTTTATAGAAACAATTTTTCAACCAAATTTTCAAAAAAAAAAAAAAACTCCGGGAATAAAGTGGCGGGAGTTTCGAAGTTGACGGCCGCTGGCCCTCTCCTCA
TTCCGGAAGCCGAAGAAACCGCGCAGAGCGTCCTCGCTCCGAAACTCCCTCAGATCCCCAGATTCGACAACAGAAACCGATGACGCCACACCGCCTCTTCACCTCTCGGCCAATTACAG

CAAATCCGAATTCGTTAATGGCCATACGTAGGCTCGGTTATCCGTTTCCTGCTCCTCTCAACCCTATCTTGAAAGCGAACGATCTTCCAAACCCTAACCTTTCGAATGGAGAAGGTTTCCG
ACGCCGACCTGTCTAGGGAGGCCGCCACTGCTGCTGATGGCGGCAGAGGAGGAGGAGATAAAGAGAGTGAGATGGACGTTGATGACCTCGTCCTTGAGCTCCTCGAGACTGTTGAGAC
CGTGCAGTCTTTTTCCGATTACCGCCGCACGCAGAGGAAAGAGTGCCATAACCTCATTCGCCGGATGAAGCTCGCAGTGCCATTGCTCGAGGAGATCCGCGATTTGGAGATCCCTGTGCC
CGACGATGTCTGCGCCAGGCTCTACAGGCTCCGGACGGCTTTCACCGCTGCCAAGAAGCTTCTCAGATGCTGTCATGACGGAAGCAAGATCTATTTGGCTTTGGAGAGCGAGGCAGTGA
TGAATAGATTTCACTTTGTTAATGAGAAATTGACTCAGGCGGTGGATGGCATGCCTTATGATGGCCTTTGTATTTCTGATGAGGCTAAGGAACAGCTTGAGCTAATGTGCTCTCAGCTAAAG
AGAGCTAAGGGGAGGACAGATACTCAAGACATGGAGCTAGCGATGGATTTGATGGTTTTGTTATCTCAAAAGGAGGAAGATCGGAATGCAAACAGTTGCATATTAGAACGCCTTGCTAAA
AAGTTAGATCTGCATTCACTCCCAGACTTGGGAGCTGAAGCAATGGCCATCAAAATGCTTATAAAAGAAAGATCTGGGCAAAATATAGAGTCCACACAGCAGATCATAAGTCTTCTGAGC
AAATTCAAACAAAATGCTGGTATTGAAGATTGCGATGGCTTAAATGAAATCATGTTGCCCAAATATCTTGAGAAATGCCCTTCCTTGATGATTCCAAATGACTTCCTTTGTCCAATATCTCTA
GAGATAATGACGGATCCTGTTATTATTGCTACCGGGCAGACATACGAGAGATCAAGCATACAAAAGTGGTTGGATGCAGGCCACCGGAACTGTCCCAAAACCAGACAGCCTCTGGCTCA
CCTTTCTCTTGCCCCTAACTACGCCCTTCGCAATGTGATTTCTCAATGGTGCATGAAGAACAAAGTTGAGTTAATGAAGAAGGACTCGTTGGTTAATTCAAAGCCTAAGGACCACAAGGA
AGAAATCGAAACTCTGGTTCAGGATCTATCCTCCTTACACCTTGATACACAGCGAAAAGCTGTTAAGAGAATTCGAATGCTTTCAAAGGAGAATCCAGAGAACAGAATTCTAATTGCCAA
ATGTGGGGGAATCCCTTCACTTGTCAGTCTTCTCTCCTACCCTGATTCAAAGATTCAGGAGAATACAGTGACTGCTCTACTGAACTTATCTATTGATGAAACAAACAAACAGCTTATAACTA
AAGCTGGGGCAATCCCACCTATCATTGGAGTTTTGAAAGGTGGAACCATGGAAGCTCGAGAGAATTCTGCGGCCGCGCTGTTCAGCCTATCCATGCTTGATGAGAACAAGGTTTTGATTG
GGGATTTGAATGGCATTCCACCATTAATAGATCTCTTGCAGAATGGAACTCTCACAGGTAAAAAAGATGCCACCATTGCTCTCTTCAATTTATGTTTAAACTCCATGAATAAGACCAGAGCC
ATAAAGGCCGGTATTGTGAAACCTTTGCTTCAAGTACTTGATGACAAGCAATTAGCCATGATCGATGAAGCTCTTTCTATTATTTTGCTCCTTTCAACACAGTCTGATGGGCGTGCTGAGAT
TGGAGAGCTATCTTTCATTGAAAAACTTGTTGGTTATATAAGAGATGGGGCACCGAAGACTAAAGAATGTGCCTTATCTGTTCTTCTTGAGCTTTGTGCACACAAGCCATCGTTTCTGTTG
GTCTCGCTCCAGTTTGGAGTTTATGAACATCTATGTGAAGTTGCAGAAAGTGGAACCCAAAGAGCTCAAAGGAAAGCAAACTCTTTGCTGCAACTTATGAGAAAAACTGGCCAAATTTT
ATGAAGATTGTATATTTCATCGCAAACTTATTACATTTATCATTTTTATTATTAAATCTTTGAATAGATTCTATTATCTTTTGGTCCATAAGTGAGTAATTTGTGTCATAAATATTGGGCTATTAAA
AATATTATTCACTGTAACATCTTTATTGCAGGGTGATGTAAAATAATCATT
>comp162502_c0_seq1
AGACTATTTTTTATGAATGCATATGTATGAGTTAGAAATATCAGAATAAATAAACAAATAAATTAATAAATAATAAAATAGATAAAGGATTCATATGATATTATGAACATGAAGAAAGCTCAAT
GTGAAAGCAGATCCAGCAGAAATTAAAAAAAAAAAAAATACATTTTAAGTAATATAATGCAAGTTCTTCAGGAAGAAAAGAGACGAGTAATTTTGTAAGTAGGAGGATTAGAGAGCGAGG
GAGGAATGATATTGAATCACTTCTCCGACTCCGCTACTCACAAAAGAATGGAAGTTTCTGCAACTCGAGAAAGAGAAATAAGAAAGACCAAAGACGATGGAGAGGGTTAGTTAGTTCAC
AAATGATCATGAGAGGAGGAGGAGGAGGCAGCAGCAGCACCCAAAAAGGACTCCAAATAATAATAATAAGGGGAAAAAACTATACAGTTTTTTTTTGTTACCTTAAAGGCTACAGTCGA
AAGTCGCCTGACCCAAATAAAGATTTCAATTGGTCCGAGTATTTCCTTCGTTGAGCGCAATATTCTCCATCTGAGAAACAAGAGAATCTTGTTTTGTGGTAGTCTGAGAATTAGGTTCAGA
TTCCGTGTTATCCTTATTTTGTCGTGGGTTAAATAACCAAATCTGCCATCCGTCACGTTTTCTGACTTTGTTGCCCTCCACTTCTAAAACATCTGAGGGCCTGATTGCATCCAATATAAATGG
TATGCTATCTGTTAATAGCTTAATCTTTAGCGGCTGTGATCTCAGTCGGTTCGGATGCGACTGAGCCGTCTCTTCAACTTCTTTACCCGCGGACCCCGGAACACATTCGGTAGGCTGTGTTC
CTTCGACACTCTGTTCAGCGGCCTTTTCTGGATCACCAGCCGACTCAGAACCAACTTCAGTAATTTGCTCGGTAGGAGGCTTTGACATGATCGAATTGATGCTATCCAGGTAGTCTTCCCA
CGGATTACTGTT
>comp162502_c0_seq5
AGACTATTTTTTATGAATGCATATGTATGAGTTAGAAATATCAGAATAAATAAACAAATAAATTAATAAATAATAAAATAGATAAAGGATTCATATGATATTATGAACATGAAGAAAGCTCAAT

GTGAAAGCAGATCCAGCAGAAATTAAAAAAAAAAAAAATACATTTTAAGTAATATAATGCAAGTTCTTCAGGAAGAAAAGAGACGAGTAATTTTGTAAGTAGGAGGATTAGAGAGCGAGG
GAGGAATGATATTGAATCACTTCTCCGACTCCGCTACTCACAAAAGAATGGAAGTTTCTGCAACTCGAGAAAGAGAAATAAGAAAGACCAAAGACGATGGAGAGGGTTAGTTAGTTCAC
AAATGATCATGAGAGGAGGAGGAGGAGGCAGCAGCAGCACCCAAAAAGGACTCCAAATAATAATAATAAGGGGAAAAAACTATACAGTTTTTTTTTGTTACCTTAAAGGCTACAGTCGA
AAGTCGCCTGACCCAAATAAAGATTTCAATTGGTCCGAGTATTTCCTTCGTTGAGCGCAATATTCTCCATCTGAGAAACAAGAGAATCTTGTTTTGTGGTAGTCTGAGAATTAGGTTCAGA
TTCCGTGTTATCCTTATTTTGTCGTGGGTTAAATAACCAAATCTGCCATCCGTCACGTTTTCTGACTTTGTTGCCCTCCACTTCTAAAACATCTGAGGGCCTGATTGCATCCAATATAAATGG
TATGCTATCTGTTAATAGCTTAATCCATTGTTATCCATGTTCGATCTCAGGAATAAATCACTGCACAAGTTCTCTGAACTGAAGTAATACTCAATTTGCTTCAATATCAAAGTGCGTTTTTGTT
CCGCAGATGGAAAAAACATAGGTTCTTGTATTGCAGGAGGTGCTGGGTTGGGAACAAATGGGTACCCTCCGAAAGGTTCGGGATGAGGGTGAGTTGGGAAAAAATACATTGGAGGTGG
AATATCAGGGTAGGCCATGGGGCCAGGAAAATGTTGGATAAGCGGAGGAGGGCCATACAATGGCACCATAGCAGGCATATGAGGTCGCATGATTGCATGGCTCGGGTGATTTCGCTGCAA
GTGGGAATCTCTAACACTCAGAGGCCTATAAGAACTCCAATCACGGAAAACACGGTCCTCATCACGCCTGCCTCCATAGCTTCCCCTTCGGTTGCTGCCACCATGCCTCCTCCGTCGATCC
CCTTGATCCACCCAGCCATGGGATTCACGAGGACGATGGTCCCAATTGCTGTTATTGCCCTTATCAGGCAACTTATCTGTCATGTTCTGAGATAATTCCGTTGAACTTGATGTTGAAGAAGA
TGATGAGGGATCACCATTACTGGAAAATCTACCACTGGTTCCAACATCATCGCCATTATTTCCACCGCCAAGAGAGTTAGAGCCCAGGGAGGGGGCCAATCCATCGAATGAAGGTCTGGT
AGAGGCTTTGGCAGATTCCGATAAAGCGGGCCAAGAAGAGGAGCCTCCCAAAACTAGCGGAGCAGTATCAATGGCTCCATTCGGCGCCCTTCCCCATACAGGCCTATTTTCCCTTTCGGA
AGCGGGACCATGGGAGGCATTGCTGCCGGCAAGAAAAGGAACAGAGTCTTGCGCGTGTTCTCGCCGATTCAGAGGAAGAACTGCTGGCTCGGGTAAGGGAATCGCGGTAGCTGCTGAA
TGAGGCGGAGCAACGGCATCCGCCTCGGATTCCCCGCGTACGGCAACGAACCCTGCGGAGTCAGAGACATTGCGGAGGACGTGTAGGTCCGAAACAAGAGACATGCTAGGGTTACAGC
TAGGGTTCCGTAGCTCTGTGTGAATGATCGGCTGGAAGAGGATTACCAGCGAAAATTTTGAGAAACTGGAATGCAGGGTTGGGGAAGGAATCAATCAGAGATTTTAAGCCTTGAGAAAA
CTCATCGCTGGCTATCGCACCGTTGCTCGCGCTCTTTCTTTCCTCCCGCATTCCCCTCCCGATCTCTCATGCAGAAACCCAAACCGCAACGAAG
>comp162537_c0_seq4
AAAAAAAAAAAAAAAAAAACAGATTTTGAAAAAGCACTTCTAAAAAATCCGCTTCGAAGGACATCGGAGAACACTTTCGCTCCCAATTCACTCTTCTTTTTCAAGAGTTTGGCCTTCCA
GCTCTGTTTTCTTACCCTGTTTCTTATCACAAATCTGCGCAAAATCCTCAGAATTCTCCTCCTTCGGGCTATCCAGCGCGCGCTTCTCTTGCTCCTCTTCTTCCTCAGCCTCCTTGCTTCCCA
AAGATGCCCCGGCAGCGCCGCCGCATCTCCTCCCCGCCGCCTTACTCTTCAACACCTTCAGCAACTCCTCCATGGCCTTCCTCTTCTCATACCTCCTGCTTTTTATCAACACTTCACTGACA
TCCGCCGGGGTTATCTCCGCCGCCTCCACCGCCGCTTCAAGCTCTCTAATCAGTATATCATTCTCTTTTCCATTCTCCAATTCATCCTCACTTATTCCCAAGTAATTCCACATCAAAATCTTTA
GCGCCGGGAAAGAACAGTAGCTCATATGGATATGCATGTCCATCCTTCCAGAACGAAGCAACGCAGGGTCGAGCTTCTCGACATGGTTCGTCGTGAACACAAAGATCTTCTCACTGCCGC
AGCAGGACCACAGCCCGTCGGTAAAGTTCAGCAAGCCGGAAAGCGTCACAGACTTTGTCGATACTGCAGCAGATGCAGGGGAGGTGAAAATATCATCCATCGAATGCGGCGGCGGATT
AGCAGCCGGGTGAGGAAGGCTGCCGCGAGTTGTGAGAGAAACAGAACAGTCTATGTCTTCGATAACAATGATGGATTTTGAGGTGGTTTTCATAAGAAGTTTTCGGAGCTCAGAGTTGG
TTTGAACTTCGGTGAGCTCGAGGTCGTAGATGTCGTAGCCGAGGAAGTTGGCCATGGCGGCGATCATACTGGACTTGCCAGTTCCCGGTGGACCATAGAGGAGATAGCCGCGCTTCCAC
GCGCGGCCGGTTTTCTGATAAAAGCTTTTTCCGGCGGCAAACTTACGGAGATCGGCAATGATGTCTTTTTTTCGGATGGGATCCATGGCGAGGGTGTCGAAGGTGGAGGGGTGTTTGAAC
GGGACCGACTCCCAAGGATAGCCGCGAGCGTCCATGGAGCCGCAGCGAGAGTTTGTGTAAAGAAGACGGTCCTGATTCCGGCGGCGGATATCGGCGGCGACTTCCATGATGTGGTCGAG
ATAGCGGGGGAGGATGAGAGGTTTGTCCTTTTTACGAATGCGGAGAGTGAAGCTGCGCTTCTCGTCGGGGAGAGAACGCCATGAGAAGGAAGGTGAGGAGCGTTGGGTTACGACGTGC
TCCCAAATGGCAGTAATGCCGGCAAAAGAGTCGACGAGGCGATCGTTGTTGGACAGGCCGAATGTGAAGTTGGAGGAGTTGACGGGGCGAGAGAGTGAGAGGCGAGAGGCGGAGAC
CGAGGCAGAGGAGGAAAGGTAGAGTTGGACGGCGCCGTAGAGCTCGTTTGTGCTGACGCCGTCGATTTCGGTAATGTCGAAGTAGCAGTAGGCAGAAAAGTTGTTGAAGATGCGCTGG
AAGAACTTAGCAACGACATGCCGGAGCTCGTGCGGTAAGACGGAGTGGAGGACGCTCTGAAAGAAAGCGAGGGCGCCGAGGAGAGAAGCCACTGAACCAAAGGTCCACACCTCCTT

CATGTTCCCCGAATAACCAAAGGTCGGATCTTTCTCCCTAGTTTTGCACGTCTTGGCAGAAAGTTGGGGGCTTTGAGCGATTTAAATAAAAAAGATCGTATCTTTTCGGGGAACATGAAGG
AGGTGTGGACCTTTGGTTCAGTGGCTTCTCTCCTCGGCGCCCTCGCTTTCTTTCAGAGCGTCCTCCACTCCGTCTTACCGCACGAGCTCCGGCATGTCGTTGCTAAGTTCTTCCAGCGCAT
CTTCAACAACTTTTCTGCCTACTGCTACTTCGACATTACCGAAATCGACGGCGTCAGCACAAACGAGCTCTACGGCGCCGTCCAACTCTACCTTTCCTCCTCTGCCTCGGTCTCCGCCTCT
CGCCTCTCACTCTCTCGCCCCGTCAACTCCTCCAACTTCACATTCGGCCTGTCCAACAACGATCGCCTCGTCGACTCTTTTGCCGGCATTACTGCCATTTGGGAGCACGTCGTAACCCAAC
GCTCCTCACCTTCCTTCTCATGGCGTTCTCTCCCCGACGAGAAGCGCAGCTTCACTCTCCGCATTCGTAAAAAGGACAAACCTCTCATCCTCCCCCGCTATCTCGACCACATCATGGAAGT
CGCCGCCGATATCCGCCGCCGGAATCAGGACCGTCTTCTTTACACAAACTCTCGCTGCGGCTCCATGGACGCTCGCGGCTATCCTTGGGAGTCGGTCCCGTTCAAACACCCCTCCACCTT
CGACACCCTCGCCATGGATCCCATCCGAAAAAAAGACATCATTGCCGATCTCCGTAAGTTTGCCGCCGGAAAAAGCTTTTATCAGAAAACCGGCCGCGCGTGGAAGCGCGGCTATCTCCT
CTATGGTCCACCGGGAACTGGCAAGTCCAGTATGATCGCCGCCATGGCCAACTTCCTCGGCTACGACATCTACGACCTCGAGCTCACCGAAGTTCAAACCAACTCTGAGCTCCGAAAAC
TTCTTATGAAAACCACCTCAAAATCCATCATTGTTATCGAAGACATAGACTGTTCTGTTTCTCTCACAACTCGCGGCAGCCTTCCTCACCCGGCTGCTAATCCGCCGCCGCATTCGATGGAT
GATATTTTCACCTCCCCTGCATCTGCTGCAGTATCGACAAAGTCTGTGACGCTTTCCGGCTTGCTGAACTTTACCGACGGGCTGTGGTCCTGCTGGGCAGTGAGAAGATCTTTGTGTTCAC
GACGAACCATGTCGAGAAGCTCGACCCTGCGTTGCTTCGTTCTGGAAGGATGGACATGCATATCCATATGAGCTACTGTTCTTTCCCGGCGCTAAAGATTTTGATGTGGAATTACTTGGGA
ATAAGTGAGGATGAATTGGAGAATGGAAAAGAGAATGATATACTGATTAGAGAGCTTGAAGCGGCGGTGGAGGCGGCGGAGATAACGCCGGCGGATGTCAGTGAAGTGTTGATAAAAA
GCAGGAGGTATGAGAAGAGGAAGGCCATGGAGGAGTTGCTGAAGGTGTTGAAGAGTAAGGCGGCGGGGAGGAGATGCGGCGGCGCTGCCGGGGCATCTTTGGGAAGCAAGGAGGCT
GAGGAAGAAGAGGAGCAAGAGAAGCGCGCGCTGGATAGCCCGAAGGAGGAGAATTCTGAGGATTTTGCGCAGATTTGTGATAAGAAACAGGGTAAGAAAACAGAGCTGGAAGGCCA
AACTCTTGAAAAAGAAGAGTGAATTGGGAGCGAAAGTGTTCTCCGATGTCCTTCGAAGCGGATTTTTTAGAAGTGCTTTTTCAAAATCT
>comp162582_c0_seq13
TTTTTTTTTTTTTTCATTATTATTATTATTTTTGGATTCCGGGTTTGTCTTTCTCTCGTCAGGGCTCTTCTTCGATTGGCGTCATGGGTTGTGAGATTGGATTTTTGTTAAGATCTGTTTGCTTTCGA
TTTATGGCACAGTGATTTCTCTTTGTTTTTGCTTACCTTGATGGATAATTGCTGTGCTTGGGAGGTTTCTTGGGCTGAGGAAGCTAGGAATTAGGCGTTTTTTTCTCTGTCTTCGCGTGGAA
CAATTTTTGTGTGTGTGTGTCTTTTGTGCGAGATATTGGATGGACTGAGGAAGGATAGGCGGTAGATATGTTTTTGGATTTGAGTTTCTGCGTTGATTTCGTTTTAAAAATTTCTCAATTCTG
CTTTTGGTCTCGGGACGAAAAGATGGATCGGTACCGGAGGGTGGAAAAACCCAGGGAGGAAACACCTATTAACGATAATGAGATCCGCATAACAACTCAGGGAAGGATGCGCAGCTACA
TCACTTATGCCACTAGCCTTCTTCAGGAAAAAGAGTCAAATGAAATTGTTTTCAAGGCAATGGGGAGAGCTATTAATAAGACTGTTGTGATTGTGGAGCTAATTAAGAGAAGGGTTGCTG
GTCTTCATCAAGATACTAAAATAGGATCCACATTCATTACTGATTCATGGGTGCCTTTAGAGGAAGGCCTACTTCCACTGGAAACTACTAGGCTTGTGTCAGTAATCGCGATTACACTCTCT
AAGCAAGAACTTGATAGGTCTTCTGCTGGATACCAATCTACTTTATCTGAAGATCAGGTGAAATTATACGCTAAATATGACAGCGAGAGAGAGAGTTATCCTGCTCGTCGAGGTAGAGGCC
GTGGCAATCTCATCAGAGGCAAGGGCAGAGGAAATGGACCTGCTATGGTAGATTCCGGGGATGGAGGTGAAGGCAACAGGAGGCGCGGATATGGCAGGGGTGGTTCCGGCCGCCGCAG
AGGCCGCGGCAACGGAGGCTCTGGTGGCCCCGTAGACACTCAGCAGGAAGCTGGTGGCCATTCGGAGGAAGCTCCTGCAGATTTAAAGGGCCAAGATCCGCAACAGAGCGGAGGGCC
GATCAGTGCAGGTAAAGGTGCTGGTGAAGCTTAATCAATCGCCAGTGACTTGTAAAGTTTTTCTTCTTTATTTGGGATCTTTTAACGGCATTCTGCATAAGATGTCAGGCTAACAACCCCTG
GCAAGGAGCTTTTAAGACTCTTTTGGACTAGTTAGCTGTCTTGCTAATTATTTTTTGTTTTTGTTTGTCCTGCTTTGATAAAGAACAGCAAGCAGCTCATTCTTTTATTTATTTTCTAAGTTTT
TGCTCCGAACTTCTATTTTTTATTTTTCCTTTAATAAATGTTTTTTTAGTTATTTTTTATTTTGAATATAAGTCAAAATATTCTTGAAAATACTCGAAACAACTTTTTAAATGTTTTCATTTTTTT
AATGCAAAACAACCCAACCACCGGCCACCACCACTGCTAGCTTCCTTTGCCGCCTTCCATTACATCTTATGTCAAAATAATATAATATTTATTTAGGTTATCAAATGTATTTTTAATTAGTATT
AAAATATTAAAAAAAAAAAAAGAAGAATGCGATCCCAAATGGCCCCTAAGTTTTTTAATACATTTGGTATTCATGTGTATCTATATTGGTTTTTCTTTGGTCCCTTGCAAGCTGTAACTAGCTC
TTCATTGCAGGTTCTGGAAAGGGATAAAATTACAAAATGGTTCCA

>comp162582_c0_seq16

TTTTTTTTTTTTTTCATTATTATTATTTTTGGATTCCGGGTTTGTCTTTCTCTCGTCAGGGCTCTTCTTCGATTGGCGTCATGGGTTGTGAGATTGGATTTTTGTTAAGATCTGTTTGCTTTCGA
TTTATGGCACAGTGATTTCTCTTTGTTTTTGCTTACCTTGATGGATAATTGCTGTGCTTGGGAGGTTTCTTGGGCTGAGGAAGCTAGGAATTAGGCGTTTTTTTCTCTGTCTTCGCGTGGAA
CAATTTTTGTGTGTGTGTGTCTTTTGTGCGAGATATTGGATGGACTGAGGAAGGATAGGCGGTAGATATGTTTTTGGATTTGAGTTTCTGCGTTGATTTCGTTTTAAAAATTTCTCAATTCTG
CTTTTGGTCTCGGGACGAAAAGATGGATCGGTACCGGAGGGTGGAAAAACCCAGGGAGGAAACACCTATTAACGATAATGAGATCCGCATAACAACTCAGGGAAGGATGCGCAGCTACA
TCACTTATGCCACTAGCCTTCTTCAGGAAAAAGAGTCAAATGAAATTGTTTTCAAGGCAATGGGGAGAGCTATTAATAAGACTGTTGTGATTGTGGAGCTAATTAAGAGAAGGGTTGCTG
GTCTTCATCAAGATACTAAAATAGGATCCACATTCATTACTGATTCATGGGTGCCTTTAGAGGAAGGCCTACTTCCACTGGAAACTACTAGGCTTGTGTCAGTAATCGCGATTACACTCTCT
AAGCAAGAACTTGATAGGTCTTCTGCTGGATACCAATCTACTTTATCTGAAGATCAGGTGAAATTATACGCTAAATATGACAGCGAGAGAGAGAGTTATCCTGCTCGTCGAGGTAGAGGCC
GTGGCAATCTCATCAGAGGCAAGGGCAGAGGAAATGGACCTGCTATGGTAGATTCCGGGGATGGAGGTGAAGGCAACAGGAGGCGCGGATATGGCAGGGGTGGTTCCGGCCGCCGCAG
AGGCCGTGGCAACGGAGGCTCTGGCGGCCCCTCAAACAATCAGCAGGAAGCTCCTGCAGATATAAAGGGTCGAGATCCGCAACAGGGCGGAGGGCCGATCAGTGCAGGTGAAGGTGC
TGGTGAAGCTTAATCAATCGCCAGTGACTTGTAAAGTTTTTCTTCTTTATTTGGGATCTTTTAACGGCATTCTGCATAAGATGTCAGGCTAACAACCCCTGGCAAGGAGCTTTTAAGACTCT
TTTGGACTAGTTAGCTGTCTTGCTAATTATTTTTTGTTTTTGTTTGTCCTGCTTTGATAAAGAACAGCAAGCAGCTCATTCTTTTATTTATTTTCTAAGTTTTTGCTCCGAACTTCTATTTTTTA
TTTTTCCTTTAATAAATGTTTTTTTAGTTATTTTTTATTTTGAATATAAGTCAAAATATTCTTGAAAATACTCGAAACAACTTTTTAAATGTTTTCATTTTTTTAATGCAAAACAACCCAACCAC
CGGCCACCACCACTGCTAGCTTCCTTTGCCGCCTTCCATTACATCTTATGTCAAAATAATATAATATTTATTTAGGTTATCAAATGTATTTTTAATTAGTATTAAAATATTAAAAAAAAAAAAG
AAGAATGCGATCCCAAATGGCCCCTAAGTTTTTTAATACATTTGGTATTCATGTGTATCTATATTGGTTTTTCTTTGGTCCCTTGCAAGCTGTAACTAGCTCTTCATTGCAGGTTCTGGAAAG
GGATAAAATTACAAAATGGTTCCA

>comp162582_c0_seq23

CACATCATATGCTAAAAAAATACGATTTTGCGTGCCTCGATCGAGCAGAAATCATTCTTGACCGTAAACTGCTCAAATTCTCGCCACGTAATGAAACAAACCCCTGCCGAAGAAGAAGAC
CATTTCGCTTCGAAGCGGATTATGAAGTGAAGCCGTTGCGGGAGCAGAATTCAGGGCCGAAAATTATTACGCAGCCAAGGTACAGGGCGGAGCAGGGCAAGGATAAAATTTTCTCATATT
GGATACCTAATTTTCTTCCCTTCCGACCTCATTGGTGTCAGTGTTTTGCGAAGAGAAGATGGATCGGTATCAGAGGGTGCTGAAGCCCAGGGAAGTTTCGCCTTCTAAGGAGAATGAAAT
TCGTATAACTTCTATGGGAAGGATGCGCGGTTATATCACTTATGCCACTAACTTGCTTCTGGACAAAGATTCGAATAAAATTGTCATCAAGGCAATGGGAAGAGCTATTAATAAGACTGTGT
TGATTGTGGAGCTAATTAAGAGAAGGGTTGCTGGTCTTCATCAAGATACTAAAATAGGATCCACATTCATTACTGATTCATGGGTGCCTTTAGAGGAAGGCCTACTTCCACTGGAAACTAC
TAGGCTTGTGTCAGTAATCGCGATTACACTCTCTAAGCAAGAACTTGATAGGTCTTCTGCTGGATACCAATCTACTTTATCTGAAGATCAGGTGAAATTATACGCTAAATATGACAGCGAGA
GAGAGAGTTATCCTGCTCGTCGAGGTAGAGGCCGTGGCAATCTCATCAGAGGCAAGGGCAGAGGAAATGGACCTGCTATGGTAGATTCCGGGGATGGAGGTGAAGGCAACAGGAGGCG
CGGATATGGCAGGGGTGGTTCCGGCCGCCGCAGAGGCCGTGGCAACGGAGGCTCTGGCGGCCCCTCAAACAATCAGCAGGAAGCTCCTGCAGATATAAAGGGTCGAGATCCGCAACAG
GGCGGAGGGCCGATCAGTGCAGGTGAAGGTGCTGGTGAAGCTTAATCAATCGCCAGTGACTTGTAAAGTTTTTCTTCTTTATTTGGGATCTTTTAACGGCATTCTGCATAAGATGTCAGGC
TAACAACCCCTGGCAAGGAGCTTTTAAGACTCTTTTGGACTAGTTAGCTGTCTTGCTAATTATTTTTTGTTTTTGTTTGTCCTGCTTTGATAAAGAACAGCAAGCAGCTCATTCTTTTATTTA
TTTTCTAAGTTTTTGCTCCGAACTTCTATTTTTTATTTTTCCTTTAATAAATGTTTTTTTAGTTATTTTTTATTTTGAATATAAGTCAAAATATTCTTGAAAATACTCGAAACAACTTTTTAAATG
TTTTCATTTTTTTAATGCAAAACAACCCAACCACCGGCCACCACCACTGCTAGCTTCCTTTGCCGCCTTCCATTACATCTTATGTCAAAATAATATAATATTTATTTAGGTTATCAAATGTATTT
TTAATTAGTATTAAAATATTAAAAAAAAAAAAGAAGAATGCGATCCCAAATGGCCCCTAAGTTTTTTAATACATTTGGTATTCATGTGTATCTATATTGGTTTTTCTTTGGTCCCTTGCAAGCT
GTAACTAGCTCTTCATTGCAGGTTCTGGAAAGGGATAAAATTACAAAATGGTTCCA

>comp162582_c0_seq25
TTTTTTCGAGCTACTTTGCCATTCTGTAATATGACGTGAACTCAAATCTATACGTTATTAATTCATACAGAAATCTGTCTATACAGTTGCTGCAGGTTTTTTTGAGTTGTCTATACAGAAATCT
ATCTTGCAGAAATATGTCTATACAGAAATCTGTCCAGCATGAAGTCAGCAATTCCTTTTGCTTGAGATTATCTTCCTTTTTTCTAGTTCAATTAAACTTTGGTTAAGATTATTTCTTACAGCTT
CCAGTACAATGACAAACAGGAGAGAATAATGCATAAATTTGATCAATAATTCAACATAACAACTTACAAAACCCTTGACCAACACATCAATAAAAGGTTACAAAACTGAACATAACAAAA
ACAACCTTTTCTGCAGTCGACACAATACGTTACAAACACAAACGGATGATTACATCAACAACAACAATTACAATGAAGACTTTCATCAAGAACATCATCCATTCAAGCATATTAATAACCT
TCGCCTCAAACACTTTGCGCTCATCGTCTATACCAAAAGATGCATCTTCAACTTCATCTATCCATCTGAAGTATCTGCAGTCGTCATCAGTTCGGTTATTCACGCACCTATAAAATTTTCTTC
CATTATTAGGTCCACGGTGGCAAGTGTACAATACACAATTCAGGTGACAGTGACAATAAACTTCAGATCGTTGGCGTCTATCTCTCGTAGCGGACGAATTCGTTGAACTCATTTGAAACGT
TTTGGAATAAACCAACCTTTTCTTACCTCGCCTGCACCTAGGACGTTCGGTCATGAACTAGGCACATCGATTGCGAGCTTCGCACGATGTCCACACGACTTATGCACGATCTCAGTTCGAT
GTTAATACGAGAATGAAACGAGGCTTCTGTCTCTGTTCTTCCACTCGCATTCGAAGCTTAGTATGTTGTTCATCTCAATCATACAACCGAACCTCTTGTCACCTGCCTTCTTCATCATCAATC
CATCAATTCGGCTTCAACCACCAAACTCCAACTAGGGTCCTCACTTCAACCAAGCTCCTTCAACTTCTGTTTGATTGAGCCCCCAACTAGCCGTCCGATTTCTGTTCGAAATTCTCCCGCA
GTTATATCTGAATTCCCTCACCCGTCTTTTCCTCTCAATCAGCCCGAATAAGGTTCGCATACTCCTTCCTCTATTCGAAACCTGCAGCCATATATCCCCCTTCTTGTACTGATCCCAACTTTCC
ACACCTCCGTTCATACACCCCAATCAGAGACCCACGTCGGAGCCCAATACAGCCATCGACACACTTCTCCTCCCTACTGCAAACGCCCAACTCCATTTCAATCCGCCAGCCTCCCAATCG
CAAATCGCACCCTCTCCCATCACTATCTCCCGATCTACCAAGCCTGCTGGCCGATTAAAGCTTGTAGCCAACCACTAGCCTCATTCATCCGAGATCCATGAACTGCCCGTCTGATCGATCCG
CAGCCGCACCCTACCTACGTGTGTCGAGCACTCCAAAGCAGCCAACCGAACTGAGCTCAACAGAGCTGAGTAGCTCTTGTACAATAGATACCAATCTACTTTATCTGAAGATCAGGTGAA
ATTATACGCTAAATATGACAGCGAGAGAGAGAGTTATCCTGCTCGTCGAGGTAGAGGCCGTGGCAATCTCATCAGAGGCAAGGGCAGAGGAAATGGACCTGCTATGGTAGATTCCGGGGA
TGGAGGTGAAGGCAACAGGAGGCGCGGATATGGCAGGGGTGGTTCCGGCCGCCGCAGAGGCCGTGGCAACGGAGGCTCTGGCGGCCCCTCAAACAATCAGCAGGAAGCTCCTGCAGA
TATAAAGGGTCGAGATCCGCAACAGGGCGGAGGGCCGATCAGTGCAGGTGAAGGTGCTGGTGAAGCTTAATCAATCGCCAGTGACTTGTAAAGTTTTTCTTCTTTATTTGGGATCTTTTA
ACGGCATTCTGCATAAGATGTCAGGCTAACAACCCCTGGCAAGGAGCTTTTAAGACTCTTTTGGACTAGTTAGCTGTCTTGCTAATTATTTTTTGTTTTTGTTTGTCCTGCTTTGATAAAGA
ACAGCAAGCAGCTCATTCTTTTATTTATTTTCTAAGTTTTTGCTCCGAACTTCTATTTTTTATTTTTCCTTTAATAAATGTTTTTTTAGTTATTTTTTATTTTGAATATAAGTCAAAATATTCTTGA
AAATACTCGAAACAACTTTTTAAATGTTTTCATTTTTTTAATGCAAAACAACCCAACCACCGGCCACCACCACTGCTAGCTTCCTTTGCCGCCTTCCATTACATCTTATGTCAAAATAATATA
ATATTTATTTAGGTTATCAAATGTATTTTTAATTAGTATTAAAATATTAAAAAAAAAAAAGAAGAATGCGATCCCAAATGGCCCCTAAGTTTTTTAATACATTTGGTATTCATGTGTATCTATAT
TGGTTTTTCTTTGGTCCCTTGCAAGCTGTAACTAGCTCTTCATTGCAGGTTCTGGAAAGGGATAAAATTACAAAATGGTTCCA
>comp162582_c0_seq28
TTTTTTCGAGCTACTTTGCCATTCTGTAATATGACGTGAACTCAAATCTATACGTTATTAATTCATACAGAAATCTGTCTATACAGTTGCTGCAGGTTTTTTTGAGTTGTCTATACAGAAATCT
ATCTTGCAGAAATATGTCTATACAGAAATCTGTCCAGCATGAAGTCAGCAATTCCTTTTGCTTGAGATTATCTTCCTTTTTTCTAGTTCAATTAAACTTTGGTTAAGATTATTTCTTACAGCTT
CCAGTACAATGACAAACAGGAGAGAATAATGCATAAATTTGATCAATAATTCAACATAACAACTTACAAAACCCTTGACCAACACATCAATAAAAGGTTACAAAACTGAACATAACAAAA
ACAACCTTTTCTGCAGTCGACACAATACGTTACAAACACAAACGGATGATTACATCAACAACAACAATTACAATGAAGACTTTCATCAAGAACATCATCCATTCAAGCATATTAATAACCT
TCGCCTCAAACACTTTGCGCTCATCGTCTATACCAAAAGATGCATCTTCAACTTCATCTATCCATCTGAAGTATCTGCAGTCGTCATCAGTTCGGTTATTCACGCACCTATAAAATTTTCTTC
CATTATTAGGTCCACGGTGGCAAGTGTACAATACACAATTCAGGTGACAGTGACAATAAACTTCAGATCGTTGGCGTCTATCTCTCGTAGCGGACGAATTCGTTGAACTCATTTGAAACGT
TTTGGAATAAACCAACCTTTTCTTACCTCGCCTGCACCTAGGACGTTCGGTCATGAACTAGGCACATCGATTGCGAGCTTCGCACGATGTCCACACGACTTATGCACGATCTCAGTTCGAT
GTTAATACGAGAATGAAACGAGGCTTCTGTCTCTGTTCTTCCACTCGCATTCGAAGCTTAGTATGTTGTTCATCTCAATCATACAACCGAACCTCTTGTCACCTGCCTTCTTCATCATCAATC

CATCAATTCGGCTTCAACCACCAAACTCCAACTAGGGTCCTCACTTCAACCAAGCTCCTTCAACTTCTGTTTGATTGAGCCCCCAACTAGCCGTCCGATTTCTGTTCGAAATTCTCCCGCA
GTTATATCTGAATTCCCTCACCCGTCTTTTCCTCTCAATCAGCCCGAATAAGGTTCGCATACTCCTTCCTCTATTCGAAACCTGCAGCCATATATCCCCCTTCTTGTACTGATCCCAACTTTCC
ACACCTCCGTTCATACACCCCAATCAGAGACCCACGTCGGAGCCCAATACAGCCATCGACACACTTCTCCTCCCTACTGCAAACGCCCAACTCCATTTCAATCCGCCAGCCTCCCAATCG
CAAATCGCACCCTCTCCCATCACTATCTCCCGATCTACCAAGCCTGCTGGCCGATTAAAGCTTATAGCCAACCACTAGCCTCTGATTTTCACAAACCTCCCTCCTATTCTCTCATTCATCCGA
GATCCATGAACTGCCCGTCTGATCGATCCGCAGCCGCACCCTACCTACGTGTGTCGAGCACTCCAAAGCAGCCAACCGAACTGAGCTCAACAGAGCTGAGTAGCTCTTGTACAATAGATA
CCAATCTACTTTATCTGAAGATCAGGTGAAATTATACGCTAAATATGACAGCGAGAGAGAGAGTTATCCTGCTCGTCGAGGTAGAGGCCGTGGCAATCTCATCAGAGGCAAGGGCAGAGG
TGCTATTAATTTGATGCTCTAAATGTCTATTATAGGATTTAGCACAATATATGAAACGATGTATTTTCATCTCTGTACAAGGAAATGGACCTGCTATGGTAGATTCCGGGGATGGAGGTGAAG
GCAACAGGAGGCGCGGATATGGCAGGGGTGGTTCCGGCCGCCGCAGAGGCCGTGGCAACGGAGGCTCTGGCGGCCCCTCAAACAATCAGCAGGAAGCTCCTGCAGATATAAAGGGTC
GAGATCCGCAACAGGGCGGAGGGCCGATCAGTGCAGGTGAAGGTGCTGGTGAAGCTTAATCAATCGCCAGTGACTTGTAAAGTTTTTCTTCTTTATTTGGGATCTTTTAACGGCATTCTG
CATAAGATGTCAGGCTAACAACCCCTGGCAAGGAGCTTTTAAGACTCTTTTGGACTAGTTAGCTGTCTTGCTAATTATTTTTTGTTTTTGTTTGTCCTGCTTTGATAAAGAACAGCAAGCAG
CTCATTCTTTTATTTATTTTCTAAGTTTTTGCTCCGAACTTCTATTTTTTATTTTTCCTTTAATAAATGTTTTTTTAGTTATTTTTTATTTTGAATATAAGTCAAAATATTCTTGAAAATACTCGAA
ACAACTTTTTAAATGTTTTCATTTTTTTAATGCAAAACAACCCAACCACCGGCCACCACCACTGCTAGCTTCCTTTGCCGCCTTCCATTACATCTTATGTCAAAATAATATAATATTTATTTAG
GTTATCAAATGTATTTTTAATTAGTATTAAAATATTAAAAAAAAAAAAGAAGAATGCGATCCCAAATGGCCCCTAAGTTTTTTAATACATTTGGTATTCATGTGTATCTATATTGGTTTTTCTTT
GGTCCCTTGCAAGCTGTAACTAGCTCTTCATTGCAGGTTCTGGAAAGGGATAAAATTACAAAATGGTTCCA
>comp162593_c0_seq8
AGTTTTTTAGTACAAAAATTTATTGCTCAAAAATTTTTTGTACTAAAAAACAATCTTAAAGAATAGTAAGAAAGTTGTTCCGAACGAAGCCTACTGAAATACAAACTGTCATCAACAGTAA
TTTCAAAAATATATATCCAATAGTTGTGAGTTTTTTCTTATTACGACAAACATAGAAGTAGTTTCTGAAGTATTTCATCTTCAATTCCCAATCAATTCCAAGATGAAGTTCTTGAAGTGCCCT
GAAGTTTTGTTGTTTATGGCAGCTATCAGACTCATTTTTCCTCTCTCTTCATACATATCTTTGATCTCCTGTAAATCTTTCTCCGCTCGAGTGACAATAATTCGAGCAAGTGTATTATCGTCAA
TTTTCTCTTTGTTGAGTGCCGATCGAAGGAGCTTTCGAAGATCGCCATTAGTCGTTTTTGTAACATCTTCCTCCAACGAACGCTTGAAAAGATCATGGTAGGCTTGCTTGACCGCCAAAAG
TTCTTTTGTTGAGTTGATACACGCCGTCTCGACGATTACTCTATAGTCTAACCCTTCCTTCAGAGCCTTATTAGCTATGATTGCTCCTCTCTGGGCAGGATTGAGCATCCAAGAGCATAATGC
TTTCTTCAGTTCTCCTGAGAGCTCATTTTCAAGTTTCTTAATGAGATTTTCGTGGTAAAGATCTTCATAAGATTGGATGATTTGTAGCCTCTGTGCTTCATCTCTATGCGCTAAAATATCGATC
AATGTTTTCTCATCTGCCTCCCTTCCTGAAATAAAAATGCATGATATATGATATGAAGGCTTAAGAAAACAATATGCTATAGTATGAAACATATAATGTTTGTCAAAAAATTTTAATGGATTTT
TGATGTAGCTAAAATGTTAAACAAGGAAAGATGCATGCTTTTGTCTGTCGTCCAGCATAATAATTTCTTAAAAGTGATAGAACATATCACTACAGAAAAATCGTCTATTACCGACAGATTCC
TGACGGATTTACTGGCGTACTATTTTTTGTCGGATTTCTATGAAATAGTTCCGATAGATTTTTAGTGAGATTTTCCAGCGGATTTTCTGAAGGATTTTAATTATTTCAATATAAAAGAGTAAGT
ACTTTAACATATTTTTCTTCCAGAGCAAAGTGGTTGCAGGTTCAGGCTTCGAACTCCACCAAAAGCACCGCGCGGGTAGGCAGGTAGCGTAGCAACCGGGCGGGTAGCGCACGCAATCT
ATTTTTTACTGTTCGGCTGCTTTTTCCGGCAGAAGAAATTCGTAGGAAAAAATTTTTCAACGGCAAATTTTACCAACGCTCGATTTACCGTCGAAAAAAATTCCGTCGGCTATTCGCCAAA
AAATTTTAATATCCGCCTGAAACTTCACAATTTTCCGCCAGAAATTCCATCGATAATGACTGATTTTTCTGTAGTGTATGCGGAAAGAGAGGGAAAGTCTGAGATATAGATAACAAAATACA
AAACCTTGGCAGGCCATCCAGAGATTCATTGCATCTTCTTTTGCAGAAGGAACTTGCAATGGAACTCTGAGC
>comp162666_c0_seq8
TCTTACTCTTATTTATATATTTTATCTGAAGTATTTACACTCTTAAACGACCACATACCAAAATTAATAATAATAATAATATAAATTATTACCAAATGGAGTACATAAAGCTACAAACAGACAAA
ATCAAGACAGCTACAGTGCAGTGATGAAGAAAAAGTAGGGTTATTTAGTAAAATTATTCTGTGCGGATACGGTCGCCGTCCGGAGGAGAATCAAAATACGTCACATCACCAGTTTGATGT

GTACCGCTGTGTGCCGGATGATATGGTGTTCTGATTCGTCTCCAAATGATCCGGTGTCTGCACAAAATAATTTTTCTGCTGCTTATAGCCAGTCAAAACCCATCTTTTCCCATGAAACAAGA
TGAACCCTCCA
>comp162700_c1_seq7
TAACCTTATTTCCCTAAAAGCTCCTCACGCCTAAGGGACCCTTGTTCGTTCCATTCGGCCGCACGCGAGAGAAGAGGGAGGAGGTGACAGGAGAGCTCAGGGAGTGGCGGTCGGCTTG
ATTTGCCGGCGAGATCTTTTAGGAAAACGTTAGAGGAAGACTTGGAGAAGCTAGAAGGTCGAACCTAAGACAAGTTCGATTCAAGGAATTGTGCGAAGAAGTTTAACGAAGGAAACCT
AAGGACACGTCACGTTGTTCTGACTCCTGCTGCGGTAGACGCTGCAGGTTCAAGATGACGATGCATATGTTCCGTCCATCGTATTATGATATCGAGTTGTTCAAGGCCTACCGGCGCAATAT
CAAAAAGCAAGTACCAGCAAAGGCACAATCTCCTGTTTGACACTATAAGATGGTCACAAGCTCTTATACGGTATAACAAGAAATAAATTGAAGTAACTTGCTTTGTAATAAACTAGTGTGT
GATAGGCTAAAACAAATTAGTTTATTACAAAGCAAGTTACTTCAATTTATTTCTTGTTATACCGTATAAGAGCTTGTGACCATCTTATAGTGTCAAACAGGAGATTGTGCCTTTGCTGGTACT
TGCTTTTTGATATTGCGCCGGTAGGCCTTGAACAACTCGATATCATAATACGATGGACGGAACATATGCATCGTCATCTTGAACCTGCAGCGTCTACCGCAGCAGGAGTCAGAACAACGTG
ACGTGTCCTTAGGTTTCCTTCGTTAAACTTCTTCGCACAATTCCTTGAATCGAACTTGTCTTAGGTTCGACCTTCTAGCTTCTCCAAGTCTTCCTCTAACGTTTTCCTAAAAGATCTCGCCG
GCAAATCAAGCCGACCGCCACTCCCTGAGCTCTCCTGTCACCTCCTCCCTCTTCTCTCGCGTGC
>comp162736_c0_seq11
TCACTCTCTCTCTCTCTCTCTCTTACAAAAAATAAAAAGAACTATTCTCAACAGATATGTTTATGGTTATAGAAAGTTGGCTTCATTTGATCAAAAGCAGCCAACAACACCGAAACAAAAA
AAACCCCGCTTCAAACCTTGTTCGAAATCTTTTTTATTTCTTGTGTAACTTCTTGTTTTATTCCTATGAAACAAAAGAAAAAACCATCTAAAAAATCCCAAACTTAAGCAACCTTTATATTG
AGGCGACGCAACCACACGGAGTGCAATATCATGTGAAATGCATCGAATCGCTTAGGCGGGTGGTTTAGTACAAAATGGTGGCGCACCGCCCTAACTCCGGCCATCAACCGCATCACCTCC
TCCTCCCCCACCGCCATTAACACCTCTTTCAATCGCGGTATATCTTCCACCGCCATCATCACAGAAAATTCCTTCCACTTCAACACATCACTGAATGGCAATACGTACCCATTTGAGATGAT
CACAGGTATACAACCAGCGTAGATGGCCTCTACCACCCGAGGGCTCGCTACCTAAAATTGTAAAGATATCTCATATCCGCTGGGGCAGAGGCAGAAGCGAGATCGCCGCATATAGGAGTA
GTAGGCATCGGCGCCGGGGGGAAGGTACTCGTGAACCTCGAGATCCGGGTCACGGCCCTTCCAGTGGCTGAGGAGGATCGGTCTGACGGGGCCGTGGACCCCTCCGGCGAAGAAGGC
GAGAAGTCGGCGTGGAGAGGATTCCGGCGGGGACGGAGAGAGAAATTCCGGGGGCATGTCACCGGAGACGAGGTTTATCTCCGGGATGCTGACGTCCTTCCGCGGCCGGAATCCCTCC
GACGTGTTGGCGTTGCAGAGCGCTCGAATGGCGTTTTTGTACAGTAATGGGTTCGCACGTGAAGCGTGGGGGCCCTGCGCAGTTGTGAAAGAAAGAACAAAATTTACGGGTAGGCTGC
AAATCAGCTTTGAGGGGTTTAAAGAAAAATATTTAGGGTATTTGATCAGATATAATGAATAATTATAGTAAGAAATAAATAATAATTTTAGATAAGTGCTAAAATATTCTTACATAAATGCAAA
TATCTTAATTAATTTATGTATAATTATCGAATATAAAGTACGGACAACATAAAGAGTCCTGTGCTGAAGTAACAATTAAAACAATTTTTACTTCGCAGTTGAGCGGGTTTGAAAACCCTTCCA
CTGGGCAAATTATTAGGTCATACATAAAAATACATTTGAATTTCTCTCTATCTATAATAAACTGCAGTCAAATCACAGTACTATACCAGTTGCGCTTTGACTGTGCTTTTAGTGTAGACAGAG
AGAAATCCGGATGATTATCTCTGTCCGGTGTCCGCACCTAATAA
>comp162736_c0_seq3
TCACTCTCTCTCTCTCTCTCTCTTACAAAAAATAAAAAGAACTATTCTCAACAGATATGTTTATGGTTATAGAAAGTTGGCTTCATTTGATCAAAAGCAGCCAACAACACCGAAACAAAAA
AAACCCCGCTTCAAACCTTGTTCGAAATCTTTTTTATTTCTTGTGTAACTTCTTGTTTTATTCCTATGAAACAAAAGAAAAAACCATCTAAAAAATCCCAAACTTAAGCAACCTTTATATTG
AGGCGACGCAACCACACGGAGTGCAATATCATGTGAAATGCATCGAATCGCTTAGGCGGGTGGTTTAGTACAAAATGGTGGCGCACCGCCCTAACTCCGGCCATCAACCGCATCACCTCC
TCCTCCCCCACCGCCATTAACACCTCTTTCAATCGCGGTATATCTTCCACCGCCATCATCACAGAAAATTCCTTCCACTTCAACACATCACTGAATGGCAATACGTACCCATTTGAGATGAT
CACAGGTATACAACCAGCGTAGATGGCCTCTACCACCCGAGGGCTCGCTACCTAAAATTGTAAAGATATCTAAACAATAAGATATAATAACATAAAAATTTCCCAAAATTTAAATGCTGACC
TCATATCCGCTGGGGCAGAGGCAGAAGCGAGATCGCCGCATATAGGAGTAGTAGGCATCGGCGCCGGGGGGAAGGTACTCGTGAACCTCGAGATCCGGGTCACGGCCCTTCCAGTGGCT

GAGGAGGATCGGTCTGACGGGGCCGTGGACCCCTCCGGCGAAGAAGGCGAGAAGTCGGCGTGGAGAGGATTCCGGCGGGGACGGAGAGAGAAATTCCGGGGGCATGTCACCGGAGA
CGAGGTTTATCTCCGGGATGCTGACGTCCTTCCGCGGCCGGAATCCCTCCGACGTGTTGGCGTTGCAGAGCGCTCGAATGGCGTTTTTGTACAGTAATGGGTTCGCACGTGAAGCGTGGG
GGCCCTGCGCAGTTGTGAAAGAAAGAACAAAATTTACGGGTAGGCTGCAAATCAGCTTTGAGGGGTTTAAAGAAAAATATTTAGGGTATTTGATCAGATATAATGAATAATTATAGTAAGA
AATAAATAATAATTTTAGATAAGTGCTAAAATATTCTTACATAAATGCAAATATCTTAATTAATTTATGTATAATTATCGAATATAAAGTACGGACAACATAAAGAGTCCTGTGCTGAAGTAAC
AATTAAAACAATTTTTACTTCGCAGTTGAGCGGGTTTGAAAACCCTTCCACTGGGCAAATTATTAGGTCATACATAAAAATACATTTGAATTTCTCTCTATCTATAATAAACTGCAGTCAAAT
CACAGTACTATACCAGTTGCGCTTTGACTGTGCTTTTAGTGTAGACAGAGAGAAATCCGGATGATTATCTCTGTCCGGTGTCCGCACCTAATAA
>comp162777_c0_seq2
TTGATTATATATTGGCACAAGTGTGGGAGATGGATTTCTGGAGTCCTAAGCAAGAAGAAAGAAAAATAAAATAAAATTTTATTTCAAATGAACAAAGATGATGAAACACAATAAATTTCCC
AATTTACAATGAATTTCACATGCTAATAGAGGATAAAAACAAAAAAAAAATAAAAAAATAATGGGTAACAGGAATAAGTATCAATAATAAAATAGTCTAAATAATTCATTGAAACTACTTAT
TATGAGACTAATAGTTAGCCGTGTCATACATCAATCCCCTCCACCGGAAGAAAAGTTGCCTTGAGTTTTGCCACTTCAAAGATAATTCATTTGGAGGGAAATACTCAAGGATATCAACAAC
TGAAAGTATTAGAGATGTTCAATCTTGTAAGCGTTGCACCAATTTTTTGTAACATGGGAATATGACCAATCTTCTTAACTTTGAGCATGCTATATGTGCCAAAGGTAAAAATTATTTTTCAGA
TGTACAATCACTGTACACCTCTGTAAATAATTTGAGGTGCCATTGTTGATCTACAACCATCTTGTAACGTTAATTAGCATCTTCAAGATATTTCTTCACCTCTTGATGAATTGGAGACACCAA
GATCATAGCCAAACAAAGATCCTCCAAGAGATCCCACGATGACCGCCAATATAAAGAAGACGGTGACTCCCCCTTCATAGAACTCCGCTCTTTTGCCATCTATACCCGGTCCCACTCCTCC
TCCAGCCATTAATCCCTAACTCCAATCTCCTCTTTACTTTCCCTAAGCAGACGACTCGAATGAAGCAAAGAGCGTAAGAAGAACAAGTGAAGGGAGCAGAGATGTAGACGGAAGAAGA
AGACAACGACGACGACTTAT
>comp162784_c1_seq2
TTTTTTTTTTTAAAAAAAAAAAAACCTTGGCGTTATTTATTTATTTCACTTATAATAATACATAGCATATAAGCGCCAAAGATGAGATTTCAGATACTTGATGCGTTATTATATGTAATGTAACCC
CAACGAGGTTCGTACATCATTGGTCTATTTCTCGGCAGTGGGCAATTCAATAAGGTCGTCCAGCACATGCGCAGTAGACCAAACCTCTGGCCCGTATATGACCGCATTGCCATTTTTCTGCA
TGACAAGCACGTACTCGCCAACTTGCCCCCTCTTCGTCTCTGAGTTTCTCCACACAGTTCTGTATTCATCATCTTTGATTTCTAGCCCCCCCTTGTGATTCAGCCTAGCAAAGCAATTTTCT
CCAAAACCTCTTGTCCTCGTATGCCACAAGATCTCCCCGTTTCCTTTCTGGAGAACCAACTCGCACTCTTTTGTCATGATGAAATTGTATCCGTCGTCGGTGAGCTTGCCATCGTTGTATAG
CACCTGGCCGGAGAATAGCACGTTGTAAACTGACGGACAATTCTGTAGATTATTCGTCGAGGAGACCATCTCATTGCTGCTATTACTGCTGCTGCTGCTTATGCTTTCCGCCGCCCGTACA
GACCAGACTTCTGGGCCGTAAATAGATACCTTGCCTTCAGGGAGGAGGATGGCAATGTATTTGCCGACCTTAGAGTTGGCATTGGAGCTTATCCACACTTGATGGCCAAGAGGGTTGCGG
ACGACAAGGCGGCCATTGTCGGTTAAGGAAAGGAAGCAGTTTTGGCCGCCGAGGACAGAAGTTTTTGAGTACCAGATGGGCTTGCCGTGGCGGTCATACAAAACTAGGTAGCAGTCAT
GCCGCATTAACAAGTTGACGCCGCTGTCCGCATAGCTGAGCTTGGCTTGCGGTTCCAGGCTCTGCCCTGTCAGCAAAACGTTAGTAACAGCCTTGCTACGGCTTGCTGATATTAGAACTAT
CAGGAGGAAGAAACATAAATGAATGATATTCAGGAAGGAATCATTGCGACCGACGGCGGCCATTACGATGAAGCTGCTGTGTATAAGATGGTTGTTTGATAAACTATGATGGAGCTTCTCT
ATTTTAGTGGAATTTATAGTTCCATGCAGAGGTTTGGATTACTATTCCATGCATGTTAATCATGGGAACTTTACCGACAAACAAATAAATGCTTGCTTCAACATCTTCCTACTATTTA
>comp162786_c0_seq7
ATCGGAATTGTGGCCAAATGAACGAGGAATACGGCAAACCCAATTGGAAGTGGCGCAAGAATCTGCAAACAAAATCCATTTTTTTCTTCAAATCGTATATCAGAAAAGATAAACTAATATA
CTCTCGCAAATCGCTTCAGATACTGTAACAAAAAGCTCTCACTCCTCCCATGCTAAACTGTTTGATCATACTACTGTTGATTCAGATTTCGTTTGGGACGGTTT
>comp162815_c0_seq4
TTCGAATCAAACTTTCGAAAGACTAGTTCCGGCTGTGAGCTGTCATCATATCCAGATACAGCGGCTTCAATGGCAGAGATTCTGATATGGCTTCAGTTGATGCCGCCATTGATTTTGACTTG

TGACGCTTGAAGAATGACCGGAATTTTTCCATCGGAGTTAGAAGATGGAAGAATTGTGGGAAGGAATCGTTTGTTCGCAAGCCAAATTTGGCGTATGATCAGATTTAGATAACGGAATAG
CATTTTACAACCATTTTATCTCTGGTACCAGCTATTATTGGGCAATAATCCTCACTGAATTCCGTTAAGATTGGACGACTAAAAGAGATGGGAGCAGCGCGCAGCAACTAAAAGGCCGGGA
AATGCGTTATTTTCAAACTTGAACGAAATATTCAAGAATAATATGATCATGATGCAGAACTTGTCAAGGAAATGGTTTGAAGCCATCTGAAAATTACGAGAGAGAATCTCCGAGATTGGCT
CAATCGATCCTTGTAATGTCCGGTGCGAGAAAGGACTTAGACATCATGACCTTAAACTCCTCAAAGTTAACGAGGCCATCACCGTCCTGATCGACGCCGTCGATCATCTTTCGGCACTGCG
CCAAAGACACCACCTCGCCGAGGTCACGCATCACGAGAGCCAACTCCTCCGCCGAGATTACACCGTTACGATCCCGATCGAATACTGAGAAAGCTTGCCGAAGGTCCTCGAGCGCCGTC
GATGGATCGACCATTTTGGTGTTCAGATCGATGAATTCTTTCAGGCTGACGAAGCCATCTCCGTCCGCATCCGCTTCACCCATCATCCGCAGTAGCTCCTCCTCCGCCAAAGGTTGCCCTA
GGCTCTCGAAGATTACTCTCAACTCAGACGATGATATCTTACCATCACCATTGGAATCAAACTTTCTGAAGACACGTTCAAGTTCGTCCATTTGCTCTGCCGTTGTATCCACATACATCGAC
TCCAATGGCGGAGATTCTGATTTGGCGCCGCTGGCTGTGGCCGGTGATTTCGACTTCCGACGCAGCTTGAAAAGTGATCGGAGTTTTCCCATCGTGTTGACAGAGAGAAGGGTCGTGGA
AAGGAATCCTTCATAAATTCATCGCTTCATTAATGTTGGCTGCCGTGCGAGCTATTATTG
>comp162815_c0_seq5
TTCGAATCAAACTTTCGAAAGACTAGTTCCGGCTGTGAGCTGTCATCATATCCAGATACAGCGGCTTCAATGGCAGAGATTCTGATATGGCTTCAGTTGATGCCGCCATTGATTTTGACTTG
TGACGCTTGAAGAATGACCGGAATTTTTCCATCGGAGTTAGAAGATGGAAGAATTGTGGGAAGGAATCGTTTGTTCGCAAGCCAAATTTGGCGTATGATCAGATTTAGATAACGGAATAG
CATTTTACAACCATTTTATCTCTGGTACCAGCTATTATTGGGCAATAATCCTCACTGAATTCCGTTAAGATTGGACGACTAAAAGAGATGGGAGCAGCGCGCAGCAACTAAAAGGCCGGGA
AATGCGTTATTTTCAAACTTGAACGAAATATTCAAGAATAATATGATCATGATGCAGAACTTGTCAAGGAAATGGTTTGAAGCCATCTGAAAATTACTTAGAAAAAAAAAAAACGAGGAAT
GAATTAACATGGCAATAATGGAGAGAAAGGATTTTGGATTTTCTGTCTAAAAGTTATAGGAGATGGTTATTTCTTTATGAGTTAAATTTATCGAATATTTTCGATCTGGGATTGATTCAATTTT
AGCCATTGCATCTTTAGGAAAAACTCTAAAGGAAATTCGTCAATTACTGACGGATTTTTTCTGGAGCTTTTTGACAGATTATTTTCCGATGGCTTTTCAACATATTTCTGGCGGAATATGTCC
GACTAATTTTTCTACATATTTTTCGGCAGCTTTTCAACCAGCTTTGGTTCCGGCGACTTTTCCGTCGGATTTTAATTTTTATATTTTAAATAAAACAATGTGACATTTAAATACTTTTGTCATTC
CAATAAATTGGTTGGGTCGTTGAAATCAAACTCACTTTTGAATCCCAGCGATGCCGAAAACACGGACAAGCAGTCGAGCAGGGTACGCAGGAGGTGGGCAGGTAGCAAGCAACCTAGT
TTTTTTAATTGATTTTTTTTTTTTATTTCTGGCGGCATTATTCCGCCAGAAAGCTGCCGAAAAAATTTATTGCTTGAAAGTTGTCGGAAAATTTTACTGCCAGAAATTAGCCGCAAGTTGTCG
TTTCCTACGTGCCTTTTACCTGCGGAAAAATATTCGCCAGAAAGCCGCCAAAAATCTTAATTTCCATCGGAAATTTTTCAATTTTTGCCTGAAAAATCCGTTGGTAATAGACGATTTTTCTG
CAGTGAAATGAAAAAAAATTAGGTATAAAAAACATATACAAATCACCCCACACGTGACAATCACTTACCGAGAGAGAATCTCCGAGATTGGCTCAATCGATCCTTGTAATGTCCGGTGCG
AGAAAGGACTTAGACATCATGACCTTAAACTCCTCAAAGTTAACGAGGCCATCACCGTCCTGATCGACGCCGTCGATCATCTTTCGGCACTGCGCCAAAGACACCACCTCGCCGAGGTC
ACGCATCACGAGAGCCAACTCCTCCGCCGAGATTACACCGTTACGATCCCGATCGAATACTGAGAAAGCTTGCCGAAGGTCCTCGAGCGCCGTCGATGGATCGACCATTTTGGTGTTCAG
ATCGATGAATTCTTTCAGGCTGACGAAGCCATCTCCGTCCGCATCCGCTTCACCCATCATCCGCAGTAGCTCCTCCTCCGCCAAAGGTTGCCCTAGGCTCTCGAAGATTACTCTCAACTCA
GACGATGATATCTTACCATCACCATTGGAATCAAACTTTCTGAAGACACGTTCAAGTTCGTCCATTTGCTCTGCCGTTGTATCCACATACATCGACTCCAATGGCGGAGATTCTGATTTGGC
GCCGCTGGCTGTGGCCGGTGATTTCGACTTCCGACGCAGCTTGAAAAGTGATCGGAGTTTTCCCATCGTGTTGACAGAGAGAAGGGTCGTGGAAAGGAATCCTTCATAAATTCATCGCTT
CATTAATGTTGGCTGCCGTGCGAGCTATTATTG
>comp162853_c0_seq2
GATCATGGCCATAGCTTGATTTGCTGCTACAGCTGAAGCATTTCTTTCCTCCTCTAGTTCCTTGTATAGAATACTCAGGGATTGCTGGTCTATTTTAATCTGTTTTTTTAAACGTTCAACTGAA
ACCTCTCGTTCAATTTCACTAACAAAACTTCCATCTGCAAACTCTGAACATGATAACTCAGCCTCAGGCTTCTTATTAATACTTGGCAATCCATTAAAGCTTAAAGTATGAGATAATTTGCA
CTCATTATCCTTTCGGGCACCATGAATGATATCATTCCAAGGAGATTGAAGCCACCTAGGAGAGAGAACTTGGGACAGACGCTGCTTCAGGTCTTCATGGATTCTAGCAGATTCCCTTGTC

AGGGCAATCTCAGTTAATTCTGTAGGCTCGTTAATCTCAACATGTGAAGTACAAGATGTTCCTAAAACATCTTCTTCTGAAGGATCTATTGTAAAATGGCAATAATGAAGTTTTAGTACAAA
AGAATTTGAACATAAAAGTATGATTAGGCAAAGCAGATCGTAATTCTGATCTTTTCTCACCTTTAACTTTTGAGATTCCTGTAGGAACTGATTTGGTTACTATTTGAGATGATGTTAGCAGAA
TTGGTCTTCCTTCAAAATGGGTCCGGCTGATTTCCTTCAAGCCATGATCATAGTCAACATTGGATTCAAGAAAAGATTCATCACAAGAACCATCCTCATCATGTTGACTCTTCAAAACTGA
AGCAGAACCAACAGAAATAACAGGATCAGGATGAATAAGCTTCTCTAGGACTATATCGTCTGTAATCAAAGATGTTTCTGGGGAATTAGTAATAGATGAAAACGACTTCAAACTTCGTTCA
AAATATTTTTCCTTGACATCTTCGGTTCTGCACGGCATAGAGGTTCGATCATCATCATCCGATAATGGAACTTCTGACTCGGGCTCTGATGTACTCTTCAACTCTCTATATTTGAAGTGAGAT
AAAAGGTCAAAACTCTGACCTCCTGGCTTATAATCTGCTAATCGTACAGAAAGAACTTCTTTTGCCTTGTTCAATCCGTCTTCAAGATGTGCCCGAGCTCGCAATTTTGCACTAGATAATG
GACTGCCATGATAATCAAACTTTTTGAAAATAGGTTTGTTCTTAGATTCTCTATATGCATAGGGTTTATTTCCTGAGTGCTGAGAACAGCATGAGCAAGGTCTTGTGAGTGGAGAACCAGCA
TCATTATCCTTGTGCAGTAATGGAACTTTCACAATCTTGTCTGAGAATGAAGTGAAAATCTGATTTCCATTATCAAGGGCCATGCTGAGTTTGCTTAGGAAAAATTTATATGTTTCCAAAGTA
TAATTCTTTTTAGCAAATGAAAGAAGACAAGTTTCACATATTTCACGTGCATCAACAAGCTTTTTGTGAATATGACAAAAGGTAAGCGATGATATCTCCAACTTATGGGTATCACAAACAA
GATCCCTATAAAATCCCAACTTCTCATCGCCTAATAGATAGCCAAACCTCGAGCACAACAAGCAGGGGGTCTGTAATTTCCAAGAACGAAATATCTTTGATGCAAAGTATAAGAAAAATGC
ATCAATTAGCAATAGAATAATCAATATCCATTCTGAGAAAGCAGAAGAGAGAGCTGCTAAAAACTGGGAAGGGCCCCCTTTTTGTTTGTTGGGAGTTGCAGCCATGATATGAAGGGCCAG
ATCAAACTCCAGAGAAAAGAGAGACTCCAAGCTCCCCAAATCCTAGCAGCATTAAGGATTGGATTCCCCAGATTAGATATCGGCTCTCGCTTGCGCCATCTCAAATGGCCTGCGACCGCG
AGACGAGCGAGAAGGCAAAGTTCGCTTTTTTCTGTAGGAAACATCCGCAACCATAAAAGTGGAAAGAGGAAGAGAGATTGGCAGGATTTGGGGGTACTATCTCGTCCTCGTACTGAAAC
ATCGGAGGCGCGAGCGGAAGAAAAGTCGGAGACCCAAAAATAGGGAAGAAATTGAAATATATTAGAGAAAAATTAACTAAATAAAATAAAAAAGAGTATTAACAACTGTACGACTGTCT
GTATGAAAACTTCTATGCGAGTACCAGATC
>comp162868_c0_seq2
CGATTTTAAGGTTATCTTAACTAAAATTATATTAAAATATCGTGATATATATAATATATAATTCAAATTCTTCATAAAAATATAATTATCTTTAAAAAAAAATATCGTTAGACGCTGCAAATTAAA
AGCTAACGGAATAACAACATAAAGAAAACAAAAATCATAATCGTATCTGACGAGTCCACGTGTCGCGAAATGACAGGGTTTGGGGATTACGTTGTTCGGGATTGCATACATGTTCGTTCA
CGACGGGCTTGTACACCGGAGATTCCCGGTGGGCCCAATCGCCGACGTGCCTTATTTCCGGCGAGTAGCCGCTGCTCATCAGATCCACCATTCGGACAAATTTGAGGGCGTTCCCTATGG
GCTTTTTATGGGACCAAAGGAATTGGAGGAGGTAGGTGGTTCTGAAGAGTTGGAGAGAGAGATAAAGAGGAGGATTAAGATGCAAAACAAATAACTCCAGCTTTATTAGCTGACGACAA
TTTGAAGTTTTTTTTTTTAATTTTATTTTTATGTATTATCATTCTTTTTTTGGGGAAAAAATTAACAGGAGTGGTTAGTTTGGTTTGGTATGTGGAAGCTTTGTAGCCGAGTCTATGATAGGAT
ATTTAAATGGATGCGTGTTTAATTTTTCTTTCATGATTTTAATTTTTTTATGTGATTGAAGCTTGGAATTTGTAGTCTTTGCTCGACTAGTGGTAGACTAT
>comp162907_c0_seq1
TTATTGGTCGCGTTTCATTCTCTTACAAAAGATGTTAAGAACTTTTCTGAAAATTATTTGAAAAAGTAGTTCAAAATTTGAGCCTGATTTCTCTCATGTTTATGAAAATTAGCAACCAATTTG
ACCTCATTTAAGTATAACCAAGTTAAATGTAATCATTATGAAGCCCTGTCTTGGTTGATATAGTTAAAGCTGAAGCCAAGCTCGATTGTAACTTTTTAAAAGCTTAATAGTCAATATAGTTTC
TATTAAGCTTTGGTTATATCTTTTCCATCAATTTGGGTTTTTATAGTAGTTGCCTAGTTAGACCAATCAGCTTAGAGTACAAAGGAGTAACTGCATCCTCTCAACGCTACAAAAACAGTTGG
AAGAACAAAACAGACCCCAAACCATTACCACCAATCCACCTCTGCTTATCAAGTAGTAGACCCAGACTTCTTGTGGTCATGATGCCTGTTGGGTGCTGGGATCATGCATGAGAAGAAGGA
TTGTTTCTTTCGCTTTTCGCCGAACAAAAGAGTTCTCAATGGAACCTTAGATTTTGCGCTCCAGTGCCTAGTGTTTTGGTTTTTTTGGTTCCCCTTGGAACCCGAGAGATTGAAGCTCTTC
CTCAATTTTGATAGAGCCTTGACAACCTCGGATTGAATTGCTTTCACATGATCCAAACCAGTTTGCAACTCCTTCGTAACTTTATTGTTTTCCTGCTGCATGTTGAGCAGCTCGCCCTGGAG
CTTTACTGCTAGGTAAGGAGTGAACTTCGAGTCCTCAATTTCAGTGTCACTCGAAGCCTTTGAAATCTCTTCCTGCATGTTGCAGAGAGTTTTGAACCTGAATTGAAGCTCTGCTTCCAGT
ATTTCATTCTGCTCTAGCCAAACTTGGAGCTCAGTATGAAACTCCCTCAGTTTCTTATCGAGCAGCGACATATCTTGTTTTAATATTTGCTTTGGGGATGCAGCAGCTGATCTAACTTCCTTA

TTATGCCCCTTGAGCATCTTCAGGTCAGCTTGCACCTCCTTGAAGGTTGTTTCAAACTTCTGTATTTGATAATAAGAAGTGCTGAATCTTAGCCAGAAGTCGAGATTCTCATCAAGAAGAG
TGTCCAGCTCAGCTCTGAACTTTTCCTCAATGGGTGAAGTTTTATGCTGCTGATCTAAATAGCATAGCTTGACTTCCTCTTCGGAGCCAATGATCTCAATTGGAGAATCTGATTTGGTTGTA
GTTACTTTAATGGTGCTTCCATTTTCTTCTTTGCTCTGCATTTGAGTTTCTTTCCGAGAACTTGCTGCACCATTGGTAGTGTGAGAGAACAGTTTCCTCCTTAAAGCTCTTATCTCCTGGTCC
TTCACAGCATTCACACTCTTCAGATCCTGAATCTGTGCCATCATGTCGAAGTGGTACTCTTTGTTCTTCTTCTCCATCTCGCTCAGTCTCTTCTTCGCATCTTTGTAATTTCGCAGAATCGAC
GTGTACTCATAGAGTAGTATTTTATGCCTCTCTTCTGGTCCCTTATTAAACAGCTCCTGCCACCCAGAAATACCTTCGTTGTCTTCAAGCTCATTATTCTCTACTATTGATGAGTCCATTCCAT
CAAGTTGTTCTACTTTTGATTTCTCTGAAGCATTAACTATCTGAGAAGAAGCTTCATTAGAATGTGAAATAGCACTAGGCTCATGCTGCTCCTTCATATGTACCTCATTCTTTTGAGCTGCAT
CACCACTATCATCTTTAGAGTTATTTCTCAGTTCTACTCCATTACTCTTCTTCTCCAATTTTTCTGTTATTTTAGAAGCTGAACTGCTATCCTTCTCCTTATGACTAGGAGACTCTAGTTTTTCT
GAAATATTATTCAGACTGTTGCAAGCCTCCACAAAATGAGTTTGAAATATCCCTTCCTCATCATGCACGTTTTTCTCAAGATCCTGAATTCTATGCAGCTTATCTTCTGCCTGCCTCACTCTC
ACATTCAATCCTTTGGAATCAGTTATCAAATTTTCTCTATCTTCCTCCAGTTCCCGAAGGTGCTTCTGAAGCTCATCAGTCTCCAACCTCAATGTCTGTATTTGCGCTGTTTGTGATGAGATT
GTTACCTCCAAGCTTATAACTTTTTCTACAAGTTCATCAATCCGTTCGGCAAGATCAACAACCGATGTGTGAGAGCTAATCTGGAAGTGCTGCTTCACCTGTAAACACATAGAGTACAGAT
CCAGCCCACCATTTTTATGGTGAAACACTTCATCGTTGTTCTTGCTTGCTTCTTTGTCAGACATCTTCTCCATTTCTGGTAAGCAATTCTCTCCCTTGAGGAACTGTAGTCTGTTTCTAGCTT
CCCTGATTCTCTTAGATTCTATGTCTGCCTCTTCACTCGATATCTTCTGTTGTTTCTGCAAGCTGACCAAGCTTTCTTCACATGATGTGATTGCCGTCGCCGCCATCAGGGCCCGGGCTTCAT
TGTCTTCGATTATGGAACTTGCACTAAACTCATCCTGTAAGGTGTATATCTCATCTTGAAAACCAGTGATCTGACTCTCAATCTCCCAGAACTTGGCAAGGCTGCTCTCATAGGAACTCTTG
ATGAATTCTTTCTCCGTCTGTAGAATGAGAATTTCTTTCTGAAGCCGATCTATCTCATCCTGCGCATTCTCCTTGGTGATATTTGAGCTTGCCTTATTGCGATTCCTCTTCAGGGTGTAGGCAT
CTCTACTTCTGTTTCTCAAGTTAATCAGGGGCAAATCAACTTGTGGCGCCTTGCGATGCTTACTGGTATCTATTGCAGTGAATTCTCTTGGCGAGCCATCGTCGTCATCGTCTCGCATTGCA
AACTGATCCGGGAAAGCTGCCGCTAGGGCATGGTTGGCCTTGTGCAGATCTCCTGAAATCCGATCGTATCGATCGGCCAAAGCTTTGTAAGCTCGATAGGCCACTTCGACGAAGTTAACC
AATTCTGGTCTCCTCTGGTAGTACATCTCCGCCTTTTTGGCGAAGGAGTCGGCGTCCACTTCTATAAGATTGATCATGTTCTTCACCATTTCTTCCATTTCTGCAAAAGATATAGGAGCTGA
AATCTTCTTTACTTGTACACAGGATATGCATTGTTCACAGGTATTATTCATCTCTTAGCTGAAATGTAGATATGTAGAATGCAGGGAAACCTCATTGATCTCTTAGCTACTTATCTTTTCCAAA
TCACCATCTTCACTTAAGAATTTTGATTTAAGAATCATGCTATTTA
>comp162908_c0_seq2
CCAGCTCAAAATACACCACCAAATAACATCTTTAATCATGCCATAAAACTGCAACCAAATAACCCCTTATACTAACAAGTTAGGGATATGATTTCTTTTGGGTTAAGTATCACTACAACACTT
ACGTAATAATGTTGACCAGTGGAAAACCTATTTTTGGAGAAGATAAGTAAAGAAATATGGAGAAAGTGCTCAGCTTTTCCTTGGTTCCATCCGTAAGCAGATCCACCACATGTTCTCTTGT
GATGATGCAAACACGTAGAGGTTTCCCTTAGATGAGTGGGCCAGGCATGCTGCTCTGGTACCATTGCAATATCTGAAGAATCATTGGAAGAATTCCTAAGTCGGGGTTAAGTGCGGGTTA
AAATTGGCGTAGGCGATTGAAATTAATTAGGTAAGAAGTTGAAGAATTCAAAGTAAACAATCTGA
>comp162909_c1_seq4
GCCTTATATTGATTTCAGTTGTATGTTTTTTGTAATTGCTGAGCTACTCACTCATTGCATTATTTAAATGATTTTAGGTAAGAAGCCTTCGATTTGAGGCTTAGTTGGGATACTAGTGTAATTTT
TATTCTACCTCTCTCGTTTATATATTCTTTTGTGACCTATAAAGGCTATTATATGTAATATTTTGAACTTTTTACCG
>comp162915_c1_seq11
AAGGGATTTTTCCCTCGTATATGTCGAGAAGCCGCCAAAATCTCCCGAATTCCCGCAAGAAATCCCGCATACCTGACATGGTTCGGCGCTGACATCGCTCAGTTCACGCGATTCCTGCTTA
AATCCCGCGACAACTCCTCGATCGTCGCCTTGTTCGTGTCTTTAAATACCGAACAAACGAGACCTTCGCTGATTGCTTCCTCCCACAGACACGACTTTCCCTGCACTCTCTTGCTCTTCTG
AAATCCCAGTCGGACCTCCCACTGCTTCCGCCAAGAATCCCTCCGATCTTCCTCCGACTGAGCTCCTTAACCCACGACAGGGCTGA

>comp162920_c1_seq1
CCGCCTCAATGCTGTGCTCCACCGCGTCGCTCTGAAAGTGCTTGCATTCTAGCGCCACCGTCGCGCCTGCATGTGGGCTATGCAGTTAGCTGAACAATTTCTCTTGGGACGAGCTTCAAA
GGTATTATGTTATGAGATTGAGCGAGCTCATGAGTGGCTTTTTATTATTGTGATTTTGATTAAAATTGAAATCCTATTATTTTCATTAAGGTTTTGTTTGGGACGGCT
>comp162920_c1_seq4
GAGTCATGTCAATTTTTGACACCCTTGGTCGCACATATTTGAATCATTTGCATCATCAAAACACAAAATAAAATAGTAGAACATATAATAATCTTTTGAATATTCAACTAGATACATCAACTT
CTCAACATGCTTAAATCCAAAAAGACCAAACTCAACTCCCATAAAAAGTTCTTCACATTAATCAATCACAAATATATCAATATAAAAGTTTCAATGATTTCAATAAAAAAACAAATCAATAT
ATCTCTAATAAACTCTTTCTGGACCAGCTACCTCGCTGGCCGAAGATTCAGAATTAGTTAATAATCATCATCGAGTGAGTAGGCCTTGAGCAGCTCGGCGCAGACAGGGAGGGCTTCGTCC
TTGAGAAAGCCCAAAGAGTTGGCATACCGCAAGTTGTTGGCTTGGCCGCTATTTTCGGTGAGCAGAATGCGCGCTTGCTCTCTGTTCTCGACGATCTCAGCGCAATCGGCTCGCGGGCTC
TTAAGAAGCTTCACGTAGCAAACCTCCTCCTCGTGGTCGTCGGGGACGCTGATGGTGTAATAGCCGCCGGCGCCGCTCACCGCCTCAATGCTGTGCTCCACCGCGTCGCTCTGAAAGTGC
TTGCATTCTAGCGCCACCGTCGCGCCTTCAATATAATCGGTGATATTGGTGACGAATCCAGCGCGACAGTTATCGCAATACACGCGGCCCTCAACTTGGAAATGGACATCGGTGGCAATGG
TGACGCCGGCGAGGAGGCAGAAGGCGGCGACGACGAGGGGCAAAAGTTGGATCTTGGCCATGGCGAAGACTCCTTTTGAATGGTGGAAAGGAAATTATGGTTGAGAGGGGTGAGAGG
AGAGAGACCGGCGGTTTTGAAAGATGATGCGGGACTCTGTTTATATAGTGCTGGGCAAGCTTCCATTTTTAGCTATTTGATGCAGTTCATACAGTTTCTAAGGCAGGGAGGGGCTCCAACT
TTCCAGTTTTTGAATGGCACCGAGGCCGCGTCGGGCTTCCGGTAACCGGTCTACGTGCGC
>comp162923_c0_seq3
AAAAAGGAAGATCTCATCATCGTCAAGCCATCTACTTCAGTTGATGAAGCTCTCGAAAAGCTAGTGGAGCATAGGATCACTGGTTTTCCTGTGATCGATGATGATTGGACATTGGTTGGAG
TTGTTTCTGATTATGATTTGCTGGCATTGAAATCCATATCAGGGTGTGGACTAACTGGTAGAAGTATGTTCCCTGAGGTGGATAGCACGTGGAAGAAATTTAATGAAATTCAGAAGTTGTCA
AGTAAAACAAATGGCAAGACCGTTGGTGAGTTGATGACACCTGCACCTCTTGTTGTTCGAGAGACATCTAATCTTGAAGCAGCTGCCAGGCTATTGCTTGAAACCAAGTACCGCAGGCT
TCCAGTAGTGGATGTTGCTGGCAAATTGATTGGAATCATCACAAGGGGAAATGTTGTTAGAGCAGCGCTAGAGCTCAGAAATGCTAGGGCAAAGAGAGCTTAAGGGAAGCAATATTTACT
TCTTATTTCGTTTGGGACAACTTATTTGCTGTTTTTTAAAACGACTTTTTAGTAAAAAAAAATTTGAATTTGAAAATTTTATACTAAAAAATTGCTTCATTTGGAAAATAAATGTTACAATTT
GGAGGCACAATAGCAGTTTCTGAGTTCTCTTCTCATTATCTTTCCTGCAGCAGTTCTAGGAATAGACTGAACAAACACCACTCTCCTCACCTTCTTGTATGGAGCCACCTTCTTAGCTACA
AATTTTACTATCTCAGCAGCTGAAATATTGCTATCATGTCTCTTAACTACAAATGCAACAGGAATCTCTCCTGCTTCCTCATCCAAATCAGATGTCACTGCAGCATCGATGATTTCAGTGTTG
GAAGTCAGCACAGCTTCCAAATCTGCTGGTGCTACCTGAAACCCCTTATACTTTATTACATCTCTTAAACGATCGACGATAAATAAGTATCCATCATGATCAAAATAACCAATATCCCCTGTC
TTCAACCAGCCATTTCTGTCGATTATAGATTCTGTCCCCTTTTCATCATTTAAGTAACCTTTCATAATAGCAGGTCCACGTAACCATAATTCTCCCATTTTGCCAGGAGGCAAAAATATTCTGT
CTTCCTGATCAACCACTTTGGCTTCCATATTTGGAGCTAAGAGCCCTGAAGATGTATATTTCTTGCAATTTTTGTTATTGAAACCGCGAGTTCCGACTGCGGTCGACTCAGTCATGCCATATC
CCTGGCCCAAAATGTTTTTAATTTTTAGATGTAAGAAAGAACAGTCATCAAAAACTAAATTTTAAAAACTTATCCATCTTATCAGTTTATTAGTGAAAGATGGACTCCATAGTCATCTTCATT
AACCAAGCAAGAGCAAAAATTATCAAATGGCTTATTTATTTGATAGAGTCTCCTTCATGTTCAAGGATTGTCTTCAATCGCATTAGGAAATTGTGAATCATAAGAAGTTAATCCATTCTGTA
GAACTAAATGTAGAAACATGTTTGTATGTCAAATCTGAAGTAAATTGGCATCTTCTGCTGGATTCAAAGTTAAAATAATTTGTATGATGATATCCTCTACTAAACATAAGCAAGTTTTTAGTG
AAGTTTAAGTGAGAATCTAAGCTTAACAACTTACAAAGCCAAATCAACCTCTTATCTAACGAGTAAAAATTTCATTGCCAGCAGCCATCACTGTTA
>comp162923_c0_seq9
AAAAAGGAAGATCTCATCATCGTCAAGCCATCTACTTCAGTTGATGAAGCTCTCGAAAAGCTAGTGGAGCATAGGATCACTGGTTTTCCTGTGATCGATGATGATTGGACATTGGTTGGAG
TTGTTTCTGATTATGATTTGCTGGCATTGAAATCCATATCAGGGTGTGGACTAACTGGTAGAAGTATGTTCCCTGAGGTGGATAGCACGTGGAAGAAATTTAATGAAATTCAGAAGTTGTCA

AGTAAAACAAATGGCAAGACCGTTGGTGAGTTGATGACACCTGCACCTCTTGTTGTTCGAGAGACATCTAATCTTGAAGCAGCTGCCAGGCTATTGCTTGAAACCAAGTACCGCAGGCT
TCCAGTAGTGGATGTTGCTGGCAAATTGATTGGAATCATCACAAGGGGAAATGTTGTTAGAGCAGCGCTAGAGCTCAGAAATGCTAGGGCAAAGAGAGCTTAAGGGAAGCAATATTTACT
TCTTATTTCGTTTGGGACAACTTATTTGCTGTTTTTTAAAACGACTTTTTAGTAAAAAAAAATTTGAATTTGAAAATTTTATACTAAAAAATTGCTTCATTTGGAAAATAAATGTTACAATTT
GGAGGCACAATAGCAGTTTCTGAGTTCTCTTCTCATTATCTTTCCTGCAGCAGTTCTAGGAATAGACTGAACAAACACCACTCTCCTCACCTTCTTGTATGGAGCCACCTTCTTAGCTACA
AATTTTACTATCTCAGCAGCTGAAATATTGCTATCATGTCTCTTAACTACAAATGCAACAGGAATCTCTCCTGCTTCCTCATCCAAATCAGATGTCACTGCAGCATCGATGATTTCAGTGTTG
GAAGTCAGCACAGCTTCCAAATCTGCTGGTGCTACCTGAAACCCCTTATACTTTATTACATCTCTTAAACGATCGACGATAAATAAGTATCCATCATGATCAAAATAACCAATATCCCCTGTC
TTCAACCAGCCATTTCTGTCGATTATAGATTCTGTCCCCTTTTCATCATTTAAGTAACCTTTCATAATAGCAGGTCCACGTAACCATAATTCTCCCATTTTGCCAGGAGGCAAAAATATTCTGT
CTTCCTGATCAACCACTTTGGCTTCCATATTTGGAGCTAAGAGCCCTGAAGATGTATATTTCTTGCAATTTTTGTTATTGAAACCGCGAGTTCCGACTGCGGTCGACTCAGTCATGCCATATC
CCTGAATCAAATCAACATGTTCAAATCTACATAAAAACTCCTTGATCAGCTTTTGACTCAGTGGAGCTGCCCCACATGAAACCTGCTTCAATGATCTCAGAACACACCCAGTGGCATCCTT
TGTCTTTATGAGAGCTGCCATTATTGGTGGAACAAGTGGAAGATGAGTTACTTCATACCTATCCATGGCTCTAATTGCCTCATGAGCATCAAATCTCTTCATAACAACAATAGTGGAACCAA
GAGATAGCAATCCAAACACAAACAATGACATTCCATACACATGAAACATTGGTATTGTAGCAAGATAAACATCCTTCCAGCTCTCCTTCTCATACTGTGAGGCCTCGAACCGCACGAAAA
GCTCAACCATGGCAATGAAGTTCCCATGAGTCAAGATGACAGCTTTACTAAATCCAGATGTGCCAGAAGTGAAGAGTATGGCTGCAGGGTCACTCTGCTGAATCACAGGCCTGAAAGTA
ATTTGTTGCTTGCAAGAAATCATCTTATCAAAAAATGTAAATTTTGACGAATCATAATTCAAATCACTTGGGACAGAAACAACAGGAACACCCAATGAATCCAAGGTTCCAACTTTCTCCA
ATTGGGCGAAAACTAGGCCTATATTCAAACCAGCGGTTAGCTTCTTGATCTCTTGGGGGCTACTGAGGGGATTCATGGTGGTAATGATGGCGCCAAGGGAGAGGACGCTAAGGAGTACGA
TAGGGAAGAAGATGGAGTTAGGAAGAAGGATGAGGACTACTTGGCCTTTCAAAATCCCAACCTGATGGAGACCAGAAACCAAAGCTTCGACCTTGGAGCGGATATCAGAGTATGAGAA
ATAGAGACCGGATAGGGAGTCAATGAGGGCCTTTTCTCCCTTGTGCGGTTTGGAGAAAAGGAAAGTGACCAAGTCTTGAAGTGGGTTTTGGGTAAGAGGAATTGGGGAGTGTTTGCTGG
TGTAAATTCCAGTTTTTGGACAGAACCAGATTGGCTTTGGATGAGAAAGAGCTTCCATGAGAAGACGAGGAGGAGGAAGAGAAATATAGAAAGTAGACGAAGAGA
>comp162932_c1_seq10
CCCCACGTCTCTCTCACTCCCTCTTATCTCAGCATTTTGGAAACCACTCAACGGCTCAACCCACTCTCTCATTCGCAAAAAACCACCACCCCATTTCCCATACAATGGACGAACTGCCCCA
GCTCCCTTTCACGGTCACTCTCTCCCTCACGCATGAGAAGCCCAGAATCGCACTCTCTACTTCCCTCTTGGACGTGAGAAGAAACCCAGGCGGACAGCCCCATCTTCCCTCTCTGGATCA
CACGAGAAGACTCAGGCAAAACCTCAGAACTCCAGCCTCGGTTCTCCCCTGCATTTTCGTGAAGACCCTAGCCCTTTCTCTGAAATTTGCATGAAGCATAGCCCACTCACCTCTCGGCAG
TTCACAGAAAATCGGCTCCTCTCCCCATTCATTCGCACGAAGCCCAGGAACTGTTCGCCCATAGAACAATTCACGAACTGCTCTCAAACCCC
>comp162938_c0_seq1
TTATTTCAATAGTCTTAAAATAAATTCAATGACTTGGAGATCCACCTTATACAAAATTTAATTAGTATATTCTAACACAATTTTGTTTTATAAATTTCAAGAGCTATCTAAACCGTAATTGTTAC
ACCCTATTTTCTAACACACTAAAAAACTAACGATAAAAGAAACGAACGAGGAACCCGCAAAAAACTTGATTAACGCGTAGGCCCCTGTGTGACTATTCCAAAAAAAAAGATAAAATAAG
TGAGGGAGCAACTTAAATACATATTTATGTAAGTCGTTAAATACTAAACAACCCCGCTTAGATTTGCTTTTGATTATTCACAATCACTTTCAAATTTTTTTGAAAATGATGAGCGATAAATCA
CAAAGTTTGCGGCTTTAACGATCTTGGAGAGGACAACCAACTCCTTTTTAACTAATAGGTCACATATCTTCTCACACTTTCTAAAGTTCATGAAAGATTGAACTTGCAAACTTTCATAGTCT
GGCGCATTGTTTAAATTTTTATGATAAACGTGTACTTATTTCATTTTATTTTTTTTTTCTTATTACTTATTCTTATATTTTTATAGGACAATTGGGGATCCTTCCTATTTGTCATGGAATAGAAATC
CTGCAAGTCAATAGATGTTCCGTAGTCCAGTAATGCTTATCAATGTTGCTTGTTTATTCAAATATCCTTCCATCTGCTTAAAAGATATAAGCTTACTTTGTATTTGAATCAGTTCTAACAAAAA
TTTTCTAAGTAGTGTATGATTAATTACTTGAGATCGTCGCCATTCTTCCAGCTGAACCTGAAAATTAACTCCCTTTACTTGCAGAATCTTTTCTGCCAGCTTTTTTTATTTTTTATGTTGAGTC
TGTTGTTGTTCTCATTTATCAACCTGAAGAGAGTATTTGTCTCAAAACGTTTGAATTATAGTCATGGCTATGTAAATAAATTTTATGCTATTATGTTTCAAATATTATAAATATTGGGAGCCCAA

ATTGAGGCTTCGTTTAATCGTTTGGGTCAGCTTTAATCCTTTTTTTTTTGTGGTTTCAAAAAATTTGTGCTAAAAAGCAATTTTATAAAACATTAAGAAAGTCTTTCAAAGGAAGCTTG
>comp162967_c1_seq10
ACGATAACATAAATCATAAAAGTACAAGAAGGCCTTAAGCATAACTTTAGTGCGTGAGAAATGATAAAAGTACCGAAAATTGAAGCGGCTGTGGTTGTACAAGCATGTATCCATTCCCATA
TATTAGGCACTTGGCTTAGCATGTTTATACAACAAAAACTTCTCAGGCTCCACATGGGGACACATAAGCATAACCTCAACATGAAAATCACTAGAATACTCATTGCTTGGTTTTAAGCAATT
AATTATAACCTATGTTTTATACATAAAGATGATATGAAAAAAATAGCACCATTAACTTTTCTTCCTTAAAAACGCACAAGAATTGATCAACTCTGCAGTTCAAGGAATCACTCCCAATGATG
ATACTTTCTTCTGGAGCTTCCCCTTGAGCTGCCTGATTTTGGCATCCACTCTTGCAGTGAAACCAATCTTGGTTTCCTGCCTCGCTTTGGATCGCTCTCTTGTCCACATTTGCTCATCCGTAA
GATCCTTCTTGAAGTACTTAATCTCCTGAATCACATGGTGATCTAGTGGATAGAACAATCTTTGGTAGGAGAGGTGAGCAAAGTAAGGAAGGTTGCAAGCTGCTGCCACAAGGAAGGTTG
TGATCCAGTATATTGGAGCTGGGCCAAGGGACTCAATTAATATGTGAAAGGCGTTCCCAGAGATGACTGGTGACATGAATCCATAGCCAAACAGGAATAGGTACCACGTGCTCACGCTTC
CCCACACAAAGAGATGCTGAATCCAGGTGAAGTGGCTCATAGTGAGGGCAATCTGCAAGTTTACTGCCCAGATAATGCAAGTGAACATGGTGGTTCCCATGGCAGCCATGTCGGCAGTTT
GCCCTTCAGACTGGAACGCTTGATCGTAGAAGATGCTGATGTTGATGAAAAATACTACAATGGATGAGTAGAGACCATTTGCCGTCCATGCAATAATCCTTGACCAATCAAAGAACAAATT
CTTGGGTCCTTGCTGGTACAAAGCTGGGAACTGAAGGCATACTTGAGAGGACACATCTTGCTCAAAAACTCCTAGTGATATGACCGGTAATGATGTGAGAATGATATTGAATAATATCATG
TACCAGTCCTCATAAACTGATTGTCCTGAAAAGCCAGTGTATGCCTCAAAATAAAACAAAGTGAGGCCAAATGCTATATTCTTGTAAAAGAAATAGCATACCATCTGTGCAATCCTTTTATA
GCACCAATGACCGTGGACAACAAGAAGTCTTTCCAGACAACGGAATTGAGCAATAGAGAAGTCGCTAGCCATCACAGCCTGCATTCCTTCCACTCCACTGATTCCAACGCCAATATCAGC
CTCTTGTATCATACCAACATCATTTGCACCATCTCCTATAGCTAAAGTAGTTTTTCCTGTTCCTTCTTTCACAAGACGTGTTACCAGTGCTTTCTGCTTTGGGGAAACCCGACAGCATATGAC
AGAAGCACAATCAACAGCTAGACCAAGAAAATGGTGCTTCATATCATCCTCCAAAGCATAACTCAAAGCTTTCCCATCAATGATTAGAGCAAATGCTGCATGAGGATCCTTCTCTAGCTTG
ACCATTCGAGAAGCATTGGTGATTTGCAGCAAAATATTATCTTTGATATTCTTCTTTGCATCTTGGTCCAGAAAATCCGTATTCATTGTTGATATGGAGATTTGTTTCAATCCTAATCGAAGCA
AGCTGCATGCAAATCCTATGTTTACGGCTGTTTCCATTTTGTCTCCTGTTAGAAGCCAGATCTTTAAACCAGCTTGTGCTAGTTTGTCTATACACTGAGGAACCCCTTTTTGTAGCTTATCCT
CAACAGCAGTTGCGCCGATAAGGACTAATTCCCGCTCCATCAAATCAGCAACTTGCTCCAGCTGAGCATCTCTATCAAGTCCAATAGCTGTCTTTGCTCTGAGAAATTCTGCATTCCATGT
TGAGTACTCTGATTCATCTAAAACTTTGTATGCCAAAGCTAAGGTACGCAGGCCTGCTTCTCCAAATTCATTTAGATGCTTCATGGTATCCGCTTCATACATTTTTCCATTCTTTGACAGTCTA
TCAAAAATGATGCTGTCTGCGCCTTTGCAGAAAAGAATTATCTGCCCAGAATCATCCCGAACAATGACAGACATTCGTTTTCTTTTGCTATTGAATTCCAGTAGATTCAGAATCTTAAATTC
CCTTTCCAATGGATTTGGATAGCTTTCTCGGATGAAAACACTTGACTGAGTCCTTTTAAAGAACTCAAAGCCAAATTCTCTCGCAGCAACAAGAAAAGCTCCTTCATCAGGTGACTCCGC
CTCGTATGTGAAGGTGCCATTTTCCTCATCATGCTCCGGAATTGCTGTGTGACAAACTGCAAGAACCCGGAAGAACATTTGAAGGGTCCCTCGACTAGGCTCCTTCACCCAATTGCCTTG
CATTAGACGGTCATCCTCAAAGCTGAATCCTTTAATCTTAGGTTTTGGATCCTTGACAACCTTGCAGGTAGTACCATCCTCCAATTGAATTACTGAAGAGTCACCCACATTGTTATCCCAAT
AATCTGCTGGGAAGCAATTGTGCCCTGGAGAACCAGAGAATTCTGAAGCGATATGCTTTGCAGCAGCAATTTCAACTTCACTCGAACCTACACCATAAGACACTCCTGCAATAGAACACT
TCAGGAAGTCCATCTGGTTGCAGGTCAATGTGCCTGTTTTATCTGATAAGATTGTATCAACCTGCCCAAGCTCTTCATTTAAATTTGAGGTTCTGGCTTCAGCTGGCTTTCCAGTATCCTCAT
CATACATCTGAATATCCTGGTTGATGAACATAGCTTGTAAGACCTTCACAACCTCTATAGAAACATAGAGAGAAACTGGAATGAGATATCCATACATGATGAGAGCAGTCACAAGATGAAA
TATGCCAGATAATGCAGGCATTAATGGGTTGTACAGAGTAGAAGCATTTTCTGGTTGAAGGTACCACCAATTCCGCATTCCAAACTTAGTGTATACTGCGTAGCCAATTGAGCTGATCAATG
AAATCAAAACAAGCAGGGTGAACAGAATGTATATGATGTAATCCATCTTCTTCTCAATGCTGCTCCGCTTTGATGGTGATTCTGTTGAATTTTGCATGACCTTACTATCATGACCCGTGAAA
ATAACCACTCCATAGATGTAAGATGTGTTCCTAAGCTTTGAATCCCTAAGAAGAATTTGGTTTGGATCCAAGGAACACACCTGCTTTTCATACTCCAAATTTCCCACAAAGGTGTATAAACT
AGGATTGGGGTCTTCACACCTAATGGTGGCATAAAAATCCTTGAAAGTTTTATCCTCATCAAAATGTAATGTCACATCCAATGCCCTCTTCACCTTCAAATTTGTCTCCCCATCCAAATTCAT
AGTCTCCACATAGCATATCCCGTCCTCATAACTCGAGGAAAGCAGAAGCAAATCAGCAGGAAAAAAGTGATCTTTCTCAACCTTCACTACATCACCAACAAAGATTTTATGCCACTTCTTG

TAACAAAAGTTCCCATCCCCTTTATGAATACTAACCTTCCTTTTATTCACCTTCATGTCCTGCATAAATCTCCTCCAATCCTCGACGGCCTCCTTAGCCATGCTCAGCCCAACTACAAACGCC
AAGGGAGCAATCATGCTCACAGCCGAGAACGGTGCAATGGGGGTGAGGGAGAGGACGGCAGCCAGGAGAAAGTAGAGATTGGCGACACGCCGGAACTGTTCAAATATGGCTTTAGGC
AGGAAGGTTATGATGTTGTACTTGGTCGTGGAAATGTAATTGGATGGATAATGGAGAGGTTTCCGACGGTGGAGGCTCGGTTGGTTACATTGCACCTTGCGGGAGAAGCCGGGGCCTTGA
AGACCATTGAATGGATTGGCGAAATCGATAAGCTCAGGGTTCGCTCGGGCACAATTGAAAGTATATATCTTGCTCCATCGGAGCTTCTCTCGCCGGAGCTTATCCCTAATTTTCTCAGCCCG
AGTCATCCTTTTATCTGCGATGAAATGCCTTCCCTCTTGCACGAAGGTGATGAGAGGAAAGGACGAACGTATGTAATTATAGATAAGAGGAAAAGACCATGAGGATGCCAGGAATCAGCA
CCCGATTTCTACCCTACAGATGAAAACTTGGGCTTCACGCAGAAGAATCTGTTGAGATGTGTCAAAATGTTCCTGAAAAGAAGGCTTATAAGAAAAAATGGATTTAACGGAGCTCAAAAT
TTCCAATTCGCGAAATGTTGGCAGAATAAAAATTGATCCGGCTTTTGAGAATAAAGAATAACACAAAGTTTCTTCAAGAATAACACAATGCAGCTCGTGCGAATAAAGAATGTCGACATC
TTTCCATTGGAGCTAAAAAATCGCTCAAATGGATGTGAAATTTTTGCAGAAACGTGAAGGAGATTTGGGTTGATGCAGAAATCAGAAGGCTTAGAGAAGAATTCGACCCGGGAATGTATA
CGAGACGCGCTGCCGACGAGAACGGAGAGGGATGGGGTGATTTCGCGGCTCAACTAACTCCAAAACTTCTGCTCGGCTTCTTTCTGCAATCTTTT
>comp162967_c1_seq19
ACGATAACATAAATCATAAAAGTACAAGAAGGCCTTAAGCATAACTTTAGTGCGTGAGAAATGATAAAAGTACCGAAAATTGAAGCGGCTGTGGTTGTACAAGCATGTATCCATTCCCATA
TATTAGGCACTTGGCTTAGCATGTTTATACAACAAAAACTTCTCAGGCTCCACATGGGGACACATAAGCATAACCTCAACATGAAAATCACTAGAATACTCATTGCTTGGTTTTAAGCAATT
AATTATAACCTATGTTTTATACATAAAGATGATATGAAAAAAATAGCACCATTAACTTTTCTTCCTTAAAAACGCACAAGAATTGATCAACTCTGCAGTTCAAGGAATCACTCCCAATGATG
ATACTTTCTTCTGGAGCTTCCCCTTGAGCTGCCTGATTTTGGCATCCACTCTTGCAGTGAAACCAATCTTGGTTTCCTGCCTCGCTTTGGATCGCTCTCTTGTCCACATTTGCTCATCCGTAA
GATCCTTCTTGAAGTACTTAATCTCCTGAATCACATGGTGATCTAGTGGATAGAACAATCTTTGGTAGGAGAGGTGAGCAAAGTAAGGAAGGTTGCAAGCTGCTGCCACAAGGAAGGTTG
TGATCCAGTATATTGGAGCTGGGCCAAGGGACTCAATTAATATGTGAAAGGCGTTCCCAGAGATGACTGGTGACATGAATCCATAGCCAAACAGGAATAGGTACCACGTGCTCACGCTTC
CCCACACAAAGAGATGCTGAATCCAGGTGAAGTGGCTCATAGTGAGGGCAATCTGCAAGTTTACTGCCCAGATAATGCAAGTGAACATGGTGGTTCCCATGGCAGCCATGTCGGCAGTTT
GCCCTTCAGACTGGAACGCTTGATCGTAGAAGATGCTGATGTTGATGAAAAATACTACAATGGATGAGTAGAGACCATTTGCCGTCCATGCAATAATCCTTGACCAATCAAAGAACAAATT
CTTGGGTCCTTGCTGGTACAAAGCTGGGAACTGAAGGCATACTTGAGAGGACACATCTTGCTCAAAAACTCCTAGTGATATGACCGGTAATGATGTGAGAATGATATTGAATAATATCATG
TACCAGTCCTCATAAACTGATTGTCCTGAAAAGCCAGTGTATGCCTCAAAATAAAACAAAGTGAGGCCAAATGCTATATTCTTGTAAAAGAAATAGCATACCATCTGTGCAATCCTTTTATA
GCACCAATGACCGTGGACAACAAGAAGTCTTTCCAGACAACGGAATTGAGCAATAGAGAAGTCGCTAGCCATCACAGCCTGCACAACGCTGAAGCATTATGAGACTATTCTCCAGCAAG
GAAAAAGGTTTTTGAACTAGGACCAGAACAGACAGGAGATTTGTACTCAGCACAATCCGTTATAGAATGGTTTGCAAGCCTGCATTCCTTCCACTCCACTGATTCCAACGCCAATATCAG
CCTCTTGTATCATACCAACATCATTTGCACCATCTCCTATAGCTAAAGTAGTTTTTCCTGTTCCTTCTTTCACAAGACGTGTTACCTAGAATAGAAGAATTGCAACAAATTGGAAAGAAATTA
TCTTTTAACCAACTAAAGTCGTGGTGTCAATAATTTAAGAACAAAAATACCAGTGCTTTCTGCTTTGGGGAAACCCGACAGCATATGACAGAAGCACAATCAACAGCTAGACCAAGAAAA
TGGTGCTTCATATCATCCTCCAAAGCATAACTCAAAGCTTTCCCATCAATGATTAGAGCAAATGCTGCATGAGGATCCTTCTCTAGCTTGACCATTCGAGAAGCATTGGTGATTTGCAGCAA
AATATTATCTTTGATATTCTTCTTTGCATCTTGGTCCAGAAAATCCGTATTCATTGTTGATATGGAGATTTGTTTCAATCCTAATCGAAGCAAGCTGCATGCAAATCCTATGTTTACGGCTGTT
TCCATTTTGTCTCCTGTTAGAAGCCAGATCTTTAAACCAGCTTGTGCTAGTTTGTCTATACACTGAGGAACCCCTTTTTGTAGCTTATCCTCAACAGCAGTTGCGCCGATAAGGACTAATTC
CCGCTCCATCAAATCAGCAACTTGCTCCAGCTGAGCATCTCTATCAAGTCCAATAGCTGTCTTTGCTCTGAGAAATTCTGCATTCCATGTTGAGTACTCTGATTCATCTAAAACTTTGTATGC
CAAAGCTAAGGTACGCAGGCCTGCTTCTCCAAATTCATTTAGATGCTTCATGGTATCCGCTTCATACATTTTTCCATTCTTTGACAGTCTATCAAAAATGATGCTGTCTGCGCCTTTGCAGAA
AAGAATTATCTGCCCAGAATCATCCCGAACAATGACAGACATTCGTTTTCTTTTGCTATTGAATTCCAGTAGATTCAGAATCTTAAATTCCCTTTCCAATGGATTTGGATAGCTTTCTCGGAT
GAAAACACTTGACTGAGTCCTTTTAAAGAACTCAAAGCCAAATTCTCTCGCAGCAACAAGAAAAGCTCCTTCATCAGGTGACTCCGCCTCGTATGTGAAGGTGCCATTTTCCTCATCATG

CTCCGGAATTGCTGTGTGACAAACTGCAAGAACCCGGAAGAACATTTGAAGGGTCCCTCGACTAGGCTCCTTCACCCAATTGCCTTGCATTAGACGGTCATCCTCAAAGCTGAATCCTTT
AATCTTAGGTTTTGGATCCTTGACAACCTTGCAGGTAGTACCATCCTCCAATTGAATTACTGAAGAGTCACCCACATTGTTATCCCAATAATCTGCTGGGAAGCAATTGTGCCCTGGAGAA
CCAGAGAATTCTGAAGCGATATGCTTTGCAGCAGCAATTTCAACTTCACTCGAACCTACACCATAAGACACTCCTGCAATAGAACACTTCAGGAAGTCCATCTGGTTGCAGGTCAATGTG
CCTGTTTTATCTGATAAGATTGTATCAACCTGCCCAAGCTCTTCATTTAAATTTGAGGTTCTGGCTTCAGCTGGCTTTCCAGTATCCTCATCATACATCTGAATATCCTGGTTGATGAACATAG
CTTGTAAGACCTTCACAACCTCTATAGAAACATAGAGAGAAACTGGAATGAGATATCCATACATGATGAGAGCAGTCACAAGATGAAATATGCCAGATAATGCAGGCATTAATGGGTTGTA
CAGAGTAGAAGCATTTTCTGGTTGAAGGTACCACCAATTCCGCATTCCAAACTTAGTGTATACTGCGTAGCCAATTGAGCTGATCAATGAAATCAAAACAAGCAGGGTGAACAGAATGTA
TATGATGTAATCCATCTTCTTCTCAATGCTGCTCCGCTTTGATGGTGATTCTGTTGAATTTTGCATGACCTTACTATCATGACCCGTGAAAATAACCACTCCATAGATGTAAGATGTGTTCCTA
AGCTTTGAATCCCTAAGAAGAATTTGGTTTGGATCCAAGGAACACACCTGCTTTTCATACTCCAAATTTCCCACAAAGGTGTATAAACTAGGATTGGGGTCTTCACACCTAATGGTGGCAT
AAAAATCCTTGAAAGTTTTATCCTCATCAAAATGTAATGTCACATCCAATGCCCTCTTCACCTTCAAATTTGTCTCCCCATCCAAATTCATAGTCTCCACATAGCATATCCCGTCCTCATAAC
TCGAGGAAAGCAGAAGCAAATCAGCAGGAAAAAAGTGATCTTTCTCAACCTTCACTACATCACCAACAAAGATTTTATGCCACTTCTTGTAACAAAAGTTCCCATCCCCTTTATGAATACT
AACCTTCCTTTTATTCACCTTCATGTCCTGCATAAATCTCCTCCAATCCTCGACGGCCTCCTTAGCCATGCTCAGCCCAACTACAAACGCCAAGGGAGCAATCATGCTCACAGCCGAGAAC
GGTGCAATGGGGGTGAGGGAGAGGACGGCAGCCAGGAGAAAGTAGAGATTGGCGACACGCCGGAACTGTTCAAATATGGCTTTAGGCAGGAAGGTTATGATGTTGTACTTGGTCGTGG
AAATGTAATTGGATGGATAATGGAGAGGTTTCCGACGGTGGAGGCTCGGTTGGTTACATTGCACCTTGCGGGAGAAGCCGGGGCCTTGAAGACCATTGAATGGATTGGCGAAATCGATAA
GCTCAGGGTTCGCTCGGGCACAATTGAAAGTATATATCTTGCTCCATCGGAGCTTCTCTCGCCGGAGCTTATCCCTAATTTTCTCAGCCCGAGTCATCCTTTTATCTGCGATGAAATGCCTTC
CCTCTTGCACGAAGGTGATGAGAGGAAAGGACGAACGTATGTAATTATAGATAAGAGGAAAAGACCATGAGGATGCCAGGAATCAGCACCCGATTTCTACCCTACAGATGAAAACTTGG
GCTTCACGCAGAAGAATCTGTTGAGATGTGTCAAAATGTTCCTGAAAAGAAGGCTTATAAGAAAAAATGGATTTAACGGAGCTCAAAATTTCCAATTCGCGAAATGTTGGCAGAATAAA
AATTGATCCGGCTTTTGAGAATAAAGAATAACACAAAGTTTCTTCAAGAATAACACAATGCAGCTCGTGCGAATAAAGAATGTCGACATCTTTCCATTGGAGCTAAAAAATCGCTCAAAT
GGATGTGAAATTTTTGCAGAAACGTGAAGGAGATTTGGGTTGATGCAGAAATCAGAAGGCTTAGAGAAGAATTCGACCCGGGAATGTATACGAGACGCGCTGCCGACGAGAACGGAGA
GGGATGGGGTGATTTCGCGGCTCAACTAACTCCAAAACTTCTGCTCGGCTTCTTTCTGCAATCTTTT
>comp162967_c1_seq23
ACGATAACATAAATCATAAAAGTACAAGAAGGCCTTAAGCATAACTTTAGTGCGTGAGAAATGATAAAAGTACCGAAAATTGAAGCGGCTGTGGTTGTACAAGCATGTATCCATTCCCATA
TATTAGGCACTTGGCTTAGCATGTTTATACAACAAAAACTTCTCAGGCTCCACATGGGGACACATAAGCATAACCTCAACATGAAAATCACTAGAATACTCATTGCTTGGTTTTAAGCAATT
AATTATAACCTATGTTTTATACATAAAGATGATATGAAAAAAATAGCACCATTAACTTTTCTTCCTTAAAAACGCACAAGAATTGATCAACTCTGCAGTTCAAGGAATCACTCCCAATGATG
ATACTTTCTTCTGGAGCTTCCCCTTGAGCTGCCTGATTTTGGCATCCACTCTTGCAGTGAAACCAATCTTGGTTTCCTGCCTCGCTTTGGATCGCTCTCTTGTCCACATTTGCTCATCCGTAA
GATCCTTCTTGAAGTACTTAATCTCCTGAATCACATGGTGATCTAGTGGATAGAACAATCTTTGGTAGGAGAGGTGAGCAAAGTAAGGAAGGTTGCAAGCTGCTGCCACAAGGAAGGTTG
TGATCCAGTATATTGGAGCTGGGCCAAGGGACTCAATTAATATGTGAAAGGCGTTCCCAGAGATGACTGGTGACATGAATCCATAGCCAAACAGGAATAGGTACCACGTGCTCACGCTTC
CCCACACAAAGAGATGCTGAATCCAGGTGAAGTGGCTCATAGTGAGGGCAATCTGCAAGTTTACTGCCCAGATAATGCAAGTGAACATGGTGGTTCCCATGGCAGCCATGTCGGCAGTTT
GCCCTTCAGACTGGAACGCTTGATCGTAGAAGATGCTGATGTTGATGAAAAATACTACAATGGATGAGTAGAGACCATTTGCCGTCCATGCAATAATCCTTGACCAATCAAAGAACAAATT
CTTGGGTCCTTGCTGGTACAAAGCTGGGAACTGAAGGCATACTTGAGAGGACACATCTTGCTCAAAAACTCCTAGTGATATGACCGGTAATGATGTGAGAATGATATTGAATAATATCATG
TACCAGTCCTCATAAACTGATTGTCCTGAAAAGCCAGTGTATGCCTCAAAATAAAACAAAGTGAGGCCAAATGCTATATTCTTGTAAAAGAAATAGCATACCATCTGTGCAATCCTTTTATA
GCACCAATGACCGTGGACAACAAGAAGTCTTTCCAGACAACGGAATTGAGCAATAGAGAAGTCGCTAGCCATCACAGCCTGCACAACGCTGAAGCATTATGAGACTATTCTCCAGCAAG

GAAAAAGGTTTTTGAACTAGGACCAGAACAGACAGGAGATTTGTACTCAGCACAATCCGTTATAGAATGGTTTGCAAGCCTAGAAATTAAATTTCATGCAAGTCGGACATTCAAAAGAA
GCGTTATGAACAAAGATTCATGTTATCAACTGAGAAACTAACTGTTATTCAAGAAGCAATATCACGAAACAAACTGGCAAACCTGCATTCCTTCCACTCCACTGATTCCAACGCCAATATC
AGCCTCTTGTATCATACCAACATCATTTGCACCATCTCCTATAGCTAAAGTAGTTTTTCCTGTTCCTTCTTTCACAAGACGTGTTACCAGTGCTTTCTGCTTTGGGGAAACCCGACAGCATAT
GACAGAAGCACAATCAACAGCTAGACCAAGAAAATGGTGCTTCATATCATCCTCCAAAGCATAACTCAAAGCTTTCCCATCAATGATTAGAGCAAATGCTGCATGAGGATCCTTCTCTAGC
TTGACCATTCGAGAAGCATTGGTGATTTGCAGCAAAATATTATCTTTGATATTCTTCTTTGCATCTTGGTCCAGAAAATCCGTATTCATTGTTGATATGGAGATTTGTTTCAATCCTAATCGAA
GCAAGCTGCATGCAAATCCTATGTTTACGGCTGTTTCCATTTTGTCTCCTGTTAGAAGCCAGATCTTTAAACCAGCTTGTGCTAGTTTGTCTATACACTGAGGAACCCCTTTTTGTAGCTTAT
CCTCAACAGCAGTTGCGCCGATAAGGACTAATTCCCGCTCCATCAAATCAGCAACTTGCTCCAGCTGAGCATCTCTATCAAGTCCAATAGCTGTCTTTGCTCTGAGAAATTCTGCATTCCA
TGTTGAGTACTCTGATTCATCTAAAACTTTGTATGCCAAAGCTAAGGTACGCAGGCCTGCTTCTCCAAATTCATTTAGATGCTTCATGGTATCCGCTTCATACATTTTTCCATTCTTTGACAG
TCTATCAAAAATGATGCTGTCTGCGCCTTTGCAGAAAAGAATTATCTGCCCAGAATCATCCCGAACAATGACAGACATTCGTTTTCTTTTGCTATTGAATTCCAGTAGATTCAGAATCTTAA
ATTCCCTTTCCAATGGATTTGGATAGCTTTCTCGGATGAAAACACTTGACTGAGTCCTTTTAAAGAACTCAAAGCCAAATTCTCTCGCAGCAACAAGAAAAGCTCCTTCATCAGGTGACT
CCGCCTCGTATGTGAAGGTGCCATTTTCCTCATCATGCTCCGGAATTGCTGTGTGACAAACTGCAAGAACCCGGAAGAACATTTGAAGGGTCCCTCGACTAGGCTCCTTCACCCAATTGC
CTTGCATTAGACGGTCATCCTCAAAGCTGAATCCTTTAATCTTAGGTTTTGGATCCTTGACAACCTTGCAGGTAGTACCATCCTCCAATTGAATTACTGAAGAGTCACCCACATTGTTATCC
CAATAATCTGCTGGGAAGCAATTGTGCCCTGGAGAACCAGAGAATTCTGAAGCGATATGCTTTGCAGCAGCAATTTCAACTTCACTCGAACCTACACCATAAGACACTCCTGCAATAGAA
CACTTCAGGAAGTCCATCTGGTTGCAGGTCAATGTGCCTGTTTTATCTGATAAGATTGTATCAACCTGCCCAAGCTCTTCATTTAAATTTGAGGTTCTGGCTTCAGCTGGCTTTCCAGTATC
CTCATCATACATCTGAATATCCTGGTTGATGAACATAGCTTGTAAGACCTTCACAACCTCTATAGAAACATAGAGAGAAACTGGAATGAGATATCCATACATGATGAGAGCAGTCACAAGA
TGAAATATGCCAGATAATGCAGGCATTAATGGGTTGTACAGAGTAGAAGCATTTTCTGGTTGAAGGTACCACCAATTCCGCATTCCAAACTTAGTGTATACTGCGTAGCCAATTGAGCTGAT
CAATGAAATCAAAACAAGCAGGGTGAACAGAATGTATATGATGTAATCCATCTTCTTCTCAATGCTGCTCCGCTTTGATGGTGATTCTGTTGAATTTTGCATGACCTTACTATCATGACCCGT
GAAAATAACCACTCCATAGATGTAAGATGTGTTCCTAAGCTTTGAATCCCTAAGAAGAATTTGGTTTGGATCCAAGGAACACACCTGCTTTTCATACTCCAAATTTCCCACAAAGGTGTAT
AAACTAGGATTGGGGTCTTCACACCTAATGGTGGCATAAAAATCCTTGAAAGTTTTATCCTCATCAAAATGTAATGTCACATCCAATGCCCTCTTCACCTTCAAATTTGTCTCCCCATCCAA
ATTCATAGTCTCCACATAGCATATCCCGTCCTCATAACTCGAGGAAAGCAGAAGCAAATCAGCAGGAAAAAAGTGATCTTTCTCAACCTTCACTACATCACCAACAAAGATTTTATGCCAC
TTCTTGTAACAAAAGTTCCCATCCCCTTTATGAATACTAACCTTCCTTTTATTCACCTTCATGTCCTGCATAAATCTCCTCCAATCCTCGACGGCCTCCTTAGCCATGCTCAGCCCAACTACA
AACGCCAAGGGAGCAATCATGCTCACAGCCGAGAACGGTGCAATGGGGGTGAGGGAGAGGACGGCAGCCAGGAGAAAGTAGAGATTGGCGACACGCCGGAACTGTTCAAATATGGCT
TTAGGCAGGAAGGTTATGATGTTGTACTTGGTCGTGGAAATGTAATTGGATGGATAATGGAGAGGTTTCCGACGGTGGAGGCTCGGTTGGTTACATTGCACCTTGCGGGAGAAGCCGGGG
CCTTGAAGACCATTGAATGGATTGGCGAAATCGATAAGCTCAGGGTTCGCTCGGGCACAATTGAAAGTATATATCTTGCTCCATCGGAGCTTCTCTCGCCGGAGCTTATCCCTAATTTTCTC
AGCCCGAGTCATCCTTTTATCTGCGATGAAATGCCTTCCCTCTTGCACGAAGGTGATGAGAGGAAAGGACGAACGTATGTAATTATAGATAAGAGGAAAAGACCATGAGGATGCCAGGAA
TCAGCACCCGATTTCTACCCTACAGATGAAAACTTGGGCTTCACGCAGAAGAATCTGTTGAGATGTGTCAAAATGTTCCTGAAAAGAAGGCTTATAAGAAAAAAATGGATTTAACGGAGCT
CAAAATTTCCAATTCGCGAAATGTTGGCAGAATAAAAATTGATCCGGCTTTTGAGAATAAAGAATAACACAAAGTTTCTTCAAGAATAACACAATGCAGCTCGTGCGAATAAAGAATGTC
GACATCTTTCCATTGGAGCTAAAAAATCGCTCAAATGGATGTGAAATTTTTGCAGAAACGTGAAGGAGATTTGGGTTGATGCAGAAATCAGAAGGCTTAGAGAAGAATTCGACCCGGGA
ATGTATACGAGACGCGCTGCCGACGAGAACGGAGAGGGATGGGGTGATTTCGCGGCTCAACTAACTCCAAAACTTCTGCTCGGCTTCTTTCTGCAATCTTTT
>comp162967_c1_seq3
ACGATAACATAAATCATAAAAGTACAAGAAGGCCTTAAGCATAACTTTAGTGCGTGAGAAATGATAAAAGTACCGAAAATTGAAGCGGCTGTGGTTGTACAAGCATGTATCCATTCCCATA

TATTAGGCACTTGGCTTAGCATGTTTATACAACAAAAACTTCTCAGGCTCCACATGGGGACACATAAGCATAACCTCAACATGAAAATCACTAGAATACTCATTGCTTGGTTTTAAGCAATT
AATTATAACCTATGTTTTATACATAAAGATGATATGAAAAAAATAGCACCATTAACTTTTCTTCCTTAAAAACGCACAAGAATTGATCAACTCTGCAGTTCAAGGAATCACTCCCAATGATG
ATACTTTCTTCTGGAGCTTCCCCTTGAGCTGCCTGATTTTGGCATCCACTCTTGCAGTGAAACCAATCTTGGTTTCCTGCCTCGCTTTGGATCGCTCTCTTGTCCACATTTGCTCATCCGTAA
GATCCTTCTTGAAGTACTTAATCTCCTGAATCACATGGTGATCTAGTGGATAGAACAATCTTTGGTAGGAGAGGTGAGCAAAGTAAGGAAGGTTGCAAGCTGCTGCCACAAGGAAGGTTG
TGATCCAGTATATTGGAGCTGGGCCAAGGGACTCAATTAATATGTGAAAGGCGTTCCCAGAGATGACTGGTGACATGAATCCATAGCCAAACAGGAATAGGTACCACGTGCTCACGCTTC
CCCACACAAAGAGATGCTGAATCCAGGTGAAGTGGCTCATAGTGAGGGCAATCTGCAAGTTTACTGCCCAGATAATGCAAGTGAACATGGTGGTTCCCATGGCAGCCATGTCGGCAGTTT
GCCCTTCAGACTGGAACGCTTGATCGTAGAAGATGCTGATGTTGATGAAAAATACTACAATGGATGAGTAGAGACCATTTGCCGTCCATGCAATAATCCTTGACCAATCAAAGAACAAATT
CTTGGGTCCTTGCTGGTACAAAGCTGGGAACTGAAGGCATACTTGAGAGGACACATCTTGCTCAAAAACTCCTAGTGATATGACCGGTAATGATGTGAGAATGATATTGAATAATATCATG
TACCAGTCCTCATAAACTGATTGTCCTGAAAAGCCAGTGTATGCCTCAAAATAAAACAAAGTGAGGCCAAATGCTATATTCTTGTAAAAGAAATAGCATACCATCTGTGCAATCCTTTTATA
GCACCAATGACCGTGGACAACAAGAAGTCTTTCCAGACAACGGAATTGAGCAATAGAGAAGTCGCTAGCCATCACAGCCTGCACAACGCTGAAGCATTATGAGACTATTCTCCAGCAAG
GAAAAAGGTTTTTGAACTAGGACCAGAACAGACAGGAGATTTGTACTCAGCACAATCCGTTATAGAATGGTTTGCAAGCCTAGAAATTAAATTTCATGCAAGTCGGACATTCAAAAGAA
GCGTTATGAACAAAGATTCATGTTATCAACTGAGAAACTAACTGTTATTCAAGAAGCAATATCACGAAACAAACTGGCAAACCTGCATTCCTTCCACTCCACTGATTCCAACGCCAATATC
AGCCTCTTGTATCATACCAACATCATTTGCACCATCTCCTATAGCTAAAGTAGTTTTTCCTGTTCCTTCTTTCACAAGACGTGTTACCTAGAATAGAAGAATTGCAACAAATTGGAAAGAAA
TTATCTTTTAACCAACTAAAGTCGTGGTGTCAATAATTTAAGAACAAAAATACCAGTGCTTTCTGCTTTGGGGAAACCCGACAGCATATGACAGAAGCACAATCAACAGCTAGACCAAGA
AAATGGTGCTTCATATCATCCTCCAAAGCATAACTCAAAGCTTTCCCATCAATGATTAGAGCAAATGCTGCATGAGGATCCTTCTCTAGCTTGACCATTCGAGAAGCATTGGTGATTTGCAG
CAAAATATTATCTTTGATATTCTTCTTTGCATCTTGGTCCAGAAAATCCGTATTCATTGTTGATATGGAGATTTGTTTCAATCCTAATCGAAGCAAGCTGCATGCAAATCCTATGTTTACGGCT
GTTTCCATTTTGTCTCCTGTTAGAAGCCAGATCTTTAAACCAGCTTGTGCTAGTTTGTCTATACACTGAGGAACCCCTTTTTGTAGCTTATCCTCAACAGCAGTTGCGCCGATAAGGACTAA
TTCCCGCTCCATCAAATCAGCAACTTGCTCCAGCTGAGCATCTCTATCAAGTCCAATAGCTGTCTTTGCTCTGAGAAATTCTGCATTCCATGTTGAGTACTCTGATTCATCTAAAACTTTGTA
TGCCAAAGCTAAGGTACGCAGGCCTGCTTCTCCAAATTCATTTAGATGCTTCATGGTATCCGCTTCATACATTTTTCCATTCTTTGACAGTCTATCAAAAATGATGCTGTCTGCGCCTTTGCA
GAAAAGAATTATCTGCCCAGAATCATCCCGAACAATGACAGACATTCGTTTTCTTTTGCTATTGAATTCCAGTAGATTCAGAATCTTAAATTCCCTTTCCAATGGATTTGGATAGCTTTCTCG
GATGAAAACACTTGACTGAGTCCTTTTAAAGAACTCAAAGCCAAATTCTCTCGCAGCAACAAGAAAAGCTCCTTCATCAGGTGACTCCGCCTCGTATGTGAAGGTGCCATTTTCCTCATC
ATGCTCCGGAATTGCTGTGTGACAAACTGCAAGAACCCGGAAGAACATTTGAAGGGTCCCTCGACTAGGCTCCTTCACCCAATTGCCTTGCATTAGACGGTCATCCTCAAAGCTGAATCC
TTTAATCTTAGGTTTTGGATCCTTGACAACCTTGCAGGTAGTACCATCCTCCAATTGAATTACTGAAGAGTCACCCACATTGTTATCCCAATAATCTGCTGGGAAGCAATTGTGCCCTGGAG
AACCAGAGAATTCTGAAGCGATATGCTTTGCAGCAGCAATTTCAACTTCACTCGAACCTACACCATAAGACACTCCTGCAATAGAACACTTCAGGAAGTCCATCTGGTTGCAGGTCAATG
TGCCTGTTTTATCTGATAAGATTGTATCAACCTGCCCAAGCTCTTCATTTAAATTTGAGGTTCTGGCTTCAGCTGGCTTTCCAGTATCCTCATCATACATCTGAATATCCTGGTTGATGAACAT
AGCTTGTAAGACCTTCACAACCTCTATAGAAACATAGAGAGAAACTGGAATGAGATATCCATACATGATGAGAGCAGTCACAAGATGAAATATGCCAGATAATGCAGGCATTAATGGGTTG
TACAGAGTAGAAGCATTTTCTGGTTGAAGGTACCACCAATTCCGCATTCCAAACTTAGTGTATACTGCGTAGCCAATTGAGCTGATCAATGAAATCAAAACAAGCAGGGTGAACAGAATG
TATATGATGTAATCCATCTTCTTCTCAATGCTGCTCCGCTTTGATGGTGATTCTGTTGAATTTTGCATGACCTTACTATCATGACCCGTGAAAATAACCACTCCATAGATGTAAGATGTGTTCC
TAAGCTTTGAATCCCTAAGAAGAATTTGGTTTGGATCCAAGGAACACACCTGCTTTTCATACTCCAAATTTCCCACAAAGGTGTATAAACTAGGATTGGGGTCTTCACACCTAATGGTGGC
ATAAAAATCCTTGAAAGTTTTATCCTCATCAAAATGTAATGTCACATCCAATGCCCTCTTCACCTTCAAATTTGTCTCCCCATCCAAATTCATAGTCTCCACATAGCATATCCCGTCCTCATAA
CTCGAGGAAAGCAGAAGCAAATCAGCAGGAAAAAAGTGATCTTTCTCAACCTTCACTACATCACCAACAAAGATTTTATGCCACTTCTTGTAACAAAAGTTCCCATCCCCTTTATGAATA

CTAACCTTCCTTTTATTCACCTTCATGTCCTGCATAAATCTCCTCCAATCCTCGACGGCCTCCTTAGCCATGCTCAGCCCAACTACAAACGCCAAGGGAGCAATCATGCTCACAGCCGAGA
ACGGTGCAATGGGGGTGAGGGAGAGGACGGCAGCCAGGAGAAAGTAGAGATTGGCGACACGCCGGAACTGTTCAAATATGGCTTTAGGCAGGAAGGTTATGATGTTGTACTTGGTCGT
GGAAATGTAATTGGATGGATAATGGAGAGGTTTCCGACGGTGGAGGCTCGGTTGGTTACATTGCACCTTGCGGGAGAAGCCGGGGCCTTGAAGACCATTGAATGGATTGGCGAAATCGAT
AAGCTCAGGGTTCGCTCGGGCACAATTGAAAGTATATATCTTGCTCCATCGGAGCTTCTCTCGCCGGAGCTTATCCCTAATTTTCTCAGCCCGAGTCATCCTTTTATCTGCGATGAAATGCC
TTCCCTCTTGCACGAAGGTGATGAGAGGAAAGGACGAACGTATGTAATTATAGATAAGAGGAAAAGACCATGAGGATGCCAGGAATCAGCACCCGATTTCTACCCTACAGATGAAAACTT
GGGCTTCACGCAGAAGAATCTGTTGAGATGTGTCAAAATGTTCCTGAAAAGAAGGCTTATAAGAAAAAATGGATTTAACGGAGCTCAAAATTTCCAATTCGCGAAATGTTGGCAGAATA
AAAATTGATCCGGCTTTTGAGAATAAAGAATAACACAAAGTTTCTTCAAGAATAACACAATGCAGCTCGTGCGAATAAAGAATGTCGACATCTTTCCATTGGAGCTAAAAAATCGCTCAA
ATGGATGTGAAATTTTTGCAGAAACGTGAAGGAGATTTGGGTTGATGCAGAAATCAGAAGGCTTAGAGAAGAATTCGACCCGGGAATGTATACGAGACGCGCTGCCGACGAGAACGGA
GAGGGATGGGGTGATTTCGCGGCTCAACTAACTCCAAAACTTCTGCTCGGCTTCTTTCTGCAATCTTTT
>comp162967_c1_seq30
ACGATAACATAAATCATAAAAGTACAAGAAGGCCTTAAGCATAACTTTAGTGCGTGAGAAATGATAAAAGTACCGAAAATTGAAGCGGCTGTGGTTGTACAAGCATGTATCCATTCCCATA
TATTAGGCACTTGGCTTAGCATGTTTATACAACAAAAACTTCTCAGGCTCCACATGGGGACACATAAGCATAACCTCAACATGAAAATCACTAGAATACTCATTGCTTGGTTTTAAGCAATT
AATTATAACCTATGTTTTATACATAAAGATGATATGAAAAAAATAGCACCATTAACTTTTCTTCCTTAAAAACGCACAAGAATTGATCAACTCTGCAGTTCAAGGAATCACTCCCAATGATG
ATACTTTCTTCTGGAGCTTCCCCTTGAGCTGCCTGATTTTGGCATCCACTCTTGCAGTGAAACCAATCTTGGTTTCCTGCCTCGCTTTGGATCGCTCTCTTGTCCACATTTGCTCATCCGTAA
GATCCTTCTTGAAGTACTTAATCTCCTGAATCACATGGTGATCTAGTGGATAGAACAATCTTTGGTAGGAGAGGTGAGCAAAGTAAGGAAGGTTGCAAGCTGCTGCCACAAGGAAGGTTG
TGATCCAGTATATTGGAGCTGGGCCAAGGGACTCAATTAATATGTGAAAGGCGTTCCCAGAGATGACTGGTGACATGAATCCATAGCCAAACAGGAATAGGTACCACGTGCTCACGCTTC
CCCACACAAAGAGATGCTGAATCCAGGTGAAGTGGCTCATAGTGAGGGCAATCTGCAAGTTTACTGCCCAGATAATGCAAGTGAACATGGTGGTTCCCATGGCAGCCATGTCGGCAGTTT
GCCCTTCAGACTGGAACGCTTGATCGTAGAAGATGCTGATGTTGATGAAAAATACTACAATGGATGAGTAGAGACCATTTGCCGTCCATGCAATAATCCTTGACCAATCAAAGAACAAATT
CTTGGGTCCTTGCTGGTACAAAGCTGGGAACTGAAGGCATACTTGAGAGGACACATCTTGCTCAAAAACTCCTAGTGATATGACCGGTAATGATGTGAGAATGATATTGAATAATATCATG
TACCAGTCCTCATAAACTGATTGTCCTGAAAAGCCAGTGTATGCCTCAAAATAAAACAAAGTGAGGCCAAATGCTATATTCTTGTAAAAGAAATAGCATACCATCTGTGCAATCCTTTTATA
GCACCAATGACCGTGGACAACAAGAAGTCTTTCCAGACAACGGAATTGAGCAATAGAGAAGTCGCTAGCCATCACAGCCTGCACAACGCTGAAGCATTATGAGACTATTCTCCAGCAAG
GAAAAAGGTTTTTGAACTAGGACCAGAACAGACAGGAGATTTGTACTCAGCACAATCCGTTATAGAATGGTTTGCAAGCCTAGAAATTAAATTTCATGCAAGTCGGACATTCAAAAGAA
GCGTTATGAACAAAGATTCATGTTATCAACTGAGAAACTAACTGTTATTCAAGAAGCAATATCACGAAACAAACTGGCAAACCTGCATTCCTTCCACTCCACTGATTCCAACGCCAATATC
AGCCTCTTGTATCATACCAACATCATTTGCACCATCTCCTATAGCTAAAGTAGTTTTTCCTGTTCCTTCTTTCACAAGACGTGTTACCAGTGCTTTCTGCTTTGGGGAAACCCGACAGCATAT
GACAGAAGCACAATCAACAGCTAGACCAAGAAAATGGTGCTTCATATCATCCTCCAAAGCATAACTCAAAGCTTTCCCATCAATGATTAGAGCAAATGCTGCATGAGGATCCTTCTCTAGC
TTGACCATTCGAGAAGCATTGGTGATTTGCAGCAAAATATTATCTTTGATATTCTGTTCCAAGCATGAAAGAAAAGCCTTCTTTGCATCTTGGTCCAGAAAATCCGTATTCATTGTTGATATG
GAGATTTGTTTCAATCCTAATCGAAGCAAGCTGCATGCAAATCCTATGTTTACGGCTGTTTCCATTTTGTCTCCTGTTAGAAGCCAGATCTTTAAACCAGCTTGTGCTAGTTTGTCTATACAC
TGAGGAACCCCTTTTTGTAGCTTATCCTCAACAGCAGTTGCGCCGATAAGGACTAATTCCCGCTCCATCAAATCAGCAACTTGCTCCAGCTGAGCATCTCTATCAAGTCCAATAGCTGTCT
TTGCTCTGAGAAATTCTGCATTCCATGTTGAGTACTCTGATTCATCTAAAACTTTGTATGCCAAAGCTAAGGTACGCAGGCCTGCTTCTCCAAATTCATTTAGATGCTTCATGGTATCCGCTT
CATACATTTTTCCATTCTTTGACAGTCTATCAAAAATGATGCTGTCTGCGCCTTTGCAGAAAAGAATTATCTGCCCAGAATCATCCCGAACAATGACAGACATTCGTTTTCTTTTGCTATTGA
ATTCCAGTAGATTCAGAATCTTAAATTCCCTTTCCAATGGATTTGGATAGCTTTCTCGGATGAAAACACTTGACTGAGTCCTTTTAAAGAACTCAAAGCCAAATTCTCTCGCAGCAACAAG

AAAAGCTCCTTCATCAGGTGACTCCGCCTCGTATGTGAAGGTGCCATTTTCCTCATCATGCTCCGGAATTGCTGTGTGACAAACTGCAAGAACCCGGAAGAACATTTGAAGGGTCCCTCG
ACTAGGCTCCTTCACCCAATTGCCTTGCATTAGACGGTCATCCTCAAAGCTGAATCCTTTAATCTTAGGTTTTGGATCCTTGACAACCTTGCAGGTAGTACCATCCTCCAATTGAATTACTG
AAGAGTCACCCACATTGTTATCCCAATAATCTGCTGGGAAGCAATTGTGCCCTGGAGAACCAGAGAATTCTGAAGCGATATGCTTTGCAGCAGCAATTTCAACTTCACTCGAACCTACAC
CATAAGACACTCCTGCAATAGAACACTTCAGGAAGTCCATCTGGTTGCAGGTCAATGTGCCTGTTTTATCTGATAAGATTGTATCAACCTGCCCAAGCTCTTCATTTAAATTTGAGGTTCTG
GCTTCAGCTGGCTTTCCAGTATCCTCATCATACATCTGAATATCCTGGTTGATGAACATAGCTTGTAAGACCTTCACAACCTCTATAGAAACATAGAGAGAAACTGGAATGAGATATCCATA
CATGATGAGAGCAGTCACAAGATGAAATATGCCAGATAATGCAGGCATTAATGGGTTGTACAGAGTAGAAGCATTTTCTGGTTGAAGGTACCACCAATTCCGCATTCCAAACTTAGTGTAT
ACTGCGTAGCCAATTGAGCTGATCAATGAAATCAAAACAAGCAGGGTGAACAGAATGTATATGATGTAATCCATCTTCTTCTCAATGCTGCTCCGCTTTGATGGTGATTCTGTTGAATTTTG
CATGACCTTACTATCATGACCCGTGAAAATAACCACTCCATAGATGTAAGATGTGTTCCTAAGCTTTGAATCCCTAAGAAGAATTTGGTTTGGATCCAAGGAACACACCTGCTTTTCATACT
CCAAATTTCCCACAAAGGTGTATAAACTAGGATTGGGGTCTTCACACCTAATGGTGGCATAAAAATCCTTGAAAGTTTTATCCTCATCAAAATGTAATGTCACATCCAATGCCCTCTTCACC
TTCAAATTTGTCTCCCCATCCAAATTCATAGTCTCCACATAGCATATCCCGTCCTCATAACTCGAGGAAAGCAGAAGCAAATCAGCAGGAAAAAAGTGATCTTTCTCAACCTTCACTACAT
CACCAACAAAGATTTTATGCCACTTCTTGTAACAAAAGTTCCCATCCCCTTTATGAATACTAACCTTCCTTTTATTCACCTTCATGTCCTGCATAAATCTCCTCCAATCCTCGACGGCCTCCT
TAGCCATGCTCAGCCCAACTACAAACGCCAAGGGAGCAATCATGCTCACAGCCGAGAACGGTGCAATGGGGGTGAGGGAGAGGACGGCAGCCAGGAGAAAGTAGAGATTGGCGACAC
GCCGGAACTGTTCAAATATGGCTTTAGGCAGGAAGGTTATGATGTTGTACTTGGTCGTGGAAATGTAATTGGATGGATAATGGAGAGGTTTCCGACGGTGGAGGCTCGGTTGGTTACATTG
CACCTTGCGGGAGAAGCCGGGGCCTTGAAGACCATTGAATGGATTGGCGAAATCGATAAGCTCAGGGTTCGCTCGGGCACAATTGAAAGTATATATCTTGCTCCATCGGAGCTTCTCTCG
CCGGAGCTTATCCCTAATTTTCTCAGCCCGAGTCATCCTTTTATCTGCGATGAAATGCCTTCCCTCTTGCACGAAGGTGATGAGAGGAAAGGACGAACGTATGTAATTATAGATAAGAGGA
AAAGACCATGAGGATGCCAGGAATCAGCACCCGATTTCTACCCTACAGATGAAAACTTGGGCTTCACGCAGAAGAATCTGTTGAGATGTGTCAAAATGTTCCTGAAAAGAAGGCTTATA
AGAAAAAATGGATTTAACGGAGCTCAAAATTTCCAATTCGCGAAATGTTGGCAGAATAAAAATTGATCCGGCTTTTGAGAATAAAGAATAACACAAAGTTTCTTCAAGAATAACACAATG
CAGCTCGTGCGAATAAAGAATGTCGACATCTTTCCATTGGAGCTAAAAAATCGCTCAAATGGATGTGAAATTTTTGCAGAAACGTGAAGGAGATTTGGGTTGATGCAGAAATCAGAAGG
CTTAGAGAAGAATTCGACCCGGGAATGTATACGAGACGCGCTGCCGACGAGAACGGAGAGGGATGGGGTGATTTCGCGGCTCAACTAACTCCAAAACTTCTGCTCGGCTTCTTTCTGCA
ATCTTTT
>comp162967_c1_seq32
ACGATAACATAAATCATAAAAGTACAAGAAGGCCTTAAGCATAACTTTAGTGCGTGAGAAATGATAAAAGTACCGAAAATTGAAGCGGCTGTGGTTGTACAAGCATGTATCCATTCCCATA
TATTAGGCACTTGGCTTAGCATGTTTATACAACAAAAACTTCTCAGGCTCCACATGGGGACACATAAGCATAACCTCAACATGAAAATCACTAGAATACTCATTGCTTGGTTTTAAGCAATT
AATTATAACCTATGTTTTATACATAAAGATGATATGAAAAAAATAGCACCATTAACTTTTCTTCCTTAAAAACGCACAAGAATTGATCAACTCTGCAGTTCAAGGAATCACTCCCAATGATG
ATACTTTCTTCTGGAGCTTCCCCTTGAGCTGCCTGATTTTGGCATCCACTCTTGCAGTGAAACCAATCTTGGTTTCCTGCCTCGCTTTGGATCGCTCTCTTGTCCACATTTGCTCATCCGTAA
GATCCTTCTTGAAGTACTTAATCTCCTGAATCACATGGTGATCTAGTGGATAGAACAATCTTTGGTAGGAGAGGTGAGCAAAGTAAGGAAGGTTGCAAGCTGCTGCCACAAGGAAGGTTG
TGATCCAGTATATTGGAGCTGGGCCAAGGGACTCAATTAATATGTGAAAGGCGTTCCCAGAGATGACTGGTGACATGAATCCATAGCCAAACAGGAATAGGTACCACGTGCTCACGCTTC
CCCACACAAAGAGATGCTGAATCCAGGTGAAGTGGCTCATAGTGAGGGCAATCTGCAAGTTTACTGCCCAGATAATGCAAGTGAACATGGTGGTTCCCATGGCAGCCATGTCGGCAGTTT
GCCCTTCAGACTGGAACGCTTGATCGTAGAAGATGCTGATGTTGATGAAAAATACTACAATGGATGAGTAGAGACCATTTGCCGTCCATGCAATAATCCTTGACCAATCAAAGAACAAATT
CTTGGGTCCTTGCTGGTACAAAGCTGGGAACTGAAGGCATACTTGAGAGGACACATCTTGCTCAAAAACTCCTAGTGATATGACCGGTAATGATGTGAGAATGATATTGAATAATATCATG
TACCAGTCCTCATAAACTGATTGTCCTGAAAAGCCAGTGTATGCCTCAAAATAAAACAAAGTGAGGCCAAATGCTATATTCTTGTAAAAGAAATAGCATACCATCTGTGCAATCCTTTTATA

GCACCAATGACCGTGGACAACAAGAAGTCTTTCCAGACAACGGAATTGAGCAATAGAGAAGTCGCTAGCCATCACAGCCTGCATTCCTTCCACTCCACTGATTCCAACGCCAATATCAGC
CTCTTGTATCATACCAACATCATTTGCACCATCTCCTATAGCTAAAGTAGTTTTTCCTGTTCCTTCTTTCACAAGACGTGTTACCTAGAATAGAAGAATTGCAACAAATTGGAAAGAAATTAT
CTTTTAACCAACTAAAGTCGTGGTGTCAATAATTTAAGAACAAAAATACCAGTGCTTTCTGCTTTGGGGAAACCCGACAGCATATGACAGAAGCACAATCAACAGCTAGACCAAGAAAAT
GGTGCTTCATATCATCCTCCAAAGCATAACTCAAAGCTTTCCCATCAATGATTAGAGCAAATGCTGCATGAGGATCCTTCTCTAGCTTGACCATTCGAGAAGCATTGGTGATTTGCAGCAA
AATATTATCTTTGATATTCTTCTTTGCATCTTGGTCCAGAAAATCCGTATTCATTGTTGATATGGAGATTTGTTTCAATCCTAATCGAAGCAAGCTGCATGCAAATCCTATGTTTACGGCTGTT
TCCATTTTGTCTCCTGTTAGAAGCCAGATCTTTAAACCAGCTTGTGCTAGTTTGTCTATACACTGAGGAACCCCTTTTTGTAGCTTATCCTCAACAGCAGTTGCGCCGATAAGGACTAATTC
CCGCTCCATCAAATCAGCAACTTGCTCCAGCTGAGCATCTCTATCAAGTCCAATAGCTGTCTTTGCTCTGAGAAATTCTGCATTCCATGTTGAGTACTCTGATTCATCTAAAACTTTGTATGC
CAAAGCTAAGGTACGCAGGCCTGCTTCTCCAAATTCATTTAGATGCTTCATGGTATCCGCTTCATACATTTTTCCATTCTTTGACAGTCTATCAAAAATGATGCTGTCTGCGCCTTTGCAGAA
AAGAATTATCTGCCCAGAATCATCCCGAACAATGACAGACATTCGTTTTCTTTTGCTATTGAATTCCAGTAGATTCAGAATCTTAAATTCCCTTTCCAATGGATTTGGATAGCTTTCTCGGAT
GAAAACACTTGACTGAGTCCTTTTAAAGAACTCAAAGCCAAATTCTCTCGCAGCAACAAGAAAAGCTCCTTCATCAGGTGACTCCGCCTCGTATGTGAAGGTGCCATTTTCCTCATCATG
CTCCGGAATTGCTGTGTGACAAACTGCAAGAACCCGGAAGAACATTTGAAGGGTCCCTCGACTAGGCTCCTTCACCCAATTGCCTTGCATTAGACGGTCATCCTCAAAGCTGAATCCTTT
AATCTTAGGTTTTGGATCCTTGACAACCTTGCAGGTAGTACCATCCTCCAATTGAATTACTGAAGAGTCACCCACATTGTTATCCCAATAATCTGCTGGGAAGCAATTGTGCCCTGGAGAA
CCAGAGAATTCTGAAGCGATATGCTTTGCAGCAGCAATTTCAACTTCACTCGAACCTACACCATAAGACACTCCTGCAATAGAACACTTCAGGAAGTCCATCTGGTTGCAGGTCAATGTG
CCTGTTTTATCTGATAAGATTGTATCAACCTGCCCAAGCTCTTCATTTAAATTTGAGGTTCTGGCTTCAGCTGGCTTTCCAGTATCCTCATCATACATCTGAATATCCTGGTTGATGAACATAG
CTTGTAAGACCTTCACAACCTCTATAGAAACATAGAGAGAAACTGGAATGAGATATCCATACATGATGAGAGCAGTCACAAGATGAAATATGCCAGATAATGCAGGCATTAATGGGTTGTA
CAGAGTAGAAGCATTTTCTGGTTGAAGGTACCACCAATTCCGCATTCCAAACTTAGTGTATACTGCGTAGCCAATTGAGCTGATCAATGAAATCAAAACAAGCAGGGTGAACAGAATGTA
TATGATGTAATCCATCTTCTTCTCAATGCTGCTCCGCTTTGATGGTGATTCTGTTGAATTTTGCATGACCTTACTATCATGACCCGTGAAAATAACCACTCCATAGATGTAAGATGTGTTCCTA
AGCTTTGAATCCCTAAGAAGAATTTGGTTTGGATCCAAGGAACACACCTGCTTTTCATACTCCAAATTTCCCACAAAGGTGTATAAACTAGGATTGGGGTCTTCACACCTAATGGTGGCAT
AAAAATCCTTGAAAGTTTTATCCTCATCAAAATGTAATGTCACATCCAATGCCCTCTTCACCTTCAAATTTGTCTCCCCATCCAAATTCATAGTCTCCACATAGCATATCCCGTCCTCATAAC
TCGAGGAAAGCAGAAGCAAATCAGCAGGAAAAAAGTGATCTTTCTCAACCTTCACTACATCACCAACAAAGATTTTATGCCACTTCTTGTAACAAAAGTTCCCATCCCCTTTATGAATACT
AACCTTCCTTTTATTCACCTTCATGTCCTGCATAAATCTCCTCCAATCCTCGACGGCCTCCTTAGCCATGCTCAGCCCAACTACAAACGCCAAGGGAGCAATCATGCTCACAGCCGAGAAC
GGTGCAATGGGGGTGAGGGAGAGGACGGCAGCCAGGAGAAAGTAGAGATTGGCGACACGCCGGAACTGTTCAAATATGGCTTTAGGCAGGAAGGTTATGATGTTGTACTTGGTCGTGG
AAATGTAATTGGATGGATAATGGAGAGGTTTCCGACGGTGGAGGCTCGGTTGGTTACATTGCACCTTGCGGGAGAAGCCGGGGCCTTGAAGACCATTGAATGGATTGGCGAAATCGATAA
GCTCAGGGTTCGCTCGGGCACAATTGAAAGTATATATCTTGCTCCATCGGAGCTTCTCTCGCCGGAGCTTATCCCTAATTTTCTCAGCCCGAGTCATCCTTTTATCTGCGATGAAATGCCTTC
CCTCTTGCACGAAGGTGATGAGAGGAAAGGACGAACGTATGTAATTATAGATAAGAGGAAAAGACCATGAGGATGCCAGGAATCAGCACCCGATTTCTACCCTACAGATGAAAACTTGG
GCTTCACGCAGAAGAATCTGTTGAGATGTGTCAAAATGTTCCTGAAAAGAAGGCTTATAAGAAAAAAATGGATTTAACGGAGCTCAAAATTTCCAATTCGCGAAATGTTGGCAGAATAAA
AATTGATCCGGCTTTTGAGAATAAAGAATAACACAAAGTTTCTTCAAGAATAACACAATGCAGCTCGTGCGAATAAAGAATGTCGACATCTTTCCATTGGAGCTAAAAAATCGCTCAAAT
GGATGTGAAATTTTTGCAGAAACGTGAAGGAGATTTGGGTTGATGCAGAAATCAGAAGGCTTAGAGAAGAATTCGACCCGGGAATGTATACGAGACGCGCTGCCGACGAGAACGGAGA
GGGATGGGGTGATTTCGCGGCTCAACTAACTCCAAAACTTCTGCTCGGCTTCTTTCTGCAATCTTTT
>comp162967_c1_seq4
ACGATAACATAAATCATAAAAGTACAAGAAGGCCTTAAGCATAACTTTAGTGCGTGAGAAATGATAAAAGTACCGAAAATTGAAGCGGCTGTGGTTGTACAAGCATGTATCCATTCCCATA

TATTAGGCACTTGGCTTAGCATGTTTATACAACAAAAACTTCTCAGGCTCCACATGGGGACACATAAGCATAACCTCAACATGAAAATCACTAGAATACTCATTGCTTGGTTTTAAGCAATT
AATTATAACCTATGTTTTATACATAAAGATGATATGAAAAAAATAGCACCATTAACTTTTCTTCCTTAAAAACGCACAAGAATTGATCAACTCTGCAGTTCAAGGAATCACTCCCAATGATG
ATACTTTCTTCTGGAGCTTCCCCTTGAGCTGCCTGATTTTGGCATCCACTCTTGCAGTGAAACCAATCTTGGTTTCCTGCCTCGCTTTGGATCGCTCTCTTGTCCACATTTGCTCATCCGTAA
GATCCTTCTTGAAGTACTTAATCTCCTGAATCACATGGTGATCTAGTGGATAGAACAATCTTTGGTAGGAGAGGTGAGCAAAGTAAGGAAGGTTGCAAGCTGCTGCCACAAGGAAGGTTG
TGATCCAGTATATTGGAGCTGGGCCAAGGGACTCAATTAATATGTGAAAGGCGTTCCCAGAGATGACTGGTGACATGAATCCATAGCCAAACAGGAATAGGTACCACGTGCTCACGCTTC
CCCACACAAAGAGATGCTGAATCCAGGTGAAGTGGCTCATAGTGAGGGCAATCTGCAAGTTTACTGCCCAGATAATGCAAGTGAACATGGTGGTTCCCATGGCAGCCATGTCGGCAGTTT
GCCCTTCAGACTGGAACGCTTGATCGTAGAAGATGCTGATGTTGATGAAAAAATACTACAATGGATGAGTAGAGACCATTTGCCGTCCATGCAATAATCCTTGACCAATCAAAGAACAAATT
CTTGGGTCCTTGCTGGTACAAAGCTGGGAACTGAAGGCATACTTGAGAGGACACATCTTGCTCAAAAACTCCTAGTGATATGACCGGTAATGATGTGAGAATGATATTGAATAATATCATG
TACCAGTCCTCATAAACTGATTGTCCTGAAAAGCCAGTGTATGCCTCAAAATAAAACAAAGTGAGGCCAAATGCTATATTCTTGTAAAAGAAATAGCATACCATCTGTGCAATCCTTTTATA
GCACCAATGACCGTGGACAACAAGAAGTCTTTCCAGACAACGGAATTGAGCAATAGAGAAGTCGCTAGCCATCACAGCCTGCACAACGCTGAAGCATTATGAGACTATTCTCCAGCAAG
GAAAAAGGTTTTTGAACTAGGACCAGAACAGACAGGAGATTTGTACTCAGCACAATCCGTTATAGAATGGTTTGCAAGCCTGCATTCCTTCCACTCCACTGATTCCAACGCCAATATCAG
CCTCTTGTATCATACCAACATCATTTGCACCATCTCCTATAGCTAAAGTAGTTTTTCCTGTTCCTTCTTTCACAAGACGTGTTACCAGTGCTTTCTGCTTTGGGGAAACCCGACAGCATATGA
CAGAAGCACAATCAACAGCTAGACCAAGAAAATGGTGCTTCATATCATCCTCCAAAGCATAACTCAAAGCTTTCCCATCAATGATTAGAGCAAATGCTGCATGAGGATCCTTCTCTAGCTT
GACCATTCGAGAAGCATTGGTGATTTGCAGCAAAATATTATCTTTGATATTCTTCTTTGCATCTTGGTCCAGAAAATCCGTATTCATTGTTGATATGGAGATTTGTTTCAATCCTAATCGAAGC
AAGCTGCATGCAAATCCTATGTTTACGGCTGTTTCCATTTTGTCTCCTGTTAGAAGCCAGATCTTTAAACCAGCTTGTGCTAGTTTGTCTATACACTGAGGAACCCCTTTTTGTAGCTTATCC
TCAACAGCAGTTGCGCCGATAAGGACTAATTCCCGCTCCATCAAATCAGCAACTTGCTCCAGCTGAGCATCTCTATCAAGTCCAATAGCTGTCTTTGCTCTGAGAAATTCTGCATTCCATG
TTGAGTACTCTGATTCATCTAAAACTTTGTATGCCAAAGCTAAGGTACGCAGGCCTGCTTCTCCAAATTCATTTAGATGCTTCATGGTATCCGCTTCATACATTTTTCCATTCTTTGACAGTCT
ATCAAAAATGATGCTGTCTGCGCCTTTGCAGAAAAGAATTATCTGCCCAGAATCATCCCGAACAATGACAGACATTCGTTTTCTTTTGCTATTGAATTCCAGTAGATTCAGAATCTTAAATT
CCCTTTCCAATGGATTTGGATAGCTTTCTCGGATGAAAACACTTGACTGAGTCCTTTTAAAGAACTCAAAGCCAAATTCTCTCGCAGCAACAAGAAAAGCTCCTTCATCAGGTGACTCCG
CCTCGTATGTGAAGGTGCCATTTTCCTCATCATGCTCCGGAATTGCTGTGTGACAAACTGCAAGAACCCGGAAGAACATTTGAAGGGTCCCTCGACTAGGCTCCTTCACCCAATTGCCTT
GCATTAGACGGTCATCCTCAAAGCTGAATCCTTTAATCTTAGGTTTTGGATCCTTGACAACCTTGCAGGTAGTACCATCCTCCAATTGAATTACTGAAGAGTCACCCACATTGTTATCCCAA
TAATCTGCTGGGAAGCAATTGTGCCCTGGAGAACCAGAGAATTCTGAAGCGATATGCTTTGCAGCAGCAATTTCAACTTCACTCGAACCTACACCATAAGACACTCCTGCAATAGAACAC
TTCAGGAAGTCCATCTGGTTGCAGGTCAATGTGCCTGTTTTATCTGATAAGATTGTATCAACCTGCCCAAGCTCTTCATTTAAATTTGAGGTTCTGGCTTCAGCTGGCTTTCCAGTATCCTCA
TCATACATCTGAATATCCTGGTTGATGAACATAGCTTGTAAGACCTTCACAACCTCTATAGAAACATAGAGAGAAACTGGAATGAGATATCCATACATGATGAGAGCAGTCACAAGATGAA
ATATGCCAGATAATGCAGGCATTAATGGGTTGTACAGAGTAGAAGCATTTTCTGGTTGAAGGTACCACCAATTCCGCATTCCAAACTTAGTGTATACTGCGTAGCCAATTGAGCTGATCAAT
GAAATCAAAACAAGCAGGGTGAACAGAATGTATATGATGTAATCCATCTTCTTCTCAATGCTGCTCCGCTTTGATGGTGATTCTGTTGAATTTTGCATGACCTTACTATCATGACCCGTGAA
AATAACCACTCCATAGATGTAAGATGTGTTCCTAAGCTTTGAATCCCTAAGAAGAATTTGGTTTGGATCCAAGGAACACACCTGCTTTTCATACTCCAAATTTCCCACAAAGGTGTATAAA
CTAGGATTGGGGTCTTCACACCTAATGGTGGCATAAAAATCCTTGAAAGTTTTATCCTCATCAAAATGTAATGTCACATCCAATGCCCTCTTCACCTTCAAATTTGTCTCCCCATCCAAATTC
ATAGTCTCCACATAGCATATCCCGTCCTCATAACTCGAGGAAAGCAGAAGCAAATCAGCAGGAAAAAAGTGATCTTTCTCAACCTTCACTACATCACCAACAAAGATTTTATGCCACTTCT
TGTAACAAAAGTTCCCATCCCCTTTATGAATACTAACCTTCCTTTTATTCACCTTCATGTCCTGCATAAATCTCCTCCAATCCTCGACGGCCTCCTTAGCCATGCTCAGCCCAACTACAAACG
CCAAGGGAGCAATCATGCTCACAGCCGAGAACGGTGCAATGGGGGTGAGGGAGAGGACGGCAGCCAGGAGAAAGTAGAGATTGGCGACACGCCGGAACTGTTCAAATATGGCTTTAG

GCAGGAAGGTTATGATGTTGTACTTGGTCGTGGAAATGTAATTGGATGGATAATGGAGAGGTTTCCGACGGTGGAGGCTCGGTTGGTTACATTGCACCTTGCGGGAGAAGCCGGGGCCTT
GAAGACCATTGAATGGATTGGCGAAATCGATAAGCTCAGGGTTCGCTCGGGCACAATTGAAAGTATATATCTTGCTCCATCGGAGCTTCTCTCGCCGGAGCTTATCCCTAATTTTCTCAGCC
CGAGTCATCCTTTTATCTGCGATGAAATGCCTTCCCTCTTGCACGAAGGTGATGAGAGGAAAGGACGAACGTATGTAATTATAGATAAGAGGAAAAGACCATGAGGATGCCAGGAATCAG
CACCCGATTTCTACCCTACAGATGAAAACTTGGGCTTCACGCAGAAGAATCTGTTGAGATGTGTCAAAATGTTCCTGAAAAGAAGGCTTATAAGAAAAAATGGATTTAACGGAGCTCAAA
ATTTCCAATTCGCGAAATGTTGGCAGAATAAAAATTGATCCGGCTTTTGAGAATAAAGAATAACACAAAGTTTCTTCAAGAATAACACAATGCAGCTCGTGCGAATAAAGAATGTCGACA
TCTTTCCATTGGAGCTAAAAAATCGCTCAAATGGATGTGAAATTTTTGCAGAAACGTGAAGGAGATTTGGGTTGATGCAGAAATCAGAAGGCTTAGAGAAGAATTCGACCCGGGAATGTA
TACGAGACGCGCTGCCGACGAGAACGGAGAGGGATGGGGTGATTTCGCGGCTCAACTAACTCCAAAACTTCTGCTCGGCTTCTTTCTGCAATCTTTT
>comp162967_c1_seq41
ACGATAACATAAATCATAAAAGTACAAGAAGGCCTTAAGCATAACTTTAGTGCGTGAGAAATGATAAAAGTACCGAAAATTGAAGCGGCTGTGGTTGTACAAGCATGTATCCATTCCCATA
TATTAGGCACTTGGCTTAGCATGTTTATACAACAAAAACTTCTCAGGCTCCACATGGGGACACATAAGCATAACCTCAACATGAAAATCACTAGAATACTCATTGCTTGGTTTTAAGCAATT
AATTATAACCTATGTTTTATACATAAAGATGATATGAAAAAAATAGCACCATTAACTTTTCTTCCTTAAAAACGCACAAGAATTGATCAACTCTGCAGTTCAAGGAATCACTCCCAATGATG
ATACTTTCTTCTGGAGCTTCCCCTTGAGCTGCCTGATTTTGGCATCCACTCTTGCAGTGAAACCAATCTTGGTTTCCTGCCTCGCTTTGGATCGCTCTCTTGTCCACATTTGCTCATCCGTAA
GATCCTTCTTGAAGTACTTAATCTCCTGAATCACATGGTGATCTAGTGGATAGAACAATCTTTGGTAGGAGAGGTGAGCAAAGTAAGGAAGGTTGCAAGCTGCTGCCACAAGGAAGGTTG
TGATCCAGTATATTGGAGCTGGGCCAAGGGACTCAATTAATATGTGAAAGGCGTTCCCAGAGATGACTGGTGACATGAATCCATAGCCAAACAGGAATAGGTACCACGTGCTCACGCTTC
CCCACACAAAGAGATGCTGAATCCAGGTGAAGTGGCTCATAGTGAGGGCAATCTGCAAGTTTACTGCCCAGATAATGCAAGTGAACATGGTGGTTCCCATGGCAGCCATGTCGGCAGTTT
GCCCTTCAGACTGGAACGCTTGATCGTAGAAGATGCTGATGTTGATGAAAAATACTACAATGGATGAGTAGAGACCATTTGCCGTCCATGCAATAATCCTTGACCAATCAAAGAACAAATT
CTTGGGTCCTTGCTGGTACAAAGCTGGGAACTGAAGGCATACTTGAGAGGACACATCTTGCTCAAAAACTCCTAGTGATATGACCGGTAATGATGTGAGAATGATATTGAATAATATCATG
TACCAGTCCTCATAAACTGATTGTCCTGAAAAGCCAGTGTATGCCTCAAAATAAAACAAAGTGAGGCCAAATGCTATATTCTTGTAAAAGAAATAGCATACCATCTGTGCAATCCTTTTATA
GCACCAATGACCGTGGACAACAAGAAGTCTTTCCAGACAACGGAATTGAGCAATAGAGAAGTCGCTAGCCATCACAGCCTGCACAACGCTGAAGCATTATGAGACTATTCTCCAGCAAG
GAAAAAGGTTTTTGAACTAGGACCAGAACAGACAGGAGATTTGTACTCAGCACAATCCGTTATAGAATGGTTTGCAAGCCTGCATTCCTTCCACTCCACTGATTCCAACGCCAATATCAG
CCTCTTGTATCATACCAACATCATTTGCACCATCTCCTATAGCTAAAGTAGTTTTTCCTGTTCCTTCTTTCACAAGACGTGTTACCAGTGCTTTCTGCTTTGGGGAAACCCGACAGCATATGA
CAGAAGCACAATCAACAGCTAGACCAAGAAAATGGTGCTTCATATCATCCTCCAAAGCATAACTCAAAGCTTTCCCATCAATGATTAGAGCAAATGCTGCATGAGGATCCTTCTCTAGCTT
GACCATTCGAGAAGCATTGGTGATTTGCAGCAAAATATTATCTTTGATATTCTGTTCCAAGCATGAAAGAAAAGCCTTCTTTGCATCTTGGTCCAGAAAATCCGTATTCATTGTTGATATGG
AGATTTGTTTCAATCCTAATCGAAGCAAGCTGCATGCAAATCCTATGTTTACGGCTGTTTCCATTTTGTCTCCTGTTAGAAGCCAGATCTTTAAACCAGCTTGTGCTAGTTTGTCTATACACT
GAGGAACCCCTTTTTGTAGCTTATCCTCAACAGCAGTTGCGCCGATAAGGACTAATTCCCGCTCCATCAAATCAGCAACTTGCTCCAGCTGAGCATCTCTATCAAGTCCAATAGCTGTCTT
TGCTCTGAGAAATTCTGCATTCCATGTTGAGTACTCTGATTCATCTAAAACTTTGTATGCCAAAGCTAAGGTACGCAGGCCTGCTTCTCCAAATTCATTTAGATGCTTCATGGTATCCGCTTC
ATACATTTTTCCATTCTTTGACAGTCTATCAAAAATGATGCTGTCTGCGCCTTTGCAGAAAAGAATTATCTGCCCAGAATCATCCCGAACAATGACAGACATTCGTTTTCTTTTGCTATTGAA
TTCCAGTAGATTCAGAATCTTAAATTCCCTTTCCAATGGATTTGGATAGCTTTCTCGGATGAAAACACTTGACTGAGTCCTTTTAAAGAACTCAAAGCCAAATTCTCTCGCAGCAACAAGA
AAAGCTCCTTCATCAGGTGACTCCGCCTCGTATGTGAAGGTGCCATTTTCCTCATCATGCTCCGGAATTGCTGTGTGACAAACTGCAAGAACCCGGAAGAACATTTGAAGGGTCCCTCGA
CTAGGCTCCTTCACCCAATTGCCTTGCATTAGACGGTCATCCTCAAAGCTGAATCCTTTAATCTTAGGTTTTGGATCCTTGACAACCTTGCAGGTAGTACCATCCTCCAATTGAATTACTGA
AGAGTCACCCACATTGTTATCCCAATAATCTGCTGGGAAGCAATTGTGCCCTGGAGAACCAGAGAATTCTGAAGCGATATGCTTTGCAGCAGCAATTTCAACTTCACTCGAACCTACACC

ATAAGACACTCCTGCAATAGAACACTTCAGGAAGTCCATCTGGTTGCAGGTCAATGTGCCTGTTTTATCTGATAAGATTGTATCAACCTGCCCAAGCTCTTCATTTAAATTTGAGGTTCTGG
CTTCAGCTGGCTTTCCAGTATCCTCATCATACATCTGAATATCCTGGTTGATGAACATAGCTTGTAAGACCTTCACAACCTCTATAGAAACATAGAGAGAAACTGGAATGAGATATCCATAC
ATGATGAGAGCAGTCACAAGATGAAATATGCCAGATAATGCAGGCATTAATGGGTTGTACAGAGTAGAAGCATTTTCTGGTTGAAGGTACCACCAATTCCGCATTCCAAACTTAGTGTATA
CTGCGTAGCCAATTGAGCTGATCAATGAAATCAAAACAAGCAGGGTGAACAGAATGTATATGATGTAATCCATCTTCTTCTCAATGCTGCTCCGCTTTGATGGTGATTCTGTTGAATTTTGC
ATGACCTTACTATCATGACCCGTGAAAATAACCACTCCATAGATGTAAGATGTGTTCCTAAGCTTTGAATCCCTAAGAAGAATTTGGTTTGGATCCAAGGAACACACCTGCTTTTCATACTC
CAAATTTCCCACAAAGGTGTATAAACTAGGATTGGGGTCTTCACACCTAATGGTGGCATAAAAATCCTTGAAAGTTTTATCCTCATCAAAATGTAATGTCACATCCAATGCCCTCTTCACCT
TCAAATTTGTCTCCCCATCCAAATTCATAGTCTCCACATAGCATATCCCGTCCTCATAACTCGAGGAAAGCAGAAGCAAATCAGCAGGAAAAAAGTGATCTTTCTCAACCTTCACTACATC
ACCAACAAAGATTTTATGCCACTTCTTGTAACAAAAGTTCCCATCCCCTTTATGAATACTAACCTTCCTTTTATTCACCTTCATGTCCTGCATAAATCTCCTCCAATCCTCGACGGCCTCCTT
AGCCATGCTCAGCCCAACTACAAACGCCAAGGGAGCAATCATGCTCACAGCCGAGAACGGTGCAATGGGGGTGAGGGAGAGGACGGCAGCCAGGAGAAAGTAGAGATTGGCGACACG
CCGGAACTGTTCAAATATGGCTTTAGGCAGGAAGGTTATGATGTTGTACTTGGTCGTGGAAATGTAATTGGATGGATAATGGAGAGGTTTCCGACGGTGGAGGCTCGGTTGGTTACATTGC
ACCTTGCGGGAGAAGCCGGGGCCTTGAAGACCATTGAATGGATTGGCGAAATCGATAAGCTCAGGGTTCGCTCGGGCACAATTGAAAGTATATATCTTGCTCCATCGGAGCTTCTCTCGC
CGGAGCTTATCCCTAATTTTCTCAGCCCGAGTCATCCTTTTATCTGCGATGAAATGCCTTCCCTCTTGCACGAAGGTGATGAGAGGAAAGGACGAACGTATGTAATTATAGATAAGAGGAA
AAGACCATGAGGATGCCAGGAATCAGCACCCGATTTCTACCCTACAGATGAAAACTTGGGCTTCACGCAGAAGAATCTGTTGAGATGTGTCAAAATGTTCCTGAAAAGAAGGCTTATAA
GAAAAAATGGATTTAACGGAGCTCAAAATTTCCAATTCGCGAAATGTTGGCAGAATAAAAATTGATCCGGCTTTTGAGAATAAAGAATAACACAAAGTTTCTTCAAGAATAACACAATGC
AGCTCGTGCGAATAAAGAATGTCGACATCTTTCCATTGGAGCTAAAAAATCGCTCAAATGGATGTGAAATTTTTGCAGAAACGTGAAGGAGATTTGGGTTGATGCAGAAATCAGAAGGCT
TAGAGAAGAATTCGACCCGGGAATGTATACGAGACGCGCTGCCGACGAGAACGGAGAGGGATGGGGTGATTTCGCGGCTCAACTAACTCCAAAACTTCTGCTCGGCTTCTTTCTGCAAT
CTTTT
>comp162977_c0_seq1
TCACTTGACTGGTTATTAAATATTTGTATTTAATATATCTCCATTATTTATCATTATTTTTCCTTCTGTATAGCCCTTGCTTAACTTTGCAATTCCTGATCCTACTTCTATCTGGTATGCTAGTTATA
TTGTATGTTAACTATGGTCGGTTTGTTGGATCAAGCATTCCACATAACCTTATTCTTGCGTTCTCTCGGGAGATACAGTATATGCTTACAAGAAGTAAGCTGCATTTTGTAAGTTTAAGGTTT
TTGGTTTGTTTGTATACATTTGACTGCTTAAACTATAGATTGGATGATTTTTCAGCTGGCTACGTTATAGCAAGTATAGATACAGAAGTTGAAGCATTTAGAAAACCATAAAGGGGAAGCGT
CATATTTTATTGCAGTTTGGGAGGCCAGCGGAAAAAGATGGATTCAGCTGCATACTTTTTTCTTTGTTTCTAACCAAAACTAAATTCTGAGTTTGACCTACAATTTGGATGTTTAAGCTTGG
GACAATGGTTTTCAAGTAGAAGGAAATCTATATTTAACCGTGTGCAGGGTCATTGTTATCCATGCACGTTGACAAATTCGTGGTTTCTAAAGGAGTATACTCTATTTCTTCAATTAGGGATTA
TCTTTCACTTTTATGAACTTATGCTTACACTTGGCATTATTCTTCTTTTATCTTGTGATTGTGCAGCCATCTCTTTTGGGTTTAGTGGAAATGTTGTCATAGCTGAAGATGCTTCAGCATCTAA
CTTAACCATCAATAATGATCA
>comp163039_c0_seq2
GTCCGCACCTAATAATTTTTCCAGTTAACTCAGGTTCTTTCTACAATTTTCAGTACATTTCCTGTAAGAACTACATATGAATCAAGCAGAACTCCCTCACCAACAGGCGAATTATTACGTCC
GGTCACCGGACATCCGGACATCCAATCCAGACGCGCTACATCACCCAAATACACCGGTGCCCAATGTATTTGGCTGACGTGACTGGGGCCCGGTGTATTTAGGTGACGTGGC
>comp163057_c1_seq1
ATACTCCAAACTCCAATCACCCAATTTTCCTCTACCTCTTATATAAAATCCGCACCTCAACCCCAAACCGCTCACCAAACCTATCATCCAATGGCTTCCCTGCTCCTCCTCATCGCCGTCCTC
CTCACCGCCATCTCGCCGTCGGAATTCGCCGCCACGGAAAACTCCTCCGATTTCATCCGTCGCAGCTGTGCTGTAACTCTCTACGCCCAACTCTGCTACAACTCCCTGAAACCATACGAAT

CCACCGTCCGGCAGAGCCCGTTAACCCTCGCCATCGCCGCCGCCAACGTCAGCCTCGCCAAGATAAAAAAAGCGTCTTTGCAGGCGGCGGAGTTGCGGCGCAGCAGCGCGGGGAGGGT
CAGCGCGGCCCTGCGTGACTGTGTTGAATTGCTGGGGACGGCGGCGGAGTTGACTAAGCAGTCCGCAGCAGAGATTCAGAAGCTTGGGCTGGCGGCGCAGTCGGGCGGGCCGAGTTTT
GCTTGGGAGGCATCCAACGCTCAGACGTGGATGAGCGCTGCCCTGACTAATGAAGATACCTGCATCGACGGATTTAACGACGCCGGCGGCGGCTCCGCCGCGGTGTTGACGGCGTTCCT
TACGAAGGTTGGGAGGGTGGAGCAGTATACAAGTAACGCTCTTGCGCTGCTTAATGCTCTTGTGGATGGTTGATTGATTCCTCTGTGGGACGTTCTTCTGTGTGGTTAATGTTATCCTAGCC
GTACAATTAACTTTGGACGGTTAATACCGGATTCCTCAAAATTTATTCTCTCTTATCCCGTTAAAATGATTAGAATCAGAACAGTGTTAAATATCTTATACATAAATTATTTTAACTGTTTGCA
AGTTGGTCTTCTCAGGTGATGGCGAGTACTGCGTGTGAAAAAATTATTATGTGCGGTGTTACGGCTTTTATTGTTCGGTATTGGTGCTTGATACCGTTATATACTGAATGACATGGTACATTC
TGATTGATTTTCAGACCATCTAGGTGTCTGCATAGAATAATTTCTCGTCCCATGAATTGTAAAAAGAAATTTACATGGCTCTGTTTGTCTAAATTTGTATTCTTCGTGTAAGCAAGAAGAAAT
AATATATTTACTCTAAAATTTCAGCTATTTAACATGAGGCAACATGGATGAAATTATTTTTACAAGAAATTTATTAGTGAAGAGTAGCTACACCCGCAAGTCCTGCCCATCCGCTCCAACGC
>comp163057_c2_seq4
AATGCAGTAGGAAATAATCAATTTTGTACAATATAAAAAAAATCTATATAATAATTAAAATTATTGAAAATTACATTACTGAACATATAAATGCAGTTCTCCAAGCTTTGGTCAAGCCACAGA
GGAAGCCCACCTTCTCCTCGCCTTTTTTGTTGTCTTTGTTTGGTCATTTGTGAGCCCACCGCAGCATTGTCCAACAATAATACCATACGAACCCATTGCCTCATGGAAGAAAGTTATAAACA
GGTAATAATAAGAAGCCACGTGCTTCTATCATCAAATCTATCTATTTCTCATCTCTCGCTCTCTGCCCGTGCACTGCACCCATTTTTCTCTAACTATATTATTTGATCAAAAACATAATACAATA
TGATCATGGGTTAACAAAGGGGCCCCACTTGTGGGGTCTGTTCGGCTCATGCACTTTAGGAACTTATTATTGCCTTTTTTAGGGTTTGGGGAAGATCATAGGAGGAGACTTCAAAATGGTA
AAATAAATAAAAGAAAAGTTATGAGAGTTCATCTTAGGATCTATTTGGAGAGAAATTATTTTGTGCAGACATCTGAACAGTCTGAAGACTAATCAGAACGTACCATGTCATTCAATATATAA
CGGTAATGAGCACCAATATCAAACAATAAAAGCCGTAGCACATAATAATTTTTCACACACACCACTCGTCATTACCTGAGAAGACCAACTTGCAAACAGTTAAAATAATTTCTGTATAAGA
TATTTAACACTGTTCTGATTGTGATCATTTTGACGGGATAAGAGAGAATAAATTTTGAGGAATCCGGTATTAACCGTCCAAAGTTAATTGTACGGCTAGGATAACATTAACCACATAGTAGA
ACGTCCCACTGAAGGAATCAATCAACCATCCACAAGAGCATTAAGCAGAGCAAGAGCGTTACTTGTATACTGCTCCACCCTCCCAACCTTCGTAACGAACACCGTCAACACCGCGGCGG
AGCCCCTGCCAGCGTCGTTAAATCCGTCGATGCAGGTATCTTCATTAGTGAGGGCAGCGCTCATCCACGTCTGAGCGTTGGATGCCTCCCAAGCAAAATTCGGCCCGCCCGACTGCGCCG
CCGGCCCAAGCTTCTGAATCTCTGATGCGGACTGCTTAGTCAACTCCGCCGCCGTCCCCAGCAATTCAACACAGTCACGCAGGGCCGCGCTGACCCTCCCCGCGCTGCTGCGCCGCAAC
TCCGCCGCCTGCAAAGACGCTTTTTTTATCTTGGCGAGGCTGACGTTGGCGGCGGCGATGGCGAGGGTTAACGGGCTCTGCCGGACGGTGGATTCGTATGGTTTCAGGGAGTTGTAGCA
GAGTTGGGCGTAGAGAGTTACAGCACAGCTGCGACGGATGAAATCGGAGGAGTTTTCCGTGGCGGCGAATTCCGACGGCGAGATGGCGGTGAGGAGGACGGCGATGAGGAGGAGCAG
GGAAGCCATTGGATG
>comp163077_c0_seq1
CCCCCTTTGCCTCGGGGTAGGAAAATCCGGGTAAGACAAGGAGGAAGAAGATGATAAAGACGATGGTGTTTTGGGTGATGAGCTCGATGAGGATGAGAAGATCACCTCATCCGATCCCC
GATCTTCATCGGAGTTCGATTTCCGTACCGATGCGGTTTCCGATTCAAAGGTTGGTTTCCGATCACAGAATTGACCGCCAATTTTCGTATCTGAAGAATTATCAGTACAACCGTTCTCCTGT
GGCCTGATGAGAATCGGGAAGATGATTCCGGTGAGGCGCCGCCGCCACCGGCGCTGTCGATTGCACGGCCGAAACTCCGGCAGGACATGGACCGCTGTGATCTGTTCCTTGGCCACAGG
CGAGAAGCAGAGAAATTGAGCAATCTTCATCTTCTTGTGAGGTCAGGTGTCGGCCATGGGTTCTTCTCTCATGACATGGCGGCTCGATCGGCAGTTTATAAGCTAGCGGGAGGGGGAAAG
GGGCTGCGAGAAGAACCCATGGCCGACACCTGACCTCACAAGAAGATGAAGATAGCTCAATTTCTCTGCTTCTCGCCTGTGGCCAAGGAACAGATCACAGCGGTCCATGTCCTGCCGGA
GTTTCGGCCGTGCAATCGACAGCGCCGGTGGCGGCGGCGCCTCACCGGAATCATCTTCCCGATTCTCATCAGGCCACAGGAGAACGGTTGTACTGATAATTCTTCAGATACGAAAATTGG
CGGTCAATTCTGTGATCGGAAACCAACCTTTGAATCGGAAACCGCATCGGTACGGAAATCGAACTCCGATGAAGATCGGGGATCGGATGAGGTGATCTTCTCATCCTCATCGAGCTCATC
ACCCAAAACACCATCGTCTTTATCATCTTCTTCCTCCTTGTCTTACCCGGATTTTCCTACCCCGAGGCAAAGGGGGGCAGTGGAAACGGGCGCGGAGAGGCGGTATGCTCTCTGTTTCTTG

GTGGTGAGCTTGATAGTGATGGTATTCTGCGGCCGGATCTGCGCAATTCTGTGGACGGCATCGGGTCTCTGCTTTCTTCCTCGCCGGAAACCGGCTACTGAGACACCTGAGCTCGGTGGT
GTGGAGACGCCAGCGTCGGCTGCATCGGCGCTGCCTCCGGCGGAGGACCGCCTGCAAAAGCGATTATGTGAGTATAACAAGAGGCGGGTTGTTCTGGAAGGATTTCTAGGAAGAAGCA
AACAAGTTTAAAGAAGGCATATAGGAATGAAATAAAGTTGGTACCCAAACGAGTGAAAATATGTTTAGTTTTTTTTTTGGATTTATGGCCATAACTTTTGCTGGTAAATCTATAAGCTAGTC
CTGCTGCTGTTTCAATTTTTTTTTTTAATGTTAATATTGTTAAATTTCTCTGTTCTCTCCAAAATAATGAAAATTCTACTTTTTTGCTTTGGAAGTGGCGCTGTAAACGAGCAGGTTTGAGCG
GACTCA
>comp163081_c2_seq1
ATGCAAAGGGAAGGGATCTAAATCTGGTGCGTCAATGAGATGCCCTGGCTGTCAGGGTTCAGGCATGAAGGTCTCTATTCGCCATCTCGGGCCATCTATGATTCAACAAATGCAGCACCC
CTGCAACGAGTGCAAAGGCACTGGCGAGACCATAGGTGAGAAAGACCGCTGCCCACAATGCAATGGCGAGAAAGTCGTCCAGGATAAGAAGGTTCTTGACGTTCTTGTTGAGAAGGGC
ATGCAAAATGGGCAAAAGATCACCTTCCCCGGAGAGGCTGATGAAGCTCCTGATTGTGTCACTGGAGACATTGTCTTTGTCCTACAACAAAAAGACCACTCCAAGTTCAAAATAAAGGG
CGATGATCACGTCGTTGAGCACACTCTCTCCCTAACCGAAGCCCTCTGTGGCTTCCAGTTCACGTTAACCCATCTTGACGGCCGGCAGCTGCTTATTAAATCAAATCCCGGAGAAGTTGTA
AAGCCTGATCAATTCAAGGCGATCAATGATGAGGGAATGCCAATGTACCAGAAGTCATTCTTGCGAGGCAAACTCTACATCCACTTCAATGTAGATCTCCCAGATTCATTGACAGCGGATC
AGTGCAAAGCTCTTGAGGGCATCCTTCCCTTGGGGATCCCGTCGCAGCTGATCGACATGGAGCTTGATGAGTGCGAA
>comp163085_c0_seq1
AGCTGTCCCAAACGAAGCCTCAATTCAAAACTTGGGAGGGGTGGATTGGCAAATTCCAAACCTAACATAATAAAATTAACCATAGAAGGTTACCTGCTATGTAAGGAATATCACATGGATT
TGAACCTACCTAATTCAACCTATTTAACAAGAGCATAGATCAATAGCCAAAGGAGAAGTAGGATTATTAATGCCTTAATCGTACTAGGGATAGGGTTTTTCCTCAATGATACTTTGATTGCTT
TCAAGAAAGCAAGTAGTAATGGTCAAAAAGGTATATTGCATCCAAATTCTAACCCATTGAGAAAAGAACATTTGGATTCACTATTTTTAAACACCATTAGCAGTCCTTGAGGTTTATAGAA
ATAGCTTGTATAGATTATTTTAAAAAAGATTGATAATGTTTCTATGGAAATGATTGCAATCATTTTATAGCTCAAACTTAATACAACAAATGTATAAAACAAAATTTTACCTGATCATCCCAAG
CCTTATCCTTATTATATATGACAGCAGCACCAAGACTACGTGCAGGATTAATGCCAGTTCCAGTGATTGGAATGGTGGCGAGGTGGACCATAAACACCGCAAATCCAATGGGAAGAGGGG
CCAAAACCTTGAACAAAACACAAAAAATGGTAAGATATTGCATAATAAGGGAATAGGTGTTTATAAAATAAAATTGCAAGATAAATTCATGAGATAGTGATTGAATTAAGAGTTGATGCAA
AGAGAAGTTCAAAGCTGATTTATTGGGTGCCCATGGTGATAAAGACCACAGAGTCTCATGCAAAAAGGAATGGAATCACTAATTGGGTCCTACTTGCTTTATCAATTTGGAGGTTCCCCCT
TCCACTTGGGGGGTCAACATCTTCTTGGCTATAACTTGTTTAAATTGTAATTGAGAAACTTCTAGTAGCTCACAACAAATTTAGAAATGATGACATGGGCAAATTGCTCATTACTTTAATCTT
TCTCTTACAGCTACAATTTTTTCCATCTTTATATGCTTATTCAACTTTTTTTTTCTTGAATAACATTGATGGTGTTGAAAATATGACTAAAAAGTAGGATTTCTTAAGTGCAGCTCATATGAAG
CATTCTAGATTGCAGATCTTTAGATCTCCAAAATTAGAAGATAGGATGAATTTCAATGTTTTTTTTGTAATTAAAGTAAAATATATATTTTACAATTCATGATCTCTATAGTTGAAAAAAAAAA
TTCTACAAACAAAAGTTAAAAATATCTTAGTTTTAAACCAAATAACATGCATCTATCTTACTTACAAAAAAAAATTTCATTGAAAACAATTATTTTGAGAACTGACTTCTAAAATAATTTTATA
AATCAATTTTGAACAACAATCTCTCATAAGCCTCCAATGTATGAAGAACATGCTCAATCAAATAATATCAAATCAAATCAAAGACTACGTAAACTGCTAGTTTAAGCCTGCAAGTCATTTTC
CATTCAGTGTATTCTTAATCTTTTATTACTCATAAGTAAGATTAGTTCTAAATAGTGATAAATGTTGTGAAAATAATAAAATATTTTCAAAAAAAAAATGTTAAAATTCATATGAGATTTAAAAG
TAAAATAAAAAAGAAGGATGATTGTTGTGTGGATGGTGCAGTAATCGGGTCCACATAGCTGTAATGAAAGCGAAAAGAAGACTACAAACGCAATACCCCAGAAATTTGTAGCCCACCTC
CGCCCTACTTCCCACACTAATTATAATAGAGAAGTCATTTTCAACCTTGACTGGATTAATATAAATTTGAGATGCTGGTACTTTGCGAGATTTATGGGAGAACTACTGAAGCATGTGAAGCT
CCTCGTATAATTTATAAAGGATCAATAGAGTTTGATTATATAATCTTATGAACTCATGTTATTAGATATCGGTGTTAAACTAATACAGATATATTTAATTATATGATTTCTTCTTTTTAAATAACAA
CAACAATTAATGAGATTAAAAGGTTTAATCTCGATAACTATTGCCATTTTTTTATAAAATATGATGAATAATAAAATAAATAAAAGCAGACGATTTCGATCATTTTTAAAAGAAAATGTTTTTT
AAAAGAATTGTCATTTTTCTCCTTTAGATGGCAGGATATTTTGGCATATTATTTTTGCAATCAGGTCGAGAGAGTGGCAATTCCTGAGCCTGCGGCTTAATTGTCTTCCTCAGCCTGTTTTTG

GTTTCTTTCCTCATCCGGTTTTTGAGATTTTTCTATCTCTGAGAATTTTATTTTGCTAAACTGTTTTCTTTCTCTAGTATTTGCTTTGGGGATTTTGTTTCTGCCCCATTTTTGGACCTTGTAGG
TTTTGGCAAGGTTTTTTTCTACTATTTTTTCTTTCTTCATTACATGTAACTAGTTTATATTAATGAAAAGGCTACGGCCATAATTTTCCCAAAAAAAAAAAAAACTCAATCCAAAGATAATTC
GTAATCAATGAATACATTGCTTC
>comp163085_c0_seq11
AGCTGTCCCAAACGAAGCCTCAATTCAAAACTTGGGAGGGGTGGATTGGCAAATTCCAAACCTAACATAATAAAATTAACCATAGAAGGTTACCTGCTATGTAAGGAATATCACATGGATT
TGAACCTACCTAATTCAACCTATTTAACAAGAGCATAGATCAATAGCCAAAGGAGAAGTAGGATTATTAATGCCTTAATCGTACTAGGGATAGGGTTTTTCCTCAATGATACTTTGATTGCTT
TCAAGAAAGCAAGTAGTAATGGTCAAAAAGGTATATTGCATCCAAATTCTAACCCATTGAGAAAAGAACATTTGGATTCACTATTTTTAAACACCATTAGCAGTCCTTGAGGTTTATAGAA
ATAGCTTGTATAGATTATTTTAAAAAAGATTGATAATGTTTCTATGGAAATGATTGCAATCATTTTATAGCTCAAACTTAATACAACAAATGTATAAAACAAAATTTTACCTGATCATCCCAAG
CCTTATCCTTATTATATATGACAGCAGCACCAAGACTACGTGCAGGATTAATGCCAGTTCCAGTGATTGGAATGGTGGCGAGGTGGACCATAAACACCGCAAATCCAATGGGAAGAGGGG
CCAAAACCGGAACATGAGAATCGCGGGCGCTGCGTTTGGGATCTGTAGCAGAGAAAACGGTATAAACTAGAACAAAAGTTCCAATAATCTCAGCAGCAAGTCCCGTCCCCTTTGAATAA
CCAGGGGCCAGCACATTAGCCCCACCATTATACCTCACATAATACGCCTTCTGAAACCCCTTCACCAGACCCACCCCACAAATCGCCCCCAGACACTGGGCCACCATGTAAAGCAAAGCC
CTCACCAGCGACACCTTCCTCGCCAAGAACAACCCGAATGTCACCGCCGGGTTTATGTGCCCGCCCGAAATCCCCGCCGTGCAGTACACCAGAATGAAGATCATCCCTCCGAAAGCCCA
GGCGATGCCGAGCACGCCGACACCAGCGCAATCAGAGTCTACAGGGAAAGCATCTTGGTCTGACTGGTGTTTGTAGCCGATGACGGTGGCGATGGTGATGTAGAGGAAGAGGAGAGTG
GCGATGAATTCGGCGATTATGGCACGGTAGAACGACCATTTGGTGAGCTCTTCGGCGTCGATTAGAGGGGCGGGAGGGGGGTCGGTGTAGTCCCTGCCGGTGAATTCGGAGCTGGGGGC
GGCGGAGGATGGTACTTCTAGGCTTTTGGCCATTTGGGTCTGATGGGCTGGACAAAGGAGAGTGAGGAGAGGATGGAATTGGGGGAATGAAGGGGTGGTATTTATTGGGTTGGAGAGTG
AGAGCTCTATCGGGAGGGAGGAAATTATTGAGGATAATAAAATTAAGTTATATGAT
>comp163085_c0_seq7
AAAGAACTTTTTCACTTAAAAAATAATAATCCATCCATATATATTTACATAAAATTTCGTATCATAATCATTTTATATTGGGCAATCATGTAAGAAGCCTGAAAAGCAAAAGGAGCTTTCATAC
GCATGCTAATATAAACTTTTTCAAAAGAAGAGTTCAACACTTTTCAAAAGACAATAAGTGATACAAGAAAGCTAACTTAGTACTCCCAAAGAAAAAAAGAAGAAGAAGATAGCTTAAAG
TCAATAAGGGGAAAGAAAATAAGAGCTCCCTCATCAAATTTGAAGCTTGCTTACAATATCTGCAACAAAGTCTAATTTCCTTAACAACAGTTCCTATTTGAAAGATGGGAGAAAGTAGCA
GTACTAATAGAAGAAGATTGCCTTCAGTAATATTAAGCTATTACACGTGAGGGTTGCTCCTGAATGATCCCAAGGCCTTGAGAGCTCCTGCCCTCAGCACATATTGATGGTATGCTGCTGCT
ATCGCCGCACCGATGAATGGCCCCACCCAAAATATCCACTGATCATCCCAAGCCTTATCCTTATTATATATGACAGCAGCACCAAGACTACGTGCAGGATTAATGCCAGTTCCAGTGATTGG
AATGGTGGCGAGGTGGACCATAAACACCGCAAATCCAATGGGAAGAGGGGCCAAAACCTTGAACAAAACACAAAAAATGGTAAGATATTGCATAATAAGGGAATAGGTGTTTATAAAAT
AAAATTGCAAGATAAATTCATGAGATAGTGATTGAATTAAGAGTTGATGCAAAGAGAAGTTCAAAGCTGATTTATTGGGTGCCCATGGTGATAAAGACCACAGAGTCTCATGCAAAAAGG
AATGGAATCACTAATTGGGTCCTACTTGCTTTATCAATTTGGAGGTTCCCCCTTCCACTTGGGGGGTCAACATCTTCTTGGCTATAACTTGTTTAAATTGTAATTGAGAAACTTCTAGTAGCT
CACAACAAATTTAGAAATGATGACATGGGCAAATTGCTCATTACTTTAATCTTTCTCTTACAGCTACAATTTTTTCCATCTTTATATGCTTATTCAACTTTTTTTTTCTTGAATAACATTGATGG
TGTTGAAAATATGACTAAAAAGTAGGATTTCTTAAGTGCAGCTCATATGAAGCATTCTAGATTGCAGATCTTTAGATCTCCAAAATTAGAAGATAGGATGAATTTCAATGTTTTTTTTGTAAT
TAAAGTAAAATATATATTTTACAATTCATGATCTCTATAGTTGAAAAAAAAAAATTCTACAAACAAAAGTTAAAAATATCTTAGTTTTAAACCAAATAACATGCATCTATCTTACTTACAAAAA
AAATTTCATTGAAAACAATTATTTTGAGAACTGACTTCTAAAATAATTTTATAAATCAATTTTGAACAACAATCTCTCATAAGCCTCCAATGTATGAAGAACATGCTCAATCAAATAATATCA
AATCAAATCAAAGACTACGTAAACTGCTAGTTTAAGCCTGCAAGTCATTTTCCATTCAGTGTATTCTTAATCTTTTATTACTCATAAGTAAGATTAGTTCTAAATAGTGATAAATGTTGTGAA
AATAATAAAATATTTTCAAAAAAAAAATGTTAAAATTCATATGAGATTTAAAAGTAAAATAAAAAAGAAGGATGATTGTTGTGTGGATGGTGCAGTAATCGGGTCCACATAGCTGTAATGAA

AGCGAAAAGAAGACTACAAACGCAATACCCCAGAAATTTGTAGCCCACCTCCGCCCTACTTCCCACACTAATTATAATAGAGAAGTCATTTTCAACCTTGACTGGATTAATATAAATTTGA
GATGCTGGTACTTTGCGAGATTTATGGGAGAACTACTGAAGCATGTGAAGCTCCTCGTATAATTTATAAAGGATCAATAGAGTTTGATTATATAATCTTATGAACTCATGTTATTAGATATCGG
TGTTAAACTAATACAGATATATTTAATTATATGATTTCTTCTTTTTAAATAACAACAACAATTAATGAGATTAAAAGGTTTAATCTCGATAACTATTGCCATTTTTTTATAAAATATGATGAATAA
TAAAATAAATAAAAGCAGACGATTTCGATCATTTTTAAAAGAAAATGTTTTTTAAAAGAATTGTCATTTTTCTCCTTTAGATGGCAGGATATTTTGGCATATTATTTTTGCAATCAGGTCGAG
AGAGTGGCAATTCCTGAGCCTGCGGCTTAATTGTCTTCCTCAGCCTGTTTTTGGTTTCTTTCCTCATCCGGTTTTTGAGATTTTTCTATCTCTGAGAATTTTATTTTGCTAAACTGTTTTCTTT
CTCTAGTATTTGCTTTGGGGATTTTGTTTCTGCCCCATTTTTGGACCTTGTAGGTTTTGGCAAGGTTTTTTTCTACTATTTTTTCTTTCTTCATTACATGTAACTAGTTTATATTAATGAAAAGG
GCTAAGGTCATAATTTTTCCCAAAAAGAAAAAAAAATATTTCTGGTAATTCCTGATAACTTTTCCTTCCTGTTTCTGCACCTTCTAATTCAAGCTTTTTGTCTTTTTTTTTTTATTTGGACATTT
TTTTAAGTACTCTTTAAGCATGCTACAGATGTCATATATAGGTTTTTGTTTTATTTATTATTTTTTAAATTATTGAATTAAATTTATTGTGATACTGAAGGAATACTTCCAAAATAGAGTTTTTAA
GTCGATATTATAATTATTGGAGTGGAAAATAAAAGTACTTATTGAAATTGGCTATATTTTTATTCTGGTGAAGTAATGAATTATTTAATATTTTTCTTCTATTATTAATTTCAATAATCTATAAGA
GAAATATTAAACTGATTGAGATGTCGTGTTTTATTTGCTTGGCTCTGTGTGAAGATTACATGACCTAAATGAGGTGGGGCGC
>comp163085_c0_seq9
AAAGAACTTTTTCACTTAAAAAATAATAATCCATCCATATATATTTACATAAAATTTCGTATCATAATCATTTTATATTGGGCAATCATGTAAGAAGCCTGAAAAGCAAAAGGAGCTTTCATAC
GCATGCTAATATAAACTTTTTCAAAAGAAGAGTTCAACACTTTTCAAAAGACAATAAGTGATACAAGAAAGCTAACTTAGTACTCCCAAAGAAAAAAAGAAGAAGAAGATAGCTTAAAG
TCAATAAGGGGAAAGAAAATAAGAGCTCCCTCATCAAATTTGAAGCTTGCTTACAATATCTGCAACAAAGTCTAATTTCCTTAACAACAGTTCCTATTTGAAAGATGGGAGAAAGTAGCA
GTACTAATAGAAGAAGATTGCCTTCAGTAATATTAAGCTATTACACGTGAGGGTTGCTCCTGAATGATCCCAAGGCCTTGAGAGCTCCTGCCCTCAGCACATATTGATGGTATGCTGCTGCT
ATCGCCGCACCGATGAATGGCCCCACCCAAAATATCCACTGATCATCCCAAGCCTTATCCTTATTATATATGACAGCAGCACCAAGACTACGTGCAGGATTAATGCCAGTTCCAGTGATTGG
AATGGTGGCGAGGTGGACCATAAACACCGCAAATCCAATGGGAAGAGGGGCCAAAACCGGAACATGAGAATCGCGGGCGCTGCGTTTGGGATCTGTAGCAGAGAAAACGGTATAAACT
AGAACAAAAGTTCCAATAATCTCAGCAGCAAGTCCCGTCCCCTTTGAATAACCAGGGGCCAGCACATTAGCCCCACCATTATACCTCACATAATACGCCTTCTGAAACCCCTTCACCAGA
CCCACCCCACAAATCGCCCCCAGACACTGGGCCACCATGTAAAGCAAAGCCCTCACCAGCGACACCTTCCTCGCCAAGAACAACCCGAATGTCACCGCCGGGTTTATGTGCCCGCCCGA
AATCCCCGCCGTGCAGTACACCAGAATGAAGATCATCCCTCCGAAAGCCCAGGCGATGCCGAGCACGCCGACACCAGCGCAATCAGAGTCTACAGGGAAAGCATCTTGGTCTGACTGGT
GTTTGTAGCCGATGACGGTGGCGATGGTGATGTAGAGGAAGAGGAGAGTGGCGATGAATTCGGCGATTATGGCACGGTAGAACGACCATTTGGTGAGCTCTTCGGCGTCGATTAGAGGG
GCGGGAGGGGGGTCGGTGTAGTCCCTGCCGGTGAATTCGGAGCTGGGGGCGGCGGAGGATGGTACTTCTAGGCTTTTGGCCATTTGGGTCTGATGGGCTGGACAAAGGAGAGTGAGGA
GAGGATGGAATTGGGGGAATGAAGGGGTGGTATTTATTGGGTTGGAGAGTGAGAGCTCTATCGGGAGGGAGGAAATTATTGAGGATAATAAAATTAAGTTATATGAT
>comp163157_c0_seq4
GCTGTGTTCTCAAGATGCAGACCGATGGAAATCTCGTAATATATAGTGGCAGCAGAGTGATTTGGGCGAGCAATACCAATCGCCAAATTGGGAATTATTATCTCCTCCTCCAGAGAGATCG
CAACGTCGTCATATACGATAGCGCTAACAATGCTATTTGGGCGACTGGAACCAACGTTGGCAATGCTGCTGTTGTCATCATCCCGCATAGCAACGGCACGACGGCGGCGGCCGGCGCGGC
GCAGAACAAGGTGAAGGAGCTGCATCCTTGAAGAATAACATCAATACAGAGACGATGAAAGAAGTATTAATTATTAGTATATATTTTGAATAAAAAGGCATTGGCTCCTGCGATGCTATTG
CCGGCCTAAACAAGTTAATTTTGCAGTGATTGTGTGTTGCTGCGGTATGAAAGACTGTATCAAATAATTAGTTTCTTATCTAAATTATATATTTCACGAGCTTTTTGCTGGTTAAATTATTGGG
TTGAGTAACGAATTTGAATGAATCTATCTGCATGTAAATAGGAACGAGAGATATAATTTGTTCAAAGGAA
>comp163176_c0_seq3
TTATTATTTTAAACAACATTTCATTAACATTCTCACTTCTTTAGCAAAAAGTTCTTTAAATGAAATAAATACTCTTAGAAAACAAAGAAAAATGAAAAATTGGATAAAAGATAAGAGAAGA

AATGAATGTTTTACAAAGTTTAGACATAAATAGCTATTTCATGATGATCATCAAAAGAACAATCCCCCTTTTGCTTCCATGAAATCACTGATCCTTTGGCTTAGTTATAGCTGATTACTGAAA
TCTTTGCCTGGAGTCCTCCTTGAACATAAACAAAAATGAATGGTTTGTTTGGGTTCAAAACCTTGAGCCATTCAGGAAGAACATATACATTCTTCTCTTGTGTTGGTGAAAGTCCCCAAAG
AGGACCTGATAGGTTCTCTATTGCCAGCTTTAGATTGGTGATTGGCTTTCCACTTTTGTTTATGATAGTCACCTTGTGCCTGTAGTATTTAACTCCTTTGTCTGAGTACCATGTATTAGTGATT
GAATGGAAGAACTCTACAGGATTCACAATAACTGGGGAAGGGCTTCTTGCTATTGTAGGAGGGTATGGTTTAGTCTCTTTGGCAGCATAACCTGTCGGAATGATTGGGGTTGGATTTTTTT
TCTCCCCTGAAAGAAATCCTTGTGAAAGGGAATGCAGCTTAGCGAATAGGCCAACAAGAGGGGCATTTCCAGCAATGGCAGGTTCAGTTTGCTCATAATTGGACCGTTGATCAACAAAC
TGATCTTGATGATCAGGACCTCCCACAAGAGCTCCTAAGATAACGTTTGGATTTCCCTTTTTGCTATCATACCATTGCTCAAAGCCTTGAATGCATCCCACAACAGCAGGAAGAACAGATT
TTGACTGAATCGAAGCACCTCTATGATGCACATTGATTGGATACTGCCCTTGGTACCCAACCAGGTAGCTCATCAGTTTTGGATTCTTTCCGAGAATGTAATCAGCCTGTGATTGAGCAAAC
TGAAGGATTTGATGAGGAGTGACTTGTCCTTCAGGGCAGTTGAGATTGGAGTTGGTTGGTGACAGATAGTCAGCATAGACCGCCATTAAAAAAGCTGAAGATGAAACATACTGCATGTTG
TTCCACTCATCAAAGTATAGCAGGCCTCCTGGTGTCATGGCAACATTATAGCCATTGTTCTTCTGAAGTGAAGAACAGAGGAAGAACTCGGCCTTGGCCTGGTACTGCTTCAATGTGGCA
GTGTACGATTCACTTCCTCCTTGCTTTAAAACCTTTGCCATAAGTATTTGCAGGCCTGCATATTTGTTGTCCCATGAGAACTCTTTAACCGCCCATTCAGTGCCTCCTAAAGAGCCAGAATT
GTCCACCACATATTTGAGATATTCTTCATCGTCTGTGGCCTCAAAAAGCCAAGCAGCAGCCCAAAGTAGCTCATCATCGTATCCTGAAGAACGATAGAATTCCCGAACAACTGGGATTGAA
TCATCATATTTTCCCCTAAATGTATCAGCAAATGTGAATAGTTGCTTGGCATGAAGCAGTAGAAGCTGCGAGTAAGAGGAATTGTAAGGTCTGAAAGCTATGGAAGCTGCAGCCAATGCA
GCAGCAGTCTCTCCAGCAACATCTGAGCCTGGATTTTCAGTATCAACCTTAAAAGCAGTTCTTGGTGTTGTCATGTCCTCTGCCCTCTCCCAGCAGTAATGGTCAGTATTTCCATCTCCAAC
CTGAGCCCATAAGAGATTGGGCTGCTTGTGAGCCTTGATGAAGTAATCAGTGCCCCACTTGATAGCATTAAGAGCATACTCCAGTTGGTTAGCAGCTGCTATTTCTTTCTCGAACTCGACC
AAACTCCAAGATAGCATTGTCAATGAATAAGCTATTGGTAGGCCAAATTTCACATGATCACCTGAGTCATAATAGCCTCCAACCAAATCCACTCCTTGCAGGAAGCCATCTTGAGTCCGGA
ATCACCACGCCACTTCACTCTCTGATCTGCCGGAAGCTTGCCAGACCTTTGAGCCTCGAAGAAGAGCAAGCTCTTGCTGAGAGCATCTCCATAGTCAAACCCACCACCAGCTACAGAAG
ACAAGGATGACAATAACAAAAAGGAAGCTAAGGCTAGAGGAAAGAGGAGATACGCCATGCCTTTTGGCTTGAAATGAAGCTGGTAGAGAGCAGACAATGCAGAGTCAGATGGGCTGTG
GAGATTGGTTTGTCTAAGTATTTTGGCGTTCTTATAGGGATCATAAGGTGAGGGAATTATTCTTCTTTTCAGATAGTTTGTCAGAGGAGCTTACGAGTGTTATGTCCTTTGAAGCTCGTCCCA
AGAGAAATTGTTCAGCTAACTGCATAGCCCACATGCAGGCGCGACGGTGGCGCTAGAATGCAAGCACTTTCAGAGCGACGCGGTGGAGCACAGCATTGAGGCGGTGAGCGGCGCCGGC
GGCTATTACACCATCAGCGTCCCCGACGACCACGAGGAGGAGGTTTGCTACGTGAAGCTTCTTAAGAGCCCGCGAGCCGATTGCGCTGAGATCGTCGAGAACAGAGAGCAAGCGCGCA
TTCTGCTCACCGAAAATAGCGGCCAAGCCAACAACTTGCGGTATGCCAACTCTTTGGGCTTTCTCAAGGACGAAGCCCTCCCTGTCTGCGCCGAGCTGCTCAAGGCCTACTCACTCGATG
ATGATTATTAACTAATTCTGAATCTTCGGCCAGCGAGGTAGCTGGTCCAGAAAGAGTTTATTAGAGATATATTGATTTGTTTTTTTATTGAAATCATTGAAACTTTTATATTGATATATTTGTGA
TTGATTAATGTGAAGAACTTTTTATGGGAGTTGAGTTTGGTCTTTTTGGATTTAAGCATGTTGAGAAGTTGATGTATCTAGTTGAATATTCAAAAGATTATTATATG
>comp163180_c0_seq7
ACAAATAACATCATTCTCCTCAAAAATGGTACGCATGTTGAGTTCATCAACAGCAGTTCTCCGTCTCCCGTTCATGGCTTCAAACGTATCCCGTTCATGGCTTCTAGTTTTCCATGGGTGAT
AGAATGTAAAATCTCTTTGGGCCACCTAGCATGCCATCTGCCTTAAGATCAGTCCGGAGACTTAGTCGTGATTCCTCTTTGTATGAGGATTTCGTTACTAGAACACAGCATCTTGCCAATAA
TTATTGGTCATAACAACAAAAACAAACAGGTCTTTAGAAGTACAAAAATCTTTAAAGAGGACGTAC
>comp163227_c0_seq12
ACGGCGGAATCTGTAACGATAAGGAAATATTCCACTGATTTCAAGCGAATAATTTCAAGAAATCAAGAACATCAAATTGAGGGAATCTTCCAATATATAAGAAAAAAAAGTTTAACAATAT
CTAACTTTATATCAAAAACAAAAAAGTACATATAGAAAGTATTCTTTCATCCAACAACAAGTTGAATGGTTGAAAGAATCTACGACGAAAACATTTCCAAGAAAACAAAAAAAAGAAAG
AAACACACCAACAGTTCACAGAAGCAGAACACCAATCTTCAACAAAAGGCCTAAATCTGAACACACACAATGTGCCACAAAGGGCCGAAAAACCCACATTCATCCTACCTAAAATCCA

CACAGAGAGCAACCTAATTCATTTGTACCTATTTTTCTACCATTCCGGCCTACGCGCTCCCCTCTGCATTCTCCTCCTCTTCTTCATTCTCTTCATCCTCTTCTTCC
>comp163228_c0_seq1
CGGGCTTGAGTCGGTCACTGTACATTATGTTCATAATTGATAGCATGCCCTTGGTCCCACCACCATTACTGGATTAAAAAACACTGAAACGAGATGGAGATTTTTCGGTTATAGATCGATCG
TTTTCTAGTTCATCTTTTGAATCCTCCCGCCAATGCCAACGATTGCGCTCGCAACCCTTTTGGGATAGAGTAGACATAGCCATTGCATCCTTAGCCATAGACATGAAAATACATATAATGATA
TATGAAG
>comp163234_c0_seq17
GCGGAGTTTGGTTCGAGGGTTAGGGGAAGAGGGGAGGAGGAGGAGTTCGTGGATGGTGAGATTAAGAGGGGATGGGGAAATTAGGGTTGGTGGGCTGTCAAGTGGATCGTCGAAGGG
GTGGGAGAGGCGAGGAATGGGCCTGGATCGGAGACGGGGTTGGGATTTGAGGGTGAGCGGAGTCGTCGGCGGTGAAGAATCCATTGCCGGCGGTGCTGGGAGAGAACTCATTAGGGT
TTTGGGGAAGGAGAAAGCCAAAGGCCTCTGTATCTCTCTCTCTAGCTCTTTCTTTCTCAAATAGTTTCCTGGAAAGTTGAAGGAGAAAGAAGGTATATAAATGTCTCTCTGTGTCGATTTC
TTTTGGGTGTTTTGTACCTTAGTTAAAAGAGGAAAAGTGCGAAGGTTCGGTTGAAGAAGGACAGATTATAGCCGTTGGAGATGGGAGTCATAGTTTTATTTGTTTACAGTTTTTTTTTTCT
GGAGTGGTGGGGTCCATTTTTTTAAATGGTAAGAGTAACGGGTCTGTGAATGCCTTAGGAATTTTTTTTTTATTTAAAAAAAATTTGTTAGGTTCTTCTGATGAGATTTAACAATTTTAAGACT
CATAATATTTAAATGTTATATGATAAAATTATGACTTTGATTTCATGAACAAATTTTAAAAATAATAAATATATTTGTTTTAA
>comp163234_c0_seq25
TGATTATAAATGCAATATTTTGTGCAATAGACAATTTAATGGATAATAGAAGCATTACTGTTTTACAAACTTGTTGAGAAGAATTTAACAAATCATAGGGAGGTAAAAAAGGCTAGTGTGAT
GATTAACAGTTTTTTTTGTAGAGAGAGTTACAATTGTGAACCAAAAAATTTGACATCTTATACAGCTTTTGCAGCAGTGTGTAGTGTGTACATACATGTAGGAGACGGTACATTATTTAGTA
TTAGCAGATAACCTTGGCGGGCGGTTCCTAACCAAGTTTCTGCACCTTTTAACTTTGCCTGTGCTTTCTCTTCTATCCTGTTTTATTGATGTTCTCTTCTTCGCCCAGTCCCCCTTCTTCACG
GCTGTCCTTGTTAACATCTTCTACTTCATATTCCTGAATGTAAGGTGACTGTTCATGATGATAGCGAAGGATAACAACAGTGATGAATGCTGCAAAGATTCGCATCTTTACACTGAATATATT
CCATAACAGAGCTGCTACTGATATAGCTACCAACTTCTTCCGAGAACATGATCTCCATGACTCTAAAACATAGCTTCTTCCTTCTTCAACTCTCTGTTGTATCTGCTGGTAATAGCAAGAGT
ACAATTTAGGCACAGTGAAGCTTGAGAAAAAACCTATTGCTAGAAGTCTCCAGGGAGTAGTGAGATGACCATATTTGGCCATGAAGAGAAGAACTGGAGCCACCTGCAACCAAAGCGA
ACACAAGTGATTCCAACAAAATAGGACAAGGAAACAGTAAACTATGTGTCCATTACTTTGAGAGTCATCACTGGTTCTCCAGAGAAAGTTTCTTGGCTTTTTGAAAGAGCAGCATTAACT
ACTGGCAAAATAATCCGAGCAACTCGAAGAAAATCCTCTTCTGTTAGCAGCATCGGTTTGGCTTTCTTCTTCTCTTGCTTTCTGTAAAATCATACTTATTTTCTGAAGGTGCAGAGTAGAAA
GAAAGAGAGAGAGAAACAAGTACCTCTGTCTAATCGAGTCGTTGAGGAATGCCAAGCACAAAATCAGAATTCCTAAATGAGATACTGCTGATACTATGCTGAACCCAACATCACTTGAG
AGAATGGAGGAGAGAAATAAAATCGATCCAAACCCAAACCAAAGACTAGATTTAGCAACATTCTTCCACATCACCAGATCCGAAACTGCTTTACATATACCTTCCAAGCTACTTTGTTCAT
CTACAGTAATGTCATCTTGTTCTGAACCTCAAAAGAAAAACTAAGTTACCATAGCACCAACAAGTAGCTTAGGAAGAAGGAAGACACGGGATCAAGCTCAGCGTTTCCTTTCGAGGAA
>comp163267_c0_seq2
CTTCAAATCTCTATGAATAAAGCTTTGATGAGCAAGACTATGCAAATACTCTACCCCCCTCGCTACATCCAGGGCGATGCTCAACCTCTTCTTCCACTCCAAAGGCTTCAAACCTTCTTCTT
TCCAGCCAAACAGGTGGCGGCTTAGCGTCCCCTGTGGCATATACTCATAAACCAAGAGCCTCTCAGACCCTTCGGCACAGTAGCCAATTAAGCCGACAAGATTAGGATGATGCAGCATCA
TTAACATTAGAACTTCCACAAGAAACTCATTACTTCCTTGTAGCCCATCTCGATTAAGTTGCTTGATTGCCACTACCTGGCCATTTTCAAGTTGGCCCTTAAAGACCCTTCCAAAACCACCT
TCGCCGATCAAATTTGCCTCCCTGAAATTTTGGGTAACCATCTCGAGTTCTTTAAAAGTAAAACTTTGTGTACAGTTGCTATTATCTGCTTTCGGAGACAACGGGTTTGTTTCATTCTCTGC
GAATTGAAGAGCCATGTAAATGTTCGATCTTTGGGGTTATAAGAAAAAGTAAAACGGGATTAAAAATAATTTAAAAGAAAAAAAAAAAAAAAACACCATCAAATAATAAAATAAGACCAA
GAATTGCCTATATATCCTGTAACAGAAGACCCTCAAAACCCATTCAAATCTCCTTCTGAAGTGTCCATAAACTAGTTTCATATCGATCCATGGCTTCTTACAACTTCATCCCAGGGCCGTATA

ACCAAAGCCCTATCGCCATTGATGAGCACAGATGGCTCTCTCTCGTTCAAACTGCTCTAAGCGAGACGATTGACGAAGAAGATGCAAAAGTTAAGGTACAGGTCTGTATCTTCTTCGTCC
CCAAAGGCTTGTTTTTTCACAAGCCAGAAGCTTTCATGCCTCAGCTGTTCGCGATTGGGCCTTACCATCACTGGGTTCCTCATCTGTATGATATGGAGCGTTATAAGCTTGCAGCTGCAAG
GAAGTTACAGTGCGACCTTAATTCTCTCTCACTGGAGGAACTTGTTTGCTGCCTTCTGAAGCATGACCAAAGGATTCGCTCTTACTATCATAAGTACCTGGATTTTAGTGGAGAGACACTT
GCCTGGATGATGGTGATAGATGCCTCCTTCTTGCTTGACTTCCTTCGCAACTTCATAGTTGATGTAGAAGCTCAGAAAGGCAAACAGAAATTATCTCAGTGGGAAACTCCAAGGATGAAG
ATGGCTTACACCATGGTCACCCGAGACATTATCATGCTTGAGAACCAGATGCCCTTCTTCTTACTTAGAAAGATCCTCCGTCGATGCCGACAATTCTCTTCTGCAAAACTTGCAGAAGAAC
AACTCTCCCAGATGCTTTTAGGCTTTGTTAAGGAGGTTTCCCCTTTCAATATAGTTCCTAGCATCGCATCCATTGATGTCAACCAATATGATCATCTTCTTCAGCTCCTCTATTTCATGATTGT
TCCCAAAGTAAACGAAGAAGAATTCGATCAGCCACACGAGATTGATTTGACCGATGTGCTTGATTTGGAGGAAGAAGATGGAAAAAAGCATGTGGGGTCTATGTGGAAGATCTTTAATTC
TGTTTGGAACTATGCCTCAAATGTGAATAAAGGAGCTACTATTGAGACAATCAAAAAGGTTCTCATCAAAGGTCCAATCAAAATCTTGATTAAGCTTCCATGGAATATCCTCACTAGCTTCC
CTGGGATCTCAATCATAAAACAACCTATTGAATCTTTTATCTGCAGTGGAAGTGTTAAGGAAAAGGATGATGATGATGTTGTTGTCCCCGATGGAAAGAATTCTGTTTCAAAGCAGCCATT
GGTTGAAGAGATAACCATACCATCAGTGACAGAGCTAACAGATGCAGGAGTCAGATTCATGCCTTCTAATGGAGATCTGACAACAATAAGGTTTGATAGGCAGCAAAAACCTGCCACCAT
ATACCTCCCAAAGGTGAGCTTAGACATAAATACAGAAGTAGTGCTAAGGAATTTGGTGGCTTATGAGGCATCGGCAGAGCCAGGGCCAATGGTCTTCACAAGATACACTGAGCTCATGAA
TGGAGTTATAGACACTGAGGAGGATGTGAAGATACTAAGGGAGAAAGGGATCATTGTGAATCGAATGAAGAGTGATAAGGAAGTAGCAGATCTTTGGAATGGAATGAGCCGTTCTGTAA
AGCTCTCAAAAGTTGAATTCATGGATAAATTTTTAGAAGATGTGAATAGTTACTATAACAGCAGGTGGAAAGTGAAGAGCAAGAGATTTATAAGCAAATATGTGTTTGGATCTTGGCAGTT
TCTTACGTTGCTTGCTGCCATTGCTCTGTTATTACTGACCTGTTTGCAGGCCTTCTGCAATGTTTATAGTTGTGGTAAACGTTGGTTCAACGTTAAAGAAGAATAGTAAATGGCCAGTTAGC
TTGTGGTATGATTTTATAAAGGAAAAGGTTCCCTACGCCATTGCTGATCAATCTACTTGGTTTGTTTTGCTTAAAATTATGTATGATAATGTGTTCAAGTGGTTAAGTGAGACGATGTTATCG
TTGGATATACATGATTGTATTTTTTTTTTTTTTTGTAGTGGACTTAAGATGAAAGATTATTTTTTGTACATCATGATAACTCAATCAA
>comp163320_c0_seq1
TGCAAGAAATAGAAGAATGAAGACCTGGAAGATTCACACAAAGAGCTTATAGGAAAGAAGATATTTGTACCAACTCAGTTGTTCAAATAATCTTATATTCAAATATTTATGAAGCTCCAAG
GAAACAATATGTATTTTACCTACACTAAAACATTAAATAATCTCATTGCCAAAGAGGCATGATTAAAGCTCCAAAAATATCCAACAGAGCCGATGCTAATGATTCAATTACAAAATATGACA
GATATTCAAATGTAGGAAGAACCATCTTCAAACACAGGCTTCCCTTAGACTCCATTGTAGCACAGAAAGAACAAACAGAATTGCAATCCTCAAGAGATTGTATGAGGAAAACTATGTAGT
TTTGTTAGCTGTTGAAGAAAGAATGGTGGCTCTGTTGGCAATGATGTCTCATCTGTAGCTAATCATCCTTTAGGAAATGAAATTTATTCTAAGAAGAACAATTAATTATCTACATTCATCTCA
ATCTCATATAGAAGAGCTCGGTAAGCTGCTCTCCAGGAATCACTTTCTGAACCGCTTCTGAAAACCATTTGCATCACCTGAAGTTCTTCTGGGTTTAATCTACGAAAGATTGACCTCAGGA
CATGCTGGATCTTATCGTCTGAAATTGAATCAACGGGTTCTAATGTGCAAATGTCAATGCCAAAAAACTTTCCTTGTGTTTCTGCTGAAGCATTTGGTTGCGAAGAGTAATTTGTTGATCCA
AATCCAAATCCACTTGGTTTTTTCTTCATAGATTTCAGGGTAGAGTTCATATTGGCTTCAATTTTTGTTGATGAACTCGACTTGGATGCCCAGTACTCCGAGCAGCGACGATGAGGATCGAG
GCATTCAATAAACTGATATATTGATGGGGACAGAAAACCAAACATTTCGAGCCCATCGATGCTCCCAGGAGGTTGAAGGGGAGGGAGTTCTTGGTTCATTAAGCTGCACTGCTTTATGATC
TCTTGGTTTAGTCTCTCTCTAATCAAATCCCAGCATTGCGAGGGAGAAGAATGAATAAAAGTATCTTCTGGATTATCTTCCAAGGTAACCTTTTGGGAAGATGGCTTTTGCATTACACCAGT
GTCTTCCAAACATTACAGTTCCAAGATGAAGTGCCTCAACAAAGTACTTGGATTTGGTTAAATGCTTCTCCCTTTCAGCATTAGATGAACTCAACCCAGTGGAATTGGTTGAGCTGCTGTT
GAAAGGCCGGTTGCCTTCAGAATTTGAATTTAATAGAAGATGTGGTTCATGTGGCTGAAGTTCAATGCCAAACAACTTTGCCGAAGACTGACTACCTTTAGAAGGAGAAGCTTCATGCCT
CCATTTGCTTTGAAAAGGAGAGGAAGGAAAAAACTCAAAAGAATTGCCAAGGGGAACTGAAGATCCACCTCCAGGGCTTGACCTCAATGGTGTACTGGATGTTGCTATGGAATCATGCT
CCATATCCTGTTCTACTGCAGTCGAAGTACAATCTTCCTCCTTAATGCTTTTAATTGTCTCGCCATTTCTATAAGGCCAGCCTTGAATTTCATTCTTATAATCTTTGGTTAAGTTTTTCTTGCTG
AGGTCCAAAAACCACTTTGGCTCCTCTTCCCATACATTATCATCATCAGAAAAGATTTTATTTTCCTTGAAAGCAATACCGTGGCGCATATTTTCAGTATATTGTGGAGTTGCTAAGTCAAGA

TTAATATGGGAGGTGCCTCTATTCAAGTTATCACTTTCCAATTCTGTTTTTACACGAGTTGTGCAAGATGTTTCTGGCTTCTGCAAACTTGCATTGGCTTCTATATCACAGTTATTTGTAGGC
AAGCTCGGCCTACTAATATCAAAAACTGCTTCCATATGTTGTGAGCATGCCATACTATTGATCTCACTAGGACGATCAGTAGCTCCACATGAATGCCCTCCACTCTTCAGACTAAAGATCTC
AACACTAGCAACGTTGATATCTACCAATCCTTTCATTGAAGTGGATGTACTTCTCTGCAATGGTACCTCGGAATCAGATTCACCTATCAGTTTTAAGCTTTCTGAATCTTCATTCATTACAATA
TTAGGTGAAACCAATCCCAGATCATATGAACCCCAATCTTGCACTGCACTTGCATACCCTTCTAAAGCTTGAACTAGAGTGTTCAACTGATCCATAGTGTAGCGAAAAAGGAAAAATTTTT
GGTTGGGTTCACAAGAACAAAGAAGCTCTGCATGGTTTAAGCAGGCATAACGATCCAATGAGCATTCACAGCCAGCAGCAGATAGGTGAAGATCATAAAAACAGAAAAAACATTCTCTC
TCCTTTGACAAATCAAAGATACTATCCATTTTCCTTGCTTTTGTAGGATCACAATGAGAATCTCTTCGTTCCTGTTCCATTTCTATTCTTTCCAGTATAGCCTTCGTCAATGTCCCTTCCTTTC
CGCAAACACGCTGCCATTTTAAGTTACTGGGTTTGTCAGATCCTAACAGAAAAATCTCCCAGAGAGCTCTGACAGCTTCTCTTGCAGCACCCATTAATAACTTGTCATGGGAAATGGATGT
CTTGCGGCGCTGTTCGCTATAAAGCTCAACCGCACACTGACCATGAGATAACCAGTCCATGGGAGCAACATTCACTGCTTCTGCACAGTTGAATCCACAATTG
>comp163349_c0_seq1
CCTATTGAATTTTGTTTCTATTAAACTTAAAGTTTGTGGGTTTGGCATTGAATCACATTTATAGACCAATATAAATAGTTCATGGACTATTTTTATCTCTCTATCGCACTTTCTCTCTCTTTCTTT
CTCTCTCTCTATAAAAATCACACACAAATTTAACAATTACAAGTAAAAATTCATGTAAAATTCACACAAAAAAGGGAAAAAACACAACAATTATGTTTTTTTTACTGAGCATCTGATTCTTT
ACTATTCATTAATGCTAGTTTCAGTCAGGTAAGTTCTCTCTGTTGGCGAACCAAGAACAAAAGGAACAAAGCCTCTGCCTCCAAAAACTGGCCGCATGAACGAATCATCAAATCTGCGCC
AATGGTGATGTACTGATACGTAGGGCTTGGCGAGGAGCATGCGAAGATTCGGTAGCCCAATAATACTATCCCCTGCTGCTTCAATCTCAGATCCCTGGCCACTTTCAAGAAGCCGGGAAA
GGAAGGATTTTGGACTTAAGGGATCCGAAGACATGCTGCTCAAATGCTTTGGTGGATGAGGAAGAAGAAGCCTGATTAGAGGCTTTGTGAGTAAGCCAAACACCACAGTACTAAACAGA
ACAACAGTGATAGTACTGGTGATCATGATAGCATTGCTTCGCAGCTGTGTGTGGCCTGCTCTTGTGAACTGGTTATATGCAAGTGCGATTGAAACTGCTCCTCTCATGAGTCCAGCCCACC
ATATTATAACTTGCTGTCTGAAGTTGATCTTCTCTGTGGAAGACTTTTTGGCAAGGTTAGACAAAAATGAGAGTGGGAAAACAAATGCAGCTCTTCCCACTAAAACCAGGCCTACAAGCA
GTGAGCTCAAACTAACTTGTTTTAGAGGGCTGTCACTAACAAATCGCCACTTCTCAATGTCCAATGCATCCATGCCAACATACAGAAAGAGAAATATCTCTGAGATAAATGACAATGTTGC
GAAAGCATGCTTTGTAGTAGTTCTAGAACTTTCTGTGACATTGTGCCAAGTATAGTGTGACATTACTATCCCACAAAAGAATACTGTGAGGATACTGCTTAGATCTAATAGTTGAGCAATCA
TGTAAGACAGATAAGCCATGAGCATCATAAGTGATACTTCACGATCAGTAGAATGTCTGCCAAAATATAACTTTTTTATGATATAAGCACTAAGCAGACCTGCCACTGCTCCAAGAATGGTG
CTCGTCACAAATAGATAGAAGAAGTTTCCAATAAGCTGAAGAACAATAACAGCATCCAAGTGAACAAGATCAAACTTCTGGATAGCATTGAAGAGCACTACTGACGTTGCATCATTGGCA
ACACCCTCACCAAAGACCAAGCTATAAAGAAATGGTGTCTCATCCTGATTGAGCACCTGTAAGGTGCAAACAGAATCTGTTGCGGAAAAGATTGCACCGATCGCAAGATAATCACCAATA
TCCAGTGAACCTATATTCAATCTTCTAAATAATCCAATTGCTCCAAATGAAATTATCGTAAAAGATATCAAGGTCCCAGCTACACCAAACATCATAATTGTCAAGAAGTTGCGAAAGAACTG
CTTCTTTTTAACCTGGAAACCAGCATTAAATATAATTGGCGGAAGCAGATATATGAAGAATAGATCCTCACTGAAAACCAGAATACGTGAGCTTTTCCCTTTTGTGGTAAGAAGAATAACA
ACTCCACTTATGAGCCCAAAGAAGAGAGCCGTTATTGATTCATTCATCCAGCGATTCTCCTCCAGCAGGTGACCAATAACAATACATGCACAGAGAAGAGCAACAAAGAGGGTAATTGAT
GCAACATAGGAATAATCAGATTTCACAAGCAACTTTGTGTTCAAAAGCAGCCCCTCCATATCCAAAGCCATCTTTCTCTTCCTCTGAAAAATAACTCCTTAAGCCTCCAAGATCACACCTT
CTTGAAAGATAAACTTCCGCACAAAGGCTCGGATCGTCTCCTGCAAAAGATTTCAGAAGCTTACACCACAATTCTTAGCATTATGTCCCTCAATCGCTTCCAAACTTTATGCACAGAAGCA
ACCCCTTCAGATCCAAATACCTCTTTCTCTTCCTCTTTATCAACGATAAACTTCTATTCAAAGGCTCAAGTCCGTCTCCGGCGAAACATTTAAAAAGCTTTCTCAATAATTATTCCCATTATC
TCCGTCAATCGCTCCACTGGTGTCTTCGCCGCCGCGGTGGATCAAAAAGTCTTCGAAGACAAAATGAAAGGCTTACAGGAATAGAGAGCGACTCTTTCCTCCCTTCTTTAATAATGTCGT
CGTCGTTTCTCAGCTCCACAGTAATTCGAGCACTTGCGAGATCATTGGATGCATTATACAATAGGGGTTTCCGATGCCCGAGCTGAGAGCGAGCGGATGATCCGACGAACTCTGGATTGCC
GTATGATTGACGCTTTATAATTAGCCAAATCTGGCTGAAAGGGCGGATTCTTCGCTTGCTCATTGTACATGTAACGGCTCCCCAATCTCAAAACCATTTGGAGGTTAAAGATTTCCCTTTCA
TCTCTCTCTCTCTCCG

>comp163391_c0_seq10
AATTTTAAACCTTAAGCTCATATACCCTTGATCCCTATTATTTACTCAATAAACATGGCCTTGCTTAATATAAATGCTTGTAATGCTCTTCCTTATCATATAGTGAAGCTTTCTCCTTACCAATTT
GCATGATAATGAATAAGAAAACTGCACAAATATTCTTAGATAGAAAAATGACAATAAAATATTGTATGCATCTTATCATACGAAAACATTACATATATTCTTAGACAATAAAAATGTATATCTT
TTACTATTTCATTTTACTCGTGAAGTAGTGGAAGGAAACCATGTCCCAGTGCTGCCATCCAAATTTTGTCCACATGAAATACATTCTCTCCACATTCATTTGCATCCCATCCCTCTAATCCAT
GAATTATTCCAGCAATACGCCATGCACTCATTACCCTTCTAGGCAGCCAATTCTCACATGAGTGCACATTTTGCAATTCATTTGGAATCTTCATGGCTGGAGTACTATAGTAAATGCAATCAT
TTCTAACTTTTTTGGGAGGAAAATAAGAATATGGAATGAATATTGTTCCATTTTGCGCCTTCCATTGCTCTACATCATCTATACCATCTCCGACTAACCAAACCTGAAAGACAAATTTTTTGA
AGTCATTTTCATGTATTATACTATCACAACTATAAAGGATGTTCACTGATATAAGGGATGAAAACCAATTATGAATGCTTAACGAATGCAAAACTTTTATACACGAATGTTTAGATAAAAAAT
AAATTAGGCATCAATATATTTGTAATATATTTATAATGTGCCCAAGTGATTTCATTGTTCCTTGATTTGTTTTATGTTGGTTGCATGAGTGCCTAAGAATGATAATTGAATCATAATGTTGAATTA
TTTTTGTGCTATTGATTGCATCAAACTCCTAGCTATTTTTTAAATGTTTATGATGAATCCTAAAAAGTTAATAATACTTATAGACTTTTCTCAATCATTAAAAATTATATTAGTTGAACCATTCTC
CCGTCATAAACTAAAGGAACTCATTACCTTAGGCATATAATTATTTGAAAGTATAAGATGACCCTGTGTAGTTCCGAACATTAATGGCTCCAACATACAGTAGTCATGCGTGTTTGTTATTAC
AACCTTTATACCTTTGTTGCATAGGCTCTGAATCACTGTGCAAGCAACCTTTGAAATGTTACCTTTCAGTAAAATTTGTTTTGTGCCTTTGGGAATGTTGTTAAGAATCACAGCAACTGCCA
AGCTACTTCCATCCACAATCCTTAATTTTAACTTTGGATACTTTTGAAGGTAAAACTCACCACTTCTATTGAGTTCTTTCACCTGATTGAAGAGCCCTAAACTTAGAACTTTAATTCCTTTGG
TTTCAGCCTGTAGAATAGACTTTTCAATCAAATTATTGATTTTCTCCTTCTCCCATGATAAGCCATATTGGAAATTGTATATTGGCATGACCCATGTTTGCATTTTGATGTTCTTCAACTTATTT
CTCTCAACAATGAAGGAAGAACTATAAAACCATGTGAGCAACATCGACATCCATGATAATGGCAACATCATCCGATCATACCACTTAGAATATTTGTATGGTTTAGAGGCCAAAGATGCAA
TTCCAAGTCGCAGATGATATATTGATTGCAAGGTGGTTGGATGTGTAAGGTGAACAACATCTATAGTCTCTTTCTTTCCTTTCAATGAGATTTCATAGACTTCATCAGTTAACTTATCCCTCG
TGTTGTACAAATAGTCATAGAATGGCATGAATAAGGAATAGTTTGTCCGAAATTGCGTGTGATGAAGAGAATGAAACGAGGAAGTGTATATGAGGTATTTCAATGGTGGAAAGATAGAAA
ACAACCACTTTGGAACCAATTCAAAGTTGCAGTGCCCCATATTGTTCATGAAGTCAATGTAGAAAACATATCCGACAAATTGTATCGTCGAGGCTGTTTCTGTATAGGCTGTAGTCAACAA
TGGGACAGCGAACAACATAGAGTAGACAATATGCTCCGCAAATGGATGAGTAACAGATGTGATTGGTTCTGTGACAATAGAGGCATGATGGTGAGAATGGTAGCGCGAGTAGAGAAAAT
GATGGTGCAATGCTCGATGAAACCAATAGTAGAGGAACTCAACTGGTCCAGCATGTAATAGAATGTTTAGAATCACACCGTTAGTTCGCCACATCGACATTTGAAAAGTAGAAGGGATCA
GGGGGAATCCCATATAGAGGAGAATTCCATTAAAGAGTATCTGGTCATCCCAGTTCCTCTCACGATCAACCTGCTCAAACTCTATACTTTTGTCAAGTATCCGGTGAGGACTTCTGGCAGTT
TGCCAGCGAGAAATGCTTATCCATATCTGATTGTGGAGCATCCTCAGCAGCATGGAAGGGAAAATCAGAAGATAAGGTATGGAGACATCCTTCATCTCCTTTGTTGCAAGTAAGTAGAAA
CTTTGTATCACCCACGGAGCTATAACCAAATACTTGAAGTTGCCGAGCCTATGCCATGGCCACTCTGTGAGCAATCCTGGTCTTGAAGCCATTAAGCTCACCAAGTTATCAACTCCAAGAG
ACGCTGCAACTATGTACACTTTCTTCTATGTAGATGGTCCTTTTAAAGCGAACTTTTATTTGGTACATTCACTGGACGTAGAGGAAGGTTC
>comp163391_c0_seq15
AATTTTAAACCTTAAGCTCATATACCCTTGATCCCTATTATTTACTCAATAAACATGGCCTTGCTTAATATAAATGCTTGTAATGCTCTTCCTTATCATATAGTGAAGCTTTCTCCTTACCAATTT
GCATGATAATGAATAAGAAAACTGCACAAATATTCTTAGATAGAAAAATGACAATAAAATATTGTATGCATCTTATCATACGAAAACATTACATATATTCTTAGACAATAAAAATGTATATCTT
TTACTATTTCATTTTACTCGTGAAGTAGTGGAAGGAAACCATGTCCCAGTGCTGCCATCCAAATTTTGTCCACATGAAATACATTCTCTCCACATTCATTTGCATCCCATCCCTCTAATCCAT
GAATTATTCCAGCAATACGCCATGCACTCATTACCCTTCTAGGCAGCCAATTCTCACATGAGTGCACATTTTGCAATTCATTTGGAATCTTCATGGCTGGAGTACTATAGTAAATGCAATCAT
TTCTAACTTTTTTGGGAGGAAAATAAGAATATGGAATGAATATTGTTCCATTTTGCGCCTTCCATTGCTCTACATCATCTATACCATCTCCGACTAACCAAACCTTAGGCATATAATTATTTGA
AAGTATAAGATGACCCTGTGTAGTTCCGAACATTAATGGCTCCAACATACAGTAGTCATGCGTGTTTGTTATTACAACCTTTATACCTTTGTTGCATAGGCTCTGAATCACTGTGCAAGCAA
CCTTTGAAATGTTACCTTTCAGTAAAATTTGTTTTGTGCCTTTGGGAATGTTGTTAAGAATCACAGCAACTGCCAAGCTACTTCCATCCACAATCCTTAATTTTAACTTTGGATACTTTTGAA

GGTAAAACTCACCACTTCTATTGAGTTCTTTCACCTGATTGAAGAGCCCTAAACTTAGAACTTTAATTCCTTTGGTTTCAGCCTGTAGAATAGACTTTTCAATCAAATTATTGATTTTCTCCT
TCTCCCATGATAAGCCATACTGCACATAAAACAAATGATTGAGTTATTTAGAATTATCAAAGAAAGAAATGAAATCTTTCAATGACTTTTATACTTGGAAATTGTATATTGGCATGACCCATG
TTTGCATTTTGATGTTCTTCAACTTATTTCTCTCAACAATGAAGGAAGAACTATAAAACCATGTGAGCAACATCGACATCCATGATAATGGCAACATCATCCGATCATACCACTTAGAATATT
TGTATGGTTTAGAGGCCAAAGATGCAATTCCAAGTCGCAGATGATATATTGATTGCAAGGTGGTTGGATGTGTAAGGTGAACAACATCTATAGTCTCTTTCTTTCCTTTCAATGAGATTTCAT
AGACTTCATCAGTTAACTTATCCCTCGTGTTGTACAAATAGTCATAGAATGGCATGAATAAGGAATAGTTTGTCCGAAATTGCGTGTGATGAAGAGAATGAA
>comp163391_c0_seq2
TCCTTTTCTCTTTTTATTTATTTTGAAATAGATTTATTCATGAGATCTGAGAAGAAATGCTGAAAAATGTTTTTTGATGATGAAACAGGTCTAAACAGCTTGAAATTTCTAATAATTGAGCTC
ATTCTAACTTAAATACTCTATGGGCTTAGTTGAACACAGGTCGCCTCATCACATTCATAGCTTCTTTTGATTTATATGTACCAAACTAAGATTCTCAGTTAGTGCATATTTATGCTACTCACCA
TCCTGAAAAGTTAGTAGAGTAAATTTATCACTATAAAGCTAATCTTGACCAAAATTTGGCCTTATATCCAAATCCAAACTTTCTTATGATGGTTTAAATTAACTTTTAATGATACAAACTGCAT
AGATTATAATAATTTACTAAACTTACATGTGATTGGTTCTGTGACAATAGAGGCATGATGGTGAGAATGGTAGCGCGAGTAGAGAAAATGATGGTGCAATGCTCGATGAAACCAATAGTAG
AGGAACTCAACTGGTCCAGCATGTAATAGAATGTTTAGAATCACACCGTTAGTTCGCCACATCGACATTTGAAAAGTAGAAGGGATCAGGGGGAATCCCATATAGAGGAGAATTCCATTA
AAGAGTATCTGGTCATC
>comp163391_c0_seq24
AATTTTAAACCTTAAGCTCATATACCCTTGATCCCTATTATTTACTCAATAAACATGGCCTTGCTTAATATAAATGCTTGTAATGCTCTTCCTTATCATATAGTGAAGCTTTCTCCTTACCAATTT
GCATGATAATGAATAAGAAAACTGCACAAATATTCTTAGATAGAAAAATGACAATAAAATATTGTATGCATCTTATCATACGAAAACATTACATATATTCTTAGACAATAAAAATGTATATCTT
TTACTATTTCATTTTACTCGTGAAGTAGTGGAAGGAAACCATGTCCCAGTGCTGCCATCCAAATTTTGTCCACATGAAATACATTCTCTCCACATTCATTTGCATCCCATCCCTCTAATCCAT
GAATTATTCCAGCAATACGCCATGCACTCATTACCCTTCTAGGCAGCCAATTCTGCAACATTATTTACATTTAGCAGTAAGATGGTTGTACAATTAAATCCAAATGAACATTTTTACTTAGAA
ATTATTGGTATCAAAGGAAGGTGAGGGAGGTGAATTGAAAGTTAAATGATTTGTCGTTGACTCAAAACCTTTAGCCATATATTTTGAAATTGTTATCTCTTTTGTTCATTTCCTTTCATTCAA
ATTCCGAGAATCCAAACATAGGGCAAGTAGCAATATTATTGAAATGCTGAATTAAAATTTATTTCATTATATAAATAGTTAGATTCTCATCATGCTTTTTAACTTGTTGTTAATGAATATATTAC
CTCACATGAGTGCACATTTTGCAATTCATTTGGAATCTTCATGGCTGGAGTACTATAGTAAATGCAATCATTTCTAACTTTTTTGGGAGGAAAATAAGAATATGGAATGAATATTGTTCCATT
TTGCGCCTTCCATTGCTCTACATCATCTATACCATCTCCGACTAACCAAACCTGAAAGACAAATTTTTTGAAGTCATTTTCATGTATTATACTATCACAACTATAAAGGATGTTCACTGATATA
AGGGATGAAAACCAATTATGAATGCTTAACGAATGCAAAACTTTTATACACGAATGTTTAGATAAAAAATAAATTAGGCATCAATATATTTGTAATATATTTATAATGTGCCCAAGTGATTTCA
TTGTTCCTTGATTTGTTTTATGTTGGTTGCATGAGTGCCTAAGAATGATAATTGAATCATAATGTTGAATTATTTTTGTGCTATTGATTGCATCAAACTCCTAGCTATTTTTTAAATGTTTATGAT
GAATCCTAAAAAGTTAATAATACTTATAGACTTTTCTCAATCATTAAAAATTATATTAGTTGAACCATTCTCCCGTCATAAACTAAAGGAACTCATTACCTTAGGCATATAATTATTTGAAAGT
ATAAGATGACCCTGTGTAGTTCCGAACATTAATGGCTCCAACATACAGTAGTCATGCGTGTTTGTTATTACAACCTTTATACCTTTGTTGCATAGGCTCTGAATCACTGTGCAAGCAACCTTT
GAAATGTTACCTTTCAGTAAAATTTGTTTTGTGCCTTTGGGAATGTTGTTAAGAATCACAGCAACTGCCAAGCTACTTCCATCCACAATCCTTAATTTTAACTTTGGATACTTTTGAAGGTA
AAACTCACCACTTCTATTGAGTTCTTTCACCTGATTGAAGAGCCCTAAACTTAGAACTTTAATTCCTTTGGTTTCAGCCTGTAGAATAGACTTTTCAATCAAATTATTGATTTTCTCCTTCTC
CCATGATAAGCCATATTGGAAATTGTATATTGGCATGACCCATGTTTGCATTTTGATGTTCTTCAACTTATTTCTCTCAACAATGAAGGAAGAACTATAAAACCATGTGAGCAACATCGACAT
CCATGATAATGGCAACATCATCCGATCATACCACTTAGAATATTTGTATGGTTTAGAGGCCAAAGATGCAATTCCAAGTCGCAGATGATATATTGATTGCAAGGTGGTTGGATGTGTAAGGT
GAACAACATCTATAGTCTCTTTCTTTCCTTTCAATGAGATTTCATAGACTTCATCAGTTAACTTATCCCTCGTGTTGTACAAATAGTCATAGAATGGCATGAATAAGGAATAGTTTGTCCGAA
ATTGCGTGTGATGAAGAGAATGAAACGAGGAAGTGTATATGAGGTATTTCAATGGTGGAAAGATAGAAAACAACCACTTTGGAACCAATTCAAAGTTGCAGTGCCCCATATTGTTCATGA

AGTCAATGTAGAAAACATATCCGACAAATTGTATCGTCGAGGCTGTTTCTGTATAGGCTGTAGTCAACAATGGGACAGCGAACAACATAGAGTAGACAATATGCTCCGCAAATGGATGAGT
AACAGATGTGATTGGTTCTGTGACAATAGAGGCATGATGGTGAGAATGGTAGCGCGAGTAGAGAAAATGATGGTGCAATGCTCGATGAAACCAATAGTAGAGGAACTCAACTGGTCCAG
CATGTAATAGAATGTTTAGAATCACACCGTTAGTTCGCCACATCGACATTTGAAAAGTAGAAGGGATCAGGGGGAATCCCATATAGAGGAGAATTCCATTAAAGAGTATCTGGTCATCCCT
GTTGTTGATGAAATGAACGTTAATTATACTTATTATATTCTATAATGCCGCAGAGTCTTTTGAAACACCGGTATTTTTGTAAGGGTGCTTCAATGAGATGACTTTACCTATGTTAAACACAAAT
GTAAGGTAGGATATTTTTGCACTCTGAAACTTTTGAAGTTGCCTCTGGTTTGGATGTTGAAGAGAGATTAACTTTGGTTTGGGTGTTGCTATAGTTAATTTGTAATTACCAAAAAATTGAAG
ATGTTCAGACATTTTTTACAATACAAGTTGGTTTGAAAGTAAGTGACACTTGTTTTCCTGCTTCAACATCTGAACCATTCATTAAATATCGTTCAACATAAAATCATATCCCCTTCGACCTCG
TCTCGCCCCGACGTCGTCCACGAAACTGCTGGCTGGTTCTCAAACCGGCATTTGCGACCGCGTAAGCCTGGCCATGTTGGTCTCGCCCTTGCCTTGTTGTAGCTGGCCCTCATGCACAGA
CCGCGGTGACTCGAGCAGGTCCTGACGGTTCTCACCGGCTCATCCACAGGCCGCAGTTGCACCCGCGCGTTGGCCTTGTCGCGCACACTTTTCTAGCCCTGGCCCTACATGCCTGCAGCT
GCAGTTTCACGTCCGCCTCGCATGCTTTCGGGTTCATATTTTGCAAACAAGTCGCGGGTCAGATTGGGTCAACCCAGTAACCCACACGGGTCGTGTCCGGGTTGACAAACCAGTTCCTCT
CACGATCAACCTGCTCAAACTCTATACTTTTGTCAAGTATCCGGTGAGGACTTCTGGCAGTTTGCCAGCGAGAAATGCTTATCCATATCTGATTGTGGAGCATCCTCAGCAGCATGGAAGG
GAAAATCAGAAGATAAGGTATGGAGACATCCTTCATCTCCTTTGTTGCAAGTAAGTAGAAACTTTGTATCACCCACGGAGCTATAACCAAATACTTGAAGTTGCCGAGCCTATGCCATGGC
CACTCTGTGAGCAATCCTGGTCTTGAAGCCATTAAGCTCACCAAGTTATCAACTCCAAGAGACGCTGCAACTATGTACACTTTCTTCTATGTAGATGGTCCTTTTAAAGCGAACTTTTATTT
GGTACATTCACTGGACGTAGAGGAAGGTTC
>comp163391_c0_seq27
AATTTTAAACCTTAAGCTCATATACCCTTGATCCCTATTATTTACTCAATAAACATGGCCTTGCTTAATATAAATGCTTGTAATGCTCTTCCTTATCATATAGTGAAGCTTTCTCCTTACCAATTT
GCATGATAATGAATAAGAAAACTGCACAAATATTCTTAGATAGAAAAATGACAATAAAATATTGTATGCATCTTATCATACGAAAACATTACATATATTCTTAGACAATAAAAATGTATATCTT
TTACTATTTCATTTTACTCGTGAAGTAGTGGAAGGAAACCATGTCCCAGTGCTGCCATCCAAATTTTGTCCACATGAAATACATTCTCTCCACATTCATTTGCATCCCATCCCTCTAATCCAT
GAATTATTCCAGCAATACGCCATGCACTCATTACCCTTCTAGGCAGCCAATTCTCACATGAGTGCACATTTTGCAATTCATTTGGAATCTTCATGGCTGGAGTACTATAGTAAATGCAATCAT
TTCTAACTTTTTTGGGAGGAAAATAAGAATATGGAATGAATATTGTTCCATTTTGCGCCTTCCATTGCTCTACATCATCTATACCATCTCCGACTAACCAAACCTGAAAGACAAATTTTTTGA
AGTCATTTTCATGTATTATACTATCACAACTATAAAGGATGTTCACTGATATAAGGGATGAAAACCAATTATGAATGCTTAACGAATGCAAAACTTTTATACACGAATGTTTAGATAAAAAAT
AAATTAGGCATCAATATATTTGTAATATATTTATAATGTGCCCAAGTGATTTCATTGTTCCTTGATTTGTTTTATGTTGGTTGCATGAGTGCCTAAGAATGATAATTGAATCATAATGTTGAATTA
TTTTTGTGCTATTGATTGCATCAAACTCCTAGCTATTTTTTAAATGTTTATGATGAATCCTAAAAAGTTAATAATACTTATAGACTTTTCTCAATCATTAAAAATTATATTAGTTGAACCATTCTC
CCGTCATAAACTAAAGGAACTCATTACCTTAGGCATATAATTATTTGAAAGTATAAGATGACCCTGTGTAGTTCCGAACATTAATGGCTCCAACATACAGTAGTCATGCGTGTTTGTTATTAC
AACCTTTATACCTTTGTTGCATAGGCTCTGAATCACTGTGCAAGCAACCTTTGAAATGTTACCTTTCAGTAAAATTTGTTTTGTGCCTTTGGGAATGTTGTTAAGAATCACAGCAACTGCCA
AGCTACTTCCATCCACAATCCTTAATTTTAACTTTGGATACTTTTGAAGGTAAAACTCACCACTTCTATTGAGTTCTTTCACCTGATTGAAGAGCCCTAAACTTAGAACTTTAATTCCTTTGG
TTTCAGCCTGTAGAATAGACTTTTCAATCAAATTATTGATTTTCTCCTTCTCCCATGATAAGCCATACACTTAGAATATTTGTATGGTTTAGAGGCCAAAGATGCAATTCCAAGTCGCAGATG
ATATATTGATTGCAAGGTGGTTGGATGTGTAAGGTGAACAACATCTATAGTCTCTTTCTTTCCTTTCAATGAGATTTCATAGACTTCATCAGTTAACTTATCCCTCGTGTTGTACAAATAGTCA
TAGAATGGCATGAATAAGGAATAGTTTGTCCGAAATTGCGTGTGATGAAGAGAATGAAACGAGGAAGTGTATATGAGGTATTTCAATGGTGGAAAGATAGAAAACAACCACTTTGGAACC
AATTCAAAGTTGCAGTGCCCCATATTGTTCATGAAGTCAATGTAGAAAACATATCCGACAAATTGTATCGTCGAGGCTGTTTCTGTATAGGCTGTAGTCAACAATGGGACAGCGAACAACA
TAGAGTAGACAATATGCTCCGCAAATGGATGAGTAACAGCAGTTCCTCTCACGATCAACCTGCTCAAACTCTATACTTTTGTCAAGTATCCGGTGAGGACTTCTGGCAGTTTGCCAGCGAG
AAATGCTTATCCATATCTGATTGTGGAGCATCCTCAGCAGCATGGAAGGGAAAATCAGAAGATAAGGTATGGAGACATCCTTCATCTCCTTTGTTGCAAGTAAGTAGAAACTTTGTATCAC

CCACGGAGCTATAACCAAATA
>comp163391_c0_seq28
AATTTTAAACCTTAAGCTCATATACCCTTGATCCCTATTATTTACTCAATAAACATGGCCTTGCTTAATATAAATGCTTGTAATGCTCTTCCTTATCATATAGTGAAGCTTTCTCCTTACCAATTT
GCATGATAATGAATAAGAAAACTGCACAAATATTCTTAGATAGAAAAATGACAATAAAATATTGTATGCATCTTATCATACGAAAACATTACATATATTCTTAGACAATAAAAATGTATATCTT
TTACTATTTCATTTTACTCGTGAAGTAGTGGAAGGAAACCATGTCCCAGTGCTGCCATCCAAATTTTGTCCACATGAAATACATTCTCTCCACATTCATTTGCATCCCATCCCTCTAATCCAT
GAATTATTCCAGCAATACGCCATGCACTCATTACCCTTCTAGGCAGCCAATTCTCACATGAGTGCACATTTTGCAATTCATTTGGAATCTTCATGGCTGGAGTACTATAGTAAATGCAATCAT
TTCTAACTTTTTTGGGAGGAAAATAAGAATATGGAATGAATATTGTTCCATTTTGCGCCTTCCATTGCTCTACATCATCTATACCATCTCCGACTAACCAAACCTTAGGCATATAATTATTTGA
AAGTATAAGATGACCCTGTGTAGTTCCGAACATTAATGGCTCCAACATACAGTAGTCATGCGTGTTTGTTATTACAACCTTTATACCTTTGTTGCATAGGCTCTGAATCACTGTGCAAGCAA
CCTTTGAAATGTTACCTTTCAGTAAAATTTGTTTTGTGCCTTTGGGAATGTTGTTAAGAATCACAGCAACTGCCAAGCTACTTCCATCCACAATCCTTAATTTTAACTTTGGATACTTTTGAA
GGTAAAACTCACCACTTCTATTGAGTTCTTTCACCTGATTGAAGAGCCCTAAACTTAGAACTTTAATTCCTTTGGTTTCAGCCTGTAGAATAGACTTTTCAATCAAATTATTGATTTTCTCCT
TCTCCCATGATAAGCCATATTGGAAATTGTATATTGGCATGACCCATGTTTGCATTTTGATGTTCTTCAACTTATTTCTCTCAACAATGAAGGAAGAACTATAAAACCATGTGAGCAACATCG
ACATCCATGATAATGGCAACATCATCCGATCATACCACTTAGAATATTTGTATGGTTTAGAGGCCAAAGATGCAATTCCAAGTCGCAGATGATATATTGATTGCAAGGTGGTTGGATGTGTAA
GGTGAACAACATCTATAGTCTCTTTCTTTCCTTTCAATGAGATTTCATAGACTTCATCAGTTAACTTATCCCTCGTGTTGTACAAATAGTCATAGAATGGCATGAATAAGGAATAGTTTGTCC
GAAATTGCGTGTGATGAAGAGAATGAAACGAGGAAGTGTATATGAGGTATTTCAATGGTGGAAAGATAGAAAACAACCACTTTGGAACCAATTCAAAGTTGCAGTGCCCCATATTGTTCA
TGAAGTCAATGTAGAAAACATATCCGACAAATTGTATCGTCGAGGCTGTTTCTGTATAGGCTGTAGTCAACAATGGGACAGCGAACAACATAGAGTAGACAATATGCTCCGCAAATGGATG
AGTAACAGCAGTTCCTCTCACGATCAACCTGCTCAAACTCTATACTTTTGTCAAGTATCCGGTGAGGACTTCTGGCAGTTTGCCAGCGAGAAATGCTTATCCATATCTGATTGTGGAGCAT
CCTCAGCAGCATGGAAGGGAAAATCAGAAGATAAGGTATGGAGACATCCTTCATCTCCTTTGTTGCAAGTAAGTAGAAACTTTGTATCACCCACGGAGCTATAACCAAATA
>comp163391_c0_seq29
AATTTTAAACCTTAAGCTCATATACCCTTGATCCCTATTATTTACTCAATAAACATGGCCTTGCTTAATATAAATGCTTGTAATGCTCTTCCTTATCATATAGTGAAGCTTTCTCCTTACCAATTT
GCATGATAATGAATAAGAAAACTGCACAAATATTCTTAGATAGAAAAATGACAATAAAATATTGTATGCATCTTATCATACGAAAACATTACATATATTCTTAGACAATAAAAATGTATATCTT
TTACTATTTCATTTTACTCGTGAAGTAGTGGAAGGAAACCATGTCCCAGTGCTGCCATCCAAATTTTGTCCACATGAAATACATTCTCTCCACATTCATTTGCATCCCATCCCTCTAATCCAT
GAATTATTCCAGCAATACGCCATGCACTCATTACCCTTCTAGGCAGCCAATTCTGCAACATTATTTACATTTAGCAGTAAGATGGTTGTACAATTAAATCCAAATGAACATTTTTACTTAGAA
ATTATTGGTATCAAAGGAAGGTGAGGGAGGTGAATTGAAAGTTAAATGATTTGTCGTTGACTCAAAACCTTTAGCCATATATTTTGAAATTGTTATCTCTTTTGTTCATTTCCTTTCATTCAA
ATTCCGAGAATCCAAACATAGGGCAAGTAGCAATATTATTGAAATGCTGAATTAAAATTTATTTCATTATATAAATAGTTAGATTCTCATCATGCTTTTTAACTTGTTGTTAATGAATATATTAC
CTCACATGAGTGCACATTTTGCAATTCATTTGGAATCTTCATGGCTGGAGTACTATAGTAAATGCAATCATTTCTAACTTTTTTGGGAGGAAAATAAGAATATGGAATGAATATTGTTCCATT
TTGCGCCTTCCATTGCTCTACATCATCTATACCATCTCCGACTAACCAAACCTGAAAGACAAATTTTTTGAAGTCATTTTCATGTATTATACTATCACAACTATAAAGGATGTTCACTGATATA
AGGGATGAAAACCAATTATGAATGCTTAACGAATGCAAAACTTTTATACACGAATGTTTAGATAAAAAATAAATTAGGCATCAATATATTTGTAATATATTTATAATGTGCCCAAGTGATTTCA
TTGTTCCTTGATTTGTTTTATGTTGGTTGCATGAGTGCCTAAGAATGATAATTGAATCATAATGTTGAATTATTTTTGTGCTATTGATTGCATCAAACTCCTAGCTATTTTTTAAATGTTTATGAT
GAATCCTAAAAAGTTAATAATACTTATAGACTTTTCTCAATCATTAAAAATTATATTAGTTGAACCATTCTCCCGTCATAAACTAAAGGAACTCATTACCTTAGGCATATAATTATTTGAAAGT
ATAAGATGACCCTGTGTAGTTCCGAACATTAATGGCTCCAACATACAGTAGTCATGCGTGTTTGTTATTACAACCTTTATACCTTTGTTGCATAGGCTCTGAATCACTGTGCAAGCAACCTTT
GAAATGTTACCTTTCAGTAAAATTTGTTTTGTGCCTTTGGGAATGTTGTTAAGAATCACAGCAACTGCCAAGCTACTTCCATCCACAATCCTTAATTTTAACTTTGGATACTTTTGAAGGTA

AAACTCACCACTTCTATTGAGTTCTTTCACCTGATTGAAGAGCCCTAAACTTAGAACTTTAATTCCTTTGGTTTCAGCCTGTAGAATAGACTTTTCAATCAAATTATTGATTTTCTCCTTCTC
CCATGATAAGCCATACACTTAGAATATTTGTATGGTTTAGAGGCCAAAGATGCAATTCCAAGTCGCAGATGATATATTGATTGCAAGGTGGTTGGATGTGTAAGGTGAACAACATCTATAGT
CTCTTTCTTTCCTTTCAATGAGATTTCATAGACTTCATCAGTTAACTTATCCCTCGTGTTGTACAAATAGTCATAGAATGGCATGAATAAGGAATAGTTTGTCCGAAATTGCGTGTGATGAAG
AGAATGAAACGAGGAAGTGTATATGAGGTATTTCAATGGTGGAAAGATAGAAAACAACCACTTTGGAACCAATTCAAAGTTGCAGTGCCCCATATTGTTCATGAAGTCAATGTAGAAAAC
ATATCCGACAAATTGTATCGTCGAGGCTGTTTCTGTATAGGCTGTAGTCAACAATGGGACAGCGAACAACATAGAGTAGACAATATGCTCCGCAAATGGATGAGTAACAGATGTGATTGGT
TCTGTGACAATAGAGGCATGATGGTGAGAATGGTAGCGCGAGTAGAGAAAATGATGGTGCAATGCTCGATGAAACCAATAGTAGAGGAACTCAACTGGTCCAGCATGTAATAGAATGTTT
AGAATCACACCGTTAGTTCGCCACATCGACATTTGAAAAGTAGAAGGGATCAGGGGGAATCCCATATAGAGGAGAATTCCATTAAAGAGTATCTGGTCATCCCTGTTGTTGATGAAATGA
ACGTTAATTATACTTATTATATTCTATAATGCCGCAGAGTCTTTTGAAACACCGGTATTTTTGTAAGGGTGCTTCAATGAGATGACTTTACCTATGTTAAACACAAATGTAAGGTAGGATATTT
TTGCACTCTGAAACTTTTGAAGTTGCCTCTGGTTTGGATGTTGAAGAGAGATTAACTTTGGTTTGGGTGTTGCTATAGTTAATTTGTAATTACCAAAAAATTGAAGATGTTCAGACATTTTT
TACAATACAAGTTGGTTTGAAAGTAAGTGACACTTGTTTTCCTGCTTCAACATCTGAACCATTCATTAAATATCGTTCAACATAAAATCATATCCCCTTCGACCTCGTCTCGCCCCGACGTC
GTCCACGAAACTGCTGGCTGGTTCTCAAACCGGCATTTGCGACCGCGTAAGCCTGGCCATGTTGGTCTCGCCCTTGCCTTGTTGTAGCTGGCCCTCATGCACAGACCGCGGTGACTCGAG
CAGGTCCTGACGGTTCTCACCGGCTCATCCACAGGCCGCAGTTGCACCCGCGCGTTGGCCTTGTCGCGCACACTTTTCTAGCCCTGGCCCTACATGCCTGCAGCTGCAGTTTCACGTCCG
CCTCGCATGCTTTCGGGTTCATATTTTGCAAACAAGTCGCGGGTCAGATTGGGTCAACCCAGTAACCCACACGGGTCGTGTCCGGGTTGACAAAC
>comp163391_c0_seq31
AATTTTAAACCTTAAGCTCATATACCCTTGATCCCTATTATTTACTCAATAAACATGGCCTTGCTTAATATAAATGCTTGTAATGCTCTTCCTTATCATATAGTGAAGCTTTCTCCTTACCAATTT
GCATGATAATGAATAAGAAAACTGCACAAATATTCTTAGATAGAAAAATGACAATAAAATATTGTATGCATCTTATCATACGAAAACATTACATATATTCTTAGACAATAAAAATGTATATCTT
TTACTATTTCATTTTACTCGTGAAGTAGTGGAAGGAAACCATGTCCCAGTGCTGCCATCCAAATTTTGTCCACATGAAATACATTCTCTCCACATTCATTTGCATCCCATCCCTCTAATCCAT
GAATTATTCCAGCAATACGCCATGCACTCATTACCCTTCTAGGCAGCCAATTCTCACATGAGTGCACATTTTGCAATTCATTTGGAATCTTCATGGCTGGAGTACTATAGTAAATGCAATCAT
TTCTAACTTTTTTGGGAGGAAAATAAGAATATGGAATGAATATTGTTCCATTTTGCGCCTTCCATTGCTCTACATCATCTATACCATCTCCGACTAACCAAACCTGAAAGACAAATTTTTTGA
AGTCATTTTCATGTATTATACTATCACAACTATAAAGGATGTTCACTGATATAAGGGATGAAAACCAATTATGAATGCTTAACGAATGCAAAACTTTTATACACGAATGTTTAGATAAAAAAT
AAATTAGGCATCAATATATTTGTAATATATTTATAATGTGCCCAAGTGATTTCATTGTTCCTTGATTTGTTTTATGTTGGTTGCATGAGTGCCTAAGAATGATAATTGAATCATAATGTTGAATTA
TTTTTGTGCTATTGATTGCATCAAACTCCTAGCTATTTTTTAAATGTTTATGATGAATCCTAAAAAGTTAATAATACTTATAGACTTTTCTCAATCATTAAAAATTATATTAGTTGAACCATTCTC
CCGTCATAAACTAAAGGAACTCATTACCTTAGGCATATAATTATTTGAAAGTATAAGATGACCCTGTGTAGTTCCGAACATTAATGGCTCCAACATACAGTAGTCATGCGTGTTTGTTATTAC
AACCTTTATACCTTTGTTGCATAGGCTCTGAATCACTGTGCAAGCAACCTTTGAAATGTTACCTTTCAGTAAAATTTGTTTTGTGCCTTTGGGAATGTTGTTAAGAATCACAGCAACTGCCA
AGCTACTTCCATCCACAATCCTTAATTTTAACTTTGGATACTTTTGAAGGTAAAACTCACCACTTCTATTGAGTTCTTTCACCTGATTGAAGAGCCCTAAACTTAGAACTTTAATTCCTTTGG
TTTCAGCCTGTAGAATAGACTTTTCAATCAAATTATTGATTTTCTCCTTCTCCCATGATAAGCCATACTGCACATAAAACAAATGATTGAGTTATTTAGAATTATCAAAGAAAGAAATGAAAT
CTTTCAATGACTTTTATACTTGGAAATTGTATATTGGCATGACCCATGTTTGCATTTTGATGTTCTTCAACTTATTTCTCTCAACAATGAAGGAAGAACTATAAAACCATGTGAGCAACATCG
ACATCCATGATAATGGCAACATCATCCGATCATACCACTTAGAATATTTGTATGGTTTAGAGGCCAAAGATGCAATTCCAAGTCGCAGATGATATATTGATTGCAAGGTGGTTGGATGTGTAA
GGTGAACAACATCTATAGTCTCTTTCTTTCCTTTCAATGAGATTTCATAGACTTCATCAGTTAACTTATCCCTCGTGTTGTACAAATAGTCATAGAATGGCATGAATAAGGAATAGTTTGTCC
GAAATTGCGTGTGATGAAGAGAATGAAACGAGGAAGTGTATATGAGGTATTTCAATGGTGGAAAGATAGAAAACAACCACTTTGGAACCAATTCAAAGTTGCAGTGCCCCATATTGTTCA
TGAAGTCAATGTAGAAAACATATCCGACAAATTGTATCGTCGAGGCTGTTTCTGTATAGGCTGTAGTCAACAATGGGACAGCGAACAACATAGAGTAGACAATATGCTCCGCAAATGGATG

AGTAACAGATGTGATTGGTTCTGTGACAATAGAGGCATGATGGTGAGAATGGTAGCGCGAGTAGAGAAAATGATGGTGCAATGCTCGATGAAACCAATAGTAGAGGAACTCAACTGGTC
CAGCATGTAATAGAATGTTTAGAATCACACCGTTAGTTCGCCACATCGACATTTGAAAAGTAGAAGGGATCAGGGGGAATCCCATATAGAGGAGAATTCCATTAAAGAGTATCTGGTCATC
CCTGTTGTTGATGAAATGAACGTTAATTATACTTATTATATTCTATAATGCCGCAGAGTCTTTTGAAACACCGGTATTTTTGTAAGGGTGCTTCAATGAGATGACTTTACCTATGTTAAACACA
AATGTAAGGTAGGATATTTTTGCACTCTGAAACTTTTGAAGTTGCCTCTGGTTTGGATGTTGAAGAGAGATTAACTTTGGTTTGGGTGTTGCTATAGTTAATTTGTAATTACCAAAAAATTG
AAGATGTTCAGACATTTTTTACAATACAAGTTGGTTTGAAAGTAAGTGACACTTGTTTTCCTGCTTCAACATCTGAACCATTCATTAAATATCGTTCAACATAAAATCATATCCCCTTCGACC
TCGTCTCGCCCCGACGTCGTCCACGAAACTGCTGGCTGGTTCTCAAACCGGCATTTGCGACCGCGTAAGCCTGGCCATGTTGGTCTCGCCCTTGCCTTGTTGTAGCTGGCCCTCATGCAC
AGACCGCGGTGACTCGAGCAGGTCCTGACGGTTCTCACCGGCTCATCCACAGGCCGCAGTTGCACCCGCGCGTTGGCCTTGTCGCGCACACTTTTCTAGCCCTGGCCCTACATGCCTGC
AGCTGCAGTTTCACGTCCGCCTCGCATGCTTTCGGGTTCATATTTTGCAAACAAGTCGCGGGTCAGATTGGGTCAACCCAGTAACCCACACGGGTCGTGTCCGGGTTGACAAACCAGTTC
CTCTCACGATCAACCTGCTCAAACTCTATACTTTTGTCAAGTATCCGGTGAGGACTTCTGGCAGTTTGCCAGCGAGAAATGCTTATCCATATCTGATTGTGGAGCATCCTCAGCAGCATGG
AAGGGAAAATCAGAAGATAAGGTATGGAGACATCCTTCATCTCCTTTGTTGCAAGTAAGTAGAAACTTTGTATCACCCACGGAGCTATAACCAAATACTTGAAGTTGCCGAGCCTATGCCA
TGGCCACTCTGTGAGCAATCCTGGTCTTGAAGCCATTAAGCTCACCAAGTTATCAACTCCAAGAGACGCTGCAACTATGTACACTTTCTTCTATGTAGATGGTCCTTTTAAAGCGAACTTTT
ATTTGGTACATTCACTGGACGTAGAGGAAGGTTC
>comp163391_c0_seq5
AATTTTAAACCTTAAGCTCATATACCCTTGATCCCTATTATTTACTCAATAAACATGGCCTTGCTTAATATAAATGCTTGTAATGCTCTTCCTTATCATATAGTGAAGCTTTCTCCTTACCAATTT
GCATGATAATGAATAAGAAAACTGCACAAATATTCTTAGATAGAAAAATGACAATAAAATATTGTATGCATCTTATCATACGAAAACATTACATATATTCTTAGACAATAAAAATGTATATCTT
TTACTATTTCATTTTACTCGTGAAGTAGTGGAAGGAAACCATGTCCCAGTGCTGCCATCCAAATTTTGTCCACATGAAATACATTCTCTCCACATTCATTTGCATCCCATCCCTCTAATCCAT
GAATTATTCCAGCAATACGCCATGCACTCATTACCCTTCTAGGCAGCCAATTCTGCAACATTATTTACATTTAGCAGTAAGATGGTTGTACAATTAAATCCAAATGAACATTTTTACTTAGAA
ATTATTGGTATCAAAGGAAGGTGAGGGAGGTGAATTGAAAGTTAAATGATTTGTCGTTGACTCAAAACCTTTAGCCATATATTTTGAAATTGTTATCTCTTTTGTTCATTTCCTTTCATTCAA
ATTCCGAGAATCCAAACATAGGGCAAGTAGCAATATTATTGAAATGCTGAATTAAAATTTATTTCATTATATAAATAGTTAGATTCTCATCATGCTTTTTAACTTGTTGTTAATGAATATATTAC
CTCACATGAGTGCACATTTTGCAATTCATTTGGAATCTTCATGGCTGGAGTACTATAGTAAATGCAATCATTTCTAACTTTTTTGGGAGGAAAATAAGAATATGGAATGAATATTGTTCCATT
TTGCGCCTTCCATTGCTCTACATCATCTATACCATCTCCGACTAACCAAACCTTAGGCATATAATTATTTGAAAGTATAAGATGACCCTGTGTAGTTCCGAACATTAATGGCTCCAACATACA
GTAGTCATGCGTGTTTGTTATTACAACCTTTATACCTTTGTTGCATAGGCTCTGAATCACTGTGCAAGCAACCTTTGAAATGTTACCTTTCAGTAAAATTTGTTTTGTGCCTTTGGGAATGTT
GTTAAGAATCACAGCAACTGCCAAGCTACTTCCATCCACAATCCTTAATTTTAACTTTGGATACTTTTGAAGGTAAAACTCACCACTTCTATTGAGTTCTTTCACCTGATTGAAGAGCCCTA
AACTTAGAACTTTAATTCCTTTGGTTTCAGCCTGTAGAATAGACTTTTCAATCAAATTATTGATTTTCTCCTTCTCCCATGATAAGCCATATTGGAAATTGTATATTGGCATGACCCATGTTTG
CATTTTGATGTTCTTCAACTTATTTCTCTCAACAATGAAGGAAGAACTATAAAACCATGTGAGCAACATCGACATCCATGATAATGGCAACATCATCCGATCATACCACTTAGAATATTTGTA
TGGTTTAGAGGCCAAAGATGCAATTCCAAGTCGCAGATGATATATTGATTGCAAGGTGGTTGGATGTGTAAGGTGAACAACATCTATAGTCTCTTTCTTTCCTTTCAATGAGATTTCATAGA
CTTCATCAGTTAACTTATCCCTCGTGTTGTACAAATAGTCATAGAATGGCATGAATAAGGAATAGTTTGTCCGAAATTGCGTGTGATGAAGAGAATGAAACGAGGAAGTGTATATGAGGTAT
TTCAATGGTGGAAAGATAGAAAACAACCACTTTGGAACCAATTCAAAGTTGCAGTGCCCCATATTGTTCATGAAGTCAATGTAGAAAACATATCCGACAAATTGTATCGTCGAGGCTGTTT
CTGTATAGGCTGTAGTCAACAATGGGACAGCGAACAACATAGAGTAGACAATATGCTCCGCAAATGGATGAGTAACAGATGTGATTGGTTCTGTGACAATAGAGGCATGATGGTGAGAAT
GGTAGCGCGAGTAGAGAAAATGATGGTGCAATGCTCGATGAAACCAATAGTAGAGGAACTCAACTGGTCCAGCATGTAATAGAATGTTTAGAATCACACCGTTAGTTCGCCACATCGACA
TTTGAAAAGTAGAAGGGATCAGGGGGAATCCCATATAGAGGAGAATTCCATTAAAGAGTATCTGGTCATCCCTGTTGTTGATGAAATGAACGTTAATTATACTTATTATATTCTATAATGCCG

CAGAGTCTTTTGAAACACCGGTATTTTTGTAAGGGTGCTTCAATGAGATGACTTTACCTATGTTAAACACAAATGTAAGGTAGGATATTTTTGCACTCTGAAACTTTTGAAGTTGCCTCTGG
TTTGGATGTTGAAGAGAGATTAACTTTGGTTTGGGTGTTGCTATAGTTAATTTGTAATTACCAAAAAATTGAAGATGTTCAGACATTTTTTACAATACAAGTTGGTTTGAAAGTAAGTGACA
CTTGTTTTCCTGCTTCAACATCTGAACCATTCATTAAATATCGTTCAACATAAAATCATATCCCCTTCGACCTCGTCTCGCCCCGACGTCGTCCACGAAACTGCTGGCTGGTTCTCAAACCG
GCATTTGCGACCGCGTAAGCCTGGCCATGTTGGTCTCGCCCTTGCCTTGTTGTAGCTGGCCCTCATGCACAGACCGCGGTGACTCGAGCAGGTCCTGACGGTTCTCACCGGCTCATCCAC
AGGCCGCAGTTGCACCCGCGCGTTGGCCTTGTCGCGCACACTTTTCTAGCCCTGGCCCTACATGCCTGCAGCTGCAGTTTCACGTCCGCCTCGCATGCTTTCGGGTTCATATTTTGCAAAC
AAGTCGCGGGTCAGATTGGGTCAACCCAGTAACCCACACGGGTCGTGTCCGGGTTGACAAAC
>comp163411_c0_seq22
TGCCTAATAAAACTAGCAGGTCTTCTTAATTCTACCATACGCATCAATCCTAAGAACAATCAATAATTGAAACTAATTGTGATTCTAGCATACGCATCAATATATTATTCGTCAAAATAGATGA
TTTTGGTTGCAAATGATGATAACGATGATGATATAGGGTTTAGATATACATGAAATACATGGGTGATTTTGTTCGAAAATATATATACAATATCCCATTTAAATTATTAGTGTATGCCAAAACTC
AACAAGGATGACTAAGTAAATCTGTGAAAAAGAACAAATTATGAGTGAAGATGTGCAGAGAGCATCTAAA
>comp163418_c0_seq15
GTCAAGAAATCTAAGTAAAACAACAAAGTTTATGCACTTATATTCAAAATTAAAAACATTTATATTTTAATTGCAAGATAACTGTTTTTTAATTAAAATACTACTCAAGCTCATTCCTTTTCCT
AAAAAAAATAAGATATTTTTATTTGGAACATACGTCCTTTAATTTTGCAGCGTAAATGGTAATTTTTGTACAGTTATGACATTTACTAAATGTGTCCCTTCAACATTCTTTTGATGTCCTCAAT
TTCATAGTTAATACCAAGTAATGGGCTTAGTCAAATCTTAAACAAGGAAAAAAAAAATTCTGAGCTTCCAGAAAATTAATTTTGCATTATCTTCTTTTTTTTTTCGTCTATGGCTTCAAGCCTT
TGAAATAAGATGTACAATCCTTTATTTTGATTGCATGGATATTTCAAAATTTTAATGCTTCTTTATAGCCTCTCATTTCAACAACGGCTGAAAATTTGGAGAGTCCTCTCTAGCTGTTCTATGT
CAGAACCTGGAACGTCTTCTATGTTGGAGCCATTCTTGTATAATTTGAAAATAGGCACTGAACTCACATTCTCTGAACTTACAATGCTAGCTTGTTCCTTCATGTTCACCTTGAGAAAAGTC
ACAGACGTGTGTTGCTTGCAGAGCTGCTCTAGATGATGCAATATTCCTTTTGAATCTTCATCTGATTGGTCATAGAATAGTGCCAGCGACAGTCCACATGATGTGATAACACTACGAAACTG
GTCTTTTGTAGTGATATGAACGGCAGAGTCATCTGCCATCTGTTTAAGGCCTTCACTTTGTCTTCGCCTGACATGCGCTTTAGCATCCTGCAAGGCTTTGTTTACTGTTTCATCTCCCGGCAT
TTCAAGAGCCAATACTTCATAATCTTTTATAGAATCTTCCCACCGATCCAACTAATACAAGATTCAAGATGTGACAACCTTTAGCTTCCAGATAAAGAAATTCATCTGACAAAGGATAAAAG
GTTCTGCTCACCTTGACATTGCAGTCAGCCCTCCTTAGCCGAGCCTTGCTGTACCTGGGCCGCAAATTAAGAACAACATTGCAATCTTCAATGGCCTTTTCCCATTGCCCAAGTTTCGACC
GGCAAGCAGCTCGGTTGCATAGTAGGATTGTATTATGGGGGTCATGGTTAAGACCACTCCCATAGGCAAGGCATGCCTCTTCAAAATTCGAGGCCTTGAAGAGATCATTACCCATTGAGCG
AGCTGATGCCACTGACCGAGCCTTTCGAGCAATAGCAGCAATCTCCCTGCTGCCAGGATCAATCCGTGCAGCCATTTGAGCTAATGAAACAGCACTCTCAAATCTTCCTGTAGCCATATCA
ACCTGTGCTCTTATTGAAAGGTGATATGCATTGCTAAGGGCGCCAAAGAACTGTGTAGCAGCATCAGTTTCGAATTTGGGTGCATCAATCATGGTTTCCTCCGCATCTTCCTGTCTGAGGA
GCATCAGAAAGGCCTCCGCTTGGTAGGCAAATACCTTCAATGCAAAAGAAAAAGGGAGAAAATACTTCTATTAGTCAGGCTGCTTAATAAAATTTTTAGTAAATTGGAACAAAAGTGTAC
CCAACCAATCGGGAGCATTTTCTTTGTCGTTTACCACTGTATCGGCTCTTAATTTTTGGGGTACACGTTGTGTGTTTTTTTTCATCAGCGACTAATATTTTTTTTTCTAAACTTTACAGGAATA
AATATTTAAAGAAACTGGAGATAAACCTGTGGAGCTGAATCAGCACCGGTGGAAATGGCAGACTGACTATCTTTTAATACAGCATTCCAGTCCCTCTGTTTCCTTGCTTCACTGCACTTGC
CAATGAAAACTTGCACAGCCTTAGCTCTAGAGATGTCTTCATGACTGGCTTCACTCTTTGCTAGCTTATAGTGATTGGCTGCTCTCTCTGGTTCACCCAATCTGAGATAGAGATTGGCAAGC
CGCTGATGCGCTCTTAAATAACAAGGATCAATCCTGATAGCCTGTCTGCACTCCTGCAAAGCTTCAAGGAGACGGCCGAGGCCGATTAGCGCTGCCGCTTTGTTGCTCCAGTAAGAAGCA
TTTTCTGGATCAATCACTATCGCTCTATCGTAGAACGCCACGGCCTCCGCATACCTCCCGTTCTTATACTCCTCGTTCCCCAACTCCTTCAGCTCTTCAGGATCAAGTCTTCTTGAAAGCGC
CCGGCACATGGACACCGGAATCTCAGCCTCATACTCAGAGCTACTTCTACGCCTCACCTCCGATTCCATCTCTTTTGCCGTCTTGGGGAGATAGTCGAGGACATCGCGGTTCGGAGTGAC
GGCGCCGGGAGCTCGGAGGTTTCCCAAATTGCTGTAGAGCATGACGTTACCAGAGGAAGCGCGGACAAGGGTATTGCTCCCCTTGGCGCGTTGGTGATCGTAGATCATACTGTCGAGATC

TGCCGATATGCTGCTGGTGGTTTGTGGCCGCACGCCGCGACCAGCGGATGGCCGATTGGTTTGATCTGGGGGACACGCGGCCGCGGCAGGCTTGGGGGAGACTAGGATCTTTGGTGTGA
CGGCGGCAGGGGATTGGTCGGGGCCAGGACGCCGCCGCTTGGTACTATGGCTGCTTCCACCAGAAAGGGAGGCATTGGCGACAATAGGGGAAGGAGAGGGGTGGCGAGGAGAGACGG
GGGCTGATCCAGCACGGCGGCGGAAGAAGGCGTTGTAGAGAATGAGCAAGCCACAGCTGGAGGAATTTTTGTCCTCATTTCCCATTCGTCGCTGTCGACGGGATGATTGCCTTGCGGAG
GGATTGGAAATTGCGTCTGGGATTTGGTTAATCGGACCTCAAAAAGGAAGATGAAGCATTCATTCTTCAAATTTGGATTTAGAAAACATTGATTTTGGTTAATCTCTGAGAATGGAGGAAG
AATAATGATCACCCGGAGGCATTGTAGGTTCAAATGAGTTGTCTCTGATCAGGGAGGCGAACATTGGCTGAAAAAGGAAGAGAGAGGAGGAAAGAAGATGACGGT
>comp163418_c0_seq16
GTCAAGAAATCTAAGTAAAACAACAAAGTTTATGCACTTATATTCAAAATTAAAAACATTTATATTTTAATTGCAAGATAACTGTTTTTTAATTAAAATACTACTCAAGCTCATTCCTTTTCCT
AAAAAAAATAAGATATTTTTATTTGGAACATACGTCCTTTAATTTTGCAGCGTAAATGGTAATTTTTGTACAGTTATGACATTTACTAAATGTGTCCCTTCAACATTCTTTTGATGTCCTCAAT
TTCATAGTTAATACCAAGTAATGGGCTTAGTCAAATCTTAAACAAGGAAAAAAAAATTCTGAGCTTCCAGAAAATTAATTTTGCATTATCTTCTTTTTTTTTTCGTCTATGGCTTCAAGCCTT
TGAAATAAGATGTACAATCCTTTATTTTGATTGCATGGATATTTCAAAATTTTAATGCTTCTTTATAGCCTCTCATTTCAACAACGGCTGAAAATTTGGAGAGTCCTCTCTAGCTGTTCTATGT
CAGAACCTGGAACGTCTTCTATGTTGGAGCCATTCTTGTATAATTTGAAAATAGGCACTGAACTCACATTCTCTGAACTTACAATGCTAGCTTGTTCCTTCATGTTCACCTTGAGAAAAGTC
ACAGACGTGTGTTGCTTGCAGAGCTGCTCTAGATGATGCAATATTCCTTTTGAATCTTCATCTGATTGGTCATAGAATAGTGCCAGCGACAGTCCACATGATGTGATAACACTACGAAACTG
GTCTTTTGTAGTGATATGAACGGCAGAGTCATCTGCCATCTGTTTAAGGCCTTCACTTTGTCTTCGCCTGACATGCGCTTTAGCATCCTGCAAGGCTTTGTTTACTGTTTCATCTCCCGGCAT
TTCAAGAGCCAATACTTCATAATCTTTTATAGAATCTTCCCACCGATCCAACTAATACAAGATTCAAGATGTGACAACCTTTAGCTTCCAGATAAAGAAATTCATCTGACAAAGGATAAAAG
GTTCTGCTCACCTTGACATTGCAGTCAGCCCTCCTTAGCCGAGCCTTGCTGTACCTGGGCCGCAAATTAAGAACAACATTGCAATCTTCAATGGCCTTTTCCCATTGCCCAAGTTTCGACC
GGCAAGCAGCTCGGTTGCATAGTAGGATTGTATTATGGGGGTCATGGTTAAGACCACTCCCATAGGCAAGGCATGCCTCTTCAAAATTCGAGGCCTTGAAGAGATCATTACCCATTGAGCG
AGCTGATGCCACTGACCGAGCCTTTCGAGCAATAGCAGCAATCTCCCTGCTGCCAGGATCAATCCGTGCAGCCATTTGAGCTAATGAAACAGCACTCTCAAATCTTCCTGTAGCCATATCA
ACCTGTGCTCTTATTGAAAGGTGATATGCATTGCTAAGGGCGCCAAAGAACTGTGTAGCAGCATCAGTTTCGAATTTGGGTGCATCAATCATGGTTTCCTCCGCATCTTCCTGTCTGAGGA
GCATCAGAAAGGCCTCCGCTTGGAATAAATATTTAAAGAAACTGGAGATAAACCTGTGGAGCTGAATCAGCACCGGTGGAAATGGCAGACTGACTATCTTTTAATACAGCATTCCAGTCC
CTCTGTTTCCTTGCTTCACTGCACTTGCCAATGAAAACTTGCACAGCCTTAGCTCTAGAGATGTCTTCATGACTGGCTTCACTCTTTGCTAGCTTATAGTGATTGGCTGCTCTCTCTGGTTC
ACCCAATCTGAGATAGAGATTGGCAAGCCGCTGATGCGCTCTTAAATAACAAGGATCAATCCTGATAGCCTGTCTGCACTCCTGCAAAGCTTCAAGGAGACGGCCGAGGCCGATTAGCGC
TGCCGCTTTGTTGCTCCAGTAAGAAGCATTTTCTGGATCAATCACTATCGCTCTATCGTAGAACGCCACGGCCTCCGCATACCTCCCGTTCTTATACTCCTCGTTCCCCAACTCCTTCAGCTC
TTCAGGATCAAGTCTTCTTGAAAGCGCCCGGCACATGGACACCGGAATCTCAGCCTCATACTCAGAGCTACTTCTACGCCTCACCTCCGATTCCATCTCTTTTGCCGTCTTGGGGAGATAG
TCGAGGACATCGCGGTTCGGAGTGACGGCGCCGGGAGCTCGGAGGTTTCCCAAATTGCTGTAGAGCATGACGTTACCAGAGGAAGCGCGGACAAGGGTATTGCTCCCCTTGGCGCGTT
GGTGATCGTAGATCATACTGTCGAGATCTGCCGATATGCTGCTGGTGGTTTGTGGCCGCACGCCGCGACCAGCGGATGGCCGATTGGTTTGATCTGGGGGACACGCGGCCGCGGCAGGCT
TGGGGGAGACTAGGATCTTTGGTGTGACGGCGGCAGGGGATTGGTCGGGGCCAGGACGCCGCCGCTTGGTACTATGGCTGCTTCCACCAGAAAGGGAGGCATTGGCGACAATAGGGGA
AGGAGAGGGGTGGCGAGGAGAGACGGGGGCTGATCCAGCACGGCGGCGGAAGAAGGCGTTGTAGAGAATGAGCAAGCCACAGCTGGAGGAATTTTTGTCCTCATTTCCCATTCGTCGC
TGTCGACGGGATGATTGCCTTGCGGAGGGATTGGAAATTGCGTCTGGGATTTGGTTAATCGGACCTCAAAAAGGAAGATGAAGCATTCATTCTTCAAATTTGGATTTAGAAAACATTGATT
TTGGTTAATCTCTGAGAATGGAGGAAGAATAATGATCACCCGGAGGCATTGTAGGTTCAAATGAGTTGTCTCTGATCAGGGAGGCGAACATTGGCTGAAAAAGGAAGAGAGAGGAGGA
AAGAAGATGACGGT
>comp163418_c0_seq17

GTCAAGAAATCTAAGTAAAACAACAAAGTTTATGCACTTATATTCAAAATTAAAAACATTTATATTTTAATTGCAAGATAACTGTTTTTTAATTAAAATACTACTCAAGCTCATTCCTTTTCCT
AAAAAAAATAAGATATTTTTATTTGGAACATACGTCCTTTAATTTTGCAGCGTAAATGGTAATTTTTGTACAGTTATGACATTTACTAAATGTGTCCCTTCAACATTCTTTTGATGTCCTCAAT
TTCATAGTTAATACCAAGTAATGGGCTTAGTCAAATCTTAAACAAGGAAAAAAAAAATTCTGAGCTTCCAGAAAATTAATTTTGCATTATCTTCTTTTTTTTTTCGTCTATGGCTTCAAGCCTT
TGAAATAAGATGTACAATCCTTTATTTTGATTGCATGGATATTTCAAAATTTTAATGCTTCTTTATAGCCTCTCATTTCAACAACGGCTGAAAATTTGGAGAGTCCTCTCTAGCTGTTCTATGT
CAGAACCTGGAACGTCTTCTATGTTGGAGCCATTCTTGTATAATTTGAAAATAGGCACTGAACTCACATTCTCTGAACTTACAATGCTAGCTTGTTCCTTCATGTTCACCTTGAGAAAAGTC
ACAGACGTGTGTTGCTTGCAGAGCTGCTCTAGATGATGCAATATTCCTTTTGAATCTTCATCTGATTGGTCATAGAATAGTGCCAGCGACAGTCCACATGATGTGATAACACTACGAAACTG
GTCTTTTGTAGTGATATGAACGGCAGAGTCATCTGCCATCTGTTTAAGGCCTTCACTTTGTCTTCGCCTGACATGCGCTTTAGCATCCTGCAAGGCTTTGTTTACTGTTTCATCTCCCGGCAT
TTCAAGAGCCAATACTTCATAATCTTTTATAGAATCTTCCCACCGATCCAACTTGACATTGCAGTCAGCCCTCCTTAGCCGAGCCTTGCTGTACCTGGGCCGCAAATTAAGAACAACATTGC
AATCTTCAATGGCCTTTTCCCATTGCCCAAGTTTCGACCGGCAAGCAGCTCGGTTGCATAGTAGGATTGTATTATGGGGGTCATGGTTAAGACCACTCCCATAGGCAAGGCATGCCTCTTC
AAAATTCGAGGCCTTGAAGAGATCATTACCCATTGAGCGAGCTGATGCCACTGACCGAGCCTTTCGAGCAATAGCAGCAATCTCCCTGCTGCCAGGATCAATCCGTGCAGCCATTTGAGC
TAATGAAACAGCACTCTCAAATCTTCCTGTAGCCATATCAACCTGTGCTCTTATTGAAAGGTGATATGCATTGCTAAGGGCGCCAAAGAACTGTGTAGCAGCATCAGTTTCGAATTTGGGT
GCATCAATCATGGTTTCCTCCGCATCTTCCTGTCTGAGGAGCATCAGAAAGGCCTCCGCTTGGAATAAATATTTAAAGAAACTGGAGATAAACCTGTGGAGCTGAATCAGCACCGGTGGA
AATGGCAGACTGACTATCTTTTAATACAGCATTCCAGTCCCTCTGTTTCCTTGCTTCACTGCACTTGCCAATGAAAACTTGCACAGCCTTAGCTCTAGAGATGTCTTCATGACTGGCTTCAC
TCTTTGCTAGCTTATAGTGATTGGCTGCTCTCTCTGGTTCACCCAATCTGAGATAGAGATTGGCAAGCCGCTGATGCGCTCTTAAATAACAAGGATCAATCCTGATAGCCTGTCTGCACTCC
TGCAAAGCTTCAAGGAGACGGCCGAGGCCGATTAGCGCTGCCGCTTTGTTGCTCCAGTAAGAAGCATTTTCTGGATCAATCACTATCGCTCTATCGTAGAACGCCACGGCCTCCGCATAC
CTCCCGTTCTTATACTCCTCGTTCCCCAACTCCTTCAGCTCTTCAGGATCAAGTCTTCTTGAAAGCGCCCGGCACATGGACACCGGAATCTCAGCCTCATACTCAGAGCTACTTCTACGCC
TCACCTCCGATTCCATCTCTTTTGCCGTCTTGGGGAGATAGTCGAGGACATCGCGGTTCGGAGTGACGGCGCCGGGAGCTCGGAGGTTTCCCAAATTGCTGTAGAGCATGACGTTACCAG
AGGAAGCGCGGACAAGGGTATTGCTCCCCTTGGCGCGTTGGTGATCGTAGATCATACTGTCGAGATCTGCCGATATGCTGCTGGTGGTTTGTGGCCGCACGCCGCGACCAGCGGATGGCC
GATTGGTTTGATCTGGGGGACACGCGGCCGCGGCAGGCTTGGGGGAGACTAGGATCTTTGGTGTGACGGCGGCAGGGGATTGGTCGGGGCCAGGACGCCGCCGCTTGGTACTATGGCT
GCTTCCACCAGAAAGGGAGGCATTGGCGACAATAGGGGAAGGAGAGGGGTGGCGAGGAGAGACGGGGGCTGATCCAGCACGGCGGCGGAAGAAGGCGTTGTAGAGAATGAGCAAGC
CACAGCTGGAGGAATTTTTGTCCTCATTTCCCATTCGTCGCTGTCGACGGGATGATTGCCTTGCGGAGGGATTGGAAATTGCGTCTGGGATTTGGTTAATCGGACCTCAAAAAGGAAGAT
GAAGCATTCATTCTTCAAATTTGGATTTAGAAAACATTGATTTTGGTTAATCTCTGAGAATGGAGGAAGAATAATGATCACCCGGAGGCATTGTAGGTTCAAATGAGTTGTCTCTGATCAG
GGAGGCGAACATTGGCTGAAAAAGGAAGAGAGAGGAGGAAAGAAGATGACGGT
>comp163418_c0_seq18
GTCAAGAAATCTAAGTAAAACAACAAAGTTTATGCACTTATATTCAAAATTAAAAACATTTATATTTTAATTGCAAGATAACTGTTTTTTAATTAAAATACTACTCAAGCTCATTCCTTTTCCT
AAAAAAAATAAGATATTTTTATTTGGAACATACGTCCTTTAATTTTGCAGCGTAAATGGTAATTTTTGTACAGTTATGACATTTACTAAATGTGTCCCTTCAACATTCTTTTGATGTCCTCAAT
TTCATAGTTAATACCAAGTAATGGGCTTAGTCAAATCTTAAACAAGGAAAAAAAAAATTCTGAGCTTCCAGAAAATTAATTTTGCATTATCTTCTTTTTTTTTTCGTCTATGGCTTCAAGCCTT
TGAAATAAGATGTACAATCCTTTATTTTGATTGCATGGATATTTCAAAATTTTAATGCTTCTTTATAGCCTCTCATTTCAACAACGGCTGAAAATTTGGAGAGTCCTCTCTAGCTGTTCTATGT
CAGAACCTGGAACGTCTTCTATGTTGGAGCCATTCTTGTATAATTTGAAAATAGGCACTGAACTCACATTCTCTGAACTTACAATGCTAGCTTGTTCCTTCATGTTCACCTTGAGAAAAGTC
ACAGACGTGTGTTGCTTGCAGAGCTGCTCTAGATGATGCAATATTCCTTTTGAATCTTCATCTGATTGGTCATAGAATAGTGCCAGCGACAGTCCTGCCAAACATAGATGCCATGAACTATG
TATTAAGAACTTAAGTAATTTACAGATTTTAGCAGTTGCATAATAACTTAACATTCTCATTAAAATGTGAATCAAAACAATAGAGAAAAAGGCTAAAGTGGAATTTGCACAATTTGTATGAT

ATCACTTTAGTTCCAACGATCTAATGTGTTCAATTTATTCTTTTAACTAATTAAAAATGATCCAAAATAGTCTTACTATTGAATTATTTTGGATCAATTTTGAGATGTCAATGGGTTATTTTGAA
TCTAGTTTAAAGAGTCGAGGAATTAAATTAGAACATTAAATTGCTTCAAATCAAAACTACCTCCTATAAATAGTCTGAATCATTTTGATCTTTTTTTCCTAAACAGTGGATGATATGAAGAGT
GGTCTCAATGTCATGCCAACCTAACAATTATCAAAATCCATTATATGAAGCAAGAAATTAAAAGAGGGGAATATTATCAAATGCATCATATATCGTCATCATTAGCCTTATACAAATACTACTT
GGGGTTAGCTACGTGCATAACATTAACATGTAGCACACAAAAATTGCATTTTCTTCACATATTACATGGTAGATGGAATGATAATCAGTAAGGCATATGGGCACAGGAAATGTGTCTCTTAT
GCAAGCACGTTTTAAATGTAACAAATCGCCAATCTCCATATGCTTTGGCCAGATGCCACATAGTAATGTAGCATTCCTTAATTTTTTTAAGAATCTCTAATGCTAATGACACGTACCACATGA
TGTGATAACACTACGAAACTGGTCTTTTGTAGTGATATGAACGGCAGAGTCATCTGCCATCTGTTTAAGGCCTTCACTTTGTCTTCGCCTGACATGCGCTTTAGCATCCTGCAAGGCTTTGT
TTACTGTTTCATCTCCCGGCATTTCAAGAGCCAATACTTCATAATCTTTTATAGAATCTTCCCACCGATCCAACTTGACATTGCAGTCAGCCCTCCTTAGCCGAGCCTTGCTGTACCTGGGCC
GCAAATTAAGAACAACATTGCAATCTTCAATGGCCTTTTCCCATTGCCCAAGTTTCGACCGGCAAGCAGCTCGGTTGCATAGTAGGATTGTATTATGGGGGTCATGGTTAAGACCACTCCC
ATAGGCAAGGCATGCCTCTTCAAAATTCGAGGCCTTGAAGAGATCATTACCCATTGAGCGAGCTGATGCCACTGACCGAGCCTTTCGAGCAATAGCAGCAATCTCCCTGCTGCCAGGATC
AATCCGTGCAGCCATTTGAGCTAATGAAACAGCACTCTCAAATCTTCCTGTAGCCATATCAACCTGTGCTCTTATTGAAAGGTGATATGCATTGCTAAGGGCGCCAAAGAACTGTGTAGCA
GCATCAGTTTCGAATTTGGGTGCATCAATCATGGTTTCCTCCGCATCTTCCTGTCTGAGGAGCATCAGAAAGGCCTCCGCTTGGTAGGCAAATACCTTCAATGCAAAAGAAAAAGGGAGA
AAATACTTCTATTAGTCAGGCTGCTTAATAAAATTTTTAGTAAATTGGAACAAAAGTGTACCCAACCAATCGGGAGCATTTTCTTTGTCGTTTACCACTGTATCGGCTCTTAATTTTTGGGGT
ACACGTTGTGTGTTTTTTTTCATCAGCGACTAATATTTTTTTTTCTAAACTTTACAGGAATAAATATTTAAAGAAACTGGAGATAAACCTGTGGAGCTGAATCAGCACCGGTGGAAATGGCA
GACTGACTATCTTTTAATACAGCATTCCAGTCCCTCTGTTTCCTTGCTTCACTGCACTTGCCAATGAAAACTTGCACAGCCTTAGCTCTAGAGATGTCTTCATGACTGGCTTCACTCTTTGC
TAGCTTATAGTGATTGGCTGCTCTCTCTGGTTCACCCAATCTGAGATAGAGATTGGCAAGCCGCTGATGCGCTCTTAAATAACAAGGATCAATCCTGATAGCCTGTCTGCACTCCTGCAAA
GCTTCAAGGAGACGGCCGAGGCCGATTAGCGCTGCCGCTTTGTTGCTCCAGTAAGAAGCATTTTCTGGATCAATCACTATCGCTCTATCGTAGAACGCCACGGCCTCCGCATACCTCCCGT
TCTTATACTCCTCGTTCCCCAACTCCTTCAGCTCTTCAGGATCAAGTCTTCTTGAAAGCGCCCGGCACATGGACACCGGAATCTCAGCCTCATACTCAGAGCTACTTCTACGCCTCACCTC
CGATTCCATCTCTTTTGCCGTCTTGGGGAGATAGTCGAGGACATCGCGGTTCGGAGTGACGGCGCCGGGAGCTCGGAGGTTTCCCAAATTGCTGTAGAGCATGACGTTACCAGAGGAAG
CGCGGACAAGGGTATTGCTCCCCTTGGCGCGTTGGTGATCGTAGATCATACTGTCGAGATCTGCCGATATGCTGCTGGTGGTTTGTGGCCGCACGCCGCGACCAGCGGATGGCCGATTGG
TTTGATCTGGGGGACACGCGGCCGCGGCAGGCTTGGGGGAGACTAGGATCTTTGGTGTGACGGCGGCAGGGGATTGGTCGGGGCCAGGACGCCGCCGCTTGGTACTATGGCTGCTTCCA
CCAGAAAGGGAGGCATTGGCGACAATAGGGGAAGGAGAGGGGTGGCGAGGAGAGACGGGGGCTGATCCAGCACGGCGGCGGAAGAAGGCGTTGTAGAGAATGAGCAAGCCACAGCT
GGAGGAATTTTTGTCCTCATTTCCCATTCGTCGCTGTCGACGGGATGATTGCCTTGCGGAGGGATTGGAAATTGCGTCTGGGATTTGGTTAATCGGACCTCAAAAAGGAAGATGAAGCATT
CATTCTTCAAATTTGGATTTAGAAAACATTGATTTTGGTTAATCTCTGAGAATGGAGGAAGAATAATGATCACCCGGAGGCATTGTAGGTTCAAATGAGTTGTCTCTGATCAGGGAGGCGA
ACATTGGCTGAAAAAGGAAGAGAGAGGAGGAAAGAAGATGACGGT
>comp163418_c0_seq24
GTCAAGAAATCTAAGTAAAACAACAAAGTTTATGCACTTATATTCAAAATTAAAAACATTTATATTTTAATTGCAAGATAACTGTTTTTTAATTAAAATACTACTCAAGCTCATTCCTTTTCCT
AAAAAAAATAAGATATTTTTATTTGGAACATACGTCCTTTAATTTTGCAGCGTAAATGGTAATTTTTGTACAGTTATGACATTTACTAAATGTGTCCCTTCAACATTCTTTTGATGTCCTCAAT
TTCATAGTTAATACCAAGTAATGGGCTTAGTCAAATCTTAAACAAGGAAAAAAAAATTCTGAGCTTCCAGAAAATTAATTTTGCATTATCTTCTTTTTTTTTTCGTCTATGGCTTCAAGCCTT
TGAAATAAGATGTACAATCCTTTATTTTGATTGCATGGATATTTCAAAATTTTAATGCTTCTTTATAGCCTCTCATTTCAACAACGGCTGAAAATTTGGAGAGTCCTCTCTAGCTGTTCTATGT
CAGAACCTGGAACGTCTTCTATGTTGGAGCCATTCTTGTATAATTTGAAAATAGGCACTGAACTCACATTCTCTGAACTTACAATGCTAGCTTGTTCCTTCATGTTCACCTTGAGAAAAGTC
ACAGACGTGTGTTGCTTGCAGAGCTGCTCTAGATGATGCAATATTCCTTTTGAATCTTCATCTGATTGGTCATAGAATAGTGCCAGCGACAGTCCACATGATGTGATAACACTACGAAACTG

GTCTTTTGTAGTGATATGAACGGCAGAGTCATCTGCCATCTGTTTAAGGCCTTCACTTTGTCTTCGCCTGACATGCGCTTTAGCATCCTGCAAGGCTTTGTTTACTGTTTCATCTCCCGGCAT
TTCAAGAGCCAATACTTCATAATCTTTTATAGAATCTTCCCACCGATCCAACTTGACATTGCAGTCAGCCCTCCTTAGCCGAGCCTTGCTGTACCTGGGCCGCAAATTAAGAACAACATTGC
AATCTTCAATGGCCTTTTCCCATTGCCCAAGTTTCGACCGGCAAGCAGCTCGGTTGCATAGTAGGATTGTATTATGGGGGTCATGGTTAAGACCACTCCCATAGGCAAGGCATGCCTCTTC
AAAATTCGAGGCCTTGAAGAGATCATTACCCATTGAGCGAGCTGATGCCACTGACCGAGCCTTTCGAGCAATAGCAGCAATCTCCCTGCTGCCAGGATCAATCCGTGCAGCCATTTGAGC
TAATGAAACAGCACTCTCAAATCTTCCTGTAGCCATATCAACCTGTGCTCTTATTGAAAGGTGATATGCATTGCTAAGGGCGCCAAAGAACTGTGTAGCAGCATCAGTTTCGAATTTGGGT
GCATCAATCATGGTTTCCTCCGCATCTTCCTGTCTGAGGAGCATCAGAAAGGCCTCCGCTTGGTAGGCAAATACCTTCAATGCAAAAGAAAAAGGGAGAAAATACTTCTATTAGTCAGGC
TGCTTAATAAAATTTTTAGTAAATTGGAACAAAAGTGTACCCAACCAATCGGGAGCATTTTCTTTGTCGTTTACCACTGTATCGGCTCTTAATTTTTGGGGTACACGTTGTGTGTTTTTTTTC
ATCAGCGACTAATATTTTTTTTTCTAAACTTTACAGGAATAAATATTTAAAGAAACTGGAGATAAACCTGTGGAGCTGAATCAGCACCGGTGGAAATGGCAGACTGACTATCTTTTAATACA
GCATTCCAGTCCCTCTGTTTCCTTGCTTCACTGCACTTGCCAATGAAAACTTGCACAGCCTTAGCTCTAGAGATGTCTTCATGACTGGCTTCACTCTTTGCTAGCTTATAGTGATTGGCTGC
TCTCTCTGGTTCACCCAATCTGAGATAGAGATTGGCAAGCCGCTGATGCGCTCTTAAATAACAAGGATCAATCCTGATAGCCTGTCTGCACTCCTGCAAAGCTTCAAGGAGACGGCCGAG
GCCGATTAGCGCTGCCGCTTTGTTGCTCCAGTAAGAAGCATTTTCTGGATCAATCACTATCGCTCTATCGTAGAACGCCACGGCCTCCGCATACCTCCCGTTCTTATACTCCTCGTTCCCCA
ACTCCTTCAGCTCTTCAGGATCAAGTCTTCTTGAAAGCGCCCGGCACATGGACACCGGAATCTCAGCCTCATACTCAGAGCTACTTCTACGCCTCACCTCCGATTCCATCTCTTTTGCCGT
CTTGGGGAGATAGTCGAGGACATCGCGGTTCGGAGTGACGGCGCCGGGAGCTCGGAGGTTTCCCAAATTGCTGTAGAGCATGACGTTACCAGAGGAAGCGCGGACAAGGGTATTGCTC
CCCTTGGCGCGTTGGTGATCGTAGATCATACTGTCGAGATCTGCCGATATGCTGCTGGTGGTTTGTGGCCGCACGCCGCGACCAGCGGATGGCCGATTGGTTTGATCTGGGGGACACGCG
GCCGCGGCAGGCTTGGGGGAGACTAGGATCTTTGGTGTGACGGCGGCAGGGGATTGGTCGGGGCCAGGACGCCGCCGCTTGGTACTATGGCTGCTTCCACCAGAAAGGGAGGCATTGG
CGACAATAGGGGAAGGAGAGGGGTGGCGAGGAGAGACGGGGGCTGATCCAGCACGGCGGCGGAAGAAGGCGTTGTAGAGAATGAGCAAGCCACAGCTGGAGGAATTTTTGTCCTCAT
TTCCCATTCGTCGCTGTCGACGGGATGATTGCCTTGCGGAGGGATTGGAAATTGCGTCTGGGATTTGGTTAATCGGACCTCAAAAAGGAAGATGAAGCATTCATTCTTCAAATTTGGATTT
AGAAAACATTGATTTTGGTTAATCTCTGAGAATGGAGGAAGAATAATGATCACCCGGAGGCATTGTAGGTTCAAATGAGTTGTCTCTGATCAGGGAGGCGAACATTGGCTGAAAAAGGA
AGAGAGAGGAGGAAAGAAGATGACGGT
>comp163418_c0_seq33
GTCAAGAAATCTAAGTAAAACAACAAAGTTTATGCACTTATATTCAAAATTAAAAACATTTATATTTTAATTGCAAGATAACTGTTTTTTAATTAAAATACTACTCAAGCTCATTCCTTTTCCT
AAAAAAAATAAGATATTTTTATTTGGAACATACGTCCTTTAATTTTGCAGCGTAAATGGTAATTTTTGTACAGTTATGACATTTACTAAATGTGTCCCTTCAACATTCTTTTGATGTCCTCAAT
TTCATAGTTAATACCAAGTAATGGGCTTAGTCAAATCTTAAACAAGGAAAAAAAAATTCTGAGCTTCCAGAAAATTAATTTTGCATTATCTTCTTTTTTTTTTCGTCTATGGCTTCAAGCCTT
TGAAATAAGATGTACAATCCTTTATTTTGATTGCATGGATATTTCAAAATTTTAATGCTTCTTTATAGCCTCTCATTTCAACAACGGCTGAAAATTTGGAGAGTCCTCTCTAGCTGTTCTATGT
CAGAACCTGGAACGTCTTCTATGTTGGAGCCATTCTTGTATAATTTGAAAATAGGCACTGAACTCACATTCTCTGAACTTACAATGCTAGCTTGTTCCTTCATGTTCACCTTGAGAAAAGTC
ACAGACGTGTGTTGCTTGCAGAGCTGCTCTAGATGATGCAATATTCCTTTTGAATCTTCATCTGATTGGTCATAGAATAGTGCCAGCGACAGTCCTGCCAAACATAGATGCCATGAACTATG
TATTAAGAACTTAAGTAATTTACAGATTTTAGCAGTTGCATAATAACTTAACATTCTCATTAAAATGTGAATCAAAACAATAGAGAAAAAGGCTAAAGTGGAATTTGCACAATTTGTATGAT
ATCACTTTAGTTCCAACGATCTAATGTGTTCAATTTATTCTTTTAACTAATTAAAAATGATCCAAAATAGTCTTACTATTGAATTATTTTGGATCAATTTTGAGATGTCAATGGGTTATTTTGAA
TCTAGTTTAAAGAGTCGAGGAATTAAATTAGAACATTAAATTGCTTCAAATCAAAACTACCTCCTATAAATAGTCTGAATCATTTTGATCTTTTTTTCCTAAACAGTGGATGATATGAAGAGT
GGTCTCAATGTCATGCCAACCTAACAATTATCAAAATCCATTATATGAAGCAAGAAATTAAAAGAGGGGAATATTATCAAATGCATCATATATCGTCATCATTAGCCTTATACAAATACTACTT
GGGGTTAGCTACGTGCATAACATTAACATGTAGCACACAAAAATTGCATTTTCTTCACATATTACATGGTAGATGGAATGATAATCAGTAAGGCATATGGGCACAGGAAATGTGTCTCTTAT

GCAAGCACGTTTTAAATGTAACAAATCGCCAATCTCCATATGCTTTGGCCAGATGCCACATAGTAATGTAGCATTCCTTAATTTTTTTAAGAATCTCTAATGCTAATGACACGTACCACATGA
TGTGATAACACTACGAAACTGGTCTTTTGTAGTGATATGAACGGCAGAGTCATCTGCCATCTGTTTAAGGCCTTCACTTTGTCTTCGCCTGACATGCGCTTTAGCATCCTGCAAGGCTTTGT
TTACTGTTTCATCTCCCGGCATTTCAAGAGCCAATACTTCATAATCTTTTATAGAATCTTCCCACCGATCCAACTTGACATTGCAGTCAGCCCTCCTTAGCCGAGCCTTGCTGTACCTGGGCC
GCAAATTAAGAACAACATTGCAATCTTCAATGGCCTTTTCCCATTGCCCAAGTTTCGACCGGCAAGCAGCTCGGTTGCATAGTAGGATTGTATTATGGGGGTCATGGTTAAGACCACTCCC
ATAGGCAAGGCATGCCTCTTCAAAATTCGAGGCCTTGAAGAGATCATTACCCATTGAGCGAGCTGATGCCACTGACCGAGCCTTTCGAGCAATAGCAGCAATCTCCCTGCTGCCAGGATC
AATCCGTGCAGCCATTTGAGCTAATGAAACAGCACTCTCAAATCTTCCTGTAGCCATATCAACCTGTGCTCTTATTGAAAGGTGATATGCATTGCTAAGGGCGCCAAAGAACTGTGTAGCA
GCATCAGTTTCGAATTTGGGTGCATCAATCATGGTTTCCTCCGCATCTTCCTGTCTGAGGAGCATCAGAAAGGCCTCCGCTTGGAATAAATATTTAAAGAAACTGGAGATAAACCTGTGGA
GCTGAATCAGCACCGGTGGAAATGGCAGACTGACTATCTTTTAATACAGCATTCCAGTCCCTCTGTTTCCTTGCTTCACTGCACTTGCCAATGAAAACTTGCACAGCCTTAGCTCTAGAGA
TGTCTTCATGACTGGCTTCACTCTTTGCTAGCTTATAGTGATTGGCTGCTCTCTCTGGTTCACCCAATCTGAGATAGAGATTGGCAAGCCGCTGATGCGCTCTTAAATAACAAGGATCAATC
CTGATAGCCTGTCTGCACTCCTGCAAAGCTTCAAGGAGACGGCCGAGGCCGATTAGCGCTGCCGCTTTGTTGCTCCAGTAAGAAGCATTTTCTGGATCAATCACTATCGCTCTATCGTAGA
ACGCCACGGCCTCCGCATACCTCCCGTTCTTATACTCCTCGTTCCCCAACTCCTTCAGCTCTTCAGGATCAAGTCTTCTTGAAAGCGCCCGGCACATGGACACCGGAATCTCAGCCTCATA
CTCAGAGCTACTTCTACGCCTCACCTCCGATTCCATCTCTTTTGCCGTCTTGGGGAGATAGTCGAGGACATCGCGGTTCGGAGTGACGGCGCCGGGAGCTCGGAGGTTTCCCAAATTGCT
GTAGAGCATGACGTTACCAGAGGAAGCGCGGACAAGGGTATTGCTCCCCTTGGCGCGTTGGTGATCGTAGATCATACTGTCGAGATCTGCCGATATGCTGCTGGTGGTTTGTGGCCGCAC
GCCGCGACCAGCGGATGGCCGATTGGTTTGATCTGGGGGACACGCGGCCGCGGCAGGCTTGGGGGAGACTAGGATCTTTGGTGTGACGGCGGCAGGGGATTGGTCGGGGCCAGGACGC
CGCCGCTTGGTACTATGGCTGCTTCCACCAGAAAGGGAGGCATTGGCGACAATAGGGGAAGGAGAGGGGTGGCGAGGAGAGACGGGGGCTGATCCAGCACGGCGGCGGAAGAAGGCG
TTGTAGAGAATGAGCAAGCCACAGCTGGAGGAATTTTTGTCCTCATTTCCCATTCGTCGCTGTCGACGGGATGATTGCCTTGCGGAGGGATTGGAAATTGCGTCTGGGATTTGGTTAATCG
GACCTCAAAAAGGAAGATGAAGCATTCATTCTTCAAATTTGGATTTAGAAAACATTGATTTTGGTTAATCTCTGAGAATGGAGGAAGAATAATGATCACCCGGAGGCATTGTAGGTTCAAA
TGAGTTGTCTCTGATCAGGGAGGCGAACATTGGCTGAAAAAGGAAGAGAGAGGAGGAAAGAAGATGACGGT
>comp163418_c0_seq39
GTCAAGAAATCTAAGTAAAACAACAAAGTTTATGCACTTATATTCAAAATTAAAAACATTTATATTTTAATTGCAAGATAACTGTTTTTTAATTAAAATACTACTCAAGCTCATTCCTTTTCCT
AAAAAAAATAAGATATTTTTATTTGGAACATACGTCCTTTAATTTTGCAGCGTAAATGGTAATTTTTGTACAGTTATGACATTTACTAAATGTGTCCCTTCAACATTCTTTTGATGTCCTCAAT
TTCATAGTTAATACCAAGTAATGGGCTTAGTCAAATCTTAAACAAGGAAAAAAAAAATTCTGAGCTTCCAGAAAATTAATTTTGCATTATCTTCTTTTTTTTTTCGTCTATGGCTTCAAGCCTT
TGAAATAAGATGTACAATCCTTTATTTTGATTGCATGGATATTTCAAAATTTTAATGCTTCTTTATAGCCTCTCATTTCAACAACGGCTGAAAATTTGGAGAGTCCTCTCTAGCTGTTCTATGT
CAGAACCTGGAACGTCTTCTATGTTGGAGCCATTCTTGTATAATTTGAAAATAGGCACTGAACTCACATTCTCTGAACTTACAATGCTAGCTTGTTCCTTCATGTTCACCTTGAGAAAAGTC
ACAGACGTGTGTTGCTTGCAGAGCTGCTCTAGATGATGCAATATTCCTTTTGAATCTTCATCTGATTGGTCATAGAATAGTGCCAGCGACAGTCCTGCCAAACATAGATGCCATGAACTATG
TATTAAGAACTTAAGTAATTTACAGATTTTAGCAGTTGCATAATAACTTAACATTCTCATTAAAATGTGAATCAAAACAATAGAGAAAAAGGCTAAAGTGGAATTTGCACAATTTGTATGAT
ATCACTTTAGTTCCAACGATCTAATGTGTTCAATTTATTCTTTTAACTAATTAAAAATGATCCAAAATAGTCTTACTATTGAATTATTTTGGATCAATTTTGAGATGTCAATGGGTTATTTTGAA
TCTAGTTTAAAGAGTCGAGGAATTAAATTAGAACATTAAATTGCTTCAAATCAAAACTACCTCCTATAAATAGTCTGAATCATTTTGATCTTTTTTTCCTAAACAGTGGATGATATGAAGAGT
GGTCTCAATGTCATGCCAACCTAACAATTATCAAAATCCATTATATGAAGCAAGAAATTAAAAGAGGGGAATATTATCAAATGCATCATATATCGTCATCATTAGCCTTATACAAATACTACTT
GGGGTTAGCTACGTGCATAACATTAACATGTAGCACACAAAAATTGCATTTTCTTCACATATTACATGGTAGATGGAATGATAATCAGTAAGGCATATGGGCACAGGAAATGTGTCTCTTAT
GCAAGCACGTTTTAAATGTAACAAATCGCCAATCTCCATATGCTTTGGCCAGATGCCACATAGTAATGTAGCATTCCTTAATTTTTTTAAGAATCTCTAATGCTAATGACACGTACCACATGA

TGTGATAACACTACGAAACTGGTCTTTTGTAGTGATATGAACGGCAGAGTCATCTGCCATCTGTTTAAGGCCTTCACTTTGTCTTCGCCTGACATGCGCTTTAGCATCCTGCAAGGCTTTGT
TTACTGTTTCATCTCCCGGCATTTCAAGAGCCAATACTTCATAATCTTTTATAGAATCTTCCCACCGATCCAACTAATACAAGATTCAAGATGTGACAACCTTTAGCTTCCAGATAAAGAAAT
TCATCTGACAAAGGATAAAAGGTTCTGCTCACCTTGACATTGCAGTCAGCCCTCCTTAGCCGAGCCTTGCTGTACCTGGGCCGCAAATTAAGAACAACATTGCAATCTTCAATGGCCTTTT
CCCATTGCCCAAGTTTCGACCGGCAAGCAGCTCGGTTGCATAGTAGGATTGTATTATGGGGGTCATGGTTAAGACCACTCCCATAGGCAAGGCATGCCTCTTCAAAATTCGAGGCCTTGAA
GAGATCATTACCCATTGAGCGAGCTGATGCCACTGACCGAGCCTTTCGAGCAATAGCAGCAATCTCCCTGCTGCCAGGATCAATCCGTGCAGCCATTTGAGCTAATGAAACAGCACTCTC
AAATCTTCCTGTAGCCATATCAACCTGTGCTCTTATTGAAAGGTGATATGCATTGCTAAGGGCGCCAAAGAACTGTGTAGCAGCATCAGTTTCGAATTTGGGTGCATCAATCATGGTTTCCT
CCGCATCTTCCTGTCTGAGGAGCATCAGAAAGGCCTCCGCTTGGTAGGCAAATACCTGTGGAGCTGAATCAGCACCGGTGGAAATGGCAGACTGACTATCTTTTAATACAGCATTCCAGT
CCCTCTGTTTCCTTGCTTCACTGCACTTGCCAATGAAAACTTGCACAGCCTTAGCTCTAGAGATGTCTTCATGACTGGCTTCACTCTTTGCTAGCTTATAGTGATTGGCTGCTCTCTCTGGT
TCACCCAATCTGAGATAGAGATTGGCAAGCCGCTGATGCGCTCTTAAATAACAAGGATCAATCCTGATAGCCTGTCTGCACTCCTGCAAAGCTTCAAGGAGACGGCCGAGGCCGATTAGC
GCTGCCGCTTTGTTGCTCCAGTAAGAAGCATTTTCTGGATCAATCACTATCGCTCTATCGTAGAACGCCACGGCCTCCGCATACCTCCCGTTCTTATACTCCTCGTTCCCCAACTCCTTCAG
CTCTTCAGGATCAAGTCTTCTTGAAAGCGCCCGGCACATGGACACCGGAATCTCAGCCTCATACTCAGAGCTACTTCTACGCCTCACCTCCGATTCCATCTCTTTTGCCGTCTTGGGGAGA
TAGTCGAGGACATCGCGGTTCGGAGTGACGGCGCCGGGAGCTCGGAGGTTTCCCAAATTGCTGTAGAGCATGACGTTACCAGAGGAAGCGCGGACAAGGGTATTGCTCCCCTTGGCGC
GTTGGTGATCGTAGATCATACTGTCGAGATCTGCCGATATGCTGCTGGTGGTTTGTGGCCGCACGCCGCGACCAGCGGATGGCCGATTGGTTTGATCTGGGGGACACGCGGCCGCGGCAG
GCTTGGGGGAGACTAGGATCTTTGGTGTGACGGCGGCAGGGGATTGGTCGGGGCCAGGACGCCGCCGCTTGGTACTATGGCTGCTTCCACCAGAAAGGGAGGCATTGGCGACAATAGG
GGAAGGAGAGGGGTGGCGAGGAGAGACGGGGGCTGATCCAGCACGGCGGCGGAAGAAGGCGTTGTAGAGAATGAGCAAGCCACAGCTGGAGGAATTTTTGTCCTCATTTCCCATTCG
TCGCTGTCGACGGGATGATTGCCTTGCGGAGGGATTGGAAATTGCGTCTGGGATTTGGTTAATCGGACCTCAAAAAGGAAGATGAAGCATTCATTCTTCAAATTTGGATTTAGAAAACATT
GATTTTGGTTAATCTCTGAGAATGGAGGAAGAATAATGATCACCCGGAGGCATTGTAGGTTCAAATGAGTTGTCTCTGATCAGGGAGGCGAACATTGGCTGAAAAAGGAAGAGAGAGGA
GGAAAGAAGATGACGGT
>comp163423_c1_seq10
CCGTGTTTAACTACTCATAAAATAAAATGTTAAAAGGAAGCAGGGTCATTACATAAACCATTAGAGTAATCAGAAACAGAATTCCTTCCAGTGAAAATTTTAGTGTAATTTACACCAATCA
GTGGTTCAAATCAGTAAATTACCATAATATTACACATGAATACTCTTCTTTAGCCTCTTCTAATTGAATTTCCTTTCCCCATCTGTACATTCATGAACTGCCATTTCTATCTTAAACCACTTAAT
CATTAAATGCGCATGAAGAATATAGAATATAAACTACAAGCTTTAGTAAACCTTTCCCAAGCCCATATAAAATTATCATCTCTTGGGTAGAACGATCAGGCAATCAGAAATCACTTCTAGAA
GCAGTCCCAAACAGTCATTCATTGTCGTCGTCGATAGATGTCCCTGATGACTCAAAGTGACCTCTATTATGAGGGAACATGGAGAAGCATTGTTGCTGGAATTCCGGAGGTGAGCTTGGTT
CAAATACATGGTGGCCGAGCTTCAAGTTCACCTCGACGATGCCGCCAATGTCTCCACCATTGCAGCATTGCCTCCTGAATAACCGGTCATGTCTGAAGCTCTCAGAATCTACAGGTGGGAT
ACCCTTGAAGAGATTGCAAGCATCCCTATGTAGAGAATCACAGATTGCTGCAGGGGTTGAAGGGTCAGTTTTCTCTGAGTTTGGATAGAAATCAGTGGAGGATAAAGGTGTTGGGGAGTT
TATTCTTGGTGTCTCAGCAAGGACTCCAGTTAGGCGTTGCTGCTCCTCAATGATTTTCTTCAGATACTTTCCCTGGGCATCTATCCTCAGCTTCAGCTGCCTTTGTACCTGAGGTAAGTCTTA
AAAGTTTGGTTCATTGAAGAGCTATACAAGTAGAATTTGTATAGTGTGCGTAGAATGAGTTTTTCAGGATTGAGACCATAGCTTTTTTAATTAGTAAATTCCGCCTTTATTCATATTACGACC
ACTATCCAGAAATGTCAAACAACAGCAATGAGACTGAGGTTTCATTTGGGGCAGCTTTCTTACCACTTTTTAAAGCAACTTTTTAGTAATTTTG
>comp163426_c4_seq1
TTTGCATGACGTATCAAGAATAAATTATAAACTCAACACTAGGGGAAAACATGAGCTGAGGGTCCTTTATTTCACATATTTGGAATCCACAAGGAGAACTATAAAAGCATGGAATAAGAAA
ATGTATGACTCTCCCACTTGATTATTCTTCATTTACCAACACGTTTGTTTTGATCGATGAAGATGCACAAGATCCCCTAAAGTGGGAATTTTTATTGGGTGCGAACACCAGACGGAGAGAA

CCATCCAATTTTCTCTCTGTCTATAGTAAAGATACAGTCAAAATGCAGCTGCTACAGTAATTGTGCTTTTACTGTAGACAGAGAAAAATTCAG
>comp163446_c0_seq4
AGAGAATCATCCGAATTTCTCTCTATTTATAGTAAAGTGCAGTGATTGTAGCAGCTGCGCTTTGACTGCACTTTTAATGTAGTCAAAAAAAAATCCAGATGATTCTCTCTATCAGATGTCCG
AACCTAATAACTTCTCGCGTAAACGGCATCAAATTGTCGCGTGCGTGACCGCTGTCCTCATTTGTGTTGTTAAAACGAGACGGAGACGAAGCCGTTCGCTTTTCTCACACTCAAATCCAG
CTAGCCAAGTCCATTTCCCTGTGCCGACTCTGCCTGATCTTGTCATCGGTTGAAGCAAGCTAGAGTCTGAAACATATAGCCTGTCACTTTACCTAGCCTTCAGCCAATCAAGCCGATTTACC
TCCTTTAACCCTCTCCACTTCTCACGTGAAAACCCCAGTTTAAGCCCACTCAAATAACACACTCCATTAAACCTTTATCAGACAAGGAGCAGAAAATAGAATCCTCCAGTGACTCGACCT
CAATGAGATCACCTTCTCCAGGCATAAGCTCTCGCGCTGATACTAGAGAAATATTCTCTATTAGAAGCAAAAAGAACAAGAGATTGAGAATAGTATAACAAGAGGCCAGAAGAGAATAAG
TTGCCATGAGAATAGAGCTTCGAATATCTGCTAGATCGGAAGAGCACAC
>comp163446_c0_seq6
AGAGAATCATCCGAATTTCTCTCTATTTATAGTAAAGTGCAGTGATTGTAGCAGCTGCGCTTTGACTGCACTTTTAATGTAGTCAAAAAAAAATCCAGATGATTCTCTCTATCAGATGTCCG
AACCTAATAACTTCTCGCGTAAACGGCATCAAATTGTCGCGTGCGTGACCGCTGTCCTCATTTGTGTTGTTAAAACGAGACGGAGACGAAGCCGTTCGCTTTTCTCACACTCAAATCCAG
CTAGCCAAGTCCATTTCCCTGTGCCGACTCTGCCTGATCTTGTCATCGGTTGAAGCCGCTAGATCCACACCGACGCAAGCTAGAGTCTGAAACATATAGCCTGTCACTTTACCTAGCCTTC
AGCCAATCAAGCCGATTTACCTCCTTTAACCCTCTCCACTTCTCACGTGAAAACCCCAGTTTAAGCCCACTCAAATAACACACTCCATTAAACCTACAAAAGCTCATTTCAAACAGCAAA
AAAAAAGTAAATCTCCTTTTACTGTGTCCATGATTATTCGCATTAGCTATTATTGTTAGATAATATATTTAGCATTATTAGTTTTTTTTAGCTAATTATGCACATTATTATTGGTGAGAGTAAAAC
AACAGAAAAAAAAAAAGAAAGTGAGAAAAAGATAT
>comp163446_c0_seq9
ATTTGATTATAAGCATGCCATATTGGGATATTATTTCATTATCCAAATAAAGCTTTAACCCTTCCAAATGTGGTCTGGGCCCCCGATTTGGACCCATTTGGGCTTGGGTTGGACCAATTTCGG
TGTTGTTCGGTTCGTCCCTTTGAGATGTTCGATTTGATTGTAAGTCTCCTTTATCAGGAACGGAGCAGAAAATAGAATCCTCCAGTGACTCGACCTCAATGAGATCACCTTCTCCAGGCAT
AAGCTCTCGCGCTGATACTAGAGAAATATTCTCTATTAGAAGCAAAAAGAACAAGAGATTGAGAATAGTATAACAAGAGGCCAGAAGAGAATAAGTTGCCATGAGAATAGAGCTTCGAAT
ATCTGCTAGATCGGAAGAGCACAC
>comp163465_c1_seq2
AATTAAGAAGTTAGATGTTTGAACCCAAATGGCTTGATTTTTCTTTTAAACATCACTTGCAGGATGCTTTTGGGACAAAACCTCACAAGAAATATCCAGTTTTGTTGAGGACTGTTGTTTTA
ACTTTGGTAATGATTAGCAGTATTTACATATGCTCAATATGTTTAAAGCAAAACGGGTTCTTGATCAGTCCCAATGTGATGAGGGTTGAAATTATGAATGAACCTTGTCGGCGCCCTGACAT
TCCTGCATCTGAACTACATTACATTCACTACCCAGAACCGACCACGTATAACAGCTTAATTGGCAGAGATGAGTGTGCATGTACCCCAGTTCGGTATTTTGCTATATTATCAATGCAAAGGT
CTGGTAGTGGTTGGTTTGAGACATTGTTAAATAGCCACATCAATATCAGCTCAAATGGTGAGATATTTTCTGTAAAAGAAAGGCGAAGCAACATTTCTACTATCACAGCCACCTTAGATAA
GGTATACAACTTGGATTGGTATAGCAGTTCCTCTAAGAATGAATGCACTGGTGCTGTTGGCTTGAAATGGATGCTTAATCAGGGTCTGATGAGGAACCATGAGGGCATAGTTGAATATTTCC
AAAATAGAGGTGTTTCTGCTATATTCCTTCTTAGAAGAAATCTTCTTCGTCGGTGGGTCTCAATTCTAGCAAATTCTCATGACCGAGAAGCAAAGCAATTGAACGGAACTCATAAAGCTCA
TGTGCATTCACGACACGAGGCTGACATTCTTGCAAGATATAAGCCCTTGATTGATGTGAATAAGTTCATTGTAGATTTCAAACAAACTAGTAAATGGTGTGCAGATGCTCTTCACTATTTCA
ATGGCACCAGGCACATTATCTTATACTATGAGGATCTCATTCTTAATCGAACAAAATTAGTGGACGTTCTTGACTTCCTAAGAATGCCACAAAGAGAGCTTAAAAGCCGCCATGTCAAGAT
CCATACAAGACCATTATCAGATCAGATTGAGAACTGGGATGTCGTTTCCCAATCACTTGAAGGAACTGAGTATGAAAGTTTCTTGCATTCCGACCATGGAAAATAGAGTGGCTAACCACAA
TCTTGCCTGCTGTAAATATTGTTGACAGCATTGGTTCTTTTGTTGATACTAGCGGATTCATTCAGACTCCTTTTTCCTGAAGAAGCAGGAAAATTTTGGCTACTTCAAAGGGTCTTTGACTG

CTAACAGAAAGGTTCTTTTAGAGTTTTCTTTAGGCTTTTCTCATCCTTGTAAGGTTTTTATATTTAGAACTGCTTTTCACAACCTAGCAAATAGCAAGGTTGCTATTCTGTTATCCATGTGCTA
AGCATTCTTTTATTTAATAAAAAAAAATGATTACTTGTTTCTCTCGAATTTGTAGTGGTTCTAATCAAATGTAACTACAAATTATAAACAAATTACAAATACTC
>comp163479_c1_seq2
GGTAGTTGCAAAATGAAGTATATAATTTTTAAAATTTTATTTCTCCATAGTACAGTCTAAAAGCTTACATAATCCTTTTCTCACAAACATAATAAAACAAATCAAGCAAAATACTTTTTATTCT
TATTAATCTCACCATAAAGTTGAACTAAAAAGTCATATCTGAGACAACACATTCATCATATATTGATGTGGCCTGCACAGAGCTTCAAGATGCTTAGCTCTCGGCATTGTAAGTCCTGACCC
TTCCGTCATGTCCACCTCTTCTGCTCCAATCCTCTTCCACTCAAAGCATTGGATTAGAATCCCCAACACTAATCCAATCTCCTTCAATGCAAGCCCTTCTCCCGGACATCTCCTCCTCCCCAT
CCCAAATGGCATCATCGTCATCTTCGACCCTTCTTCTTCCTTCGCATTTTGAAATCTTTCGGGGAGAAATTTGGTCGGTTCAGACCAAATCGCAGGATCTCTATGGATTTTATGAACATTTAC
TAAGAGCAGAGTTCCTCGGTCCACGGCGTAGCCTCCGATCTCGCAGTCCTCTCTTGCTACGTGAGGGATGAGGAGAGGGGCGACGGGATACAGACGGAAGGTTTCGTTGATGATGCAGT
GGAGGTATGGAAGTTTGATGAGATCTGATTCTTCGAGGAGGCGATCATGTCCGATTTGGTCATCGATCTCTTGCCTCGCCTTCTCTAATTTGTCGGGATTGTTTAGTAAGAGAGACATCGAC
CATTCTATTGTGTTTGATGAAGTGTCTGTGCCCGCTGCGATTAAGCTATTAATAAGCGATTTAATGAAGACATCAGTGTACTGCTCCGGCTGGTCGTCCTGCAAAGAGAGCAGAGCATCAA
TCATGGTTTTATGAATCTTCTGCTTCTGCTTCAATCCCCCTTCTTTTTCTTCTTCTTCCTCCTCTGCCCTTCTTCTCCTCTTGTGCTCTTCTATAAGTGACTGAACAATTTCATCCCTGTACTTG
TGATACCTCGCCAGCTTCTTATCCACTTCTCGACGTGCAAACCACCCGATCGCCGCCGGCAAAAAGTCTCCGACGTTGTTCGCGCCGGTAAGCGAGAAGATCTTCTCTATCACGTCCTTG
AAATTATGCGCCTCCTCCTTTTCACCTTTCTCCCCATAATACCTCTTCCCTGTTACACATTTGATAATCGATCAACGAAAATTGATAACGAATTTCGAGTTAAAAGTTCAGAACCTGCTATAA
TCCTCATCATGACATTCATTGCAAGCCCGAACAAGCTTGAACTGATCTCCACTTTCCCCGAGGAATGGAAGAGGCTTCGGGCCAGGGATCGAGCCTCGTCCGCCCGAATATCGGAGAAG
AAGGCGAGGCGGGGGCCCGAAAGAGCTTCGACGGTGGCGATTCGGCGCATTTCGCGCCAGTGGGGTCCGTATGCGGCGAAGGGGAGGCTGGTGTGGTTGTAGGTTATGACGGCCATGG
AAGGGAAATGAGGGCGGTCTGCGAGTGCGATGTCGTTGGTGGTGAAGCATTCATTGGCGATGGAGGCGGAGGAGACGATGAGGGCGGGATGGTTTCCGAAGCGGAGGAGGAGGATCG
GGCCGTGCTGGGTGGAGAGACTTGCAAGAGATTGGTGGAGGGGTTTCTGGAGAAGGTGGAGGTGGCCGAGGATGGGAAACGAATTAGGAGGGCTTGGAGGCGGCTTCTTCTTCTCCCT
CTTCTTGTTCTTGTTGGTTATGAAGAGTTTGATTACGAGGAGGAAGGAGAGGAAGAGAGCTAAGCTGGTGAGGATCTTCATGGCGTCCATGGCGGGAGGAGGAAGAAGGGGTTCTGTAT
GGGAATCGGTGGCTTTAGCTTGTTTAATTTATAGGCAAAATTCTTAAACAGAACGTATTAATAATTTAATTTAAAATTAAAGGGAAAAAAAGTATATTATAATATATATTTTTTAAATATATTTG
ATTTGATGTGCCTTTTAATAGTGAATTATATGACTGAAATAAATTTTACTTATGTTAACAAAATATGAAGAACAAGAATTTGGCTGGATTTATGTGAATAACTAGATAAGAATCATGTGAATGT
GAATCTACTTTAATTTTTGTTCTTAATGTGATCAATGGTGCAGGCGTTAAGAAAGGAGCCACTA
>comp163500_c0_seq2
AAAGATAAAAAGTATATTGAATTTTAAATAATAAATATACTCAACACCAATCTACTTGATCTTGAGTTGACTGGCATTGCAAGTAAATTTATGGATCTAAATAGTTAGTGCATAGGGTAAAAA
AAATAAATATGCTAGAATAGAAGTAGTAAAGAGATAATAATATTTGTTATCATCAAAATTATAAGCTCAAAATTCCAAATTAAAACACATAAAATATAAAAATATAGATTCTCATGATTATAGA
TCTCATGAATCCTTCGTGAATTGAGCTCATACATTTTTACCTTTGCCCTAGAAATTGGAACACTTACCATGTGCTACATCAGCTTACCTCAAAGTGTGTACATAAACGTCAATCTAAATCATA
GCCAAAAATCATTACTGTTCAAATAAGTTAAAAAATAAAAGCTACGGGAAAACTACTCCACGCCCTCCCAAATTTGGTACAAAAAAACCTTCATTGGAAGAAGATCTCCAAATTCTTTTT
GTCGATTCTGCATCCCAGATGGAAACAATTCTGCAACAGTATTCGTCAAATGATGTCATATCAAGGATATTAATTGCTACAATGGGGATGGTTAAGAAATGTAAGAGAAAAATCATTTCACC
AGTAAAGACGAAAAGCCATCAAGCTCATCTGAATTCATCAGATGATTAGGTCAAAAGCAATAGGCCTGCATAAGGCTTCCCACAAATAAGACATGTTTCTATTTGATTTTGTGCAGACATT
TTGTTAAACATTTACAAGGTAATCTTCATTCTTTATGGATAAATCGAAATACCATACTTCATTGTTCGTTTTCCTCTTTGCAGTGAAATAATGGAGTCACATCTCAGCTTGTTTGGTAGCCTCC
TGCAATTCAGCTAACTCCTGCTGATCCGGTACAATCTTCTTCCTCTTTTCCGTCTCCTTCTTTATAAGCTCGCGAAAAGTAGACAAAGATTCGAGTACCTTTGCTGCTTCAATTTCATCCCTT
TTATCTGAATTCCCAATCTCAACCTTCTTGACAAATGCATTAAGACTATCCACATGTTCGCTAGCCTGCTTTAGAACACTCTGCATATCTGTCCGTCTCTCCATGGTCGAATCCTCCAGCACC

TGCATCTCATTCACAGCCCTCTGGTTCTCCTCTCGCTCCACCATGCGCTCATGAGCGTTACGGCCGAACATCTGAACAGCTTCCATGACTTTCAGCTGAGCCCATAGCTTCGTCGCTTTAAC
ATGGTCTTCCAGCTTACGGATCCGGCGAATAAGGTCCATGATCATCTCCGTCTTCTGGTCCTGAAGCTCCTTATCTTCACGCTTCTCATCCTCAATTCTCATAGCCTCCAGTCTTTTGAGCAC
CAACTGCACCACGTCCTCTGCCTCTTCCTCCACTTCCTGCTGCCTTTGCCTGCCCTGCATCTTTAGCTCAGAGAAACAATTATTAGTTATGTTCTGTTTCGGAAAAAAAAATTAAGTGACGAC
GGTGAATCAAAACGTTCCATGCTTTCTAATGGATGGGCTGCAGCACCATAGACAGAAGCTGGTTCAGAATCTCCACTCACCGCAAAAGGCAAAGCGGCACCAGGCGTGAATGCGCCCGC
AGTACTAGCTGCTTGGAGATTACCAGGAGGCGAACGGGAAGGGAATTTAATGGAGCTTGCACGGCGAATGCGGAGGCGGCTAATGAGGAGGCACCATATGGCCTCACCGCGCGAGAGG
TTTGCGCTGATCTGCTGCAGAAGGCAAACCATGGCAGCGAGAGAATACTCCTCGAGGTGGCGGAGGTCAGAGCAGGCGCCGGCAAGAGCGTTCCAGTCATCGGGAGTGTTGGCAGCTG
CCGTGTTCAGGAACTCGAGTGCGTTCTGCATGATATTAGATAGTGGGTCCACGCCGCCGTAGTAATGGCCCTTGCAAAGAACGGCCCGAAGCGCCATGGCAGCGTCGTATCCGAAGGAG
ACCAAACGGGAGATAACCTCCTTGTAGACGCGATCGAGGTAATTGATAAGAATCTCCTCGAGCTGCTCCTCGGAGAAGTTGCCGCACCCATTTTCATCAAATCCGTAATTGTAAAGACTAG
GGCCAGGGTTAAACCAGGGGCCTCGATTGTGTTTGATGCTAGGGTTCGCAACACGGGTAGGATCAGACAAGGGAACGGCAGTAGGAGAAGCACCGGGGGGGTGTTTGAGGACTCCGG
AGTCTGAAGGGTTCATCGGGACCGCCGCCGCCGGGGGGGCTTGGCGCTTCCCGTCAATGGCACCAGCACTGAGGGGCTTCTTACTACCTTTGCGGCCCATGGTAGTCGATCTGCAGCAA
TTGCAGGTTCAGGGGACGGTGGTTGATAAACCTTACGCATTTCATGTCTTTTTGGTTGACAACCCCTCCCTCCAGATCCTAACTTGGCGCAATTGAAAAGCTCCATGGATCTCTCCGAATC
TCTTGGTCAGCTCCTACATATAAGAAAGCAAGATTTACTACAGATTGTTAGAATAACTCTAACTTGTTAAAGATA
>comp163500_c0_seq3
AAAGATAAAAAGTATATTGAATTTTAAATAATAAATATACTCAACACCAATCTACTTGATCTTGAGTTGACTGGCATTGCAAGTAAATTTATGGATCTAAATAGTTAGTGCATAGGGTAAAAA
AAATAAATATGCTAGAATAGAAGTAGTAAAGAGATAATAATATTTGTTATCATCAAAATTATAAGCTCAAAATTCCAAATTAAAACACATAAAATATAAAAATATAGATTCTCATGATTATAGA
TCTCATGAATCCTTCGTGAATTGAGCTCATACATTTTTACCTTTGCCCTAGAAATTGGAACACTTACCATGTGCTACATCAGCTTACCTCAAAGTGTGTACATAAACGTCAATCTAAATCATA
GCCAAAAATCATTACTGTTCAAATAAGTTAAAAAATAAAAGCTACGGGAAAACTACTCCACGCCCTCCCAAATTTGGTACAAAAAAACCTTCATTGGAAGAAGATCTCCAAATTCTTTTT
GTCGATTCTGCATCCCAGATGGAAACAATTCTGCAACAGTATTCGTCAAATGATGTCATATCAAGGATATTAATTGCTACAATGGGGATGGTTAAGAAATGTAAGAGAAAAATCATTTCACC
AGTAAAGACGAAAAGCCATCAAGCTCATCTGAATTCATCAGATGATTAGGTCAAAAGCAATAGGCCTGCATAAGGCTTCCCACAAATAAGACATGTTTCTATTTGATTTTGTGCAGACATT
TTGTTAAACATTTACAAGGTAATCTTCATTCTTTATGGATAAATCGAAATACCATACTTCATTGTTCGTTTTCCTCTTTGCAGTGAAATAATGGAGTCACATCTCAGCTTGTTTGGTAGCCTCC
TGCAATTCAGCTAACTCCTGCTGATCCGGTACAATCTTCTTCCTCTTTTCCGTCTCCTTCTTTATAAGCTCGCGAAAAGTAGACAAAGATTCGAGTACCTTTGCTGCTTCAATTTCATCCCTT
TTATCTGAATTCCCAATCTCAACCTTCTTGACAAATGCATTAAGACTATCCACATGTTCGCTAGCCTGCTTTAGAACACTCTGCATATCTGTCCGTCTCTCCATGGTCGAATCCTCCAGCACC
TGCATCTCATTCACAGCCCTCTGGTTCTCCTCTCGCTCCACCATGCGCTCATGAGCGTTACGGCCGAACATCTGAACAGCTTCCATGACTTTCAGCTGAGCCCATAGCTTCGTCGCTTTAAC
ATGGTCTTCCAGCTTACGGATCCGGCGAATAAGGTCCATGATCATCTCCGTCTTCTGGTCCTGAAGCTCCTTATCTTCACGCTTCTCATCCTCAATTCTCATAGCCTCCAGTCTTTTGAGCAC
CAACTGCACCACGTCCTCTGCCTCTTCCTCCACTTCCTGCTGCCTTTGCCTGCCCTGCATCGCAAAAGGCAAAGCGGCACCAGGCGTGAATGCGCCCGCAGTACTAGCTGCTTGGAGATT
ACCAGGAGGCGAACGGGAAGGGAATTTAATGGAGCTTGCACGGCGAATGCGGAGGCGGCTAATGAGGAGGCACCATATGGCCTCACCGCGCGAGAGGTTTGCGCTGATCTGCTGCAGA
AGGCAAACCATGGCAGCGAGAGAATACTCCTCGAGGTGGCGGAGGTCAGAGCAGGCGCCGGCAAGAGCGTTCCAGTCATCGGGAGTGTTGGCAGCTGCCGTGTTCAGGAACTCGAGT
GCGTTCTGCATGATATTAGATAGTGGGTCCACGCCGCCGTAGTAATGGCCCTTGCAAAGAACGGCCCGAAGCGCCATGGCAGCGTCGTATCCGAAGGAGACCAAACGGGAGATAACCTC
CTTGTAGACGCGATCGAGGTAATTGATAAGAATCTCCTCGAGCTGCTCCTCGGAGAAGTTGCCGCACCCATTTTCATCAAATCCGTAATTGTAAAGACTAGGGCCAGGGTTAAACCAGGG
GCCTCGATTGTGTTTGATGCTAGGGTTCGCAACACGGGTAGGATCAGACAAGGGAACGGCAGTAGGAGAAGCACCGGGGGGGTGTTTGAGGACTCCGGAGTCTGAAGGGTTCATCGGG
ACCGCCGCCGCCGGGGGGGCTTGGCGCTTCCCGTCAATGGCACCAGCACTGAGGGGCTTCTTACTACCTTTGCGGCCCATGGTAGTCGATCTGCAGCAATTGCAGGTTCAGGGGACGGT

GGTTGATAAACCTTACGCATTTCATGTCTTTTTGGTTGACAACCCCTCCCTCCAGATCCTAACTTGGCGCAATTGAAAAGCTCCATGGATCTCTCCGAATCTCTTGGTCAGCTCCTACATAT
AAGAAAGCAAGATTTACTACAGATTGTTAGAATAACTCTAACTTGTTAAAGATA

>comp163512_c1_seq2

GAAGGGCTACGCTGGGCAGGGGTCCCTCGTTCTTTAGCTATTGCGAATTCGAAGAAGCGAAGCGATTAATGGCGTTGGGGAGAAGAATAGCTCACCGATTCCTCGATGGGGCGAAGGGC
TACGCTGGGCAGATTAGATCCGGCGCCGGCGATGTTGGGAAGGCAGCGCGAGGCTCTGTTTTCCGTGGGGTTTGTCCTGACTCCGGCGACCGTTGGATACAGTGTCGGCCCGTTTTCCAG
GCGGCGGTACCGCCTGACAGGATATCGATGCCTTTTGGTTTGAATCTTTTTGATCGGATATGGGGGCAGAATGGTGATTGTATGAGGCGGGAGATGGTGGCTTCACCGCCGCTGGTGTCGC
CGACTGCTGGTGAGGAGGAAGAGGAGAAACGGGCTGCAATGGGAATGACGGTGAAGGAGGTAAGGAAAGTGCTTAAGGCGTCGCAGATGGAGGTGGTGAGGTCGAGGTTGAGGGCG
ATTCCGAAGAGCTTTGTGGCTACATCGGAGTTCTTGCGAATCTGTTGCGAGGTTTCGGGGGAGGAAAAGGGACCGGAGCTTGCGCGGTCTCTGGATGAGTCCGCGGCGGTAATTGTTATC
GGAAACGTCGTTTTTGTCAGGCCCGATCAGGTAGCAAAAGCAATCGAGCGCACAATCCCCCTTGCTCTCTCCCCTCTAACTGACCGAATGAAGGAGGAGCTAAGAAAGCTGGAACAGC
AGAAGGCTACGATCGACAAGCTCGCAGAGGCCAAAGCAAGAAGGGAATTATGGTTTGGCCTAGCTTTCCTGGCCGCTCAAACCGCCGGGTTCATGAGGCTAACGTTCTGGGAGCTTTCA
TGGGACGTCATGGAACCCATCTGCTTCTACGTCACTTCCATGTACTTCATGGCCGGCTACGTCTTCTTCCTGAGGACTTCCACAGAACCTTCCTTCGAAGGTTTCTTCAAGAGTCGCTTTG
CCACGAAGCAGAAGCGGCTCATGAAGGCTCGCAATTTTGATCTCGGCAGGTTTAATGAACTCAAGGAAGCCTGTCGTTCAAGCGAGGCGCCTTCAGAAATGGAGCTGGTGGCTTCTTCT
TCCTGTAAGTAATTACCCTTTCTCTTCTGCAAAGAAGGGAAGCCGAGTATTCTTTTTGATCTGCGGATTGTTTTCTGTTACCGGAGTTCAATCAATTAGGCCTGCATTGGCTGAAATAGGAG
GAGCAATTTTTTGTATTGATCATCTTATTATCTACCATTAGAACTATAGAGTATGAAGTGGGGGAAATTTGAGGGAACGATGTAGTAGATATAGGCATTTTTTTCCTTAAAGAAACACTGTTA
TTGCGGCGATTATCACTGTTATATTATGATGTTTTTTTTTTTTTGTTCAACCATTATAGTGAATTTATTAGTGGATATTTTCAATTATTGTATCAGTTCTTTTTTGTGCAATCAAAAAGAGCAATCT
TCCTTTTTCTTGGAATTGTAAATCTTGGAAGTGTCAAAATTTGACGCAACTTGGAACT

>comp163512_c1_seq5

GAAGGGCTACGCTGGGCAGGGGTCCCTCGTTCTTTAGCTATTGCGAATTCGAAGAAGCGAAGCGATTAATGGCGTTGGGGAGAAGAATAGCTCACCGATTCCTCGATGGGGCGAAGGGC
TACGCTGGGCAGATTAGATCCGGCGCCGGCGATGTTGGGAAGGCAGCGCGAGGCTCTGTTTTCCGTGGGGTTTGTCCTGACTCCGGCGACCGTTGGATACAGTGTCGGCCCGTTTTCCAG
GCGGCGGTACCGCCTGACAGGATATCGATGCCTTTTGGTTTGAATCTTTTTGATCGGATATGGGGGCAGAATGGTGATTGTATGAGGCGGGAGATGGTGGCTTCACCGCCGCTGGTGTCGC
CGACTGCTGGTGAGGAGGAAGAGGAGAAACGGGCTGCAATGGGAATGACGGTGAAGGAGGTAAGGAAAGTGCTTAAGGCGTCGCAGATGGAGGTGGTGAGGTCGAGGTTGAGGGCG
ATTCCGAAGAGCTTTGTGGCTACATCGGAGTTCTTGCGAATCTGTTGCGAGGTTTCGGGGGAGGAAAAGGGACCGGAGCTTGCGCGGTCTCTGGATGAGTCCGCGGCGGTAATTGTTATC
GGAAACGTCGTTTTTGTCAGGCCCGATCAGGAATTAAAGTTAAGGGTTCCGTGTTTGAAAATGATAGCCAAGGATGAAGAGACCTCAGACCAGTAGCAAAAGCAATCGAGCGCACAATC
CCCCTTGCTCTCTCCCCTCTAACTGACCGAATGAAGGAGGAGCTAAGAAAGCTGGAACAGCAGAAGGCTACGATCGACAAGCTCGCAGAGGCCAAAGCAAGAAGGGAATTATGGTTTG
GCCTAGCTTTCCTGGCCGCTCAAACCGCCGGGTTCATGAGGCTAACGTTCTGGGAGCTTTCATGGGACGTCATGGAACCCATCTGCTTCTACGTCACTTCCATGTACTTCATGGCCGGCTA
CGTCTTCTTCCTGAGGACTTCCACAGAACCTTCCTTCGAAGGTTTCTTCAAGAGTCGCTTTGCCACGAAGCAGAAGCGGCTCATGAAGGCTCGCAATTTTGATCTCGGCAGGTTTAATGA
ACTCAAGGAAGCCTGTCGTTCAAGCGAGGCGCCTTCAGAAATGGAGCTGGTGGCTTCTTCTTCCTGTAAGTAATTACCCTTTCTCTTCTGCAAAGAAGGGAAGCCGAGTATTCTTTTTGA
TCTGCGGATTGTTTTCTGTTACCGGAGTTCAATCAATTAGGCCTGCATTGGCTGAAATAGGAGGAGCAATTTTTTGTATTGATCATCTTATTATCTACCATTAGAACTATAGAGTATGAAGTG
GGGGAAATTTGAGGGAACGATGTAGTAGATATAGGCATTTTTTTCCTTAAAGAAACACTGTTATTGCGGCGATTATCACTGTTATATTATGATGTTTTTTTTTTTTTGTTCAACCATTATAGTGA
ATTTATTAGTGGATATTTTCAATTATTGTATCAGTTCTTTTTTGTGCAATCAAAAAGAGCAATCTTCCTTTTTCTTGGAATTGTAAATCTTGGAAGTGTCAAAATTTGACGCAACTTGGAACT

>comp163540_c1_seq4

ATCAGGTGTATCTTGGGATGAAACGAAGAAAATGATAGTGATGGGTCAAGAAGAGTATGCATCACATATTCAGGTATTATTTTAAGCACATAAATTAAATTTATATGTTGGGGTTAACAACTT
ATTTTGTATTCCTTACAGGCTTTTCCCAAGGATGCTGCTTATTTAAATATTGCAATTGCAAATTATGTTGAATTGGAGATAGTATGTGGTATTGGACATGCAACTGGAGAGTGGGCGAAATCT
GGAAATTCTAAAACTCCACTTGGAACGCAACAATTACATTTAAGCGATGATGAATCCCCAAAATTTATGGATGTTGGTACAGATGGACCAATGGATATTGAGTGCTCTGATATGCAAGAGA
AAGAGAATAATACCGGAACAACAAAGAAACCTCAACCTTCATCTGCTATAGGAGGCGGCGTGAAAAAGAAGTCTAAAAAATCAACTGTCGATCACGACATTCGTGAGTATATTTGTTTGA
TGGCAGAAAGTGTGAATGAAGTTGCTAACGCAATGAAGAGCTCAACAACATCTCCAAAACAAATTCAGAATTCGAAGGCTAGATGGATGCGT
>comp163541_c0_seq2
ATTGTATAACTTTCTATTTTCTGCTAGAATTGTCTGATAGTTTTCAGCAGCATCTAACAACACTTTTAGTCTAAACCCCATGCTGTTCAATTCTTCTGTCCATTGCCTTTGAGAATTATTGACT
TCTACTTTGATGGACTCTGATGATGTCCTCAAGCCCTTTAGTGATTGTAGTTGAAAAGAAATGAAATTATGGAACACATTCTCCTTTTTAGTCCAATTATGGAACTTAAACTCAGAAAATGC
CTTAAGTTCATTCATCCTCTTACTCGACTCTGCTAGCAAGAATTCAGCCTCTTTTGTTCTTTCTTGTAATCTGGCTTCATTTTGCTTTGCCTTGGCATCTAATTGCTGGTAATGATCTTCGTAA
GTGCTATTTATTGCTTCCAATTCCTTTTTAAATTCTAAAATCATTCTATCATTATTTTCCTTCTCTTTCATCAACTTCAATGTTTGCTGTTCCTCCTGTTTCTTACTCTCCTCGATTGCAGTTTTC
TTTACTTGTGCTTCTTCATTTGTTCCAGATGCCAGTGTCTCAAGCACTCTGATTCTTGATTGATACTTCTCTTCGCGTGCTTTAATCAGATCATTTTGATTACGAATATGCTCTGTCTGGGTTG
ATATCCGGCGCTCAATCTCTTGCACAACTTTTCTCAGTAAACTCGCTACACGCAGAGGCATCTCTCCATTCTTCCTATCGATGCTTTCATCCAGAATTCTGTTAATGACACTTAGAAGTGATT
GGGTTGGAGCATTATCTAAACCATTTGATTTAATGATTTCAGAAATCTTGGCTGCAGGAATATCTGAATAACGTCCATGCTTTAGCTGGAACACCTCATGAAACTTATGGCCCCCATGACGC
AGTGAGGCGGTTTGCTCTGGCACAGCAGAACTATGCCAAACGCGATGAAATATTGATTCTGAATACTTATTTTTGTTCTCCTCTTCTCCAGTTGGAGAATTTTTCCCACTTCGGACAAGATT
TCCTTCGCAAACATCCATAGCACCTCTATCATCTTTTGATGTGAACCACTTCTTTCTTGTCTTGTTTCCACAATTGGCTGTCCCATGAATGTCACCTTCTCCTAGTTTAGAATTGAAGTGATC
CCTAAGGGACAAAAGACAATCAAGCACTGCAGAAATGGAGCCATTTTCCAAATCATCTACAGTAAAACGTGGCAGGCCCATTCCCTCTACAGCTGAAAGAAATCTCCTGACTTTCTCCCG
CCGCTGCTCGAATGAAATATAAGCACCTCGCTGATTCTCCAAGGCACTAGGATCCTGCCGCTTCTCAACGCCGCATAAAACGGTTCCATCAAAAAGCAGAGCCCTTAGTTCTTCATCGGAT
GCCTCCAAGGGAATGTTGAGACTCGGAAACAAATTATTTAGCCATTCTACAACTCTCCGCCTCTTGCCTGATGCATCTGCAGCACTCATGCTTCCAATGCCGTCCGATTCACAATCCTCTCC
TTCCAGATTTAAGTTAGCTTCTTCTTCGAATTTCGACATTTCACCGTATTCTCTCCCTTAAGCTGCAAATCCGCCAACTCACAGCGACGTTCAAAGTACCAACAAATATAAATCTCCCAGGA
GCTTCAGCAGCATTCTCAAGCAACCGCAGATTTGGCAATGCAACCCAAATTAATTAACTGCTGCCTGCGTAGAATAAGAACCACAAGACGAAACCAAAGAAGCTGGATCGCCGAAACCC
TAGCTATTGACCAGCGTGGGATCGGTAATGTTGGTTCCG
>comp163542_c0_seq3
TTTTTTATTTTTTTAAAGTCAACTCTTTTTTTTTTTTAATTAAATATTTTGCTACACAATCGATGTTTGAAATATTTTTTCCTTTTCTTGCAACATCCAAACCCGAGAAAAATTATTTCATGTGA
AGTCCGGACGGTAAGACTGTCCGGATGTGAGTGCACATAGTATTTGTACACACTTTATAAAAAAAAATTAAATTATGTATAAATTTATACTTA
>comp163570_c1_seq5
ATTTTTTTTTTTTTTTTTTTAATTTCATTTCACTACAACTGAACCCAAACAAAAAGAGGAAAATCATACAATTAGTACACAAAAACAAAAACCAAATAAAACAAATCAAGTCGTGGATCCA
GAGAAAGGGAAAGCAGGTGACTAGCGGTTCAACAAAATAAAATCAAAATTTTGAGCAAAACACAGAATCAAATGCTAAACTTAAGGAATGCATCTGAAAGATGGAAAACCATATAAAA
CAAATCAAAACACAACCCTTTCTATCATCTTCTCCGTCTTCAGCGAACAATACCGGGATTTGTTCCATGGCAACTGGCCACAGGGACAGATTTTTAAAAAAGAAAGGGAAAGCCAGTGA
AGAGTGCTTTCGGGCATGAGCTGGAGGTTGAAAACAAACAAAATAGGTAAGAAAAATTTTAAGACGAACCTTCTTCAAGCTATGAAGAGCAGCTTGTGCGACAGTTTCTGCTGACCTGC
TCCGCCGTAGCTCCGAATGCGAGATTTTTAGGGCAAGGAAGTATCTGGGGCAAGAGCGGGAGGCGAAAATCATGTTTAAGCTTTTTTTTTTCTCGGGTTTGGGGGGCACGTGCCCCGGCA
AGCCCCCCCTAAATCTGCCGTTGCTGCCAACAGATCAAGATGAATGGAACTGGTACATGTAATATTCTTAAATTTGTAATTTAATTCACTCAAAATAATTAGATAAAAAATGTCATCAAACG

TGATTTTTAAAGCAAAACATGAATTTTATATAGGCTATAATTAAATTATTTTGGGACATATTAATACTACGTACAAGCTAGAAAGAGAAACATGCATGAAAATGATACAACCCCACCTGCTAC
ACAAATTACTTCACCGCCACCGCCTCACCCGCATCCACCGCTGCCACCGCCGGCAACAATTGCAGCTAAGATAGGACCAGTACTGGAATAACGACTACCACCGTTGTTTTTACCAGCTGC
TTTCAGCCGGCTGTTGCGACCTCCAACTCCGCCGCCGCCATAGTAAACGACGTTCTGACTCTGCTTCGGCTTCTTTCTAAATCCTGCGCAGGCGAAGACTATGAAGGCTATAATTGCTGCC
GCGATGCAAAGCATGAAAAGAGCTCCGGATATTGTAGAGCCGCACCACTCTTTGCTTGCGTTGGCGAATTCCTTCAATATTCGTGCCATGTTGTTGGTTCGAATAAAACTGGAAGCAGGC
GATTTTATAGGCTTTTCATTACAGTTTCAGAAATTTCACAGAAGTTCTTCGAGGAGTTTAATA
>comp163619_c0_seq13
AAATTCAATCATTGAGCAAATCCTTCTAATTTACACACACACAAAAAAAAGGATTAACATCCCTTCAAAAACTAACCACTATTGATCATAACAAGCCAAATAAGATTAAACAAAGCTGGTAA
TTAACCCTGAATTATCCATCCTTTTGCCTAATTCCTCCATTATTAGCTTATCCTTCTCAAATGTACTTAGCAAAAAGGATTCACAAAGCTGGTAATTAGCCATGAATTATTCATCTTTCTACGT
GATTCGTCCATCATTACCTTATCCAGTTTCTCAAAGTTTTCAGCTTTGAGGGGCACCCATCCATGAATTGCCTCACCAGCTACGGCTGTGAGCCATCTCGGCCATCCCGCGGCGGCTTGTTC
CCCTTCGATGTGCTTGTGAAGGTTCTCCACTCTGACGACAACCGCTTTAGGGCTTTCCACGACCGCTTTCACCGTTACCTTCGGCTCCTCGATCTTCTCTTTATCCTCCGCCTCATCCTCCT
TTGGCCTCTCCCTCGACGATATCTCCGAAACTGCCTCCGATCTTCGGTTATCCCTGAGAATGCCTGACGAATCCAGCACGGGCGTCACCGACACGACATTCTTCGACGACGAGCAGCCCA
TCGCCGCCGGATCGCCGGCCGGGGATTGGCAGCACCGGATTATCCAACGAGTTGGGGATTGGGAGGAACCGAGAGGGGATAAAGGCGGAGAAGACAGCAAGATGTCCGCGGTGGAAC
ACGGGGACAAATCCCAAAAAAAGCAAAGAAAGGCGAGAACTTTATATCCTATTCTAGGCTTTTGATGGTATCGAAATCTTCGCGAAATTAATGGGAGATGAACGGAGCAGAAGCTTTTAG
GAGTAATGCCGCTTCTGCGAGATTCTCGCGGGAAGGCCCTCGAGGAATCTCTCTTTCATTTCCCTGATGGGGAAACAAAATATAGCCGTTGTG
>comp163619_c0_seq2
TCAAAAAAAAAAAAAAAGAAGAGAGAGCGGGAACAAATAAAAGAAAAGAAAAGAAAATAGAAAATCAAAGCTATGCATAAGTAGCAGTCGCTGTTTTCCATCCTTCTCTAATATGAAAA
CTGGAACATCTTGTGAGAAAAATGGAAACATATTTAGCCTCGTTCCTATCAATGTAACAGTCTTTACCAAAGTGTTTTGTCGGGCAATTTATAACAGATAGGATCCGTTGCAATGTACAGCA
GGAGCTTCTTAAGTATAAGCAACAGCTTTGATTCACTGAACTTGATTTCTTCCAGCCACTGTATTTAAAGACAATTTTGAGTGTAGATCTCGTTGGAACCATGATTTTCGAACGGCTTTCCG
GATTTGGCGATCATGTTTTTCTAACAGTTCATCAACCTTTTGGGCTTTAGACAACAATGGCCCTGAAAACTCCACCCTGCCTCCATCATGGTATCCCAGCATACTGCGCTTTGATTGCATTG
TGCCATTTCTTGAGAAATCGTGTGAATGAAATCTGGAAGAATCGAATGAATCTGGGTGTTCCAGTTGTGCCCAATTTTTCATCGCTGCTTTCTTTGCCAATTTATACTGCTCATTGCGCCTG
TGATGATCAAGTGGATAATGTGCAGTATTTCCATTAGCTTGGCCATCAAAACCAGAACTAACTCTGGCTCTGATAGCAGCTGGCAGCTCCGGTGCACCATTATTATGGTTCCTTGAGGTAGT
TTTAATATGAGTTTTAGCAAATGGACGATCTTCCCGCAGTTTCTTATCCCATGCAAGGCCATTAGAATGATTAACATGCAAAGGACCTGAGAAAGGAATGCTCCCTTGAGAAATATTCTTAA
TATGTCGACCTCGATCAGAAGGATTTAGTGCTGCCATTGGCTGAAGATCTACAAAGAGTCTAGAACCACCATTAACTCTGGGACAAGGTAACTTGTCGCCACTGCTATCAGTTCTATGGTT
AATTTTTCGATGCTCTGCCTCTTGGTGGTTTGATAATCTACTCAAGCCGCTAAGTTCTTGTGTATCCCTATGAACCCTTAATGGTCTCCTCGTCACTTCTGTGACACGTGCTCTACCGTTGCC
CTTTGGAATCCTCCTGTTAGCATCCTCTTGGATTTTTGCATCAATTTCTTTGGTAGGTGGGTATTTTGGCAAACTTGATGGTTCACACGAAAGAGGCATTGCTGAGAAATACTGTGGAAATG
ACAAGAAATAAAGTATTAAGTCTAACAGTAAGTACAAAAGAAAATGAAATGACAGTAGCAGTTAAGGGAGTTGATATATGAAGATTCTTGAAAAATTATAGCTGACATTATACATGGACGA
AAGGCCAAAACTGATTCATACCTCAGATGCCAGAGCAGACTCA
>comp163619_c0_seq5
TCAAAAAAAAAAAAAAAGAAGAGAGAGCGGGAACAAATAAAAGAAAAGAAAAGAAAATAGAAAATCAAAGCTATGCATAAGTAGCAGTCGCTGTTTTCCATCCTTCTCTAATATGAAAA
CTGGAACATCCTGTTTGGTTAACATCTCAGAATCAGCAAAAAAAATATGACAAATGAATGAAATTTTTTATATTTTTTTCATCCTTAACATATTTAAGGCTGCTAAACTCACTTGTGAGAAAAA
TGGAAACATATTTAGCCTCGTTCCTATCAATGTAACAGTCTTTACCAAAGTGTTTTGTCGGGCAATTTATAACAGATAGGATCCGTTGCAATGTACAGCAGGAGCTTCTTAAGTATAAGCAA

CAGCTTTGATTCACTGAACTTGATTTCTTCCAGCCACTCGTTGGAACCATGATTTTCGAACGGCTTTCCGGATTTGGCGATCATGTTTTTCTAACAGTTCATCAACCTTTTGGGCTTTAGAC
AACAATGGCCCTGAAAACTCCACCCTGCCTCCATCATGGTATCCCAGCATACTGCGCTTTGATTGCATTGTGCCATTTCTTGAGAAATCGTGTGAATGAAATCTGGAAGAATCGAATGAAT
CTGGGTGTTCCAGTTGTGCCCAATTTTTCATCGCTGCTTTCTTTGCCAATTTATACTGCTCATTGCGCCTGTGATGATCAAGTGGATAATGTGCAGTATTTCCATTAGCTTGGCCATCAAAAC
CAGAACTAACTCTGGCTCTGATAGCAGCTGGCAGCTCCGGTGCACCATTATTATGGTTCCTTGAGGTAGTTTTAATATGAGTTTTAGCAAATGGACGATCTTCCCGCAGTTTCTTATCCCAT
GCAAGGCCATTAGAATGATTAACATGCAAAGGACCTGAGAAAGGAATGCTCCCTTGAGAAATATTCTTAATATGTCGACCTCGATCAGAAGGATTTAGTGCTGCCATTGGCTGAAGATCTA
CAAAGAGTCTAGAACCACCATTAACTCTGGGACAAGGTAACTTGTCGCCACTGCTATCAGTTCTATGGTTAATTTTTCGATGCTCTGCCTCTTGGTGGTTTGATAATCTACTCAAGCCGCTA
AGTTCTTGTGTATCCCTATGAACCCTTAATGGTCTCCTCGTCACTTCTGTGACACGTGCTCTACCGTTGCCCTTTGGAATCCTCCTGTTAGCATCCTCTTGGATTTTTGCATCAATTTCTTTGG
TAGGTGGGTATTTTGGCAAACTTGATGGTTCACACGAAAGAGGCATTGCTGAGAAATACTCAGATGCCAGAGCAGACTCAGCACTTCCACGCCTTCGAGGTTCTATAGATAGAAATCTTT
CTAATAATTTGTAGACAGATTGTGGCAAATTTCTGAAAGTCTCATGCAGGTGACCCTCATAAGGACGATGGGGCTTGAAAATTATTTCATGAGGTAACTTGGAATTCTTCCAGTATTCATCT
GATGGGGAACCACATAGTTTAAAGATTTTGTGCAGTTGCTCAACCTCAGTTCTTCCTTGCAAAATGGGCTTCTTGAAGAACATCTCTGCAAATACACAACCAACACTCCACATGTCTACTG
ATGCTCCATAATCGACGGAGCCCAACAGAAGCTCAGGTGGACGGTACCATAGTGTTACAACACGACTAGTTAGCGGTTGCTTATTTTCAGGTGGCATGAAGTTTGCCAGACCAAAATCAG
CAATTTTTAAGATTCCATCATTGTTAACTAAGAGGTTTGCACCCTTAATATCTCGATGGATGATTCCTCGCGAATGACAATGTTCAAGGCCAGATAGCAATTGTTTCATATAACACTTCACCT
GCACGCACAAATTGCCATTTTTCATTGAAAGTT
>comp163619_c0_seq9
TCAAAAAAAAAAAAAAGAAGAGAGAGCGGGAACAAATAAAAGAAAAGAAAAGAAAATAGAAAATCAAAGCTATGCATAAGTAGCAGTCGCTGTTTTCCATCCTTCTCTAATATGAAAA
CTGGAACATCCTGTTTGGTTAACATCTCAGAATCAGCAAAAAAATATGACAAATGAATGAAATTTTTTATATTTTTTTCATCCTTAACATATTTAAGGCTGCTAAACTCACTTGTGAGAAAAA
TGGAAACATATTTAGCCTCGTTCCTATCAATGTAACAGTCTTTACCAAAGTGTTTTGTCGGGCAATTTATAACAGATAGGATCCGTTGCAATGTACAGCAGGAGCTTCTTAAGTATAAGCAA
CAGCTTTGATTCACTGAACTTGATTTCTTCCAGCCACTGTATTTAAAGACAATTTTGAGTGTAGATCTCGTTGGAACCATGATTTTCGAACGGCTTTCCGGATTTGGCGATCATGTTTTTCTA
ACAGTTCATCAACCTTTTGGGCTTTAGACAACAATGGCCCTGAAAACTCCACCCTGCCTCCATCATGGTATCCCAGCATACTGCGCTTTGATTGCATTGTGCCATTTCTTGAGAAATCGTGT
GAATGAAATCTGGAAGAATCGAATGAATCTGGGTGTTCCAGTTGTGCCCAATTTTTCATCGCTGCTTTCTTTGCCAATTTATACTGCTCATTGCGCCTGTGATGATCAAGTGGATAATGTGC
AGTATTTCCATTAGCTTGGCCATCAAAACCAGAACTAACTCTGGCTCTGATAGCAGCTGGCAGCTCCGGTGCACCATTATTATGGTTCCTTGAGGTAGTTTTAATATGAGTTTTAGCAAATG
GACGATCTTCCCGCAGTTTCTTATCCCATGCAAGGCCATTAGAATGATTAACATGCAAAGGACCTGAGAAAGGAATGCTCCCTTGAGAAATATTCTTAATATGTCGACCTCGATCAGAAGG
ATTTAGTGCTGCCATTGGCTGAAGATCTACAAAGAGTCTAGAACCACCATTAACTCTGGGACAAGGTAACTTGTCGCCACTGCTATCAGTTCTATGGTTAATTTTTCGATGCTCTGCCTCTT
GGTGGTTTGATAATCTACTCAAGCCGCTAAGTTCTTGTGTATCCCTATGAACCCTTAATGGTCTCCTCGTCACTTCTGTGACACGTGCTCTACCGTTGCCCTTTGGAATCCTCCTGTTAGCAT
CCTCTTGGATTTTTGCATCAATTTCTTTGGTAGGTGGGTATTTTGGCAAACTTGATGGTTCACACGAAAGAGGCATTGCTGAGAAATACTCAGATGCCAGAGCAGACTCAGCACTTCCACG
CCTTCGAGGTTCTATAGATAGAAATCTTTCTAATAATTTGTAGACAGATTGTGGCAAATTTCTGAAAGTCTCATGCAGGTGACCCTCATAAGGACGATGGGGCTTGAAAATTATTTCATGAG
GTAACTTGGAATTCTTCCAGTATTCATCTGATGGGGAACCACATAGTTTAAAGATTTTGTGCAGTTGCTCAACCTCAGTTCTTCCTTGCAAAATGGGCTTCTTGAAGAACATCTCTGCAAAT
ACACAACCAACACTCCACATGTCTACTGATGCTCCATAATCGACGGAGCCCAACAGAAGCTCAGGTGGACGGTACCATAGTGTTACAACACGACTAGTTAGCGGTTGCTTATTTTCAGGT
GGCATGAAGTTTGCCAGACCAAAATCAGCAATTTTTAAGATTCCATCATTGTTAACTAAGAGGTTTGCACCCTTAATATCTCGATGGATGATTCCTCGCGAATGACAATGTTCAAGGCCAGA
TAGCAATTGTTTCATATAACACTTCACCTGTGATTCGGTGAATTTCACATCTGGTGATGATGATAATCCAGAAAGATCATGTTCCATATATTCAAACACCAGATATAAGCTACAAGATAACCG
AGAGGTGATCAAACCTTCCAGTTTCATAACATTTGGATGGTCGAGTCGACGAAGTATCTCTATTTCTCGTGCCATGAACTTGACACTTTCTGGCTCAAAATTGTCAAATCTCACCTTCTTCA

GGGCAACAACTTTTCCATTTTCAAGATCCCTTGCTCTAAACACACTGCTGTAAGTGCCTTGCCCTTATCCAGTTTCTCAAAGTTTTCAGCTTTGAGGGGCACCCATCCATGAATTGCCTCA
CCAGCTACGGCTGTGAGCCATCTCGGCCATCCCGCGGCGGCTTGTTCCCCTTCGATGTGCTTGTGAAGGTTCTCCACTCTGACGACAACCGCTTTAGGGCTTTCCACGACCGCTTTCACC
GTTACCTTCGGCTCCTCGATCTTCTCTTTATCCTCCGCCTCATCCTCCTTTGGCCTCTCCCTCGACGATATCTCCGAAACTGCCTCCGATCTTCGGTTATCCCTGAGAATGCCTGACGAATCC
AGCACGGGCGTCACCGACACGACATTCTTCGACGACGAGCAGCCCATCGCCGCCGGATCGCCGGCCGGGGATTGGCAGCACCGGATTATCCAACGAGTTGGGGATTGGAGGAACCGA
GAGGGGATAAAGGCGGAGAAGACAGCAAGATGTCCGCGGTGGAACACGGGGACAAATCCCAAAAAAAGCAAAGAAAGGCGAGAACTTTATATCCTATTCTAGGCTTTTGATGGTATCG
AAATCTTCGCGAAATTAATGGGAGATGAACGGAGCAGAAGCTTTTAGGAGTAATGCCGCTTCTGCGAGATTCTCGCGGGAAGGCCCTCGAGGAATCTCTCTTTCATTTCCCTGATGGGGA
AACAAAATATAGCCGTTGTG
>comp163696_c0_seq1
AATTTTTATGAGTTGAAAAATAGATATTCTCACCATTTATATAACTCAAAACTCATTAATTTCATAGCTGTGAACTTTCTTTCTTGAAGTCCTTCTACCACTCACACTCCAGTGCACCATAAC
AATGACACATCAATAATCTCAGAAGCTCACATGAACCAAATTTTTTATTTTCTCACACAATGAAGCTTCTAATGACATTCCATTTGGAATATGCAAGCTTTTTATTACCTTCATATAAACCTTT
TATATACTTTCCAATTTCCAAAATACAATGCAAACACCAAATCCAAAGAACAATAACAAGTTGCTAACAGTACATCTTAACATAACAAAGCCTTGATTTTGTCATTCCCAGATGCAGAAGTA
AAATGCAGTCTTCTTATTTCCCTTCAACGAAGGAGACTAAAGCATCCCTGTAAACACCATGAGTGAATTTTTTTAGAACATCTTCGAGTTTAGTTCCATGTCTCTTCAGATACTCTTCTTTG
ATTTCCTCTATATCAACATCAGACCTCGACAAAATCACTCGACTGAGAGCTTCTTTTGAGTCATTATCTGCTCCATCTTTTAAAGCCTTGTTTATCAACTGAAAACCAAAGTGATAAAGGTG
AAAAGCAATGAATTTGGAACTGATTAAAGATTATTTATTTCATGAATATTAGATTGTTTACTGTTAAACCGGTCCAGCCATTTCGCATAGAACCGGACGGCGGTCCAAACGCCCAAGCGGA
GCATTTTAGGAAGGATTTTTGAAGGATTTGGGAGGGAAATTTGAATATAGGGACTGTTTAGGGATTTCCTT
>comp163696_c0_seq11
AATTTTTATGAGTTGAAAAATAGATATTCTCACCATTTATATAACTCAAAACTCATTAATTTCATAGCTGTGAACTTTCTTTCTTGAAGTCCTTCTACCACTCACACTCCAGTGCACCATAAC
AATGACACATCAATAATCTCAGAAGCTCACATGAACCAAATTTTTTATTTTCTCACACAATGAAGCTTCTAATGACATTCCATTTGGAATATGCAAGCTTTTTATTACCTTCATATAAACCTTT
TATATACTTTCCAATTTCCAAAATACAATGCAAACACCAAATCCAAAGAACAATAACAAGTTGCTAACAGTACATCTTAACATAACAAAGCCTTGATTTTGTCATTCCCAGATGCAGAAGTA
AAATGCAGTCTTCTTATTTCCCTTCAACGAAGGAGACTAAAGCATCCCTGTAAACACCATGAGTGAATTTTTTTAGAACATCTTCGAGTTTAGTTCCATGTCTCTTCAGATACTCTTCTTTG
ATTTCCTCTATATCAACATCAGACCTCGACAAAATCACTCGACTGAGAGCTTCTTTTGAGTCATTATCTGCTCCATCTTTTAAAGCCTTGTTTATCAACTTGCTGAAATATACAGGAGCTGAA
TTGATGCTCTCAACTGTATCTATTATGTATGGCTCATCTCCAAGATCCTGCTCATTGCATGTAGAAATCAGTATATTTAAATTGTTCAAATTACAAATATGCAAAACAAATAAAAATTTTGACT
GCTTCAAGCAAAAGTAGGGACTAATGTCATCCACATGTAAGATATCTTAAACTTAAGCGAGGAGAAAACAAGACACCTCTTCTATGGACTTTCCAAAAAGTTCCTTGTAGAATTTGAAGG
TTACTCTTAGTTGCGACTTGCTCCTCGTAGTAAGGATCCTCACTACATCCTCATTCACTATGAGGTTCTCTGGTGTTTTTTTCTGAATGGCATTATGGAGAGCTTTGGCCTCTGATTTTGCTAT
CTCCTCGTGCACATGAGGGCCTTCATATCTGTAGGAGCTCACAAGCCTGACCAGAAACTATATCCATGAAATCATAGATAAAAGCTTGGTTAGAAACTAATGTTTTCGCCATTGCATAAGTT
TCTGTAACATTTTGAAAATTGCATGAAGCCATTAATCCCAAGAGGAACCATACCTCTAAAACATCGCGAACAATGGGTGGATCAAACATCGGGGAGCTAGGGGCACATGCACCTACTAGC
TCTAGTCGACCCCACTTAGTTACCTAATTGAGTAGGGTTGTCAATTTTTGACACGACCCACCAACCCGACCCGAAAAATCTGGGTTAGAGTCGACCCGTTTAACAAATGGGTCGGGTTCG
GATTTCAATTTTTGACCCATTTAAAAAACAAGTAGGGTCTGGGTCAACCCAGAACCCGAAATTGACCCGAGAATACCCGGATACAATCAAATGTGACCCAAGATTGCTCCGCCCATTTTAA
TTTTTCCCGCGTGGGAGGGAACCGAGGTAATCCACCCTTTCTAAACCTACTCCTGAATAAATGCCAGCTCATTACAAAGTTGACTATGTGTAGATCATGATTACATTCCCTAAATTAGGTAA
GCTAATACTTTTTTCTCCTTGAGGGGTACAGATAAATTACTTCAGTCCTTTTTAGGGGATGTTATTGGTTCAGTCCCATACAACAAAAAAAGGGAAATTTCACAACCAGTAACCAAATTAG
GCAATCCACAAGCAAACATAAATATAAATAACAGAAATTTCAACATTTTCAAGGTCATTGACCTCACTGCAATATGAAATATGAGGCATTGTGGAGCATTACTTACATTAACATGGCTTCCA

GAGATGCTATAGGCAACTTCTTCCTCAAGGGAATGATGAAATAAGGCATGATATGCCTTCCTTGCCCCTAAAAGCTCGGCTGCTGATCGAGTGCAAGCAATTTCAACAAGAATTGTAGGG
GAATGACCTTTGTGGATTACTTGGTGAGCCCAACGAGCATCCCTTTCCCATGGATGCATTGCCCATTGAAAAGTCAAGTTCTTAAACCTCGCATATTCGAACTCGAGAAAATGAATGTAAT
CAAGGTCTACTCGTGCAAAGCTTCCATTATCAGCCTTAAATAGACCACCAAAGTTTTTTCTGAACTGGGCCAGTGTTTCTGGCCGGCGGCGCCATCTCCCCATTGCCGCCACCACAGACTT
CTCTTCCACTCCAAGCCCTCCCAATCCTGCGAGGGCCTTGGTGAGTTCCTTGCATTCCTCAGCTAAGATATGAGCCATGGTTGGTTTCTTCTCCTTTTCTTCTTCAACTCCGCAAATCG
>comp163696_c0_seq24
AATTTTTATGAGTTGAAAAATAGATATTCTCACCATTTATATAACTCAAAACTCATTAATTTCATAGCTGTGAACTTTCTTTCTTGAAGTCCTTCTACCACTCACACTCCAGTGCACCATAAC
AATGACACATCAATAATCTCAGAAGCTCACATGAACCAAATTTTTTATTTTCTCACACAATGAAGCTTCTAATGACATTCCATTTGGAATATGCAAGCTTTTTATTACCTTCATATAAACCTTT
TATATACTTTCCAATTTCCAAAATACAATGCAAACACCAAATCCAAAGAACAATAACAAGTTGCTAACAGTACATCTTAACATAACAAAGCCTTGATTTTGTCATTCCCAGATGCAGAAGTA
AAATGCAGTCTTCTTATTTCCCTTCAACGAAGGAGACTAAAGCATCCCTGTAAACACCATGAGTGAATTTTTTTAGAACATCTTCGAGTTTAGTTCCATGTCTCTTCAGATACTCTTCTTTG
ATTTCCTCTATATCAACATCAGACCTCGACAAAATCACTCGACTGAGAGCTTCTTTTGAGTCATTATCTGCTCCATCTTTTAAAGCCTTGTTTATCAACTTGCTGAAATATACAGGAGCTGAA
TTGATGCTCTCAACTGTATCTATTATGTATGGCTCATCTCCAAGATCCTCTTCTATGGACTTCCCAAACAGTTCCTTGTAGAATTTGAATGTTACTCTTAGTTGCGACTTGCTCCTCGTAGTAA
GGATCCTCACTACATCCTCATTCACTATGAGGTTCTCTGGTGTTTTTTTCTGAATGGCATTATGGAGAGCTTTGGCCTCTGATTTTGCTATCTCCTCGTGCACATGAGGGCCTTCATATCTGTA
GGAGCTCACAAGCCTGACCAGAAACTATATCCATGAAATCATAGATAAAAGCTTGGTTAGAAACTAATGTTTTCGCCATTGCATAAGTTTCTGTAACATTTTGAAAATTGCATGAAGCCATT
AATCCCAAGAGGAACCATACCTCTAAAACATCGCGAACAATGGGTGGATCAAACATCGGGGAGCTAGGGGCACATGCACCTACTAGCTCTAGTCGACCCCACTTAGTTACCTAATTGAGT
AGGGTTGTCAATTTTTGACACGACCCACCAACCCGACCCGAAAAATCTGGGTTAGAGTCGACCCGTTTAACAAATGGGTCGGGTTCGGATTTCAATTTTTGACCCATTTAAAAAACAAGT
AGGGTCTGGGTCAACCCAGAACCCGAAATTGACCCGAGAATACCCGGATACAATCAAATGTGACCCAAGATTGCTCCGCCCATTTTAATTTTTCCCGCGTGGGAGGGAACCGAGGTAATC
CACCCTTTCTAAACCTACTCCTGAATAAATGCCAGCTCATTACAAAGTTGACTATGTGTAGATCATGATTACATTCCCTAAATTAGGTAAGCTAATACTTTTTTCTCCTTGAGGGGTACAGAT
AAATTACTTCAGTCCTTTTTAGGGGATGTTATTGGTTCAGTCCCATACAACAAAAAAAGGGAAATTTCACAACCAGTAACCAAATTAGGCAATCCACAAGCAAACATAAATATAAATAACA
GAAATTTCAACATTTTCAAGGTCATTGACCTCACTGCAATATGAAATATGAGGCATTGTGGAGCATTACTTACATTAACATGGCTTCCAGAGATGCTATAGGCAACTTCTTCCTCAAGGGAA
TGATGAAATAAGGCATGATATGCCTTCCTTGCCCCTAAAAGCTCGGCTGCTGATCGAGTGCAAGCAATTTCAACAAGAATTGTAGGGGAATGACCTTTGTGGATTACTTGGTGAGCCCAAC
GAGCATCCCTTTCCCATGGATGCATTGCCCATTGAAAAGTCAAGTTCTTAAACCTCGCATATTCGAACTCGAGAAAATGAATGTAATCAAGGTCTACTCGTGCAAAGCTTCCATTATCAGC
CTTAAATAGACCACCAAAGTTTTTTCTGAACTGGGCCAGTGTTTCTGGCCGGCGGCGCCATCTCCCCATTGCCGCCACCACAGACTTCTCTTCCACTCCAAGCCCTCCCAATCCTGCGAG
GGCCTTGGTGAGTTCCTTGCATTCCTCAGCTAAGATATGAGCCATGGTTGGTTTCTTCTCCTTTTCTTCTTCAACTCCGCAAATCG
>comp163696_c0_seq28
AATTTTTATGAGTTGAAAAATAGATATTCTCACCATTTATATAACTCAAAACTCATTAATTTCATAGCTGTGAACTTTCTTTCTTGAAGTCCTTCTACCACTCACACTCCAGTGCACCATAAC
AATGACACATCAATAATCTCAGAAGCTCACATGAACCAAATTTTTTATTTTCTCACACAATGAAGCTTCTAATGACATTCCATTTGGAATATGCAAGCTTTTTATTACCTTCATATAAACCTTT
TATATACTTTCCAATTTCCAAAATACAATGCAAACACCAAATCCAAAGAACAATAACAAGTTGCTAACAGTACATCTTAACATAACAAAGCCTTGATTTTGTCATTCCCAGATGCAGAAGTA
AAATGCAGTCTTCTTATTTCCCTTCAACGAAGGAGACTAAAGCATCCCTGTAAACACCATGAGTGAATTTTTTTAGAACATCTTCGAGTTTAGTTCCATGTCTCTTCAGATACTCTTCTTTG
ATTTCCTCTATATCAACATCAGACCTCGACAAAATCACTCGACTGAGAGCTTCTTTTGAGTCATTATCTGCTCCATCTTTTAAAGCCTTGTTTATCAACTTGCTGAAATATACAGGAGCTGAA
TTGATGCTCTCAACTGTATCTATTATGTATGGCTCATCTCCAAGATCCTGCTCATTGCATGTAGAAATCAGTATATTTAAATTGTTCAAATTACAAATATGCAAAACAAATAAAAATTTTGACT
GCTTCAAGCAAAAGTAGGGACTAATGTCATCCACATGTAAGATATCTTAAACTTAAGCGAGGAGAAAACAAGACACCTCTTCTATGGACTTTCCAAAAAGTTCCTTGTAGAATTTGAAGG

TTACTCTTAGTTGCGACTTGCTCCTCGTAGTAAGGATCCTCACTACATCCTCATTCACTATGAGGTTCTCTGGTGTTTTTTTCTGAATGGCATTATGGAGAGCTTTGGCCTCTGATTTTGCTAT
CTCCTCGTGCACATGAGGGCCTTCATATCTGTAGGAGCTCACAAGCCTGACCAGAAAATTAACATGGCTTCCAGAGATGCTATAGGCAACTTCTTCCTCAAGGGAATGATGAAATAAGGC
ATGATATGCCTTCCTTGCCCCTAAAAGCTCGGCTGCTGATCGAGTGCAAGCAATTTCAACAAGAATTGTAGGGGAATGACCTTTGTGGATTACTTGGTGAGCCCAACGAGCATCCCTTTCC
CATGGATGCATTGCCCATTGAAAAGTCAAGTTCTTAAACCTCGCATATTCGAACTCGAGAAAATGAATGTAATCAAGGTCTACTCGTGCAAAGCTTCCATTATCAGCCTTAAATAGACCAC
CAAAGTTTTTCTGAACTGGGCCAGTGTTTCTGGCCGGCGGCGCCATCTCCCCATTGCCGCCACCACAGACTTCTCTTCCACTCCAAGCCCTCCCAATCCTGCGAGGGCCTTGGTGAGTT
CCTTGCATTCCTCAGCTAAGATATGAGCCATGGTTGGTTTCTTCTCCTTTTCTTCTTCAACTCCGCAAATCG
>comp163696_c0_seq4
CCTATCATTTACCTTGCTGAAATATACAGGAGCTGAATTGATGCTCTCAACTGCATCTATTATGTATGGCTCATCTCCAAGATCCTGCTCATTGCATGTACAAATCATAATTAAACTGAGCTCA
AATTACAAATATGCAAAACAAATAAATTTTTTGACCGCTTCAAGCAAAACTAGGGACTAATGTCATCAACATGTAAGATATCTTAAACTTAAGCGAGGAGAAAACAAAACACCTCTTCTAT
GGACTTCCCAAACAGTTCCTTGTAGAATTTGAATGTTACTCTTAGTTGCGACTTGCTCCTCGTAGTAAGGATCCTCACTACATCCTCATTCACTATGAGGTTCTCTGGTGTTTTTTTCTGAAT
GGCATTATGGAGAGCTTTGGCCTCTGATTTTGCTATCTCCTCGTGCACATGAGGGCCTTCATATCTGTAGGAGCTCACAAGCCTGACCAGAAAATTAACATGGCTTCCAGAGATGCTATAG
GCAACTTCTTCCTCAAGGGAATGATGAAATAAGGCATGATATGCCTTCCTTGCCCCTAAAAGCTCGGCTGCTGATCGAGTGCAAGCAATTTCAACAAGAATTGTAGGGGAATGACCTTTG
TGGATTACTTGGTGAGCCCAACGAGCATCCCTTTCCCATGGATGCATTGCCCATTGAAAAGTCAAGTTCTGTAAAGAGGTGTCTTAATAAGCACTTATAGAAATTCTGAGTTCATGTAGTTT
CTCAACATTTTGAAACGTTAAAGAAGAACCTCCTCTCATGGGGGAATTATTGGGTTCGGACACCAGACACAGCTTACCGTTCGGTATTTCACAGTACCAAATAATACTGAGCTCGGTACTT
CACAGTACCGACTTGGCACTGCTCAGTACCGAGTAGGGGGTGATG
>comp163696_c0_seq7
AATTTTTATGAGTTGAAAAATAGATATTCTCACCATTTATATAACTCAAAACTCATTAATTTCATAGCTGTGAACTTTCTTTCTTGAAGTCCTTCTACCACTCACACTCCAGTGCACCATAAC
AATGACACATCAATAATCTCAGAAGCTCACATGAACCAAATTTTTTATTTTCTCACACAATGAAGCTTCTAATGACATTCCATTTGGAATATGCAAGCTTTTTATTACCTTCATATAAACCTTT
TATATACTTTCCAATTTCCAAAATACAATGCAAACACCAAATCCAAAGAACAATAACAAGTTGCTAACAGTACATCTTAACATAACAAAGCCTTGATTTTGTCATTCCCAGATGCAGAAGTA
AAATGCAGTCTTCTTATTTCCCTTCAACGAAGGAGACTAAAGCATCCCTGTAAACACCATGAGTGAATTTTTTTAGAACATCTTCGAGTTTAGTTCCATGTCTCTTCAGATACTCTTCTTTG
ATTTCCTCTATATCAACATCAGACCTCGACAAAATCACTCGACTGAGAGCTTCTTTTGAGTCATTATCTGCTCCATCTTTTAAAGCCTTGTTTATCAACTTGCTGAAATATACAGGAGCTGAA
TTGATGCTCTCAACTGTATCTATTATGTATGGCTCATCTCCAAGATCCTCTTCTATGGACTTCCCAAACAGTTCCTTGTAGAATTTGAATGTTACTCTTAGTTGCGACTTGCTCCTCGTAGTAA
GGATCCTCACTACATCCTCATTCACTATGAGGTTCTCTGGTGTTTTTTTCTGAATGGCATTATGGAGAGCTTTGGCCTCTGATTTTGCTATCTCCTCGTGCACATGAGGGCCTTCATATCTGTA
GGAGCTCACAAGCCTGACCAGAAAATTAACATGGCTTCCAGAGATGCTATAGGCAACTTCTTCCTCAAGGGAATGATGAAATAAGGCATGATATGCCTTCCTTGCCCCTAAAAGCTCGGC
TGCTGATCGAGTGCAAGCAATTTCAACAAGAATTGTAGGGGAATGACCTTTGTGGATTACTTGGTGAGCCCAACGAGCATCCCTTTCCCATGGATGCATTGCCCATTGAAAAGTCAAGTT
CTTAAACCTCGCATATTCGAACTCGAGAAAATGAATGTAATCAAGGTCTACTCGTGCAAAGCTTCCATTATCAGCCTTAAATAGACCACCAAAGTTTTTCTGAACTGGGCCAGTGTTTCT
GGCCGGCGGCGCCATCTCCCCATTGCCGCCACCACAGACTTCTCTTCCACTCCAAGCCCTCCCAATCCTGCGAGGGCCTTGGTGAGTTCCTTGCATTCCTCAGCTAAGATATGAGCCATGG
TTGGTTTCTTCTCCTTTTCTTCTTCAACTCCGCAAATCG
>comp163702_c1_seq7
CACGCTCTTGTAGCGTGGTGGGGGCAACCTTAAGTACCACGTTCCGACATCGCCGACAGAGTCCTCGACCTCGACATATGCACCGCCGATATTGCTACGTAAGGTATAAAATTCCAAATCC
CATTTTTAGTGTTTGCGGCGGACCGGAGAACCACTTATACACGCTCTCGTAGCACGGTAGAAGGGACCTCGGTGCGAGGCATACAACGAGATGGGTAAGCCCTATACACGCTCTTGTAGC

GTGGTGGGGAAAAGCCACTCGTGTTTTCATATCTATACTTTGGAGCCCCAATACACTCTCTTGTAACGTGGTGGGATGAAAAGATGTACGGCCCGGTGT

>comp163727_c0_seq2

TCTTTTTGCTTGTATATTGTTCTAATACCATTTGTCATAGCATTGATGCTTATCCATTGAAGTTTTATCCATTTTGGTCTATACAACCTCATGATTTTGTTCCTAGGACGATCATCTATCCTAGGA
TTGATCTAGCATGAGCATACATTACCTTAGAATTCTTATGAAATCACCAACCATTACATCAAAGGCACTTGAAGGGTTAAAAGCTCAATTCAACTATTCTTAATAGAGAGGTTCAAACTCTC
AACCTCAAGATTTTACTTAGCTTTGAGATTTGCTCACTCTCGCTAACCAATTGCTTCATTACTGATAGGAATCTGATCTCCACAGCCAAAAACAACAATAACGAAGAGATAACACAACAAA
AATCAGCAAGAAAGAATGAGATTATGGACTAATTTTAATGATATCGACACAATAAACCAAGATTATTACTCCATTTGGTCAAATTCCGAGAAATCTAATGCAGAAAAATACCATGCTGCCTT
TTCTGGATCATATCAAAATTACTTTTAAATTCATGAGAAATTTTTTCAGGCTGAATATGTTCTTATATGACGGTTCTCTGATTTTTAACAGGCCTTGGAGGCGCTTAATCAGCAGGAAGAGAC
TAAGAAGTTATGCTTGAGGAGGACACAAACAGGTCCCTGGTAGAAGTAAC

>comp163731_c2_seq11

AAAGATGTCGGCGCCGGCTCCGGCCAGACGGTCAAATTCGCACGCCGTACATCCAGCGGCCGCTACATTAGCCTCTCTCGTGACGATATCGAACTTTCCGGCGACCTCTCCGGCGATTAC
ATGAACTACAGTGTTCACATTCCGCCAACTCCCGATAACCAGCCCATGGATGAGCCCGTGGAGGCGCAGTCCGTCGCCGTGAAAGCAGAGGAGCAATACGTGTCTAACTCGCTTTTCAC
CGGTGGATTTAATAGCGAGACAAGGGCCCACCTCATGGACAAGGTGATCGACTCTGTGGTTACCCACCCTCAAATGGCTGGCTCCAAAGGATCCTCCCCATGCGCCATGCCCACATGCGA
CGGCAAGCTCATTACGGACGAACGCGGCAACGATATTGACCCATGCGATTGCCACTTCAAAATTTGCCGCGATTGCTACCTCGACGCTCGAAAGGACACCGGGAATTGCCCTGGCTGCA
AAGAACCCTACTCCGCCACCGGTGACAATGACGATGAAGCCCCTGTCGACTTCCCCGCCTCTTCCACGGCCCCTGCCGCACCGGCATTACCATCTCCAACGTCAAAGCCCGGCCACCAC
AGCTTCTTAGAGCCAACCAAATCATTGTTGGCGCGCGGCCAAGCAGGGGCCTCTGACTTTGACCATAACAGATGGCTTTTTGAGACGCAGGGGACATACGGGTACGGAAATGCATTCTG
GCCAAAAGGCGGTGGCAATGGCGGCGGAGGCTATGATGAGGATGGGGAGGATGATGATGATGAAAAGGAAATGGGCGATGGGATGCCGGAGATGTCGGAGAAGCCATGGAAGCCGTTG
ACGAGGGTTGTTCCGATGCCGACGGGGATTATTCAGCCGTATAGGTTGTTGATGCTGGTGAGATTCGTGGCTTTGTTTTTCTTCTTGGGGTGGAGGATACAGCATCCAAATGAGGAGGCAC
TTTGGCTGTGGGGCATGTCGGTGGTATGCGAGGTTTGGTTTGCTTTCTCATGGATTTTGGATCAGGTTCCTAAATTGCATCCGATTAATCGCGTCACGGATCTAAAGGTACTAAAAGAAAA
GTTCGAGACGCCATCGCCGGCTAATCCCACCGGTCGCTCCGACCTCCCTGCCACCGACTTTTTCGTCTCAACCGCCGATCCCGACAAAGAGCCACCGCTCGTCACCGCCAACACCATCCT
CTCCATCCTAGCCGTGGACTACCCGATCGAAAAGATCGCCTGCTACATCTCCGACGACGGAGGCGCTCTCCTCACCTTCAAGACTATGGCTGAGGCGGCGAGCTTCACGAACATATGGGT
ATCGTTCTGCCGCAAACACAACAACAAACAGAGAAATCCGGATAACAAAGAATCTGAGTTGTTTTCCTTTTTTTTTTTTTAATGAATCCCTGCCAACAACGCTATGTCTAGATTTATCGAA
CGATAAATTTCTTCTTCAATCTCTTAATTCATAGTATAAATATGTTCTCCAAACAATGGAATTTTGATTTGCAAAGGCTATCTCTCCGTCATATCTTCCTGGTAAGTATGATTAGTGTTCG

>comp163769_c0_seq4

CTTTAGGAGGTTCTATTTGAAAATAATAAATAAGTTGGGCTATATAAAGGGACGGCTTCAAGCCTTGCAAAGCATAAACACAGTTAGTTTGTTATCTCATTAGATTGAGGTGGGAGGAGGA
AGTGGCTTTACGATAAGCTCCCAAACAGCCAACCATGGCTGATCAAGGCCAGGTGAGGCTGTTGCCATGCCTATTACTGGCATTGGTGGTTAGCTTGACCGCCATGACTGTAGAGGCAGA
CTCTAATAAATATATCTACTCATCTCCGCCGCCACCATATCACTATAAATCTCCTCCACCGCCATCTCCCTCTCCGCCTCCTCCA

>comp163822_c2_seq9

AAAGGAGTCAAACTAGAAGCTAAACCATTTCAAAATTCATATTTAGTTTCTTACATAAAACATAGAATTATCAACAGCATATGGAACGCAAATTATAACTAAGAGACAAAGAAATGCCAGC
GTATATATTCCCTTATCAACTGATTAATGCGCTGTCTTATTGGTTAAGATCATGTTCTATAGAATCATAGTTTCCAAAACATAAACGTACGTAAGGCTTGAGCAGCGGTCTTTTTCCTCCTACC
TGGTTCTGCCCGAAGAAACTTAACCACGCGCGTGAAGCCCGAGCGACTTCCGATCCTACAAGCGGAGAAGAAGGCGAAGGAGTAGTAGAAGAAGGCGAAGATTCCTTCTTCATGGGAA
GCCCTAGAGACTTCCGATCTGACATGAGAAGAAGAAGCCGAAGAAGCTCCCAAATAAATTGAAGGAGAGGAATTCGAAAAGGCGTAAAATCAGACGGTGCGGATTAAACAAAAACAA

CGACAAGATCGCTTATGGCAGAAACAAAACACAACATATAAATTTCGGCGGAATTTCAGCTTAAATTTTCGGCGGGCGGGTTTTGGACGGAATATTTAAATGTTGATGAAAATAAATAATT
TTAAAAGTAATATTATTAAATAATTTATTTTTAGATAACGTTGTTAAATAAATAATTTTAACATTGTTAAATTAA
>comp163885_c0_seq2
AATTGAAATCGTGAAGAAGAGCAAAAACCTGAAAATTAGAAGAGAAGCGAGCAGTTAGGAAAGGAGCAAGCGATGAGCAGCGGCCGATCCGTCGACGACAAAATCTCCAAAGAACTC
AAATCGCTTTCGCTCCGTGGCCCCGCCGGAGGCAGCGGCAGAGGCGGAGGGATTTTGCTGGATGAGGAGGATGTGGCAATCCGGTTTGCTAACGTGAGGAAGAAGGGCTTCAAGCTCT
GGGAGAGGGTGAAAGGACGAATGATCAACATACTGGACGGTCTAGAACTCCACACCGGCGTATTCTCTGCCGCCGAGCAGCAGAGGATCGTCGATTGCGTCTATGATTTCCAGGAAAAG
GGGCGTAAGGGGAAGCTTAGAGAGCGCACATATTTAGAGCCTAGAAAGTGGATGCGAGGTAAAGGACGTGTCACGATACAATTTGGTTGTTGTTACAATTATGCATTGGACAGGAATGGG
AATCCCCCTGGCATAATCCGTGATGAGGTAGTCGATCCTATCCCACCATTATTGAAACAAATGATCAAGAGAATGGTTGCTTGGCATGTTCTCCCACCATCATGCATCCCCAACAGCTGCAT
TGTCAACATTTATGACAAGGATGTCTGCATTTCTCCTCACATTGATCACCATGATTTTGTTCGGCCATTTTGCACTGTCTCTTTCTTGAGTGAATGCAATATCGTTTTTGGAACGCAATTCAA
GACAAATGGACCAGGAGATTTCTCAGG
>comp163926_c0_seq3
GCAAAATACAAAACCACAAACATAAGTAGGATTAGGATTACCTACCATGTATAGAACATTTTTTCAAACTAAAATGATAGAAAATTTGTTCTTTAATTTCCTACAATTTAACAGTATCAATAA
TATAAATAAAAACTCAAAATAAAGATAAAAGAAGCAATCCTCCATCAAAATGAATCAATGTTGAACCGACACAATGGACATAAGCCAGATATTTCGAACCACCTACTAATACAAATAAGAT
GAAATCTGTGAATGCAACAAGGCGTGCAAGCCACTTCCTCCCCCACCCTCATCTCATGCAAACAAATAGAGCAAGCATCATTCTCCTTCTCCGCCATTGCCACATTCTTATAAACATTCTTC
GCCAACAAATTCATCCTTCTATCGCGAAGCAACTCCGGATCCACATCATCTAATTCTTCAATATAAACATACTCTTCCTCCTCTATATTGATCTCGACTAGGACAACCACTTGCAACATCTTC
AACCCTTCAGCTCCAGCACAATCGCACACCAACCTAGTGATGGTGCTTGAGATCCTGTCTCTTAATATTTCAGCATCCTCTTGATCAACCAATGTATCCAATGGAATAGGCTCCTTCTCTTC
GATCAAGTCTCTAAGAGATGATGGATTGCAGTCGGAGAATTTATGCCTTGACCTTGCAACAATATCATCATCGCCCATTCTCTCCCCGTTGATATTGTAGACGAGGCTGAGGAGGAGATATG
GTTCTCCAGCATTATCGTCAATGTCAGAAATGTTAAAATTTCTATTGTAGAAGATATGGATGTTGTCGTTGATATGGATTATTGAAACCATTGCTATAAATATGTTATATGTTAAAGAACTCCT
CGATCTGGTTTGATTGTACCA
>comp163937_c0_seq3
GAAACGGTCTAATGATCTCCCTTCTCTTTGGTTATGGGATAAAAGTGGCATTGTTATCAAACAAACAGCGTATTGGATGATAAATGATATTCAAAACCCTTTTGGCGCAGGATTGTCTTCAT
TCTCTATATTTGCATTTAATGTGGAGGAGGAGAAAGGACAAATCATAACCAAGCCTAATACTACATTCATAACCTCCAACAATCGTTGTGAGTGGAAGATGATTCCTTATGAGGAGTGGTTG
TGTTTGGTGCACATAAGTGGAAATAATATCAATGTTTGGATGCTGATGGTGACAACATGTGATGAGAATATTCAACAATGGAAGTGGAAGGGGTCATACTCTGCAGAATATATTAAAAATC
GTAAATTTGCTTTTGGAGATGGCATTCCAAATGAGAAAGTTGTGTTTTTGGAAACACTTTATAGAGAATTATGGTTTTATCTTCACGAACGAGTATTGGGAGCAAAATATATGTATCCTCTG
ACATTAAATTTCAAGATGTATATGGACGGAAATTTTTAACGCTACTTA
>comp163961_c0_seq3
TAATTTTCAAAATAATTTTCTGTTTAGCAAATCGTTTAAATAGATAGAGCGAAATGTCAGAAAGCAAAGCAATTGCTGAAATAATTCTGAATCAACAATCCAATATCAGATTCTTAGATAAA
AATCGATATTTCCTTTTGACAAATTTATGATTTTTATACCTTTAGCAGAACAACTGAAGAACAGATGTCCCAGATTTTTGACTCTCCGGCGAGCTCGACTCACGGACGACCTCCGCCTGCA
AATCACAGGCAGCGGCTTCATTCGTTTCCAATGTGAAACTCAGCTTAGCCCTCGGCTCGTACACATTGTTTCCAACATCTTCCATAGAAATGGGAAGCCAATCTGATATCATGCAAGAATA
ACAGCAGCTTATCGGCGTAGGAAAAATCGCTGGCATAAACTGTAAAACAGAAGGCACCGATCCCTCCTCCATTTTTGGCTTCTTCTCTATGACAGCCTCATCGCTCTCCCTTGCCCGCTTC
CTGGACTCCCCTTCGCCGGAGCTCAACCCCAGAGAAAGCTCTAGCCTGACATCCACACCATCATCTTCCACCACCGGTGGCGATTCCATCGACGTCGGGGCCGGCGCACTCCTCTCTAAT

AGTCCCATCAAATCCAACTCCATTCTGTCGATCTCTCTCCCTACCAAATCAAAAACCAGTTTTTTTTCTCTGATACTTAAACGATGGACGGAAAAAATCTTCTTTTATGTGGCGCCACCTCT
CCTCAGAACCGTCAAGACGTGGTAGGATTATATTAATATTAAGCTGCATATTTATAAATAGCACCCATGATCTCCCGCGGACAGCTCATTGGTAGAGGCCAAGAAATTCTTCCCGCTCATATT
CGAATCACCTAACATTTCCAAAAAAAAAAAAAAAATCTTAACGGTGGGTGTTACTTCACTCTGTCAGCTAATTCCCCAAAAAGGATCTAAAGCTGGAATGGCAATATCTTGGCTAGGATATA
TGAAGAGGTCGGAGTCCAAATGTCCAATGTATTTTGCGCTTTCCATGTAACTTTTTTTTTCTTTTTGTTATTTTAGCTTATAAATTTACTATATTTGCATTCAATGTTGCCATCACTGATTGCA
AGAATTTGAAGGCCAGAAACCGAACCCAGAAGCGTTGACAGTGGTGCTTGAACTTTATATCAGAAGAATTCAAAGGATTATCTTATTATCTAGTTAACTAATAGATTATAGAATATCTTGTT
ATAAGTTCGGTAAGTTCGATCTTTATTAAACCTTTATAT
>comp163961_c0_seq6
GAATGCACCACATCACGTAGTACACAACGGTACCGAGCACCAAGCAAAAAAAATAACGTGGCGCGTTTTGATTGTTCTCCAAACCGTCCAATGTCAGCACATAATAATTTGTTAATTAATA
GCCAAAACTTTTACGAAAATCACTCTCGCCCTCAAATAAAATGGCCAAATGCTCATCGGTGCTATGTTAGGGGCTTCGAATGTGAGCCATCGACAAAATTAGTGCTATTTAAAATTATGCA
GCTTAATAATAACATAACCCTACCTCGTGTCGACAGCTCTGAGGAGAGGTGGCGCCATATAAAAGAAGATCTTTTCCGTCTATCATTTAAGCATTAGCGCAAAAAAATTGGTTTTTGATTTG
GTAGGGAGAGCGATCGACAGATGGAGTTGGATTTGATGGGACTATTAGAGAGGAGTGCGCCGGCCCCGACGTCGATGGAATCGCCACCGGTGGTGGAAGATGATGGTGTGGATGTCAGG
CTAGAGCTTTCTCTGGGGTTGAGCTCCGGCGAAGGGGAGTCCAGGAAGCGGGCAAGGGAGAGCGATGAGGCTGTCATAGAGAAGAAGCCAAAAATGGAGGAGGGATCGGTGCCTTCT
GTTTTACAGTTTATGCCAGCGATTTTTCCTACGCCGATAAGCTGCTGTTATTCTTGCATGATATCAGATTGGCTTCCCATTTCTATGGAAGATGTTGGAAACAATGTGTACGAGCCGAGGGCT
AAGCTGAGTTTCACATTGGAAACGAATGAAGCCGCTGCCTGTGATTTGCAGGCGGAGGTCGTCCGTGAGTCGAGCTCGCCGGAGAGTCAAAAATCTGGGACATCTGTTCTTCAGTTGTT
CTGCTAAAGGTATAAAAATCATAAATTTGTCAAAAGGAAATATCGATTTTTATCTAAGAATCTGATATTGGATTGTTGATTCAGAATTATTTCAGCAATTGCTTTGCTTTCTGACATTTCGCTC
TATCTATTTAAACGATTTGCTAAACAGAAAATTATTTTGAAAATTAACTAAGTGATTTCTTCTTAAGGTGGGCTTAGCAATGTTGTTAGAGTAATGATTCATTTGTGTTTTCAAGATATTAAAA
AAAAAA
>comp163963_c0_seq1
GCAATATAAATACATAGTTTTTTTTTTAATTTATCTTGAATTATATATTAATCTGTTAAAATATATTGGAAAGTAATTAAATAATTAAATAATAAATAGCTTTATGCGGCGCGGATGGGGGCGAA
CAAGCTGTGCCGCCTGCTAGAGGCAAATCTCGTACGGGTCCCGCAAAGATTTCCTGCGGCCACGTCAGCTGTCTCATTCTATGGTTCTTTTGCGAGTCTCTCCAACATATTCTCCGAAGTC
CATATTGACAACTCAGCGAACGTTTGAGGCTAATGGCCTTGTCGTAATTTTTTTGTAGTTGAATGGCATACTTTGACTGCTATAACGTCTTCGTGCATTTAACCTCCGGTAAAAAATAAATA
AACTAATAAAGAAGCCGCGGACGGGTGAAGAAATCCGGACAGCAGCGCTGTAAAATCTTTCTCTCTCCGCTAATTGTTGACGATTCTCGCTCTCTGCAACTACCCGCTCTCATAAGACAA
GATCGAAGAGGAAGAAAAGGATGAGAGGACTGGAGGAATTTCTTCGAATGGTAGGAAGAAGAGAAGAGTATCAGTTCGATATTGGATCTCTTCGACCTCTGCACCATTGGTGATAAATA
ATACGTTGGCTGCTGCTTTCTAGAACGCTTACTTGATTTTGTTAATTATTGAAGACTAGAAGTTGGGTTATTTCTTTTCATTAGCTGTGAACATTCTTTCTTGAAATATACATTGGCATCCTCT
AGAATCATGATACCAAATTCGGCAAATCTTTTAACGATGAACTCGGATGAAATGACGGATTTTCCACAAAATGAAAAAATAATTTCTCGGGTGATGAGTTCTCCTGGCCTGATTTCGGACC
TGGGAAGTATACAAAATGATGCTGATTCAGGCAGTTTGACATCTGTATCACGTCAAAGAATAATAATAGTTGCCAATTTTCTCCCACTTCATTTTTTGAAGGAGAAACAAACTGGAAAGTG
GTGCTTTTCATGGGACGAAGACTCTCTTCTACTGCAGCTTAAAGATGGTTTATCTTCTGAAACTGAAGTCATTTATGTTGGGAGTCTGAAAGTTGATGTTGATGTTAGCGAGCAGGAAGAA
ATTGCTCAAAAGCTTTTGGATGATTACAAGTGCGTCCCAACGTTCCTTCCAATTGACCTTCAAAAGAATTTCTATCATGGTTTCTGTAAGCAACAGTTGTGGCCACTTTTCCATTACATGCT
TCCCATCTGTCTCAACAAGGGTGACCTCTTTGATCGGTCTCTTTTTCAAGCTTATGTTTCAGTCAATAGAATATTTGCTGACAAGGTGATGGAAGTGATTAATTCAGATGATGACTATGTATG
GGTTCATGATTATCACCTAATGCTTCTTCCCACATTCTTGAGGAAGAGACTAAACCGAATCAAGCTTGGGTTCTTCCTCCACACCCCGTTTCCTTCCTCAGAAATTTATCGGACTCTACCTG
TCAGAGATGACATTCTTAGAGGATTGCTTAATTCTGATCTAATTGGATTCCATACATTTGATTATGCCCGGCACTTTCTTTCATGCTGTAGTCGGATGCTAGGGCTGAACTATGAGTCTAAGC

GCGGTCACATTGGAATTGAGTATGGTGGTCGTACCGTTGGCATTAAAATTCTTCCTGTTGGTGTTCACATTGGACGGCTTCAATCTCTATTAAGTCTTCCTTCTACGATTGGCAAGATTCAA
GAAATTGAGCAGAAGTTCAAGGGGCAAAAGTTGCTAGTAGGTGTAGATGATATGGACATCTTCAAAGGCATCAGCTTGAAGTTGCTGGCATTTGAGTTGCTACTCGATAGGAATCCTGAT
CTACGAGGAAAAGTGGTACTTGTTCAAATTGTAAATCTGGCAAGGAGCTCTGGTAATGATGTGAAGGATGCAAGAGCAGAAACAATGTCATTGGCAGAAAGAATCAACCTTCGGTATGG
CAACCAAATCTACAATCCTGTGGTACTTATTGATTACGCTATCCCCTTTCATGAAAAGATTGCTTTCTATGTTGCTGCTGAGTGCTGCATTGTAAATGCCTTAAGGGATGGAATGAATTTGGT
TCCATATGAATATGTAGTTTGTCGACAAGGAACTGAGGAGATGGATAAATTAAGAGATATTGGCAGAGGTTCTCCACGAACCAGTACACTTATTGTTTCTGAATTTGTGGGATGTTCTCCAT
CCTTGAGTGGAGCTTTCAGGGTAAACCCTTGGAGCCTTGAGGATGTGGCTGATGCTTTGTATCAGGCCATTGACATGAACGAGTCTGAAAGACAGCTCCGGCATGAAAAGCATTACCGCT
ATGTGAGCTCTCATCATGTGTCGTATTGGGCGCGCAGCTTCACACAGGATCTTGAGAGAGCATGCAAAGATCACTACAACCAAAGGTGCTGGGGTGTTGGTCTTGGTTTAGGTTTCAGGG
TTATTGCCCTTTCTCCTGGTTTCAGGAAACTATCTGTTGGCCATGTTGTCTCGTCCCACCGCAGATCCAATAGGAGAGCAATCTTCTTAGATTATGATGGAACACTAATGCCAGAAACATCT
ATAGTAAAAGATCCAAGCAGTGATATTATTTCTATGCTAAAGACTCTGTGCAGTGACCCTAGAAATAATGTGTTTATTGTTAGTGGAAGAGGGCGGGCTCCTCTTGCCAACTGGTTTTCATC
ATGTGAAGCACTTGGAATTGCTGCTGAACATGGCTATTTCATCAGGTGGAATGCGTCTTCTAATTGGGAATCTAGTCCAGTTGTGGTCGATCCAGATTGGATGAACATAGCAGAACCTGTC
ATGACTTTGTATACAGAGGCAACTGATGGATCCTACATAGAAGTGAAGGAGAGTGCTTTGGTCTGGCATCATCAATATGCTGATCCTGTCTTTGGCTCATGCCAAGCAAAGGAATTGTTGG
ACCATCTTGAGAATGTTCTTGCTAATGACCCGGTGGTTGTTAAACGTGGGAATCTTATTGTGGAAGTTAAACCTCAAGGAGTCGGTAAAGGACTGGTTGTGGAGAAGCTGCTTAAGACGC
TTATAAATGATGGAAAACCACCAGATTTTTTGATGTGCATTGGTGATGATCGATCTGACGAGGACATGTTTGAAAGCATTATTTCTACAGCCTCTAGTTCACTTTTTCCTGTAGTTCCTGAA
GTGTTTGCCTGTACTGTTGGCCAGAAGCCAAGTAAAGCCAAGTATTTTGTCAATGATACCATGGAAGTGATCAACCTGCTGGAAGCCATTGCAGATTCTTCTTCTCAAAGAAGCAAGGAT
TTAAAAAAATTGGTTTCGTTTGAAGGGTCAATGGAGTCACAAGATCTGCCTATTGTTTCTGCATGAGAAGATTATTGGTTCTCTTGTACATTCATTACTCTTTTCTGTGGCTCATAGATTCTG
TGACTCTGACTCTAACTATTGTGAAGGACAACTGAAGAGATACAAATAGAAGCAGAGGATTAATTAAACTTTCGCTGACTCTACTATGCCTTTTGTAGTGGTTTTTTTTTTCTTTTTTTTTGT
TTGTAAAGGTGACAGTATGATTGACATGATTGTTCATTGACATTAAAGGAATATTTAATAT
>comp163977_c0_seq10
AGTATCGATCCAAGGCAAGACAAATATAACCAGAAGTACAGATATTTGATCTTTACATACAGGAAATCACTTCATCATACTTTATGACTGAATCTTTTCTGAAAATCTGCCACAATTCTCAG
ATGAGAAGGAAAAAAATATTATGACCACTATATCTCTATTAGAACAGTACTATATGCGTTTACTGTTAAGCACTTGAAATTATTGTATCCTTTAGAAGCAAGGAGGAGGAAATACGTGTCCTA
TGGCTTTTGCAATTCCAACGTTCGTGCATGCGGTCAAGGGGACGGTAGGGGCGAGGCCTGGGCTGGGTGGAAGAGGCTTCAGGTTAATGTTCTCGAGTGTTATGTGGTCGCAAGGCACA
AAGGAGCTGCAGACGAGGTTGATGGCGACATCAGTAGCTGAAGTTCCCCTTATGTTTTGGAACCTCACGTTGTCGATCTTTACTCTGGAAGCTGGCTTTTCTTCATCGCAAGAATTGGGA
CAATACTCCTGGTCAATAATAATCGGATTGCTAACCTCCTTCATCACAATATCCGAAAACAATATATTCGTAGCAGATATCCGAGGCGAATCTCTCCACGTCTTAATCCTCACCCCGTTCGTT
GTTCCAGCAAGCGTGCAATTCAGAACTCTTATTGCATTCACTCCCTTATCTTCACTGTTCTTTCCCAAGCTCCCAACACTGATGCCATGGCCGGGCCCACACCTGACTTTGCTGACCGTTAT
GTTTGAACTTCCAGGTCCGATGGAGACACAGTCGTCACCGGAGGAGATTCTGGACTTTGAGATTCGGATATCGGATGAATATGCGATGTGGATTCCGTCGGTGTTGGGGCTGTTTCCGGG
AGCGGTGATGTGAACGCCGAGGACGGTGACGTACTGCGATTGGCCTATAACCATGTGGAAGAACTTGGAGTCGACAAGAGTGATGGCACGAATCTTGGCGTTGATCACGTGAAGAAACT
TTAAAGTCGGTGGAAGGCTATTTCCATGCTTATGCGGCCATATTGCACGACCCTGACCATCAAATGTGCCTAATCCAGTGACCAACAGGTTGCTCACATGCCGAAAGGCAATCCATTCCTT
GTGCTTGAAGTTCCCTAGATTAATCGGAGCCCGAACCAGGCCCTTCACCTGAACCACCATAGGACTCTTGCAGGGTCCCCTGAAGGACACTGGCCCTACCAAGAAGGTACCCTCCGGAA
TCAGAACCCTACACCTAACCCTAAAGCTACCATACCATCCACAATCTGTTCCACAGGCTTCATTCCATGCCTTAGAAAAAGCTTGGGCTCCATCGCTTTTACCGTTAGGGGAGGCTCCGTA
GCGAAGAACGTTAAATATTTTGTCTGTAAAGACGACTCCATCACAATTGTTGAGATGTGCTACTGCGCAGAGAAGGATGAACAGCAAACTGCATGATTTCATGGTTTCACTGTTGCTGCT
GTTCTTGCCTTGTTCTCTTCCTCCCTTTGATCTCTCCCTTTTTAATTTTTTTTTTTATTTTTTTTTCCTTTTTTTTTTCAGCAACCAGGGCTTAATGTTCTCACAGAACTTATAATTGGATACATA

TATCCTGGAAGGCCACTTGCTAACGTTGCATTCAAGACATATGGCTATATTAGCATGACTCAAGCAATCACCTTCCTTGCAGATTTCAAGCTAGGCCATTACATGAAGATTCCACCAAAGTC
CATGTTTTTGGTTCAGGTAATGCATAGATCTTGTATTTTATTGTACAAGATCTGTTAAATCTCTCTATTTATAGGATTTAAAAGATGTAAGAATTTCTCCCTCACATAATTCTTAAATTTTAAAA
TAAATAATCAAATAATTTATCTGACTTTCAGAAAATTTTATGGTTGTCATACAAAAATAACTTTCAGATTTGAACTCTCTCAAAATTTACTAAACCTGATTTTACAGATTTTACTAATCCCATT
TGAGTTAAATACTAATCGTAGCCAAACGGCCCCAAAGCCATCAAAATCAGAACTTTCACGATGAGAACAAAATCAAAATCAAAATCGAAACCGAGTAAAAAACTTGATTGCATGTTTGC
AGAATAACATCATCATCAGGGAAAGTAATCATAAATTACCTCTGTGGATTTTCAGCTGGTAGGCACCATCGTGGCCTCCTCCGTCTACTTCGGCACCGCCTGGTGGCTTCTTGAAACAATC
GAAAACATCTGCGACCCATCAAAGCTCCCCGAAGGCAGCCCCTGGACTTGCCCCGGCGATACCGTCTTCTTCAACGCTTCCATCATCTGGGGCGTCGTCGCCCCTCGCCGCATGTTCGGC
AATCTCGGCCTCTACACCAAAATGAACTACTTCTTCCTCATCGGCATCATTGCCCCCTTCCCCATCTGGCTCCTCTACCGCTTATACCCCAACAAGAAATGGCTCAGGCTCATCAACATGCC
CATCATCCTCGGCGCCACAAGCGCAATGCCGCCGGCCAGGGCGGTTAATTACATCATGTGGGGGACGGTGGGAATCTTCTTCAGCTTTGTGGTTTACAGGAGGTATAAAGGGTGGTGGGC
GAGGCATAACTATGTTTTATCGGCGGGATTGGATGCTGGAGTGGCGTTCATGGCGACTCTGACTTATTTTTCTCTGCAAATTAAGAATATTGATGGGGTGAATTGGTGGGGGTTGCCGTTGA
ACGATCATTGCCCTTTGGCGAGTTGTCCGACCGCTCCGGGGGTGAAGGTTCCGAACTGTCCTGTGCATTAGGATTAAGGCATTTCGTCGAACAGTTGGCGGGCGAGTATACAGGAATGTG
TTTACACCAGTTCATTGTGTTTTATTTTTGTTGCTCTTGTATTTAGAATATATTGACACACTCGAATGCTGAAAGTCCGTAATTTTTTTTTGCAATGATGTTTTTGAGGGATTTACATAAATACC
ACTTTATTTTATGCTATTTACAAGTTTAACACTTAAAACATTAATATTACATGCGTATCACCCATATACAATTTTAATATCAAATATATCACTCCT

>comp163977_c0_seq7

AATAATAATGAAATATTTAAAATTGAAAGAGAGTGATATAAGATGTTTTGCTTGTAAATTACTCTATAAATAAAATAAATGTGAATTCAATATATATCATTTACGGTGATTTCAGTGGAAGAGA
ACGAGCACCCGATTGAAGAAGTTCGGCTTACAGTCGACCCCGCCGACAACCCTAACCTCCCAGTCCTGACCTTCCGAACATGGCTTCTCGGATCAATCTCCTGCGTAATGCTCGCTTTTG
CCAACAAATTCTTCGGCTACAGAACGAATCAGCTTACCATTGGTCCAGTCTGCGTTCAAATCGTCTCGCTGCCCATTGGCAGATTCCTCGCGGCTTCTTTGCCGAAGAAGAACATCAAGG
TTCCTTTCACCAAATGGACTTTCTCCCTCAATCCTGGGCCATTCAATATGAAGGAGCATTGTCTCATCACCATTTTTGCCAACTCTGGTGCCGGCGGCGTCTATTCCATACACATTGTCACCA
TTGTTAGGGCTTTCTATCGCAGAAATATCAATCCTGTTGTTGCCTTCTTGTTGTCACAAACCACTCAGGTGCTAGGTTATGGTTGGGCTGGAATTTTCAGAAAGTATCTGGTGGAATCTCCA
TATATGTGGTGGCCTGCAAATCTTGTTCAAGTTTCCCTCTTCAAGTAGTGAATCTTCATCATTAAGTTAATTCAATTTTCATAATCCTCTTGCTTTTATGCTAAAATAAATAATCAAATCTTTTT
CTTTTGTTTTTTTAAGAACACTGCATCAAAAAGAGAAGAGAAGAAAGGGCAGCCTGACAAAATTTCAATTCTTCGTAGTAATCTTTGTATTCAGCTTTGCATACTATGCCATTCCCGGCTAT
CTCTTCCCAGCCATCTCCACAGTCTCTGTCCTCTGCTTGATATTTAAGGAGTCGATCACCATGCAACAAATTGGATCAGGGATGAAAGGGCTTGGCATTGGCTCCTTCGGTCTTGATTGGT
CAACAGTTGCTGGTTTTCTTGGTAGCCCATTGGGAACACCTGCAAGTGCAATCTTTAACATAATGGTAGGCTACTTCATTGCCATCTATATAATGCTTCCATTTGCCTATTGGACCAACATAT
ACAGGGCAAAGAATTTTCCAATGATCTCGTCCCATGTCTTCGACTCTTCTGCGCAACCGTACAACACGACACGAATTATTAATAGCAAGACTTTCACCTTGAATGTTGAAGAACAGGAGA
GTTACAGCAAGATAAACATGAGTATTATGTTTGCTTTGACATATGGCTTAGGATTTGCAACCTTGACATCATCGGTCATGCATGTTGCTCTCCACAATGGAAAGGATATTTGGCAGTCATGG
AGACAAGCCAGAGATTCCTCTAAGGAGAAGAAATTAGATGTTCATGCTAGATTGATGAAGAATTATGATGCTATACCTCAATGGTGGTTCCATGTTCTCTTGATTTTGATGATTCTACTCTCA
ATTTTCACTTGTGTGGGATTTGGTGGAGCTTTGCAACTTCCATGGTGGGGAATATTATTGGGTTGTGCTATGGCTTTCTTCTTCACATTACCTATTGGAGTCATTACAGCAACTACAAACCAG
TCACCAGGGCTCAATATCATCACAGAGTACATCATTGGTCTAATTCTACCTGGAAAGCCCCTAGCAAATGTGACATTCAAGACATATGGCTATATTAGCATGACTCAAGCACTCACATTTCT
CAGTGATTTCAACCTTGGACACTATATGAAGATTCCCCCCAGATCTATGTTCATCTCACAGTTGACAGGGACCGTGATCGCAGCGACGGTCAACTTTGGAACTGCATGGTTTCTTTTAGAT
TCAGTTGAGAACATATGTGACACTGATAAACTACCAGCTGACAGTCCATGGACATGCCCAGGGGACGATGTATTCTACAATGCATCCATCATATGGGGACTTGTTGGCCCTATGCGAATGTT
CGGAAAACTCGGTCTCTACTCTGCTTTGAACTACTTCTTCCTTGCTGGATTCTTGCTACCCATTCCTTTCTGGTGGATTTCTAAGGTCTTCCCTAACTGCAAATGGCTCAAATACATCCATTT
ACCTCTCATTATTGGAGGTGCTGGTGGTATCCCTCCTGCACACACAGTGAACTATATCACCTGGGGTGCTGTTGGCATCTTCTTCAACTACTACATTTACAACAAATATAAGGAATGGTGGG

CAAGGCACACCTACGTAATGTCGGCCGCCCTCGATGCCGGAGTCGCTTTCATGGGAGTTCTCATCTTCTTTGCCTTACAATACAATGATATTGGTGGAATTAGTTGGGTGGGAGGCGATGC
AAGTGACTTCTGTCCCCTTGCAAATTGCCCTACAGCTCCTGGAGTGGTCGCCAAAGGATGCCCAGTGATAGCCTGAGATATGATTACTATGTTTCATTAAGCACTTACTAGTTGGTGATAAG
CTTGCATGACTTGTTGTTCTAGTACTCCCAAGTCATTGTACATATAATCGTTCATTTTTTATTTTTTCTTTGTTTCCTGAAAACTTATATTCAAGCCAATGTTCTTTGTATGTTCTGTGGAATTT
GTTATTTATAGCATTTGAAGTCTATGTGAATATATTCTACGTATGTATGGTCTCTATAGTTCCTCATTAGAGTCCAACATTCCTTAGCTATTTATGATCATGATATACAATATTTGGATGCC
>comp163984_c1_seq2
TTGCTAAGAGCCTAAGAAGAGAAGAAAATAGAGAGCTTATATTCTTATTGAAGGAATGGCTTCCATTCTTCCATTAGATTGCTTCATTTTCTTCTTCTTCTTCTTAGCTACTTCCTCCATCGC
CATTAACATCAGAGACTATGGGGCGGTTCCTGATGGCTCAAAGGACAGCACCAAGAATATGGTATTCAGTTTTGTGAGCAATGCAAACATAAGCCGCATATCATCAATTAACAGCAAATAC
TTTCACATGCTCGTTTTCGGCAGCAACAACATAACATTCGACTCCATAACTATCTCTGCCCCTGGCGACAGCCCCAACACCGACGGCATCCACATTGCCGTCTCCCAGAACATTATCGTTC
AAAACTCGGTTATTGGCACCGGCGATGATTGTGTTTCCGTTGGTGATAAGACTCATAATCTCACCATCTCGGGGGTCCGATGTGGGCCGGGCCATGGTATTAGCGTCGGCAGTCTTGGAAG
GAACCGCAATGAAGAAGATGTTGTTGGGCTCACTATAAGAAATTGTAGCTTTATAGACACTCAAAATGGGGTGAGGATTAAGACATGGCAGAATGGATTGAACAAACTTCATGTTAGAGA
TCTTGTGTTTGAAGATATTATGATGGAGGATGTGGAGAATCCTATTGTTATTGACCAGAAGTATTGCCCTTCAAGGACTTGTCCTGACAATTTGAGGCCGTCGAGTGTGAAGATAAGAAAT
GTGAAGTTCAACAATATTGTGGGCACATCGACGAGCCAAACGGCGATCAACTTGGTGTGTAGTGCATCCAATCCTTGCGAGGGGGTTGAGCTGAACCACATTGATCTCGAGTTCAATGGT
GTTGGTGAGGCCAAAGCTACTTGTATCAACGTGAAGGCACCTAAGGTCATCGGCCTCGTCAAACCGGATAATTGTGCTTAGTTAATTATTGTTGCAACATTTCTATATTTGCATGCTGTGAA
GTGTGAGAGAGAACTTTTGCATTGTCTCTATTTTTATGTATTATCCCATTATATGTATGCTTATTGTGCCAAATAATATTAAGAAGAAGAGGATTGTTCAAAAAAGAAAAAA
>comp163984_c3_seq2
AAAAAAAAAAAAACACATAAATTTTGAACAATCCTCTTCTTCTTAATATTATTTGGCACAATAAGCATACATATAATGGGATAATACATAAAAATAGAGACAATGCAAAAGTTCTCTCTCAC
ACTTCACAGCATGCAAATATAGAAATGTTGCAACAATAATTAACTAAGCACAATTATCCGGTTTGACGAGGCCGATGACCTTAGGTGCCTTCACGTTGATACAAGTAGCTTTGGCCTCACC
AACACCATTGAACTCGAGATCTATGTGATTCAGCTCAACCCCCTCGCAAGGATTGGATGCACTACACACCAAGTTGATCGCCGTTTGGCTCGTCGATGTGCCCACAATATTGTTGAACTTC
ACATTTCTTATCTTCACACTCGACGGCCTCAACATAGGAAAAGGCCTGCAAAGAGATTTGTGATTCTGGCGGCACTTGTTCTTCGTCCAGGCATCAGCACCCTGACCATCGACCTTACCTG
GGCCGAAGAGAATGAGCCCATTGATATACTGAAATTCAATCCAGTTGGGCTTATACAAAGCTAGATTTTTCTTTGCAATCAAAGTTCCGATCAGGTGAATGGTTACCGGGCTCTTACATGG
GCCCCTGAACATTAACGGCCCAATTAAGTATCTTCCCATTGGGATCTCAATACTTGCATTGCTCTCAGCTGCGCATGCATTATTCCAAGCCTTTTGTAGTGCCTTGGTGCTGTCCTTTGAGCC
ATCAGGAACCGCCCCATAGTCTCTGATGTTAATGGCGATGGAGGAAGTAGCTAAGAAGAAGAAGAAGAAAATGAAGCAATCTAATGGAAGAATGGAAGCCATTCCTTCAATAAGAATATA
AGCTCTCTATTTTCTTCTCTTCTTAGGCTCTTAGCAAAG
>comp163984_c3_seq3
AAAAAAAAAAAAACACATAAATTTTGAACAATCCTCTTCTTCTTAATATTATTTGGCACAATAAGCATACATATAATGGGATAATACATAAAAATAGAGACAATGCAAAAGTTCTCTCTCAC
ACTTCACAGCATGCAAATATAGAAATGTTGCAACAATAATTAACTAAGCACAATTATCCGGTTTGACGAGGCCGATGACCTTAGGTGCCTTCACGTTGATACAAGTAGCTTTGGCCTCACC
AACACCATTGAACTCGAGATCTATGTGATTCAGCTCAACCCCCTCGCAAGGATTGGATGCACTACACACCAAGTTGATCGCCGTTTGGCTCGTCGATGTGCCCACAATATTGTTGAACTTC
ACATTTCTTATCTTCACACTCGACGGCCTCAAATTGTCAGGACAAGTCCTTGAAGGGCAATACTTCTGGTCAATAACAATAGGATTCTCCACATCCTCCATCATAATATCTTCAAACACAAG
ATCTCTAACATGAAGTTTGTTCAATCCATTCTGCCATGTCTTAATCCTCACCCCATTCTGAGTGTCTCTAAAGCTGCAATTTCTTATAGTGAGCCCAACAACATCTTCTTCATTGAGGTTCCT
TCCAAGACTGCCAATGCTAATACCATGGCCTGGCCCACATCGAACTCCCGAGATGGTGAGATTATGAGTCTTATCACCAACGGAAACACAATCATCGCCGGTGCCAATAACCGAGTTTTG
AACGATAATGTTCTGGGAGACGGCAATGTGGATGCCGTCGGTGTTGGGGCTGTCGCCAGGGGCAGAGATAGTTATGGAGTCGAATGTTATGTTGTTGCTGCCGAAAACGAGCATGTGAA

AGTATTTGCTGTTAATTGATGATATGCGGCTTATGTTTGCATTGCTCACAAAACTAAATACCATATTCATAGGGAAAGGCCTGCAAAGAGATTTGTGATTCTGGCGGCACTTGTTCTTCGTCC
AGGCATCAGCACCCTGACCATCGACCTTACCTGGGCCGAAGAGAATGAGCCCATTGATATACTGAAATTCAATCCAGTTGGGCTTATACAAAGCTAGATTTTTCTTTGCAATCAAAGTTCC
GATCAGGTGAATGGTTACCGGGCTCTTACATGGGCCCCTGAACATTAACGGCCCAATTAAGTATCTTCCCATTGGGATCTCAATACTTGCATTGCTCTCAGCTGCGCATGCATTATTCCAAG
CCTTTTGTAGTGCCTTGGTGCTGTCCTTTGAGCCATCAGGAACCGCCCCATAGTCTCTGATGTTAATGGCGATGGAGGAAGTAGCTAAGAAGAAGAAGAAGAAAATGAAGCAATCTAATG
GAAGAATGGAAGCCATTCCTTCAATAAGAATATAAGCTCTCTATTTTCTTCTCTTCTTAGGCTCTTAGCAAAG
>comp163991_c0_seq5
GAATACAAAAACAAATGATATAAAAAGGAAGAATGAAATTCTATTTCATTTAGCTTTATAAACAACGAATTAAAGAACCAAATTAAAAATAAATGATTTGTTTGCTAAAAAAAAAACTACT
AGTATACAAAGAAATATCTCATACTGATAAACATCTTATATTCATGTGCCCATTTGCAAAGATCGCTACAAAATCTTCTTTTCACTTGTACCTCATGTGACCATAGATTATCTCCTTCCAAAGC
TTTAGTTCCACATGAAAAATTTTCACGAGTAAATGTCAATGAAGGTAATTTTTGCCATATCATATTCCTAAAGATCTGTGAAGCAAGTTTTCGACACTGATGTGTAGTATTTGATATCTCGTT
CAAAAGATCAATAGCATGCAAGCGAGTTGATTCATCATGAAACAACAATATTCCTCTAATATATTTAGAAAGAATATGTCCTTGACTTGCAGCTAATTGAAGATGATGCAAACCAATATCAA
ATCTTCTTAGATTGAACATTTCCTCCAATCCAAGCAAGAAGAGAGCATTGACATTTTCACTCTCAGCACAACTATGAAGAAATCGTAGATAGCTTTGTTTATCTTGCCAATACATGATCCAG
TCTTCTACATTCATATATTTTCCTACTCTTTTTGATGAACTTGTTGCATGAAAATCCTTGCATATTTGTCTGAAATATCGAATGTCTCTGCTAGGTGTTGGAGAGTCTGCAAGAGCTTCTAGCA
AAATTTCCTCTAAAAGATCAGGTGGTAGGGAAAAAATGCTAGACTCTCTTCTACTTTCCTTCATGATCTTTTTGTTCTTCATGTTTGCTCCTTGAAGCTTGCAGATTGAATGCTTCAACAAT
GGCTGTTTAAGACTATAGAAGAGTTGAAGAACGGATGATGAGAAAGAAGGTTGAGAAATCTTAAAGATTTACTAAAAATTAATGAATATAATTATGTT
>comp164043_c0_seq8
CCGAAGAAGCTCTTCCCCTTACTGAACTTAACAAAAGTCTCTTCTAATAGCAGCAGTGCGGTAAAGAGTTTCTCCTTCGATCCTGCCTTGATTTTCTCATCCTCATTCCACATCAACACCTG
AATCTGAGTAATGAACGTGTCGTCGATGTAGGTGGCCCAGAAGTGAGAGGTGACGCGGTCGTAGGCGTCGGCAGGAAGGATGGGAAAGCCTGAAGAGGACCAGACTTGATCGATGTAT
TCAATAATCAAAAGGGAGTGGCAGATGGGCTTGCCGCCATGGATGAGTACAGGGAACCTCTTATAGACAGGGTTAGATTTAAGAAGAAGCTCACTCTTCTTGGTGGGATCTTCTTCGAGA
AACTCGTACTTGATCACCTTCAGATTTAGAGCGATTCTTGCTCTCGCGACCAAGGGGCTCTCCCACGTCCCGATGAGCTTTACATCTTCTGCTGCCATTATTTATTTCCTTGAAATTTCAGAT
CTGAAACTTGATAAATGAAGAGAGAGAGCCGAATGAGCGATTAGGAATGTTGCTTACTGGTATTTAAAGCTCGAAATCAGGAGAATTTATACGGTACAAGTGGACGACGAAGCATAGATG
TAAGAAAATTTTCAGTCT
>comp164106_c0_seq1
GAAATGATCCCCACATATTTACTGGCAAATATAATAACACATTTTGCAAAAAGCCTTGCCATGTTAGGAAAAGCGTAACAGTTACTATCAACATTTTTAGTTTTGCAGCACCTGTAAGCCTA
ACGGTGTCGAGAAACACCATAGCTGCTGATGAAATAAACCAACGGTCAAATACTACGACTCTACATCACAGCAAAAAAATCAACTTCCTGCAACAGACTCTTGCTTCCACTGTTTAGTAC
AAGTCCAGAAACTTGTGAGCATAGTAGTGTCTATCTGCACTACTAATTTGATAATAACATGAAGCTTGATCAATAATTTCACTAATAAATTTGAAAATTTCTGGTTAGAGCAATCTTGAGAA
CCTATCTATAATCAGCACTGTGTACATGGCTAACTAGAAGCAGGGAGAATGTAATAGAAGAGAGCTAAATGATACAGCATTACTAGGTATACAAGAAAAAAAGAATGGATGCAAATATTAT
AAACATACAGGCAGGGCAGGGCCAACTTTGCACAAAATGCATCTTTTAAAATCTACCTAATAACCTAACAAAACTGCTGCTTCATTTGCGCACTGGAATCTGATTTCAAATAGGAGATTTT
GCAAGTGCAAGAGTATGCCACCACCCACAAGAAACATTCATGATGCGGAGGTTCTCGATTGGGACCTCAGAGGGTATGTTCCGGTCAATAGAATCACCAAGGCCAAGCTGGCCATATTTG
TTCCATCCCCAGCAAAAAACTCTGCTATCCTCTGTAACTATGGCACTGTGGCGAGCACCACAGGAAACAATTTTCGCATTCATACTCTCTAATGATGGGGCATCCAATAGTTTGGGAAGTG
TCTCTGCTTGATCTAGACCGGTTCCTAGCTGTCCAAACTGATTTCCGCCAAATGCATAGACACCACCATCAACTGAAATACACAGTGTGTGCCATAAACCAGCAGCAACTCCGAGCAAAC
TGACGCCCAAAAGAGAAGAAACACATTTTGGACTGAGTTCATCATCTGTAGATCCATTCCCGCACTGTCCATAAAGTCCCCAACCAAATGTAAGTACTGCACCACTATCAGTCACTACTG

CACTATGACGACCTCCACATGCAATAGCCTTCACACAGCTTCCAATAACCTTGTAAGCATGCCCTTCCGAACTTGTATTTCCTTTACTGGAAGCTGATGCTCTATCTTTAACATAAGAAGCT
GATTCAATGCATGGTACAAGATGGGGGGAAGAAACCATGCGTGTTCGAGAACCCAAACCCAGTTGGCCTTCACCGCCATATCCCCAACCCCACACCTGACCAACATCTGACAATGCAAG
TGTATGTCGACCACCAGCAGATACTGCTGTAATCTTCACTCCCACGCCAAGTGTTACAAGACAGGGAAGTGCTGAAAGAGTTTCATCACTTGGTGATGAGCTCTCAGGACCTTGTTTAGC
TGAAGATAACCTTCTGCGTTTTGCAGTATCCTCACTGCCTTTGCCATCATAATTAACTATTGTTCCACTAATTGATCTGGAGCCGTGTAATGTGGAGCTCACTTGCTCATTCGGAAAAGCGC
TTTGTCTTTCATCCTTCTCTAAACTTCCTATAGCAGGTTGTTCTCCAATCACCTTCCCAGTTGGAACGCACTCTTTCCAGCCCCATGTGTACACCTCCCCACATGCTGTAATTGCAACACAG
TGAGCCCATCCTGCAGCTGCTTTCACTATCTCTGCTTCAGCAGGAAGAGGAAATGCCTCTGGAATTTCCTCGTGCTTTCCAGACGTAAGGTAACTCTGGCCCATATCATCCGTAGATCCCC
ATGTGATGAGCTTGCCCGATTCTGAAATAGCCATAGCGAAGCCGCATCCACCCCCGGAGACGTCCCTCCACGCTTCGCCGGTGGCAGATTCTGGTCGGCGAACTGGAACCGGATGCAGTA
GCGGCGATCTATCCGGAGAGACTCCAGGAAGATAGCCCCACATCAGTACCACCTCTTTTTCCTTCTCTTTCTCCTCCACCTTACCCAGCTTCCGCTTGTCCTCCTCCATCTCCTCTGCTCCC
CCGCTACCATTCATTCTCCACCTCCCTTCTCTTTATCAACTACGTATCCACCACTTCTCCAAGAAGAAAAGCTCTGTCTTCTCCTTTCTTTCTTTAGCTTGTCGAACTTGGTTCGCCTCAGCA
CGAGCTCTCGCATATGGAAGAGAAAGACAATACGAGAAAGCGAAATCAAGCAAAAATGAAAAATAAAAGAGCAGTCGACGAGCCCTAATTCGTTCGAATCATTTGCTTGGAGAACGTA
AGAGCCACGTAGGATATCAGTCTGGTGGTCCCGTACA
>comp164106_c0_seq11
GAAATGATCCCCACATATTTACTGGCAAATATAATAACACATTTTGCAAAAAGCCTTGCCATGTTAGGAAAAGCGTAACAGTTACTATCAACATTTTTAGTTTTGCAGCACCTGTAAGCCTA
ACGGTGTCGAGAAACACCATAGCTGCTGATGAAATAAACCAACGGTCAAATACTACGACTCTACATCACAGCAAAAAAATCAACTTCCTGCAACAGACTCTTGCTTCCACTGTTTAGTAC
AAGTCCAGAAACTTGTGAGCATAGTAGTGTCTATCTGCACTACTAATTTGATAATAACATGAAGCTTGATCAATAATTTCACTAATAAATTTGAAAATTTCTGGTTAGAGCAATCTTGAGAA
CCTATCTATAATCAGCACTGTGTACATGGCTAACTAGAAGCAGGGAGAATGTAATAGAAGAGAGCTAAATGATACAGCATTACTAGGTATACAAGAAAAAAAGAATGGATGCAAATATTAT
AAACATACAGGCAGGGCAGGGCCAACTTTGCACAAAATGCATCTTTTAAAATCTACCTAATAACCTAACAAAACTGCTGCTTCATTTGCGCACTGGAATCTGATTTCAAATAGGAGATTTT
GCAAGTGCAAGAGTATGCCACCACCCACAAGAAACATTCATGATGCGGAGGTTCTCGATTGGGACCTCAGAGGGTATGTTCCGGTCAATAGAATCACCAAGGCCAAGCTGGCCATATTTG
TTCCATCCCCAGCAAAAAACTCTGCTATCCTCTGTAACTATGGCACTGTGGCGAGCACCACAGGAAACAATTTTCGCATTCATACTCTCTAATGATGGGGCATCCAATAGTTTGGGAAGTG
TCTCTGCTTGATCTAGACCGGTTCCTAGCTGTCCAAACTGATTTCCGCCAAATGCATAGACACCACCATCAACTGAAATACACAGTGTGTGCCATAAACCAGCAGCAACTCCGAGCAAAC
TGACGCCCAAAAGAGAAGAAACACATTTTGGACTGAGTTCATCATCTGTAGATCCATTCCCGCACTGTCCATAAAGTCCCCAACCAAATGTAAGTACTGCACCACTATCAGTCACTACTG
CACTATGACGACCTCCACATGCAATAGCCTTCACACAGCTTCCAGTAAAGGAAATACAAGTTCGGAAGGGCATGCTTACAAGGTTATTGGAAGCTGTGTGAAGGCTATTGCATGTGGAGG
TCGTCATAGTGCAGTAGTGACTGATAGTGGTGCAGTACTTACATTTGGTTGGGGACTTTATGGACAGTGCGGGAATGGATCTACAGATGATGAACTCAGTCCAAAATGTGTTTCTTCTCTTT
TGGGCGTCAGTTTGCTCGGAGTTGCTGCTGGTTTATGGCACACACTGTGTATTTCAGTTGATGGTGGTGTCTATGCATTTGGCGGAAATCAGTTTGGACAGCTAGGAACCGGTCTAGATCA
AGCAGAGACACTTCCCAAACTATTGGATGCCCCATCATTAGAGAGTATGAATGCGAAAATTGTTTCCTGTGGTGCTCGCCACAGTGCCATAGTTACAGAGGATAGCAGAGTTTTTTGCTGG
GGATGGAACAAATATGGCCAGCTTGGCCTTGGTGATTCTATTGACCGGAACATACCCTCTGAGGTCCCAATCGAGAACCTCCGCATCATGAATGTTTCTTGTGGGTGGTGGCATACTCTTG
CACTTGCAAAATCTCCTATTTGAAATCAGATTCCAGTGCGCAAATGAAGCAGCAGTTTTGTTAGGTTATTAGGTAGATTTTAAAAGATGCATTTTGTGCAAAGTTGGCCCTGCCCTGCCTGT
ATGTTTATAATATTTGCATCCATTCTTTTTTTCTTGTATACCTAGTAATGCTGTATCATTTAGCTCTCTTCTATTACATTCTCCCTGCTTCTAGTTAGCAATGTACACAGTGCTGATTATAGATAG
GTTCTCAAGATTGCTCTAACCAGAAATTTTCAAATTTATTAGTGAAATTATTGATCAAGCTTCATGTCATTATCAAATTAGTAGTGCAGATAGACACTACTATGCTCACAAGTTTCTGGACTT
GTACTAAACACTGGCAAGAGTCTGTTGCAGGAAGTTTCTTCTTTTGCTGTGATGTAGAGTCGGAGTATTTGACCGTTGGTTTATTTCATCAGCAGCTATGGTGTTTCTCGACACCGTTAGG
CTTACAGGTGCTGCAAAACTAAAAATGTTGATAGTAACTGTTACGCTTTTCCTAACATGGCAAGGCTTTTTGCAAAATGTGTTATTATATTTGCCAGTAAATATGTGGGGATCATTTCAGTCT

GAATTCTTTCATTATTCGAATTTATTGATGCCTAATAATCTTAATTTTTCGCAGGTGTTTTTA
>comp164106_c0_seq18
GAAATGATCCCCACATATTTACTGGCAAATATAATAACACATTTTGCAAAAAGCCTTGCCATGTTAGGAAAAGCGTAACAGTTACTATCAACATTTTTAGTTTTGCAGCACCTGTAAGCCTA
ACGGTGTCGAGAAACACCATAGCTGCTGATGAAATAAACCAACGGTCAAATACTACGACTCTACATCACAGCAAAAAAATCAACTTCCTGCAACAGACTCTTGCTTCCACTGTTTAGTAC
AAGTCCAGAAACTTGTGAGCATAGTAGTGTCTATCTGCACTACTAATTTGATAATAACATGAAGCTTGATCAATAATTTCACTAATAAATTTGAAAATTTCTGGTTAGAGCAATCTTGAGAA
CCTATCTATAATCAGCACTGTGTACATGGCTAACTAGAAGCAGGGAGAATGTAATAGAAGAGAGCTAAATGATACAGCATTACTAGGTATACAAGAAAAAAAGAATGGATGCAAATATTAT
AAACATACAGGCAGGGCAGGGCCAACTTTGCACAAAATGCATCTTTTAAAATCTACCTAATAACCTAACAAAACTGCTGCTTCATTTGCGCACTGGAATCTGATTTCAAATAGGAGATTTT
GCAAGTGCAAGAGTATGCCACCACCCACAAGAAACATTCATGATGCGGAGGTTCTCGATTGGGACCTCAGAGGGTATGTTCCGGTCAATAGAATCACCAAGGCCAAGCTGGCCATATTTG
TTCCATCCCCAGCAAAAAACTCTGCTATCCTCTGTAACTATGGCACTGTGGCGAGCACCACAGGAAACAATTTTCGCATTCATACTCTCTAATGATGGGGCATCCAATAGTTTGGGAAGTG
TCTCTGCTTGATCTAGACCGGTTCCTAGCTGTCCAAACTGATTTCCGCCAAATGCATAGACACCACCATCAACTGAAATACACAGTGTGTGCCATAAACCAGCAGCAACTCCGAGCAAAC
TGACGCCCAAAAGAGAAGAAACACATTTTGGACTGAGTTCATCATCTGTAGATCCATTCCCGCACTGGATATTGAGCACACAGGTAAAAATTACAAAAGAGCAAACTGAATGATTAGTCA
TTCCATGTGACCTCAAGATTTAAGAGAAAATTATGAAGGATTAAAAGTCAATATCTCCATGATACAAGGAACCTCATCTTCAACAATATACTTGCTTTATCTCCATCCTTTCTACTATACCTAC
ATATTAATCCTGTCCATAAAGTCCCCAACCAAATGTAAGTACTGCACCACTATCAGTCACTACTGCACTATGACGACCTCCACATGCAATAGCCTTCACACAGCTTCCAGTAAAGGAAATA
CAAGTTCGGAAGGGCATGCTTACAAGGTTATTGGAAGCTGTGTGAAGGCTATTGCATGTGGAGGTCGTCATAGTGCAGTAGTGACTGATAGTGGTGCAGTACTTACATTTGGTTGGGGAC
TTTATGGACAGGTATAGTAGAAAGGATGGAGATAAAGCAAGTATATTGTTGAAGATGAGGTTCCTTGTATCATGGAGATATTGACTTTTAATCCTTCATAATTTTCTCTTAAATCTTGAGGTC
ACATGGAATGACTAATCATTCAGTTTGCTCTTTTGTAATTTTTACCTGTGTGCTCAATATCCAGTGCGGGAATGGATCTACAGATGATGAACTCAGTCCAAAATGTGTTTCTTCTCTTTTGGG
CGTCAGTTTGCTCGGAGTTGCTGCTGGTTTATGGCACACACTGTGTATTTCAGTTGATGGTGGTGTCTATGCATTTGGCGGAAATCAGTTTGGACAGCTAGGAACCGGTCTAGATCAAGCA
GAGACACTTCCCAAACTATTGGATGCCCCATCATTAGAGAGTATGAATGCGAAAATTGTTTCCTGTGGTGCTCGCCACAGTGCCATAGTTACAGAGGATAGCAGAGTTTTTTGCTGGGGAT
GGAACAAATATGGCCAGCTTGGCCTTGGTGATTCTATTGACCGGAACATACCCTCTGAGGTCCCAATCGAGAACCTCCGCATCATGAATGTTTCTTGTGGGTGGTGGCATACTCTTGCACT
TGCAAAATCTCCTATTTGAAATCAGATTCCAGTGCGCAAATGAAGCAGCAGTTTTGTTAGGTTATTAGGTAGATTTTAAAAGATGCATTTTGTGCAAAGTTGGCCCTGCCCTGCCTGTATGT
TTATAATATTTGCATCCATTCTTTTTTTCTTGTATACCTAGTAATGCTGTATCATTTAGCTCTCTTCTATTACATTCTCCCTGCTTCTAGTTAGCAATGTACACAGTGCTGATTATAGATAGGTTC
TCAAGATTGCTCTAACCAGAAATTTTCAAATTTATTAGTGAAATTATTGATCAAGCTTCATGTCATTATCAAATTAGTAGTGCAGATAGACACTACTATGCTCACAAGTTTCTGGACTTGTAC
TAAACACTGGCAAGAGTCTGTTGCAGGAAGTTTCTTCTTTTGCTGTGATGTAGAGTCGGAGTATTTGACCGTTGGTTTATTTCATCAGCAGCTATGGTGTTTCTCGACACCGTTAGGCTTAC
AGGTGCTGCAAAACTAAAAATGTTGATAGTAACTGTTACGCTTTTCCTAACATGGCAAGGCTTTTTGCAAAATGTGTTATTATATTTGCCAGTAAATATGTGGGGATCATTTCAGTCTGAATT
CTTTCATTATTCGAATTTATTGATGCCTAATAATCTTAATTTTTCGCAGGTGTTTTTA
>comp164106_c0_seq28
ATTTCAGCCAAATTCAAGATGCCAGAACCCCAGGAATACTTGAATGGTCTTGTATCTAGACAATGATACATTCCCTAACGTCATTTGCTCCCACTTAAGTTGGAATTCATTTTTGGGTTAATC
ATGAATTCGTTTTCTAATGTTATTTGCTTACTCCCTTCTTGAATTTGCAGATAGTGGTGCAGTACTTACATTTGGTTGGGGACTTTATGGACAGGTATAGTAGAAAGGATGGAGATAAAGCA
AGTATATTGTTGAAGATGAGGTTCCTTGTATCATGGAGATATTGACTTTTAATCCTTCATAATTTTCTCTTAAATCTTGAGGTCACATGGAATGACTAATCATTCAGTTTGCTCTTTTGTAATTT
TTACCTGTGTGCTCAATATCC
>comp164106_c0_seq3

GAAATGATCCCCACATATTTACTGGCAAATATAATAACACATTTTGCAAAAAGCCTTGCCATGTTAGGAAAAGCGTAACAGTTACTATCAACATTTTTAGTTTTGCAGCACCTGTAAGCCTA
ACGGTGTCGAGAAACACCATAGCTGCTGATGAAATAAACCAACGGTCAAATACTACGACTCTACATCACAGCAAAAAAATCAACTTCCTGCAACAGACTCTTGCTTCCACTGTTTAGTAC
AAGTCCAGAAACTTGTGAGCATAGTAGTGTCTATCTGCACTACTAATTTGATAATAACATGAAGCTTGATCAATAATTTCACTAATAAATTTGAAAATTTCTGGTTAGAGCAATCTTGAGAA
CCTATCTATAATCAGCACTGTGTACATGGCTAACTAGAAGCAGGGAGAATGTAATAGAAGAGAGCTAAATGATACAGCATTACTAGGTATACAAGAAAAAAAGAATGGATGCAAATATTAT
AAACATACAGGCAGGGCAGGGCCAACTTTGCACAAAATGCATCTTTTAAAATCTACCTAATAACCTAACAAAACTGCTGCTTCATTTGCGCACTGGAATCTGATTTCAAATAGGAGATTTT
GCAAGTGCAAGAGTATGCCACCACCCACAAGAAACATTCATGATGCGGAGGTTCTCGATTGGGACCTCAGAGGGTATGTTCCGGTCAATAGAATCACCAAGGCCAAGCTGGCCATATTTG
TTCCATCCCCAGCAAAAAACTCTGCTATCCTCTGTAACTATGGCACTGTGGCGAGCACCACAGGAAACAATTTTCGCATTCATACTCTCTAATGATGGGGCATCCAATAGTTTGGGAAGTG
TCTCTGCTTGATCTAGACCGGTTCCTAGCTGTCCAAACTGATTTCCGCCAAATGCATAGACACCACCATCAACTGAAATACACAGTGTGTGCCATAAACCAGCAGCAACTCCGAGCAAAC
TGACGCCCAAAAGAGAAGAAACACATTTTGGACTGAGTTCATCATCTGTAGATCCATTCCCGCACTGTCCATAAAGTCCCCAACCAAATGTAAGTACTGCACCACTATCAGTCACTACTG
CACTATGACGACCTCCACATGCAATAGCCTTCACACAGCTTCCAGTAAAGGAAATACAAGTTCGGAAGGGCATGCTTACAAGGTTATTGGAAGCTGTGTGAAGGCTATTGCATGTGGAGG
TCGTCATAGTGCAGTAGTGACTGATAGTGGTGCAGTACTTACATTTGGTTGGGGACTTTATGGACAGGTATAGTAGAAAGGATGGAGATAAAGCAAGTATATTGTTGAAGATGAGTGCGGG
AATGGATCTACAGATGATGAACTCAGTCCAAAATGTGTTTCTTCTCTTTTGGGCGTCAGTTTGCTCGGAGTTGCTGCTGGTTTATGGCACACACTGTGTATTTCAGTTGATGGTGGTGTCTA
TGCATTTGGCGGAAATCAGTTTGGACAGCTAGGAACCGGTCTAGATCAAGCAGAGACACTTCCCAAACTATTGGATGCCCCATCATTAGAGAGTATGAATGCGAAAATTGTTTCCTGTGG
TGCTCGCCACAGTGCCATAGTTACAGAGGATAGCAGAGTTTTTTGCTGGGGATGGAACAAATATGGCCAGCTTGGCCTTGGTGATTCTATTGACCGGAACATACCCTCTGAGGTCCCAATC
GAGAACCTCCGCATCATGAATGTTTCTTGTGGGTGGTGGCATACTCTTGCACTTGCAAAATCTCCTATTTGAAATCAGATTCCAGTGCGCAAATGAAGCAGCAGTTTTGTTAGGTTATTAG
GTAGATTTTAAAAGATGCATTTTGTGCAAAGTTGGCCCTGCCCTGCCTGTATGTTTATAATATTTGCATCCATTCTTTTTTTCTTGTATACCTAGTAATGCTGTATCATTTAGCTCTCTTCTATTA
CATTCTCCCTGCTTCTAGTTAGCAATGTACACAGTGCTGATTATAGATAGGTTCTCAAGATTGCTCTAACCAGAAATTTTCAAATTTATTAGTGAAATTATTGATCAAGCTTCATGTCATTATC
AAATTAGTAGTGCAGATAGACACTACTATGCTCACAAGTTTCTGGACTTGTACTAAACACTGGCAAGAGTCTGTTGCAGGAAGTTTCTTCTTTTGCTGTGATGTAGAGTCGGAGTATTTGA
CCGTTGGTTTATTTCATCAGCAGCTATGGTGTTTCTCGACACCGTTAGGCTTACAGGTGCTGCAAAACTAAAAATGTTGATAGTAACTGTTACGCTTTTCCTAACATGGCAAGGCTTTTTGC
AAAATGTGTTATTATATTTGCCAGTAAATATGTGGGGATCATTTCAGTCTGAATTCTTTCATTATTCGAATTTATTGATGCCTAATAATCTTAATTTTTCGCAGGTGTTTTTA
>comp164106_c0_seq41
GAAATGATCCCCACATATTTACTGGCAAATATAATAACACATTTTGCAAAAAGCCTTGCCATGTTAGGAAAAGCGTAACAGTTACTATCAACATTTTTAGTTTTGCAGCACCTGTAAGCCTA
ACGGTGTCGAGAAACACCATAGCTGCTGATGAAATAAACCAACGGTCAAATACTACGACTCTACATCACAGCAAAAAAATCAACTTCCTGCAACAGACTCTTGCTTCCACTGTTTAGTAC
AAGTCCAGAAACTTGTGAGCATAGTAGTGTCTATCTGCACTACTAATTTGATAATAACATGAAGCTTGATCAATAATTTCACTAATAAATTTGAAAATTTCTGGTTAGAGCAATCTTGAGAA
CCTATCTATAATCAGCACTGTGTACATGGCTAACTAGAAGCAGGGAGAATGTAATAGAAGAGAGCTAAATGATACAGCATTACTAGGTATACAAGAAAAAAAGAATGGATGCAAATATTAT
AAACATACAGGCAGGGCAGGGCCAACTTTGCACAAAATGCATCTTTTAAAATCTACCTAATAACCTAACAAAACTGCTGCTTCATTTGCGCACTGGAATCTGATTTCAAATAGGAGATTTT
GCAAGTGCAAGAGTATGCCACCACCCACAAGAAACATTCATGATGCGGAGGTTCTCGATTGGGACCTCAGAGGGTATGTTCCGGTCAATAGAATCACCAAGGCCAAGCTGGCCATATTTG
TTCCATCCCCAGCAAAAAACTCTGCTATCCTCTGTAACTATGGCACTGTGGCGAGCACCACAGGAAACAATTTTCGCATTCATACTCTCTAATGATGGGGCATCCAATAGTTTGGGAAGTG
TCTCTGCTTGATCTAGACCGGTTCCTAGCTGTCCAAACTGATTTCCGCCAAATGCATAGACACCACCATCAACTGAAATACACAGTGTGTGCCATAAACCAGCAGCAACTCCGAGCAAAC
TGACGCCCAAAAGAGAAGAAACACATTTTGGACTGAGTTCATCATCTGTAGATCCATTCCCGCACTGGATATTGAGCACACAGGTAAAAATTACAAAAGAGCAAACTGAATGATTAGTCA
TTCCATGTGACCTCAAGATTTAAGAGAAAATTATGAAGGATTAAAAGTCAATATCTCCATGATACAAGGAACCTCATCTTCAACAATATACTTGCTTTATCTCCATCCTTTCTACTATACCTAC

ATATTAATCCTGTCCATAAAGTCCCCAACCAAATGTAAGTACTGCACCACTATCAGTCACTACTGCACTATGACGACCTCCACATGCAATAGCCTTCACACAGCTTCCAATAACCTTGTAAG
CATGCCCTTCCGAACTTGTATTTCCTTTACTGGAAGCTGATGCTCTATCTTTAACATAAGAAGCTGATTCAATGCATGGTACAAGATGGGGGGAAGAAACCATGCGTGTTCGAGAACCCAA
ACCCAGTTGGCCTTCACCGCCATATCCCCAACCCCACACCTGACCAACATCTGACAATGCAAGTGTATGTCGACCACCAGCAGATACTGCTGTAATCTTCACTCCCACGCCAAGTGTTACA
AGACAGGGAAGTGCTGAAAGAGTTTCATCACTTGGTGATGAGCTCTCAGGACCTTGTTTAGCTGAAGATAACCTTCTGCGTTTTGCAGTATCCTCACTGCCTTTGCCATCATAATTAACTA
TTGTTCCACTAATTGATCTGGAGCCGTGTAATGTGGAGCTCACTTGCTCATTCGGAAAAGCGCTTTGTCTTTCATCCTTCTCTAAACTTCCTATAGCAGGTTGTTCTCCAATCACCTTCCCA
GTTGGAACGCACTCTTTCCAGCCCCATGTGTACACCTCCCCACATGCTGTAATTGCAACACAGTGAGCCCATCCTGCAGCTGCTTTCACTATCTCTGCTTCAGCAGGAAGAGGAAATGCC
TCTGGAATTTCCTCGTGCTTTCCAGACGTAAGGTAACTCTGGCCCATATCATCCGTAGATCCCCATGTGATGAGCTTGCCCGATTCTGAAATAGCCATAGCGAAGCCGCATCCACCCCCGG
AGACGTCCCTCCACGCTTCGCCGGTGGCAGATTCTGGTCGGCGAACTGGAACCGGATGCAGTAGCGGCGATCTATCCGGAGAGACTCCAGGAAGATAGCCCCACATCAGTACCACCTCT
TTTTCCTTCTCTTTCTCCTCCACCTTACCCAGCTTCCGCTTGTCCTCCTCCATCTCCTCTGCTCCCCCGCTACCATTCATTCTCCACCTCCCTTCTCTTTATCAACTACGTATCCACCACTTCT
CCAAGAAGAAAAGCTCTGTCTTCTCCTTTCTTTCTTTAGCTTGTCGAACTTGGTTCGCCTCAGCACGAGCTCTCGCATATGGAAGAGAAAGACAATACGAGAAAGCGAAATCAAGCAAA
AATGAAAAATAAAAGAGCAGTCGACGAGCCCTAATTCGTTCGAATCATTTGCTTGGAGAACGTAAGAGCCACGTAGGATATCAGTCTGGTGGTCCCGTACA
>comp164106_c0_seq50
GAAATGATCCCCACATATTTACTGGCAAATATAATAACACATTTTGCAAAAAGCCTTGCCATGTTAGGAAAAGCGTAACAGTTACTATCAACATTTTTAGTTTTGCAGCACCTGTAAGCCTA
ACGGTGTCGAGAAACACCATAGCTGCTGATGAAATAAACCAACGGTCAAATACTACGACTCTACATCACAGCAAAAAAATCAACTTCCTGCAACAGACTCTTGCTTCCACTGTTTAGTAC
AAGTCCAGAAACTTGTGAGCATAGTAGTGTCTATCTGCACTACTAATTTGATAATAACATGAAGCTTGATCAATAATTTCACTAATAAATTTGAAAATTTCTGGTTAGAGCAATCTTGAGAA
CCTATCTATAATCAGCACTGTGTACATGGCTAACTAGAAGCAGGGAGAATGTAATAGAAGAGAGCTAAATGATACAGCATTACTAGGTATACAAGAAAAAAAGAATGGATGCAAATATTAT
AAACATACAGGCAGGGCAGGGCCAACTTTGCACAAAATGCATCTTTTAAAATCTACCTAATAACCTAACAAAACTGCTGCTTCATTTGCGCACTGGAATCTGATTTCAAATAGGAGATTTT
GCAAGTGCAAGAGTATGCCACCACCCACAAGAAACATTCATGATGCGGAGGTTCTCGATTGGGACCTCAGAGGGTATGTTCCGGTCAATAGAATCACCAAGGCCAAGCTGGCCATATTTG
TTCCATCCCCAGCAAAAAACTCTGCTATCCTCTGTAACTATGGCACTGTGGCGAGCACCACAGGAAACAATTTTCGCATTCATACTCTCTAATGATGGGGCATCCAATAGTTTGGGAAGTG
TCTCTGCTTGATCTAGACCGGTTCCTAGCTGTCCAAACTGATTTCCGCCAAATGCATAGACACCACCATCAACTGAAATACACAGTGTGTGCCATAAACCAGCAGCAACTCCGAGCAAAC
TGACGCCCAAAAGAGAAGAAACACATTTTGGACTGAGTTCATCATCTGTAGATCCATTCCCGCACTCATCTTCAACAATATACTTGCTTTATCTCCATCCTTTCTACTATACCTGTCCATAAA
GTCCCCAACCAAATGTAAGTACTGCACCACTATCAGTCACTACTGCACTATGACGACCTCCACATGCAATAGCCTTCACACAGCTTCCAATAACCTTGTAAGCATGCCCTTCCGAACTTGT
ATTTCCTTTACTGGAAGCTGATGCTCTATCTTTAACATAAGAAGCTGATTCAATGCATGGTACAAGATGGGGGGAAGAAACCATGCGTGTTCGAGAACCCAAACCCAGTTGGCCTTCACC
GCCATATCCCCAACCCCACACCTGACCAACATCTGACAATGCAAGTGTATGTCGACCACCAGCAGATACTGCTGTAATCTTCACTCCCACGCCAAGTGTTACAAGACAGGGAAGTGCTGA
AAGAGTTTCATCACTTGGTGATGAGCTCTCAGGACCTTGTTTAGCTGAAGATAACCTTCTGCGTTTTGCAGTATCCTCACTGCCTTTGCCATCATAATTAACTATTGTTCCACTAATTGATCT
GGAGCCGTGTAATGTGGAGCTCACTTGCTCATTCGGAAAAGCGCTTTGTCTTTCATCCTTCTCTAAACTTCCTATAGCAGGTTGTTCTCCAATCACCTTCCCAGTTGGAACGCACTCTTTCC
AGCCCCATGTGTACACCTCCCCACATGCTTATGCATAAGAACTTTGATAAATTAAATCTTTTCCAGGTTATATTTAAAAGCATTTATACAAAATCTAAACAAAATTGTTACCACACCTCCTTA
CATGATCCTATTCTAATAATAGGTAAAATCTGAGCACTCCAATTAAATATTGAATCTTGAATTTACAGTAAGTAGAAAAAAAAAAACCTGTAATTGCAACACAGTGAGCCCATCCTGCAGCTG
CTTTCACTATCTCTGCTTCAGCAGGAAGAGGAAATGCCTCTGGAATTTCCTCGTGCTTTCCAGACGTAAGGTAACTCTGGCCCATATCATCCGTAGATCCCCATGTGATGAGCTTGCCCGAT
TCTGAAATAGCCATAGCGAAGCCGCATCCACCCCCGGAGACGTCCCTCCACGCTTCGCCGGTGGCAGATTCTGGTCGGCGAACTGGAACCGGATGCAGTAGCGGCGATCTATCCGGAGA
GACTCCAGGAAGATAGCCCCACATCAGTACCACCTCTTTTTCCTTCTCTTTCTCCTCCACCTTACCCAGCTTCCGCTTGTCCTCCTCCATCTCCTCTGCTCCCCCGCTACCATTCATTCTCCA

CCTCCCTTCTCTTTATCAACTACGTATCCACCACTTCTCCAAGAAGAAAAGCTCTGTCTTCTCCTTTCTTTCTTTAGCTTGTCGAACTTGGTTCGCCTCAGCACGAGCTCTCGCATATGGAA
GAGAAAGACAATACGAGAAAGCGAAATCAAGCAAAAATGAAAAATAAAAGAGCAGTCGACGAGCCCTAATTCGTTCGAATCATTTGCTTGGAGAACGTAAGAGCCACGTAGGATATCA
GTCTGGTGGTCCCGTACA
>comp164106_c0_seq8
GAAATGATCCCCACATATTTACTGGCAAATATAATAACACATTTTGCAAAAAGCCTTGCCATGTTAGGAAAAGCGTAACAGTTACTATCAACATTTTTAGTTTTGCAGCACCTGTAAGCCTA
ACGGTGTCGAGAAACACCATAGCTGCTGATGAAATAAACCAACGGTCAAATACTACGACTCTACATCACAGCAAAAAAATCAACTTCCTGCAACAGACTCTTGCTTCCACTGTTTAGTAC
AAGTCCAGAAACTTGTGAGCATAGTAGTGTCTATCTGCACTACTAATTTGATAATAACATGAAGCTTGATCAATAATTTCACTAATAAATTTGAAAATTTCTGGTTAGAGCAATCTTGAGAA
CCTATCTATAATCAGCACTGTGTACATGGCTAACTAGAAGCAGGGAGAATGTAATAGAAGAGAGCTAAATGATACAGCATTACTAGGTATACAAGAAAAAAAGAATGGATGCAAATATTAT
AAACATACAGGCAGGGCAGGGCCAACTTTGCACAAAATGCATCTTTTAAAATCTACCTAATAACCTAACAAAACTGCTGCTTCATTTGCGCACTGGAATCTGATTTCAAATAGGAGATTTT
GCAAGTGCAAGAGTATGCCACCACCCACAAGAAACATTCATGATGCGGAGGTTCTCGATTGGGACCTCAGAGGGTATGTTCCGGTCAATAGAATCACCAAGGCCAAGCTGGCCATATTTG
TTCCATCCCCAGCAAAAAACTCTGCTATCCTCTGTAACTATGGCACTGTGGCGAGCACCACAGGAAACAATTTTCGCATTCATACTCTCTAATGATGGGGCATCCAATAGTTTGGGAAGTG
TCTCTGCTTGATCTAGACCGGTTCCTAGCTGTCCAAACTGATTTCCGCCAAATGCATAGACACCACCATCAACTGAAATACACAGTGTGTGCCATAAACCAGCAGCAACTCCGAGCAAAC
TGACGCCCAAAAGAGAAGAAACACATTTTGGACTGAGTTCATCATCTGTAGATCCATTCCCGCACTGGATATTGAGCACACAGGTAAAAATTACAAAAGAGCAAACTGAATGATTAGTCA
TTCCATGTGACCTCAAGATTTAAGAGAAAATTATGAAGGATTAAAAGTCAATATCTCCATGATACAAGGAACCTCATCTTCAACAATATACTTGCTTTATCTCCATCCTTTCTACTATACCTAC
ATATTAATCCTGGAAAGCAGTTAATATTAAGGTGGAGTAGGACACAACATAAGGTAGTAATCAATGATTCAAAGGGTTGTACATCAAAATTATTTTGCTCTATTCTTATAGTCAATGTCAATC
TAGAAGTAATATTCAAGATGACAAAATAGCTAAAGGTTCACAATATGCTCTGCAGTCATAAAGCAGAAATCAACCCATCATACTAAATTTGATCGGAAGTAAATTGACAAAAGCAAAAGA
AGTTAAGTAGTTCAGCACAGAGCTACTGTGCTATCCAAAATATATTCTCCTGAAAGGAGAAGAAAATGACCGTCAGCAAATTATCTCACTGTACAAAAGCAAGGTTGGACTCAGATTTAC
CACAGCAAATTAAATCAATCGGCTACCGAAATGTATATGTTGGGATTACTCCAAAACTTTAATGATCTTAATCCTTATTATATTTCATATGAGAGAACAATTTGGAGAATGTTTTAAAATCAA
GTGCTCGGCAGGACAGGTTAGAAAGGTGTCAAAATAAATATTACCGATCATCAAAATTAAGTACAAAGTTA
>comp164106_c0_seq9
GAAATGATCCCCACATATTTACTGGCAAATATAATAACACATTTTGCAAAAAGCCTTGCCATGTTAGGAAAAGCGTAACAGTTACTATCAACATTTTTAGTTTTGCAGCACCTGTAAGCCTA
ACGGTGTCGAGAAACACCATAGCTGCTGATGAAATAAACCAACGGTCAAATACTACGACTCTACATCACAGCAAAAAAATCAACTTCCTGCAACAGACTCTTGCTTCCACTGTTTAGTAC
AAGTCCAGAAACTTGTGAGCATAGTAGTGTCTATCTGCACTACTAATTTGATAATAACATGAAGCTTGATCAATAATTTCACTAATAAATTTGAAAATTTCTGGTTAGAGCAATCTTGAGAA
CCTATCTATAATCAGCACTGTGTACATGGCTAACTAGAAGCAGGGAGAATGTAATAGAAGAGAGCTAAATGATACAGCATTACTAGGTATACAAGAAAAAAAGAATGGATGCAAATATTAT
AAACATACAGGCAGGGCAGGGCCAACTTTGCACAAAATGCATCTTTTAAAATCTACCTAATAACCTAACAAAACTGCTGCTTCATTTGCGCACTGGAATCTGATTTCAAATAGGAGATTTT
GCAAGTGCAAGAGTATGCCACCACCCACAAGAAACATTCATGATGCGGAGGTTCTCGATTGGGACCTCAGAGGGTATGTTCCGGTCAATAGAATCACCAAGGCCAAGCTGGCCATATTTG
TTCCATCCCCAGCAAAAAACTCTGCTATCCTCTGTAACTATGGCACTGTGGCGAGCACCACAGGAAACAATTTTCGCATTCATACTCTCTAATGATGGGGCATCCAATAGTTTGGGAAGTG
TCTCTGCTTGATCTAGACCGGTTCCTAGCTGTCCAAACTGATTTCCGCCAAATGCATAGACACCACCATCAACTGAAATACACAGTGTGTGCCATAAACCAGCAGCAACTCCGAGCAAAC
TGACGCCCAAAAGAGAAGAAACACATTTTGGACTGAGTTCATCATCTGTAGATCCATTCCCGCACTGTCCATAAAGTCCCCAACCAAATGTAAGTACTGCACCACTATCAGTCACTACTG
CACTATGACGACCTCCACATGCAATAGCCTTCACACAGCTTCCAATAACCTTGTAAGCATGCCCTTCCGAACTTGTATTTCCTTTACTGGAAGCTGATGCTCTATCTTTAACATAAGAAGCT
GATTCAATGCATGGTACAAGATGGGGGGAAGAAACCATGCGTGTTCGAGAACCCAAACCCAGTTGGCCTTCACCGCCATATCCCCAACCCCACACCTGACCAACATCTGACAATGCAAG

TGTATGTCGACCACCAGCAGATACTGCTGTAATCTTCACTCCCACGCCAAGTGTTACAAGACAGGGAAGTGCTGAAAGAGTTTCATCACTTGGTGATGAGCTCTCAGGACCTTGTTTAGC
TGAAGATAACCTTCTGCGTTTTGCAGTATCCTCACTGCCTTTGCCATCATAATTAACTATTGTTCCACTAATTGATCTGGAGCCGTGTAATGTGGAGCTCACTTGCTCATTCGGAAAAGCGC
TTTGTCTTTCATCCTTCTCTAAACTTCCTATAGCAGGTTGTTCTCCAATCACCTTCCCAGTTGGAACGCACTCTTTCCAGCCCCATGTGTACACCTCCCCACATGCTTATGCATAAGAACTTT
GATAAATTAAATCTTTTCCAGGTTATATTTAAAAGCATTTATACAAAATCTAAACAAAATTGTTACCACACCTCCTTACATGATCCTATTCTAATAATAGGTAAAATCTGAGCACTCCAATTAA
ATATTGAATCTTGAATTTACAGTAAGTAGAAAAAAAAAAACCTGTAATTGCAACACAGTGAGCCCATCCTGCAGCTGCTTTCACTATCTCTGCTTCAGCAGGAAGAGGAAATGCCTCTGGA
ATTTCCTCGTGCTTTCCAGACGTAAGGTAACTCTGGCCCATATCATCCGTAGATCCCCATGTGATGAGCTTGCCCGATTCTGAAATAGCCATAGCGAAGCCGCATCCACCCCCGGAGACGT
CCCTCCACGCTTCGCCGGTGGCAGATTCTGGTCGGCGAACTGGAACCGGATGCAGTAGCGGCGATCTATCCGGAGAGACTCCAGGAAGATAGCCCCACATCAGTACCACCTCTTTTTCCT
TCTCTTTCTCCTCCACCTTACCCAGCTTCCGCTTGTCCTCCTCCATCTCCTCTGCTCCCCCGCTACCATTCATTCTCCACCTCCCTTCTCTTTATCAACTACGTATCCACCACTTCTCCAAGAA
GAAAAGCTCTGTCTTCTCCTTTCTTTCTTTAGCTTGTCGAACTTGGTTCGCCTCAGCACGAGCTCTCGCATATGGAAGAGAAAGACAATACGAGAAAGCGAAATCAAGCAAAAATGAAA
AATAAAAGAGCAGTCGACGAGCCCTAATTCGTTCGAATCATTTGCTTGGAGAACGTAAGAGCCACGTAGGATATCAGTCTGGTGGTCCCGTACA
>comp164139_c0_seq2
TTGCTATTTATCTTCCTAATTGGAAACCCTGAAATTTAAACCAATTTGAGAAATATAGCGCAAAATAACCAAAACATCGCTTCATAGAAGCTCAAATTCTGAGAACAACTCAAGCTTTCTAA
ATTAGGAAGATAAATAGCAACAAACTCAGTCAAGTGTCCATCCCTCACCTCTCAATTTTCCAGCTCTCTTTATCCAAAAATAGAAAGCAAAGGCAACCACACATACCTAACAAATTTCCCT
TTATATAAACCAGCATCCATGTCCTTTCTCCATCTCAGAAGGGGAAGATAAATCAATCAAAATAAAGAGAAAGAAGAGAAATAGAAAAGCCGATCATGACGATGACAAAGTTCGAACTCA
TTGGCACCCTCTGCCTCATTTCTTCTATTGCTCTAGTCTCCTCCAGTGACACCTTCTCCATTCAAGGCCGTGTCTATTGTGATACTTGTCGTGTTGGCTTCGAAACTAACATCACCGAATACA
TCTCAGGTGCTAAAGTTAAGGTCGAGTGCACGCATTTTACTACAAATAAGGTTGAACATCATAACGAAGGTGTAACAGATTCCACCGGCTCCTACAAGATTAATGTGGTAGATGGTCACG
AAGATGAGATTTGTGAGGTCGTGCTCGTCGAAAGCTCCCTCGAAAATTGCAAGGAGATCGAGATTGGCAGAGATAGGGCGCAAGTATTGCTCGCGAATGATGGTGGCATAAGTGGCAAG
GCAAGGCATGCAAACTCACTTGGCTTCCTCAGGAATGAGCCTCTTCCTTCCTGTGCAAAGCTTCTTAAAGAGTACTATGGCCTGGGAGAGGAAGAGTAGTTAAAGCAAGTTATAATTAAG
AATTTTGGGCTAATGCGTTCTCATTTCTATTTACAACATCTCTTCTAAGCCATTTGTGCTCGTTTCTACACTTTTTCTATTCTTCATGAGACTAACGTTTGCAGCAAATAGTTGATATAATTAAT
TTGCTTTGACATATAATGGAACAAAGCATTAAAATGTATTAGAAGTTACAGATCCTTATAACTTATTGAGCAAACAAAATTCTTGTCGACATGCTATTTGAATCAAATTCATGCTTTGTATTG
ATTATGATTGAAATAGGTTAAGAGAGCGCATGTTCTCTTAGATCCTATTAATTGATGGATGAAATAAATGTGAAATTGAGTAGTTGTTG
>comp164139_c1_seq1
AATCAATACAAAGCATAAATTTGATTCAAATAGCATGTCAACAAAAATTTTGTTTGCTCAATAAGTTATAAGGATCTGTAACTTCTAATACATTTTAATGCTTTGTTCCATTATATGTCAAAGC
AAATTAATTATATCAACTATTTGCTGCAAACGTTAGTCTCATGAAGAATAGAAAAAGTGTAGAAACGAGCACAAATGACTTAGAAGAGATGTTGTAAATAGAAATGAGAACGCATTAGCC
CAAAATTCTTAATTATAACTTGCTTTAACTACTCTTCCTCTCCCAGGCCATAGTACTCTTTAAGAAGCTTTGCACAGGAAGGAAGAGGCTCATTCCTGAGGAAGCCAAGTGAGTTTGCATG
CCTTGCCTTGCCACTTATGCCACCATCATTCGCGAGCAATACTTGCGCCCTATCTCTGCCAATCTCGATCTCCTTGCAATTTTCGAGGGAGCTATCGACGAGCACGACCTCACAAATCTCAT
CTTCGTGACCATCTACCACATTAATCTTGTAGGAGCCGGTGGAATCTGTTACACCTTCGTTATGATGTTCAACCTTATTTGTAGTAAAATGCGTGCACTCGACCTTAACTTTAGCACCTGCCA
TCATAAACACCACAGATCTATAATGCTCAATTACTTATTACTCAATTGATCAATTGATCTGTCTTTCAGTTGATTATATAAATGTGAGTACCTGAGATGTATTCGGTGATGTTAGTTTCGAAGC
CAACACGACAAGTATCACAATAGACACGGCCTTGAATGGAGAAGGTGTCACTGGAGGAGACTAGAGCAATAGAAGAAATGAGGCAGAGGGTGCCAATGAGTTCGAACTTTGTCATCGT
CATGATCGGCTTTTCTGTCTCTTCTCTCTCTTTATTTTGATTGATTTATCTTCCCCTTCTGAGATGGAGAAAGGACATGGATGCTGGTTTATATAAAGGGAAATTTGTTAGGTATGTGTGGTTG
CCTTTGCTTTCTATTTTTGGATAAAGAGAGCTGGAAAATTGAGAGGTGAGGGATGGACACTTGACCTAGTTTGTTGCTATTTATCTTCCTAATTTAGAAACCTTGAGTGGTTCTCATAATTT

GAGCTTCTATGAAGAGATATTTTGGTTAATTTTGAGCTATAATTCTCAAATTGATATAAATTTCAGGGTTTCCAATTATAGCCATATTGTCATTGTTAAAATGGAATCAACTATTCTTTGATCAT
TTGTAGATATATACAAAATAAGCTTATTTAAATGATGATAAAATGATGAAATTTA
>comp164179_c0_seq7
CTAATTCCCACATGATGAAAAAAAAAGTAAAAAGTCTCCAGAAGAAAATAATCCTATTTGACCTCTATACATTTCTAACATCAAGAAATAGAAGAATCTCAGATCTCGAAATGAGATATATT
TTGTCGATCCATCAACAAAAACCTGGATCGACAAGGCAAATTCATCGCCATTCGGGACTAAAAAGCCGGAGGTGGACGAAATGCAAGGAAACCATAAAGCAGCGGAGCAATAAGCAAA
GAAAAAAGAATGCTTCCCTGAGATTGTTGGTGGCTAAGAGGCCAACAGCGCCACGGAGACCAAAAAAAAAAGCAATCCACTGAAACGCGCCGGAGAGACGGCAAGGGCAAAGACCT
AGCGGAGCGGGCGAAGCGTCAGGAGGTGGCGTTGTCGCAAGAGTTCACTTGAAATTTGGCATTCGGTTTTGGC
>comp164205_c0_seq3
ACAATGGAGAAAAAGGATTCATGCAGCCGGCCCACGTAGTAAGATAACGGCTTGATGTTGTAATTGGTGTAATTCAACTATGTAAATATTGAGGGATAATTGCACTTACATTGCACCATAAT
AGAAGATTTTCATTGTTATGCTTGCAAGGAGAGCTTTCCTTGGGACAGATTGTCCCTTATACGGGAAATAGATGGCATAAAGACCAACAACAGAAGAGATTAACACTGATATAGCTGATAA
AGCCCCCATAGCGACACTGGGATCCTTTGGAAATATACAAATTACACCATCTTTTCCTGGTATTGGCGTTCCAGCTGCAGGCTTCTTCAATTCTGCTACTATTCCAAGAACAAAAGATAAAC
AACCGAAGAAAAAAATTGTGGCAGCGAACTGTGTGGTCTGATTTAATGCCATTTCTCTCTCTTTTGATCCTCAAAG
>comp164205_c1_seq3
TCTTTACATAAAAGCCAATCGCCTTCTTCCTTTCACCAAGGCTTTTCTCCTGCATGTGAGGTCCATTCGACAATCTTCAAAGCTCTTTTGAGGATCAAAACAGAGAGAAATGGCATTAAAT
CAGACCACACAGTTCGCTGCCACAATTTTTTTCTTCGGTTGTTTATCTTTTGTTCTTGGAATAGTAGCAGAATTGAAGAAGCCTGCAGCTGGAACGCCAATACCAGGAAAAGATGGTGTAA
TTTGTATATTTCCAAAGGATCCCAGTGTGGCTTTGGGGGCTTTATCAGCTATATCAGTGTTAATCTCTTCTGCTGTTGGTCTTTATGCCATTTATTTCCCCTATAAGGGACAATCTGTCCCAAG
GAAAGCTCTCCTTGCAAGCATAACAATGAAAATCTTCTATTATGGTGCAATAGTCCTAACCATAACAGGAGTTGGAGCTACCCTCTTCAACACAATCAATGAAGGGCTTATTCATGCAAGA
AATGTGCATGAAGATCTGGAATATCTTTGCCCAACTGCAAAAACCGGTCTCTTCGGTGGCGCATCCTTCTTAAACCTTGACGCTAGTCTCTTCTGGCTCGTATGTCAGATGCTTGTTAGAAA
TGCTCGAGAGGATTACTTCGATTACGAGGAAGAAGGAGAAATAAAGGATGATTACATTCAGATTAGTAACAATTGATGACGAGAAGGAGGAGACAAGCATGATTTAAAGTCCCATGAGG
GTGACAATGAGACCATCAATGTACATATATAACTAATTTCTTATTTATTCATTCCTCTTTTTTTCTTATTATGTCTAAACATTTTATTCGAAGTTGTTATGTAATGATGTTGTATTTAACTAAGTT
GTGGATTCTTAATTATAAATAGTTAGTGTCTTAATGTATGCTTGGGGGATGGAATGATG
>comp164237_c3_seq12
TATTGGTTTAGAAAGAGAGGTATGGGGGACGAGAGAAACTGAACCCAAGTTTGCATCTCTCTCTCTCTCTCTCTATCGTATCTCTCCCTCTCCTGAATCTTTCTTTTTACATCATGTCTCAAT
AGTTTTCTCGATCTACCTCCCTCTCTCTATTTTTAAGTCCACAACAAACCCATGAACTAAATTCAACTCTGCTATTCTTAAGTCCGCAGCAAACCCAAGAACTAAACTCAATTATGCTATCA
ACCTGATTTTGCCGGCAATGGCTGTAAGTTCTCGGCGCAACAGCCATTTTCATGAGCTCGCTAAAATGGCAATCATGGAACAAAAAGAAAAATTCAATGGCAAGGTGAAGCATGTTAAAC
CATCTCCAACCGAACTCCATATCACCGTATGCCGTCCGCTATTTGTTCCATTCGAA
>comp164244_c1_seq28
AAAGGCGGGCCTCAAGGCGAGCCTTATATTGCGCAATTTTAAACCTTGCTTTATCACTTTGACTGAGTTTCACATCTCCCCTGTTCATGTTGGCAATAAACTGATAATTAATATCATCGACAA
TCTATTTTTTCTCTCTTCCTGCTAGGGCATTGTTAGGCTATGCTTGGCTGCTTCACCAGCCTGGATGGGCTAGCCGAGATCATCTTTCCATATGGTTATGCAGTATCAGAACATTTTATTTATA
AATGTCAAGTGATGCATAAGATATTTGTGCACTTGGCCTCAATCCGTGCGATACATGTAAATTAGTCCTGACATGTAAATAAGTATTTAAGACTTTAGATACAACAGGTGGCGAAAATAATG
TTGATATTATCGTAAGTCATACAAATGTTGTTTGAATAAGTTCCTTACTGCTGCAGGTTATTAACTTGTTTGTATTTCCAGTACCATTAAATGATCTTCATGAAGCAGGTAGATTGGGTTCTTT

ACAATTGCCAACAGGGCCATGGATATGAATGGGTGGTTTGAAGCATATGTTATTATCTGCAGTGCCTGCACCAGACTCCAATGACTTGTGAAAAAGTAGGTAGGCTACAGAACGTTCTATT
GCATTTGTGCTAATCGATACACCGTAGATTTCAGGAGGCCTCTTTGAATCTTTGGCACCCTGAAGTCCTATCATAGAGCTACTTCCAATGCTGACATCTTTTGCAGTGCCATAGTAATGGCTT
TTTTCAGCACTTCTTGCCAAACGATACAAACTGTCCCTTATGCACAGCTTAGTTCTAACACCCGATCTGTAATCATCTGAAGCTGCTGAAAATTGGCTGCTTTTAGAGACACGTCATCAGG
AAAAGTACTCGTCATGCAAGAATTGTTTTGAAAAGTTGAAGATTCTATATCAACCGCTTGTAGATTTTGCCTTATATAGGCTGGTTTTTTATCACCGTCAACTTCAACGTTAAATTTATATTTT
CTTTGAACTGAAGATTCACTTGCTGCAGCATGTTGTTCTGAAGGTCTATTCATTACCAATGGAGAAGCAAACTTCCGACATGCATATATTTTTTCGTTTTTCTCTATTGGCATTGACGGCATG
GAAAAGGTCCCAGGTGAAATTCCTACGGACTGCGTGGCGTGTCTTGACAGATCGTTTGAGGACACATAACCAGGTAAAACACTGGATGTAAGTGTTGTCTGTCCTGGTAGAACGTGTTG
AAGTTTAAGGATGTCGCTCATCTGTGCTTGAGGATTGTAGGAATGTAAGCACTTGGAAGAATAAGATTCTGAGAAAAGGTTTTGCGTTGAAATAACTTGAAGAGAACAAGGCTGCGGAG
CTGACACCACAGAAGAAATAAGTTCCTGAACTTGGACATTCTGTATCAAGCTACAGCCACTTAATGAACTTTGCACGGATCCTAAAGTTGGATTGATTTTCTTCGTTGTGGTTTCACAAAG
GTCCTTAAGAGTCATATTAATCATGTCAGGATATCTGCATGCATTCCCATAGCCAACTTGTTCAAGTGTTGAACCTTTTTCACCAATCCCAGCATGAAGATACGCCAGTTCAGATAGTGCCG
ATTTACCCTTCCTTTCAGCATCAGAATACATTGGAAAAGAACTACAGCGGGTATCAGAGGGAGCCTCAGTGTTAATGGCCGATGGTTCAATTCCAGGCAAAATATCGTCGCACAAACCATG
AGAAGATATATTTTTAAAAGCACTAAACTCTGAAACTGAAGATAGTACGACTGATTGTAAAGTATCTTCCATACCATGAAAGCCATGTGAACTAGATGAGAACCATGAGATATCATCTTCGC
TGACAGTGCTCCCTTGTACAAATGTGGAACCAAGGTTTCTGAGCATTCCGTCAAAATCCTCAAGATCTTCAACATTAGGACAATCATAAAATAGAGGATCATTCTCTCCTTCCCTTCGTTCC
ATTGTCGGTACACTAACGTTGGCAGAGGAAATATTACCAGTTTTTTTGTGATGTATAGAACCATTTTTCCAAGGAAAAGGATAATCTGAGCATAATGTGGTGTTTCCTATGCCATTTTCACCC
TCTCCAAAAGAACTAACGGAGATTTCAGCCCCTTGGCAATCTAAAAGGGTGTTGCCTGTAGTGCACTTTGTAAAGCCTAATGCTTGAGCCTTTTTTTCAGATATAAGAGACCGTGAAAGTT
TTGGAATATTGGAACCTTCCTCTTTCAAAGCAGAGGGAAAAAGTCTTCCATCTTGTCCTGGAAGAGAAGGCACATTTGCTGTACCTTCACGATCTATATTTGTTTTGACATCACTTGCTGC
CTCATATCGTGGCTTCTTACGTGAATCACTTAAAGTTACAGATTCAAGAGCATGTTCATCTTGAAAAGGAAGAAATTTATTAACACCACCATCTACATCGTCGAAAATATCCCGAACCTCGT
ATCCGTTCCAATGTGACATCACTCTCCCTCTCTCTATCCTGGCCCGAAGCTGTACCAATCAACCGGATCCAATCTCATATTTGAGTCCAGAAGCTTCAGCCGGAGGCAGTATCAAAATCTAG
AACGCTCCTCCCGGCGCTTCTCTCTTTCTCTCTCCCCCTTTCCCCCACTCTTTCTCTCTCTCTCCCTCCCTCCCTTTCTCTCTATCTTTATATTATATTATGTATTTTGAGTTGAATAAGCTAAA
CCATAAATTGCATTTAGATTAAGCCAATTGATTTAGCATATTTGAAATCCGCCAAAATATTTGGCGATTTTGCTCTGTTTTAAATTATTTTTTTAGTATGCTTCTTAAGC
>comp164244_c1_seq42
AAAGGCGGGCCTCAAGGCGAGCCTTATATTGCGCAATTTTAAACCTTGCTTTATCACTTTGACTGAGTTTCACATCTCCCCTGTTCATGTTGGCAATAAACTGATAATTAATATCATCGACAA
TCTATTTTTTCTCTCTTCCTGCTAGGGCATTGTTAGGCTATGCTTGGCTGCTTCACCAGCCTGGATGGGCTAGCCGAGATCATCTTTCCATATGGTTATGCAGTATCAGAACATTTTATTTATA
AATGTCAAGTGATGCATAAGATATTTGTGCACTTGGCCTCAATCCGTGCGATACATGTAAATTAGTCCTGACATGTAAATAAGTATTTAAGACTTTAGATACAACAGGTGGCGAAAATAATG
TTGATATTATCGTAAGTCATACAAATGTTGTTTGAATAAGTTCCTTACTGCTGCAGGTTATTAACTTGTTTGTATTTCCAGTACCATTAAATGATCTTCATGAAGCAGGTAGATTGGGTTCTTT
ACAATTGCCAACAGGGCCATGGATATGAATGGGTGGTTTGAAGCATATGTTATTATCTGCAGTGCCTGCACCAGACTCCAATGACTTGTGAAAAAGTAGGTAGGCTACAGAACGTTCTATT
GCATTTGTGCTAATCGATACACCGTAGATTTCAGGAGGCCTCTTTGAATCTTTGGCACCCTGAAGTCCTATCATAGAGCTACTTCCAATGCTGACATCTTTTGCAGTGCCATAGTAATGGCTT
TTTTCAGCACTTCTTGCCAAACGATACAAACTGTCCCTTATGCACAGCTTAGTTCTAACACCCGATCTGTAATCATCTGAAGCTGCTGAAAATTGGCTGCTTTTAGAGACACGTCATCAGG
AAAAGTACTCGTCATGCAAGAATTGTTTTGAAAAGTTGAAGATTCTATATCAACCGCTTGTAGATTTTGCCTTATATAGGCTGGTTTTTTATCACCGTCAACTTCAACGTTAAATTTATATTTT
CTTTGAACTGAAGATTCACTTGCTGCAGCATGTTGTTCTGAAGGTCTATTCATTACCAATGGAGAAGCAAACTTCCGACATGCATATATTTTTTCGTTTTTCTCTATTGGCATTGACGGCATG
GAAAAGGTCCCAGGTGAAATTCCTACGGACTGCGTGGCGTGTCTTGACAGATCGTTTGAGGACACATAACCAGGTAAAACACTGGATGTAAGTGTTGTCTGTCCTGGTAGAACGTGTTG
AAGTTTAAGGATGTCGCTCATCTGTGCTTGAGGATTGTAGGAATGTAAGCACTTGGAAGAATAAGATTCTGAGAAAAGGTTTTGCGTTGAAATAACTTGAAGAGAACAAGGCTGCGGAG

CTGACACCACAGAAGAAATAAGTTCCTGAACTTGGACATTCTGTATCAAGCTACAGCCACTTAATGAACTTTGCACGGATCCTAAAGTTGGATTGATTTTCTTCGTTGTGGTTTCACAAAG
GTCCTTAAGAGTCATATTAATCATGTCAGGATATCTGCATGCATTCCCATAGCCAACTTGTTCAAGTGTTGAACCTTTTTCACCAATCCCAGCATGAAGATACGCCAGTTCAGATAGTGCCG
ATTTACCCTTCCTTTCAGCATCAGAATACATTGGAAAAGAACTACAGCGGGTATCAGAGGGAGCCTCAGTGTTAATGGCCGATGGTTCAATTCCAGGCAAAATATCGTCGCACAAACCATG
AGAAGATATATTTTTAAAAGCACTAAACTCTGAAACTGAAGATAGTACGACTGATTGTAAAGTATCTTCCATACCATGAAAGCCATGTGAACTAGATGAGAACCATGAGATATCATCTTCGC
TGACAGTGCTCCCTTGTACAAATGTGGAACCAAGGTTTCTCCAAAGACAAATATTAAGTTAAAGTAGAGAGCATGCAACCTAATAAAGCTAATGACATTGTAAATTATAAGTCGTTGCAAA
GTTACCTGAGCATTCCGTCAAAATCCTCAAGATCTTCAACATTAGGACAATCATAAAATAGAGGATCATTCTCTCCTTCCCTTCGTTCCATTGTCGGTACACTAACGTTGGCAGAGGAAATA
TTACCAGTTTTTTTGTGATGTATAGAACCATTTTTCCAAGGAAAAGGATAATCTGAGCATAATGTGGTGTTTCCTATGCCATTTTCACCCTCTCCAAAAGAACTAACGGAGATTTCAGCCCC
TTGGCAATCTAAAAGGGTGTTGCCTGTAGTGCACTTTGTAAAGCCTAATGCTTGAGCCTTTTTTTCAGATATAAGAGACCGTGAAAGTTTTGGAATATTGGAACCTTCCTCTTTCAAAGCA
GAGGGAAAAAGTCTTCCATCTTGTCCTGGAAGAGAAGGCACATTTGCTGTACCTTCACGATCTATATTTGTTTTGACATCACTTGCTGCCTCATATCGTGGCTTCTTACGTGAATCACTTAA
AGTTACAGATTCAAGAGCATGTTCATCTTGAAAAGGAAGAAATTTATTAACACCACCATCTACATCGTCGAAAATATCCCGAACCTCGTATCCGTTCCAATGTGACATCACTCTCCCTCTCT
CTATCCTGGCCCGAAGCTGTACCAATCAACCGGATCCAATCTCATATTTGAGTCCAGAAGCTTCAGCCGGAGGCAGTATCAAAATCTAGAACGCTCCTCCCGGCGCTTCTCTCTTTCTCTC
TCCCCCTTTCCCCCACTCTTTCTCTCTCTCTCCCTCCCTCCCTTTCTCTCTATCTTTATATTATATTATGTATTTTGAGTTGAATAAGCTAAACCATAAATTGCATTTAGATTAAGCCAATTGATT
TAGCATATTTGAAATCCGCCAAAATATTTGGCGATTTTGCTCTGTTTTAAATTATTTTTTTAGTATGCTTCTTAAGC
>comp164261_c0_seq12
CTAGCAAACCATCTAAAGTGTGTGTAGAAGGTCCTGTGGCAGGGGTAGGTCCTAAGGTGAGTGATTAGTTAAAGTTGTCTAAAATTCTAAGTTTGTTAAGGCTTAAGGGGAATTTATGTTA
GAATTTAAATATTAATATACTAAGAATTTTATTTAAATATATTTTAAGGGTTAATTGATTACTATCTTTGTTTCACACCGAGGCCGTGAAGTTTATTTCACCTTCTTTGGGACTAAGTTAATAGC
CTCGTAGGTCTGGGTTACCTAATAACAATCGGGGTTAATTAAGTTATTTTAAGTTAGTTAAAATTAAGGGAATAGATTAATATTAAGGGGAGTTGCTTATTTATTTTAAAGGATTGATTAATAA
TTTTATTAACCTATTAAGAAAATTCTGGAGTTTGGGGGAATTAATAGATTTTGAAGGTTATCCAAATATGTCGACTAAGGGGTCATAACTTGACTTTTGAAATTAGGGAGAAAATTGAACAT
TTCAGATTTTTAATAAATAGTAAACCTTATTTTGAGTTGCTAGACAGATGACACACTCAATTTAGAGTTTTCTATGAAAAGTTATGATTTTTAGAAGTTTTAACCCGGTTTTGACCTAAGGTT
GACCAAAATGTTGACTTTTTTACTTTGACCAGGTTTTGGTGGTTTAAATTTTTTGGAAAATACTAGATTTTAATTAGAG
>comp164261_c0_seq15
ATCCTCCTTGGGGCTAACTTAATAACCTCGTAGGCCTGGGTTACCCAGTAGCAATCGGGGTTGATTAAGTTATTTAAATTGGTTGGAGTTAAAGGAGTGGATTGATATTAAAGGGAGTTGGT
TACTGATTTTAATGGGTTGGTTGAGATTTTTATTATCCTATTAAAAAAATTCTGGAATTTGGGAGAGTTAATGAATTTTTGAAGTTATCCAGATATGCTAACTAAGGGGTCAAAACTTGACTT
TTAAAATTAGAGTTAATCTTAAGGGTTTCAGATTTTTAATAAATACTAGACCTAATTATGAGTTGTTATACATGTGGCACACTCAATTTGGAGTTTTCTATGAAAAGTTATGATTTTTAGAAGT
TTTAACCCGATTTTGACTTAGAGTTGACCAAAATGTTGACCTTTTGACTTTGACCAGATTTTGAGGGTTTAAAATTTTTGGAAAATACTAGCTTTTAATTAGAGTCATTAGCCACTGAGTTT
GATTGATTTAGAATTTTTTACAATTAGTTATGAATTTTCTAAGTTCAGGTAATTAAGGGAATTTCTAAATTTCGTAATAAATGGGTTGTTTTAAAAGTTTAATCCAAACCAGCTCCAAATTGA
TTGGAATCACTTCCAAAGGTTAGGTATGACCTAAGAAAGTGATCCCAAGTGTTTGGAGAAAATTTTGAATTTTTAATAATTTTTTATTATTTAATTTATTTTTTTCTTTTTTATATATTTTTTCTT
TTATGTTTTATTTATTTATTTATTCACTTTTTATTTTTTTTTATTTAT
>comp164268_c0_seq9
ATGATCACCCATCTGGGTCACTGACGGGTATATTAATAAAAGAGGTTACAAAGAACAAAAAAAACTTTATAAAGTAAATTGAAAGTTATAACAAAATATATTTAAAAGACAACAAAACAA
ACTAAAAAAAATGTGAGGCGGTCCCAATCGGCCTCAGGTGTGGCTCGAACTGACAACTGCAGGCAAAGAGTAAGTCATAGCAAGAAAAGCTCAGGGGCCCGAAGGAAGCCACAGGAA

ACGCCACCGGTTGCCGTCGGACCAGAGAACAGTCAGCAGGGTGCGGGATGATGTCAGGCTAATTGCGTGACTGCAATCTAGCAGTACGATGAGGCCGCCCAGGGGGTAAGGCAACGAG
CGATCGGTGTAATGATGAACATTACGAGATGAAGCACAAAGGGAAGGAGTAATGAAGCATTTCAAAATTTTTCCTGTGAGGGAACTTTTCCCAAAAGAAGGAATAGTTCATGCTCCATTA
TCTTTATCCTCATCATGTGAAAGAAACAACGTGAGAAGAAGCTTGAGTTTTTGCTCTGCTAGGGTTTGCATGGTGCCCTAGTTTTGTGCGTTTCCACCATCCCGCCGGGGGCCATCCCCCA
TGGCTGAGTTCCCGCCAGAATTTTGCCGAAGTAGCCCCTT
>comp164342_c0_seq6
ACATTGTTCTCAGTTTCTTGTAGGGAAATTTCATTGCTTTTACTTGCAGGTAAAAGAGCCACCGAGTCGACATTAATATTGTTCTCAGTTTCTTGTCCGGAAATTTCAGAGCTTTTACTTTC
AAGTAAAAGAACCCCCGAGTCGACATTGTTATTGTTCTCAGTTTCAATAATCTCCGTGAACTGTAGCAGAGCTTTCTCGTCTTCTTCAACTTGTGCTCGAATATTCTGCTCTGAAACTCTAG
GAAGAATCTCCTTCTCTAAAGATTCCTTGCTTCCACCAGGTGGTGAGTGAGCCTCTGAAGCAACGTTGATGGACCCAATTTCCTTACCTAATGATGATCCAGCATGCTGCTTCCAGTCTTC
TGTAAGAATGAACTGCGGAGAAGATATAGCTCTTTGATGTTTGAACAAGGGAGTCAAAATATCAGTTTTTACAGAGTTATCAGATATCCCAACTTCATTTGGTTGAATATTTAGCTCCAAGG
CTTTGGTTGTATCAGAAGGCTTCATCTTTATCACCTTTTTTTTCGTTTTATGGATGGCAGGTTGACCTCTCACAAGATCTTCCACAGTTTCTAAAGCAAATCTATTTGCCATGAACCTTCTGA
AAGTGAGACGTTTTTCATCACGTTCAGGCTCATGCTTCCCCCCATACTTGCATAAATTATGACATGAATCCGTCGGTTCTCTGAGATACCGAGAGCGGACATAGCCAAAACTATTTGAACG
GGACATTGCATGACTACATTTTGTCTTTGCCCCATCTTTTGAGGCGCTTATTTCTTTTTCCACGAATGCTTTTTCCATTGGAGAAAGAATAGGCTTAGAACTTCATTAGTAGGGGTGAAGGA
TTCCAACAGAAAGATAACTTGAGCCCTTTCCTAAACCATTGAATAAAGCACAACCACATGTCGAGATCCACGCAATGAAATCCTTAACTCACACCGATTCCAGAAAAGGCAAAAAGGGA
TAAATAATAGTCAAGGTGACGCCTTTCGGACGATTCCCTTAACGAGAAAGTGAAGAAATGGGTGTTCATTTGAGGACTTCAACCAGTGTACAAGACAAGGCTCAGCCTCCTCCACCATCT
CCACCTTCTTCTTCCCCCTCTCTTGCAGAACGTGCAAGCAACGAAGGAGCCCTTTATTTTCTTGGAATGGCCGAATTTGTGCCAGAGTTTTGAGAAAGGAGGCTAACGGAGAGCGCGAG
AAATGGAGGGGAAAGTAACGGTTCGGACTTCATAAGATCCATGAAATGGTTAGGACTTCGGTTGGTTAGCTGCCAATGATTTTGATCCGCCAACGCTTCTTGCCCGAACTTTGTCTCTGTT
CGGTCCAAAATTAACTACGGTTGTTCTCATCTCCTTCTCATGGCGTGTATGGTTTGGACACCGTTTTAAGACCCAGCTTTAGAACCTTACGGATGGAGAGGAGGTGGAGGGTGCTGTTGTG
GGGGAAACTTTTATATGGCGAAGGGGTTGGAATGATGGAAGCACGGAATTAAGAGGACTTTTTGGTTGGCAGGTTATCATTAGCTTAATTTAGTAGTAATTACGAATGTTTTTTG
>comp164342_c0_seq7
ACATTGTTCTCAGTTTCTTGTAGGGAAATTTCATTGCTTTTACTTGCAGGTAAAAGAGCCACCGAGTCGACATTAATATTGTTCTCAGTTTCTTGTCCGGAAATTTCAGAGCTTTTACTTTC
AAGTAAAAGAACCCCCGAGTCGACATTGTTATTGTTCTCAGTTTCAATAATCTCCGTGAACTGTAGCAGAGCTTTCTCGTCTTCTTCAACTTGTGCTCGAATATTCTGCTCTGAAACTCTAG
GAAGAATCTCCTTCTCTAAAGATTCCTTGCTTCCACCAGGTGGTGAGTGAGCCTCTGAAGCAACGTTGATGGACCCAATTTCCTTACCTAATGATGATCCAGCATGCTGCTTCCAGTCTTC
TGTAAGAATGAACTGCGGAGAAGATATAGCTCTTTGATGTTTGAACAAGGGAGTCAAAATATCAGTTTTTACAGAGTTATCAGATATCCCAACTTCATTTGGTTGAATATTTAGCTCCAAGG
CTTTGGTTGTATCAGAAGGCTTCATCTTTATCACCTTTTTTTTCGTTTTATGGATGGCAGGTTGACCTCTCACAAGATCTTCCACAGTTTCTAAAGCAAATCTATTTGCCATGAACCTTCTGA
AAGTGAGACGTTTTTCATCACGTTCAGGCTCATGCTTCCCCCCATACTTGCATAAATTATGACATGAATCCGTCGGTTCTCTGAGATACCGAGAGCGGACATAGCCAAAACTATTTGAACG
GGACATTGCATGACTACATTTTGTCTTTGCCCCATCTTTTGAGGCGCTTATTTCTTTTTCCACGAATGCTTTTTCCATTGGAGAAAGAATAGGCTTAGAACTTCATTAGTAGGGGTGAAGGA
TTCCAACAGAAAGATAACTTGAGCCCTGCAATGCCAAAGAAAGCACACCTGTAAGATTAGCAGAATGGGAGCATATTGTAATAGTATATGACTATCATTCGAACTAAAAAGAACAGGCAA
GAAGACAATGGAAACCTAACAGGACATTCCTCCATTAATAATGGTTTAGATCAAAACAAGGAGATATTCAAGGGGGAAAAACAGTTCTGTCATGTAGAGTTCTCATCTGCTTTCATTTGAT
TAGAGTAAGCTGAAGTTAACATTACATGAAGTACTCCAGAGATTGAAGGAAGAAGAAGAATTCATAAAGCCAGCCGAAAATAGATGGGACAAAGCTAATAATGATGATCAACTTTCACTG
GACGGATTAAAGCCTATAAGATCTATACAAGCAAATTAGCAAAATATGTCAA
>comp164344_c1_seq4

TTTTCTCTACAATTCTCTGAATTTAATATGGTATTAGAGAAAGGCATTCAGTTCTTCAAAAACAAGAGCGTGAGAGAGGGAGAGCTGTTTCTTGCGTTCTGTTCTTTCTCGCGTGCGTCCA
GGGCAGCCAAACAAGTGGAGGGCTTCCCCTTTGCGGTCTTCTACATGCGTCTCTCGTGTGTGCCTACCTAGGGGGCAAATAAGTGAAGAAGAGGATCAAGAGGAGCGAGCAGGAATTG
AGGAATACAGGTTCCCACAACCAGCCTTCATTACTAGAATTATGAGGAATATAGGTTCCCACCACTGACATTCATTGCCAAAATTCTGAGAAATACTAGTCCTTCCACCAACCTTTATTGCC
GAAATTTTAAGGAATATTGGTCCTTCTGGTTCCTTCGTGCGTCCAAGACCGAGGATAGATCGAGCGGGAGGGCTCCTGTTGCATGCAAAGTCCAATGTCAACTGAGCATAATCAAGAGAA
GTCGAGCACAAAACCTCTTGAGTATGTATGGAAAGAGTTGAGAGTTCCTTCTCGTGAGGAACTAGGGCCAGGACAAGCAAAAGTGCGAGACCAGCATCCTTCCTGCAATCAGACCAGA
GGGATCAAAGGCCTTGCGCGCGCCTGTACAGGGTCGATCGAACGGGAGTGTTCGGGTGGAGTCAGATGATTGATTTGCATTGGGCTGATGGCTGGCTCGATCAGCAGGAGGAGATTTGC
GTGGCATGTGCTAAGCCGCTTGATACGTGTTTTTCTGCTGCGGTCCTTGAAGCTTGCGCAACCTTAAAAACATATTTTTTCCTACTGTCATGCCTTCGTTGTCGGCCTTCCCTCGTGGTTGC
TATCTTATCTATAACCAATTTAGCCTTCGTTGCTACCCTTTGCCACCGTAATGTTTGAAGCTAATTTCTCCCAAAGTTTTGACCAAATAGAGGACTCCGTGGTGACTAATTACCAAGCTGAG
AATGACATATATGTATTCCTCAATTTAAGAGGAGTGTTAAAATCTCAACCATCCCTTGGCCCTTGATCCCTTAGCCAGCATGAAGAT
>comp164354_c0_seq14
TATTTTGGCATATCATTTAGTTTGTTATCTACATTGTTTTAAGCATTTCCATGTTAATTGGCTGAATATTGGTTTTCTTGTAATTTCTTAGAAGCTGATGACTATCAAATGCAACGCTTTTTGTTA
TGTAACCATTTCTCTCACAATAAAAAACGAGATCTCACTACTGTTATGCAAAATATATATTACATCTATTATCAATTTCATGATGCCATTAGGACTGGCTGTAAGCAAAGGATTCAACAAAGT
TATGTAGTGATTATAATTCTTGTAGGTTATTGCTAGGAAGTACATGAAGATTTCAAGTAAACCCTAATATAAAAACAGCGAGAAAACTCCACAAATTCAATGGAAAGTCAAAGAAAACAAT
AGTTTTATTGTAAGAATCAAACTAACAGAAGAAAAAATTGTAAGATACAATTGATCCTCAATTCAAGAGGAGGATCTCTCTCCCAATCCCTAGACGCCACTAGAGTCCAAGATACCAAAC
TCCAAGAACAAGAACAACCTAAATAATTTGCATAACCCTAATCCTCCCTCCCTATTTAAAGCAAATAAACCCACCTCCTTAGCCCCCCCTCCTATTTTAAATAGCTAGTGGACCCCACACTC
CTTGGGTGGCAAAACAAAGGGGGGCGGACTGCTTGGTTCACCTCCTATAAAATTGGACCAGCCCTTTATCCAAGGCCGGGCTAGACTGGTCTAACAATTGCTTGCTTGGCTGTTGCTGTC
TTTAGTATCATCTTCTCATTAATTTCTATTACCAACCCATAATATTTTAACAAGTTTTAGTTTTTAATTATTTTCTATTTTCAATGTAATAAATATGTATTTAAAATTGATAGCATGTTTTCCCCGG
AGTTTGTATGCTTGTTGAATGAGTAGGTCGTATATTGTTGGAGACCTAATATTAGAAACTCTAAACCTTTGAACTTCGTCTTCGCATTCCCATAGATACCCCTTGTTGGACTCTTCAGTGTCT
CAAACTCAACTTCTTTAATAGTATAAGAATTTTCAAATAAAAATCTAAGAATTAGCTTAATGGGCATTTATGTCCATCTAGCTTATCGACTTTTCCCATTTTTCTAGTTTGGACTGTTTTGTTC
TTTCTTGTTCACAATTTTTCCTTAACAGGTAGATAGAGTGTATTTGAGCACGCCTGGAAAGATAGCCATCATCGACCATGAGAAGAAAAGAACCCTTGTTCTGAGGAAGGAAGGCCTTCC
TGATGCAGCTCTTTGGAACCCATGGGATAAGAAGGCTAAGGCTCTCCCCGATTTTGGCGATGAGGATTTCCAGGTCATGCTATGCTTGGATTCTGCAGCTGTCGAGAATCCCATCACTCTC
AAACCATCAGAAGAATGGATGGGCCGCCAAGAACTCTCCACCGTGCTGTCAAGCTACTTCAGTGGACAGCTCGACCCTCGCCAAGTTCTCGCTGGAAAATAATCATCATCGCATTCTATT
TTGCCTGGACAATCATAAAAGAACCTCAACCTTTAGTCAAGCTGCTTCAGTGGACAACTCAAACGTTTACAAAGTTCTTGCTGGCTTCTAACAGGTCATTGCATTCTGTTTTAGTATTTAG
CATGCAGAGGTTCATGAAATTGTGCATAGTTTCATATATTCATACTTTTTGTTTATCTGCTGGTATGTTTTCCTGTTATGGTGTCATTGGACTTATGATAGAATATTTCAGATCAGTCAGTAGAG
AATACCTTTCTCAGTTGCATCATTATTCATTTTGTTTAGGAAAATTTGAGCTTTTGTATTTTTCTACAATGAGTGTTCTCATATTCATTATGATCATTCACTAACGCTTCATTTGTGACAAA
>comp164359_c2_seq5
AAAAAATGCAGATTTTTATGCGTTTTTATGTTCCAAACTTTTATGGGAATCGAGTAGATGCTTTTCTTTTTTTTATTATTTTTGTTTCATGTCCCAAACTGTTTTAAGAATGAGATATTGGCATC
CAAAACTTGTTATGCGCTTTGATTTGGAAAAGAGCAATGGTGACTATTCATGAGCCTTTTATAAAGTAATATTTAGGGATGTGTTAGAGAACTTTCATGAGTTTTAAAGTTGGGTTTGCTTG
CTTGTAGATTCAGATGCTATTTTATGACAATTTCAATTGTTGAAAACGAATTGTTCGAATACGAGATGGTTTGATTCATATTGAAAGGTTTCTTAATCTTATTTTGGAATGGTTTGTGACATGC
TTAAGAGTATTCATATGTCTTTTCTCTTAATGATGGGTATTCTTAGTCATCTTGTAAAATGTCTTTGAGAGTGTTTATGAATGGTTTTATGGTCGAAAGCTTTCTTTTGCAAGCCGATTTGTGT
CTAGTGGTTTTGTTTTAAAGGCTTTTATACGACGTTTTTTTTTATGAAAGCTTTACAACGAGGGGTTGTATACAACTTTCTTGTTGGAAGGTTTTGGGTTTCATATCCCACTCCAAATGAAA

GGAGGTGCTTGATTATGGGTTGTCGGGTAGAGTTTTGTGTCGAAACAAAATGAAAAGAAAGGTGATTTTGGGTAGAGGGCTTTTGGAGAGTTTTGTGTAGTAATTCTCTTAAGAGTTCTT
GTGAGCTATTTTGG
>comp164459_c0_seq1
GCGGAGGACGAAGTCCACCGACCCCGATATGCTTTCCCTGCCATAATAATAATAACAGATTATTATAATATTCTAGCATATATTTTACGGGAAGAAAAGAGGCTAACATCAACTCTCTCACTT
TGCAAACACGAAAGCTCATGCGATCAACCCCTCATCCTGTCCTCTAATCCTTCTACCTTCTCAAGTCTCTCCATCCTTGTTTCTATCTTTTAAATCTCGCGATATCGAAACCTTAATCTTAGG
AATGGCAATGTTAACGAACGGGACTAAGTCGGCGTCGGATTTCGCGAGGGTGACGGTGCCAGTTTACGTAGGGGACCTGCACCCTGACGTTACCAATGAGAACCTCATGGAGCTCTTCT
CTACCATCGGGGAAGTTGATTCCGTTCGAGTTTGCCGAGATTTTCACACCGGCTGCTCTCGGGGATACGGTTACGTCAATTACATCTCCTGTCAGGATGCAATAAATGCTATTGAGAAATTG
AACCATACTGTGTTGTGTGGAAAGCCAATTAGGGTTATGAAGTCACATCATGATCCAGATTTAAGAAACAGTGGGATTGGAAATGTATTTGTGAAGAACCTAAGTAGATCAATTGACTCTG
TCAAGCTTAAGGAGATGTTTTTGAAATTTGGGACTATCTCGTCCTGTAAAGTGGCTACTGAAGATGGAAAGAGCAAAGGATTTGGATTTGTCCAGTTTGATTCTCAAGTTTCTGCAAATAT
GGCCATTGAAAATCTTAATGGTATAATAGTTGATGGAAAGAAAATCTATGTGGGTAAATTCATCAAAAAGAATGATAGAAAATTACCAAATCCGGAACTGAAGTTCACTAACTTGTACATG
AAGAATCTGGACAAAGTCATGACAAAAGAACTTATTGAACTGAAGTTTTCTGAGTTTGGAAAGGTTTTTAATGTGGCAATTGAAAAGGATGAAGAAGGAAACCCAAAAGGTTTTGGGTT
TGTCAACTTTGCAAATCCTGAGAGTGCCAAGAAGGCGGTGGAAGCAATGAATGGACTTCAACTAGGATCCAAGAAGCTGTATGTTGCAAGGGCTCAAAAAAAGGCAGAGCGGAAAACA
TTTCTGTGTCATACGTTTCAGGACAAACGCAATGAACAAATTAAAAAAAATTCGGCTTCAAATCTATATGTTAAGAACATTGCTGATAATATAGACGATAGTATATTGTGGGAGCATTTCAG
TCAATTCGGTACTATTACTTCAGCTAAAGTTATGCACGATGATAAAGGAAACAGTAAAGGTTTTGGATTTGTTTGTTTCAGCACTCAGGAGGAAGCTTCCAAAGCTTTGAACACTCTTCAT
GGACACTTGTTTTATGGTAAACCTCTTTATGTTGCAATTGCCCAAAGGAAAGAGGAAAGACAAGCACAATTGTTGCGTCAATATTCTCAGCGAATGGCTGGAGTGACAGGCTTAGATGCT
GATGTTGTCCCTACTAGCTATCCTCAAGTTTACTATAGCCCTTCTAAGGTTATGAAATGTTTTCCTTCTCAACAATATCTCATGTATAAATCATTTGGGTTGAGATCGGGATGTCAGCCAAGT
GGATTTGTTCGTCCATCTAGACCAGTGCTTCAACGGAATTCGTCTTCTATTTTTTCAAGTACTTGGCAGCAAAGACCAAACTGGGCTGAAATGAATGCAGATTTGCTGCCTCAATCTGGGA
AATCTATTCTATCCTTTTCTCACTTGCTCCAACTTAACCATTCTTTCAATTCTAATGGACAACAGACAAATAATGTACACTACGAAAGACAGGAAGATATGAATGATTTAAAACGGACTCTT
CCTGCTTCTTCCAACACCGAAAGGATGGAGATGCTCAATAGTATGCTCGCTACCACTCCACCCCAGCAACAGAAACAAATGCTTGGTCAACACCTCTTCCCGTTGGTTCAGAAACTCAAG
GCGGAGTTGGCGGCTAAAATTACTGGAATGTTGCTACAGATAGATAACTCGGAATTACTGCTGTTGCTGGAATCACCTGAAGCGCTTGCAGCAAAGGTGGAAGAGGCTGCTGGAATTTTG
GAGCTCTCCAAGACCAAAATGAGCAGCCAGGGTGCTATTAGTTCTAACAATTTTTCATCTGCTGAGATTGCGGTCAATTAATTGATAATAATTCTTAATAAAAGATTTCGATGACACGATTA
ATAATAGATAATAAATTATGTGAAAATATTCTGATATTAATCAGAATGCACTAAAAAATTATTAACTTTATATTAAAATTAGCATTATTTATAAAATATT
>comp164459_c0_seq2
CCGGGAGAGTTTTGTGGCCCATCAGAGCTCCGTCGTGACTGAGCCTGTGGCGAGAGAGAGAGTCTGCTGATACATTACTATATTAGACAAAATAGAGAGAGAAGATACCGCGGGTGAGG
TGGACGAGGTCGCATCGGCCCCCGTCTTCTTTTCCCACCCTCCATATATTAATAATCTCTTCCAGAGAAGAAAGAACTCCCGCGCTCATGCAATCGGTTCCTCATCACTTCCTCTTCTTGTT
CTTGTATTCCTCCTTTCTTCTTCGACTCTGAATCGATCGTCGGCAGAGAAACCCTAACTCTACTTGTAATGGCAACGGATACAACGCCGACTGCGGTGGCGGTTTCGCCGGTTCCCGCACT
GTACATCGGGGACCTACACCAGGATGTAACTGATCCGAACCTTTTCGAGCTTTTCTCAACCATTGGCGATATCGCTTCTGTTCGCGTCTGCCGGGATTCCGTCTCCGGGTGCTCGCTTGGCT
ACGGTTATGTCAATTATATGTCTGAGCAGGATGCTGAAAATGCTATCGAGAAATTAAACTACAGTGTTTTGTGCGGGAAGCCCATTAGGATTATGTGGTCACACAAGGATCCAGTTGCTAG
GAACAGCGGGGTTGGAAATATATTTGTGAAGAACCTAAGTGGATCCATTGACAATTTGAAACTTCAAGCTATGTTTGGGAAATTCGGGACTATCTTGTCCAGCAAAGTGGCTTTGCAGGA
TGGAAAGAGCAAAGGTTATGGCTTTGTCCAGTTTGATTCGCAAGATTCTGCAAACGCTGCCATAGAAAATCTGAACGGTACAACAGTTGATGGCAAGAAACTCTTTGTGAATAAGTTCAT
AAAAAAGAGTGAGCGAATTCCCTGCAATCTTGAAGCCAAGTATACGAACCTGTACATGAAAAACTTGGATCAGGATATAACAGAAGAACTTATTGAACTAAAGTTTTCAGAGTATGGAAA

AATCTTTAATGTGAAGATTGCAAAGGATGAAGCAGGAAACTCAAGAGGATTTGGGTTTGTAAACTTTGAAAACCCTGAGGATGCTAAAAAGGCAGTGGAGGCAATGAATGGACTTCGGC
TGGGATCAAAGAAACTATATGTTGCAAGGGCCCAAAAGAAGGATGAGCGACTACAAATTTTGCAGCATACGTTCGAGGAAAAACGGAATGAACAGATTAGAAATAATATGGCTTCAAAT
CTGTATATTAAGAACATTGATGATTGTGTTGATGACAATGCTCTGTGGGAGTATTTCAGTCAATGCGGTACTGTTGCCTCGGCAAAAGTTATGCACGATGAGAAAGGAAACAGTAAAGGTT
TCGGGTTTGTTTGTTATACTACTCCAGAGGAGGCTTCAAATGCAGTGAATACTCTTCATGGAAGCATGTTCCATGGAAAACCTTTATATGTTGCAATTGCCCAAAGGAAAGAGGAAAGAG
CAGAACAGTTGCGTCTTCAGTATGCCCAAAGAATGAATGGTTTGGCTGGTTCAGCAACCGCTGTAGTCCCTACTGTTTATCCCACACTTTACTACAGCCCTCCGGGAGTTATGCCACAAAT
TTCTGCCCGACAAGGTATTGTATATCAACCTTTTGGGCTGAGACCAGGATGGCGGCCAAGTGGATTTGTTCCCCCATCTAGGCCAACTTTTCAGCCAATGCCGCTTCCAATTATTCCTAATA
CTCCTAGACAACAAAGGCAAAACAGGGGTCGGATGAATGGACATTTTCTGCCTCAATCTGGGCAATCTATGCCATACATGGCTCCGTTGCAACAGCCTAACCATTCTCTGAATTATTTGAA
AGATAATAATAGCCAAGAGTTTGTACAAAATGGAAGGCGGTGGGGCATGAATAATTTGCCTCTTGGTCTTTCTGCTTCATCTAATCCACAAGGGATGGAGCAGCTCAGTAGCAGGCTCGCT
GCAGCTCCACTACAGCAACAGAAACAGATGCTCGGCGAACGCCTATTCCCATTGGTCCAGAAACTCAAGGCAGATCTGGCTGCTAAAATAACGGGGATGTTGTTGGAGATGGATAACTC
CGAATTGCTATTGCTGTTGGAGTCGCCTGATGCCCTCGCTGCAAAGGTGGAAGAAGCTGTTCAAGTATTGAAACTCTCCAAAACCCAGGTGAGTGGTCGCGTGCCTCTTCACCCTAACAA
TTTCTTGCCTTCAGAGGTTTCCGTAAATTGAGAGGGATTCGTGATTCTGAAAATTACCATTCTTTACTAAGTTTCTTTGTTAGTCTTATGATTTTTGGGCGAGAGAGTTCTCCTCCATATGGA
TATAGACATATAGTTTTAGAATTTTCCCTTTGGATTTTTCCTGATCAAATTTCTTTTCTGGACCTTTTTTTTTTTTGTTGGACCAATTATTTAGCGTTTTTCCAAGTACCGTTGTAATTTGAGTT
CGCTCTACACATATTTTCCTTAAATTGGAAGTTTAATTTCTTCTTCTTCAGCAGGCCGTATTAATTCTAACATTGCATTGCTTCT
>comp164459_c0_seq6
GCCTGCTGAAGAAGAAGAAATTAAACTTCCAATTTAAGGAAAATATGTGTAGAGCGAACTCAAATTACAACGGTACTTGGAAAAACGCTAAATAATTGGTCCAACAAAAAAAAAAAAAGG
TCCAGAAAAGAAATTTGATCAGGAAAAATCCAAAGGGAAAATTCTAAAACTATATGTCTATATCCATATGGAGGAGAACTCTCTCGCCCAAAAATCATAAGACTAACAAAGAAACTTCGT
AAAGAATGGTAATTTTCAGAATCACGAATCCCTCTCAATTTACGGAAACCTCTGAAGGCAAGAAATTGTTAGGGTGAAGAGGCACGCGACCACTCACCTGGGTTTTGGAGAGTTTCAAT
ACTTGAACAGCTTCTTCCACCTTTGCAGCGAGGGCATCAGGCGACTCCAACAGCAATAGCAATTCGGAGTTATCCATCTCCAACAACATCCCCGTTATTTTAGCAGCCATATCTGCCTTGA
GTTTCTGGACCAATGGGAATAGGCGTTCGCCGAGCATCTGTTTCTGTTGCTGTAGTGGAGCTGCAGCGAGCCTGCTACTGAGCTGCTCCATCCCTTGTGGATTAGATGAAGCAGAAAGAC
CAAGAGGCAAATTATTCATGCCCCACCGCCTTCCATTTTGTACAAACTCTTGGCTATTATTATCTTTCAAATAATTCAGAGAATGGTTAGGCTGTTGCAACGGAGCCATGTATGGCATAGATT
GCCCAGATTGAGGCAGAAAATGTCCATTCATCCGACCCCTGTTTTGCCTTTGTTGTCTAGGAGTATTAGGAATAATTGGAAGCGGCATTGGCTGAAAAGTTGGCCTAGATGGGGGAACAA
ATCCACTTGGCCGCCATCCTGGTCTCAGCCCAAAAGGTTGATATACAATACCTTGTCGGGCAGAAATTTGTGGCATAACTCCCGGAGGGCTGTAGTAAAGTGTGGGATAAACAGTAGGGA
CTACAGCGGTTGCTGAACCAGCCAAACCATTCATTCTTTGGGCATACTGAAGACGCAACTGTTCTGCTCTTTCCTCTTTCCTTTGGGCAATTGCAACATAAAGAGGTTTACCATAAAACAA
GTGTCCATGAAGAGTGTTCAAAGCTTTGGAAGCTTCCTCCTGAGTGCTGAAACAAACAAATCCAAAACCTTTACTGTTTCCTTTATCATCGTGCATAACTTTAGCTGAAGTAATAGTACCG
AATTGACTGAAATGCTCCCACAATATACTATCGTCTATATTATCAGCAATGTTCTTAACATATAGATTTGAAGCCGAATTTTTTTTAATTTGTTCATTGCGTTTGTCCTGAAACGTATGACACA
GAAATGTTTTCCGCTCTGCCTTTTTTTGAGCCCTTGCAACATACAGCTTCTTGGATCCTAGTTGAAGTCCATTCATTGCTTCCACCGCCTTCTTGGCACTCTCAGGATTTGCAAAGTTGACA
AACCCAAAACCTTTTGGGTTTCCTTCTTCATCCTTTTCAATTGCCACATTAAAAACCTTTCCAAACTCAGAAAACTTCAGTTCAATAAGTTCTTTTGTCATGACTTTGTCCAGATTCTTCAT
GTACAAGTTAGTGAACTTCAGTTCCGGATTTGGTAATTTTCTATCATTCTTTTTGATGAATTTACCCACATAGATTTTCTTTCCATCAACTATTATACCATTAAGATTTTCAATGGCCATATTTG
CAGAATCTTGAGAATCAAACTGGACAAATCCAAATCCTTTGCTCTTTCCATCTTCAGTAGCCACTTTACAGGACGAGATAGTCCCAAATTTCAAAAACATCTCCTTAAGCTTGACAGAGTC
AATTGATCTACTTAGGTTCTTCACAAATACATTTCCAATCCCACTGTTTCTTAAATCTGGATCATGATGTGACTTCATAACCCTAATTGGCTTTCCACACAACACAGTATGGTCCAGATTTAA
GAAACAGTGGGATTGGAAATGTATTTGTGAAGAACCTAAGTAGATCAATTGACTCTGTCAAGCTTAAGGAGATGTTTTTGAAATTTGGGACTATCTCGTCCTGTAAAGTGGCTACTGAAGA

TGGAAAGAGCAAAGGATTTGGATTTGTCCAGTTTGATTCTCAAGTTTCTGCAAATATGGCCATTGAAAATCTTAATGGTATAATAGTTGATGGAAAGAAAATCTATGTGGGTAAATTCATCA
AAAAGAATGATAGAAAATTACCAAATCCGGAACTGAAGTTCACTAACTTGTACATGAAGAATCTGGACAAAGTCATGACAAAAGAACTTATTGAACTGAAGTTTTCTGAGTTTGGAAAG
GTTTTTAATGTGGCAATTGAAAAGGATGAAGAAGGAAACCCAAAAGGTTTTGGGTTTGTCAACTTTGCAAATCCTGAGAGTGCCAAGAAGGCGGTGGAAGCAATGAATGGACTTCAACT
AGGATCCAAGAAGCTGTATGTTGCAAGGGCTCAAAAAAAGGCAGAGCGGAAAACATTTCTGTGTCATACGTTTCAGGACAAACGCAATGAACAAATTAAAAAAAATTCGGCTTCAAATC
TATATGTTAAGAACATTGCTGATAATATAGACGATAGTATATTGTGGGAGCATTTCAGTCAATTCGGTACTATTACTTCAGCTAAAGTTATGCACGATGATAAAGGAAACAGTAAAGGTTTTG
GATTTGTTTGTTTCAGCACTCAGGAGGAAGCTTCCAAAGCTTTGAACACTCTTCATGGACACTTGTTTTATGGTAAACCTCTTTATGTTGCAATTGCCCAAAGGAAAGAGGAAAGACAAG
CACAATTGTTGCGTCAATATTCTCAGCGAATGGCTGGAGTGACAGGCTTAGATGCTGATGTTGTCCCTACTAGCTATCCTCAAGTTTACTATAGCCCTTCTAAGGTTATGAAATGTTTTCCTT
CTCAACAATATCTCATGTATAAATCATTTGGGTTGAGATCGGGATGTCAGCCAAGTGGATTTGTTCGTCCATCTAGACCAGTGCTTCAACGGAATTCGTCTTCTATTTTTTCAAGTACTTGGC
AGCAAAGACCAAACTGGGCTGAAATGAATGCAGATTTGCTGCCTCAATCTGGGAAATCTATTCTATCCTTTTCTCACTTGCTCCAACTTAACCATTCTTTCAATTCTAATGGACAACAGAC
AAATAATGTACACTACGAAAGACAGGAAGATATGAATGATTTAAAACGGACTCTTCCTGCTTCTTCCAACACCGAAAGGATGGAGATGCTCAATAGTATGCTCGCTACCACTCCACCCCA
GCAACAGAAACAAATGCTTGGTCAACACCTCTTCCCGTTGGTTCAGAAACTCAAGGCGGAGTTGGCGGCTAAAATTACTGGAATGTTGCTACAGATAGATAACTCGGAATTACTGCTGTT
GCTGGAATCACCTGAAGCGCTTGCAGCAAAGGTGGAAGAGGCTGCTGGAATTTTGGAGCTCTCCAAGACCAAAATGAGCAGCCAGGGTGCTATTAGTTCTAACAATTTTTCATCTGCTG
AGATTGCGGTCAATTAATTGATAATAATTCTTAATAAAAGATTTCGATGACACGATTAATAATAGATAATAAATTATGTGAAAATATTCTGATATTAATCAGAATGCACTAAAAAATTATTAACT
TTATATTAAAATTAGCATTATTTATAAAATATT
>comp164473_c1_seq2
TACCAAAAGATGTGATCTTTGCCCATGCAAATGTAACTAATGACATTCTATCAATCAAAAATATAGAATTTCTTTCTCCTCCTTTTATTCTTCTGATTTTTTCACCATATTGTGTTTAGAATTCT
ACAGCACAATTGCAAAGACAAAAGCAACAACTACAACCAAAATATAGCTTAGATGAGCGCATTATTTCCAATTACACACCTGGAAATCAACATTTCTAGCTGAAGTGGGCAAAGCAGTTG
CAAAATACTGAAGCTCGGTAAGCACTCCAAAGCCAAAATCCATCAAGGTTAGTTTTCCAAAGTTCAGGAGAGCTGGCATCTGGGTCTGTTGGAGGAAACAACACAGCTATGTGCAAGAA
ATACTGACAGTTTAGAAACAATAGAAACAACAAAATAAGATTACTGCTGTTGACCTCGATTTTGGCTCGAATATGCTCGACGACCTTCGATTGATTCAATTAGACC
>comp164513_c0_seq2
ATATTTCCTTAAAAAACAGAATGGCGAAAGAGAAAAAATAACATATTTATGCAAAAAATGCTACTAAATATTACCAAAAAAATGCATATCCAATATTTTAAAAAAACTTATAATTCAAAAAT
ATTCTTGTTACATGCTTCTTAAAATATTAACATATCCAATTTATTAAAGTGATGGTGTGAACCATATTGAAGGGGGCCAGTTAGGCTGTGAATTAGAATCAAGGAAATATTGGAAGCAATCAT
TATGAAGATCATAAAGTCCAGCATCACGAGTAGCATCATCTTGTGTCATGATGTCCGCATGATCGGCAAAGTATATGCAGTTTGGTCTAATTCCAAGAGCTACTGTGTGATGTAATGAAATT
GTTTGGTTGTAACCTATAAACAAAGAGAAATCCCCCAAGTCTGTCATGGAGATCCATTGAGAGGAAGAACAAGAAGAAGCATTAATAGCCGGCACAAACTTAAAGACAGAGATTTTGGT
TGTCTTCTTTGTATATCTTTCATCAACCTCCATACCACTGATCTCCCTCTTAATTTTTAGAAGAGTTCCTGATAAAAATGCAATGTAGTTCCTGCAATAAGAGTCAGTGGAGTACGGATCTTG
TATTGCTTGGCAAACAAATTTAACATTTTTTCCTTCATGGTCAAATGCCAATACTGCTGAGAAATGTGTGATTGCATAGAACATTTGCTCTTCTTCATGAAAGTAAACATCTTCAAACTCTTC
AAATTGGTCAACGTGGTAAGGAGGCAGTTGTGGCCCGAGAACCCAAGCATGGTCACCAGGCCTTGCCAAAGCTAGTAATTTTATAAAGCCGTAAATAACAACAATGGTACCGAATGGGT
CGCTAGAGGAAATGGCAATCTTCTGGAAATATAGATCACGGAAACTTTCTGCATCAATGACATATGATGGATGCTTTTCATCATAATAATACTTGATATGACCATCGAGGTCGTATTCGGGTT
TGATCCTTTGCCGTTGATCTTCGGGGATGGTGAAGAGGGATGGAAGTGATATTTTAGCTTTTGTTAGAGGATTGAAGAGGCAAGGTTGCAAATAAGTTAGGTCAAGGGTCACGAGCCATC
CATCCTTGGAGCCATAGCAGCGACGGCCAGAGATGTGGTTACCAATGGAGGAGAAGCGACGATTGCAGCCGATGCCGCCTTCTTCTTTTTTGGTGGTAAGATCGCAGAAAGTAAGTGTG
TCAGAGGCCTCTCCGGCATTGTTGGGAAGAAGCAACCATGGGGATGGAGTACGTTGAATTCTAAAGAGATCATTCTTGGCAAGAGCGAAATTCCACGATGTGCATGTGGCAGCGAATCG

GAGGAAGTCTACAATGGGAAAGTGCGATGAGAGGCAATGGAGGAGATCTATGGGGAGATCAGACCATGCCATATTATATGTTATGAAATGGAAAGGGAAGAAAGAAGAGGCCAGCCTCC
TTGCACTGCATTGCTTAAACAAAAATAAATAGAGTTGGAATTAAATGTCCAATCTCTGGAGATATATAGCCTGGC
>comp164513_c0_seq5
AAACCAGATTGAAGGGGGCCAATTACGCTGCGAATCGGAGTCAAGGAAAAACTGGAAGCAATCATTATGGAGATCAAAAAGTCCAACATCATGAGTAATGTCATCTTGCGTCGTGTTCTC
CGCATGGTCTGTAAAGTATATGCAATTTGGTTTAATTCCAGGAGCTACTGTGTGATGCAATGAAAAGGTTTGGTTATAACCTATAAACAAAGAGCAACCCCCCAAGTCCCTGATAGAGATC
CATTTTGAGGAAGAACAAGACGAAGCATCAACAGCCGGCACAAACTTAAAAATCGAGATATTGGTTGTCTTATATGTAAACTTTTCATTGGCCTCCAAACAAGTACAATCGATCTCCCTAG
TAATTTTTAGAAGAGTTCCCGACAAAAATGCAATGTAGTTCCTTTGCTCTTCTTCATGAAAGTAAACATCTTCAAACTCTTCAAATTGGTCAACGTGGTAAGGAGGCAGTTGTGGCCCGAG
AACCCAAGCATGGTCACCAGGCCTTGCCAAAGCTAGTAATTTTATAAAGCCGTAAATAACAACAATGGTACCGAATGGGTCGCTAGAGGAAATGGCAATCTTCTGGAAATATAGATCACG
GAAACTTTCTGCATCAATGACATATGATGGATGCTTTTCATCATAATAATACTTGATATGACCATCGAGGTCGTATTCGGGTTTGATCCTTTGCCGTTGATCTTCGGGGATGGTGAAGAGGGA
TGGAAGTGATATTTTAGCTTTTGTTAGAGGATTGAAGAGGCAAGGTTGCAAATAAGTTAGGTCAAGGGTCACGAGCCATCCATCCTTGGAGCCATAGCAGCGACGGCCAGAGATGTGGTT
ACCAATGGAGGAGAAGCGACGATTGCAGCCGATGCCGCCTTCTTCTTTTTTGGTGGTAAGATCGCAGAAAGTAAGTGTGTCAGAGGCCTCTCCGGCATTGTTGGGAAGAAGCAACCATG
GGGATGGAGTACGTTGAATTCTAAAGAGATCATTCTTGGCAAGAGCGAAATTCCACGATGTGCATGTGGCAGCGAATCGGAGGAAGTCTACAATGGGAAAGTGCGATGAGAGGCAATGG
AGGAGATCTATGGGGAGATCAGACCATGCCATATTATATGTTATGAAATGGAAAGGGAAGAAAGAAGAGGCCAGCCTCCTTGCACTGCATTGCTTAAACAAAAATAAATAGAGTTGGAAT
TAAATGTCCAATCTCTGGAGATATATAGCCTGGC
>comp164565_c0_seq17
TTAATTTCAACATAAATAATCTATTAGAGACAACTATGACTCATACATGGGCAGAACAACAACAAAAAAAAACGAATAAAAAAATAAACATGGCACTGTAAGCTTAATAATATAAGATTTG
TCACAAAGTTAACAATCTCTTATGCTTAATATATAGCAGAAAGGATAAGTATAAAACCCCGCAGAAAACACCAGATAATATAAGCACCCAGTTGAAGTTGGAGGGACTGTCAAACACAGA
ATTCTGAAAGTTCATCCCAAATACACCTGTAACAACTGCAAAAATGGTTACAACAAAAGTGGCTCCGGTGAGCAGCAACTCAAATTGAATCAACTGATTTCGCACATTGTCCAGTTTGAT
GTTGATAAAATCCTCAGTATCATCTATATACTCTTTCAGAGATGATAATTTGCTTAGTGTATTATCTGTGACAACAAAATATGCTTCCAAAAGCATCTCCAATTCTTCAACATGACTCCCATTA
TTGAATGAACTCCTGGAGCTGTCCTCCTTGCTTGGGCTGATAATACTAGTGGAAAATGTTGGTGAAACTGGAGCAGACCTTGAGAAACCAGTGTTGCAGAATTCAAGACTGCTCGAATAT
CGGAGAGTGGATTGAAATGCTTCGATTCCATCCTCCTTCTGGGTAAGATACATCTCAGCCATGTCACCATCATCATCCATGAGATGTTCTATTTCATCGCGAATCTGAAATGTTGATCTAAAG
TTTTCAATCCCAAAACAAGAATAAGATTACTAATTACTGCATACCTTCTGAACTCTGTAGGTTAAAGCAAGAAGATGGCTTTTTAGCCTGCGAACATGCTCTAGATTTATGGTGCTAATTGT
GGTTGCTAGCTCATCAAGCACAGGATATATCTCAATTTCCAGTTCAGTGGCCTGAGATTCAAGTGATGCTGAAGCCAACTCAAGAGCAAGTTCCAAAGCCTTGAATTCAAAAGGAAGATC
ATCTGCACATCAAATGTAGCTAGCTAATTTCTTAAGAATTTTATGAAGTGATGAAAGCCAACAAAAAAGATAACTTTGTTACCATTTTGATCTTTGCTTGCCTGAAGTCTTTGGCCCAATTC
TGATTCATAGTACAGAACACTGCTATCCAAAGAATTCATGAGTAAAACCTCATCCGCCATGATTATGCATCTAATTTGCTCCAGGCTGACTACAATTGCTTTCTCTCGACCAAGAATCGTTG
AGGGGTAGACAAACAAGGGATCTAAGAGCCTGAGATCTCGAGCTGGAAGCTGGCAGCGCCTCATCAGTGTGAATTTATCAAATGCCAAAGTTTGAGATTTTCCACTACGGTCTATCTGTA
TCCATGAACGGCTGTTGAAACCTCTTTTCTTCATAGCTGAAGCTCCAAATCCCTGAAGAAAATGGTTTCCAGCTCCATCTGGCTTTGGTATTTCGTCATAAGAAGCCGTTTCTGGAATGCC
TGATTCTCCGAGCTTAGAGTACGACTTCACCATTGGAAAAATGCTTTCTCAATTCTTATTAGATAAAGATCAAATCCAGCTTAAACCATTATATTTTCCCACAAAGAAGGGAGAACAAAAA
AGAAAATTGCAAGTCCAGCTAAAATGCATTCCCATCTTTGATTTATTCCACAAAGGGATCCATACTTCCATTAAAAAAAACGAAGAAAAG
>comp164611_c0_seq14
TTAATTAGGTTACGAGTTCTGCTGAATTAACTGAAGAAAGATGCTTAACCAGTCACTGTTTGCTGGTGTGGCACCATCAAGATTCACAAACTTTGAAAGGAGTTAACTCTTTTATATATTTT

CATATAAATAGAATATTATTCTTCTTTTCTTTTTCTTATATATAGATCATGAGCATTGTCGTGGGCCAATTTATTTATAATATATATAGTCCTAGCTACTCCTTAAGGATTGAGAATCTTGATGATG
CTGCTTCGGCAATAAATGACTTTAATATCAATCGCGCGCATATTCTATATGATCTTTTCTCCTTTCTTCCATCTGATCTAATGAACAGGTAAAACCTCTCAACATTTTTGTTCTGTCTTTTATTT
TTGTTCTTGGAACTTGTTTATTTAACGAGTTATGAGTTATATGCAATAGGATTTTCCTGATCAGTTCTTGCGTGTACAATACTGTACATTCAACTAATTAACATTTTCTTTAAAAAAAAAAAA
AATCAAATCCTTACCTACTCCCTACCGTCCCACGTCGCACGATCCAGCGCCGCTCGTCGCACGTCGAAACCATACCGAGCGTCACGTCTCGCACGTCGCTCACATGACTCGTTCGAGTAG
TTAAAAAGCCGATGGTACGAGGCCTTTGGCCTCTTCTCTCTTCTGCTCTCGCCACACTCCCGCTCCTTTTCTTTGACCCCTTCGTGGCTTGTTCGACCTCGCTTGCTCGACGACGTACCCTT
CCTGTTAGATCGGAAGTCACGACATTTTGAGCGGAGGCAAGAACAGGAGTCGCTCGCTGTGAAGGAACTTTCTACCTTTCGTGCGCTTCTTCTCCACCTCCCGACGCCGTCCCCTTTCCG
TTTGGATCGGAAGTTAGACATTTTG
>comp164612_c0_seq3
CTGATACATTTTGTTTGAATTTGCCAATGTAGAAAGTAAATTGAACTCATTCTATAAAAGAATAAATAAAATGATAAAAGAACAATTAGCAACAACTCAAGAAGAAACAACAAAAGAAAC
AAAAAATATCACTAAACCTACTTGCTAGCTTCAATTCTCTACACACAGAAGTGTGCCCTAAACAAAACTTGTTACTTTCCAGATCCTGAAGGGAGCAAAACTCTACTGATTTACTGTGATC
ACCTACATAGTAAGAAGGAACCATAGGCGAGAAAATGGAAGAAAATGATTGGTCTTAAAGCATGGATGCTTCTAGAAGCAGAAAATATATAGAAACTTAAATAATAATAATAAAAATAATA
ATAAATTACCAGTTAAGTTGTTGCAACTGATGGTTTCTTCAAGAATTTCTGGCACATGGTAGTACCATGAAATGTAAGAAGTGGGTGTTCTAAGAACTCTAATCTATCAGAAGATGTAATTT
CTGTGAAACCAAAAGCGGACGTCCACATGTTCAGAAGTTCAGGAATAGCAGGTAGCATTAGCCTTTCCACTCCTAATGAACAAAGCAACTTCTGCAATTCATTCATTAGAATGCGACACA
TTCCTTGTCGGCGAAATTTCACACGGGTGCCGATAAATGGCATTTCTGCAAGCCTTTCACCATGTATTCTTAAAGTAGCCACACTTATAAGCTCATCTCCATCCATTAACATCATGGTATAAA
ACCCCCAAAAGTTCAATCTATTCAACTCAGAAATGCTATTGAAAATGATATCCGTAGCAAGGTCACTCTGAGTACGTGCCTCAAACAAAGGCTCAAAACATTCATGCAAGACATCAAGTG
CAATATGGAGCATTCCTGAATTCTCAACTACAGATTCATCATCAAGTTTGCCAAAATCTTCTATATTTGATCTCCAAATAGTCCAATACAATCCTTTCACATGACTAGGATTCGGGTTCCCAA
GAAGTGATTGAAGATGAGAAATAATCTTCGAACATGCTTTACTGCAAGTCCAAGTCCCAGTTGGGCACTCTTTTAGCTCCAACCCTTGTTTTTGTAAGCAACCGACATGATATTGTTGTTC
ACATTGATCACAATAAATCCCTGAATTATGGTTGAAATTCTCTGCATCAAAATTAAACTTGCTAAGACCACAAATTCCACATTGACATGATGGGCAAAACCATTTACCTTCAGGCACATCCT
CTAGACCAATACAAGTTGGATGAAACGCTGATGGACAAAGATCACATAACATTATCAGACCACCATCTCGACAGACAGTGCATATATCATCAGCTTCATGATTGGAAAAGTCTCTTCTTAAT
CTTGAATGTTGGAATTTATTTGGCTTCAAAGCACTTAGAATCTGCTTTTGGCAATCTAACAACGACCTACCATCTTGTAATATGATATTGGTAGAAGGTCTTGAAGTTCTGCTTCCAGCATG
AGCTCCAAACTTGGAAAGAGGAAACACCTTTAGGCAACACCTACATTTAATTCCATCGTGAGTTATCCAACCCTCTTTCATTACGTGGCCATCTGCTTTTCTCACAAAGGCTACCTTCTGC
CTTGGCAACACAACATCTTTATCAATCAACCAGGACATAACTGTTTTAGCAACTTGATCTTTAGAAGATAGAAACATGGCCAAGCGAGATCTTTTGCCTGAACGTAGCACAGACGATGTG
AACCTTCCGCTAGATGGGTTTTGACTGCTAGAAGCACCGAAAGCATCCATCTTCATAGCATGTTTTTGGTAACAATAAATTCTCCTCTTCTTTAGCTCTCTCTTTTTGTCTAAATGCTCCTTA
GAATTAAACTCATTAGAATCAGATAAATTAGAGGAAGAGAAAAGTTTTTTTTCTAATATTCCTTTTTGCATAACATGAGATTTCTGACACTCTATTTTTCTCTTCTTTAGCTCACACTTTTCTT
CTAAATGCACCTTATAATCAAATTGACCAGAATCACACAAATTGGAGGAAGATGAAGATTCTTGTACCCTTTCTTCTTTTTGCTTCATAAGAGATTTTTTATGCTCAAATTTTCTCTTCTTTA
GCTTCCTCTTTTTTCCAAAATACTCAGAATTAAATTCATCAACAGCAACCAAATTAAAGGAAGATAAAGATCCTTGCCCGAATCTCTCTTTTTGCATCACAAGAGATTTTTGATTATTGGGG
GTTATTAAACCTGAATAACATGGCAGTTCATGAGCTTCCTCCTTGCTAGGAGTAATACTAGACATATTATTTGATGCACCAATGAAACATTTTTCATCATTAATACATTCAAATGACATTTTCT
GGGTGTTTTCACGATGTACTTCTTCTAGGTATCCCTTACAGGCAGAATAAAGAGATATATAGGTCTTCCCATTCGGAGAGGTGTAGCGTAATTCCTGCCTCACCTTCCTGTTAGTTTGCCAA
AATTTCCAACCCATTTTCAAGAGATAATCCTTAGTTTTTGACCTCAGTTCCTTTACATTAGTAGATGAAAGCTTTGACTTGTCTTTTAGCATTTTAATATAGTCAATGTATGCTTTTATAACAC
CTGATAAATGTAGTGTTTTTGCATTCTCATATATGTCATTTTCATTTGAATCCAGAGTTTCAGTATTCATCCCCAAATAATTACCGGAAGCTTGTGCCTTTTCTCCAATATATTCAGGTGACTTC
AACTGAATAACCTTACAGTTAGTCAAACCAGAAGAATCTGATTCACCATAGAGCAACTCTGCAGTTTGCTTGTTTTCAGAATGACTAGCATCAATGGGATGCATTATCTGGTCTTGTTTTAG

AGAGATGAAATCTGCTTTTGTAGGAGAAGTATCCAGCAAATGATTGCCCGAGTCAATGTCCTTTGAGTTGCCTTTATCTCTAGCTTTACCACTCATCTTTTCGTTCTCCTTCAACAAATACTT
GAGAACCTTTATAAGAGAGTAATAGATATTTTGTTCAGGTGAAATATATCGAAATCTTAATATAGTGTCTCTTTTACATTCTATCCTCCAACCAATAGACACAAGATGTTTCCTCAATTTTACC
GTTTGAATTCGAGGATATCCCAGCTTCACCTTTTTCATGGAGTGCTTACTGAATTCTGTATGAGAAGAAACGGACAGTTGCAGTAAATCATGGCAGAATTCACCTTCAAGTTTCACAGGTT
TCCATGCCCTTGATGTTATGCTTTTCTTATCGTCATGGGTTTCAAGTGTAAATTTAATGTCACAAACACTAGATGCCACAATTTTACTATCCCCTTCATTATTCCTAAACTTTTCATCTTCCTGA
TGTTCATCCACCACCTTTATTTCTTCACCAGATATAAATTTCTGCACTGAATCCAAAATGCGAGGCTCTAGATGTTTCTGTTTTTGAATATCCTGAATTTTATTTGAAGTTGTTATACAGCATT
TGCCAGCATGACTAGTTGATGCCAGAGAAAATTTCCCCACATTAGTAGATCTTCCCACTCTTTCATGTGCCAAACACTCACTGAAATTTTGTACAGAGGAAGAAGAGTGAGTATCAGGTA
AGCTACTACTAGCAATTGATTTTGTCGCTCGAATCACATCTAATACCAACTCTTTCCATACGAGCTTTGATCCAGAATACCAAACCTTAATCTTCTCACGGAAAGTATTTGTAGCTTTCAGGC
GAGGCCATATCTCCTTGATTTCTGATAGCACAATATCTTTTTGCCTAATTTTTTTCATGATCTGAAGGAACAACCACAGACCTCGATGTCTCCATTGCCCAGAAACACAATTCCACTCTTGG
GTCAATCGAATCTCAGAGGCAAAAATGAAACTTTGATCACTCATGTCTGGAAAGAAAACCAACAATTTACTAGAATCAACAGTTCGACCAATCAAAACTCCTTCCCTCCAGGAATCTTTG
ATGGAAACATCAACACACTGCCCAAAAATTATTTCAGATTTTTTAATGTCATGGATAGGAGGCAAAGGCCTTATCATTCTATTGTAGCTTTCCTCCACCGCAATACAGTTTTCTGATGCAGC
TTCTATTCTAGAGATTGGTTGAATAATTCTAGAATAATCGCCATTATCCATCATATGATTTAATTTAACTATGCGGCAGCAACTCTGACAATCTGTGATCACACCGGTAAACCAACATGCATG
AGACTCTTCAAAAAAACGAAGTACCTCTACTTTCTCTCCTACTAGAAGCTTGTTGCCTTCGTCGCCATTGTTTGACTGCATCCTCCAAGTATTCACCTCGTTGGAGATGAACGCCATTATCA
GAATCGGGAGATCAGACAGGGTTT
>comp164612_c0_seq5
CTGATACATTTTGTTTGAATTTGCCAATGTAGAAAGTAAATTGAACTCATTCTATAAAAGAATAAATAAAATGATAAAAGAACAATTAGCAACAACTCAAGAAGAAACAACAAAAGAAAC
AAAAAATATCACTAAACCTACTTGCTAGCTTCAATTCTCTACACACAGAAGTGTGCCCTAAACAAAACTTGTTACTTTCCAGATCCTGAAGGGAGCAAAACTCTACTGATTTACTGTGATC
ACCTACATAGTAAGAAGGAACCATAGGCGAGAAAATGGAAGAAAATGATTGGTCTTAAAGCATGGATGCTTCTAGAAGCAGAAAATATATAGAAACTTAAATAATAATAATAAAAATAATA
ATAAATTACCAGTTAAGTTGTTGCAACTGATGGTTTCTTCAAGAATTTCTGGCACATGGTAGTACCATGAAATGTAAGAAGTGGGTGTTCTAAGAACTCTAATCTATCAGAAGATGTAATTT
CTGTGAAACCAAAAGCGGACGTCCACATGTTCAGAAGTTCAGGAATAGCAGGTAGCATTAGCCTTTCCACTCCTAATGAACAAAGCAACTTCTGCAATTCATTCATTAGAATGCGACACA
TTCCTTGTCGGCGAAATTTCACACGGGTGCCGATAAATGGCATTTCTGCAAGCCTTTCACCATGTATTCTTAAAGTAGCCACACTTATAAGCTCATCTCCATCCATTAACATCATGGTATAAA
ACCCCCAAAAGTTCAATCTATTCAACTCAGAAATGCTATTGAAAATGATATCCGTAGCAAGGTCACTCTGAGTACGTGCCTCAAACAAAGGCTCAAAACATTCATGCAAGACATCAAGTG
CAATATGGAGCATTCCTGAATTCTCAACTACAGATTCATCATCAAGTTTGCCAAAATCTTCTATATTTGATCTCCAAATAGTCCAATACAATCCTTTCACATGACTAGGATTCGGGTTCCCAA
GAAGTGATTGAAGATGAGAAATAATCTTCGAACATGCTTTACTGCAAGTCCAAGTCCCAGTTGGGCACTCTTTTAGCTCCAACCCTTGTTTTTGTAAGCAACCGACATGATATTGTTGTTC
ACATTGATCACAATAAATCCCTGAATTATGGTTGAAATTCTCTTCAGGCACATCCTCTAGACCAATACAAGTTGGATGAAACGCTGATGGACAAAGATCACATAACATTATCAGACCACCAT
CTCGACAGACAGTGCATATATCATCAGCTTCATGATTGGAAAAGTCTCTTCTTAATCTTGAATGTTGGAATTTATTTGGCTTCAAAGCACTTAGAATCTGCTTTTGGCAATCTAACAACGAC
CTACCATCTTGTAATATGATATTGGTAGAAGGTCTTGAAGTTCTGCTTCCAGCATGAGCTCCAAACTTGGAAAGAGGAAACACCTTTAGGCAACACCTACATTTAATTCCATCGTGAGTTAT
CCAACCCTCTTTCATTACGTGGCCATCTGCTTTTCTCACAAAGGCTACCTTCTGCCTTGGCAACACAACATCTTTATCAATCAACCAGGACATAACTGTTTTAGCAACTTGATCTTTAGAAG
ATAGAAACATGGCCAAGCGAGATCTTTTGCCTGAACGTAGCACAGACGATGTGAACCTTCCGCTAGATGGGTTTTGACTGCTAGAAGCACCGAAAGCATCCATCTTCATAGCATGTTTTTG
GTAACAATAAATTCTCCTCTTCTTTAGCTCTCTCTTTTTGTCTAAATGCTCCTTAGAATTAAACTCATTAGAATCAGATAAATTAGAGGAAGAGAAAAGTTTTTTTTCTAATATTCCTTTTTGC
ATAACATGAGATTTCTGACACTCTATTTTTCTCTTCTTTAGCTCACACTTTTCTTCTAAATGCACCTTATAATCAAATTGACCAGAATCACACAAATTGGAGGAAGATGAAGATTCTTGTACC
CTTTCTTCTTTTTGCTTCATAAGAGATTTTTTATGCTCAAATTTTCTCTTCTTTAGCTTCCTCTTTTTTCCAAAATACTCAGAATTAAATTCATCAACAGCAACCAAATTAAAGGAAGATAAA

GATCCTTGCCCGAATCTCTCTTTTTGCATCACAAGAGATTTTTGATTATTGGGGGTTATTAAACCTGAATAACATGGCAGTTCATGAGCTTCCTCCTTGCTAGGAGTAATACTAGACATATTA
TTTGATGCACCAATGAAACATTTTTCATCATTAATACATTCAAATGACATTTTCTGGGTGTTTTCACGATGTACTTCTTCTAGGTATCCCTTACAGGCAGAATAAAGAGATATATAGGTCTTCC
CATTCGGAGAGGTGTAGCGTAATTCCTGCCTCACCTTCCTGTTAGTTTGCCAAAATTTCCAACCCATTTTCAAGAGATAATCCTTAGTTTTTGACCTCAGTTCCTTTACATTAGTAGATGAA
AGCTTTGACTTGTCTTTTAGCATTTTAATATAGTCAATGTATGCTTTTATAACACCTGATAAATGTAGTGTTTTTGCATTCTCATATATGTCATTTTCATTTGAATCCAGAGTTTCAGTATTCATC
CCCAAATAATTACCGGAAGCTTGTGCCTTTTCTCCAATATATTCAGGTGACTTCAACTGAATAACCTTACAGTTAGTCAAACCAGAAGAATCTGATTCACCATAGAGCAACTCTGCAGTTT
GCTTGTTTTCAGAATGACTAGCATCAATGGGATGCATTATCTGGTCTTGTTTTAGAGAGATGAAATCTGCTTTTGTAGGAGAAGTATCCAGCAAATGATTGCCCGAGTCAATGTCCTTTGAG
TTGCCTTTATCTCTAGCTTTACCACTCATCTTTTCGTTCTCCTTCAACAAATACTTGAGAACCTTTATAAGAGAGTAATAGATATTTTGTTCAGGTGAAATATATCGAAATCTTAATATAGTGT
CTCTTTTACATTCTATCCTCCAACCAATAGACACAAGATGTTTCCTCAATTTTACCGTTTGAATTCGAGGATATCCCAGCTTCACCTTTTTCATGGAGTGCTTACTGAATTCTGTATGAGAAG
AAACGGACAGTTGCAGTAAATCATGGCAGAATTCACCTTCAAGTTTCACAGGTTTCCATGCCCTTGATGTTATGCTTTTCTTATCGTCATGGGTTTCAAGTGTAAATTTAATGTCACAAACA
CTAGATGCCACAATTTTACTATCCCCTTCATTATTCCTAAACTTTTCATCTTCCTGATGTTCATCCACCACCTTTATTTCTTCACCAGATATAAATTTCTGCACTGAATCCAAAATGCGAGGCT
CTAGATGTTTCTGTTTTTGAATATCCTGAATTTTATTTGAAGTTGTTATACAGCATTTGCCAGCATGACTAGTTGATGCCAGAGAAAATTTCCCCACATTAGTAGATCTTCCCACTCTTTCATG
TGCCAAACACTCACTGAAATTTTGTACAGAGGAAGAAGAGTGAGTATCAGGTAAGCTACTACTAGCAATTGATTTTGTCGCTCGAATCACATCTAATACCAACTCTTTCCATACGAGCTTT
GATCCAGAATACCAAACCTTAATCTTCTCACGGAAAGTATTTGTAGCTTTCAGGCGAGGCCATATCTCCTTGATTTCTGATAGCACAATATCTTTTTGCCTAATTTTTTTCATGATCTGAAGG
AACAACCACAGACCTCGATGTCTCCATTGCCCAGAAACACAATTCCACTCTTGGGTCAATCGAATCTCAGAGGCAAAAATGAAACTTTGATCACTCATGTCTGGAAAGAAAACCAACAA
TTTACTAGAATCAACAGTTCGACCAATCAAAACTCCTTCCCTCCAGGAATCTTTGATGGAAACATCAACACACTGCCCAAAAATTATTTCAGATTTTTTAATGTCATGGATAGGAGGCAAA
GGCCTTATCATTCTATTGTAGCTTTCCTCCACCGCAATACAGTTTTCTGATGCAGCTTCTATTCTAGAGATTGGTTGAATAATTCTAGAATAATCGCCATTATCCATCATATGATTTAATTTAAC
TATGCGGCAGCAACTCTGACAATCTGTGATCACACCGGTAAACCAACATGCATGAGACTCTTCAAAAAAACGAAGTACCTCTACTTTCTCTCCTACTAGAAGCTTGTTGCCTTCGTCGCCA
TTGTTTGACTGCATCCTCCAAGTATTCACCTCGTTGGAGATGAACGCCATTATCAGAATCGGGAGATCAGACAGGGTTT
>comp164646_c0_seq1
GAAGGTGAGGGATTTATCGAGGGAGTGAATGTGAGAATGAAGGTAGTGGAGAGTGTTAATTCGGATATACAGCCGCTTCGTTCCTCGGCTCGTCGACGGCCGTGGGTGGTGAGTTTCTCC
CGCCGGAGTTCCCGCAGCCGGGCCTACACCGCCGACCACGGCCTGTCCGTTGTCCATTCCAGCTGTGCACGGCTTCGCTTTCTTCCACAGCTTCGTTAATTTCGAGTCCTGATTGCATCGC
GTTAATGGCGGCAACAACGGCACGTAGCTCTGCCGATTCCCGCATGAAGCAACGAAGGTGGCATACTCCTTGAAGATCGCTTCAAGCCCCTCGGCCAAATCGTGCACCATTTCATCTCTC
GCCGTCATCGGAATCTGGAAGAATTCATCGACGGTCACCTTCGCCAATTTTATCAGATCCACCGCCGACAGTGTGAATGGCTCGCTCTTCGATTTCGGCATCCAGTTCTCTGTTTCTTTGGC
TCGTTTAACACTCTCCCTCCCAATCCGAAGCCTCTCATCCACCCAAGTCTTCAAGAGTCCAACAGCAATGGTGTCGATCTCGTACGGAACCATCTCTCTAAGAAGATTATTCTTGTTTCCAT
CATCGTTATCTGCAGCGTCTTCACCGACCATCTGAATTAGCAGCTTTTCAAGCCTGCTGGCGCAATGGAGAACGCGCACAAGGTTGTTGGTGAGGCTATTCACCTTAGCAAGATATTGCTT
CAGTACAACTCCGAAGCAATTATGCAACGTCACCAACGCTATTCCGGTGGCCGCCGGGCACCATTGTCGCAGCACAGGACTGAACTTCTCTCTCTCCGTCGCGGCGAGTGCCTCCGTGTC
TTTCGCCAACTCGATCAAGATATCGCTGGGATCCTCGTCCACCTCCACGATCATGCTATCGACATGATCATGCCCTGTCTCGAACACCTTCGTAAAGGCATTGCGCAGAGATCTTTGAATAT
AGAATTCAACCCGGCTGGAAACAGAGCCAGATGAGGTTTCTCTCTCTACCAGTCCCGGCGTGTTACCTGCCAACCCGGGGAGGTCTTCGACGATGATCTTTGTTGTTGATAGCGCAAGCG
AAAGAATTTTCTCCATGTTTTCCAGTGTTCCCTTGTCGAAATTCTCGTGGTAGTCCAGTAGCCTTTTCTCTGTCCACCCCTGCATCGAAACCAGAACGCCGGAGAGAACCTTGATATAACT
CGGTTCCCGATCTACCACTCTCTTCACGTCGGTTGCCACTTCGAACAACATGTTCAACGTCGCCTTGATCAAGTCCGGTTCGATCTGCCCGGTGACGACGTACTGCTGGAACAAAATCCA
GGTGAAGCAAAGATTGTGGATACTTCGGTTGATTCCTAAAGTAGGCCATGTTTTCTTAATTAGCTCTAGAAGTTCGTCGACCTCATCGAGCACTACGGTTTCTTCGCGCATGTCGAATATGG

ATTTAAGCAGGGCGAGGTAAAGATGAACGTTGACGGGGAATCCGTCGGCCCAGTGGATGCTTCCGGCGCTGGATGTTTTGGAGCTGCGCCAGGCAAGAGCAGTCACGGCGCTTGTAAG
ATTTCGCATTGCTTCGGAGTTTTTGCTTGTATCGATGGGCTTTACCTCGCTATCCATTAGAACTTGGCGGAATCGGAGAGCGGCGGCATTGAGTCGGTC
>comp164646_c0_seq2
GAAGGTGAGGGATTTATCGAGGGAGTGAATGTGAGAATGAAGGTAGTGGAGAGTGTTAATTCGGATATACAGCCGCTTCGTTCCTCGGCTCGTCGACGGCCGTGGGTGGTGAGTTTCTCC
CGCCGGAGTTCCCGCAGCCGGGCCTACACCGCCGACCACGGCCTGTCCGTTGTCCATTCCAGCTGTGCACGGCTTCGCTTTCTTCCACAGCTTCGTTAATTTCGAGTCCTGATTGCATCGC
GTTAATGGCGGCAACAACGGCACGTAGCTCTGCCGATTCCCGCATGAAGCAACGAAGGTGGCATACTCCTTGAAGATCGCTTCAAGCCCCTCGGCCAAATCGTGCACCATTTCATCTCTC
GCCGTCATCGGAATCTGGAAGAATTCATCGACGGTCACCTTCGCCAATTTTATCAGATCCACCGCCGACAGTGTGAATGGCTCGCTCTTCGATTTCGGCATCCAGTTCTCTGTTTCTTTGGC
TCGTTTAACACTCTCCCTCCCAATCCGAAGCCTCTCATCCACCCAAGTCTTCAAGAGTCCAACAGCAATGGTGTCGATCTCGTACGGAACCATCTCTCTAAGAAGATTATTCTTGTTTCCAT
CATCGTTATCTGCAGCGTCTTCACCGACCATCTGAATTAGCAGCTTTTCAAGCCTGCTGGCGCAATGGAGAACGCGCACAAGGTTGTTGGTGAGGCTATTCACCTTAGCAAGATATTGCTT
CAGTACAACTCCGAAGCAATTATGCAACGTCACCAACGCTATTCCGGTGGCCGCCGGGCACCATTGTCGCAGCACAGGACTGAACTTCTCTCTCTCCGTCGCGGCGAGTGCCTCCGTGTC
TTTCGCCAACTCGATCAAGATATCGCTGGGATCCTCGTCCACCTCCACGATCATGCTATCGACATGATCATGCCCTGTCTCTAACACCTTGGTAAAGACAGCATTTTAGTTGTCCCTGTAGC
CTTTCCTGCTTCTTTTCATTTGGTCTATGTTCTTTCATTTGGTCTCTGTTCTTATAAAAAGACTCTTTTGTAATAATGAGAGAGATTGGAAAGTGACCTTTTCTTCATTGGTTTGCCTGTGTTG
GTGTTTTGTCTGTATCCTGAATCCTCTTGATTCAGGTGGTGGTTCCTGTTCATCTCTCTCTACAGAGAGTTATTCTGTTATCTCATCCCGTTCAAAGAATTTCTGTTTTACCGAGGAATACTTT
GTTCTCTCTTCTGGCGGGGTCAGTGGATCTAATAAAACTGGCGAAAGTGACCTTCAATGAATTCTTCCAGATTCCGATGACGGCGAGAGATGAAATGGTGCACGATTTGGCCGAGGGGCT
TGAAGCGATCTTCAAGGAGTATACCACCTTCGTTGCTTCATGCGGGAATCGGCAGAGCTACGTGCCGTTGTTGCCGCCATTAACGCGATGCAATCAGGACTCGAAATTAACGAAGCTGTG
GAAGAAAGCGAAGCCGTGCACAGCTGGAATGGACAACGGACAGGCCGTGGTCGGCGGTGTAGGCCCGGCTGCGGGAACTCCGGCGGGAGAAACCCACCACCCACGGCCGTCGACGA
GCCGAGGAACGAAGCGGCTGTATATCCGAATTAACACTCTCCACTACCTTCATTCTCACATTCACTCCCTCGATAAATCCCTCACCTTC
>comp164646_c0_seq4
TCGGAGTTTTTGCTTGTATCGATGGGCTTTACCTCGCTATCCATTAGAACTTGGCGGAATCGGAGAGCGGCGGCATTGAGTCGGTCAAGGGGAAGAGATGGGTGGAGCAGAAGCCCGAC
TTCGAGGATCTTGAGTTGCCTCCTTTGCCATTGATGATACTCTTGGCTATCATTGAATTCCGATGGTTTCAGATGGCGGAGGAGCTCAAGCGGGAGGATGATGGTCTCCGCTCTTCTCCCAT
TCTGACCGACGAAGCTTCTCATGAGGATTTTTCTCAGCCTGTTATCGCTCTGCTCCGTAACGCGCATCTGCCGCCGCATGATCTCGGCGGAGGTCATCGGCCGCTTGATCCTTCCGGGAGA
GCTGGGAACAAAGTTTCTTCCCGGGGAATTTGGACTGGCGGCATGAAGATTTGCAACCGATCTCAGCGCCATGGAATTCATCGCGCGCATTGGAGGCGGCCGTCGGGCTTTCAGCCCGA
GCGTGCTCTTCACCCGACTGGTCACCACCATCATCCCCGTGCCGCCCTTGGGGCCGACTACCGCCGGCTGCTGCGTATAGGGGGCCTCCACCACATCACCCCAGCTTCCAACGTAGGCGA
TGGCTCCCCTGTTTCCGAAGCCAGGGGACGAGCGGCAGGACATGAAGAAAATCTCGTAGGCGGTTTCGCGGAGGTCGGAGCGGCGGAGTGTGCCGAGGCGGGCGCCGAAGGGCCAGT
TGATTTCGCTGTCCCCGGACGATATGGAGGCGAGGGATTCATTGTTGGGGGAGATGGAGGAGCGGGGGGACGCGGGTGGAGCCATGAACTAGCTAGTAAGGGGCGGCCAGCTGCGGCG
ACGGCCGCGTTAGCTGAGGAACATGGCCACCATCGCATGGATCTTGTTCGGGCCTGGCCGCCCCTAAATTGAAAAATGGTGAGCATGAG
>comp164670_c0_seq1
TTTTCCACAATAATTCTATACTCACAATAAGTTTTCTATTTTTCTAAGTTCAAATATGGTCATGTTTGAGCATCCCTTTCGTTTGTGTTTTGCTTTCCAAATTGTTACTGACCATTTCATGATTC
TGATGGAAATGCAGCCCAATCTTCACCCACTTGGCTGGAAGGACCTCAGTTTGGAAGGCCATATCGCCGGAGGTGCTTCAGGCTTCATTCAACACCTCGCCGGAAATGGAGCAGCTCTTC
AGGTCTAAGAGGAGCTCTGAGGAGATCTTCTTCGCTCCTCCCAACTAAAAAACAAGAAAGATATGCCTATGAGCTTTATATGCCGCACCTTTTACTGGTTTTTCCTTCCTGCAAGTAATGA
ACTGGTTATTTGTTCTGTTATTCCACTAGTTCACCCCTTCTGGTGGACTGTAACTTAATGACTATTGTGTTTTTATTTCATATTATTCATGAATATTTCTGCTTTTGCTGCAGCAGTTGAGAGA

GCTGATTAGTCTCATACAAA
>comp164672_c0_seq14
TTGTATCTTTTAATTTAGATTCTAGGACATTACATATCATCCCAGTTGTCATAGGGATTTGTATAGTCTATAAAGTACTTTATAGTTCATGTGATGCTATTGATGTATTTGAATTTTGAACATTTT
GTTTTGGTTATTAACTTTTCTTATTATCACTAGAATGTTTGCTGTTTATCTGCAAAAATCAAAATAGTGATTTACCTCTTGTTGGAATTGTTTTCTTCCCTGTGCTGCTTCTGCTCTTAAGACC
TTAATGGCAACTGGTGTATGATCCAGATATGCCTTGTACACAGGTCCATAGCCACCTTCACCAATCTTATTTTCTGCAGTGAAGTCATTAGTTGCTGCTTTAATCTCATCAATGGTGTATCTC
CTGTAGCGGGTGTCAGTTTGAGTTAGTGCATCCAATGCTTTCTTCTTTTCTTCAGCCTCATGCCTAGCTTTTCTTTCTGCATTTTGTCTCTTATGTGCTTCGAGCTCAGCAATCTTTCGTGCA
GTTTCAGCTGCTTCAACGGCAGCTCTACATTTTACTTTCTCCATTTCAGCCATAGCAAGAGCTGCTTCTTCTGCATGTCTTAATTCCTCAAACTTTTGGGCTTCTTCAATTTTCCATTGTTGA
AGCTCCATTGCCTTCTGTTTAGCTGTTACAGCTTCTTTGCAGGCATTGCTATACATCTCCATTGTTTGCTTTAGCTCAAGTTTCAGCCTTCTCATTTCAGCCTCAAGTTCTCGATTCACACCA
GTAACTGAGTGTCGTTTGCTTGTAGGACTATCAACAGAGGCTGAAGAAAATTCAAGAGAATTTCCAATGTTCATTGATTGGAAGCTGTGTCCTTGTATGTCAATGTCATCTGAAAATGAGC
TTCCATAATCAACAGAACTTCCAGACAGGTTAGCAAAATCGAGCTTCTCCATGAACAGATTGTTGGGTGGCGGTGCTGAACGAGCTATGGAAAGTGGCCTATCTCTTGCAGAAGTATTCA
GATAATCAGGCATAAGATCTATTGATCTTCGTTCTGTCCTTTCATGCAAGGGGGTTGGTGGACCTGAAACTCTCCAGCTCCTCCTTGAATATGATGATCTACTGCTTGAATCTTCCTGGTCA
GGTAGGACTAGTGAAGTTGCTGTGATGCTCAATTGCCTTGGTGGAATAACGTTAGGAGTTGAGTTTCTTGCTGCTCGGATGCTAGTTGTTTTCCCTTTGGAAAGGACATACACAGCACAG
AAGTCTGGTGCTGATTTCATCAGGCTACTTGGGACATCAAAACTTCTGAGTTTTTTCATGATGCTCCTATTTGAAGCCCCAACGACTATGTTTTGGATGGAGCTGGCATTGATGTAATCAAC
TATGGCCTTAGAAATATCAGCACCCTCCAGGACTACCTCCTTTAGCTGCATCTGCAAATTCCAAAAAACAACATCTACAAAATGAAAGAGGAAGGGTACCTACTAGAGGATTTGCTCCGG
ACATGAATGAGAACAACAATCCTTGTTTTAATGAGATGATCAACGGCCCATTTCACGGCATGCTGGCTGTTCTTGTCCTTGTCGATGGCCACCGCAGTTGGGCAGATTTCTGATACAGGGC
TTCGGTCGCCCATCAGCTCCGACCACAAGAGACTGAGCTGCAAAGGTACGCTTATCGACCACAGGCCACCTGTAATGACTTCGTTCGCTTGTTCTTTATGCCTGCATTGTAGGAGATGAAT
GATGGTATGAAGGGAGAACAAAGAGGAGAAGAAGATAAGATCTGTCTTTTTTTTCCTCTCTTCCTCTTTCTTCTTACCGTCATTGCTTTCCCCTCTTTTTTGGGAACAAAATTGTAGCTTCC
TGGAATCACGGAATTGTATAGCTTAACTTTGCCAAACTGCTAACGGCTGAGGACCAAAGAAGGGCCAGCCGTTGAGTCTGGCCACATATTTAAGGCAGAGAATAGGTTGGTTGGAGGAG
CTAAGGACATTTTCTATTTTTGTATTTAATCTATTGATATTTTTGAAAAAAATATTCTGAAAATAAATTGAATAGGAGGAAGATTG
>comp164672_c0_seq19
GATAAATTCTATAATAAATTCACTAATATTACAAATACATGAGAAAAGAAGGAGCACATAACGAAACAAATAGGTGCAACATTTTTAGAAGTTTTTTGCAATGCGGCAAATTAAGCTTTAAG
CCCAAGCCATGATAAAGTCTTATGGTCAGCCACATTTCAGATATTTGATACCTTTATATTTGTATTACAAAGGTTTGGGGAGTAAGGCAATAGGAAGATCTGGCTTATCCAATCCATTTAGGC
TGAAAAATATAAAAACTTCAACATGAATGATTTTCATTTCCCACAGTCAATTGAAAACCATCAAATTACATTGAACTTCACACTAAACCGAAAAGCATTAACATACAAAACCAATTCATTAT
ATAATTCAAATTTACTCATCCTGAACATCACCATCAGATGCTGATTTCGACCTTGCTACAACGCCTGAATTGCTTGCAACCTTTTCCTTCTGCATAAGCAACCTTGCTCGTGTCTGTGGATTG
TTGTAGCTATGTGGTCTTGGTGGAAAGTTGGACCTTGTTCTACTATGGTGGTGGCTAGTTTTATATTCATTTCCAATGTTTCTTAGTCTGTTAAGCTCTGGCAAGACTTCTGCTCCAAGATCG
GGCCTATCTTTCCTCCTTAATTCAGCACATTTTAATGCAAGTTTTGCAAAATCAAGAGTTTCATCCATAGGCCAATCAGTGATTGATGGATCAAGCAACTGTTCAAATGTTCCTTTTTCAATT
GCTCTCTCAACATGATGAGTGAGACCCATTGGTGGCTTTGCTGTGATAAGTTGAAGAAGCATTACTCCAAGGGAGTAAACATCGGACTTAGCTCCAAGCATTCCAGTTTGTTGGTATTCAG
GATCAATGTAGCAGAATGTTCCTGCTGCTGAAGTCATGTGGTATTGTGTGACGCTATCAGCTATAGATGGTGGGACAAGTCTGGCTAGACCAACATCACTGATCTTGCTAACATAATTTCGA
TCTAATAGGATATTAGCTGGCTTGAGATCTCGATGAACAAGTGGTTCTGGTTTGGTCTGGTGCAAGAATAATAGTGCTGTGGCTATCTCTGCGGCTATTTTAAATCGAACATTCCATGGAATT
GGTGGGGTGTTTCGTCGTTGGAAGAGCCTATCCTCTAAGCTCCCATTGTCCATGAACTCATATACTAGGCATCCATATTCTGGGCAAGCGCCGAGCAATAGTACCATGTTGGGATGTCGAAT
GCAACTTAAAACTTCAACCTCTTGTTGAAATTGTTTCCTTCCTTGTGTTGCATCAGGTTTCAAAATCTTAATGGCAACAGGTGTATGATCCAAATAACCCTTGTAGACAGGTCCATAGCCAC

CTTCACCAATCTTATTTTCAACAGCAAAGTTATTACTAGCAGCTTCAATCTCATCAATTGTGTATTTCCTGTATCGTATATCATTATAAGCAAGCTCTTCCAAGGCTTTCTTCTTCTCCATTGCC
TCACGTCGCGCTTTAATCTCAGCATTCTTTCTCTTCTGTGATTCAAGCTCTGCAATCCTTTGTGCAGCTTCAGCAGCTTCAATGGCGGCTTTGCATTTTGCCTTCTCCATTTCAGCTATTGCA
AGGGCTGTTTCTTCAGCATGCCTTAATTCCTCAAATTTCCTTGATTCTTCCAATTTCCATTGTTGAAGCTCCATAGCCTTCTGCTTAGCTGTAATTGCTTCTTTGCAAGCAGTGCTGTACATC
TCCATTGTTTGCTTTAACTCAAGTTTCAACTTTCTCATCTCATTTTCAAGCTCTCGATTTTGACTAGCAATGGGAGAGCGACTACTCCTTGGGCTATCTATGGAAGCTGATGAATGTTGAAA
GGAGTCTCCGAAATCCATAGAATTGAAGCTAAGTCCCACCGGTAAATCAATTTCATCTGAAAACGTGTCAATACAATCAGTAGACCTTCGAGGCCAATTTGAGGAATCGAGATTGTCCATG
AATTGATTATTGGGGCTTGAACTAGCCATGGAAAGAGGAACATCCTTTAAAGTTGTCTTCATATAATCAGGTAATCTATCAAGTGATATTCTTTCTGTACTTTCATGTAGAGGAGTTGCTGGA
GCTGAACTTTTCCAGCTTCCCCTTGCAGGTGTCCTAGCTGAATCTTCATGGTCCGGTGAAAATTTTGGAGATGCTAGGAGGGGCAATTGCCTAGGAGGAAAGGTAGTAGAAGGAGGTGG
ACTTTTTGCTGATCGAACTGTGGTTGGCTTACCCTTTGAAATGACATAAACAGTACAGAAGTCTGGTGCAGATTTAATAAGGCAGCTTGGAACATCAGGATGTCTAAATTTTTTCATGAGC
AAACTCTTATTAGAGATGCCAACAACAATGTTTTGAATATGATTAGCTTTGATGTAATCAATTATGGCCTTCGAAATATCAACGCCTTCAAGTACTATCTCCTTCAACTGCATCCCTTTGCAA
GCACAAAATCCCCTGTAAGGAAGAAACAATTGATTTATCTCTGCATCTGCTTGATCTCTGGTGACTTCAGTGCTAACTTCTGTCATGGAGGATTTGTTCCTGACATGGATGATGTTGATGAT
CGGTGAGCGGACGAGATTGTTGATGGCCCATTTCATGGCATATTTACTATTTTTGTCTTTGTCGATGGCCACGGCGGTCGAGCTCGAACCCGACAAGCTCGTGCCGTCCTCCATGATCTCTA
CAGGTTTACTTCAGATCATCTCATTTAGATTATTTTCTGCTTGCCTTTTAAACTAGAATTATCATTGAAGAAATGAAAATTTTTTTGATAGAAATTAAGGAAAAGAGGAAGAGGAAGATCTT
TCTCTCCTCTTTTTTTTTTTTAACAAAATACCTGAATTTTTCTGGCAACAATAT
>comp164672_c0_seq26
TTGTATCTTTTAATTTAGATTCTAGGACATTACATATCATCCCAGTTGTCATAGGGATTTGTATAGTCTATAAAGTACTTTATAGTTCATGTGATGCTATTGATGTATTTGAATTTTGAACATTTT
GTTTTGGTTATTAACTTTTCTTATTATCACTAGAATGTTTGCTGTTTATCTGCAAAAATCAAAATAGTGATTTACCTCTTGTTGGAATTGTTTTCTTCCCTGTGCTGCTTCTGCTCTTAAGACC
TTAATGGCAACTGGTGTATGATCCAGATATGCCTTGTACACAGGTCCATAGCCACCTTCACCAATCTTATTTTCTGCAGTGAAGTCATTAGTTGCTGCTTTAATCTCATCAATGGTGTATCTC
CTGTAGCGGGTGTCAGTTTGAGTTAGTGCATCCAATGCTTTCTTCTTTTCTTCAGCCTCATGCCTAGCTTTTCTTTCTGCATTTTGTCTCTTATGTGCTTCGAGCTCAGCAATCTTTCGTGCA
GTTTCAGCTGCTTCAACGGCAGCTCTACATTTTACTTTCTCCATTTCAGCCATAGCAAGAGCTGCTTCTTCTGCATGTCTTAATTCCTCAAACTTTTGGGCTTCTTCAATTTTCCATTGTTGA
AGCTCCATTGCCTTCTGTTTAGCTGTTACAGCTTCTTTGCAGGCATTGCTATACATCTCCATTGTTTGCTTTAGCTCAAGTTTCAGCCTTCTCATTTCAGCCTCAAGTTCTCGATTCACACCA
GTAACTGAGTGTCGTTTGCTTGTAGGACTATCAACAGAGGCTGAAGAAAATTCAAGAGAATTTCCAATGTTCATTGATTGGAAGCTGTGTCCTTGTATGTCAATGTCATCTGAAAATGAGC
TTCCATAATCAACAGAACTTCCAGACAGGTTAGCAAAATCGAGCTTCTCCATGAACAGATTGTTGGGTGGCGGTGCTGAACGAGCTATGGAAAGTGGCCTATCTCTTGCAGAAGTATTCA
GATAATCAGGCATAAGATCTATTGATCTTCGTTCTGTCCTTTCATGCAAGGGGGTTGGTGGACCTGAAACTCTCCAGCTCCTCCTTGAATATGATGATCTACTGCTTGAATCTTCCTGGTCA
GGTAGGACTAGTGAAGTTGCTGTGATGCTCAATTGCCTTGGTGGAATAACGTTAGGAGTTGAGTTTCTTGCTGCTCGGATGCTAGTTGTTTTCCCTTTGGAAAGGACATACACAGCACAG
AAGTCTGGTGCTGATTTCATCAGGCTACTTGGGACATCAAAACTTCTGAGTTTTTTCATGATGCTCCTATTTGAAGCCCCAACGACTATGTTTTGGATGGAGCTGGCATTGATGTAATCAAC
TATGGCCTTAGAAATATCAGCACCCTCCAGGACTACCTCCTTTAGCTGCATCTGCAAATTCCAAAAAACAACATGTGCATTGCAACCATCAAATTAATTAAAATATATCATCACTTGTGATTG
TTTTCAGCTACAAAATGAAAGAGGAAGGGTACCTACTAGAGGATTTGCTCCGGACATGAATGAGAACAACAATCCTTGTTTTAATGAGATGATCAACGGCCCATTTCACGGCATGCTGGC
TGTTCTTGTCCTTGTCGATGGCCACCGCAGTTGGGCAGATTTCTGATACAGGGCTTCGGTCGCCCATCAGCTCCGACCACAAGAGACTGAGCTGCAAAGGTACGCTTATCGACCACAGGC
CACCTGTAATGACTTCGTTCGCTTGTTCTTTATGCCTGCATTGTAGGAGATGAATGATGGTATGAAGGGAGAACAAAGAGGAGAAGAAGATAAGATCTGTCTTTTTTTTCCTCTCTTCCTCT
TTCTTCTTACCGTCATTGCTTTCCCCTCTTTTTTGGGAACAAAATTGTAGCTTCCTGGAATCACGGAATTGTATAGCTTAACTTTGCCAAACTGCTAACGGCTGAGGACCAAAGAAGGGCC
AGCCGTTGAGTCTGGCCACATATTTAAGGCAGAGAATAGGTTGGTTGGAGGAGCTAAGGACATTTTCTATTTTTGTATTTAATCTATTGATATTTTTGAAAAAAATATTCTGAAAATAAATTG

AATAGGAGGAAGATTG
>comp164690_c0_seq2
ACTCCACCCCATAGTTAAATTCCTGTTTAAACAGATAAGCCAAAAAGTAATTAGAAGAAGGGCACATGATGCGTGATGCTGATGGGATCAAGATTACTTTTTGAAATCCAATCCGAAGGAA
ATTAGTATACATGAAAAGCAGGCATGGTTACATAAAAAGAGCATGATGAATCCTGCAGCAAACAAAGGTTAGAGCAAAGAACAGAGAAGTGAGCAATCAGTTCTTCATTATCTTCCTTGC
CATGATCAAGCTACAGTATATGCTTAAATAAACTAGCCAGTTAGTTACAAACCAACGGCTGTGATTAACAGCAGAAAATAAACGATTGAGATTGCTCAGAGCAGGTGTAGTGAGGAAGTG
GCGCAGTCATAGCACATGGAGAGGTAAGCCACCAGCTCTATATATGAGAAAGCTTATCACATGTGCTTATCTTATCATCCCCCCTCAAGCTAGCAGTGGGAGGCCCAATGGTAAGCTTGGA
ACGAAGAG
>comp164701_c1_seq2
TTTTTATTTTTTATTTTTTATTAAATATATTAATTTATGCAATAGTAGCAATAGAGCATCAATGGCTGATGGAGGAGAGATTATTCTATGCGGTATCTAAATACATCCATATATCAATCTGGACGT
GCCACATCATTCAATGCACTGTGCATTGGGTGACATAGCGCGTCCGAATTGACCTCCGGACTCGTATGAACACCGCAGGTAATAATTTTTCCTGATGGAGAGGTAATATCAACGGTTAGTT
GTTTTTGATTATTTAGGTAGGAATAATTTTGAGAAAGTTCTTCGGCTCTCAAAAGAAGTTGTTCTAATTTTAATAATATTTTCAAAACTATAAGGAAATAGTAATTAACTAAATTATAAATAGT
TTTGGTTGACTTCATTTTGATTCTTCCAACTTCTTATAGGCTAACTAATCTTCTAGCTATTACTATCATTTCAAGTAATACTTACTATTTTCGAGCAAAATAATATAGGCAGAGCAAAAAGAAA
AGAACGAAGAGCTCAGAACGGAGATGAAGAAAATTCTTATAAATGCTACAGATCCATTAAAATCACTTGAACTGATTGATTCAATTCAACGGCTTGGAGTGGCATATCATTTTGAGAAGG
AAATCAACGACATATTACGTAGAGTTCATAAGATCAACATCATTGATGATGATCTATATGTAGTTGCACTTCGTTTTCGACTATTACGACAGCAAAGATATAACATAAGTTCTGATGTATTCAA
TAGGTTTTTGGATGATAAAGGAGATTTCATGGCTTGCTTATGCAACAATGTGAAAGCTTTACTAAGCTTATATGAATCTGCTTATCTTGGATTTCCTGATGAAGAAATACTAGAGAAAGCTA
AAATTTTTTCAAGGTTTCACTTGAAGTCATTGATCTGCAAGATGGAGCCATCTTTTGCTTTGATGGTGACAAGTGCTTTGGAATTGCCATTGGTCAGAACACCTGATCGGCTCAAGGCTAG
AAATTACATAGAAATTTATGACAAGTACAAATTGTGCAATCAAAAACTACTTGATTTTGCAAAATTTGATTTCAATATATTGCAAGCTACTTATCAAGAGGAGCTTCGAATCATATCTGAGTG
GTGGAAAGAGTTAGGCTTGTTCAAAGACTTACCATTTATACGTGATCAAATAGTAGAATCCTACTTCTGGATGCTTACAACCTACTGTGAACACTGCTATTCACATGCTAGAGTTATTATGA
CAAAGCTGATGGCCCTTGCTGTGACAACAGATGATATTTATGATATTTATGGGACACTAGAAGAGCTCGAGTTATTCACCAATGCAATAGAAAGCTGGAATCTTGAAAAAGTTAGAACGTT
GCCAGAATATATGCAACGATGCTTCCTTGCGATTTACAATACCTTCAAGGATATCGAGGATGAGTTAGCTCCTGAGCACAATTCTTTTCGAGTTCATTACCTTAGAGATGAGTTGAAGCGAA
TGGCTCATGCGTACCTTCAAGAAACCAAATGGGCGAGCGAGTGCTATATTCCTAAGCTTGAGGAATATGTTAAAGTAACATTTATTACTTCTGGGTGCATATTTATCACGTGTGCTTCATATG
TTGGAATGAAGGAATTTATCTCAAAGGATATTTTTGACTGGGTTATCAGCCCTTCTGAGATTATCAAATCTTTTTGTGCAATTGGGAGGCTTATGAATGATATTGGTTCATATCAAAATGAAC
AAAAAAGGAATCATCTTGCTTCATCAGTTCAATGTTATATCAAAGAGTATGGTTGCTCTGAAGTGGAAGCTTGCAAGAAGCTTAAGGAGATGGTGGAAGAACATTGCATGATTATAAATAG
AGAATTTGTGTCAACAAAGAACATCCCATTGCCATTAATTAGGCCAATCTTAAACTTGTCACGTATCTATGATTTTTACTACAAGGACACACGTGACATATATACCGACTCCTCAGAGTTCAT
GAAGAAAAGTATCATTGAAGTGCTCGTTGATCCGATTTTGATTTATAGGAACGATTGAAACTTTTCTTACATAATGGGAAATCTGTGTGACTACCTACAAATAATATTTCAAGTGCATTGTTG
GAATCGTGCGTGTCTCTCTAAACTGCTTGAATGCTTTTTTGTATTAGTCTTTTACTTCATCATTGTCAAATTAAATGTGATTTTTAAGTTTTAATAAAGAATATGTATGGTTTCTGAGGATAATT
TGAGCTAAAACTTCCTCAAAAGCATATTTTAATTA
>comp164701_c3_seq1
GAAATCTTGCAAGTTTTATATGTTGTCTTCTAAACCTTTAATTAAAATATGCTTTTGAGGAAGTTTTAGCTCAAATTATCCTCAGAAACCATACATATTCTTTATTAAAACTTAAAAATCACAT
TTAATTTGACAATGATGAAGTAAAAGACTAATACAAAAAAGCATTCAAGCAGTTTAGAGAGACACGCACGATTCCAACAATGCACTTGAAATATTATTTGTAGGTAGTCACACAGATTTCC
CATTATGTAAGAAAAGTTTCAATCGTTCCTATAAATCAAAATCGGATCAACGAGCACTTCAATGATACTTTTCTTCATGAACTCTGAGGAGTCGGTATATATGTCACGTGTGTCCTTGTAGTA

AAAATCATAGATACGTGACAAGTTTAAGATTGGCCTAATTAATGGCAATGGGATGTTCTTTGTTGACACAAATTCTCTATTTATAATCATGCAATGTTCTTCCACCATCTCCTTAAGCTTCTTG
CAAGCTTCCACTTCAGAGCAACCATACTCTTTGATATAACATTGAACTGATGAAGCAAGATGATTCCTTTTTTGTTCATTTTGATATGAACCAATATCATTCATAAGCCTCCCAATTGCACAA
AAAGATTTGATAATCTCAGAAGGGCTGATAACCCAGTCAAAAATATCCTTTGAGATAAATTCCTTCATTCCAACATATGAAGCACACGTGATAAATATGCACCCAGAAGTAATAAATGTTAC
TTTAACATATTCCTCAAGCTTAGGAATATAGCACTCGCTCGCCCATTTGGTTTCTTGAAGGTACGCATGAGCCATTCGCTTCAACTCATCTCTAAGGTAATGAACTCGAAAAGAATTGTGCT
CAGGAGCTAACTCATCCTCGATATCCTTGAAGGTATTGTAAATCGCAAGGAAGCATCGTTGCATATATTCTGGCAACGTTCTAACTTTTTCAAGATTCCAGCTGTAATTATTTATTAAATCAAT
ATTTTTAGCTTAGAAATGTGGCAAATTAATTACTAGATTATAATGTTGGTTTGTTTTTTAATTTCTTTTTTACCTTTCTATTGCATTGGTGAATAACTCGAGCTCTTCTAGTGTCCCATAAATATC
ATAAATATCATCTGTTGTCACAGCAAGGGCCATCAGCTTTGTCATAATAACTCTAGCATGTGAATAGCAGTGTTCACAGTAGGTTGTAAGCATCCAGAAGTAGGATTCTACTATTTGATCAC
GTATAAATGGTAAGTCTTTGAACAAGCCTAACTCTTTCCACCACTCAGATATGATTCGAAGCTCCTCTTGATAAGTAGCTTGCAATATATTGAAATCAAATTTTGCAAAATCAAGTAGTTTTT
GATTGCACAATTTGTACTTGTCATAAATTTCTATGTAATTTCTAGCCTTGAGCCGATCAGGTGTTCTGACCAATGGCAATTCCAAAGCACTTGTCACCATCAAAGCAAAAGATGGCTCCATC
TTGCAGATCAATGACTTCAAGTGAAACCTTGAAAAAATTTTAGCTTTCTCTAGTATTTCTTCATCAGGAAATCCAAGATAAGCAGATTCATATAAGCTTAGTAAAGCTTTCACATTGTTGCAT
AAGCAAGCCATGAAATCTCCTTTATCATCCAAAAACCTATTGAATACATCAGAACTTATGTTATATCTTTGCTGTCGTAATAGTCGAAAACGAAGTGCAACTACATATAGATCATCATCAATG
ATGTTGATCTTATGAACTCTACGTAATATGTCGTTGATTTCCTTCTCAAAATGATATGCCACTCCAAGACGTTGAATTGAATCAATCAGCTCAAGTGATTTTAATGGATCAATAGCATTTATAA
GAATTTTCTTCATCTCTACTCTGAGCTCTTCATTCTTCTCTTTTTGCTCTGCCAGATTGGTATTTGATTGTAATGGTGAATAGTTGATGAAATAATCTCCCCAAACAGTTGGGTGAAAATCTG
CAGATTGGCGAGAAGTTGTCAGAGGTACAGGAAGAGCCTCCATTAAAGTCACTCTACTTAGAAAGGTAGAGATGCTTAGAGAAGCTAATTTTTATGGCTAAATGTATTGATGGTGTGAAC
AATTTATAATAGCAAGGGCGAGATGGAGCGATGGAAAGGCCAAAATTTAAGAAATAAAAGGACAGATATTTTCATTAAATGCAGACCAAATTATTTGTTTATTCTCCACGACAATAATTGAT
GGTAGATCCAAGCAATAAACACGAAATAAACATGAAGTATTATCCTATTAAAATAAGATAGTTTTTATGACTTTAGCTCTCATCAATATTTTTTAATGACCTTCCAATGCCACAACGTAGGAA
AATTTGACTCTTAGGCTTGCTC
>comp164701_c3_seq2
GAAATCTTGCAAGTTTTATATGTTGTCTTCTAAACCTTTAATTAAAATATGCTTTTGAGGAAGTTTTAGCTCAAATTATCCTCAGAAACCATACATATTCTTTATTAAAACTTAAAAATCACAT
TTAATTTGACAATGATGAAGTAAAAGACTAATACAAAAAAGCATTCAAGCAGTTTAGAGAGACACGCACGATTCCAACAATGCACTTGAAATATTATTTGTAGGTAGTCACACAGATTTCC
CATTATGTAAGAAAAGTTTCAATCGTTCCTATAAATCAAAATCGGATCAACGAGCACTTCAATGATACTTTTCTTCATGAACTCTGAGGAGTCGGTATATATGTCACGTGTGTCCTTGTAGTA
AAAATCATAGATACGTGACAAGTTTAAGATTGGCCTAATTAATGGCAATGGGATGTTCTTTGTTGACACAAATTCTCTATTTATAATCATGCAATGTTCTTCCACCATCTCCTTAAGCTTCTTG
CAAGCTTCCACTTCAGAGCAACCATACTCTTTGATATAACATTGAACTGATGAAGCAAGATGATTCCTTTTTTGTTCATTTTGATATGAACCAATATCATTCATAAGCCTCCCAATTGCACAA
AAAGATTTGATAATCTCAGAAGGGCTGATAACCCAGTCAAAAATATCCTTTGAGATAAATTCCTTCATTCCAACATATGAAGCACACGTGATAAATATGCACCCAGAAGTAATAAATGTTAC
TTTAACATATTCCTCAAGCTTAGGAATATAGCACTCGCTCGCCCATTTGGTTTCTTGAAGGTACGCATGAGCCATTCGCTTCAACTCATCTCTAAGGTAATGAACTCGAAAAGAATTGTGCT
CAGGAGCTAACTCATCCTCGATATCCTTGAAGGTATTGTAAATCGCAAGGAAGCATCGTTGCATATATTCTGGCAACGTTCTAACTTTTTCAAGATTCCAGCTTTCTATTGCATTGGTGAATA
ACTCGAGCTCTTCTAGTGTCCCATAAATATCATAAATATCATCTGTTGTCACAGCAAGGGCCATCAGCTTTGTCATAATAACTCTAGCATGTGAATAGCAGTGTTCACAGTAGGTTGTAAGC
ATCCAGAAGTAGGATTCTACTATTTGATCACGTATAAATGGTAAGTCTTTGAACAAGCCTAACTCTTTCCACCACTCAGATATGATTCGAAGCTCCTCTTGATAAGTAGCTTGCAATATATTG
AAATCAAATTTTGCAAAATCAAGTAGTTTTTGATTGCACAATTTGTACTTGTCATAAATTTCTATGTAATTTCTAGCCTTGAGCCGATCAGGTGTTCTGACCAATGGCAATTCCAAAGCACTT
GTCACCATCAAAGCAAAAGATGGCTCCATCTTGCAGATCAATGACTTCAAGTGAAACCTTGAAAAAATTTTAGCTTTCTCTAGTATTTCTTCATCAGGAAATCCAAGATAAGCAGATTCAT
ATAAGCTTAGTAAAGCTTTCACATTGTTGCATAAGCAAGCCATGAAATCTCCTTTATCATCCAAAAACCTATTGAATACATCAGAACTTATGTTATATCTTTGCTGTCGTAATAGTCGAAAAC

GAAGTGCAACTACATATAGATCATCATCAATGATGTTGATCTTATGAACTCTACGTAATATGTCGTTGATTTCCTTCTCAAAATGATATGCCACTCCAAGACGTTGAATTGAATCAATCAGCT
CAAGTGATTTTAATGGATCAATAGCATTTATAAGAATTTTCTTCATCTCTACTCTGAGCTCTTCATTCTTCTCTTTTTGCTCTGCCAGATTGGTATTTGATTGTAATGGTGAATAGTTGATGAAA
TAATCTCCCCAAACAGTTGGGTGAAAATCTGCAGATTGGCGAGAAGTTGTCAGAGGTACAGGAAGAGCCTCCATTAAAGTCACTCTACTTAGAAAGGTAGAGATGCTTAGAGAAGCTAA
TTTTTATGGCTAAATGTATTGATGGTGTGAACAATTTATAATAGCAAGGGCGAGATGGAGCGATGGAAAGGCCAAAATTTAAGAAATAAAAGGACAGATATTTTCATTAAATGCAGACCAA
ATTATTTGTTTATTCTCCACGACAATAATTGATGGTAGATCCAAGCAATAAACACGAAATAAACATGAAGTATTATCCTATTAAAATAAGATAGTTTTTATGACTTTAGCTCTCATCAATATTTT
TTAATGACCTTCCAATGCCACAACGTAGGAAAATTTGACTCTTAGGCTTGCTC
>comp164701_c3_seq6
TTTTTGTTCATTCTGAAAGATCAAATACAATAAAATTACTGATGAATACAATATATGATCAACATGAAAATTTAATGAATTTTTGAATTTTTTTTTGACAAAATTCCTTTTAGCTATACATTTTT
AAAATATCACAAATGGTATCAATCAATATGTCTCATAAGTTTATCATTTTTCAAAACAAAATTTCCAACTATAAAAGATAAAATATATCCTTAATATCAAATCCATAAAACTTACTTGATATGA
ACCAATATCATTCATAAGCCTCCCAATTGCACAAAAAGATTTGATAATCTCAGAAGGGCTGATAACCCAGTCAAAAATATCCTTTGAGATAAATTCCTTCATTCCAACATATGAAGCACACG
TGATAAATATGCACCCAGAAGTAATAAATGTTACTTTAACATATTCCTCAAGCTTAGGAATATAGCACTCGCTCGCCCATTTGGTTTCTTGAAGGTACGCATGAGCCATTCGCTTCAACTGG
AATTCAAATGAATAATCAAAACAAAGGCAACAAGATATAGATTAGTGAATAATGATGTTAATCTTATTGGTTTAATTATATGATCAAACTAGAAACAAAACTATTTTTAAAATATTAAATATTA
ATACATATCTACTTTCAATAATATATGCATATATAACCACAATTAATTTATTACCTCATCTCTAAGGTA
>comp164708_c0_seq10
TACATCCGATAACCGCACCTAATAATATTCCCGATCAAATGGCAGGCAACAACTTCAAAATTTGTACCAAGGCTCAAATAAAAAAGCTAAGGCTACTGCATATGGGAATCAAAACAAACAT
AACAGGTTTCCTTCAATAGCGCAGATACTCAAAGCTTAAAACAGTAAGCACAGGTTAGGTAGTTAAATAAATTTATGAAATAAAAATATTGACCATTTGTTCTGAGACAATGGCATCAACA
AAGTCAATCTCCAATGACAGATAATTAGTACCCAGATTAGATTTCTGCTCTATTCACAGATAGGAAGTATTGCAGATGTTTATATAAAGCCGTCCAGAAATTACATAATAAATAATAAGCAAA
CGATATGAAAAACTAATAGTTCCAACTGAATGAGTTTCTCAAGCACAGTTCTAAGCTTTCGTCATCTGGCCGACCTGAGTATTCATATAATTCATGCCTTCAGTAAAAGAGCCTTGCTGAAG
CATCTTCATATCCCCTTGCATGCTTGAACCAGATAGGTTTCCACCTTGAAGACCACCTGCATCTCTCTTACCAGAGCTCTCTGCTCCCTTAGCAGAACCCTTGCCATCTCCCTCTATCTGCCA
TGGCGTCGCATCCATTCCACAGAACCACCAGATAAACAAAAAAAGCCAACAAAAATCGCAATCAGATTAACGATTACAAAAGGGCAAAAGAAGAAGAATGACCTATCACCCCGCCAAC
CACCAATTCCGATCTATCATTCTGAAAAGAAGTAAAAGCTCACATAAATGGTCACAGAGTAATCACCTCTTTATACTTCTGCGAATACAACCTATGCGGTTTAATGTAGTCTTCAAACCGGT
CTAGTGTTGTCATTGCACACAGTACATCGTCCTCAGTAATTTTCTTCCTCTTTTCCCTCAGGCACTTATCTCTAGCCTCGCTGGTGATGAAACTGATGAACTCGAAAACGCACTCCTGCATG
GTCTCCTTCGCGTCCTTGGCAATCTTTCCTTTGGGACGGAGCGCCTTCTTCATGATGCGGCTGATGAAGGCGATGGGAAGGAGCCTATCCAGCTCACGGACATTGGATCTCGGGCTCTGG
TCGCCACTCTCGTGGCTTCCGCCGCAAGGGCTTGCTGGCACCTCTGCCGCCATAGGCGGTACCCTAGATCCGCGACGGCACGAGAACGAATCATAAACGTGAACGCTCGTCTGCGTAGC
GGAACCCTAGACAATCGATCGCTTAGTTCAAAAATCGTACCTCGCAAAACAATAGAGCGCGCGAACGAGGCTGAG
>comp164708_c0_seq11
TACATCCGATAACCGCACCTAATAATATTCCCGATCAAATGGCAGGCAACAACTTCAAAATTTGTACCAAGGCTCAAATAAAAAAGCTAAGGCTACTGCATATGGGAATCAAAACAAACAT
AACAGGTTTCCTTCAATAGCGCAGATACTCAAAGCTTAAAACAGTAAGCACAGGTTAGGTAGTTAAATAAATTTATGAAATAAAAATATTGACCATTTGTTCTGAGACAATGGCATCAACA
AAGTCAATCTCCAATGACAGATAATTAGTACCCAGATTAGATTTCTGCTCTATTCACAGATAGGAAGTATTGCAGATGTTTATATAAAGCCGTCCAGAAATTACATAATAAATAATAAGCAAA
CGATATGAAAAACTAATAGTTCCAACTGAATGAGTTTCTCAAGCACAGTTCTAAGCTTTCGTCATCTGGCCGACCTGAGTATTCATATAATTCATGCCTTCAGTAAAAGAGCCTTGCTGAAG
CATCTTCATATCCCCTTGCATGCTTGAACCAGATAGGTTTCCACCTTGAAGACCACCTGCATCTCTCTTACCAGAGCTCTCTGCTCCCTTAGCAGAACCCTTGCCATCTCCCTCTATCTCTTT

ATACTTCTGCGAATACAACCTATGCGGTTTAATGTAGTCTTCAAACCGGTCTAGTGTTGTCATTGCACACAGTACATCGTCCTCAGTAATTTTCTTCCTCTTTTCCCTCAGGCACTTATCTCT
AGCCTCGCTGGTGATGAAACTGATGAACTCGAAAACGCACTCCTGCATGGTCTCCTTCGCGTCCTTGGCAATCTTTCCTTTGGGACGGAGCGCCTTCTTCATGATGCGGCTGATGAAGGC
GATGGGAAGGAGCCTATCCAGCTCACGGACATTGGATCTCGGGCTCTGGTCGCCACTCTCGTGGCTTCCGCCGCAAGGGCTTGCTGGCACCTCTGCCGCCATAGGCGGTACCCTAGATCC
GCGACGGCACGAGAACGAATCATAAACGTGAACGCTCGTCTGCGTAGCGGAACCCTAGACAATCGATCGCTTAGTTCAAAAATCGTACCTCGCAAAACAATAGAGCGCGCGAACGAGG
CTGAGGGGGAGTTCTAAAACTCGTGATCTTCCCTTTTTCGG
>comp164708_c0_seq12
TACATCCGATAACCGCACCTAATAATATTCCCGATCAAATGGCAGGCAACAACTTCAAAATTTGTACCAAGGCTCAAATAAAAAAGCTAAGGCTACTGCATATGGGAATCAAAACAAACAT
AACAGGTTTCCTTCAATAGCGCAGATACTCAAAGCTTAAAACAGTAAGCACAGGTTAGGTAGTTAAATAAATTTATGAAATAAAAATATTGACCATTTGTTCTGAGACAATGGCATCAACA
AAGTCAATCTCCAATGACAGATAATTAGTACCCAGATTAGATTTCTGCTCTATTCACAGATAGGAAGTATTGCAGATGTTTATATAAAGCCGTCCAGAAATTACATAATAAATAATAAGCAAA
CGATATGAAAAACTAATAGTTCCAACTGAATGAGTTTCTCAAGCACAGTTCTAAGCTTTCGTCATCTGGCCGACCTGAGTATTCATATAATTCATGCCTTCAGTAAAAGAGCCTTGCTGAAG
CATCTTCATATCCCCTTGCATGCTTGAACCAGATAGGTTTCCACCTTGAAGACCACCTGCATCTCTCTTACCAGAGCTCTCTGCTCCCTTAGCAGAACCCTTGCCATCTCCCTGCCAAAATT
AGAGCAAAATTAGTACTGGAGAACTGCAAGAATGAGCATCTTAAACGTTAAACGTTTGTCATCGGGCTATCTTTAACAACTAAGGCAGCATGTATGTAGTGCTTAAATTTTAAATTGGCCA
TGTAAAATATCACATAAAAATTGATGCGCCTTTTCGACATGTAAGCAGTACATTACTTTTACATTAAACATGTAAGCAACCACATCTAGAAATGCACAATGACCAATAAAATCATAAAACTAT
AGCATTAATTCTACTTTAATAGAACACTGCCAGGGCCCGGATTATATACCATAATTTTCCTTTCGGTGGAAACCCATAAATATTAACTCCTTAACACGCAAATGGGAGACAGTATAGTGCCAT
AAGGCATCACAGTATAAATAATAGAACTGCTATTGAAGAAAATAAAAAAAACCGATTACAGAGGGGAGAGATTAGTTTGATTACCTCTATCTGCCATGGCGTCGCATCCATTCCACAGAAC
CACCAGATAAACAAAAAAAGCCAACAAAAATCGCAATCAGATTAACGATTACAAAAGGGCAAAAGAAGAAGAATGACCTATCACCCCGCCAACCACCAATTCCGATCTATCATTCTGAA
AAGAAGTAAAAGCTCACATAAATGGTCACAGAGTAATCACCTCTTTATACTTCTGCGAATACAACCTATGCGGTTTAATGTAGTCTTCAAACCGGTCTAGTGTTGTCATTGCACACAGTACA
TCGTCCTCAGTAATTTTCTTCCTCTTTTCCCTCAGGCACTTATCTCTAGCCTCGCTGGTGATGAAACTGATGAACTCGAAAACGCACTCCTGCATGGTCTCCTTCGCGTCCTTGGCAATCTT
TCCTTTGGGACGGAGCGCCTTCTTCATGATGCGGCTGATGAAGGCGATGGGAAGGAGCCTATCCAGCTCACGGACATTGGATCTCGGGCTCTGGTCGCCACTCTCGTGGCTTCCGCCGCA
AGGGCTTGCTGGCACCTCTGCCGCCATAGGCGGTACCCTAGATCCGCGACGGCACGAGAACGAATCATAAACGTGAACGCTCGTCTGCGTAGCGGAACCCTAGACAATCGATCGCTTAG
TTCAAAAATCGTACCTCGCAAAACAATAGAGCGCGCGAACGAGGCTGAGGGGGAGTTCTAAAACTCGTGATCTTCCCTTTTTCGG
>comp164708_c0_seq13
TACATCCGATAACCGCACCTAATAATATTCCCGATCAAATGGCAGGCAACAACTTCAAAATTTGTACCAAGGCTCAAATAAAAAAGCTAAGGCTACTGCATATGGGAATCAAAACAAACAT
AACAGGTTTCCTTCAATAGCGCAGATACTCAAAGCTTAAAACAGTAAGCACAGGTTAGGTAGTTAAATAAATTTATGAAATAAAAATATTGACCATTTGTTCTGAGACAATGGCATCAACA
AAGTCAATCTCCAATGACAGATAATTAGTACCCAGATTAGATTTCTGCTCTATTCACAGATAGGAAGTATTGCAGATGTTTATATAAAGCCGTCCAGAAATTACATAATAAATAATAAGCAAA
CGATATGAAAAACTAATAGTTCCAACTGAATGAGTTTCTCAAGCACAGTTCTAAGCTTTCGTCATCTGGCCGACCTGAGTATTCATATAATTCATGCCTTCAGTAAAAGAGCCTTGCTGAAG
CATCTTCATATCCCCTTGCATGCTTGAACCAGATAGGTTTCCACCTTGAAGACCACCTGCATCTCTCTTACCAGAGCTCTCTGCTCCCTTAGCAGAACCCTTGCCATCTCCCTGCCAAAATT
AGAGCAAAATTAGTACTGGAGAACTGCAAGAATGAGCATCTTAAACGTTAAACGTTTGTCATCGGGCTATCTTTAACAACTAAGGCAGCATGTATGTAGTGCTTAAATTTTAAATTGGCCA
TGTAAAATATCACATAAAAATTGATGCGCCTTTTCGACATGTAAGCAGTACATTACTTTTACATTAAACATGTAAGCAACCACATCTAGAAATGCACAATGACCAATAAAATCATAAAACTAT
AGCATTAATTCTACTTTAATAGAACACTGCCAGGGCCCGGATTATATACCATAATTTTCCTTTCGGTGGAAACCCATAAATATTAACTCCTTAACACGCAAATGGGAGACAGTATAGTGCCAT
AAGGCATCACAGTATAAATAATTGAACCGTTCTCGAAGAAAATAAACAAACCGATTACAGGGGGAGTGGTTAGATTGATTACCTCTGAAAAGAACTAAAAGCTCACAGAAATGGTCACA

GAGTAATCACCTCCCTATACTTCTTCAAATACAACTTAAGCGGTTCAATGTAGTCGTCAAACCCTAGTGTCACCATTGCCAACAGTATATCGTCCCCATTAATTGTCTTCCTCTTCTCCCTCA
GGCACTTTTCGCTAGCCTCGCTGGTGATGAAAGCGATGAACTCGGAAACGCACTCCTGCACGCTATCCTTCGCGTACTTGGCAACCTTTCCATTGTGAGGGACAGCCTTCCTCATGATGC
GGCTGATGTTGGCCATGGGAAGGAGCCTATCCTGCTCACGGGCAAAGGATCTCGGACTCTCGTCGTCACTCTCGTGGGTTCCGCCGCAATGGCTTGCTGGCATCTCCTCCGCCATAGGCG
GTACCCTCGCAAAACAATAGAGCGCGCGAACGAGGCTGAGTGGGAGTTCTATAACTCGGGATCTTCCCCTTTTCGGTTGCTTCGCGTTGGGGAGAGCAGGTGGGCGATCGGACGCTGGA
GGCTGGAGAATCGAGGAAGGAGGAGACGACTGGG
>comp164708_c0_seq15
TCAAAGCTTAAAACAGTAAGCACAGGTTAGGTAGTTAAATAAATTTATGAAATAAAAATATTGACCAATGGCCTCATCAAAGTCACTCTTCAAAGACAAGATAATTCGTACCCAGATTAGA
TTTCTGCTCTATTCACAGATAGGAAGTATTGCAGATGTTTATATAAATCCGTCCAGAAATTACATATTAAAAAATAAGCAAACGAAATAAAAAACTAATAGTTCCAACTGAATGAGTTTCTC
AAGCACAGTTCTAAGCTTTCGTCCTCTTGTTGACCTGAACCAGAAGCATCTACTAAAAGATGCAGAAGGCCCATTTAGCTGGAGAGGTCTCCAGGATGATACTGAGTATTCATATAATTCA
TGCCTTCAGTAAAAGAGCCTTGCTGAAGCATCTTCATATCCCCTTGCATGCTTGAACCAGATAGGTTTCCACCTTGAAGACCACCTGCATCTCTCTTACCAGAGCTCTCTGCTCCCTTAGC
AGAACCCTTGCCATCTCCCTCTATCTGCCATGGCGTCGCATCCATTCCACAGAACCACCAGATAAACAAAAAAAGCCAACAAAAATCGCAATCAGATTAACGATTACAAAAGGGCAAAA
GAAGAAGAATGACCTATCACCCCGCCAACCACCAATTCCGATCTATCATTCTGAAAAGAAGTAAAAGCTCACATAAATGGTCACAGAGTAATCACCTCTTTATACTTCTGCGAATACAACC
TATGCGGTTTAATGTAGTCTTCAAACCGGTCTAGTGTTGTCATTGCACACAGTACATCGTCCTCAGTAATTTTCTTCCTCTTTTCCCTCAGGCACTTATCTCTAGCCTCGCTGGTGATGAAAC
TGATGAACTCGAAAACGCACTCCTGCATGGTCTCCTTCGCGTCCTTGGCAATCTTTCCTTTGGGACGGAGCGCCTTCTTCATGATGCGGCTGATGAAGGCGATGGGAAGGAGCCTATCCA
GCTCACGGACATTGGATCTCGGGCTCTGGTCGCCACTCTCGTGGCTTCCGCCGCAAGGGCTTGCTGGCACCTCTGCCGCCATAGGCGGTACCCTAGATCCGCGACGGCACGAGAACGAA
TCATAAACGTGAACGCTCGTCTGCGTAGCGGAACCCTAGACAATCGATCGCTTAGTTCAAAAATCGTACCTCGCAAAACAATAGAGCGCGCGAACGAGGCTGAGGGGGAGTTCTAAAAC
TCGTGATCTTCCCTTTTTCGG
>comp164708_c0_seq16
TCAAAGCTTAAAACAGTAAGCACAGGTTAGGTAGTTAAATAAATTTATGAAATAAAAATATTGACCAATGGCCTCATCAAAGTCACTCTTCAAAGACAAGATAATTCGTACCCAGATTAGA
TTTCTGCTCTATTCACAGATAGGAAGTATTGCAGATGTTTATATAAATCCGTCCAGAAATTACATATTAAAAAATAAGCAAACGAAATAAAAAACTAATAGTTCCAACTGAATGAGTTTCTC
AAGCACAGTTCTAAGCTTTCGTCCTCTTGTTGACCTGAACCAGAAGCATCTACTAAAAGATGCAGAAGGCCCATTTAGCTGGAGAGGTCTCCAGGATGATACTGAGTATTCATATAATTCA
TGCCTTCAGTAAAAGAGCCTTGCTGAAGCATCTTCATATCCCCTTGCATGCTTGAACCAGATAGGTTTCCACCTTGAAGACCACCTGCATCTCTCTTACCAGAGCTCTCTGCTCCCTTAGC
AGAACCCTTGCCATCTCCCTGCCAAAATTAGAGCAAAATTAGTACTGGAGAACTGCAAGAATGAGCATCTTAAACGTTAAACGTTTGTCATCGGGCTATCTTTAACAACTAAGGCAGCAT
GTATGTAGTGCTTAAATTTTAAATTGGCCATGTAAAATATCACATAAAAATTGATGCGCCTTTTCGACATGTAAGCAGTACATTACTTTTACATTAAACATGTAAGCAACCACATCTAGAAAT
GCACAATGACCAATAAAATCATAAAACTATAGCATTAATTCTACTTTAATAGAACACTGCCAGGGCCCGGATTATATACCATAATTTTCCTTTCGGTGGAAACCCATAAATATTAACTCCTTA
ACACGCAAATGGGAGACAGTATAGTGCCATAAGGCATCACAGTATAAATAATAGAACTGCTATTGAAGAAAATAAAAAAAACCGATTACAGAGGGGAGAGATTAGTTTGATTACCTCTATC
TGCCATGGCGTCGCATCCATTCCACAGAACCACCAGATAAACAAAAAAAGCCAACAAAAATCGCAATCAGATTAACGATTACAAAAGGGCAAAAGAAGAAGAATGACCTATCACCCCG
CCAACCACCAATTCCGATCTATCATTCTGAAAAGAAGTAAAAGCTCACATAAATGGTCACAGAGTAATCACCTCTTTATACTTCTGCGAATACAACCTATGCGGTTTAATGTAGTCTTCAAA
CCGGTCTAGTGTTGTCATTGCACACAGTACATCGTCCTCAGTAATTTTCTTCCTCTTTTCCCTCAGGCACTTATCTCTAGCCTCGCTGGTGATGAAACTGATGAACTCGAAAACGCACTCCT
GCATGGTCTCCTTCGCGTCCTTGGCAATCTTTCCTTTGGGACGGAGCGCCTTCTTCATGATGCGGCTGATGAAGGCGATGGGAAGGAGCCTATCCAGCTCACGGACATTGGATCTCGGGC
TCTGGTCGCCACTCTCGTGGCTTCCGCCGCAAGGGCTTGCTGGCACCTCTGCCGCCATAGGCGGTACCCTAGATCCGCGACGGCACGAGAACGAATCATAAACGTGAACGCTCGTCTGC

GTAGCGGAACCCTAGACAATCGATCGCTTAGTTCAAAAATCGTACCTCGCAAAACAATAGAGCGCGCGAACGAGGCTGAGGGGGAGTTCTAAAACTCGTGATCTTCCCTTTTTCGG
>comp164708_c0_seq2
TCAAAGCTTAAAACAGTAAGCACAGGTTAGGTAGTTAAATAAATTTATGAAATAAAAATATTGACCAATGGCCTCATCAAAGTCACTCTTCAAAGACAAGATAATTCGTACCCAGATTAGA
TTTCTGCTCTATTCACAGATAGGAAGTATTGCAGATGTTTATATAAATCCGTCCAGAAATTACATATTAAAAAATAAGCAAACGAAATAAAAAACTAATAGTTCCAACTGAATGAGTTTCTC
AAGCACAGTTCTAAGCTTTCGTCCTCTTGTTGACCTGAACCAGAAGCATCTACTAAAAGATGCAGAAGGCCCATTTAGCTGGAGAGGTCTCCAGGATGATACTGAGTATTCATATAATTCA
TGCCTTCAGTAAAAGAGCCTTGCTGAAGCATCTTCATATCCCCTTGCATGCTTGAACCAGATAGGTTTCCACCTTGAAGACCACCTGCATCTCTCTTACCAGAGCTCTCTGCTCCCTTAGC
AGAACCCTTGCCATCTCCCTCTATCTCTTTATACTTCTGCGAATACAACCTATGCGGTTTAATGTAGTCTTCAAACCGGTCTAGTGTTGTCATTGCACACAGTACATCGTCCTCAGTAATTTT
CTTCCTCTTTTCCCTCAGGCACTTATCTCTAGCCTCGCTGGTGATGAAACTGATGAACTCGAAAACGCACTCCTGCATGGTCTCCTTCGCGTCCTTGGCAATCTTTCCTTTGGGACGGAGC
GCCTTCTTCATGATGCGGCTGATGAAGGCGATGGGAAGGAGCCTATCCAGCTCACGGACATTGGATCTCGGGCTCTGGTCGCCACTCTCGTGGCTTCCGCCGCAAGGGCTTGCTGGCACC
TCTGCCGCCATAGGCGGTACCCTAGATCCGCGACGGCACGAGAACGAATCATAAACGTGAACGCTCGTCTGCGTAGCGGAACCCTAGACAATCGATCGCTTAGTTCAAAAATCGTACCTC
GCAAAACAATAGAGCGCGCGAACGAGGCTGAGGGGGAGTTCTAAAACTCGTGATCTTCCCTTTTTCGG
>comp164722_c0_seq10
GGGCTTGCTTTTGTGGTGGTTTGTGCTATATTAATCTAAATGATGCGTAATATTAGAGCATAAACATCTAATAAACTCAAACTGCTGTGATTGAAATCAGCTCAATCAATTAGTTTATAGAAG
TATGATCAGCTTCTACATATTTATTTCATTTGCAGGAAGGTTCTCTTAAGGCACCACCCCAAACAACTACTACAAAGTTATCTTACCAATAATAGAAAAGAATCAAAGGATCAAGTCTATCA
CCATGCGGGGTTTCATGATTTATTAAGCTTTAAAGTTTTATTCCAATTCCAAATGCCAATATGAAACTATGCTAACCAGACAGCTTTTTCCTCATAGACAAGCTCTCTTTCCACTGCTTTCCT
CTTGGCCTGTGTTATTCCTCTCCTCGGTTTCCACCAAGTGCCTGTTGAATGTTGCAACTTCTGATCCATTGTTGATGCTACTTCGTGCATTTTCCCTGGAAATGTTGCAAGTAGATGATGGA
GTGAATACCTTCCTGTTGAGGTCGATCTGCAGTCCAGGAGCTTCCTCATTTCCTGAATTTGTGGTCCAGTTGCCATCCAAATGCAGTGGGGAGAAACAGTGCGGCAGCAACTGCCGATAA
AATAGCTCTTCAAAAGCAATCGAGTCCTGACAATTGTGTAGAATAAATTAAAATAGAGCACAAATCACTTGCAAGCATGTACTGAATCACAATTTTAGTTTTATTAGATGACAACCTGTTTG
AGACCTTCAGTACTAGATTTGCTAATCCCCGGAGTATCCCCAAACTCCTGAATAATGGACGGGCCTTGTTCACCTTGTTCATTGATTCCTTTGGCTTCTTCCTTGTTCAAAGCTGCATCTTTC
TCTACTTCACTACTGGGCGATGTGTTAGAATCACAGGAGGACGGATCATGTTTCTTTTTGCCTGGAACATGATCAACATCATGGATTCCACAAATTGCTGTTTCTGCGCACTTGGTAGATTT
TGGAGACCTTGCAGATTTTGTGCTTTGGCAGGTATCTGGTGATGAAGAGATCTCTAATAATGCAGAGGAAGGTTGTATCGCTATGTTTGTCTCCTGCTGTTCGATTTGTTGAAATTTCCGCT
TATGAGTTTGGCTAAAATTTCCTTTAGAGTCACTGTTATCTTCAGGAACTTGGGCCGACTCTGTGGCTTGCACCATTACTTGGGTAAATGCGTGGTTTGGCAGAGAAGGAGATTGCAATAG
CCCATGAACCCTCCACCAAGCAGCTGCAGCGGCAATTGTGGCTGTTACAATAGCCGATATGCCAGAAGATGCGCTAGATTGTTTCTCTGGAGCTTCGCCAACTAACTTGCTATTTGATTGC
ATAGAAAAATCCACATAGGCAGGTGGCAGTGAAGCAGCTGCAATGCTAGCTGCTTGATGAACTGCTGGATTTTGTGACAAGGCAGATACAAGGAGACTTGGAAAGTAGTTGGTTAAGTT
TATAAGTGCACTAAATGGATTGTCATCATTACTGAACGGGTTATGAAGTGAGATGGATGGAACCGCCTGATGGATGGATGAAATTGAGTTTTTTGTCCGATGTATTGTTGTTCTTGTAGAAT
CAAGTGACAGGTTAAGATCTGCATTTGCTTCTAGTTGAGTTGACACTGATTTTGAGATATCTTCATCAGAATATTTTGTACCTACAAATTGAGCAGGAATATTTTCATGGTTTTCACCACCTT
GTAGATTGTCCTTCCATAATGTAAATGGCATCTCCATATGGCCTAGTACTTGCCATTTCTCTTCAGTAGTGAAACCAAACCGAGAATCTGTACTCATTCCAAAAATTTGTAAATCAGAATCAT
TTTTTCTCAGTTCTTTTGTTATTCTACTTCTAGTTTCTTTCAACTCTTCCAATGTTGGCATCTGTTCTAAAGGTTCTGAAGTGCTATTAAGATGAAAATAGATAGAAGCGGATGAAGTGCCTT
CTCCAAATGCCTGCTTTGTATTTTGTTCAATTGCAACTTTCACTGGTTCCTTAATCTTAGTATCCATTGCCACTTCATCAGTTCCTAGCGAAACAAAAGCCTGTGACAAATTTTTCTCTTTTA
CTTCCACAAATGGACTTTGTGAGGCTGCACCGGTCTTCCGAGGATATGAACTACATGGTTTTCGTTTAGGCCTTGGGGGAGGAATTTCTATGTCATGAGGATGTCCAGGTGACATGCCTCC
AGTCCCATTCTCCAACTTTAAAAAAAACTTCTGCGCATGACTTCTTATCTGAACAGCAGTCTTTGTACCGATATGCTCTTGAATACGCTGCCATGCCCTGCCATGCAGCCTGAGCGCTTCCA

AGAACCTGTTATGCTCCTCGTCTGTCCACTTCTCTCGCTGCTTCGTAATCGTGTAAGGTTTTCTCATCTTAGCCAGCTCCCTCACTGGGCTCATTCCCATTACTGATTCCGAAATGTAAATGA
ACCACGGATGAGACAAACGGTTTCAAATCAACCCTAACTCATATAAGTAAGAAACGGTTAAATTCTTCATGCATGTTCCCAACTGAAATGTCGGGCACCTAATGCATTCACTGATATGCAG
GGAGTAACAGGACAACAAACAAGCCCAAAAGAAAAAAGAAAGGAAAACGCCAAGAAGAGGCAGCGGTCACTCACCAAAGAAACCTTAGAAACAGGAAAGAAACTCCGGCGGTCGC
AGCCTCATGTCTATCTCTCTATTCCTCTCCTCCCTTGATCTCCTTCGCCTCACATTAGCACCCAGTGCAATCCTATCTACAATCGCCCATCTTGCCATCGTGGGTTTATGTACAAGTAGAAACA
TGGAGGCTGTTATGGCCGGCGAAGGGAGCTGTCGATTCGGCATGCCTAACTCCAGAACCCTCGCTCTCTCTCCCTCTCTCCTCGCAGCTCCACCCTAACTCTCTCTACAGTAAGGTCGCA
AACCATAAGAGTAGGCCAGGACAGGAGGAATCTGAGCCACAACTTTTTGGCATGGAGATTTCGGGCGAATGGTGAGCGAACTGGCGAAGCAGAGCGAGATTCGATGATCAATTCT
>comp164722_c0_seq5
GGGCTTGCTTTTGTGGTGGTTTGTGCTATATTAATCTAAATGATGCGTAATATTAGAGCATAAACATCTAATAAACTCAAACTGCTGTGATTGAAATCAGCTCAATCAATTAGTTTATAGAAG
TATGATCAGCTTCTACATATTTATTTCATTTGCAGGAAGGTTCTCTTAAGGCACCACCCCAAACAACTACTACAAAGTTATCTTACCAATAATAGAAAAGAATCAAAGGATCAAGTCTATCA
CCATGCGGGGTTTCATGATTTATTAAGCTTTAAAGTTTTATTCCAATTCCAAATGCCAATATGAAACTATGCTAACCAGACAGCTTTTTCCTCATAGACAAGCTCTCTTTCCACTGCTTTCCT
CCTCGGTTTCCACCAAGTGCCTGTTGAATGTTGCAACTTCTGATCCATTGTTGATGCTACTTCGTGCATTTTCCCTGGAAATGTTGCAAGTAGATGATGGAGTGAATACCTTCCTGTTGAGG
TCGATCTGCAGTCCAGGAGCTTCCTCATTTCCTGAATTTGTGGTCCAGTTGCCATCCAAATGCAGTGGGGAGAAACAGTGCGGCAGCAACTGCCGATAAAATAGCTCTTCAAAAGCAATC
GAGTCCTGTTTGAGACCTTCAGTACTAGATTTGCTAATCCCCGGAGTATCCCCAAACTCCTGAATAATGGACGGGCCTTGTTCACCTTGTTCATTGATTCCTTTGGCTTCTTCCTTGTTCAA
AGCTGCATCTTTCTCTACTTCACTACTGGGCGATGTGTTAGAATCACAGGAGGACGGATCATGTTTCTTTTTGCCTGGAACATGATCAACATCATGGATTCCACAAATTGCTGTTTCTGCGC
ACTTGGTAGATTTTGGAGACCTTGCAGATTTTGTGCTTTGGCAGGTATCTGGTGATGAAGAGATCTCTAATAATGCAGAGGAAGGTTGTATCGCTATGTTTGTCTCCTGCTGTTCGATTTGT
TGAAATTTCCGCTTATGAGTTTGGCTAAAATTTCCTTTAGAGTCACTGTTATCTTCAGGAACTTGGGCCGACTCTGTGGCTTGCACCATTACTTGGGTAAATGCGTGGTTTGGCAGAGAAG
GAGATTGCAATAGCCCATGAACCCTCCACCAAGCAGCTGCAGCGGCAATTGTGGCTGTTACAATAGCCGATATGCCAGAAGATGCGCTAGATTGTTTCTCTGGAGCTTCGCCAACTAACTT
GCTATTTGATTGCATAGAAAAATCCACATAGGCAGGTGGCAGTGAAGCAGCTGCAATGCTAGCTGCTTGATGAACTGCTGGATTTTGTGACAAGGCAGATACAAGGAGACTTGGAAAGTA
GTTGGTTAAGTTTATAAGTGCACTAAATGGATTGTCATCATTACTGAACGGGTTATGAAGTGAGATGGATGGAACCGCCTGATGGATGGATGAAATTGAGTTTTTTGTCCGATGTATTGTTG
TTCTTGTAGAATCAAGTGACAGGTTAAGATCTGCATTTGCTTCTAGTTGAGTTGACACTGATTTTGAGATATCTTCATCAGAATATTTTGTACCTACAAATTGAGCAGGAATATTTTCATGGT
TTTCACCACCTTGTAGATTGTCCTTCCATAATGTAAATGGCATCTCCATATGGCCTAGTACTTGCCATTTCTCTTCAGTAGTGAAACCAAACCGAGAATCTGTACTCATTCCAAAAATTTGTA
AATCAGAATCATTTTTTCTCAGTTCTTTTGTTATTCTACTTCTAGTTTCTTTCAACTCTTCCAATGTTGGCATCTGTTCTAAAGGTTCTGAAGTGCTATTAAGATGAAAATAGATAGAAGCGG
ATGAAGTGCCTTCTCCAAATGCCTGCTTTGTATTTTGTTCAATTGCAACTTTCACTGGTTCCTTAATCTTAGTATCCATTGCCACTTCATCAGTTCCTAGCGAAACAAAAGCCTGTGACAAA
TTTTTCTCTTTTACTTCCACAAATGGACTTTGTGAGGCTGCACCGGTCTTCCGAGGATATGAACTACATGGTTTTCGTTTAGGCCTTGGGGGAGGAATTTCTATGTCATGAGGATGTCCAGG
TGACATGCCTCCAGTCCCATTCTCCAACTTTAAAAAAAACTTCTGCGCATGACTTCTTATCTGAACAGCAGTCTTTGTACCGATATGCTCTTGAATACGCTGCCATGCCCTGCCATGCAGCC
TGAGCGCTTCCAAGAACCTGTTATGCTCCTCGTCTGTCCACTTCTCTCGCTGCTTCGTAATCGTGTAAGGTTTTCTCATCTTAGCCAGCTCCCTCACTGGGCTCATTCCCATTACTGATTCCG
AAATGTAAATGAACCACGGATGAGACAAACGGTTTCAAATCAACCCAAAGAAACCTTAGAAACAGGAAAGAAACTCCGGCGGTCGCAGCCTCATGTCTATCTCTCTATTCCTCTCCTCCC
TTGATCTCCTTCGCCTCACATTAGCACCCAGTGCAATCCTATCTACAATCGCCCATCTTGCCATCGTGGGTTTATGTACAAGTAGAAACATGGAGGCTGTTATGGCCGGCGAAGGGAGCTG
TCGATTCGGCATGCCTAACTCCAGAACCCTCGCTCTCTCTCCCTCTCTCCTCGCAGCTCCACCCTAACTCTCTCTACAGTAAGGTCGCAAACCATAAGAGTAGGCCAGGACAGGAGGAAT
CTGAGCCACAACTTTTTGGCATGGAGATTTCGGGCGAATGGTGAGCGAACTGGCGAAGCAGAGCGAGATTCGATGATCAATTCT
>comp164734_c1_seq6

GGGAGCTCGCTGGCTGTGAAGACAGGCATGGGCGAAACTAGCGATCGTAGCACAGAAGATGTAAGTGAATTAAAGGAACTGGGTGAGGACATGCGAGCTAGAAAGAAGAAAAGATTA
CGAAAAGATCTTATCAGTTCGACAGATTGGCGTGGTATCTGCTACTTCTGTCGGGTTCCACCCCACATGGATCCGGCTAACCTCCGCCAAATCCTCTCTAAGTTTGGAGATTTACAGAGGA
TATACCTCGTTCCCCAAGGAGATACACGAAAAAGAATGAGGTACAGTTTGAATGTC
>comp164741_c0_seq11
CTCAAAGATGAGCATCTTTAGCTTCCTCACCTTCGGGTCACCCCCGTTGCCACTGAATCCTCCATATCCTCTCGTTTCGCGAAGAGGAGGTGCTCCCCAGTTGTCATGATCTTCCCTCCGA
CGATCATACCCAAGCCCTTCCTCAGATTCCCCTCTACTCCTTTCATCTTCAGCACCCCGGCGGTAAAATTCATCTCTTCTCCCCAAAAATTCAGCACCCCTTCATAGCCCATGCTCATCTCTT
CTCTAGAAAGTGGTTCTAGCGGATGCATTCCATCATTCCCCTCAAGGATCTCCACTTCCGGATTCTCCCTCCCCCTAAATGGGTTCGGATTCCCGCATCTCCCATTGTCGCCGGTGGTTTCC
CTTGCTTCCGATGTCATCGGATTTTGTTTGCCCTCAAGCATCTTCTTCATCATATCTTTCAAGGCGGTGAACCTCTCATCCATCTTTTCATGGATGGAAGTAAATTGGTTTTGAAAATCAGCG
GTTCTTTCCTCAAAATCGGTCTTCAGTTGGCCTATTTCTCCCTC
>comp164753_c0_seq3
AAAGCGATTGTCTCACCTTGAGGCTACGCTCATTTGTCTACTTCTAGGGTTGTCGGAAGAAGAAGAACCACACAAGAGATGAAACCACTCCGAGGGAGACGGAGCCGGCGATCCGTAG
TGATGAAGAGGACGGCGTAGGATGTGGAGGTGGCGTGGAGTTCCAACCGTCAATGGTCCACTCGTAGTCCGGTCGTTGTCTATCGATGACTTGGATGAGGGTCGGTAGGATCCGATCCA
AATAAGCTCCAACAGTGTCGTCGTCGGAGTCAAATCCCACGTCGAAGAAGGGCGATTGTAGAACGTTCAGATTACCGTGGGCATCAAACACAAGTCCCCATTCTAGCTCGGGATCCGCC
ATGCGACATGTAGGTGAAGATGGGTTGTCGCCACAAGAAGCTCTCCAATGGGGAAGATGAGGAGGTCGGCGGGTTGTAGAGAGGAGGGGGAAGCCGGCGGTAGAGCTAGGGTGAGAG
GAGGGATTAGTTTTAGAAAACTGAAATTAATTTCAGTTTAAGGGAATTGGGAGGAAAATTTGAGATTTGATTTTCCAATTAAGAGGCAATTAAGGCATTAAAGAAATTT
>comp164756_c1_seq4
TGCTCAAAAGCAATATATAAAATTATTTAAAATTGTATTACATCAAATAAAAACATGTAAAAACAACTCCATGCATTAATAGTTGCCTTCCCCAACATGAGAAAAGAAAATCTACAAAAAAA
CTAGGCACTTCTTACCCTCTAGAAGAGAACCCTCCAGAGATATGGAATCAGTTCCTCCCTCTATTACAGTTTGAATCAGCAAGAGAATATTATTATTATATGATCCATCTCAACATGAAATCA
TTAGGACATAGCTGAATTTAAACTTTAGTCCCCAACCAAATACTAACACAAGAAGATCATCAAATAGAAGACAATGGTGGTGAAAAGCCAAGAACTTGCTTTCCTATCTTTCATTGATCCT
CTATTTTCAGATAAAATTTTTTAAGTGCTTTAGCTGAGGCAAAAACAAATTATATGACCTTTTTAACTCTTATGTTAACATATGCATATTAGCTCAGATAAAGTTAGATAGACTGTGGAATTCT
GAAGAGATGTTCCTCACCACTTACCTCATCAACTTTCGTCACCCATGCACTCTTGCTCCTCGACCGCCTCCTCGAAGCTCCTCTTACCTTCCTCTCCTGCCGTAAACCCTTCGAATCCTTCA
TGACGAATTCGGCACTCTTCCCTATCATTGTCACTTCCATTCCCTTCCCTATAGGATGCTTTGAAGACCTCACAGCACTTTTATCCTGTAAAGACCTCCATTCCCCTCCCAAGCCTGATTTTT
CACCTAGCAAGTTATTGGCAACCACAACAGACTTGGCAGGATTCATATCAAGCAGTTTGAGCAAGCCTGTGTAGGATTCAGACTTGCAGTCCACTACAGAGAAGTCGACATTCTCGCACT
CGGGCAATAGCTTATAGGGATCTCCTACTTTGAACTCGACGATGTCGGTGAGGCCGGAATCCTCGATGACCTGTTTTGATTCAACCAGAGACTCTGACTGTGGGAGAATGCAAACAAGCC
GGCCGCCGGTTTGCCGAGCAGCTACAGCAAGTGCAACTGTTGATTGAGATGCTTGTGGAGATACTTCAACAATGTGTTGAGCACTCATGCCCGCAGCCAAAGCTGATAGGAACTGAGTG
CTTTCTGGTTCAATTGGGCTGTTAGACTCCTTCACATCTTTACACAGCTTAACCGTGTCAAGGTAGGCATTGGCGGCAAATTGAGGAGACCAAACCATGATAGCGAGCCAGATCAAAACA
ATCACACTCTCTTCTCAATATCTTCCTTCATAAAGCACTTTAAAGGGAGAGAAAAGAAGGAAGGAGAAGGTTGTAGAGACAATGGGAAGAGGCGAAAAGGGAGAAAAAGGGGTTTTAG
TTGGATTTTTCTTTGTTTCTCGCTAATAACTCAGCGAAGGCGAGGGATGCAACGAACAATTAATACCTAACGGAAAATTCAAGACATGGCGGCGAAAAGAACAGCTTCGACCCCAAAGTA
TCTCCGGCCAGCGGCGGCCCGCAGGTCTCGGTAGAGAGCATGAAAGGGAAAACGTTTTGGTTGTTTTGCCTTTGCGGTGCGGATTCCCTTCACATCGTTTTTGTTAACGGTACTTTT
>comp164770_c0_seq1
TTTTCTCTCTCCCAATTTAATTCCAATCTCCACCAAATCAGCCCCTAATCCTCTCCTAATTGCCGCCCCTAGCCATTAATTGATTAATTTGAATTTTCAAATTAATTTCCCTCCAAATCCACTTA

AATCTGAATTTAATTTCAGATTTTAGGTTGGATTTGGATGGGGAGAGATTTAAAATTTCAAATTTCAAATTTCAAACAAAGAGGAATAAAGGAGGGAAGAGGTAATTAATATCCTCTAATCT
AGGGCTTAATTAGAGAACGAAATAAATTAGAGAAGGGGAGGGAAGGGGGGCCGGCTAAATGAGGGAAAAGGGGAGCGTCCACATTCAATGTATGTGGTTCTTTGCATTGATTGTTGATT
GAGAAATTCAAATATGAATAAATTTCAGATTTGCATCAATTGGGGGGCTTCTTAAAAAATAGTTTTACTAATTAGGAAAGTATTTGAAATTCAAA
>comp164786_c2_seq11
TTTCAAAAAAAAAAAAAAAAATCTAATGATTTTTATTTTTCCTCTTAAAATGTAATAGTGGAAATAAACTTCGACTAGTTTAATGGATTTTCCGTGCGGAGTTTATAATCATTTATGCTATTTA
GCCAGAGTAGAGGATGATAATAATATATATTAGTCTAATGAACTGAGTTGGCTGTTGTGGCAACTCGTTTCCTGATCCTTAGACGACGAACTGTGTGAATTGGTTCTTCTTGTTTGGTTTCTT
CGGAGTTATGGTTATTTGTTGTGGCGAGATTCTCTTGAATGAGCTTTTCATTACGTGGAGGAGGAGGATGGTGAGAAGAAGTACTGGACCTTGTAGAGGCAGGGCCTTGCGCTGGTTTGG
ATAAGGCCGAGGAAGGAGAGATTGAGCTGCTGTCAGTCTTCACCTTCTCTGGTTTCTTGATTAAAACGGGGTTGGTTTGTTCAGTCTTCTGCTGCTGGGAAGCTAATGGTTGCTCTGTTTC
GTTAGCTGGTGCTGTAGATGCATGATCAGCGGGTCTTGGTTCCTCAACAGGGTGTACTGTAATGGATGATGGTTGCGAGACAACATATTGCAAGATCGTCAAAAACCATGACCGTCCATTA
CTCAAAACATTTTGCCAATAATGGGCAGCTAAATCGTTAGCTCGTGTTCTCAACTCCAGTAGAGTACGATCAATCTTATTTTCATGTTTAGACATATAAGGCTGGAAGAAGGTGCTGTAAAT
ATATGTCGTCCCCTTTGTTTTAGGATGCCAAAGATATACAAAGAAAACCAATTTAAATTCGCCATACAGAGGCAACCATGAAATGAGAGCATCTCCAACTCTCTCAAAAACAGCCAGAAC
AGCAACTAAAATCCAGTACTGGCACCAAAACAGCAGCTGCTCAATTTCCGGCCTATTCAATTCCACAGTCTTGTAGCATTCATATGCCGGATAGGCATATCCAAGAATCATGATAGAAACT
CTGGTGAGAAAGCCTCCCATCATTGCGACCAATTTAACATTTCCTCCCCCTGAAGCAACTACTATATGGCTTCTCTAAAGCCCGGATCTCTTTCAACAAAGACGGAGATGCAGCAGAGGA
GGTATGGCCGGCCAAAGACCGAACCCGAAAAGATTCGATGACTTGCGATCGGCTTATCGCGCCCTTCTTGGCCTTACCAGGGGTTCTCTCTTTATGGCCGGCCATGTGGACATATATAGCG
AGGCGGCAGGCCTCGAATGGCGAATCGCCGCGCCTATTTTTCGGAGAGGAGAGAGAGAGAG
>comp164786_c2_seq14
ATCATGATTCTTGGATATGCCTATCCGGCATATGAATGCTACAAGACTGTGGAATTGAATAGGCCGGAAATTGAGCAGCTGCTGTTTTGGTGCCAGTACTGGATTTTAGTTGCTGTTCTGGC
TGTTTTTGAGAGAGTTGGAGATGCTCTCATTTCATGGTTGCCTCTGTATGGCGAATTTAAATTGGTTTTCTTTGTATATCTTTGGCATCCTAAAACAAAGGGGACGACATATATTTACAGCAC
CTTCTTCCAGCCTTATATGTCTAAACATGAAAATAAGATTGATTGTACTCTACTGGAGTTGAGAACACGAGCTAACGATTTAGCTTCCCAGTATTGGCAAAATGTTTTGAGCAATGGACGGT
CATGGTTTTTGACGATCTTGCAGTATGTTGTCTCGCAACCATCATCAACTACGGAACCAAGACACGCTGATCATGCATCTACAGCACCAGCTAACGAAACAGAGCAACCATTAGCTTCCCA
GCAGCAGAAGACTGAACAAACCAACCCCGTTTTAATCAAGAAACCAGAGAAGGTGAAGACTGACAGCAGCTCAATCTCTCCTTCCTCGGCCTTATCCAAACCAGCGCAAGGCCCTGCC
TCTACAAGGTCCAGTACTTCTTCTCACCATCCTCCTCCTCCACGTAATGAAAAGCTCATTCAAGAGAATCTCGCCACAACAAATAACCATAACTCCGAAGAAACCAAACAAGAAGAACCA
ATTCACACAGTTCGTCGTCTAAGGATCAGGAAACGAGTTGCCACAACAGCCAACTCAGTTCATTAGACTAATATATATTAGTCTAATGAACTGAGTTGGCTGTTGTGGCAACTCGTTTCCT
GATCCTTAGACGACGAACTGTGTGAATTGGTTCTTCTTGTTTGGTTTCTTCGGAGTTATGGTTATTTGTTGTGGCGAGATTCTCTTGAATGAGCTTTTCATTACGTGGAGGAGGAGGATGGT
GAGAAGAAGTACTGGACCTTGTAGAGGCAGGGCCTTGCGCTGGTTTGGATAAGGCCGAGGAAGGAGAGATTGAGCTGCTGTCAGTCTTCACCTTCTCTGGTTTCTTGATTAAAACGGGG
TTGGTTTGTTCAGTCTTCTGCTGCTGGGAAGCTAATGGTTGCTCTGTTTCGTTAGCTGGTGCTGTAGATGCATGATCAGCGGGTCTTGGTTCCTCAACAGGGTGTACTGTAATGGATGATGG
TTGCGAGACAACATATTGCAAGATCGTCAAAAACCATGACCGTCCATTACTCAAAACATTTTGCCAATAATGGGCAGCTAAATCGTTAGCTCGTGTTCTCAACTCCAGTAGAGTACGATCA
ATCTTATTTTCATGTTTAGACATATAAGGCTGGAAGAAGGTGCTGTAAATATATGTCGTCCCCTTTGTTTTAGGATGCCAAAGATATACAAAGAAAACCAATTTAAATTCGCCATACAGAGG
CAACCATGAAATGAGAGCATCTCCAACTCTCTCAAAAACAGCCAGAACAGCAACTAAAATCCAGTACTGGCACCAAAACAGCAGCTGCTCAATTTCCGGCCTATTCAATTCCACAGTCTT
GTAGCATTCATATGCCGGATAGGCATATCCAAGAATCATGATAGAAACTCTGGTGAGAAAGCCTCCCATCATTGTGAGTGATTTAGCATTTCCTCCCATTGTAGCAATTACTTTATAACTTCT
CTAAAAATCTAAAATATTTGAAAAAAAAAAGCATTATCAGAATGAAAGGAATCAGGAAATAAGGAAAAAAAAAATGAATCACTCAAGCTTTTTCAAAATCACAATATCGTAGAAGAATATAA

TCATATATCAATTGAAATCACAACTGACCAATGCTTAGAAAGCGAAGAAATCTGATCAAAGAATACAAATATATAATTATATTCTTCTACGATATTGTGATTTTGAAAAAGCTTGAGTGATTC
ATTTTTTTTTCCTTATTTCCTGATTCCTTTCATTCTGATAATGCTTTTTTTTTCAAATATTTTAGATTTTTAGAGAAGTTATAAAGTAATTGCTACAATGGGAGGAAATGCTAAATCACTCACA
ATGATGGGAGGCTTTCTCACCAGAGTTTCTGTGTAAGTGATTTTGAGTTGAATGGTTTATGATGAGGTTGTTGTAGCCTGTTGTAACTTCTGTTTGACATATCACCATTGTTTATTTTACTCAT
ACAACATGTTTTTGTGCCAATTTTGGTTGGTGTTTGTGCTTTGTATTCTTTCTCTCACTCGATGTGTCTACAAACACAAAGTACATTAAAATTTTCTCTGTTTCTTGGTTCTCAGGCTTTTATT
TGTTGTAATGTCAGTTTATGAGAGGGTTGGAGATGCCCTCATTTCATTGTAATAAGCATATCCATTTAGTCAGTTATGTATTGTATGATGAATAGCTGGCGTAGGAAAATGGAAAACGAATTT
GGGTTAGGATATTATGATAAGGATCAATCAAAGTGCACATATAAGGG
>comp164786_c2_seq8
TTTCAAAAAAAAAAAAAAAAAATCTAATGATTTTTATTTTTCCTCTTAAAATGTAATAGTGGAAATAAACTTCGACTAGTTTAATGGATTTTCCGTGCGGAGTTTATAATCATTTATGCTATTTA
GCCAGAGTAGAGGATGATAATAATATATATTAGTCTAATGAACTGAGTTGGCTGTTGTGGCAACTCGTTTCCTGATCCTTAGACGACGAACTGTGTGAATTGGTTCTTCTTGTTTGGTTTCTT
CGGAGTTATGGTTATTTGTTGTGGCGAGATTCTCTTGAATGAGCTTTTCATTACGTGGAGGAGGAGGATGGTGAGAAGAAGTACTGGACCTTGTAGAGGCAGGGCCTTGCGCTGGTTTGG
ATAAGGCCGAGGAAGGAGAGATTGAGCTGCTGTCAGTCTTCACCTTCTCTGGTTTCTTGATTAAAACGGGGTTGGTTTGTTCAGTCTTCTGCTGCTGGGAAGCTAATGGTTGCTCTGTTTC
GTTAGCTGGTGCTGTAGATGCATGATCAGCGGGTCTTGGTTCCTCAACAGGGTGTACTGTAATGGATGATGGTTGCGAGACAACATATTGCAAGATCGTCAAAAACCATGACCGTCCATTA
CTCAAAACATTTTGCCAATAATGGGCAGCTAAATCGTTAGCTCGTGTTCTCAACTCCAGTAGAGTACGATCAATCTTATTTTCATGTTTAGACATATAAGGCTGGAAGAAGGTGCTGTAAAT
ATATGTCGTCCCCTTTGTTTTAGGATGCCAAAGATATACAAAGAAAACCAATTTAAATTCGCCATACAGAGGCAACCATGAAATGAGAGCATCTCCAACTCTCTCAAAAACAGCCAGAAC
AGCAACTAAAATCCAGTACTGGCACCAAAACAGCAGCTGCTCAATTTCCGGCCTATTCAATTCCACAGTCTTGTAGCATTCATATGCCGGATAGGCATATCCAAGAATCATGATAGAAACT
CTGGTGAGAAAGCCTCCCATCATTGTGAGTGATTTAGCATTTCCTCCCATTGTAGCAATTACTTTATAACTTCTCTAAAAATCTAAAATATTTGAAAAAAAAAGCATTATCAGAATGAAAGG
AATCAGGAAATAAGGAAAAAAAAATGAATCACTCAAGCTTTTTCAAAATCACAATATCGTAGAAGAATATAATCATATATCAATTGAAATCACAACTGACCAATGCTTAGAAAGCGAAGA
AATCTGATCAAAGAATACAAATATATAAAAATATGTCCTAGAAATTCCGAACTATAAATGATCATTACAGTTTAGTAAAACTCTTCATTTACAAAGGCGAAGGAGGAAATTTTAAGCAAAAT
GATGTAGAGAATAAGCGAAGTGTGGGTCTCCGAAAGGATTCTG
>comp164815_c0_seq1
AAGGCTGGATCCAGACTTTCAATTCTGTTTGTGGCAAGAATTACTTTAACATCACCCCTTGGGTCAAAACCATCTAATTGATTCAACAATTCCAACATAGTGCGTTGTATCTCAAGTTCTCC
TTCTGAATAAGCATCATATCTTTTAGTTCCAATTGCATCGATCTCATCAATAAACACTATTGATGGAGAAAGATCATCAGCAAATCTAAAGAGTTCCCTTACTAATTTTGGACCATCACCCAA
GTACCTCTGGATCAATTCACTTCCGACAACACGCAAGAATGTGGCAGAAGTTGTTTTGGCCACGGCTTTGGCAAGCAAAGTTTTCCCAGTTCCGGGCTCTCCATAGAGTATGAC
>comp164882_c1_seq1
AAAAAAAAAAAAAAAAAATTCAAAGCTTTGTCTCTCAGATGAAAGAATAGAAAACAATTAGACCACTCGAATGGTGCCTGGAGACAATGTTGATAAGTTGCTTTGAGATTGAGGCGGCG
CTATACTGAGCTCCAAATCATTAGTGGCAGTACTCGTTGTTGCAGGATTAACCAAAGTTTGAGGTGTGGCTGAAGATGCAGATAGTTTGAGTAAACTGGCAGGTTGTGGTTGATCCTGAGT
AACAATTGTTGAAATGAAGGTAAAGGTGGGGTGGCCCACATCCTCGCTTCCTTTCCTGCCCTTCTCTCCTTCTCGATGACTTCGACAACCCACCATCTGATAGAAACCCGTCATCGCCGGC
TCCATGATACCCCGATGAGTTCGAGCCGCACGCCCCCAGGTTCATCATGCTCTTGCTCTTCAACAAACCTTTCGTCATCGTCTGTTCCATCGCTTCCCTCTTCAAATCCCTCGTCTCCATCT
CTTGCTCCCCCTTCCACCTCTCTCCATCCACGATCCTTACTCCAAAAAGCTTCAATCCCTTCTCCTCCTCCATAGCGCCCTAATCTCCCCTCGCTCGCTCTCGAGAGCGCGCGCTCTCTCTC
TTCTTATCGTCCTCTTCCTTCTCGGAGAGAGGAAATAAATCAGAGGAGAGCCGCACTAGTGCTCGCTTTCGGATCTGGTTTTATACTGATATCCAAATCCGCAAATAGAAACTGCCAACGA
TGTTCCGCGAAAGTGC

>comp164895_c0_seq1
GGCTTATAGCATTTTCATTTCTTAAACTGCACTGTATACATTCATCACTTCTAAAAGAACTTTCAGGACATGCTGCTAAAAAAAAAAAAATATTAATCAAGAACAAAATAATCATCAATTCAAT
ATAAATCTTCTGGAAAAAAAAAATAAAAAAAGAAAGTGTCGCCAATGATTCTTCTGCAACTTTTGTTAAAATCATTACTTGTCTACTGCCTTCATTTCATACTTTCTTAAGTTTCTTTACCTTT
GCCATCAATGCCTTGAGTACTATCGAGCATTCAAGGAACTATAGGTGAAGGAAATGTATTCATATTTACATTATTATGTAAGTTGATATATTTCTGTAGTAGTTTTCTGTTCCAGAAGAAATTT
ACAGATAAGAGCTGTTCATACTGTTACTTTTTCCTGGAAAGTACTTACCTCCTCTTTACAGCAATCATGCTTGGTAGATGCTTCAACAGGTTCATTTTGCTGAGGTAGAACAGCTCTCCGTT
TGTTTCATAAAGGTTTCTCCTTCTATAGAACATGAAACTATGACTTTCTCTGAAGATGTGATTTAGCTTTTAAGAGAGCTTTTGCCACTTCCTCATCTGCTGGAAATTCCCTCGTCAAAATCT
GATAATCTTGTGCTGATGCCTCCCACTTCTCAAGCTTAGCAAAGCAATCAGCTCTTCGAAGTCTAGCCTTAGCATAGGAAGGTTTCAAATTAAGAACCGTATTGCAATCTTCAATTGCTTTC
TCCCATTGTCCAAGTTTGGAATGACAGGCAGCTCGGTTGCAGAGAAGAATGGCATTCAAGGGATCATGATGAAGCCCTTCTCCATAAGCAGTGCATGCTTCTGAATACTTTGAAAACTTG
AAGAACTCATTGCCTTTGTTTCGAGCTGATGCGACGGCTTTTACTCTCCTAACTACAGCACTGATTTCTCTGTTGTTCTGGTGTTTATCAGCCCTTTCCACTAATGCAACCGCTTCATTGAA
CCTTCCTGAAGCTAAATTAACCTCTGCTTGAATCATGAGGATGTATCCATTCACTGCTGAACCAAAGAAATTTATGATTGCATCAGTGTCAAACTTGGGTGCATTCTTAAGAACTGAGTCA
GCCTCTTCATGCCTGCCAAGTCTTAGAAGCGCTTCTGCTTGTAATGCAAATACTTTTGGAGCTGAATCTGCACCACTAGAAATGGCAGATTGGGATTCTTTAAGCATGCTCTGCCACTCCTT
CAGTTTTCGAGCCTCGCTGCATCTTAGAATATGCATTTGGAGATTTGTTGCTCTAGTGATATCATCAGTATCAGCCACTTTTCGAGATTGGTTGTAGTGATGAACTGCCTTTTCGGCTTCCCC
TAACCTGAGATATAAAGTCGCTAACCGATAATGAGCTCTCGAATATGAAGGATCAATCCTTACTGCCTCCTTACACTCTTTGATAGCTTCAATGAGCCTACCCATTCCAGCAAGTGCTGCCG
CCTTATTGTTCCAATAAGAAGCCTTCTCAGGATCAAGCATGATCGCCTTGTTATAGAGAGCTAATGCCTCACTGAGCCTCCCAGCATTGAACTCCTCATTGCCCATCTCCTTCAACTTCTCA
GGATCCGTTCTCTTTGACAAAGCCCTGCACAACACCGGTGGTTTTGAGGCCTTCTGTTCTGCTTTGCTCCTCAAAATGTTGCCCATTCCCCCATTGATGCCACTATTCTTTCCCCAGATGTT
GCCTAATTGGCCATACACCATTACATTGCTAGAAGAAGCTCGCACCAATTTGCTGTTACCCTTTGCCTTCTGATAGTCATAGATCATCATGTCAAGCTCCCCTGAGAGGCCATTACCAGGCG
TGGTAACCTGCTTTCGCTTCTCCGGAGCTGCTGCTGGCTTCGACTTGTGTACCACAAATTGGGAGGCATTTAGATGACTCCTTCGTTGAGGATGGCTGGAGCTCGGTGGTCGAGTCTTCG
CTGGGGAAGATGGTGTTGCAGGCTTGCGGTTGGGAATTGATTTCTTCGATGAAGACCGATTATGGCGGCCAAAGAGCAAGCTTAGCAAGTTGCAGCCTTTGATCTTCTTCTGGTCACTGC
TAACCTCGGTCTTCATCTCCTCCTTCAGTGGTTCTAAGGAGATAGATGGATCCAGAAGGAATTGGCTGCAAAGCTTGGAGCTTTATTGAGGATTTGGAATGCCTCGGCAAAAAGGAGGCG
AGAAGAAAGGATTACATTGATCGGGATGGGAAAAATAAGAGAACGTCGTGCGTTCATGAAAAAGGGAAAAAGGAAAAAACCTAAACAAAAAAAACCAAAAAAAAGATCGTTGAAAG
AGTAAAACAGCCGATGTATCTTACCAGAAATACCATTGTGAGGGGGAGTCTCAAACAAGCCTCCTTTC
>comp164907_c0_seq16
ATAATAATAATAATATGACTTTCATTAGACATTCTTCACAAATTCCTGGATATGATACTAGTTTTGCTTTTGTTTTCCTTATTTCTTACTTCTAAATACATGAAAGATCAAAATGTTTCCCTTGA
AATTCTATAAAAATCCTCCACATTATGCATGTGACTTCTGCTAGAAAAATGAAAAAAGATGGCCGGAGGCTGTAAGTGCCCTGAGGAAAAATAAAATAAAATAAAATATCCAAACAAACC
ATTCATGGATGGACTGCAATGTGCAAAAACTAATATAAAATCTCTGACATTGGTTGATTTTTAGGCTTATGAAAAAGCCTATGACCAATCTTTTCTTATCTCGAAGTAATAGCTCATTTCCAA
ATAACATTTACCATCATCATCCACAAACTTTGTTTTAGCAGAATAGGATCCCCTTGCAAATATGCCAGAAGGGGTGGTATCCTCTTCTAGCTCATAGGTGTATGGCTCCTTCTGAGGGCTGA
AAGTTCCCAGCATCACCTTTGTGTTCTCAACTCTCACTCCAGTCTTCCACACAGTGTTCGTGTACCGGAGTCCTGATACAATGTTATTTGAGACAGTGAAGGAGAATTTGAGTCGGTAGCG
ACTGCTGTCCTTAAGAGTAAATGCATAGCCCTTCGCATTTGGGACGAAAGGGATAGGTAGAACCAGGTCTGGTCTATCAGGAGTGATGATTGTGAGACTCTGGATCTTCACTTCTGGATCT
GGATTCTCTCCAACTTCAGACAAATCAACGCCGGCAAGCAGCTGCTCTTTCCATTTCCTCAGGCTTTCATCATCCTTGTCCATCTCAAGCTGTTCCTTGAGAGCCAATTGAGGGCCGAGCG
CCACATGTGCGTCTTTCCGATCATTGTCGTCGTCCTCGGCATCTTCGTCGTCCTCCTCGCCACCGTCGGTGGAGTAAAAAGAAGCACTGCTCATCTTCCTTTTGAGTTCGTTATCCGAATCG
CCCGGCTCCTCCCCCTCCTTCAAGCTCAATGTCACATCTTCTGATGCCGGCGTCGACATTTCTATCTTTTTCTCTCCTTCCATGCTTAAAAAACTGGTTCCTTTGCTTCTCGTTTCAGGGAA

AGACGAGGCAGGGGACGATGGGAGAGGGAAAGATCTTAAGAATAGAAGAAGATAGTAAGGGCTTCTGTTTCTCTATTTTTATAGAAAAGCTGTTATGCGTCTCATAGGATCTGCAAAAAT
GGCGGAGAGACAACCAAATGAGGGGGAAGAGTTCTCTGCCTATGTGGCTTCCGACATTTAAGATCTGTG
>comp164907_c0_seq18
CTCTCTCTCTCTCTCTCTTCTCTTTCTTCACCTCCCTCTCTCCCTTTCTCTACTCTTTGAATCCTCAACAAGATATTTTTATTATAAAAATGAAGTTTTGGGCCTAGTGTTTTGGCACTCAATT
CCTCAGCTCCGCTATGCTCCGAGAGAATCTTCATATGTGCCTCAGCACTGAGGTGAAGAACACATTGTATTCCTCCATTTCTCCATTTTCTTTAGAGCTCAAAATTGGGGCCTCGACAACGT
TGATGTTGTGGTGCCCACAAAGCAACTTCTGAGCTGGGATTCTAGAAGTCGGAGAAACATGCTAACAATGGAAGGGAAATTACCCTAAACATCTGCAACTTCCATTCTTTATATCAGAGG
AATGCAAAAGAGAGAAATGCTTACATATATATATTTATACACTTACAGCTCTCTGCAGAGATTAACAGATATTTTCTACAGTTCTAAATGTACGTGAAATGAGATGTTGTTAGTTTGCCCAAT
CTTATGCCCTATCGATCACAATGACGAACTAACCCTTGGATATTTATTTCCCAAAGTCTTAGCATATTTGGAAAGCATTTTTTTGTGACCACCTAATTATTTGGCAAGGTTACTATTTCAGAA
AAACTTTTCCTCCAAGCTGTGGGAAACAGAAGATTTTCATGAAAGCAATCAACCTAACGTGCTGTCATGTCGTACAAATGGCTTAGATGCTAATGAATCATGATCCACTTGTTTTATTTGGC
ATGCAATAGGGTAAAATCACATGTCTAAAGGACTAAATTAATCACCTAAAAATAATTAATATGCTTTATATAAGTTCAAGCAAAGCATGTACTTAAATGCCTTTTACAAGATTTTAACTAACC
TCTCAACATCAATGGCTAAGAAAATTGAGGAAACACTTGCTCAAGAATATTTTTTTTTCTAAGTTTAATTGGGGATAAGATTAGAAAGAGTTGCCTTAGATGGCTTGAATACATAAAATGTT
AGCCTTCCGATTATCCAG
>comp164907_c0_seq19
TTCGTGCCCGATCAAGCTAATTTTTTGACGGGTATCAGAAAGTATTTGACGGCATAAATAATCCGTCAGCTGAACCTAGCGAGCTCACCTTCCGTCTTTGGGCCCACACTTGCTTTGCCCTA
CTGAAAAAATGAGTTTGTAATAGGGGGATTCAAATTCGGAATGGGGATTCTTAATCAAGCTAATCAACTAACCACCATAAATCTCTGTGGGTAAATTACATAAATTTCATTTGAGTTTATGA
AAATAAAAAACAGTATATTTGCCTCAAAAGCTGCGTTACGATGAACCAGCACGTCTAAATTGTAAAGCATAGACGGAAAGCCTGTCACCGTCTAGACTTTTCTGGAGGGATTAAAAAGAT
ATTATTCCTCGTGGCTAACTGGTTCCAGCGGACTACAACAATAGTTTCCATGCAAGCTTGCGTTTCTTAAGAAGCGTTCAAGATCGTTACACGTTCCCCTAGAGCTATATATAATGAACCTT
GATCAATTTCTATGACGTTAAACATCAATATATGCAATAATTAAACATCGTTAATTAAGCCTAATGAAGACAAGGTACTTGTTATTCTTCTTTATAAAGACCTCACCAACAACTTCCTGCCGT
CCTCACCACCCCCCTCTCTCTCTTCCACACACTCTTTCCTCTCTCATATCATACCATACTACAAACCTAACTTTCTCTCTCTTCCGCCGACTCTGCCATGGTGGAAGAATCGAGCAACATGG
ATAAGAGGATAAACGGAAACCAGGAGGCTGAGGTGAGGAAGGGGCCATGGACTATGGAAGAGGACCTCATCCTCATAAACTATATAGCCAACCATGGAGAGGGAGTATGGAATAATCTT
GCCAGATCTGCAGGTGATTGAGATGCAAATATTTCTTGAAGGATTTTTATTTTTTGAGTTTGAGAATGATTTGATTGATTAAGGTGATATATATATTGGTGGGTTTTATGAGAAGGTTTGAAG
AGAACAGGGAAGAGCTGCCGGCTTCGATGGCTGAACTATCTGAGGCCAGACGTGAGGAGGGGGAACATAACGCCGGAGGAGCAGCTGCTGATCATGGAGCTGCATGCCAGATGGGGA
AACAGGTGAGCTTCTTCTTACATGCATGCCCTGCATCCTCTTTAAGCTGAGCTGGGATTCTAGAAGTCGGAGAAACATGCTAACAATGGAAGGGAAATTACCCTAAACATAGCTCTCTGCA
GAGATTAACAGATATTTTCTACAGTTCTAAATGTACGTGAAATGAGATGTTGTTAGTTTGCCCAATCTTATGCCCTATCGATCACAATGACGAACTAACCCTTGGATATTTATTTCCCAAAGT
CTTAGCATATTTGGAAAGCATTTTTTTGTGACCACCTAATTATTTGGCAAGGTTACTATTTCAGAAAAACTTTTCCTCCAAGCTGTGGGAAACAGAAGATTTTCATGAAAGCAATCAACCTA
ACGTGCTGTCATGTCGTACAAATGGCTTAGATGCTAATGAATCATGATCCACTTGTTTTATTTGGCATGCAATAGGGTAAAATCACATGTCTAAAGGACTAAATTAATCACCTAAAAATAATT
AATATGCTTTATATAAGTTCAAGCAAAGCATGTACTTAAATGCCTTTTACAAGATTTTAACTAACCTCTCAACATCAATGGCTAAGAAAATTGAGGAAACACTTGCTCAAGAATATTTTTTTT
TCTAAGTTTAATTGGGGATAAGATTAGAAAGAGTTGCCTTAGATGGCTTGAATACATAAAATGTTAGCCTTCCGATTATCCAG
>comp164907_c0_seq21
TTCGTGCCCGATCAAGCTAATTTTTTGACGGGTATCAGAAAGTATTTGACGGCATAAATAATCCGTCAGCTGAACCTAGCGAGCTCACCTTCCGTCTTTGGGCCCACACTTGCTTTGCCCTA
CTGAAAAAATGAGTTTGTAATAGGGGGATTCAAATTCGGAATGGGGATTCTTAATCAAGCTAATCAACTAACCACCATAAATCTCTGTGGGTAAATTACATAAATTTCATTTGAGTTTATGA

AAATAAAAAACAGTATATTTGCCTCAAAAGCTGCGTTACGATGAACCAGCACGTCTAAATTGTAAAGCATAGACGGAAAGCCTGTCACCGTCTAGACTTTTCTGGAGGGATTAAAAAGAT
ATTATTCCTCGTGGCTAACTGGTTCCAGCGGACTACAACAATAGTTTCCATGCAAGCTTGCGTTTCTTAAGAAGCGTTCAAGATCGTTACACGTTCCCCTAGAGCTATATATAATGAACCTT
GATCAATTTCTATGACGTTAAACATCAATATATGCAATAATTAAACATCGTTAATTAAGCCTAATGAAGACAAGGTACTTGTTATTCTTCTTTATAAAGACCTCACCAACAACTTCCTGCCGT
CCTCACCACCCCCCTCTCTCTCTTCCACACACTCTTTCCTCTCTCATATCATACCATACTACAAACCTAACTTTCTCTCTCTTCCGCCGACTCTGCCATGGTGGAAGAATCGAGCAACATGG
ATAAGAGGATAAACGGAAACCAGGAGGCTGAGGTGAGGAAGGGGCCATGGACTATGGAAGAGGACCTCATCCTCATAAACTATATAGCCAACCATGGAGAGGGAGTATGGAATAATCTT
GCCAGATCTGCAGGTTTGAAGAGAACAGGGAAGAGCTGCCGGCTTCGATGGCTGAACTATCTGAGGCCAGACGTGAGGAGGGGGAACATAACGCCGGAGGAGCAGCTGCTGATCATG
GAGCTGCATGCCAGATGGGGAAACAGGTGAGCTTCTTCTTACATGCATGCCCTGCATCCTCTTTAAGCTGAGCTGGGATTCTAGAAGTCGGAGAAACATGCTAACAATGGAAGGGAAATT
ACCCTAAACATAGCTCTCTGCAGAGATTAACAGATATTTTCTACAGTTCTAAATGTACGTGAAATGAGATGTTGTTAGTTTGCCCAATCTTATGCCCTATCGATCACAATGACGAACTAACC
CTTGGATATTTATTTCCCAAAGTCTTAGCATATTTGGAAAGCATTTTTTTGTGACCACCTAATTATTTGGCAAGGTTACTATTTCAGAAAAACTTTTCCTCCAAGCTGTGGGAAACAGAAGA
TTTTCATGAAAGCAATCAACCTAACGTGCTGTCATGTCGTACAAATGGCTTAGATGCTAATGAATCATGATCCACTTGTTTTATTTGGCATGCAATAGGGTAAAATCACATGTCTAAAGGAC
TAAATTAATCACCTAAAAATAATTAATATGCTTTATATAAGTTCAAGCAAAGCATGTACTTAAATGCCTTTTACAAGATTTTAACTAACCTCTCAACATCAATGGCTAAGAAAATTGAGGAAA
CACTTGCTCAAGAATATTTTTTTTTCTAAGTTTAATTGGGGATAAGATTAGAAAGAGTTGCCTTAGATGGCTTGAATACATAAAATGTTAGCCTTCCGATTATCCAG
>comp164907_c0_seq22
ATAATAATAATAATATGACTTTCATTAGACATTCTTCACAAATTCCTGGATATGATACTAGTTTTGCTTTTGTTTTCCTTATTTCTTACTTCTAAATACATGAAAGATCAAAATGTTTCCCTTGA
AATTCTATAAAAATCCTCCACATTATGCATGTGACTTCTGCTAGAAAAATGAAAAAAGATGGCCGGAGGCTGTAAGTGCCCTGAGGAAAAATAAAATAAAATAAAATATCCAAACAAACC
ATTCATGGATGGACTGCAATGTGCAAAAACTAATATAAAATCTCTGACATTGGTTGATTTTTAGGCTTATGAAAAAGCCTATGACCAATCTTTTCTTATCTCGAAGTAATAGCTCATTTCCAA
ATAACATTTACCATCATCATCCACAAACTTTGTTTTAGCAGAATAGGATCCCCTTGCAAATATGCCAGAAGGGGTGGTATCCTCTTCTAGCTCATAGGTGTATGGCTCCTTCTGAGGGCTGA
AAGTTCCCAGCATCACCTTTGTGTTCTCAACTCCAACTTCAGACAAATCAACGCCGGCAAGCAGCTGCTCTTTCCATTTCCTCAGGCTTTCATCATCCTTGTCCATCTCAAGCTGTTCCTT
GAGAGCCAATTGAGGGCCGAGCGCCACATGTGCGTCTTTCCGATCATTGTCGTCGTCCTCGGCATCTTCGTCGTCCTCCTCGCCACCGTCGGTGGAGTAAAAAGAAGCACTGCTCATCTT
CCTTTTGAGTTCGTTATCCGAATCGCCCGGCTCCTCCCCCTCCTTCAAGCTCAATGTCACATCTTCTGATGCCGGCGTCGACATTTCTATCTTTTTCTCTCCTTCCATGCTTAAAAAACTGGT
TCCTTTGCTTCTCGTTTCAGGGAAAGACGAGGCAGGGGACGATGGGAGAGGGAAAGATCTTAAGAATAGAAGAAGATAGTAAGGGCTTCTGTTTCTCTATTTTTATAGAAAAGCTGTTAT
GCGTCTCATAGGATCTGCAAAAATGGCGGAGAGACAACCAAATGAGGGGGAAGAGTTCTCTGCCTATGTGGCTTCCGACATTTAAGATCTGTG
>comp164907_c0_seq5
TTCGTGCCCGATCAAGCTAATTTTTTGACGGGTATCAGAAAGTATTTGACGGCATAAATAATCCGTCAGCTGAACCTAGCGAGCTCACCTTCCGTCTTTGGGCCCACACTTGCTTTGCCCTA
CTGAAAAAATGAGTTTGTAATAGGGGGATTCAAATTCGGAATGGGGATTCTTAATCAAGCTAATCAACTAACCACCATAAATCTCTGTGGGTAAATTACATAAATTTCATTTGAGTTTATGA
AAATAAAAAACAGTATATTTGCCTCAAAAGCTGCGTTACGATGAACCAGCACGTCTAAATTGTAAAGCATAGACGGAAAGCCTGTCACCGTCTAGACTTTTCTGGAGGGATTAAAAAGAT
ATTATTCCTCGTGGCTAACTGGTTCCAGCGGACTACAACAATAGTTTCCATGCAAGCTTGCGTTTCTTAAGAAGCGTTCAAGATCGTTACACGTTCCCCTAGAGCTATATATAATGAACCTT
GATCAATTTCTATGACGTTAAACATCAATATATGCAATAATTAAACATCGTTAATTAAGCCTAATGAAGACAAGGTACTTGTTATTCTTCTTTATAAAGACCTCACCAACAACTTCCTGCCGT
CCTCACCACCCCCCTCTCTCTCTTCCACACACTCTTTCCTCTCTCATATCATACCATACTACAAACCTAACTTTCTCTCTCTTCCGCCGACTCTGCCATGGTGGAAGAATCGAGCAACATGG
ATAAGAGGATAAACGGAAACCAGGAGGCTGAGGTGAGGAAGGGGCCATGGACTATGGAAGAGGACCTCATCCTCATAAACTATATAGCCAACCATGGAGAGGGAGTATGGAATAATCTT
GCCAGATCTGCAGGTTTGAAGAGAACAGGGAAGAGCTGCCGGCTTCGATGGCTGAACTATCTGAGGCCAGACGTGAGGAGGGGGAACATAACGCCGGAGGAGCAGCTGCTGATCATG

GAGCTGCATGCCAGATGGGGAAACAGGTGAGCTTCTTCTTACATGCATGCCCTGCATCCTCTTTAAGCTGAGCTGGGATTCTAGAAGTCGGAGAAACATGCTAACAATGGAAGGGAAATT
ACCCTAAACATCTGCAACTTCCATTCTTTATATCAGAGGAATGCAAAAGAGAGAAATGCTTACATATATATATTTATACACTTACAGCTCTCTGCAGAGATTAACAGATATTTTCTACAGTTCT
AAATGTACGTGAAATGAGATGTTGTTAGTTTGCCCAATCTTATGCCCTATCGATCACAATGACGAACTAACCCTTGGATATTTATTTCCCAAAGTCTTAGCATATTTGGAAAGCATTTTTTTG
TGACCACCTAATTATTTGGCAAGGTTACTATTTCAGAAAAACTTTTCCTCCAAGCTGTGGGAAACAGAAGATTTTCATGAAAGCAATCAACCTAACGTGCTGTCATGTCGTACAAATGGCT
TAGATGCTAATGAATCATGATCCACTTGTTTTATTTGGCATGCAATAGGGTAAAATCACATGTCTAAAGGACTAAATTAATCACCTAAAAATAATTAATATGCTTTATATAAGTTCAAGCAAA
GCATGTACTTAAATGCCTTTTACAAGATTTTAACTAACCTCTCAACATCAATGGCTAAGAAAATTGAGGAAACACTTGCTCAAGAATATTTTTTTTCTAAGTTTAATTGGGGATAAGATTA
GAAAGAGTTGCCTTAGATGGCTTGAATACATAAAATGTTAGCCTTCCGATTATCCAG
>comp164919_c0_seq3
TGAAGCTGGGAGCTTAGATGTTTTAGTTCATAGAAATCAGTCAGTGAGTCAGCACAGTAGTTGAAATCGGATGGGACTACAGGTGGCAATGGCGTTCGGCTGGAGCAGTGAGGAGGATG
CGGCTACCGCAAGAGCTCAACTGGAGGTGTCTTCGGGCTAGGGTCGGGAGCAGGCGTCGGGTCAAGGAGTTGCAGTTTGGACTCGAAGGGAAGGGCAGATCTTGCGTGGATTTCGGTT
TGAAGAGATGGAGAGGCAGCGGCTGGAGCTTGAGTTAAGAGCAGGTCGGTTCGTGGAGTTCTTCAAGCAGCAAGAGATCGGGCAACAGCCGAGATATCTGGGTGAGGATCGAAAGTC
GGGCGTGGTCTTGAGGTCGGGTATGGCTGCTGGAGGAAGATTGGGTTTGGCGAACTCGACAGCACCTCGGAATAAAGGAGGGAGACGGAGCTTGGTGTCGGTAGGTGAGGAGGAACT
GCGGCTCTGGAGTATTCAAGGGCCTGGAGGGAGAGTGGATCAAATATGGAGGATGAAATCGATTGGAGAGGATGGAGGTCCGGCTTGGGTCTAGAGCGAAATCGGGGCTGCTGGAGGT
CGGAAGAGGCTTCGGAAGGCTACAGGAATCGGAAGCAGGGAGAAGTCGGAGGTTTGAGGACTCGTTAGGCCTGGTTTGAGACAGCTTAATTTTATTTTCAGTTTTGAAATGGCATAATTT
CTTTTTTTTGACTCTTTGAATGCTTTTTTTGACTGTTTGAATGCTTTTTTGAAAATTCAATTTATTTATTGTTTTTAAAAACGTTATGGTATTAGTTTCTACCTTTAAAACCTACATTTCTTGAT
GTTTTTGAAAAAACTTTAATAAAAAAAAATGGAGGTTTTAAACGGAGAAGCTAATACCATAACACTTCTCAAAACAATAAAGAAATGGAGTTTCCTAAAAAGCATGCATCACATTCAAACA
GCGGAAAAAAAAAATTATGACGTTTTGAAATTGGAGAAGGGAAAATTAAGCTGTTTCAAACGAGGCCTTAATAGCGAGGCAGATCGCAGGATTTAGGCAGGATTTCTGATCGAGACTCAA
AGGGCGCCGAATGTACAGTCTTGCAGATATTCAAAATGCTCGTGTTATCATGGATGTTCTTGTTGAAATGCTAAATGCTTTAGATCCGAGAAATAAAGAGGATGTCAAGCAGGAGGTGATC
GTTGAACTTGTTGAAAAGTGTCGTCTATATAGGCAAAGGGTGGTTCATCTTGTTAACTCTACTTCGAACGAGAAATTACTTCGTGAAGGGCTTGCTTTAAATGATGATCTACAAAATGTTCT
ATCAAAATATGATGCTATTGTTATTGAAGTTTCTAAAAAACCAAAGAAGCAGAACTCTCTCCATGATTTGGTAGATCTTCATGATAAAAATCAGGATGCTGACAATGAATCACATCGAAGGT
CAAATGCTCCTCTTCAACAGCTCTTGCTACCTGCTCCTCCTGGACCAAATGATATACCAATTACTTCTCCAAAATCATGTCCTCAATTAGATCTTCTTAGCGGCGAGGAGTTCAATACAACT
TCAACAGATAACTCATTAGCTCTTGTGCCAGCAAATGAAACAACTGCTCCTTCGGCAAATGACCAAAATATGCTAGTTCTAACAGGCATTTATTCTCCAAGGAACTGTGGAAATAACTGTA
CTGCCAATATAGCTTTTCAAGCTGAACAAGATTATACTGTTGCATCTCTGTTTCAGCAGCAACAGCAGTCTATATCTTACTTAGATGGGAGTGCCATGAACCATGGACAATCTATACATGAG
CACGCTTCATTTGTTCAAAAAGCTCAGCCAAGTCATGGGGATCTTTCATGGAATAATCATAATGTGGATACACAGCAACAAGCACGTATCGGAGAAGTCGATCAAGGTGGAGCTCTCCCC
CCACCTCCTTGGGAGATTGGACTGGCTGAAAGTAGCCAGCCGTTGACCATACAACAGCAGCATTTCCAAAATGAGCAGACACAAGAAATTATTACATATTCTACAGGAATTCAGCGAAAC
CAACCCATGGGAATGTTTCCTCCTCATGATCAAGCTTACCATCTCTCAAATGCATATTCTCAGTCCATGCAGGGTTATAATTACGGCCAACAGTCAGAATCTCACTCTTACTCAAACACCAG
AGAGCTCTCTCATAAATTGAACGGGCTTGGCTTGCAAGATAGTTACATGAATGCAGGTCATTACTACCAAATGCCTTCTACTTCATCTTCTTCTTACTTGCAACAGCGATTCAAGGAGGATG
ACAATTTGTTTGGTGATCTTGTGAGCATTGCAAAGTCAAAGACGAATAGACCTGCTAAGTGATTGAAGACATTTAGCGACTGTGAATTTGGATTTCTTTTAATTTTAACTAATTCTTTCCAT
TTTGCTGTCCAATGTATCTGCATATTAATTTTATTACATTAACCTCAATTGTTT
>comp165001_c0_seq9
CGCTGGCATGCATGTCCTCCTCCTATGAAGGATGATGGCAATATTTTGCTTGGGCTTTTTGCATTGACAACGAGAGAGAGCACGCTGGCTGGTCGGCGGCCATGAACGCGGTCGTGACCG

TTTATTTGCTCGCTCCCTCTTGCTGTCATCCACTTCGCCCTGTAAAATGGCTGCCAGGAAATTTCGGAGCCGTGGCTCTCTTACCAGGAGATTTCTACTGTATGAAAGGTAAGCCTAATAAA
TTTTATAAAATTTCTTGTATATTTATATATCTA
>comp165048_c1_seq1
GCCCTTCCATGTTTAGCCGCCATCGGTGCCCGAAGTGGGGCCTCCATAACCTCAATGGTAACTTCGCCGCAGACCCTGTTGAGATCCGCGAGGTATGGGACTCCAACCTGGAGGAGGAA
CTCTCTCTCATCCGCTCTATTGTCGCCGACTACCCCTTCGTCTCCATGGACACCGAATTCCCCGGCGTTGCAATTCTCCCCGTCGTCACTTCTCCGAATTCAATAACCAAATTCAATTACCA
GACTCTCCAACTCAACGTCAACATCCTCAAACTCATCCAACTCGGCCTCACCTTCTTTGACTCTGCAGGCCACCTCCCTTCCTTCGGCTCTCCCCCTCGCCCTTGCATTTGGCAGTTCAAT
TTCCGCGAGTTCGACGCAGCAAGAGACATCTTTGACCGCAGCTCAATCGAGCTTCTCCGCTGCAGCGGCATTGACTTAGAAAAGAATCATTGCCTAGGCGTCACTGCTAATCGCTTTTCC
AAACTCATCATCTCTTCCGGCGTCGTGCAAAATGATTCCGTTCGATGGATCACTTTCAATGGTCGCTTTGACTTAGGATTTCTTCTCAAAACGTTAACTTGCCATGATCTACCGGACTCTCA
GGAGGATTTCTACCGTTCTCTACGGTTGTATTTTCCTAAAGTCTATGACATCAAGAATATTATGTGGTTCCGCAACAACTTCCCTGACGGGCTGAAAAAGGTCGCAGAGGAGCTTGGGGTG
AAGAGAGCGGGAACCTGCCATCAGACTGGATCGGATAGTTTGCTCGCCGCAGCCGTCTTCAAGAGGTTGAAAGATCATTTTATTGTTGGATCTCTTGAGAGATATGTTGGAGTTTTACGTG
GTCTTAGCTCGGATTGAACATGATCCATTATTTGCAATAATAAAATTTTTGTAATGAAAATAAATTTGGGCCATCTCCTATTATTTTCTTTGGGAATTTTTTTGGTGGCCTATTGAATATTGGGT
TTTCTATATTTTTTTAATAAAATTTATTACTT
>comp165048_c1_seq6
GCCCTTCCATGTTTAGCCGCCATCGGTGCCCGAAGTGGGGCCTCCATAACCTCAATGGTAACTTCGCCGCAGACCCTGTTGAGATCCGCGAGGTATGGGACTCCAACCTGGAGGAGGAA
CTCTCTCTCATCCGCTCTATTGTCGCCGACTACCCCTTCGTCTCCATGGACACCGAATTCCCCGGCGTTGCAATTCTCCCCGTCGTCACTTCTCCGAATTCAATAACCAAATTCAATTACCA
GACTCTCCAACTCAACGTCAACATCCTCAAACTCATCCAACTCGGCCTCACCTTCTTTGACTCTGCAGGCCACCTCCCTTCCTTCGGCTCTCCCCCTCGCCCTTGCATTTGGCAGTTTAATT
TCCGTGAGTTCGACGCAGCAAGCGACATTTGCGACCGCAGCTCGATCGAGCTTCTCCGCCGCAGCGGCATGGACTTAGAAAAGAATCACCACATCGGCATCACCGCTGATCGCTTTTCC
GAACTCATCATCTCTTACGGCATCGTGCAGAATTCCGTCCAATGGGTCACTTTCCACAGTTGCTTCGACTTAGGATTTCTTCTCAAAACATTAACTTGCCGCAATCTACCGAACTATCATGC
GGATTTCTACCGTTTGCTAGAGTTGTATTTTCCTAAATTCTATGACATCAAGTTTTTGATGATGTTCTGCAACAACCTCCATGGCGGGCTCAACGATGTCGCAGAGGATCTTGGTATGAATAT
AGTCGAAACTCGCCACCAATCTGGATTGGATAGTTTGCTCGCTGCTGCCGTCTTCAGGAAGTTGAAAGTTAGTTTTTTTCATGGATCTGTCGAGAGATACGCTGGAGTTTTATATGACCTTC
AGACGTGATTGAACATGATCTATTATTAGCAATAATAAATTAAAAGAAAAAATTAACTAATAGTTAATTTTTTTTTTTAAGGAAAATAAATTTGGGCCATCCCCAAGTAATTGAATATTGGTTT
TTCTATGTTTTTTAAATAAAATGTATTGCTT
>comp165048_c1_seq8
TTTATTTTATGTGTAACGATTAAAACATTATATATATAGACTAATGAGCAAATTTCGGTGAAGGCATGGACAAGAGCATTTGGTGAAGAGGAATTTGTTAGTGATTTTTGGATTTTAATTACAT
CCGCCCAAAATGGTGGAATTGCAGGAATTACGGGTCAACAGAGGAGAATAACTATTTATAGGATTTAAAGTTTTCTTTTTAAACCCTATTGCTAAACAATTCCTTCAAAAAAACAATTCCTT
CTCCCTTCACAACTATTTGAACATGACTTCGGCGCCTCAGATGCTTTATGGTGACTATGCCGCTGCCGTCGATGCCGTCGACTCTGTTGAGATTCGCGAGGTATGGAATTTTAATCTGGAGG
AGGAATTCTTTCTCATCAGCTCTATTGTCGCCGACTACCCCTTCGTCGCCATAGACAGCGAGTTCCCTGGCGTCCCACTTCGCCCCGTGATTAGTTCTCCGACTTCATTCACCGAGTTCAAT
TACCAAACTCTGAAAATCAACGTCAATTTCCTCAAACTCATCCAAGTCGGCCTGACCTTCTTCGACTCTTCAGGCCGCCTCCCTCTCGTCGGCTCTCCCCCTCGCCCTTGCATTTGGCAGT
TCAACTTTCGCGAATTCAATGCTGCAAGCGACTTCTTCGCCCCTGACTCCATCGAGCTTCTCCGCCGTAGTGGAATAAACTTCGAAAAGAATCACTGCCTCGGCGTCGCTGCCAATCGCT
TTGCCGAACTCATGATCGCTTCTGGCGTCGTGCTGAATGACACCGTCCAATGGATCACTTTCCATGGCTGCTATGACTTCGGTTATCTTCTCAAAATGTTAACTTGCCGCAATCTGCCGGAC
TCTCAGGAGGATTTCTTCCATTCTCTGCGGCTGTATTTTCCAAACATCTATGACATCAAGCATTTGACGAAGATCTGCTCTAACTTCTATGGCGGGCTGAACAAGCTCGCAGGGCTACTTGG

GGTGAAGAGGATGGGAACCTGTCACCAGGCTGGATCAGATAGTTTGCTCACTGCTGCCACGTTCACAAAGTTGAAATCTTGTTTAGCAAATGGATCTGTACAGAGATGTGCTGGAGTTTT
ATATGGTCTCGGTGTTGATTGAACCATGCATATGCCATGTCTAGTATAAGTAAAGTAAAAGAAAAAAATCATTAGTATTTAAATTTTTTGTAAAAATAAAAATAAATTTGGGATCAAATGAAT
TTTCATTTTTGTTATTTGTAACATTGTGAAATGTTACAAATAATAGAATACATGAATTTAAATTTTATATCAGATGCAATAGCTCTTATAACTATTTAGATGA
>comp165055_c1_seq10
GCAAAGAGCTATTGAGGGCTACTAGCTGAGGAGTTTTCTGATAGACTTTCATAAGCCATCGATAATTATAAACTGGCATCCAAACATTGTATATCATGATCATAAATGGCTAAGGAATGTTG
GACTCTAATGAGGAACTATAGAGACCATACATACGTAGAATATATTCACATAGACTTCAAATGCTATAAATAACAAATTCCACAGAACATACAAAGAACATTGGCTTGAATATAAGTTTTCA
GGAAACAAAGAAAAAATAAAAAATGAACGATTATATGTACAATGACTTGGGAGTACTAGAACAACAAGTCATGCAAGCTTATCACCAACTAGTAAGTGCTTAATGAAACATAGTAATCAT
ATCTCAGGCTATCACTGGGCATCCTTTGGCGACCACTCCAGGAGCTGTAGGGCAATTTGCAAGGGGACAGAAGTCACTTGCATCGCCTCCCACCCAACTAATTCCACCAATATCATTGTAT
TGTAAGGCAAAGAAGATGAGAACTCCCATGAAAGCGACTCCGGCATCGAGGGCAGCCGACATTACGTAGGTGTGCCTTGCCCACCATTCCTTATATTTGTTGTAAATGTAGTAGTTGAAG
AAGATGCCAACAGCACCCCAGGTGATATAGTTCACTGTGTGTGCAGGAGGGATACCACCAGCACCTCCAATAATGAGAGGTAAATGGATGTATTTGAGCCATTTGCAGTTAGGGAAGACC
TTAGAAATCCACCAGAAAGGAATGGGTAGCAAGAATCCAGCAAGGAAGAAGTAGTTCAAAGCAGAGTAGAGACCGAGTTTTCCGAACATTCGCATAGGGCCAACAAGTCCCCATATGAT
GGATGCATTGTAGAATACATCGTCCCCTGGGCATGTCCATGGACTGTCAGCTGGTAGTTTATCAGTGTCACATATGTTCTCAACTGAATCTAAAAGAAACCATGCAGTTCCAAAGTTGACC
GTCGCTGCGATCACGGTCCCTGTCAACTGTGAGATGAACATAGATCTGGGGGGAATCTTCATATAGTGTCCAAGGTTGAAATCACTGAGAAATGTGAGTGCTTGAGTCATGCTAATATAGC
CATATGTCTTGAATGTCACATTTGCTAGGGGCTTTCCAGGTAGAATTAGACCAATGATGTACTCTGTGATGATATTGAGCCCTGGTGACTGGTTTGTAGTTGCTGTAATGACTCCAATAGGTA
ATGTGAAGAAGAAAGCCATAGCACAACCCAATAATATTCCCCACCATGGAAGTTGCAAAGCTCCACCAAATCCCACACAAGTGAAAATTGAGAGTAGAATCATCAAAATCAAGAGAACA
TGGAACCACCATTGAGGTATAGCATCATAATTCTTCATCAATCTAGCATGAACATCTAATTTCTTCTCCTTAGAGGAATCTCTGGCTTGTCTCCATGACTGCCAAATATCCTTTCCATTGTGG
AGAGCAACATGCATGACCGATGATGTCAAGGTTGCAAATCCTAAGCCATATGTCAAAGCAAACATAATACTCATGTTTATCTTGCTGTAACTCTCCTGTTCTTCAACATTCAAGGTGAAAG
TCTTGCTATTAATAATTCGTGTCGTGTTGTACGGTTGCGCAGAAGAGTCGAAGACATGGGACGAGATCATTGGAAAATTCTTTGCCCTGTATATGTTGGTCCAATAGGCAAATGGAAGCATT
ATATAGATGGCAATGAAGTAGCCTACCATTATGTTAAAGATTGCACTTGCAGGTGTTCCCAATGGGCTACCAAGAAAACCAGCAACTGTTGACCAATCAAGACCGAAGGAGCCAATGCCA
AGCCCTTTCATCCCTGATCCAATTTGTTGCATGGTGATCGACTCCTTAAATATCAAGCAGAGGACAGAGACTGTGGAGATGGCTGGGAAGAGATAGCCGGGAATGGCATAGTATGCAAAG
CTGAATACAAAGATTACTACGAAGAATTGAAATTTTGTCAGGCTGCCCTTTCTTCTCTTCTCTTTTTGATGCAGTGTTTTGAAGAGGGAAACTTGAACAAGATTTGCAGGCCACCACATAT
ATGGAGATTCCACCAGATACTTTCTGAAAATTCCAGCCCAACCATAACCTAGCACCTGAGTGGTTTGTGACAACAAGAAGGCAACAACAGGATTGATATTTCTGCGATAGAAAGCCCTAA
CAATGGTGACAATGTGTATGGAATAGACGCCGCCGGCACCAGAGTTGGCAAAAATGGTGATGAGACAATGCTCCTTCATATTGAATGGCCCAGGATTGAGGGAGAAAGTCCATTTGGTGA
AAGGAACCTTGATGTTCTTCTTCGGCAAAGAAGCCGCGAGGAATCTGCCAATGGGCAGCGAGACGATTTGAACGCAGACTGGACCAATGGTAAGCTGATTCGTTCTGTAGCCGAAGAAT
TTGTTGGCAAAAGCGAGCATTACGCAGGAGATTGATCCGAGAAGCCATGTTCGGAAGGTCAGGACTGGGAGGTTAGGGTTGTCGGCGGGGTCGACTGTAAGCCGAACTTCTTCAATCGG
GTGCTCGTTCTCTTCCACTATCCACCCTTCGGCAAGCTCGAGCTTTGGCTGATGAGAGAGACCGACGATACAGTCGCCCATCGTTTTGTCCTTCTCCTTCTTCCTTCTCCTTCTCGGATTCT
CTTCCTTTATTTAAATACAAAGCGTTGCGTATTTTTATACTCGATACGGTTTTTTGTCAATGCTTTTTTTAGACGCTTACGGTTATAACTCGGCGAACGATATTTTGCCTCGTAGCATTCAGCT
CGTCCGTGGTGATCTGTTCGTTAACTAGAACGGTTT
>comp165062_c1_seq1
TTTTTGTCACAACCACCATTTTCTATAAAAGGGTTACAGTGTGGCAAAGATTAGCACGTAAATAATAATAAGCCATTGAATGCTTGATCATTATCATGTACTTAAAGGTATTAATCCAATTATT
GTTGAATCATGTAATCTCTTAAATTTAGAAAGCTAATAATCACAATTTTTTACTTTCTTAATTCGCCTATAATTGTATATATTTTTTGTGACTTATTTTTTAACATGGATGGATGAAAATTATATC

CACAATCTCCTGATTCGACTTAAAAATATTGAAAGAGGCACATGAATAAAATTAAATAAAGCTTTACATAAAAACTTGATAAAGAAACAAAGGATAGTGAGAAAGAGCTTGGTCCTGTAG
AAGAAAAAAGAACGTACCATAATGGTAAGAGGGGAGGCGTACATGCAAGTGCCGAAGATGACACAGAGGATGCCGACAATCATCGACCTCTTCTCGTGACTATGACCCACCGTGAGCA
CAACTGACACAACCACCGCCATGAAAGCCAATTCCCCTACTAAGATCTTCAAAACCTTCAACCTCAGATCCTTAGGGGCGTAAATGAAGAAGATCGTAAGATAAGCTCCCTCAAGCACAA
TACCAATGCCATTTATGGTAACTACGAGCATTGAATTGGGGTGGACGATGGGTAAGCCATAGAAAACCCAGAGCATGCAGTTGAGCAGAGTCGCAACATACGGGATTGGTGAGAACTGCT
CTACTGCATTCTTTCTCCATATTTTTATGAAAGTCGGTAAAGGCGAAAGAAATAGTCTAAATGATACTACATTTCCTATCCCGATCCCATTTTCCCAATACAATTTTTAAGAAATCGGAAAGG
AAAAGTGAAATTAATCAAAGGAAAATTGTTATGCCGCGCCGAAGTTACCGATGATCCCCACGATGTTACGGACAGCATCGGTGCTCAC
>comp165062_c1_seq10
AAAAATAATAATAATAATAATTGATTAGAAATAATAATTTTTGAATTAATATTCTGACCTTTGAATTCGATAAAAATTCCAACTATTATCAAATTCCCAAAATGATTACCTTCCCTACGACAAC
AATAGAGCTTGAAAGAAATCAAGATTTAAAACAGTGAATGCGCGGCTAATAGAAAGAGCTATCCATTAACTATCGCTTGATTCATCTTAAAAGTGGATATTACAAAAAAAATGCAACTCTT
GGACCTCCTTTTCCTTTGGGAGCTTGAAGCCATTAGTCTTCCTTTCAGGCCATTTCAACCTCCTGTCTTTTCCTGTCCTCATTCTGTTGCTCAACGATCGGGGTACTTCGGCAATAACATCC
GTACAACACTAGCTGGAGGAATCCGAGCAGCGCTCCGAGCCCGTTCGGTAGCTGCACGAGGGTCACCACATCAGTCTTCAACAAATTTTAACAGCAGAAGATTAAGAAAGAAAAAATA
GTATAGTTGTCAGGAAAGAAATGTGTTACCGTAATGAAAATGTCGAACTTCAAAAGTGCATAGGCGGTCCAGCATACGCCATTCAGAAAGCTAACTAAGGAAAGCCAGAACGGCATGTA
CTTCACGCTTTTTGTCACGACCACCATTTTCATAACGGTAAGAGGGGAGGCATACATGCAAGTGCCGAAGATGACACAGAGGATGCCGACAATCATCGACCTCTTCTCGTGACTATGACC
CACCGTGAGCACAACTGACACAACCACCGCCATGAAAGCCAATTCCCCCATTAAGATCTTCAAAACCTTCAACCTCAGATCCTTAGGGGCGTAAATGAAGAAGATCGTAAGATAAGCTCC
CTCAAGCACAATACCAATGCCATTTATGGTAACTACGAGCATTGAATTGGGGTGGACGATGGGTAAGCCATAGAAAACCCAGAGCATGCAGTTGAGCAGAGTCGCAACATACGGGATTGG
TGAGAACTGCTCTACTGCATTCTTTCTCCATATTTTTATGAAAGTGGGTAACGGTGAAAGAAACAGTCCGAATGATATCACATTCCCGATTATCCCAATGACGTTACGGACAACGTCTGTGC
TCACCATCTTTGTTGGAATATTGAGAAAACAGATGGACGCAGAAAAAGAGATGAGTTGTGGGGAAGAAGAGGAGCAGAGGTT
>comp165062_c1_seq13
AAAAATAATAATAATAATAATTGATTAGAAATAATAATTTTTGAATTAATATTCTGACCTTTGAATTCGATAAAAATTCCAACTATTATCAAATTCCCAAAATGATTACCTACCCTACGACAAC
AACAGAGCTTAAAAGAAATCAAGATTTAAAGCAATGAATGAGCGGCTAATAGATAGAGCTATCCATTAGCAATCGCTTGATCCATCTTGAACGTGGATATTACACAAGCAAGAGAGAAGC
TAGCTAGTGCACTATCCACTCCACCCAACATCAAAGAGAAAAAAAATCAGCTACTCTTGGATTTCCTTTTCCTTTGGGAGCTTGAAGCCATTAGTCTTCACTTCAGGCCATTTCAACCTCC
TGCCTTTTCTTCTTCTCCTCATTCTGTTGCTCAAAGGTCGGGGTACTTCGGCAATAACATCCGTACAGCACCAGCTGGAGGAATCCGAGTAGCGCTCCGAGCCCGTTCGGTAGCTGCACG
AGGGTCACCACATCAGTCTTCAACAAATTTTAACAGCAGAAGATTAAGAAAGAAAAAATAGTATAGTTGTCAGGAAAGAAATGTGTTACCGTAATGAAAATGTCGAACTTCAAAAGTGC
ATAGGCGGTCCAGCATACGCCATTCAGAAAGCTAACTAAGGAAAGCCAGAACGGCATGTACTTCACGCTTTTTGTCACGACCACCATTTTCATAACGGTAAGAGGGGAGGCATACATGCA
AGTGCCGAAGATGACACAGAGGATGCCGACAATCATCGACCTCTTCTCGTGACTATGACCCACCGTGAGCACAACTGACACAACCACCGCCATGAAAGCCAATTCCCCCATTAAGATCTT
CAAAACCTTCAACCTCAGATCCTTAGGGGCGTAAATGAAGAAGATCGTAAGATAAGCTCCCTCAAGCACAATACCAATGCCATTTATGGTAACTACGAGCATTGAATTGGGGTGGACGAT
GGGTAAGCCATAGAAAACCCAGAGCATGCAGTTGAGCAGAGTCGCAACATACGGGATTGGTGAGAACTGCTCTACTGCATTCTTTCTCCATATTTTTATGAAAGTCGGTAAAGGCGAAAG
AAATAGTCTAAATGATACTACATTTCCTATCCCGATCCCATTTTCCCAATACAATTTTTAAGAAATCGGAAAGGAAAAGTGAAATTAATCAAAGGAAAATTGTTATGCCGCGCCGAAGTTAC
CGATGATCCCCACGATGTTACGGACAGCATCGGTGCTCAC
>comp165062_c1_seq14
AAAAATAATAATAATAATAATTGATTAGAAATAATAATTTTTGAATTAATATTCTGACCTTTGAATTCGATAAAAATTCCAACTATTATCAAATTCCCAAAATGATTACCTACCCTACGACAAC

AACAGAGCTTAAAAGAAATCAAGATTTAAAGCAATGAATGAGCGGCTAATAGATAGAGCTATCCATTAGCAATCGCTTGATCCATCTTGAACGTGGATATTACACAAGCAAGAGAGAAGC
TAGCTAGTGCACTATCCACTCCACCCAACATCAAAGAGAAAAAAAATCAGCTACTCTTGGATTTCCTTTTCCTTTGGGAGCTTGAAGCCATTAGTCTTCACTTCAGGCCATTTCAACCTCC
TGCCTTTTCTTCTTCTCCTCATTCTGTTGCTCAAAGGTCGGGGTACTTCGGCAATAACATCCGTACAGCACCAGCTGGAGGAATCCGAGTAGCGCTCCGAGCCCGTTCGGTAGCGTAATGA
AAATGTCGAACTTCAAAAGTGCATAGGCGGTCCAACATACGCCATTGAGAAAGCTAACTAAAGAAAGCCAGAACGGCATGTACTTCACGCTTTTTGTCACAACCACCATTTTCATAACGG
TAAGGGGGGAGGCGTACATGCAAGTGCCAAAGATGACACAGAGGATGCCGACCATCATCGTCCTCTTCTCATGAGTATGACCCACCGTGAGCACAACGGACACCACCACCGCCATGAAA
GCCAGTTCCCCTGCTAGGAACTTCAAAACCTTCAACCTCAAATCCTTAGGGGCATAAAGAAAGAAGATTGTAAGATAAGCACCCTCAAGCACAATACCAGTGCCATTTATGGTAACAACG
AGTATTGAATTTGGGTGGACGATGGGTAAGCCATAGAAAACCCAGAGCATGCAGTTGAGCAGAGTCGCAACATACGGGATTGGTGAGAACTGCTCTACTGCATTCTTTCTCCATATTTTTA
TGAAAGTCGGTAATGGAGAAAGAAACAGTCCGAATGATATCACATTCCCGATTATCCCAACGACGTTACGGACAGTGTCTGTGCTCACCATCTTTGTTGGAATATTGAGAAAACAGATGG
ACGCAGAAAAAGAGATGAGTTGTGGGGAAGAAGAGGAGCAGAGGTT
>comp165062_c1_seq5
CTGATAAAGGGTTACAGTGTGTCTCGTAAATAATAATAAGCCATCGAATGCTTGATCGTTATCATCTACTTAAACGTATTAATTCAACTATTATTGAATCATATAATATCTTAAATTTAGAAAGC
TAATAGTCATGTTTTTTTACTTTCTTAATTCACCTATAATTGTATATATTTTTTGTGACTTATTTTTTAACATGGATGGATGAAAATTATATCCACAATCTCCCGATTCGTCTTAAAAATATTGAA
AGAGGCACAGGAATAAAATTAAACAAAGCTTTACATAAAAACTTGACAAAGAAACAAAGGATAATGAGAAAGAGCTTGGTCTTGTAGAAGAAAATAAGAACTTACCATAACGGTAAGG
GGGGAGGCGTACATGCAAGTGCCAAAGATGACACAGAGGATGCCGACCATCATCGTCCTCTTCTCATGAGTATGACCCACCGTGAGCACAACGGACACCACCACCGCCATGAAAGCCA
GTTCCCCTGCTAGGAACTTCAAAACCTTCAACCTCAAATCCTTAGGGGCATAAAGAAAGAAGATTGTAAGATAAGCACCCTCAAGCACAATACCAGTGCCATTTATGGTAACAACGAGTA
TTGAATTTGGGTGGACGATGGGTAAGCCATAGAAAACCCAGAGCATGCAGTTGAGCAGAGTCGCAACATACGGGATTGGTGAGAACTGCTCTACTGCATTCTTTCTCCATATTTTTATGAA
AGTCGGTAATGGAGAAAGAAACAGTCCGAATGATATCACATTCCCGATTATCCCAACGACGTTACGGACAGTGTCTGTGCTCACCATCTTTGTTGGAATATTGAGAAAACAGATGGACGC
AGAAAAAGAGATGAGTTGTGGGGAAGAAGAGGAGCAGAGGTT
>comp165062_c1_seq8
AAAAATAATAATAATAATAATTGATTAGAAATAATAATTTTTGAATTAATATTCTGACCTTTGAATTCGATAAAAATTCCAACTATTATCAAATTCCCAAAATGATTACCTTCCCTACGACAAC
AATAGAGCTTGAAAGAAATCAAGATTTAAAACAGTGAATGCGCGGCTAATAGAAAGAGCTATCCATTAACTATCGCTTGATTCATCTTAAAAGTGGATATTACAAAAAAAATGCAACTCTT
GGACCTCCTTTTCCTTTGGGAGCTTGAAGCCATTAGTCTTCCTTTCAGGCCATTTCAACCTCCTGTCTTTTCCTGTCCTCATTCTGTTGCTCAACGATCGGGGTACTTCGGCAATAACATCC
GTACAACACTAGCTGGAGGAATCCGAGCAGCGCTCCGAGCCCGTTCGGTAGCGTAATGAAAATGTCGAACTTCAAAAGTGCATAGGCGGTCCAGCATACGCCATTCAGAAAGCTAACTA
AGGAAAGCCAGAACGGCATGTACTTCACGCTTTTTGTCACGACCACCATTTTCATAACGGTAAGAGGGGAGGCATACATGCAAGTGCCGAAGATGACACAGAGGATGCCGACAATCATC
GACCTCTTCTCGTGACTATGACCCACCGTGAGCACAACTGACACAACCACCGCCATGAAAGCCAATTCCCCCATTAAGATCTTCAAAACCTTCAACCTCAGATCCTTAGGGGCGTAAATG
AAGAAGATCGTAAGATAAGCTCCCTCAAGCACAATACCAATGCCATTTATGGTAACTACGAGCATTGAATTGGGGTGGACGATGGGTAAGCCATAGAAAACCCAGAGCATGCAGTTGAGC
AGAGTCGCAACATACGGGATTGGTGAGAACTGCTCTACTGCATTCTTTCTCCATATTTTTATGAAAGTCGGTAAAGGCGAAAGAAATAGTCTAAATGATACTACATTTCCTATCCCGATCCC
ATTTTCCCAATACAATTTTTAAGAAATCGGAAAGGAAAAGTGAAATTAATCAAAGGAAAATTGTTATGCCGCGCCGAAGTTACCGATGATCCCCACGATGTTACGGACAGCATCGGTGCTC
AC
>comp165086_c2_seq6
CCTTTCTTCGGGCTTGAACACCGGCTTGGTTGATCGCACTTTTGGAACTTTTACTTTTGAAAGTATACTTTATATCTTTCCAATATTTATCTCGAGGCATGGCAAGGGGATATAGAAGGGGG

AGAAAAAGAGAGAGAGGCTTTACTTTTCAATAATTTCATCCAAAACTATCGATCTCTATCACAAACAATTTTTATTTTTTATTTTTTTGCTTTCCAACAAATACTCAAAGCAACTCATTCTCC
AACAATCCTACTTTTCATCTCTTTTTCTGAAAATTTACATAGTTTCACTTTCAAAAACATACACTTCTACATTTAAAACATACAATCTCCACTTCTTACTTTTCATACACCTCAAGTTCCATTT
TTAAAACACATATTTTTCACAAAAATCATACACTTCATCTCTAACACAAGACACTTTACCTTTAAACTTCCACAAGCATCTCCCTTCAAACTTACATTTT
>comp165090_c1_seq4
AAATTCTTTTTTAATATTTTTTAGTCTCATTAGCATGAAAATAATTAAGGAATGATAAATAAAAAGAAACGTGACTTAATTAGAAAGGAAACAAGACTAACTAGAAATTTTGAGTTTTTTCT
CTTAATTTCCCATTCATTTTCATATACATACTCATTCTAATTACAACCACCAGCCCCAAAGCCAATTCTCAAGTTTTTTATATCATGCACTATATTGAATGTGCGTAACTGCACATTACCAATAA
TCACCTTATCACTAGCATATTTATTGGCAGCGAAGGCTAAGCATTGTTTAGATTTAGTGGTCCCGAGAAGTATCCCTGAGATATCTAATTTAATAATAACCCCGCCATCAAATTCAAACGTAA
TCTGCGGCACCTTCACGTTCACGTTTGGGTAGTTAGTAAAGTTATAGCATGTGTCAAGTATGCCCCATGACGGCGCCCTCGGGTAATTGGCCATGTATTTACGGAAAGTGCTGCGGATGCC
ATGGTAGGTAATGGGCGGCAGTCGGCTAATCACTGTGCCAGAGTCCAACATGGCTTTTTTGCCGGAGGCCAACATGGTTCTAGGGGATTTGAACACTGTAGGTGAATGGGCAGACAAATC
ACCCCCAACAGTAATAGAAATTAGCTTGACGAAGTAGAAGGATGGCATCATTTCGCTGGTTATCATCGGTGTATATTGCACATGAATGGCATCAATCCCTACATCACTCCCTAGTGAAAGAT
ATCCAATTATGCCTGGCTTTGATGGTAGACAATATGAGAATACCAAGTCATATGATTGAGCTGTCTGGGAAATAATTGAAAGAGGACCCCGGCCGAAGCCTAATATCCCATCTACCTTCGGT
ACGCTGAAGCTGTTGTTTTGTCCGCAGCCATAACGAAAATTCTTTATAATATCATAGGAGAATTGTAAGGTGTCTTGAACGAGGTAGCCTTTAGTGTAAGAACCATCGCGGTATTGTACCTC
ATAGAGACAATTGTCGGTCCAGGAGCAATCATATGTATTTAGTTGGCTACAATAGTCGGAGTGGCAGCGGATGGATGCATAGGTGGAGGACTCGAAAGGGTTATATATTGGGTCTTTTTGG
GGATAACACTTCTCGCAGGGTATACATTGCATCCAAGTGACATCGCTGCCGGTGTCAAAAACAAGTGAGTATGGGGAGGGAGGGCTGCCTAGGTATATGGTGACGATAAATTTGTGGGTA
CCAAAAAAAAACGCCTTCTTCTATTGGAACTGTTGCTGAAACAGAGCTGCCAAGTGGGTTGAGATGGTATTCTCCGGTAAGCCATCGCACTGATACTAGAGAATTCTTCTCTATTAGATCTA
TTAGAAGAAAAAAGAAGAAG
>comp165090_c1_seq8
AAATTCTTTTTTAATATTTTTTAGTCTCATTAGCATGAAAATAATTAAGGAATGATAAATAAAAAGAAACGTGACTTAATTAGAAAGGAAACAAGACTAACTAGAAATTTTGAGTTTTTTCT
CTTAATTTCCCATTCATTTTCATATACATACTCATTCTAATTACAACCACCAGCCCCAAAGCCAATTCTCAAGTTTTTTATATCATGCACTATATTGAATGTGCGTAACTGCACATTACCAATAA
TCACCTTATCACTAGCATATTTATTGGCAGCGAAGGCTAAGCATTGTTTAGATTTAGTGGTCCCGAGAAGTATCCCTGAGATATCTAATTTAATAATAACCCCGCCATCAAATTCAAACGTAA
TCTGCGGCACCTTCACGTTCACGTTTGGGTAGTTAGTAAAGTTATAGCATGCGTCGAGTATGATCCATGACAACGCTCTCAGGTAATTGATCATGCCTTTTTCGAAAAATGCTGTGGATGCC
GGAGAAGATAATTTAAAATGGATTTTCTGCAAGAGGCCAAGATGGTTCTAGGGGCGCTGAACACACTAGGTGAAAAGGCAAGCCATTCACGCCAAATAGAAATTAGATTGACGTAGTAG
AAAGATGGCCTCCTTTGGTTGGTTATCATCGGTGTATATAGCACAAGAGTGAGATATTTTCCTAGTACAAGATATCCAATTATGCCTGACTTTGATGGTAGACAATATAAGAATACCAAGTCA
TATGATTGAGCTGTCTGGGAAATAATTGAAA
>comp165090_c1_seq9
AAATTCTTTTTTAATATTTTTTAGTCTCATTAGCATGAAAATAATTAAGGAATGATAAATAAAAAGAAACGTGACTTAATTAGAAAGGAAACAAGACTAACTAGAAATTTTGAGTTTTTTCT
CTTAATTTCCCATTCATTTTCATATACATACTCATTCTAATTACAACCACCAGCCCCAAAGCCAATTCTCAAGTTTTTTATATCATGCACTATATTGAATGTGCGTAACTGCACATTACCAATAA
TCACCTTATCACTAGCATATTTATTGGCAGCGAAGGCTAAGCATTGTTTAGATTTAGTGGTCCCGAGAAGTATCCCTGAGATATCTAATTTAATAATAACCCCGCCATCAAATTCAAACGTAA
TCTGCGGCACCTTCACGTTCACGTTTGGGTAGTTAGTAAAGTTATAGCATGTGTCAAGTATGCCCCATGACGGCGCCCTCGGGTAATTGGCCATGTATTTACGGAAAGTGCTGCGGATGCC
ATGGTAGGTAATGGGCGGCAGTCGGCTAATCATTCTGCTAGAGTCCAACATGGCTTTTCTGCCAGGGGCCAACATGGTTCTAGGGGAGTTTTCGACCAATCACCCCCAACAGAAATAGA
AATTAGCTTGACGAAGTAGAAGGATGGCATCATTTCGCTGGTTATCATCGGTGTATATTGCACATGAATGGCATCATCACTCCCTAGTGTAAGATATCCAATTATGCCTGACTTTGATGGTAG

ACAATATGAGAATACCAAGTCATATGATTGAGCTGTCTGGGAAATAATTGAAACTGGCCCCCGGCCGAAGCCTAATATCCCATCTACCTTCGGTACGCTGAGGCTATTGTTTTGTCCGCAGC
CATAACGGAAATTCTTTATAATATCATAGGAGAATTGTAAGGTGTCTTTAACGAGGTAGCCTTTAGTGAAAGAACCATCGCGGTATTGTACCTCGTAGAGACAATTGTCGGTCCTGGAGCAA
TCATATGTATTTAGTTGGCTACAATAGTCGTTGTAGCAGCGGATGGATGCATAGGTAGAGGACTCGAAAGGGTTATATATTGGGTCATTTTGGGGATAACACTTATCGCAGGGTATACATTGG
ATCCAAGTGACATCGCTGCCGGTGTCAAAAACAAGTGAGTATGGCAAGGGAGGGGTGCCTAGGTATATGGTGACGATAAATTTGTGGGTACCAAAAAAAATGCCTTCTTCTATTGGAACT
GTTGCTGAAAGAGAGCTGCCAAGTGGGTTGATGCCAGCGGAGTTGTTTGGCAGCAATGACTCAACATCAATGAGATGGTATTCTCTGGCACGCCATCGCACTGATACTAGAGAATTCTTC
TCTATTAGATCTATTAGAAGAAAAAAGAAGAAG
>comp165091_c1_seq5
CAAACAAAATATAAATATAACATAACAACATAATATGTATGTTTAACAAATGCAGTATGATGATAGCATTTCGTACTTTTTTACTCTATGTACGCATTAATACAATATTAATCAAACAAAAGAC
GATCATGTCTATGCAATTAGCATTCAAAATCATATGATAATGACAGATTAATATTTTGATTGTTGCGTCCTGAAGTATTCATGGCAATAGAGAAATATAAAGTTTAACGTCTCAGCTTTGTACG
AAGACCCGACATGCATGCAAATAAATAGAGACCGGGTGGAGTTTTCGGTGTCACTTGGTGATCGGGTGGAGTTTCGGCATCACTTGAAAGTCGGGCAGAATTCTAGCATCATTTGGAAGT
CGGGCGGAACTCCGGCGGTGGTTGAGGAGGAGTTTTTTTCCTCCTATTCTTCTCTTATATTCCTCTTCTTCTTCTTACTTGGGTCCCCTCTCAAAATAAATGAGGTGCTATTTATAGTTTTCT
GAGAGTGGTATGACCCGTAAATAGGAGGAGGATCAAGGGCAAATGTAGA
>comp165104_c1_seq1
TGTTTTGAGAATCCAATGCTTTATTTCCAACCAATTTATTCACATTTGACAACTTAACAAGTAGATTGTGCATGCCTTGGTGATATCTTATTGTTCATCAATATAAAATTTACATCTGATATTAA
CACAAATATGTTACAATGACTCTGATTCTGGGATGACAAATAATACAACAACTTAAATGTATAAAAGCGTACCTTCTTCAATGCGAGTGAATGCAGGAGCTCCATAGTATGGTCAAAGAAC
AACTTAAGATTTTGTATGGGACGGTTTTTTTAATGCTTCTTAAAGTAACTTTTTAATGCAAAAGTTTTAATTTTTGTACTGAAAAGTTGCTTTAAGAAGAAATAAAAAAAATCGTCCCAAACG
AAAC
>comp165108_c0_seq5
AAACTGAAAATCTACACAAATGCTGATGATGTATGAAATAATGCATATATAAAAGAGAGCTGATTTTATAAATATAAAATTTACAGGTTACATCCCAGGGTCTGCAGTAAAGTAAAGGTGGC
CAGGCCCCACAGACAACAAGCCTGGGTTCTAAGCCTATAAGAGAGTAGAATTAAATATAACTCTGCTTATGGTCTTCAGGCTCCAAGTCTTCTATGGAATTCCAAAAAGGAACATCCTCCT
GATGAAGGGTATTACTTTTTGGAGCCCCATTTGTATATATATAAGCAGTTATTTTTAATTCCTTTACAGATTCAAGACACTTTACAGACTCAATCTATATACAATAGATCTAAGAAAATAAAGC
TTTACATTTCTCTTTATAAGTACATCAGTCCCTATGAACATTCTACTTTTGCAGGAATATAAAAGACAGGAAAGTAAAAGCAGGTATGTAATTATGCATGTTATGAGTGCATTGTGAACAGTT
ATATATGCAGGAAAGTAAAGACAGAAAAGTAAAGTACACGGCCCTCACTTATTCACTCCGCTCTCTGGTGCACTGACAGAAAAATAAATATGCATATTATGAGGTACAGGAAAGTAAGCA
AACACACATGGGTGCCCTCTAGGCAAGACACCCGCAAACAAACCACAACAAACCACCGGCCTAGCCTAGGCTAAAGTTGGTTGTCCTAGTAAGCATCTATGTGTGTGAAAGAGGTCGTG
TGGCTTGGATAGGTCTTATAAGAATTTATGTTTGAATTGTGTGTATTATTAAGGTTTATTTATTAAAGGCCAAAAGAAAATTTAATTAATGTTGAAGTAGTGTGTATTCTATTTTTAAAATTATT
CCAATTTTAGAAGTTAAAGTTACTTGATTTAGTTTTAAAGAACTTATAGATTGGTAACTTCCTAAGTGTAGGAGATTATCAATTTATAAGTTCTAGGACTTTCAGAATGAACACCCATCTTATA
TGGAAGACAGACATTCATTTGAGAGTCCCTAAATTTTTATTAGAGAGAGAGAAAGGGACCTTACAAATGCAATGTGACATACCTTCCAAAAGGAGTCGAGTATGCCACATAAGGTCGGTG
TATTATCAAAAATTTGAAGATCTCCAAATCCAAATGTATTGTGCCTTCCTAAAAGGAGCCGTGCACAATACATGGAATATCTTTTGGTTATTTTGATGTATTAAGGGTCTTTTGAAATCAAAA
TGTCTAAAGCCCTCCAAAAAGAATAAAGCTTTAGGCATAAAAGACCTTAATCCATTTAGTCTAAATTAATAATCAAATTTGATTGA
>comp165152_c0_seq5
TTGTTAGAGGAGGATGAAGCCAAGTAGACATTGTTATCAAAGACTTGAAAAACACTGGGGGGAGCGCAAACAAACTTCAATGTGGATGGTGCAAAGGTGGTAAGGAGACGATGGAGAA

GCAAAAGACATAAGAATTTTCCACATATTCAAAGGCAAAGCCAAGTAAATTATTGCGAGCAAGACGGAGGCAATATCAACAATATATTACGATACACCCCTGAGTACCTTTTCGTTAACAA
AGGAGGACGATCTAAAGGAGGTGTATGCTCTTTAAGGTGAATTATTGATTGCAAAAGAATCAGGTGAATAAATGACAAGATAAAATAGAAATTGTGGAGAACGATAAGATTAACTCTACAT
AAGCGACTACATTAAATAACTGTATGACCTACAAGGGTAGAGGTTGGAGCATATAGAAGAAATGTTATGTTCTATAGCCCTAAAATAGCTTGCTCACTAAAGCAAGCTACAAGTGAAATCC
ATGCAGCTCAACGTCTTTACAATCACATGAGGTCCTGAGAAGGAAATCGTGAATTTTTAAGAGTAAATTCTTTCCTATAAAATATGATTAGAAAAAGT
>comp165211_c2_seq15
CTAAAAGATTGAATAAATATATAAAAACATATTATCAGTCATGATCACATTGCCACACAAGGCCAACTAGAGAATATCATCAGGAACTCTCTCAACAATGTTCATTTCTATATATTTGGAAAG
ACTTCTCAATTTGAGAGTTAGTGATAACAACCTTGATTAATAAAAACAAATAGAATCTGAAAAGCTGATGTAATGGACCAATGGTCCTCCACAAAGTGCCCTAGACTCATCTCAAATGTCC
ATTATGGGCCATGAAGCTTTGAAAAAAAGTTAATTTGGAAGCAAATAGACAATCAGTTTTTTTCCTGAATGGAGTAGCATGAAAGCATAAACTCTTAAGAATATTTGAAATAAGACTTAG
GATGGAGTTTAGTGTGTTTTCAAAGAAAATTTAGGTATATGACTGCTCTTCTTTTGTAGTTTTGGGTCGCTCTTCATTTTCATGATAAGTATTAAATGTCAATATTATCATATTCATTATCTAAA
CTGTTTGGGATCATCTTTCAAAATCATCATTCTTCATCCCTTACATTATGTGATATAAAAAGTGGTTAACTTAGAATACTTACTACATCATAGCCCGTGTCCAGTGATATCCGACCGTAACACC
TTAATTTGGAAGAAATAGTGGCGATTGAACTCCTTAAGCTTTAGCATCCATTTTATCTTCCATTGTTACTCGCAAACCACATAAGCAGAAAAATATAAACAGAAAGAAGAATATTCTAAAGG
CTTTGACCATAACGTTGTCTCTTTCC
>comp165211_c2_seq16
TCTACTTGTTTTTAAAAACTCTTGTTCCAACTAAAATTTTAAATGTGCTTTATCATTTTTCACAAAAAATACTAAGTTTTTACATCGCTAACATCTTGATGGCAATTCATGTAATTTGGGGAAT
CATAAATATCAGTATATCCATATAAATTCTCCAACACCTTCAGGCATTGCTATCACAATCTTCTTCTAGTAGAATGAGATTAATTATCTTAAGAGAACTAAACTACATTGACATATTAATAGCAT
GTAATAAAACAATGGCACGACAAATCGGACAATTGTTATTTCTGCAGAGCCACAAGTAAATACAATCTGTATGAAATAAATGTCGGCATGATGGCAATTGGCTAACCTCCTCTCCTTCATCA
AATGGTGACAAACAAATCATGCACTCTAGTGCAGCTTCCTCTTCCTCTCCCTTAACCCCCACTTCATTCTTGTGATATATGAACGTTGGAATCCAACACCGCATTTCTAAAGCATTCGGCCC
ATGGTCGAGTAAGCCGCACACTGAGAGGAGTGGGCGTCGAAGCCAAGTGCAGCAACCCATTGTGATAGCACTGATAACGAGGAGTGAGATGGAGACAAGTGCAAGGCTAAAGAGCAA
GTAGTCGTAGTTGAGTTTGTCATTGGATGGAGGAGAGCAAAATGGTGCCATTTTGATAGTTGCTACATAAGTAACATCAACTTTTAGAAATTAGGAAATTTATTCTCTTTGCTACTCATCCTT
CCTTAGAGGTCACCATAAATAACACCTATAAGAAAATAATTTCTCCTTAAAAAATTTACAAAATAAATTTAAATTAAAATTCTTCTTCTCTTTTTAAACCATTCTAAGTTCGCTCTTTCCCTTT
CTCTCTC
>comp165211_c2_seq22
TTTTAAACGCTATTGTAATTATAAGAAAAACTCTATGAATGTTATGAGAAGTTTAATAAAATTACATGTAAATAAAATAAGAAAATATACTCTTCTTATTTGAGAGTTAGTGATAAAATGCTAC
GACAAATTGGACAGTTGCTATGTGAGTTGAGCCAAAAGTCAATACAAGCTGCATGAAACAAGTGTTTGCATGATGTCAATTGGCTAATCTCCTCTCCTTCATCAAATGGTGACAAACAAA
TCATGCACTCTAGTGCAGCTTCCTCTTCCTCTCCCTTAACCCCCACTTCATTCTTGTGATATATGAACGTTGGAATCCAACACCGCATTTCTAAAGCATTCGGCCCATGGTCGAGTAAGCCG
CACACTGAGAGGAGTGGGCGTCGAAGCCAAGTGCAGCAACCCATTGTGATAGCACTGATAACGAGGAGTGAGATGGAGACAAGTGCAAGGCTAAAGAGCAAGTAGTCGTAGTTGAGTT
TGTCATTGGATGGAGGAGAGCAAAATGGTGCCATTTTGATAGTTGCTACATAAGTAACATCAACTTTCTTAGCCTTCACTTAGTCTATTCTATATTTTTCATCAAGCTTGGCCGCCCAAATCT
GCCGCCGGAGCCTCTTCCTATCGGCTCCCGTACTTCCGCCCTCTCCCCTTCAGTTTGGCAGATCAA
>comp165215_c2_seq21
AAAGCTGCTTTAAGAAACAATAAAAAAGCTGTCCCAAACAATTATTAGTGGAATCAATGGAAGTATGATACAAATAATTGGCAAGCTTATCATCACCGCCATTGTCATCATTATCATAATCA
TTAGCCTTATCGCGACTATTCTTGACTTGAAACTCGAGATTTGATCAAGTTTGCTACATGATGTGCAACCCAGATGGAACCGGCCTCGGAGCACGAAAAGGAATGAGTATGTCCTTCCCTT

TCTATTGACAATGGAGCACCAGGCACCTGTCTTGAAATCTCTTCAAACAATGACAATGGACCCCAGTGATCATCAACGCCGAACAAAAATGCAATTTGTTTCTTTTTTTCTTTCATGAAGC
TCCAATCTGGCTCCTCTGAAAGCTTCTCAAACTCTGTCTTTGCCATGAATAAGACATTGCGCATTGTGTGGTACTTCATGAGGCAAGTGCAAGCAGCATCAACTGCTGTTATAGAACATGA
TGGACCAAGAAGTCTACTTACGAGAGCTCCCGAAATTCTCGTGGGAAAAAACCCCAACAAGGCCACAAGAAAGCTGAAGGTTGCACTCAAAAGGGAAGACCTTGCAAGCACCCCAAT
CATGTACTGTGTTGACGAACTTGTGTTTAAAGCCAAAAATGGGTACAGAAAAATGGAAAATATCTCCTGTTCCGGGAATCTCTTGAGAACTTCAATGCATACATACGAACCAATAGAATGT
CCAATCAATATTATTGGAAGATGTTTATTCTGGAGTTCTTTGATGAAACCCACCTTGTGATTTATTTGCTCTTGTAATGAGAACATCCTTCCATATGCCATGTCCTGTCTTGAATGGGATATAT
GCCCAATTGCTGTAATAGATGCGCAACCCCCAAGTTGTTCGTAGAGAGCTTCAACAAAGTCCTTGTAAAAAGAAGCGACGCCTGGATTCCCTGGTATAACGACAACATTCAAAGAGGCC
TCTTCAGACTCTACTTCGAGCAGTTCAGTCGAGAATCCAGAGACGAGACAATTTCTTGCAGTGGCCCTCTTTCTGTAAAGAGATGGCAATTCCTCATCATCCATTGAGCAACTTTTGTACC
CTGCAGCAGATTTCATCCTAGAAAGCAACAGCGTTCTTTGATGTTAAATTTTCTACGGCCGAGAAGGCAGACCAAAAGGCAAAAGACACTTCTCTTCAATTGAGGAAGAATAGGGCAGG
AAAGGCGAAAGTTGCAGCGAATAGAGGAGAGAGTTATGGGCGAAATCGATGGTGGCATGTGAAGCAGGCTAGCAGCTGCTGGCTTTATACTGCATTCTGTCCTCTCCAACTCCCTTCAA
AAGTCCAGCGTTGCTCGGAGAAAAATGTAATAGAGGAGTGAGTTGAGTTTGGTATGCTCAAGTGATTGATTGAGTTGAGTTTGATTTGTTTTGATTAATTATGGGACAAA
>comp165215_c2_seq3
TATATTCTCCAATTTATACTATTGTGAAGCATATAAAGTCAAATTTCTCTAATATTACATGGTTATTTGGCCAGTAAATAAGTAATCCCACATTAAAGTTTATAATATACAGATTGGTCTCCCTAA
TTAAACATTTTGAAGCTTAAAAGGTACATTAAAACCATACTAGTTTAGCAATTAAATGGATATTTGTATAAAAGGAAGGAAATAAAGGCAGTATTCACCTCTTCAAACAATGACAATGGAC
CCCAGTGATCATCAACGCCGAACAAAAATGCAATTTGTTTCTTTTTTTCTTTCATGAAGCTCCAATCTGGCTCCTCTGAAAGCTTCTCAAACTCTGTCTTTGCCATGAATAAGACATTGCGC
ATTGTGTGGTACTTCATGAGGCAAGTGCAAGCAGCATCAACTGCTGTTATAGAACATGATGGACCAAGAAGTCTACTTACGAGAGCTCCCGAAATTCTCGTGGGAAAAAACCCCAACAA
GGCCACAAGAAAGCTGAAGGTTGCACTCAAAAGGGAAGACCTTGCAAGCACCCCAATCATGTACTGTGTTGACGAACTTGTGTTTAAAGCCAAAAATGGGTACAGAAAAATGGAAAAT
ATCTCCTGTTCCGGGAATCTCTTGAGAACTTCAATGCATACATACGAACCAATAGAATGTCCAATCAATATTATTGGAAGATGTTTATTCTGGAGTTCTTTGATGAAACCCACCTTGTGATTT
ATTTGCTCTTGTAATGAGAACATCCTTCCATATGCCATGTCCAGGGGAAAAAAAATTGAGCCTGCGTAGCAACACGCGGGCAAGTGAACAGCAGATGAGCTCTTATAGATCTATTTCCCAG
TCATTTCTCTTCTTCCTTCTCCCACTCCTCTGTTCAGATTCACATTCACCTCAGATATAAGCATCAACTGAGATACCACAAACGCCTTCTTATCATTTTCTATCTGTTCTTCAAGAAAATGATC
TCTTGGTTAATTTTCTTCCATGTTGCTTCTGTCCTGTCTTGAATGGGATATATGCCCAATTGCTGTAATAGATGCGCAACCCCCAAGTTGTTCGTAGAGAGCTTCAACAAAGTCCTTGTAAA
AAGAAGCGACGCCTGGATTCCCTGGTATAACGACAACATTCAAAGAGGCCTCTTCAGACTCTACTTCGAGCAGTTCAGTCGAGAATCCAGAGACGAGACAATTTCTTGCAGTGGCCCTC
TTTCTGTAAAGAGATGGCAATTCCTCATCATCCATTGAGCAACTTTTGTACCCTGCAGCAGATTTCATCCTAGAAAGCAACAGCGTTCTTTGATGTTAAATTTTCTTCTCTTCAATTGAGGA
AGAATAGGGCAGGAAAGGCGAAAGTTGCAGCGAATAGAGGAGAGAGTTATGGGCGAAATCGATGGTGGCATGTGAAGCAGGCTAGCAGCTGCTGGCTTTATACTGCATTCTGTCCTCTC
CAACTCCCTTCAAAAGTCCAGCGTTGCTCGGAGAAAAATGTAATAGAGGAGTGAGTTGAGTTTGGTATGCTCAAGTGATTGATTGAGTTGAGTTTGATTTGTTTTGATTAATTATGGGACA
AA
>comp165215_c2_seq7
TATATTCTCCAATTTATACTATTGTGAAGCATATAAAGTCAAATTTCTCTAATATTACATGGTTATTTGGCCAGTAAATAAGTAATCCCACATTAAAGTTTATAATATACAGATTGGTCTCCCTAA
TTAAACATTTTGAAGCTTAAAAGGTACATTAAAACCATACTAGTTTAGCAATTAAATGGATATTTGTATAAAAGGAAGGAAATAAAGGCAGTATTCACCTCTTCAAACAATGACAATGGAC
CCCAGTGATCATCAACGCCGAACAAAAATGCAATTTGTTTCTTTTTTTCTTTCATGAAGCTCCAATCTGGCTCCTCTGAAAGCTTCTCAAACTCTGTCTTTGCCATGAATAAGACATTGCGC
ATTGTGTGGTACTTCATGAGGCAAGTGCAAGCAGCATCAACTGCTGTTATAGAACATGATGGACCAAGAAGTCTACTTACGAGAGCTCCCGAAATTCTCGTGGGAAAAAACCCCAACAA
GGCCACAAGAAAGCTGAAGGTTGCACTCAAAAGGGAAGACCTTGCAAGCACCCCAATCATGTACTGTGTTGACGAACTTGTGTTTAAAGCCAAAAATGGGTACAGAAAAATGGAAAAT

ATCTCCTGTTCCGGGAATCTCTTGAGAACTTCAATGCATACATACGAACCAATAGAATGTCCAATCAATATTATTGGAAGATGTTTATTCTGGAGTTCTTTGATGAAACCCACCTTGTGATTT
ATTTGCTCTTGTAATGAGAACATCCTTCCATATGCCATGTCCTGGTAGAAAAAGTAACCCATATATCATGGTATTTCATAAATGAAGAGTATTCTTCAATAAGAAATTATATGAAATATAAAAA
TCCAACAGCAACTCTTTGTATGATAAAAAGCTTATAGTGTGCATTCTACATAGAATTACAGCAATAGTGCTATTATTAGCTAAATTTAAGAAGAATGACAAGTAGAAAAGCTCTGTCCAGCG
CATCATTCCAATATTGATGCATTTTTGTACATGTAATATCTTGAAAAACTTCTCTTATTGCTCATTTTATAAGGAAAAAGTTTAGATCGGGTGCTCCAACAGCAAATTAAGCCCAGCACCTAA
TCTGTTTCCATGAACAAATAGCCAAATGCAAAGAAGTGATTAGTTGGAAAAGCCAAACCAGGGGAAAAAAAATTGAGCCTGCGTAGCAACACGCGGGCAAGTGAACAGCAGATGAGCT
CTTATAGATCTATTTCCCAGTCATTTCTCTTCTTCCTTCTCCCACTCCTCTGTTCAGATTCACATTCACCTCAGATATAAGCATCAACTGAGATACCACAAACGCCTTCTTATCATTTTCTATCT
GTTCTTCAAGAAAATGATCTCTTGGTTAATTTTCTTCCATGTTGCTTCTGTCCTGTCTTGAATGGGATATATGCCCAATTGCTGTAATAGATGCGCAACCCCCAAGTTGTTCGTAGAGAGCTT
CAACAAAGTCCTTGTAAAAAGAAGCGACGCCTGGATTCCCTGGTATAACGACAACATTCAAAGAGGCCTCTTCAGACTCTACTTCGAGCAGTTCAGTCGAGAATCCAGAGACGAGACAA
TTTCTTGCAGTGGCCCTCTTTCTGTAAAGAGATGGCAATTCCTCATCATCCATTGAGCAACTTTTGTACCCTGCAGCAGATTTCATCCTAGAAAGCAACAGCGTTCTTTGATGTTAAATTTT
CTTCTCTTCAATTGAGGAAGAATAGGGCAGGAAAGGCGAAAGTTGCAGCGAATAGAGGAGAGAGTTATGGGCGAAATCGATGGTGGCATGTGAAGCAGGCTAGCAGCTGCTGGCTTTAT
ACTGCATTCTGTCCTCTCCAACTCCCTTCAAAAGTCCAGCGTTGCTCGGAGAAAAATGTAATAGAGGAGTGAGTTGAGTTTGGTATGCTCAAGTGATTGATTGAGTTGAGTTTGATTTGTT
TTGATTAATTATGGGACAAA
>comp165268_c0_seq6
TTCATTTTTAAGTTGAATTACACGGGACCGATTTGTTGGCTGCAGGCGATTCTCTAGGGTTATCCAAACCTCATGAGCAGTAGATAAAGAGAGAACATAAGGTAGCACAGATGCAGAAATA
GTGGACAACAAGGCAGAAACTAAGTTTTGGTCAACTAGTTTCCACAGTCGGAATTCCTGAGAATCTGCTTCTGTCGCAGGCGGCAAAATGCTACCAGTCAAGTAACCTTCAAAACCATT
AGCTGAGAACAGTTTGAACACTTGCAAACGCCAAATAGGGTAGTTTTCATTGGTCAGTGAAGTAGAAACAATATTCTTCAAGTTGTTCATCAGAAACTTCAAGGAAGATGGAATGGAGA
GCTCTGTCATTGAAGTAGTGAGAGAAGCTGCTGGTGATGCAGCCGAGGAAGCCGAAATCTGTTCAGCCATTCAGAGAAAAAAAAAAAACTGATAGTGAAGCAGGATGAAGCCCTCAGA
GCTCTGATACCATGAAATCAACCTGCAATAAAGAAGGCATAGAAGAACAGAGAAGGAAATGGCTTTAAAAATTGTATTTGCTTTCTTACTTCAGCTACACAGTAAAGGCTTCAATATAAAG
AAGCCAAAGGGGACAGAGGCTGCACAAGCCGCCTGGACTGGTACAACCCAGTCTGAGCGAAACAGAGAAGGTGCAACCTTAACTAATCATGATTGGTGGAGTAGCATAACTAAAGTGC
TCTTGCAATATATTCTAACAGTCCCCTTCAAACTTATCATTTGAGGAGCAAATGGTAAGTTTGTCTCTTAAAAGAGAAAATCTGAGAGGTGGTAATGGTTTAGTTAGAATATCAGCCAACTG
ATCCTGGGAGTGGACATGTAGAATAGAGATAGCTCCTTGTTGAATGTGCTGACTGATAAAGTGATAATCTATCTCAATATGCTTCGTGCGAGCATGAAAAACAGGATTATGAGCTAAGGCA
ATCGCCGAAGTGTTATCACAATAGACTCTTGTAGGAGATGTTTGTGGCAAGGATATATCGTCTAGTAAGCGACGTAACCATAGAATGTCGGATGTAGCAGAGGAGAGTGCTCGATATTCCG
CCTCGGTCGAAGACTTGGCAACAGTGACTTGCTTTTTAACACACCAAGAGATAAGTGTTGGACCAAGATAAGAACAGAAGCCAGTAACCGATTTG
>comp165283_c0_seq1
ATTCCCCTTTTGGAGTGCAATTCACTGTAAAAAGTTAAGGAAAAAAAGAACTACGATAAAACAATTCCAAGTGGTCTATATCTGGTTCAAAATAACACAAAGTATTTCCAACTACATATTA
CTAAATTTACAGTGTGTAGATAAATCAGCACCTCAAAATCTGGATCATTGTAAAAATTATAACTAACAATACAGATAAAAAAAAAAACAATGCGCCGGATTTTCTATATCGTAATTCCTAGTT
TATGGCATGCTATTTTGGTATTTCATAATGCAAATCAGTGTGATGGAACAGCTGAGCCTGTGTACCCCACTAGTATGGGCAAAAAAAAGATTAATTCTCCCTTTAGTTGCTTATCATAGAAAA
TCAAAATTGTGAGGGAATTAGGAAAGTTTATTTATGGAATGGGGGCTGATTTGAATTCAAATTTGGAAGGGAATTAGGAAGGTTTATTTAGAGAAACAGTGCTGATTTGAATTCAGATTTT
GGAGGAAATTAGAAAGGTTTATGTAAGGAAAGATGCCGATTTAATTCAGATTTGTGCGGGATTTAGGAAGATGTATTTAGGATTAATTTGGTTATTTAACTTTCATTATGTAAATTGTTTTGT
TGTAGTTTTCTTTGGAGTTGTTCATAGAGGCGCTTAGGGTTTGTGGAGAGTCGGTCCATCTCTCGAATAGTATATCAACTTGTATTTTGGTTTGTTATGCCGATATCTCATTATTTCTCGCTGA
TTTCTGTTTTTTGGGCTGAAGCGATAAACACAACTTATTATGTGTTAAATAAGGTGAATGTTAGAGTCAAACTAGATAAGACACCCTATGGATCCTTAAGGGTAAGACACCCAATCTTTCCC

ACCTCTATTTCTTTGGTTGTAAGGTTTTCATTTATAATAACGGGAAGTCACAATTAGGGAAATTTGACCAAAATTTGATGAAGGTGTTCTTAGGATATTCAAGTATTGGTAAATTCTTTAAGG
TGTTTAATAAAAGGACATTTGTGGTGGAGGAGATTACTCATATAGTGTTTGATGAATCTGATCCTAAGGAGTTTAGAATTGAGGATGAAGATGGTGTTATAGAGATTTCCAATGGAGCTAAA
AATTTGGAGAAGAGGGTAAAGTCCTAAATCACTGAAGAAGTAGGTTGGATTATGTAAAGAGTATAAAATTCAAATCTAGAGGAAAGGGTTGATCATACACACACTTTGCATAGGGATTGG
AGATATTCTTTGAGTCATCCTAAAGAACTAATCTTAGGAGACCCTTCTGAAGGTGTTAGGACTAGGCATGGACTTAGGAAGGAAGTGAACCACTCTGCCTTTATCT
>comp165311_c1_seq15
TTCTTCTATTAATCAAATTCAAGAAAATTATTTGAATTATAAGTAAATGAGATATCTATTTATACATAATGCAAAGTGAAAAAAAGAAGGTGCAATACAATAATTTTCTTCCATTGCTAGAAAT
ATGCATGCTTCTATAGCTTAGCATTTTCTTTAGGTTGGTAACATTGTGATATCCTCTATTCAAAGAAGATTTGATAACTTGGAAGAAGACAAGATCAACTGAAGCCTATAAATGCAACGCAA
GCCTTCTTCTTTAGTTGGTTCTGTCGAAATCTTTGTTCGGCTAGCTTTCTCACAGCCATTAGTTTTTCTTTTTGTTTTAGGTACTAGTGACCACATGCACTTTCCTTCTCTGCTATTACAGTAC
TTCAACCAATTCTTCCCTTCGCTTTTTGCTTTCATAATTGTGGCTTCTTCTTGGTACTCTGAGTTGTATATAGCACAGCATGCAGAAGTAATTCTTTGTTGCCTTCTCTGGAGGGACAGAACA
TTCATAGAGTTTCCACCATTGGGAGTTACAAAAAATTGTATAAATATATGGTGTAATTTCACCTGGAAGAAGATTATATCAGGGTAGTGCTTCTGAATTAGTTGGCCAATGACTTTCATCCGC
CTAATCACCTCTACATCTTCACGGGACCAGACATTATAACTCATTACCTTGATCAATCTGGAAGCGGTGTCATGATAACCCTCATCACCTTTATGACCTTGTACTTCAAATCCAGATCTGTCC
AGAACCCCATCCTTTCGGGATTTGTTCTCAGATGAATCAGAAGATGAGAGGCTCCATACACCAAACGCAAGGAGAGCAGTTCCTGCTATAACTGCACCAACTTTGAGTGCTGAGATTGGT
GTAGAAGTTTGAGAGGATCTACTCTTAGAATTACCCATTAATTAATCCTTTCTTATTTCCTCCTCACTCATCCTCTCCTTTCTCTGCAATCACACATGGCTGAGAGTGTTCTTGTAAATGTAA
GGTAAAGTTGGAAAGCATATTTGATGTGATGACTATTCCTAGTCC
>comp165311_c1_seq19
TTCTTCTATTAATCAAATTCAAGAAAATTATTTGAATTATAAGTAAATGAGATATCTATTTATACATAATGCAAAGTGAAAAAAAGAAGGTGCAATACAATAATTTTCTTCCATTGCTAGAAAT
ATGCATGCTTCTATAGCTTAGCATTTTCTTTAGGTTGGTAACATTGTGATATCCTCTATTCAAAGAAGATTTGATAACTTGGAAGAAGACAAGATCAACTGAAGCCTATAAATGCAACGCAA
GCCTTCTTCTTTAGTTGGTTCTGTCGAAATCTTTGTTCGGCTAGCTTTCTCACAGCCATTAGTTTTTCTTTTTGTTTTAGGTACTAGTGACCACATGCACTTTCCTTCTCTGCTATTACAGTAC
TTCAACCAATTCTTCCCTTCGCTTTTTGCTTTCATAATTGTGGCTTCTTCTTGGTACTCTGAGTTGTATATAGCACAGCATGCAGAAGTAATTCTTTGTTGCCTTCTCTGGAGGGACAGAACA
TTCATAGAGTTTCCACCATTGGGAGTTACAAAAAATTGTATAAATATATGGTGTAATTTCCTGAAAGACAATCAAGTTAAATTCGATCATAACTATCCTTGATTAGTATGAGGTATCTATCTTC
AAGGCTCAAACGAACTTACACCTGGAAGAAGATTATATCAGGGTAGTGCTTCTGAATTAGTTGGCCAATGACTTTCATCCGCCTAATCACCTCTACATCTTCACGGGACCAGACATTATAA
CTCATTACCTTGATCAATCTGGAAGCGGTGTCATGATAACCCTCATCACCTTTATGACCTTGTACTTCAAATCCAGATCTGTCCAGAACCCCATCCTTTCGGGATTTGTTCTCAGATGAATCA
GAAGATGAGAGGCTCCATACACCAAACGCAAGGAGAGCAGTTCCTGCTATAACTGCACCAACTTTGAGTGCTGAGATTGGTGTAGAAGTTTGAGAGGATCTACTCTTAGAATTACCCATT
AATTAATCCTTTCTTATTTCCTCCTCACTCATCCTCTCCTTTCTCTGCAATCACACATGGCTGAGAGTGTTCTTGTAAATGTAAGGTAAAGTTGGAAAGCATATTTGATGTGATGACTATTCCT
AGTCC
>comp165315_c1_seq14
CCCAAACACCCCGATGATGGCCGTGTTGAATGCAGATCTCAACCGGAGGGGTTCCCAAATACTTTCAATTGTCTCCCAAAAGTCCATTCCAAATACTTTCAAACTCTACCTAAGATATCCA
CCAAAAATGCCAAAACTGCACAAAAATCCCAAAACTCCACAAAAATCCCAAAAAATTCAGAAAAATCTCAAAACTCCAAAATCCCAAAAATATTTAAAATTGAAATAAATCCATCAACG
CAGTTAAAGCAAAATCCATTCGCCCTCCGCAACAGTTTGTCGAG
>comp165329_c0_seq8
GCATCATAACGATTCCATCCCCAAGCAGGAGGCGATGCATAAGGAGATTGGGGATGGAATCTTCTCTGAAAAGAAGAATGATTGTGAATGCAACACCATATCTCCTGCTCACAAAAGATA

GAGTTTATATTCTATGGTTTGCTCTTTTGGGGGTGCTATATGGTCACATATCAAGCGATTGGGAATGCCAACGGCGCAACCGTTGTGAGTCCACATTTCACCAGTATCCACCATGCAAGAGA
GTTCAAAAAGCACGGCGGTGACCTCAGAGAAAATGCTAGGGCAATAAATAGGGCTTATTTCGAGGGCGATGCAATCAGAGGTGAAATGTTCAACATTGACATTGTTGTCGCGGCAGATTT
TGAGTTGGGCGATGTCCTAAGATCCATTTCCTTCTTGGTCACCATCAAGAGATCGTTGCATGTAGCGTATGAAGCTGGGGTCTCTAAGCTTGTGGCCATGGTGATTGGTGACAAGGAAAAC
ATTCATGAACCAAACACTATCGGCTGAAATGGTGGCTTTGGAGATGATGAGGTCAAGATCGCTCAAGGCTTGAACTATTTGTAAGAGAATGCCATGCCCATTCTCACCTTCCACCCTGAGA
AGAGTACAATCCTCGAATGTTTCATTGTCAACGCACACACAGAGAGGAGGTATGCATTCAGAGTCAAAGTTGGAACAACCTTCAGCCATCACTATCTCTGAAGCTCACTTAGTAGATAGG
AAAGTAGTGGGCGGAGGAAGAAGAAGACGAAGGAGAAGCCTGTCCGGGAACTGAGAAGAAGAAATAATTAAGAAGATTAATGGTATTTGGTAGGAGAAGCAGAAGCTCTAATCAATCT
GCTATTTATAATTAATCTCTCTGAACTTCAGCTATTTGTTAGGACGAAAATTCCGTTGATTAGGACTCCTAATAAGCCTCACTATCCCCCTCTATTTCTCCTCATTTCCTGATTTCCGTTGCCG
GAAGAAGTCCCACGCCACCATCTATTTCTCCTCATTTT
>comp165378_c0_seq2
ATTATCAAACCTACACAGAACTACTTGGCTTAATACATATCAATATGGCTTTGTATTTTCGCCCTCCCATGCTTTCGTACAGTAAGAAGCAGCCAGTGCTCTTTAGCTTTTCTTCCACGCCAA
CATTTCTAATTCTTCAAGGAAGTATTACAAGATTCCATCCCATAAGCTGCCTCTCAACAGCTAATACAAAAACTTCTCCTGCTCGTCGCACAGCAAATTTTCAGCCAACCATTTGGGATGAT
AACTACATCCAATCGCTTCCAGTTCATTTCATGGAGGAGAAGTATAAACACCAAAGAGATAAATTGAAGGAGGAAGTCAGGTGTTTGATTGGCCAACAACATGAACTTGTTAAACAGCTT
GAACTTGTGGATGCCCTACGACAGTTAGGAATTGATTATCATTTTAATTCAGATATCACAAATTTGTTGAGTAATATTAGTTCATCAACGGAAAATATATATAACTTGATAAAGAATAACAATC
TTTATGGCTCTGCATTGCTCTTTAGGCTTCTAAGGGAGCATGGCATCAATAATGCTGCAATACTAAGGTATGTACCAACATGTGCATGCATATAAAATATTCATTAAAGGGTTATTTAGTTCAG
TGTAAATATAATTATAAAATTTAGGTTCAAGCAATATTCTCCCTAATTTCTTGGGTATAATAGAGTCATAAATTCAATTCCATCTTTATGACTTCCTTTAAAAGAAAATCTATATTTTATCTCCAT
GTGATATTTTATTTATGGCTAGTCTTATTTCTTGTTTTTTTCCAATGGACTTATGCCCTTGGATCTATAAATAATATTGTTCTCTACTTCTAAGTAAATAATATTTATGATATGTTTCCATCCTTAA
AAAAATCCGATATAAATCTAAATAAAATGTTACCTAAACAACAATATTGAGATAACTATATCATAAAAGACACTCTATGTCATTAAATTCTATTGCAATCTTATCTAGAATGCCAATAGGGATA
ATGATGTATCATTTTTTTCTTATTGTAACTAATTTTAGCTATTAGTTAGCAAGACTAATATTTAGCAATTCATTAATTTTATGTACATCAAGATGCTTTCGGAATAGGACTAAAATGCTAATGAAT
AAAATAATTTACCTTATTGAACGTCAAATATGATATTAATTCTATTGCATTTTATAATAAGTATGCTTAATAAAATCCTTTTTAATTATTTCGTAGAGTTGATACTCTGATAAGCTGCTTCAAAAA
AGTGAGAGAAAGCTTCAATCCCAATCATCAATATGATGTTAAAGAAATGCTCAGTTTGTATGAGGCTTCCTACCTTGCTATGGAAGGAGAGGAAGAACTGGATGAGGCAGGAAAGTTTGC
TATGGAGCACTTAAGACGTCTTGATAGATCTTTACTAAGTCCACAACTTATTGAAGAAATAGATCATGCCTTGGAGCTACCCTTGCAT
>comp165378_c0_seq5
ATTATCAAACCTACACAGAACTACTTGGCTTAATACATATCAATATGGCTTTGTATTTTCGCCCTCCCATGCTTTCGTACAGTAAGAAGCAGCCAGTGCTCTTTAGCTTTTCTTCCACGCCAA
CATTTCTAATTCTTCAAGGAAGTATTACAAGATTCCATCCCATAAGCTGCCTCTCAACAGCTAATACAAAAACTTCTCCTGCTCGTCGCACAGCAAATTTTCAGCCAACCATTTGGGATGAT
AACTACATCCAATCGCTTCCAGTTCATTTCATGGAGGAGAAGTATAAACACCAAAGAGATAAATTGAAGGAGGAAGTCAGGTGTTTGATTGGCCAACAACATGAACTTGTTAAACAGCTT
GAACTTGTGGATGCCCTACGACAGTTAGGAATTGATTATCATTTTAATTCAGATATCACAAATTTGTTGAGTAATATTAGTTCATCAACGGAAAATATATATAACTTGATAAAGAATAACAATC
TTTATGGCTCTGCATTGCTCTTTAGGCTTCTAAGGGAGCATGGCATCAATAATGCTGCAATACTAAGTTGATACTCTGATAAGCTGCTTCAAAAAAGTGAGAGAAAGCTTCAAGCCCAATC
ATCAATATGATGTTAAGGAAATGATCAGTTTGTATGAGGCTTCCTACCTTGCTATGGAAGGAGAGGAAGAACTGGATGAGGCAGGAAAGTTTGCTATGGAGCACTTAAGACGTCTTGATA
GATCTTTACTAAGTCCACAACTTATTGAAGAAATAGATCATGCCTTGGAGCTACCCTTGCAT
>comp165378_c1_seq1
CAAAATAATATTAAGATCATTACATTCTAGAAAGCTATTTTAAGAAGCAGTTAGAAAGCCGTCTCAAACGAAGCCTTAGTTTAAAAGAAAAATCATTTTGATTTTTTTTTTTTTTCAAATTTG

GTATCCATACCTTCATATCAAAGTACAAGAATTTATACTTGAATATAGATTAATTATGATTGAATAAAAAAAAAATTATGTGCATATCATCTAATAACATTGCTGATAATAATAATTAGCATATGC
CAATTTTAAGAGTAGATGTAATATAAAATCTCTTCATAATTACTCAAATAATTTAAATCATAAGCCATCACCATGAAATGAACTGGAAGCGATTGGATGTAGTTATCATCCCAAATGGTTGGC
TGAAAATTTGCTGTGCGACGAGCAGGAGAAGTTTTTGTATTAGCTGGTGAGAGGCAGCTTATGGGATGGAATCTTGTAATACTTCCTTGAAGAATTAGAAATGTTGGCGTGGAAGAAAAG
CTAAAGAGCACTGGCTGCTTCTTACTGTACGAAAGCATGGGAGGGCGAAAATACAAAGCCATATTGATATGTATTAAGCCAAGTAGTTCTGTGTAGGTTTGATAATAGATGGGAACCCCCT
ATGCCTATTTAT
>comp165383_c0_seq8
ACCCTTCAAAAACACACACAAGTTACTTCCCACAAACACATACTCTTTTCATCTTCAAAACTCGCACAAGTTATACTTTTTTAAAATTCATGTATTTTACTATTTCAAAGTTCACACACTTTA
TCTTACTCTTACATTTCAAACTTAAACACTAAACTCGTTTCACTTTTCACCTTTCAAAATCACAAAATTCACTTCTTAAACTTTACACCCATTTTCACTTTTTAACATATAAACACTTTACTTT
CCATACCTTTATACACTTTCCTTTGCAATTTTCCATACATTTTACCTTCCAACACATGAGCACACGATTCACCTTTCAAGATATAAACTTTTCACCTTTCTAAATCACACAATTTTCCACTTTT
CCAAAATTCACTCAAACTTTGCCTTTTGAAACTTTTACTTTTGAAAGTACACTTTGTAATCTTCTAACATGTATCTCGAGGTGTGGGAAAGGGGAAGAAGAAGAAAAGTGAGAGAGAGGT
AGAGAGAAAGAGAGCTTTTGATTTTCAACACTTTCATCTTCTACACATTTTAAAACATCATTTGATATACCAGACTTTATTTCTCACAATCCAACCCTTTCTTTTCATTTTTACACATTTCATC
TTTTAAGGTAAACATACACCTTACTTCTCACATGAACAATTCCACCTTTCTAAAACACAATCACAAGCCCCATTTATTATTTCTCACACATATCATCTTACTCCTCACACTGGCACACTTCAT
CATTCAAAGATCACATACTATACTTTTATATTTTCTAAAGTTACACAATTCACCTTTCAGGACATACACTTTTTCACATAAAACACTTCTCAATATGAATACATTTCACCTT
>comp165431_c1_seq3
CATATTATAGAACATAAATAACGAATTTTAACTACACATATAAGTTCAAGGACGATGAAGAGAGGTAGAGTCATATAAAATGTAGGCGATAATTTTTTTCAGCTTTTTAAAATAATTTTATAGT
ACAAATATTAAAATTTTTGTACTAAAAAATTATTTTAAGAAGCAATAAGAAAGTCATCTCAAACGAAGTCTTAGTCCATTCTAGATCGGATAAAGCATTGTCTTCCTTTTTCTTTTTATATAAT
TATTTTTCACTCTGCAAGAAGAAGAGCGAGCCCAATCCTCCACCCAGATTCCTAATTTGCAGATTCACCATTGGATAAAGTAGGATTTTGTGGTAATACCCATTTGTCCCAGTCACGGCGTT
TTCTGATTTTATCACCCTGCACTTCCACATTGCCCGAGAATTCCATGCTGTCTGATATGAATTGTTCATCGCTGGTCAACGGTGCGATGTAATCTAGCATAAAACTGTACGATCTTAAATAAT
TATATACGAGATTTGCTTCCTTCATTAAAGTCTCAACTCTCCTAAACCCAGCAATCAAACTTATTGGAACCCAACCGTTCGCATCCATCTGTCTCTTCAGAAATATATCTGTTTCCAAATTTC
CCGAACTGAAGTAGTACTCAATCTGCTTCCTCAACCTAGTTCGCATATTATACAACCAACTGAAAATTTTTGATTGTTGTGCTGCATTAATGTCTTGGAGAGGAATAAAATATCCAACATGT
GGGTGGGTTGGATGGTAATACGCACAATGAGGAGCATATGGGAAGACATTATTGAGGCCTTGAGCTGAAGAGCTGGTGGTGGGCTCGGGGTTGTCAAAGAAAGGCACTGGGAAAGTTG
GCGGAGGGACCGTACCATATGCATGCAGGTTAGGTTGTCCGAAATCTATATGAGGAGCCATAACATTTGGTGCTTGATAAGGATTCGTTTCATTGAAAGTTCCACGATTGTTATTTAATCTC
CCAATATTTCTATTAGGAGGTCGCACTTCATTGGAAGGTATTCTTCTCCATGCCACCCGCTCAGTAGGGGCATTGTAATTTCTGCGGACTGAGGATGATGCTGAATGGTTCCCTCTTGGCAA
GTTGACCGGTCTGCTGACGATCGATGGGTCAGCCTCAACCTCTTCATTGAAGGCTCTTCCCAACGCCGCCAATTGGTTTCCTTTGGCAATCGCATTGTCGTTGCCTATGGCGGAAGAAGA
GGAGGTTGAATGGTTCTCACTAGACATAGCCAATCTTTAGAGGGGATACTTCTGGCTCTTAGATTGATGACTGAGATTATTGGTTGGTGGAATGAATCGAAGGAGGAATAAGTATCGTAGA
GAAGAATTACGTTATTGATTGATGTATTTGTTCTGTGATGGTTGGCTTCTTC
>comp165431_c1_seq4
TCCCAGTCACGGCGTTTTCTGATTTTATCACCCTGCACTTCCACATCGTCCGAGAATCTTAAGCTATCTGATATGAATTGTTCATTGCTAGTCAACCGTGCGATATGATCTGGCATTAAATTTA
ACGATCTTAAATAATTATATACGAGATTTGCTTCCTTCATTAAAGTCTCAACCTGCAAAATAGCATTGCAAATACAACATTTGTAAAAGGATAAAACTTGAGATAAAGTGCATGAAATAATA
AAGTTTAAGATAAATATACTC
>comp165431_c1_seq8

ATGTGGGTGGGTTGGATAGTAATTATACGGAGGACAGTAAGAAGCATCTGAAAATCATATAACAACATTAACTAAACTTCACATCAAGTTGGTAGAAAATGATGTGCAATTCAATAAAAAG
ACTACAGCACAATGTATGTCTTACATGGGAAGACATTGAGACTTTGAAATGAAGAGCTGGTGGAGGGCTCGGGGTTGTCAAAGAAAGGCACTGGGAAAGTTGGCGGAGGGACCGTACC
ATATGCATGCAGGTTAGGTTGTCCGAAATCTATATGAGGAGCCGTAAAATTTGGTGCTCGATAAGGACTCGTTTCAATATTGAAAGTTCCATGATTGTTATTTAACCTCCCAATATTTCTATTA
GGAGGTCGCACTTCATTGGAAGGTATTCTTCTCCATGCCACCCGCTCAGTAGGGGCATTGTAATTTCTGCGGACTGAGGATGATGCTGAATGGTTCCCTCTTGGCAAGTTGACCGGTCTGC
TGACGATCGATGGGTCAGCCTCAACCTCTTCATTGAAGGCTCTTCCCAACGCCGCCAATTGGTTTCCTTTGGCAATCGCATTGTCGTTGCCTATGGCGGAAGAAGAGGAGGTTGAATGGT
TCTCACTAGACATAGCCAATCTTTAGAGGGGATACTTCTGGCTCTTAGATTGATGACTGAGATTATTGGTTGGTGGAATGAATCGAAGGAGGAATAAGTATCGTAGAGAAGAATTACGTTAT
TGATTGATGTATTTGTTCTGTGATGGTTGGCTTCTTC
>comp165462_c0_seq5
CCAAATAACTTCATTGATACAAGCGTACTGAAAAGTGTTTAATAATATTTCTATGAAATGCATGCTTAAAATTAAGAAATAATATCTCTATGAAACGTGTTCCTACTCTGACCAGATCATTCAT
GGTAAACAATAATACAATATTACTCGAGTAAAAAAGGTAATAAATGTTGGTACAGTTTTCATCCACATGAGAACTCAAATGGGCAGTAAACATTGGAAAAGTTATTACGTCCGGATAAAAA
ACCGAAAATCCGAACGAATACCGAGCGATACCGAGTGCTGTATCGAGCATGGTACCAAGCACCCAAGTGATGTAGCGCTTTGTTATTGGTTTCTGGACGCATTAGGTGTCCGAACACAAT
AATTACTCTTGCAGAAGTGTATGTACCATTTATGTTTGCTGTGTTGATTCTTAAAGACTGGTGCAGTATCTTCCTGTTATTCAGAATTCAAGTTATAGTTAGTCATCATCAATTTTATTAGGTTT
CCTTTGTTTTTTTTTTATTGTTAATGAAAGCTTACCTTACATGGACAATGGAGAGGATATTTGAGGAGCATTTTTATGAGAAATATTTTATTAAATAGCCCTTTTTGTACATTGTGGAATTATTC
GAGAGAATGTTCATGTAATTTGGCAAAACATAAAGAAGCCTGTTTTTGGAAGTTCTTGAAAATGGAGAGGATTTGCAAGTTCTTGAAAGCTGATATTTGCTTCTACCATTTACCTCAGTAG
AAAAGCTGTAATAGAATTATGTATACCATATGGTATATGATATTTGCCCTAAATGAG
>comp165473_c1_seq3
CAAACTAAGGCTTAATCAAATTAGATTAATTTCTTAGAATAAGTACTTGCTTTAACTAGCAAAGTTTAACAAGACTGCTACAAACTGACCAGTTTTTAAAGAGAAATTCATTCCTCAAAAG
GCCATCCTAAGGCTTACTTACACAACTTTAATTAACTGCTCTTTCTGCTACTTCACTCTATCAACCACTTGCTTTGAATACCTCACAGCTCTTTGTCATAAATTGGAACACCAAAGAGCTTA
ACAGTACAGTCATGGCCCTCCTCTTCCTCCCCCCTAATGGTTACTGAGCACTGAGGCTGTCCTGTGGTAGCCATCTCTATGAGCATTCCATGCCTGTATGTGTGCAGTGGGCTTACATGTTG
CTGTAGGTAGTGAATGATATCATCATAGAGCTTCTTTAAATGATTGAGCTCTGATAAGAGGAGAGAGTTCCTCCTTCTCAGCCTTAGGTTGTCCTCGGAGAGGGCCAAGAGGAAGCTGGT
GTCGTCGAGGGTGGACGAAAGCCGCGGTGGCGGCTCGACGATGACTTCTGTGTAACACTGGAAAGATGGCTGCGAGTAATGAAAGCAATGTTGTTGTTGATGATGGTGATGATGAATAT
GATGATTCTTCCTTCTGTGAATTTCTAAGAGAAGATGCTTCTCTCCTTTTTTGAAGAACTCATTAGCAAACTCCCACCTTTCTGCAACTATCTTTCTGAATCCATAGGTGTTGAGCTGCCTAA
CAAAGCTAGAGAAGTTGTTGTGTTTGAAGTATTTGGGAAGGAGATCTTTGCAAAATCTGGAGTTCTCCATACTATAAATGTGTTGTTATCTTCTCCCCAAGAGACAATGTGATCAGTACAT
GGATCATCAACAAGATGGTAGGTTTTGGTGAGGAATGGAGCTGGAATTGGCTTGTGAGAGTTCACAGGAACTACCATGTCATCACATGATTTTTCTACCAAGAAAGCCATGGAGAGGAA
GAAGAGAATGGAAGCTAGCAGCTCTCTGGCTACTACATCTAGCTATCCTGCTTTGAGTATATATGCATTTGTATTTGTAGCGCTCATCTCTCTCTCTCTCTCATCTCTCTCTCTCTCTCTCTGA
CATTTTGTTTTTGGGATTTATACATACCTAAC
>comp165498_c1_seq6
CGGAAAATTTCCAAACAAGTGCAAGTCCAACATCAGGATTGTTTTAACTCCAACAGATCAAGTATATCAAGAGTCATAGAATTCAGACCCGATTGTCTTAAGAGCTTTAAAACCTTAAAA
AACTATATTCAACAAATATAATCAAACCAAAGCAGCCATAAAATACAGATTCAGCGTAGAAAAGGAAAAAAAAATCTTACAGCGCAACACTTCGCGAATCTCAAGCGGCTCGAGTACTAG
CTCTTTCAGCTGGCAGACTTCGCAGTTCATGGTTCTGATGACTTGACCTAGGTTAACGATCAGAATAGATGGCAGAAATATATATGGACGCGAGACCGACTAGGTAAATCAGGGTTTTGGG
CGCAGGCGGAGCTAAAAGTAGCTCGGAGTGAAGAGGAGGAGACCTCTTTCGACCTTCGCTATCGTGGAGCACTAACCTGGTATTACATGTCCTCTTGCGTGACGGTATTGTGCTGAAAA

GCAAAAAAAGCCTGTAATTGACTGCTCGGCCGACCAGGGTGGCGATGAATTTCTGGCGGACTCACGAACGTCTTCGAAGAACTGTTTAGCAACAGGAGTTCTCTGTTTATATAGCGAAC
GTGAGAAGAGAGATAATGGTGGAGAAAAGGGACTAAAATGGAGGTGGCCGGTAATGAGGGAAATGGTGGGGAAGAAGGTGGTGGTGGTGAAAATGGAAAGAAAAGAGAGAAATATG
GAGGGAAGTGGTGAGATAAAGAGGGTGAAAAATGAATGAAAATGGAGGCGGGGTGCAAGAGAAGAAGATGATGCAAAATAAAATGGTGGGTGTCTGGGAAGAGAAGAAAAAGAATA
AGAAGAAGAAGAAGAAAAAGAGATAGAGAGAGAGAGAGAGGAAGAAACTGATTGAATACTAGTTGGAAGCACAGGTTGTGTATTAAGGAACTTTTATTTGGTCTGGTCACAGGACGTA
ACTCGGTATAACTCGGTATTGCTCGGTATTGGTCT
>comp165531_c5_seq7
ACTCAAACCCGAAGTTCCACTCGGCTTGCAAGTGAGAACGGGTGATCGCCGCAAGAGGATCTCCAATGGGAGGGAGAGAGGGGCGGCGGCTTGGAGAGGGGAGAGGATTTGAGCCGT
GGGTTTAAAGGAGATGTGGGATCTCGGTTGGGAGGGAGAGAAGAGAGAAGTGGTGGCTGTTTTGTGTGAAGAGAAAAAACTGAATTCTCAATTCAGTTTTGAATAGGAGGAAAATAAC
GGATTTGAATTTCAAGTTCAGGTTTTGAAATCCAAATTCGGTTTAGAGGGAGAGTGGAATAAAAGAAATATCAGGAGATGATTAGGTGGCAATTAGAGAGAGAATTAAGGGACAAGGGT
GGCCGGCCATATAGTAAGGAGATAGGCTTTGGAATTTAGAAAGTGGGGCCCACGTTTGGATAAATAATTCTCTCAATCAAATCTAAAATTATAATTTCAGATTTGATTATATTTTAAGTAACT
CCCAAATTGAGTTATTAAGGAGGAATAGAATTTGAATTTCAAAAGAAGCATTTATCCCCTTAAATGTCTTTCTTAATTGGATGAAGACCAATTAGGAAATTAACTTAATTAAGGGTAATTTG
AAATTCAAACACGTTTGGGTCCAAGGGGGAAGATCCGGCGGCAGGAGTTTTTGCTGGACGGTGGGCTAGGGGTCACGGTGGGCGCTGGACGGGGAAGTGGGGTCGGCAGACGGGCTA
GCAGCGACGGGATTAAATTGGCTGGCGGCCAATTCTCTGGCCAAGCCCACAGGGCCGGCGGCAAGGAAAATTGAAGAGGGAGTTCTAGGGCTTGGAAGCAGTAATATTTATTTAGAGAA
AATTCAGAAATTCTTGACTCACTCACCTCTTCTTCAAACTCTCAATGGGAAGTAATAAGAATTCCGGAACTCACACAGAGATCCACGAAATTTCCATCAAGAACTCGCGGAGAGAACTAG
GGGAATGGAGAACTCACAAAGAGATCAA
>comp165552_c1_seq1
TGCCAACTTTGAGGACAACAGTCTGCAAAAAAATGTGCAATCGAGAATGTCAAATGAGATTTGCTAGTTTGAACAATATAGACAAATTTCTTTCTATATATAATAAATTAGATTGAGGTTAT
TAAGCATAGAGGAAGTTAAGGTACATATAGGCTAGGCTAGACTTTACTAAAGATTGTACCGAACTGTGTTTTCATTAATAAAACTTTGTTTCTTTTCCCTTCATATGGTATCAGAGCAAGAAT
AACCCATTTGGACCATCTCTCTTCCTCTTCTTCCATCATCTTCGTTCCTCTCTTAGGTTTCGGCTGAGATAATCTAGTATTAGGCTTTCTCTAGTCGAACATCAAACATGTGCATGGCGTCGT
TCGGTCGTTCCAGTCGAATGCCAAAATTTTCGCTATTTGCTTGCTGGTGAACAGTGCGACGCTGTATTTTGGAGTGAAAATTGAG
>comp165554_c0_seq15
GGGATATTCGATGCAACTAAGTTAAAGATTAGTGTTACAATTGTTAGCATCTTTCTTTTTTGGGAAAAAAAGTTAGCATGTTTCGGCTTACATTAAGCTTTTAAAATGCGTAGAATCTCTATC
TCCATATATATATCTCTCTCTCTTTCTTCTTATCTCTCTTTGAAAGAAAACTAAGAGTCATTGAGGCCCGACGTTGGTGGCGTTGGAAGCCATGAGTTCTCCCATGAAATTCTGTCCCGAATG
CAACAATCTACTCTACCCAAAAGAAGATAAGAAGCGCAAGATCCTACTCAGTGCCTGCCGCACCTGTAGTTACACGGTTCCGTATCACAGAAAATTCTGACTTGTGATCAGAAATATATAT
ATCTATATATTCGTATTGAATAAATGAATTGATAAATAAATTACAGGAGGTGGCGGAAAGTAACTGCGTGTACAGAAATGTGATAAATCATGATTCAGATTATGAGCGACCTCAAGATATGCG
AGATGCTTATGCAGATCCCACACTTCCTCGAGAAAAGGGCGTCAAATGTCCTGCCTGCAACCATCCAAAAGTTGCATTCTCCTTGGTTTGCTCTTCTTCATTCATTAATTTAAATTGATTCT
TGCTCATGCATGCCATTAATGCCCTTAATTCGTTGATGCCTTAAGAAATAGGTACTAAAGACACGCTAGACTGCTAAAATTTAATTTATTTTTTTTGAATGAGTGTATAAATTTCATGTGTATT
TAATTTGGTAAGTTATTATACCATTATTTACTTAATATATACAGCATTATTAAATTTATTAGGAGCATAGTTTTATATTTTTAATAATAAATGTATCATATATTGATGTATTATTATGTGTGTGCCCCC
ACATTTATTATGTGTTATATTATAG
>comp165554_c0_seq19
GATTATTCTCTAATTTAATATACGCTATAATATAACACATAATAAATGTGGGGGCACACACATAATAATACATCAATATATGATACATTTATTATTAAAAATATAAAACTATGCTCCTAATAAATTT

AATAATGCTGTATATATTAAGTAAATAATGGTATAATAACTTACCAAATTAAATACACATGAAATTTATACACTCATTCAAAAAAAATAAATTAAATTTTAGCAGTCTAGCGTGTCTTTAGTAC
CTATTTCTTAAGGCATCAACGAATTAAGGGCATTAATGGCATGCATGAGCAAGAATCAATTTAAATTAATGAATGAAGAAGAGCAAACCAAGGAGAATGCAACTTTTGGATGGTTGCAGG
CAGGACATTTGACGCCCTTTTCTCGAGGAAGTGTGGGATCTGCATAAGCATCTCGCATATCTTGAGGTCGCTCATAATCTGAATCATGATTTATCACATTTCTGTACACGCAGTTACTTTCCG
CCACCTCCGTGTAACTACAGGTGCGACAGGCACTGAGTAGGATCTTGCGCTTCTTATCTTCTTTTGGGTAGAGTAGATTGTTGCATTCGGGACAAAATTTCATAGAAACACTCATGGCTTC
CAAAGCCATCAACTTCAAGCCCGAATGCCAATTATTTTTTTTTTTCACAGAGAGAGAGATGAAGAGGGAAAGAAAAGCGACACAATTAGAGATAGAGATTATGTGCCGTTTGAAAGGCTT
A
>comp165554_c0_seq24
TTCAAACGGCACATAATCTCTATCTCTAATTGTGTCGCTTTTCTTTCCCTCTTCATCTCTCTCTCTGTGAAAAAAAAAAATAATTGGCATTCGGGCTTGAAGTTGATGGCTTTGGAAGCCATG
AGTGTTTCTATGAAATTTTGTCCCGAATGGTAGAATGCATTACCTCTCCTCCCTCTGCATTGTCCTATATGGCGCTTTCTCATTTCATTGTTCTGCATGTTTTAATTACGCTTAACAGAAATTAT
ATAAACTTCTATGAATCTTTATCTGTCTAATTAAATGCAGCAACAATCTACTCTACCCAAAAGAAGATAAGAAGCGCAAGATCCTACTCAGTGCCTGCCGCACCTGTAGTTACACGGAGGT
GGCGGAAAGTAACTGCGTGTACAGAAATGTGATAAATCATGATTCAGATTATGAGCGACCTCAAGATATGCGAGATGCTTATGCAGATCCCACACTTCCTCGAGAAAAGGGCGTCAAATG
TCCTGCCTGCAACCATCCAAAAGTTGCATTCTCCTTGATTAACCCGAGGAAAAGCCTAGCACGGATGAATGCTTGCTGCAACCCGAGCTGCCGTCATAACTGGAGGGATGATGATGAAAG
CATTGCTTAATGATTGATTGCAGTTTAATAATTATATTTACAAATAATAGAGAATTAAAAAATAAAATAAGATTCGGATGGGCTATCTATCATTTTAATCGTTCCCCATAAGAACTTGATCTAAG
GAATTTGTTTTAGTGAGTAACAGGAGACCATTACAATTTGTTTGATTGTTTTGGCATGGTGTGTCAATTTTTATTAAGAAAATATTTCAAGAAATCCTACATATAATCATTTTCAAGAAAATG
AAAAAAAAAAAGTAATAATAATAATAAAATTACCTCCATTGAAGTCTTCGTGTTCTGTATGATCAAGCTAATATATAATTTTAAACTTTCCATGTATGTTTAAAATTATATATTAGCTTGATCAT
ACAGAACACGAAGACTTCAATGGAGGTAATTTTATTATTATTATTACTTTTTTTTTTTCATTTTCTTGAAAATGATTATATGTAGGATTTCTTGAAATATTTTGTTAATAAAAATTGACACATGT
ATCATGCCAAAACAATCAAACAAATTGTAATGGTCTCCTGTTACTCACTAAAACAAATTCCTTAGATCAAGTTCTTATGGGGAACGATTAAAATGATAGATAGCCCATCCGAATCTTATTTTA
TTTTTTAATTCTCTATTATTTGTAAATATAATTATTAAACCGCAATCAATCATTAAGCAATGCTTTCATCATCATCCCTCCAGTTATGACGGCAGCTCGGGTTGCAGCAAGCATTCATCCGTGC
TAGGCTTTTCCTCGGGTTAATCAAGGAGAATGCAACTTTTGGATGGTTGCAGGCAGGACATTTGACGCCCTTTTCTCGAGGAAGTGTGGGATCTGCATAAGCATCTCGCATATCTTGAGGT
CGCTCATAATCTGAATCATGATTTATCACATTTCTGTACACGCAGTTACTTTCCGCCACCTCCTGTAATTTATTTATCAATTCATTTATTCAATACGAATATATAGATATATATATTTCTGATCACA
AGTCAGAATTTTCTGTGATACGGAACCGTGTAACTACAGGTGCGACAGGCACTGAGTAGGATCTTGCGCTTCTTATCTTCTTTTGGGTAGAGTAGATTGTTGCTGCATTTAATTAGACAGAT
AAAGATTCATAGAAGTTTATATAATTTCTGTTAAGCGTAATTAAAACATGCAGAACAATGAAATGAGAAAGCGCCATATAGGACAATGCAGAGGGAGGAGAGGTAATGCATTCTACCATTC
GGGACAAAATTTCATAGAAACACTCATGGCTTCCAAAGCCATCAACTTCAAGCCCGAATGCCAATTATTTTTTTTTTCACAGAGAGAGAGATGAAGAGGGAAAGAAAAGCGACACAATT
AGAGATAGAGATTATGTGCCGTTTGAAAGGCTTA
>comp165554_c0_seq27
TTCAAACGGCACATAATCTCTATCTCTAATTGTGTCGCTTTTCTTTCCCTCTTCATCTCTCTCTCTGTGAAAAAAAAAAATAATTGGCATTCGGGCTTGAAGTTGATGGCTTTGGAAGCCATG
AGTGTTTCTATGAAATTTTGTCCCGAATGGTAGAATGCATTACCTCTCCTCCCTCTGCATTGTCCTATATGGCGCTTTCTCATTTCATTGTTCTGCATGTTTTAATTACGCTTAACAGAAATTAT
ATAAACTTCTATGAATCTTTATCTGTCTAATTAAATGCAGCAACAATCTACTCTACCCAAAAGAAGATAAGAAGCGCAAGATCCTACTCAGTGCCTGCCGCACCTGTAGTTACACGGTTCC
GTATCACAGAAAATTCTGACTTGTGATCAGAAATATATATATCTATATATTCGTATTGAATAAATGAATTGATAAATAAATTACAGGAGGTGGCGGAAAGTAACTGCGTGTACAGAAATGTGA
TAAATCATGATTCAGATTATGAGCGACCTCAAGATATGCGAGATGCTTATGCAGATCCCACACTTCCTCGAGAAAAGGGCGTCAAATGTCCTGCCTGCAACCATCCAAAAGTTGCATTCTC
CTTGATTAACCCGAGGAAAAGCCTAGCACGGATGAATGCTTGCTGCAACCCGAGCTGCCGTCATAACTGGAGGGATGATGATGAAAGCATTGCTTAATGATTGATTGCAGTTTAATAATTA

TATTTACAAATAATAGAGAATTAAAAAATAAAATAAGATTCGGATGGGCTATCTATCATTTTAATCGTTCCCCATAAGAACTTGATCTAAGGAATTTGTTTTAGTGAGTAACAGGAGACCATT
ACAATTTGTTTGATTGTTTTGGCATGGTGTGTCAATTTTTATTAAGAAAATATTTCAAGAAATCCTACATATAATCATTTTCAAGAAAATGAAAAAAAAAAAGTAATAATAATAATAAAATTA
CCTCCATTGAAGTCTTCGTGTTCTGTATGATCAAGCTAATATATAATTTTAAACTTTCCATGTATGTTTAAAATTATATATTAGCTTGATCATACAGAACACGAAGACTTCAATGGAGGTAATT
TTATTATTATTATTACTTTTTTTTTTTCATTTTCTTGAAAATGATTATATGTAGGATTTCTTGAAATATTTTGTTAATAAAAATTGACACATGTATCATGCCAAAACAATCAAACAAATTGTAATG
GTCTCCTGTTACTCACTAAAACAAATTCCTTAGATCAAGTTCTTATGGGGAACGATTAAAATGATAGATAGCCCATCCGAATCTTATTTTATTTTTTAATTCTCTATTATTTGTAAATATAATTAT
TAAACCGCAATCAATCATTAAGCAATGCTTTCATCATCATCCCTCCAGTTATGACGGCAGCTCGGGTTGCAGCAAGCATTCATCCGTGCTAGGCTTTTCCTCGGGTTAATCAAGGAGAATGC
AACTTTTGGATGGTTGCAGGCAGGACATTTGACGCCCTTTTCTCGAGGAAGTGTGGGATCTGCATAAGCATCTCGCATATCTTGAGGTCGCTCATAATCTGAATCATGATTTATCACATTTC
TGTACACGCAGTTACTTTCCGCCACCTCCTGTAATTTATTTATCAATTCATTTATTCAATACGAATATATAGATATATATATTTCTGATCACAAGTCAGAATTTTCTGTGATACGGAACCGTGTA
ACTACAGGTGCGACAGGCACTGAGTAGGATCTTGCGCTTCTTATCTTCTTTTGGGTAGAGTAGATTGTTGCTGCATTTAATTAGACAGATAAAGATTCATAGAAGTTTATATAATTTCTGTTA
AGCGTAATTAAAACATGCAGAACAATGAAATGAGAAAGCGCCATATAGGACAATGCAGAGGGAGGAGAGGTAATGCATTCTACCATTCGGGACAAAATTTCATAGAAACACTCATGGCTT
CCAAAGCCATCAACTTCAAGCCCGAATGCCAATTA
>comp165554_c0_seq44
TTCAAACGGCACATAATCTCTATCTCTAATTGTGTCGCTTTTCTTTCCCTCTTCATCTCTCTCTCTGTGAAAAAAAAAAATAATTGGCATTCGGGCTTGAAGTTGATGGCTTTGGAAGCCATG
AGTGTTTCTATGAAATTTTGTCCCGAATGGTAGAATGCATTACCTCTCCTCCCTCTGCATTGTCCTATATGGCGCTTTCTCATTTCATTGTTCTGCATGTTTTAATTACGCTTAACAGAAATTAT
ATAAACTTCTATGAATCTTTATCTGTCTAATTAAATGCAGCAACAATCTACTCTACCCAAAAGAAGATAAGAAGCGCAAGATCCTACTCAGTGCCTGCCGCACCTGTAGTTACACGGAGGT
GGCGGAAAGTAACTGCGTGTACAGAAATGTGATAAATCATGATTCAGATTATGAGCGACCTCAAGATATGCGAGATGCTTATGCAGATCCCACACTTCCTCGAGAAAAGGGCGTCAAATG
TCCTGCCTGCAACCATCCAAAAGTTGCATTCTCCTTGATTAACCCGAGGAAAAGCCTAGCACGGATGAATGCTTGCTGCAACCCGAGCTGCCGTCATAACTGGAGGGATGATGATGAAAG
CATTGCTTAATGATTGATTGCAGTTTAATAATTATATTTACAAATAATAGAGAATTAAAAAATAAAATAAGATTCGGATGGGCTATCTATCATTTTAATCGTTCCCCATAAGAACTTGATCTAAG
GAATTTGTTTTAGTGAGTAACAGGAGACCATTACAATTTGTTTGATTGTTTTGGCATGGTGTGTCAATTTTTATTAAGAAAATATTTCAAGAAATCCTACATATAATCATTTTCAAGAAAATG
AAAAAAAAAAAGTAATAATAATAATAAAATTACCTCCATTGAAGTCTTCGTGTTCTGTATGATCAAGCTAATATATAATTTTAAACTTTCCATGTATGTTTAAAATTATATATTAGCTTGATCAT
ACAGAACACGAAGACTTCAATGGAGGTAATTTTATTATTATTATTACTTTTTTTTTTTCATTTTCTTGAAAATGATTATATGTAGGATTTCTTGAAATATTTTGTTAATAAAAATTGACACATGT
ATCATGCCAAAACAATCAAACAAATTGTAATGGTCTCCTGTTACTCACTAAAACAAATTCCTTAGATCAAGTTCTTATGGGGAACGATTAAAATGATAGATAGCCCATCCGAATCTTATTTTA
TTTTTTAATTCTCTATTATTTGTAAATATAATTATTAAACCGCAATCAATCATTAAGCAATGCTTTCATCATCATCCCTCCAGTTATGACGGCAGCTCGGGTTGCAGCAAGCATTCATCCGTGC
TAGGCTTTTCCTCGGGTTAATCAAGGAGAATGCAACTTTTGGATGGTTGCAGGCAGGACATTTGACGCCCTTTTCTCGAGGAAGTGTGGGATCTGCATAAGCATCTCGCATATCTTGAGGT
CGCTCATAATCTGAATCATGATTTATCACATTTCTGTACACGCAGTTACTTTCCGCCACCTCCGTGTAACTACAGGTGCGACAGGCACTGAGTAGGATCTTGCGCTTCTTATCTTCTTTTGGG
TAGAGTAGATTGTTGCTGCATTTAATTAGACAGATAAAGATTCATAGAAGTTTATATAATTTCTGTTAAGCGTAATTAAAACATGCAGAACAATGAAATGAGAAAGCGCCATATAGGACAAT
GCAGAGGGAGGAGAGGTAATGCATTCTACCATTCGGGACAAAATTTCATAGAAACACTCATGGCTTCCAAAGCCATCAACTTCAAGCCCGAATGCCAATTATTTTTTTTTTCACAGAGAG
AGAGATGAAGAGGGAAAGAAAAGCGACACAATTAGAGATAGAGATTATGTGCCGTTTGAAAGGCTTA
>comp165587_c0_seq2
TCCTTGATAGCTCATGGTCATCAGCTAATACATTACAACAAAACAGACTCACTAAAAATATATCTTAACTTTTCGCTGAACTTTTATGGTACATAGATCATCCGAGTTCCTTTTGGCAATCAT
TGTATACATCAGTAATGCCTTTTAGCTAGTTTACTGTATAATTCACATCAAATTTATTTACTACGAGAAATACTTGAATCATATACATCTTTAGGCCTCATGCGAACAACAAAATTCCAATGTG

TTTTTAGGATCTTGAATGTAGTAGATTGGTTCAACCTTTCCTTGTCGTTGTAATTGT
>comp165589_c1_seq1
CAACTTATCGAAATAAGTTGCTAATCCAAGTTTCGTTAGGACAACTCTTATTTTTTTCTAAAACAATTTTTTTAGTAAAAAAAATTATTTCATGAAATTGTTGTACTAAAAAAACCGTAAAAA
TACTATCATAAATAAAACCTAGTATAAGAAGTTGCAAACTTACTGAGGTATGACATTGACTATTTGTTGGTGGTTATTTGAACAACGTATACTAGCTCTCAAGAACCCTAAACTTATACTGGC
TTAATCAATTTGAACCAAATGAAACTGAGTTTAAGGGACCCAAATCACTGGTTCAATCGCATGAAACGTTCAAGCTGGCTCACATATTTTCTGCAAGGTTTGAAGCATTCAAAACGAACA
TGGAACTATAATAACCCTTCTCGCTTTTTTCTCCCAAATCATAAAGCAAACCGAGTATTTAACACTGACACCTCCACGTGCACTACAACCCACTTAATCCCTTCCATTAATTATATTTCTTTT
CTTGAGTTTCATACATAATAATAATAAATAAAATCGCATAAAGTGCACATAGTACATAGCCACAGACATCAACGACACCCAATCCCAAGCGAACGGCCAAGATTTCCCAACCGTTGCAAAT
TGATCACTGGCAGGCCTGTAACGGCCAGCCACAGAGACAGTCATTATTGGAGAACGACGCCGCTTCGAGGTGATCGAACGGCGAACCCGTGGGAATCCGACCGCACAGATGGTTATGAC
TTAGATCAAGGTGGCCAACGAACGCCGCCGTTAGCAAAGTCTTTGGCACATCTCCGGTGAGTTTATTGTACGAGAGATCCATCGCCGTAAAGTACGATCTCGATGTGAACGCATCAGGAA
TCGAACCCTCAAGGCCGTTTCTGCTTAGATTCAGAATACCCAATCCCTTGCTCCCCAAAACCTCCGCCGGAATCCCGCCGGTGAGGAGGTTGCTGTCGAGGTAGAGCGAGGAGAGCACC
GGCATCGATCCGAAAGATGCTGGGATTGACCCCGAGATATTGTTATCGGAGAGGTCGAGGTCGGCGAGGCGGTTCATGTAACTGATTGAGGTCGGAATCACGCCAGAGAGCTGGTTCCG
GCCGAGGAGAGCCCTGCTAAGCATTTTCAAATTGCCGAAGTCGGAAGGGATCGAACCGGTTAAGAGGTTATTGCCGAGGTTAAGGTGCATTAATCCTGTAATCTGGGTTAGGGATTTGGG
GATTGAGCCAGAAATCTGGTTGTCGGCGACGTTGAGGACAGTGAGTTGGGAGAATTTGCCAATGTCGGCAGGTAAAGGGCCGGATAGGCGGTTACCGACGAGATCTAGAATGCGTAGGG
AGGTCAGAGTGGTTATGCAAGGGGGAATGGGGCCAGAGATCTGCTTCCAGTCAGCAATGATGAGAGATGAGAGGCTTTTGAGGCGGCAGATGGCTGGCGAGATGTGGCCGGTCATGAG
GCCGGATCGTCCGGCGCGTGTAATGATTGGGTCTTCGGATTCCCCGCGGAGAATAAGATCGGCGACGCGGCCGGTCGACGGTTCGCAGGACACCCCGTACCATTGAGAGCAGCAGTCGC
TGCCGGTCCAGGAGGAGAAAACACCGATGTAGGGGTCGGAGAGGGCTGATCGGAACTCGAGAAGCGCAGCTCGGTCAGCGGCCGAGCAAGAAGCGGCGGTGGCAGTGCTAACAGCG
AAAGCAAGTAGAAGGTAGAAGAAGAGGAAGTGGTAGTAGTGGGAAGAAGAAGAACAGGCATCCATTATCGCTTCCGCTCGG
>comp165672_c0_seq1
GATATTTGCATAAAACTCTTCATGTGAATTGGGATACACTGTAGTCTTATTATAAAAATCCTATTATTAATAGTAATTTATATACCAAGAGTTTCATAAATCATAACAAACTCAAGCATTAATAT
CGGAAATTTAACGTATTATCCTTTTCAAACAAAATAAAATTAGAAACGGAGTTTTTTACAAGTTTCCATAGTTCTGCTGAAAGATATATGCATGCACTGATACAGAAGAAAACTTTCATGGC
TTCTCAAGTGCAAAGAAATTATTTTTTATGCACCAAAACTAAGTTTTTTTCCCCTATAAAAGAAAAAAAACAAAAAAAATCAATCCTTCAAGCGACCAATGCTTTCCTCTACTTCAGTTAT
AAGTGCAGAAAGTGAAATTATTTAAGGTGGATTATCTTACATATGGGCTGTGAAGAATTTGAAATCATGGTGATGATGAACTCTTAGCTGCTTAAGCCATGAGTCTGCTGCACTGTAAAGG
GTCATAAGCTTTTCGTGATCCGAATTTTCAGAGTTTAGCCAGATATTTTCCCGCATCCTATAGGTTGCAAGGCCAAATGGAGGAAGGCTAATAGTGTTTTTGGTATCCTTGGAACTCCCATC
TTTCGTCGTAGAAATTTTCGACTTGTCTTCTTCAGAGTCGATGAATGTGTCATCTGCAATTTAAATGCAAAAAATGAATGAATATGTAGAAAACAAGGAGCCTGAATTGATTATGTAGGAG
GATTGGTCTCTGAATCATATAAATTCTGTGTTCTATGATGTTATGTTTTGATTATTCTTTTTCTTTTTTTTATTCCTACGGTAATCGTGTGGCAGACCCTCTTCAGTAGACATCCTTGCCTTGAT
CTTATTGTTAAAAGTAGCATAATAGGCCCTAAATCTACACGATCAATATGCCATAGCTGCCACCTTTGTTGCCCAATGGTGTAAATATTTTGCTCATTTACGTATGATTGATTTCTCTTTTCATT
GAGGTTACTTGTTCAAGCAAACTCAACTATGTATGTTCAATACCTCTATCATAGACCTATGTACTGGGTGTTTAATCGCACATATTGAGTGTGATAAGACACTAACAGTAATTGTTTTTGAAT
ATATAGCAAAACGTCACAATTTCTATGACTCTAAGGGAGTTACTAAAAACATGGGATTATATACTAAGCTTCAATTCGTCACAAGATTTAGGAAGATCTTAACCTTGATTTTGTTTTGGGCTT
GCCAAAGATGAACAACGACGTTGATGTTAGGGAATCGGCTTAGTCAGCTAGCCGTACTATACTTTCTTGGTTTTAGCTATGTATTAGACCTCTATATATTGGGGTTATCTGATGTATCTGCTG
GATAATAATAATATACACTTTTCTCCTTCTTTCCTCTTTGTTCCGTCGCTCTTCCGGTCCCTCTGGTTCAGTTCCGTCCAAGAACCTTCACCTGGTATCAGAGCTCAAACGCTCCACAGTGTC
TTCCTGATCCCATGGCAAAAGAGGACTCAGCCAGCTCTCGTTGGACAACATCAAATGTCTCTAATCAAGAAACCGAGATGGTCATCCCTTATCATCTCAAATTCCTAATCTCAAATGTCAA

GAACCTGGTTCCCACTCAACTCA
>comp165672_c0_seq11
GATATTTGCATAAAACTCTTCATGTGAATTGGGATACACTGTAGTCTTATTATAAAAATCCTATTATTAATAGTAATTTATATACCAAGAGTTTCATAAATCATAACAAACTCAAGCATTAATAT
CGGAAATTTAACGTATTATCCTTTTCAAACAAAATAAAATTAGAAACGGAGTTTTTACAAGTTTCCATAGTTCTGCTGAAAGATATATGCATGCACTGATACAGAAGAAAACTTTCATGGC
TTCTCAAGTGCAAAGAAATTATTTTTTATGCACCAAAACTAAGTTTTTTTCCCATATGGGCTGTGAAGAATTTGAAATCATGGTGATGATGAACTCTTAGCTGCTTAAGCCATGAGTCTGCT
GCACTGTAAAGGGTCATAAGCTTTTCGTGATCCGAATTTTCAGAGTTTAGCCAGATATTTTCCCGCATCCTATAGGTTGCAAGGCCAAATGGAGGAAGGCTAATAGTGTTTTTGGTATCCTT
GGAACTCCCATCTTTCGTCGTAGAAATTTTCGACTTGTCTTCTTCAGAGTCGATGAATGTGTCATCTTGGAAAGCAGATGAGATAGTATGAAAGGTCAAAAATGAAGCATACAGATCTTTC
TCATAACAACGAGCAGGAATATAACAGATAGGGTACCAGGCGACAGACATCCAACTAGCAGGTGAAAGATCTGCACTCTTAAATGACATTAAGCCTGGAAACCTTCTCGAAAGCGCATTT
ACTTTATTCATTAGTGGAAATCTTCGACAGGGAGGGCTAGATTCCATATACTTAAAGTACAAGTAACCCAACCGATCAGTTGGCCATGAACCTTCCTGATCAAAATAAGAATCTTCAGATGT
AACATCCCATGACCTTGATGGCTTCTCGCTCTCACTGTCATCACTCCATGAATCACTCTCGCTTTCCTCAACCATATTTCTCGCAGAAGCCAGAGATTCACTGGTGTAAATCTGGATAGCAG
ATAAGTAGGGAACATAGTACTGAAGTACAGTTTCGCCGTTGGGGCAGATGATCGGGATGCCTGTTCCGAAGATACTCCATTCATTGTACTGATCCCAGAGGTCTCGAAGATTAAAGTATTC
AACTTCTTCCTTGCCAATTGGCTGCCATAGACTGTTCAAATCCTTGAGGCTTGTCTTAGGGAGAGAATGTGAGCCGACTGAAACCAAGGTTCGGTCAAGAAAGCATTGCAGATTAGAATG
ACTTGTAAGTTTTGAAGCCATACTTCTCTTCCTATTTCTTATCAAAAGGGCTTTCTCTCTCTTCACCCGTCTTTCGCACTTTTCCTCTCGTCTCTCCTCTTGTATCTATACTAATCTTTAGTCTA
TTTTTAGAAACTCTTAATCTCTCTCATGAATGCTGTCTCTTTCTCTTCCCACCGCACCTCGCTAACGATTTCCTTCCAAAAAGCTGCACAAAAATACAGAAATACTACACCGTAACTGAGTT
ATGATCTCATCTAGACCACATCAGTCCTCCCACCATTCAAAAAATTTCTCTTTTCATCGGTTCTCTGCGAAGAGCAAGCCGTAAAACTAGGGAAGAAGATAGAAGTAAAAAAATATGACTC
TTTTGCTCTCGCTCCTCTGTCTCTCTCTCTCTCTC
>comp165672_c0_seq8
GATATTTGCATAAAACTCTTCATGTGAATTGGGATACACTGTAGTCTTATTATAAAAATCCTATTATTAATAGTAATTTATATACCAAGAGTTTCATAAATCATAACAAACTCAAGCATTAATAT
CGGAAATTTAACGTATTATCCTTTTCAAACAAAATAAAATTAGAAACGGAGTTTTTACAAGTTTCCATAGTTCTGCTGAAAGATATATGCATGCACTGATACAGAAGAAAACTTTCATGGC
TTCTCAAGTGCAAAGAAATTATTTTTTATGCACCAAAACTAAGTTTTTTTCCCCTATAAAAGAAAAAAAACAAAAAAAAATCAATCCTTCAAGCGACCAATGCTTTCCTCTACTTCAGTTAT
AAGTGCAGAAAGTGAAATTATTTAAGGTGGATTATCTTACATATGGGCTGTGAAGAATTTGAAATCATGGTGATGATGAACTCTTAGCTGCTTAAGCCATGAGTCTGCTGCACTGTAAAGG
GTCATAAGCTTTTCGTGATCCGAATTTTCAGAGTTTAGCCAGATATTTTCCCGCATCCTATAGGTTGCAAGGCCAAATGGAGGAAGGCTAATAGTGTTTTTGGTATCCTTGGAACTCCCATC
TTTCGTCGTAGAAATTTTCGACTTGTCTTCTTCAGAGTCGATGAATGTGTCATCTTGGAAAGCAGATGAGATAGTATGAAAGGTCAAAAATGAAGCATACAGATCTTTCTCATAACAACGA
GCAGGAATATAACAGATAGGGTACCAGGCGACAGACATCCAACTAGCAGGTGAAAGATCTGCACTCTTAAATGACATTAAGCCTGGAAACCTTCTCGAAAGCGCATTTACTTTATTCATTA
GTGGAAATCTTCGACAGGGAGGGCTAGATTCCATATACTTAAAGTACAAGTAACCCAACCGATCAGTTGGCCATGAACCTTCCTGATCAAAATAAGAATCTTCAGATGTAACATCCCATGA
CCTTGATGGCTTCTCGCTCTCACTGTCATCACTCCATGAATCACTCTCGCTTTCCTCAACCATATTTCTCGCAGAAGCCAGAGATTCACTGGTGTAAATCTGGATAGCAGATAAGTAGGGAA
CATAGTACTGAAGTACAGTTTCGCCGTTGGGGCAGATGATCGGGATGCCTGTTCCGAAGATACTCCATTCATTGTACTGATCCCAGAGGTCTCGAAGATTAAAGTATTCAACTTCTTCCTTG
CCAATTGGCTGCCATAGACTGTTCAAATCCTTGAGGCTTGTCTTAGGGAGAGAATGTGAGCCGACTGAAACCAAGGTTCGGTCAAGAAAGCATTGCAGATTAGAATGACTTGTAAGTTTT
GAAGCCATACTTCTCTTCCTATTTCTTATCAAAAGGGCTTTCTCTCTCTTCACCCGTCTTTCGCACTTTTCCTCTCGTCTCTCCTCTTGTATCTATACTAATCTTGTATATTCATTAGAGGATAT
AGTTTACCCACACGCGTACTTAGTCTATTTTTAGAAACTCTTAATCTCTCTCATGAATGCTGTCTCTTTCTCTTCCCACCGCACCTCGCTAACGATTTCCTTCCAAAAAGCTGCACAAAAAT
ACAGAAATACTACACCGTAACTGAGTTATGATCTCATCTAGACCACATCAGTCCTCCCACCATTCAAAAAATTTCTCTTTTCATCGGTTCTCTGCGAAGAGCAAGCCGTAAAACTAGGGAA

GAAGATAGAAGTAAAAAAATATGACTCTTTTGCTCTCGCTCCTCTGTCTCTCTCTCTCTCTC
>comp165672_c0_seq9
GATATTTGCATAAAACTCTTCATGTGAATTGGGATACACTGTAGTCTTATTATAAAAATCCTATTATTAATAGTAATTTATATACCAAGAGTTTCATAAATCATAACAAACTCAAGCATTAATAT
CGGAAATTTAACGTATTATCCTTTTCAAACAAAATAAAATTAGAAACGGAGTTTTTTACAAGTTTCCATAGTTCTGCTGAAAGATATATGCATGCACTGATACAGAAGAAAACTTTCATGGC
TTCTCAAGTGCAAAGAAATTATTTTTTATGCACCAAAACTAAGTTTTTTTCCCCTATAAAAGAAAAAAAACAAAAAAAATCAATCCTTCAAGCGACCAATGCTTTCCTCTACTTCAGTTAT
AAGTGCAGAAAGTGAAATTATTTAAGGTGGATTATCTTACATATGGGCTGTGAAGAATTTGAAATCATGGTGATGATGAACTCTTAGCTGCTTAAGCCATGAGTCTGCTGCACTGTAAAGG
GTCATAAGCTTTTCGTGATCCGAATTTTCAGAGTTTAGCCAGATATTTTCCCGCATCCTATAGGTTGCAAGGCCAAATGGAGGAAGGCTAATAGTGTTTTTGGTATCCTTGGAACTCCCATC
TTTCGTCGTAGAAATTTTCGACTTGTCTTCTTCAGAGTCGATGAATGTGTCATCTTGGAAAGCAGATGAGATAGTATGAAAGGTCAAAAATGAAGCATACAGATCTTTCTCATAACAACGA
GCAGGAATATAACAGATAGGGTACCAGGCGACAGACATCCAACTAGCAGGTGAAAGATCTGCACTCTTAAATGACATTAAGCCTGGAAACCTTCTCGAAAGCGCATTTACTTTATTCATTA
GTGGAAATCTTCGACAGGGAGGGCTAGATTCCATATACTTAAAGTACAAGTAACCCAACCGATCAGTTGGCCATGAACCTTCCTGATCAAAATAAGAATCTTCAGATGTAACATCCCATGA
CCTTGATGGCTTCTCGCTCTCACTGTCATCACTCCATGAATCACTCTCGCTTTCCTCAACCATATTTCTCGCAGAAGCCAGAGATTCACTGGTGTAAATCTGGATAGCAGATAAGTAGGGAA
CATAGTACTGAAGTACAGTTTCGCCGTTGGGGCAGATGATCGGGATGCCTGTTCCGAAGATACTCCATTCATTGTACTGATCCCAGAGGTCTCGAAGATTAAAGTATTCAACTTCTTCCTTG
CCAATTGGCTGCCATAGACTGTTCAAATCCTTGAGGCTTGTCTTAGGGAGAGAATGTGAGCCGACTGAAACCAAGGTTCGGTCAAGAAAGCATTGCAGATTAGAATGACTTGTAAGTTTT
GAAGCCATACTTCTCTTCCTATTTCTTATCAAAAGGGCTTTCTCTCTCTTCACCCGTCTTTCGCACTTTTCCTCTCGTCTCTCCTCTTGTATCTATACTAATCTCTTCCATGCCAAAAAGAAAA
AAGAAAACATAAGGCCAAAAAGGGACGTTTAAAAATAGAACATCAAGGAACAAAAAGAAATAGAACAGGAAGGAACAAGAAGAAAAATGTATTTCTCGCTCTTCCCATTTGTAAGAAA
CTAACTTCTTTCTCTTCAGCAGAGCAGAAGAGGAAGAAGAGCAAGAAGACAGCAAAAAGAAGAAGATGAAAGATGCCGGGGAAGAAAAAAGAAGGATAATTACAATGCAAATTCTTA
GATAACGTACTGTATATTCATTAGAGGATATAGTTTACCCACACGCGTACTTAGTCTATTTTTAGAAACTCTTAATCTCTCTCATGAATGCTGTCTCTTTCTCTTCCCACCGCACCTCGCTAAC
GATTTCCTTCCAAAAAGCTGCACAAAAATACAGAAATACTACACCGTAACTGAGTTATGATCTCATCTAGACCACATCAGTCCTCCCACCATTCAAAAAATTTCTCTTTTCATCGGTTCTCT
GCGAAGAGCAAGCCGTAAAACTAGGGAAGAAGATAGAAGTAAAAAAATATGACTCTTTTGCTCTCGCTCCTCTGTCTCTCTCTCTCTCTC
>comp165683_c0_seq2
CGCGCTCAACTAAATAACCCAAAAATCCAACGTGGCATCCCTCCCTCCACCCATTTAATTTGACTTAGTCAACACCCTCTCGAACACCACACATAAAAAAAAAAATAACAAAAATTAATTTC
CCAGCTGCCAGAATTACCATCCCCACATCAAATAACAAAAAAAAAAACTAAAAAAAAAAACACCACCAATTCCCCCACGAACGGCGGCCAGCGCTCGTCCGTTCGCATCCATGGCCGCT
GTTCTCCTCCTTCTTCTCCTCTCTCTTCCCTCCATCTCCCTCTCCCTCACCGATGCCGACGCCCTCCTCCTCCTCAAATCCTCCTTCCAAAACCCCGAACCCTCCCTCTCCTCCTGGTTCACC
GGTTCACCCAACAGCTCCTCCCCTTGCGCCCTCCCTCGATGGACCGGCGTCCTCTGCTTCAACGGCATCATCACTGGTCTACGTCTCGGCCATCTCAACCTTACCGGCACCATAAATATCC
AAGCTCTCACCCATTTCCCTTCCATCCGATCCATTAGCTTCGTCAACAACTCCCTTTCCGGCCCAATCCCCGATTTCTCCCGCTTAGACGCTCTCAAATCACTTTACCTTTCCCATAATCGCT
TCTCTGGTCCCATCCCGCTCAACCTTTTTTCCACTTTGGATCATTTAAAGAAACTCTGGATCAACGACAATGATTTCTCTGGCCCTGTTCCGTTTTCTTTATCGAAAGCTAAGAGTTTGTTG
GAGCTTCATATTGAAGATAATGGCTTTTCTGGAGAGATGCCGGATTTAAAGCTGCCATTGTTGCAGTCTTTTAATGCGTCCAATAATGAGTTGAGTGGACCTGTTCCGGTTTCTCTTGTGCG
GTTTGGGGGGTCGGCGTTTGAGGGTAATGAGAGATTATGTGGGCCGCCGATGCCCGGTCCAGCCTGCCCGGTGCCTACGCCTGCGCCAGCGCCGGTGCCGAAGGAAGAAATAATTGCTC
CGAAGGAAGAAATAATTGCGCCGGCGCCGAAGGTGATGCCGAAGCTGAGGCCCTTTGTTGCCGGTGGTGCCAATAGAGGACATTTTGTGGGTATTGTGGTCGGATTGGTGTTAGTTTTGG
TGCTGTTGGCGATTGGATTTGCCGCCGTTAAGAGACGAAGAAAAGTTAGCGACTTGGAGTCTGCCGGTGGACGAAAGGCGGCTGCATTCGATATGGAGAAGGGTGCGGTGACGGCGGT
GGCGGCGGCGAGCCCGGTGAATGACGGGAACGAGCATGGTTCTAGTGGGCACAAGCGCTACGGGTTGGCCCGGGGATCTGGTGGTGGTGGAGGTGGAAGCAGCGGCGCCGTGCTGGT

GATGGTGAACAGCCAAAGGGAGGCATTCGGGCTGCCGGACTTGATGAAGTCGGCGGCGGAGGTGCTCGGAAATGGAACGTTGGGCTCAGCTTACAAGGCCATTATGGCCGGCGGCCAC
GCGGTTGCAGTGAAGAGGATGAGGGAGCTGAGCCGACTAAACAAGGAGTTGTTTGATGGAGAGATGAGGCGTTTCGGACGGCTTAACCATCCCAACGTTCTAACCCCGCTCGCTTACCA
TTTCCGGAAAGAGGAGAAGCTTATAGTATATGACTACGTTCCAAAGGGAAGCCTCCTTTATATACTCCATGGCGATCGAGGAGCGGATCACGCTGCCTTAGACTGGCCAACTCGTCTAAAA
ATCGTCCTCGGCATCGCGCGAGGAATGGCGTATCTCCACGCCGAATTGGTCTCCTACGAAGTCCCACACGGCAATCTCAAGACTGCTAACGTCCTCATCTCCCCGACTTTTGACCCTCTAA
TCGCCGATTACGGCCTCATCGCCCTCGTCAACCCCGACGTCGCCCCGACCGTCCTCTTCTCTTTCAAATCCCCCGAAGCCCAACATCTCCGCCACGTGTCTCCGAAATCCGACGTCTTCTG
CTTTGGCGTCGTTCTCCTCGAAATTCTGACGGGTAAGTTTCCGTCTCAGTACTTGAATAACACCAAGGGTGGTACGGATGTCGTGCAGTGGACGGCGTCTGCGATCTCCGATCATCGGGA
GGCTGAGCTTATAGATCCGGTGATTACGGCGAAGGCGGCGCCGACGATGGAGATGGTGAGGTTACTTCGGGTTGGTGCGGCGTGTACACAGGTTAGTCCGGATGAGAGACCGGAGATGA
AGGAGGCGGCGGAGCTTGTTGAGGAAATTGCGGTTGCTGTGGCGGGCGCGTCGGTGGGGGCTGGGGGATCGAGCCGGGAGGAGGCGGCGGGTTCGGTGGGAACCGATGGCTCGTTGC
ATGCCGGTTCATCATCGTGAGTTATCATAGTCTTTTGATTCTGATCATGCGGTGTATATTCGTTCAATTTTTTTAATTTTATATGAGGATCTTGGTTTGACTTTTATTTATTTTCCATTAATGCTC
GACTCACAAACAGACATAATAGGTTGTCTCAATTTTCGTGCCTGGTTTTGTTAAAAAATTTGATTTTGTTAAAACAAATTGACTATACCACCC
>comp165708_c0_seq3
AAATGTTTTATTTTGTTTAGCGGAAATGCTCAAATTTATTAAAAACTTATTAATTTAAATATTGAAAAGAATATTACTTGGAATAAGCCTTAATTTAGACTTTTTCTTTGATTGCAAGTGTGAT
ACGTTGGCCTCCGAGGAGTTTGCACCTCCAACTTGAATGTTCGTAATTGCATTAAGGAAAGAAGCTAAAGTATTGCTCCATTTGTCTTCATCACCCCGAATCACTCCACACATATGTAGGG
AAAGCTTTGTCTTCAGGCATTATAAAATCCTCTTGCAAATCTACTTCACAAATTCTATTCCACACAAATCCATTTCATCAGAGAGTGAGGAGATAGAGAGGAGAAGAAGTGAAGATGTTGT
AGGAAGATTAGGTGGGGTGTAGGAGTATAGGAATAGGGGTGTCAATATATGCACGACTCAAAAACCCGACGCGTTCGAAGCCGCATCCTTCCCCGCATTAGCTCTCATAATCGCCCTCAC
GACCTCACCCTTGCCTGCCCGTGCGACCTTTCCCTTGCTTGCCCTCAAGACCTTGCCCTCACCTTCACCCTCTCCCTCGCCCATACTTATGTCCTAGTCCTAGCCCTCGAGGCCTTTCCCTC
ACTCTCACCCATGAGGCCTTATCCTTGCCCGCACGTGCTCGCATAGTCGCCCTCACCCCTCAATGCCTCGCCCACACAGTTGCACTCGCCCTAGCTACCTCGCCCACTCCAGCTCTATCTC
ACACTCGCCCTCTCATGCTTGCACAAAAACACTTATCGCCGCCCTCTCTCGCCCTCGTCGTTTCATGCTCGCGCTCAGCCTCTCCCTTACAATCGCCCTTACCCTTGCCCTCTTAGCATGCT
CACACTTGCCCATTGCACTCAACTATCTCCACCAAAAACATCTTTTGACACCCCTAAGTAGGTGTAGAGGTATGTGTTAGCAGGGTAGGGTTAGAGGTCAGTATAGGTAGTAGGGGTAGG
AGTAGAGGTGTGGGTAGGTAGTATGCACTACAGGAAAAATGACAATTTCCGGCGACCATTTCCGTCGGAAAAATGGCTAAAATCCG
>comp165727_c0_seq6
AAAACATTTTAATATACATATAAATTTATTATATTTATCACAAAATCATTAAAAAAACTATAAATTAACACCTAAAGTAACATTGTAAGGAAATTTCATAAATTTCAAAGCTATTTTCACAATTT
AGACTAAAATCTACCCTAAAAAATATAATAATAATGAAATAAAATTTAACCAAATCATGCCTTAAACTAGCAGTCTGATGTACCCGAAATGAAACCTCTGCGTCTCTTGTAGTCACTGATGC
TGAGTGTGAATCCTTTACTTTTTCCATACTCGTCAGGAACTGCATGCCCAATGGTTGCTTCTATCTTCCGCTTCCTCTCCATGAAGCAACGTTGAACACCCTCAGCTCCTTGAAATTTGTTC
AGAGCAGCCAACGAAATCGGCAAGTCAAAATTATGCAGAGCCATTAGTTCTTCACCATGATCAACCGGAGGTCTGGTGTCAATCAGTTTCCCTTCATCTTTAGCCTCCTCCATGGCTGTCA
CAAGGGCTGCTTCGCCAGCTTCTTCAGGCTTGTTAGTATCATGATCAACATTTGTGGTTTCTTCATTACTATAAATCACACACTGAAGAAGAGGCTTAGTCCATTTTCTGGCGGAAGGATCA
TAACCTTCTTCACCAAACTTTAAGTTTGTATAAACTGGCTTCATTTCTGATGCTTTTTTATAATTCTCAGCTCTATTTTTGCGATTCATCTCCAATAACCTGAAAGATCTTGCATCAGCTGGCT
TAATCTGTCTAGACAAATCCTCCAACTCCTTCAGCTTCGCACGCAACCTATCTGCCTCACTTTCTTTCCCAGCACTCTCTGCAGTTTCAATGGAAGCCTTCAAACGGATCTTTTCGACTGCA
ACATTGATTGGCCTTGAAGAAGATGATTTATTTACCTCTATCATCTTCTTCACAGTTCTCGGTGAATATACAAAAGTGTTCGTCTTTTGAATTTCTTCCTTCTTCTCATCAACTTCTTGCAGGC
AAGGAATGTGCCTTCCATTACGTTCCATTTCTCTCAGCCATTCCTTGAATTCTTCCTCTGTTGGTGGAGAGTTAGAAACCTTAATCATTTGCCATCTAGCTGCTGAAGCTTCGCTGCCCCATA
TACAGTTCAGCCATTTGCAAGTGGTTCGACCTTCGAATTCATACAGTCGATTTTGGTTTGTTGCATCGACATTATTGACTACACAAAGCCTGTATTTTGGACCGTTGCGAGTCATCCCAATA

CCAACTCTAACAAAACATCCAGCAACTATTTTCTCAAAGAAAGGTTCCATATGCCATTTAGCCAGCTTTGATCTACGAATGGTTATTTCTTTTACATCTTCATACCTGATTGGTCTCGACTTA
GATGCCATTAAATCATCTGAGTTATCATCAATGTCGTTATCTTCAGATAATTTGTCTTCTTTTCCATCATCACTATTGTCACTTGAATCAAATTTAGGAACAGTGATTGCAAGTGAATACTTGC
GGTAAGAATTTGGTTCTTGCTGCCTCATCCTCTTAGCTCGTAGCTCATTTAATGCGTCGATCTTTGCAGCTGGTTTCTCTGTTCTGACAGAAGATTGGCAATGAGAAGGAAAAGTTGGAGG
GAACTCTTTTTCATAATTCTCCACCTTAGTTGATGAGTCTCTTCTTCTTTTCTTCAAGCGATAATCATCTATTTTTTCACTCCTGGTTGCAAGAATCAACTCTCGATTTAGCTCTGAAAGCTTA
CTCATCTCTTCCTTATCTGCTTCATCTTTGTACAAATCACTTCCAATAGATAGTGCACTATCAGATTCATCACCATTAAACCTTCTATCTTTGTCATTCTCCCAGTTTCTCATATTGCCTTTCTC
TGGAGGGTCTCCAACACTCTTCTTCAGAGAGAATCTTGATCCAGAGTGTTTCCACTTATTGAACTTTGAGTTATCAGAATCATTTTCATTGGATCCACTACCAGAAGCTTCTCCTCGCCTTC
TTGAATGAGCATTGCTTTGGTTCTTCTTTGTTGGTGAGCTAGTTCGACCGGCAGCCTCAAGCAGCAAACTATCCAAATCTGCCATGACTTCAAAAGCTTCCTCTTTGAAGATCGGCGATCA
GTGGCGGCGGAGGCGAGAAGACGTCGTAAGGGATTATCGGAAGCCAAAAGCGCAATTTCAGGACTTGCAGAACTGTGTAAATGTAGCCTTCAAGAATCGTCAGTATACGCATAAGGTCA
CCGCAGAAGCGTTGAAAACAGCCGTTGAGCTCATCGGAGGGCGGCACCGGCGACAGGGAGAAGAGACAAATCCTGATGAATGGATTTGAGGATGCTCTGTAACTATTTATAGAGGTTTT
TTTTTTTTTGGACGATCAGTAGTTGGAATCGGTCTGATGGGAGAGCAGTTTTGTGTAACAGAATTCTGTTTATAGTGGAAAAAAATCCGGATTCAACTAGTGGAATGGGTTAATTTACTAAT
TTAGTGTCACTTATATTGATTAA
>comp165731_c0_seq1
CAAAAAAGTAGACTTCCATACCAAGATGTATTTCAAAATAATTTGAAGAAATATGGATTATCTTTAAAACGACATTCTCTTTCTTCTATAACATCATCTTTCAGAAGAAGTTTCATTGCCACC
ATTTGTGAGCTTCCTTGAGGTTGATGCTGCATTCGAATTTCTTGCATGAATTGCCTCATCTCAGTACGTAATGAGTTGACCTCCTCTTTGAGATTTACTATTTGACTTAAATTGCTTCCAACA
CTACTCCATACTGAACACCCTTTCGTTAGCCTCCATATTCGCATCCTCTGGTGTAAGTCCATCCTTAGCCAAAAGCTCACAAGCTTGTATCCATCTCTGATTAGCATCTTCAGATGACCAGGT
GCCATCTTTACGCAATCTGTTAACTCTCCATAATAATAATCTATCCTTGATAGCCATCTCTTGTTGCCTTCGAACAGAACTCACACGGCCCATAATGTGAGGACAAGTCTGTTTCTTTGCATT
TGCAGCATTTATAGCGGCCCTCTCTTGTTACATATGAATCTAACTCTATGTTGATTTGTTATGAAATTTTTAATTAAATATAATGATGTTACTAACCTTGTCTTTTAGTTGTGACCACTTGGTGA
CTAGATGACACCACTATGCAGCATCAACACTATCTGGAATAGACTCCGTAATTTCCTCGATAGGACGACAATCCCAATGCTCAGCTTTTAAATTAGTCTTATAGTTACGCCACTTAACTCCA
AGGGATTGAAGAATCTAGTGACGTATATGTCCAGGAAATG
>comp165792_c0_seq1
CCAATGCAGGGTAGGGCTATATATATCCAAAGATTGCACATTTGATTCCAACTTCTCTTTCATTTATTTATGCATTCTAGTGCTAGAAGGCTTCACAACTCCTTTCATCGCTTCCCATCACATT
GAATATAGTATTATGGCATGGTCTGATCTTCCCATAGATCTCCTCTATTGCATCTCATCACACTTGCTCGTTGTAGACTTCCTCCGATTCTCCGCTGTCTGCACCTCATGGAATTTCGGTCTAG
CTAATAATGCACTCTTTAGAATAAAATGTTGCCCCTCGCCATGGTTGTGCCTTCCCAGCAATGTCGGAGAGGACTCTGACACACTCACATTCTATGATCTTACCATGAAAAAAGAAGGAAA
CGTCGACAACTTCCTTCACTTATCTTCCCTCGGTAGCCACATCTTTGGTCGTCGTTGCTTTGGCTCCAAGGATGGATGGCTGGTGACCCTTGACAAAACTGATATGCAACCTTGCCTATTCA
ACCCTCTAACAAAAGCTGAAATATCCCTCCCATCCCTCTTGACCATCCCCGAAGATCAATGGCATAGGATCAAGCCCCAATACGCCCTTGACGGTCATGTCGAATTTTATTATGATGAAAAG
CATCCAATCTTTTCCGCAGATGTGGAAATTTTTCGTGATCTTTATTTCCAGAAGATTGTCCTTTCCTCTAACGACCGATTTGGTACTGCTGTTGTTGTTTATGGCCATTCAAAACAGTTAGCT
TTGGCAAAGCCATGCGACCATGCTTGGGTTCTCGGCCCACAACGGCCTCCTTACCATATCGACCAATTTGAAGAGTTTGAAGATGTTTACTTTCATGAAGAAGAGCAAATGTTCTATGCAA
TCACACATTTCTCAATGGTTCTGGCCTTCGACCTCAAAGGACAAAATATTGAAGTGATATGCCCACCGATACAAGACCCGTTAACAACAAATTATGATTGCAAGAACTATATTTTCTTTTTA
TTGGGCAGCCTTTTAAAAATCGAGAGAGAGACATCAGAATTTAGTATACGAAATCATAGATGTTATATCTGGATCTGGGTGAACCATTTTACTATGAAAAGATAGCTAGGATCGCGGTTTTC
AAGTTAGGGCCATATTTTACTTCTTCTTCTGGCTCATTCTCTCAATGGATCCATGTCGACGATTTGGATGGTTTTTTCATATTTATAGGTTGTAACCATACATTCGCATTGCATCACAAGGTTG
CTCCTGGAATTAGACCGAATTGTGTATACTTTAATGATCAATGGGATGCAATGTCACCAAAGAAAGTTGCTCGTGATTTTGGTCTTTATGATCTTCAAAATCATTGCTTCGAATATTTTGTTT

ACCCTGATTCACAACCTAAATTGTCTCCTTCAATATGGTTTATGCCATCATTTTCATAAATGAAAAATCTTAGTATTTTTAAGAAGCATGTACCGTGAGTATCTTTGAATTATGAAATTATTTTC
ATTATTTGTAAGCCATTTTTCAGCTAATA
>comp165792_c0_seq2
CCAATGCAGGGTAGGGCTATATATATCCAAAGATTGCACATTTGATTCCAACTTCTCTTTCATTTATTTATGCATTCTAGTGCTAGAAGGCTTCACAACTCCTTTCATCGCTTCCCATCACATT
GAATATAGTATTATGGCATGGTCTGATCTTCCCATAGATCTCCTCTATTGCATCTCATCACACTTGCTCGTTGTAGACTTCCTCCGATTCTCCGCTGTCTGCACCTCATGGAATTTCGGTCTAG
CTAATAATGCACTCTTTAGAATAAAATGTTGCCCCTCGCCATGGTTGTGCCTTCCCAGCAATGTCGGAGAGGACTCTGACACACTCACATTCTATGATCTTACCATGAAAAAAGAAGGAAA
CGTCGACAACTTCCTTCACTTATCTTCCCTCGGTAGCCACATCTTTGGTCGTCGTTGCTTTGGCTCCAAGGATGGATGGCTGGTGACCCTTGACAAAACTGATATGCAACCTTGCCTATTCA
ACCCTCTAACAAAAGCTGAAATATCCCTCCCATCCCTCTTGACCATCCCCGAAGATCAATGGCATAGGATCAAGCCCCAATACGCCCTTGACGGTCATGTCGAATTTTATTATGATGAAAAG
CATCCAATCTTTTCCGCAGATGTGGAAATTTTTCGTGATCTTTATTTCCAGAAGATTGTCCTTTCCTCTAACGACCGATTTGGTACTGCTGTTGTTGTTTATGGCCATTCAAAACAGTTAGCT
TTGGCAAAGCCATGCGACCATGCTTGGGTTCTCGGCCCACAACGGCCTCCTTACCATATCGACCAATTTGAAGAGTTTGAAGATGTTTACTTTCATGAAGAAGAGCAAAGGTTCTACGCG
ATCACACATTTCTCGGCAGTAATTGCATTTGATCTCGATGGAAATAATGTTAAAATAGTTTGCCAAGCAATAGAAGACCCCCATGACACAAACTCTTCTTGTAGGAACTACATTGCATTTTT
GTCGGGAACTCTTCTAAAAATTACTAGGGAGATCGATTGTACTTGTTTGGAGGCCAATGAAAAGTTTACATATAAGACAACCAATATCTCGATTTTTAAGTTTGTGCCGGCTGTTGATGCTT
CGTCTTGTTCTTCCTCAAAATGGATCTCTATCAGGGACTTGGGGGGTTGCTCTTTGTTTATAGGTTATAACCAAACCTTTTCATTGCATCACACAGTAGCTCCTGGAATTAAACCAAATTGC
ATATACTTTACAGACCATGCGGAGAACACGACGCAAGATGACATTACTCATGATGTTGGACTTTTTGATCTCCATAATGATTGCTTCCAGTTTTTCCTTGACTCCGATTCGCAGCGTAATTG
GCCCCCTTCAATCTGGTTT
>comp165792_c3_seq2
CGAGTCTATTTTTTATGATGTCTTCATAATATAAAAATAAAAAATTTAGGATCAAATCATAATGTAAGAAGCAATTTTTTTAAATAAATTCTATATTTTTAAATTTTAAATAAATTTTACACAC
GAATTCATGACTGTGCTGGATGTGCTGGCTTTGGAATTTAAGAACTTTATGATCTTAAGCAAGAAGAAACATTGTTATGAATTTTGTTCTGTATTCAGACAAACATGGCAGCGATTATTTGG
ATAGGACTCTTTTTCTTTGGCTATAAATAGTAAATACTATATAAGAAGAATTTTTATAGCATAAGAACATACATCATTTCCCAAAAAAGAATATCATACAATAATTTCATCCTATAATCCTATTC
TTGCTCACGTCCAGTCAAATCCAAGGAAAAATTGTCATTTGTTTCTTCTACAACAGATTTAGAAAGTTCAAAACCTCTATCTCTCTAAACTAGGTCACGCTCCTCTTTTTCTCTATATAAAA
GGCTAGAGAGCATCATAGCTAACAAAAACAACACAATATGACTTGGTCCGATCTCCCGATAGATCTCCTCCATTGCATCTCCTCACATTTGCGTATCGTTGACATCCTCCGCTTTTCTGGTG
TATGCACCTCATGGAATTTAGTTATTTCTAATAATGCACTCCTAGGCATTCATTATCGTCCCTCCCCGTGGCTGCTTCTTCCTAGTGATGTCGGAGAGACCTCTGACACACTCACCTTTCACG
ATCTTACCATCGAAGAAGAAGAAGAAGATGGCGTCAACATCCATTATCGCTTCTCCTCCCTCACGGGCAACATCTTCGGCCGTCGTTGCATTGGCTCTAAAGATGGATGGCTTGTGACACT
TGATAAGAAAGATCTACGGCCTCGTATCTTCAACCCTCTCACAAAAGCTGAAATATCCTTCCCATCGCTCTTCACCATCCCTGATGATAAATATCATACGATCAAGCCCGAATATGCTTCAG
ACGGCTCTATCGAGTTATCTTATAAAGAAATTTGTCGAGGCCTTTGCACATACACAAAATATTTCTGCAATGTATATTTTCAAAAGATTGTCATCTCCTCTAATGATCCACATGGAACTGCAA
TTGTAATCTATGGTTATGATAAATTGTTGGCTTTAGCAAGGCCTGGTGATCGAGCTTGGGTTCTTGGCCCACAGCTGCTTCCTTACAACACCAATGATCATGATGAATTTGAAGATGTGTGC
TATCACGAAGAAGAACAAAGGTTCTACGCCATAACACGTTTCTCGAGGGTGCTAGCCTTCGACCATAATGGACAAAATGTTGAAATGATATGCCAGCCAATACAGGATGAACGCCCCATA
CAAGAATATTGCATGAACTATATTTTCTTTTTAATGGGAACTCTTCTAAAAATAGAGAGAGAGATCAATATTTTTACTCGGCCTGTTGATGAAAGGTACGCTGGGAAGACAATTAGAATCTC
AGTTTTGAAGTTAGTCCAGGATGTTGTTGCAACTTCTTCTTCATTCTCTCAATGGACCACCATCAAAGACTTGGGGGGGTTTTCCTTATTTGTGGGTTGTAACCAGACATTCTCATTGCATC
ATACGACTGTTCCTGAAATTAAACCAAACTGTATATACTTCTGTGATCATTTGGATGAATCGTCACAAGATGATGTTTATCATGATGCTGGGTTTTTTGATCTTCAGAATGATTGCTTTCACTA
TTTTTTTGACTATGATTCACAACCTAACTGGCCTC

>comp165807_c1_seq10
GATTTTAAAAGCCTTGCTATCAAATGTCAATTTGTTAACCCAAGTGATTTTGGACATTCCCAAAGCTAAATACGCGAACTAAGCCTATTTTTTTGGGAAAAAATACGGGAACTAATCCGATC
CGCAACAAAACAATCCTCCTCATATCATTGCCGTACAAAATCATAGAAAGGCATTATGATCAACGGTCAGAAATAATTACAGCAATTAATCCGCACATAAACAAAGCCAGCCAATTAGCGC
TCACCTCGTCTCACCTATTTATAACCTTTCGCTACCCACCGTTCGTCATTCGCCGCTCTATTCGTCTCGGATTTGTTTTCGATTAGCGATTCCAATGGCTCCAAAGGCGGAGAAGAAGCTCG
CTGAGAAGAAGCCCGTCGCCGAGAAGTCGGTGGAGGAGAAGGAGAAGAAGGTAGAGGTGTCCGAGAAGGCCGACAAAACGCCGGCGGGAAAGAAACCGAGGGCGGGAAAGAGCCT
TCCTGCAACAAAAGAAGGCGTCGCTCCTGGCGATAAGAAGAAGAAGAAGGCCAAGAAGTCCTCCGAGACTTACAAGATTTATATCTTTAAGGTTTTGAAGCAGGTTCACCCAGACATCG
GGATCTCTAGCAAGGCGATGAGCATCATGAACTCCTTCATTAACGACATCTTCGAGAAACTTGCTCAGGAGTCTTCGCGACTCGCCCGATACAATAAGAAGCCGACTATCACATCTCGGG
AGATCCAGACTTCGGTTCGCCTCGTTCTACCCGGTGAGCTCGCTAAGCACGCGGTTTCTGAGGGCACCAAGGCTGTTACCAAGTTCACCAGTTCTTGATCTGGTTTGTTAATTCTACGGTC
TTGATGTGTAATTTTCTTGTTTTTCTGGGAATGCTTCTTTTAAACTTTCTGGTCCTAAGATTCTGTAATTAGTAGTAGAACATGTTTCGCATGTCTACGTTTTAATTTGTTTTTGGTTATACTTT
CGCAGCGGAAACATTTTCTATGAAAAGTCTTTTCTGGTAGCTGCTTGATTATTTCTGTTATTGAATATTTCGATCCTCGTAATTGTTTATGCTAATTCTTTTTATGTTTTAAGTATGGATT
>comp165807_c1_seq5
GCACCAGAGAAACCCGGATCCAACGCTAGCTTTCTTCTTTCCAATCGTCTTCTCGATCAACCGAATTCGTTTTCCCTTTCTCTTTTATCTTATTTCCATGGCGCCAAAGGCCGAGAAGAAGC
CGGCGGAGAAGAAACCGGCACCAGAGAAGCCCGCGGCCGAAGAAAAACCAGCGGAGAAGAAGCCGGCCGAGAAGAAACCGAAGGCCGAGAAGCGGTTGCCTGCGAAAGATGGAG
GCGACAAGAAGAAGAAGAAGAAGGCCAAGAAGTCCTCCGAGACTTACAAGATTTATATCTTTAAGGTTTTGAAGCAGGTTCACCCAGACATCGGGATCTCTAGCAAGGCGATGAGCATC
ATGAACTCCTTCATTAACGACATCTTCGAGAAACTTGCTCAGGAGTCTTCGCGACTCGCCCGATACAATAAGAAGCCGACTATCACATCTCGGGAGATCCAGACTTCGGTTCGCCTCGTTC
TACCCGGTGAGCTCGCTAAGCACGCGGTTTCTGAGGGCACCAAGGCTGTTACCAAGTTCACCAGTTCTTGATCTGGTTTGTTAATTCTACGGTCTTGATGTGTAATTTTCTTGTTTTTCTGG
GAATGCTTCTTTTAAACTTTCTGGTCCTAAGATTCTGTAATTAGTAGTAGAACATGTTTCGCATGTCTACGTTTTAATTTGTTTTTGGTTATACTTTCGCAGCGGAAACATTTTCTATGAAAAG
TCTTTTCTGGTAGCTGCTTGATTATTTCTGTTATTGAATATTTCGATCCTCGTAATTGTTTATGCTAATTCTTTTTATGTTTTAAGTATGGATT
>comp165808_c1_seq4
GTGGATGAGGTAGACGCCGATGCTTGAGTCGCCATGCGAGAAGCCCGGGATCAACTGTCTACTGATACCATGAAGAAACCCAATGAGGGTTCAAGAAGCTCGAAGATGAACAGGAAAG
AAATGTAAATTGTTATTCAAAACATTAGTTTGATTACAGAAGCTGAGAAATATAAATAGAGAGAAGGAGAAGTAATCCACGGCTAGTACACGTGTCAAACTCCTATTCGAGATACAAACTA
TTTATTTATTTATTACATTCTTCTAACACCAACAAGCTTTGGCTCATTGAAGTTTTTTTTTTCTTTGAACAACTTATCTCGTAGTTTCATTCAAATTCGTTCCCTCAACCCAATAATTTTACTTG
TAAAAAGCTCGTGAAATATATCATTTAGAAAAGAAACTAATTATTTGATACAATCATTCATACTGCAGCAGCAACACACAATCAACGGAAAATTAACTTGCTTAATTAGGCAATAGCATCGC
AGGAGCCAATGCCTTTTTATTCAAAATATATACTAACAATACTTCATTCATCTTCACTGTATTAATGTTACTCGTTGTTCAAGGATACAGCTCCTTCACCTTGTTCTGTGCAGCGCCGGATGTC
GCCGCTGTGCCGTTGCTATGCGGGATGACGAAAACATCAGCATTGCCAACGTTAGTTCCAGTCGCCCAAATAGCGTTGTTAGCGTTATCGTATATGACGACGTTGCGATCTCTCTGGAGGA
TGAGATAATAATCACCGTCTTGGCGATTGGTGTTGCTCGCCCAGATAGCATCGCCGTTACTATATATTACGAGATTGCCATCGGTTTGCATGGTGAGAACACATTCGGAGGCCTGTCCGTTG
GTCCCCGAGGCCCAGACGGCTCCGCCGTTATCGTAGAGAACGAGGTTACAATCTTGTTGCATGATGAATAAGTAGGTGCCTTCTGCGAGTGATTGTCCAGGGTTAAGGGATTGGCCGGCG
TTCAGGCGGTCGTCGGCGGAGACAGGAGTGGTTAGGAGGCTAAATAAGGTGGCGACGAAGAGGAG
>comp165826_c0_seq17
GATTGTGGAGCTAATTAAGAGAAGAGTCGCTGGTCTTCATCAAAATACTGCAGTCGGGTCTTTAGACATAACTGATACATGGGAGCCATTAGAAGAAGGCTTACTTCCACTTGAAACGAC
AAGGCATGTATCAATGATCAGCATAACCCTCTCTAAAATGGAGCTGGATAAGTTATCTGCTGGATATCAATCTCCTTTACCGGCAGATCAGGTGAAACCATCTGCTGAATATGATTATGAGG

GAGAGGTTTCTCCTGCTGGTCGAGGAAGAGGCCGTGTTGGTCATGGTAGAGGAAGGGGAAGAGAAAATGGACCTATGATGGTAGATTATGGTGAACGGAATTGGGACAATAGAGAGCG
TGGATTTGGCAGGGGAGGTTGGGGACGTGGTAGAGGCCGTGGTTTCAGAGGTTGGGGCAGAGGAGGAGGCTACGGTGGTCGTTCCAATTATCAGCAAGACAATGCTGTCTACATTGAG
GATGCTACTCATGCTCCCGTTCAGGGCCGAGGTCGAGGAGGTGGCCGACGGCAAATTCGTCAAGGAAGGGGCCGCGATGCGAGGTCAAATGGTCCCATCAATGCAGTTGCTGGCGAAG
CTTAGTAAATCATTGGTTGCATGCAAGATTTTCTTCTTTATAAAGCCTTGGATGCCTGCTCAGTCCAGAGTGTTCTTTCTTACTTTAGTTTTCAATTTTTTAGATAAAAAACTCACAGGTTAA
CCATAGTCGGTTTTAAGTTGTCAGGATAAAACCTGGTAATGAGTTCTTATGACTCTATAGGATTAGTTAGATGTCATCTCATGTCTTTCCTTTAATGTTTGATTGTAGCATTCAACCTGTAGAG
CAGCACATTCTTTTATTATTATTATTTTATGCTTAAACTTTCTGGCTTGAGTAATCATTATGTTTAGTTAATGAA
>comp165830_c0_seq1
TGCCTTTATCTTTGATTAAATCTCTACCGCTACTTTTTAACAACATGACATTTATGTAAATTCCTTTTCTACTACTTCATCTAGTATAAATGTATCTACTGTATCCAGTATTAACCCCCAATTTTC
TAGTTGGCTTCTTCTTAACAAGAATCTAGCAGTTGCTCTCTTCTCCACAATCTCCTCATCCTTGTTGCCCTTCATTATTAACTTGGATTCCTTCCATTTCATCTGGACAATTCTTGAATGCCGA
CTATTCTCTACAAATCGTTCTAGAGTTATATAACTCAAGAATGAACTTCACTCCCTCACTATGCAAAATCAGACCATGATTGATTATCTCTCCACTGTAAAACATTTACTTCACTCCCTCACTA
TGCGAAATCAGTCCATGATTGATTCTCTCCACTATAAAACATTTGGTGGACACATTGCAACTGCTGGTGGTACCATCGACACGAAGATATTATCCAATATATCTTAAATGGTGTACCTACTAA
TTATCAAGCCTTCAAAGCGACCATCCGAACAAAGCTCACACCCATTTCTTTGGATGATTTATGTGCTCTTCTCTGTAGTGA
>comp165849_c0_seq4
TTTCTTTCCTATCTTTTAGCTATTGTATATCTCATTATCTTCTTCCTTCATACAAACCCGATTCTAATTTTCTTTTGTTTTCAGCACTATAAATAGTATTAAAAAAAATCTATATTCATAAACCAAT
AAGAAAAAGCCTCAAGATCTATCCTCCCATCAAATATTAACATTTGAACTTTTAGTTGTCATCATCCAAATCATAAATATGAAAAAAGTCTAGTAGTAGCTGTTGACCCTCATACTAGTGTC
GGGGTCGAGTCGACCGCGCAGGTGGGTTTTAAAAGA
>comp165866_c2_seq1
TTGTGTTACAAATAAATCTCTAATTACCGATGAATAGCTGTCAATTCTAAAATAGATGGCTTCCTCTACCTATAAAAACTCCACGCAAGAGAAGAAGCCAACCATCACAGAACAAATACAT
CAATCAATAACGTAATTCTTCTCTACGATACTTATTCCTCCTTCGATTCATTCCACCAACCAATAATCTCAGTCATCAATCTAAGAGCCAGAAGTATCCCCTCTAAAGATTGGCTATGTCTAGT
GAGAACCATTCAACCTCCTCTTCTTCCGCCATAGGCAACGACAATGCGATTGCCAAAGGAAACCAATTGGCGGCGTTGGGAAGAGCCTTCAATGAAGAGGTTGAGGCTGACCCATCGAT
CGTCAGCAGATCGGTCAACTTGCCAAGAGGGAACCATTCAGCATCATCCTCAGTCCGCAGAAATTACAATGCCCCTACTGAGCGGGTGGCATGGAGAAGAATACCTTCCAATGAAGTGC
GACCTCCTAATAGAAATATTGGGAGGTTAAACAACAATCGTGGAACTTTCAATGAAACGAGTCCTTATCAAACACGAAATGTTATGACCCCTCATATGGAGTTCCGACAACCTAACATGCA
GGCATATGGTGCAGTCCCTCTGCCAATTTTCCCTGTGCCATTCATTGGCAACTCCGAGCCCTCCACCAGCTCTTCAGCTCAAGGCCTCAATAATGTCTTCCCATATGCTCCTCATTGTGCGT
ATTACCATCCAACCCACCCACATGTTGGATATTTTATTCCTCTCCAAGACATTAATGCAGCACAACAATCAAAAATTTTCAGTTGGTTGTATAATATGCGAACTAGGTTGCGAAAGCAGATT
GAGTACTACTTCAGCTCCGGAAATTTGGAAACAGATATTTTTCTGAAGAGACAGATGGATGCTAACGGTTGGGTTCCAATAACTTTAATTGCTTGGTTTAGGAGAGTTCAGACTTTAATGA
AGGAAGCACATCTCGTATATAATTATTTAAGATGGTTAAATTTTATGCCAGATCATATCGCACGGTTGACTAGCAATGAACAATTCATATCAGATAGCTTAAGATTCTCGGACGATGTGGAAG
TGCAGGGTGATAAAATCAGAAAACGCCATGAATGGGAGAAATGGGTATCACCTAAAAATCCTACTTAATCCAATGCTGAATCTGCCGACCAGACTCAAATGACTCAAAATTAGGAATATCT
GTGGAGGATTATGCTCGCTCCTCTTCTTCTTCTACAGAAACTAAACTTGAAGAATATCAGTTTTCTTGATGACTATTTTGTTCGATATATAAAACAAAGCACAAAAAAGCATTAAACAATGT
CTTATCCGATCTATATGGCGTGGAGTGGATCACATCTTTCTTCTTAGTGTTTATGATAAGTAATATTGGCTATTGGTAAGGTGAGTGCAGGGTAAGAAAAAATAAAGCCAAATAAGAACAAA
TAATTCGGCCAAAGAAACTTTCAGACCGAATGGAGCTACGCATTTCAGCCTTGGTTTTTTAAGTTATTTCTTGTTTAGAATATATTATTACTTGTCTTATGTAATAAATAATATTGTTTGTCTTG
ATGTGACTTTGGGAGGACAAAAGAGTTGGGACTAAATTTCTCATAAATCTTGTATGCCTCTGCCTGCCTTCATTGTGCTCAAACTTATGTGTGTTGTTAAAATCCTTTATTGATGTTCTATAA

AGGATTTTAACAACACACATAAGTTTGAGCACAATGAAGGCAGGCAGAGGCATACAAGATTTATGAGAAATTTAGTCCCAACTCTTTTGTCCTCCCAAAGTCACATCAAGACAAACAATA
TTATTTATTACATAAGACAAGTAATAATATATTCTAAACAAGAAATAACATAAAAAACCAAGGCTGAAATGGGCAGCTCCAGTCGGTCTGAAAGTTTCTTTGGCCGAATTATTTGTTCTTATT
TGGCTTTATTTTTTCTTACCCTGCACTCACCTTACCAATAGCCAATATTACTTATCATAAACACTAAGAAGAAAGATGTGATCCACTCCACGCCATATAGATCGGATAAGACATTGTTTAATG
CTTTTTTGTGCTTTGTTTTATATATCGAACAAAATAGTCATCAAGAAAACTGATATTCTTCAAGTTTAGTTTCTGTAGAAGAAGAAGAGGAGCGAGCATAATCCTCCACAGATATTCCTAATT
TTGAGTCATTTGAGGTTGGTCGGCAGATTCAGCATTGGATTAAGTAGGATTTTTAGGTGATACCCATTTCTCCCATTCATGGCGTTTTCTGATTTTATCACCCT
>comp165866_c2_seq2
TTGTGTTACAAATAAATCTCTAATTACCGATGAATAGCTGTCAATTCTAAAATAGATGGCTTCCTCTACCTATAAAAACTCCACGCAAGAGAAGAAGCCAACCATCACAGAACAAATACAT
CAATCAATAACGTAATTCTTCTCTACGATACTTATTCCTCCTTCGATTCATTCCACCAACCAATAATCTAAAAAAGCTCAATCCTTCTCCATTCAGTCATCAATCTAAGAGCTAGACATATCCC
CTCTAAAGATTGACTATGCCTAGTGAGGACCATTCAGCCTCCTCCTCTTCCGCCGTAGGCAACAACAATGTGTCTGCCAAAGGAAACCAATATGCGGTGTTGGGAAGACCCTTCAATGAA
GCGGTTGAGGCTGACCCATCGATCGTCAGCAGATCGGTCAACTTGCCAAGAGGGAACCATTCAACATCACCCTCAGTCCGGGAAAATTGCAATGCACCTGTTGAGCGAGTGGCATGGAG
AAGAATACCTTCCAATGAAGTGCGACCTCCTAATAGAAATATTGGGAGATTAAATAACAATCGTGGAACTTTCAATGAAACGAGTCCTTATCAAACACGAAATGTTATGACCCCTCATATG
GAGTTCCGACAACCTAACATGCAGGCATATGGTGCAGTCCCTCTGCCAATTTTCCCTGTGCCATTCATTGGCAACTCCGAGCCCTCCACCAGCTCTTCAGCTCAAGGCCTCAATAATGTCT
TCCCATATGCTCCTCATTGTGCGTATTACCATCCAACCCACCCACATGTTGGATATTTTATTCCTCTCCAAGACATTAATGCAGCACAACAATCAAAAATTTTCAGTTGGTTGTATAATATGCG
AACTAGGTTGAGGAAGCAGATTGAGTACTACTTCAGTTCGGGAAATTTGGAAACAGATATATTTCTGAAGAGACAGATGGATGCGAACGGTTGGGTTCCAATAAGTTTGATTGCTGGGTT
TAGGAGAGTTGAGACTTTAATGAAGGAAGCAAATCTCGTATATAATTATTTAAGATCGTACAGTTTTATGCTAGATTACATCGCACCGTTGACCAGCGATGAACAATTCATATCAGACAGCA
TGGAATTCTCGGGCAATGTGGAAGTGCAGGGTGATAAAATCAGAAAACGCCGTGACTGGGACAAATGGGTATTACCACAAAATCCTACTTTATCCAATGGTGAATCTGCAAATTAGGAAT
CTGGGTGGAGGATTGGGCTCGCTCTTCTTCTTGCAGAGAGCGTAAATTGAAAAATAATTATATAAAAAGAAAAAGGAAGACAATGCTTTATCCGATCTAGAATGGACTAAGACTTCGTTTG
AGATGACTTTCTTATT
>comp165886_c0_seq5
TTTGATTGGATAGCTCTCAAATGGCTTTGCAAGCAACAGCTTTTTCTTTTTTCCCTTCATTAATTTGTATCTTTTCTAACGGTCGATCAAACACAAATTAAAAAACAGAAATCAAACCCCTC
CCAAACGAATTAACAGCAAAATCAAACGCTACAGCTTTGCAATTCCCTTCAGTTATACAAGCCATAACGCACAGGAATGGTGACTCCCCCTCTCTCATTATTTATCCACTCAGTGCCCTGTA
TGAAGTCCGCCACCGTGAACCTCGCCGCCGACCTCTTCAGTTTCCTCACGCCCTTCCACTTCACCCTCCCATCAAACTTCGCCCCTGGCCCTGTGTTGTTATACTCCCCGTAAAATAGAGT
ATTGAGCGCGAAATCCCCCTCCCATGGCAAATACCCATCGGGATGTATAACATCGCTGATCTCCGTCTCCATGATCACATGCCGCGCGTACTCCTTCCACGGCCTGCCGAGGTAGCTCCTGA
TCTTGTTCTTGAAAGGCAACAGCTTGTCGTCGCCGATGATCTTGCATTTGTGGAGGACAAAGCCGGTGGTTTCGTGGCGGTCGACGCGGCCGTGGGCGAGGACGATGTTCTGTTGGTTG
TCGAGGGGGCGACGGATGACCATGAGGCAGTTTTGGAAGACAGAGGCGGAGTCGCCGAAGATGAAATCGACGGTGCCGGCGATGACGCAGCTACGGTAGAATTGTCGCTTGGCTTGGG
CGTAGAGCGTGTCTTGGTAGCCTTCGAAGCGGCATTCGAAGAAGATGGAACGATCGGATTGGACCCGGATTGCCACGGCTTGGTGCTTGATGGCTCCGGCGGTGTTGCGGACGCCAAAG
CCAATGCCCATGAAGTTGTCGCCGGAGACCACAAACGTAGCAGTCATGAAAGTTCTGACGCCATCGACGAAATTCTTCTCTCCAGTGATGATCGTCTTCGCACTTCCATCGCCGTAAACG
GTCAAGTTCGGCATTTTCTTCGTGACATTTACTGTCTCCTCGTACACTCCCTCTTTAACATAGATCACATACCTTAATCATCATTTTAATACGCTCCTCAGTATTAAAAAATTAATTTACCTCT
TAATTCATTAATAATTAAGAAATTTAACCTTCCAGTGTACTCGTTCGGAATCTTCGCCAACGCCATTGAAATCGTCGTGTAATCGCCGGTCCCATCCTTAGCCACCGTAACATTTGGCTTTAG
ATTCGTATTGACCTGCCTGAGAACCCTTCTCTCATCCTCATGAACCCACGAAGGAATGCCCTCATCGTCAAATTGGTGCAGTTCTTCTTTCTCAGCGAGCAGCTTCCTTCCACTAACGGTC
AATCCCGGAACCTCCAGCATTTCAAGAAAGCTCGATAGCTCCTTAACAATTGCAAGAGAGTTGCTCGTTAGCTCCCTTGCTGTCTTCAACGTCTTTTTCAGCTTCACCTTAAGCTCACCGT

CAGGGAAACCGTCAATGCAGGTTTGTTGATACGACATGACAGAGCTCAGCCATGTTCTGATGTCGCGCGACTTCCTGGGGATATGTTCAATGCCGGTGGAGACGATGTCGTCGAGCGAAC
GTTTGATCTCGTCCTTGGAGTCCTCGAACAGCTGCCGGCAGTCTTCGACGGCGCCACGAACTCTCGGGTCCTTGTCGGCTAGGAGGCGGGTGCGGCGGAAGGCCGTGCTGATCTCGTCG
AGAACAACGGCGACGGCGGCCTTGACGAGTTCTTTGGGGTCGGTGGTGTTCTTCTTCACGACTTTGTTGAGGCTGGAGACGCAGGTTTGTTTGTAATCGGTGGATTCGCACATTACCTGA
ATGGACTTGGCGGTGGAATGCAGTTGCGTGGCGGGGGAACCGGCAGTGGCGGATGTGACGATGTGGGAGGAAGGAGTGTCTTTCTCTGGGCTGTAGAGCGTGCAGGCGGTGCCGACGA
CGGCGAGGAGGAGAATGAGAGAAGCGATGGAGCCGGCGATCCGCCTGTAGCGGCGCTTCCGCTGTTCTTCGTCGGAGAAGGCTGCCATGGCGGGAGAGATGGTAATGGAAGTTTTTAT
GGAAAGACACGCACGTAGTTTTATTATTATCTTCCTCTTCTCCCCGCCTAGCTAGTCTTTCTCCGAGCTTTTT
>comp165886_c0_seq6
TTTGATTGGATAGCTCTCAAATGGCTTTGCAAGCAACAGCTTTTTCTTTTTTCCCTTCATTAATTTGTATCTTTTCTAACGGTCGATCAAACACAAATTAAAAAACAGAAATCAAACCCCTC
CCAAACGAATTAACAGCAAAATCAAACGCTACAGCTTTGCAATTCCCTTCAGTTATACAAGCCATAACGCACAGGAATGGTGACTCCCCCTCTCTCATTATTTATCCACTCAGTGCCCTGTA
TGAAGTCCGCCACCGTGAACCTCGCCGCCGACCTCTTCAGTTTCCTCACGCCCTTCCACTTCACCCTCCCATCAAACTTCGCCCCTGGCCCTGTGTTGTTATACTCCCCGTAAAATAGAGT
ATTGAGCGCGAAATCCCCCTCCCATGGCAAATACCCATCGGGATGTATAACATCGCTGATCTCCGTCTCCATGATCACATGCCGCGCGTACTCCTTCCACGGCCTGCCGAGGTAGCTGGGG
AAGGAACCCTTGGCCGCCTCCAGGTCGTCGGTGGCGGTGATGCGGCAGGATTGGAGGACGATGCCCGTGGGTTCGTTCGGGTCGGAGCGGCCTTGGGCCGTCACCATGTTCTTCTGGTT
CAAGCCGGGTCGCCGGGCCTGGATATCGCAGTCTTGAAAGACGGCGGCCGCGTTGCCGAAGATGAAATCGACGGTGCCTTGGATGAGACAGGAGCGGAAGAACTGACGAAGGGAGTG
GACGTAGAGGGTGTCTTGGTGGGCGATGATGACGCAGTTGTAGAAGGCGGAGAGGTCGGACCCGACCCGGAGTGCCACGGCTTGGTGCTTTGATGGGCCGGCTGTGTTCTCGATGCGG
AGATCGCGGGCCAGGAAACCCGAGCCCACTGCGGCTAAGGTGGCGGAACGGAAGGTGGTCCAGCCGTCGACGACATTCCGGCTTCCGGTGATCACCGTCTTCGTCCTCCCATCCCCAA
CAAACATTATATTCGTCTTATTCTTCGGCACCTCTACGTTCTCCGCGTAAACCCCGGCCTTTATTTTTATGACAAATCTTCGCTTGCTCTTCACCGGCGCCGCCGCCACCGCTTCCGTAATGG
TTCTAAAATTCCCGCTCCCGTCCACCGCCACCGTCAAATCTGCCGTCGTGTCCGCTGACTGAAGCTGCTGCCTGTCCCTCGCCGTCATCCATTCCGGAAATCCATCATGACGGCTCGCTAA
CTTCCGCCCCTCTGTCGCCGCCGCCGCCGCCATGTCGCCGTCCGTCATATTCCTTATCATGGCGAGAGCGTTTTCGCACATGCGCTCGATGTAGGTAAGCCCTCGAAGGATCCCCAACCGA
AGCCGGCGGTCATCGCTGGCGTGTGAGAAGCCGTCGGCGCAGGACTCCTGGTTGGTAATGGCACCGCTGATCATGATCTTCAGGTCGGCGGCATGGCGGGAGAGTGATTTGGAGCTTGC
AAAGCCCGGCGAGTAAGATCGGAGATCGGCGTCGGCGCGACGGAGCTCGTCGAGCGTTTCGTCGAACATCTCGAGACAGTCTTGAACGGCGGCGCGTTCGCGGGAGGTGAGACTTGGT
CGGGCGGAGATGATTTTGGCTCCGGCGATTGAGCGGCGGACGGCGGCGAGGGTGAGGTTTAGGGAGGCGTGTATTACGTCTTTAGAGGAGGAGATGGAATGGAGAAGCGAGGCGGAGG
CGGCGATGGAGGAGATGCAGAGCTCAGGATGGCGGGCGGAGACGCAGGAGGAACGGAGGAATGTGTGGGAAGGTGTGACGGCTGCGGCGGCGGCGATTGAGATGATGGCGGCGAGT
AGGAGTATGGTAGCATTGGGAATAAAAGGTCTCATTTTCAGATAGAGCTTAAGGGAAGGGATAGGGAATTTGCGTGAGGACTGAAGAGATTATAAAGGTGATGGTGATGACGGGGAGGG
AATGTGGGTAGGCTTGGAGGATTTTTTTAGGATTGAAAAGATTTG
>comp165906_c0_seq6
TTTTTCTGCACTCTTTCTCCCTTTGCGTCTCCTTCTTCTCTTCGTCTGCCTCCACGCCTTCTCTTTCCTCCATCATTCTATTATCCGTCTCGCAGATATCTTCTTTGCTCCGATTCATGGCGGAT
AAGAACATATTGGTGACGGGAGGGGCGGGCTACATAGGGAGCCACACGGTGTTGCAGCTCCTCCAGGAAGGGTTCCGCGTCGTGGCTGTCGACAACCTCTACAATTCTTCCGAGGTCGC
CATCAAGAGGGTTGCAGAGCTTGCTGGTGACGCGAAGAAGAATCTCGCGTTTGAACGGGTTGATCTTCGAGATAGAGAAGCATTGGACACTGTTTTTGCTTCTACAAAATTCGATGCTGT
TATTCATTTTGCTGGCTTGAAGGCTGTAGGCGAAAGTGTCCATAAGCCTTTGCTTTATTATAACAACAATCTTATTGGTACAATAACTCTCTTAGAAGTGATGGCTGCTCATGACTGCAAAA
ATCTCATTTTTTCATCATCTGCAACTGTTTATGGATGGCCAAAGCAAGTTCCCTGCACGGAAGAGTTCTCTCCTAGCGCGGCAAACGTTTATGGACGAACCAAGCTTATCATTGAAGATATT
TGCCGTGACTTACAAAAATCAGACTCTGATTGGAGGATAATATTGCTTAGATATTTTAATCCTGTTGGTGCTCATCCCAGTGGAGATATTGGTGAAGATCCTCGTGGAATACCTAACAACCT

CATGCCTTTTATACAACAAGTTGCTGTTGGGAGGAGACCTGCACTTAGTGTTTATGGAAACGATTATAATACCAGTGATGGAACTGGGGTTCGTGACTATATTCATGTTGTGGATCTAGCGG
ATGGACATATTGCAGCTCTCCAGAAGCTTTTCCAGGACCCAAACATAGGTTGTGAGGTGTACAACCTTGGGACTGGCAAGGGAACTTCTGTGTTGGAAATGGTTGCAGCTTTTGAGAAG
GCCTATGGAAAGATGTTCATTTCTTGTGCAGAAAATCCCTTTAGTAATGGCTGCAAGGAGGTCCGGTGATGCAGAAATTGTTTATGCCTCAACTAAGAAGGCAGAGAGGGAGTTGAATTG
GAGAGCCAAGTATGGAATTGATGAGATGTGCCGTGATCAGTGGAACTGGGCAAGAAAAAATCCTTGGGGTTATGGATCGCCTGAATCTGAGAGTTGAGGATGCTTTATGGCACTCGTTTT
TACATAACTTTTATCTATTTGCAGCCTGTGTTAATAAAAGAAATGCTAATTTCTGGTTCTTTTTGTGTAAATGTTTTCAAAGAGGCATAAAGGAGTCCAGCCCAGTGCTTTGCTGATTGGCTG
CACTATTGTAAAGGGCATCCAGCTGATGTAAGTCCAGGTACAGGCCATTGAAAAATCCTATTAAGCTTAAAATAATAATTCAGATATTCTGACCATTCTTCGTGCTATCAGAGCAAAAAGAT
AAACAAGCAGAGAAATTATTTTTTT
>comp165912_c1_seq3
CTTATATCTAACTTTGGAGAATCATCATTAGTAAAGAGATTTTTGAATCTTGAAATTTCTACACTTGTTTGATTTTATATGGCAACCACGTATATAATTATGTTGAAAAACTGTCGGAACATTT
TTATATTTCATATATAAGCCTTGCCTTGATTCAATATAATGTTTGTTCTATGTCGAGTAGTTGGAATGTTTTCTTAAAATTTATATTTCAAAGAAGCTCTCACAAGCTCCAATACCTACCCCATT
TGCATAGCCCGGTAAGATAGAATTCCAATATTTGAAATCCCGCACCAGCGTCCTCGAGAAGGGCTCCTTAGAAACAGTCATATCTCCAAGTTCAATGATGTCACATAGGATTACATTATGCT
ATTACAATTGTTTAAAAATATGAGCAACACTTCTCACATCACCATTAGTAATATAAGCTTTTATTATTGTAGTTCACTCCACAATATTTCATTCAGGTAAATCCTCAAACATCTCATATACTATC
CCTGCATCTAATTTACTTACGTACATATTGATTAGGACAGTTTGAACAAATAAGTTTGGTCTACATTTCTCATTTATTATTTTCACAATTGAAGGTGCTTCTTCGTTAATGCCATTACCAAGGC
TCCTAATGGTGTTGGTAATTTGTATCTGCATGGCTTTCACATAGAGCTATTGTGATTAACTTGAAGAAACCAAAACTTGTTCCAATATCCAACATTGTAGCAGAATGGATGGATGGTTTATGC
AAATATTGCCTTCCGATCTTGTTCTGTTTCTATTGTTCCAGACATTTATTCACAGCAAGATCATGAGATGGAAGTTT
>comp165969_c0_seq3
ACCTGTTGCACATTCATCATACAAACACATAAAAAAATGTTTTGCAGCTTTATTTAATCAATGCCTAGAGCGGGTTAAAATTGTAATCTGATTCAAAGATTACACATCCATAGAGCTGCATA
GAGAGAAAAAAAAAATACTCACCATATCTCAGTGGTAAAAATTGGATGAAAACAAGTAAGAAATTAAGCAAAAATGGCAACTTAATACACACACAGATATGATACGAACCCTGAGCATA
ACGATAAACAGAAATTCAATGTCCGCCTTGCTTTCCCCGTTATTACATGCGTGCTAGAGTTTCCACTTGATGTTACAGAGCCGCCAAGCTCCTCTACAATCTTTGTAAGCTTTGATTTCTTAT
CATCATCAGCAATATTCATGAACATGATTCTTGGGCAGTTTCCTTTAATATCACGCCTAGTACTTTCCTGTGCTAACCTTTCCCCTTGAGAATGAGTTGTCAAATTAGAATGGGTGACCAAAT
TAGGAATTTCTTTGGACAACTGAGGACATCCCAAAGCTGCCAAAGATTGCTCACTGAAACAATCATAATTTCCTTCATCATGAAGAGAGCCACAATTTTGATCGGAAAACGAAGAATCAT
CAGGCACATGACTTCCTTTAACTCTCTTAGACTTCAAAGAGGAAACAGAAGCTTCCCATACTAGCCTCTTTCCTCTTAGCCGAGAAGAGAAGAATCTTTCATCAGCAACATCTTTTGCATT
TATCATATCTTTCAAAGAAAAATCTTCCTGATCAGTTTCCTTGACAGATGAAGTGTTTGAATTAGAAATTGCGCCAAGTCTAGAAGGTTCACTAAATCTGCATCTTATCATGTTGGATTTGG
AAGATATTTTTTTTCAACATGTTGAGAGGACTCCATGAAAGTAACAATATCCTTCTTAAGATGTGCAACCATAAGTGTAGCATCTCCAGGAAAGCACAATGCCCTCCTAATATCAGCTTTT
ACTGGCAGTTTGCGAGCTTTTCCTTTGCCATCTACCACTCCTACCGTTATGAAACCCTGTTTATGCCAAAATTGCTCAGATTCCATATCAGCCCAACAAAATAATGTTGAAACCCCAACTCT
TCTAAGTCTTTCTTTCAAAGGAAGGGCATCGGTTGCATGATGTATGAAGAGTTGAAAGAAAGACTTAGAAGAGTTGGGGTTTCAACATTATTTTGTTGGGCTGATATGGAATCTGAGCAAT
TTTGGCATAAACAGGGTTTCATAACGGTAGGAGTGGTAGATGGCAAAGGAAAAGCTCGCAAACTGCCAGTAAAAGCTGATATTAGGAGGGCATTGTGCTTTCCTGGAGATGCTACACTTA
TGGTTGCACATCTTAAGAAGGATATTGTTACTTTCATGGAGTCCTCTCAACATGTTGAAAAAAAAAATATCTTCCAAATCCAACATGATAAGATGCAGATTTAGTGAACCTTCTAGACTTGGC
GCAATTTCTAATTCAAACACTTCATCTGTCAAGGAAACTGATCAGGAAGATTTTTCTTTGAAAGATATGATAAATGCAAAAGATGTTGCTGATGAAAGATTCTTCTCTTCTCGGCTAAGAG
GAAAGAGGCTAGTATGGGAAGCTTCTGTTTCCTCTTTGAAGTCTAAGAGAGTTAAAGGAAGTCATGTGCCTGATGATTCTTCGTTTTCCGATCAAAATTGTGGCTCTCTTCATGATGAAGG
AAATTATGATTGTTTCAGTGAGCAATCTTTGGCAGCTTTGGGATGTCCTCAGTTGTCCAAAGAAATTCCTAATTTGGTCACCCATTCTAATTTGACAACTCATTCTCAAGGGGAAAGGTTAG

CACAGGAAAGTACTAGGCGTGATATTAAAGGAAACTGCCCAAGAATCATGTTCATGAATATTGCTGATGATGATAAGAAATCAAAGCTTACAAAGATTGTAGAGGAGCTTGGCGGCTCTG
TAACATCAAGTGGAAACTCTAGCACGCATGTAATAACGGGGAAAGCAAGGCGGACATTGAATTTCTGTTTATCGTTATGCTCAGGGTGATCCGTGATCTGCAATGTATGATCGAGCCATCA
AAGACCATATTTCTTACTTGTGAAGAAGACATGGAGCAGGCTCTTCTTGCAGCAAAAATGGGGGTCCTAACTTTTAGTAGTGACTGGCTTATGAACTGTGTAATGAGACAGGAACTTGAG
CTGGCTGCTCATCAGTTTGCAGAGTCACTCTAGAAATCAAAATTTAGTATAAATTTTTTTGATCTTAGTATATTTTGTGCCCTGATATAAGAGGCTTGCAATAGAGGCCAGTATAGCTGTGAT
ATATTATGATAGAAACGCAAAAATCTCTTTATTCTGTATTAGTTGGATTATAGTCAAGCAAAACTCATTAGTATGGCTTTTTCAAAGTAGATTCAAGCTTCGTTTG
>comp165969_c0_seq4
CAAATGAAGCTTGAATCTACTTCGAAAAAAAAATTCTAATGAGTTTTGTTTTATTATAATCCAACTAATACAGCATAAAGAGATTTTTGCGTTTCTATCATAATATATCACAGCTATACTGGCCT
CTATTGCATGCCTCTTATATCAGGGCACAAAATATACTAAGATCAAAAAAATTTATACTAAATTTTGATTTCTAGAGTGACTCTGCAAACTGATGAGCAGCCAGCTCAAGTTCCTGTCTCATT
ACACAGTTCATAAGCCAGTCACTACTAAAAGTTAGGACCCCCATTTTTGCTGCAAGAAGAGCCTGCTCCATGTCTTCTTCACAAGTAAGAAATATGGTCTTTGATGGCTCGATCATACATT
GCAGATCACGGATCACACTGCCACCAGCAGATGATATGATGGATGAAAGGTCATTGACGGATGGTTGGATATGCTTAGAAAGGCAGACTTGATATCCTCTGAGTAATGAATGAGGATTTGC
TCTTGCTCTAATAACCGCATCACTTAGTTTACATTTATACTTAACATGATAGTCCTCATCTTCTAATATATGCTGAGATTCATCTAAGAACTTGCCTTCTCGGAAGCTAGATTTCAACCAATTG
GCAGAAACTATCCAAGTCCCTGAGCATAACGATAAACAGAAATTCAATGTCCGCCTTGCTTTCCCCGTTATTACATGCGTGCTAGAGTTTCCACTTGATGTTACAGAGCCGCCAAGCTCCT
CTACAATCTTTGTAAGCTTTGATTTCTTATCATCATCAGCAATATTCATGAACATGATTCTTGGGCAGTTTCCTTTAATATCACGCCTAGTACTTTCCTGTGCTAACCTTTCCCCTTGAGAATG
AGTTGTCAAATTAGAATGGGTGACCAAATTAGGAATTTCTTTGGACAACTGAGGACATCCCAAAGCTGCCAAAGATTGCTCACTGAAACAATCATAATTTCCTTCATCATGAAGAGAGCC
ACAATTTTGATCGGAAAACGAAGAATCATCAGGCACATGACTTCCTTTAACTCTCTTAGACTTCAAAGAGGAAACAGAAGCTTCCCATACTAGCCTCTTTCCTCTTAGCCGAGAAGAGAA
GAATCTTTCATCAGCAACATCTTTTGCATTTATCATATCTTTCAAAGAAAAATCTTCCTGATCAGTTTCCTTGACAGATGAAGTGTTTGAATTAGAAATTGCGCCAAGTCTAGAAGGTTCAC
TAAATCTGCATCTTATCATGTTGGATTTGGAAGATATTTTTTTTTCAACATGTTGAGAGGACTCCATGAAAGTAACAATATCCTTCTTAAGATGTGCAACCATAAGTGTAGCATCTCCAGGAA
AGCACAATGCCCTCCTAATATCAGCTTTTACTGGCAGTTTGCGAGCTTTTCCTTTGCCATCTACCACTCCTACCGTTATGAAACCCTGTTTATGCCAAAATTGCTCAGATTCCATATCAGCCC
AACAAAATAATGTTGAAACCCCAACTCTTCTAAGTCTTTCTTTCAAAGGAAGGGCATCGGTTGCATGATGTATGAAGAGTTGAAAGAAAGACTTAGAAGAGTTGGGGTTTCAACATTATT
TTGTTGGGCTGATATGGAATCTGAGCAATTTTGGCATAAACAGGGTTTCATAACGGTAGGAGTGGTAGATGGCAAAGGAAAAGCTCGCAAACTGCCAGTAAAAGCTGATATTAGGAGGGC
ATTGTGCTTTCCTGGAGATGCTACACTTATGGTTGCACATCTTAAGAAGGATATTGTTACTTTCATGGAGTCCTCTCAACATGTTGAAAAAAAAATATCTTCCAAATCCAACATGATAAGAT
GCAGATTTAGTGAACCTTCTAGACTTGGCGCAATTTCTAATTCAAACACTTCATCTGTCAAGGAAACTGATCAGGAAGATTTTTCTTTGAAAGATATGATAAATGCAAAAGATGTTGCTGA
TGAAAGATTCTTCTCTTCTCGGCTAAGAGGAAAGAGGCTAGTATGGGAAGCTTCTGTTTCCTCTTTGAAGTCTAAGAGAGTTAAAGGAAGTCATGTGCCTGATGATTCTTCGTTTTCCGAT
CAAAATTGTGGCTCTCTTCATGATGAAGGAAATTATGATTGTTTCAGTGAGCAATCTTTGGCAGCTTTGGGATGTCCTCAGTTGTCCAAAGAAATTCCTAATTTGGTCACCCATTCTAATTT
GACAACTCATTCTCAAGGGGAAAGGTTAGCACAGGAAAGTACTAGGCGTGATATTAAAGGAAACTGCCCAAGAATCATGTTCATGAATATTGCTGATGATGATAAGAAATCAAAGCTTAC
AAAGATTGTAGAGGAGCTTGGCGGCTCTGTAACATCAAGTGGAAACTCTAGCACGCATGTAATAACGGGGAAAGCAAGGCGGACATTGAATTTCTGTTTATCGTTATGCTCAGGGACTTG
GATAGTTTCTGCCAATTGGTTGAAATCTAGCTTCCGAGAAGGCAAGTTCTTAGATGAATCTCAGCATATATTAGAAGATGAGGACTATCATGTTAAGTATAAATGTAAACTAAGTGATGCGG
TTATTAGAGCAAGAGCAAATCCTCATTCATTACTCAGAGGATATCAAGTCTGCCTTTCTAAGCATATCCAACCATCCGTCAATGACCTTTCATCCATCATATCATCTGCTGGTGGCAGTGTGA
TCCGTGATCTGCAATGTATGATCGAGCCATCAAAGACCATATTTCTTACTTGTGAAGAAGACATGGAGCAGGCTCTTCTTGCAGCAAAAATGGGGGTCCTAACTTTTAGTAGTGACTGGCT
TATGAACTGTGTAATGAGACAGGAACTTGAGCTGGCTGCTCATCAGTTTGCAGAGTCACTCTAGAAATCAAAATTTAGTATAAATTTTTTTGATCTTAGTATATTTTGTGCCCTGATATAAGA
GGCTTGCAATAGAGGCCAGTATAGCTGTGATATATTATGATAGAAACGCAAAAATCTCTTTATTCTGTATTAGTTGGATTATAGTCAAGCAAAACTCATTAGTATGGCTTTTTCAAAGTAGAT

TCAAGCTTCGTTTG
>comp165985_c1_seq2
TTGTCCCATAATTAATCAAAACAAATCAAACTCAACTCAATCAATCACTTGAGCATACCAAACTCAACTCACTCCTCTATTACATTTTTCTCCGAGCAACGCTGGACTTTTGAAGGGAGTT
GGAGAGGACAGAATGCAGTATAAAGCCAGCAGCTGCTAGCCTGCTTCACATGCCACCATCGATTTCGCCCATAACTCTCTCCTCTATTCGCTGCAACTTTCGCCTTTCCTGCCCTATTCTTC
CTCAATTGAAGAGAAGTGTCTTTTGCCTTTTGGTCTGCCTTCTCGGCCGTAGAAAATTTAACATCAAAGAACGCTGTTGCTTTCTAGGATGAAATCTGCTGCAGGGTACAAAAGTTGCTC
AATGGATGATGAGGAATTGCCATCTCTTTACAGAAAGAGGGCCACTGCAAGAAATTGTCTCGTCTCTGGGTATCATTTTCCGTTTTTTTTTTTCCCTTCTTACTGATAAGTAGTGTTTTTGTT
ATGCTAAAGCTGGTTTTTAGATATATTGATGCGAAAGCCGATGAAAGGTATTTTTTTTTTTTTTTTTGTGGGAAGCAGATTCTCGACTGAACTGCTCGAAGTAGAGTCTGAAGAGGCCTCT
TTGAATGTTGTCGTTATACCAGGGAATCCAGGCGTCGCTTCTTTTTACAAGGACTTTGTTGAAGCTCTCTACGAACAACTTGGGGGTTGCGCATCTATTACAGCAATTGGGCATATATCCCA
TTCAAGACAGGACATGGCATATGGAAGGATGTTCTCATTACAAGAGCAAATAAATCACAAGGTGGGTTTCATCAAAGAACTCCAGAATAAACATCTTCCAATAATATTGATTGGACATTCT
ATTGGTTCGTATGTATGCATTGAAGTTCTCAAGAGATTCCCGGAACAGGAGATATTTTCCATTTTTCTGTACCCATTTTTGACTTTAAACACAAGTTCGTCAACACAGTACATGATTGGGGT
GCTTGCAAGGTCTTCCCTTTTGAGTGCAACCTTCAGCTTTCTTGTGGCCTTGTTGGGGTTTTTTCCCACGAGAATTTCGGGAGCTCTCGTAAGTAGACTTCTTGGTCCATCATGTTCTATAA
CAGCAGTTGATGCTGCTTGCACTTGCCTCATGAAGTACCACACAATGCGCAATGTCTTATTCATGGCAAAGACAGAGTTTGAGAAGCTTTCAGAGGAGCCAGATTGGAGCTTCATGAAAG
AAAAAAAGAAACAAATTGCTTTTTTGTTCGGCGTTGATGATCACTGGGGTCCATTGTCATTGTTTGAAGAGATTTCAAGACAGGTGCCTGGTGCTCCATTGTCAATAGAAAGGGAAGGAC
ATACTCATTCCTTTTCGTGCTCCGAGGCCGGTTCCATCTGGGTTGCACATCATGTAGCAAACTTGATCAAATCTCGAGTTTCAAGTCAAGAATAGTCGCGATAAGGCTAATGATTATGATAA
TGATGACAATGGCGGTGATGATAAGCTTGCCAATTATTTGTATCATACTTCCATTGATTCCACTAATAATTGTTTTGGACAGCTTTTTTATTG
>comp165985_c1_seq20
TTGTCCCATAATTAATCAAAACAAATCAAACTCAACTCAATCAATCACTTGAGCATACCAAACTCAACTCACTCCTCTATTACATTTTTCTCCGAGCAACGCTGGACTTTTGAAGGGAGTT
GGAGAGGACAGAATGCAGTATAAAGCCAGCAGCTGCTAGCCTGCTTCACATGCCACCATCGATTTCGCCCATAACTCTCTCCTCTATTCGCTGCAACTTTCGCCTTTCCTGCCCTATTCTTC
CTCAATTGAAGAGAAGGATGAAATCTGCTGCAGGGTACAAAAGTTGCTCAATGGATGATGAGGAATTGCCATCTCTTTACAGAAAGAGGGCCACTGCAAGAAATTGTCTCGTCTCTGGGT
ATCATTTTCCGTTTTTTTTTTTCCCTTCTTACTGATAAGTAGTGTTTTTGTTATGCTAAAGCTGGTTTTTAGATATATTGATGCGAAAGCCGATGAAAGGTATTTTTTTTTTTTTTTTTGTGGG
AAGCAGATTCTCGACTGAACTGCTCGAAGTAGAGTCTGAAGAGGCCTCTTTGAATGTTGTCGTTATACCAGGGAATCCAGGCGTCGCTTCTTTTTACAAGGACTTTGTTGAAGCTCTCTA
CGAACAACTTGGGGGTTGCGCATCTATTACAGCAATTGGGCATATATCCCATTCAAGACAGGACATGGCATATGGAAGGATGTTCTCATTACAAGAGCAAATAAATCACAAGGTGGGTTTC
ATCAAAGAACTCCAGAATAAACATCTTCCAATAATATTGATTGGACATTCTATTGGTTCGTATGTATGCATTGAAGTTCTCAAGAGATTCCCGGAACAGGTCTTCCCTTTTGAGTGCAACCT
TCAGCTTTCTTGTGGCCTTGTTGGGGTTTTTTCCCACGAGAATTTCGGGAGCTCTCGTAAGTAGACTTCTTGGTCCATCATGTTCTATAACAGCAGTTGATGCTGCTTGCACTTGCCTCATG
AAGTACCACACAATGCGCAATGTCTTATTCATGGCAAAGACAGAGTTTGAGAAGCTTTCAGAGGAGCCAGATTGGAGCTTCATGAAAGAAAAAAAGAAACAAATTGCTTTTTTGTTCGG
CGTTGATGATCACTGGGGTCCATTGTCATTGTTTGAAGAGATTTCAAGACAGGTGCCTGGTGCTCCATTGTCAATAGAAAGGGAAGGACATACTCATTCCTTTTCGTGCTCCGAGGCCGGT
TCCATCTGGGTTGCACATCATGTAGCAAACTTGATCAAATCTCGAGTTTCAAGTCAAGAATAGTCGCGATAAGGCTAATGATTATGATAATGATGACAATGGCGGTGATGATAAGCTTGCCA
ATTATTTGTATCATACTTCCATTGATTCCACTAATAATTGTTTTGGACAGCTTTTTTATTG
>comp165985_c1_seq25
TTGTCCCATAATTAATCAAAACAAATCAAACTCAACTCAATCAATCACTTGAGCATACCAAACTCAACTCACTCCTCTATTACATTTTTCTCCGAGCAACGCTGGACTTTTGAAGGGAGTT
GGAGAGGACAGAATGCAGTATAAAGCCAGCAGCTGCTAGCCTGCTTCACATGCCACCATCGATTTCGCCCATAACTCTCTCCTCTATTCGCTGCAACTTTCGCCTTTCCTGCCCTATTCTTC

CTCAATTGAAGAGAAGGATGAAATCTGCTGCAGGGTACAAAAGTTGCTCAATGGATGATGAGGAATTGCCATCTCTTTACAGAAAGAGGGCCACTGCAAGAAATTGTCTCGTCTCTGGGT
ATCATTTTCCGTTTTTTTTTTTCCCTTCTTACTGATAAGTAGTGTTTTTGTTATGCTAAAGCTGGTTTTTAGATATATTGATGCGAAAGCCGATGAAAGGTATTTTTTTTTTTTTTTTTGTGGG
AAGCAGATTCTCGACTGAACTGCTCGAAGTAGAGTCTGAAGAGGCCTCTTTGAATGTTGTCGTTATACCAGGGAATCCAGGCGTCGCTTCTTTTTACAAGGACTTTGTTGAAGCTCTCTA
CGAACAACTTGGGGGTTGCGCATCTATTACAGCAATTGGGCATATATCCCATTCAAGACAGGACATGGCATATGGAAGGATGTTCTCATTACAAGAGCAAATAAATCACAAGGTGGGTTTC
ATCAAAGAACTCCAGAATAAACATCTTCCAATAATATTGATTGGACATTCTATTGGTTCGTATGTATGCATTGAAGTTCTCAAGAGATTCCCGGAACAGGAGATATTTTCCATTTTTCTGTAC
CCATTTTTGACTTTAAACACAAGTTCGTCAACACAGTACATGATTGGGGTGCTTGCAAGGTCTTCCCTTTTGAGTGCAACCTTCAGCTTTCTTGTGGCCTTGTTGGGGTTTTTCCCACGA
GAATTTCGGGAGCTCTCGTAAGTAGACTTCTTGGTCCATCATGTTCTATAACAGCAGTTGATGCTGCTTGCACTTGCCTCATGAAGTACCACACAATGCGCAATGTCTTATTCATGGCAAAG
ACAGAGTTTGAGAAGCTTTCAGAGGAGCCAGATTGGAGCTTCATGAAAGAAAAAAAGAAACAAATTGCTTTTTTGTTCGGCGTTGATGATCACTGGGGTCCATTGTCATTGTTTGAAGA
GGTGAATACTGCCTTTATTTCCTTCCTTTTATACAAATATCCATTTAATTGCTAAACTAGTATGGTTTTAATGTACCTTTTAAGCTTCAAAATGTTTAATTAGGGAGACCAATCTGTATATTATA
AACTTTAATGTGGGATTACTTATTTACTGGCCAAATAACCATGTAATATTAGAGAAATTTGACTTTATATGCTTCATAATAGTATAAATTGGAGAATCTA
>comp165985_c1_seq3
TTGTCCCATAATTAATCAAAACAAATCAAACTCAACTCAATCAATCACTTGAGCATACCAAACTCAACTCACTCCTCTATTACATTTTTCTCCGAGCAACGCTGGACTTTTGAAGGGAGTT
GGAGAGGACAGAATGCAGTATAAAGCCAGCAGCTGCTAGCCTGCTTCACATGCCACCATCGATTTCGCCCATAACTCTCTCCTCTATTCGCTGCAACTTTCGCCTTTCCTGCCCTATTCTTC
CTCAATTGAAGAGAAGGATGAAATCTGCTGCAGGGTACAAAAGTTGCTCAATGGATGATGAGGAATTGCCATCTCTTTACAGAAAGAGGGCCACTGCAAGAAATTGTCTCGTCTCTGGAT
TCTCGACTGAACTGCTCGAAGTAGAGTCTGAAGAGGCCTCTTTGAATGTTGTCGTTATACCAGGGAATCCAGGCGTCGCTTCTTTTTACAAGGACTTTGTTGAAGCTCTCTACGAACAAC
TTGGGGGTTGCGCATCTATTACAGCAATTGGGCATATATCCCATTCAAGACAGGACAGAAGCAACATGGAAGAAAATTAACCAAGAGATCATTTTCTTGAAGAACAGATAGAAAATGATA
AGAAGGCGTTTGTGGTATCTCAGTTGATGCTTATATCTGAGGTGAATGTGAATCTGAACAGAGGAGTGGGAGAAGGAAGAAGAGAAATGACTGGGAAATAGATCTATAAGAGCTCATCT
GCTGTTCACTTGCCCGCGTGTTGCTACGCAGGCTCAATTTTTTTTCCCCTGGTTTGGCTTTTCCAACTAATCACTTCTTTGCATTTGGCTATTTGTTCATGGAAACAGATTAGGTGCTGGGCT
TAATTTGCTGTTGGAGCACCCGATCTAAACTTTTTCCTTATAAAATGAGCAATAAGAGAAGTTTTTCAAGATATTACATGTACAAAAATGCATCAATATTGGAATGATGCGCTGGACAGAGC
TTTTCTACTTGTCATTCTTCTTAAATTTAGCTAATAATAGCACTATTGCTGTAATTCTATGTAGAATGCACACTATAAGCTTTTTATCATACAAAGAGTTGCTGTTGGATTTTTATATTTCATATA
ATTTCTTATTGAAGAATACTCTTCATTTATGAAATACCATGATATATGGGTTACTTTTTCTACCAGGACATGGCATATGGAAGGATGTTCTCATTACAAGAGCAAATAAATCACAAGGTGGGT
TTCATCAAAGAACTCCAGAATAAACATCTTCCAATAATATTGATTGGACATTCTATTGGTTCGTATGTATGCATTGAAGTTCTCAAGAGATTCCCGGAACAGGTCTTCCCTTTTGAGTGCAA
CCTTCAGCTTTCTTGTGGCCTTGTTGGGGTTTTTCCCACGAGAATTTCGGGAGCTCTCGTAAGTAGACTTCTTGGTCCATCATGTTCTATAACAGCAGTTGATGCTGCTTGCACTTGCCTC
ATGAAGTACCACACAATGCGCAATGTCTTATTCATGGCAAAGACAGAGTTTGAGAAGCTTTCAGAGGAGCCAGATTGGAGCTTCATGAAAGAAAAAAAGAAACAAATTGCTTTTTTGTT
CGGCGTTGATGATCACTGGGGTCCATTGTCATTGTTTGAAGAGATTTCAAGACAGGTGCCTGGTGCTCCATTGTCAATAGAAAGGGAAGGACATACTCATTCCTTTTCGTGCTCCGAGGCC
GGTTCCATCTGGGTTGCACATCATGTAGCAAACTTGATCAAATCTCGAGTTTCAAGTCAAGAATAGTCGCGATAAGGCTAATGATTATGATAATGATGACAATGGCGGTGATGATAAGCTTG
CCAATTATTTGTATCATACTTCCATTGATTCCACTAATAATTGTTTTGGACAGCTTTTTTATTG
>comp166029_c0_seq1
GAAGAGTAATATAAAATGTAAACAATCAACAAGAAAAATTCATTAAATTTTCAGAATTATTACATATCAACAAGGTTCTACATTGAGAACCTTTATAAATGGATATGTTCCGTAAAAAAAAT
GTCATCATAGGACATATCATCATCATATCATCAATTGTTAAGTCCAAAAATAAGCCCTTCATAGGGCATTTTCCTCTCCTTTCCGTTATCACCAAGGCTGAAATAAATCGTCTTCATCCTCAGA
AACACCTGGCAAGTCAGCAAACTATCAGATCCCGCCTGATGTGCTTTCCCCACCGCACGCTTCACATTTAGCTTCTCCGCCACCTTCTCCAGTCCCCCGTAAAGCCCATCACATCTTCTTAT

CAAGCACTTCACATCATATATGCGGCCACCAAAAAACCATTTTGTCAGCGCCATGAACTCCTCCATGGTCTCTGGCAGCATCACCCGCATGTTCGTCGTCAGCTGAAATAGAGCTTTGAAC
AAGATTACGAAATCGTAGCATCCGTGGAAAGCTACCCATGTCGCTTTGGAGAAGTCACCGACGAGGCCCGCATCAAGCAGAAGGCGGGCGAGCCAGGCGGAGGGGATGCCAACCTGTC
GATTCCAGGTAAGATTTATCCCAGACGACTCGAGAAGGGAGATTGTGCCGGGAGCGTGAGGGTGGCTGCGGAGGTCGAAGTCTTGGAGTTCGAACTGCCAGGTGGCGGAGGAGGTGCC
GCCGTCGCTGATAGTTATGCCGACTTGAGTGGGGCGGATGACGTTGGCATTCAGTTTAAGGACAGAGTAGCGCTCGGTAGGGGAGAGCTGCGTGTGATGCTTAGAGTGTCGTAAAATGC
AGCCGAGTGTGAACTCAGTGTCGAAGAAGACGAAGGGGGAGCGGCAACGGCGAGAAAATTGGATGAAGGAGGCCGATTCGATGTAGAAGTTTCGAATATCGACGTGATGAAGAGCAG
CCATGAGAGCTTTATTGCGGAATTTTTGGAAGCAGTACAAATGTCGAATG
>comp166083_c0_seq3
CCTTTTGAAAGCAAAAGGAATATTCACCATAGCTTTCAGGAGTTGTTCTGCAGGTCCAAGGAAAACCGAGTCATCGTTAAATAGCCTAAGCTTCAGCTCCTCGTCCGAGGTCGGTGCCAT
TCTCAACAATGTTTGTAGTAGCTCTATTGGAAGCTCGTTTCCTTCCATTAATTCATCACAAACTTCTTCTATTTTCACATTATGTGCCTTTAAAGAGATGGCCAAATTCTGCGATTTCTTCTGC
TCAAGAATCTGTATGCGTTGACCAGAAGAATCATTTCCAAAATCCCTTTTAGAATCATTCCTGCTCTTATCAGCAGCATTGTACCCAAACAAAGTTTCTATCATCTCCTCATTAAACTGGAAT
GATCCAGCTTTAAGCTGATGCCAGACCATGGATTGATCAGGGTTGGCCATGACCTTATCCCAGAAGAATGGCTTCAGCTTGGTCTTGGCACTGTCAGTATCAGCATTTTCTTTTGAAGAAT
CATTTTGCATGGGATAAGAATTTGCATAAGAACTTGAATTTTCTTGTGGAAGACAGGAAGAGCCTTTTGGCGGCAGTGGCGGCCCTCCTTTGCGAGGGGGAGGAGCAGGCTTTGGTTTAG
GACGCGGAGGAGGTGGGGCCGGAGGAGGCGCAGAAGTTCTTCCAGGAGGTGGCTTGAGAGGTGGTGGAGGCGGCACTCCTGCCGGAGAAGGATTGAGAGGTGCATTTGTAAGCTCAC
TTGAACTTGAAGCTACAGAATGCCCATCAGATAAGGTAGCATTTTGGTTCGGGTCAGCTTTAAAAGATGAACCAAACTTATCTTTGTACAAACCAAAAGAATTCTGCGATGAACCAGAGA
AGTCACTAAGGCTTAAGGACAAAATTGGTCTCTCATCCTTTTGCCCATTGACAGTCTGATACTTGCTTCCAGCATATCTATTACAGCAACAAAAGCAGAATGCAGCAAGTGTAAAAGTAGC
TGCTGCAGTTACTACCACTGCAATAATAATTGTTTGAAAACTGTTGCTTGCAGCAGAAAGATGAGGTATTGGATGAACTTGGGGCTCAAGGAAGTGATCTGATGATGGAGTTTGAGAAAA
ATCCCCATGATCATCTTCAGGCAAGGGGGGTTCATTGGGAGGTAATGACAGAACAGGAGCAAGCCGTTTAAATCCGATAGCGGGGGTTGGGGCTGGAGGTAGAGCTACGACTGGAGCCA
GAGCTGAGCTTCGAGTTTGGATTCGATGACTTGGACTTAGAGTTGGAGCTGGAGATGGGTCAGAGGGCCTTTTGAAAGAGAATCTTGCTCGAGCATTCTTGAAAAATGAGTAGCAGGCC
TGTGCATTAGGCTGATATCTGGGAAAACAGGAAGGATATTTTGCTTGATCAGACAGTCAATAAGAGAGCGCTTCATGTCAGGGGTAAGGTGAGTGAGGGTCTCTTGCATGTCTTTCCATAC
ATGTTCAGTGGAAGAATCCAGAATGCTATGTATTATTAGCAAATCTGAAGCAATCATGACTGATTTTGTTCCCAACAAATCAAGCATGCAATTTGACCATACATGTTCCATCTGCATTTCATT
TCTTCTCCCGTACCTGACAAATCCGTTACCATCGTATCCTCGGATCCAAAGAACAATCCATCGCCCGCTCGCCTTCCTCTCGAACCCCTCGGTACGGCAAATACTAGGAAAAAAACCGCGC
ACGCGGCTAAAAGAACATCACTTCTCATAGCCATCATACATCGGAACGAGCTCTCAGAGAGGATCAGCCTTTATCAACTCATCAATAGCTGCAATATGTTCTTCCGATCAAAATCCTATGCA
ATCCAATCTCGGAAAGACCCCGAATAACCTTTCCTCTATAATGGAAAAAATGGCTGATTCCGAATGCAGAAGCCACTGCGCGAAATGCGAAGAAAAGGGTCGAATATCAGAGGAGGAAA
TGAACGAAGCCTTTGATCCTCTTCCGGCGAGTTCGTCGGGAAATGGCCTTCCGACGTGGTAGGAAATCAAGACGTGCCCGAGATCTAGGTTTCGACGTTTCGTCGCGGACGACGAGCTG
GTGATAAATAGCGCATTTTGATTCTCTTCTATAGGTTC
>comp166110_c0_seq5
TTTATGGATGATATAATTTAATACTTTTCTCAAGGAGTGCCAAATTGAGATTCAGTACTTCATGACAAAAACAAAGGTGCATATGCTCTTTATTTTGTAAGACCAAAACATCAGGAATCTGTT
AATAATTTAACATGCTCTTTCTATAGTAATACAAATCAAGTAAACCAGTGCCTGTAACCAATCCTTCAGTCTGCATAGCCACTAATATACATATAAGAGCCCAGGGTTCAAATCCTAATTAAA
GCTGGAACATCAAACAATAAATAGTTTCCCTATTCTAACTAGCCAGTTTGAATATGTTTAGATAGAGCTCTTATTGTTGGTATGTTGTTGTTTATCAACTGCAGCAACCATCTGATTTTGCTG
CATCATTTGTGGTCCATAAGTGCAAATGTTCATCACCTGAGGAGGCATTTGCATCTGAGGAAAGCCAAAGTAGCTTGAATATTGTTCAGGGATATGAAAGTTATGCATATGGGTAGCAAAA
TTAATAGAATTTGGGCCTTGAAGAGGGCAAACATGTGGTTGGTTTGATATAGAAGTTCCAAGTGTAGTAGACTGAATATCAGATGATACTTTTGGTGGTTGAACTGGATTTGGTATTGATGT

CATTGAATGATGATTCATTGCATTGCCCATGCATGACATCAATACCAAATCCAGTTCAACCACCAAAAGTATCATCTGATATTCAGTCTACTACACTTGGAACTTCTATATCAAACCAACCAC
ATGTTTGCCCTCTTCAAGGCCCAAATTCTATTAATTTTGCTACCCATATGCATAACTTTCATATCCCTGAACAATATTCAAGCTACTTTGGCTTTCCTCAGATGCAAATGCCTCCTCAGGTGAT
GAACATTTGCACTTATGGACCACAAATGATGCAGCAAAATCAGATGGTTGCTGCAGTTGATAAACAACAACATACCAACAATAAGAGCTCTATCTAAACATATTCAAACTGGCTAGTTAGA
ATAGGGAAACTATTTATTGTTTGGTGTTCCAGCTTTAATTAGGATTTGAACCCTGGGCTCTTATATGTATATTAGTGGCTATGCAGACTGAAGGATTGGTTACAGGCACTGGTTTACTTGATT
TGTATTACTATAGAAAGAGCATGTTAAATTATTAACAGATTCCTGATGTTTTGGTCTTACAAAATAAAGAGCATATGCACCTTTGTTTTTGTCATGAAAAAAAA
>comp166155_c0_seq3
TTTTTATAACAGCACTTATTAGTGTGAAAAAATAAATTTAAATGATTTAACTGATCAGTTTACAAATGTTAAAACAAAGCATTACAAGTTTGTTTCCCAAGGACAAGGATGCAAACTCCCAT
GCCGGGCCAAATATAAAGAAGACAAAAATAGGCTACCTTCTACAATTATAAGCTACAGATCTTCTCATCAGTTAAATTGATTAGAGTGGGCTAACCATAGTAACGGCTAAAATATTTAAATT
ACAAGCAACAAAGGTCAGCACTTACGTAACAATAAGGAGATTGTTTTTGAAGCCATTATTGTTGGTAATAGGACGTAAGAGATCGATTGCCCAAACACCATCAACAATGAATTTGATCTCA
TAGCGTCCAGGATAGAGCTTCAAGCACAAAGAAAACACCCCAGTATTGTTCTTTTCCATCCTCCACTGACTTGTCCATCCATCGAAGGACCCAGTAAGAAGAACTTCTGAGGCAGAGTTA
GGCCAGACTATGCATACAGAGCGCAAAATACTCAGGGACATTTCAGCAGCAGCAAGCCTCAGTTGTTTAACTTCAATAACTTTTTGAAATTCAATGACCTCAAGTGCAATCTTGCCTTCCA
TAACAGCCAGCTTAGCTCTTGTTGAACGTAATATATCTCTGGCATTCTTTATTTCATTCTGCTGGAATTCCAAAGCATCAGAAAGAGAATGCAGCTGCTCAAGTAAACTTTCTTGGTCTTGA
TCCGTGTTGCATGCAATTAAAACTTCTTTCCATCCACAACTGGTGAATATAAGAGATGCATGAATTGGATGCATCCTTTTCATCATAAGTCAGCATACAGCATAAAGGCGCAGGAGATTGAA
AAACTGCCACCTCACGTTCCAGTTTGGATCCACCGAAACCTGTGCCAATTAAGGTTAGAATTTGAAAAATGCTCCATGAATTTCCTCCTCAACCTGCCAAGATCCATGCCCCATGCTTCTA
CCTCAAAGCCAAAGCACCAACTGCACTCCTTGATTTCTGCCTCTGAGCCAGTAAGACCACCAACGGCTCCTGCCTAATTCCATCGCCTCACCGCCGCATCATGGATCCGTGGGATCCTTG
CTAATTCGGAGAATGATTGAACTTATTTTTATTCTCTTATTTCAAATGCTTCCTATTAGAAATTCTCCTGGGATTATATAAATTATGTACATGCTGGAATGAGATGCAATCAATCCTCTAATTCT
TAGCATGGTATCAAAGCTTACCGATTTTTTCCTTTCCTGGTTTTCCCAAAGTTGTCGCCTTCATTTCGTATTGTCATAATCTTGTAGCCTTTATCACTGCATTCTCTTCCTTTCCTTGTATTCTT
CATATAGTTTGTAGCTTTTATCACTATAGTCTTTTTATGCCCTTTTTGTTGCAAACTAATTACTACCACAAGTTTCCCTCTCCATGTCCCTTCATCATGGGTTCCTTTGGAAATGAAGATGACT
ATTCAAGTTCAACAGGAGGATTGCAAAATATCCATTCAATATATCAATTGAATGGAAAGAACAATCTCCAGTGCTCACAATTGGTTCACACATCCTTAAAGAGATAATAAAATGTGAGTCAT
TTACATAGCATCTGATCTGAGCAAAAGGCTATCCCACGTTTAATTTTTGGGATGAACATGGCTTTATGGTGATATCCTGATTGAAGAACTCAATAGTTCCTAAGATATGTGACATAAGCATAT
TCCTTCATTCAACAAAAAATATGGATACACTAAATCAAATGTATTATAAAATCTATGATCTGTACAAATTTATGAAATGAAGAACAAAATCTCAT
>comp166155_c0_seq6
TTTTTATAACAGCACTTATTAGTGTGAAAAAATAAATTTAAATGATTTAACTGATCAGTTTACAAATGTTAAAACAAAGCATTACAAGTTTGTTTCCCAAGGACAAGGATGCAAACTCCCAT
GCCGGGCCAAATATAAAGAAGACAAAAATAGGCTACCTTCTACAATTATAAGCTACAGATCTTCTCATCAGTTAAATTGATTAGAGTGGGCTAACCATAGTAACGGCTAAAATATTTAAATT
ACAAGCAACAAAGGTCAGCACTTACGTAACAATAAGGAGATTGTTTTTGAAGCCATTATTGTTGGTAATAGGACGTAAGAGATCGATTGCCCAAACACCATCAACAATGAATTTGATCTCA
TAGCGTCCAGGATAGAGCTTCAAGCACAAAGAAAACACCCCAGTATTGTTCTTTTCCATCCTCCACTGACTTGTCCATCCATCGAAGGACCCAGTAAGAAGAACTTCTGAGGCAGAGTTA
GGCCAGACTATGCATACAGAGCGCAAAATACTCAGGGACATTTCAGCAGCAGCAAGCCTCAGTTGTTTAACTTCAATAACTTTTTGAAATTCAATGACCTCAAGTGCAATCTTGCCTTCCA
TAACAGCCAGCTTAGCTCTTGTTGAACGTAATATATCTCTGGCATTCTTTATTTCATTCTGCTGGAATTCCAAAGCATCAGAAAGAGAATGCAGCTGCTCAAGTAAACTTTCTTGGTCTTGA
TCCGTGTTGCATGCAATTAAAACTTCTTTCCATCCACAACTGGTGAATATAAGAGATGCATGAATTGGATGCATCCTTTTCATCATAAGTCAGCATACAGCATAAAGGCGCAGGAGATTGAA
AAACTGCCACCTCACGTTCCAGTTTGGATCCACCGAAACCTGAGGCAATCAGGAATAATTGCCATTCAGTTTCCAGTAGCATTTTATAATTATCAAATTATTTAGGTTAATTTGAGATTAATT
AAGATTTTAATTACATCATTATTATTTCATTAGGAATGACCTGTGCCAATTAAGGTTAGAATTTGAAAAATGCTCCATGAATTTCCTCCTCAACCTGCCAAGATCCATGCCCATGCTTCTACCT

CAAAGCCAAAGCACCAACTGCACTCCTTGATTTCTGCCTCTGAGCCAGTAAGACCACCAACGGCTCCTGCCTAATTCCATCGCCTCACCGCCGCATCATGGATCCGTGGGATCCTTGCTAA
TTCGGAGAATGATTGAACTTATTTTTATTCTCTTATTTCAAATGCTTCCTATTAGAAATTCTCCTGGGATTATATAAATTATGTACATGCTGGAATGAGATGCAATCAATCCTCTAATTCTTAGC
ATGGTATCAAAGCTTACCGATTTTTTCCTTTCCTGGTTTTCCCAAAGTTGTCGCCTTCATTTCGTATTGTCATAATCTTGTAGCCTTTATCACTGCATTCTCTTCCTTTCCTTGTATTCTTCATAT
AGTTTGTAGCTTTTATCACTATAGTCTTTTTATGCCCTTTTTGTTGCAAACTAATTACTACCACAAGTTTCCCTCTCCATGTCCCTTCATCATGGGTTCCTTTGGAAATGAAGATGACTATTCA
AGTTCAACAGGAGGATTGCAAAATATCCATTCAATATATCAATTGAATGGAAAGAACAATCTCCAGTGCTCACAATTGGTTCACACATCCTTAAAGAGATAATAAAATGTGAGTCATTTACA
TAGCATCTGATCTGAGCAAAAGGCTATCCCACGTTTAATTTTTGGGATGAACATGGCTTTATGGTGATATCCTGATTGAAGAACTCAATAGTTCCTAAGATATGTGACATAAGCATATTCCTT
CATTCAACAAAAAATATGGATACACTAAATCAAATGTATTATAAAATCTATGATCTGTACAAATTTATGAAATGAAGAACAAAATCTCAT
>comp166177_c3_seq3
AAAAAAAAAAAAAAAATCATAATAATAAGGCATGGTTGGTATGAATAATTACACTTGCGGGACAAGGCAACAAGTAATACTTTACAGACAATTATTTTTTATAACAAATAATACTTTCAAGC
TAGAACCCAAAATATTAGCAACTTTGAAATCTTATGATTGAAATATTATGTCTGTTTTTATTTGAAGTATTGGCAAAATGTGAGGATTATTAATAAAAAGATTTTTAAAAAAATATTTCAATTC
TCAGCTGCTCTGGTTTCTG
>comp166194_c0_seq2
TATCTTTCATAAACTTGCCTCTCCATCTCTTCAACATCCTCTTGATCCTCCGGCATCATATTCAATGGACGATGTGCTCTTCTACCACGACTTTCATCAGGCAAGTCAGTTCCAGCATCATTAA
TAAAATCGTCTTCGGCCTCCTCATCTTCCTCTTCCTCATCGTCACTATCGACCTGGGCCTCAAGGTCGAAGAACTCAGAACCCCTCTTCCGATATTTCTGGCCGCGGCCACCGTATAGCTCC
TCATTCTCGTCTTCTTCATCTTTGTATTCCTTGTCATGATCGTCCTCTATGGCTGCATCGTCAATGAAATCGGACCGAACCCGCTTCCTCCCACCTGTCCTCCTCCTGTAGTCTTCGTTCTCTT
CTTCCTCTATCTCTTCATCATCCTCCTCCTCTTCATAATCGTCTTCTCGGCGACGAGACATCCCGTTACTCCCTCCGCTTTAAGATTTGACGTGATTTGCGAAATCCTGAGTGGTTGGATCAA
CAACAGCGAATCCTTCCTTTCGAATTGTTGGGAAAGATTTATATAAATTTTGCTAGCAAAAATAAGCTTTAAGTTTGTTCAGCAAAACCGCCAAAGCGGGAGAGGCGGAGGGGGAAAGC
GGGAGAGGCGGAGGGGTTTGAAACCGAAAGCGGG
>comp166210_c1_seq1
GGGTGTGCTTGACTAACTTGACCGCACCTTATTGTCAAATTGGCTCTTTTCAAACGGAGCAAACCAGAATCTGAGCAATGTGAGCTGACTGAATATGCCATAAAATTACCAATTCCCTCTT
ATATTTTTTAGACGTGATTAGTGCTTTTGATATTACTTTTGAACCTCTTTTACCATCATTTTTTTGGGGGGGAGTTTCTCGTGATACTACATGGAGAAATGAGAAATTGATGTAGACTTATACA
TTCTTTGCAATATAAGCCAAAGATAAACATAACTTATTAATATACTGTGTAAATATATGCAAAAACCATAGCCAGTTTTGTTTTCAAGCAGCAAATAAACAGTAGAAAAAAAAAAAAAAAA
CGTTTGAAACAAGATGCCATTAGTAAAGATCATCCAAGGTTAAGTTCTCCATGGATCCCAACTTAGGTGTAGATTCAGCTGAGTAATTTTCCAGTTTCTCATCAGGAACAGTCATTAACAA
GTCCGCAGAGAAGGCACAATTGAGACCAAATGCCATATTCATTCCATCTCCTAACGGTAAATACAGATCAGAATATCTTGCTCTAGCCACCACTATATCAGTAACAATGGAATCAAAGATTC
CATCTTCTATTCCAGGAAGTGAAGCAAGAACTGGCTCTTGCATTCCAGCCTTCTTAGCTCGAATGACATATCTATCTTTCTCATTATCCTTGTAAAGCATGATGAAATCTCCTGATATAAGTC
CATGGCTCCTTACAAACTCACCAGTATTCTCCAATATGTACATTCTGCTCTTGTTGTTAGGCCAAAACCTTCATTAGAAATACAGAGGAATCACTTTTAGTTTTGGCTATTTCTGTTTCTGAA
ATATGAATTCAAACCTGAACTTAAAATTCCAAACTCGAGCTGTGTCCAAATCGATCATTGGCAATGAGATGCCTTCTCTTACAGTGAGGATTGGAAGATTGGCTTCTGTCTCTTTCTTAGGA
AGAACAACTCTTCCGAGCGAGCCGACATCACTGTTCCTGAGTTCCTTCTGGAGAAGGAATCTCAAACCATTCATGGCTAGGGGTTCTGGTGGAGTGGTGACTGAGCGAGGTAGTATAGG
CGGTGAGGAAAAGCCGGCGGCGGTGGCGGCGCGGCGGTGTCCGGTGGA
>comp166210_c1_seq2
GGGTGTGCTTGACTAACTTGACCGCACCTTATTGTCAAATTGGCTCTTTTCAAACGGAGCAAACCAGAATCTGAGCAATGTGAGCTGACTGAATATGCCATAAAATTACCAATTCCCTCTT

ATATTTTTTAGACGTGATTAGTGCTTTTGATATTACTTTTGAACCTCTTTTACCATCATTTTTTTGGGGGGGAGTTTCTCGTGATACTACATGGAGAAATGAGAAATTGATGTAGACTTATACA
TTCTTTGCAATATAAGCCAAAGATAAACATAACTTATTAATATACTGTGTAAATATATGCAAAAACCATAGCCAGTTTTGTTTTCAAGCAGCAAATAAACAGTAGAAAAAAAAAAAAAAAA
CGTTTGAAACAAGATGCCATTAGTAAAGATCATCCAAGGTTAAGTTCTCCATGGATCCCAACTTAGGTGTAGATTCAGCTGAGTAATTTTCCAGTTTCTCATCAGGAACAGTCATTAACAA
GTCCGCAGAGAAGGCACAATTGAGACCAAATGCCATATTCATTCCATCTCCTAACGGTAAATACAGATCAGAATATCTTGCTCTAGCCACCACTATATCAGTAACAATGGAATCAAAGATTC
CATCTTCTATTCCAGGAAGTGAAGCAAGAACTGGCTCTTGCATTCCAGCCTTCTTAGCTCGAATGACATATCTATCTTTCTCATTATCCTTGTAAAGCATGATGAAATCTCCTGATATAAGTC
CATGGCTCCTTACAAACTCACCAGTATTCTCCAATATGTACATTCTGCTCTTGTTGTTAGGCCAAAACCTGAACTTAAAATTCCAAACTCGAGCTGTGTCCAAATCGATCATTGGCAATGAG
ATGCCTTCTCTTACAGTGAGGATTGGAAGATTGGCTTCTGTCTCTTTCTTAGGAAGAACAACTCTTCCGAGCGAGCCGACATCACTGTTCCTGAGTTCCTTCTGGAGAAGGAATCTCAAA
CCATTCATGGCTAGGGGTTCTGGTGGAGTGGTGACTGAGCGAGGTAGTATAGGCGGTGAGGAAAAGCCGGCGGCGGTGGCGGCGCGGCGGTGTCCGGTGGA
>comp166247_c1_seq1
CTGATTCAATTGTCAACAGATAAATAACAAAGAGAGAAGAACCTGAGCTTTAATGCTGAACTTGAAATGCTTTCATCAAAAGCTTAAAAATGTAGCCTAGCTAGTACAGGTAAGAAAGAT
TAATAAATGATCATAGCTCACAGCCTCGTAATTCTATCTTTTCTAGACACATGCTTTTAATTTGTACTACTTTATACTCTTTTGATATGAATGATTTGCATCGCAATAACAATTCAACACATAAT
GAGTTACACTTTCTCACTAATTTCTCCAAACTCTGGATTTAACTTACAAATCCTTTCTTGGTGTTAAATGAAAACGAATAAAAACAATGGAAACCCTGCTCTCCATCTTTACAGTTCCTCCC
TCTTCACTATTTCCTAATTACTTAAAATGAAACAAAATAATAAGAGCAATTAAAAGTACATCATAACAAAAAAATCAACTAATCGAAAAGGGGAAAAAACTCACAAACTCTAGTCGCAAT
CGACCTTAAGATCTGACGGTGGAAGAAAAGAATCCATCGCAAGGCCATGCAGATTGAAATCGGCCTCCTCAATCGTCCAACACTCCTCCAACCGGCGCTTATGATTAATCGATCCCTCAC
CGTATCTGAACAGAGTGACGACGGTTCGGCCATTGTGAGCGATATTGATGCCATCAATGTAGCGATAATCGTCAATGATGGATTCCATAAAAGTTTCCCAGAATATGGAAGAATCATTGTTG
ATGCTTTTGCAGCTGATCTGGGTGGAGGCGGAGATGCGACTGCAGCCAGATTTCATACGGAGGAGATGAGAGTCTTCCAGCTGCATAAGGAGACCAGTGCGTTGGCTAAAGTAGCCCCA
AACGGTGTGGCGGATAATGTCGAAGCTGGATGCGCTGCGGGATTTGAGGACATTGGGGCTGACTTCGAGCTTTAGAATGAAGCATTCTTCGCCGTTTATGATTTTTTCGCCGATGCAGACT
GCGTCGACGAAGAGATTTGCCGTTGAGCGCGGGTCTAGGCCCTGTAGAGATCTCCGGAGAGGACGCGGCGGGCCACGCGAAGCGTGCGATTGCTCCGAGCAGGACTGGCGCCAGGCG
AGCTTGCCATTACAGCCCGCGCTCATCTTACAGCCGGCCATAATGAGCTCAAAGAACCAGAGCTCGGGATTTTTCTGCCATAACACGAAGCCTCCGATCTCGCTTTTGCCATTCCTTGTTAT
GGACGGAGGAACGGCGACAGCGGCGGAGGAGTTATCGTTGCCCATGTGGAACTCCGACGAGCTCATCCGCACCTTCCCAACCACATACATACTCTGCACCGACGTTAGCGCCGCCTGCC
CGCCGGTCGCCGCTATGTATTGTTGTAGTATGTACTTGGCCGTTGAGGCTTGAATGGAGGATTCACAGACGGAGCGGTTACTGAATGCGGTGTCGAACGGCACGGGCATAGGGATGAGCG
GCGAGCCGATGACGCTGAGGAGGAGCTGCAGCTCGGCGGTCCGGCCTGAAAGCATCAGCGGCGGGCTGTCAGTGGCTTTGTTGAAAGCTTGCGACTTGAGCCAAGCGTAGTGATTGTC
TTGGTCGGTCGATGACGACGACGATCCTCCCTTCCGGTTGGGAAACATCTCCTCCGGTAAAGGAACCTCGAGGACGGTGTCAAGGGCATCTTCTCGCTCTAGATTTGGGCAAAGCTTCCT
CATATGTAGAGAGGAAATAGATTGATCCATCGTTAAATATATCGATCGATGGATCGTCGGAGAGGATGGTATAAATATAGGGATAAATGTTTTGGAAGATGAAGGAAACTTGCTTTTTTTGC
GCCTTGGCTACCAAATCGTTGT
>comp166269_c1_seq1
TATTTCTCCAATCTTCCCCCTATTCAATTTATTTTCAGAATATTTTTTTCAAAAATATCAATAGATTAAATACAAAAATAGAAAATGTCCTTAGCTCCTCCAACCAACCTATTCTCTGCCTTAAA
TATGTGGCCAGACTCAACGGCTGGCCCTTCTTTGGTCCTCAGCCGTTAGCAGTTTGGCAAAGTTAAGCTATACAATTCCGTGATTCCAGGAAGCTACAATTTTGTTCCCAAAAAAGAGGG
GAAAGCAATGACGGTAAGAAGAAAGAGGAAGAGAGGAAAAAAAAGACAGATCTTATCTTCTTCTCCTCTTTGTTCTCCCTTCATACCATCATTCATCTCCTACAATGCAGGCATAAAGAA
CAAGCGAACGAAGTCATTACAGGTGGCCTGTGGTCGATAAGCGTACCTTTGCAGCTCAGTCTCTTGTGGTCGGAGCTGATGGGCGACCGAAGCCCTGTATCAGAAATCTGCCCAACTGC
GGTGGCCATCGACAAGGACAAGAACAGCCAGCATGCCGTGAAATGGGCCGTTGATCATCTCATTAAAACAAGGATTGTTGTTCTCATTCATGTCCGGAGCAAATCCTCTAAGACGGAAG

CTGGGCCGGAAACCACAAGAGATCAAGCAGATGAAGAAGTAAATCAGCTGTTTCTCCCTTATAGGGGGTTTACTGCTCGCAAAGGGATGCAGCTAAAGGAGGTAGTCCTGGAGGGTGCT
GATATTTCTAAGGCCATAGTTGATTACATCAATGCCAGCTCCATCCAAAACATAGTCGTTGGGGCTTCAAATAGGAGCATCATGAAGTCTATCCAACACACCGCACATCATTTTATCTTCAA
CGCAATATTGAGCCTTTCAAACCTTACTAACATCAGTTCTTCTAATATGCAGAAAACTCAGAAGTTTTGATGTCCCAAGTAGCCTGATGAAATCAGCACCAGACTTCTGTGCTGTGTATGTC
CTTTCCAAAGGGAAAACAACTAGCATCCGAGCAGCAAGAAACTCAACTCCTAACGTTATTCCACCAAGGCAATTGAGCATCACAGCAACTTCACTAGTCCTACAAAGCATTTTAGGTTTT
ATAACAGGATAAGACCTCACTTCCCGTTTCTGCATCATACAGTAGATCATCATATTCAAGGAGGAGCTGGAGAGTTTCAGGTCCACCAACCCCCTTGCATGAAAGGACAGAACGAAGATC
AATAGATCTTATGCCTGATTATCTGAATACTTCTGCAAGAGATAGGCCACTTTCCATAGCTCGTTCAGCACCGCCACCCAACAATCTGTTCATGGAGAAGCTCGATTTTGCTAACCTGTCTG
GAAGTTCTGTTGATTATGGAAGCTCATTTTCAGATGACATTGACATACAAGGACACAGCTTCCAATCAATGAACATTGGAAATTCTCTTGAATTTTCTTCAGCCTCTGTTGATAGTCCTACA
AGCAAACGACACTCAGTTACTGGTGTAAGTGCAAGAAAATGATTGAAGTCATCATTTGTAATGTGACGTTCATTTTTAGGCATCGTCTAATCTGTTTTTTTAGAATCTTAG
>comp166269_c1_seq20
TATTTCTCCAATCTTCCCCCTATTCAATTTATTTTCAGAATATTTTTTTCAAAAATATCAATAGATTAAATACAAAAATAGAAAATGTCCTTAGCTCCTCCAACCAACCTATTCTCTGCCTTAAA
TATGTGGCCAGACTCAACGGCTGGCCCTTCTTTGGTCCTCAGCCGTTAGCAGTTTGGCAAAGTTAAGCTATACAATTCCGTGATTCCAGGAAGCTACAATTTTGTTCCCAAAAAAGAGGG
GAAAGCAATGACGGTAAGAAGAAAGAGGAAGAGAGGAAAAAAAAGACAGATCTTATCTTCTTCTCCTCTTTGTTCTCCCTTCATACCATCATTCATCTCCTACAATGCAGGCATAAAGAA
CAAGCGAACGAAGTCATTACAGGTGGCCTGTGGTCGATAAGCGTACCTTTGCAGCTCAGTCTCTTGTGGTCGGAGCTGATGGGCGACCGAAGCCCTGTATCAGAAATCTGCCCAACTGC
GGTGGCCATCGACAAGGACAAGAACAGCCAGCATGCCGTGAAATGGGCCGTTGATCATCTCATTAAAACAAGGATTGTTGTTCTCATTCATGTCCGGAGCAAATCCTCTAGTAGGTACCC
TTCCTCTTTCATTTTGTAGATGTTGTTTTTTGGAATTTGCAGATGCAGCTAAAGGAGGTAGTCCTGGAGGGTGCTGATATTTCTAAGGCCATAGTTGATTACATCAATGCCAGCTCCATCCAA
AACATAGTCGTTGGGGCTTCAAATAGGAGCATCATGAAGTCTATCCAACACACCGCACATCATTTTATCTTCAACGCAATATTGAGCCTTTCAAACCTTACTAACATCAGTTCTTCTAATATG
CAGAAAACTCAGAAGTTTTGATGTCCCAAGTAGCCTGATGAAATCAGCACCAGACTTCTGTGCTGTGTATGTCCTTTCCAAAGGGAAAACAACTAGCATCCGAGCAGCAAGAAACTCAA
CTCCTAACGTTATTCCACCAAGGCAATTGAGCATCACAGCAACTTCACTAGTCCTACAAAGCATTTTAGGTTTTATAACAGGATAAGACCTCACTTCCCGTTTCTGCATCATACAGTAGATC
ATCATATTCAAGGAGGAGCTGGAGAGTTTCAGGTCCACCAACCCCCTTGCATGAAAGGACAGAACGAAGATCAATAGATCTTATGCCTGATTATCTGAATACTTCTGCAAGAGATAGGCC
ACTTTCCATAGCTCGTTCAGCACCGCCACCCAACAATCTGTTCATGGAGAAGCTCGATTTTGCTAACCTGTCTGGAAGTTCTGTTGATTATGGAAGCTCATTTTCAGATGACATTGACATAC
AAGGACACAGCTTCCAATCAATGAACATTGGAAATTCTCTTGAATTTTCTTCAGCCTCTGTTGATAGTCCTACAAGCAAACGACACTCAGTTACTGGTGTGAATCGAGAACTTGAGGCTG
AAATGAGAAGGCTGAAACTTGAGCTAAAGCAAACAATGGAGATGTATAGCAATGCCTGCAAAGAAGCTGTAACAGCTAAACAGAAGGCAATGGAGCTTCAACAATGGAAAATTGAAGA
AGCCCAAAAGTTTGAGGAATTAAGACATGCAGAAGAAGCAGCTCTTGCTATGGCTGAAATGGAGAAAGTAAAATGTAGAGCTGCCGTTGAAGCAGCTGAAACTGCACGAAAGATTGCT
GAGCTCGAAGCACATAAGAGACAAAATGCAGAAAGAAAAGCTAGGCATGAGGCTGAAGAAAAGAAGAAAGCATTGGATGCACTAACTCAAACTGACACCCGCTACAGGAGATACACC
ATTGATGAGATTAAAGCAGCAACTAATGACTTCACTGCAGAAAATAAGATTGGTGAAGGTGGCTATGGACCTGTGTACAAGGCATATCTGGATCATACACCAGTTGCCATTAAGGTCTTAA
GAGCAGAAGCAGCACAGGGAAGAAAACAATTCCAACAAGAGGTTGAAGTCTTGAGCTGCATCCGTCATCCTAACATGGTCTTACTACTTGGCGCCTGCCCTGAGAATGGATGCTTAGTG
TACGAGTTCATGGACAATGGGAGCTTAGATGACAGGCTCTTCCGAAAAGGAAACACCCCACCAATTCCATGGAGCACAAGATTCAAAATTGCAGCTGAAATAGCTACTGCACTCTTGTTC
CTCCATCAGGCAAAACCTGAGCCGCTGGTCCACCGTGATCTAAAGCCAGCAAATATCCTTCTAGACCGAAACTATGTCAGCAAGATCAGCGATGTCGGTCTAGCAAGGCTTGTGCCACCA
TGTGTGGCTGACACTGTAACACAGTACCACATGACATCAACTGCAGGAACATTCTGCTATATCGACCCTGAGTACCAACAAACTGGGATGCTCGGCGTCAAGTCTGATATTTACTCTCTAG
GAATAATGCTGCTTCAGATGATAACGGCTAAGCCTCCAATGGGCCTCACCCACCATGTGGAGAGGGCAATTGAGAAAGGAACATTTGCAAGGCTTCTTGATCCAGCAATAACTGATTGGC
CAGTAGAAGATGCACTTTCCTATGCTAAGCTTGCCTTGAAATGTGCTGAACTGAGGAGAAAGGACCGGCCTGATCTTGCAACAGTAGTCTTGCCAGAGCTGAACAAACTAAGAAAACTT

GGACGCGACCATGAAGCTAACAGACGACATAAAGGAAAAAGCTGCAGGACTGGCAGCAATTTCAGCACTCCACCAAGGCCACACAGCAACAACAATTCACAGCCACGGACAGGGCCA
CTCATGCAGCAGGAACAGGTTGCAAGCAACGCTGGTGTTACAGCAAAGTCGACATCACCAAGTGATGATGATGTCAAAGCTGGATAAAAGCACAGGATTCAAAACAAAAGAAGGTTTG
CATAAGCTAAACGTTTTGGATGGATGTTACAGGCTTAGGATGGATATTAAAGGTGAAGAAAATTATAATTTTATGGCTGATTATAGTGTAGGGCATGTCAGCTGTAAGTTATTCATATTTAATT
TTTTTTCCTTCTCCAATTAATAGAATCTGTATTAAAAAAATTATGGAAGAATCATTGGTTAATCTTAATTCACAATTGCCTGAAGTTTGCTAACTTGGCTTCATGGAAGACCTCAGATGAAAC
AAAACTTGAAGACTGTCAGTTCATTCAAATCAATAAATGAAGAGAAAATTGCTATACAACGGAATTCAGCAAGGCCGACATGTTGAAATCAGGGATTTTGTAAAATTAGAAACTTGGCTT
GGATAAATTTGTCCCAGGTGCCATATATCACAAATGAAATATCAAAGTAACATGCGTATGGTATGATACACTGCTCCAGCGGATCCTAAGAACTTTTAGGAAATTTGTTAAGTCAGAATGAT
TCTAACAAAATGTAGGGGTTAGGCCATGCCTGAAGATGTGAGATTCAAATATTGCTGCCAAAATGGCTTTTGAAGAGGAATTGAGTGATGCATTCTCCAATAAAACTATCCCCAAGCTTTG
GTTTGATCAAGCATGTGAGTGTTGAAAGGGATTTATTTGAATCATTTGCTTATATTTATAATTTTTATTCGTTTCCTACAATTTAGAAAGTAGATGCTATTTTCTATGAGGTTGTACTATTTCAA
TAGAGTAGTTTCTAGATTTTGGACAATTGATGCTGCTTTAAATTAGTAAAATTTAATTTTATGATTGACTTAGGGTTCGTTTGGGACAACGGTTGCTTTTTATAAAGTGATAGCAAAGCTGTT
GTTTAGAAAAAAAACTTTTTAAAAATTGCTAGAAATCAACTTTCTAATACAAAAATTTATTGAAAGTAGTCAAAAGTAGTGACCAAATAAAAATTGTTATTGATGTCTAAAAAATAAAACA
GTAAAAAAATTGCCCCAAATAAGCCTTAGAATTTAATGTTTCCTATATCAGACTATTACTTTTTTTAAATGAGAACCTATTTGAATCATTATAACTAATCTAATAAGGGTTGAATGAAATACCC
CCACCCCGACCACCACCTCTAGTCGGAAGTCTTCCACCTGTTTAATCCGAGTTTCGAACATCCAGTAGCGGTTCTCCACCTCCTATGGCTATAGGTCCTTCTGTGTCGAATCTTACCACCAA
TCTCCACATTCTAGCTACTATTTCTCTCCATTCAGAAGATATATTGTCCCATTCTCAAATTTCATGGAAACAATGGAATTTCACCCTATTTTTATAGGAAAATTGATAAAGAATTAGAGGAAAT
GGCAAAATATATCATTTAGAGTGTCACACTCT
>comp166269_c1_seq25
TATTTCTCCAATCTTCCCCCTATTCAATTTATTTTCAGAATATTTTTTTCAAAAATATCAATAGATTAAATACAAAAATAGAAAATGTCCTTAGCTCCTCCAACCAACCTATTCTCTGCCTTAAA
TATGTGGCCAGACTCAACGGCTGGCCCTTCTTTGGTCCTCAGCCGTTAGCAGTTTGGCAAAGTTAAGCTATACAATTCCGTGATTCCAGGAAGCTACAATTTTGTTCCCAAAAAAGAGGG
GAAAGCAATGACGGTAAGAAGAAAGAGGAAGAGAGGAAAAAAAAGACAGATCTTATCTTCTTCTCCTCTTTGTTCTCCCTTCATACCATCATTCATCTCCTACAATGCAGGCATAAAGAA
CAAGCGAACGAAGTCATTACAGGTGGCCTGTGGTCGATAAGCGTACCTTTGCAGCTCAGTCTCTTGTGGTCGGAGCTGATGGGCGACCGAAGCCCTGTATCAGAAATCTGCCCAACTGC
GGTGGCCATCGACAAGGACAAGAACAGCCAGCATGCCGTGAAATGGGCCGTTGATCATCTCATTAAAACAAGGATTGTTGTTCTCATTCATGTCCGGAGCAAATCCTCTAGTAGGTACCC
TTCCTCTTTCATTTTGTAGATGTTGTTTTTTGGAATTTGCAGATGCAGCTAAAGGAGGTAGTCCTGGAGGGTGCTGATATTTCTAAGGCCATAGTTGATTACATCAATGCCAGCTCCATCCAA
AACATAGTCGTTGGGGCTTCAAATAGGAGCATCATGAAAAAACTCAGAAGTTTTGATGTCCCAAGTAGCCTGATGAAATCAGCACCAGACTTCTGTGCTGTGTATGTCCTTTCCAAAGGG
AAAACAACTAGCATCCGAGCAGCAAGAAACTCAACTCCTAACGTTATTCCACCAAGGCAATTGAGCATCACAGCAACTTCACTAGTCCTACCTCACTTCCCGTTTCTGCATCATACAGTA
GATCATCATATTCAAGGAGGAGCTGGAGAGTTTCAGGTCCACCAACCCCCTTGCATGAAAGGACAGAACGAAGATCAATAGATCTTATGCCTGATTATCTGAATACTTCTGCAAGAGATAG
GCCACTTTCCATAGCTCGTTCAGCACCGCCACCCAACAATCTGTTCATGGAGAAGCTCGATTTTGCTAACCTGTCTGGAAGTTCTGTTGATTATGGAAGCTCATTTTCAGATGACATTGACA
TACAAGGACACAGCTTCCAATCAATGAACATTGGAAATTCTCTTGAATTTTCTTCAGCCTCTGTTGATAGTCCTACAAGCAAACGACACTCAGTTACTGGTGTGAATCGAGAACTTGAGG
CTGAAATGAGAAGGCTGAAACTTGAGCTAAAGCAAACAATGGAGATGTATAGCAATGCCTGCAAAGAAGCTGTAACAGCTAAACAGAAGGTGAGAAAAACTCTCCCGCTATTCCATTAA
AATAAGATATTTGTAACCTTAAGCGCCAAGATTAAGTTTGTAAAATCTTGTAATTGTTTGGATGCAGGCAATGGAGCTTCAACAATGGAAAATTGAAGAAGCCCAAAAGTTTGAGGAATTA
AGACATGCAGAAGAAGCAGCTCTTGCTATGGCTGAAATGGAGAAAGTAAAATGTAGAGCTGCCGTTGAAGCAGCTGAAACTGCACGAAAGATTGCTGAGCTCGAAGCACATAAGAGAC
AAAATGCAGAAAGAAAAGCTAGGCATGAGGCTGAAGAAAAGAAGAAAGCATTGGATGCACTAACTCAAACTGACACCCGCTACAGGAGATACACCATTGATGAGATTAAAGCAGCAAC
TAATGACTTCACTGCAGAAAATAAGATTGGTGAAGGTGGCTATGGACCTGTGTACAAGGCATATCTGGATCATACACCAGTTGCCATTAAGGTCTTAAGAGCAGAAGCAGCACAGGGAAG

AAAACAATTCCAACAAGAGGTAAATCACTATTTTGATTTTTGCAGATAAACAGCAAACATTCTAGTGATAATAAG

>comp166269_c1_seq64

TATTTCTCCAATCTTCCCCCTATTCAATTTATTTTCAGAATATTTTTTTCAAAAATATCAATAGATTAAATACAAAAATAGAAAATGTCCTTAGCTCCTCCAACCAACCTATTCTCTGCCTTAAA
TATGTGGCCAGACTCAACGGCTGGCCCTTCTTTGGTCCTCAGCCGTTAGCAGTTTGGCAAAGTTAAGCTATACAATTCCGTGATTCCAGGAAGCTACAATTTTGTTCCCAAAAAAGAGGG
GAAAGCAATGACGGTAAGAAGAAAGAGGAAGAGAGGAAAAAAAAGACAGATCTTATCTTCTTCTCCTCTTTGTTCTCCCTTCATACCATCATTCATCTCCTACAATGCAGGCATAAAGAA
CAAGCGAACGAAGTCATTACAGGTGGCCTGTGGTCGATAAGCGTACCTTTGCAGCTCAGTCTCTTGTGGTCGGAGCTGATGGGCGACCGAAGCCCTGTATCAGAAATCTGCCCAACTGC
GGTGGCCATCGACAAGGACAAGAACAGCCAGCATGCCGTGAAATGGGCCGTTGATCATCTCATTAAAACAAGGATTGTTGTTCTCATTCATGTCCGGAGCAAATCCTCTAAGACGGAAG
CTGGGCCGGAAACCACAAGAGATCAAGCAGATGAAGAAGTAAATCAGCTGTTTCTCCCTTATAGGGGGTTTACTGCTCGCAAAGGGATGCAGCTAAAGGAGGTAGTCCTGGAGGGTGCT
GATATTTCTAAGGCCATAGTTGATTACATCAATGCCAGCTCCATCCAAAACATAGTCGTTGGGGCTTCAAATAGGAGCATCATGAAAAAACTCAGAAGTTTTGATGTCCCAAGTAGCCTGA
TGAAATCAGCACCAGACTTCTGTGCTGTGTATGTCCTTTCCAAAGGGAAAACAACTAGCATCCGAGCAGCAAGAAACTCAACTCCTAACGTTATTCCACCAAGGCAATTGAGCATCACA
GCAACTTCACTAGTCCTACAAAGCATTTTAGGTTTTATAACAGGATAAGACCTCACTTCCCGTTTCTGCATCATACAGTAGATCATCATATTCAAGGAGGAGCTGGAGAGTTTCAGGTCCA
CCAACCCCCTTGCATGAAAGGACAGAACGAAGATCAATAGATCTTATGCCTGATTATCTGAATACTTCTGCAAGAGATAGGCCACTTTCCATAGCTCGTTCAGCACCGCCACCCAACAATC
TGTTCATGGAGAAGCTCGATTTTGCTAACCTGTCTGGAAGTTCTGTTGATTATGGAAGCTCATTTTCAGATGACATTGACATACAAGGACACAGCTTCCAATCAATGAACATTGGAAATTC
TCTTGAATTTTCTTCAGCCTCTGTTGATAGTCCTACAAGCAAACGACACTCAGTTACTGGTGTGAATCGAGAACTTGAGGCTGAAATGAGAAGGCTGAAACTTGAGCTAAAGCAAACAA
TGGAGATGTATAGCAATGCCTGCAAAGAAGCTGTAACAGCTAAACAGA

>comp166298_c0_seq3

TTAAAAAAAAAAAAAAAAAAACCCTTCAAAACATTATTTTCACAGAAAATTATATAAAAATTGGGAACAGCGAACAACAACTCAACAGTTTAAATATCAAGTTCTTTGGCCGTCAACAGC
AGTAAATGCAATTCCAGCAGCTGCAAAGAGAGGGGAAATTGGAGGCAGACCCAAAACTCCCCAGCTTATGAAATGAAAACCCCCAGTGGATGAAAAAAGGGTGGGGGAAACCTTAATC
AGATTGATTCAGTAGAGACTCAGAAGAAATTGCAGAATTTGATTGAGGGTCCCTATACTCATCATTATCAGGAGGAGGAGCTTCGTCCCTCACCTCAGATTTCATGATTTTAGGAGCTTCC
AACGACGTTAATGTCGCTTGCGGGTTCCTCTGGTTAGGAAATAAAGGAAAATGATCCGGTATAGACATTTCTGGTAATGAGTAGTTATAATAGCTTATCCAGGGATGATCAAATCTGCAAGC
TGAGCCATAGCTGCAGGTTCCATACATGCTGTAGAATGAGCAAACCGCATGGCCAGGTCTCATAGGGAGGCCAAAGGGTCCAAGAGTGTTGGTAGCTGATGAGAATCTCTCTCTCGGGTG
ATGGTATTTGCAAGTTGAACCATATTTACAGCTTCCAGTTTTCAAGTAATACTGGCATTCGGGTTGATCGGGTCGCTCCGGGAAATTTTCGACGAAGCTGGGACTTGGATGGGCGTTGTTC
TTGTACTTTGGTGTCTGAGTGTGCCCAACTATATCAGGAGATGGAACCTGACTCACATTACCCGTGTAATTGGTCCATCCCTGCTGCCCAGGAATGGTCCCTGAGGTCGGAGGAATAATAA
CAGGCATGTAAGCTGGCAGGCATTGCATGCGAGGATTGGACATATAGGGTGGCCCGTTAGATAATGGCCAAGCTGCAATTCCACCAGCAAAAGGCATGCTAGGTGCTGGCGCCACAGATG
ATAATGGAGACCCATAAGCGGAGGACCCATTTAGAGCCAGAGGAAATATGGCGCCTGATGCAGGCTGGGGATGGTCAAACTTGCAAGCAACTCCAAATTTGCAGGTTCCAGTTCTCATGT
AATATGAGCAGGATTTCTCATCCTGACGCATTGGAAAGCCTAAAATGTTAAATCCTAATGCCCGTGCATCTTGTCTGTCACGTGGATGATGATATTTGCATGTTGCACCGAACTTGCACATC
CCTGTTCTTAGAAAGTACTGACAATCAGGCTGACCAACTCTTTCTGGAAGTTCACCTTTTGCCCGAGTCCCCTGCCCAACGTAAGTTGGGTGATTATAGCGGCAGCTGCTTCCATATCCAC
ACGATCCTGTCTTCATGTAGTGCATGCAATCAGGCTCTCCAGGGCGATCGGGATATGGATTTGCATACGCATCAACTTCATCATAATTATCCTCAACTTTTAACCGCCGCATAGATTCATCTAT
ATTATGATTCACGGGGGAGGCATTCGAATACGTCGGAAGTGCATTGTTCTGAAACTCCCGTGTATCCGGCATCGGTAACGCGATCAAAGCTCGACTTCCTCTTTCAAAGGACACAAACAA
CTTCCTTCCACCACCAAAACCGTCCCACAAAAGCAAAAAACTATGGAATCCCCAGATCAAATCAGCACTATCCCTGTCTGGAAATCTCGATCTCTTCAAGCCCCAAATCCACTCGTCTATT
ACCTCGGATCATCGGATGCTTCTCCACTCTTGCATTCAGCCATCAAACATCCAAGTCGGCGAGGATCGACAAGGAATCTAACGCTCTCCTGCTCAGATGCTTCCGCCTAAAAAGCATCGAA

TTCGAAGATCACCCCAGACATGTCTGCAAATCTCGATGGTTCTCCCCCTCTCTCTCTCTCTCTCCCTCTTCCCCATTCTCGCACCTTTCTCCCCCTCGCCAGAACTCCAGTCGCCGGCGTTT
CCTTCTCCCCTACAATGAGGCTGCTTCGCAAGCAACATCACCAGATTCCGTCCACTTCGTCCCCAAAGCCCTCGAAGCCCCCGTCGCGATCTTCCTCCGTCGCTCGATCAGCGGCCTCTTA
CCTCCTCCGCGAGCAGCGCCTCGTCTTCCTAATCCTCGGCATCCTCATCGCCTCCTCCTTCTTCCTCCTCCAAGCACTCCTCTTCTCCAGGCCCTCCCAAACTTCCCCTCGCCACATCTCGC
CTCCCCAGTCCTCTTCTCACTTCTCCTCCGCCGTTGTAGCGAGGCCTATGATACCGATTGGGCTGAGGAAACCATCATTGCGGGTTCTGGTAACGGGCGGGTCGGGTTTTGTTGGCAGCCA
TTTGGTGGACCGATTGATCGATAGGGGGGATAGCGTGATCGTCATCGACAATTTCTATACGGGGAGAAAGGGGAATGCCGTCCATCATATTGGCAATCCAAGGTACGAGTTGATAAGACAC
GATGTCGTCGAGCCAATCCTCGTTGAAGTCGACCAGATTTACCATTTGGCTTTCCCGGCGTCTCCCGTTCATTACAAGCGTAATCCGATCAAGACAATCAAGACAAATGTGATCGGAACCT
TGAACATGTTGGGCTTGGCAAAGAGAACTGGAGCTCGGTTTTTATTGACAAGCACTAGTGAGGTTTACGGAGACGCACTTGAGTACCCGCAGAAGGAGAATTATTGGGGAAATGCAAAT
CCTATTGGTGTTAGAGGCTGTTATGAAGAAGGGAAACGAGTTGCTGAAACTTTAGCCATGGACTACCATCGTAGCGCTGGTGTGCAGGTACGCATTGCACGTATTTTCAATACATATGGGC
CTCGTATGAGCATTGATGATGGACGTGTAATCAGCAACTTTGTTGCCCAGGCACTCCGAAGAGAACCACTGACTATCTATGGTGATGGAAATCAAACACGAAGTTTCCAGTATGTTTCTGA
CTTGATTGCTGGATTAATCAAGCTGATGGATGGTGAAAATGTTGGACCTTTTAATCTTGGAAACCCGGGAGAATTTACTATGTTAGATCTGGTTGAGGTTATCAAGGAAACAATTGATCCA
AGTGCGACAGTAGTTTTCAAACCCAAAACGGCTGATGATCCCCATAGAAGAAAGCCTGATATCACCAAGGCCAAGCAGCTACTGAACTGGGAACCAAAGGTTCCACTCAGAGAAGGTC
TCTCAATAATGGCCAAAGACTTCCGCAGAAGGATTCAAAACGAGTAAAACTGAGCCAAAGACATCGAAGTACAACAACCTTGCGATCATGAGAGAAGACAAGGGCTGACTATCTAATTT
TATTTTCATTTTACTTTCTTTTCACAATCTATTCTTTATTCTTGAAATATAAGGGGCCCTTTGTGATCACTTTATGGTTTTGCTTTATTTATATTTAATATAAAAAAAAATTAGTATAAA
>comp166298_c0_seq8
GATTGCTCTGATTATAGTCAATGCATTGTACTTGGTTCACTTGTTGATTAATAAGAATTTGAAAGTAAAATTGAGACTAAAGGCTTCAGTTGGTAATTTGCCGTCTTTTTTGGCACCCAGCCA
TATCCCTGCAAATAGATCCTGTATTTACTGTTCATGGATTCATGAGCATGGATTATGCACTTGTGCAGTAGGTTGTGCTCTGCTGATATGCTAGCTTAGTTTTTTGATGCATTTGCTATCTACCT
TTTTTTTTCTTGAAAGCAAAGATGCTCTTCTTCTACGTATGGTATTTTTTTTTAAAGGTTGCAAGCATCAATTAAAAGTCAGACACAAAACTAATTACAAATAAATAGGCATGAGATCCATTA
CAAATAAGAATGGAACGAGGCACCAAATTTTATGTAGGAGCTAATGAATATTTAATTTAAAAATGTCACGACCCACACTCAAACTTAAGACCTCTACAAGAACTAAGGAGCACACTGCTT
GAGCTTCTTTTGGTTTGTGTTTGATAGGATGAGTACTGGTAAATGCTTAAATTGAAGCTTAATTTGACAGGTGCAAGTAAACATTTCTACTGAATCATCATCTTATTCATCCTTATCCTATTTT
CATTTATGTAGGCTTTGTAATATTAGGCTTTGTAATATCCTTCTTATTAATATTTTATTTCTAGTTTTGTGAGCTTAGTTAGCTTTATCTATTTTGGACAAAGCATATTAGTTTAACCTGAACTGT
TGAGGGGAAGTGGAGAGTAGTTTTTTTCAGTATAGTCATTGTTGTCTGCCTCATAAGAACCACTTAAGGCTGTTCGTATCTTTCCTTATAAGAACTTAAATAGAAGATTGTAGAAGGTTTGC
CACGAAATACTATGGGTCTTGAATATGCTATTCGGCATAAGTATTTAGTAGATCTGTAAACCTACTTTGAATTGTCAAATGCACTGGTGAGAGATTTTTAAATAAATAAATTTTGTTTCTTGC
AGCATGCTATATGTCAAGCATGTTAAAGCTTAAGCAAAAGAGTCTAGGAAGCTTCGAGAAAATGTAACTTTAAATGATGTGCACTATGCATGCTAACGGTATCCCTGTCATGAGAGTTTAG
GGATTTGACATGCTGGTATTCTATATTTTTGACTAAACCCAGTGACCGGGAAATACTACTTTTGGCATTTGAAAATTTAAAGCATCATACCGTTACTT
>comp166301_c1_seq15
ATTAAAAAGGATAAATTAAAGAATGATCATGTTCGCGGAAATATTACAGTGAAATCTATTAAAGATTAGGTGGTTGAAAATAAGCTTAGGTGATTTGAACATATGCAATCAAGGGTAGAAA
GTATGCCTAGTAGGCAAGTTGATAAAATTAGTGTCAAAGACTAATGAAAAAGAAGGATAAGTAAGATTTATGGATAGAAGATGTTCTTAAAGATTTGCAAAAAGTGTGCTTGAATAATATG
AAAATCATGCATGTTTTTATTTTGGTCAAGTTGTCTTATAATATTTTGTTTTTCCTTGTATATTTGATGCATAGGTTACTATAAACAGAGTGGCTGGAGCGAAGTGGGGTCCTTGCAGTGGCC
AAGCATGCATAGCAATTGATTATTTGCTCGTGGAAGAGCAATTCGCCCCGCTTTTGGTTGATTTACTCAAGGCAAAGATCAAGAAAACGTACAAAGATCCTGAAAACTCTGCAAGGATTG
TTCACAAGCAACATTTTCAAAGACTGAGCAACTTTCTCAACGACTCGGCAGTTGCTGCCTCTGTTGTGTATGGTGGTTCCTTCAATGCTGAAAGCCTGTTAATCGAGCCCACCATTCTTCT
AAATCCTCCTTTAGACTCTGAGATCATGAGAGAGGAGATTTTTGGACCCTTGCTTCCTGTAATCACAGTAAGATATGAAGCTAAGTGTTATTTGAAAAGAAATATAAATCTTCTATCATTCAT

GTTGTCTAAATGCCATTATTTTTGGATATTAGCATTATTTGGATTGTAGTTTTGTTGGTCAAATGTTCTGTGTTTGATTTACGGTTTATGTCCATAATAATAGTAGCGTTATCTAGATATTTGAC
TTATTTGGATGTTATCTCTATCTAAATTACTCTGACAATCATACTTCAATGCAGAAGATAATGTCATACTAAACACATTTCATTTTTAGAAAGCAAAACACAGCATTACATACAGAAATATTAA
AACCAAACAGCCACTGAAAATCATTTAAGCTTCACACAGCTGCTCATCTCATTCACAGCTAAAGAAGATTGAAGATAGCATTGAGTTTCTGAAGGTAAGGCCGAAGCCTCTCGTCATTTA
CGCCTTCACCGTCGATGAGAAGTTGAAGAGGAGAGTCATCGAAGAAACATCATCAGGAAGCATTACTTTTAATGATGCAATGTTACAATTTGCAGTAGATTCATTGCCATTTGGTGGAACT
GGTGAAAGCGGTTTCGGAAAGTACCACGGAAAGTTCTCCTTCGACCAGTTCAGCCATGGAAAAGCCATATTGAGAAGAAGCTTTCTCATAGAACTCTCATGCAGATACCCTCCTTGGAA
CGAGTGGAAGCTCCAGTTCATCAGGTATATTTACAGGTTTTACTACATTGGGATTATACTAATGTTACTTGGACTCAAGAGATGAGATAATCGACTAAAAAATTCATGTATGGTAGCTTTTTA
TGTAGCCAAGTCTGGGAATTCTGTGGGTGAACCGTTAAGTCTTATGTATTTGTTATTTTCTATAACTTTGTTGGTGGTTTGTTATTAAGCAAGTCTGGATTTCTGT
>comp166301_c1_seq17
ATTAAAAAGGATAAATTAAAGAATGATCATGTTCGCGGAAATATTACAGTGAAATCTATTAAAGATTAGGTGGTTGAAAATAAGCTTAGGTGATTTGAACATATGCAATCAAGGGTAGAAA
GTATGCCTAGTAGGCAAGTTGATAAAATTAGTGTCAAAGACTAATGAAAAAGAAGGATAAGTAAGATTTATGGATAGAAGATGTTCTTAAAGATTTGCAAAAAGTGTGCTTGAATAATATG
AAAATCATGCATGTTTTTATTTTGGTCAAGTTGTCTTATAATATTTTGTTTTTCCTTGTATATTTGATGCATAGGTTACTATAAACAGAGTGGCTGGAGCGAAGTGGGGTCCTTGCAGTGGCC
AAGCATGCATAGCAATTGATTATTTGCTCGTGGAAGAGCAATTCGCCCCGCTTTTGGTTGATTTACTCAAGGCAAAGATCAAGAAAACGTACAAAGATCCTGAAAACTCTGCAAGGATTG
TTCACAAGCAACATTTTCAAAGACTGAGCAACTTTCTCAACGACTCGGCAGTTGCTGCCTCTGTTGTGTATGGTGGTTCCTTCAATGCTGAAAGCCTGTTAATCGAGCCCACCATTCTTCT
AAATCCTCCTTTAGACTCTGAGATCATGAGAGAGGAGATTTTTGGACCCTTGCTTCCTGTAATCACACTAAAGAAGATTGAAGATAGCATTGAGTTTCTGAAGGTAAGGCCGAAGCCTCT
CGTCATTTACGCCTTCACCGTCGATGAGAAGTTGAAGAGGAGAGTCATCGAAGAAACATCATCAGGAAGCATTACTTTTAATGATGCAATGTTACAATTTGCAGTAGATTCATTGCCATTT
GGTGGAACTGGTGAAAGCGGTTTCGGAAAGTACCACGGAAAGTTCTCCTTCGACCAGTTCAGCCATGGAAAAGCCATATTGAGAAGAAGCTTTCTCATAGAACTCTCATGCAGATACCC
TCCTTGGAACGAGTGGAAGCTCCAGTTCATCAGGTATATTTACAGGTTTTACTACATTGGGATTATACTAATGTTACTTGGACTCAAGAGATGAGATAATCGACTAAAAAATTCATGTATGGT
AGCTTTTTATGTAGCCAAGTCTGGGAATTCTGTGGGTGAACCGTTAAGTCTTATGTATTTGTTATTTTCTATAACTTTGTTGGTGGTTTGTTATTAAGCAAGTCTGGATTTCTGT
>comp166301_c1_seq19
ATTAAAAAGGATAAATTAAAGAATGATCATGTTCGCGGAAATATTACAGTGAAATCTATTAAAGATTAGGTGGTTGAAAATAAGCTTAGGTGATTTGAACATATGCAATCAAGGGTAGAAA
GTATGCCTAGTAGGCAAGTTGATAAAATTAGTGTCAAAGACTAATGAAAAAGAAGGATAAGTAAGATTTATGGATAGAAGATGTTCTTAAAGATTTGCAAAAAGTGTGCTTGAATAATATG
AAAATCATGCATGTTTTTATTTTGGTCAAGTTGTCTTATAATATTTTGTTTTTCCTTGTATATTTGATGCATAGGTTACTATAAACAGAGTGGCTGGAGCGAAGTGGGGTCCTTGCAGTGGCC
AAGCATGCATAGCAATTGATTATTTGCTCGTGGAAGAGCAATTCGCCCCGCTTTTGGTTGATTTACTCAAGGCAAAGATCAAGAAAACGTACAAAGATCCTGAAAACTCTGCAAGGATTG
TTCACAAGCAACATTTTCAAAGACTGAGCAACTTTCTCAACGACTCGGCAGTTGCTGCCTCTGTTGTGTATGGTGGTTCCTTCAATGCTGAAAGCCTGTTAATCGAGCCCACCATTCTTCT
AAATCCTCCTTTAGACTCTGAGATCATGAGAGAGGAGATTTTTGGACCCTTGCTTCCTGTAATCACACTAAAGAAGATTGAAGATAGCATTGAGTTTCTGAAGGTAAGGCCGAAGCCTCT
CGTCATTTACGCCTTCACCGTCGATGAGAAGTTGAAGAGGAGAGTCATCGAAGAAACATCATCAGGAAGCATTACTTTTAATGATGCAATGTTACAAGTATTCTTCTTTTTTCTTCCTCTAA
CTTTCTTAATTTTAGAAGCAGTAAATGCTTAACCATTTGAGAAAAAAACTTGTTGCACAGTTTGCAGTAGATTCATTGCCATTTGGTGGAACTGGTGAAAGCGGTTTCGGAAAGTACCACG
GAAAGTTCTCCTTCGACCAGTTCAGCCATGGAAAAGCCATATTGAGAAGAAGCTTTCTCATAGAACTCTCATGCAGATACCCTCCTTGGAACGAGTGGAAGCTCCAGTTCATCAGGTATAT
TTACAGGTTTTACTACATTGGGATTATACTAATGTTACTTGGACTCAAGAGATGAGATAATCGACTAAAAAATTCATGTATGGTAGCTTTTTATGTAGCCAAGTCTGGGAATTCTGTGGGTGA
ACCGTTAAGTCTTATGTATTTGTTATTTTCTATAACTTTGTTGGTGGTTTGTTATTAAGCAAGTCTGGATTTCTGT
>comp166301_c1_seq2

ATTAAAAAGGATAAATTAAAGAATGATCATGTTCGCGGAAATATTACAGTGAAATCTATTAAAGATTAGGTGGTTGAAAATAAGCTTAGGTGATTTGAACATATGCAATCAAGGGTAGAAA
GTATGCCTAGTAGGCAAGTTGATAAAATTAGTGTCAAAGACTAATGAAAAAGAAGGATAAGTAAGATTTATGGATAGAAGATGTTCTTAAAGATTTGCAAAAAGTGTGCTTGAATAATATG
AAAATCATGCATGTTTTTATTTTGGTCAAGTTGTCTTATAATATTTTGTTTTTCCTTGTATATTTGATGCATAGGTTACTATAAACAGAGTGGCTGGAGCGAAGTGGGGTCCTTGCAGTGGCC
AAGCATGCATAGCAATTGATTATTTGCTCGTGGAAGAGCAATTCGCCCCGCTTTTGGTTGATTTACTCAAGGCAAAGATCAAGAAAACGTACAAAGATCCTGAAAACTCTGCAAGGATTG
TTCACAAGCAACATTTTCAAAGACTGAGCAACTTTCTCAACGACTCGGCAGTTGCTGCCTCTGTTGTGTATGGTGGTTCCTTCAATGCTGAAAGCCTGTTAATCGAGCCCACCATTCTTCT
AAATCCTCCTTTAGACTCTGAGATCATGAGAGAGGAGATTTTTGGACCCTTGCTTCCTGTAATCACAGTAAGATATGAAGCTAAGTGTTATTTGAAAAGAAATATAAATCTTCTATCATTCAT
GTTGTCTAAATGCCATTATTTTTTGGATATTAGCATTATTTGGATTGTAGTTTTGTTGGTCAAATGTTCTGTGTTTGATTTACGGTTTATGTCCATAATAATAGTAGCGTTATCTAGATATTTGAC
TTATTTGGATGTTATCTCTATCTAAATTACTCTGACAATCATACTTCAATGCAGAAGATAATGTCATACTAAACACATTTCATTTTTAGAAAGCAAAACACAGCATTACATACAGAAATATTAA
AACCAAACAGCCACTGAAAATCATTTAAGCTTCACACAGCTGCTCATCTCATTCACAGCTAAAGAAGATTGAAGATAGCATTGAGTTTCTGAAGGTAAGGCCGAAGCCTCTCGTCATTTA
CGCCTTCACCGTCGATGAGAAGTTGAAGAGGAGAGTCATCGAAGAAACATCATCAGGAAGCATTACTTTTAATGATGCAATGTTACAAGTATTCTTCTTTTTTCTTCCTCTAACTTTCTTAA
TTTTAGAAGCAGTAAATGCTTAACCATTTGAGAAAAAAACTTGTTGCACAGTTTGCAGTAGATTCATTGCCATTTGGTGGAACTGGTGAAAGCGGTTTCGGAAAGTACCACGGAAAGTTCT
CCTTCGACCAGTTCAGCCATGGAAAAGCCATATTGAGAAGAAGCTTTCTCATAGAACTCTCATGCAGATACCCTCCTTGGAACGAGTGGAAGCTCCAGTTCATCAGGTATATTTACAGGTT
TTACTACATTGGGATTATACTAATGTTACTTGGACTCAAGAGATGAGATAATCGACTAAAAAATTCATGTATGGTAGCTTTTTATGTAGCT
>comp166301_c1_seq7
ATTAAAAAGGATAAATTAAAGAATGATCATGTTCGCGGAAATATTACAGTGAAATCTATTAAAGATTAGGTGGTTGAAAATAAGCTTAGGTGATTTGAACATATGCAATCAAGGGTAGAAA
GTATGCCTAGTAGGCAAGTTGATAAAATTAGTGTCAAAGACTAATGAAAAAGAAGGATAAGTAAGATTTATGGATAGAAGATGTTCTTAAAGATTTGCAAAAAGTGTGCTTGAATAATATG
AAAATCATGCATGTTTTTATTTTGGTCAAGTTGTCTTATAATATTTTGTTTTTCCTTGTATATTTGATGCATAGGTTACTATAAACAGAGTGGCTGGAGCGAAGTGGGGTCCTTGCAGTGGCC
AAGCATGCATAGCAATTGATTATTTGCTCGTGGAAGAGCAATTCGCCCCGCTTTTGATAACCCTTCTGTATGCGAAAAGCCATACTACATTAAACAAAAAATTCATATTATTCTAAATTAAAA
GCATTCAATTTATGATTATCTAATCATTACCCATTCACATTTCAAATGTATGCTTGAATTGATAGGTATCTTGATCAATCTTAAGTCAAACTTTACAGTCGGTTATCAGTCCAAATATTGTAAGA
GTTATTATCATTTGCATTATTTACATAGCAGATATGTGCTTAGAAAACCTTATTAAAACAATTCTCATCATCTCAGATATCTTCACAGTTTCCATCACTCCACCTCACATGAAAGGTTTTTAAG
AACATTAAGGGCTCATTTGGTTCCACGTAATTCCTCCGTTTTTTGGTTTGAAACGGAAAACTCAGTTGACGGAACATGTTTGGATGTGA
>comp166313_c4_seq1
TTGCTCTGTGTCAAAAACTATAGAGGAACAACTAAATCTTCTTGGTTCGAAAATATTAGAAATATCTTTTCTTTATTAGATTTAGAATTGAATTTAATTGTTAATAGGACACAATAGAAATGA
CATTTCTTTATCAGATTTAGATGATAATTTGACTCTTAATAAAATACAATAGAGAAAAAAGATTGATGTAACTGACCCCACAATGGGATAAATATTTGTTGTTGTTGTAGACCGAAGAACCA
CAATAACATCTGCTTATTAAGGTTTTGAACACAGTACATGAAGTTCCCGCCAAAGTAAGGTGTGGAGAACGATTAATATATGCAACATTATTGCTGTCTTATTTTCATGACTCACAGTGCTG
ATTAAATGTCAACTAACTACTAATAATGTTGAACTTGTGAATGCCATGTCAATCTCCGTAAACCCAAAATTTGGATACAAGTAGACATTTGGTGTGGAACTTAAGCTTCGTTTGGGACAACT
TCTTTGTTACTTCTTAGAGCAACTTTTTATTACAAAAATTTAAAATTTTATAC
>comp166341_c0_seq3
CCAAGCTTCCTTGCATCACCAACATTGACATACATATCAACTATATATGTTTCTCTATAATACACAAATAAAATTAGATCTTATATCATTAATCTGAATCTAGACAAAACAGAAAGGATCATCG
ACCAAATCATAAATGATCCACAAGTCTCATTAATAGATGTCACACGATCTCGCTGGATCTGTAAAACTCCAAGATCATCACAGCGGCCGTACATAGATTCCCATCTTCCATACTTTTCCCAA
GCAATCAAAAGAAAGAGGTAGCCGATTGGATGCATGGTTTGATAGATCTGGTGACAGGTTCTTCTCAAAAGCTGGAGGATAACTTCGACCTGTACGCTCATTACAAGTAGATAAGCGGTT

GCATGCATGGTTTGATCTTGCTTGCTTTGTTGGTTAATCGTGTTTGTTGTCTTCTTGATGGACATTTGCATGTTTAGTTCCAGTATTAAAAAAAAAAAAAGAAACAGAAGAGGACGCGGTT
ACTGATGGAGAAAACCATCTCAATTTATTGAAAGATCAACGGCTGTGATAATGCGGTTTAAGTTCTACGATTAGGATGAATCCACGCTAATGTTTGTCCTGGTAACAGGCATCTAATTAAAA
AAAAGGTGGTGTTTGCAGGACCGCCTTCCACGTGGCTTAATGTTCTCTAGTTCACCACGTGGAAAACAAATTGGGCGGGTTACAGTGGTTTAAGTTTAGATATGGTGGGATGTTGCT
>comp166344_c0_seq1
ATCGAGAGAGGTAACTTGGCATATATTTAGAGCTTTATTTTTGACTTTCGAAAATATATCTTCTATATTTCATTAGTTTTCTTAGGTGACGAGCTCTCGATTTACAATATCATATATTTGGCACA
ATAGTGTGTTCACGAGTTTAAGTTTGAGCCTCTTCTAAGACTCTTGATGGCATATAATATGAAGAAGAAAAGTTTATTGGAGAATATGCATTTGTTTGCTAGATTCAAAATTATGTTGTTATC
TTTCTACAAGGAATTGGTGAATCTTGAGGTGCTGTTCTGCTTATTGAACTCCCTTCCTTCTTTATTTGCTCAAACTGCATGTGAAAGTGGTGTTAGCAAGTGGTTGGAGAGCAATTATTGGG
CTTCATAAGTGGAAGTGTAGTTGTTTGGAGTGTTTCTTTGGCATAACGTGTAGGAAATTTTACGAAAGCTTTGTGAATTGTTTTGTCTTGAGAAGTTTTACTATATGTTTTGCGAATGTTTTA
TAATAGAGA
>comp166351_c1_seq1
TTTTTGTGCTAGAAAGCCGCTTTACGAAGCAGAAAGAAAGCCGTACCAAATGAAGCCTAAGTCTAAAATATCTTCAAATCCATCCCCCACCCATTTTAATGAGAATATTGTAAAGATCAAA
CAACTTCACATAAATTTTAACCAAACAGAGAAATGTCATTTATTTGTCATACAATATGTACAACACAAGTGCTGCTATCAAACAGCTCAATTGTTAACACATTTAAAGGAATATAGTTCAGC
AATCTGTTAGATCTTCATTGTAAGATTTCAAAATTAAATGAAGGTGTCATAATTATTAGGTTGTTTATTACCCAATAATGAGGACGTAGAAGGGAAAGGTTGGCCAATAAATATAGAAGACT
AACTTTTGATTCTAAGTAAAAATGAATAGGATAGAGAGATTGATGGCCTCAATGAGCAGCTGGAGGAAGATGCTAAATGCCTTGAACGTCTCCAGCTACAGTTGCTTGATGAGCGAAGCA
AGAGAGCTGATGTTGAGAGGGAGAATAGTATGTTGCAGAATCAGATTACCATGCTCATGACCTTGCTTGAAGAAAACCAAGCAATGGAAGATGAAGCAGCAGAAGGGGATGAAGCTTCC
CTTCCCTGAAATTCATGGATGTTCTTTAATTTTATGATAAGGCAGCAGCTCCAGAGACTCATTTGTTACCTGTTTAATAGATTTACAGCAAAGTGTTATTTGTTATTGCAATCTAGAAGAATA
AGAGATGGGTATCAATATACTTATTTTGTTCTTTTTATTACTTAAAATGTGCAGTGTTACAGTTACATGCATATGTATTTATTGTAAGCAGTTCAGAATATTTTATAATATTCATTTGATGTATGA
AAGTGGTGGAAACATATATTTTTCATATTTCTTTTGATGTGGATTGACCATTGTGCATATTTTGTAAGCCGCACTTAAGCGCGGTATAACTTAGATCATCTTCAACCGAACTGCATAATATACG
ATGGTATGCCATCCGCTATTCGCTCCATTCGAACGCTAGATTGC
>comp166356_c0_seq2
GACCGTTTGGAATTGGGAATGGTCGGGTTTTGATAAATAAGTGATTGAGATCTAATATTGAAGGTCGAGATAGAATGATCATGGAGGAATGGATTGTTCATGGCTTTGGAACTTCTTGACG
AGAATAAATGAATAGCGGAGATGGGCTTTGAGTGCTTTGATGGAGGAGGAGGTGTATGTACGTGTTTGGGGTTGAGGTGAGCACTTAAGGGGTTAAGGCTTCTAAGGTTTTGGAAGAGG
TGAGAGGTGGTTCGACTTCGGTGGAGGAAGGAGGCCATGGCGGATTGGGGCTGCGTTGGAGGAGGAATACAAATAAAAAT
>comp166361_c1_seq2
TTGATCCATAGAAAACTCATCTTATGGCAAAAGAATATAAATCTAAATCTTATTCTAAAATTAAAACAAAAAGAAAAAGTAGAATCTACATTCTTTGATTTCTTCCTAAAGAGGAGAATCAA
AATATATTTCTTCTATTAATTCCTTGTTACCTTTATTCAGGCAAATCGCAATGTCGAACTGAAAGTTGCAGGTGGTATCGGTTAGGACTTGCGTTGGGTGAGCAGATCGGAGAGGGAGTTGC
GAACTACAGGTCTGGAATGCGAGCTTAGAAGCGACGGGCAGAGGATTTCGCCAATCAGAGTGAGGAAGTCCGTGAGAATGAGAAGGAGAGCAGGTTTTTTTTGAATGGGACCTGGGGT
TCGTATGAACTGGGAAGAGAGGGAGGTTTCTGTCGTATGAACTGCAGTAGGGAGAAGGAGAAGAGGGTAGTTCGGGTTGGAGGCCTGCGGCAAAGGCTCTCAGTCCGTATGAATGGAG
AGGAGAAGGCTTTATTTTGTGTGGGTAGAGAGCGTGAGAGTAGTTAGCGAATGCCAGGAAAAGGCCTTGGCTTC
>comp166386_c0_seq14
AACGAAATAAATAGAAGAAAATATTTTTACAACATTTACAACAGGCTTATTTCTTATGCAATCGTATGCAACAAAACCAAAAACATTGGACATCCTTGAGGTTAACAATTGAATAATATAAA

ATTAATTAGAGGAAAAAAGTATATATGTGGCAGTATTCCTTCACCCATTCTCCAATTATGTTGTGAAAAGCAAGTAAACGAGACCCTTCAGGTATTCAACTATTTCGGATCCTCCTAGTTTC
GAAGTTCCATAAACTCTGTCCACAAATGTTATCGGAACCTTTATCTCAAATATTTATGATAAACTTTCTTATCCTCAGTTCTCCGGTTATCCATATCTAGGTGAAAGAATTTGAAGCCTAATGA
TTGGTGAGATTATTCTGTGTTGACACCGAATACAGCTCTACATCAGGATACCAACCAGAAAGAGCCACGTCAGTGATACCGAGCTGCAGGTTTAGGTATCCGGACGGGGATCAGCTTCCG
CGCAGAATAATTTTTCCCAAACGAAGCCTAAATCTTTATACATTTTGCATCGTGATACGTAGCACATTATCTCTATGAACTTCAATTTA
>comp166388_c0_seq3
AGAATTGAGGGCTTATCCCATTTTCATTAAAATGAAATAAATTACATGTGTAGCTTATAAAAAGAACCAAAAAAGGTGCAAAAGCACCAGGCAAAAAAGGCTACAAAACCCTACAAGGA
GTTGAGGAAAAAACAGCTATAACTCCAACAAAATTATACCACCACAAAAAATAACCTCAGAAAAACAACCATAAAAAACCAGAAATACATTCAGATAGATACTTCCCCCATCACGGGCTT
AGGAATTGCTGGTGCTCTAACTTGTTTGCAGTAGCCCCCAGTATTTCCATCCAAAGGGAGAAAGAAGGCAATTCTATCATCCTTGCTCATATAAACAAACTCATCCATATCGATGAAACCAT
CGCAACTTTGAAGCAGTAGTAAGAAGAGATTCGTAGTAGGGACGTTTACTGACAGAGTATCCAACCAGAGAAAGGTTCCATTCCGCGGCTGTTGCATCTGTAAAATCCTTGTTAAAAGGA
ACAATTTCTTTCGGAGTTTCGATGAGAGAAATCGGGATAAAGGGCTTAGATATTTCTGGAGTAGAAATAATGTTTGACCAGAGTCGGTCCAGGACGAGAGGTGCAACGGGAGGTGGAGG
TGCCAAGCGGGCGGAAGTGGAGAGGGGAGGAGGGATCCATGTGGAGAGGAGCGCGTGGTACCACCGGCGAAGGGCAAGCCAAAAATTGGCGGCTAGGGCAAAATGAAG
>comp166401_c0_seq8
ATATGACGTGGCGGCTGCCATGAATGATTCTTGGGAGGACATGGAGATTTGGAACTATTACCAGAGCTTGACATGGAAGGCTAGAGATTTTCGATTTCATGTCCAAGAATTATGTGAAGTAT
ACAATGAAAACAAGTAAAATGATTCATTTGCAGCAAAGATCTGTTGTGCAAGATTGACTCAGAGGGAAAGGCAAAAAGTCATATGGTGCTCGTGTGTTGCTGTTAAGGATTATGGAGAG
GAGGATTCATACTATTATGAATATGAAGATTATATAATGTGAGATATATGAGTTTAGAACTTACAGTGTTATATATATCTTCTCCTCTACCAATTTTTGAGTATTGTAGTTGTGCAAGATTAAGT
AACAATTAACATTATATGAAGATTCAAAGAGAGAATTTATGAATATAATGTAATTTTGATGGTACACTTTTTTATTATATTATTTATCATTTTGTATAACAAGTGTAACAAACAAAAAATTTAAA
ACCGCCGATGCCGGTCCCAAATTCAGGTGAAAAATATTGTGTTTTTTTAGTATTGATAATTTGTGCCCCACACCAAAAAAAACCTAGCTCCACCACTGGGTGGAAGGGATCAAGATGGATT
CATCACCAGTGCAGCATCAAAGTTTTCAATGGAAAAAAGATGACCAGGGAGTTGACAAGAAAGAGAGAGGATACTCCTCTTCATTCAGATGTCAGGGTAGGGTATCTAGCTTCTGTTAA
GAAGATGGTTTCAAGGAAAGCAAAAAAAGCTCTGATTGATTTGTTGTTTAAGCAGAATCTTCTAGGATAAACGCTAGATGATTCTTTTAGGGATTATGATAAATTTTGGAGCTGCTATAGCT
GATCTTGTTGAAG
>comp166419_c0_seq4
CCAGTTGAAGCTGGCAACAAGGAACTGCATTACGTGAATGTGCTTCCCTCAATTGGTTTAAATTTCTACAGCATCCTTCAGCACGACACTTGTGATGACAAGAAATGCAGTTGACAAGAT
TGTTACTCGTATGCACACACCCATTAATCGTTGACAGAAATGTTGAAGCCATATGGTTAGAAAACTAGAATCGAAAATTCTTTAGAGATATTGGTGAGTGACAAGGCACACATATATGTTGC
ATGCAAGATTCTTATTCAATATCTTCTCAATAACTTTCAAGATCTTTCTTAACCAAGCTACTTAGCACCAATATTTTTTATTTTATTTTGGTGTACAATGTAATAGATTGGACTCTATATGTGGT
ACTGATCTTGAAGAAGATTTTCATCCGTTTTTTAATATTATTTTACCTTCAAATAGTGGCAGACCCCCACTTTAGAAGCAAGAAAATTTTTTTGTAGCTGAAACTCCTACTCTTGGTGATAAA
TGTTAAAGAAATGGTGTGTTTCCACTTTCCTCATTCCCACAGATGCTTTCTGTAATATGTGTT
>comp166425_c0_seq2
AAGCTGCTTCAAAAAGCAACTAAAAAGCCGCTCCAAACGAAGCCTTAATAAACAATAAAAAAACACCCAAATGAAGCCAAAATCTACAATGCAACCACACATCTTCAGATTAAGAATAA
TAATAAAAAAAGACAACGATGAATCATCAAGTTAGGTGATTTCAGATTATCACAATTTACAACAGTAAGGGTGAGAACACTGTGAAATAAATAACATGCCTATTTGATATTAACACTTAATT
AAGATTCCTCATCATCATCAGAGCTGAAATCCTCCAAGCGCCGGTCTTTGATTGCAGGAAATATCGACTGCCGGGCCGACATTGATTTCATTATTGTATTATCCTTGCTTCTTGATGTACTCG
TCACTTTCCTCTGTGCTTCTCTGACTTCTTTGCAGGCCTTATCCAAAACCTCAAGGAAATCCCTCACTGTGACAAACAATCTCAACCCCTCGTCTCTTCCAGCATTCCCATGGAAATAGTC

AGTGTGCTGCAGCTTTCTTGACATTCTCAAGTTCATAGCTGAGGCTTGAAACAGCCCTGAGACCAAGGTGACGGTACTGATCTTCAGATTCTTCAGGAATTTCGTCGCTGAAGTCGACAG
AGTTAAAGCTAGAAATTGTAATGCTGGAGTTACTCCTACTATCTCTTGCTATTTGCACAGCACGCCTGCCTTCGGAGCGGATGATCTCTTGAACAACAAAATGTAGTAGTGTGGTCTTTCCA
TCGGCGCCTTTAACGTCTGAAAGTTTCAAAAGTGTGTCAAGCTTGAATGCCTCTGCTCCTCCACGGTATGTACCATCATTCATGCGGTTCCCAGTTTTGAGAACAGCTTCCAACAATTTTA
AAAATAAACGACTGTTTTGGAGATCATTGCAGGCTTCTTCCACAGTAGCAAGTGATTCCTTCATACTTGAGGATTCCTCTTCGAATGAAGTCATGAAAAGCAATGCATCGATCCTTTTGAA
AGCAAAAGGAATA
>comp166439_c1_seq3
TCCTTAAAGTTTTACCAAAAGTCAAATAACATAAAGCTCAATAATATAAAATAAATTATAATAAAAAATTACATAAATCTCAATAATGAAAAATCGAATGATATAATAAAAAAAAGATCATCA
TATGGACAAAGCCAAGGGCACCTCTCTTTCATGCGACCCCAGGGAAGAATCGCTCGCTGCGCCCTCCGCCGTCCCATTCACCGCATCGCCGACATCCTGTGTTCCGATCACCGTTGTCTC
GCCGAACCTCACCCGCCTCCCTTCTCCTGGTGAACACACCTGCGACTCCACCGGAGCCGCCACCGCTTCCGTCGGCGGTGACTTGTAGCAGGGCATGATGGTGCGGTACAAGCAGGTCA
AGATCCAAAAGATCCAGGGAATGGCGATTAGGGCCATTCCGACGATGGGAAACCAGCCGGTCTCGTCGGTCTCCGGCTGGGTTATATAGAGGACAAGGAAGATGCCACCGGAGAGGATT
AACAGAAAGAAAAAGCAGGTAATGAACCAGATGCAAAGATCCTCTGCCTTTCCCTCCGGTGGCGGCATTTCCTAGGGATCGGCCCCGCGCCACCGTCACCACGGCGCGTCTTTATCTTG
CTTGGCTTATCAGATCTTAGTTATTAAGTCACTAATATAACTGTATTGCTTATTTCTCCATGGTGTTCATCTTCTTACTCGATGTGCTTTGATTTGCGAAGGAACGAGGAGAGGGCTTCGTCG
CCGGTAGGTGAGATCGAGATGGGGAAGGATGAGAGAGCTGAAATATTCAAATGGTATGATGCCATGGGAGGAGGGATTGAAGGACAGAGAAGAGCGGAGAAACGTCGGGGAGTTAAC
AACAGCCTTTTGTTTCGATCTGTTTCTGCCTTGCTATTATGAGAAATGGTTTTTTTATAAATAAAATAAAGTTTAAAGGTTAGATTTGCAAATAAATAAATATTATTTTATATGTCTGTAAAGCA
TAAATTTGTTTTTTGAAGAAGCTTTTATTACTAAAA
>comp166457_c0_seq4
GTGACCACACCTCAAGGCCAGAAAATATAGACTGCAGTGTATCTGGATCTCGCTAAATAATAATGAACAGCTCAAGGCCACAAGTTTAATAATATGAATCATCCTTTATAATAATCGCAATT
CATTCACTTAGTTGGCAAATCATTTATGTACAACAGTGCTAGCAAGTTCAGGAGATACCAGACCTTTCAAAGACTGGAGATATTTACAGCAAAATTTAGGAAGATGATAGTCGTATGTACA
ATGATATTTACATAAGAATTGCCCATATATATAGGCCATATATACAGGTTTTATAGAATCATAAAAAGAAGTAAAAAGAGGAACCAGAGCTTCGCAGGAGGTCCTGATTATAGTTCCTTAATT
ACTCAGCATTTTGTGTCTTGCAGCGAAGTATTTGATTATCAACACCAGGATGGATGATCCTTGGTCAATCCAATGCACTCCAATATTCAGTTCCAAACATGGCTTGCTTATTCTTTTTTTCTT
TTTTGTATTTTGTAAGTAAGTCTTTATATGATATCATGGAAGTTCGATAAGATGACAGGGGAAGCATCTCAAAAGGACAAGCCCAGGATGAGTTGAGGACAATCAAATGCCTAATCTGAAG
AAAGCCACCTTTGAATGTACTCAATCCCCTCTGACATAAGAAAAATGCAAAAAACAGATTAGCAACCAATGCCATTCCACAATAACAAACCAAAGAAAAAATAACAAGTAGATGAAGCA
TATTAGCAGCACAGACTTCTTCCTAAAGAGAAACCTGTAAGTACCATATATTCAATAATAATTACTACAACTCTTAAAAGAAATCAACAATATTCCAATAGCACATCTCATAACTTTTGAAGA
AATGTTATTCAAGGATTTGAACGAAAGTAGATAATGCCATAAATCATAAGCAACTAAAACTAAAGCTTTTGGGAATTGAATATAATCGCTAAGATCATGTCCAACCGAAAAACTAATTTTCT
ACTCCAAAACGCAATAACACTTCAAAATGAAGTAATTCATCAATAATTGAACTCTATTTCTTAACCCAAAAAAAAAAAAGAATATTCCAT
>comp166464_c1_seq4
AACTAACTTTAAAAATGAAAGTCAGATCAGCCAAAAATCAGTTCATTTCAGAAACAAAAAATTGGAATTATCATAACATAAGAAACAATGTGCAAGTTCAATTCAATTTAAGTTTAAATTA
ATCCATAATAGATGTTTGAAACTAGCAGTAGAAAATGGAGAGAATTTTGTTGTTATTGCAACGGTTTCTCCAATTGGATCGGTTGCAAAGAAGATGAAGAAGCAGGGTGGTATTTATGAGA
AGTTGAAGCAGTTAGATTGTGAGAGATTTATAGATCGTATAAAGAAGATTTGATGTTTGATTTGAAGATCACGTTGTGGATAAAGCCTTTATGTTTGTAAAGATTTGCCACTTTTGACGGAG
TTATTGTCCGCTGTAGTTTTATTTTTTCATTTTTCCCTAAAATAAAACTCTTGTTAAAGATTATTAAGTTGATGAGAAATTCGTTTTAGTAAACATAAGAAATTTTACAGATTTAGAAAACTGC
AGCAA

>comp166472_c0_seq18
AAAAGCACTCTACTACATTACCTGCGGCTCGGTGAACTTGATTCCAGGTGTTGCAACAAGTCCTGCAAGATCATGATCCATATATTCAAATACAAGATACAAATTGCATGACAATCTTGATG
TAACTATTGCTTCAAGCTTCACAATATTTTGATGATTGAGTCTCCGCAGAATTTGAATTTCCCTTGCCATGAAACGGACACTTTCAGAATCCATGTTAACAAACCTCACTTTCTTGAGTGCA
ACAATTTTACCAGTTTCAAGGTCACGGGCTTTGTACACGCTGCTGTATGTTCCTTGTCCAATCTTGTCTAATTTCTCAAACGACTCAGCTCTGCGAGGGAGCCAGCCATTCACTGCTTCTG
GTGCAACATTCGTAAGCCATGCAGGCCAACCTGCAGCCACATGCTCACCAGAGAACCCATCGGGCACACTAAAATTTTCCATAGCCAATCCAAAGTCACCCATCTTCGGATCAGTATCTG
CCCCATGCACCCTAAAATCCACTGTAGCCCTCCTATGGTGCCCGGCCAAGTTCTGATTCCTATCAACAGGAGCCAAAGAACCCCTATCTCCTTCATCTGTCTGCACAGGAATGGGAGCAAC
CACATTCTCTTGTGATTTTGTTGCAGGCCTTTTACCACTCCCGCAGCTTCTAGTGGCAGCATCAGCATCAAACAATATAGCTGAAACATCCTTCCTCAAAGGAGTAACTAGCCGTGAGAGG
GATCTGCTTGAGCACTTCTTTATACCTTCAACAATCTCATCTGTTTCAATCCCTCTAGAACAAATGCAGCCCATATGAACTAGAAAGCCCCACCCTTCTCTCAATGTAAAAAATCAAAATTG
CTAATTGAATATGTAGATTCATCAAGCCTTCTTACGGAAGACCATTCTTCTGAACCTTATAAAACCTCTAATTCCTAGAACTTCAATCAGAGATCGCCCCCTTTCGAATATGTGTCTCAAATC
TCAAAAAATCAACAGCAGAACCCTCAGCTCTTCAAAATCCAAGCCAGAAACCAATCGAGGATCGTCGGAATCTGATCAAAATCCCACCTTGCGCCGAAATTTTCAGAATGAGGGTTTGT
TCCCGTGTTTCTAGCTCAATACTGGCTTATCCCTTCTCCCTGTTGGGATCCCTTCATCATCATCTCTCTCTTCTTTCTCTCTCGTTTCTGGAAATGAAATGAGAAAATGTAGGAGGTTGGTAA
CGAGAAGGACATAAACTCTATACAAAATTAGCAAAAAGCAGCAAAAGCTTTTATCGAAGACGGTGAGGAGAAGAAATAATTGAAGATGAAAGGG
>comp166472_c0_seq2
CCAGTTTATGTTAGGGAAGAAAAGGCTTCAGACAGGTTCACACTATAAGTGCTTCTTGGTCTTGACCTTATCAAGCCGAGCTTTTCGGACAGCTTGCTGGATTTGCCTCTCATGCTCTTTC
AGCAACTCTTCCATATTCCCACCAGGGGGCATTAATGGCCCTGAGTATTGGATTTTTCTGCTTTTGCCACCATAACCTATAGTAGTTTCCTGGCCTCCAGTCATTACATCTCTCTTTTGAGTA
GAGAAAGTTCTATCTAGCACACGCTGCTTCTCCTTCGGCTCAGCAGCATCAAGCCTACCATGCCGGGAGCTCATACTACTTGTATTGGCGAACTCCACGCCACCACTTGGAGGTATGCAA
GATTTCTGTGATCTCAACTGAGCACCATTCGGTACCAAAACAGTACTGGATGCACGATTAATCATCCTCATATCATCTTTCTTAATCCATGAGGATCCAAATGCACTTGTTTGCATTGTTGAA
TTAGAGTTAGGTAAGTCATTCTGAGCTACTCTGGGAGGATCAATGGGAAACCCTGACCCAGCTTCTTCTAGAGCTCTGTACTTATCACTTGCGCTTTTGGGATTTGTTTGGCCTTGCCATCT
CTGCAGGTTCCCATTAGCATCCTGCCCTGGCTTAGGTTTGTGGTCAGCTGCAACTTTATTTTCGCGAGCTGCAGCTCTTTGCCTTCTAGCTTCCTCATCCCGAAGCTTAGCATCATACTCCTT
GCTAGGTGGATATGTGGGCAAGCTTGAAGGATCACAGGCTAGAGGATTTGTTGTGAAAAATTTGCTTTGAAGGGCAGAAGATGCAGTTCCTCGATCCTCTGGTTGAATTGCAAGAAGAGT
ATCAAGAAGAGCCAAAGTTGACGGAGGGCAATCCCTGAATGTCTCTTCAATTCGGCGAGGGTATTGACGCTGAGGTTTGAAAATAGTAGCATGGGGCAACTTTGGTTTTCTCCAATAGTC
TTCTGGAGGAGATCCACAGAGTTTGAAAATTTTATGCAATTGTTCTACCTCAGTTCTTCCTGGCATGATTGGCTTTCCAACAAGTAATTCTGCAAGGATACAACCACTACTCCATAAATCAA
CAGCAACACCATATTCAGTGGCACCCAACAAGAGCTCAGGAGGCCGATACCATAGAGTCACAACACGGCTTGTTAAGGACTGATTCTGGCCAGGTTTGAAAAATGTTGCCAGGCCAAAA
TCCGCTATTTTAAGGATGCCATCATTGTCAATCAGAAGATTAGAGCCCTTGATATCTCGATGTAAAATTCCATGGCTATGGCAGTATTCAAGCCCACGAAATAGTTGTTGCATGTAGCACTTA
ATCTGTGGCTCGGTGAACTTGATGCCGGGTGTTGAAGCAAGTCCGGCAAGATCATGATCCATATATTCAAATACAAGATATAAGTTGCATGACATTCTTGATGTAACTATTCCGACGAGCTT
TACAATATTAGGATGATCGAGTTTACGCAAAATATGAATTTCTCTTGCCATGAAACGAACACTTTCGGGATCCATATTAACAAAACGCACTTTCTTGAGAGCAACCATTTTTCCAGTTTGAA
GGTCGCGAGCTTTATACACAGTGCTATAAGTTCCCTGTCCAATCTTATTTAATTTCTCAAATGAATCTGCCCTTCGAGGCAGCCAACCATTCACTGCTTCTGGTGCAACACTGATGAGCCAC
GCCGGCCAACCAATAGCCACATGCTCACCGGAAAAACCATTTGGCACATCAAAGATCCCTGAGTTCGACCCACCATTGCCCTTCGTAGTCAATATATTTGATTGAAGATTTTCACCATTCA
CTCTGACATCAACAGTCCTGTTTCTCTGGTGCCCGACAGACATCCCTGTTCTATCTGAAGAGACAACCACACTATTCTTCTCACCTCCATTAGATTGTCTAGGAAAAGGGGGCATCAAATT
CTCTAGTAGCTTAACTGCAGGTCTCGTGCTGGCCCCAATGCTTCTAGTAGCAGCAGCTTCAGCTTCAATGAACAAAGCAGCATCCTTCTGTGAATGAGTAACAAGCTGTTTTAAGGATTTT
CTTGACCCCTTGTTTAAGTCCTCAGCAATTTCTTCTGTTTCAATTCCTTTCGAGCAAATGCAGCCCATAAGGAAGAAGCAGAAAGTGCCACGCTTTTCCCCCAATTATAAAACCAGGCTGC

AGACGGAAAAAGGAGATGCATCAAGCCTAATAGCAGAATTTTATTCTTCAGAAGGTTGAAATCCAGAGACGAACCTAATGGTTTCCTAGCTTCTGACCTTCATGAAAGAAAAAATCACCC
TTTCTTTCAAAAAGAAAGTTTTCTTATAAAACTGAAACTCCAAATCTTCCTCGATAAATGTAATACCACACCCCCACCCTCAAAACCAGATAAGAAACCCGGCGGGGATTGACGGAAGAC
GATCAAAATCTCAATTTGGCACGGGGGTTTCCAGAAAATGGACCCCGGCCGATGTTTCTGGAGCCCTCTTTTGATCTATCAGGTATTCCAGATCAAAGAACCCGGCCGGCGTTTCTGCAG
CTCTCTTTTGATCTATCAGCTTCCCAAAGGCCTATGTTCATCCTTTCTTCCCATCTCTCTCTCTCTCCGCGTATCTTCTTCACAATCCCAATTCTGGCAATGGAAAGGAGGAAAAATCGCTAT
GTTGT
>comp166472_c0_seq29
CACAACCATGATTACAGGATGACAGGGATAGTCCTTCATTACAGAAAATACCAACAAATGAGCTTTCAAATTATGAGAAAAGAGAGTATATTACTGAGCAAGTGCTTTAAATAAAGCTTGA
TAAATCATATTGAAGGAACTAACACAAAGTTAATTAAAAACTTTGAAAAACAATATGATGCAGTAAATGAAAGCACTTGATAGCCACTCAACTCACTTTGCTTTGAAGGGCAGAAGATGC
AGTTCCTCGATCCTCTGGTTGAATTGCAAGAAGAGTATCAAGAAGAGCCAAAGTTGACGGAGGGCAATCCCTGAATGTCTCTTCAATTCGGCGAGGGTATTGACGCTGAGGTTTGAAAAT
AGTAGCATGGGGCAACTTTGGTTTTCTCCAATAGTCTTCTGGAGGAGATCCACAGAGTTTGAAAATTTTATGCAATTGTTCTACCTCAGTTCTTCCTGGCATGATTGGCTTTCCAACAAGTA
ATTCTGCAAGGATACAACCACTACTCCATAAATCAACAGCAACACCATATTCAGTGGCACCCAACAAGAGCTCAGGAGGCCGATACCATAGAGTCACAACACGGCTTGTTAAGGACTGAT
TCTGGCCAGGTTTGAAAAATGTTGCCAGGCCAAAATCCGCTATTTTAAGGATGCCATCATTGTCAATCAGAAGATTAGAGCCCTTGATATCTCGATGTAAAATTCCATGGCTATGGCAGTAT
TCAAGCCCACGAAATAGTTGTTGCATGTAGCACTTAATCTGTGGCTCGGTGAACTTGATGCCGGGTGTTGAAGCAAGTCCGGCAAGATCATGATCCATATATTCAAATACAAGATATAAGT
TGCATGACATTCTTGATGTAACTATTCCGACGAGCTTTACAATATTAGGATGATCGAGTTTACGCAAAATATGAATTTCTCTTGCCATGAAACGAACACTTTCGGGATCCATATTAACAAAAC
GCACTTTCTTGAGAGCAACCATTTTTCCAGTTTGAAGGTCGCGAGCTTTATACACAGTGCTATAAGTTCCCTGTCCAATCTTATTTAATTTCTCAAATGAATCTGCCCTTCGAGGCAGCCAA
CCATTCACTGCTTCTGGTGCAACACTGATGAGCCACGCCGGCCAACCAATAGCCACATGCTCACCGGAAAAACCATTTGGCACATCAAAGATCCCTGAGTTCGACCCACCATTGCCCTTC
GTAGTCAATATATTTGATTGAAGATTTTCACCATTCACTCTGACATCAACAGTCCTGTTTCTCTGGTGCCCGACAGACATCCCTGTTCTATCTGAAGAGACAACCACACTATTCTTCTCACC
TCCATTAGATTGTCTAGGAAAAGGGGGCATCAAATTCTCTAGTAGCTTAACTGCAGGTCTCGTGCTGGCCCCAATGCTTCTAGTAGCAGCAGCTTCAGCTTCAATGAACAAAGCAGCATCC
TTCTGTGAATGAGTAACAAGCTGTTTTAAGGATTTTCTTGACCCCTTGTTTAAGTCCTCAGCAATTTCTTCTGTTTCAATTCCTTTCGAGCAAATGCAGCCCATAAGGAAGAAGCAGAAAG
TGCCACGCTTTTCCCCCAATTATAAAACCAGGCTAATGGTTTCCTAGCTTCTGACCTTCATGAAAGAAAAAATCACCCTTTCTTTCAAAAAGAAAGTTTTCTTATAAAACTGAAACTCCAA
ATCTTCCTCGATAAATGTAATACCACACCCCCACCCTCAAAACCAGATAAGAAACCCGGCGGGGATTGACGGAAGACGATCAAAATCTCAATTTGGCACGGGGGTTTCCAGAAAATGGA
CCCCGGCCGATGTTTCTGGAGCCCTCTTTTGATCTATCAGGTATTCCAGATCAAAGAACCCGGCCGGCGTTTCTGCAGCTCTCTTTTGATCTATCAGCTTCCCAAAGGCCTATGTTCATCCT
TTCTTCCCATCTCTCTCTCTCTCCGCGTATCTTCTTCACAATCCCAATTCTGGCAATGGAAAGGAGGAAAAATCGCTATGTTGT
>comp166472_c0_seq3
TTTTCTTTGTTTTTACCTTGTCTAAACGAGCTTTGCGCACAGCCTGCTGGATTTGCCTCTCATGCTCTTTAAGCAGTTCTTCTATGTTACCACCTGGGGGCATTAATGGCCCTGAGTAGTGAA
TTCTTTTGTTTTTTCCACCGTAACTCATGACAGCTTCCTTGGCTCCAACCTTGTCATCTCCCTTATAGGTAGAAGAAGGTCTATCTAGTACATGCTGCTTCTCAGATGACTCAGCAACATCA
AGCCTATTATACGTGGAGCTCATATTGCTCTTTGAATTGGCAAAGTCCATGCCACCACTATTCTGAGGCATATATGATCTCTGAGATCGTAATCGTGGAACGTTGGGTGCCCACATAGTACTA
GATGCTTGGTTAGGAATCGTCCTGATATCATCGTTCTTCCCAAATGCACTGGGGTGCAATGTAGTGTTGGAATGAGACAAGCCATGTGGAGCTCCTCTGGGAGGATCAATGGGGAACCCT
GAACCAACTTCTTCTAAAGGACTGTACTTCTCACTCGTACTTTTGGTATTTGATTGCTCACGCCACTTCTTTGGGTCTCCGTTGGCTTCTTGCCCGGTCATAGGTTTATTATCCAATGGGCCT
TTCCTTCCAGAATCAAATGGCTTGCCTTTAAGAGCTGCAGCTTTTTGCTTCCCTGTGCAGAGCCAAAGATGCTGTTCCTCGATCCTCTGGTTGAATTGCGAGAAGGGTATCAAGAAGAGA
CAATGTTGAAGGAGGAAACTCCCTGAATGTCTCTGCAATACGCCGAGGGTAGGGGTGATGAGGTTTAAAAATAGTAGACTGCGACAACTTAGATTTTTTCCAGAAGTCTTCTGGTGGGGA

TCCACAAAGTTTGAAGATTTTATGTAGTTGTTCAACCTCTGTTCTTCCTGGCAGGATAGGTTTCCCAGCAAGCAATTCTGCAAGGATGCAACCAGCACTCCATAAATCAACAGCAACACCA
TATTCAGTAGCACCAAGCAAAAGCTCAGGAGGCCGGTACCATAGAGTCACCACACGACTTGTTAAGGACTGGTTCTGATCAGGATCGAAAATCGTAGCCAGGCCAAAGTCTGCTATTCTG
AGGATTCCATTGCTGTCAACCAGAAGGTTAGATCCCTTGATGTCGCGATGTAAAATTTTGCGTTTATGGCAATGTTCGAGGCCACGAAGTAATTGTTGCATAAAGCACTTAACCTGCGGCT
CGGTGAACTTGATTCCAGGTGTTGCAACAAGTCCTGCAAGATCATGATCCATATATTCAAATACAAGATACAAATTGCATGACAATCTTGATGTAACTATTGCTTCAAGCTTCACAATATTTT
GATGATTGAGTCTCCGCAGAATTTGAATTTCCCTTGCCATGAAACGGACACTTTCAGAATCCATGTTAACAAACCTCACTTTCTTGAGTGCAACAATTTTACCAGTTTCAAGGTCACGGGC
TTTGTACACGCTGCTGTATGTTCCTTGTCCAATCTTGTCTAATTTCTCAAACGACTCAGCTCTGCGAGGGAGCCAGCCATTCACTGCTTCTGGTGCAACATTCGTAAGCCATGCAGGCCAA
CCTGCAGCCACATGCTCACCAGAGAACCCATCGGGCACACTAAAATTTTCCATAGCCAATCCAAAGTCACCCATCTTCGGATCAGTATCTGCCCCATGCACCCTAAAATCCACTGTAGCCC
TCCTATGGTGCCCGGCCAAGTTCTGATTCCTATCAACAGGAGCCAAAGAACCCCTATCTCCTTCATCTGTCTGCACAGGAATGGGAGCAACCACATTCTCTTGTGATTTTGTTGCAGGCCT
TTTACCACTCCCGCAGCTTCTAGTGGCAGCATCAGCATCAAACAATATAGCTGAAACATCCTTCCTCAAAGGAGTAACTAGCCGTGAGAGGGATCTGCTTGAGCACTTCTTTATACCTTCA
ACAATCTCATCTGTTTCAATCCCTCTAGAACAAATGCAGCCCATATGAACTAGAAAGCCCCACCCTTCTCTCAATGTAAAAAATCAAAATTGCTAATTGAATATGTAGATTCATCAAGCCTT
CTTACGGAAGACCATTCTTCTGAACCTTATAAAACCTCTAATTCCTAGAACTTCAATCAGAGATCGCCCCCTTTCGAATATGTGTCTCAAATCCTGAAGCAAGAATTAAATGACTTATGAAT
CAATATGCAGACGACCTAAAACAGAATCTAAACAGAAGGCCAAGCAGATACAATTACTCAAAAAATCAACAGCAGAACCCTCAGCTCTTCAAAATCCAAGCCAGAAACCAATCGAGGAT
CGTCGGAATCTGATCAAAATCCCACCTTGCGCCGAAATTTTCAGAATGAGGGTTTGTTCCCGTGTTTCTAGCTCAATACTGGCTTATCCCTTCTCCCTGTTGGGATCCCTTCATCATCATCTC
TCTCTTCTTTCTCTCTCGTTTCTGGAAATGAAATGAGAAAATGTAGGAGGTTGGTAACGAGAAGGACATAAACTCTATACAAAATTAGCAAAAAGCAGCAAAAGCTTTTATCGAAGACGG
TGAGGAGAAGAAATAATTGAAGATGAAAGGG
>comp166504_c0_seq3
CTTAGGCGTATGTGAATCTCCCTCACCCTCAGGTCGCCTAGATTGAGTTATTTGGACTTCCGAGACTTTTGTGGATATCTTCAATTTATGTGCGTGTGACCTCCCTGCATGAGTGGATAGTCC
CACCTTGAATAGATATTCCTCGTCTAAGCATCTACTAAGAGAGTGCATCTCATAACGAAGAGCTTCAATATCAGGCAGCTTCAACATACTCAACAAGTCCTTTTCTCCGTCGGTATAAATAC
GCCCCATCCGAGATAAGCATTCTACAGACAACACCGCTCCATGTTCTCTGAAAAAT
>comp166504_c1_seq8
GTGACAGTGTACGAGTTTAGTTTGCTAGCGGGGTTGCGATTTCCGCCGCTGCCTGAACTGCTTGATATCCTGACGGCTTGTGGAATGAGTTTGTCTCAATTCTACTATCGGGCTATGTCGGT
CATAGTGGGTCTGATCGCATTCTTCAGAAACCATGGAGCGGTATTGTCTGTAGAATGCTTATCCTGGATGGGGCGTATCTATACCGACCCACAGGGTCGAATTTCCTTTAGATCCAACTGGT
TGGAAGTTCGTACTCGGGATCCATCCAAAGATTGTATTAGCGATTTTTTCTTTGTGCAGAATGATTGGCGCCTCCAAGAAAGATGGGGAAAGTTGAAGGAGCTACCTATTCCCCTTCATATT
GGAGCAGAAGACTTGTTGAATATGTTAAAGCTTCCTGATATCATTGTTGGCTTTTGTCACCTTAGGAGCCTTCTTCGAACCCTCTGAACACGCAGTAGGACGATCTTCCACAACGGAAACA
TCTTTGGTAGAAGTGTGATCACCCATTTTGATTGCCTCACGAGAATCCACTAGAATGAAAAATTCCGTAACTCCTGCACGAGAGGAGGGGCGCTAATTCGAGCATGGAAAATTTCCCAAT
CGCAAATAGCGGAAGCCTCAACCTCCAGTAAGCCGGAGGGGGAGGAAGA
>comp166504_c1_seq9
ATCACCAAGTCATTAGGAAAATGATACTTTTTTCCTAAAAGAGATGAAGGAGTCTTCGGTAATGGTAGAAACCACATCGTCGGCTTTTTTCGCCTTAGAAGCCTTCTTCGAACCCTCTGGA
CGCGCAATAGGACGATCTTCCACGACGGAAACATCTTTGGTAGAAGTGCGATCACCCATTTTGATTACCTCACGAGAATCCACCTGGAAGAAAAATCCCGTAACTCCTACACGCGAGGAG
GGGCGCTAATTCG
>comp166516_c0_seq15

GCCACATAGATGCAAATGAATGCTACCACCAACCCGGCGTAGCTTCTTGACAACACCGTGTGTGCTCCGGTCACTCCGAACTTAATTCCGATGAGCAATCCCACAATTACCTGGCTGATAA
ACATTTGAACGCCGCCTTCTAAAAGCAGAGCACGTCGCCCCAACTTGTCTACTGATACAATCGACACAGATGTGGCAACCAAGTTCACCAAGCCGGTGATAACAGAAGATATGAGGGAA
GCCTGGTCGCCGAAGCCTATGGTTTTGAAGAGCACAGGAGCATAGAACATGATCGCGTTTATGCCTGTGATCTGTTGAAAAAAAGGGATAAGTATTGCCATTGTTAGTTGTGGTCGATACC
TACGCTCTGTGATCTTTTTCCATGGATGCTTCACCGCCTTGGAGTCGTTACTGGCGATTACAAGATCATGGAATTCCTCATGTATTTGATCAGTGCCACGCACTTTGCGGAGCATGGCCTTG
GCCGCCTCCTCATGCCCGCGTTCAATTAGAGAGTTTGGGGTCTCAGGGAGAGCCAATGCGCCAATCGCGATAATGGCGCCGGGCACGGAAGCAAGCGCAAGGCTCACGCGCCAGCTCCA
TCCCCCTTTGATCTGGGCCATCCAGAAGTTGATGAGATTTGCGGCGAGGATACCAATTGTGATCATCATCTGGAAGCCAATATTGAGCATGCCGCGGAGCCTAGCTGGAGCCATCTCCGAA
AGGTAGACAGGCACTGATTGGTTGGCGAAACCGACGCCGATGCCGAGGAGGATGCGGCCGAGGATTAGCATCAGCAAGTTCACTGCCGCGCCGTTGAGGGTTGCGCCGATAAGGAAGA
TGAGGCCGCCGCCGAACATGGACCATTTGCGACCGAACACCCTTGTAACAGTCGCGGCGAAGAAGGAGGCGATAAGTGCCGCCAGATACAAAGACGACGTGAATAGCGTGAGGAGCTC
GCTATTGAATTTACAGTATTCATTAGTCGAGACATCCTCCTTTTCCATATGGTATACCGATGGGAAGAATCGGATAAGAAAAGAATCCATTGATGTAACTCCACCGGAGATTCCAAGATCATA
CCCAAATATGAGGCCGCCGGTGGCGGCGACGAAACACGCCAGGAATACAAAAACTGTCAACTTGGCGGGGTAATCCTTCGGGTCGGCCATTATTGATATGGACAGATGGGACTTTAATTT
TGTTTAGGAAATCAATATATCGCTAACCGAAACAGAATTGAAGAATTATAGTAGAGGAACGAGAGGAGGTAACAAAGTAATTGATTTCCGGGAGCTAAAGATCGATCAATGGAGGAGGAA
AGTTTTGGATTGCATGATCTCTCTTCTTCGTCCA
>comp166547_c0_seq1
TAAATCCTCTAAAAGAATTCAACATAAGATTTCTATTCCCCACTAGAAAACGAGAAACAACTAATTATTAATCTAGAAAAAAAAAAGCAAATACACAGATCGAAGATAGACTTGAAAAGG
CGATGAAGAAACCATATTGAATACTAGTTTCGGTTTCTCATTATTGTCATTGGCTTACAAAAACATCAAAAGCTTCTGTTGTAAACTAGTTTAATTAGGGTTTCCTCATGATTCTGTAAGAGG
CTTTCGAGCCAAGAAGAAGTAAATGGCGGTGAAGATCTCCTTCCTTCCACCAGCTGCGAGACCATCCGCAGCTTTCTCTAACAATGACGAAACTCTCAAACTTCCTGCAGGAGCAAGGC
GCACAAATTCCAGCACCCGAACCATTACTCTTGTCATAAAACGTCCAATAGCTGTCGAGGGGAAGCTACTCAAGGAGAAATGGCTCGTATCCATAGGCAAATACCATGGCAAAGGTGAAT
CAGTAGCAAAATCCTTCTCCCAAATGACCTCAAATCCAGCTTTTTGTAAGGCATCAAGGCATTGATGTGTTGATCTTATGTCAGGAAGCCCATTACCAATCTCAATCTCAGCCTTTAT
>comp166547_c0_seq10
TAAATCCTCTAAAAGAATTCAACATAAGATTTCTATTCCCCACTAGAAAACGAGAAACAACTAATTATTAATCTAGAAAAAAAAAAGCAAATACACAGATCGAAGATAGACTTGAAAAGG
CGATGAAGAAACCATATTGAATACTAGTTTCGGTTTCTCATTATTGTCATTGGCTTACAAAAACATCAAAAGCTTCTGTTGTAAACTAGTTTAATTAGGGTTTCCTCATGATTCTGTAAGAGG
CTTTCGAGCCAAGAAGAAGTAAATGGCGGTGAAGATCTCCTTCCTGGACAAATAAGCACACACCTTCCACCAGCTGCGAGACCATCCGCAGCTTTCTCTAACAATGACGAAACTCTCAA
ACTTCCTGCAGGAGCAAGGCGCACAAATTCCAGCACCCGAACCATTACTCTTGTCATAAAACGTCCAATAGCTGTCGAGGGGAAGCTACTCAAGGAGAAATGGCTCGTATCCATAGGCA
AATACCATGGCAAAGGTGAATCAGTAGCAAAATCCTTCTCCCAAATGACCTCAAATCCAGCTTTTTGTAAGGCATCAAGGCATTGATGTGTTGATCTTATGTCAGGAAGCCCATTACCAAT
CTCAATCTCAGCCTTTAT
>comp166547_c1_seq1
CTTACAAAAAGCTGGATTTGAGGTCATTTGGGAGAAGGATTTTGCTACTGATTCGCCTTTGCCATGGTATTTGCCTATGGATACGAGCCATTTCTCCTTGAGTAGCTTCCCCTCGACAGCTA
TTGGACGTTTTATGACAAGAGTAATGGTTCGGGTGCTGGAATTTGTGCGCCTTGCTCCTGCAGGAAGTATGAGAGTTTCGTCATTGTTAGAGAAAGCTGCGGATGGTCTCGCAGCTGGTG
GAAGGAAGGAGATCTTCACCGCCATTTACTTCTTCTTGGCTCGAAAGCCTCTTACAGAATCATGAGGAAACCCTAATTAAACTAGTTTACAACAGAAGCTTTTGATGTTTTTGTAAGCCAA
TGACAATAATGAGAAACCGAAACTAGTATTCAATATGGTTTCTTCATCGCCTTTTCAAGTCTATCTTCGATCTGTGTATTTGCTTTTTTTTTTCTAGATTAATAATTAGTTGTTTCTCGTTTTCT
AGTGGGGAATAGAAATCTTATGTTGAATTCTTTTAGAGGATTTAATTATATATATATTGTCATTTGCTTAAATTGTTCTTATATGTTATCTATTTTTGGAAAAGTTATTATGCGCGGTGATTGGAC

CAT
>comp166555_c0_seq5
CAGCAAAAAAATTATCCCAAAAGAAACATTTGTCAAAAGACCAAACAATCATGTAATGAGAGAAACACCATTTACCTTCCCATGCTATGATGTCTTTACCAAAGAGATGCAGAAAAAAAC
TATTAGCTGCAGCACAAAAACTGAGAGATAGTTGGATATGACCGCTAATTGATTTTAACAAATATAACAACTTCTTTCATGTGAAAAATGAATGGTTGAGAAGGCTCTGGCAAGCAAAAC
AGAGATCAGTGAAGACAACCAGTAGCATCATTCTTCAGCTTCGTTTTGTGGTATGGAGTTCATTGTCTCCAGGTCTCTCTTGAACTTGTCTTTTAGTTTTTCTATCTCGATGAGGCGTGATT
GAGCTTGCACAATTTCACACAGAAGAGAATCCTCAGTACAACGGAGAAGTGATGTCCAATGTTGCAATAGATCAGATTCTGTAGCTTTTGCTTTCTTGTGTCGAGATTTAGAGGTTTCACC
AGTTTTCTCATGTCTGAGAATTTTCCCTTCTAGTATTCTAGCATGTCTTCTGCTAGACTTCAGTGCTAAAAGAAAAAAAAAAACATTTAAACAAAGCGAGAATACACAGTAAAACCGTAAA
CAAGAAAAGAAAGCTATCCAAATAGAGAGTAAAAATGATACTAATTTTAATGGATACAAGCTTTCTGCTCAAGAAAAAAAATAAACAGATTTACCACTCAATCGCCCTCTAGTATCCTGGT
ACATAAGATCTGACCACATATAAGCAG
>comp166555_c1_seq4
TTGGAGAGAGATTAGCTCATGAAAAGTTCCAAAATGTAAGCCTGTGTGGTAGTTGTCCATGCTCTTCGTGTATCAAAGGTGAACCACACATTCCGTCACTTGTTTCCCTTATTTATACAATA
ATAGTGACTCTATTAATCTAATGGTTTCTTTCCTGCTAATTATTCAAACACTTTGCCCTTTTGTAATTTACCATTTACAGGTGTATTTTGCAATTTGTGCCTAACTTTAACCTATTTTTATATTAT
CAGCTGCTTATATGTGGTCAGATCTTATGTACCAGGATACTAGAGGGCGATTGAGTGCACTGAAGTCTAGCAGAAGACATGCTAGAATACTAGAAGGGAAAATTCTCAGACATGAGAAAA
CTGGTGAAACCTCTAAATCTCGACACAAGAAAGCAAAAGCTACAGAATCTGATCTATTGCAACATTGGACATCACTTCTCCGTTGTACTGAGGATTCTCTTCTGTGTGAAATTGTGCAAGC
TCAATCACGCCTCATCGAGATAGAAAAACTAAAAGACAAGTTCAAGAGAGACCTGGAGACAATGAACTCCATACCACAAAACGAAGCTGAAGAATGATGCTACTGGTTGTCTTCACTGA
TCTCTGTTTTGCTTGCCAGAGCCTTCTCAACCATTCATTTTTCACATGAAAGAAGTTGTTAAATTTGTTAAAATCAATTAGCGGTCATATCCAACTATCTCTCAGTTTTTGTGCTGCAGCTAA
TAGTTTTTTTCTGCATCTCTTTGGTAAAGACATCATAGCATGGGAAGGTAAATGGTGTTTCTCTCATTAAAAA
>comp166570_c2_seq8
CAAACAGCTGATATACTTACAAAGCCATTATCAACCACTAGATTCAAACTGTTACGGGACAAGCTCACTGTTTCAGCCGAACCATTGGCTTGAGGGGGGATATTAGAGACATTACAAATAC
CGCATATATTTAGTTAATTGTAATTTGCTTTAATATACATTTTACAAGTTTGCCATGTATCTAACTGCTTATATTATAAATACAATCACCGCTGCTCCGTAAGGAGCATCCTGTTTTCTCCTTCCT
CTTCTGTGTTAATCTGTTCTCTGTTCTCCTGTTCTTGAATATTCACATAAAAAGCATCAATGGTTCGTTTTCCCCTTTACTCATCTCCCGTGACTCACGAGCCACGCTCTCGAGAATGGCTTT
CGTTAGGCCTCCCCTGAACAAAATTTCTGGCTCCGACACTGGCTCTAGTTGCTTTGAAGGAATTGAAGAAGGGGAAAAGAAGAGAATAACATTGTAAGAAAATTGAATTGAAATGGCTT
TCATTCACATATTTGGTTCATAATAAATTAAGTAGAAACTAGTATCAAGAAGAAAAAAAAAGCAAAAACAAAAT
>comp166573_c0_seq1
CATCATATAGACTTTGTTTATTAAACTAAGCAAAGTCCATATAAGAAAGATGAGCTCATCCAAGCCCTCTTGCATGTTCAAAACCAAAGTTGTAAGATAAATTTCTCACCTCTTATAGCTATG
ATCTCGATCAATTGCTATACAAAAAGGTATTAAAAAATATACTAAACATCCCCTATAGCCCATAACCAAGTACTCATCCTTTGAGAACTTCTCTCATATACATAAAGCATTCCATACAGTAAA
CATCTTAATAGTAAAGCCCACAGCAACAAAACATAACCCATCCCTTCCAGATTCCATGAAATGATGTTACCATCTAGGCCATGAAGTCACGTATACACGATAAATTATTGTGTGGAGATCGA
ATGCAGAATGCTGTCCGTAAATCAATCAGAATGTGCCACATCAGCAGTACATAGCAGCGATATCGATCGGTACCGAACAGTACCGACCCACGGTACTGAGCGAAAGGGTGATGTGGCGCA
TTCTGATTGTTCTTCGGACGTCGTTCGATGTCCACAGAGAATAATTAATCATGTTTTATAGCATTATTGCAACTAATACTAAGCATCTCCATTGATCGAAACCTGGAAAATAAGCTTGAATTT
CAGGTTGCTGGTTTTTATCCAACTCCTTTAACAAATTTTAGATATTAAGTACTAGTGAATGAAAGACCATTGATGCCCTATTTTCCTAAATTAGCCTTGTGAAAGTTTGTTCATCTACTGAAG
AATTCTATAAAGTCTTTTTAGATTCTTGCTCAGGATGCTTTGGGTTCCAGGAGACTTCACTGGTACCTACCTTGTTTTTGTCTATTTTTGAGTCTATTGATGAATAATTAAGAGGCTTTTTATG

TAAGCTTGGTAATGACTTGTGCCTGTGTTAAGAAAAGCTATGTGAAGTTGTAAAGAATTATTTCATGTGATTTAGGTTTCGTTTG
>comp166609_c0_seq1
TTTTAAGTGGGAGATGAATAAATGCTATATTTGGCAAATCATCGCTATCAAATTGAGCTTGTGAAAGAAATGAGAATTTTGAAATAGATTAGGCGCAATTAGAAAGAATAGATAAAGAAAT
AACTATATGAGAAGAATGATGAATCTTAAAATGTATTAAAAATATACAACAACATCAAGCTTTTGTCTCACTACGTGGGGCCGGTGCGGCGGCTCCTGCACACTTATCGTTGTATCTAGGTC
ATACAGCGTGTCGCTTCCA
>comp166627_c3_seq20
GTTGCTTCTTTTCTCATTAGAAAAGAGCCTCAAATCAAACCATGTCAGCACCACTTCCTGCTTCTGTTCCAAAACCAATCTCAGAATTTGCTGTGAAGAAAACTTGCTTTCAGTTCCAGCG
AAGAAGATTAGTATTCTATGGTTTGATTGGTGCTGGGATGGGTTTATCGGCATCTTCTGATGAAGCTAGTGCGGCTAAGAGGCGGCCGCCGCCGCCGCCGCCGCCGCCAACCTTTCTTTGC
TATTTTTAGGTGCCTTGCTTGCTGGGAAATGCAGCCTGGCATAGAGGGGCTTCGGCGCCCTCGTAACTCGATCAAATCCTCTTACACAAACAAAACCTCTCCAAAAGCACTGCTGTATTCC
TTCTGTTGTGAGACCTCTGAATATTGTTGGTATTGAAGTTCCAAAACAAACAGTTCTTCTAGGTAGGTTCCATATCCAGTCTCTTGGAATGTCAATTGGGTTACGGCTCGTATGGTGATCAG
CAAATACCTCATTAAC
>comp166629_c0_seq4
ATTACAAAACCCAAAACACTAAAGCAAAATTTCCATAGATAAGAAAAGTATAACAATAATTTCAATGAAAAAAGGACTAAAAATGCTCATGAAAAGTAAATCAAGCATAGAAACTACATA
GGCTTACATGTCTAACAACAGCTCAGTAAAGCATTTAACTTGGAATAAACTCCGGTGCTGAACGCTGAAGGAAGGAATCCACAGTCAAGTAATACAGATGGCTAAAATATTATCCTCTGC
AAGTTTCTTCTCAATTTCATCTTGATCCTTTCTTGTGAAGCCACTAGCTGCGAGTTCCTTGTTCAAGAAACCAGCAGACTTCTCTATGGACCAAAATGACGGCCTACCATTCTCTTCAAGAT
TAGAATCAGAAACAGCCACCTTACACACTTTCTCATCCAAATTATCCACGACAGCAGACTTAACCGGGACAACCAACTCCTCAGAATTTAAAGACTGACCAACCAAAACAGAAGGGGCA
GACCTCACAGACACCAAAGGAGCCTCACGAGGAAGGCCCTCAACGCTATTACAAACACGAATGGAAGTCAAATTCATCCCACAAGGAGTAAAAGGTTGAGCGTCTAGGAATAAAATAG
CAGCAAACTCAACACGATTAGCATCTCCACCCACCTCAGTTTCCACAGCAATTTCCACAACTTCTAAACCAACCAAAACAGGGGAATCACAAACAGGCACAGGGCTCACCACCTGATTA
GAGCAGAAAGGAGAAAGAGGAGCACAAACAATACCACCTAAACTATCAATATCA
>comp166633_c2_seq1
CCCAAACCCGAATTTTTTTGGGTCAGATTCGGGTCAGCTTTTCGGGTCAGGTCCAATATTGACACCCATAATGCTTGATTTGAGGTAAAGTGAGTATAAAATATTCTATATGTAAGAGTTTTT
AAAATATTGATGGTTGGTTTGAAGAAAAATGAATGTAAAGTAAAATAAATATAATGTAAATCATTTTTGACCTAAACTAGCTCGATTTTGAGCGACAACTATTTACACTGCAAGTAAGCTCG
CTTGAACACCGTCTTCACATGCCAACCAACACTGTTAGTCGGCGCCACCAACCGCCGGCCATCACCTGCCTCCAACTGCCTTTACTTGCCGCTGGCCATCCCACCACCGGCCATCACAGT
CACCAGCCACCACCCACCACAAGCCCTCCACCCGCAGTCATTCGTCACTAGTCTCCGACATCATCTTTGGACTTTATACTTCATTCATTTTACTTCAAACAAAACAAAAAAAATAATAATCT
CGTGTATTTCTTTTACACTCATCCAAACAATTCCAAAATATTTATTTTACACTGCATTCCTTTTTACATTTTATTCTTTTTTTAGGCAACCAAACAACCCCTAAATGTTAGAGATGAAAAGGTA
AAAGCAGAATTATCTGAGAGTAGGGAACTATGCCACTGGTGATAAAATGTTTAGTAAAATATATCATCATAGTTTTATGAAAAATCTATAATATCTTTGCTTACCGATGATACTTCTCATATTA
AATCATCATTCAGATTGAAGATGATGCTGTTAGGCGTCTATAGGCTTTGGATGCTAGACCATTGGATCTCCCGTTTAATATTATATCATGGTGTGGATAGATTTGAATTATTTTGATAAGTTTGT
CTTTATAGTTAAATTCAAATTAAATAATTATGATAAGAATTATCATTTAGTTTAAACTTATTTCTATTTGTTAAAATGGATAGGGACGGGTTATTACCTCA
>comp166651_c1_seq17
AATGATATATTTTCTCATTGAGACCTCGCTTCTAGTTGTAAGAACAGTCTCTTTGAAGACATAAATGTTTCTTGTACTTTTCACAGGGTCCCATCAGATAGCATGGGTGTTACCACTGTGATT
TCTTTAATAATCATGAATATTTTATATGATGATTGTTGTTCTCAGTGGCAAGTGTGTTCCCTTGTCACTTCTATTACTGTTCCCTTAATGTGAGTTTTTTTTT

>comp166657_c0_seq2
GAGAGGGAGAGGGAGAGGAAGAGGAAGTGGAAGAGGAGGGAGAAGATCGTTGTTGGGCTTTCTAGTATAAGTGGGTGGGTAGGCTATGGTTTGAGAGTGAGGAAGTTGTAAGGGAAG
GAGAAGAGTGAAGTAGTGTGAATGTTTTTTAAGAGAGTGGAGGGCTGCTTTTTTGTGTATTTTGCTTTATAAGAGGGGAGGCTGATGAGTTGTGTTCTTTTCTTGCATGGGAGGTTAAATC
GATGGTCCAAAGTGGATTTTCTTGCAGTAAAAAGTGGAATGCATGTGCTGACAAGTAGCATCAAAGGACCCGCAGCTTTGTTTAGCTCCTTCTTCATTCATTTTCATATGACCAAAATTCTA
TGCACTGACTCATATGACCAGATCATGTAAAATATAATTTATTAGTCCATTTTTTATTTTTTTTGGGAGTTTAATATGTTAGAAAATGGATGATTTGCTATATTAAAAAATCACTTTTTAGATAA
ATAAAATACAAATGCAATATAAATTTTCATGCATTTATGGTGCATCACTTTCTCTCTCTAAAATGCCAATCAAATCTTATCACTACAGGAAATTTAATGAATAACTGTGGAACTTCCGATGCC
GGAATTAGGCCAGTGGTTAAACCATCGGTCTGATCTCCGCTATTTACTGACAGTCTGACCACTGATAAAAAAGACCAAATTGCCGAAACTTCTCGGGGGTCAAATGATAGTCGGAAGTTC
CGCCGGTATTATGTTTTCATGATGTATTTATAGGCGGCTTAGCCATCTTTTTTAAGTGAAAAATTGATGTATACCGTCAGAAACTCGCCGGATTTTTTCAAAACAGCCGAAAAATCGACATAT
ACCACATGTAATGAATGATTTTCCTATAATGTATATAATTTTCAATCAAATAAAGAGAACATTTGGACTCAAATCTCTCATTCTCTTAAAAGTAGTCCAACATAGATGACAATTATAAATCCAA
AAGTGACAAATCTTCAATAGAAATCAAGTTATGCAAAGGAAAGATTATGATTAAAGTAACAAACCTTAACTAGCCAAACCATATTTTACATGATTAAAATCACATGTCCTATAGAAGCAGC
CTATGTGTCATATGCATATAGCTTCTTCAAGCACTTAACATCCTATTCTTACAGTATCCAAAAGAAAAAAAAAAACAATAGA
>comp166709_c3_seq7
AAGGTTGCGAACCACTAACGTGGTGATTGCTTGAGCCCGTAGAGAGCCTTCTTGAGGCGACAGACATAGTCCGGATGAGTTTGGTCGATGAATCCAACGGGTTGCTTCATATAGACGGTG
TGGCTAAGTGTTCCATGGAGAAACGCATTGGAGACGTCGAGCTGATGAATTTGCCATTGATGATGGAGGGCAATCACAAGAAGAATCCGAACAGTTGGTATTTTGGCGACGGGACTGAA
AGTTTCGGCATAATCAACGCCTTGTTCTTGGTTGAAGCCTTGCGCAACTAGTCTAGCTTTGTATCGAGCAATCGTTCCGTCAGCAAGATATTTGGTACGAAAG
>comp166713_c0_seq6
CGCCGCGAAGAAATTAAAGCATCCCTGATGGAGTCTGAGAAGGGGAAGGGCGTTGCTACAAATGACTCTGTGGACAACGAGGGCCGCCTCTGGAACTCCTTTATGGAGAAGCACTTGC
GATCTCTTACCCAAACTCTCCATGAGAAGGAGCTCGTTGATCTCCTCGTCAAGCTCGGTATGTCATTCCCTCAGATTGCCAAAGAAATTAGAATTACAGCGAGACAATACCTTGTAGGTCA
AAGACTTGTTGTTTATGGTTTGCATGAGGAAACTACTACAGAAACTTTGTGTGATGCCTTTTCCTCATATGGCGAAATTGC
>comp166713_c0_seq8
AGGAACGCACTTGCTGCCCTGCCTTGCCTTTTATTGCAAAACCATCCCAATCTTGTTTGCTTCTCTTCCTAAAGCATCCCTGATGGAGTCTGATCAGAAGGGGAAGGGCGCTGCTACAAA
GGACGACTCTGTGGACAACGAGCTAGTCCCCGGCCGGAACTCCTTGATGGAGAGACACTTGCGATCTCTTACCGAAACTCTCCATGAGAAGGAGCTCGTTGATCTCCTCGTCAAGCTCG
GCATGACATTCCCTCAGGCTGCTGAAGAAATTAGAGATTCAGCTAGACTATACCTTGCAAATCAAAGACTTGCAGTTTATGGTTTGCCTGAGAAAACTACTTCAGAAACTTTGTGTGATGC
CTTTTCCTCATATGGCGAAATTGCGGAAGGACATGTTATGCTTGACAGTGCAACTGGAAAGTCTCGTCGTTTTGGCTTCATTACATTCAAAAAGAAAAATTCTCTTCAAAAAGCACTGGA
AAAAGAAAACACATTAGTTGATAGCGGTCAAGCTTTCCACCATCCTCCAAATGAAAGATTCGACGGTGCAATTGCGACTGCCGATGTAGAATTCAGAAGACTGTTCGTTGGAAATATTCC
TATGAATATACCAACTAAAAAGTTGCTCGGTTTCTTTGCAAAGTATGGGGAAATAGAAGAAGGTTCTGTTATATATGATCATGAAACGGGAGAATCAAAAGGATATGGGTTTGTGACATTC
AAGACGTATGAGGATGCAACGAACGCCATCAATGATCCAGATAAACTCCTTGGAACGAACCAACTCTTTGTGAAGCGTGCACATAAATACGAGCCGAAGAATCTGTAGCCTAAGCTCCTT
ACGCCGGCGATTGCCGGCCTGAAGCATAGCTTACATATACGTACTATTTTGCTTAACGGTAACCATTTTATTGGAGTTAGAAACCTGAAGCTGTTAACCTCTTGTCACTAATAGAGTTCGAT
GATGTTGTAGAATAATGGTCAGCTACTAAAGATTAATGGAGA
>comp166729_c1_seq1
CAATACTGAACTATATATATATATATATATAATTTGGTTATATTTAATAAAAAAAATTATTATCCAATTATTATGCCTTGGCTATAACAATTAAGATCAATCCATCACTCCTTTATATTGCAATTAGTTA

GATAAATTTCATAATAGAGAAATAACACATTTTATTAACTTGAGGTAACAATAATAAAAAGCATATATCACTAGTAAAAAAATAAGCTTTTCACTGTATTTGCGTTCGCTACAAAGACTTTAT
GTTCACAGAAAACGTGGATTGTGAAAGTCATTCAAATAGATGGAATCAAGATCCTTAAAGAGCCTATTTCAGGAGTATCATAACGACCATCACAAAGGTCAAGTCCCCCCGATAAAAGC
AGTAATCTTTCGATTATTTCCAAAAGCTTGTGTATCAACCAACACACATTTCTGATGATGAGTGAAAAGTGTCCCCACAACCGCTAGCCTCAAACAAAAGCTAAGGAAGCAACTGCCACC
GCACAGACACACAAAAGCATCAAACTGTAGCTTCCCACGCCACACTCCTATCACTCAAAGCTCGGCACTTTCTTATGACAAAATCCTTTTCTACCGGTTTCACATTGAAATTTATCTTCTTT
GCTTCCATCAGAGCAAGAAGGCAGAAAAAGCAATGCAAACAGAACTAGTAGATAATTTAGAGAAACAAGACCATTCATCCAACAAATATTGTATATGCCTCTCGCAGGAAGAGAGTTAA
GTTGAAATAATCTAGTCTAATAGATGATAATGCAGCTGCGCAGATACAATTGCATCAGGGAGAGAAAAGTTGGTTTCACCTGAAACAAGGCAATATCAACATAATCGCAAAAAATCCTTAT
TATAGTCAAATTTCTTTACCTACTTTCATTAAAAATGCAAGGAGAGAATAAACACTATATCCAGCTGCAGGTTGTCATTCTTCTTTATCAAGTTGTGCCTATTCCTTTGAGGCATAATACAAA
CACCTGATGTGCATAAACCTACTCTCATGAGGTCATTAATGGCTGTACCAAGAAAATTTCCACACCTGCAAGCTTAAATTTTTCTGAGTCAAAAAAATTGCACAACCAAATCCAATCATTT
GGTCTCTCAGTAAGCTCTTTGTAAGGTTTCTTTCTTGCTAACACTCCACCACCAAGCTTTTTATAATCTATATAGGCCTTCGAGCGAAAATCTCCTACTCATCGTGTGCATCACACAATCC
>comp166775_c1_seq3
CCACAATGCAATCCTGCGACAGCGATCGGCAATGTCCATTCCCCTCTTCTTCTCGCACTGGTGATACATGACCGCCGAGTTGATGCCGGCCGCTGTGTGCGGCGTGACCTTCATCATTCCT
ATTCGTTGCCTTGGCGTTTTTCGTTTACTTCAATCTCTTTCTCTTTACACTTTTTTCTTTTTGATTCTCAATCTTTTGCTTGAATCTCTTTCATTCAGACTTTAGTTAAAAGCAAACTGATGGAT
ATAGGTAGAATGAACTGGAGTCCCAGTCTCTTAATAAAAGGTTTAAAATTTCAATCCAAATCTGACTCAATCAAACCTGTAGGATGTAACATGAAAAACACTTGATTCGTCTAGCACAATA
CCATCACATAAAAGTTTGTACCGAAGACTCATCAAAAGAAATTCCAATGCAGTGCAGCTTCAACAAATTATTAAAAAAAAAGAACAAGCCTAACCGGTGCAATGTCAAACTCAAATTCTT
AATATCGAATTTTTGTTTTACCTCCTTCCTGTATCTATTTAAGGCTTAGCTTCTTTTGTATTCTCTGCCGTCTTCTTCAATCTTGTTGAAGCCTTTTTCTCCCTGTTTTATAAGCTTCAAATACT
GAAGGAATGATATTTCATGGGTTTATATTACTAATTGGTTTCAATGGGCTTTAATTTGGGTAGCTTCTTGATCCTTGCTTATCTAACAGCGATTGTGAGGTTGGTACCTTTTGCTTCTTGTCTC
GATGGGTCCTTTGGTTCAAGATGTGTTCGTTCTATGATCACTGATCAGTTTAAGTTAG
>comp166781_c0_seq10
TTATTATTGGAAAATCACTTTAAAAACACAGAAATTTGTATCATTAAGATCCTTATGAACTAAACAAAAAAATTACATAACAAAAAGTACAATTAGGACTTTTACGGCAATCTCAAAGCATT
TAGAAATAAATCCAACACAATAATTTCTATGACAGAATACGAAATGAGTAATGCCAAATTTGATTCAGAGTATCGTCGCAACAAACAATAATTATTTCCAAATTACAATTAGCAGACAATAT
CCCCCACACGAACAAAAATTCAGAACTATCAAACCAGATGAGAACATAAGAACCCATCTCCCTTTTATTCTGGCTTCGAGAAGAAATCGTTGACAGATGGCATGATCTGAGGCTCTTTATT
GCGCACATATTTTCTAAGGCCGTGTTCTCTATATCTCTCCCCTTCGGCGAGATTGTCGAGCACTCCGGCTGTTCTTTTTTCCTTGCGCACCCTGACATCAGGATTGGTCGTTATGTTCCTCAC
TAATTGCATCACACAAATACCAACAACAGTTCCAACCGCTGCAAACAGCGGATAGACCTAACCATTCAATTAGAAAAAATTAGATTAATAAGATTTAAATTTCGTTTGAGACTATTTTCTTA
CTGCTTATTAAGATACTTTTTTAACTAAAAAAAGTATCTTTAATAACAGTAAGAAAGCAGTCTCAAATAGGAGATATTATTCTGTG
>comp166781_c0_seq20
AAAAAAAAAAAAAAAAACAATTATCCTAATTTCTAAATACGTAATTCATTTCACTAAAGTAAACAAGATTACCAAAACACAAATACATCACATTCAAATTTGAGCTCTCAAACTAAACAGA
AAAAAATTACATAACAAAAAAAAAAAATACAATCAGGATTTTTATGGCAATCTCAAAGCATTTAGACATAAATCCAGCACAATAAGTTCTTATGATACAATACGAAAAGAGTAATGTCAGT
TTTGATTCAGAGTACCGTCGCAACAAACTACAATTATGTCCAAATTACAATAAACAGACGATATCCCCCACACGAACTAAAATTCAGAACTATCAAACCAGATGAGAACATAAGAACCCAT
CTCCCTTTTATTCTGGCTTCGAGAAGAAATCGTTGACAGATGGCATGATCTGAGGCTCTTTATTGCGCACATATTTTCTAAGGCCGTGTTCTGCATATTTCTCCCCTTCGGCGTGATTGTCGA
GCACTCCAGCTGCTCGATTTTCCTTGAGCACCCTGACATCAGGATTCGTCGTTATGTTCCTCATTAATTGCATCACACAAATACCTACAGCAGTTCCAACTGCTGCAAACAGCGGATAGAC
CTCAGGCCTCAACCATCTTTTGACTGTCGATGAAGCCATTGGGGCACGCAAGAGAAGCGAATATCAGAAGAGAAGAGGGGAATAGAAGAAGAGAGAAATATATGAAGCAAAAACTTGC

AAAAGACGAGAAATAAATATTGCAAGATTGCATTTAAGGGAGAGAAAGAGGTCAATTTTAGTATGAGAGCCGTTTCGGGTTTGGGCTGGCTTCGCCTGAACCACTAGCTGTACACGTTTT
CTTTGTTATTCTCCTCTTACGGGGACCGGAAATCGGAGTAGAAAATAAGGACTTGATCTGAATTTGTGGTCGGGGTGTTTGTAATTAGGGATGGGTGAGGTTGGGTGGCTTTGAAAATATG
GAAGGAAAGCTTGACTTGCTTTTTGGGGTTTTTTTATTGTTAATGCAGATACAAGGCCACAAAATCTACGGATTGAAATTTCTAGAACCCGTGGAGTTTGACTTCTGTTTCAATTTCTAGG
AAAAAGAAGTTATTGAAGATGACTAATTTAACATTAAAATTTAAAATATACTGGAGTTATATTGGCTCGTAAGTTGGCTAAATTTTATAGTTGATGAGTCAATTTTGTGTCAGCGTTGAGGTG
CGAGCAGGCGAGCACGCAGGTGCGGCCGTGCAGGGTGAGTGTGTGCCACGCTTGCGTGAGTGTGGGGTAGCACATCGTGTGCGAGTGCTGAGGGAGACATGGCGGGATCATGTGAAG
CAAGATTGGACTAGATTGACCCGGATCAAACCCAATTTTCAAACGGATTGAAAAACGAAACTCGAAGTTAGCACATTTGTTAAGGTCGACCCAAACTAGAATTTTCTAGGTTAGATTCGG
ATCGGGTTACCGAGTCGGGTCAATAATTGACATCCATATAGTAGGATAAAGACTTGATTTTATTGTTATAGGTTATAAAATCCAATTTCATGATGAGTCAAACTTACATTAACTTTTGTAATAA
AGAGAACATGATGATATAATATTAGGATTGATCCTAAAAGTTTAGAAAGAAATTGGGGCCCTACTAAAAAGTTTTGGCTAGAAATAGAGATTTAATGAAGAATTACCTTCACCATACTTGTA
CATTTGTTTAATTCTCTTATTGATATTTTTTTTTCAAAAAAAAATAAGAATCAATATTACCCTACTTCATTTCATTAGTGTGAGCTGGATGTGCGCATGGATCCTTTTCTATGTCTTCCAAGTGCT
AAGAGATCAATTTAATTTTCACATTATTTTTCACTAGTGCTACATATGAATTGATTGTTCGACAAATTACTTGTAACTTAGCTAGATTATAGCTAGTTCACTAGTGCTACATTTTTTAAGATTGG
CTTGAATCTTTATTTGGTTTATTTTTACATAA
>comp166781_c0_seq22
AAAAAAAAAAAAAAAAACAATTATCCTAATTTCTAAATACGTAATTCATTTCACTAAAGTAAACAAGATTACCAAAACACAAATACATCACATTCAAATTTGAGCTCTCAAACTAAACAGA
AAAAAATTACATAACAAAAAAAAAAAATACAATCAGGATTTTTATGGCAATCTCAAAGCATTTAGACATAAATCCAGCACAATAAGTTCTTATGATACAATACGAAAAGAGTAATGTCAGT
TTTGATTCAGAGTACCGTCGCAACAAACTACAATTATGTCCAAATTACAATAAACAGACGATATCCCCCACACGAACTAAAATTCAGAACTATCAAACCAGATGAGAACATAAGAACCCAT
CTCCCTTTTATTCTGGCTTCGAGAAGAAATCGTTGACAGATGGCATGATCTGAGGCTCTTTATTGCGCACATATTTTCTAAGGCCGTGTTCTCTATATCTCTCCCCTTCGGCGAGATTGTCGA
GCACTCCGGCTGTTCTTTTTTCCTTGCGCACCCTGACATCAGGATTGGTCGTTATGTTCCTCACTAATTGCATCACACAAATACCAACAACAGTTCCAACCGCTGCAAACAGCGGATAGAC
CTAACCATTTAATTAGAAAAAATAGATCAATAAGACTAAAAAATTTCTTCCCCAAAGATTAGAAATACAAAAAAAAAACACCATCATAATCTAATCAAATATTCATAACATAAAATAAAAGA
GACAAATCGAAATAATAGCTGGATACCTCAGGCCTCAACCATCTTTTGACTGTCGATGAAGCCATTGGGGCACGCAAGAGAAGCGAATATCAGAAGAGAAGAGGGGAATAGAAGAAGA
GAGAAATATATGAAGCAAAAACTTGCAAAAGACGAGAAATAAATATTGCAAGATTGCATTTAAGGGAGAGAAAGAGGTCAATTTTAGTATGAGAGCCGTTTCGGGTTTGGGCTGGCTTCG
CCTGAACCACTAGCTGTACACGTTTTCTTTGTTATTCTCCTCTTACGGGGACCGGAAATCGGAGTAGAAAATAAGGACTTGATCTGAATTTGTGGTCGGGGTGTTTGTAATTAGGGATGGG
TGAGGTTGGGTGGCTTTGAAAATATGGAAGGAAAGCTTGACTTGCTTTTTGGGGTTTTTTTATTGTTAATGCAGATACAAGGCCACAAAATCTACGGATTGAAATTTCTAGAACCCGTGGA
GTTTGACTTCTGTTTCAATTTCTAGGAAAAAGAAGTTATTGAAGATGACTAATTTAACATTAAAATTTAAAATATACTGGAGTTATATTGGCTCGTAAGTTGGCTAAATTTTATAGTTGATGA
GTCAATTTTGTGTCAGCGTTGAGGTGCGAGCAGGCGAGCACGCAGGTGCGGCCGTGCAGGGTGAGTGTGTGCCACGCTTGCGTGAGTGTGGGGTAGCACATCGTGTGCGAGTGCTGAG
GGAGACATGGCGGGATCATGTGAAGCAAGATTGGACTAGATTGACCCGGATCAAACCCAATTTTCAAACGGATTGAAAAACGAAACTCGAAGTTAGCACATTTGTTAAGGTCGACCCAA
ACTAGAATTTTCTAGGTTAGATTCGGATCGGGTTACCGAGTCGGGTCAATAATTGACATCCATATAGTAGGATAAAGACTTGATTTTATTGTTATAGGTTATAAAATCCAATTTCATGATGAGT
CAAACTTACATTAACTTTTGTAATAAAGAGAACATGATGATATAATATTAGGATTGATCCTAAAAGTTTAGAAAGAAATTGGGGCCCTACTAAAAAGTTTTGGCTAGAAATAGAGATTTAAT
GAAGAATTACCTTCACCATACTTGTACATTTGTTTAATTCTCTTATTGATATTTTTTTTTCAAAAAAAAATAAGAATCAATATTACCCTACTTCATTTCATTAGTGTGAGCTGGATGTGCGCATG
GATCCTTTTCTATGTCTTCCAAGTGCTAAGAGATCAATTTAATTTTCACATTATTTTTCACTAGTGCTACATATGAATTGATTGTTCGACAAATTACTTGTAACTTAGCTAGATTATAGCTAGTT
CACTAGTGCTACATTTTTTAAGATTGGCTTGAATCTTTATTTGGTTTATTTTTACATAA
>comp166806_c1_seq1

AAAAAATCGCCTCAAACCAAGCCTAAAATCATAAACCCAATTCAAGCAACTAACAAAACTTCCTCCATAACATGCCCTTGTGCCATAAAAGAAGAACTTTTACGTCACCATCACTTAGCT
GTTAATATCTTGACAGCTGAAGCATTACAAATACGGTGTAATGCCACAATAGAATCACCAGACTTGCAGAGTCCGGTTGCCTTCGCATGCCGAAGAGCAAAGTCAAGCGCTTCCTCTGTT
GCTTCAGTATGAGAAGGCTTTGCTGATCCTTCACTTAATACTGGAATCACTCCTCTATAAACAAGGCTGTGCCTTGCCGGAGCTTCGTCGCTGCAGTACCAGTCGAAAGAATCACTATTTAT
CTTTATCTCAGGAACCACCACAGACAAAATGGGCACTTTTGGCCTGTACTTAGCCACCAGTCTTGAAGCAGTCCCTCCTCTCGTCAGAACAAGAATAAGAGCAGCTCTGACAGACTTTG
CAAGTCGAACGGCAGACGAAGCAAGGCTCTCCAATGGAGTACTTGGCGACTTTGTTACAGTCGTGAGTGCTTTAAACACAGCGTCATAATCCATAAATGATTCTGCTTCCTCGCGTATCCT
CGCCATGATCTCAACAGCAAGAACAGGGTAAGCACCCGCTGCTGTCTCGCCGCTGAGCATGACACAATTAGTGCCATCAAGAACAGCATTGGCGACATCTGTAGCTTCTGCTCTGGTGG
GACGAGGAGACCTGATCATAGATTCCAGCATTTGGGTTGCAGTCACCACTGGCTTCCCTTGAACGTTGCATTTGTGGATCATTAGCTTCTGCGCAAGGAAGATTTTCTCAATTGGTATCTC
CATGCCCAGGTCTCCTCTGGCCACCATGAAAGCATCAGAATTTTCAAGTATATCATCAAAATTCGCCACCCCTTCTTGGTTTTCAACCTTCGACATCAGCATGACAGACTTTGCATGATCAC
CCAGAAGCTCTCTAACTGCAACCATGTCAGAACCTTTTCGAACAAAGGAGAGGGCGATAATGTCGATTTTGTTCGGGATGCCCCATTTGAAGATATCTTCTTTGTCTTTCTCAGTTAAGGT
GGGAAGATCAACAACAACTCCTGGAAGATTTACGTTCTTTCTCTCACCGAGAACAGCAGCATTTTCGCAGCGGCAGTGAACAAGACCTTGCTCCTTATCGCAGCTTATCACATTAAGTGA
AATTGTGCCATCAGAACAGAGGATGATACTTCCAGGTTTTACATCCTCCGCCATTTTCTTGTAGCTCATTGATATCATGGTTTGGTCACCCTCTATACTATAATCTGTTGTTATGGTGATCTCT
TGATTTTTCTCAAGCTTTATGGGCTTTCCATCTTTCAAGAATCCTGTTCGGATCTCAGGACCCTTTGTATCGAGCATTACAGCGCAGAGTATTCCTGTTCTTTCCATGGCAACCCTGAGATTA
TCTAGGGTTTCCTGATGGTACTCATGGGATCCATGGGAGAAATTGAATCGCGCGACGCTCATTCCGGCCCTAAGCAATTTCTCGATCATCTCAATCGATCGCGATGCAGGGCCGAGCGTAC
AAACGATCTTCGTCTTCGGCCTTCTCATCACTTGATCTTCCGCAACGGGGCTGAAAATCTTCGACGTCTCCATTGTTGATGATCTCAAAGAACAAATAGAGGAGAAAGTTGAGAACTTTA
AGGGGAAGCTTAATATATATAGGGAGAGGGTTAGTTATTTTTGTTAAGAGAAACAAAATTTTGCTAAATTTGAAAAGTTCCGCGTACAATAGAAAATAGAAAACACAATTCTTCTTGGCCT
ATT
>comp166839_c2_seq1
CGACCAATCATTTTCCTTTTGCAATGGTGGTTTTTATTTTGAAATTCCCCGTCTCGATTGGTTGTCGGCATTCTATAAATGGGTAGCAGGAGGGAATGGAGGGACAGCCGCACTTTCGGCG
CGTAGCCTGTAAGGAGATTCCGTTGCCGTTGCCGCCTCTCCTTTCCTTCTTCAATCTCCTCTTCCTACCTTACCTTGTCTTAGCTCTTCCTATCCGGTGCTTCCTCGTTCTTCTACTGCGCCG
CGGAAGACAGGGAGGCCACTGCTGCTTTCTTGCCTCAGCGTGTCGCGGAGGAGGGAGAGGGACGTTGGGTGATGGCGATGATGGTGGAGGTCGGAGAGGATGGGGCTCGGAACGATG
AGAAGAGGCCGGCTGTTGAGGTGTCGGATGAAACTGCGCACCAGATTAGCACAGATCCTTGGTATCAAGTTGGGTTTGTTCTCACAACGGGGATCAACAGCTCTTACGTATTGGGGTATT
CAGGATCAATCATGGTTCCTTTGGGATGGATAGGTGGTGTAGTTGGTTTACTTTTAGCCGCTGCAATATCCTTGAATGCCAATGTTCTTATTGCTCGTCTTCATAATGTCGGAGGTAAAAGGC
AAATCAGATACAGAGATCTTGCTGGTCACATATATGGAAGGAAAATGTATTGTCTTACATGGGCCCTCCAATATGTGAATCTTTTCATGATCAACACCGGTTATATCATTTTGGCCGGGCAAG
CACTCAAGGCTATTTATGTTCTGTACCGGGATGATGGTGCTTTGAAGCTCCCATACTGTATAGCAATTGCTGGAGTTGTTTGTGCTCTTTTTGCCTTTGGAATACCTCATCTATCTGCTTTGA
GGATATGGCTTGGATTCTCTACGTTCTTCAGTTTTGTATATATTGTCATAGCGTTTGTGCTCTCACTTCGGGATGGATTGAACAGTCAGAAAAGGGATTACAGCATTCCTGCATCACACTGG
GACAGGATATTCAGTACCATTGGTGCAATTGCTAATCTTGTTTTTGCGTACAATACTGGGATGCTGCCTGAAATACAGGCAACAATAAAAGAACCTGCCATTAAAAATATGGAGAAAGCCC
TATGGTTTCAATTTACAGTTGGTGGTTTGCCTTTATATGCTGTCACTTTTATGGGTTATTGGGCGTATGGTTCCTCAACTTCGAGCTATCTCCTCAACAGCGTTCATGGACCAGTTTGGGTCA
AGACAGTAGCCAACGTTGCATCCTTTCTCCAGACAGTAATTGCTTTACATATTTTTGCCAGTCCCATGTATGAGTACCTGGACACAAAGTATGGGAGAGGAGGGAGCCCCTTCGCTCTAAA
GAATATTTTATTTCGGATCATCGTTAGAGGTGGGTACCTCACCATAAACACACTCCTCTCAGCTCTTCTTCCATTCCTCGGAGACTTCATGAGCCTGACTGGGGCTTTGAGCACATTCCCAC
TCACCTTTGTTCTCGCAAACCACATGTATCTAATGGCAAATATGAAGAAACTCTCAGTTCCTCGAAAGGCTTGGCATTGGCTGAATGTTTTAGGCTTCAGCTTCTTGGCTCTCATTGCTGCT
ATTTCTGCAGTGAGGCTAGTAACGGTCGATTCGAAAACTTATCATCTCTTCGCTGATATTTGAAAATATATTTTTGTTTGTACGATTTGTTTTTTCGGCATTTGAGAAATTTTCTTCCTTTTGA

AGTACAATATTTCGATCATTATCGCCGATCTGTTTCAAACTATTATCCATAAAATTGATGTATTCTATCAACACTTCAGTGGCAATCAAAGCTCACAACCCACAATCTAATGAGAGATTATTAC
GTGCGGATGTTGAACGGGTTCAGTGGAAAATGAGGAGGCGACACGTCATGTAATGCACTGTGCATTGGACGATATGACATCTTCTCATTTGCCTCCGAACATGTTCGACATCACGTAATAA
TTTTTTCAAGCCAAGAGACGTTGGGTCGGTCAGAAGCATTCAGGTTTTTTTGTTGTTGTTAGCTTGTACTATGAATTTTTTTTGTCCTAAAGCAATCTAATGCAAAAGTTTCAATGCAATTA
CTAATTTGTAGCCTATATGATTGTAACTCTGCTTTCGAAGAATGATATTAAAGAAATTCCTTAAA
>comp166894_c0_seq10
AGAGATGACCGGATCTGGGGAGCCGTTTGCCGAACTGGATCTGTTGTCGGCGAGAGTAGATGTCTCGGATTGAGCCATTGAACAGGATAGGATCAGCGACCTGCTCTGATACCATGTAGA
AACTCTTAGGACAGAGCTTCTTGATTAAAGAAATAGAGAATACAGAAGGGAAATAGAAAGTGCAAAGCAGAGAAAGAAGAATTTATTATTATACAGATAACCGAGTACAGCCTGTTTAAA
TAGAGAAAATAGGTAACATATAAAGCCATATAAATGGCTATACAATTATTTAGATAGATTGCTCTAATATCCCCCCACAAACAGATGATTGTTCTACAATTGTAAGTTTGTCTCGTAGTAGTTG
AAAACGAGGAGTCGATAGAGCTTTTGTGAAAATATCTGCCGTTTGATCAATAGTGCTAATAGGTATAATCCGAATCGTGTTTTGACGAATGTGATCTCTAAGAAATCTATGATCAATCTCGA
TGTGTTTTGTGCGAGCATGAAACACCGGATTGTTGGCAATTGCTATAGCTGAAGTATTGTCGCATCTAATGTCGGTCGGTGATGTTTGAGGAACGCCAAAGTCGGTAAGAAGACGCCTAA
GCCAAATGATGTCGGATGCAAGTGCGGCGAGGGCTCGGTACTCAGATTCTGTGGAGGAACGGGCAACGGTTGTTTGCTTTTTGACTGTCCAAGAAATGAGCGTGTTTCCTAAGAACGAA
CAGTAGCCTGAGGTAGACTTGCGTGAAACTGGATCACCTGCCCAATCCGCATCTTCTCTTGTAAGAATTGGATATATTCGATGACCTCCAGCAAAAATGATGCTTTATCTCTTTTCTGATCG
CTATTTGGCAAGAGTCCTATAAGCATTTGAAATCTGTCAAAAACAGGTTGAGTGTTCATTTAGCTACACATTCCAGAAGCAATCTGCTTAAATGAAAGCATAATTATGCAACTTATCTGGTT
GAGATAAAATTGTAGTCTCACGAAAAAGCAGTGCTAATTAGTTCCTTTGACGTCCATTTACCCTTGAAAAATGCATATTGCATGAGCATGTCATCAGGTTATCAGCAAACTGTGATCTAGGA
GGCTCTTCCATTAGAGGCTAATCCTAGGTTTAGCTATCATTTATTTTACTTCTACGTCTTTGTTCTATGGCAGAATGCTTCAATCGTGGGGTGCTTGCCTTCTGCTCGTTGCTTTTCCCATCAA
GTTTCAAGGTCAGCTCTTTTTTAGAAGAGCCTTCTCCCTTTCCGAACTCCTCTTCATCTTCGTCGTCCTCTTCAAACCCCATGTTGAATCTCGAGAAAGACTCCATGAATCGATTCTTCTTC
TAGAATCAGCTGTTCCTAAGTTTCTTATGCTCTCAAGACTGCTAGAGAGGTAGAATCGTGGAGAGGAAAAAATTGCTAGTAGAAACCCTAGAAAGCGAATGCAGGAGGTACTGGCGAGT
AGGTTCGATGCGAGTGACTCGAAGTCTCGAAGCTTTGGAGCTTCCAGCGGAAAGTGTAATAGCCGCGGCCT
>comp166902_c1_seq5
AATGACTCTTGAGCGATTGGTTGGTTGAAGCCTCTTTTCCAAAGTGTCCCAGATTTCGTGTGTAGTTTGCAGCGTCAATACATAAGAAAGGATATTCGGTGAGATAGTGGAGAACAATGCC
GCAGCAAGATTTTGATCAGTGAGTTGCCATAGTTAATAGTTTGGATTCAAACTAATGGTTCCAGATGAATCGGAAATGTGTTTTACTGGACAAACATCAGATCCGGTGAGAAAACCATCAA
AACCGTTGGCGGAAAAGATTTTAAAAACCTGGAGCTTCCAAATGGCGTGTTTGTCGCTGTTCAGCTGGATGTTGATGATATGCTTGATATTTGAGATTATGAACTTCAGAGACACGGGAAT
GATGATATCTGTTGCGGCTGTAACTGTAGAAGCGGCTTGAGGAGCCGAAGAGCTTTGGCTGTCTACCATAGAGGAATCTCAGTCACACTATTGAGCCTGGAGCTCTGATACCATGAAATGA
TCTGCAAAATAGATGTTAGAAAAACAGAGGAAGAAGAAGAGGTTTGGTTGAAGGTTTCATATATTCCCTTAGTCAACAATTCCTGTATACATTGGTATATATTAAGAAGAAGGGAACAGGT
TGTTAGAGTTTCAGAAGGAGCAGGTGCCCAGACGCGGCGATGGCAGAAAACCATTTTTTGTAATGATTCGCTGAGGAGGAAGTGGAGCAGTAGCCGAATATAAGCAGGTGTGCTTGTAT
AAGTTGAATGGCGTATATGATCTGTTAACAGCCCCCCGCAAACTTACGGTTCAGGTGGTTGAATGGTAAGTTTGTCTCTGAGCATTTTGAAGCGAGCGAGAGCTAATGGTTTGGTGAGTAT
GTCGGCAGGTTGATCCACTGAGTTTATGTGATGTACAGTGATCTCGTTGGATAGAATACGCTCTCTGATGAAATGGTAGTCAATTTCAATGTGCTTCGTTCTGGCATGAAAAACAGGGTTG
TTTGCAAGTGCCAGGGCTGATGTATTGTTGCAGGAGAGAGCTGTAAGAGTTGATGATGAAGCTTCAAAATCAGTGAGCAAGCGACGAAGCCATATGACATCTGATGTTGCAGAAGCAAG
AGCTCTATATTCGGCTTCAGTGGACGATTTAGCAACCGTAGGTTGTTTCTTTACACACCAAGAGACTAAGGTGTCGCCCAAGTAAGTACAGAATCCGGAGATGGATTTTCTGTCATTAGAA
TCGGATGCCCAGTCAGAGTCAGAGAAAGAAGTGAGTTGGAGATTTCCTCTTGTAATTGGCAATCCGAAATGATGGTTCCCCGAATATAACGCAGCAGCCTCTTAAGAGCGGTAAAGTCTT
GCTCAGTCGGATTGTGCATATGTTGGCAAATCATGTTAGTAGCATATGCAATATCCGGCCTCGTGATGGTTAAATACTGCAATGATCCAGCTAATTGCCTGTATAGTTTGGCATCTGAGTTGA

GGAAATTGTTGCTGTTGGATGAGGGCTTAAGAGCAATAGGTGTTTGAGAGGATTGACAATCCTTCAAACCTGCTGATTGTAAGATGTCTTGGGCGTATTGTGCTTGATGCAATAAGTATCC
GGTAGGTTGGCGTAGTACCTGAATTCCCAAGAAGAAGGAGATGGGGCCGAGTTCCTTTAAGGAGAACTGAAGTTGTAGCTTGGTGATGAGTGCAGAAATACTGTCTTTGTTGTTGCCGG
AGATTAAGAGGTCACCCACATACATAAGTATGTAGAGATGAGTGCTGCCTTGCTTGTAGACTAGTAGAGAGGGGTCGGACTTGCTGAAGCTGAATCCGAGGGATTGAAGGAAACCGGTA
AATGTATGAAACCATTGTCTAGGTGCTTGTTTAAGCCCGTAGATGGTTTTGTTCAGCTTGCATACATAATCGGGACGATGACTGTCTATGTAGCCTTGAGGTTGCTTCATATATA
>comp166944_c0_seq9
TTTTTGGGGGACATCATATATAATCCTAAATTTTTATTAAATATATATAAAAAAAACATTTATATTATATATTTATCACTGAAGGATAATATAGATCTGCACACACAACTTAAATGTACAACAGA
GAATTATATATTGAAAAAAAGATAGGGATTTTTATATACATAACACCTAAAACTATTGAAAATACATATCTAACACTCAAACATTAATATTTATTTATTTACCACTATGTTACACATACTGTCAC
ATTCTGTCACTCTCAACAACCACCTTCATTGTAGTTACCTCCAGCGGCGGCATCCAGCTCAATAGCGGCCACATGGACCATCTACATCCAGCCAGGACCACCGGCGACCGCGTGTGGGCT
CGCACTGGTTGTTCCTTCGACGCCTCTTGAAACAACAATTGTGAAATCGGCGACTACTGCAACACACTCTCCTGCACCGCCTACAGCTCGCCGCCGAGCACCTTGGCCGAGTTCACCCTC
[truncated: 426,708 more chars]
